# Detection of bacterial pathogens from clinical specimens using conventional microbial culture and *16S* metagenomics: a comparative study

**DOI:** 10.1186/s12879-017-2727-8

**Published:** 2017-09-19

**Authors:** Lalanika M. Abayasekara, Jennifer Perera, Vishvanath Chandrasekharan, Vaz S. Gnanam, Nisala A. Udunuwara, Dileepa S. Liyanage, Nuwani E. Bulathsinhala, Subhashanie Adikary, Janith V. S. Aluthmuhandiram, Chrishanthi S. Thanaseelan, D. Portia Tharmakulasingam, Tharaga Karunakaran, Janahan Ilango

**Affiliations:** 1 Dean and Chair Professor of Microbiology, Faculty of Medicine, University of Colombo, P.O. box 271, Colombo, Sri Lanka; 2 Department of Chemistry, Faculty of Science, University of Colombo, Colombo, Sri Lanka; 3 Credence Genomics Pvt. Ltd, 12 – 3/2, Sunethradevi Road, Kohuwala, Nugegoda Sri Lanka

## Abstract

**Background:**

Infectious disease is the leading cause of death worldwide, and diagnosis of polymicrobial and fungal infections is increasingly challenging in the clinical setting. Conventionally, molecular detection is still the best method of species identification in clinical samples. However, the limitations of Sanger sequencing make diagnosis of polymicrobial infections one of the biggest hurdles in treatment. The development of massively parallel sequencing or next generation sequencing (NGS) has revolutionized the field of metagenomics, with wide application of the technology in identification of microbial communities in environmental sources, human gut and others. However, to date there has been no commercial application of this technology in infectious disease diagnostic settings.

**Methods:**

Credence Genomics Rapid Infection Detection™ test, is a molecular based diagnostic test that uses next generation sequencing of bacterial *16S* rRNA gene and fungal *ITS1* gene region to provide accurate identification of species within a clinical sample. Here we present a study comparing *16S* and *ITS1* metagenomic identification against conventional culture for clinical samples. Using culture results as gold standard, a comparison was conducted using patient specimens from a clinical microbiology lab.

**Results:**

Metagenomics based results show a 91.8% concordance rate for culture positive specimens and 52.8% concordance rate with culture negative samples. 10.3% of specimens were also positive for fungal species which was not investigated by culture. Specificity and sensitivity for metagenomics analysis is 91.8 and 52.7% respectively.

**Conclusion:**

*16S* based metagenomic identification of bacterial species within a clinical specimen is on par with conventional culture based techniques and when coupled with clinical information can lead to an accurate diagnostic tool for infectious disease diagnosis.

**Electronic supplementary material:**

The online version of this article (10.1186/s12879-017-2727-8) contains supplementary material, which is available to authorized users.

## Introduction

In nature and in human disease, bacterial microorganisms are found in complex communities. The need for further understanding the role of different microorganisms on human health led to the inception of the Human Microbiome Project (HMP) [1], which uses metagenomics (i.e. the genetic material within a given sample) to characterize the composition of the microbial community in the human body.

Bacterial infection is among the top ten most common causes of death worldwide [2]. Microbial flora in clinical specimens obtained from different parts of the human body includes a variety of different organisms both pathogenic and non-pathogenic. Traditionally, diagnosis of bacterial or fungal infections relied solely on culture based techniques and culture has been considered the gold standard of pathogen detection. However, some organisms may not be easily detectable by conventional culture methods used in most laboratories due to many factors. In a conventional clinical microbiology laboratory setting, microbial culture of most specimens will be carried out under aerobic conditions. Clinical specimens are not routinely investigated for a variety of pathogens e.g. fungi, anaerobes or rickettsial pathogens unless specifically requested or indicated by the clinical history. Standard culture techniques rely largely on morphological and biochemical characterisation for identification, which can lead to decreased specificity. Also, only a fraction of organisms can be successfully cultured in a multipathogen sample due mostly to various factors such as fastidious growth requirements, non-viable organisms or inhibition of pathogenic organisms due to bacteriocin production by other microbes present in clinical specimens [3, 4]. These factors make accurate diagnosis and treatment of infections a challenge.

### *16S* rRNA and *ITS1*

In 1985, Pace et al. published a new revolutionary method of bacterial characterisation [5]. The *16S* rRNA gene is a universal gene found in all bacterial chromosomes. In identifying the presence of conserved and variable regions in the *16S* rRNA gene for use in phylogenetic identification, this technique has opened up an entirely new horizon for bacterial identification. Since the first introduction of this technique, *16S* rRNA based characterisation of bacterial species has been universally accepted as an accurate method of bacterial identification, far superior to morphological or biochemical identification [6, 7]. In much the same way, *ITS1* (internal transcribed spacer 1) of the *18S* rRNA gene has emerged as a useful genomic marker for identification of fungal species [8]. Similar to *16S* rRNA, the *18S* rRNA gene is ubiquitous among fungal species and contains a mixture of highly conserved regions interspersed with variable genetic regions that facilitates metagenomics identification of fungal species. However, despite the accuracy of *16S* rRNA and *ITS1* based detection, clinical application has been severely limited due to the limitations of Sanger sequencing. Sanger sequencing is the “classical” DNA sequencing technique and uses chain termination method to identify the sequence of bases within a DNA molecule. However, the application of Sanger sequencing is limited to an amplified product of a single DNA molecule. Thus Sanger sequencing limits the identification of pathogens in polymicrobial specimens encountered in clinical settings which will contain DNA molecules from different bacterial species. As a result, application of Sanger sequencing requires clinical isolates being cultured in vitro*,* extending the limitations of culture based identification to this technique [5].

### Next generation sequencing based species identification using *16S* rRNA

Next generation sequencing (NGS) takes DNA sequencing technology to the next level. By parallel sequencing processes, NGS allows the simultaneous sequencing of different DNA fragments while delivering accurate identification results. The combination of a universal gene based species identification and NGS gave rise to a new field known as “metagenomics”, where microbial diversity within a sample is defined using the genetic material present [9]. Therefore NGS has contributed to the studying of clinical specimens with a multitude of organisms such as the gut flora and has proven to be a useful tool for microbiome analysis [10]. However, use of metagenomics in clinical microbiology settings with respect to clinical utility has not been comprehensively studied [5, 8].

In this study, results of bacterial culture and metagenomic *16S* rRNA gene testing were compared to determine the specificity and sensitivity of metagenomics relative to aerobic bacterial culture in a clinical setting.

#### Credence rapid infection detection™ (credence RID™)

Credence Rapid Infection Detection™ is a two-part diagnostic test, that uses partial *16S* rRNA and *ITS1* gene region of bacteria and fungi (respectively) to identify the microbial composition in a clinical sample, combining molecular amplification with metagenomics identification of species. The V1-V2 region of the *16S* rRNA gene has enough variability to provide species based identification for use in clinical diagnosis in previous research publications and similarly, the *ITS1* gene region in fungi, due to its variability among species, is used for clinical diagnosis [11, 12].The amplified gene regions are sequenced using the Ion Torrent Personal Genome Machine (PGM) using the Ion PGM™ HiQ™ OT2 and Ion PGM™ HiQ™ Sequencing kits. Credence Rapid Infection Detection™ uses semi-conductor based NGS for rapid detection of bacterial or fungal detection in clinical specimens.

The test is carried out in two phases: First, preliminary testing for the presence or absence of bacterial or fungal DNA is carried using fusion primers targeting the V1-V2 region of the bacterial *16S* rRNA gene and fungal *ITS1* gene region. Custom barcoded primer pools were used for amplification of *16S* rRNA and *ITS1* gene regions (Primer sequences are available in Additional file 1: Table S1). All primers included universal primer sequences fused with a key sequence, barcode and adaptor sequences as required for analysis on the Ion Torrent platform. The presence of bacteria or fungi (or both) was recorded within 24 h of receipt of specimen. Once the presence of bacteria/fungi is confirmed, the amplification products are purified and sequenced. The sequencing data is analysed using proprietary bioinformatics pipelines to identify the composition of organisms within the sample. For the purposes of this study, results obtained using this test method shall hereafter be referred as “metagenomics analysis/results/workflow”.

## Material and methods

### Ethics statement

Application for ethical review was submitted to SIDCER accredited Ethical Review Committee, Faculty of Medicine, University of Colombo (Reference EC-16-134). Study protocol was approved on 18th August 2016.

### Specimen collection

Clinical specimens received from the Microbiology department of Nawaloka Metropolis Laboratory, Nawaloka Hospital, Colombo, an ISO 15189 accredited laboratory was used for the comparative study. The laboratory used standard aerobic culture methods for processing clinical specimens and they conformed to standard protocols published for clinical microbiology laboratories for detection of pathogens and interpretation of results [13, 14]. Samples from non-sterile sites were incubated overnight. Sterile fluids were cultured on agar plates (blood, chocolate and Maconkeys agar) and BHI broth. Plates were incubated for 48 h; if no growth was observed at 24 h BHI broth was sub-cultured for upto 5 days. Blood cultures were incubated for 5 days routinely or upto 14 days where enteric fever or Brucellosis is suspected (or as per request from physician). Species identification for Gram negative bacilli were carried out using RapID™ system (remel, Thermo Fisher Scientific).

The remaining or left over specimens (that had been processed for bacterial culture) were stripped of patient identification details and coded before being included into the study. A total of 103 specimens were transferred in batches of 10–12 in ice to the laboratory for metagenomic *16S* /*ITS1* analysis. The samples were selected from the sample entry register using a random number table over ten consecutive days. The specimens tested are listed in Additional file 2: Table S2. If there was no remaining sample in the selected specimen, the number was skipped and next number in the table was used for selecting the specimen. The researchers in the NGS laboratory were blinded to the microbial culture results. Metagenomic *16S*/*ITS1* identification was carried out using the Credence Genomics Rapid Infection Detection™ test.

The following steps were used in the workflow leading to species identification using NGS.

### DNA extraction

As the first step DNA extraction of fungal and bacterial DNA from each specimen was carried out using the QIAAmp^®^ DNA Mini kit (Qiagen) according to manufacturer’s instructions. Each batch of specimens were extracted with negative buffer control (extraction control).

### Library preparation

The presence or absence of DNA was confirmed through PCR amplification of the bacterial *16S* gene V1-V2 region and fungal *ITS1* gene region. PCR reactions were prepared in a laminar flow PCR work station with all material UV irradiated prior to use. 12.5ul of Platinum^®^ PCR supermix (Invitrogen) and 2.5ul of each primer pool was added to a final reaction volume of 25ul. PCR was conducted using 12.5 μM of each primer and 3.75 μl of template DNA.

PCR Amplification was carried out using the following cycle conditions:

95 °C for 5 min, 10 cycles at 95 °C for 30s, 58 °C for 30s and 72 °C for 60s, followed by 35 cycles for 95 °C for 30s, 68 °C for 30s and 72 °C for 60s and one cycle at 72 °C for 10 min for *16S* amplification; and 95 °C for 5 min, 10 cycles at 95 °C for 30s, 55 °C for 30s and 72 °C for 60s, followed by 35 cycles for 95 °C for 30s, 68 °C for 30s and 72 °C for 60s and one cycle at 72 °C for 10 min for *ITS1* amplification respectively. Specimens were run in batches of 10 with positive and negative buffer controls for bacterial and fungi respectively. PCR products were run on a 2% agarose gel and visualized using ethidium bromide. Specimens were run in batches of 10 with 50 bp ladder, extraction (negative buffer controls), positive controls and PCR blank. *E coli* ATCC No.25922 and. *C. albicans* ATCC No 10231 strains were used as positive controls.

Where PCR inhibition was observed this was confirmed by conducting a PCR reaction with the specimen and 1ul of positive control added. No amplification even in the presence of positive control was taken as confirmation of PCR inhibition.

Final library products were purified using 0.9X of Agencourt AMPure (Beckman Coulter) beads according to manufacturer’s instructions, eluted in low TE and quantified using the Qubit dsDNA HS Assay kit (Invitrogen).

Specimen were pooled in batches of 20 and sequenced using the Ion Torrent PGM™ platform. The sequence data was analysed using a proprietary bioinformatics pipeline that maps the reads sequenced to a phylogenetic tree with the entire microbial profile within the sample tested**.**

### Semi-conductor sequencing

Template preparation and sequencing of final libraries was conducted on the Ion OneTouch 2 system and Ion PGM using Ion PGM^™^ Hi-Q^™^ OT2 Kit (Thermo Fisher Scientific) and Ion PGM^™^ Hi-Q^™^ Sequencing Kit (Thermo Fisher Scientific) on the Ion Torrent Personal Genome Machine (PGM) according to manufacturer’s instructions. Barcoded bacterial and fungal libraries were multiplexed on a single chip on a 400 bp run to obtain sequencing data. Specimens were run in batches of 20 on an Ion 318^™^v2 chip (Thermo Fisher Scientific).

### Data processing and bioinformatics

Data was analysed using Credence Genomics proprietary bioinformatics pipeline for analysis of clinical isolates. Reads obtained from sequencing run were trimmed, removing barcode and adaptor sequences. After trimming, quality control parameters (Phred Quality Score cut off and minimum read length) for all sequence data were checked. A minimum of 2000 reads per specimen were selected, with cut offs at Phred quality score 16; fragment length > 300 bp for bacterial reads and >200 bp for fungal reads. FASTQ formats generated were mapped to the NCBI-RefSeq (26:09:2016) database using Credence Infectious Panel Pipeline 1.1.0 (Credence Genomics). FASTA files and phylogenies generated from the bioinformatics pipelines are available in Additional files 3 and 4 respectively.

### Phylogenetic output & relative abundance

**Fig. 1:**
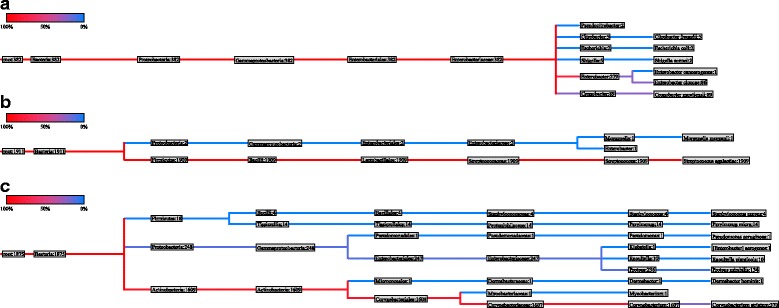
Phylogenetic tree of urine specimen (**a**) nasogastric aspiration specimen (**b**) and pus swab (**c**). Numbers displayed next to the species in the final branches of the phylogenetic tree indicate the number of reads successfully aligned to the reference 16S rRNA sequence of this species

Results of metagenomic analyses were compiled according to results from phylogenetic mapping. Relative abundance for each organism was calculated based on number of reads mapped for each species as a percentage of the root read value, with the species with the largest percentage of reads classified as the species of highest abundance. However, relative abundance data was not used for the final analysis of the specimens. Figure 1 shows the phylogenetic trees from 3 different samples.

### Analysis of culture negative specimens

When culture negative results were positive for *16S* PCR results, the libraries generated were subjected to metagenomic analysis to determine the species of the bacteria detected.

## Results

**Fig. 2:**
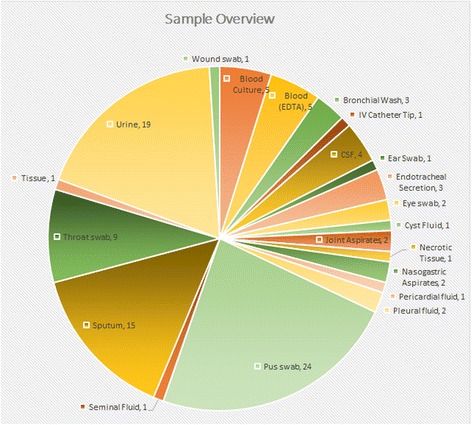
The types and numbers of different clinical specimens used in the analysis (*n* = 103)

Total number of specimens and specimen types analysed in this study are shown in Fig. 2. For the purpose of this validation, bacterial culture results where available were considered as the base line for comparison. Culture negative specimens where PCR based detection was positive were reviewed by a medical microbiologist for possible correlation of clinical data with metagenomic analysis. Final analysis of results was possible for a total of 97 specimens out of the 103 samples compared (Fig. 2).

### Assessment criteria

Specimens were considered positive for bacteria/fungi if expected bands were observed in the PCR reaction (>300 bp and >200 bp respectively). Library contamination was identified by the presence of bands in the extraction controls or PCR blank. Contaminated specimen(s) were excluded from the final analysis. Positive samples were sequenced and bioinformatics analysis carried out. Specimens which did not pass the quality parameters for bioinformatics analysis were considered conflicts in the final analysis.

For the purposes of the validation, culture results of specimens were provided the default status as accurate and the results of the metagenomic analysis were compared against the culture results. As a result, it was expected that at least culture negative results would show discrepancies when compared against the metagenomic results (due purely to the likely presence of anaerobic bacteria, slow growing organisms, fastidious organisms etc. which would not have been detected during conventional bacterial culture). However, these too were considered conflicts in the final analysis of results.

Where species/genera of culture isolates were detected in the metagenomic analysis, this result was designated as a “match”. The results of comparisons were designated as “conflicts” when species (or species classification as described in the culture report i.e. “coliform organisms”, “*Staphylococcus* spp.”, etc.) isolated in culture were not detected in metagenomic testing or when culture negative specimens were positive for bacterial species in the metagenomic workflow. The species identified in the metagenomic workflow were assessed for clinical significance by a microbiologist in order to verify the significance of the positive metagenomic results based on clinical history.

From the 103 specimens received for analysis, 5 blood culture specimens (P8, P9, P18, P19 and P52) showed signs of inhibition after the first DNA extraction and PCR reactions were resolved on the agarose gel (i.e. absence of amplified bands and primer dimers). Other specimen processed in the same batch with the blood culture specimens showed no sign of inhibition. After PCR inhibition was confirmed (as described in *Methods*), based on a literature review on PCR inhibitors in blood culture, it was concluded that inhibition was possibly due to components in the blood culture medium [15]. One specimen (P56) could not be analysed in the metagenomic workflow due to contamination and was excluded from the analysis process, resulting in a final comparative analysis of 97 specimens.

### Overview of results comparison

Of the 97 specimens processed using metagenomic analysis, 36 specimens were reported as no bacterial growth (NBG) and were culture negative; 61 specimens were culture positive. A comprehensive list with individual specimen results, culture to NGS comparison, is available in Additional file 2: Table S2. Fig. 1 shows an example of the final format of the phylogenetic tree mapped after bioinformatics analysis.

Comparison of PCR/metagenomics results with bacterial culture outcomes are shown in Table 1. Of the 61 culture positive specimens, 60 were positive at PCR level (Table 1) and 56 of these metagenomics results matched with culture results either at species level (*n* = 3) or genus level (*n* = 53). Of the culture negative specimens processed using the metagenomic workflow, 52.8%(*n* = 19/36) matched with culture results (Table 2).

**Table 1:** Comparison of culture results vs PCR results

Total Specimens	103			
NGS processed specimens	97			
		Total Specimens	Bacterial PCR Results	
			Negative	Positive
Culture results	No Bacterial Growth	36	19	17
	Culture Positive	61	1	60

**Table 2:** Comparison of culture results and bacterial metagenomic results

	Culture Negatives (%)	Culture Positives (%)	Total Specimens
Matches with metagenomic results	19 (52.8%)	56 (91.9%)	75 (77.3%)
Conflicts with metagenomic results	17 (47.2%)	5 (8.1%)	22 (22.7%)
Total	36 (100%)	61 (100%)	97 (100%)

#### Analysis of culture positive results

A total of 61 specimens were culture positive and of these 56 had species that were identified by the metagenomics workflow, giving a match rate of 56/61 (91.8%) (see Table 2). Details of specimens with conflicting results are shown in Table 3. For conciseness, where metagenomic workflow identified a large number of detected species, only species of the highest abundance and/or clinical relevance are displayed.

**Table 3:** The details of culture positive specimens with conflicting results

Specimen Type	Culture Results	Metagenomic Results (no. of species within the genus)
Sputum (P25)^c a^	Coliforms	*Porphyromonas pasteri* ^b^ *Streptococcus* species. (9) *Prevotella* species (10)
Throat Swab (P29)^a^	*Moraxella spp*	*Prevotella* species (9) *Prevotella melaninogenica* ^b^
Pus Swab (P70)	Coagulase negative *Staphylococcus* species	*Streptococcus* ^b^ *Filifactor alocis* *Prevotella intermedia*
Pus Swab (P75)	*Acinetobacter spp*	*Prevotella bivia* ^b^ *Peptoniphilus indolicus* *Finegoldia magna* *Dialister micraerophilus* *Veillonella montpellierensis* *Streptococcus anginosus* *Ureaplasma parvum*
Pus Swab (P97)	Coliform Organisms	(−)

^a^Samples in which all species detected in metagenomics workflow are not displayed due to the large number of species identified. See Additional file 2 for all species identified per sample

^b^species of highest abundance

^c^fungal species detected

#### Analysis of culture negative results

A total of 36 culture negative specimens were received and analysed by metagenomic workflow. Of these, 19 specimens were also negative from the metagenomic workflow, which left 17 conflicting specimens where PCR based detection was positive but culture negative (hereafter referred to as ‘PPCN’ specimens) (Table 4). As with Table 3, where a large number of species were identified in the metagenomic workflow only species of highest abundance and/or clinical relevance is displayed.

**Table 4:** Metagenomic results of PCR Positive, Culture Negative (PPCN) specimens

SRN	Specimen Type	NGS results
P1^c^	Urine	Enterococcus faecalis*, *Enterococcus phoeniculicola*
P5^c^	Urine	Methylobacterium longum*, *Paracoccus sphaerophysae, Burkholderiale, Paracoccus sphaerophysae, Aquabacterium parvum, Bacillus amyloliquefaciens, Kytococcus aerolatus*
P14^d^	Urine	Morganella morganii*, Stenotrophomonas maltophilia
P23^d^	CSF	*Ralstonia* ^a^
P24^d^	Pleural Fluid	***Tessaracoccus*** *
P30^d^	Pus Swab	Finegoldia magna*, *Pseudomonas aeruginosa* (low abudance), *Staphylococcus aureus* (low abundance)
P34^d^	Urine	Prevotella bivia*, *Streptococcus infantis*
P37^b, d^	Endo tracheal secretion	*Streptococcus* ^a^, *Streptococcus parasanguinis, Streptococcus mitis, Raoultella planticola, Porphyromonas gingivalis*
P40^d^	Pus Swab	*Rhizobiales* ^a^, ***Corynebacterium, Prevotella bivia, Corynebacterium tuberculostearicum***
P51^d^	Pus Swab	Corynebacterium*, ***Jonquetella anthropi***, *Staphylococcus* (low abundance)
P53^d^	Cyst fluid	*Micrococcus luteus* ^a^, *Streptococcus* spp., *Staphylococcus* spp.
P54^d^	CSF	*Mycoplasma hominis*, *Comamonas denitrificans*
P57^d^	Wound Swab	*Phyllobacteriaceae* ^a^, *Sphingopyxis fribergensis*, *Staphylococcus* spp. (9), ***Corynebacterium*** (6)
P59^d^	Pleural Fluid	*Staphylococcus aureus* ^a^, *Sphingopyxis fribergensis*
P60^d^	Sputum	*Comamonas denitrificans* ^a^, *Acinetobacter baumannii*, *Streptococcus* spp. (5), *Staphylococcus* spp. (5)
P67^d^	Pus Swab	*Staphylococcus aureus* ^a^, *Burkholderia multivorans*
P81^b, d^	Seminal Fluid	Prevotella bivia*, *Staphylococcus intermedius*, *Staphylococcus petrasii*, *Lactobacillus fermentum*

Anaerobic species are highlighted in **bold** text ^*^

^a^species of highest abundance

^b^fungal species detected

^c^Sequencing output did not pass the QC parameters for bioinformatics analysis

^d^Samples in which all species identified in the metagenomic analysis are not displayed due to the large number of species identified. See Additional file 2 for all species identified per sample

### Fungal results

As standard practice, Credence Genomics metagenomic workflow also uses *ITS1* region of fungal DNA for detection and identification of fungal species. 10 specimens were positive for fungal identification by metagenomics analysis. The species isolated from fungal metagenomics are listed in Table 5 (only species of highest abundance displayed). However, these specimens were not tested by fungal culture in a routine Microbiology laboratory.

**Table 5:** Total fungal species identified using ITS1 metagenomic workflow

Specimen type (number of specimens with identified species)	Fungal NGS Species
Throat Swab (1), Sputum (1), Seminal fluid (1),	*Candida albicans* ^a^
Sputum (1), Bronchial wash (1)	*Candida tropicalis* ^a^
Throat Swab (1)	*Lomatium nevadense* ^a^
Endotracheal secretion (1)	*Saccharomyces cerevisiae* ^a^
Pus Swab (1)	*Uwebraunia* ^a^
Pus Swab (1)	*Candida orthopsilosis* ^a^

^a^ species of highest abundance

## Discussion

Next Generation Sequencing (NGS) based identification of bacterial and fungal species has been widely available for the last decade and increasingly attention has been focused on clinical applications of this technique [16, 10]. The versatility of NGS technology allows application of *16S* identification to uncultured clinical samples, resulting in fast, comprehensive analysis of the microbial profile within a clinical sample. One of the greatest advantages of this technology is the universal coverage of all medically relevant bacteria and fungi in a single test. In this study we describe a commercial diagnostic product offered by Credence Genomics Pvt. Ltd. as part of routine clinical diagnostics for universal detection and identification of fungal and bacterial clinical species and validation of the said product against conventional bacterial culture.

In order to establish a baseline accuracy for validation of this product, bacterial culture specimens were compared against the results of the partial *16S* based metagenomic identification.

### Results concordance for bacterial metagenomic results

60/61 culture positive specimens were also positive for bacteria in the metagenomics analysis library preparation (PCR). 56/60 specimens were matches when sequenced and analysed; giving a total concordance of 56/61 for culture positive specimens.

The conflicting results in P25, P29, P70, P75 and P97 (see Table 3) cannot be resolved without further validation or querying the microbiological identification process. While the absence of metagenomic identified species among culture isolates may be attributed to decreased sensitivity, slow growth, growth inhibition, microbial interaction or even the growth conditions; the failure of the metagenomics workflow to identify culture species/isolates cannot be explained since conventional wisdom indicates that PCR based testing would be more sensitive than culture. However, since identification of species in conventional culture requires visual identification, we can speculate that human error could account for misidentification of morphologically similar organisms leading to the conflicts seen here.

The conflict seen with P97 is also difficult to explain as the metagenomics results of the specimen shows negative whereas culture has isolated coliforms from this sample. While it is possible to argue that the culture maybe showing a false positive due to contamination, it is equally likely that the bacterial load present in the sample may be below the analytical sensitivity threshold of the metagenomic test, which results in a false negative from the metagenomics analysis.

The minor conflict is seen with the metagenomics results culture results in specimen P58 is due to the difference in the coagulase activity of the species. Other instances where culture isolates were reported as coagulase negative *Staphylococcus* species were correctly reflected in metagenomic results. Out of a total of 8 Staphylococcus isolates identified by culture, 6 correlated correctly with metagenomics detected species. Given the sensitivity of metagenomic identification, it is extremely likely that this conflict in reporting of coagulase activity is due to the limitations of the tube test used to detect coagulase activity, as absence of appropriate plasma controls or shortened reaction time may have led to culture based misidentification [17]. However, as per the assessment criteria, the culture isolates match at genus level with the metagenomic analysis and is therefore designated as matches.

Out of 17 PPCN (PCR positive, culture negative) specimens (see Table 4), 2 (P1, P5) failed to meet the quality parameters for successful bioinformatics analysis (i.e. less than 2000 reads of >300 bp at Phred quality score 16) and were designated as conflicts. Though these specimens showed successful amplification of expected bacterial fragment at the library preparation step, the concentration of the purified library was too low for successful sequencing and subsequent bioinformatics analysis. Metagenomic results for 8 specimens (P14, P24, P30, P34, P40, P51, P57 and P81) can be readily explained as to why these specimens were reported as culture negative. These specimens predominantly contain anaerobic bacteria which would not grow in standard aerobic culture media as was used in this case. Additionally, *M. hominis* detected in another specimen (P54), is a fastidious organism, which would typically take up to 4 days to grow and lacks a cell wall which makes Gram staining and morphological identification difficult [18, 19]. Therefore, these 9 metagenomic workflow results in context can be attributed as false negatives in culture. Furthermore, fungal metagenomics showed that 2 specimens (P37 and P81) had fungal species (*Saccharomyces cerevisiae* and *Candida albicans* respectively). Inhibition of bacterial culture growth due to the presence of fungal species is a distinct possibility, and can be considered as an example of the limitation of bacterial culture. Therefore, out of a total of 17 PPCN, the discrepancies of 10 specimens (P14, P24, P30, P34, P37, P40, P51, P54, P57 and P81) can be attributed to the limitations of standard culture methods.

Literature review of *16S* sensitivity against culture negative specimens, shows on average that 50% of culture negative specimens will be reported as positive by metagenomics/*16S* PCR analysis [20–22]. The metagenomic results of culture negative specimens reported in this study, were further analysed for clinical validity by the clinical microbiologist. It was finally concluded, that results of PPCN specimens may be due to increased sensitivity of the metagenomics work flow, which resulted in unviable or low bacterial load being detected. But in the absence of clinical follow up in real time, it is not possible to confirm the NGS findings at this point in time and is considered a limitation of this study.

### Final concordance values for bacterial Metagenomics

Based on the analysis conducted, it was found that metagenomics results have a concordance rate/positive predictive value of 91.8% (56/61) when compared with culture positive specimens. It covers a wider range of aerobic and anaerobic bacteria and provided species level identification where conventional culture could not. However, it should be noted that metagenomics results can give a wide range of organism and microbial profile can be difficult to interpret. Some of the matching specimen have a large number of bacterial species identified and though culture isolates will also be present in the metagenomics results, in some cases it will be present at a very low abundance (i.e. the percentage of read sequenced from this organism relative to all reads sequenced within the sample is low,

Using stringent comparison criteria for all specimens analysed in the study, the concordance rate was 77.3% (*n* = 75/97) for metagenomics vs. culture comparison. However, based on the limitation of conventional aerobic culture and already documented error rates of false negatives in culture (i.e. assuming at least 50% of culture negatives as false negatives attributes another 10 specimens as matches), concordance rate is likely closer to 87.6% (*n* = 85/97). Based on clinical review the conflicts in the PPCN specimens can be attributed to the higher sensitivity of metagenomic-based identification of species commonly detected in respective clinical settings in relation to each particular specimen. When PCR results of PPCN specimens are included in the analysis in addition to culture positive specimens the concordance rate increased to 94.8%.

Though there was no corresponding fungal culture data for validation of fungal results, it is important to note that 10 of 97 specimens (10.3%) were positive for fungal detection which would routinely be missed in a standard clinical setting. The primary fungal species detected was *Candia albicans* with other less common *Candida* species (see Table 5) and clinical sites being mainly throat swabs and endotracheal secretions which may have been ignored during culture interpretation at the Microbiology work bench.

### Specificity and sensitivity

By using culture results as the base line for the presence or absence of disease, the specificity of and sensitivity of the Credence RID™ test can be calculated, using the assessment criteria as mentioned above, the sensitivity and specificity of the test is 91.8% (*n* = 56/61) and 52.8% (19/36) respectively.

## Conclusions

### Commercial clinical Metagenomics-based bacterial identification

The profile of bacteria within a clinical specimen can vary widely based on treatment history of the patient and site of sampling (Fig. 1). As evidenced by the specimens in this study, non-sterile clinical sites, such as throat, respiratory tract or skin can often demonstrate more than 20 different bacterial species. Therefore, clinical interpretation of metagenomic based results of bacterial and fungal identification requires careful analysis of symptoms and the clinical relevance of each organism identified. This can make the application of metagenomic as the sole clinical diagnostic tool challenging, especially where clinical details on the patient’s condition are not available. Therefore, application of metagenomics analysis in the clinical setting will require thorough knowledge on the patient’s condition and clinical history. When compared to conventional diagnostic tests which provide a straight yes/no result, metagenomic diagnostics is far more complex. However, if specimen collection and clinical correlation as carefully conducted, metagenomics diagnostics can be universally applied for elimination of a suspected pathogen, for confirmation of a suspected pathogen or for a broad screening of where there is no suspected pathogen.

The commercial metagenomic diagnostic product offered by Credence Rapid Infection Detection™ has proven to be clinically applicable and has the ability to identify a superior range of organisms. The use of the universal *16S* rRNA for bacterial species identification has already been established as more accurate than culture [6, 7]. Its application has previously been hindered by the limitations of sanger sequencing and inability to design a rapid method of detection, which has now been addressed with the commercial availability of parallel sequencing platforms.

The outcome of this study shows that clinical metagenomics is at very least comparable to bacterial culture. However, the natural limitations in attributing culture as the default for the gold standard of diagnostic detection has resulted in a culture biased outcome for this study. The limitations of the culture technique cannot be ignored in a routine diagnostic setting as most specimens will be processed by aerobic culture methods unless specifically indicated, providing a relatively inaccurate diagnostic test result. This would naturally result in false negative cultures where anaerobic pathogens are present in the specimen as demonstrated by the metagenomic based results of this study. The additional limitations of growth conditions, growth rates of organisms or even bacteriocins produced by polymicrobial growth can easily alter the results of bacterial culture testing.

In comparison, a culture independent test such as *16S* based metagenomic identification is not limited by these considerations and can rapidly identify a wide range of organisms. Based on the wide range of organisms identified and the speed and high throughput capacity of metagenomics, it is clear that it can be a powerful clinical tool for diagnosis. The ability of the test to provide information on relative abundance of the varied organisms in the specimens adds further value to the report.

With regard to the clinical application of this test, a detailed output of microbial composition of a specimen provides an opportunity to the infectious diseases specialists to analyse the patient situation in a more comprehensive manner. However, this wider coverage of bacterial species naturally leads to complex reporting profiles which requires close correlation with patient symptoms and clinical judgement for application.

### Limitations of the credence rapid infection detection™ test

Out of a total 103 culture specimen received for analysis in the NGS laboratory, 6 specimens were excluded from the final analysis. 5 of these specimens were blood culture specimens. Processing of these specimens showed PCR inhibition, possibly due to the presence of sodium polyanetholesulfonate which is a known inhibitor of PCR. [15]. This has implications for clinical application of this diagnostic test as specimens that are already submitted for blood culture cannot be analysed without significant changes made to the test process. To mitigate this limitation, blood specimens must be collected into sterile EDTA containers instead, for analysis.

Secondly, the chance of incidental contamination of the specimen upon collection is very high, and indeed has been observed by the Credence Genomics on routine clinical testing (anecdotal evidence). Collection of blood, CSF and other sterile fluids can easily be contaminated by bacterial skin flora if the collection process is not carried out aseptically or the specimen is collected from a catheter/cannula site. The presence of contaminating bacteria, especially skin flora while not affecting the sequencing output (contaminating bacteria will simply be displayed along with all other bacterial species in the specimen), can affect clinical application of the test as the abundance values would be skewed due to the presence of the contaminating bacteria. Furthermore, the library preparation process is highly susceptible to contamination by amplicons and rigorous procedure must be used to ensure that all specimens pass the necessary quality controls.

Thirdly, the limitations in the curation of the bioinformatics databases can have an impact on the results outcome. The Credence RID™ test uses the NCBI Refseq, a rapidly expanding, curated database with *16S* sequences curated for 17,654 bacterial species and *ITS1* sequences curated for 5365 fungi species [23]. However, database curation is a long and tedious process and new variations in existing species classifications or novel species identification can take a long time. Therefore, the results of the test are limited by the accuracy and scope of the existing Refseq database.

### Limitations of this study

As a comparative study using bacterial culture as the standard for comparison there are a number of limitations to be addressed. Firstly, this study only uses the culture results from a clinical microbiology lab where only aerobic culture is routinely conducted. As explained previously, clinical microbiology labs in Sri Lanka will only routinely culture for aerobic bacteria unless specifically requested for a wider range by the physician. As a result, the culture isolates and culture negative specimen can be hypothesized to indicate the presence on anaerobic species or slow growing, fastidious organisms. Furthermore, species based identification in bacterial culture is also limited due to inaccuracy of biochemical testing and growth inhibition/competition that can occur in polymicrobial specimens. This hampers the interpretation of metagenomic based analysis, particularly in the case of conflicts in culture isolates and culture negative specimen.

Secondly, there is no information on the fungal culturing for these specimens. The presence of fungal organisms as detected by the metagenomic workflow can inhibit the growth of bacterial culture. However, without fungal culture results, there is no independent verification of the fungal results from the metagenomic analysis.

The above limitations, results in a biased measurement of culture vs. metagenomic identification of bacteria. For a truly accurate representation of the sensitivity and accuracy of metagenomic identification, a clinical correlational study with patient follow up and treatment would have to be conducted to ensure that the outcome of the metagenomic analysis is a true representation of bacterial flora within a specimen.

## Additional files

Additional file 1: Primer sequences. Primer sequences used for amplification of the bacterial *16S* rRNA V1—V2 region and fungal *ITS1* region respectively (Barcode and adaptor sequences are not included). (DOCX 12 kb)

Additional file 2: Details of specimen type, culture results and metagenomic results. All specimens analysed are described in this table. *Specimens where metagenomic results match culture results*; reference number cells highlighted in green. *Specimens where metagenomic results conflict with culture results*; reference number cells highlighted in red. *Specimens that were excluded from the final analysis*; reference number cells highlighted in black. *Specimen where metagenomic result cell is highlighted in light grey*; all species identified in the metagenomic workflow are not listed due to the large number of species identified. Species listed are based on species that match culture results, abundance (i.e. high abundance) and clinical relevance (i.e. low abundance). *Specimens where metagenomics results conflict with culture negative results*; reference number cells in white. *Genus (n);* multiple species of this genus identified in the metageomics workflow. The genus and number of species within that genus (n) displayed. *species of highest abundance. Bold species that match culture results. (DOCX 27 kb)

Additional file 3: QC passed FASTA files generated from the Credence Infectious Panel Pipeline 1.1.0. Unaligned BAM files generated from the sequencing run were trimmed of barcode and adaptor sequences and this quality checked for the following QC parameters: Phred quality score 16; fragment length > 300 bp for bacterial reads and >200 bp for fungal reads respectively and final FASTA sequences obtained. Specimen files are named as follows: Additional file 3_B/F_Specimen number (e.g. Additional file 3_B_P1 – fasta files of bacterial sequences within specimen P1; Additional file 3_F_P1- fasta files of fungal sequences within specimen P1). (PDF 37575 kb)

Additional file 4: Final phylogenies generated from the Credence Infectious Panel Pipeline 1.1.0**.** Phylogenies generated from the quality checked FASTA files for each specimen is available here. Specimen files are named as follows: Additional file 4_B/F_Specimen number (e.g. Additional file 4_B_P1– phylogeny of bacterial sequences within specimen P1; Additional file 4_F_P1 – phylogeny of fungal sequences within specimen P1). (PDF 620 kb)

## Abbreviations

BHI: Brain, heart, infusion agar
Credence RID^TM^: Credence Rapid Infection Detection^TM^
HMP: Human Microbioime Project
NBG: No bacterial growth
NCBI: National Centre for Biotechnology Information
NGS: Next generation sequencing
OT2: One Touch Two^TM^
PCR: Polymerase chain reaction
PGM: Personal Genome Machine^TM^
PPCN: PCR positive, culture negative
SIDCER: Strategic Initiative for Developing Capacity in Ethical Review
