# Supplementary material for: Detection of bacterial pathogens from clinical specimens using conventional microbial culture and 16S metagenomics: a comparative study
Source: BMC Infect Dis. 2017 Sep 19;17:631. doi: 10.1186/s12879-017-2727-8 (PMC5606128; doi:10.1186/s12879-017-2727-8)
Supplement: Supplementary file 1 — Primer sequences. Primer sequences used for amplification of the bacterial 16S rRNA V1—V2 region and fungal ITS1 region respectively (Barcode and adaptor sequences are not included). (DOCX 12 kb) [file 12879_2017_2727_MOESM1_ESM.docx]

# **Additional File 1**

| **Region** | **Primer** | **Primer Sequence (5’ to 3’)** | **Barcoded (Y/N)** |
| --- | --- | --- | --- |
| *16S* rRNA gene V2 region | V2F | AGAGTTTGATCCTGGCTCAG | Y |
| *16S* rRNA gene V1 region | V1R | CTGCTGCC/IDEOXYL/CCCGTAGGAG | Y |
| *ITS1* region | *ITS1*F | TCCGTAGGTGAACCTGCGG | Y |
| *ITS1* region | *ITS2*R | GCTGCGTTCTTCATCGATGC | Y |
