# Supplementary material for: Detection of bacterial pathogens from clinical specimens using conventional microbial culture and 16S metagenomics: a comparative study
Source: BMC Infect Dis. 2017 Sep 19;17:631. doi: 10.1186/s12879-017-2727-8 (PMC5606128; doi:10.1186/s12879-017-2727-8)
Supplement: Supplementary file 2 — Details of specimen type, culture results and metagenomic results. All specimens analysed are described in this table. Specimens where metagenomic results match culture results; reference number cells highlighted in green. Specimens where metagenomic results conflict with culture results; reference number cells highlighted in red. Specimens that were excluded from the final analysis; reference number cells highlighted in black. Specimen where metagenomic result cell is highlighted in light grey; all species identified in the metagenomic workflow are not listed due to the large number of species identified. Species listed are based on species that match culture results, abundance (i.e. high abundance) and clinical relevance (i.e. low abundance). Specimens where metagenomics results conflict with culture negative results; reference number cells in white. Genus (n); multiple species of this genus identified in the metageomics workflow. The genus and number of species within that genus (n) displayed. *species of highest abundance. Bold species that match culture results. (DOCX 27 kb) [file 12879_2017_2727_MOESM2_ESM.docx]

# **Additional File 2:**

**Details of specimen type, culture results and metagenomic results**

All specimens analysed are described in this table.

*Specimens where metagenomic results match culture results*; reference number cells highlighted in green.

*Specimens where metagenomic results conflict with culture results*; reference number cells highlighted in red.

*Specimens that were excluded from the final analysis*; reference number cells highlighted in black.

*Specimen where metagenomic result cell is highlighted in light grey*; all species identified in the metagenomic workflow are not listed due to the large number of species identified. Species listed are based on species that match culture results, abundance (i.e. high abundance) and clinical relevance (i.e. low abundance).

*Specimens where metagenomics results conflict with culture negative results*; reference number cells in white.

*Genus (n);* multiple species of this genus identified in the metageomics workflow. The genus and number of species within that genus (n) displayed.

*species of highest abundance

**Bold** species that match culture results

| **Reference**  **Number** | **Specimen**  **Type** | **Culture results** | **NGS results** | |
| --- | --- | --- | --- | --- |
|  |  |  | **Bacterial Results** | **Fungal results** |
| P1 | Urine | *NBG* | *Enterococcus faecalis *, Enterococcus phoeniculicola* | *(-)* |
| P2 | Urine | *NBG* | *(-)* | *(-)* |
| P3 | Urine | *NBG* | *(-)* | *(-)* |
| P4 | Urine | *NBG* | *(-)* | *(-)* |
| P5 | Urine | *NBG* | *Methylobacterium longum *, Paracoccus sphaerophysae, Burkholderiale, Paracoccus sphaerophysae, Aquabacterium parvum, Bacillus amyloliquefaciens, Kytococcus aerolatus* | *(-)* |
| P6 | Pus Swab | *Diptheriodes spp, Staphylococcus spp* | *Prevotella bivia *, Prevotella timonensis, Prevotella buccalis, Prevotella bergensis,*  ***Corynebacterium glucuronolyticum*** *(low abundance),* ***Staphylococcus aureus*** *(low abundance)* | *(-)* |
| P7 | Pus Swab | *Coagulase negative Staphylococcus species* | ***Staphylococcus epidermis, Staphylococcus hominis, Staphylococcus sp****. (low abundance)* | *(-)* |
| P8 | Blood Culture | *Group A beta-hemolytic Streptococcus* | *Excluded from final analysis due to PCR inhibition* | |
| P9 | Blood Culture | *NBG* | *Excluded from final analysis due to PCR inhibition* | |
| P10 | Sputum | *Pseudomonas spp* | ***Pseudomonas aeruginosa****, Staphylococcus aureus, Elizabethkingia *, Stenotrophomonas maltophilia* | *(-)* |
| P11 | Urine | *Coliform organisms* | ***Escherichia coli, Enterobacter cloacae*** ******, Shigella sonnei, Citrobacter freundii, Enterobacter cancerogenus, Cronobacter muytjensii*** | *(-)* |
| P12 | Sputum | *Normal Respiratory Tract Flora* | ***Streptococcus spp. (10), Klebsiella pneumoniae, Staphylococcus spp. (3), Raoultella planticola*** *** | *(-)* |
| P13 | Nasogastric Aspirate | *Group B beta-hemolytic Streptococcus* | ***Streptococcus agalactiae*** **, Morganella morganii, Enterobacter* | *(-)* |
| P14 | Urine | *NBG* | *Morganella morganii *, Stenotrophomonas maltophilia* | *(-)* |
| P15 | Pus Swab | *Coliform organisms* | *Staphylococcus epidermidis *,* ***Proteus mirabili****s, Corynebacterium tuberculostearicum* | *(-)* |
| P16 | Throat Swab | *Normal Respiratory Tract Flora* | ***Streptococcus spp. (8)*** ******, Prevotella spp. (10) (low abundance)*** | *(-)* |
| P17 | Pus Swab | *Group A beta-hemolytic Streptococcus* | ***Streptococcus pyogenes*** **,* ***Streptococcus pseudopneumoniae*** | *Candida orthopsilosis*, Candida parapsilosis* |
| P18 | Blood Culture | *Pseudomonas spp* | *Excluded from final analysis due to PCR inhibition* | |
| P19 | Blood Culture | *Coliform organisms* | *Excluded from final analysis due to PCR inhibition* | |
| P20 | Pus Swab | *Coliform organisms* | *Corynebacterium falsenii *, Corynebacterium resistens, Corynebacterium tuberculostearicum,* ***Escherichia coli, Enterobacter cloacae*** | *(-)* |
| P21 | Pus Swab | *Pseudomonas spp* | *Achromobacter *, Rhizobium freirei,* ***Pseudomonas stutzeri (low abundance)*** | *(-)* |
| P22 | Throat Swab | *Normal Respiratory Tract Flora* | ***Streptococcus spp. (8)*** *** | *Candida albicans *, Candida orthopsilosis, Saccharomyces cerevisiae, Aspergillus gracilis* |
| P23 | CSF | *NBG* | *Ralstonia ** | *(-)* |
| P24 | Pleural Fluid | *NBG* | *Tessaracoccus ** | *(-)* |
| P25 | Sputum | *Coliform organisms* | *Porphyromonas pasteri *, Streptococcus spp. (9), Prevotella spp. (10)* | *Candida albicans *, Candida orthopsilosis, Candida parapsilosis, Candida metapsilosis, Saccharomyces cerevisiae, Aspergillus gracilis, Aspergillus penicillioides, Penicillium oxalicum, Sterigmatomyces halophilus* |
| P26 | Sputum | *Moraxella spp.* | *Streptococcus spp. (5) *, Prevotella spp. (6),* ***Moraxella catarrhalis*** | *Candida tropicalis *, Candida parapsilosis, Candida albicans, Candida zeylanoides, Sterigmatomyces halophilus* |
| P27 | Urine | *Coliform organisms* | ***Enterobacter*** ******, Escherichia coli, Shigella boydii, Shigella sonnei, Citrobacter koser, Citrobacter freundii****, Fenollaria massiliensis, Leptotrichia goodfellowii, Fusobacterium nucleatum, Cronobacter muytjensii* | *(-)* |
| P28 | Urine | *Coliform organisms* | ***Enterobacter*** **, Mycoplasma hominis, Prevotella timonensis* | *(-)* |
| P29 | Throat Swab | *Heavy growth of Morexella species* | *Prevotella spp. (9), Prevotella melaninogenica ** | *(-)* |
| P30 | Pus Swab | *NBG* | *Finegoldia magna *, Pseudomonas aeruginosa (low abudance), Staphylococcus aureus (low abundance)* | *(-)* |
| P31 | Pus Swab | *Coliform organisms* | *Achromobacter *, Achromobacter insolitus, Pseudomonas aeruginosa,* ***Providencia stuartii****, Staphylococcus epidermidis* | *(-)* |
| P32 | CSF | *NBG* | *(-)* | *(-)* |
| P33 | Urine | *Coliform organisms* | ***Raoultella planticola*** ******, Klebsiella pneumoniae*** | *(-)* |
| P34 | Urine | *NBG* | *Prevotella bivia *, Streptococcus infantis* | *(-)* |
| P35 | Sputum | *Normal Respiratory Tract Flora* | ***Prevotella histicola*** ******, Prevotella spp. (12), Streptococcus spp. (6)*** | *(-)* |
| P36 | Sputum | *Normal Respiratory Tract Flora* | ***Neisseria spp. (5), Prevotella spp. (6), Streptococcus spp. (2)*** | *(-)* |
| P37 | Endo tracheal secretion | *NBG* | *Streptococcus *, Streptococcus parasanguinis, Streptococcus mitis, Raoultella planticola, Porphyromonas gingivalis* | *Saccharomyces cerevisiae*, Candida africana, Saccharomyces paradoxus, Candida albicans, Candida metapsilosis, Aspergillus gracilis* |
| P38 | Bronchial wash | *Acinetobacter Species* | *Achromobacter *,* ***Acinetobacter baumannii****, Staphylococcus petrasii* | *Candida tropicalis*, Candida albicans, Saccharomyces paradoxus, Saccharomyces cerevisiae,Candida africana* |
| P39 | Pus Swab | *Pseudomonas Species* | *Corynebacterium *, Corynebacterium striatum, Mycobacterium, Proteus mirabilis,* ***Pseudomonas aeruginosa (low abundance)****, Staphylococcus aureus (low abundance)* | *(-)* |
| P40 | Pus Swab | *NBG* | *Rhizobiales *, Corynebacterium, Prevotella bivia, Corynebacterium tuberculostearicum* | *(-)* |
| P41 | Urine | *Coliform organisms* | ***Enterobacter*** ******, Escherichia coli, Prevotella timonensis, Cronobacter muytjensii*** | *(-)* |
| P42 | Sputum | *Normal Respiratory Tract Flora* | ***Streptococcus spp. (6), Streptococcus*** **,* ***Prevotella spp. (11), Tropheryma whipplei, Micrococcales, Streptococcus parasanguinis*** | *(-)* |
| P43 | Necrotic Tissue | *Aerobic Spore Bearers* | ***Bacillus cereus*** **, Staphylococcus aureus, Neisseria meningitidis* | *(-)* |
| P44 | Throat Swab | *No Bacterial Pathogen* | ***Streptococcus*** **,* ***Streptococcus spp. (6)*** | *(-)* |
| P45 | Pus Swab | *Coliform organisms* | ***Raoultella planticola*** ******, Klebsiella pneumoniae, Escherichia coli*** | *(-)* |
| P46 | Urine | *Coliform organisms* | ***Enterobacter*** ******, Shigella dysenteriae*** | *(-)* |
| P47 | Urine | *Pseudomonas Species* | ***Pseudomonas aeruginosa*** **, Enterococcus faecalis, Streptococcus anginosus* | *(-)* |
| P48 | Pericardial Fluid | *NBG* | *(-)* | *(-)* |
| P49 | Sputum | *Normal Respiratory Tract Flora* | ***Streptococcus*** **,* ***Streptococcus spp. (6), Haemophilus influenzae*** | *(-)* |
| P50 | Sputum | *Heavy growth of No haemolytic Strepto coccus* | ***Streptococcus*** **, Streptococcus spp. (6), Streptococcus parasanguinis, Prevotella spp. (7)* | *(-)* |
| P51 | Pus Swab | *NBG* | *Corynebacterium *, Jonquetella anthropi, Staphylococcus (low abundance)* | *(-)* |
| P52 | Blood Culture | *-* | *Excluded from final analysis due to PCR inhibition* | |
| P53 | Cyst Fluid | *NBG* | *Micrococcus luteus *, Streptococcus spp., Staphylococcus spp.* | *(-)* |
| P54 | CSF | *NBG* | *Mycoplasma hominis*, Comamonas denitrificans* | *(-)* |
| P55 | ET Secretion | *NBG* | *(-)* | *(-)* |
| P56 | IV Catheter Tip | *NBG* | *Excluded from final analysis due to contamination* | |
| P57 | Wound Swab | *NBG* | *Phyllobacteriaceae *, Sphingopyxis fribergensis, Staphylococcus spp. (9), Corynebacterium (6)* | *(-)* |
| P58 | Pus Swab | *Coagulase negative Staphylococcus species* | ***Staphylococcus*** *aureus *, Sphingopyxis terrae, Pannonibacter phragmitetus, Rothia aeria* | *(-)* |
| P59 | Pleural Fluid | *NBG* | *Staphylococcus aureus *, Sphingopyxis fribergensis* | *(-)* |
| P60 | Sputum | *NBG* | *Comamonas denitrificans *, Acinetobacter baumannii, Streptococcus spp. (5), Staphylococcus spp. (5)* | *(-)* |
| P61 | Urine | *Coliform organisms* | ***Proteus mirabilis*** **, Prevotella bivia, Pseudomonas aeruginosa* | *(-)* |
| P62 | Urine | *Coliform organisms* | ***Enterobacter cloacae*** **,* ***Escherichia coli, Shigella dysenteriae*** | *(-)* |
| P63 | Sputum | *Pseudomonas Species* | *Streptococcus *, Streptococcus peroris,* ***Pseudomonas aeruginosa (low abundance)*** | *(-)* |
| P64 | Pus Swab | *Coagulase negative Staphylococcus species* | *Staphylococcus aureus *,* ***Staphylococcus saccharolyticus*** | *(-)* |
| P65 | Pus Swab | *Coliform organisms* | ***Raoultella planticola*** **,* ***Klebsiella pneumoniae*** | *(-)* |
| P66 | Throat Swab | *No Bacterial Pathogen* | ***Streptococcus parasanguinis*** **,* ***Streptococcus spp. (10)*** | *(-)* |
| P67 | Pus Swab | *NBG* | *Staphylococcus aureus *, Burkholderia multivorans* | *(-)* |
| P68 | Eye Swab | *NBG* | *(-)* | *(-)* |
| P69 | Throat Swab | *No Bacterial Pathogen* | ***Streptococcus parasanguinis*** **,* ***Streptococcus spp. (3), Staphylococcus epidermidis*** | *(-)* |
| P70 | Pus Swab | *Coagulase negative Staphylococcus species* | *Streptococcus *, Filifactor alocis, Prevotella intermedia* | *(-)* |
| P71 | Ear Swab | *Pseudomonas Species* | *Achromobacter *,* ***Pseudomonas aeruginosa (low abundance)****, Comamonas denitrificans* | *(-)* |
| P72 | Pus Swab | *NBG* | *(-)* | *(-)* |
| P73 | Joint Aspirate (knee) | *NBG* | *(-)* | *(-)* |
| P74 | Sputum | *Normal Respiratory Tract Flora* | ***Neisseria*** **,* ***Porphyromonas*** | *(-)* |
| P75 | Pus Swab | *Acinetobacter species* | *Prevotella Bivia *, Peptoniphilus indolicus, Finegoldia magna, Dialister micraerophilus, Veillonella montpellierensis, Streptococcus anginosus, Ureaplasma parvum* | *(-)* |
| P76 | Throat Swab | *No Bacterial Pathogen* | ***Prevotella*** **,* ***Prevotella histicola, Megasphaera micronuciformis, Streptococcus peroris*** | *Lomatium nevadense*, Osmorhiza claytonii, Laserpitium petrophilum, Convolvulus farinosus, Vigna angularis, Caulanthus anceps, Alternaria tenuissima, Candida, Saturnispora diversa* |
| P77 | Nasogastric Aspirate | *Group B beta-hemolytic Streptococcus* | *Streptococcus *,* ***Streptococcus agalactiae****,* ***Streptococcus pneumoniae, Streptococcus mitis****, Lactobacillus iners, Staphylococcus epidermidis, Staphylococcus aureus* | *Not sequenced.* |
| P78 | Tissue | *Coliform organisms* | *Streptococcus pyogenes *, Streptococcus parasanguinis,* ***Enterococcus faecalis****, Staphylococcus aureus, Corynebacterium falsenii,* ***Proteus mirabilis****, Bacteroides fragilis, Prevotella bivia* | *(-)* |
| P79 | CSF | *NBG* | *(-)* | *(-)* |
| P80 | Sputum | *(H)coliform organisms* | *Streptococcus parasanguinis *,* ***Enterococcus faecium****, Staphylococcus haemolyticus, Staphylococcus petrasii, Prevotella bivia, Micrococcus luteus* | *(-)* |
| P81 | Seminal Fluid | *NBG* | *Prevotella bivia *, Staphylococcus intermedius, Staphylococcus petrasii, Lactobacillus fermentum* | *Candida albicans ** |
| P82 | Sputum | *(H)Acinetobacter species* | ***Acinetobacter baumannii*** **, Staphylococcus petrasii, Mycoplasma salivarium, Streptococcus anginosus, Streptococcus lutetiensis, Kurthia gibsonii,Kurthia gibsonii, Alloprevotella rava, Phocaeicola abscessus* | *(-)* |
| P83 | ET Secretion | *NBG* | *(-)* | *(-)* |
| P84 | Urine | *(H)coliform organisms* | ***Raoultella planticola*** **,* ***Klebsiella pneumoniae, Prevotella bivia, Enterococcus faecalis*** | *(-)* |
| P85 | Pus Swab | *Staphylococcus aureus* | ***Staphylococcus aureus*** **, Streptococcus pyogenes, Sphingomonas leidyi, Pannonibacter phragmitetus* | *Uwebraunia *, Aspergillus gracilis* |
| P86 | Eye Swab | *Staphylococcus aureus* | ***Staphylococcus aureus*** **, Staphylococcus petrasii, Streptococcus pyogenes, Morganella morganii, Proteus mirabilis, Pannonibacter phragmitetus, Azorhizobium caulinodans* | *(-)* |
| P87 | Urine | *Coliform organisms* | ***Enterobacteriaceae*** **,* ***Citrobacter freundii, Enterobacter cloacae, Shigella boydii, Shigella sonnei*** | *(-)* |
| P88 | Urine | *Coliform organisms* | ***Enterobacteriaceae*** **,* ***Enterobacter cloacae, Escherichia coli, Pseudocitrobacter faecalis, Citrobacter freundii, Shigella flexneri*** | *(-)* |
| P89 | Pus Swab | *Staphylococcus aureus* | ***Staphylococcus aureus*** **, Streptococcus pyogenes, Enterococcus faecalis, Sphingopyxis fribergensis* | *(-)* |
| P90 | Pus Swab | *Coliform organisms* | *Achromobacter *,* ***Enterococcus faecalis****, Pseudomonas aeruginosa, Bacteroides fragilis,* ***Proteus mirabilis****, Bordetella flabilis* | *(-)* |
| P91 | Bronchial wash | *Pseudomonas species* | *Xanthomonadaceae *,* ***Stenotrophomonas maltophilia*****(previously known as Pseudomonas maltophilia), Stenotrophomonas daejeonensis* | *(-)* |
| P92 | Joint Aspiration (iliac joint) | *NBG* | *(-)* | *(-)* |
| P93 | Pus Swab | *Coagulase negative Staphylococcus species* | *Anaerococcus provenciensis *, Anaerococcus octavius, Anaerococcus pacaensis, Finegoldia magna, S****taphylococcus lugdunensis, Staphylococcus haemolyticus****, Corynebacterium appendicis, Dermabacter hominis, Pseudoclavibacter alba* | *(-)* |
| P94 | Bronchial wash | *Pseudomonas species* | ***Pseudomonas aeruginosa*** **, Acinetobacter baumannii, Stenotrophomonas pavanii, tenotrophomonas maltophilia, Comamonas testosteroni, Comamonas kerstersii, Bacteroides fragilis, Mycoplasma salivarium* | *(-)* |
| P95 | Throat Swab | *NBG* | *(-)* | *(-)* |
| P96 | Throat Swab | *No Bacterial Pathogen* | *Stenotrophomonas *, Stenotrophomonas pavanii, Stenotrophomonas maltophilia,* ***Streptococcus spp. (7), Niesseria spp. (2****), Staphylococcus aureus* | *(-)* |
| P97 | Pus Swab | *Coliform organisms* | *(-)* | *(-)* |
| P98 | IV Catheter Tip | *NBG* | *(-)* | *(-)* |
| P99 | Blood(EDTA) | *NBG* | *(-)* | *(-)* |
| P100 | Blood(EDTA) | *NBG* | *(-)* | *(-)* |
| P101 | Blood(EDTA) | *NBG* | *(-)* | *(-)* |
| P102 | Blood(EDTA) | *NBG* | *(-)* | *(-)* |
| P103 | Blood(EDTA) | *NBG* | *(-)* | *(-)* |
