# Supplementary material for: Detection of bacterial pathogens from clinical specimens using conventional microbial culture and 16S metagenomics: a comparative study
Source: BMC Infect Dis. 2017 Sep 19;17:631. doi: 10.1186/s12879-017-2727-8 (PMC5606128; doi:10.1186/s12879-017-2727-8)
Supplement: Supplementary file 4 — Final phylogenies generated from the Credence Infectious Panel Pipeline 1.1.0. Phylogenies generated from the quality checked FASTA files for each specimen is available here. Specimen files are named as follows: Additional file 4_B/F_Specimen number (e.g. Additional file 4_B_P1– phylogeny of bacterial sequences within specimen P1; Additional file 4_F_P1 – phylogeny of fungal sequences within specimen P1). (PDF 620 kb) [file 12879_2017_2727_MOESM4_ESM.pdf]

Additional File 4_B_P1……4
Additional file 4_B_P5……5
Additional file 4_B_P6……6
Additional file 4_B_P7……7
Additional file 4_B_P10……8
Additional file 4_B_P11……9
Additional file 4_B_P12……10
Additional file 4_B_P13……11
Additional file 4_B_P14……12
Additional file 4_B_P15……13
Additional file 4_B_P16……14
Additional file 4_B_P17……15
Additional file 4_B_P20……16
Additional file 4_B_P21……17
Additional file 4_B_P22……18
Additional file 4_B_P23……19
Additional file 4_B_P24……20
Additional file 4_B_P25……21
Additional file 4_B_P26……22
Additional file 4_B_P27……23
Additional file 4_B_P28……24
Additional file 4_B_P29……25
Additional file 4_B_P30……26
Additional file 4_B_P31……27
Additional file 4_B_P33……28
Additional file 4_B_P34……29
Additional file 4_B_P35……30
Additional file 4_B_P36……31
Additional file 4_B_P37……32

Additional file 4_B_P38………33
Additional file 4_B_P39………34
Additional file 4_B_P40………35
Additional file 4_B_P41………36
Additional file 4_B_P42………37
Additional file 4_B_P43………38
Additional file 4_B_P44………39
Additional file 4_B_P45………40
Additional file 4_B_P46………41
Additional file 4_B_P47………42
Additional file 4_B_P49………43
Additional file 4_B_P50………44
Additional file 4_B_P51………45
Additional file 4_B_P53………46
Additional file 4_B_P54………47
Additional file 4_B_P57………48
Additional file 4_B_P58………49
Additional file 4_B_P59………50
Additional file 4_B_P60………51
Additional file 4_B_P61………52
Additional file 4_B_P62………53
Additional file 4_B_P63………54
Additional file 4_B_P64………55
Additional file 4_B_P65………56
Additional file 4_B_P66………57
Additional file 4_B_P67………58
Additional file 4_B_P69………59
Additional file 4_B_P70………60
Additional file 4_B_P71………61

Additional file 4_B_P74……62
Additional file 4_B_P75……63
Additional file 4_B_P76……64
Additional file 4_B_P77……65
Additional file 4_B_P78……66
Additional file 4_B_P80……67
Additional file 4_B_P81……68
Additional file 4_B_P82……69
Additional file 4_B_P84……70
Additional file 4_B_P85……71
Additional file 4_B_P86……72
Additional file 4_B_P87……73
Additional file 4_B_P88……74
Additional file 4_B_P89……75
Additional file 4_B_P90……76
Additional file 4_B_P91……77
Additional file 4_B_P93……78
Additional file 4_B_P94……79
Additional file 4_B_P96……80
Additional file 4_F_P17……81
Additional file 4_F_P22……82
Additional file 4_F_P25……83
Additional file 4_F_P26……84
Additional file 4_F_P37……85
Additional file 4_F_P38……86
Additional file 4_F_P76……87
Additional file 4_F_P81……88
Additional file 4_F_P85……89

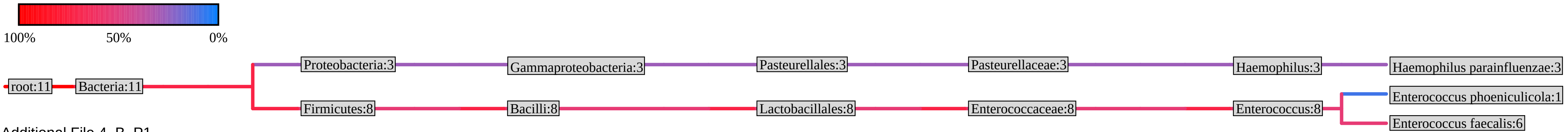

Additional File 4_B_P1

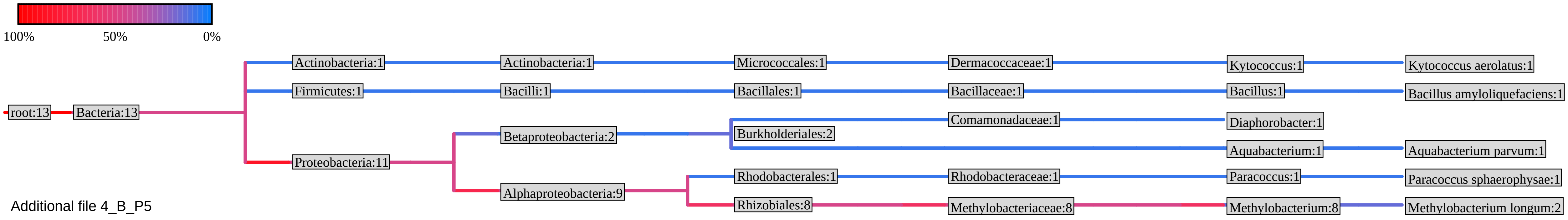

Additional file 4_B_P5

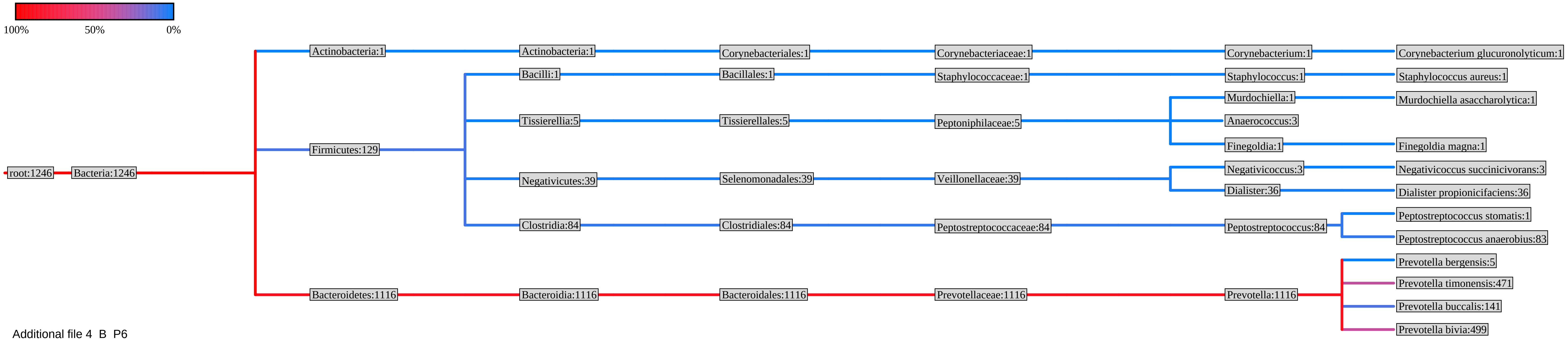

Additional file 4_B_P6

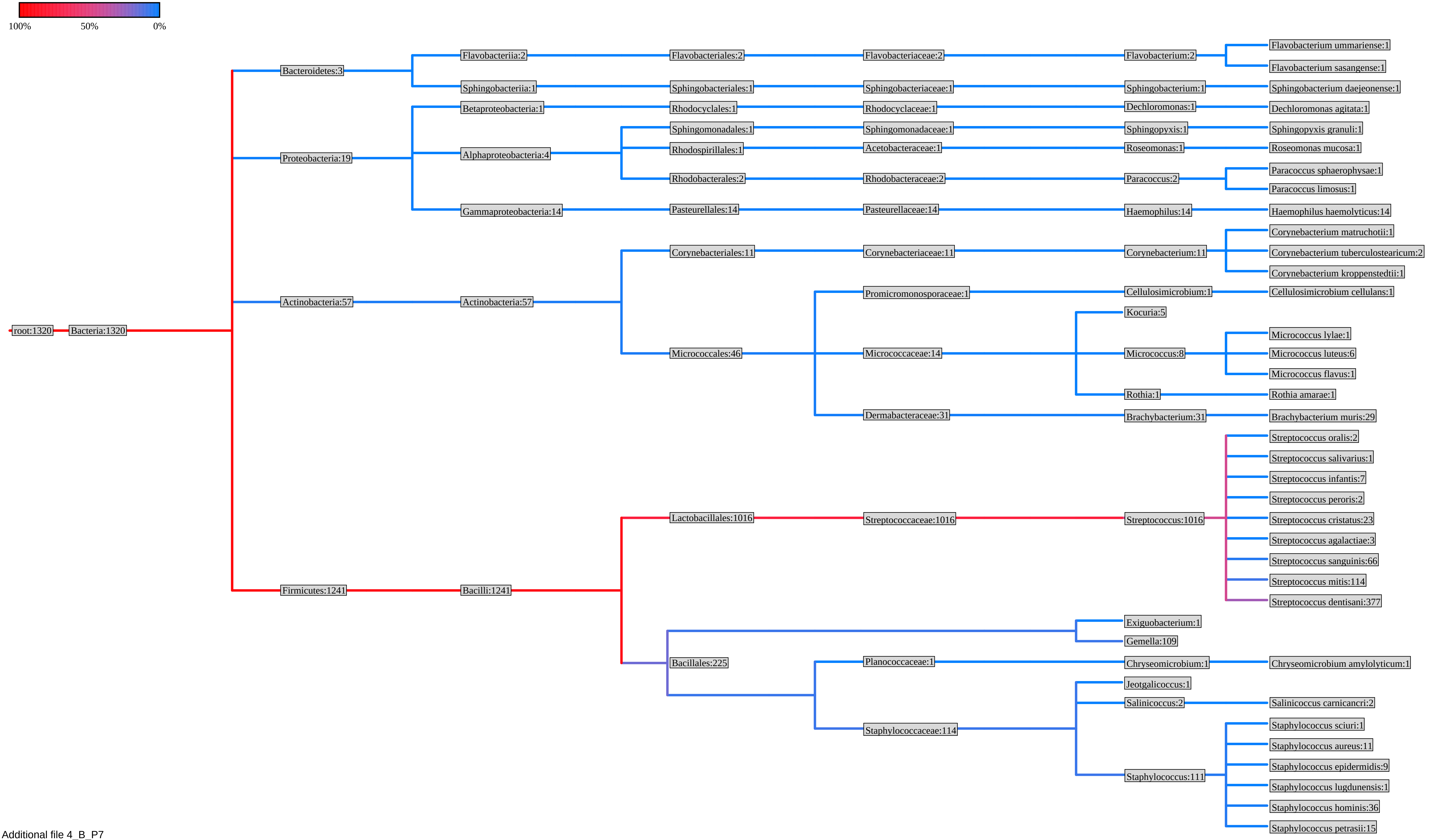

Additional file 4_B_P7

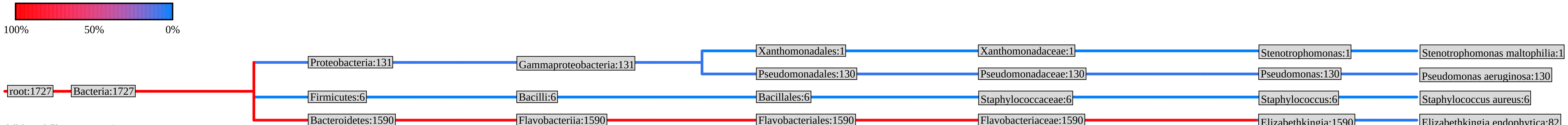

Additional file 4_B_P10

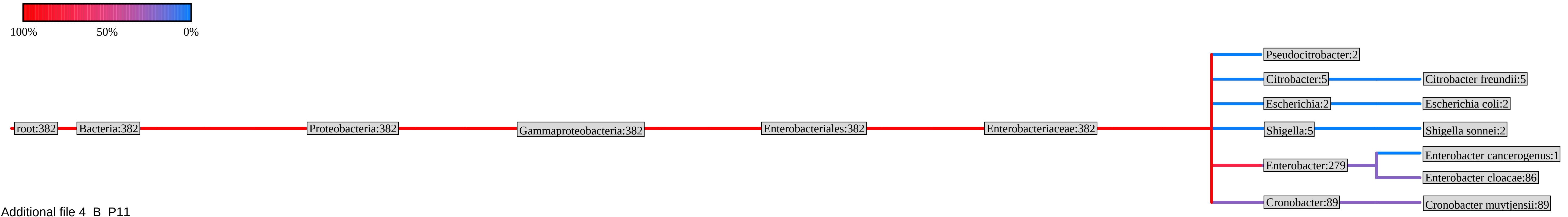

Additional file 4_B_P11

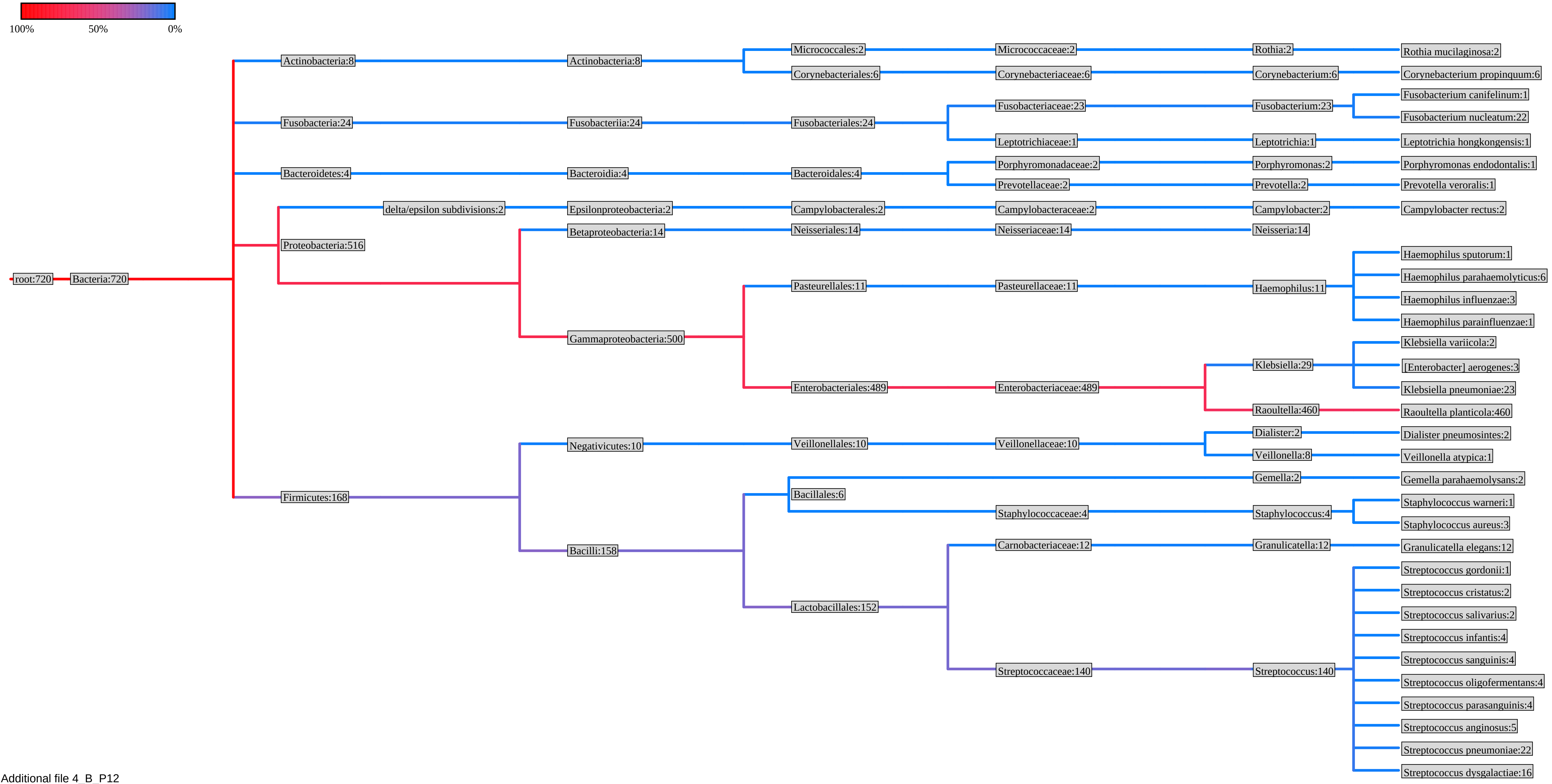

Additional file 4_B_P12

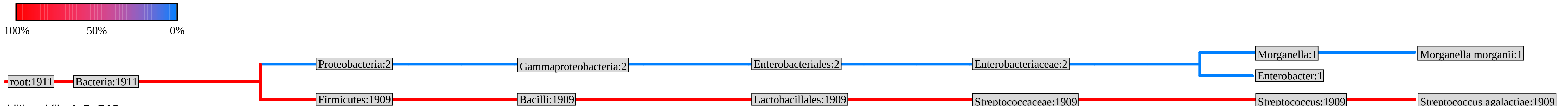

Additional file 4_B_P13

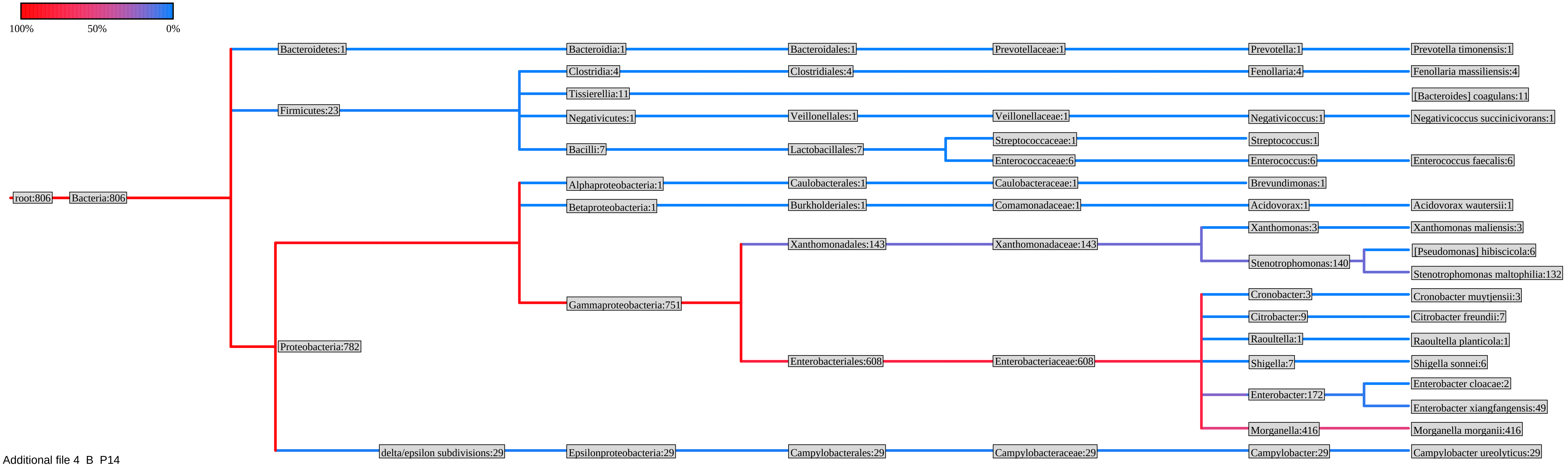

Additional file 4_B_P14

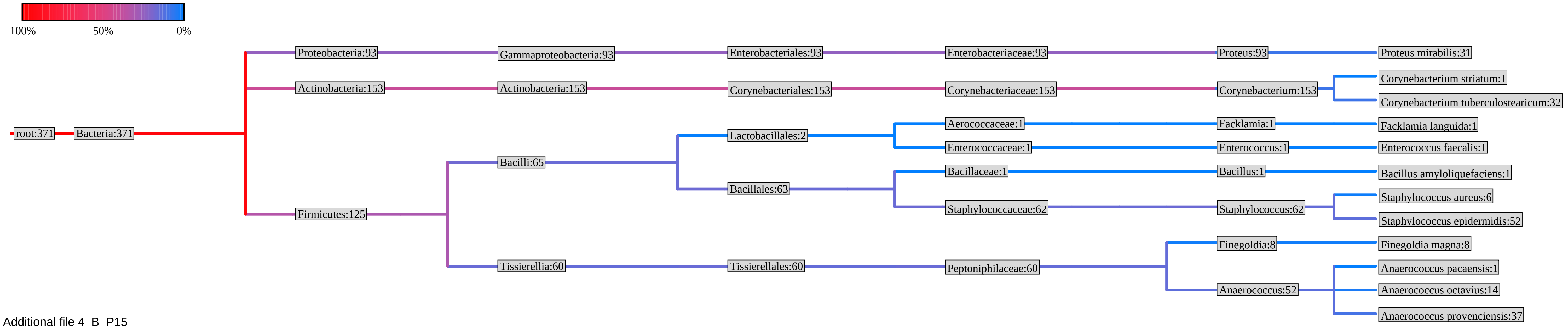

Additional file 4_B_P15

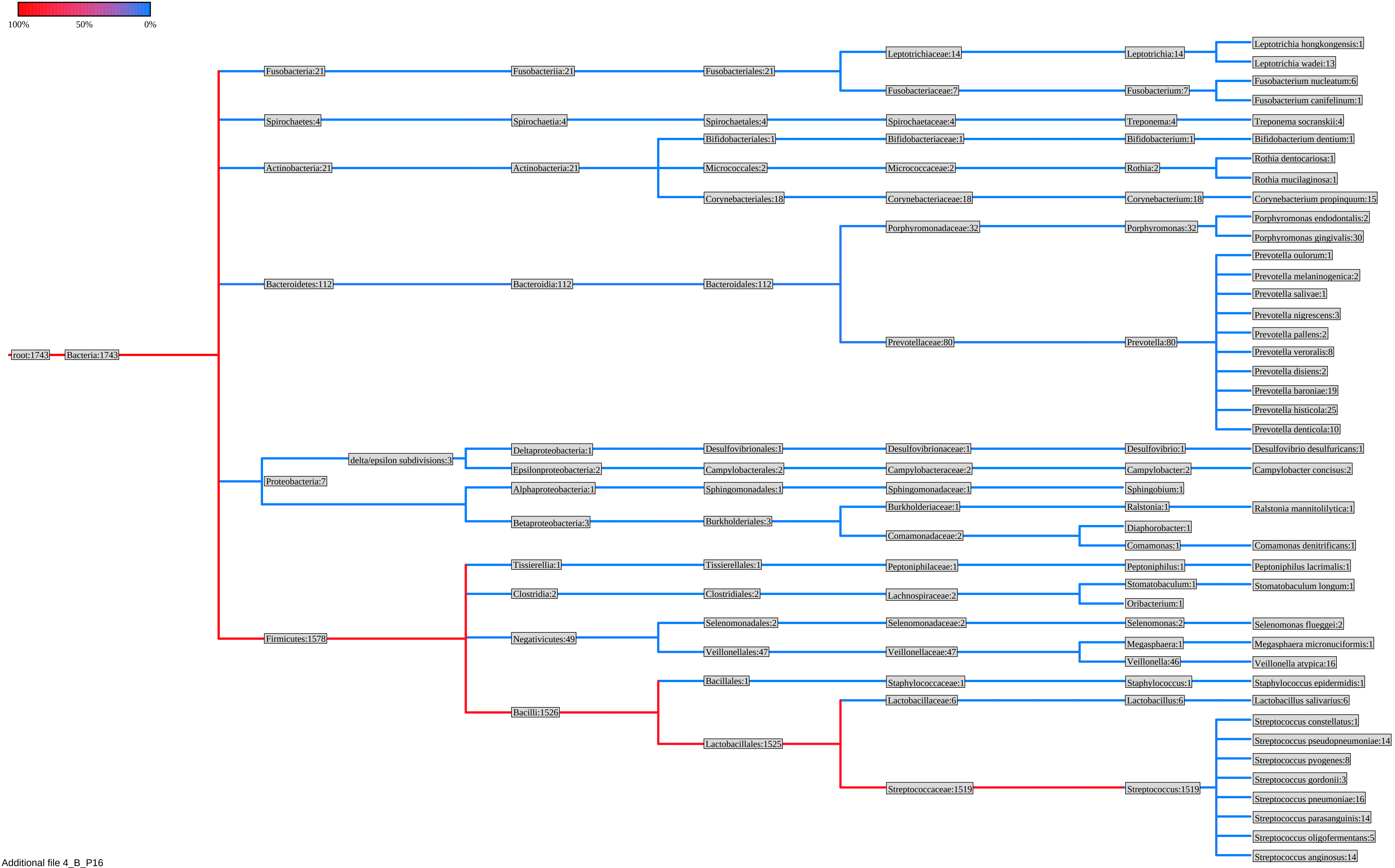

Additional file 4_B_P16

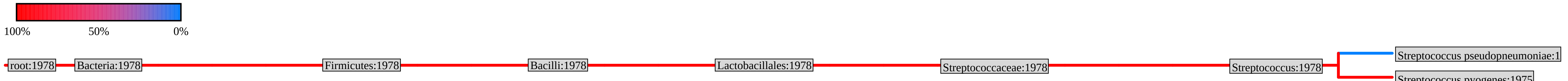

Additional file 4_B_P17

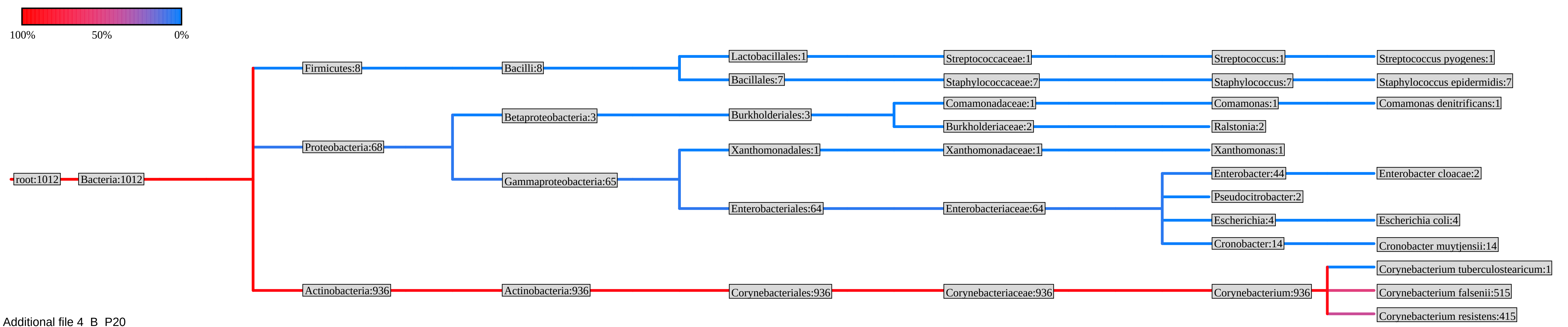

Additional file 4_B_P20

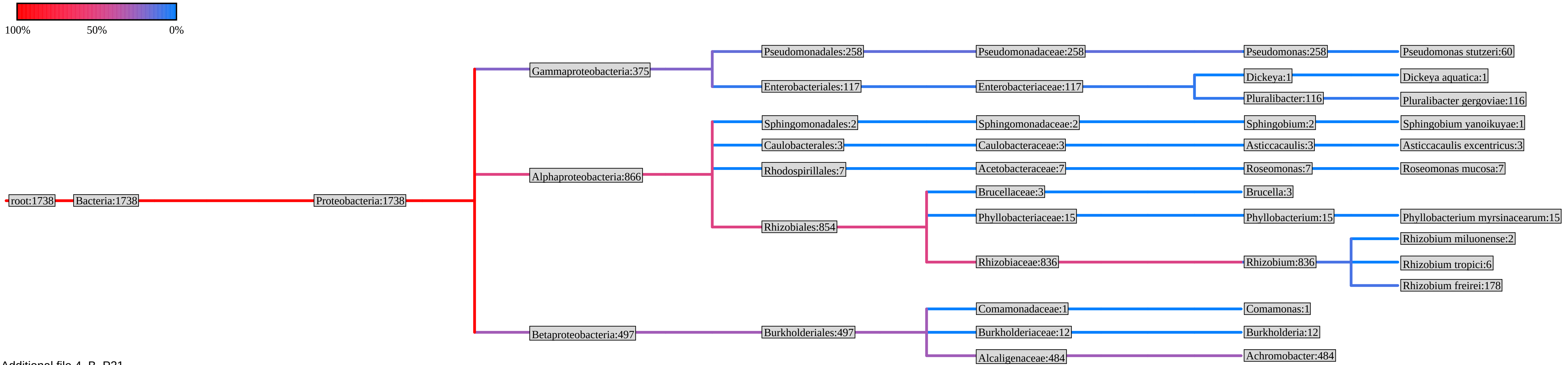

Additional file 4_B_P21

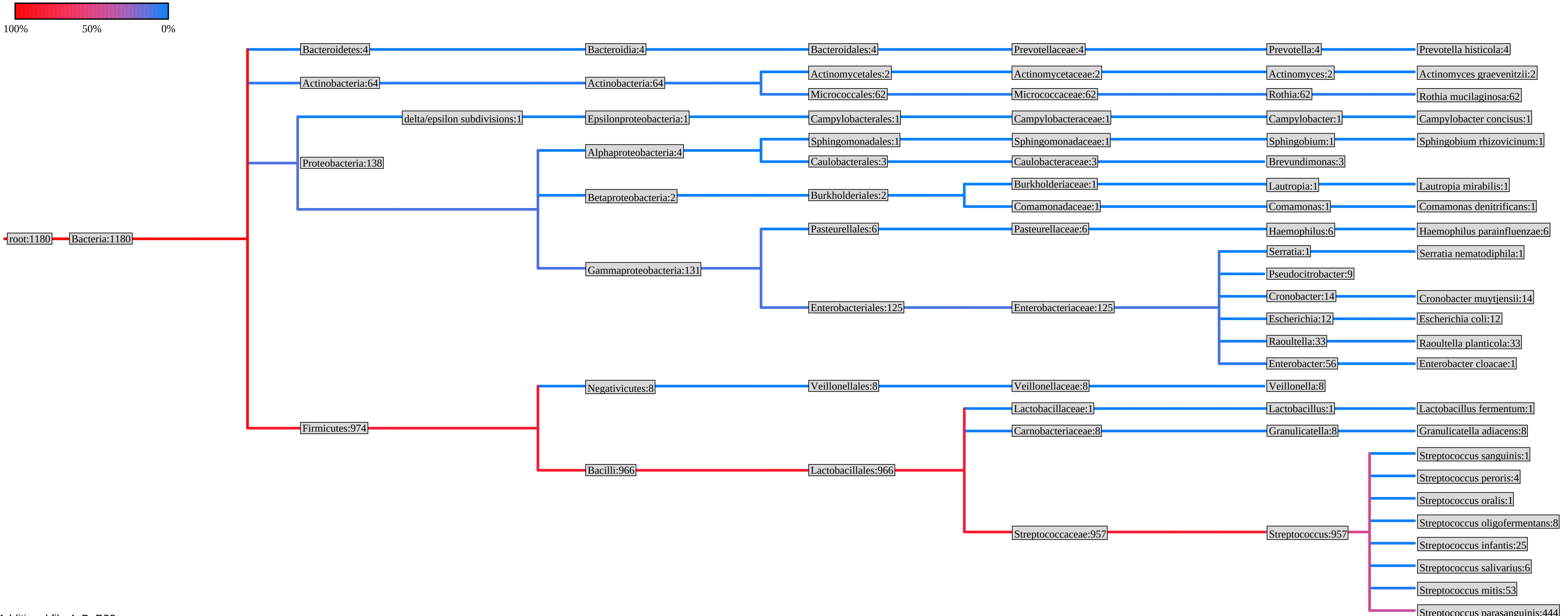

Additional file 4_B_P22

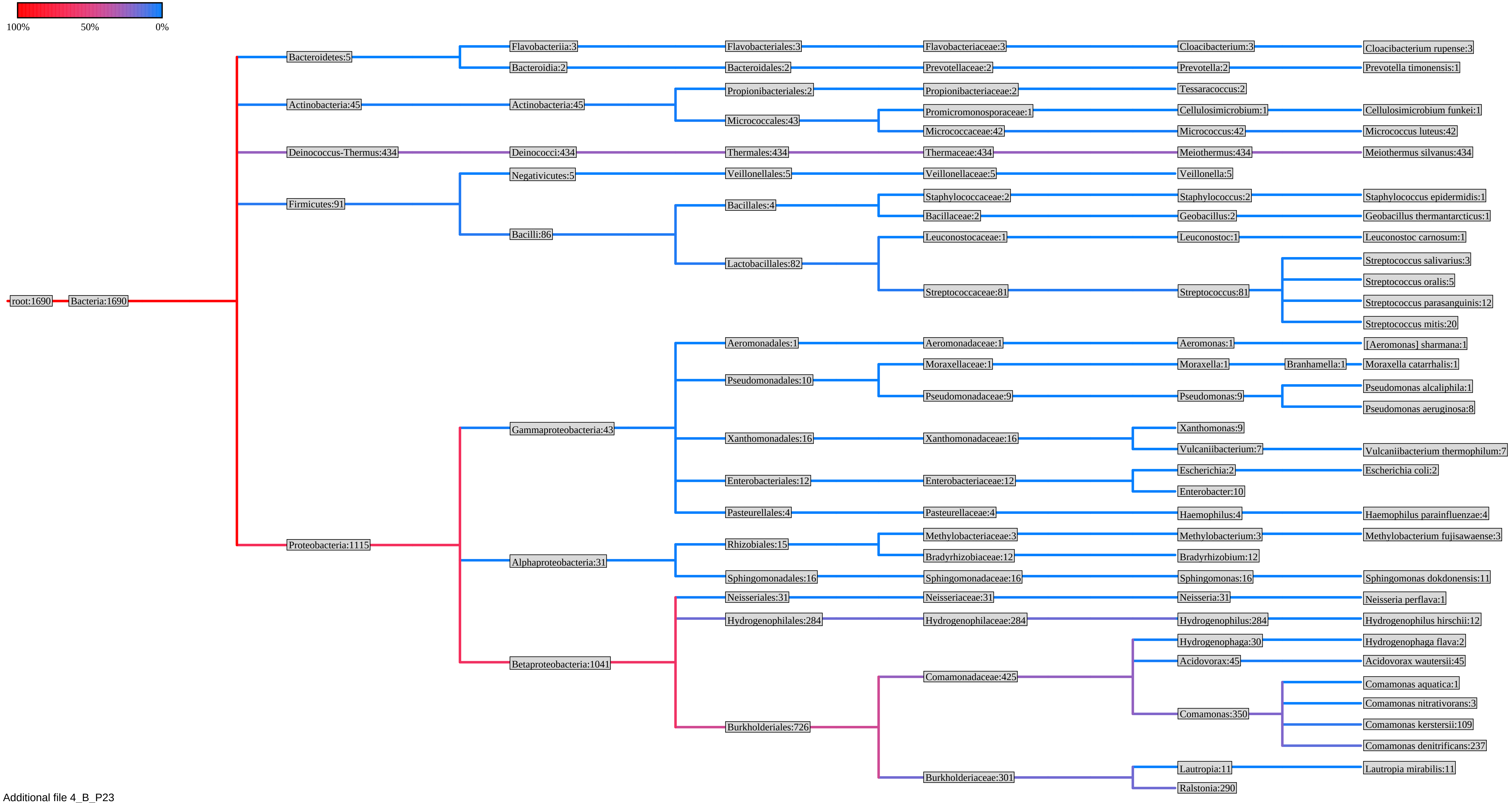

Additional file 4_B_P23

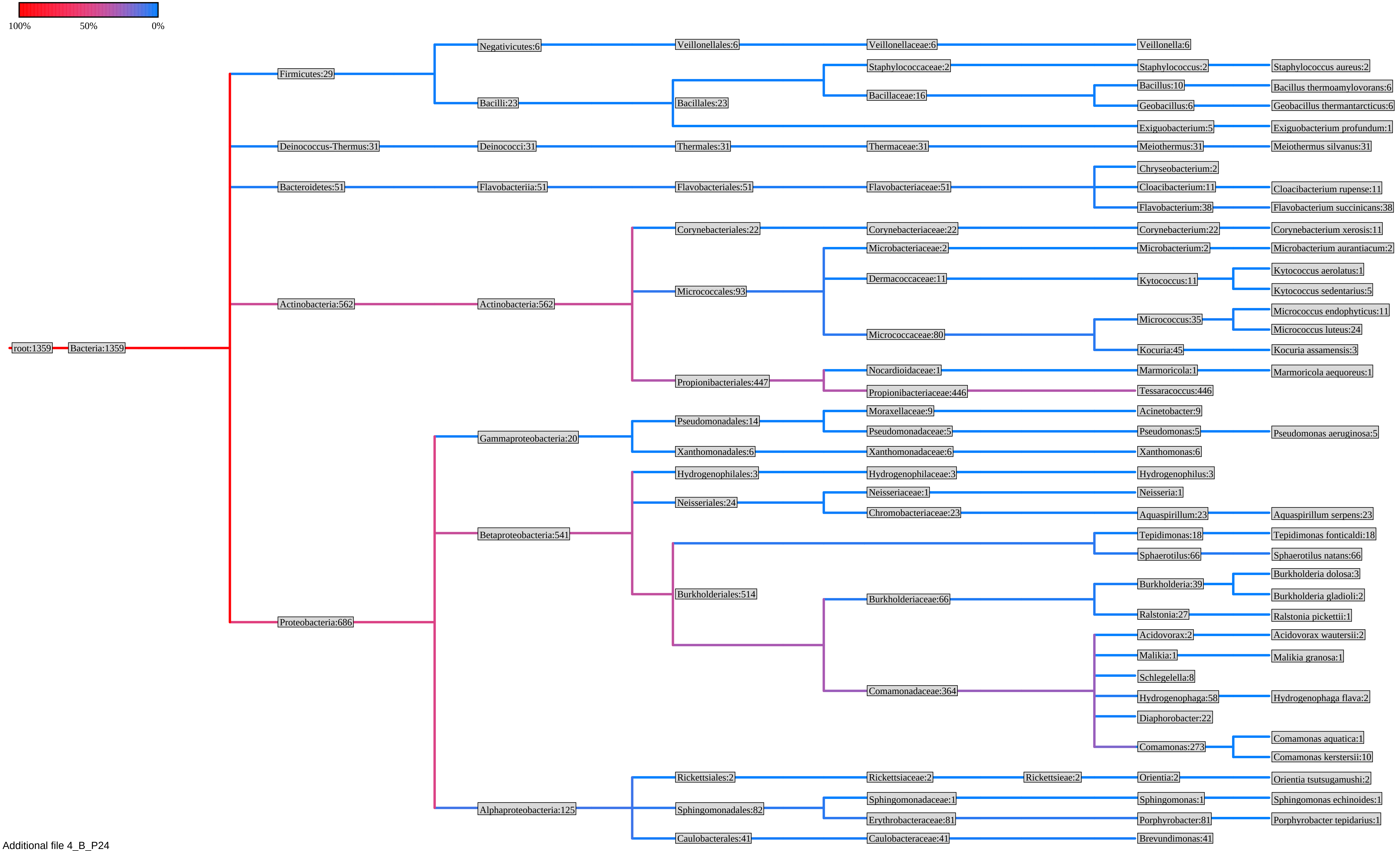

Additional file 4_B_P24

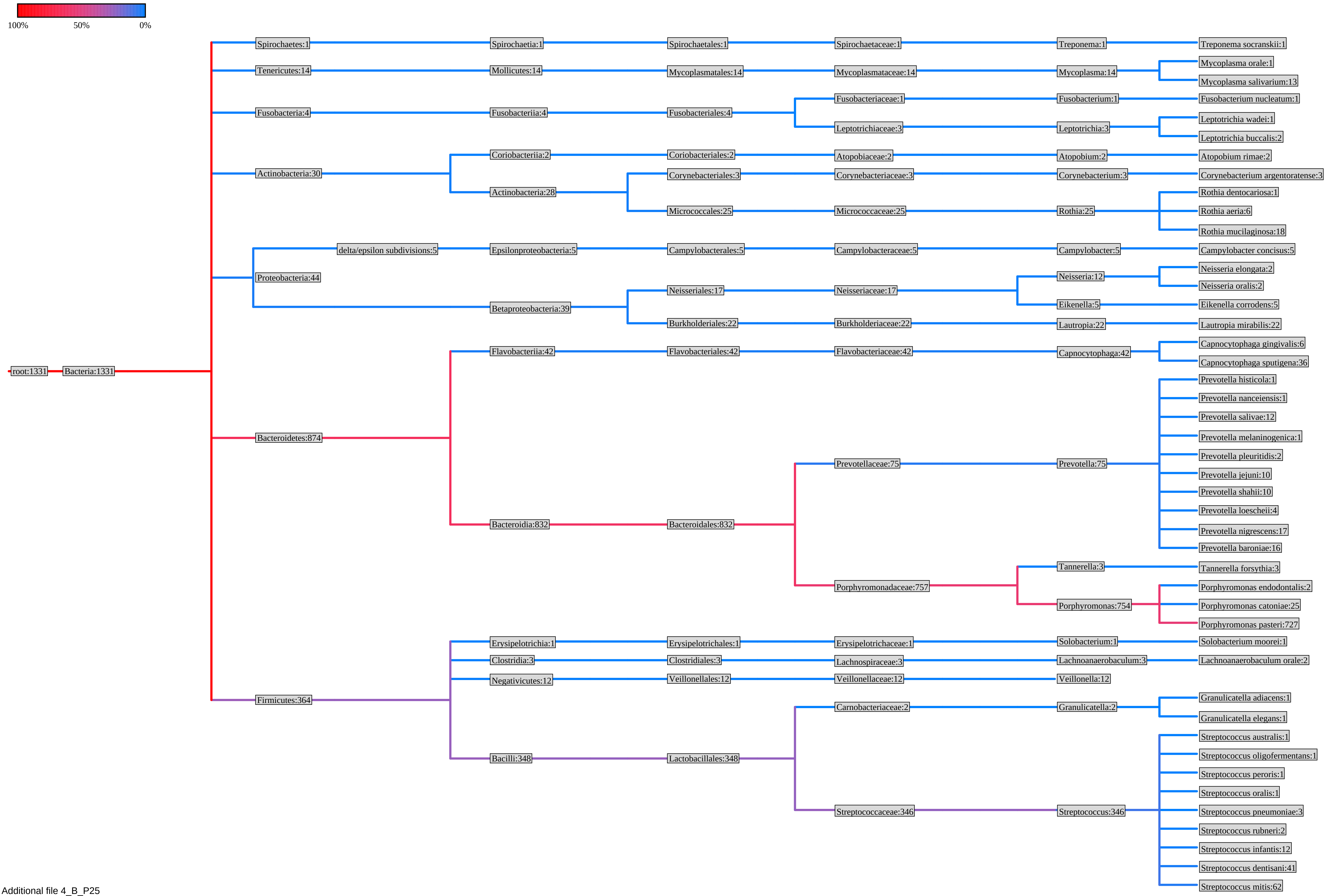

Additional file 4_B_P25

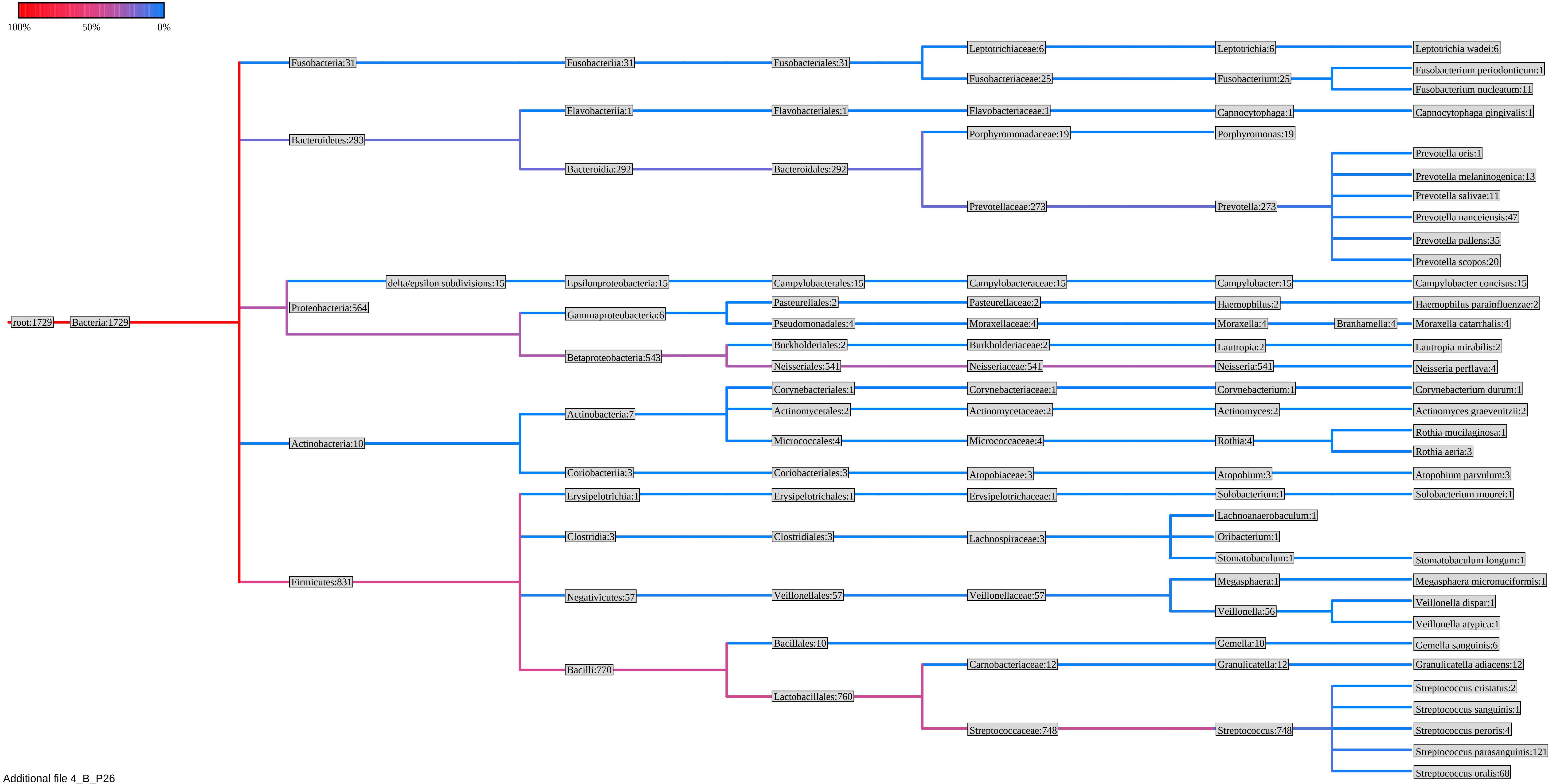

Additional file 4_B_P26

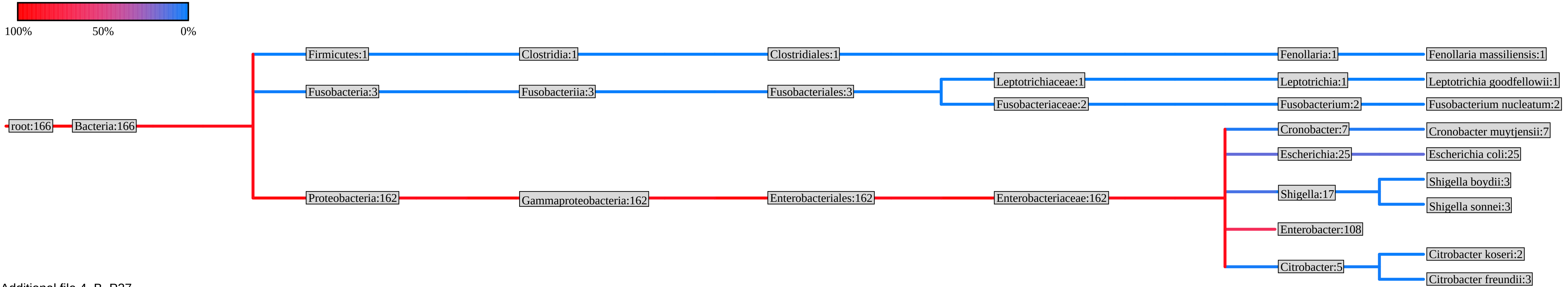

Additional file 4_B_P27

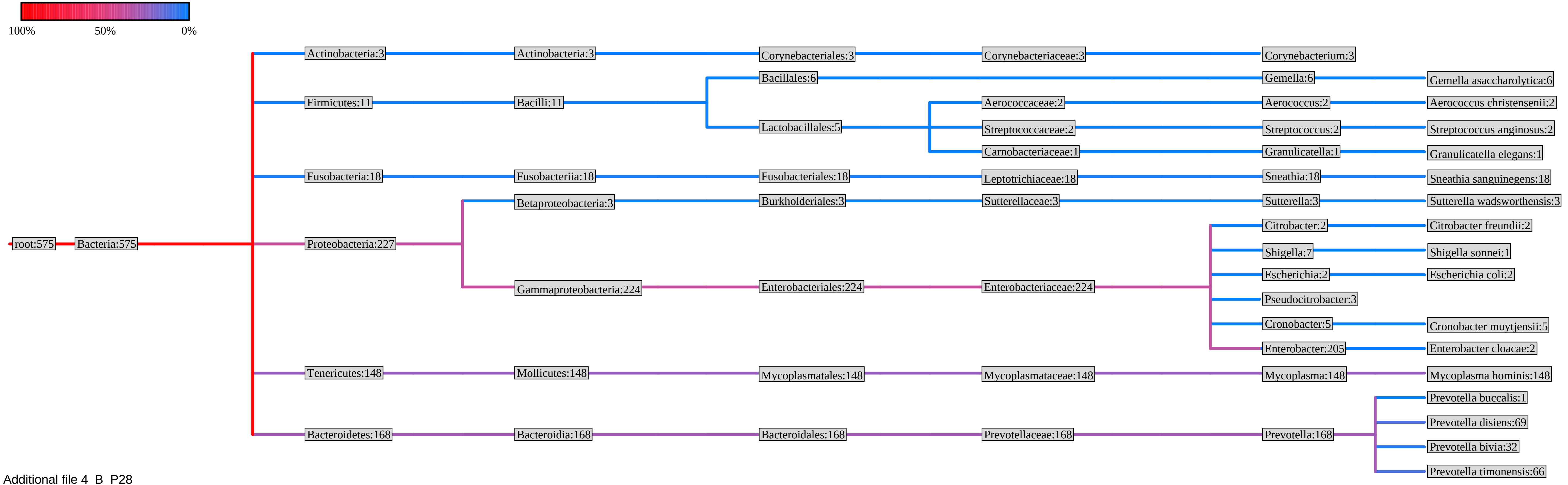

Additional file 4_B_P28

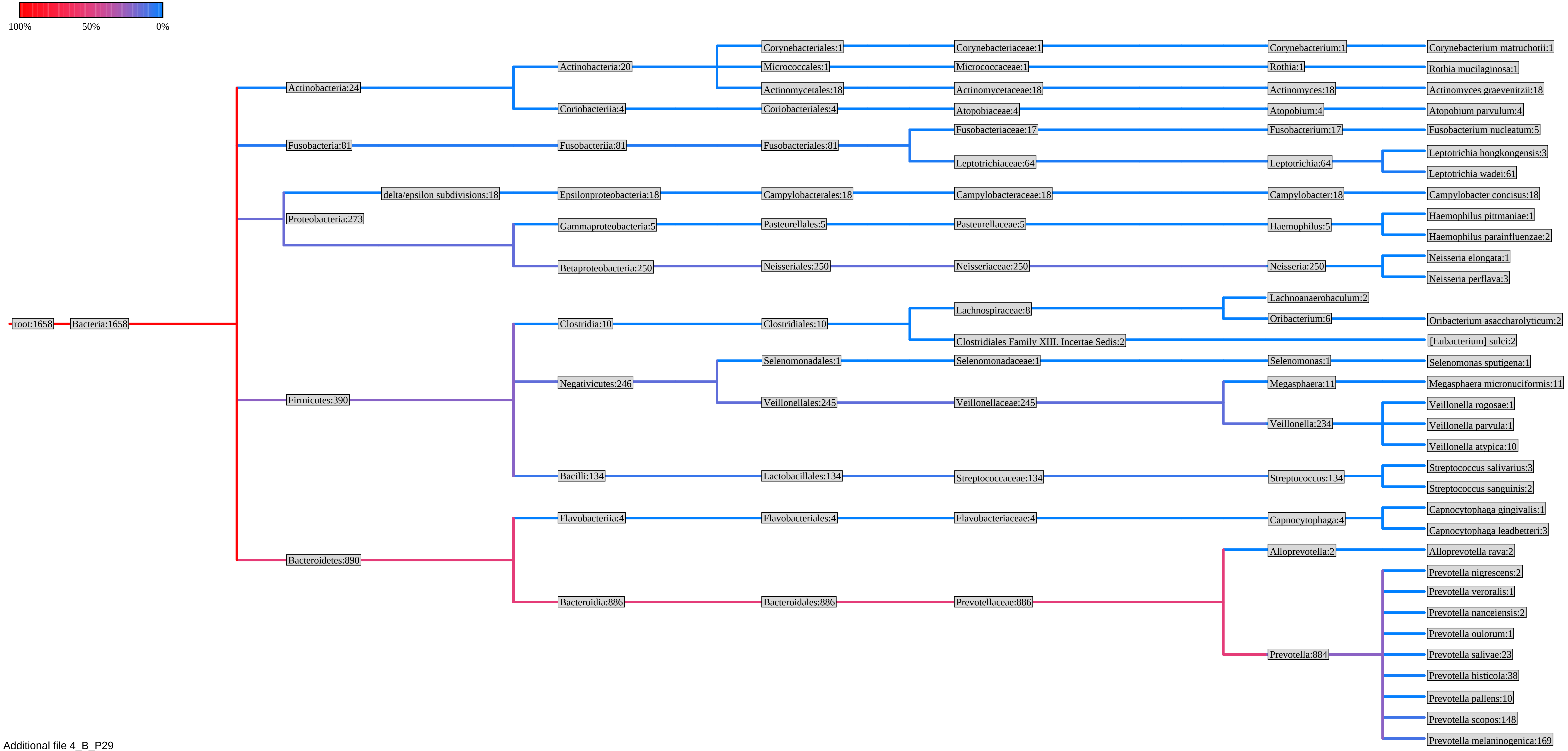

Additional file 4_B_P29

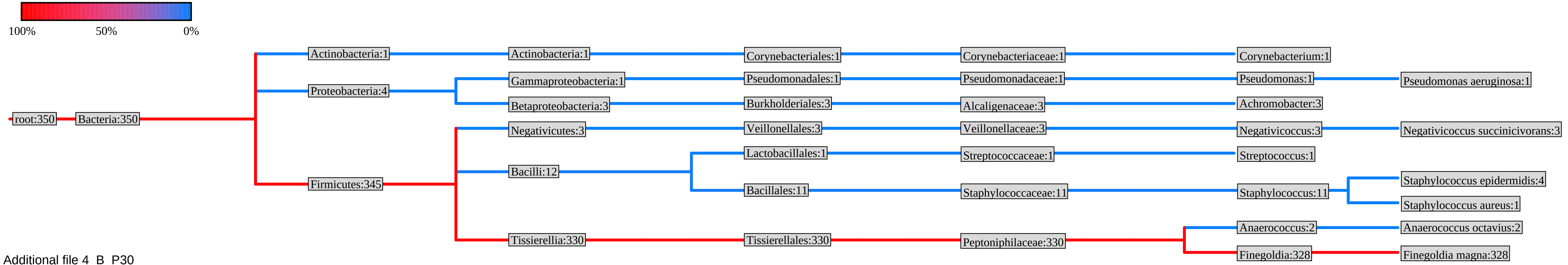

Additional file 4_B_P30

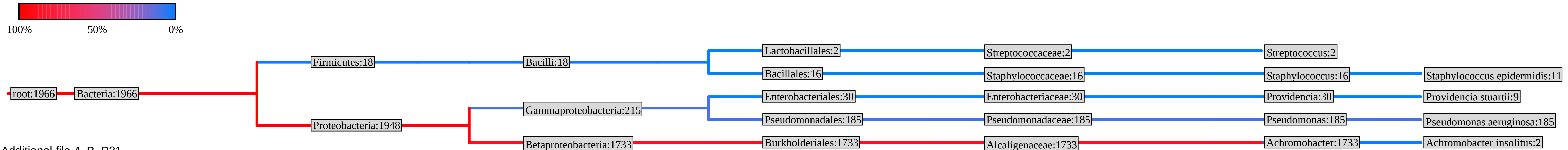

Additional file 4_B_P31

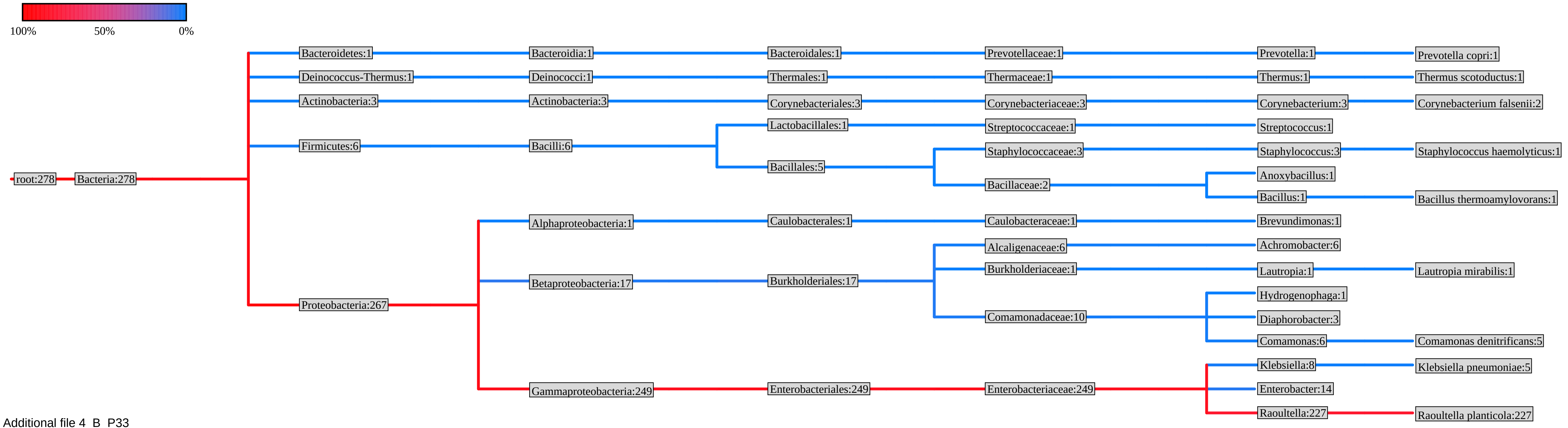

Additional file 4_B_P33

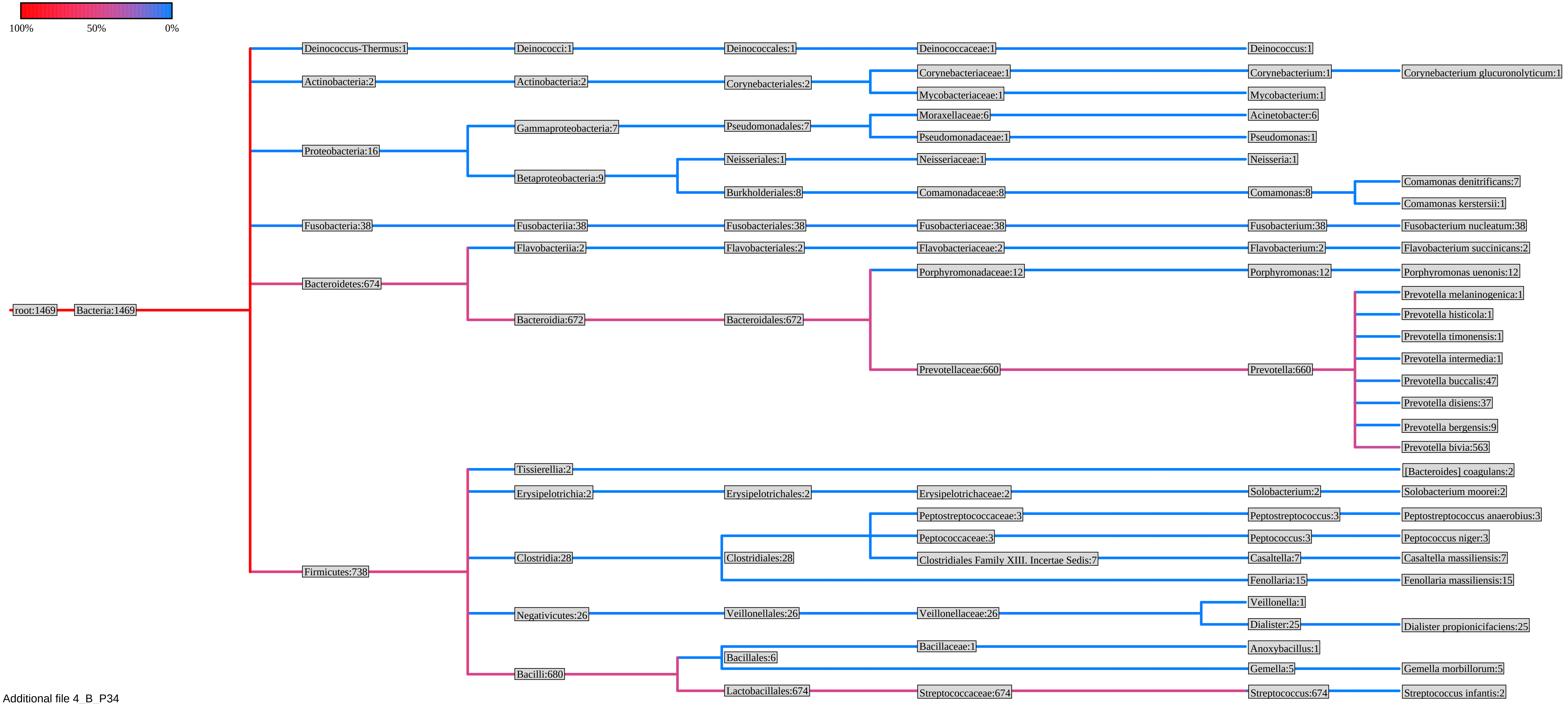

Additional file 4_B_P34

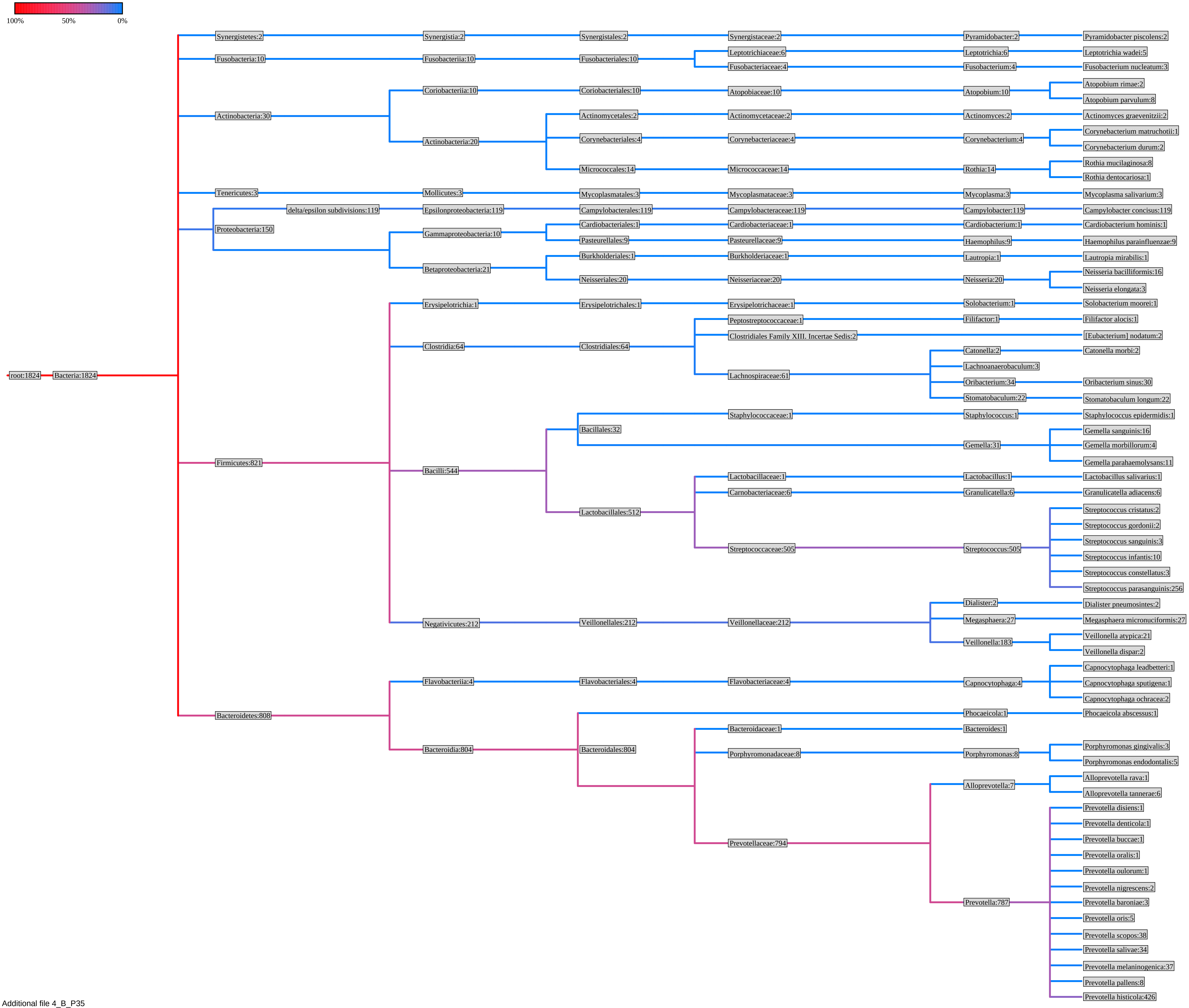

Additional file 4_B_P35

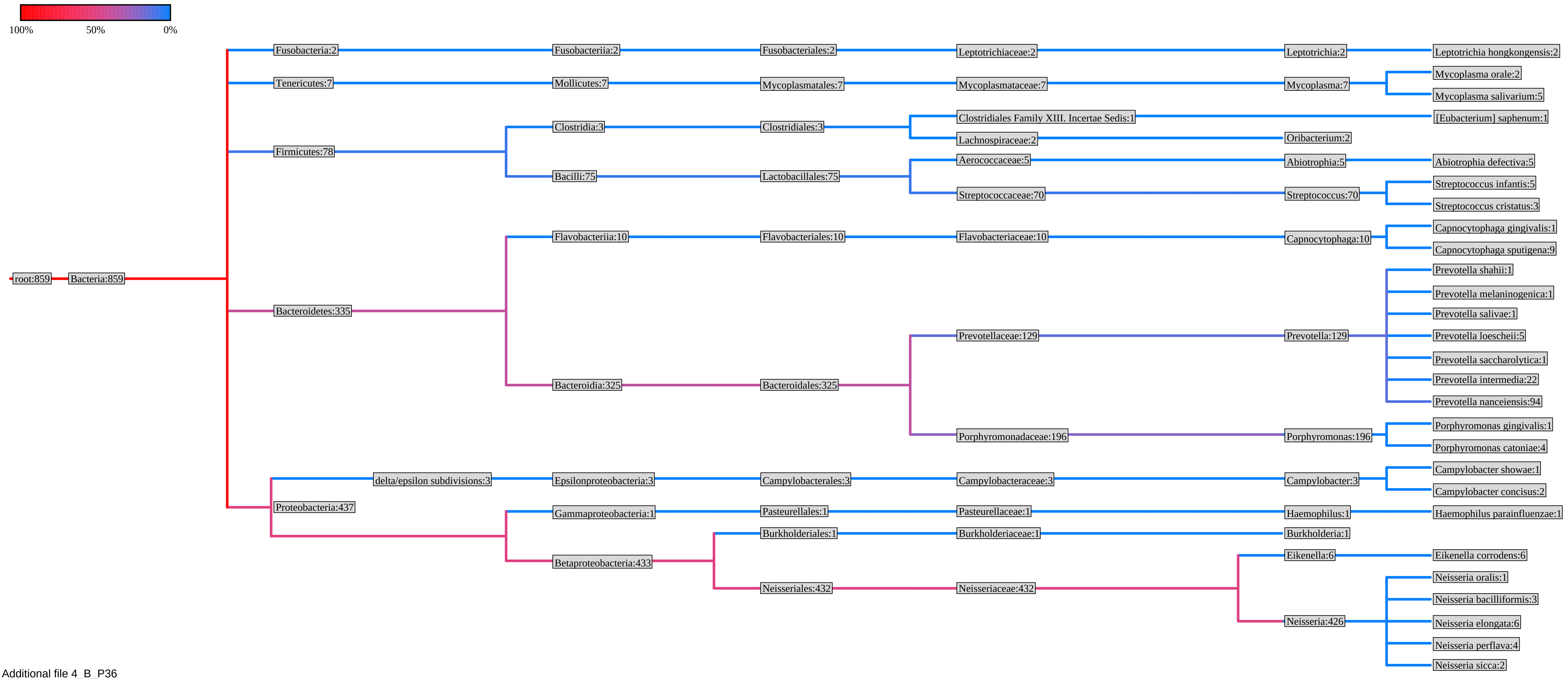

Additional file 4_B_P36

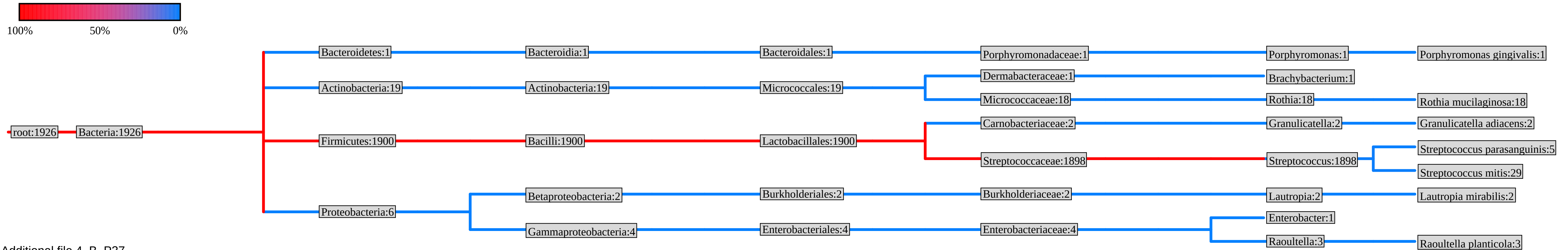

Additional file 4_B_P37

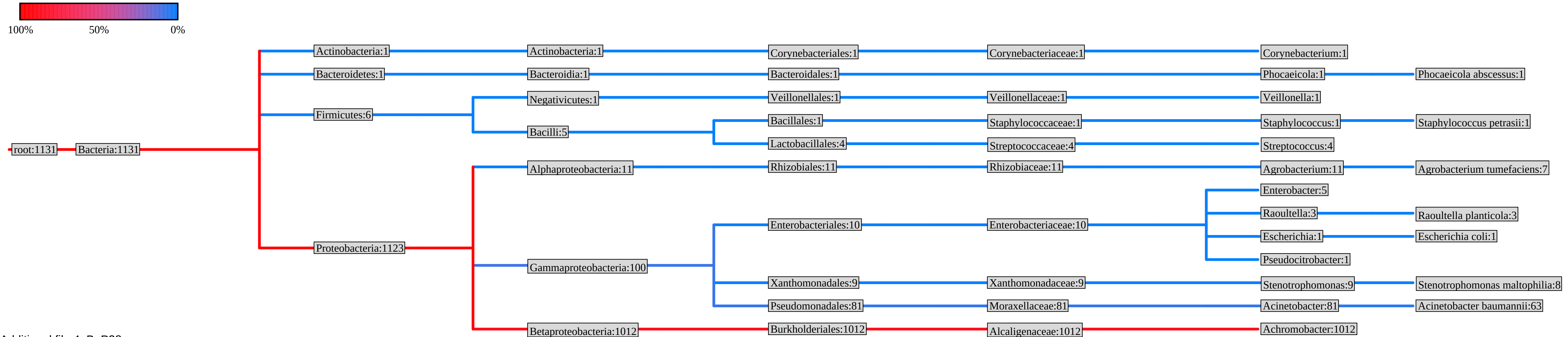

Additional file 4_B_P38

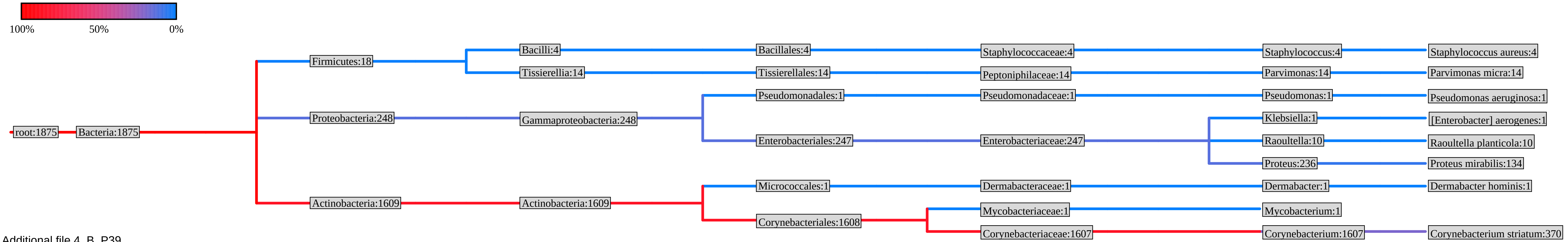

Additional file 4_B_P39

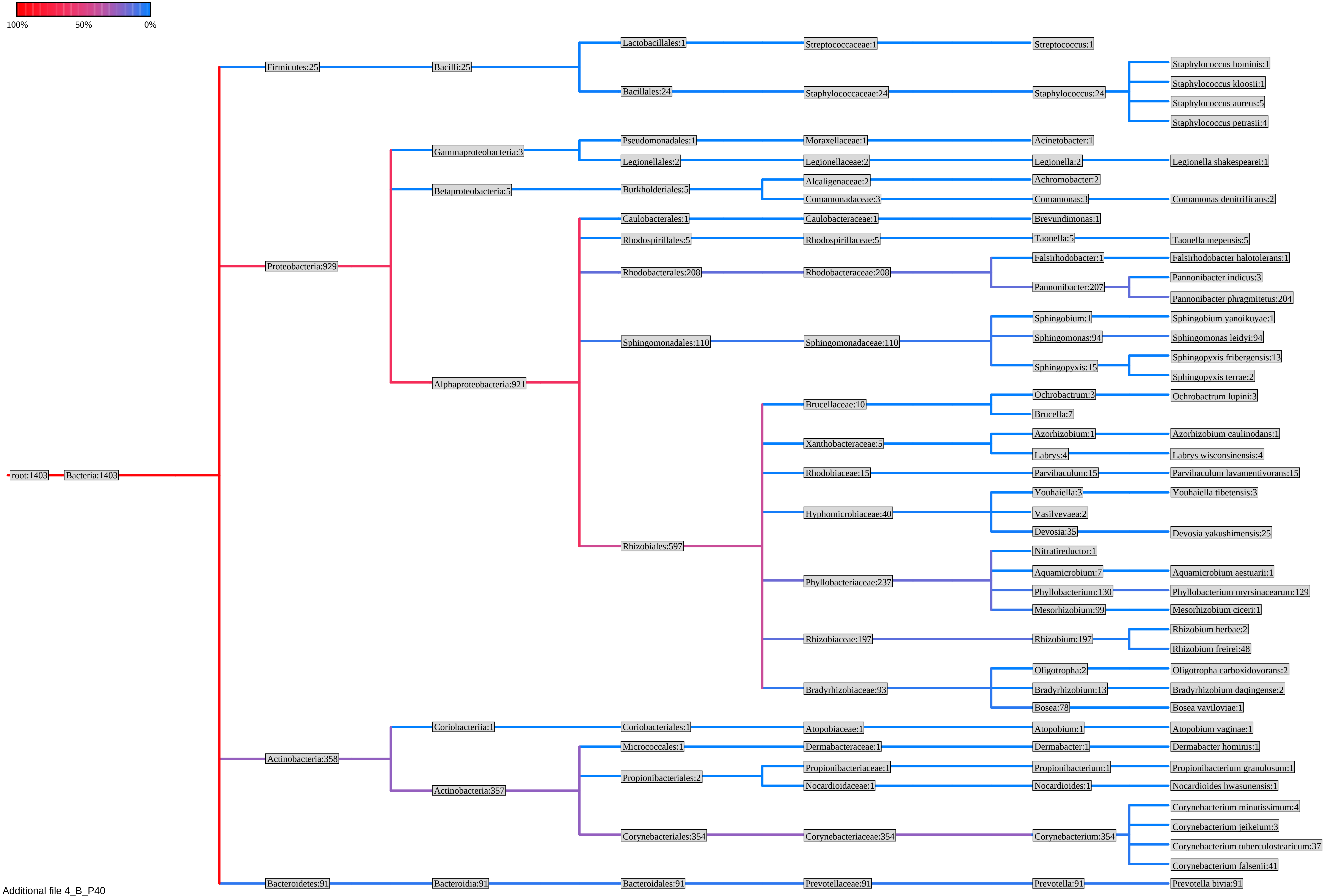

Additional file 4_B_P40

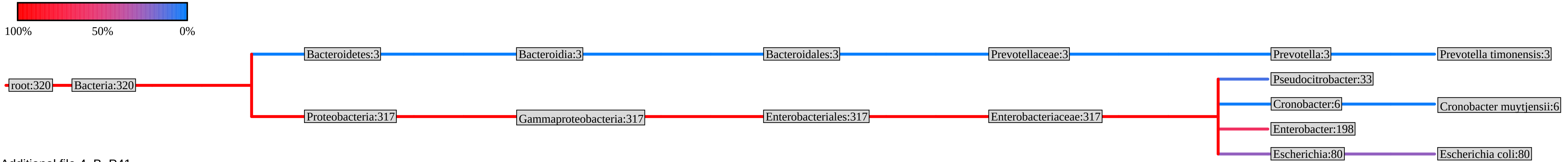

Additional file 4_B_P41

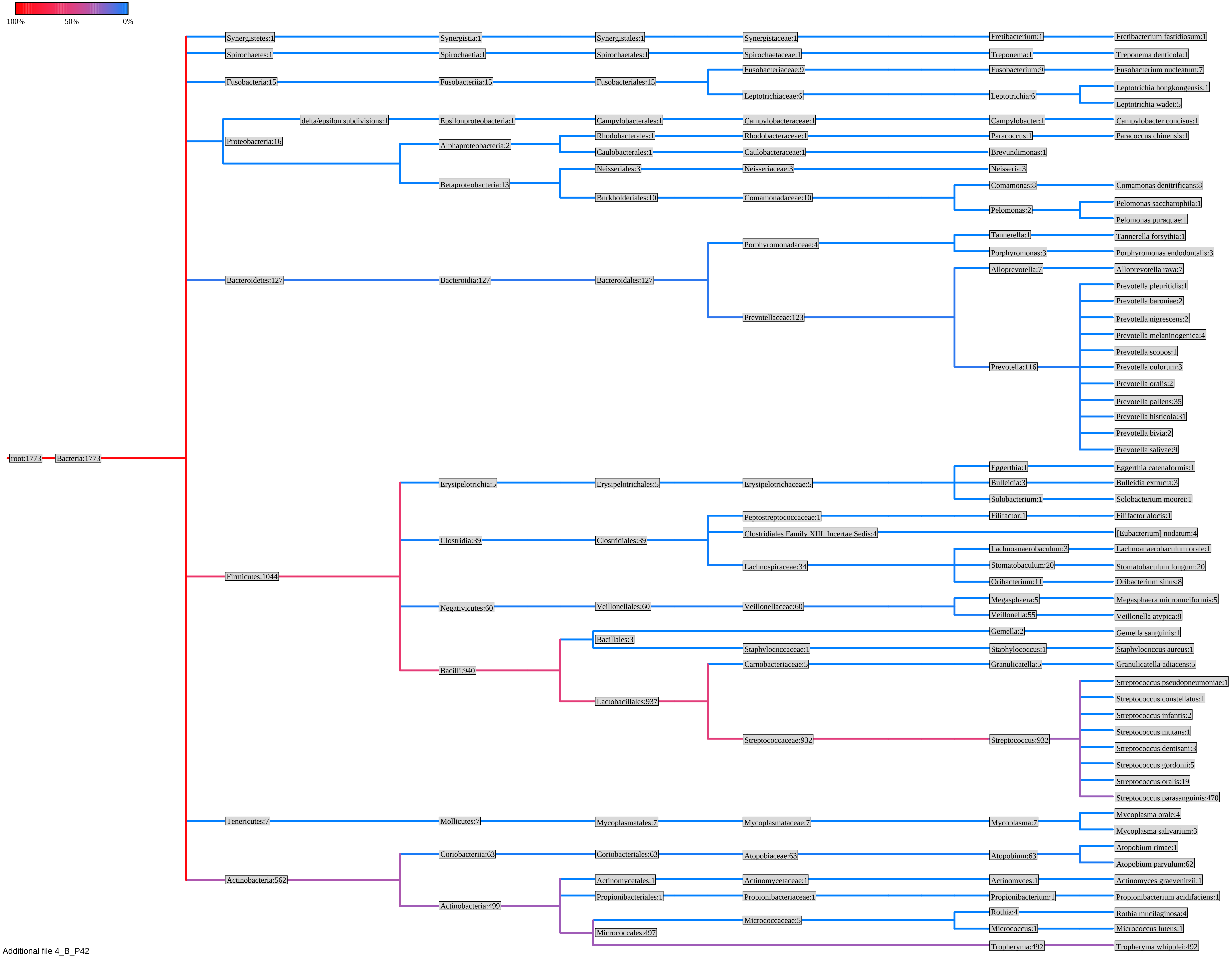

Additional file 4_B_P42

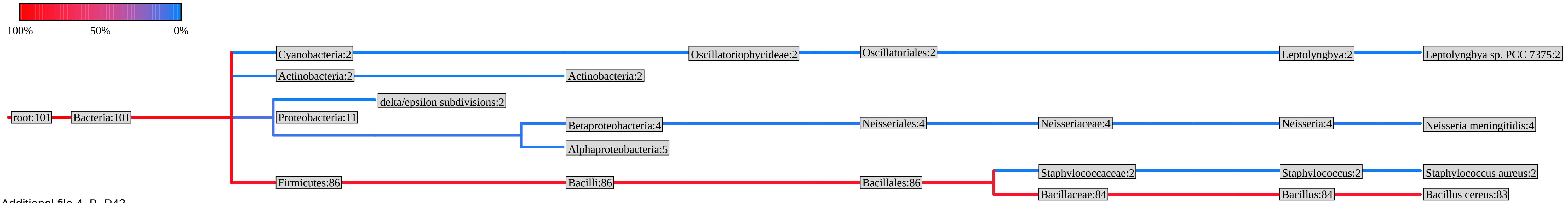

Additional file 4_B_P43

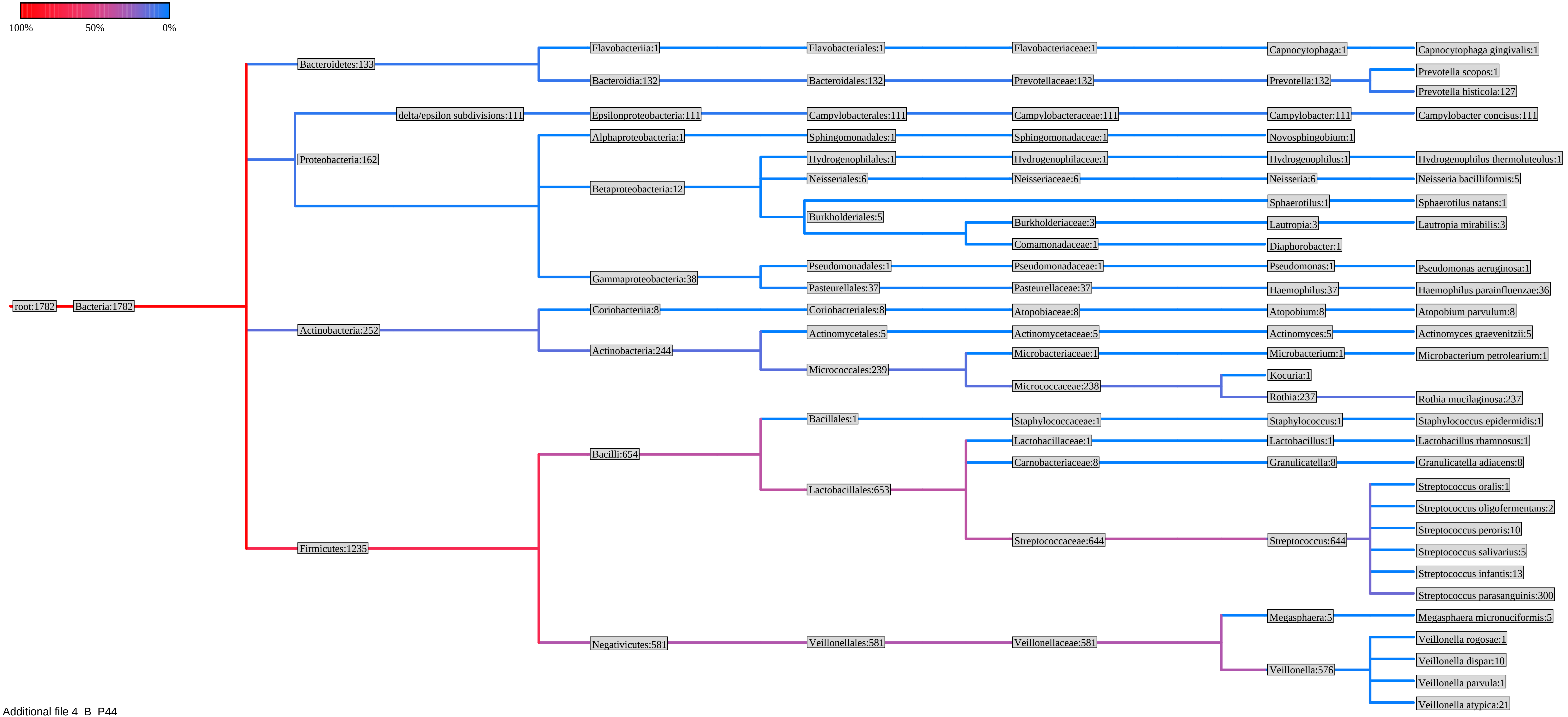

Additional file 4_B_P44

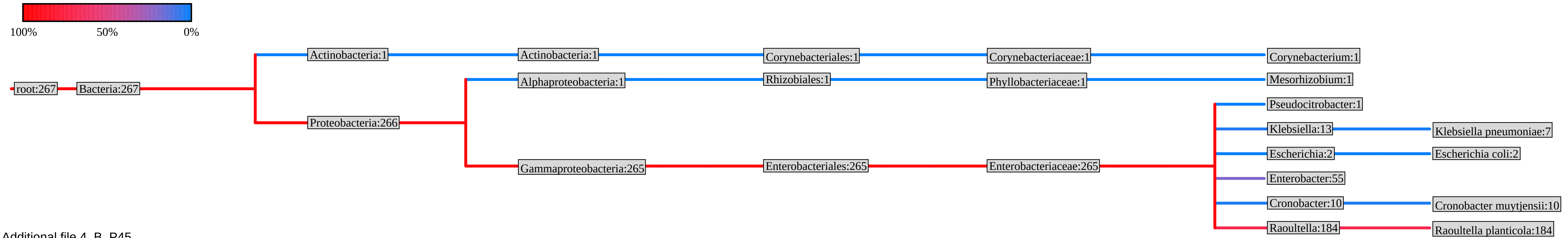

Additional file 4_B_P45

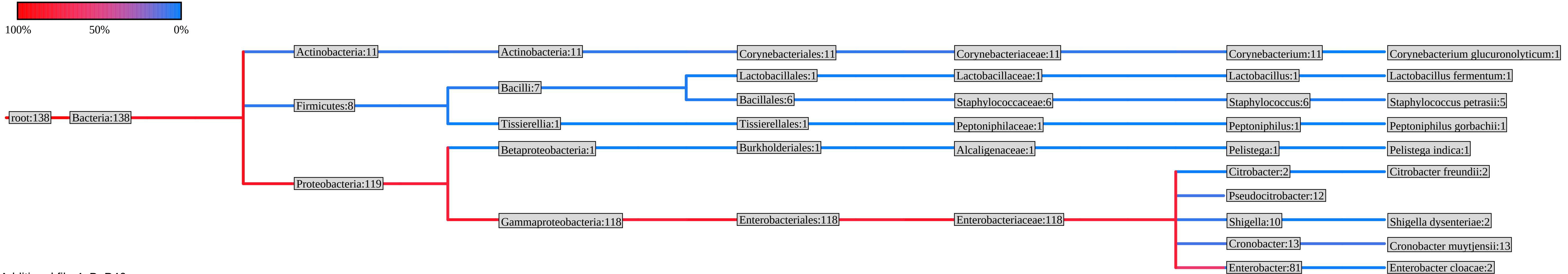

Additional file 4_B_P46

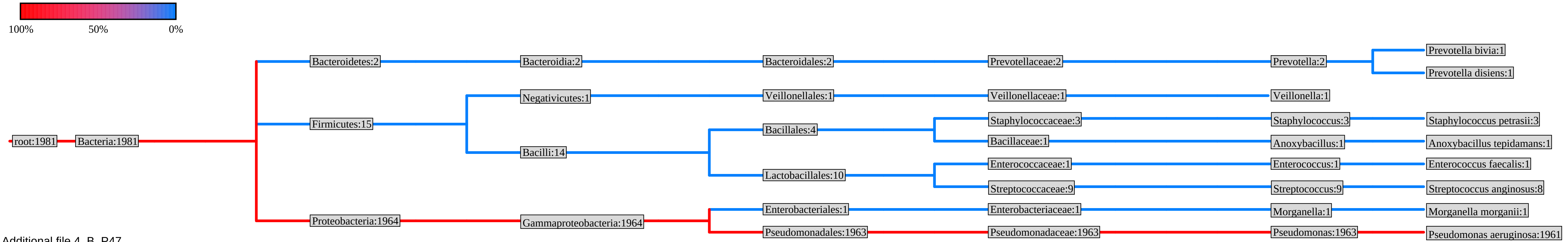

Additional file 4_B_P47

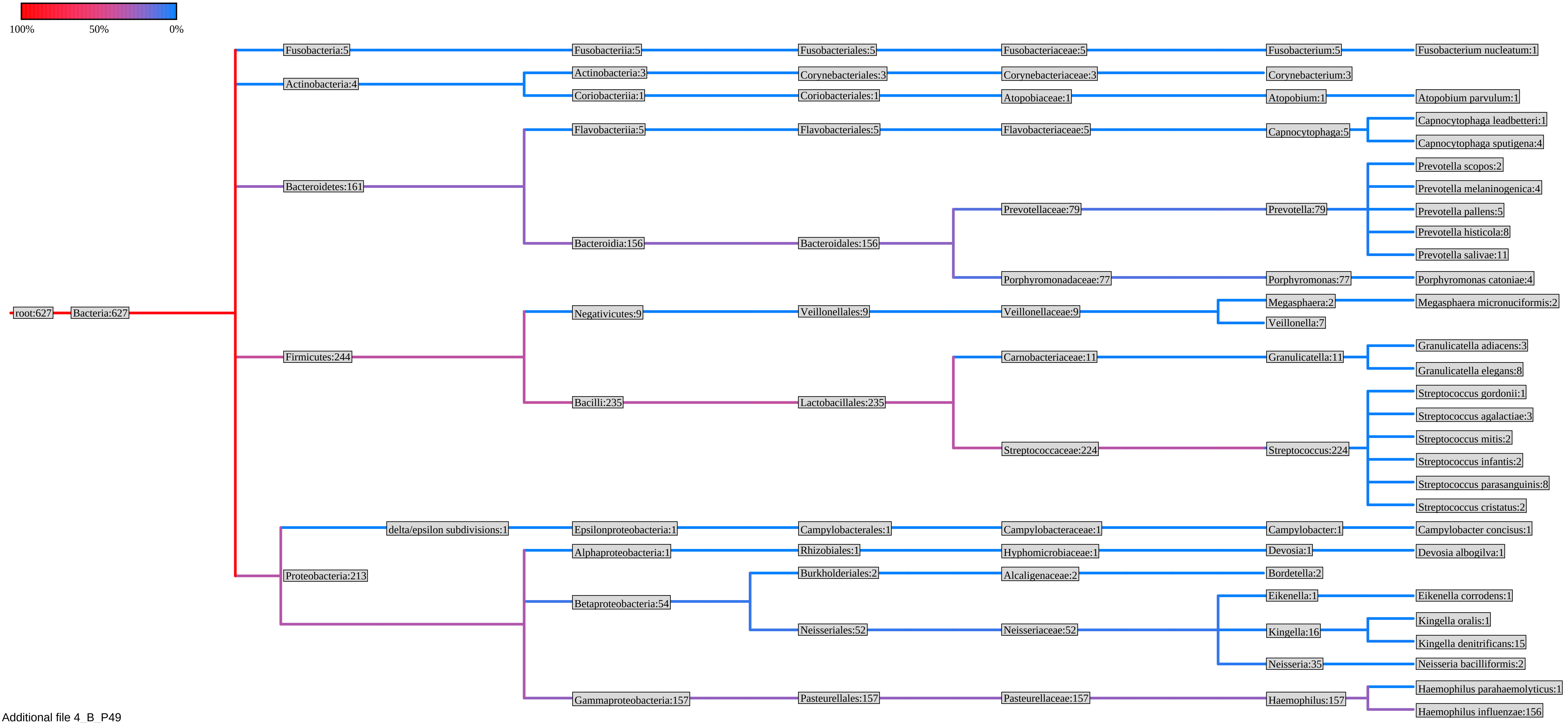

Additional file 4_B_P49

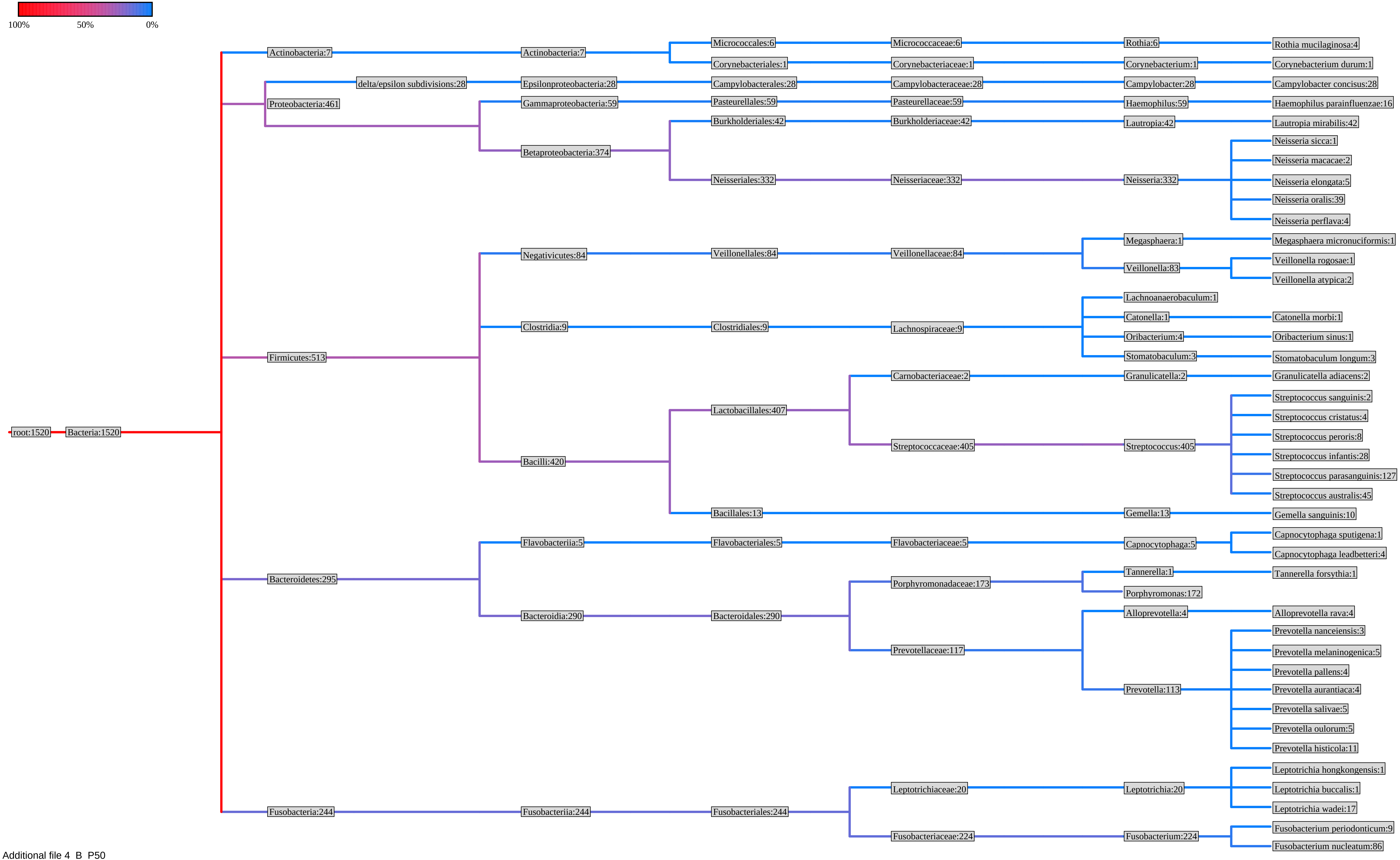

Additional file 4 B P50

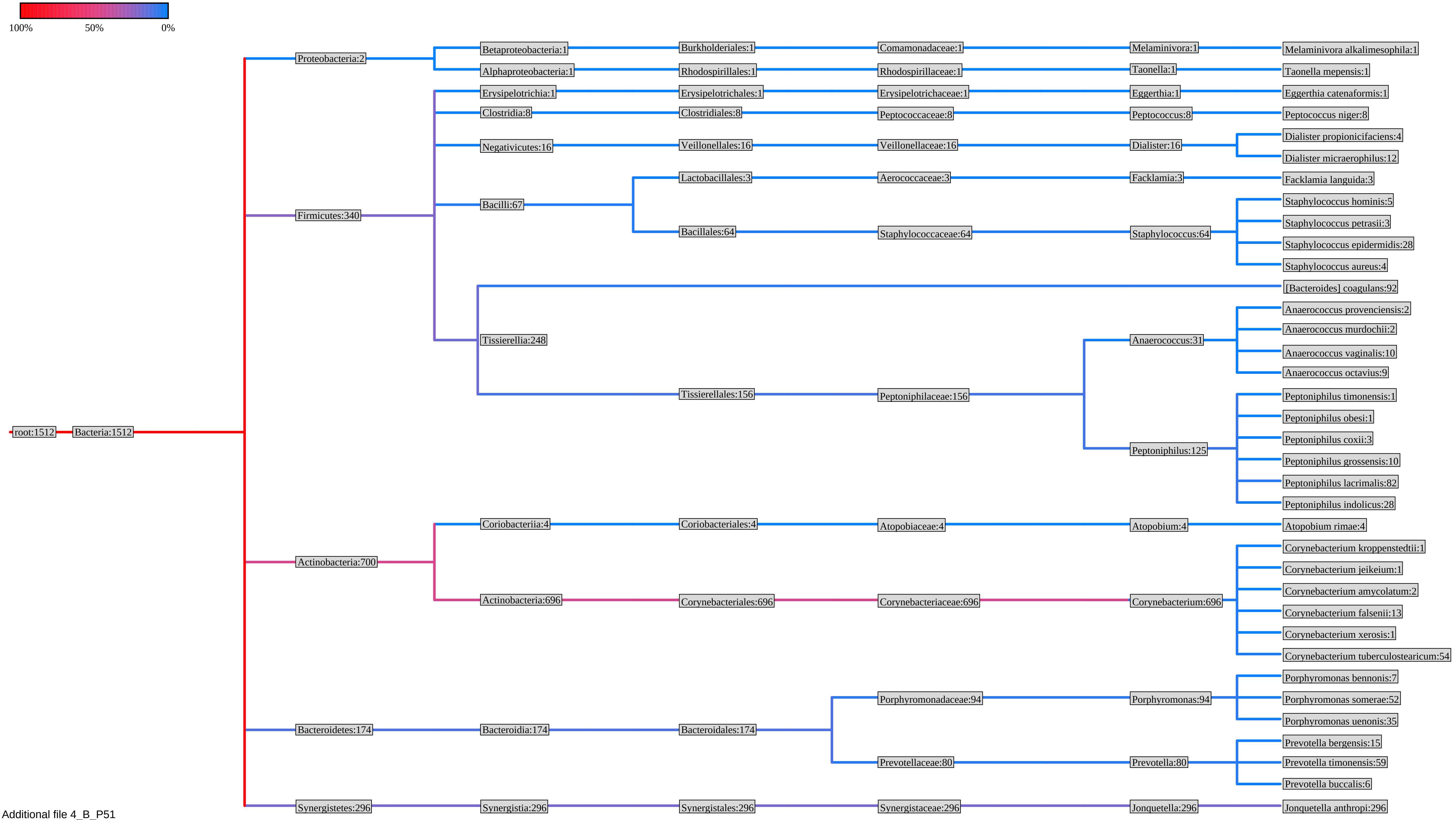

Additional file 4_B_P51

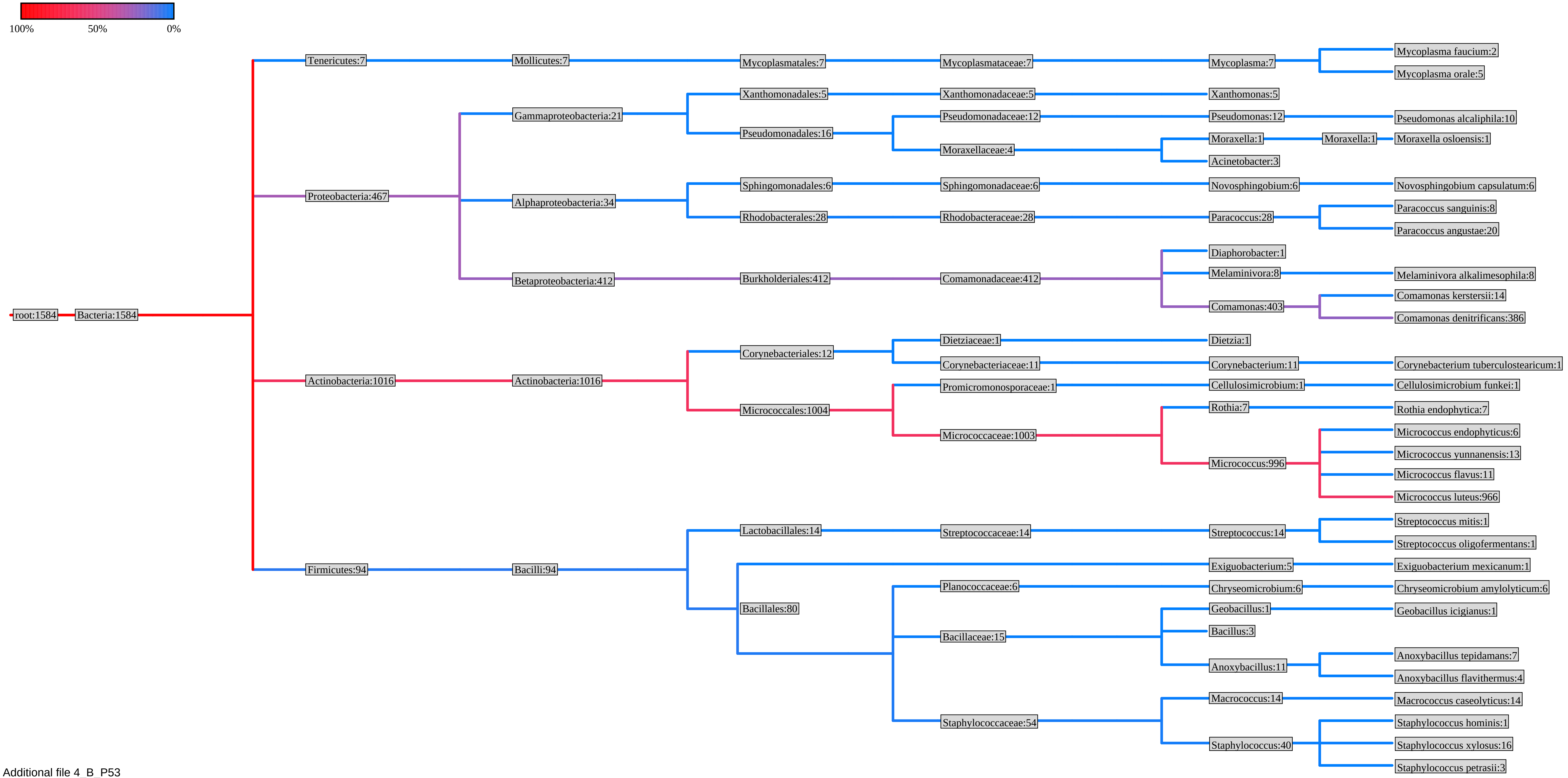

Additional file 4_B_P53

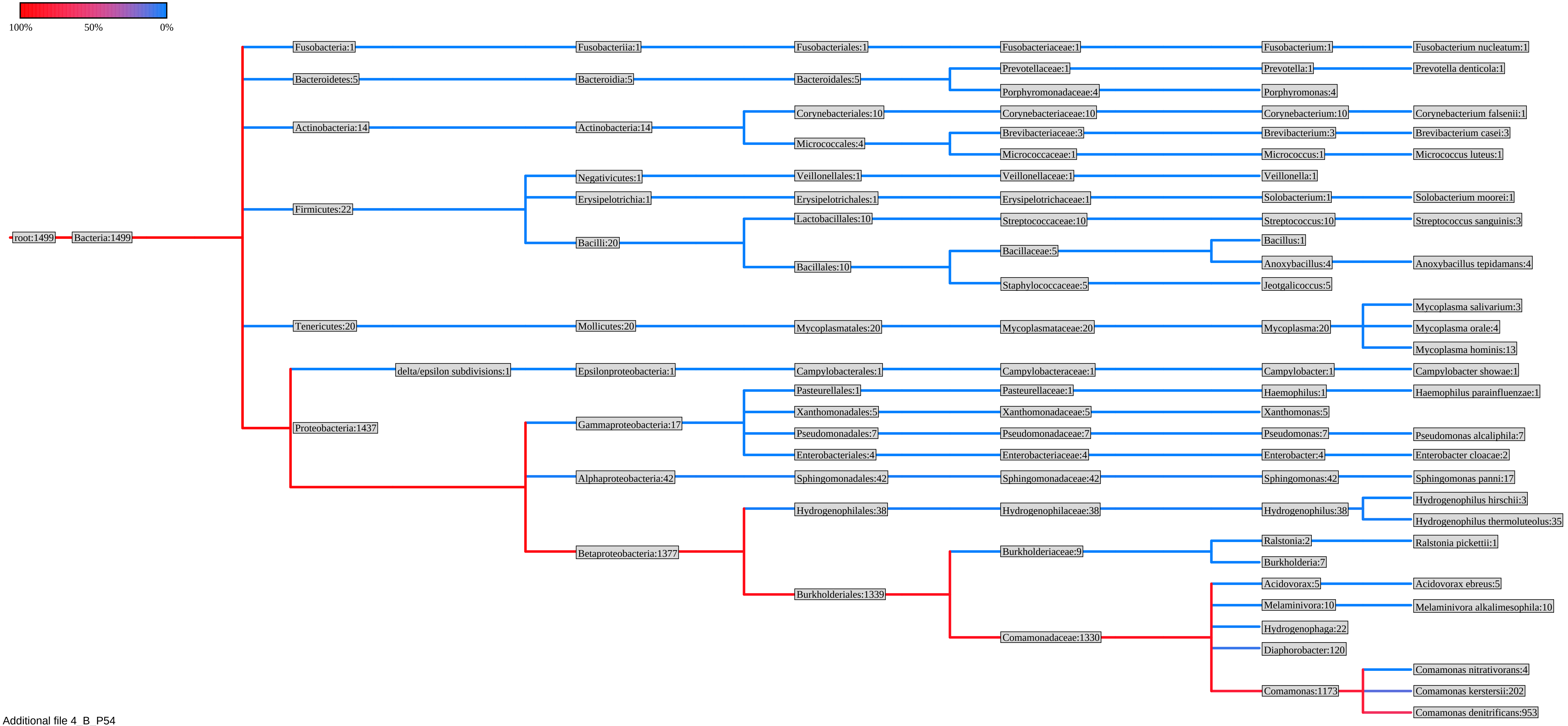

Additional file 4_B_P54

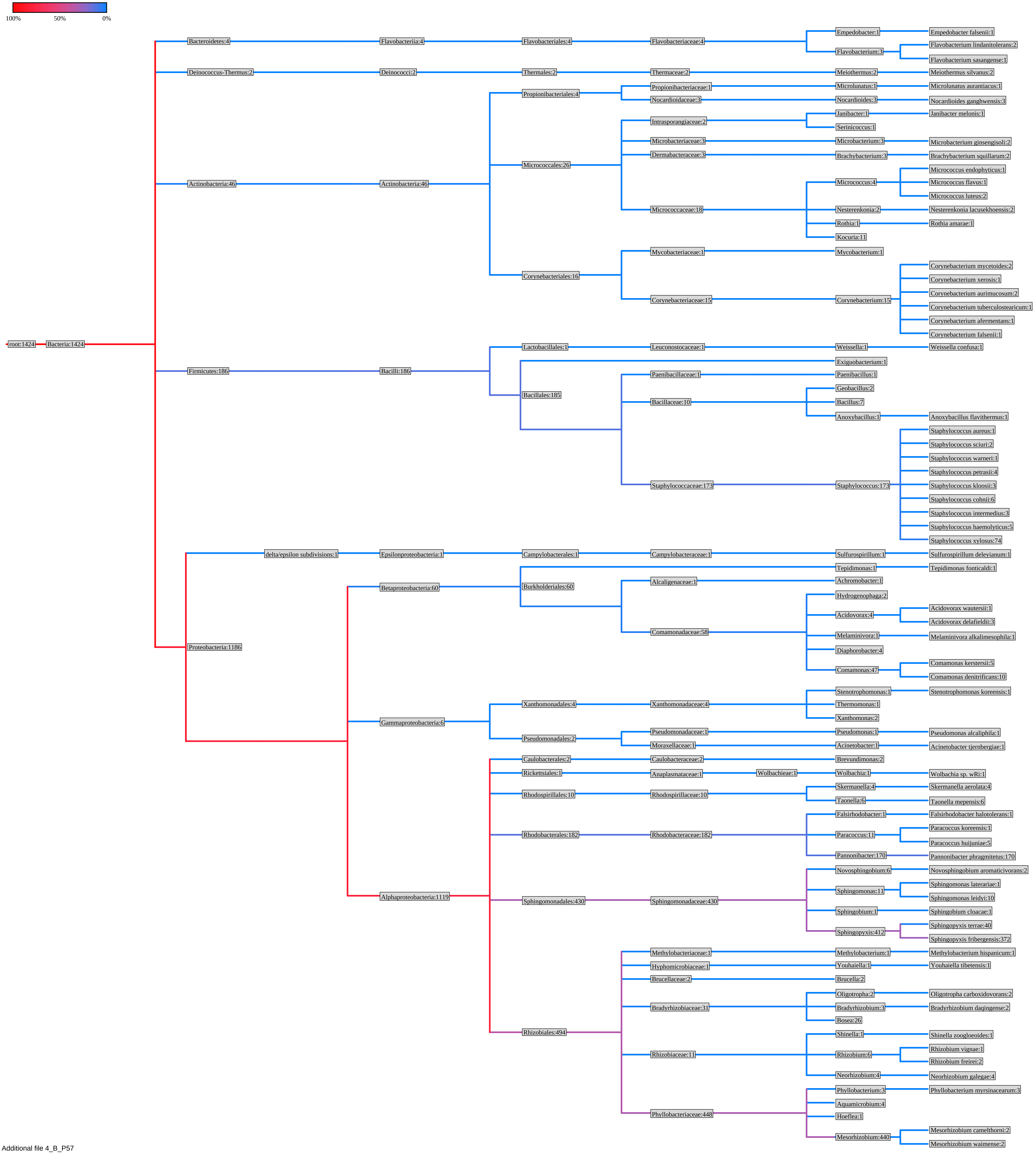

Additional file 4_B_P57

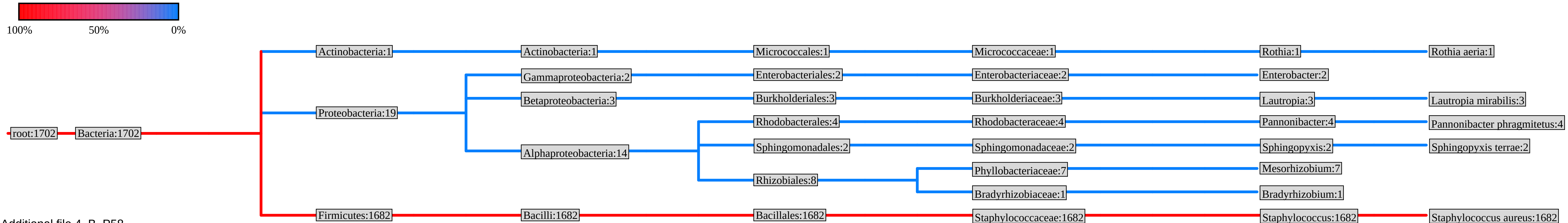

Additional file 4_B_P58

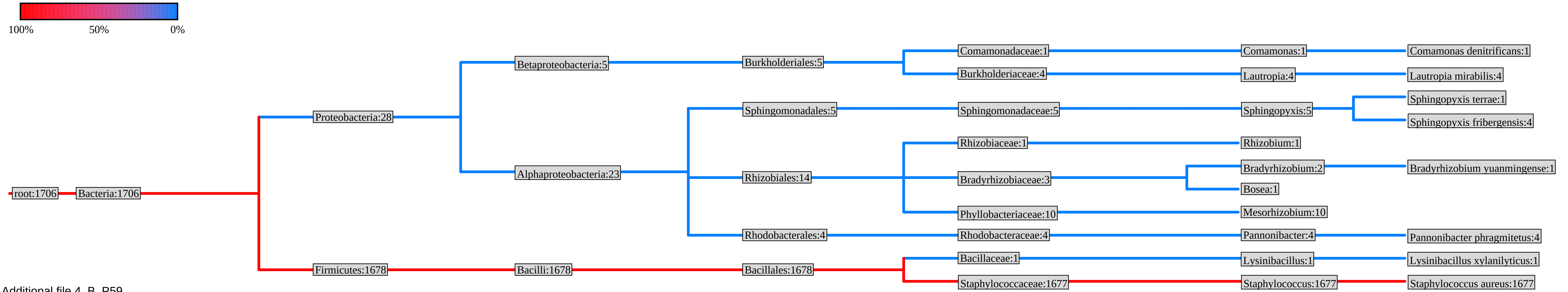

Additional file 4_B_P59

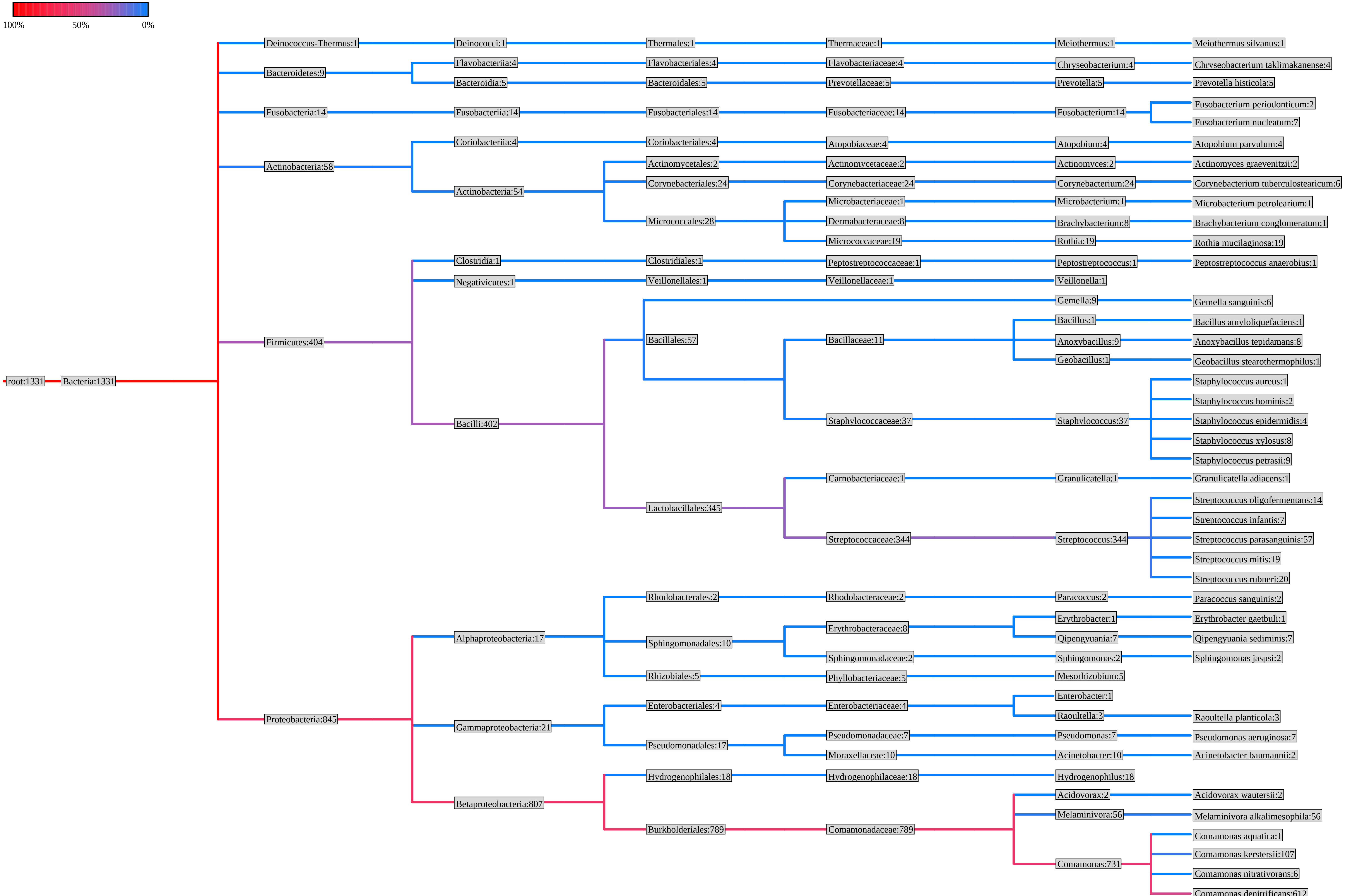

Additional file 4_B_P60

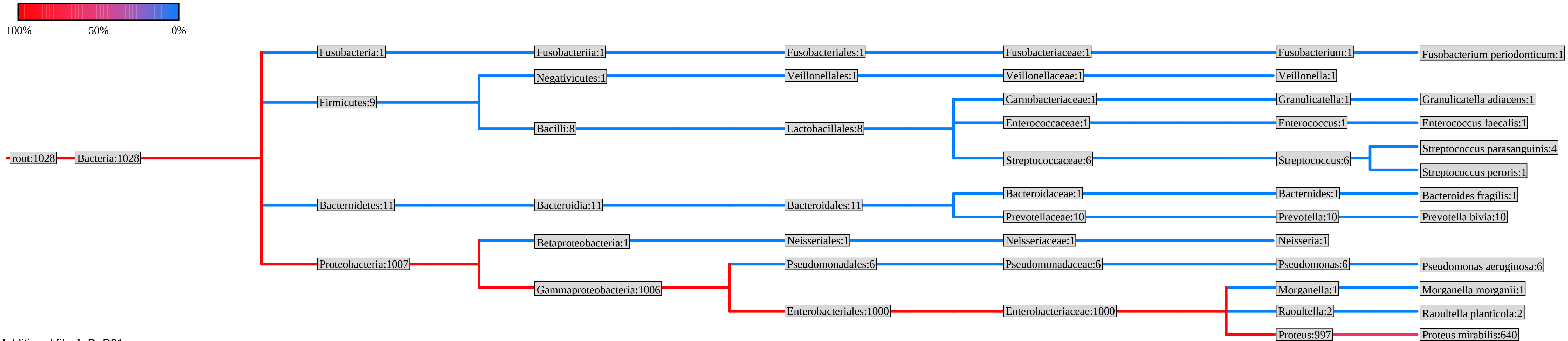

Additional file 4_B_P61

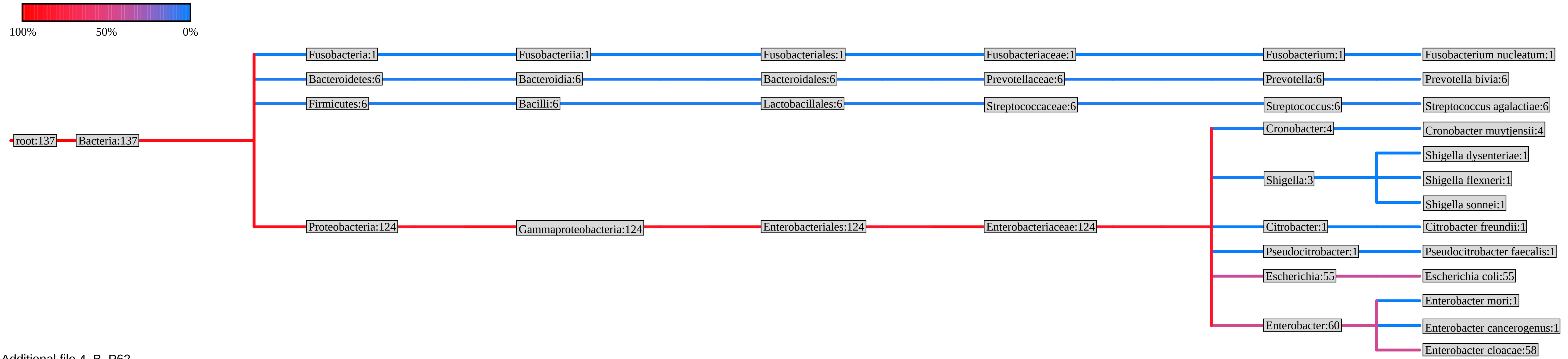

Additional file 4_B_P62

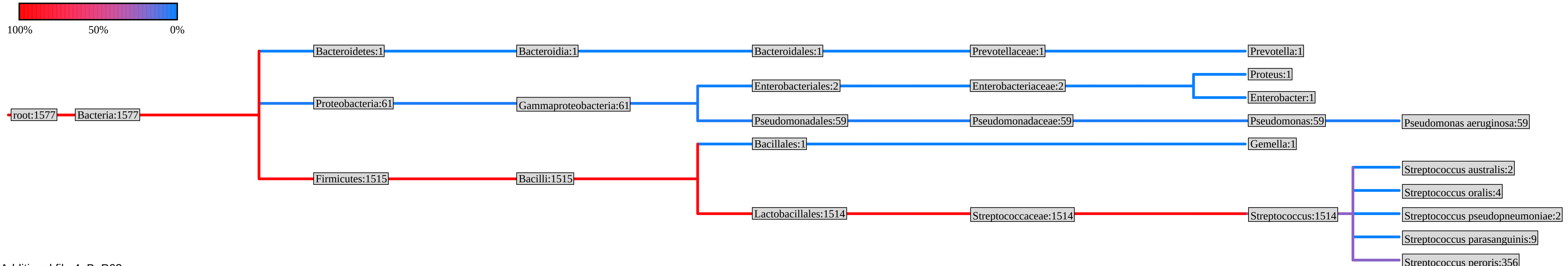

Additional file 4_B_P63

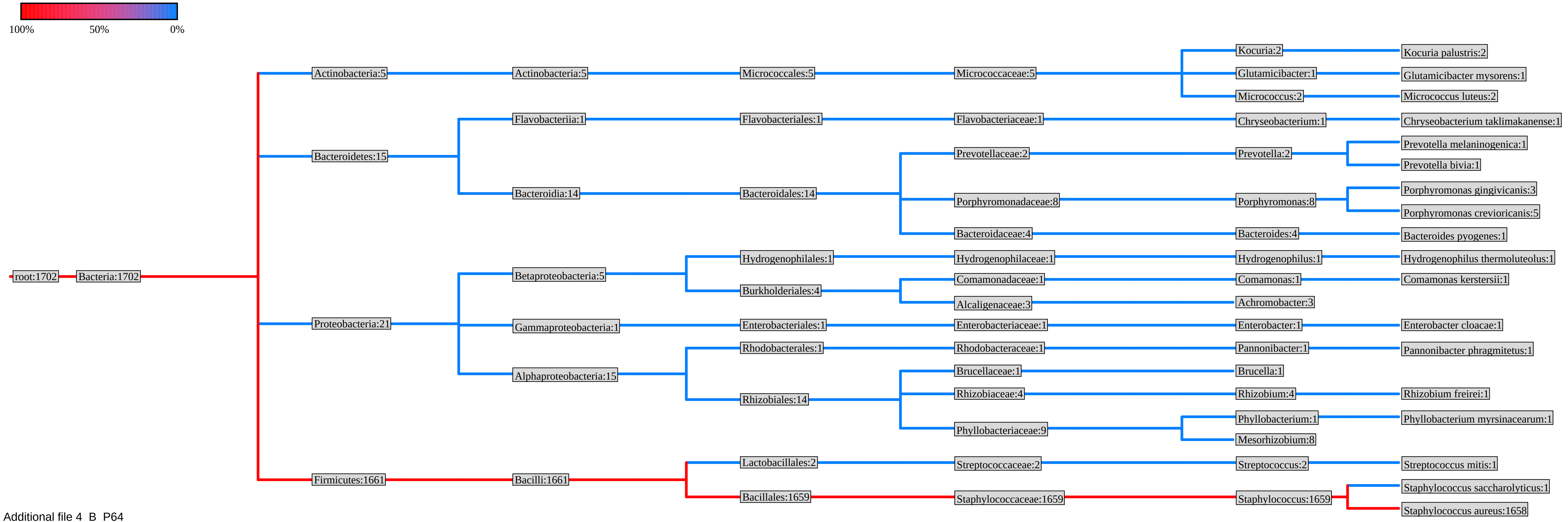

Additional file 4_B_P64

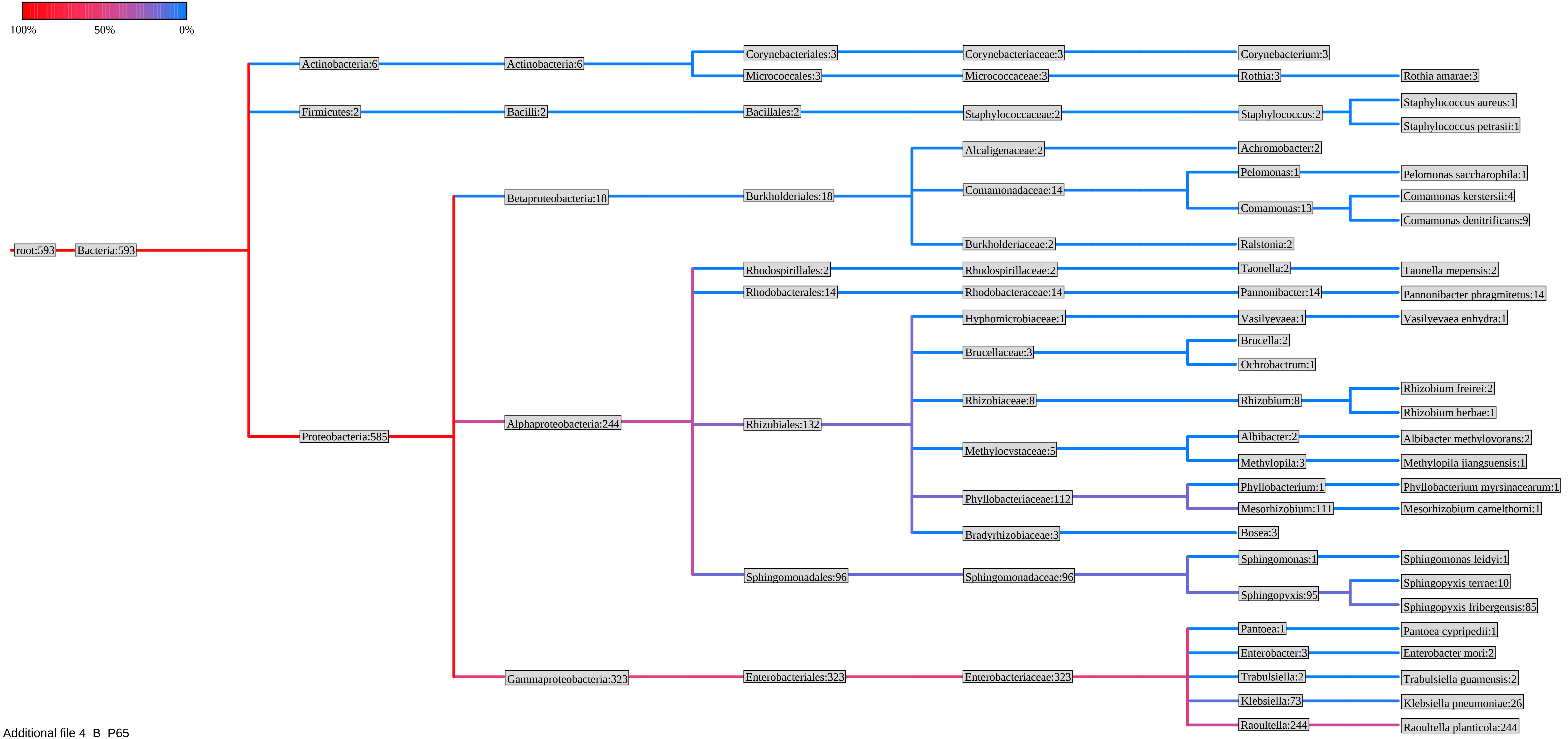

Additional file 4_B_P65

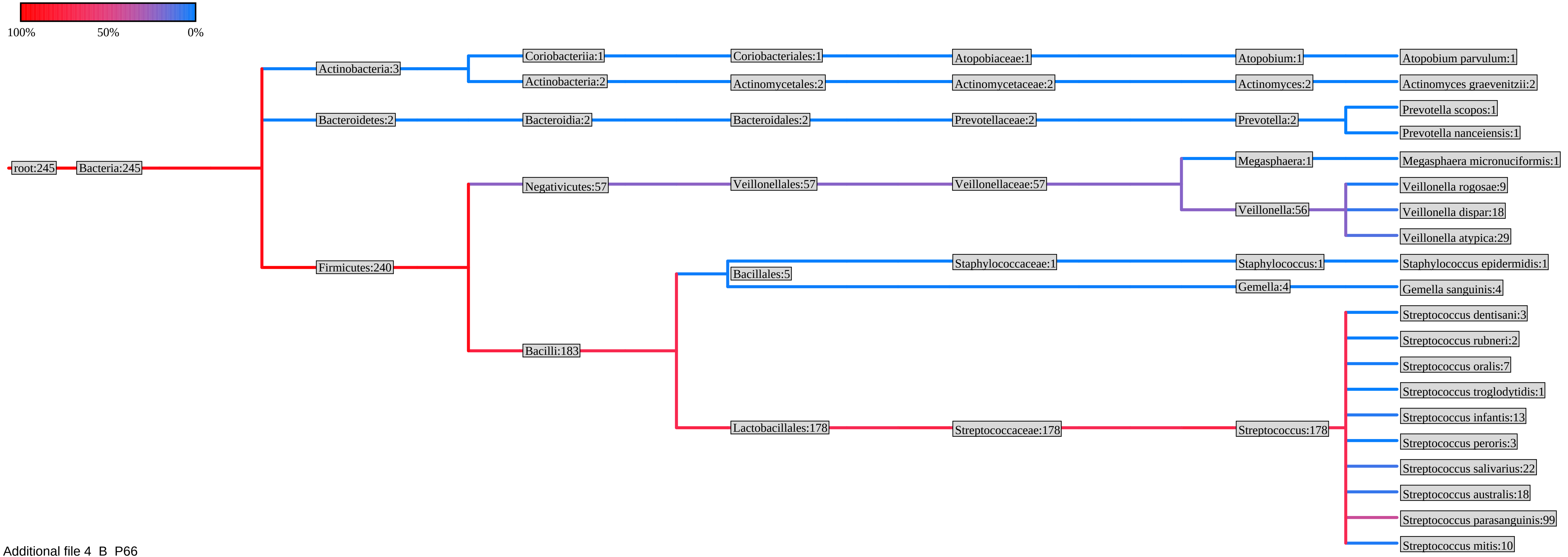

Additional file 4_B_P66

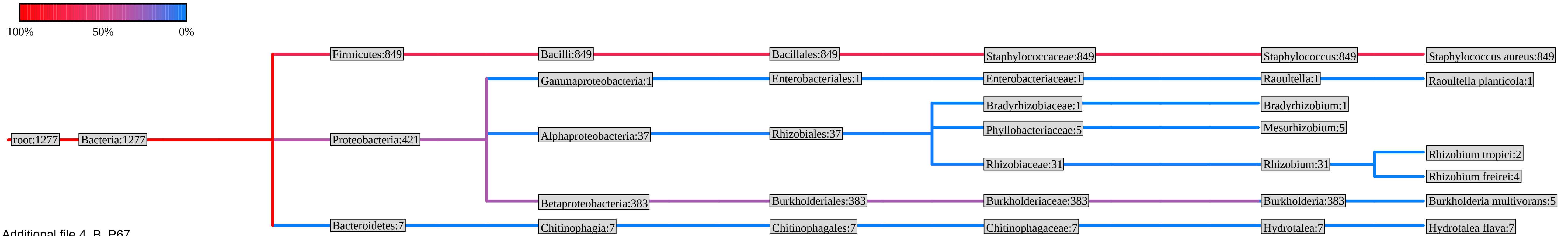

Additional file 4_B_P67

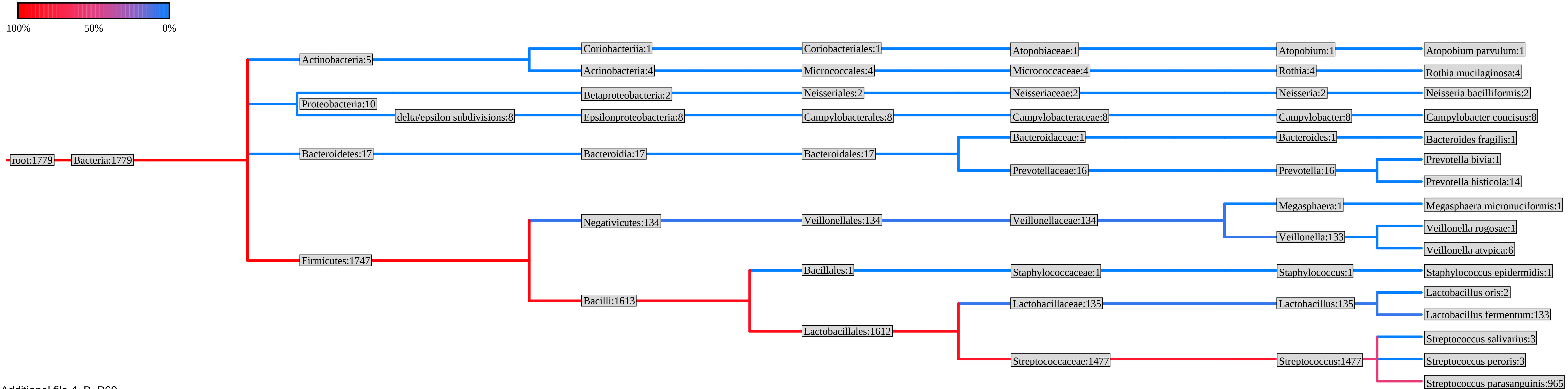

Additional file 4_B_P69

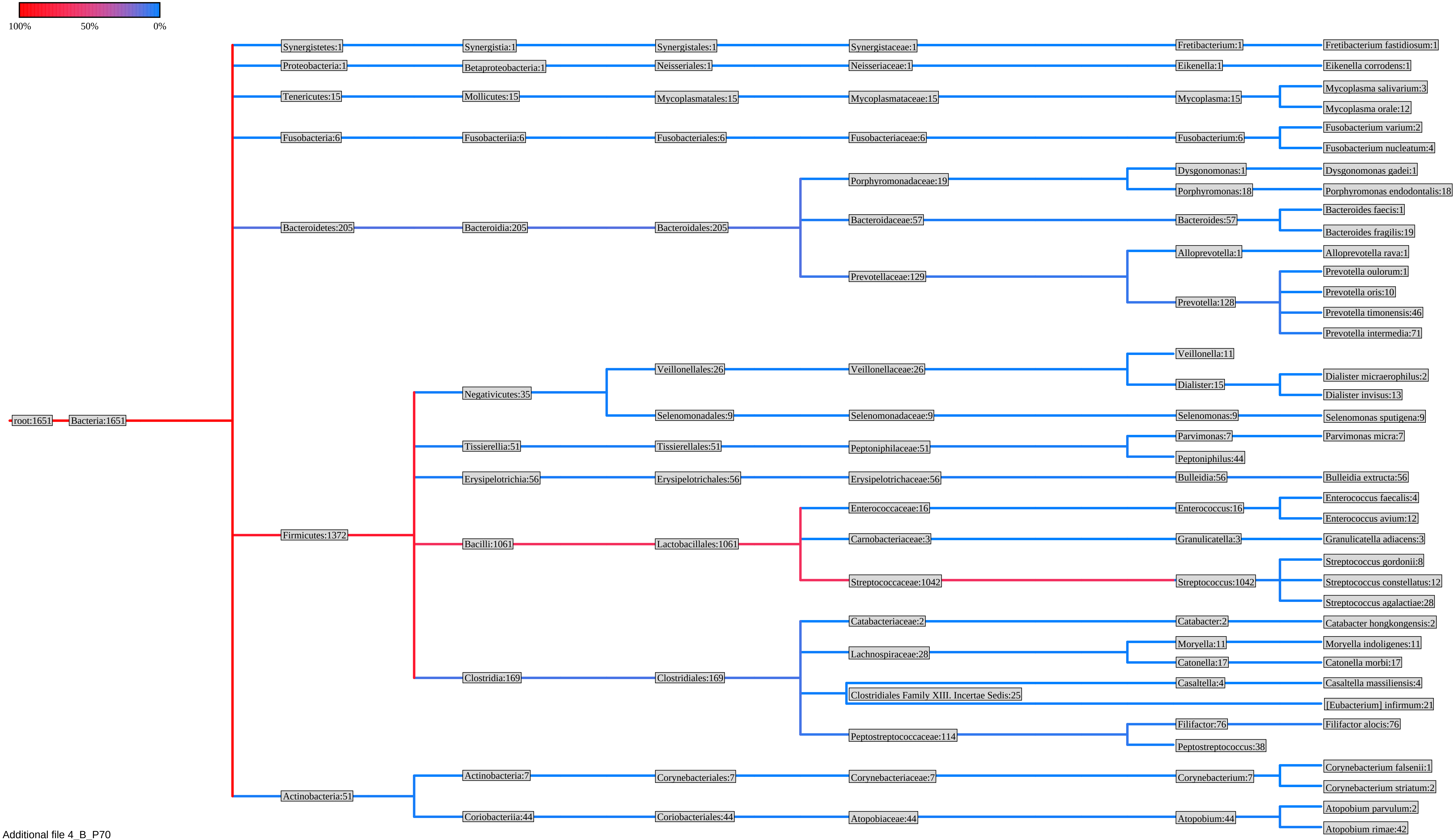

Additional file 4_B_P70

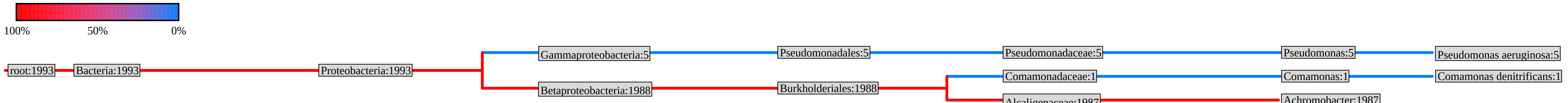

Additional file 4_B_P71

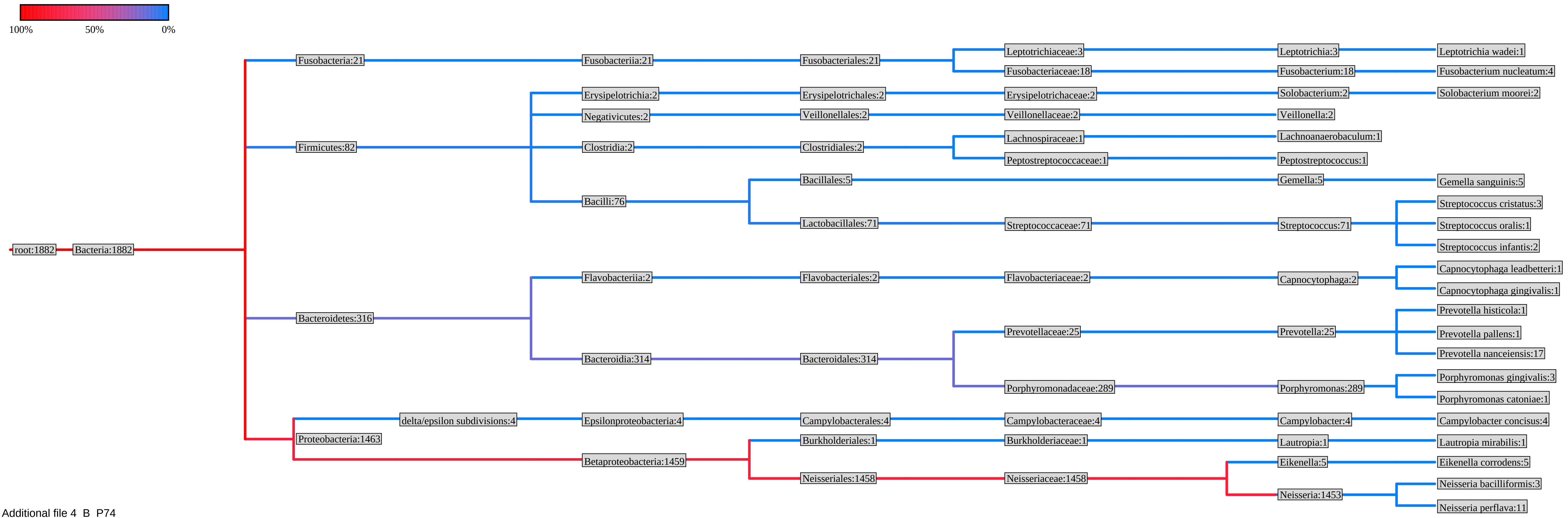

Additional file 4_B_P74

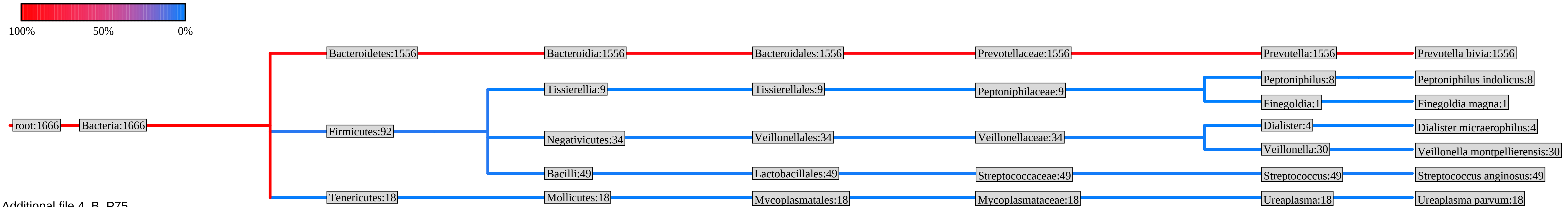

Additional file 4_B_P75

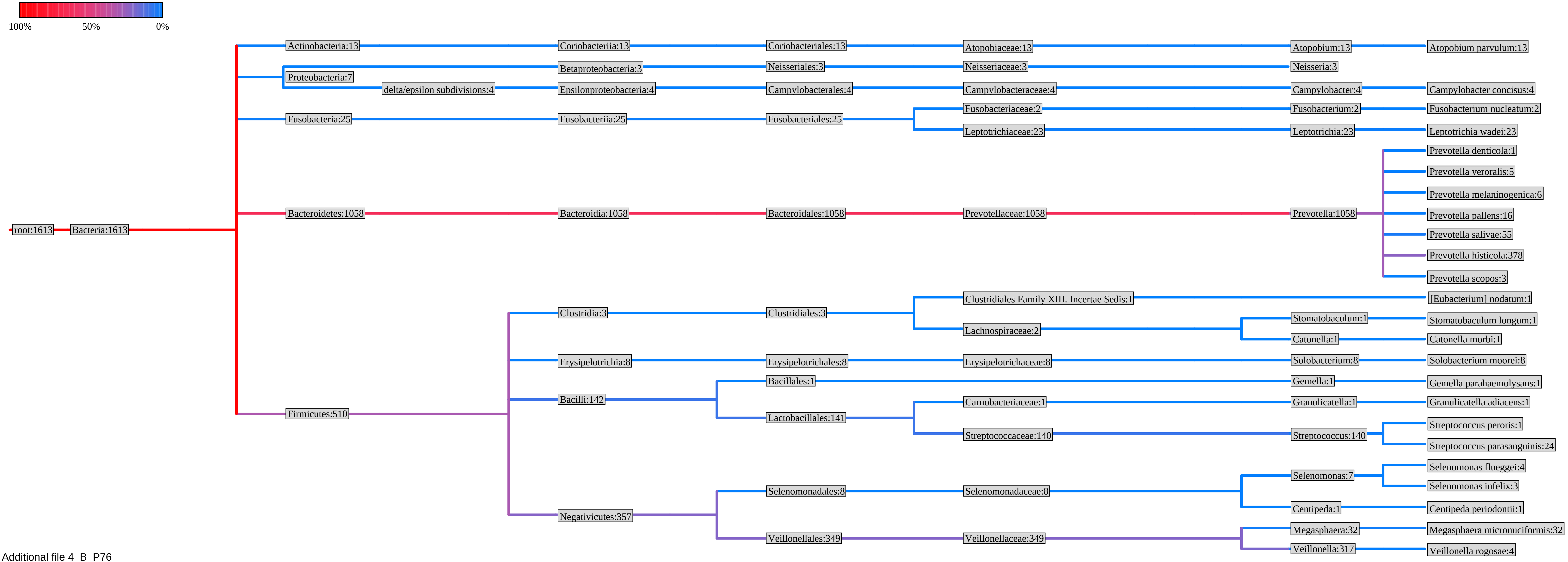

Additional file 4_B_P76

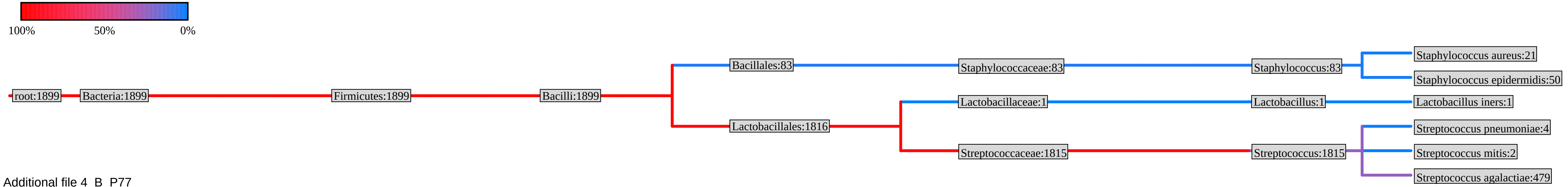

Additional file 4_B_P77

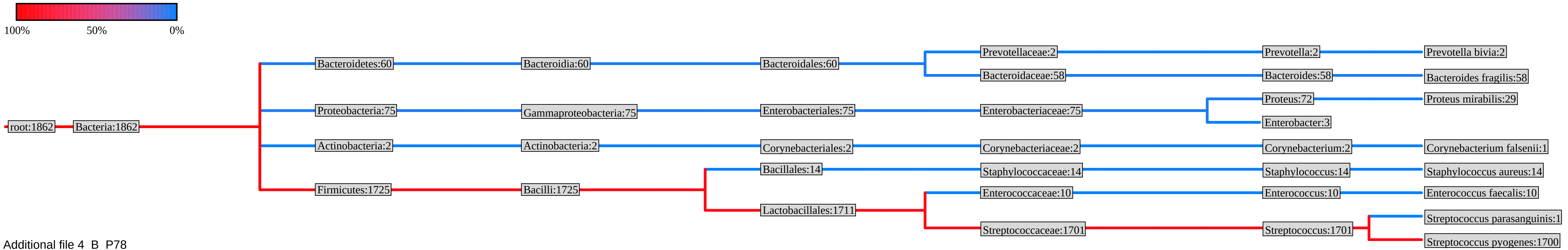

Additional file 4_B_P78

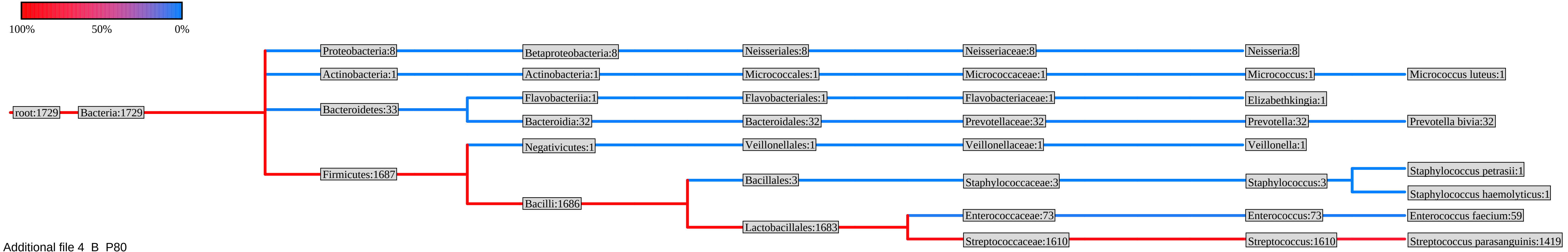

Additional file 4_B_P80

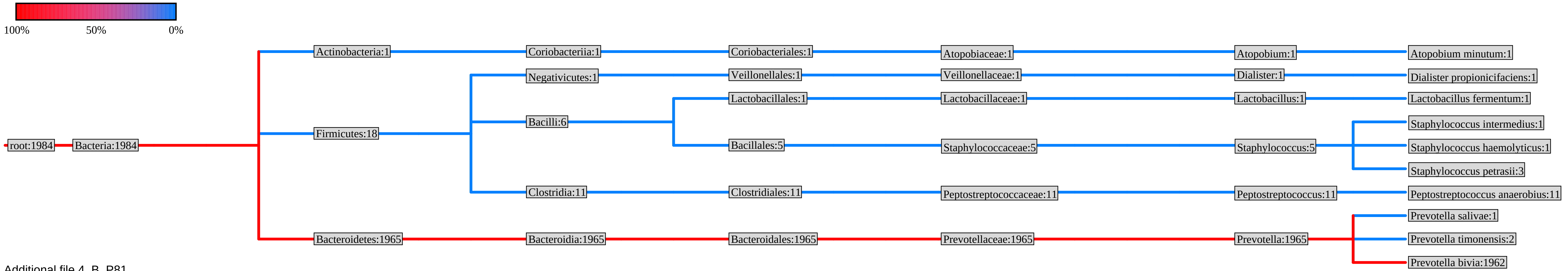

Additional file 4_B_P81

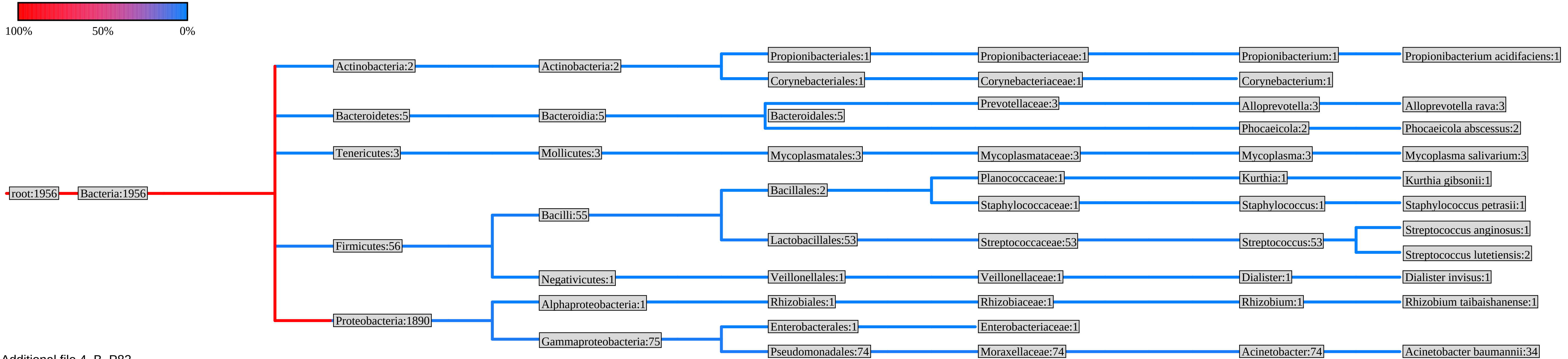

Additional file 4_B_P82

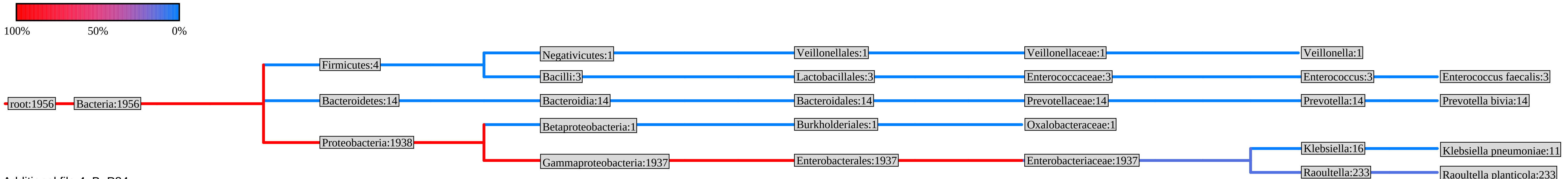

Additional file 4_B_P84

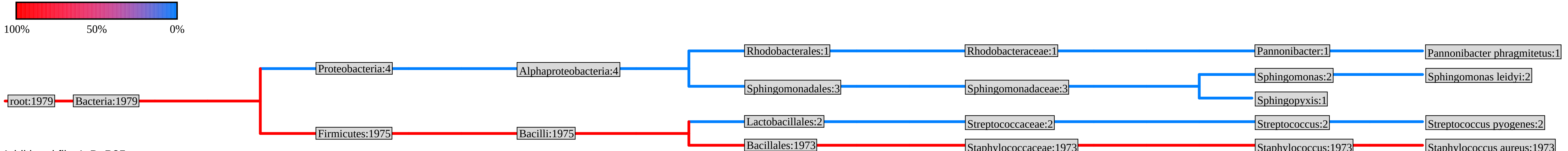

Additional file 4_B_P85

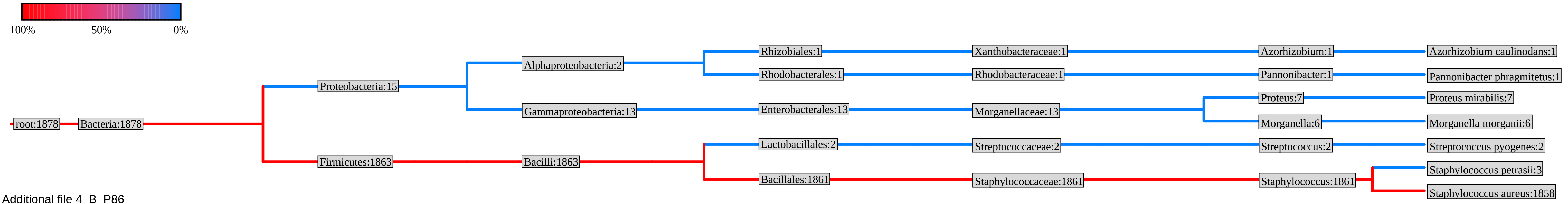

Additional file 4_B_P86

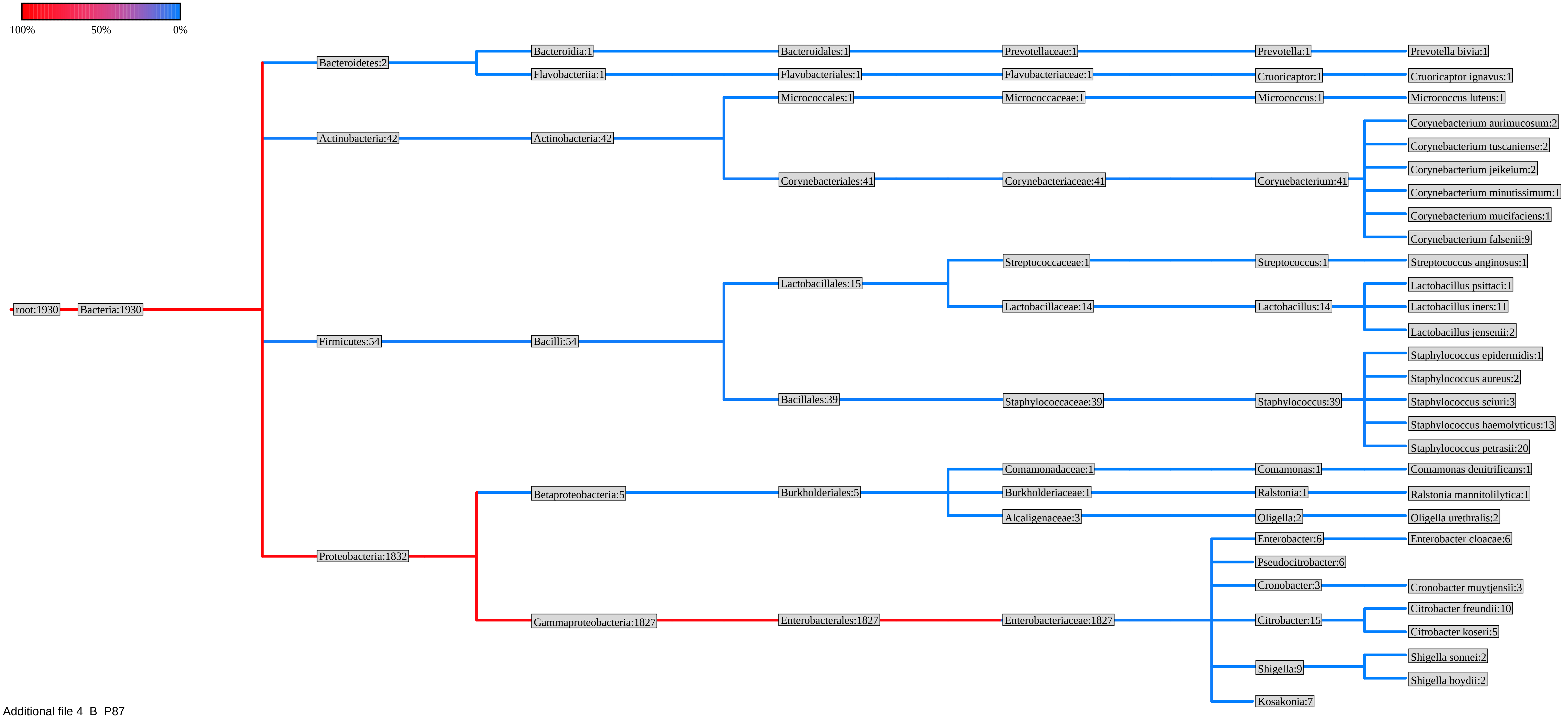

Additional file 4_B_P87

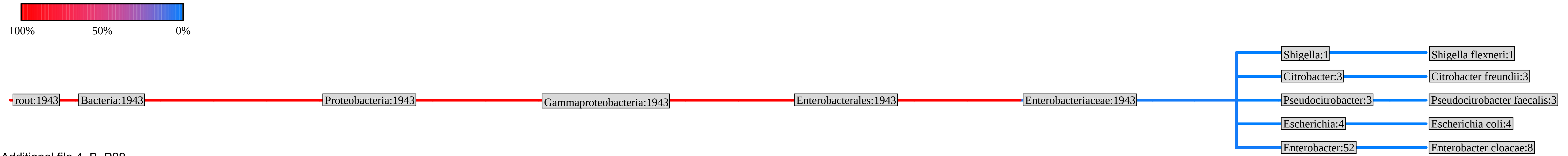

Additional file 4_B_P88

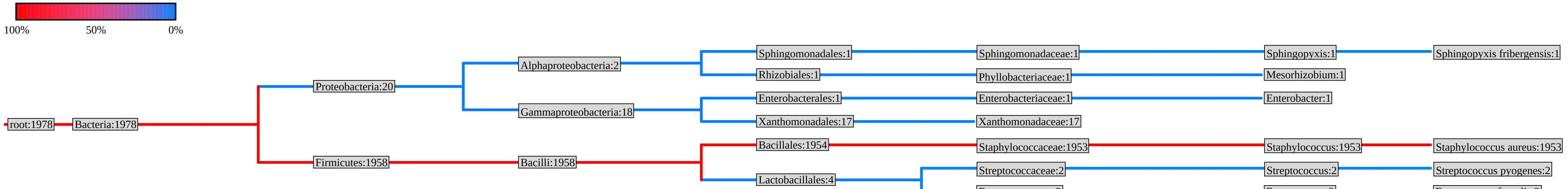

Additional file 4_B_P89

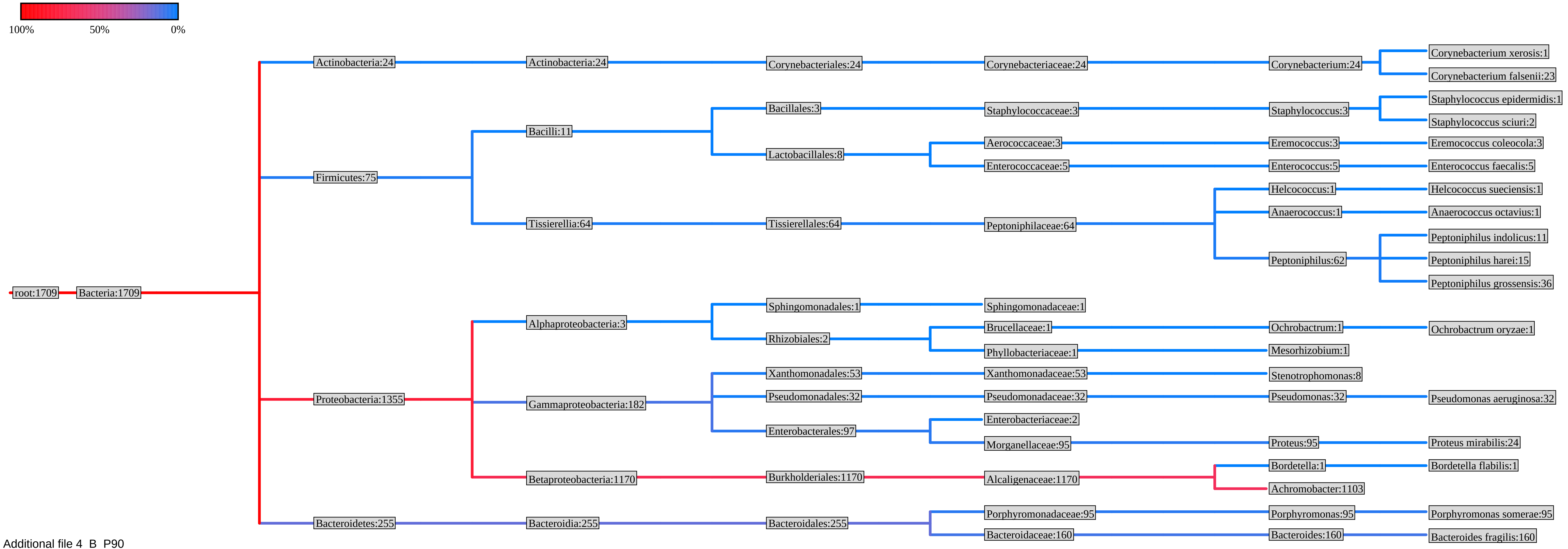

Additional file 4_B_P90

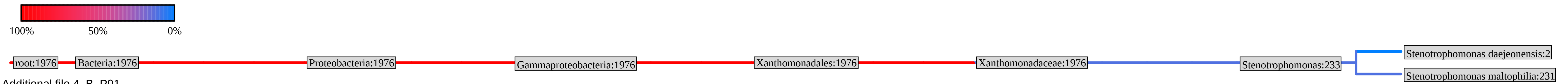

Additional file 4_B_P91

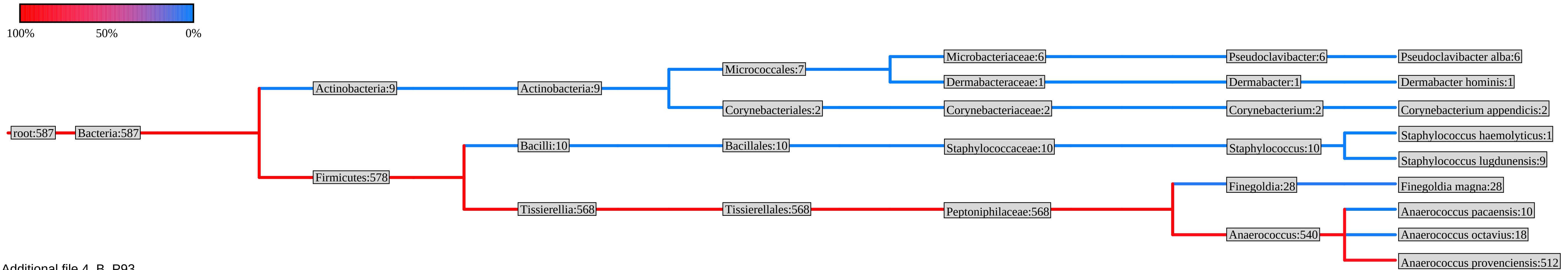

Additional file 4_B_P93

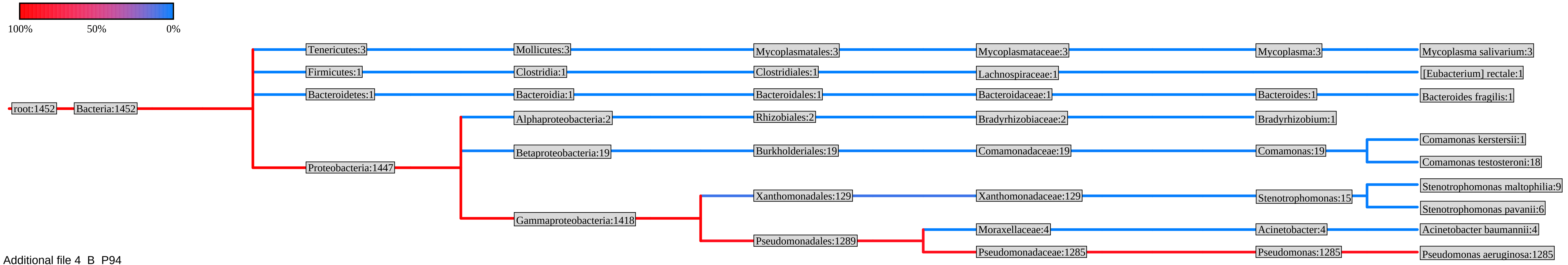

Additional file 4_B_P94

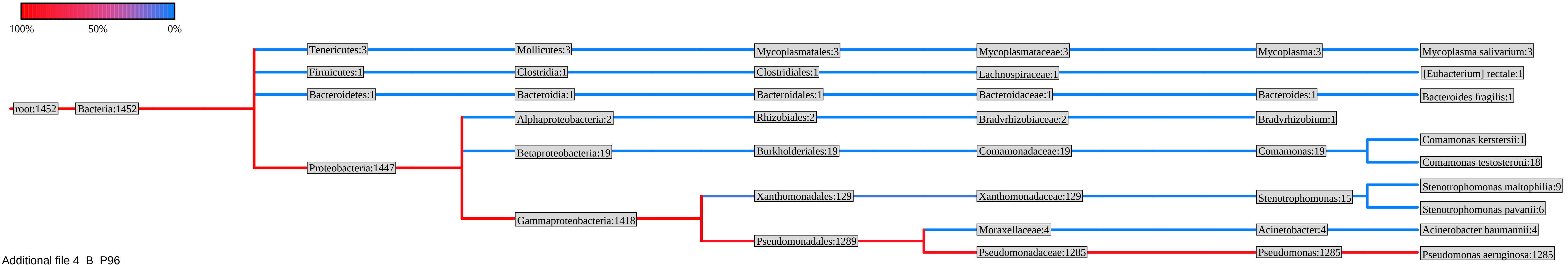

Additional file 4_B_P96

Additional file 4_F_P17

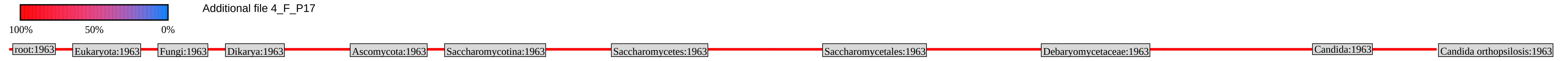

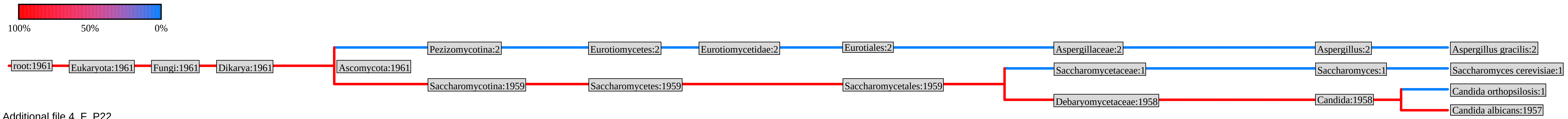

Additional file 4_F_P22

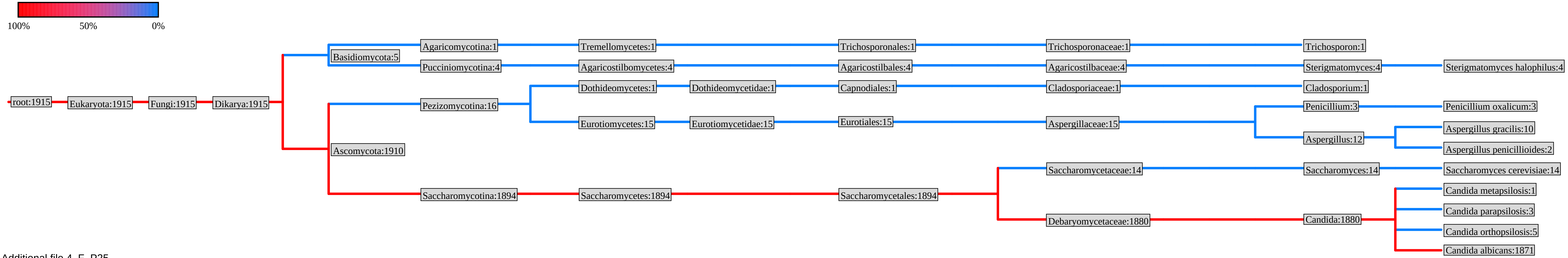

Additional file 4_F_P25

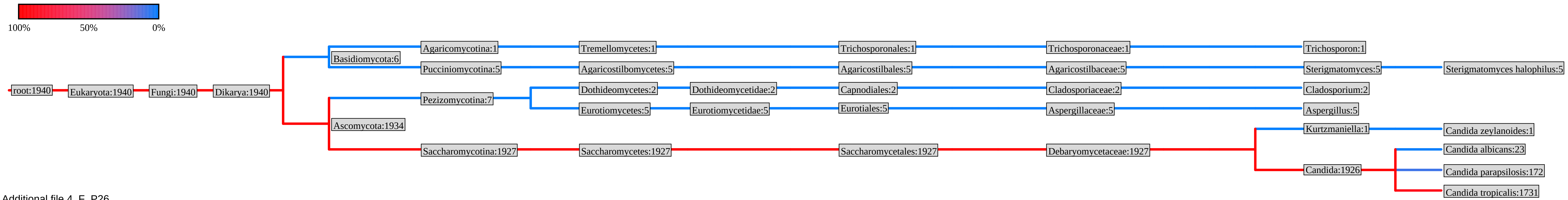

Additional file 4_F_P26

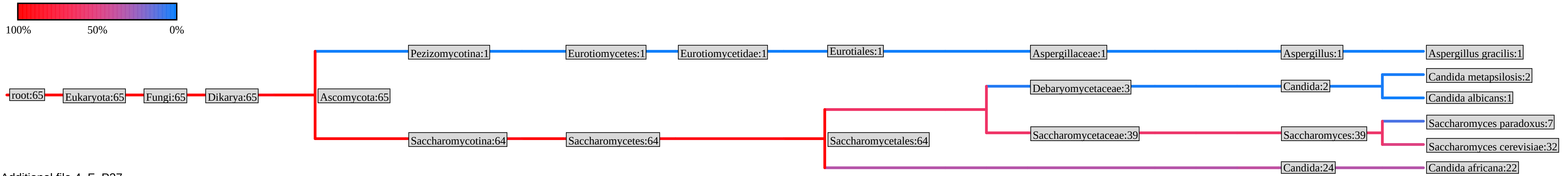

Additional file 4_F_P37

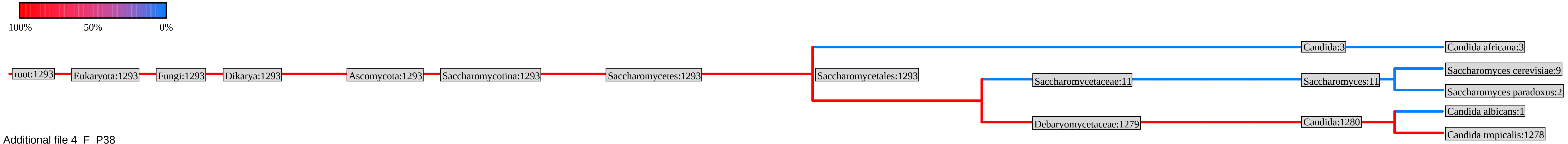

Additional file 4_F_P38

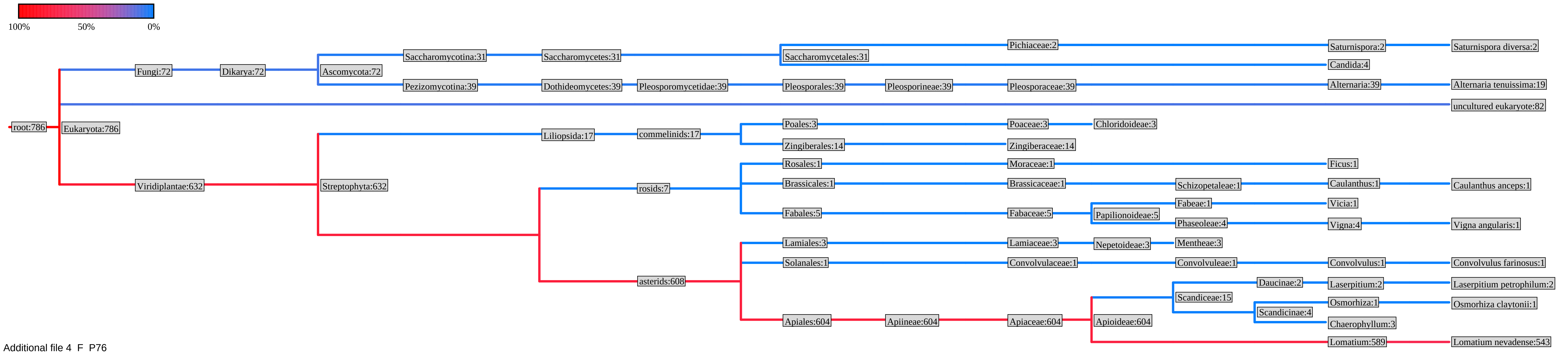

Additional file 4_F_P76

Additional file 4_F_P81

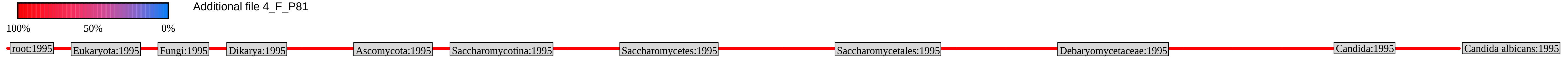

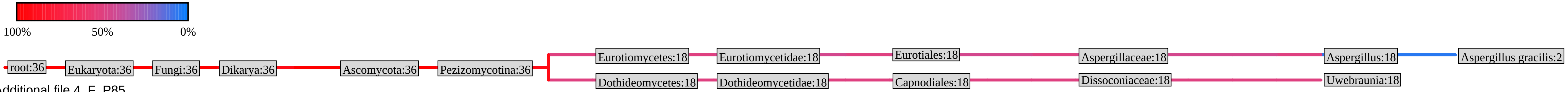

Additional file 4_F_P85
