# Supplementary material for: Detection of bacterial pathogens from clinical specimens using conventional microbial culture and 16S metagenomics: a comparative study
Source: BMC Infect Dis. 2017 Sep 19;17:631. doi: 10.1186/s12879-017-2727-8 (PMC5606128; doi:10.1186/s12879-017-2727-8)
Supplement: Supplementary file 3 — QC passed FASTA files generated from the Credence Infectious Panel Pipeline 1.1.0. Unaligned BAM files generated from the sequencing run were trimmed of barcode and adaptor sequences and this quality checked for the following QC parameters: Phred quality score 16; fragment length > 300 bp for bacterial reads and >200 bp for fungal reads respectively and final FASTA sequences obtained. Specimen files are named as follows: Additional file 3_B/F_Specimen number (e.g. Additional file 3_B_P1 – fasta files of bacterial sequences within specimen P1; Additional file 3_F_P1- fasta files of fungal sequences within specimen P1). (PDF 37575 kb) [file 12879_2017_2727_MOESM3_ESM.pdf]

Contents

Bacteria 3

Additional file 3_B_P1 3

Additional file 3_B_P5 33

Additional file 3_B_P6 54

Additional file 3_B_P7 168

Additional file 3_B_P10 280

Additional file 3_B_P11 503

Additional file 3_B_P12 726

Additional file 3_B_P13 949

Additional file 3_B_P14 1171

Additional file 3_B_P15 1394

Additional file 3_B_P16 1621

Additional file 3_B_P17 1844

Additional file 3_B_P20 2067

Additional file 3_B_P21 2289

Additional file 3_B_P22 2511

Additional file 3_B_P23 2734

Additional file 3_B_P24 2956

Additional file 3_B_P25 3179

Additional file 3_B_P26 3403

Additional file 3_B_P27 3627

Additional file 3_B_P28 3849

Additional file 3_B_P29 4072

Additional file 3_B_P30 4300

Additional file 3_B_P31 4544

Additional file 3_B_P33 4766

Additional file 3_B_P34 4989

Additional file 3_B_P35 5212

Additional file 3_B_P36 5440

Additional file 3_B_P37 5662

Additional file 3_B_P38 5885

Additional file 3_B_P39........6107
Additional file 3_B_P40........6329
Additional file 3_B_P41........6552
Additional file 3_B_P42........6774
Additional file 3_B_P43........6998
Additional file 3_B_P44........7114
Additional file 3_B_P45........7348
Additional file 3_B_P46........7571
Additional file 3_B_P47........7793
Additional file 3_B_P49........8015
Additional file 3_B_P50........8238
Additional file 3_B_P51........8462
Additional file 3_B_P53........8688
Additional file 3_B_P54........8912
Additional file 3_B_P57........9135
Additional file 3_B_P58........9359
Additional file 3_B_P59........9584
Additional file 3_B_P60........9810
Additional file 3_B_P61........10034
Additional file 3_B_P62........10258
Additional file 3_B_P63........10483
Additional file 3_B_P64........10709
Additional file 3_B_P65........10934
Additional file 3_B_P66........11158
Additional file 3_B_P67........11401
Additional file 3_B_P69........11633
Additional file 3_B_P70........11860
Additional file 3_B_P71........12090
Additional file 3_B_P74........12312
Additional file 3_B_P75........12534
Additional file 3_B_P76........12758
Additional file 3_B_P77........12991
Additional file 3_B_P78........13213

Additional file 3_B_P80....13437
Additional file 3_B_P81....13662
Additional file 3_B_P82....13884
Additional file 3_B_P84....14107
Additional file 3_B_P85....14329
Additional file 3_B_P86....14552
Additional file 3_B_P87....14774
Additional file 3_B_P88....14997
Additional file 3_B_P89....15220
Additional file 3_B_P90....15442
Additional file 3_B_P91....15666
Additional file 3_B_P93....15888
Additional file 3_B_P94....16131
Additional file 3_B_P96....16355
Fungi....16586
Additional file 3_F_P17....16586
Additional file 3_F_P22....16763
Additional file 3_F_P25....16922
Additional file 3_F_P26....17085
Additional file 3_F_P37....17243
Additional file 3_F_P38....17406
Additional file 3_F_P76....17574
Additional file 3_F_P81....17682
Additional file 3_F_P85....17840

Bacteria

Additional file 3_B_P1

>1
CTGCTGCCGCCCTGAGGAGTCTGGGCTGTGTCTCAGTCCCAGTGTGGCCGATCACCCTCTCAGGTCGGCTATG
CATCGTGGCCTTGGTGAGCCGTTACCTCACCAACTAGCTAATGCACCGCGGGTCCATCCATCAGCGACACCCG
AAAGCGCCTTTCACTCTTTATGCCATGCGCATAAACTGTTTATGCGGTATTTAGCCACCTTGTTTTTTCCAAG

TCGTTTACTCCCCCTCTGATTGGGTAGGTTTACCCACGTGTTTACTTCACCCGTCCGCCACTCCTCTTTTCCA
ATTTGAGTGCAGCACTCGGGAGGAAAGAAGCGTTCGTACTTG
>2
CTGCTGCCGCCCTAGGAGTCTGGGCCGTGTCTCAGTCCCAGTGTGGCCGATCACCCTCTCAGGTCGGCTATGC
ATCGTGGCCTTTGGGTGAGCCGTTACCTCACCAACTAGCTAATGCACCGCGGGTCCATCCATCAGCGACACCC
GAAAGCGCCTTTTCACTCTTTATGCCATGCGGCATAAACTGTTTATGCGGTATTTAGCACCTGTTTTTCCAAG
TCGTTTATCCCCCTCTGATGGGTAGGTTTACCCACGTGTTTACTTCACCCGTCCGCCACTCCTCTTTTCCAAT
TTGAGTGCAAGCACTCGGGAGGAAAGGA
>3
CTGCTGCCGCCCGTAGGAGTCTGGGCCGTGTCTCAGTCCCAGTGTGGCCGATCACCCTCTCAGGTCGGCTATG
CATCGTGGCCTTGGTGAGCCGTTACCTCACCAACTAGCTAATTGCACCGCGGGTCCATCCATCAGCGACACCC
GAAAGCGCCTTTTCACTTCTTTTATTGCCATTGCGGCATTAAAAACTTGTTTTTATTGCGGTTATTTTTAGCA
CCCTTTTGTTTTCCCAAAAGTGTTTTATTTCCCCTTCTGATTGGGGTTAGGGGTTTTACCCCACGTGTTTACT
TTCACCCCCGTCC
>4
CTGCTGCCGCCCGTAGGAGTCTGGGCCGTGTCTCAGTCCCAGTGTGGCCGATCACCCTCTCAGGTCGGCTATG
CATCGTGGCCTTGGTGAGCCGTTACCTCACCAACTAGCTAATGCACCGCGGGTCCATCCATCAGCGACACCCG
AAAGCGCCTTTTTCACTCTTTATGCCATGCGGCATAAACTGTTTATGCGGTATTTTAGCACCTGTTTTTTTCC
AAGTCGTTTTATCCCCCTCTGATGGGTAGGTTTACCCACGTGCTACTTCACCCGTCCGCCACTCCTCTTTTCC
AATCGAGTTGCAAGCACT
>5
CTGCTGCCGCCCGTAGGAGTCTGGGCCGTGTCTCAGTCCCAGTGTGGCCGATCACCCTCACAGGTCGGCTATG
CAATCGTGGCCTTGGTGAGCCGTTACCTCACCAACTAGCTAATGCACCGCGGGTCCATCCATCAGCGACACCC
GAAAGCGCCTTTTCACTCTTTATGCCATGCGGCATAAACTGTTTATGCGGTATTTAGCACCTGTTTTTTCCAA
GTCGTTTATCCCCCTCTGATGGGTAGGTTTACCCACGTGTTTACTCACCCGTCCGCCACTCCTCCTTCCAATC
GAGTGCAAGCACTCGGGAGGAAAGAAGTCGTTTCGACTTGACATGTATTTTAGGCACACCGCCAGCGTTCGTC
CTGACGTCCAAG
>6
CTGCTGCGCCCGTAGGAGTCTGGGCCGTGTCTCAGTCCCAGTGTGGCTGGTCATCCGCTCAGACCAGCTAGAG
ATCGTCGGCTTGGTGAGCCTTTACCTCACCAACTACCTAATCCCACTTGGGCTCATCCTATGGCATGTGGCCC
GAAGGTCCCACACTTTAATCTTTCGATACTACGCGGTATTAGCTACAGTTTCCCGTAGTTATCCCCCTCCATA
AGCCAGATTCCCAAGCATTACTCACCCGTCCGCCACTGTATCAAAGAAGCAAGCTTCTCTATGTTACCGTTCG
ACTTGCATGTGTTAAGCCTGACCGTCCATCGT
>7
CTGCTGCCGCCCGTAGGAGTCTGGGCCGTGTCTCAGTCCCAGTGTGGCCGATCACCCTCTCAGGTCGGCTATG
CATCGTGGCCTTGGTGAGCCGTTACCTCACCAACTAGCTAATGCACCGCGGGTCCATCCATCAGCGACACCCG
AAAGCGCCTTTTCACTCTTTATGCCATGCGCATAAACTGTTTATGCGGTATTTTAGCACCTGTTTTTTCCAAG
TCGTTTTATCCCCCTCTGATGGGTAGGTTTTACCCACGTGTTTTACTCACCCGTCCGCCACTCCTCTTTTTCC
AATTTTGAGTGCAAGCACTCGGG
>8
CTGCTGCCGCCCGTAGGAGTCTGGGCCGTGTCTCAGTCCCAGTGTGGCTGGTCATCCTCTCAGACCAGCTAGA
GATCGTCGGCTTGGTGAGCCTTTACCCCACCAACTACCTAATCCCACTTGGGCTCATCCTATGGCATGTGGCC
CGAAGGTCCCACACTTTTAATCTTTCGATATTACGCGGTATCAGCTACAGTTTTCCCCGTAGTTTATCCCCCC
TCCATAAGCCAGATTTCCCAAGCATTTACTCACCCGTCCGCCACTCGTCATCAAAGAAAGCAAGCTTTCTCTA
TGTTTACC
>9
CTGCTGCCGCCCGTAGGAGTCTGGGCCGTGTCTCAGTCCCAGTGTGGCCGATCACCCTCTCAGGTCGGCTATG
CATCGTGGCCTTGGTGAGCCGTTACCTCACCAACTAGCTAATGCACCGCGGGGTCCATCCATCAGCGACACCC
GAAAGCGCCTTTTCACTCTTTATAGCCATGCGGCATAAACTGTTTATGCGGTATTTAGCCACCTTGTTTTTTC
CAAGTCGTTTACTCCCCCTCGATGGGTAGGTTTACCCACGTGTTTACTTCACCCGTTCCGCCACTCCTCTTTT
CCAATTTGAGTTGCAAGCACTCGG
>10
CTGCTGCCGCCCGTAGGAGTCTGGGCCGTGTCTCAGTCCCAGTGTGGCTGGTCGTCCTCTCAAACCAGCTGCG
GATCGTCGCCTTGGTGAGCCGTTACCTCACCAACTAGCTTAATCCGATTATCGGCCGCTCCAATTAGTGACGA

GGTCTTTGCGATCCCCCCCCCCTTTTTCCCCCCCGTAGGGCGTATGCGGTATTTTAGCTACGCTTTTCGCGTA
GTTTATTCCCCCGCTACTGGGCACGTTTCCGATACATTTACTCACCCGTTTCGCCACTCGCCATCCAGGGAGC
AAGCTTCGTTCTGA
>11
CTGCTGCCGCCCGTAGGAGTCTGGGCCGTGTCTCAGTCCCAGTGTGGCCGATCACCCTCTCAGGTCGGCTATG
CATCGTGGCCTTGGTGAGCCGTTACCTCACCAACTAGCTAATGCACCGCGGGTCCATCCATCAGCGACACCCG
AAAGCGCCTTTTCACTCTTTATGCCATGCGGCATAAACTGTTTATGCGGTATTTTAGCCACCTTGTTTTTTCC
AAGTCGTTTACTCCCCCCTCTGATGGGTAGGTTTACCCACGTGTTTACTTCACCCGTCCGCCACTCCGTCTTT
TCCAATCGAGTTGCAAGCACTCGGGAGGAAAGAAGCGTTTCGACTTGACATGTATTTTAGGACGCCGCC
>12
CTGCTGCCGCCCGTAGGAGTCTGGGCCGTGTCTCAGTCCCAGTGTGGCCGATCACCCTCTCAGGTCGGCTATG
CATCGTGGCCTTGGTGAGCCGTTACCTCACCAACTAGCTAATGCACCGCGGGTCCATCCATCAGCGACACCCG
AAAGCGCCTTTCACGTCTTATGCCATGCGGCATAAACTGTTATGCGGTATTAGCCACCTGTTTTTCCAAGTCG
TTATCCCCCTCTGATGGGTAGGTTACCCACGTGTTACTCACCCGTCCGCCACTCCTCTTTCCAATTGAGTGCA
AGCACTCGGGAGGAAAGAAGCGTTCGACTTGACATGTA
>13
CTGCTGCCGCCCGTAGGAGTCTGGGCCGTGTCTCAGTCCCAGTGTGGCCGATCACCCTCTCAGGTCGGCTATG
CATCGTGGCCTTGGTGAGCCGTTACCTCACCAACTAGCTAATGCACCGCGGGTCCATCCATCAGCGACACCCG
AAAGCGCCTTTCACTCTTATGCCATGCGGCATAAACTGTTATGCGGTATTAGCACCTGTTTTTCCAAGTCGTT
ATCCCCCTCTGATGGGTAGGTTACCCACGTGTTACTCACCCGTCCGCCACTCCTCTTTCCAATTGAGTGCAGC
ACTCGGGAGAAAGAAGCGTTCGACTTGCATGTATTAGGCACGCCGCCAGCGTTCGTCCTGAGCCAGGATCAAA
CATCT
>14
CTGCTGCCGCCCGTAGGAGTCTGGGCCGTGTCTCAGTCCCAGTGTGGCCGATCACCCTCTCAGGTCGGCTATG
CATCGTGGCCTTGGTGAGCCGTTACCTCACCAACTAGCTAATGCACCGCGGGTCCATCCATCAGCGACACCCG
AAAGCGCCTTTTCACTCTTATGCCATGCGGCATAAACTGTTTATGCGGTATTTAGCCACCTGTTTTTTCCAAG
TCGTTTACTCCCCCTCTGATGGGTAGGTTTACCCACGTGTTTACTCACCCGTCCGCCACTCCTCTTTTCCAAT
TTGAATGCAAGCACTCAGGAGGAAAGAAGCGTTCGACTTGCATGTATTAGGCACGCCGCCAGCGTTCGTCCTG
AGCCAGGATCAAACTCT
>15
CTGCTGCCGCCCGTAGGAGTCTGGGCCGTGTCTCAGTCCCAGTGTGGCCGATCACCCTCTCAGGTCGGCTATG
CATCGTGGCCTTGGTGAGCCGTTACCTCACCAACTAGCTAATGCACCGCGGGTCCATCCATCAGCGACACCCG
AAAGCGCCTTTTCACTCTTATGCCATGCGCATAAACTGTTTATGCGGTATTTAGCACCTGTTTTTCCAAGTCG
TTTATCCCCCTCTGATGGGTAGGTTTACCCACGTGTTTACTCACCCGTCCGCCACTCCTCTTTTCCAATTTGA
GTGCAAGCACTCGGGAGAAAGAAGCGTTCGACTTGCATGTATTTTAGGCACGCCGCCAGCGTTCGCCTGTAGC
CAGGCA
>16
CTGCTGCCGCCCGTAGGAGTCTGGGCCGTGTCTCAGTCCCAGTGTGGCTGGTCATCCTCTCAGACCAGCTAGA
GATCGTCGGCTTGGTGAGCCTTTACCTCACCAACTACCTAATCCCACTTGGGCTCATCCTATGGCATGTGGCC
CGAAGGTCCCACACTTTAATCTTTCGACTATTACGCGGTATTAGCTACAGTTTCCCGTAGTTATCCCCCTCCA
TAAGCCAGATTCCCAAGCATTACTCACCCGTCCGCCACTCGTCATCAAGGAAGCAAGCTTCTCTATGTTACCG
TTCGACTTGCGTGTGTTAAGCCTGCCGCCAGCGTTCAATCTGAGCCAGGATCAAACTCT
>17
CTGCTGCCGCCCGTAGGAGTCTGGGCCGTGTCTCAGTCCCAGTGTGGCTGGTCATCCTCTCAGACCAGCTAGA
GATCGTCGGCTTGGTGAGCCTTTACCTCACCAACTACCTAATCCCACTTGGGCTCATCCTATGGCATGTGGCC
CGAAGGTCCCACACTTTAATCTTTCGATATTACGCGGTATTAGCTACAGTTTCCCGTAGTTATCCCCCCTCCA
ATAAGCCAGATTCCCAAGCATTACTCACCCGTCCGCCACTCGTCATCAAAGAAGCAAGCTTCTTTATGTTACC
GTTCGACTTGCATGTGTTAAGCCTGCCGCCAGCGTTCAATCTGAGCCAGGATCAAACTCT
>18
CTGCTGCCGCCCGTAGGAGTCTGGGCCGTGTCTCAGTCCCAGTGTGGCCGATCACCCTCTCAGGTCGGCTATG
CATCGTGGCCTTGGTGAGCCGTTACCTCACCAACTAGCTAATGCACCGCGGGCCCATCCATCAGCGACACCCG
AAAGCGCCTTTTCACTCTTATGCCATGCGCATAAACTGTTTATGCGGTATTTAGCCACCTGTTTTTCCAAGTC
GTTTTATCCCCCTCTGATGGGTAGGTTTACCCACGTGTTACTCACCCGTCCGCCACTCCTCTTTCCAATTGAG
TGCAGCACTCGGGAGAAAGAAGCGTTCGTACTTGCATGTATTTTAGGCACGCCGCCAGCGTTCGCCTGA

>19
CTGCTGCCGCCCGTAGGAGTCTGGGCCGTGTCTCAGTCCCAGTGTGGCCGATCACCCTCTCAGGTCGGCTATG
CATCGTGGCCTTGGTGAGCCGTTACCTCACCAACTAGCTAATGCACCGCGGGTCCATCCATCAGCGACACCCG
AAAGCGCCTTTTCACTCTTATGCCATGCGCATAAACTGTTATGCGGTATTAGCCACCTGTTTTTCCAAGTCGT
TATCCCCCTCTGATGGGTAGGTTACCCACGTGTTACTCACCCGTCCGCCACTCCTCTTTCCAATTGAGTGCAG
CACTCGGGAGGAAAGAAGTCGTTCGTACTTGCATGTATTAGGCACGCCGCCAGCGTTCGTCCTGAGCCAGATC
GAAACTCT
>20
CTGCTGCCGCCCGTAGGAGTCTGGGCCGTGTCTCAGTCCCAGTGTGGCTGGTCATCCTCTCAGACCAGCTAGA
GATCGTCGGCTTGGTGAGCCTTTACCCCACCAACTACCTAATCCCACTTGGGCTCATTCCTATGGCATGTGGC
CCGAAGGTCCCACACTTTAATCTTTTCGATATTTACGCGGTATTTAGCTACAGTTTTTCCCGTAGTTTATTCC
CCCTCCATTAAGCCAGATTTTCCCCCAAGCATTTTACTTCACCCGTTCCGCCACACGTCATCAAAGAAGCAAG
CTTTCTCTTATTGTTTTTACCGTTTCGACTTTGCATTGTGTTTTTAAGC
>21
CTGCTGCCGCCCGTAGGAGTCTGGGCCGTGTCTCAGTCCCAGTGTGGCTGGTCATCCTCTCAGACCAGCTAGA
GATCGTCGGCTTGGTGAGCCTTTACCCCACCAACTACCTAATCCCACTTGGGCTCATCCTATGGCATGTGGCC
CGAAGGTCCCACACTTTTAATCTTTTCGACTATTTACGCGGTTATTTTAGCTACAGTTTTCCCGTAGTTTATT
CCCCCTCCATTAAGCCAGATTTCCCAAGCATTTTACTTCACCCGTTCCGCCACTCGTTCATTCAAAGAAGCAA
GCTTTCTCTTATTGTTTTACCGTTTCGACTTTGCATTG
>22
CTGCTGCCGCCCGTAGGAGTCTGGGCCGTGTCTCAGTCCCAGTGTGGCCGATCACCCTCTCAGGTCGGCTATG
CATCATGGCCTTGGTGAGCCGTTACCTCACCAACTAGCTAATGCACCGCGGGTCCATCCATCAGCGACACCCG
AAAGCGCCTTTCACTCTTACTGCCATGCGGCATAAACCGTTATGCGGTATTACGCACCTGTTTTCCAAGTGTT
ATCCCCCCTCTGATGGGTAGGTTACCCACGTGTTACTCACCCGTCCGCCACTCCTCTTTCCAATTGAGTGCAG
CACTCGGGAGGAAAGAAGCGTTCGACTTGC
>23
CTGCTGCCGCCCGTAGGAGTCTGGGCCGTGTCTCAGTCCCAGTGTGGCTGGTCGTCCTCTCAAACCAGCTACG
GATCGTCGCCTTGGGTGAGCCATTACCTCACCAACTAGCTAATCCGATATCGGCCGCTCCAATAGTGAGAGGT
CTTGCACCGATCCCCCCCCTTTTCCCCCGGTAGGGCGTATGCGGTATTAGCTACGCTTTCGCTCGTAGTTATC
CCCCGGCTACTCGGGCACGTTCCGATACATTACTCACCCGTTCGCCACTCGCCATCCAGAGAGCAAGCTCTCT
GATGCTGCCGTTCGACTTGCATGTAGG
>24
CTGCTGCCGCCCGTAGGAGTCTGGGCCGTGTCTCAGTCCCAGTGTGGCTGGTCATCCTCTCAGACCAGCTAGA
GATCGTCGGCTTGTGAGCCTTTACCTCACCAACTACCTAATCCCACTTGGGCTCATCCTATGGCATGTGGCCC
GAAGGTCCCACACTTTAATCTTTCGATATTATCGCGGTATTAGCTACAGTTTTCCCGTAGTTATCCCCCTCCC
ATAAGCCAGTATTTCCCAAGCATTTACTCACCCGTCCGCCACTCGTCATCAAAGAAGCAAGCTTTCTCTATGT
TTTACCGTTTCGACTTGCATGTGTTTAAGCCTGCCGCCAGCGTTCAATCAG
>25
CTGCTGCCGCCCTAGGAGTCTGGGCCGTGTCTCAGTCCCAGTGTGGCTGGTCATCCTCTCAGACCAGCTAGAG
ATCGTCGGCTTGGGTGAGCCTTTACCTCACCAACTACCTAATCCCACTTGGGCTCATCCTATGGTATGTGGCC
CGAAGGTCCCACACTTAATCTTTCGATATTTACGCGGTATTAGCTACAGTTTCCCGTAGTTATCCCCCTCCAT
AAGCCAGATTTCCCAAGCATTTACTCACCCGTCCGCCACTCGTCATCAAAGAAGCAAGCTTTCTCTATGTTTA
CCGTTTCGACTTGCATGTGTTTAAGCCTGCCGCCAGCCGTT
>26
CTGTGCCGCCCGTAGGAGTCTGGGCCGTGTCTCAGTCCCAGTGTGGCTGGTCGTCCTCTCAAACCAGCTACGG
ATCGTCGCCTTGGTGAGCCGTTACCTCACCAACTAGCTAATCCGATATCGGCCGCTCCAATTAGTGAGAGGTC
TTTACGATCCCCCCCTTTCCCCCGTTAGGGCGATGCGGTTATTTAGCTTACGCTTTTCGCGTAGTTTATTCCC
CCCCGCTTACTTGGGCACGTTTCCGATACATTTACTTCACCCGTTTCGCCACTCGCCATCCAGAGAGCAAGCT
TCTCTGATGCTGCCGTTTTCGACTTTGCATTGTTGTTAAGGCATCCCGCTAGCGTTTTCAATTCTGAGCCAGG
ATCAAACTTCTT
>27
CTGCTGCCGCCCGTAGGAGTCTGGGCCGTGTCTCAGTCCCAGTGTGGCCGATCACCCTCTCAGGTCGGCTATG
CATCGTGGCCTTGGTGAGCCGTTACCTCACCAACTAGCTAATGCACCGCGGGTCCATCCATCAGCGACACCCG
AAAGCGCCTTTTCACTCTTTATTGCCATGCGCATAAACTGTTTTATGCGGTATTTTAGCCACCTTGTTTTTTT

CCAAGTCGTTTTATTCCCCCTCTGATTGGGTAGGTTTTACCCACGTGTTTTACTTCACCCGTTCCGCCACTCC
TCTTTTTCCAATTTTGAGTTGCAGCACTCGGGAGGAAAGAAGCGTTTCGACTTGCATGTATTTTAGGCACGTC
CG
>28
CTGCTGCCGCCCGTAGGAGTCTGGGCCGTGTCTCAGTCCCAGTGTGGCCGATCACCCTCTCAGGTCGGCTATG
CATCGTGGCCTTGGTGAGCCGTTACCCACCAACTAGCTAATGCACCGCGGGTCCATCCATCAGCGACACCCGA
AAGCGCCTTTTCACTCTTTATGCCATGCGCATAAACTGTTTATGCGGTATTTAGCACCTGTTTTTCCAAGTGT
TTTATCCCCCTCTGATGGGTAGGTTTACCCACGTGTTTACTCACCCGTCCGCCACTCCTCTTTTTCCAATTTT
GAGTGCAAGCACTCGGGAGGAAAGAAGTCGTTCGTACTT
>29
CTGCTGCCGCCCGTAGGAGTAAGGGCCGTGTCTCAGTCCCCTTTGTGGCCGATCACCCTCTTCAGGCCGGCTA
CCCATCATCGCCTTGGTGAGCCGTTTACCTCTCCAACTAGCTAATGGGACGCAAAGCTCTTCTCACAGCGCAT
ATTAGCTTTTTCATAATTTCTAGGATGCCCTAAAATTCATAATATCAGGTATTTTAGCATTTTCGTTTTTTCC
AAATTGTTTTGTTCCCTTATTCTTGTTGAGGCAAGTTTTTCTTTTTTTTACGCGTTTTACTTCACCCGTTCCG
CCACCCCCCAAACGAAGTTTTCAAGTTTTTGACTTTTGCATTGTTGTTTTTTTTTTTTAAGCATTTTTTCTTGT
TCAGCGTTTTTCATCC
>30
CTGCTGCCGCCCGTAGGAGTCTGGGCCGTGTCTCAGTCCCAGTGTGGCTGGTCATCCTCTCAGACCAGCTAGA
GATCGTCGGCTGGTGAGCCTTTACCCCACCAACTACCTAATCCCACTTGGGCTCATCCTATGGCATGTGGCCC
GAAGGTCCCACACTTTAATCTTTCGATATTACGCGGTATTTAGCTACATGTTTTCCCGTACGTTTATCCCCCT
CCATAAGCCAGATTTCCCAAGCATTTACCCACCCGTCCGCCACTCGTCATCAAAGAAGCAAGCTTCTCTATGT
TTACCGTTTCGACTTGCATGTCGTTTAAGCCTGACCTGCCAGCGTTC
>31
CTGCTGCCGCCCGTAGGAGTCTGGGCCGTGTCTCAGTCCCAGTGTGGCTGGTCATCCTCTCAGACCAGCTAGA
GATCGTCGGCTTGGTGAGCCTTTACCCACCAACTACCTAATCCCACTTGGGCTCATTCCTATGGCATGTGGCC
CGAAGGTCCCACACTTTTAATCTTTTCGATATTTACGCGGTATTTAGCTACAGTTTTTCCCGTAGTTTATTCC
CCCTCCCATTAAGCCAGATTTTCCCAAGCATTTTACTTCACCCGTTCCGCCACTCGTCATCAAAGAAGCAAGC
TTTCTCTTATTGTTTTACCGTTTCGACTTTGCATTGTGTTTTAAGCCTGACCGCCAGCGTTTCAATCTGAGCC
AGGATCAAACTCT
>32
CTGCTGCCGCCCGTAGGAGTAAGGGCCGTGTCTCAGTCCCCTTTGTGGCCGATCACCCTCTCAGGCCGGCTAC
CCATCATCGCCTTGGTGAGCCGTTACCTCTCCAACTAGCTAATGGGACGCAAAGCTCTCTCACAGCGCATATA
GCTTTTCATAATTTTCTAGGATGCCCTAAAATTCAATAATTATTCAGGTATTTTTAGCATTTTTCGTTTTTTT
TCCAAATTGTTTTTGTTCCCTTATTCTTGTTGAGGCAAGTTTTTCTTTTTTTTACGCGTTTTACTTCACCCGT
TCCGCCACCCAAACCGAAGTTTTC
>33
CTGCTGCCGCCCGTAGGAGTCTGGGCCGTGTCTCAGTCCCAGTGTGGCCGATCACCCTCTCAGGTCGGCTATG
CATCGTGGCCTTTGGTGAGCTGTTTACCTCACCAACTAGCTTAATGCACCGCGGGTCCATCCATCAGCGACAC
CCCCGAAGCGCCTTTTTTCACTCTTTTATTGCCATTGCGGCATTAAAAACTTGTTTTTTTATTGCGGTTATTT
TTTAGCACCTTTGTTTTTTCCAAGTTCGTTTTTTTATTCCCCCCCTTCTGATTGGGGGGTTAGGTTTTTTACC
CCCCACGTTGTTTTTTACTTCACCCCCCGTTCCCCGCCCATCCCCTCTTTTTCCATTTTTGAGT
>34
CTGCTGCCGCCCGTAGGAGTCTGGGCCGTGTCTCAGTCCCAGTGTGGCCGATCACCCTCTCAGGTCGGCTATG
CATCGTGGCCTTGGTGAGCCGTTACCTCACCAACTAGCTAATGCACCGCGGGTCCATCCATCAGCGACACCCG
AAAGCGCCTTTCACTCTTTATGCCATGCGCATAAACTGTTTATGCGGTATTTAGCACCTGTTTTTCCAAGTCG
TTTTATCCCCCTCTGATGGGTAGGTTTACCCACGTGTTTACTTCACCCGTCCGCCACTCCTCTTTTCCAATTT
TGAGTGCAAGCACTCGGGAGGAAAGAAGCGTTCGACTTGACATGTATTTTAGGCACGCCGCCAGCGTCGTCCC
TGTACGCCAGGCAGAACATCT
>35
CTGCTGCCGCCCTAGGAGTCTGGGCCGTGTCTCAGTCCCAGTGTGGCCGATCACCCTCTCAGGTCGGCTATGC
ATCGTGGCCTTGGTGAGCCGTTACCTCACCAACTAGCTAATGCACCGCGGGTCCATCCATCAGCGACACCCGA
AAGCGCCTTTCACTCTTTATGCCATTGCGGCATTAAACTGTTTATTGCGGTATTTAGCCACCTTGTTTTTTCC
AAGTCGTTTTATTCCCCCTCGATTGGGGTTAGGTTTACCCACGTGTTTACTTCACCCGTCCGCCACTCCTCTT
TCCAATTTTGAGT

>36
CTGCTGCCGCCCGTAGGAGTCTGGGCCGTGTCTCAGTCCCAGTGTGGCCGATCACCCTCTCAGGTCGGCTATG
CATCGTGGCCTTGGTGAGCCGTTACCTCACCAACTAGCTAATGCACCGCGGGTCCATCCATCAGCGACACCCG
AAAGCGCCTTTCACTCTTATGCCATGCGGCATAAACTGTTTATGCGGTATTTAGCACCTGTTTTTTCCAAGTC
GTTTATCCCCCTCTGATGGGTAGGTTTACCCACGTGTTTACTTCACCCGTCCGCCACTCCTCTTTTCCAATTT
GAGTGCAAGCACTCGGGAGGAAAGAAGCGTTCGTACTTGACATGTA
>37
CTGCTGCCGCCCGTAGGAGTCTGGGCCGTGTCTCAGTCCCAGTGTGGCCGATCACCCTCTCAGGTCGGCTATG
CATCGTGGCCTTGGTGAGCCGTTACCTCACCAACTAGCTAATGCACCGCGGGTCCGTCCATCAGCGACACCCG
AAAGCGCCTTTCACTCTTTATGCCATGCGGCATAAACTGTTTATGCGGTATTTAGCACCTTGTTTTTTCCAAG
TCGTTTATCCCCCTCTGATGGGTAGGTTTACCCACGTGTTTACTTCACCCGTCCGCCACTCCTCTTTTCCAAT
TTGAGTGCAAGCACTCGGGAGGAAAGGAAGC
>38
CTGCTGCCGCCGTAGGAGTCTGGGCCGTGTCTCAGTCCCAGTGTGGCTGGTCATCCTCTCAGACCAGCTAGAG
ATCGTCGGCTTGGTGAGCCTTTACCTCACCAACTACCTAATCCCACTTGGGCTCATCCTATGGCATGTGGCCC
GAAGGTCCCACACTTTCATCTCCCGATTTCTACGCGGTATTTAGCTACAGTTTTTCCCATAGTTTTATCCCCC
CCTTCCATAAGCCAGATTTCCCAAGCATTTTACTCACCCGTCCGCCACTCGTCATTCAAAGAAGCAAGCTTTC
TCTATGTTTTT
>39
CTGCTGCCGCCCGTAGGAGTCTGGGCCGTGTCCCAGTCCCAGTGTGGCTGGTCATCCTCTCAGACCAGCTAGA
GATCGTCGGCTTGGTGAGCCTTTACCTCACCAACTACCTAATCCCACTTGGGCTCATCCTATGGCATGTGGCC
CGAAGGTCCCACACTTTCATCTCCCGATTCTACGCGGTATTAGCTACAGTTTCCCATAGTTATCCCCCTCCAT
AAGCTAGATTCCCAAGCATTACTCACCCGTCCGCCACTCGCCATCAAAGAAGCAAGCTTCTCTATGTTACCGT
TCGACTTGCATGTGTTAAGCCTGCCGCCAGCGTTCAATCTGAGCCAGGATCAAACTCT
>40
CTGCTGCCGCCCTAGGAGTCTGGGCCGTGTCTCAGTCCCAGTGTGGCTGGTCATCCTCTCAGACCAGCTAGAG
ATCGTCGGCTTGGTGAGCCTTTACCCCACCAACTACCTAATCCCACTTGGGCTCATCCTATGGCATGTGGCCC
GAAGGTCCTACACTTTTAATCTTTTCGATATTTACGCGGTATTTAGCTACAGTTTTTCCCGTAGTTTATTCCC
CCTCCATAAGCCAGATTTCCCAAGCATTTACTCACCCGTCCGCCACTCGTCATTCAAAGAAGCAAGCTTTCTC
TATGTTTTACCGTTTCGACTTTGCATTGTGTTTTAAGCCTGCCGCCAGCGTTTCAATCTGAGCCAGGATCAAA
CTCT
>41
CTGCTGCCGCCCGTAGGAGTCTGGGCCGTGTCTCAGTCCAGTGTGGCCGATCACCCTCTCAGGTCGGCTATGC
ATCGTGGCCTTGGTGAGCCGTTACCTCACCAACTAGCTAATGCACCGCGGGTCCATCCATCAGCGACACCCGA
AAGCGCCTTTCGCTCATGCCATGCGGCATAAACTGTTTATGCGGTATTTTAGCACCTGTTTTTTCCAAGTCGT
TTATCCCCCTCTGATGGGTAGTTTACCCACGTGTTTACTCACCCGTCCGCCACTCCTCTTTTCCAATTTGATG
TGCAAGCTACTCGGGAGGAAAGAAGCGTTCGGTACTTGACAT
>42
CTGCTGCCGCCCGTAGGAGTCTGGGCCGTGTCTCAGTCCCAGTGTGGCCGATCACCCTCTCAGGTCGGCTATG
CATCGTGGCCTTGGTGAGCCGTTACCTCACCAACTAGCTAATGCACCGCGGGTCCATCCATCAGCGACACCCG
AAAGCGCCTTTTCACTCTTTATGCCATGCGCATAAACTGTTTATGCGGTATTTAGCACCTGTTTTTTCCAAGT
CGTTTTATCCCCCTCTGATGGGTAGGTTTACCCACGTGTTTACTTCACCCGTCCGCCACTCCTCTTTTCCAAT
TTTGAGTGCAAGCACTCGGGAGGAAAGAAGCGTTCGTACTTGACATG
>43
CTGCTGCCGCCCGTAGGAGTCTGGGCCGTGTCTCAGTCCCAGTGTGGCCGATCACCCTCTCAGGTCGGCTATG
CATCGTGGCCTTGGTGAGCCGTTACCTCACCAACTAGCTTAATGCACCGCGGGTCCATCCATCAGCGACACCC
GAAAGCGCCTTTTTCACTTCTTTTATTTAGCCATTGCGGCATTAAAAACTTGTTTTTTATTGCGGTTATTTTT
AGCACCTTTGTTTTTCCAAAAGTTGTTTTTATTCCCCCTTCTGATTGGGGTTAGGGTTTTACCCCACGTGTTT
TACTTACCCCGTTCCCGCCCCATCCC
>44
CTGCGCCGCCCGTAGGAGTCTGGGCCGTGTCTCAGTCCCAGTGTGGCTGGTCGTCCTCTCAAACCAGCTACGG
ATCGTCGCCTTGGTGAGCCGTTACCTCACCAACTAGCTTAATCCGATTACGGCCGCTCCAATAGTTGAGAGGT
CTTTGCGATCCCCCCCCCCTTTTTCCCCCCCCGTAGGGCGTATGCGGTATTTAGCTTACGCTTTTCGCGTAGT

TTATCCCCCGCTACTTGGGCACGTTTCCGATACATTTACTTCACCCGTTTCGCCACTCGCCATCCAGAGAGCA
AGCTCTCTATGCTTGCCTGGTTTTCGACTTTTTGCGTTAA
>45
CTGCTGCCGCCCGTAGGAGTCTGGGCCGTGTCTCAGTCCCAGTGTGGCCGATCACCCTCTCAGGTCGGCTATG
CATTCGTGGCCTTGGTGAGCCGTTTACCTCACCAACTAGCTAATGCACCGCGGGTCCATCCATCAGCGACACC
CGAAAGCGCCTTTTCACTCTTATGCCATGCGGCATAAACTGTTATGCGGTATTAGCACCTGTTTTTCCAAGTC
GTATCCCCCTCTGATGGGTAGGTTACCCACGTGTTTTACTCACCCGTCCGCCACTCCTCTTTTTCCAATTGAG
TGCAAGCACTCGGGAGGAAAAGAA
>46
CTGCTGCCGCCCGTAGGAGTCTGGGCCGTATCTTCAGTCCCAATGTGGCCGCACACCCTCTCAGGCCGGCTAC
CCGTCGCCGCCTTGGTAGGCCATTACCCCACCAACAAGCTGATTAGGCCGCAAGCTCATCCCACACCAGAAAA
ACCTTTCCACCACACCAACCACGATGCGGTCCTATTAGGGTTATTTAGACCCAGTTTTTTTCCCAAGCTTTT
TATTCCCCCCCCAGTGCAGGGCAGATCACTTTTACGTGTTTTACTCACCCGTTTTCGCCACTTCGAGCACCCC
CAGCCAAGCCTGGGGCCTTTTTTCCGCTTTTCGGACTTTTGACATTGGTGTTTTTTAAGCACGCCGCCA
>47
CTGCTGCCGCCCGTAGGAGTCTGGGCCGTGTCTCAGTCCCAGTGTGGCCGATCACCCTCTCAGGTCGGCTATG
CATCGTGGCCTTTGGTGAGCCGTTACCTCACCAACTAGCTAATGCACCGCGGGTCCATCCATCAGCGACACCC
GAAAGCGCCTTTCACTCTTTATGCCATGCGCATAAACTGTTTATGCGGTATTTAGCCACCTTGTTTTTCCAAG
TCGTTTATCCCCCTCTGATGGGTAGGTTACCCACGTGTTTACTCACCCGTCCGCCACTCCTCTTTCCAATTTG
AGTGCAAGCACTCGGGAGGAAAGAAGCGT
>48
CTGCTGCCGCCCGTAGGAGTCTGGGCCGTGTCTCAGTCCCAGTGTGGCCGATCACCCTCTCAGGTCGGCTATG
CATCGTGGCCTTGGTGAGCCGTTACCTCACCAACTAGCTAATGCACCGCGGGTCCATCCATCAGCGACACCCG
AAAGCGCCTTTTCACTCTTTATGCCATGCGGCATAAACTGTTTATGCGGTATTTAGCACCTGTTTTTCCAAGT
CGTTTATCCCCCTCTGATGGGTAGGTTTACCCACGTGTTTACTCACCCGTCCGCCACTCCTCTTTTCCAATTT
GAGTGCAAGCACTCGGGAGGAAAGAAGCGTTCGTACT
>49
CTGCTGCCGCCCTAGGAGTCTGGGCCGTGTCTCAGTCCCAGTGTGGCTGGTCGTCCTCTCAAACCAGCTAGAG
ATCGTCGGCTTGGTGAGCCTTTACCTCACCAACTACCTAATTCCCCACTTGGGCTTCATCCTATTGGCATGTG
GCCCCGAAGGTCCCCACACTTTTAATCTTTTCGATATTTACGCGGTATTTAGCTACAGTTTTCCCCCGTAGTT
TATTCCCCCCTCCCATAAGCCAGATTTCCCCCAAGCATTTACTCACCCCGTTCCCGCCACTCGTCATCAAAGA
AGCAAGCTTTCTTCTTATTGTTTACCGTTTCGACTTTGCATTGTGTTTAAGCCTGCCGCCAGCGTTTCAATCC
GAGCCAGGATCAAACTCT
>50
CTGCTGCCGCCCTAGGAGTCTGGGCCGTGTCTCAGTTCCCAGTGTGGCTGGTCATCCTCTCAGACCAGCTAGA
GATCGTCGGCTTTGTGAGCCTTTACCTCACCAACTTACCTAATTCCCCACTTGGGCTTCATCCTATTGGCATG
TGGCCCGAAGGTCCCCCACACTTTTAATCTTTTCGATATTTACGCGGTATTTTAGCTACAGTTTTCCCCGTAG
TTTATTCCCCCCTCCCATTAAGCCAGATTTCCCCCAAGCATTTACTCACCCCGTTCCCGCCACTCGTCATTCA
AAGAAGCAAGCTTTCTTCTATTGTTTACCGTTTCGACTTTGCATTGTGTTTAAGCCTTGACCGCCAGCGTTTC
AATCTGAGCCAGGATTCAAACTTCT
>51
CTGCTGCCGCCCGTAGGAGTCTGGGCCGTGTCTCAGTCCCAGTGTGGCCGATCACCCTCTCAGGTCGGCTATG
CATCGTGGCCTTGGTGAGCCGTTACCTCACCAACTAGCTAATGCACCGCGGGTCCATCCATCAGCGACACCCG
AAAGCGCCTTTTCACTCTTTTATGCCATGCGGCATAAACTGTTTATGCGGTATTTTAGCACCTGTTTTTTTCCA
AGTCGTTTTATCCCCCCCCTCTGATGGGTAGGTTTACCCACGTGTTTTACTCACCCGTCCGCCACTCCTCTTT
TTCCAATTTTGAGTTGCAAGCACTCGGGAGGAAAGAAGCGTTTTCGACTTT
>52
CTGCTGCCGCCCGTAGGAGTCTGGGCCGTGTCTCAGTCCCAGTGTGGCTGGTCATCCTCTCAGACCAGCTAGA
GATCGTCGGCTTGGTGAGCCTTTACCTCACCAACTACCTAATCCCACTTGGGCTCATCCTATTGGCATGTGGC
CCGAAGGTCCCACACTTTTTAATCTTTTCGATATTTACGCGGTATTTTAGCTACAGTTTTTCCCGTAGTTTTA
TCCCCCTCCATAAGCCAGATTTCCCAAGCATTTTACTCACCCGTTCCGCCACTTCGTCATCAAAAGAAGCAAG
CTTTTCTCTATGTTTTACCGTTTTCGACTTTTGCAGTGTTTTAAG
>53

CTGCTGCCGCCCGTAGGAGTCTGGGCCGTGTCTCAGTCCCAGTGTGGCCGATCACCCTCTCAGGTCGGCTATG
CATTCGTGGCCTTGGTTGAGCCGTTACCTTCACCAACTAGCTTAATTGCACCGCGGGGGTTCCATTCCATCAG
CGACACCCCCCGAAAAAAGCGCCTTTTTTCACTTCTTTTTATTTGCCCATTGCGGCATTAAAAACTTGTTTTA
TTGCGGGGTTATTTTAGCACCCCTTTGTTTTCCCCAAAAGTTGTTTTATTCCCCCTTTTCTGATTGGGGGTTA
GGGGTTTTTACCCCACGTTGTTTTTACT
>54
CTGCTGCCGCCCGTAGGAGTCTGGGCCGTGTCTTCAGTCCCAGTGTGGCCGATCACCCTCTTCAGGTCGGCTA
TGCATTCGTGGCCTTGGTTGAGCCGTTTACCTCACCAACTAGCTTAATTGCACCGCGGGGGTTCCATCCATTC
AGCGACACCCCCGAAAAAAGCGCCCTTTTTTCACTTCTTTTTATTTGCCCCATTGCGGCATTAAAACTTGTTT
TATTGCGGGGTTATTTTAGCACCCCTTTTGTTTTCCCCAAAAGTTTGTTTTATTTCCCCTTCTTGATTGGGGT
TAGGGGTTTTAC
>55
CTGCTGCCGCCCGTAGGAGTCTGGGCCGTGTCTCAGTCCCAGTGTGGCTGGTCATCCTCTCAGACCAGCTAGA
GATCGTCGGCTTGGTGAGCCTTTACCTCACCAACTACCTAATTCCCACTTGGGCTTCATTCCTATTGGCATGT
GGCCCGAAGGTCCCACACTTTTAATCTTTTCGATTATTTACGCGGTTATTTAGCTACAGTTTTTCCCGTACGT
TTATTCCCCCTCCCATTAAGCCAGATTTCCCCCAAGCATTTACTTCACCCGTTCCGCCACTTCGTCATTCAAA
GAAGCAAGCTTTTCTTTTTATTGTTTACCGTTTTCGACTTGCATTGTTGTTTTAAGCCTTGCCGCCAGCGTTT
CAATTCTTGAGCCAGGATCAAACTCTT
>56
CTGCTGCCGCCCGTAGGAGTCTGGGCCGTGTCTCAGTCCCAGTGTGGCCGATCACCCTCTCAGGTCGGCTATG
CATCGTGGCCTTGGTGAGCCGTTACCTCACCAACTAGCTAATGCACCGCGGGTCCATCCATCAGCGACACCCG
AAAGCGCCTTTCACTCTTATGCCATGCGGCATAAACTGTTTATGCGGTATTTAGCACCTGTTTTTTCCAAGTG
TTTATCCCCCTCTGATGGGTAGGTTTACCCACGTGTTTACTCACCCGTCCGCCACTCCTCTTTCCAATTTGAG
TGCAAGCACTCGGGA
>57
CTGCTGCCGCCCGTAGGAGTCTGGGCCGTGTCTCAGTCCCAGTGTGGCCGATCACCCTCTCAGGTCGGCTATG
CATCGTGGCCTTTGGTGAGCCGTTACCTCACCAACTAGCTAATGCACCGCGGGTCATCCATCAGCGACACCCG
AAAGCGCCTTTTCACTCTTTATGCCATGCGGCATAAACTGTTTATGCGGTATTTAGCACCTGTTTTCCAAGTC
GTTTATCCCCCTCTGATGGGTAGGTTTACCCACGTGTTTACTCACCCGTCCGCCACTCCTCTTTCCAATTTGA
GTGCAAGCACGTCGGGAGGAAAGGAAGACGTTCGGACGT
>58
CTGCTGCCGCCCGTAGGAGTCTGGGCCGTGTCTCAGTCCCAGTGTGGCTGGTCTCCTCTCAAACCAGCTACGG
ATCGTCGCCTTGGTGAGCCATTACCTCACCAACTAGCTAATCCGATATCGGCCGCTCCAATAGTGAGAGGTCT
TTGCGATTCCCCCCCTTTTCCCCCCGTAGGGCGTATGCGGTTATTTAGCTTACGCTTTTCGCGTAGTTTATTC
CCCCCCGCTACTTGGGCACGTTCCCGATTACATTTACTTCACCCCCGTTTCGCCACTCGCCATTCCAGAGAGC
AAGCTCTCTTGATTGCTTTGCCCCCGTTTGACTTGCATTG
>59
CTGCTGCCGCCCGTAGGAGTCTGGGCCGTGTCTCAGTCCCAGTGTGGCCGATCACCCTCTCAGGTCGGCTATG
CATCGTGGCCTTGGTGAGCCGTTACCTCACCAACTAGCTAATTGCACCGCGGGTCCATCCATCAGCGACACCC
CGAAAGCGCCTTTTCACTTCTTTTATTTGCCATTTGCGGCATTTAAAAACTTGTTTTTTATTTGCGGTTATTT
TTAGCACCCTTTGTTTTTCCCCAAAAGTTGTTTTTATTCCCCCTTCTGATTTGGGGGTTAGGGGTTTTTACCC
CCACGTGTTTACTT
>60
CTGCTGCCGCCCGTAGGAGTCTGGGCCGTGTCTCAGTCCCAGTGTGGCTGGTCATCCTCTCAGACCAGCTAGA
GATCGTCGGCTTGGTGAGCCTTTACCTCACCAACTACCTAATCCCACTTGGGCTTCATCCTATTGGCATGTGG
CCCGAAGGTCCCACACTTTTAATCTTTTCGATATTTACGCGGTATTTTAGCTACAGTTTTTCCCGTAGTTTTA
TCCCCCTCCATAAGCCAGATTTCCCAAGCATTTTACTCACCCGTTCCGCCACTTCGTCATTCAAAGAAGCAAG
CTTTTCTTTTTTATGTTTACCGTTTTCGACTTTGCATTGTTGTTTTAAGCCTTGCCGCCAGCGTTTTCAATTC
TTGAGCCAGGA
>61
CTGCTGCCGCCCGTAGGAGTCTGGGCCGTGTCTCAGTCCCAGTGTGGCCGATCACCCTCTCAGGTCGGCTATG
CATTCGTGGCCTTGGTGAGCCGTTTACCTTCACCAACTAGCTTAATGCACCGCGGGGGTCCATCCATTCAGCG
ACACCCCCGAAAAAGCGCCCTTTTTCACTTCTTTTTATTTGCCCCATTGCGGCATTAAAAACTTGTTTTTATT

GCGGTATTTTTAGCACCCTTTGTTTTTCCCCAAAAAGTTGTTTTTATTCCCCCTTCTGATTGGGGTTAGGGTT
TTACCCCACGTTGTTTTACTTAC
>62
CTGCTGCCGCCCGTAGGAGTCTGGGCCGTGTCTCAGTCCCAGTGTGGCCGATCACCCTCTCAGGTCGGCTATG
CATCGTGGCCTTGGTGAGCCGTTACCTTCACCAACTAGCTTAATGCACCGCGGGTCCATCCATCAGCGACACC
CCGAAAAGCGCCTTTTTCACTTCTTTTATTGCCATTGCGGCATTTAAAAACTTGCTTATTGCGGGTTATTTTT
AGCACCCCTTTGTTTTCCCCAAAAGTTTGTTTTATTTTCCCCCTTTCTTGATTGGGGTTAGGGGTTTTACCCC
ACGTGTTT
>63
CTGCTGCCGCCCGTAGGAGTCTGGGCCGTGTCTCAGTCCCAGTGTGGCTGGTCGTCCTCTCAAACCAGCTACG
GATCGTCGCCTTTGGGTGAGCCATTTACCTCACCAACTAGCTAATGCACCGCGGGTCCATCCATCAGCGACAC
CCGAAAGCGCCTTTTTCACTCTTTATGCCATGCGGCATAAACTGTTTTATGCGGTATTTTAGCACCTGTTTTT
TCCAAGTGTTTTATCCCCCCCCTCTGATGGGTAGGTTTTACCCACGTGTTTTACTCACCCGTCCGCCACTCCT
CTTTTTCCAATTTTGAGTTGCAGCACTCGGGAGGAAAGAAGCGTTTTCGACTTGCA
>64
CTGCTGCCGCCCGTAGGAGTCTGGGCTGTGTCTCAGTCCCAGTGTGGCCGATCACCCTCTCAGGTCGGCTATG
CATCGTGGCCTTGGTGAGCCGTTACCTCACCAACTAGCTTAATGCACCGCGGGTCCATCCATCAGCGACACCC
GAAAGCGCCTTTTCACTCTTTATGCCATGCGCATAAACTGTTTTATGCGGTATTTAGCACCTTGTTTTTTCCA
AGTCGTTTTATTCCCCCCCCTCTGATTGGGTTAGGTTTTACCCACGTGTTTTACTTCACCCGTTCCGCCACCC
TCCTTTCTTACATTTTGAGTTGCAAGCACTCGGGAGAA
>65
CTGCTGCCGCCCGTAGGAGTCTGGCCGTGTCTCAGTCCCAGTGTGGCCGATCACCCTCTCAGGTCGGCTATGC
ATCGTGGCCTTGGTGAGCCGTTACCTCACCAACTAGCTAATGCACCGCGGGTCCATCCATCAGCGACACCCGA
AAGCGCCTTTTTCACTCTTTATGCCATGCGGCATAAACTGTTTATGCGGTATTTAGCACCTGTTTTTTCCAAG
TCGTTTTATCCCCCTCTGATGGGTAGGTTTACCCACGTGTTTTACTCACCCGTCCGCCACTCCTCTTTTTCCA
ATTTTGAGTGCAAGCACTCGGGAGGAAAGAAGCGTTTCGACTTGCATGTATTTTAGGCACGTCCGCCAGCGTT
CGCCTGAGCCAGATCAAACTCT
>66
CTGCTGCCGCCCGTAGGAGTCTGGGCCGTGTCTCAGTCCCAGTGTGGCCGATCACCCTCTCAGGTCGGCTATG
CATCGTGGCCTTGGTGAGCCGTTACCTCACCAACTAGCTAATGCACCGCGGGTCCATCCATCAGCGACACCCG
AAAGCGCCTTTTCACTCTTTATGCCATGCGGCATAAACTGTTTTATGCGGTATTTAGCACCTGTTTTTTCCAA
GTCGTTTTATCCCCCTCTGATGGGTAGGTTTACCCACGTGTTTTACTCACCCGTCCGCCACTCCTCTTTTTCC
AATCGAGTGCAAGCACTCGGGAGGAAAGAAGT
>67
CTGCTGCCGCCCGTAGGAGTCTGGGCCGTGTCTCAGTCCCAGTGTGGCCGATCACCCTCTCAGGTCGGCTATG
CATCGTGGCCTTGGTGAGCCGTTACCTCACCAACTAGCTAATGCACCGCGGGTCCATCCATCAGCGACACCCG
AAAGCGCCTTTTTCACTCTTTATGCCATGCGGCATAAACTGTTTATGCGGTATTTAGCACCTGTTTTTTCCAA
GTCGTTTTATCCCCCTCTGATGGGTAGGTTTACCCACGTGTTTTACTCACCCGTCCGCCACTCCTCTTTTTCC
AATTTTGAGTGCAAGCACTCGGGAGGAAAGAAGCGTTCGTACTTGCATGTATTTTAGGCACGCCGCCAGCGTT
CGTCCTGAGCCAGGATCAAACTCT
>68
CTGCTGCCGCCCGTAGGAGTCTGGGCCGTGTCTCAGTCCCAGTGTGGCCGATCACCCTCTCAGGTCGGCTATG
CATCGTGGCCTTGGTGAGCCGTTTACCTCACCAACTAGCTAATGCACCGCGGGTCCATCCATCAGCGACACCC
GAAAGCGCCTTTTTCACTCTTTATGCCATGCGGCATAAACTGTTTATGCGGTATTTAGCACCTGTTTTTTCCA
AGTGTTTTATCCCCCCTCTGATGGGTAGGTTTACCCACGTGTTTTACTCACCCGTCCGCCACTCCTCTTTTTC
CAATTTGAGTGCAAGCACTCGGGAGGAAAGAAGCGTTTCGACTTGCATGTATTTTAGGCACGCCGCCAGCGTT
CGCCTGTGCCAGGCACGAAA
>69
CTGCTGCCGCCCGTAGGAGTCTGGGCCGTGTCTCAGTCCCAGTTGTGGCCGATCACCCTCTTCAGGTCGGCTA
TGCATTCGTGGCCTTGGTTGAGCCGTTTACCTCACCAACTAGCTTAATTGCACCGCGGGGGTTCCATCCATCA
GCGACACCCCCCGAAAAAAGCGCCTTTTTTTTCACTTCTTTTTATTGCCATTGCGGCATTAAAAAACTTGTTT
TTATTGCGGGGTTATTTTTAGCACCCCTTTGTTTTTCCCCAAAAAGTTTGTTTTTTATTTCCCCCCTTTCTTG
ATTGGGGGGGGTTAGG
>70

CTGCTGCCGCCCGTAGGAGTCTGGGCCGTGTCTCAGTCCCAGTGTGGCCGATCACCCTCTCAGGTCGGCTATG
CATCGTGGCCTTGGTGAGCCGTTACCTCACCAACTAGCTAATGCACCGCGGGTCCATCCATCAGCGACACCCG
AAAGCGCCTTTCACTCTTATGCCATGCGGCATAAACTGTTTATGCGGTATTTAGCCACCTGTTTTTCCAAGTC
GTTTATCCCCCTCTGATGGGTAGGTTTACCCACGTGTTTACTCACCCGTCCGCCACTCCTCTTTCCAATTTGA
GTGCAAGCACTCGGGAGGAAAGAAGCGTTCGA
>71
CTGCTGCCGCCCGTAGGAGTCTGGGCCGTGTCTCAGTCCCAGTGTGGCTGGTCATCCTCTCAGACCAGCTAGA
GATCGTCGGCTTGTGAGCCTTTACCTCACCAACTACCTAATCCCACTTGGGCTCATCCTATGGCATGTGGCCC
GAAGGTCCCACACTTTAATCTTTCGATATTATCGCGGTATTAGCTACAGTTTTCCCGTACGTTATCCCCCTCC
ATAAGCCAGATTTCCCAAGCATTTACTCACCCGTCCGCCACTCGTCATCAAAGAAGCAAGCTTTCTCTATGTT
TACCGTTCGACTTGCATGTGTTTTAAG
>72
CTGCTGCCGCCCGTAGGAGTCTGGGCCGTGTCTCAGTCCCAGTGTGGCCGATCACCCTCTCAGGTCGGCTATG
CATCGTGGCCTTGTGAGCCGTTACCTCACCAACTAGCTAATGCACCGCGGGTCCATCCATCAGCGACACCCGA
AAGCGCCTTTCACTCTTATGCCATGCGCATAAACTGTTTATGCGGTATTTAGCCACCTGTTTTTCCAAGTCGT
TTTATCCCCCTCTGATGGGTAGGTTTACCCACGTGTTTACTCACCCGTCCGCCACTCCTCTTTCCAATTTGAG
TGCAAGCACTCGGGAGGAAAGAAGCGTTCGACTTGACATG
>73
CTTGCTGCCGCCCTAGGAGTCTGGGCCGTGTCTCAGTTCCCAGTGTGGCTGGTCATCCTCTCAGACCAGCTAG
AGATCGTCGGCTGTGAGCCTTTACCTCACCAACTACCTAATCCCCACTTGGGCTTCATCCTATGGCATGTGGC
CCCGAAGGTCCCCACACTTTAATCTTTCGATATTTACGCGGTATTTAGCTACAGTTTTCCCCGTAGTTTATTC
CCCCCTCCCCATAAGCCAGATTTCCCCCAAGCATTTACTCACCCCGTTCCCGCCACTCGTCATCAAAGAAGCA
AGCTTTCTCTTATTGTTTACCGTTTCGACTTGCATTGTGTTTAAGCCTGCCGCCAGCGTTCAATCTGAGCCAG
GATCAAACTCT
>74
CTGCTGCCGCCCGTAGGAGTCTGGGCCGTGTCTCAGTCCCAGTGTGGCTGGTCATCCTCTCAGACCAGCTAGA
GATCGTCGGCTTGGTGAGCCTTTACCTCACCAACTACCTAATCCCACTTGGGCTCATCCTATGGCATGTGGCC
CGAAGGTCCCACACTTTAATCTTTCGACTATTATCGCGGTATTTAGCTACAGTTTTCCCGTACGTTATCCCCC
TCCCATAAGCCAGATTTCCCAAGCATTTACTCACCCGTCCGCCACTCGTCATCAAAGAAGCAAGCTTTCTCTT
ATGTTTACCGTTTCGACTTTGCATGTCGTTTTAAGCCTGA
>75
CTGCTGCCGCCCGTAGGAGTCTGGGCCGTGTCTCAGTCCCAGTGTGGCCGATCACCCTCTCAGGTCGGCTATG
CATCGTGGCCTTTGGTGAGCCGTTACCTCACCAACTAGCTAATGCACCGCGGGTCCATCCATCAGCGACACCC
GAAAGCGCCTTTCACTCTTTATGCCATGCGCATAAACTGTTTATGCGGTATTTAGCACCTTGTTTTTCCAAGT
CGTTTTATCCCCCTCTGATGGGTAGGTTTACCCACGTGTTTACTCACCCGTCCGCCACTCCTCTTTCCAATTT
GAGTGCAAGCACTCGGGAGGAAAGGA
>76
CTGCTGCCGCCCTAGGAGTCTTGGGCCGTGTCTCAGTCCCAGTGTGGCTGGTCGTCCTCTCAAACCAGCTACG
GATCGTCGCCTTTGGTGAGCCATTACCTCACCAACTAGCTAATCCGATATCGGCTTGCTCAATAGTGAGAGGT
CTTGCGATCCCCCCCCTTTCCCCGTACGGCGTATGCGGTATTTAGCTACGCTTTCGCGTAGTTATCCCCCGC
TACTGGGCACTGTTCCGATACATTTACTCACCCGCTCGCCACTCGCCATCCAGAGAGCAAGCTCTCTGATGCT
GCCGTTTCGACTTGCATGTA
>77
CTGCTGCCGCCCGTAGGAGTCTGGGCCGTGTCTCAGTCCCAGTGTGGCCGATCACCCTCTCAGGTCGGCTATG
CATCGTGGCCTTGGTGAGCCGTTACCTCACCAACTAGCTAATGCACCGCGGGTCCATCCATCAGCGACACCCG
AAAGCGCCTTTTCACTCTTTATGCCATGCGGCATAAACTGTTTATGCGGTATTTTAGCACCTTGTTTTTTCCA
AGTCGTTTTATTCCCCCTCTGATGGGTAGGTTTTACCCACGTGTTTACTTCACCCGCCCGCCACTCCTCTTTC
CAATCGAGTGCAAGCTA
>78
CTGCTGCCGCCCGTAGGAGTCTGGGCCGTGTCTCAGTCCCAGTGTGGCCGATCACCCTCTCAGGTCGGCTATG
CATCGTGGCCTTGGTGAGCCGTTACCTCACCAACTAGCTAATGCACCGCGGGTCCATCCATCAGCGACACCCG
AAAGCGCCTTTTCACTCTTTATGCCATGCGGCATAAACTGTTTATGCGGTATTTTAGCACCTGTTTTTTCCAA
GTCGTTTTATCCCCCTCTGATGGGTAGGTTTACCCACGTGTTTACTTCACCCGTCCGCCACTCCTCTTTTCCA
ATTTTGAGTGCAAGCACTCGGGAGAAAAGAGTCGTCGTGTACATGTATTTTAGGCACGCCGCCA

>79
CTGCTGCCGCCCGTAGGAGTCTGGGCCGTGTCTCAGTCCCAGTGTGGCTGGTCATCCTCTCAGGCCAGCTTAG
AGATCGTCGGCTTGGTGAGCCTTTACCTCACCAACTACCTAATCCCACTTGGGCTTCATCCTATGGCATGTGG
CCCGAAGGTCCCACACTTTTAATCTTTTCGATATTTACGCGGTATTTTAGCTACAGTCTCCCGTTAGTTTATC
CCCCTCCATAAGCCAGATTTCCCAAGCATTTTACTTCACCCGTTCCGCCACTTCGTCATTCAAAAAGAAAGCA
AGCTTTTCTCTTATTGTTTTACCGTTTTCGACTTTTGCATTGTGTTTT
>80
CTGCTGCCGCCCGTAGGAGTCTGGGCCGTGTCTCAGTCCCAGTGTGGCCGATCACCCTCTCAGGTCGGCTATG
CATCGTGGCCTTGGTGAGCCGTTACCTCACCAACTAGCTAATGCACCGCGGGTCCATCCATCAGCGACACCCG
AAAAGCGCCTTTTTCACTCTTTTATTGCCATGCGGCATAAACTGTTTTATGCGGTATTTTAGCACCTGTTTTT
TTCCAAGTCGTTTTATCCCCCTCTGATGGGTAGGTTTTACCCACGTGTTTTACTCACCCGTCCGCCACTCCTC
TTTTTCCAATTTGAGTGCAAGCACTCGGGAGGAAAGAAGCGTTTCGTACTTGCA
>81
CTGCTGCCGCCCGTAGGAGTCTGGGCCGTGTCTCAGTCCCAGTGTGGCTGGTCGTCCTCTCAAACCAGCTACG
GATCGTCGCCTTTGTGAGCCATTACCTCACCAACTAGCTAATCCGATATCGGCCGCTCCAATAGTGAGAGGTC
TTTGCGATCCCCCCCCTTTTCCCCCGTAGGGCGTATGCGGTATTTTAGCTACGCTTTTCGCGTAGTTTTATCC
CCCCGGCTACTGGGCACGTTCCGATACATTACTCACCCGTTCGCCACTCGCCAATCCCAGAGAGCAAGCTCTC
TGATGCTGCCGTTCGACTTGTCATGTA
>82
CTGCTGCCGCCCGTAGGAGTCTGGGCCGTGTTCTCAGTCCCAGTGTGGCTGGTCGTCCTTCTCAAACCAGCTA
CGGATCGTCGCCTTGGTGAGCCGTTACCTCACCAACTAGCTTAATTCCGATTATCGGCCGCTTCCAATTAGTG
GGAGGTTCTTTTGCGATTCCCCCCCCCCCTTTTTTCCCCCCCCCCGTAGGGCGTATGCGGTATTTTAGCTT
ACGCTTTTTCGCGTTAGTTTATTCCCCCCCCCGCTTACTGGGCACGTTTTCCGATTACATTTTACTTCACCCG
TTTTCGCCACTTCGCCATTCCAGAGAGCAAGCTCTTCTTGATTGCTGCC
>83
CTGCTGCCGCCCGTAGGAGTCTGGGCCGTGTCTCAGTCCCAGTGTGGCTTGGTTCGTCCTCTCAAACCAGCTA
CGGATCGTCGCCTTGGTGAGCCATTTACCTCACCAACTAGCTAATCCGATATCGGCCGCTCCAATAGTGAGAG
GTTCTTTTGCGATCCCCCCCCCTCCTTCCCCCCCCCGGTAGGGCGTATGCGGTTATTTTAGCTACGCTTTTT
CGCGTAGTTTTATCCCCCCCCCGCTACTGGGCACGTTTCCGATACATTTTACTCACCCCCGTTTTCGCCACTC
GCCATCCAGAGAGCAAGCTCTCTTGATGCTGCCGTTTTTCGACTTTGCATGTGTTAAGGCATTCCCCCCCGCT
TAGCGTTTTTCAATTCGTTGAGCCAGGATCAAAAAAACTCT
>84
CTGCTGCCGCCCTAGGAGTCTGGGCCGTGTCTCAGTCCCAGTGTGGCCGATCACCCTCTCAGGTCGGCTATGC
ATCGTGGCCTTGGTGAGCCGTTACCTCACCAACTAGCTAATGCACCGCGGGTCCATCCATCAGCGACACCCGA
AAGCGCCTTTCACTCTTTATGCCATGCGCATAAACTGTTTATGCGGTATTTAGCCACCTTGTTTTTCCAAGTC
GTTTATCCCCCTCTGATGGGTAGGTTTACCCACGTGTTTACTTCACCCGTCCGCCACTCCTCTTTCCAATTTG
AGTGCAAGCACTCGGGAGGAAAGAAGCGTTCGTA
>85
CTGCTGCCGCCCGTAGGAGTCTGGGCCGTGTCTCAGTCCCAGTGTGGCCGATCACCCTCTCAGGTCGGCTATG
CATCGTGGCCTTGGTGAGCCGTTACCTCACCAACTAGCTAATGCACCGCGGGTCCATCCATCAGCGACACCCG
AAAGCGCCTTTCACTCTTATGCCATGCGCATAAACTGTTTATGCGGTATTTAGCACCTGTTTTTCCAAGTCGT
TTATCCCCCTCTGATGGGTAGGTTTACCCACGTGTTACTCACCCGTCCGCCACTCCTCTTTCCAATTTGATGT
GCAAGCACTCGGGAGGAAAGAAGCGTTCGTACGTTGAC
>86
CTGCTGCCGCCCGTAGGAGCTGGGCCGCGTCTCAGTCCAGTGTGGCTGGTCATCCTCTCAGACCAGCTAGAGA
TCATCGGCTGGCGAGCCTTTACCTCACCAACTACCTAATCCCACTTGGGCTCATCCTATGGTATGTGGCCCGA
AGGTCCCACACTTAATCTTCGATATTACGCGGTATTAGCTACAGTTTCCCGTACGTTATCCCCTCCCATAAGC
CAGATTTCCCAAGCATTTACTCACCCGTCCGCCACTCGTCATCAAAGAAGCAAGCTTTCTCTTATGTTTACCG
TTTCGACTTGCATTGTCGTTTAAGCCTGCCGCCAGCGTTCAACTCT
>87
CTGCTGCCGCCCTAGGAGTCTGGGCCGTGTCTCAGTCCCAGTGTGGCTGGTCATCCTCTCAGACCAGCTAGAG
ATCGTCGGCTTGGTGAGCCTTTACCTCACCAACTACCTAATTCCCACTTGGGCTTCATCCTATTGGCATGTGG
CCCGAAGGTCCCACACTTTAATCTTTCGATATTTACGCGGTATTTAGCTACAGTTTTCCCGTACGTTTATTCC

CCCCTCCATTAAGCCAGATTTCCCCCAAGCATTTACTCACCCGTTCCGCCACTCGTCATTCGAAGAAGCAAGC
TTTCTTCTAT
>88
CTGCTTGCCGCCCTAGGAGTCTGGGCCGTGTCTCAGTCCCAGTGTGGCTGGTCGTCCTCTCGAACCAGCTACG
GATCGTCGCCTTGGTGAGCCATTACCTCACCAACTAGCTAATCCGATATCGGCCGCTCCAATTAGTGAGAGGT
CTTTGCGATCCCCCCCCCCTTTTCCCCCGTAGGGCGTATGCGGTATTTAGCTACGCTTTCGCGTAGTTTATTC
CCCCCCGCTACTGGGCACGTTCCGATACATTTACTCACCCGTTTCGCCACTCGCCATCCAGAGAGCAAGCTCT
CTTGATTGCTTGCCGTTTCGACTTGCATTGTGTAAGGCCAAT
>89
CTGCTGCCGCCCGTAGGAGTCTGGGCCGTGTCTCAGTCCCAGTGTGGCCGATCACCCTCTCAGGTCGGCTATG
CATCGTGGCCTTTGGTGAGCCGTTACCTCACCAACTAGCTAATGCACCGCGGGTCCATCCATCAGCGACACCT
GAAAGCGCCTTTTCACTCTTTATGCCATGCGGCATAAACTGTTTATGCGGTATTTAGCACCTTGTTTTTC
CAAGTCGTTTTATCCCCCTCTGATTGGGTAGGTTTACCCACGTGTTTTACTTCACCCGTCCGCCACTCCTCTT
TTCCAATTCGAGTGCAAGCACT
>90
CTGCTGCCGCCCGTAGGAGTCTGGGCCGTGTCTCAGTCCCAGTGTGGCCGATCACCCCTCTGAGCGGCTATGC
ATCGTGGCCTTGGTGAGCCGTTACCTCACCAACTAGCTAATGCACCGCGGGTCCATCCATCGACGACACCCGA
AAGCGCCTTTTCACTCTTTATGCCATGCGGCATAAACTGTTTATGCGGTATTTAGTCCACCTGTTTTTTCCAA
GTCGTTTTATCCCCCTCTGATGGGTAGGTTTACCCACGTGTTTTACTCACCCGTCCGCCACTCCTCTTTCCAA
TTTTGAGTGCAAGACTCGGGA
>91
CTGCTGCCGCCCGTAGGAGTCTGGGCCGTATCTCAGTCCCAATGTGGCCGCACACCCTCTCAGGCCGGCTACC
CGTCGCCGCCTTGGTAGGCCATTACCCACCAACAAGCTGATAGGCCGCAAGCTCATCCCACACCAGAAAAACC
TTTCCACCACACCAACCACGATGCGGTCCTATAGGGTTATTTAGACCCAGTTTTTCCCAAGCTTTATTCCCCC
AGTGCAGGGCAGATCCACTTTACGTGTTTACTCACCCGTTTCGCCACTCGAGCACCCCAGCAAGCTGGGGCCT
TTTTCCGTTTTCGACTTTGCATTGTGTTTTTAAGCACGCCGCCAGCGTTTCATCCTGAG
>92
CTGCTGCCGCCCGTAGGAGTCTGGGCCGTGTCTCAGTCCCAGTGTGGCTGGTCATCCTCTCAGACCAGCTAGA
GATCGTCGGCTTGGTGAGCCTTTACCTCACCAACTACCTAATCCCACTTGGGCTTCATCCTATTGGTATGTGG
CCCGAAGGTCCCACACTTTTAATCTTTTCGATTATTTACGCGGTTATTTAGCTACAGTTTTTCCCGTAGTTTA
TTCCCCCTCCATTAAGCCAGATTTTCCCAAGCATTTTACTTCACCCGTTCCGCCACTTCGTTCATTCAAAGAA
GCAAGCTTTTCTTCTTATTG
>93
CTGCTGCCGCCCTAGGAGTCTGGGCCGTGTCTCAGTCCCAGTGTGGCTGGTCATCCTCTTCAGACCAGCTAGA
GATTCGTCGGCTTGGTGAGCCTTTACCTCACCAACTTACCTTAATTCCCACTTGGGCTCATTCCTATTGGCAT
TGTGGCCCCCGAAGGTCCCGCACTTTTCATCTCCCGATTTTCTACGCGGTATTTTAGCTTACAGTTTTTCCCG
TAGTTTTACTTCCCCCCCCTTCCATTAAGCTTAGATTTTCCCCCAAGCATTTTACTTCACCCGTCCGCCACTT
CGTCATTCAAAGAAGCAAGCTTTTCTTTTTTTTTATTGTTTTTACCGTTTTCGACTTTTGCATTGTTGTTTT
>94
CTGCTGCCGCCCGTAGGAGTCTGGGCCGTGTCTCAGTCCCAGTGTGGCTGGTCGTCCTCTCAAACCAGCTACG
GATCGTCGCCTTGGTGAGCCATTTACCTCACCAACTAGCTAATCCGATATCGGCCGCTCCAATAGTGAGAGGT
CTTTTGCTCGATCCCCCCCCCCCCTTTTTCCCCCCCCCGTAGGGCGTATGCGGTATTTTAGCTACGCTTTTCG
CGTAGTTTTATCCCCCGCTACTTGGGCACGTTTCCGATACATTTACTTCACCCGTTTCGCCACTCGCCATCCC
AGAGAGCA
>95
CTGCTGCCGCCCGTAGGAGTCTGGGCCGTGTCTCAGTCCCAGTGTGGCCGATCACCCTCTCAGGTCGGCTATG
CATCGTGGCCTTGGTGAGCCGTTACCTCACCAACTAGCTAATGCACCGCGGGTCCATCCATCAGCGACACCCG
AAAGCGCCTTTCACTCTTTATGCCATGCGGCATAAACTGTTTATGCGGTATTTAGCACCTGTTTTTCCAAGTC
GTTTATTCCCCCTCTGATTGGGTAAGTTTACCCACGTGTTTACTTCACCCGTTCCGCCACTCCTCTTTTCCAA
TTTTGAGTGCAAGCACTCGGGAGGAAAGAAGCGTTC
>96
CTGCTGCCGCCCGTAGGAGTCTGGGCCGTGTCTCAGTCCCAGTGTGGCCGATCACCCTCTCAGGTCGGCTATG
CATCGTGGCCTTGTGAGCCGTTACCTCACCAACTAGCTAATGCACCGCGGGTCCATCCATCAGCGACACCCGA
AAGCGCCTTTCACTCTTTATGCCATGCGGCATAAACTGTTTATTGCGGTATTTAGCCACCTTGTTTTTTCCAA

GTCGTTTATCCCCCTCTGATGGGTAGGTTTACCCACGTGTTTACTTCACCCGTCCGCCACTCCTCTTTCCAAT
TTGAGTGCAAGCACTCGGGAGGAAAGAAGCGTTCGACTTGACATGT
>97
CTGCTGCCGCCCGTAGGAGTCTGGGCCGTGTCTCAGTCCCAGTGTGGCCGATCACCCTCTCAGGTCGGCTATG
CATCGTGGCCTTGGTGAGCCGTTACCTCACCAACTAGCTAATGCACCGCGGGTCCATCCATCAGCGACACCCG
AAAGCGCCTTTCACTCTTATGCCATGCGGCATAAACTGTTTATGCGGTATTTTAGCACCTGTTTTTCCAAGTC
GTTTATCCCCCTCTGATGGGTAGGTTTACCCACGTGTTTACTTCACCCGTCCGCCACTCCTCTTTCCAATTTT
GAGTGCAAGCACTCGGGAGGAAAGAAGCGTTCGACTTGACA
>98
CTGCTGCCGCCCGTAGGAGTCTGGGCCGTGTCTCAGTCCCAGTGTGGCCGATCACCCTCTCAGGTCGGCTATG
CATCGTGGCCTTGTGAGCCGTTACCTCACCAACTAGCTAATGCACCGCGGGTCCATCCATCAGCGACACCCGA
AAGCGCCTTTCACTCTTTATGCCATGCGCATAAACTGTTTATGCGGTATTTAGCCACCTGTTTTTCCAAGTCG
TTTATCCCCCTCTGATGGGTAGGTTTACCCACGTGTTTACTTCACCCGTCCGCCACTCCTCTTTCCAATTTGA
GTGCAAGCACTCGGGAGGAAAGAAGC
>99
CTGCTGCCGCCCGTAGGAGTCTGGGCCGTGTCTCAGTCCCAGTGTGGCTGGTCATCCTCTCAGACCAGCTAGA
GATCGTCGGCTTGGTGAGCCTTTACCCCACCAACTACCTAATCCCACTTGGGCTCATCCTATGGCATGTGGCC
CGAAGGTCCCACACTTTTAATCTTTTCGATATTTACGCGGTATTTAGCTACAGTTTTTCCCGTAGTTTATTCC
CCCTCCATAAGCCAGATTTCCCAAGCATTTACTCACCCGTTCCGCCACTCGTCATTCAAAGAAGCAAGCTTTC
TCTTATTGTTTTACCGTTTCGACTTT
>100
CTGCTGCCGCCCGTAGGAGTCTGGGCCGTGTCTTCAGTCCCAGTGTGGCCGATCACCCTTCTCAGGTCGGCTA
TTGCATCGTGGCCTTGGTTGAGCCGTTACCTTCACCAACTAGCTTAATTGCACCGCGGGTCCATCCATTCAGC
GACACCCCCGAAAAGCGCCTCTTACTTCTTTTTATTGCATTGCGGCATTAAAAAACTTGTTTTTTTATTGCGG
TTATTTTTTAGCACCCTTTTGTTTTTTCCCCAAAAGTTGTTTTTTATTTCCCCCCTTCTTGATTGGGGGTTAG
GTTTTTACCCCCACGTTGT
>101
CTGCTGCCGCCCGTAGGAGTCTGGGCCGTGTCTCAGTCCCAGTGTGGCCGATCACCCTCTCAGGTCGGCTATG
CATTCGTGGCCTTGGTGAGCCGTTACCTCACCAACTAGCTTAATTGCACCGCGGGTCCATCCATTCAGCGACA
CCCCCGAAAAAGCGCCTTTTTTTCACTTCTTTTTATTGCCATTGCGGCATTTAAAAAACTTGTTTTTTATTGC
GGGTTATTTTTTAGCACCCTTTTGTTTTTCCCCAAAAAGTTTGTTTTTATTTCCCCCTTTCTTGATTTTTGGG
GGTTAGGGTTTTTACCCCCACGTTGTTTTACTTTTCACCCCCGTTCCCCGCCCCACTTCCC
>102
CTGATGCCGCCCGTAGGAGTCTGGGCCGTGTCTCAGTCCCAGTGTGGCTGGTCATCCTCTCAGACCAGCTAGA
GATCGTCGGCTTGGTGAGCCTTTACCTCACCAACTACCTATCCCACTTGGGCTCATCCTATGGTATGTGGCCC
GAAGGTCCCACACTTTTAATCTTTTCGATATTTACGCGGTATTTTAGCTACAGTTTTTCCCGTAGTTTATCCC
CCTCCATAAGCCAGATTTCCCAAGCATTTTACTCACCCGTTCCGCCACTCGTCATCAAAGAAGCAAGCTTTTC
TCTATTGTTTTACCGTTTTCGACTTTGCATGTGTTTTAAGCCTGCCGC
>103
CTGCTGCCGCCCGTAGGAGTCTGGGCCGTGTCTCAGTCCCAGTGTGGCCGATCACCCTCTCAGGTCGGCTATG
CATCGTGGCCTTGGTGAGCCGTTACCTCACCAACTAGCTAATGCACCGCGGGTCCATCCATCAGCGACACCCG
AAAGCGCCTTTTCACTCTTTATTGCCATGCGGCATAAACTGTTTTATGCGGTATTTTAGCACCTGTTTTTTTT
CCAAGTGTTTTATCCCCCCTCTGATGGGTAGGTTTTACCCACGTGTTTTACTCACCCGTCCGCCACTCCTCTT
TTCCAATTTGAGTGCAAGCA
>104
CTGCTGCCGCCCGTAGGAGTCTGGGCCGTGTCTCAGTCCCAGTGTGGCTGGTCATCCTCTCAGACCAGCTAGA
GATCGTCGGCTTGTGAGCCTTTACCTCACCAACTACCTAATCCCACTTGGGCTCATCCTATGGCATGTGGCCC
GAAGGTCCCACACTTTTAATCTTTTCGATATTTACGCGGATTTTAGCTACAGTTTTTCCCGTAGTTTTATCCC
CCTCCATAAGCCAGATTTTCCCAAGCATTTTACTCACCCGCCGCCACTCGTCATCAAAAAAAGCAAGCTTCTC
TATGTTTTACCGTTTTCGACTTTGCATGTGTTTTAAGCCTTGCCGCCAGCGTTCAATCTTGAGCAGGATCAAA
CTCT
>105
CTGCTGCCGCCCGTAGGAGTCTGGGCCGTGTCTCAGTCCCAGTGTGGCCGATCACCCTCTCAGGTCGGCTATG
CATCGTGGCCTTGGTGAGCCGTTACCTCACCAACTAGCTAATGCACCGCGGGTCCATCCATCAGCGACACCCG

AAAGCGCCTTTTTCACTCTTTATGCCATGCGGCATAAACTGTTTATGCGGTATTTTAGCACCTGTTTTTTCCA
AGTCGTTTTATCCCCCCTCTGATGGGTAGGTTTTACCCACGTGTTTTACTCACCCGTCCGCCACTCCTCTTTT
TCCAATTTTGAGTGCAAGCACTCGGGAGAAAGAAGCGTTTTCGACTTGCATGTATTTTAGGCACGTCCCGCCA
GCCGTTTTCGTTCCCTGAGCCAGGATCAAACTCCTTT
>106
CTGCTGCCGCCCGTAGGAGTCTGGGCCGTGTCTCAGTCCCAGTGTGGCCGATCACCCTCTCAGGTCGGCTATG
CATCGTGGCCTTGGTGAGCCGTTACCTCACCAACTAGCTAATGCACCGCGGGTCCATCCATCAGCGACACCCG
AAAGCGCCTTTTTCACTCTTTATGCCATGCGGCATAAACTGTTTATGCGGTATTTAGCACCTGTTTTTTCCAA
GTGTTTTATCCCCCCTCTGATGGGTAGGTTTTACCCACGTGTTTTACTCACCCGTCCGCCACTCCTCTTTTTC
CAATTTTGAGTG
>107
CTGCTGCCGCCCGTAGGAGTCTGGGCCGTATCTCAGTCCCAATGTGGCCGCACACCCTCTCAGGCCGGCTACC
CGTCGCCGCCTTGGTAGGGCCATTACCCCCACCAACAAGCTGATAGGCCGCAAGCTCATCCCACACCAGAAAA
ACCTTTTCCACCACACCAACCACGATGCGGTCCTATAGGGTTATTTAGACCCAGTTTTTCCCAAGCTTTTATC
CCCCAGTGCAGGGCAGATCACTTTTACGTTGTTTACTCACCCGTTTTCGCCACTCGAGCACCCCCAGCAAGCT
GGGGCCTTTTTTCCGTTTTTCGACTTTTGCATGTTGTTTTTTAAGCACGCCGCCAGCGTTTTCATCCTTG
>108
CTGCTGCCGCCCGTAGGAGTCTGGGCCGTGTCTCAGTCCCAGTGTGGCTGGTCATCCTCTCAGACCAGCTAGA
GATCGTCGGCTGGTGAGCCTTTACCTCACCAACTACCTAATCCCACTTGGGCTTCATCCTATGGCATGTGGCC
GAAGGTCCCACACTTTAATCTTTCGATATTTACGCGGTATTTAGCTACAGTTTTCCCGTAGTTATTCCCCTCC
ATAAGCCAGATTTCCCAAGCATTTACTCACCCGTCCGCCACTCGTCATCAAAGAAGCAAGCTTTCTCTTATTG
TTTACCGTTTCGACTTTGCATTGTGTTTTAAGCCTGACC
>109
CTGCTGCCGCCCGTAGGAGTCTGGGCCGTGTCTCAGTCCCAGTGTGGCCGATCACCCTCTCAGGTCGGCTATT
GCATCGTGGCCTTGGTGAGCCGTTTACCTCACCAACTAGCTTAATTGCACCGCGGGTTCCATCCATTCAGCGA
CACCCCCGAAAAAGCGCCTTTTTTTCACTTCTTTTTATTTGCCATTTGCGGCATTTAAAAAACTTGTTTTTTA
TTTTTGCGGGGTTTATTTTTAGCACCCTTTTGTTTTTCCCCAAAAAGTTTGTTTTATTTTCCCCCTTTCTTTG
ATTTTGGGGTTTAGGGGTTTTACCCCACGTGT
>110
CTGCTGCCGCCCGTAGGAGTCTGGGCCGTGTCTCAGTCCCAGTGTGGCCGATCACCCTTCTCAGGTTCGGCTA
TGCATTCGTGGCCTTGGTGAGCCGTTACCTTCACCAACTAGCTTAATTGCACCGCGGGTCCATCCATCAGCGA
CACCCCCGAAAAAGCGCCTTTTTTTCACTTCTTTTTATTTGCCATTTGCGGCATTTAAAAAACTTGTTTTTAT
TTGCGGGTTATTTTTAGCAACCCTTTTGTTTTCCCCAAAAGTTTGTTTTATTTTCCCCTTTCTTGATTTGGGT
TTAGGGTTTACCCACGTGTTTACTTTAC
>111
CTGCTGCCGCCCGTAGGAGTCTGGGCCGTGTCTCGGTCCCAGTGTGGCTGGTCATCCTCTCAGACCAGCTAGA
GATTCGTCGGCTTTGGTGAGCCTTTACCTCACCAACTACCTAATTCCCACTTTGGGCTTCATCCTATTGGCAT
GTGGCCCGAAGGTCCCACACTTTTTAATCTTTTTCGATATTTTACGCGGTTATTTTAGCTACAGTTTTTCCCG
TAGTTTTATTCCCCCTCCCATTAAGCCAGATTTTCCCAAGCATTTTACTTCACCCGTTCCGCCACTTCGTTCA
TTCAAAGAAGCAAGCTTTTCTTCTTATTGTTTTTACCGTTTTCGACTTTTGCATTGTGTTTTTAAGCCTTGCC
GCCAGCGTTTTCAATTCTTGAGCCAGGATCAAACTTCTT
>112
CTGCTGCCGCCCGTAGGAGTCTGGGCCGTGTCTCAGTCCCAGTGTGGCTGGTCGTCCTCTCAAACCAGCTACG
GATCGTCGCCTTGTGAGCCATTACCTCACCAACTAGCTAATCCGATATCGGCCGCTCCAATAGTGAGAGGTCT
TTGCGATCCCCCCCCCTTTTCCCCCGTAGGGCGTATGCGGTATTTAGCTACGCTTTTCGCGTAGTTTATCCCC
CGCTACTGGGCACGTTCCGATACATTTACTCACCCGTTTCGCCACTCGCCATCCAGAGAGCAAGCTCTCTGAT
GCTGCCGTTTCGACTTTGCATGTGGTA
>113
CTGCTGCCGCCCGTAGGAGTCTGGGCCGTGTCTCAGTCCCAGTGTGGCTGGTCGTCCTCTCAAACCAGCTACG
GATCGTCGCCTTGTGAGCCGTTACCCCACCAACTAGCTAATCCGATATCGGCCGCTCCAATCAGTGAGAGGTC
TTTACGATCCCCCCTTCCCCGTAGGGCGTACTGCGGTATTAGCTACGCTTTCGCGTAGTTATCCCCGCTACTG
GGCACGTTTCCGATACATTTACTCACCGTTTCGCCACTCGCCATCCAGAGAGCAAGCTCTCTGATGCTGCCGT
TTCGACTTGCATGTGTAAGGCATCCCGCTAGCGTTTCAATCTGA
>114

CTGCTGCCGCCCTAGGAGTCTGGGCCGTGTCTCAGTCCCAGTGTGGCTGGTCATCCTCTCAGACCAGCTAGAG
ATCGTCGGCTTGGTGAGCCTTTACCTCACCAACTACCTAATCCCACTTGGGCTCATCCTATGGCATGTGGCCC
GAAGGTCCCACACTTTTAATCTTTCGATATTACGCGGTATTAGCTACAGTTTTCCCGTAGTTATCCCCCTCCA
TAAGCCAGATTTCCCAAGCATTTACTCACCCGTCCGCCACTCGTCATCAAAGAAGCAAGCTTTTCTCTATGT
TTTACCGTTTTCGACTTTGCAGTGTTTTAA
>115
CTGCTGCCGCCCGTAGGAGTCTGGGCCGTGTCTCAGTCCCAGTGTGGCCGATCACCCTCTCAGGTCGGCTATG
CATCGTGGCCTTGGTGAGCCGTTACCTCACCAACTAGCTAATGCACCGCGGGCCCATCCATCAGCGACACCCG
AAAGCGCCTTTTCACTCTTTATGCCATGCGGCATAAACTGTTTATGCGGTATTTAGCACCTGTTTTTTCCAAG
TCGTTTTATCCCCCTCTGATGGGTAGGTTTTACCCACGTGTTTTACTCACCCGTCCGCCACTCCTCTTTTTCC
AATTTTGAGTGCAAGCACTCGGGAGGAAAGAAGCGTTTTCGACTTGCATGTATTTTAGGCACGCCGCCAGCGT
TCGCCCTGTGCCAGG
>116
CTGCTGCCGCCCGTAGGAGGTATGGGCCGTGTCTCGAGTCCCAAGTGTGGCCGATCACCCTCTCAGGTCGGCT
ATGCATTCGTGGCCTTGGTGGAGCCGTTGACCTCACCAACTAGCTAATGCACCGCGGGTCCATCCATCAGCGA
CACCCGAAAGCGCCTTTTTTCACTCTTTTATGCCATGCGGCATAAACTGTTTTATGCGGTATTTTAAGCACCT
GTTTTTTTCCAAGTGTTTTTATCCCCCCTCTGATGGGTAGGTTTTACCCACGTGTTTTTACTCACCCGTCCGC
CACTCCTCTTTTCC
>117
CTGCTGCCGCCCTAGGGAGTCTGGGCCGCGTCTCAGTCCCAGTGTGGCCGATCACCCTCTCAGGTCGGCTATG
CATCGTGGCCTTTGGTGAGCCGTTACCTCACCAACTAGCTAATGCACCGCGGGTCCATCCATCAGCGACACCC
GAAAGCGCCTCTCACTTCTTTATCGCATGCGGCATAAACTGTTTATGCGGTATTTTAGCACCTTGTTTTTCCA
AGTCGTTTATTCCCCCCCTGATTGGGTAGGTTTACCCACGTGTTTACTCACCCGTCCGCCACTCCTCTTTTCC
AATTTGAGTGCAAGCACTCGGGAGGAAAGAAGCGTTCGACTTGACATGTAT
>118
CTGCTGCCGCCCGTAGGAGTCTGGGCCGTGTCTCAGTCCCAGTGTGGCCGATCACCCTCTCAGGTCGGCTATG
CATCGTGGCCTTGGTGAGCCGTTACCTCACCAACTAGCTAATGCACCGCGGGTCCATCCATCAGCGACACCCG
AAAGCGCCTTTCACTCTTTATGCCATGCGGCATAAACTGTTTATGCGGTATTTAGCACCTGTTTTTCCAAGTC
GTTTATCCCCCTCTGATGGGTAGGTTTACCCACGTGTTACTCACCCGTCCGCCACTCCTCTTTCCAATCGAGT
GCAAGCACTCGGGAGGAAAGAAGCGTTCGACTTGCATGTATTTAGGCACGCCGCCAGCGTTCGTCCTG
>119
CTGCTGCCGCCCTAGGAGTCTGGGCCGTGTCTCAGTCCCAGTGTGGCTGGTCATCCTCTCAGACCAGCTAGAG
ATCGTCGGCTTGGTGAGCCTTTACCTCACCAACTACCTAATTCCCACTTGGGCTTCATCCTATGGCATGTGGC
CCGAAGGTCCCACACTTTAATCTTTCGATATTTACGCGGTATTTAGCTACAGTTTTCCCGTACGTTTATTCCC
CCCTCCCATTAAGCCAGATTTCCCCCAAGCATTTACTCACCCGTTCCGCCACTCGTCATTCAAAGAAGCAAGC
TTTCTTCTAT
>120
CTGCTGCCGCCCGTAGGAGTCTGGGGCCGTGTCTCAGTCCCAGTGTGGCCGATCACCCTCTCAGGTCGGCTAT
GCATGTGGCCTTGGTGAGCCGTTACCTCACCAACTAGCTAATGCACCGCGGGTCCATCCATCAGCGACACCCG
AAAGCGCCTTTCACTCTTTATGCCATGCGGCATAAACTGTTTATGCGGTATTTAGCCACCTGTTTTTCCAAGT
CGTTTATCCCCCTCTGATGGGTAGGTTTACCCACGTGTTACTTCACCCGTCCGCCACTCCTCTTTCCAATTTG
AGTGCAAGCACTCGGGAGGAAAGAAGCGTTCGACTTGACAT
>121
CTGCTGCCGCCCTAGGAGTCTGGGCCGTGTCTCAGTCCCAGTGTGGCTGGTCATCCTCTCAGACCAGCTAGAG
ATCGTCGGCTTGGCGAGCCTTTTACCCACCAACTACCTAATCCCACTTGGGCTCATCCTATGGCATGTGGCCC
GAAGGTCCCACACTTTAATCTTTTCGACTATTACGCGGTATTTAGCTACAGTTTTTCCCGTACGTTATCCCCC
TCCCATAAGCCAGATTTCCCCAAGCATTTTACTCACCCGTCCGCCACTCGTCATCAAAGAAGCAAAGCTTTCT
CTATGTTTTACCGTTTCGACTTGCATGTGTTTTAAGCCTGCCGCCAGCCG
>122
CTGCTGCCGCCCGTAGGAGCTGGGCCGTGTCTCAGTCCCAGTGTGGCTGGTCATCCTCTCAGACCAGCTAGAG
ATCGTCGGCTTGGTGAGCCTTTACCTCACCAACTACCTAATTCCCACTTGGGCTTCATCCTATTGGCATGTGG
CCCGAAGGTCCCACACTTTTAATCTTTTCGATATTTACGCGGTATTTAGCTACAGTTTTCCCGTACGTTTATT
CCCCCTCCATAAGCCAGATTTCCCCCAAGCATTTACTCACCCGTTCCGCCACTCGTCATTCAAAGAAGCAAGC
TTTCTTCTTATTGTTTACCGTTTCGACTTTGCATTGTGTTTAAGCCTTGCCGCCAGCGTTTCAATCG

>123
CTGCTGCCGCCCGTAGGAGTCTGGGCCGTGTCTCAGTCCAGTGTGGCCGATCACCCTTCTCAGGTCGGCTATG
CATCGTGGCCTTGGTGAGCCGTTACCTCACCAACTAGCTTAATGCACCGCGGGTCCATCCATCAGCGACGACC
CGAAAGCGCCTTTTTTCACGTTCTTTTATTTAGCCATTTGGCGGCATTAAAAAACTTTGTTTTTTAATTTGCG
GTTAGTTTTTAGCACCCTTTTGTTTTCCCAAAAGTCGTTTTATTTCCCCTTTCTGATTTGGGGTTAGGGTTTT
ACCCACGTGTTTATTTAC
>124
CTGCTGCCGCCCGTAGGAGTCTGGGCCGTGTCTCAGTCCCAGTGTGGCCGATCACCCTCTCAGGTCGGCTATG
CATTCGTGGCCTTGGTGAGCCGTTACCTCACCAACTAGCTTAATGCACCGCGGGTTCCATCCATCAGCGACAC
CCGAAAGCGCCTTTTTTCACTTCTTTTTATTGCCATTGCGGCATTAAAAAACTTGTTTTTTTATTGCGGTTAT
TTTTTTAGCACCTTTGTTTTTTTCCCAAAAGTTTGTTTTTTTATTCCCCCCCTTTCTTGATTGGGGGGTTAGG
TTTTTTACCCCCCACGTTGTTTTTTACTTTTTCAC
>125
CTGCTGCCGCCCGTAGGAGTCTGGGCCGTGTCTCAGTCCCAGTGTGGCCGATCACCCTCTCAGGTCGGCTATG
CATCGTGGCCTTGGTGAGCCGTTACCTCACCAACTAGCTAATGCACCGCGGGTCCATCCATCAGCGACACCCG
AAAGCGCCTTTTCACTCTTTATGCCATGCGGCATAAACTGTTTATGCGGTATTTTAGCACCTGTTTTTTCCAA
GTCGTTTTATCCCCCTCTGATGGGTAGGTTTTACCCACGTGTTTTACTTCACCCGTCCGCCACTCCTCTTTTC
CAATCGAGTGCAAGCACTCGGGAGGAAAGAAGCGTTTCGTACTTGCATGTATTTTAGGCACGCCGCCAGCGTT
C
>126
CTGCTGCCGCCCGTAGGAGTCTGGGCCGTGTCTCAGTCCCAGTGTGGCCGATCACCCTCTCAGGTCGGCTATG
CATCGTGGCCTTGGTGAGCCGTTACCTCACCAACTAGCTAATGCACCGCGGGTCCATCCATCAGCGACACCCG
AAAGCGCCTTTTCACTCTTTATGCCATGCGGCATAAACTGTTTATGCGGTATTTTAGCACCTGTTTTTTCCAA
GTCGTTTTATCCCCCTCTGATGGGTAAGTTTTACCCACGTGTTTTACTCACCCGTCCGCCACTCCTCTTTTCC
AATCGAGTGCAAGCACTCGGGAGGAAAGAAGCGTTTCGACTTGCATGTATTTTAGGCACGCCGCCAGCGTTCG
TTCCTGAGCCAGGATCAAACTCTTT
>127
CTGCTGCCGCCCGTAGGAGTCTGGGCCGTATCTCAGTCCCAATGTGGCGCACACCCTCTCAGGCCGGCTACCC
GTCGCCGCCTTTGGTAGCCATTACCCCACCAACAAGCTGATAGGCCGCAAGCTCATCCCACACCAGAAAAACC
TTTCCACCACACCAACCACGATGCGGTCCTATAGGGTATTAGACCCAGTTTTCCCCAAGCTTTATCCCCCCAG
TGCAGGGCAGATCACTTTACGTGTTACTCACCCGTTTCGCCACTCGAGCACCCCAGCAAGCTGGGGCCTTTTC
CGTTTCGACTTGCAGTGTTTTAAGCACGCCGTCCAGCGTTCACCTGAGCCAGGA
>128
CTGCTGCCGCCCGTAGGAGTCTGGGCCGTGTCTCAGTCCCAGTGTGGCCGATCACCCTCTCAGGTCGGCTATG
CATCGTGGCCTTGGTGAGCCGTTACCTCACCAACTAGCTAATGCACCGCGGGTCCATCCATCAGCGACACCCG
AAAGCGCCTTTTCACTCTTTATGCCATGCGGCATAAACTGTTTATGCGGTATTTTAGCACCTGTTTTTTCCAA
GTCGTTTTATCCCCCTCTGATGGGTAGGTTTTACCCACGTGTTTTACTTCACCCGTCCGCCACTCCTCTTTTT
CCAATTTTGAGTGCAAGCACTCGGGAGGAAAGAAGCGTTTCGACTTGCATGTATTTTAGGCACGCCGCCAGCG
TTCGCCTGTGTCCAGGCATCGAAACATCTT
>129
CTGCTGCCGCCCGTAGGAGTCTGGGCCGTGTCTCAGTCCCAGTGTGGCTGGTCGTCCTCTCAAACCAGCTACG
GATCGTCGCCTTGGTGAGCCATTTACCTCACCAACTAGCTAATCCGATATCGGCCGCTCCAATAGTGAGAGGT
CTTTGCGATCCCCCCCCTTTTCCCCCGTAGGGCGTATGCGGTATTTAGCTACGCTTTTCGCGTAGTTTATCCC
CCGCTACTGGGCACGTTCCGATACATTTACTCACCCGTTTCGCCACTCGCCATCCAGAGAGCAAGCTCTCTGA
TGCTGCCGTTTTCGACTTTGCATGTGTAAGGCCATCCC
>130
CTGCTGCCGCCCGTAGGAGTCTGGGCCGTGTCTCAGTCCCAGTGTGGCCGATCACCCTCTCAGGTCGGCTATG
CATCGTGGCCTTGGTGAGCCGTTACCTCACCAACTAGCTAATCCGATATCGGCCGCTCCAATAGTGAGAGGTC
TTTGCGATCCCCCCCCTTTTCCCCCGTAGGGCGTATGCGGTATTAGCTACGCTTTTCGCGTAGTTATCCCCCG
CTACTCGGGCACGTTCCGATACATTACTCACCCGTTCGCCACTCGCCATCCAGAGAGCAAGCTCTCTGATGCT
GCCGTTCGACTTGCATGTGTAAGGCATCCCCGCTAGCGTTTTCAATCTGAGCCAAGG
>131
CTGCTGCCGCCCGTAGGAGTCTGGGCCGTGTCTCAGTCCCAGTGTGGCCGATCACCCTCTTCAGGTCGGCTAT
GCATTCGTGGCCTTGGTTGAGCCGTTTACCTTCACCAAACTAGCTTAATTGCACCGCGGGGGTCCCATCCATC

AGCGACACCCCCCGAAAAAAGCGCCCTTTTTTCACTTCTTTTTATTTGCCCCATTGCGGCATTAAAACTTGTT
TTATTGCGGGGTTATTTTAGCAACCCTTTGTTTCCCAAAGTTGTTTATTCCCTTCTGATTGGGTTTAGGGTTT
ACCCACGTGTTTACTTTACCCCGTCCCGC
>132
CTGCTGCCGCCCGTAGGAGTCTGGGCCGGTGTCTCAGTCCCAGTGTGGCTGGTCGTCCTCTCAAACCAGCTAC
GGATTGTCGCCTTGGTGAGCCATTTACCTCACCAACTAGCTAATCCGATATCGGCCGCTCCAATTAGTGAGAG
GTCTTTTGCGATCCCCCCCCCCTTTTTTCCCCCCCCCCGTAGGGCGTATGCGGTATTTTAGCTACGCTTTTTC
GCGTAGTTTTATCCCCCCGCTACTTGGGCACGTTTCCGATACATTTTACTCACCCGTTTTCGCCACTTCGCCA
TCCCAGAGA
>133
CTGCTGCCGCCGTAGGAGTCTGGGCCGTGTCTCAGTCCCAGTGTGGCCGATCACCCTCTCAGGTCGGCTATGC
ATTCGTGGCCTTGGTGAGCCGTTTACCTCACCAACTAGCTTAATTGCACCGCGGGTTCCATCCATCAGCGACA
CCCGAAAGCGCCTTTTTTTCACTCTTTTATTGCCATTGCGGCATTAAACTGTTTTATGCGGTATTTTAGCACC
TTGTTTTTTTTTCCAAGTTGTTTTTATTCCCCCCTTCTTGATTGGGTTAGGTTTTACCCACGTTGTTTTTACT
TCACCCGTTCCGCCACTTCCTTCTTTTTTCC
>134
CTGCTGCCGCCCTAGGAGTCTGGGCCGTGTCTCAGTCCCAGTGTGGCCGATCACCCTCTCAGGTCGGCTATGC
ATCGTGGCCTTGGTGAGCCGTTACCTCACCAACTAGCTAATGCACCGCGGGTCCATCCATCAGCGACACCCGA
AAGCGCCTTTTCACTCTTTATGCCATGCGGCATAAACTGTTTATGCGGTATTTAGCACCTGTTTTTTTCCAAG
TGTTTTACTCCCTCTGATGGGTAGGCTACCCACGTGTTTTACTTCACCCGTCCGCCACTCCTCTTTTTTCCAA
TTTTGAGTGCAAGCACTCGGGAGGGAAGAAGCGTTTCGACTTGCATGTATTTTAGGCACGCCGCCAGCGTTTC
GTCCTGAGCCAGGATCAAACTCCTT
>135
CTGCTGCCGCCCGTAGGAGTCTGGGCCGTGTCTCAGTCCCAGTGTGGCCGATCACCCTCTCAGGTCGGCTATG
CATCGTGGCCTTGGTGAGCCGTTACCTCACCAACTAGCTAATGCACCGCGGGTCCATCCATCAGCGACACCCG
AAAGCGCCTTTTCACTCTTTATGCCATGCGGCATAAACTGTTTATGCGGTATTTTAGCACCTGTTTTTTCCAA
GTCGTTTTATCCCCCTCTGATGGGTAGGTTTACCCACGTGTTTTACTTCACCCGTCCGCCACTCCTCTTTTTC
CAATTTTGAGTGCAAGCACTCGGGAGGAAAGAAGCGTTCGGTAC
>136
CTGCTGCCGCCCTAGGAGTCTGGGCCGTGTCTCAGTCCCAGTGTGGCTGGTCGTCCTCTCAAACCAGCTACGG
ATCGTCGCCTTGGTGAGCCATTACCTCACCAACTAGCTAATCTGATATCGGCCGCTCCAATAGTGAGAGGTCT
TTGCGATCCCCCCCTTTTCCCCCGTAGGGCGTATGCGGTATTTAGCTACGCTTCGCTCGTAGTTTATCCCCCG
CTACTGGGCACGTTCCGATACATTTACTCACCCGTTTCGCCACTCGCCATCCAGAGAGCAAGCTCTCTGATGC
TGCCGTTTCGACTTGCGATGTAGTA
>137
CTGCTGCCGCCCGTAGGAGTCTGGGCCGTGTCTCAGTCCCAGTGTGGCCGATCACCCTCTCAGGTCGGCTATG
CATCGTGGCCTTGGTGAGCCGTTACCTCACCAACTAGCTAATGCACCGCGGGTCCATCCATCAGCGACACCCG
AAAGCGCCTTTCACTCTTATGCCATGCGGCATAAACTGTTTATGCGGTATTTAGCACCTGTTTTTCCAAGTCG
TTTATCCCCCTCTGATGGGTAGGTTTACCCACGTGTTTACTCACCCGTCCGCCACTCCTCTTTCCAATTTGAG
TGCAAGCACTCGGGAGGAAAGAAGACG
>138
CTGCTTGCCGCCCGTAGGGGTCTGGGCCGTGTCTCAGTCCCAGTGTGGCTGGTCATCCTCTCAGACCAGCTAG
AGATCGTCGGCTTGGTGAGCCTTTACCTCACCAACTACCTAATCCCACTTGGGCTCATCCTATGGCATGTGGC
CCGAAGGTCCCACACTTTTAATCTTTTCGATATTTACGCGGTATTTTAGCTACAGTTTTTCCCGTACGTTTAT
TCCCCCTCCATAAGCCAGATTTCCCAAGCATTTACTCACCCGTTCCGCCACTCGTCATTCAAAGAAGCAAGCT
TTCTCTTATTGTTTTACCGTTTTCGACTTTTGCATTGTGTTTTAAGCCTTGCCGCCAGCGTTTCAATCTG
>139
CTGCTGCCGCCCGTAGGAGTCTGGGCCGTGTCTCAGTCCCAGTGTGGCTGGTCGTCCTCTCAAACCAGCTACG
GATCGTCGCCTTGGTGAGCCATTACCTCACCAACTAGCTAATCCGATATCGGCCGCTCCAATAGTGAGAGGTC
TTTGCTCGATCCCCCCCTTTTCCCCCCGTAGGGCGTATGCGGTATTTAGCTACGCTTTCGCTCGTAGTTTATT
CCCCCGCTACTGGGCACGTTCCGATTACATTTACTTCACCCGTTTCGCCACTCGCCATCCAGAGAGCAAGCTC
TCTTGATGCTTGCCGTTTCGACTTGCAT
>140

CTGCTTGCCGCCCGTAGGAGTCTGGGCCGTGTCTCAGTCCCAGTGTGGCCGATCACCCTCTTCAGGTCGGCTA
TGCATTCGTGGCCTTGGTTGAGCCGTTTACCTCACCAACTAGCTTAATTGCACCGCGGGTCCATCCATTCAGC
GACACCCGAAAGCGCCTTTTTTCACTTCTTTTTATTTGCCATTGCGGCATTAAAAACTTGTTTTTTATTTGCG
GTTATTTTTTAGCACCTTTTGTTTTTTCCAAAAGTTGTTTTTTATTTCCCCCCTTTCTTGATTTGGGGGTTAG
GTTTTTACCCCCACGTGTTTTTACTTTACCCCCGTTCCCGC
>141
CTGCTGCCGCCCGTAGGAGTCTGGGCCGTGTCTCAGTCCCAGTGTGGCCGATCACCCTCTCAGGTCGGCTATG
CATTCGTGGCCTTGGTGAGCCGTTACCTCACCAACTAGCTAATGCACCGCGGGTCCATCCATCAGCGACACCC
GAAAGCGCCTTTTCACTCTTTATTGCCATTGCGGCATTAAACTGTTTTATGCGGTATTTTAGCACCTTGTTTT
TTTTCCAAGTTGTTTATTCCCCCCCTTCTGATTGGGTTAGGTTTACCCACGTTGTTTTACTTCACCCGTTCCG
CCACTCCTCTTTTC
>142
CTGCTGCCGCCCGTAGGAGTCTGGGCCGTGTCTCAGTCCCAGTGTGGCCGATCACCCTCTCAGGTCGGCTATG
CATCGTGGCCTTTGGTGAGCCGTTACCTCACCAACTAGCTTAATGCACCGCGGGTCCATCCATTCAGCGACAC
CCCGAAAAAGCGCCTTTTTCACTTCTTTTATTGCCATTGCGGCATTAAAAACTTGTTTTTTATTGCGGTTATT
TTTAGCACCCTTTGTTTTTCCCAAAAGTTGTTTTTATTTCCCCCTTCTTGATTTTGGGGTTAGGGTTTTACCC
CACGTGTTTT
>143
CTGCTGCGCCCGTAGGAGTCTGGGCCGTGTCTCAGTCCCAGTGTGGCCGATCACCCTCTCAGGTCGGCTATGC
ATCGTGGCCTTGGTGAGCCGTTACCTCACCAACTAGCTTAATGCACCGCGGGTCCATCCATCAGCGACACCCG
AAAGCGCCTTTTCACTCTTTATTGCCATTGCGGCATTAAAACTTGTTTTATTGCGGTATTTTAGCCACCTTGT
TTTTTTCCAAGTCGTTTTATTCCCCCTCTGATTGGGTTAGGTTTACCCCACGTGTTTTACTTCACCCGTTCCG
CCACTCCTCTTTTCCAATTTGAGTTGCAAGCACTCGGGAGGAAAGAAGCGTTTCGTCGTTGA
>144
CTGCTGCCGCCCGTAGGAGTCTGGGCCGTGTCTCAGTCCCAGTGTGGCTGGTCATCCTCTCAGGTCGGCTATG
CATTCGTGGCCTTGGCGAGCCGTTTACCTCACCAACTAGCTTAATTGCACCGCGGGTCCATCCATTCAGCGAC
ACCCGAAAGCGCCTTTTTCACTTCTTTTTATTGCCATTGCGGCATTAAAAACTTGTTTTTTATTGCGGTTATT
TTTTTAGCACCTTTGTTTTTTTCCAAAAGTTGTTTTTTTATTCCCCCCCTTCTTGATTGGGGGGTTAGGTTTT
TTACCCCCCACGTTGTTTTTTACTTTACCCCCGTTCCCGCCCCATTCCCTCTTTTTTCCATTTTTGAGTTGCA
AGACTC
>145
CTGCTGCCGCCCGTAGGAGTCTGGGCCGTGTCTCAGTCCCAGTGTGGCTGGTCGTCCTCTCAAACCAGCTACG
GATCGTCGCCTTGGTGAGCCACTTACCTCACCAACTAGCTAATCCGATATCGGCCGCTCCAATAGTGAGAGGT
CTTTGCGATCCCCCCCCCTTTTCCCCCCGTAGGGCGTATGCGGTATTTAGCTACGCTTTCGCGTAGTTTATCC
CCCGCTACTGGGCACGTTCCGATTACATTTACTCACCCGTTTCGCCACTCGCCATCCAGAGAGCAAGCTCTAC
TGATCGTGCT
>146
CTGCTGCCGCCCGTAGGAGTCTGGGCCGTGTCTCAGTCCCAGTGTGGCCGATCACCCTCTCAGGTCGGCTATG
CATCGTGGCCTTTGGTGAGCCGTTACCTCACCAACTAGCTTAATGCACCGCGGGTCCATCCATCAGCGACACC
CGAAAGCGCCTTTTCACTCTTTATTGCCATTGCGGCATTAAACTGTTTTATTGCGGTATTTTAGCACCTTGTT
TTTTCCAAGTCGTTTTATTCCCCCCCTCTGATTGGGTTAGGTTTACCCACGTGTTTACTTCACCCGTTCCGCC
ACTCCTCTTTTCCAATCGA
>147
CTGCTGCCGCCCGTAGGAGTCTGGGCCGTGTCTCAGTCCAGTGTGGCCGATCACCCTCTCAGGTCGGCTATGC
ATCGTGGCCTTGGTGAGCCGTTACCTCACCAACTAGCTAATGCACCGCGGGTCCATCCATCAGCGACACCCGA
AAGCGCCTTTCACTCTTTATGCCATGCGGCATAAACTGTTTATTGCGGTATTTAGCACCTTGTTTTTTCCAAG
TCGTTTTATCCCCCTCTGATTGGGTAGGTTTACCCACGTGTTTACTTCACCCGTCCGCCACTCCTCTTTTCCA
ATTTTGAGTGCAAGCACTCGGGAGAAAGAAGA
>148
CTGCTGCCGCCCGTAGGAGTCTGGGCCGTGTCTCAGTCCCAGTGTGGCCGATCACCCTCTCAGGTCGGCTATG
CATCGTGGCCTTGGTGAGCCGTTACCTCACCAACTAGCTAATGCACCGCGGGTCCATCCATCAGCGACACCCG
AAAGCGCCTTTTCACTCTTTATGCCATGCGGCATAAACTGTTTTATGCGGTATTTTAGCCACCTTGTTTTTTC
CAAGTCGTTTTATTCCCCCTCTGATGGGTAGGTTTTACCCACGTGTTTTACTCACCCGTTCCGCCACTCCTCT

TTTCCAATTTTGAGTTGCAAGCACTCGGGAGGAAAGAAGCGTTTCGTACTTGCATGTATTTTAGCACGCCGCC
AGCGTTTCGTTCCTGAGCCAGCATCGA
>149
CTGCTGCCGCCCGTAGGAGTCTGGGCCGTATCTCAGTCCCAATGTGGCGCACACCCTCTCAGGCCGGCTACCC
GTCGCCGCCTTGGTAGCCATTACCCCACCAACAAGCTGATAGGCCGCAAGCTCATCCCACACCAGAAAAACCT
TTTCCACCACACCAACCACGATGCGGTCCTATAGGGTTATTTAGACCCAGTTTTTCCCAAGCTTTTATTCCCC
CAGTGCAGGGCAGATCACTTTTACGTTGTTTACTCACCCGTTTTCGCCACTCGAGCACCCCCAGCAAGCTGGG
GCCTTTTTTCCGTTTTCGACTTTGCATTGGTTGTTTTTTAAGCACGCCGCCAGCGTTTCATCCTG
>150
CTGCTGCCGCCCGTAGGAGTCTGGGCCGTGTCTCAGTCCCAGTGTGGCCGATCACCCTCTCAGGTCGGCTATG
CATCGTGGCCTTGGTGAGCCGTTACCTCACCAACTAGCTAATGCACCGCGGGTCCATCCATCAGCGACACCCG
AAAGCGCCTTTTCACTCTTTATTGCCATGCGGCATAAACTGTTTTATTGCGGTACTTTAGCACCTTGTTTTTT
CCAAGTTCGTTTTATTCCCCCTCTGATTGGGTAGGTTTTACCCCACGTGTTTTACTTCACCCGTTCCGCCACT
TCCTCTTTTTCCAATTTTGAGTTGCAAGCACTCGGGAGGAAAGAAGCGTTTTCGACTTGCATGTATTTTAGGC
ACGCCGCCAGCGTTTCGTTCCTGAGCCAGGAT
>151
CTGCTGCCGCCCGTAGGAGTCTGGGCCGTGTCTCAGTCCCAGTGTGGCTGGTCATCCTCTCAGACCAGCTAGA
GATCGTCGGCTTGGGTGAGCCTTTACCTCACCAACTACCTAATCCCACTTGGGCTCATCCTATGGCATGTGGC
CCGAAGGTCCCACACTTTAATCTTTTCGATATTACGCGGTATTTAGCTACAGTTTTTCCCGTAGTTAGCCCCC
TCCATAAGCCAGATTTCCCAAGCATTTACTCACCCGTCCGCCACTGTCATCAAAGAAGCAAGCTTTTCTTTTT
AGTACCGTTTCGTACTTGCATGTG
>152
CTGCTGCCGCCTGTAGGAGTCTGGGCCGTGTCTCAGTCCCAGTGTGGCCGATCACCCTCTCAGGTCGGCTATG
CATCGTGGCCTTGGTGAGCCGTTACCTCACCAACTAGCTAATGCACCGCGGGTCCATCCATCAGCGACACCCG
AAAGCGCCTTTTCACTCTTTATGCCATGCGGCATAAACTGTTTATGCGGTATTTTAGCACCTGTTTTTTCCAA
GTGTTTTATCCCCCCTCTGATGGGTAGGTTTTACCCACGTGTTTTACTCACCCGTCCGCCACTCCTCTTTTTC
CAATTTTGAGTGCAAGCACTCGGGGAGGAAAGAAGCGTTCGTACTTGCATGTATTTTAGGCACGCCGCCAGCG
TTCGTCCCT
>153
CTGCTGCCGCCCGTAGGAGTCTGGGCCGTGTCTCAGTCCCAGTGTGGCTGGTCATCCTCTCAGACCAGCTAGA
GATCGTCGGCTTGGTGAGCCTTTACCCCACCAACTACCTAATCCCACTTGGGCTCATCCTATGGCATGTGGCC
CGAAGGTCCCACACTTTTAATCTTTTCGATATTTACGCCGGTATTTAGCTACAGTTTTCCCGTAGTTTATCCC
CCTCCATTAAGCCAGATTTCCCAAGCATTTACTCACCCGTCCGCCACTCGTCATTCAAAGAAGCAAGCTTTCT
CTTATTGTTTTACCGTTTCGACTTTGCATTGTGTTTAAGCCTGCCGCCAGCGTTTCAATCTGAGCCAGGATCA
AACTCT
>154
CTGCTGCCGCCCGTAGGAGTCTGGGCCGTGTCTCAGTCCCAGTGTGGCCGATCACCCTCTCAGGTCGGCTATG
CATCGTGGCCTTTGGTGAGCCGTTACCTCACCAACTAGCTAATGCACCGCGGGTCCATCCATCAGCGACACCC
GAAAGCGCCTTTCACTCTTTATGCCATGCGCATAAACTGTTTATGCGGTATTTAGCCACCTTGTTTTTTCCAA
GTCGTTTATCCCCCTCTGATGGGTAGGTTTACCCACGTGTTTACTCACCCGTCCGCCACTCCTCTTTCCAATT
TGAGTGCAAGCACTCGGGAGGAAAGAAGTCGTTCGTACTTGACATGTATTTTAGG
>155
CTGCTGCCGCCCGTAGGAGTCTGGGCCGTGTCTCAGTCCCAGTGTGGCCGATCACCCTCTCAGGTCGGCTATG
CATCGTGGCCTTGGTGAGCCGTTACCTCACCAACTAGCTAATGCACCGCGGGTCCATCCATCAGCGACACCCG
AAAGCGCCTTTTCACTCTTTATGCCATGCGGCATAAACTGTTTATGCGGTATTTAGCACCTGTTTTTCCAAGT
CGTTTATCCCCCTCTGATGGGTAGGTTTACCCACGTGTTTACTCACCCGTCCGCCACTCCTCTTTTCCAATTT
TGAGTGCAAGCACTCGGGAGGAAAGAAGCGTTCGTACTTGACATGTATTTTAC
>156
CTGCTGCCGCCCTAGGAGTCTGGGCCGTGTCTCAGTCCCAGTGTGGCTGGTCATCCTCTCAGACCAGCTAGAG
ATCGTCGGCTTTGGTGAGCCTTTACCTCACCAACTACCTAATTCCCACTTGGGCTTCATCCTATTGGCATGTG
GCCCGAAGGTCCCACACTTTTAATCTTTTCGATATTTTACGCGGTTATTTAGCTACAGTTTTTCCCGTAGTT
TATTCCCCCCTCCATTAAGCCAGATTTTCCCCCAAGCATTTTACTTCACCCGTTCCGCCACTTCGTCATTCAA
AGAAAGCAAGCTTTTCTTCTTATTGTTTTACCGTTTTCGACTTTTGCATTGTGTTTTAAGCCTTGCCGCCAGC
GTTTTCAATTCTGAGCCAGGATT

>157
CTGCTGCCGCCCTAGGAGTCTGGGCCGCGTCTCAGTCCCAGTGTGGCTGGTCATCCTCTCAGACCAGCTAGAG
ATCGTCGGCTTGGTGAGCCTTTACCTCACCAACTACCTAATTCCCACTTGGGCTTCATCCTATTGGCATGTGG
CCCGAAGGTTCCCACACTTTTAATCTTTTCGATATTTACGCGGTATTTTAGCTACAGTTTTCCCGTACGTTTA
TTCCCCCCTCCCATTAAGCCAGATTTCCCCCAAGCATTTACTTCACCCGTTCCGCCACTTCCTCTTTTCCAAT
CGAGTGCAAGCACTCGGGAGGAAAGAAGCGTTTCGACTTGCATTGTATTTTAGGCACGCCGCCAGCGTTTCGT
TCCTGAGCCAGGATTCA
>158
CTGTTGCCGCCCGTAGGAGTCTGGGCCGTGTCTCAGTCCCAGTTGTGGCTGGTCGTTCCTCTCAAACCAGCTA
CGGATCGTCGCCTTGGTGAGCCATTACCTCACCAACTAGCTAATCCGATATCGGCCGCTCCAATAGTGAGAGG
TCTTGCGATCCCCCCCTTTTCCCCCCGTAGGGCGTATGCGGTATTTAGCTACGCTTTCGCGTAGTTATCCCCG
CTTACTTCGGGCACGTTCGATACATTACTTCACCGTTCGCCACTCGCCATCCAGAGAGCAAGCTCTCTGATGC
TTGCGTTCGACTTGCATGTGTTAAGGCATCCCGCTAGCGTTCAATCTGAGCCAGATTCAAACTCT
>159
CTGCTGCCGCCCGTAGGAGTCTGGGCCGTGTCTCAGTCCCAGTGTGGCCGATCACCCTCTCAGGTCGGCTATG
CATCGTGGCCTTTGGTGAGCCGTTACCTCACCAACTAGCTAATGCACCGCGGGTCCATCCATCAGCGACACCC
GAAAGCGCCTTTTCACTCTTTATGCCATGCGGCATAAACTGTTTATGCGGTATTTAGCACCTGTTTTTTCCAA
GTGTTTTATCCCCCTCTGATGGGTAGGTTTTACCCACGTGTTTTACTCACCCGTCCGCCACTCCTCTTTTCCA
ATCGAGTGCAAGCACTCGGGAGGAAAGAAGCGTTCGACTTGCATGTATTTTAGGCACGCCGCCAGCGTTCGTC
CTGTAGCCAAGGCATCGAAACAATCTTT
>160
CTGCTGCCGCCCGTAGGAGTCTGGGCCGTGTCTCAGTCCCAGTGTGGCTGGTCGTCCTCTCAAACCAGCTACG
GATCGTCGCCTTGGTGAGCCATTTACCTCACCAACTAGCTAATCCGATATCGGCCGCTCCAATAGTGAGAGGT
CTTGCGATCCCCCCCTTTCCCCCGTAGGGCGTATGCGGTATTTAGCTACGCTTCGCGTAGTTTATCCCCCGCT
ACTGGGCACGTTCCGATACATTACTCACCCGTTCGCCACTCGCCATCCAGAGAGCAAGCTCTCTGATGCTGCC
GTTCGACTTGCATGTGTAAGGTCCATCC
>161
CTGCTGCCGCCCGTAGGAGTCTGGGCCGTGTCTCAGTCCCAGTGTGGCTGGTCATCCTCTCAGACCAGCTAGA
GATCGTCGGCTTGGTGAGCCTTTACCCCCCACCAACTAGCTAATTGCACCGCGGGTCCATCCATCAGCGACAC
CCCCGAAAGCGCCTTTTTCACTCTTTATGCCATGCGCATAAACTTGTTTTATGCGGTATTTTAGCACCTTGTT
TTTTTCCAAGTTGTTTTTATTCCCCCCCCCCTTCTTGATTGGGTTAGGTTTTACCCCCCACGTTGTTTTACTT
CACCCCCGTTCCCCGCCACTTCCTTCTTTTTTCCAATTTTGAGTTGCAAGCACTCGGGAGGAAAGAAGCGTTT
TCGACTTTGCATTGTATTTTAGGCACGCCGCCA
>162
CTGCTGCGCCCGTAGGAGTCTGGGCCGTGTTTCAGTCCCAGTGTGGCTGGTCATCCTCTCAGACCAGCTAGAG
ATCGTCGGCTTTGGGTGAGCCTTTACCTCACCAACTACCTAATCCCACTTGGGCTCATCCTATGGCATGTGGC
CCGAAGGTCCCACACTTTTAATCTTTTCGATATTTACGCGGTATTTAGCTACAGTTTTTCCCGTAGTTTATCC
CCCTCCATAAGCCAGATTTCCCAAGCATTTACTCACCCGTCCGCCACTCGTCATCAAAGAAGCAAGCTTTTCT
CTATGTTTTACCGTTTTCGACTTTTGCAGTGTTTTAAGCCTGCCGCCATACGTTTTCAATCTGAGCCAGGATC
AAACTC
>163
CTGCTGCCGCCCGTAGGAGTCTGGGCCGTGTCTCAGTCCCAGTGTGGCCGATCACCCTCTCAGGTCGGCTATG
CATTCGTGGCCTTGGTGAGCCGTTACCTTCACCAACTAGCTTAATGCACCGCGGGGGTCCATTCCATTCAGCG
ACACCCCCCGAAAAAGCGCCTTTTTCACTTCTTTTTATTTGCCATTGCGGCATTAAAAAACTTGTTTTTTTAT
GCGGTTATTTTTTAGCACCCCTTTGTTTTTTCCCCAAAAGTTGTTTTTTTATTCCCCCCCTTCTTGATTGGGG
GGGTTAGGTTTTTACCCCCCCACGTTGTTTTTTTACTTCACCCC
>164
CTGCTGCCGCCCGTAGGAGTCTGGGCCGTGTCTCAGTCCCAGTGTGGCTGGTCGTCCTCTCAAACCAGCTACG
GATCGTCGCCTTGGTGAGCCATTACCTCACCAACTAGCTAATCCGATATCGGCCGCTCCAATAGTGAGAGGTC
TTGCGATCCCCCCCTTTTCCCCCGTAGGGCGTATGCGGTATTAGCTACGCTTCGCGTAGTTATCCCCCGCTAC
TGGGCACGTTCCGATACATTACTCACCCGTTCGCCACTCGCCATCCAGAGACGCAAGCTCTCTGATGCTGCCG
TTCGACTTGCATGTGTTAAG
>165

CTGCTTGCCGCCCGTAGGAGTCTGGGCCGTGTCTCAGTCCCAGTGTGGCCGATCACCCTCTCAGGTCGGCTAT
GCATTCGTGGCCTTTGGTGAGCCGTTACCTCACCAACTAGCTTAATTGCACCGCGGGTTCCATCCATTCAGCG
ACACCCGAAAGCGCCTTTTTTCACTTCTTTTATTTGCCATTGCGGCATTTAAAAAACTTGTTTTTTATTGCG
GTTATTTTTTTAGCACCTTTGTTTTTTCCCCAAAAGTTTGTTTTTTATTTCCCCCCCTTTCTTGATTGGGGGG
TTAGGTTTTTTACCCCCCAC
>166
CTGCTGCCGCCCGTAGGAGTCTGGGCCGTGTCTCAGTCCCAGTGTGGCCGATCACCCTCTCAGGTCGGCTATG
CATTCGTGGCCTTGGTGAGCCGTTACCCTCACCAACTAGCTTAATGCACCGCGGGTTCCATCCATTCAGCGAC
ACCCCCCGAAAAAGCGCCTTTTTTCACTTCTTTTATTGCCCATTGCGGCATTAAAAACTTGTTTTTATTGCG
GTTATTTTTAGCACCCCTTTGTTTTCCCCAAAAGTTTGTTTTTATTTCCCCCTTTCTTGATTTGGGGTTTAGG
GTTTTACCCCACGTTGTTTTACTTTCACCCCGTTTCC
>167
CTGCTGCCGCCCGTAGGAGTCTGGGCCGTGTCTCAATCCCAGTGTGGCCGATCACCCTCTCAGGTCGGCTATG
CATCGTGGCCTTGGTGAGCCGTTACCTCACCAACTAGCTTAATGCACCGCGGGTCCATCCATCAGCGACACCC
CGAAAGCGCCTTTTCACTCTTTATTGCCATTGCGCATTAAACTGTTTTATTGCGGTATTTTAGCACCTTGTTT
TTTTCCAAGTCGTTTTATCCCCCACTTGATGGGTTAGGTTTTACCCACGTTGTTTTACTTCACCCGTCCGCCT
ACATCCTCTTTTTCCAATTCGAGTGCAAGCACTTTCGGGAGGAAAGAAGCGTTTTCGACTTGCAATTGT
>168
CTGCTGCCGCCCGTAGGAGTCTGGGCCGTGTCTCAGTCCCAGTGTGGCCGATCACCCTCTCAGGTCGGCTATG
CATCGTGGCCTTGGTGAGCCGTTACCTCACCAACTAGCTAATGCACCGCGGGTCCATCCATCAGCGACACCCG
AAAGCGCCTTTTCACTCTTTATGCCATGCGGCATAAACTGTTTTATGCGGTATTTAGCACCTGTTTTTTCCAA
GTCGTTTTATCCCCCCCTCTGATGGGTAGGTTTTACCCACGTGTTTTACTCACCCGTCCGCCACTCCTCTTTT
CCAATTTGAGTGCAAGCACTCGGGAGGAAAGAAGCGTTCGTACTTGCATGTATTTTAGGCACGCCGCCAGCGT
TCGTCCTGTAGCCAGGCA
>169
CTGCTGCCGCCCGTAGGAGTCTGGGCCGTGTCTCAGTCCCAGTGTGGCCGATCACCCTCTCAGGTCGGCTATG
CATCGTGGCCTTGGTGAGCCGTTACCTCACCAACTAGCTAATGCACCGCGGGTCCATCCATCAGCGACACCCG
AAAGCGCCTTTTCACTCTTTATGCCATGCGGCATAAACTGTTTATGCGGTATTTTAGCACTTTGTTTTTTCCA
AGTGTTTATCCCCCCCTCTGATTGGGTAGGTTTTACCCACGTGTTTACTTCACTCGTCCGCCTACTCCTCTTT
TTCCAATTTTGAGTGACAAGCACTCGGGAGGAAAGAAGCGTTTTCGACTTCGCATGTTATTTTAGGCACGCCG
CCAGCGTTTCGTCCTTGAGCCAGGCATCAAACATCCTTT
>170
CTGCTGCCGCCCGTAGGAGTCTGGGCCGTGTCTCAGTCCCAGTGTGGCTGGTCGTCCTCTCAAACCAGCTACG
GATCGTCGCCTTTGTGAGCCATTACCTCACCAACTAGCTAATCCGATATCGGCCGCTCCAATAGTGAGAGGTC
TTGCGATCCCCCCCTTTCCCCCGTAGGGCGTATGCGGTATTAGCTACGCTTTCGCTCGTAGTTATCCCCCGGC
TACTGGGCACGTTCCGATACATTACTCACCCGTTCGCCACTCGCCATCCAGAGAGCAAGCTCTCTGATGCTGC
CGTTCGACTTGCATGTGTAAGGCATCCCGTCTAGCGTTCAAATCTGAGCCAGGATCAAAACTC
>171
CTGCTGCCGCCCGTAGGAGTAAGGGCCGTGTCTCAGTCCCCTTTGTGGCCGATCACCCTCTCAGGCCGGCTAC
CCATCATCGCCTTGGTGAGCCGTTTACCTCTTCCAACTAGCTTAATGGGACGCAAAGCTTCTCTCACAGCGCA
TATAGCCTTTCATAACCTAGGATGCCCTAAAGTCATAATATCAGGTATTTTAGCATTTTCGTTTTTTCCAAAT
GTTTTGTCCCTATCTGTGAGGCAAGTTTTCTTTTTTTTACGCGTTTTACTCACCCGTCCGCCACCCAAACCGA
AGTTTTCAAGTTTTGACTTTTTGC
>172
CTGCTGCCGCCCGTAGGAGTCTGGGCCGTGTCTCAGTCCCAGTGTGGCCGATCACCCTCTCAGGTCGGCTATG
CATCGTGGCCTTTGGTGAGCCGTTACCTCACCAACTAGCTAATGCACCGCGGGTCCATCCATCAGCGACACCC
GAAAGCGCCTTTTCACTCTTTATTGCCATTGCGGCATTAAAACTTGTTTTATTGCGGTTATTTAGCACCTTGT
TTTTTCCAAGTGTTATTCCCCCCCTTCTGATTGGGTTAGGTTTACCCACGTGTTTTACTTCACCCGTTCCGCC
ACTCCTTC
>173
CTGCTGCGCCCGTAGGAGTCTGGGCCGTGTCTCAGTCCCAGTGTGGCCGATCACCCTCTCAGGTCGGCTATGC
ATTGTGGCCTTTGGTGAGCCGTTACCTCACCAACTAGCTAATCCGATATCGGCCGCTCCAATAGTGAGAGGTC
TTTACGATCCCCCCCTTTCCCCCGTAGGGCGTATGCGGTATTTAGCTTACGCTTTCGCGTAGTTATCCCCCGC

TACTTGGGCACGTTTCCGATACATTTACTCACCGTTTCGCCACTCGCCATCCAGAGAGAAGCTCTCATGATGC
TGACTGTC
>174
CTGCTGCCGCCCGTAGGAGTCTGGGCCGTGACTCAGTCCCAGTGTGGCCGATCACCCTCTCAGGTCGGCTATG
CATCGTGGCCTTGGTGAGCCGTTTACCTCACCAACTAGCTAATGCACCGCGGGTCCATCCATCAGCGACACCC
GAAAGCGCCTTTTCACTCTTTATGCCATGCGGCATAAACTGTTTATGCGGTATTTTAGCACCTGTTTTTTCCA
AGTCGTTTATCCCCCTCTGATGGGTAGGTTTTACCCACGTGTTTTACTTCACCCGTCCGCCACTCCTCTTTTT
CCAATTTTGAGTGCAAGCACTCGGGAGGAAAGAAGCGTTTCGACTTGCATGTATTTTAGGCACGCCGCCAGCG
TTCGCCTGAGCCAGGATCGAAACATCCT
>175
CTGCTGCCGCCCGTAGGAGTCTGGGCCGTGTCTCAGTCCCAGTGTGGCCGATCACCCTCTTCAGGTCGGCTAT
GCATTCGTGGCCTTGGTGAGCCATTTACCTCACCAACTAGCTAATTGCACCGCGGGTCCATCCATCAGCGACA
CCCGAAAGCGCCTTTTTCACTTCTTTTATGCCATGCGGCATAAACTTGTTTTATTGCGGTATTTTAGCACCTT
GTTTTTTCCAAGTTGTTTTATTCCCCCTTCTGATTGGGTTAGGTTTTACCCACGTTGTTTTACTTCACCCGTT
CCGCCACTTCCTTCTTTTTCCAATTTTGAGTTGCAAGCACTCGGGAGGAAAGAAGCGTTTTCGACTTGACATT
GT
>176
CTGCTGCCGCCCTAGGAGTCTGGGCCGTGTCTCAGTCCCAGTGTGGCTGGTCGTCCTCTCAAACCAGCTACGG
ATCGTCGCCTTGGTGAGCCATTTACCTCACCAACTAGCTAATCCGATATCGGCCGCTCCAATTAGTGAGAGGT
CTTTGCGATCCCCCCCCCTTTTCCCCCCGTAGGGCGTATGCGGTATTTTAGCTACGCTTTTCGCGTAGTTTTA
TTCCCCCCCCGCTACTTGGGCACGTTTCCGATTACATTTACTTCACCCGTTTCGCCACTCGCCATTCCAGAGA
GCAAGCTCTCTTGATTGCTTGCCGTTTTCGACTTTGCATTGTGTTAAGGCCGTCATT
>177
CTGCTGCCGCCCGTAGGAGTCTGGTCCGTGTCTCAGTACCAGTGTGGGGGATCACCCTCTCAGGCCCCCTAAA
GATCGTAGACTTGGTTGAGCCATTACCTCACCAACTATCTAATCTTGCGCGTGCCCATCTCTATCCACCGGAG
TTTTCAATAAAAAACGATGCCGTCTCTTTATATTTATGGGGTATTTAATTCTTTCCTTTTCGAGAGGCTATCC
CCCACGAGATAAAGGCAGGTTTTCGCACACGTGTTTCCGCACCCGTACGCCGCTCTTCTTGGTTTCCCGAAAC
GAACCATTACCGCCTTCGGCTTTGCATTGTGTTTAGGCCTTCCCGCTTAGCGTTTCATTCCTTGAGCCAGGAT
CAAACTCTT
>178
CTGCTTGCCGCCCGTAGGAGTCTGGGCCGTGTCTCAGTCCCAGTGTGGCCGATCACCCTCTCAGGTCGGCTAT
GCATCGTGGCCTTTGGTGAGCCGTTACCTCACCAACTAGCTAATGCACCGCGGGTCCATCCATCAGCGACACC
CGAAAGCGCCTTTTCACTCTTTATTGCCATTGCGGCATTAAAACTTGTTTATTGCGGTTATTTTAGCACCTTG
TTTTTTTCCAAGTTCGTTTTATTCCCCCCCTTCTTGATTGGGTTAGGTTTTACCCCCACGTTGTTTTACTTCA
CCCGTTCCGCCACTTCCTCTTTTCCAATTTTGAGTTGCAAGCACTCGGGAGGAAAGAAG
>179
CTGCTGCCGCCCTAGGAGTCTGGGCCGTGTCTCAGTCCCAGTGTGGCTGGTCATCCTCTCAGACCAGCTAGAG
ATCGTCGGCTTTGGTGAGCCTTTACCTCACCAACTACCTAATCCCACTTGGGCTCATCCTATTGGCATGTGGC
CCGAAGGTCCCACACTTTTAATCTTTTCGATATTTACGCGGTTATTTAGCTACAGTTTTCCCGTAGTTTATTC
CCCCCTCCCATTAAGCCAGATTTCCCAAGCATTTACTTCACCCGTCCGCCACTCGTTCATCAAAGAAGCAAGC
TTTTCTTTTTTATTGTTTACCGTTTTCGACTTTGCATTGTTGTTTTAAGCC
>180
CTGCTGCCGCCCGTAGGAGTCTGGGGCCGTGTCTTCAGTCCCAGTTGTGGCCGATCACCCTCTTCAGGTTCGG
CTATTGCATTCGTGGCCCTTGGTTGAGCCGTTTACCCCTTCACCAACTAGCTTAATTGCACCCGCGGGGGTTC
CCCATTCCCATTCAGCGACACCCCCCGAAAAAAGCGCCCCTTTTTTCACTTTCTTTTTATTTTTGCCCCCATT
TTGCCGGGCCATTTTAAAACTTTTGTTTTATTTTGCCGGGGTTTTATTTTAAGCCCAACCCCTTTTGGTTTCC
CCAAAAGTT
>181
CTGCTGCCGCCCGTAGGAGTCTGGGGCCGTGTCTCAGTCCCAGTGTGGCCGATCACCCTCTCAGGTCGGCTAT
GCATCGTGGCCTTGGTGAGCCGTTACCTCACCAACTAGCTAATGCACCGCGGGTCCATCCATCAGCGACACCC
GAAAGCGCCTTTTCACTCTTTATGCCATGCGGCATAAACTGTTTATGCGGTATTTTAGCACCTGTTTTTTCCA
AGTCGTTTTATCCCCCTCTGATGGGTAGGTTTTACCCCCACGTGTTTTACTTCACCCGTCCGCCACTCCTCTT
TTTCCAATTTTGAGTGCAAGCACTCGGGAGGAAAGAAGCGTTTCGACTTGCATGTATTTTAGGCACGCCGCCA
GCGTTCGCCTGAGCCAGGATCAAACTCT

>182
CTGCTGCCGCCCGTAGGAGTCTGGGCCGTATCTCAGTCCCAATGTGGCGCACACCCTCTCAGGCCGGCTACCC
GTCGCCGCCTTGGTAGCCATTACCCCACCAACAAGCTGATAGGCCGCAAGCTCATCCCACACCAGAAAAACCT
TTCCACCACACCAACCACGATGCGGTCCTATGGGGTATTAGACCCAGTTTCCCAAGCTTTTATCCCCCCAGTG
CAGGGCAGATCACTTACGTGTTACTCACCCGTTCGCCACTCGAGCACCCCCAGCAAGCTGGGGCCTTCCCGTT
CGACTTGCATGTGTTTTAAGCACGCCGCCAGCGTTCATCCTGAGCCAGGATCAAACTCT
>183
CTGCTGCCGCCCGTAGGAGTCTGGGCCGTGTCTCAGTCCCAGTTGTGGCTTGGTCATCCTTCTTCAGACCAGC
TTAGAGATTCGTTCGGCTTTGGTTGAGCCTTTTACCTCACCAGCTTACCTTAATTCCCACTTGGGCTTCATTC
CTTATGGCATGTGGCCCGAAGGTTCCCACACTTTTAATTCTTTTCGATTATTTACGCGGTTATTTAGCTTAC
AGTTTTTCCCGTTAGTTTATTCCCCCTCCATTAAGCCAGATTTTCCCAAGCATTTACTCACCCGTTCCGCCAC
TTCGTCATTCAAAGAAGCAAGCTTTCTTCTTATTGTTTTACCGTTTTCGACTTTGCATTGTGTTTTAAGCCTT
GCCGCCAGCGTTTTCAATCTGAGCCAGGAT
>184
CTGCTGCCGCCCTAGGAGTCTGGGCCGTGTCTCAGTCCCAGTGTGGCTGGTCATCCTCTCAGACCAGCTAGAG
ATCGTCGGCTTTGGGTGAGCCTTTACCTCACCAACTTACCTAATTCCCACTTTGGGCTTCATTCCTATTGGTT
ATGTGGCCCGAAGGTTCCCCCACACTTTTAATTCTTTTTCGATTATTTACGCGGTATTTTAGCTACAGTTTTT
CCCCCGTTAGTTTTATTCCCCCTTCCATTAAGCCAGATTTTCCCAAGCATTTTACTTCACCCGTTCCCCGCCA
CTTCGTCATTCAAAGAAGCAAGCTTTTCTTCTTATTGTTTTTACCGTTTTCGACTTTTGCATTGTGTTTTTAA
GCCTTGCCGCCAGCGTTTTCAATTCTGAGCCAGGATTCAAACTTCTT
>185
CTGCTGCCGCCCTAGGAGTCTGGGCCGTGTCTCAGTCCCAGTGTGGCCGATCACCCTCTCAGGTCGGCTATGC
ATTCGTGGCCTTGGTGAGCCGTTACCCCACCAACCAGCTAATGCACTGCGGGTCCATCCATCAGCGACACCCG
AAAGCGCCTTTTTCACTCTTTATGCCATGCGGCATAAACTGTTTATGCGGTATTTAGCACCTTGTTTTTTCCA
AGTGTTTTATCCCCCTCTGATGGGGTAGGTTTTACCCACGTGTTTTACTCACCCGTCCGCCACTCCTCTTTTC
CAATTTTGAGTTGCAAGCACTCGGGAGGAAAGAAGCGTTGTACT
>186
CTGCTGCCGCCCGTAGGAGTCTGGGCCGTGTCTCAGTCCCAGTGTGGCCGATCACCCTCTCAGGTCGGCTATG
CATCGTGGCCTTGGTGAGCCGTTACCTCACCAACTAGCTAATGCACCGCGGGTCCATCCATCAGCGACACCCG
AAAGCGCCTTTTTCACTCTTTATTGCCATGCGGCATAAACTGTTTTATGCGGTATTTTAGCACCTTGTTTTTT
TCCAAGTCGTTTTATCCCCCTCTGATGGGTAGGTTTTACCCACGTGTTTTACTTCACCCGTCCGCCACTCCTC
TTTTTCCAATTTTGAGTTGCAAGCACTCGGGAGGAAAGAAGCGTTTCGTACTTGCATGTATTT
>187
CTGCTGCCGCCCGTAGGAGTCTGGGCCGTGTCTCAGTCCCAGTGTGGCCGATCACCCTCTCAGGTCGGCTATG
CATCGTGGCCTTGGTGAGCCGTTTACCTCACCAACTAGCTAATGCACCGCGGGTCCATCCATCAGCGACACCC
GAAAGCGCCTTTTTCACTCTTTATTGCCATTGCGGCATAAACTGTTTTATTGCGGTATTTTAGCACCTTGTTT
TTTTCCAAGTTCGTTTTATTCCCCCTTCTGATTGGGTTAGGTTTTACCCACGTGTTTTACTTCACCCGTTCCG
CCACTCCTCTTTTTCCAATTTTGAGTTGCAAGCACTCGGGAGAAAGAAGCGTTTCGACTTGCATGTATTTTAG
CACGCCGCCAGCGTTTCGTCCTGAGCCAG
>188
CTGCTGCCGCCCGTAGGAGTCTGGGCCGTATCTCAGTCCCAATGTGGCCGCACACCCTCTCAGGCCGGCTACC
CGTCGCCGCCTTGGTAGGCCATTACCCCACCAACAAGCTGATAGGCCGCAAGCTCATCCCACACCAGAAAAAC
CTTTCCACCACACCAACCACGATGCGGTCCTATAGGGTATTAGACCCAGTTTTTCCCAAGCTTTATCCCCCAG
TGCAGGGCAGATCACTTACGTGTTACTCACCCGTTCGCCACTCGAGCACCCCAGCAAGCTGGGGCCTTTTCCG
TTTCGACTTGCATGGTGTTTTAAGCACGCCGCCAGCGTTCATCCTGAGCCAGGATCAAACTCT
>189
CTGCTGCCGCCCTAGGAGTCTGGGCCGTGTCTCAGTCCCAGTGTGGCCGATCACCCTCTCAGGTCGGCTATGC
ATCGTGCCTTGGTGAGCCGTTTACCTCACCAACTAGCTTAATTGCACCGCGGGTCCATCCATCAGCGACACCC
GAAAGCGCCTTTTTTCACTCTTTATGCCATGCGGCATAAACTGTTTTATGCGGTATTTTAGCACCTGTTTTTT
TCCAAGTCGTTTTATCCCCCTCTGATGGGTAGGTTTTACCCACGTGTTTTACTTCACCCGTCCGCCACTCCTC
TTTTTCCAATTTTGAGTTGCAAGCACTCGGGAGGAAAGAAGCGTTTCGACTTGCATGTATTTTTAGGCACGCC
GTCCAGCGTTTCGTCCTGAGCC
>190

CTGCTGCCGCCCGTAGGAGTCTGGGCCGTGTCTCAGTCCCAGTGTGGCCGATCACCCTCTCAGGTCGGCTATG
CATCGTGGCCTTGGTGAGCCGTTTACCTCACCAACTAGCTTAATTGCACCGCGGGTCCATCCATCAGCGACAC
CCGAAAGCGCCTTTTTCACTCTTTTATGCCATGCGGCATAAACTGTTTTATGCGGTATTTTAGCACCTGTTTT
TTTCCAAGTCGTTTTATTCCCCCTCTGATGGGTAGGTTTTACCCACGTGTTTTACTTCACCCGTCCGCCACTC
CTCTTTTTCCAATTTTGAGTTGCAGCA
>191
CTGCTGCCGCCCGTAGGAGTCTGGGCCGTGTCTCAGTCCCAGTGTGGCTGGTCGTCCTCTCGAACCAGCTACG
GATCGTCGCCTTTGGTGAGCCATTACCTCACCAACTAGCTAATTCCGATATCGGCCGCTCCAATTAGTGAGAG
GTCTTTGCTCGATCCCCCCCCTTTTCCCCCGTAGGGCGTATGCGGTTATTTAGCTTACGCTTTCGCGTAGTTT
ATTCCCCCGCTACTTGGGCACGTTTCCGATTACATTTACTTCACCCCGTTTCGCCACTCGCCATTCCAGAGAG
CAAGCTCTCTTGATTGCTTGCCGTTTTCGACTTTGCATTGTGTT
>192
CTGCTGCCGCCCGTAGGAGTCTGGGCCGTGTCTTCAGTCCCAGTGTGGCCGATCACCCTCTTCAGGTCGGCTA
TGCATCGTGGCCTTGGTGAGCCGTTTACCTCACCAACTAGCTTAATTGCACCGCGGGTCCATCCATCAGCGAC
ACCCCGAAAGCGCCTTTTTCACTTCTTTTATTGCCATTGCGGCATTAAACTTGTTTTTATTGCGGTTATTTTT
AGCACCTTGTTTTTTTTTTCCAAGTTTGTTTTTTATTCCCCCCCCCTTCTTGATTGGGTTAGGTTTTTACCCA
CGTTGTTTTTACTTCACCCGTTCCCGCCACTTCCCTTCTTTTTCCAATTTTTGAGTTGCAAGCACTTCGGGAG
GAAGA
>193
CTGCTGCCGCCCGTAGGAGTCTGGGCCGTGTCTCAGTCCCAGTGTGGCTGGTCGTCCTCTCAAACCAGCTACG
GATCGTCGCCTTTGGTGAGCCGTTACCTCACCAACTAGCTAATCCGATATCGGCCGCTCCAATAGTGAGAGGT
CTTGCGATCCCCCCCCTTTTCCCCCGTAGGGCGTATGCGGTATTAGCTACGCTTTCGCTCGTAGTTATCCCCC
GCTACTGGGCACGTTCCGATACATTACTCACCCGTTCGCCACTCGCCATCCAGAGAGCAAGCTCTCTGATGCT
GCCGTTCGACTTGCATGTGTAAAGGCCGATCCC
>194
CTGCTGCCGCCCGTAGGAGTCTGGGGCCGTGTCTCAGTCCCAGTGTGGCCGATCACCCTCTCAGGTCGGCTAT
GCATCGTGGCCTTGGTGAGCCGTTACCTCACCAACTAGCTAATGCACCGCGGGTCCATCCATCAGCGACACCC
GAGAGCGCCTTTTTCACTCTTTATGCCATGCGGCATAAAACTGTTTATGCGGTATTTAGCACCTGTTTTTTCC
AAGTGTTTTATCCCCCTCTGATGGGTAGGTTTTACCCACGTGTTTACTCACCCGTCCGCCACTCCTCTTTTCC
AATCGAGTGCAAGCACTCGGGAGGAAAGA
>195
CTGCTGCCGCCCGTAGGAGTCTGGGCCGTGTCTCAGTCCCAATGTGGCCGTACACCCTCTCAGGCCGGCCACC
CGTCGTCGCCTTTGGTAGGCCATTACCCCCACCAACAAGCTGATAGGCCGCGGGCTCATCTTGCACCGAAAAA
CTTTCACCACACACACTAAAGACATGGTCCTATCCGGTATTTTAGACCCCCCAGTTTTTTCCCAGGCTTTATC
CCGAGGTTGCAAGGCAGATCACCCACGTGTTTACTCACCCCCGTTTTCGCCCCACTTCGAGTACCTTCTGCAA
GCAGAGGCCTTTTTTCCGTTTTCGACTTTGCATGTGTTTTTTAAGCACGCCGCCAGCGTTTCGTTCCTGAGCC
AGGATTCAAACT
>196
CTGCTGCCGCCCGTAGGAGTCTGGGCCGTGTCTCAGTCCCAGTGTGGCCGATCACCCTCTCAGGTCGGCTATG
CATCGTGGCCTTGGTGAGCCGTTACCTCACCAACTAGCTAATGCACCGCGGGTCCATCCATCAGCGACACCCG
AAAGCGCCTTTTCACTCTTATGCCATGCGGCATAAACTGTTTATGCGGTATTTAGCACCTGTTTTTTCCAAGT
CGTTTTATCCCCCCTCTGATGGGTAGGTTTACCCACGTGTTTACTCACCCGTCCGCCACTCCTCTTTTTCC
AATCGAGTGCAGCACTCGGGAGAAAGAAGCGTTCGTACTTTGACATGGTATTTT
>197
CTGCTGCCGCCCGTAGGAGTCTGGGCCGTGTCTCAGTCCCAGTGTGGCCGATCACCCTCTCAGGTCGGCTATG
CATTCGTGGCCTTGGTTGAGCCGTTTACCTTCACCAAACTAGCTTAATTGCACCGCGGGGGTTCCCCATTCCA
TTCAGCGACACCCCCCGAAAAAAGCGCCCTTTTTTCACTTCTTTTTATTTGCCCCATTGCGGCATTAAAACTT
TGTTTTATTTGCGGGGTTATTTTAGCACCCCTTTGTTTCCCCAAAAGTTTGTTTTATTTCCCCTTTCTTTTGA
TTTGGGGTTAGGGGTTTTACCCC
>198
CTGCTGCCGCCCGTAGGAGTCTGGGCCGTGTCTCAGTCCCAGTGTGGCCGATCACCCTCTCAGGTCGGCTATG
CATTCGTGGCCTTGGTGAGCCGTTTACCTTCACCAACTAGCTTAATTGCACCGCGGGGGTCCATCCATTCAGC
GACACCCCCCGAAAAAAGCGCCTTTTTTTCACTTCTTTTTTATTTGCCCCATTTGCGGCATTAAAAACTTGTT

TTTATTGCGGGGTTATTTTTAGCACCCCTTTTGTTTTCCCCAAAAGTTTGTTTTATTTCCCCCTTCTTGATTG
GGGGTTAGGGGTTTTTACCCCCACGTTGTTTTTTA
>199
CTGCTGCCGCCCGTAGGAGTCTGGGCCGTGTCTCAGTCCCAGTGTGGCCGATCACCCTCTCAGGTCGGCTTAT
GCATGTGGCCTTGGTGAGCCGTTACCTCACCAACTAGCTTAATTGCACCGCGGGTCCATCCATCAGCGACACC
CCCGAAAGCGCCTTTTTCACTTCTTTATTGCCATTGCGGCATTAAACTTGTTTTATTGCGGTTATTTTAGCAC
CTTGTTTTTTTCCAAGTTCGTTTTATTCCCCCTTCTTGATTGGGTTAGGTTTTACCCCCACGTTGTTTTACTT
CACCCCCGTTCCGCCACTTCCTTCTTTTTCCAATTTTGAGTTGCAAGCACTCGGGAGGAAATGAAGACG
>200
CTGCTGCCGCCCTAGGAGTCTGGGCCGTGTCTCAGTCCCAGTGTGGCTGGTCATCCTCTCAGACCAGCTAGAG
ATCGTCGGCTTGGTGAGCCTTTACCCCACCAACTACCTAATCCCACTTGGGCTCATCCTATGGCATGTGGCCC
GAAAGTCCCACACTTTTAATCTTTTCGATATTTACGCGGTATTTAGCTACAGTTTTTCCCGTAGTTTATTCCC
CCTCCCATAAGCCAGATTTCCCAAGCATTTACTCACCCGTTCCGCCACTTCGTCATCAAAGAAGCAAGCTTTC
TCTTATTGTTTTACCGTTTCGACTTTGCATTGTGTTTTAAGCCTTGACCGCCAGCCGT
>201
CTGCTGCCGCCCGTAGGAGTCTGGGCCGTGTCTCAGTCCCAGTGTGGCCGATCACCCTCTCAGGTCGGCTATG
CATGTGGCCTTGGTGAGCCGTTACCTCACCAACTAGCTAATGCACCGCGGGTCCATCCATCAGCGACACCCGA
AAGCGCCTTTTCACTCTTTATTGCCATGCGGCATAAACTGTTTATGCGGTATTTAGCACCTTGTTTTTTCCAA
GTCGTTTTATCCCCCCTCTGATGGGTAGGTTTTACCCACGTGTTTTACTTCACCCGTTCCGCCACTCCTCTTT
TTCCAATTTTGAGTTGCAAGCACTCGGGAGGAAAGAAGCGTTTCGTACTTGAC
>202
CTGCTGCCGCCCGTAGGAGTCTGGGCCGTGTCTCAGTCCCAGTGTGGCTGGTCGTCCTCTCAAACCAGCTACG
GATCGTCGCCTTGGTGAGCCATTACCTCACCAACTAGCTAATCCGATATCGGCCGCTCCAATAGTGAGAGGTC
TTGCGATCCCCCCCCTTTTCCCCCGTAGGGCGTATGCGGTATTTAGCTACGCTTTCGCGTAGTTTATCCCCCC
GGCTACTGGGCACGTTCCGATACATTACTCACCCGTTCGCCACTCGCCATCCAGAGAGCAAGCTCTCTGATGC
TGCCGTTTCGACTTGCATGTGTAAGGTCCG
>203
CTGCTGCCGCCCGTAGGAGTCTGGGCCGTGTCTCAGTCCCAGTTGTGGCTGGTCATCCTCTCAGACCAGCTAG
AGATCGTCGGCTTGGTGAGCCTTTACCTCACCAACTACCTAATCCCACTTGGGCTCATCCTATGGCATGTGGC
CCGAAGGTCCCACACTTTCATCTCCCGATTTCTACGCGGTATTTAGCTTACAGTTTTTCCCGTAGTTTTATCC
CCCTCCCATAAAGCTAGATTTTCCCCAAGCATTTTACTCACCCGTCCGCCACTCGTCATCAAAAGAAGCAAGC
TTTTCTTT
>204
CTGCTGCCGCCCGTAGGAGTCTGGGCCGTGTCTCAGTCCCAGTGTGGCCGATCACCCTCTCAGGTCGGCTATG
CATCGTGGCCTTGGTGAGCCGTTACCTCACCAACTAGCTAATGCACCGCGGGTCCATCCATCAGCGACACCCG
AAAGCGCCTTTTCACTCTTTATGCCATGCGGCATAAACTGTTTATGCGGTATTTTAGCACCTGTTTTTTCCAA
GTCGTTTTATCCCCCTCTGATGGGTAGGTTTTACCCACGTGTCACTCACCCGTCCGCCACTCCTCTTTTCCAA
TCGAGTGCAAGCACTCGGGAGAAAGAAGCGTTCGTACTTGCAT
>205
CTGCTGCCGCCCTAGGAGTCTGGGCCGTGTCTCAGTCCCAGTGTGGCCGATCACCCTCTCAGGTCGGCTATGC
ATCGTGGCCTTGGTGAGCCGTTACCTCACCAACTAGCTAATGCACCGCGGGTCCATCCATCAGCGACACCCGA
AAGCGCCTTTTTCACTCTTTATGCCATGCGGCATAAACTGTTTTATGCGGTATTTTAGCACCTGTTTTTTCCA
AGTGTTTTATCCCCCCTCTGATGGGTAGGTTTTACCCACGTGTTTTACTCACCCGTCCGCCACTCCTCTTTTC
CAATCGAGTGCAAGCACTCGGGAGGAAAGAAGCGTTCGTACTTGCATGTATTTTAGGCACGCCGCCAGCGTTC
GTCCTGCGCCAG
>206
CTGCTGCCGCCCGTAGGAGTCTGGGCCGTGTCTCAGTCCCAGTGTGGCCGATCACCCTCTCAGGTCGGCTATG
CATCGTGGCCTTGGTGAGCCGTTACCTCACCAACTAGCTAATGCACCGCGGGTCCATCCATCAGCGACACCCG
AAAGCGCCTTTTTCACTCTTTATGCCATGCGGCATAAACTGTTTATGCGGTATTTTAGCACCTGTTTTTTTCC
AAGTGTTTTATCCCCCCTCTGATGGGTAGGTTTTACCCACGTGTTTTACTCACCCGTCCGCCACTCCTCTTTT
TCCAATTTTGAGTTGCAAGCACTCGGGAGAAAGAAGCGTTTCGACTTGCATGTATTTTAGC
>207
TCTGCTGCCGCCCGTAGGAGTCTGGGCCGTGTCTCAGTCCCAGTGTGGCCGATCACCCTCTCAGGTCGGCTAT
GCATCGTGGCCTTGGTGAGCCGTTACCTCACCAACTAGCTAATGCACCGCGGGTCCATCCATCAGCGACACCC

GAAAGCGCCTTTTCACTCTTATGCCATGCGCATAAACTGTTTATGCGGTATTTAGCACCTGTTTTTCCAAGTC
GTTTTATCCCCCTCTGATGGGTAGGTTTACCCACGTGTTTACTCACCCGTCCGCCACTCCTCTTTTCCAATTT
TGAGTGCAAGCACTCGGGAGGAAAGAAGCGTTCGTACTTGCATGTATTTTAGGCACGCCGCCAGCGTTCGCCT
GTGTCCAGGCAT
>208
CTGCTGCCGCCCGTAGGAGTCTGGGCCGTGTCTCAGTCCCAGTGTGGCTGGTCGTCCTCTCAAACCAGCTACG
GATCGTCGCCTTGGTGAGCCATTACCTCACCAACTAGCTAATCCGATATCGGCCGCTCCAATAGTGAGAGGTC
TTTTGCGATCCCCCCCTTTTCCCCCGTAGGGCGTATGTGGTATTAGCTACGCTTTCGCGTAGTTTATCCCCCG
CTACTGGGCACGTTCCGATACATTACTCACCCGTTTCGCCACTCGCCAATCCAGAGAGCAAGCTCTCTGATGC
TGCCGTTTTCGACTTGCATTGTA
>209
CTGCTGCCGCCCGTAGGAGTCTGGGCCGTGTCTCAGTCCCAGTGTGGCCGATCACCCTCTCAGGTCGGCTATG
CATCGTGGCCTTGGTGAGCCGTTTACCTCACCAACTAGCTAATGCACCGCGGGTCCATCCATCAGCGACACCC
GAAAGCGCCTTTTCACTCTTTATTGCCATTGCGGCATTAAACTTGTTTATTGCGGTTATTTTAGCACCTTGTT
TTTTTTCCAAGTTGTTTTATTCCCCCTTCTTGATTGGGTTAGGTTTTTACCCACGTTGTTTTACTTCACCCGT
TCCGCCACTCCTCTTTTCCAATT
>210
CTGCTGCCGTCCCGTAGGAGTCTGGGCCGTGTCTCAGTCCCAGTGTGGCCGATCACCCTCTCAGGTCGGCTAT
GCATCGTGGCCTTGGTGAGCCGTTACCTCACCAACTAGCTAATTGCACCGCGGGTCCATCCATCAGCGACACC
CCCGAAAGCGCCTTTTTCACTCTTTTATGCCATGCGGCATAAACTGTTTTATGCGGTATTTTAGCACCTGTTT
TTTTCCAAGTCGTTTTATCCCCCTCTGATGGGTAGGTTTTACCCACGTGTTTTACTTCACCCGTCCGCCACTC
CTCTTTTTCCAATTTCGAGTTGCAGCACTCGGGAGGAAAGAAGCGTTTTCGACTTGCATTGTATTTTAG
>211
CTGCTGCCGCCCGTAGGAGTCTGGGCCGTGTCTCAGTCCCAGTGTGGCCGATCACCCTCTCAGGTCGGCTATG
CATCGTGGCCTTGGTGAGCCGTTACCTCACCAACTAGCTAATGCACCGCGGGTCCATCCATCAGCGACACCCC
CGAAAGCGCCTTTTTCACTCTTTATGCCATGCGGCATAAACTGCTATGCGGTATTTTAGCACCTGTTTTTTTC
CAAGTGTTTTATCCCCCCTCTGATGGGTAGGTTTTACCCACGTGTTTTACTTCACCCGTCCGCCACTCCTCTT
TTTCCAATTTTGAGTTGCAGCACTCGGGAGAAAGAAGTCG
>212
CTGCTGCCGCCCGTAGGAGTCTGGGCCGTGTCTCAGTCCCAGTGTGGCTGGTCGTCCTCTCAAACCAGCTACG
GATCGTCGCCTTGGTGAGCCATTTACCTCACCAACTAGCTAATCCGATATCGGCCGCTCCAATAGTGAGAGGT
CTTTGCTCGATCCCCCCCCTTTTCCCCCCGTAGGGCGTATGCGGTATTTAGCTACGCTTTCGCGTAGTTTATC
CCCCGCTACTGGGCACGTTCCGATACATTACTCACCCGTTTCGCCACTCGCCATCCAGAGAGCAAGCTCTCTG
ATGCTGCCGTTCGACTTGTCAT
>213
CTGCTGCCGCCCGTAGGAGTCTGGGCCGTGTCTCAGTCCCAGTGTGGCCGATCACCCTCTCAGGTCGGCTATG
CATGTGGCCTTGGTGAGCCGTTACCTCACCAACTAGCTAATGCACCGCGGGTCCATCCATCAGCGACACCCGA
AAGCGCCTTTTCACTCTTTATGCCATGCGGCATAAACTGTTTATGCGGTATTTAGCACCTGTTTTTTTCCAAG
TGTTTTATCCCCCCTCTGATGGGTAGGTTTTACCCACGTGTTTACTCACCCGTCCGCCACTCCTCTTTTCCAA
TTTCGAGTGCAAGCACTCGGA
>214
CTGCTGCCGCCCGTAGGAGTCTGGGCCGTGTCTCAGTCCCAGTGTGGCTGGTTCGTCCTCTCAAACCAGCTAC
GGATCGTCGCCTTGGTGAGCCATTTACCTCACCAACTAGCTAATCCGATATCGGCCGCTCCAATTAGTGAGAG
GTCTTTTGCTCGATTCCCCCCCCCCCTTTTTCCCCCGTAGGGCGTATGCGGTATTTAGCTTACGCTTTTCGCG
TAGTTTTATTCCCCCCCCGCTACTTGGGCACGTTTCCGATTACATTTTACTTCACCCGTTTCGCCACTCGCCA
ATCCAGAGAGC
>215
CTGCTGCCGCCCGTAGGAGTCTGGGCCGTGTCTCAGTCCCAGTGTGGCCGATCACCCTCTCAGGTCGGCTATT
GCATCGTGGCCTTGGTGAGCCGTTACCTCACCAACTAGCTTAATTGCACCGCGGGTTCCATCCATTCAGCGAC
ACCCGAAAGCGCCTTTTTTCACTCTTTTATTGCCATGCGGCATAAAACTGTTTTATGCGGTATTTTTAGCACC
TTGTTTTTTTTTCCAAGTTTGTTTTTATTCCCCCCTTCTGATTGGGTTAGGTTTTTACCCCACGTTGTTTTTA
CTTCACCCGTTCCGCCACTTCCTCTTTTTTCCAATTTTGAG
>216

CTGCTGCCGCCCCGTAGGAGTCTGGGGCCGTGTCTCAGTCCCCAGTGTGGCTGGTCATCCTCTCAGACCAGCT
AGAGATCGTCGGCTTGGTGGGCCTTTACCTCACCAACTACCTAATCCCACTTGGGCTCATCCTATGGTATGTG
GCCCGAAGGTCCCACACTTTAATCTTTCGATATTACGCGGTATTAGCTACAGTTTCCCGTAGTTATCCCCCTC
CATAAGCCAGATTCCCAAGCATTACTCACCCGTCCGCCACTCGTCATCAAAGAAGCAAGCTTCTCTATGTTAC
CGTTCGACTTGCATGTGTTAAGCCTGCCCGCCAGCGTTCAATCGAGCCAGGAT
>217
CTGCTGCCGCCCTAGGAGTCTGGGCCGTGTCTCAGTCCCAGTGTGGCCGATCACCCTCTCAGGTCGGCTATGC
ATCGTGGCCTTGGTGAGCCGTTACCTCACCAACTAGCTAATGCACCGCGGGTCCATCCATCAGCGACACCCGA
AAGCGCCTTTCACTCTTATGCCATGCGGCATAAACTGTTTATGCGGTATTTAGCACCTGTTTTTTCCAAGTCG
TTTATCCCCCTCTGATGGGTAGGTTTACCCACGTGTTTACTCACCCGTCCGCCACTCCTCTTTTCCAATTTGA
GTGCAAGCACTCGGGAGGAAAGAAGCGTTCGTACTTGACATGTATTTTAGGCACGGCC
>218
CTGCTGCCGCCCTAGGAGTCTGGGCCGTGTCTCAGTCCCAGTGTGGCTGGTCATCCTCTCAGACCAGCTAGAG
ATCGTCGGCTTTGGTGAGCCTTTACCCCACCAACTACCTAATCCCACTTGGGCTCATCCTATGGCATGTGGCC
CGAAGGTCCCACACTTTTAATCTTTTCGATATTTACGCAGTATTTAGCTACAGTTTTCCCGTAGTTTATTCCC
CCTCCATAAGCCAGATTTCCCAAGCATTTACTCACCCGTTCCGCCACTCGTCATCAAAGAAGCAAGCTTTCTC
TTATTGTTTTACCGTTTCGACTTTGCATTGTGTTTTAAGCCTGCCGCCAGCGTTTTCAATCTGAGCCAGGATC
AAACTCT
>219
CTGCTGCCGCCCGTAGGAGTCTGGGCCGTGTCTCAGTCCCAGTGTGGCCGATCACCCTCTCAGGTCGGCTATT
GCATCGTGGCCTTTGGTGAGCCGTTACCTTCACCAACTAGCTTAATGCACCGCGGGTCCATCCATCAGCGACA
CCCGAAAGCGCCTTTTTCACTCTTTATTGCCATGCGGCATTAAAACTTGTTTTATTGCGGTTATTTTAGCACC
TTGTTTTTTTTCCAAGTTGTTTTATTCCCCCTTCTGATTGGGTTAGGTTTTACCCACGTTGTTTTACTTCACC
CGTTCCGCCACTTCCTCTTTTTCCAATTTTGAGTTGCAAGCACTCGGGAGGAAAGAAGACGT
>220
CTGCTGCCGCCTGTAGGAGTCTGGGCCGTGTCTCAGTCCCAGTGTGGCCGATCACCCTCTCAGGTCGGCTATG
CATCGTGGCCTTGGTGAGCCGTTACCTCACCAACTAGCTAATGCACCGCGGGTCCATCCATCAGCGACACCCG
AAAGCGCCTTTTTCACTCTTTATGCCATGCGGCATAAAACTGTTTTATGCGGTATTTTAGCACCTGTTTTTTT
CCAAGTGTTTTATCCCCCCCTCTGATTGGGTAGGTTTTACCCACGTGTTTTACTTCACCCGTTCCGCCACTCC
TCTTTTCCAATTTTGAGTGCAAGCACTCGGGAGAAAGAA
>221
CTGCTGCCGCCCGTAGGAGTCTGGGCCGTGTCTCAGTCCCAGTGTGGCCGATCACCCTCTCAGGTCGGCTATG
CATCGTGGCCTTGGGTGAGCCGTTACCTCACCAACTAGCTAATGCACCGCGGGTCCATCCATCAGCGACACCC
GAAAGCGCCTTTTTCACTCTTTATGCCATGCGGCATAGACTGTTTTATTGCGGTATTTTACGCACCTGTTTTT
TTCCAAGTGTTTTATCCCCCTCTGATGGGTAGGTTTTACCCACGTGTTTTACTCACCCGTCCGCCACTCCTCT
TTTCCAATCGA
>222
CTGCTGCCGCCCGTAGGAGTCTGGGCCGTGTCTCAGTCCCAGTGTGGCCGATCACCCTCTTCAGGTCGGCTAT
GCATTCGTGGCCTTGGTTGAGCCGTTTACCTTCACCAACTAGCTTAATTGCACCGCGGGGGGTTCCCCATTCC
ATTCAGCGACACCCCCCGAAAAAAGCGCCTTTTTTCACTTCTTTTTATTGCCATTGCGGCATTAAAAAACTT
GTTTTTTATTGCGGTTATTTTTTAGCACCTTTGTTTTTTCCCCAAAAAGTTTGTTTTTTATTCCCCCCCTTCT
TGATTGGGGGGTTAGGTTTTTTACCCCCCACGTTGTTTTTTACTTTACCCCCCCGTTCCCGCCCCCATTTCCC
CTCTT
>223
CTGCTGCCGCCCGTAGGAGTCTGGGGCCGTGTCTCAGTCCCAGTGTGGCCGATCACCCTCTCAGGTCGGCTAT
GCATCGTGGCCTTGGTGAGCCGTTACCTCACCAACTAGCTAATGCACCGCGGGTCCATCCATCAGCGACACCC
GAAAGCGCCTTTTTCACTCTTTATGCCATGCGGCATAAAACTGTTTTATGCGGTATTTTAGCACCTGTTTTTT
TCCAAGTGTTTTATCCCCCTCTGATGGGTAGGTTTTACCCACGTGTTTTACTCACCCGTCCGCCACTCCTCT
TTTCCAATTTTGAGTGCA
>224
CTGCTGCCGCCCGTAGGAGTTCTGGGCCGTGTCTCAGTCCCAGTGTGGCCGATCACCCTCTCAGGTCGGCTAT
GCATCGTGGCCTTGGTGAGCCGTTACCTCACCAACTAGCTAATGCACCGCGGGTCCATCCATCAGCGACACCC
GAAAGCGCCTTTTTCACTCTTTTATGCCATGCGGCATAAACTGTTTTATGCGGTATTTTAGCACCTGTTTTTT

TCCAAGTTCGTTTTATCCCCCCTCTGATGGGTAGGTTTTACCCACGTGTTTTACTCACCCGTCCGCCACTCCT
CTTTTTCCAATTTTGA
>225
CTGCTGCCGCCCGTAGGAGTCTGGGCCGTGTCTTCAGTCCCAGTGTGGCCGATCACCCTCTTCAGGTCGGCTA
TGCATTCGTGGCCTTGGTTGAGCCCGTTTACCTTCACCAAACTAGCTTAATTGCACCGCGGGGGTTCCATTCC
ATTCAGCGACACCCCCCCCGAAAAAAAGCGCCTTTTTTTTTCACTTCTTTTTTATTGCCATTGCGGCATTAAA
AAAACTTGTTTTTTTATTGCGGTTTATTTTTTTTTTTTTTAGCACCTTTGTTTTTTTTTTTTTTCTTAACGTT
TTGTTTTTTTTACT
>226
CTGCTGCCGCCCGTAGGAGTCTGGGCCGTGTCTCAGTCCCAGTGTGGCTGGTCACCCTCTTCAGACTAGCTTA
GAGATTCGTCGGCTTTGGTTGAGCCTTTACCCCCACCAACTACCTTAATTCCCACTTGGGCTTCATTCCTATT
GGCATTGTGGCCCGAAGGTCCCACACTTTTTAATCTTTTTCGATTATTTACGCGGTATTTTAGCTACAGTTTT
TTCCCGTAGTTTTATTCCCCCTCCATTAAGCCAGATTTTCCCAAGCATTTTACTTCACCCGTTCCGCCACTTC
GTCATTCAAAGAA
>227
CTGCTGCCGCCCGTAGGAGTCTGGGCCGTGTCTCAGTCCCAGTGTGGCCGATCACCCTCTCAGGTCGGCTATG
CATCGTGGCCTTGGTGAGCCGTTACCTCACCAACTAGCTAATGCACCGCGGGTCCATCCATCAGCGACACCCG
AAAGCGCCTTCACTCTTATGCCATGCGGCATAAACTGTTATGCGGTATTAGCACCTGTTTTCCAAGTGTTATC
CCCTCTGATGGGTAGGTTACCCACGTGTTACTCACCCGTCCGCCACTCCTCTTCCATTCGAGTGCAAGCACTC
GGAGAAGGA
>228
CTGCTGCCGCCCTAGGAGTCTGGGCCGTGTCTCAGTCCCAGTGTGGCTGGTCATCCTCTTCAGACCAGCTAGA
GATCGTCGGCTTGGTGAGCCTTTACCTCACCAACTACCTTAATTCCCACTTTGGGCTTCATCCTATTGGTATG
TGGCCCGAAGGTCCCACACTTTTTAATCTTTTCGATATTTACGCGGTATTTTAGCTACAGTTTTTCCCGTAGT
TTTATCCCCCTCCATTAAGCCAGATTTTCCCCCAAGCATTTACTCACCCGTTCCGCCACTTCGTCATTCAAAG
AAGCAAGCTTTCTCTTATTGTTTTACCGTTTTCGACTTTTGCATTGCCGTTTTA
>229
CTGCTGCCGCCCGTAGGAGTCTGGGCCGTGTCTCAGTCCCAGTGTGGCCGATCACCCTCTCAGGTCGGCTATG
CATGTGGCCTTGGTGAGCCGTTACCTCACCAACTAGCTAATGCACCGCGGGTCCATCCATCAGCGACACCCGA
AAGCGCCTTTTCACTCTTTATGCCATGCGGCATAAACTGTTTTATGTCGGTATTTTAGCACCTGTTTTTTTCC
AAGTCGTTTTATCCCCCCCCTCTGATGGGTAGGTTTTACCCACGTGTTTTACTTCACCCGTCCGCCACTCCTC
TTTTTCCAA
>230
CTGCTGCCGCCCGTAGGAGTCTGGGCCGTGTCTTCAGTCCCAGTGTGGCCGATCACCCTCTTCAGGTCGGCTA
TGCATTCGTGGCCCTTGGTTGAGCCCGTTTACCTTCACCAAACTAGCTTAAATTGCACCGCGGGGGTTCCCCA
TTCCATCAGCGACACCCCCCCCGAAAAAAGCGCCCCTTTTTTTTCACTTCTTTTTTATTTGCCATTGCGGCAT
TAAAAAAAACTTGTTTTTTTATTTGCGGTTTATTTTTTTAGCACCCCTTTGTTTCTTTCCCCCAAAAGTTTTG
TTTTTTTATTCCCCCCCCT
>231
CTGCTGCCCCCGTAGGAGTCTGGGCCGTGTCTCAGTCCCAGTGTGGCTGGTCATCCTTCTCAGACCAGCTAGA
GATCGTTCGGCTTGGTGAGCCTTTTACCTTCACCAACTTACCTTAATTCCCACTTTGGGCTTCATCCTATTGG
CATTGTGGCCCGAAGGTCCCACACTTTTTTAATCTTTTTCGATTATTTTACGCGGTATTTTAGCTACAGTTTT
TCCCGTAGTTTTATTCCCCCTCCATAAGCCAGATTTTCTTCAAGCATTTTACTTCACCCGTTCCGCCACTTCG
TTCATTCAAAGAAGCAAGCTTTTCTTTTT
>232
CTGCTGCCGCCCGTAGGAGTCTGGGCCGTGTCTCAGTCCCAGTTGTGGCCGATCACCCTCTCAGGTCGGCTAT
GCATTCGTGGCCTTGGTGAGCCGTTTACCTTCACCAACTAGCTTAATTGCACCGCGGGGGTCCATTCCATTCA
GCGACACCCCCCGAAAAAAGCGCCCTTTTTTCACTTCTTTTTATTTGCCCCATTTGCGGGCATTAAAACTTTG
TTTTATTTGCCGGGGTTTATTTAGCCCAACCCTTTGGTTTCCCAAAGGTTTGTTTATTTCCCTTTCCTTTGAT
TTGGGTTAGGGTTTACCCACGTGTTTA
>233
CTGCTGCCGCCCGTAGGAGTCTGGGCCGTGTCTCAGTCCCAGTGTGGCCGATCACCCTCTCAGGTCGGCTATT
GCATCGTGGCCTTGGTTGAGCCGTTTACCTTCACCAACTAGCTTAATTGCACCGCGGGTCCATTACCATTCAG
CGACACCCCCCGAAAAAAAGCGCCTTTTTTTCACTTCTTTTTTATTTTGCCCCCATTTGCGGCATTTAAAACT

TTGTTTTATTTGCCGGGGTTTATTTAGCCCAACCCTTTGTTTCCCAAAGTTGTTTATTTCCCTTTCTTGATTT
GGGTTAGGGTTTACCCACGTGTTTACTTTACC
>234
CTGCTGCCGCCCGTAGGGGTCTGGGCCGTGTCTTCAGTCCCAGTGTGGCTGGTCGTCCTTCTCAAACCAGCTA
CGGATCGTCGCCTTGGTGAGCCGTTACCTCACCAACTAGCTTAATCCGATTATCGGCCGCTCCAATTAGTTGA
GAGGTCTTTACGATTCCCCCCCCTTTTCCCCCGTAGGGCGTATGCGGTATTTAGCTTACGCTTTTCGCGTAGT
TTATCCCCCCCGCTTACTTGGGCACGTTTCCGATACATTTACTTCACCCGTTTTCGCCACTGCCATCCAGAGA
GCAAGCTCTCTGATGCTTGCGTTTCGACTTTGCATTGTGTTAAGGCATCCCCCGCTAGCTGTTTTCAATTCTG
AGCCAGGA
>235
CTGCTGCCGCCCGTAGGAGTCTGGGCCGTGTCTCAGTCCCAGTGTGGCCGATCACCCTCTCAGGTCGGCTATG
CATTCGTGGCCTTGGTTGAGCCGTTTACCTCACCAACTAGCTTAATTGCACCGCGGGGGTTCCCCATCCATCA
GCGACACCCCCCGAAAAAAGCGCCCTTTTTTCACTTCTTTTTTATTGCCCATTGCGGCATTAAAAACTTGTTT
TTATTGCGGGGTTATTTTAGCACCCCTTTGTTTTCCCCAAAAGTTTGTTTTATTTCCCCCTTTCTTGATTGGG
GTTAGGGGTTTTACCCCACGTTGTTTTACTT
>236
CTGCTGCCGCCCGTAGGAGTCTGGGCCGTGTCTCAGTCCCAGTGTGGCTGGTCATCCTCTCAGACCAGCTAGA
GATCGTCGGCTTGGTGAGCCTTTACCCCACCAACTACCTAATCCCACTTGGGCTCATCCTATGGCATGTGGCC
CGAAGGTCCCACACTTTTAATCTTTTCGATATTTACGCGGTATTTTAGCTACAGTTTTTCCCGTAGTTTATTC
CCCCTCCATAAGCCAGATTTCCCAAGCATTTACTCACCCGTTCCGCCACTCGTCATTCAAAGAAGCAAGCTTT
TCTCTATGTTTTACCGTTTCGACTTTGCATTGTGTTTTAAGCCTTGCCGCCAGCGTTTCAATCTGAGCCA
>237
CTGCTGCCGCCCGTAGGAGTCTGGGCCGTGTCTCAGTCCCAGTGTGGCCGATCACCCTCTCAGGTCGGCTATT
GCATCGTGGCCTTGGTTGAGCCGTTTACCTCACCAACTAGCTTAATTGCACCGCGGGGGTTCCATCCATCAGC
GACACCCCCGAAAAAAGCGCCTTTTTTTCACTTCTTTTTATTGCCATTGCGGCATTAAAAAACTTGTTTTTA
TTGCGGTTATTTTTTAGCACCTTTGTTTTTTCCAAAAGTTGTTTTTTATTCCCCCCCTTCTTGATTGGGGGGT
TGGGGGTTTTTACCCCCACGCGTTTTTACTTACCCCCCGTTCC
>238
CTGCTGCCGCCCGTAGGAGTCTGGGCCGTGTCTTCAGTCCCAGTTGTGGCCGATCACCCTTCTCAGGTCGGCT
ATGCATTCGTGGCCTTTGGTTGAGCCGTTTACCTTCACCAACTAGCTTAATGCACCGCGGGTCCATCCATCAG
CGACACCCCCGAAAAAAGCGCCTTTTTTTCACTTCTTTTTATTTGCCATTGCGGCATTAAAAAACTTGTTTTT
TATTGCGGTTATTTTTAGCACCTTTGTTTTTCCAAAAAGTTTGTTTTTTATTCCCCCCTTCTTGATTGGGGGG
TTAGGTTTTTTACCCCCCACGTTGTTTTTTTACTACC
>239
CTGCTGCCGCCCGTAGGAGTCTGGGCCGTGTCTTCAGTCCCAGTGTGGCCGATCACCCTTCTCAGGTCGGCTA
TGCATTCGTGGCCTTGGTTGAGCCGTTTACCTTCACCAACTAGCTTAATTGCACCGCGGGTCCATCCATCAGC
GACACCCCCGAAAAAAGCGCCTTTTTTTTCACTTCTTTTTATTGCCATTGCGGCATTAAAAAACTTGTTTTTT
ATTGCGGTTATTTTTAGCACCTTTGTTTTTCCCCAAAAGTTTGTTTTTTATTCCCCCCTTCTTGATTGGGGGG
TTAGGTTTTTTAC
>240
CTGCTGCCGCCCGTAGGAGTCTGGGCCGTGTTCTCAGTCCCAGTGTGGCCGATCACCCTCTCAGGTCGGCTAT
GCATTCGTGGCCTTGGTGAGCCGTTTACCTCACCAACTAGCTTAATTGCACCGCGGGTTCCATCCATTCAGCG
ACACTTCGAAAAAAGCGCCTTTTTTTTCACTTCTTTTTTATTGCCATTGCGGCATTAAAAAAACTTGTTTTTT
TTTTTATGCGGTTATTTTTTTAGCACCTTTGTTTTTTTTTTTTTAAGTTTGTTTTTTTTATTCCCCCCCCCCC
TTTCTTGATTGGGTTAGGTT
>241
CTGCTCCGCCCGTAGGAGTCTGGGCCGTGTCTCAGTCCCAGTGTGGCTGGTCGTCCTCTCAAACCAGCTACGG
ATCGTCGCCTTGGTGAGCCGTTACCTCACCAACTAGCTAATCCGATATCGGCCGCTCCAATAGTGAGAGGTCT
TTACGATTCCCCCCCTTTCCCCCGCAGGGCGTATGCGGTATTAGCTACGCTTTTCGCGTAGTTATCCCCCGCC
GTACTCGGGCACGTTCCGATACATTACTCACCCGTTCGCCACTCGCCATCCCAGAGAGCAAAGCCTCTCTGAT
GCTTGCCGTTCGACGTACGTCTTGACATTT
>242
CTGCTGCCGCCCGTAGGAGTCTGGGCCGTGTCTCAGTCCCAGTGTGGCCGATCACCCTTCTCAGGTCGGCTAT
GCATTCGTGGCCTTGGTTGAGCCGTTTACCTTCACCAAACTTAGCTTAATTGCACCGCGGGGGTCCATCCATT

CAGCGACACCCCCGAAAAAAGCGCCTTTTTTTCACTTCTTTTTATTGCCATTGCGGCATTAAAAACTTGTTTT
TATTGCGGGGTTATTTTAGCACCCTTTGTTTTCCCCAAAAGTTGTTTTATTTCCCCTTCTTGATTGGGGTTAG
GGGTTTTACCCCACGTGTTT

Additional file 3_B_P5

>1
CTGCTGCCGCCCGTAGGAGTCTGGGCCGTGTCTCAGTCCCAGTGTGGCTGGTCATCCTCTCAGACCAGCTATG
GATCGTCGCCTTGTGGGCCATTACCCCACCAACTAGCTAATCCAACGCGGGCCGATCTTTCAGCGATAAATCC
TTTCCCCTTAAGGGCGTATCCGGTATTAGCACAAGTTTCCCTGAGTTATTCCGAACTGAAAGGTACGTTCCCA
CGCGTTACTCACCCGTCCTGCCACTGCTGTATTTTGCTACAACCGTTTTCGACTTGCATGTGTTTAAGCCTGC
CGCCAGCGTTCGTTCTGAGCCAGG
>2
CTGCTGCCGCCCGTAGGAGTCTGGGCCGTGTCTCAGTCCCAGTGTGGCTGATCATCCTCTCAGACCAGCTAAG
GATCGTCGCCTTGGTGCGCCTTTACCACACCAACTAGCTAATCCTACGCGGGCTCATCTCAGGGCGATAAATC
TTTTGGTCTTTTTCGACATCATCCGGTATTTAGCACCCCTTTTCGGGGAGTTTATTTTCCGAACCCCAAGGCA
GATTCCCACGCGTTACGCACCCGTGCGCCACTATGCCCGAAGGCATCGTTTCGACTTTGCATGTGTTTTAGGC
ATGCCGCCAGCGTTTCGTTTCTTGAGCCAGGATCAAACTTCT
>3
CTGCTGCCGCCCGTAGGAGTTTGGGCCGTGTCTCAGTCCCAATGTGGCTGATCATCCTCTCAGACCAGCTACT
GATTCGTCGCCTTGGTGAGCCCTTTACCTCACCAACTAGCTAATCAGACGCGGGCCGCTCTAAAGGCGATAAA
TCTTTCCCCCGAAGGGCACATCCGGTATTTTAGCTCAAGTTTTTCCCTGAGTTTTATTTTCCGAACCTAAAGG
CACGTTTCCCACGTGTTTACTCACCCGTCCGCCACTAACTCCGAAGAGTTTTCGTTTCGACTTTGCATGTTGT
TTTAGGCCTTGCCGCCAGCGTTTTCGCTTCTTGAGCCAGGATCAAACTTCTT
>4
CTGCTGCCGCCCGTAGGAGTCTGGACCGTGTCTCAGTTCCAGTGTGGCCGATCACCCTCTCAGGTCGGCTATG
CATCGTTGCCTTGGTAAGCCGTTACCTTACCAACTAGCTAATGCAGCGCGGATCCATCTATAAGTGACAGCAA
GACCGTCTTTTTTACTTTTGAACCATGCGGTTTCAAAATATTTATCCGGTATTTAGCTCCGGTTTTTCCCGAA
GTTTATCCCAGTCTTTTATAGGTAGGTTTTATCCACGTGTTTTACTCACCCGTCCGCCGCTAACATCAGAGAA
GCAAGCTTTCTCGTCCGTTTCGCTCGATTTTGCATTGTATTTTAGGCACGCCGCCAGCGTTTCACCTGAGCCA
GGACAACATCT
>5
GCTGCCGCCCGTAGGAGTCTGGGCCGTGTCTCAGTCCCAGTGTGGCCGGTCGCCCTCTCAGGCCGGCTACCCG
TCATCGTCTTGGTGAGCCATCACCTCACCAACAAACTGATAGGCCGCGAGTCCATCCCCCACCGATAATCTTT
CCAGCACCCACCATGCGATGAGTGCTCGTATCCCGGTTATTTAGCCACGATTTTTCCCGAGGTTTATCCCGAA
GTTGGGGGGCAGGTTTACTCACGTGTTTACTTCACCCGTTTTCGCCACTTAATCGGGTGGTGCAAGCACCACC
GTTCATTCGTTTCGACTTTGCATGTGTTTTTAAGCACGCCGCCAGCGTTTC
>6
CTGCTGCCGCCCGTAGGAGTCTGGGCCGTGTCTCAGTCCCAGTGTGGCTGATCATCCTCTCAGACCAGCTACT
GATCGTCGCCTTGGTAGGCCGTTACCCCACCAACTAGCTAATCAGCGCGGGCCGATCTTCCGGCAGTAAACCT
TTTCCCCAAAAGGGCGTATCCGGTATTAGCCCTAGTTTTTCCCAGGGTTTATTCCAGACCAGAAGGCACGTTC
CCACGCGTTACTCACCCGTCCGCCGCTGACCCCGAAGGGCCCGCTCGACTTGCATGTGTTAAGCCTGCCGCCA
GCGTTCGCTCTGAGCCAGGATCAAACTCT
>7
CTGCTGCCGCCCGTAGGAGTTTTGGGCCGTGTCTCAGTCCCAATGTGGCTGATCATCCTCTCAGACCAGCTAC
TGATCATCGCCTTGGTGAGCCTTTACCTCACCAACTAGCTAATCAGACGCGGGCCGCTCTAAAGGCGATAAAT
CTTTCCCCCGAAGGGCACATCCGGTATTTTAGCTCAAGTTTTCCCTGAGTTTATTTTCCGAACCTAAAGGCAC
GTCCCCACGTGTTTACTCACCCGTCCGCCACTAACTACCGAAGAGTTTTCGTTTTCGACTTTGCATGTTGTTT
TAGGCCTTGCCGCCAGCGTTTTCGCTTCTTGAGCCAGGATCAAACTTCTT
>8
CTGCTGCCGCCCGTAGGAGTTTTGGGCCGTGTCTCAGTCCCAATGTGGCTGATCATCCTCTCAGACCAGCTAC
TGATCGTCGCCTTGGTGAGCCTTTACCTCACCAACTAGCTAATCAGACGCGGGCCGCTCTAAAGGCGATAAAT
CTTTCCCCCGAAGGGCACATCCGGTATTTAGCTCAAGTTTTTCCCTGAGTTTTATTTCCGAACCTAAAGGCAC
GTTTCCCACGTGTTTACTCACCCGTCCGCCACTAACTCCGAAGAGTTTTCGTTTCGACTTGCACGTGTTTAGG
CCTGCCGCCAGCGTTTCGCTTCTGAGCCAGGATCAAACTTCT
>9
CTGCTTGCCGCCCTAGGAGTTTTGGGCCGTGTCTCAGTCCCAATGTGGCTGATCATCCTCTCAGACCAGCTAC
TGATCGTCGCCTTGGTGAGCCTTTACCTCACCAACTAGCTAATTCAGACGCGGGCCGCTCTTAAAGGCGATAA

ATCTCTCCCCCGAAGGGCACATCCGGTATTTAGCTTCAAGTTTTTCCCTGAGTTTTATTTTCCGAACCTAAAG
GCACGTTTCCCACGTTGTTTACTCACCCGTTCCGCCACTAACTTCCGAAGAGTTTTCGTTTCGACTTGCATTG
TTGTTTTAGGCCTTGCCGCCAGCGTTTCGCTTCTTGAACCAGGATCAAACTTCT
>10
CTGCTGCCGCCCGTAGGAGTCTGGGCCGTGTCTCAGTCCCAGTGTGGCTGGTCGTCCTCTCAGACCAGCTACA
GATCGCAGGCTTTGGTAAGCCTTTACCCCACCAACTACCTAATCTGCCATCAGCCGCTCTAGTAGCACAAGGC
CCGAAGGTCCCCTGCTTTTCATCCGTAGATCTCATTGCGGTTATTTTAGCTACTTCTTTTTCGAGTAGTTTTA
TTCCCCCCCCCACTACTAGGCACGTTTCCGATGTTATTTTACTTCACCCGTTTCGCCACTCGTCAGCATCCGA
AGACCTGTTTACCGTTTCGACTTTGCA
>11
CTGCTGCCGCCCGTAGGAGTCTGGGCCGTGTCTCAGTCCCAGTGTGGCTTGATCATCCTCTCAGACCAGCTAA
GGATTCGTCGCCTTGGTTGCGCCTTTACCACACCAACTAGCTAATCCTACGCGGGCTCATCCTCTGGCGATAA
ATCTTTTTGGACTTTTCGTCATCATCTGGTATTTTAGCAGTTCGTTTTTTTCCAACTTGTTTTTTATTTTCCG
AACCAAAAAGGGCAGATTTTCCCACGCGTTTACGCACCCGTGCGCCACTTAGACCCCCGAAGATCTTGTTTTT
CGACTTTTGCATGTGTT
>12
CTGCTGCCGCCCGTAGGAGTCTGGGCCGTGTCTCAGTCCCAGTGTGGCTGATCATCCTCTCAGACCAGCTACT
GATCGTCGCCTTGGTAGGCCGTTACCCCACCAACTAGCTAATCAGACGCGGGCCGATCTTTCCGGCAGTAAAC
CTTTTCCCCAAAAGGGCGTATCCGGTATTTAGCCCTAGTTTTTTCCCAGGGTTTATTTTCCAGACCAGAAGG
CACGTTCCCCACGCGTTACTCACCCGTCCGCCCCTGACCCCGAAGGGCCCGCTCGACTTTGCAGTGTTTTAAG
CCTGCCGCCAGCG
>13
CTGCTGCCGCCCGTAGGAGTCTGGGCCGTGTCTCAGTCCCAGTGTGGCTGATCATCCTCTCAGACCAGCTACA
GATCGTCGGCTTGGTAAGCTTTATCCCACCAACTACCTAATCTGCCATCGGCCGCTCCGTCCGCGCGAGGTCC
GAAGATCCCCTGCTTTCATCCGTAGATCGTATGCGGTATTTAGCAAAGCTTTTCGCCTCGTTTACCCCCCCCC
ACGATCGGGCACGTTCGATGTATTTACTCACCCGTTCGCCACTCGTCAGCATCCGAAGACCTGTTACCGTTCG
ACTTGCATGTGTAAGGCATGCCGCCAGCGTTCAATCTGAGCCAGGATCAAACTCT
>14
CTGCTGCCGCCCGTAGGAGTCTGGGCCGTGTCTCAGTCCCAGTGTGGCTGATCATCCTCTCAGACCAGCTACT
GATCGTCGCCTTGGTAGGCCGTTACCCCACCAACTAGCTAATCAGACGCGGGCCGATCTTTCCGGCAGTAAAC
CTTTTTCCCCAAAAGGGCGTATCCGGTATTAGCCCTAGTTTTTTCCCAGGGTTTATTTTCCAGACCAGAAGGC
ACGTTCCCACGCGTTACTCACCCGTCCGCCGCTGACCCCGAAGGGCCCGCTCGACTTGCATGTGTTAAGCCTG
CCGCCAGCGTTCGCTCTGAGCCAGGATCAAACTCT
>15
CTGCTGCCGCCCGTAGGAGTCTGGGCCGTGTCTCAGTCCCAGTGTGGCCGGTCGCCCTCCCAGGCCGGCTACC
CGTCGTCGCCTTGGTAGGCCATTTACCCCCACCAACAAGCTGATAGGACGCGAGTCCATCCACCACCGATAAA
TCTTTTTCCACCAACACCCCCCATGCAAGGGTTTTGGCGTTATCCAGTTATTTTAGACGCCATTTTTTTCCAG
CGCTTTTATCCCAGAGTTGATGGGCAGGTTTTTACTCACGTTGTTTTTACTTCACCCCCCGTTTTTCGCCACT
TGATCCACGAGTTGCAAGCACCCCCCGCTTTTTCACCGTTTTTCGAC
>16
CTGCTGCCGCCCGTAGGAGTCTGGACCGTGTCTCAGTTCCAGTGTGGCTGGTCATCCTCTCAGACCAGCTAGG
GATCGTCGCCTAGGTGAGCCGTTACCCCACCTACTAGCTAATCCCATCTGGGCACATCTGATGGCAAGAGGCC
CGAAGGTCCCCCTCTTTTGGTCTTGCGACGTTATGCGGTATTTAGCTACCGTTTTTCCAGTAGTTATCCCCCC
TCCCATCAGGCAGTTTTTCCCCAGACATTTACTCACCCGTCCGCCACTCGTCACGAGCAGCAAGCTGCTTTCC
TGTTTACCGTTCGACTTGCATGTGTTTACGGCCTGCCGCCAGCGT
>17
CTGCTGCCGCCCGTAGGAGTCTGGACCGTGTCTCAGTTCCAGTGTGGCTGGTCATCCTCTCAGACAGCTAGGG
ATCGTCGCCTAGGTGAGCCGTTACCCCACCTACTAGCTAATCCCATCTGGGTTCATCTGATGGCAAGAGGCCC
GAAGGTCCCCCTCTTTTGGTCTTGCGACGTTATGCGGTATTTAGCTACCGTTTTCCAGTAGTTATCCCCCTCC
ATCAGGCAGTTTTTCCCAGACATTACTCACCCGTCCGCCGCTCGTCACCCAGGAGCAAGCTCCCTGTGTTACC
GCCCGACTTGCATGTGTTTAGGCCTGCCGCCAGCGTTCAATCTGACGCCAGGCAT
>18
CTGCTGCCGCCCGTAGGAGTCTGGGCCGTGTCTCAGTCCCAGTGTGGCTGATCGTCCTCTCAGACCAGCTACC
GATCGCAGGCTTGGTAGGCCTTTACCCCACCAACTACCTAATCGGACGCAGGCTGCTCCCAAGGCGCCCTAAG

GCTTTGACCCACAGGCCCCATGCGGTATTAGCCCCAGTTTTCCCAGGGTTGTCCCCCACCTCAGGATACATTC
CTACGCGATACTCATCCGTCCGCCGCTGATGGCCGAAACCACCCGCGCGACTTGCATAGTCGTTAAGCATGCC
GCCAGCGTTCGCTCTGAGCCAGGATCAAACTCT
>19
CTGCTGCCGCCCGTAGGAGTCTGGGCCGTGTCTCAGTCCCAGTGTGGCTGGTCATCCTCTCAGACCAGCTATG
GATCGTCGCCTTGGTGGGCCATTTACCCCACCAACTAGCTAATCCAACGCGGGCCGATCTTTTCAGCGATAAA
TTCTTTTTTCCCCCCCTTTTAAGGGCGTATCCGGTTATTTTAGCACAAGTTTCCCCCTGAGTTTTTATTTTCC
GAACTTGAAAGGTACGTTTTTCCCGACGCGTTTACTTCACCCGTCTTGCCACTTGCTTGTATTTTTTGCTTAC
AACCGTTTTTTTCGACTTTTG
>20
CTGCTGCCGCCCGTAGGAGTCTGGACCGTGTCTCAGTTCTGGTGTGGCTGGTCGTCCTCTCAGGCCAGCTACA
GATCGTCGCCTTGGTGGGCCTTTACCCCACCAACTAGCTAATCTGATATCGGCCGCTCCAATCGCGCGAGGTC
TTGCGATCCCCCGCTTTTCATCCGTAGATCGTATGCGGTATTTAGCACAGCTTTTCGCTGCGTTTATCCCCCC
ACGACTGGGCACGTTCCGATATATTACTCACCCGTTTTCGCCACTCGCCACCAGGATTTGCTCCCGTGCTGCC
GTTTTCGACTTTGCATGTGTAAGGCATGCCGCCAGCGTTCAATCTGAGCCAGGATCAAACTCT
>21
CTGCTGCCGCCCGTAGGAGTTTGGACCGTGTCTCAGTTCCAATGTGGGGGACCTTCCTCTCAGAACCCCTATC
CATCGAAGGCTTGGTGGGCCGTTACCTCACCAACAACCTAATGGAACGCATCCCCATCCTTTACCGGAATCCT
TTTAATAATGAAACCATGCGGAATCATTTATGCCATCGGGTATTTAATCTTTTCTTTTCGAAAGGCTATCCCC
CGAGTAAAGGGCAGGTTTGGATACGTGTTTACTCACCCGTGCGCCGGTCGCCGGCAAAGAAAGCAAGCTTTTC
TTTCCCGATGCCCCTTCGACTTTTGCATGTGTTTTAAGCCTTGTAGCTAGCGTTTCATCCCTGAGCCAGGATC
AAACTCT
>22
CTGCTGCCGCCCGTAGGAGTCTGGACCGTGTCTCAGTTCCAGTGTGGCCGATCACCCTCTCAGGTCGGCTACG
CATCGTTGCCTTGGTAAGCCGTTACCTTACCAACTAGCTAATGCGGCGCGGATCCATCTATAAGTGACAGCAA
GACCGTCTTTCACTGTTGAACCATGCGGTTCAACATGTTTATCCGGTATTTAGCTCCGGTTTTTCCCCGAAGT
TATCCCAGTCTTTATAGGTAGGTTTATCCACGTGTTTACTCACCCGTCCACCGCTAACGTCAGAGGAGCAAGC
TCCTCGTCTGTTTTCGCTCGACTTGCATGTATTTTAGGCACGCCGCCAGCGTTCATCCTGAGCCAGGATCAAA
CTCT
>23
CTGCTGCCGCCCGTAGGAGTTTTGGGCCGTGTCTCAGTCCCAATGTTGGCTGATCATCCCTCTCAGACCAGCT
ACTGATCGTCGCCTGGTGAGCCTTTACCTCACCAACTAGCTAATTCAGACGCGGGCCGCTCTAAAGGCGATAA
ATCTTTCCCCCCCGAAGGGCACATCCGGTTATTTAGCTTCAAGTTTTCCCTGAGTTTTATTTCCGAACCTAAA
GGCACGTTCCCACGTGTTTACTCACCCGTTCCGCCACTAACTCCGAAGAGTTTCGTTTCGACTTGCATTGTTG
TTTAGGCCGCCGCCAGCGTTTCGCTTCTGAGCCAGGAT
>24
CTGCTGCCGCCCGTAGGAGTCTGGGCCGTGTCTCAGTCCCAGTTGTGGCTGATCATCCTCTCAGACCAGCTAT
GATCGTCGCCTGGTAGGCCGTTTACCCCACCAACTAGCTAATCAGGCGCGGGCCGATCTTCCGGCAGTAAACC
TTTCCCCCCAAAGGGCGTATCCGGTATTTAGCCCTAGTTTTTCCCAGGGTTTATTTCCAGACCAGAAGGCACG
TTCCCACGCGTTACTCACCCGTCCGCCGCTGACCCCCGAAGGGCCCGCTCGACTTGCATGTGTTTAAGCCTGC
CGCCAGCG
>25
CTGCTTGCCGCCCGTAGGAGTTTTGGGCCGTGTCTCAGTCCCAATGTGGCTGATCATCCTCTCAGACCAGCTA
CTGATCGTCGCCTTTGGTGAGCCTTTACCTCACCAACTAGCTAATCAGACGCGGGCCGCTCTAAAGGCGATAA
ATCTTTCCCCCGAAGGGCACATCCGGTATTTAGCTCAAGTTTTTCCCTGAGTTTATTTCCGAACCTAAAGGCA
CGTTTCCCACGTGTTTACTCACCCGTTCCGCCACTAACTTCCGAAGAGTTTTCGTTTTCGACTTTGCATGTTG
TTTTAGGCCTTGCCGCCAGCGTTTCGCTTCTGAGCCAGGATCAAACTTCT
>26
CTGCTGCCGCCCGTAGGAGTCTGGGCCGTGTCTCAGTCCCAGTGTGGCTTGATCATCCTCTCAGACCAGCTAC
AGATCGTCGGCTTGGTAAGCTTTTATCCCACCAACTACCTAATCTGCCATCGGCCGCTCCGTCCGCGCGAGGT
CCGAAGATCCCCTGCTTTTCATCCGTAGATCGTATGCGGTATTTTAGCAAAGCTTTTTCGCCTCGTTTATCCC
CCACGATCAGGCACGTTTCCGATGTTATTTACTCACCCGTTTCGCCACTTCGTCAGCATCCGAAGACCTGTTT
TACCGTTTTCGACTTTGCATGTGTAAGGCATGCGCCAGCGTTCAATCTGACCAGGATCAAACTCT
>27

CTGCTGCCGCCCGTAGGAGTCTGGGCCGTGTCTCAGTCCCAGTGTGGCTGATCATCCTCTCAGACCAGCTACT
GATCGTCGCCTTGGTAGGCCGTTACCCCACCAACTAGCTAATCAGACGCGGGCCGATCTTCCGGCAGTAAACC
TTTCCCCAAAAGGGCGTATCCGGTATTAAGCCCTAGTTTTTCCCAGGGTTATTCCAGACCAGAAGGCACGTTC
CCACGCGTTACTCACCCGTCCGCCGCTGACCCCGAAGGGCCCGCTCGACTTGCATGTGTTAAGCCTGCCGCCA
GCGTTCGCTACTGATG
>28
CTGCTGCCGCCCGTAGGAGTCTGGGCCGTGTCTCAGTCCCAGTGTGGCTTGATCATTCCTCTCAGACCAGCTA
CTGATCGTCGCCTTGGTAGGCCGTTACCCCACCAACTAGCTAATTCAGACGCGGGCCGATCTTTCCGGCAGTA
AACCTTTTTCCCCAAAAGGGCGTATCCGGTATTTTAGCCCTAGTTTTTTCCCAGGGTTTATTTTCCAGACCA
GAAGGCACGTTTCCCACGCGTTTACTCACCCGTCCGCCGCTGACCCCGAAGGGCCCGCTCGACTTTTGCATGT
TGTTTAAGCCT
>29
CTGCTGCCGCCCGTAGGAGTCTGGACCGTGTCTCAGTTCTGGTGTGGCTGGTCGTCCTCTCAGGCCAGCTACA
GATCGTCGCCTTGGTGGGCCTTTACCCCACCAACCTAGCTAATCTGATATCGGCCGCTCCAATCGCGCGAGGT
CTTTTGCGATCCCCCGCTTTTCATCCGTAGATCGTATGCGGTATTTAGCACAGCTTTTCGCTGCGTTTATCCC
CCACGACTCGGGCACGTTCCGATATATTTACTCACCCGTTTCGCCACTCGCCACCAGGATTTTGCTCCCGTGC
TGCCGTTTCGACTTTGCATGTGTAGGGCATGCCGCCAGCGTTTCAATCTTGAACCAGG
>30
CTGCTGCCGCCCGTAGGAGTCTGGACCGTGTCTCAGTTCTGGTGTGGCTTGGTCGTCCTCTCAGGCCAGCTAC
AGATCGTCGCCTTGGTGGGCCTTTACCCCACCAACTAGCTAATCTGATATCGGCCGCTCCAATCGCGCGAGGT
CTTTGCGATCCCCCGCTTTTCATCCGTAGATCGTATGCGGTATTTAGCACAGCTTTCGCTGCGTTTATCCCCC
ACGACTGGGCACGTTCCGATATTATTTACTCACCCGTTTCGCCACTCGCCACCAGGATTTTGCTCCCGTGCTG
CCGTTTTCGACTTTTGCATTGTT
>31
CTGCTGCCGCCCGTAGGAGTCTGGACCGTGTCTCAGTTCTGGTGTGGCTGGTCGTCCTCTCAGGCCAGCTACA
GATCGTCGCCTTGTGGGCCTTTACCCCACCAACTAGCTAATCTGATATCGGCCGCTCCAATCGCGCGAGGTCT
TGCGATCCCCCGCTTTTCATCCGTAGATCGTATGCGGTATTAGCACAGCTCTCGCTGCGTTATCCCCCACGAC
TGGGCACGTTCCGATATATTACTCACCCGTTCGCCACTCGCCACCAGGATTGTTCCCGTGCTGCCGTTCGACT
TGCATGTGTAACGGCA
>32
CTGCTGCCGCCCGTAGGAGTCTGGGCCGTGTCTCAGTCCCAGTGTGGCTGATCATCCTCTCAAACCAGCTATG
GATCGTCGGCTTGGTAGGCCATTACCCCACCAACTACCTAATCCAACGCGGGCTAATCTTTCGGCGATAAATC
TTTTCCCCCAGGGGGCGTATGCGGTATTACCCTCAGTTTTTCCCAAGACTATTCCGCACCGAAAGGCATATTC
CCACGTGTTACTCACCCGTCCGCCGCTCACCCGAAGGCGCGCTCGACTTGCATGTGTTAGGCCTGCCGCCAGC
GTTCGTTCTGAGCCAGGATCAAACTCT
>33
CTGCTGCCGCCCGTAGGAGTTTGGGCCGGCTGTCTTCAGTCCCAATTCGATGGCTGATCAATTCCTCTCAGAC
CAGCTACTGATTCGTCGCCTTGGTGAGCCTTTACCTCACCAACTAGCTAATTCAGACGCGGGCCGCTCTAAAG
GCGATAAATCTTTTTCCCCCGAAGGGCACATCCGGTATTTTAGCTCAAGTTTTTTCCCCGAGTTTTATTTCCG
AACCTAAAGGCCACGTTTCCCACGTTGTTTTACTCACCCGTTCCGCCACTAACTTCCGAAGAGTTTTTCGTTT
TCGACTTTTGCATGTTGTTTTTAGGCCTTGCCGCCAGCGTTTTCGCTTCTTGAGCCAGGATTCAAACTTCTT
>34
CTGCTGCCGCCCGTAGGAGTTTTGGGCCGTGTCTCAGTCCCAATGTGGCTGATCATCCCTCTCAGACCAGCTA
CTGATCGTCGCCTGGTGAGCCTTTACCTCACCAACTAGCTAATCAGACGCGGGCCGCTCTAAAGGCGATAAAT
CTTTCCCCCCCGAAGGGCACATCCGGTTATTTAGCTTCAAGTTTTTCCCTGAGTTTATTTCCGAACCTAAAGG
CACGTTCCCACGTGTTTACTCACCCGTTCCGCCACTTAACTTCCGAAGAGTTTCGTTTCGACTTGCATGTTGT
TTAGGCCTTGCCGCCAGCGTTTCGCTTCTGAGCCAGGA
>35
CTGCTGCCGCCCGTAGGAGTCTGGGCCGTGTCTCAGTCCCAGTGTGGCTGATCATCCTCTCAGACCAGCTACT
GATCGTCGCCTTGGTAGGCCGTTTACCCCACCAACTAGCTAATCAGACGCGGGCCGATCTTCCGGCAGTAAAC
CTTTTCCCCAAAAGGGCGTATCCGGTTATTTAGCCCTAGTTTTTTCCCAGGGTTTATTTTCCAGACCAGAAGG
CACGTTTCCCACGCGTTACTCACCCGTCCGCCGCTGACCCGAAGGGCCCGCTCGACTTTGCATGTTGTTTAAG
CCTGCGCCAG
>36

CTGCTGCCGCCCGTAGGAGTTTGGGCCGTGTCTCAGTCCCAATGTGGCTTGATCATTCCTCTCAGACCAGCTA
CTGATCGTCGCCTTTGGTGAGCCTTTTACCTCACCAACTAGCTAATCAGACGCGGGCCGCTCTTAAAGGCGAT
AAATCTTTTTCCCCCCCCCGAAGGGCACATCCGGTATTTTAGCTTCAAGTTTTTTTTCCCTGAGTTTTTATTT
TTCCGAACCTAAAGGCACGTTTCCCACGTTGTTTACTCACCCGTTCCGCCACTTAACTTCCGAAGAGTTTTT
TCGTTTTTCGACTTTTGCATGTTGTTTTTTAGGCCTTGCCGCCAGCGTTTTTTCGCTTCTTGAGCCAGGATCA
AAAAACTTCTTATCACCGACTTGCCCCCCCCATTAGAGAGGCTGAGACTTGCAAGGCACACAGGGGGGGGGAT
AGG
>37
CTGCTGCCGCCCGTAGGAGTCTGGGCCGTGTCTCAGTCCCAGTGTGGCTGATCATCCTCTCAGACCAGCTAAG
GATCGTCGCCTTGGTGCGCCTTTACCACACCAACTAGCTAATCCTACGCGGGCTCATCTCAGGGCGATAAATC
TTTTGGTCTTTTTCGACATCATCCGGTATTTTAGCACCCCTTTTTTCGGGGAGTTTTATTTTCCGAACCCCAA
GGCAGATTTCCCACGCGTTACGCACCCGTTGCGCCACTATGCCCGAAGGCATCGTTTCGACTTTGCATTGTGT
TTTAGGCATGCCGCCAGCGTTTTCGTTTTCTTGAGCCAGGATCAAACTTCTT
>38
CTGCTGCCGCCCGTAGGAGTCTGGGCCGTGTCTCAGTCCCAGTGTGGCTTGATCATCCTCTCAGACCAGCTAC
TGATCGTCGCCTTGGTTAGGCCGTTACCCCACCAACTAGCTAATCAGACGCGGGCCGATCTTTCCGGCAGTAA
ACCTTTTCCCCAAAAGGGCGTTATCCGGTTATTTAGCCCTAGTTTTTTCCCAGGGTTTTATTTTTCCAGACCA
GAAGGCACGTTTCCCACGCGTTTACTCACCCCCGTCCGCCGCTTGACCCCCCCCGAAGGGCCCCCCGCTCGAC
TTTTGCATGTTGTTTTTAAGCCTTGCCGCCAGCGTTTCGCTACTT
>39
CTGCTGCCGCCCGTAGGAGTTTTGGACCGTGTCTCAGTTCCAATGTGGGGGACCTTCCTCTCAGAACCCCTAT
CCATCGAAGGCTTGGTGGGCCGTTACCTCACCAACAACCTAATGGAACGCATCCCCATCCTTTTACCGGAATC
CTTTTTAATAATGAAACCATGCGGAATCATTATGCCATCGGGTATTAATCTTTCTTTTTCGAAAGGCTATCCC
CGAGTAAAGGGCAGGTTTTGGATACGTGTTACTCACCCGTGCGCCGGTCGCCGGCAAAGAAAGCAAGCTTTTC
TTTCCCGATGCCCCTCGACTTGCATGTGTTTTAAGCCTGTAGCTAGCGTTCATCCCTGAGCCAAGGATCAAAC
TCT
>40
CTGCTGCCGCCCGTAGGAGTTTTGGGCCGTGTCTCAGTCCCAATGTGGCTGATCATCCTCTCAGACCAGCTAC
TGATCGTCGCCTTGGTGAGCCTTTACCTCACCAACTAGCTAATCAGACGCGGGCCGCTCTAAAGGCGATAAAT
CTTCCCCCGAAGGGCACATCCGGTATTTAGCTCAAGTTTTCCCTGAGTTTATGCCGAACCTAAAGGCACGTTC
CCACGTGTTACTCACCCGTCCGCCACTAACTCCGAAGAGTTTCGTTTCGACTTGCATTGTGTTTAGGCCCGCC
GCCAGCGTTCGCTCTGAGCCAGGATCAAACTCT
>41
CTGCTTGCCGCCCGTAGGAGTTTTGGGCCGTGTCTCAGTCCCAATGTGGCTGATCATTCCTCTCAGACCAGCT
ACTGATCGTCGCCTTTGGTGAGCCTTTACCTCACCAACTAGCTAATCAGACGCGGGCCGCTCTAAAGGCGATA
AATCTTTCCCCGAAGGGCACATCCGGTATTTAGCTTCAAGTTTTTCCCCTGAGTTTTATTTTCCGAACCTTAA
AGGCACGTTTCCCACGTTGTTTACTCACCCGTTCCGCCACTAACTTCCGAAGAGTTTTTCGTTTTCGACTTTG
CATTGTTGTTTTTTAGGCCTTGCCGCCAGCGTTTTCGCTCTTGAGCCAGGATCAAACTTCT
>42
CTGCTGCCGCCCGTAGGAGTTTTGGGCCGTGTCCCAGTCCCAATGTGGCTGATCATCCTCTCAGACCAGCTAC
TGATCGTCGCCTGTGAGCCTTTACCTCACCAACTAGCTAATCAGACGCGGGCCGCTCTAAAGGCGATAAATCT
TTCCCCCGAAGGGCACATCCGGTATTTAGCTCAAGTTTCCCTGAGTTATTTCCGAACCTAAAGGCACGTTCCC
ACGTGTTACTCACCCGTCCGCCACTAACTCCGAAGAGTTTCGTTTCGACTTGCATGTGTTTAGGCCCGCCGCC
AGCGTTCGACTCTGAGCCAGGATCAAACCTCT
>43
CTGCTGCCGCCCGTAGGAGTTTTGGGCCGTGTCTCAGTCCCAATGTGGCTGATCATCCTCTTCAGACCAGCTA
CTGATCGTCGCCTTGGTGAGCCTTTACCTCACCAACTAGCTAATCAGACGCGGGCCGCTCTAAAGGCGATAAA
TCTTCCCCCCGAAGGGCACATCCGGTATTTAGCTTCAAGTTTTTCCCTGAGTTTTTATTTTCCGAACCTAAAG
GCACGTTTCCCACGTTGTTTACTCACCCCCGTTCCGCCACTAACTTCCGAAAAGAGTTTTTTCGTTTTCGACT
TGCATTGTTTGTTTTTTAGGGGCCCTTTGCCCCCGCCCCAGCGTTTTCGCTTCTTGAGC
>44
CTGCTGCCGCCCTAGGAGTCTGGGCCGTGTCTCAGTCCCAGTGTGGCTGGTCATCCTCTCAGACCAGCTATGG
ATCGTCGCCTTGGTGGGCCATTACCCCACCAACTAGCTAATCCAACGCGGGCCGATCTTTCAGCGATAAATTC
TTTCCCCCTTTAAGGGCGTATCCGGTATTTAGCACAAGTTTTCCCTGAGTTTATTTTCCGAACTGAAAGGTAC

GTTTCCCACGCGTTACTTCACCCGTCCTGCCACTTGCTTGTATTTTTGCTTACAACCGTTTTTCGACTTGCAT
TGTGTTTTTAAGCCTTTGCCCCGCCAGCGTTCGTTTTCTTGAGCCAAG
>45
CTGCTGCCGCCCGTAGGAGTTTGGGCCGTGTCTCAGTCCCAATGTGGCTGATCATCCTCTCAGACCAGCTACT
GATCGTCGCCTTGGTGAGCCTTTACCTCACCAACTAGCTAATCAGACGCGGGCCGCTCTAAAGGCGATAAATC
TTTCCCCCCCGAAGGGCACATCCGGTATTTAGCTCAAGTTTTCCCTGAGTTTATTTCCGAACCTAAAGGCAC
GTTTCCCACGTGTTTACTCACCCGTTCCGCCACTAACTCCGAAGAGTTTTCGTTTCGACTTTGCATGTTGTTT
TAGGCCTTGCCGCCAGCGTTTTCGCTTCTGAGCCAGGATCAAACTTCTT
>46
CTGCTGCCGCCCGTAGGAGTCTGGACCGTGTCTCAGTTCCAGTGTGGCTGGTCATCCTCTCAGACCAGCTAGG
GATCGTCGCCTAGGTGAGCCGTTACCCCACCTACTGGCTAATCCCATCTGGGTTCATCTGATGGCAAGAGTCC
CGAAGGTCCCCCTCTTTTTGGTCTTGCGACGTTATGCGGTATTAGCTACCGTTTTTCCAGTAGTTATCCCCCC
TCCATCAGGCAGTTTTTTCCCAGACATTTACTCACCCGTCCGCCGCTCGTCACCCAGGAGCAAGCTCCCTGTG
TACCGCCCGACTTGCATGTGTTTTAGGCCTGCCGCCAGCGTTCAATCTGAGCCAGGATCAAACTCT
>47
CTGCTGCCGCCCGTAGGAGTTTTGGGCCGTGTCTCAGTCCCAATGTGGCTGATCATCCTCTCAGACCAGCTAC
TGATCGTCGCCTTTGGTGAGCCTTTACCTCACCAACTAGCTAATCAGACGCGGGCCGCTCTAAAGGCGAATAA
ATCTTTCCCCCCCCGAAGGGCACATCCGGTATTTAGCTCAAGTTTTTCCCTGAGTTTATTTCCGAACCTAAAG
GCACGTTTCCCACGTGTTTACTCACCCGTCCGCCATAACTCCGAAGAGTTTTCGTTTCGACTTGCATGTGTTT
TAGGCCCGCCGCCAGCGTTTTCGCTCTGAGCCAGAATCAAAACTCT
>48
CTGCTGCCGCCCGTAGGAGTCTGGGCCGTGTCTCAGTCCCAGTGTGGCTGATCATCCTCTCAGACCAGCTACT
GATCGTCGCCTTGGTAGGCCGTTACCCCACCAACTAGCTAATCAGACGCGGGCCGATCTTCCGGCAGTAAACC
TTTCCCCAAAAGGGCGTATCCGGTATTAGCCCTAGTTTTTCCCAGGGTTTATTTCCAGACCAGAAGGCACGTT
CCCACGCGTTACTCACCCGTCCGCCGCTGACCCCGAAGGGCCCGCTCGACTTTGCATGTGTTTAAGCCTGCGC
CAGCGTTCGCTCTGACGACCAGGAT
>49
CTGCTGCCGCCCGTAGGAGTTTTGGGCCGTGTCTCAGTCCCAATGTGGCTGATCATCCCTCTCAGACCAGCTA
CCTGATCGTCGCCTGGTGAGCCCTTTACCTCACCAACTAGCTAATCAGACGCGGGCCGCTCTAAAGGCGATAA
ATCTTTCCCCCGAAGGGCACATCCGGTATTTAGCTCAAGTTTTTCCCTGAGTTTATTTCCGAACCTAAAGGCA
CGTTCCCACGTGTTTACTCACCCGTCCGCCACTAACTCCGAAGAGTTTCGTTTCGACTTGCATGTGTTTTAGG
CCTGCCGCCAGCGTTCGACTCTGAGCCAGGATCAAACTCT
>50
CTGCTGCCGCCCGTAGGAGTTTGGGCCGTGTCTCAGTCCCAATGTGGCTGATCATCCTCTCAGACCAGCTACT
GATCGTCGCCTTGGTGAGCCTTTACCTCACCAACTAGCTAATCAGACGCGGGCCGCTCTAAAGGCGATAAATC
TTTCCCCCGAAGGGCACATCCGGTATTAGCTCAAGTTTTCCCTGAGTTTATTTCCGAACCTAAAGGCACGTTC
CCACGTGTTACTCACCCGTCCGCCACTAACTCCGAAGAGTTTCGTTTCGACTTGCATGTGTTTTAGGCCTGCC
GCCAGCGTTCGCTCTGAGCCAGGATCAAACTCT
>51
CTGCTGCCGCCCGTAGGAGTTTTGGGCCGTGTCTCAGTCCCAATGTGGCTGATCATCCTCTCAGACCAGCTAC
TGATTCGTCGCCTTGGTGAGCCTTTACCTCACCAACTAGCTAATCAGACGCGGGCCGCTCTAAAGGCGATAAA
TCTTTCCCCCGAAGGGCACATCCGGTTATTTTAGCTTCAAGTTTTTCCCTGAGTTTTATTTTCCGAACCTAAA
GGCACGTTCCCACGTGTTACTTCACCCCCGTTCCGCCACTAACTTCCGAAGAGTTTTCGTTTCGACTTGCATT
GTTGTTTAGGCCCCGCCCCGCCCAGCGTTCGCTTCTGAGCCCCAGGGATCAAACT
>52
CTGCTGCCGCCCGTAGGAGTCTGGGCCGTGTCTCAGTCCCAGTGTGGCTTGATCATTCCTCTCAGACCAGCTA
CTTGATCGTCGCCTTGGTAGGCCGTTACCCCACCAACTAGCTAATCAGACGCGGGCCGATCTTTCCGGCAGTA
AACCTTTTTCCCCAAAAGGGCGTATCCGGTATTAGCCCTAGTTTTTCCCAGGGTTTATTTCCAGACCAGAAGG
CACGTTCCCACGCGTTACTCACCCGTCCGCCGCCTGACCCCGAAGGGCCCGCTCGACTTTGCATGTGTTTAAG
CCTGCCGCCAGCGTTTCGCTACTGA
>53
CTGCTGCCGCCCGTAGGAGTTTTGGGCCGTGTCTCAGTCCCAATGTGGCTGATCATCCTCTCAGACCAGCTAC
TGATCGTCGCCTTGGTGAGCCTTTACCTCACCAACTAGCTAATCAGACGCGGGCCGCTCTAAAGGCGATAAAT
CTTTCCCCCGAAGGGCACATCCGGTATTTAGCTCAAGTTTTCCCTGAGTTTATTTCCGAACCTAAAGGCACGT

TCCCACGTGTTACTCACCCGTCCGCCACTAACTCCGAAGAGTTTCGTTCGACTTGCATGTGTTTAGGCCTGCC
GCCAGCGTTCGCTCTGAGCCAGGATCAAACTCT
>54
CTGCTGCCGCCCGTAGGAGTTTTGGGCCGTGTCTCAGTCCCAATGTGGCTGATCATCCTCTCAGACCAGCTAC
TGATCGTCGCCTTGGTGAGCCTTTACCTCACCAACTAGCTAATCAGACGCGGGCCGCTCTAAAGGCGATAAAT
CTTTCCCCCCCGAAGGGCACATCCGGTATTTAGCTCAAGTTTTCCCTGAGTTTATTTCCGAACCTAAAGGCAC
GTTCCCACGTGTTTACTCACCCGTCCGCCACTAACTCCGAAGAGTTTTCGTTTCGACTTGCATGTGTTTTAGG
CCTGCCGCCAGCGTTTTCGCTCTGAGCCAGGATCAAACTCT
>55
CTGCTGCCGCCCGTAGGAGTCTGGACCGTGTCTCAGTTCCAGTGTGGCTGGTCATCCTCTCAGACCAGCTAGG
GATCGTCGCCTAGGTGAGCCGTTACCCCACCTACTAGCCAATCCCATCTGGGCACATCTGATGGCAAGAGGCC
CGAAGGTCCCCCTCTTTTGGTCTTGCGACGTTATGCGGTATTAGCTACCGTTTTCCAGTAGTTATCCCCCCTC
CATCAGGCAGTTTTTTCCCCAGACATTACTCACCCGTCCGCCACTCGTCACCCGAGAGCAAGCTCTCTGTGCT
ACCGTTCGACTTGCATGTGTTAGGCCTGCCGCCAGCGTTCAATCTGAGCCAGGATCAAACTCT
>56
CTGCTGCCGCCCGTAGGAGTCTGGGCCGTGTCTCAGTCCCAGTGTGGCTGATCATCCTCTCAGACCAGCTACT
GATCGTCGCCTTGGTAGGCCGTTACCCCACCAACTAGCTAATCAGACGCGGGCCGATCTTCCGGCAGTAAACC
TTTCCCCAAAAGGGCGTATCCGGTATTAGCCCTAGTTTTTCCCAGGGTTATTCCAGACCAGAAGGCACGTTCC
CACGCGTTACTCACCCGTCCGCCGCTGACCCCGAAGGGCCCGCTACGACTTGCATGTGTTAAGCCTGCCGCCA
GCGTTGCTACTGACGACCAGGATCAA
>57
CTGCTGCCGCCCGTAGGAGTATGGACCGTGTCTCAGTTCCATTGTGGGTGATCGTCCTCTCGAACCACCTACC
GATCGTAGCCTTTGGTAGGCCGTTACCCTACCAACTAGCTAATCGGACGCGGGCTCATCTTAAAGCGGATTTA
CTCCTTTTAACCTATAAAACTGTCGAAATATAGGCTTAATGCGGTATTAATTAGCCTTTCGGCCAGCTATCCC
CCATTTTTAAGGCAGATTCCCACGCGTTACTCACCCGTCCGCCACTAGGTATTGCTACCTCGTTCGACTTGCA
TGTGTGAAGCACACCGCCAGCGTTCGTCCTGAGCCAGGATCAAACTCT
>58
CTGCTGCCGCCCGTAGGAGTTTGGGCCGTGTCTCAGTCCCAATGTGGCTGATCATCCTCTCAGACCAGCTACT
GATCGTCGCCTTGGTGAGCCTTTACCTCACCAACTAGCTAATCAGACGCGGGCCGCTCTAAAGGCGATAAATC
TTTTTCCCCCGAAGGGCACATCCCGGTATTTTAGCTCAAGTTTTTCCCTGAGTTTATTTCCGAACCTAAAGGC
ACGTTTCCCACGTGTTTTACTCACCCGTCCGCCACTAACTCCGAAGAGTTTTCGTTTTCGACTTTGCATGTGT
TTTAGGCCTGCCGCCAGCGTTTTCGCTCTGACCAGGATCAAATCT
>59
CTGCTGCCGCCCGTAGGAGCTCAGGGCTTTGGAATCCCAGAACCAGCTTTCCCCCTACCTGTGTGAGCCTCTT
TTGAGCCTCAGTTTCCTCATCTGTAAAATACAGGGTTTGGGCTGGGCACAGCGGCTCATTGCCTGTAATCCCA
GCACTTTGGAGGCCGAGGCGGGCGGAGTCACTTTTGAGATCAGGAGTTTCGAGACCAGCCTTGGCCAACATTG
GTTGAAAAAACCCCCCCCCCCCATTCTTCTTACTTAAAAAAAAAAAATTACAAAAAAAAAAAATTTTTAGCCG
GGCATTTGGTTGGTTGCATTGTTCTTGTTAATTCCCCCCATTCTTTACTTCTT
>60
CTGCTGCCGCCCGTAGGAGTCTGGGCCGTGTCTCAGTCCCAGTGTGGCTGATCATCCTCTCAGACCAGCTAAG
GATCGTCGCCTGGTGCGCCTTTACCACACCAACTAGCTAATCCTACGCGGGCTCATCCTCTGGCGATAGATCT
TTGGACTTCGTCATCACCGGTATTAGCAGTCGTTTTTCCAACTGTTTATTTCCGAACCAAAGGGCAGATTTCC
CACGCGTTACGCACCCGTGCGCCCACTAGACCCGAAGATCTCGTTTCGACTTGCATTGTGTTTTAGGCATGCC
GCCAGCGAT
>61
CTGCTGCCGCCCGTAGGAGTCTGGACCGTGTCTCAGTTCCAGTGTGGCTGGTCATTCCTCTCAGACCAGCTAG
GGATCGTCGCCTAGGTGAGCCGTTACCCCACCTACTAGCTAATCCCATCTGGGCACATCTGATTGGCAAGAGG
CCCGAAGGTCCCCCTCTTTTTGGTCTTGCGACGTTATGCGGTATTTAGCTTACCGTTTTCCAGTAGTTTATTC
CCCCTCCATTCAGGCAGTTTTTCCCAGACATTTACTCACCCGTCCGCCACTCGTCACCCGAGAGCAAGCTCTC
TTGTGCTTACCGTTTCGACTTGCATTGTGTTTAGGC
>62
CTGCTGCCGCCCGTAGGAGTTTTTGGGCCGTGTCTCAGTCCCAATGTGGCTGATCATCCTCTCAGACCAGCTA
CTGATGTCGCCTTTGGTGAGCCTTTACCTCACCAACTAGCTAATCAGACGCGGGCCGCTCTAAAGGCGATAAA
TCTTTCCCCCGAAGGGCACATCCGGTATTTAGCTTCAAGTTTTCCCTGAGTTTATTTCCGAACCTAAAGGCAC

GTTCCCACGTGTTACTCACCCGTTCCGCCACTAACTTCCGAAGAGTTTTCGTTTCGACTTGCATTGTTGTTTT
AGGCCTTGCCGCCAGCGTTCGCTCTGAGCCAGGATTCAACTTCT
>63
CTGCTGCCGCCCGTAGGAGTCTGGGGCCGTGTCTCAGTCCCAGTGTGGCCGGTCGCCCCTCTCAGGCCGCTAC
CCGTCGTCGCTTGGTAGGCCATTACCCCACCAACAAGCTGATAGGACGCGAGTCCATCCACCACCGATAAATC
TTTCCACCAACACCCCATGCAAGGGTTGGTCGGTATCCAGTATTAGACGCCGTTTCCAGCGCTTATCCCAGAG
TGATGGGCAGGTTACTCACGTGTTACTCACCCGTTCGCCACTGATCCACGAGTGCAAGCACCCGCTTTCACCG
TTTCGACTTGCATGTGTTTAAGCACGGCCGCCAGCGTTCGTCCTGAGCCAGGATCAAACTCT
>64
CTGCTGCCGCCCGTAGGAGTCTGGGCCGTGTCTCAGTCCCAGTGTGGCTGATCGTCCTCTCAGACCAGCTACC
GATCGCAGGCTTGGTAGGCCTTTACCCACCAACTACCTAATCGGACGCAGGCTGCTCCCAAGGCGCCCTAAGG
CTTTGACCCACAGGCCCCATGCCGGTATTAGCCCAGTTTTCCCAGGGTTGTCCCCCACCTCAGGATACATTCC
TACGCGATACTCACCCGTCCGCCGCTGATGGCCGAAACCACCCGCGCGACTTGCATGTGTTAAGCATGCCGCC
AGCGTTCGCTCTGAGCCAGGATCAAACTCT
>65
CTGCTGCCGCCCGTAGGAGTCTGGGCCGTGTCTCAGTCCCAGTGTGGCTGATCATCCTCTCAGACCAGCTACT
GATCGTCGCCTTGGTAGGCCGTTACCCCACCAACTAGCTAATCAGGCGCGGGCCGATCTTTCCGGCAGTAAAC
CTTTTCCCCAAAAGGGCGTATCCGGTATTTAGCCCTAGTTTTTCCCAGGGTTTATTTTCCAGACCAGAAGGC
ACGTTTCCCACGCGTTACTCACCCGTCCGCCGCTGACCCCGAAGGGCCCGCTCGACTTTGCATGTTGTTTAAG
CCTGCCGCCAGCGTTTCGCTCTGAGCCAGGA
>66
CTGCTGCCGCCCGTAGGAGTTTGGACCGTGTCTCAGTTCCAATGTGGGGACCTTCCTCTCAGAACCCTATCCA
TCGAAGGCTTTGGTGGGCCGTTGACCTTCACCAACAACCTAATGGAACGCATCCCCATTCCTTTTTACCGGAA
TTCCTTTTTTTAATTAATTGAAACCATGCGGAATTCATTTTATGCCATCGGGTTATTTTAATTCTTTTTCTTT
TTTCGAAAGGCTATCCCCGAGTTAAAGGGCAGGTTTTGGATACGTTGTTTTACTTCACCCGTTGCGCCGGTTC
GCCAGGCAAAGAAAGCAAGCTTTTTTCTTTTTCCCGATTGCCCCCCCCTTCGACTTTTTGCATTGTTGTTTTT
TAAGCCTTG
>67
CTGCTGCCGCCCGTAGGAGTATGGCCCGTGTCGCAGTGCCATTCTTTGGGGGGTCGCGCTCTCACGCCCCCCT
ATCCGTCATAGCCTTTGGTGAGCTTTTACCTCACCAACTAGCTGATAGACCGCAGACCATTTCATAAAGCGGC
TTGCGCCTTTACCCTTTGCGGGACTATGGGAAATTACCCCGTCTTTTCGACGAGCTATGCCCCACTTTACGGT
TATGTTATCTTACGTTGTTTACTTAAGCCGTTTCGCCGGTTGCCTTTGCGGCCCCTTTGACTTTTGCATTGCC
GGTTATTCCACATTTTTCCAGCGTTTCACCCTTGAGCCAGGATTCAAACTTCTT
>68
CTGCTGCCGCCCGTAGGAGTCTGGACCGTGTCTCAGTTCCAGTGTGGCTGGTCATCCTCTCAGACCAGCTAGG
GATCGTCGCCTAGGTGAGCCGTTACCCCCCACCTACTTAGCTAATCCCATCTGGGCACATCTGATTGGCAAGA
GGCCCGAAGGTCCCCCTCTTTTTGGTCTTGCGACGTTATGCGGTATTTTAGCTTACCGTTTTTTCCAGTAGT
TTATTCCCCCCCTTCCATCAGGCAGTTTTTTTCCCCCCAGACATTTTACTCACCCGTCCGCCGCTCGTCACCG
AGCGAGCATGTCTTGCTTTTTTCC
>69
CTGCTGCCGCCCGTAGGAGTTTTGGGCCGTGTCTCAGTCCCAATGTGACTGATCATCCTCTCAGACCAGCTAC
TGATCGTCGCCTTGGTGAGCCTTTACCTCACCAACTAGCTAATCAGACGCGGGCCGCTCTAAAGGCGATAAAT
CTTTCCCCCGAAGGGCACATCCGGTATTTAGCTCAAGTTTTCCCTGAGTTTATTTCCGAACCTAAAGGCACGT
TCCCACGTGTTACTCACCCGTCCGCCACTAACTCCGAAGAGTTTTCGTTTCGACTTGCATGTGTTTAGGCCTG
CCGCCAGCGTTTCGCTCTGAGCCAGGATCAAACTCT
>70
CTGCTGCCGCCCGTAGGAGTTTGGGCCGTGTCTCAGTCCCAATGATGGCTGATCATCCTCTTCAGACCAGCTA
CTGATTCGCGCCTTGGTGAGCCTTTACCTCACCAACTAGCTAATTCAGACGCGGGCCGCTCTAAAGGCGATAA
ATCTTTCCCCCCCGAAGGGCACATCCGGTTATTTTAGCTTCAAGTTTTTTTCCCTGAGTTTTTATTTTTCCGA
ACCTTAAAAAGGCACGTTTTCCCCACGTTGTTTTTACTCACCCCCCCGTTTCCCCGCCCCACTAACTTCCGAA
AAAGAGTTTTTTTCGTTTTTTTCGA
>71
CTGCTGCCGCCCGTAGGAGTCTGGGCCGTGTCTCAGTCCCAGTGTGGCTGATCATCCTCTCAGACCAGCTACT
GATCGTCGCCTTGGTAGGCCGTTACCCCACCAACTAGCTAATCAGACGCGGGCCGATCTTTCCGGCAGTAAAC

CTTTTTCCCCCAAAAGGGCGTATCCGGTATTAGCCCTAGTTTTTCCCAGGGTTTATTTCCAGACCAGAAGGCA
CGTTCCCACGCGTTACTCACCCGTCCGCCGCTGACCCCGAAGGGCCCGCTACGACTTTGCATGTGTTTAAGCC
CTGCCGCCAGCGTTTCGCTCTGAGCCAGGATCAAACTCT
>72
CTGCTGCCGCCCTAGGAGTTTTGGACCGTGTCTCAGTTCCAATGTGGGGGACCTTCCTCTTCAGAACCCCTAC
TGATCGTTGCCTTTGGTGGGCCGTTACCCCCGCCAACAAGCTAATTCAGACGCATCCCCATCCATTAACCGAT
AAATCTTTTTACTTTCACATCTGATGCCGTCAGTGAAGACCATAAAGGTATTTAGTCTGCCTTTTTCGGCAGG
TTTATTCCCTTTAGTCATTGGGAAGGTTTTGGATTACGCGTTTACTTCACCCGTTGCGCCGGTCGACGTCCAA
AAAGTGCAAGCAACTTTAA
>73
CTGCTGCCGCCCGTAGGAGTCTGGGCCGTGTCTCAGTCCCAGTGTGGCTGGTCATCCTCTCAGACCAGCTATG
GATCGTCGCCTTGTGGGCCATTTACCCCACCAACTAGCTAATCCAACGCGGGCCGATCTTTCAGCGATAAATC
TTTTCCCCTTTAAGGGCGTATCCGGTATTTAGCACAAGTTTTCCCTGAGTTTATTTCCGAACTGAGAGGTACG
TTTCCCACGCGTTACTCACCCGTCTGCCACTGCTTGTATTTTGCTACAACCGTTTCGACTTGCATGTGTTTTA
AGCCTTGCCGCCAGCGTTCGTTTCTTGAGCCAGGATCAAACTCT
>74
CTGCTGCCGCCCGTAGGAGTCGTGGGCCGTGTCTCAGTCCCAGTGTGGCTGGTCATCCTCTCAGACCAGCTAT
GGATCGTCGCCTTTGGTGGGCCATTACCCCACCAACTAGCTAATCCAACGCGGGCCGATCTTTCAGCGATAAA
TCTTTTCCCCTTTAAGGGCGTATCCGGTATTTAGCACAAGTTTCCCTGAGTTTATTTCCGAACTGAAAGGTA
CGTTTCCCACGCGTTACTCACCCGTCTGCCACTGCTTGTATTTTGCTACAACCGTTTTCGACTTTGCATGTGT
TTTAAGCCTTGCCGCCAGCGTTTCGTTTCTTGAGCCAGGATCAAACATCTT
>75
CTGCTGCCGCCCGTAGGAGTTTGGGCCGTGTCTCAGTCCCAATGTGGCTGATCATCCTCTCAGACCAGCTACT
GATCGTCGCCTTGGTGAGCCTTTACCTCACCAACTAGCTAATCAGACGCGGGCCGCTCTAAAGGCGATAAATC
TTTCCCCCCCGAAGGGCACATCCGGTATTTAGCTCAAGTTTTTCCCTGAGTTTTATTTCCGAACCTAAAGGCA
CGTTTCCCACGTGTTTACTCACCCGTTCCGCCACTAACTTCCGAAGAGTTTTTCGTTTTCGACTTTGCATGTT
GTTTTAGGCCTTGCCGCCAGCGTTTTCGCTTCTGAGCCAGGATCAAACTTCTT
>76
CTGCTGCCGCCCGTAGGAGTTTTGGGCCGTGTCTCAGTCCCAATGTGGCTGATCATCCTCTCAGACCAGCTAC
TGATCGTCGCCTTTGGTGAGCCTTTACCTCACCAACTAGCTAATCAGACGCGGGCCGCTCTAAAGGCGATAAA
TCTTTTCCCCCGAAGGGCACATCCGGTATTTTAGCTCAAGTTTTTTCCCTGAGTTTATTTCCGAACCTAAAG
GCACGTTTCCCACGTGTTTTACTCACCCGTCCGCCACTAACTCCGAAGAGTTTTCGTTTTCGACTTGCATGTG
TTTTAGGCCTGCCGCCAGCGTTTTCGCTCTGAGCCAGGATCAAACTCT
>77
CTGCTGCCGCCCGTAGGAGTCTGGGCCGTGTCTCAGTCCCAGTGTGGCTGATCATCCTCTCAGACCAGCTAAG
GATCGTCGCCTTGGGTGCGCCTTTACCACACCAACTAGCTAATCCTACGCGGGCTCATCTCAGGGCGATAAAT
CTTTTGGTCTTTTCGACATCATCCGGTATTTTAGCACCCCTTTTTCGGGGAGTTTATTTCCGAACCCCAAGGC
AGATTCCCACGCGTTACGCACCCGTGCGCCACTATGCCCGAAGGCATCGTTTCGACTTGCATGTGTTTTAGGC
ATGCCGCCAGCGTTTTCGTTTTCTGAGCCAGGATCAAACTCT
>78
CTGCTGCCGCCCGTAGGAGTTTGGGCCGTGTCTCAGTCCCAATGTGGCTGATCATCCTCTCAGACCAGCTACT
GATCGTCGCCTTGGTGAGCCTTTTACCTCACCAACTAGCTAATCAGACGCGGGCCGCTCTAAAGGCGATAAAT
CTTTTTCCCCCCCCCGAAGGGCACATCCGGTATTTAGCTCAAGTTTTTTCCCTGAGTTTTATTTTCCGAACC
TAAAGGCACGTTTCCCCACGTGTTTTACTCACCCGTCCGCCACTAACTCCGAAGAGTTTTCGTTTTCGACTTT
GCATGTTGTTTTTAGGCCTTGCCGCCAGCGTTTTTCGCTCTGAGCCAGGATCAAACTTCTT
>79
CTGCTGCCGCCCGTAGGAGTCTGGGGCCGTGTCTCAGTCCCAGTGTGGCTGATCATCCTCTCAGACCAGCTAA
GGATCGTCGCCTTGGTGCGCCTTTACCACACCAACTAGCTAATCCTACGCGGGCTCATCTCAGGGCGATAAAT
CTTTGGTCTTTCGACATCATCCGGTATTAGCACCCCTTTCGGGGGAGTTATTTCCGAACCCCAAGGCAGATTC
CCACGCGTTACGCACCCGTGCGCCACTATGCCCGAAGGCATCGTTCGACTTGCATATGTTTAGGCATGCCGCC
AGCGTTCGTTTCTGAGCCAGGATCAAACTCT
>80
CTGCTTGCCGCCCCGTAGGGAGTCTTGGGGCCGTTGTCTTCAGTTCCCCAGTTGTGGCTTGATTCATCCCTCT
CAGACCAGCTATGATCGTCGCCTTTGGTAGGCCGTTTACCCCCACCAACTAGCTAATCAGACGCGGGCCGATC

TTTCCGGCAGTTAAACCTTTCCCCCCCCAAAAGGGCGTATCCGGTATTAGCCCCTAGTTTTTCCCAGGGTTTA
TTTTCCAGACCAGAAGGCACGTTTCCCACGCGTTTACTCACCCCCGTCCGCCGCTGACCCCCCGAAGGGCCCC
CGCTCGACT
>81
CTGCTGCCGCCCGTAGGAGTTTGGGCCGCGTCTCAGTCCCAATGTGGCTGATCATCCTCTCAGACCAGCTACT
GATCGTCGCCTTTGGTGAGCCTTTACCTCACCAACTAGCTAATCAGACGCGGGCCGCTCTAAAGGCGATAAAT
CTTTTCCCCCGAAGGGCACATCCGGTATTTAGCTCAAGTTTCCCCTGAGTTTTATTCCGAACCTAAAGGCAC
GTTCCCACGTGTTACTCACCCGTCCGCCACTAACTCCGAAGAGTTTTCGTTTCGATTGCATGTGTTTTAGGCC
TGCCGCCAGCGTTTCGCTCTGAGCCAGGATCAAACTCT
>82
CTGCTGCCGCCCGTAGGAGTCTGGGCCGTGTCTCAGTCCCAGTGTGGCCGGTCGCCCTCTCAGGCCGGCTACC
CGTCGTCGCCTTGGTAGGCCATTACCCCACCAACAAGCTGATAGGACGCGAGTCCATCCACCACCGATAAATC
TTTTCCACCAACACCCCATGCAAGGGTTTGGTCGGTATCCAGTATTAGACGCCGTTTTTCCAGCGCTTATCCC
AGAGTGATGGGCAGGTTTACTCACGTGTTTACTCACCCGTTTCGCCACTGATCCACGAGTGCAAGCACCCGCA
TCACCTGTTCGATCTTGCATGTGTTTAAGCACGCCGCCAGCGTTCGTCCTGAGCCAAGGATAAACTCCT
>83
CTGCTGCCGCCCGTAGGAGTCTGGGCCGTGTCTCAGTCCCAGTGTGGCTGATCATCCTCTCAGACCAGCTACT
GATCGTCGCCTTGGTAGGCCGTTACCCCACCAACTAGCTAATCAGACGCGGGCCGATCTTTCCGGCAGTAAAC
CTTTTCCCCAAAAGGGCGTATCCGGTATTTAGCCCTAGTTTTTCCCAGGGTTTTATTTCCAGATCAGAAGGC
ACGTTCCCACGCGTTACTCACCCGTCCGCCGCTGACCCCGAAGGGCCCGCTCGACTTGCATGTGTTTAAGCCT
GCCGCCAGCGTTCGCTCTGAGCCAGGATCAAAACCTCT
>84
CTGCTGCCGCCCGTAGGAGTCTGGACCGTGTCTCAGTTCCAGTGTGGCCGATCACCCCTCTCAGGTCGGCTAT
GCATCGTTGCCTTGGTAAGCCGTTACCTTACCAACTAGCTAATGCAGCGCGGATCCATCTATAAGTGACAGCA
AGACCGTCTTTTTACTTTTGAACCATGCGGTTTCAAAAAATATTTTATCCGGTATTTAGCTCCGGTTTTTTCC
CGAAGTTTATCCCAGTCTTTTATAGGTAGGTTTTATCCACGTGTTTTACTCACCCGTCCGCCGCTAACATCAG
AGAAGCAAGCTTTCTCGTCCGTTTCGCTCGATTTTGCATTGTATTTTAGGCACGCCGC
>85
CTGCTGCCGCCCGTAGGAGTTTGGGCCGTGTCTCAGTCCCAATCGTGGCTGATCATTCCTCTCAGACCAGCTA
CTGATCGTCGCCTTGGTGAGCCTTTTACCTCACCAACTAGCTAATCAGACGCGGGCCGCTCTAAAGGCGATAA
ATCTTTTTCCCCCCCCCCGAAGGGCACATCCGGTATTTTTAGCTCAAGTTTTTTCCCTGAGTTTTATTTTCCG
AACCTAAAGGCACGTTTTCCCCACGTGTTTTACTCACCCGTCCGCCACTAACTCCGAAGAGTTTTCGTTTTCG
ACTTTTGCATGTGTTTTAGGCCTGCCGCCAGCGTTTTCGCTCTGAGCCAGGATCAAACTCT
>86
CTGCTGCCGCCCGTAGGAGTCTGGACCGTGTCTCAGTTCTGGTGTGGCTGGTCGTCCTCTCAGGCCAGCTACA
GATCGTCGCCTTGGTGGGCCTTTACCCCACCAACTAGCTAATCTGATATCGGCCGCTCCAATCGCGCGAGGTC
TTTGCGATCCCCCGCTTTTCATCCGTAGACCGTATGCGGTATTTAGCACAGCTTTTCGCTGCGTTTATCCCCC
ACGACTCGGGCACGTTTCCGATATATTTACTCACCCGTTTTCGCCACTCGCCACCAGGATTTTGCTCCCGTGC
TGCCGTTTTCGACT
>87
CTGCTGCCGCCCGTAGGAGTCTGGGCCGTGTCTCAGTCCCAGTGTGGCTGATCATCCTCTCAGACCAGCTACT
GATCGTCGCCTTGGTAGGCCGTTACCCCACCAACTAGCTAATCAGACGCGAGCCGATCTTCCGGCAGTAAACC
TTTCCCCCCAAAAGGGCGTATCCGGTTATTTAGCCCTAGTTTTCCCAGGGTTTATTTCCACGACCAGAAGGCA
TTGTTTCCCACGCGTTACTCACCCGTCCGCCGCTGACCCCCCGAAGGGCCCCCGCTCGACTTGCATGTGTTTA
AGCCTGCCGCCAGCGTTCGCTCTGAGCCAGGATCAA
>88
CTGCTGCCGCCCGTAGGAGTCTGGGCCGTGTCTCAGTCCCAGTGTGGCTGGTCATCCTCTCAGACCAGCTATG
GATCGTCGCCTTGTGGGCCATTACCCCACCAACTAGCTAATCCAACGCGGGCCGATCTTTCAGCGATAAATCT
TTTCCCCTTTAAGGGCGTATCCGGTATTAGCACAAGTTTTCCCTGAGTTTATTTCCGAACTGAAAGGTACGTT
TCCCACGCGTTACTCACCCGTCTGCCACTGCTTGTATTTTGCTACAACCGTTTTCGACTTGCATGCGTTTAAG
CCTTGCCGCCAGCGTTCGTTTCTTGAGCCAGGATCAAACTCTT
>89
CTGCTGCCGCCCGTAGGAGTCTGGGCCGTGTCTCAGTCCCGGTGTGGCTGATCATCCTCTCAGACCAGCTACT
GATTCGTCGCCTTGGTAGGCCGTTACCCCACCAACTAGCTAATTCAGACGCGGGCCGATCTTCCGGCAGTAAA

CCTTTTCCCCCCAAAAAGGGCGTATCCGGTTATTTAGCCCCCTAGTTTTTTTCCCAGGGTTTTATTTTCCAGA
CCAGAAGGCACGTTTCCCACGCGTTACTCACCCCCGTCCGCCGCTGACCCCCCCGAAGGGCCCCCGCTCGACT
TTGCATGTGTTTTAAGCCTTGCCGCCAGCGTTTCGCTCTTGAGCCAGGATCAAACTCTT
>90
CTGCTGCCGCCCGTACGGAGTCTGGGCCGTGTCTCAGTCCCAGTGTGGCTGATCGTCCTCTCAGACCAGCTAC
AGATCGTAGGCTTTGGTTAAGCTTTTATCCCACCAACTACCTTAATCTGCCATCGGCCGCTCTAGTAGCACAA
GGCCCGAAGGTCCCCTGCTTTTTCACCATAGAGTCCTCATGCGGTATTAGCCACTCTTTTTCGAGTAGTTTTA
TCCCCCCACTACTAGGCACGTTCCCGATGTATTACTCGACCCGTTCGCCACTCGTCAGCATCCCGAAAGACCT
GTTTACCGTTTCGACTTTGACAGTGTAAGGCATGCCGCCAGCGTTTTCAATCGCGAGCCA
>91
CTGCTGCCGCCCGTAGGAGTATGGACCGTGTCTTCAGTTCCATTGTGGGTGATCGTCCTCTCGAACCACCTAC
CGATCGTAGCCTTGGTAGGCCGTTACCCTACCAACTAGCTAATCGGACGCGGGCTCATTCTTTAAAACGGATT
TACTCCTTTTAACCTTATAAAACTGTCGAAATATAGGCTTTATGCGGTTATTTAATTTAGCCTTTTCGGCCAG
CTTATCCCCCATTTTTAAGGCAGATTTCCCACGCGTTACTTCACCCGTCCGCCACTAGGTATTTTGCTACCTT
CGTTTCGACTTTGCATGTTGTGAAGCACACCGCCAGCGTTCGTCCTTGA
>92
CTGCTGCCGCCCGTAGGAGTTTTGGGCCGTGTCTCAGTCCCAATGTGGCTGATCATCCTCTCAGACCAGCTAC
TGATCGTCGCCTTTGGTGAGCCTTTACCTCACCAACTAGCTAATTCAGACGCGGGCCGCTCTAAAGGCGATAA
ATCTTTCCCCCGAAGGGCACATCCGGTATTTATGACTTCAAGTTTTTCCCTGAGTTTTATTTTCCGAACCTAA
AGGCACGTTTCCCACGTGTTTACTCACCCGTTCCGCCACTAACTTCCGAAGAGTTTTCGTTTTCGACTTGCAT
TGTTGTTTTTAGGCCTTGCCGCCAGCGTTTCGCTTCTTGAGCCAGGATCAAACTTCT
>93
CTGCTGCCGCCCGTAGGAGTTTTTGGGCCGTGTCTCAGTCCCAATGTGGCTGATCATCCTCTCAGACCAGCTA
CTGATCGTCGCCTTTGGTGAGCCTTTACCTCACCAACTAGCTAATCAGACGCGGGCCGCTCTAAAGGCGATAA
ATCTTTCCCCCGAAGGGCACATCCGGTATTTAGCTTCAAGTTTTTCCCTGAGTTTTATTTTCCGAACCTTAAA
GGCACGTTTCCCACGTTGTTTACTCACCCGTTCCGCCACTAACTTCCGAAGAGTTTTCGTTTCGACTTGCATT
GTTGTTTTAGGCCTTGCCGCCAGCGTTTCGCTTCTTGAGCCAGGATTCAAACTTCTT
>94
CTGCTGCCGCCCGTAGGAGTCTGGGCCGTGTCTCAGTCCCAGTGTGGCTGATCATCCTCTCAGACCAGCTAAG
GATCGTCGCCTTGGTGCGCCTTTACCACACCAACTAGCTAATCCTACGCGGGCTCATCTCAGGGCGATAAATC
TTTTGGTCTTTTCGACATCATCCGGTATTTAGCACCCCTTTTCGGGGAGTTTATTTTCCGAACCCCAAGGCAG
ATTCCCACGCGTTACGCACCCGTGCGCCACTATGCCCGAAGGCATCGTTTCGACTTTGCATTGTTGTTTAGGC
AGTGCCGCCAGCGTTTCGTTTCTTGAGCCAGGATTCAAACTTCT
>95
CTGCTGCCGCCCGTAGGAGTTTTGGGCCGTGTCTCAGTCCCAATGTGGCTGATCATCCTCTCAGACCAGCTAC
TGATCGTCGCCTTGGTGAGCCTTTACCTCACCAACTAGCTAATCAGACGCGGGCCGCTCTAAAGGCGATAAAT
CTTCCCCCGAAGGGCACATCCGGTATTTAGCTCAAGTTTTTCCCTGAGTTTTATTTCCGAACCTAAAGGCACG
TTTCCCACGTGTTTACTCACCCGTTCCGCCACTAACTCCGAAGAGTTTTCGTTTCGACTTTGCATGTTGTTTT
AGGCCTTGCCGCCAGCGTTTCGCTTCTGAGCCAGGATCAAACTTCT
>96
CTGCTGCCGCCCGTAGGAGTCTGGGCCGTGTCTCAGTCCCAGTGTGGCTGATCATCCTCTCAGACCAGCTCTG
ATCGTCGCCTTGGTAGGCCGTTACCCCACCAACTAGCTAATCAGACGCGGGCCGATCTTCCGGCAGTAAACCT
TTTCCCCAAAAGGGCGTATCCGGTATTAGCCCTAGTTTTTCCCAGGGTTTATTTCCAGACCAGAAGGCACGTT
CCCACGCGTTACTCACCCGTCCGCCGCTGACCCCGAAGGGCCCGCTCGACTTTGCATGTGTTAAGCCTGCGCC
AGCGTTCGCTCTGAGCCAGGATCAAACTC
>97
CTGCTGCCGCCCGTAGGAGTCTGGGGCCGTGTCTCAGTCCCAGTGTGGCTGATCATCCTCTCAGACCAGCTAC
TGATCGTCGCCTTGGTAGGCCGTTACCCCACCAACTAGCTAATCAGGCGCGGGCCGATCTTCCGGCAGTAAAC
CTTTTCCCCAAAAGGGCGTATCCGGTATTAGCCCTAGTTTTTCCCAGGGTTTATTTCCAGACCAGAAGGCACG
TTCCCACGCGTTACTCACCCGTCCGCCGCTGACCCCGAAGGGCCCGCTCGACTTGCATGTGTTAAGCCTGCCG
CCAGCGTTCGCTCTGAGCCAGGATCAAACTCT
>98
CTGCTGCCGCCCGTAGGAGTCTGGGGCCGTGTCTCAGTCCCAGTGTGGCTGATCATCCTCTCAGACCAGCTCA
GATCGTCGGCTTGGTAAGCTTTTATCCCACCAACTACCTAATCTGCCATCGGCCGCTCCGTCCGCGCGAGGTC

CGAAGATCCCCTGCTTTCATCCGTAGATCGTATGCGGTATTAGCAAAGCTTTTCGCCTCGTTATCCCCCACGG
ATCGGGCACGTTCCGATGTATTACTCACCCGTTCGCCACTCGTCAGCATCCGAAGACCTGTTACCGTTTCGAC
TTGCATGTGTAAGGCATGCCGCCAGCGTTCAATCTGACCAGGATCAAACTCT
>99
CTGCTGCCGCCCGTAGGAGTCTGGGCCGTGTCTCAGTCCTAGTGTGGCTGATCATCCTCTCAGACCAGCTACT
GATTCGTCGCCTTGGTAGGCCGTTACCCCACCAACTAGCTAATCAGACGCGGGCCGATCTTTCCGGCAGTAAA
CCTTTTCCCCAAAAGGGCGTATCCGGTATTTAGCCCTAGTTTTTCCCAGGGTTTATTTCCAGACCAGAAGGC
ACGTTTCCCACGCGTTACTCACCCGTCCGCCGCTGACCCCGAAGGGCCCGCTCGACTTTGCATGTGTTTAAGC
CTGCGCCAGCGTTTCGCTCTGAGCCAGGATCAAAACTCT
>100
CTGCTTGCCGCCCGTAGGAGTTTTGGGGCCGTGTCTCAGTCCCAATGTGGCTGATCATCTTCTCAGACCAGCT
ACTGATCGTCGCCTTGGTGAGCCTTTACCTCACCAACTAGCTAATTCAGACGCGGGCCGCTCTAAAGGCGATA
AATCTTCCCCCCCGAAGGGCACATCCGGTATTTAGCTCAAGTTTTCCCTGAGTTTATTTCCGAACCTAAAGGC
ACGTTTCCCACGTGTTTACTCACCCGTCCGCCACTAACTCCGAAGAGTTTTCGTTTCGACTTGCATGTTGTTT
TAGGCCTTGCCGCCAGCGTTTCGCTTCTTGAGCCAGGATCAAACTTCT
>101
CTGCTGCCGCCCGTAGGAGTCTGGGGCCGTGTCTCAGTCCCAGTGTGGCTGGTCATCCTCTCAGACCAACTAC
GAATCGTTGCCTTTGGTAGGCCTTTTACCCCACCAACTAGCTAATCCGACGTAGGCTCAACCCAGAGCGCAAG
GCCCTTTTCAGGTCCCCTGCTTTTTGATTTTTCTCAATATCATGCGGTATTTTAGCCCGAGTTTTTCCTCGGG
TTTATCCCCCTCCTCCAGGACAGATTTCCTACGTATTTACTCACCCGTCCGCCACTCGCCACCCATAAAGACC
GAAATCTTACTGTGCTGCCGTTTCGACTTGCATGTGTTTTAGGCATGCCGCCAGCGTTCAACTCTGAGC
>102
CTGCTGCCGCCCGTAGGAGTCTGGGCCGTGTCTCAGTCCCAGTGTGGCCGGTCGCCCTCTCAGGCCGGCTACC
CGTCGTCGCCTTGGTAGGCCATTTACCCCCACCAACAAGCTGATAGGACGCGAGTCCATCCACCACCGATAAA
TCTTTTCCACCAACACCCCATGACAAGGGTTTGGTCGTTATCCAGTTATTTTAGACGCCGTTTTTTTCCAGCG
CTTTTTATTCCCCAGAGTGATTGGGGGCAGGTTTTTACTTCACGTTGTTTTACTTTCACCCCGTTTTCGCCCC
ACTTGATTCCCCACGAGTGCAAAAGCACCCCGCCTTTTCACCCCGTTTTGACCTTGCATTGT
>103
CTGCTGCCGCCCTAGGAGTCTGGACCGTGTCTCAGTTCCAGTGTGACTGATCATCCTCTCAGACCAGTTACGG
ATCGTCGCCTTGGTAGGCCTTTACCCCACCAACTAGCTAATCCGACCTAGGCTCATCTGATAGCGTGAGGTCC
GAAGATCCCCCACTTTCTCCCTCAGGACGTATGCGGTTATTTAGCGCCCGTTTTTCCGGACGTTATTCCCCAC
TACCAGGCAGATTTCCCTAGGCATTTTACTCACCCGTTCCGCCGCTGAATCCAGGAGCAAGCTCCCTTCATCC
GCTCGACTTGCATTGTGTTTTAGGCCTGCCGCCAGCGTTTCAATTCTTGAGCCAGGATCAAACATCTT
>104
CTGCTGCCGCCCGTAGGAGTTTTGGGCCGTGTCTCAGTCCCAATGTGGCTGATCATCCTCTCAGACCAGCTAC
TGATCGTCGCCTTTGGTGAGCCTTTACCTCACCAACTAGCTAATCAGACGCGGGCCGCTCTAAAGGCGATAAA
TCTTTCCCCCCCGAAGGGCACATCCGGTATTTAGCTCAAGTTTTTCCCTGAGTTTATTTCCGAACCTAAAGGC
ACGTTTCCCACGTGTTTACTCACCCGTCCGCCACTAACTCCGAAGAGTTTTCGTTTTCGACTTTGCATGTTGT
TTTAGGCCTTGCCGCCAGCGTTTCGCTTCTGAGCCAGGATCAAACTTCT
>105
CTGCTGCCGCCCGTAGGAGTCTGGGCCGTGTCTCAGTCCCAGTGTGGCTGATCATCCTCTCAGACCAGCTACT
GATCGTCGCCTTGGTAGGCCGTTACCCCACCAACTAGCTAATCAGACGCGGGCCGATCTTCCGGCAGTAAACC
TTTCCCCAAAAGGGCGTATCCGGTATTAGCCCTAGTTTTTCCCAGGGTTTATTTCCAGACCAGAAGGCACGGT
TCCCACGCGTTACTCACCCGTCCGCCGCTGACCCCGAAGGGCCCGCTCGACTTGCATGTGTTAAGCCTGCCGC
CAGCGTTC
>106
CTGCTGCCGCCCGTAGGAGTTTTGGGCCGTGTCTCAGTCCCAATGTGGCTGATCATCCTCTCAGACCAGCTAC
TGATCGTCGCCTTTGGTGAGCCTTTACCTCACCAACTAGCTAATCAGACGCGGGCCGCTCTAAAGGCGATAAA
TCTTTCCCCCCCGAAGGGCACATCCGGTATTTAGCTCAAGTTTTTCCCTGAGTTTATTTCCGAACCTAAAGGC
ACGTTTCCCACGTGTTTACTCACCCGTCCGCCACTAACTCCGAAGAGTTTTCGTTTTCGACTTGCATGTGTTT
TAGGCCTTGCCGCCAGCGTTTTCGCTCTGAGCCAGGATCAAACTCT
>107
CTGCTGCCGCCCGTAGGAGTCTGGGCCGTGTCTCAGTCCCAGTGTGGCTGGTCATCCTCTCAGGACCAGCTAT
GGATCGTCGCCTTGGTGGGCATTACCCACCAACTAGCTAATCCAACGCGGGCCGATCTTTTCAGCGATAAATC

TTTTTTCCCCTTTAAGGGCGTATCCGGTATTTTAGCACAAGTTTTTCCCTGAGTTTTATTTCCGAACTGAAAG
GTACGTTTTCCCACGCGTTTACTCACCCGTCTGCCACTGCTGTATTTTGCTACAACCGTTTTCGACTTTGCAT
GTGTTTTAAG
>108
CTGCTGCCGCCCGTAGGAGTCTGGGCCGTGTCTCAGTCCCAGTGTGGCCGATCACCCTCTCAGGTCGGCTACG
CATCGTTGCCTTTGGTGAGCCGTTACCTCACCAACTAGCTAATGCGACGCGGGTCCATCCATAAGTGACAGCC
GAAGCCGCCGTTCAATTTCGAACCATGCGGTTTCAAAATGTTTATCCGGTATTTAGCCCCGGTTTTTCCCGGA
GTTACTCCCAGTCTTTATTGGGCAGGTTTACCCACGTGTTTACTCACCCGTCCGCCGCTAACTTTCATAAGAG
CAAGCTCTTAATCCGTTTTCGCTCGACTTGACATGTATTTTAGGCACGCCGCCAGCGTTCATCCTGAGC
>109
CTGCTGCCGCCCGTAGGAGTCTGGGCCGTGTCTCAGTCCCAGTGTGGCTGGTCATCCTCTCAGACCAGCTATG
GATCGTCGCCTTTGGTGGGCCATTACCCCACCAACTAGCTAATCCAACGCGGGCCGATCTTTCAGCGAATAAA
TCTTTCCCCTTTAAGGGCGTATCCGGTATTAGCACAAGTTTCCCTGAGTTTATTTCCGAACTGAAAGGTACGT
TCCCACGCGTTACTCACCGTCTGCCACTGCTTGTATTTGCTACAACCGTTTTCGACTTGCATGTGTTTTAAGC
CTTGCCGCCAGCGTTCGTTTCTTGAGCCAGGATCAAACATCTT
>110
CTGCTGCCGCCCGTAGGAGTTTTGGGCCGTGTCTCAGTCCCAATGTGGCTGATCATCCTCTCAGACCAGCTAC
TGATCGTCGCCTTGGTGAGCCTTTACCTCACCAACTAGCTAATCAGACGCGGGCCGCTCTAAAGGCGATAAAT
CTTCCCCCGAAGGGCACATCCGGTATTAGCTCAAGTTTCCCTGAGTTTATTTCCGAACCTAAAGGCACGTTCC
CACGTGTTACTCACCCGTCCGCCATAACTCCGAAGAGTTTCGTTTCGACTTGCATGTGTTTAGGCCCGCCGCC
AGCGTTCGCTCTGAGCCAGGATCAAACTCT
>111
CTGCTGCCGCCCGTAGGAGTTTTGGGCCGTGTCTCAGTCCCAATGTGGCTGATCATCCTCTCAGACCAGCTAC
TGATCGTCGCCTTGGTGAGCCTTTACCTCACCAACTAGCTAATCAGACGCGGGCCGCTCTAAAGGCGATAAAT
CTTTCCCCCGAAGGGCACATCCGGTATTTAGCTCAAGTTTTCCCTGAGTTTATTTCCGAACCTAAAGGCACGT
TCCCACGTGTTTACTCACCCGTCCGCCACTAACTCCGAAGAGTTTTCGTTTCGACTTGCATTGTTGTTTTAGG
CCTTGCCGCCAGCGTTTCGCTTCTGAGCCAGGATCAAACTTCT
>112
CTGCTGCCGCCCGTAGGAGTCTGGACCGTGTCTCAGTTCCAGTGTGGCTGGTCATCCTCTCAGACCAGCTAGG
GATCGTCGCCTAGGTGAGCCGTTACCCCACCTACTAGCTAATCCCATCTGGGCACATCTGATGGCAAGAGGCC
CGAAGGTCCCCCTCTTTTGGTCTTGCGACGTTATGCGGTATTTTAGCTACCGTTTTTCCAGTAGTTTATTCCC
CCTTCCCATCAGGCAGTTTTTCCCCAGACATTTACTCACCCGTCCGCCACTCGTCACCCGAGAGCAAGCTCTC
TGTGCTTACCGTTTCG
>113
CTGCTGCCGCCCGTAGGAGTCTGGACCGTGTTCTTCAGTTCTTGGTTGTGGCTTGGTTCGTCCTCTTCAGGCC
AGCTACAGATCGTCGCCTTTGGTGGGCCTTTACCCCCACCAACTAGCTTAATTCTTGATTATCGGCCGCTTCC
AATCGCGCGAGGTCTTTGCGATCCCCCCGCTTTTTCATTCCGTAGATTCGTATTGCGGTTATTTTTAGCACAG
CTTTTTCGCTTGCGTTTTTATTCCCCCCACGACTTCGGGGGCACGTTTCCCGATTACTTTTTATTTCACCCCC
GTTTTCGCCCCACGCCCCACCCCAGGGGATTTTGCCCTC
>114
CTGCTGCCGCCCGTAGGAGTTTTGGGCCGTGTCTCAGTCCCAATCGTGGCGTGATTCATTCCCCTCACGACCA
GCTACTTGATTCGTCGCCTTTGGTTGAGCCTTTTACCTCACCAACTAGCTAATTCAGACGCGGGCCGCTCTTA
AAGGCGATAAATTCTTTTCCCCCCCCCGAAGGGCACATCCGGTTATTTTTTAGCTTCAAGTTTTTTTTCCCCC
TGAGTTTTTTATTTTTCCGAACCTTAAAAAGGCACGTTTTCCCCCACGTTGTTTTTTACTCACCCCCCGTTCC
CCGCCCCACTTAACTTCCCGAAAAGAGTTTTTTCGTTTTTTCGACTTTTGCATTT
>115
CTGCTGCGCCCGTAGGAGTCTGGGCCGTGTCTCAGTCCCAGTGTGGCTGATCATCCTCTTCAGACCAGCTACT
GATCGTTCGCCTTGGTAGGCCGTTACCCCACCAACTAGCTAATCAGACGCGGGCCGATCTTCCGGCAGTAAAC
CTTTTCCCCAAAAGGGCGTATCCGGTATTAGCCCTAGTTTTTCCCAGGGTTTATTTCCAGACCAGAAGGCACG
TTCCCACGCGTTACTCACCCGTCCGCCGCTGACCCCGAAGGGCCCGCTCGACTTGCATGTGTTTAAGCCTGCG
CCAGCGTTCGCTCTGAGCCAGGATCAAACTCT
>116
CTGCTGCCGCCCGTAGGAGTTTGGGCCGTGTCTCAGTCCCAATGTGGCTGATCATCCTCTCAGACCAGCTACT
GATCGTCGCCTTGGTGAGCCTTTACCTCACCAACTAGCTAATCAGACGCGGGCCGCTCTAAAGGCGATAAATC

TTTCCCCCCCCGAAGGGCACATCCGGTATTTAGCTCAAGTTTTTCCCTGAGTTTATTTCTGAACCTAAAGGCA
CGTTCCCACGTGTTTACTCACCCGTCCGCCACTAACTCCGAAGAGTTTTCGTTTCGACTTGCATGTGTTTTAG
GCCTGCCGCCAGCGTTTTCGCTCTGAGCCAGGATCAAACTCT
>117
CTGCTGCCGCCCGTAGGAGTCTGGGGCCGTGTCTCAGTCCCAGTGTGGCTGATCATCCTCTCAGACCAGCTAC
TGATCGTCGCCTTGGTAGGCCGTTACCCCACCAACTAGCTAATCAGACGCGGGCCGATCTTCCGGCAGTAAAC
CTTTCCCCAAAAGGGCGTATCCGGTTATTTAGCCCTAGTTTTTCCCAGGGTTTATTTCCAGACCAGAAGGCAC
GTTCCCACGCGTTACTCACCCGTCCGCCGCTGACCCCGAAGGGCCCGCTACGACTTGCATGTGTTTAAGCCTG
CCGCCAGCGTTCGCTCTGAGCCA
>118
CTGCTTGCCGCCCGTAGGAGTTTTGGGCCGTGTCTCAGTCCCAATGTGGCTGATCATTCCTCTCAGACCAGCT
ACTGATTCGTCGCCTTTGGTGAGCCTTTACCTCACCAACTAGCTAATTCAGACGCGGGCCGCTCTAAAGGCGA
TAAATCTTTCCCCCCGAAGGGCACATCCGGTTATTTTAGCTTCAAAGTTTTTTCCCCTGAGTTTTTATTTTTT
TTTTTCCGCACCTAAAAGGCACGTTTCCCACGTTGTTTTACTTCACCCCCGTTCCGCCCCACTTAACTTCCGA
AAAGAGTTTTTTTCGTTTTTCGACTTTGCATTTGTTTGTTTTTTAGGGGGCCCCTTTGCCCCCGCCCCAAGCG
TTTCGCTTTCTTGAGCCCCCGAATTCAAAAAAC
>119
CTGCTGCCGCCCTAGGAGTTTTGGACCGTGTCTCAGTTCCAATGTGGGGGACCTTTCCTCTCAGAACCCCTAT
CCATCGAAGGCTTGGTGGGCCGTTACCTCACCAACGACCTAATGGAACGCATCCCCCATCCTTTACCGGAATC
CTTTTAATAATGAAACCATGCGGAATCATTTATGCCATCGGGTATTTAATCTTTTCTTTTTCGAAAGGCTATC
CCCCCGAGTAAAGGGCAGGTTTTGGATACGTTGTTTTACTCACCCGTGCGCCGGTCGCCGGCAAAGAAAGCAA
GCTTTTTCTTTTCCCCCGATGCCCCCCCTTCGACTTTGCATGTTGTTTTAAGCCTTGTTAGCTAGCGTTTTCA
TTCCCTTGAGCCAGGATCAAACTCTT
>120
CTGCTGCGCCCGTAGGAGTCTGGGCCGTGTCTCAGTCCCAGTGTGGCTGATCATCCTTCTCAGACCAGCTCTG
ATCGTCGCCTTGGTAGGCCGTTACCCCACCAACTAGCTAATCAGACGCGGGCCGATCTTTCCGGCAGTAAACC
TTTTCCCCAAAAGGGCGTATCCGGTATTAGCCCTAGTTTTTCCCAGGGTTTATTTCCAGACCAGAAGGCACGT
TCCCACGCGTTACTCACCCGTCCGCCGCTGACCCCGAAGGGCCCGCTCGACTTGCATGTGTTTAAGCCTGCCG
CCAGCGTTCGCTCTGAGCCAGGATCAAACTCTA
>121
CTGCTGCCGCCCGTAGGAGTTTGGGCCGTGTCTCAGTCCCAATGTGGCTGATCATCCTCTCAGACCAGCTACT
GATCGTCGCCTTGGTGAGCCTTTACCTCACCAACTAGCTAATCAGACGCGGGCCGCTCTAAAGGCGATAAATC
TTTCCCCCCCGAAGGGCACATCCGGTATTTAGCTCAAGTTTTTCCCTGAGTTTATTTCCGAACCTAAAGGCAC
GTTCCCACGTGTTTACTCACCCGTCCGCCACTAACTCCGAAGAGTTTTCGTTTCGACTTGCATGTGTTTTAG
GCCTGCCGCCAGCGTTTTCGCTCTGAGCCAGGATCAAACTCT
>122
CTGCTGCCGCCCGTAGGAGTTTTGGACCGTGTCTCAGTTCCAATGTGGGGGACCTTTCCTCTCAGAACCCCTA
TCCATCGAAGGCTTTGGTGGGCCGTTACCTCACCAACAACCTAATGGAACGCATCCCCATCCTTTTACCGGAA
TCCTTTTTAATAATGAAACCATGCGGAATCATTTTATGCCATCGGGTATTTTAATCTTTTTCTTTTTCGAAAG
GCTATCCCCGAGTAAAAGGGCAGGTTTTGGATACGTGTTTTACTCACCCGTGCGCCGGTCGCCGGCGAAGAAA
AGCAAGCTTTTTCTTTTCCCGATGCCCCCCCTTCGACTTTTGCATGTTGTTTTTAAGCCTTGTAGCTAGCGTT
TTTCATCCCTG
>123
CTGCTGCCGCCCGTAGGAGTCTGGGGCCGTGTCTCAGTCCCAGTGTGGCTGATCATCCTCTCAGACCAGCTCA
GATCGTCGGCTTGGTAAGCTTTTATCCCACCAACTACCTTAATCTGCCATCGGCCGCTCCGTCCGCGCGAGGT
CCGAAGATCCCCTGCTTTTCATCCGTAGATCGTATGCGGTATTTAGCAAAGCTTTTCGCCTCGTTTATCCCCC
ACGATCGGGCACGTTCCGATGTTATTTACTCACCCGTTTCGCCACTCGTCAGCATCCGAAGACCTGTTTACCG
TTTTCGACTTGCATGTGTTAAGGCATGCCGACCAG
>124
CTGCTGCCGCCCGTAGGAGTTTTGGGCCGTGTCTCAGTCCCAATGTGGCTGATCATCCTCTCAGACCAGCTAC
TGATCGTCGCCTTGGTGAGCCTTTACCTCACCAACTAGCTAATCAGACGCGGGCCGCTCTAAAGGCGATAAAT
CTTCCCCCCCGAAGGGCACATCCGGTATTTAGCTTCAAGTTTTTCCCTGAGTTTTATTTCCGAACCTTAAAGG
CACGTTTCCCACGTGTTTACTTCACCCGTTCCGCCACTTAACTTCCGAAGAGTTTTCGCTTCGACTTTGCATT
GTTGTTTTAGGCCTTGCCGCCAGCGTTTCGCTTCTTGAGCCAGGATTCAAACTTCTT

>125
CTGCTGCCGCCCGTAGGAGTTTTGGGCCGTGTCTCAGTCCCAATGTGGCTGATCATTCCTCTCAGACCAGCTA
CTGATCGTCGCCTTGGTGAGCCTTTACCTCACCAACTAGCTAATCAGACGCGGGCCGCTCTAAAGGCGATAAA
TCTTTCCCCCGAAGGGCACATCCGGTATTTAGCTCAAGTTTTTCCCTGAGTTTATTTCCGAACCTAAAGGCAC
GTTTCCCACGTGTTTACTCACCCGTCCGCCACTAACTCCGAAGAGTTTTCGTTTTCGACTTTGCATTGTTGTT
TTAGGCCTTGCCGCCAGCGTTTCGCTTCTTGAGCCAGGATCAAACTTCTT
>126
CTGCTGCCGCCCGTAGGAGTCTGGGCCGTGTCTCAGTCCCAGTGTGGCTGATCATTCCTCTCAGACCAGCTAC
TGATCGTCGCCTTGGTAGGCCGTTACCCACCAACTAGCTAATCAGACGCGGGCCGATCTTCCGGCAGTAAACC
TTTCCCCAAAAGGGCGTATCCGGTATTAGCCCTAGTTTTTCCCAGGGTTTATTTCCAGACCAGAAGGCACGTT
CCCACGCGTTACTCACCCGTCCGCCGCTGACCCCGAAGGGCCCGCTACGACTTGCATGTGTTAAGCCTGCGCC
AGCGTTCGCTCTGATGACCA
>127
CTGCTGCCGCCCGTAGGAGTCTGGGCCGTGTCTCAGTCCCAGTGTGGCCGGGTCGCCCTCTCAGGCCGGCTAC
CCGTCGTCGCCTTGGTAGGCCATTACCCCACCAACAAGCTGATTAGGACGCGAGTCCATCCACCACCGATAAA
TCTTTTCCACCAACACCCCATGCAAGGGTTTGGTCGTTATCCAGTTATTTAGACGCCGTTTTTCCAGCGCTTT
ATTCCCAGAGTGATTGGGCAGGTTTTACTCACGTGTTTTACTTCACCCCCGTTTTCGCCACTTGATTCCACGA
GTGCAAGCACCCGCTTTTCACCGTTTTCGACTTTGCATTGTGTTTTAAGCACGCCGCCAGCGTTTCGTTCCTG
AGCCAGGATTCAAACTTCTT
>128
CTGCTGCCACCCCGTAGGAGTCTGGGCCGTGTCTCAGTCCCAGTGTGGCTGATCATCCTCTCAGACCAGCTAC
TGATCGTCGCCTTGGTAGGCCGTTACCCCACTAACTAGCTAATCAGACGCGGGCCGATCTTTCCGGCAGTAAA
CCTTTTCCCCAAAAGGGCGTATCCGGTATTTAGCCCTAGTTTTTTCCCAGGGTTTATTTCCAGACCAGAAGGC
ACGTTCCCACGCGTTACTCACCCGTCCGCCGCTGACCCCCCGAAGGGCCCGCTCGACTTTGCATGTGTTTTAA
GCCTGCCGCCAGCGTTCGCCCTGAGCCAGGATCAAACCTCT
>129
CTGCTGCCGCCCTAGGAGTCTGGGCCGTATCTCAGTCCCAATGTGGCCGTCCACCCTCTCAGGCCGGCTACCC
CTCGCCGCCTTGGTAGGCCATTACCCCCACCAACAAGCTGATTAGGCCGCGAGCTCATCCTACACCGAAAAAA
ACTTCCAACCATCACACTAAAAAAATGGCTCCTATCCGGTATTTAGACCCAGTTTTTCCCAGGCTTTATCCCG
AAGTGCAGGGCAGATCACCCACGTGTTTACTCACCCCCGTTTCGCCACTCGAGTACCCTGCAAGCAGGGCCTT
TTTCCGTTTTCGACTTTGCATTGTGTTTTAAGCACGCCGCCAGCGTTTCGTTCCTTGAGCCAGGATCAAACTC
TT
>130
CTGCTGCCGCCCGTAGGAGTCTGGGCCGTGTCTCAGTCCCAGTGTGGTTTGATCATCCTCTCGGACCAGCTAC
TGATCATCGCCTTGGTAAGCTATTGCCTCACCAACTAGCTAATCAGACGCGAGCCCATCCTCGGGCGGATTTC
CTCCTTTTTGCTCCTCAGCCTACGGGGTATTAGCAGCCGTTTTTCCAGCTGTTTGTTTCCCCTCCCAAGGGCA
GGTTCTTACGCGTTACTCACCCGTCCGCCACTGGAAACACCACTTCCCGTCCGACTTGCATGTGTTTTAAGCA
TGCCGCCAGCGTTCATCCTGAGCCAGGATCAAATCTCT
>131
CTGCTGCCGCCCGTAGGAGTCTGGGGCCGTGTCTCAGTCCCAGTGTGGCTGATCATCCTCTTCAGACCAGCTC
AGATCGTCGGCTTGGTTAAGCTTTTATCCCACCAACTACCTTAATCTGCCATCGGCCGCTCCGTCCGCGCGAG
GTCCGAAGATCCCCTGCTTTTCATCCGTAGATCGTATGCGGTATTAGCAAAGCTTTTCGCCTCGTTTATCCCC
CCCCACGATCGGGCACGTTCCGATGTATTTACTCACCCGTTCGCCACTCGTCAGCATCCGAAGACCTGTTTAC
CGTTTTCGACTTGCATGTGTAAGGCATGCCGCCAGCGTTCAATCTGACCAGGATCAAACTCT
>132
CTGCTGCCGCCCGTAGGAGTCTGGGCCGTGTCTCAGTCCCAGTGTGGCTGATCATCCTCTCGGACCAGCTACT
GATCTCGCCTTGGTAAGCTATTTGCCTCACCAACTAGCTAATCAGACGCGAGCCCATCCTCGGGCGGATTTCC
TCCTTTTTGCTCCTCAGCCTACGGGGGTATTAGCAGCCGTTTTTCCAGCTGTTTGTTTTCCCCCTCCCAAGGG
CAGGTTCTTACGCGTTTACTACCCGTCCGCCATGGAAACACCACTTCCCGTCCGACTTGCATGTGTTTTAAGC
ATGCCGCCAGCGTTTCATCCTGAACCAGGATCAAACTCT
>133
CTGCTGCCGCCCGTAGGAGTCTGGACCGTGTCTCAGTTCCAGTGTGGCTGGTCATCCTCTCAGACCAGCTAGG
GATCGTCGCCTAGGTGAGCCGTTACCCCACCTACTAGCTAATCCCATCTGGGCACATCTGATGGCAAGAGGCC
CGAAGGTCCCCCTCTTTTGGTCTTGCGACGTTATGCGGTATTTAGCTACCGTTTTTCCAGTAGTTTATTCCCC

CTCCATCAGGCAGTTTTTTCCCAGACATTTACTCACCCGTTCCGCCACTCGTCAACGAAGCAGCAAGCTGCTT
TTCCTGTTTT
>134
CTGCTGCCGCCCGTAGGAGTTTTGGGCCGTGTCTCAGTCCCAATGTGGCTGATCATCCTCTCAGACCAGCTAC
TGATCGTCGCCTTTGGTGAGCCTTTACCTCACCAACTAGCTAATCAGACGCGGGCCGCTCTAAAGGCGATAAA
TCTTTCCCCCGAAGGGCACATCCGGTATTTAGCTTCAAGTTTTTCCCTGAGTTTTATTTCCGAACCTAAAGGC
ACGTTCCCACGTGTTTACTCACCCGTTCCGCCACTAACTTCCGAAGAGTTTTCGTTTCGACTTGCATTGTTGT
TTTAGGCCTTGCCGCCAGCGTTTTCGCTTCTTGAGCCAGGATTCAAACTTCTT
>135
CTGCTGCCGCCCGTAGAGTTTGGGCCGGTGTCTCAGTCCCAATGTGGCTGATCATCCTCTCAGACCAGCTACT
GATCGTCGCCTTTGGTGAGCCTTTACCTCACCAACTAGCTAATCAGACGCGGGCCGCTCTAAAGGCGATAAAT
CTTTCCCCCGAAGGGCACATCCGGTATTTAGCTCAAGTTTTCCCTGAGTTTTATTTCCGAACCTAAAGGCAC
GTTTCCCACGTGTTTACTTCACCCGTCCGCCACTAACTTACCGAAGAGTTTTTCGTTTTCGACTTTGCATTGT
TGTTTTAGGCCTTGCCGCCAGCGTTTCGCTTCTTGAGCCAGGATCAAACTTCTT
>136
CTGCTGCCGCCCGTAGGAGTCTGGGCCGTGTCTCAGTCCCAGTGTGGCTGGTCATCCTCTCAGACCAGTTATG
GATCGTCGCCTTGGTGGGCCATTTACCCCACCAACCAGCTAATCCAACGCGGGCCGATCTTTTTCAGCGATAA
ATCTTTTTTCCCCTTTTAAGGGCGTATCCGGTATTAGCACAAGTTTTTCCCTGAGTTATTCCGAACTGAAAGG
TACGTTCCCACGCGTTACTCACCCGTCTGCCACTGCTGTATTGCTACAACCGTTCGACTTGCATGTGTTAAGC
CTGCGCCAG
>137
CTGCTGCCGCCCGTAGGAGTTTGGGCCGGTGTCTCAGTCCCAATCGTGGCTGATCATCCTCTCAGACCAGCTA
CTGATTCGCGCCTTGGTGAGCCTTTACCTCACCAACTAGCTAATCAGACGCGGGCGCTCTAAAGGCGATAAAT
CTTTTTCCCCGAAGGGCACATCCGGTATTAGCTCAAGTTTTTCCCTGAGTTATTCCGAACCTAAAGGCACGTT
CCCCACGTGTTACTCACCCGTTCCGCCACTAACTCTGAAGAGTTTTCGTTTTCGACTTGCATGTGTTTTAGGC
CTGCCGCCAGCGTTTTCGCTCTGAGCCAGGATCAAACT
>138
CTGCTGCCGCCCGTAGGAGTTTGGGCCGTGTCTCAGTCCCAATCGTGGCTGATCATCCTCTTCAGACCAGCTA
CTGATTCGTCGCCTTGGTTGAGCCTTTTACCTCACCAACTAGCTAATCAGACGCGGGCCGCTCTAAAGGCGAT
AAATCTTTTTTCCCCCGAAGGGCACATCCGGTATTTTAGCTCAAGTTTTTTCCCTGAGTTTTATTTCCGAACC
TAAAGGCACGTTTCCCCACGTGTTTTACTCACCCGTCCGCCACTAACTCCGAAGAGTTTTCGTTTTCGACTTT
TGCATGTGTTTTTAGGCCCGCCGCCAGCGTTTTTCGCTCTGAGCCAGGATCAAACTCTT
>139
CTGCTGCCGCCCGTAGGAGTTTGGGCCGGTGTCTCAGTCCCAATGTGGCTGATCATCCTCTTCAGACCAGCTA
CTGATTCGTCGCCTTGGTTGAGCCTTTTACCTCACCAACTAGCTAATTCAGACGCGGGCCGCTCTAAAGGCGA
TAAATCTTTTTTTCCCCCGAAGGGCACATCCGGTATTTTAGCTCAAGTTTTTTTCCCTGAGTTTTATTTTCCG
AACCTAAAAGGCCACGTTTTCCCCACGTGTTTTACTCACCCGTCCGCCACTAACTCCGAAGAGTTTTCGTTTT
CGACTTTTGCATGTGTTTTTAGGCCCGCCGCCAGCGTTTTTCGCTCTGAGCCAGGATCAAAACCTCT
>140
CTGCTGCCGCCCGTAGGAGTTTTGGGGCCGTGTCTCAGTCCCAATGTGGCTGATCATCCTCTCAGACCAGCTA
CTGATGTCGCCTTGGTGAGCCTTTACCTCACCAACTAGCTAATCAGACGCGGGCCGCTCTAAAGGCGATAAAT
CTTTCCCCCCCGAAGGGCACATCCGGTATTTAGCTTCAAGTTTTCCCTGAGTTTATTTCCGAACCTAAAGGC
ACGTTTCCCACGTGTTTACTCACCCGTCCGCCACTAACTTCCGAAGAGTTTTCGTTTCGACTTGCATTGTTGT
TTTAGGCCTTGCCGCCAGCGTTTCGCTTCTTGAGCCAGGA
>141
CTGCTGCCGCCCGTAGGAGTTTTTGGGGCCGTGTCCCAGTCCCAATGTGGCTGATCATCCTCTCAGACCAGCT
ACTGATCGTCGCCTGTGAGCCTTTTACCTCACCAACTAGCTAATCAGACGCGGGCCGCTCTAAAGGCGATAAA
TCTTTCCCCCCCGAAGGGCACATCCGGTATTTAGCTTCAAGTTTTCCCTGAGTTTATTTCCGAACCTAAAGG
CACGTTTCCCACGTGTTTACTCACCCGTTCCGCCACTAACTTCCGAAGAGTTTTCGTTTCGACTTGCATTGTT
GTTTTAGGCCTTGCCG
>142
CTGCTGCCGCTCGTAGGAGTTTTGGGCCGTGTCTCAGTCCCAATGTGGCTGATCATCCTCTCAGACCAGCTAC
TGATCGTCGCCTTGGTGAGCCTTTACCTCACCAACTAGCTAATCAGACGCGGGCCGCTCTAAAGGCGATAAAT
CTTCCCCCGAAGGGCACATCCGGTATTTAGCTTCAAGTTTTCCCTGAGTTTTATTTCCGAACCTAAAGGCACG

TTTCCCACGTGTTTACTCACCCGTTCCGCCACTTAACTTCCGAAGAGTTTTCGTTTCGACTTGCATTGTTGTT
TTAGGCCCGCCGCCAGCGTTTCGCTTCTTGAGCCAGGATTCAAACTTCT
>143
CTGCTGCCGCCCGTAGGAGTTTTGGGCCGTGTCTCAGTCCCAATGTGGCTGATCATCCCTCTCAGACCAGCTA
CTGATTCGTCGCCTTGGTGAGCCTTTACCTCACCAACTAGCTAATCAGACGCGGGCCGCTCTAAAGGCGATAA
ATCTTTCCCCCCGAAGGGCACATCCGGTATTTAGCTTCAAGTTTTCCCTGAGTTTATTTCCGAACCTAAAGGC
ACGTTTCCCACGTGTTTACTCACCCGTTCCGCCACTTAACTTCCGAAGAGTTTTCGTTTCGACTTGCATTGTT
GTTTTAGGCCCGCCGCCAGCGTTTCGCTTCTTGAGCCAGGATTCAAACTTCTT
>144
CTGCTGCCGCCCGTAGGAGTTTTGGGCCGTGTCTCAGTCCCAATGTTGGCTGATCATCCTCTCAGACCAGCTA
CTGATGTCGCCTTTGGTGAGCCTTTACCTCACCAACTAGCTAATCAGACGCGGGCCGCTCTAAAGGCGATAAA
TCTTTTCCCCGAAGGGCACATCCGGTATTTTAGCTCAAGTTTTTCCCTGAGTTTTATTTTCCGAACCTTAAAG
GCACGTTTCCCACGTGTTTTACTCACCCGTTCCGCCACTAACTTCCGAAGAGTTTTTCGTTTTCGACTTTGCA
TTGTTGTTTTTAGGCCTTGCCGCCAGCGTTTTCGCTTCTTGAGCCAGGATCAAACTTCTT
>145
CTGCTGCCGCCCGTAGGAGTTTGGGCCGGTGTCTCAGTCCCAATCGTGGCTGATCATCCTCTTCAGACCAGCT
ACTGATCGTCGCCTTGGTGAGCCTTTTACCTCACCAACTAGCTAATCAGACGCGGGCCGCTCTAAAGGCGATA
AATCTTTTTCCCCCCCCCCGAAGGGCACATCCGGTATTTTTAGCTCAAGTTTTTTTCCCTGAGTTTTATTTTC
CGAACCTAAAGGCACGTTTTCCCCACGTGTTTTACTCACCCGTCCGCCACTAACTCCGAAGAGTTTTTCGTTT
TCGACTTTTGCATGTTGTTTTTAGGCCTTGCCGCCAGCGTTTTTCGCTCT
>146
CTGCTGCCGCCCGTAGGAGTCTGGGCCGTGTCTCAGTCCCAGTGTGGCCGGTCGCCCTCTCAGGCCGGCTACC
CGTCGTCGCCTTGGTAGGCCATTACCCCACCAACAAGCTGATAGGACGCGAGTCCATCCACCACCGATAAATC
TTTTCCACCAACACCCCATGCAAGGGTTTGGCGTATCCAGTTATTTAGACGCCGTTTTTCCCAGCGCTTTTAT
CCCAGAGTGATTGGGCAGGTTTTACTCACGTGTTTTACTTCACCCGTTTCGCCACTTGATCCACGAGTGCAAG
CACCCGCTTTCACCGTTTCGAC
>147
CTGCTGCCGCCCGTAGGAGTCTGGGCCGTGTCTCAGTCCCAGTGTGGCTGATCATCCTCTCAGACCAGCTATG
ATCGTCGCCTTGGTAGGCCGTTACCCCACCAACTAGCTAATCAGACGCGGGCCGATCTTCCGGCAGTAAACCT
TTCCCCAAAAGGGCGTATCCGGTATTTAGCCCTAGTTTTTCCCAGGGTTTATTTCCAGACCAGAAGGCACGTT
CCCACGCGTTACTCACCCGTCCGCCGCTGACCCCGAAGGGCCCGCTACGACTTGCATGTGTTTAAGCCTGCGC
CAGCGTTCGCTCTGAGCCAGGATCAAACTCTA
>148
CTGCTTGCCGCCCGTAGGAGTCTGGGCCGTGTCTCAGTCCCAGTGTGGCTTGATCATTCCTCTCAGACCAGCT
ACTGATTCGTCGCCTTGGTAGGCCGTTACCCCACCAACTAGCTAATCAGACGCGGGCCGATCTTCCGGCAGTA
AACCTTTCCCCAAAAGGGCGTATCCGGTTATTTAGCCCTAGTTTTTCCCAGGGTTTATTTCCAGACCAGAAGG
CACGTTCCCACGCGTTACTCACCCGTCCGCCCCTGACCCCGAAGGGCCCGCTCGACTTGCATGTGTTTTAAGC
CTTGCCGCCAGCGTTCGCTTCCGAGCCAGGATCAAACTCTT
>149
CTGCTGCCGCCCGTAGGAGTTTTGGGGCCGTGTCTCAGTCCCAATGTGGCTGATCATTCCTCTCAGACCAGCT
ACTGATGTCGCCTTTGGTGAGCCTTTACCTCACCAACTAGCTTAATTCAGACGCGGGCCGCTCTTAAAGGCGA
TAAATCTTTTCCCCCCCCGAAGGGCACATCCGGTTATTTTAGCTTCAAGTTTTTCCCTGAGTTTTATTTTCCG
AACCTAAAGGCACGTTTCCCACGTGTTTACTTCACCCGTTCCGCCACTTAACTTCCGAAGAGTTTTTCGTTTT
CGACTTTGCATTGTTGTTTTTAGGCCTTGCCGCCAGCGTTTTCGCTTCTTGAGCCAGGATTCAAACTTCTT
>150
CTGCTGTGCCCGTAGGAGTTTTGGGCCGTGTCTCAGTCCCAATGTGGCTGATCATCCTCTCAGACCAGCTACT
GATCGTCGCCTTGGCGAGCCTTTACCTCGCCAACTAGCTAATCAGACGCGGGCCGCTCTAAAGGCGATAAATC
TTTCCCCCGAAGGGCACATCCGGTATTTAGCTCAAGTTTTTCCCTGAGTTTTATTTCCGAACCTAAAGGCACG
TTTCCCACGTGTTTACTCACCCGTCCGCCACTAACTCCGAAGAGTTTTCGTTTTCGACTTTGCATGTTGTTTT
AGGCCTTGCCGCCAGCGTTTTCGCTTCTGAGCCAGGATCAAACTCTT
>151
CTGCTGCCGCCCGTAGGAGTCTGGGCCGTGTCTCAGTCCCAGTGTGGCTGGTCGTCCTCTCAGACCAACTACA
GATCGTTGCCTTGGTAGGCCTTTACCCCACCAACTAGCTAATCTGACATCGGCCGCTCCAATAGCGAGAGGTC
TTTTGCGATCCCCCCCCTTTTCACCCTCAGGTCGTATGCGGTATTAATCCGGCTTTCGCCGGGCTATCCCCCA

CTACTGGGCACGTTCCGATGCATTACTCACCCGTTCGCCACTCGTCAGCATCCGAAGACCTGTTACCGTTCGA
CTTGCATGTGTAAGGCATGCGCCAGCGTTCAATCTGAGCCAGAT
>152
CTGCTGCCGCCCGTAGGAGTTTGGGCCGTGTCTCAGTCCCAATGTGGCTGATCATCCTCTCAGACCAGCTACT
GATCGTCGCCTTTGGTGAGCCTTTTACCTCACCAACTAGCTAATCAGACGCGGGCCGCTCTAAAGGCGATAAA
TCTTTTTCCCCCGAAGGGCACATCCGGTATTAGCTCAAGTTTTTCCCTGAGTTTATTCCGAACCTAAAGGGCA
CGTTCCCACGTGTTTACTCACCCGTCCGCCACTAACTCCGAAGAGTTTTCGTTTTCGACTTGCATGTGTTTTA
GGCCTGCCGCCAGCGTTTTCGCTCTGAGCCAGGATCAAACTCT
>153
CTGCTGCCGCCCTAGGAGTCTGGGCCGTGTCTCAGTCCCAGTGTGGCTTGGTCATCCTCTCAGACCAGCTATG
GATCGTCGCCTTGGTGGGCCATTTACCCCCACCAACTAGCTAATCCAACGCGGGCCGATCTTTTCAGCGATAA
ATCTTTTCCCCCCTTTAAGGGCGTATCCGGTTATTTAGCACAAGTTTTCCCTGAGTTTATTTCCGAACTGAAA
GGTACGTTTCCCACGCGTTACTTCACCCGTCTGCCACTGCTTGTATTTTGCTTACAACCGTTTTCGACTTGCA
TTGTGTTTTAAGCCTTGCCGCCAGCGTTTCGTTTTCTTGAGCCAGGATCAAACTCTT
>154
CTGCTGCCGCCCGTAGGAGTCTGGGCCGTGTCTCAGTCCCAGTGTGGCGGTCGCCCTCTCAGGCCGGCTACCC
GTCGTCGCCTTGGTAGCCATTACCCCACCAACAAGCTGATAGGACGCGAGTCCATCCACCACCGATAAATCTT
TTCCACCAACACCCCATGCAAGGGTTGGTCGTATCCAGTGTTAGACGCCGTTTTTCCAGCGCTTTATCCCAGA
GTGATGGGCAGGTTTACTCACGTGTTTACTCACCCGTTTCGCCACTGATCCACGAGTGCAAGCACCCGCTTT
TCACCGTTTTCGACTTTGCATGTGTTTTAAGCA
>155
CTGCTGCCGCCCGTAGGAGTTTTGGGGCCGTGTCTCAGTCCCAATGTGGCTGATCATCCTCTCAGACCAGCTA
CTGATGTCGCCTTGGTGAGCCTTTACCTCACCAACTAGCTAATCAGACGCGGGCCGCTCTAAAGGCGATAAAT
CTTTTCCCCCGAAGGGCACATCCGGTATTAGCTCAAGTTACCCTGAGTTATTCCGAACCTAAAGGCACGTTCC
CACGTGTTACTCACCCGTCCGCCACTAACTCCGAAGAGTTTTCGTTTCGACTTGCATGTGTTTTAGGCCTGCC
GCCAGCGTTTTCGCTCTGAGCCAGGATCAAAACTCT
>156
CTGCTGCCGCCCGTAGGAGTTTTGGGGCCGTGTCTCAGTCCCAATGTGGCTGATCATCCTCTCAGACCAGCTA
CTGATCGTCGCCTTGGTGAGCCTTTACCTCACCAACTAGCTAATCAGACGCGGGCCGCTCTAAAGGCGATAAA
TCTTTTCCCCCGAAGGGCACATCCGGTATTTAGCTCAAGTTTTTCCCTGAGTTTATTTCCGAACCTAAAGGCA
CGTTTCCCACGTGTTTACTCACCCGTCCGCCACTAACTCCGAAGAGTTTTCGTTTTCGACTTTGCATGTGTTT
TAGGCCTGCCGCCAGCGTTTTCGCTCTGAGCCAGGATCAAACTCT
>157
CTGCTGCCGCCCTAGGAGTCTGGGCCGTGTCTCAGTCCCAGTGTGGCCGGTCGCCCTCTCAGGCCGGGCTACC
CTCGTCGCCTTGGTAGGCCATTTACTCCACCAACAAGCTGATAGGACGCGAGTCCATCCACCACCGATAAATC
TTTTTCCACCGACACCCCATGCAAGGGTTTGGTCGTATCCAGTATTTTAGACGCCGTTTTTCCAGCGCTTTTA
TCCCCCAGAGTGATGGGCAGGTTTTACTCACGTGTTTTACTTCACCCGTTTCGCCACTTGATTCCACGAGTTG
CAAGCACCCCCGCTTTTCACCGTTTTCGACTTTGCATTGTCTTTTAAGCACGCCGCCAGCGTTTTCGTCCTAG
CCAGGACAA
>158
CTGCTGCCGCCCTAGGAGTCTGGGCCGTGTCTCAGTCCCAGTGTGGCTGATCATCCTCTCGGACCAGCTACTG
ATCATCACCTTGGTAAGCTATTGCCTCACCAACTAGCTAATCAGACGCGAGCCCCTCCTCGGGCGGATTCCTC
CTTTTTTGCTCCTCAGCCTACGGGGTATTAGCAGCCGTTTTCCAGCTGTTGTTCCCCTCCCAAGGGCAGGTTC
TTACGCGTTACTCACCCGTCCGCCACTGGAAACACACTTCCCGTCCGACTTGCATGTGTTTTAACGCATGCCG
CCAGCGTTCATCCTGACCAGGATCAAACCTCT
>159
CTGCTGCCGCCCGTAGGAGTCTGGGCCGTGTCTCAGTCCCAGTGTGGCCGGTCGCCCTCTCAGGCCGGCTACC
CCGTCGTCGCCTTGGTTAGGCCATTACCCCCCACCCAACAAGCTGATAGGACGCGAGTCCATCCACCACCGAT
AAATCTTTTTTTCCACCAACACCCCATGCAAGGGTTTTGGTCGTTATCCAGTTATTTTAGACGCCGTTTTTTT
CCAGCGCTTTTATCCCAGAGTTGATTGGGCAGGTTTTTACTTCACGTTGTTTTTTACTTCACCCGTTTTTTCG
CCACTTGATTCCACGAGTTGCAAGCACCCGCTTTTTCACCGTTTTTCGACTTTTTGCATTGTTGTTTTTTTAA
GCACGCCGCCAGCGTTTTTCGTTCCTTGACCAGGATTCAAACTTCTTATTCACCGACTTGCCCCCCA
>160

CTGCTGCCGCCCGTAGGAGTTTGGGCCGTGTCTCAGTCCCAATGTGGCTGATCATCCTCTCAGACCAGCTACT
GATCGTCGCCTTGGTGAGCCTTTACCTCACCAACTAGCTAATCAGACGCGGGCCGCTCTAAAGGCGATAAATC
TTTTTCCCCCGAAGGGCACATCCGGTATTTAGCTCAAGTTTTTCCCTGAGTTTTATTTCCGAACCTAAAGGCA
CGTTTCCCACGTGTTTTACTCACCCGTCCGCCACTAACTCCGAAGAGTTTTTCGTTTTCGACTTTTGCATGTG
TTTTAGGCCTGCCGCCAGCGTTTTTCGCTCTGAGCCAGGATCAAACTCT
>161
CTGCTGCCGCCCTAGGAGTCTGGGCCGTGTCTTCAGTCCCAGTGTGGCTTGATCATCCTCTCAGACCAGCTAC
TGATTCGTCGCCTTGGTTAGGCCGTTACCCCACCAACTAGCTTAATTCAGACGCGGGCCGATCTTTCCGGCAG
TTAAACCTTTTTCCCCAAAAGGGCGTTATCCGGTTATTTAGCCCTAGTTTTTTTCCCAGGGTTTTTATTTTTC
CAGACCAGAAGGCACGTTTCCCACGCGTTTACTTCACCCCCCGTTCCGCCGCTTGACCCCCCCCCCGAAGGGC
CCCCCCGCTCGACTTTTGCATTGTTGT
>162
CTGCTGCCGCCCGTAGGAGTTTTGGGCCGTGTCTCAGTCCCAATGTTGGCTGATCATCCTCTCAGACCAGCTA
CTGATCGTTCGCCTTGGTGAGCCTTTTACCTCACCAACTAGCTAATCAGACGCGGGCCGCTCTAAAGGCGATA
AATCTTTTCCCCCGAAGGGCACATCCGGTATTTAGCTCAAGTTTTTTCCCTGAGTTTTATTTCCGAACCTAAA
GGCACGTTTCCCACGTGTTTACTCACCCGTCCGCCACTAACTTCCGAAGAGTTTTCGTTTTCGACTTTGCATT
GTTGTTTTAGGCCTTGCCGCCAGCGTTTCATCTTCTTGAGCCAGGATTCAAACTTCTT
>163
CTGCTGCCGCCCGTAGGAGTTTTGGGCCGTGTCTCAGTCCCAATGTGGCTGATCATCCTCTCAGACCAGCTAC
TGATCGTCGCCTTGGTGAGCCTTTTACCTCACCAACTAGCTAATCAGACGCGGGCCGCTCTAAAGGCGATAAA
TCTTTTTCCCCGAAGGGCACATCCGGTATTTTAGCTCAAGTTTTTTCCCTGAGTTTTATTTCCGAACCTAAAG
GCACGTTTCCCACGTGTTTTACTCACCCGTCCGCCACTAACTCCGAAGAGTTTTTCGTTTTCGACTTTTGCAT
GTTGTTTTTAGGCCTTGCCGCCAGCGTTTTTCGCTTCTGAGCCAGGATCAAACTTCTT
>164
CTGCTGCCGCCCGTAGGAGTCTGGGCCGTGTCTCAGTCCCAGTGTGGCTGATCATCCTCTCAGACCAGCTACT
GATCGTCGCCTTGGTAGGCCGTTACCCCACCAACTAGCTAATCAGACGCGGGCCGATCTTTCCGGCAGTAAAC
CTTTTTTCCCCAAAAGGGCGTATCCGGTATTTTAGCCCTAGTTTTTTCCCAGGGTTTTATTTTCCAGACCAGA
AGGCACGTTTCCCACGCGTTTACTCACCCCCGTCCGCCGCTGACCCCGAAGGGCCCCCCGCTCGACTTTTGCA
TGTGTTTTAAGCCTGCGCCA
>165
CTGCTGCCGCCCGTAGGAGTCTGGGCCGTGTCTCAGTCCCAGTGTGGCTGGTCGTCCTCTCAGACCAGCTACA
GATCGCAGGCTTTGGTTAAGCCTTTTACCCCCACCAACTACCTTAATTCTTGCCATCAGCCGCTTCTTAGTTA
GCACAAGGCCCGAAGGTCCCCTGCTTTTTCACCGTTAGATCTTCATTGCGGTTATTTTAGCTTACTTCTTTTT
TCGAGTAGTTTTATTCCCCCCCCCACTTACTTAGGCACGTTTCCGATTGTTATTTTACTTCACCCGTTTTCGC
CACTTCGTTCAGCATTCCCGAAGACCTTGTTTTACCGTTTTCGACTTTTACATTGTTGTTAAAGCATTGCCG
>166
CTGCTGCCGCCCGTAGGAGTTTTGGGCCGTGTCTCAGTCCCGATGTGGCTGATCATCCTCTCAGACCAGCTAC
TGATCGTCGCCTTTGGTGAGCCTTTACCTCACCAACTAGCTAATCAGACGCGGGCCGCTCTAAAGGCGATAAA
TCTTTTCCCCCCCGAAGGGCACATCCGGTATTTAGCTTCAAGTTTTTCCCTGAGTTTTATTTCCGAACCTAAA
GGCACGTTTCCCACGTGTTTACTTCACCCGTTCCGCCACTAACTCCGAAGAGTTTTCGTTTTCGACTTTGCAT
TGTTGTTTTAGGCCTTGCCGCCAGCGTTTTCGCTTCTTGAGCCAGGATTCAAACTCTT
>167
CTGCTGCCGCCCGTAGGAGTTTTTGGGCCGTGTCTCAGTCCCAATGTGGCTGATCATTCCTCTCAGACCAGCT
ACTGATTCGTCGCCTTTGGTGAGCCTTTACCTCACCAACTAGCTAATTCAGACGCGGGCCGCTCTAAAGGCGA
TAAATCTTTTCCCCCCCGAAGGGCACATCCGGTATTTAGCTCAAGTTTTTCCCTGAGTTTATTTCCGAACCTA
AAGGCACGTTTCCCACGTGTTTACTCACCCGTTCCGCCACTAACTCCGAAGAGTTTTCGTTTTCGACTTGCAT
TGTTGTTTAGGCCTTGCCGCCAGCGTTTCGCTTCTTGAGCCAGGATCAAATCTTCT
>168
CTGCTGCCGCCCGTAGGAGTTTGGACCGTGTCTCAGTTCCAATGTGGGGGACCTTCCTCTTCAGAACCCCTAT
CCATCGAAGGCTTGGTGGGCCGTTACCTCACCAACAACCTAATTGGAACGCATCCCCATTCCTTTTACCGGAA
TCCTTTTTTAATTAATTGAAAAACCATGCGGAATTCATTTTTTTATGCCATCGGGGGTTATTTTTAATTCTTT
TTTTCTTTTTTTCGAAAAAGGCTATCCCCCCCGAGTTAAAAAAGGGGGGCAGGTTTTTTGGATACGTTTGTTT
TTTACTTCACCCCCCGTTGCGCCCCGGTTCGCCGGC
>169

CTGCTGCCGCCGTAGGAGTCTGGGCCGTGTCTCAGTCCCAGTGTGGCTGATCATCCTCTCGGACCAGCTACTG
ATCATCGCCTTGGTAAGCTATTGCCTCACCAACTAGCTAATCAGACGCGAGCCCCTCCTCGGGCGGATTCCTC
CTTTTTGCTCCTCAGCCTACGGGGTATTAGCAGCCGTTTTCCAGCTGTTTGTCCCCCTCCCAAGGGCAGGTTC
TTACGCGTTACTCACCCGTCCGCCACTGGAAACACCACTTCCCGTCCGACTTGCATGTGTTTAAGCATGCCGC
CAGCGTTCATCCTGAGCCAGGATCAAACTCT
>170
CTGCTGCCGCCCGTAGGAGTCTGGGCCGTGTCTCAGTCCCAGTGTGGCTGGTCATCCTCTCAGACCAGCTATG
GATCGTCGCCTTGGTGGGCCATTTACCCCACCAACTAGCTAATCCAACGCGGGCCGATCTTTTCAGCGATAAA
TCTTTTTCCCCTTTAAGGGCGTATCCGGTATTAGCACAAGTTTTCCCTGAGTTTATTTCCGAACTGAAAGGTA
CGTTTCCCACGCGTTACTCACCCGTCTGCCACTGCTGTATTTTGCTACAACCGTTTCGACTTGCATGTGTTTA
AGCCTGCCGCCAGCGTTCGTTTCTGAGCCAGGATAAACATCT
>171
CTGCTGCCGCCCGTAGGAGTTTGGACCGTGTCTCAGTTTCCAATGTGGGGGACCTTCCTCTCAGAACCCCTAT
TCCATCGAAGGCTTGGTGGGCCGTTACCTTCACCCAACAACCTAATTGGAACGCATCCCCATTCCTTTTACCG
GAATCCTTTTTTTAATTAATTGAAAAACCATGCGGAATTCATTTTTTTATTGCATCGGGGGTATTTTTTAAAA
TTCTTTTTTTCTTTTTTTCGAAAAAGGCTATCCCCCCCCGAGTTAAAAAAAGGGGGCAGTTTTTGGATACGTT
GTTTTTACTCACCCCCCGTGCGCCGGTTCGCCGGCAAAAAAGAAAAAAAGCAAGCTTTTTTTCTTTTTTTCCC
CCCCGATTGCCCCCCCTTCGATTTTTTTGCATTGTTTGTTTTTTAAAAGCCCCTTTGTTGGCTTAGC
>172
CTGCTGCCGCCCGTAGGAGTTTGGGCCGGTGTCTCAGTCCCAATGTGGCTGATCAATCCTCTTCAGACCAGCT
ACTGATTCGTCGCCTTGGTTGAGCCTTTTACCTCACCAACTAGCTAATCAGACGCGGCCGCTCTAAAGGCGAT
AAATCTTTTTCCCCCCCCCCGAAGGGCACATCCGGTATTTTAGCTCAAGTTTTTTTCCCTGAGTTTTATTTCC
GAACCTAAAGGCACGTTTTCCCACGTGTTTTACTCACCCGTCCGCCACTAACTCCGAAGAGTTTTTCGTTTTC
GACTTTTGCATGTGTTTTTAGGCCCTGCCGCCAGCGTTTTTCGCTCTGAGCCAGGATCAAAAC
>173
CTGCTTGCCGCCCGTAGGAGTTTTGGGCCGTGTCTCAGTCCCAATGTGGCTGATCATTCCTCTCAGACCAGCT
ACTGATTCGTCGCCTTTGGTGAGCCTTTACCTCACCAACTAGCTAATTCAGACGCGGGCCGCTCTTAAAGGCG
ATAAATCTTTTCCCCCGAAGGGCACATCCGGTATTTTAGCTCAAGTTTTTCCCTGAGTTTTATTTCCGAACCT
AAAGGCACGTTTCCCACGTGTTTACTCACCCGTTCCGCCACTAACTCCGAAGAGTTTTCGTTTCGACTTTGCA
TGTTGTTTTAGGCCTTGCCGCCAGCGTTTTCGCTTCTTGAGCCAGGATCAAACTTCTT
>174
CTGCTGCCGCCCGTAGGAGTCTGGGCCGTGTCTCAGTCCCAGTGTGGCTTGATCATCCTCTCAGACCAGCTAC
TGATCGTCGCCTTGGTAGGCCGTTACCCCACCAACTAGCTAATCAGACGCGGGCCGATCTTCCGGCAGTAAAC
CTTTTCCCCAAAAGGGCGTATCCGGTATTTAGCCCTAGTTTTTCCCAGGGTTTACTTTTCCACGACCAGAAG
GCACGTTCCCACGCGTTACTCACCCGTCCGCCGCTGACCCCGAAGGGCCCGCTAGCCGACTTGCATGTGTTTA
AGCCTGCGCCAG
>175
CTGCTGCCGCCCGTACGGAGTCTGGACCGTGTCTCAGTTCCAGTGTGGCTGGTCATCCTCTTCAGACCAGCTA
GGGATCGTCGCCTAGGTGAGCCGTTACCCCCCACCTACTAGCTAATCCCATCTGGGCACATCTGATTGGCAAA
GAGGCCCGAAGGTCCCCCCCCTCTTTTTGGCTTTGCGACGTTATGCGGTTATTTTTAGCACCGTTTTTTTCCA
GTAGTTTTATTCCCCCCTTCCATCAGGCAGTTTTTTTTCCCAGACATTTTACTCACCCGTCCGCCACTCGTCA
GCGAAGCAGCAAGCTGCTTTTCCTGTTTTACCGTTTTCGACTTTTGCATGTGTTTTTAGGCCTGCCGCCAGCG
TTT
>176
CTGCTGCCGCCCGTAGGAGTCTGGGCCGTGTCTCAGTCCCAGTGTGGCTGGTCGTCCTCTCAGACCAGCTACA
GATCGCAGGCTTTGGTTAAGCCTTTACCCCACCAACTACCTAATCTGCCATCAGCCGCTCTAGTAGCACAAGG
CCCGAAGGTCCCCTGCTTTTTCATCCGTAGATCTCATGCGGTATTTTAGCTACTCTTTTTTCGAGTAGTTTTT
ATCCCCCCACTACTAGGCACGTTTTCCCGATGTATTTTACTCACCCCGTTTTCGCCACTCGTTCAGCATCCCG
AAGACCTGTTACCGTT
>177
CTGCTGCCGCCCGTAGGAGTTTGGGCCGGTGTCTTCAGTTCCCAATTCGTTGGCTGATTCATCCTCTTCAGAC
CAGCTACTGATTCGTCGCCTTGGTTGAGCCTTTTACCTCACCAACTAGCTAATTCAGACGCGGGCCGCTCTTA
AAAGGCGATAAAAATCTTTTTCCCCCCGAAAGGGGGCACATCCGGTATTTTTAGCTTCAAAAGTTTTTTCCCC
CCTGAGTTTTTTATTTTTTCCGAAAACCTAAAAAAAGGCACGTTTTTCCCCCCACGTTGTTTTTACTCACCCC

CCGTTCCGCCACTAACTTCCGAAGAGTTTTTTTCGTTTTTTCGACTTTTTGCATGTTGTTTTTTAGGCCTTGC
CGCCAGCGTTTTTTCGCTTCTTGAGCCAGGATTCAAACTTCTT

Additional file 3_B_P6

>1
CTGCTGCCGCCCGTAGGAGTTTGGACCGTGTCTCAGTTCCAATGTGGGGGACCTTCCTCTCAGAACCCCTACT
GATCGTCGCCTTGGTGGGCCGTTACCCCGCCAACAAGCTAATCAGACGCATCCCCATCCATAACCGCTAAACC
TTTAATCGTTATTTGATGTCAGCAAACGATATCATACGGTATTAGGCCGTCTTTCAACGGGTTATCCCGTAGT
TATGGGAAGGTTGGATACGCGTTACTCACCCGTGCGCCAGTCGTCATCAAGGAAAGCAAGCTTCCTCATGATT
ACCCTGCGACTTGCATGTGTTAAGCCTGTAGCTAGCGTTCATCCGTGAGCCAAGGATCAAACTCT
>2
CTGCTGCCGCCCGTAGGAGTTTGGACCGTGTCTCAGTTCCAATGTGGGGACCTTCCTCTCAGAACCCCTACTG
ATCGTCGCCTTGGTGGGCCGTTACCCCGCCAACAAGCTAATCAGACGCATCCCCATCCATAACCGATAAATCT
TTATTCATAATCTCATGAGATCAAACGAATACATAAGGTATTAGTCCAACTTCGCTGGGTTATCCCTTAGTTA
TGGGCAGGTTGGATACGCGTTACCTCACCCGTGCGCCGGTCGCCGACATAAATAGCCAAGCTATCTATTCGCT
GCCCCTCGACTTGCATGTGTTAAGCCTATAGCTAGCGTTCATCCTGAGCCAGGATCAAACTCT
>3
CTGCTGCCGCCCGTAGGAGTTTGGACCGTGTCTCAGTTCCAATGTGGGGGACCTTCCTCTCAGAACCCCTACT
GATCGTTGCCTTGGTGGGCCGTTACCCCGCCAACAAGCTAATCAGACGCATCCCATCCATAACCGATAAATCT
TTATTCGTAATCTCATGAGATCAAACGAATACATAAGGTATTAGTCCAACTTCGCTGGGTTATCCCTTAGTTA
TGGGCAGGTTGGATACGCGTTACTCACCCGTGCGCCGGTCGCCGACATAAATAGCAAGCTATCTATTCGCTGC
CCCTCGACTTGCATGTGTTAAGCCTATAGCTAGCGTTCTCCTGAGCCAGGATCAAACTCT
>4
CTGCTGCCGCCCGTAGGAGTTTTGGACCGTGTCTCAGTTCCAATGTGGGGGACCTTCCTCTCAGAACCCCTAC
TGATCGTTGCCTTTGGTGGGCCGTTACCCCGCCAACAAGCTAACTCAGACGCATCCCCATCCATAACCGATAA
ATCTTTATTCATAATCTCATGAGATCAAACGAATACATAAGGTATTAGTCCAACTTCGCTGGGTTATCCTTAG
TTATGGGCAGGTTGGATACGCGTTACCTCACCGTGCGCCGGTCGCCGACATAAATAGCCAAGCTATCTATTCG
CTGCCCCTCGACTTGCATGTGTTAACGCCTATAGCTAGCGTTCATCCTGAGCCAGGATCAAACTCT
>5
CTGCTGCCGCCCGTAGGAGTTTGGACCGTGTCTCAGTTCCAATGTGGGGGACCTTCCTCTCAGAACCCCTACT
GATCGTCGCCTTTGGTGGGCCGTTACCCCCGCCAACAAGCTAATCAGACGCATCCCCATCCATAACCGCTAAA
CCTTTAATCGTTATTTGATGTCAGCAAACGATATCATACGGTATTAGGCCGTCTTTCAACGGGTTATCCCGTA
GTTATGGGAAGGTTGGATACGCGTTACTCACCCGTGCGCCAGTCGTCATCAAGGAAAGCAAGCTTCCCTCACT
GTTACCCTGCGACTTGCATGTGTTAAGCCTGTAGCTAGCGTCATCCAGTGACGCCA
>6
CTGCTGCCGCCCGTAGGAGTTTGGGCCGTATCTCAGTCCCAATGTGGCCGCACACCCTCTCAGGCCGGCTACC
CGTCGCCGCCTTGGTAGGCCATTACCCCACCAACAAGCTGATAGGCCGCAAGCTCATCCCACACCAGAAAAAC
CTTTCCACCACACCAACCACGATGCGGTCCTATAGGGTATTAGACCCAGTTTCCCAAGCTTATCCCCCCAGTG
CAGGGCAGATCACTTACGTGTTACTCACCCGTTCGCCACTCGAGCACCCCCAGCAAGCTGGGGCCCTTCCGTT
CGACTTGCATGTGTTAAGCACGCCGCCAGCGTTCATCCTGAGCCAGGATCAAACTCT
>7
CTGCTGCCGCCCGTAGGAGTTTGGACCGTGTCTCAGTTCCAATGTGGGGGACCTTCCTCTCAGAACCCCTACT
GATCGTTGCCTTGGTGGGCCGTTACCCCACCAACAAGCTAATCAGACGCATCCCCATCCATAACCGATAAATC
TTTATTCGTAATCTCATGAGATCAAACGAATATATAAGGTATTAGTCCAACTTCGCTGGGTTATCCCTTAGTT
ATGGGCAGGTTGGATACGCGTTACTCACCCGTGCGCCGGTCGCCGACATAAATAGCAAGCTATCTATTCGCTG
CCCCTCGACTTGCATGCGTTAAGCCTATAGCTAGCGTTCATCCTGAGCCAGGATCAAACTCT
>8
CTGCTGCCGCCCGTAGGAGTTTGAACCGTGTCTCAGTTCCAATGTGGGGGACCTTCCTCTCAGAACCCCTACT
GATCGTTGCCTTGGTGGGCCGTTACCCCACCAACAAGCTAATCAGACGCATCCCCATCCATAACCGATAAATC
TTTATTCGTAATCTCATGAGATCAAACGAATATATAAGGTATTAGTCCAACTTCGCTGGGTTATCCCTTAGTT
ATGGGCAGGTTGGATACGCGTTACTCACCCGTGCGCCGGTCGCCGACATAAATAGCAAGCTATCTATTCGCTG
CCCCTCGACTTGCATGTGTTAAGCCTATAGCTAGCGTTCATCCTGAGCCAGGATCAAACTCT
>9
CTGCTGCCGCCCGTAGGAGTTTGGACCGTGTCTCAGTTCCAATGTGGGGGACCTTCCTCTCAGAACCCCTACT
GATCGTCGCCTTGGTGGGCCGTTACCCCCGCCAACAAGCTAATCAGACGCATCCCCCATCCATAACCGCTAAA
CCTTTTAATCGTTTATTTTGATGTCAGCAAACGATATCATACGGTATTTAGGCCGTCTTTTCAACGGGTTTAT

CCCGTAGTTTATGGGAAGGTTGGATACGCGTTACTCACCCGTGCGCCAGTCGTCATCAAGGAAAGCAAGCTTT
CCCTTCATGTTTACCCTGCGACTTGCATGTGTTTAAGCCTG
>10
CTGCTGCCGCCCGTAGGAGTTGAACCGTGTCTCAGTTCCAATGTGGGGGACCTTCCTCTCAGAACCCCTACTG
ATCGTTGCCTTGGTGGGCCGTTACCCCACCAACAAGCTAATCAGACGCATCCCCATCCATAACCGATAAATCT
TTATTCGTAATCTCATGAGATCAAACGAATATATAAGGTATTAGTCCAACTTCGCTGGGTTATCCCTTAGTTA
TGGGCAGGTTGGATACGCGTTACTCACCCGTGCGCCGGTCGCCGACATAAATAGCAAGCTATCTATTCGCTGC
CCCTCGACTTGCATGTGTTAAGCCTATAGCTAGCGTTCATCCTGAGCCAGGATCAAACTCT
>11
CTGCTGCCGCCCGTAGGAGTTTGGACCGTGTCTCAGTTCCAATGTGGGGGACCTTCCTCTCAGAACCCCTACT
GATCGTCGCCTTGGTGGGCCGTTACCCCGCCAACAAGCTAATCAGACGCATCCCCATCCATAACCGCTAAACC
TTTAATCGTTATTTGATGTCAGCAAACGATATCATACGGTATTAGGCCGTCTTTCAACGGGTTATCCCGTAGT
TATGGGAAGGTTGGATACGCGTTACTCACCCGTGCGCCAGTCGTCATCAAGGAAAGCAAGCTTTCCCTCATGA
TTTACCCTGCGACTTGCATGTGTTAAGCCTGTAGCTAGCGTTCATCCCGTGAGCCAAGGATCAAACC
>12
CTGCTGCCGCCCTAGGAGTTTGGACCGTGTCTCAGTTCCAATGTGGGGGACCTTCCTCTCAGAACCCCTACTG
ATCGTCGCCTTGGTGGGCCGTTACCCCGCCAACAAGCTAATCAGACGCATCCCCATCCATAACCGCTAACCTT
TAATCGTTTATTTTGATGTCAGCAAACGATATCATACGGTATTTAGGCCGTCTTTTCAACGGGTTATCCCGTA
GTTTATGGGAAGGTTGGATACGCGTTACTCCACCCGTGCGCCGGTCGCCGACATAAATAGCCAAGCTATCTAT
TCGCTGCCCCCCCTCGACTTGCATGTGTTTAAGCCTATAGCTAGCGTTCATCCTGAGCCAGGATCAAACTCCT
>13
CTGCTGCCGCCCGTAGGAGTTTTGGACCGTGTCTCAGTTCCAATGTGGGGGACCTTCCTCTCAGAACCCCTAC
TGATCGTTGCCTTGGTGGGCCGTTACCCCGCCAACAAGCTAATCAGACGCATCCCCATCCATAACCGATAAAT
CTTTATTCGTAATCTCATGAGATCAAACGAATACATAAGGTATTAGTCCAACTTCGCTGGGTTATCCCTTAGT
TATGGGCAGGTTGGATACGCGTTACTCACCCGTGCGCCGGTCGCCGACATAAATAGCAAGCTATCTATTCGCT
GCCCCTCGACTTGCATGTGTTTAAGCCTATAGCTAGCGTTCATCCTGAGCCAGGATCAAACTCT
>14
CTGCTGCCGCCCGTAGGAGTTGAACCGTGTCTCAGTTCCAATGTGGGGGACCTTCCTCTCAGAACCCCTACTG
ATCGTTGCCTTGTGGGCCGTTACCCCACCAACAAGCTAATCAGACGCATCCCCATCCATAACCGATAAATCTT
TATTCGTAATCTCATGAGATCAAACGAATATATAAGGTATTAGTCCAACTTCGCTGGGTTATCCCTTAGTTAT
GGCAGGTTGGATACGCGTTACCTCACCGTGCGCCGGTCGCCGACATAAATAGCAAGCTATCTATTCGCTGCCC
CTCGACTTGCATGTGTTAAGCCTATAGCTAGCGTTCATCCTGAGCCAGGATCAAACTCT
>15
CTGCTGCCGCCCGTAGGAGTTTTGGACCGTGTCTCAGTTCCAATGTGGGGGACCTTCCTCTCAGAACCCCTAC
TGATCGTCGCCTTTGGTGGGCCGTTACCCCGCCAACAAGCTAATCAGACGCATCCCCATCCATAACCGCTAAA
CCTTTAATCGTTATTTGATGTCAGCAAACGACGTCATACGGTATTAGTCCGTCTTTCAACGGGTTATCCCGTG
GTCATGGGAAGGTTGGATACGCGTTACTCACCCGTGCGCCAGTCGTCGTCAAGGAAAGCAAGCTTTCCTCACG
TTACCCTGCGACTTGCATGTGTTAAGCCTGTAACTAGCGTTCATCCTGAGCCAGGATCAAACCTCT
>16
CTGCTGCCGCCCGTAGGAGTTTGGACCGTGTCTCAGTTCCAATGTGGGGGACCTTCCTCTCAGAACCCCTACT
GATCGTTGCCTTGGTGGGCCGTTACCCCGCCAACAAGCTAATCAGACGCATCCCCATCCATAACCGATAAATC
TTTATTCGTAATCTCATGAGATCAAACGAAATACATAAGGTATTAGTCCAACTTCGCTGGGTTATCCCTTAGT
TATGGGCAGGTTGGATACGCGTTACCTCACCCGTGCGCCGGTCGCCGACATAAATAGCAAGCTATCTATTCGC
TGCCCCTCGACTTGCATGTGTTTAAGCCTATAGCTAGCGTTCATCCTGAGCCAGGATCAAACTCT
>17
CTGCTGCCGCCCGTAGGAGTTTGGAACCGTGTCTCAGTCCCAATGTGGGGGACCTTCCTCTCAGAACCCCTAC
TGATCGTTGCCTTGGTGGGCCGTTACCCCGCCAACAAGCTAATCAGACGCATCCCCATCCATAACCGATAAAT
CTTTATTCGTAATCATCATGAGATCAAACGAAATACATAAGGTATTAGTCCAACTTCGCTGGGTTATCCCTTA
GTTACGGGCAGGTTGGATACGCGTTACTCACCGTGCGCCGGTCGCCGACATAAATAGCCAAGCTATCTATTCG
CTGCCCCTCGACTTGCATGTGTTAAGCCTATAGCTAGCGTTCATCCTGAGCCAGGATCAAACTCT
>18
CTGCTGCCGCCTGTAGGAGTTTGGACCGTGTCTCAGTTCCAATGTGGGGGACCTTCCTCTCAGAACCCCTACT
GATCGTCGCCTTTGGTGGGCCGTTACCCCGCCAACAAGCTAATCAGACGCATCCCCATCCATAACCGCTAAAC
CTTTAATCGTTATTTTGATGTCAGCAAACGATATCATACGGTATTAGGCCGTCTTTCAACGGGTTATCCCGTA

GTTATGGGAAGGTTGGATACGCGTTACTCACCCGTGCGCCAGTCGTCATCAAGGAAAGCAAGCCTTTCCTCAT
GTTTTACCCTGCGAACTTGCATGTGTTAACGCCTGTAGCCAGCGTTCATCCTGAGCCAGGATCAAACTCT
>19
CTGCTGCCGCCCGTAGGAGTTTTGGACCGTGTCTCAGTTCCAATGTGGGGGACCTTCCTCTCAGAACCCCTAC
TGATCGTTGCCTTTGGTGGGCCGTTACCCCGCCAACAAGCTAATCAGACGCATCCCCATCCATAACCGATAAA
TCTTTATTCATAATCTCATGAGATCAAACGAATACATAAGGTATTAGTCCAACTTCGCTGGGTTATCCCTTAG
TTATGGGCAGGTTGGATACGCGTTACTCACCCGTGCGCCGGTCGCCGACATAAATAGCAAGCTATCTATTCGC
TGCCCCTCGACTTGCATGTGTTAAGCCTATAGCTAGCGTTCATCCTGAGCCAGGATCAAACTCT
>20
CTGCTGCCGCCCGTAGGAGTTTTGGACCGTGTCTCAGTTCCAATGTGGGGGACCTTCCTCTCAGAACCCCTAC
TGATCGTCGCCTTTGGTGGGCCGTTACCCCGCCAACAAGCTAATCAGACGCATCCCCATCCATAACCGCTAAA
CCTTTAATCGTTATTTGATGTCAGCAAACGACGTCATACGGTATTAGTCCGTCTTTCAACGGGTTATCCCGTG
GTCATGGGAAGGTTGGATACGCGTTACTCACCCGTGCGCCAGTCGTCATCAAGGAAAGCCAAGCTTTCCTCAT
GTTACCCTGCGACTTGCATGTCGTTAAGCCTGTAGCTAGCGTTCAATCCTGAGCCAGGATCAAACCTCT
>21
CTGCTGCCGCCCGTAGGAGTTTGGACCGTGTCTCAGTTCCAATGTGGGGGACCTTCCTCTCAGAACCCCTACT
GATCGTCGCCTTTGGTGGGCCGTTACCCCGCCAACAAGCTAATCAGACGCATCCCCATCCATAACCGCTAAAC
CTTTAATCGTTATTTGATGTCAGCAAACGATATCATACGGTATTAGGCCGTCTTTCAACGGGTTATCCCGTAG
TTATGGGAAGGTTGGATACGCGTTACTCACCCGTGCGCCAGTCGTCATCAAGGAAAGCAAGCTTTCCCTCATG
TTACCCTGCGACTTGCATGTGTTAAGCCTGTAGCTAGCGTTCATCCTGAGCCAGGATCAAACTCT
>22
CTGCTGCCGCCCGTAGGAGTTTGGACCGTGTCTCAGTTCCAATGTGGGGGACCTTCCTCTCAGAACCCCTACT
GATCGTCGCCTTGGTGGGCCGTTACCCCGCCAACAAGCTAATCAGACGCATCCCCATCCATAACCGCTAAACC
TTTAATCGTTATTTGATGTCAGCAAACGATATCATACGGTATTAGGCCGTCTTTCAACGGGTCATCCCGTAGT
TATGGGAAGGTTTGGATACGCGTTACTCACCCGTGCGCCAGTCGTCATCAAGGAAAGCAAGCTTTTCCTCATG
TTTACCCCTGCGACTTGCATGTGTTTAAGCCTGTAGCTAGCGTTCATCCTGAGCCAGGATCAAACTCT
>23
CTGCTGCCGCCCTAGGAGTTTGGACCGTGTCTCAGTTCCAATGTGGGGACCTTCCTCTCAGAACCCCTACCTG
ATCGTTGCCTTGGGTCGGGCCGTTACCCCACCAACAAGCTAATCAGACGCATCCCCATCCATAACCGATAAAT
CTTTATTCGTAATCTCATGAGAGTCAAACGAAATACATAAGGTATTAGTCCAACTTCGCTGGGTTATCCCTTA
GTTATGGGCAGGTTGGATACGCGTTACCTCACCTGCGCGGTCCGCCGACATAAATAGCCAAGCTATCTATTCG
CTGCCCCTCGACTTGCATGTGTTTAACGCCTATAGCTAGCGTTCATCCTGAGCCAGGATCAAACTCT
>24
CTGCTGCCGCCCGTAGGAGTTTGGACCGTGTCTCAGTTCCAATGTGGGGGACCTTCCTCTCAGAACCCCTACT
GATCGTTGCCTTTGGTGGGCCGTTACCCCGCCAACAAGCTAATCAGACGCATCCCCATCCATAACCGATAAAT
CTTTATTCGTAATCTCATGAGATCAAACGAATACATAAGGTATTAGTCCAACTTCGCTGGGTTATCCCTTAGT
TTATGGGCAGGTTTGGATACGCGTTACTCACCCGTGCGCCGGTCGCTGACATAAATAGCAAGCTATCTATTCG
CTGCCCCCCCTCGACTTGCATGTGTTTAAGCCTATAGCTAGCGTTCATCCTGAGCCAGGATCAAACTCT
>25
CTGCTGCCGCCCGTAGGAGTTTGAACCGTGTCTCAGTTCCAATGTGGGGGACCTTCCTCTCAGAACCCCTACT
GATCGTTGCCTTGGGTGGGCCGTTACCCCACCAACAAGCTAATCAGACGCATCCCCATCCATAACCGATAAAT
CTTTATTCGTAATCTCATGAGATCAAACGAATATATAAGGTATTAGTCCAACTTCGCTGGGTTATCCCTTGAG
TTATGGGCAGGTTGGATACGCGTTACTCACCCGTGCGCCGGTCGCCGACATAAATAGCAAGCTATCTATTCGC
TGCCCCTCGACTTGCATGTGTTAAGCCTATAGCTAGCGTTCATCCTGAGCCAGGATCAAACTCT
>26
CTGCTGCCGCCCGTAGGAGTTTGGACCGTGTCTCAGTTCCAATGTGGGGGACCTTCCTCTCAGAACCCCTACT
GATCGTTGCCTTGGTGGGCCGTTACCCCCGCCAACAAGCTAATCAGACGCATCCCATCCATAACCGATAAATC
TTTATTTCGTAATCTCATGAGATCAAACGAATACATAAGGTATTTAGTCCAACTTCGCTGGGTTTATCCCTTA
GTTTATGGGCAGGTTTGGATACGCGTTACTCACCCGTGCGCCGGTCGCCGACATAAATAGCAAGCTATCTATT
CGCTGCCCCCCCCTCGACTTGCATGTGTTTTAAGCCTTATAGCTAGCGTTCATCCTGAGCCAGGATCAAACTC
T
>27
CTGCTGCCGCCCGTAGGAGTTTGGACCGTGTCTCAGTTCCAATGTGGGGGACCTTCCTCTCAGAACCCCTACT
GATCGTTGCCTTGGGTGGGCCGTTACCCCACCAACAAGCTAATCAGACGCATCCCCATCCATAACCGATAAAT

CTTTATTCGTAATCTCATGAGATCAAACGAAATATATAAGGTATTAGTCCAACTTCGCTGGGTTATCCCTTAG
TTATGGGCAGGTTGGATACGCGTTACTCACCCGTGCGCCGGTCGCCGACATAAATAGCAAGCTATCTATTCGC
TGCCCCTCGACTTGCATGTGTTAAGCCTATAGCTAGCGTTCATCCTGAGCCAGGATCAAACTCT
>28
CTGCTGCCGCCCGTAGGAGTTTGGACCGTGTCTCAGTTCCAATGTGGGGGACCTTCCTCTCAGAACCCCTACT
GATCGTAGCCTTGGTGGGCCGTTACCCCGCCAACAAGCTAATCAGACCGCATCCCCATCCATAACCGCTAAAC
CTTTAATCGTTATTTGATGTCAGCAAACGATATCATACGGTATTAGGCCGTCTTTCAACGGGTTATCCCGTAG
TTATGGGAAGGTTGGATACGCGTTACTCACCCGTGCGCCAGTCGTCATCAAGGAAAGCAAGCCTTTCCTCATG
TTACCCTGCCGGTACTT
>29
CTGCTGCCGCCCGTAGGAGTTTGGACCGTGTCTCAGTTCCAATGTGGCCGATCACCCTCTCAGGTCGGCTACT
GATCGTCGCCTTGGTGGGCAGTTATCTCACCAACTAGCTAATCAGACGCGGGTCCATCCTATACCGCTAACAC
TTTGATATATCTACGATGCCGTAAATATATATTATCATGTATTAGTAAACTTTTCAGTATGTTTATCCATGTG
TATAGGGCAGGTTACCCACGCGTTACTCACCCGTCCGCCGCTCATCTTTCATTTAATGCAAGCATCAAATCAG
ACGCGCTCGACTTGCATGTGTTAGGCACGCCGCCAGCGTTCATCCTGAGTCAGGATCAAACTCT
>30
CTGCTGCCGCCCGTAGGAGTTTTGGACCGTGTCTCAGTTCCAATGTGGGGGACCTTCCTCTCAGAACCCCTAC
TGATCGTCGCCTTTGGTGGGCCGTTACCCCGCCAACAAGCTAATCAGACGCATCCCCATCCATAACCGCTAAA
CCTTTAATCGTTATTTGATGTCAGCAAACGATATCATACGGTATTAGGCCGTCTTTCAACGGGTTATCCCGTA
GTTATGGGAAGGTTGGATACGCGTTACTCACCCGTGCGCCAGTCGTCATCAAGGAAAGCAAGCTTTCCTCATG
TTACCCTGCGACATGCATGTGTTAAGCCTGTAGCTAGCGTTCATCCCTGAGCCAGGCATCAAACTCT
>31
CTGCTGCCGCCCGTAGGAGTTTTGGACCGTGTCTCAGTTCCAATGTGGGGGACCTTCCTCTCAGAACCCCTAC
TGATCGTCGCCTTTGGTGGGCCGTTACCCCGCCAACAAGCTAATCAGACGCATCCCCATCCATAACCGCTAAA
CCTTTAATCGTTATTTGATGTCAGCAAACGATATCATACGGTATTAGGCCGTCTTTCAACGGGTTATCCCGTA
GTTATGGGAAGGTTGGATACGCGTTACTCACCCGTGCGCCAGTCGTCATCAAGGAAAGCAAGCTTTCCCTCAT
GATTACCCTGCGTACATGCATGTGTAAGCCTGTAGCTAGCGTTCATCCCGTGACG
>32
CTGCTGCCGCCCGTAGGAGTTTGGACCGTGTCTCAGTTCCAATGTGGCCGATCACCCTCTCAGGTCGGCTACT
GATCGTCGCCTTGGTGGGCAGTTATCTCACCAACTAGCTAATCAGACGCGGGTCCATCCTATACCGCTAACAC
TTTGATATATCTACGATGCCGTAAATATATATTATCATGTATTAGTAAACTTTTCAGTATGTTATCCATGTGT
ATAGGGCAGGTTACCCACGCGTTACTCACCCCGTCCGCCGCTCATCTTTCATTAATGCAAGCATCAAATCAGA
CGCGCTCGACTTGCATGTGTTAGGCACGCCGCCAGCGTTCATCCTGAGCCAGGATCAAACTCT
>33
CTGCTGCCGCCCGTAGGAGTTTGGACCGTGTCTCAGTTCCAATGTGGGGGACCTTCCTCCTCAGAACCCCTAC
TGATCGTCGCCTTGGTGGGCCGTTACCCCGCCAACAAGCTAATCAGACGCATCCCCATCCATAACCGCTAAAC
CTTTAATCGTTATTTGATGTCAGCAAACGATATCATACGGTATTAGGCCGTCTTTCAACGGGTTATCCCGTAG
TTATGGGAAGGTTGGATACGCGTTACTCACCCGTGCGCCAGTCGTCATCAAGGAAAGCAAGCTTTCCCTCATG
TTACCCTGCGACGACTGC
>34
CTGCTGCCGCCCGTAGGAGTTTGGACCGTGTCTCAGTTCCAATGTGGGGGACCTTCCTCTCAGAACCCCTACT
GATCGTCGCCTTTGGTGGGCCGTTACCCCGCCAACAAGCTAATCAGACGCATCCCCATCCATAACCGCTAAAC
CTTTAATCGTTATTTGATGTCAGCAAACGACGTCATACGGTATTAGTCCGTCTTTCAACGGGTTATCCCGTGG
TCATGGGAAGGTTGGATACGCGTTACTCACCCGTGCGCCAGTCGTCGTCAAGGAAAGCAAGCTTTCCTCACGT
TACCCTGCGACTTGCATGTGTTAAGCCTGTAGCCTAGCGTTCATCCTGAGCCAGGATCAAACCTCT
>35
CTGCTGCCGCCCTAGGAGTTTGGACCGTGTCTCAGTTCCAATGTGGGGGACCTTCCTCTCAGAACCCCTACTG
ATCGTCGCCTTTGGTGGGCCGTTACCCCGCCAACAAGCTAATCAGACGCATCCCCATCCATAACCGCTAAACC
TTTAATCGTTATTTGATGTCAGCAAACGATATCATACGGTATTAGGCCGTCTTTCAACGGGTTATCCCGTAGT
TATGGGAAGGTTGGATACGCGTTACTCACCCGTGCGCCAGTCGTCATCAAGAAAGCAAGCCTTCCCTCAGTGT
TACCCTGCGACTTGCATGTGTTAAGCCTGTAGCTAGCGTCATCCTGAGCCAGGCATCAAACTCT
>36
CTGCTGCCGCCCGTAGGAGTTTGGACCGTGTCTCAGTTCCAATGTGGGGGACCTTCCTCTCAGAACCCCTACT
GATCGTTGCCTTGGTGGGCCGTTACCCCGCCAACAAGCTAATCAGACGCATCCCCATCCATAACCGATAAATC

TTTATTCATAATCTCATGAGATCAAACGAACACATAAGGTATTAGTCCAACTTCGCTGGGTTTTATCCCTTAG
TTATGGGCAGGTTGGATACGCGTTACTCACCCGTGCGCCGGTCGCCGACATAAATAGCAAGCTATCTATTCGC
TGCCCCTCGACTTGCATGTGTTTAACGCCTATAGCTAGCGTTCATCCTGAGCCAGGATCAAACTCT
>37
CTGCTGCCGCCCGTAGGAGTTTGGACCGTGTCTCAGTTCCAATGTGGGGGACCTTCCTCTCAGAACCCCTACT
GATCGTCGCCTTGGTGGGCCGTTACCCCGCCAACAAGCTAATCAGACGCATCCCCATCCATAACCGCTAAACC
TTTAATCGTTTATTTGATGTCAGCAAACGATATCATACGGTATTAGGCCGTCTTTCAACGGGTTTATCCCGTA
GTTTATGGGAAGGTTGGATACGCGTTACTCACCCGTGCGCCAGTCGTCATCAAGGAAAGCAAGCTTCCCTCAT
GACTTACCCTGCCGGACTTGCATGTGTTAAGCCTTGTAGCTAGCGTCATCCTGAGCCAGGATCAAACTCT
>38
CTGCTGCCGCCCGTAGGAGTTTGGACCGTGTCTCAGTTCCAATGTGGGGGACCTTCCTCTCAGAACCCCTACT
GATCGTCGCCTTTGGTGGGCCGTTACCCCCGCCAACAAGCTAATCAGACGCATCCCCATCCATAACCGCTAAA
CCTTTAATCGTTTATTTTGATGTCAGCAAACGATATCATACGGTATTTAGGCCGTCTTTTCAACGGGTTTATC
CCGTGGTCATGGGAAGGTTTGGATACGCGCTACTCACCCGTGCGCCAGTCGTCGTCAAGGGAAGCAAGCTTCC
CCTTCACGTTACCCTGCGACTTTGCATGTGTTTAAGCCTTGTAGCTAGCGTCATCCTGAGCCAGGATCAAACT
CTATCACCGACTGCCCCCCAGTAGAAGAGG
>39
CTGCTGCCGCCCGTAGGAGTTTGGACCGTGTCTCAGTTCCAATGTGGGGGACCTTCCTCTCAGAACCCCTACT
GATCGTTGCCTTGGTCGGGCCGTTACCCCACCAACAAGCTAATCAGACGCATCCCCATCCATAACCGATAAAT
CTTTATTTCGTAATCTCATGAGATCAAACGAATATATAAGGTATTTAGTTCCAACTTGCGCTGGGTTATCCTA
GTTTATGGGCAGGTTTGGATACGCGTTACCTCGACCGTGCGCCGGTCGCCGACATAAATAGCAAGCTATCTAT
TCGCTGCCCCCCTTCGACTTGCATGTGTTTTAAGCCTTATAGCTAGCGTTCATCCCTTGAGCCAGGATCAAAC
TCT
>40
CTGCTGCCGCCCGTAGGAGTTTGGACCGTGTCTCAGTTCCAATGTGGGGGACCTTCCTCTCAGAACCCCTACT
GATCGTCGCCTTGGTGGGCCGTTACCCCGCCAACAAGCTAATCGGACGCATCCCCATCCATAACCGCTAAACC
TTTAATCGTTATTTGATGTCAGCAAACGACGTCATACGGTATTAGTCCGTCTTTCAACGGGTTATCCCGTGGT
CATGGGAAGGTTGGATACGCGTTACTCACCCGTGCGCCAGTCGTCGTCAAGGAAAGCAAGCTTTCCTCACGTT
ACCCTGCGACTTGCATGTGTTTAAGCCTGTAGCTAGCGTTCATCCTGAGCCAGGATCAAACTCT
>41
CTGCTGCCGCCCGTAGGAGTTTGGACCGTGTCTCAGTTCCAATGTGGGGGACCTTCCTCTCAGAACCCCTACT
GATCGTCGCCTTGGTGGGCCGTTACCCCGCCAACAAGCTAATCAGACGCATCCCCATCCATAACCGATAAATC
TTTATTCGTAATCTCATGAGATCAAACGAATACATAAGGTATTAGTCCAACTTCGCTGGGTTATCCCTTAGTT
ATGGGCAGGTTTGGATACGCGTTACTCACCCGTGCGCCGGTCGCCGACATAAATAGCAAGCTATCTATTCGCT
GCCCCCCCTCGACTTGCATGTGTTTAAGCCTATAGCTAGCGTTCATCCTGAGCCAGGATCAAACTCT
>42
CTGCTGCCGCCCGTAGGAGTTTGGACCGTGTCTCAGTTCCAATGTGGGGGACCTTCCTCTCAGAACCCCTACT
GATCGTCGCCTTGGTGGGCCGTTACCCCGCCAACAAGCTAATCAGACGCATCCCCATCCATAACCGCTAAACC
TTTAATCGTTTATTTTGATGTCAGCAAACGATATCATACGGTATTTAGGCCGTCTTTTCAACGGGTTTATCCC
CGTAGTTTATGGGAAGGTTGGATACGCGTTACTCACCCGTGCGCCAGTCGTCATCAAGGAAAGCAAGCTTTTC
CCTCATGATTTT
>43
CTGCTGCCGCCCGTAGGAGTTGAACCGTGTCTCAGTTCCAATGTGGGGGACCTTCCTCTCAGAACCCCTACTG
ATCGTTGCCTTGGTGGGCCGTTACCCCACCAACAAGCTAATCAGACGCATCCCCATCCATAACCGATAAATCT
TTATTCGTAATCTCATGAGATCAAAGCGAATATATAAGGTATTAGTCCAACTTCGCTGGGTTATCCCTTAGTT
ATGGGCAGGTTGGATACGCGTTACTCACCCGTGCGCCGGTCGCCGACATAAATAGCAAGCTATCTATTCGCTG
CCCCTCGACTTGCATGTGTTAAGCCTATAGCTAGCGTTCATCCTGAGCCAGGATCAAACTCT
>44
CTGCTGCCGCCCGTAGGAGTTTTGGACCGTGTCTCAGTTCCAATGTGGGGGACCTTCCTCTCAGAACCCCTAC
TGATCGTCGCCTTGGTGGGCCGTTACCCCGCCAACAAGCTAATCAGACGCATCCCCATCCATCACCGCTAAAC
CTTTAATCGTTTATTTTGATGTCAGCAAACGATGTCATACGGTATTTAGTCCGTCTTTTCAACGGGTTTATCC
CGTGGTCATGGGAAGGTTTGGATACGCGTTACTCACCCGTGCGCCAGTCGTCGTCAAGGGAAGCCAAGCTTCC
CTTCACGTTACCCTGCGACTTTGCATGTGTTTAAGCCTTGTAGCTAGCGTCATCCTTGAGCCAGGATCAAACT
CT

>45
CTGCTGCCCCCGTAGGAGTTTTGGACCGTGTCTCAGTTCCAATGTGGGGGACCTTCCTCTCAGAACCCCTACT
GATCGTCGCCTTTGGTGGGCCGTTACCCCGCCAACAAGCTAATCAGACGCATCCCCATCCATAACCGCTAAAC
CTTTAATCGTTATTTGATGTCAGCAAACGATATCATACGGTATTAGGCCGTCTTTCAACGGGTTATCCCGTAG
TTATGGGAAGGTTGGATACGCGTTACTCACCCGTGCGCCAGTCGTCGTCAAGGAAAGCAAGCTTTCCTCACGT
TACCCTGCGACTTGCATGTGTTAAGCCTGTAGCTAGCGTCATCCTGAGCCAGGATCAACTCT
>46
CTGCTGCCGCCCGTAGGAGTTTGGACCGTGTCTCAGTTCCAATGTGGGGGACCTTCCTCTCAGAACCCCTACT
GATCGTCGCCTTGGTGGGCCGTTACCCCGCCAACAAGCTAATCAGACGCATCCCCATCCATAACCGCTAAACC
TTTAATCGTTATTTGATGTCAGCAAACGATATCATACGGTATTAGGCCGTCTTTCAACGGGTTATCCCGTAGT
TATGGGAAGGTTGGATACGCGTTACTCACCCGTGCGCCAGTCGTCATCAAGGAAAGCAAGCTTTCCCTCATGT
TACCCTGCGAACATGCATGTGTTAAGCCTGTAGCTAGCGTTCATCCCTGAGCAGGATCAAACTGCCGTAT
>47
CTGCTGCCGCCCGTAGGAGTTTTGGACCGTGTCTCAGTTCCAATGTGGGGGACCTTCCTCTCAGAACCCCTAC
TGATCGTCGCCTTTGGTGGGCCGTTACCCCGCCAACAAGCTAATCAGACGCATCCCCATCCATAACCGCTAAC
CTTTAATCGTTATTTGATCGTCAGCAAACGATATCATACGGTATTAGGCCGTCTTTCAACGGGTTATCCCGTA
GTTATGGGAAGGCTGGATACGCGTTACCTCACCCGTGCGCCAGTCGTCGTCAAGGGAAGCAAGCTTTCCCTCA
CGTTACCCTGCGACTTGCATGTGTTTAAGCCTGTAGCCTAGCGTTCAATCCTGAGCCAGGATCAAACCTCT
>48
CTGCTGCCGCCCGTAGGAGTTTTGGACCGTGTCTCAGTTCCAATGTGGGGGACCTTCCTCTCAGAACCCCTAC
TGATCGTTGCCTTGGTGGGCCGTTACCCCGCCAACAAGCTAATCAGACGCATCCCCATCCATAACCGATAAAT
CTTTATTCGTAATCTCATGAGATCAAACGAATACATAAGGTATTAGTCCAACTTCGCTGGGTTATCCCTTAGT
TATGGGCAGGTTGGATACGCGTTACTCACCCGTGCGCCGGTCGCCGACATAAATAGCAAGCTATCTATTCGCT
GCCCCTCGACTTGCATGTGTTAAGCCTATAGCTAGCGTTCATCCTGAGCCAGGATCAAACTCT
>49
CTGCTGCCGCCCGTAGGAGTTTGAACCGTGTCTCAGTTCCAATGTGGGGGACCTTCCTCTCAGAACCCCTACT
GATCGTTGCCTTGGTGGGCCGTTACCCCACCAACAAGCTAATCAGACGCATCCCCATCCATAACCGATAAATC
TTTATTCGTAATCTCATGAGATCAAACGAATATATAAGGTATTAGTCCAACTTCGCTGGGTTATCCCTTAGTT
ATGGGCAGGTTGGATACGCGTTACTCACCCGTGCGCCGGTCGCCGACATAAATAGCAAGCTATCTATTCGCTG
CCCCTCGACTTGCATGTGTTAAGCCTATAGCTAGCGTTCATCCTGAGCCAGGATCAAACTCT
>50
CTGCTGCCGCCCGTAGGAGTTTGGACCGTGTCTCAGTTCCAATGTGGGGGACCTTCCTCTCAGAACCCCTACT
GATCGTCGCCTTGGTGGGCCGTTACCCCGCCAACAAGCTAATCAGACGCATCCCCATCCATAACCGCTAAACC
TTTAATCGTTATTTTGATGTCAGCAAACGATGTCATACGGTATTAGTCCGTCTTTTCAACGGGTTATCCCGTG
GTCATGGGAAGGTTGGATACGCGTTACTCACCCGTGCGCCAGTCGTCGTCAAGGGAAGCAAGCTTCCCTCACG
TTACCCTGCGACTTGCATGTGTTAAGCCTGTAGCTAGCGTCATCCCTGAGCCAAGGCATCAAACAT
>51
CTGCTGCCGCCCGTAGGAGTTTGGACCGTGTCTCAGTTCCAATGTGGGGGACCTTCCTCTCAGAACCCCTACT
GATCGTCGCCTTTGGTGGGCCGTTACCCCGCCAACAAGCTAATCAGACGCATCCCCATCCATAACCGCTAACC
TTTAATCGTTATTTTGATGTCAGCAAACGATATCATACGGTATTAGGCCGTCTTTCAACGGGTTATCCCGTAG
TTATGGGAAGGTTGGATACGCGTTACTCACCCGTGCGCCAGTCGTCGTCAAGGGAAGCAAGCTTCCCTCACGT
TACCCTGCGACTTGCATGTGTAACGCCTGTAGCTAGCGTCATCCTGA
>52
CTGCTGCCGCCCGTAGGAGTTTGGACCGTGTCTCAGTTCCAATGTGGGGGACCTTCCTCTCAGAACCCCTACT
GATCGTTGCCTTTGGTGGGCCGTTACCCCGCCAACAAGCTAATCAGACGCATCCCCATCCATAACCGATAAAT
CTTTATTCGTAATCTCATGAGATCAAACGAATACATAAGGTATTAGTCCAACTTCGCTGGGTTATCCCTTAGT
TATGGGCAGGTTGGATACGCGTTACTCACCCGTGCGCCGGTCGCCGACATAAATAGCAAGCTATCTATTCGCT
GCCCCTCGACTTGCATGTGTTAAGCCTATAGCTAGCGTTCATCCTGAGCCAGGATCAAACTCT
>53
CTGCTGCCGCCCTAGGAGTTGGACCGTGTCTCAGTTCCAATGTGGGGACCTTCCTCTCAGAACCCCTACTGAT
CGTCGCCCTTTGGGTGGGCCGTTACCCCGCCAACAAGCTAATCAGACGCATCCCCCATCCATAACCGCTAAAC
CTTTAATCGTTATTTGATGTCAGCAAACGATATCATACGGTATTAGGCCGTCTTTCAACGGGTTATCCCGTAG
TTATGGGAAGGTTGGATACGCGTTACTCACCCGTGCGCCAGTCGTCATCAAGGAAAGCAAGCTTTCCTCATGT
TACCCTGCGTACATGCATGTGTTAAGCCTGTACGCTAGCTCGTTATCC

>54
CTGCTGCCGCCCGTAGGAGTTTGGACCGTGTCTCAGTTCCAATGTGGGGGACCTTCCTCTCAGAACCCCTACT
GATCGTTGCCTTGGTGGGCCGTTACCCCGCCAACAAGCTAATCAGACGCATCCCCATCCATAACCGATAAATC
TTTATTCGTAATCTCATGAGATCAAACGAATACATAAGGTATTAGTCCAACTTCGCTGGGTTATCCCTTAGTT
ATGGGCAGGTTGGATACGCGTTACTCACCCGTGCGCCGGTCGCCGACATAAATAGCAAGCTATCTATTCGCTG
CCCCTCGACTTGCATGTGTTAAGCCTATAGCTAGCGTTCATCCTGAGCCAGGATCAAACTCT
>55
CTGCTGCCGCCCGTAGGAGTTTTGGACCGTGTCTCAGTTCCAATGTGGGGGACCTTCCTCTCAGAACCCCTAC
TGATCGTTGCCTTGGTGGGCCGTTACCCCGCCAACAAGCTAATCAGACGCATCCCCATCCATAACCGATAAAT
CTTTATTCGTAATCTCAATGAGATCAAACGAATACATAAGGTATTAGTCCAACTTCGCTGGGTTATCCCTTAG
TTATGGGCAGGTTTGGATACGCGTTACTCACCCGTGCGCCGGTCGCCGACATAAATAGCAAGCTATCTATTCG
CTGCCCCCCTCGACTTGCATGTGTTTAAGCCTATAGCTAGCGTTCATCCTGAGCCAGGATCAAACTCT
>56
CTGCTGCCGCCCGTAGGAGTTTGGACCGTGTCTCAGTTCCAATGTGGGGGACCTTCCTCTCAGAACCCCTACT
GATCGTCGCCTTTGGTGGGCCGTTACCCCGCCAACAAGCTAATCAGACGCATCCCCATCCATAACCGCTAAAC
CTTTAATCGTTATTTGATGTCAGCAAACGATATCATACGGTATTAGGCCGTCTTTCAACGGGTTATCCCGTAG
TTATGGGAAGGTTGGATACGCGTTACTCACCCGTGCGCCAGTCGTCATCAAGGAAAGCAAGCCTTTCCTCATG
TTACCCTGCGACATGCATGTGTTAAGCCTGTAGCTAGCGTTCATCCCGTGAGCCAAGGCATCAAATC
>57
CTGCTGCCGCCCGTAGGAGTTTGGACCGTGTCTCAGTTCCAATGTGGGGGACCTTCCTCTCAGAACCCCTACT
GATCGTTGCCTTGGTGGGCCGTTACCCCGCCAACAAGCTAATCAGACGCATCCCCATCCATAACCGATAAATC
TTTATTCGTAATCATCATGAGATCAAACGAATACATAAGGTATTTAGTCCAACTTCGCTGGGTTTATCCCTTA
GTTTATGGGCAGGTTTGGATACGCGTTACTCACCCGTGCGCCAGTCGTCATCAAGGAAAGCAAGCTTTTCCTC
ATGTTTACCCTGCGACTTGCATGTGTTTAAGCCTGTAGCTAGCGTTCATCCTGAGCCAGATCAAACCTCT
>58
CTGCTGCCGCCCGTAGGAGTTTGGACCGTGTCTCAGTTCCAATGTGGGGGACCTTCCTCTCAGAACCCCTACT
GATCGTTGCCTTGGTGGGCCGTTACCCCACCAACAAGCTAATCAGACGCATCCCCATCCATAACCGATAAATC
TTTATTCGTAATCTCATGAGATCAAACGAATACATAAGGTATTAGTCCAACTTCGCTGGGTTATCCCTTAGTT
ATGGGCAGGTTGGATACGCGTTACTCACCCGTGCGCCGGTCGCCGACATAAATAGCAAGCTATCTATTCGCTG
CCCCTCGACTTGCATGTGTTTAAGCCTATAGCTAGCGTTCATCCTGAGCCAGGATCAAACTCT
>59
CTGCTGCCGCCCGTAGGAGTTTGGACCGTGTCTCAGTTCCAATGTGGGGGACCTTCCTCTCAGAACCCCTACT
GATCGTTGCCTTGGTCGGGCCGTTACCCCACCAACAAGCTAATCAGACGCATCCCCATCCATAACCGATAAAT
CTTTATTCGTAATCTCATGAGATCAAACGAATACATAAGGTATTAGTCCAACTTCGCTGGGTTATCCCTTAGT
TATGGGCAGGTTTGGATACGCGTTACCTCACCCGTGCGCCGGTCGCCGACATAAATAGCAAGCTATCTATTCG
CTGCCCCTCGACTTGCATGTGTTTAACGCCCATAGCTAGCGTTCATCCTGAGCCAGGATCAAACTCT
>60
CTGCTGCCGCCCTAGGAGTTTGGACCGTGTCTCAGTTCCAATGTGGCCGATCACCCTCTCAGGTCGGCTACTG
ATCGTCGCCTTGGTGGGCAGTTATCTCACCAACTAGCTAATCAGACGCGGGTCCATCCTATACCGCTAACACT
TTGATATATCTACGATGCCGTAAATATATATTATCATGTATTAGTAAACTTTTCAGTATGTTTATCCATGTGT
ATAGGGCAGGTTACCCACGCGTTACTCACCCGTCCGCCGCTCATCTTTCATTTAATGCAAGCATCAAATCAGA
CGCGCTCGACTTGCATGTGTTAGGCACGCCGCCAGCGTTCATCCTGAGCCAGGATCAAACTT
>61
CTGCTGCCGCCCGTAGGAGTTTTGGACCGTGTCTCAGTTCCAATGTGGGGGACCTTCCTCTCAGAACCCCTAC
TGATCGTCGCCTTTGGTGGGCCGTTACCCCGCCAACAAGCTAATCAGACGCATCCCCATCCATAACCGCTAAA
CCTTTAATCGTTATTTGATGTCAGCAAACGATATCATACGGTATTAGGCCGTCTTTCAACGGGTTATCCCGTA
GTTATGGGAAGGTTGGATACGCGTTACTCACCCGTGCGCCAGTCGTCATCAAGGAAAGCAAGCTTTCCCTCAT
GTTACCCTGCGACTTGCATGTGTTAAGCCTGTAGCTAGCGTTCATCCCGTGTAGC
>62
CTGCTGCCGCCCGTAGGAGTTTGGGCCGTGTCTCAGTCCCAATGTGGCCGTACACTCTCTCAAGCCGGCTACT
GATCGTCGCCTTGGTGAGCTTTTATCTCACCAACTAGCTAATCAGACGCAAGTCCATCTTACACCGATAAATC
TTTGGTCACTCTATCATGCGATAAATTGACTTTATAGGGTATTTATTCTTTCGTTTTCCAAAGGCTATCCCCT
TGTGTAAGGCAGGTTACTCACGTGTTACTCACCCGTTCGCCACTAATCCACTTAAAATCATTTCCGAAGAAT

CAACTAAGTTTCATCGTTCGACTTGCATGTGTTTATGCACGCCGCCAGCGTTCGTCCTGAGCCAGGATCAAAC
TCT
>63
CTGCTGCCGCCCGTAGGAGTCTGGACCGTGTCTCAGTTCCAGTGTGGCCGTTCATCCTCTTAGACCGGCTACT
GATCGTTGGTTTGGTGGGCCGTTACCCCACCAACTGCCTAATCAGACGCAAACCCCTCTTCAGGCGATAGCTT
ACAGGTAGAGGCTACCCTTTCTTCCACAGGTCATGCGGCCCGTGGAACGTATTCGGTATTAGCAGTCGTTTCC
GTCTGTTTGTCCCCATCCTGAAGGCAGGTTGTTTTACGTGTTACTCACCCGTTTGCCACTGGATTCGATAAAG
AGCAAGCTCTTCATCTCTTCCCGTTCGACTTGCATGTGTTAAGCACGCCGCCAGCGTTCGTCCTGAGCCAGGA
TCAAACTCT
>64
CTGCTGCCGCCCGTAGGAGTCTGGACCGTGTCTCAGTTCCAGTGTGGCCGTTCATCCTCTCAGACCGGCTACT
GATCGTTGGTTTGGTGGGCCGTTACCCCACCAACTGCCTAATCAGACGCAAACCCCTCTTCAGGCGATAGCTT
TACAGGTAGAGGCTACCCTTTCTTCCCACAGGTCATGCGGCCCGTGGAACGTATTTCGGTATTAGCAGTCGTT
TTCCGTCTGTTTGTCCCCATCCTGAAGGCAGGTTTGTTTTACGTGTTACTCACCCGTTTTGCCACTGGATTTC
GATAAAGAGCAAGCTCTTCATCTTCTTCCCGTTCGACTTGCATGTGTTTAAGCACGCCGCCAGCGTTCGTCCT
GAGCCAGGATCAAACTCT
>65
CTGCTGCCGCCCGTAGGAGTTTGGACCGTGTCTCAGTTCCAATGCGGGGGACCTTCCTCTCAGAACCCCTACT
GATCGTTGCCTTGGTGGGCCGTTACCCCGCCAACAAGCTAATCAGACGCATCCCCATCCATAACCGCTAAACC
TTTAATCGTTATTTTGATGTCAGCAAACGATATCATACGGTATTTAGGCCGTCTTTCAACGGGTTTATCCCGT
AGTTTATGGGAAGGTTGGACACGCGTTACTCACCCGTGCGCCAGTCGTCATCAAGGAAAGCAAGCTTTCCCTC
ATGTTTACCCTGCGACTTGCATGTGTTTAAGCCTGTAGCTAGCGTTCATCCTGAGCCAGGATCAAACTCT
>66
CTGCTGCCGCCCGTAGGAGTTTGGACCGTGTCTCAGTTCCAATGTGGGGGACCTTCCTCTCAGAACCCCTACT
GATCGTCGCCTTGGTGGGCCGTTACCCCGCCAACAAGCTAATCAGACGCATCCCCATCCATCACCGCTAAACC
TTTTAATCGTTTATTTTGATGTCAGCAAACGATGTCATACGGTATTTAGTTCCGTCTTTTTTCAACGGGTTTT
ATTCCCCCCGTGGTTCATGGGAAGGTTTGGATACGCGTTACTCACCCGTGCGCCAGTCGTCGTCAAGGGAGCA
AGCTTTCC
>67
CTGCTGCCGCCCGTAGGAGTTTTGGGACCGTGTCTCAGTTCCAATGTGGGGGGACCTTTCCTCTCAGAACCCC
TACTGATCGTCGCCTTTGGTGGGCCGTTACCCCGCCAACAAGCTAATCAGACGCATCCCCATCCATAACCGCT
AAACCTTTAATCGTTATTTGATGTCAGCAAACGATATCATACGGTATTAGGCCGTCTTTCAACGGGTTATCCC
GTAGTTATGGGAAGGTTGGATACGCGTTACTCACCCGTGCGCCAGTCGTCATCAAGGAAAGCAAGCCTTTCCT
CATGTTACCCTGCGACTTGCATGTGTTAAGCCTGTAGCTAGCGTTCATCCCTGAGCCAGGATCAAACTCT
>68
CTGCTGCCGCCCGTAGGAGTTTTGGACCGTGTCTCAGTTCCAATGTGGGGGACCTTCCTCTCAGAACCCCTAC
TGATCGTTGCCTTGGTGGGCCGTTACCCCGCCAACAAGCTAATCAGACGCATCCCCATCCATAACCGATAAAT
CTTTATTCATAATCTCATGAGATCAAACGAATACATAAGGTATTAGTCCAACTTCGCTGGGTTATCCCTTAGT
TATGGGCAGGTTGGATACGCGTTACTCACCCGTGCGCCGGTCGCCGACATAAATAGCAAGCTATCTATTCGCT
GCCCCTCGACTTGCATGTGTTAAGCCTATAGCTAGCGTTCATCCTGAGCCAGGATCAAACTCT
>69
CTGCTGCCGCCCGTAGGAGTCTGGGCCGTATCTCAGTCCCAATGTGGCCGCACACCCTCTCAGGCCGGCTACC
CGTCGCCGCCTTGGTAGGCCATTACCCCACCAACAAGCTGATAGGCCGCAAGCTCATCCCACACCAGAAAAAC
CTTTCCACCACACCAACCACGATGCGGTCCTATAGGGTATTAGACCCAGTTTCCCAAGCTTATCCCCCAGTGC
AGGGCAGATCACTTACGTGTTACTCACCCGTTCGCCACTCGAGCACCCTGCAAGCAAGGGCCTTTCCGTTCGA
CTTGCATGTGTTAAGCACGCCGCCAGCGTTCATCCTGAGCCAGGATCAAACTCT
>70
CTGCTGCCGCCCGTAGGAGTTTGGACCGTGTCTCAGTTCCAATGTGGGGGACCTTCCTCTCAGAACCCCTACT
GATCGTCGCCTTGGTGGGCCGTTACCCCGCCAACAAGCTAATCAGACGCATCCCCATCCATAACCGCTAAACC
TTTAATCGTTATTTTGATGTCAGCAAACGATATCATACGGTATTAGGCCGTCTTTTCAACGGGTTATCCCGTA
GTTTATGGGAAGGTTGGATACGCGTTACTCACCCGTAGCGCCAGTCGTCATCAAGGAAAGCAAGCTTTCCCTC
ATGTTTAC
>71

CTGCTGCCGCCCTAGGAGTCTGGACCGTGTCTCAGTTCCAGTGTGGCCGTTCATCCCCTCAGACCGGCCTACT
GATCGTTGGTTTGGTGGGCCGTTACCCCACCAACTGCCTAATCAGACGCAAACCCCTCTTCAGGCGATAGCTT
TACAGGTAGAGGCTACCCTTTCTTCACAGGTCATGCGACCCGTTGGAACGTATTCGGTATTAGCAGTCGTTTC
CGTCTGTTTGTCCCCATCCTGAAGGCAGGTTGTTTACGTGTTACTCACCCGTTTGCCACTGGATTCGATAAA
CGAGCAAGCTCTTCATCTCTTCCCGTTCGACTTGCATGTCGTTAAGCACGCCGCCAGCGTTCGT
>72
CTGCTGCCGCCCGTAGGAGTTTTGGACCGTGTCTCAGTTCCAATGTGGGGGACCTTCCTCTCAGAACCCCTAC
TGATCGTTGCCTTGGTGGGCCGTTACCCCGCCAACAAGCTAATCAGACGCATCCCCATCCATAACCGATAAAT
CTTTATTCGTAATCTCATGAGATCAAACGAAATACATAAGGTATTAGTCCAACTTCGCTGGGTTATCCCTTAG
TTATGGGCAGGTTGGATACGCGTTACTCACCGTGCGCCGGTCGCCGACATAAATAGCAAGCTATCTATTCGCT
GCCCCTCGACTTGCATGTGTTAAGCCTATAGCTAGCGTTCATCCTGAGCCAGGATCAAACTCT
>73
CTGCTGCCGCCCGTAGGAGTCTGGGCCGTATCTCAGTCCCAATGTGGCCGCACACCCTCTCAGGCCGGCTACC
CGTCGCCGCCTTGGTAGGCCATTACCCCACCAACAAGCTGATAGGCCGCAAGCTCATCCCACACCAGAAAAAC
CTTTCCACCACACCAACCACGATGCGGTCCTATAGGGTATTAGACCCAGTTTCCCAAGCTTATCCCCCAGTGC
AGGGCAGATCACTTACGTGTTACTCACCCGTTCGCCACTCGAGCACCCCAGCCAAGCTGGGGCCTTTCCGTTC
GACTTGCATGTGTTAACGCACGCCGCCAGCGTTCTCCTGAGCCAGGATCAACGCT
>74
CTGCTGCCGCCCGTAGGAGTTGGACCGTGATCTCAGTTCCAACTGTGGCCCGATCACCCTCTCAGGTCGGCTA
CTGAATCGTCGCCTTGGTGGGCAGTTATCTCACCAACTAGCTAATCAGACGCGGGTCCACCCTATACCGCTAA
CACTTTGATATATCTACGATGCCGTAAATATATATTATCATGTATTAGTAAACTTCTCAGTATGTTATCCATG
TGTATAGGGCAGGTTACCCACGCGTTACTCACCCGTCCGCCGCTCATCTTTCATTAATGCAAGCATCAAATCA
GACGCGCTCGACTTGCATGTGTTAGGCACGCCGCCAGCGTTCATCCTGAGCCAGGATCAAACTCT
>75
CTGCTGCCGCCCGTAGGAGTTTGGACCGTGTCTCAGTTCCAATGTGGGGGACCTTCCTCTCAGAACCCCTACT
GATCGTCGCCTTGGTGGGCCGTTACCCCGCCAACAAGCTAATCAGACGCATCCCCATCCATAACCGCTAAACC
TTTAATCGTTATTTGATGTCAGCAAACGATATCATACGGTATTAGGCCGTCTTTCAACGGGTTATCCCGTAGT
TATGGGAAGGTTGGATACGCGTTACTCACCCGTGCGCCAGTCGTCATCAAGGAAAGCAAGCTTTCCCTCATGT
TACCCTGCGACTTGCATGTGTTAAGCCTGTAGCTAGCGTTCATCCGTGAGCCAAGGATCAAACTCT
>76
CTGCTGCCGCCCGTAGGAGTTTTGGACCGTGTCTCAGTTCCAATGTGGGGGACCTTCCTCTCAGAACCCCTAC
TGATCGTTGCCTTGGTGGGCCGTTACCCCGCCAACAAGCTAATCAGACGCATCCCCATCCATAACCGATAAAT
CTTTATTCATAATCTCATGAGAATCAAACGAATACATAAGGTATTAGTCCAACTTCGCTGGGTTATCCCTTAG
TTATGGGCAGGTTGGATACGCGTTACTCACCCGTGCGCCGGTCCGCCGACATAAATAGCAAGCTATCTATTCG
CTGCCCCCCTCGACTTGCATGTGTTAACGCCTATAGCTAGCGTTCATCCTGAGCCAGGATCAAACTCT
>77
CTGCTGCCGTCCGTAGGAGTTTTGGACCGTGTCTCAGTTCCAATGTGGGGGACCTTCCTCTCAGAACCCCTAC
TGATCGTCGCCTTTGGTGGGCCGTTACCCCGCCAACAAGCTAATCAGACGCATCCCCATCCATAACCGCTAAA
CCTTTAATCGTTATTTGATGTCAGCAAACGATATCATACGGTATTAGGCCGTCTTTCAACGGGTTATCCCGTA
GTTATGGGAAGGTTGGATACGCGTTACTCACCCGTGCGCCAGTCGTCATCAAGGAAAGCAAGCTTTCCCTCAT
GTTACCCTGCGACTTGCATGTGTTAAGCCTGTAGCTAGCGTTCAATCCCGTAGAGCCAAGGCATCAAAC
>78
CTGCTGCCGCCCGTAGGAGTTTGGACCGTGTCTCAGTTCCAATGTGGGGGACCTTCCTCTCAGAACCCCTACT
GATCGTCGCCTTGGTGGGCCGTTACCCCGCCAACAAGCTAATCAGACGCATCCCCATCCATAACCGCTAAACC
TTTAATCGTTATTTGATGTCAGCAAACGATATCATACGGTATTAGGCCGTCTTTCAACGGGTTATCCCGTAGT
TATGGGAAGGTTGGATACGCGTTACTCACCCGTGCGCCAGTCGTCATCAAGGAAAGCAAGCTTTCCCTCATGT
TACCCTGCGACATGCATGTGTTAAGCCTGTAGCTAGCGTTCATCCCTGAGCCAAGCTATCAAACCTCT
>79
CTGCTGCCGCCCGTAGGAGTTTGGACCGTGTCTCAGTTCCAATGTGGGGGACCTTCCTCTCAGAACCCCTACT
GATCGTCGCCTTTGGTGGGCCGTTACCCCGCCAACAAGCTAATCACGACGCATCCCCCATCCATCACCGCTAA
ACCTTTAATCGTTATTTGAGTCAGCAAACGATGTCATACGGTATTAGTCCGTCTTTCAACGGGTTATCCCGTG
GTCATGGGAAGGTTGGATACGCGTTACCTCACCCGTGCGCCAGTCGTCGTCAAGGAAAGCAAGCTTTCCCTCA
CGTTACCTGCGACGTTAGCCG
>80

CTGCTGCCGCCCGTAGGAGTTTGGACCGTGTCTCAGTTCCAATGTGGCCGATCACCCTCTCAGGTCGGCTACT
GATCGTCGCCTTGGTGGGCAGTTATCTCACCAACTAGCTAATCAGACGCGGGTCCATCCTATACCGCTAACAC
TTTGATATATCTACGATGCCGTAAATATATATTATCATGTATTAGTAAACTTTTCAGTATGTTTATCCATGTG
TATAGGGCAGGTTACCCACGCGTTACTCACCCGTCCGCCGCTCATCTTTCATAATGCAAGCATCAAATCAGAC
GCGCTCGACTTGCATGTGTTAGGCACGCCGCCAGCGTTCATCCTGAGCCAGGATCAAACTCT
>81
CTGCTGCCGCCCGTAGGAGTTTGGACCGTGTCTCAGTTCCAATGTGGGGGACCTTCCTCTCAGAACCCCTACT
GATCGTTGCCTTGGTGGGCCGTTACCCCGCCAACAAGCTAATCAGACGCATCCCCATCCATAACCGATAAATC
TTTATTCATAATCTCATGAGATCAAACGAATACATAAGGTATTAGTCCAACTTCGCTGGGTTATCCCTTAGTT
ATGGGCAGGTTGGATACGCGTTACTCACCCGTGCGCCGGTCGCCGACATAAATAGCAAGCTATCTATTCGCTG
CCCCTCGACTTGCATGTGTTAAGCCTATAGCTAGCGTTCATCCTGAGCCAGGATCAAACTCT
>82
CTGCTGCCGCCCGTAGGAGTTTGGACCGTGTCTCAGTTCCAATGTGGGGGACCTTCCTCTCAGAACCCCTACT
GATCGTCGCCTTTGGTGGGCCGTTACCCCGCCAACAAGCTAATCAGACGCATCCCCATCCATAACCGCTAAAC
CTTTAATCGTTATTTGATGTCAGCAAACGATATCATACGGTATTAGGCCGTCTCTCAACGGGTTATCCCGTAG
TTATGGGAAGGTTGGATACGCGTTACTCACCCGTGCGCCAGTCGTCATCAAGGAAAGCAAGCTTTCCTCATGT
TTACCCTGCGACTTGCATGTGTTTAAGCCTGTAGCTAGCGTTCATCCTGAGCCAGGATCAAACTCT
>83
CTGCTGCCGCCCGTAGGAGTTTGAACCGTGTCTCAGTTCCAATGTGGGGGACCTTCCTCTCAGAACCCCTACT
GATCGTTGCCTTGGTGGGCCGTTACCCCACCAACAAGCTAATCAGACGCATCCCCATCCATAACCGATAAATC
TTTATTCGTAATCTCATGAGATCAAACGAATATATAAGGTATTAGTCCAACTTCGCTGGGTTATCCCTTAGTT
GTGGGCAGGTTGGATACGCGTTACTCACCCGTGCGCCGGTCGCCGACATAAATAGCAAGCTATCTATTCGCTG
CCCCTCGACTTGCATGTGTTAAGCCTATAGCTAGCGTTCATCCTGAGCCAGGATCAAACTCT
>84
CTGCTGCCGCCCGTAGGAGTTTGGACCGTGTCTCAGTTCCAATGTGGGGGACCTTCCTCTCAGAACCCCTACT
GATCGTTGCCTTGGTGGGCCGTTACCCCGCCAACAAGCTAATCAGACGCATCCCCATCCATAACCGATAAATC
TTTATTCGTAATCTCATGAGATCAAACGAATACATAAGGTATTAGTCCAACTTCGCTGGGTTATCCCTTAGTT
ATGGGCCAGGTTGGATACGCGTTACTCACCCGTGCGCCGGTCGCCGACATAAATAGCAAGCTATCTATTCGCT
GCCCCTCGACTTGCATGTGTTAAGCCTATAGCTAGCGTTCATCCTGAGCCAGGATCAAACTCT
>85
CTGCTGCCGCCCGTAGGAGTTTGGACCGTGTCTCAGTTCCAATGTGGGGGACCTTCCTCTCAGAACCCCTACT
GATCGTCGCCTTTGGTGGGCCGTTACCCCGCCAACAAGCTAATCAGACGCATCCCCATCCATAACCGCTAAAC
CTTTTAATCGTTATTTTGATGTCAGCAAACGATATCATACGGTATTTAGGCCGTCTTTTCAACGGGTTTATCC
CGTAGTTTATGGGAAGGTTGGATACGCGTTACTCACCCGTGCGCCAGTCGTCATCAAGGAAAGCAAGCTTTCC
CTCATGTTTTACCCTGCGAACTTA
>86
CTGCTGCCGCCCGTAGGAGTTTGGACCGTGTCTCAGTTCCAATGTGGGGGACCTTCCTCTCAGAACCCCTACT
GATCGTCGCCTTTGGTGGGCCGTTACCCCGCCAACAAGCTAATCAGACGCATCCCCATCCATAACCGCTAAAC
CTTTAATCGTTATTTGATGTCAGCAAACGACGTCATACGGTATTAGTCCGTCTTTCAACGGGTTATCCCGTGG
TCATGGGAAGGTTGGATACGCGTTACTCACCCGTGCGCCAGTCGTCGTCAAGGAAAGCAAGCTTTCCTCACGT
TACCCTGCGACTTGCATGTGTTAAGCCTGTAGCTAGCGTTCATCCTGAGCCAGGATCAAACTCT
>87
CTGCTGCCGCCCGTAGGAGTTTTGGACCGTGTCTCAGTTCCAATGTGGGGGACCTTCCTCTCAGAACCCCTAC
TGATCGTCGCCTTTGGTGGGCCGTTACCCCGCCAACAAGCTAATCAGACGCATCCCCATCCATAACCGCTAAA
CCTTTAATCGTTTATTTTGATGTCAGCAAACGATATCATACGGTATTTAGGCCGTCTTTTCAACGGGTTTATC
CCGTAGTTTATGGGAAGGTTTGGATACGCGTACTCACCCGTGCGCCAGTCGTCATCAAAGGAAAGCAAGCTTT
TCCCTCATGCTT
>88
CTGCTGCCGCCCGTAGGAGTTTTGGACCGTGTCTCAGTTCCAATGTGGGGGACCTTCCTCTCAGAACCCCTAC
TGATCGTCGCCTTTGGTGGGCCGTTACCCCGCCAACAAGCTAATCAGACGCATCCCCATCCATCACCGCTAAA
CCTTTAATCGTTTATTTTGATGTCAGCAAACGATGTCATACGGTATTTAGTCCGTCTTTTCAACGGGTTTATC
CCGTGGTCATGGGAAGGTTTGGATACGCGTTACTCACCCGTGCGCCAGTCGTCGTCAAGGGAAGCAAGCTTTC
CCTCACGTTACCCTGCGACTTTGCATGTGTTTAAGCCTTGTAGCTAGCGTTCATCCGTTGAGCCAGGCATTCA
ACTCT

>89
CTGCTGCCGCCCTAGGAGTTTTGGACCGTGTCTCAGTTCCAATGTGGGGGACCTTCCTCTCAGAACCCCTACT
GATCGTCGCCTTTGGTGGGCCGTTACCCCGCCAACAAGCTAATCAGACGCATCCCCATCCATAACCGCTAAAC
CTTTAATCGTTATTTGATGTCAGCAAACGATATCATACGGTATTAGGCCGTCTTTCAACGGGTTATCCCGTAG
TTATGGGAAGGTTGGATACGCGTTACTCACCCGTGCGCCAGTCGTCATCAAGGAAAGCAAGCTTTCCCTCATG
TTACCCTGCGACTTGCATGTGTTAAGCCTGTAGCTAGCGTTCATCCCTGAGCCAAGGATCAAACTCT
>90
CTGCTGCCGCCCGTAGGAGTTTGGACCGTGTCTCAGTTCCAATGTGGGGGACCTTCCTCTCAGAACCCCTACT
GATCGTCGCCTTGGTGGGCCGTTACCCCGCCAACAAGCTAATCAGACGCATCCCCATCCATAACCGCTAAACC
TTTAATCGTTATTTTGATGTCAGCAAACGATATCATACGGTATTAGGCCGTCTTTCAACGGGTTATCCCGTAG
TTATGGGAAGGTTGGATACGCGTTACTCACCCGTGCGCCAGTCGTCATCAAGGAAAGCAAGCTTTCCCTCATG
ATTACCCTGGCGACATGCATGTGTTAAGCCTGTAGCTAGCGTTCATCCCTGAGCCAAGGATCG
>91
CTGTGCCGCCCGTAGGAGTTTGGACCGTGTCTCAGTTCCAATGTGGGGGACCTTCCTCTCAGAACCCCTACTG
ATCGTTGCCTTTGGTGGGCCGTTACCCCGCCAACAAGCTAATCAGACGCATCCCCATCCATAACCGATAAATC
TTTATTCGTAATCTCATGAGATCAAACGAATATATAAGGTATTAGCCCAACTTCGCTGGGTTTATCCCTTAGT
TTATGGGCAGGTTTGGATACGCGTTACTCACCCGTGCGCCGGTCGCCGACATAAATAGCAAGCTATCTATTCG
CTGCCCCTTCGACTTGCATGTGTTTAACGCCTTATAGCTAGCGTTCACCTTGAGCCAGGATCAACTCT
>92
CTGCTGCCGCCCGTAGGAGTTTGGACCGTGTCTCAGTTCCAATGTGGGGGACCTTCCTCTCAGAACCCCTACT
GATCGTTGCCTTTGGTGGGCCGTTACCCCGCCAACAAGCTAATCAGACGCATCCCCATCCATAACCGATAAAT
CTTTATTCGTAATCTCATGAGATCAAACGAATACATAAGGTATTAGTCCAACTTCGCTGGGTTATCCCTTAGT
TATGGGCAGGTTGGATACGCGTTACCTCACCGTGCGCCGGTCGCCGACATAAATAGCAAGCTATCTATTCGCT
GCCCCTCGACTTGCATGTGTTAAGCCTATAGCTAGCGTTCATCCTGAGCCAGGATCAAACTCT
>93
CTGCTGCCGCCCGTAGGAGTTTTGGACCGTGTCTCAGTTCCAATGTGGGGGACCTTCCTCTCAGAACCCCTAC
TGATCGTCGCCTTTGGTGGGCCGTTACCCCGCCAACAAGCTAATCAGACGCATCCCCATCCATAACCGCTAAA
CCTTTAATCGTTATTTGATGTCAGCAAACGATATCATACGGTATTAGGCCGTCTTTCAACGGGTTATCCCGTA
GTTATGGGAAGGTTGGATACGCGTTACTCACCCGTGCGCCAGTCGTCATCAAGGAAAGCAAGCTTTCCCTCAT
GTTACCCTGCGTAACTTGCATGTGTTAAGCCTGTAGCTAGCGTTCATCCGTGAGCCAAGGCATCAAACTCT
>94
CTGCTGCCGCCCGTAGGAGTTTGGACCGTGTCTCAGTTCCAATGTGGGGGACCTTCCTCTCAGAACCCCTACT
GATCGTCGCCTTGGTGGGCCGTTACCCCGCCAACAAGCTAATCAGACGCATCCCCATCCATCACCGCTAAACC
TTTAATCGTTTATTTGATGTCAGCAAACGACGTCATACGGTATTAGTCCGTCTTTCAACGGGTTTATCCCGTG
GTCATGGGAAGGTTTGGATACGCGTTACTCACCCGTGCGCCAGTCGTCGTCAAGGAAAGCAAGCTTTTCCTCA
CGTTACCCTGCGACTTGCATGTGTTTAAGCCTGTAGCTAGCGTTCATCCTGAGCCAGGATCAAACTCT
>95
CTGCTGCCGCCCGTAGGAGTTTGGACCGTGTCTCAGTTCCAATGTGGCCGATCACCCTCTCAGGTCGGCTACT
GATCGTCGCCTTGGTGGGCAGTTATCTCACCAACTAGCTAATCAGACGCGGGTCCATCCTATACCGCTAACAC
TTTGATATATCTACGATGCCGTAAATATATATTTATCATGTATTAGTAAACTTTCAGTATGTTTTATCCATGT
GTATAGGGCAGGTTTACCCACGCGTTACTCACCCGTCCGCCGCTCATCGTTTCAATTTAAGCAAGCATCAAAT
CAGACGCGCTCGACTGCATG
>96
CTGCTGCCGCCCGTAGGAGTTTGGACCGTGTCTCAGTTCCAATGTGGGGGACCTTCCTCTCAGAACCCCTACT
GATCGTTCGCCTTTGGTGGGCCGTTACCCCGCCAACAAGCTAATCAGACGCATCCCCATCCATAACCGCTAAA
CCTTTAATCGTTATTTGATGTCAGCAAACGATATCATACGGTATTAGGCCGTCTTTCAACGGGTTATCCCGTA
GTTATGGGAAGGTTGGATACGCGTTACTCACCCGTGCGCCAGTCGTCATCAAGGAAAGCAAGCTTTCCCTCAT
GTTACCCTGCGACTTGCATGTGTTAAGCCTGTAGCTAGCGTTCATCCCTGAGCCAAGGATCAAACTCT
>97
CTGCTGCCGCCCGTAGGAGTTTGGACCGTGTCTCAGTTCCAATGTGGGGGACCTTCCTCTCAGAACCCCTAC
TGATCGTTGCCTTTGGTGGGCCGTTACCCCGCCAACAAGCTAATCAGACGCATCCCCATCCATAACCGATAAA
TCTTTATTCATAATCTCATGAGATCAAACGAATACATAAGGTATTAGTCCAACTTCGCTGGGTTATCCCTTAG
TTATGGGCAGGTTGGATACGCGTTACTCACCCGTGCGCCGGTCGCCGACATAAATAGCAAGCTATCTATTCGC
TGCCCCTCGACTTGCATGTGTTAAGCCTATAGCTAGCGTTCATCCTGAGCCAGGATCAAACTCT

>98
CTGCTGCCGCCCGTAGGAGTTTTGGACCGTGTCTCAGTTCCAATGTGGGGGACCTTCCTCTCAGAACCCCTAC
TGATCGTCGCCTTTGGTGGGCCGTTACCCCGCCAACAAGCTAATCAGACGCATCCCCATCCATAACCGCTAAA
CCTTTAATCGTTATTTGATGTCAGCAAACGATATCATACGGTATTTAGGCCGTCTTTCAACGGGTTTATCCCG
TAGTTTATGGGAAGGTTGGATACGCGTTACTCACCCGTGCGCCAGTCGTCATCAAGGAAAGCAAGCTTTCCTC
ATGTTTACCCTGCGACTTGCATGTGTTTAAGCCTGTAGCTAGCGTTCATCCTGAGCCAGGATCAAACTCT
>99
CTGCTGCCGCCCGTAGGAGTTTTGGACCGTGTCTCAGTTCCAATGTGGGGGACCTTCCTCTCAGAACCCCTAC
TGATCGTTGCCTTTGGTGGGCCGTTACCCCGCCAACAAGCTAATCAGACGCATCCCCATCCATAACCGATAAA
TCTTTTATTCATAATCTCATGAGATCAAACGAATACATAAGGTATTTAGTCCAACTTCGCTGGGTTTATCCCT
TAGTTTATGGGCAGGTTTGGATACGCGTTACTCACCCGTGCGCCGGTCGCCGACATAAATAGCAAGCTATCTA
TTCGCTGCCCCCCCTCGACTTGCATGTGTTTTAAGCCTTATAGCTAGCGTTCATCCTGAGCCAGGATCAAACT
CT
>100
CTGCTGCCGCCCGTAGGAGTTTGGACCGTGTCTCAGTTCCAATGTGGGGGACCTTCCTCTCAGAACCCCTACT
GATCGTCGCCTTGGTGGGCCGTTACCCCGCCACAAGCTAATCAGACGCATCCCCATCCATAACCGCTAAACCT
TTAATCGTTATTTGATGTCAGCAAACGATATCATACGGTATTAGGCCGTCTTTCAACGGGTTATCCCGTAGTT
ATGGGAAGGTTGGATACGCGTTACTCACCCGTGCGCCAGTCGTCATCAAGGAAAGCAAGCTTTCCCTGACTGT
TACCCTGCGACTGCATGTGTTAAGCCTGTAGCTAGCGTCATCCCTGAGCAA
>101
CTGCTGCCGCCCGTACGGAGTTTGGACCGTGTCTCAGTTCCAATGTGGGGGACCTTCCTCTCAGAACCCCTAC
TGATCGTCGCCTTGGTGGGCCGTTACCCCGCCAACAAGCTAATCAGACGCATCCCCATCCATAACCGCTAAAC
CTTTAATCGTTTATTTTGATGTCAGCAAACGATATCATACGGTATTTAGGCCGTCTTTTCAACGGGTTTATCC
CGTAGTTTATGGGAAGGTTGGATACGCGTTACTCACCCGTGCGCCAGTCGTCATCAAGGAAAGCAAGCTTTCC
CTTCATGTTTACCCTGCGACTTGCATGTGTTTAAGCCTTGTAGCTAGCGTCATCCTGAGCCAGGATCAAACTC
T
>102
CTGCTGCGCCCGTAGGAGTTTGGACCGTGTCTCAGTTCCAATGTGGGGGACCTTCCTCTCAGAACCCCTACTG
ATCGTTGCCTTGGTGGGCCGTTACCCCGCCAACAAGCTAATCAGACGCATCCCCATCCATAACCGATAAATCT
TTATTCGTAATCTCATGAGATCAAACGAATACATAAGGTATTAGTCCAACTTCGCTGGGTTATCCCTTAGTTA
TGGGCAGGTTGGATACGCGTTACTCACCGTGCGCCGGTCGCCGACATAAATAGCAAGCTATCTATTCGCTGCC
CCTCGACTTGCATGTGTTAAGCCTATAGCTAGCGTTCATCCTGAGCCAGGATCAAACTCT
>103
CTGCTGCCGCCCGTAGGAGTTTGGACCGTGTCTCAGTTCCAATGTGGGGGACCTTCCTCTCAGAACCCCTAC
TGATCGTCGCCTTTGGTGGGCCGTTACCCCGCCAACAAGCTGATCAGACGCATCCCCATCCATAACCGCTAAA
CCTTTAATCGTTATTTGATGTCAGCAAACGATATCATACGGTATTAGGCCGTCTTTCAACGGGTTATCCCGTA
GTTATGGGAAGGTTGGATACGCGTTACTCACCCGTGCGCCAGTCGTCATCAAGGAAAGCAAGCTTTCCTCATG
TTACCCTGCGACTTGCATGTGTTAAGCCTGTAGCTAGCGTTCATCCCTGAGCAAGATCAAACCTCT
>104
CTGCTGCCGCCCGTAGGAGTTTGGACCGTGTCTCAGTTCCAATGTGGGGGACCTTCCTCTCAGAACCCCTACT
GATCGTTGCCTTGGTGGGCCGTTACCCCGCCAACAAGCTAATCAGACGCATCCCCATCCATAACCGATAAATC
TTTATTCATAATCTCATGAGATCAAACGAATACATAAGGTATTAGTCCAACTTCGCTGGGTTATCCCTTAGTT
ATGGGAAGGTTGGATACGCGTTACTCACCCGTGCGCCAGTCGTCATCAAGGAAAGCAAGCTTTCCCTCATGAT
TACCCTGCGACATGCATGTGTTAAGCCTGTAGCTAGCGTTCATCCTGAGCCAGGATCAAACTCT
>105
CTGCTGCCGCCCGTAGGAGTCTGGACCGTGTCTCAGTTCCAGTGTGGCCGTTCATCCTCTCAGACCGGCTACT
GATCGTTGGTTTGGTGGGCCGTTACCCCCACCAACTGCCTAATCAGACGCAAACCCCTCTTTCAGGCGATAGC
TTTACAGGTAGAGGCTACTTCTTTTCTTTCCACAGGTCATTGCGACCCGTTGGAACGTATTTCGGTTATTTTA
GCAGTCGTTTTTCCGTCTTGTTTTGTCCCCCCCATCTTGAAGGCAGGTTTTGTTTTTACGTGTTTACTCACCC
GTTTTTGCCACTGGATTTTCGATAAAGAGCAAGCTCTTTCATTCTTCTTTTCCCGTTTCGACTTGCATGTGTT
TAAGCACGCCGCCAGCGTTCGCCTGAGCCAGGATCAAACTCT
>106
CTGCTGCCGCCCGTAGGAGTTTGGACCGTGTCTCAGTTCCAATGTGGGGGACCTTCCTCTCAGAACCCCTACT
GATCGTCGCCTTGGTGGGCCGTTACCCCCGCCAACAAGCTAATCAGACGCATCCCCCATCCATAACCGCTAAA

CCTTTTAATCGTTTATTTTGATGTCAGCAAACGATATCATACGGTATTTAGGCCGTCTTTTTCAACGGGTTTA
TCCCGTTAGTTTATGGGAAGGTTTGGATACGCGTTACTCACCCGTGCGCCAGTCGTCATTCAAGGAAAGCAAG
CTTTTCCTCATGCT
>107
CTGCTGCCGCCCGTAGGAGTTTGGACCGTGTCTCAGTTCCAATGTGGGGGACCTTCCTCTCAGAACCCCTACT
GATCGTTGCCTTGGTGGGCCGTTACCCCGCCAACAAGCTAATCAGACGCATCCCCATCCATAACCGATAAATC
TTTATTCGTAATCTCATGAGATCAAACGAATACATAAGGTATTTAGTCCAACTTCGCTGGGTTTATCCCTTAG
TTATGGGCAGGTTTGGATACGCGTTACTCACCCGTGCGCCGGTCGCCGACATAAATAGCAAGCTATCTATTCG
CTGCCCCCCTCGACTTGCATGTGTTTAAGCCTATAGCTAGCGTTCATCCTTGAGCCAGGATCAAACTCT
>108
CTGCTGCCGCCCGTAGGAGTTTGGACCGTGTCTCAGTTCCAATGTGGGGGACCTTCCTCTCAGAACCCCTACT
GATCGTTGCCTTTGGTGGGCCGTTACCCCGCCAACAAGCTAATCAGACGCATCCCCCGTCCATAACCGATAAA
TCTTTTATTTCATAATCTCATGAGATCAAACGAATACATAAGGTATTTAGTCCAACTTCGCTGGGTTTATCCC
TTAGTTTATGGGCAGGTTTGGATACGCGTTACTCACCCGTGCGCCGGTCGCCGACATAAATAGCAAGCTATCT
ATTCGCTGCCCCCCCTCGACTTGCATGTGTTTAAGCCTATAGCTAGCGTTCATCCTGAGCCAGGATCAAACTC
T
>109
CTGCTGCCGCCCGTAGGAGTTTGGACCGTGTCTCAGTTCCAATGTGGGGGACCTTCCTCTCAGAACCCCTACT
GATCGTCGCCTTTGGTGGGCCGTTACCCCGCCAACAAGCTAATCAGACGCATCCCCATCCATAACCGCTAAAC
CTTTAATCGTTATTTGATGTCAGCAAACGATATCATACGGTATTAGGCCGTCTTTCAACGGGTTATCCCATAG
TTATGGGAAGGTTGGATACGCGTTACTCACCCGTGCGCCAGTCGTCATCAAGGAAAGCAAGCTTTCCTCATGT
TACCCTGCGACATGCATGTGTTAAGCCTGTAGCTAGCGTTCATCCTGAGCCAGGATCAAACTCT
>110
CTGCTGCCGCCCGTAGGAGTTTTGGACCGTGTCTCAGTTCCAATGTGGGGGGACCTTCCTCTCAGAACCCCTA
CTGATCGTCGCCTTTGGTGGGCCGTTACCCCGCCAACAAGCTAATCAGACGCATCCCCATCCATCACCGCTAA
ACCTTTAATCGTTATTTGATGTCAGCAAACGATGTCATACGGTATTAGTCCGTCTTTCAACGGGTTATCCCGT
GGTCATGGGAAGGTTGGATACGCGTTACTCACCCGTGCGCCAGTCGTCGTCAAGGGAAGCAAGCTTCCCTCAC
GTTACCCTGCGACTTGCATGTGTTAAGCCTGTAGCTAGCGTTCATCCCTGAGCCAAGGCATCAAACTCT
>111
CTGCTGCCGCCCGTAGGAGTTTTGGACCGTGTCTCAGTTCCAATGTGGGGGACCTTCCTCTCAGAACCCCTAC
TGATCGTCGCCTTTGGTGGGCCGTTACCCCGCCAACAAGCTAATCAGACGCATCCCCATCCATAACCGCTAAA
CCTTTAATCGTTATTTGATGTCAGCAAACGATATCATACGGTATTAGGCCGTCTTTCAACGGGTTATCCCGTA
GTTATGGGAAGGTTGGATACGCGTTACTCACCCGTGCGCCAGTCGTCATCAAGGAAAGCAAGCTTTCCCTCAT
GTTACCCTGCCGAACTTGCATGTGTTAAGCCTGTAGCTAGCGTTCATCCCGTGAGCCAAGGCATCAAACTCT
>112
CTGCTGCCGCCCGTAGGAGTTTGGACCGTGTCTCAGTTCCAATGTGGGGGACCTTCCTCTCAGAACCCCTACT
GATCGTTGCCTTTGGTGGGCCGTTACCCCGCCAACAAGCTAATCAGACGCATCCCCATCCATAACCGATAAAT
CTTTATTCATAATCTCATGAGATCAAACGAATACATAAGGTATTAGTCCAACTTCGCTGGGTTATCCCTTAGT
TATGGGCAGGTTGGATACGCGTTACCTCACCCGTGCGCCGGTCGCCGACATAAATAGCCAAGCTATCTATTCG
CTGCCCCTGACTTGCATGTGTTAACGCCTGTAGCTAGCGTTCATCCTGAGCCAGGATCAAACTCT
>113
CTGCTGCCGCCCGTAGGAGTTTGGACCGTGTCTCAGTTCCAATGTGGGGGACCTTCCTCTCAGAACCCCTACT
GATCGTCGCCTTTGGTGGGCCGTTACCCCGCCAACAAGCTGATCAGACGCATCCCCATCCATAACCGCTAAAC
CTTTAATCGTTATTTGATGTCAGCAAACGATATCATACGGTATTAGGCCGTCTTTCAACGGGTTATCCCGTAG
TTATGGGAAGGTTGGATACGCGTTACTCACCCGTGCGCCAGTCGTCATCAAGGAAAGCAAGCTTTCCTCATGT
TACCCTGCGACTTGCATGTGTTAAGCCTGTAGCTAGCGTCATCCTGAGCCAGGATCAAGCTCT
>114
CTGCTGCCGCCCGTAGGAGTTTGGACCGTGTCTCAGTTCCAATGTGGGGGACCTTCCTCTCAGAACCCCTACT
GATCGTCGCCTTTGGTGGGCCGTTACCCCGCCAACAAGCTAATCACGACGCATCCCCATCCATCACCGCTAAA
CCTTTAATCGTTATTTGATGTCAGCAAACGATGTCATACGGTATTAGTCCGTCTTTCAACGGGTTATCCCGTA
GTTATGGGAAGGTTGGATACGCGTTACTCACCCGTGCGCCAGTCGTCATCAAGGAAAGCAAGCTTTCCCTCAT
GTTGACCCTGCCGAACT
>115

CTGCTGCCGCCCGTAGGAGTTTGGACCGTGTCTCAGTTCCAATGTGGGGGACCTTCCTCTCAGAACCCCTCAC
TGATCGTCGCCTTTGGTGGGCCGTTACCCCGCCAACAAGCTAATCAGACGCATCCCCATCCATCACCGCTAAA
CCTTTAATCGTTATTTGATGTCAGCAAACGATGTCATACGGTATTAGTCCGTCTTTCAACGGGTTATCCCGTG
GTCATGGGAAGGTTGGATACGCGTTACTCACCCGTGCGCCAGTCGTCGTCAAGGAAAGCAAGCTTTCCCTCAT
GTTACCCTGCGACTTGCATGTGTTAAGCCTGT
>116
CTGCTGCCGCCCGTAGGAGTTTGGACCGTGTCTCAGTTCCAATGTGGGGGACCTTCCTCTCAGAACCCCTACT
GATCGTCGCCTTTGGTGGGCCGTTACCCCGCCAACAAGCTAATCAGACGCATCCCCATCCATCACCGCTAAAC
CTTTAATCGTTATTTGATGTCAGCAAACGATGTCATACGGTATTAGTCCGTCTTTCAACGGGTTATCCCGTGG
TCATGGGAAGGTTGGATACGCGTTACTCACCCGTGCGCCAGTCGGTCGTCAAGGGAAGCAAGCTTCCCCTCAC
GTTACCCTGCGACTTGCATGTGTTAAGCCTGTAGCTAGCGTTCATCCTGAGCCAGGCATCAAACTCT
>117
CTGCTGCCGCCCGTAGGAGTTTTGGACCGTGTCTCAGTTCCAATGTGGGGGACCTTCCTCTCAGAACCCCTAC
TGATCGTCGCCTTGGTGGGCCGTTACCCCGCCAACAAGCTAATCAGACGCATCCCCATCCATAACCGCTAACC
TTTAATCGTTATTTGATGTCAGCAAACGATATCATACGGTATTAGGCCGTCTTTCAACGGGTTATCCCGTAGT
TATGGGAAGGTTGGATACGCGTTACTCACCCGTGCGCCAGTCGTCATCAAGGAAAGCAAGCTTTCCCTCATGT
TACCCTGCGACTTGCATGTGTTAAGCCTGTAGCTAGCGTTCATCCCGTGAGCCAGGATCAAACTCT
>118
CTGCTGCCGCCCGTAGGAGTTTTGGACCGTGTCTCAGTTCCAATGTGGGGGACCTTCCTCTCAGAACCCCTAC
TGATCGTCGCCTTTGGTGGGCCGTTACCCCGCCAACAAGCTAATCAGACGCATCCCCATCCATAACCGCTAAA
CCTTTAATCGTTATTTGATGTCAGCAAACGATATCATACGGTATTTAGGCCGTCTTTCAACGGGTTATCCCGT
AGTTATGGGAAGGTTGGATACGCGTTACTCACCCGTGCGCCAGTCGTCATCAAGGAAAGCAAGCTTTCCTCAT
GTTTACCCTGCGACTTGCATGTGTTTAAGCCTTGTAGCTAGCGTTCATCCTGAGCCAGGATCAAACTCT
>119
CTGCTGCCGCCCGTAGGAGTTTTGGACCGTGTCTCAGTTCCAATGTGGGGGACCTTCCTCTCAGAACCCCTAC
TGATCGTCGCCTTTGGTGGGCCGTTACCCCGCCAACAAGCTAATCAGACGCATCCCCATCCATCACCGCTAAA
CCTTTAATCGTTATTTGATGTCAGCAAACGATGTCATACGGTATTAGTCCGTCTTTCAACGGGTTATCCCGTA
GTTATGGGAAGGTTGGATACGCGTTACTCACCCGTGCGCCAGTCGTCATCAAGGAAAGCAAGCTTTCCCTCAT
GTTACCCTGCCGACTTGCATGTGTTAAGCCTGTAGCTAGCGTTCATCCCTGAGCCAAGGCATCAA
>120
CTGCTGCCGCCCGTAGGAGTTTGGACCGTGTCTCAGTTCCAATGTGGCCGATCACCCTCTCAGGTCGGCTACT
GATCGTCGCCTTGGTGGGCAGTTATCTCACCAACTAGCTAATCAGACGCGGGTCCATCCTATACCGCTAACAC
TTTGATATATCTACGATGCCGTAAATATATATTTATCATGTATTTAGTTAAACTTTTCAGTATGTTTATCCAT
GTGTATAGGGCAGGTTTACCCACGCGTTACTCACCCGTCCGCCGCTCATCTTTCATTTTAATGCAAGCATCAA
ATCAGACGCGCTCGACTTGCATGTGTTTAGGCACGCCGCCAGCGTTCATCCTGAGCCAGGATCAAACTCT
>121
CTGCTGCCGCCCGTAGGAGTTTGGACCGTGTCTCAGTTCCAATGTGGGGGACCTTCCTCTCAGAACCCCTACT
GATCGTTGCCTTGGTGGGCCGTTACCCCGCCAACAAGCTAATCAGATGCATCCCCATCCATAACCGATAAATC
TTTATTCATAATCTCATGAGAATCAAACGAATACATAAGGTATTAGTCCAACTTCGCTGGGTTATCCCTTAGT
TATGGGCAGGTTGGATACGCGTTACCTCACCCGTGCGCCGGTCGCCGACATAAATAGCAAGCTATCTATTCGC
TGCCCCTCGACTTGCATGTGTTAACGCCTATAGCTAGCGTTCTCCTGAGCC
>122
CTGCTGCCGCCCGTAGGAGTCTGGACCGTGTCTCAGTTCCAGTGTGGCCGTTCATCCTCTCAGACCGGCTACC
TGATCGTTGGTTTGGTGGGCCGTTACCCCACCAACTGCCCTAATCAGACGCAAACCCCTCTTCAGGCGATAGC
TTACAGGTAGAGGCTACCCTTTCTTCACAGGTCATGCGACCCGTGGAACGTATTCGGTATTAGCAGTCGTTTC
CGTCTGTTGTCCCCATCCTGAAGGCAGGTTGTTTACGTGTTACTCACCCGTTTGCCACTGGATTCGATAAGAG
CAAGCTCTTCA
>123
CTGCTGCCGCCCGTAGGAGTTTGAACCGTGTCTCAGTTCCAATGTGGGGGACCTTCCTCTCAGAACCCCTACT
GATCGTTGCCTTGGTGGGCCGTTACCCCGCCAACAAGCTAATCAGACGCATCCCCATCCATAACCGATAAATC
TTTATTTCATAATCTCATGAGATCAAACGAATACATAAGGTATTTAGTTCCAACTTCGCTGGGTTTATCCCTT
AGTTTATGGGCAGGTTTGGATACGCGTTACTCACCCGTGCGCCGGTCGCCGACATAAATAGCAAGCTTATCTA
TTCGCTGCCCCCCCCTTCGACTTGCATGTGTTTTAAGCCTTATAGCTAGCGTTCATCCTTGAGCCAGGATCAA
ACTCT

>124
CTGCTGCCGCCCGTAGGAGTTTGGACCGTGTCTCAGTTCCAATGTGGGGGACCTTCCTCTCAGAACCCCTACT
GATCGTCGCCTTTGGTGGGCCGTTACCCCGCCAACAAGCTAATCAGACGCATCCCCATCCATAACCGATAAAT
CTTTATTCATAATCTCATGAGAATCAAACGAATACATAAGGTATTAGTCCAACTTCGCTGGGTTATCCCTTAG
TTATGGGCAGGTTGGATACGCGTTACCTCACCGTGCGCCGGTCGCCGACATAAATAGCAAGCTATCTATTCGC
TGCCCCTCGACTTGCATGTGTTAAGCCTATAGCTAGCGTTCATCCTGAGCCAGGATCAAACTCT
>125
CTGCTGCCGCCCGTAGGAGTTTGGACCGTGTCTCAGTCCACATGTGGGGGACCTTTCCTCTCAGAACCCCTAC
TGATCGTCGCCTTTGGTGGGCCGTTACCCCGCCAACAAGCTAATCAGACGCATCCCCATCCATAACCGCTAAA
CCTTTAATCGTTATTTGATGTCAGCAAACGATATCATACGGTATTAGGCCGTCTTTCAACGGGTTATCCCGTA
GTTATGGGAAGGTTGGATACGCGTTACTCACCCGTGCGCCAGTCGTCATCAAGGAAAGCAAGCTTTCCCTCAT
GTTACCCTGCGACTTGCATGTGTTAAGCCTGTAGCTAGCGTTCATCCTGAGCCAGGATCAAACTCT
>126
CTGCTGCCGCCCGTAGGAGTTTGGACCGTGTCTCAGTTCCAATGTGGGGGACCTTCCTCTCAGAACCCCTACT
GATCGTCGCCTTGGTGGGCCGTTACCCCGCCAACAAGCTAATCAGACGCATCCCCATCCATAACCGCTAAACC
TTTAATCGTTTATTTTGATGTCAGCAAACGATATCATACGGTATTTAGGCCGTCTTTTCAACGGGTTTATCCC
GTAGTTTATGGGAAGGTTGGATACGCGTTACTCACCCGTGCGCCAGTCGTCATCAAGGAAAGCAAGCCTTTCC
TTCATGTTTACCCTGCGACTTGCATGTGTTTTAAGCCTGTAGCTAGCGTTCATCCTGAGCCAGGATCAAACTC
T
>127
CTGCTGCCGCCCGTAGGAGTTTGAACCGTGTCTCAGTTCCAATGTGGGGGACCTTCCTCTCAGAACCCCTACT
GATCGTTGCCTTGGTGGGCCGTTACCCCACCAACAAGCTAATCAGACGCATCCCCATCCATAACCGATAAATC
TTTATTCGTAATCTCATGAGATCAAACGAATATATAAGGTATTAGTCCAACTTCGCTGGGTTATCCCTTAGTT
ATGGGCAGGTTGGATACGCGTTACTCACCCGTGCGCCGGTCGCCGACATAAATAGCAAGCTATCTATTCGCTG
CCCCTCGACTTGCATGTGTTAAGCCTATAGCTAGCGTTCATCCTGAGCCAGGATCAAACTCT
>128
CTGCTGCCGCCCGTAGGAGTTTTGGACCGTGTCTCAGTTCCAATGTGGGGGACCTTCCTCTCAGAACCCCTAC
TGATCGTCGCCTTTGGTGGGCCGTTACCCCGCCAACAAGCTAATCAGACGCATCCCCATCCATAACCGCTAAA
CCTTTAATCGTTATTTGGTGTCAGCAAACGATATCATACGGTATTAGGCCGTCTTTCAACGGGTTATCCCGTA
GTTATGGGAAGGTTGGATACGCGTTACTCACCCGTGCGCCAGTCGTCGTCAAGGAAAGCAAGCTTTCCTCACG
TTACCCTGCGAACGCTTGCATTGTGATAAGGCCTAGACGTCAGCGTTCATCCGTGCGCA
>129
CTGCTGCCGCCCGTAGGAGTTTGAACCGTGTCTCAGTTCCAATGTGGGGGACCTTCCTCTCAGAACCCCTACT
GATCGTTGCCTTGTGGGCCGTTACCCCACCAACAAGCCTAATCAGACGCATCCCCATCCATAACCGATAAATC
TTTATTCGTAATCTCATGAGATCAAACGAATATATAAGGTATTAGTCCAACTTCGCTGGGTTATCCCTTAGTT
ATGGGCAGGTTGGATACGCGTTACTCACCCGTGCGCCGGTCGCCGACATAAATAGCAAGCTATCTATTCGCTG
CCCCTCGACTTGCGTGTGTTAAGCCTATAGCTAGCGTTCATCCTGAGCCAGGATCAAACTCT
>130
CTGCTGCCGCCCGTAGGAGTTTGGACCGTGTCTCAGTTCCAATGTGGGGGACCTTCCTCTCAGAACCCCTACT
GATCGTCGCCTTTGGTGGGCCGTTACCCCGCCAACAAGCTAATCAGACGCATCCCCATCCATAACCGCTAAAC
CTTTAATCGTTATTTGATGTCAGCAAACGATATCATACGGTATTAGGCCGTCTTTCAACGGGTTATCCCGTAG
TTATGGGAAGGTTGGATACGCGTTACTCACCCGTGCGCCAGTCGTCATCAAGGAAAGCAAGCTTTCCCTCATG
TTACCCTGCGACATGCATGTGTTAAGCCTGTAGCTAGCGTTCATCCGTGAGCCAAGGATCAACTCT
>131
CTGCTGCCGCCCGTAGGAGTTTGGACCGTGTCTCAGTTCCAATGTGGCCGATCACCCTCTCAGGTCGGCTACT
GATCGTCGCCTTGGTGGGCAGTTATCTCACCAACTAGCTAATCAGACGCGGGTCCATCCTATACCGCTAACAC
TTTGATATATCTACGATGCCGTAAATATATATTATCATGTATTAGTAAACTTTTCAGTATGTTTATCCATGTG
TATAGGGCAGGTTTACCCACGCGTTACTCACCCGTCCGCCGCTCATCTTTCATTTAATGCAAGCATCAAATCA
GACGCGCTCGACTTGCATGTGTTTAGGCACGCCGCCAGCGTTCATCCTGAGCCAGGATCAAACTCT
>132
CTGCTGCCGCCCTAGGAGTTTGAACCGTGTCTCAGTTCCAATGTGGGGGACCTTCCTCTCAGAACCCCTACTG
ATCGTTGCCTTGGTGGGCCGTTACCCCACCAACAAGCTAATCAGACGCATCCCCATCCATAACCGATAAATCT
TATTCGTAATCTCATGAGATCAAAACGAAATACATAAGGTATTAGTCCAACTTCGCTGGGTTTATCCCTTAGT

TTATGGGCAGGTTTGGATACGCGTTACTCACCCGTGCGCCGGTCGCCGACATAAATAGCAAGCTATCTATTCG
CTGCCCCCCCCTCGACTTGCATGTGTTTTAAGCCTATAGCTAGCGTTCATCCCGAGCCAGGATCAAACTCT
>133
CTGCTGCCGCCCGTAGGAGTTTTGGACCGTGTCTCAGTTCCAATGTGGGGGACCTTCCTCTCAGAACCCCTAC
TGATCGTTGCCTTGGTGGGCCGTTACCCCGCCAACAAGCTAATCAGACGCATCCCCATCCATAACCGCTAAAC
CTTTAATCGTTATTTTGATGTCAGCAAACGATATCATACGGTATTAGGCCGTCTTTTCAACGGGTTATCCCGT
AGTTTATGGGAAGGTTGGATACGCGTTACTCACCCGTGCGCCAGTCGTCCAGTCAAGGAAAGCAAGCTTTCCC
TCATGCTTTTATCCAGCCCGAGTTCGACTGTGTAAGCCT
>134
CTGCTGCCGCCCGTAGGAGTTTGAACCGTGTCTCAGTTCCAATGTGGGGGACCTTCCTCTCAGAACCCCTACT
GATCGTTGCCTTGGTGGGCCGTTACCCCGCCAACAAGCTAATCAGACGCATCCCCATCCATAACCGATAAATC
TTTATTCATAATCTCATGAGATCAAACGAATACATAAGGTATTAGTCCAACTTCGCTGGGTTTATCCCTTAGT
TTATGGGCAGGTTTGGATACGCGTTACCTCGACCCGTGCGCCGGTCGCCGACATAAATAGCCAAGCTATCTAT
TCGCTGCCCCCCCTTCGACTTGCATGTGTTTAAGCCTATAGCTAGCGTTCATCCTGAGCCAGGATCAAACTCT
>135
CTGCTGCCGCCCGTAGGAGTTTTGGACCGTGTCTCAGTTCCAATGTGGGGGACCTTCCTCTCAGAACCCCTAC
TGATCGTCGCCTTTGGTGGGCCGTTACCCCGCCAACAAGCTAATCAGACGCATCCCCATCCATCACCGCTAAA
CCTTTAATCGTTATTTGATGTCAGCAAACGATGTCATACGGTATTAGTCCGTCTTTCAACGGGTTATCCCGTG
GTCATGGGAAGGTTGGATACGCGTTACTCACCCGTGCGCCAGTCGTCGTCAAGGGAAGCAAGCTTCCCTCACG
TTACCCTGCGACTTGCATGTGTTAAGCCTGTAGCTAGCGTTCATCCTGAGCCAGGATCAAACTCT
>136
CTGCTGCCGCCCGTAGGAGTTTGGACCGTGTCTCAGTTCCAATGTGGGGGACCTTCCTCTCAGAACCCCTACT
GATCGTTGCCTTGGTGGGCCGTTACCCCGCCAACAAGCTAATCAGACGCATCCCCATCCATAACCGATAAATC
TTTATTCGTAATCTCATGAGATCAAACGAAATACATAAGGTATTAGTCCAACTTCGCTGGGTTATCCCTTAGT
TATGGGCAGGTTGGATACGCGTTACTCACCGTGCGCCGGTCGCCGACATAAATAGCAAGCTATCTATTCGCTG
CCCCTCGACTTGCATGTGTTTAAGCCTATAGCTAGCGTTCATCCTGAGCCAGGATCAAACTCT
>137
CTGCTGCCGCCCGTAGGAGTTTGGACCGTGTCTCAGTTCCAATGTGGGGGACCTTCCTCTCAGAACCCCTACT
GATCGTCGCCTTGGTGGGCCGTTACCCCGCCAACAAGCTAATCAGACGCATCCCCATCCATAACCGATAAATC
TTTTATTTCATAATCTCATGAGATCAAACGAATACATAAGGTATTTAGTTCCAACTTTCGCTGGGTTTATCCC
TTAGTTTAGGGCAGGTTTTGGATACGCGTTACTCACCCCCGTGCGCCGGTCGCCGACGTAAATAGCAAGCTAT
CTTATTTCGCTGCCCCCCCCCTTCGATTGCATTGTGTTTTAAGCCTTATAGCTAGCGTTCATCCTTGAGCCAGG
ATCAAACTCTATCACCGACTGCCCCCCCATAGAGAGGTGAGACTGCCAAGGCACACAGGGGATAGG
>138
CTGCTGCCGCCCGTAGGAGTTTGGACCGTGTCTCAGTTCCAATGTGGGGGACCTTCCTCTCAGAACCCCTACT
GATCGTCGCCTTGGTGGGCCGTTACCCCGCCAACAAGCTAATCAGACGCATCCCCATCCATAACCGCTAAACC
TTTAATCGTTATTTTGATGTCAGCAAACGATATCATACGGTATTTAGGCCGTCTTTTCAACGGGTTATCCCGT
AGTTTATGGGAAGGTTGGATACGCGTTACTCACCCGTGCGCCAGTCGTCATCAAGGAAAGCAAGCTTTCCCTC
ATGTTACCCTGCGACGACTGACAT
>139
CTGCTGCCGCCCGTAGGAGTTTGGACCGTGTCTCAGTTCCAATGTGGGGGACCTTCCTCTCAGAACCCCTACT
GATCGTCGCCTTTGGTGGGCCGTTACCCCGCCAACAAGCTAATCAGACGCATCCCCATCCATAACCGCTAAAC
CTTTAATCGTTTATTTTGATGTCAGCAAACGATATCATACGGTATTTAGGCCGTCTTTTCAACGGGTTTATCC
CGTAGTTTATGGGAAGGTTTGGATACGCGTTACTCACCCGTGCGCCAGTCGTCATCAAGGAAAGCAAGCTTTC
CTTCATGTTTTACCCTGCGACTTGCATTGTGTTTAAGCCTTGTAGCTAGCGTCATCCTGAGCCAGGATCAAAC
TCGT
>140
CTGCTGCCGCCCGTAGGAGTTTGGACCGTGTCTCAGTTCCAATGTGGGGGACCTTCCTCTCAGAACCCCTACT
GATCGTTGCCTTGGTGGGCCGTTACCCCGCCAACAAGCTAATCAGACGCATCCCCATCCATAACCGATAAATC
TTTATTCATAATCTCATGAGATCAAACGAATACATAAGGTATTAGTCCAACTTCGCTGGGTTATCCCTTAGTT
ATGGGCAGGTTTGGATACGCGTTACTCACCCGTGCGCCGGTCGCCGACATAAATAGCAAGCTATCTATTCGCT
GCCCCCCTCGACTTGCATGTGTTTAAGCCTATAGCTAGCGTTCATCCTGAGCCAGGATCAAACTCT
>141

CTGCTGCCGCCCGTAGGAGTTTGAACCGTGTCTCAGTTCCAATGTGGGGGACCTTCCTCTCAGAACCCCTACT
GATCGTTGCCTTGGTGGGCCGTTACCCCACCAACAAGCTAATCAGACGCATCCCCATCCATAACCGATAAATC
TTTATTCGTAATCTCATGAGATCAAACGAAATATATAAGGTATTAGTCCAACTTCGCTGGGTTATCCCTTAGT
TTATGGGCAGGTTTGGATACGCGGTTACTCACCCGTGCGCCGGTCGCCGACATAAATAGCAAGCTATCTATTT
CGCTGCCCCTCGACTTGCATGTGTTTAAGCCTATAGCTAGCGTTCATCCTGAGCCAGGATCAAACTCT
>142
CTGCTGCCGCCCGTAGGAGTTTGGACCGTGTCTCAGTTCCAATGTGGGGGACCTTCCTCTCAGAACCCCTACT
GATCGTTGCCTTGGTGGGCCGTTACCCCGCCAACAAGCTAATCAGACGCATCCCCATCCATAACCGATAAATC
TTTATTCATAATCTCATGAGATCAAACGAATACATAAGGTATTTAGTCCAACTTCGCTGGGTTATCCCTTAGT
TTATGGGCAGGTTTGGATACGCGTTACTCACCCGTGCGCCGGTCGCCGACATAAATAGCAAGCTATCTATTCG
CTGCCCCTCGACTTGCATGTGTTTAAGCCTATAGCTAGCGTTCATCCTGAGCCAGGATCAAACTCT
>143
CTGCTGCCGCCCGTAGGAGTTTTGGACCGTGTCTCAGTTCCAATGTGGGGGACCTTCCTCTCAGAACCCCTAC
TGATCGTTGCCTTTGGTGGGCCGTTACCCCGCCAACAAGCTAATCAGACGCATCCCCATCCATAACCGATAAA
TCTTTATTCATAATCTCATGAGATCAAACGAATCATAAGGTATTAGTCCAACTTCGCTGGGTTATCCCTTAGT
TATGGGCAGGTTGGATACGCGTTACTCACCCGTGCGCCGGTCGCCGACATAAATAGCAAGCTATCTATTCGCT
GCCCCTCGACTTGCATGTGTTAACGCCTATAGCTAGCGTTCATCCTGAGCCAGGCATCAAACTCT
>144
CTGCTGCCGCCCGTAGGAGTTTGGACCGTGTCTCAGTTCCAATGTGGGGGACCTTCCTCTCAGAACCCCTACT
GATCGTCGCCTTTGGTGGGCCGTTACCCCGCCAACAAGCTAATCAGACGCATCCCCATCCATAACCGCTAAAC
CTTTAATCGTTATTTGATGTCAGCAAACGATATCATACGGTATTAGGCCGTCTTTCAACGGGTTATCCCGTAG
TTATGGGAGGGTTGGATACGCGTTACTCACCCGTGCGCCAGTCGTCATCAAGGAAAGCAAGCTTTCCCTCATG
TTACCCTGCGACTTGCATGTGTTAAGCCTGTAGCTAGCGTTCATCCCTGAGCCAAGGATCAAACTCT
>145
CTGCTGCCGCCCGTAGGAGTTTTGGACCGTGTCTCAGTTCCAATGTGGGGGACCTTCCTCTCAGAACCCCTAC
TGATCGTCGCCTTGGTGGGCCGTTACCCCGCCAACAAGCTAATCAGACGCATCCCCATCCATAACCGCTAAAC
CTTTAATCGTTTATTTTGATGTCAGCAAACGATATCATACGGTATTTAGGCCGTCTTTCAACGGGTTTATCCC
GTGGTCATGGGAAGGTTGGATACGCGTTACTCACCCGTGCGCCAGTCGTCGTCAAGGGAAGCAAGCTTCCCTT
CACGTTACCCTGCGACTTGCATGTGTTTAAGCCTTGTAGCTAGCGTTCATCCCGTGACGCAAGGCATCAAACT
CCT
>146
CCTGCCGCCCGTAGGAGTTTGGACCGTGTCTCAGTTCCAATGTGGGGGACCTTCCTCTCAGAACCCCTACTGA
TCGTCGCCTTTGGTGGGCCGTTACCCCGCCAACAAGCTAATCAGACGCATCCCCATCCATCACCGCTAAACCT
TTAATCGTTATTTGATGTCAGCAAACGATGTCATACGGTATTAGTCCGTCTTTCAACGGGTTATCCCGTGGTC
ATGGGAAGGTTGGATACGCGTTACTCACCCGTGCGCCAGTCGTCGTCAAGGGAAGCAAGCTTCCCTCACGTTA
CCCTGCGACTTGCATGTGTTAAGCCTGTAGCTAGCGTTCATCCTGAGCCAGGATCAAACTCT
>147
CTGCTGCCGCCCGTAGGAGTTTTGGACCGTGTCTCAGTTCCAATGTGGGGGACCTTCCTCTCAGAACCCCTAC
TGATCGTCGCCTTTGGTGGGCCGTTACCCCGCCAACAAGCTAATCAGACGCATCCCCCATCCCATCAACCGCT
AAACCTTTAATCGTTATTTGATGTCAGCAAACGATGTCATACGGTATTAGGCCGTCTTTCAACGGGTTATCCC
GTAGTTATGGGAAGGTTGGATACGCGTTACTCACCCGTGCGCCAGTCGTCATCAAGGAAAGCAAGCTTTCCCT
CATGTTACCCTGCGACTTCGCATGTGTTAAGCCTGTAGCTAGCGTTCATCCCTGACGCCAAGGCATCAA
>148
CTGCTGCCGCCCGTAGGAGTTTGGACCGTGTCTCAGTTCCAATGTGGGGGACCTTCCTCTCAGAACCCCTACT
GATCGTCGCCTTGGTGGGCCGTTACCCCGCCAACAAGCTAATCAGACGCATCCCCATCCATAACCGCTAAACC
TTTAATCGTTATTTGATGTCAGCAAACGATATCATACGGTATTAGGCCGTCTTTCAACGGGTTATCCCGTAGT
TATGGGAAGGTTGGATACGCGTTACTCACCCGTGCGCCAGTCGTCATCAAGGAAAGCAAGCTTTCCTCATGTT
TACCCTGCGACATGCATGTGTTAAGCCTGTAGCTAGCGTTCATCCTGAGCCAGGATCAAACTCT
>149
CTGCTGCCGCCCGTAGGAGTTTTGGACCGTGTCTCAGTTCCAATGTGGGGGACCTTCCTCTCAGAACCCCTAC
TGATCGTCGCCTTGGTGGGCCGTTACCCCGCCAACAAGCTAATCAGACGCATCCCCATCCATAACCGCTAAAC
CTTTAATCGTTATTTGATGTCAGCAAACGATATCATACGGTATTTAGGCCGTCTTTCAACGGGTTATCCCGTA
GTTTATGGGAAGGTTGGATACGCGTTACTCACCCGTGCGCCAGTCGTCATCAAGGAAAGCAAGCTTTCCTCAC
TGTTTACCCTGCGAACTTGCATGTGTTAAGCCTTGTAGCTAGC

>150
CTGCTGCCGCCCGTAGGAGTTTGGACCGTGTCTCAGTTCCAATGTGGGGGACCTTCCTCTCAGAACCCCTAC
TGATCGTCGCCTTGGTGGGCCGTTACCCCCGCCAACAAGCTAATCAGACGCATCCCCATCCATAACCGCTAAA
CCTTTAATCGTTTATTTTGATGTCAGCAAACGATATCATTACGGTATTTAGGCCGTCTTTTCAACGGGTTTAT
TCCCGTAGTTTATGGGAAGGTTGGATACGCGTTACTCACCCGTGCGCCAGTCGTCATTCAAGGAAAGCAAGCC
TTTCCCTTC
>151
CTGCTGCCGCCCGTAGGAGTTTGGACCGTGTCTCAGTTCCAATGTGGGGGACCTTCCTCTCAGAACCCCTACT
GATCGTCGCCTTGGTGGGCCGTTACCCCGCCAACAAGCTAATCAGACGCATCCCCATCCATCACCGCTAAACC
TTTAATCGTTATTTGATGTCAGCAAACGATGTCATACGGTATTAGTCCGTCTTTCAACGGGTTATCCCGTGGT
CATGGGAAGGTTGGATACGCGTTACTCACCCGTGCGCCAGTCGTCGTCAAGGGAAGCAAGCTTCCCTCACGTT
ACCCTGCGACTTGCATGTGTTAAGCCTAGTAGCTAGCGTTCATCCCTGAGCCAAGGCATCAAAC
>152
CTGCTGCCGCCCGTAGGAGTTTGGACCGTGTCTCAGTTCCAATGTGGCCGATCACCCTCTCAGGTCGGCTACT
GATCGTCGCCTTGGTGGGCAGTTATCTCACCAACTAGCTAATCAGACGCGGGTCCATCCTATACCGCTAACAC
TTTGATATATCTACGATGCCGTAAATATATTATTTTATTCATGTTATTTAGTTAAACTTTTTCAGTATGTTTT
ATCCATGTTGTTATTAGGGCAGGTTTTACCCCCCCACGCGTTACTCACCCCCCCGTCCGCCGCTCATCTTTCA
TTTTAATGCAAGCATCAAATCAGACGCGCTCGACTTGCATGTGTTTAGGCACGCCGCCAGCGTTCATCCTGAG
CCAGGATCAAACCTCT
>153
CTGCTGCCGCCCGTAGGAGTTTGGACCGTGTCTCAGTTCCAATGTGGGGGACCTTCCTCTCAGAACCCCTACT
GATCGTTGCCTTGGTGGGCCGTTACCCCGCCAACAAGCTAATCAGACGCATCCCCATCCATAACCGATAAATC
TTTTATTCGTAATCATCATGAGATCAAACGAATACATAAGGTATTTAGTCCAACTTCGCTGGGTTATCCCTTA
GTTTATGGGCAGGTTTGGATACGCGTACTCACCGTGCGCCGGTCGCCGACATAAATAGCAAGCTATCTATTTC
GCTGCCCCCCCTCGACTTGCATGTGTTTTAAGCCTATAGCTAGCGTTCATCCTAGAGCCAAGGATCAAACTCC
AT
>154
CTGCTGCCGCCCGTAGGAGTTTGAACCGTGTCTCAGTTCCAATGTGGGGGACCTTCCTCTCAGAACCCCTACT
GATCGTTGCCTTGGTGGGCCGTTACCCCGCCAACAAGCTAATCAGACGCATCCCCATCCATAACCGATAAATC
TTTATTCATAATCTCATGAGATCAAACGAATACATAAGGTATTTAGTCCAACTTCGCTGGGTTTATCTCTTTA
GTTTATGGGCAGGTTTGGATACGCGTTACTCACCCGTGCGCCGGTCGCCGACATAAATAGCAAGCTATCTATT
CGCTGCCCCCCCTCGACTTGCATGTGTTTTAAGCCTATAGCTAGCGTTTCATCCTGAGCCAG
>155
CTGCTGCCGCCCGTAGGAGTTTGAACCGTGTCTCAGTTCCAATGTGGGGGACCTTCCTCTCAGAACCCCTACT
GATCGTTGCCTTGGTGGGCCGTTACCCCACCAACAAGCTAATCAGACGCATCCCCATCCATAACCGATAAATC
TTATTCGTAATCTCATGAGATCAAACGAAATATATAAGGTATTAGTCCAACTTCGCTGGGTTATCCCTTAGTT
TTATGGGCAGGTGGATACGCGTTACGTCACCCGTGCGCCGGTCGCCGACATAAATAGCAAGCTATCTATTCGC
TGCCCCTCGACTTGCATGTGTTTAAGCCTATAGCTAGCGTTCATCCTGAGCCAGGATCAAACTCT
>156
CTGCTGCCGCCCGTAGGAGTTTGGACCGTGTCTCAGTTCCAATGTGGGGGACCTTCCTCTCAGAACCCCTACT
GATCGTTGCCTTTGGTGGGCCGTTACCCCGCCAACAAGCTAATCAGACGCATCCCCATCCATAACCGATAAAT
CTTTATTCGTAATCTCATGAGATCAAACGAATACATAAGGTATTAGTCCAACTTCGCTGGGTTATCCCTTAGT
TATGGGCAGGTTGGATACGCGTTACTCACCCGTGCGCCGGTCGCCGACATAAATAGCAAGCTATCTATTCGCT
GCCCCTCGACTTGCATGTGTTAAGCCTATAGCTAGCGTTCATCCTGAGCCAGGATCAAACTCT
>157
CTGCTGCCGCCCGTAGGAGTTTGGACCGTGTCTCAGTTCCAATGTGGGGGACCTTCCTCTCAGAACCCCTACT
GATCGTTGCCTTGGTGGGCCGTTACCCCGCCAACAAGCTAATCAGACGCATCCCCATCCATAACCGATAAATC
TTTATTCGTAATCATCATGAGATCAAACGAAATACATAAGGTATTTAGTCCAACTTCGCTGGGTTTATCCCTT
AGTTTATGGGCAGGTTTGGATACGCGTTACTCACCCGTGCGCCGGTCGCCGACATAAATAGCAAGCTATCTAT
TCGCTGCCCCCCCTCGACTTGCATGTGTTTTAAGCCTATAGCTAGCGTTCATCCTGAGCCAGGATCAAACTCT
>158
CTGCTGCCGCCCGTAGGAGTTTGGACCGTGTCTCAGTTCCAATGTGGGGGACCTTCCTCTCAGAACCCCTACT
GATCGTCGCCTTGGTGGGCCGTTACCCCGCCAACAAGCTAATCAGACGCATCCCCATCCATAACCGATAAATC
TTTATTCATAATCTCATGAGATCAAACGAATACATAAGGTATTAGTCCAACTTCGCTGGGTTATCCCTTAGTT

ATGGGCAGGTTGGATACGCGTTACTCACCCGTGCGCCGGTCGCCGACATAAATAGCAAGCTATCTATTCGCTG
CCCCTCGACTTGCATGTGTTAAGCCTATAGCTAGCGTTCATCCTGAGCCAGGATCAAACTCT
>159
CTGCTGCCGCCCGTAGGAGTTTGGACCGTGTCTCAGTTCCAATGTGGGGGACCTTCCTCTCAGAACCCCTACT
GATCGTCGCCTTGGTGGGCCGTTACCCCGCCAACAAGCTAATCAGACGCATCCCCATCCATAACCGATAAATC
TTTATTCGTAATCTCATGAGATCAAACGAATACATAAGGTATTTAGTCCAACTTCGCTGGGTTTATCCCTTAG
TTTATGGGCAGGTTTGGATACGCGTACTCACCCGTGCGCCGGTCGCCGACATAAATAGCAAGCTATCATTCGC
TGCCCCTTCGACTGCATGTGTTTAAGCCTATAGCTAGCGTTCATCCTTGAGCCAGGATCAAACTCT
>160
CTGCTGCCGCCCGTAGGAGTTTTGGACCGTGTCTCAGTTCCAATGTGGGGGACCTTCCTCTCAGAACCCCTAC
TGATCGTTGCCTTGGTCGGGCCGTTACCCCGCCAACAAGCTAATCAGACGCATCCCCATCCATAACCGATAAA
TCTTTATTCGTAATCTCATGAGATCAAACGAATACATAAGGTATTTAGTCCAACTTCGCTGGGTTATCCCTTA
GTTTATGGGCAGGTTTGGATACGCGTTACCTCACCCGTGCGCCGGTCGCCGACATAAATAGCAAGCTTATCTA
TTCGCTGCCCCCCTCGACTTGCATGTGTTTAAGCCTTATAGCTAGCGTTCATCCTGAGCCAGGATCAAACTCT
>161
CTGCTGCCGCCCGTAGGAGTTTGGACCGTGTCTCAGTTCCAATGTGGGGGACCTTCCTCTCAGAACCCCTACT
GATCGTCGCCTTTGGTGGGCCGTTACCCCGCCAACAAGCTAATCAGACGCATCCCCATCCATAACCGCTAAAC
CTTTAATCGTTATTTGATGTCAGCAAACGATATCATACGGTATTAGGCCGTCTTTCAACGGGTTATCCCGTAG
TTATGGGAAGGTTGGATACGCGTTACTCACCCGTGCGCCAGTCGTCATCAAGGAAAGCAAGCTTCCCTCATGT
TACCCTGCGACTTGCATGTGTTAAGCCTGTAGCTAGCGTTCATCCGTGACGCCAAGGCA
>162
CTGCTGCCGCCCGTAGGAGTTTTGGACCGTGTCTCAGTTCCAATGTGGGGGACCTTCCTCTCAGAACCCCTAC
TGATCGTTGCCTTTGGTGGGCCGTTACCCCGCCAACAAGCTAATCAGACGCATCCCCATCCATAACCGATAAA
TCTTTATTCGTAATCTCATGAGATCAAACGAATACATAAGGTATTAGTCCAACTTCGCTGGGTTATCCCTTAG
TTATGGGCAGGTTGGATACGCGTTACTCACCCGTGCGCCGGTCGCCGACATAAATAGCAAGCTATCTATTCGC
TGCCCCTCGACTTGCATGTGTTAAGCCTATAGCTAGCGTTCATCCTGAGCCAGGATCAAACTCT
>163
CTGCTGCCGCCCTAGGAGTTTGGACCGTGTCTCAGTTCCAATGTGGGGGACCCTTCCTCTCAGAACCCCTACT
GATCGTTGCCTTGGGTGGGCCGGTTACCCCGCCAACAAGCTAATCAGACGCATCCCCATCCATAACCGATAAA
TCTTTATTCATAATCTCATGAGATCAAACGAATACATAAGGTATTAGTCCAACTTCGCTGGGTTATCCCTTAG
TTATGGGCAGGTTGGATACGCGTTACTCACCCGTGCGCCGGTCGCCGACATAAATAGCAAGCTATCTATTCGC
TGCCCCCCTCGACTTGCATGTGTTTAAGCCTATAGCTAGCGTTCATCCTGAGCCAGGATCAAACTCT
>164
CTGCTGCCGCCCGTAGGAGTTTGGACCGTGTCTCAGTTCCAATGTGGGGGACCTTCCTCTCAGAACCCCTACT
GATCGTCGCCTTGGTGGGCCGTTACCCCGCCAACAAGCTAACTCACGACGCATCCCCCATCATAACCGCTAAA
CCTTTTAATCGTTATTTTGATGTCAGCAAACGACGTCATACGGTATTTAGTCCGTCTTTCAACGGGTTTATCC
CGCGGTCATGGGAAGGTTTGGATACGCGTTACTCACCCGTGCGCCAGTCGTCGTCAAGGAAGCAAGCTTTTCC
TCACGTTACCCTGCGACTTGCATGTGTTTTAAGCCTGTTAGCTAGCGTTCAATCCTGAGCCAGGATCAAACTC
T
>165
CTGCTGCCGCCCTAGGAGTTTGAACCGTGTCTCAGTTCCAATGTGGGGGACCTTCCTCTCAGAACCCCTACTG
ATCGTTGCCTTGGTGGGCCGTTACCCCACCAACAAGCTAATCAGACGCATCCCCATCCATAACCGATAAATCT
TTATTTCGTAATCTCATGAGATCAAACGAATACATAAGGTATTTAGTCCAACTTCGCTGGGTTTATCCCTTAG
TTTATGGGCAGGTTTGGATACGCGTTACTCACCCGTGCGCCGGTCGCCGACATAAATAGCAAGCTATCTATTC
GCTGCCCCCCCTCGACTTGCATGTGTTTAAGCCTTATAGCTAGCGTTCATCCTGAGCCAGGATCAAACTCT
>166
CTGCTGCCGCCCGTAGGAGTTTGGACCGTGTCTCAGTTCCAATGTGGGGGACCTTCCTCTCAGAACCCCTACC
TGATCGTTGCCTTGGTGGGCCGTTACCCCGCCAACAAGCTAATCAGACGCATCCCCATCCATAACCGATAAAT
CTTTATTCGTAATCTCATGAGATCAAACGAATACATAAGGTACTAGTCCAACTTCGCTGGGTTTATCCCTTAG
TTATGGGCAGGTTTGGATACGCGTTACCTCACCCGTGCGCCGGTCGCCGACATAAATAGCCAAGCTATCTATT
CGCTGCCCCCCCTCGACTTGCATGTGTTTAAGCCTATAGCTAGCGTTCATCCCTGAGCCAGGATCAAACTCT
>167
CTGCTGCCGCCCGTAGGAGTTTGGACCGTGTCTCAGTTCCAATGTGGGGGACCTTCCTCTCAGAACCCCTACT
GATCGTTGCCTTGGTGGGCCGTTACCCCGCCAACAAGCTAATCAGACGCATCCCCATCCATAACCGATAAATC

TTTATTCATAATCTCATGAGATCAAACGAATACATAAGGTATTAGTCCAACTTCGCTGGGTTATCCCTTAGTT
ATGGGCAGGTTTGGATACGCGTTACTCACCCGTGCGCCGGTCGCCGACATAAATAGCAAGCTATCTATTCGCT
GCCCCCCCTCGACTTGCATGTGTTTAAGCCTATAGCTAGCGTTCATCCTGAGCCAGGATCAAACTCT
>168
CTGCTGCCGCCCGTAGGAGTTTGGACCGTGTCTCAGTTCCAATGTGGGGGACCTTCCTCTCAGAACCCCTACT
GATCGTCGCCTTGGTCGGGCCGTTACCCCGCCAACAAGCTAATCAGACGCATCCCCATCCATCACCGCTAAAC
CTTTAATCGTTATTTGATGCAGCAAACGATGTCATACGGTATTAGGCCGTCTTTCAACGGGTTATCCCGTAGT
TATGGGAAGGTTGGATACGCGTTACTCACCCGTGCGCCAGTCGTCCATCAAGGGAAGCAAGCTTCCCTCACGT
TACCCTGCGACTTGCATGTGTTAACGCCTGTAGCTAGCGTCAT
>169
CTGCTGCCGCCCGTAGGAGTTTTGGACCGTGTCTCAGTTCCAATGTGGGGGACCTTCCTCTCAGAACCCCTAC
TGATCGTCGCCTTGGTGGGCCGTTACCCCGCCAACAAGCTAATCAGACGCATCCCCATCCATAACCGCTAAAC
CTTTAATCGTTTATTTTGATGTCAGCAAACGACGTCATACGGTATTTAGTCCGTCTTTTCAACGGGTTTATCC
CCGTGGTCATTGGGAAGGTTTGGATACGCGTTACTCACCCCGTGCGCCAGTCGTCGTCAAGGAAAGCAAGCTT
TTCCTCACGTTACCCCCTTGCGACTTGCATGTGTTTTAAGCCTGTTAGCTTAGCGTTCATCCTGAGCCAGGAT
CAAACTCT
>170
CTGCTGCCGCCCTAGGAGTTTTGGACCGTGTCTCAGTTCCAATGTGGGGGACCTTCCTCTCAGAACCCCTACT
GATCGTTGCCTTGGTGGGCCGTTACCCCGCCAACAAGCTAATCAGACGCATCCCCATCCATAACCGATAAATC
TTTATTCATAATCTCATGAGATCAAACGAATACATAAGGTATTAGTCCAACTTCGCTGGGTTATCCCTTAGTT
ATGGGCAGGTTGGATACGCGTTACTCACCCGTGCGCCGGTCGCCGACATAAATAGCAAGCTATCTATTCGCTG
CCCCCCTCGACTTGCATGTGTTTAAGCCTATAGCTAGCGTTCATCCTGAGCCAGGATCAAACTCT
>171
CTGCTGCCGCCCGTAGGAGTTTGGACCGTGTCTCAGTTCCAATGTGGGGGACCTTCCTCTCAGAACCCCTACT
GATCGTCGCCTTGGTGGGCCGTTACCCCGCCAACAAGCTAATCAGACGCATCCCCATCCATAACCGCTAAACC
TTTAATCGTTATTTGATGTCAGCAAACGATATCATACGGTATTAGGCCGTCTTTCAACGGGTTATCCCGTAGT
TATGGGAAGGTTGGATACGCGTTACTCACCCGTGCGCCAGTCGTCATCAAGGAAAGCAAGCTTCCTCATGTTA
CCCTGCGTACGTACGCAC
>172
CTGCTGCCGCCCGTAGGAGTTTGGACCGTGTCTCAGTTCCAATGTGGGGGACCTTCCTCTCAGAACCCCTACT
GATCGTCGCCTTGGTGGGCCGTTACCCCGCCAACAAGCTAATCAGACGCATCCCCATCCATAACCGCTAAACC
TTTAATCGTTATTTGATGTCAGCAAACGATATCATACGGTATTAGGCCGTCTTTCAACGGGTTATCCCGTAGT
TATGGGAAGGTTGGATACGCGTTACTCACCCGTGCGCCAGTCGTCATCAAGGAAAGCAAGCCTTTCCTCATGT
TACCCTGCGACTTGCATGTGTTAAGCCTGTAGCTAGCGTTCATCCCGTGAGCCAAGG
>173
CTGCTGCCGCCCGTAGGAGTTTGGACCGTGTCTCAGTTTCCAATGTGGGGGACCTTCCTCTCAGAACCCCTAC
TGATCGTTGCCTTGGTGGGCCGTTACCCCGCCAACAAGCTAATCAGACGCATCCCCATCCATAACCGATAAAT
CTTTATTCGTAATCTCATGAGATCAAACGAATACATAAGGTATTAGTCCAACTTCGCTGGGTTTATCCCTTAG
TTATGGGCAGGTTTGGATACGCGTTACTCACCCGTGCGCCGGTCGCCGACATAAATAGCAAGCTATCTATTTC
GCTGCCCCCCTCGACTTGCATGTGTTTAAGCCTATAGCTAGCGTTCATCCTGAGCCAGGATCAAACTCT
>174
CTGCTGCCGCCCTAGGAGTTTGAACCGTGTCTCAGTTCCAATGTGGGGGACCTTCCTCTCAGAACCCCTACTG
ATCGTTGCCTTGGTCGGGCCGTTACCCCACCAACAAGCTAATCAGACGCATCCCCATCCATAACCGATAAATC
TTTATTCGTAATCTCATGAGATCAAACGAATACATAAGGTATTAGTCCAACTTCGCTGGGTTATCCCTTAGTT
ATGGGCAGGTTTGGATACGCGTTACTCACCCGTGCGCCGGTCGCCGACATAAATAGCAAGCTATCTATTCGCT
GCCCCTCGACTTGCATGTGTTTAAGCCTATAGCTAGCGTTCATCCTGAGCCAGGATCAAACTCT
>175
CTGCTGCCGCCCGTAGGAGTTTGGACCGTGTCTCAGTTCCAATGTGGGGGACCTTCCTCTCAGAACCCCTACT
GATCGTTGCCTTGGTGGGCCGTTACCCCGCCAACAAGCTAATCAGACGCATCCCCATCCATAACCGATAAATC
TTTATTCATAATCTCATGAGATCAAACGAATACATAAGGTATTAGTCCAACTTTCGCTGGGTTATCCCTTAGT
TATGGGCAGGTTTGGATACGCGTTACTCACCCGTGCGCCGGTCGCCGACATAAATAGCAAGCTATCTATTCGC
TGCCCCCCTCGACTTGCATGTGTTTAAGCCTATAGCTAGCGTTCATCCTGAGCCAGGATCAAACTCT
>176

CTGCTGCCGCCCGTAGGAGTTTGGACCGTGTCTCAGTTCCAATGTGGGGGACCTTCCTCTCAGAACCCCTACT
GATCGTCGCCTTGGTGGGCCGTTACCCCGCCAACAAGCTAATCAGACGCATCCCCATCCATAACCGCTAAACC
TTTAATCGTTTATTTTGATGTCAGCAAACGATATCATACGGTATTTAGGCCGTCTTTTCAACGGGTTTATCCC
GTAGTTTATGGGAAGGTTGGATACGCGTTACTCACCCGTGCGCCAGTCGTCATCAAGGAAAGCAAGCTTTCCC
TCATGTTTTACCCTGCGACTTGC
>177
CTGCTGCCGCCCGTAGGAGTTTGGACCGTGTCTCAGTTCCAATGTGGGGGACCTTCCTCTCAGAACCCCTACT
GATCGTCGCCTTTGGTGGGCCGTTACCCCGCCAACAAGCTAATCAGACGCATCCCCATCCATAACCGCTAAAC
CTTTAATCGTTATTTGATGTCAGCAAACGATATCATACGGTATTAGGCCGTCTTTCAACGGGTTATCCCGTAG
TTATGGGAAGGTTGGATACGCGTTACTCACCCGTGCGCCAGTCGTCATCAAGGAAAGCAAGCTTTCCTCATGT
TACCCTGCGACTTGCATGTGTTAAGCCTGTAGCTAGCGTTCATCCTGAGCCAGGATCAAACTCT
>178
CTGCTGCCGCCCGTAGGAGTTTGGACCGTGTCTCAGTTCCAATGTGGGGGACCTTCCTCTCAGAACCCCTACT
GATCGTTGCCTTGGGTGGGCCGGTTACCCCGCCAACAAGCTAATCAGACGCATCCCCATCCATAACCGATAAA
TCTTTTATTTCATAATCTCATGAGATCAAACGAATACATAAGGTATTTAGTTCCAACTTCGCTGGGTTTATCC
CTTTAGTTTATGGGCAGGTTTTGGATACGCGTTACTCACCCGTGCGCCGGTCGCCGACATAAATAGCAAGCTA
TCTATTCGCTGCCCCCCCCTCGACTTTGCATGTGTTTTAAGCCTTATAGCTAGCGTTCATCCTGAGCCAGGAT
CAAACTCT
>179
CTGCTGCCGCCCGTAGGAGTTTGGACCGTGTCTCAGTTCCAATGTGGGGGACCTTCCTCTCAGAACCCCTACT
GATCGTCGCCTTGGTGGGCCGTTACCCCGCCAACAAGCTAATCAGCGCATCCCCATCCATAACCGCTAAACCT
TTAATCGTTATTTGATGTCAGCAAACGATATCATACGGTATTAGGCCGTCTTTCAACGGGTTATCCCGTAGTT
ATGGGAAGGTTGGATACGCGTTACGTCACCCGTGCGCCAGTCGTCATCAAGGAAAGCAAGCTTCCTCATGTTA
CCCTGCGACTGCATTTGTGTAAGCCTGTAGCTAGCGTCATCCTTGAGCCAGGATCAAACTCT
>180
CTGCTGCCGCCCGTAGGAGTTTGGACCGTGTCTCAGTTCCAATGTGGGGGACCTTCCTCTCAGAACCCCTACT
GATCGTCGCCTTTGGTGGGCCGTTACCCCGCCAACAAGCTAATCAGACGCATCCCCATCCATAACCGCTAAAC
CTTTAATCGTTATTTGATGTCAGCAAACGATATCATACGGTATTAGGCCGTCTTTCAACGGGTTATCCCGTAG
TTATGGGAAGGTTGGATACGCGTTACTCACCCGTGCGCCAGTCGTCATCAAGGAAAGCAAGCTTTCCCTCATG
TTACCCTGCCGACGATGACAT
>181
CTGCTGCCGCCCGTAGGAGTTTTGGACCGTGTCTCAGTTCCAATGTGGGGGACCTTCCTCTCAGAACCCCTAC
TGATCGTCGCCTTTGGTGGGCCGTTACCCCGCCAACAAGCTAATCAGACGCATCCCCATCCATAACCGCTAAA
CCTTTAATCGTTATTTGATGTCAGCAAACGATATCATACGGTATTAGGCCGTCTTTCAACGGGTTATCCCGTA
GTTATGGGAAGGTTGGATACGCGTTACTCACCCGTGCGCCAGTCGTCATCAAGGAAAGCAAGCTTTCCCTCAT
GTTACCCTGCCGAACTTGCATGTGTTAAGCCTGTAGCTAGCGTTCATCCTGAGCCAGGATCAACTCAT
>182
CTGCTGCCGCCCGTAGGAGTTTGGACCGTGTCTCAGTTCCAATGTGGGGGACCTTCCTCTCAGAACCCCTACT
GATCGTCGCCTTTGGTGGGCCGTTACCCCGCCAACAAGCTAATCAGACGCATCCCCATCCATAACCGCTAAAC
CTTTAATCGTTATTTGATGTCAGCAAACGATATCATACGGTATTAGGCCGTCTTTCAACGGGTTATCCCGTAG
TTATGGGAAGGTTGGATACGCGTTACTCACCCGTGCGCCAGTCGTCATCAAGGAAAGCAAGCTTCCTCATGTT
ACCCTGCGACTGCATGTGTAAGCCTGTAGCTAGCGTCATCCTGAGCAGGCATCAACGA
>183
CTGCTGCCGCCCGTAGGAGTTTTGGACCGTGTCTCAGTTCCAATGTGGGGGACCTTTCCTCTCAGAACCCCTA
CTGATCGTCGCCTTTGGTGGGCCGTTACCCCGCCAACAAGCTAATCAGACGCATCCCCATCCATAACCGCTAA
ACCTTTAATCGTTATTTGATGTCAGCAAACGATATCATACGGTATTAGGCCGTCTTTCAACGGGTTATCCCGT
AGTTATGGGAAGGTTGGATACGCGTTACTCACCCGTGCGCCAGTCGTCATCAAGGAAAGCAAGCTTTCCCTCA
CTGTTACCCTGCGTACTTGCATGTGTTAAGCCTGTAGCTAGCGTTCATCCTGAGCCAGGATCAAACTCT
>184
CTGCTGCCGCCCGTAGGAGTTTTGGACCGTGTCTCAGTTCCAATGTGGGGGACCTTCCTCTCAGAACCCCTAC
TGATCGTTGCCTTGGTGGGCCGTTACCCCGCCAACAAGCTAATCAGACGCATCCCCATCCATAACCGATAAAT
CTTTATTCATAATCTCATGAGATCAAACGAATACATAAGGTATTTAGTCCAACTTCGCTGGGTTTATCCCTTA
GTTATGGGCAGGTTTGGATACGCGTTACTCACCCGTGCGCCGGTCGCCGACATAAATAGCAAGCTATCTATTC
GCTGCCCCTCGACTTGCATGTGTTTAAGCCTATAGCTAGCGTTCATCCTGAGCCAGGATTTCAAACTCT

>185
CTGCTGCCGCCCGTAGGAGTTTGGACCGTGTCTCAGTTCCAATGTGGGGGACCTTCCTCTCAGAACCCCTACT
GATCGTTGCCTTGGTGGGCCGTTACCCCGCCAACAAGCTAATCAGACGCATCCCCATCCATAACCGATAAATC
TTTATTCGTAATCATCATGAGATCAAACGAAATACATAAGGTATTAGTCCAACTTCGCTGGGTTATCCTTAGT
TATGGGCAGGTTGGATACGCGTTACCTCACCGTGCGCCGGTCGCCGACATAAATAGCCAAGCTATCTATTCGC
TGCCCCCCTCGACTTGCATGTGTTTAACGCCTATAGCTAGCGTTCA
>186
CTGCTGCCGCCCGTAGGAGTTTGGACCGTGTCTCAGTTCCAATGTGGGGGACCTTCCTCTCAGAACCCCTACT
GATCGTTGCCTTTGGTGGGCCGTTACCCCGCCAACAAGCTAATCAGACGCATCCCCATCCATAACCGCTAAAC
CTTTAATCGTTATTTGATGTCAGCAAACGATATCATACGGTATTAGGCCGTCTTTCAACGGGTTATCCCGTAG
TTATGGGAAGGTTGGATACGCGTTACTCACCCGTGCGCCAGTCGTCATCAAGGAAAGCAAGCTTTCCCTCATG
TTACCCTGCGACATGCATGTGTTAAGCCTGTAGCTAGCGTTCATCCGTGACGCCAAG
>187
CTGCTGCCGCCCGTAGGAGTTTGAACCGTGTCTCAGTTCCAATGTGGGGGACCTTCCTCTCAGAACCCCTACT
GATCGTTGCCTTGGTGGGCCGTTACCCCACCAACAAGCTAATCAGACGCATCCCCATCCATAACCGATAAATC
TTTATTCGTAATCTCATGAGATCAAACGAAATACATAAGGTATTAGTCCAACTTCGCTGGGTTATCCCTTAGC
TTATGGGCAGGTTGGATACGCGTTATTCACCCGTGCGCCGGTCGCCGACATAAATAGCAAGCTATCTATTCGC
TGCCCCTCGACTTGCATGTGTTAAGCCTATAGCTAGCGTTCATCCTGAGCCAGGATCAAACTCT
>188
CTGCTGCCGCCCGTAGGAGTTTGAACCGTGTCTCAGTTCCAATGTGGGGGACCTTCCTCTCAGAACCCCTACT
GATCGTTGCCTTGGTGGGCCGTTACCCCACCAACAAGCTAATCAGACGCATCCCCATCCATAACCGATAAATC
TTTATTCGTAATCTCATGAGATCAAACGAATATATAAGGTATTAGTCCAACTTCGCTGGGTTATCCCTTAGTT
TATGGGCAGGTTGGATACGCGTTACTCACCCGTGCGCCGGTCGCCGACATAAATAGCAAGCTATCTATTCGCT
GCCCCCCTCGACTTGCATGTGTTTAAGCCTTATAGCTAGCGTTCATCCTGAGCCAGGATCAAACTCT
>189
CTGCTGCCGCCCGTAGGAGTTTTGGACCGTGTCTCAGTTCCAATGTGGGGGACCTTCCTCTCAGAACCCCTAC
TGATCGTTGCCTTGGTGGGCCGTTACCCCGCCAACAAGCTAATCAGACGCATCCCCATCCATAACCGATAAAT
CTTTATTCATAATCTCATGAGATCAAACGAATACATAAGGTATTAGTCCAACTTCGCTGGGTTATCCCTTAGT
TATGGGCAGGTTTGGATACGCGTTACTCACCCGTGCGCCGGTCGCCGACATAAATAGCAAGCTATCTATTCGC
TGCCCCCCTCGACTTGCATGTGTTTAAGCCTATAGCTAGCGTTCATCCTGAGCCAGGATCAAACTCT
>190
CTGCTGCCGCCCGTAGGAGTTTGAACCGTGTCTCAGTTCCAATGTGGGGGACCTTCCTCTCAGAACCCCTACT
GATCGTTGCCTTGGTGGGCCGTTACCCCACCAACAAGCTAATCAGACGCATCCCCATCCATAACCGATAAATC
TTTATTCGTAATCTCATGAGATCAAACGAATATATAAGGTATTAGTCCAACTTCGCTGGGTTATCCCTTAGTT
TATGGGCAGGTTTGGATACGCGTTACTCACCCGTGCGCCGGTCGCCGACATAAATAGCAAGCTATCTATTCGC
TGCCCCCCCTCGACTTGCATGTGTTTAAGCCTATAGCTAGCGTTCATCCTGAGCCAGGATCAAACTCT
>191
CTGCTGCCGCCCGTAGGAGTTTGGACCGTGTCTCAGTTCCAATGTGGGGGACCTTCCTCTCAGAACCCCTACT
GATCGTCGCCTTTGGTGGGCCGTTACCCCGCCAACAAGCTAATCAGACGCATCCCCATCCATAACCGCTAAAC
CTTTAATCGTTATTTGATGTCAGCAAACGATATCATACGGTATTAGGCCGTCTTTCAACGGGTTATCCCGTAG
TTATGGGAAGGTTGGATACGCGTTACTCACCCGTGCGCCAGTCGTCATCAAGGAAAGCAAGCCTTTCCTCATG
TTACCCTGCGACTTGCATGTGTAAGCCTGTAGCTAGCGTTCATCCTGAGCCAAGGCATCCAAACTCT
>192
CTGCTGCCGCCCGTAGGAGTTTTGGACCGTGTCTCAGTTCCAATGTGGGGGACCTTCCTCTCAGAACCCCTAC
TGATCGTCGCCTTTGGTGGGCCGTTACCCCGCCAACAAGCTAATCAGACGCATCCCCATCCATCACCGCTAAA
CCTTTAATCGTTATTTGATGTCAGCAAACGATGTCATACGGTATTAGTCCGTCTTTCAACGGGTTATCCCGTA
GTTATGGGAAGGTTGGATACGCGTTACTCACCCGTGCGCCAGTCGTCATCAAGGAAAGCAAGCTTTCCCTCAC
GTTACCCTGCGACTTGCATGTGTTAAGCCTGTAGCTAGCGTTCATCCCTGAGCCAAGGATCAAACTCT
>193
CTGCTGCCGCCCGTAGGAGTTTTGGACCGTGTCTCAGTTCCAATGTGGGGGACCTTCCTCTCAGAACCCCTAC
TGATCGTCGCCTTTGGTGGGCCGTTACCCCGCCAACAAGCTAATCAGACGCATCCCCATCCATAACCGCTAAA
CCTTTAATCGTTATTTGATGTCAGCAAACGATATCATACGGTATTAGGCCGTCTTTCAACGGGTTATCCCGTA
GTTATGGGAAGGTTGGATACGCGTTACTCACCCGTGCGCCAGTCGTCATCAAGGAAAGCAAGCTTTCCCTCAT
GTTTACCCTGCGACTTGCATGTGTTAAGCCTGTAGCTAGCGTTCATCCTGAGCCAGGATCAAACTCT

>194
CTGCTGCCGCCCGTAGGAGTTTTGGACCGTGTCTCAGTTCCAATGTGGGGGACCTTCCTCTCAGAACCCCTAC
TGATCGTCGCCTTGGTGGGCCGTTACCCCGCCAACAAGCTAATCAGACGCATCCCCATCCATAACCGATAAAT
CTTTATTTCATAATCTCATGAGATCAAACGAATACATAAGGTATTTAGTCCAACTTCGCTGGGTTTATCCCTT
AGTTTATGGGCAGGTTTGGATACGCGTTACTCACCCGTGCGCCGGTCGCCGACATAAATAGCAAGCTATCTTA
TTCGCTGCCCCCCTCGACTTGCATGTGTTTAAGCCTTATAGCTAGCGTTCATCCTTGAGCCAGGATCAAACTC
T
>195
CTGCTGCCGCCCGTAGGAGTTTGGACCGTGTCTCAGTTCCAATGTGGGGGACCTTCCTCTCAGAACCCCTACT
GATCGTTGCCTTGGTGGGCCGTTACCCCGCCAACAAGCTAATCAGACGCATCCCCATCCATAACCGATAAATC
TTTATTCGTAATCTCATGACGATCAAGACGAATACATAAGGTATTAGTCCAACTTCGCTGGGTTTATCCCTTA
GTTTATGGGCAGGTTTGGATACGCGTTACTCACCCGTGCGCCGGTCGCCGACATAAATAGCAAGCTATCTATT
CGCTGCCCCCCCTTCGACTTGCATGTGTTTAAGCCTATAGCTAGCGTTCATCCTGAGCCAGGATCAAACTCT
>196
CTGCTGCCGCCCGTAGGAGTTTTGGACCGTGTCTCAGTTCCAATGTGGGGGACCTTCCTCTCAGAACCCCTAC
TGATCGTTGCCTTTGGTGGGCCGTTACCCCGCCAACAAGCTAATCAGACGCATCCCCATCCATAACCGATAAA
TCTTTATTCGTAATCTCATGAGATCAAACGAAATACATAAGGTATTTAGTCCAACTTCGCTGGGTTTATCCCT
TAGTTTATGGGCAGGTTTGGATACGCGTTATTTCACCCGTGCGCCGGTCGCCGACATAAATAGCAAGCTTATC
TATTCGCTGCCCCCCCTTCGACTTGCATGTGTTTAAGCCTTATAGCTAGCGTTCATCCTGAGCCAGGATCAAA
CTCT
>197
CTGCTGCCGCCCGTAGGAGTTTTGGACCGTGTCTCAGTTCCAATGTGGGGGACCTTCCTCTCAGAACCCCTAC
TGATCGTCGCCTTTGGTGGGCCGTTACCCCGCCAACAAGCTAATCAGACGCATCCCCATCCATAACCGCTAAA
CCTTTAATCGTTATTTGATGTCAGCAAACGATATCATACGGTATTAGGCCGTCTTTCAACGGGTTATCCCGTA
GTTATGGGAAGGTTGGATACGCGTTACTCACCCGTGCGCCAGTCGTCATCAAGGAAAGCAAGCTTTCCCTCAT
GTTACCCTGCGACTTGCATGTGTTAAGCCTGTAGCTAGCGTTCATCCGTGAGCCAAGGATCAACTCT
>198
CTGCTGCCGCCCGTAGGAGTTTTGGACCGTGTCTCAGTTCCAATGTGGGGGACCTTCCTCTCAGAACCCCTAC
TGATCGTCGCCTTTGGTGGGCCGTTACCCCGCCAACAAGCTAATCAGACGCATCCCCATCCATAACCGCTAAA
CCTTTAATCGTTATTTGATGTCAGCAAACGATATCATACGGTATTAGGCCGTCTTTCAACGGGTTATCCCGTA
GTTATGGGAAGGTTGGATACGCGTTACTCACCCGTGCGCCAGTCGTCATCAAGGAAAGCAAGCTTTCCCTCAT
GTTACCCTGCGACTTGCATGTGTTAAGCCTGTAGCTAGCGTTCATCCCGTGAGCCAAGGATCAAACTGCT
>199
CTGCTGCCGCCCGTAGGAGTTTGGACCGTGTCTCAGTTCCAATGTGGGGGACCTTCCTCTCAGAACCCCTACT
GATCGTTGCCTTTGGTGGGCCGTTACCCCGCCAACAAGCTAATCAGACGCATCCCCATCCATAACCGATAAAT
CTTTATTCGTAATCTCATGAGATCAAACGAAATACATAAGGTATTAGTCCAACTTCGCTGGGTTATCCCTTAG
TTATGGGCAGGTTGGATACGCGTTACTCACCCGTGCGCCGGTCGCCGACATAAATAGCAAGCTATCTATTCGC
TGCCCCTCGACTTGCATGTGTTAAGCCTATAGCTAGCGTTCATCCTGAGCCAGGATCAAACTCT
>200
CTGCTGCCGCCCGTAGGAGTTTGAACCGTGTCTCAGTTCCAATGTGGGGGACCTTCCTCTCAGAACCCCTACT
GATCGTTGCCTTGGTGGGCCGTTACCCCGCCAACAAGCTAATCAGACGCATCCCCATCCATAACCGATAAATC
TTTATTCATAATCTCATGAGATCAAACGAATACATAAGGTATTAGTCCAACTTCGCTGGGTTATCCCTAGTTA
TGGGCAGGTTGGATACGCGTTACCTCACCCGTGCGCCGGTCGCCGACATAAATAGCAAGCTATCTATTCGCTG
CCCCTCGACTTGCATGTGTTAAGCCTATAGCTAGCGTTCATCCTGAGCCAGGATCAAACTCCT
>201
CTGCTGCCGCCCGTAGGAGTTTTGGACCGTGTCTCAGTTCCAATGTGGGGGACCTTTCCTCTCAGAACCCCTA
CTGATCGTCGCCTTTGGTGGGCCGTTACCCCGCCAACAAGCTAATCAGACGCATCCCCATACCATAACCGCTA
ACCTTTAATCGTTATTTGATGTCAGCAAACGATATCATACGGTATTAGGCCGTCTTTCAACGGGTTATCCCGT
AGTTATGGGAAGGTTGGATACGCGTTACTCACCCGTGCGCCAGTCGTCATCAAGGAAAGCAAGCTTCCTCATG
TTACCCTGCCGACATGCATGTGTTAAGCCT
>202
CTGCTGCCGCCCTAGGAGTTTTGGACCGTGTCTCAGTTCCAATGTGGGGGACCTTCCTCTCAGAACCCCTACT
GATCGTTGCCTTTGGTGGGCCGTTTACCCCGCCAACAAGCTAATCAGACGCATCCCCATCCATAACCGATAAA
TCTTTATTCGTAATCTCATGAGATCAAACGAATACATAAGGTATTAGTCCAACTTCGCTGGGTTATCCCTTAG

TTATGGGCAGGTTGGATACGCGTTACTCACCCGTGCGCCGGTCGCCGACATAAATAGCCAAGCTATCTATTCG
CTGCCCCTCGACTTGCATGTGTTAAGCCTATAGCTAGCGTTCATCCTGAGCCAGGATCAAACTCT
>203
CTGCTGCCGCCCGTAGGAGTTTTGGACCGTGTCTCAGTTCCAATGTGGGGGACCTTCCTCTCAGAACCCCTAC
TGATCGTCGCCTTTGGTGGGCCGTTACCCCGCCAACAAGCTAATCAGACGCATCCCCATCCATAACCGCTAAA
CCTTTAATCGTTATTTGATGTCAGCAAACGATATCATACGGTATTAGGCCATCTTTCAACGGGTTATCCCGTA
GTTTATGGGAAGGTTGGATACGCGTTACTCACCCGTGCGCCAGTCGTCATCAAGGAAAGCAAGCTTCCTCATG
TTTACCCTGCGTACATGCATGTGTTAACGCCTGTACGTC
>204
CTGCTGCCGCCCGTAGGAGTTTTGGACCGTGTCTCAGTTCCAATGTGGGGGACCTTCCTCTCAGAACCCCTAC
TGATCGTCGCCTTTGGTGGGCCGTTACCCCGCCAACAAGCTAATCAGACGCATCCCCATCCATAACCGCTAAA
CCTTTAATCGTTTATTTGATGTCAGCAAACGATATCATACGGTATTAGGCCGTCTTTCAACGGGTTTATCCCG
TAGTTATGGGAAGGTTGGATACGCGTTACTCACCCGTGCGCCGGTCGCCGACATAAATAGCCAAGCTTATCTT
ATTCGCTTGCCCCTTCGACTTGCATGT
>205
CTGCTGCCGCCCGTAGGAGTTTGGACCGTGTCTCAGTTCCAATGTGGGGGACCTTTCCTCTCAGAACCCCTAC
TGATCGTCGCCTTTGGTGGGCCGTTACCCCGCCAACAAGCTAATCAGACGCATCCCCATCCATCACCGCTAAA
CCTTTAATCGTTATTTGATGTCAGCAAACGATGTCATACGGTATTAGTCCGTCTTTCAACGGGTTATCCCGTG
GTCATGGGAAGGTTGGATACGCGTTACTCACCCGTGCGCCAGTCGTCGTCAAGGGAAGCAAGCTTCCCTTCAC
GTTACCCTGCGACTTGCATGTGTTAAGCCTGTAGCTAGCGTTCATCCTGAGCCAGGATCAAACTCT
>206
CTGCTGCCGCCCGTAGGAGTTTTGGACCGTGTCTCAGTTCCAATGTGGGGGACCTTCCTCTCAGAACCCCTAC
TGATCGTCGCCTTTGGTGGGCCGTTACCCCGCCAACAAGCTAATCAGACGCATCCCCATCCATAACCGCTAAC
CTTTAATCGTTATTTGATGTCAGCAAACGATATCATACGGTATTAGGCCGTCTTTCAACGGGTTATCCCGTAG
TTATGGGAAGGTTGGATACGCGTTACTCACCCGTGCGCCAGTCGTCATCAAGGAAAGCAAGCCTTTCCCTCAC
GTGTTTATCCCAGCCCGTAGTTCGAACTGG
>207
CTGCTGCCGCCCGTAGGAGTTTTGGACCGTGTCTCAGTTCCAATGTGGGGGACCTTCCTCTCAGAACCCCTAC
TGATCGTCGCCTTGGTGGGCCGTTACCCCGCCAACAAGCTAATCAGACGCATCCCCATCCATAACCGCTAAAC
CTTTAATCGTTATTTGATGTCAGCAAACGATATCATACGGTATTAGGCCGTCTTTCAACGGGTTATCCCGTAG
TTTATGGGAAGGTTGGATACGCGTTACTCACCCGTGCGCCAGTCGTCATCAAGGAAAGCAAGCTTTCCCTCAT
GTTTACCCTGCGACTTGCATGTGTTAAGCCTGTAGCTAGCGTTCATCCTGAGCCAGGATCAAACTCT
>208
CTGCTGCCGCCCGTAGGAGTTTTGGACCGTGTCTCAGTTCCAATGTGGGGGACCTTCCTCTCAGAACCCCTAC
TGATCGTCGCCTTTGGTGGGCCGTTACCCCGCCAACAAGCTAATCAGACGCATCCCCATCCATAACCGCTAAA
CCTTTAATCGTTATTTGATGTCAGCAAACGATATCATACGGTATTAGGCCGTCTTTCAACGGGTTATCCCGTA
GTTATGGGAAGGTTGGATACGCGTTACTCACCCGTGCGCCAGTCGTCATCAAGGAAAGCAAGCTTTCCTCATG
TTACCCTGCGACTTGCATGTGTTAAGCCTGTAGCTAGCGTTCATCCCTGAGCCAAGGATCAAACTCT
>209
CTGCTGCCGCCCGTAGGAGTTTGGACCGTGTCTCAGTTCCAATGTGGGGGACCTTCCTCTCAGAACCCCTACT
GATCGTCGCCTTGGTGGGCCGTTACCCCCGCCAACAAGCTAATCAGACGCATCCCCATCCATAACCGCTAAAC
CTTTAATCGTTTATTTTGATGTCAGCAAACGATATCATACGGTATTTAGGCCGTCTTTTCAACGGGTTTATCC
CGTAGTTTATGGGAAGGTTGGATACGCGTTACTCACCCGTGCGCCAGTCGTCATTCAAGGAAAGCAAGCTTTT
CCCTCATGTTTTACCCTGCGACTTGCATTGTGTTTAAGCCTTGTA
>210
CTGCTGCCGCCCGTAGGAGTTTGGACCGTGTCTCAGTTCCAATGTGGGGGACCTTCCTCTCAGAACCCCTAC
TGATCGTTGCCTTTGGTGGGCCGTTACCCCGCCAACAAGCTAATCAGACGCATCCCCATCCATAACCGATAAA
TCTTTATTCATAATCTCATGAGATCAAACGAATACATAAGGTATTAGTCCAACTTCGCTGGGTTATCCCTTAG
TTATGGGCAGGTTTGGATACGCGTTACTCACCCGTGCGCCGGTCGCCGACATAAATAGCAAGCTATCTATTCG
CTGCCCCCCTCGACTTGCATGTGTTTAAGCCTATAGCTAGCGTTCATCCTGAGCCAGGATCAAACTCT
>211
CTGCTGCCGCCCGTAGGAGTTTGGACCGTGTCTCAGTTCCAATGTGGGGGACCTTCCTCTCAGAACCCCTACT
GATCGTCGCCTTTGGTGGGCCGTTACCCCGCCAACAAGCTAATCAGACGCATCCCCATCCATAACCGCTAAAC
CTTTAATCGTTATTTGATGTCAGCAAACAATATCATACGGTATTAGGCCGTCTTTCAACGGGTTATCCCGTAG

TTATGGGAAGGTTGGATACGCGTTACTCACCCGTGCGCCAGTCGTCATCAAGGAAAGCAAGCTTTCCCTCATG
ATTACCCTGCGGTACTTCGCATGTGTTAAGCCTGTAGCTAGCGTCATCCTGAGCCAAGGATCAAACTCT
>212
CTGCTGCCGCCCGTAGGAGTTTTGGACCGTGTCTCAGTTCCAATGTGGGGGACCTTCCTCTCAGAACCCCTAC
TGATCGTTGCCTTTGGTGGGCCGTTACCCCGCCAACAAGCTAATCAGACGCATCCCCATCCATAACCGATAAA
TCTTTATTCGTAATCTCATGAGATCAAACGAAATACATAAGGTATTAGTCCAACTTCGCTGGGTTATCCCTTA
GTTATGGGCAGGTTGGATACGCGTTACCTCACCGTGCGCCGGTCGCCGACATAAATAGCAAGCTATCTATTCG
CTGCCCCTCGACTGCATGTGTTAAGCCTATAGCTAGCGTCATCCTGAGCCAGGATCAAACTCT
>213
CTGCTGCCGCCCGTAGGAGTTTGGACCGTGTCTCAGTTCCAATGTGGGGGCCTTCCTCTCAGAACCCCTACTG
ATCGTCGCCTTGGTGGGCCGTTACCCCGCCAACAAGCTAATCAGACGCATCCCCATCCATAACCGCTAAACCT
TTAATCGTTATTTGATGTCAGCAAACGATATCATACGGTATTAGGCCGTCTTTCAACGGGTTATCCCGTAGTT
ATGGGAAGGTTGGATACGCGTTACTCACCCGTGCGCCAGTCGTCATCAAGGAAAGCAAGCTTTCCCTGACTGT
TACCCTGCGACTTGCATGTGTTAAGCCTGTAGCTAGCGTTCATCCCGTGAGCCAA
>214
CTGCTGCCGCCCGTAGGAGTTTGGACCGTGTCTCAGTTCCAATGTGGGGGACCTTCCTCTCAGAACCTCTACT
GATCGTTGCCTTTGGTGGGCCGTTACCCCGCCAACAAGCTAATCAGACGCATCCCCATCCATAACCGATAAAT
CTTTTATTTCGTAATCTCATGAGATCAAACGAATACATAAGGTATTTAGTTCCAACTTTCGCTGGGTTTATCC
CTTTAGTTTATGGGCAGGTTTGGATACGCGTTACTCACCCGTGCGCCGGTCGCCGACATTAAATAGCAAGCTA
TCTTATTTCGCTGCCCCCCCTCGACTTTGCAT
>215
CTGCTGCCGCCCGTAGGAGTTTGGACCGTGTCTCAGTTCCAATGTGGGGGACCTTCCTCTCAGAACCCCTACT
GATCGTTGCCTTGGTGGGCCGTTACCCCGCCAACAAGCTAATCAGACGCATCCCCATCCATAACCGATAAATC
TTTTATTCGTAGTCTCATGAGATCAAACGAATACATAAGGTATTTAGTTCCAACTTTCGCTGGGTTTATCCCT
TAGTTTATGGGCAGGTTTGGATACGCGTTACTCACCGTGCGCCGGTCGCCGACATTAAATAGCAAGCTATCTA
TTTCGCTTGCCCCCCCCTCGACTTTGCATGTGTTTTAAGCCTTATAGCTAGCGTTCATCCTGAGCCAGGATCA
AACTCT
>216
CTGCTGCCGCCCTAGGAGTTTGAACCGTGTCTCAGTTCCAATGTGGGGGACCTTCCTCCCAGAACCCCTACTG
ATCGTTGCCTTGTGGGCCGTTACCCCACCAACAAGCCTAATCAGACGCATCCCCATCCATAACCGATAAATCT
TTATTCGTAATCTCATGAGATCAAACGAATATATAAGGTATTAAGTCCAACTTCGCTGGGTTATCCCTTAGTT
ATGGGCAGGTTGGATACGCGTTACTCACCCGTGCGCCGGTCGCCGACATAAATAGCAAGCTATCTATTCGCTG
CCCCTCGACTTGCATGTGTTAACGCCTATAGCTAGCGTTCATCCTGAGCCAGGATCAAACTCT
>217
CTGCTGCCGCCCGTAGGAGTCTGGGCCGCATCTCAGTCCCAATGTGGCCGCACACCCTCTCAGGCCGGCTACC
CGTCGCCGCCTTGGTAGGCCATTACCCCACCAACAAGCTGATAGGCCGCAAGCTCATCCCACACCAGAAAAAC
CTTCCACCACACCAACCACGATGCGGTCCTATAGGGTATTTAGACCCAGTTTCCCAAGCTTTATCCCCCCAGT
GCAGGGCAGATCACTTACGTGTTACTCACCCGTTTCGCCACTCGAGCACCCCAGCAAGCTGGGGCCTTTCCGT
TCGACTTGCATTGTGTTTAAGCACGCCGCCAGCGTTCACCT
>218
CTGCTGCCGCCCGTAGGAGTTTGGACCGTGTCTCAGTTCCAATGTGGGGGACCTTCCTCTCAGAACCCCTACT
GATCGTCGCCTTTGGTGGGCCGTTACCCCGCCAACAAGCTAATCAGACGCATCCCCATCCATAACCGCTAAAC
CTTTAATCGTTATTTTGATGTCAGCAAACGATATCATACGGTATTTAGGCCGTCTTTTCAACGGGTTTATCCC
GTAGTTTATGGGAAGGTTGGATACGCGTTACTCACCCGTGCGCCAGTCGTCATCAAGGAAAGCAAGCTTTCCC
TCATGACT
>219
CTGCTGCCGCCCGTAGGAGTTTGGACCGTGTCTCAGTTCCAATGTGGGGGACCTTCCTCTCAGAACCCCTACT
GATCGTCGCCTTGGTGGGCCGTTACCCCGCCAACAAGCTAATCAGACGCATCCCCATCCATAACCGCTAAACC
TTTAATCGTTATTTTGATGTCAGCAAACGATATCATACGGTATTTAGGCCGTCTTTTCAACGGGTTATCCCGT
AGTTTATGGGAAGGTTGGATACGCGTTACTCACCCGTGCGCCAGTCGTCATCAAGGAAAGCAAGCTTTCCCTC
ATGTTTACCCTGCGACATGCATGTGTTAAGCCTGTAGCTAGCGTTCATCCCGTGAGCCAAGGCATC
>220
CTGCTGCCGCCCGTAGGAGTTTGAACCGTGTCTCAGTTCCAATGTGGGGGACCTTCCTCTCAGAACCCCTACT
GATCGTTGCCTTGGTGGGCCGTTACCCCACCAACAAGCTAATCAGACGCATCCCCATCCATAACCGATAAATC

TTTATTCGTAATCTCATGAGATCAAACGAATATATAAGGTATTAGTCCAGCTTTCGCTGGGTTATCCCTTAGT
TATGGGCAGGTTGGATACGCGTTACTCACCCGTGCGCCGGTCGCCGACATAAATAGCAAGCTATCTATTCGCT
GCCCCTCGACTTGCATGTGTTAAGCCTATAGCTAGCGTTCATCCTGAGCCAGGATCAAACTCT
>221
CTGCTGCCGCCCGTAGGAGTTTGGACCGTGTCTCAGTTCCAATGTGGGGGACCTTCCTCTCAGAACCCCTACT
GATCGTTGCCTTTGGTGGGCCGTTACCCCGCCAACAAGCTAATCAGACGCATCCCCATCCATAACCGATAAAT
CTTTATTCATAATCTCATGAGATCAAACGAATACATAAGGTATTAGTCCAACTTCGCTGGGTTATCCCTTAGT
TATGGGAAGGTTGGATACGCGTTACTCACCCGTGCGCCAGTCGTCATCAAGGAAAGCAAGCTTTCCCTCATGT
TACCCTGCGACTTGCATGTGTTAAGCCTGTAGCTAGCGTTCATCCCTGAGCCAGGATCAACTCT
>222
CTGCTGCCGCCCGTAGGAGTTTGGACCGTGTCTCAGTTCCAATGTGGGGGACCTTCCTCTCAGAACCCCTACT
GATCGTCGCCTTGGTGGGCCGTTACCCCGCCAACAAGCTAATCAGACGCATCCCCATCCATAACCGCTAAACC
TTTAATCGTTTATTTGATGTCAGCAAACGATATCATACGGTATTTAGGCCGTCTTTTCAACGGGTTTATCCCG
TAGTTTATGGGAAGGTTGGATACGCGTTACTCACCCGTGCGCCAGTCGTCATCAAGGAGAGCAAGCTTTTCCT
CATTGTTTACCCTGCGACTTGCATGTGTTTTAAGCCTATTAGCTAGCGTTCAATCCTTGAGCCAGGATCAAAC
TCT
>223
CTGCTGCCGCCCGTAGGAGTTTTGGACCGTGTCTCAGTTCCAATGTGGGGGACCTTCCTCTCAGAACCCCTAC
TGATCGTCGCCTTGGTGGGCCGTTACCCCGCCAACAAGCTAATCAGACGCATCCCCATCCATAACCGCTAAAC
CTTTAATCGTTATTTGATGTCAGCAAACGATATCATACGGTATTAGGCCGTCTTTCAACGGGTTATCCCGTAG
TTATGGGAAGGTTGGATACGCGTTACTCACCCGTGCGCCAGTCGTCATCAAGGAAAGCAAGCTTCCCTCATGT
TACCCTGCGACTTGCATGTGTTAAGCCTGTAGCTAGCGTTCATCCGTGAGCCAAGGCATCAACTCT
>224
CTGCTGCCGCCTCGGAGGTTAGGTTGACCGTGTCTCAGTTCCAATGTGGGGGACCTTCCTCTCAGAACCCCTA
CTGATCGTCGCCTTTGGTGGGCCGTTACCCCGCCAACAAGCTAATCAGACGCATCCCCATCCATCAACCGCTA
AACCTTTAATCGTTATTTTGATGTCAGCAAACGATGTCATACGGTATTAGGCCGTCTTTCAACGGGTTATCCC
GTGTATGGGAAGGTTGGATACGCGTTACTCACCCGTGCGCCAGTCGTCGTCAAGGGAAGCAAGCTTCCCTCAC
GTACCTTGCCGAG
>225
CTGCTGCCGCCCGTAGGAGTTTGAACCGTGTCTCAGTTCCAATGTGGGGGACCTTCCTCTCAGAACCCCTACT
GATCGTTGCCTTGGTGGGCCGTTACCCCACCAACAAGCTAATCAGACGCATCCCCATCCATAACCGATAAATC
TTTATTCGTAATCTCATGAGATCAAACGAATATATAAGGTATTAGTCCAACTTCGCTGGGTTATCCCTTAGTT
TATGGGCAGGTGGATACGCGTTACGTCACCCGTGCGCCGGTCGCCGACATAAATAGCAAGCTATCTATTCGCT
GCCCCTCGACTTGCATGTGTTTAAGCCTATAGCTAGCGTTCATCCTGAGCCAGGATCAAACTCT
>226
CTGCTGCCGCCCGTAGGAGTTTGGACCGTGTCTCAGTTCCAATGTGGCCGATCACCCTCTCAGGTCGGCTACT
GATCGTCGCCTTGGTGGGCAGTTATCTCACCAACTAGCTAATCAGACGCGGGTCCATCCTATACCGCTAACAC
TTGATATATCTACGATGCCGTAAATATATTATTTTATCATGTATTTAGTAAACTTTCAGTATGTTTATCCATG
TTGTATAGGGCAGGTTTACCCACGCGTTACTCACCCGTCCGCCGCTCATTCTTTCATTTTAATGCAAGCATCA
AATCAGACGCGCTCGACTTGCATGTGTTTAGGCACGCCGCCAGCGTCATCCTGAGCCAGGATCAAACTCT
>227
CTGCTGCCGCCCGTAGGAGTTTGGACCGTGTCTCAGTTCCAATGTGGGGGACCTTCCTCTCAGAACCCCTACT
GATCGTCGCCTTGGTGGGCCGTTACCCCGCCAACAAGCTAATCAGACGCATCCCCATCCATCACCGCTAAACC
TTTTAATCGTTTATTTTGATGTCAGCAAACGATATCATACGGTTATTTAGGCCGTCTTTTCAACGGGTTTATT
CCCGTAGTTTATGGGAAGGTTTGGATACGCGTTACTCACCCGTGCGCCAGTCGTCACCAAGGAAAGCAAGCTT
TTCCTCATGTTACCCTTGCGACTTTGCATGTGTTTAAGCCTGTTAGCTTAGCGTTCATTCCTGAGCCAGGATC
AAACTCT
>228
CTGCTGCCGCCCGTAGGAGTTTGGACCGTGTCTCAGTTCCAATGTGGCCGATCACCCTCTCAGGTCGGCTACT
GATACGTCGCCTTGGTGGGCAGTTATCTCACCAACTAGCTAATCAGACGCGGGTCCACCCTATACCGCTAACA
CTTTGATATATCTACGATGCCGTAAATATATATTATCATGTAGTTTAGTAAACTTTTCAGTATGTTATCCATG
TGTATAGGGCAGGTTACCCACGCGTTACTCACCCGTCCGCCGCTCATCTTCATTTAATGCAAGCATCAAATCA
GACGCGCTCGACTTGCATGTCGTTTAGGCACGCCGCCAGCGTTCATCCTGAGCCAGGATCAAACCTCT
>229

CTGCTGCCGCCCGTAGGAGTTTGGACCGTGTCTCAGTTCCAATGTGGGGGACCTTCCTCTCAGAACCCCTACT
GATCGTTGCCTTTGGTGGGCCGTTACCCCGCCAACAAGCTAATCAGACGCATCCCCATCCATAACCGATAAAT
CTTTATTCGTAATCTCATGAGATCAGACGAATACATAAGGTATTAGTCCAACTTCGCTGGGTTATCCCTTAGT
TATGGGCAGGTTGGATACGCGTTACTCACCCGTGCGCCGGTCGCCGACATAAATAGCAAGCTATCTATTCGCT
GCCCCTCGACTTGCATGTGTTTAACGCCTATAGCTAGCGTTCATCCTGAGCCAGGATCAAACTCT
>230
CTGCTGCTGCCCGTAGGAGTTTTGGACCGTGTCTCAGTTCCAATGTGGGGGACCTTTCCTCTCAGAACCCCTA
CTGATCGTCGCCTTTGGTGGGCCGTTACCCCGCCAACAAGCTAATCAGACGCATCCCCATCCATAACCGCTAA
ACCTTTAATCGTTATTTGATGTCAGCAAACGATATCATACGGTATTAGGCCGTCTTTCAACGGGTTATCCCGT
AGTTATGGGAAGGTTGGATACGCGTTACTCACCCGTGCGCCAGTCGTCATCAAGGAAAGCAAGCCTTTCCTCA
TGTTACCCTGCGACTTGCATGTGTTAAGCCTGTAGCTAGCGTTCATCCCTGAGCCAGGATCAAACTCT
>231
CTGCTGCCGCCCTAGGAGTTTGGACCGTGTCTCAGTTCCAATGTGGCCGATCACCCTCTCAGGTCGGCTACTG
ATCGTCGCCTTGGTGGGCAGTTATCTCACCAACTAGCTAATCAGACGCGGGTCCATCCTATACCGCTAACACT
TTGATATATCTACGATGCCGTAAATATATATTATCATGTATTAGTAAACTTTTCAGTATGTTATCCATGTGTA
TAGGGCAGGTTACCCACGCGTTACTCACCCGTCCGCCGCTCATCTTTCATCAATGCAAGCATCAAATCATACG
CGCTCGACTTGCATGTGTTAGGCACGCCGCCAGCGTTCATCCTGAGCCAGGATCAAACTCT
>232
CTGCTGCCGCCCGTAGGAGTCTGGACCGTGTCTCAGTTCCAGTGTGGCCGTTTCATCCTCTTAGACCGGCTAC
TGATCGTTGGTTTGGTGGGCCGTTACCCCACCAACTGCCTAATCAGACGCAAACCCCTCTTCAGGCGATAGCT
TACAGGTGGAGGCTACCCTTTCTTCCACAGGTCATGCGGCCCGTGGAACGTATTCGGTATTAGCAGTCGTTTC
CGTCTGTTGTCCCCATCCTGAAGGCAGGTTGTTTTACGTGTTACTCACCCGTTTGCCACTGGATTCGATAAAG
AGCAAGCTCTTCATCCCTTCCCGTTCGACTTGCATGTGTTAAGCACGCCGCCAGCGTTCGTCCTGAGCCAGGA
TCAAACTCT
>233
CTGCTGCCGCCCGTAGGAGTTTTGGACCGTGTCTCAGTTCCAATGTGGGGGACCTTCCTCTCAGAACCCCTAC
TGATCGTTGCCTTGGTGGGCCGTTACCCCGCCAACAAGCTAATCAGACGCATCCCCATCCATAACCGATAAAT
CTTTTATTTCATAATCTCATGAGATCAAACGAATACATAAGGTATTTAGTCCAACTTCGCTGGGTTTATCCCT
TAGTTTATGGGCAGGTTTGGATACGCGTTACTCACCCGTGCGCCGGTCGCCGACATAAATAGCAAGCTATCTT
ATTCGCTGCCCCCCCTCGACTTGCATGTGTTTAAGCCTTATAGCTAGCGTTCATCCTTGAGCCAGGATCAAAC
TCT
>234
CTGCTGCCGCCCGTAGGAGTTTGGACCGTGTCTCAGTTCCAATGTGGGGGACCTTCCTCTCAGAACCCCTACC
TGATCGTCGCCTTGGTGGGCCGTTACCCCGCCAACAAGCTAATCAGACGCATCCCCATCCATAACCGCTAAAC
CTTTAATCGTTTATTTTGATGTCAGCAAACGATATCATACGGTATTTAGGCCGTCTTTTCAACGGGTTTATCC
CGTAGTTTATGGGAAGGTTGGATACGCGTTACTCACCCGTGCGCCAGTCGTCCATTCAAGGAAAAGCAAGCTT
TTCCCCCTTGA
>235
CTGCTGCCGCCCGTAGGAGTTTGGACCGTGTCTCAGTTCCAATGTGGGGGACCTTCCTCTCAGAACCCCTACT
GATCGTCGCCTTGGTGGGCCGTTACCCCGCCAACAAGCTAATCAGACGCATCCCCATCCATAACCGCTAAACC
TTTAATCGTTTATTTTGATGTCAGCAAACGATATCATACGGTATTTAGGCCGTCTTTTCAACGGGTTTATCCC
GTAGTTTATGGGAAGGTTTGGATACGCGTTACCACCCGTGCGCCAGTCGTCATCAAGGAAAGCAAGCTTTTTC
CTCATGTTTACCCTTGCGACATTGCAGTGTTTAAGCCTGTTAGCTTAGCGTTCATCCTGAGCCAGGATCAAAC
TTCT
>236
CTGCTGCCGCCCGTAGGAGTTTTGGACCGTGTCTCAGTTCCAATGTGGGGGACCTTCCTCTCAGAACCCCTAC
TGATCGTCGCCTTTGGTGGGCCGTTACCCCGCCAACAAGCTAATCAGACGCATCCCCATCCATAGCCGATAAA
TCTTTATTCGTAATCATCATGAGAGTCAAACGAAATACATAAGGGTATTAGTCCAACTTCGCTGGGTTATCCC
TTAGTTATGGGCAGGTTGGATACGCGTTACCTCACCGTGCGCCGGTCGCCGACATAAATAGCAAGCTATCTAT
TCGCTGCCCCTCGACTTGCATGTGTTAAGCCTATAGCTAGCGTCATCCTGAGCCAGGATCAAACTCT
>237
CTGCTGCCGCCCGTAGGAGTTTGGACCGTGTCTCAGTTCCAATGTGGGGGACCTTCCTCTCAGAACCCCTACT
GATCGTCGCCTTGGTGGGCCGTTACCCCGCCAACAAGCTAATCAGACGCATCCCCATCCATAACCGCTAAACC
TTTAATCGTTTATTTTGATGTCAGCAAACGATATCATACGGTATTTAGGCCGTCTTTTCAACGGGTTTATCCC

GTAGTTTATGGGAAGGTTGGATACGCGTTACTCACCCGTGCGCCAGTCGTCATCAAGGAAAGCAAGCTTTTCC
CTTCATGTTTTACCCTGCGACTTGCATTGTGTTTAAGCCTTGTAGCTAGCGTCATCCTGAGCCAGGATCAAAC
TCT
>238
CTGCTGCCCCCGTAGGAGTTTGGACCGTGTCTCAGTTCCAATGTGGGGGACCTTCCTCTCAGAACCCCTACTG
ATCGTCGCCTTTGGTGGGCCGTTACCCCGCCAACAAGCTAATCAGACGCATCCCCATCCATCACCGCTAAACC
TTTAATCGTTATTTGATGTCAGCAAACGATGTCATACGGTATTAGTCCGTCTTTTCAACGGGTTATCCCGTGG
TCATGGGAAGGTTGGATACGCGTTACTCACCCGTGCGCCAGTCGTCGTCAAGGGAAGCAAGCTTCCCTCACGT
TACCTGCGACTTTGCACGTGATTAAGCC
>239
CTGCTGCCGCCCGTAGGAGTTTTGGACCGTGTCTCAGTTCCAATGTGGGGGACCTTCCTCTCAGAACCCCTAC
TGATCGTCGCCTTTGGTGGGCCGTTACCCCGCCAACAAGCTAATCAGACGCATCCCCATCCATAACCGCTAAA
CCTTTAATCGTTATTTGATGTCAGCAAACGATATCATACGGTATTAGGCCGTCTTTCAACGGGTTATCCCGTA
GTTATGGGAAGGTTGGATACGCGTTACTCACCCGTGCGCCAGTCGTCATCAAGGAAAGCAAGCTTTCCCTCAT
GTTACCCTGCGACATGCATGTGTTAAGCCTGTAGCTAGCGTTCATCCTGAGCCAGGATCAACCTCT
>240
CTGCTGCCGCCCGTAGGAGTCTGGGCCGTATCTCAGTCCCAATGTGGCCGCACACCCTCTCAGGCCGGCTACC
CGTCGCCGCCTTGGTAGGCCATTACCCCACCAACAAGCTGATAGGCCGCAAGCTCATCCCACACCAGAAAAAC
CTTTCCACCACACCAACCACGATGCGGTCCTATAGGGTATTAGACCCAGTTTCCCCAAGCTTATCCCCCAGTG
CAGGGCAGATCACTTACGTGTTACTCACCCGTTCGCCACTCGAGCACCCCAGCAAGCTGGGGCCTTTCCGTCG
ACTGCATGTGTTAAGCACGCCGCCAGCGTTCATCCTGAGCCAGGATCAAACTCT
>241
CTGCTGCCGCCCGTAGGAGTCTGGGCCGTATCTCAGTCCCAATGTGGCCGCACACCCTCTCAGGCCGGCTACC
CGTCGCCGCCTTGGTAGGCCATTACCCCACCAACAAGCTGATAGGCCGCAAGCTCATCCCACACCAGAAAAAC
CTTTCCACCACACCAACCACGATGCGGTCCTATAGGGTATTAGACCCAGTTTCCCAAGCTTTATCCCCCAGTG
CAGGGCAGATCACTTACGTGTTACTCACCCGTTCGCCACTCGAGCACCCCAGCAAGCTGGGGCCTTTCCGTTC
GACTTGCATGTGTTTTAAGCACGCCGCCAGCGTCACCTGAG
>242
CTGCTGCCGCCCGTAGGAGTTTGGACCGTGTCTCAGTTCCAATGTGGGGGACCTTCCTCTCAGAACCCCTACT
GATCGTCGCCTTTGGTGGGCCGTTACCCCGCCAACAAGCTAATCAGACGCATCCCCATCCATAACCGATAAAT
CTTTATTCATAATCTCATGAGATCAAACGAATACATAAGGTATTTAGTCCAACTTCGCTGGGTTTATCCCTTA
GTTATGGGCAGGTTTGGATACGCGTTACTCACCCGTGCGCCGGTCGCCGACATAAATAGCAAGCTATCTATTC
GCTGCCCCCCCTCGACTTGCATGTGTTTAAGCCTATAGCTAGCGTTCATCCTGAGCCAGGATCAAACTCT
>243
CTGCTGCCGCCCGTAGGAGTTTGGACCGTGTCTCAGTTCCAATGTGGCCGATCACCCTCTCAGGTCGGCTACT
GATCGTCGCCTTGGTGGGCAGTTATCTCACCAACTAGCTAATCAGACGCGGGTCCATCCTATACCGCTAACAC
TTTGATATATCTACGATGCCGTAAATATATATTATCATGTATTAGTAAACTTTTCAGTATGTTATCCATGTGT
ATAGGGCAGGTTACCCACGCGTTACTCACCCGTCCGCCGCTCATCTTTCATTAATGCAAGCATCAAACCAGAC
GCGCTCGACTTGCATGTGTTAGGCACGCCGCCAGCGTTCATCCTGAGCCAGGATCAAACTCT
>244
CTGCTGCCGCCCGTAGGAGTTTGGACCGTGTCTCAGTTCCAATGTGGGGGACCTTCCTCTCAGAACCCCTACT
GATCGTCGCCTTGGTGGGCCGTTACCCCGCCAACAAGCTAATCAGACGCATCCCCATCCATAACCGCTAAACC
TTTAATCGTTATTTGATGTCAGCAAACGATATCATACGGTATTAGGCCGTCTTTCAACGGGTTATCCCGTAGT
TATGGGAAGGTTGGATACGCGTTACTCACCCGTGCGCCAGTCGTCATCAAGGAAAGCAAGCTTTCCCGTCATG
TTTACCCTGCGAACTTGCATGTGTTAAGCCTTGTAGCTAAGCGTTCATCCGTGAGCCAAGGCATCAAACTC
>245
CTGCTGCCGCCCGTAGGAGTCTGGGCCGTACCTCAGTCCCAATGTGGCCGCACACCCTCTCAGGCCGGCTACC
CGTCGCCGCCTTGGTAGGCCATTACCCCACCAACAAGCTGATAGGCCGCAAGCTCATCCCACACCAGAAAAAC
CTTCCACCACACCAACCACGATGCGGTCCTATAGGGTATAGACCCAGTTTCCCAAGCTTTATCCCCCAGTGCA
GGGCAGATCACTTACGTGTTACTCACCCGTTCGCCACTCGAGCACCCCAGCAAGCTGGGGCCTTTCCGTCGAC
TTGCATGTGTTTAAGCACGCCGCCAGCGTCATCCTGAGCCAGGATCAAACTCT
>246
CTGCTGCCGCCCGTAGGAGTTTGGACCGTGTCTCAGTTCCAATGTGGGGACCTTCCTCTCAGAACCCCTACTG
ATCGTCGCCTTTGGTGGGCCGTTACCCCGCCAACAAGCTAATCAGACGCATCCCCATCCATAACCGCTAAACC

TTTAATCGTTATTTGATGTCAGCAAACGATATCATACGGTATTAGGCCGTCTTTCAACGGGTTATCCCGTAGT
TATGGGAAGGTTGGATACGCGTTACTCACCCGTGCGCCAGTCGTCATCAAGGAAAGCAAGCTTTCCCTCATGT
TACCCTGCGACTTGCATGTGTTAAGCCTGTAGCTAGCGTTCATCCTGAGCCAGGATCAAACTCT
>247
CTGCTGCCGCCCGTAGGAGTTTTGGACCGTGTCTCAGTTCCAATGTGGGGGACCTTCCTCTCAGAACCCCTAC
TGATCGTTGCCTTTGGTGGGCCGTTACCCCGCCAACAAGCTAATCAGACGCATCCCCATCCATAACCGATAAA
TCTTTATTCGTAATCTCATGAGATCAAACGAATACATAAGGTATTAGTCCAACTTCGCTGGGTTATCCCTTAG
TTATGGGCAGGTTGGATACGCGTTACTCACCCGTGCGCCGGTCGCCGACATAAATAGCAAGCTATCTATTCGC
TGCCCCTCGACTTGCATGTGTTAAGCCTATAGCTAGCGTTCATCCTGAGCCAGGATCAAACTCT
>248
CTGCTGCCGCCCGTAGGAGTTTGGACCGTGTCTCAGTTCCAATGTGGGGGACCTTTCTCTCAGAACCCCTACT
GATCGTCGCCTTTGGTGGGCCGTTACCCCGCCAACAAGCTAATCAGACGCATCCCCATCCATAACCGCTAAAC
CTTTAATCGTTATTTGATGTCAGCAAACGATATCATACGGTATTAGGCCGTCTTTCAACGGGTTATCCCGTAG
TTATGGGAAGGTTGGATACGCGTTACTCACCCGTGCGCCAGTCGTCATCAAGGAAAGCAAGCTTTCCTCATGT
TTACCCTGCGACTTGCATGTGTTAAGCCTGTAGCTGGCGTTCATCCTGAGCCAGGATCAAACTCT
>249
CTGCTGCCGCCCGTAGGAGTTTGGACCGTGTCTCAGTTCCAATGTGGCCGATCACCCTCTCAGGTCGGCTACT
GATCGTCGCCTTGGTGGGCAGTTATCTCACCAACTAGCTAATCAGACGCGGGTCCATCCTATACCGCTAACAC
TTTGATATATCTACGATGCCGTAAATATATATTTTATCATGTATTTAGTAAACTTTTCAGTATGTTTATCCAT
GTGTATAGGGCAGGTTTACCCACGCGTTACTCACCCGTCCGCCGCTCATCTTCATTTAATGCAAGCATCAAAT
CAGACGCG
>250
CTGCTGCCGCCCGTAGGAGTTTGGACCGTGTCTCAGTTCCAATGTGGGGGACCTTCCTCTCAGAACCCCTACT
GATCGTCGCCTTTGGTGGGCCGTTACCCCGCCAACAAGCTAATCAGACGCATCCCCATCCATAACCGCTAAAC
CTTTAATCGTTATTTTGATGTCAGCAAACGATATCATACGGTATTAGGCCGTCTTTTCAACGGGTTATCCCGT
AGTTATGGGAAGGTTGGATACGCGTTACTCACCCGTGCGCCAGTCGTCATCAAGGAAAGCAAGCTTCCTCATG
TTACCCTGCCGGAACTTGCATGTGTTAAGCCTGTAGCTAGCGTTCATCCCTGAGCAGGATCAAA
>251
CTGCTGCCGCCCGTAGGAGTTTGGACCGTGTCTCAGTTCCAATGTGGGGGACCTTCCTCTCAGAACCCCTACT
GATCGTTGCCTTGGTAGGCCGTTACCCCGCCAACAAGCTAATCAGACGCATCCCCATCCATAACCGCTAAACC
TTTAATCGTTATTTTGATGTCAGCAAACGATATCATACGGTATTTAGGCCGTCTTTTCAACGGGTTATCCCGT
AGTTTATGGGAAGGTTGGATACGCGTTACTCACCCGTGCGCCAGTCGTCGTCAAGGAAAGCAAGCTTTCCCTC
ATGTTTACCCTGCGACTTGCATGTGTAAGCCTTGTAGCTAGCGTTCATCCCTGAGCCAGGATCAAACTCT
>252
CTGCTGCCGCCCGTAGGAGTTTTGGACCGTGTCTCAGTTCCAATGTGGGGGACCTTCCTCTCAGAACCCCTAC
TGATCGTCGCCTTTGGTGGGCCGTTACCCCGCCAACAAGCTAATCAGACGCATCCCCATCCATAACCGATAAA
TCTTTATTCATAATCTCATGAGATCAAACGAATACATAAGGTATTAGTCCAACTTCGCTGGGTTATCCCTTAG
TTATGGGCAGGTTGGATACGCGTTACTCACCCGTGCGCCAGTCGTCATCAAGGAAAGCAAGCCTTTCCTCATG
TTACCCTGCGACTTGCATGTGTTAAGCCTGTAGCCTAGCGTTCATCCTGAGCCAGGATCAAACTCT
>253
CTGCTGCCGCCCTAGGAGTTTGGACCGTGTCTCAGTTCCAATGTGGGGGACCTTCCTCTCAGAACCCCTACTG
ATCGTTGCCTTTGGTGGGCCGTTACCCCGCCAACAAGCTAATCAGACGCATCCCCATCCATAACCGATAAATC
TTTATTCGTAATCTCATGAGATCAAACGAAATACATAAGGTATTAGTCCAACTTCGCTGGGTTATCCCTTAGT
TATGGGCAGGTTGGATACGCGTTACCTCACCGTGCGCCGGTCGCCGACATAAATAGCCAAGCTATCTATTCGC
TGCCCCTCGACTTGCATGTGTTAAGCCTATAGCTAGCGTTCATCCTGAGCCAGGATCAAACTCT
>254
CTGCTGCCGCCCGTAGGAGTTTTGGACCGTGTCTCAGTTCCAATGTGGGGGACCTTCCTCTCAGAACCCCTAC
TGATCGTTCGCCTTTGGTGGGCCGTTACCCCGCCAACAAGCTAATCAGACGCATCCCCATCCATAACCGCTAA
ACCTTTAATCGTTATTTGATGTCAGCAAACGATATCATACGGTATTAGGCCGTCTTTCAACGGGTTATCCCGT
AGTTATGGGAAGGTTGGATACGCGTTACTCACCCGTGCGACCAGTCGTCCATCAAGGAAAGCAAGCTTTCCCT
CATGTTTACCCTGCGACATGCATGTGTTAAGCCTGTAGCTAGCGTTCATCCCTGAGCCAAGGATCAAACTGCC
T
>255

CTGCTGCCGCCCGTAGGAGTTTGAACCGTGTCTCAGTTCCAATGTGGGGGACCTTCCTCTCAGAACCCCTACT
GATCGTTGCCTTGGTGGGCCGTTACCCCACCAACAAGCTAATCAGACGCATCCCCATCCATAACCGATAAATC
TTTATTCGTAATCTCATGAGATCAAACGAATATATAAGGTATTAGTCCAACTTCGCTGGGTTATCCCTTAGTT
ATGGGCAGGTTGGATACGCGTTACTCACCCGTGCGCCGGTCGCCGACATAAATAGCAAGCTATCTATTCGCTG
CCCCCCTCGACTTGCATGTGTTAAGCCTATAGCTAGCGTTCATCCTGAGCCAGGATCAAACTCT
>256
CTGCTGCCGCCCGTAGGAGTTTGGACCGTGTCTCAGTTCCAATGTGGGGGACCTTCCTCTCAGAACCCCTACT
GATCGTCGCCTTTGGTGGGCCGTTACCCCGCCAACAAGCTAATCAGACGCATCCCCATCCATAACCGCTAAAC
CTTTAATCGTTATTTGATGTCAGCAAACGACGTCATACGGTATTAGTCCGTCTTTCAACGGGTTATCCCGTGG
TCATGGGAAGGTTGGATACGCGTTACTCACCCGTGCGCCAGTCGTCGTCAAGGAAAGCAAGCTTTCCTCACGT
TACCCTGCGACTTGCATGTGTTTAAGCCTATAGCTAGCGTTCATCCTGAGCCAGGATCAAACTCT
>257
CTGCTGCCGCCCGTAGGAGTTTGGACCGTGTCTCAGTTCCAATGTGGGGGACCTTCCTCTCAGAACCCCTACT
GATCGTTGCCTTGGTGGGCCGTTACCCCGCCAACAAGCTAATCAGACGCATCCCCATCCATAACCGATAAATC
TTTATTCATAATCTCATGAGATCAAACGAATACATAAGGTATTAGCCCAACTTCGCTGGGTTATCCCTTAGTT
ATGGGCAGGTTGGATACGCGTTACTCACCCGTGCGCCGGTCGCCGACATAAATAGCAAGCTATCTATTCGCTG
CCCCTCGACTTGCATGTGTTAAGCCTATAGCTAGCGTTCATCCTGAGCCAGGATCAAACTCT
>258
CTGCTGCCGCCCGTAGGAGTTTTGGACCGTGTCTCAGTTCCAATGTGGGGGACCTTCCTCTCAGAACCCCTAC
TGATCGTCGCCTTGGTGGGCCGTTACCCCGCCAACAAGCTAATCAGACGCATCCCCATCCATAACCGCTAAAC
CTTTAATCGTTATTTTGATGTCAGCAAACGATATCATACGGTATTTAGGCCGTCTTTTCAACGGGTTTATCCC
GTGAGTTATGGGAAGGTTGGATACGCGTTACTCACCCGTGCGCCAGTCGTCAGTCAAGGAAAGCAAGCTTTCC
CTCACGTGA
>259
CTGCTGCCGCCCGTAGGAGTTTGGACCGTGTCTCAGTTCCAATGTGGGGGACCTTCCTCTCAGAACCCCTACT
GATCGTTGCCTTGGTGGGCCGTTACCCCGCCAACAAGCTAATCAGACGCATCCCCATCCATAACCGATAAATC
TTTATTCGTAATCTCATGAGATCAAACGAATACATAAGGTATTTAGTCCAACTTCGCTGGGTTATCCCTTAGT
TTATGGGCAGGTTTGGATACGCGTTACTCACCCGTGCGCCGGTCGCCGACATAAATAGCAAGCTATCTATTCG
CTGCCCCCCCTCGACTTGCATGTGTTTTAAGCCTATAGCTAGCGTTCATCCTGAGCCAGGATCAAACTCT
>260
CTGCTGCCGCCCGTAGGAGTTTGGACCGTGTCTCAGTTCCAATGTGGGGGACCTTCCTCTCAGAACCCCTACT
GATCGTTGCCTTTGGTGGGCCGTTACCCCGCCAACAAGCTAATCAGACGCATCCCCATCCATAACCGATAAAT
CTTTTATTTCGTAATCTCATGAGATCAAACGAATACATAAGGTATTTAGTTCCAACTTCGCTTGGGTTTATCC
CTTAGTTTATGGGCAGGTTTTGGATACGCGTTACTCACCCGTGCGCCGGTCGCCGACATAAATAGCAAGCTAT
CTATTCGCTGCCCCCCCCTCGACTTGCATTGTGTTTTAAGCCTTATAGCTAGCGTTCATCCTGAGCCAGGATC
AAACTCT
>261
CTGCTGCCGCCCGTAGGAGTTTGGACCGTGTCTCAGTTCCAATGTGGGGGACCTTCCTCTCAGAACCCCTACT
GATCGTCGCCTTTGGTGGGCCGTTACCCCGCCAACAAGCTAACTCAGACGCATCCCCATCCATAACCGCTAAC
CGTTTAATCGTTTATTTTGATGTCAGCAAACGATATCATACGGTATTTAGGCCGTCTTTTCAACGGGTTTATC
CCGTAGTTTATGGGAAGGTTGGATACGCGTTACTCACCCGTGCGCCAGTCGTCATCAAGGAAAGCCAAGCTTC
CCTTCACGTTAACCCTGCGACTTTGCATGTGTTTAAGCCTTGTAGCTAGCGTCA
>262
CTGCTGCCGCCCGTAGGAGTTTTGGACCGTGTCTCAGTTCCAATGTGGGGGACCTTCCTCTCAGAACCCCTAC
TGATCGTCGCCTTTGGTGGGCCGTTACCCCGCCAACAAGCTAATCAGACGCATCCCCATCCATCACCGCTAAA
CCTTTAATCGTTATTTTGATGTCAGCAAACGATGTCATACGGTATTAGTCCGTCTTTCAACGGGTTATCCCGT
GGTCATGGGAAGGTTGGATACGCGTTACTCACCCGTGCGCCAGTCGTCGTCAAGGGAAGCAAGCTTCCCTCAC
GTTACCCTGCGACTTGCATGTGTTAAGCCTGTAGCTAGCGTTCATCCTGAGCCAGGATCAAACTCT
>263
CTGCTGCCGCCCTAGGAGTTTCGGACCGTGTCTCAGTTCCAATGTGGGGGACCTTCCTCTCAGAACCCCTACT
GATCGTCGCCTTGGTGGGCCGTTACCCCGCCAACAAGCTAATCAGACGCATCCCCATCCATAACCGCTAAACC
TTTAATCGTTTATTTTGATGTCAGCAAACGATATCATACGGTATTTAGGCCGTCTTTTCAACGGGTTTATCCC
GTAGTTTATGGGAAGGTTGGATACGCGTTACTCACCCGTGCGCCAGTCGTCATCAAGGAAAGCAAGCTTTCCC
TCATGTTTACCCTGCGACTGCATGTGTTTAAGCCTTGTAGCTAGCGTCATCCTGAGCCAGGATCAAACTCT

>264
CTGCTGCCGCCCGTAGGAGTTTGGACCGTGTCTCAGTTCCAATGTGGGGGACCTTCCTCTCAGAACCCCTACT
GATCGTCGCCTTGGTGGGCCGTTACCCCCGCCAACAAGCTAATCAGACGCATCCCCCATCCATAACCGCTAAA
CCTTTAATCGTTTATTTTGATGTCAGCAAACGATATCATACGGTATTTAGGCCGTCTTTTCAACGGGTTTATC
CCCGTAGTTTATGGGAAGGTTTGGATACGCGTTACTCACCCCGTGCGCCAGTCGTCATCAAGGAAAGCAAGCT
TTTCCCTTCATGTTTACCCTGCGACTTGCATTGTGTTTAAGCCTTGTAGCTAGCGTCATCCTGAGCCAGGATC
AAACTCTATCACCCCGACTGCCCCCCATAGAGAGGTGAGAGCTGCCGAAAGGCACACAGGGGATAGG
>265
CTGCTGCCGCCCGTAGGAGTTTTGGACCGTGTCTCAGTTCCAATGTGGGGGACCTTCCTCTCAGAACCCCTAC
TGATCGTTGCCTTTGGTGGGCCGTTACCCCGCCAACAAGCTAATCAGACGCATCCCCATCCATAACCGATAAA
TCTTTATTCGTAATCTCATGAGATCAAACGAAATACATAAGGTATTTAGTCCAACTTCGCTGGGTTTATCCCT
TAGTTTATGGGCAGGTTTGGATACGCGTTACTCACCCGTGCGCCGGTCGCCGACATAAATAGCAAGCTATCTA
TTCGCTGCCCCTCGACTTGCATGTGTTTAAGCCTATAGCTAGCGTTCATCCTGAGCCAGGATCAAACTCT
>266
CTGCTGCCGCCCGTAGGAGTTTGGACCGTGTCTCAGTTCCAATGTGGGGGACCCTTCCTCTCAGAACCCCTAC
TGATCGTCGCCTTTGGGTGGGCCGTTACCCCGCCAACAAGCTAATCAGACGCATCCCCATCCATAACCGCTAA
CCTTTAATCGTTTATTTGATGTCAGCAAACGATATCATACGGTATTTAGGCCGTCTTTCAACGGGTTTATCCC
GTAGTTTATGGGAAGGTTGGATACGCGTTACTCACCCGTGCGCCAGTCGTCATCAAGGAAAGCAAGCTTTCCC
TCATGTTTACCCTGCGACTTGCATGTGTTAAGCCTTGTAGCTAGCGTTCATCCTGAGCCAGGATCAAACTCT
>267
CTGCTGCCGCCCGTAGGAGTTTGGACCGTGTCTCAGTTCCAATGTGGGGGACCTTCCTCTCAGAACCCCTACT
GATCGTCGCCTTTGGTGGGCCGTTACCCCGCCAACAAGCTAATCAGACGCATCCCCATCCATAACCGCTAAAC
CTTTAATCGTTATTTTGATGTCAGCAAACGATATCATACGGTATTAGGCCGTCTTTTCAACGGGTTATCCCGT
AGTTATGGGAAGGTTGGATACGCGTTACTCACCCGTGCGCCAGTCGTCATCAAGGAAAGCAAGCTTTCCCTCA
TGTTTACCCTGCGACTGCATGTGTTAAGCCTGTAGCTAGCGTTCATCCCTGAGCCAAGGATCAAACT
>268
CTGCTGCCGCCCGTAGGAGTTTTGGACCGTGTCTCAGTTCCAATGTGGGGGACCTTCCTCTCAGAACCCCTAC
TGATCGTCGCCTTTGGTGGGCCGTTACCCCGCCAACAAGCTAATCAGACGCATCCCCATCCATAACCGCTAAA
CCTTTAATCGTTATTTGATGTCAGCAAACGACGTCATACGGTATTAGTCCGTCTTTCAACGGGTTATCCCGTG
GTCATGGGAAGGTTGGATACGCGTTACTCACCCGTGCGCCAGTCGTCGTCAAGGAAAGCAAGCTTTCCTCACG
TTACCCTGCGACTTGCATGTGTTAAGCCTGTAGCTAGCGTTCATCCTGAGCCAGGATCAAACTCT
>269
CTGCTGCCGCCCGTAGGAGTTTTGGACCGTGTCTCAGTTCCAATGTGGGGGACCTTCCTCTCAGAACCCCTAC
TGATCGTGCCTTTGGTGGGCCGTTACCCCGCCAACAAGCTAATCAGACGCATCCCCATCCATAACCGCTAAAC
CTTTAATCGTTATTTGATGTCAGCAAACGATATCATACGGTATTAGGCCGTCTTTCAACGGGTTATCCCGTAG
TTATGGGAAGGTTGGATACGCGTTACTCACCCGTGCGCCAGTCGTCATCAAGGAAAGCAAGCTTTCCCTCATG
TTACCCTGCGACTTGCATGTGTTAAGCCTGTAGCTAGCGTTCATCCCTGAGCCAAGGATCAAAC
>270
CTGCTGCCGCCCGTACGGAGTTTGGACCGTGTCTCAGTTCCAATGTGGGGGACCTTCCTCTCAGAACCCCTAC
TGATCGTTGCCTTGGTGGGCCGTTACCCCGCCAACAAGCTAATCAGACGCATCCCCATCCATAACCGATAAAT
CTTTATTCATAATCTCATGAGATCAAACGAATACATAAGGTATTAGTCCAACTTCGCTGGGTTATCCCTTAGT
TATGGGCAGGTTTGGATACGCGTTACTCACCCGTGCGCCGGTCGCCGACATAAATAGCAAGCTATCTATTCGC
TGCCCCTCGACTTGCATGTGTTTAAGCCTATAGCTAGCGTTCATCCTGAGCCAGGATCAAACTCT
>271
CTGCTGCCGCCCGTAGGAGTTTTGGACCGTGTCTCAGTTCCAATGTGGGGGACCTTCCTCTCAGAACCCCTAC
TGATCGTCGCCTTTGGTGGGCCGTTACCCCGCCAACAAGCTAATCAGACGCATCCCCATCCATAACCGCTAAA
CCTTTAATCGTTATTGATGTCAGCAAACGATATCATACGGTATTAGGCCGTCTTTCAACGGGTTATCCCGTAG
TTATGGGAAGGTTGGATACGCGTTACTCACCCGTGCGCCAGTCGTCGTCAAGGGAAGCAAGCTTCCCTCACGT
TACCCTGCGACTGCATGTGTTAAGCCTGTAGCTAGCGTTCATCCTGAGCCAGGATCAAACTCGT
>272
CTGCTGCCGCCCTAGGAGTTTGAACCGTGTCTCAGTTCCAATGTGGGGGACCTTCCTCTCAGAACCCCTACTG
ATCGTTGCCTTGGTGGGCCGTTACCCCGCCAACAAGCTAATCAGACGCATCCCCATCCATAACCGATAAATCT
TTATTCGTAATCTCATGAGATCAAACGAATACATAAGGTATTAGTCCAACTTCGCTGGGTTATCCCTTAGTTA

TGGGCAGGTTGGATACGCGTTACTCACCCGTGCGCCGGTCGCCGACATAAATAGCAAGCTATCTATTCGCTGC
CCCTCGACTTGCATGTGTTAAGCCTATAGCTAGCGTTCATCCTGAGCCAGGATCAAACTCT
>273
CTGCTGCCGCCCGTAGGAGTTTGGACCGTGTCTCAGTTCCAATGTGGGGGACCTTCCTCTCAGAACCCCTACT
GATCGTTGCCTTTGGTGGGCCGTTACCCCGCCAACAAGCTAATCAGACGCATCCCCATCCATAACCGATAAAT
CTTATTCGTAATCTCATGAGATCAAACGAAATACATAAGGTATTAGTCCAACTTCGCTGGGTTATCCCTTAGT
TATGGGCAGGTTGGATACGCGTTACTCACCCGTGCGCCGGTCGCCGACATAAATAGCAAGCTATCTATTCGCT
GCCCCTCGACTTGCATGTGTTAAGCCTATAGCTAGCGTTCATCCTGAGCCAGGATCAAACTCT
>274
CTGCTGCCGCCCGTAGGAGTTTTGGACCGTGTCTCAGTTCCAATGTGGGGGACCTTCCTCTCAGAACCCCTAC
TGATCGTCGCCTTTGGTGGGCCGTTACCCCGCCAACAAGCTAATCAGACGCATCCCCATCCATAACCGATAAA
TCTTTATTCATAATCTCATGAGATCAAACGAATACATAAGGTATTAGTCCAACTTCGCTGGGTTATCCCTTAG
TTATGGGCAGGTTGGATACGCGTTACTCACCCGTGCGCCGGTCGCCGACATAAATAGCAAGCTATCTATCGCT
GCCCCTCGACTTGCATGTGTTAAGCCTATAGCTAGCGTTCATCCTGAGCCAGGATCAAACTCT
>275
CTGCTGCCGCCCGTAGGAGTTTTGGACCGTGTCTCAGTTCCAATGTGTGGGACCTTCCTCTCAGAACCCCTAC
TGATCGTCGCCTTGGTGGGCCGTTACCCCACCAACTGCCTAATCAGACGCAAACCCCTCTTCAGGCGATAGCT
TACAGGTAGAGGCTACCCTTTCTTCCACAGGTCATGCGGCCCGTGGAACGTATTCGGTATTAGCAGTCGTTTT
CCGTCTGTTTGTCCCCATCCTGAAGGCAGGTTGTTTTACGTGTTACTCACCCGTTTGCCACTGGATTCGATAA
AGAGCAAGCTCTTCATCTCTTCCCGTTCGACTGCATGTGTTAAGCACGCCGCCAGCGTTCGTCCTGAGCCAGG
ATCAAACTCT
>276
CTGCTGCCGCCCGTAGGAGTTTTGGACCGTGTCTCAGTTCCAATGTGGGGGACCTTCCTCTCAGAACCCCTAC
TGATCGTCGCCTTTGGTGGGCCGTTACCCCGCCAACAAGCTAATCAGACGCATCCCCATCCATAACCGCTAAA
CCTTTAATCGTTATTTGATGTCAGCAAACGATATCATACGGTATTAGGCCGTCTTTCAACGGGTTATCCCGTA
GTTATGGGAAGGTTGGATACGCGTTACTCACCCGTGCGCCAGTCGTCATCAAGGAAAGCAAGCTTTCCCTCAT
GTTACCCTGCCGACATGCATGTGTTAAGCCTTAGTACGCTGACTC
>277
CTGCTGCCGCCCGTAGGAGTTGAACCGTGTCTCAGTTCCAATGTGGGGGACCTTCCTCTCAGAACCCCTACTG
ATCGTTGCCTTGGTGGGCCGTTACCCCACCAACAAGCTAATCAGACGCATCCCCATCCATAACCGCTAAACCT
TTAATCGTTATTTGATGTCAGCAAACGATATCATACGGTATTAGGCCGTCTTTCAACGGGTTATCCCTTAGTT
ATGGGCAGGTTGGATACGCGTTACTCACCCGTGCGCCGGTCGCCGACATAAATAGCAAGCTATCTATTCGCTG
CCCCTCGACTTGCATGTGTTTAAGCCTATAGCTAGCGTTCATCCTGAGCCAGGATCAAACTCT
>278
CTGCTGCCGCCCGTAGGAGTTTTGGACCGTGTCTCAGTTCCAATGTGGGGGACCTTCCTCTCAGAACCCCTAC
TGATCGTCGCCTTTGGTGGGCCGTTACCCCGCCAACAAGCTAATCAGCGCATCCCCATCCATAACCGCTAAAC
CTTTAATCGTTATTTGATGTCAGCAAACGATATCATACGGTATTAGGCCGTCTTTCAACGGGTTATCCCGTAG
TTATGGGAAGGTTGGATACGCGTTACTCACCCGTGCGCCAGTCGTCATCAAGGAAAGCAAGCTTTCCTCATGT
TACCCTGCCGACATGCATGTGTTAAGCCTGTAGCTAGCGTTCATCCGTGACGCAAGCA
>279
CTGCTGCCGCCCGTAGGAGTTTTGGACCGTGTCTCAGTTCCAATGTGGGGGACCTTTCCTCTCAGAACCCCTA
CTGATCGTCGCCTTTGGTGGGCCGTTACCCCGCCAACAAGCTAATCAGACGCATCCCCATCCATAACCGCTAA
ACCTTTAATCGTTATTTGATGTCAGCAAGACGATATCATACGGTATTAGGCCGTCTTTCAACGGGTTATCCCG
TAGTTATGGGAAGGTTGGATACGCGTTACTCACCCGTGCGCCAGTCGTCATCAAGGAAAGCAAGCTTTCCCTC
ATGTTACCCTGCGACTTGCATGTGTTAAGCCTATAGCTAGCGTTCATCCCTGAGCCAAGGCATCAAATC
>280
CTGCTGCCGCCCGTAGGAGTCTGGGCCGTATCTCAGTCCCAATGTGGCCGCACACCCTCTCAGGCCGGCTACC
CGTCGCCGCCTTGGTAGGCCATTACCCCACCAACAAGCTGATAGGCCGCAAGCTCATCCCACACCAGAAAAAC
CTTTCCACCACACCAACCACGATGCGGTCCTATAGGGTATTAGACCCAGTTTCCCAAGCTTATCCCCCAGTGC
AGGGCAGATCACTTACGTGTTACTCACCCGTTCGCCACTCGAGCACCCCAGCAAGCTGGGGCCTTTCCGTTCG
ACTTGCATGTGTTTAAGCACGCCGCCAGCGTTCACCTGAGCCAGGATCAACTCT
>281
CTGCTGCCGCCCGTAGGAGTTTGGACCGTGTCTCAGTTCCAATGTGGGGGACCTTCCTCTCAGAACCCCTACT
GATCGTTGCCTTGGTGGGCCGTTACCCCGCCAACAAGCTAATCAGACGCATCCCCATCCATAACCGATAAATC

TTTTATTTCATAATCTCATGAGATCAAACGAATACATAAGGTATTTAGTCCAACTTCGCTTGGGTTTATCCCT
TAGTTTATGGGCAGGTTTGGATACGCGTTACTCACCCGTGCGCCGGTCGCCGGCATAAATAGCAAGCTATCTA
TTTCGCTGCCCCCCCCTCGACTTGCATGTGTTTTAAGCCTTATAGCTAGCGTTCATCCTTGAGCCAGGATCAAA
CTCT
>282
CTGCTGCCGCCCGTAGGAGTTTGGACCGTGTCTCAGTTCCAATGTGGGGGACCTTCCTCTCAGAACCCCTACT
GATCGTCGCCTTTGGTGGGCCGTTACCCCGCCAACAAGCTAATCAGACGCATCCCCATCCATAACCGCTAAAC
CTTTAATCGTTTATTTTGATGTCAGCAAACGATATCATACGGTATTTAGGCCGTCTTTTCAACGGGTTTATCC
CGTAGTTTATGGGAAGGTTTGGATACGCGTTACTCACCCGTGCGCCAGTCGTCATCAAGGAAAGCAAGCTTTT
CCCTTCATGATTTT
>283
CTGCTGCCGCCCGTAGGAGTTTGGACCGTGTCTCAGTTCCAATGTGGGGGACCTTCCTCTCAGAACCCCTACT
GATCGTCGCCTTGGTGGGCCGTTACCCCGCCAACAAGCTAATCAGACGCATCCCCATCCATAACCGCTAAACC
TTTAATCGTTTATTTTGATGTCAGCAAACGATATCATACGGTATTTAGGCCGTCTTTTCAACGGGTTTATCCC
GTAGTTTATGGGAAGGTTGGATACGCGTTACTCACCCGTGCGCCAGTCGTCATCAAGGAAAGCAAGCTTTTCC
CGTCATGATTTTACCCTGCGACATGCATGTGTT
>284
CTGCTGCCGCCCGTAGGAGTTTGGACCGTGTCTCAGTTCCAATGTGGGGGACCTTCCTCTCAGAACCCCTACT
GATCGCTCGCCTTGGTGGGCCGTTACCCCGCCAACAAGCTAATCAGACGCATCCCCATCCATCAACCGCTAAA
CCTTTAATCGTTTATTTTGATGTCAGCAAACGATATCATACGGTATTTAGGCCGTCTTTTCAACGGGTTTATC
CCGTAGTTTATGGGAAGGTTGGATACGCGTTACTCACCCGTGCGCCAGTCGTCATCAAGGAAAGCAAGCTTTT
CCTCATGATTTTGCCCTGCGACTTGCATTGTGTTTAAGCCTGTAGC
>285
CTGCTGCCGCCCGTAGGAGTTTGGACCGTGTCTCAGTTCCAATGTGGGGGACCTTCCTCTCAGAACCCCTACT
GATCGTTGCCTTGGTGGGCCGTTACCCCGCCAACAAACTAATCAGACGCATCCCCATCCATAACCCGATAAAT
CTTTATTCATAATCTCATGAGATCAAACGAATACATAAGGTATTAGTCCAACTTCGCTGGGTTATCCCTTAGT
TTATGGGCAGGTTGGATACGCGTTACTCACCGTGCGCCGGTCGCCGACCATAAATAGCAAGCTATCTATTCGC
TGCCCCTCGACCTTGCATGTGTTTTAACGCCTATAGCTAGCGTTCACCTGAGCCAGGATCAAACTCT
>286
CTGCTGCCGCCCGTAGGAGTTTGGACCGTGTCTCAGTTCCAATGTGGGGGACCTTCCTCTCAGACGCCCTACT
GATCGTCGCCTTTGGTGGGCCGTTACCCCGCCAACAAGCTAATCAGACGCATCCCCATCCATCACCGCTAAAC
CTTTAATCGTTTATTTTGATGTCAGCAAACGATATCATACGGTATTTAGGCCGTCTTTTCAACGGGTTTATCC
CGTAGTTTATGGGAAGGTTGGATACGCGTTACTCACCCGTGCGCCAGTCGTCATCAAGGAAAGCAAGCTTTCC
TCATGTTTTAC
>287
CTGCTGCCGCCCGTAGGAGTTTGGACCGTGTCTCAGTTCCAATGTGGGGGACCTTCCTCTCAGAACCCCTACT
GATCGTTGCCTTGGTGGGCCGTTACCCCGCCAACAAGCTAATCAGACGCATCCCCATCCATAACCGATAAATC
TTTATTCATAATCTCATGAGATCAAACGAATACATAAGGTATTAGTCCAACTTCGCTGGGTTATCCCTAGTTA
TGGGCAGGTTTGGATACGCGTTACTCACCCGTGCGCCGGTCGCCGACATAAATAGCAAGCTATCTATTCGCTG
CCCCTCGACTTGCATGTGTTTAAGCCTATAGCTAGCGTCATCCTGAGCCAGGATCAAACTCT
>288
CTGCTGCCGCCCGTAGGAGTTTGGACCGTGTCTCAGTTCCAATGTGGGGGACCTTCCTCTCAGAACCCCTACT
GATCGTTGCCTTGGTGGGCCGTTACCCCGCCAACAAGCTAATCAGACGCATCCCCCATCCATAACCGATAAAT
CTTTATTTCGTAATCTCATGAGATCAAACGAATACATAAGGTATTTAGTCCAACTTCGCTGGGTTTATCCCTT
AGTTTATGGGCAGGTTTGGATACGCGTTACTCACCCGTGCGCCGGTCGCCGACATAAATAGCAAGCTATCTAT
TCGCTGCCCCCCCCTCGACTTGCATGTGTTTAAGCCTTATAGCTAGCGTTCATCCTGAGCCAGGATCAAACTC
T
>289
CTGCTGCCGCCCGTAGGAGTTTGGACCGTGTCTCAGTTCCAATGTGGGGGACCTTCCTCTCAGAACCCCTACT
GATCGTTGCCTTGGTGGGCCGTTACCCCGCCAACAAGCTAATCAGACGCATCCCCATCCATAACCGATAAATC
TTTATTCGTAATCTCATGAGATCAAACGAATACATAAGGTATTAGTCCAACTTCGCTGGGTTATCCCTTAGTT
ATGGGCAGGTTGGATACGCGTTACCTCACCCGTGCGCCGGTCGCCGACATAAATAGCCAAGCTATCTATTCGC
TGCCCCTCGACTTGCATGTGTTTAAGCCTATAGCTAGCGTCATCCTGAGCCAGGATCAAACTCT
>290

CTGCTGCCGCCCGTAGGAGTTTGGACCGTGTCTCAGTTCCAATGTGGGGGACCTTCCTCTCAGAACCCCTACT
GATCGTTGCCTTGGTGGGCCGTTACCCCGCCAACAAGCTAATCAGACGCATCCCCATCCATAACCGATAAATC
TTTATTCATAATCTCATGAGATCAAACGAATACATAAGGTATTAGTCCAACTTCGCTGGGTTATCCCTTAGTT
ATGGGCAGGTTGGATACGCGTTACTCACCCGTGCGCCGGTCGCCGACATAAATAGCAAGCTATCTATTCGCTG
CCCCCCTCGACTTGCATGTGTTAAGCCTATAGCTAGCGTTCATCCTGAGCCAGGATCAAACTCT
>291
CTGCTGCCGCCCGTAGGAGTTTGGACCGTGTCTCAGTTCCAATGTGGGGGACCTTCCTCTCAGAACCCCTACT
GATCGTTGCCTTGGTGGGCCGTTACCCCGCCAACAAGCTAATCAGACGCATCCCCATCCATAACCGATAAATC
TTTATTCGTAATCTCATGACGATCAAACGAAATACATAAGGTATTAGTCCAACTTCGCTGGGTTATCCCTTAG
TTATGGGCCAGGTTGGATACGCGTTACTCACCCGTGCGCCGGTCGCCGACATAAATAGCAAGCTATCTATTCG
CTGCCCCTCGACTTGCATGTGTTAAGCCTATAGCTAGCGTTCATCCTGAGCCAGGATCAAACTCT
>292
CTGCTGCCGCCCGTAGGAGTTTGGACCGTGTCTCAGTTCCAATGTGGGGGACCTTCCTCTCAGAACCCCTACT
GATCGTTGCCTTGGTGGGCCGTTACCCCGCCAACAAGCTAATCAGACGCATCCCCATCCATAACCGATAAATC
TTTATTCATAATCTCATGAGATCAAACGAATACATAAGGTATTAGTCCAACTTCGCTGGGTTATCCCTTAGTT
ATGGGCAGGTTGGATACGCGTTACTCACCCGTGCGCCGGTCGCCGACATAAATAGCAAGCTATCTATTCGCTG
CCCCTCGACTTGCATGTGTTAAGCCTATAGCTAGCGTTCATCCTGAGCCAGGATCAAACTCCT
>293
CTGCTGCCGCCCGTAGGAGTTTGGGCCGTGTCTCAGTCCCAATGTGGCCGTACACTCTCTCAAGCCGGCTACT
GATCGTCGCCTTGGTGAGCTTTTATCTCACCAACTAGCTAATCAGACGCAAGTCCATCTTACACCGATAAATC
TTTGGTCACTCTATCATGCGATAAATTGACTTTATAGGGTATTATTCTTCGTTTCCAAAGGCTAATCCCCTTG
TGTAAGGCAGGTTACTCACGTGTTACTCACCCGTTCGCCACTAATCCACTTAAAATCATTCCGAAGAAATCAA
CTAAGTTTCATCGTTCGACTTGCATGTGTTATGCACGCCGCCAGCGTTTATCCTGAGCCAGGATCAAACTCT
>294
CTGCTGCCGCCCGTAGGAGTTTTGGACCGTGTCTCAGTTCCAATGTGGGGGACCTTCCTCTCAGAACCCCTAC
TGATCGTCGCCTTTGGTGGGCCGTTACCCCGCCAACAAGCTAATCAGACGCATCCCCATCCATAACCGCTAAA
CCTTTAATCGTTATTTGATGTCAGCAAACGACGTCATACGGTATTAGGCCGCCTTTCAACGGGTTATCCCGTA
GTTATGGGAAGGTTGGATACGCGTTACTCACCCGTGCGCCAGTCGTCATCAAGGAAAGCAAGCTTCCTCATGT
TTACCCTGCGACTTGCATGCGTTAAGCCTGTAGCTAGCGTTCATCCTGAGCCAGGATCAAACTCT
>295
CTGCTGCCGCCCTAGGAGTTTGAACCGTGTCTCAGTTCCAATGTGGGGGACCTTCCTCTCAGAACCCCTACTG
ATCGTTGCCTTGGTGGGCCGTTACCCCACCAACAAGCTAATCAGACGCATCCCCATCCATAACCGATAAATCT
TTTATTTCGTTAATCTCATTGAGATCAAACGAATACATAAGGTTATTTTAGTTCCAACTTCGCTGGGTTTTAT
TCCCTTTAGTTTATGGGCAGGTTTGGATACGCGTTACTCACCCGTGCGCCGGTCGCCGACATTAAATAGCAAG
CTTATCTTATTCGCTGCCCCCCCTTCGACTTTGCATGTGTTTTAAGCCTTATAGCTAGCGTTCATCCTGAGCC
AGGATTCAAACTCTT
>296
CTGCTGCCGCCCGTAGGAGTCTGGACCGTGTCACAGTTCCAGTGTGGCCGTTCATCCTCTCAGACCGGCTACT
GATCGTTGGTTTGGTGGCCGTTACCCCACCAACTGCCTAATCAGACGCAAACCCCTCTTCAGGCGATAGCTTT
ACAGGTAGAGGCTACCCTTTCTTTCCCACAGGTCATGCGGCCCGTGGAACGTATTTCGGTATTTAGCAGTCGT
TTTTCCGTCTGTTTTGTCCCCCCCATCCTGAAGGCAGGTTTGTTTTTACGTGTTACTCACCCGTTTTTGCCAC
TGGATTTCGATAAAGAGCAAGCTCTTCATCTCTTCCCGTTTCGACTTGCATGTGTTTAAGCACGCCGCCAGCG
TTCGTCCTGAGCCAGGATCAAACTCT
>297
CTGCTGCCGCCCGTAGGGGTTTGGACCGTGTCTCAGTTCCAATGTGGGGGACCTTCCTCTCAGAACCCCTACT
GATCGTCGCCTTTGGTGGGCCGTTACCCCGCCAACAAGCTAATCAGACGCATCCCCATCCATAACCGCTAAAC
CTTTAATCGTTATTTGATGTCAGCAAACGATATCATACGGTATTAGGCCGTCTTTCAACGGGTTATCCCGTAG
TTATGGGAAGGTTGGATACGCGTTACTCACCCGTGCGCCAGTCGTCATCAAGGAAAGCAAGCTTCCTCATGTT
ACCCTGCGACTTGCATGTGTTAAGCCTGTAGCTAGCGTTCATCCTGAGCCAGGATCAAACTCT
>298
CTGCTGCCGCCCGTAGGAGTTTGGACCGTGTCTCAGTTCCAATGTGGGGGACCTTCCTCTCAGAACCCCTACT
GATCGTCGCCTTTGGTGGGCCGTTACCCCGCCAACAAGCTAATCAGACGCATCCCCATCCATAACCGCTAAAC
CTTTAATCGTTATTTGATGTCAGCAAAGCGATATCACGGTATTAGGCCGTCTTTCAACGGGTTATCCCGTAGT

TATGGGAAGGTTGGATACGCGTTACTCACCCGTGCGCCAGTCGTCATCAAGGAAAGCAAGCTTTCCCTGACTG
TTACCCTGCGAACGTTC
>299
CTGCTGCCGCCCGTAGGAGTTTGGACCGTGTCTCAGTTCCAATGTGGGGGACCTTCCTCTCAGAACCCCTACT
GATCGTCGCCTTTGGTGGGCCGTTACCCCGCCAACAAGCTAATCAGACGCATCCCCATCCATAACCGCTAAAC
CTTTAATCGTTATTTTGATGTCAGCAAACGATATCATACGGTATTAGGCCGTCTTTCAACGGGTTATCCCGTA
GTTATGGGAAGGTTGGATACGCGTTACTCACCCGTGCGCCAGTCGTCATCAAGGAAAGCAAGCCTTTCCTCAT
GTTTACCCTGCGACTTGCATGTGTTAAGCCTTGTAGCTAGCGTCATCCTGAGCCAGGATCAAACTCT
>300
CTGCTGCCGCCCGTAGGAGTTTTGGACCGTGTCTCAGTTCCAATGTGGGGGACCTTCCTCTCAGAACCCCTAC
TGATCGTCGCCTTTGGTGGGCCGTTACCCCGCCAACAAGCTAATCAGACGCATCCCCATCCATAACCGCTAAA
CCTTTAATCGTTATTTGATGTCAGCAAACGATATCATACGGTATTAGGCCGTCTTTCAACGGGTTATCCCGTA
GTTATGGGAAGGTTGGATACGCGTTACTCACCCGTGCGCCAGTCGTCATCAAGGAAAGCAAGCCTTTCCTCAT
GTTACCCTGCGACTTGCATGTGTTAAGCCTGTAGCTAGCGTTCATCCTGAGCCAGGATCAAACTCT
>301
CTGCTGCCGCCCGTAGGAGTTTGGACCGTGTCTCAGTTCCAATGTGGGGGACCTTCCTCTCAGAACCCCTACT
GATCGTCGCCTTGGTGGGCCGTTACCCCCGCCACAAGCTAATCAGACGCATCCCCCATCCATAACCGCTAAAC
CTTTTAATCGTTTATTTTGATTGTCAGCAAACGATATCATACGGTATTTAGGCCGTCTTTTCAACGGGTTTAT
TCCCGTAGTTTATGGGAAGGTTGGATACGCGTTACTCACCCGTGCGCCAGTCGTCATCAAGGAAAAGCAAGCT
TTCCCTTCATGTTT
>302
CTGCTGCCGCCCGTAGGAGTTTGGACCGTGTCTCAGTTCCAATGTGGGGGACCTTCCTCTCAGAACCCCTACT
GATCGTCGCCTTGGTGGGCCGTTACCCCGCCAACAAGCTAATCAGACGCATCCCCATCCATAACCGCTAAACC
TTTAATCGTATTTTGATGTCAGCAAACGATATCATACGGTATTTAGGCCGTCTTTTCAACGGGTTTATCCCGT
AGTTTATGGGAAGGTTGGATACGCGTTACTCACCCGTGCGCCAGTCGTCATCAAGGAAAGCAAGCTTTCCCTT
CATGTTTTACCCTGCGACTTGCATTGTGTTTAAGCCTTGTAGCTAGCGTCATCCCTGAGCCAGGATTCAAACT
CT
>303
CTGCTGCCGCCCGTAGGAGTTTCGGTGACCGTGTCTCAGTTCCAATGTGGGGGACCTTCCTCTCAGAACCCCT
ACTGATCGTCGCCTTTGGTGGGCCGTTACCCCGCCAACAAGCTAATCAGACGCATCCCCATCCATAACCGATA
AATCTTTATTCGTAATCTCATGAGATCAAACGAATACATAAGGTATTAGTCCAACTTCGCTGGGTTTATCCCT
TAGTTATGGGCAGGTTTGGATACGCGTTACCTCACCGTGCGCCGGTCGCCGACATAAATAGCAAGCTATCTAT
TCGCTGCCCCCCTCGACTTGCATGTGTTTAAGCCTTGTAGCTAGCGTTCATCCTTGAGCCAGGATCAAACTCC
T
>304
CTGCTGCCGCCCGTAGGAGTTTTGGACCGTGTCTCAGTTCCAATGTGGGGGACCTTCCTCTCAGAACCCCTAC
TGATCGTCGCCTTTGGTGGGCCGTTACCCCGCCAACAAGCTAATCAGACGCATCCCCATCCATAACCGCTAAA
CCTTTAATCGTTATTTGATGTCAGCAAACGATATCATACGGTATTAGGCCGTCTTTCAACGGGTTATCCCGTA
GTTATGGGAAGGTTGGATACGCGTTACTCACCCGTGCGCCAGTCGTCATCAAGGAAAGCAAGCCTTTCCTCAT
GTTACCCTGCGACTTGCATGTGTTAAGCCTGTAGCTAGCGTTCATCCCTGAGCAAGGCATC
>305
CTGCTGCCGCCCGTAGGAGTTTGAACCGTGTCTCAGTTCCAATGTGGGGGACCTTCCTCTCAGAACCCCTACT
GATCGTTGCCTTGGTGGGCCGTTACCCCGCCAACAAGCTAATCAGACGCATCCCCATCCATAACCGATAAATC
TTTATTCGTAATCTCATGAGATCAAACGAATACATAAGGTATTAGTCCAACTTCGCTGGGTTATCCCTTAGTT
ATGGGCAGGTTGGATACGCGTTACTCGCCCGTGCGCCGGTCGCCGACATAAATAGCAAGCTATCTATTCGCTG
CCCCCCTCGACTTGCATGTGTTAAGCCTATAGCTAGCGTTCATCCTGAGCCAGGATCAAACTCT
>306
CTGCTGCCGCCCGTAGGAGTTTGGACCGTGTCTCAGTTCCAATGTGGGGGACCTTCCTCTCAGAACCCCTACT
GATCGTCGCCTTGGTGGGCCGTTACCCCGCCAACAAGCTAATCAGACGCATCCCCATCCATAACCGCTAAACC
TTTAATCGTTTATTTTGATGTCAGCAAACGATATCATACGGTATTTAGGCCGTCTTTTCAACGGGTTTATCCC
CGTAGTTTATGGGAAGGTTGGATACGCGTTACTCACCCGTGCGCCAGTCGTCATCAAGGAAAGCAAGCTTTTC
CCCCTTGA
>307

CTGCTGCCGCCCGTAGGAGTTTGAACCGTGTCTCAGTTCCAATGTGGGGGACCTTCCTCTCAGAACCCCTACT
GATCGTTGCCTTGGTGGGCCGTTACCCCACCAACAAGCTAATCAGACGCATCCCCATCCATAACCGATAAATC
TTTATTCGTAATCTCATGAGATCAAACGAATATATAAGGTATTAGTCCAACTTCGCTGGGTTATCCCTTAGTT
ATGGGCAGGTTGGATACGCGTTACTCACCCGTGCGCCGGTCGCCGACATAAATAGCAAGCTATCTATTCGCTG
CCCCTCGACTTGCATGTGTTAAGCCTATAGCTAGCGTTCTCCTGAGCCAGGATCAAACTCT
>308
CTGCTGCCGCCCGTAGGAGTTTTGGACCGTGTCTCAGTTCCAATGTGGGGGACCTTCCTCCCAGAACCCCTAC
TGATCGTCGCCTTTGGTGGGCCGTTACCCCGCCAACAAGCTAATCAGACGCATCCCCATCCATAACCGCTAAA
CCTTTAATCGTTATTTTGATGTCAGCAAACGACGTCATACGGTATTTAGTCCGTCTTTCAACGGGTTTATCCC
GTAGTTTATGGGAAGGTTTGGATACGCGTTACTCACCCGTGCGCCAGTCGTCATCAAGGAAAGCAAGCTTTCC
TCATGTTTACCCTGCGACATGCATGTGTTTAAGCCTGTAGCTAGCGTTCATCCTGAGCCAGGATCAAACTCT
>309
CTGCTGCCGCCCGTAGGAGTTTGGACCGTGTCTCAGTTCCAATGTGGGGGACCTTCCTCTCAGAACCCCTACT
GATCGTCGCCTTTGGTGGGCCGTTACCCCGCCAACAAGCTAATCAGACGCATCCCCATCCATAACCGCTAAAC
CTTTAATCGTTATTTGATGTCAGCAAACGATATCATACGGTATTAGGCCGTCTTTTCAACGGGTTATCCCGTA
GTTATGGGAAGGTTGGATACGCGTTACTCACCCGTGCGCCAGTCGTCATCAAGGAAAGCAAGCTTTCCCTCGA
TGTTTACCCTGCGACTTGCATGTGTAAGCCTGTAGCTAGCGTTCATCCCTGAGCCAAGGATCAAACTCT
>310
CTGCTGCCGCCCGTAGGAGTTTTGGGCCGTGTCTCAGTCCCAATGTGGCCGTACACTCTCTCAAGCCGGCTAC
TGATCGTCGCCTTGGTGAGCTTTTATCTCACCAACTAGCTAATCAGACGCAAGTCCATCTTACACCGATAAAT
CTTTTGGTCACTCTATCATGCGATAAATTTGACTTTATAGGGTATTTTATTTCTTTCGTTTTTCCAAAGGCTA
TCCCCTTTGTGTAAGGCAGGTTTACTCACGTGTTACTCACCCGTTTCGCCACTAATCCACTTTAAAATCATTT
CCGAGGAAATCAACTAAGTTTTCATCGTTTCGACTTGCATGTGTTTATGCACGCCGCCAGCGTTTTATCCTGA
GCCAGGATCAAACTCT
>311
CTGCTGCCGCCCGTAGGAGTTTTGGACCGTGTCTCAGTTCCAATGTGGGGGACCTTCCTCTCAGAACCCCTAC
TGATCGTTGCCTTGGTGGGCCGTTACCCCGCCAACAAGCTAATCAGACGCATCCCCATCCATAACCGATAAAT
CTTTATTCATAATCTCATGAGATCAAACGAATACATAAGGTATTAGTCCAACTTCGCTGGGTTATCCCTTAGT
TTATGGGCAGGTTTGGATACGCGTTACTCACCCGTGCGCCGGTCGCCGACATAAATAGCAAGCTATCTATTCG
CTGCCCCTCGACTTGCATGTGTTTAAGCCTATAGCTAGCGTTCATCCTGAGCCAGGATCAAACTCT
>312
CTGCTGCCGCCCGTAGGAGTTTGGACCGTGTCTCAGTTCCAATGTGGGGGACCTTCCTCTCAGAACCCCTACT
GATCGTCGCCTTGGTGGGCCGTTACCCCGCCAACAAGCTAATCAGACGCATCCCCATCCATAACCGCTAACCT
TTAAATCGTTATTTGATGTCAGCAAACGATATCATACGGTATTAGGCCGTCTTTTCAACGGGTTATCCCGTAG
TTTATGGGAAGGTTGGATACGCGTTACTCACCCGTGCGCCAGTCGTCATCAAGGAAAGCAAGCTTTCCCTCAT
GTTTACCCTGCGACTTGCATGTGTTAAGCCTGTAGCTAGCGTTCATCCTGAGCCAGGATCAAACTCT
>313
CTGCTGCCGCCCGTAGGAGTTTGGACCGTGTCTCAGTTCCAATGTGGGGGACCTTCCTCTCAGAACCCCTACT
GATCGTCGCCTTTGGTGGGCCGTTACCCCGCCAACAAGCTAATCAGACGCATCCCCATCCATAACCGCTAAAC
CTTTAATCGTTATTTTGATGTCAGCAAACGATATCATACGGTATTAGGCCGTCTTTCAACGGGTTGTCCCGTA
GTTTATGGGAAGGTTGGATACGCGTTACTCACCCGTGCGCCAGTCGTCATCAAGGAAAGCAAGCTTTCCCTGA
CTGATTTT
>314
CTGCTGCCGCCCGTAGGAGTTAGGACCGTGTCTCAGTTCCTATGTGGGGGGCCTTCCTCTCAGAACCCCTACC
GATCGAAGCCTTGGTGGGCCGTTACCTCACCAACAAGCTAATCGGACGCATCCCCATCCATAAGCGGCAAGCC
TTTGGTTCAAATCTGATGCCGTCTTTGAACAACATAGGGGATTAGTCCGCATTTCTGCGGGTTTATACCCTAC
TTATGGGCAGGTTGGATACGCGTTACTCACCCGTGCGCCGGTCGACGTCCAAACGGTGCAAGCACCAAATGCC
GTTTCCCCTCGACTTGCATGTGTTTAAGCCTATAGCTAGCGTTCATCCTGAGCCAAGGATCAAACTCT
>315
CTGCTGCCGCCCGTAGGAGTTTTGGACCGTGTCTCAGTTCCAATGTGGGGGACCTTCCTCTCAGAACCCCTAC
TGATCGTTGCCTTTGGTGGGCCGTTACCCCGCCAACAAGCTAATCAGACGCATCCCCATCCATAACCGATAAA
TCTTATTCGTAATCTCATGAGATCAAACGAAATACATAAGGTATTAGTCCAACTTCGCTGGGTTATCCCTTAG
TTATGGGCAGGTTGGATACGCGTTACTCACCCGTGCGCCGGTCGCCGACATAAATAGCAAGCTATCTATTCGC
TGCCCCTCGACTTGCATGTGTTAAGCCTATAGCTAGCGTTCATCCTGAGCCAGGATCAAACTCT

>316
CTGCTGCCGCCCGTAGGAGTTTTGGACCGTGTCTCAGTTCCAATGTGGGGGACCTTCCTCTCAGAACCCCTAC
TGATCGTTGCCTTTGGTGGGCCGTTACCCCGCCAACAAGCTAATCAGACGCATCCCCATCCATAACCGATAAA
TCTTTATTCGTAATCTCATGAGATCAAACGAAATACATAAGGTATTAGTCCAACTTCGCTGGGTTATCCCTTA
GTTATGGGCAGGTTGGATACGCGTTACTCACCCGTGCGCCGGTCGCCGACATAAATAGCAAGCTATCTATTCG
CTGCCCCTCGACTTGCATGTGTTAAGCCTATAGCTAGCGTTCATCCTGAGCCAGGATCAAACTCT
>317
CTGCTGCCGCCCTAGGAGTTTGAACCGTGTCTCAGTTCCAATGTGGGGGACCTTCCTCTCAGAACCCCTACTG
ATCGTTACCTTGTGGGCCGTTACCCCACCAACAAGCTAATCAGACGCATCCCCATCCATAACCGATAAATCTT
TATTCATAATCTCATGAGATCAAACGAATACATAAGGTATTAGTCCAACTTCGCTGGGTTATCCCTTAGTTAT
GGGCAGGTTGGATACGCGTTACTCACCCGTGCGCCAGTCGTCATCAAGGAAAGCAAGCTTTCCTCATGTTACC
CTGCGACATGCATGTGTTAAGCCTGTAGCTAGCGTTCATCCTGAGCCAGGATCAAACTCT
>318
CTGCTGCCGCCCGTAGGAGTTTTGGACCGTGTCTCAGTTCCAATGTGGGGGACCTTCCTCTCAGAACCCCTAC
TGATCGTCGCCTTTGGTGGGCCGTTACCCCGCCAACAAGCTAATCAGACGCATCCCCATCCATAACCGCTAAA
CCTTTAATCGTTATTTGATGTCAGCAAACGATATCATACGGTATTAGGCCGTCTTTCAACGGGTTATCCCGTA
GTTATGGGAAGGTTGGATACGCGTTACTCACCCGTGCGCCAGTCGTCATCAAGGAAAGCAAGCTTTCCTCATG
TTACCCTGCGACATGCATGTGTTAAGCCTGTAGCTAGCGTTCATCCCGTGAGCCAAGGCATCAAAC
>319
CTGCTGCCGCCCGTAGGAGTTTGGACCGTGTCTCAGTTCCAATGTGGGGGACCTTCCTCTCAGAACCCCTACT
GATCGTCGCCTTTGGTGGGCCGTTACCCCGCCAACAAGCTAATCAGACGCATCCCCATCCATAACCGCTAAAC
CTTTAATCGTTATTTTGATGTCAGCAAACGATATCATACGGTATTTAGGCCGCCTTTCAACGGGTTTATCCCG
TAGTTTATGGGAAGGTTTGGATACGCGTTACTCACCCGTGCGCCAGTCGTCATCAAGGAAAGCAAGCTTTCCT
CATGTTTACCCTGCGACTTGCATGTGTTTAAGCCTGTAGCTAGCGTTCATCCTGAGCCAGGATCAAACTCT
>320
CTGCTGCCGCCCGTAGGAGTTTTGGACCGTGTCTCAGTTCCAATGTGGGGGACCTTCCTCTCAGAACCCCTAC
TGATCGTCGCCTTTGGTGGGCCGTTACCCCGCCAACAAGCTAATCAGACGCATCCCCATCCATAACCGCTAAA
CCTTTAATCGTTATTGATGTCAGCAAACGACGTCATACGGTATTAGTCCGTCTTTCAACGGGTTATCCCGTGG
TCATGGGAAGGTTGGATACGCGTTACTCACCCGTGCGCCAGTCGTCGTCAAGGAAAGCAAGCTTCCTCACGTA
CCCTGCGACTTGCATGTGTTAAGCCTGTAGCTAGCGTCATCCTGAGCCAGGATCAAACTCT
>321
CTGCTGCCGCCCGTAGGAGTTTGGACCGTGTCTCAGTTCCAATGTGGGGGACCTTCCTCTCAGAACCCCTACT
GATCGTTGCCTTGGTGGGCCGTTACCCCGCCAACAAGCTAATCAGACGCATCCCCATCCATAACCGCTAAACC
TTTAATCGTTTATTTTGATGTCAGCAAACGATGTCATACGGTATTTAGTCCGTCTTTTCAACGGGTTTATCCC
GTGGTCATGGGAAGGTTGGATACGCGTTACTCACCCGTGCGCCAGTCGTCGTCAAGGAAAGCCAAGCTTTCCT
TCACGTTACCCTGCGACTTTGCACG
>322
CTGCTGCCGCCCGTAGGAGTTTTGGACCGTGTCTCAGTTCCAATGTGGGGGGACCTTCCCTCTCAGAACCCCT
ACTGATCGTCGCCTTTGGTGGGCCGTTACCCCGCCAACAAGCTAATCAGACGCATCCCCATCCATAACCGCTA
AACCTTTAATCGTTATTTGATGTCAGCAAACGATATCATACGGTATTAGGCCGTCTTTCAACGGGTTATCCCG
TAGTTATGGGAAGGTTGGATACGCGTTACTCACCCGTGCGCCAGTCGTCATCAAGGAAAGCAAGCTTTCCCTC
ATGTTACCCTGCGACACTGCTG
>323
CTGCTGCCGCCCGTAGGAGTTTTGGACCGTGTCTCAGTTCCAATGTGGGGGACCTTCCTCTCAGAACCCCTAC
TGATCGTCGCCTTGGTGGGCCGTTACCCCACCAACAAGCTAATCAGACGCATCCCCATCCATAACCGATAAAT
CTTTATTCGTAATCTCATGAGATCAAACGAATACATAAGGTATTAGTCCAACTTCGCTGGGTTATCCCGTAGT
TATGGGAAGGTTGGATACGCGTTACTCACCCGTGCGCCAGTCGTCATCAAGGAAAGCAAGCTTTCCTCATGTT
ACCCTGCGACTTGCATGTGTTAAGCCTGTAGCTAGCGTTCATCCTGAGCCAGGATCAAACTCT
>324
CTGCTGCCGCCCGTAGGAGTTTGGACCGTGTCTCAGTTCCAATGTGGGGGACCTTCCTCTCAGAACCCCTACT
GATCGTCGCCTTGGTGGGCCGTTACCCCGCCAACAAGCTAATCAGACGCATCCCCATCCATCACCGCTAAACC
TTTAATCGTTATTTTGATGTCAGCAAACGATGTCATACGGTATTTAGTCCGTCTTTTCAACGGGTTTATCCCG
TGGTCATGGGAAGGTTGGATACGCGTTACTCACCCGTGCGCCAGTCGTCGTCAAGGGAAGCAAGCTTCCCCTG
ACCGTTACCTGCGACCTTGCATGTGTTAAGCCTGTAGCTAGCGTCATCCCTGAGCA

>325
CTGCTGCCGCCCGTAGGAGTTTGGACCGTGTCTCAGTTCCAATGTGGGGGACCTTCCTCTCAGAACCCCTACT
GATCGTCGCCTTGGTGGGCCGTTACCCCGCCAACAAGCTAATCAGACGCATCCCCATCCATAACCGCTAAACC
TTTAATCGTTATTTGATGTCAGCAAACGATGTCATACGGTATTAGGCCGTCTTTCAACGGGTTATCCCGTAGT
TATGGGAAGGTTGGATACGCGTTACTCACCCGTGCGCCAGTCGTCATCAAGGAAAGCAAGCTTTCCCTCATGT
TTACCCTGCGACTTGCATGTGTTAAGCCTGTAGCTAGCGTTCATCCTGAGCCAGGATCAAACTCT
>326
CTGCTGCCGCCCGTAGGAGTTTTGGACCGTGTCTCAGTTCCAATGTGGGGGACCTTCCTCTCAGAACCCCTAC
TGATCGTCGCCTTTGGTGGGCCGTTACCCCGCCAACAAGCTAATCAGACGCATCCCCATCCATAACCGCTAAA
CCTTTAATCGTTATTTGATGTCAGCAAACGATATCATACGGTATTAGGCCGTCTTTCAACGGGTTATCCCGTA
GTTATGGGAAGGTTGGATACGCGTTACTCACCCGTGCGCCAGTCGTCATCAAGGAAAGCAAGCTTTCCCTCAT
GTTACCCTGCGACTTGCATGTGTTAAGCCTGTAGCTAGCGTTCATCCCTGAGCAGGCATCAAACTCT
>327
CTGCTGCCGCCCGTAGGAGTCTGGACCGTGTCTCAGTTCCAGTGTGGCCGTTCATCCTCTCAGACCGGCTACT
GATCGTTGGTTTGGTGGGCCGTTACCCCACCAACTGCCTAATCAGACGCAAACCCCTCTTCAGGCGATAGCTT
ACAGGTAGAGGCTACCCTTTCTTCCACAGGTCATGCGACCCGTGGAACGTATTCGGTATTAGCAGTCGTTTCC
GTCTGTTGTCCCCATCCTGAAGGCAGGTTGTTTACGTGTTACTCACCCGTTTGCCACTGGATTCGATAAAGAG
CAAGCTCTTCATCT
>328
CTGCTGCCGCCCGTAGGAGTTTGGACCGTGTCTCAGTTCCAATGTGGGGGACCTTCCTCTCAGAACCCCTACT
GATCGTTGCCTTTGGTGGGCCGTTACCCCGCCAACAAGCTAATCAGACGCATCCCCATCCATAACCGATAAAT
CTTTATTCATAATCTCATGAGATCAAACGAATACATAAGGTATTAGTCCAACTTCGCTGGGTTATCCCTTAGT
TATGGGCAGGTTGGATACGCGTTACTCACCCGTGCGCCGGTCGCCGACATAAATAGCAAGCTATCTATTCGCT
GCCCCTCGACTTGCATGTGTTAAGCCTATAGCTAGCGTTCATCCTGAGCCAGGATCAAACTCT
>329
CTGCTGCCGCCCGTAGGAGTTTTGGACCGTGTCTCAGTTCCAATGTGGGGGACCTTTCCTCTCAGAACCCCTA
CTGATCGTCGCCTTTGGTGGGCCGTTACCCCGCCAACAAGCTAATCAGACGCATCCCCATCCATAACCGCTAA
ACCTTTAATCGTTATTTGATGTCAGCAAACGATATCATACGGTATTAGGCCGTCTTTCAACGGGTTATCCCGT
AGTTATGGGAAGGTTGGATACGCGTTACTCACCCGTGCGCCAGTCGTCATCAAGGAAAGCAAGCTTTCCCTCA
TGTTTTACCCTGCCGTACATGCATGTGTTAAGCCTTTGTAGCTAGCTGTTC
>330
CTGCTGCCGCCCTAGGAGTCTGGACCGTGTCTCAGTTCCAGTGTGGCCGTTTCATCCTCTCAGACCGGCTACT
GATCGTTGGTTTGGTGGGCCGTTACCCCACCAACTGCCTAATCAGACGCAAACCCCTCTTCAGGCGATAGCTT
TACAGGTAGAGGCTACCCTTTCTTTCCACAGGTCATGCGACCCGTGGAACGTATTTCGGTATTAGCAGTCGTT
TCCGTCTGTTTGTCCCCATCCTGAAGGCAGGTTTGTTTTACGTGTTACTCACCCGTTTGCCACTGGATTTCGA
TAAAGAGCAAGCTCTTCATCTCTTCCCGTTCGACTTGCATGTGTTTAAGCACGCCGCCAGCGTTCGTCCTGAG
CCAGGATCAAACTCT
>331
CTGCTGCCGCCCGTAGGAGTTTTGGACCGTGTCTCAGTTCCAATGTGGGGGACCTTCCTCTCAGAACCCCTAC
TGATCGTCGCCTTTGGTGGGCCGTTACCCCGCCAACAAGCTAATCAGACGCATCCCCATCCATAACCGCTAAA
CCTTTAATCGTTATTTGATGTCAGCAAACGATATCATACGGTATTAGGCCGTCTTTCAACGGGTTATCCCGTA
GTTATGGGAAGGTTGGATACGCGTTACTCACCCGTGCGCCAGTCGTCATCAAGGAAAGCAAGCTTTCCCTCAT
GTTACCCTGCGACTTGCATGTGTTAAGCCTGTAGCTAGCGTTCATCCCATGAGCCAGGCATCAACTCT
>332
CTGCTGCCGCCCGTAGGAGTTTTGGACCGTGTCTCAGTTCCAATGTGGGGGACCTTCCTCTCAGAACCCCTAC
TGATCGTCGCCTTGGTGGGCCGTTACCCCGCCAACAAGCTAATCAGACGCATCCCCATCCATAACCGCTAAAC
CTTTAATCGTTATTTTGATGTCAGCAAACGATATCATACGGTATTTAGGCCGTCTTTTCAACGGGTTTATCCC
GTAGTTTATGGGAAGGTTGGATACGCGTTACTCACCCGTGCGCCAGTCGTCATCAAGGAAAGCAAGCTTTCCT
CATGTTTACCCTGCGACTTGCATGTGTTTAAGCCTGTAGCTAGCGTTCATCCTGAGCCAGGATCAAACTCT
>333
CTGCTGCCGCCCGTAGGAGTTTTGGACCGTGTCTCAGTTCCAATGTGGGGGACCTTCCTCTCAGAACCCCTAC
TGATCGTCGCCTTTGGTGGGCCGTTACCCCGCCAACAAGCTAATCAGACGCATCCCCATCCATCACCGCTAAA
CCTTTAATCGTTATTTGATGTCAGCAAACGATGTCATACGGTATTAGTCCGTCTTTCAACGGGTTATCCCGTG

GTCATGGGAAGGTTGGATACGCGTTACTCACCCGTGCGCCAGTCGTCGTCAAGGGAAGCAAGCCTTCCCTCAC
GTTACCCTGCGACTTGCATGTGTTAAGCCTGTAGCTAGCGTCATCCCTGAGCCAGGCATCAAACTCT
>334
CTGCTGCCGCCCGTAGGAGTTTTGGACCGTGTCTCAGTTCCAATGTGGGGGACCTTCCTCTCAGAACCCCTAC
TGATCGTCGCCTTGGTGGGCCGTTACCCCGCCAACAAGCTAATCAGACGCATCCCCATCCATCACCGCTAAAC
CTTTTAATCGTTTATTTTGATGTCAGCAAACGATGTCATACGGTATTTAGTCCGTCTTTTTCAACGGGTTTAT
TCCCGTGGTCATGGGAAGGTTTGGATACGCGTTACTCACCCGTGCGCCAGTCGTCGTCAAGGGAAGCAAGCTT
CCCTTCACGTACCCTGCGACTTTGCATGTGTTTTAAGCCTGTAGCTAGCGTTCATCCTGAGCCAAGGATCAAA
CTCT
>335
CTGCTGCCGCCCGTAGGAGTTTTGGACCGTGTCTCAGTTCCAATGTGGGGGACCTTCCTCTCAGAACCCCTAC
TGATCGTTGCCTTGGTGGGCCGTTACCCCACCAACAAGCCTAATCAGACGCATCCCCATCCATAACCGATAAA
TCTTTATTCGTAATCTCATGAGATCAAACGAATATATAAGGTATTAGTCCAACTTCGCTGGGTTATCCCTTAG
TTTATGGGCAGGTTGGATACGCGTTACTCACCCGTGCGCCAGTCGTCATCAAGAAAGCAAGCTTTCCTCATGT
TACCCTGCGACTTGCATGTGTTAAGCCTGTAGCTAGCGTTCATCCTGAGCCAGGATCAAACTCT
>336
CTGCTGCCGCCCGTAGGAGTTTGGACCGTGTCTCAGTTCCAATGTGGGGGACCTTCCTCTCAGAACCCCTACT
GATCGTTGCCTTGGTGGGCCGTTACCCCGCCAACAAGCTAATCAGACGCATCCCCATCCATAACCGATAAATC
TTTATTCATAATCTCATGAGATCAAACGAATACATAAGGTATTAGTCCAACTTCGCTGGGTTATCCCTTAGTT
TATGGGCAGGTTTGGATACGCGTTACCTCACCGTGCGCCGGTCGCCGACATAAATAGCAAGCTATCTATTCGC
TGCCCCCCCCTCGACTTGCATGTGTTTTAAGCCTATAGCTAGCGTTCATCCTGAGCCAGGATCAAACTCT
>337
CTGCTGCCGCCCGTAGGAGTTTGGACCGTGTCTCAGTTCCAATGTGGGGGACCTTCCTCTCAGAACCCCTACT
GATCGTTGCCTTGGTGGGCCGTTACCCCGCCAACAAGCTAATCAGACGCATCCCCATCCATAACCGATAAATC
TTTATTCGTAATCTCATGAGATCAAACGAATACATAAGGTATTAGTCCAACTTCGCTGGGTTATCCCTTAGTT
TATGGGCAGGTTTGGATACGCGTTACTCACCCGTGCGCCGGTCGCCGACATAAATAGCAAGCTATCTATTCGC
TGCCCCTCGACTTGCATGTGTTTAAGCCTTATAGCTAGCGTTCATCCTGAGCCAGGATCAAACTCT
>338
CTGCTGCCGCCCGTAGGAGTTTGGACCGTGTCTCAGTTCCAATGTGGGGGACCTTCCTCTCAGAACCCCTACT
GATCGTCGCCTTTGGTGGGCCGTTACCCCGCCAACAAGCTAATCAGACGCATCCCCATCCATAACCGCTAAAC
CTTTAATCGTTATTTGATGTCAGCAAACGATATCATACGGTATTAGGCCGTCTTTCAACGGGTTATCCCGTAG
TTATGGGAAGGTTGGATACGCGTTACTCACCCGTGCGCCAGTCGTCATCAAGGAAAGCAAGCTTTCCCTCATG
TTACCCTGCGACATGCATGTGTTAAGCCTGTAGCTAGCGTTCATCCTGAGCCAGGATCAAACTCT
>339
CTGCTGCCGCCCGTAGGAGTTTTGGACCGTGTCTCAGTTCCAATGTGGCCGATCACCCTCTCAGGTCGGCTAC
TGATCGTCGCCTTGGTGGGCAGTTATCTCACCAACTAGCTAATCAGACGCGGGTCCATCCTATACCGCTAACA
CTTGATATATCTACGATGCCGTAAATATATATTTATCATGTATTTAGTAAACTTTTCAGTATGTTTATCCATG
TGTATAGGGCAGGTTTACCCACGCGTTACTCACCCGTCCGCCGCTCATCTTCATCAATGCAAGCATCAAATCA
TACGCGCTCGACTTGCATGTGTAGGCACGCCGCCAGCGTTCATCCTGAGCCAGGATCAAACTCT
>340
CTGCTGCCGCCCGTAGGAGTTTTGGACCGTGTCTCAGTTCCAATGTGGGGGACCTTCCTCTCAGAACCCCTAC
TGATCGTTGCCTTTGGTGGGCCGTTACCCCGCCAACAAGCTAATCAGACGCATCCCCATCCATAACCGATAAA
TCTTTATTCGTAATCTCATGAGATCAAACGAAATACATAAGGTATTAGTCCGACTTCGCTGGGTTATCCCTTA
GTTATGGGCAGGTTGGATACGCGTTACTCACCCGTGCGCCGGTCGCCGACATAAATAGCAAGCTATCTATTCG
CTGCCCCTCGACTTGCATGTGTTAAGCCTATAGCTAGCGTTCATCCTGAGCCAGGATCAAACTCT
>341
CTGCTGCCGCCCGTAGGAGTTTTGGACCGTGTCTCAGTTCCAATGTGGGGGACCTTCCTCTCAGAACCCCTAC
TGATCGTCGCCTTTGGTGGGCCGTTACCCCGCCAACAAGCTAATCAGACGCATCCCCATCCATAACCGCTAAA
CCTTTAATCGTTATTTGATGTCAGCAAACGATATCATACGGTATTAGGCCGTCTTTCAACGGGTTATCCCGTA
GTTATGGGAAGGTTGGATACGCGTTACTCACCCGTGCGCCAGTCGTCATCAAGGAAAGCAAGCTTTCCCTCAT
GTTACCCTGCGGAACTTGCATGTGTTAAGCCTGTAGCTAGCGTTCATCCCGTGAGCCAGGCATCAAACATC
>342
CTGCTGCCGCCCGTAGGAGTTTGGACCGTGTCTCAGTTCCAATGTGGCCGATCACCCTCTCAGGTCGGCTACT
GATCGTCGCCTTGGTGGGCAGTTATCTCACCAACTAGCTAATCAGACGCGGGTCCATCCTATACCGCTAACAC

TTTGATATATCTACGATGCCGTAAATATATATTATCATGTATTAGTAAACTTTTCAGTATGTTATCCATGTGT
ATAGGGCAGGTTACCCACGCGTTACTCACCCGTCCGCCGCTCATCTTCATCAATGCAAGCATCAAATCATACG
CGCTCGACTTGCATGTGTTAGGCACGCCGCCAGCGTTCATCCTGAGCCAGGATCAAACTCT
>343
CTGCTGCCGTCCGTAGGAGTTTTGGACCGTGTCTCAGTTCCAATGTGGGGGACCTTCCTCTCAGAACCCCTAC
TGATCGTCGCCTTGGTGGGCCGTTACCCCGCCAACAAGCTAATCAGACGCATCCCCATCCATCACCGCTAAAC
CTTTTAATCGTTATTTTGATGTCAGCAAACGATGTCATACGGTATTTAGTCCGTCTTTTCAACGGGTTTATCC
CGTGGTCATGGGAAGGTTGGATACGCGTTACTCACCCGTGCGCCAGTCGTCGTCAAGGGAAGCAAGCTTCCCT
CACGTTACCCTGCGACTTGCCATGTGTTAAGCCTTTGTAGCTAGCGTCATCCTGAGCCAGGATCAAACTCT
>344
CTGCTGCCGCCCGTAGGAGTTTGGACCGTGTCTCAGTTCCAATGTGGGGGACCTTCCTCTCAGAACCCCTACT
GATCGTGCCTTTGGTGGGCCGTTACCCCGCCAACAAGCTAATCAGACGCATCCCCATCCATAACCGCATAACC
TTTAATCGTTATTTGATGTCAGCAAACGATATCATACGGTATTAGGCCGTCTTTCAACGGGTTATCCCGTAGT
TATGGGAAGGTTGGATACGCGTTACTCACCCGTGCGCCAGTCGTCATCAAGGAAAGCAAGCTTCCTCATGTTA
CCCTGCGACTGCACT
>345
CTGCTGCCGCCCGTAGGAGTTTGGACCGTGTCTCAGTTCCAATGTGGGGGACCTTCCTCTCAGAACCCCTACT
GATCGTTGCCTTGGTGGGCCGTTACCCCGCCAACAAGCTAATCAGACGCATCCCCATCCATCACCGCTAAACC
TTTAATCGTTATTTGATGTCAGCAAACGATGTCATACGGTATTAGTCCGTCTTTCAACGGGTTATCCCGTGGT
CATGGGAAGGTTGGATACGCGTTACTCACCCGTGCGCCAGTCGTCGTCAAGGGAAGCAAGCTTCCCTCACGTT
ACCCTGCGACTTGCATGTGTTAAGCCTGTAGCTAGCGTTCATCCTGAGCCAGGATCCAAACTCGT
>346
CTGCTGCCGCCCGTAGGAGTTTTGGACCGTGTCTCAGTTCCAATGTGGGGGACCTTCCTCTCAGAACCCCTAC
TGATCGTCGCCTTTGGTGGGCCGTTACCCCGCCAACAAGCTAATCAGACGCATCCCCATCCATCACCGCTAAC
CTTTAATCGTTTATTTGATGTCAGCAAACGATATCATACGGTATTTAGGCCGTCTTTCAACGGGTTTATTCCC
GTAGTTTATGGGAAGGTTGGATACGCGTTACTCACCCGTGCGCCAGTCGTCATCAAGGAAAGCAAGCTTCCTT
CATGTTTACCCTGCGACTTGCATGTCGTTTAAGCACGCCGCCAGCCGGTTTC
>347
CTGCTGCCGCCCGTAGGAGTTTTGGACCGTGTCTCAGTTCCAATGTGGGGGACCTTCCTCTCAGAACCCCTAC
TGATCGTCGCCTTGGTGGGCCGTTACCCCGCCAACAAGCTAATCAGACGCATCCCCATCCATAACCGCTAAAC
CTTTAATCGTTTATTTTGATGTCAGCAAACGATATCATACGGTATTTAGGCCGTCTTTTCAACGGGTTTATCC
CGTGGTCATGGGAAGGTTGGATACGCGTTACTCACCCGTGCGCCAGTCGTCGTCAAGGGAAGCAAGCTTCCCC
TTCACGTTACCCTGCGAACTTTGCCATGTGTTTAACGCCTTGTAGCTAGCGTTCATCCCTGAGCCAAGGATCA
AACTCT
>348
CTGCTGCCGCCCGTAGGAGTTTTGGACCGTGTCTCAGTTCCAATGTGGGGGACCTTCCTCTCAGAACCCCTAC
TGATCGTCGCCTTTGGTGGGCCGTTACCCCGCCAACAAGCTAATCAGACGCATCCCCATCCATCACCGCTAAA
CCTTTAATCGTTATTTGATGTCAGCAAACGATGTCATACGGTATTAGTCCGTCTTTCAACGGGTTATCCCGTG
GTCATGGGAAGGTTGGATACGCGTTACTCACCCGTGCGCCAGTCGTCGTCAAGGGAAGCAAGCTTCCCTCACG
TTACCCTGCGACTTGCATGTGTTAAGCCTGTAGCTAGCGTTCATCCCGTGAGCCAAGGCATCAAACT
>349
CTGCTGCCGCCCGTAGGAGTTTGGACCGTGTCTCAGTTCCAATGTGGGGGACCTTCCTCTCAGAACCCCTACT
GATCGTCGCCTTGGTGGGCCGTTACCCCGCCAACAAGCTAATCAGACGCATCCCCATCCATCACCGCTAAACC
TTTAATCGTTTATTTTGATGTCAGCAAACGATGTCATACGGTATTTAGTCCGTCTTTTCAACGGGTTTATCCC
GTGGTCATGGGAAGGTTGGATACGCGTTACTCACCCGTGCGCCAGTCGTCGTCAAGGGAAGCAAGCTTCCCTT
CACCGGTTAAC
>350
CTGCTGCCGCCCGTAGGAGTTTTGGACCGTGTCTCAGTTCCAATGTGGGGGACCTTCCTCTCAGAACCCCTAC
TGATCGTTGCCTTTGGTGGGCCGTTACCCCGCCAACAAGCTAATCAGACGCATCCCCATCCATAACCGATAAA
TCTTTATTCGTAATCTCATGAGATCAAACGAATACATAAGGTATTAGTCCAACTTCGCTGGGTTATCCCTTAG
TTATGGGCAGGTTGGATACGCGTTACTCACCCGTGCGCCGGTCGCCGACATAAATAGCAAGCTATCTATTCGC
TGCCCCTCGACTTGCATGTGTTAAGCCTATAGCTAGCGTTCATCCTGAGCCAGGATCAACGACTAT
>351

CTGCTGCCGCCCGTAGGAGTTTGGACCGTGTCTCAGTTCCAATGTGGGGGACCTTCCTCTCAGAACCCCTACT
GATCGTTGCCTTGGTGGGCCGTTACCCCGCCAACAAGCTAATCAGACGCATCCCCATCCATAACCGATAAATC
TTTATTCATAATCTCATGAGATCAAACGAATACATAAGGTATTAGTCCAACTTCGCTGGGTTATCCCTTAGTT
ATGGGCAGGTTTGGATACGCGTTACTCACCCGTGCGCCGGTCGCCGACATAAATAGCAAGCTATCTATTCGCT
GCCCCCCTCGACTTGCATGTGTTTAAGCCTATAGCTAGCGTTCATCCTGAGCCAGGATCAAACTCT
>352
CTGCTGCCGCCCGTAGGAGTTTGGACCGTGTCTCAGTTCCAATGTGGGGGACCTTCCTCTCAGAACCCCTACT
GATCGTTGCCTTGGTGGGCCGTTACCCCGCCAACAAGCTAATCAGACGCATCCCCATCCATAACCGATAAATC
TTTATTCGTAATCTCATGAGATCAAACGAATACTAAGGTATTAGTCCAACTTCGCTGGGTTATCCCTTAGTTA
TGGGCAGGTTGGATACGCGTTACTCACCCGTGCGCCGGTCGCCGACATAAATAGCAAGCTATCTATTCGCTGC
CCCTCGACTTGCATGTGTTAAGCCTATAGCTAGCGTTCATCCTGAGCCAGGATCAAACTCT
>353
CTGCTGCCGCCCGTAGGAGTTTTGGACCGTGTCTCAGTTCCAATGTGGGGGACCTTCCTCTCAGAACCCCTAC
TGATCGTTGCCTTGGTAGGCCGTTACCCCACCAACAAGCTAATCAGACGCATCCCCCATCCATAACCGATAAA
TCTTTATTCGTAATCTCATGAGATCAAACGAATATATAAGGTATTAGTCCAACTTCGCTGGGTTATCCCTTAG
TTATGGGCAGGTTGGATACGCGTTACTCACCCGTGCGCCGGTCGCCGACATAAATAGCAAGCTATCTATTCGC
TGCCCCTCGACTTGCATGTGTTAAGCCTATAGCTAGCGTTCATCCTGAGCCAGGATCAAACTCT
>354
CTGCTGCCGCCCGTAGGAGTTTGGACCGTGTCTCAGTTCCAATGTGGGGGACCTTCCTCTCAGAACCCCTACT
GATCGTTGCCTTGGTGGGCCGTTACCCCGCCAACAAGCTAATCAGACGCATCCCCATCCATAACCGATAAATC
TTTATTCGTGAATCTCATGAGATCAAACGAATACATAAGGTATTAGTCCAACTTCGCTGGGTTATCCCTTAGT
TATGGGCAGGTTTGGATACGCGTTACTCACCCGTGCGCCGGTCGCCGACATAAATAGCAAGCTATCTATTCGC
TGCCCCCCCTCGACTTGCATGTGTTTTAAGCCTATAGCTAGCGTTCATCCTGAGCCAGGATCAAACTCT
>355
CTGCTGCCGCCCGTAGGAGTTTTGGACCGTGTCTCAGTTCCAATGTGGCCGATCACCCTCTCAGGTCGGCTAC
TGATCGTCGCCTTGGTGGGCAGTTATCTCACCAACTAGCTAATCAGAACGCGGGTCCATCCTATACCGCTAAC
ACTTTGATATATCTACGATGCCGTAAATATATATTATCATGTATTAGTAAACTTTTCATATGTTATCCATGTG
TATAGGGCAGGTTACCCACGCGTTACTCACCCGTCCGCCGCTCATCTTCATCAATGCAAGCATCAAATCATAC
GCGCTCGACTTGCATGTGTTAGGCACGCCGCCAGCGTTCATCCTGAGCCAGGATCAAACTCT
>356
CTGCTGCCGCCCGTAGGAGTTTGGACCGTGTCTCAGTTCCAATGTGGGGGACCTTCCTCTCAGAACCCCTACT
GATCGTTGCCTTGGTGGGCCGTTACCCCGCCAACAAGCTAATCAGACGCATCCCCATCCATAACCGATAAATC
TTTATTTCATAATCTCATGAGATCAAACGAATACATAAGGTATTTAGTCCAACTTCGCTGGGTTTATCCCTTA
GTTTATGGGCAGGTTTGGATACGCGTTACTCACCCGTGCGCCGGTCGCCGACATAAATAGCAAGCTATCTATT
TCGCTGCCCCCCCCTCGACTTGCATGTGTTTTAAGCCTATAGCTAGCGTTCATCCTGAGCCAGGATCAAACTC
T
>357
CTGCTGCCGCCCGTAGGAGTTTGAACCGTGTCTCAGTTCCAATGTGGGGGACCTTCCTCTCAGAACCCCTACT
GATCGTTGCCTTTGGTGGGCCGTTACCCCGCCAACAAGCTAATCAGACGCATCCCCATCCATAACCGATAAAT
CTTTATTCGTAATCTCATGAGATCAAACGAAATACATAAGGTATTAGTCCAACTTCGCTGGGTTATCCCTTAG
TTATGGGCAGGTTTGGATACGCGTTACTCACCCGTGCGCCGGTCGCCGACATAAATAGCAAGCTATCTATTCG
CTGCCCCTCGACTTGCATGTGTTTAAGCCTATAGCTAGCGTTCATCCTGAGCCAGGATCAAACTCT
>358
CTGCTGCCGCCCGTAGGAGTTTGGACCGTGTCTCAGTTCCAATGTGGGGGACCTTCCTCTCAGAACCCCTACT
GATCGTCGCCTTGGTGGGCCGTTACCCCGCCAACAAGCTAATCAGACGCATCCCCATCCATCACCGCTAAACC
TTTAATCGTTATTTTGATGTCAGCAAACGATATCATACGGTATTAGGCCGTCTTTCAACGGGTTATCCCGTAG
TTATGGGAAGGTTGGATACGCGTTACTCACCCGTGCGCCAGTCGTCATCAAGGAAAGCAAGCTTTCCCTCATG
TTTACCCTGCGACTTGCATGTGTTAAGCCTGTAGCTAGCGTTCATCCTGAGCCAGGATCAAACTCT
>359
CTGCTGCCGCCCGTAGGAGTTTGGACCGTGTCTCAGTTCCAATGTGGGGGACCTTCCTCTCAGAACCCCTACT
GATCGTCGCCTTGGTGGGCCGTTACCCCGCCAACAAGCTAATCAGACGCATCCCCATCCATAACCGCTAAACC
TTTAATCGTTATTTGATGTCAGCAAACGATATCATACGGTATTAGGCCGTCTTTCAACGGGTTATCCCGTAGT
TATGGGAAGGTTGGATACGCGTTACTCACCCGTGCGCCAGTCGTCATCAAGGAAGCAAGCATTCCCTTCATGA
TTGCCATGCCGACGTTGCACT

>360
CTGCTGCCGCCCGTAGGAGTTTGGACCGTGTCTCAGTTCCAATGTGGGGGACCTTCCTCTCAGAACCTCTACT
GATCGTTGCCTTGGTGGGCCGTTACCCCACCAACAAGCTAATCAGACGCATCCCCATCCATAACCGATAAATC
TTTATTCGTAATCTCATGAGATCAAACGAATATATAAGGTATTAGTCCAACTTCGCTGGGTTATCCCTTAGTT
TATGGGCAGGTTTGGATACGCGTTACTCACCCGTGCGCCGGTCGCCGACATAAATAGCAAGCTATCTATTCGC
TGCCCCCCTCGACTTGCATGTGTTTAAGCCTATAGCTAGCGTTTCATCCTGAGCCAGGTCAAACTCT
>361
CTGCTGCCGCCCGTAGGAGTTTGGACCGTGTCTCAGTTCCAATGTGGGGGACCTTCCTCTCAGAACCCCTACT
GATCGTTGCCTTTGGTGGGCCGTTACCCCGCCAACAAGCTAATCAGACGCATCCCCATCCATAACCGATAAAT
CTTTTATTTCGTAATCTCATGAGATCAAACGAAATACATAAGGTATTTAGTCCAACTTCGCTGGGTTTATCCT
TTAGTTTATGGGCAGGTTTGGATACGCGTTACTCACCCGTGCGCCGGTAGCCGACATAAATAGCAAGCTATCT
ATTTCGCTGCCCCCCCTCGACTTGCATGTGTTTTAAGCCTGTAGCTAGCGTTCATCCTGAGCCAGGATCAAAC
TCT
>362
CTGCTGCCGCCCGTAGGAGTTTGGACCGTGTCTCAGTTCCAATGTGGGGGACCTTCCTCTCAGAACCCCTACT
GATCGTCGCCTTGGTGGGCCGTTACCCCGCCAACAAGCTAATCAGACGCATCCCCATCCATCACCGCTAAACC
TTTAATCGTTATTTTGATGTCAGCAAACGATATCATACGGTATTAGGCCGTCTTTTCAACGGGTTATCCCGTA
GTTATGGGAAGGTTGGATACGCGTTACTCACCCGTGCGCCGGTCGCCGACATAAATAGCAAGCTATCTATTCG
CTGCCCCTCGACTTGCATGTGTTTAAGCCTATAGCTAGCGTTCATCCTGAGCCAGGATCAAACTCCT
>363
CTGCTGCCGCCCGTAGGAAGTTTGGACCGTGTCTCAGTTCCAATGTGGGGGACCTTCCTCTCAGAACCCCTAC
TGATCGTCGCCTTTGGTGGGCCGTTACCCCGCCAACAAGCTAATCAGACGCATCCCCATCCATCACCGCTAAA
CCTTAATCGTTATTTTGATGTCAGCAAACGATGTCATACGGTATTTAGTCCGTCTTTTCAACGGGTTATCCCG
TGGTCATGGGAAGGTTGGATACGCGTACTCACCCGTGCGCCAGCGTCGTCAAGGGAAGCAAGCTCCCTCACGT
ACCCTGCGACTGCATGTGTAAGCCTGTAGCTAGCGTCATCCTGAGCCAGGATCAACTCT
>364
CTGCTGCCGCCCGTAGGAGTTTTGGACCGTGTCTCAGTTCCAATGTGGGGGACCTTCCTCTCAGAACCCCTAC
TGATCGTCGCCTTTGGTGGGCCGTTTACCCCGCCAACAAGCTAATCAGACGCATCCCCATCCATCACCGCTAA
ACCTTTAATCGTTTATTTGATGTCAGCAAACGATGTCATACGGTATTAGTCCGTCTTTTCAACGGGTTATCCC
GTGGTCATGGGAAGGTTGGATACGCGTTACTCACCCGTGCGCCGGTCGCCGACATAAATAGCAAGCTATCTAT
CGCTGCCCCTCGACTTGCATGTGTTTAAGCCTTATAGCTAGCGTTCACCTGAGCCAGGATCAAACTCCT
>365
CTGCTGCCGCCCGTAGGAGTTTTGGACCGTGTCTCAGTTCCAATGTGGGGGACCTTTCCTCTCAGAACCCCTA
CTGATCGTCGCCTTTGGTGGGCCGTTACCCCGCCAACAAGCTAATCAGACGCATCCCCATCCATAACCGCTAA
ACCTTTAATCGTTATTTGATGTCAGCAAACGATATCATACGGTATTAGGCCGTCTTTCAACGGGTTATCCCGT
AGTTATGGGAAGGTTGGATACGCGTTACTCACCCGTGCGCCAGTCGTCATCAAGGAAAGCAAGCTTTCCCTCA
TGTTACCCTGCGAACTTGCATGTGTTAAGCCTGTAGCTAGCGTTCATCCCTGAGCAAGGCATCAAACT
>366
CTGCTGCCGCCCGTAGGAGTTTTGGACCGTGTCTCAGTTCCAATGTGGCCGATCACCCTCCTCGAGTCGGCTA
CTGATCGTCGCCTTGGTGGGCAGTTATCTCACCAACTAGCTAATCAGACGCGGGTCCATCCTATACCGCTAAC
ACTTTGATATATCTACGATGCCGTAAATATATATTTATCATGTATTTAGTAAACTTTTCAGTATGTTTATCCA
TGTGTATAGGGCAGGTTTACCCCCACGCGTTACTCACCCCGTCCGCCGCTCATCTTTCATTTAATGCAAGCAT
CAAATCAGACGCGCTCGACTTGCATGTGTTAGGCACGCCGCCAGCGTTCATCCTGAGCCAGGATCAAACTCT
>367
CTGCTGCCGCCCGTAGGAGTTTTGGACCGTGTCTCAGTTCCAATGTGGGGGACCTTCCTCTCAGAACCCCTAC
TGATCGTCGCCTTTGGTGGGCCGTTACCCCGCCAACAAGCTAATCAGACGCATCCCCATCCATAACCGCTAAA
CCTTTAATCGTTATTTGATGTCAGCAAACGATATCATACGGTATTAGGCCGTCTTTCAACGGGTTATCCCGTA
GTTATGGGAAGGTTGGATACGCGTTACTCACCCGTGCGCCAGTCGTCATCAAGGAAAGCAAGCCTTTCCCTAG
ACTGTTACCCTGCGAACTTGCATGTGTTAAGCCTGTAGCTAGCGTTCATCCGTGACGCCAAGGCAT
>368
CTGCTGCCGCCCGTAGGAGTTTGGACCGTGCCTCAGTTCCAATGTGGGGGACCTTCCTCTCAGAACCCCTACT
GATCGTTGCCTTGGTGGGCCGTTACCCCGCCAACAAGCTAATCAGACGCATCCCCATCCATAACCGATAAATC
TTTATTCGTAATCTCATGAGATCAAACGAATACATAAGGTATTAGTCCAACTTCGCTGGGTTTATCCCTTAGT

TATGGGCAGGTTTGGATACGCGTTACTCACCCGTGCGCCGGTCGCCGACATAAATAGCAAGCTATCTATTCGC
TGCCCCCCTCGACTTGCTGATGTTGTAAGCCTATAGCTAGCGTTCATCCTTGAGCCAGGATCAAACTCT
>369
CTGCTGCCGCCCGTAGGAGTCTGGGCCGTATCTCAGTCCCAATGTGGCCGCACACCCTCTCAGGCCGGCTACC
CGTCGCCGCCTTGGTAGGCCATTACCCCACCAACAAGCTGATAGGCCGCAAGCTCATCCCACACCAGAAAAAC
CTTTCCACCACACCAACCACGATGCGGTCCTATAGGGTATAGACCCAGTTTCCCAAGCTTATCCCCCAGTGCA
GGGCAGATCCACTTACGTGTACTCACCCGTTCGCCACTCGAGCACCCCAGCAAGCTGGGCTCCGTTCGACTAC
TGCGTAGCT
>370
CTGCTGCCGCCCGTAGGAGTTTGGACCGTGTCTCAGTTCCAATGTGGGGGACCTTCCTCTCAGAACCCCTACT
GATCGTCGCCTTGGTGGGCCGTTACCCCGCCAACAAGCTAATCAGACGCATCCCCATCCATAACCGATAAATC
TTTATTCGTAATCTCATGAGATCAAACGAAATACATAAGGTATTAGTCCAACTTCGCTGGGTTATCCCTTAGT
TATGGGCAGGTTGGATACGCGTTGCTCACCCGTGCGCCGGTCGCCGACATAAATAGCAAGCTATCTATTCGCT
GCCCCTCGACTTGCATGTGTTTAAGCCTATAGCTAGCGTTCATCCTGAGCCAGGATCAAACTCT
>371
CTGCTGCCGCCCGTAGGAGTTTTGGACCGTGTCTCAGTTCCAATGTGGGGGACCTTCCTCTCAGAACCCCTAC
TGATCGTTGCCTTTGGTGGGCCGTTACCCCGCCAACAAGCTAATCAGACGCATCCCCATCCATAACCGATAAA
TCTTTATTCATAATCTCATGAGATCAAACGAATACATAAGGTATTAGTCCAACTTCGCTGGGTTATCCCTTAG
TTATGGGCAGGTTGGATACGCGTTACTCACCCGTGCGCCGGTCGCCGACATAAATAGCCAAGCTATCTATTCG
CTGCCCCTCGACTTGCATGTGTTAACGCCTATAGCTAGCGTTCATCCTGAGCCAGGATCAAACTCT
>372
CTGCTGCCGCCCGTAGGAGTTTGGACCGTGTCTCAGTCCCAATGTGGGGGACCTTCCTCTCAGAACCCCTACT
GATCGTTGCCTTGGTGGGCCGTTACCCCGCCAACAAGCTAATCAGACGCATCCCCATCCATAACCGATAAATC
TTTATTCGTAATCTCATGAGATCAAACGAATACATAAGGTATTAGCCCAACTTCGCTGGGTTATCCCTTAGTT
ATGGGCAGGTTGGATACGCGTTACTCACCCGTGCGCCGGTCGCCGACATAAATAGCAAGCTATCTATTCGCTG
CCCCTCGACTTGCATGTGTTAAGCCTATAGCTAGCGTTCATCCTGAGCCAGGATCAAACTCT
>373
CTGCTGCCGCCCGTAGGAGTTTGGACCGTGTCTCAGTTCCAATGTGGGGGACCTTCCTCTCAGAACCCCTACT
GATCGTTGCCTTGGTGGGCCGTTACCCCGCCAACAAGCTAATCAGACGCATCCCCATCCATAACCGATAAATC
TTTATTTCATAATCTCATGAGATCAAACGAATACATAAGGTATTTAGTCCAACTTTCGCTGGGTTTATCCCTT
AGTTTATGGGCAGGTTTGGATACGCGTTACTCACCCGTGCGCCGGTCGCCGACATAAATAGCAAGCTATCTAT
TTCGCTGCCCCCCCTCGACTTGCATGTGTTTTAAGCCTTATAGCTAGCGTTCATCCTGAGCCAGGATCAAACT
CT
>374
CTGCTGCCGCCCGTAGGAGTTTGGACCGTGTCTCAGTTCCAATGTGGGGGACCTTCCTCTCAGAACCCCTACT
GATCGTCGCCTTTGGTGGGCCGTTACCCCGCCAACAAGCTAATCAGACGCATCCCCATCCATAACCGCTAAAC
CTTTTAATCGTTTTATTTTGATGTCAGCAAACGATATCATACGGTATTTAGGCCGTCTTTTCAACGGGTTTAT
TCCCGTAGTTTTATGGGAAGGTTTGGATACGCGTTACTCACCCGTGCGCCAGTCGTCATCAAGGAAAGCAAGC
TTTTCCCTTCATGTTTTACCCTGCGACATGCATGTGTTTTAAGCCTTGTAG
>375
CTGCTGCCGCCCGTAGGAGTTTGGACCGTGTCTCAGTTCCAATGTGGGGGACCTTCCTCTCAGAACCCCTACT
GATCGTTGCCTTGGTGGGCCGTTACCCCACCAACAAGCCTAATCAGACGCATCCCCATCCATAACCGATAAAT
CTTTTATTTCGTAATCTCATGAGATCAAACGAATATATAAGGTATTTAGTCCAACTTCGCTGGGTTATCCCTT
AGTTTATGGGCAGGTTTGGATACGCGTTACTCACCCGTGCGCCGGTCGCCGACATAAATAGCAAGCTATCTAT
TCGCTGCCCCCCTTCGACTTGCATGTGTTTAAGCCTTATAGCTAGCGTTCATCCTTGAGCAGGATCAAACTCT
>376
CTGCTGCCGCCCGTAGGAGTTTGAACCGTGTCTCAGTTCCAATGTGGGGGACCTTCCTCTCAGAACCCCTACT
GATCGTTGCCTTGGTGGGCCGTTACCCCACCAACAAGCTAATCAGACGCATCCCCATCCATAACCGATAAATC
TTTATTCGTAATCTCATGAGATCAAACGAATATATAAGGTATTAGTCCAACTTCGCTGGGTTATCCCTTAGTT
ATGGGCAGGTTGGATACGCGTTACTCACCCGTGCGCCGGTCGCCGACATAAATAGCAAGCTATCTATTCGCTG
CCCCTCGACTTGCATGTGTTAAGCCTATAGCTAGCGTTCATCCTGAGCCAGGATCAAACTCT
>377
GCCGCCCGTAGGAGTTTGGGCCGTGTCTCAGTCCCAATGTGGCCGTACACTCTCTCAAGCCGGCTACTGATCG
TCGCCTTGGTGAGCTTTTATCTCACCAACTAGCTAATCAGACGCAAGTCCATCTTACACCGATAAATCTTTGG

TCACTCTATCATGCGATAAATTGACTTTATAGGGTATTATTCTTCGTTTCCAAAGGCTATCCCCTTGTGTAAG
GCAGGTTACTCACGTGTTACTCACCCGTTCGCCACTAATCCACTTAAAATCATTCCGAAGAAATCAACTAAGT
TTCATCGTTCGACTTGCATGTGTTATGCACGCCGCCAGCGTTCGTCCTGAGCCAGGATCAAACTCT
>378
CTGCTGCCGCCCGTAGGAGTTTGGACCGTGTCTCAGTTCCAATGTGGGGGACCTTCCCTCTCAGAACCCCTAC
TGATCGTTGCCTTGGTGGGCCGTTACCCCGCCAACAAGCTAATCAGACGCATCCCCATCCATAACCGATAAAT
CCTTTATTCATAATCTCATGAGATCAAACGAATACATAAGGTATTAGTCCAACTTCGCTGGGTTATCCCTTAG
TTATGGGCAGGTTGGATACGCGTTACCTCGACCCGTGCGCCGGTCGCCGACATAAATAGCCAAGCTATCTATT
CGCGTCCCCTCGACTTGCATGTGTTAACGCCTATAGCTAGCGTTCATCCTGAGCCAGGATCAAACTCCT
>379
CTGCTGCCGCCCGTAGGAGTTTGAACCGTGTCTCAGTTCCAATGTGGGGGACCTTCCTCTCAGAACCCCTACT
GATCGTTGCCTTGGTGGGCCGTTACCCCACCAACAAGCTAATCAGACGCATCCCCATCCATAACCGATAAATC
TTTATTCATAATCTCATGAGATCAAACGAATACATAAGGTATTAGTCCAACTTCGCTGGGTTATCCCTTAGTT
TATGGGCAGGTTTGGATACGCGTTACTCACCCGTGCGCCGGTCGCCGACATAAATAGCAAGCTATCTATTCGC
TGCCCCTCGACTTGCATGTGTTTAAGCCTATAGCTAGCGTTCATCCTGAGCCAGGATCAAACTCT
>380
CTGCTGCCGCCCGTAGGAGTTTGGACCGTGTCTCAGTTCCAATGTGGGGGACCTTCCTCTCAGAACCCCTACT
GATCGTCGCCTTTGGTGGGCCGTTACCCCGCCAACAAGCTAATCAGACGCATCCCCATCCATAACCGCTAAAC
CTTTAATCGTTATTTTGATGTCAGCAAACGATATCATACGGTATTTAGGCCGTCTTTTCAACGGGTTTATCCC
GTAGTTTATGGGAAGGTTGGATACGCGTTACTCACCCGTGCGCCAGTCGTCATCAAGGAAAGCAAGCTTTCCC
TTCAGTGTTTTACCCTGCGACTTGCATGTGTTTAAGCCTTGTAGCTAGCGTCATCCTGAGCCAGGATCAAACT
CT
>381
CTGCTGCCGCCCGTAGGAGTTTGGACCGTGTCTCAGTTCCAATGTGGGGGACCTTCCTCTCAGAACCCCTACT
GATCGTCGCCTTGGTGGGCCGTTACCCCGCCAACAAGCTAATCAGACGCATCCCCATCCATAACCGCTAAACC
TTTAATCGTTATTTGATGTCAGCAAACGATATCATACGGTATTAGGCCGTCTTTTCAACGGGTTATCCCGTAG
TTATGGGAAGGTTGGATACGCGTTACTCACCCGTGCGCCAGTCGTCATCAAGGAAAGCAAGCTTCCTCATGTT
ACCCTGCGACTGCATGTGTAAGCCTGTAGCTAGCGTCATCCTGAGCCAGGATCAAACTCT
>382
CTGCTGCCGCCCGTAGGAGTTTGGACCGTGTCTCAGTTCCAATGTGGGGGACCTTCCTCTCAGAACCCCTACT
GATCGTCGCCTTGGTGGGCCGTTACCCCGCCAACAAGCTAATCAGACGCATCCCCATCCATAACCGCTAAACC
TTTAATCGTTATTTTGATGTCAGCAAACGATGATCATACGGTATTAGGCCGTCTTTTCAACGGGTTATCCCGT
GAGTTATGGGAAGGTTGGATACGCGTTACTCACCCGTGCGCCAGTCGTCATCAAGGAAAGCAAGCTTCCCTCA
CGTGACTTG
>383
CTGCTGCCGCCCGTAGGAGTTTGGACCGTGTCTCAGTTCCAATGTGGGGGACCTTCCTCTCAGAACCCCTACT
GATCGTCGCCTTGGTGGGCCGTTACCCCGCCAACAAGCTAATCAGACGCATCCCCATCCATAACCGCTAAACC
TTTAATCGTTATTTGATGTCAGCAAACGATATCATACGGTATTAGGCCGCCTTTCAACGGGTTATCCCGTGGT
CATGGGAAGGTTGGATACGCGTTACTCACCCGTGCGCCAGTCGTCGTCAAGGAAAGCAAGCTTTCCTCATGTT
ACCCTGCGACTTGCATGTGTTAAGCCTGTAGCTAGCGTTCATCCTGAGCCAGGATCAAACTCT
>384
CTGCTGCCGCCCGTAGGAGTTTGAACCGTGTCTCAGTTCCAATGTGGGGGACCTTCCTCTCAGAACCCCTACT
GATCGTCGCCTTGGTGGGCCGTTACCCCGCCAACAAGCTAATCAGACGCATCCCCATTCATAACCGATAAATC
TTTATTCGTAATCTCATGAGATCAAAACGAATACATAAGGTATTAGTCCAACTTCGCTGGGTTATCCCTTAGT
TATGGGCAGGTTGGATACGCGTTACTCACCCGTGCGCCGGTCGCCGACATAAATAGCAAGCTATCTATTCGCT
GCCCCTCGACTTGCATGTGTTAAGCCTATAGCTAGCGTTCATCCTGAGCCAGGATCAAACTCT
>385
CTGCTGCCGCCCGTAGGAGTTTGGACCGTGTCTCAGTTCCAATGTGGGGGACCTTCCTCTCAGAACCCCTACT
GATCGTCGCCTTTGGTGGGCCGTTACCCCGCCAACAAGCTAATCAGACGCATCCCCATCCATAACCGCTAAAC
CTTTAATCGTTATTTGATGTCAGCAAACGATATCATACGGTATTAGGCCGTCTTTCAACGGGTTATCCCGTAG
TTATGGGAAGGTTGGATACGCGTTACTCACCCGTGCGCCAGTCGTCATCAAGGAAAGCAAGCTTTCCCTCATG
ATTACCCTGCCGACTTGCATGTGTTAAGCCTAGTAGCTGACGTTCATCCCGTAGCGCA
>386

CTGCTGCCGCCCGTAGGAGTTTGGACCGTGTCTCAGTTCCAATGTGGGGGACCTTCCTCTCAGAACCCCTACT
GATCGTCGCCTTGGTGGGCCGTTACCCCGCCAACAAGCTAATCAGACGCATCCCCATCCATAACCGCTAAACC
TTTAATCGTTTATTTGATGTCAGCAAACGACGTCATACGGTATTTAGTCCGTCTTTCAACGGGTTATCCCGTG
GTCATGGGAAGGTTTGGATACGCGTTACTCACCCGTGCGCCAGTCGTCGTCAAGGAAAGCAAGCTTTCCTCAC
GTTACCCTGCGACTTGCATGTGTTTAAGCCTGTAGCTAGCGTTCATCCTGAGCCAGGATCAAACTCT
>387
CTGCTGCCGCCCTAGGAGTTTTGGACCGTGTCTCAGTTCCAATGTGGGGGACCTTCCTCTCAGAACCCCTACT
GATCGTCGCCTTTGGTGGGCCGTTACCCCGCCAACAAGCTAATCAGACGCATCCCCATCCATAACCGCTAACC
TTTAATCGTTATTTGATGTCAGCAAACGATATCATACGGTATTAGGCCGTCTTTCAACGGGTTATCCCGTAGT
TTATGGGAAGGTTGGATACGCGTTACTCACCCGTGCGCCAGTCGTCGTCAAGGGAAGCAAGCTTCCCTCACGT
TACCCTGCGACTTGCATGTGTTAACGCCTGTAGTCTAGCGTTCATCCCTGAGCAAGGCATCAAATC
>388
CTGCTGCCGCCCGTAGGAGTTTTGGACCGTGTCTCAGTTCCAATGTGGGGGACCTTTCCTCTCAGAACCCCTA
CTGATCGTCGCCTTTGGTGGGCCGTTACCCCGCCAACAAGCTAATCAGACGCATCCCCATCCATAACCGCTAA
ACCTTTAATCGTTATTTGATGTCAGCAAACGACGTCATACGGTATTAGTCCGTCTTTCAACGGGTTATCCCGT
GGTCATGGGAAGGTTGGATACGCGTTACTCACCCGTGCGCCAGTCGTCATCAAGGAAAGCAAGCTTTCCTCAT
GTTACCCTGCGACATGCATGTGTTAAGCCTGTAGCTAGCGTTCATCCTGAGCCAGGATCAAACTCT
>389
CTGCTGCCGCCCGTAGGAGTTTTGGACCGTGTCTCAGTTCCAATGTGGGGGACCTTCCTCTCAGAACCCCTAC
TGATCGTCGCCTTGGTGGGCCGTTACCCCGCCAACAAGCTAATCAGACGCATCCCCATCCATAACCGCTAAAC
CTTTAATCGTTTATTTTGATGTCAGCAAACGATATCATACGGTATTTAGGCCGTCTTTTCAACGGGTTTATCC
CGTAGTTTATGGGAAGGTTTGGATACGCGTTACTCACCCGTGCGCCAGTCGTCATCAAGGAAAGCAAGCTTTC
CCTTCATGTTTTGACCCTGCCGG
>390
CTGCTGCCGCCCGTAGGAGTTTGGACCGTGTCTCAGTTCCAATGTGGGGGACCTTCCTCTCAGAACCCCTACT
GATCGTCGCCTTGGTGGGCCGTTACCCCGCCAACAAGCTAATCAGACGCATCCCCATCCATAACCGCTAAACC
TTTAATCGTTTATTTTGATGTCAGCAAACGATATCATACGGTATTTAGGCCGTCTTTTCAACGGGTTTATCCC
GTAGTTTATGGGAAGGTTGGATACGCGTTACTCACCCGTGCGCCAGTCGTCATCAAGGAAAGCAAGCTTTTCC
CTCATGTTTTACCCTGCGAACT
>391
CTGCTGCCGCCCGTAGGAGTTTTGGACCGTGTCTCAGTTCCAATGTGGGGGACCTTCCTCTCAGAACCCCTAC
TGATCGTTGCCTTTGGTGGGCCGTTACCCCGCCAACAAGCTAATCAGACGCATCCCCATCCATAACCGATAAA
TCTTTATTCATAATCTCATGAGATCAAACGAATACATAAGGTATTAGTCCAACTTCGCTGGGTTATCCCTTAG
TTATGGGCAGGTTGGATACGCGTTACTCACCCGTGCGCCGGTCGCCGACATAAATAGCAAGCTATCTATTCGC
TGCCCCTCGACTTGCATGTGTTAAGCCTATAGCTAGCGTTCATCCTGAGCCAGGATCAAACTCT
>392
CTGCTGCCGCCCGTAGGAGTTTGGACCGTGTCTCAGTTCCAATGTGGGGGACCTTCCTCTCAGAACCCCTACT
GATCGTTGCCTTGGTGGGCCGTTACCCCGCCAACAAGCTAATCAGACGCATCCCCATCCATAACCGATAAATC
TTTATTCGTAATCTCATGAGATCAAACGAATACATAAGGTATTTAGTCCAACTTCGCTGGGTTTATCCCTTAG
TTTATGGGCAGGTTTGGATACGCGTTACTCACCCGTGCGCCGGTCGCCGACATAAATAGCAAGCTATCTATTC
GCTGCCCCTCGACTTGCATGTGTTTAAGCCTATAGCTAGCGTTCATCCTTGAGCCAGGATCAAACTCCT
>393
CTGCTGCCGCCCGTAGGAGTTTGGACCGTGTCTCAGTTCCAATGTGGGGGACCTTCCTCTCAGAACCCCTACT
GATCGTTGCCTTGGTGGGCCGTTACCCCGCCAACAAGCTAATCAGACGCATCCCCATCCATAACCGATAAATC
TTTATTCGTAATCTCATGAGATCAAACGAATACATAAGGTATTAGTCCAACTTCGCTGGGTTATCCCTTAGTT
ATGGGCAGGTTGGATACGCGTTACTCACCCGTGCGCCGGTCGCCGACATAAATAGCAAGCTATCTATTCGCTG
CCCCTCGACTTGCATGTGTTAAGCCTATAGCTAGCGTTCATCCTGAGCCAGGATCAAACTCT
>394
CTGCTGCCGCCCGTAGGAGTTTGAACCGTGTCTCAGTTCCAATGTGGGGGACCTTCCTCTCAGAACCCCTACT
GATCGTTGCCTTGGTGGGCCGTTACCCCACCAACAAGCTAATCAGACGCATCCCCATCCATAACCGATAAATC
TTTATTCGTAATCTCATGAGATCAAACGAAATATATAAGGTATTAGTCCAACTTCGCTGGGTTATCCCTTAGT
TTTATGGGCAGGTTGGATACGCGTTACTCACCCGTGCGCCGGTCGCCGACATAAATAGCAAGCTATCTATTCG
CTGCCCCCCCTCGACTTGCATGTGTTTAAGCCTATAGCTAGCGTTCATCCTGAGCCAGGATCAAACTCT
>395

CTGCTGCCGCCCGTAGGAGTTTGGACCGTGTCTCAGTTCCAATGTGGGGGACCTTCCTCTCAGAACCCCTACT
GATCGTCGCCTTGGTGGGCCGTTACCCCGCCAACAAGCTATCAGACGCATCCCCATCCATAACCGCTAAACCT
TTAATCGTTATTTGATGTCAGCAAACGACGTCATACGGTATTAGTCCGTCTTTCAACGGGGTTATCCCGTGGT
CATGGGAAGGTTGGATACGCGTTACTCACCCGTGCGCCAGTCGTCGTCAAGGAAAGCAAGCTTTCCTCACGTA
CCCTGCGACTTGCATGTGTTAAGCCTGTAGCCTAGCGTTCACCCTGAGCCAGGATCAAACCTCT
>396
CTGCTGCCGCCCGTAGGAGTTTGGACCGTGTCTCAGTTCCAATGTGGGGGACCTTCCTCTCAGAACCCCTACT
GATCGTCGCCTTTGGTGGGCCGTTACCCCGCCAACAAGCTAATCAGACGCATCCCCATCCATAACCGCTAAAC
CTTTAATCGTTATTTTGATGTCAGCAAACGACGTCATACGGTATTAGTCCGTCTTTCAACGGGTATCCCGTGG
TCATGGGAAGGTTTGGATACGCGTTACTCACCCGTGCGCCAGTCGTCGTCAAGGAAAGCAAGCTTTCCTCACG
TTACCCTGCGACTTGCATGTGTTTAAGCCTGTAGCTAGCGTTCATCCTGAGCCAGGATCAAACTCT
>397
CTGCTGCCGCCCGTAGGAGTTTGGACCGTGTCTCAGTTCCAATGTGGGGGACCTTCCTCTCAGAACCCCTACT
GATCGTCGCCTTTGGTGGGCCGTTACCCCGCCAACAAGCTAATCAGACGCATCCCCATCCATCACCGCTAAAC
CTTTAATCGTTTATTTTGATGTCAGCAAACGATGTCATACGGTATTTAGTCCGTCTTTTCAACGGGTTATCCC
GTGGTCATGGGAAGGTTGGATACGCGTTACTCACCCGTGCGCCAGTCGTCGTCAAGGGAAGCAAGCTTCCCTT
CACGTTACCCTGCGACTTGCATGTGTTTAAGCCTGTAGCTAGCGTCATCCTGAGCCAGGATCAAACTCT
>398
CTGCTGCCGCCCGTAGGAGTTTGAACCGTGTCTCAGTTCCAATGTGGGGGACCTTCCTCTCAGAACCCCTACT
GATCGTTGCCTTTGGTGGGCCGTTACCCCGCCAACAAGCTAATCAGACGCATCCCCATCCATAACCGATAAAT
CTTTATTCGTAATCTCATGAGATCAAACGAAATACATAAGGTATTAGTCCAACTTCGCTGGGTTATCCCTTAG
TTATGGGCAGGTTGGATACGCGTTACTCACCCGTGCGCCGGTCGCCGACATAAATAGCAAGCTATCTATTCGC
TGCCCCTCGACTTGCATGTGTTAAGCCTATAGCTAGCGTTCATCCTGAGCCAGGATCAAACTCT
>399
CTGCTGCCGCCCGTAGGAGTCTGGGCCGTATCTCAGTCCCAATGTGGCCGCACACCCTCTCAGGCCGGCTACC
CCGTCGCCGCCTTGGTAGGCCATTACCCCCACCAACAAGCTGATAGGCCGCAAGCTCATCCCACACCAGAAAA
ACCTTCCACCACACCAACCACGATGCGGTCCTATAGGGTATTAGATCCAGTTTCCCAAGCTATCCCCCAGTGC
AGGGCAGATCACTTACGTGTTACTCACCCGTCGCCACTCGAGCACCCAGCAAGCTGGGGCCTTTCCGTTCGAC
TTGCATGTGTTAAGCACGCCGCCAGC
>400
CTGCTGCCGCCCGTAGGAGTTTTGGACCGTGTCTCAGTTCCAATGTGGGGGACCTTCCTCTCAGAACCCCTAC
TGATCGTTGCCTTGGTGGGCCGTTACCCCGCCAACAAGCTAATCAGACGCATCCCCATCCATAACCGATAAAT
CTTATTCATAATCTCATGAGATCAAACGAATACATAAGGTATTTAGTCCAACTTCGCTGGGTTTATCCCTTAG
TTTATGGGCAGGTTTGGATACGCGTTACTCACCCGTGCGCCGGTCGCCGACATAAATAGCAAGCTATCTATTC
GCTGCCCCCCTTCGACTTGCATGTGTTTAAGCCTTATAGCTAGCGTCATCCTTG
>401
CTGCTGCCGCCCGTAGGAGTTTGGACCGTGTCTCAGTTCCAGTGTGGCCGTTCATCCTCTCAGACCGGCTACT
GATCGTTGGTTTGGTGGGCCGTTACCCCACCAACTGCCTAATCAGACGCATCCCCATCCATAACCGCTAAACC
TTTTAATCGTTTATTTTGATGTCAGCAAACGATATCATACGGTATTTAGGCCGTCTTTTCAACGGGTTTATTC
CCGTAGTTTATGGGAAGGTTTGGATACGCGTTACTCACCCGTGCGCCGGTCGCCGACATAAATAGCAAGCTAT
CTATTTCGCTGCCCCCCCTCGACTTGCATGTGTTTAAGCCTATAGCTAGCGTTCATCCTGAGCCAGGATCAAA
CTCT
>402
CTGCTGCCGCCCGTAGGAGTTTGAACCGTGTCTCAGTTCCAATGTGGGGGACCTTCCTCTCAGAACCCCTACT
GATCGTTGCCTTGGTGGGCCGTTACCCCACCAACAAGCTAATCAGACGCATCCCCATCCATAACCGATAAATC
TTTATTCGTAATCTCATGAGATCAAACGAAATATATAAGGTATTAGTCCAACTTCGCTGGGTTATCCCTTAGT
TTATGGGCAGGTTTGGATACGGCGTTACTCGACCCGTGCGCCGGTCGCCGACATAAATAGCAAGCTATCTATT
CGCTGCCCCCCTCGACTTGCATGTGTTTAAGCCTATAGCTAGCGTTCATCCTGAGCCAGGATCAAACTCT
>403
CTGCTGCCGCCCGTAGGAGTTTGGACCGTGTCTCAGTTCCAATGTGGGGGACCTTCCTCTCAGAACCCCTACT
GATCGTTGCCTTGGTGGGCCGTTACCCCGCCAACAAGCTAATCAGACGCATCCCCATCCATAACCGATAAATC
TTTATTCGTAATCTCATGAGATCAAACGAATACATAAGGTATTTAGTCCAACTTCGCTGGGTTTATCCCTTAG
TTTATGGGCAGGTTTGGATACGCGTTACTCACCCGTGCGCCGGTCGCCGACATAAATAGCAAGCTATCTATTT
CGCTGCCCCCCCCTCGACTTGCATGTGTTTTAAGCCTATAGCTAGCGTTCATCCTGAGCCAGGATCAAACTCT

>404
CTGCTGCCGCCCGTAGGAGTTTGGACCGTGTCTCAGTTCCAATGTGGGGGACCTTCCTCTCAGAACCCCTACT
GATCGTCGCCTTTGGTGGGCCGTTACCCCGCCAACAAGCTAATCAGACGCATCCCCATCCATAACCGCTAAAC
CTTTAATCGTTATTTGATGTCAGCAAACGATATCATACGGTATTAGGCCGTCTTTTCAACGGGTTATCCCGTA
GTTATGGGAAGGTTGGATACGCGTTACTCACCCGTGCGCCAGTCGTCATCAAGGAAAGCAAGCTTCCTCATGT
TACCCTGCGACTTGCATGTGTTAAGCCTGTAGCTAGCGTTCATCCTGAGCCAAGGATCAAACTCG
>405
CTGCTGCCGCCCGTAGGAGTTTTGGGACCGTGTCTCAGTTCCAATGTGGGGGACCTTCCTCTCAGAACCCCTA
CTGATCGTCGCCTTTGGTGGGCCGTTACCCCGCCAACAAGCTAATCAGACGCATCCCCATCCATAACCGAATA
AATCTTTATTCATAATCTCATGAGATCAAACGAATACATAAGGTATTAGTCCAACTTCGCTGGGTTATCCCTT
AGTTATGGGCAGGTTGGATACGCGTTACCTCACCGTGCGCCGGTCGCCGACATAAATAGCCAAGCTATCTATT
CGCTGCCCCTCGACTTGCATGTGTTTAACGCCTATAGCTAGCGATTCATCCTG
>406
CTGCTGCCGCCCGTAGGAGTTGAACCGTGTCTCAGTTCCAATGTGGGGGACCTTCCTCTCAGAACCCCTACTG
ATCGTTGCCTTGGTGGGCCGTTACCCCACCAACAAGCTAATCAGACGCATCCCCATCCATAACCGCTAAACCT
TTAATCGTTATTTGATGTCAGCAAACGACGTCATACGGTATTAGTCCGTCTTTCAACGGGTTTATCCCGTGGT
CATGGGAAGGTTTGGATACGCGTTACTCACCCGTGCGCCAGTCGTCGTCAAGGAAAGCAAGCTTTCCTCACGT
TACCCTTGCGACTGCATGTGTTTAAGCCTGTAGCTAGCGTTATCCTGAGCCAGGATCAAACTCT
>407
CTGCTGCCGCCCGTAGGAGTTTGGACCGTGTCTCAGTTCCAATGTGGGGGACCTTCCTCTCAGAACCCCTACT
GATCGTCGCCTTTGGTGGGCCGTTACCCCGCCAACAAGCTAATCAGACGCATCCCCATCCATAACCGCTAAAC
CTTTAATCGTTATTTGATGTCAGCAAACGATATCATACGGTATTAGGCCGTCTTTCAACGGGTTATCCCGTAG
TTATGGGAAGGTTGGATACGCGTTACTCACCCGTGCGCCAGTCGTCAGTCAAGGAAAGCAAGCTTTCCCTCAC
GTGACCTTGTCCATGCCGTAGTGG
>408
CTGCTGCCGCCCTAGGAGTTTGGACCGTGTCTCAGTTCCAATGTGGGGGACCTTCCTCTCAGAACCCCTACTG
ATCGTCGCCTTGGTGGGCCGTTACCCCCGCCAACAAGCTAATCAGACGCATCCCCCATCCATAACCGCTAAAC
CTTTAATCGTTATTTGATTTGTCAGCAAACGATATCATACGGTATTTAGGCCGTCTTTTCAACGGGTTTAGTC
CCGTAGTTTATGGGAAGGTTGGATACGCGTTACTCACCCGTGCGCCAGTCGTCATCAAGGAAAAGCAAGCTTT
CCCTTCACT
>409
CTGCTGCCGCCCGTAGGAGTTTGGACCGTGTCTCAGTTCCAATGTGGGGGACCTTCCTCTCAGAACCCCTACT
GATCGTTGCCTTTGGTGGGCCGTTACCCCGCCAACAAGCTAATCAGACGCATCCCCATCCATAACCGATAAAT
CTTTATTCGTAATCTCATGAGATCAAACGAATACATAAGGTATTAGTCCAACTTCGCTGGGTTATCCCTTAGT
TATGGGCAGGTTGGATACGCGTTACTCACCCGTGCGCCGGTCGCCGACATAAATAGCAAGCTATCTATTCGCT
GCCCCCTCGACTTGCATGTGTTAAGCCTATAGCTAGCGTTCATCCTGAGCCAGGATCAAACTCT
>410
CTGCTGCCGCCCGTAGGAGTTTTGGACCGTGTCTCAGTTCCAATGTGGGGGACCTTCCTCTCAGAACCCCTAC
TGATCGTCGCCTTTGGTGGGCCGTTACCCCGCCAACAAGCTAATCAGACGCATCCCCATCCATCACCGCTAAA
CCTTTAATCGTTATTTGATGTCAGCAAACGATGTCATACGGTATTAGTCCGTCTTTCAACGGGTTATCCCGTG
GTCATGGGAAGGTTGGATACGCGTTACTCACCCGTGCGCCAGTCGTCGTCAAGGGAAGCAAGCTTCCCTCACG
TTACCCTGCGACTTGCATGTGTTAAGCCTGTAGCTAGCGTTCATCCCGTGAGCCAAGGCAT
>411
CTGCTGCCGCCCGTAGGAGTTTGGACCGTGTCTCAGTTCCAATGTGGGGGACCTTCCTCTCAGAACCCCTACT
GATCGTTGCCTTGGTGGGCCGTTACCCCGCCAACAAGCTAATCAGACGCATCCCCATCCATAACCGATAAATC
TTTATTCGTAATCTCATGAGATCAAACGAAATACATAAGGTATTAGTCCAACTTCGCTGGGTTATCCCTTAGT
TATGGGCAGGTTGGATACGCGTTACTCACCCGTGCGCCGGTCGCCGACATAAATAGCAAGCTATCTATTCGCT
GCCCCTCGACTTGCATGTGTTAAGCCTATAGCTAGCGTTCATCCTGAGCCAGGATCAAACTCT
>412
CTGCTGCCGCCCGTAGGAGTTTGGACCGTGTCTCAGTTCCAATGTGGGGGACCTTCCTCTCAGAACCCCTACT
GATCGTTGCCTTGGTGGGCCGTTACCCCGCCAACAAGCTAATCAGACGCATCCCCATCCATAACCGATAAATC
TTTATTCGTAATCTCATGAGATCAAACGAATACATAAGGTATTTAGTCCAACTTCGCTGGGTTTATCCCTTAG
TTTATGGGCAGGTTTGGATACGCGTTACTCACCCGTGCGCCGGTCGCCGACATAAATAGCAAGCTATCTATTC
GCTGCCCCCCTCGACTTGCATGTGTTTAAGCCTATAGCTAGCGTTCATCCTGAGCCAGGATCAAACTCT

>413
CTGCTGCCGCCCGTAGGAGTTTGGACCGTGTCTCAGTTCCAATGTGGGGGACCTTCCTCTCAGAACCCCTACT
GATCGTCGCCTTTGGTGGGCCGTTACCCCGCCAACAAGCTAATCAGACGCATCCCCATCCATCACCGCTAAAC
CTTTAATCGTTATTTGATGTCAGCAAACGATATCATACGGTATTAGGCCGTCTTTCAACGGGTTTATCCCGT
AGTTTATGGGAAGGTTGGATACGCGTTACTCACCCGTGCGCCAGTCGTCATCAAGGAAAGCAAGCTTTCCCTC
ATGTTTACCCTGCGACTTGCGATGTGTTTAAGCCTTGTAGCTAGCGTTCATCCTGAGCCAGGATCAAACTCT
>414
CTGCTGCCGCCCGTAGGAGTTTGGACCGTGTCTCAGTTTCCAATGTGGGGGACCTTCCTCTCAGAACCCCTAC
TGATCGTTGCCTTTGGTGGGCCGTTACCCCGCCAACAAGCTAATCAGACGCATCCCCATCCATAACCGATAAA
TCTTTTATTCATAATCTCATGAGATCAAACGAATACATAAGGTATTTAGTCCAACTTTCGCTGGGTTATCCCT
TAGTTTATGGGCAGGTTTGGATACGCGTTACTCACCCGTGCGCCGGTCGCCGACATAAATAGCAAGCTATCTA
TTCGCTGCCCCCCTCGACTTGCATGTGTTTAAGCCTTATAGCTAGCGTTTCATCCTGAGCCAAGGATCAAACT
CT
>415
CTGCTGCCGCCCGTAGGAGTTTGGACCGTGTCTCAGTTCCAATGTGGGGGACCTTCCTCTCAGAACCCCTACT
GATCGTCGCCTTTGGTGGGCCGTTACCCCGCCAACAAGCTAATCAGACGCATCCCCATCCATAACCGCTAAAC
CTTTAATCGTTATTTGATGTCAGCAAACGATATCATACGGTATTAGGCCGTCTTTCAACGGGTTATCCCGTAG
TTATGGGAAGGTTGGATACGCGTTACTCACCCGTGCGCCAGTCGTCATCAAGGAAAGCAAGCTTTCCTCATGT
TACCCTGCGACATGCATGTGTTAAGCCTGTAGCTAGCGTTCATCCCGTGAGCCAAGGCATCAAATC
>416
CTGCTGCCGCCCGTAGGAGTTTTGGACCGTGTCTCAGTTCCAATGTGGGGGACCTTCCTCTCAGAACCCCTAC
TGATCGTCGCCTTTGGTGGGCCGTTACCCCGCCAACAAGCTAATCAGACGCATCCCCATCCATAACCGCTAAA
CCTTTAATCGTTATTTGATGTCAGCAAACGATATCATACGGTATTAGGCCGTCTTTCAACGGGTTATCCCGTA
GTTATGGGAAGGTTGGATACGCGTTACTCACCCGTGCGCCAGTCGTCATCAAGGAAAGCAAGCTTTCCCTCAT
GTTACCCTGCGAACTTGCATGTGTTAAGCCTGTAGCTAGCGTTCATCCTGAGCCAAGGCATCAAACTCT
>417
CTGCTGCCGCCCGTAGGAGTTTGGACCGTGTCTCAGTTCCAATGTGGGGGACCTTCCTCTCAGAACCCCTACT
GATCGTCGCCTTTGGTGGGCCGTTACCCCGCCAACAAGCTAATCAGACGCATCCCCATCCATCACCGCTAAAC
CTTTAATCGTTATTTGATGTCAGCAAACGATGTCATACGGTATTAGTCCGTCTTTCAACGGGTTATCCCGTGG
TCATGGGAAGGTTGGATACGCGTTACTCACCCGTGCGCCGGTCGCCGACATAAATAGCAAGCTATCTATTCGC
TGCCCCTCGACTTGCATGTGTTAAGCCTATAGCTAGCGTTCATCCTGAGCCAGGATCAAACTCT
>418
CTGCTGCCGCCCGTAGGAGTTTTGGACCGTGTCTCAGTTCCAATGTGGGGGACCTTCCTCTCAGAACCCCTAC
TGATCGTCGCCTTTGGTGGGCCGTTACCCCGCCAACAAGCTAATCAGACGCACCCCCATCCATAACCGCTAAA
CCTTTAATCGTTATTTGATGTCAGCAAACGATATCATACGGTATTAGGCCGTCTTTCAACGGGTTATCCCGTA
GTTATGGGAAGGTTGGATACGCGTTACTCACCCGTGCGCCAGTCGTCATCAAGGAAAGCAAGCTTTCCCTCAT
GTTACCCTGCGACTTGCATGTGTTAAGCCTGTAGCTAGCGTTCATCCTGAGCCAGGATCAAACTCT
>419
CTGCTGCCGCCCGTAGGAGTTTGAACCGTGTCTCAGTTCCAATGTGGGGGACCTTCCTCTCAGAACCCCTACT
GATCGTTGCCTTGGTGGGCCGTTACCCCACCAACAAGCTAATCAGACGCATCCCCATCCATAACCGATAAATC
TTTATTCATAATCTCATGAGATCAAACGAATACATAAGGTATTAGTCCAACTTCGCTGGGTTATCCCTTAGTT
ATGGGCAGGTTGGATACGCGTTACTCACCCGTGCGCCGGTCGCCGACATAAATAGCAAGCTATCTATTCGCTG
CCCCTCGACTTGCATGTGTTAAGCCTATAGCTAGCGTTCATCCTGAGCCAGGATCAAACTCT
>420
CTGCTGCCGCCCGTAGGAGTTTGGACCGTGTCTCAGTTCCAATGTGGGGGACCTTCCTCTCAGAACCCCTACT
GATCGTCGCCTTTGGTGGGCCGTTACCCCGCCAACAAGCTAATCAGACGCATCCCCATCCATAACCGCTAACC
TTTAATCGTTATTTGATGTCAGCAAACGATATCATACGGTATTAGGCCGTCTTTCAACGGGTTATCCCGTAGT
TATGGGAAGGTTGGATACGCGTTACTCACCCGTGCGCCAGTCGTCATCAAGGAAAGCAAGCTTTCCCTCATGA
TTACCCTGCGACTTGCATGTGTTAAGCCTGTAGCTAGCGTTCATCCTGAGCCAAGGCATCAAACTCT
>421
CTGCTGCCGCCCGTAGGAGTTTGGACCGTGTCTCAGTTTCCAATGTGGGGGACCTTCCTCTCAGAACCCCTAC
TGATCGTTGCCTTGGTGGGCCGTTACCCCGCCAACAAGCTAACGTCAGAACGCATCCCCATCCATAACCGATA
AATCTTTTATTCATAATCTCATGAGATCAAACGAATACATAAGGTATTAGTCCAACTTTCGCTGGGTTATCCT
TAGTTTATGGGCAGGTTTGGATACGCGTTACTCACCCGTGCGCCGGTCGCCGACATAAATAGCAAGCTATCTA

TTTCGCTGCCCCCCTCGACTTTGACATTGTGTTTAAGCCTATAGCTAGCGATTTCATCCTTAGAGCCAAGGAC
AAACTCCT
>422
CTGCTGCCGCCCGTAGGAGTTTGGACCGTGTCTCAGTTCCAATGTGGGGGACCTTCCTCTCAGAACCCCTACT
GATCGTTGCCTTGGTGGGCCGTTACCCCGCCAACAAGCTAATCAGACGCATCCCCATCCATAACCGCTAAACC
TTTAATCGTTATTTTGATGTCAGCAAACGATATCATACGGTATTTAGGCCGTCTTTTCAACGGGTTATCCCGT
AGTTTATGGGAAGGTTGGATACGCGTTACTCACCCGTGCGCCAGTCGTCATCAAGGAAAGCAAGCTTTCCCTC
ACTGATTTTACCCTGCGACTTGCATGTGTTTTAAGCCTGTAGCTAGCGTTCATCCTGAGCCAAGGATCAAACT
CT
>423
CTGCTGCCGCCCGTAGGAGTTTGGACCGTGTCTCAGTTCCAATGTGGGGGACCTTCCTCTCAGAACCCCTACT
GATCGTTGCCTTGGTGGGCCGTTACCCCGCCAACAAGCTAATCAGACGCATCCCCATCCATAACCGATAAATC
TTTATTCATAATCTCATGAGATCAAACGAATACATAAGGTATTAGTCCAACTTCGCTGGGTTATCCCTTAGTT
ATGGGCAGGTTTGGATACGCGTTACTCACCCGTGCGCCGGTCGCCGACATAAATAGCAAGCTATCTATTCGCT
GCCCCCCCTCGACTTGCATGTGTTTAAGCCTATAGCTAGCGTTCATCCTGAGCCAGGATCAAACTCCT
>424
CTGCTGCCGCCCGTAGGAGTCTGGGCCGTATCTCAGTCCCAATGTGGCCGCACACCCTCTCAGGCCGGCTACC
CGTCGCCGCCTTGGTAGGCCATTACCCCACCAACAAGCTGATAGGCCGCAAGCTCATCCCACACCAGAAAAAC
CTTTCCACCACACCAACCACGATGCGGTCCTATAGGGTATTAGACCCAGTTTCCCAAGCTTTATCCCCCAGTG
CAGGGCAGATCACTTACGTGTTACTCACCCGTTCGCCACTCGAGCACCCCAGCAAGCTGGGGCCTTTCCGTTT
CGACTTGCATGTGTTTTAAGCACGCCGCCAGCGTTCACCTGAGCCAGGATCAAACTCT
>425
CTGCTGCCGCCCGTAGGAGTTTGGACCGTGTCTCAGTTCCAATGTGGGGGACCTTCCTCTCAGAACCCCTACT
GATCGTCGCCTTTGGTGGGCCGTTACCCCGCCAACAAGCTAATCAGACGCATCCCCATCCATAACCGCTAAAC
CTTTTAATCGTTTTATTTTTGATGTCAGCAAACGATATCATACGGTATTTTAGGCCGTCTTTTTCAACGGGTT
TTATTCCCGTAGTTTTATGGGAAGGTTTGGATACGCGTTTACTCACCCGTGCGCCAGTCGTCATCAAGGAAAG
CAAGCTTTTCCTTCATGT
>426
CTGCTGCCGCCCGTAGGAGTCTGGGCCGTATCTCAGTCCCAATGTGGCCGCACACCCTCTCAGGCCGGCTACC
CGTCGCCGCCTTGGTAGGCCATTACCCCACCAACAAGCTGATAGGCCGCAAGCTCATCCCACACCAGAAAAAC
CTTTCCACCACACCAACCACGATGCGGTCCTATAGGGTATTAGACCCAGTTTTCCCAAGCTTTATCCCCCAGT
GCAGGGCAGATCACTTACGTGTTACTCACCCGTTCGCCACTCGAGCACCCCAGCCAAGCTGGGGCCTTTCCGT
TCGACTTGCATGGTGTTTTAAGCACGCCGCCAGCGTTCACCTGAGCCAGGATCAAACTCT
>427
CTGCTGCCGCCCGTAGGAGTTTGGACCGTGTCTCAGTTCCAATGTGGGGGACCTTCCTCTCAGAACCCCTACT
GATCGTCGCCTTAGTGGGCCGTTACCCCGCCAACAAGCTAATCAGACGCATCCCCATCCATAACCGCTAAACC
TTTAATCGTTATTTGATGTCAGCAAACGATATCATACGGTATTAGGCCGTCTTTCAACGGGTTATCCCGTAGT
TATGGGAAGGTTGGATACGCGTTACTCACCCGTGCGCCAGTCGTCATCAAGGAAAGCAAGCTTTCCTCATGTT
TACCCTGCGACTTGCATGTGTTTAAGCCTTGTAGCTAGCGTCATCCTGAGCCAGGATCAAACTCT
>428
CTGCTGCCGCCCTAGGAGTTTTGGACCGTGTCTCAGTTCCAATGTGGGGGACCTTCCTCTCAGAACCCCTACT
GATCGTCGCCTTTGGTGGGCCGTTACCCCGCCAACAAGCTAATCAGACGCATCCCCATCCATAACCGCTAAAC
CTTTAATCGTTTATTTTGATGTCAGCAAACGATATCATACGGTATTTAGGCCGTCTTTTCAACGGGTTTATCC
CGTAGTTTATGGGAAGGTTGGATACGCGTTACTCACCCGTGCGCCAGTCGTCATCAAGGAAAGCAAGCTTTCC
CTCATGTTTACCCTGCGACTTGCATGTGTTTAAGCCTTGTAGCTAGCGTTCATCCTGAGCCAGGATCAAACTC
T
>429
CTGCTGCCGCCCGTAGGAGTTTGGACCGTGTCTCAGTTCCAATGTGGGGGACCCTTCCTCTCAGAACCCCTAC
TGATCGTCGCCTTGGTGGGCCGTTACCCCGCCAACAAGCTAATCAGACGCATCCCCATCCATAACCGCTAAAC
CTTTAATCGTTATTTGATGTCAGCAAACGATATCATACGGTATTAGGCCGTCTTTCAACGGGTTATCCCGTAG
TTATGGGAAGGTTGGATACGCGTTACTCACCCGTGCGCCAGTCGTCATCAAGGAAAGCAAGCTTTCCTCATGT
TTACCCTGCGACTTGCATGTGTTAAGCCTGTAGCTAGCGTTCATCCTGAGCCAGGATCAAACTCT
>430

CTGCTGCCGCCCGTAGGAGTTTGGACCGTGTCTCAGTTCCAATGTGGCCGATCACCCTCTCAGGTCGGCTACT
GATCGTCGCCTTGGTGGGCAGTTATCTCACCAACTAGCTAATCAGACGCGGGTCCATCCTATACCGCTAACAC
TTTGATATATCTACGATGCCGTAAATATATATTATCATGTATTAGTAAACTTTTCAGTATGTTTATCCATGTG
TATAGGGCAGGTTACCCACGCGTTACTCACCCGTCCGCCGCTCATCTTCATTAATGCAAGCATCAAATCAGAC
GCGCTCGACTTGCATGTGTTAGGCACGCCGCCCAGCGTTCGCCAGC
>431
CTGCTGCCGCCCGTAGGAGTTTTGGACCGTGTCTCAGTTCCAATGTGGGGGACCTTCCTCTCAGAACCCCTAC
TGATCGTCGCCTTTGGTGGGCCGTTACCCCGCCAACAAGCTAATCAGACGCATCCCCATCCATAACCGCTAAA
CCTTTAATCGTTATTTGATGTCAGCAAACGATACCATACGGTATTAGGCCGTCTTTCAACGGGTTATCCCGTG
GTCATGGGAAGGTTGGATACGCGTTACTCACCCGTGCGCCAGTCGTCGTCAAGGAAAGCAAGCTTTCCTCACG
TTACCCTGCGACTTGCATGTGTTAAGCCTGTAGCTAGCGTCATCCTGAGCCAGGATCAAACTCT
>432
CTGCTGCCGCCCGTAGGAGTTTGGACCGTGTCTCAGTTCCAATGTGGGGGACCTTCCTCTCAGAACCCCTACT
GATCGTCGCCTTTGGTGGGCCGTTACCCCGCCAACAAGCTAATCAGACGCATCCCCATCCATAACCGCTAAAC
CTTTAATCGTTATTTGATGTCAGCAAACGATATCATACGGTATTAGGCCGTCTTTCAACGGGTTAGTCCCGTA
GTTATGGGAAGGTTGGATACGCGTTACTCACCCGTGCGCCAGTCGTCATCAAGGAAAGCAAGCTTTCCCTCAT
GTTACCCTGCCGAACGTTGCA
>433
CTGCTGCCGCCCGTAGGAGTTTGGACCGTGTCTCAGTTCCAATGTGGGGGACCTTCCTCTCAGAACCCCTACT
GATCGTCGCCTTGGTGGGCCGTTACCCCGCCAACAAGCTAATCAGACGCATCCCCATCCATAACCGCTAAACC
TTTAATCGTTATTTGATGTCAGCAAACGATATCATACGGTATTAGGCCGTCTTTCAACGGGTTATCCCGTAGT
TATGGGAAGGTTGGATACGCGTTACTCACCCGTGCGCCAGTCGTCATCAAGGAAAGCAAGCTTTCCTCATGTT
ACCCTGCGACATGCATGTGTTAAGCCTAGTAGCTGACGTTCATCCCTAGCGCAAG
>434
CTGCTGCCGCCCGTAGGAGTTTGGACCGTGTCTCAGTTCCAATGTGGGGGACCTTCCTCTCAGAACCCCTACT
GATCGTCGCCTTGGTGGGCCGTTACCCCGCCAACAAGCTAATCAGACGCATCCCCATCCATCACCGCTAAACC
TTTAATCGTTATTTTGATGTCAGCAAACGATGTCATACGGTATTAGTCCGTCTTTTCAACGGGTTATCCCGTG
TCATGGGAAGGTTGGATACGCGTTACTCACCCGTGCGCCAGTCGTCGTCAAGGGAAGCAAGCTTCCCTCACGT
TACCTGCCGACCTTGCATGTGTTAAGCCTGTAGCTAGCGTTCATCCTGAGCCAGGATCAAACTCT
>435
CTGCTGCCGCCCGTAGGAGTTTGGACCGTGTCTCAGTTCCAATGTGGGGGACCTTCCTCTCAGAACCCCTACT
GATCGTCGCCTTTGGTGGGCCGTTACCCCGCCAACAAGCTAATCAGACGCATCCCCATCCATAACCGCTAAAC
CTTTAATCGTTATTTTGATGTCAGCAAACGATATCATACGGTATTAGGCCGTCTTTTCAACGGGTTATCCCGT
AGTTATGGGAAGGTTGGATACGCGTTACTCACCCGTGCGCCAGTCGTCATCAAGGAAAGCAAGCTTCCCTCAT
GTTACCTGCCGATGCATGTGTTAAGCCTGTAGCTAGCGTTCATCCCGTGAGCCAAGG
>436
CTGCTGCCGCCCGTAGGAGTTGTGACCGTGTCTCAGTTCCAGTGTGGCCGTTCATCCTCTCAGACCGGCTACT
GATCGTTGGTTTGGTGGGCCGTTACCCCACCAACTGCCTAATCAGACGCAAACCCCTCTTCAGGCGATAGCTT
ACAGGTAGAGGCTACCCTTTCTTCCACAGGTCATGCGCACCGTGGAACGTATTCGGTATTAGCAGTCGTTTTC
CGTCTGTTTGTCCCCATCCTGAAGGCAGGTTGTTTTTACGTGTTACTCACCCGTTTGCCACTGGATTCGATAA
AGAGCAAGCTCTTCATCTCTTCCCGTTCGACTTGCATGTGTTAAGCACGCCGCCAGCGTTCGTCCTGAGCCAG
GATCAAACTCT
>437
CTGCTGCCGCCCGTAGGAGTTTTGGACCGTGTCTCAGTTCCAACGTGGGGGACCTTCCTCTCAGAACCCCTAC
TGATCGTTGCCTTGGTGGGCCGTTACCCCGCCAACAAGCTAATCAGACGCATCCCCATCCATAACCGCTAAAC
CTTTAATCGTTATTTGATGTCAGCAAACGATATCATACGGTATTAGGCCGTCTTTCAACGGGTTATCCCGTAG
TTATGGGAGGGTTGGATACGCGTTACTCACCCGTGCGCCAGTCGTCCATCAAGGAAAGCAATGCATTCCCGTT
CCATGCTTGA
>438
CTGCTGCCGCCCGTAGGAGTTTGGACCGTGTCTCAGTTCCAATGTGGGGGACCTTCCTCTCAGAACCCCTACT
GATCGTTGCCTTTGGTGGGCCGTTACCCCGCCAACAAGCTAATCAGACGCATCCCCATCCATAACCGATAAAT
CTTTATTCGTAATCTCATGAGATCAAACGAAATACATAAGGTATTAGTCCAACTTCGCTGGGTTATCCCTTAG
TTATGGGCAGGTTGGATACGCGTTACTCACCCGTGCGCCGGTCGCCGACATAAATAGCAAGCTATCTATTCGC
TGCCCCTCGACTTGCATGTGTTAAGCCTATAGCTAGCGTTCATCCTGAGCCAGGATCAAACTCT

>439
CTGCTGCCGCCCGTAGGAGTTTGGACCGTGTCTCAGTTCCAATGTGGGGGACCTTCCTCTCAGAACCCCTACT
GATCGTTGCCTTTGGTGGGCCGTTACCCCGCCAACAAGCTAATCAGACGCATCCCCATCCATAACCGATAAAT
CTTTATTCGTAATCTCATGAGATCAAACGAATACATAAGGTATTAGTCCAACTTCGCTGGGTTATCCCTTAGT
TATGGGCAGGTTGGATACGCGTTACTCACCCGTGCGCCGGTCGCCGACATAAATAGCAAGCTATCTATTCGCT
GCCCCTCGACTTGCATGTGTTTAAGCCTATAGCTAGCGTTCATCCTGAGCCAGGATCAAACTCT
>440
CTGCTGCCGCCCGTAGGAGTCTGGGCCGTATCTCAGTCCCAATGTGGCCGCACACCCTCTCAGGCCGGCTACC
CGTCGCCGCCTTTGGTAGGCCATTACCCCACCAACAAGCTGATAGGCCGCAAGCTCATCCCACACCAGAAAAA
CCTTTCCACCACACCAACCACGATGCGGTCCTATAGGGTATTAGACCCAGTTTCCCAAGCTTACTCCCCCAGT
GCAGGGCAGATCACTTACGTGTTACTCACCCGTTCGCCACTCGAGCACCCCAGCAAGCTGGGGCCTTTCCGTT
CGACTTGCATGTGTTAAGCACGCCGCCAGCGTTCATCCTGAGCCAGGATCAAACTCT
>441
CTGCTGCCGCCCGTAGGAGTTTGGACCGTGTCTCAGTTCCAATGTGGGGGACCTTCCTCTCAGAACCCCTACT
GATCGTCGCCTTTGGTGGGCCGTTACCCCGCCAACAAGCTAATCAGACGCATCCCCATCCATCACCGCTAAAC
CTTTAATCGTTATTTGATGTCAGCAAACGATGTCATACGGTATTAGTCCGTCTTTCAACGGGTTATCCCGTGG
TCATGGGAAGGTTGGATACGCGTTACTCACCCGTGCGCCAGTCGTCGTCAAGGGAAGCAAGCTTCCCTCACGT
TACCCTGCGACTTGCCATGTGTTAAGCCTGTAGCTAGCGTTCATCCGTGAGCCAAGGCATCAAACTCT
>442
CTGCTGCCGCCCGTAGGAGTTTTGGACCGTGTCTCAGTTCCAATGTGGGGGACCTTCCTCTCAGAACCCCTAC
TGATCGTTGCCTTGGTGGGCCGTTACCCCACCAACAAGCTAATCAGACGCATCCCCATCCATAACCGATAAAT
CTTTATTCGTAATCTCATGAGATCAAACGAATATATAAGGTATTAGTCCAACTTCGCTGGGTTATCCCTTAGT
TATGGGCAGGTTGGATACGCGTTACTCACCCGTGCGCCGGTCGCCGACATAAATAGCAAGCTATCTATTCGCT
GCCCCTCGACTTGCATGTGTTAAGCCTATAGCTAGCGTTCATCCTGAGCCAGGATCAAACTCT
>443
CTGCTGCCGCCCGTAGGAGTTTTGGACCGTGTCTCAGTTCCAATGTGGGGGACCTTCCTCTCAGAACCCCTAC
TGATCGTCGCCTTTGGTGGGCCGTTACCCCGCCAACAAGCTAATCAGACGCATCCCCATCCATAACCGCTAAA
CCTTTAATCGTTATTTTGATGTCAGCAAACGATATCATACGGTATTAGGCCGTCTTTCAACGGGTTATCCCGT
AGTTTATGGGAAGGTTGGATACGCGTTACTCACCCGTGCGCCAGTCGTCATCAAGGAAAGCAAGCCTTTCCTC
ATGTTACCCTGCGAACTTGCATGTGTTAAGCCTGTAGCTAGCGTTCATCCCTGAGCAAGGCATC
>444
CTGTTGCCGCCCGTAGGAGTTTGGACCGTGTCTCAGTTCCAATGTGGGGGACCTTCCTCTCAGAACCCCTACT
GATCGTTGCCCTTGGTGGGCCGTTACCCCACCAACAAGCTAATCAGACGCATCCCCATCCATAACCGATAAAT
CTTTATTCGTAATCTCATGAGATCAAACGAATATATAAGGTATTAAGTCCAACTTCGCTGGGTTATCCCTTAG
TTAGTGGGCAGTTGGATGACGCGTTACTCACCGTGCGCCGGTCGCCGACATAAATAGCAAGCTATCTATTCGC
TGCCCCTCGACTTGCATGTGTTAACGCCTATAGCTAGCGTTCACCTGAGCCAGGATCAAACTCT
>445
CTGCTGCCGCCCGTAGGAGTCTGGGCCGTATCTCAGTCCCAATGTGGCCGCACACCCTCTCAGGCCGGCTACC
CGTCGCCGCCTTGGTAGGCCATTACCCCACCAACAAGCTGATAGGCCGCAAGCTCATCCCACACCAGAAAAAC
CTTTCCACCACACCAACCACGATGCGGTCCTATAGGGTATTAGACCCAGTTTCCCAAGCTTATCCCCCAGTGC
AGGGCAGATCACTTACGTGTTACTCACCCGTTCGCCACTCGAGCACCCCAGCAAGCTGGGGCCTTTCCGTTCG
ACTTGCATGTGTTTAAGCACGCCGCCAGCGTTCACCTGAGCCAGGATCAAACTCT
>446
CTGCTGCCGCCCGTAGGAGTTTTGGACCGTGTCTCAGTTCCAATGTGGGGGACCTTCCTCTCAGAACCCCTAC
TGATCGTTGCCTTTGGTGGGCCGTTACCCCGCCAACAAGCTAATCAGACGCATCCCCATCCATAACCGATAAA
TCTTTATTCATAATCTCATGAGATCAAACGAATACATAAGGTATTAGTCCAACTTCGCTGGGTTATCCCTTAG
TTATGGGCAGGTTGGATACGCGTTACTCACCCGTGCGCCGGTCGCCGACATAAATAGCCAAGCTATCTATTCG
CTGCCCCTCGACTTGCATGTGTTAACGCCTATAGCTAGCGTTCATCCTGAGCCAGGATCAAACTCT
>447
CTGCTGCCGCCCGTAGGAGTTTGGACCGTGTCTCAGTTCCAATGTGGGGGACCTTCCTCTCAGAACCCCTACT
GATCGTCGCCTTGGTGGGCCGTTACCCCCGCCAACAAGCTAATCAGACGCATCCCCATCCATAACCGCTAAAC
CTTTAATCGTTTATTTTGATGTCAGCAAACGATATCATACGGTATTTAGGCCGTCTTTTCAACGGGTTTATTC
CCGTAGTTTATGGGAAGGTTGGATACGCGTTACTCACCCGTGCGCCAGTCGTCATCAAGGAAAGCAAGCTTTT

CCCTTCATGTTTTACCCTGCGACTTGCATTGTGTTTAAGCCTTGTAGCTAGCGTCATCCTGAGCCAGGATCAA
ACTCT
>448
CTGCTGCCGCCCGTAGGAGTCTGGGCCGTATCTCAGTCCCAATGTGGCCGCACACCCTCTCAGGCCGGCTACC
CGTCGCCGCCTTGGTAGGCCATTACCCCACCAACAAGCTGATAGGCCGCAAGCTCATCCCACACCAGAAAAAC
CTTTCCACCACACCAACCACGATGCGGTCCTATAGGGTATTAGACCCAGTTTCCCAAGCTTATCCCCCAGTGC
AGGGCAGATCACTTACGTGTTACTCACCCGTTCGCCACTCGAGCACCCCAGCAAGCTGGGGCCTTTCCGTTCG
ACTTGCATGTGTTAAGCACGCCGCCAGCGTTCATCCTGAGCCAGGATCAAACTCT
>449
CTGCTGCCGCCCGTAGGAGTTTGGACCGTGTCTCAGTTCCAATGTGGGGGACCTTCCTCTCAGAACCCCTACT
GATCGTCGCCTTTGGTGGGCCGTTACCCCGCCAACAAGCTAATCAGACGCATCCCCATCCATAACCGCTAAAC
CTTTAATCGTTTATTTTGATGTCAGCAAACGATATCATACGGTATTTAGGCCGTCTTTTCAACGGGTTTATCC
CGTAGTTTATGGGAAGGTTGGATACGCGTTACTCACCCGTGCGCCAGTCGTCATCAAGGAAAGCAAGCTTTCC
CGTTCACTGTTTACCCTGCGACATGCATGTGTTAAGC
>450
CTGCTGCCGCCCGTAGGAGTTTGGACCGTGTCTCAGTTCCAATGTGGGGGACCTTCCTCTCAGAACCCCTACT
GATCGTCGCCTTTGGTGGGCCGTTACCCCGCCAACAAGCTAATCAGACGCATCCCCATCCATAACCGATAAAT
CTTTATTCATAATCTCATGAGATCAAACGAATACATAAGGTATTTAGTCCAACTTCGCTGGGTTTACCCCCCT
TAGTTATGGGCAGGTTTGGATACGCGTTACTCACCCGTGCGCCGGTCGCCGACATAAATAGCAAGCTATCTAT
TCGCTGCCCCCCCTTCGACTTGCATGTGTTTAAGCCTTATAGCTAGCGTTCATCCTTGAGCCAGGATCAAACT
CT
>451
CTGCTGCCGCCCGTAGGAGTTTGGACCGTGTCTCAGTTCCAATGTGGGGGACCTTCCTCTCAGAACCCCTACT
GATCGTCGCCTTTGGTGGGCCGTTACCCCGCCAACAAGCTAATCAGACGCATCCCCATCCATAACCGCTAACC
TTTTAATCGTTTTATTTTTGATGTCAGCAAACGATATCATACGGTATTTTAGGCCGTCTTTTTCAACGGGTTT
TATCCCGTAGTTTTATGGGAAGGTTTGGATACGCGTTACTCACCCGTGCGCCAGTCGTCATCAAGGAAAGCAA
TGCCATTTTC
>452
CTGCTGCCGCCCGTAGGAGTTTTGGACCGTGTCTCAGTTCCAATGTGGGGGACCTTCCTCTCAGAACCCCTAC
TGATCGTCGCCTTTGGTGGGCCGTTACCCCGCCAACAAGCTAATCAGACGCATCCCCCATCCATAACCGCTAA
ACCTTTTAATCGTTTATTTTGATGTCAGCAAACGATATCATACGGTATTTAGGCCGTCTTTTCAACGGGTTTA
TCCCGTAGTTTATGGGAAGGTTGGATACGCGTTACTCACCCGTGCGCCAGTCGTCATCAAGGAAAGCAAGCTT
TCCCTTCATGTTTTACCCTGCGACTTGCATTGTGTTTAAGCCTTGTAGCTAGCGTTCATCCTGAGCCGGAATC
AAACTCT
>453
CTGCTGCCGCCCGTAGGAGTTTGGACCGTGTCTCAGTTCCAATGTGGGGGACCTTCCTCTCAGAACCCCTACT
GATCGTTGCCTTGGTGGGCCGTTACCCCGCCAACAAGCTAATCAGACGCATCCCCATCCATAACCGATAAATC
TTTATTCGTAATCTCATGAGATCAAACGAATACATAAGGTATTTAGTCCAACTTCGCTGGGTTTATCCCTTAG
TTTATGGGCAGGTTTGGATACGCGTTACTCACCCGTGCGCCGGTCGCCGACATAAATAGCAAGCTATCTATTC
GCTGCCCCCCCTCGACTTGCATGTGTTTAAGCACGCCGCCAGCGTTCGTCCTGAGCCAGGATCAAACTCT
>454
CTGCTGCCGCCCGTAGGAGTTTGGACCGTGTCTCAGTTCCAATGTGGGGGACCTTCCTCTCAGAACCCCTACT
GATCGTTGCCTTGGTGGGCCGTTACCCCGCCAACAAGCTAATCAGACGCATCCCCATCCATAACCGCTAAACC
TTTAATCGTTATTTGATGTCAGCAAACGACGTCATACAGTATTAGTCCGTCTTCAACGGGTTATCCCGTGGTC
ATGGGAAGGTTGGATACGCGTTACTCACCCGTGCGCCAGTCGTCGTCAAGGAAAGCAAGCTTCCTCACGTTAC
CCTGCGACTTGCATGTGTTAAGCCTGATAGCTAGCGTCATCCTGAGCCA
>455
CTGCTGCCGCCCGTAGGAGTTTGGACCGTGTCTCAGTTCCAATGTGGGGGACCTTCCTCTCAGAACCCCTACT
GATCGTTGCCTTGGTGGGCCGTTACCCCGCCAACAAGCTAATCAGACGCATCCCCATCCATAACCGATAAATC
TTTTATTTCATAATCTCATGAGATCAAACGAATACATAAGGTATTTAGTTCCAACTTCGCTGGGTTTATCCCT
TAGTTTATGGGCAGGTTTTGGATACGCGTTACTCACCCGTGCGCCGGTCGCCGACATAAATAGCAAGCTTATC
TATTTCGCTGCCCCCCCCTTCGACTTGCATGTGTTTTAAGCCTTATAGCTAGCGTTCACCCCC
>456

CTGCTGCCGCCCGTAGGAGTTTGGACCGTGTCTCAGTTCCAATGTGGGGGACCTTCCTCTCAGAACCCCTACT
GATCGTCGCCTTGGTGGGCCGTTACCCCGCCAACAAGCTAATCAGACGCATCCCCATCCATAACCGCTAAACC
TTTAATCGTTATTTGATGTCAGCAAACGATATCATACGGTATTAGGCCGTCTTTCAACGGGTTATCCCGTAGT
TATGGGAAGGTTGGATACGCGTTACTCACCCGTGCGCCAGTCGTCATCAAGGAAAGCAAGCTTTCCTCATGTT
ACCCTGCGACTTGCATGTGTTAAGCCTGTAGCTAGCGTCATCCAGTCTAGCA
>457
CTGCTGCCGCCCGTAGGAGTTTGGACCGGTGTCTCAGTTCCAATGTGGGGGACCTTCCTCTCAGAACCCCTAC
TGATCGTCGCCTTGGGTGGGCCGTTACCCCGCCAACAAGCTAATCAGACGCATCCCCATCCATAACCGCTAAA
CCTTTAATCGTTATTTTGATGTCAGCAAACGATATCATACGGTATTAGGCCGTCTTTTCAACGGGTATCCCGT
AGTTTATGGGAAGGTTGGATACGCGTACTCACCCGTGCGCCAGTCGTCATCAAGGAAAGCAAGCTTCCTCATG
TTTTACCCGTGCGACTGCATGTGTTAAGCCTGTAGCTAGCGTTCATCCGTGAGCCAGGATCAAACTCT
>458
CTGCTGCCGCCCGTAGGAGTTTGGACCGTGTCTCAGTTCCAATGTGGGGGACCTTCCTCTCAGAACCCCTACT
GATCGTCGCCTTGGTGGGCCGTTACCCCGCCAACAAGCTAATCAGACGCATCCCCATCCATAACCGCTAAACC
TTTAATCGTTTATTTTGATGTCAGCAAACGATATCATACGGTATTTAGGCCGTCTTTTCAACGGGTTTATCCC
GTAGTTTATGGGAAGGTTGGATACGCGTTACTCACCCGTGCGCCAGTCGTCATCAAGGAAAGCAAGCTTTCCC
TTCATGTTTACCCTGCGACTTGCATGTGTTTAAGCCTTGTAGCTAGCGTTCATCCTGAGCCAGGATCAAACTC
T
>459
CTGCTGCCGCCCGTAGGAGTTTGGACCGTGTCTCAGTTCCAATGTGGGGGACCTTCCTCTCAGAACCCCTACT
GATCGTCGCCTTTGGTGGGCCGTTACCCCGCCAACAAGCTAATCAGACGCATCCCCATCCATAACCGCTAAAC
CTTTAATCGTTTATTTTGATGTCAGCAAACGATATCATACGGTATTTAGGCCGTCTTTTCAACGGGTTTATCC
CGTAGTTTATGGGAAGGTTGGATACGCGTTACTCACCCGTGCGCCAGTCGTCGTCAAGGAAAGCAAGCTTTCC
TTCACGTTACCCTGCGACTTGCATGTGTTTAAGCCTTGTAGCTAGCGTCATCCTGAGCCAGGATCAACTCT
>460
CTGCTGCCGCCCGTAGGAGTTTGGACCGTGTCTCAGTTCCAATGTGGGGGACCTTCCTCTCAGAACCCCTACT
GATCGTCGCCTTGGTGGGCCGTTACCCCGCCAACAAGCTAATCAGACGCATCCCCATCCATAACCGCTAAACC
TTTAATCGTTATTTTGATGTCAGCAAACGACGTCATACGGTATTTAGTCCGTCTTTCAACGGGTTTATCCCGT
GGTCATGGGAAGGTTTGGATACGCGTTACTCACCCGTGCGCCAGTCGTCGTCAAGGAAAGCAAGCTTTTCCTC
ACGTTACCCTTGCGACTGCATGTGTTTTAAGCCTGTAGCTAGCGTCATCCTGAGCCAGGATCAAACTCT
>461
CTGCTGCCGCCCGTAGGAGTTTGGACCGTGTCTCAGTTCCAATGTGGGGGACCTTCCTCTCAGAACCCCTACT
GATCGTCGCCTTTGGTGGGCCGTTACCCCGCCAACAAGCTAATCAGACGCATCCCCATCCATAACCGCTAAAC
CTTTAATCGTTTATTTTGATGTCAGCAAACGATATCATACGGTATTTAGGCCGTCTTTTCAACGGGTTTATCC
CGTAGTTTATGGGAAGGTTTGGATACGCGTTACTCACCCGTGCGCCAGTCGTCATCAAGGAAAGCAAGCTTTC
CCTTCATGTTTACCCTGCGACATGCATGCGTTAAGCCTTGTAGCTAGCGTTCATCCTGAGCCAGGATCAAACT
CT
>462
CTGCTGCCGCCCGTAGGAGTTTGAACCGTGTCTCAGTTCCAATGTGGGGGACCTTCCTCTCAGAACCCCTACT
GATCGTTGCCTTGGTGGGCCGTTACCCCACCAACAAGCTAATCAGACGCATCCCCATCCATAACCGATAAATC
TTTATTTCGTAATCTCATGAGATCAAACGAAATATATAAGGTATTTAGTCCAACTTCGCTGGGTTATCCCTTA
GTTTTATGGGCAGGTTTGGATACGCGTTACTCACCCGTGCGCCGGTCGCCGACATAAATAGCAAGCTATCTAT
TCGCTGCCCCCCCTCGACTTGCATGTGTTTAAGCCTATAGCTAGCGTTCATCCTGAGCCAGGATCAAACTCT
>463
CTGCTGCCGCCCGTAGGAGTTTGGACCGTGTCTCAGTTCCAATGTGGGGGACCTTCCTCTCAGAACCCCTACT
GATCGTCGCCTTGGTGGGCCGTTACCCCGCCAACAAGCTAATCAGACGCATCCCCATCCATAACCGCTAAACC
TTTAATCGTTATTTGATGTCAGCAAACGATGATCATACGGTATTAGCCGTCTTTCAACGGGTTATCCCGTGTA
TGGGAAGGTTGGATACGCGTTACTCACCCGTGCGCCAGTCGTCGTCAAGGGAAGCAAGCATTCCCTCACGTTA
CCTTGCCGA
>464
CTGCTGCCGCCCGTAGGAGTTTGGACCGTGTCTCAGTTCCAATGTGGGGGACCTTCCTCTCAGAACCCCTACT
GATCGTCGCCTTTGGTGGGCCGTTACCCCGCCAACAAGCTAATCAGACGCATCCCCATCCATCACCGCTAAAC
CTTTAATCGTTATTTGATGTCAGCAAACGATGTCATACGGTATTAGTCCGTCTTTCAACGGGTTATCCCGTGG

TCATGGGAAGGTTGGATACGCGTTACTCACCCGTGCGCCAGTCGCCGTCAAGGGAAGCAAGCTTCCCTCACGT
TACCCTGCGACTTGCATGTGTTAAGCCTGTAGCTAGCGTTCATCCTGAGCCAGGATCAAACTCT
>465
CTGCTGCCGCCCGTAGGAGTTTGGACCGTGTCTCAGTTCCAATGTGGGGGACCTTCCTCTCAGAACCCCTACT
GATCGTCGCCTTGGTGGGCCGTTACCCCGCCAACAAGCTAATCAGACGCATCCCCATCCATAACCGCTAAACC
TTTAATCGTTATTTGATGTCAGCAAACGATATCATACGGTATTAGGCCGTCTTTCAACGGGTTATCCCGTAGT
TATGGGAAGGTTGGATACGCGTTACTCACCCGTGCGCCAGTCGTCATCAAGGAAAGCAAGCTTTCCTCATGTT
ACCCTGCGACATGCATGTGTTAAGCCTGTACGCTAGCGTTATCCCGTGAGCCAAGATCA
>466
CTGCTGCCGCCCGTAGGAGTTTGAACCGTGTCTCAGTTCCAATGTGGGGGACCTTCCTCTCAGAACCCCTACT
GATCGTTGCCTTGGTGGGCCGTTACCCCACCAACAAGCTAATCAGACGCATCCCCATCCATAACCGATAAATC
TTTATTTCGTAATCTCATGAGATCAAACGAATACATAAGGTATTTAGTCCAACTTCGCTGGGTTTATCCCTTA
GTTATGGGCAGGTTTGGACACGCGTTACTCACCCGTGCGCCGGTCGCCGACATAAATAGCAAGCTATCTATTC
GCTGCCCCCCTCGACTTGCATGTGTTTAAGCCTTATAGCTAGCGTTCATCCTGAGCCAGGATCAAACTCT
>467
CTGCTGCCGCCCGTAGGAGTTTGGACCGTGTCTCAGTTCCAATGTGGGGGACCTTCCTCTCAGAACCCTACTG
ATCGTTGCCTTGGTGGGCCGTTACCCCGCCAACAAGCTAATCAGACGCATCCCCATCCATAACCGATAAATCT
TTATTCGTAATCTCATGAGATCAAACGAAATACATAAGGTATTAGTCCAACTTCGCTGGGTTATCCCTTAGTT
ATGGGCAGGTTGGATACGCGTTACTCACCCGTGCGCCGGTCGCCGACATAAATAGCAAGCTATCTATTCGCTG
CCCCTCGACTTGCATGTGTTTAAGCCTATAGCTAGCGTTCATCCTGAGCCAGGATCAAACTCT
>468
CTGCTGCCGCCCGTAGGAGTTTGGACCGTGTCTCAGTTCCAATGTGGGGGACCTTCCTCTCAGAACCCTACTG
ATCGTCGCCTTGGTGGGCCGTTACCCCGCCAACAAGCTAATCAGACGCATCCCCATCCATAACCGATAAATCT
TTATTCGTAATCTCATGAGATCAAACGGAATACATAAGGTATTAGTCCAACTTCGCTGGGTTATCCCTTAGTT
ATGGGCAGGTTGGATACGCGTTACTCACCCGTGCGCCGGTCGCCGACATAAATAGCAAGCTATCTATTCGCTG
CCCCTCGACTTGCATGTGTTTAACGCCTATAGCTAGCGTTCATCCTGAGCCAGGATCAAACTCT
>469
CTGCTGCCGCCCGTAGGAGTTTGGACCGTGTCTCAGTTCCAATGTGGGGGACCTTCCTCTCAGAACCCCTACT
GATCGTTGCCTTTGGTGGGCCGTTACCCCGCCAACAAGCTAATCAGACGCATCCCCATCCATAACCGATAAAT
CTTTATTCATAATCTCATGAGAATCAAACGAATACATAAGGTATTAGTCCAACTTCGCTGGGTTATCCCTTAG
TTATGGGCAGGTTGGATACGCGTTACTCACCCGTGCGCCGGTCGCCGACATAAATAGCAAGCTATCTATTCGC
TGCCCCTCGACTTGCATGTGTTAAGCCTATAGCTAGCGTTCATCCTGAGCCAGGATCAAACTCT
>470
CTGCTGCCGCCCGTAGGAGTTTGGACCGTGTCTCAGTTCCAATGTGGGGGACCTTCCTCTCAGAACCCCTACT
GATCGTCGCCTTTGGTGGGCCGTTACCCCGCCAACAAGCTAATCAGACGCATCCCCATCCATAACCGCTAAAC
CTTTAATCGTTATTTGATGTCAGCAAACGATATCATACGGTATTAGGCCGTCTTTCAACGGGTTATCCCGTAG
TTATGGGAAGGTTGGATACGCGTTACTCACCCGTGCGCCAGTCGTCATCAAGGAAAGCAAGCTTTCCTCATGT
TACCCTGCGACTTGCATGTGTTAAGCCTGTAGCTAGCGTTCATCCCTGAGCCAGGATCAAACTCT
>471
CTGCTGCCGCCCGTAGGAGTTTGGACCGTGTCTCAGTTCCAATGTGGGGGACCTTCCTCTCAGAACCCCTACT
GATCGTCGCCTTTGGTGGGCCGTTACCCCGCCAACAAGCTAATCAGACGCATCCCCATCCATAACCGCTAACC
TTTAATCGTTTATTTGATGTCAGCAAACGATATCATACGGTATTTAGGCCGTCTTTCAACGGGTTTATCCCGT
AGTTTATGGGAAGGTTGGATACGCGTTACTCACCCGTGCGCCAGTCGTCATCAAGGAAAGCAAGCTTTCCCTT
CACTGTTTT
>472
CTGCTGCCGCCCTAGGAGTTTGGACCGTGTCTCAGTTCCAATGTGGCCGATCACCCTCTCAGGTCGGCTACTG
ATCGTCGCCTTGGTGGGCAGTTATCTCACCAACTAGCTAATCAGACGCGGGTCCATCCTATACCGCTAACACT
TTGATATATCTACGATGCCGTAAATATATATTTATCATGTATTAGTAAACTTTTCAGTATGTTTATCCATGTG
TATTAGGGCAGGTTTACCCACGCGTTACTCACCCGTCCGCCGCTCATTCTTTCATCAATGCAAGCATCAAATC
ATACGCGCTCGACTTGCATGTGTTAGGCACGCCGCCAGCGTTCATCCTGAGCCAGGATCAAACTCT
>473
CTGCTGCCGCCCGTAGGAGTTTTGGACCGTGTCTCAGTTCCAATGTGGGGGACCTTCCTCTCAGAACCCCTAC
TGATCGTCGCCTTTGGTGGGCCGTTACCCCGCCAACAAGCTAATCAGACGCATCCCCATCCATCACCGCTAAA
CCTTTAATCGTTATTTGATGTCAGCAAACGATGTCATACGGTATTAGGCCGTCTTTCAACGGGTTATCCCGTA

GTTATGGGAAGGTTGGATACGCGTTACTCACCCGTGCGCCAGTCGTCATCAAGGAAAGCAAGCATTTCCCTCA
TGATTTTACCCTGCGTACTTGCAATGTG
>474
CTGCTGCCGCCCGTAGGAGTTTGGACCGTGTCTCAGTTCCTATGTGGGGGCCTTCCTCTCAGAACCCCTACT
GATCGTCGCCTTGGTGGGCCGTTACCCCGCCAACAAGCTAATCAGACGCATCCCCATCCATAACCGCTAAACC
TTTTAATCGTTTATTTTGATGTCAGCAAACGATATCATACGGTTATTTAGGCCGTCTTTTCAACGGGTTTATT
CCCGTAGTTTATGGGAAGGTTTGGATACGCGTTACTCACCCGTGCGCCAGTCGTCATCAGGGAAAGCAAGCTT
TTCCTCATGCTTTT
>475
CTGCTGCCGCCCGTAGGAGTTTTGGACCGTGTCTCAGTTCCAATGTGGGGGACCTTCCTCTCAGAACCCCTAC
TGATCGTTGCCTTTGGTGGGCCGTTACCCCGCCAACAAGCTAATCAGACGCATCCCCATCCATAACCGATAAA
TCTTTATTCATAATCTCATGAGATCAAACGAATACATAAGGTATTAGTCCAACTTCGCTGGGTTATCCCTTAG
TTATGGGCAGGTTGGATACGCGTTACTCACCCGTGCGCCGGTCGCCGACATAAATAGCAAGCTATCTATTCGC
TGCCCCCCTCGACTTGCATGTGTTAAGCCTATAGCTAGCGTTCATCCTGAGCCAGGATCAAACTCT
>476
CTGCTGCGCCCGTAGGAGTTTGGACCGTGTCTCAGTTCCAATGTGGGGGACCTTCCTCTCAGAACCCCTACTG
ATCGTTGCCTTGGTGGGCCGTTACCCCGCCAACAAGCTAATCAGACGCATCCCCATCCATAACCGATAAATCT
TTATTCGTAATCTCATGAGATCAAACGAATACATAAGGTATTAGTCCAACTTCGCTGGGTTATCCCTTAGTTA
TGGGCAGGTTGGATACGCGTTACTCACCCGTGCGCCGGTCGCCGACATAAATAGCAAGCTATCTATTCGCTGC
CCCTCGACTTGCATGTGTTAAGCCTATAGCTAGCGTTCATCCTGAGCCAGGATCAAACTCT
>477
CTGCTGCCGCCCGTAGGAGTCTGGACCGTGTCTCAGTTCCAGTGTGGCCGTTCATCCTCTCAGACCGGCTATT
GATCATCGTCTTGGTAGGCCGTTACCCCACCAACTAACTAATCAAACGCAAACCCCTCCTGCGGCGGTAGCTT
TCTTTCGAGAGGCCACCTTTCCTGTCGGTTTCATGCGAAACCGGAGTCTATTGGGTATTAGCAGTCGTTTTCC
GTCTGTTTGTCCCCATCCGTTGGGCAGGTTTGTTTTACGCGTTACTCACCCGTTTGCCACTAAGATTTACAAG
AGCAAGCTCTCGTATCTCTCCGTTCGACTTGCATGTGTTTAGGCACGCCGCCAGCGTTCGTCCTGAGCCAGGA
TCAAACTCT
>478
CTGCTGCCGCCCGTAGGAGTTTTGGACCGTGTCTCAGTTCCAATGTGGGGGACCTTCCTCTCAGAACCCCTAC
TGATCGTTGCCTTTGGTGGGCCGTTACCCCGCCAACAAGCTAATCAGACGCATCCCCATCCATAACCGATAAA
TCTTTATTTCGTAATCTCATGAGATCAAACGAATACATAAGGTATTTAGTCCAACTTCGCTGGGTTTATCCCT
TAGTTTATGGGCAGGTTTGGATACGCGTTACTCACCCGTGCGCCGGTCGCCGACATAAATAGCAAGCTATCTA
TTCGCTGCCCCTCGACTTGCATGTGTTTAAGCCTTATAGCTAGCGTTCATCCTGAGCCAGGATCAAACTCT
>479
CTGCTGCCGCCCGTAGGAGTCTGGACCGTGTCTCAGTTCCAATGTGGCCGTTCATCCTCTCAGAACCCCTACT
GATCGTCGCCTTGGTGGGCCGTTACCCCGCCAACAAGCTAATCAGACGCATCCCCATCCATCACCGCTAAACC
TTTAATCGTTATTTGATGTCAGCAAACGATGTCATATGGTATTAGTCCGTCTTTCAACGGGTTATCCCGTGGT
CATGGGAAGGTTGGATACGCGTTACTCACCCGTGCGCCAGTCGTCGTCAAGGGAAGCAAGCTTCCCTCACGTT
ACCCTGCGACTTGCATGTGTTTAAGCCTGTAGCTTAGCGTTCATCCTGAGCCAGGATCAAACTCT
>480
CTGCTGCCGCCCGTAGGAGTTTGGACCGTGTCTCAGTTCCAATGTGGGGGACCTTCCTCTCAGAACCCCTACT
GATCGTCGCCTTGGTGGGCCGTTACCCCGCCAACAAGCTAATCAGACGCATCCCCATCCATCACCGCTAAACC
TTTAATCGTTATTTTGATGTCAGCAAACGATGTCATACGGTATTAGTCCGTCTTTTCAACGGGTTATCCCGTG
GTCATGGGAAGGTTGGATACGCGTTACTCACCCGTGCGCCAGTCGTCGTCAAGGGAAGCAAGCTTCCCTCACG
TTACCCTGCGACTTGCATGTGTTAAGCCTGTAGCTAGCGTTCATCCCTGAGCAAGGCATCAACTCT
>481
CTGCTGCCGCCCGTAGGAGTTTGAACCGTGTCTCAGTTCCAATGTGGGGGACCTTCCTCTCAGAACCCCTACT
GATCGTTGCCTTGGTGGGCCGTTACCCCACCAACAAGCTAATCAGACGCATCCCCATCCATAACCGATAAATC
TTTATTTCGTAATCTCATGAGATCAAACGAATATATAAGGTATTTAGTTCCAACTTCGCTGGGTTATCCCTTA
GTTTTATGGGCAGGTTTGGATACGCGTTACTCACCCGTGCGCCGGTCGCCGACATAAATAGCAAGCTATCTAT
TTCGCTGCCCCCCCCTCGACTTTGCATTGTGTTTTAAGCCTTATAGCTAGCGTTTCATCCTGAGCCAGGATCA
AACTCT
>482

CTGCTGCCGCCCGTAGGAGTTTGGACCGTGTCTCAGTTCCAATGTGGGGGACCTTCCTCTCAGAACCCCTACT
GATCGTCGCCTTGGTGGGCCGTTACCCCGCCAACAAGCTAATCAGACGCATCCCCATCCATAACCGCTAAACC
TTTAATCGTTATTTGATGTCAGCAAACGATATCATACGGTATTAGGCCGTCTTTCAACGGGTTATCCCGTAGT
TATGGGAAGGTTGGATACGCGTTACTCACCCGTGCGCCAGTCGTCATCAAGGAAAGCAAGCTTCCCTGACTGT
TACCCTGCGACTTGCATGTGTAAGCCTGTAGCTAGCGTTCATCCGTGAGCCAAGGATCAAACCT
>483
CTGCTGCCGCCCGTAGGAGTCTGGACCGTGTCTCAGTTCCAGTGTGGCCGTTCATCCTCTCAGACCGGCTACT
GATCGTTGGTTTGGTGGGCCGTTACCCCACCAACTGCCTAATCAGACGCAAACCCCTCTTCAGGCGATAGCTT
TACAGGTAGAGGCTACCCTTTTCTTTCCACAGGTCATGCGGCCCGTGGAACGTATTTCGGTATTTAGCAGTCG
TTTTTCCGCTGTTTGTCCCCCCATCCTGAAGGCAGGTTTGTTTTTTACGTGTTACTCACCCGTTTTTGCCACT
GGATTTTCGATAA
>484
CTGCTGCCGCCCGTAGGAGTTGAACCGTGTCTCAGTTCCAATGTGGGGACCTTCCTCTCAGAACCCCTACTGA
TCGTTGCCTTGGTGGGCCGTTACCCCACCAACAAGCTAATCAGACGCATCCCCATCCATAACCGCTAAACCTT
TAATCGTTATTTTGATGTCAGCAAACGATGTCATACGGTATTTAGTCCGTCTTTTCAACGGGTTTATCCCGTG
GTCATGGGAAGGTTGGATACGCGTTACTCACCCGTGCGCCAGTCGTCGTCAAGGGAAGCAAGCTTCCCTCACG
TACCCTGCGACTTGCATGTGTTTAAGCCTTGTAGCTAGCGTCATCCTGAGCCAAGGATCAACTCT
>485
CTGCTGCCGCCCGTAGGAGTTTGGACCGTGTCTCAGTTCCAATGTGGGGGACCTTCCTCTCAGAACCCCTACT
GATCGTCGCCTTGGTGGGCCGTTACCCCGCCAACAAGCTAATCAGACGCATCCCCATCCATAACCGCTAAACC
TTTAATCGTTATTTGATGTCAGCAAACGATATCATACGGTATTAGGCCGTCTTTTCAACGGGTTATCCCGTAG
TTATGGGAAGGTTGGATACGCGTTACTCACCCGTGCGCCAGTCGTCATCAAGGAAAGCAAGCTTTCCTCATGT
TACCCTGCGACATGCATGTGTTAAGCCTGTAGCTAGCGTTCATCCGTGAGCCAAGGATCAACTCT
>486
CTGCTGCCGCCCGTAGGAGTTTGGACCGTGTCTCAGTTCCAATGTGGGGGACCTTCCTCTCAGAACCCCTACT
GATCGTCGCCTTGGTGGGCCGTTACCCCGCCAACAAGCTAATCAGACGCATCCCCATCCATCACCGCTAAACC
TTTTAATCGTTTATTTTGATGTCAGCAAACGATGTCATACGGTATTTAGTCCGTCTTTTCAACGGGTTTATCC
CGTGGTCATGGGAAGGTTGGATACGCGTTACTCACCCGTGCGCCAGTCGTCGTCAAGGGAAGCAAGCTTCCCT
TCACGTACCCTGCGACTTGCATGTGTTAAGCCTTGTAGCTAGCGTCATCCTGAGCCAGGATCAAACTCT
>487
CTGCTGCCGCCCGTAGGAGTTTGGACCGTGTCTCAGTTCCAATGTGGGGGACCTTCCTCTCGGAACCCCTACT
GATCGTCGCCTTGGTGGGCCGTTACCCCGCCAACAACGCTAATCAGACGCATCCCCATCCATAACCGCTAAAC
CTTTAATCGTTATTTGATGTCAGCAAACGATATCATACGGTATTAGGCCGTCTTCAACGGGTTATCCCGTAGT
TATGGGAAGGTTGGATACGCGTACTCACCCGTGCGCCAGCGTCATCAAGGAAAGCAAGCTTCCTCATGTTACC
CTGCGACTGCATGTGTAAGCCTGTAGCTAGCGTCATCCTGAGCCAGGATCAACTCT
>488
CTGCTGCCGCCCGTAGGAGTTTGGACCGTGTCTCAGTTCCAATGTGGGGGACCTTCCTCTCAGAACCCCTACT
GATCGTCGCCTTGGTGGGCCGTTACCCCGCCAACAAGCTAATCAGACGCATCCCCATCCATAACCGCTAAACC
TTTAATCGTTATTTGATGTCAGCAAACGACGTCATACGGTATTAGTCCGTCTTTCAACGGGTTATCCCGTGGT
CATGGGAAGGTTGGATACGCGTTACTCACCCGTGCGCCAGTCGTCGTCAAGGAAAGCAAGCTTTCCTCACGTT
ACCCTGCGACTTGCATGTGTTTAAGCCTGTAGCTAGCGTTCATCCTGAGCCAGGATCAAACTCT
>489
CTGCTGCCGCCCGTAGGAGTTTGGACCGTGTCTCAGTTCCAATGTGGGGGACCTTCCTCTCAGAACCCCTACT
GATCGTCGCCTTTGGTGGGCCGTTACCCCGCCAACAAGCTAATACAGACGCATCCCCATCCATAACCGCTAAA
CCTTTAATCGTTATTTGATGTCAGCAAACGATGTCATACGGTATTAGTCCGTCTTTCAACGGGTTATCCCGTG
GTCATGGGAAGGTTGGATACGCGTTACTCACCCGTGCGCCAGTCGTCCGTCAAGGGAAGCAAGCTTCCCCTCA
CGTTACCCTGCGACTTGCATGTGTTAAGCCTGTAGCTAGCGTTCATCCCTGAGCAAGGCATCA
>490
CTGCTGCCGCCCGTAGGAGTTTTGGACCGTGTCTCAGTTCCAATGTGGGGGACCTTCCTCTCAGAACCCCTAC
TGATCGTCGCCTTTGGTGGGCCGTTACCCCGCCAACAAGCTAATCAGACGCATCCCCATCCATCACCGCTAAA
CCTTTAATCGTTATTTGATGTCAGCAAACGACGTCATACGGTATTAGTCCGTCTTTCAACGGGTTATCCCGTG
GTCATGGGAAGGTTGGATACGCGTTACTCACCCGTGCGCCAGCGTCATCAAGGAAAGCAAGCTTCCTCATGTT
ACCCTGCGACTTGCATGTGTTAAGCCTGTAGCTAGCGTTCATCCTGAGCCAGGATCAAACTCT
>491

CTGCTGCCGCCCGTAGGAGTTTGGACCGTGTCTCAGTTCCAATGTGGGGGACCTTCCTCTCAGAACCCCTACT
GATCGTTGCCTTTGGTGGGCCGTTACCCCGCCAACAAGCTAATCAGACGCATCCCCATCCATAACCGATAAAT
CTTTATTCGTAATCTCATGAGATCAAACGAAATACATAAGGTATTAGTCCAACTTCGCTGGGTTATCCCTTAG
TTATGGGCAGGTTGGATACGCGTTACCTCACCCGTGCGCCGGTCGCCGACATAAATAGCAAGCTATCTATTCG
CTGCCCCTCGACTTGCATGTGTTAAGCCTATAGCTAGCGTTCATCCTGAGCCAGGATCAAACTCT
>492
CTGCTGCCGCCCGTAGGAGTTTGGACCGTGTCTCAGTTCCAATGTGGGGGACCTTCCTCTCAGAACCCCTACT
GATCGTCGCCTTTGGTGGGCCGTTACCCCGCCAACAAGCTAATCAGACGCATCCCCATCCATAACCGCTAAAC
CTTTAATCGTTATTTGATGTCAGCAAACGATATCATACGGTATTAGGCCGTCTTTCAACGGGTTATCCCGTAG
TTATGGGAAGGTTGGATACGCGTTACTCACCCGTGCGCCAGTCGTCATCAAGGAAAGCAAGCTTTCCCTCATG
ATTGCCCATGCCGTACATGACATGTGTTAAGCCTAGGTACG
>493
CTGCTGCCGCCCGTAGGAGTTTGGACCGTGTCTCAGTTCCAATGTGGGGGACCTTCCTCTCAGAACCCCTACT
GATCGTCGCCTTGGTGGGCCGTTACCCCGCCAACAAGCTAATCAGACGCATCCCCATCCATAACCGCTAAACC
TTTAATCGTTATTTGATGTCAGCAAACGATATCATACGGTATTAGGCCGTCTTTCAACGGGTTATCCCGTGGT
CATGGGAAGGTTGGATACGCGTTACTCACCCGTGCGCCAGTCGTCGTCAAGGGAAGCAAGCTTCCCTCACGTT
ACCCTGCGACTTGCATGTGTTAAGCCTGTAGCTAGCGTTCATCCCTGAGCAAGGCATCGAAA
>494
CTGCTGCCGCCCGTAGGAGTTTTGGACCGTGTCTCAGTTCCAATGTGGGGGACCTTCCTCTCAGAACCCCTAC
TGATCGTCGCCTTTGGTGGGCCGTTACCCCGCCAACAAGCTAATCAGACGCATCCCCATCCATAACCGATAAA
TCTTTATTCGTAATCTCATGAGATCAAACGAAATACATAAGGTATTAGTCCAACTTCGCTGGGTTATCCCTTA
GTTATGGGCAGGTTGGATACGCGTTACTCACCCGTGCGCCGGTCGCCGACATAAATAGCAAGCTATCTATTCG
CTGCCCCTCGACTTGCATGTGTTAAGCCTATAGCTAGCGTTCATCCTGAGCCAGGATCAAACTCT
>495
CTGCTGCCGCCCGTAGGAGTTTGGACCGTGTCTCAGTTCCAATGTGGGGGACCTTCCTCTCAGAACCCCTACT
GATCGTCGCCTTTGGTGGGCCGTTACCCCGCCAACAAGCTAATCAGACGCATCCCCATCCATAACCGCTAAAC
CTTTAATCGTTATTTGATGTCAGCAAACGATATCATACGGTATTAGGCCGTCTTTCAACGGGTTATCCCGTAG
TTATGGGAAGGTTGGATACGCGTTACTCACCCGTGCGCCAGTCGTCATCAAGGAAAGCAAGCTTTCCCTCATG
TTACCCTGCGACTTGCATGTGTTAAGCCTGTAGCTAGCGTTCATCCGTGAGCCAAGGCATCAAACTCTAGACT
AGCC
>496
CTGCTGCCGCCCGTAGGAGTTTGGACCGTGTCTCAGTTCCAATGTGGGGGACCTTCCTCTCAGAACCCCTACT
GATCGTCGCCTTGGTGGGCCGTTACCCCGCCAACAAGCTAATCAGACGCATCCCCATCCATCACCGCTAAACC
TTTAATCGTTATTTGATGTTAGCAAACGATGTCATACGGTATTAGTCCGTCTTTCAACGGGTTAGTCCCGTAG
TTATGGGAAGGTTGGATACGCGTTACTCACCCGTGCGCCGGTCGCCGACATAAATAGCAAGCTATCTATTCGC
TGCCCCTCGACTTGCATGTGTTAAGCCTATAGCTAGCGTTCATCCTGAGCCAGGATCAAACTCT
>497
CTGCTGCCGCCCGTAGGAGTTTGGACCGTGTCTCAGTTCCAATGTGGGGGACCTTCCTCTCAGAACCCCTACT
GATCGTTGCCTTGGTGGGCCGTTACCCCGCCAACAAGCTAATCAGACGCATCCCCATCCATAACCGATAAATC
TTATTCATAATCTCATGAGATCAAACGAATACATAAGGTATTAGTCCAACTTCGCCGGGTTATCCCTTAGTTA
TGGGCAGGTTGGATACGCGTTACCGTCACCCGTGCGCCGGTCGCCGACATAAATAGCAAGCTATCTATTCGCT
GCCCCTCGACTTGCATGTGTTAAGCCTATAGCTAGCGTTCATCCTGAGCCAGGATCAAACTCT
>498
CTGCTGCCGCCCGTAGGAGTTTTGGACCGTGTCTCAGTTCCAATGTGGGGGACCTTCCTCTCAGAACCCCTAC
TGATCGTCGCCTTGGTGGGCCGTTACCCCACCAACTGCCTAATCAGACGCAAACCCCTCTTCAGGCGATAGCT
TACAGGTAGAGGCTACCCTTTCTTCCACAGGTCATGCGACCCGTGGAACGTATTCGGTATTAGCAGTCGTTTC
CGTCTGTTGTCCCCATCCTGAAGGCAGGTTGTTTACGTGTTACTCACCCGTTTGCCACTGGATTCGATAAAGA
GCAAGCTCTTCATCTCTTCCCGTCGACTGCATGTGTTAGCACGCCGCCAGCGTCGTCCTGAGCCAGGATCAAA
CTCT
>499
CTGCTGCCGCCCGTAGGAGTTTGGACCGTGTCTCAGTTCCAATGTGGGGGACCTTCCTCTCAGAACCCCTACT
GATCGTCGCCTTTGGTGGGCCGTTACCCCGCCAACAAGCTAATCAGACGCATCCCCATCCATCACCGCTAAAC
CTTTAATCGTTATTTGATGTCAGCAAACGATGTCATACGGTATTAGTCCGTCTTTCAACGGGTTATCCCGTGG

TCATGGGAAGGTTGGATACGCGTTACTCACCCGTGCGCCAGTCGTCGTCAAGGAAAGCAAGCTTCCTCACGTT
ACCCTGCGACTGCATGTGTTAAGCCTGTAGCTAGCGTCATCCTGAGCCAGGATCAAACTCT
>500
CTGCTGCCGCCCGTAGGAGTTTGGACCGTGTCTCAGTTCCAATGTGGGGGACCTTCCTCTCAGAACCCCTACT
GATCGTTGCCTTGGTGGGCCGTTACCCCGCCAACAAGCTAATCAGACGCATCCCCATCCATCACCGCTAAACC
TTTAATCGTTTATTTTGATGTCAGCAAACGATGTCATACGGTTATTTAGTCCGTCTTTTCAACGGGTTTATTC
CCCGTGGTCATGGGAAGGTTTGGATACGCGTTACTCACCCGTGCGCCAGTCGTCGTCAAGGGAAGCAAGCTTC
CCTTCACGTTACCTGCGACCTTTGCATGTGTTTAAGCCTTGTAGCTAGCGTCATCCGTTGAGCCAAGGCATTC
AAAC
>501
CTGCTGCCGCCCGTAGGAGTTTTGGACCGTGTCTCAGTTCCAATGTGGGGGACCTTCCTCTCAGAACCCCTAC
TGATCGTTGCCTTGGTGGGCCGTTACCCCGCCAACAAGCTAATCAGACGCATCCCCATCCATAACCGATAAAT
CTTTATTCGTAATCTCATGAGATCAAACGAATACATAAGGTATTTAGTCCAACTTCGCTGGGTTTATCCCTTA
GTTATGGGCAGGTTTGGATACGCGTTACTCACCCGTGCGCCGGTCGCCGACATAAATAGCAAGCTATCTATTC
GCTGCCCCTTCGACTTGCATGTGTTTAAGCCTTATAGCTAGCGTTCATCCTTGAGCCAGGATCAAACTCT
>502
CTGCTGCCGCCCGTAGGAGTTTTGGACCGTGTCTCAGTTCCAATGTGGGGGACCTTCCTCTCAGAACCCCTAC
TGATCGTTGCCTTTGGTGGGCCGTTACCCCGCCAACAAGCTAATCAGACGCATCCCCATCCATAACCGATAAA
TCTTTATTCGTAATCTCATGAGATCAAACGAATACATAAGGTATTAGTCCAACTTCGCTGGGTTATCCCTTAG
TTATGGGCAGGTTGGATACGCGTTACTCACCCGTGCGCCGGTCGCCGACATAAATAGCAAGCTATCTATTCGC
TGCCCCTCGACTTGCATGTGTTAAGCCTATAGCTAGCGTTCATCCTGAGCCAGGATCAAACTCT
>503
CTGCTGCCGCCCGTAGGAGTTTGGACCGTGTCTCAGTTCCAATGTGGGGGACCTTCCTCTCAGAACCCCTACT
GATCGTCGCCTTTGGTGGGCCGTTACCCCGCCAACAAGCTAATCAGACGCATCCCCATCCATCACCGCTAAAC
CTTTAATCGTTATTTGATGTCAGCAAACGATGTCATACGGTATTAGTCCGTCTTTCAACGGGTTATCCCGTGT
TCATGGGAAGGTTGGATACGCGTTACTCACCCGTGCGCCAGTCGTCGTCAAGGGAAGCAAGCTTCCCTCACGT
TACCCTTGCCGACGTAGCT
>504
CTGCTGCCGCCCGTAGGGGTCTGGACCGTGTCTCAGTTCCAATGTGGGGGACCTTCCTCTCAGAACCCCTACT
GATCGTCGCCTTGGTGGGCCGTTACCCCGCCAACAAGCTAATCAGACGCATCCCCATCCATAACCGCTAAACC
TTTAATCGTTATTTGATGTCAGCAAACGACGTCATACGGTATTTAGTCCGTCTTTCAACGGGTTTATCCCGTG
GTCATGGGAAGGTTTGGATACGCGTTACTCACCCGTGCGCCAGTCGTCGTCAAGGAAAGCAAGCTTTCCTCAT
GTTACCCTGCGACATGCATGTGTTTAAGCCTGTAGCTAGCGTTCATCCTGAGCCAGGATCAAACTCT
>505
CTGCTGCCGCCCGTAGGAGTTTTGGACCGTGTCTCAGTTCCAATGTGGGGGACCTTCCTCTCAGAACCCCTAC
TGATCGTTGCCTTTGGTGGGCCGTTACCCCGCCAACAAGCTAATCAGACGCATCCCCATCCATAGCCGCTAAA
CCTTTAATCGTTATTTGATGTCAGCAAACGACGTCATACGGTATTAGTCCGTCTTTCAACGGGTTATCCCGTG
GTCATGGGAAGGTTGGATACGCGTTACTCACCCGTGCGCCAGTCGTCATCAAGGAAAGCAAGCTTTCCTCATG
TCACCCTGCGACTTGCATGTGTTAAGCCTGTAGCTAGCGTTCATCCTGAGCCAGGATCAAACTCT
>506
CTGCTGCCGCCCGTAGGAGTTTGGACCGTGTCTCAGTTCCAATGTGGGGGACCTTCCTCTCAGAACCCCTACT
GATCGTCGCCTTTGGTGGGCCGTTACCCCGCCAACAAGCTAATCAGACGCATCCCCATCCATAACCGCTAAAC
CTTTAATCGTTATTTGATGTCAGCAAACGACGTCATACGGTATTAGTCCGTCTTTCAACGGGTTATCCCGTGG
TCATGGGAAGGTTGGATACGCGTTACTCACCCGTGCGCCAGTCGTCGTCAAGGAAAGCAAGCTTTCCTCACGT
TACCCTGCGACTTGCATGTGTTTAAGCCTGTAGCTAGCGTTCATCCTGAGCCAGGATCAAACTCT
>507
CTGCTGCCGCCCGTAGGAGTTTGGACCGTGTCTCAGTTCCAATGTGGGGGACCTTCCTCTCAGAACCCCTACT
GATCGTCGCCTTTGGTGGGCCGTTACCCCCGCCAACAAGCTAATCAGACGCATCCCCCATCCATCACCGCTAA
ACCTTTAATCGTTTATTTTGATGTCAGCAAACGATGTCATACGGTATTTAGTCCGTCTTTTCAACGGGTTTAT
CCCCCGTGGTCATGGGAAGGTTGGATACGCGTTACTCACCCGTGCGCCAGTCGTCGTCAAGGGAAGCAAGCTT
CCCTTCACGTTACCCTGCGACTTTGCATGTGTTTAAGCCTTGTAGCTAGCGTCATCCGTGAGCCAAGGCATCA
AACTCT
>508

CTGCTGCCGCCCGTAGGAGTTTGGACCGTGTCTCAGTTCCAATGTGGGGGACCTTCCTCTCAGAACCCCTACT
GATCGTCGCCTTTGGTGGGCCGTTACCCCGCCAACAAGCTAACTCAGACGCATCCCCATCCATAACCGCTAAA
CCTTTAATCGTTATTTGATGTCAGCAAACGATATCATACGGTATTAGGCCGTCTTTCAACGGGTTATCCCGTA
GTTATGGGAAGGTTGGATACGCGTTACTCACCCGTGCGCCAGTCGTCCATCAAGGAAAGCAAGCTTTCCCTGA
CTGTTACCCTGCGAACTTGCATGTGTTAAGCCTGTAGCTAGCGTTCATCCC
>509
CTGCTGCCGCCCGTAGGAGTTTTGGACCGTGTCTCAGTTCCAATGTGGGGGACCTTCCTCTCAGAACCCCTAC
TGATCGTCGCCTTGGTGGGCCGTTACCCCGCCAACAAGCTAATCAGACGCATCCCCATCCATCACCGCTAAAC
CTTTAATCGTTATTTGATGTCAGCAAACGATGTCATACGGTATTAGTCCGTCTTTCAACGGGTTATCCCGTGG
TCATGGGAAGGTTGGATACGCGTTACTCACCCGTGCGCCAGTCGTCGTCAAGGGAAGCAAGCTTCCCTCACGT
TACCCTGCGACTTGCATGTGTTAAGCCTGTAGCTAGCGTTCATCCGTGAGCCAAGGATCAACTCT
>510
CTGCTGCCGCCCGTAGGAGTTTGGACCGTGTCTCAGTTCCAATGTGGGGGACCTTCCTCTCAGAACCCCTACT
GATCGTCGCCTTGGTGGGCCGTTACCCCGCCAACAAGCTAATCAGACGCATCCCCATCCATCACCGCTAAACC
TTTAATCGTTATTTGATGTCAGCAAACGATGTCATACGGTATTAGTCCGTCTTTTCAACGGGTTATCCCGTGG
TCATGGGAAGGTTGGATACGCGTTACTCACCCGTGCGCCAGTCGTCGTCAAGGGAAGCAAGCTTCCCTCACGT
TACCCTGCGACTTGCATGTGTTAAGCCTGTAGCTAGCGTTCATCCGTGAGCCAAGGATCAAACTCT
>511
CTGCTGCCGCCCGTAGGAGTTTGGACCGTGTCTCAGTTCCAATGTGGGGGACCTTCCTCTCAGAACCCCTACT
GATCGTTGCCTTGGTGGGCCGTTACCCCGCCAACAAGCTAATCAGACGCATCCCCATCCATAACCGATAAATC
TTTATTCGTAATCTCATGAGATCAAACGAATACATAAGGTATTAGTCCAACTTCGCTGGGTTATCCCTTAGTT
ATGGGCAGGTTGGATACGCGTTACTCACCCGTGCGCCGGTCGCCGACATAAATAGCAAGCTATCTATTCGCTG
CCCCTCGACTTGCATGTGTTTAAGCCTATAGCTAGCGTTCATCCTGAGCCAGGATCAAACTCT
>512
CTGCTGCCGCCCGTAGGAGTTTGGACCGTGTCTCAGTTCCAATGTGGCCGATCACCCTCTCAGGTCGGCTACT
GATCGTCGCCTTGGTGGGCAGTTATCTCACCAACTAGCTAATCAGACGCGGGTCCATCCTATACCGCTAACAC
TTTGATATATCTACGATGCCGTAAATATATATTATCATGTATTAGTAAACTTTTCAGTATGTTATCCATGTGT
ATAGGGCAGGTTACCCACGCGTTACTCACCCGTCCGCCGCTCATCTTTCATTAATGCAAGCATCAAATCAGAC
GCGCTCGACTTGCATGTGTTAGGCACGCCGCCAGCGTTCATCCTGAGCCAGGATCAAACTCT
>513
CTGCTGCCGCCCGTAGGAGTTTGGACCGTGTCTCAGTTCCAATGTGGGGGACCTTCCTCTCAGAACCCCTACT
GATCGTCGCCTTGGTGGGCCGTTACCCCGCCAACAAGCTAATCAGACGCATCCCCATCCATAACCGCTAAACC
TTTAATCGTTATTTGATGTCAGCAAACGATATCATACGGTACTAGGCCGTCTTTCAACGGGTTATCCCGTAGT
TATGGGAAGGTTGGATACGCGTTACTCACCCGTGCGCCAGTCGTCATCAAGGAAAGCAAGCTTTCCCTCATGT
TACCCTGCGACTTGCATGTGTTAAGCCTGTAGCTAGCGTTCATCCCTGAGCCAAGGATCAAACTCT
>514
CTGCTGCCGCCCGTAGGAGTTTGGACCGTGTCTCAGTTCCAATGTGGGGGACCTTCCTCTCAGAACCCCTACT
GATCGTCGCCTTGGTGGGCCGTTACCCCGCCAACAAGCTAATCAGACGCATCCCCATCCATAACCGCTAAACC
TTTTAATCGTTATTTTGATGTCAGCAAACGATATCATACGGTATTTAGGCCGTCTTTTTCAACGGGTTTATCC
CGTAGTTTTATGGGAAGGTTGGATACGCGTTACTCACCCGTGCGCCAGTCGTCATCAAGGAAAGCAAGCTTTC
CCTTCGAT
>515
CTGCTGCCGCCCGTAGGAGTTTGGACCGTGTCTCAGTTCCAATGTGGGGGACCTTCCTCTCAGAACCCCTACT
GATCGTTGCCTTGGTGGGCCGTTACCCCGCCAACAAGCTAATCAGACGCATCCCCATCCATAACCGCTAAACC
TTTTAATCGTTTATTTTGATGTCAGCAAACGATATCATACGGTATTTAGGCCGTCTTTTCAACGGGTTTATCC
CGTGGTTCATGGGAAGGTTGGATACGCGTTACTCACCCGTGCGCCAGTCGTCATCAAGGAAAGCAAGCTTTTC
CCTTCATGT
>516
CTGCTGCCGCCCGTAGGAGTTTGGACCGTGTCTCAGTTCCAATGTGGGGGACCTTCCTCTCAGAACCCCTACT
GATCGTCGCCTTGGTGGGCCGTTACCCCGCCAACAAGCTAATCAGACGCATCCCCATCCATAACCGCTAAACC
TTTAATCGTTATTTGATGTCAGCAAACGATATCATACGGTATTAGGCCGTCTTTCAACGGGTTATCCCGTAGT
TATGGGAAGGTTGGATACGCGTTACTCACCCGTGCGCCAGTCGTCATCAAGGAAAGCAAGCTTTCCCTGACTG
TTTACCCTGCGAACTTGCATGTGTTAAGCCTGTAGCTAGCGTTCATCCCTGAGCCAAGATTCAAACTCT
>517

CTGCTGCCGCCCGTAGGAGTTTGGACCGTGTCTCAGTTCCAATGTGGGGGACCTTCCTCTCAGAACCCCTACT
GATCGTCGCCTTTGGTGGGCCGTTACCCCGCCAACAAGCTAATCAGACGCATCCCCATCCATAACCGCTAAAC
CTTTAATCGTTATTTGATGTCAGCAAACGATATCATACGGTATTAGGCCGTCTTTCAACGGGTTATCCCGTAG
TTATGGGAAGGTTGGATACGCGTTACTCACCCGTGCGCCAGTCGTCATCAAGGAAAGCAATGCTTCCCTCATG
TTACCCTGCGACTTGCATGTGTTAAGCCTGTAGCTAGCGTTCATCCTGAGCCAGGATCAAACTCT
>518
CTGCTGCCGCCCGTAGGAGTCTGGACCGTGTCTCAGTTCCAGTGTGGCCGTTCATCCTCTCAGACCGGCTACT
GATCGTTGGTTTGGTGGGCCGTTACCCCACCAACTGCCTAATCAGACGCAAACCCCTCTTCAGGCGATAGCTT
ACAGGTAGAGGCTACCCTTTCTTCCACAGGTCATGCGACCCGTGGAACGTATTCGGTATTTAGCAGTCGTTTT
CCGTCTGTTTGTCCCCATCCTGAAGGCAGGTTGTTTTTACGTGTTACTCACCCGTTTTGCCACTGGATTCGAT
AAAGAGCAAGCTCTTCATCTCTTCCCGTTCGACTTGCATGTGTTTAAGCACGCCGCCAGCGTTCGCCTGAGCC
AGGATCAAACTCT
>519
CTGCTGCCGCCCGTAGGAGTTTGGACCGTGTCTCAGTCCAATGTAGGGGACCTTCCTCTCAGAACCCCTACT
GATCGTTGCCTTTGGTGGGCCGTTACCCCGCCAACAAGCTAATCAGACGCATCCCCATCCATAACCGATAAAT
CTTTATTCATAATCTCATGAGATCAAACGAATACATAAGGTATTTAGTCCAACTTCGCTGGGTTTATCCCTTA
GTTTATGGGCAGGTTTGGATACGCGTTACCTCGACCCGTGCGCCGGTCGCCGACATAAATAGCAAGCTATCTA
TTCGCTGCCCCTCGACTTGCATGTGTTTAAGCCTATAGCTAGCGTTCATCCTGAGCCAGGATCAAACTGC
>520
CTGCTGCCGCCCGTAGGAGTTTGGACCGTGTCTCAGTTCCAATGTGGGGGACCTTCCTCTCAGAACCCCTACT
GATCGTTGCCTTTGGTGGGCCGTTACCCCGCCAACAAGCTAATCAGACGCATCCCCATCCATAACCGATAAAT
CTTTATTTCGTAATCTCATGAGATCAAACGAAATACATAAGGTATTTAGTCCAACTTCGCTGGGTTTATCCCT
TAGTTTATGGGCAGGTTTGGATACGCGTTACTCACCCGTGCGCCGGTCGCCGACATAAATAGCAAGCTATCTA
TTCGCTGCCCCCCCTTCGACTTGCATGTGTTTTAAGCCTTATAGCTAGCGTTCATCCTGAGCCAGGATCAAAC
TCT
>521
CTGCTGCCGCCCGTAGGAGTTTGGACCGTGTCTCAGTTCCAATGTGGCCGATCACCCTCTCAGGTCGGCTACT
GATCGTCGCCTTGGTGGGCAGTTATCTCACCAACTAGCTAATCAGACGCGGGTCCATCCTATACCGCTAACAC
TTGATATATCTACGATGCCGTAAATATATATTTATCATGTATTTAGTTAAACTTTTCAGTATGTTTATCCATG
TGTATAGGGCAGGTTTACCCACGCGTTACTCACCCGTCCGCCGCTCATCTTTCATTTTAATGCAAGCATCAAA
TCAGACGCGCTCGACTTGCATGTGTTTAGGCACGCCGCCAGCGTTCATCCTGAGCCAGGATCAAACTCT
>522
CTGCTGCCGCCCGTAGGAGTTTGGACCGTGTCTCAGTTCCAATGTGGGGGACCTTCCTCTCAGAACCCCTACT
GATCGTCGCCTTTGGTGGGCCGTTACCCCGCCAACAAGCTAATCAGACGCATCCCCATCCATAACCGCTAAAC
CTTTTAATCGTTTATTTTGATGTCAGCAAACGATATCATACGGTATTTAGGCCGTCTTTTTCAACGGGTTTAT
CCCCCGTAGTTTATGGGAAGGTTTGGATACGCGTTACTCACCCGTGCGCCAGTCGTCATCAAGGAAAGCAAGC
TTTTCCCTTCATGT
>523
CTGCTGCCGCCCGTAGGAGTTTGGACCGTGTCTCAGTTCCAATGTGGGGGACCTTCCTCTCAGAACCCCTACT
GATCGTCGCCTTTGGTGGGCCGTTACCCCGCCAACAAGCTAATCAGACGCATCCCCATCCATAACCGCTAAAC
CTTTAATCGTTATTTTGATGTCAGCAAACGATATCATACGGTATTAGGCCGTCTTTTCAACGGGTTATCCCGT
AGTTTATGGGAAGGTTGGATACGCGTTACTCACCCGTGCGCCAGTCGTCATCAAGGAAAGCAAGCTTCCTCAT
GTTACCCTGCGACTGCATGTGTTAAGCCTGTAGCTAGCGTTCATCCCGTGAGCCAAGGATCAAACTCT
>524
CTGCTGCCGCCCGTAGGAGTTTGGACCGTGTCTCAGTTCCAATGTGGGGGACCTTCCTCTCAGAACCCCTACT
GATCGTCGCCTTGGTGGGCCGTTACCCCGCCAACAAGCTAATCAGACGCATCCCCATCCATAACCGCTAAACC
TTTAATCGTTATTTGATGTCAGCAAACGATATCATACGGTATTAGGCCGTCTTTCAACGGGTTATCCCGTAGT
TATGGGAAGGTTGGATACGCGTTACTCACCCGTGCGCCAGTCGTCATCAAGGAAAGCAAGCTTTCCCTCATGT
TACCCTGCGACTTGCATGTGTTAAGCCTGTAGCTAGCGTTCATCCCTGAGCCAAGGCATCAA
>525
CTGCTGCCGCCCGTAGGAGTTTGGACCGTGTCTCAGTTCCAATGTGGGGGACCTTCCTCTCAGAACCCCTAC
TGATCGTCGCCTTTGGTGGGCCGTTACCCCGCCAACAAGCTAATACAGACGCATCCCCATCCATCACCGCTAA
ACCTTTAATCGTTATTTGATGTCAGCAAACGATGTCATACGGTATTAGTCCGTCTTTTCAACGGGTTATCCCG

TGGTCATGGGAAGGTTGGATACGCGTTACTCACCCGTGCGCCAGTCGTCGTCAAGGGAAGCAGCCTCCCCTCG
ACGTTACCCTGCGACTTGCACGTGTTGAAG
>526
CTGCTGCCGCCCGTAGGAGTTTGGACCGTGTCTCAGTTCCAATGTGGGGGACCTTCCTCTCAGAACCCCTACT
GATCGTCGCCTTGGTGGGCCGTTACCCCGCCAACAAGCTAATCAGACGCATCCCCATCCATCACCGCTAAACC
TTTAATCGTTATTTGATGTCAGCAAACGATGTCATACGGTATTAGTCCGTCTTTCAACGGGTTATCCCGTGGT
CATGGGAAGGTTGGATACGCGTTACTCACCCGTGCGCCAGTCGTCGTCAAGGGAAGCAAGCTTCCCTCACGTT
ACCCTGCGACTTGCACGTGTTGAAGCTTAGACG
>527
CTGCTGCCGCCCGTAGGAGTTTGAACCGTGTCTCAGTTCCAATGTGGGGGACCTTCCTCTCAGAACCCCTACT
GATCGTTGCCTTGGGTGGGCCGTTACCCCACCAACAAGCTAATCAGACGCATCCCCATCCATAACCGATAAAT
CTTTATTCGTAATCTCATGAGATCAAACGAAATATATAAGGTATTAGTCCAACTTCGCTGGGTTATCCCTTAG
TTATGGGCAGGTTGGATACGGCGTTACTCGACCCGTGCGCCGGTCGCCCGACATAAATAGCAAGCTATCTATT
CGCTGCCCCTCGACTTGCATGTGTTAAGCCTATAGCTAGCGTTCATCCTGAGCCAGGATCAAACTCT
>528
CTGCTGCCGCCCGTAGGAGTTTTGGACCGTGTCTCAGTTCCAATGTGGGGGACCTTCCTCTCAGAACCCCTAC
TGATCGTCGCCTTTGGTGGGCCGTTACCCCGCCAACAAGCTAATCAGACGCATCCCCATCCATAACCGCTAAA
CCTTTAATCGTTATTTGATGTCAGCAAACGATATCATACGGTATTAGGCCGTCTTTCAACGGGTTATCCCGTA
GTTATGGGAAGGTTGGATACGCGTTACTCACCCGTGCGCCAGTCGTCATCAAGGAAAGCAAGCTTTCCCTCAT
GTTACCCTGCGACATGCATGTGTTAAGCCTGTAGCTAGCGTTCATCCTGAGCCAGGATCAAACTCGT
>529
CTGCTGCCGCCCGTAGGAGTTTTGGACCGTGTCTCAGTTCCAATGTGGGGGACCTTCCTCTCAGAACCCCTAC
TGATCGTTGCCTTGGTGGGCCGTTACCCCGCCAACAAGCTAATCAGACGCATCCCCATCCATAACCGATAAAT
CTTTATTCATAATCTCATGAGAATCAAACGAATACATAAGGTATTAGTCCAACTTCGCTGGGTTATCCCTTAG
TTATGGGCAGGTTGGATACGCGTTACTCACCCGTGCGCCGGTCGCCGACATAAATAGCAAGCTATCTATTCGC
TGCCCCTCGACTTGCATGTGTTAAGCCTATAGCTAGCGTTCATCCTGAGCCAGGATCAAACTCT
>530
CTGCTGCCGCCCGTAGGAGTTTGAACCGTGTCTCAGTTCCAATGTGGGGGACCTTCCTCTCAGAACCCCTACT
GATCGTTGCCTTGTGGGCCGTTACCCCACCAACAAGCTAATCAGACGCATCCCCATCCATAACCGATAAATCT
TATTCGTAATCTCATGAGATCAAACGAAATACATAAGGTATTAGTCCAACTTCGCTGGGTTATCCCTTAGTTA
TGGGCAGGTTGGATACGCGTTACCTCACCCGTGCGCCGGTCGCCGACATAAATAGCAAGCTATCTATTCGCTG
CCCCTCGACTTGCATGTGTTAAGCCTATAGCTAGCGTTCATCCTGAGCCAGGATCAAACTCCT
>531
CTGCTGCCGCCGTAGGAGTTTGGACCGTGTCTCAGTTCCAATGTGGCCGATCACCCTCTCAGGTCGGCTACTG
ATCGTCGCCTTGGTGGGCAGTTATCTCACCAACTAGCTAATCAGACGCGGGTCCATCCTATACCGCTAACACT
TTGATATATCTACGATGCCGTAAATATATATTATCATGTATTAGTAAACTTTTCAGTATGTTATCCATGTGTA
TAGGGCAGGTTACCCACGCGTTACTCACCCGTCCGCCGCTCATCTTCATCAATGCAAGCATCAAATCATACGC
GCTCGACTTGCATGTGTTAGGCACGCCGCCAGCGTTCATCCTGAGCCAGGATCAAACTCT
>532
CTGCTGCCGCCCGTAGGAGTTTTGGACCGTGTCTCAGTTCCAATGTGGGGGACCTTCCTCTCAGAACCCCTAC
TGATCGTTGCCTTGGTGGGCCGTTACCCCGCCAACAAGCTAATCAGACGCATCCCCATCCATAACCGATAAAT
CTTTATTCGTAATCTCATGAGATCAAACGAAATACATAAGGTATTAGTCCAACTTCGCTGGGTTATCCCTTAG
TTATGGGCAGGTTGGATACGCGTTACTCACCCGTGCGCCGGTCGCCGACATAAATAGCAAGCTATCTATTCGC
TGCCCCTCGACTTGCATGTGTTAAGCCTATAGCTAGCGTTCATCCTGAGCCAGGATCAAACTCT
>533
CTGCTGCCGCCCGTAGGAGTTTGGACCGTGTCTCAGTTCCAATGTGGGGGACCTTCCTCTCAGAACCCCTACT
GATCGTCGCCTTGGTGGGCCGTTACCCCGCCAACAAGCTAATCAGACGCATCCCCATCCATCACCGCTAAACC
TTTAATCGTTATTTGATGTCAGCAAACGATATCATACGGTATTAGACCGTCTTTCAACGGGTTATCCCGTAGT
TATGGGAAGGTTGGATACGCGTTACTCACCCGTGCGCCAGTCGTCATCAAGGAAAGCAAGCTTTCCTCATGTT
ACCCTGCGACTTGCATGTGTTAAGCCTGTAGCTAGCGTTCATCCTGAGCCAGGATCAAACTCT
>534
CTGCTGCCGCCCGTAGGAGTTTGGACCGTGTCTCAGTTCCAATGTGGGGGACCTTCCTCTCAGAACCCCTACT
GATCGTCGCCTTGGTGGGCCGTTACCCCGCCAACAAGCTAATCAGACGCATCCCCATACCATAACCGCTAAAC
CTTTAAATCGTTATTTTGATGTCAGGCAAACGATATCATACGGTATTAGGCCGTCTTTTCAACGGGTTATCCC

GTAGTTATGGGAAGGTTGGATACGCGTACTCACCCGTGCGCCAGTCGTCATCAAGGAAAGCAAGCTTTCCCTC
ATGTTACCCGTGCCGTAGA
>535
CTGCTGCCGCCCGTAGGAGTTTGGACCGTGTCTCAGTTCCAATGTGGGGGACCTTCCTCTCAGAACCCCTACT
GATCGTCGCCTTGGTGGGCCGTTACCCCGCCAACAAGCTAATCAGACGCATCCCCATCCATAACCGCTAAACC
TTTAATCGTTATTTTGATGTCAGCAAACGATATCATACGGTATTAGGCCGTCTTTTCAACGGGTTATCCCGTA
GTTATGGGAAGGTTGGATACGCGGTTACTCACCCGTGCGCCAGTCGTCATCAAGGAAAGCAAGCTTCCTCATG
TTACCCTGCGGACTTGCATGTGTTAAGCCTAGTAGCTAGCGTTCATCCCTGGAGGCCAGGATCAAACTCGT
>536
CTGCTGCCGCCCGTAGGAGTTTGGACCGTGTCTCAGTTCCAATGTGGGGGACCTTCCTCTCAGAACCCCTACT
GATCGTCGCCTTGGTGGGCCGTTACCCCGCCAACAAGCTAATCAGACGCATCCCCATCCATAACCGCTAAACC
TTTAATCGTTATTTTTGATGTCAGCAAACGATGTCATACGGTATTAGGCCGTCTTTTCAACGGGTTAGTCCCG
TAGTTTATGGGAAGGTTGGATACGCGTTACTCACCCGTGCGCCAGTCGTCATCAAGGAAAAGCAAGCTTTCCT
CATGTTTACCCTG
>537
CTGCTGCCGCCCGTAGGAGTTTGGACCGTGTCTCAGTTCCAATGTGGGGGACCTTCCTCTCAGAACCCCTACT
GATCGTTGCCTTTGGTGGGCCGTTACCCCGCCAACAAGCTAATCAGACGCATCCCCATCCATAACCGATAAAT
CTTTATTCATAATCTCATGAGATCAAACGAATACATAAGGTATTAGTCCAACTTCGCTGGGTTATCCCTTAGT
TATGGGCAGGTTGGATACGCGTTACTCGACCCGTGCGCCGGTCGCCGACGTAAATAGCAAGCTATCTATCGCT
GCCCCTCGACTTGCATGTGTTAAGCCTATAGCTAGCGTTCATCCTGAGCCAGGATCAAACTCGT
>538
CTGCTGCCGCCCGTAGGAGTTTTGGACCGTGTCTCAGTTCCAATGTGGGGGACCTTTCCTCTCAGAACCCCTA
CTGATCGTTGCCTTTGGTGGGCCGTTACCCCGCCAACAAGCTAATCAGACGCATCCCCATCCATAACCGATAA
ATCTTTATTCGTAATCTCATGAGATCAAACGAATACATAAGGTATTAGTCCAACTTCGCTGGGTTATCCCTTA
GTTATGGGCAGGTTGGATACGCGTTACTCACCCGTGCGCCGGTCGCCGACATAAATAGCAAGCTATCTATTCG
CTGCCCCTCGACTTGCATGTGTTAAGCCTATAGCTAGCGTTCATCCTGAGCCAGGATCAAACTCCT
>539
CTGCTGCCGCCCGTAGGAGTTTTGGACCGTGTCTCAGTTCCAATGTGGGGGACCTTCCTCTCAGAACCCCTAC
TGATCGTCGCCTTTGGTGGGCCGTTACCCCGCCAACAAGCTAATCAGACGCATCCCCATCCATAACCGCTAAA
CCTTTAATCGTTTATTTGATGTCAGCAAACGATATCATACGGTATTTAGGCCGTCTTTCAACGGGTTTATCCC
GTAGTTTATGGGAAGGTTGGATACGCGTTACTCACCCGTGCGCCAGTCGTCATCAAGGAAAGCAAGCTTTCCC
TTCACTGATTTT
>540
CTGCTGCCGCCCGTAGGAGTTTGGACCGTGTCTCAGTTCCAATGTGGGGGACCTTCCTCTCAGAACCCCTACT
GATCGCCGCCTTGGTGGGCCTGTTACCCCGCCAACAAGCTAATCAGACGCATCCCCATACCATAACCGCTAAC
CTTTTAATCGTTTTATTTTGATTGCAGCAAACGATATCATACGGTTATTTTAGGCCGTCTTTTTCAACGGGTT
TTATCCCCCGTAGTTTTATGGGAAGGTTTGGATACGCGTTACTCACCCGTGCGCCAGTCGTCATTTCAAGGAA
AGCAAGCCTTTTC
>541
CTGCTGCCGCCCGTAGGAGTTTGGACCGTGTCTCAGTTCCAATGTGGGGGACCTTCCTCTCAGAACCCCTAC
TGATCGTCGCCTTTGGTGGGCCGTTACCCCGCCAACAAGCTAATCAGACGCATCCCCATCCATAACCGCTAAA
CCTTTAATCGTTATTTGATGTCAGCAAACGATATCATACGGTATTAGGCCGTCTTTCAACGGGTTATCCCGTA
GTTATGGGAAGGTTGGATACGCGTTACTCACCCGTGCGCCAGTCGTCATCAAGGAAAGCAAGCTTTCCCTCAT
GTTACCCTGCGGCAACTTGCATGTGTTAAGCCTGTAGCTAGCGTTCATCCCGTGACGCAA
>542
CTGCTGCCGCCCGTAGGAGTTTGGACCGTGTCTCAGTTCCAATGTGGGGGACCTTCCTCTCAGAACCCCTACT
GATCGTCGCCTTTGGTGGGCCGTTACCCCGCCAACAAGCTAATCAGACGCATCCCCATCCATCAACCGCTAAA
CCTTTAATCGTTATTTGATGTCAGCAAACGATGTCATACGGTATTAGGTCCGTCTTTCAACGGGTTATCCCGT
GTATGGGAAGGTTGGATACGCGTTACTCACCCGTGCGCCAGTCGTCAGTCAAGGAAAGCAAGCTTCCCTCACG
TACCTTGTCC
>543
CTGCTGCCGCCCGTAGGAGTTTGGACCGTGTCTCAGTTCCAATGTGGGGGACCTTCCTCTCAGAACCCCTACT
GATCGTTGCCTTGGTGGGCCGTTACCCCGCCAACAAGCTAATCAGACGCATCCCCATCCATAACCGATAAATC
TTTATTCGTAATCTCATGAGATCAAACGAATACATAAGGTATTTAGTCCAACTTTCGCTGGGCTATCCCTTTA

GTTATGGGCAGGTTTGGATACGCGTTACTCACCCGTGCGCCGGTCGCCGACATAAATAGCAAGCTATCTATTC
GCTGCCCCCCCTCGACTTGCATGTGTTTAAGCCTTATAGCTAGCGTTCATCCTGAGCCAGGATCAAACTCT
>544
CCTGCTGCCGCCCGTAGGAGTTTGGACCGTGTCTCAGTTCCAATGTGGGGGACCTTCCTCTCAGAACCCCTAC
TGATCGTTGCCTTGGTGGGCCGTTACCCCGCCAACAAGCTAATCAGACGCATCCCCATCCATAACCGATAAAT
CTTTATTCATAATCTCATGAGATCAAACGAATACATAAGGTATTAGTCCAACTTCGCTGGGTTATCCCTTAGT
TTATGGGCAGGTTTGGATACGCGTTACTCACCCGTGCGCCGGTCGCCGACATAAATAGCAAGCTATCTATTCG
CTGCCCCCCCTCGACTTGCATGTGTTTAAGCCTATAGCTAGCGTTCATCCTGAGCCAGGATCAAACTCT
>545
CTGCTGCCGCCCGTAGGAGTTTGGACCGTGTCTCAGTTCCAATGTGGGGGACCTTCCTCTCAGAACCCCTACT
GATCGTTGCCTTTGGTGGGCCGTTACCCCGCCAACAAGCTAATCAGACGCATCCCCATCCATAACCGATAAAT
CTTTATTCATAATCTCATGAGATCAAACGAATACATAAGGTATTAGTCCAACTTCGCTGGGTTATCCCTTAGT
TATGGGCAGGTTGGATACGCGTTACTCACCCGTGCGCCGGTCGCCGACATAAATAGCAAGCTATCTATTCGCT
GCCCCTCGACTTGCATGTGTTAAGCCTATAGCTAGCGTTCATCCTGAGCCAGGATCAAACTCT
>546
CTGCTGCCGCCCGTAGGAGTTTGGACCGTGTCTCAGTTCCAATGTGGGGGACCTTCCTCTCAGAACCCCTACT
GATCGTCGCCTTGGTGGGCCGTTACCCCGCCAACAAGCTAATCAGACGCATCCCCATCCATAACCGCTAAACC
TTTAATCGTTATTTTGATGTCAGCAAACGATATCATACGGTATTAGGCCGTCTTTTCAACGGGTTTATCCCGT
AGTTTATGGGAAGGTTGGATACGCGTTACTCACCCGTGCGCCAGTCGTCATCAAGGAAAGCAAGCTTTCCCTC
ATGATTTACCCTGCGACTTGCATGTGTTTAAGCCTTGTAGCTAGCGTTCATCCCTGAGCCAGGATCAAACTCT
>547
CTGCTGCCGCCCGTAGGAGTTTTGGACCGTGTCTCAGTTCCAATGTGGGGGACCTTCCTCTCAGAACCCCTAC
TGATCGTTGCCTTTGGTCGGGCCGTTACCCCGCCAACAAGCTAATCAGACGCATCCCCATCCATAACCGATAA
ATCTTTATTCGTAATCATCATGAGAGTCAAACGAAATACATAAGGTATTAGTCCAACTTCGCTGGGTTATCCC
TTAGTTATGGGCAGGTTGGATACGCGTTACCTCACCCGTGCGCCGGTCGCCGACATAAATAGCAAGCTATCTA
TTCACTGCCCCTCGACTTGCATGTGTTTAAGCCTATAGCTAGCGTTCATCCTGAGCCAGGATCAAACTCT
>548
CTGCTGCCGCCCGTAGGAGTTTGGACCGTGTCTCAGTTCCAATGTGGGGGACCTTCCTCTCAGAACCCCTACT
GATCGTCGCCTTTGGTGGGCCGTTACCCCGCCAACAAGCTAATCAGACGCATCCCCATCCATCAACCGCTAAA
CCTTTAATCGTTATTTGATGTCAGCAAACGATGTCATACGGTATTAGGCCGTCTTTCAACGGGTTATCCCGTA
GTTATGGGAAGGTTGGATACGCGTTACTCACCCGTGCGCCAGTCGTCATCAAGGAAAGCAAGCTTTCCCTCAT
GTTACCCTGCGACTTGCATGTGTTAAGCCTGTAGCTAGCGTTCATCCTGAGCCAGGATCAAACTCT
>549
CTGCTGCCGCCCGTAGGAGTTTGGACCGTGTCTCAGTTCCAATGTGGGGGACCTTCCTCTCAGAACCCCTACT
GATCGTGCCTTTGGTGGGCCGTTACCCCGCCAACAAGCTAATCAGACGCATCCCCATCCATCACCGCTAAACC
TTTAATCGTTATTTGATGTCAGCAAACGATGTCATACGGTATTAGTCCGTCTTTCAACGGGTTATCCCGTGTA
TGGGAAGGTTGGATACGCGTTACTCACCCGTGCGCCAGTCGTCAGTCAAGGAAAGCAAGCTTTCCTCACGTTA
CCTTGCCGATGCCGTAGT
>550
CTGCTGCCGCCCGTAGGAGTTTGGACCGTGTCTCAGTTCCAATGTGGGGGACCTTCCTCTCAGAACCCCTACT
GATCGTTGCCTTGGTGGGCCGTTACCCCACCAACAAGCTAATCAGACGCATCCCCATCCATAACCGATAAATC
TTTATTCGTAATCTCATGAGATCAAACGAATATATAAGGTATTAGTCCAACTTCGCTGGGTTATCCCTTAGTT
ATGGGCAGGTTGGATACGCGTTACTCACCCGTGCGCCGGTCGCCGACATAAATAGCAAGCTATCTATTCGCTG
CCCCTCGACTTGCATGTGTTAAGCCTATAGCTAGCGTTCATCCTGAGCCAGGATCAAACTCT
>551
CTGCTGCCGCCCGTAGGAGTTTTGGACCGTGTCTCAGTTCCAATGTGGGGGACCTTCCTCTCAGAACCCCTAC
TGATCGTCGCCTTGGTGGGCCGTTACCCCGCCAACAAGCTAATCAGACGCATCCCCATCCATCACCGCTAAAC
CTTTAATCGTTATTTGATGTCAGCAAACGATGTCATACGGTATTAGTCCGTCTTTCAACGGGTTATCCCGTGG
TCATGGGAAGGTTGGATACGCGTTACTCACCCGTGCGCCAGTCGTCGTCAAGGGAAGCAAGCTTCCCTCACGT
TACCCTGCGACTTGCATGTGTTAAGCCTGTAGCTAGCGTCATCCTGAGCCAGGATCAAACTCT
>552
CTGCTGCCGCCCGTAGGAGTTTGGACCGTGTCTCAGTTCCAATGTGGGGGACCTTCCTCTCAGAACCCCTACT
GATCGTGCCTTTGGTGGGCCGTTACCCCGCCAACAAGCTAATCAGACGCATCCCCATCCATAACCGCTAAACC
TTTAATCGTTATTTGATGTCAGCAAACGATATCATACGGTATTAGGCCGTCTTTCAACGGGTTATCCCGTAGT

TATGGGAAGGTTGGATACGCGTTACTCACCCGTGCGCCAGTCGTCATCAAGGAAAGCAAGCTTTCCTCATGTT
ACCCTGCGACTTGCATGTGTTAAGCCTGTAGCTAGCGTTCATCCTGAGCCAGGATCAAACTCT
>553
CTGCTGCCGCCCGTAGGAGTTTGGACCGTGTCTCAGTTCCAATGTGGGGGACCTTCCTCTCAGAACCCCTACT
GATCGTCGCCTTGGTGGGCCGTTACCCCGCCAACAAGCTAATCAGACGCATCCCCATCCATAACCGCTAAACC
TTTAATCGTTATTTTGATGTCAGCAAACGATATCATACGGTATTAGGCCGTCTTTTCAACGGGTTATCCCGTA
GTTATGGGAAGGTTGGATACGCGTTACTCACCCGTGCGCCAGTCGTCATCAAGGAAAGCAAGCTTTCCTCATG
TTACCCTGCGACATGCATGTGTTAAGCCTGTAGCTAGCGTTCATCCTGAGCCAAGGATCAAACTCT
>554
CTGCTGCCGCCCGTAGGAGTTTGGACCGTGTCTCAGTTCCAATGTGGGGGACCTTCCTCTCAGAACCCCTACT
GATCGTCGCCTTGGTGGGCCGTTACCCCGCCAACAAGCTAACTACAGACGCATCCCCATCCATCACCGCTAAA
CCTTTAATCGTTATTTGATGTCAGCAAACGATATCATACGGTATTAGGCCGTCTTTCAACGGGTTATCCCGTA
GTTATGGGAAGGTTGGATACGCGTTACTCACCCGTGCGCCAGTCGTCATCAAGGAAAGCAAGCTTCCTCATGT
TACCCTGCGACATGCATGTGTTAAGCCTGTAGCTAGCGTTCAATCCCGTGAG
>555
CTGCTGCCGCCCGTAGGAGTTTGGACCGTGTCTCAGTTCCAATGTGGGGGACCTTCCTCTCAGAACCCCTACT
GATCGTCGCCTTTGGTGGGCCGTTACCCCGCCAACAAGCTAATCAGACGCATCCCCATCCATAACCGCTAAAC
CTTTAATCGTTATTTTGATGTCAGCAAACGATATCATACGGTATTTAGGCCGTCTTTTCAACGGGTTTATCCC
GTAGTTTATGGGAAGGTTGGATACGCGTTACTCACCCGTGCGCCAGTCGTCATCAAGGAAAGCAAGCTTCCTC
ATGTTTACCCTGCGACTGCATGTGTTTTAAGCCTGTAGCTAGCGTTCATCCCGTGAGCCAAG
>556
CTGCTGCCGCCCGTAGGAGTTTGGACCGTGTCTCAGTTCCAATGTGGCCGATCACCCTCTCAGGTCGGCTACT
GATCGTCGCCTTGGTGGGCAGTTATCTCACCAACTAGCTAATCAGACGCGGGTCCATCCTATACCGCTAACAC
TTTGATATATCTACGATGCCGTAAATATATATTTATCATGTATTTAGTAAACTTTTCAGTATGTTTATCCATG
TGTATAGGGCAGGTTTACCCACGCGTTACTCACCCGTCCGCCGCTCATCTTTCATTTAATGCAAGCATCAAAT
CAGACGCGCTCGACTTGCATGTATTAGGCACGCCGCCAGCGTTCATCCTGAGCCAGGATCAAACTCT
>557
CTGCTGCCGCCCGTAGGAGTTTGGACCGTGTCTCAGTTCCAATGTGGGGGACCTTCCTCTCAGAACCCCTACT
GATCGTCGCCTTGGTGGGCCGTTACCCCGCCAACAAGCTAATCAGACGCATCCCCATCCATAACCGCTAAACC
TTTAATCGTTATTTGATGTCAGCAAACGATATCATACGGTATTAGGCCGTCTTTTCAACGGGTTATCCCGTAG
TTATGGGAAGGTTGGATACGCGTTACTCACCCGTGCGCCAGTCGTCATCAAGGAAAGCAAGCTTTCCTCATGT
TACCCTGCGACATGCATGTGTTAAGCCTTAGTACGCCGA
>558
CTGCTGCCGCCCGTAGGAGTTTGGACCGTGTCTCAGTTCCAATGTGGGGGACCTTCCTCTCAGAACCCCTACT
GATCGTCGCCTTTGGTGGGCCGTTACCCCGCCAACAAGCTAATCAGACGCATCCCCATCCATAACCGCTAAAC
CTTTAATCGTTATTTGATGTCAGCAAACGACGTCATACGGTATTAGTCCGTCTTTCAACGGGTTATCCCGTGG
TCATGGGAAGGTTGGATACGCGTACTCACCCGTGCGCCAGTCGTCGTCAAGGAAAGCCAAGCTTTCCTCACGT
TACCCTGCGGCTTGCATGTGTTAAGCCTGTAGCTAGCGTTCATCCTGAGCCAGGATCAAACTCT
>559
CTGCTGCCGCCCGTAGGAGTTTGGACCGTGTCTCAGTTCCAATGTGGGGGACCTTCCTCTCAGAACCCCTACT
GATCGTCGCCTTGGTGGGCCGTTACCCCGCCAACAAGCTAATCAGACGCATCCCCATCCATAACCGCTAAACC
TTTAATCGTTATTTTGATGTCAGCAAACGATATCATACGGTATTAGGCCGTCTTTTCAACGGGTTATCCCGTA
GTTATGGGAAGGTTGGATACGCGTTACTCACCCGTGCGCCAGTCGTCATCAAGGAAAGCAAGCTTCCTCATGT
TACCCTGCGACTTGCATGTGTTAAGCCTGTAGCTAGCGTTCATCCTGAGCCAGGATCAAACTCT
>560
CTGCTGCCGCCCGTAGGAGTTTGGACCGTGTCTCAGTTCCAATGTGGGGGACCTTCCTCTCAGAACCCCTACT
GATCGTCGCCTTGGTGGGCCGTTACCCCGCCAACAAGCTAATCAGACGCATCCCCATCCATAACCGCTAAACC
TTTAATCGTTTATTTTGAGTCAGCAAACGATATCATACGGTATTTAGGCCGTCTTTTCAACGGGTTTAGTCCC
GTAGTTTATGGGAAGGTTGGATACGCGTTACTCACCCGTGCGCCAGTCGTCATCAAGGAAAAGCAAGCTTTCC
CTTCGACTGT
>561
CTGCTGCCGCCCGTAGGAGTTTGGACCGTGTCTCAGTTCCAATGTGGGGGACCTTCCTCTCAGAACCCCTACT
GATCGTTGCCTTGGTGGGCCGTTACCCCGCCAACAAGCTAATCAGACGCATCCCCATCCATCACCGCTAAACC
TTTAATCGTTATTTTGATGTCAGCAAACGATGTCATACGGTATTAGTCCGTCTTTTCAACGGGTTATCCCGTG

GTCATGGGAAGGTTGGATACGCGTTACTCACCCGTGCGCCAGTCGTCGTCAAGGGAAGCAAGCTTCCCTCACG
TTACCCTGCGACTTGCATGTGTTAAGCCTGTAGCTAGCGTTCATCCCTGAGCCAAGGATCAACTCT
>562
CTGCTGCCGCCCGTAGGAGTTTGGACCGTGTCTCAGTTCCAATGTGGGGGACCTTCCTCTCAGAACCCCTACT
GATCGTCGCCTTTGGTGGGCCGTTACCCCGCCAACAAGCTAATCAGACGCATCCCCATCCATAACCGCTAAAC
CTTTAATCGTTTATTTTGATGTCAGCAAACGATATCATACGGTATTTAGGCCGTCTTTTCAACGGGTTTATCC
CGTAGTTTATGGGAAGGTTGGATACGCGTTACTCACCCGTGCGCCAGTCGTCATCAAGGAAAGCAAGCTTTCC
CTTCATGTTTACCCTGCGACTTGCATTGTGTTTAAGCCTTGTAGCTAGCGTCATCCTGAGCCAGGATTCAAAC
TCT
>563
CTGCTGCCGCCCGTAGGAGTTTGGACCGTGTCTCAGTTCCAATGTGGGGGACCTTCCTCTCAGAACCCCTACT
GATCGTTGCCTTTGGTGGGCCGTTACCCCGCCAACAAGCTAATCAGACGCATCCCCATCCATAACCGATAAAT
CTTTATTCGTAATCTCATGAGATCAAACGAAATACATAAGGTATTTAGTCCAACTTCGCTGGGTTTATCCCTT
AGTTTATGGGCAGGTTTGGATACGCGTTACTCACCCGTGCGCCGGTCGCCGACATAAATAGCAAGCTATCTAT
TCGCTGCCCCTCGACTTGCATGTGTTTAAGCCTATAGCTAGCGTTCATCCTGAGCCAGGATCAAACTCT
>564
CTGCTGCCGCCCGTAGGAGTTTGGACCGTGTCTCAGTTCCAATGTGGGGGACCTTCCTCTCAGAACCCCTACT
GATCGTCGCCTTTGGTCGGGCCGTTACCCCGCCAACAAGCTAACTCAGACGCATCCCCATCCATAACCGCTAA
ACCTTTTAATCGTTATTTTTGATGTCAGCAAACGATATCATACGGTATTTAGGCCGTCTTTTCAACGGGTTTA
TCCCGTAGTTTATGGGAAGGTTCGGATACGCGTTACTCACCCGTGCGCCAGTCGTCATCAAGGAAAGCAAGCT
TTCCTTCATGTTTACCCTGCGACATGCATGTGTTTTAAGCCTGT
>565
CTGCTGCCGCCCGTAGGAGTTTGGACCGTGTCTCAGTTCCAATGTGGGGGACCTTCCTCTCAGAACCCCTACT
GATCGTCGCCTTGGTGGGCCGTTACCCCGCCAACAAGCTAATCAGACGCATCCCCATCCATAACCGCTAAACC
TTTAATCGTTATTTTGATGTCAGCAAACGATATCATACGGTATTTAGGCCGTCTTTTCAACGGGTTTATCCCG
TAGTTTATGGGAAGGTTGGATACGCGTTACTCACCCGTGCGCCAGTCGTCATCAAGGAAAAGCAAGCTTTCCC
TCATGTTT
>566
CTGCTGCCGCCCGTAGGAGTTTTGGACCGTGTCTCAGTTCCAATGTGGGGGACCTTCCTCTCAGAACCCCTAC
TGATCGTTGCCTTGGTGGGCCGTTACCCCACCAACAAGCTAATCAGACGCATCCCCATCCATAACCGATAAAT
CTTTATTCGTAATCTCATGAGATCAAACGAAATACATAAGGTATTAGTCCAACTTCGCTGGGTTATCCCTTAG
TTATGGGCAGGTTGGATACGCGTTACTCACCCGTGCGCCGGTCGCCGACATAAATAGCAAGCTATCTATTCGC
TGCCCCTCGACTTGCATGTGTTAAGCCTATAGCTAGCGTTCATCCTGAGCCAGGATCAAACTCT
>567
CTGCTGCCGCCCGTAGGAGTTTGGACCGTGTCTCAGTTCCAATGTGGGGGACCTTCCTCTCAGAACCCCTACT
GATCGTCGCCTTTGGTGGGCCGTTACCCCGCCAACAAGCTAATCAGACGCATCCCCATCCATAACCGCTAAAC
CTTTAATCGTTATTTGATGTCAGCAAACGATATCATACGGTATTAGGCCGTCTTTCAACGGGTTATCCCGTAG
TTATGGGAAGGTTGGATACGCGTTACTCACCCGTGCGCCAGTCGTCATCAAGGAAAGCAAGCTTTCCTCATGT
TACCCTGCGACTTGCATGTGTTAAGCCTGTAGCTAGCGTTCATCCTGAGCCAGGATCAAACTCT
>568
CTGCTGCCGCCCGTAGGAGTTTGGACCGTGTCTCAGTTCCAATGTGGCCGATCACCCTCTCAGGTCGGCTACT
GATCGTCGCCTTGGTGGGCAGTTATCTCACCAACTAGCTAATCAGACGCGGGTCCATCCTATACCGCTAACAC
TTTGATATATCTACGATGCCGTAAATATATATTATCATGTATTAGTAAACTTTTCAGTATGTTATCCATGTGT
ATAGGGCAGGTTACCCACGCGTTACTCACCCGTCCGCCGCTCATCTTTCATTAATGCAAGCATCAAATCAGAC
GCGCTCGACTTGCATGTGTTAGGCACGCCGCCAGCGTTCATCCTGAGCCAGGATCAAACTCT
>569
CTGCTGCCGCCCATAGGAGTTTGGACCGTGTCTCAGTTCCAATGTGGGGGACCTTCCTCTCAGAACCCCTACT
GATCGTCGCCTTTGGTGGGCCGTTACCCCGCCAACAAGCTAATCAGACGCATCCCCATCCATCACCGCTAAAC
CTTTAATCGTTATTTGATGTCAGCAAACGATGTCATACGGTATTAGTCCGTCTTTCAACGGGTTATCCCGTGG
TCATGGGAAGGTTGGATACGCGTTACTCACCCGTGCGCCAGTCGTCGTCAAGGGAAGCAAGCTTCCCTCACGT
TACCCTGCGACTTGCATGTGTTAAGCCTGTAGCTAGCGTTCATCCTGAGCCAGGATCAAACTCT
>570
CTGCTGCCGCCCGTAGGAGTTTTGGACCGTGTCTCAGTTCCAATGTGGGGGACCTTCCTCTCAGAACCCCTAC
TGATCGTTGCCTTTGTGGGCCGTTACCCCGCCAACAAGCTAATCAGACGCATCCCCATCCATAACCGATAAAT

CTTTATTCGTAATCTCATGAGATCAAACGAAATACATAAGGTATTAGTCCAACTTCGCTGGGTTATCCCTTAG
TTATGGGCAGGTTGGATACGCGTTACTCACCCGTGCGCCGGTCGCCGACATAAATAGCAAGCTATCTATTCGC
TGCCCCTCGACTTGCATGTGTTAAGCCTATAGCTAGCGTTCATCCTGAGCCAGGATCAAACTCT
>571
CTGCTGCCGCCCGTAGGAGTTTGGACCGTGTCTCAGTTCCAATGTGGGGGACCTTCCTCTCAGAACCCCTACT
AATCGTTGCCTTGGTGGGCCGTTACCCCGCCAACAAGCTAATCAGACGCATCCCCATCCATAACCGATAAATC
TTTATTCGTAATCTCATGAGATCAAACGAAATACATAAGGTATTAGTCCAACTTCGCTGGGTTATCCCTTAGT
TATGGGCAGGTTGGATACGCGTTACTCACCCGTGCGCCGGTCGCCGACATAAATAGCAAGCTATCTATTCGCT
GCCCCTCGACTTGCATGTGTTAACGCCTATAGCTAGCGTTCATCCTGAGCCAGGATCAAACTCT
>572
CTGCTGCCGCCCGTAGGAGTTTGGACCGTGTCTCAGTTCCAATGTGGGGGACCTTCCTCTCAGAACCCCTACT
GATCGTCGCCTTGGTGGGCCGTTACCCCGCCAACAAGCTAATCAGACGCATCCCCATCCATAACCGCTAAACC
TTTAATCGTTATTTGATGTCAGCAAACGATATCATACGGTATTAGGCCGTCTTTCAACGGGTTATCCCGTAGT
TATGGGAAGGTTGGATACGCGTTACTCACCCGTGCGCCAGTCGTCAGTCAAGGAAAGCAAGCTTCCTCATGTT
ACCCTGCGACTGCATGTGTTAAGCTGTACGCTAGCGTATCCGTGAGCCCAGGATCAAACCTA
>573
CTGCTGCCGCCCGTAGGAGTTTGGACCGTGTCTCAGTTCCAATGTGGGGGACCTTCCTCTCAGAACCCCTACT
GATCGTTGCCTTTGGTGGGCCGTTACCCCGCCAACAAGCTAATCAGACGCATCCCCATCCATAACCGATAAAT
CTTTATTCATAATCTCATGAGAATCAAACGAATACATAAGGTATTAGTCCAACTTCGCTGGGTTATCCCTTAG
TTATGGGCAGGTTGGATACGCGTTACTCACCCGTGCGCCGGTCGCCGACATAAATAGCCAAGCTATCTATTCG
CTGCCCCTCGACTTGCATGTGTTAAGCCTGTAGCTAGCGTTCATCCTGAGCCAGGATCAAACTCT
>574
CTGCTGCCGCCCGTAGGAGTTTGGACCGTGTCTCAGTTCCAATGTGGCCGTTCATCCTCTCAGACCGGCTACT
GATCGTCGCCTTTGGTGGGCTGTTATCTCACCAACTAGCTAATCAGACGCGGGTCCATCCTATACCGCTAACA
CTTTGATATATCTACGATGCCGTAAATATATATTATCATGTATTAGTAAACTTTTCAGTATGTTATCCATGTG
TATAGGGCAGGTTACCCACGCGTTACTCACCCGTCCGCCGCTCATCTTTCATTTAATGCAAGCATCAAATCAG
ACGCGCTCGACTTGCATGTGTTAGGCACGCCGCCAGCGTTCATCCTGAGCCAGGATCAAACTCT
>575
CTGCTGCCGCCCTAGGAGTTTGAACCGTGTCTCAGTTCCAATGTGGGGGACCTTCCTCTCAGAACCCCTACTG
ATCGTTGCCTTGGTGGGCCGTTACCCCCACCAACAAGCTAATCAGACGCATCCCCCATCCATAACCGATAAAT
CTTTATTTCGTAATCTCATGAGATCAAACGAATACATAAGGTATTTAGTCCAACTTCGCTGGGTTTATCCCCT
TAGTTTATGGGCAGGTTTGGATACGCGTTACTCACCCCGTGCGCCGGTCGCCGACATAAATAGCAAGCTATCT
TATTCGCTGCCCCCCCTTCGACTTGCATTGTGTTTAAGCCTTATAGCTAGCGTTCATCCTTGAGCCAGGATCA
AACTCT
>576
CTGCTGCCGCCCGTAGGAGTTTGGACCGTGTCTCAGTTCCAATGTGGCCGATCACCCTCTCAGGTCGGCTACT
GATCGTCGCCTTGGTGGGCAGTTATCTCACCAACTAGCTAATCAGACGCGGGTCCATCCTATACCGCTAACAC
TTTGATATATCTACGATGCCGTAAATATATATTTATCATGTATTTAGTAAACTTTTCAGTATGTTTATCCATG
TGTATAGGGCAGGTTTACCCACGCGTTACTCACCCGTCCGCCGCTCATCTTTCATTTAATGCAAGCATCAAAT
CAGACGCGCTCGACTTGCATGTGTTTAGGCACGCCGCCAGCGTTCATCCTGAGCCAGGATCAAACTCT
>577
CTGCTGCCGCCCGTAGGAGTTTTGGACCGTGTCTCAGTTCCAATGTGGCCGTTCATCCTCTCAGACCAGCTAC
TGATCGTCGCCTTGGTGGGCTGTTATCTCACCAACTAGCTAATCAGACGCGAGCCCATCTATGACCGATAAAT
CTTTGACCGTTGAAACATGTGTTTTTCTACGATTTTATGCGGTATTAATCCCGGTTTTCCCGAGGCTATCCCA
CTGTCATAGGCAAGTTGCTCACGCGTTACTCACCCGTTCGCCGCTCATCTTCATTTAATGCAAGCATCAAATC
AGACGCGCTCGACTTGCATGTGTTAGGCACGCCGCCAGCGTTCATCCTGAGCCAGGATCAAACTCT
>578
CTGCTGCCGCCCGTAGGAGTTTGGACCGTGTCTCAGTTCCAATGTGGGGGACCTTCCTCTCAGAACCCCTACT
GATCGTTGCCTTGGTGGGCCGTTACCCCGCCAACAAGCTAATCAGACGCATCCCCATCCATAACCGATAAATC
TTTATTCGTAATCTCATGAGATCAAACGAAATACATAAGGTATTAGTCCAACTTCGCTGGGTTATCCCTTAGT
TATGGGCAGGTTGGATACGCGTTACCTCACCCGTGCGCCGGTCGCCGACATAAATAGCAAGCTATCTATTCGC
TGCCCCTCGACTTGCATGTGTTAAGCCTATAGCTAGCGTTCATCCTGAGCCAGGATCAAACTCT
>579

CTGCTGCCGCCCGTAGGAGTTTGGACCGTGTCTCAGTTCCAATGTGGCCGATCACCCTCTCAGGTCGGCTACT
GATCGTCGCCTTGGTGGGCAGTTATCTCACCAACTAGCTAATCAGACGCGGGTCCATCCTATACCGCTAACAC
TTTGATATATCTACGATGCCGTAAATATATATTATCATGTATTAGTAAACTTTTCAGTATGTTATCCATGTGT
ATAGGGCAGGTTACCCACGCGTTACTCACCCGTCCGCCGCTCATCTTCATTAATGCAAGCATCAAATCAGACG
CGCTCGACTTGCATGTGTTAGGCACGCCGCCAGCGTTCATCCTGAGCCAGGATCAAACTCT
>580
CTGCTGCCGCCCGTAGGAGTTTTGGACCGTGTCTCAGTTCCAATGTGGGGGACCTTCCTCTCAGAACCCCTAC
TGATCGTCGCCTTTGGTGGGCCGTTATCCCGCCAACAAGCCTAATCAGACGCATCCCCATCCATAACCGCTAA
ACCTTTAATCGTTATTTGATGTCAGCAAACGATATCATACGGTATTAGGCCGTCTTTCAACGGGTTATCCCGT
AGTTATGGGAAGGTTGGATACGCGTTACTCACCCGTGCGCCAGTCGTCATCAAGGAAAGCAAGCTTCCTCATG
TTACCCTGCGACTTGCATGTGTTAAGCCTGTAGCTAGCGTCATCCTGAGCCAGGCATCAAACTCT
>581
CTGCTGCCGCCCGTAGGGGTTTGGACCGTGTCTCAGTTCCAATGTGGGGGACCTTCCTCTCAGAACCCCTACT
GATCGTTGCCTTTGGTGGGCCGTTACCCCGCCAACAAGCTAATCAGACGCATCCCCATCCATAACCGATAAAT
CTTTATTCGTAATCTCATGAGATCAAACGAATACATAAGGTATTAGTCCAACTTCGCTGGGTTATCCCTTAGT
TATGGGCAGGTTGGATACGCGTTACTCACCCGTGCGCCAGTCGTCATCAAGGAAAGCAAGCTTTCCTCATGTT
ACCCTGCGACTTGCATGTGTTTAAGCCTGTAGCTAGCGTTCATCCTGAGCCAGGATCAAACTCT
>582
CTGCTGCCGCCCGTAGGAGTTTGGACCGTGTCTCAGTTCCAATGTGGGGGACCTTCCTCTCAGAACCCCTACT
GATCGTCGCCTTTGGTGGGCCGTTACCCCGCCAACAAGCTAATCAGACGCATCCCCATCCATAACCGCTAAAC
CTTTAATCGTTTATTTTGATGTCAGCAAACGATATCATACGGTATTTAGGCCGTCTTTCAACGGGTTTATCCC
GTAGTTTATGGGAAGGTTGGATACGCGTTACTCACCCGTGCGCCAGTCGTCATCAAGGAAAGCAAGCTTTCCC
TCATGATTTACCCTGCGACTTGCATGTGTTTAAGCCTTGTAGCTAGCGTCATCCTGAGCCAGGATCAAACTCT
>583
CTGCTGCCGCCCGTAGGAGTTTGGACCGTGTCTCAGTTCCAATGTGGGGGACCTTCCTCTCAGAACCCCTACT
GATCGTCGCCTTGGTGGGCCGTTACCCCGCCAACAAGCTAATCAGACGCATCCCCATCCATAACCGATAAATC
TTTATTCGTAATCTCATGAGATCAAACGAATACATAAGGTATTAGTCCAACTTCGCTGGGTTATCCCTTAGTT
ATGGGCAGGTTGGATACGCGTTACTCACCCGTGCGCCGGTCGCCGACATAAATAGCAAGCTATCTATTCGCTG
CCCCTCGACTTGCATGTGTTAAGCCTATAGCTAGCGTTCATCCTGAGCCAGGATCAAACTCT
>584
CTGCTGCCGCCCGTAGGAGTTTTGGACCGTGTCTCAGTTCCAATGTGGGGGACCTTCCTCTCAGAACCCCTAC
TGATCGTCGCCTTTGGTGGGCCGTTACCCCGCCAACAAGCTAATCAGACGCATCCCCATCCATAACCGCTAAA
CCTTTAATCGTTATTTGATGTCAGCAAACGATATCATACGGTATTAGGCCGTCTTTCAACGGGTTATCCCGTA
GGTATGGGAAGGTTGGATACGCGTTACTCACCCGTGCGCCAGTCGTCATCAAGGAAAGCAAGCTTCCTCACGT
TACCCTGCGACTTGCATGTGTTAAGCCTGTAGCTAGCGTTCATCCTGAGCCAGGATCAAACTCT
>585
CTGCTGCCGCCCGTAGGAGTTTGGACCGTGTCTCAGTTCCAATGTGGGGGACCTTCCTCTCAGAACCCCTACT
GATCGTCGCCTTGGTGGGCCGTTACCCCGCCAACAAGCTAATCAGACGCATCCCCATCCATAACCGCTAAACC
TTTAATCGTTATTTTGATGTCAGCAAACGATATCATACGGTATTAGGCCGTCTTTTCAACGGGTTATCCCGTA
GTTTATGGGAAGGTTGGATACGCGTTACTCACCCGTGCGCCAGTCGTCATCAAGGAAGCAAGCTTCCCTGATG
TTTTACCCTGCGACTTG
>586
CTGCTGCCGCCCGTAGGAGTTTGGACCGTGTCTCAGTTCCAATGTGGGGGACCTTCCTCTCAGAACCCCTACT
GATCGTCGCCTTGGTGGGCCGTTACCCCGCCAACAAGCTAATCAGACGCATCCCCATCCATAACCGCTAAACC
TTTAATCGTTTATTTTGATGTCAGCAAACGACGTCATACGGTATTTAGTTCCGTCTTTCAACGGGTTTATCCC
GTGGTCATGGGAAGGTTTGGATACGCGTTACTCACCCGTGCGCCAGTCGTCGTCAAGGAAAGCAAGCTTTTCC
TCACGTTACCCCCTTGCGACTTGCATGTGTTTTAAGCCTGTTAGCTTAGCGTTCATCCTGAGCCCCAGGATCA
AAACCTCTATCACCGACTGCCCCCCCATAGAGAGGCTGAGGACTGCCAAGGCACACAGGGGATAGG
>587
CTGCTGCCGCCCGTAGGAGTTTGAACCGTGTCTCAGTTCCAATGTGGGGGACCTTCCTCTCAGAACCCCTACT
GATCGTTGCCTTGGTGGGCCGTTACCCCACCAACAAGCTAATCAGACGCATCCCCATCCATAACCGATAAATC
TTTATTCGTAATCTCATGAGATCAAACGAAATATATAAGGTATTAGTCCAACTTCGCTGGGTTATCCCTTGAG
TTTATGGGCAGGTTTGGATACGCGTTACGTCACCCGTGCGCCGGTCGCCGACATAAATAGCAAGCTATCTATT

CGCTGCCCCCCCTCGACTTTGCATGTGTTTTAAGCCTTATAGCTAGCGTTCATCCTGAGCCAGGATCAAACTC
T
>588
CTGCTGCCGCCCGTAGGAGTTTGGACCGTGTCTCAGTTCCAATGTGGGGGACCTTCCTCTCAGAACCCCTACT
GATCGTCGCCTTTGGTGGGCCGTTACCCCGCCAACAAGCTAATCAGACGCATCCCCGTCCATAACCGCTAAAC
CTTTAATCGTTATTTGATGTCAGCAAACGACGTCATACGGTATTAGTCCGTCTTTCAACGGGTTATCCCGTGG
TCATGGGAAGGTTGGATACGCGTTACTCACCCGTGCGCCAGTCGTCGTCAAGGAAAGCAAGCTTTCCTCACGT
TACCCTGCGACTTGCATGTGTTTAAGCCTGTAGCTAGCGTTCATCCTGAGCCAGGATCAAACTCT
>589
CTGCTGCCGCCCGTAGGAGTTTTGGACCGTGTCTCAGTTCCAATGTGGGGGACCTTCCTCTCAGAACCCCTAC
TGATCGTCGCCTTTGGTGGGCCGTTACCCCGCCAACAAGCTAATCAGACGCATCCCCATCCATAACCGATAAA
TCTTTATTCATAATCTCATGAGATCAAACGAATACATAAGGTATTAGTCCAACTTCGCTGGGTTATCCCTTAG
TTATGGGCAGGTTGGATACGCGTTACTCACCCGTGCGCCGGTCGCCGACATAAATAGCAAGCTATCTATTCGC
TGCCCCTCGACTTGCATGTGTTAACGCCTATAGCTAGCGTTCATCCTGAGCCAGGATCAAACTCT
>590
CTGCTGCCGCCCGTAGGAGTTTGGACCGTGTCTCAGTTCCAATGTGGGGGACCTTCCTCTCAGAACCCCTACT
GATCGTTGCCTTGGTGGGCCGTTACCCCGCCAACAAGCTAATCAGACGCATCCCCATCCATAACCGCTAAACC
TTTAATCGTTATTTGATGTCAGCAAACGACGTCATACGGTATTAGTCCGTCTTTCAACGGGTTATCCCGTGGT
CATGGGAAGGTTGGATACGCGTTACTCACCCGTGCGCCAGTCGCGTCAAGGAAAGCAAGCTTTCCTCACGTAC
CCTGCGACTTGCATGTGTAAGCCTATAGCTAGCGTCATCCTGAGCCAGGATCAAACTCT
>591
CTGCTGCCGCCCGTAGGAGTTTTGGGACCGTGTCTCAGTTCCAATGTGGGGGACCTTCCTCTCAGAACCCCTA
CTGATCGTCGCCTTTGGTGGGCCGTTACCCCGCCAACAAGCTAATCAGACGCATCCCCATCCATAACCGCTAA
ACCTTTAATCGTTATTTGATGTCAGCAAACGATGTCATACGGTATTAGGCCGTCTTTCAACGGGTTATCCCGT
AGTTATGGGAAGGTTGGATACGCGTTACTCACCCGTGCGCCAGTCGTCATCAAGGAAAGCAAGCTTTCCCTGA
CTGTTACCCTGCGACTTGCATGTGTTAAGCCTGTAGCTAGCGTCATCCTGAGCAGGATCAACTCT
>592
CTGCTGCCGCCCGTAGGGGTTTGGACCGTGTCTCAGTTCCAATGTGGGGGACCTTCCTCTCAGAACCCCTACT
GATCGTTGCCTTGGTGGGCCGTTACCCCGCCAACAAGCTAATCAGACGCATCCCCATCCATAACCGATAAATC
TTTATTCGTAATCTCATGAGATCAAACGAATACATAAGGTATTAGTCCAACTTCGCTGGGTTATCCCTTAGTT
ATGGGCAGGTTGGGATACGCGGTTACTCACCCGTGCGCCGGTCGCCGACATAAATAGCAAGCTATCTATTCGC
TGCCCCTCGACTTGCATGTGTTAACGCCTATAGCTAGCGTTCATCTGAGCCAGGATCAACTCCT
>593
CTGCTGCCGCCCGTAGGAGTTTGGACCGTGTCTCAGTTCCAATGTGGGGGACCTTCCTCTCAGAACCCCTACT
GATCGTTGCCTTGGTGGGCCGTTACCCCGCCAACAAGCTAATCAGACGCATCCCCATCCATAACCGATAAATC
TTTATTCGTAATCTCATGAGATCAAACGAAATACATAAGGTATTAGTCCAACTTCGCTGGGTTATCCCTTAGT
TATGGGCAGGTTGGATACGCGTTACTCACCCGTGCGCCGGTCGCCGACATAAATAGCAAGCTATCTATTCGCT
GCCCCTCGACTTGCATGTGTTAAGCCTATAGCTAGCGTTCATCCTGAGCCAGGATCAAACTCT
>594
CTGCTGCCGCCCGTAGGAGTTTGGACCGTGTCTCAGTTCCAATGTGGGGGACCTTCCTCTCAGAACCCCTACT
GATCGTCGCCTTTGGTGGGCCGTTACCCCGCCAACAAGCTAATCAGACGCATCCCCATCCATAACCGCTAAAC
CTTTAATCGTTATTTTGATGTCAGCAAACGATATCATACGGTATTAGGCCGTCTTTTCAACGGGTTATCCCGT
AGTTATGGGAAGGTTGGATACGCGTTACTCACCCGTGCGCCAGTCGTCATCAAGGAAAGCAAGCTTTCCCTCA
TGTTACCCTGCGACTTGCATGTGTTAAGCCTGTAGCTAGCGTTCATCCCTGAGCCAGGATCAAACTCGT
>595
CTGCTGCCGCCCGTAGGAGTTTTGGACCGTGTCTCAGTTCCAATGTGGGGGACCTTCCTCTCAGAACCCCTAC
TGATCGTTGCCTTTGGTGGGCCGTTACCCCGCCAACAAGCTAATCAGACGCATCCCCATCCATAACCGATAAA
TCTTTATTCGTAATCTCATGAGATCAAACGAAATACATAAGGTATTAGTCCAACTTCGCTGGGTTATCCCTTA
GTTATGGGCAGGTTGGATACGCGTTACTCACCCGTGCGCCGGTCGCCGACATAAATAGCAAGCTATCTATTCG
CTGCCCCTCGACCTTGCATGTGTTAAGCCTATAGCTAGCGTTCATCCTGAGCCAGGATCAAACTCT
>596
CTGCTGCCGCCCGTAGGAGTTTGGACCGTGTCTCAGTTCCAATGTGGGGGACCTTCCTCTCAGAACCCCTACT
GATCGTTGCCTTGGTGGGCCGTTACCCCGCCAACAAGCTAATCAGACGCATCCCCATCCATAACCGATAAATC
TTTATTCGTAATCTCATGAGATCAAACGAATACATAAGGTATTAGTCCAACTTCGCTGGGTTATCCCTTAGTT

ATGGGCAGGTTGGATACGCGTTACTCACCCGTGCGCCGGTCGCCGACATAAATAGCAAGCTATCTATTCGCTG
CCCCTCGACTTGCATGTGTTAAGCCTATAGCTAGCGTTCATCCTGAGCCAGGATCAAACTCT
>597
CTGCTGCCGCCCGTAGGAGTTTGGACCGTGTCTCAGTTCCAATGTGGGGGACCTTCCTCTCAGAACCCCTACT
GATCGTTGCCTTTGGTGGGCCGTTACCCCGCCAACAAGCTAATCAGACGCATCCCCATCCATAACCGATAAAT
CTTTATTCGTAATCTCATGAGATCAAACGAAATACATAAGGTATTAGTCCAACTTCGCTAGGTTATCCCTTAG
TTATGGGCAGGTTGGATACGCGTTACTCACCCGTGCGCCGGTCGCCGACATAAATAGCAAGCTATCTATTCGC
TGCCCCTCGACTTGCATGTGTTAAGCCTATAGCTAGCGTTCATCCTGAGCCAGGATCAAACTCT
>598
CTGCTGCCGCCCGTAGGAGTTTTGGACCGTGTCTCAGTTCCAATGTGGGGGACCTTCCTCTCAGAACCCCTAC
TGATCGTTGCCTTTGGTGGGCCGTTACCCCGCCAACAAGCTAATCAGACGCATCCCCATCCATAACCGATAAA
TCTTTATTCATAATCTCATGAGAATCAAACGAATACATAAGGTATTAGTCCAACTTCGCTGGGTTATCCCTTA
GTTTATGGGCAGGTTTGGATACGCGTTACTCACCCGTGCGCCGGTCGCCGACATAAATAGCAAGCTATCTATT
CGCTGCCCCTCGACTTGCATGTGTTTAAGCCTATAGCTAGCGTTCATCCTGAGCCAGGATCAAACTCT
>599
CTGCTGCCGCCCGTAGGAGTTTTGGACCGTGTCTCAGTTCCAATGTGGGGGACCTTCCTCTCAGAACCCCTAC
TGATCGTTGCCTTGGTGGGCCGTTACCCCGCCAACAAGCTAATCAGACGCATCCCCATCCATAACCGATAAAT
CTTTATTTCATAATCTCATGAGATCAAACGAATACATAAGGTATTTAGTCCAACTTCGCTGGGTTTATCCCTT
AGTTTATGGGCAGGTTTGGATACGCGTTACTCACCCGTGCGCCGGTCGCCGACATAAATAGCAAGCTATCTAT
TCGCTGCCCCCCCTCGACTTGCATGTGTTTAAGCCTTATAGCTAGCGTTCATCCTGAGCCAGGATCAAACTCT
>600
CTGCTGCCGCCCGTAGGAGTTTGGACCGTGTCTCAGTTCCAATGTGGGGGACCTTCCTCTCAGAACCCCTACT
GATCGTCGCCTTTGGTGGGCCGTTACCCCGCCAACAAGCTAATCAGACGCATCCCCATCCATAACCGATAAAT
CTTTATTCATAATCTCATGAGATCAAACGAATACATAAGGGTATTAGTCCAACTTCGCTGGGTTACTCCTAGT
TATGGGCAGGTTGGATACGCGTTACTCACCCGTGCGCCGGTCGCCGACATAAATAGCCAAGCTATCTATTCGC
TGCCCCTCGACTTGCATGTGTTAACGCCTATAGCTAGCGTTCATCCTGAGCCAGGATCAAACTCT
>601
CTGCTGCCGCCCGTAGGAGTTTGAACCGTGTCTCAGTTCCAATGTGGGGGACCTTCCTCTCAGAACCCCTACT
GATCGTTGCCTTGGGTGGGCCGTTACCCCACCAACAAGCTAATCAGACGCATCCCCATCCATAACCGATAAAT
CTTTATTCGTAATCTCATGAGATCAAACGAATATATAAGGTATTAGTCCAACTTCGCTGGGTTATCCCTTAGT
TATGGGCAGGTTGGATACGCGTTACTCACCCGTGCGCCGGTCGCCGACATAAATAGCAAGCTATCTATTCGCT
GCCCCCCTCGACTTGCATGTGTTTAAGCCTATAGCTAGCGTTCATCCGTGACGCAGGATCAAACTCT
>602
CTGCTGCCGCCCGTAGGAGTTTTGGACCGTGTCTCAGTTCCAATGTGGGGGACCTTCCTCCTCAGAACCCCTA
CTGATCGTCGCCTTTGGTGGGCCGTTACCCCGCCAACAAGCTAATCAGACGCATCCCCATCCATCACCGCTAA
ACCTTTAATCGTTATTTGATGTCAGCAAACGATGTCTACGGTATTAGTCCGTCTTTCAACGGGTTATCCCGTG
GTCATGGGAAGGTTGGATACGCGTTACTCACCCGTGCGCCAGTCGTCGTCAAGGGAAGCAAGCTTCCCTCACG
TACCCTGCGACTTGCATGTGTTAAGCCTGTAGCTAGCGTCATCCTGAGCCAGGCATCAAACTCT
>603
CTGCTGCCGCCCGTAGGAGTTTTGGACCGTGTCTCAGTTCCAATGTGGGGGACCTTCCTCTCAGAACCCCTAC
TGATCGTCGCCTTTGGTGGGCCGTTACCCCGCCAACAAGCTAATCAGACGCATCCCCATCCATAACCGCTAAA
CCTTTAATCGTTATTTGATGTCAGCAAACGATATCATACGGTATTAGGCCGTCTTTCAACGGGTTATCCCGTA
GTTATGGGAAGGTTGGATACGCGTTACTCACCCGTGCGCCAGTCGTCATCAAGGAAAGCAAGCTTTCCCTCAT
GATTTACCCTGCGAC
>604
CTGCTGCCGCCCGTAGGAGTTAGGACCGTGTCTCAGTTCCTATGTGGGGGCCTTCCTCTTAGCAACCCCTACC
GATCGAAGCCTTGGTGAGCCGTTTACCTCACCAACAAGCTTAATCGGAACGCATCCCCCATCCATAAGCGGCA
AGCCTTTTGGTTTCAAATCTGATTGCCGTTCTTTGAACAACATAGGGGATTTTAGTTCCGCATTTTTCTTGCG
GGTTTTATTACCCCCTACTTTATGGGCAGGTTTTGGATACGCGTTACTTCACCCCGTGCGCCGGTCGCCGACA
TAAATAGCAAGCTTATCTATTCGCTGCCCCCCCCTTCGACTTGCATTGTGTTTTAAGCCTATAGCAGCGTTCA
TCCTTGAGCC
>605
CTGCTGCCGCCCGTAGGAGTTTGGACCGTGTCTCAGTTCCAATGTGGGGGACCTTCCTCTCAGAACCCCTACT
GATCGTCGCCTTGGTGGGCCGTTACCCCCCGCCAACAAGCTAATCAGACGCATCCCCCATCCATAACCGCTAA

ACCTTTAATCGTTTATTTTGATGTCAGCAAACGATATCATACGGTATTTAGGCCGTCTTTTTCAACGGGTTTA
TTCCCCCGTAGTTTATGGGAAGGTTTGGATACGCGTTACTCACCCGTGCGCCAGTCGTCATCAAGGAAAGCAA
GCTTTTCCCCCTTG
>606
CTGCTGCCGCCCGTAGGAGTTTTGGACCGTGTCTCAGTTCCAATGTGGGGGACCTTCCTCTCAGAACCCCTAC
TGATCGTTGCCTTGGTGGGCCGTTACCCCGCCAACAAGCTAATCAGACGCATCCCCATCCATAACCGATAAAT
CTTTATTCGTAATCTCATGAGATACAAACGAAATATCATAAGGTATTAGTCCAACTTCGCTGGGTTATCCCTT
AGTTATGGGCAGGTTGGATACGCGTTACTCACCCGTGCGCCGGTCGCCGACATAAATAGCAAGCTATCTATTC
GCTGCCCCTCGACTTGCATGTGTTTAAGCCTATAGCTAGCGTTCACCTGAGCCAGGATCAAACTCT
>607
CTGCTGCCGCCCGTAGGAGTTGAACCGTGTCTCAGTTCCAATGTGGGGGACCTTCCTCTCAGAACCCCTACTG
ATCGTTGCCTTGGTGGGCCGTTACCCCACCAACAAGCCTAATCAGACGCATCCCCATCCATAACCGATAAATC
TTTTATCGTAATCTCATGAGATCAAACGAATATATAAGGTATTAAGTCCAACTTTCGCTGGGTTTATCCCTTA
GTTTATGGGCAGGTTTGGATACGCGTTACTCACCCGTGCGCCGGTCGCCGACATAAATAGCGAGCTATCTATT
CGCTGCCCCCCCCTCGACTTGCATGTGTTTTAAGCCATAGCTAGCGTTTCATCCTGAGCCAGGATCAAACTCT
>608
CTGCTGCCGCCCGTAGGAGTTTGGACCGTGTCTCAGTTCCAATGTGGCCGATCACCCTCTCAGGTCGGCTACT
GATCGTCGCCTTGGTGGGCAGTTATCTCACCAACTAGCTAATCAGACGCGGGTCCATCCTATACCGCTAACAC
TTTGATATATCTACGATGCCGTAAATATATATTTATCATGTATTTAGTAAACTTTTCAGTATGTTTATCCATG
TGTATAGGGCAGGTTTACCCACGCGTTACTCACCCGTCCGCCGCTCATCTTTCATTTTAATGCAAGCATCAAA
TCAGACGCGCCACTTGCATGTGTTTAGGCACGCCGCCAGCGTTCATCCTGAGCCAGGATCAAACTCT
>609
CTGCTGCCGCCCGTAGGAGTTTGGACCGTGCCTCAGTTCCAATGTGGGGGACCTTCCTCTCAGAACCCCTACT
GATCGTTGCCTTGGTGGGCCGTTACCCCGCCAACAAGCTAATCAGACGCATCCCCATCCATCACCGCTAAACC
TTTAATCGTTATTTGATGTCAGCAAACGATGTCATACGGTATTAGTCCGTCTTTTCAACGGGTTATCCCGTGG
TCATGGGAAGGTTGGATACGCGTTACTCACCCGTGCGCCGGTCGCCGACATAAATAGCAAGCTATCTATTCGC
TGCCCCTCGACTTGCATGTGTTAAGCCTATAGCTAGCGTTCATCCTGAGCCAGGATCAAACTCT
>610
CTGCTGCCGCCCGTAGGAGTTTGAACCGTGTCTCAGTTCCAATGTGGGGGACCTTCCTCTCAGAACCCCTACT
GATCGTTGCCTTGTGGGCCGTTACCCCACCAACAAGCTAATCAGACGCATCCCCATCCATAACCGATAAATCT
TTATTCGTAATCTCATGAGATCAAACGAAATATATAAGGTATTAGTCCAACTTCGCTGGGTTATCCCTTAGTT
ATGGGCAGGTTGGATACGCGTTACTCACCCGTGCGCCGGTCGCCGACATAAATAGCAAGCTATCTATTCGCTG
CCCCTCGACTTGCATGTGTTAAGCCTATAGCTAGCGTTCATCCTGAGCCAGGATCAAACTCT
>611
CTGCTGCCGCCCGTAGGAGTTTGGACCGTGTCTCAGTTCCAATGTGGCCGATCACCCTCTCAGGTCGGCTACT
GATCGTCGCCTTGGTGGGCAGTTATCTCACCAACTAGCTAATCAGACGCGGGTCCATCCTATACCGCTAACAC
TTTGATATATCTACGATGCCGTAAATATATATTATCATGTATTAGTAAACTTTTCAGTATGTTATCCATGTGT
ATAGGGCAGGTTACCCACGCGTTACGTCACCCGTCCGCCGCTCATCTTTCATTAATGCAAGCATCAAATCAGA
CGCGCTCGACTTGCATGTGTTAGGCACGCCGCCAGCGTTCATCCTGAGCCAGGATCAACTCT
>612
CTGCTGCCGCCCGTAGGAGTTTGGACCGTGTCTCAGTTCCAATGTGGGGGACCTTCCTCTCAGAACCCCTACT
GATCGTTGCCTTGGTGGGCCGTTACCCCGCCAACAAGCTAATCAGACGCATCCCCATCCATAACCGATAAATC
TTTATTCGTAATCTCATGAGATCAAACGAAATACATAAGGTATTAGTCCAACTTCGCTGGGTTATCCCTTAGT
TTATGGGCAGGTTTGGATACGCGTTACTCACCCGTGCGCCGGTCGCCGACATAAATAGCAAGCTATCTATTCG
CTGCCCCCCCTCGACTTGCATGTGTTTTAAGCCTATAGCTAGCGTTCATCCTGAGCCAGGATCAAACTCT
>613
CTGCTGCCGCCCGTAGGAGTTTTGGACCGTGTCTCAGTTCCAATGTGGGGGACCTTCCTCTCAGAACCCCTAC
TGATCGTCGCCTTTGGTGGGCCGTTACCCCGCCAACAAGCTAATCAGACGCATCCCCATCCATCACCGCTAAA
CCTTTAATCGTTATTTGATGTCAGCAAACGATGTCATACGGTATTAGTCCGTCTTTCAACGGGTTATCCCGTG
GTCATGGGAAGGTTGGATACGCGTTACTCACCCGTGCGCCAGTCGTCGTCAAGGGAAGCAAGCTTCCCTCACG
TTACCCTGCGACTTGCATGTGTTAAGCCTGTAGCTAGCGTTCATCCTGAGCCAGGATCAAACTCT
>614
CTGCTGCCGCCCGTAGGAGTTTGAACCGTGTCTCAGTTCCAATGTGGGGGACCTTCCTCTCAGAACCCCTACT
GATCGTTGCCTTGGTGGGCCGTTACCCCACCAACAAGCTAATCAGACGCATCCCCATCCATAACCGATAAATC

TTTATTCGTAATCTCATGAGATCAAACGAAATATATAAGGTATTAGTCCAACTTCGCTGGGTTATCCCTTAGT
TATGGGCAGGTTGGATACGCGTTACGTCACCCGTGCGCCGGTCGCCGACATAAATAGCAAGCTATCTATTCGC
TGCCCCTCGACCTTGCATGTGTTAAGCCTATAGCTAGCGTTCATCCTGAGCCAGGATCAAACTCT
>615
CTGCTGCCGCCCGTAGGAGTTTGGACCGTGTCTCAGTTCCAATGTGGGGGACCTTCCTCTCAGAACCCCTACT
GATCGTCGCCTTGGTGGGCCGTTACCCCGCCAACAAGCTAATCAGACGCATCCCCATCCATAACCGCTAAACC
TTTAATCGTTATTTGATGTCAGCAAACGATATCATACGGTATTAGGCCGTCTTTCAACGGGTTATCCCGTAGT
TATGGGAAGGTTGGATACGCGTTACTCACCCGTGCGCCAGTCGTCATCAAGGAAAGCAAGCTTTCCCTCATGT
TACCCTGCGGTAACTTGCATGTGTTAAGCCTGTAGCTAGCGTTCATCCCGTGAGCCAAGGCATCAAATCATCC
TAGTA
>616
CTGCTGCCGCCCGTAGGAGTTTTGGACCGTGTCTCAGTTCCAATGTGGGGGACCTTCCTCCTCAGAACCCCTA
CTGATCGTCGCCTTTGGTGGGCCGTTACCCCGCCAACAAGCTAATCAGACGCATCCCCATCCATAACCGCTAA
ACCTTTAATCGTTATTTGATGTCAGCAAACGATATCATACGGTATTAGGCCGTCTTTCAACGGGTTATCCCGT
AGTTATGGGAAGGTTGGATACGCGTTACTCACCCGTGCGCCAGTCGTCATCAAGGAAAGCAAGCTTTCCCTCA
TGTTACCCTGCGACTGCATGGTGTTTAAGCCTGTAGCTAGCGTCATCCCTGAGCCAGGATCAACTCAT
>617
CTGCTGCCGCCCGTAGGAGTTTGGACCGTGTCTCAGTTCCAATGTGGGGGACCTTCCCTCTCAGAACCCCTAC
TGATCGTTGCCTTGGTGGGCCGTTACCCCGCCAACAAGCTAATCAGACGCATCCCCATCCATAACCGATAAAT
CTTTATTCGTAATCTCATGAGATCAAACGAAATACATAAGGTATTAGTCCAACTTCGCTGGGTTATCCCTTAG
TTATGGGCAGGTTGGATACGCGTTACTCACCCGTGCGCCGGTCGCCGACATAAATAGCAAGCTATCTATTCGC
TGCCCCTCGACTTGCATGTGTTAAGCCTATAGCTAGCGTTCATCCTGAGCCAGGATCAAACTCT
>618
CTGCTGCCGCCCGTAGGAGTTTGGACCGTGTCTCAGTTCCAATGTGGGGGACCTTCCTCTCAGAACCCCTACT
GATCGTTGCCTTGGTGGGCCGTTACCCCACCAACAAGCTAATCAGACGCATCCCCATCCATAACCGATAAATC
TTTATTCATAATCTCATGAGATCAAACGAATACATAAGGTATTAGTCCAACTTCGCTGGGTTATCCCTTAGTT
ATGGGCAGGTTGGATACGCGTTACTCACCCGTGCGCCGGTCGCCGACATAAATAGCAAGCTATCTATTCGCTG
CCCCTCGACTTGCATGTGTTAAGCCTATAGCTAGCGTTCATCCTGAGCCAGGATCAAACTCT
>619
CTGCTGCCGCCCGTAGGAGTTTTGGACCGTGTCTCAGTTCCAATGTGGGGGACCTTCCTCTCAGAACCCCTAC
TGATCGTTGCCTTTGGTGGGCCGTTACCCCGCCAACAAGCTAATCAGACGCATCCCCATCCATAACCGCTAAA
CCTTTAATCGTTATTTGATGTCAGCAAACGATATCATACGGTATTAGGCCGTCTTTCAACGGGTTATCCCGTA
GTTATGGGAAGGTTGGATACGCGTTACTCACCCGTGCGCCAGTCGTCATCAAGGAAAGCAAGCTTTCCCTCAT
GTTACCCTGCCGTAAC
>620
CTGCTGCCGCCCGTAGGAGTTTGGACCGTGTCTCAGTTCCAATGTGGGGGACCTTCCTCTCAGAACCCCTACT
GATCGTCGCCTTTGGTGGGCCGTTACCCCGCCAACAAGCTAATCAGACGCATCCCCATCCATAACCGCTAAAC
CTTTAATCGTATTTGATTGTCAGCAAACGATATCATACGGTATTAGGCCGTCTTTCAACGGGTTATCCCGTAG
TTATGGGAAGGTTGGATACGCGTTACTCACCCGTGCGCCAGTCGTCATCAAGGAAAGCAAGCTTTCCTCATGT
TACCCTGCGACTTGCATGTGTTAAGCCTGTAGCTAGCGTTCATCCTGAGCCAGGATCAAACTCT
>621
CTGCTGCCGCCCGTAGGAGTTTGGACCGTGTCTCAGTTCCAATGTGGGGGACCTTCCTCTCAGAACCCCTACT
GATCGTCGCCTTTGGTGGGCCGTTACCCCGCCAACAAGCTAATCAGACGCATCCCCATCCATAACCGCTAAAC
CTTTAATCGTTATTTGATGTCAGCAAACGATATCATACGGTATTAGGCCGTCTTTCAACGGGTTATCCCGTAG
TTATGGGAAGGTTGGATACGCGTTACTCACCCGTGCGCCAGTCGTCATCAAGGAAAGCAAGCTTTCCCTCATG
TTACCCTGCCGACTTGCATGTGTTAAGCCTGTAGCTAGCGTTCATCCTGAGCCAGGATCAAACTCT
>622
CTGCTGCCGCCCGTAGGAGTTTTGGACCGTGTCTCAGTTCCAATGTGGGGGACCTTCCTCTCAGAACCCCTAC
TGATCGTCGCCTTTGGTGGGCCGTTACCCCGCCAACAAGCTAATCAGACGCATCCCCATCCATAACCGCTAAA
CCTTTAATCGTTATTTGATGTCAGCAAACGATATCATACGGTATTAGGCCGTCTTTCAACGGGTTATCCCGTA
GTTATGGGAAGGTTGGATACGCGTTACTCACCCGTGCGCCAGTCGTCATCAAGGAAAAGCAAGCTTCCCTTCA
TGTTACCCTGCCGACTTGCATGTGTTAAGCCTGTAGCTAGCGTTCATCCTGAGCCAGGCATCAAAT
>623

CTGCTGCCGCCCGTAGGAGTTTTGGACCGTGTCTCAGTTCCAATGTGGGGGACCTTTCCTCTCAGAACCCCTA
CTGATCGTCGCCTTTGGTGGGCCGTTACCCCGCCAACAAGCTAATCAGACGCATCCCCATCCATCACCGCTAA
ACCTTTAATCGTTTATTTGATGTCAGCAAACGATGTCATACGGTATTTAGTCCGTCTTTTCAACGGGTTTATC
CCGTGGTCATGGGAAGGTTTGGATACGCGTTACTCACCCGTGCGCCAGTCGTCATCAAGGAAAGCAAGCTTTC
CCTTCATGAT
>624
CTGCTGCCGCCCGTAGGAGTTTTGGACCGTGTCTCAGTTCCAATGTGGGGGACCTTTCCTCTCAGAACCCCTA
CTGATCGTCGCCTTGGTGGGCCGTTACCCCGCCAACAAGCTAATCAGACGCATCCCCATCCATAACCGCTAAA
CCTTTAATCGTTTATTTTGATGTCAGCAAACGATTATTCATACGGTTATTTAGGCCGTCTTTTCAACGGGTTT
ATTCCCCGTAGTTTATGGGAAGGTTCGGATACGCGTTACTCACCCGTGCGCCAGTCGTCATTCAAGGAAAGCA
AGCCTTTTCCC
>625
CTGCTGCCGCCCGTAGGAGTTTTGGACCGTGTCTCAGTTCCAATGTGGGGGACCTTCCTCTCAGAACCCCTAC
TGATCGTTGCCTTTGGTGGGCCGTTACCCCGCCAACAAGCTAATCAGACGCATCCCCATCCATAACCGATAAA
TCTTTATTTCATAATCTCATGAGATCAAACGAATACATAAGGTATTTAGTCCAACTTCGCTGGGTTTATCCCT
TAGTTATGGGCAGGTTTGGATACGCGTTACTCACCCGTGCGCCGGTCGCCGACATAAATAGCAAGCTATCTAT
TCGCTGCCCCCCTTCGACTTGCATGTGTTTAAGCCTTATAGCTAGCGTTCATCCTGAGCCAGGATCAAACTCT
>626
CTGCTGCCGCCCTAGGAGTTTGGACCGTGTCTCAGTTCCAATGTGGGGGACCTTTCCTCTCAGAACCCCTACT
GATCGTCGCCTTTGGTGGGCCGTTACCCCGCCAACAAGCTAATCAGACGCATCCCCATACCATAACCGCTAAC
CTTTAATCGTTATTTGATGTCAGCAAACGACGTCATACGGTATTTAGTCCGTCTTTCAACGGGTTATCCCGTG
GTCATGGGAAGGTTTGGATACGCGTTACTCACCGTGCGCCAGTCGTCGTCAAGGAAAGCAAGCTTTCCTCACG
TTACCCTGCGACTTGCATGTGTTTAAGCCTGTAGCTAGCGTTCATCCTGAGCCAGGATCAAACTCT
>627
CTGCTGCCGCCCGTAGGAGTCTGGACCGTGTCTCAGTTCCAGTGTGGCCGTTTCATCCTCTCAGACCGGCTAC
TGATCGTTGGTTTGGTGGGCCGTTACCCCACCAACTGCCTAATCAGACGCAAACCCCTCTTCAGGCGATAGCT
TTACAGGTAGAGGCTACCCTTTCTTCCACAGGTCATGCGCACCGTGGAACGTATTCGGTATTAGCAGTCGTTT
CCGTCTGTTTGTCCCCATCCTGAAGGCAGGTTTGTTTTACGTGTTACTCACCCGTTTGCCACTGGATTCGATA
AAGAGCAAGCTCTTCATCTCTTCCCGTTCGACTTGCATGTGTTAAGCACGCCGCCAGCGTTCGCCTGAGCCAG
GATCAACTCT
>628
CTGCTGCCGCCCTAGGAGTTTTGGACCGTGTCTCAGTTCCAATGTGGGGGACCTTCCTCTCAGAACCCCTACT
GATCGTTGCCTTGGTGGGCCGTTACCCCGCCAACAAGCTAATCAGACGCATCCCCATCCATAACCGATAAATC
TTTATTCATAATCTCATGAGATCAAACGAATACATAAGGTATTAGTCCAACTTCGCTGGGTTTATCCCTTAGT
TATGGGCAGGTTTGGATACGCGTTACTCACCCGTTCGCCACTCTCATTTAGTAATCTTCCGCCGAGGCTTTCT
TGTCTACTAAATCCCGTTTCGACTTGCATGTGTTTAAGCACGCCGCCAGCGTTCGTCCTGAGCCAGGATCAAA
CTCT
>629
CTGCTGCCGCCCGTAGGAGTTTGGACCGTGTCTCAGTTCCAATGTGGCCGATCACCCTCTCAGGTCGGCTACT
GATCGTCGCCTTGGTGGGCAGTTATCTCACCAACTAGCTAATCAGACGCGGGTCCATCCTATACCGCTAACAC
TTTGATATATCTACGATGCCGTAAATATATATTATCATGTATTAGTAAACTTTTCAGTATGTTATCCATGTGT
ATAGGGCAGGTTACCCACGCGTTACTCACCCGTCCGCCGCTCATCTTTCATTAATGCAAGCATCAAATCAGAC
GCGCTCGACTTGCATGTGTTAGGCACGCCGCCAGCGTTCATCCTGAGCCAGGATCAAACTCT
>630
CTGCTGCTGCCCGTAGGAGTTTTGGACCGTGTCTCAGTTCCAATGTGGGGGACCTTCCTCTCAGAACCCCTAC
TGATCGTTGCCTTTGGTGGGCCGTTACCCCGCCAACAAGCTAATCAGACGCATCCCCATCCATAACCGATAAA
TCTTTATTCATAATCTCATGAGAATCAAACGAATACATAAGGTATTAGTCCAACTTCGCTGGGTTATCCCTTA
GTTATGGGCAGGTTGGATACGCGTTACTCACCCGTGCGCCGGTCGCCGACATAAATAGCAAGCTATCTATTCG
CTGCCCCTCGACTTGCATGTGTTAAGCCTATAGCTAGCGTTCACCCTGAGCCAGGATCAAACTCT
>631
CTGCTGCCGCCCGTAGGAGTTTGGACCGTGTCTCAGTTCCAATGTGGGGGACCTTCCTCTCAGAACCCCTACT
GATCGTTGCCTTGGTGGGCCGTTACCCCACCAACAAGCTAATCAGACGCATCCCCATCCATAACCGATAAATC
TTTTATTTCGTAATCTCATGAGATCAAACGAATATATAAGGTATTTAGTTCCAACTTCGCTGGGTTTATCCCT
TGAGTTTATGGGCAGGTTTGGATACGCGTTACCTCACCGTGCCGCCGGTCGCCGACATAAATAGCAAGCTATC

TATTTCGCTGCCCCCCCTCGACTTGCATGTGTTTTAAGCCTTATAGCTAGCGTTTCATCCTGAGCCAGGATCA
AACTCT
>632
CTGCTGCCGCCCGTAGGAGTTTGGACCGTGTCTCAGTCCCAATGTGGCCGCACACCCTCTCAGGCCGGCTACC
CGTCGCCGCCTTGGTAGGCCATTACCCCACCAACAAGCTGATAGGCCGCAAGCTCATCCCACACCAGAAAAA
CCTTTCCACCACACCAACCACGATGCGGTCCTATAGGGTATTTAGACCCAGTTTCCCAAGCTTATCCCCCAGT
GCAGGGCAGATCACTTACGTGTTACTCACCCGTTCGCCACTCGAGCACCCCAGCAAGCTGGGGCCTTTCCGTT
CGACTTGCATGTGTTTTAAGCACGCCGCCAGCGTTCACCTGAGCCAGGACAA
>633
CTGCTGCCGCCCGTAGGAGTTTTGGACCGTGTCTCAGTTCCAATGTGGGGGACCTTCCTCTCAGAACCCCTAC
TGATCGTCGCCTTTGGTGGGCCGTTACCCCGCCAACAAGCTAATCAGACGCATCCCCATCCACAACCGCTAAC
CTTTAATCGTTATTTGATGTCAGCAAACGATATCATACGGTATTAGGCCGTCTTTCAACGGGTTATCCCGTAG
TTATGGGAAGGTTGGATACGCGTTACTCACCCGTGCGCCAGTCGTCATCAAGGAAAGCAAGCTTTCCCTGACT
GTTACCCTGCGACTTGCATGTGTTAAGCCTGTAGCTAGCGTTCATCCCGTGAGCAAGGCATC
>634
CTGCTGCCGCCCGTAGGAGTTTGAACCGTGTCTCAGTTCCAATGTGGGGGACCTTCCTCTCAGAACCCCTACT
GATCGTTGCCTTGGTGGGCCGTTACCCCGCCAACAAGCTAATCAGACGCATCCCCATCCATAACCGATAAATC
TTTATTCATAATCTCATGAGATCAAACGAATACATAAGGTATTAGTCCAACTTCGCTGGGTTATCCCTTAGTT
ATGGGCAGGTTGGATACGCGTTACTCACCCGTGCGCCGGTCGCCGACATAAATAGCAAGCTATCTATTCGCTG
CCCCTCGACTTGCATGTGTTTAAGCCTATAGCTAGCGTTCATCCTGAGCCAGGATCAAACTCT
>635
CTGCTGCCGCCCGTAGGAGTTTGGACCGTGTCTCAGTTCCAATGTGGGGGACCCTCCTCTCAGAACCCCTACT
GATCGTCGCCTTTGGTGGGCCGTTACCCCGCCAACAAGCTAACTCAGACGCATCCCCATCCATAACCGCTAAA
CCTTTAATCGTTTATTTTGATGTCAGCAAACGATATCATACGGTATTTAGGCCGTCTTTTCAACGGGTTTATC
CCGTAGTTTATGGGAAGGTTGGATACGCGTTACTCACCCGTGCGCCAGTCGTCATCAAGGAAAGCAAGCTTTC
CCTTCATGTTTTACCCTGCGACTTGCATTGTGTTTAAGCCTTGTAGCTAGCGTCAT
>636
CTGCTGCCGCCCGTAGGAGTTTGGACCGTGTCTCAGTTCCAATGTGGGGGACCTTCCTCTCAGAACCCCTACT
GATCGTTGCCTTGGTGGGCCGTTACCCCGCCAACAAGCTAATCAGACGCATCCCCATCCATAACCGATAAATC
TTTATTCATAATCTCATGAGATCAAACGAATACATAAGGTATTAGTCCAACTTCGCTGGGTTATCCCTTAGTT
ATGGGCAGGTTGGATACGCGTTACTCACCCGTGCGCCGGTCGCCGACATAAATAGCAAGCTATCTATTCGCTG
CCCCTCGACTTGCATGTGTTAAGCCTATAGCTAGCGTTCATCCTGAGCCAGGATCAAACTCT
>637
CTGCTGCCGCCCGTAGGAGTTTGGACCGTGTCTCAGTTCCAATGTGGGGGACCTTCCTCTCAGAACCCCTACT
GATCGTTGCCTTGGTGGGCCGTTACCCCGCCAACAAGCTAATCAGACGCATCCCCATCCATAACCGCTAAACC
TTTTAATCGTTTATTTTGATGTCAGCAAACGACGTCATACGGTATTTAGTCCGTCTTTCAACGGGTTTATCCC
GTGGTCATGGGAAGGTTTGGATACGCGTTACTCACCCGTGCGCCAGTCGTCGTCAAGGAAAGCAAGCTTTTCC
TCACGTTACCCCCTTGCGACTTTGCATGTGTTTTAAGCCTATAGCTTAGCGTTCATCCTGAGCCAGGATCAAA
CTCT
>638
CTGCTGCCGCCCGTAGGAGTTTGGACCGTGTCTCAGTTCCAATGTGGGGGACCTTCCTCTCAGAACCCCTACT
GATCGTCGCCTTTGGTGGGCCGTTACCCCGCCAACAAGCTAATCAGACGCATCCCCATCCATAACCGCTAAAC
CTTTAATCGTTATTTGATGTCAGCAAACGATATCATACGGTATTAGGCCGTCTTTTCAACGGGTTATCCCGTA
GTTATGGGAAGGTTGGATACGCGTTACGCACCCGTGCGCCAGTCGTCATCAAGGAAAGCAAGCTTCCTCATGA
TTTTACCCTGCC
>639
CTGCTGCCGCCCGTAGGAGTTTGAACCGTGTCTCAGTTCCAATGTGGGGGACCTTCCTCTCAGAACCCCTACT
GATCGTTGCCTTGGTGGGCCGTTACCCCACCAACAAGCTAATCAGACGCATCCCCATCCATAACCGATAAATC
TTTTATTTCGTAATCTCATGAGATCAAACGAAATACATAAGGTTATTTTAGTCCAACTTTCGCTGGGTTTATC
CCTTAGTTTATGGGCAGGTTTTGGATACGCGTTACTCACCCGTGCGCCGGTCGCCGACATAAATAGCAAGCTA
TCTTATTTCGCTGCCCCCCCTTCGACTTTGCATGTGTTTTAAGCCTTATAGCTAGCGTTCATCCTTGAGCCAG
GATCAAACTCTATCACCGACTGCCCCCCCATAGAGAGGCTGAGACTGCCAAGGCACACAGGGGATAGG
>640

CTGCTGCCGCCCGTAGGAGTTTGGACCGTGTCTCAGTTCCAATGTGGGGGACCTTCCTCTCAGGTCGGCTACT
GATCGTCGCCTTGGTGGGCAGTTATCTCACCAACTAGCTAATCAGACGCGGGTCCATCCTATACCGCTAACAC
TTTGATATATCTACGATGCCGTAAATAATATATTAATCATGTATTAGTAAACTTTTCAGTATGTTATCCATGT
GTATAGGGCAGGTTACCCACGCGTTACTCACCCGTCCGCCGCTCATCTTTCATTAATGCAAGCATCAAATCAG
ACGCGCTCGACTTGCATGTGTTAGGCACGCCGCCAGCGTTCATCCTGAGCCAGGATCAAACTCT
>641
CTGCTGCCGCCCGTAGGAGTTTGGACCGTGTCTCAGTTCCAATGTGGGGGACCTTCCTCTCAGAACCCCTACT
GATCGTCGCCTTTGGTGGGCCGTTACCCCGCCAACAAGCTAATCAGACGCACCCCCATCCATAACCGCTAAAC
CTTTAATCGTTATTTGATGTCAGCAAACGATATCATACGGTATTAGGCCGTCTTTTCAACGGGTTATCCCGTA
GTTATGGGAAGGTTGGATACGCGTTACTCACCCGCGCGCCAGTCGTCATCAAGGAAAAGCAAGCTTTCCCTCA
TGTTACCCTGCGACTTGCATGTGTTAAGCCTGTAGCTAGCGTTCATCCTGAGCCAGGATCAAACTCT
>642
CTGCTGCCGCCCGTAGGAGTTTGGACCGTGTCTCAGTTCCAATGTGGGGGACCTTCCTCTCAGAACCCCTACT
GATCGTTGCCTTGGTGGGCCGTTACCCCGCCAACAAGCTAATCAGACGCATCCCCATCCATAACCGATAAATC
TTTATTCGTAATCTCATGAGATCAAACGAAATACATAAGGTATTAGTCCAACTTCGCTGGGTTATCCCTTAGT
TTATGGGCAGATTGGATACGCGTTACTCACCCGTGCGCCGGTCGCCGACATAAATAGCAAGCTATCTATTCGC
TGCCCCCCTCGACTTGCATGTGTTTAAGCCTATAGCTAGCGTTCATCCTGAGCCAGGATCAAACTCT
>643
CTGCTGCCGCCCGTAGGAGTTGTGACCGTGTCTCAGTTCCAGTGTGGCCGTTCATCCTCTTAGACCGGCTACT
GATCGTTGGTTTGGTGGGCCGTTACCCCACCAACTGCCTAATCAGACGCAAACCCCTCTTCAGGCGATAGCTT
ACAGGTAGAGGCTACCCTTTTCTTCCACAGGTCATGCGGCCACGTGGAACGTATTTCGGTATTTAGCAGTCGT
TTTTCCGTCTGTTTGTCCCCCCATCCTGAAGGCAGGTTGTTTTTTACGCGTTACTCACCCGTTTTGCCACTGG
ATTTCGATAAAGAGCAAGCTCTTCATCTCTCCCGTCGACTTGCATGTGTTTAAGCACGCCGCCAGCGTCGTCC
TGAGCCAGGATCAAACTCT
>644
CTGCTGCCGCCCGTAGGAGTTTGGACCGTGTCTCAGTTCCAATGTGGGGGACCTTCCTCTCAGAACCCCTACT
GATCGTCGCCTTTGGTGGGCCGTTACCCCGCCAACAAGCTAATCAGACGCATCCCCATCCATAACCGCTAAAC
CTTTAATCGTTATTTGATGTCAGCAAACGATATCATACGGTATTAGGCCGTCTTTCAACGGGTTATCCCGTAG
TTATGGGAAGGTTGGATACGCGTTACTCACCCGTGCGCCAGTCGTCATCAAGGAAAGCAAGCTTTCCCTCATG
TTACCCTGCGACTTGCATGTGTTAAGCCTGTAGCTAGCGTTCATCCCGTGAGCCAGATCAAACATCGT
>645
CTGCTGCCGCCCGTAGGAGTTTGGACCGTGTCTCAGTTCCAATGTGGGGGACCTTCCTCTCAGAACCCCTACT
GATCGTCGCCTTTGGTGGGCCGTTACCCCGCCAACAAGCTAATCAGACGCATCCCCATCCATCACCGCTAAAC
CTTTAATCGTTATTTGATGTCAGCAAACGATGTCATACGGTATTAGTCCGTCTTTCAACGGGTTATCCCGTGG
TCATGGGAAGGTTGGATACGCGTTACTCACCCGTGCGCCAGTCGTCGTCAAGGGAAGCAAGCTTCCCTCACGT
TACCCTGCGACTTGCATGTGTTAAGCCTGTAGCTAGCGTCATCCTGAGCCAGGATCAAACTCT
>646
CTGCTGCCGCCCGTAGGAGTTTGGACCGTGTCTCAGTTCCAATGTGGGGGACCTTCCTCTCAGAACCCCTACT
GATCGTCGCCTTTGGTGGGCCGTTACCCCGCCAACAAGCTAATCAGACGCATCCCCATCCATAACCGCTAAAC
CTTTAATCGTTATTTGATGTCAGCAAACGATATCATACGGTATTAGGCCGTCTTTCAACGGGTTATCCCGTAG
TTATGGGAAGGTTGGATACGCGTTACTCACCCGTGCGCCAGTCGTCATCAAGGAAAGCAAGCTTTCCCTCATG
TTACCCTGCGACTTGCATGTGTTAAGCCTGTAGCTAGCGTTCATCCTGAGCCAGGATCAAACTCT
>647
CTGCTGCCGCCCGTAGGAGTTTGGACCGTGTCTCAGTTCCAATGTGGGGGACCTTCCTCTCAGAACCCCTACT
GATCGTCGCCTTGGTGGGCCGTTACCCCGCCAACAAGCTAATCAGACGCATCCCCATCCATAACCGCTAAACC
TTTTAATCGTTTATTTTGATGTCAGCAAACGATATCATACGGTATTTAGGCCGTCTTTTCAACGGGTTATCCC
GTAGTTTATGGGAAGGTTGGATACGCGTTACTCACCCGTGCGCCAGTCGTCATCAAGGAAAAGCAAGCTTTCC
CTTCATGTTTAC
>648
CTGCTGCCGCCCGTAGGAGTTTAGACCGTGTCTCAGTTCCAATGTGGGGGACCTTCCTCTCAGAACCCCTACT
GATCGTCGCCTTTGGTGGGCCGTTACCCCGCCAACAAGCTAATCAGACGCATCCCCATCCATAACCGATAAAT
CTTATTCATAATCTCATGAGATCAAACGAATACATAAGGTATTAGTCCAACTTCGCTGGGTTATCCCTTAGTT
ATGGGCAGGTTGGATACGCGTTACTCACCCGTGCGCCGGTCGCCGACATAAATAGCAAGCTATCTATTCGCTG
CCCCTCGACTTGCATGTGTTAAGCCTATAGCTAGCGTTCATCCTGAG

>649
CTGCTGCCGCCCGTAGGAGTTTGGACCGTGTCTCAGTTCCAATGTGGGGGACCTTCCTCTCAGAACCCCTACT
GATCGTCGCCTTGGTGGGCCGTTACCCCGCCAACAAGCTAATCAGACGCATCCCCATCCATAACCGCTAAACC
TTTAATCGTTATTTGATGTCAGCAAACGATATCATACGGTATTAGGCCGTCTTTCAACGGGTTATCCCGTAGT
TATGGGAAGGTTGGATACGCGTTACTCACCCGTGCGCCAGTCGTCATCAAGGAAAGCAAGCATTTCCCTTCGA
TGTTACCCTGCCGACGTTGC
>650
CTGCTGCCGCCCTAGGAGTTGAACCGTGTCTCAGTTCCAATGTGGGGGACCTTTCCTCTCAGAACCCCTACTG
ATCGTTGCCTTGGTGGGCCGTTACCCCACCAACAAGCTAATCAGACGCATCCCCATCCATAACCGATAAATCT
TTATTCGTAATCTCATGAGATCAAACGAAATATATAAGGTATTAGTCCAACTTCGCTGGGTTATCCCTTAGTT
ATGGGCAGGTTGGATACGCGTTACTCACCCGTGCGCCGGTCGCCGACATAAATAGCAAGCTATCTATTCGCTG
CCCTCGACTTGCATGTGTAGCTATAG
>651
CTGCTGCCGCCCGTAGGAGTTTGGACCGTGTCTCAGTTCCAATGTGGGGACCTTCCTCTCAGAACCCCTACTG
ATCGTTGCCTTTGGTGGGCCGTTACCCCGCCAACAAGCTAATCAGACGCATCCCCATCCATAACCGATAAATC
TTTATTCGTAATCTCATGAGATCAAACGAATACATAAGGTATTAGTCCAACTTCGCTGGGTTTATCCCTTAGT
TATGGGCAGGTTTGGATACGCGTTACTCACCCGTGCGCCGGTCGCCGACATAAATAGCAAGCTATCTATTCGC
TGCCCCTCGACTTGCATGTGTTTAAGCCTATAGCTAGCGTTCATCCTGAGCCAGGATCAAACTCT
>652
CTGCTGCCGCCCGTAGGAGTTTGGACCGTGTCTCAGTTCCAATGTGGGGGACCTTCCTCTCAGAACCCCTACT
GATCGTTGCCTTGGTGGGCCGTTACCCCACCAACAAGCTAATCAGACGCATCCCCATCCATAACCGATAAATC
TTTATTCGTAATCTCATGAGATCAAACGAATACATAAGGTATTAGTCCAACTTCGCTGGGTTATCCCTTAGTT
ATGGGCAGGTTGGATACGCGTTACTCACCCGTGCGCCGGTCGCCGACATAAATAGCAAGCTATCTATTCGCTG
CCCCTCGACTTGCATGTGTTAAGCCTATAGCTAGCGTTCATCCTGAGCCAGGATCAAACTCT
>653
CTGCTGCCGCCCTAGGAGTTTTGGACCGTGTCTCAGTTCCAATGTGGGGGACCTTCCTCTCAGAACCCCTACT
GATCGTTGCCTTGGTGGGCCGTTACCCCGCCAACAAGCTAATCAGACGCATCCCCATCCATAACCGATAAATC
TTTATTTCGTAATCTCATGAGATCAAACGAAATACATAAGGTATTTAGTCCAACTTCGCTGGGTTTATCCCTT
AGTTTATGGGCAGGTTTGGATACGCGTTACTCACCCGTGCGCCGGTCGCCGACATTAAATAGCAAGCTTATCT
ATTCGCTGCCCCCCTTCGACTTGCATGTGTTTAAGCCTTATAGCTAGCGTTCATCCTTGAGCCAGGATCAAAC
TCT
>654
CTGCTGCCGCCCGTAGGAGTTTGGACCGTGTCTCAGTTCCAATGTGGGGGACCTTCCTCTCAGAACCCCTACT
GATCGTTGCCTTGGTGGGCCGTTACCCCGCCAACAAGCTAATCAGACGCATCCCCATCCATAACCGATAAATC
TTTATTCGTAATCTCATGAGATCAAACGAATATATAAGGTATTAGTCCAACTTCGCTGGGTTATCCCTTAGTT
ATGGGCAGGTTGGATACGCGTTACTCACCCGTGCGCCGGTCGCCGACATAAATAGCAAGCTATCTATTCGCTG
CCCCTCGACTTGCATGTGTTTAAGCCTGTAGCTAGCGTTCATCCTGAGCCAGGATCAAACTCT
>655
CTGCTGCCGCCCGTAGGAGTTTGGACCGTGTCTCAGTTCCAATGTGGCCGATCACCCTCTCAGGTCGGCTACT
GATCGTCGCCTTGGTGGGCAGTTATCTCACCAACTAGCTAATCAGACGCGGGTCCATCCTATACCGCTAACAC
TTGATATATCTACGATGCCGTAAATATATATTTATCATGTATTTAGTAAACTTTTCAGTATGTTTATCCATGT
GTATAGGGCAGGTTTACCCACGCGTTACTCGCCCCCGTCCGCCGCTCATCTTTTCATCAATGCAAGCATCAAA
TCATACGCGCTCGACTTGCATGTGTTTAGGCACGCCGCCAGCGTTCATCCTGAGCCAGGATCAAACTCT
>656
CTGCTGCCGCCCGTAGGAGTTTGGACCGTGTCTCAGTTCCAATGTGGGGGACCTTCCTCTCAGAACCCCTACT
GATCGTTGCCTTGGTGGGCCGTTACCCCGCCAACAAGCTAATCAGACGCATCCCCATCCATAACCGATAAATC
TTTATTCGTAATCTCATGAGATCAAACGAAATACATAAGGTATTAGTCCAACTTCGCTGGGTTATCCCTTAGT
TATGGGCAGGTTGGATACGCGTTACTCACCCGTGCGCCGGTCGCCGACATAAATAGCAAGCTATCTATTCGCT
GCCCCTCGACTTGCATGTGTTAAGCCTATAGCTAGCGTTCATCCTGAGCCAGGATCAAACTCT
>657
CTGCTGCCGCCCGTAGGAGTTTTGGACCGTGTCTCAGTTCCAATGTGGGGGACCTTCCTCTCAGAACCCCTAC
TGATCGTCGCCTTGGTGGGCCGTTACCCCGCCAACAAGCTAATCAGACGCATCCCCATCCATAACCGCTAAAC
CTTTAATCGTTATTTGATGTCAGCAAACGATATCATACGGTATTAGGCCGTCTTTCAACGGGTTATCCCGTAG

TTATGGGAAGGTTGGATACGCGTTACTCACCCGTGCGCCAGTCGTCATCAAGGAAAGCAAGCTTTCCTCATGT
TACCCTGCGACTTGCATGTGTTAAGCCTGTAGCTAGCGTTCATCCTGAGCCAGGATCAAACTCT
>658
CTGCTGCCGCCCGTAGGAGTTTGGACCGTGTCTCAGTTCCAATGTGGGGGACCTTCCTCTCAGAACCCCTACT
GATCGTCGCCTTGGTGGGCCGTTACCCCGCCAACAAGCTAATCAGACGCATCCCCATCCATAACCGCTAAACC
TTTAATCGTTTATTTTGATGTCAGCAAACGATATCATACGGTATTTAGGCCGTCTTTTCAACGGGTTTATCCC
GTAGTTTATGGGAAGGTTGGATACGCGTTACTCACCCGTGCGCCAGTCGTCATCAAGGAAAGCAAGCTTTCCT
CATGTTTACCCTGCGACTTGCATTGTGTTTAAGCCTTGTAGCTAGCGTTCATCCCTTGAGCCAAGGATTCAAA
CTCT
>659
CTGCTGCCGCCCGTAGGAGTTTGGACCGTGTCTCAGTTCCAATGTGGGGGACCTTCCTCTCAGAACCCCTACT
GATCGTTGCCTTTGGTGGGCCGTTACCCCCGCCAACAAGCTAATCAGACGCATCCCCCCATCCATAACCGATA
AATCTTTTATTTCATTAATCTCATGAGACCAAACGAATACATAAGGTATTTAGTTCCAACTTTCGCTTGGGTT
TATCCCTTAGTTTATGGGCAGGTTTTGGATACGCGTTACTTCACCCGTGCGCCGGTCGCCGACATAAATAGCA
AGCTATCTTATTTCGCTGCCCCCCCTTCGACTTTGCATTGTTGTTTTAAGCCTTATAGCTAGCGTTCATCCTT
GAGCCAGGATCAAACTCTATCACCGACTGCCCCCCCATAGAGAGGCTGAGACTGCCAAGGCACACAGGGATAG
G
>660
CTGCTGCCGCCCGTAGGAGTTTTGGACCGTGTCTCAGTTCCAATGTGGGGGACCTTCCTCTCAGAACCCCTAC
TGATCGTCGCCTTTGGTGGGCCGTTACCCCGCCAACAAGCTAATCAGACGCATCCCCATCCATAACCGCTAAA
CCTTTTAATCGTTTATTTTGATGTCAGCAAACGATATCATACGGTATTTAGGCCGTCTTTTCAACGGGTTTAT
CCCGTAGTTTATGGGAAGGTTTGGATACGCGTTACTCACCCGTGCGCCAGTCGTCATCAGGGAAAAGCAAGCT
TTTCCCTTCATGTTTTACCCTGCGACTTGCATTGTGTTTAAGCCTTGTAGCTAGCGTCATCCTGAGCCAGGAT
CAAACTCT
>661
CTGCTGCCGCCCGTAGGAGTTTTGGACCGTGCCTCAGTTCCAATGTGGGGGACCTTCCTCTCAGAACCCCTAC
TGATCGTCGCCTTTGGTGGGCCGTTACCCCGCCAACAAGCTAATCAGACGCATCCCCATCCATCACCGCTAAA
CCTTTAATCGTTATTTGATGTCAGCAAACGATGTCATACGGTATTAGTCCGTCTTTCAACGGGTTATCCCGTG
GTCATGGGAAGGTTGGATACGCGTTACTCACCCGTGCGCCAGTCGTCGTCAAGGGAAGCAAGCTTCCCTCACG
TTACCCTGCGACTTGCATGTGTTAAGCCTATAGCTAGCGTTCATCCTGAGCCAAGGATCCAACTCT
>662
CTGCTGCCGCCCGTAGGAGTTTGGACCGTGTCTCAGTTCCAATGTGGGGGACCTTCCTCTCAGAACCCCTACT
GATCGTCGCCTTTGGTGGGCCGTTACCCCGCCAACAAGCTAATCAGACGCATCCCCATCCATAACCGCTAAAC
CTTTAATCGTTATTTGATGTCAGCAAACGATATCATACGGTATTAGGCCGTCTTTCAACGGGTTATCCCGTAG
TTATGGGAAGGTTGGATACGCGTTACTCACCCGTGCGCCAGTCGTCATCAAGGAAAGCAAGCTTTCCTCATGT
TACCCTGCGACTTGCATGTGTTAAGCCTGTAGCTAGCGTTCATCCTGAGCCAGGATCAAACTCT
>663
CTGCTGCCGCCCGTAGGAGTTTGGACCGTGTCTCAGTTCCAATGTGGGGGACCTTCCTCTCAGAACCCCTACT
GATCGTTGCCTTGGTGGGCCGTTACCCCGCCAACAAGCTAATCAGACGCATCCCCATCCATAACCGATAAATC
TTTATTCGTAATCTCATGAGATCAAACGAATACATAAGGTATTAGTCCAACTTCGCTGGGTTATCCCTTAGTT
TATGGGCAGGTTTGGATACGCGTTACTCACCCGTGCGCCGGTCGCCGACATAAATAGCAAGCTATCTATTCGC
TGCCCCCCCTCGACTTGCATGTGTTTAAGCCTATAGCTAGCGTTCATCCTGAGCCAGGATCAAACTCT
>664
CTGCTGCCGCCCGTAGGAGTTTTGGACCGTGTCTCAGTTCCAATGTGGGGGACCTTCCTCTCAGAACCCCTAC
TGATCGTCGCCTTGGTGGGCCGTTACCCCGCCAACAAGCTAATCAGACGCATCCCCATCCATAACCGCTAAAC
CTTTTAATCGTTATTTTTGATGTCAGCAAACGATATCATACGGTATTTAGGCCGTCTTTTCAACGGGTTTATC
CCGTAGTTTATGGGAAGGTTGGATACGCGTTACTCACCCGTGCGCCAGTCGTCATCAAGGAAAGCAAGCTTTT
CCCTTCATGTT
>665
CTGCTGCCGCCCGTAGGAGTTTGGACCGTGTCTCAGTTCCAATGTGGGGGACCTTCCTCTCAGAACCCCTACT
GATCGTTGCCTTTGGTGGGCCGTTACCCCGCCAACAAGCTAATCAGACGCATCCCCATCCATAACCGATAAAT
CTTTATTCATAATCTCATGAGATCAAACGAATACATAAGGTATTAGTCCAACTTCGCTGGGTTATCCCTTAGT
TATGGGCAGGTTGGATACGCGTTACTCACCCGTGCGCCGGTCGCCGACATAAATAGCAAGCTATCTATTCGCT
GCCCCTCGACTTGCATGTGTTAAGCCTATAGCTAGCGTTCATCCTGAGCCAGGATCAAACTCT

>666
CTGCTGCCGCCCGTAGGAGTCTGGACCGTGTCTCAGTTCCAGTGTGGCCGTTCATCCTCTCAGACCGGCTACT
GATCGTTGGTTTTGGTGGGCCGTTACCCCCCACCAACTGCCTAATCAGACGCAAACCCCCTCTTTCAGGCGAT
AGCTTTTACAGGTAGAGGCTACCTTTTTTCTTTCCACAGGTCATGCGGCCCCCGTGGAACGTATTTCGGTATT
TTAGCAGTCGTTTTTTTCCGTCTGTTTTGCCCCCCCATCCCTGAAGGCAGGTTTGTTTTTACGTTGTTACTC
ACCCGTTTTGCC
>667
CTGCTGCCGCCCGTAGGAGTTTGGACCGTGTCTCAGTTCCAATGTGGGGGACCTTCCTCTCAGAACCCCTACT
GATCGTTGCCTTGGTGGGCCGTTACCCCACCAACAAGCTAATCAGACGCATCCCCATCCATAACCGATAAATC
TTTATTCATAATCTCATGAGATCAAACGAATACATAAGGTATTAGTCCAACTTCGCTGGGTTATCCCTTAGTT
TATGGGCAGGTTTGGATACGCGTTACGTCACCCGTGCGCCGGTCGCCGACATAAATAGCAAGCTATCTATTCG
CTGCCCCTCGACTTGCATGTGTTTTAAGCCTATAGCTAGCGTTCATCCTGAGCCAGGATCAAACTCT
>668
CTGCTGCCGCCCGTAGGAGTTTGGACCGTGTCTCAGTTCCAATGTGGGGGACCTTCCTCTCAGAACCCCTACT
GATCGTTGCCTTGGTGGGCCGTTACCCCGCCAACAAGCTAATCAGACGCATCCCCATCCATAACCGATAAATC
TTTATTTCGTAATCTCATGAGAGTCAAACGAAATACATAAGGTATTAGTCCAACTTCGCTGGGTTATCCCTTA
GTTTATGGGCAGGTTGGATACGCGTTACTCACCCGTGCGCCGGTCGCCGACATAAATAGCAAGCTATCTATTC
GCTGCCCCTCGACTTGCATGTGTTTAAGCCTATAGCTAGCGTTCATCCTGAGCCAGGATCAAACTCT
>669
CTGCTGCCGCCCGTAGGAGTTTGAACCGTGTCTCAGTTCCAATGTGGGGGACCTTCCTCTCAGAACCCCTACT
GATCGTTGCCTTGGTGGGCCGTTACCCCACCAACAAGCTAATCAGACGCATCCCCATCCATAACCGATAAATC
TTATTCGTAATCTCATGAGATCAAACGAAATACATAAGGTATTAGTCCAACTTCGCTGGGTTTATCCCTTAGT
TTATGGGCAGGTTTGGATACGCGTTACTCACCCGTGCGCCGGTCGCCGACATAAATAGCAAGCTATCTATTCG
CTGCCCCCCTCGACTTGCATGTGTTTTAAGCCTATAGCTAGCGTTCATCCTGAGCCAGGATCAAACTCT
>670
CTGCTGCCGCCCGTAGGAGTTTGGACCGTGTCTCAGTTCCAATGTGGGGGACCTTCCTCTCAGAACCCCTACT
GATCGTCGCCTTTGGTGGGCCGTTACCCCGCCAACAAGCTAATCAGACGCATCCCCATCCATCACCGCTAAAC
CTTTAATCGTTATTTGATGTCAGCAAACGATGTCATACGGTATTAGTCCGTCTTTCAACGGGTTATCCCGTGG
TCATGGGAAGGTTGGATACGCGTTACTCACCCGTGCGCCAGTCGTCGTCAAGGGAAGCAAGCTTCCCTCACGT
TACCCTGCGACTTGCATGTGTTAAGCCTGTAGCTAGCGTCATCCTGAGCCAGGATCAAAT
>671
CTGCTGCCGCCCGTAGGAGTTTGGACCGTGTCTCAGTTCCAATGTGGGGGACCTTCCTCTCAGAACCCCTACT
GATCGTCGCCTTTGGTGGGCCGTTACCCCGCCAACAAGCTAATCAGACGCATCCCCATCCATAACCGCTAAAC
CTTTAATCGTTATTTGATGTCAGCAAACGATATCATACGGTATTAGGCCGTCTTTCAACGGGTTATCCCGTAG
TTATGGGAAGGTTGGATACGCGTTACTCACCCGTGCGCCAGTCGTCATCAAGGAAAGCAAGCTTTCCCTCATG
TTACCCTGCGACTTGCATGTGTTAAGCCTGTAGCTAGCGTTCATCCTGAGCCAGGATCAAACTCT
>672
CTGCTGCCGCCCGTAGGAGTTTGGACCGTGTCTCAGTTCCAATGTGGGGGACCTTCCTCTCAGAACCCCTACT
GATCGTTGCCTTTGGTGGGCCGTTACCCCGCCAACAAGCTAATCAGACGCATCCCCATCCATAACCGATAAAT
CTTTATTCATAATCTCATGAGATCAAACGAATACATAAGGTATTAGTCCAACTTCGCTGGGTTATCCCTTAGT
TATGGGCAGGTTGGATACGCGTTACTCACCCGTGCGCCGGTCGCCGACATAAATAGCAAGCTATCTATTCGCT
GCCCCTCGACTTGCATGTGTTAAGCCTATAGCTAGCGTTCTCCTGAGCCAGGATCAAACTCT
>673
CTGCTGCCGCCCGTAGGAGTTTTGGACCGTGTCTCAGTTCCAATGTGGGGGACCTTCCTCTCAGAACCCCTAC
TGATCGTCGCCTTTGGTGGGCCGTTACCCCGCCAACAAGCTAATCAGACGCATCCCCATCCATCACCGCTAAA
CCTTTAATCGTTATTTGATGTCAGCAAACGATGTCATACGGTATTTAGTCCGTCTTTCAACGGGTTATCCCGT
GGTCATGGGAAGGTTTGGATACGCGTTACTCACCCGTGCGCCAGTCGTCGTCAAGGGAAGCAAGCTTCCCTTC
ACGTTACCCTGCGACTTGCATGTGTTAAGCCTTGTAGCTAGCGTTCATCCTGAGCCAAGGATCAAACTCT
>674
CTGCTGCCGCCCGTAGGAGTTTGGACCGTGTCTCAGTTCCAATGTGGGGGACCTTCCTCTCAGAACCCCTACT
GATCGTCGCCTTTGGTGGGCCGTTACCCCGCCAACAAGCTAATCAGACGCATCCCCATCCATAACCGCTAAAC
CTTTAATCGTTTATTTTGATTGTCAGCAAACGATATCATACGGTTATTTAGGCCGTCTTTTCAACGGGTTTAT
TCCCGTAGCTTTATGGGAAGGTTTGGATACGCGTTACTCACCCGTGCGCCAGTCGTCATTCAAGGAAAGCAAG
CTTTTCCCTTCATGAC

>675
CTGCTGCCGCCCTAGGAGTTTTGGACCGTGTCTCAGTTCCAATGTGGGGGACCTTCCTCTCAGAACCCCTACT
GATCGTCGCCTTTGGTGGGCCGTTACCCCGCCAACAAGCTAATTAGACGCATCCCCATCCATAACCGCTAAAC
CTTTAATCGTTATTTGATGTCAGCAAACGATATCATACGGTATTAGGCCGTCTTTCAACGGGTTATCCCGTAG
TTATGGGAAGGTTGGATACGCGTTACTCACCCGTGCGCCAGTCGTCATCAAGGAAAGCAAGCTTCCTCATGTT
ACCCTGCCGACTTGCATGTGTTAAGCCTAGTAGCTGCGTTCATCCTGAGCCAGGATCAAACTCT
>676
CTGCTGCCGCCCGTAGGAGTTTGGACCGTGTCTCAGTTCCAATGTGGGGGACCTTCCTCTCAGAACCCCTACT
GATCGTCGCCTTTGGTGGGCCGTTACCCCGCCAACAAGCTAATCAGACGCATCCCCATCCATCACCGCTAAAC
CTTTAATCGTTTATTTTGATGTCAGCAAACGATGTCATACGGTATTTAGTCCGTCTTTTCAACGGGTTATTCC
CGTGGTCATGGGAAGGTTTGGATACGCGTTACTCACCCGTGCGCCAGTCGTCGTCAAGGGAAGCAAGCTTCCC
TTCACGTTACCCTGCGACTTGCATGTGTTAAGCCTTGTAGCTAGCGTCATCCTTGAGCCAGGATTCAAACTCC
GT
>677
CTGCTGCCGCCCGTAGGAGTTTTGGACCGTGTCTCAGTTCCAATGTGGGGGACCTTTCCTCTCAGAACCCCTA
CTGATCGTCGCCTTTGGTGGGCCGTTACCCCGCCAACAAGCTAATCAGACGCATCCCCATCCATAACCGCTAA
ACCTTTAATCGTTATTTGATGTCAGCAAACGATATCATACGGTATTAGGCCGTCTTTCAACGGGTTATCCCGT
AGTTATGGGAAGGTTGGATACGCGTTACTCACCCGTGCGCCAGTCGTCATCAAGGAAAGCAAGCTTTCCTCAT
GTTACCCTGCGACTTGCATGTGTTAAGCCTGTAGCTAGCGTTCATCCCTGAGCCAGGATCAAACTCT
>678
CTGCTGCCGCCCGTAGGAGTTAGGACCGTGTCTCAGTTCCTATGTGGGGGGCCTTCCTCTCAGAACCCCTACC
GATCGAAGCCTTGGTGAGCCGTTACCTCACCAACAAGCTAATCGGACGCATCCCCATCCATAAGCGGCAAGCC
TTTGGTTTCAAATCTGATGCCGTCTTTGAACAACATAGGGGATTTAGTCCGCATTTTCTGCGGGTTTATACCC
TACTTTATGGGCAGGTTTGGATACGCGTTACTCACCCGTGCGCCGGTCGACGTCCAAATGGTGCAAGCACCAA
ATGCCGTTTTCCCCTCGACTTGCATGTGTTTAAGCCTGTAGCTAGCGTTTCATCCTGAGCCAGGATCAAACTC
T
>679
CTGCTGCCGCCCGTAGGAGTTTTGGACCGTGTCTCAGTTCCAATGTGGGGGACCTTCCTCTCAGAACCCCTAC
TGATCGTTGCCTTTGGTGGGCCGTTACCCCGCCAACAAGCTAATCAGACGCATCCCCATCCATAACCGATAAA
TCTTTATTCGTAATCTCATGAGATCAAACGAATACATAAGGTATTTAGTCCAACTTCGCTGGGTTTATCCCTT
AGTTTATGGGCAGGTTTGGATACGCGTTACTCGACCCGTGCGCCGGTCGCCGACATAAATAGCAAGCTATCTA
TTCGCTGCCCCCCTCGACTTGCATGTGTTTAAGCCTTATAGCTAGCGTTCATCCTGAGCCAGGATCAAACTCT
>680
CTGCTGCCCGCCCGTAGGAGTTTGAACCGTGTCTCAGTTCCAATGTCGGGGGACCTTCCTCTCAGAACCCCTA
CCTGATCGTTGCCTTGGGTGGGCCGTTACCCCACCAACAAGCTAATCAGACGCACCCCCATCCATAACCGATA
AATCTTTATTTCATAATCTCATGAGATCAAACGAATACATAAGGTATTTAGTCCAACTTCGCTGGGTTTATCC
CTTAGTTTATGGGCAGGTTTGGATACGCGTTACTCACCCGTGCGCCGGTCGCCGACATAAATAGCAAGCTATC
TATTCGCTGCCCCCCTCGACTTGCATGTGTTTAAGCCTTATAGCTAGCGTTCATCCTTGAGCCAGGATCAAAC
TCT
>681
CTGCTGCCGCCCGTAGGAGTTTGGACCGTGTCTCAGTTCCAATGTGGGGGACCTTCCTCTCAGAACCCCTACT
GATCGTTGCCTTGGGTGGGCCGTTACCCCACCAACAAGCTAATCAGACGCATCCCCATCCATAACCGATAAAT
CTTTATTCGTAATCTCATGAGATCAAACGAATATATAAGGTATTAGTCCAACTTCGCTGGGTTATCCCTTAGT
TATGGGCAGGTTGGATACGCGTTACTCACCCGTGCGCCGGTCGCCGACATAAATAGCAAGCTATCTATTCGCT
GCCCCTCGACTTGCATGTGTTAAGCCTATAGCTAGCGTTCATCCTGAGCCAGGATCAAACTCT
>682
CTGCTGCCGCCCGTAGGAGTTTGGACCGTGTCTCAGTTCCAATGTGGGGGACCTTCCTCTCAGAACCCCTACT
GATCGTCGCCTTTGGTGGGCCGTTACCCCGCCAACAAGCTAATCAGACGCATCCCCATCCATAACCGCTAAAC
CTTTAATCGTTATTTGATGTCAGCAAACGATATCATACGGTATTAGGCCGTCTTTCAACGGGTTATCCCGTAG
TTATGGGAAGGTTGGATGCGCGTTACTCACCCGTGCGCCAGTCGTCATCAAGGAAAGCAAGCTTTCCTCATGT
TTACCCTGCGACTTGCATGTGTTTAAGCCTGTAGCTAGCGTTCATCCTGAGCCAGGATCAAACTCT
>683
CTGCTGCCGCCCGTAGGAGTTTGGACCGTGTCTCAGTTCCAATGTGGGGGACCTTCCTCTCAGAACCCCTACT
GATCGTCGCCTTTGGTGGGCCGTTACCCCGCCAACAAGCTAATCAGACGCATCCCCATCCATAACCGCTAAAC

CTTTAATCGTTATTTGATGTCAGCAAACGATATCATACGGTATTAGGCCGTCTTTCAACGGGTTATCCCGTAG
TTATGGGAAGGTTGGATACGCGTTACTCACCCGTGCGCCAGTCGTCATCAAGGAAAGCAAGCTTTCCCTCATG
TTACCCTGCCGAACTTGCATGTGTTAAGCCTGTAGCTAGCGTTCATCCCGTGACGCAA
>684
CTGCTGCCGCCCGTAGGAGTTTGGACCGTGTCTCAGTTCCAATGTGGGGGACCTTCCTCTCAGAACCCCTACT
GATCGTTGCCTTGGTGGGCCGTTACCCCGCCAACAAGCTAATCAGACGCATCCCCATCCATAACCGATAAATC
TTTATTCGTAATCATCATGAGATCAAACGAAATACATAAGGTATTAGTCCAACTTCGCTGGGTTATCCCTTAG
TTATGGGCAGGTTGGATACGCGTTACTCACCCGTGCGCCGGTCGCCGACATAAATAGCAAGCTATCTATTCGC
TGCCCCTCGACTTGCATGTGTTTAAGCCTATAGCTAGCGTTCATCCTGAGCCAGGATCAAACTCT
>685
CTGCTGCCGCCCGTAGGAGTCTGGACCGTGTCTCAGTTCCAGTGTGGCCGTTCATCCTCTCAGACCGGCTATT
GATCATCGTCTTGGTAGGCCGTTACCCCACCAACTAACTAATCAAACGCAAACCCCTCCTGCGGCGGTAGCTT
CTTCGAGAGGCCACCTTTCCTGTCGGTTTCATGCGAAACCGGAGTCTATTGGGTATTAGCAGTCGTTTTCCGT
CTGTTGTCCCCATCCGTTGGGCAGGTTGTTTACGCGTTACTCACCCGTTTGCCACTAAGATTTACAAGAGCA
AGCTCTCGTATCTCTCCGTTCGACTTGCATGTGTTAGGCACGCCGCCAGCGTTCGTCCTGAGCCAGGATCAAA
CTCT
>686
CTGCTGCCGCCCGTAGGAGTTTGGACCGTGTCTCAGTTCCAATGTGGGGGACCTTCCTCTCAGAACCCCTACT
GATCGTCGCCTTGGTGGGCCGTTACCCCGCCAACAAGCTAATCAGACGCATCCCCATCCATAACCGCTAAACC
TTTTAATCGTTTATTTTGATGTCAGCAAACGATATCATACGGTATTTAGGCCGTCTTTTCAACGGGTTTATCC
CGTGAGTTTATGGGAAGGTTGGATACGCGTTACTCACCCGTGCGCCAGTCGTCAGTCAAGGAAAGCAAGCCTT
TTCCCTTCA
>687
CTGCTGCCGCCCGTAGGAGTTTGGACCGTGTCTCAGTTCCAATGTGGGGGACCTTCCTCTCAGAACCCCTACT
GATCGTTGCCTTGGTGGGCCGTTACCCCGCCAACAAGCTAATCAGAACGCATCCCCATCCATAACCGATAAAT
CTTATTCGTAATCTCATGAGATCAAACGAATACATAAGGTATTAGTCCAACTTCGCTGGGTTATCCTAGTTTA
TGGGCAGGTTTGGATACGCGTTACCTCACCGTGCGCCGGTCGCCGACATAAATAGCAAGCTATCTATTTCGCT
GCCCCCCCTCGACTTGACATGTGTTTTAAGCCTATAGCTAGCGTCATCCTGAGCCAGGATCAAACTCT
>688
CTGCTGCCGCCCGTAGGAGTTTGGACCGTGTCTCAGTTCCAATGTGGGGGACCTTCCTCTCAGAACCCCTACT
GATCGTTGCCTTGGTGGGCCGTTACCCCGCCAACAAGCTAATCAGACGCATCCCCATCCATAACCGATAAATC
TTTTATTCATAATCTCATGAGATCAAACGAATACATAAGGTATTAGTCCAACTTCGCTGGGTTATCCCTTAGT
TTTATGGGCAGGTTTGGATACGCGTTACTCACCCGTGCGCCGGTCGCCGACATAAATAGCAAGCTATCTATTT
CGCTGCCCCCCCCTCGACTTTGCAGTGTTTTAAGCCTATAGCTAGCGTCATCCTGAGCCAGGATCAAACTCT
>689
CTGCTGCCGCCCGTAGGAGTTTGGACCGTGTCTCAGTTCCAATGTGGGGGACCTTCCTCTCAGAACCCCTACT
GATCGTCGCCTTTGGTGGGCCGTTACCCCGCCAACAAGCTAATCAGACGCATCCCCATCCATAACCGCTAAAC
CTTTAATCGTTATTTGATGTCAGCAAACGACGTCATACGGTATTAGTCCGTCTTTCAACGGGTTATCCCGTGG
TCATGGGAAGGTTGGATACGCGTTACTCACCCGTGCGCCAGTCGTCGTCAAGGAAAGCAAGCTTTCCTCACGT
TACCCTGCGACTTGCATGTGTTAAGCCTGTAGCTAGCGTTCATCCTGAGCCAGGATCAAACTCT
>690
CTGCTGCCGCCCGTAGGAGTTTGGACCGTGTCTCAGTTCCAATGTGGGGGACCTTCCTCTCAGAACCCCTACT
GATCGTCGCCTTTGGTGGGCCGTTACCCCGCCAACAAGCTAATCAGACGCATCCCCATCCATAACCGCTAAAC
CTTTAATCGTTATTTGATGTCAGCAAACGATATCATACGGTATTAGGCCGTCTTTCAACGGGTTATCCCGTAG
TTATGGGAAGGTTGGATACGCGTTACTCACCCGTGCGCCAGTCGTCATCAAGGAAAGCAAGCTTTCCCTCATG
TTACCCTGCGACTTGCATGTGTTAAGCCTGTAGCTAGCGTTCATCCTGAGCCAGGATCAAACTCT
>691
CTGCTGCCGCCCGTAGGAGTTTTGGACCGTGTCTCAGTTCCAATGTGGGGGACCTTCCTCTCAGAACCCCTAC
TGATCGTCGCCTTTGGTGGGCCGTTACCCCGCCAACAAGCTAATCAGACGCATCCCCATCCATAACCGCTAAA
CCTTTAAATCGTTATTTGATGTCAGCAAACGATATCATACGGTATTAGGCCGTCTTTCAACGGGTTATCCCGT
AGTTATGGGAAGGTTGGATACGCGTTACTCACCCGTGCGCCAGTCGTCCATCAAGGAAAGCAAGCTTTCCCTC
ATGTTACCC
>692

CTGCTGCCGCCCGTAGGAGTTTGGACCGTGTCCCAGTTCCAATGTGGGGGACCTTCCTCTCAGAACCCCTACT
GATCGTTGCCTTTGGTGGGCCGTTACCCCGCCAACAAGCTAATCAGACGCATCCCCATCCATAACCGCTAAAC
CTTTAATCGTTATTTGATGTCAGCAAACGATATCATACGGTATTAGGCCGTCTTTCAACGGGTTATCCCGTAG
TTATGGGAAGGTTGGATACGCGTTACTCACCCGTGCGCCAGTCGTCATCAAGGAAAGCAAGCTTTCCCTCATG
TTACCCTGCGACTTGCATGTGTTAAGCCTGTAGCTAGCGTTCATCCCTGAGCCAGGATCAAACTCT
>693
CTGCTGCCGCCCGTAGGAGTTTGGACCGTGTCTCAGTTCCAATGTGGGGGACCTTCCTCTCAGAACCCCTACT
GATCGTTGCCTTGGTGGGCCGTTACCCCGCCAACAAGCTAATCAGACGCATCCCCATCCATGACCGATAAATC
TTTATTCATAATCTCATGAGATCAAACGAATACATAAGGTATTAGTCCAACTTCGCTGGGTTATCCCTTAGTT
ATGGGCCAGGTTGGATACGCGTTACTCGACCCGTGCGCCGGTCGCCGACATAAATAGCCAAGCTATCTATTCG
CTGCCCCTCGACTTGCATGTGTTAACGCCTATAGCTAGTGTCATCCTGAGCCAGGATCAAACTCT
>694
CTGCTGCCGCCCGTAGGAGTTTGGACCGTGTCTCAGTTCCAATGTGGGGGACCTTCCTCTCAGAACCCCTACT
GATCGTCGCCTTGGTGGGCCGTTACCCCGCCAACAAGCTAATCAGACGCATCCCCATCCATAACCGCTAAACC
TTTAATCGTTATTTGATGTCAGCAAACGATATCATACGGTATTAGGCCGTCTTTCAACGGGTTATCCCGTAGT
TATGGGAAGGTTGGATACGCGTTACTCACCCGTGCGCCAGTCGTCATCAAGGAAAGCAAGCTTTCCCTCATGT
TACCCTGCGACTTGCATGTGTTAAGCCTGTAGCTAGCGTCATCCTGAGCCAGGATCAAACTCT
>695
CTGCTGCCGCCCGTAGGAGTTTGGACCGTGTCTCAGTTCCAATGTGGGGGACCTTCCTCTCAGAACCCCTACT
GATCGTTGCCTTGGTGGGCCGTTACCCCGCCAACAAGCTAGTCAGACGCATCCCCATCCATAACCGATAAATC
TTTATTCATAATCTCATGAGATCAAACGAATACATAAGGTATTAGTCCAACTTCGCTGGGTTATCCCTTAGTT
ATAGGCAGGTTGGATACGCGTTACCTCACCCGTGCGCCGGTCGCCGACATAAATAGCCAAGCTATCTATTCGC
TGCCCCTCGACTGCATGTGTTAAGCCTATAGCTAGC
>696
CTGCTGCCGCCCGTAGGAGTTTGGACCGTGTCTCAGTTCCAATGTGGGGGACCTTCCTCTCAGAACCCCTACT
GATCGTCGCCTTTGGTGGGCCGTTACCCCGCCAACAAGCTAATCAGACGCATCCCCATCCATCACCGCTAAAC
CTTTAATCGTTATTTGATGTCAGCAAACGATGTCATACGGTATTAGTCCGTCTTTCAACGGGTTATCCCGTGG
TCATGGGAAGGTTGGATACGCGTTACTCACCCGTGCGCCAGTCGTCGTCAAGGGAAGCAAGCTTTCCCTGACT
GTTACCCTGCGACTTGCATGTGTTAAGCCTATAGCTAGCGTTCATCCCTGAGCCAGGATCAAACTCT
>697
CTGCTGCCGCCCGTAGGAGTTTGGACCGTGTCTCAGTTCCAATGTGGGGGACCTTCCTCTCAGAACCCCTACT
GATCGTTGCCTTTGGTGGGCCGTTACCCCGCCAACAAGCTAATCAGACGCATCCCCATCCATAACCGATAAAT
CTTTATTCGTAATCTCATGAGATCAAACGAAATACATAAGGTATTAGTCCAACTTCGCTGGGTTATCCCTTAG
TTATGGGCAGGTTGGATACGCGTTACTCACCCGTGCGCCGGTCGCCGACATAAATAGCAAGCTATCTATTCGC
TGCCCCTCGACTTGCATGTGTTAAGCCTATAGCTAGCGTTCATCCTGAGCCAGGATCAAACTCT
>698
CTGCTGCCGCCCGTAGGAGTTTGGACCGTGTCTCAGTTCCAATGTGGGGGACCTTCCTCTCAGAACCCCTACT
GATCGTCGCCTTTGGTGGGCCGTTACCCCGCCAACAAGCTAATCAGACGCATCCCCATCCATCACCGCTAAAC
CTTTAATCGTTTATTTGATGTCAGCAAACGATGTCATACGGTATTAGTCCGTCTTTCAACGGGTTATCCCGTG
GTCATGGGGAGGTTGGATACGCGTTACTCACCCGTGCGCCAGTCGTCGTCAAGGGAAGCAAGCTTCCCTCACG
TTACCCTGCGACTTGCATGTGTTTAAGCCTGTAGCTAGCGTTCATCCTGAGCCAGGATCAAACTCT
>699
CTGCTGCCGCCCGTAGGAGTTTGGACCGTGTCTCAGTTCCAATGTGGGGGACCTTCCTCTCAGAACCCCTACT
GATCGTTGCCTTGGTGGGCCGTTACCCCGCCAACAAGCTAATCAGACGCATCCCCATCCATAACCGATAAATC
TTTATTCGTAATCTCATGAGATCAAACGAAATACATAAGGTATTAGTCCAACTTCGCTGGGTTATCCCTTAGT
TATGGGCAGGTTGGATACGCGTTACCTCACCGTGCGCCGGTCGCCGACATAAATAGCAAGCTATCTATTCGCT
GCCCCTCGACTTGCATGTGTTTAAGCCTATAGCTAGCGTTCATCCTGAGCCAGGATCAAACTCT
>700
CTGCTGCCGCCCGTAGGAGTTTGGACCGTGTCTCAGTTCCAATGTGGGGGACCTTCCTCTCAGAACCCCTACT
GATCGTTGCCTTGGTGGGCCGTTACCCCGCCAACAAGCTAATCAGACGCATCCCCATCCATAACCGATAAATC
TTTATTTCATAATCTCATGAGATCAAACGAATACATAAGGTATTTAGTCCAACTTCGCTGGGTTTATTCCCTT
AGTTTATGGGCAGGTTTGGATACGCGTTACTCACCCGTGCGCCGGTCGCCGACATAAATAGCAAGCTTATCTT
ATTCGCTGCCCCCCCTTCGACTTGCATGTGTTTAAGCCTATAGCTAGCGTTCATCCTTGAGCCAGGATCAAAC
TCT

>701
CTGCTGCCGCCCGTAGGAGTTTGGACCGTGTCTCAGTTCCAATGTGGGGGACCTTCCTCTCAGAACCCCTACT
GATCGTCGCCTTGGTGGGCCGTTACCCCGCCAACAAGCTAATCAGACGCATCCCCATCCATAACCGCTAAACC
TTTAATCGTTATTTTGATGTCAGCAAACGATATCATACGGTATTTAGGCCGTCTTTTCAACGGGTTTATCCCG
TAGTTTATGGGAAGGTTGGATACGCGTTACTCACCCGTGCGCCAGTCGTCATCAAGGAAAGCAAGCCTTTCCT
CATGTTTACCCTGCGACTTGCATGTGTTTAAGCCTGTAGCTAGCGTTCATCCCGTGAGCCAGGATCAAACTCT
>702
CTGCTGCCGCCCGTAGGAGTTTTGGACCGTGTCTCAGTTCCAATGTGGGGGACCTTCCTCTCAGAACCCCTAC
TGATCGTTGCCTTGGTGGGCCGTTACCCCACCAACAAGCTAATCAGACGCATCCCCATCCATAACCGATAACC
TTTAATCGTTATTTGATGTCAGCAAACGATATCATACGGTATTAGGCCGTCTTTCAACGGGTTATCCCGTAGT
TATGGGAAGGTTGGATACGCGTTACTCACCCGTGCGCCAGTCGTCATCAAGGAAAGCAAGCTTTCCCTCATGT
TACCCTGCGACATGCATGTGTTAAGCCTGTAGCTAGCGTCATCCTGAGCCAGGATCAAACTCT
>703
CTGCTGCCGCCCGTAGGAGTTTGGACCGTGTCTCAGTTCCAATGTGGCCGATCACCCTCTCAGGTCGGCTACT
GATCGTCGCCTTGGTGGGCAGTTATCTCACCAACTAGCTAATCAGACGCGGGTCCATCCTATACCGCTAACAC
TTTGATATATCTACGATGCCGTAAATATATATTATCATGTATTAGTAAACTTTTCAGTATGTTATCCATGTGT
ATAGGGCAGGTTACCCACGCGTTACTCACCCGTCCGCCGCTCATCTTCATTTAATGCAAGCATCAAATCAGAC
GCGCTCGACTTGCATGTGTTAGGCACGCCGCCAGCGTTCATCCTGAGCCAGGATCAAACTCT
>704
CTGCTGCCGCCCGTAGGAGTTTTGGACCGTGTCTCAGTTCCAATGTGGGGGACCTTCCTCTCAGAACCCCTAC
TGATCGTCGCCTTTGGTGGGCCGTTACCCCGCCAACAAGCTAATCAGACGCATCCCCATCCATAACCGCTAAA
CCTTTAATCGTTATTTGATGTCAGCAAACGATATCATACGGTATTAGGCCGTCTTTCAACGGGTTATCCCGTA
GTTATGGGAAGGTTGGATACGCGTTACTCACCCGTGCGCCAGTCGTCATCAAGGAAAGCAAGCTTTCCTCATG
TTACCCTGCGACTTGCATGTGTTAAGCCTGTAGCTAGCGTTCATCCTGAGCCAGGATCAAACTCT
>705
CTGCTGCCGCCCGTAGGAGTTAGGACCGTGTCTCAGTTCCTATGTGGGGGGCCTTCCTCTCAGAACCCCTACC
GATCGAAGCCTTGGTGAGCCGTTACCTCACCAACAAGCTAATCGGACGCATCCCCATCCATAAGCGGCAAGCC
TTTGGTTCAAATCTGATGCCGTCTTTGAACAACATAGGGGATTTAGTCCGCATTTTCTGCGGGTTTATACCCT
ACTTTATGGGCAGGTTTGGATACGCGTTACTCACCCGTGCGCCGGTCGACGTCCAAATGGTGCAAGCACCAAA
TGCCGTTTTCCCCCCCTCGACTGCATGTGTTTAAGCCTGTAGCTAGCGTTTCATCCTGAGCCAGGATCAAACT
CT
>706
CTGCTGCCGCCCGTAGGAGTTTGGACCGTGTCTCAGTTCCAATGTGGGGGACCTTCCTCTCAGAACCCCTACT
GATCGTCGCCTTGGTGGGCCGTTACCCCGCCAACAAGCTAATCAGACGCATCCCCATCCATAACCGCTAAACC
TTTAATCGTATTTGATGTCAGCAAACGATATCATACGGTATTAGGCCGTCTTTCAACGGGTTATCCCGTAGTT
ATGGGAAGGTTGGATACGCGTTACTCACCCGTGCGCCAGTCGTCATCAAGGAAAGCAAGCTTTCCTCATGTTA
CCCTGCCGTAACGTA
>707
CTGCTGCCGCCCGTAGGAGTTTGGACCGTGTCTCAGTTCCAATGTGGGGGACCTTCCTCTCAGAACCCCTACT
GATCGTTGCCTTGGTGGGCCGTTACCCCGCCAACAAGCTAATCAGACGCATCCCCATCCATAACCGATAAATC
TTTATTCGTAATCTCATGAGATCAAACGAATACATAAGGTATTAGTCCAACTTCGCTGGGTTATCCCTTAGTT
TATGGGCAGGTTTGGATACGCGTTACCTCACCCGTGCGCCGGTCGCCGACATAAATAGCAAGCTATCTATTTC
GCTGCCCCCCCTCGACTTGCATGTGTTTTAAGCCTATAGCTAGCGTTCAT
>708
CTGCTGCCGCCCGTAGGAGTTTGGACCGTGTCTCAGTTCCAATGTGGGGGACCTTCCTCTCAGAACCCCTACT
GATCGTCGCCTTGGTGGGCCGTTACCCCGCCAACAAGCTAATCAGACGCATCCCCATCCATAACCGCTAAACC
TTTAATCGTTATTTGATGTCAGCAAACGATATCATACGGTATTAGGCCGTCTTTCAACGGGTTATCCCGTAGT
TATGGGGAAGGTTGGATACGCGTTACTCACCCGTGCGCCAGTCGTCATCAAGGAAAGCAAGCTTTCCCTCATG
TTACCCTGCGACTTGCATGTGTAAGCCTGTAGCTAGCGTCATCCTGAGCCAGGATCAAACTCT
>709
CTGCTGCCGCCCGTAGGAGTTTGGACCGTGTCTCAGTTCCAATGTGGGGGACCTTCCTCTCAGAACCCCTACT
GATCGTTGCCTTGGTGGGCCGTTACCCCGCCAACAAGCTAATCAGACGCATCCCCATCCATAACCGATAAATC
TTTATTCGTAATCTCATGAGATCAAACGAAATACATAAGGTATTTAGTTCAACTTCGCTGGGTTTATCCCTTA

GTTTATGGGCAGGTTTGGATACGCGTTACTCACCCGTGCGCCGGTCGCCGACATAAATAGCAAGCTATCTATT
CGCTGCCCCCCCTCGACTTGCATGTGTTTAAGCCTATAGCTAGCGTTCATCCTGAGCCAGGATCAAACTCT
>710
CTGCTGCCGCCCGTAGGAGTTTGGACCGTGTCTCAGTTCCAATGTGGGGACCTTCCCCTCAGAACCCCTACTG
ATCGTCGCCTTTGGTGGGCCGTTACCCCGCCAACAAGCTAATCAGACGCATCCCCATCCATAACCGCTAAACC
TTTAATCGTTATTTGATGTCAGCAAACGATATCATACGGTATTAGGCCGTCTTTCAACGGGTTATCCCGTAGT
TATGGGAAGGTTGGATACGCGTTACTCACCCGTGCGCCAGTCGTCATCAAGGAAAGCAAGCTTTCCCTCATGT
TACCCTGCGAACTTGCATGTGTTAAGCCTGTAGCTAGCGTTCATCCCGTGAGCCAAGGATCAAACTCT
>711
CTGCTGCCGCCCGTAGGAGTTTGGACCGTGTCTCAGTTCCAATGTGGGGGACCTTCCTCTCAGGACCCCTACT
GATCGTCGCCTTGGTGGGCCGTTACCCCGCCAACAAGCTAATCAGACGCATCCCCATCCATAACCGCTAAACC
TTTAATCGTTATTTGATGTCAGCAAACGATATCATACGGTATTAGGCCGTCTTTCAACGGGTTATCCCGTAGT
TATGGGAAGGTTGGATACGCGTTACTCACCCGTGCGCCAGTCGTCATCAAGGAAAGCAAGCTTTCCCTCATGT
TACCCTGCGACGTCGTGACA
>712
CTGCTGCCGCCCGTAGGAGTTTGGACCGTGTCTCAGTTCCAATGTGGGGGACCTTCCTCTCAGAACCCCTACT
GATCGTTGCCTTGGTGGGCCGTTACCCCGCCAACAAGCTAATCAGACGCATCCCCATCCATAACCGATAAATC
TTTATTCATAATCTCATGAGATCAAACGAATACATAAGGTATTAGTCCAACTTCGCTAGGTTATCCCTTAGTT
ATGGGCAGGTTGGATACGCGTTACTCACCCGTGCGCCGGTCGCCGACATAAATAGCAAGCTATCTATTCGCTG
CCCCTCGACCTTGCATGTGTTAAGCCTATAGCTAGCGTTCATCCTGAGCCAGGATCAAACTCT
>713
CTGCTGCCGCCCGTAGGAGTTTGGACCGTGTCTCAGTTCCAATGTGGGGGACCTTCCTCTCAGAACCCCTACT
GATCGTCGCCTTGGTGGGCCGTTACCCCGCCAACAAGCTAATCAGACGCATCCCCATCCATAACCGCTAAACC
TTTAATCGTTATTTGATGTCAGCAAACGATATCATACGGTATTAGGCCGTCTTTCAACGGGTTATCCCGTAGT
TATGGGAAGGTTGGATACGCGTTACTCACCCGTGCGCCAGTCGTCATCAAGGAAAGCAAGCCTTCCTCATGTT
ACCCTGCGACATGCATGTGTTAAGCCTGTAGCTAGCGTTCATCCCTGACGGCAAGA
>714
CTGCTGCCGCCCGTAGGAGTCTGGACCGTGTCTCAGTTCCAGTGTGGCCGTTCATCCTCTCAGACCGGCTACT
GATCGTTGGTTTGGTGGGCCGTTACCCCACCAACTGCCTAATCAGACGCAAACCCCTCTTCAGGCGATAGCTT
ACAGGTAGAGGCTACCCTTTCTTCCACAGGTCATGCGACCCGTGGAACGTATTCGGTATTAGCAGTCGTTTTC
CGTCTGTTGTCCCCATCCTGAAGGCAGGTTGTTTTACGTGTTACTCACCCGTTTGCCACTGGATTCGATAAAG
AGCAAGCTCTTCATCTCTTCCCGTCGACTGCATGTGTTAAGCACGCCGCCAGCGTTCGTCC
>715
CTGCTGCCGCCCGTAGGAGTTTTGGACCGTGTCTCAGTTCCAATGTGGGGGACCTTCCTCTCAGAACCCCTAC
TGATCGTTGCCTTTGGTGGGCCGTTACCCCGCCAACAAGCTAATCAGACGCATCCCCATCCATAACCGATAAA
TCTTTATTCGTAATCTCATGAGATCAAACGAAATACATAAGGTATTAGTCCAACTTCGCTGGGTTATCCCTTA
GTTATGGGCAGGTTGGATACGCGTTACTCACCCGTGCGCCGGTCGCCGACATAAATAGCAAGCTATCTATTCG
CTGCCCCTCGACTTGCATGTGTTAAGCCTATAGCTAGCGTTCATCCTGAGCCAGGATCAAACTCT
>716
CTGCTGCCGCCCGTAGGAGTTTGGACCGTGTCTCAGTTCCAATGTGGGGGACCTTCCTCTCAGAACCCCTACT
GATCGTCGCCTTGGTGGGCCGTTACCCCGCCAACAAGCTAATCAGACGCATCCCCATCCATAACCGCTAAACC
TTTAATCGTTATTTGATGTCAGCAAACGATATCATACGGTATTAGGCCGTCTTTCAACGGGTTATCCCGTAGT
TATGGGAAGGTTGGATACGCGTTACTCACCCGTGCGCCAGTCGTCATCAAGGAAAGCAAGCTTTCCTCATGTT
ACCCTGCGACTTGCATGTGTTAAGCCTGTAGCTAGCGTTCATCCTGAGCCAGGATCAAACTCT
>717
CTGCTGCCGCCCGTAGGAGTTTGGACCGTGTCTCAGTTCCAATGTGGGGGACCTTCCTCTCAGAACCCCTACT
GATCGTCGCCTTGGTAGGCCGTTACCCCGCCAACAAGCTAATCAGACGCATCCCCATCCATAACCGCTAAACC
TTTAATCGTTATTTGATGTCAGCAAACGATATCATACGGTATTAGGCCGTCTTTCAACGGGTTATCCCGTAGT
TATGGGAAGGTTGGATACGCGTTACTCACCCGTGCGCCAGTCGTCATCAAGGAAAGCAAGCTTTCCTCATGTT
ACCCTGCGACTTGCATGTGTTAAGCCTGTAGCTAGCGTTCATCCTGAGCCAGGATCAAACTCT
>718
CTGCTGCCGCCCGTAGGAGTTTGGACCGTGTCTCAGTTCCAATGTGGGGGACCTTCCTCTCAGAACCCCTACT
GATCGTTGCCTTGGTGGGCCGTTACCCCGCCAACAAGCTAATCAGACGCATCCCCATCCATAACCGATAAATC
TTTATTCGTAATCTCATGAGATCAAACGAAATACATAAGGTATTAGTCCAACTTCGCTGGGTTATCCCTAGTT

ATGGGCAGGTTGGATACGCGTTACTCACCCGTGCGCCGGTCGCCGACATAAATAGCAAGCTATCTATTCGCTG
CCCCTCGACTTGCATGTGTTAAGCCTATAGCTAGCGTTCATCCTGAGCCAGGATCAAACTCT
>719
CTGCTGCCGCCCGTAGGAGTTTGGACCGTGTCTCAGTTCCAATGTGGGGGACCTTCCTCTCAGAACCCCTACT
GATCGTCGCCTTTGGTGGGCCGTTACCCCGCCAACAAGCTAATCAGACGCATCCCCATCCATCACCGCTAAAC
CTTTAATCGTTATTTGATGTCAGCAAACGATGTCATACGGTATTAGTCCGTCTTTCAACGGGTTATCCCGTGG
TCATGGGAAGGTTGGATACGCGTTACTCACCGTGCGCCAGTCGTCATCAAGGAAAGCAATGCATTCCCTTCAC
TGATTGCCCA
>720
CTGCTGCCGCCCGTAGGAGTTTGGACCGTGTCTCAGTTCCAATGTGGCCGATCACCCTCTCAGGTCGGCTACT
GATCGTCGCCTTGGTGGGCAGTTATCTCACCAACTAGCTAATCAGACGCGGGTCCATCCTATACCGCTAACAC
TTGATATATCTACGATGCCGTAAATATATATTATCATGTATTAGTAAACTTTTCAGTATGTTATCCATGTGTA
TAGGGCAGGTTACCCACGCGTTACTCACCCGTCCGCCGCTCATCTTCATTAATGCAAGCATCAAATCAGACGC
GCTCGACTTGCATGTGTTAGGCACGCCGCCAGCGTTCATCCTGAGCCAGGATCAAACTCT
>721
CTGCTGCCGCCCGTAGGAGTTTTGGACCGTGTCTCAGTTCCAATGTGGGGGACCTTCCTCTCAGAACCCCTAC
TGATCGTTGCCTTTGGTGGGCCGTTACCCCGCCAACAAGCTAATCAGACGCATCCCCATCCATAACCGATAAA
TCTTTATTCGTAATCTCATGAGATCAAACGAAATACATAAGGTATTAGTCCAACTTCGCTGGGTTATCCCTTA
GTTATGGGCAGGTTGGATACGCGTTACTCACCCGTGCGCCGGTCGCCGACATAAATAGCAAGCTATCTATTCG
CTGCCCCTCGACTTGCATGTGTTAACGCCTATAGCTAGCGTTCATCCTGAGCCAGGATCAAACTCT
>722
CTGCTGCCGCCCGTAGGAGTTTTGGACCGTGTCTCAGTTCCAATGTGGGGGACCTTCCTCTCAGAACCCCTAC
TGATCGTTGCCTTGGTGGGCCGTTACCCCGCCAACAAGCTAATCAGACGCATCCCCATCCATAACCGATAAAT
CTTTATTCGTAATCTCATGAGATCAAACGAAATACATAAGGTATTAGTCCAACTTCGCTGGGTTATCCCTTAG
TTATGGGCAGGTTGGATACGCGTTACTCACCCGTGCGCCGGTCGCCGACATAAATAGCAAGCTATCTATTCGC
TGCCCCTCGACTTGCATGTGCTAAGCCTATAGCCTAGCGTTCATCCTGAGCCAGGATCAAACCTCAT
>723
CTGCTGCCGCCCGTAGGAGTTTGGACCGTGTCTCAGTTCCAATGTGGGGGACCTTCCTCTCAGAACCCCTACT
GATCGTTGCCTTGGTGGGCCGTTACCCCGCCAACAAGCTAATCAGACGCATCCCCATCCATAACCGCTAAACC
TTTAATCGTTTATTTTGATGTCAGCAAACGATATCATACGGTATTTAGGCCGTCTTTTCAACGGGTTTATCCC
GTAGTTTATGGGAAGGTTGGATACGCGTTACTCACCCGTGCGCCAGTCGTCATCAAGGAAAGCAAGCATTTCC
CTTTCAGTGACTTTT
>724
CTGCTGCCGCCCGTAGGAGTTTGGACCGTGTCTCAGTTCCAATGTGGGGGACCTTTCCTCTCAGAACCCCTAC
TGATCGTCGCCTTTGGTGGGCCGTTACCCCGCCAACAAGCTAATCAGACGCATCCCCATCCATAACCGCTAAA
CCTTTAATCGTTTATTTTGATGTCAGCAAACGATATCATACGGTATTTAGGCCGTCTTTTCAACGGGTTTATT
CCCGTAGTTTATGGGAAGGTTGGATACGCGTTACTCACCCGTGCGCCAGTCGTCATCAAGGAAAGCAAGCATT
TCCCTTTC
>725
CTGCTGCCGCCCGTAGGAGTTTTGGACCGTGTCTCAGTTCCAATGTGGGGGACCTTCCTCTCAGAACCCCTAC
TGATCGTCGCCTTTGGTGGGCCGTTACCCCGCCAACAAGCTAATCAGACGCATCCCCATCCATAACCGCTAAA
CCTTTAATCGTTATTTGATGTCAGCAAACGATATCATACGGTATTAGGCCGTCTTTCAACGGGTTATCCCGTA
GTTATGGGAAGGTTGGATACGCGTTACTCACCCGTGCGCCAGTCGTCATCAAGGAAAGCAAGCTTTCCCTCAT
GTTACCCTGCGAACTTGCATGTGTTAAGCCTGTAGCTAGCGTTCATCCCTGAGCAGGCATCAAACATGCCT
>726
CTGCTGCCGCCCGTAGGAGTTTGGACCGTGTCTCAGTTCCAATGTGGGGGACCTTCCTCTCAGAACCCCTACT
GATCGTCGCCTTTGGTGGGCCGTTACCCCGCCAACAAGCTAATCAGACGCATCCCCATCCATAACCGCTAAAC
CTTTAATCGTTATTTGATGTCAGCAAACGATATCATACGGTATTAGGCCGTCTTTCAACGGGTTATCCCGTAG
TTATGGGAAGGTTGGATACGCGTTACTCACCCGTGCGCCAGTCGTCATCAAGGAAAGCAAGCTTTCCCTCATG
TTTACCCTGCGACATGCATGTGTTAAGCCTGTAGCTAGCGTCATCCTGAGCCAAGGCATCAAC
>727
CTGCTGCCGCCCGTAGGAGTTTGGACCGTGTCTCAGTTCCAATGTGGGGGACCTTCCTCTCAGAACCCCTACT
GATCGTCGCCTTGGTGGGCCGTTACCCCGCCAACAAGCTAATCAGACGCATCCCCATCCATAACCGCTAAACC
TTTAATCGTTATTTTGATGTCAGCAAACGATATCATACGGTATTTAGGCCGTCTTTTCAACGGGTTATCCCGT

AGTTTATGGGAAGGTTGGATACGCGTTACTCACCCGTGCGCCAGTCGTCATCAAGGAAAGCAAGCTTTCCCTC
ACTGATTTGACCCTGCGACATGCATGTGTTAAGCCTTGTAGCTAGCGGCATCCCTGAGCCAAGGA
>728
CTGCTGCCGCCCGTAGGAGTTTGGACCGTGTCTCAGTTCCAATGTGGGGGACCTTCCTCTCAGAACCCCTACT
GATCGTCGCCTTGGTGGGCCGTTACCCCGCCAACAAGCTAATCAGACGCATCCCCATCCATAACCGCTAAACC
TTTAATCGTTATTTGATGTCAGCAAACGATATCATACGGTATTAGGCCGTCTTTCAACGGGTTATCCCGTAGT
TATGGGAAGGTTGGATACGCGTACTCACCCGTGCGCCAGTCGTCATCAAGGAAAGCAAGCCTTTCCTCATGTT
ACCCGATGCCGTA
>729
CTGCTGCCGCCCGTAGGAGTTTGGACCGTGTCTCAGTTCCAATGTGGGGGACCTTCCTCTCAGAACCCCTACT
GATCGTTGCCTTGGTGGGCCGTTACCCCGCCAACAAGCTAATCAGACGCATCCCCATCCATAACCGATAAATC
TTTATTCGTAATCTCATGAGATCAAACGAATACATAAGGTATTAGTCCAACTTCGCTGGGTTATCCCTTAGTT
ATGGGCAGGTTGGATACGCGTTACTCACCCGTGCGCCGGTCGCCGACATAAATAGCAAGCTATCTATTCGCTG
CCCCTCGACTTGCATGTGTTAAGCCTATAGCTAGCGTTCATCCTGAGCCAGGATCAAACTCT
>730
CTGCTGCCGCCCGTAGGAGTTTTGGACCGTGTCTCAGTTCCAATGTGGGGGACCTTCCTCTCAGAACCCCTAC
TGATCGTCGCCTTTGGTGGGCCGTTACCCCGCCAACAAGCTAATCAGACGCATCCCCATCCATAACCGCTAAA
CCTTTAATCGTTTATTTTGATGTCAGCAAACGATATCATACGGTATTTAGGCCGTCTTTTTCAACGGGTTTAT
TCCCGTAGTTTTATGGGAAGGTTTGGATACGCGTTACTCACCCGTGCGCCAGTCGTCATCAAGGAAAGCAAGC
TTTCCTTCATGTTTACCCTGCGACATGCATT
>731
CTGCTGCCGCCCGTAGGAGTTTTGGACCGTGTCTCAGTTCCAATGTGGGGGACCTTCCTCTCAGAACCCCTAC
TGATCGTCGCCTTTGGTGGGCCGTTACCCCCGCCAACAAGCTAATCAGACGCATCCCCCATCCATAACCGCTA
AACCTTTAATCGTTTATTTTGATTGTCAGCAAACGATATCATACGGTATTTAGGCCGTCTTTTCAACGGGTTT
ATTCCCCGTAGTTTATGGGAAGGTTGGATACGCGTTACTCACCCGTGCGCCAGTCGTCATCAAGGAAAAGCAA
GCTTTTCCCCCTTGATGT
>732
CTGCTGCCGCCCGTAGGAGTCTGGACCGTGTCTCAGTTCCAGTGTGGCCGTTCATCCTCTTAGACCGGCTACT
GATCGTTGGTTTGGTGGGCCGTTACCCCACCAACTGCCTAATCAGACGCAAACCCCTCTTCAGGCGATAGCTT
ACAGGTAGAGGCTACCCTTTCTTCCACAGGTCATGCGGCCCATGGAACGTATTCGGTATTAGCAGTCGTTTCC
GTCTGTTGTCCCCATCCTGAAGGCAGGTTGTTTACGTGTTACTCACCCGTTTGCCACTGGATTCGATAAAGAG
CAAGCTCTTCATCTCTTCCCGTTCGACTTGCATGTGTTAAGCACGCCGCCAGCGTTCGTCCTGAGCCAGGATC
AAACTCT
>733
CTGCTGCCGCCCGTAGGAGTTTTGGACCGTGTCTCAGTTCCAATGTGGGGGACCTTCCTCTCAGAACCCCTAC
TGATCGTCGCCTTTGGTGGGCCGTTACCCCGCCAACAAGCTAATCAGACGCATCCCCATCCATAACCGCTAAA
CCTTTAATCGTTATTTGATGTCAGCAAACGATATCATACGGTATTAGGCCGTCTTTCAACGGGTTATCCCGTA
GTTATGGGAAGGTTGGATACGCATTACTCACCCGTGCGCCAGTCGTCATCAAGGAAAGCAAGCTTTCCTCATG
TTACCCTGCGACTTGCATGTGTTAAGCCTGTAGCTAGCGTTCATCCTGAGCCAGGATCAAACTCT
>734
CTGCTGCCGCCCGTAGGAGTTTGGACCGTGTCTCAGTTCCAATGTGGGGGACCTTCCTCTCAGAACCCCTACT
GATCGTCGCCTTTGGTGGGCCGTTACCCCGCCAACAAGCTAATCAGACGCATCCCCATCCATAACCGCTAAAC
CTTTAATCGTTATTTGATGTCAGCAAACGATATCATACGGTATTAGGCCGTCTTTCAACGGGTTATCCCGTAG
TTATGGGAAGGTTGGATACGCGTTACTCACCCGTGCGCCAGTCGTCATCAAGGAAAGCAAGCTTTCCCTCATA
TTACCCTGCGACTTGCATGTGTTAAGCCTGTAGCTAGCGTTCATCCGTGAGCCAAG
>735
CTGCTGCCGCCCGTAGGAGTTTGAACCGTGTCTCAGTTCCAATGTGGGGGACCTTCCTCTCAGAACCCCTACT
GATCGTTGCCTTGGTGGGCCGTTACCCCACCAACAAGCTAATCAGACGCATCCCCATCCATAACCGATAAATC
TTTATTCGTAATCTCATGAGATCAAACGAAATACATAAGGTATTAGTCCAACTTCGCTGGGTTATCCCTTAGT
TATGGGCAGGTTGGATACGCGTTACTCACCCGTGCGCCGGTCGCCGACATAAATAGCAAGCTATCTATTCGCT
GCCCCCCTCGACTTGCATGTGTTTAAGCCTATAGCTAGCGTTCATCCTGAGCCAGGATCAAACTCT
>736
CTGCTGCCGCCCGTAGGAGTTTGGACCGTGTCTCAGTTCCAATGTGGGGGACCTTCCTCTCAGAACCCCTACT
GATCGTCGCCTTGGTGGGCCGTTACCCCGCCAACAAGCTAATCAGACGCATCCCCATCCATAACCGCTAAACC

TTTAATCGTTATTTTGATGTCAGCAAACGATATCATACGGTATTAGGCCGTCTTTTCAACGGGTTATCCCGTA
GTTATGGGAAGGTTGGATACGCGTTACTCACCCGTGCGCCAGTCGTCATCAAGGAAAGCAAGCTTTCCCTCAT
GATTTACCCTGCGATTGCATGTGTTAAGCCTGTAGCTAGCGTTATCCTGAGCCCAAGGATCAACTCGT
>737
CTGCTGCCGCCCGTAGGAGTTTGGACCGTGTCTCAGTTCCAATGTGGGGGACCTTCCTCTCAGAACCCCTACT
GATCGTCGCCTTTGGTGGGCCGTTACCCCGCCAACAAGCTAATCAGACGCATCCCCATCCATAACCGCTAAAC
CTTTTAATCGTTATTTTGATGTCAGCAAACGATATCATACGGTATTTAGGCCGTCTTTTCAACGGGTTATCCC
GTAGTTTATGGGAAGGTTGGATACGCGTTACTCACCCGTGCGCCAGTCGTCATCAGGGAAAGCAAGCTTCCTC
ATGATTTTACCCTGCGACTTGCATGTGTTTTAAGCCTG
>738
CTGCTGCCGCCCGTAGGAGTTTGGACCGTGTCTCAGTTCCAATGTGGGGGACCTTCCTCTCAGAACCCCTACT
GATCGTCGCCTTGGTGGGCCGTTACCCCGCCAACAAGCTAATCAGACGCATCCCCATCCATAACCGCTAAACC
TTTTAATCGTATTTTGATGTCAGCAAACGATATCATACGGTATTTTAGGCCGTCTTTTTCAACGGGTTTATCC
CGTAGTTTATGGGAAGGTGGATACGCGTTACTCACCCGTGCGCCAGTCGTCATCAAGGAAAGCAAGCTTTTCC
CTTCATGA
>739
CTGCTGCCGCCCGTAGGAGTTTGGACCGTGTCTCAGTTCCAATGTGGGGGACCTTCCTCTCAGAACCCCTACT
GATCGTCGCCTTGGTGGGCCGTTACCCCGCCAACAAGCTAATCAGACGCATCCCCATCCATAACCGCTAAACC
TTTTAATCGTTTATTTTTGAGTCAGCAAACGATATCATACGGTATTTTAGGCCGTCTTTTTTCAACGGGTTTT
ATCCCGTAGTTTTATGGGAAGGTTGGATACGCGTTTACTCACCCGTGCGCCAGTCGTCATCAAGGAAAGCAAG
CATTTTGCCC
>740
CTGCTGCCGCCCGTAGGAGTTTTGGACCGTGTCTCCGTCTCAATGTGGGGGACCTTCCTCTCAGAACCCCTAC
TGATCGTCGCCTTTGGTGGGCCGTTACCCCGCCAACAAGCTAATCAGACGCATCCCCATCCATAACCGCTAAA
CCTTTAATCGTTATTTGATGTCAGCAAACGATATCATACGGTATTAGGCCGTCTTTCAACGGGTTATCCCGTA
GTTATGGGAAGGTTGGATACGCGTTACTCACCCGTGCGCCAGTCGTCATCAAGGAAAGCAAGCTTTCCCTCAT
GATTGACCCTGCCGACTTGCATGTGTTAAGCCTGTAGCTAGCGTTCATCCGTGAGCCAAGGATCAAACTCT
>741
CTGCTGCCGCCCGTAGGAGTTTGGACCGTGTCTCAGTTCCAATGTGGGGGACCTTCCTCTCAGAACCCCTACT
GATCGTCGCCTTTGGTGGGCCGTTACCCCGCCAACAAGCTAATCAGACGCATCCCCATCCATAACCGCTAAAC
CTTTAATCGTTATTTGATGTCAGCAAACGATATCATACGGTATTAGGCCGTCTTTCAACGGGTTATCCCGTAG
TTATGGGAAGGTTGGATACGCGTTACTCACCCGTGCGCCAGTCGTCATCAAGGAAAGCAAGCTTTCCCTCATG
TTACCCTGCGACTTGCATGTGTTAAGCCTGTAGCTAGCGTCATCCTGAGCCAAGGATCAACTCT
>742
CTGCTGCGCCCGTAGGAGTTTGGACCGTGTCTCAGTTCCAATGTGGGGGACCTTCCTCTCAGAACCCCTACTG
ATCGTCGCCTTTGGTGGGCCGTTACCCCGCCAACAAGCTAATCAGACGCATCCCCATCCATAACCGCTAAACC
TTTAATCGTTATTTGATGTCAGCAAACGATATCATACGGTATTAGGCCGTCTTTCAACGGGTTATCCCGTAGT
TATGGGAAGGTTGGATACGCGTTACTCACCCGTGCGCCAGTCGTCATCAAGGAAAGCAAGCTTTCCCTCATGT
TACCCTGCCGTACTGC
>743
CTGCTGCCGCCCGTAGGAGTTTGGACCGTGTCTCAGTTCCAATGTGGGGGACCTTCCTCTCAGAACCCCTACT
GATCGTTGCCTTGGTGGGCCGTTACCCCGCCAACAAGCTAATCAGACGCATCCCCATCCATAACCGATAAATC
TTTATTCGTAATCTCATGAGATCAAACGAAATACATAAGGTATTAGTCCAACTTCGCTGGGTTATCCCTTAGT
TATGGGCAGGTTGGATACGCGTTACTCACCCGTGCGCCGGTCGCCGACATAAATAGCAAGCTATCTATTCGCT
GCCCCTCGACTTGCATGTGTTAAGCCTATAGCTAGCGTTCATCCTGAGCCAGGATCAAACTCT
>744
CTGCTGCCGCCCGTAGGAGTTTGGACCGTGTCTCAGTTCCAATGTGGGGGACCTTCCTCTCAGAACCCCTACT
GATCGTCGCCTTTGGTGGGCCGTTACCCCGCCAACAAGCTAATCAGACGCATCCCCATCCATAACCGCTAAAC
CTTTAATCGTTATTTGATGTCAGCAAACGATATCATACGGTATTAGGCCGTCTTTCAACGGGTTATCCCGTAG
TTATGGGAAGGTTGGATACGCGTTACTCACCCGTGCGCCAGTCGTCATCAAGGAAAGCAAGCTTTCCCTCATG
TTACCCTGCGACCTGCATGTGTTAAGCCTGTAGCTAGCGTTCATCCTGAGCCAGGATCAACTCT
>745
CTGCTGCCGCCCGTAGGAGTTTGGACCGTGTCTCAGTTCCAATGTGGGGGACCTTCCTCTCAGAACCCCTACT
GATCGTCGCCTTTGGTGGGCCGTTACCCCGCCAACAAGCTAATCAGACGCATCCCCATCCATAACCGCTAAAC

CTTTAATCGTTATTTGATGTCAGCAAACGATATCATACGGTATTAGGCCGTCTTTCAACGGGTTATCCCGTAG
TTATGGGAAGGTTGGATACGCGTTACTCACCCGTGCGCCAGTCGTCATCAAGGAAAGCAAGCTTTCCCTCATG
TTACCCTGCGACTTGCATGTGTTAAGCCTGTAGCTAGCGTCATCCTGAGCCAGGATCAACTCT
>746
CTGCTGCCGCCCGTAGGAGTTTGGACCGTGTCTCAGTTCCAATGTGGGGGACCTTCCTCTCAGAACCCCTACT
GATCGTTGCCTTGGTGGGCCGTTACCCCGCCAACAAGCTAATCAGACGCATCCCCATCCATAACCGATAAATC
TTTATTCGTAATCTCATGAGATCAAACGAATACATAAGGTATTAGTCCAACTTCGCTGGGTTATCCCTTAGTT
ATGGGCAGGTTGGATACGCGTTACTCACCCGTGCGCCGGTCGCCGACATAAATAGCAAGCTATCTATTCGCTG
CCCCTCGACCTTGCATGTGTTAAGCCTATAGCTAGCGTTCATCCTGAGCCAGGATCAAACTCT
>747
CTGCTGCCGCCCGTAGGAGTTTGGACCGTGTCTCAGTTCCAATGTGGGGACCTTCCTCTCAGAACCCCTACTG
ATCGTTGCCTTGGTGGGCCGTTACCCCGCCAACAAGCTAATCAGACGCATCCCCATCCATAACCGATAAATCT
TTATTCGTAATCTCATGAGATCAAACGAAATACATAAGGTATTAGTCCAACTTCGCTGGGTTATCCCTTAGTT
ATGGGCAGGTTGGATACGCGTTACTCACCCGTGCGCCGGTCGCCGACATAAATAGCAAGCTATCTATTCGCTG
CCCCTCGACTTGCATGTGTTAAGCCTATAGCTAGCGTTCATCCTGAGCCAGGATCAAACTCT
>748
CTGCTGCCGCCCGTAGGAGTTTGGACCGTGTCTCAGTTCCAATGTGGGGGACCTTCCTCTCAGAACCCCTACT
GATCGTTGCCTTGGTGGGCCGTTACCCCGCCAACAAGCTAATCAGACGCATCCCCATCCATAACCGATAAATC
TTTATTCATAATCTCATGAGATCAAAACGAATACATAAGGTATTAGTCCAACTTCGCTGGGTTATCCCTTAGT
TATGGGCAGGTTGGATACGGCGTTACTCACCCGTGCGCCGGTCGCCGACATAAATAGCAAGCTATCTATTCGC
TGCCCCTCGACTTGCATGTGTTTAAGCCTATAGCTAGCGTTCATCCTGAGCCAGGATCAAACTCT
>749
CTGCTGCCGCCCTAGGAGTTTGAACCGTGTCTCAGTTCCAATGTGGGGGACCTTCCTCTCAGAACCCCTACTG
ATCGTTGCCTTGGTGGGCCGTTACCCCACCAACAAGCTAATCAGACGCATCCCCATCCATAACCGATAAATCT
TTATTTCGTAATCTCATGAGATCAAACGAATATCATAAGGTATTTAGTCCAACTTCGCTGGGTTTATCCCTTA
GTTATGGGCAGGTTTGGATACGCGTTACTCACCCGTGCGCCGGTCGCCGACATTAAATAGCAAGCTATCTATT
CGCTGCCCTCGACTTGCATGTGTTTAAGCCTATAGCTAGCGTTCATCCTTGAGCCA
>750
CTGCTGCCGCCCGTAGGAGTTTTGGACCGTGTCTCAGTTCCAATGTGGGGGACCTTTCCTCTCAGAACCCCTA
CTGATCGTTGCCTTGGTGGGCCGTTACCCCGCCAACAAGCTAATCAGACGCATCCCCATCCATAACCGATAAA
TCTTTATTCGTAATCTCATGAGATCAAACGAAATACATAAGGTATTAGTCCAACTTCGCTGGGTTATCCCTTA
GTTATGGGCAGGTTGGATACGCGTTACTCACCCGTGCGCCGGTCGCCGACATAAATAGCAAGCTATCTATTCG
CTGCCCCTCGACTTGCATGTGTTTAAGCCTATAGCTAGCGTTCATCCTGAGCCAGGATCAAACTCT
>751
CTGCTGCCGCCCGTAGGAGTTGAACCGTGTCTCAGTTTCCAATGTGGGGGACCTTCCTCTCAGAACCCCTACT
GATCGTTGCCTTGGTGGGCCGTTACCCCGCCAACAAGCTAATCAGACGCATCCCCATCCATAACCGATAAATC
TTTATTCATAATCTCATGAGATCAAACGAATACATAAGGTATTAGTCCAACTTCGCTGGGTTTATCCCTTAGT
TATGGGCAGGTTTGGATACGCGTTACTCACCCGTGCGCCGGTCGCCGACATAAATAGCAAGCTATCTATTCGC
TGCCCCCCTTCGACTTGCATGTGTTTAAGCCTATAGCTAGCGTTCACCTTAGAGCCAGGATCAAACTCTT
>752
CTGCTGCCGCCCGTAGGAGTTTGGACCGTGTCTCAGTTCCAATGTGGGGGACCTTCCTCTCAGAACCCCTACT
GATCGTTGCCTTGGTGGGCCGTTACCCCGCCAACAAGCTAATCAGACGCATCCCCATCCATAACCGATAAATC
TTATTCGTAATCTCATGAGATCAAACGAAATACATAAGGTATTAGTCCAACTTCGCTGGGTTATCCCTTAGTT
ATGGGCAGGTTGGACGTACGCGTTACTCACCCGTGCGCCGGTCGCCGACATAAATAGCAAGCTATCTATTCGC
TGCCCCTCGACTTGCATGTGTTAAGCCTATAGCTAGCGTCATCCTGAGCCAGGATCAACTCT
>753
CTGCTGCCCCCGTAGGAGTTTTGGACCGTGTCTCAGTTCCAATGTGGGGGACCTTCCTCTCAGAACCCCTACT
GATCGTCGCCTTGGTGGGCCGTTACCCCGCCAACAAGCTAATCAGACGCATCCCCACTCATCACCGTAAACCT
TTAATCGTTATTTGATGTCAGCAAACGATATCATACGGTATTAGGCCGTCTTTTCAACGGGTTATCCCGTAGT
TATGGGAAGGTTGGATACGCGTTACTCACCCGTGCGCCAGTCGTCATCAAGGAAAGCAGCTTCCTCATGTTAC
CCTGCGACTGCATGTGTTAAGCCTGTAGCTAGCGTCATCCTGACGCAG
>754
CTGCTGCCGCCCGTAGGAGTTTGGACCGTGTCTCAGTTCCAATGTGGGGGACCTTCCTCCTCAGAACCCCTAC
TGATCGTTGCCTTGGTGGGCCGTTACCCCGGCCAACAAGCTAATCAGACGCATCCCCATCCATGACCGATAAA

TCTTTATTCATAATCTCATGAGATCAAACGAATACATAAGGTATTAGTCCAACTTCGCTGGGTTATCCCTTAG
TTATGGGCAGGTTGGATACGCGTTACTCACCCGTGCGCCGGTCGCCGACATAAATAGCAAGCTATCTATTCGC
TGCCCCCCTCGACTTGCATGTGTTTAAGCCTATAGCTAGCGTTCATCCTGAGCCAGGATCAAACTCT
>755
CTGCTGCCGCCCGTAGGAGTTTTGGACCGTGTCTCAGTTCCAATGTGGGGGACCTTCCTCTCAGAACCCCTAC
TGATCGTTGCCTTTGGTGGGCCGTTACCCCGCCAACAAGCTAATCAGACGCATCCCCATCCATAACCGATAAA
TCTTTATTCGTAATCTCATGAGATCAAACGAAATACATAAGGTATTAGTCCAACTTCGCTGGGTTATCCCTTA
GTTATGGGCAGGTTGGATACGCGTTACTCACCCGTGCGCCGGTCGCCGACATAAATAGCAAGCTATCTATTCG
CTGCCCCTCGACTTGCATGTGTTAAGCCTATAGCTAGCGTTCTCCTGAGCCAGGATCAAACTCT
>756
CTGCTGCCGCCCGTAGGGGTTTGGACCGTGTCTCAGTTCCAATGTGGGGGACCTTTCCTCTCAGAACCCCTAC
TGATCGTCGCCTTTGGTGGGCCGTTACCCCGCCAACAAGCTAATCAGACGCATCCCCATCCATAACCGCTAAA
CCTTTAATCGTTATTTGATGTCAGCAAACGATATCATACGGTATTAGGCCGTCTTTCAACGGGTTATCCCGTA
GTTATGGGAAGGTTGGATACGCGTTACTCACCCGTGCGCCAGTCGTCATCAAGGAAAGCAAGCTTTCCTCATG
TTACCCTGCGACTTGCATTGTGTTAAGCCTGTAGCTAAGCGTTTCATCCATGAGCCAGGATCAAACTCT
>757
CTGCTGCCGCCCGTAGGAGTTTTGGACCGTGTCTCAGTTCCAATGTGGGGGACCTTCCTCTCAGAACCCCTAC
TGATCGTCGCCTTGGTGGGCCGTTACCCCGCCAACAAGCTAATCAGACGCATCCCCATCCATAACCGCTAAAC
CTTTAATCGTTATTTTGATGTCAGCAAACGATATCATACGGTATTAGGCCGTCTTTCAACGGGTTATCCCGTA
GTTTATGGGAAGGTTGGATACGCGTTACTCACCCGTGCGCCAGTCGTCATCAAGGAAAGCAAGCTTTCCCTCA
TGTTACCCTGCGAACTTGCATGTGTTAAGCCTGTAGCTAGCGTCATCCTGAGCCAGGATCAAACTCT
>758
CTGCTGCCGCCCGTAGGAGTTTGGACCGTGTCTCAGTTCCAATGTGGGGGACCTTCCTCTCAGAACCCCTACT
GATCGTTGCCTTGGTGGGCCGTTACCCCGCCAACAAGCTAATCAGACGCATCCCCATCCATAACCGATAAGTC
TTTATTCATAATCTCATGAGATCAAACGAATACATAAGGTATTAGTCCAACTTCGCTGGGTTATCCCTTAGTT
ATGGGCAGGTTGGATACGCGTTACTCACCCGTGCGCCGGTCGCCGACATAAATAGCAAGCTATCTATTCGCTG
CCCCTCGACTTGCATGTGTTAAGCCTATAGCTAGCGTTCATCCTGAGCCAGGATCAAACTCT
>759
CTGCTGCCGCCCGTAGGAGTTTGGACCGTGTCTCAGTTCCAATGTGGGGGACCTTCCTCTCAGAACCCCTACT
GATCGTTGCCTTGGTGGGCCGTTACCCCGCCAACAAGCTAATCAGACGTATCCCCCATCCATAACCGATAAAT
CTTTTATTTCATAATCTCATGAGATCAAACGAATACATAAGGTATTTAGTCCAACTTCGCTGGGTTTATCCCT
TTAGTTTATGGGCAGGTTTGGATACGCGTTACTCACCCGTGCGCCGGTCGCCGACATAAATAGCAAGCTATCT
ATTCGCTGCCCCCCCTCGACTTGCATGTGTTTTAAGCCTTATAGCTAGCGTTCATCCTGAGCCAGGATCAAAC
TCT
>760
CTGCTGCCGCCCGTAGGAGTTTGGACCGTGTCTCAGTTCCAATGTGGGGGACCTTCCTCTCAGAACCCCTACT
GATCGTCGCCTTGGTGGGCCGTTACCCCGCCAACAAGCTAATCAGACGCATCCCCATCCATAACCGCTAAACC
TTTTAATCGTTTATTTTGATGTCAGCAAACGATATCATACGGTATTTAGGCCGTCTTTTTCAACGGGTTTATC
CCGTAGTTTATGGGAAGGTTTGGATACGCGTTACTCACCCGTGCGCCAGTCGTCATCAAGGAAAGCAAGCTTT
TCCTCATGTTTTACCCTGCGACTTGCATTGTGTTTAAGCCTTTGTAGCTAGCGT
>761
CTGCTGCCGCCCGTAGGAGTTTTGGACCGTGTCTCAGTTCCAATGTGGGGGACCTTCCTCTCAGAACCCCTAC
TGATCGTCGCCTTTGGTGGGCCGTTACCCCGCCAACAAGCTAATCAGACGCATCCCCATCCATAACCGCTAAA
CCTTTAATCGTTATTTGATGTCAGCAAACGATATCATACGGTATTAGGCCGTCTTTCAACGGGTTATCCCGTA
GTTATGGGAAGGTTGGATACGCGTTACTCACCCGTGCGCCAGTCGTCATCAAGGAAAGCAAGCTTTCCCTCAT
GTTACCCTGCGACTTGCATGTGTTAAGCCTGTAGCTAGCGTTCATCCCTGAGCCAGGATCAAACTCT
>762
CTGCTGCCGCCCGTAGGAGTTTGGACCGTGTCTCAGTTCCAATGTGGGGGACCTTCCTCTCAGAACCCCTACT
GATCGTCGCCTTTGGTGGGCCGTTACCCCGCCAACAAGCTAATCAGACGCATCCCCATCCATAACCGCTAAAC
CTTTAATCGTTTATTTTGATGTCAGCAAACGATATCATACGGTATTTAGGCCGTCTTTTCAACGGGTTTATCC
CGTAGTTTATGGGAAGGTTGGATACGCGTTACTCACCCGTGCGCCAGTCGTCATCAAGGAAAGCAAGCTTTCC
CTTCATGTTTACCCTGCGA
>763

CTGCTGCCGCCCGTAGGAGTTTGAACCGTGTCTCAGTTCCAATGTGGGGGACCTTCCTCTCAGAACCCCTACT
GATCGTCGCCTTTGGTGGGCCGTTACCCCGCCAACAAGCTAATCAGACGCATCCCCATCCATAACCGCTAAAC
CTTTTAATCGTTATTTTGATGTCAGCAAACGATATCATACGGTATTTAGGCCGTCTTTTCAACGGGTTTATCC
CGTAGTTTATGGGAAGGTTGGATACGCGTTACTCACCCGTGCGCCAGTCGTCATCAAGGAAAGCAAGCTTTTC
CCTTCATGATTTT
>764
CTGCTGCCGCCCGTAGGAGTTTGGACCGTGTCTCAGTTCCAATGTGGGGGACCTTCCTCTCAGAACCCCTACT
GATCGTTGCCTTGGTGGGCCGTTACCCCGCCAACAAGCTAATCAGACGCATCCCCATCCATAACCGATAAATC
TTTATTCGTAATCTCATGAGATCAAACGAAATACATAAGGTATTAGTCCAACTTCGCTGGGTTATCCCTTAGT
TATGGGCAGGTTGGATACGCGTTACTCACCCGTGCGCCGGTCGCCGACATAAATAGCAAGCTATCTATTCGCT
GCCCCCCTCGACTTGCATGTGTTAAGCCTATAGCTAGCGTTCATCCTGAGCCAGGATCAAACTCT
>765
CTGCTGCCGCCCGTAGGAGTTTGGACCGTGTCTCAGTTCCAATGTGGGGGACCTTCCTCTCAGAACCCCTACT
GATCGTTGCCTTGGTGGGCCGTTACCCCGCCAACAAGCTAATCAGACGCATCCCCATCCATAACCGATAAATC
TTTATTCGTAATCTCATGAGATCAAACGAAATACATAAGGTATTAGTCCAACTTCGCTGGGTTATCCCTTAGT
TATGGGCAGGTTGGATACGCGTTACTCACCCGTGCGCCGGTCGCCGACATAAATAGCAAGCTATCTATTCGCT
GCCCCCCTCGACTTGCATGTGTTTAAGCCTATAGCTAGCGTTCATCCTGAGCCAGGATCAAACTCT
>766
CTGCTGCCGCCCGTAGGAGTTTGGACCGTGTCTCAGTTCCAATGTGGGGGACCTTCCTCTCAGAACCCCTACT
GATCGTTGCCTTGGTGGGCCGTTACCCCGCCAACAAGCTAATCAGACGCATCCCCATCCATAACCGATAAATC
TTTATTCATAATCTCATGAGAATCAAACGAATACATAAGGTATTAGTCCAACTTCGCTGGGTTATCCCTTAGT
TATGGGCAGGTTGGATACGCGTTACTCACCCGTGCGCCGGTCGCCGACATAAATAGCAAGCTATCTATTCGCT
GCCCCTCGACTTGCATGTGTTAAGCCTATAGCTAGCGTTCATCCTGAGCCAGGATCAAACTCT
>767
CTGCTGCCGCCCGTAGGAGTTTGGACCGTGTCTCAGTTCCAATGTGGGGGACCTTCCTCTCAGAACCCCTACT
GATCGTCGCCTTTGGTGGGCCGTTACCCCGCCAACAAGCTAATCAGACGCATCCCCATCCATAACCGCTAAAC
CTTTTAATCGTTATTTTGATGTCAGCAAACGATATCATACGGTATTTAGGCCGTCTTTTCAACGGGTTTATCC
CGTGTTATGGGAAGGTTGGATACGCGTTACTCACCCGTGCGCCCAGTCGTCGTCAAGGGAAAGCAAGCTTCCC
TCACGTACCTTGT
>768
CTGCTGCCGCCCGTAGGAGTTTGAACCGTGTCTCAGTTCCAATGTGGGGGACCTTCCTCTCAGAACCCCTACT
GATCGTCGCCTTGGTGGGCCGTTACCCCGCCAACAAGCTAATCAGACGCATCCCCATCCATCACCGCTAAACC
TTTAATCGTTATTTTGATGTCAGCAAACGATGTCATACGGTATTAGTCCGTCTTTTCAACGGGTTATCCCGTG
GTCATGGGAAGGTTGGATACGCGTTACTCACCCGTGCGCCAGTCGTCGTCAAGGGAAGCAAGCTTCCCTCACG
TTACCCTGCGACTGCATGTGTAAGCCTGTAGCTAGCGTCATCCTGAGCCAGGATCAACTCT
>769
CTGCTGCCGCCCGTAGGAGTTTTGGACCGTGTCTCAGTTCCAATGTGGGGGACCTTCCTCTCAGAACCCCTAC
TGATCGTCGCCTTTGGTGGGCCGTTACCCCGCCAACAAGCTAATCAGACGCATCCCCATCCATAACCGCTAAA
CCTTTAATCGTTATTTTGATGTCAGCAAACGATATCATACGGTATTAGGCCGTCTTTTCAACGGGTTATCCCG
TAGTTATGGGAAGGTTGGATACGCGTTACTCACCCGTGCGCCAGTCGTCATCAAGGAAAGCAAGCTTTCCTCA
TGTTACCCTGCGACTTGCATGTGTTAAGCCTGTAGCTAGCGTTCATCCTGAGCCAGGATCAACTCT
>770
CTGCTGCCGCCCGTAGGAGTTTGGACCGTGTCTCAGTTCCAATGTGGGGGACCTTCCTCTCAGAACCCCTACT
GATCGTCGCCTTTGGTGGGCCGTTACCCCGCCAACAAGCTAATCAGACGCATCCCCATCCATCACCGCTAAAC
CTTTAATCGTTATTTGATGTCAGCAAACGATGTCATACGGTATTAGTCCGTCTTTCAACGGGTTATCCCGTAG
TTATGGGAAGGTTGGATACGCGTTACTCACCCGTGCGCCAGTCGTCATCAAGGAAAGCAAGCTTTCCTCATGT
TACCCTGCGACTTGCATGTGTTAAGCCTGTAGCTAGCGTTCATCCCTGAGCCAGGATCAAACTCT
>771
CTGCTGCCGCCCGTAGGAGTTTGGACCGTGTCTCAGTTCCAATGTGGGGGACCTTCCTCTCAGAACCCCTACT
GATCGTCGCCTTGGTGGGCCGTTACCCCGCCAACAAGCTAATCAGACGCATCCCCATCCATAACCGCTAAACC
TTTAATCGTTATTTGATGTCAGCAAACGATATCATACGGTATTAGGCCGTCTTTCAACGGGTTATCCCGTAGT
TATGGGAAGGTTGGATACGCGTTACTCACCCGTGCGCCAGTCGTCATCAAGGAAAGCAAGCTTCCCTGACTGT
TACCCATGCGACTTGCATGTGTTAAGCCTGTAGCTAGCGTTCATCCTGAGCCAGGATCAAACTCT
>772

CTGCTGCCGCCCGTAGGAGTTTGGACCGTGTCTCAGTTCCAATGTGGGGGACCTTCCTCTCAGAACCCCTACT
GATCGTTGCCTTTGGTGGGCCGTTACCCCGCCAACAAGCTAATCAGACGCATCCCCATCCATAACCGATAAAT
CTTTATTCGTAATCTCATGAGATCAAACGAAATACATAAGGTATTAGTCCAACTTCGCTGGGTTATCCCTTAG
TTATGGGCAGGTTGGATACGCGTTACTCACCCGTGCGCCGGTCGCCGACATAAATAGCAAGCTATCTATTCGC
TGCCCCTCGACTTGCATGTGTTAAGCCTATAGCTAGCGTTCATCCTGAGCCAGGATCAAACTCT
>773
CTGCTGCCGCCCGTAGGAGTTGAACCGTGTCTCAGTTCCAATGTGGGGGACCTTCCTCTCAGAACCCCTACTG
ATCGTTGCCTTGGTGGGCCGTTACCCCGCCAACAAGCTAATCAGACGCATCCCCATCCATAACCGCTAAACCT
TTTAATCGTTTATTTTTGATGTCAGCAAACGATGTCATACGGTATTTAGTCCGTCTTTTTCAACGGGTTATCC
CGTAGTTATGGGAAGGTTGGATACGCGTTACTCACCCGTGCGCCAGTCGTCATCAAGGAAAAGCAAGCTTTCC
CTCATGTTACCCTGCCGACATGCATGTGTTAAGCCTAGTACGCTGCGTTATCCCG
>774
CTGCTGCCGCCCGTAGGAGTTTGGACCGTGTCTCAGTTCCAATGTGGGGGACCTTCCTCTCAGAACCCCTACT
GATCGTCGCCTTTGGTGGGCCGTTACCCCGCCAACAAGCTAATCAGACGCATCCCCATCCATAACCGATAAAT
CTTTATTCGTAATCTCGTGAGATCAAACGAAATACATAAGGTATTAGTCCAACTTCGCTGGGTTATCCCTTAG
TTATGGGCAGGTTGGATACGCGTTACTCACCCGTGCGCCGGTCGCCGACATAAATAGCAAGCTATCTATTCGC
TGCCCCTCGACCTTGCATGTGTTAAGCCTATAGCTAGCGTTCATCCTGAGCCAGGATCAAACTCT
>775
CTGCTGCCGCCCGTAGGAGTTTTGGACCGTGTCTCAGTTCCAATGTGGGGGACCTTCCTCTCAGAACCCCTAC
TGATCGTTGCCTTGGTGGGCCGTTACCCCGCCAACAAGCTAATCAGACGCATCCCCATCCATAACCGATAAAT
CTTTACTCATAATCTCATGAGATCAAACGAATACATAAGGTATTAGTCCAACTTCGCTGGGTTATCCCTTAGT
TATGGGCAAGTTGGATACGCGTTACTCACCCGTGCGCCGGTCGCCGACATAAATAGCAAGCTATCTATTCGCT
GCCCCTCGACTTGCATGTGTTTAACGCCTATAGCTAGCGTTCATCCTGAGCCAGGATCAAACTCT
>776
CTGCTGCCGCCCGTAGGAGTTTTGGACCGTGTCTCAGTTCCAATGTGGGGGACCTTCCTCTCAGAACCCCTAC
TGATCGTCGCCTTTGGTGGGCCGTTACCCCGCCAACAAGCTAATCAGACGCATCCCCATCCATAACCGCTAAA
CCTTTAATCGTTATTTGATGTCAGCAAACGATATCATACGGTATTAGGCCGTCTTTCAACGGGTTATCCCGTA
GTTTATGGGAAGGTTGGATACGCGTTACTCACCCGTGCGCCAGTCGTCATCAAGGAAAGCAAGCCTTTCCTCA
TGTTTACCCTGCGACTTGCATGTGTTTAAGCCTTGTAGCTAGCGTCATCCTGAGCCAGGATCAAACTCT
>777
CTGCTGCCGCCCGTAGGAGTTTGGACCGTGTCTCAGTTCCAATGTGGGGGACCTTCCTCTCAGAACCCTCCAC
TGATCGTCGCCTTTGGTGGGCCGTTACCCCGCCAACAAGCTAATCAGACGCATCCCCATCCATAACCGCTAAA
CCTTTAATCGTTATTTGATGTCAGCAAACGATATCATACGGTATTAGGCCGTCTTTCAACGGGTTATCCCGTA
GTTATGGGAAGGTTGGATACGCGTTACTCACCCGTGCGCCAGTCGTCATCAAGGAAAGCAAGCTTCCCTCATG
TTACCCTGCGACTGCATGTGTTAAGCCTGTAGCTAGCCGTCAT
>778
CTGCTGCCGCCCGTAGGGGTTTGGACCGTGTCTCAGTTCCAATGTGGGGGACCTTCCTCTCAGAACCCCTACT
GATCGTTGCCTTGGTGGGCCGTTACCCCGCCAACAAGCTAATCAGACGCATCCCCATCCATAACCGATAAATC
TTTATTCATAATCTCATGAGATCAAACGAATACATAAGGTATTAGTCCAACTTCGCTGGGTTATCCTTAGTTA
TGGGCAGGTTGGATACGCGTTACTCACCCGTGCGCCGGTCCGCCGACATAAATAGCAAGCTATCTATTCGCTG
CCCCTCGACTTGCATGTGTTAACGCCTATAGCTAGCGTTCTCCTGAGCCAGGATCAAACTCCT
>779
CTGCTGCCGCCCGTAGGAGTTTGGACCGTGTCTCAGTTCCAATGTGGGGGACCTTCCTCTCAGAACCCCTACT
GATCGTTGCCTTTGGTGGGCCGTTACCCCGCCAACAAGCTAACTCAGACGCATCCCCATCCATAACCGCTAAA
CCTTTAATCGTTATTTGATGTCAGCAAACGACGTCATACGGTATTAGTCCGTCTTTCAACGGGTTATCCCGTG
GTCATGGGAAGGTTGGATACGCGTTACTCACCCGTGCGCCAGTCGTCATCAAGGAAAGCAAGCTTTCCTCATG
TTACCCTGCGACTTGCATGTGTTAAGCCTGTAGCTAGCGTTCATCCTGAGCCAGGATCAAACTCT
>780
CTGCTGCCGCCCGTAGGAGTTTTGGACCGTGTCTCAGTTCCAATGTGGGGGACCTTCCTCTCAGAACCCCTAC
TGATCGTCGCCTTTGGTGGGCCGTTACCCCGCCAACAAGCTAATCAGACGCATCCCCATACCATAACCGCTAA
CCTTTAATCGTTATTTGATGTCAGCAAACGATATCATACGGTATTAGGCCGTCTTTCAACGGGTTATCCCGTA
GTTATGGGAAGGTTGGATACGCGTTACTCACCCGTGCGCCAGTCGTCATCAAGGAAAGCAAGCTTTCCCTCAT
GTTACCCTGCGACTTGCATGTGTTAAGCCTGTAGCTAGCGTTCATCCCTGAGCCAAGGATCAAACTGCCTAGT
>781

CTGCTGCCGCCCGTAGGAGTTTTGGACCGTGTCTCAGTTCCAATGTGGGGGACCTTCCTCTCAGAACCCCTAC
TGATCGTCGCCTTTGGTGGGCCGTTACCCCCGCCAACAAGCTAATCAGACGCATCCCCATCCATAACCGCTAA
ACCTTTTAATCGTTTATTTTGATGTCAGCAAACGATATCATACGGTATTTAGGCCGTCTTTTCAACGGGTTTA
TCCCGTAGTTTATGGGAAGGTTTGGATACGCGTTACTCACCCGTGCGCCAGTCGTCATCAAGGAAAGCAAGCT
TTTCCCTTTTATGTTTACCCTTGCGACTTG
>782
CTGCTGCCGCCCGTAGGAGTTTGAACCGTGTCTCAGTTCCAATGTGGGGGACCTTCCTCTCAGAACCCCTACT
GATCGTTGCCTTGGGTGGGCCGTTACCCCACCAACAAGCTAATCAGACGCATCCCCATCCATAACCGATAAAT
CTTTATTCGTAATCTCATGAGATCAAACGAAATATATAAGGTATTAGTCCAACTTCGCTGGGTTATCCCTTAG
TTTATGGGCAGGTTTGGATACGCGTTACTCACTCGTGCGCCGGTCGCCGACATAAATAGCAAGCTATCTATTC
GCTGCCCCTCGACTTGCATGTGTTTAAGCCTATAGCTAGCGTTCATCCTGAGCCAGGATCAAACTCT
>783
CTGCTGCCGCCCGTAGGAGTCTGGGCCGTATCTCAGTCCCGATGTGGCCGCACACCCTCTCAGGCCGGCTACC
CGTCGCCGCCTTGGTAGGCCATTACCCCACCAACAAGCTGATAGGCCGCAAGCTCATCCCACACCAGAAAAAC
CTTTCCACCACACCAACCACGATGCGGTCCTATAGGGTATTAGACCCAGTTTCCCAAGCTTACTCCCCCAGTG
CAGGGCAGATCCACTTACGTGTTACTCACCCGTTCGCCACTCGAGCACCCCAGCAAGCTGGGGCCTTTCCGTT
CGACTTGCATGTGTTAAGCACGCCGCCAGCGTCATCCTGAGCCAGGATCAACTCT
>784
CTGCTGCCGCCCGTAGGAGTTTGGACCGTGTCTCAGTTCCAATGTGGGGGACCTTCCTCTCAGAACCCCTACT
GATCGTCGCCTTTGGTGGGCCGTTACCCCGCCAACAAGCTAATCAGACGCATCCCCATCCATAACCGCTAAAC
CTTTAATCGTTATTTGATGTCAGCAAACGACGTCATACGGTATTAGTCCGTCTTTCAACGGGTTATCCCGTGG
TCATGGGAAGGTTGGATACGCGTTACTCACCCGTGCGCCAGTCGTCGTCAAGGAAAGCAAGCTTCCTCATGTT
ACCCTGCGACTTGCATGTGTTAAGCCTGTAGCTAGCGTTCATCCTGAGCCAGGATCAAACTCT
>785
CTGCTGCCGCCCGTAGGAGTTTGGACCGTGTCTCAGTTCCAATGTGGGGGACCTTCCTCTCAGAACCCCTACT
GATCGTTGCCTTTGGTGGGCCGTTACCCCGCCAACAAGCTAATCAGACGCATCCCCATCCATAACCGATAAAT
CTTTATTCGTAATCTCATGAGATCAAACGAATACATAAGGTATTAGTCCAACTTCGCTGGGTTATCCCTTAGT
TATGGGCAGGTTGGATACGCGTTACTCACCCGTGCGCCGGTCGCCGACATAAATAGCAAGCTATCTATTCGCT
GCCCCTCGACTTGCATGTGTTAAGCCTATAGCTAGCGTTCATCCTGAGCCAGGATCAAACTCT
>786
CTGCTGCCGCCCGTAGGAGTTTGGACCGTGTCTCAGTTCCAATGTGGGGGACCTTCCTCTCAGAACCCCTACT
GATCGTCGCCTTGGTGGGCCGTTACCCCACCAACAAGCTAATCAGACGCATCCCCATCCATAACCGCTAAACC
TTTAATCGTTATTTGATGTCAGCAAACGATATCATACGGTATTAGGCCGTCTTTCAACGGGTTAGTCCCGTAG
TTATGGGAAGGTTGGATACGCGTTACTCACCCGTGCGCCAGTCGTCATCAAGGAAAGCAAGCTTTCCCTCATG
TTACCCTGCGACTTGCATGTGTTAAGCCTGTAGCTAGCGTTCATCCCTGAGCAGGATCAAACTCT
>787
CTGCTGCCGCCCGTAGGAGTTTGAACCGTGTCTCAGTTCCAATGTGGGGGACCTTCCTCTCAGAACCCCTACT
GATCGTTGCCTTGGTGGGCCGTTACCCCGCCAACAAGCTAATCAGACGCATCCCCATCCATAACCGATAAATC
TTTATTCGTAATCATCATGAGATCAAACGAAATACATAAGGTATTTAGTCCAACTTCGCTGGGTTTATCCCTT
AGTTTATGGGCAGGTTTGGATACGCGTTACTCACCCGTGCGCCGGTCGCCGACATAAATAGCAAGCTATCTAT
TTCGCTGCCCCCCCTCGACTTTGCATGTGTTTTAAGCCTATAGCTAGCGTTCATCCTGAGCCAGGATCAAACT
CT
>788
CTGCTGCCGCCCGTAGGAGTTTTGGACCGTGTCTCAGTTCCAATGTGGGGGACCTTCCTCTCAGAACCCCTAC
TGATCGTCGCCTTTGGTGGGCCGTTACCCCGCCAACAAGCTAATCAGACGCATCCCCATCCATAACCGCTAAA
CCTTTAATCGTTATTTTGATGTCAGCAAACGATGTCATACGGTATTTAGTCCGTCTTTTCAACGGGTTATCCC
GTGGTCATGGGAAGGTTGGATACGCGTTACTCACCCGTGCGCCAGTCGTCGTCAAGGGAAGCAAGCTTCCCCT
GACCGTTACCCTGCGACTTGCATGTGTTAAGCCTTGTAGCTAGCGTCATCCTGAGCCAGGATCAAACCTCT
>789
CTGCTGCCGCCCGTAGGAGTTTGGACCGTGTCTCAGTTCCAATGTGGGGGACCTTCCTCTCAGAACCCCTACT
GATCGTCGCCTTTGGTGGGCCGTTACCCCGCCAACAAGCTAATCAGACGCATCCCCATCCATAACCGCTAAAC
CTTTAATCGTTATTTGATGTCAGCAAACGATATCATACGGTATTAGGCCGTCTTTTCAACGGGTTATCCCGTA
GTTATGGGAAGGTTGGATACGCGTTACTCACCCGTGCGCCAGTCGTCATCAAGGAAAGCAAGCTTCCTCATGT
TACCCTGCGACTTGCATGTGTTAAGCCTGTAGCTAGCGTTCATCCGTGAGCCAAGGATCAAACTCT

>790
CTGCTGCCGCCCGTAGGAGTTTGAACCGTGTCTCAGTTCCAATGTGGGGGACCTTCCTCTCAGAACCCCTACT
GATCGTCGCCTTTGGTGGGCCGTTACCCCGCCAACAAGCTAATCAGACGCATCCCCATCCATAACCGCTAAAC
CTTTAACCGTTATTTGATGTCAGCAAACGATATCATACGGTATTAGGCCGTCTTTCAACGGGTTATCCCGTAG
TTATGGGAAGGTTGGATACGCGTTACTCACCCGTGCGCCAGTCGTCATCAAGGAAAGCAAGCTTTCCCGTCAT
GTTACCCTGCGACTTGCATGTGTTAAGCCTGTAGCTAGCGTCATCCTGAGCCAGGATCAAACTGCCA
>791
CTGCTGCCGCCCGTAGGAGTTTGGACCGTGTCTCAGTTCCAATGTGGGGGACCTTCCTCTCAGAACCCCTACT
GATCGTCGCCTTGGTGGGCCGTTACCCCGCCAACAAGCTAATCAGACGCATCCCCATCCATAACCGCTAAACC
TTTAATCGTTATTTGATGTCAGCAAACGATATCATACGGTATTAGGCCGTCTTTCAACGGGTTATCCCGTAGT
TATGGGAAGGTTGGATACGCGTTACTCACCCGTGCGCCAGTCGTCATCAAGGAAAGCAAGCTTTCCCTCATGT
TACCCTGCGACTTGCATGTGTTAAGCCTGTAGCTAGCGTTCATCCGTGAGCCAAGGATCAACTC
>792
CTGCTGCCGCCCGTAGGAGTTTGGACCGTGTCTCAGTTCCAATGTGGGGGACCTTCCTCTCAGAACCCCTACT
GATCGTTGCCTTTGGTGGGCCGTTACCCCGCCAACAAGCTAATCAGACGCATCCCCATCCATAACCGATAAAT
CTTTATTCGTAATCTCATGAGATCAAACGAAATACATAAGGTATTAGTCCAACTTCGCTGGGTTATCCCTTAG
TTATGGGCAGGTTGGATACGCGTTACTCACCCGTGCGCCGGTCGCCGACATAAATAGCAAGCTATCTATTCGC
TGCCCCTCGACTTGCATGTGTTAAGCCTATAGCTAGCGTTCATCCTGAGCCAGGATCAAACTCT
>793
CTGCTGCCGCCCGTAGGAGTTTGGACCGTGTCTCAGTTCCAATGTGGGGGACCTTCCTCTCAGAACCCCTACT
GATCGTCGCCTTTGGTGGGCCGTTACCCCGCCAACAAGCTAATCAGACGCATCCCCATCCATAACCGATAAAT
CTTTATTCGTAATCTCATGAGATCAAACGAAATACATAAGGTATTAGTCCAACTTCGCTGGGTTATCCCTTAG
TTATGGGCAGGTTGGATACGCGTTACTCACCCGTGCGCCGGTCGCCGACATAAATAGCAAGCTATCTATTCGC
TGCCCCTCGACTTGCATGTGTTAAGCCTATAGCTAGCGTTCATCCTGAGCCAGGATCAAACTCT
>794
CTGCTGCCGCCCGTAGGAGTTTGGACCGTGTCTCAGTTCCAATGTGGGGGACCTTCCTCTCAGAACCCCTACT
GATCGTTGCCTTGGTAGGCCGTTACCCCGCCAACAAGCTAATCAGACGCATCCCCATCCATAACCGATAAATC
TTTATTCATAATCTCATGAGATCAAACGAATACATAAGGTATTAGTCCAACTTCGCTGGGTTATCCCTTAGTT
ATGGGCAGGTTGGATACGCGTTACTCACCCGTGCGCCGGTCGCCGACATAAATAGCAAGCTATCTATTCGCTG
CCCCTCGACTTGCATGTGTTAAGCCTATAGCTAGCGTTCATCCTGAGCCAGGATCAAACTCT
>795
CTGCTGCCGCCCGTAGGAGTTTGGACCGTGTCTCAGTTCCAATGTGGGGGACCTTCCTCTCAGAACCCCTACT
GATCGTTGCCTTGGTGGGCCGTTACCCCGCCAACAAGCTAATCAGACGCATCCCCATCCATAACCGATAAATC
TTTATTCATAATCTCATGAGATCAAACGAATACATAAGGTATTAGTCCAACTTCGCTGGGTTATCCCTTAGTT
ATGGGCAGGTTGGATACGCGTTACTCACCCGTGCGCCGGTCGCCGACATAAATAGCAAGCTATCTATTCGCTG
CCCCTCGACTTGCATGTGTTAAGCCTATAGCTAGCGTTCATCCTGAGCCAGGATCAAACGTCT
>796
CTGCTGCCGCCCGTAGGAGTTTGGACCGTGTCTCAGTTCCAATGTGGGGGACCTTCCTCTCAGAACCCCTACT
GATCGTCGCCTTTGGTGGGCCGTTACCCCGCCAACAAGCTAATCAGACGCATCCCCATCCATCACCGCTAAAC
CTTTAATCGTTATTTGATGTCAGCAAACGATGATCATACGGTATTAGCCGTCTTTCAACGGGTTATCCCGTGT
ATGGGAAGGTTGGATACGCGTTACTCACCCGTGCGCCAGTCGTCGTCAAAGGAAAGCAAGCTTTCCCTCACGT
TACCTTGCCGATGCCGTACG
>797
CTGCTGCCGCCCGTAGGAGTTTGGACCGTGTCTCAGTTCCAATGTGGGGGACCTTCCTCTCAGAACCCCTACT
GATCGTTGCCTTGGTCGGGCCGTTACCCCACCAACAAGCTAATCAGACGCATCCCCATCCATAACCGATAAAT
CTTTATCGTAATCTCATGAGATCAAACGAATATATAAGGTATTAGTCCAACTTCGCTGGGTTATCCCTTAGTT
ATGGGCAGGTTGGATACGCGTTACTCACCCGTGCGCCGGTCGCCGACATAAATAGCAAGCTATCTATTCGCTG
CCCCTCGACTTGCATGTGTTAACGCCCATAGCTAGC
>798
CTGCTGCCGCCCGTAGGAGTTTGGACCGTGTCTCAGTTCCAATGTGGGGGACCTTCCTCTCAGAACCCCTACT
GATCGTTGCCTTTGGTGGGCCGTTACCCCGCCAACAAGCTAATCAGACGCATCCCCATCCATAACCGATAAAT
CTTTATTCGTAATCTCATGAGATCAAACGAAATACATAAGGTATTAGTCCAACTTCGCTGGGTTATCCCTTAG
TTATGGGCAGGTTGGATACGCGTTACTCACCCGTGCGCCGGTCGCCGACATAAATAGCAAGCTATCTATTCGC
TGCCCCTCGACTTGCATGTGTTAAGCCTATAGCTAGCGTTCATCCTGAGCCAGGATCAAACTCT

>799
CTGCTGCCGCCCGTAGGAGTTTGGACCGTGTCTCAGTTCCAATGTGGGGGACCTTCCTCTCAGAACCCCTACT
GATCGTCGCCTTGGTGGGCCGTTACCCCGCCAACAAGCTAATCAGACGCATCCCCATCCATCACCGCTAAACC
TTTAATCGTTTATTTGATGTCAGCAAACGATGTCATACGGTATTTAGTCCGTCTTTCAACGGGTTTATCCCGT
GGTCATGGGAAGGTTGGATACGCGTTACTCACCCGTGCGCCAGTCGTCGTCAAGGGAAGCAAGCTTCCCTCAC
GTTACCCTGCGACTTGCATGTGTTAAGCCTGTAGCTAGCGTTCATCCTGAGCCAGGATCAAACTCT
>800
CTGCTGCCGCCCGTAGGAGTTTGGACCGTGTCTCAGTTCCAATGTGGGGGACCTTCCTCTCAGAACCCCTACT
GATCGTCGCCTTGGTGGGCCGTTACCCCGCCAACAAGCTAATCAGACGCATCCCCATCCATAACCGCTAAACC
TTTAATCGTTATTTGATGTCAGCAAACGATATCATACGGTATTAGGCCGTCTTTCAACGGGTTATCCCGTAGT
TATGGGAAGGTTGGATACGCGTTACTCACCCGTGCGCCAGTCGTCATCAAGGAAAGCAAGCTTTCCCTCACTG
TTTGACCCATG
>801
CTGCTGCCGCCTGTAGGAGTTTGGACCGTGTCTCAGTTCCAATGTGGGGGACCTTCCTCTCAGAACCCCTACT
GATCGTTGCCTTTGGTGGGCCGTTACCCCGCCAACAAGCTAATCAGACGCATCCCCATCCATAACCGATAAAT
CTTTATTCATAATCTCATGAGATCAAACGAATACATAAGGTATTAGTCCAACTTCGCTGGGTTATCCCTTAGT
TATGGGCAGGTTGGATACGCGTTACTCACCCGTGCGCCGGTCGCCGACATAAATAGCAAGCTATCTATTCGCT
GCCCCTCGACTTGCATGTGTTAACGCCTATAGCTAGCGTTCATCCTGAGCCAGGATCAAACTCT
>802
CTGCTGCCGCCCGTAGGAGTTTGGACCGTGTCTCAGTTCCAATGTGGCCGATCACCCTCTCAGGTCGGCTACT
GATCGTCGCCTTGGTGGGCAGTTATCTCACCAACTAGCTAATCAGACGCGGGTCCATCCTATACCGCTAACAC
TTTGATATATCTACGATGCCGTAAATATATATTATCATGTATTAGTAAACTTTTCAGTATGTTATCCATGTGT
ATAGGGCAGGTTACCCACGCGTTACTCACCCGTCCGCCGCTCCATCTTCATTTAATGCAAGCATCAAATCAGA
CGCGCTCGACTTGCATGTCGTAGGCACGCCGCCAGCGTCATCCTGAGCCAGGATCAAACCTCT
>803
CTGCTGCCGCCCGTAGGAGTTTGGACCGTGTCTCAGTTCCAATGTGGGGGACCTTCCTCTCAGAACCCCTACT
GATCGTCGCCTTTGGTGGGCCGTTACCCCGCCAACAAGCTAATCAGACGCATCCCCATCCACTAACCGCTAAA
CCTTTAATCGTTATTTGATGTCAGCAAACGATATCATACGGTATTAGGCCGTCTTTCAACGGGTTATCCCGTA
GTTATGGGAAGGTTGGATACGCGTTACTCACCCGTGCGCCAGTCGTCATCAAGGAAAGCAAGCTTTCCCTCAT
GTTACCCTGCGACTTGCATGTGTTAAGCCTGTAGCTAGCGTTCATCCCTGAGCAAGGCA
>804
CTGCTGCCGCCCGTAGGAGTTTGGACCGTGTCTCAGTTCCAATGTGGGGGACCTTCCTCTCAGAACCCCTACT
GATCGTTGCCTTGGTGGGCCGTTACCCCGCCAACAAGCTAATCAGACGCATCCCCATCCATAACCGATAAATC
TTTATTTCATAATCTCATGAGATCAAACGAATACATAAGGTATTTAGTCCAACTTCGCTGGGTTTATCCCTTA
GTTATGGGCAGGTTTGGATACGCGTTACTCACCCGTGCGCCGGTCGCCGACATAAATAGCAAGCTATCTATTC
GCTGCCCCCCTCGACTTGCATGTGTTTAAGCCTTATAGCTAGCGTTCATCCTTGAGCCAGGATCAAACTCT
>805
CTGCTGCCGCCCGTAGGAGTTTTGGACCGTGTCTCAGTTCCAATGTGGGGGACCTTCCTCTCAGAACCCCTAC
TGATCGTCGCCTTTGGTGGGCCGTTACCCCGCCAACAAGCTAATCAGACGCATCCCCATCCATCACCGCTAAC
CTTTAATCGTTATTTGATGTCAGCAAACGATATCATACGGTATTAGGCCGTCTTTCAACGGGTTATCCCGTAG
TTATGGGAAGGTTGGATACGCGTTACTCACCCGTGCGCCAGTCGTCATCAAGGAAAGCAAGCTTTCCCTCATG
TTACCCTGCGACTTGCATGTGTTAAGCCTGTAGCTAGCGTTCATCCCTGAGCAAGG
>806
CTGCTGCCGCCCGTAGGAGTTAGGACCGTGTCTCAGTTCCTATGTGGGGGGCCTTCCTCTCAGAACCCCTACC
GATCGAAGCCTTGGTGAGCCGTTACCTCACCAACAAGCTAATCGGACGCATCCCCATCCATAAGCGGCAAGCC
TTTGGTTCAAATCTGATGCCGTCTTTGAACAACATAGGGGATTAGTCCGCATTTCTGCGGGTTATACCCTACT
TATGGGCAGGTTGGATACGCGTTACTCACCCGTGCGCCGGTCGACGTCCAAACGGTGCAAGCACCAAATGCCG
TTTCCCCTCGACTTGCATGTGTTAAGCCTGTAGCTAGCGTTCATCCTGAGCCAGGATCAAACTCT
>807
CTGCTGCCGCCCGTAGGAGTTTTGGACCGTGTCTCAGTTCCAATGTGGGGGACCTTCCTCTCAGAACCCCTAC
TGATCGTCGCCTTTGGTGGGCCGTTACCCCGCCAACAAGCTAATCAGACGCATCCCCATCCATAACCGCTAAA
CCTTTAATCGTTATTTGATGTCAGCAAACGATATCATACGGTATTAGGCCGTCTTTCAACGGGTTATCCCGTA
GTTATGGGAAGGTTGGATACGCGTTACTCACCCGTGCGCCAGTCGTCATCAAGGAAAGCAAGCTTTCCTCATG
TTACCCTGCGACTTGCATGTGTTAAGCCTGTAGCTAGCGTTCATCCTGAGCCAGGATCAAACTCT

>808
CTGCTGCCGCCCGTAGGAGTTTGGACCGTGTCTCAGTTCCAATGTGGGGGACCTTCCTCTCAGAACCCCTACT
GATCGTCGCCTTGGTGGGCCGTTACCCCGCCAACAAGCTAATCAGACGCATCCCCATCCATAACCGCTAAACC
TTTTAATCGTTTATTTTGATTGTCAGCAAACGATATCATACGGTATTTAGGCCGTCTTTTCAACGGGTTTATT
CCCGTAGTTTTATGGGAAGGTTGGATACGCGTTACTCACCCGTGCGCCAGTCGTCATCAAGGAAAGCAAGCTT
TCCCTTCAT
>809
CTGCTGCCGCCCGTAGGAGTTTGGACCGTGTCTCAGTTCCAATGTGGGGGACCTTCCTCTCAGAACCCCTACT
GATCGTGCCTTGGTGGGCCGTTACCCCGCCAACAAGCTAATCAGACGCATCCCCCCTCCATAACCGCTAAACC
TTTAATCGTTTATTTTGATGTCAGCAAACGATATCATACGGTATTTAGGCCGTCTTTTCAACGGGTTTATCCC
GTAGTTTATGGGAAGGTTGGATACGCGTTACTCACCCGTGCGCCAGTCGTCATCAAGGAAAGCAAGCTTTCCC
TTCATGTTTTACCCTGCGACTGCATGTGTTTAAG
>810
CTGCTGCCGCCCGTAGGAGTTTGGGCCGTGTCTCAGTCCCAATGTGGCCGTACACTCTCTCAAGCCGGCTACT
GATCGTCGCCTTGGCGAGCTTTTATCTCACCAACTAGCTAATCAGACGCAAGTCCATCTTACACCGATAAATC
TTTGGTCACTCTATCATGCGATAAATTGACTTTATAGGGTATTATTCTTCGTTTTCCAAAGGCTATCCCCTTG
TGTAAGGCAGGTTACTCACGTGTTACTCACCCGTTCGCCACTAATCCACTTAAAATCATTCCGAAGAAATCAA
CTAAGTTTCATCGTTCGACTTGCATGTGTTATGCACGCCGCCAGCGTTTATCCTGAGCCAGGATCAAACTCT
>811
CTGCTGCCGCCCGTAGGAGTTTGGACCGTGTCTCAGTTCCAATGTGGGGGACCTTCCTCTCAGAACCCCTACT
GATCGTCGCCTTTGGTGGGCCGTTACCCCGCCAACAAGCTAATCAGACGCATCCCCATCCATAACCGCTAAAC
CTTTAATCGTTATTTTGATGTCAGCAAACGATATCATACGGTATTAGGCCGTCTTTTCAACGGGTTATCCCGT
AGTTATGGGAAGGTTGGATACGCGTTACTCACCCGTGCGCCAGTCGTCATCAAGGAAAGCAAGCTTCCCTCAT
GTTTACCTGCGACTTGCATGTGTTAAGCCTGTAGCTAGCGTTCATCCTGAGCCAGGATCAAACTCT
>812
CTGCTGCCGCCCGTAGGAGTTTGGACCGTGTCTCAGTTCCAATGTGGGGGACCTTCCTCTCAGAACCCCTACT
GATCGTCGCCTTTGGTGGGCCGTTACCCCGCCAACAAGCTAATCAGACGCATCCCCATCCATCACCGCTAAAC
CTTTTAATCGTTTATTTTGATGTCAGCAAACGATGTCATACGGTATTTAGCCGTCTTTTCAACGGGTTTATCC
CGTGGTCATGGGAAGGTTGGATACGCGTTACTCACCCGTGCGCCAGTCGTCGTCAAGGGAAAGCAAGCTTTCC
CCTTCACGTTACCTTGCCGACCGTAGCG
>813
CTGCTGCCGCCCGTAGGAGTTTGGACCGTGTCTCAGTTCCAATGTGGGGGACCTTCCTCTCAGAACCCCTACT
GATCGTTGCCTTGGTGGGCCGTTACCCCGCCAACAAGCTAATCAGACGCATCCCCATCCATAACCGATAAATC
TTTATTCATAATCTCATGAGAATCAAACGAATACATAAGGTATTAGTCCAACTTCGCTGGGTTATCCCTTAGT
TATGGGCAGGTTGGATACGCGTTACTCACCGTGCGCCGGTCGCCGACATAAATAGCAAGCTATCTATTCGCTG
CCCCTCGACTTGCATGTGTTTTAAGCCTATAGCTAGCGTTCATCCTGAGCCAGGATCAAACTCT
>814
CTGCTGCCGCCCGTAGGAGTTTGGACCGTGTCTCAGTTCCAATGTGGGGGACCTTCCTCTCAGAACCCCTACT
GATCGTCGCCTTTGGTGGGCCGTTACCCCGCCAACAAGCTAATCAGACGCATCCCCATCCATAACCGCTAAAC
CTTTAATCGTTATTTTGATGTCAGCAAACGATATCATACGGTATTTAGGCCGTCTTTTCAACGGGTTTATCCC
GTAGTTTATGGGAAGGTTGGATACGCGTTACTCACCCGTGCGCCAGTCGTCATCAAGGAAAAGCCAAGCTTTC
CTTCGACGTTACC
>815
CTGCTGCCGCCCGTAGGAGTTTGGACCGTGTCTCAGTTCCAATGTGGGGGACCTTCCTCTCAGAACCCCTACT
GATCGTTGCCTTTGGTGGGCCGTTACCCCGCCAACAAGCTAATCAGACGCATCCCCATCCATAACCGATAAAT
CTTTATTCATAATCTCATGAGATCAAACGAATACATAAGGTATTAGTCCAACTTCGCTGGGTTATCCCTTAGT
TTATGGGCAGGTTTGGATACGCGTTACTCACCCGTGCGCCGGTCGCCGACATAAATAGCAAGCTATCTATTTC
GCTGCCCCCCCTCGACTTGCATGTGTTTTAAGCCTATAGCTAGCGTTCATCCTGAGCCAGGATCAAACTCT
>816
CTGCTGCCGCCCGTAGGAGTTTGGACCGTGTCTCAGTTCCAATGTGGGGGACCTTCCTCTCAGAACCCCTACT
GATCGTTGCCTTTGGTGGGCCGTTACCCCGCCAACAAGCTAATCAGACGCATCCCCATCCACTAACCGAATAA
ATCTTTATTCATAATCTCATGAGAATCAAACGAATACATAAGGTATTAGTCCAACTTCGCTGGGTTATCCCTT
AGTTTATGGGCAGGTTTGGATACGCGTTACTCACCCGTGCGCCGGTCGCCGACATAAATAGCAAGCTATCTAT
TCGCTGCCCCCCTCGACTTGCATGTGTTTAAGCCTATAGCTAGCGTTCATCCTGAGCCAGGATCAAACTCT

>817
CTGCTGCCGCCCGTAGGAGTTTGGACCGTGTCTCAGTTCCAATGTGGGGGACCTTCCTCTCAGAACCCCTACT
GATCGTCGCCTTTGGTGGGCCGTTACCCCGCCAACAAGCTAATCAGACGCATCCCCATCCATAACCGCTAAAC
CTTTAATCGTTATTTTGATGTCAGCAAACGATATCATACGGTATTAGGCCGTCTTTTCAACGGGTTATCCCGT
AGTTATGGGAAGGTTGGATACGCGTTACTCACCCGTGCGCCAGTCGTCATCAAGGAAAGCAAGCTTTCCCTCA
TGTTACCCTGCGACTTGCATGTGTTAAGCCTGTAGCCAGCGTCATCTGAGCCAGGATCCAACTCGT
>818
CTGCTGCCGCCCGTAGGAGTTTGGACCGTGTCTCAGTTCCAATGTGGCCGATCACCCTCTCAGGTCGGCTACT
GATCGTCGCCTTGGTGGGCAGTTATCTCACCAACTAGCTAATCAGACGCGGGTCCATCCTATACCGCTAACAC
TTTGATATATCTACGATGCCGTAAATATATATTATCATGTATTAGTAAACTTTTCAGTATGTTATCCATGTGT
ATAGGGCAGGTTACCCACGCGTTACTCACCCGTCCGCCGCTCATCTTTCATTAATGCAAGCATCAAGTCAGAC
GCGCTCGACTTGCATGTGTTAGGCACGCCGCCAGCGTTCATCCTGAGCCAGGATCAAACTCT
>819
CTGCTGCCGCCCGTAGGAGTTTGGACCGTGTCTCAGTTCCAATGTGGGGGACCTTCCTCTCAGAACCCCTACT
GATCGTTGCCTTGGTGGGCCGTTACCCCACCAACAAGCTAATCAGACGCATCCCCATCCATAACCGATAAATC
TTTATTCGTAATCTCATGAGATCAAACGAATACATAAGGTATTAGTCCAACTTCGCTGGGTTATCCCTTAGTT
ATGGGCAGGTTGGATACGCGTTACTCACCCGTGCGCCGGTCGCCGACATAAATAGCAAGCTATCTATTCGCTG
CCCCTCGACTTGCATGTGTTAAGCCTATAGCTAGCGTTCATCCCGAGCCAGGATCAAACTCT
>820
CTGCTGCCGCCCGTAGGAGTTTGGACCGTGTCTCAGTTCCAATGTGGGGGACCTTCCTCTCAGAACCCCTACT
GATCGTCGCCTTGGTGGGCCGTTACCCCGCCAACAAGCTAATCAGACGCATCCCCATCCATAACCGCTAAACC
TTTAATCGTTATTTGATGTCAGCAAACGATATCATACGGTATTAGGCCGTCTTTCAACGGGTTATCCCGTAGT
TATGGGAAGGTTGGATACGCGTTACTCACCCGTGCGCCAGTCGTCATCAAGGAAAGCAAGCTTTCCTCATGTT
ACCCTGCGACTTGCATGTGTTAAGCCTGTAGCTAGCGTTCATCCTGAGCCAGGATCAAACTCT
>821
CTGCTGCCGCCCGTAGGAGTTTGGACCGTGTCTCAGTTCCAATGTGGGGGACCTTCCTCTCAGAACCCCTACT
GATCGTCGCCTTGGTGGGCCGTTACCCCGCCAACAAGCTAATCAGACGCATCCCCATCCATAACCGCTAAACC
TTTAATCGTTATTTGATGTCAGCAAACGATATCATACGGTATTAGGCCGTCTTTCAACGGGTTATCCCGTAGT
TATGGGAAGGTTGGATACGCGTTACTCACCCGTGCGCCAGTCGTCAGTCAAGGAAAGCAAGCTTTCCCTGACT
GTTACCCTGCGAATTGCA
>822
CTGCTGCCGCCCGTAGGAGTTTGGACCGTGTCTCAGTTCCAATGTGGGGGACCTTCCTCTCAGAACCCCTACT
GATCGTCGCCTTTGGTGGGCCGTTACCCCGCCAACAAGCTAATCAGACGCATCCCCATCCATCACCGCTAAAC
CTTTAATCGTTATTTGATGTCAGCAAACGATGTCATACGGTATTAGTCCGTCTTTCAACGGGTTATCCCGTGG
TCATGGGAAGGTTGGATACGCGTTACTCACCCGTGCGCCAGTCGTCGTCAAGGGAAGCAAGCTTCCCTCACGT
TACCCTGCGACTTGCATGTGTTAAGCCTGTAGCTAGCGTTCATCCGTGAGCCAAGGATCAAACTCT
>823
CTGCTGCCGCCCGTAGGAGTTTGGACCGTGTCTCAGTTCCAATGTGGGGGACCTTCCTCTCAGAACCCCTACT
GATCGTTGCCTTGGTGGGCCGTTACCCCGCCAACAAGCTAATCAGACGCATCCCCATCCATAACCGATAAATC
TTTATTCATAATCTCATGAGATCAAACGAATACATAAGGTATTAGTCCAACTTCGCTGGGTTATCCCTTAGTT
ATGGGCAGGTTGGATACGCGTTACTCACCCGTGCGCCGGTCGCCGACATAAATAGCAAGCTATCTATTCGCTG
CCCCTCGACTTGCATGTGTTAAGCCTATAGCTAGCGTTCATCCTGAGCCAGGATCAAACTCT
>824
CTGCTGCCGCCCGTAGGAGTTTGGACCGTGTCTCAGTTCCAATGTGGGGGACCTTCCTCTCAGAACCCCTACT
GATCGTTGCCTTGGTGGGCCGTTACCCCGCCAACAAGCTAATCAGACGCATCCCCATCCATAACCGATAAATC
TTTATTCATAATCTCATGAGATCAAACGAATACATAAGGTATTAGTCCAACTTCGCTGGGTTATCCCTTAGTT
ATGGGCAGGTTGGATACGCGTTACTCACCCGTGCGCCGGTCGCCGACATAAATAGCAAGCTATCTATTCGCTG
CCCCTCGACTTGCATGTGTTAACGCCTATAGCTAGCGTTCATCCTGAGCCAGGATCAAACTCT
>825
CTGCTGCCGCCCGTAGGAGTTTGGACCGTGTCTCAGTTCCAATGTGGCCGATCACCCTCTCAGGTCGGCTACT
GATCGTCGCCTTGGTGGGCAGTTATCTCACCAACTAGCTAATCAGACGCGGGTCCATCCTATACCGCTAACAC
TTTGATATATCTACGATGCCGTAAATATATAATTATCATGTATTAGTAAACTTTTCAGTATGTTATCCATGTG
TATAGGGCAGGTTACCCACGCGTTACTCACCCGTCCGCCGCTCATCTTCATTAATGCAAGCATCAAATCAGAC
GCGCTCGACTTGCATGCGTTAGGCACGCCGCCAGCGTTCATCCTGAGCCAGGATCAAACTCT

>826
CTGCTGCCGCCCGTAGGAGTTTGAACCGTGTCTCAGTTCCAATGTGGGGGACCTTCCTCTCAGAACCCCTACT
GATCGTCGCCTTTGGTGGGCCGTTACCCCGCCAACAAGCTAATCAGACGCATCCCCATCCATAACCGCTAAAC
CTTTAATCGTTATTTGATGTCAGCAAACGATATCATACGGTATTAGGCCGTCTTTCAACGGGTTATCCCGTAG
TTATGGGAAGGTTGGATACGCGTTACTCACCCGTGCGCCAGTCGTCATCAAGAAAGCAAGCTTTCCCTCATGT
TACCCTGCCGACATGCA
>827
CTGCTGCCGCCCGTAGGAGTTTTGGACCGTGTCTCAGTTCCAATGTGGGGGACCTTCCTCTCAGAACCCCTAC
TGATCGTTGCCTTGGTGGGCCGTTACCCCGCCAACAAGCTAATCAGACGCATCCCCATCCATAACCGATAAAT
CTTTATTCATAATCTCATGAGATCAAACGAATACATAAGGTATTAGTCCAACTTCGCTGGGTTATCCCTTAGT
TATGGGCAGGTTGGATACGCGTTACTCACCCGTGCGCCGGTCGCCGACATAAATAGCAAGCTATCTATTCGCT
GCCCCTCGACTTGCATGTGTTAAGCCTATAGCTAGCGTTCTCCTGAGCCAGGATCAAACTCT
>828
CTGCTGCCGCCCGTAGGAGTTTGGACCGTGTCTCAGTTCCAATGTGGGGGACCTTCCTCTCAGAACCCCTACT
GATCGTTGCCTTGGTGGGCCGTTACCCCACCAACAAGCTAATCAGACGCATCCCCATCCATAACCGATAAATC
TTTATTCGTAATCTCATGAGATCAAACGAATACATAAGGTATTAGTCCAACTTCGCTGGGTTATCCCTTAGTT
ATGGGCAGGTTGGATACGCGTTACTCACCCGTGCGCCGGTCGCCGACATAAATAGCAAGCTATCTATTCGCTG
CCCCTCGACTTGCATGTGTTAAGCCTATAGCTAGCGTTCATCCTGAGCCAGGATCAAACTCT
>829
CTGCTGCCGCCCGTAGGAGTTTTGGACCGTGTCTCAGTTCCAATGTGGGGGACCTTCCTCTCAGAACCCCTAC
CTGATCGTCGCCTTTGGTGGGCCGTTACCCCGCCAACAAGCTAATCAGACGCATCCCCATCCATAACCGCTAA
ACCTTTAATCGTTATTTGATGTCAGCAAACGATATCATACGGTATTAGGCCGTCTTTCAACGGGTTATCCCGT
AGTTATGGGAAGGTTGGATACGCGTTACTCACCCGTGCGCCAGTCGTCATCAAGGAAAGCAAGCTTTCCCTCA
TGTTACCCTGCCGATTGCACTGTG
>830
CTGCTGCCGCCCGTAGGAGTTTGGACCGTGTCTCAGTTCCAATGTGGGGGACCCTTCCTCTCAGAACCCCTAC
TGACTCGTCGCCTTGGGTGGGCCGTTACCCCGCCAACAAGCTAATCAGACGCATCCCCATCCATAACCGCTAA
ACCTTTAATCGTTATTTGATGTCAGCAAACGATATCATACGGTATTAGGCCGTCTTTCAACGGGTTATCCCGT
AGTTATGGGAAGGTTGGATACGCGTTACTCACCCGTGCGCCAGTCGTCATCAAGGAAAGCAAGCTTTCCCTCA
TGTTACCCTGCGACATGCATGTGTTAAGCCTGTAGCTAGCGTTCATCCCGTGACGCAA
>831
CTGCTGCCGCCCGTAGGAGTTTTGGACCGTGTCTCAGTTCCAATGTGGGGGACCTTCCTCTCAGAACCCCTAC
TGATCGTCGCCTTTGGTGGGCCGTTACCCCGCCAACAAGCTAATCAGACGCATCCCCATCCATAACCGCTAAA
CCTTTAATCGTTATTTGATGTCAGCAAACGACGTCATACGGTATTAGTCCGTCTTTCAACGGGTTATCCCGTG
GTCATGGGAAGGTTGGATACGCGTTACTCACCCGTGCGCCAGTCGTCGTCAAGGAAAGCAAGCTTTCCTCACG
TTACCCTGCGACATGCATGTGTTAAGCCTGTAGCTAGCGTTCATCCTGAGCCAGGATCAAACTCT
>832
CTGCTGCCGCCCGTAGGAGTTTGGACCGTGTCTCAGTTCCAATGTGGGGGACCTTCCTCTCAGAACCCCTACT
GATCGTCGCCTTTGGTGGGCCGTTACCCCGCCAACAAGCTAATCAGACGCATCCCCATCCATAACCGCTAAAC
CTTTAATCGTTATTTGATGTCAGCAAACGATATCATACGGTATTAGGCCGTCTTTTCAACGGGTTATCCCGTC
GTTATGGGAAGGTTGGATACGCGTTACTCACCCGTGCGCCAGTCGTCATCAAGGAAAGCAAGCCTTTCCTCAT
GTTACCCTGCGACTTGCATGTGTTAAGCCTGTAGCTAGCGTCATCCTGAGCCAGGATCAAACTCT
>833
CTGCTGCCGCCCGTAGGAGTTTGGACCGTGTCTCAGTTCCAATGTGGGGGACCTTCCTCTCAGAACCCCTACT
GATCGTTGCCTTGGTGGGCCGTTACCCCGCCAACAAGCTAATCAGACGCATCCCCATCCATAACCGATAAATC
TTTATTCGTAATCTCATGAGATCAAACGAAATACATAAGGTATTAGTCCAACTTCGCTGGGTTATCCCTTAGT
TATGGGCAGGTTGGATACGCGTTACCTCACCCGTGCGCCGGTCGCCGACATAAATAGCAAGCTATCTATTCGC
TGCCCCTCGACTTGCATGTGTTAAGCCTATAGCTAGCGTTCATCCTGAGCCAGGATCAAACTCT
>834
CTGCTGCCGCCCGTAGGAGTTTGGACCGTGTCTCAGTTCCAATGTGGGGGACCTTCCTCTCAGAACCCCTACT
GATCGTCGCCTTGGTGGGCCGTTACCCCGCCAACAAGCTAATCAGACGCATCCCCATCCATAACCGCTAAACC
TTTTAATCGTTTTATTTTTGATGTCAGCAAACGATATCATACGGTATTTTAGGCCGTCTTTTTCAACGGGTTT
ATCCCGTAGTTTTATGGGAAGGTTGGATACGCGTTACTCACCCGTGCGCCAGTCGTCATCAAGGAAAAGCAAG
CTTTTCCTTCGATGTTTTACCCTGCGACATGCATGTGTT

>835
CTGCTGCCGCCCGTAGGAGTTTGGACCGTGTCTCAGTTCCAATGTGGGGGACCTTCCTCTCAGAACCCCTACT
GATCGTCGCCTTTGGTGGGCCGTTACCCCGCCAACAAGCTAATCAGACGCATCCCCATCCATAACCGCTAAAC
CTTTAATCGTTATTTGATGTCAGCAAACGATATCATACGGTATTAGGCCGTCTTTCAACGGGTTATCCCGTAG
TTATGGGAAGGTTGGATACGCGTTACTCACCCGTGCGCCAGTCGTCATCAAGGAAAGCAAGCTTTCCTCATGT
TACCCTGCGACTTGCATGTGTTAAGCCTGTAGCTAGCGTTCATCCTGAGCCAGGATCAAACTCT
>836
CTGCTGCCGCCCGTAGGAGTTTGGACCGTGTCTCAGTTCCAATGTGGGGGACCTTCCTCTCAGAACCCCTACT
GATCGTCGCCTTTGGTGGGCCGTTACCCCGCCAACAAGCTAATCAGACGCATCCCCATCCATAACCGCTAAAC
CTTTAATCGTTATTTGATGTCAGCAAACGATATCATACGGTATTAGGCCGTCTTTCAACGGGTTATCCCGTAG
TTATGGGAAGGTGGATACGCGTTACTCACCCGTGCGCCAGTCGTCCATCAAGGAAAGCAAGCTTCCCTGACTG
TTACCCTGCGACTGCATGTGTTAAGCCTGTAGCTAGCGTTCATCCCGTGAGCCAGGATCAAACAT
>837
CTGCTGCCGCCCGTAGGAGTTTGGACCGTGTCTCAGTTCCAATGTGGCCGATCACCCTCTCAGGTCGGCTACT
GATCGTCGCCTTGGTGGGCAGTTATCTCACCAACTAGCTAATCAGACGCGGGTCCATCCTATACCGCTAACAC
TTGATATATCTACGATGCCGTAAATATATATTTATCATGTATTTAGTAAACTTTTCAGTATGTTTATCCATGT
GTATAGGGCAGGTTACCCACGCGTTACTCACCCGTCCGCCGCTCATCTTTCATCAATGCAAGCATCAAATCAT
ACGCGCTCGACTTGCATGTGTTTAGGCACGCCGCCAGCGTTCATCCTGAGCCAGGATCAAACTCT
>838
CTGCTGCCGCCCGTAGGAGTTTGGGCCGTGTCTCAGTCCCAATGTGGCCGTACACTCTCTCAAGCCGGCTACT
GATCGTCGCCTTGGTGTAGCTTTATTCTCACCAACTAGCTAATCAGACGCAAGTCCATCTTACACCGATAAAT
CTTTGGTCACTCTATCATGCGATAAATTGACTTTATAGGGTATTATTCTTCGTTTCCAAAGGCTACTCCCTTG
TGATGAAGCAGGTTACTCACGTGTTACTCACCCGTTCGCCACTAATCCACTTAAAATCATTCCGAAGAAATCA
ACTAAGTTTCATCGTTCGACTTGCATGTGTTATGCACGCCGCCAGCGTTTATCCTGAGCCAGGATCAAACTCT
>839
CTGCTGCCGCCCGTAGGAGTTTGGACCGTGTCTCAGTTCCAATGTGGGGGACCTTCCTCTCAGAACCCCTACT
GATCGTTGCCTTGGTGGGCCGTTACCCCGCCAACAAGCTAATCAGACGCATCCCCATCCATAACCGATAAATC
TTTATTCGTAATCTCATGAGATCAAACGAAATACATAAGGTATTAGTCCAACTTCGCTGGGTTATCCCTTAGT
TATGGGCAGGTTGGATACGCGTTACTCACCCGTGCGCCGGTCGCCGACATAAATAGCAAGCTATCTATTCGCT
GCCCCTCGACTTGCATGTGTTAAGCCTATAGCTAGCGTTCATCCTGAGCCAGGATCAAACTCT
>840
CTGCTGCCGCCCGTAGGAGTTTGGACCGTGTCTCAGTTCCAATGTGGGGGACCTTCCTCTCAGAACCCCTACT
GATCGTTGCCTTGGTGGGCCGTTACCCCGCCAACAAGCTAATCAGAACGCATCCCCATCCATAACCGATAAAT
CTTTATTCATAATCTCATGAGATCAAACGAATACATAAGGTATTAGTCCAACTTCGCTGGGTTATCCCTTAGT
TATGGGCAGGTTGGATACGCGTTACTCACCCGTGCGCCGGTCGCCGACATAAATAGCAAGCTATCTATTCGCT
GCCCCTCGACTTGCATGTGTTAACGCCTATAGCTAGCGTTCATCCTGAGCCAGGATCAAACTCT
>841
CTGCTGCCGCCCGTAGGAGTTTGGACCGTGTCTCAGTTCCAATGTGGGGGACCTTCCTCTCAGAACCCCTACT
GATCGTTGCCTTGGTGGGCCGTTACCCCGCCAACAGGCTAATCAGACGCATCCCCATCCATAACCGATAAATC
TTTATTCGTAATCTCATGAGATCAAACGAAATACATAAGGTATTAGTCCAACTTCGCTGGGTTATCCCTTAGT
TATGGGCAGGTTGGATACGCGTTACTCACCCGTGCGCCGGTCGCCGACATAAATAGCAAGCTATCTATTCGCT
GCCCCTCGACTTGCATGTGTTAAGCCTATAGCTAGCGTTCATCCTGAGCCAGGATCAAACTCT
>842
CTGCTGCCGCCCGTAGGAGTTTGGACCGTGTCTCAGTTCCAATGTGGGGGACCTTCCTCTCAGAACCCCTACT
GATCGTTGCCTTGGTCGGGCCGTTACCCCACCAACAAGCTAATCAGACGCATCCCCATCCATAACCGATAAAT
CTTTATTCGTAATCTCATGAGATCAAAACGAATACATAAGGTATTAGTCCAACTTCGCTGGGTTATCCCTTAG
TTTATGGGCAGGTTTGGATACGCGTTACTCACCCGTGCGCCGGTCGCCGACATAAATAGCAAGCTATCTATTC
GCTGCCCCCCCTCGACTTGCATGTGTTTAAGCCTATAGCTAGCGTTCATCCTGAGCCAGGATCAAACTCT
>843
CTGCTGCCGCCCGTAGGAGTTTGGACCGTGTCTCAGTTCCAATGTGGGGGACCTTCCTCTCAGAACCCCTACT
AATCGTTGCCTTGGTGGGCCGTTACCCCGCCAACAAGCTAATCAGACGCATCCCCCATCCATAACCGATAAAT
CTTTTATTTCATAATCTCATGAGATCAAAAACGAATACATAAGGTATTTAGTCCAACTTTCGCTGGGTTTATC
CCTTAGTTTTATGGGCAGGTTTTGGATACGCGTTACTCACCCGTGCGCCGGGTCGCCGACATAAAAATAGCA

AGCTATCTATTTCGCTGCCCCCCCTCGACTTTGCATGTGTTTTAAGCCTATAGCTAGCGTTCATCCTGAGCCA
GGATCAAACTCTATCAACCGACTGCCCCCCCATAGAGAGGCTGAGACTGCCAAGGCACACAGGGGATAGG
>844
CTGCTGCCGCCCGTAGGAGTTTGGACCGTGTCTCAGTTCCAATGTGGGGGACCTTCCTCTCAGAACCCCTACT
GATCGTCGCCTTGGTGGGCCGTTACCCCGCCAACAAGCTAATCAGACGCATCCCCATCCATCACCGCTAAACC
TTTAATCGTTATTGATGTCAGCAACGACGTCATACGGTATTAGTCCGTCTTTCAACGGGTATCCCGTGGTCAT
GGGAAGGTTGGACTACGCGTTACTCACCCGTGCGCCAGTCGTCGTCAAGGAAAGCCAAGCTTTCCTCCACGTT
ACCCTGCGACTTGCATGTCGTTAAGCCGTGT
>845
CTGCTGCCGCCCGTAGGAGTTTTGGACCGTGTCTCAGTTCCAATGTGGGGGACCTTCCTCTCAGAACCCCTAC
TGATCGTCGCCTTTGGTGGGCCGTTACCCCGCCAACAAGCTAATCAGACGCATCCCCATCCATCAACCGCTAA
ACCTTTAATCGTTTATTTTGATGTCAGCAAACGATGTCATACGGTATTTAGGCCGTCTTTTCAACGGGTTTAT
CCCGTGTTAGGGAAGGTTGGATACGCGTTACTCACCCGTGCGCCAGTCGTCGGTCAAGGGAAGCAAGCTCCCT
CACGTTACCCTGCGAGCTTTGCATGTGTTTAACGCCTTGTAGCTAGCGTTCATCCCCGTGACG
>846
CTGCTGCCGCCCGTAGGAGTTTGGACCGTGTCTCAGTTCCAATGTGGGGGACCTTCCTCTCAGAACCCCTACT
GATCGTTGCCTTGGTGGGCCGTTACCCCCGCCAACAAGCTAATCAGACGCATCCCCCATCCATAACCGATAAA
TCTTTTATTTCATAATCTCATGAGATCAAACGAATACATAAGGTATTTAGTTCCAACTTCGCTGGGTTTATCC
CTTAGTTTATGGGCAGGTTTGGATACGCGTTACTCACCCGTGCGCCGGTCGCCGACATAAATAGCAAGCTATC
TATTCGCTGCCCCCCCCTTCGACTTGCATGTGTTTTAAGCCTTATAGCTAGCGTTCATCCTTGAGCCAGGATCA
AACTCTATCACCGACTGCCCCCCCATAGAGAGGCTGAGACTGCCAAGGCACACAGGGGATAGG
>847
CTGCTGCCGCCCGTAGGAGTCTGGGCCGTATTTCAGTCCCAATGTGGCCGCACACCCTCTCAGGCCGGCTACC
CGTCGCCGCCTTGGTAGGCCATTACCCCCCACCAACAAGCTGATAGGCCGCAAGCTCATCCCACACCAGAAAA
ACCTTTCCACCACACCAACCACGATGCGGTCCTATAGGGTATTAGACCCAGTTTCCCAAGCTTTATCCCCCCC
AGTGCAGGGCAGATCACTTACGTGTTACTCACCCGTTCGCCACTCGAGCACCCCAGCAAGCTGGGGCCTTTCC
GTTTCGACTTGCATGTGTTTAAGCACGCCGCCAGCGTCATCCTGAGCCAGGATCAAACTCT
>848
CTGCTGCCGCCCGTAGGAGTTTGGACCGTGTCTCAGTTCCAATGTGGGGGACCTTCCTCTCAGAACCCCTACT
GATCGTTGCCTTTGGTGGGCCGTTACCCCGCCAACAAGCTAATCAGACGCATCCCCATCCATAACCGATAAAT
CTTTATTCGTAATCTCATGAGATCAAACGAATACATAAGGTATTAGTCCAACTTCGCTGGGTTACTCCTAGTT
ATGGGCAGGTTGGATACGCGTTACTCACCCGTGCGCCGGTCGCCGACATAAATAGCAAGCTATCTATTCGCTG
CCCCTCGACTTGCATGTGTTAAGCCTATAGCTAGCGTTCATCCTGAGCCAGGATCAAACTCT
>849
CTGCTGCCGCCCGTAGGAGTTTGGACCGTGTCTCAGTTCCAATGTGGGGGACCTTCCTCTCAGAACCCCTACT
GATCGTTGCCTTGGTGGGCCGTTACCCCGCCAACAAGCTAATCAGACGCATCCCCATCCATAACCGATAAATC
TTTATTCGTAATCATCATGAGAGTCAAACGAAATACATAAGGTATTAGTCCAACTTCGCTGGGTTATCCCTTA
GTTATGGGCAGGTTTGGATACGCGTTACTCACCCGTGCGCCGGTCGCCGACATAAATAGCAAGCTATCTATTC
GCTGCCCCCCCTCGACTTGCATGTGTTTAAGCCTATAGCTAGCGTTCATCCTGAGCCAGGATCAAACTCT
>850
CTGCTGCCGCCCGTAGGAGTTTGGACCGTGTCTCAGTTCCAATGTGGGGGACCTTCCTCTCAGAACCCCTACT
GATCGTTGCCTTGGTGGGCCGTTACCCCGCCAACAAGCTAATCAGACGCATCCCCATCCATAACCGATAAATC
TTTATTCGTAATCTCATGAGATCAAACGAATACATAAGGTATTAGTCCAACTTCGCTGGGTTATCCCTTAGTT
ATGGGCAGGTTGGATACGCGTTACTCACCCGTGCGCCGGTCGCCGACATAAATAGCAAGCTATCTATTCGCTG
CCCCTCGACTTGCATGTGTTAAGCCTATAGCTAGCGTTCATCCTGAGCCAGGATCAAACTCT
>851
CTGCTGCCGCCCGTAGGAGTTTGGACCGTGTCTCAGTTCCAATGTGGGGGACCTTCCTCTCAGAACCCCTACT
GATCGTCGCCTTGGTGGGCCGTTACCCCGCCAACAAGCTAATCAGACGCATCCCCATCCATAACCGCTAAACC
TTTAATCGTTATTTGATGTCAGCAAACGACGTCATACGGTATTTAGTCCGTCTTTCAACGGGTTTATCCCGTG
GTCATGGGAAGGTTTGGATACGCGTTACTCACCCGTGCGCCAGTCGTCGTCAAGGAAAGCAAGCTTTCCTCAC
GTTACCCCCCCGCGACTTGCATGTGTTTAAGCCTGTAGCTAGCGTCACCTGAGCCAGGATCAACTCT
>852
CTGCTGCCGCCCGTAGGAGTTTTGGACCGTGTCTCAGTTCCAATGTGGGGGACCTTCCTCTCAGAACCCCTAC
TGATCGTCGCCTTTGGTGGGCCGTTACCCCCGCCAACAAGCTAATCAGACGCATCCCCCATCCATAACCGCTA

AACCTTTTAATCGTTTATTTTGATGTCAGCAAACGATATCATACGGTATTTAGGCCGTCTTTTCAACGGGTTT
ATTCCCCGTAGTTTATGGGAAGGTTTGGATACGCGTTACTCACCCGTGCGCCAGTCGTCATCAAGGAAAGCAA
GCTTTTCCTCATGTTT
>853
CTGCTGCCGCCCGTAGGGGGTTTGGACCGTGTCTCAGTTCCAATGTGGGGGACCTTCCTCTCAGAACCCCTAC
TGATCGTTGCCTTGGTGGGCCGTTACCCCGCCAACAAGCTAATCAGACGCATCCCCATCCATCACCGCTAAAC
CTTTAATCGTTATTTGATGTCAGCAAACGATGTCATACGGTATTAGTCCGTCTTTTCAACGGGTTATCCCGTG
GTCAGGGAAGGTTGGATACGCGTTACTCACCCGTGCGCCAGTCGTCGTCAAGGGAAGCAAGCTTCCCTCACGT
ACCCTGCGACTGCATGTGTAAGCCTTGTAGCTAGCGTCATCCCTGAGCCA
>854
CTGCTGCCGCCCGTAGGAGTTTGGACCGTGTCTCAGTTCCAATGTGGCCGATCACCCTCTCAGGTCGGCTACT
GATCGTCGCCTTGGTGGGCAGTTATCTCACCAACTAGCTAATCAGACGCGGGTCCATCCTATACCGCTAACAC
TTGATATATCTACGATGCCGTAAATATATATTATCATGTATTAGTAAACTTTCAGTATGTTATCCATGTGTAT
AGGGCAGGTTACCCACGCGTTACTCACCCGTCCGCCGCTCATCTTCATTAATGCAAGCATCAAATCAGACGCG
CTCGACTTGCATGTGTTAGGCACGCCGCCAGCGTCATCCTGACCAGGATCAAACTCT
>855
CTGCTGCCGCCCGTAGGAGTTTTGGACCGTGTCTCAGTTCCAATGTGGGGGACCTTCCTCTCAGAACCCCTAC
TGATCGTCGCCTTTGGTGGGCCGTTACCCCGCCAACAAGCTAATCAGACGCATCCCCATCCATAACCGCTAAA
CCTTTAATCGTTATTTGATGTCAGCAAACGATATCATACGGTATTAGGCCGTCTTTCAACGGGTTATCCCGTA
GTTATGGGAAGGTTGGATACGCGTTACTCACCCGTGCGCCAGTCGTCATCAAGGAAAGCAAGCTTCCTCATGT
TACCCTGCGACTGCATGTGTTAAGCCTGTAGCTAGCGTTCATCCCGTGAGCCCAAGGATCAAACTCT
>856
CTGCTGCTGCCCGTAGGAGTTTGGACCGTGTCTCAGTTCCAATGTGGGGGACCTTCCTCTCAGAACCCCTACT
GATCGTCGCCTTTGGTGGGCCGTTACCCCGCCAACAAGCTAACTCAGACGCATCCCCCATCCATAACCGCTAA
ACCTTTAATCGTTATTTGATGTCAGCAAACGATATCATACGGTATTAGGCCGTCTTTTCAACGGGTTATCCCG
TAGTTATGGGAAGGTGGATACGCGTTACTCACCGTGCGCCGGTCGCCGACATAAATAGCCAAGCTATCTATTC
GCTGCCCCCCCTCGACTTGCATGTGTTTTAAGCCTATAGCAGCGTC
>857
CTGCTGCCGCCCGTAGGAGTTTGGACCGTGTCTCAGTTCCAATGTGGGGGACCTTCCTCTCAGAACCCCTACT
GATCGTTGCCTTGGTGGGCCGTTACCCCGCCAACAAGCTAATCAGACGCATCCCCATCCATAACCGATAAATC
TTTATTCGTAATCTCATGAGATCAAACGAATACATAAGGTATTAGTCCAACTTCGCTGGGTTATCCCTTAGTT
ATGGGCAGGTTGGATACGCGTTACTCACCCGTGCGCCGGTCGCCGACATAAATAGCAAGCTATCTATTCGCTG
CCCCCCCTCGACTTGCATGTGTTTTAAGCCTATAGCTAGCGTTCATCCTGAGCCAGGATCAAACTCT
>858
CTGCTGCCGCCCGTAGGAGTTTGGACCGTGTCTCAGTTCCAATGTGGGGGACCTTCCTCTCAGAACCCCTACT
GATCGTTGCCTTTGGTGGGCCGTTACCCCGCCAACAAGCTAATCAGACGCATCCCCATCCATAACCGATAAAT
CTTTATTCGTAATCTCATGAGATCAAACGAAATACATAAGGTATTAGTCCAACTTCGCTGGGTTATCCCTTAG
TTATGGGCAGGTTGGATACGCGTTACTCACCCGTGCGCCGGTCGCCGACATAAATAGCAAGCTATCTATTCGC
TGCCCCTCGACTTGCATGTGTTAAGCCTAGTAGCTAGCGTTCATCCTGAGCCAGGATCAAACTCT
>859
CTGCTGCCGCCCGTAGGAGTTTGGACCGTGTCTCAGTTCCAATGTGGCCGATCACCCTCTCAGGTCGGCTACT
GATCGTCGCCTTGGTGGGCAGTTATCTCACCAACTAGCTAATCAGACGCGGGTCCATCCTATACCGCTAACAC
TTTGATATATCTACGATGCCGTAAATATATATTATCATGTATTAGTAAACTTTTCAGTATGTTATCCATGTGT
ATAGGGCAGGTTACCCACGCGTTACTCACCCGTCCGCCGCTCATCTTTCATTAATGCAAGCATCAAATCAGAC
GCGCTCGACTTGCATGTGTTAGGCACGCCGCCAGCGTTCATCCTGAGCCAGGATCAAACTCT
>860
CTGCTGCCGCCCGTAGGAGTTTGGACCGTGTCTCAGTTCCAATGTGGGGGACCTTCCTCTCAGAACCCCTACT
GATCGTTGCCTTGGTGGGCCGTTACCCCGCCAACAAGCTAATCAGACGCATCCCCATCCATAACCGATAAATC
TTTATTCGTAATCTCATGAGATCAAACGAAATACATAAGGTATTAGTCCAACTTCGCTGAGTTATCCCTTAGT
TATGGGCAGGTTGGATACGCGTTACTCACCCGTGCGCCGGTCGCCGACATAAATAGCAAGCTATCTATTCGCT
GCCCCTCGACTTGCATGTGTTAAGCCTATAGCTAGCGTTCATCCTGAGCCAGGATCAAACTCT
>861
CTGCTGCCGCCCGTAGGAGTTTGGACCGTGTCTCAGTTCCAATGTGGGGGACCTTCCTCTCAGAACCCCTACT
GATCGTTGCCTTGGTGGGCCGTTACCCCGCCAACAAGCTAATCAGACGCATCCCCATCCATAACCGATAAATC

TTTATTCGTAATCTCATGAGATCAAACGAAATACATAAGGTATTAGTCCAACTTCGCTGGGTTATCCCTTAGT
TATGGGCAGGTTGGATACGTGTTACTCACCCGTGCGCCGGTCGCCGACATAAATAGCAAGCTATCTATTCGCT
GCCCCTCGACCTTGCATGTGTTAAGCCTATAGCTAGCGTTCATCCTGAGCCAGGATCAAACTCT
>862
CTGCTGCCGCCCGTAGGAGTTTGGACCGTGTCTCAGTTCCAATGTGGGGGACCTTCCTCTCAGAACCCCTACT
GATCGTTGCCTTTGGTGGGCCGTTACCCCGCCAACAAGCTAATCAGACGCATCCCCATCCATAACCGATAAAT
CTTTATTCATAATCTCATGAGATCAAACGAATACATAAGGTATTAGTCCAAACTTCGCTGGGTTATCCCTTAG
TTATGGGCAGGTTGGATACGCGTTACTCACCCGTGCGCCGGTCGCCGACATAAATAGCAAGCTATCTATTCGC
TGCCCCTCGACTTGCATGTGTTAGGCCTATAGCTAGCGTTCATCCTGAGCCAGGATCAAACTCT
>863
CTGCTGCCGCCCGTAGGAGTTTGGACCGTGTCTCAGTTCCAATGTGGGGGACCTTCCTCTCAGAACCCCTACT
GATCGTCGCCTTTGGTGGGCCGTTACCCCGCCAACAAGCTAATCAGACGCATCCCCATCCATAACCGCTAAAC
CTTTAATCGTTATTTGATGTCAGCAAACGATATCATACGGTATTAGGCCGTCTTTCAACGGGTTATCCCGTGG
TCATGGGAAGGTTGGATACGCGTTACTCACCCGTGCGCCAGTCGTCGTCAAGGGAAGCAAGCTTCCCCTGACC
GTTACCTGCGAACC
>864
CTGCTGCCGCCCGTAGGAGTTGAACCGTGTCTCAGTTCCAATGTGGGGGACCTTCCTCTCAGAACCCCTACTG
ATCGTCGCCTTTGGTGGGCCGTTACCCCGCCAACAAGCTAATCAGACGCATCCCCATCCATAACCGCTAAACC
TTTAATCGTTATTTGATGTCAGCAAACGATATCATACGGTATTAGGCCGTCTTTCAACGGGTTATCCCGTAGT
TATGGGAAGGTTGGATACGCGTTACTCACCCGTGCGCCAGTCGTCATCAAGGAAAGCAAGCTTCCCTGACTGT
TACCCTGCGACTTGCATGTGTTAAGCCTGTAGCTAGCGTTCATCCGTGAGCCAAGGATCAAACTCTATCGAGC
CGA
>865
CTGCTGCCGCCCGTAGGAGTTTGGACCGTGTCTCAGTTCCAATGTGGGGGACCTTCCTCTCAGAACCCCTACT
GATCGTCGCCTTTGGTGGGCCGTTACCCCGCCAACAAGCTAATCAGACGCATCCCCATCCATAACCGCTAAAC
CTTTAATCGTTATTTGATGTCAGCAAACGATATCATACGGTATTAGGCCGTCTTTCAACGGGTTATCCCGTAG
TTATGGGAAGGTTGGATACGCGTTACTCACCCGTGCGCCAGTCGTCATCAAGGAAAGCAAGCTTTCCCTCACT
GTTACCCTGCGTACTGCACTGTGTTGAA
>866
CTGCTGCCGCCCGTAGGAGTTTTGGACCGTGTCTCAGTCCCAATGTGGGGGACCTTCCTCTCAGAACCCCTAC
TGATCGTCGCCTTTGGTGGGCCGTTACCCCGCCAACAAGCTAATCAGACGCATCCCCATCCATAACCGCTAAA
CCTTTAATCGTTATTTTGATGTCAGCAAACGATATCATACGGTATTTAGGCCGTCTTTCAACGGGTTTATCCC
GTAGTTTATGGGAAGGTTGGATACGCGTTACTCACCCGTGCGCCAGTCGTCATCAAGGAAAAGCAAGCTTTCC
CTCATGTTTACCCTGCGACTTGCATGTGTTAAGCCTGTAGCTAGCGTTCATCCTGAGCCAGGATCAAACTCT
>867
CTGCTGCCGCCCGTAGGAGTTTGGACCGTGTCTCAGTTCCAATGTGGGGGACCTTCCTCTCAGAACCCCTACT
GATCGTTGCCTTGGTGGGCCGTTACCCCGCCAACAAGCTAATCAGACGCATCCCCATCCATAACCGATAAATC
TTATTCGTAATCTCATGAGATCAAACGAAATACATAAGGTATTAGTCCAACTTCGCTGGGTTATCCCTTAGTT
ATGGGCAGGTTGGATACGGCGTTACTCACCCGTGCGCCGGTCGCCGACATAAATAGCAAGCTATCTATTCGCT
GCCCCTCGACTTGCATGTGTTAAGCCTATAGCTAGCGTTCATCCTGAGCCAGGATCAAACTCT
>868
CTGCTGCCGCCCGTAGGAGTTTGGACCGTGTCTCAGTTCCAATGTGGGGGACCTTCCTCTCAGAACCCCTACT
GATCGTTGCCTTGGTGGGCCGTTACCCCGCCAACAAGCTAATCAGACGCATCCCCATCCATAACCGCTAAACC
TCTAATCGTTATTTGATGTCAGCAAACGATATCATACGGTATTAGGCCGTCTTTCAACGGGTTAGTCCCGTAG
TTATGGGAAGGTTGGATATGCGTTACTCACCCGTGCGCCAGTCCGTCATCAAGGAAAGCAAGCTTTCCTCATG
TTACCCTGCGACATGCATGTGTTAAGCCTGTAGCTAGCGTTCATCCTGAGCCAGGATCAAACTCT
>869
CTGCTGCCGCCCGTAGGAGTTTGGACCGTGTCTCAGTTCCAATGTGGGGGACCTTCCTCTCAGAACCCCTACT
GATCGTCGCCTTGGTGGGCCGTTACCCCGCCAACAAGCTAATCAGACGCATCCCCATCCATAACCGCTAAACC
TTTAATCGTTATTTGATGTCAGCAAACGATATCATACGGTATTAGGCCGTCTTTCAACGGGTTATCCCGTAGT
TATGGGAAGGTTGGATACGCGTTACTCACCCGTGCGCCAGTCGTCATCAAGGAAAGCAAGCTTCCTCATGTTA
CCCTGCGACTTGCATGTGTTAAGCCTGTAGCTAGCGTTCATCCCGTGAGCCAA
>870

CTGCTGCCGCCCGTAGGAGTTTGGACCGTGTCTCAGTTCCAATGTGGGGGACCTTCCTCTCAGAACCCCTACT
GATCGTCGCCTTGGTGGGCCGTTACCCCGCCAACAAGCTAATCAGACGCATCCCCATCCATAACCGCTAAACC
TTTAATCGTTTATTTTGATGTCAGCAAACGATGATCATACGGTATTTAGGCCGTCTTTTCAACGGGTTTATCC
CGTGAGTATGGGAAGGTTGGATACGCGTTACTCACCCGTGCGCCAGTCGTCAGTCAAGGAAGCAAGCTTCCTT
CAGTGACC
>871
CTGCTGCCGCCCGTAGGAGTTTGGACCGTGTCTCAGTTCCAATGTGGGGGACCTTCCTCTCAGAACCCCTACT
GATCGTCGCCTTTGGTGGGCCGTTACCCCGCCAACAAGCTAATCAGACGCATCCCCATCCATAACCGCTAAAC
CTTTAATCGTTATTTGATGTCAGCAAACGATATCATACGGTATTAGGCCGTCTTTCAACGGGTTATCCCGTAG
TTATGGGAAGGTTGGATACGCGTTACTCACCCGTGCGCCAGTCGTCATCAAGGAAAGCAAGCTTTCCCTCATG
TTACCCTGCGACTTGCATGTGTTAAGCCTGTAGCTAGCGTTCATCCTGAGCCAGGATCAAACTCT
>872
CTGCTGCCGCCCGTAGGAGTTTTGGACCGTGTCTCAGTTCCAATGTGGGGGACCTTCCTCTCAGAACCCCTAC
TGATCGTTGCCTTGGTGGGCCGTTACCCCGCCAACAAGCTAATCAGACGCATCCCCATCCATAACCGATAAAT
CTTTATTCATAATCTCATGAGATCAAACGAATACATAAGGTATTAGTCCAACTTCGCTGGGTTATCCCTTAGT
TATGGGCAGGTTGGATACGCGTTACTCACCCGTGCGCCAGTCGTCATCAAGGAAAGCAAGCTTTTCCTCATGT
TTACCCTGCGACTTGCATGTGTTTAAGCCTGTAGCTAGCGTTCATCCTGAGCCAGGATCAAACTCT
>873
CTGCTGCCGCCCGTAGGAGTTTGGACCGTGTCTCAGTTCCAATGTGGGGGACCTTCCTCTCAGAACCCCTACT
GATCGTCGCCTTGGTGGGCCGTTACCCCGCCAACAAGCTAATCAGACGCATCCCCATCCATAACCGCTAAACC
TTTAATCGTTTATTTTGATGTCAGCAAACGATATCATACGGTATTTAGGCCGTCTTTTCAACGGGTTTATCCC
GTAGTTTATGGGAAGGTTTGGATACGCGTTACTCACCCGTGCGCCAGTCGTCATCAAGGGAAGGCAAGCTTTT
CCCTTCATGT
>874
CTGCTGCCGCCCGTAGGAGTTTGGACCGTGTCTCAGTTCCAATGTGGGGGACCTTCCTCTCAGAACCCCTACT
GATCGTTGCCTTGGTGGGCCGTTACCCCACCAACAAGCTAATCAGACGCATCCCCATCCATAACCGATAAATC
TTTATTCGTAATCTCATGAGATCAAACGAAATATATAAGGTATTAGTCCAACTTCGCTGGGTTATCCCTTGAG
TTTTATGGGCAGGTTTGGATACGGCGTTACTCACCCGTGCGCCGGTCGCCGACATAAATAGCAAGCTATCTAT
TCGCTGCCCCCCCTCGACTTGCATGTGTTTTAAGCCTATAGCTAGCGTTCATCCTGAGCCAGGATCAAACTCT
>875
CTGCTGCCGCCCGTAGGAGTTTGGACCGTGTCTCAGTTCCAATGTGGGGGACCTTCCTCTCAGAACCCCTACT
GATCGTCGCCTTTGGTGGGCCGTTACCCCGCCAACAAGCTAATCAGACGCATCCCCATCCATAACCGCTAAAC
CTTTAATCGTTATTTTGATGTCAGCAAACGATATCATACGGTATTAGGCCGTCTTTTCAACGGGTTATCCCGT
AGTTATGGGAAGGTTGGATACGCGTTACTCACCCGTGCGCCAGTCGTCATCAAGGAAAGCAAGCTTTCCTCAT
GTTTACCCTGCGACTTGCATGTGTTAAGCCTGTAGCTAGCGTTCATCCTGAGCCAAGGATCAAACTCT
>876
CTGCTGCCGCCCGTAGGAGTTTGGACCGTGTCTCAGTTCCAATGTGGGGGACCTTCCTCTCAGAACCCCTACT
GATCGTCGCCTTGGTGGGCCGTTACCCCGCCAACAAGCTAATCAGACGCATCCCCATCCATAACCGATAAATC
TTTATTCGTAATCTCATGAGATCAAACGAAATACATAAGGTATTAGTCCAACTTCGCTGGGTTATCCCTTAGT
TATGGGCAGGTTGGATACGCGTTACTCACCCGTGCGCCGGTCGCCGACATAAATAGCAAGCTATCTATTCGCT
GCCCCTCGACTTGCATGTGTTAAGCCTATAGCTAGCGTTCATCCTGAGCCAGGATCAAACTCT
>877
CTGCTGCCGCCCGTAGGAGTTTGGACCGTGTCTCAGTTCCAATGTGGGGGACCTTCCTCTCAGAACCCCTAC
TGATCGTCGCCTTTGGTGGGCCGTTACCCCGCCAACAAGCTAATCAGACGCATCCCCATCCATAACCGCTAAA
CCTTTAATCGTTATTTGATGTCAGCAAACGATATCATACGGTATTAGGCCGTCTTTCAACGGGTTATCCCGTA
GTTATGGGAAGGTTGGATACGCGTTACTCACCCGTGCGCCAGTCGCCATCAAGGAAAGCAAGCTTCCTCATGT
TTACCCTGCGACTTGCATGTGTTAAGCCTTGTAGCTAGCGTC
>878
CTGCTGCCGCCCGTAGGAGTTTGGACCGTGTCTCAGTTCCAATGTGGGGGACCTTCCTCTCAGAACCCCTACT
GATCGTCGCCTTTGGTGGGCCGTTACCCCGCCAACAAGCTAATCAGACGCATCCCCATCCATAACCGCTAAAC
CTTTAATCGTTATTTGATGTCAGCAAACGATATCATACGGTATTAGGCCGTCTTTCAACGGGTTATCCCGTGA
GTTATGGGAAGGTTGGATACGCGTTACTCACCCGTGCGCCAGTCGTCAGTCAAGGAAAGCAAGCTTCCCTCAC
GTGACTTTG
>879

CTGCTGCCGCCCGTAGGAGTTTGGACCGTGTCTCAGTTCCAATGTGGGGGACCTTCCTCTCAGAACCCCTACT
GATCGTCGCCTTTGGTGGGCCGTTACCCCGCCAACAAGCTAATCAGACGCATCCCCATCCATAACCGCTAAAC
CTTTAATCGTTATTTGATGTCAGCAAACGATATCATACGGTATTAGGCCGTCTTTCAACGGGTTATCCCGTAG
TTATGGGAAGGTTGGATACGCGTTACTCACCCGTGCGCCAGTCGTCATCGAGGAAAGCAAGCTTTCCTCATGT
TACCCTGCGACTTGCATGTGTTAAGCCTGTAGCTAGCGTTCATCCTGAGCCAGGATCAACTCT
>880
CTGCTGCCGCCCGTAGGAGTTTGGACCGTGTCTCAGTTCCAATGTGGGGGACCTTCCTCTCAGAACCCCTACT
GATCGTTGCCTTGGTCGGGCCGTTACCCCGCCAACAAGCTAATCAGACGCATCCCCATCCATAACCGATAAAT
CTTTATTCATAATCTCATGAGATCAAACGAATACATAAGGTATTAGTCCAACTTCGCTGGGTTATCCCTTAGT
TATGGGCAGGTTGGATACGCGTTACTCACCCGTGCGCCGGTCGCCGACATAAATAGCAAGCTATCTATTCGCT
GCCCCTCGACTTGACATGTGTTAAGCCTATAGCTAGCGTTCATCCTGAGCCAGGATCAAACTCT
>881
CTGCTGCCGCCCGTAGGAGTTGAACCGTGATCTCAGTTCCAACTGTGGGGGACCCTTCCTCTCAGGAACCCCT
ACTGACTCGAGTTGCCCTTGGGTGGGCCGGTTACCCCACCAACAAGCTAATCAGACGCATCCCCATCCATAAC
CGATAAATCTTTATTCGTAATCTCATGAGATCAAACGAAATATATAAGGTATTAGTCCAACTTCGCTGGGTTA
TCCCTTAGTTTTATGGGCAGGTTGGATACGCGTTACTCACCCGTGCGCCGGTCGCCGACATAAATAGCAAGCT
ATCTATTCGCTGCCCCCCTCGACTTGCATGTGTTTAAGCCTATAGCTAGCGTTCATCCTGAGCCAGGATCAA
ACTCT
>882
CTGCTGCCGCCCGTAGGAGTTTGGACCGTGTCTCAGTTCCAATGTGGGGGGCCTTCCTCTCAGAACCCCTACT
GATCGTTGCCTTGGTGGGCCGTTACCCCGCCAACAAGCTAATCAGACGCATCCCCATCCATAACCGATAAATC
TTTATTCGTAATCTCATGAGATCAAACGAATACATAAGGTATTAGTCCAACTTCGCTGGGTTATCCCTTAGTT
ATGGGCAGGTTGGATACGCGTTACTCACCCGTGCGCCGGTCGCCGACATAAATAGCAAGCTATCTATTCGCTG
CCCCTCGACTTGCATGTGTTAAGCCTATAGCTAGCGTTCACCCTGAGCCAGGATCAAACTCT
>883
CTGCTGCCGCCCGTAGGAGTTTGGACCGTGTCTCAGTTCCAATGTGGCCGATCACCCTCTCAGGTCGGCTACT
GATCGTCGCCTTGGTGGGCAGTTATCTCACCAACTAGCTAATCAGACGCGGGTCCATCCTATACCGCTAACAC
TTTGATATATCTACGATGCCGTAAATATATATTATCATGTATTAGTAAACTTTTTCAGTATGTTTATCCATGT
GTATAGGGCAGGTTACCCACGCGTTACTCACCCGTCCGCCGCTCATCTTTCATTTAATGCAAGCATCAAATCA
GACGCGCTCGACTTGCATGTGTTAGGCACGCCGCCAGCGTTCATCCTGAGCCAGGATCAAACTCT
>884
CTGCTGCCGCCCGTAGGAGTTTGGACCGTGTCTCAGTTCCAATGTGGGGGACCTTCCTCTCAGAACCCCTACT
GATCGTCGCCTTTGGTGGGCCGTTACCCCGCCAACAAGCTAATCAGACGCATCCCCATCCATAACCGCTAACC
TTTAATCGTTATTTGATGTCAGCAAACGATATCATACGGTATTAGGCCGTCTTTCAACGGGTTATCCCGTAGT
TATGGGAAGGTTGGATACGCGTTACTCACCCGTGCGCCAGTCGTCATCAAGGAAAGCAAGCTTTCCCTGACTG
TTACCCTGCCGAACTTGCATGTGTTAAGCCTGTAGCTAGCGTCATCCTGAGCCAGGATCAAACTCT
>885
CTGCTGCCGCCCGTAGGAGTTTGGACCGTGTCTCAGTTCCAATGTGGGGGACCTTCCTCTCAGAACCCCTACT
GATCGTCGCCTTTGGTGGGCCGTTACCCCGCCAACAAGCTAATCAGACGCATCCCCATCCATCACCGCTAAAC
CTTTAATCGTTATTTGATGTCAGCAAACGATGTCATACGGTATTAGTCCGTCTTTCAACGGGTTATCCCGTGT
ATGGGAAGGTGGATACGCGTTACTCACCCGTGCGCCAGTCGTCGTCAAGGGAAAGCAAGCATTCCCCTTCGAC
TACCTGGTCG
>886
CTGCTGCCGCCCGAGGAGTTTGGACCGTGTCTCAGTTCCAATGTGGGGGACCTTCCTCGTCAGAACCCCTACT
GATCGTCGCCTTGGTGGGCCGTTACCCCGCCAACAAGCCTAATCAGACGCATCCCCATCCATAACCGCTAAAC
CTTTAATCGTTATTTGATGTCAGCAAACGACGTCATACGGTATTAGTCCGTCTTTCAACGGGTTATCCCGTGG
TCATGGGAAGGTTGGATACGCGTTACTCACCCGTGCGCCAGTCGTCGTCAAGGAAAGCAAGCTTCCTCACGTT
ACCCTGCGACTTGCATGTGTTAAGCCTGTAGCTAGCGTTATCCTGAGCCAGGATCAAACTCT
>887
CTGCTGCCGCCCGTAGGAGTTTGGACCGTGTCTCAGTTCCAATGTGGCCGATCACCCTCTCAGGTCGGCTACT
GATCGTCGCCTTGGTGGGCAGTTATCTCACCAACTAGCTAATCAGACGCGGGTCCATCCTATACCGCTAACAC
TTTGATGTATCTACGATGCCGTAAATATATATTATCATGTATTAGTAAACTTTTCAGTATGTTATCCATGTGT
ATAGGGCAGGTTACCCACGCGTACGTCACCCGTCCGCCGCTCATCTTTCATCAATGCAAGCATCAAATCATAC
GCGCTCGACTTGCATGTGTTAGGCACGCCGCCAGCGTTCATCCTGAGCCAGGATCAAACTCT

>888
CTGCTGCCGCCTGTAGGAGTTTGGACCGTGTCTCAGTTCCAATGTGGGGGACCTTCCTCTCAGAACCCCTACT
GATCGTTGCCTTTGGTGGGCCGTTACCCCGCCAACAAGCTAATCAGACGCATCCCCATCCATAACCGATAAAT
CTTTATTCATAATCTCATGAGATCAAACGAATACATAAGGTATTAGTCCAACTTCGCTGGGTTATCCCTTAGT
TTTATGGGCAGGTTTTGGGATACGCGTTACTCACCCGTGCGCCGGTCGCCGACATAAATAGCAAGCTATCTAT
TCGCTGCCCCCCCTCGACTTGCATGTGTTTTAAGCCTATAGCTAGCGTTCATCCTGAGCCAGGATCAAACTCT
>889
CTGCTGCCGCCCGTAGGAGTCTGGACCGTGTCTCAGTTCCAGTGTGGCCGTTCATCCTCTCAGACCCGGCTAC
TGATCGTTGGTTTGGTGGGCCGTTACCCCACCAACTGCCTAATCAGACGCAAACCCCTCTTTCAGGCGATAGC
TTTACAGGTAGAGGCTACCCTTTCTTTCCACAGGTCATGCGGCCCGTGGAACGTATTTCGGTATTTAGCAGTC
GTTTTCCGTCTGTTTGTCCCCCCATCCTTGAAGGCAGGTTTGTTTTTACGTGTTACTCACCCGTTTTGCCACT
GGATTTCGATAAAGAGCAAGCTCTTTCATCTCTTTCCCG
>890
CTGCTGCCGCCCGTAGGAGTTGAACCGTGTCTCAGTTCCAATGTGGGGGACCTTTCCTCTCAGAACCCCTACT
GATCGTCGCCTTTGGTGGGCCGTTACCCCGCCAACAAGCTAATCAGACGCATCCCCATCCATAACCGCTAACC
TTTAATCGTTATTTGATGTCAGCAAACGATATCATACGGTATTAGGCCGTCTTTCAACGGGTTATCCCGTAGT
TATGGGAAGGTTGGATACGCGTTACTCACCCGTGCGCCAGTCGTCATCAAGGAAAGCAAGCTTTCCCTCACTG
ATTGCCCATGCGTAACTTGCATGTGTTAAGCCTGTAGCTAGCGTTCATCCCTGAGCAA
>891
CTGCTGCCGCCCGTAGGAGTTAGGACCGTGTCTCAGTTCCTATGTGGGGGGCCTTCCTCTCAGAACCCCTACC
GATCGAAGCCTTGGTGAGCCGTTACCTCACCAACAAGCTAATCGGACGCATCCCCATCCATAAGCGGCAAGCC
TTTGGTTCAAATCTGATGCCGTCTTTGAACAACATAGGGGATTAGTCCGCATTTCTGCGGGTTATACCCTACT
TATGGGCAGGTTGGATACGCGTTACTCACCCGTGCGCCGGTCGACGTCCAAATGGTCGCAAGCACCAAATGCC
GTTTCCCCTCGACTTGCATGTGTTAAGCCTGTAGCTAGCGTTCATCCTGAGCCAGGATCAAACTCT
>892
CTGCTGCCGCCCGTAGGAGTTTGGACCGTGTCTCAGTTCCAATGTGGGGGACCTTCCTCTCAGAACCCCTACT
GATCGTCGCCTTGGTGGGCCGTTACCCCGCCAACAAGCTAATCAGACGCATCCCCATCCATAACCGCTAAACC
TTTTAATCGTTTATTTTGATGTCAGCAAACGATATCATACGGTATTTAGGCCGTCTTTTCAACGGGTTTATCC
CGTAGTTTATGGGAAGGTTGGATACGCGTTACTCACCCGTGCGCCAGTCGTCATCAAGGAAAGCAAGCTTTCC
CTTCATGA
>893
CTGCTGCCGCCCGTAGGAGTTTGGACCGTGTCTCAGTTCCAATGTGGGGGACCTTCCTCTCAGAACCCCTACT
GATCGTTGCCTTGGTGGGCCGTTACCCCGCCAACAAGCTAATCAGACGCATCCCCATCCATAACCGATAAATC
TTTATTCGTAATCTCATGAGATCAAACGAATACATAAGGTATTAGTCCAACTTCGCTGGGTTATCCCTTAGTT
ATGGGCAGGTTGGATACGCGTTACTCACCCGTGCGCCGGTCGCCGACATAAATAGCAAGCTATCTATTCGCTG
CCCCCCTCGACTTGCATGTGTTTAAGCCTATAGCTAGCGTTCATCCTGAGCCAGGATCAAACTCT
>894
CTGCTGCCGCCCGTAGGAGTCTGGACCGTGTCTCAGTTCCAATGTGGGGGACCTTCCTCTCAGAACCCCTACT
GATCGTTGCCTTTGGTGGGCCGTTACCCCGCCAACAAGCTAATCAGACGCATCCCCATCCATAACCGATAAAT
CTTTATTCGTAATCTCATGAGATCAAACGAATACATAAGGTATTAGTCCAACTTCGCTGGGTTATCCCTTAGT
TATGGGCAGGTTGGATACGCGTTACTCACCCGTGCGCCGGTCGCCGACATAAATAGCAAGCTATCTATTCGCT
GCCCCTCGACTTGCATGTGTTTAAGCCTATAGCTAGCGTTCATCCTGAGCCAGGATCAAACTCT
>895
CTGCTGCCGCCCGTAGGAGTTTGGGACCGTGTCTCAGTTCCAATGTGGGGGACCTTCCTCCTCAGAACCCCTA
CGTGATCGTCGCCTTGGTCGGGCCGTTACCCCGGCCAACAAGCGTAATCAGACGCATCCCCCATCCATAACCG
CTAACCTTTAATCGTTTATTTTGATTGTCAGCAAACGATATCATACGGTATTTAGGCCGTCTTTTCAACGGGT
TTATTCCCGTAGTTTATGGGAAGGTTGGATACGCGTTACTCACCCGTGCGCCAGTCGTCATCAAGGAAAGCAA
GCTTTCCCTTCATGATTTTGCCCATGCCGACTTGCATTGTGTTTAAGCCTTGTAG
>896
CTGCTGCCGCCCGTAGGAGTTTGGACCGTGTCTCAGTTCCAATGTGGGGGACCTTCCTCTCAGAACCCCTACT
GATCGTTGCCTTTGGTGGGCCGTTACCCCGCCAACAAGCTAATCAGACGCATCCCCATCCATAACCGATAAAT
CTTTATTCATAATCTCATGAGATCAAACGAATACATAAGGTATTAGTCCAACTTGCGCTGGGTTATCCCTTAG
TTATGGGCAGGTTTGGATACGCGTTACCTCACCGTGCGCCGGTCGCCGACATAAATAGCAAGCTATCTATTCG
CTGCCCCCCTCGACTTGCATGTGTTTAAGCCTATAGCAGCGTCATCCTGA

>897
CTGCTGCCGCCCGTAGGAGTTTGGACCGTGTCTCAGTTCCAATGTGGGGGACCTTCCTCTCAGAACCCCTACT
GATCGTTGCCTTGGTGGGCCGTTACCCCGCCAACAAGCTAATCAGGCGCATCCCCATCCATAACCGATAAATC
TTTATTCGTAATCTCATGAGATCAAACGAATACATAAGGTATTAGTCCAACTTCGCTGGGTTATCCCTTAGTT
ATGGGCAGGTTGGATACGCGTTACTCGACCGTGCGCCGGTCGCCCGACATAAATAGCAAGCTATCTATTCGCT
GCCCCCCTCGACTTGCATGTGTTAAGCCTATAGCTAGCGTTCTCCTGAGCCAGGATCAACATCT
>898
CTGCTGCCGCCCGTAGGAGTTTGGGCCGTGTCTCAGTCCCAATGTGGCCGTACACTCTCTCAAGCCGGCTACT
GATCGTCGCCTTGGTGAGCTTTTATCTCACCAACTAGCTAATCAGACGCAAGTCCATCTTACACCGATAAATC
TTTGGTCACTCTATCATGCGATAAATTGACTTTATAGGGTATTATTCTTCGTTTCCAAAGGCTATCCCCTTGT
GTAAGGCAGGTTACTCACGTGTTACTCACCCGTTCGCCACTAATCCACTTAAAATCATTCCGAAGAAATCAAC
TAAGTTTCATCGTTCGACTTGCATGTGTTATGCACGCCGCCAGCGTTTATCCTGAGCCAGGATCAAACTCT
>899
CTGCTGCCGCCCGTAGGAGTTTGGACCGTGTCTCAGTTCCAATGTGGGGGACCTTCCTCTCAGAACCCCTACT
GATCGTTGCCTTTGGTGGGCCGTTACCCCGCCAACAAGCTAATCAGACGCATCCCCATCCATAACCGATAAAT
CTTTATTCGTAATCTCATGAGATCAAACGAATACATAAGGTATTAGTCCAACTTCGCTGGGTTATCCCTTAGT
TATGGGCAGGTTGGATACGCGTTACTCACCCGTGCGCCGGTCGCCGACATAAATAGCAAGCTATCTATTCGCT
GCCCCTCGACTTGCATGTGTTAAGCCTATAGCTAGCGTTCATCCTGAGCCAGGATCAAACTCT
>900
CTGCTGCCGCCCGTAGGAGTTTGGACCGTGTCTCAGTTCCAATGTGGGGGACCTTCCTCTCAGAACCCCTACT
GATCGTCGCCTTGGTGGGCCGTTACCCCGCCAACAAGCTAATCAGACGCATCCCCATCCATAACCGCTAAACC
TTTAATCGTTTATTTTGATGTCAGCAAACGATATCATACGGTATTTAGGCCGTCTTTTCAACGGGTTATCCCG
TAGTTTATGGGAAGGTTGGATACGCGTTACTCACCCGTGCGCCAGTCGTCATCAAGGAAAGCAAGCTTTCCCT
CATGTTTACCCTGCGACTTGCATGTGTTTAAGCCTGTAGCTAGCGTTCATCCTGAGCCAGGATCAAACTCT
>901
CTGCTGCCGCCCGTAGGAGTTTGGACCGTGTCTCAGTTCCAATGTGGGGGACCTTCCTCTCAGAACCCCTACT
GATCGTCGCCTTGGTGGGCCGTTACCCCGCCAACAAGCTAATCAGACGCATCCCCATCCATAACCGCTAAACC
TTTTAATCGTTTATTTTGATGTCAGCAAACGATATCATACGGTATTTAGGCCGTCTTTTCAACGGGTTTATCC
CGTAGTTTATGGGAAGGTTGGATACGCGTTACTCACCCGTGCGCCAGTCGTCATCAAGGAAAAGCAAGCTTTC
CCTCATGATTTA
>902
CTGCTGCCGCCCGTAGGAGTTTGGACCGTGTCTCAGTTCCAATGTGGGGGACCTTCCTCTCAGAACCCTACTG
ATCGTTGCCTTGGTGGGCCGTTACCCCGCCAACAAGCTAATCAGACGCATCCCCATCCATAACCGATAAATCT
TTATTCGTAATCTCATGAGATCAAACGAAATACATAAGGTATTAGTCCAACTTCGCTGGGTTATCCCTTAGTT
ATGGGCAGGTTGGATACGCGTTACTCACCCGTGCGCCGGTCGCCGACATAAATAGCAAGCTATCTATTCGCTG
CCCCTCGACTTGCATGTGTTAAGCCTATAGCTAGCGTTCATCCTGAGCCAGGATCAAACTCT
>903
CTGCTGCCGCCCGTAGGAGTTTGGACCGTGTCTCAGTTCCAATGTGGGGGACCTTCCTCTCAGAACCCCTACT
GATCGTTGCCTTTGGTGGGCCGTTACCCCGCCAACAAGCTAATCAGACGCATCCCCATCCATAACCGCTAAAC
CTTTAATCGTTATTTGATGTCAGCAAACGATATCATACGGTATTAGGCCGTCTTTCAACGGGTTATCCCGTAG
TTATGGGAAGGTTGGATACGCGTTACTCACCCGTGCGCCAGTCGTCATCAAGGAAAGCAAGCTTTCCCTCATG
TTACCCTGCGACTTGCATGTGTTAAGCCTGTAGCTAGCGTTCATCCTGAGCCAGGCATCAAACTCT
>904
CTGCTGCCGCCCGTAGGAGTTTGGACCGTGTCTCAGTTCCAATGTGGCCGATCACCCTCTCAGGTCGGCTACT
GATCGTCGCCTTGGTGGGCAGTTATCTCACCAACTAGCTAATCAGACGCGGGTCCATCCTATACCGCTAACAC
TTGATATATCTACGATGCCGTAAATATATATTATCATGTATTAGTAAACTTTTCAGTATGTTATCCATGTGTA
TAGGGCAGGTTACCCACGCGTTACTCACCCGTCCGCCGCTCATCTTTCATTTAATGCAAGCATCAAATCAGAC
GCGCTCGACTTGCATGTGTTAGGCACGCCGCCAGCGTTCATCCTGAGCCAGGATCAAACTCT
>905
CTGCTGCCGCCCGTAGGAGTTTGGACCGTGTCTCAGTTCCAATGTGGGGGACCTTCCTCTCAGAACCCCTACT
GATCGTTGCCTTGGTGGGCCGTTACCCCGCCAACAAGCTAATCAGACGCATCCCCATCCATAACCGATAAATC
TTTATTCGTAATCTCATGAGATCAAACGAAATACATAAGGTATTAGTCCAACTTCGCTGGGTTATCCCTTAGT
TATGGGCAGGTTGGATACGCGTTACTCACCCGTGCGCCGGTCGCCGACATAAATAGCAAGCTATCTATTCGCT
GCCCCTCGACTTGCATGTGTTAAGCCTATAGCTGGCGTTCATCCTGAGCCAGGATCAAACTCT

>906
CTGCTGCCGCCCGTAGGAGTTTGGACCGTGTCTCAGTTCCAATGTGGGGGACCTTCCTCTCAGAACCCCTACT
GATCGTCGCCTTTGGTGGGCCGTTACCCCGCCAACAAGCTAATCAGACGCATCCCCATCCATAACCGCTAAAC
CTTTAATCGTTATTTGATGTCAGCAAACGACGTCATACGGTATTAGTCCGTCTTTCAACGGGTTATCCCGTGG
TCATGGGAAGGTTGGATACGCGTTACTCACCCGTGCGCCAGTCGTCGTCAAGGAAAGCAAGCTTTCCTCACGT
TACCCTGCGACTTGCATGTGTTAAGCCTGTAGCTAGCGTTCATCCTGAGCCAGGATCAAACTCT
>907
CTGCTGCCGCCCGTAGGAGTTTGGGCCGTGTCTCAGTCCCAATCGTGGCCGTACACTCTCTCAAGCCGGCTAC
TGATCGTCGCCTTGGTGAGCTTTATCTCACCAACTAGCTAATCAGACGCAAGTCCATCTTTACACCGATAAAT
CTTTTGGTCACTCTATCATGCGATAAATTTGACTTTATAGGGTATTTTATTTCTTTCGTTTTTCCAAAGGCTA
TCCCCCTTTGTGTAAGGCAGGTTTACTCACGTGTTACTTCACCCCCGTTTCGCCACTAATCCACTTTAAAAAA
TCATTTCCGAAGAAATCAACTAAGTTTTCATTCGTTTCGACTTGCATGTGTTTATTGCACGCCGCCAGCGTTT
TATCCTGAGCCAGGATCAAACTCT
>908
CTGCTGCCGCCCGTAGGAGTTTGGACCGTGTCTCAGTTCCAATGTGGGGGACCTTCCTCTCAGAACCCCTACTG
ATCGTCGCCTTTGGTGGGCCGTTACCCCGCCAACAAGCTAATCAGACGCATCCCCATCCATAACCGCTAAACC
TTTAATCGTTATTTGATGTCAGCAAACGATATCATACGGTATTAGGCCGTCTTTCAACGGGTTATCCCGTAGT
TATGGGAAGGTTGGATACGCGTTACTCACCCGTGCGCCAGTCGTCATCAAGGAAAGCAAGCTTTCCCTGACTG
TTACCCTGCGAACTTGCATGTGTTAAGCCTGTAGCTAAGCGTTCATCCC
>909
CTGCTGCCGCCCGTAGGAGTTTGGACCGTGTCTCAGTTCCAATGTGGGGGACCTTCCTCTCAGAACCCCTACT
GATCGTCGCCTTGGTGGGCCGTTACCCCGCCAACAAGCTAATCAGACGCATCCCCATCCATAACCGCTAAACC
TTTAATCGTTATTTTGATGTCAGCAAACGATATCATACGGTATTAGGCCGTCTCTCAACGGGTTATCCCGTAG
TTATGGGAAGGTTGGATACGCGTTACTCACCCGTGCGCCAGTCGTCATCAAGGAAAGCAAGCTTTTCCTCATG
TTTACCCTGCGACTTGCATGTGTTTTAAGCCTGTAGCTAGCGTTCATCCTGAGCCAGGATCAAACTCT
>910
CTGCTGCCGCCCGTAGGAGTTTGGACCGTGTCTCAGTTCCAATGTGGGGGACCTTCCTCTCAGAACCCCTACT
GATCGTCGCCTTGGGTGGGCCGTTACCCCGCCAACAAGCTAATCAGACGCATCCCCATCCATAACCGCTAAAC
CTTAATCGTTATTTTGATGTCAGCAAACGACGTCATACGGTATTAGTCCGTCTTTCAACGGGTATCCCGTGGT
CATGGGAAGGTTGGATACGCGTTACTCACCCGTGCGCCAGTCGTCGTCAAGGAAAGCAAGCTTTTCCTCACGT
TACCCTGCGACTTGCATGTGTTTTAAGCCTGTAGCTAGCGTTCATCCTGAGCCAGGATCAAACTCT
>911
CTGCTGCCGCCCGTAGGAGTTTGGACCGTGTCTCAGTTCCAATGTGGGGGACCTTCCTCTCAGAACCCCTACT
GATCGTTGCCTTGGTGGGCCGTTACCCCGCCAACAAGCTAATCAGACGCATCCCCATCCATAACCGATAAATC
TTTATTCGTAATCTCATGAGATCAAAACGAAATACATAAGGTATTTAGTCCAACTTCGCTGGGTTATCCCTTA
GTTTTATGGGCAGGTTTGGATACGGCGTTACCTCACCCGTGCGCCGGTCGCCGACATAAATAGCAAGCTATCT
ATTTCGCTGCCCCCCCCTCGACTTTGCATGTGTTTTAAGCCTTATAGCTAGCGTTCATCCTGAGCCAGGATCA
AACTCT
>912
CTGCTGCCGCCCGTAGGAGTTTGAACCGTGTCTCAGTTCCAATGTGGGGGACCTTCCTCTCAGAACCCCTACT
GATCGTTGCCTTGGTGGGCCGTTACCCCGCCAACAAGCTAATCAGACGCATCCCCATCCATAACCGCTAAACC
TTTAATCGTTATTTTGATGTCAGCAAACGATATCATACGGTATTTAGGCCGTCTTTTCAACGGGTTATCCCGT
AGTTATGGGAAGGTTGGATACGCGTTACTCACCCGTGCGCCAGTCGTCATCAAGGAAAGCAAGCTTTCCCTCA
TGTTTACCCTGCGTAACTGCGC
>913
CTGCTGCCGCCCGTAGGAGTTTGAACCGTGTCTCAGTTCCAATGTGGGGGACCTTCCTCTCAGAACCCCTACT
GATCGTTGCCTTGGTGGGCCGTTACCCCACCAACAAGCTAATCAGACGCATCCCCATCCATAACCGATAAATC
TTTATTCGTAATCTCATGAGATCAAACGAAATATATAAGGTAATTAGTCCAACTTCGCTGGGTTATCCCTTGA
GTTTATGGGCAGGTTTGGATACGGCGTTACTCACCCGTGCGCCGGTCGCCGACATAAATAGCAAGCTATCTAT
TCGCTGCCCCTCGACTTGCATGTGTTTAAGCCTATAGCTAGCGTTCATCCTGAGCCAGGATCAAACTCT
>914
CTGCTGCCGCCCGTAGGAGTTTGGACCGTGTCTCAGTTCCAATGTGGGGGACCTTCCTCTCAGAACCCCTACT
GATCGTCGCCTTTGGTGGGCCGTTACCCCGCCAACAAGCTAATCAGACGCATCCCCATCCATAACCGCTAAAC
CTTTAATCGTTATTTGATGTCAGCAAACGATATCATACGGTATTAGGCCGTCTTTCAACGGGTTATCCCGTAG

TTATGGGAAGGTTGGATACGCGTTACTCACCCGTGCGCCAGTCGTCATCAAGGAAAGCAAGCTTTCCTCATGT
TACCCTGCGACATGCATGTGTTAAGCCTGTAGCTAGCGTTCATCCTGAGCCAGGATCCAAACTCGT
>915
CTGCTGCCGCCCGTAGGAGTTTGGACCGTGTCTCAGTTCCAATGTGGGGGACCTTCCTCTCAGAACCCCTACT
GATCGTCGCCTTTGGTGGGCCGTTACCCCGCCAACAAGCTAATCAGACGCATCCCCATCCATAACCGCTAAAC
CTTTAATCGTTATTTGATGTCAGCAAACGACGTCATACGGTATTAGTCCGTCTTTCAACGGGTTATCCCGTAG
TTATGGGAAGGTTGGATACGCGTTACTCACCCGTGCGCCAGTCGTCATCAAGGAAAGCAAGCTTTCCTCATGT
TACCCTGCGACTTGCATGTGTTAAGCCTGTAGCTAGCGTTCATCCTGAGCCAGGATCAAACTCT
>916
CTGCTGCCGCCCGTAGGAGTTTGGACCGTGTCTCAGTTCCAATGTGGGGGACCTTCCTCTCAGAACCCCTACT
GATCGTCGCCTTTGGTGGGCCGTTACCCCGCCAACAAGCTAATCAGACGCATCCCCATCCATAACCGCTAAAC
CTTTAATCGTTATTTGATGTCAGCAAACGATATCATACGGTATTAGGCCGTCTTTCAACGGGTTATCCCGTAG
TTATGGGAAGGTTGGATACGCGTTACTCACCCGTGCGCCAGTCGTCATCAAGGAAAGCAAGCTTTCCTCACTG
TTACCCTGCG
>917
CTGCTGCCGCCCGTAGGAGTTTGGACCGTGTCTCAGTTCCAATGTGGGGGACCTTCCTCTCAGAACCCCTACT
GATCGTTGCCTTGGTGGGCCGTTACCCCGCCAACAAGCTAATCAGACGCATCCCCATCCATAACCGATAAATC
TTTATTCGTAATCATCATGAGATCAAAACGAAATACATAAGGTATTAGTCCAACTTCGCTGGGTTATCCCTTA
GTTATGGGCAGGTTGGATACGCGTTACTCACCCGTGCGCCGGTCGCCGGCATAAATAGCAAGCTATCTATTCG
CTGCCCCTCGACTTGCATGTGTTAAGCCTATAGCTAGCGTTCATCCTGAGCCAGGATCAAACTCT
>918
CTGCTGCCGCCCGTAGGAGTTTGGACCGTGTCTCAGTTCCAATGTGGGGGACCTTCCTCTCAGAACCCCTACT
GATCGTCGCCTTTGGTGGGCCGTTACCCCGCCAACAAGCTAATCAGACGCATCCCCATCCATAACCGCTAAAC
CTTTAATCGTTATTTGATGTCAGCAAACGATATCATACGGTATTAGGCCGTCTTTTCAACGGGTTATCCCGTA
GTTATGGGAAGGTTGGATACGCGTTACTCACCCGTGCGCCAGTCGTCATCAAGGAAAAGCAAGCTTTCCCTCA
TGTTACCCTGCGACTTGCATGTGTTAAGCCTGTAGCTAGCGTCATCCTGAGCCAGGATCAAACTCT
>919
CTGCTGCCGCCCGTAGGAGTTTGGACCGTGTCTCAGTTCCAATGTGGGGGACCTTCCTCTCAGAACCCCTACT
GATCGTCGCCTTGGTGGGCCGTTACCCCGCCAACAAGCTAATCAGACGCATCCCCATCCATAACCGCTAAACC
TTTAATCGTTATTTTGATGTCAGCAAACGATATCATACGGTATTAGGCCGTCTTTTCAACGGGTTATCCCGTA
GTTATGGGAAGGTTGGATACGCGTTACTCACCCGTGCGCCAGTCGTCATCAAGGAAAAGCAAGCTTTCCCTCA
TGTTACCCTGCCGACTTGCATGTGTTAAGCCTGTAGCTAGCGTTCATCCCTGAGCCAGGATCAAACGTCAGCT
AGCAA
>920
CTGCTGCCGCCCGTAGGAGTTTGGACCGTGTCTCAGTTCCAATGTGGCCGATCACCCTCTCAGGTCGGCTACT
GATCGTCGCCTTGGTGGGCAGTTATCTCACCAACTAGCTAATCAGACGCGGGTCCATCCTATACCGCTAACAC
TTTGATATATCTACGATGCCGTAAATATATATTATCATGTATTAGTAAACTTTTCAGTATGTTATCCATGTGT
ATAGGGCAGGTTACCCACGCGTTACTCACCCCGTCCGCCGCTCATCTTCATTAATGCAAGCATCAAATCAGAC
GCGCTCGACTTGCATGTGTTAGGCACGCCGCCAGCGTTCATCCTGAGCCAGGATCAAACTCT
>921
CTGCTGCCGCCCGTAGGAGTTTGGACCGTGTCTCAGTTCCAATGTGGGGGACCTTCCTCTCAGAACCCCTACT
GATCGTCGCCTTTGGTGGGCCGTTACCCCGCCAACAAGCTAATCAGACGCATCCCCATCCATAACCGCTAAAC
CTTTAATCGTTTATTTTGATGTCAGCAAACGATATCATACGGTATTTAGGCCGTCTTTTCAACGGGTTTATCC
CGTAGTTTATGGGAAGGTTGGATACGCGTTACTCACCCGTGCGCCAGTCGTCATCAAGGAAAGCAAGCTTTCC
CTCATGATTTAC
>922
CTGCTGCCGCCCGTAGGAGTTTTGGACCGTGTCTCAGTTCCAATGTGGGGGACCTTCCTCTCAGAACCCCTAC
TGATCGTTGCCTTTGGTGGGCCGTTACCCCGCCAACAAGCTAATCAGACGCATCCCCATCCATAACCGATAAA
TCTTTATTCGTAATCTCATGAGATCAAACGAAATACATAAGGTATTAGTCCAACTTCGCTGGGTTATCCCTTA
GTTATGGGCAGGTTGGATACGCGTTACTCACCCGTGCGCCGGTCGCCGACATAAATAGCAAGCTATCTATTCG
CTGCCCCTCGACTTGCATGTGTTAAGCCTATAGCTAGCGTTCATCCTGAGCCAGGATCAAACTCT
>923
CTGCTGCCGCCCGTAGGAGTTTGGACCGTGTCTCAGTTCCAATGTGGGGGACCTTCCTCTCAGAACCCCTACT
GATCGTTGCCTTTGGTGGGCCGTTACCCCGCCAACAAGCTAATCAGACGCATCCCCATCCATAACCGATAAAT

CTTTATTCATAATCTCATGAGATCAAACGAATACTAAGGTATTAGTCCAACTTCGCTGGGTTATCCTAGTTAT
GGGCAGGTTGGATACGCGTTACTCACCGTGCGCCGGTCGCCGACATAAATAGCAAGCTATCTATTCGCTGCCC
CTCGACTTGCATGTGTTAACGCCTTATAGCTAGCGTTCATCCTGAGCCAAGGATCAAACTCT
>924
CTGCTGCCGCCCGTAGGAGTTTGGACTGTGTCTCAGTTCTAATGTGGGGGACCTTCCTCTCAGAACCCCTACT
GATCGTCGCCTTTGGTGGGCCGTTACCCCGCCAACAAGCTAATCAGACGCATCCCCATCCATAACCGCTAAAC
CTTTAATCGTTATTTGATGTCAGCAAACGATATCATACGGTATTAGGCCGTCTTTCAACGGGTTATCCCGTA
GTTATGGGAAGGTTGGATACGCGTTACTCACCCGTGCGCCAGTCGTCATCAAGGAAAGCAAGCTTTCCCTCAT
GTTACCCTGCCGACTTGCATGTGTTAAGCCTGTAGCTAGCGTTCATCCCGTGAGCCAGGATCAAACTCT
>925
CTGCTGCCGCCCGTAGGAGTTTTGGGCCGTGTCTCAGTCCCAATGTGGCCGTACACTCTCTCAAGCCGGCTAC
TGATCGTCGCCTGGTGAGCTTTTATCTCACCAACTAGCTAATCAGACGCAAGTCCATCTTACACCGATAAATC
TTTGGTCACTCTATCATGCGATAAATTGACTTTATAGGGTATTATTCTTCGTTTCCAAAGGCTATCCCCTTGT
GTAAGGCAGGTTACTCACGTGTTACTCACCCGTTCGCCACTAATCCACTTAAAATCATTCCGAAGAAATCAAC
TAAGTTTCATCGTTCGACTTGCATGTGTTATGCACGCCGCCAGCGTTTATCCTGAGCCAGGATCAAACTCT
>926
CTGCTGCCGCCCGTAGGAGTTTGGACCGTGTCTCAGTTCCAATGTGGGGGACCTTCCTCTCAGAACCCCTACT
GATCGTTGCCTTGGTGGGCCGTTACCCCGCCAACAAGCTAATCAGACGCATCCCCATCCATAACCGATAAATC
TTTATTCATAATCTCATGAGATCAAACGAATACATAAGGTATTAGTCCAACTTCGCTGGGTTATCCCTTAGTT
ATGGGCAGGTTGGATACGCGTTACTCACCCGTGCGCCGGTCGCCGACATAAATAGCAAGCTATCTATTCGCTG
CCCCTCGACTTGCATGTGTTAAGCCTATAGCTAGCGTTCATCCTGAGCCAGGATCAAACTCT
>927
CTGCTGCCGCCCGTAGGAGTTTGGACCGTGTCTCAGTTCCAATGTGGGGGACCTTCCTCTCAGAACCCCTACT
GATCGTCGCCTTTGGTGGGCCGTTACCCCGCCAACAAGCTAATCAGACGCATCCCCATCCATAACCGCTAAAC
CTTTAATCGTTATTTGATGTCAGCAAACGATATCATACGGTATTAGGCCGTCTTTCAACGGGTTATCCCGTAG
TTATGGGAAGGTTGGATACGCGTTACTCACCCGTGCGCCAGTCGTCATCAAGGAAAGCAAGCTTTCCTCATGT
TACCCTGCGACTTGCATGTGTTAAGCCTGTAGCTAGCGTTCATCCTGAGCCAGGATCAACTCT
>928
CTGCTGCCGCCCGTAGGAGTTTGGACCGTGTCTCAGTTCCAATGTGGGGGACCTTCCTCTCAGAACCCCTACT
GATCGTTGCCTTTGGTGGGCCGTTACCCCGCCAACAAGCTAATCAGACGCATCCCCATCCATAACCGATAAAT
CTTATTCGTAATCTCATGAGATCAAACGAAATACATAAGGTATTAGTCCAACTTCGCTGGGTTATCCCTTAGT
TATGGGCAGGTTGGATACGCGTTACTCACCCGTGCGCCGGTCGCCGACATAAATAGCAAGCTATCTATTCGCT
GCCCCTCGACTTGCATGTGTTAAGCCTATAGCTAGCGTTCATCCTGAGCCAGGATCAAACTCT
>929
CTGCTGCCGCCCGTAGGAGTTTGGACCGTGTCTCAGTTCCAATGTGGGGGACCCTTCCTCTCAGAACCCCTAC
TGATCGTTGCCTTGGGTGGGCCGGTTACCCCACCAACAACGCTAATCAGACGCATCCCCATCCATAACCGATA
AATCTTTATTCGTAATCTCATGAGATCAAACGAATATATAAGGTATTAGTCCAACTTCGCTGGGTTATCCCTT
AGTTATGGGCAGGTTGGATACGCGTTACTCACCCGTGCGCCGGTCGCCGACATAAATAGCAAGCTATCTATTC
GCTGCCCCCCTCGACTTGCATGTGTTTAAGCCTATAGCTAGCGTTCATCCTGAGCCAGGATCAAACTCT
>930
CTGCTGCCGCCCGTAGGAGTTTGGACCGTGTCTCAGTTCCAATGTGGGGGACCTTCCTCTCAGAACCCCTACT
GATCGTCGCCTTTGGTGGGCCGTTACCCCGCCAACAAGCTAATCAGACGCATCCCCATCCATAACCGCTAAAC
CTTTAATCGTTATTTGATGTCAGCAAACGATATCATACGGTATTAGGCCGTCTTTCAACGGGTTATCCCGTAG
TTATGGGCAGGTTGGATACGCGTTACCTCACCCGTGCGCCGGTCGCCGACATAAATAGCAAGCTATCTATTCG
CTGCCCCTCGACTTGCATGTGTTAAGCCTATAGCTAGCGTTCATCCTGAGCCAGGATCAAACTCT
>931
CTGCTGCCGCCCGTAGGAGTTTGGACCGTGTCTCAGTTCCAATGTGGGGGACCTTCCTCTCAGAACCCCTACT
GATCGTTGCCTTGGTGGGCCGTTACCCCGCCAACAAGCTAATCAGACGCATCCCCATCCATAACCGATAAATC
TTTATTCGTAATCTCATGAGATCAAACGAATACATAAGGTATTAGTCCAACTTCGCTGGGTTATCCCTTAGTT
ATGGGCAGGTTTGGATACGCGTTACTCACCCGTGCGCCGGTCGCCGACATAAATAGCAAGCTATCTATTCGCT
GCCCCTCGACTTGCATGTGTTTAAGCCTATAGCTAGCGTTCATCCTGAGCCAGGATCAAACTCT
>932
CTGCTGCCGCCCGTAGGAGTTTGGACCGTGTCTCAGTTCCAATGTGGGGGACCTTCCTCTCAGAACCCCTACT
GATCGTTGCCTTGGTGGGCCGTTACCCCGCCAACAAGCTAATCAGACGCATCCCCATCCATAACCGATAAATC

TTTATTCGTAATCTCATGAGATCAAACGAAATACATAAGGTATTAGTCCAACTTCGCTGGGTTATCCCTTAGT
TATGGGCAGGTTGGATACGCGTTACTCACCCGTGCGCCGGTCGCCGACATAAATAGCAAGCTATCTATTCGCT
GCCCCTCGACTTGCATGTGTTAAGCCTATAGCTAGCGTTCATCCTGAGCCAGGATCAAACTCT
>933
CTGCTGCCGCCCGTAGGAGTTTGAACCGTGTCTCAGTTCCAATGTGGGGGACCTTCCTCTCAGAACCCCTACT
GATCGTTGCCTTTGGTGGGCCGTTACCCCGCCAACAAGCTAATCAGACGCATCCCCATCCATAACCGCTAAAC
CTTTAATCGTTATTTGATGTCAGCAAACGATATCATACGGTATTAGGCCGTCTTTCAACGGGTTATCCCGTAG
TTATGGGAAGGTTGGATACGCGTTACTCACCCGTGCGCCAGTCGTCATCAAGGAAAGCAAGCTTTCCCTCATG
TTACCCTGCGACTTGCATGTGTTAAGCCTGTAGCTAGCGTTCATCCTGAGCCAGGATCAAACTCT
>934
CTGCTGCCGCCCGTAGGAGTTTGGACCGTGTCTCAGTTCCAATGTGGGGGACCTTCCTCTCAGAACCCCTACT
GATCGTGCCTTTGGTGGGCCGTTACCCCGCCAACAAGCTAATCAGACGCATCCCCATCCATAACCGCTAAACC
TTTAATCGTTATTTGATGTCGCAAACGATATCATACGGTATTAGGCCGTCTTTCAACGGGTTATCCCGTAGTT
ATGGGAAGGTTGGATACGCGTTACTCACCCGTGCGCCAGTCGTCATCAAGGAAAGCAAGCTTTCCCTCATGTT
ACCCTGCGACGTTCGCAT
>935
CTGCTGCCGCCCGTAGGAGTTTGGACCGTGTCTCAGTTCCAATGTGGGGGACCTTCCTCTCAGAACCCCTACT
GATCGTTGCCTTGGTGGGCCGTTACCCCACCAACAAGCTAATCAGACGCATCCCCATCCATAACCGATAAATC
TTTATTCGTAATCTCATGAGATCAAACGAAATATATAAGGTATTAGTCCAACTTCGCTGGGTTATCCCTTAGT
TATGGGCAGGTTGGATACGCGTTACGTCACCCGTGCGCCGGTCGCCGACATAAATAGCAAGCTATCTATTCGC
TGCCCCTCGACTTGCATGTGTTAAGCCTATAGCTAGCGTTCATCCTGAGCCAGGATCAAACTCT
>936
CTGCTGCCGCCCGTAGGAGTTTGGACCGTGCCTCAGTTCCAATGTGGGGGACCTTCCTCTCAGAACCCCTACT
GATCGTTGCCTTGGTGGGCCGTTACCCCGCCAACAAGCTAATCAGACGCATCCCCATCCATAACCGATAAATC
TTTATTCGTAATCTCATGAGATCAAACGAATACATAAGGTATTAGTCCAACTTCGCTGGGTTATCCCTTAGTT
ATGGGCAGGTTGGATACGCGTTACTCACCCGTGCGCCGGTCGCCGACATAAATAGCAAGCTATCTATTCGCTG
CCCCTCGACTTGCATGTGTTAAGCCTATAGCTAGCGTTCATCCTGAGCCAGGATCAAACTCT
>937
CTGCTGCCGCCCGTAGGAGTTTGGACCGTGTCTCAGTTCCAATGTGGGGGACCTTCCTCTCAGAACCCCTACT
GATCGTTGCCTTTGGTGGGCCGTTACCCCGCCAACAAGCTAATCAGACGCATCCCCATCCATAACCGATAAAT
CTTTATTCATAATCTCATGAGATCAAACGAATACATAAGGTATTAGTCCAACTTCGCTGGGTTATCCCTTAGT
TATGGGCAGGTTGGATACGCGTTACTCACCCGTGCGCCGGTCGCCGACATAAATAGCAAGCTATCTATTCGCT
GCCCCTCGACTTGCATGTGTTAAGCCTATAGCTAGCGTTCATCCTGAGCCAGGATCAAACTCT
>938
CTGCTGCCGCCCGTAGGAGTTTGGACCGTGTCTCAGTTCCAATGTGGGGGACCTTCCTCTCAGAACCCCTACT
GATCGTTGCCTTGGTGGGCCGTTACCCCGCCAACAAGCTAATCAGACGCATCCCCATCCATAACCGATAAATC
TTTATTCGTAATCTCATGAGAGTCAAACGAAATACATAAGGTATTAGTCCAACTTCGCTGGGTTATCCCTTAG
TTATGGGCAGGTTGGATACGCGTTACTCACCCGTGCGCCGGTCGCCGACATAAATAGCAAGCTATCTATTCGC
TGCCCCTCGACTTGCATGTGTTAAGCCTATAGCTAGCGTTCATCCTGAGCCAGGATCAAACTCT
>939
CTGCTGCCGCCCGTAGGAGTTTGGACCGTGTCTCAGTTCCAATGTGGGGGACCTTCCTCTCAGAACCCCTACT
GATCGTCGCCTTTGGTGGGCCGTTACCCCGCCAACAAGCTAATCAGACGCATCCCCATCCATAACCGCTAAAC
CTTTAATCGTTATTTGATGTCAGCAAACGACGTCATACGGTATTAGTCCATCTTTCAACGGGTTATCCCGTGG
TCATGGGAAGGTTGGATACGCGTTACCTCACCCGTGCGCCAGTCGTCATCAAGGAAAGCAAGCTTTCCCTCAT
GTTGACCCTGCCGACTGCATGTGTTAAGCCTGTAGCTAGCGTTCATCCCGTG
>940
CTGCTGCCGCCCGTAGGAGTTTGGACCGTGTCTCAGTTCCAATGTGGGGACCTTCCTCTCAGAACCCCTACTG
ATCGTCGCCTTTGGTGGGCCGTTACCCCGCCAACAAGCTAATCAGACGCATCCCCATCCATAACCGCTAAACC
TTTTAATCGTTATTTTGATGTCAGCAAACGATATCATACGGTATTTAGGCCGTCTTTTTCAACGGGTTTATCC
CGTAGTTTATGGGAAGGTTGGATACGCGTTACTCACCCGTGCGCCAGTCGTCATCAAGGAAAAGCAAGCTTTC
CCTTTCAT
>941
CTGCTGCCGCCCGTAGGAGTTTGGACCGTGTCTCAGTTCCAATGTGGGGGACCTTCCTCTCAGAACCCCTACT
GATCGTCGCCTTTGGTGGGCCGTTACCCCGCCAACAAGCTAATCAGACGCATCCCCATCCATAACCGCTAAAC

CTTTAATCGTTATTTTGATGTCAGCAAACGATATCATACGGTATTTAGGCCGTCTTTTCAACGGGTTATCCCG
TAGTTTTATGGGAAGGTTGGATACGCGTTACTCACCCGTGCGCCAGTCGTCATCAAGGAAAGCAAGCTTTCCC
TTTGACTG
>942
CTGCTGCCGCCCGTAGGAGTTTGGACCGTGTCTCAGTTCCAATGTGGGGGACCTTCCTCTCAGAACCCCTACT
GATCGTTGCCTCGGTGGGCCGTTACCCCGCCAACAAGCTAATCAGACGCATCCCCATCCATAACCGCTAAACC
TTTAATCGTTATTTGATGTCAGCAAACGATATCATACGGTGTTAGGCCGTCTTTCAACGGGTTATCCCGTAGT
TATGGGAAGGTTGGATACGCGTTACTCACCCGTGCGCCAGTCGTCATCAAGGAAAGCAAGCTTTCCTCATGTT
ACCCTGCGACTTGCATGTGTTAAGCCTGTAGCTAGCGTTCATCCTGAGCCAGGATCAAACTCT
>943
CTGCTGCCGCCCGTAGGAGTTTGGACCGTGTCTCAGTTCCAATGTGGGGGACCTTCCTCTCAGAACCCCTACT
GATCGTCGCCTTGGTGGGCCGTTACCCCGCCAACAAGCTAATCAGACGCATCCCCATCCATAACCGCTAAACC
TTTAATCGTTATTTGATGTCAGCAAACGATATCATACGGTATTAGGCCGTCTTTCAACGGGTTATCCCGTAGT
TATGGGAAGGTTGGATACGCGTTACTCACCCGTGCGCCAGTCGTCATCAAGGAAAAGCAAGCTTTCCCTCATG
TTACCCTGCCGGAACTTG
>944
CTGCTGCCGCCCGTAGGAGTTTGGACCGTGTCTCAGTTCCAATGTGGCCGATCACCCTCTCAGGTCGGCTACT
GATCGTCGCCTTGGTGGGCAGTTATCTCACCAACTAGCTAATCAGACGCGGGTCCATCCTATACCGCTAACAC
TTGATATATCTACGATGCCGTAAATATATATTATCATGTATTAGTAAACTTTTCAGTATGTTATCCATGTGTA
TAGGGCAGGTTACCCACGCGTTACTCACCCGTCCGCCGCTCATCTTTCATCAATGCAAGCATCAAATCATACG
CGCTCGACTTGCATGTGTTAGGCACACCGCCAGCGTTCATCCGAGCCAGGATCAAACTCT
>945
CTGCTGCCGCCCGTAGGAGTTTGGACCGTGTCTCAGTTCCAATGTGGGGGACCTTCCTCTCAGAACCCCTACT
GATCGTCGCCTTGGTGGGCCGTTACCCCGCCAACAAGCTAATCAGACGCATCCCCATCCATCACCGCTAAACC
TTTAATCGTTATTTGATGTCAGCAAACGATGTCATACGGTATTAGTCCGTCTTTCAACGGGTTATCCCGTGGT
CATGGGAAGGTTGGATACGCGTTACCGTCACCCGTGCGCCAGTCGTCGTCAAGGGAAGCAAGCTTCCCTCACG
TACCCTGCGACTTGCACTGTG
>946
CTGCTGCCGCCCGTAGGAGTTTTGGACCGTGTCTCAGTTCCAATGTGGCCGATCACCCTCTCAGGTCGGCTAC
TGATCGTCGCCTTGGTGGGCAGTTATCTCACCAACTAGCTAATCAGACGCGGGTCCATCCTATACCGCTAACA
CTTTGATATATCTACGATGCCGTAAATATATATTTATCATGTATTTAGTAAACTTTTCAGTATGTTTATCCAT
GTGTATAGGGCAGGTTTACCCACGCGTTACTCACCCGTCCGCCGCTCATCTTTCATCAATGCAAGCATCAAAT
CATACGCGCTCGACTTGCATGTGTTTAGGCACGCCGCCAGCGTTCATCCTGAGCCAGGATCAAACTCT
>947
CTGCTGCCGCCCGTAGGAGTTTGGACCGTGTCTCAGTTCCAATGTGGGGGACCTTCCTCTCAGAACCCCTACT
GATCGTTGCCTTGGTGGGCCGTTACCCCGCCAACAAGCTAATCAGACGCATCCCCATCCATAACCGATAAATC
TTTATTCATAATCTCATAGATCAAACGAAATACATAAGGTATTAGTCCAACTTCGCTGGGTTATCCCTTAGTT
ATGGGCAGGTTGGATACGCGTTACCTCACCCGTGCGCCGGTCGCCGACATAAATAGCAAGCTATCTATTCGCT
GCCCCTCGACTTGCATGTGTTTAAGCCTATAGCTAGCGTTCATCCTGAGCCAGGATCAAACTCT
>948
CTGCTGCCGCCCGTAGGAGTTTTGGACCGTGTCTCAGTTCCAATGTGGGGGACCTTCCTCTCAGAACCCCTAC
TGATCGTCGCCTTTGGTGGGCCGTTACCCCGCCAACAAGCTAATCAGACGCATCCCCATCCATAACCGCTAAA
CCTTTAATCGTTATTTTGATGTCAGCAAACGATATCATACGGTATTTAGGCCGTCTTTTCAACGGGTTTATCC
CGTAGTTTATGGGAAGGTTGGATGCGCGTTACTCACCCGTGCGCCAGTCGCCATCAAGGAAAGCAAGCTTTTC
CTCATGTTTACCCTGCGACTTGCATTGTGTTTTAAGCCTGTAGCTAGCGTTCATCCTGAGCCAGGATCAAACT
CT
>949
CTGCTGCCGCCCGTAGGAGTTTGGACCGTGTCTCAGTTCCAATGTGGGGGACCTTCCTCTCAGAACCCCTACT
GATCGTCGCCTTTGGTGGGCCGTTACCCCGCCAACAAGCTAATCAGACGCATCCCCATCCATAACCGATAAAT
CTTTATTTCATAATCTCATGAGATCAAACGAATACATAAGGTATTAGTCCAACTTCGCTGGGTTATCCCTTAG
TTTATGGGCAGGTTTGGATACGCGTTACTCACCCGTGCGCCGGTCGCCGACATAAATAGCAAGCTATCTATTC
GCTGCCCCCCCTTCGACTTGCATGTGTTTAAGCCTTATAGCTAGCGTTCATCCTGAGCCAGGATCAAACTCT
>950

CTGCTGCCGCCCGTAGGAGTTTGGACCGTGTCTCAGTTCCAATGTGGGGGACCTTCCTCTCAGAACCCCTACT
GATCGTCGCCTTTGGTGGGCCGTTACCCCGCCAACAAGCTAATCAGACGCATCCCCATCCATCACCGCTAAAC
CTTTAATCGTTATTTTGATGTCAGCAAACGATGATCATACGGTATTTAGCCGTCTTTTCAACGGGTTTATCCC
GTGTATGGGAAGGTTGGATACGCGTTACTCACCCGTGCGCCAGTCGTCGTCAAGGAAGCAAGCTTCCCTCACG
TGACCTTG
>951
CTGCTGCCGCCCGTAGGAGTTTGAACCGTGTCTCAGTTCCAATGTGGGGGACCTTCCTCTCAGAACCCCTACT
GATCGTTGCCTTGGTGGGCCGTTACCCCGCCAACAAGCTAATCAGACGCATCCCCATCCATCACCGCTAAACC
TTTAATCGTTATTTGATGTCAGCAAACGATGTCATACGGTATTAGTCCGTCTTTCAACGGGTTATCCCGTGGT
CATGGGAAGGTTGGATACGCGTTACTCACCCGTGCGCCAGTCGTCGTCAAGGGAAGCAAGCTTCCCTCACGTA
CCTGCGACCTGCATGTGTTAAGCCTGTAGCTAGCGTCATCCTGAGCCAGGATCAAACTCT
>952
CTGCTGCCGCCCGTAGGAGTTTTGGACCGTGTCTCAGTTCCAATGTGGGGGACCTTCCTCTCAGAACCCCTAC
TGATCGTTGCCTTGGTCGGGCCGTTACCCCGCCAACAAGCTAATCAGACGCATCCCCATCCATAACCGATAAA
TCTTTATTCATAATCTCATGAGATCAAACGAATACATAAGGTATTAGTCCAACTTCGCTGGGTTATCCTAGTT
ATGGGCAGGTTGGATACGCGTTCTCACCCGTGCGCCAGTCGTCATCAAGGAAAGCAAGCTTTCCTCATGTTAC
CCTGCGACTTGCATGTGTTAAGCCTGTAGCTAGCGTTCATCCTGAGCCAGGATCAAACTCT
>953
CTGCTGCCGCCCGTAGGAGTTTGGACCGTGTCTCAGTTCCAATGTGGGGGACCTTCCTCTCAGAACCCCTACT
GATCGTCGCCTTTGGTGGGCCGTTACCCCGCCAACAAGCTAATCAGACGCATCCCCATCCATAACCGCTAAAC
CTTTAATCGTTATTTGATGTCAGCAAACGATATCATACGGTATTAGGCCGTCTTTCAACGGGTTATCCCGTAG
TTATGGGAAGGTTGGATACGCGTTACTCACCCGTGCGCCAGTCGTCATCAAGGAAAGCAAGCTTCCTCATGTT
ACCCTGCGACTTGCATGTGTTAAGCCTGTAGCTAGCGTTCATCCTGAGCCAGGATCAAACTCT
>954
CTGCTGCCGCCCGTAGGAGTCTGGACCGTGTCTCAGTTCCAGTGTGGCCGTTCATCCTCCCAGACCGGCTACT
GATCGTTGGTTTGGTGGGCCGTTACCCCACCAACTGCCTAATCAGACGCAAACCCCTCTTCAGGCGATAGCTT
ACAGGTAGAGGCTACCCTTTCTTCCACAGGTCATGCGACCCGTGGAACGTATTCGGTATTTAGCAGTCGTTTT
CCGTCTGTTTGTCCCCATCCTGAAGGCAGGTTGTTTTTACGTGTTACTCACCCGTTTTGCCACTGGATCGATA
AAGAGCAAGCTCTTCATCTCTTCCCGTCGACTTGCATGTGTTTAAGCACGCCGCCAGCGTCGTCCTGAGCCAG
GATCAAACTCT
>955
CTGCTGCCGCCCGTAGGAGTTTGGACCGTGTCTCAGTTCCAATGTGGGGGACCTTCCTCTCAGAACCCCTACT
GATCGTTGCCTTTGGTGGGCCGTTACCCCGCCAACAAGCTAATCAGACGCATCCCCATCCATAACCGATAAAT
CTTTATTCGTAATCTCATGAGATCAAACGAATACATAAGGTATTAGTCCAACTTCGCTGGGTTATCCCTTAGT
TATGGGCAGGTTGGATACGCGTTACTCACCCGTGCGCCGGTCGCCGACATAAATAGCAAGCTATCTATTCGCT
GCCCCTCGACTTGCATGTGTTAAGCCTATAGCTAGCGTTCATCCTGAGCCAGGATCAAACTCT
>956
CTGCTGCGCCCGTAGGAGTTTGGACCGTGTCTCAGTTCCAATGTGGGGGACCTTCCTCTCAGAACCCCTACTG
ATCGTCGCCTTTGGTGGGCCGTTACCCCGCCAACAAGCTAATCAGACGCATCCCCATCCATCACCGCTAAACC
TTTAATCGTTATTTGATGTCAGCAAACGATGCCATACGGTATTAGTCCGTCTTTCAACGGGTTATCCCGTGGT
CATGGGAAGGTTGGATACGCGTTACTCACCCGTGCGCCAGTCGTCGTCAAGGGAAGCAAGCTTCCCTCACGTT
ACCCTGCGACTTGCATGTGTTAAGCCTGTAGCTAGCGTTCATCCTGAGCCAGGATCAAACTTCT
>957
CTGCTGCCGCCCGTAGGAGTTTGAACCGTGTCTCAGTTCCAATGTGGGGGACCTTCCTCTCAGAACCCCTACT
GATCGTTGCCTTTGGTGGGCCGTTACCCCGCCAACAAGCTAATCAGACGCATCCCCATCCATAACCGATAAAT
CTTTATTCGTAATCTCATGAGATCAAACGAATACATAAGGTATTAGTCCAACTTCGCTGGGTTATCCCTTAGT
TATGGGCAGGTTGGATACGCGTTACTCACCCGTGCGCCGGTCGCCGACATAAATAGCAAGCTATCTATTCGCT
GCCCCTCGACCTTGCATGTGTTAAGCCTATAGCTAGCGTTCATCCTGAGCCAGGATCAAACTCT
>958
CTGCTGCGCCCGTAGGAGTTTGGACCGTGTCTCAGTTCCAATGTGGGGGACCTTCCTCTCAGAACCCCTACTG
ATCGTCGCCTTGGTGGGCCGTTACCCCGCCAACAAGCTAATCAGACGCATCCCCATCCATAACCGCTAAACCT
TTAATCGTTATTTGATGTCAGCAAACGATATCATACGGTATTAGGCCGTCTTTCAACGGGTTATCCCGTAGTT
ATGGGAAGGTTGGATACGCGTTACTCACCCGTGCGCCAGTCGTCATCAAGGAAAGCAAGCTTTCCTCATGTTA
CCCTGCGACTTGCATGTGTTAAGCCTGTAGCTAGCGTTCATCCTGAGCCAGGATCAAACTCT

>959
CTGCTGCCGCCCGTAGGAGTTTGGACCGTGTCTCAGTTCCAATGTGGGGGACCTTCCTCTCAGAACCCCTACT
GATCGTTGCCTTGGTGGGCCTGTTACCCCGCCAACAAGCTAATCAGACGCATCCCCATCCATAACCGATAAAT
CTTATTCATAATCTCATGAGATCAAACGAATACATAAGGTATTAGTCCAACTTCGCTGGGTTATCCCTTAGTT
ATGGGCAGGTTGGATACGCGTTACTCACCGTGCGCCGGTCGCCGACATAAATAGCAAGCTATCTATTCGCTGC
CCCTCGACTTGCATGTGTTAAGCCTATAGCTAAGCGTTCATCC
>960
CTGCTGCCGCCCGTAGGAGTTTTGGACCGTGTCTCAGTTCCAATGTGGGGGACCTTCCTCTCAGAACCCCTAC
TGATCGTCGCCTTTGGTGGGCCGTTACCCCGCCAACAAGCTAATCAGACGCATCCCCATCCATAACCGCTAAA
CCTTTAATCGTTATTTGATGTCAGCAAACGATATCATACGGTATTAGGCCGTCTTTCAACGGGTTATCCCGTA
GTTATGGGAAGGTTGGATACGCGTTACTCACCCGTGCGCCAGTCGTCATCAAGGAAAGCAAGCTTTCCCTCAT
GTTACCCTGCCGACTTGCATGTGTTAAGCCTGTAGCTAGCGTTCATCCCTGAGCCAAGGCATCAAACTCT
>961
CTGCTGCCGCCCGTAGGAGTTTTGGACCGTGTCTCAGTTCCAATGTGGGGGACCTTCCTCTCAGAACCCCTAC
TGATCGTTGCCTTGGTGGGCCGTTACCCCGCCAACAAGCTAATCAGACGCATCCCCATCCATAACCGATAAAT
CTTTATTCGTAATCTCATGAGATCAAACGAAATACTAAGGTATTAGTCCAACTTCGCTGGGTTATCCCTAGTT
ATGGGCAGGTTGGATACGCGTTACCTCACCCGTGCGCCGGTCGCCGACATAAATAGCAAGCTATCTATTCGCT
GCCCCTCGACTTGCATGTGTTAACGCCTATAGCTAGCGTTCATCCTGAGCCAGGATCAAACTCT
>962
CTGCTGCCGCCCGTAGGAGTTTGGACCGTGTCTCAGTTCCAATGTGGCCGATCACCCTCTCAGGTCGGCTACT
GATCGTCGCCTTGGTGGGCAGTTATCTCACCAACTAGCTAATCAGACGCGGGTCCACCCTATACCGCTAACAC
TTTGATATATCTACGATGCCGTAAATATATATTATCATGTATTAGTAAACTTTTCAGTATGTTATCCATGTGT
ATAGGGCAGGTTACCCACGCGTTACTCACCCGTCCGCCGCTCATCTTTCATTAATGCAAGCATCAAATCAGAC
GCGCTCGACTTGCATGTGTTAGGCACGCCGCCAGCGTTCATCCTGAGCCAGGATCAAACTCT
>963
CTGCTGCCGCCCGTAGGAGTTTTGGACCGTGTCTCAGTTCCAATGTGGGGGACCTTCCTCTCAGAACCCCTAC
TGATCGTCGCCTTTGGTGGGCCGTTACCCCGCCAACAAGCTAATCAGACGCATCCCCATCCATAACCGCTAAA
CCTTTAATCGTTATTTGATGTCAGCAAACGATATCATACGGTATTAGGCCGTCTTTCAACGGGTTATCCCGTA
GTTATGGGAAGGTTGGATACGCGTTACTCACCCGTGCGCCAGTCGTCATCAAGGAAAGCAAGCTTCCTCATGT
TACCCTGCGACTTGCATGTGTTAAGCCTGTAGCTAGCGTTCATCCTGAGCCAGGATCAAACTCT
>964
CTGCTGCCGCCCGTAGGAGTTTGGACCGTGTCTCAGTTCCAATGTGGGGGACCTTCCTCTCAGAACCCCTACT
GATCGTCGCCTTTGGTGGGCCGTTACCCCGCCAACAAGCTAATCAGACGCATCCCCATCCATAACCGCTAAAC
CTTAATCGTTATTTGATGTCAGCAAACGATATCATACGGTATTAGGCCGTCTTTCAACGGGTTATCCCGTAGT
TATGGGAAGGTTGGATACGCGTACTCACCCGTGCGCCAGTCGTCATCAAGGAAAGCAAGCTTCCTCACGTACC
CTGCGACTGCATGTGTTAAGCCTGTAGCTAGCGTTCATCCGTGAGCCAAGG
>965
CTGCTGCCGCCCGTAGGAGTTTGGACCGTGCCTCAGTTCCAATGTGGGGGACCTTCCTCTCAGAACCCCTACT
GATCGTTGCCTTTGGTGGGCCGTTACCCCGCCAACAAGCTAATCAGACGCATCCCCATCCATAACCGATAAAT
CTTTATTCATAATCTCATGAGATCAAACGAATACATAAGGTATTAGTCCAACTTCGCTGGGTTATCCCTTAGT
TATGGGCAGGTTGGATACGCGTTACCTCACCCGTGCGCCGGTCGCCGACATAAATAGCAAGCTATCTATTCGC
TGCCCCTCGACTTGCATGTGTTAAGCCTATAGCTAGCGTTCATCCTGAGCCAGGATCAAACTCT
>966
CTGCTGCCGCCCGTAGGAGTTTGGACCGTGTCTCAGTTCCAATGTGGGGGACCTTCCCTCTCAGAACCCCTAC
TGATCGTCGCCTTGGTGGGCCGTTACCCCGCCAACAAGCTAATCAGACGCATCCCCATCCATAACCGCTAAAC
CTTTAATCGTTATTTGATGTCAGCAAACGATATCATACGGTATTAGGCCGTCTTTCAACGGGTTATCCCGTAG
TTATGGGAAGGTTGGATACGCGTTACTCACCCGTGCGCCAGTCGTCATCAAGGAAAGCAAGCTTTCCCTCATG
TTTACCCTGCGTACGTGCG
>967
CTGCTGCCGCCCGTAGGAGTTTGGACCGTGTCTCAGTTCCAATGTGGGGGACCTTCCTCTCAGAACCCCTACT
GATCGTCGCCTTGGTGGGCCGTTACCCCGCCAACAAGCTAATCAGACGCATCCCCATCCATCACCGCTAAACC
TTTAATCGTTATTTGATGTCAGCAAACGATGTCATACGGTATTAGTCCGTCTTTCAACGGGTTATCCCGTGGT
CATGGGAAGGTTGGATACGCGTTACTCACCCGTGCGCCAGTCGTCGTCAAGGGAAGCAAGCTTCCCTCACGTT
ACCCTGCGACTTGCATGTGTTAAGCCTGTAGCTAGCGTTCATCCTGAGCCAGGATCAAACTCT

>968
CTGCTGCCGCCCGTAGGAGTTTGGACCGTGTCTCAGTTCCAATGTGGGGGACCTTCCTCTCAGAACCCCTACT
GATCGTTGCCTTTGGTGGGCCGTTACCCCGCCAACAAGCTAATCAGACGCATCCCCATCCATAACCGATAAAT
CTTATTCGTAATCTCATGAGATCAAACGAAATACATAAGGTATTAGTCCAACTTCGCTGGGTTATCCCTTAGT
TATGGGCAGGTTGGATACGCGTTACTCACCCGTGCGCCGGTCGCCGACATAAATAGCAAGCTATCTATCGCTG
CCCCCCTCGACTTGCATGTGTTTAAGCCTATAGCTAGCGTTCATCCTGAGCCAGGATCAAACTCT
>969
CTGCTGCCGCCCGTAGGAGTTTGGACCGTGTCTCAGTTCCAATGTGGGGGACCTTCCTCTCAGAACCCCTACT
GATCGTCGCCTTTGGTGGGCCGTTACCCCGCCAACAAGCTAATCAGACGCATCCCCATCCATAACCGCTAAAC
CTTTAATCGTTATTTTGATGTCAGCAAACGATATCATACGGTATTAGGCCGTCTTTTCAACGGGTTATCCCGT
AGTTATGGGAAGGTTGGATACGCGTTACTCACCCGTGCGCCAGTCGTCATCAAGGAAAGCAAGCTTTCCCTCA
TGTTACCCTGCGACATGCATGTGTTAAGCCTTGTACGCTAGCGTTATCCCTGAGCCAGGATCAACTCT
>970
CTGCTGCCGCCCGTAGGAGTTTGGACCGTGTCTCAGTTCCAATGTGGCCGATCACCCTCTCAGGTCGGCTACT
GATCGTCGCCTTGGTGGGCAGTTATCTCACCAACTAGCTAATCAGACGCGGGTCCATCCTATACCGCTAACAC
TTTGATATATCTACGATGCCGTAAATATATATTTTATCATGTATTTTAGTTAAACTTTTTTCAGTATGTTTTA
TCCATGTGTATTAGGGCAGGTTTTACCCCCCACGCGTTACTCACCCCCCCGTCCGCCCCGCTCATCTTTTCAT
TTAATGCAAGCATCAAAAATCAGACGCGCTCGACTTTGCATGTGTTTTAGGCACGCCCGCCCCAGCGTTCATC
CTGAGCCCCAGGATCAAAAACTCT
>971
CTGCTGCCGCCCGTAGGGGTTTGGACCGTGTCTCAGTTCCAATGTGGGGGACCTTCCTCTCAGAACCCCTACT
GATCGTCGCCTTGGTGGGCCGTTACCCCGCCAACAAGCTAATCAGACGCATCCCCATCCATAACCGCTAAACC
TTTAATCGTTATTTGATGTCAGCAAACGATATCATACGGTATTAGGCCGTCTTTCAACGGGTTATCCCGTAGT
TATGGGAAGGTTGGATACGCGTTACTCACCCGTGCGCCAGTCGCATCAAGGAAAGCAAGCTTCCTCATGTTAC
CCTGCGACTTGG
>972
CTGCTGCCGCCCGTACGGAGTTTGGACCGTGTCTCAGTTCCAATGTGGGGGACCTTCCTCTCAGAACCCCTAC
TGATCGTTGCCTTTGGTCGGGCCGTTACCCCGCCAACAAGCTAATCAGAACGCATCCCCATCCATAACCGATA
AATCTTTATTCGTAATCTCATGAGATCAAACGAAATACATAAGGTATTAGTCCAACTTCGCTGGGTTATCCCT
AGTTATGGGCAGGTTGGATACGCGTTACTCACCCGTGCGCCGGTCGCCGACATAAATAGCAAGCTATCTATTC
GCTGCCCCTCGACCTTG
>973
CTGCTGCCGCCCGTAGGAGTTTGGACCGTGTCTCAGTTCCAATGTGGGGGACCTTCCTCTCAGAACCCCTACT
GATCGTCGCCTTTGGTGGGCCGTTACCCCGCCAACAAGCTAATCAGACGCATCCCCATCCATCACCGCTAAAC
CTTTAATCGTTATTTGATGTCAGCAAACGATATCATACGGTATTAGGCCGTCTTTCAACGGGTTATCCCGTAG
TTATGGGAAGGTTGGATACGCGTTACTCACCCGTGCGCCGGTCGCCCGACATAAATAGCAAGCTATCTATTCG
CTGCCCCTCGACTTGCATGTGTTAAGCCTA
>974
CTGCTGCCGCCGTAGGAGTTTGGACCGTGTCTCAGTTCCAATGTGGGGGACCTTCCTCTCAGAACCCCTACTG
ATCGTCGCCTTTGGTGGGCCGTTACCCCGCCAACAAGCTAATCAGACGCATCCCCATCCATAACCGCTAAACC
TTTAATCGTTATTTGATGTCAGCAAACGACGTCATACGGTATTAGTCCGTCTTTCAACGGGTTATCCCGTGGT
CATGGGAAGGTTGGATACGCGTTACTCACCCGTGCGCCAGTCGTCGTCAAGGGAAGCAAGCTTCCCTCACGTT
ACCCTGCGACTTGCATGTGTTAAGCCTGTAGCTAGCGTTCATCCTGAGCCAGGATCAAACTCT
>975
CTGCTGCCGCCCGTAGGAGTTTGGACCGTGTCTCAGTTCCAATGTGGGGGACCTTCCTCTCAGAACCCCTACT
GATCGTTGCCTTGGTGGGCCGTTACCCCGCCAACAAGCTAATCAGACGCATCCCCATCCATAACCGATAAATC
TTTATTCATAATCTCATGAGATCAAACGAATACATAAGGTATTAGTCCAACTTCGCTGGGTTATCCCTTAGTT
ATGGGCAGGTTGGATACGCGTTACTCACCCGTGCGCCGGTCGCCGACATAAATAGCAAGCTATCTATTCGCTG
CCCCTCGACTTGCATGTGTTAAGCCTATAGCTAGCGTTCATCCTGAGCCAGGATCAAACTCT
>976
CTGCTGCCGCCCGTAGGAGTTTGGACCGTGTCTCAGTTCCAATGTGGGGGACCTTCCTCTCAGAACCCCTACT
GATCGTCGCCTTTGGTGGGCCGTTACCCCGCCAACAAGCTAATCAGACGCATCCCCATCCATCACCGCTAAAC
CTTTAATCGTTATTTGATGTCAGCAAACGATGTCATACGGTATTAGTCCGTCTTTCAACGGGTTATCCCGTGG

TCATGGGAAGGTTGGATACGCGTTACTCACCCGTGCGCCAGTCGTCGTCAAGGGAAGCAAGCTTCCCTCACGT
TACCCTGCGACTTGCATGTGTTAAGCCTGTAGCTAGCGTTCATCCGTGAGCCAAGGATCAAACCT
>977
CTGCTGCCGCCCGTAGGAGTTTGGACCGTGTCTCAGTTCCAATGTGGCCGATCACCCTCTCAGGTCGGCTACT
GATCGTCGCCTTGGTGGGCAGTTATCTCACCAACTAGCTAATCAGACGCGGGTCCATCCTATACCGCTAACAC
TTTGATATATCTACGATGCCGTAAATATATATTATCATGTATTAGTAAACTTTTCAGTATGTTATCCATGTGT
ATAGGGCAGGTTACCCACGCGTTACTCACCCGTCCGCCGCTCATCTTTCATTAATGCAAGCATCAAATCAGAC
GCGCTCGACTTGCATGTGTTAGGCACGCCGCCAGCGTTCATCCTGAGCCAGGATCAAACTCT
>978
CTGCTGCCGCCCGTAGGAGTTTGGACCGTGCCTCAGTTCCAATGTGGGGGACCTTCCTCTCAGAACCCCTACT
GATCGTCGCCTTTGGTGGGCCGTTACCCCGCCAACAAGCTAATCAGACGCATCCCCATCCATAACCGCTAAAC
CTTTAATCGTTATTTGATGTCAGCAAACGATATCATACGGTATTAGGCCGTCTTTCAACGGGTTATCCCGTAG
TTATGGGAAGGTTGGATACGCGTTACTCACCCGTGCGCCAGTCGTCCATCAAGGAAAAGCAAGGCTTTCCCTC
GACTGATTTGA
>979
CTGCTGCCGCCCGTAGGAGTTTGACCGTGTCTCAGTTCCAATGTGGGGGACCTTCCTCTCAGAACCCCTACTG
ATCGTTGCCTTGGTGGGCCGTTACCCCGCCAACAAGCTAATCAGACGCATCCCCATCCATAACCGATAAATCT
TTATTCGTAATCTCATGAGATCAAACGAAATACATAAGGTATTAGTCCAACTTCGCTGGGTTATCCCTTAGTT
ATGGGCAGGTTGGATACGCGTTACTCACCCGTGCGCCGGTCGCCGACATAAATAGCAAGCTATCTATTCGCTG
CCCCCCCTCGACTTGCATGTGTTTAAGCCTATAGCTAGCGTTCATCCTGAGCCAGGATCAAACTCT
>980
CTGCTGCCGCCCGTAGGAGTTTGGACCGTGTCTCAGTTCCAATGTGGGGGACCTTCCTCTCAGAACCCCTACT
GATCGTCGCCTTGGTGGGCCGTTACCCCGCCAACAAGCTAATCAGACGCATCCCCATCCATAACCGCTAAACC
TTTAATCGTTATTTGATGTCAGCAAACGATATCATACGGTATTAGGCCGTCTTTCAACGGGTTATCCCGTAGT
TATGGGAAGGTTGGATACGCGTTACTCACCCGTGCGCCAGTCGTCATCAAGGAAAGCAAGCTTTCCTCATGTT
ACCCTGCGACTTGCATGTGTTAAGCACGCCGCCAGCGTTCGTCCCTGAGCCAGGATCAAACTCT
>981
CTGCTGCCGCCCGTAGGAGTTTGGACCGTGTCTCAGTTCCAATGTGGGGGACCTTCCTCTCAGAACCCCTACT
GATCGTTGCCTTGGTGGGCCGTTACCCCGCCAACAAGCTAATCAGACGCATCCCCATCCATAACCGATAAATC
TTTATTCATAATCTCATGAGATCAAACGAATACATAAGGTATTAGTCCAACTTCGCTGGGTTATCCCTAGTTA
TGGGCAGGTTGGATACGCGTTACTCACCCGTGCGCCGGTCGCCGACATAAATAGCCAAGCTATCTATTCGCTG
CCCCTCGACTTGCATGTGT
>982
CTGCTGCCGCCCGTAGGAGTTTGGACCGTGTCTCAGTTCCAATGTGGGGGACCTTCCTCTCAGAACCCCTACT
GATCGTCGCCTTTGGTGGGCCGTTACCCCGCCAACAAGCTAATCAGACGCATCCCCATCCATCAACCGCTAAA
CCTTTAATCGTTATTTGATGTCAGCAAACGATATCATACGGTATTAGGCCGTCTTTCAACGGGTTATCCCGTA
GTTATGGGAAGGTTGGATACGCGTTACTCACCCGTGCGCCAGTCGTCATCAAGGAAAGCAAGCTTTCCTCATG
TTACCCTGCGACTTGCATGTGTTAAGCCTGTAGCTAGCGTTCATCCTGAGCCAGGATCAAACTCT
>983
CTGCTGCCGCCCGTAGGAGTTGAACCGTGTCTCAGTTCCAATGTGGGGGACCTTCCTCTCAGCAACCCCTACT
GATCGTTGCCTTGGTGGGCCGTTACCCCACCCAACAAGCTAATCAGACGCATCCCCATCCATAACCGCTAAAC
CTTTAATCGTTTATTTTGAGTCAGCAAACGACGTCATACGGTATTTAGTCCGTCTTTCAACGGGTTTTATCCC
GTGGTCATGGGAAGGTTTGGATACGCGTTACTCACCCGTGCGCCAGTCGTCGTCAAGGAAAGCAAGCTTTCCT
CACGTTACCCCTGCGACTTGCATGTGTTTAAGCCTGTAGCTAGCGTCATCCTGAGCCAGGATCAAACTCT
>984
CTGCTGCCGCCCGTAGGAGTTTGACCGTGTCTCAGTTCCAATGTGGGGGACCTTCCTCTCAGAACCCCTACTG
ATCGTCGCCTTTGGTGGGCCGTTACCCCGCCAACAAGCTAATCAGACGCATCCCCATCCATAACCGCTAAACC
TTTAATCGTTATTTGATGTCAGCAAACGATGTCATACGGTATTAGGCCGTCTTTTCAACGGGTTATCCCGTGT
TAGGGAAGGTTGGATACGCGTTACTCACCCGTGCGCCAGTCGTCGTCAAGGGAAGCAAGCTTCCCTCACGTAC
CTTGTCCGACCGTAGC
>985
CTGCTGCCGCCCGTAGGAGTTTGGACCGTGTCTCAGTTCCAATGTGGGGGACCTTCCTCTCAGAACCCCTACT
GATCGTCGCCTTGGTGGGCCGTTACCCCGCCAACAAGCTAATCAGACGCATCCCCATCCATAACCGCTAAACC
TTTAATCGTTTATTTGATGTCAGCAAACGATATCATACGGTATTTAGGCCGTCTTTCGACGGGTTATCCCGTA

GTTTATGGGAAGGTTGGATACGCGTTACTCACCCGTGCGCCAGTCGTCATCAAGGAAAGCAAGCTTTCCCTCA
TGTTTACCCTGCGACTTGCATGTGTTTAAGCCTTGTAGCTAGCGTCATCCTGAGCCAGGATCAAACTCT
>986
CTGCTGCCGCCCGTAGGAGTTTGGACCGTGTCTCAGTTCCAATGTGGGGGACCTTCCTCTCAGAACCCCTACT
GATCGTCGCCTTGGTGGGCCGTTACCCCGCCAACAAGCTAATCAGACGCATCCCCATCCATAACCGCTAAACC
TTTTAATCGTTTATTTTGATGTCAGCAAACGATATCATACGGTATTTAGGCCGTCTTTTCAACGGGTTTATCC
CGTAGTTTATGGGAAGGTTGGATACGCGTTACTCACCCGTGCGCCAGTCGTCATCAAGGAAAGCAAGCTTTCC
TCATGATTTTACCCTGCGACTTGCATGTGTTTAAGCCTGTAGCTAGCGTTCATCCTGAGCCAGGATCAAACTC
T
>987
CTGCTGCCGCCCGTAGGAGTTTGGACCGTGTCTCAGTTCCAATGTGGGGGACCTTCCTCTCAGAACCCCTACT
GATCGTTGCCTTTGGTGGGCCGTTACCCCGCCAACAAGCTAATCAGACGCATCCCCATCCATAACCGATAAAT
CTTTATTCGTAATCTCATGAGATCAAACGAAATACATAAGGTATTAGTCCAACTTCGCTGGGTTATCCCTTAG
TTATGGGCAGGTTGGATACGCGTTACTCACCCGTGCGCCGGTCGCCGACATAAATAGCAAGCTATCTATTCGC
TGCCCCTCGACTTGCATGTGTTAAGCCTATAGCTAGCGTTCATCCTGAGCCAGGATCAAACTCT
>988
CTGCTGCCGCCCGTAGGAGTTTGGACCGTGTCTCAGTTCCAATGTGGGGGACCTTCCTCTCAGAACCCCTACT
GATCGTCGCCTTTGGTGGGCCGTTACCCCGCCAACAAGCTAATCAGACGCATCCCCATCCATAACCGCTAAAC
CTTTAATCGTTATTTGATGTCAGCAAACGATATCATACGGTATTAGGCCGTCTTTCAACGGGTTATCCCGTAG
TTATGGGAAGGTTGGATACGCGTTACTCACCCGTGCGCCAGTCGTCATCAAGGAAAGCAAGCTTTCCTCATGT
TACCCTGCGACTGCATGTGTTAAGCCTGTAGCTAGCGTTCATCCTGAGCCAGGATCAACTCT
>989
CTGCTGCCGCCCGTAGGAGTTTGGACCGTGTCTCAGTTCCAATGTGGGGGACCTTCCTCTCAGAACCCCTACT
GATCGTCGCCTTTGGTGGGCCGTCCTACCCCGCCAACAAGCTAATCAGACGCATCCCCCATCCATCACCGCTA
AACCTTTAATCGTTATTTGATGTCAGCAAACGATGTCATACGGTATTAGTCCGTCTTTTTCAACGGGTTATCC
CGTGGTCATGGGAAGGTTGGATACGCGTTACTCACCCGTGCGCCAGTCGTCGTCAAGGGAAGCAAGCTTTCCT
CATGTTACCCTGCGACATGCATGTGTTAAGCTGTACGCTAGCGTATCCTGAGCCAGGATCAACTCAT
>990
CTGCTGCCGCCCGTAGGAGTTTGGACCGTGTCTCGTACGTCAATGTGGGGGACCTTCCTCTCAGAACCCCTAC
TGATCGTCGCCTTGGTGGGCCGTTACCCCGCCAACAAGCTAATCAGACGCATCCCCATCCATAACCGCTAAAC
CTTTAATCGTTATTTGATGTCAGCAAACGATATCATACGGTATTAGGCCGTCTTTCAACGGGTTATCCCGTAG
TTATGGGAAGGTTGGATACGCGTTACTCACCCGTGCGCCAGTCGTCATCAAGGAAAGCAAGCTTCCTCATGTT
ACCCTGCGACTGCGACTGTAT
>991
CTGCTGCCGCCCGTAGGAGTTTGGACCGTGTCTCAGTTCCAATGTGGGGGACCTTCCTCTCAGAACCCCTACT
GATCGTCGCCTTGGTGGGCCGTTACCCCGCCAACAGGCTAATCAGACGCATCCCCATCCATAACCGCTAAACC
TTTAATCGTTATTTGATGTCAGCAAACGATATCATACGGTATTAGGCCGTCTTTCAACGGGTTATCCCGTAGT
TATGGGAAGGCTGGGATACGCGTTACTCACCCGTGCGCCAGTCGTCATCAAGGAAAGCAAGCTTTCCTCATGT
TACCCTGCGACATGCATGTGTTAAGCCTGTAGCTAGCGTTCATCCTGAGCCAGGATCAAACTCT
>992
CTGCTGCCGCCCGTAGGAGTTTGGACCGTGTCTCAGTTCCAATGTGGGGGACCTTCCTCTCAGAACCCCTACT
GATCGTCGCCTTGGTGGGCCGTTACCCCGCCAACAAGCTAATCAGACGCATCCCCATCCATCACCGCTAAACC
TTTAATCGTTATTTTTGAGTCAGCAAACGATGTCATACGGTATTAGGCCGTCTTTTCAACGGGTTATCCCGTA
GTTATGGGAAGGTTGGATACGCGTTACTCACCCGTGCGCCGGTCGCCGACATAAATAGCAAGCTATCTATTCG
CTGCCCCTCGACTTGCATGTGTTAAGCCTATAGCTAAGCGTTCATCCTGAGCCAAGGATCAAACTCCT
>993
CTGCTGCCGCCCGTAGGAGTTTGGACCGTGTCTCAGTTCCAATGTGGGGGACCTTCCTCTCAGAACCCCTACT
GATCGTCGCCTTGGTGGGCCGTTACCCCGCCAACAAGCTAATCAGACGCATCCCCATCCATCACCGCTAAACC
TTTAATCGTTATTTGATGTCAGCAAACGATATCATACGGTATTAGGCCGTCTTTCAACGGGTTATCCCGTAGT
TATGGGAAGGTTGGATACGCGTTACTCACCCGTGCGCCAGTCGTCATCAAGGAAAGCAAGCTTTCCTCATGTT
ACCCTGCGACATGCATGTGTTAAGCCTAGTAGCTGCGTTCATCCTAAGC
>994
CTGCTGCCGCCCGTAGGAGTTTGGACCGTGTCTCAGTTCCAATGTGGGGGACCTTCCTCTCAGAACCCCTACT
GATCGTCGCCTTGGTGGGCCGTTACCCCGCCAACAAGCTAATCAGACGCATCCCCATCCATAACCGCTAAACC

TTTAATCGTTATTTGATGTCAGCAAACGATATCATACGGTATTAGGCCGTCTTTCAACGGGTTATCCCGTAGT
TATGGGAAGGTTGGATACGCGTTACTCACCCGTGCGCCAGTCGTCATCAAGGAAAAGCAAGCTTTCCCTCATG
TTACCCTGCCGAACTTGGCAC
>995
CTGCTGCCGCCCGTAGGAGTTTTGGACCGTGTCTCAGTTCCAATGTGGGGGACCTTCCTCTCAGAACCCCTAC
TGATCGTCGCCTTTGGTGGGCCGTTACCCCGCCAACAAGCTAATCAGACGCATCCCCATCCATAACCGCTAAA
CCTTTAATCGTTATTTGATGTCAGCAAACGATATCATACGGTATTAGGCCGTCTTTCAACGGGTTATCCCGTA
GTTATGGGAAGGTTGGATACGCGTTACTCACCCGTGCGCCAGTCGTCATCAAGGAAAGCAAGCTTTCCCTCAT
GTTACCCTGCCGACTTGCATGTGTTAAGCCTGTAGCTAGCGTTCATCCTGAGCCAGGATCAAACTCT
>996
CTGCTGCCGCCCGTAGGAGTTTGGACCGTGTCTCAGTTCCAATGTGGGGGACCTTCCTCTCAGAACCCCTACT
GATCGTTGCCTTGGTGGGCCGTTACCCCGCCAACAAGCTAATCAGACGCATCCCCATCCATAACCGATAAATC
TTTATTCATAATCTCATGAGATCAAACGAATACATAAGGTATTAGTCCAACTTCGCTGGGTTATCCCTTAGTT
ATGGGCAGGTTGGATACGCGTTACTCACCCGTGCGCCGGTCGCCGACATAAATAGCAAGCTATCTATTCGCTG
CCCCTCGACTTGCATGTGTTAAGCCTATAGCTAGCGTTCATCCTGAGCCAGGATCAAACTCT
>997
CTGCTGCCGCCCGTAGGAGTTTGGAACCGTGTCTCAGTTCCAATGTGGGGGACCTTCCTCTCAGAACCCCTAC
TGATCGTTGCCTTGGTGGGCCGTTACCCCGCCAACAAGCTAATCAGACGCATCCCCATCCATAACCGATAAAT
CTTTATTCGTAATCTCATGAGATCAAACGAAATACATAAGGTATTAGTCCAACTTCGCTGGGTTATCCCTTAG
TTATGGGCAGGTTGGATACGCGTTACTCACCCGTGCGCCGGTCGCCGACATAAATAGCAAGCTATCTATTCGC
TGCCCCTCGACTTGCATGTGTTAAGCCTATAGCTAGCGTTCATCCTGAGCCAGGATCAAACTCT
>998
CTGCTGCCGCCCGTAGGAGTTTGGACCGTGTCTCAGTTCCAATGTGGGGGACCTTCCTCTCAGAACCCCTACT
GATCGTCGCCTTGGTGGGCCGTTACCCCGCCAACAAGCTAATCAGACGCATCCCCATCCATAACCGCTAAACC
TTTAATCGTTATTTGATGTCAGCAAACGATATCATACGGTATTAGGCCGTCTTTCAACGGGTTATCCCGTAGT
TATGGGAAGGTTGGATACGCGTTACTCACCCGTGCGCCAGTCGTCATCAAGGAAAGCAAGCTTTCCCTCATGT
TACCCTGCCGACTTGCATGTGTTAAGCTGTACGCTAGCGTTATCCCTGAGCCCAAGGATCAAACGT
>999
CTGCTGCCGCCCGTAGGAGTTTGGACCGTGTCTCAGTTCCAATGTGGGGGACCTTCCTCTCAGAACCCCTACT
GATCGTCGCCTTGGTGGGCCGTTACCCCGCCAACAAGCTAATCAGACGCATCCCCATCCATAACCGCTAAACC
TTTAATCGTTATTTGATGTCAGCAAACGATATCATACGGTATTAGGCCGTCTTTCAACGGGTTATCCCGTAGT
TATGGGAAGGTTGGATACGCGTTACTCACCCGTGCGCCAGTCGTCAGTCAAGGGGAAAGCAAGCTTTCCCCTC
ACGTTGACCTTTGTCCCAAT
>1000
CTGCTGCCGCCCGTAGGAGTTTGGACCGTGTCTCAGTTCCAATGTGGGGGACCTTCCTCTCAGAACCCCTACT
GATCGTTGCCTTTGGTGGGCCGTTACCCCGCCAACAAGCTAATCAGACGCATCCCCATCCATAACCGATAAAT
CTTTATTCATAATCTCATGAGATCAAACGAATACATAAGGTATTAGTCCAACTTTGCTGGGTTATCCCTTAGT
TATGGGCAGGTTGGATACGCGTTACTCACCCGTGCGCCGGTCGCCGACATAAATAGCAAGCTATCTATTCGCT
GCCCCTCGACCTTGCATGTGTTAAGCCTATAGCTAGCGTTCATCCTGAGCCAGGATCAAACTCT

Additional file 3_B_P7

>1
CTGCTGCCGCCCGTAGGAGTCTGGGCCGTGTCTCAGTCCCAGTGTGGCCGGTCGCCCTCTCAGGCCGGCTACC
CGTCATCGCCTTGGTGAGCCATTACCTCACCAACAAACTGATAGGCCGCGAGTCCATCCCCCACCGAAAAACT
TCCACAACACACCATGCGGCGCGAAGTCATATCCCGGTATTACGCCCCGATTTCCCGAGGTTATCCCGAAGAG
GGGGGCAGGTTACGTCACGTGTTACTCACCCGTTCGCCACTAATCAGACAAGGCAAGCCCTGTCGTCATCGTT
CGACTGCATGTGTTAAGCACGCCGCCAGCGTTCGTCCTGAGCCAGGATCAAACTCT
>2
CTGCTGCCGCCCGTAGGAGTCTGGGCCGTGTCTCAGTCCCAGTGTGGCCGGTCGCCCTCTCAGGCCGGCTACC
CGTCATCGCCTTGGTGAGCCATTACCTCACCAACAAACTGATAGGCCGCGAGTCCATCCCCCACCGAAAAACT
TCCACAACACACCATGCGGCGCGAAGTCATATCCGGTATTACGCCCCGATTTCCCGAGGTTATCCCGAAGAGG
GGGGCAGGTTACGTCACGTGTTACTCACCCGTTCGCCACTAATCAGACAAGGCAAGCCCTGTCGTCATCGTTC
GACTGCATGTGTTAAGCACGCCGCCAGCGTTCGTCCTGAGCCAGGATCAAACTCT
>3
CTGCTGCCGCCCGTAGGAGTCTGGGCCGTGTCTCAGTCCCAGTGTGGCCGATCACCCTCTCAGGTCGGCTATG
TATCGTCGCCTTGGTGAGCCGTTACCCCACCAACTAGCTAATACAACGCAGGTCCATCTGGTAGTGGTGCAAT
TGCACCTTTTAAGCAAATGTCATGCAACATCTACTCTTATGCGGTATTAGCTATCGTTTCCAATAGTTATCCC
CCGCTACCAGGCAGGTTACCTACGCGTACTCACCCGTTCGCAACTCATCCGGAGAAGCAAGCTCCTCCTTCAG
CGTTCTACTTGCATGTATTAGGCACGCCGCCAGCGTTCGTCCTGAGCCAGGATCAAACTCT
>4
CTGCTGCCGCCCGTAGGAGTCTGGGCCGTGTCTCAGTCCCAGTGTGGCCGATCACCCTCTCAGGTCGGCTATG
TATCGTCGCCTTGGTGAGCCCTTACCCCACCAACTAGCTAATACAACGCAGGTCCATCTGGTAGTGATGCAAT
TGCACCTTTCAAACAGTTGTCATGCAACAACTGCTATTATGCGGTATTTAGCTATCGTTTTCCAATAGTTATC
CCCCGCTACCAGGCAGGTTACCTACGCGTACTCACCCGTTCGCAACTCATCCAGAGAAGCAAGCTCCTCCTTC
AGCGTTCTACTTGCATGTATTAGGCACGCCGCCAGCGTTCGTCCTGAGCCAGGATCAAACTCT
>5
CTGCTGCCGCCCGTAGGAGTCTGGGCCGTGTCTCAGTCCCAGTGTGGCCGATCACCCTCTCAGGTCGGCTATG
TATCGTCGCCTTGGTGAGCCCTTACCCCACCAACTAGCTAATACAACGCAGGTCCATCTGGTAGTGATGCAAT
TGCACCTTTCAAACAGTTGTCATGCAACAACTGCTATTATGCGGTATTAGCTATCGTTTCCAATAGTTATCCC
CCGCTACCAGGCAGGTTACCTACGCGTACTCACCCGTTCGCAACTCATCCGGAGAAGCAAGCTCCTCCTTCAG
CGTTCTACTTGCATGTATTAGGCATGCCGCCAGCGTTCGTCCTGAGCCAGGATCAAACTCT
>6
CTGCTGCCGCCCGTAGGAGTCTGGGCCGTGTCTCAGTCCCAGTGTGGCCGATCACCCTCTCAGGTCGGCTATG
TATCGTCGCCTTGGTGAGCCGTTACCCCACCAACTAGCTAATACAACGCAGGTCCATCTGGTAGTGATGCAAT
TGCACCTTTTAAGTAAATGTCATGCAACATCTACTCTTATGCGGTATTAGCTATCGTTTCCAATAGTTATCCC
CCGCTACCAGGCAGGTTACCTACGCGTTACTCACCCGTTCGCAACTCATCCAGAAGAGCAAGCTCCTCCTTCA
GCGTTCTACTTGCATGTATTAGGCACGCCGCCAGCGTTCGTCCTGAGCCAGGATCAAACTCT
>7
CTGCTGCCGCCCGTAGGAGTCTGGGCCGTGTCTCAGTCCCAGTGTGGCCGATCACCCTCTCAGGTCGGCTATG
TATCGTCGCCTTGGTGAGCCGTTACCCCACCAACTAGCTAATACAACGCAGGTCCATCTGGTAGTGGTGCAAT
TGCACCTTTTAAGCAAATGTCATGCAACATCTACTCTTATGCGGTATTAGCTATCGTTTCCAATAGTTATCCC
CCGCTACCAGGCAGGTTACCTACGCGTTACTCACCCGTTCGCAACTCATCCAGAGAAGCAAGCTCCTCCTTCA
GCGTTCTACTTGACATGTATTACGGCAGTCGCCAGCCGTTGTC
>8
CTGCTGCCGCCCGTAGGAGTCTGGGCCGTGTCTCAGTCCCAGTGTGGCCGATCACCCTCTCAGGTCGGCTATG
TATCGTCGCCTTGGTGAGCCGTTACCCCCACCAACTAGCTAATACAACGCAGGTCCATCTGGTAGTGGTGCAA
TCGCACCTTTTAAGCAAATGTCATGCAACATCTACTCTTATGCGGTATTAGCTATCGTTTTCCAATAGTTATC
CCCCCGCTACCAGGCAGGTTACCTACGCGTTACTCACCCGTTCGCAACTCATCCGGAGAAGCAAGCTCCTCCT
TCAGCGTTCTACTTGCATGTATTTAGGCACGCCGCCAGCGTTCGTCCTGAGCCAGGATCAAACTCT
>9
CTGCTGCCGCCCGTAGGAGTCTGGGCCGTGTCTCAGTCCCAGTGTGGCCGATCACCCTCTCAGGTCGGCTATG
TATCGTCGCCTTGGTGAGCCCTTACCCCACCAACTAGCTAATACAACGCAGGTCCATCTGGTAGTGATGCAAT
TGCACCTTTCAAACAGTTGTCATGCAACAACTGCTATTATGCGGTATTAGCTATCGTTTCCAATAGTTATCCC

CCGCTACCAGGCAGGTTACCTACGCGTTACTCACCCGTTCGCAACTCATCCAGAGAAGCAAGCTCCTCCTTCA
GCGTTCTACTTGCATGTATTAGGCACGCCGCCAGCGTTCGTCCTGAGCCAGGATCAAACTCT
>10
CTGCGCCGCCCGTAGGAGTCTGGGCCGTGTCTCAGTCCCAGTGTGGCCGATCACCCTCTCAGGTCGGCTATGT
ATCGTCGCCTTGGTCGAGCCCTTACCCCACCAACTAGCTAATACAACGCAGGTCCATCTGGTAGTGATGCAAT
TGCACCTTTCAACAGTTGTCATGCAACAACTGCTACTTATGCGGTATTACGCTATCGTTTCCAATAGTTATCC
CCCGCCTACCAGGCAGGTTACCTACGCGTTACTCACCCGTTTCGCAACTCATCCAGAGAAGCAAGCTCCCTCC
TTTCAGCGTTCTACTTGCATGTATTTAGGCACGCCGCCAGCGTCGTCCTGAGCCAGGATCAAACTCT
>11
CTGCTGCCGCCCGTAGGAGTCTGGGCCGTGTCTCAGTCCCAGTGTGGCCGATCACCCTCTCAGGTCGGCTATG
TATCGTCGCCTTGGTGAGCCCTTACCCCACCAACTAGCTAATACAACGCAGGTCCATCTGGTAGTGATGCAAT
TGCACCTTTCAAACAGTTGTCATGCAACAACTGCTATTATGCGGTATTAGCTATCGTTTCCAATAGTTATCCC
CCGCTACCAGGCAGGTTACCTACGCGTTACTCACCCGTTCGCAACTCATCCAGAGAAGCAAGCTCCTCCTTCA
GCGTTCTACTTGCCATGTATTAGGCACGCCGCCAGCGTTCGTCCTGAGCCAGGATCAAACTCT
>12
CTGCTGCCGCCCGTAGGAGTCTGGGCCGTGTCTCAGTCCCAGTGTGGCCGATCACCCTCTCAGGTCGGCTATG
TATCGTCGCCTTGGTGAGCCGTTACCCCACCAACTAGCTAGTACAACGCAGGTCCATCTGGTAGTGGTGCAAT
TGCACCTTTTAAGCAAATGTCATGCAACATCTACTCTTATGCGGTATTAGCTATCGTTTCCAATAGTTATCCC
CCGCTACCAGGCAGGTTACCTACGCGTTACTCACCCGTTCGCAACTCATCCGGAGAAGCAAGCTCCTCCTTCA
GCGTTCTACTTGCATGTATTAGGCACGCCGCCAGCGTTCGTCCTGAGCCAGGATCAAACTCT
>13
CTGCTGCCGCCCGTAGGAGTCTGGGCCGTGTCTCAGTCCCAGTGTGGCCGATCACCCTCTCAGGTCGGCTATG
TATCGTCGCCTTGGTGAGCCGTTACCCCACCAACTAGCTAATACAACGCAGGTCCATCTGGTAGTGGTGCAAT
TGCACCTTTTAAGCAAATGTCATGCAACATCTACTCTTATGCGGTATTAGCTATCGTTTCCAATAGTTATCCC
CCGCACCAGGCAGGTACCTACGCGTTACTCACCCGTTCGCAACTCATCCGGAGAAGCAAGCTCCTCCTTCAGC
GTTCTACTTGCATGTATTAGGCACGCCGCCAGCGTTCGTCCTGAGCCAGGATCAAACTCT
>14
CTGCTGCCGCCCGTAGGAGTCTGGGCCGTGTCTCAGTCCCAGTGTGGCCGATCACCCTCTCAGGTCGGCTATG
TATCGTCGCCTTGGTGAGCCGTTACCCCACCAACTAGCTAATACAACGCAGGTCCATCTGGTAGTGGTGCAAT
TGCACCTTTTAAGCAAATGTCATGCAACATCTACTCTTTATGCGGTATTTAGCTATTCGTTTTTCCAATAGTT
TATCCCCCCCGCTACCAGGCAGGTTACCTTACGCGTTACTCACCCGTTTCGCAACTCATCCGGAGAAGCAAGC
TCCTCCTTCAGCGTT
>15
CTGCTGCCGCCCGTAGGAGTCTGGACCGTGTCTCAGTTCCAGTGTGGCTGATCACCCTCTCAGGTCGGCTACG
CATCGTTGCCTTGGTAAGCCGTTACCTTACCAACTAGCTAATGCGGCGCGGATCCATCTATAAGTGACAGCAA
AACCGTCTTTCACTATGAACCATGCGGTTCAATATATTATCCGGTATTAGCTCCGGTTTCCCGAAGTTATCCC
AGTCTTATAGGTAGGTTATCCACGTGTTACTCACCCGTCCGCCGCTAACGTCAAAGGAGCAAGCTCCTTATCT
GTTCGCTCGACTGCATGTATTAGGCACGCCGCCAGCGTCATCCTGAGCCAGGATCAAACTCT
>16
CTGCTGCCGCCCGTAGGAGTCTGGGCCGTGTCTCAGTCCCAGTGTGGCCGATCACCCTCTCAGGTCGGCTATG
TATCGTCGCCTTGGTGAGCCCTTACCCCCCACCAACTAGCTAATACAACGCAGGTCCATCTGGTAGTGATGCA
ATTGCACCTTTTCAAACAGTTTGTCATGCAACAACTGCTATTTATGCGGTTATTTAGCTATCGTTTTTCCAAT
AGTTTATCCCCCCCGCTACCAGGCAGGTTTACCTTACGCGTTACTCACCCCCGTTTCGAACTCATCCAGAGAA
GCAAGCTCCTCCTTTCAGCGTCTACTTGCATTGTATTTAGGCACGCCGCCAGCGTCGTCCTGAGCCAGGATCA
AACTCT
>17
CTGCTGCCGCCCGTAGGAGTCTGGGCCGTGTCTCAGTCCCAGTGTGGCCGATCACCCTCTCAGGTCGGCTATG
TATCGTCGCCTTGGTGAGCCGTTACCTCACCAACTAGCTAATACAACGCAGGTCCATCTGGTAGTGATGCAAC
TGCACCTTTTAATTGACTATCATGCAATAATCAATTTTATGCGGTATTAGCCTATCGTTTTCCAATAGTTACT
CCCCCGCTACCAGGCAGGTTACCTACGCGTTACTCACCCGTTCGCAACTCATCCAGAGAAGCAAGCTCCTCCT
TCAGCGTTCTACTTGCATGTATTAGGCACGCCGCCAGCGTTCGTCCTGAGCCAGGATCAAACTCT
>18
CTGCTGCCGCCCGTAGGAGTCTGGGCCGTGTCTCAGTCCCAGTGTGGCCCGATCACCCTCTCAGGTCGGCTAT
GTATCGTCGCCTTGGGTGAGCCCTTACCCCCACCAACTAGCTAATACAACGCAGGTCCATCTGGTAGTGATGC

AATTGCACCTTTCAAACAGTTGTCATGCAACAACTGCTATTTATGCGGTATTTAGCTATCGTTTTCCAATAGT
TTATCCCCCCCGCTACCAGGCAGGTTACCTTACGCGTTACTCACCCCCGTTCGAACTCATCCGGAGAAGCAAG
CTCCTCCTCAGCGTTCTACTTGCATGTATTTAGGCACGCCGCCAGCGTTCGTCCTGAGCCCCAGGATCAAACT
CTTATCACCGACTGCCCCCCCATAGAGAGGCTGAGACTGCCAAGGCACACAGGGGATAGG
>19
CTGCTGCCGCCCGTAGGAGTCTGGGCCGTGTCTCAGTCCCAGTGTGGCCGATCACCCTCTCGGGTCGGCTATG
CATCGTCGCCTTGGTAGGCCTTTACCCCACCAACTAGCTAATGCACCGCAAAGCCATCTCATAGTGTTAGCAA
AACCAACTTTTAAACATCAACCATGCAGTTAATGCTGTTTATCCGGTATTTAGCTATCGTTTTCCAATAGTTT
GTCCCAGTCTATGAGGCAGGTTTCCTTTACGTGTTACTCACCCATTCGCCGCTAAGTTTTCGTCGCAGCACCA
AAACTAAACGTCTCGACTGCA
>20
CTGCTGCCGCCCGTAGGAGTCTGGACCGTGTCTCAGTTCCAGTGTGGCCGATCACCCTCTCAGGTCGGCTACG
TATCGTTGCCTTGGTAAGCCGTTACCTTACCAACTAGCTAATACGGCGCAGGTCCATCTATAAGTGATAGCAA
AGCCATCTTTCACTATCGAACCATGCGGTTTCGAAATATTTATCCGGTATTAGCTCCGGTTTTTCCCGAAGTT
ATCCCAGTCTTATAGGTAGGTTTTACCTACGTGTTTACTCACCCGTCCGCCTCTAACGTCAAAGGAGCAAGCT
CCTCGTCTGTTCGCTCGACTTGCATGTATTAGGCACGCCGCCAGCGTTCATCCTGAGCCAGGATCAAACTCT
>21
CTGCTGCCGCCCGTAGGAGTCTGGGCCCGTGTCTCAGTCCCAGTGTGGCCGATCACCCTCTCAGGTCGGCTAT
GCATCGTCGCCTTGGTAGGCCCTTTTACCCCCCACCCAACTAGCTAATGCACCGCAAAGCCCATCTCATAGTG
TTAGCAAAACCAACTTTTAAACATCAACCATGCAGTTAATTGCTGTTTATCCGGTTATTTAGCTTATCGTTTC
CCCAATTAGTTTGTCCCCCAGTCTTATGAGGCAGGTTTCTTTACGTGTTACTCACCCCCGTTCGCCGCTAAGT
TTTGTCGTGACAGC
>22
CTGCTGCCGCCCGTAGGAGTCTGGGCCGTGTCTCAGTCCCAGTGTGGCCGATCACCCTCTCAGGTCGGCTATG
TATCGTCGCCTTGGTGAGCCCTTACCCCACCAACTAGCTAATACAACGCAGGTCCATCTGGTAGTGATGCAAT
TGCACCTTTCAACAGTTGTCATGCAACAACTGCTATTATGCGGTATTAGCTATCGTTTCCAATAGTTATCCCC
CGCTACCAGGCAGGTTACCTACGCGTACTCACCCGTTCGCAACTCATCCAGAGAAGCAAGCTCCTCCTTCAGC
GTTCTACTTGCATGTATTAGGCACGCCGCCAGCGTTCGTCCTGAGCCAGGATCAAACTCT
>23
CTGCTGCCGCCCGTAGGAGTCTGGGCCGTGTCTCAGTCCCAGTGTGGCCGATCACCCTCTCAGGTCGGCTATG
TATCGTCGCCTTTGGTGAGCCCTTACCCCACCAACTAGCTAATACAACGCAGGTCCATCTGGTAGTGATGCAA
TTGCACCTTTCAAACAGTTTGTCATGCAACAACTGCTATTATGCGGTATTAGCTATCGTTTCCAATAGTTATC
CCCCGCTACCAGGCAGGTTACCTACGCGTACTCACCCGTTCGCAACTCATCCAGAGAAGCAAGCCCCTCCTTC
AGCGTCTACTTCGCATGTATTAGGCACGCCGCCAGCGTTCGTCCTGAGCCAGGATCAAACTCT
>24
CTGCTGCCGCCCGTAGGAGTCTGGGCCGTGTCTCAGTCCCAGTGTGGCCGATCACCCTCTCAGGTCGGCTATG
TATCGTCGCCTTGGTGAGCCCTTACCCCACCAACTAGCTAATACAACGCAGGTCCATCTGGTAGTGATGCAAT
TGCACCTTTCAAACAGTTGTCATGCAACAACTGCTATTATGCGGTATTAGCTATCGTTTCCAATAGTTATCCC
CCGCTACCAGGCAGGTTACCTACGCGTACTCACCCGTTCGCAACTCATCCAGAGAAGCAAGCTCCTCCTTCAG
CGTTCTACTTGCATGTATTAGGCACGCCGCCAGCGTTCGTCCTGAGCCAGGATCAAACTCT
>25
CTGCTGCCGCCCGTAGGAGTCTGGGCCGTGTCTCAGTCCCAGTGTGGCCGATCACCCTCTCAGGTCGGCTATG
TATCGTCGCCTTGGTGAGCCCTTACCCCACCAACTAGCTAATACAACGCAGGTCCATCTGGTAGTGATGACAT
TGCACCTTTCAAACAGTTGTCATGCAACAACTGCTATTATGCGGTATTAGCTATCGTTTCCAATAGTTATCCC
CCGCTACCAGGCAGGTTACCTACGCGTTACTCACCCGTTCGCAACTCATCCAGAGAAGCAAGCTCCTCCTTCA
GCGTTCTACTTGCATGTATTAGGCACGCCGCCAGCGTTCGTCCTGAGCCAGGATCAACTCT
>26
CTGCTGCCGCCCGTAGGAGTCTGGGCCGTGTCTCAGTCCCAGTGTGGCCGATCACCCTCTCAGGTCGGCTATG
CATCGTCGCCTTGGTAGGCCTTTACCCCACCAACTAGCTAATGCACCGCAAAGCCATCTCATAGTGTTAGCAT
AACCAACTTCAAACATCAACCATGCAGTTAATGCTGTTATCCGGTATTAGCTATCGTTTCCAATAGTTGTCCC
AGTCTATAAGGCAGGTTCTTTACGTGTTACTCACCCGTTCGCCGCTAGTTTCGTCGACAGCACCAAACTAACG
TCTCGACTGACTTGCTGATAGCAGC
>27

CTGCTGCCGCCCGTAGGAGTCTGGGCCGTGTCTCAGTCCCAGTGTGGCCGATCACCCTCTCAGGTCGGCTATG
TATCGTCGCCTTGGTGAGCCCTTACCCCACCAACTAGCTAATACAACGCAGGTCCATCTGGTAGTGACGCAAT
TGCACCTTTCAAACAGTTGTCATGCAACAACTGCTTTATGCGGTATTAGCTATCGTTTCCAATAGTTATCCCC
CGCTACCAGGCAGGTTACCTACGCGTTACTCACCCGTTCGCAACTCATCCAGAGAAGCAAGCTCCTCCTTCAG
CGTTCTACTTGCATGTATTAGGCACGCCGCCAGCGTTCGTCCTGAGCCAGGATCAAACTCT
>28
CTGCTGCCGCCCGTAGGAGTCTGGGCCGTGTCTCAGTCCCAGTGTGGCCGATCACCCTCTCAGGTCGGCTACG
TATCGTCGCCTTGGTGAGCCCTTACCCCACCAACTAGCTAATACAACGCAGGTCCATCTGGTAGTGATGCAAT
TGCACCTTTCAAACAGTTGTCATGACAACAACTGCTATTATGCGGTATTAGCTATCGTTTCCAATAGTTATCC
CCCGCTACCAGGCGGGTTACCTACGCGTTACTCACCCGTTTCGCAACTCATCCAGAGAAGCAAGCTCCTCCTT
CAGCGTTCTACTTGCATGTATTTAGGCACGCCGCCAGCGTTCGTCCTGAGCCAGGATCAAACTCT
>29
CTGCTGCCGCCCGTAGGAGTCTGGGCCGTGTCTCAGTCCCAGTGTGGCCGATCACCCTCTCAGGTCGGCTATG
TATCGTCGCCTTGGTGAGCCCTTACCCCACCAACTAGCTAATACAACGCAGGTCCATCTGGTAGTGATGCAAT
TGCACCTTTCAAACAGTTGTCATGCAACAACTGCTATTATGCGGTATTAGCTATCGTTTCCAATAGTTACTCC
CCCGCTACCAGGCAGGTTACCTACGCGTTACTCACCCGTTCGCAACTCATCCAGAGAAGCAAGCTCCTCCTTC
AGCGTTCTACTTGCATGTATTAGGCACGCCGCCAGCGTTCGTCCTGAGCCAGGATCAAACTCT
>30
CTGCTGCCGCCCGTAGGAGTCTGGGCCGTGTCTCAGTCCCAGTGTGGCCGATCACCCTCTCAGGTCGGCTATG
TATCGTCGCCTTGGTGAGCCCTTACCCCACCAACTAGCTAATACAACGCAGGTCCATCTGGTAGTGATGCAAT
TGCACCTTTCAAACAGTTGTCATGCAACAACTGCTATTATGCGGTATTAGCTATCGTTTCCAATAGTTATCCC
CCGCTACCAGGCAGGTTACCTACGCGTACTCACCCGTTCGCAACTCATCCGGAGAAGCAAGCTCCTCCTTCAG
CGTTCTACTTGCATGTATTAGGCACGCCGCCAGCGTTCGTCCTGAGCCAGGATCAAACTCT
>31
CTGCTGCCGCCCGTAGGAGTCTGGGCCGTGTCTCAGTCCCAGTGTGGCCGATCACCCTCTCAGGTCGGCTATG
TATCGTCGCCTTGGCGAGCCGTTACCTCACCAACTAGCTAATACAACGCAGGTCCATCTGGTAGTGATGCAAT
TGCACCTTTCAAACAGTTGTCATGACAACACTGCTATTATGCGGTATTAGCTATCGTTTCCAAATACGTTATC
CCCCGCTACCGGGCAGGTTACCTACGCGTTACTCACCCGTTCGCAACTCATCCAGAGACGCAAGCTCCTCCTT
CAGCGTTCTACTGCATGTATTACGGCACGCCGCCAGCGTTCGTCCCGAGCCAGGATCAAACTCT
>32
CTGCTGCCGCCCGTAGGAGTCTGGACCGTGTCTCAGTTCCAGTGTGGCCGATCACCCTCTCAGGTCGGCTACG
TATCGTTGCCTTGGTAAGCCGTTACCTTACCAACTAGCTAATACGGCGCAGGTCCATCTATAAGTGATAGCAA
AGCCATCTTTCACTATCGAACCATGCGGTTCGAAATAATTATCCGGTATTAGCTCCGGTTTCCCGAAGTATCC
CAGTCTTATAGGTAGGTTACCTACGTGTTACTCACCCGTCCGCCGCTAACGTCAAAGGCAGCAAGCTCCTCGT
CTGTTCGCTCGACTT
>33
CTGCTGCCGCCCGTAGGAGTCTGGGCCGTGTCTCAGTCCCAGTGTGGCCGATCACCCTCTCAGGTCGGCTATG
TATCGTCGCCTTGGTGAGCCCTTACCCCACCAACTAGCTAATACAACGCAGGTCCATCTGGTAGTGATGCAAT
CGCACCTTTCAAACAGTTGTCATGCAACAACTGCTATTATGCGGTATTAGCTATCGTTTCCAATAGTTATCCC
CCGCTACCAGGCAGGTTACCTACGCGTTACTCACCCGTTCGCAACTCATCCAGAGAAGCAAGCTCCTCCTTCA
GCGTTCTACTTGCATGTATTAGGCACGCCGCCAGCGTCGTCCTGAGCCAGGATCAAACTCT
>34
CTGCTGCCGCCCGTAGGAGTCTGGGCCGTGTCTCAGTCCCAGTGTGGCCGATCACCCTCTCAGGTCGGCTATG
TATCGTCGCCTTGGTGAGCCGTTACCCCACCAACTAGCTAATACAACGCAGGTCCATCTGGTAGTGGTGCAAT
TGCACCTTTTAAGCAAATGTCATGCAACATCTACTCTTATGCGGTATTAGCTATCGTTTCCAATAGTTATCCC
CCGCTACCAGGCAGGTTACCTACGCGTTACTCACCCGTTCGCAACTCATCCGGAGAAGCAAGCTCCTCCTCAG
CGTTCTACTTGCATGTATTAGGCACGCCGCCAGCGTTCGTCCTGAGCCAGGATCAAACTCT
>35
CTGCTGCCGCCCGTAGGAGTCTGGGCCGTGTCTCAGTCCCAGTGTGGCCGATCACCCTCTCAGGTCGGCTATG
TATCGTCGCCTTGGTGAGCCGTTACCCCACCAACTAGCTAATACAACGCAGGTCCATCTGGTAGTGGTGCAAT
TGCACCTTTTAAGCAAATGTCATGCAACATCTACTCTTATGCGGTATTAGCTATCGTTTTCCAATAGTTTATC
CCCCGCTACCAGGCAGGTTACCTACGCGTTACTCACCCGTTCGCAACTCATCCGGAGAAGCAAGCTCCTCCTC
AGCGTTCTACTGCATGTATTTAGGCACGCCGCCAGCGTTCGTCCTGAGCCAGGATCAAACTCT
>36

CTGCTGCCGCCCGTAGGAGTCTGGGCCGTGTCTCAGTCCCAGTGTGGCCGATCACCCTCTCAGGTCGGCTATG
TATCGTCGCCTTGGTGAGCCGTTACCTCACCAACTAGCTAATACAACGCAGGTCCATCTGGTAGTGATGCAAT
TTGCACCTTTCAAACAGTTGTCATGCAACAACTGCTATTTATGCGGTATTAGCTATCGTTTTCCAATAGTTTA
TCCCCCGCTACCAGGCAGGTTACCTACGCGTTACTCACCCGTTTCGCAACTCATCCGGAGAAGCAAGCTCCTC
CTTCAGCGTTCTACTTGCATGTATTTAGGCACGCCGCCAGCGTTCGTCCTGAGCCAGGATCAAACTCT
>37
CTGCTGCCGCCCGTAGGAGTCTGGGCCGTGTCTCAGTCCCAGTGTGGCCGATCACCCTCTCAGGTCGGCTATG
TATCGTCGCCTTGGTGAGCCCTTACCCCACCAACTAGCTAATACAACGCAGGTCCATCTGGTAGTGATGCAAT
TGCACCTTTCAAACAGTTGTCATGCAACAACTGCTATTATGCGGTATTAGCTATCGTTTCCAATAGTTATCCC
CCGCTACCAGGCAGGTTACCTACGCGTTACTCACCCGTTCGCAACTCATCCAGAGAAGCAAGCTCCTCCTTCA
GCGTTCTACTGCATGTATTAGGCACGCCGCCAGCGTCGTCCTGAGCCAGGATCAACTCT
>38
CTGCTGCCGCCCGTAGGAGTCTGGGCCGTGTCTCAGTCCCAGTGTGGCCGATCACCCTCTCAGGTCGGCTATG
CATCGTCGCCTTGGTAGGCTTTTACCCCACCAACTAGCTAATGCACCGCAAAGCCATCTCATAGTGTTAGCAT
AACCAACTTTCAAACATCAACCATGCAGTTAATGCTGTTATCCGGTATTAGCTATCGTTTCCAATACGTTGTC
CCAGTCTATGAGGCAGGTTCTTTACGTGTTACTCACCCGTTCGCCGCTAAGTTTCCTCAGCTGCAAGCACCAG
AAAACTTCGACTCGACTTGCCAGTAGTATAGGCACGGCCGATCCAGCGTCCGTC
>39
CTGCTGCCGCCCGTAGGAGTCTGGGCCGTGTCTCAGTCCCAGTGTGGCCGATCACCCTCTCAGGTCGGCTATG
TATCGTCGCCTTGGTGAGCCCTTACCCCACCAACTAGCTAATACAACGCAGGTCCATCTGGTAGTGATGCAAT
TGCACCTTTCAAACAGTTGTCATGCAACAACTGCTATTATGCGGTATTAGCTATCGTTTCCAATAGTTATCCC
CCGCTACCAGGCAGGTTACCTACGCGTACTCACCCGTTCGCAACTCATCCAGAGAAGCAAGCTCCTCCTTCAG
CGTTCTACTTGCATGTATTAGGCACGCCGCCAGCGTTCGTCCTGAGCCAGGATCAAACTCT
>40
CTGCTGCCGCCCGTAGGAGTCTGGGCCGTGTCTCAGTCCCAGTGTGGCCGATCACCCTCTCAGGTCGGCTATG
TATCGTCGCCTTGGTGAGCCGTTACCCCACCAACTAGCTAATACAACGCAGGTCCATCTGGTAGTGGTGCAAT
TGCACCTTTTAAGCAAATGTCATGCAACATCTACTCTTATGCGGTATTAGCTATCGTTTCCAATAGTTACCCC
CCGCTACCAGGCAGGTTACCTACGCGTACTCACCCGTTCGCAACTCATCCGGAGAAGCAAGCTCCTCCTTCAG
CGTTCTACTTGCATGTATTAGGCACGCCGCCAGCGTTCGTCCTGAGCCAGGATCAAACTCT
>41
GCCCGTAGGAGTCTGGACCGTGTCTCAGTTCCAGTGTGGCCGATCACCCTCTCAGGTCGGCTACGTATCGTTG
CCTTGGTAAGCCGTTACCTTACCAACTAGCTAATACGGCGCAGGTCCATCTATAAGTGATAGCAAAGCCATCT
TTCACTATCGAACCATGCGGTTCGAAATATTATCCGGTATTAGCTCCGGTTTCCCGAAGTTATCCCAGTCTTA
TAGGTAGGTTACCTACGTGTTACTCACCCGTCCGCCGCTAACGTCAAAGGAGCAAGCTCCTCGTCTGTTCGCT
CGACTTGCATGTATTAGGCACGCCGCCAGCGTTCATCCTGAGCCAGGATCAAACTCT
>42
CTGCTGCCGCCCGTAGGAGTCTGGGCCGTGTCCCAGTCCCAGTGTGGCCGATCACCCTCTCAGGTCGGCTATG
TATCGTCGCCTTGGTGAGCCCTTACCCCACCAACTAGCTAATACAACGCAGGTCCATCTGGTAGTGATGCAAT
TGCACCTTTCAAACAGTTGTCATGCAACAACTGCTATTTATGCGGTGTTAGCTTATTCGTTTTCCAATAGTTT
ATCCCCCGCTACCAGGCAGGTTTACCTACGCGTTACTCACCCGTTTCGCAACTCATCCGGAGAAGCAAGCTCC
TCCTTCAGCGTTCTACTTGCATGTTATTTAGGCACGCCGCCAGCGTTCGTCCTGAGCCAGGATCAAACTCT
>43
CTGCTGCCGCCGTAGGAGTTTTGGGCCGTGTCTCAGTCCCAATGTGGCCGATCACCCTCTCAGGTCGGCTATG
CATCGTTGCCTTGGTGAGCCGTTACCTCACCAACTAGCTAATGCACCGCGGGTCCATCCACCAGTGACGCAAA
AGCGCCTTTCAAATAGAAACCATGCGGTTTTTCTATTTTGTTTTATACGGTATTTACGCACCTGTTTTTCCAA
GTGTTTATCCCCTTTCTGATGGGCAGGTTTACCCACGTGTTACTCACCCGTTTCGCCACTCCTTTCTTTCGGT
GGAGCAAGCTCCGGTGAAAAAAAAAGCGTCGACTTGCATGTATTTAGGCACGCCGCCAGCGTTCGCCTGAGCC
AGGATCAAACTCT
>44
CTGCTGCCGCCCGTAGGAGTCCGGGCCGTGTCTCAGTCCCAGTGTGGCCGATCACCCTCTCAGGTCGGCTATG
TATCGTCGCCTTGGTGAGCCGTTACCCCACCAACTAGCTAATACAACGCAGGTCCATCTGGTAGTGGTGCAAT
TGCACCTTTTAAGCAAATGTCATGCAACATCTACTCTTATGCGGTATTAGCTATCGTTTTCCAATAGTTTATC
CCCCCGCTACCAGGCAGGTTACCTACGCGTTACTCACCCGTTCGCAACTCATCCGGAGAAGCAAGCTCCTCCT
CAGCGTTCTACTTGCATGTATTTAGGCACGCCGCCAGCGTTCGTCCTGAGCCAGGATCAAACTCT

>45
CTGCTGCCGCCCGTAGGAGTCTGGGCCGTGTCTCAGTCCCAGTGTGGCCGATCACCCTCTCAGGTCGGCTATG
TATCGTCGCCTCGGTGAGCCGTTACCTCACCAACTAGCTAATACAACGCAGGTCCATCTGGTAGTGATGCAAT
TGCACCTTTTAATCGACTATCATGCAATAATCAATTTTATGCGGTATTAGCCTATCGTTTCCAATAGTTACTC
CCCCGCTACCAGGCAGGTTACCTACGCGTTACTCACCCGTTCGCAACTCATCCAGAGAAGCAAGCTCCTCCTT
CAGCGTTCTACTTGCATGTATTAGGCACGCCGCCAGCGTTCGTCCTGAGCCAGGATCAAACTCT
>46
CTGCTGCCGCCCGTAGGAGTCTGGACCGTGTCTCAGTTCCAGTGTGGCCGATCACCCTCTCAGGTCGGCTACG
TATCGTTGCCTTGGTAAGCCGTTACCTTACCAACTAGCTAATACGGCGCAGGTCCATCTATAAGTGATAGCAA
AGCCATCTTTCACTATCGAACCATGCGGTTCGAAATATTATCCGGTATTAGCTCCGGTTTCCCGAAGTTATCC
CAGTCTTATAGGTAGGTTACCTACGTGTTACTCACCCGTCCGCCGCTAACGTCGAAGGAGCAAGCTCCTCGTC
TGTTCGCTCGACTTGCATGTATTAGGCACGCCGCCAGCGTTCATCCTGAGCCAGGATCAAACTCT
>47
CTGCTGCCGCCCGTAGGAGTCTGGGCCGTGTCTCAGTCCCAGTGTGGCCGATCACCCTCTCAGGTCGGCTATG
TATCGTCGCCTTGGTGAGCCGTTACCTCACCAACTAGCTAATACAACGCAGGTCCATCTGGTAGTGATGCAAT
TGCACCTTTTAATTGACTATCATGCAATAATCAATTTTATGCGGTATTAGCCTATCGTTTCCAATAGTTACTC
CCCCGCTACCAGGCAGGTTACCTACGCGTTACTCACCCGTTCGCAACTCATCCAGAGAAGCAAGCTCCTCCTT
CAGCGTTCTACTTGCATGTATTAGGCACGCCGCCAGCGTTCGTCCTGAGCCAGGATCAAACTCT
>48
CTGCTGCCGCCCGTAGGAGTCTGGGCCGTGTCTCAGTCCCAGTGTGGCCGATCACCCTCTCAGGTCGGCTATG
TATCGTCGCCTTGGTGAGCCGTTACCTCACCAACTAGCTAATACAACGCAGGTCCATCTGGTAGTGATGCAAT
TTGCACCTTTTAATTGACTATCATGCAATAATCAATTTTATGCGGTATTAGCCTATCGTTTCCAATAGTTACT
CCCCCGCTACCAGGCAGGTTACCTACGCGTTACTCACCCGTTCGCAACTCATCCAGAGAAGCAAGCTCCTCCT
TCAGCGTTCTACTTGCATGTATTAGGCACGCCGCCAGCGTTCGTCCTGAGCCAGGATCAAACTCT
>49
CTGCTGCCGCCCGTAGGAGTCTGGGCCGTGTCTCAGTCCCAGTGTGGCCGATCACCCTCTCAGGTCGGCTATG
TATCGTCGCCTTGGTGAGCCCTTACCCCACCAACTAGCTAATACAACGCAGGTCCATCTGGTAGTGATGCAAT
TGCACCTTTCAAACAGTTGTCATGCAACAACTGCTATTATGCGGTATTAGCTATCGTTTCCAATAGTTATCCC
CCGCTACCAGGCAGGTTACCTACGCGTTACTCACCCGTTCGCAACTCATCCAGAGAAGCAAGCTCCTCCTCAG
CGTTCTACTTGCATGTATTAGGCACGCCGCCAGCGTCGTCCTGAGCCAGGATCAACTCT
>50
CTGCTGCCGCCCGTAGGAGTCTGGGCCGTGTCTCAGTCCCAGTGTGGCCGATCACCCTCTCAGGTCGGCTATG
TATCGTCGCCTTGGTGAGCCCTTACCCCACCAACTAGCTAATACAACGCAGGTCCATCTGGTAGTGATGCAAT
TGCACCTTTCAAACAGTTGTCATGCAACAACTGCTATTATGCGGTATTAGCTATCGTTTCCAATAGTTATCCC
CCGCTACCAGGCAGGTTACCTACGCGTTACTCACCCGTTCGCAACTCATCCAGAGAAGCAAGCTCCTCCTTCA
GCGTTCTACTTGCCATGTATTAGGCACGCCGCCAGCGTTCGTCCTGAGCCAGGATCAAACTCT
>51
CTGCTGCCGCCCGTAGGAGTCTGGGCCGTGTCTCAGTCCCAGTGTGGCCGATCACCCTCTCAGGTCGGCTATG
TATCGCCGCCTTGGTGAGCCGTTACCTCACCAACTAGCTAATACAACGCAGGTCCATCTGGTAGTGATGCAAT
TTGCACCTTTTAAGCAAATGTCATGCAACATCTACTATTATGCGGTATTACGCTATCGTTTTCCAATAGTTAT
CCCCCGCTACCAGGCAGGTTACCTACGCGTTACTCACCCGTTCGCAACTCATCCAGAGAAGCAAGCTCCTCCT
TCAGCGTTCTACTTGCATGTATTAGGCACGCCGCCAGCGTTCGTCCTGAGCCAGGATCAAACTCT
>52
CTGCTGCCGCCCGTAGGAGTCTGGGCCGTGTCTCAGTCCCAGTGTGGCCGATCACCCTCTCAGGTCGGCTATG
TATCGTCGCCTTGGTGAGCCCTTACCCCACCAACTAGCTAATACAACGCAGGTCCATCTGGTAGTGATGCAAT
TGCACCTTTCAAACAGTTGTCATGCAACAACTGCTATTATGCGGTATTAGCTATCGTTTTCCAATAGTTATCC
CCCGCTACCAGGCAGGTTACCTACGCGTTACTCACCCGTTCGCAACTCATCCAGAGAAGCAAGCTCCTCCTCA
GCGTTCTACTTGCCATGTATTAGGCACGCCGCCAGCGTCGTCCTGAGCCAGGATCAAACTCT
>53
CTGCTGCCGCCCGTAGGAGTCTGGGCCGTGTCTCAGTCCCAGTGTGGCCGATCACCCTCTCAGGTCGGCTATG
TATCGTCGCCTTGGTGAGCCGTTACCCCACCAACTAGCTAATACAACGCAGGTCCATCTGGTAGTGGTGCAAT
TGCACCTTTTAAGCAAATGTCATGCAACATCTACTCTTATGCGGTATTAGCTATCGTTTCCAATAGTTATCCC
CCGCTACCAGGCAGGTTACCTACGCGTTACTCACCCGTTCGCAACTCATCCGGAGAAGCAAGCTCCTCCTTCA
GCGTTCTACTTGCATGTATTAGGCACGCCGCCAGCGTTCGTCCTGAGCCAGGATCAAACTCT

>54
CTGCTGCCGCCCGTAGGAGTCTGGGCCGTGTCTCAGTCCCAGTGTGGCCGATCACCCTCTCAGGTCGGCTATG
TATCGTCGCCTTGGTGAGCCCTTACCCCACCAACTAGCTAATACAACGCAGGTCCATCTGGTAGTGATGCAAT
TGCACCTTTCAAACAGTTGTCATGCAACAACTGCTATTATGCGGTATTAGCTATCGTTTCCAATAGTTATCCC
TCGCTACCAGGCAGGTTACCTACGCGTACTCACCCGTTCGCAACTCATCCAGAGAAGCAAGCTCCTCCTTCAG
CGTTCTACTTGCATGTATTAGGCACGCCGCCAGCGTTCGTCCTGAGCCAGGATCAAACTCT
>55
CTGCTGCCGCCCGTAGGAGTCTGGGCCGTGTCTCAGTCCCAGTGTGGCCGATCACCCTCTCAGGTCGGCTATG
TATCGTCGCCTTGGTGAGCCGTTACCTCACCAACTAGCTAATACAACGCAGGTCCATCTGGTAGTGATGCAAT
TTGCACCTTTTAATTGACTATCATGCAATAATCAATTTTATGCGGTATTAGCCTATCGTTTCCAATAGTTATC
CCCCGCTACCAGGCAGGTTACCTACGCGTTACTCACCCGTTCGCAACTCATCCAGAGAAGCAAGCTCCTCCTT
CAGCGTTCTACTTGCATGTATTAGGCACGCCGCCAGCGTTCGTCCTGAGCCAGGATCAAACTCT
>56
CTGCTGCCGCCCGTAGGAGTCTGGGCCGTGTCTCAGTCCCAGTGTGGCCGATCACCCTCTCAGGTCGGCTATG
TATCGTCGCCTTGGTGAGCCCTTACCCCACCAACTAGCTAATACAACGCAGGTCCATCTGGTAGTGATGCAAT
TGCACCTTTCAAACAGTTGTCATGCAACAACTGCTATCATGCGGTATTAGCTATCGTTTCCAATAGTTATCCC
CCGCTACCAGGCAGGTTACCTACGCGTACTCACCCGTTCGCAACTCATCCAGAGAAGCAAGCTCCTCCTTCAG
CGTTCTACTTGCATGTATTAGGCACGCCGCCAGCGTTCGTCCTGAGCCAGGATCAAACTCT
>57
CTGCTGCCGCCCGTAGGAGTCTGGGCCGTGTCTCAGTCCCAGTGTGGCCGATCACCCTCTCAGGTCGGCTATG
TATCGTCGCCTTGGTGAGCCCTTACCCCACCAACTAGCTAATACAACGCAGGTCCATCTGGTAGTGATGCAAT
TTGCACCTTTCAAACAGTTGTCATGCAACAACTGCTATTATGCGGTATTAGCTATCGTTTCCAATAGTTATCC
CCCGCTACCGGGCAGGTTACCTACGCGTTACTCACCCGTTCGCAACTCATCCAGAGAACGCAAGCTCCTCCTT
CAGCGTTCTACTTGCATGTATTACGGCAGCCGCCAGCGTTCGTCCTGA
>58
CTGCTGCCGCCCGTAGGAGTCTGGGCCGTGTCTCAGTCCCAGTGTGGCCGATCACCCTCTCAGGTCGGCTATG
TATCGTCGCCTTGGTGAGCCGTTACCCCCCACCAACTAGCTAATACAACGCAGGTCCATCTGGTAGTGGTGCA
ATTGCACCTTTTAAGCAAATGTCATGCAACATCTACTCTTTATGCGGTATTTAGCTATCGTTTTCCAATAGTT
ATCCCCCCGCTACCAGGCAGGTTACCTACGCGTTACTCACCCGTTCGCAACTCATCCGGAGAAGCAAGCTCCT
CCTTCAGCGTTCTACTTGCATGTATTTAGGCACGCCGCCAACGTTCGTCCTGAGCCAGGATCAAACTCT
>59
CTGCTGCCGCCCGTAGGAGTCTGGGCCGTGTCTCAGTCCCAGTGTGGCCGATCACCCTCTCAGGTCGGCTACG
CATCGTTGCCTTGGTGGGCCGTTACCCCGCCAACCAGCTAATGCGCCGCAGGCCCATCCATCAGCGACAGCCG
AAACCGTCTTTCAACTCCCCCACCCCGCGGTGGAGAGGATTTATCCGGTATTTACGCCACGGTTTTTCCCGCA
GTTATCCCCCCGGCTGATGGGCAGGTTTGCCTACGTGTTTACTCACCCCGTCCGCCACTCGATCAGAGGGAGC
AAGCTCCCTCCTCTCGCGTTCGACTTGCATGTATTTAGGCACGCCGCCAGCGTTCATCCTGAGCCAGGATCAA
ACTCT
>60
CTGCTGCCGCCCGTAGGAGTCTGGGCCGTGTCTCAGTCCCAGTGTGGCCGATCACCCTCTCAGGTCGGCTGTG
TATCGTCGCCTTGGTGAGCCGTTACCTCACCAACTAGCTAATACAACGCAGGTCCATCTGGTAGTGATGCAAT
TTGCACCTTTTAATTGACTATCATGCAATAATCAATTTTATGCGGTATTAGCCTATCGTTTTCCAATAGTTAC
TCCCCCGCTACCAGGCAGGTTACCTACGCGTTACTCACCCGTTTCGCAACTCATCCAGAGAAGCAAGCTCCTC
CTTCAGCGTTCTACTTGCATGTATTTAGGCACGCCGCCAGCGTTCGTCCTGAGCCAGGATCAAACTCT
>61
CTGCTGCCGCCCGTAGGAGTCTGGGCCGTGTCTCAGTCCCAGTGTGGCCGATCACCCTCTCAGGTCGGCTATG
TATCGTCGCCTTGGTGAGCCGTTACCCCACCAACTAGCTAATACAACGCAGGTCCATCTGGTAGTGGTGCAAT
TGCACCTTTTAAGCAAATGTCATGCAACATCTACTCTTATGCGGTATTAGCTATCGTTTTCCAATAGTTATCC
CCCGCTACCAGGCAGGTTACCTACGCGTTACTCACCCGTTCGCAACTCATCCGGAGAAGCAAGCTCCTCCTTC
AGCGTTCTACTTGCATGTATTTAGGCACGCCGCCAGCGTTCGTCCTGAGCCAGGATCAAACTCT
>62
CTGCTGCCGCCCGTAGGAGTCTGGGCCGTGTCTCAGTCCCAGTGTGGCCGATCACCCTCTCAGGTCGGCTATG
TATCGTCGCCTTGGTGAGCCGTTACCCCACCAACTAGCTAATACAACGCAGGTCCATCTGGTAGTGGTGCAAT
TGCACCTTTTAAGCAAATGTCATGCAACATCTACTCTATGCGGTATTAGCTATCGTTTCCAATAGTTATCCCC

CGCTACCAGGCAGGTACCTACGCGTACTCACCCGTTCGCAACTCATCCGGAGAAGCAAGCTCCTCCTCAGCGT
TCTACTGCATGTATTTAGGCACGCCGCCAGCGTTCGTCCTGAGCCAAGGATCAAACTCT
>63
CTGCTGCCGCCCGTAGGAGTCTGGGCCGTGTCTCAGTCCCAGTGTGGCCGATCACCCTCTCAGGTCGGCTATG
TATCGTCGCCTTGGTGAGCCGTTACCCCACCAACTAGCTAATACAACGCAGGTCCATCTGGTAGTGGTGCAAT
TGCACCTTTTAAGCAAATGTCATGCAACATCTACTCTTATGCGGTATTAGCTATCGTTTCCAATAGTTATCCC
CCGCTACCAGGCAGGTTACCTACGCGTACTCACCCGTTCGCAACTCATCCGGAGAAGCAAGCTCCTCCTTCAG
CGTTCTACTTGCATGTATTAGGCACGCCGCCAGCGTTCGTCCTGAGCCAGGATCAAACTCT
>64
CTGCTGCCGCCCGTAGGAGTCTGGGCCGTGTCTCAGTCCCAGTGTGGCCGATCACCCTCTCAGGTCGGCTATG
TATCGTCGCCTTGGTGAGCCGTTACCTCACCAACTAGCTAATACAACGCAGGTCCATCTGGTAGTGATGCAAT
TTGCACCTTTTAATTGACTATCATGCAATAATCAATTTTATGCGGTATTAGCCTATCGTTTCCAATAGTTATC
CCCCGCTACCAGGCAGGTTACCTGCGCGTTACTCACCCGTTCGCAACTCATCCAGAGAAGCAAGCTCCTCCTT
CAGCGTTCTACTTGCATGTATTAGGCACGCCGCCAGCGTTCGTCCTGAGCCAGGATCAAACTCT
>65
CTGCTGCCGCCCGTAGGAGTCTGGGCCGTGTCTCAGTCCCAGTGTGGCCGATCACCCTCTCAGGTCGGCTATG
TATCGTCGCCTTGGTGAGCCGTTACCTCACCAACTAGCTAATACAACGCAGGTCCATCTGGTAGTGATGCAAT
TTGCACCTTTTAATTGACTATCATGCAATAATCAATTTTATGCGGTATTAGCCTATCGTTTCCAATAGTTATC
CCCCGCTACCAGGCAGGTTACCTACGCGTTACTCACCCGTTCGCAACTCATCCAGAGAAGCAAGCTCCTCCTT
CAGCGTTCTACTTGCATGTATTACGGCACGCCGCCAGCGTTCGTCCTGAGCCAGGATCAAACTGCTA
>66
CTGCTGCCGCCCGTAGGAGTCTGGGCCGTGTCTTAGTCCTAGTGTGGCCGATCACCCTCTCAGGTCGGCTATG
TATTGTCGCCTTGGTGAGCCCTTACCCCACCAACTAGCTAATACAACGCAGGTCCATCTGGTAGTGATGCAAT
TTGCACCTTTCAAACAGTTGTCATGCAACAACTGCTATTATGCGGTATTTAGCTATCGTTTTCCAATAGTTTA
TCCCCCGCTACCAGGCAGGTTACCTACGCGTTACTCACCCGTTCGCAACTCATCCAGAGAAGCAAGCTCCTCC
TTTCAGCGTTCTACTTGCATGTATTTAGGCACGCCGCCAGCGTTCGTCCCGAGCCAGGATCAAACTCTTT
>67
CTGCTGCCGCCCGTAGGAGTCTGGGCCGTGTCTCAGTCCCAGTGTGGCCGATCACCCTCTCAGGTCGGCTATG
TATCGTCGCCTTGGTGAGCCGTTACCCCACCAACTAGCTAATACAACGCAGGTCCATCTGGTAGTGGTGCAAT
TGCACCTTTTAAGCAAATGTCATGCAACATCTACTCTTATGCGGTATTAGCTATCGTTTTCCAATAGTTATCC
CCCGCTACCAGGCAGGTTACCTACGCGTTACTCACCCGTTCGCAACTCATCCGGAGAAGCAAGCTCCTCCTTC
AGCGTTCTACTTGCATGTATTTAGGCACGCCGCCAGCGTTCGTCCTGAGCCAGGATCAAACTCT
>68
CTGCTGCCGCCCGTAGGAGTCTGGGCCGTGTCTCAGTCCCAGTGTGGCCGATCACCCTCTCAGGTCGGCTATG
TATCGTCGCCTTGGTGAGCCGTTACCTCACCAACTAGCTAATACAACGCAGGTCCATCTGGTAGTGATGCAAT
TTGCACCTTTTAATTGACTATCATGCAATAATCAATTTTATGCGGTATTAGCCTATCGTTTCCAATAGTTACT
CCCCCGCTACCAGGCAGGTTACCTACGCATTACTCACCCGTTCGCAACTCATCCAGAGAAGCAAGCTCCTCCT
TCAGCGTTCTACTTGCATGTATTAGGCACGCCGCCAGCGTTCGTCCTGAGCCAGGATCAAACTCT
>69
CTGCTGCCGCCCGTAGGAGTCTGGGCCGTGTCTCAGTCCCAGTGTGGCCGATCACCCTCTCAGGTCGGCTATG
CATCGTCGCCTTGGTAGGCCTTTACCCCACCAACTAGCTAATGCACCGCAAAGCCATCTCATAGTGTTAGCAA
AACCAACTTTTAAACATCAACCATGCAGTTAATGCTGTTATCCGGTATTAGCTATCGTTTTCCAATAGTTTGT
CCCAGTCTATGAGGCAGGTTCTTTACGTGTTACTCACCCGTTCGCCGCTAAGTTGTCGTCGACAGCAACCAGT
AAACTACGCAACT
>70
CTGCTGCCGCCCGTAGGAGTCTGGGCCGTGTCTCAGTCCCAGTGTGGCCGATCACCCTCTCAGGTCGGCTATG
TATCGTCGCCTTGGTGAGCCCTTACCCCACCAACTAGCTAATACAACGCAGGTCCATCTGGTAGTGATGCAAT
TGCACCTTTCAAACAGTTGTCATGCAACAACTGCTATTATGCGGTATTAGCTATCGTTTTCCAATAGTTATCC
CCCGCTACCAGGCAGGTTACCTACGCGTTACTCACCCGTTCGCAACTCATCCAGAGAAGCAAGCTCCTCCTTC
AGCGTTCTACTGCATGTATTAGGCACGCCGCCAGCGTCGTCCTGAGCCAGGATCAAACTCT
>71
CTGCTGCCGCCCGTAGGAGTCTGGGCCGTGTCTCAGTCCCAGTGTGGCCGATCACCCTCTCAGGTCGGCTATG
TATCGTCGCCTTGGTGAGCCCTTACCCCACCAACTAGCTAATACAACGCAGGTCCATCTGGTAGTGATGCAAT
TGCACCTTTCAAACAGTTGTCATGCAACAACTGCTATTATGCGGTATTTAGCTATCGTTTTCCAATAGTTTAT

CCCCCGCTACCAGGCAGGTTACCTACGCGTTACTCACCCGTTCGCAACTCATCCAGAGAAGCAAGCTCCTCCT
CAGCGTTCTACTTGCATGTATTAGGCACGCCGCCAGCGTCGTCCTGAGCCAGGATCAAACTCT
>72
CTGCTGCCGCCCGTAGGAGTCTGGGCCGTGTCTCAGTCCCAGTGTGGCCGATCACCCTCTCAGGTCGGCTATG
TATCGTCGCCTTGGTGAGCCCTTACCCCACTAACTAGCTAATACAACGCAGGTCCATCTGGTAGTGATGCAAT
TGCACCTTTCAAACAGTTGTCATGCAACAACTGCTATTATGCGGTATTTAGCTATCGTTTTTCCAATAGTATC
CCCCGCTACCAGGCAGGTTACCTACCGCGTTACTCACCCGTTCGCAACTCATCCGGAGAAGCAAGCTCCTCCT
CAGCGTTCTACTGCATGTATTTAGGCACGCCGCCAGCGTTCGTCCTGAGCCAGGATCAAACTCT
>73
CTGCTGCCGCCCGTAGGAGTCTGGGCCGTGTCTCAGTCCCAGTGTGGCCGATCACCCTCTCAGGTCGGCTATG
TATCGTCGCCTTGGTGAGCCGTTACCTCACCAACTAGCTAATACAACGCAGGTCCATCTGGTAGTGATGCAAT
TTGCACCTTTTAATTGACTATCATGCAATAATCAATTTTATGCGGTATTAGCCTATCGTTTCCAATAGTTACT
CCCCCGCTACCAGGCAGGTTACCTACGCGTTACTCACCCGTTCGCAGCTCATCCAGAGAAGCAAGCTCCTCCT
CAGCGTTCTACTTGCATGTATTAGGCACGCCGCCAGCGTTCGTCCTGAGCCAGGATCAAACTCT
>74
CTGCTGCCGCCCGTAGGAGTCTGGGCCGTGTCTCAGTCCCAGTGTGGCCGATCACCCTCTCAGGTCGGCTATG
TATCGTCGCCTTGGTGAGCCGTTACCTCACCAACTAGCTAATACAACGCAGGTCCATCTGGTAGTGATGCAAT
TGCACCTTTTAATTGACTATCATGCAATAATCAATTTTATGCCGGTATTAGCCTATCGTTTCCAATAGTTACT
CCCCCGCTACCAGGCAGGTTACCTACGCGTTACTCACCCGTTCGCAACTCATCCAGAGAAGCAAGCTCCTCCT
TCAGCGTTCTACTTGCATGTATTACGGCACGCCGCCAGCGTTCGTCCTGAGCCAGGATCAAACTCT
>75
CTGCTGCCGCCCGTAGGAGTCTGGGCCGTGTCTCAGTCCCAGTGTGGCCGATCACCCTCTCAGGTCGGCTGCG
TATCGTCGCCTTGGTGAGCCGTTACCTCACCAACTAGCTAATACAACGCAGGTCCATCTGGTAGTGATGCAAT
TGCACCTTTTAATTGACTATCATGCAATAATCAATTTTATGCGGTATTAGCTATCGTTTCCAATAGTTATCCC
CCGCTACCAGGCAGGTTACCTACGCGTTACTTACCCGTTCGCAACTCATCCAGAGAAGCAAGCTCCTCCTTCA
GCGTTCTACTTGCATGTATTAGGCACGCCGCCAGCGTTCGTCCTGAGCCAGGATCAAACTCT
>76
CTGCTGCCGCCCGTAGGAGTTTGGGCCGTGTCTCAGTCCCAATGTGGCCGATCCACCCTCTCAGGCTCGGCTA
TGCATCACGGCCTTTGGTGAGCCGTTACCTCACCAACTAGCTAATGCACCGCGGGTCCATCCATCAGCAGAAG
CCCGAAGCCTCTTTTCCTCTTCAAACCCATGCGGTTCGAAGACCTATGCCGGTTTTAGCATCCGTTTCCGAAT
GTTACTCCCCCTCTGATGGGCAGGTTACCCCACGTGTTACTCACTCGTTCGCCACTAGATTGACCAGTGCAAG
CACCGGTCGCTCTCGTTCGACTGCATGTACTTAGGCACGCCGCCAGCGTCGTCCTGAGCCAGGATCAACTCT
>77
CTGCTGCCGCCCGTAGGAGTCTGGGCCGTGTCTCAGTCCCAGTGTGGCCGATCACCCTCTCAGGTCGGCTATG
TATCGTCGCCTTGGTGAGCCCTTACCCCCACCAACTAGCTAATACAACGCAGGTCCATCTGGTAGTGATGCAA
TTGCACCTTTCAAACAGTTGTCATGCAACAATTGCTATTTATGCGGTATTTAGCTATCGTTTTCCAATAGTTT
ATCCCCCCGCTACCAGGCAGGTTACCTACGCGTTACTCACCCCGTTCGCAACTCATCCGGAGAAGCAAGCTCC
TCCTCAGCGTTCTACTTGCATGTATTTAGGCACGCCGCCAGCGTTCGTCCTGAGCCAGGATCAAACTCT
>78
CTGCTGCCGCCCGTAGGAGTCTGGGCCGTGTCTCAGTCCCAGTGTGGCCGATCACCCTCTCAGGTCGGCTATG
TATCGTCGCCTTGGTGAGCCCTTACCCCACCAACTAGCTAATACAACGCAGGTCCATCTGGTAGTGATGCAAT
TGCACCTTTCAAACAGTTGTCATGCAACAACTGCTATTATGCGGTATTAGCTATCGTTTTCCAATAGTTATCC
CCCGCTACCAGGCAGGTTACCTACGCGTTACTCACCCGTTCGCAACTCATCCGGAGAAGCAAGCTCCTCCTTC
AGCGTTCTACTTGCATGTATTAGGCACGCCGCCAGCGTTCGTCCTGGGCCAGGATCAAACTCT
>79
CTGCTGCCGCCCGTAGGAGTCTGGGCCGTGTCTCAGTCCCAGTGTGGCCGATCACCCTCTCAGGTCGGCTATG
CATCGTCGCCTTGGTAGGCCTTTACCCCACCAACTAGCTAATGCACCGCAAAGCCATCTCATAGTGTTAGCAA
AACCAACTTTTAAACATCAACCATGCAGTTAATGCTGTTATCCGGTATTAGCTATCGTCTCCAATAGTTGTCC
CAGTCTATGAGGCAGGTTCTTTACGTGTTACTCACCCGTTCGCCGCTAAGTTTCGTCGGCAGCACAAAACTCC
AACTACGCTCGCATGCATG
>80
CTGCTGCCGCCCGTAGGAGTCTGGGCCGTGTCTCAGTCCCAGTGTGGCCGATCACCCTCTCAGGTCGGCTATG
TATCGTCGCCTTGGTGAGCCGTTACCTCACCAACTAGCTAATACAACGCAGGTCCATCTGGTAGTGATGCAAT
TGCACCTTTTAATTGACTATCATGCAATAATCAATTTTATGCGGTATTTAGCCTATCGTTTTCCAATAGTTTA

CTCCCCGCTACCAGGCAGGTTACCTACGCGTTACTCACCCGTTCGCAACTCATCCAGAGAAGCAAGCTCCTCC
TTCAGCGTTCTACTTGCATGTATTTAGGCACGCCGCCAGCGTTCGTCCTGAGCCAGGATCAAACTCT
>81
CTGCTGCCGCCCGTAGGAGTCTGGACCGTGTCTCAGTTCCAGTGTGGCTGGTCATCCTCTCAGACCAGCTAGA
GATCGCAGGCTTGGTAGGCCTTTACCCCACCAACTACCTAATCCCACTTGGGCTCATCTTATGGCATGCGGCC
TTACGGTCCCGCACTTCGTCTCTCGACACTACGCGGTATTAGCTACAGTTTCCCGTAGTTATCCCCCTCCATA
AGCCAGATTCCCAAGCATTACTCACCCGTCCGCCACTCGTCAGCAAGAAAGCAAGCTTTCTCCTGCTACCGTT
CGACTTGCATGTGTTTAAGCCTGCCGCCAGCGTTCAATCTGAGCCAGGATCAAACTCT
>82
CTGCTGCCGCCCGTAGGAGTCTGGACCGTGTCTCAGTTCCAGTGTGGCCGATCACCCTCTCAGGTCGGCTACG
TATCGTTGCCTTGGTAAGCCGTTACCTTACCAACTAGCTAATACGGCGCAGGTCCATCTATAAGTGATAGCAA
AGCCATCTTTCACTATCGAACCATGCGGTTCGAAATATTATCCGGTATTAGCTCCGGTTTTCCCGAAGTTATC
CCAGTCTTATAGGTAGGTTTACCTACGTGTTACTCACCCGTCCGCCGCTAACGTCAAAGGAGCAAGCTCCTCG
TCTGTTCGCTCGACTTGCATGTATAGGCACGCCGCCAGCGTCATCCTGAGCCAGGATCAAACTCT
>83
CTGCTGCCGCCCGTAGGAGTCTGGGCCGTGTCTCAGTCCCAGTGTGGCCGATCACCCTCTCAGGTCGGCTATG
TATCGTCGCCTTGGTGAGCCGTTACCTCACCAACTAGCTAATACAACGCAGGTCCATCTGGTAGTGATGCAAT
TGCACCTTTTAATTGACTATCATGCAATAATCAATTTTATGCGGTATTAGCCTATCGTTTCCAATAGTTACTC
CCCCGCTACCAGGCAGGTTACCTACGCGTTACTCACCCGTTCGCAACTCATCCAGAGAAGCAAGCTCCTCCTT
CAGCGTTCTACTTGCATGTATTAGGCACGCCGCCAGCGTTCGTCCTGAGCCAGGATCAAACTCT
>84
CTGCTGCCGCCCGTAGGAGTCTGGGCCGTGTCTCAGTCCCAGTGTGGCCGATCACCCTCTCAGGTCGGCTATG
TATCGTCGCCTTGGTGAGCCGTTACCTCACCAACTAGCTAATACAACGCAGGTCCATCTGGTAGTGATGCAAT
TTGCACCTTTTAATTGACTATCATGCAATAATCAATTTTATGCGGTATTAGCCTATCGTTTCCAATAGTTACT
CCCCGCTACCAGGCAGGTTACCTACGCGTTACTCACCCGTTCGCAACTCATCCAGAGAAGCAAGCTCCTCCTT
CAGCGTTCTACTTGCATGTATTACGGCACGCCGCCAGCGTTCGTCCTGAGCCAGGATCAAACTCT
>85
CTGCTGCGCCCTAGGAGTCTGGACCGTGTCTCAGTTCCAGTGTGGCCGATCACCCTCTCAGGTCGGCTACGCA
TCGTTGCCTTGGTAAGCTGTTACCTTACCAACTAGCTAATGCGGCGCGGATCCATCTATAAGTGACAGCAAAA
CCGTCTTTCACTATTGAACCATGCGGTTCAATATATTTATCCGGTATTTAGCTCCGGTTTTCCCGAAGTTACC
CCAGTCTTATAGGTAGGTTATCCACGTGTTACTCACCCGTCCGCCGCTAACGTTAGAGGAGCAAGCTCCTCGT
CTGTCGCTCGACTTGCATGTATTAGGCACGCCGCCAGCGTTCATCCTGAGCCAGGATCAAACTCT
>86
CTGCTGCCGCCCGTAGGAGTCTGGGCCGTGTCTCAGTCCCAGTGTGGCCGATCACCCTCTCAGGTCGGCTATG
TATCGTCGCCTTGGTGAGCCGTTACCCCACCAACTAGCTAATACAACGCAGGTCCATCTGGTAGTGGTGCAAT
TGCACCTTTTAAGCAAATGTCATGCAACATCTACTCTTATGCGGTATTAGCTATCGTTTTCCAATAGTTATCC
CCCGCTACCAGGCAGGTTACCTACGCGTTACTCACCCGTTCGCAACTCATCCGGAGAAGCAAGCTCCTCCTTC
AGCGTTCTACTTGCATGTATTTAGGCACGCCGCCAGCGTTCGTCCTGAGCCAGGATCAAACTCT
>87
CTGCTGCCGCCCGTAGGAGTCTGGGCCGTGTCTCAGTCCCAGTGTGGCCGATCACCCTCTCAGGTCGGCTATG
TATCGTCGCCTTGGTGAGCCGTTACCCCCACCAACTAGCTAATACAACGCAGGTCCATCTGGTAGTGGTGCAA
TTGCACCTTTTAAGCAAATGTCATGCAACATCTACTCTTTATGCGGTATTTAGCTATCGTTTTCCAATAGTTT
ATCCCCCCTCTACCAGGCAGGTTACCTTACGCGTTACTCACCCGTTTCGCAACTCATCCAGAGAAGCAAGCTC
CTCCTTTCAGCGTTCTACTTGCATTGTATTTAGGCACGCCGCCAGCGTTCGCCTGAGCCAGGATCAAACTCT
>88
CTGCTGCCGCCCGTAGGAGTCTGGGCCGTGTCTCAGTCCCAGTGTGGCCGATCACCCTCTCAGGTCGGCTATG
TATCGTCGCCTTGGTGAGCCGTTACCTCACCAACTAGCTAATACAACGCAGGTCCATCTGGTAGTGATGCAAT
TTGCACCTTTTAATTGACTATCATGCAATAATCAATTTTATGCGGTATTAGCCTATCGTTTCCAATAGTTACT
CCCCCGCTACCAGGCAGGTTACCTACGCGTTACTCACCCGTTCGCAACTCATCCAGAGAAGCAAGCTCCTCCT
TCAGCGTTCTACTTGCATGTATTAGGCACGCCGCCAGCGTTCGTCCTGAGCCAGGATCAAACTCT
>89
CTGCTGCCGCCCGTAGGAGTCTGGGCCGTGTCTCAGTCCCAGTGTGGCCGATCACCCTCTCAGGTCGGCTATG
CATCGTCGCCTTGGTAGGCTTTTACCCCACCAACTAGCTAATGCACCGCAAAGCCATCTCATAGTGTTAGCAT
AACCAACTTCAAACATCAACCATGCAGTTAATGCTGTTATCCGGTATTAGCTATCGTTTCCAATAGTTGTCCC

AGTCTATGAGGCAGGTTCTTTACGTGTTACTCACCCGTTCGCCGCTAAGTTCTCGCGCGACCAGCAACGAATA
AACTCGCTCGTAC
>90
CTGCTGCCGCCCGTAGGAGTCTGGGCCGTGTCTCAGTCCCAGTGTGGCCGATCACCCTCTCAGATCGGCTATG
TATCGTCGCCTTGGTGAGCCGTTACCTCACCAACTAGCTAATACAACGCAGGTCCATCTGGTAGTGATGCAAT
TTGCACCTTTTAATTGACTATCATGCAATAATCAATTTTATGCGGTATTAGCTATCGTTTTCCAATAGTTATC
CCCCGCTACCAGGCAGGTTACCTACGCGTTACTCACCCGTTCGCAACTCATCCAGAGAAGCAAGCTCCTCCTT
CAGCGTTCTACTTGCATGTATTTAGGCACGCCGCCAGCGTTCGTCCTGAGCCAGGATCAAACTCT
>91
CTGCTGCCGCCCGTAGGAGTCTGGGCCGTGTCTCAGTCCCAGTGTGGCCGATCACCCTCTCAGGTCGGCTATG
TATCGTCGCCTTGGTGAGCCGTTACCCCCCACCAACTAGCTAATACAACGCAGGTCCATCTGGTAGTGGTGCA
ATTGCACCTTTCAAACAGCTGTCATGCGACAACTGCTATTTATGCGGTATTTAGCTATCGTTTTCCAATAGTT
TATCCCCCCGCTACCAGGCAGGTTACCTACGCGTTACTCACCCGTTCGCAACTCATCCAGAGAAGCAAGCTCC
TCCTTCAGCGTTCTACTTGCATGTATTTAGGCACGCCGCCAGCGTTCGCCTGAGCCAGGATCAAACTCT
>92
CTGCTGCCGCCCGTAGGAGTCTGGGCCGTGTCTCAGTCCCAGTGTGGCCGATCACCCTCTCAGGTCGGCTATG
TATCGTCGCCTTGGTGAGCCGTTACCTCACCAACTAGCTAATACAACGCAGGTCCATCTGGTAGTGATGCAAT
TGCACCTTTTAATTGACTATCATGCAATAATCAATTTTATGCGGTATTAGCCTATCGTTTCCAATAGTTATCC
CCCGCTACCAGGCAGGTTACCTACGCGTTACTCACCCGTTCGCAACTCATCCAGAGAAGCAAGCTCCTCCTTC
AGCGTTCTACTTGCATGTATTAGGCACGCCGCCAGCGTTCGTCCTGAGCCAGGATCAAACTCT
>93
CTGCTGCCGCCCGTAGGAGTCTGGGCCGTGTCTCAGTCCCAGTGTGGCCGATCACCCTCTCAGGTCGGCTATG
TATCGTCGCCTTGGTGAGCCGTTACCCCACCAACTAGCTAATACAACGCAGGTCCATCTGGTAGTGGTGCAAT
TGCACCTTTTAAGCAAATGTCATGCAACATCTACTCTTATGCGGTATTAGCTATCGCTTCCAATAGTTATCCC
CCGCTACCAGGCAGGTTACCTACGCGTTACTCACCCGTTCGCAACTCATCCAGAGAAGCAAGCTCCTCCTTCA
GCGTTCTACTTGCATGTATTAGGCACGCCGCCAGCGTTCGTCCTGAGCCAGGATCAAACTCT
>94
CTGCTGCCGCCCGTAGGAGTCTGGGCCGTGTCTCAGTCCCAGTGTGGCCGATCACCCTCTCAGGTCGGCTATG
TATCGTCGCCTTGGTGAGCCCTTACCCCACCAACTAGCTAATACAACGCAGGTCCATCTGGTAGTGATGCAAT
TGCACCTTTCAAACAGTTGTCATGCAACAACTGCTATTATGCGGTATTAGCTATCGTTTTCCAATAGTTATCC
CCCGCTACCAGGCAGGTTACCTACGCGTTACCCACCCGTTCGCAACTCATCCAGAGAAGCAAGCTCCTCCTTC
AGCGTTCTACTTGCATGTATTAGGCACGCCGCCAGCGTTCGTCCTGAGCCAGGATCAAACTCT
>95
CTGCTGCCGCCCGTAGGAGTCTGGGCCGTGTCTCAGTCCCAGTGTGGCCGATCACCCTCTCAGGTCGGCTATG
TATCGTCGCCTTGGTGAGCCCTTACCCCACCAACTAGCTAATACAACGCAGGTCCATCTGGTAGTGATGCAAT
TGCACCTTTCAAACAGTTGTCATGCAACAACTGCTATTATGCGGTATTTAGCTATCGTTTTCCAATAGTTTAT
CCCCCGCTACCAGGCAGGTTACCTACGCGTTACTCACCCGTTCGCAACTCATCCAGAGAAGCAAGCTCCTCCT
TCAGCGTTCTACTTGCATGTATTAGGCACGCCGCCAGCGTTCGTCCTGAGCCAGGATCAAACTCT
>96
CTGCTGCCGCCCGTAGGAGTCTGGGCCGTGTCTCAGTCCCAGTGTGGCCGATCACCCTCTCAGGTCGGCTATG
TATCGTCGCCTTGGTGAGCCCTTACCCCACCAACTAGCTAATACAACGCAGGTCCATCTGGTAGTGATGCAAT
TGCACCTTTCAAACAGTTGTCATGCAGCAACTGCTATTTATGCGGTATTTAGCTATCGTTTTCCAATAGTTTA
TCCCCCCCGCTACCAGGCAGGTTACCTACGCGTTACTCACCCGTTCGCAACTCATCCAGAGAAGCAAGCTCCT
CCTTTCAGCGTTCTACTGCATGTATTTAGGCACGCCGCCAGCGTCGTCCTGAGCCAGGATCAAACTCT
>97
CTGCTGCCGCCCGTAGGAGTCTGGGCCGTGTCTCAGTCCCAGTGTGGCCGATCACCCTCTCAGGTCGGCTATG
TATCGTCGCCTTGGTGAGCCGTTACCTCACCAACTAGCTAATACAACGCAGGTCCATCTGGTAGTGATGCAAT
TGCACCTTTTAATTGACTATCATGCAATAATCAATTTTATGCGGTATTAGCCTATCGTTTCCAATAGTTACTC
CCCCGCTACCAGGCAGGTTACCTACGCGTTACTCACCCGTTCGCAACTCATCCAGAGAAGCAAGCTCCTCCTT
CAGCGTTCTACTTGCATGTATTAGGCTACGCCGCCAGCGTTCGTCCTGAGCCAGGATCAAACTCT
>98
CTGCTGCCGCCCGTAGGAGTCTGGGCCGTGTCTCAGTCCCAGTGTGGCCGATCACCCTCTCAGGTCGGCTATG
TATCGTCGCCTTGGTGAGCCGTTACCCCACCAACTAGCTAATACAACGCAGGTCCATCTGGTAGTGGTGCAAT
TGCACCTTTTAAGCAAATGTCATGCAACATCTACTCTTATGCGGTATTAGCTATCGTTTCCAATAGTTATCCC

CCGCTACCAGGCAGGTTACCTACGCGTTACTCACCCGTTCGCAACTCATCCGGAGAAGCAAGCTCCTCCTTCA
GCGTTCTACTTGCATGTATTAGGCACGCCGCCAGCGTTCGTCCTGAGCCAGGATCAAACTCT
>99
CTGCTGCCGCCCGTAGGAGTCTGGGCCGTGTCTCAGTCCCAGTGTGGCCGATCACCCTCTCAGGTCGGCTATG
TATCGTCGCCTTGGTGAGCCCTTACCCCACCAACTAGCTAATACAACGCAGGTCCATCTGGTAGTGATGCAAT
TGCACCTTTCAACAGTTGTCATGCAACAACTGCTATTATGCGGTATTAGCTATCGTTTCCAATAGTTATCCCC
CGCTACCAGGCAGGTTACCTACGCGTACTCACCCGTTCGCAACTCATCCAGAGAAGCAAGCTCCTCCTTCAGC
GTTCTACTGCATGTATTAGGCACGCCGCCAGCGTCGTCCTGAGCCAGGATCAAACTCT
>100
CTGCTGCCGCCCGTAGGAGTCTGGGCCGTGTCTCAGTCCCAGTGTGGCCGATCACCCTCTCAGGTCGGCTATG
TATCGTCGCCTTGGTCGAGCCCGTTACCCCACCAACTAGCTAATACAACGCAGGTCCATCTGGTAGTGGTGCA
ATTTGCACCTTTTAAGCAAATGTCAAGCAACATCTACTCTTATGCGGTATTAGCTATCGTTTCCAATAGTTAT
CCCCCGCTACCAGGCAGGTTACCTACGCGTACTCACCCGTTCGCAACTCATCCGGAGAAGCAAGCTCCTCCTC
AGCGTTCTACTGCATGTATTAGGCACGCCGCCAGTGTTCGTCCTGAGCCAGGATCAAACTCT
>101
CTGCTGCCGCCCGTAGGAGTCTGGGCCGTGTCTCAGTCCCAGTGTGGCCGATCACCCTCTCAGGTCGGCTATG
TATCGTTGCCTTGGTGAGCCGTTACCTCACCAACTAGCTAATACAACGCAGGTCCATCTGGTAGTGGTGCAAT
TGCACCTTTCAAGCAGCTATCATGCGATATCTACTGTTATGCGGTATTACGCTATCGTTTTCCAATAGTTATC
CCCCGCTACCAGGCAGGTTACCTACGCGTTACTCACCCGTTCGCAACTCATCCGGAAAGAGCAAGCTCCTTCC
TTCAGCGTTCTACTTGCATGTATTAGGCACGCCGCCAGCGTTCGTCCTGAGCCAGGATCAAACTCT
>102
CTGCTGCCGCCCGTAGGAGTCTGGGCCGTGTCTCAGTCCCAGTGTGGCCGATCACCCTCTCAGGTCGGCTATG
TATCGTCGCCTTGGTGAGCCGTTACCTCACCAACTAGCTAATACAACGCAGGTCCATCTGGTAGTGATGCAAT
TGCACCTTTTAATTGACTATCATGCAATAATCAATTTTATGCGGTATTAGCCTATCGTTTCCAATAGTTACTC
CCCCGCTACCAGGCAGGTTACCTACGCGTTACTCACCCGTTCGCAACTCATCCAGAGAAGCAAGCTCCTCCTT
CAGCGTTCTACTTGCATGTATTAGGCACGCCGCCAGCGTTCGTCCTGAGCCAGGATCAAACTCT
>103
CTGCTGCCGCCCGTAGGAGTCTGGGCCGTGTCTCAGACCCAGTGTGGCCGATCACCCTCTCAGGTCGGCTATG
TATCGTCGCCTTGGTGAGCCGTTACCTCACCAACTAGCTAATACAACGCAGGTCCATCTGGTAGTGATGCAAT
TGCACCTTTTAATTGACTATCATGCAATAATCAATTTTATGCGGTATTTAGCCTATCGTTTTCCAATAGTTAC
TCCCCCGCTACCAGGCAGGTTACCTACGCGTTACTCACCCGTTCGCAACTCATCCAGAGAAGCAAGCTCCTCC
TTCAGCGTTCTACTTGCATGTATTTAGGCACGCCGCCAGCGTTCGTCCTGAGCCAGGATCAAACTCT
>104
CTGCTGCCGCCCGTAGGAGTCTGGGCCGTGTCTCAGTCCCAGTGTGGCCGATCACCCTCTCAGGTCGGCTATG
CATCGTCGCCTTGGTAGGCCTTTACCCCACCAACTAGCTAATGCACCGCAAAGCCATCTCATAGTGTTAGCAA
AACCAACTTTTAAACATCAACCATGCAGTTAATGCTGTTATCCGGTATTAGCTATCGTTTTCCAATAGTTTGT
CCCAGTCTATGAGGCAGGTTCTTTACGTGTTTACTCACCCGTTTCGCCGCTAAGTTTCGTCGCGACACGAACA
CCAAACTACGAC
>105
CTGCTGCCGCCCGTAGGAGTCTGGGCCGTGTCTCAGTCCCAGTGTGGCCGATCACCCTCTCAGGTCGGCTATG
TATCGCCGCCTTGGTGAGCCCTTACCCCACCAACTAGCTAATACAACGCAGGTCCATCTGGTAGTGATGCAAT
TGCACCTTTCAAACAGTTGTCATGCAACAACTGCTATTTATGCGGTATTTAGCTATCGTTTTTCCAATAGTTT
ATCCCCCCGCTACCAGGCAGGTTACCTTACGCGTTACTCACCCGTTCGCAACTCATCCGGAGAAGCAAGCTCC
TCCTTCAGCGTTCTACTTGCCATGTATTTAGGCACGCCGCCAGCGTTCGTCCTGAGCCAGGATCAAACTCT
>106
CTGCTGCCGCCCGTAGGAGTCTGGGCCGTGTCTCAGTCCCAGTGTGGCCGATCACCCTCTCAGGTCGGCTATG
CATCGCCGCCTTGGTAGGCCTTTACCCCACCAACTAGCTAATGCACCGCAAAGCCATCTCATAGTGTTAGCAA
AACCAACTTTTAAACATCAACCATGCAGTTAATGCTGTTATCCGGTATTAGCTATCGTTTTCCAATAGTTTGT
CCCAGTCTATGAGGCAGGTTTCTTTACGTGTTACTCACCCGTTTCGCCGCTAAGTTTTCGTCGGCACACGAAA
CTCCAAAACTACGCTC
>107
CTGCTGCCGCCCGTAGGAGTCTGGGCCGTGTCTCAGTCCCAGTGTGGCCGATCACCCTCTCAGGTCGGCTATG
TATCGTCGCCTTGGTGAGCCCTTACCCCACCAACTAGCTAATACAACGCGGGTCCATCTGGTAGTGATGCAAT
TGCACCTTTCAAACAGTTGTCATGCAACAACTGCTATTATGCGGTATTTAGCTATCGTTTTCCAATAGTTTAT

CCCCCCGCTACCAGGCAGGTTACCTACGCGTTACTCACCCGTTCGCAACTCATCCAGAGAAGCAAGCTCCTCC
TTTCAGCGTTCTACTTGCATGTATTTAGGCACGCCGCCAGCGTTCGTCCTGAGCCAGGATCAAACTCT
>108
CTGCTGCCGCCCGTAGGAGTCTGGGCCGTGTCTCAGTCCCAGTGTGGCCGATCACCCTCTCAGGTCGGCTATG
TATCGTCGCCTTGGTGAGCCCTTACCCCACCAACTAGCTAATACAACGCAGGTCCATCTGGTAGTGATGCAAT
TGCACCTTTCAAACAGTTGTCATGCAACAACTGCTATTATGCGGTATTAGCTATCGTTTCCAATAGTTATCCC
CCGCTACCAGGCAGGTTACCTACGCGTTACTCACCCGTTCGCAACTCATCCAGAGAAGCAAGCTCCTCCTTCA
GCGTTCTACTTGCATGTATTAGGCACGCCGCCAGCGTCGTCCTGAGCCAGGATCAAACTCT
>109
CTGCTGCCGCCCGTAGGAGTCTGGGCCGTGTCTCAGTCCCAGTGTGGCCGATCACCCTCTCAGGTCGGCTATG
TATCGTCGCCTTGGTGAGCCCTTACCCCACCAACTAGCTAATACAACGCAGGTCCATCTGGTAGTGATGCAAT
TGCACCTTTCAAACAGTTGTCATGCAACAACTGCTATTATGCGGTATTAGCTATCGTTTCCAATAGTTATCCC
CCGCTACCAGGCAGGTTACCTACGCGTTACTCACCCGTTCGCAACTCATCCAGAGAAGCAAGCTCCTCCTTCA
GCGTTCTACTTGCATGTATTAGGCACGCCGCCAGCGTTCGTCCTGAGCCAGGATCAAACTCT
>110
CTGCTGCCGCCCGTAGGAGTCTGGGCCGTGTCTCAGTCCCAGTGTGGCCGATCACCCTCTCAGGTCGGCTATG
CATCGTCGCCTTGGTAGGCCTTTACCCCACCAACTAGCTAATGCACCGCAAAGCCATCTCATAGTGTTAGCAA
AACCAACTTTTAAACATCAACCATGCAGTTAATGCTGTTATCCGGTATTAGCTATCGTTTTCCAATAGTTTGT
CCCAGTCTATGAGGCAGGTTTCTTTACGTGTTTACTCACCCGTTTCGCCGCTAAGTTGTCGTCGCAGCAACCA
GAAACTACGTCGAACTCGACTTGCATGTATAGGCACGCCGCC
>111
CTGCTGCCGCCCGTAGGAGTCTGGGCCGTGTCTCAGTCCCAGTGTGGCCGATCACCCTCTCAGGTCGGCTATG
TATCGTCGCCTTGGTGAGCCCTTACCCCACCAACTAGCTAATACAACGCAGGTCCATCTGGTAGTGATGCAAT
TGCACCTTTCAAACAGTTGTCATGCAACAACTGCTATTATGCGGTATTTAACTATCGTTTTCCAATAGTTATC
CCCCCGCTACCAGGCAGGTTACCTACGCGTTACTCACCCGTTCGCAACTCATCCGGAGAAGCAAGCTCCTCCT
TCAGCGTTCTACTTGCCATGTATTAGGCACGCCGCCAGCGTTCGTCCTGAGCCAGGATCAAACTCT
>112
CTGCTGCCGCCCGTAGGAGTCTGGGCCGTGTCTCAGTCCCAGTGTGGCCGATCACCCTCTCAGGTCGGCTATG
TATCGTCGCCTTGGTGAGCCGTTACCCCACCAACTAGCTAATACAACGCAGGTCCATCTGGTAGTGGTGCAAT
TGCACCTTTTAAGCAAATGTCATGCAACATCTACTCTTATGCGGTATTAGCTATCGTTTCCAATAGTTATCCC
CCGCTACCAGGCAGGTTACCTACGCGTACTCACCCGTTCGCAACTCATCCGGAGAAGCAAGCTCCTCCTCAGC
GTTCTACTGCATGTATTAGGCACGCCGCCAGCGTTCGTCCTGAGCCAGGATCAAACTCT
>113
CTGCTGCCGCCCGTAGGAGTCTGGGCCGTGTCTCAGTCCCAGTGTGGCCGATCACCCTCTCAGGTCGGCTATG
TATCGTCGCCTTGGTGAGCCGTTACCCCACCAACTAGCTAATACAACGCAGGTCCATCTGGTAGTGGTGCAAT
TGCACCTTTTAAGCAAATGTCATGCAACATCTACTCTTATGCGGTATTAGCTATCGTTTCCAATAGTTATCCC
CCGCTACCAGGCAGGTTACCTACGCGCTACTCACCCGTTCGCAACTCATCCAGAGAAGCAAGCTCCTCCTTCA
GCGTTCTACTTGCATGTATTAGGCACGCCGCCAGCGTTCGTCCTGAGCCAGGATCAAACTCT
>114
CTGCTGCCGCCCGTAGGAGTCTGGGCCGTGTCTCAGTCCCAGTGTGGCCGATCACCCTCTCAGGTCGGCTATG
TATCGTCGCCTTGGTGAGCCCTTACCCCACCAACTAGCTAATACAACGCAGGTCCATCTGGTAGTGATGCAAT
TGCACCTTTCAAACAGTTGTCATGCAACAACTGCTATTATGCGGTATTAGCTATCGTTTCCAATAGTTATCCC
CCGCTACCAGGCAGGTTACCTACGCGTTACTCACCCGTTCGCAACTCATCCAGAGAAGCAAGCTCCTCCTTCA
GCGTTCTACTTGCATGTATTAGGCACGCCGCCAGCGTTCGTCCTGAGCCAGGATCAAACTCT
>115
CTGCTGCCGCCCGTAGGAGTCTGGGCCGTGTCTCAGTCCCAGTGTGGCCGATCACCCTCTCAGGTCGGCTATG
TATCGTCGCCTTGGTGAGCCCTTACCCCACCAACTAGCTAATACAACGCAGGTCCATCTGGTAGTGATGCAAT
TGCACCTTTCAAACAGTTGTCATGCAACAACTGCTATTATGCGGTATTAGCTATCGTTTCCAATAGTTATCCC
CCGCTACCAGGCAGGTTACCTACGCGTTACTCACCCGTTCGCAACTCATCCAGAGAAGCAAGCTCCTCCTTCA
GCGTTCTACTTGCCATGTATTACGGCACGCCGCCAGCGTTCGTCCTGAGCCAGGATCAAACTCT
>116
CTGCTGCCGCCCGTAGGAGTCTGGGCCGTGTCTCAGTCCCAGTGTGGCCGATCACCCTCTCAGGTCGGCTATG
TATCGTCGCCTTGGTGAGCCGTTACCTCACCAACTAGCTAATACAACGCAGGTCCATCTGGTAGTGATGCAAT
TTGCACCTTTAATTGACTATCATGCAATAATCAATTTTATGCGGTATTAGCCTATCGTTTTCCAATAGTTACT

CCCCCCGCTACCAGGCAGGTTACCTACGCGTTACTCACCCGTTCGCAACTCATCCAGAGAAGCAAGCTCCTCC
TTCAGCGTTCTACTTGCATGTATTAGGCACGCCGCCAGCGTTCGTCCTGAGCCAGGATCAAACTCT
>117
CTGCTGCCGCCCGTAGGAGTCTGGGCCGTGTCTCAGTCCCAGTGTGGCCGATCACCCTCTCAGGTCGGCTATG
TATCGTCGCCTTGGTGAGCCCTTACCCCACCAACTAGCTAATACAACGCAGGTCCATCTGGTAGTGATGCAAT
TGCACCTTTCAAACAGTTGTCATGCAACAACTGCTATTATGCGGTATTAGCTATCGTTTCCAATAGTTATCCC
CCGCTACCAGGCAGGTTACCTACGCGTACTCACCCGTTCGCAACTCATCCAGAGAAGCAAGCTCCTCCTCAGC
GTCTACTGCATGTATTAGGCACGCCGCCAGCGTCGTCCTGAGCCAGGATCAACTCT
>118
CTGCTGCCGCCCGTAGGAGTCTGGGCCGTGTCTCAGTCCCAGTGTGGCCGATCACCCTCCCAGGTCGGCTATG
TATCGTCGCCTTGGTGAGCCCTTACCCCACCAACTAGCTAATACAACGCAGGTCCATCTGGTAGTGATGCAAT
TGCACCTTTCAAACAGTTGTCATGCAACAACTGCTATTTATGCGGTATTTAGCTATCGTTTTCCAATAGTTAT
TCCCCCGCTACCAGGCAGGTTACCTACGCGTTACTCACCCGTTCGCAACTCATCCGGAGAAGCAAGCTCCTCC
TTCAGCGTCTA
>119
CTGCTGCCGCCCGTAGGAGTCTGGGCCGTGTCTCAGTCCCAGTGTGGCCGATCACCCTCTCAGGTCGGCTATG
TATCGTCGCCTTGGTGAGCCCTTACCCCACCAACTAGCTAATACAACGCAGGTCCATCTGGTAGTGATGCAAT
TGCACCTTTCAAACAGTTGTCATGCAACAACTGCTATTATGCGGTATTAGCTATCGTTTCCAATAGTTATCCC
CCGCTACCAGGCAGGTTACCTACGCGTTACTCACCCGTTCGCAACTCATCCAGAGAAGCAAGCTCCTCCTTCA
GCGTTCTACTTGCATGTATTACGGCACGCCGCCAGCGTTCGTCCTGAGCCAGGATCAAACTCT
>120
CTGCTGCCGCCCGTAGGAGTCTGGACCGTGTCTCAGTTCCAGTGTGGCCGATCACCCTCTCAGGTCGGCTACG
CATCGTTGCCTTGGTAAGCCGTTACCTTACCAACTAGCTAATGCGGCGCGGATCCATCTATAAGTGACAGCAA
AACCGTCTTTCACTATTGAACCATGCGGTTCAATATATTATCCGGTATTAGCTCCGGTTTCCCGAAGTTATCC
CAGTCTTATAGGTAGGTTATCCACGTGTTACTCACCCGTCCGCCGCTAACGTCAGAGGAGCAAGCTCCTCGTC
TGTTCGCTCGACTTGCATGTATTAGGCACGCCGCCAGTGTTCATCCTGAGCCAGGATCAAACTCT
>121
CTGCTGCCGCCCGTAGGAGTCTGGGCCGTGTCTCAGTCCCAGTGTGGCCGATCACCCTCTCAGGTCGGCTATG
TATCGTCGCCTTGGTGAGCCGTTACCCCACCAACTAGCTAATACAACGCAGGTCCATCTGGTAGTGGTGCAAT
TGCACCTTTTAAGCAAATGTCATGCAACATCTACTCTTATGCGGTATTAGCTATCGTTTCCAATAGTTATCCC
CCGCTACCAGGCAGGTTACCTACGCGTACTCACCCGTTCGCAACTCATCCGGAGAAGCAAGCTCCTCCTTCAG
CGTTCTACTTGCATGTATTAGGCACGCCGCCAGCGTTCGTCCTGAGCCAGGATCAAACTCT
>122
CTGCTGCCGCCCGTAGGAGTCTGGGCCGTGTCTCAGTCCCAGTGTGGCCGATCACCCTCTCAGGTCGGCTATG
TATCGTCGCCTTGGTGAGCCGTTACCCCACCAACTAGCTAATACAACGCAGGTCCATCTGGTAGTGGTGCAAT
TGCACCTTTTAAGCAAATGTCATGCAACATCTACTCTTATGCGGTATTAGCTATCGTTTTCCAATAGTTATCC
CCCGCTACCAGGCAGGTTACCTACGCGTTACTCACCCGTTCGCAACCCATCCGGAGAAGCAAGCTCCTCCTCA
GCGTTCTACTTGCATGTATTAGGCACGCCGCCAGCGTTCGTCCTGAGCCAGGATCAAACTCT
>123
CTGCTGCCGCCCGTAGGAGTCTGGGCCGTGTCTCAGTCCCAGTGTGGCCGATCACCCTCTCAGGTCGGCTATG
CATCGTCGCCTTGGTAGGCCTTTACCCCACCAACTAGCTAATGCACCGCAAAGCCATCTCATAGTGTTAGCAA
AACCAACTTTTAAACATCAACCATGCAGTTAATGCTGTTATCCGGTATTTAGCTATCGTTTTCCAATAGTTTG
TCCCAGTCTATGAGGCAGGTTTCTTTACGTGTTACTCACCCGTTCGCCGCTAAGTTCTTCCGGTGCAAGCACC
AAAAAACTTCGCTCGACTTGCATGTATTAGGCACGCCGCCAGCGTTCGTCCTGAGCCAGGATCAAACTCT
>124
CTGCTGCCGCCCGTAGGAGTCTGGGCCGTGTCTCAGTCCCAGTGTGGCCGATCACCCTCTCAGGTCGGCTATG
TATCGTCGCCTTGGTGAGCCCTTACCCCCACCAACTAGCTAATACAACGCAGGTCCATCTGGTAGTGATGCAA
TTGCACCTTTCAAACAGTTGTCATGCAACAACTGCTATTATGCGGTATTTAGCTATCGTTTTCCAATAGTTAT
CCCCCCGCTACCAGGCAGGTTACCTACGCGTTACTCACCCGTTCGCAACTCATCCAGAGAAGCAAGCTCCTCC
TTTCAGCGTTCTACTTGCATGTATTTAGGCACGCCGCCAGCGTTCGTCCTGAGCCAGGATCAAACTCTT
>125
CTGCTGCCGCCCGTAGGAGTCTGGGCCGTGTCTCAGTCCCAGTGTGGCCGATCACCCTCTCAGGTCGGCTATG
CATCGTCGCCTTGGTAGGCCTTTACCCCACCAACTAGCTAATGCACCGCAAAGCCATCTCATAGTGTTAGCAA
AACCAACTTTTAAACATCAACCATGCAGTTAATGCTGTTTATCCGGTATTTAGCTATCGTTTTCCAATAGTTT

GTCCCAGTCTATGAGGCAGGTTTCTTTACGTGTTACTCACCCGTTTCGCCGCTAAGTTGTCGCGACAGCAACC
AGAAACTAC
>126
CTGCTGCCGCCCTAGGAGTCTGGACCGTGTCTCAGTTCCAGTGTGGCCGATCACCCTCTCAGGTCGGCTACGT
ATCGTTGCCTTGGTAAGCCGTTACCTTACCAACTAGCTAATACGGCGCAGGTCCATCTATAAGTGATAGCAAA
GCCATCTTTCACTATCGAACCATGCGGTTCGAAATATTATCCGGTATTAGCTCCGGTTTCCCGAAGTTATCCC
AGTCTTATAGGTAGGTTACCTACGTGTTACTCACCCGTCCGCCGCTAACGCCAAAGGAGCAAGCTCCTCGTCC
GTGTTCGCTACGACTTGCGTGTATAGGCACGCC
>127
CTGCTGCCGCCCGTAGGAGTCTGGGCCGTGTCTCAGTCCCAGTGTGGCCGATCACCCTCTCAGGTCGGCTATG
TATCGTCGCCTTGGTGAGCCCTTACCCCACCAACTAGCTAATACAACGCAGGTCCATCTGGTAGTGATGCAAT
TGCACCTTTCAAACAGTTGTCATGCAACAACTGCTATTATGCGGTATTAGCTATCGTTTCCAATAGTTATCCC
CCGCTACCAGGCAGGTTACCTACGCGTTACTCACCCGTTCGCAACTCATCCAGAGAAGCAAGCTCCTCCTCAG
CGTTCTACTGCATGTATTAGGCACGCCGCCAGCGTCGTCCTGAGCCAGGATCAAACTCT
>128
CTGCTGCCGCCCGTAGGAGTCTGGGCCGTGTCCCAGTCCCAGTGTGGCCGATCACCCTCTCAGGTCGGCTATG
TATCGTCGCCTTGGTGAGCCCTTACCCCCACCAACTAGCTAATACAACGCAGGTCCATCTGGTAGTGATGCAA
TTGCACCTTTCAAACAGTTGTCATGCAACAACTGCTATTATGCGGTATTACGCTATCGTTTCCAATAGTTATC
CCCCGCCTACCAGGCAGGTTACCTACGCGTTACTCACCCGTTCGCAACTCATCCAGAGAAGCAAGCTCCTCCT
CAGCGTTCTACTGCATGTATTAGGCACGCCGCCAGCGTCGTCTG
>129
CTGCTGCCGCCCGTAGGAGTCTGGGCCGTGTCTCAGTCCCAGTGTGGCTGGTCGTCCTCTCAGACCAGCTACG
GATCGTCGCCTTGGTAAGCCTTTACCCCACCAACTAGCTAATCCGACATCGGCCGCTCCAATAGCGCGAGGCC
TTTTCGGTCCCCCCGCTTTCACCCTCAGGTCGTATGCGGTATTAATCCGGCTTCGCCGGGCTATCCCCCACTA
CTGGGCACGTTCCGATGCATTACTCACCCGTTCGCCACTCGCCGCCAGGCCGAAGCCCGCGCTGCCGTTCGAC
TTGCATGTGTAAAGCATGCCGCCAGCGTTCAATCTGAGCCAGGATCAAACTCT
>130
CTGCTGCCGCCCGTAGGAGTCTGGGCCGTGTCTCAGTCCCAGTGTGGCCGATCACCCTCTCGGGTCGGCTATG
TATCGTCGCCTTGGTGAGCCCTTACCCCACCAACTAGCTAATACAACGCAGGTCCATCTGGTAGTGATGCAAT
TGCACCTTTCAAACAGTTGTCATGCAACAACTGCTATTATGCGGTATTAGCTATCGTTTTCCAATAGTTATCC
CCCGCTACCAGGCAGGTTACCTACGCGTTACTCACCCGTTCGCAACTCATCCAGAGAAGCAAGCTCCTCCTTC
AGCGTTCTACTTGCATGTATTAGGCACGCCGCCAGCGTTCGTCCTGAGCCAGGATCAAACTCT
>131
CTGCTGCCGCCCGTAGGAGTCTGGGCCGTGTCTCAGTCCCAGTGTGGCCGATCACCCTCTCAGGTCGGCTATG
CATCGTCGCCTTGGTGAGCCCTTACCCCACCAACTAGCTAATACAACGCAGGTCCATCTGGTAGTGATGCAAT
TGCACCTTTCAAACAGTTGTCATGCAACAACTGCTATTATGCGGTATTAGCTATCGTTTTCCAATAGTTATCC
CCCGCTACCAGGCAGGTTACCTACGCGTTACTCACCCGTTCGCAACTCATCCGGAGAAGCAAGCTCCTCCTTC
GAGCGTTCTACTTGCATGTATTAGGCACGCCGCCAGCGTTCGCCCTGAGCCAGGATCAAACTCT
>132
CTGCTGCCGCCCGTAGGAGTCTGGGCCGTGTCTCAGTCCCAGTGTGGCCGATCACCCTCTCAGGTCGGCTATG
CATCGTCGCCTTGGTAGGCCTTTACCCCACCAACTAGCTAATGCACCGCAAAGCCATCTCATAGTGTTAGCAA
AACCAACTTTAAACATCAACCATGCAGTTAATGCTGTTATCCGGTATTAGCTATCGTTTCCAATAGTTGTCCC
AGTCTATGAGGCAGGTTCTTTACGTGTTACTCACCCGTTCGCCGCTAAGTTCGCGTCGACACGCGAACCAAGC
TAAACTAACGTCGACTTCGGCA
>133
CTGCTGCCGCCCGTAGGAGTCTGGGCCGTGTCTCAGTCCCAGTGTGGCCGATCACCCTCTCAGGTCGGCTATG
TATCGTCGCCTTGGTGAGCCGTTACCCCACCAACTAGCTAATACAACGCAGGTCCATCTGGTAGTGGTGCAAT
TGCACCTTTTAAGCAAATGTCATGCAACATCTACTCTTTATGCGGTATTTAGCATCGTTTTCCAATAGTTTAT
CCCCCCCGCTGCCAGGCAGGTTTACCTACGCGTTACTCACCCGTTTCGCAACTCATCCGGAGAAGCAAGCTCC
TCCTTCAGCGTTCTACTTGCATGTATTTAGGCACGCCGCCAGCGTTCGTTCCTGAGCCAGGATCAAACTCTT
>134
CTGCTGCCGCCCGTAGGAGTCTGGGCCGTGTCTCAGTCCCAGTGTGGCCGATCACCCTCTCAGGTCGGCTATG
TATCGTCGCCTTGGTGAGCCGTTACCCCACCAACTAGCTAATACAACGCAGGTCCATCTGGTAGTGGTGCAAT
TGCACCTTTTAAGCAAATGTCATGCAACATCTACTCTTATGCGGTATTAGCTATCGTTTCCAATAGTTATCCC

CCGCTACCAGGCAGGTTACCTACGCGTTACTCACCCGTTCGCAACTCATCCGGAGAAGCAAGCTCCTCCTTCA
GCGTTCTACTTGCATGTATTAGGCACGCCGCCAGCGTTCGTCCTGAGCCAGGATCAAACTCT
>135
CTGCTGCCGCCCGTAGGAGTCTGGGCCGTGTCTCAGTCCCAGTGTGGCCGATCACCCTCTCAGGTCGGCTATG
TATCGTCGCCTTGGTGAGCCCTTACCCCACCAACTAGCTAATACAACGCAGGTCCATCTGGTAGTGATGCAAT
TGCACCTTTCAAACAGTTGTCATGCAACAACTGCTATTATGCGGTATTAGCTATCGTTTCCAATAGTTATCCC
CCGCTACCAGGCAGGTTACCTACGCGTTACTCACCCGTTCGCAACTCATCCAGAGAAGCAAGCTCCTCCTTCA
GCGTTCTACTTGCATGTATTAGGCACGCCGCCAGCGTTCGTCCTGAGCCAGGATCAAACTCT
>136
CTGCTGCCGCCCGTAGGAGTCTGGGCCGTGTCTCAGTCCCAGTGTGGCCGATCACCCTCTCAGGTCGGCTATG
TATCGTCGCCTTGGTGAGCCGTTACCTCACCAACTAGCTAATACAACGCAGGTCCATCTGGTAGTGATGCAAT
TGCACCTTTTAATTGACTATCATGCAATAATCAATTTTATGCCGGTATTAGCCTATCGTTTCCAATAGTTACT
CCCCCGCTACCAGGCAGGTTACCTACGCGTACTCACCCGTTCGCAACTCATCCAGAGAAGCAAGCTCCTCCTC
AGCGTTCTACTTGCATGTATTACGGCACGCCGCCAGCGTCGTCCTGAGCCAGGATCAAACTCT
>137
CTGCTGCCGCCCGTAGGAGTCTGGGCCGTGTCTCAGTCCCAGTGTGGCCGATCACCCTCTCAGGTCGGCTATG
TATCGTCGCCTTGGTGAGCCCTTACCCCACCAACTAGCTAATACAACGCAGGTCCATCTGGTAGTGATGCAAT
TGCACCTTTCAAACAGTTGTCATGCAACAACTGCTATTATGCGGTATTAGCTATCGTTTCCAATAGTTATCCC
CCGCTACCAGGCAGGTTACCTACGCGTACTCACCCGTTCGCAACTCATCCAGAGAAGCAAGCTCCTCCTTCAG
CGTTCTACTTGCATGTATTAGGTACGCCGCCAGCGTTCGTCCTGAGCCAGGATCAAACTCT
>138
CTGCTGCCGCCCGTAGGAGTCTGGGCCGTGTCTCAGTCCCAGTGTGGCCGATCACCCTCTCAGGTCGGCTATG
TATCGTCGCCTTGGTGAGCCCTTACCCCACCAACTAGCTAATACAACGCAGGTCCATCTGGTAGTGGTGCAAT
TGCACCTTTTAAGCAAATGTCATGCAACATCTACTCTTATGCGGTATTAGCTATCGTTTCCAATAGTTATCCG
CTACCAGGCAGGTTACCTACGCGTTACTCACCCGTTCGCAACTCATCCGGAGAAGCAAGCTCCTCCTCAGCGT
TCTACTTGCATGTATTAGGCACGCCGCCAGCGTTCGTCCTGAGCCAGGATCAAACTCT
>139
CTGCTGCCGCCCGTAGGAGTCTGGGCCGTGTCTCAGTCCCAGTGTGGCCGATCACCCTCTCAGGTCGGCTATG
TATCGTCGCCTTGGTGAGCCGTTACCCCACCAACTAGCTAATACAACGCAGGTCCATCTGGTAGTGATGCAAT
GCACCTTTTAAGCAAATGTCATGCAACATCTACTCTTATGCGGTATTAGCTATCGTTTCCAATAGTTATCCCC
CGCTACCAGGCAGGTTACCTACGCGTTACTCACCCGTTCGCAACTCATCCAGAGAAGCAAGCTCCTCCTTCAG
CGTTCTACTGCATGTATTAGGCACGCCGCCAGCGTCGTCCTGAGCCAGGATCAAACTCT
>140
CTGCTGCCGCCCGTAGGAGTCTGGGCCGTGTCTCAGTCCCAGTGTGGCCGATCACCCTCTCAGGTCGGCTATG
TATCGTCGCCTTGGTGAGCCCTTACCCCACCAACTAGCTAATACAACGCAGGTCCATCTGGTAGTGATGCAAT
TGCACCTTTCAAACAGTTGTCATGCAACAACTGCTATTATGCGGTATTAGCTATCGTTTTCCAATAGTTATCC
CCCGCTACCAGGCAGGTTACCTACGCGTTACTCACCCGTTCGCAACTCATCCGGAGAAGCAAGCTCCTCCTTC
AGCGTTCTACTTGCATGTATTAGGCACGCCGCCAGCGTTCGTCCTGAGCCAGGATCAAACTCT
>141
CTGCTGCCGCCCGTAGGAGTCTGGGCCGTGTCTCAGTCCCAGTGTGGCCGATCACCCCTCTCAGGTCGGCTAT
GTATCGTCGCCTTGGTGAGCCGTTACCCCACCAACTAGCTAATACAACGCAGGTCCATCTGGTAGTGGTGCAA
TTGCACCTTTTAAGCAAATGTCATGCAACATCTACTCTTATGCGGTATTAGCTATCGTTTCCAATAGTTATCC
CCCGCTACCAGGCAGGTTACCTACGCGTACTCACCCGTTCGCAACTCATCCAGAGAAGCAAGCTCCTCCTTCA
GCGTTCTACTGCATGTATTAGGCACGCCGCCAGCGTTCGTCCTGAGCCAGGATCAAACTCT
>142
CTGCTGCCGCCCGTAGGAGTCTGGGCCGTGTCTCAGTCCCAGTGTGGCCGATCACCCTCTCAGGTCGGCTATG
TATCGTCGCCTTGGTGAGCCCTTACCCCACCAACTAGCTAATACAACGCAGGTCCATCTGGTAGTGATGCAAT
TGCACCTTTCAAACAGTTGTCATGCAACAACTGCTATTATGCGGTATTAGCTATCGTTTCCAATAGTTATCCC
CCGCTACCAGGCAGGTTACCTACGCGTTACTCACCCGTTCGCAACTCATCCAGAGAAGCAAGCTCCTCCTCAG
CGTTCTACTTGCATGTATTAGGCACGCCGCCAGCGTTCGTCCTGAGCCAGGATCAAACTCT
>143
CTGCTGCCGCCCGTAGGGGTCTGGGCCGTGTCTCAGTCCCAGTGTGGCCGATCACCCTCTCAGGTCGGCTATG
TATCGTCGCCTTGGTGAGCCGTTACCCCCACCAACTAGCTAATACAACGCAGGTCCATCTGGTAGTGGTGCAA
TTGCACCTTTTAAGCAAATGTCATGCAACATCTACTCTTATGCGGTATTAGCTATCGTTTCCAATAGTTATCC

CCCGCTACCAGGCAGGTTACCTACGCGTTACTCACCCGTTCGCAACTCATCCGGAGAAGCAAGCTCCTCCTTC
AGCGTTCTACTTGCATGTATTAGGCACGCCGCCAGCGTTCGTCCTGAGCCAGGATCAAACTCT
>144
CTGCTGCCGCCCGTAGGAGTCTGGGCCGTGTCTCAGTCCCAGTGTGGCCGATCACCCTCTCAGGTCGGCTATG
TATCGTCGCCTTGGTGAGCCCTTACCCCACCAACTAGCTAATACAACGCAGGTCCATCTGGTAGTGATGCAAT
TGCACCTTTCAAACAGTTGTCATGCAACAACTGCTATTATGCGGTATTAGCTATCGTTTCCAATAGTTATCCC
CCGCTACCAGGCAGGTTACCTACGCGTTACTCACCCGTTCGCAACTCATCCGGAGAAGCAAGCTCCTCCTTCA
GCGTTCTACTTGCCATGTATTAGGCACGCCGCCAGCGTTCGTCCTGAGCCAGGATCAACTCT
>145
CTGCTGCCGCCCGTAGGAGTCTGGGCCGTGTCTCAGTCCCAGTGTGGCCGATCACCCTCTCAGGTCGGCTATG
TATCGTCGCCTTGGTGAGCCCTTACCCCACCAACTAGCTAATACAACGCAGGTCCATCTGGTAGTGATGCAAT
TGCACCTTTCAAACAGTTGTCATGCAACAACTGCTATTATGCGGTATTAGCTATCGTTTTCCAATAGTTATCC
CCCGCTACCAGGCAGGTTACCTACCGCGTTACTCACCCGTTCGCAACTCATCCAGAGAAGCAAGCTCCTCCTT
CAGCGTTCTACTTGCCATGTATTAGGCACGCCGCCAGCGTTCGTCCTGAGCCAGGATCAAACTCT
>146
CTGCTGCCGCCCTAGGAGTCTGGGCCGTGTCTCAGTCCCAGTGTGGCCGCATCACCCTCTCAGGTCGGCTATG
TATCGTCGCCTTGGTCGAGCCCTTACCCCACCAACTAGCTAATACAACGCAGGTCCATCTGGTAGTGATGCAA
TTGCACCTTTCAACAGTTGTCATGCAACAACTGCTATTTATGCGGTATTTAGCTATCGTTTTCCAATAGTTTA
TCCCCCCGCTACCAGGCAGGTTACCTTACGCGTTACTCACCCGTTCGCAACTCATCCGGAGAGCAAGCTCCTC
CTCAGCGTTCTACTTGCATGTATTTAGGCACGCCGCCAGCGTTCGTCCTGAGCCAGGATCAAACTCTT
>147
CTGCTGCCGCCCGTAGGAGTCTGGGCCGTGTCTCAGTCCCAGTGTGGCCGATCACCCTCTCAGGTCGGCTATG
TATCGTCGCCTTGGTGAGCCGTTACCCCACCAACTAGCTAATACAACGCAGGTCCATCTGGTAGTGATGCAAT
GCATCTTTTAAGCAAATGTCATGCAACATCTACTATTATGCGGTATTAGCTATCGTTTTCCAATAGTTATCCC
CCGCTACCAGGCAGGTTACCTACGCGTTACTCACCCGTTCGCAACTCATCCAGAGAAGCAAGCTCCTCCTCAG
CGTCTACTTGCATGTATTAGGCACGCCGCCAGCGTCGTCCTGAGCCAGGATCAACTCT
>148
CTGCTGCCGCCCGTAGGAGTCTGGGCCGTGTCTCAGTCCCAGTGTGGCCGATCACCCTCTCAGGTCGGCTATG
TATCGTCGCCTTGGTGAGCCGTTACCTCACCAACTAGCTAATACAACGCAGGTCCATCTGGTAGTGATGCAAT
TTGCACCTTTAATTGACTATCATGCAATAATCAATTTTATGCGGTATTAGCCTATCGTTTCCAATAGTTACTC
CCCCGCTACCAGGCAGGTTACCTACGCGTTACTCACCCGTTCGCAACTCATCCAGAGAAGCAAGCCTCCTCCT
TCAGCGTTCACTTGCATGTATTAGGCACACCGCCAGCGTTCGTCCTGAGCCAGGATCAAACTCT
>149
CTGCTGCCGCCCGTAGGAGTCTGGGCCGTGTCTCAGTCCCAGTGTGGCCGATCACCCTCTCAGGTCGGCTATG
TATCGTCGCCTTGGTGAGCCGTTACCCCACCAACTAGCTAATACAACGCAGGTCCATCTGGTAGTGGTGCAAT
TGCACCTTTTAAGCAAATGTCATGCAACATCTACTCTTATGCGGTATTAGCTATCGTTTCCAATAGTTATCCC
CCGCTACCAGGCAGGTTACCTACGCGTTACTCACCCGTTCGCAACTCATCCGGAGAAGCAAGCTCCTCCTTCA
GCGTTCTACTTGCATGTATTAGGCACGCCGCCAGCGTTCGTCCTGAGCCAGGATCAAACTCT
>150
CGCTGCCGCCCGTAGGAGTCTGGGCCGTGTCTCAGTTCCAGTGTGGCCGATCACCCTCTCAGGTCGGCTACGT
ATCGTTGCCTTGGTAAGCCGTTCCTTACCAACTAGCTAATACGGCGCAGGTCCATCTATAAGTGATAGACAAA
GCCATCTTCACTATCAACCTGCGGTTCGAAATATTATCCGGTATTAGCTCCGGTTTCCCGAAGTTATCCCAGT
CTTATAGGTAGGTTTACCTACGTGTTACTCACCCGTCCGCCGCTAACATCAAAGGAGCAAGCTCCTCGTCTGT
TCGCTCGACTTGCATGTATTAGGCACGCCGCCAGCGTTCATCCTGAGCCAGGATCAAACCTCT
>151
CTGCTGCCGCCCGTAGGAGTCTGGGCCGTGTCTCAGTCCCAGTGTGGCCGATCACCCTCTCAGGTCGGCTATG
TATCGTCGCCTTGGTGAGCCGTTACCCCACCAACTAGCTAATACAACGCAGGTCCATCTGGTAGTGGTGCAAT
TGCACCTTTTAAGCAAATGTCATGCAACATCTACTCTTATGCGGTATTAGCTATCGTTTCCAATAGTTATCCC
CCGCTACCAGGCAGGTTACCTACGCGTTACTCACCCGTTCGCAACTCATCCAGAGAAGCAAGCTCCTCCTTCA
GCGTTCTACTTGCATGTATTAGGCACGCCGCCAGCGTTCGTCCTGAGCCAGGATCAAACTCT
>152
CTGCTGCCGCCCGTAGGAGTCTGGGCCGTGTCTCAGTCCCAGTGTGGCCGGTCGCCCTCTCAGGCCGGCTACC
CGTCATCGCCTTGGTGAGCCATTACCTCACCAACAAACTGATAGGCCGCGAGTCCATCCCCCACCGAAAAACT
TTCCACAACACACCATGCGGTGCGAAGTCATATCCGGTATTACGCCCCGATTTCCCGAGGTTATCCCGAAGAG

GGGGGCAGGTTACTCACGTGTTACTCACCCGTTCGCCACTAATCGGACAGGGCAAGCCCTGTCGTCATCGTTC
GACTTGCATGTGTTAAGCACGCCGCCAGCGTTCGTCCTGAGCCAGGATCAAACTCT
>153
CTGCTGCCGCCCGTAGGAGTCTGGGCCGTGTCTCAGTCCCAGTGTGGCCGATCACCCTCTCAGGTCGGCTATG
TATCGTCGCCTTGGTGAGCCCTTACCCCACCAACTAGCTAATACAACGCAGGTCCATCTGGTAGTGATGCAAT
TGCACCTTTCAAACAGTTGTCATGCAACAACTGCTATTATGCGGTATTAGCTATCGTTTCCAATAGTTATCCC
CCGCTACCAGGCAGGTTACCTACGCGTACTCACCCGTTCGCAACTCATCCGGAGAAGCAAGCTCCTCCTTCAG
CGTTCTACTTGCATGTATTAGGCACGCCGCCAGCGTTCGTCCTGAGCCAGGATCAAACTCT
>154
CTGCTGCCGCCCGTAGGAGTCTGGGCCGTGTCTCAGTCCCAGTGTGGCCGGTCGCCCTCTCAGGCCGGCTACC
CGTCATCGCCTTGGGTGAGCCATTACCTCACCAACAAACTGATAGGCCGCGAGTCCATCCCCCACCGAAAAAC
TTCCACAACACACCATGCGGTGCGAAGTCATATCCCGGTATTAGCCCCGATTTCCCGAGGTTATCCCGAAGAG
GGGGGCAGGTTACTCACGTGTTACTCACCCGTTCGCCACTAATCGGACAGGGCAAGCCCTGTCGTCATCGTTC
GACTTGCATGTGTTAAGCACGCCGCCAGCGTTCGTCCTGAGCCAGGATCAAACTCT
>155
CTGCTGCCGCCCGTAGGAGTCTGGGCCGTGTCTCAGTCCCAGTGTGGCCGATCACCCTCTCAGGTCGGCTATG
TATCGTCGCCTTGGTGAGCCGTTACCCCACCAACTAGCTAATACAACGCAGGTCCATCTGGTAGTGGTGCAAT
TGCACCTTTCAAACAGCTGTCATGCAACAACTGCTATTTATGCGGTATTTAGCTATCGTTTTCCAATAGTTAT
CCCCCCGCTACCAGGCAGGTTACCTACGCGTTACTCACCCGTTCGCAACTCATCCAGAGAAGCAAGCTCCTCC
TTCAGCGTTCTACTTGCATGTATTTAGGCACGCCGCCAGCGTTCGTCCTGAGCCAGGATCAAACTCT
>156
CTGCTGCCGCCCGTAGGAGTCTGGGCCGTGTCTCAGTCCCAGTGTGGCCGATCACCCCCTCAGGTCGGCTATG
TATCGTCGCCTTGGTGAGCCGTTACCTCACCAACTAGCTAATACAACGCAGGTCCATCTGGTAGTGATGCAAT
TGCACCTTTTAATTGACTATCCTGCAATAATCAATTTTATGCGGTATTAGCTATCCGTTTCCAATAGTTATCC
CCCGCTACCAGGCAGGTTACCTACGCGTACTCACCCGTTCGCAACTCATCCAGAGAAGCAAGCTCCTCCTTCA
GCGTTCTACCTT
>157
CTGCTGCCGCCCGTAGGAGTCTGGACCGTGTCTCAGTTCCAGTGTGGCCGATCACCCTCTCAGGTCGGCTATG
CATCGTTGCCTTGGTAAGCCGTTACCTTACCAACTAGCTAATGCAGCGCGGATCCATCTATAAGTGACAGCAA
GACCGTCTTTCACTTTTTGAACCAGCGGTTTCAAAATATTATCCGGTATTTAGCTCCGGTTTTTCCCGAAGTT
ATCCCAGTCTTTATAGGTAGGTTTATCCACGTGTTTACTCACCCGTCCGCCGCTAACATCAGAGAAGCAAGCT
CTCGTCCGTTCGCTCGACTGCATGTATTTAGGCACGCCGCCAGCGTCATCCTGAGCCAGGATCAAACTCT
>158
CTGCTGCCGCCCGTAGGAGTCTGGGCCGTGTCTCAGTCCCAGTGTGGCCGATCACCCTCTCAGGTCGGCTATG
TATCGTCGCCTTGGTGAGCCGTTACCCCACCAACTAGCTAATACAACGCAGGTCCATCTGGTAGTGGTGCAAT
TGCACCTTTTAAGCAAATGTCATGCAACATCTACTCTTATGCGGTATTAGCTATCGTTTCCAATAGTTATCCC
CCGCTACCAGGCAGGTTACATACGCGTTACTCACCCGTTCGCAACTCATCCGGAGAAGCAAGCTCCTCCTTCA
GCGTTCTACTTGCATGTATTAGGCACGCCGCCAGCGTTCGTCCTGAGCCAGGATCAAACTCT
>159
CTGCTGCCGCCCGTAGGAGTCTGGGCCGTGTCTCAGTCCCAGTGTGGCCGATCACCCTCCTCAGGTCGGCTAT
GTATCGTCGCCTTGGTGAGCCGTTACCCCACCAACTAGCTAATACAACGCAGGTCCATCTGGTAGTGATGCAA
TTGCATCTTTTAAGCAAATGTCATGCAACATCTACTATTATGCGGTATTAGCTATCGTTTCCAATAGTTATCC
CCCGCTACCAGGCAGGTTACCTACGCGTTACTCACCCGTTCGCAACTCATCCGGAGAAGCAAGCTCCTCCTTC
AGCGTTCTACTTGCATGTATTAGGCACGCCGCCAGCGTTCGTCCTGAGCCAGGATCAAACTCT
>160
CTGCTGCCGCCCGTAGGAGTCTGGGCCGTGTCTCAGTCCCAGTGTGGCCGATCACCCTCTCAGGTCGGCTATG
TATCGTCGCCTTGGTGAGCCCTTACCCCACCAACTAGCTAATACAACGCAGGTCCATCTGGTAGTGATGCAAT
TGCACCTTTCAAACAGTTGTCATGCAACAACTGCTATTTATGCGGTATTTAGCTATCGTTTTCCAATAGTTAT
CCCCCGCTACCAGGCAGGTTACCTACGCGTTACTCACCCGTTCGCAACTCATCCAGAGAAGCAAGCTCCTCCT
TTCAGCGTTCTACTGCATGTATTAGGCACGCCGCCAGCGTCGTCCTGAGCCAGGATCAAACTCT
>161
CTGCTGCCGCCCGTAGGAGTTTGGCCGGTGTCTCAGTCCCAATCGTGGCTGATCATCCTCCTCAGACCAGCTA
CTGATCGTCGCCTTGGTGAGCCTTTACCTCACCAACTAGCTAATCAGACGCGGGCCGCTCTAAAGGCGATAAA
TCTTTCCCCCCGAAGGGCACATTCGGTATTTAGCACAAGTTTCCCTGAGTTTATTTCCGAACCTAAAGGCACG

TTCCCACGTGTTACCTCACCCGTCCGCCACTAAGTCCGAAGACTTCGTTTCGACTTGCATGTGTTAGGCCTGC
CGCCAGCGTTCATCCTGAGCCAGGATCAAACTCT
>162
CTGCTGCCGCCCGTAGGAGTCTGGACCGTGTCTCAGTTCCAGTGTGGCCGATCACCCTCTCAGGCGGCTACGT
ATCGTTGCCTTGGTAAGCCGTTACCTTACCAACTAGCTAATACGGCGCAGGTCCATCTATAAGTGATAGCAAA
GCCATCTTTCACTATCGAACCATGCGGTTCGAAATATTATCCGGTATTAGCTCCGGTTTTCCCGAAGTTATCC
CAGTCTTATAGGTAGGTTTACCTACGTGTTACTCACCCGTCCGCCGCTAACGTCAAAGGAGCAAGCTCCTCGT
CTGTTCGCTCGACTTGCATGTATTAGGCACGCCGCCAGCGTCATCCTGAGCCAGGATCAAACTCT
>163
CTGCTGCCGCCCGTAGGAGTCTGGGCCGTGTCTCAGTCCCAGTGTGGCCGGTCGCCCTCTCAGGCCGGCTACC
CGTCATCGCCTTGGTGAGCCATTACCTCACCAACAAACTGATAGGCCGCGAGTCCATCCCCCACCGAAAAACT
TCCACAACACACCATGCGGTGCGAAGTCATATCCCGGTATTACGCCCCGATTTCCCGAGGTTATCCCGAAGAG
GGGGGCAGGTTACTCACGTGTTACTCACCCGTTCGCCACTAATCGACAGGGCAAGCCCTGTCGTCATCGTTCG
ACTTGCATGTGTTAAGCACGCCGCCAGCGTTCGTCCTGAGCCAGGATCCAAACTCT
>164
CTGCTGCCGCCCGTAGGAGTCTGGGCCGTGTCTCAGTCCCAGTGTGGCCGATCACCCTCTCAGGTCGGCTATG
TATCGTCGCCTTGGTGAGCCGTTACCCCACCAACTAGCTAATACAACGCAGGTCCATCTGGTAGTGGTGCAAT
TGCACCTTTTAAGCAAATGTCATGCAACATCTACTCTTATGCGGTATTAGCTATCGTTTCCAATAGTTGTCCC
CCGCTACCAGGCAGGTTACCTACGCGTACTCACCCGTTCGCAACTCATCCGCTCGGTGCAAGCACCAAGCTTC
AGCGTTCTACTTGCATGTATTAGGCACGCCGCCAGCATTCGTCCTGAGCCAGGATCAAACTCT
>165
CTGCTGCCGCCCGTAGGAGTCTGGGCCGTGTCTCAGTCCCAGTGTGGCCGATCACCCTCTCAGGTCGGCTATG
TATCGTCGCCTTGGTGAGCCCTTACCCCACCAACTAGCTAATACAACGCAGGTCCATCTGGTAGTGATGCAAT
TGCACCTTTCAAACAGTTGTCATGCAACAACTGCTATTATGCGGTATTTAGCTATCGTTTTCCAATAGTTTAT
CCCCCCGCTACCAGGCAGGTTACCTACGCGTTACTCACCCGTTTCGCAACTCATCCGGAGAAGCAAGCTCCTC
CTCAGCGTTCTACTTGCCATGTATTTAGGCACGCCGCCAGCGTTCGTTCCTGAGCCAGGATCAAACTCT
>166
CTGCTGCCGCCCGTAGGAGTCTGGGCCGTGTCTCAGTCCCAGTGTGGCCGATCACCCTCCTCGAGTCGGCTAT
GTATCGTCGCCTTGGTGAGCCCTTACCCCACCAACTAGCTAATACAACGCAGGTCCATCTGGTAGTGATGCAA
TTGCACCTTTCAAACAGTTGTCATGCAACAACTGCTATTATGCGGTATTAGCTATCGTTTCCAATAGTTATCC
CCCGCTACCAGGCAGGTTACCTACGCGTTACTCACCCGTTCGCAACTCATCCAGAGAAGCAAGCTCCTCCTTC
AGCGTTCTACTTGCATGTATTAGGCACGCCGCCAGCGTTCGTCCTGAGCCAGGATCAAACTCT
>167
CTGCTGCCGCCCGTAGGAGTCTGGGCCGTGTCTCAGTCCCAGTGTGGCCGATCACCCTCTCAGGTCGGCTATG
TATCGTCGCCTTGGTGAGCCGTTACCCCACCAACTAGCTAATACAACGCAGGTCCATCTGGTAGTGATGCAAT
TGCACCTTTTAAGTAAATGTCATGCAACATCTACTCTTATGCGGTATTAGCTATCGTTTTCCAATAGTTTATC
CCCCGCTACCAGGCAGGTTACCTACGCGTTACTCACCCGTTCGCAACTCATCCAGAAGAGCAAGCTCCTCCTT
TCAGCGTTCTACTTGCATGTATTTAGGCACGCCGCCAGCGTTCGTCCTGAGCCAGGATCAAACTCT
>168
CTGCTGCCGCCCGTAGGAGTCTGGGCCGTGTCTCAGTCCCAGTGTGGCCGATCACCCTCTCAGGTCGGCTATG
TATCGTCGCCTTGGTGAGCCGTTACCTCACCAACTAGCTAATACAACGCAGGTCCATCTGGTAGTGATGCAAT
TGCACCTTTTAATTGACTATCATGCAATAATCAATTTTATGCGGTATTAGCCTATCGTTTCCAATAGTTATCC
CCCGCTACCAGGCAGGTTACCTACGCGTTACTCACCCGTTCGCAACTCATCCAGAGAAGCAAGCTCCTCCTTC
AGCGTTCTACTTGCATGTATTAGGCACGCCGCCAGCGTTCGTCCTGAGCCAGGATCAAACTCT
>169
CTGCTGCCGCCGTAGGAGTCTGGACCGTGTCTCAGTTCCAGCTGTGGCCGATCACCCTCTCAGGTCGGCTACG
TATCGTCGCCTTGGGTAAGCCGTTACCTTACCAACTAGCTAATACGGCGCGGGTCCATCTATAAGTGATAGCA
AAACCATCTTTCACTATCGAACCATGCGGTTCGAAATATTATCCGGTATTAGCTCCGGTTTCCCGAAGTTATC
CCAGTCTTATAGGTAGGTTACCCACGTGTTACTCACCCGTCCGCCGCTAACGTCAAAGGAGCAAGCTCCTTGT
CTGTTCGCTCGACTTGCATGTATTAGGCACGCCGCCAGCGTTCATCCTGAGCCAGGATCAAACTCT
>170
CTGCTGCCGCCCGTAGGAGTCTGGACCGTGTCTCAGTTCCAGTGTGGCCGATCACCCTCTCAGGTCGGCTACG
TATCGTTGCCTTGGTAAGCCGTTACCTTACCAACTAGCTAATACGGCGCAGGTCCATCTATAAGTGATAGCAA
AGCCATCTTTCACTATCGAACCATGCGGTTTCGAAATATTATCCGGTATTAGCTCCGGTTTTTCCCGAAGTTA

TCCCAGTCTTATAGGTAGGTTTTACCTACGTGTTTACTCATCCGTCCGCCGCTAACGTCGAAGGAGCAAGCTC
CTCGTCTGTTTCGCTCGACTGCATGTATTAGGCACGCCGTTCAGCGTTCGCCTGAGCCAGGATCAAACTCT
>171
CTGCTGCCGCCCGTAGGAGTCTGGGCCGTGTCTCAGTCCCAGTGTGGCCGATCACCCTCTCAGGTCGGCTATG
TATCGTCGCCTTGGTGAGCCCTTACCCCACCAACTAGCTAATACAACGCAGGTCCATCTGGTAGTGATGCAAT
TGCACCTTTCAAACAGTTGTCATGCAACAACTGCTATTATGCGGTATTAGCTATCGTTTCCAATAGTTATCCC
CCGCTACCAGGCAGGTTACCTACGCGTTACTCACCCGTTCGCAACTCATCCAGAGAAGCAAGCTCCTCCTTCA
GCGTTCTACTTGCATGTATTAGGCACGCCGCCAGCGTTCGTCCTGAGCCAGGATCAAACTCT
>172
CTGCTGCCGCCCGTAGGAGTCTGGGCCGTGTCTCAGTCCCAGTGTGGCCGATCACCCTCTCAGGTCGGCTATG
TATCGTCGCCTTGGTGAGCCGTTACCTCACCAACTAGCTAATACAACGCAGGTCCACCTGGTAGTGATGCAAT
TGCACCTTTTAATTGACTATCATGCAATAATCAATTTTATGCGGTATTAGCTATCGTTTCCAATAGTTATCCC
CCGCTACCAGGCAGGTTACCTACGCGTACTCACCCGTTCGCAACTCATCCAGAGGAGCAAGCTCCTCCTCAGC
GTTCTACTGCATGTATTAGGCACGCCGCCAGCGTCGTCCTGAGCCAGGATCAAACTCT
>173
CTGCTGCCGCCCGTAGGAGTCTGGGCCGTGTCTCAGTCCCAGTGTGGCCGATCACCCTCTCAGGTCGGCTATG
TATCGTCGCCTTGGTGAGCCGTTACCTCACCAACTAGCTAATACAACGCAGGTCCATCTGGTAGTGATGCAAT
TGCACCTTTTAATTGACTATCATGCAATAATCAATTTTATGCGGTATTAGCCTATCGTTTCCAATAGTTATCC
CCCGCTACCAGGCAGGTTACCTACGCGTTACTCACCCGTTCGCAACTCATCCAGAGAAGCAAGCTCCTCCTTC
AGCGTTCTACTTGCATGTATTAGGCACGCCGCCAGCGTTCGTCCTGAGCCAGGATCAAACTCT
>174
CTGCTGCCGCCCGTAGGAGTCTGGGCCGTGTCTCAGTCCCAGTGTGGCCGATCACCCTCTCAGGTCGGCTATG
TATCGTCGCCTTGGTGAGCCCTTACCCCACCAACTAGCTAATACAACGCAGGTCCATCTGGTAGTGATGCAAT
TGCACCTTTCAAACAGTTGTCATGCAACAACTGCTATTATGCGGTATTAGCTATCGTTTCCAATAGTTATCCC
CCGCTACCAGGCAGGTTACCTACGCGTTACTCACCCGTTCGCAACTCATCCGGAGAAGCAAGCTCCTCCTTCA
GCGTTCTACTTGCATGTATTAGGCACGCCGCCAGCGTTCGTCCTGAGCCAGGATCAAACTCT
>175
CTGCTGCCGCCCGTAGGAGTCTGGGCCGTGTCTCAGTCCCAGTGTGGCCGATCACCCTCTCAGGTCGGCTATG
TATCGTCGCCTTGGTGAGCCCTTACCCCACCAACTAGCTAATACAACGCAGGTCCATCTGGTAGTGATGCAAT
TGCACCTTTCAAACAGTTGTCATGCAACAACTGCTATTATGCGGTATTAGCTATCGTTTTCCAATAGTTATCC
CCCCGCTACCAGGCAGGTTACCTACGCGTTACTCACCCGTTCGCAACTCATCCAGAGAAGCAAGCTCCTCCTT
CAGCGTTCTACTGCCATGTATTAGGCACGCCGCCAGCGTCGTCCTGAGCCAGGATCAAACTCT
>176
CTGCTGCCGCCCGTAGGAGTCTGGGCCGTGTCTCAGTCCCAGTGTGGCCGATCACCCTCTCAGGTCGGCTATG
TATCGTCGCCTTGGTGAGCCCTTACCCCACCAACTAGCTAATACAATGCAGGTCCATCTGGTAGTGATGCAAT
TGCACCTTTCAAACAGTTGTCATGCAACAACTGCTATTTATGCGGTATTTAGCTATCGTTTTCCAATAGTTTA
TCCCCCCGCTACCAGGCAGGTTACCTTACGCGTTACTCACCCGTTTCGCAACTCATCCGGAGAAGCAAGCTCC
TCCTCAGCGTTCTACTTGCATGTATTTAGGCACGCCGCCAGCGTTCGTCCTGAGCCAGGATCAAACTCT
>177
CTGCTGCCGCCCGTAGGAGTCTGGGCCGTGTCTCAGTCCCAGTGTGGCCGATCACCCTCTCAGGTCGGCTATG
CATCGTCGCCTTGGTAGGCCTTTACCCCACCAACTAGCTAATGCACCGCAAAGCCATCTCATAGTGTTAGCAA
AACCAACTTTTAAACATCAACCATGCAGTTAATGCTGTTATCCGGTATTAGCCTATCGTTTTCCAATAGTTTT
GTCCCAGTCTATGAGGCAGGTTTCCTTTACGTGTTACTCACCCGTTTCGCCGCTAAGTTTCGTTCGCGACACG
AAACATCC
>178
CTGCTGCCGCCCGTAGGAGTCTGGGCCGTGTCTCAGTTCCAGTGTGGCCGATCACCCTCTCAGGTCGGCTATG
TATCGTCGCCTTGGTGAGCCCTTACCCCACCAACTAGCTAATACAACGCAGGTCCATCTGGTAGTGATGCAAT
TGCACCTTTCAAACAGTTGTCATGCAACAACTGCTATTATGCGGTATTTAGCTATCGTTTTCCAATAGTTTAT
CCCCCGCTACCAGGCAGGTTACCTACCGCGTTACTCACCCGTCGCAACTCATCCAGAGAAGCAAGCTCCTCCT
CAGCGTCTACTGCATGTATTTAGGCACGCCGCCAGCGTCGTCCTGAGCCAGGATCGAACTCT
>179
CTGCTGCCGCCCGTAGGAGTCTGGGCCGTGTCTCAGTCCCAGTGTGGCCGATCACCCTCTCAGGTCGGCTATG
TATCGTCGCCTTGGTGAGCCCTTACCCCACCAACTAGCTAATACAACGCAGGTCCATCTGGTAGTGATGCAAT
TGCACCTTTCAAACAGTTGTCATGCAACAACTGCTATTATGCGGTATTAGCTATCGTTTTCCAATAGTTATCC

CCCGCTACCAGGCAGGTTACCTACCGCGTTACTCACCCGTTCGCAACTCATCCAGAGAAGCAAGCTCCTCCTC
AGCGTCTACTGCATGTATTAGGCACGCCGCCAGCGTCGTCCTGAGCCAGGATCGAAACTCT
>180
CTGCTGCCGCCCGTAGGAGTCTGGGCCGTGTCTCAGTCCCAGTGTGGCCGATCACCCTCTCAGGTCGGCTATG
TATCGTCGCCTTGGTGAGCCGTTACCCCACCAACTAGCTAATACAACGCAGGTCCATCTGGTAGTGGTGCAAT
TGCACCTTTTAAGCAAATGTCATGCAACATCTACTCTTATGCGGTATTAGCTATCGTTTTCCAATAGTTATCC
CCCGCTACCAGGCAGGTTACCTACGCGTTACTCACCCGTTCGCAACTCATCCAGAAGAGCAAGCTCCTCCTTC
AGCGTTCTACTGCATGTATTAGGCACGCCGCCAGCGTTCGTCCTGAGCCAGGATCAAACTCT
>181
CTGCTGCCGCCCGTAGGAGTCTGGGCCGTGTCTCAGTCCCAGTGTGGCCGATCACCCTCTCAGGTCGGCTATG
TATCGTCGCCTTGGTGAGCCGTTACCTCACCAACTAGCTAATACAACGCAGGTCCATCTGGTAGTGATGCAAT
TGCACCTTTTAATTGACTATCATGCAATAATCAATTTTATGCGGTATTAGCCTATCGTTTCCAATAGTTACTC
CCCCGCTACCAGGCAGGTACCTACGCGTTACTCACCCGTTCGCAACTCATCCAGAGAAGCAAGCTCCTCCTTC
AGCGTTCTACTTGCCATGTATTAC
>182
CTGCTGCCGCCCTAGGAGTCTGGGCCGTGTCTCAGTCCCAGTGTGGCCGATCACCCTCTCAGGTCGGCTATGT
ATCGTCGCCTTGGTGAGCCCTTACCCCACCAACTAGCTAATACAACGCAGGTCCATCTGGTAGTGATGCAATT
GCACCTTTCAAACAGTTGTCATGCAACAACTGCTATTATGCGGTATTAGCTATCGTTTCCAATAGTTATCCCC
CGCTACCAGGCAGGTTACCTACGCGTACTCACCCGTTCGCAACTCATCCAGAGAAGCAAGCTCCTCCTTCAGC
GTTCTACTTGCCATGTATTAGGCACGCCGCCAGCGTTCGTCCTGAGCCAGGATCAAACTCT
>183
CTGCTGCCGCCCGTAGGAGTCTGGGCCGTGTCTCAGTTCCAGTGTGGCCGATCACCCTCTCAGGTCGGCTACG
TATCGTTGCCTTGGTAAGCCGTTACCTTACCAACTAGCTAATACGGCGCAGGTCCATCTATAAGTGATAGCAA
AGCCATCTTTCACTATCGAACCATGCGGTTTCGAAATATTTATCCGGTATTAGCTCCGGTTTTTCCCGAAGTT
ATCCCAGTCTTATAGGTAGGTTTATCCACGTGTTACTCACCCGTCCGCCGCTAACGTCAGAGGAGCAAGCTCC
TCGTCTGTTTCGCTCGACTTGCATGTATTTAGGCACGCCGCCAGCGTCGTCCTGAGCAGGCATC
>184
CTGCTGCCGCCCGTAGGAGTCTGGGCCGTGTCTCAGTCCCAGTGTGGCCGATCACCCTCTCAGGTCGGCTATG
TATCGTCGCCTTGGTGAGCCGTTACCCCACCAACTAGCTAATACAACGCAGGTCCATCTGGTAGTGATGCAAT
TGCACCTTTCAAACAGTTGTCATGCAACAACTGCTATTATGCGGTATTAACTATCGTTTCCAATAGTTATCCC
CCGCTACCAGGCAGGTTACCTACGCGTTACTCACCCGTTCGCAACTCATCCAGAGAAGCAAGCTCCTCCTTCA
GCGTTCTACTTGCATGTATTAGGCACGCCGCCAGCGTTCGTCCTGAGCCAGGATCAAACTCT
>185
CTGCTGCCGCCCGTAGGAGTCTGGGCCGTGTCTCAGTCCCAGTGTGGCCGATCACCCTCTCAGGTCGGCTATG
CATCGTCGCCTTGGTAGGCCTTTACCCCCCACCAACTAGCTAATGCACCGCAAAGCCATCTCATAGTGTTAGC
AAAACCAACTTTAAACATCAACCATGCAGTTAATGCTGTTTATCCGGTATTTAGCTATCGTTTTCCAATAGTT
TGTCCCAGTCTATGAGGCAGGTTTCTTTACGTGTTTACTCACCCGTTTCGCCGCTAAGTTTCGTCGCGACACG
ACACCAACT
>186
CTGCTGCCGCCCGTAGGAGTCTGGACCGTGTCTCAGCTCCAGTGTGGCTGGTCATCCTCTCAGACCAGCTAGA
GATCGCAGGCTTGGTAGGCCTTTACCCCACCAACTACCTAATCCCACTTGGGCTCATCTTATGGCATGCGGCC
TTACGGTCCCGCACTTCGTCTCTCGACACTACGCGGTATTAGCTACAGTTTCCCGTACGTTATCCCCCTCCAT
AAGCCAGATTCCCAAGCATTTACTCACCCGTCCGCCACTCGTCAGCAAGAAAGCAAGCTTTCTCCTGCTACCG
TTCGACTTGCATGTGTTAAGCCTGCCGCCAGCGTTCAATCTGAGCCAGGATCAAACTCT
>187
CTGCTGCCGCCCGTAGGAGTCTGGGCCGTGTCTCAGTCCCAGTGTGGCCGATCACCCTCTCAGGTCGGCTATG
TATCGTCGCCTTGGTGAGCCGTTACCCCACCAACTAGCTAATACAACGCAGGTCCATCTGGTAGTGGTGCAAT
TGCACCTTTTAAGCAAATGTCATGCAACATCTACTCTTATGCGGTATTTAGCTATCGTTTTCCAATAGTTATC
CCCCCGCTACCAGGCAGGTTACCTACGCGTTACTCACCCGTTCGCAACTCATCCGGAGAAGCAAGCTCCTCCT
TCAGCGTTCTACTTGCATGTATTTAGGCACGCCGCCAGCGTTCGTCCTGAGCCAGGATCAAACTCT
>188
CTGCTGCCGCCCGTAGGAGTCTGGACCGTGTCTCAGTTCCAGTGTGGCCGATCACCCTCTCAGGTCGGCTACG
TATCGTTGCCTTGGTAAGCCGTTACCTTACCAACTAGCTAATACGGCGCAGGTCCATCTATAAGTGATAGCAA
AGCCATCTTTCACTATCGAACCATGCGGTTCGAAATATTATCCGGTATTAGCTCCGGTTTTTCCCGAAGTTAT

CCCAGTCTTATAGGTAGGTTTTACCTACGTGTTACTCACCCGTCCGCCGCTAACGTCGAAGGAGCAAGCTCCT
CGTCTGTTCGCTCGACTTGCATGTATTAGGCACGCCGCCAGCGTTCATCCTGAGCCAGGATCAAACTCT
>189
CTGCTGCCGCCCGTAGGAGTCTGGGCCGTGCCTCAGTCCCAGTGTGGCCGATCACCCTCTCAGGTCGGCTATG
TATCGTCGCCTTGGTGAGCCGTTACCCCACCAACTAGCTAATACAACGCAGGTCCATCTGGTAGTGGTGCAAT
TGCACCTTTTAAGCAAATGTCATGCAACATCTACTCTTATGCGGTATTAGCTATCGTTTTCCAATAGTTATCC
CCCCGCTACCAGGCAGGTTACCTACGCGCTACTCACCCGTTCGCAACTCATCCGGAGAAGCAAGCTCCTCCTC
AGCGTTCTACTTGCATGTATTAGGCACGCCGCCAGCGTTCGTCCTGAGCCAGGATCAAACTCT
>190
CTGCTGCCGCCCGTAGGAGTCTGGGCCGTGTCTCAGTCCCAGTGTGGCCGATCACCCTCTCAGGTCGGCTATG
TATCGTCGCCTTGGTGAGCCGTTACCTCACCAACTAGCTAATACAACGCAGGTCCATCTGGTAGTGATGCAAT
TTGCACCTTTTAATTGACTATCATGCAATAATCAATTTTATGCGGTATTAGCCTATCGTTTTCCAATAGTTAC
TCCCCCGCTACCAGGCAGGTTACCTACGCGTTACTCACCCGTTCGCAACTCATCCAGAGAAGCAAGCTCCTCC
TTCAGCGTTCTACTTGCATGTATTAGGCACGCCGCCAGCGTTCGTCCTGAGCCAGGATCAAACTCT
>191
CTGCTGCCGCCCTAGGAGTCTGGGCCGTGTCTCAGTCCCAGTGTGGCCGATCACCCTCTCAGGTCGGCTATGT
ATCGTCGCCTTGGTGAGCCCTTACCCCACCAACTAGCTAATACAACGCAGGTCCATCTGGTAGTGATGCAATT
GCACCTTTCAAACCAGTTGTCATGCAACAACTGCTATTATGCGGTATTAGCTATCGTTTCCAATAGTTATCCC
CCGCTACCAGGCAGGTTACCTACGCGTACTCACCCGTTCGCAACTCATCCAGAGAAGCAAGCTCCTCCTTCAG
CGTTCTACTGCATGTATTAGGCACGCCGCCAGCGTCGTCCTGAGCCAGGATCAAACTCT
>192
CTGCTGCCGCCCGTAGGAGTCTGGGCCGTGTCTCAGTCCCAGTGTGGCCGATCACCCTCTCAGGTCGGCTATG
CATCGTCGCCTTGGTAGGCCTTTACCCCACCAACTAGCTAATGCACCGCAAAGCCATCTCATAGTGTTAGCAA
AACCAACTTTTAAACATCAACCATGCAGTTTAATGCTGTTATCCGGTATTAGCTATCGTTTCCAATAGTTGTC
CCAGTCTATGAGCAGGTTCTTTACGTGTTACTCACCCGTTCGCCGCTAAGTTCGTCGTCACACAAACTCAACT
CGCTGCTGCATTAG
>193
CTGCTGCCGCCCGTAGGAGTCTGGGCCGTGTCTCAGTCCCAGTGTGGCCGATCACCCTCTCAGGTCGGCTATG
TATCGTCGCCTTGGTGAGCCGTTACCCCACCAACTAGCTAATACAACGCAGGTCCATCTGGTAGTGGTGCAAT
TGCACCTTTTAATTGACTATCATGCAATAATCAATTTTATGCGGTATTTAGCTATCGTTTTCCAATAGTTTAT
CCCCCCGCTACCAGGCAGGTTTACCTACGCGTTACTCACCCGTTTCGCAACTCATCCAGAGAAGCAAGCTCCT
CCTTTCAGCGTTCTACTTGCAGTATTTAGGCACGCCGCCAGCGTTCGTCCTGAGCCAGGATCAAACTCT
>194
CTGCTGCCGCCCGTAGGAGTCTGGGCCGTGTCTCAGTCCCAGTGTGGCCGATCACCCTCTCAGGTCGGCTATG
TATCGTCGCCTTGGTGAGCCCTTACCCCACCAACTAGCTAATACAACGCAGGTCCATCTGGTAGTGATGCAAT
TGCACCTTTCAAACAGTTGTCATGCAACAACTGCTATTATGCGGTATTTAGCTATCGTTTTCCAATAGTTTAT
CCCCCCGCTACCAGGCAGGTTACCTACGCGTTACTCACCCGTTTCGCAACTCATCCAGAGAAGCAAGCTCCTC
CTTTCAGCGTTCTACTGCATGTATTTAGGCACGCCGCCAGCGTCGTCCTGAGCCAGGATCAAACTCT
>195
CTGCTGCCGCCCGTAGGAGTCTGGGCCGTGTCTCAGTCCCAGTGTGGCCGATCACCCTCTCAGGTCGGCTATG
TATCGTCGCCTTGGTGAGCCCTTACCCCCACCAACTAGCTAATACAACGCAGGTCCATCTGGTAGTGATGCAA
TTGCACCTTTTCAAACAGTTGTCATGCAACAACCGCTATTTATGCGGCATTTAGCTATCGTTTTCCAATAGTT
TATCCCCCCGCTACCAGGCAGGTTACCTACGCGTTACTCACCCGTTTCGCAACTCATCCAGAGAAGCAAGCTC
CTCCTTTCAGCGTTCTACTGCATTGTATTTAGGCACGCCGCCAGCGTTCGCCTGAGCCAGGATCAAACTCTT
>196
CTGCTGCCGCCCGTAGGAGTCTGGGCCGTGTCTCAGTCCCAGTGTGGCCGATCACCCTCTCAGGTCGGCTATG
TATCGTCGCCTTGGTGAGCCCTTACCCCACCAACTAGCTAATACAACGCAGGTCCATCTGGTAGTGATGCAAT
TGCACCTTTCAAACAGTTGTCATGCAACAACTGCTATTATGCGGTATTAGCTATCGTTTCCAATAGTTATCCC
CCGCTACCAGGCAGGTTACCTACGCGTTACTCACCCGTTCGCAACTCATCCAGAGAAGCAAGCTCCTCCTCAG
CGTTCTACTTCGCATGTATTAGGCACGCCGCCAGCGTTCGTCCTGAGCCAGGATCAACTCT
>197
CTGCTGCCGCCCGTAGGAGTCTGGGCCGTGTCTCAGTCCCAGTGTGGCCGATCACCCTCTCAGGTCGGCTATG
TATCGTCGCCTTGGTGAGCCCTTACCCCACCAACTAGCTAATACAACGCAGGTCCATCTGGTAGTGATGCAAT
TGCACCTTTCAAACAGTTGTCATGCAACAACTGCTATTTATGCGGTATTTAGCTATCGTTTTTCCAATAGTTT

ATCCCCCCCGCTACCAGGCAGGTTTACCTACGCGTTACTCACCCGTTTCGCAACTCATCCAGAGAAGCAAGCT
CCTCCTTTCAGCGTTCTACTTGCATTGTATTTAGGCACGCCGCCAGCGTCGTCCTGAGCCAGGATCAAACTCT
>198
CTGCTGCCGCCCGTAGGAGTCTGGGCCGTGTCTCAGTCCCAGTGTGGCCGATCACCCTCTCAGGTCGGCTATG
TATCGTCGCCTTGGTGAGCCGTTACCTCACCAACTAGCTAATACAACGCAGGTCCATCTGGTAGTGATGCAAT
TTGCACCTTTTAATTGACTATCATGCAATAATCAATTTTATGCGGTATTAGCCTATCGTTTCCAATAGTTACT
CCCCCGCTACCAGGCAGGTTACCTACGCGTTACTCACCCGTTCGCAACTCATCCGGAGAAGCAAGCTCCTCCT
TCAGCGTTCTACTTGCATGTATTAGGCACGCCGCCAGCGTTCGTCCCGAGCCAGGATCAAACTCT
>199
CTGCTGCCGCCCGTAGGAGTCTGGGCCGTGTCTCAGTCCCAGTGTGGCCGATCACCCTCTCAGGTCGGCTATG
TATCGTCGCCTTGGTGAGCCGTTACCTCACCAACTAGCTAATACAACGCAGGTCCATCTGGTAGTGATGCAAT
TTGCACCTTTTAATTGACTATCATGCAATAATCAATTTTATGCGGTATTAGCCTATCGTTTTCCAATAGTTAC
TCCCCCGCTACCAGGCAGGTTGCCTACGCGTTACTCACCCGTTCGCAACTCATCCAGAGAAGCAAGCTCCTCC
TTCAGCGTTCTACTTGCATGTATTAGGCACGCCGCCAGCGTTCGTCCTGAGCCAGGATCAAACTCT
>200
CTGCTGCCGCCCGTAGGAGTCTGGGCCGTGTCTCAGTCCCAGTGTGGCCGATCACCCTCTCAGGTCGGCTATG
TATCGTCGCCTTGGTGAGCCCTTACCCCACCAACTAGCTAATACAACGCAGGTCCATCTGGTAGTGATGCAAT
TGCACCTTTCAAACAGTTGTCATGCAACAACTGCTATTATGCGGTATTTAGCTATCGTTTTCCAATAGTTATC
CCCCCGCTACCAGGCAGGTTACCTACGCGTTACTCACCCGTTCGCAACTCATCCAGAGAAGCAAGCTCCTCCT
TCAGCGTTCTACTTGCATGTATTAGGCACGCCGCCAGCGTCGTCCTGAGCCAGGATCAAACTCT
>201
CTGCTGCCGCCCGTAGGAGTCTGGACCGTGTCTCAGTTCCAGTGTGGCTGGTCATCCTCTCAGACCAGCTAGA
GATCGCAGGCTTGGTAGGCCTTTACCCCACCAACTACCTAATCCCACTTGGGCTCATCTTATGGCATGCGGCC
TTACGGTCCCGCACTTTCGTCTCTCGACACTACGCGGTATTAGCTACAGTTTCCCGTACGTTATCCCCTCCCA
TAAGCCAGATTCCCAAGCATTTACTCACCCGTCCGCCACTCGTCAGCAAGAAAGCAAGCTTTTCTCCTGCTAC
CGTTCGACTTGCATGTGTTTAAGCCTGCCGCCAGCGTTCAATCTGAGCCAGGATCAAACTCT
>202
CTGCTGCCGCCCGTAGGAGTCTGGGCCGTGTCTCAGTCCCAGTGTGGCCGGTCACCCTCTCAGGCCGGCTACC
CCGTCGACGCCTTGGTGAGCCGTTACCTCACCAACTAGCTGATAGGCCCGCGAGCCCATCCCGGACCCGAAGT
TCTTTCCAGCTGCTGAAGATGCCTTCGCAGCTCGTATCCCGGTTATTTAGCAGCTGTTTTTTCCAACTGTTTA
TCCCCCAGAGTCCGGGGCAGGTTTGCACGTGTTACTCACCCCCCGTTTCGCACTGATCCGAAGAGCAAGCTCT
TTCTTTCTCCGTTTCG
>203
CTGCTGCCGCCCGTAGGAGTCTGGGCCGTGTCTCAGTCCCAGTGTGGCCGATCACCCTCTCAGGTCGGCTATG
TATCGTCGCCTTGGTGAGCCGTTACCCCCCACCAACTAGCTAATACAACGCAGGTCCATCTGGTAGTGGTGCA
ATTGCACCTTTTAAGCAAATGTCATGCAACATCTACTCTTATGCGGTATTTAGCTATCGTTTTCCAATAGTTT
ATCCCCCCCGCTACCAGGCAGGTTACCTACGCGTTACTCACCCGTTTCGCAACTCATCCGGAGAAGCAAGCTC
CTCCTTCAGCGTTCTACTTGCATGTATTTAGGCACGCCGCCAGCGTCGTCCTGAGCCAGGATCAAACTCT
>204
CTGCTGCCGCCCGTAGGAGTCTGGGCCGTGTCTCAGTCCCAGTGTGGCCGATCACCCTCTCAGGTCGGCTATG
TATCGTCGCCTTGGTGAGCCCTTACCCCACCAACTAGCTAATACAACGCAGGTCCATCTGGTAGTGATGCAAT
TGCACCTTTCAAACAGTTGTCATGCAACAACTGCTATTATGCGGTATTAGCTATCGTTTCCAATAGTTATCCC
CCGCTACCAGGCAGGTTACCTACGCGTTACTCACCCGTTCGCAACTCATCCAGAGAAGCAGCTCCTCCTTCAG
CGTTCTACTTGCATGTATTAGGCACGCCGCCAGCGTTCGTCCTGAGCCAGGTCAAACTCT
>205
CTGCTGCCGCCCGTAGGAGTCTGGGCCGTGTCTCAGTCCCAGTGTGGCCGATCACCCTCTCAGGTCGGCTATG
TATCGTCGCCTTGGTGAGCCCTTACCCCACCAACTAGCTAATACAACGCAGGTCCATCTGGTAGTGATGCAAT
TGCACCTTCCAAACAGTTGTCATGCAACAACTGCTATTATGCGGTATTAGCTATCGTTTTCCAATAGTTATCC
CCCGCTACCAGGCAGGTTACCTACGCGTTACTCACCCGTTCGCAACTCATCCAGAGAAGCAAGCTCCTCCTCA
GCGTTCTACTGCATGTATTAGGCACGCCGCCAGCGTCGTCCTGAGCCAGGATCAAACTCT
>206
CTGCTGCCGCCCGTAGGAGTCTGGGCCGTGTCTCAGTCCCAGTGTGGCCGATCACCCTCTCAGGTCGGCTATG
TATCGTCGCCTTGGTGAGCCCTTACCCCACCAACTAGCTAATACAACGCAGGTCCATCTGGTAGTGATGCAAT
TGCACCTTTCAAACAGTTGTCATGCAACAACTGCTATTATGCGGTATTAGCTATCGTTTTCCAATAGTTATCC

CCCGCTACCAGGCAGGTTACCTACGCGTTACTCACCCGTTCGCAACTCATCCAGAGAAGCAAGCTCCTCCTTC
AGCGTTCTACTTGCATGTATTAGGCACGCCGCCAGCGTTCGTCCTGAGCCAGGATCAAACTCT
>207
CTGCTGCCGCCCGTAGGAGTCTGGGCCGTGTCTCAGTCCCAGTGTGGCCGATCACCCTCTCAGGTCGGCTATG
TATCGTCGCCTTGGTGAGCCCTTACCCCACCAACTAGCTAATACAACGCAGGTCCATCTGGTAGTGATGCAAT
TGCACCTTTCAAACAGTTGTCATGCAACAACTGCTATTATGCGGTATTAGCTATCGTTTCCAATAGTTATCCC
CCGCTACCAGGCAGGTTACCTACGCGTTACTCACCCGTTCGCAACTCATCCAGAGAAGCAAGCTTCTCCTTCA
GCGTTCTACTTGCATGTATTAGGCACGCCGCCAGCGTCGTCCTGAGCCAGGATCAAACTCT
>208
CTGCTGCCGCCCGTAGGAGTCTGGGCCGTGTCTCAGTCCCAGTGTGGCCGATCACCCTCTCAGGTCGGCTATG
TATCGTCGCCTTGGTGAGCCCTTACCCCACCAACTAGCTAATACAACGCAGGTCCATCTGGTAGTGATGCAAT
TGCACCTTTCAAACAGTTGTCATGCAACAACTGCTATTATGCGGTATTAGCTATCGTTTCCAATAGTTATCCC
CCGCTACCAGGCAGGTTACCTACGCGTTACTCACCCGTTCGCAACTCATCCAGAGAAGCAAGCTCCTCCTCAG
CGTTCTACTTGCATGTATTAGGCACGCCGCCAGCGTCGTCCTGAGCCAGGATCAACTCT
>209
CTGCTGCCGCCCGTAGGAGTCTGGGCCGTGTCTCAGTCCCAGTGTGGCCGATCACCCCTCTCAGGTCGGCTAT
GTATCGTCGCCTTGGTGAGCCGTTACCCCACCAACTAGCTAATACAACGCAGGTCCATCTGGTAGTGGTGCAA
TTGCACCTTTTAAGCAAATGTCATGCAACATCTACTCTTATGCGGTATTAGCTATCGTTTCCAATAGTTATCC
CCCGCTACCAGGCAGGTTACCTACGCGTACTCACCCGTTCGCAACTCATCCGGAGAAGCAAGCTCCTCCTTCA
GCGTTCTACTTGCATGTATTAGGCACGCCGCCAGCGTTCGTCCTGAGCCAGGATCAAACTCT
>210
CTGCTGCCGCCCGTAGGAGTCTGGGCCGTGTCTCAGTCCCAGTGTGGCCGATCCCCCTCTCAGGTCGGCTATG
TATCGTCGCCTTGGTGAGCCGTTACCTCACCAACTAGCTAATACAACGCAGGTCCATCTGGTAGTGATGCAAT
TTGCACCTTTTAATTGACTATCATGCAATAATCAATTTTATGCGGTATTAGCCTATCGTTTCCAATAGTTACT
CCCCCGCTACCAGGCAGGTTACCTACGCGTACTCACCCGTTCGCAACTCACTCCAGAGAAGCAAGCCTCCTCC
TTCAGCGTTCTACTGCATGTATTAGGCACGCCGCCAGCGTTCGTCCTGAGCCAGGCATCGA
>211
CTGCTGCCGCCCGTAGGAGTCTGGACCGTGTCTCAGTTCCAGTGTGGCTGGCCATCCTCTCAGACCAGCTAGA
GATCGCAGGCTTGGTAGGCCTTTACCCACCAACTACCTAATCCCACTTGGGCTCATCTCATGGCATGCGGCCT
TGCGGTCCCGCACTTCATCTCCCGATACTACGCGGTACTTAGCTACAGTTTCCCGTAGTTATCCCCTCCCATA
AGCCGACTTCCCAAGCATTACTCACCCGTCCGCCACTCGTCAGCAAGAAAGCCAAGCTTTCTCCTGCTACCGT
TCGACTTGCATGTGTTAAGCCTGCCGCCAGC
>212
CTGCTGCCGCCCGTAGGAGTCTGGGCCGTGTCTCAGTCCCAGTGTGGCCGATCACCCTCTCAGGTCGGCTATG
TATCGTCGCCTTGGTGAGCCCTTACCCCACCAACTAGCTAATACAACGCAGGTCCATCTGGTAGTGATGCAAT
TGCACCTTTCAACAGTTGTCATGCAACAACTGCTATTTATGCGGTATTTAGCTATCGTTTTCCAATAGTTATC
CCCCCGCTACCAGGCAGGTTACCTACGCGTTACTCACCCGTTCGCAACTCATCCAGAGAAGCAAGCTCCTCCT
TTCAGCGTTCTACTTGCATTGTATTTAGGCACGCCGCCAGCGTTCGTCCTGACGCCAGGCA
>213
CTGCTGCCGCCCGTAGGAGTCTGGGCCGTGTCTCAGTCCCAGTGTGGCTGATCATCCTCTCAAACCAGCTATG
GATCGTCGGCTTGGTAGGCCATTACCCCACCAACTACCTAATCCAACGCGGGCTAATCTCTTGGCGATAAATC
TTTCCCCCAAAGGGCACATACGGTATTACTCCCAGTTTCCCGAGGCTATTCCGTACCAAGAGGCATATTCCCA
CGCGTTACTCACCCGTCCGCCGCTAGACCCGAAGATCTCGCTCGACTTGCATGTGTTAGGCCTGCCGCCAGCG
TTCGTTCTGAGCCAGGATCAAACTCT
>214
CTGCTGCCGCCCGTAGGAGTCTGGGCCGTGTCTCAGTCCCAGTGTGGCCGATCACCCTCTCAGGTCGGCTATG
TATCGTCGCCTTGGTGAGCCGTTACCCCACCAACTAGCTAATACAACGCAGGTCCATCTGGTAGTGATGCAAT
TGCACCTTTTAAGTAAATGTCATGCAACATCTACTCTTATGCGGTATTAGCTATCGTTTCCAATAGTTATCCC
CCGCTACCAGGCAGGTTACCTACGCGTTACTCACCCGTTCGCAACTCATCCAGAAGAGCAAGCTCCTCCTTCA
GCGTTCTACTTGCATGTATTACGGCACGCCGCCAGCGTCGTCCTGAGCCAGGATCAAACTCT
>215
CTGCTGCCGCCCGTAGGAGTCTGGGCCGTGTCTCAGTCCCAGTGTGGCCGATCACCCTCTCAGGTCGGCTATG
TATCGTCGCCTTGGTGAGCCCTTACCCCACCAACTAGCTAATACAACGCAGGTCCATCTGGTAGTGATGCAAT
TGCACCTTTTAATTGACTATCATGCAATAATCAATTTTATGCGGTATTAGCTATCGTTTTCCAATAGTTTATC

CCCCGCTACCAGGCAGGTTACCTTACGCGTTACTCACCCGTTTCGCAACTCATCCAGAGAAGCAAGCTCCTCC
TTCAGCGTTCTACTTGCATGTATTAGGCACGCCGCCAGCGTCGTCCTGAGCCAGGATCAACTCT
>216
CTGCTGCCGCCCGTAGGAGTCTGGGCCGTGTCTCAGTCCCAGTGTGGCCGATCACCCTCTCAGGTCGGCTATG
TATCGTCGCCTTGGTGAGCCGTTACCCCACCAACTAGCTAATACAACGCAGGTCCATCTGGTAGTGATGCAAT
TGCACCTTTTAAGTAAATGTCATGCAACATCTACTCTTATGCGGTATTAGCTATCGTTTCCAATAGTTATCCC
CCGCTACCAGGCAGGTTACCTACGCGTTACTCACCCGTTCGCAACTCATCCAGAAGAGCAAGCTCCTCCTTCA
GCGTTCTACTTGCATGTATTAGGCACGCCGCCAGCGTTCGTCCTGAGCCAGGATCAAACTCT
>217
CTGCTGCCGCCCGTAGGAGTCTGGGCCGTGTCTCAGTCCCAGTGTGGCCGATCACCCTCTCAGGTCGGCTATG
TATCGTCGCCTTGGTGAGCCCTTACCCCACCAACTAGCTAATACAACGCAGGTCCATCTGGTAGTGATGCAAT
TGCACCTTTCAAACAGTTGTCATGCAACAACTGCTATTATGCGGTATTAGCTATCGTTTCCAATAGTTATCCC
CCGCTACCAGGCAGGTTACCTACGCGTTACTCACCCGTTCGCAACTCATCCGGAGAAGCAAGCTCCTCCTCAG
CGTTCTACTTGCCATGTATTAGGCACGCCGCCAGCGTTCGTCCTGAGCCAGGATCAAACTCT
>218
CTGCTGCCGCCCGTAGGAGTCTGGGCCGTGTCTCAGTCCCAGTGTGGCCGATCACCCTCTCAGGTCGGCTATG
TATCGTCGCCTTGGTGAGCCCTTACCCCACCAACTAGCTAATACAACGCAGGTCCATCTGGTAGTGATGCAAT
TGCACCTTTCAAACAGTTGTCATGCAACAACTGCTATTATGCGGTATTAGCTATCGTTTTCCAATAGTTATCC
CCCGCTACCAGGCAGGTTACCTACGCGTTACTCACCCGTTCGCAACTCATCCAGAGAAGCAAGCTCCTCCTCA
GCGTTCTACTTGCATGTATTTACGGCAGCCGCCACCGTCGTCCTCGA
>219
CTGCTGCCGCCCGTAGGAGTCTGGGCCGTGTCTCAGTCCCAGTGTGGCCGATCCACCCTCTCAGGTCGGGCTA
TGTATCGTTGCCTTGGTGGAGCCGTTGACCTCACCAACTAGCTAATACAACGCAGGTCCATCTGGTAGTGATG
CAATTTGCACCTTTTAAGCAAATGTCATGCAACATCTACTATTATGCGGTATTACGCTATCGTTTCCAATAGT
TATCCCCCGCTACCAGGCAGGTTACCTACGCGTTACTCACCCGTTCGCAACTCATCCGCTCGGTGCAAGCACC
AAGCTTCAGCGTTCTACTTGCATGTATTAGGCACGCCGCCAGCGTTCGTCCTGAGCCAGGATCAAACTCCT
>220
CTGCTGCCGCCCGTAGGAGTCTGGGCCGTGTCTCAGTCCCAGTGTGGCCGATCACCCTCTCAGGTCGGCTATG
CATCGTCGCCTTGGTAGGCCTTTACCCCACCAACTAGCTAATGCACCGCAAAGCCATCTCATAGTGTTAGCAA
AACCAACTTTTAAACATCAACCATGCAGTTAATGCTGTTTATCCGGTATTTAGCTATTCGTTTTTCCAATAGT
TTTGTCCCAGTCTATGAGGCAGGTTTTCTTTTACGTGTTTACTCACCCGTTTCGCCGCTAAGTTTTCGTTCGC
GAGCACGAAACACCAAACT
>221
CTGCTGCCGCCCGTAGGAGTCTGGGCCGTGTCTCAGTCCCAGTGTGGCCGATCACCCTCTCAGGTCGGCTATG
TATCGTCGCCTTGGTGAGCCGTTACCTCACCAACTAGCTAATACAACGCAGGTCCATCTGGTAGTGATGCAAT
TGCACCTTTTAATTGACTATCATGCAATAATCAATTTTATGCCGGTATTAAGCCTATCGTTTTCCAATAGTTT
ACTCCCCCGCTACCAGGCAGGTTACCTACGCGTTACTCACCCGTTCGCAACTCATCCAGAGAAGCAAGCTCCT
CCTTCAGCGTTCTACTGCATGTATTAGGCACGCCGCCAGCGTTCGTCCTGAGCCAGGATCAAACTCT
>222
CTGCTGCCGCCCGTAGGAGTCTGGGCCGTATCTCAGTCCCAATGTGGCCGTCCACCCTCTCAGGCCGACTACC
CGTCGTCGCCTTGGTGAGCCATTACCTCACCAACAAGCTGATAGGCCGTGAGCCCATCCAAGACCAGTAAACC
CTTTCCACACACCACCATGCGGTGACATGTAGCATCCAGTATTAGACCCAGTTTCCCGGGCTTATCCCAGAGT
CAAGGGCAGGTTACTCACGTATTACTCACCCGTTCGCCACTCATCCCACACCAGCAAGCTGGTGCTTCAGCCG
TTCGACTTGCATGTGTTAAGCACGCCGCCAGCGTTCGTCCGAGCCAGGATCAAACTCT
>223
CTGCTGCCGCCCGTAGGAGTCTGGGCCGTGTCTCAGTCCCAGTGTGGCCGATCACCCTCTCAGGTCGGCTATG
TATCGTCGCCTTGGTGAGCCCTTACCCCACCAACTAGCTAATACAACGCAGGTCCATCTGGTAGTGATGCAAT
TGCACCTTTCAAACAGTTGTCATGCAACAACTGCTATTATGCGGTATTAGCTATCGTTTCCAATAGTTATCCC
CCGCTACCAGGCAGGTTACCTACGCGTTACTCACCCGTTCGCAACTCATCCAGAGAAGCAAGCCTCCTCCTTC
AGCGTTCTACTTGCCATGTATTAGGCACGCCGCCAGCGTTCGTCCTGAGCCAGGATCAACTCT
>224
CTGCTGCCGCCCGTAGGAGTCTGGGCCGTGTCTCAGTCCCAGTGTGGCCGATCACCCTCTCAGGTCGGCTATG
TATCGTCGCCTTGGTGAGCCCTTACCCCACCAACTAGCTAATACAACGCAGGTCCATCTGGTAGTGATGCAAT
TGCACCTTTCAAACAGTTGTCATGCAACAACTGCTATTATGCGGTATTAGCTATCGTTTCCAATAGTTATCCC

CCGCTACCAGGCAGGTTACCTACGCGTTACTCACCCGTTCGCAACTCATCCAGAGAAGCAAGCTCCTCCTTCA
GCGTTCTACTTGCATGTATTAGGCACGCCGCCAGCGTTCGTCCTGAGCCAGGATCAAACTCT
>225
CTGCTGCCGCCCGTAGGAGTCTGGGCCGTGTCTCAGTCCCAGTGTGGCCGATCACCCTCTCAGGTCGGCTATG
TATCGTCGCCTTGGTGAGCCGTTACCCCACCAACTAGCTAATACAACGCAGGTCCATCTGGTAGTGGTGCAAT
TGCACCTTTTAAGCAAATGTCATGCAACATCTACTCTTATGCGGTATTAGCTATCGTTTCCAATAGTTATCC
CCCGCTACCAGGCAGGTTACCTACGCGTTACTCACCCGTTCGCAACTCATCCGGAGAAGCAAGCTCCTCCTCA
GCGTTCTACTTGCCATGTATTAGGCACGCCGCCAGCGTTCGTCCTGAGCCAGGATCAAACTCT
>226
CTGCTGCCGCCCGTAGGAGTCTGGGCCGTGTCTCAGTCCCAGTGTGGCCGATCACCCTCTCAGGTCGGCTATG
TATCGTCGCCTTGGTGAGCCCTTACCCCACCAACTAGCTAATACAACGCAGGTCCATCTGGTAGTGATGCAAT
TGCACCTTTCAAACAGTTGTCATGCAACAACTGCTATTATGCGGTATTAGCTATCGTTTCCAATAGTTATCC
CCCCGCTACCAGGCAGGTTACCTACGCGTTACTCACCCGTTCGCAACTCATCCAGAGAAGCAAGCTCCTCCTT
CAGCGTTCTACTTGCATGTATTAGGCACGCCGCCAGCGTTCGTCCTGAGCCAGGATCAAACTCT
>227
CTGCTGCCGCCCGTAGGAGTCTGGGCCGTGTCTCAGTCCCAGTGTGGCCGATCACCCTCTCAGGTCGGCTATG
TATCGTCGCCTTGGTGAGCCCTTACCCCACCAACTAGCTAATACAACGCAGGTCCATCTGGTAGTGATGCAAT
TGCACCTTTCAAACAGTTGTCATGCAACAACTGCTATTATGCGGTATTAGCTATCGTTTCCAATAGTTATCCC
CCGCCTACCAGGCAGGTTACCTACCGCGTTACTCACCCGTTCGCAACTCATCCGGAGAAGCAAGCTCCTCCTT
CAGCGTTCTACTTGCCATGTATTAGGCACGCCGCCAGCGTTCGTCCTGAGCCAGGATCAAACTCT
>228
CTGCTGCCGCCCGTAGGAGTCTGGGCCGTGTCTCAGTCCCAGTGTGGCCGGTCGCCCTCTCAGGCCGGCTACC
CGTCATCGCCTTGGTGAGCCATTACCTCACCAACAAACTGATAGGCCGCGAGTCCATCCCCCACCGAAGAACT
TTCCACAACACACCATGCGGTGCGAAGTCATATCCGGTATTAGCCCCGATTTCCCGAGGTTATCCCGAAGAGG
GGGGCAGGTTACGTCACGTGTTACTCACCCGTTCGCCACTAATCGGACAGGGCAAGCCCTGTCGTCATCGTTC
GACTTGCATGTGTTAAGCACGCCGCCAGCGTTCGTCCTGAGCCAGGATCAAACTCT
>229
CTGCTGCCGCCCGTAGGAGTCTGGGCCGTGTCTCAGTCCCAGTGTGGCCGATCACCCTCTCAGGTCGGCTATG
TATCGTCGCCTTGGTGAGCCCTTACCCCACCAACTAGCTAATACAACGCAGGTCCATCTGGTAGTGATGCAAT
TGCACCTTTCAAACAGTTGTCATGCAACAACTGCTATTATGCGGTATTTAGCTATCGTTTTTCCAATAGTTAT
CCCCCGCTACCAGGCAGGTTACCTACCGCGTTACTCACCCGTTCGCAACTCATCCAGAGAAGCAAGCTCCTCC
TCAGCGTTCTACTTGCATGTATTAGGCACGCCGCCAGCGTCGTCCTGAGCCAGGATCAAACTCT
>230
CTGCTGCCGCCCGTAGGAGTCTGGACCGTGTCTCAGTTCCAGTGTGGCCGATCACCCTCTCAGGTCGGCTACG
TATCGTTGCCTTGGTAAGCCGTTACCTTACCAACTAGCTAATACGGCGCGGATCCATCTATAAGTGACAGCAA
AGCCGCCTTCACTATGAACCATGCGGTTCAATATGTTATCCGGTATTAGCTCCGGTTTTTCCCGAAGTTATC
CCAGTCTTATACGGTAGGTTTATCCACGTGTTACTCACCCGTCCGCCGCTAACGTCAAAGGAGCAAGCTCCTT
ATCTGTTCGCTCAACTTGCATGTATTTAGGCACGCCGCCAGCGTCATCCTGAGCCAGGATCAAACTCT
>231
CTGCTGCCGCCCGTAGGAGTCTGGACCGTGTCTCAGTTCCAGTGTGGCCGATCACCCTCTCAGGTCGGCTACG
TATCGTTGCCTTGGTAAGCCGTTACCTTACCAACTAGCTAATACGGCGCAGGTCCATCTATAAGTGATGGCAA
AGCCATCTTTCACTATCGAACCATGCGGTTCGAAATATTATCCGGTATTAGCTCCGGTTTTTCCCGAAGTTAT
CCCAGTCTTATAGGTAGGTTTACCTACGTGTTACTCACCCGTCCGCCGCTAACGTCAAAGGAGCAAGCTCCTC
GTCTGTTCGCTCGACTTGCATGTATTAGGCACGCCGCCAGCGTTCATCCTGAGCCAGGATCAAACTCT
>232
CTGCTGCCGCCCGTAGGAGTCTGGGCCGTGTCTCAGTCCCAGTGTGGCCGATCACCCTCTCAGGTCGGCTATG
CATCGTCGCCTTGGTAGGCCTTTTACCCCACCAACTAGCTAATGCACCGCAAAGCCATCTCATAGTGTTAGCA
AAACCAACTTTTAAACATCAACCATGCAGTTAATGCTGTTATCCGGTATTAGCTATCGTTTCCAATAGTTATC
CCCCGCTACCAGGCAGGTTACCTACGCGTACTCACCCGTTCGCAACTCATCCAGAGAAGCAAGCTCCTCCTTC
AGCGTTCTACTTGCATGTATTAGGCACGCCGCCAGCGTTCGTCCTGAGCCAGGATCAAACTCT
>233
CTGCTGCCGCCCTAGGAGTCTGGACCGTGTCTCAGTTCCAGTGTGGCTGGTCATCCTCTCAGACCAGCTAGAG
ATCGCAGGCTTGGTAGGCCTTTACCCCACCAACTACCTAATCCCACTTGGGCTCATCTTATGGCATGCGGCCT
TACGGTCCCGCACTTCGTCTCTCGACACTACGCGGTATTAGCTACAGTTTCCCGTAGTTATCCCCCTCCATAA

GCCAGATTCCCAAGCATTACTCACCCGTCCGCCACTCGTCAGCAAGAAAGCAAGCTTTCTCCTGCTACCGTTC
GACTTGCATGTGTTAAGCCTGCCGCCAGCGTTCAATCTGAGCCAGGATCAAACTCT
>234
CTGCTGCCGCCCGTAGGAGTCTGGGCCGTGTCTCAGTCCCAGTGTGGCCGATCACCCTCTCAGGTCGGCTATG
CATCGTCGCCTTGGTAGGCCTTTACCCCCCACCAACTAGCTAATGCACCGCAAAGCCATCTCATAGTGTTAGC
AAAACCAACTTTTAAACATCAACCATGCAGTTAATGCTGTTTATCCGGTATTTAGCTATCGTTTTCCAATAGT
TTGTCCCCCAGTCTTATGAGGCAGGTTTCTTTACGTGTTTACTCACCCCGTTTCGCCGCTAAGTTTTCGTCGT
CGACACGAACA
>235
CTGCTGCCGCCCGTAGGAGTCTGGGCCGTGTCTCAGTCCCAGTGTGGCCGATCACCCTCTCAGGTCGGCTATG
TATCGTCGCCTTGGTGAGCCGTTACCCCACCAACTAGCTAATACAACGCAGGTCCATCTGGTAGTGGTGCAAT
TGCACCTTTTAAGCAAATGTCATGCAACATCTACTCTTATGCGGTATTAGCTATCGTTTCCAATAGTTATCCC
CCGCTACCAGGCAGGTTACCTACGCGTTACTCACCCGTTCGCAACTCATCCGGAGAAGCAAGCTCCTCCTTCA
GCGTTCTACTTGCATGTATTAGGCACGCCGCCAGCGTTCGTCCTGAGCCAGGATCAAACTCT
>236
CTGCTGCCGCCCGTAGGAGTCTGGGCCGTGTCTCAGTCCCAGTGTGGCCGATCACCCTCTCAGGTCGGCTATG
TATCGTCGCCTTGGTGAGCCCTTACCCCACCAACTAGCTAATACAACGCAGGTCCATCTGGTAGTGATGCAAT
TGCACCTTTCAAACAGTTGTCATGCAACAACTGCTATTATGCGGTATTAGCTATCGTTTCCAATAGTTATCCC
CCGCTACCAGGCAGGTTACCTGCGCGTACTCACCCGTTCGCAACTCATCCGGAGAAGCAAGCTCCTCCTTCAG
CGTTCTACTTGCCATGCATTACGGCACGCCGCCAGCGTTCGTCCTGAGCCAGGATCAAACTCT
>237
CTGCTGCCGCCCGTAGGAGTCTGGGCCGTGTCTCAGTCCCAGTGTGGCCGATCACCCTCTCAGGTCGGCTATG
TATCGTCGCCTTGGTGAGCCCTTACCCCACCAACTAGCTAATACAACGCAGGTCCATCTGGTAGTGATGCAAT
TGCACCTTTCAAACAGTTGTCATGCAACAACTGCTATTATGCGGTATTAGCTATCGTTTCCAATAGTTATCCC
CCGCTACCAGGCAGGTTACCTACGCGTACTCACCCGTTCGCAACTCATCCGGAGAAGCAAGCTCCTCCTTCAG
CGTTCTACTTGCATGTATTAGGCACGCCGCCAGCGTTCGTCCTGAGCCAGGATCAAACTCT
>238
CTGCTGCCGCCCGTAGGAGTCTGGACCGTGTCCCAGTTCCAGTGTGGCCGATCACCCTCTCAGGTCGGCTACG
TATCGTTGCCTTGGTAAGCCGTTACCTTACCAACTAGCTAATACGGCGCGGATCCATCTATAAGTGACAGCAA
GGCCGTCTTTCACTATTGAACCATGCGGTTCAAAATCTTATCAGGTATTAGCTCCGGTTTTCCCGAAGTTATC
CCAGTCTTATAGGTAGGTTATCCACGTGTTACTCACCCGTCCGCCGCTAACGTCAAAGGAGCAAGCTCCTCAT
CTGTTCGCTCGACTTGCATGTATTTAGGCACGCCGCCAGCGTTCGTCCTGAGCCAGGATCAAACTCT
>239
CTGCTGCCGCCCGTAGGAGTCTGGGCCGTGTCTCAGTCCCAGTGTGGCCGATCACCCTCTCAGGTCGGCTATG
TATCGTCGCCTTGGTGAGCCGTTACCTCACCAACTAGCTAATACAACGCAGGTCCATCTGGTAGTGATGCAAT
TTGCACCTTTTAATTGACTATCATGCAATAATCAATTTTATGCGGTATTAGCCTATCGTTTTCCAATAGTTAC
TCCCCCGCTACCAGGCAGGTTACCTACGCGTTACTCACCCGTTCGCAACTCATCCAGAGAAGCAAGCTCCTCC
TTCAGCGTTCTACTGCATGTATTAGGCACGCCGCCAGCGTTCGTCCTGAGCCAGGATCAAACTCT
>240
CTGCTGCCGCCCGTAGGAGTCTGGGCCGTGTCTCAGTCCCAGTGTGGCCGATCACCCTCTCAGGTCGGCTATG
TATCGTCGCCTTGGTGAGCCGTTACCTCACCAACTAGCTAATACAACGCAGGTCCATCTGGTAGTGATGCAAT
TGCACCTTTTAATTGACTATCATGCAATAATCAATTTTATGCGGTATTAGCCTATCGTTTCCAATAGTTACTC
CCCCGCTACCAGGCAGGTTACCTACGCGTTACTCACCCGTTCGCAACTCATCCAGAGAAGCAAGCTCCTCCTT
CAGCGTTCTACTTGCATGTATTAGGCATGCCGCCAGCGTTCGTCCTGAGCCAGGATCAAACCTCT
>241
CTGCTGCCGCCCGTAGGAGTCTGGGCCGTGTCTCAGTCCCAGTGTTGGCCGATCACCCTCTCAGGTCGGCTAT
GTATCGTCGCCTTGGTGAGCCCTTACCCCACCAACTAGCTAATACAACGCAGGTCCATCTGGTAGTGATGCAA
TTGCACCTTTCAAACAGTTGTCATGCAACAACTGCTATTATGCGGTATTAGCTATCGTTTCCAATAGTTATCC
CCCGCTACCAGGCAGGTTACCTACGCGTTACTCACCCGTTCGCAACTCATCCAGAGAAGCAAGCTCCTCCTTC
AGCGTTCTACTTGCATGTATTAGGCACGCCGCCAGCGTTCGTCCTGAGCCAGGATCAAACTCT
>242
CTGCTGCCGCCCGTAGGAGTCTGGGCCGTGTCTCAGTCCCAGTGTGGCCGATCACCCTCTCAGGCCGGCTATG
TATCGTCGCCTTGGTGAGCCCTTACCCCACCAACTAGCTAATACAACGCAGGTCCATCTGGTAGTGATGCAAT
TGCACCTTTTCAAACAGTTGTCATGCAACAACTGCTATTTATGCGGTATTTAGCTATCGTTTTCCAATAGTTT

ATCCCCCCGCTACCAGGCAGGTTACCTACGCGTTACTCACCCGTTCGCAACTCATCCAGAGAAGCAAGCTCCT
CCTTTCAGCGTTCTACTTGCATGTATTTAGGCACGCCGCCAGCGTTCGCCTGAGCCAGGATCAAACTCT
>243
CTGCTGCCGCCCGTAGGAGTCTGGGCCGTGTCTCAGTCCCAGTGTGGCCGATCACCCTCTCAGGTCGGCTATG
TATCGTCGCCTTGGTGAGCCGTTACCCCACCAACTAGCTAATACAACGCAGGTCCATCTGGTAGTGATGCAAT
TGCACCTTTTAAGTAAATGTCATGCAACATCTACTCTTATGCGGTATTAGCTATCGTTTCCAATAGTTATCCC
CCGCTACCAGGCAGGTTACCTACGCGTTACTCACCCGTTCGCAACTCATCCAGAAGAGCAAGCTCCTCCTCAG
CGTTCTACTTGCATGTATTAGGCACGCCGCCAGCGTCGTCCTGAGCCAGGATCAAACTCT
>244
CTGCTGCCGCCCGTAGGAGTCTGGGCCGTGTCTCAGTCCCAGTGTGGCCGATCACCCTCTCAGGTCGGCTATG
TATCGTCGCCTTGGTGAGCCGTTACCCCACCAACTAGCTAATACAACGCAGGTCCATCTGGTAGTGGTGCAAT
TGCACCTTTTAAGCAAATGTCATGCAACATCTACTCTTATGCGGTATTAGCTATCGTTTCCAATAGTTATCCC
CCGCTACCAGGCAGGTTACCTACGCGTTACTCACCCGTTCGCAACTCATCCGGAGAAGCCAAGCTCCTCCTTC
AGCGTTCTACTTGCATGTATTAGGCACGCCGCCAGCGTTCGTCCTGAGCCAGGATCAAACTCT
>245
CTGCTGCCGCCCGTAGGAGTCTGGGCCGTGTCTCAGTCCCAGTGTGGCCGATCACCCTCTCAGGTCGGCTATG
TATCGTCGCCTTGGTGAGCCCTTACCCCACCAACTAGCTAATACAACGCAGGTCCATCTGGTAGTGATGCAAT
TGCACCTTTCAAACAGTTGTCATGCAACAACTGCTATTATGCGGTATTAGCTATCGTTTCCAATAGTTATCCC
CCGCTACCAGGCAGGTTACCTACGCGTACTCACCCGTTCGCAACTCATCCGGAGAAGCCAAGCTCCTCCTTCA
GCGTTCTACTTGCCATGTATTAGGCACGCCGCCAGCGTTCGTCCTGAGCCAGGATCAAACTCT
>246
CTGCTGCCGCCCGTAGGAGTCTGGGCCGTGTCTCAGTCCCAGTGTGGCCGATCACCCTCTCAGGTCGGCTATG
TATCGTCGCCTTGGTGAGCCCTTACCCCACCAACTAGCTAATACAACGCAGGTCCATCTGGTAGTGATGCAAT
TGCACCTTTCAAACAGTTGTCATGCAACAACTGCTATTATGCGGTATTTAGCTATCGTTTTCCAATAGTTATC
CCCCGCTACCAGGCAGGTTACCTACGCGTTACTCACCCGTTCGCAACTCATCCAGAGAAGCAAGCTCCTCCTT
CAGCGTTCTACTTGCATGTATTTAGGCACGCCGCCAGCGTCGTCCTGAGCCAGGATCAAACTCT
>247
CTGCTGCCGCCCGTAGGAGTCTGGGCCGTGTCTCAGTCCCAGTGTGGCCGATCACCCTCTCAGGTCGGCTATG
TATCGTCGCCTTGGTGAGCCGTTACCCCACCAACTAGCTAATACAACGCAGGTCCATCTGGTAGTGGTGCAAT
TGCACCTTTTAAGCAAATGTCATGCAACATCTACTCTTATGCGGTATTAGCTATCGTTTCCAATAGTTATCCC
CCGCTACCAGGCAGGTTACCTACGCGTTACTCACCCGTTCGCAACTCATCCGGAGAAGCAAGCTCCTCCTTCA
GCATTCTACTTGCATGTATTAGGCACGCCGCCAGCGTTCGTCCTGAGCCAGGATCAAACTCT
>248
CTGCTGCCGCCCGTAGGAGTCTGGGCCGTGTCTCAGTCCCAGTGTGGCCGATCACCCTCTCAGGTCGGCTATG
TATCGTCGCCTTGGTGAGCCCTTACCCCACCAACTAGCTAATACAACGCAGGTCCATCTGGTAGTGATGCAAT
TGCACCTTTCAAACAGTTGTCATGCAACAACTGCTATTATGCGGTATTTAGCTATCGTTTTCCAATAGTTATC
CCCCGCTACCAGGCAGGTTACCTACCGCGTTACTCACCCGTTCGCAACTCATCCAGAGAAGCAAGCTCCTCCT
CAGCGTTCTACTTGCCATGTATTAGGCACGCCGCCAGCGTTCGTCCGTGAC
>249
CTGCTGCCGCCCGTAGGAGTCTGGGCCGTGTCTCAGTCCCAGTGTGGCCGATCACCCTCTCAGGTCGGCTATG
TATCGTCGCCTTGGTGAGCCCTTACCCCACCAACTAGCTAATACAACGCAGGTCCATCTGGTAGTGATGCAAT
TGCACCTTTTAAGCAAATGTCATGCAACATCTACTCTTTATGCGGTATTTAGCTATCGTTTTTCCAATAGTTT
ATCCCCCCCGCTACCAGGCAGGTTACCTACGCGTACTCACCCGTTCGCAACTCATCCGGAGAAGCAAGCTCCT
CCTTCAGCGTTCTCTGCATGTATTTAGGCACGCCGCCAGCGTCGTCCTGAGCCAGGATCAACTCT
>250
CTGCTGCCGCCCGTAGGAGTCTGGGCCGTGTCTCAGTCCCAGTGTGGCCGATCACCCTCTCAGGTCGGCTATG
CATCGTCGCCTTGGTAGGCCTTTACCCCCCCACCAACCAGCTAATGCACCGCAAAGCCATCTCATAGTGTTAG
CAAAACCAACTTTAAACATCAACCATGCAGTTAATGCTGTTTTATCCGGTATTTTAGCTATTCGTTTTTCCAA
TAGTTTTGTCCCCCCAGTCTTATTGAGGCAGGTTTTCTTTTACGTGTTTACTCACCCCCCGTTTTCGCCGCTA
AGTTTCGTCGCGACACA
>251
CTGCTGCCGCCCGTAGGAGTCTGGGCCGTGTCTCAGTCCCAGTGTGGCCGATCACCCTCTCAGGTCGGCTATG
CATCGTCGCCTTGGTAGGCCTTTACCCCACCAACTAGCTAATGCACCGCAAAGCCATCTCATAGTGTTAGCAA
AACCAACTTTTAAACATCAACCATGCAGTTAATGCTGTTATCCGGTATTAGCTATCGTTTTCCAATAGTTTGT

CCCAGTCTATGAGGCAGGTTCTTTACGTGTTACTCACCCGTTCGCCGCTAAGTTGTCGTCGCAGCGAACCAGC
AAACTAACTCGACTCGCTTGC
>252
CTGCTGCCGCCCGTAGGAGTCTGGACCGTGTCTCAGTTCCAGTGTGGCCGATCACCCTCTCAGGTCGGCTACG
TATCGTTGCCTTGGTAAGCCGTTACCTTACCAACTAGCTAATACGGCGCAGGTCCATCTATAAGTGATAGCAA
AGCCATCTTTCACTATCGAACCATGCGGTTCGAAATATTATCCGGTATTAGCTCCGGTTTCCCGAAGTTATCC
CAGTCTTATAGGTAGGTTACCTACGTGTTACTCACCCGTCCGCCGCTAACGTCAAAGGAGCAAGCTCCTCGTC
TGTTCGCTCGACTGCACGTATAGGACGCCGCCAGCGTCATCCTGAGCCAGGATCAACTCT
>253
CTGCTGCCGCCCGTAGGAGTCTGGACCGTGTCTCAGTTCCAGTGTGGCCGATCACCCTCTCAGGTCGGCTACG
TATCGTTGCCTTGGTAAGCCGTTACCTTACCAACTAGCTAATACGGCGCAGGTCCATCTATAAGTGATAGCAA
AGCCATCTTTCACTATCGAACCATGCGGTTTCGAAATAATTATCCGGTATTAGCTCCGGTTTTTCCCGAAGTT
ATCCCAGTCTTATAGGTAGGTTTTACCTACGTGTTTACTCACCCGTCCGCCGCTAACGTCGAAGGAGCCAAGC
TCCTCGTCGTGTTTCGCTCGACTTGCCATGTATAGGCACGCCGCCAGCGTCATCCTGAGCCAGGATCAACTCT
>254
CTGCTGCCGCCCGTAGGAGTCTGGGCCGTGTCTCAGTCCCAGTGTGGCCGATCACCCTCTCAGGTCGGCTATG
TATCGTCGCCTTGGTGAGCCGTTACCCCACCAACTAGCTAATACAACGCAGGTCCATCTGGTAGTGGTGCAAT
TGCACCTTTTAAGCAAATGTCATGCAACATCTACTCTTATGCGGTATTAGCTATCGTTTCCAATAGTTATCCC
CCGCTACCAGGCAGGTTACCTACGCGTTACTCACCCGTTCGCAACTCATCCGGAGAAGCAAGCTCCTCCTTCA
GCGTTCTACTTGCATGTATTAGGCACGCCGCCAGCGTTCGTCCTGAGCCAGGATCAAACTCT
>255
CTGCTGCCGCCCGTAGGAGTCTGGGCCGTGTCTCAGTCCCAGTGTGGCCGATCACCCTCTCAGGTCGGCTATG
TATCGTCGCCTTGGTGAGCCCTTTACCCCCACCAACTAGCTAATACAACGCAGGTCCATCTGGTAGTGATGCA
ATTGCACCTTTTCAAACAGTTGTCATGCAACAACTGCTATTTATGCGGTATTTAGCTATCGTTTTTCCAATAG
TTTATTCCCCCCCGCTACCAGGCAGGTTTACCTACGCGTTACTCACCCGTTTCGCAACTCATCCGGAGAAGCA
AGCTCCTCCTTCAGCGTT
>256
CTGCTGCCGCCCGTAGGAGTCTGGACCGTGTCTCAGTTCCAGTGTGGCCGATCACCCTCTCAGGTCGGCTATG
TATCGTCGCCTTGGTGAGCCCTTACCCCACCAACTAGCTAATACAACGCAGGTCCATCTGGTAGTGATGCAAT
TGCACCTTTTCAAACAGTTGTCATGCAACAACTGCTATTTATGCGGTATTTTAGCTATCGTTTTTCCAATAGT
TTATCCCCCCCGCTACCAGGCAGGTTTACCTACGCGTTACTCACCCGTTTCGCAACTCATCCAGAGAAGCAAG
CTCCTCCTTCAGCGTCTACTGCATGTATTTAGGCACGCCGCCAGCGTCGTCTGAGCCAGGATCAAACTCT
>257
CTGCTGCCGCCCGTAGGAGTCTGGGCCGTGTCTCAGTCCCAGTGTGGCCGATCACCCTCTCAGGTCGGCTATG
TATCGTCGCCTTGGTGAGCCCTTACCCCACCAACTAGCTAATACAACGCAGGTCCATCTGGTAGTGATGCAAT
TGCACCTTTCAAACAGTTGTCATGCAACAACTGCTATTATGCGGTATTAGCTATCGTTTCCAATAGTTATCCC
CCGCTACCAGGCAGGTTACCTACGCGTTACTCACCCGTTCGCAACTCATCCAGAGAAGCAAGCTCCTCCTTCA
GCGTTCTACTTGCCATGTATTAGGCACGCCGCCAGCGTTCGTCCTGAGCCAGGATCAAACTCT
>258
CTGCTGCCGCCCGTAGGAGTCTGGGCCGTGTCTCAGTCCCAGTGTGGCCGATCACCCTCTCAGGTCGGCTATG
TATCGTCGCCTTGGTGAGCCCTTACCCCACCAACTAGCTAATACAACGCAGGTCCATCTGGTAGTGATGCAAT
TGCACCTTTCAAACAGTTGTCATGCAACAACTGCTATTATGCGGTATTAGCTATCGTTTCCAATAGTTATCCC
CCGCTACCAGGCAGGTTACCTACCGCGTTACTCACCCGTTCGCAACTCATCCAGAGAAGCAAGCTCCTCCTTC
AGCGTTCTACTTGCATGTATTAGGCACGCCGCCAGCGTTCGTCCTGAGCCAGGATCAAACTCT
>259
CTGCTGCCGCCCGTAGGAGTCTGGGCCGTGTCTCAGTCCCAGTGTGGCCGATCACCCTCTCAGGTCGGCTATG
TATCGTCGCCTTGGTGAGCCGTTACCTCACCAACTAGCTAATACAACGCAGGTCCATCTGGTAGTGATGCAAT
TGCACCTTTTAATTGACTATCATGCAATAATCAATTTTATGCGGTATTAGCCTATCGTTTCCAATAGTTATCC
CCCGCTACCAGGCAGGTTACCTACGCGTTACTCACCCGTTCGCAACTCATCCAGAGAAGCAAGCTCCTCCTTC
AGCGTTCTACTTGCCATGTATTAGGCGCGCCGCCAGCGTTCGCTCGTCGCAAGG
>260
CTGCTGCCGCCCGTAGGAGTCTGGGCCGTGTCTCAGTCCCAGTGTGGCTGATCATCCTCTCAGACCAGCTACG
GATCGTCGCCTTGGTAGGCCTTTACCCCACCAACTAGCTAATCCGACGCGGGCTCATCCCCAGGCGATAAATC
TTTGATCTTATCGATATCATCCGGTATTAGCAGCAGTTTCCCGCTGTTATCCCAGACCTGAGGGCAGATTCCC

ACGCGTTACGCACCCGTGCGCCACTAAGGCCGAAGCCTTCGTCGACTTGCATGTGTTAGGCATGCGCCAGCGT
TCGTTCCTGAGCCAGGACAATCTGT
>261
CTGCTGCCGCCCGTAGGAGTCTGGGCCGTGTCTCAGTCCCAGTGTGGCCGATCACCCTCTCAGGTCGGCTATG
TATCGTCGCCTTGGTGAGCCTTTACCCCACCAACTAGCTAATACAACGCAGGTCCATCTGGTAGTGATGCAAT
TGCACCTTTCAAACAGTTGTCATGCAACAACTGCTATTATGCGGTATTTAGCTATCGTTTTCCAATAGTTATC
CCCCGCTACCAGGCAGGTTACCTACGCGTTACTCACCCGTTCGCAACTCATCCGGAGAAGCAAGCTCCTCCTT
CAGCGTTCTACTTGCATGTATTTAGGCACGCCGCCAGCGTTCGTCCTGACGCCAGGATCAAACTCTT
>262
CTGCTGCCGCCCGTAGGAGTCTGGGCCGTGTCTCAGTTCCCAGTGTGGCCGATCACCCTCTCAGGTCGGCTAT
GTATCGTCGCCTTGGTGAGCCGTTACCCCACCAACTAGCTAATACAACGCAGGTCCATCTGGTAGTGGTGCAA
TTGCACCTTTTAAGCAAATGTCATGCAACATCTACTCTTATGCGGTATTAGCTATCGTTTCCAATAGTTATCC
CCCGCTACCAGGCAGGTTACCTACGCGTTACTCACCCGTTCGCAACTCATCCAGAGAAGCAAGCTCCTCCTTC
AGCGTTCTACTTGACATGTATTAGGCACGCCGCCAGCGTTCGTCCTAGACGCC
>263
CTGCTGCCGCCCGTAGGAGTCTGGGCCGTGTCTCAGTTCCAGTGTGGCCGATCACCCTCTCAGGTCGGCTATG
CATCGTTGCCTTGGTAAGCCGTTACCTTACCAACTAGCTAATGCAGCGCGGATCCATCTATAAGTGACAGCAA
GACCGTCTTTCACTTTAACCATGCGGTTCAAAATATTATCCGGTATTAGCTCCGGTTTCCCGAAGTTATCCCA
GTCTTATAGGTAGGTTATCCACGTGTTACTCACCCGTCCGCCGCTAACATCAGAGAAGCAAGCTTCTCGTCCG
TTCGCTCGACTTGCATGTATTAGGCACGCCGCCAGCGTTCATCCTGAGCCAGGATCAACTCT
>264
CTGCTGCCGCCCGTAGGAGTCTGGACCGTGTCTCAGTTCCAGTGTGGCCGATCACCCTCTCAGGTCGGCTACG
TATCGTTGCCTTGGTAAGCCGTTACCTTACCAACTAGCTAATACGGCGCAGGTCCATCTATAAGTGATAGCAA
AGCCATCTTTCACTATCGAACCATGCGGTTCGAAATATTATCCGGTATTAGCTCCGGTTTTCCCGAAGTTATC
CCAGTCTTATAGGTAGGTTACCTACGTGTTACTCACCCGTCCGCCGCTAACGTCGAAGGAGCAAGCCTCCTCG
TCTGTTCGCTCGACTTGCAGTGTACTCAGGCAC
>265
CTGCTGCCGCCCGTAGGAGTCTGGGCCGTGTCTCAGTCCCAGTGTGGCCGATCACCCTCTCAGGTCGGCTATG
CATCGTCGCCTTGGTAGGCCTTTACCCCACCAACTAGCTAATGCACCGCAAAGCCATCTCATAGTGTTAGCAA
AACCAACTTTTAAACATCAACCATGCAGTTAATGCTGTTATCCGGTATTAGCTATCGTTTCCAATAGTTGTCC
CAGTCTATGAGGCAGGTTCTTTACGTGTTACTCACCCGTTCGCCGCTAAGTTGTCGTCGACAGCAACCAGAAA
CTACGTCGAACTCG
>266
CTGCTGCCGCCCGTAGGAGTCTGGGCCGTGTCTCAGTCCCAGTGTGGCCGATCACCCTCTCAGGTCGGCTATG
TATCGTCGCCTTGGTGAGCCGTTACCCCACCAACTAGCTAATACAACGCAGGTCCATCTGGTAGTGGTGCAAT
TGCACCTTTTAAGCAAATGTCATGCAACATCTACTCTTATGCGGTATTAGCTATCGTTTCCAATAGTTATCCC
CCGCTACCAGGCAGGTTACCTACGCGTACTCACCCGTTCGCAACTCATCCGGAGAAGCAAGCTCCTCCTTCAG
CGTTCTACTTGCATGTATTAGGCACGCCGCCAGCGTTCGTCCTGAGCCAGGATCAAACTCT
>267
CTGCTGCCGCCCGTAGGAGTCTGGGCCGTGTCTCAGTCCCAGTGTGGCCGATCACCCTCTCAGGTCGGCTATG
TATCGTCGCCTTGGTCGAGCCCGTTCACCCCACCAACTAGCTAATACAACGCAGGTCCATCTGGTAGTGGTGC
AATTTGCACCTTTAGGCAAATGTCATGCAACATCTACTCTTATGCGGTATTTGGCTATCGTTTTCCAATAGTT
TATCCCCCCGCTACCAGGCAGGTTTACCTACGCGTTACTCACCCGTTTCGCGACTCATCCGGAGAAGCAAGCT
CCTCCTTCAGCGTTCTACTTGCATGTATTTAGGCACGCCGCCAGCGTTCGTCCTGAGCCAGGATCAAACTCTT
>268
CTGCTGCCGCCCGTAGGAGTCTGGGCCGTGTCTCAGTCCCAGTGTGGCCGATCACCCTCTCAGGTCGGCTATG
TATCGTCGCCTTGGTGAGCCGTTACCCCACCAACTAGCTAATACAACGCAGGTCCATCTGGTAGTGGTGCAAT
TGCACCCTTTAAGCAAATGTCATGCAACATCTACTCTTATGCGGTATTAGCTATCGTTTCCAATAGTTATCCC
CCCGCTACCAGGCAGGTTACCTACGCGTTACTCACCCGTTCGCAACTCATCCGGAGAAGCAAGCTCCTCCTCA
GCGTTCTACTTGCATGTATTTACGGCACGCCGCCAGCGTTCGTCCTGAGCCAGGATCAAACTCTT
>269
CTGCTGCCGCCCGTAGGAGTCTGGGCCGTGTCTCAGTCCCAGTGTGGCCGATCACCCTCTCAGGTCGGCTATG
TATCGTCGCCTTGGTGAGCCCTTACCCCACCAACTAGCTAATACAACGCAGGTCCATCTGGTAGTGATGCAAT
TGCACCTTTCAAACAGTTGTCATGCAACAACTGCTATTATGCGGTATTTAGCTATCGTTTTCCAATAGTTATC

CCCCGCTACCAGGCAGGTTTACCTACGCGTTACTCACCCGTTTCGCAACTCATCCAGAGAAGCAAGCTCCTCC
TTTCAGCGTTCTACTTGCATTGTATTTAGGCACGCCGCCAGCGTTCGTCCTGAGCCAGGATCAAACTCT
>270
CTGCTGCCGCCCGTAGGAGTCTGGGCCGTGTCTCAGTCCCAGTGTGGCCGATCACCCTCTCAGGTCGGCTATG
TATCGTCGCCTTGGTGAGCCCTTACCCCACCAACTAGCTAATACAACGCAGGTCCATCTGGTAGTGATGCAAT
TGCACCTTTCAAACAGTCGTCATGCAACAACTGCTATTATGCGGTATTAGCTATCGTTTCCAATAGTTATCCC
CCGCTACCAGGCAGGTTACCTACGCGTTACTCACCCGTTCGCAACTCATCCAGAGAAGCAAGCTCCTCCTCAG
CGTTCTACTGCATGTATTACGGCACGCCGCCAGCGTCGTCCTGAGCCAGGATCAAACTCT
>271
CTGCTGCCGCCCGTAGGAGTCTGGGCCGTGTCTCAGTCCCAGTGTGGCCGATCACCCTCTCAGGTCGGCTATG
TATCGTCGCCTTGGTGAGCCGTTACCTCACCAACTAGCTAATACAACGCAGGTCCATCTGGTAGTGATGCAAT
TTGCACCTTTAATTGACTATCATGCAATAATCAATTTTATGCGGTATTAGCCTATCGTTTCCAATAGTTACTC
CCCCGCTACCAGGCAGGTTACCTACGCGTTACTCACCCGTTCGCAACTCATCCAGAGAAGCAAGCTCCTCCTC
AGCGTTCTACTTGCATGTATTAGGCACGCCGCCAGCGTTCGTCCTGAGCCAGGATCAAACTCT
>272
CTGCTGCCGCCCGTAGGAGTCTGGACCGTGTCTCAGTTCCAGTGTGGCTGGTCATCCTCTCAGACCAGCTAGA
GATCGCAGGCTTGGTAGGCCTTTACCCACCAACTACCTAATCCCACTTGGGCTCATCTCATGGCATGCGGCCT
TACGGTCCCGCACTTCGTCTCTCGACACTACGCGGTATTAGCTACAGTTTCCCGTACGTTATCCCCCTCCATA
AGCCAGATTCCCAAGCATTACTCACCCGTCCGCCACTCGTCAGCAAGAAAGCAAGCTTTCTCCTGCTACCGTT
CGACTTGCATGTGTTAAGCCTGCCGCCAGCGTTCAATCTGAGCCAGGATCAAACTCT
>273
CTGCTGCCGCCCGTAGGAGTCTGGGCCGTGTCTCAGTCCCAGTGCGGCCGGTCGCCCTCTCAGGCCGGCTACC
CGTCATCGCCTTGGGTGAGCCATTACCTCACCAACAAACTGATAGGCCGCGAGTCCATCCCCCACCGAAAACT
TCCACAACACACCATGCGGTGCGAAGTCATATCCGGTATTACGCCCCGATTTTCCCGAGGTTATCCCGAAGAG
GGGGGCAGGTTACGTCACGTGTTACTCACCCGTTCGCCACTAATCGGACAGGGCAAGCCCTGTCGTCATCGTT
CGACTTGCATGTGTTTAAGCACGCCGCCAGCGTTCGTCCTGAGCCAGGATCAAACTCT
>274
CTGCTGCCGCCCGTAGGAGTCTGGGCCGTGTCTCAGTCCCAGTGTGGCCGGTCGCCCTCTCAGGCCGGCTACC
CGTCATCGCCTTGGTGAGCCATTACCTCACCAACAAACTGATACGGCCGCGAGTCCATCCCCCACCGAAAAAC
TTTCCACAACACACCATGCGGTGCGAAGTCATATCCGGTATTTAGCCCCGATTTTCCCGAGGTTATCCCGAAG
AGGGGGCAGGTTACTCACGTGTTACTCACCCGTTCGCCACTAATCGGACAGGGCAAGCCCTGTCGTCATCGTT
TCGACTTGCATGTGTTTAAGCACGCCGCCAGCGTTCGTCCTGAGCCAGGATCAAACTCT
>275
CTGCTGCCGCCCGTAGGAGTCTGGACCGTGTCTCAGTTCCAGTGTGGCCGATCACCCTCTCAGGTCGGCTACG
TATCGTTGCCTTGGTAAGCCGTTACCTTACCAACTAGCTAATACGGCGCAGGTCCATCTATAAGTGATAGCAA
AGCCATCTTCACTATCGAACCATGCGGTTCGAAATATTATCCGGTATTAGCTCCGGTTTCCCGAAGTTATCCC
AGTCTTATAGGTAGGTTACCTACGTGTTACTCACCCGTCCGCCGCTAACGTCAAAGGAGCAAGCTCCTCGTCC
TGTTCGCTCGACTTGCATGTATTAGGCACGCCGCCAGGCGTTCATCCTGAGCCAGGATCAAACTCT
>276
CTGCTGCCGCCCGTAGGAGTCTGGGCCGTGTCTCAGTCCCAGTGTGGCCGATCACCCTCTCAGGTCGGCTATG
TATCGTCGCCTTGGTGAGCCCTTACCCACCCAACTAGCTAATACAACGCAGGTCCATCTGGTAGTGATGACAA
TTGCACCTTTTACAACAAGTTGTCATGCAACAACTGCTATTATGCGGTATTAGCTATCGTTTCCAATAGTTAT
CCCCCGCTACCAGGCAGGTTACCTACCGCGTTACTCACCCGTTCGCAACTCATCCGGAGAAGCAAGCTCCTCC
TCAGCGTTCTACTTGCATGTATTAGGCACGCCGCCAGCGTTCGTCCTGAGCCAGGATCAAACTCT
>277
CTGCTGCCGCCCGTAGGAGTTTGGGCCGTGTCTCAGTCCCAATGTGGCCGATCACCCTCTCAGGTCGGCTATG
CATCACGGCCTTGGTGAGCCGTTACCTCACCAACTAGCTAATGCACCGCGGGTCCATCCATCAGCAGAAGCCG
AAGCCTCTTTTTCCTTCTTCAGTCATGCGACTAAAGAACCTATGCGGTTTTTAGCCATCCGTTTTCCGAATGG
TTATCCCCCCCTCTGATGGGCAGGTTACCCACGTGTTACTCACCCGTTCGCCACTAGATTGACCAGTGCAAGC
ACCGGTCGCTCTCGTTCGACTTGCATGTACTTAGGCACGCCGACCAGCGTTCGTCCTGAGCCAGGATCAAACC
TCT
>278
CTGCTGCCGCCCGTAGGAGTCTGGGCCGTGTCTCAGTCCCAGTGTGGCCGATCACCCTCTCAGGTCGGCTATG
TATCGTCGCCTTGGTGAGCCGTTACCTCACCAACTAGCTAATACAACGCAGGTCCATCTGGTAGTGATGCAAT

TTGCACCTTTTAATTGACTATCATGCAATAATCAATTTTATGCGGTATTAGCCTATCGTTTCCAATAGTTACT
CCCCCGCTACCAGGCAGGTTACCTACGCGTTACTCACCCGTTCGCAACTCATCCAGAGAAGCAAGCTCCTCCT
TCAGCGTTCTACTTGCATGTATTAGGCACGCCGCCAGCGTTCGTCCTGAGCCAGGATCAAACTCT
>279
CTGCTGCCGCCCGTAGGAGTCTGGGCCGTGTCTCAGTCCCAGTGTGGCCGATCACCCTCTCAGGTCGGCTATG
TATCGTCGCCTTGGTGAGCCGTTACCCCACCAACTAGCTAATACAACGCAGGTCCATCTGGTAGTGATGCAAT
TTGCACCTTTTAAGTAAATGTCATGCAACATCTACTCTTATGCGGTATTAGCTATCGTTTTCCAATAGTTATC
CCCCGCTACCAGGCAGGTTACCTACGCGTTACTCACCCGTTCGCAACTCATCCAGAAGAGCAAGCTCCTCCTT
CAGCGTTCTACTTGCATGTATTAGGCACGCCGCCAGCGTTCGTCCTGAGCCAGGATCAAACTCT
>280
CTGCTGCCGCCCGTAGGAGTCTGGGCCGTGTCTCAGTCCCAGTGTGGCCGATCACCCTCTCAGGTCGGCTATG
TATCGTCGCCTTGGTGAGCCCTTACCCCACCAACTAGCTAATACAACGCAGGTCCATCTGGTAGTGATGCAAT
TGCACCTTTCAAACAGTTGTCATGCAACAACTGCTATTATGCGGTATTAGCTATCGTTTCCAATAGTTATCCC
CCGCTACCAGGCAGGTTACCTACGCGTTACTCACCCGTTCGCAACTCATCCGGAGAAGCAAGCTCCTCCTTCA
GCGTTCTACTTGCCATGTATTAGGCACGCCGCCAGCGTTCGTCCTGAGCCAGGATCAAACTCT
>281
CTGCTGCCGCCCGTAGGAGTCTGGGCTGTGTCTCAGTCCCAGTGTGGCCGATCACCCTCTCAGGTCGGCTATG
TATCGTCGCCTTGGTGAGCCCTTACCCCACCAACTAGCTAATACAACGCAGGTCCATCTGGTAGTGATGCAAT
TGCACCTTTCAAACAGTTGTCATGCAACAACTGCTATTTATGCGGTTATTTAGCTATCGTTTTTCCAATAGTT
TATCCCCCCGCTACCAGGCAGGTTTACCTACGCGTTACTCACCCGTTCGCAACTCATCCAGAGAAGCAAGCTC
CTCCTTTCAGCGTTCTACTTGCATTGTATTTAGGCACGCCGCCAGCGTTCGTCCTGAGCCAGGATCAAACTCT
T
>282
CTGCTGCCGCCCGTAGGAGTCTGGACCGTGTCTCAGTTCCAGTGTGGCCGATCACCCTCTCAGGTCGGCTACG
TATCGTCGCCTTGGTGAGCCGTTACCTCACCAACTAGCTAATACAACGCAGGTCCATCTGGTAGTGATGCAAT
TGCACCTTTTAATTGACTATCATGCAATAATCAATTTTATGCGGTATTAGCTATCGTTTCCAATAGTTATCCC
CCGCTACCAGGCAGGTTACCTACGCGTTACTCACCCGTTCGCAACTCATCCAGAGAAGCAAGCTGCTCCTTCA
GCGTCTACTGCATGTATTAGGCTACGCCGCCAGCGTCGTCCTGAGCCAGGATCAAACTCT
>283
CTGCTGCCGCCCGTAGGAGTCTGGGCCGTGTCTCAGTCCCAGTGTGGCCGATCACCCTCTCAGGTCGGCTATG
TATCGTCGCCTTGGTGAGCCGTTCACCCCACCAACTAGCTAATACAACGCAGGTCCATCTGGTAGTGGTACAA
TTGCACCTTTTAAGCAAATGTCATGCAACATCTACTCTTATGCGGTATTAGCTATCGTTTCCAATAGTTATCC
CCCGCCTACCAGGCAGGTTACCTACGCGTTACTCACCCGTTCGCAACTCATCCGGAGAAGCAAGCTCCTCCTT
CAGCGTTCTACTTGCCATGTATTAGGCACGCCGCCAGCGTTCGTCCTGAGCCAGGATCAAACTCT
>284
CTGCTGCCGCCCGTAGGAGTCTGGGCCGTGTCTCAGTCCCAGTGTGGCCGATCACCCTCTCAGGTCGGCTATG
TATCGTCGCCTTGGTGAGCCGTTACCTCACCAACTAGCTAATACAACGCAGGTCCATCTGGTAGTGATGCAAT
TGCACCTTTTAATTGACTATCATGCAATAATCAATTTTATGCGGTATTAGCCTATCGTTTTCCAATAGTTACT
CCCCCGCTACCAGGCAGGTTACCTACGCGTTACTCACCCGTTCGCAACTCATCCAGAGAAGCAAGCTCCTCCT
TCAGCGTTCTACTGCATGTATTTAGGCACGCCGCCAGCGTTCGTCCTGAGCCAGGATCAAACTCT
>285
CTGCTGCCGCCCGTAGGAGTCTGGGCCGTGTCTCAGTCCCAGTGTGGCCGATCACCCTCTCAGGTCGGCTATG
TATCGTCGCCTTGGTGAGCCGTTACCTCACCAACTAGCTAATACAACGCAGGTCCATCTGGTAGTGATGCAAT
TGCACCTTTTAATTGACTATCATGCAATAATCAATTTTATGCGGTATTAGCTATCGTTTCCAATAGTTACTCC
CCCGCTACCAGGCAGGTTACCTACGCGTTACTCACCCGTTCGCAACTCATCCAGAGAAGCAAGCTCCTCCTTC
AGCGTTCTACTTGCATGTATTAGGCACGCCGCCAGCGTTCGTCCTGAGCCAGGATCAAACTCT
>286
CTGCTGCCGCCCGTAGGAGTCTGGGCCGTGTCTCAGTCCCAGTGTGGCCGATCACCCTCTCAGGTCGGCTATG
CATCGTCGCCTTGGTAGGCCTTTACCCCACCAACTAGCTAATGCACCGCAAAGCCATCTCATAGTGTTAGCAA
AACCAACTTTTAAACATCAACCATGCAGTTAATCGCTGTTATCCGGTATTAGCTATCGTTTCCAATAGTTGTC
CCAGTCTATGAGGCAGGTTCTTTACGTGTTACTCACCCGTTCGCCGCTAAGTTCTCGTCGCAGCGAACCAGCA
ACGTAACTCGAACTCGCCTTGCCAT
>287

CTGCTGCCGCCCGTAGGAGTCTGGGCCGTGTCTCAGTCCCAGTGTGGCCGATCACCCTCTCAGGTCGGCTATG
TATCGTCGCCTTGGTGAGCCGTTACCCCACCAACTAGCTAATACAACGCAGGTCCATCTGGTAGTGGTGCAAT
TGCACCTTTTAAGCAAATGTCATGCAACATCTACTCTTATGCGGCATTAGCTATCGTTTCCAATAGTTATCCC
CCGCTACCAGGCAGGTTACCTACGCGTTACTCACCCGTTCGCAACTCATCCGGAGAAGCAAGCTCCTCCTCAG
CGTTCTACTTGCATGTATTAGGCACGCCGCCAGCGTTCGTCCTGAGCCAGGATCAAACTCT
>288
CTGCTGCCGCCCGTAGGAGTCTGGGCCGTGTCTCAGTCCCAGTGTGGCCGATCACCCTCTCAGGTCGGCTATG
TATCGTCGCCTTGGTGAGCCCTTACCCCACCAACTAGCTAATACAACGCAGGTCCATCTGGTAGTGATGCAAT
TGCACCTTTCAAACAGTTGTCATGCAACAACTGCTATTATGCGGTATTTAGCTATCGTTTTTCCAATAGTTAT
CCCCCGCTACCAGGCAGGTTACCTACCGCGTTACTCACCCGTTCGCAACTCATCCAGAGAAGCAAGCTCCTCC
TCAGCGTCTACTGCATGTATTAGGCACGCCGCCAGCGTCGTCCTGCG
>289
CTGCTGCCGCCCGTAGGAGTCTGGGCCGTGTCTCAGTCCCAGTGTGGCCGATCACCCTCTCAGGTCGGCTATG
TATCGTCGCCTTGGTGAGCCGTTACCTCACCAACTAGCTAATACAACGCAGGTCCATCTGGTAGTGATGCAAT
TGCACCTTTTAATTGACTATCATGCAATAATCAATTTTATGCGGTATTAGCCTATCGTTTCCAATAGTTACTC
CCCCGCTACCAGGCAGGTTACCTACGCGTTACTCACCCGTTCGCAACTCATCCAGAGAAGCAAGCTCCTCCTT
CAGCGTTCTACTTGCATGTATTAGGCACGCCGCCAGCGTTCGTCCTGAGCCAGGATCAAACTCT
>290
CTGCTGCCGCCCGTAGGAGTCTGGGCCGTGTCTCAGTCCCAGTGTGGCCGATCACCCTCTCAGGTCGGCTATG
CATCGTCGCCTTGGTAGGCTTTTACCCCACCAACTAGCTAATGCACCGCAAAGCCATCTCATAGTGTTAGCAA
AACCAACTTTCAAACATCAACCATGCAGTTAATGCTATTTATCCGGTATTTAGCTTATTCGTTTTCCAATAGT
TTGTCCCAGTCTATTGAGGCAGGTTTCTTTACGTGTTACTCACCCCCGTTTCGCCGCTAAGTGTCGTCGACAG
CGAACCACAACGTAACGTCACT
>291
CTGTTGCCGCCCGTAGGAGTCTGGGCCGTGTCTCAGTCCCAGTGTGGCCGACCACCCTCTCAGGTCGGCTATG
TATCGTCGCCTTGGTGAGCCGTTACCTCACCAACTAGCTAATACAACGCAGGTCCATCTGGTAGTGATGCAAT
TGCACCTTTAAGCAAATGTCATGCAACATCTACTTATTTATGCGGTATTTAGCTATCGTTTTCCAATAGTTTA
TCCCCCCGCTACCAGGCAGGTTTACCTACGCGTACTCACCGTTCGCAACTCATTCCAGAGAAGCAAGCTCCTC
CTTCAGCGTCTACTTGCATGTATTAGGCACGCCGCCAGCGTCGTCCTGAGCCAGGATCAAACTCTT
>292
CTCTGCCGCCCGTAGGAGTCTGGGCCGTGTCTCAGTCCCAGTGTGGCCGATCACCCTCTCAGGTCGGCTATGC
ATCGTCGCCTTGGTAGGCCTTTACCCCCCACCAACTAGCTAATGCACCGCAAAGCCATCTCATAGTGTTAGCA
AAACCAACTTTTAAACATCAACCATGCAGTTAATGCTGTTTATCCGGTATTTAGCTATCGTTTTCCAATAGTT
TGTCCCCCAGTCTATTGAGGCAGGTTTCTTTACGTGTTACTCACCCCCGTTTCGCCGCTAAGTTTCGTCGCGA
CACGCAAACCA
>293
CTGCTGCCGCCCGTAGGAGTCTGGGCCGTGTCTCAGTCCCAGTGTGGCCGATCACCCTCTCAGGTCGGCTATG
TATCGTCGCCTTGGTGAGCCGTTACCCCACCAACTAGCTAATACAACGCAGGTCCATCTGGTAGTGGTGCAAT
TGCACCTTTCAAACAGCTGTCATGCAACAACTGCTATTATGCGGTATTAGCTATCGTTTCCAATAGTTATCCC
CCGCTACCAGGCAGGTTACCTACGCGTACTCACCCGTTCGCAACTCATCCAGAGAAGCAAGCTCCTCCTTCAG
CGTTCTACTTGCATGTATTAGGCACGCCGCCAGCGTTCGTCCTGAGCCAGGATCAAACTCT
>294
CTGCTGCCGCCCGTAGGAGTCTGGGCCGTGTCTCAGTCCCAGTGTGGCCGATCACCCTCTCAGGTCGGCTATG
TATCGTCGCCTTGGTGAGCCGTTACCTCACCAACTAGCTAATACAACGCGAGGTCCATCTGGTAGTGATGCAA
TTTGCACCTTTTAAGCAAATGTCATGCAACATCTACTATTATGCGGTATTACGCTATCGTTTCCAATAGTTAT
CCCCCGCTACCAGGCAGGTTACCTACGCGTACTCACCCGTTCGCAACTCATCCGCTCGGTGCAAGCACCAAGC
TTCAGCGTTCTACTTGCATGTATTACGGCACGCCGCCAGCGTTCGTCCTGAGCCAGGATCAAACTCCT
>295
CTGCTGCCGCCCGTAGGAGTCTGGGCCGTATCTCAGTCCCAGTGTGGCCGATCACCCTCTCAGGTCGGCTATG
TATCGTCGCCTTGGTGAGCCCTTACCCCACCAACTAGCTAATACAACGCAGGTCCATCTGGTAGTGATGCAAT
TGCACCTTTCAAACAGTTGTCATGCAACAACTGCTATTATGCGGTATTAGCTATCGTTTCCAATAGTTATCCC
CCGCTACCAGGCAGGTTACCTACGCGTTACTCACCCGTTCGCAACTCATCCAGAGAAGCAAGCTCCTCCTTCA
GCGTTCTACTTCGCATGTATTAGGCACGCCGCCAGCGTTCGTCCCGAGCCAGGATCAAACTCT
>296

CTGCTGCCGCCCGTAGGAGTCTGGGCCGTGTCTCAGTCCCAGTGTGGCCGATCACCCTCTCAGGTCGGCTATG
TATCGTCGCCTTGGTGAGCCCTTACCCCACCAACTAGCTAATACAACGCAGGTCCATCTGGTAGTGATGCAAT
TGCACCTTTCAAACAGTTGTCATGCAACAACTGCTATTATGCGGTATTAGCTATCGTTTCCAATAGTTATCCC
CCGCTACCAGGCAGGTTACCTACGCGTTACTCACCCGTTCGCAACTCATCCAGAGAAGCAAGCTCCTCCTTCA
GCGTTCTACTTGCATGTATTAGGCACGCCGCCAGCGTTCGTCCTAAGCCAGGATCAAACTCT
>297
CTGCTGCCGCCCGTAGGAGTCTGGGCCGTGTCTCAGTCCCAGTGTGGCCGATCACCCTCTCAGGTCGGCTATG
TATCGTCGCCTTGGTGAGCCGTTACCCCACCAACTAGCTAATACAACGCAGGTCCATCTGGTAGTGGTGCAAT
TGCACCTTTTAAGCAAATGTCATGCAACATCTACTCTTATGCGGTATTAGCTATCGTTTCCAATAGTTATCCC
CCGCTACCAGGCAGGTTACCTACGCGTTACTCACCCGTTCGCAACTCATCCGGAGAAGCAAGCTCCTCCTTCA
GCGTTCTACTTGCATGTATTAGGCACGCCGCCAGCGTTCGTCCTGAGCCAGGATCAAACTCT
>298
CTGCTGCCGCCCGTAGGAGTCTGGGCCGTGTCTCAGTCCCAGTGTGGCCGATCACCCTCTCAGGTCGGCTATG
TGTCGTCGCCTTGGTGAGCCGTTACCCCACCAACTAGCTAATACAACGCAGGTCCATCTGGTAGTGGTGCAAT
TGCGCCTTTTAAGCAAATGTCATGCAACATCTACTCTTATGCGGTATTAGCTATCGTTTTCCAATAGTTATCC
CCCCGTTACCAGGCAGGTTACCTACGCGTTACTCACCCGTTCGCAACTCATCCGGAGAAGCAAGCCCCTCCTT
CAGCGTTCTACTTGCATGTATTTAGGCACGCCGCCAGCGTTCGTCCTGAGCCAGGATCAAACTCT
>299
CTGCTGCCGCCCGTAGGAGTCTGGGCCGTGTCTCAGTCCCAGTGTGGCCGATCACCCTCTCAGGTCGGCTATG
TATCGTCGCCTTGGTGAGCCGTTACCTCACCAACTAGCTAATACAACGCAGGTCCATCTGGTAGTGGTGCAAT
TTGCACCTTTTAATTGACTATCATGCAATAATCAATTTTATGCGGTATTAGCCTATCGTTTCCAATAGTTACT
CCCCCGCTACCAGGCAGGTTACCTACGCGTTACTCACCCGTTCGCAACTCATCCAGAGAAGCAAGCTCCTCCT
TCAGCGTTCTACTTGCATGTATTTAGGCACGCCGCCAGCGTTCGTCCTGAGCCAGGATCAAACTCT
>300
CTGCTGCCGCCCGTAGGAGTCTGGGCCGTGTCTCAGTCCCAGTGTGGCCGATCACCCTCTCAGGTCGGCTATG
TATCGTCGCCTTGGTGAGCCCTTACCCCACCAACTAGCTAATACAACGCAGGTCCATCTGGTAGTGATGCAAT
TGCACCTTTCAAACAGTTGTCATGCAACAACTGCTATTATGCGGTATTAGCTATCGTTTCCAATAGTTATCCC
CCGCTACCAGGCAGGTTACCTACGCGTTACTCACCCGTTCGCAACTCATCCGGAGAAGCAGGCTCCTCCTTCA
GCGTTCTACTTGCATGTATTAGGCACGCCGCCAGCGTTCGTCCTGAGCCAGGATCAAACTCT
>301
CTGCTGCCGCCCGTAGGAGTCTGGGCCGTGTCTCAGTCCCAGTGTGGCCGATCACCCTCTCAGGTCGGCTATG
TATCGTCGCCTTGGTGAGCCCTTACCCCACCAACTAGCTAATACAACGCAGGTCCATCTGGTAGTGATGCAAT
TGCACCTTTCAAACAGTTGTCATGCAACAACTGCTATTATGCGGTATTTAGCTATCGTTTTCCAATAGTTATC
CCCCGCTACCAGGCAGGTTACCTACGCGTTACTCACCCGTTCGCAACTCATCCAGAGAAGCAAGCTCCTCCTT
TCAGCGTTCTACTTGCATGTATTTAGGCACGCCGCCAGCGTCGTCCTGAGCCAGGATCAAACTCT
>302
CTGCTGCCGCCCGTAGGAGTCTGGGCCGTGTCTCAGTCCCAGTGTGGCCGATCACCCTCTCAGGTCGGCTATG
TATCGTCGCCTTGGTGGGCCGTTACCTCACCAACTAGCTAATACAACGCAGGTCCATCTGGTAGTGATGCAAT
TGCACCTTTTAATTGACTATCATGCAATAATCAATTTTATGCGGTATTAGCTATCGCTTCCAATAGTTATCCC
CCGCTACCAGGCAGGTTACCTACGCGTTACTCACCCGTTCGCAACTCATCCAGAGAAGCAAGCTCCTCCTTCA
GCGTTCTACTTGCATGTATTAGGCACGCCGCCAGCGTTCGTCCTGAGCCAGGATCAAACTCT
>303
CTGCTGCCGCCCGTAGGAGTCTGGACCGTGTCTCAGTTCCAGTGTGGCCGATCACCCTCTCAGGTCGGCTACG
TATCGTTGCCTTGGTAAGCCGTTACCTTACCAACTAGCTAATACGGCGCAGGTCCATCTATAAGTGATAGCAA
AGCCATCTTTCACTATCGAACCATGCGGTTCGAAATATTATCCGGTATTAGCTCCGGTTTTCCCGAAGTTATC
CCAGTCTTATAGGTAGGTTACCTACGTGTTACTCACCCGTCCGCCGCTAACGTCAAAGGAGCAAGCTCCTCGT
CTGTTCGCTCGACTTGCATGTATTAGGCACGCCGCCAGCGTTCATCCTGAGCCAGGATCAAACTCT
>304
CTGCTGCCGCCCGTAGGAGTCTGGACCGTGTCTCAGTTCCAGTGTGGCCGATCACCCTCTCAGGTCGGCTACG
TATCGTTGCCTTGGTAAGCCGTTACCTTACCAACTAGCTAATACGGCGCAGGTCCATCTATAAGTGATAGCAA
AGCCATCTTTCACTATCGAACCATGCGGTTTCGAAATATTATCCGGTATTAGCTCCGGTTTTTCCCGAAGTTA
TCCCAGTCTTATAGGTAGGTTTTACCTACGTGTTTACTCACCCGTCCGCCGCTAACGTCAAAGGAGCAAGCTC
CTCGTCTGTTTCGCTCGACTTGCATGTATTAGGCACGCCGCCAGCGTTCATCCTGAGCCAGGATCAAACTCT
>305

CTGCTGCCGCCCGTAGGAGTCTGGGCCGTGTCTCAGTCCCAGTGTGGCCGATCACCCTCTCAGGTCGGCTATG
TATCGTCGCCTTGGTGAGCCGTTACCTCACCAACTAGCTAATACAACGCAGGTCCATCTGGTAGTGATGCAAT
TTGCACCTTTTAATTGACTATCATGCAATAATCAATTTTATGCGGTATTTAGCCTATCGTTTTCCAATAGTTA
CTCCCCCGCTACCAGGCAGGTTACCTACGCGTTACTCACCCGTTCGCAACTCATCCGGAGAAGCAAGCTCCTC
CTTCAGCGTTCTACTTGCATGTATTTAGGCACGCCGCCAGCGTTCGTCCTGAGCCAGGATCAAACTCT
>306
CTGCTGCCGCCCGTAGGAGTCTGGGCCGTGTCTCAGTCCCAGTGTGGCCGATCACCCTCTCAGGTCGGCTATG
TATCGTCGCCTTGGTGAGCCCTTACCCCACCAACTAGCTAATACAACGCAGGTCCATCTGGTAGTGATGCAAT
TGCACCTTTTCAAACAGTTTGTCATGCAACAACTGCTATTTATGCGGTATTTAGCCATCGTTTTTCCAATAGT
TATCCCCCCCGCTACCAGGCAGGTTACCTTACGCGTTACTCACCCGTTTCGCAACTCATCCGGAGAAGCAAGC
TCCTCCTCAGCGTTCTACTTGCATGTATTTAGGCACGCCGCCAGCGTTCGTCCTGAGCCAGGGATCAAAGCTC
T
>307
CTGCTGCCGCCCGTAGGAGTCTGGGCCGTGTCTCAGTCCCAGTGTGGCCGATCACCCTCTCAGGTCGGCTATG
TATCGTCGCCTTGGTGAGCCCTTACCCCACCAACTAGCTAATACAACGCAGGTCCATCTGGTAGTGATGCAAT
TGCACCTTTTCAAACAGTTTGTCATGCAACAACTGCTATTTATGCGGTATTTTAGCTATCGTTTTTCCAATAG
TTTTATCCCCCCCGCTACCAGGCAGGTTACCTTACGCGTTACTCACCTGTTTCGCAACTCATCCAGAGAAGCA
AGCTCCTCCTTTCAGCGTTCTACTTGCATTGTATTAGGCACGCCGCCAGCGTTCGTCCTGAGCCAGGATCAAA
CTCT
>308
CTGCTGCCGCCCGTAGGAGTCTGGGCCGTGTCTCAGTCCCAGTGTGGCCGATCACCCTCTCAGGTCGGCTATG
TATCGTCGCCTTGGTGAGCCCTTACCCCACCAACTAGCTAATACAACGCAGGTCCATCTGGTAGTGATGCAAT
TGCACCTTTCAAACAGTTGTCATGCAACAACTGCTATTATGCGGTATTTAGCTATCGTTTTTCCAATAGTTTA
TCCCCCGCTACCAGGCAGGTTACCTACGCGTTACTCACCCGTTCGCAACTCATCCGGAGAAGCAAGCTCCTCC
TCAGCGTTCTACTTGCATGTATTTAGGCACGCCGCCAGCGTTCGTCCTGAGCCAGGATCAAACTCT
>309
CTGCTGCCGCCCGTAGGAGTCTGGGCCGTGTCTCAGTCCCAGTGTGGCCGATCACCCTCTCAGGTCGGCTATG
TATCGTCGCCTTGGTGAGCCGTTACCCCACCAACTAGCTAATACAACGCAGGTCCATCTGGTAGTGGTGCAAT
TGCACCTTTCAAACAGCTGTCATGCAACAACTGCTATTATGCGGTATTAGCTATCGTTTCCAATAGTTATCCC
CCGCTACCAGGCAGGTTACCTACGCGTTACTCACCCGTTCGCAACTCATCCAGAGAAGCAAGCTCCTCCTTCA
GCGTTCTACTTGCATGTATTAGGCACGCCGCCAGCGTTCGTCCTGAGCCAGGATCAAACTCT
>310
CTGCTGCCGCCCGTAGGAGTCTGGGCCGTGTCTCAGTCCCAGTGTGGCCGATCACCCTCTCAGGTCGGCTATG
TATCGTTGCCTTGGTGAGCCGTTACCTCACCAACTAGCTAATACAACGCAGGTCCATCTGGTAGTGGTGCAAT
TGCACCTTTTAAGCAGCTATCATGCGATATCTACTGTTATGCGGTATTACGCTATCGTTTTCCAATAGTTATC
CCCCGCTACCAGGCAGGTTACCTACGCGTACTCACCCGTTCGCAACTCATCCGGAAAGAGCAAGCTCCTTCCT
CAGCGTTCTACTGCATGTATTAGGCACGCCGCCAGCGTTCGTCCTGAGCCAGGATCAAACTCT
>311
CTGCTGCCGCCCGTAGGAGTCTGGGCCGTGTCTCAGTCCCAGTGTGGCCGATCACCCTCTCAGGTCGGCTATG
TATCGTCGCCTTGGTGAGCCCTTACCCCACCAACTAGCTAATACAACGCAGGTCCATCTGGTAGTGATGCAAT
TGCACCTTTCAAACAGTTGTCATGCAACAACTGCTATTATGCGGTATTAGCTATCGTTTTCCAATAGTTATCC
CCCGCTACCAGGCAGGTTACCTACGCGTTACTCACCCGTTCGCAACTCATCCGGAGAAGCAAGCTCCTCCTCA
GCGTTCTACTTGCATGTATTAGGCACGCCGCCAGCGTTCGTCCTGAGCCAGGATCAAACTCT
>312
CTGCTGCCGCCCGTAGGAGTCTGGGCCGTGTCTCAGTCCCAGTGTGGCCGATCACCCTCTCAGGTCGGCTATG
TATCGTCGCCTTGGTGAGCCGTTACCTCACCAACTAGCTAATACAACGCAGGTCCATCTGGTAGTGATGCAAT
TTGCACCTTTTAATTGACTATCATGCAATAATCAATTTTATGCGGTATTAGCCTATCGTTTTCCAATAGTTAT
CCCCCGCTACCAGGCAGGTTACCTACGCGTTACTCACCCGTTCGCAACTCATCCAGAGAAGCAAGCTCCTCCT
TCAGCGTTCTACTTGCATGTATTAGGCACGCCGCCAGCGTTCGTCCTGAGCCAGGATCAAACTCT
>313
CTGCTGCCGCCCGTAGGAGTCTGGGCCGTGTCTCAGTCCCAGTGTGGCCGATCACCCTCTCAGGTCGGCTATG
TATCGTCGCCTTGGTGAGCCCTTACCCCACCAACTAGCTAATACAACGCAGGTCCATCTGGTAGTGATGCAAT
TGCACCTTTCAAACAGTTGTCATGCAACAACTGCTATTTATGCGGTACTAGCTATCGTTTTTCCAATAGTTTA

TCCCCCCCGCTACCAGGCAGGTTACCTTACGCGTTACTCACCCGTTCGCAACTCATCCAGAGAAGCAAGCTCC
TCCTTTCAGCGTTCTACTTGCATTGTATTAGGCACGCCGCCAGCGTTCGCCTGAGCCAGGATCAAACTCT
>314
CTGCTGCCGCCCGTAGGAGTCTGGGCCGTGTCTCAGTCCCAGTGTGGCCGATCACCCTCTCAGGTCGGCTATG
TATCGTCGCCTTGGTGAGCCGTTACCCCCCACCAACTAGCTAATACAACGCAGGTCCATCTGGTAGTGGTGCA
ATTGCACCTTTTAAGCAAATGTCATGCAACATCTACTCTTTATGCGGTATTTAGCTATCGTTTTCCAATAGTT
TATCCCCCCCGCTACCAGGCAGGTTACCTACGCGTTACTCACCCGTTTCGCAACTCATCCGGAGAAGCAAGCT
CCTCCTTCAGCGTTCTACTGCATGTATTTAGGCACGCCGCCAGCGTTCGTCCTGAGCCAGGATCAAACTCT
>315
CTGCTGCCGCCCGTAGGAGTCTGGGCCGTGTCTCAGTCCCAGTGTGGCCGATCACCCTCTCAGGTCGGCTATG
TATCGTCGCCTTGGTGAGCCGTTACCCCACCAACCAGCTAATACAACGCAGGTCCATCTGGTAGTGGTGCAAT
TGCACCTTTTAAGCAAATGTCATGCAACATCTACTCTTATGCGGTATTAGCTATCGTTTCCAATAGTTATCCC
CCGCTACCAGGCAGGTTACCTACGCGTTACTCACCCGTTCGCAACTCATCCGGAGAAGCAAGCTCCTCCTTCA
GCGTTCTACTTGCATGTATTAGGCACGCCGCCAGCGTTCGTCCTGAGCCAGGATCAAACTCT
>316
CTGCTGCCGCCCGTAGGAGTCTGGGCCGTGTCTCAGTCCCAGTGTGGCCGATCACCCTCTCAGGTCGGCTATG
TATCGTCGCCTTGGTGAGCCCTTACCCCACCAACTAGCTAATACAACGCAGGTCCATCTGGTAGTGATGCAAT
TGCACCTTTCAAACAGTTGTCATGCAACAACTGCTATTATGCGGTATTAGCTATCGTTTTCCAATAGTTATCC
CCCGCTACCAGGCAGGTTACCGACCGCGTTACTCACCCGTTCGCAACTCATCCAGAGAAGCAAGCTCCTCCTC
AGCGTCTACTGCATGTATAGGCTACGCCGCCAGCGT
>317
CTGCTGCCGCCCGTAGGAGTCTGGGCCGTGTCTCAGTCCCAGTGTGGCCGATCACCCTCTCAGGTCGGCTATG
TATCGTCGCCTTGGTGAGCCCTTACCCCACCAACTAGCTAATACAACGCAGGTCCATCTGGTAGTGATGCAAT
TGCACCTTTCAAACAGTTGTCATGCAACAACTGCTATTATGCGGTATTAGCTATCGTTTCCAATAGTTATCCC
CCGCTACCAGGCAGGTTACCTACCGCGTACTCACCCGTTCGCAACTCATCCGGAGAAGCAAGCTCCTCCTCAG
CGTCTACTGCATGTATTAGGCACGCCGCCAGCGTCGTCCTGAGCCAGGATCAAACTCT
>318
CTGCTGCCGCCCGTAGGAGTCTGGACCGTGTCTCAGTTCCAGTGTGGCCGATCACCCTCTCAGGTCGGCTACG
TATCGTTGCCTTGGTAAGCCGTTACCTTACCAACTAGCTAATACGGCGCAGGTCCATCTATAAGTGATAGCAA
AGCCATCTTTCACTATCGAACCATGCGGTTCGAAATATTATCCGGTATTAGCTCCGGTTTTCCCGAAGTATCC
CAGTCTTATAGGTAGGTTTACCTACGTGTTACTCACCCGTCCGCCGCTAACGTCGAAGGAGCAAGCTCCTCGT
CTGTTCGCTCGACTTGCATGTATTAGGCACGCCGCCAGCGTTCATCCTGAGCCAGGATCAAACTCT
>319
CTGCTGCCGCCCGTAGGAGTCTGGGCCGTGTCTCAGTCCCAGTGTGGCCGATCACCCTCTCAGGTCGGCTATG
TATCGTCGCCTTGGTGAGCCCTTACCCCACCAACTAGCTAATACAACGCAGGTCCATCTGGTAGTGATGCAAT
TGCACCTTTCAAACAGTTGTCATGCAACAACTGCTATTATGCGGTATTAGCTATCGTTTCCAATAGTTATCCC
CCGCTACCAGGCAGGTTACCTACGCGTTACTCACCCGTTCGCAACTCATCCAGAGAAGCAAGCTCCTCCTTCA
GCGTTCTACTTGCATGTATTAGGCACGCCGCCAGCGTTCGTCCTGAGCCAGGATCAAACTCT
>320
CTGCTGCCGCCCGTAGGAGTCTGGGCCGTGTCTCAGTCCCAGTGTGGCCGATCACCCTCTCAGGTCGGCTATG
TATCGTCGCCTTGGTGAGCCGTTACCCCACCAACTAGCTAATACAACGCAGGTCCATCTGGTAGTGGTGCAAT
TGCACCTTTTAAGCAAATGTCATGCAACATCTACTCTTATGCGGTATTAGCTATCGTTTCCAATAGTTATCCC
CCGCTACCAGGCAGGTTACCTACGCGTTACTCACCCGTTCGCAACTCATCCGGAAAGAGCAAGCTCCTTCCTT
CAGCGTTCTACTTGCATGTATTAGGCACGCCGCCAGCGTTCGTCCTGAGCCAGGATCAAACTCT
>321
CTGCTGCCGCCCGTAGGAGTCTGGGCCGTGTCTCAGTCCCAGTGTGGCCGATCACCCTCTCAGGTCGGCTATG
CATCGTCGCCTTGGTAGGCCTTTACCCCACCAACTAGCTAATGCACCGCAAAGCCATCTCATAGTGTTAGCAA
AACCAACTTTTAAACATCAACCATGCAGTTAATGCTGTTATCCGGTATTAGCTATCGTTTCCAATAGTTGTCC
CAGTCTATGAGGCAGGTTTCTTTACGTGTTACTCACCCGTTCGCCGCTACGTTGTCGTCGAGCAGCAAACCAG
CAACTAACTC
>322
CTGCTGCCGCCCGTAGGAGTCTGGGCCGTGTCTCAGTCCCAGTGTGGCCGATCACCCTCTCAGGTCGGCTATG
TATCGTCGCCTTGGTGAGCCCTTACCCCACCAACTAGCTAATACAACGCAGGTCCATCTGGTAGTGATGCAAT
TGCACCTTTCAAACAGTTGTCATGCAACAACTGCTATTATGCGGTATTAGCTATCGTTTTCCAATAGTTATCC

CCCGCTACCAGGCAGGTTACCTACGCGTTACCTCACCCGTTCGCAACTCATCCAGAGAAGCAAGCTCCTCCTT
CAGCGTCCTACTTGCATGTATTAGGCACGCCGCCAGCGTTCGTCCTGAGCCAAGATCAAACTCT
>323
CTGCTGCCGCCCGTAGGAGTCTGGGCCGTGTCTCAGTCCCAGTGTGGCCGATCACCCTCTCAGGTCGGCTATG
TATCGTCGCCTCGGTGAGCCGTTACCCCACCAACTAGCTAATACAACGCAGGTCCATCTGGTAGTGGTGCAAT
TGCACCTTTTAAGCAAATGTCATGCAACATCTACTCTTATGCGGTATTAGCTATCGTTTCCAATAGTTATCCC
CCGCTACCAGGCAGGTACCTACGCGTACTCACCCGTTCGCAACTCATCCAGAGAAGCAAGCTCCTCCTCAGCG
TTCTACTGCATGTATTAGGCACGCCGCCAGCGTCGTCCTGAGCCAGGATCAAACTCT
>324
CTGCTGCCGCCCGTAGGAGTCTGGGCCGTGTCTCAGTCCCAGTGTGGCCGATCACCCTCTCAGGTCGGCTATG
TATCGTCGCCTTGGTGAGCCCTTACCCCACCAACTAGCTAATACAACGCAGGTCCATCTGGTAGTGATGCAAT
TGCACCTTTCAAACAGTTGTCATGCAACAACTGCTATTATGCGGTATTAGCTATCGTTTCCAATAGTTATCCC
CCGCTACCAGGCAGGTTACCTACGCGTTACTCACCCGTTCGCAACTCATCCAGAGAAGCAAGCTCCTCCTTCA
GCGTTCTACTTGCATGTATTAGGCACGCCGCCAGCGTCGTCCTGAGCCAGGATCAAACTCT
>325
CTGCTGCCGCCCGTAGGAGTCTGGGCCGTGTCTCAGTCCCAGTGTGGCCGATCACCCTCTCAGGTCGGCTATG
TATCGTCGCCTTGGTGAGCCCTTACCCCACCAACTAGCTAATACAACGCAGGTCCATCTGGTAGTGATGCAAT
TGCACCTTTCAAACAGTTGTCATGCAACAACTGCTATTATGCGGTATTAGCTATCGTTTCCAATAGTTATCCC
CCGCTACCAGGCAGGTTACCTACGCGTTACTCACCCGTTCGCAACTCATCCAGAGAAGCAAGCTCCTCCTTCA
GCGTTCTACTTGCATGTATTAGGCACGCCGCCAGCGTTCGTCCTGAGCCAGGATCAAACTCT
>326
CTCTGCCGCCCGTAGGAGTCTGGGCCGTGTCTCAGTCCCAGTGTGGCCGATCACCCTCTCAGGTCGGCTATGT
ATCGTCGCCTTGGTGAGCCGTTACCTCACCAACTAGCTAATACAACGCAGGTCCACCTGGTAGTGATGCAATT
GCACCTTTTAATTGACTATCATGCAATAATCAATTTTATGCGGTATTAGCTATCGTTTCCAATAGTTATCCCC
CGCTACCAGGCAGGTTACCTACGCGTTACTCACCCGTTCGCAACTCATCCAGAGAAGCAAGCTCCTCCTTCAG
CGTTCTACTTGCATGTATTAGGCACGCCGCCAGCGTTCGTCCTGAGCCAGGATCAAACTCT
>327
CTGCTGCCGCCCGTAGGAGTCTGGGCCGTGTCTCAGTCCCAGTGTGGCCGATCACCCTCTCAGGTCGGCTATG
TATCGTCGCCTTGGTGAGCCGTTACCTCACCAACTAGACTAATACAACGCAGGTCCATCTGGTAAGTGATGCA
ATTTGCACCTTTTAATTGACTATCATGCAATAATCAACTTTTATGTCGGTATTAGCCTATCGTTTCCAATAGT
TACTCCCCCGCTACCAGGCAGGTTACCTACGCGTTACTCACCCGTTCGCAACTCATCCAGAGAAGCAAGCTCC
TCCTTCAGCGTTCTACTTGCATGTATTACGGCAGCCGCCA
>328
CTGCTGCCGCCCGTAGGAGTCTGGGCCGTGTCTCAGTCCCAGTGTGGCCGATCACCCTCTCAGGTCGGCTATG
TATCGTCGCCTTGGTGAGCCGTTACCCCACCAACTAGCTAATACAACGCAGGTCCATCTGGTAGTGATGCAAT
TGCACCTTTCAAACAGTTGTCATGCAACAACTGCTATTATGCGGTATTAGCTATCGTTTCCAATAGTTATCCC
CCGCTACCAGGCAGGTTACCTACGCGTTACTCACCCGTTCGCAACTCATCCAGAGAAGCAAGCTCCTCCTTCA
GCGTTCTACTTGCATGTATTAGGCACGCCGCCAGCGTTCGTCCTGAGCCAGGATCAAACTCT
>329
CTGCTGCCGCCCGTAGGAGTCTGGACCGTGTCTCAGTTCCAGTGTGGCCGATCACCCTCTCAGGTCGGCTACG
TATCGTCGCCTTGGTAAGCCGTTACCTTACCAACTAGCTAATACGGCGCGGGTCCATCTATAAGTGATAGCAA
AACCATCTTTCACTATCGAACCATGCGGTTCGAAATATTATCCGGTATTAGCTCCGGTTTCCCGAAGTTATCC
CAGTCTTATAGGTAGGTTACCCACGTGTTACTCACCCGTCCGCCGCTAACGTCAAAGGAGCAAGCTCCTTGTC
TGTTCGCTCGACTTGCATGTATAGGCACGCCGTCCAGCGTCATCCTGAGCCAGGATCAAACTCT
>330
CTGCTGCCGCCCGTAGGAGTCTGGGCCGTGTCTCAGTCCCAGTGTGGCCGATCACCCTCTCAGGTCGGCTATG
TATCGTCGCCTTGGTGAGCCGTTACCTCACCAACTAGCTAATACAACGCAGGTCCATCTGGTAGTGATGCAAT
TGCACCTTTTAATTGACTATCATGCAATAATCAATTTTATGCGGTATTAGCCTATCGTTTCCAATAGTTACTC
CCCCGCTACCAGGCAGGTTACCTACGCGTTACTCACCCGTTCGCAACTCATCCAGAGAAGCAAGCTCCTCCTT
CAGCGTTCTACTTGCATGTATTAGGCACGCCGCCAGCGTTCGTCCTGAGCCAGGATCAAACTCT
>331
CTGCTGCCGCCCGTAGGAGTCTGGTCCGTGTCTCAGTACCAGTGTGGGGGATTCTCCTCTCAGAGCCCCTAGA
CATCGTCGCCTTGGTGAGCCGTTACCTCACCAACTAGCTAATGTCACGCGAGCCCATCCATATCCTATAAATA
TTTAACTACTTGATGATGCCATCTCGTAGTCTTATGCGGTGTTAATCCGAATTTCTTCGGGCTACTCCCCCCT

GATATGGGTAGGTTTGCTCACGCGTTACGCACCCGTGCGCCACTCTCACAATCTTTAGCAAGCTAAAAATTGG
ATCCCGTTTCGACTGCATGTATTAGGCCTGCCGCTAGCGTTCATCCTGAGCCAGGATCAAACTCT
>332
CTGCTGCCGCCCGTAGGAGTCTGGGCCGTGTCTCAGTCCCAGTGTGGCCGATCACCCTCTCAGGTCGGCTATG
TATCGTCGCCTTGGTGAGCCGTTACCTCACCAACTAGCTAATACAACGCAGGTCCATCTGGTAGTGATGCAAT
TTGCACCTTTTAATTGACTATCATGCAATAATCAATTTTATGCGGTATTAGCCTATCGTTTCCAATAGTTACT
CCCCCGCTACCAGGCAGGTTACCTACGCGTTACTCACCCGTTCGCAACTCATCCAGAGAAGCAAGCTCCTCCT
TCAGCGTTCTACTTGCATGTATTAGACACGCCGCCAGCGTTCGTCCTGAGCCAGGATCAAACTCT
>333
CTGCTGCCGCCCGTAGGAGTCTGGGCCGTGTCTCAGTCCCAGTGTGGCCGATCACCCTCTCAGGTCGGCTATG
TATCGTCGCCTTGGTGAGCCGTTACCCCACCAACTAGCTAATACAACGCAGGTCCATCTGGTAGTGGTGCAAC
TGCACCTTTTAAGCAAATGTCATGCAACATCTACTCTTTATGCGGTATTTAGCTATCGTTTTTCCAATAGTTT
ATCCCCCCCGCTACCAGGCAGGTTACCTACGCGTTACTCACCCGTTCGCAACTCATCCGGAGAAGCAAGCTCC
TCCTTCAGCGTTCTACTTGCATGTATTTAGGCACGCCGCCAGCGTTCGTCCTGAGCCAGGATCAAACTCT
>334
CTGCTGCCGCCCGTAGGAGTCTGGGCCGTGTCTCAGTCCCAGTGTGGCCGATCACCCTCTCAGGTCGGCTATG
TATCGTCGCCTTGGTGAGCCCTTACCCCACCAACTAGCTAATACAACGCAGGTCCATCTGGTAGTGATGACAA
TTGCACCTTCCAAACAGTTGTCATGCAACAACTGCTATTATGCGGTATTTAGCTATCGTTTTCCAATAGTTTA
TCCCCCGCTACCAGGCAGGTTACCTACGCGTTACTCACCCGTTCGCAACTCATCCAGAGAAGCAAGCTCCTCC
TCAGCGTTCTACTTGCATGTATTTAGGCACGCCGCCAGCGTCGTCCTGAGCCAGGATCAAACTCT
>335
CTGCTGCCGCCCGTAGGAGTCTGGGCCGTGTCTCAGTCCCAGCGTGGCCGATCACCCTCTCAGGTCGGCTATG
TATCGTCGCCTTGGTGAGCCGTTACCCCACCAACTAGCTAATACAACGCAGGTCCATCTGGTAGTGGTGCAAT
TGCACCTTTTAAGCAAATGTCATGCAACATCTACTCTTATGCGGTATTAGCTATCGTTTTCCAATAGTTTATC
CCCCCGCTACCAGGCAGGTTACCTACGCGTTACTCACCCGTTCGCAACTCATCCGGAGAAGCGAGCTCCTCCT
TCAGCGTTCTACTTGCATGTATTTAGGCACGCCGCCAGCGTTCGTCCTGAGCCAGGATCAAACTCT
>336
CTGCTGCCGCCCGTAGGAGTCTGGGCCGTGTCTCAGTCCCAGTGTGGCCGATCACCCTCTCAGGTCGGCTATG
TATCGTCGCCTTGGTGAGCCGTTACCCCCCACCAACTAGCTAATACAACGCAGGTCCATCTGGTAGTGGTGCA
ATTGCACCTTTTAAGCAAATGTCATGCAACATCTACTCTTTATGCGGTATTTAGCTATCGTTTTCCAATAGTT
TATCCCCCCGCTACCAGGCAGGTTACCTTACGCGTTACTCACCCGTTTCGCAACTCATCCGGAGAAGCAAGCT
CCTCCTTCAGCGTTCTACTTGCATGTATTTAGGCACGCCGCCAGCGTTCGTCCTGAGCCAGGATCAACT
>337
CTGCTGCCGCCCGTAGGAGTCTGGGCCGTGTCTCAGTCCCAGTGTGGCCGATCACCCTCTCAGGTCGGCTATG
TATCGTCGCCTTGGTGAGCCGTTACCCCACCAACTAGCTAATACAACGCAGGTCCATCTGGTAGTGGTGCAAT
TGCACCTTTTAAGCAAATGTCATGCAACATCTACTCTTATGCGGTATTAGCTATCGTTTCCAATAGTTATCCC
CCGCTACCAGGCAGGTTACCTACGCGTTACTCACCCGTTCGCAACTCATCCGGAGAAGCAAGCTCCTCCTTCA
GCGTTCTACTTGCATGTATTAGGCACGCCGCCAGCGTTCGTCCTGAGCCAGGATCAAACTCT
>338
CTGCTGCCGCCCGTAGGAGTCTGGGCCGTGTCTCAGTCCCAGTGTGGCCGATCACCCTCTCAGGTCGGCTATG
TATCGTCGCCTTGGTGAGCCCTTACCCCACCAACTAGCTAATACAACGCAGGTCCATCTGGTAGTGATGCAAT
TGCACCTTTCAAACAGTTGTCATGCAACAACTGCTATTATGCGGTATTTAGCTATCGTTTTCCAATAGTTTAT
CCCCCGCTACCAGGCAGGTTACCTACGCGTTACTCACCCGTTCGCAACTCATCCAGAGAAGCAAGCTCCTCCT
TTCAGCGTTCTACTTGCATGTATTAGGCACGCCGCCAGCGTTCGATCCTGAGCCAGGAG
>339
CTGCTGCCGCCCGTAGGAGTCTGGACCGTGTCTCAGTTCCAGTGTGGCCGATCGCCCTCTCAGGTCGGCTACG
TATCGTTGCCTTGGTAAGCCGTTACCTTACCAACTAGCTAATACGGCGCAGGTCCATCTATAAGTGATAGCAA
AGCCATCTTTCACTATCGAACCATGCGGTTCGAAATATTATCCGGTATTAGCTCCGGTTTCCCGAAGTTATCC
CAGTCTTATACGGTAGGTTACCTACGTGTTACTCACCCGTCCGCCGCTAACGTCGAAGGAGCCAAGCTCCTCG
TCTGTTCGCTCGACTTGCATGTATTAGGCACGCCGCCAGCGTCATCCTGA
>340
CTGCTGCCGCCCGTAGGAGTCTGGGCCGTGTCTCAGTCCCAGTGTGGCCGATCACCCTCTCAGGTCGGCTATG
TATCGTCGCCTTGGTGAGCCGTTACCCCACCAACTAGCTAATACAACGCAGGTCCATCTGGTAGTGGTGCAAT
TGCACCTTTTAAGCAAATGTCATGCAACATCTACTCTTATGCGGTATTAGCTATCGTTTCCAATAGTTATCCC

CCGCTACCAGGCAGGTTACCTACGCGTACTCACCCGTTCGCAACTCATCCGGAGAAGCAAGCTCCTCCTCCAG
CGTCTACTTGCATGTATTAGGCACGCCGCCAGCGTTCGTCCTGAGCCAGGATCAAACTCT
>341
CTGCTGCCGCCCGTAGGAGTCTGGGCCGTGTCTCAGTCCCAGTGTGGCCGATCACCCTCTCAGGTCGGCTATG
TATCGTCGCCTTGGTGAGCCGTTACCTCACCAACTAGCTAATACAACGCAGGTCCATCTGGTAGTGATGCAAT
TTGCACCTTTTAATTGACTATCATGCAATAATCAATTTTATGCGGTATTAGCCTATCGTTTCCAATAGTTATC
CCCCGCTACCAGGCAGGTTACCTACGCGTTACTCACCCGTTCGCAACTCATCCAGAGAAGCAAGCTCCTCCTT
CAGCGTTCTACTTGCATGTATTACGGCACGCCGCCAGCGTTCGTCCTGAGCCAGGATCAAACTCT
>342
CTGCTGCCGCCCGTAGGAGTCTGGACCGTGTCTCAGTTTCCAGTTGTGGCCGATCACCCTCTCAGGTCGGCTA
TGCATCGTTGCCTTGGTAAGCCGTTACCTTACCAACTAGCTAATGCAGCGCGGATCCATCTATAAGTTGACAG
CAAGAACCGTCTTTTACTTTTGAACCATGCGGTTCAAAATATTATCCGGTATTAGCTCCGGTTTCCCGAAGTT
ATCCCAGTCTTATAGGTAGGTTATCCACGTTGTTACTCACCCGTCCGCCGCTAACATCAGAGAAGCAAGCTTC
TCGTCCGTTCGCTCGACTTGCATGTATTAGGCACGCCGCCACGTTCATCCTGAGCCAAGGATCAAACTCT
>343
CTGCTGCCGCCCGTAGGAGTCTGGGCCGTGTCTCAGTCCCAGTGTGGCCGATCACCCTCTTCAGGTCGGCTAT
GTATCGTCGCCTTGGTGAGCCGTTACCTCACCAACTAGCTAATACAACGCAGGTCCATCTGGTAGTGATGCAA
TTGCACCTTTTAATTGACTATCATGCAATAATCAATTTTATGCGGTATTAGCCTATCGTTTTCCAATAGTTAC
TCCCCCGCTACCAGGCAGGTTACCTACGCGTTACTCACCCGTTCGCAACTCATCCAGAGAAGCAAGCTCCTCC
TCAGCGTTCTACTGCATGTATTAGGCACGCCGCCAGCGTTCGTCCTGAGCCAGGATCAAACTCT
>344
CTGCTGCCGCCCGTAGGAGTCTGGGCCGTGTCTCAGTCCCAGTGTGGCCGATCACCCTCTCAGGTCGGCTATG
TATCGTCGCCTTGGTGAGCCCTTACCCCACCAACTAGCTAATACAACGCAGGTCCATCTGGTAGTGATGCAAT
TGCACCTTTCAAACAGTTGTCATGCAACAACTGCTATTATGCGGTATTAGCTATCGTTTCCAATAGTTATCCC
CCGCTACCAGGCAGGTTACCTACGCGTTACTCACCCGTTCGCAACTCATCCAGAGAAGCAAGCCTCCTCCTCA
GCGTTCTACTTGCATGTATTAGGCACGCCGCCAGCGTTCGTCCTGAGCCAGGATCAAACTCT
>345
CTGCTGCCGCCCGTAGGAGTCTGGGCCGTGTCTCAGTCCCAGTGCGGCCGATCACCCTCTCAGGTCGGCTATG
TATCGTCGCCTTGGTGAGCCCTTACCCCACCAACTAGCTAATACAACGCAGGTCCATCTGGTAGTGATGCAAT
TGCACCTTTCAAACAGTTGTCATGCAACAACTGCTACTATGCGGTATTAGCTATCGTTTCCAATAGTTATCCC
CCGCTACCAGGCAGGTTACCTACCGCGTTACTCACCCGTTCGCAACTCATCCAGAGAAGCAAGCCTCCTCCTT
CCAGCGTTCTACTTGCCATGTATTAGGCACGCCGCCAGCGTCGTCCCGAGCCCAGGATCAAACTCCT
>346
CTGCTGCCGCCCGTAGGAGTCTGGGCCGTGTCTCAGTCCCAGTGTGGCCGATCACCCTCTCAGGTCGGCTATG
TATCGTCGCCTTGGTGAGCCGTTACCTCACCAACTAGCTAATACAACGCAGGTCCATCTGGTAGTGATGCAAT
TGCACCTTTTAATTGACTATCATGCAATAATCAATTTTATGCGGTATTAAGCCTATCGTTTCCAATAGTTATC
CCCCGCTACCAGGCAGGTTACCTACGCGTTACTCACCCGTTCGCAACTCATCCAGAGAAGCAAGCTCCTCCTT
CAGCGTTCTGCTTGCATGTATTAGGCACGCCGCCAGCGTTCGTCCTGAGCCAGGATCAAACTCT
>347
CTGCTGCCGCCCGTAGGAGTCTGGGCCGTGTCTCAGTCCCAGTGTGGCCGATCACCCTCTCAGGTCGGCTATG
TATCGTCGCCTTGGTGAGCCGTTACCTCACCAACTAGCTAATACAACGCAGGTCCATCTGGTAGTGATGCAAT
TGCACCTTTTAATTGACTATCATGCAATAATCAATTTTATGCGGTATTTAGCCTATCGTTTTCCAATAGTTTA
CTCCCCCGCTACCAGGCAGGTTTACCTACGCGTTACTCACCCGTTTCGCAACTCATCCAGAGAAGCAAGCTCC
TCCTTCAGCGTTCTACTTGCATGTATTTAGGCACGCCGCCAGCGTTCGTCCTGAGCCAGGATCAAACTCT
>348
CTGCTGCCGCCCGTAGGAGTCTGGGCCGTGTCTCAGTCCCAGTGTGGGGGATCTCCCTCTCAGGACCCCTAAA
GATCATCGCCTTGGTAAGCCGTTACCTTACCAACTAGCTAATCTTTGTGCGTGCCCATCCTTTATCCACCGTA
GCTTTTCAATATCAAATGATGCCACCTGATATATTTATTAGGGTTATTTAATCATTCCTTTTCGAATGGCTAT
CCCCTTTGATTAAGGGTTAGGTTTGCACACATGTTTTACGCACCCGTACGCCGCTCTCTTCTAGTTAGCAAGC
TACCAAATACCGCTCGACTTGCATGTGTTTTAGGCCTTCCGCTAGCGTTCACCTTGAGCCAGGATCAAACTCT
>349
CTGCTGCCGCCCGTAGGAGTCTGGGCCGTGTCTCAGTCCCAGTGTGGCCGATCACCCTCTCAGGTCGGCTATG
TATCGTCGCCTTGGTGAGCCGTTACCCCACCAACTAGCTAATACAACGCAGGTCCATCTGGTAGTGATGCAAT
TGCACCTTTTAAGTAAATGTCATGCAACATCTACTCTTTATGCGGTATTTAGCTATTCGTTTTCCAATAGTTA

TCCCCCCGCTACCAGGCAGGTTACCTTACGCGTTACTCACCCGTTCGCAACTCATCCAGAAGAGCAAGCTCCT
CCTTCAGCGTTCTACTTGCATGTATTTAGGCACGCCGCCAGCGTCGTCCTGAGCCAGGATCAAACTCT
>350
CTGCTGCCGCCCGTAGGAGTCTGGGCCGTGTCTCAGTCCCAGTGTGGCCGATCACCCTCTCAGGTCGGCTATG
TATCGTCGCCTTGGTGAGCCGTTACCCCACCAACTAGCTAATACAACGCAGGTCCATCTGGTAGTGGTGCAAT
TGCACCTTTTAAGCAAATGTCATGCAACATCTACTCTTTATGCGGTATTTAGCTATCGTTTTCCAATAGTTT
ATCCCCCGCTACCAGGCAGGTTACCTACGCGTACTCACCCGTTCGCAACTCATCCGGAGAAGCAAGCTCCTCC
TTCAGCGTCTACTTGCATGTATTTAGGCACGCCGCCAGCGTTCGTCCTGAGCCAGGATCAAACTCT
>351
CTGCTGCCGCCCGTAGGAGTCTGGGCCGTGTCTCAGTCCCAGTGTGGCCGATCACCCTCTCAGGTCGGCTATG
TATCGTCGCCTTGGTGAGCCGTTACCCCACCAACTAGCTAATACAACGCAGGTCCATCTGGTAGTGGTGCAAT
TGCACCTTTTAAGCAAATGTCATGCAACATCTACTCTTATGCGGTATTAGCTATCGTTTTCCAATAGTTTATC
CCCCGCTACCAGGCAGGTTACCTACGCGTACTCACCCGTTCGCAACTCATCCGGAGAAGCAAGCTCCTCCTCA
GCGTTCTACTTGCATGTATTTAGGCACGCCGCCAGCGTTCGTCCTGAGCCAGGATCAAACTCT
>352
CTGCTGCCGCCCGTAGGAGTCTGGGCCGTGTCTCAGTCCCAGTGTGGCCGATCACCCTCTCAGGTCGGCTATG
CATCGTCGCCTTGGTAGGCCTTTACCCCACCAACTAGCTAATGCACCGCAAAGCCATCTCATAGTGTTAGCAA
AACCAACTTTTAAACATCAACCATGCAGTTAATGCTGTTATCCGGTATTTAGCTATCGTTTCCAATAGTTTG
TCCCAGTCTATGAGGCAGGTTTCTTTACGTGTTACTCACCCGTTCGCCGCTAAGTTGTCGTCGCAGCGAACCA
GCAAACTAAC
>353
CTGCTGCCGCCCGTAGGAGTCTGGGCCGTGTCTCAGTCCCAGTGTGGCCGATCACCCTCTCAGGTCGGCTATG
TATCGTCGCCTTGGTGAGCCCTTACCCCACCAACTAGCTAATACAACGCAGGTCCATCTGGTAGTGATGCAAT
TGCACCTTTCAAACAGTTGTCATGCAACAACTGCTATTATGCGGTATTAGCTATCGTTTCCAATAGTTATCCC
CCGCTACCAGGCAGGTTACCTACGCGTACTCACCCGTTCGCAACTCATCCAGAGAAGCAAGCTCCTCCTCAGC
GTTCTACTGCATGCATTAGGCACGCCGCCAGCGTCGTCCTGAGCCAGGATCAAACTCT
>354
CTGCTGCCGCCCGTAGGAGTCTGGGCCGTGTCTCAGTCCCAGTGTGGCCGATCACCCTCTCAGGTCGGCTATG
TATCGTCGCCTTGGTGAGCCGTTACCCCACCAACTAGCTAATACAACGCAGGTCCATCTGGTAGTGGTGCAAT
TGCACCTTTTAAGCAAATGTCATGCAACATCTACTCTTATGCGGTATTAGCTATCGCTTCCAATAGTTATCCC
CCGCTACCAGGCAGGTTACCTACGCGTTACTCACCCGTTCGCAACTCACCGGAGAACGCAAGCTCCCTCCTTC
AGCGTTCTACTTGCAGTATTACGGCAGCCGC
>355
CTGCTGCCGCCCGTAGGAGTCTGGGCCGTGTCTCAGTCCCAGTGTGGCCGATCACCCTCTCAGGTCGGCTATG
TATCGTCGCCTTGGTGAGCCCTTACCCCACCAACTAGCTAATACAACGCAGGTCCATCTGGTAGTGATGCAAT
TGCACCTTTCAAACAGTTGTCATGCAACAACTGCTATTATGCGGTATTAGCTATCGTTTCCAATAGTTACCTC
CCCGCTACCAGGCAGGTTACCTACGCGTTACTCACCCGTTCGCAACTCATCCGGAGAAGCAAGCTCCTCCTTC
AGCGTTCTACTTGCATGTATTAGGCACGCCGCCAGCGTTCGTCCTGAGCCAGGATCAAACTCT
>356
CTGCTGCCGCCCGTAGGAGTCTGGGCCGTGTCTCAGTCCCAGTGTGGCCGATCACCCTCTCAGGTCGGCTATG
TATCGTCGCCTTGGTGAGCCGTTACCCCACCAACTAGCTAATACAACGCAGGTCCATCTGGTAGTGGTGCAAT
TGCACCTTTTAAGCAAATGTCATGCAACATCTACTCTTATGCGGTATTAGCTATCGTTTTCCAATAGTTTATC
CCCCCCGCTACCAGGCAGGTTACCTACGCGTTACTCACCCGTTCGCAACTCATCCGGAGAAGCAAGCTCCTCC
TCAGCGTTCTACTGCATGTATTTAGGCACGCCGCCAGCGTCGTCCTGAGCCAGGATCAAACTCT
>357
CTGCTGCCGCCCGTAGGAGTCTGGGCCGTGTCTCAGTCCCAGTGTGGCCGATCACCCTCTCAGGTCGGCTATG
CATCGTCGCCTTGGTAGGCCTTTACCCCACCAACTAGCTAATGCACCGCAAAGCCATCTCATAGTGTTAGCAA
AACCAACTTTTAAACATCAACCATGCAGTTAATGCTGTTTATCCGGTATTTAGCTATCGTTTTCCAATAGTTT
TGTCCCAGTCTATGAGGCAGGTTTCTTTACGTGTTTACTCACCCGTTTCGCCGCTAAGTTTTCGTTCGGCACA
CGAAAACTCCAAAAGCTAC
>358
CTGCTGCCGCCCGTAGGGGTCTGGGCCGTGTCTCAGTCCCAGTGTGGCCGGTCGCCCTCTCAGGCCGGCTACC
CGTCATCGCCTTGGTCGAGCCATTACCTCACCAACAAACTGATAGGCCGCGAGTCCATCCCCCACCGAAAAAC
TTCCACAACACACCATGCGGCGCGAAGCCATATCCGGTATTAGCCCCGATTTTCCCGAGGTTTATCCCGAAGA

GGGGGGCAGGTTACTCACGTGTTACGTCACCCGTTCGCCACTAATCAGACAAGGCAAGCCCTGTCGTCATCGT
TCGACTGCATGTGTTTAAGCACGCCGCCAGCGTCGTCCTGAGCCAGGATCAAACTCT
>359
CTGCTGCCGCCCGTAGGAGTCTGGGCCGTGTCTCAGTCCCAGTGAGGCCGATCACCCTCTCAGGTCGGCTATG
TATCGTCGCCTTGGTGAGCCGTTACCTCACCAACTAGCTAATACAACGCAGGTCCATCTGGTAGTGATGCAAT
TGCACCTTTTAATTGACTATCATGCAATAATCAATTTTATGCGGTATTAGCCTATCGTTTTCCAATAGTTTAC
TCCCCCGCTACCAGGCAGGTTACCTACGCGTTACTCACCCGTTCGCAACTCATCCAGAGAAGCAAGCTCCTCC
TCAGCGTTCTACTTGCATGTATTTAGGCACGCCGCCAGCGTTCGTCCTGAGCCAGGATCAAACTCT
>360
CTGCTGCCGCCCGTAGGAGTCTGGGCCGTGTCTCAGTCCCAGTGTGGCCGATCACCCTCTCAGGTCGGCTATG
TATCGTCGCCTTGGTGAGCCCTTACCCCACCAACTAGCTAATACAACGCAGGTCCATCTGGTAGTGATGCAAT
TGCACCTTTCAAACAGTTGTCATGCAACAACTGCTATTATGCGGTATTTAGCTATCGTTTTCCAATAGTTATC
CCCCCGCTACCAGGCAGGTTACCTACGCGTTACTCACCCGTTCGCAACTCATCCAGAGAGGCAAGCTCCTCCT
TCAGCGTTCTACTTGCATGTATTTAGGCACGCCGCCAGCGTTCGTCCTGAGCCAGGATCAAACTCT
>361
CTGCTGCCGCCCGTAGGAGTCTGGGCCGTGTCTCAGTCCCAGTGTGGCCGATCACCCTCTCAGGTCGGCTATG
TATCGTCGCCTTGGTGAGCCCTTACCCCACCAACTAGCTAATACAACGCAGGTCCATCTGGTAGTGATGCAAT
TGCACCTTTCAAACAGTTGTCATGCAACAACTGCTATTATGCGGTATTAGCTATCGTTTTCCAATAGTTTATC
CCCCGCTACCAGGCAGGTTACCTACGCGTTACTCACCCGTTCGCAACTCATCCAGAGAAGCAAGCTCCTCCTC
AGCGTTCTACTTGCATGTATTAGGCACGCCGCCAGCGTTCGTCCTGAGCCAGGATCAAACTCT
>362
CTGCTGCCGCCCGTAGGAGTCTGGGCCGTGTCTCAGTCCCAGTGTGGCCGATCACCCTCTCAGGTCGGCTGTG
TATCGTCGCCTTGGTGAGCCCTTACCCCACCAACTAGCTAATACAACGCAGGTCCATCTGGTAGTGATGCAAT
TGCACCTTTTAATTGACTATCATGCAATAATCAATTTTATGCGGTATTAGCTATCGTTTTCCAATAGTTTATC
CCCCGCTACCAGGCAGGTTACCTACGCGTTACTCACCCGTTCGCAACTCATCCAGAGAAGCAAGCTCCTCCTT
CAGCGTTCTACTTGCATGTATTAGGCACGCCGCCAGCGTTCGTCCTGAGCCAGGATCAAACTCT
>363
CTGCTGCCGCCCGTAGGAGTCTGGGCCGTGTCTCAGTCCCAGTGTGGCCGATCACCCTCTCAGGTCGGCTATG
TATCGTCGCCTTGGTGAGCCGTTACCCCACCAACTAGCTAATACAACGCAGGTCCATCTGGTAGTGGTGCAAT
TGCACCTTTTAAGCAAATGTCATGCAACATCTACTCTTATGCGGTATTAGCTATCGTTTCCAATAGTTATCCC
CCGCTACCAGGCAGGTTACCTACGCGTTACTCACCCGTTCGCAACTCATCCAGAAGAGCAAGCTCCTCCTTCA
GCGTTCTACTTGCCATGTATTAGGCACGCCGCCAGCGTCGTCCTGAGCCAGGATCAAACTCT
>364
CTGCTGCCGCCCGTAGGAGTCTGGGCCGTGTCTCAGTCCCAGTGTGGCCGATCACCCTCTCAGGTCGGCTATG
TATCGTCGCCTTGGTGAGCCCTTACCCCACCAACTAGCTAATACAACGCAGGTCCATCTGGTAGTGATGCAAT
TGCACCTTTCAAACAGTTGTCATGCAACAACTGCTATTATGCGGTATTAGCTATCGTTTTCCAATAGTTATCC
CCCGCTACCAGGCAGGTTACCTACGCGTTACTCACCCGTTCGCAACTCATCCAGAGAAGCAAGCTCCTCCTTC
AGCGTTCTACTTGCCATGTATTTAGGCACGCCGCCAGCGTTCGTCCTGAGCCAGGATCAAACTCT
>365
CTGCTGCCGCCCGTAGGAGTCTGGACCGTGTCTCAGTTCCAGTGTGGCCGATCACCCTCTCAGGTCGGCTACG
TATCGTTGCCTTGGTAAGCCGTTACCTTACCAACTAGCTAATACGGCGCAGGTCCATCTATAAGTGATAGCAA
AGCCATCTTTCACTATCGAACCATGCGGTTTCGAAATATTTATCCGGTATTTAGCTCCGGTTTTTCCCCCGA
AGTTATCCCCCAGTCTTTATTAGGTAGGTTTTACCCCTACGTGTTTACTCACCCGTCCGCCGCTAACGTCAAA
GGAGCAAGCTCCTCGTCTGTTTCGCTCGACTTGCATGTATTAGGCACGCCGCCAGCGTCATCCTGAGCCAGGA
TCAAACTCT
>366
CTGCTGCCGCCCGTAGGAGTCTGGGCCGTGTCTCAGTCCCAGTGTGGCCGATCACCCTCTCAGGTCGGCTATG
TATCGTCGCCTAGGTGAGCCGTTACCTCACCTACTAGCTAATACAACGCAGGTCCATCTTGTAGTGGAGCAAT
TGCCCCTTTCAAATAAATGACATGTGTCATCCATTGTTATGCGGTATTAGCTATCCGTTTCCAATAGTTATCC
CCCGCTACAAGGCAGGTTACCTACGCGTTACTCACCCGTTCGCAACTCATCCAAGAAGAGCAAGCTCCTCTCT
TCAGCGTTCTACCTTGCATGTATTAGGCACGCCGCCAGCGTTCGTCCTGAGCCAGGATCAAACTCT
>367
CTGCTGCCGCCCGTAGGAGTCTGGGCCGTGTCTCAGTCCCAGTGTGGCCGATCACCCTCTCAGGTCGGCTATG
TATCGTCGCCTTGGTGAGCCGTTACCTCACCAACTAGCTAATACAACGCAGGTCCATCTGGTAGTGATGCAAT

TTGCACCTTTTAATTGACTATCATGCAATAACCAATTTTATGCGGTATTAGCTATCCGTTTCCAATAGTTATC
CCCCGCTACCAGGCAGGTTACCTACGCGTTACTCACCCGTTCGCAACTCATCCAGAGAAGCAAGCTCCTCCTT
CAGCGTTCTACTTGCATGTATTAGGCACGCCGCCAGCGTTCGTCCTGAGCCAGGATCAAACTCT
>368
CTGCTGCCGCCCGTAGGAGTCTGGGCCGTGTCTCAGTCCCAGTGTGGCCGATCACCCTCTCAGGTCGGCTATG
CATCGTCGCCTTGGTAGGCCTTTACCCCACCAACTAGCTAATGCACCGCAAAGCCATCTCATAGTGTTAGCAA
AACCAACTTTTAAACATCAACCATGCAGTTAATGCTGTTTATCCGGTATTTAGCTATTCGTTTTCCAATAGTT
TGTCCCAGTCTATGAGGCAGGTTTCTTTACGTGTTTACTCACCCGTTTCGCCGCTAAGTTTTCGTTCGCGACA
CAAACATCCAAACTACGCTTCGCATGGCATTGAGTAGGCTCAGCCGTCCG
>369
CTGCTGCCGCCCGTAGGAGTCTGGGCCGTGTCTCAGTCCCAGTGTGGCCGATCACCCTCTCAGGTCGGCTATG
TATCGTCGCCTTGGTGAGCCCTTACCCCACCAACTAGCTAATACAACGCAGGTCCATCTGGTAGTGATGCAAT
TGCACCTTTCAAACAGTTGTCATGCAACAACTGTTATTATGCGGTATTAGCTATCGTTTCCAATAGTTATCC
CCCGCTACCAGGCAGGTTACCTACGCGTTACTCACCCGTTCGCAACTCATCCAGAGAAGCAAGCTCCTCCTTC
AGCGTTCTACTTGCATGTATTAGGCACGCCGCCAGCGTCGTCCTGAGCCAGGATCAAACTCT
>370
CTGCTGCCGCCCGTAGGAGTCTGGGCCGTGTCTCAGTCCCAGTGTGGCCGATCACCCTCTCAGGTCGGCTATG
TATCGTCGCCTTGGTGAGCCGTTACCCCACCAACTAGCTAATACAACGCAGGTCCATCTGGTAGTGATGCAAT
TGCACCTTTTAAGTAAATGTCATGCAACATCTACTCTTATGCGGTATTAGCTATCGTTTCCAATAGTTATCCC
CCGCTACCAGGCAGGTTACCTACGCGTTACTCACCCGTTCGCAACTCATCCAGAAGAGCAAGCTCCTCCTCAG
CGTTCTACTGCATGTATTAGGCACGCCGCCAGCGTCGTCCTGAGCCAGGATCAAACTCT
>371
CTGCTGCCGCCCGTAGGAGTCTGGGCCGTGTCTCAGTCCCAGTGTGGCCGATCACCCTCTCAGGTCGGCTATG
CATCGTCGCCTTGGTAGGCCTTTACCCCACCAACTAGCTAATGCACCGCAAAGCCATCTCATAGTGTTAGCAA
AACCAACTTTTAAACATCAACCATGCAGTTAATGCTGTTATCCGGTATTAGCTATCGTTTTCCAATAGTTTGT
CCCAGTCTATGAGGCAGGTTTCTTTACGTGTTACTCACCCGTTCGCCGCTAAGTTTCGTCGTGACACGAAACA
TCCAAAACT
>372
CTGCTGCCGCCCGTAGGAGTCTGGGCCGTGTCTCAGTCCCAGTGTGGCCGATCACCCTCTCAGGTCGGCTATG
TATCGTCGCCTTGGTGAGCCGTTACCTCACCAACTAGCTAATACAACGCAGGTCCATCTGGTAGTGATGCAAT
TGCACCTTTTAATTGACTATCATGCAATAATCAATTTTATGCGGTATTTAGCCTATCGTTTTCCAATAGTTTA
CTCCCCCCGCTACCAGGCAGGTTACCTACGCGTTACTCACCCGTTCGCAACTCATCCAGAGAAGCAAGCTCCT
CCTTCAGCGTTCTACTTGCATGTATTTAGGCACGCCGCCAGCGTTCGTCCTGAGCCAGGATCAAACTCT
>373
CTGCTGCCGCCCGTAGGAGTCTGGGCCGTGTCTCAGTCCCAGTGTGGCCGATCACCCTCTCAGGTCGGCTATG
TATCGTCGCCTTGGTGAGCCCTTACCCCACCAACTAGCTAATACAACGCAGGTCCATCTGGTAGTGATGCAAT
TGCACCTTTCAAACAGTTGTCATGCAACAACTGCTATTTATGCGGTATTTAGCTATCGTTTTCCAATAGTTTA
TCCCCCCGCTACCAGGCAGGGTACCTACGCGTTACTCACCCGTTCGCAACTCATCCAGAGAAGCAAGCTCCTC
CTTCAGCGTTCTACTTGCATTGTATTAGGCACGCCGCCAGCGTTCGTCCTGAGCCAGGATCAAACTCA
>374
CTGCTGCCGCCCGTAGGAGTCTGGGCCGTGTCTCAGTCCCAGTGTGGCCGATCACCCTCTCAGGTCGGCTACG
TATCGTTGCCTTGGTAAGCCGTTACCTTACCAACTAGCTAATACGGCGCAGGTCCATCTATAAGTGATAGCAA
AGCCATCTTTCACTATCGAACCATGCGGTTCGAAATATTATCCGGTATTAGTCTCCGGTTTCCCGAAGTTATC
CCAGTCTTATAGGTAGGTTACCTACGTGTTACTCACCCGTCCGCCGCTAACGTCAAAGGAGCCAAGCTCCTCG
TCTGTTCGCTCGACTTGCATGTATTAGGCACGCCGCCAGCGTTCATCCTGAGCCAGGATCAAACTCT
>375
CTGCTGCCGCCCGTAGGAGTCTGGGCCGTGTCTCAGTCCCAGTGTGGCCGATCACCCTCTCAGGTCGGCTATG
TATCGTCGCCTTGGTGAGCCGTTACCTCACCAACTAGCTAATACAACGCAGGTCCATCTGGTAGTGATGCAAT
TTGCACCTTTAATTGACTATCATGCAATAATCAATTTTATGCCGGTATTAGCCTATCGTTTTCCAATAGTTAC
TCCCCCGCTACCAGGCAGGTTACCTACGCGTTACTCACCCGTTCGCAACTCACCCAGAGAAGCAAGCTCCTCC
TCAGCGTTCTACTTGCATGTATTAGGCACGCCGCCAGCGTTCGTCCTGAGCCAGGATCAAACTCT
>376
CTGCTGCCGCCCGTAGGAGTCTGGGCCGTGTCTCAGTCCCAGTGTGGCCGATCACCCTCTCAGGTCGGCTATG
TATCGTCGCCTTGGTGAGCCGTTACCCCACCAACTAGCTAATACAACGCAGGTCCATCTGGTAGTGGTGCAAT

TGCACCTTTTAAGCAAATGTCATGCAACATCTACTCTTATGCGGTATTAGCTATCGTTTCCAATAGTTATCCC
CCGCTACCAGGCAGGTTACCTACGCGTACTCACCCGTTCGCAACTCATCCGGAGAAGCAAGCTCCTCCCTTCA
GCGTTCTACTTGCATGTATTAGGCACGCCGCCAGCGTTCGTCCTGAGCCAGGATCAAACTCT
>377
CTGCTCGCCGCCCGTAGGAGGTCTGGGCCGTGTCTCAGTCCCAGTGTGGCCGATCACCCTCTCAGGTCGGCTA
TGTATCGTCGCCTTCGGTGAGCCCTTACCCCACCAACTAGCTAATACAACCGCAGGTCCATCTGGTAGTGATG
CAATTTGCACCTTTCAAACAGTTGTCATGCAACAACTGCTATTATGCGGTATTAGCTATCGTTCCAATAGTTA
CTCCCCGCTACCAGGCAGGTTACCTACGCGTTACTCACCCGTTCGCAACTCATCCAGAGAAGCAAGCTCCTCC
TCAGCGTTCTACTTGCATGTATTAGGCACGCCGCCAGCGTCGTCCTAAGCCAGGATCAACTCT
>378
CTGCTGCCGCCCGTAGGAGTCTGGGCCGTGTCTCAGTCCCAGTGTGGCCGATCACCCTCTCAGGTCGGCTATG
TATCGTCGCCTTGGTGAGCCGTTACCTCACCAACTAGCTAATACAACGCAGGTCCATCTGGTAGTGATGCAAT
TGCACCTTTCAAACAGTTGTCATGCAACAACTGCTATTATGCGGTATTAGCTATCGTTTCCAATAGTTATCCC
CCGCTACCAGGCAGGTTACCTACGCGTTACTCACCCGTTCGCAACTCATCCAGAGAAGCAAGCTCCTCCTTTC
AGCGTTCTACTTGACATGTATTTAGGCACGCCGCCAGCGTTCGTCCTGAGCCAGGATCAAACTCT
>379
CTGCTGCCGCCCGTAGGAGTTTTGGGCCGTGTCTCAGTCCCAATGTGGCCGATCACCCTCTCAGGTCGCTATG
CATCACGGCCTTGGTGAGCCGTTACCTCACCAACTAGCTAATGCACCGCGGGTCCATCCATCAGCAGAAGCCG
AAGCCTCTTTTCCTCTTCAAACCATGCGGTTCGAAGACCTATGCCGGTTTTAGCATCCGTTTCCGAATGTTAC
TCCCCTCTGATGGGCAGGTTACCCCACGTGTTACTCGACCCGTTCGCCACTAGACTTGACCAGTGCAAGCACC
GGTCGCTCTCGTTCGACTGCATGC
>380
CTGCTGCCGCCCGTAGGAGTCTGGGCCGTGTCTCAGTCCCAGTGTGGCCGATCACCCTCTCAGGTCGGCTATG
TATCGTCGCCTTGGTGAGCCCTTACCCCACCAACTAGCTAATACAACGCAGGTCCATCTGGTAGTGATGCAAT
TGCACCTTTCAACAGTTGTCATGCAACAACTGCTATTATGCGGTATTTAGCTATCGTTTTCCAATAGTTATCC
CCCGCTACCAGGCAGGTTACCTACGCGTTACTCACCCGTTCGCAACTCATCCAGAGAAGCAAGCTCCTCCTTC
AGCGTTCTACTTGCATGTATTAGGCACGCCGCCAGCGTTCGTCCTGACGCCAGGCAT
>381
CTGCTGCCGCCCGTAGGAGTCTGGACCGTGTCTCAGTTCCAGTGTGGCCGATCACCCTCTCAGGTCGGCTATG
TATCGTCGCCTTGGTGAGCCGTTACCCCACCAACTAGCTAATACAACGCAGGTCCATCTGGTAGTGATGCAAT
TGCACCTTTCAAACAGTTGTCATGCAACAACTGCTATTATGCGGTATTTAGCTATCGTTTTCCAATAGTTTAT
CCCCCGCTACCAGGCAGGTTACCTACGCGTTACTCACCCGTTTCGCAACTCATCCAGAGAAGCAAGCTCCTCC
TTCAGCGTTCTACTTGCCATTGTATTTAGGCACGCCGCCAGCGTTCGTCCTGAGCCAGGATCAAACTCT
>382
CTGCTGCCGCCCGTAGGAGTCTGGTCCGTGTCTCAGTACCAGTGTGGGGGATAAACCTCTCAGTTCCCTACCC
ATCGTCGCCTTGGTGAGCCGTTACCTCACCAACAAGCTAATGGGACGCATGCCTATCCTATACCGAATTTCTT
TCCTCACCACTTGATGCCAAGCTGTGAGCATACGCAGTATTAGTCCGTCTTCGACGGGTTATCCTCCTGTATA
GGGCAAGTTGCATACGCGTTACGCACCCGTGCGCCGGTCGCCACCCAGATAGCAAGCTACCTTGTGCTGCCCC
TCGACTTGCATGTGTTAAGCCTATCGCTAGCGTTCATCCTGAGCCAGGATCAAACTCT
>383
CTGCTGCCGCCCGTAGGAGTCTGGGCCGTGTCTCAGTCCCAGTGTGGCCGATCACCCTCTCAGGTCGGCTATG
TATCGTCGCCTTGGTGAGCCCTTACCCCACCAACTAGCTAATACAACGCAGGTCCATCTGGTAGTGATGCAAT
TGCACCTTTCAAACAGTTGTCATGCAACAACTGCTATTATGCGGTATTAGCTATCGTTTCCAATAGTTATCCC
CCGCTACCAGGCAGGTTACCTACGCGTTACTCACCCGTTCGCAACTCATCCAGAGAAGCAAGCTCCTCCTTCA
GCGTTCTACTTGCATGTATTAGGCACGCCGCCAGCGTTCGTCCTGAGCCAGGATCAAACTCT
>384
CTGCTGCCGCCCGTAGGAGTCTGGGCCGTGTCTCAGTCCCAGTGTGGCTGATCATCCTCTCAGACCAGCTATG
GATCGTCGCCTTGGTAGGCCTTTACCCCACCAACTAGCTAATCCAACGCGGGCTCATCCATCTCCGATAAATC
TTTCTCCCGAAGGACGTATACGGTATTAGCACACGTTTCCATGCGTTATTCCGTAGAGATGGGTAGATTCCCA
CGCGTTACTCACCCGTCTGCCGCTCCCCTTGCGGGGCGCTCGACTTGCATGTGTTAAGCCTGCCGCCAGCGTT
CGTTCTGAGCCAGGATCAAACTCT
>385
CTGCTGCCGCCCGTAGGAGTCTGGGCCGTGTCTCAGTCCCAGTGTGGCCGATCACCCTCTCAGGTCGGCTATG
CATCGTCGCCTTGGTAGGCTTTTACCACCAACTAGCTAATGCACCGCAAAGCCATCTCATAGTGTTAGCAAAA

CCAACTTTCAAACATCAACCATGCAGTTAATGCTAATTTATCCGGTATTTAGCTATTCGTTTTCCAATAGTTT
GTCCCCCAGTCTTATTGAGGCAGGTTTCTTTACGTGTTTACTCACCCCCGTTTCGCCGCTAAGTTTCGTTCGC
GACACAAAACTCCAAA
>386
CTGCTGCCGCCCGTAGGAGTCTGGGCCGTGTCTCAGTCCCAGTGTGGCCGATCACCCTCTCAGGTCGGCTATG
TATCGTCGCCTTGGTGAGCCGTTACCCCACCAACTAGCTAATACAACGCAGGTCCATCTGGTAGTGGTGCAAT
TGCACCTTTTAAGCAAATGTCATGCAACATCTACTCTTATGCGGTATTAGCTATCGTTTCCAATAGTTAATCC
CCCGCTACCAGGCAGGTTACCTACGCGTTACTCACCCGTTCGCAACTCATCCAGAGAAGCAAGCTCCTCCTCA
GCGTTCTACTGCATGTATTAGGCACGCCGCCAGCGTCGTCCTGAGCCAGGATCAAACTGCT
>387
CTGCTGCCGCCCGTAGGAGTCTGGGCCGTGTCTCAGTCCCAGTGTGGCCGATCACCCTCTCAGGTCGGCTATG
TATCGTCGCCTTGGTGAGCCCTTACCCCACCAACTAGCTAATACAACGCAGGTCCATCTGGTAGTGATGCAAT
TGCACCTTTCAAACAGTTGTCATGCAACAACTGCTATTATGCGGTATTAGCTATCGTTTCCAATAGTTATCCC
CCGCTACCAGGCAGGTTACCTACGCGTTACTCACCCGTTCGCAACTCATCCAGAGAAGCAAGCTCCTCCTTCA
GCGTTCTACTTGCATGTATTAGGCACGCCGCCAGCGTCGTCCTGAGCCAGGATCAAACTCT
>388
CTGCTGCCGCCCGTAGGAGTCTGGGCCGTGTCTCAGTCCCAGTGTGGCCGATCACCCTCTCAGGTCGGCTATG
TATCGTCGCCTTGGTGAGCCCTTACCCCACCAACTAGCTAATACAACGCAGCTCATCTGGTAGTGATGCAATT
GCACCTTTCAAACAGTTGTCATGCAACAACTGCTATTAGCGGTATTAGCTATCGTTTCCAATAGTTATCCCCC
GCTACCAGGCAGGTTACCTACGCGTTACTCACCCGTTCGCAACTCATCCAGAGAAGCAAGCTCCTCCTCAGCG
TTCTACTGCATGTATTAGGCACGCCGCCAGCGTCGTCCTGAGCCAGGATCAACGTCT
>389
CTGCTGCCGCCCGTAGGAGTCTGGGCCGTGTCTCAGTCCCAGTGTGGCCGATCACCCTCTCAGGTCGGCTATG
TATCGTCGCCTTGGTGAGCCGTTACCCCACCAACTAGCTAATACAACGCAGGTCCATCTGGTAGTGATGCAAT
TGCACCTTTCAAACAGTTGTCATGCAACAACTGCTATTATGCGGTATTAGCTATCGTTTCCAATAGTTATCCC
CCGCTACCAGGCAGGTTACCTACGCGTTACTCACCCGTTCGCAACTCATCCAGAGAAGCAAGCTCCTCCTTCA
GCGTTCTACTTGCATGTATTAGGCACGCCGCCAGCGTTCGTCCTGAGCCAGGATCAAACTCT
>390
CTGCTGCCGCCCGTAGGAGTCTGGACCGTGTCTCAGTTCCAGTGTGGCCGATCACCCTCTCAGGTCGGCTACG
TATCGTTGCCTTGGTAAGCCGTTACCTTACCAACTAGCTAATACGGCGCAGGTCCATCTATAAGTGATAGCAA
AGCCATCTTTCACTATCGAACCATGCGGTTCGAAATATTATCCGGTATTAGCTCCGGTTTTTCCCGAAGTTAT
CCCAGTCTTATAGGTAGGTTTTACCTACGTGTTACTCACCCGTCCGCCGCTAACGTCAAAGGAGCAAGCTCCT
CGTCTGTTTCGCTCGACTTGCATGTATTAGGCACGCCGCCAGCGTTCATCCTGAGCCAGGATCAAACTCT
>391
CTGCTGCCGCCCCGTAGGAGTCTGGGCCGTGTCTCAGTCCCAGTGTGGCCGATCACCCTCTCAGGTCGGCTAT
GTATCGTCGCCTTGGTGAGCCCTTACCCCACCAACTAGCTAATACAACGCAGGTCCATCTGGTAGTGATGCAA
TTGCACCTTTCAAACAGTTGTCATGCAACAACTGCTATTATGCGGTATTAGCTATCGTTTTCCAATAGTTATC
CCCCGCTACCAGGCAGGTTACCTACGCGTACTCACCCGTTCGCAACTCATCCGGAGAAGCAAGCTCCTCCTCA
GCGTTCTACTGCATGTATTTAGGCACGCCGCCAGCGTTCGTCCTGAGCCAGGATCAAACTCT
>392
CTGCTGCCGCCCGTAGGAGTCTGGGCCGTGTCTCAGTTCCCAGTGTGGCTGATCATCCTCTCAAACCAGCTAT
GGATCGTCGGTTTGGTAGGCCATTACCCCCACCAACTACCTAATCCAACGCGGGCTAATCTCTTGGCGATAAA
TCTTTCCCCCAAAGGGCACATACGGTATTACTCCCAGTTTTTCCCGAGGCTATTCCGTACCAAGAGGCATATT
TCCCCACGCGTTACTCACCCGTCCGCCGCTAGACCCGAAGATCTCGCTCGACTTGCATGTGTTTAGGCCTGCC
GCCAGCGTTCGTTTCTGAGCCAGGATCAAACTCT
>393
CTGCTGCCGCCCGTAGGAGTCTGGGCCGTGTCTCAGTCCCAGTGTGGCCGATCACCCTCTCAGGTCGGCTATG
TATCGTCGCCTTGGTGAGCCGTTACCCCACCAACTAGCTAATACAACGCAGGTCCATCTGGTAGTGATGCAAC
TGCACCTTTCAAACAGTTGTCATGCAACAACTGCTATTATGCGGTATTTAGCTATCGTTTTCCAATAGTTATC
CCCCGCTACCAGGCAGGTTACCTACGCGTACTCACCCGTTCGCAACTCATCCAGAGAAGCAAGCTCCTCCTCA
GTGTTCTACTGCATGTATTTAGGCACGCCGCCAGCGTCGTCCTGAGCCAGGATCAAACTCT
>394
CTGCTGCCGCCCGTAGGAGTCTGGGCCGTGTCTCAGTCCCAGTGTGGCCGATCACCCTCTCAGGTCGGCTATG
TATCGTCGCCTTGGTGAGCCCTTACCCCCACCAACTAGCTAATACAACGCAGGTCCATCTGGTAGTGATGCAA

TTGCACCTTTCAAACAGTTGTCATGCAACAACTGCTATTTATGCGGTATTTAGCTATCGTTTTTCCAATAGTT
TATCCCCCCGCTACCAGGCAGGTTACCTACGCGTTACTCACCCGTTTCGCAACTCATCCAGAGAAGCAAGCTC
CTCCTTTCAGCGTTCTACTTGCATTGTATTAGGCACGCCGCCAGCGTTGTCCTGAGCCCCAGGATCAAACTCT
>395
CTGCTGCCGCCCGTAGGAGTCTGGGCCGTGTCTCAGTCCCAGTGTCGCCGATCACCCTCTCAGGTCGGCTATG
TATCGTCGCCTTGGTGAGCCCTTACCCCACCAACTAGCTAATACAACGCAGGTCCATCTGGTAGTGATGCAAT
TGCACCTTTTCAAACAGTTGTCATGCAACAACTGCTATTTATGCGGTATTTAGCTATCGTTTTTCCAATAGTT
TATCCCCCCGCTACCAGGCAGGTTACCTACGCGTTACTCACACGTTCGCAACTCATCCAGAGAAGCAAGCTC
CTCCTCAGCGTTCTACTTGCATGTATTTAGGCACGCCGCCAGCGTTCGTCCGAGCCAGGATCAAACTCT
>396
CTGCTGCCGCCCGTAGGAGTCTGGGCCGTGTCTCAGTCCCAGTGTGGCCGATCACCCTCTCAGGTCGGCTATG
TATCGTCGCCTTGGTGAGCCGTTACCTCACCAACTAGCTAATACAACGCAGGTCCATCTGGTAGTGATGCAAT
TGCACCTTTTAATTGACTATCATGCAATAATCAATTTTATGCGGTATTAGCCTATCGTTTCCAATAGCTATCC
CCCGCTACCAGGCAGGTTACCTACGCGTTACTCACCCGTTCGCAACTCATCCAGAGAAGCAAGCTCCTCCTTC
AGCGTTCTACTTGCATGTATTTACGGCAGCC
>397
CTGCTGCCGCCCGTAGGAGTCTGGGCCGTGTCTCAGTTCCAGTGTGGCCGATCACCCTCTCAGGTCGGCTACG
TATCGTTGCCTTGGTGAGCCGTTACCCCACCAACTAGCTAATACAACGCAGGTCCATCTGGTAGTGGTGCAAT
TGCACCTTTTAAGCAAATGTCATGCAACATCTACTCTTATGCGGTATTAGCTATCGTTTTCCAATAGTTATCC
CCCGCTACCAGGCAGGTTACCTACGCGTTACTCACCCGTTCGCAACTCATCCGGAGAAGCAAGCTCCTCCTTC
AGCGTTCTACTTGCATGTATTTAGGCACGCCGCCAGCGTCCGTCCTGAGCCAGGATCAAACTCT
>398
CTGCTGCCGCCCGTAGGAGTCTGGGCCGTGTCTCAGTCCCAGTGTGGCCGATCACCCTCTCAGGTCGGCTATG
TATCGTCGCCTTGGTGAGCCGTTACCCCACCAACTAGCTAATACAACGCAGGTCCATCTGGTAGTGGTGCAAT
TGCACCTTTTAAGCAAATGTCATGCAACATCTACTCTTATGCGGTATTAGCTATCGTTTCCAATAGTTATCCC
CCGCTACCAGGCAGGTTACCTACGCGTTACTCACCCGTTCGCAACTCATCCGGAGAAGCAAGCTCCTCCTTCA
GCGTTCTACTTGCATGTATTAGGCACGCCGCCAGCGTTCGTCCTGAGCCAGGATCAAACTCT
>399
CTGCTGCCGCCCGTAGGAGTCTGGGCCGTGTCTCAGTCCCAGTGTGGCCGATCACCCTCTCAGGTCGGCTATG
TATCGTCGCCTTGGTGAGCCGTTACCTCACCAACTAGCTAATACAACGCAGGTCCATCTGGTAGTGATGCAAT
TTGCACCTTTTAATTGACTATCATGCAATAATCAATTTTATGCGGTATTAGCCTATCGTTTCCAATAGTTACT
CCCCCGCTACCAGGCAGGTTACCTACGCGTTACTCACCCGTTCGCAACTCATCCAGAGAAGCAAGCTCCTCCT
TCAGCGTTCTACTTGCATGTATTAGGCACGCCGCCAGCGTTCGTCCTGAGCCAGGATCAAACTCT
>400
CTGCTGCCGCCCGTAGGAGTCTGGGCCGTGTCTCAGTCCCAGTGTGGCCGATCACCCTCTCAGGTCGGCTATG
TATCGTCGCCTTGGTGAGCCGTTACCCCACCAACTAGCTAATACAACGCAGGTCCATCTGGTAGTGGTGCAAT
TGCACCTTTTAAGCAAATGTCATGCAACATCTACTCTTATGCGGTATTAGCTATCGTTTCCAATAGTTATCCC
CCGCTACCAGGCAGGTTACCTACGCGTTACTCACCCGTTCGCAACTCATCCGGAGAAGCAAGCTCCTCCTTCA
GCGTTCTACTGCATGTATTAGGCACGCCGCCAGCGTTCGTCCTGAGCCAGGATCAAACTCT
>401
CTGCTGCCGCCCGTAGGAGTCTGGGCCGTGTCTCAGTCCCAGTGTGGCCGATCACCCTCTCAGGTCGGCTATG
TATCGTCGCCTTGGTGAGCCCTTACCCCACCAACTAGCTAATACAACGCAGGTCCATCTGGTAGTGATGCAAT
TGCACCTTTCAAACAGTTGTCATGCAACAACTGCTATTATGCGGTATTAGCTATCGTTTCCAATAGTTATCCC
CCGCTACCAGGCAGGTTACCTACGCGTTATTCACCCGTTCGCAACTCATCCGGAGAAGCAAGCTCCTCCTTCA
GCGTTCTACTTGCATGTATTAGGCACGCCGCCAGCGTTCGTCCTGAGCCAGGATCAAACTCT
>402
CTGCTGCCGCCCGTAGGAGTCTGGGCCGTGTCTCAGTCCCAGTGTGGCCGATCACCCTCTCAGGTCGGCTATG
TATCGTCGCCTTGGTGAGCCCTTACCCCACCAACTAGCTAATACAACGCAGGTCCATCTGGTAGTGATGCAAT
TGCACCTTTCAAACAGTTGTCATGCAACAACCGCTATTATGCGGTATTAGCTATCGTTTCCAATAGTTATCCC
CCGCTACCAGGCAGGTTACCTACGCGTTACTCACCCGTTCGCAACTCATCCAGAGAAGCAAGCTCCTCCTTCA
GCGTTCTACTTGCCATGTATTAGGCACGCCGCCAGCGTTCGTCCTGAGCCAGGATCAAACTCT
>403
CTGCTGCCGCCCGTAGGAGTCTGGGCCGTGTCTCAGTCCCAGTGTGGCCGATCACCCTCTCAGGTCGGCTATG
TATCGTTGCCTTGGTGAGCCGTTACCTCACCAACTAGCTAATACAACGCAGGTCCATCTGGTAGTGGTGCAAT

TGCACCTTTCAAGCAGCTATCATGCGATATCTACTGTTTATGCGGTATTTACGCTATCGTTTTCCAATAGTTT
ATCCCCCCCGCTACCAGGCAGGTTACCTACGCGTTACTCACCCGTTTCGCAACTCATCCGGAAAGAGCAAGTT
CCTTTCCTTCAGCGTTCTACTTGCATGTATTTAGGCACGCCGCCAGCGTTCGTCCTGAGCCAGGATCAAACTC
T
>404
CTGCTGCCGCCCGTAGGAGTCTGGACCGTGTCTCAGTTCCAGTGTGGCCGATCACCCTCTCAGGTCGGCTACG
TATCGTTGCCTTGGTAAGCCGTTACCTTACCAACTAGCTAATACGGCGCGGATCCATCTATAAGTGACAGCAA
GGCCGTCTTTCACTATTGAACCATGCGGTTCAAAATCTTATCAGGTATTAGCTCCGGTTTTCCCGAAGTTATC
CCAGTCTTATAGGTAGGTTATCCACGTGTTACTCACCCGTCCGCCGCTAACGTCAAAGGAGCAAGCTCCTCAT
CTGTTCGCTCGACTTGCATGTATTAGGCACGCCGCCAGCGTTCATCCTGAGCCAGGATCAAACTCT
>405
CTGCTGCCGCCCGTAGGAGTCTGGGCCGTGTCTCAGTCCCAGTGTGGCCGATCACCCTCTCAGGTCGGCTATG
TATCGTCGCCTTGGTGAGCCGTTACCTCACCAACTAGCTAATACAACGCAGGTCCATCTGGTAGTGATGCAAT
TGCACCTTTTAATTGACTATCATGCAATAATCAATTTTATGCCGGTATTAGCCTATCGTTTCCAATAGTTACT
CCCCCGCTACCAGGCAGGTTACCTACGCGTACTCACCCGTTCGCAACTCATCCAGAGAAGCAAGCTCCTCCTT
CAGCGTTCTACTTGCATGTATTAGGCACGCCGCCAGCGTTCGTCCTGAGCCAGGATCAACTCT
>406
CTGCTGCCGCCCGTAGGAGTCTGGACCGTGTCTCAGTTCCAGTGTGGCCGATCACCCTCTCAGGTCGGCTACG
TATCGTTGCCTTGGTAAGCCATTACCTTACCAACTAGCTAATACGGCGCGGGTCCATCTATAAGTGATAGCAA
AACCATCTTTCACTTTAGAACCATGCGGTTCTAAATGTTATCCGGCATTAGCCCCGGTTTTCCCGGAGTTATT
CCAGTCTTATAGGTAGGTTTTACCCACGTGTACTCACCCGTCCGCCGCTAACGTCAAAGGAGCAAGCTCCTAT
CTGTTCGCTCGACTTGCATGTATAGGCACGCCGCCAGCGTCGAT
>407
CTGCTGCCGCCCGTAGGAGTCTGGGCCGTGTCTCAGTCCCAGTGTGGCCGATCACCCTCTCAGGTCGGCTATG
TATCGTCGCCTTGGTGAGCCCTTACCCCACCAACTAGCTAATACAACGCAGGTCCATCTGGTAGTGATGCAAT
TGCACCTTTCAAACAGTTGTCATGCAACAACTGCTATTATGCGGTATTTAGCTATCGTTTTCCAATAGTTATC
CCCCGCTACCAGGCAGGTTACCTACCGCGTTACTCACCCGTTCGCAACTCATCCAGAGAAGCAAGCTCCTCCT
TCAGCGTTCTACTTGCCATGTATTTAGGCACGCCGCCAGCGTTCGTCCTGAGCCAGGATCAAACTCT
>408
CTGCTGCCGCCCGTAGGAGTCTGGGCCGTGTCTCAGTCCCAGTGTGGCCGATCACCCTCTCAGGTCGGCTATG
TATCGTCGCCTTGGTGAGCCCTTACCCCACCAACTAGCTAATACAACGCAGGTCCATCTGGTAGTGATGCAAT
TGCACCTTTCAAACCAGTTGTCATGCAACAACTGCTATTATGCGGTACTAGCTATCGTTTTCCAATAGTTATC
CCCCCGCTACCAGGCAGGTTACCTACGCGTTACTCACCCGTTCGCAACTCATCCAGAGAAGCAAGCTCCTCCT
CAGCGTTCTACTGCATGTATTAGGCACGCCGCCAGCGTCGTCCTGAGCCAGGATCAAACTCT
>409
CTGCTGCCGCCCGTAGGAGTCTGGGCCGTGTCTCAGTCCCAGTGTGGCCGATCACCCTCTCAGGTCGGCTATG
TATCGTCGCCTTGGTGAGCCCTTACCCCACCAACTAGCTAATACAACGCAGGTCCATCTGGTAGTGATGCAAT
TGCACCTTTCAAACAGTTGTCATGCAACAACTGCTATTATGCGGTATTAGCTATCGTTTTCCAATAGTTATCC
CCCCGCTACCAGGCAGGTTACCTACGCGTTACTCACCCGTTCGCAACTCATCCAGAGAAGCAAGCTCCTCCTC
AGCGTTCTACTTGCATGTATTAGGCACGCCGCCAGCGTTCGTCCTGAGCCAGGATCAAACTCT
>410
CTGCTGCCGCCCGTAGGAGTCTGGGCCGTGTCTCAGTCCCAGTGTGGCCGATCACCCTCTCAGGTCGGCTACG
CATCGTTGCCTTGGTGGGCCGTTACCCCGCCAACCAGCTAATGCGCCGCAGGCCCATCCATCAGCGACAGCCG
AAACCGTCTTTCAACTCCCCACCCCGCGGTGGAGAGGATTATCCGGTATTAGCCACGGTTTTCCCGCAGTTAT
CCCCGGCTGATGGGCAGGTTTGCCTACGTGTTACTCACCCGTCCGCCACTCGATCAGAGGGAGCAAGCTCCCT
CCTCTCGCGTCGACTTGCAGTATTAGGCACGCCGCCAGCGTTCATCCTGAGCCAGGATCAAACTCT
>411
CTGCTGCCGCCCGTAGGAGTCTGGGCCGTGTCTCAGTCCCAGTGTGGCCGATCACCCTCTCAGGTCGGCTATG
TATCGTCGCCTTGGTGAGCCGTTACCCCACCAACTAGCTAATACAACGCAGGTCCATCTGGTAGTGGTGCAAT
TGCACCTTTTAAGCAAATGTCATGCAACATCTACTCTTATGCGGTATTAGCTATCGTTTCCAATAGTTATCCC
CCGCTACCAGGCAGGTTACCTACGCGTTACTCACCCGTTCGCAACTCATCCGGAGAAGCAAGCTCCTCCTTCA
GCGTTCTACTTGCATGTATTAGGCACGCCGCCAACGTTCGTCCTGAGCCAGGATCAAACTCT
>412

CTGCTGCCGCCCGTAGGAGTCTGGGCCGTGTCTCAGTCCCAGTGTGGCCGATCACCCTCTCAGGTCGGCTATG
TATCGTCGCCTTGGTGAGCCGTTACCCCACCAACTAGCTAATACAACGCAGGTCCATCTGGTAGTGGTGCAAT
TGCACCTTTCAAACAGCTGTCATGCAACAACTGCTATTATGCGGTATTTAGCTATCGGTTTCCAATAGTTATC
CCCCGCTACCAGGCAGGTTACCTACGCGTTACTCACCCGTTCGCAACTCATCCAGAGAAGCAAGCTCCTCCTC
AGCGTTCTACTTGCATGTATTAGGCACGCCGCCAGCGTTCGTCCTGAGCCAGGATCAAACTCT
>413
CTGCTGCCGCCCGTAGGAGTCTGGGCCGTGTCTCAGTCCCAGTGTGGCCGATCACCCTCTCAGGTCGGCTATG
TATCGTCGCCTTGGTGAGCCGTTACCCCACCAACTAGCTAATACAACGCAGGTCCATCTGGTAGTGGTGCAAT
TGCACCTTTTAAGCAAATGTCATGCAACATCTACTCTTATGCGGTATTAGCTATCGTTTCCAATAGTTATCCC
CCGCTACCAGGCAGGTTACCCACGCGTACTCACCCGTTCGCAACTCATCCGGAGAAGCAAGCTCCTCCTTCAG
CGTTCTACTTGCATGTATTAGGCACGCCGCCAGCGTTCGTCCTGAGCCAGGATCAAACTCT
>414
CTGCTGCCGCCCGTAGGAGTCTGGGCCGTGTCTCAGTCCCAGTGTGGCCGATCACCCTCTCAGGTCGGCTATG
TATCGTCGCCTTGGTGAGCCCTTACCCCACCAACTAGCTAATACAACGCAGGTCCATCTGGTAGTGATGCAAT
TGCACCTTTCAAACAGTTGTCATGCAACAACTGCTATTATGCGGTATTAGCTATCGTTTCCAATAGTTATCCC
CCGCTACCAGGCAGGTTACCTACCGCGTTACTCACCCGTTCGCAACTCATCCAGAGAAGCAAGCTCCTCCTTC
AGCGTTCTACTTGCATGTATTAGGCACGCCGCCAGCGTTCGTCCTGAGCCAGGATCAACTCT
>415
CTGCTGCCGCCCGTAGGAGTCTGGGCCGTGTCTCAGTCCCAGTGTGGCCGATCACCCTCTCAGGTCGGCTATG
TATCGTCGCCTTGGTGAGCCGTTACCCCACCAACTAGCTAATACAACGCAGGTCCATCTGGTAGTGGTGCAAT
TGCACCTTTTAAGCAAATGTCATGCAACATCTACTCTTATGCGGTATTAGCTATCGTTTCCAATAGTTATCCC
CCGCTACCAGGCAGGTTACCTACGCGTACTCACCCGTTCGCAACTCATCCGGAGAAGCAAGCTCCTCCTCAGC
GTTCTACTTGCATGTATTAGGCACGCCGCCAGCGTTCGTCCTGAGCCAGGATCAAACTCT
>416
CTGCTGCCGCCCGTAGGAGTCTGGGCCGTGTCTCAGTCCCAGTGTGGCCGATCACCCTCTCAGGTCGGCTATG
TATCGTCGCCTTGGTGAGCCCTTACCCCACCAACTAGCTAATACAACGCAGGTCCATCTGGTAGTGATGCAAT
TGCACCTTTCAACAGTTGTCATGCAACAACTGCTATTATGCGGTATTAGCTATCGTTTCCAATAGTTATCCCC
CGCTACCAGGCAGGTTACCTACGCGTACTCACCCGTTCGCAACTCATCCGGAGAAGCAAGCTCCTCCTTCAGC
GTTCTACTTGCCATGTATTAGGCACGCCGCCAGCGTTCGTCCTGAGCCAGGATCAAACTCT
>417
CTGCTGCCGCCCGTAGGAGTCTGGGCCGTGTCTCAGTCCCAGTGTGGCCGATCACCCTCTCAGGTCGGCTATG
TATCGTCGCCTTGGTGAGCCCTTACCCCACCAACTAGCTAATACAACGCAGGTCCATCTGGTAGTGATGCAAT
TGCACCTTTCAAACAGTTGTCATGCAACAACTGCTATTATGCGGTATTAGCTATCGTTTCCAATAGTTATCCC
CCGCTACCAGGCAGGTTACCTACGCGTTACTCACCCGTTCGCAACTCATCCGGAGAAGCAAGCTCCTCCTTCA
GCGTTCTACTTGCATGTATTAGGCACGCCGCCAGCGTTCGTCCTGAGCCAGGATCAAACTCT
>418
CTGCTGCCGCCCGTAGGAGTCTGGGCCGTGTCTCAGTCCCAGTGTGGCCGATCACCCTCTCAGGTCGGCTATG
TATCGTCGCCTTGGTGAGCCCTTACCCCACCAACTAGCTAATACAACGCAGGTCCATCTGGTAGTGATGCAAT
TGCACCTTTCAAACAGTTGTCATGCAACAACTGCTATTATGCGGTATTAGCTATCGTTTCCAATAGTTATCCC
CCGCTACCAGGCAGGTTACCTACGCGTTACTCACCCGTTCGCAACTCATCCAGAGAAGCAAGCTCCTCCTTCA
GCGTTCTACTTGCATGTATTAGGCACGCCGCCAGCGTTCGTCCTGAGCCAGGATCAAACTCT
>419
CTGCTGCCGCCCGTAGGAGTCTGGGCCGTGTCTCAGTCCCAGTGTGGCCGATCACCCTCTCAGGTCGGCTATG
TATCGTCGCCTTGGTGAGCCCTTACCCCACCAACTAGCTAATACAACGCAGGTCCATCTGGTAGTGATGCAAT
TGCACCTTTCAAACAGTTGTCATGCAACAACTGCTATTATGCGGTATTAGCTATCGTTTTCCAATAGTTATCC
CCCGCTACCAGGCAGGTTACCTACGCGTTACTCACCCGTTCGCAACTCATCCGGAGAAGCAAGCTCCTCCTCA
GCGTTCTACTTGCATGTATTAGGCACGCCGCCAGCGTTCGTCCTGAGCCAGGATCAACTCT
>420
CTGCTGCCGCCCGTAGGAGTCTGGGCCGTGTCTCAGTCCCAGTGTGGCCGATCACCCTCTCAGGTCGGCTATG
TATCGTCGCCTTGGTGAGCCGTTACCCCACCAACTAGCTAATACAACGCAGGTCCATCTGGTAGTGGTGCAAT
TGCACCTTTTAAGCAAATGTCATGCAACATCTACTCTTATGCGGTATTAGCTATCGTTTCCAATAGTTATCCC
CCCGCTACCAGGCAGGTTACCTACGCGTTACTCACCCGTTCGCAACTCATCCGGAGAAGCAAGCTCCTCCTTC
AGCGTTCTACTTGCATGTATTAGGCACGCCGCCAGCGTTCGTCCTGAGCCAGGATCAAACTCT
>421

CTGCTGCCGCCCGTAGGAGTCTGGGCCGTGTCTCAGTCCCAGTGTGGCCGATCACCCTCTCAGGTCGGCTATG
TATCGTCGCCTTGGTGAGCCGTTACCCCACCAACTAGCTAATACAACGCAGGTCCATCTGGTAGTGGTGCAAT
TGCACCTTTTAAGCAAATGTCATGCAACATCTACTCTTATGCGGTATTAGCTATCGTTTCCAATAGTTATCCC
CCGCTACCAGGCAGGTTACCTACGCGTTACTCACCCGTTCGCAACTCATCCAGAGAAGCAAGCTCCTCCTTCA
GCGTTCTACTTGCATGTATTTAGGCACGCCGCCAGCGTTCGTCCTGAGCCAGGATCAAACTCT
>422
CTGCTGCCGCCCGTAGGAGTCTGGGCCGTGTCTCAGTCCCAGTGTGGCCGATCACCCTCTCAGGTCGGCTATG
TATCGTCGCCTTGGTGAGCCCTTACCCCACCAACTAGCTAATACAACGCAGGTCCATCTGGTAGTGATGCAAT
TGCACCTTTCAAACAGTTGTCATGCAACAACTGCTATTTATGCGGTATTTAGCTATCGTTTTCCAATAGTTTA
TCCCCCGCTACCAGGCAGGTTTACCTACGCGTTACTCACCCGTTCGCAACTCATCCAGAGAAGCAAGCTCCTC
CTTTCAGCGTTCTACTTGCATTGTATTTAGGCACGCCGCCAGCGTTCGCCTGAGCCAGGATCAAACTCT
>423
CTGCTGCCGCCCGTAGGAGTCTGGGCCGTGTCTCAGTCCCAGTGTGGCCGATCACCCTCTCAGGTCAGCTATG
TATCGTCGCCTTGGTGAGCCCTTACCTCACCAACTAGCTAATACAACGCAGGTCCATCTGGTAGTGATGCAAT
TGCACCTTTTTAAGCAAATGTCATGCAACATCTACTATTTATGCGGTATTTAGCTATCGTTTTTCCAATAGTT
TATCCCCCCGCTACCAGGCAGGTTACCTTACGCGTTACTCACCCGTTCGCAACTCATCCGCTCGGTGCAAGCA
CCAAGCTTTCAGCGTTTCTACTTTTGCATGTATTTAGGCACGCCGCCAGCGTTCGTCCTGAGCCAGGATCAAA
CTCTT
>424
CTGCTGCCGCCCGTAGGAGTCTGGGCCGTGTCTCAGTTCCCAGTGTGGCCGATCACCCTCTCAGGTCGGCTAT
GTATCGTCGCCTTGGTGAGCCGTTACCCCCCACCAACTAGCTAATACAACGCAGGTCCATCTGGTAGTGGTGC
AATTGCACCTTTTAAGCAAATGTCATGCAACATCTACTCTTTATGCGGTTATTAGCATCGTTTTCCAATTAGT
TTATCCCCCCGCTACCAGGCAGGTTACCTTACGCGTTACTCACCCGTTTCGCAACTCATCCGGAGAAGCAAGC
TCCTCCTTCAGCGTTCTACTTGCATTGTTATTTAGGCACGCCGCCAGCGTTCGTCCTGAGCCAGGATCAAACT
CTT
>425
CTGCTGCCGCCCGTAGGAGTCTGGGCCGTGTCTCAGTCCCAGTGTGGCCGATCACCCTCTCAGGTCGGCTATG
TATCGTCGCCTTGGTGAGCCCTTACCCCACCAACTAGCTAATACAACGCAGGTCCATCTGGTAGTGATGCAAT
TGCACCTTTCAAACAGTTGTCATGCAACAACTGCTATTATGCGGTATTAGCTATCGTTTTCCAATAGTTATCC
CCCGCTACCAGGCAGGTTACCTACGCGTTACTCACCCGTTCGCAACTCATCCGGAGAAGCAAGCTCCTCCTCA
GCGTTCTACTTGCCATGTATTAGGCACGCCGCCAGCGTTCGTCCTGAGCCAGGATCAAACTCT
>426
CTGCTGCCGCCCGTAGGAGTCTGGGCCGTGTCTCAGTCCCAGTGTGGCCGATCACCCTCTCAGGTCGGCTATG
TATCGTCGCCTTGGTGAGCCGTTACCCCCCACCAACTAGCTAATACAACGCAGGTCCATCTGGTAGTGGTGCA
ATTGCACCTTTTAAGCAAATGTCATGCAACATCTACTCTTATGCGGTATTTAGCTATCGTTTTCCAATAGTTA
TCCCCCCGCTACCAGGCAGGTTACCTACGCGTTACTCACCCGTCGCAACTCATCCGGAGAAGCAAGCTCCTCC
TTCAGCGTTCTACTTGCATGTATTTAGGCACGCCGCCAGCGTTCGTCCTGAGCCAGGATCAAACTCT
>427
CTGCTGCCGCCCGTAGGAGTCTGGGCCGTGTCTCAGTCCCAGTGTGGCCGGTCGCCCTCTCAGGCCGGCTACC
CGTCATCGCCTTGGTGAGCCATTACCTCACCAACAAACTGATAGGCCGCGAGTCCATCCCCCACCGAAAACTT
CCACAACACACCATGCGGTGCGAAGTCATATCCGGTATTTACGCCCCGATTTTCCCGAGGTTATCCCGAAGAG
GGGGGCAGGTTACGTCACGTGTTACTCACCCGTTCGCCACTAATCGGACAGGGCAAGCCCTGTCGTCATCGTT
TCGACTTGCATGTGTTTAAGCACGCCGCCAGCGTTCGTCCTGAGCCAGGATCAAACTCT
>428
CTGCTGCCGCCCGTAGGAGTCTGGGCCGTGTCTCAGTCCCAGTGTGGCCGATCACCCTCTCAGGTCGGCTATG
TATCGTCGCCTTGGTGAGCCGTTACCTCACCAACTAGCTAATACAACGCAGGTCCATCTGGTAGTGATGCAAT
TGCACCTTTTAATTGACTATCATGCAATAATCAATTTATGCGGTATTAGCCTATCGTTTTCCAATAGTTACTC
CCCCGCTACCAGGCAGGTTACCTACGCGTTACTCACCCGTTCGCAACTCATCCAGAGAAGCAAGCTCCTCCTT
CAGCGTTCTACTTGCATGTATTAGGCACGCCGCCAGCGTTCGTCCTGAGCCAGGATCAACTCT
>429
CTGCTGCCGCCCGTAGGAGTCTGGGCCGTGTCTCAGTCCCAGTGTGGCCGATCACCCTCTCAGGTCGGCTATG
TATCGTCGCCTTGGTGAGCCGTTACCTCACCAACTAGCTAATACAACGCAGGTCCATCTGGTAGTGATGCAAT
TGCACCTTTTAATTGACTATCATGCAATAATCAATTTTATGCGGTATTTAGCCTATCGTTTTCCAATAGTTTA

CTCCCCCCGCTACCAGGCAGGTTACCTACGCGTTACTCACCCGTTCGCAACTCATCCAGAGAAGCAAGCTCCT
CCTCGGCGTTCTACTTGCATGTATTTAGGCATGCCGCCAGCGTTCGTCCTGAGCCAGGATCAAACTCT
>430
CTGCTGCCGCCCGTAGGAGTCTGGGCCGTGTCTCAGTCCCAGTGTGGCCGATCACCCTCTCAGGTCGGCTATG
TATCGTCGCCTTGGTGAGCCGTTACCTCACCAACTAGCTAATACAACGCAGGTCCATCTGGTAGTGATGCAAT
TGCACCTTTTAATTGACTATCCATGCAATAATCAATTTTATGCGGTATTAGCCTATCGTTTCCAATAGTTACT
CCCCCGCTACCAGGCAGGTTACCTACGCGTTACTCACCCGTTCGCAACTCATCCAGAGAAGCAAGCTCCTCCT
TCAGCGTTCTACTTGCATGTATTACGGCAGCCGCCAGCGTTCG
>431
CTGCTGCCGCCCGTAGGAGTCTGGGCCGTGTCTCAGTCCCAGTGTGGCCGATCACCCTCTCAGGTCGGCTATG
TATCGTCGCCTTGGTGAGCCGTTACCTCACCAACTAGCTAATACAACGCAGGTCCATCTGGTAGTGATGCAAT
TGCACCTTTTAATTGACTATCATGCAATAATCAATTTTATGCGGTATTTAGCCTATCGTTTTCCAATAGTTTA
CTCCCCCCCGCTACCAGGCAGGTTACCTACGCGTTACTCACCCGTTCGCAACTCATCCAGAGAAGCAAGCTCC
TCCTCAGCGTTCTACTGCATGTATTTAGGCACGCCGCCAGCGTCGTCCTGAGCCAGGATCAAACTCT
>432
CTGCTGCCGCCCGTAGGAGTCTGGGCCGTGTCTCAGTCCCAGTGTGGCCGATCACCCTCTCAGGTCGGCTATG
CATCGTCGCCTTGGTAGGCCTTTTACCCCACCAACTAGCTAATGCACCGCAAAGCCATCTCATAGTGTTAGCA
TAACCAACTTTCAAACATCAACCATGCAGTTAATGCTGTTATCCGGTATTAGCTATCGTTTCCAATAGTTGTC
CCAGTCTATAAGGCAGGTTCTTTACGTGTTACTCACCCGTTCGCCGCTAAGTTCTCGCGCACAGCACAAGTAA
ACTCGCATGCGACGT
>433
CTGCTGCCGCCCGTAGGAGTCTGGGCCGTGTCTCAGTCCCAGTGTGGCCGATCACCCTCTCAGGTCGGCTATG
TATCGTCGCCTTGGTGAGCCCTTACCCCACCAACTAGCTAATACAACGCAGGTCCATCTGGTAGTGATGCAAT
TGCACCTTTCAAACAGTTGTCATGCAACAACTGCTATTATGCGGTATTAGCTATCGTTTTCCAATAGTTATCC
CCCGCTACCAGGCAGGTTACCTACCGCGTTACTCACCCGTTCGCAACTCATCCGGAGAAGCAAGCTCCTCCTT
CAGCGTTCTACTTGCCATGTATTAGGCGCGCCGCCAGCGTTCGTCCTGAGCCAGGATCAAACTCT
>434
CTGCTGCCGCCCGTAGGAGTCTGGGCCGTGTCTCAGTCCCAGTGTGGCCGATCACCCTCTCAGGTCGGCTATG
TATCGTCGCCTTGGTGAGCCCTTACCCCACCAACTAGCTAATACAACGCAGGTCCATCTGGTAGTGATGCAAT
TGCACCTTTTCAAACAGTTGTCATGCAACAACTGCTATTATGCGGTATTTAGCTATCGTTTTTCCAATAGTTA
TCCCCCGCTACCAGGCAGGTTACCTACCGCGTTACTCACCCGTTCGCAACTCATCCAGAGAAGCAAGCTCCTC
CTCAGCGTTCTACTGCATGTATTTAGGCACGCCGCCAGCGTCGTCCTGAGCCAGGATCAACTCT
>435
CTGCTGCCGCCCGTAGGAGTCTGGGCCGTGTCTCAGTCCCAGTGTGGCCGATCACCCTCTCAGGTCGGCTATG
TATCGTCGCCTTGGTGAGCCGTTACCCCACCAACTAGCTAATACAACGCAGGTCCATCTGGTAGTGGTGCAAT
TGCACCTTTTAAGCAAATGTCATGCAACATCTACTCTTATGCGGTATTAGCTATCGTTTCCAATAGTTATCCC
CCGCTACCAGGCAGGTTACCTACGCGTACTCACCCGTTCGCAACTCATCCGGAGAAGCAAGCTCCTCCTTCAG
CGTCTACTTGCATGTATTAGGCACGCCGCCAGCGTTCGTCCTGAGCCAGGATCAAACTCT
>436
CTGCTGCCGCCCGTAGGAGTCTGGGCCGTGTCTCAGTCCCAGTGTGGCCGATCACCCTCTCAGGTCGGCTATG
TATCGTCGCCTTGGTGAGCCGTTACCCCACCAACTAGCTAATACAACGCAGGTCCATCTGGTAGTGGTGCAAT
TGCACCTTTTAAGCAAATGTCATGCAACATCTACTCTTATGCGGTATTAGCTATCGTTTCCAATAGTTATCCC
CCGCTACCAGGCAGGTTACCTACGCGTTACTCACCCGTTCGCAACTCATCCGGAGAAGCAAGCTCCTCCTCAG
CGTTCTACTTGCATGTATTAGGCACGCCGCCAGCGTTCGTCCTGAGCCAGGATCAAACTCT
>437
CTGCTGCCGCCCGTAGGAGTCTGGGCCGTGTCTCAGTCCCAGTGTGGCCGATCACCCTCTCAGGTCGGCTATG
TATCGTCGCCTTGGTGAGCCCTTACCCCACCAACTAGCTAATACAACGCAGGTCCATCTGGTAGTGATGCAAT
TGCACCTTTCAAACAGTTGTCATGCAACAACTGCTATTATGCGGTATTAGCTATCGTTTCCAATAGTTATCCC
CCGCTACCAGGCAGGTTACCTACCGCGTACTCACCCGTTCGCAACTCATCCGGAGAAGCAAGCTCCTCCTCAG
CGTTCTACTTGCCATGTATTAGGCACGCCGCCAGCGTTCGTCCGTGACG
>438
CTGCTGCCGCCCGTAGGAGTCTGGACCGTGTCTCAGTTCCAGTGTGGCCGATCACCCTCTCAGGTCGGCTACG
TATCGTTGCCTTGGTAAGCCGTTACCTTACCAACTAGCTAATACGGCGCAGGTCCATCTATAAGTGATAGCAA
AGCCATCTTTCACTATCGAACCATGCGGTTCGAAATATTATCCGGTATTAGCTCCGGTTTCCCGAAGTTATCC

CAGTCTTATACGGTAGGTTACCTACGTGTTACTCACCCGTCCGCCGCTAACCGTCAAAGGAGCAAGCTCCTCA
TCTGTTCGCTCGACTTGCATGTATTAGGCACGCCGTCCGCGTTCAAGCTCTGCCCAGGAGACAACTCATAT
>439
CTGCTGCCGCCCGTAGGAGTCTGGGCCGTGTCTCAGTCCCAGTGTGGCCGATCACCCTCTCAGGTCGGCTATG
TATCGTCGCCTTGGTGAGCCCTTACCCCACCAACTAGCTAATACAACGCAGGTCCATCTGGTAGTGATGCAAT
GCACCTTTCAAACAGTTGTCATGCAACAACTGCTATTATGCGGTATTAGCTATCGTTTCCAATAGTTATCCCC
CGCTACCAGGCAGGTTACCTACGCGTACTCACCCGTTCGCAACTCATCCAGAGAGCAAGCTCCTCCTCAGCGT
TCTACTGCATGTATTAGGCACGCCGCCAGCGTCGTCCGTG
>440
CTGCTGCCGCCCGTAGGAGTCTGGGCCGTGTCTCAGTCCCAGTGTGGCCGATCACCCTCTCAGGTCGGCTATG
CATCGTCGCCTTGGTGAGCCCTTACCCCACCAACTAGCTAATACAACGCAGGTCCATCTGGTAGTGATGCAAT
TGCACCTTTCAAACAGTTGTCATGCAACAACTGCTATTATGCGGTATTAGCTATCGTTTTCCAATAGTTATCC
CCCGCTACCAGGCAGGTTACCTACGCGTTACTCACCCGTTCGCAACTCATCCAGAGAAGCAAGCTCCTCCTTC
AGCGTTCTACTTGCATGTATTAGGCACGCCGCCAGCGTTCGTCCTGAGCCAGGATCAAACTCT
>441
CTGCTGCCGCCCGTAGGAGTCTGGACCGTGTCTCAGTTCCAGTGTGGCCGATCACCCTCTCAGGTCGGCTACG
TATCGTCGCCTTGGTGAGCCGTTACCTCACCAACTTAGCTAATACAACGCAGGTCCATCTGGTAGTGATGCAA
TTGCACCTTTTAATTCGACTATCATGCAATAATCAATTTTATGCGGTATTAGCTATCGTTTCCAATAGTTATC
CCCCGCTACCAGGCAGGTTACCTACGCGTTACTCACCCGTTCGCAACTCATCCAGAGAAGCAAGCTCCTCCTC
AGCGTTCTACTTGCATGTATTAGGCACGCCGCCAGCGTTCGTCCTGAGCCAGGATCAAACTCT
>442
CTGCTGCCGCCCGTAGGAGTCTGGGCCGTGTCTCAGTCCCAGTGTGGCCGATCACCCTCTCAGGTCGGCTATG
TATCGTCGCCTTGGTGAGCCGTTACCTCACCAACTAGCTAATACAACGCAGGTCCATCTGGTAGTGATGCAAT
TGCACCTTTTAATTGACTATCATGCAATAATCAATTTTATGCGGTATTAGCCTATCGTTTCCAATAGTTACTC
CCCCGCTACCAGGCAGGTTACCTACGCGTTACTCACCCGTTCGCAACTCCATCCAGAGAAGCAAGCTCCCCCT
TCAGCGTTCTACTTGCATGTATTAGGCACGCCGCCAGCGTTCGTCCTGAGCCAGGCATCAAACTCT
>443
CTGCTGCCGCCCGTAGGAGTCTGGGCCGTGTCTCAGCCCCAGTGTGGCCGATCACCCTCTCAGGTCGGCTATG
CATCGTCGCCTTGGTAGGCCTTTACCCCACCAACTAGCTAATGCACCGCAAAGCCATCTCATAGTGTTAGCAA
AACCAACTTTCAAACATCAACCATGCAGTTAATGCTGTTATCCGGTATTAGCTATCGTTTCCAATAGTTGTCC
CAGTCTATGAGGCAGGTTCTTTACGTGTTACTCACCCGTTCGCCGCTAAGTTGTCGTCGACAGCAACCAGAAA
CTACTCGACTGCTTGCATGTATTAG
>444
CTGCTGCCGCCCGTAGGAGTCTGGGCCGTGTCTCAGTCCCAGTGTGGCCGATCACCCTCTCAGGTCGGCTATG
TATCGTCGCCTTGGTGAGCCGTTACCCCACCAACTAGCTAATACAACGCAGGTCCATCTGGTAGTGGTGCAAT
TGCACCTTTTAAGCAAATGTCATGCAACATCTACTCTTATGCGGTATTAGCTATCGTTTTCCAATAGTTATCC
CCCCGCTACCAGGCAGGTTACCTACGCGTTACTCACCCGTTCGCAACTCATCCGGAGAAGCAAGCTCCTCCTC
AGCGTTCTACTTGCATGTATTAGGCACGCCGCCAGCGTCGTCCTGAGCCAGGATCAACTCT
>445
CTGCTGCCGCCCGTAGGAGTCTGGGCCGTGTCTCAGTCCCAGTGTGGCCGATCACCCTCTCAGGTCGGCTATG
TATCGTCGCCTTGGTGAGCCCTTACCCCACCAACTAGCTAATACAACGCAGGTCCATCTGGTAGTGATGCAAT
TGCACCTTTCAAACAGTTGTCATGCAACAACTGCTATTATGCGGTATTAGCTATCGTTTCCAATAGTTATCCC
CCGCTACCAGGCAGGTTACCTACGCGTTACTCACCCGTTCGCAACTCATCCAGAGAAGCAAGCTCCTCCTTCA
GCGTTCTACTTGCATGTATTAGGCACGCCGCCAGCGTTCGTCCTGAGCCAGGATCAAACTCT
>446
CTGCTGCCGCCCGTAGGAGTCTGGACCGTGTCTCAGTTCCAGTGTGGCCGATCACCCTCTCAGGTCGGCTACG
TATCGTTGCCTTGGTAAGCCGTTACCTTACCAACTAGCTAATACGGCGCAGGTCCATCTATAAGTGATAGCAA
AGCCATCTTCACTATCGAACCATGCGGTTCGAAATATTATCCGGTATTAGCTCCGGCTTCCCCGAAGTTATCC
CAGTCTTATAGGTAGGTTACCTACGTGTTACTCACCCGTCCGCCGCTAACGTCGAAGGAGCAGCTCCTCGTCT
GTTCGCTCGACTTGCATGTATTAGGCACGCCGCCAGCGTTCATCCTGAGCCAGGATCAAACTCT
>447
CTGCTGCCGCCCGTAGGAGTCTGGACCGTGTCTCAGTTCCAGTGTGGCCGATCACCCTCTCAGGTCGGCTACG
TATCGTTGCCTTGGTAAGCCGTTACCTTACCAACTAGCTAATACGGCGCAGGTCCATCTATAAGTGATAGCAA
AGCCATCTTTCACTATCGAACCATGCGGTTCGAAATATTATCCGGTATTAGCTCCGGTTTCCCGAAGTTATCC

CAGTCTTATAGGTAGGTTACCTACGTGTTACTCACCCGTCCGCCGCTAACGTCAAAGGAGCAAGCTCCTCGTC
TGTTCGCTCGACTTGCATGTATTAGGCACGCCGCCAGCGTTCATCCTGAGCCAGGACAACTCTA
>448
CTGCTGCCGCCCGTAGGAGTCTGGGCCGTGTCTCAGTCCCAGTGTGGCCGATCACCCTCTCAGGTCGGCTATG
TATCGTCGCCTTGGTGAGCCGTTACCCCACCAACTAGCTAATACAACGCAGGTCCATCTGGTAGTGGTGCAAT
TGCACCTTTTAAGCAAATGTCATGCAACATCTACTCTTTATGCGGTATTTAGCTATCGTTTTCCAATAGTTAT
CCCCCGCTACCAGGCAGGTTACCTACGCGTTACTCACCCGTTCGCAACTCATCCGGAGAAGCAAGCTCCTCCT
TCAGCGTTCTACTTGCATGTATTTAGGCACGCCGCCAGCGTTCGTCCTGAGCCAGGATCAAACTCT
>449
CTGCTGCCGCCCGTAGGAGTCTGGGCCGTGTCTCAGTCCCAGTGTGGCCGATCACCCTCTCAGGTCGGCTATG
TATCGTCGCCTTGGTGAGCCGTTACCTCACCAACTAGCTAATACAACGCAGGTCCATCTGGTAGTGATGCAAT
TTGCACCTTTTAATTGACTATCATGCAATAATCAATTTTATGCGGTATTAGCCTATCGTTTCCAATAGTTACT
CCCCCGCTACCAGGCAGGTTACCTACGCGTTACTCACCCGTTCGCAACTCATCCAGAGAAGCAAGCTCCTCCT
TCAGCGTTCTACTTGCATGTATTAGGCACGCCGCCAGCGTTCGTCCTGAGCCAGGATCAAACTCT
>450
CTGCTGCCGCCCGTAGGAGTCTGGGCCGTGTCTCAGTCCCAGTGTGGCCGATCACCCTCTCAGGTCGGCTATG
TATCGTCGCCTTGGTGAGCCCTTACCCCACCAACTAGCTAATACAACGCAGGTCCATCTGGTAGTGATGCAAT
TGCACCTTTCAAACAGTTGTCATGCAACAACTGCTATTATGCGGTATTAGCTATCGTTTTCCAATAGTTATCC
CCCGCTACCAGGCAGGTTACCTACGCGTTACTCACCCGTTCGCAACTCATCCAGAGAAGCAAGCTCCTCCTCA
GCGTTCTACTTGCATGTATTAGGCACGCCGCCAGCGTCGTCCTGAGCCAGGATCAACTCT
>451
CTGCTGCCGCCCGTAGGAGTCTGGACCGTGTCTCAGTTCCAGTGTGGCCGATCACCCTCTCAGGTCGGCTACG
CATCGTTGCCTTGGTAAGCCGTTACCTTACCAACTAGCTAATGCGGCGCGGATCCATCTATAAGTGACAGCAA
AACCGTCTTTCACTATTGAACCATGCGGTTCGATATATTATCCGGTATTAGCTCCGGTTTCCCGAAGTTATCC
CAGTCTTATAGGTAGGTTATCCACGTGTTACTCACCCGTCCGCCGCTAACGTCAGAGGAGCAAGCTCCTCGTC
TGTTCGCTCGACTTGCATGTATTAGGCACGCCGCCAGCGTTCATCCTGAGCCAGGATCAAACTCT
>452
CTACTGCCGCCCCGTAGGAGTCTGGGCCGTGTCTCAGTCCCAGTGTGGCCGATCACCCTCTCAGGTCGGCTAT
GTATCGTCGCCTTGGTGTAGCCCTACCCCATCCAACTAGCTAATACAACGCAGGTCCATCTGGTAGTGATGCA
ATTGCACCTTTCAAACAGTTGTCATGCAACAACTGCTATTATGCGGTATTAGCTATCGTTTCCAATAGTTATC
CCCGCCTACCAGGCAGGTTACCTACGCGTTACTCACCCGTTCGCAACTCATCCAGAGAAGCAAGCTCCTCCTT
CAGCGTTCTACTTGCATGTATTAGGCACGCCGCCAGCGTTCGTCCTGAGCCAGGATCAAACTCT
>453
CTGCTGCCGCCCGTAGGAGTCTGGGCCGTGTCTCAGTCCCAGTGTGGCCGATCACCCTCTCAGGTCGGCTATG
TATCGTCGCCTTGGTGAGCCCTTACCCCACCAACTAGCTAATACAACGCAGGTCCATCTGGTAGTGATGCAAT
TGCACCTTTTCAAACGTAGTTCATCGAGCAACAACTGCTATTATGCGGTATTTAGCTATCGTTTTCCAATAGT
TATCCCCCGCTACCAGGCAGGTTACCTACGCGTTACTCACCCGTTCGCAACTCATCCAGAGAAGCAAGCTCCT
CCTCAGCGTCTACTGCATGTATTAGGCACGCCGCCAGCGTCGTCCTGAGCCAGGATCAAACTCT
>454
CTGCTGCCGCCCGTAGGAGTCTGGACCGTGTCTCAGTTCCAGTGTGGCCGATCACCCTCTCAGGTCGGCTACG
TATCGTTGCCTTGGTAAGCCGTTACCTTACCAACTAGCTAATACGGCGCAGGTCCATCTATAAGTGATAGCAA
AGCCATCTTTCACTATCGAACCATGCGGTTCGAAATATTATCCGGTATTAGCTCCGGTTTCCCGAAGTTATCC
CAGTCTTATACGGTAGGTTACCTACGTGTTACTCACCCGTCCGCCGCTAACGTCAAAGGCAGCAAGCTCCTCG
TCTGTTCGCTCGACTTGCATGTATTAGGCACGCCGCCAGCGTCATCCTGAGCCAGGATCAACTCT
>455
CTGCTGCCGCCCGTAGGAGTCTGGGCCGTGTCTCAGTCCCAGTGTGGCCGATCACCCTCTCAGGTCGGCTATG
TATCGTCGCCTTGGTGAGCCGTTACCTCACCAACTAGCTAATACAACGCAGGTCCATCTGGTAGTGATGCAAT
TTGCACCTTTTAATTGACTATCATGCAATAATCAATTTTATGCGGTATTAGCCTATCGTTTCCAATAGTTATC
CCCCGCTACCAGGCAGGTTACCTACGCGTTACTCACCCGTTCGCAACTCATCCAGAGAAGCAAGCTCCTCCTT
CAGCGTTCTACTTGCCATGTATTACGGCACGCCGCCAGCGTTCGTCCTGACGCCAAGGC
>456
CTGCTGCCGCCCGTAGGAGTCTGGGCCGTGTCTCAGTCCCAGTGTGGCCGATCACCCTCTCAGGTCGGCTATG
TATCGTCGCCTTGGTGAGCCGTTACCCCACCAACTAGCTAATACAACGCAGGTCCATCTGGTAGTGGTGCAAT
TGCACCTTTTAAGCAAATGTCATGCAACATCTACTCTTATGCGGTATTAGCTATCGTTTTCCAATAGTTTATC

CCCCGCTACCAGGCAGGTTACCTACGCGTTACTCACCCGTTCGCAACTCATCCGGAGAAGCAAGCTCCTCCTT
CAGCGTTCTACTTGCATGTATTAGGCACGCCGCCAGCGTTCGTCCTGAGCCAGGATCAAACTCT
>457
CTGCTGCCGCCCGTAGGAGTCTGGGCCGTGTCTCAGTCCCAGTGTGGCCGATCACCCTCTCAGGTCGGCTATG
TATCGTCGCCTTGGTGAGCCGTTACCTCACCAACTAGCTAATACAACGCAGGTCCATCTGGTAGTGATGCAAT
TTGCACCTTTTAATTGACTATCATGCAATAATCAATTTTATGCGGTATTTAGCCTATCGTTTTCCAATAGTTT
ACTCCCCCCGCTACCAGGCAGGTTACCTACGCGTTACTCACCCGTTCGCAACTCATCCAGAGAAGCAAGCTCC
TCCTCAGCGTTCTACTTGCATGTATTTAGGCACGCCGCCAGCGTTCGTCCTGAGCCAGGATCAAACTCT
>458
CTGCTGCCGCCCGTAGGAGTCTGGGCCGTGTCTCAGTCCCAGTGTGGCCGATCACCCTCTCAGGTCGGCTATG
TATCGTCGCCTTGGTGAGCCCTTACCCCACCAACTAGCTAATACAACGCAGGTCCATCTGGTAGTGATGCAAT
TGCACCTTTCAAACAGTTGTCATGCAACAACTGCTATTATGCGGTATTAGCTATCGTTTCCAATAGTTATCCC
CCGCTACCAGGCAGGTTACCTACGCGTTACTCACCCGTTCGCAACTCATCCAGAGAAGCAAGCTCCTCCTTCA
GCGTTCTACTTGCATGTATTAGGCACGCCGCCAGCGTTCGTCCTGAGCCAGGATCAAACTCT
>459
CTGCTGCCGCCCGTAGGAGTCTGGGCCGTGTCTCAGTCCCAGTGTGGCCGATCACCCTCTCAGGTCGGCTATG
TATCGTCGCCTTGGTGAGCCCTTACCCCACCAACTAGCTAATACAACGCAGGTCCATCTGGTAGTGATGCAAT
TGCACCTTTTACAACAAGTTGTCATGCAACAACTGTCTATTATGCGGTATTAGCTATCGTTTCCAATAGTTAT
CCCCCGCTACCAGGCAGGTTACCTACGCGTTACTCACCCGTTCGCAACTCATCCAGAGAAGCAAGCTCCTCCT
TCAGCGTTCTACTTGCATGTATTAGGCACGCCGCCAGCGTTCGTCCTGAGCCAGGATCAAACTCT
>460
CTGCTGCCGCCCGTAGGAGTCTGGGCCGTGTCTCAGTCCCAGTGTGGCCGATCACCCTCTCAGGTCGGCTATG
TATCGTCGCCTTGGTGAGCCGTTACCTCACCAACTAGCTAATACAACGCAGGTCCATCTGGTAGTGATGCAAT
TTGCACCTTTTAATTGACTATCATGCAATAATCAATTTTATGCGGTATTAAGCCTATCGTTTCCAATAGTTAT
CCCCCGCTACCAGGCAGGTTACCTACGCGTTACTCACCCGTTCGCAACTCATCCAGAGAAGCAAGCTCCTCCT
TCAGCGTTCTACTTGCATGTATTAGGCACGCCGCCAGCGTTCGTCCTGAGCCAGGATCAAACTCT
>461
CTGCTGCCGCCCGTAGGAGTCTGGGCCGTGTCTCAGTCCCAGTGTGGCCGATCACCCTCTCAGGTCGGCTATG
TATCGTCGCCTTGGTGAGCCCTTACCCCACCAACTAGCTAATACAACGCAGGTCCATCTGGTAGTGATGCAAT
TGCACCTTTTCAAACAGTTGTCATGCAACAACTGCTATTTATGCGGTATTTTAGCTATCGTTTTTCCAATAGT
TTATCCCCCCCCGCTACCAGGCAGGTTACCTACGCGTACTCACCCGTTCGCAACTCATCCGGAGAGCAAGCTC
CTCCTCAGCGTCTACTTGCCATGTATTTAGGCACGCCGCCAGCGTTCGTCCTGAGCCAGGATCAAACTCT
>462
CTGCTGCCGCCCGTAGGAGTCTGGGCCGTGCCTCAGTCCCAGTGTGGCCGATCACCCTCTCAGGTCGGCTATG
TATCGTCGCCTTGGTGAGCCGTTACCCCACCAACTAGCTAATACAACGCAGGTCCATCTGGTAGTGGTGCAAT
TGCACCTTTTAAGCAAATGTCATGCAACATCTACTCTTATGCGGTATTAGCTATCGTTTTCCAATAGTTTATC
CCCCGCTACCAGGCAGGTTACCTACGCGTACTCACCCGTCGCAACTCATCCGGAGAAGCAAGCTCCTCCTCAG
CGTCTACTTGCATGTATTAGGCACGCCGCCAGCGTCGTCCTGAGCCAGGATCAAACTCT
>463
CTGCTGCCGCCCGTAGGAGTCTGGGCCGTGTCTCAGTCCCAGTGTGGCCGATCACCCTCTCAGGTCGGCTATG
TATCGTCGCCTTGGTGAGCCGTTACCCCACCAACTAGCTAATACAACGCAGGTCCATCTGGTAGTGGTGCAAT
TGCACCTTTTAAGCAAATGTCAAGCAACATCTACTCTTTATGCGGTATTTAGCTATCGTTTTCCAATAGTTTA
TCCCCCGCTACCAGGCAGGTTACCTACGCGTTACTCACCCGTTCGCAACTCATCCGGAGAAGCAAGCTCCTCC
TTCAGCGTTCTACTTGCATGTATTTAGGCACGCCGCCAGCGTTCGTCCTGAGCCAAGGATCAAACTCT
>464
CTGCTGCCGCCCGTAGGAGTCTGGGCCGTGTCTCAGTCCCAGTGTGGCCGATCACCCTCTCAGGTCGGCTATG
CATCGTCGCCTTGGTAGGCCTTTACCCCACCAACTAGCTAATGCACCGCAAAGCCATCTCATAGTGTTAGCAA
AACCAACTTTTAAACATCAACCATGCAGTTAGTGCTGTTTTATCCGGTATTTTAGCTATTCGTTTTTTCCCAA
TAGTTTTGTCCCAGTCTTATTGAGGCAGGTTTTCTTTACGTGTTTACTCACCCGTTTTCGCCGCTAAGTTTTC
GTCGCAGCA
>465
CTGCTGCCGCCCGTAGGAGTCTGGGCCGTGTCTCAGTCCCAGTGTGGCCGATCACCCTCTCAGGTCGGCTATG
TATCGTCGCCTTGGTGAGCCGTTACCTCACCAACTAGCTAATACAACGCAGGTCCATCTGGTAGTGATGCAAT
TTGCACCTTTAATTGACTATCATGCAATAATCAATTTTATGCGGTATTAGCCTATCGTTCCAATAGTTATCCC

CCGCTACCAGGCAGGTTACCTACGCGTACTCACCCGTTCGCAACTCATCCAGAGAAGCAAGCTCCTCCTTCAG
CGTTCTACTTGCATGTATTAGGCACGCCGCCAGCGTCGTCCTGAGCCAGGCATGAA
>466
CTGCTGCCGCCCGTAGGAGTCTGGGCCGTGTCTCAGTCCCAGTGTGGCCGATCACCCTCTCAGGTCGGCTATG
TATCGTCGCCTTGGTGAGCCGTTACCCCACCAACTAGCTAATACAACGCAGGTCCATCTGGTAGTGGTGCAAT
TGCACCTTTTAAGCAAATGTCATGCAACATCTACTCTTATGCGGTATTAGCTATCGTTTTCCAATAGTTATCC
CCCGCTACCAGGCAGGTTACCTACGCGTTACTCACCCGTTCGCAACTCATCCGGAGAAGCAAGCTCCTCCTTC
AGCGTTCTACTTGCATGTATTTAGGCACGCCGCCAGCGTTCGTCCTGAGCCAGGATCAAACTCT
>467
CTGCTGCCGCCCGTAGGAGTCTGGGCCGTGTCTCAGTCCCAGTGTGGCCGATCACCCTCTCAGGTCGGCTATG
CATCGTCGCCTTGGTAGGCCTTTACCCCCACCAACTAGCTAATGCACCGCAAAGCCATCTCATAGTGTTAGCA
AAACCAACTTTTAAACATCAACCATGCAGTTAATGCTGTTTATCCGGTATTTAGCTATTCGTTTTCCAATTAG
TTTGTCCCAGTCTTATGAGGCAGGTTTCTTTACGTGTTTACTCACCCCCGTTTCGCCGCTAAGTTGTCGTCGC
AGCGAACCACAAACGTA
>468
CTGCTGCCGCCCGTAGGAGTCTGGGCCGTGTCTCAGTCCCAGTGTGGCCGATCACCCTCTCAGGTCGGCTATG
TATCGTCGCCTTGGTGAGCCCTTACCCCACCAACTAGCTAATACAACGCAGGTCCATCTGGTAGTGATGCAAT
TGCACCTTTCAAACAGTTGTCATGCAACAACTGCTATTATGCGGTATTTAGCTATCGTTTTCCAATAGTTTAT
CCCCCCGCTACCAGGCAGGTTACCTACGCGTTACTCACCCGTTCGCAACTCATCCGGAGAAGCAAGCTCCTCC
TTCAGCGTTCTACTTGCATGTATTTAGGCACGCCGCCAGCGTTCGTCCTGAGCCAGGATCAAACTCT
>469
CTGCTGCCGCCCGTAGGAGTCTGGGCCGTGTCTCAGTCCCAGTGTGGCCGATCACCCTCTCAGGTCGGCTATG
TATCGTCGCCTTGGTGAGCCCTTACCCCACCAACTAGCTAATACAACGCAGGTCCATCTGGTAGTGATGCAAT
TGCACCTTTCAAACAGTTGTCATGCAACAACTGCTATTATGCGGTATTTAGCTATCGTTTTCCAATAGTTATC
CCCCCGCTACCAGGCAGGTTACCTACGCGTTACTCACCCGTTCGCAACTCATCCGGAGAAGCAAGCTCCTCCT
TCAGCGTTCTACTTGCATGTATTTAGGCACGCCGCCAGCGTTCGTCCTGAGCCAGGATCAAACTCT
>470
CTGCTGCCGCCCGTAGGAGTCTGGGCCGTGTCTCAGTCCCAGTGTGGCCGATCACCCTCTCAGGTCGGCTATG
CATCGTCGCCTTGGTAGGCCTTTACCCCACCAACTAGCTAATGCACCGCAAAGCCATCTCATAGTGTTAGCAA
AACCAACTTTAAACATCAACCATGCAGTTAATGCTGTTATCCGGTATTAGCTATCGTTTCCAATAGTTGTCCC
AGTCTATGAGGCAGGTTCTTTACGTGTTACTCACCCGTTCGCCGCTAAGTTCGTCGTCGACAGCAACCGAAAC
ACGTCGACTCG
>471
CTGCTGCCGCCCGTAGGAGTCTGGGCCGTGTCTCAGTCCCAGTGTGGCCGATCACCCTCTCAGGTCGGCTATG
TATCGTCGCCTTGGTGAGCCGTTACCTCACCAACTAGCTAATACAACGCAGGTCCATCTGGTAGTGATGCAAT
TTGCACCTTTTAATTGACTATCATGCAATAATCAATTTTATGCGGTATTTAGCCTATCGTTTTCCAATAGTTA
CTCCCCCGCTACCAGGCAGGTTACCTACGCGTTACTCACCCGTTCGCAACTCATCCGGAGAAGCAAGCTCCTC
CTTCAGCGTTCTACTTGCATGTATTTAGGCACGCCGCCAGCGTTCGTCCTGAGCCAGGATCAAACTCT
>472
CTGCTGCCGCCCGTAGGAGTCTGGGCCGTGTCTCAGTCCCAGTGTGGCCGATCACCCTCTCAGGTCGGCTATG
CATCGTCGCCTTGGTAGGCCTTTACCCCACCAACTAGCTAATGCACCGCAAAGCCATCTCATAGTGTTAGCAA
AACCAACTTTTAAACATCAACCATGCAGTTAATGCTGTTATCCGGTATTAGCTATCGTTTTCCAATAGTTGTC
CCAGTCTATGAGGCAGGTTCTTTACGTGTTACTCACCCGTTCGCCGCTAAGTTCGTCGTCGACAGCAACCAGC
TAACTAACTCGACTC
>473
CTGCTGCCGCCCGTAGGAGTCTGGGCCGTGTCTCAGTCCCAGTGTGGCCGATCACCCTCTCAGGTCGGCTATG
CATCGTCGCCTTGGTAGGCCTTTACCCCCACCAACTAGCTAATGCACCGCAAAGCCATCTCATAGTGTTAGCA
TAACCAACTTTCAAACATCGACCATGACAGTTAATGCTGTTTATCCGGTTATTTAGCTTATCGTTTTCCAATA
GTTTGCCCAGTCTATGAGGCAGGTTTCTTTACGTGTTTACTCACCCGTTCGCCGCTAAGTTTTCGTCGCAGCA
CCAAAACTAACG
>474
CTGCTGCCGCCCGTAGGAGTCTGGGCCGTGTCTCAGTCCCAGTGTGGCCGATCACCCTCTCAGGTCGGCTATG
TATCGTCGCCTTGGTGAGCCCTTACCCCACCAACTAGCTAATACAACGCAGGTCCATCTGGTAGTGATGCAAT
TGCACCTTTTCAAACAGTTTGTCATGCAACAACTGCTATTTATGCGGTATTTAGCTATCGTTTTTCCAATAGT

TTATCCCCCCCGCTACCAGGCAGGTTTACCTACGCGTTACTCACCCGTTTCGCAACTCATCCGGAGAAGCAAG
CTCCTCCTTCAGCGTTCTACTTGCATGTATTTAGGCACGCCGCCAGCGTTCGTTCCTGAGCCAGGATCAAAC
TCT
>475
CTGCTGCCGCCCGTAGGAGTCTGGGCCGTGTCTCAGTCCCAGTGTGGCCGACCACCCTCTCAGGTCGGCTATG
TATCGTCGCCTTGGTGAGCCCTTACCCCACCAACTAGCTAATACAACGCAGGTCCATCTGGTAGTGATGCAAT
TGCACCTTTTAAGTAAATGTCATGCAACATCTACTCTTATGCGGTATTAGCTATCGTTTCCAATAGTTATCCC
CCGCTACCAGGCAGGTTACCTACGCGTTACTCACCCGTTCGCAACTCATCCAGAAGAGCAAGCTCCTCCTTCA
GCGTTCTACTTGCATGTATTACGGCACGCCGCCAGCGTCGTCCTGAGCCAGGATCAAACTCT
>476
CTGCTGCCGCCCGTAGGAGTCTGGGCCGTGTCTCAGTCCCAGTGTGGCCGATCACCCTCTCAGGTCGGCTATG
TATCGTCGCCTTGGTGAGCCCTTACCCCACCAACTAGCTAATACAACGCAGGTCCATCTGGTAGTGATGCAAT
TGCACCTTTCAAACAGTTGTCATGCAACAACTGCTATTATGCGGTATTAGCTATCGTTTCCAATAGTTATCCC
CCGCTACCAGGCAGGTTACCTACGCGTTACTCACCCGTTCGCAACTCATCCAGAGAAGCAAGCTCCTCCTTCA
GCGTTCTACTTGCATGTATTAGGCACGCCGCCAGCGTTCGTCCTGAGCCAGGATCAAACTCT
>477
CTGCTGCCGCCCGTAGGAGTCTGGACCGTGTCTCAGTTCCAGTGTGGCCGATCACCCTCTCAGGTCGGCTACG
TATCGTTGCCTTGGTAAGCCGTTACCTTACCAACTAGCTAATACGGCGCAGGTCCATCTATAAGTGATAGCAA
AGCCATCTTTCACTATCGAACCATGCGGTTCGAAATATTATCCGGTATTAGCTCCGGTTTTCCCGAAGTTATC
CCAGTCTTATAGGTAGGTTTACCTACGTGTTACTCACCCGTCCGCCGCTAACGTCAAAGGAGCAAGCTCCTCG
TCTGTTCGCTACGACTTGCATGTATTAGGCACGCCGCCAGCGTCATCCTGAGCCAGGATCAAACTCT
>478
CTGCTGCCGCCCGTAGGAGTCTGGGCCGTGTCTCAGTCCCAGTGTGGCCGATCACCCTCTCAGGTCGGCTATG
TATCGTCGCCTTGGTGAGCCGTTACCCCACCAACTAGCTAATACAACGCAGGTCCATCTGGTAGTGGTGCAAT
TGCACCTTTTAAGCAAATGTCATGCAACATCTACTCTTTATGCGGTATTTAGCTATCGTTTTCCAATAGTTTA
TCCCCCCGCTACCAGGCAGGTTACCTACGCGTTACTCACCCGTTCGCAACTCATCCGGAGAAGCAAGCTCCTC
CTGCAGCGTTCTACTGCATGTATTTAGGCACGCCGCCAGCGTTCGTCCTGAGCCAGGATCAAACTCT
>479
CTGCTGCCGCCCGTAGGAGTCTGGGCCGTGTCTCAGTCCCAGTGTGGCCGGTCACCCTCTCAGGCCGGCTACC
CGTCGTCGCCTTGGTGAGCCATTACCTCACCAACAAGCTGATAGGCCGTGAGCCCATCCAAGACCAGTAAACC
CTTTTCCACACACCACCATGCGGTGACATGTAGCATCCAGTATTTAGACCCAGTTTTTCCCGGGCTTTATCCC
AGAGTCAAGGGCAGGTTACTCACGTATTTACTCACCCGTTTCGCCACTCATCCACACCAGCAAGCTGGTGCTT
TTCAGCGTTTCGACTTGCATGTGTTTAAGCACGCCGCCAGCGTTCGTCCTGAGCCAGGATCAAACTCT
>480
CTGCTGCCGCCCGTAGGAGTCTGGGCCGTGTCTCAGTCCCAGTGTGGCCGATCACCCTCTCAGGTCGGCTATG
TATCGTCGCCTTGGTGAGCCGTTACCCCACCAACTAGCTAATACAACGCAGGTCCATCTGATAGTGGTGCAAT
TGCACCTTTTAAGCAAATGTCATGCAACATCTACTCTTATGCGGTATTAGCTATCGTTTCCAATAGTTATCCC
CCGCTACCAGGCAGGTTACCTACGCGTTACTCACCCGTTCGCAACTCATCCAGAGAAGCAAGCTCCTCCTTCA
GCGTTCTACTTGCATGTATTAGGCACGCCGCCAGCGTTCGTCCTGAGCCAGGATCAAACTCT
>481
CTGCTGCCGCCCGTAGGAGTCTGGGCCGTGTCTCAGTCCCAGTGTGGCCGATCACCCTCTCAGGTCGGCTATG
CATCGTCGCCTTGGTAGGCCTTTACCCCACCAACTAGCTAATGCACCGCAAAGCCATCTCATAGTGTTAGCAA
AACCAACTTTTAAACATCAACCATGCAGTTAATGCTGTTATCCGGTATTAGCTATCGTTTTCCAATAGTTTTG
TCCCAGTCTATGAGGCAGGTTTCTTTACGTGTTTACTCACCCGTTTCGCCGCTAAGTTGTCGCGCAGCGAACC
AGCAACGAACTCGAACTCGCATTGC
>482
CTGCTGCCGCCCGTAGGAGTCTGGGCCGTGTCTCAGTCCCAGTGTGGCCGATCACCCTCTCAGGTCGGCTATG
TATCGTCGCCTTGGTGAGCCCTTACCCCACCAACTAGCTAATACAACGCAGGTCCATCTGGTAGTGATGCAAT
TGCACCTTTCAAACAGTTGTCATGCAACAACTGCTATTATGCGGTATTAGCTATCGTTTCCAATAGTTATCCC
CCGCTACCAGGCAGGTTACCTACCGCGTACTCACCCGTTCGCAACTCGTCCGGAGAAGCAAGCTCCTCCTTCA
GCGTTCTACTTGCCATGTATTAGGCACGCCGCCAGCGTTCGTCCTGAGCCAGGATCAAACTCT
>483
CTGCTGCCGCCCGTAGGAGTCTGGGCCGTGTCTCAGTCCCAGTGTGGCCGGTCGCCCTCTCAGGCCGGCTACC
CGTCATCGTCTTGGTGAGCCATCACCTCACCAACAAACTGATAGGCCGCGAGTCCATCCCCCACCGATAAATC

TTTCCAACACCCACCATGCGATGAGTGCTCATATCCGGTATTAGCCACGATTTCCCGAGGTTATCCCGAAGTG
GAGGGCAGGTTACTCACGTGTTACTCACCCGTTCGCCACTAATCAGGCGGTGCAAGCACCACCGTCATCGTTC
GACTTGCATGTGTTAAGCACGCCGCCAGCGTTCGTCCTGAGCCAGGATCAAACTCT
>484
CTGCTGCCGCCCGTAGGAGTCTGGGCCGTGTCTCAGTCCCAGTGTGGCCGATCACCCTCTCAGGTCGGCTATG
CATCGTCGCCTTGGTAGGCCTTTACCCCACCAACTAGCTAATGCACCGCAAAGCCATCTCATAGTGTTTAGCA
AAACCAACTTTTAAACATCAACCATGCAGTTAATGCTGTTTATCCGGTATTTTAGCTATTCGTTTTTCCAAT
TAGTTTTGTCCCCAGTCTTATTGAGGCAGGTTTTCTTTACGTGTTTACTCACCCGTTTTCGCCGCTAAGTTTT
CGTTCGCGACACG
>485
CTGCTGCCGCCCGTAGGAGTCTGGGCCGTGTCTCAGTCCCAGTGTGGCCGATCACCCTCTCAGGTCGGCTATG
TATCGTCGCCTTGGTGAGCCCTTACCCCACCAACTAGCTAATACAACGCAGGTCCATCTGGTAGTGATGCAAT
TGCACCTTTCAAACAGTTGTCATGCAACAACTGCTATTATGCGGTATTTAGCTATCGTTTTCCAATAGTTATC
CCCCGCTACCAGGCAGGTTACCTACCGCGTTACTCACCCGTTCGCAACTCATCCGGAGAAGCAAGCTCCTCCT
CAGCGTTCTACTGCCATGTATTTAGGCACGCCGCCAGCGTTCGTCCTGAGCCAGGATCAAACTCT
>486
CTGCTGCCGCCCGTAGGAGTCTGGGCCGTGTCTCAGTCCCAGTGTGGCCGATCACCCTCTCAGGTCGGCTATG
TATCGTCGCCTTGGTGAGCCCTTACCCCACCAACTAGCTAATACAACGCAGGTCCATCTGGTAGTGATGCAAT
TGCACCTTTCAAACAGTTGTCATGCAACAACTGCTATTATGCGGTATTTAGCTATCGTTTTCCAATAGTTTAT
CCCCCGCTACCAGGCAGGTTACCTACCGCGTTACTCACCCGTTCGCAACTCATCCGGAGAAGCAAGCTCCTCC
TCAGCGTTCTACTTGCCATGTATTTAGGCACGCCGCCAGCGTTCGTCCTGAGCCAGGATCAAACTCT
>487
CTGCTGCCGCCCGTAGGAGTCTGGGCCGTGTCTCAGTCCCAGTGTGGCCGATCACCCTCTCAGGTCGGCTATG
TATCGTCGCCTTGGTGAGCCGTTACCCCACCAACTAGCTAATACAACGCAGGTCCATCTGGTAGTGGTGCAAT
TGCACCTTTTAAGCAAATGTCATGCAACATCTACTCTTATGCGGTATTAGCTATCGTTTTCCAATAGTTATCC
CCCGCTACCAGGCAGGTTACCTACGCGTTACTCACCCGTTCGCAACTCATCCGGAGAAGCAAGCTCCTCCTTC
AGCGTTCTACTTGCATGTATTAGGCACGCCGCCAGCGTTCGTCCTGAGCCAGGATCAAACTCT
>488
CTGCTGCCGCCCGTAGGAGTTTGGGCCGTATCTCAGTCCCAATCGTGGCCGGTCACCCTCTCAGGCCGGCTAC
CCGTCGAAGCCTTGGTGAGCCACTACCTCACCAACAAGCTGATAGGCCGCGAGTCCATCCCAGACCAGAAAAC
CTTTCCAACAAACCCCATGCGAGGAAAGCTGAATATCCGGTATTAGCACCTGTTTTCCAGAAGTTATCCCAGA
GACTGGGGCAGGTTACTCACGTGTTACTCACCCGTTCGCCACTCGTGTACCCCCCCGAAGAGGCCTTACCGTT
TCGACTTGCATGTGTTTAAGCACGCCGCCAGCGTTCGTCCTGAGCCAGGATCAAACTCT
>489
CTCTGCCGCCCGTAGGAGTCTGGGCCGTGTCTCAGTCCCAGTGTGGCCGATCACCCTCTCAGGTCGGCTATGT
ATCGTCGCCTTGGTGAGCCGTTACCTCACCAACTAGCTAATACAACGCAGGTCCGTCTGGTAGTGATGCAATT
GCACCTTTAATTGACTATCATGCAATAATCAATTTATGCGGTATTAGCTATCGTTTCCAATAGTTATCCCCCG
CTACCAGGCAGGTACCTACGCGTACTCACCCGTTCGCAACTCATCCAGAGAAGCAAGCTCCTCCTCAGCGTTC
TACTGCATTGTA
>490
CTGCTGCCGCCCGTAGGAGTCTGGGCCGTGTCTCAGTCCCAGTGTGGCCGATCACCCTCTCAGGTCGGCTATG
TATCGTCGCCTTGGTGAGCCGTTACCTCACCAACTAGCTAATACAACGCAGGTCCATCTGGTAGTGATGCAAT
TGCACCTTTTAATTGACTATCATGCAATAATCAATTTTATGCGGTATTAGCCTATCGTTTTCCAATAGTTATC
CCCCGCTACCAGGCAGGTTACCTACGCGTACTCACCCGTTCGCAACTCATCCAGAGAAGCAAGCTCCTCCTTC
AGCGTTCTACTTGCATGTATTAGGCACGCCGCCAGCGTCGTCCTGAGCCAGGATCAAACTCT
>491
CTGCTGCCGCCCGTAGGAGTCTGGGGCCGTGTCTCAGTCCCAGTGTGGCCGATCACCCTCTCAGGTCGGCTAT
GTATCGTCGCCTTGGTGAGCCGTTACCCCACCAACTAGCTAATACAACGCAGGTCCATCTGGTAGTGGTGCAA
TTGCACCTTTTAAGCAAATGTCATGCAACATCTACTCTTATGCGGTATTAGCTATCGTTTCCAATAGTTATCC
CCCGCTACCAGGCAGGTTACCTACGCGTTACTCACCCGTTCGCAACTCATCCGGAGAAGCAAGCTCCTCCTCA
GCGTTCTACTTGCATGTATTTAGGCACGCCGCCAGCGTTCGTCCTGAGCCAGGATCAAACTCT
>492
CTGCTGCCGCCCGTAGGAGTCTGGGCCGTGTCTCAGTCCCAGTGTGGCCGATCACCCTCTCAGGTCGGCTATG
TATCGTCGCCTTGGTGAGCCGTTACCCCACCAACTAGCTAATACAACGCAGGTCCATCTGGTAGTGGTGCAAT

TGCACCTTTTAAGCAAATGTCATGCAACATCTACTCTTATGCGGTATTAGCTATCGTTTCCAATAGTTATCCC
CCGCTACCAGGCAGGTTACCTACGCGTTACTCACCCGTTCGCAACTCATCCGGAGAAGCAAGCTCCTCCTTCA
GCGTTCTACTGCACGTATTAGGCACGCCGCCAGCGTTCGTCCTGAGCCAGGATCAAACTCT
>493
CTGCTGCCGCCCGTAGGAGTCTGGGCCGTATCTCAGTCCCAATGTGGCCGTCCACCCTCTCAGGCCGGCTACC
CGTCGCCGCCTTGGTAGGCCATTACCCCACCAACAAGCTGATAGGCCGCGAGCTCATCCTACACCGAAAAAAC
TTCCAACCATCACACTAAAAATGGCTCCTATCCGGTATTAGACCCAGTTTCCCAGGCTTATCCCGAAGTGCAG
GGCAGATCACCCACGTGTTACTCACCCGTTCGCCACTCGAGTACCCTGCAAGCAGGGCCTTTCCGTTCGACTT
GCATGTGTTAAGCACGCCGCCAGCGTTCGTCCTGAGCCAGGATCAAACTCT
>494
CTGCTGCCGCCCGTAGGAGTTTGGGCCGTGTCTCAGTCCCAATGTGGCCGATCACCCTCTCAGGTCGGCTATG
CATCACGGCCTTTGGTGAGCCGTTACCTCACCAACTAGCTAATGCACCGCGGGTCCATCCATCAGCAGAAGCC
CGAAGCCTCTTTTTCCTCTTCAAACCATGCGGTTCGAAGACCTATGCGGTTTTTAGCATCCGTTTTCCGAATG
TTACTCCCCTCTGATGGGCAGGTTACCCCACGTGTTACTCGACCCGTTCGCCACTAGATTTGACCAGTGCAAG
CACCGGTCGCTCTCGTTCGACTGGCATGTATAGGCACGCCGCCAGCGTTCGTCCTGAGCCAGGATCAAACTCT
>495
CTGCTGCCGCCCGTAGGAGTCTGGGCCGTGTCTCAGTCCCAGTGTGGCCGATCACCCTCTCAGGTCGGCTATG
CATCGTCGCCTTGGTGAGCCCTTACCCCACCAACTAGCTAATACAACGCAGGTCCATCTGGTAGTGATGCAAT
TGCACCTTTCAAACAGTTGTCATGCAACAACTGCTATTTATGCGGTATTTAGCTATCGTTTTCCAATAGTTTA
TCCCCCCGCTACCAGGCAGGTTACCTACGCGTTACTCACCCGTTTCGCAACTCATCCGGAGAAGCAAGCTCCT
CCTTCAGCGTTCTACTTGCATGTATTTAGGCACGCCGCCAGCGTTCGTCCTGAGCCAGGATCAAACTCT
>496
CTGCTGCCGCCCGTAGGAGTCTGGGCCGTGTCTCAGTCCCAGTGTGGCCGATCACCCTCTCAGGTCGGCTATG
CATCGTCGCCTTGGTAGGCTTTACCCCCACCAACTAGCTAATGCACCGCAAAGCCATCTCATAGTGTTAGCAA
AACCAACTTTCAAACATCAACCATGCAGTTAATGCTATTTATCCGGTATTTAGCTATTCGTTTTTCCAATAGT
TTGTCCCAGTCTATGAGGCAGGTTTCTTTACGTGTTTACTCACCCGTTTCGCCGCTAACGTTGTTCGCGACAG
CAACCAGAA
>497
CTGCTGCCGCCCGTAGGAGTCTGGACCGTGTCTCAGTTCCAGTGTGGCCGATCACCCTCTCAGGTCGGCTATG
CATCGTTGCCTTGGTAAGCCGTTACCTTACCAACTAGCTAATGCAGCGCGGATCCGTCTATAAGTGACAGCAA
GACCGTCTTTTACTTTTGAACCATGCGGTTCAAAATATTATCCGGTATTAGCTCCGGTTTCCCGAAGTTATCC
CAGTCTTATAGGTAGGTTATCCACGTGTTACTCACCCGTCCGCCGCTAACATCAGAGAAGCAAGCTTCTCGTC
CGTTCGCTCGACTTGCATGTATTAGGCACGCCGCCAGCGTTCATCCTGAGCCAGGATCAAACTCT
>498
CTGCTGCCGCCCGTAGGAGTCTGGGCCGTGTCTCAGTCCCAGTGTGGCCGATCACCCTCTCAGGTCGGCTATG
TATCGTCGCCTTGGTGAGCCGTTACCCCACCAACTAGCTAATACAACGCAGGTCCATCTGGTAGTGGTGCAAT
TGCACCTTTTAAGCAAATGTCATGCAACATCTACTCTTATGCGGTATTAGCTATCGTTTCCAATAGTTATCCC
CCGCTACCAGGCAGGTTACCTACGCGTTACTCACCCGTTCGCAACTCATCCGGGGAAGCAAGCTCCTCATTCA
GCGTTCTACTTGACATGTATTAGGCACGCCGCCAGCGTTCGTCCTGAGCCAGGATCAAACTCT
>499
CTGCTGCCGCCTGTAGGAGTCTGGGCCGTGTCTCAGTCCCAGTGTGGCCGGTCGCCCTCTCAGGCCGGCTACC
CGTCATCGCCTTGGTGAGCCATTACCTCACCAACAAACTGATAGGCCGCGAGTCCATCCCCCCACCGAAAAAC
TTTCCACAACACACCATGCGGTGCGAAGTCATATCCGGTATTTACGCCCCGATTTTCCCGAGGTTATCCCGAA
GAGGGGGGCAGTTACGTCACGTGTTACTCACCCGTTCGCCACTAATCGGACAGGGCAAGCCCTGTCGTCATCG
TTTCGACTTGCAGTGTTAAGCACGCCGCCAGCGCTCGTCCTGC
>500
CTGCTGCCGCCCGTAGGAGTCTGGGCCGTGTCTCAGTCCCAGTGTGGCCGATCACCCTCTCAGGTCGGCTATG
TATCGTCGCCTTGGTGAGCCGTTACCTCACCAACTAGCTAATACAACGCAGGTCCATCTGGTAGTGATGCAAT
TTGCACCTTTAATTGACTATCATGCAATAATCAATTTTATGCGGTATTTAGCCTATCGTTTTCCAATAGTTTA
CTCCCCCGCTACCAGGCAGGTTACCTACGCGTTACTCACCCGTTCGCAACTCATCCGGAGAAGCAAGCTCCTC
CTTCAGCGTTCTACTTGCATGTATTTAGGCACGCCGCCAGCGTTCGTCCTGAGCCAGGATCAAACTCT
>501
CTGCTGCCGCCCGTAGGAGTCTGGGCCGTGTCTCAGTCCCAGTGTGGCCGATCACCCTCTCAGGTCGGCTATG
TATCGTCGCCTTGGTGAGCCGTTACCTCACCAACTAGCTAATACAACGCAGGTCCATCTGGTAGTGATGCAAT

TGCACCTTTTAATTGACTATCATGCAATAATCAATTTATGCGGTATTTAGCCTATCGTTTTCCAATAGTTTAC
TCCCCCCCGCTACCAGGCAGGTTACCTACGCGTTACTCACCCGTTTCGCAACTCATCCAGAGAAGCAAGCTCC
TCCTTTCAGCGTTCTACTTGCATGTATTTAGGCACGCCGCCAGCGTTCGTCCTGAGCCAGGATCAAACTCT
>502
CTGCTGCCGCCCGTAGGAGTCTGGGCCGTGTCTCAGTCCCAGTGTGGCCGATCACCCTCTCAGGTCGGCTATG
TATCGTCGCCTTGGTGAGCCGTTACCCCACCAACTAGCTAATACAACGCAGGTCCATCTGGTAGTGGTGCAAT
TGCACCTTTTAAGCAAATGTCATGCAACATCTACTCTTATGCGGTATAGCTATCGTTTTCCAATAGTTTATCC
CCCCCGCTACCAGGCAGGTTACCTACGCGTTACTCACCCGTTCGCAACTCATCCGGAGAAGCAAGCTCCTCCT
CAGCGTTCTACTGCATGTATTTAGGCACGCCGCCAGCGTCGTCCTGAGCCAGGATCAAACTCT
>503
CTGCTGCCGCCCGTAGGAGTCTGGGCCGTGTCTCAGTCCCAGTGTGGCCGATCACCCTCTCAGGTCGGCTATG
CATCGTCGCCTTGGTAGGCCTTTACCCCACCAACTAGCTAATGCACCGCAAAGCCATCTCATAGTGTTAGCAA
AACCAACTTTTAAACATCAACCATGCAGTTAATGCTGTTATCCGGTATTAGCTATCGTTTCCAATAGTTGTCC
CAGTCTATGAGGCAGGTTCTTTACGTGTTACTCACCCGTTCGCCGCTAAGTTCTTCCGGTGCAAGCACCAAAA
AACTTCGCTCGACTTGCATGTATTAGGCACGCCGCCAGCGTTCGTCCTGAGCCAGGATCAAACTCT
>504
CTGCTGCCGCCCGTAGGAGTCTGGGCCGTGTCTCAGTCCCAGTGTGGCCGATCACCCTCTCAGGTCGGCTATG
TATCGTCGCCTTGGTGAGCCGTTACCTCACCAACTAGCTAATACAACGCAGGTCCATCTGGTAGTGATGCAAT
TGCACCTTTTAATTGACTATCATGCAATAATCAATTTTATGCGGTATTTTAGCCTATCGTTTTTCCAATAGTT
TACTCCCCCCCCGCTACCAGGCAGGTTACCTACGCGTTACTCACCCGTTTCGCAACTCATCCGGAGAAGCAAG
CTCCTCCTCAGCGTTCTACTTG
>505
CTGCTGCCGCCCGTAGGAGTCTGGGCCGTGTCTCAGTCCCAGTGTGGCCGATCACCCTCTCAGGTCGGCTATG
TATCGTCGCCTTGGTGAGCCCTTACCCCACCAACTAGCTAATACAACGCAGGTCCATCTGGTAGTGATGCAAT
TGCACCTTTCAAACAGTTGTCATGCAACAACTGCTATTATGCGGTATTAGCTATCGTTTCCAATAGTTATCCC
CCGCTACCAGGCAGGTTACCTACGCGTTACTCACCCGTTCGCAACTCATCCAGAGAAGCAAGCTCCTCCTTCA
GCGTTCTACTTGCCATGTATTAGGCACGCCGCCAGCGTTCGTCCTGAGCCAGGATCAAACTCT
>506
CTGCTGCCGCCCGTAGGAGTCTGGACCGTGTCTCAGTTCCAGTGTGGCCGATCACCCTCTCAGGTCGGCTACG
TATCGTTGCCTTGGTAAGCCGTTACCTTACCAACTAGCTAATACGGCGCAGGTCCATCTATAAGTGATAGCAA
AGCCATCTTTCACTATCGAACCATGCGGTTCGAAATATTATCCGGTATTAGCTCCGGTTCCCGAAGTTATCCC
AGTCTTATAGGTAGGTTACCTACGTGTTACTCACCCGTCCGCCGCTAACGTCAAAGGAGCAAGCTCCTCGTCT
GTTCGCTCGACTTGCATGTATTAGGCACGCCGCCCAGCGTTCATCCTGAGCCAGGATCAAACTCT
>507
CTGCTGCCGCCCGTAGGAGTCTGGGCCGTGTCTCAGTCCCAGTGTGGCCGATCACCCTCTCAGGTCGGCTATG
TATCGTCGCCTTGGTGAGCCGTTACCCCACCAACTAGCTAATACAACGCAGGTCCATCTGGTAGTGGTGCAAT
TGCACCTTTTAAGCAAATGTCATGCAACATCTACTCTTATGCGGTATTAGCTATCGTTTTCCAATAGTTATCC
CCCGCTACCAGGCAGGTTACCTACGCGTTACTCACCCGTTCGCAACTCATCCGGAGAAGCAAGCTCCTCCTTC
AGCGTTCTACTTGCATGTATTTAGGCACGCCGCCAGCGTTCGTCCTGAGCCAGGATCAAACTCT
>508
CTGCTGCCGCCCGTAGGAGTCTGGGCCGTGTCTCAGTCCCAGTGTGGCCGATCACCCTCTTAGGTCGGCTATG
CATCGTCGCCTTGGTAGGCCTTTACCCCACCAACTAGCTAATGCACCGCAAAGCCATCTCATAGTGTTAGCAA
AACCAACTTTTAAACATCAACCATGCAGTTAATGCTGTTATCCGGTATTAGCTATCGTTTTCCAATAGTTTGT
CCCAGTCTATGAGGCAGGTTCTTTACGTGTTACTCACCCGTTCGCCGCTAAGTTTCGTTCGCGACACGAACAC
CAAAACTACGCTTCGCTGCATTGAGTAGGCAGCGCC
>509
CTGCTGCCGCCCGTAGGAGTCTGGGCCGTGTCTCAGTCCCAGTGTGGCCGATCACCCTCTCAGGTCGGCTATG
TATCGTCGCCTTGGTGAGCCCTTACCCCACCAACTAGCTAATACAACGCAGGTCCATCTGGTAGTGATGCAAT
TGCACCTTTCAAACAGTTGTCATGCAACAACTGCTATTATGCGGTATTAGCTATCGTTTTCCAATAGTTATCC
CCCGCTACCAGGCAGGTTACCTACGCGTTACTCACCCGTTCGCAACTCATCCAGAGAAGCAAGCTCCTCCTTC
AGCGTTCTACTTGCCATGTATTAGGCACGCCGCCAGCGTTCGTCCTGAGCCAGGATCAAACTCT
>510
CTGCTGCCGCCCGTAGGAGTCTGGGGCCGTGTCTCAGTCCCAGTGTGGCCGATCACCCTCTCAGGTCGGCTAT
GTATCGTCGCCTTGGTGAGCCGTTACCCCACCAACTAGCTAATACAACGCAGGTCCATCTGGTAGTGATGCAA

TTGCACCTTTTAAGTAAATGTCATGCAACATCTACTCTTATGCGGTATTAGCTATCGTTTTCCAATAGTTATC
CCCCGCTACCAGGCAGGTTACCTACGCGTTACTCACCCGTTCGAACTCATCCAGAAGAGCAAGCTCCTCCTCA
GCGTTCTACTGCATGTATTAGGCACGCCGCCAGCGTCGTCCTGAGCTAGGATCAAACTCT
>511
CTGCTGCCGCCCGTAGGAGTCTGGGCCGTGTCTCAGTCCCAGTGTGGCCGATCACCCTCTCAGGTCGGCTATG
TATCGTCGCCTTGGTGAGCCCTTACCCCACCAACTAGCTAATACAACGCAGGTCCATCTGGTAGTGATGCAGT
TGCACCTTTCAAACAGTTGTCATGCAACAACTGCTATTATGCGGTATTAGCTATCGTTTCCAATAGTTATCCC
CCGCTACCAGGCAGGTTACCTACGCGTTACTCACCCGTTCGCAACTCATCCAGAGAAGCAAGCTCCTCCTTCA
GCGTTCTACTTGCCATGTATTAGGCACGCCGCCAGCGTTCGTCCTGAGCCAGGATCAAACTCT
>512
CTGCTGCCGCCCGTAGGAGTCTGGGCCGTGTCTCAGTCCCAGTGTGGCCGATCACCCTCTCAGGTCGGCTATG
TATCGTCGCCTTGGTGAGCCGTTACCTCACCAACTAGCTAATACAACGCAGGTCCATCTGGTAGTGATGCAAT
TGCACCTTTTAATTGACTATCATGCAATAATCAATTTTATGCGGTATTAGCCTATCGTTTCCAATAGTTATCC
CCCGCTACCAGGCAGGTTACCTACGCGTTACTCACCCGTTCGCAACTCATCCAGAGAAGCAAGCTCCTCCTTC
AGCGTTCTACTTGCATGTATTAGGCACGCCGCCAGCGTTCGTCCTGAGCCAGGATCAAACTCT
>513
CTGCTGCCGCCCGTAGGAGTCTGGGCCGTGTCTCAGTCCCAGTGTGGCCGATCACCCTCTCAGGTCGGCTATG
TATCGTCGCCTTGGTGAGCCGTTACCCCACCAACTAGCTAATACAACGCAGGTCCATCTGGTAGTGGTGCAAT
TGCACCTTTTAAGCAAATGTCATGCAACATCTACTCTTATGCGGTATTAGCTATCGTTTCCAATAGTTATCCC
CCGCTACCAGGCAGGTTACCTACGCCGTTACTCACCCGTTCGCAACTCATCCGGAGAAACAAGCTCCTCCTTC
AGCGTTCTACTTGCATGTATTAGGCACGCCGCCAGCGTTCGTCCTGAGCCAGGATCAAACTCT
>514
CTGCTGCCGCCCGTAGGAGTCTGGGCCGTGTCTCAGTCCCAGTGTGGCCGATCACCCTCTCAGGTCGGCTATG
TATCGTCGCCTTGGTGAGCCCTTACCCCACCAACTAGCTAATACAACGCAGGTCCATCTGGTAGTGATGCAAT
TGCACCTTTCAAACAGTTGTCATGCAACAACTGCTATTATGCGGTATTTAGCTATCGTTTTTCCAATAGTTTA
TCCCCCGCTACCAGGCAGGTTACCTACCGCGTTACTCACCCGTTCGCAACTCATCCAGAGAAGCAAGCTCCTC
CTCAGCGTTCTACTTGCATGTATTAGGCACGCCGCCAGCGTCGTCCTGAGCCAGGATCAAACTCT
>515
CTGCTGCCGCCCGTAGGAGTCTGGGCCGTGTCTCAGTCCCAGTGTGGCCGATCACCCTCTCAGGTCGGCTATG
TATCGTCGCCTTGGTGAGCCGTTACCTCACCAACTAGCTAATACAACGCAGGTCCATCTGGTAGTGATGCAAT
TGCACCTTTTAATTGACTATCATGCAATAATCAATTTTATGCGGTATTAGCTATCGTTTCCAATAGTTATCCC
CCGCTACCAGGCAGGTTACCTACGCGTACTCACCCGTTCGCAACTCATCCAGAGAAGCAAGCTCCTCCTTCAG
CGTTCTACTTGCATGTATTAGGCACGCCGCCAGCGTTCGTCCTGAGCCAGGATCAAACTCT
>516
CTGCTGCCGCCCGTAGGAGTCTGGGCCGTGTCTCAGTCCCAGTGTGGCCGATCACCCTCTCAGGTCGGCTATG
TATCGTCGCCTTGGTGAGCCCTTACCCCACCAACTAGCTAATACAACGCAGGTCCATCTGGTAGTGATGCAAT
TGCACCTTTCAAACAGTTGTCATGCAACAACTGCTATTATGCGGTATTTAGCTATCGTTTTCCAATAGTTATC
CCCCGCTACCAGGCAGGTTACCTACCGCGTTACTCACCCGTTCGCAACTCATCCAGAGAAGCAAGCTCCTCCT
TCAGCGTTCTACTTGCCATGTATTAGGCACGCCGCCAGCGTCGTCCTGA
>517
CTGCTGCCGCCCGTAGGAGTCTGGGCCGTGTCTCAGTCCCAGTGTGGCCGATCACCCTCTCAGGTCGGCTATG
TATCGTCGCCTTGGTGAGCCGTTACCTCACCAACTAGCTAATACAACGCAGGTCCATCTGGTAGTGATGCAAT
TGCACCTTTTAATTGACTATCATGCAATAATCAATTTTATGCGGTATTAGCCTATCGTTTCCAATAGTTACTC
CCCCGCTACCAGGCAGGTTACCTACGTGTTACTCACCCGTTCGCAACTCATCCAGAGAAGCAAGCTCCTCCTT
CAGCGTTCTACTTGCATGTATTAGGCACGCCGCCAGCGTTCGTCCTGAGCCAGGATCAAACCTCT
>518
CTGCTGCCGCCCGTAGGAGTCTGGGCCGTGTCTCAGTCCCAGTGTGGCCGATCACCCTCTCAGGTCGGCTATG
TATCGTCGCCTTGGTGAGCCGTTACCTCACCAACTAGCTAATACAACGCAGGTCCATCTGGTAGTGATGCAAT
TTGCACCTTTTAATTGACTATCATGCAATAATCAATTTTATGCGGTATTTAGCCTATCGTTTTCCAATAGTTA
CTCCCCCCGCTACCAGGCAGGTTACCTACGCGTTACTCACCCGTTCGCAACTCATCCAGAGAAGCAAGCTCCT
CCTTCAGCGTTCTACTTGCATGTATTTAGGCACGCCGCCAGCGTTCGTCCTGAGCCAGGATCAAACTCT
>519
CTGCTGCCGCCCGTAGGAGTCTGGGCCGTGTCTCAGTCCCAGTGTGGCCGATCACCCTCTCAGGTCGGCTATG
TATCGTCGCCTTGGTGAGCCGTTACCCCACCAACTAGCTAATACAACGCAGGTCCATCTGGTAGTGATGCAAT

TGCACCTTTTAATTGACTATCATGCAATAATCAATTTTATGTGGTATTAGCTATCGTTTCCAATAGTTATCCC
CCGCTACCAGGCAGGTTACCTACGCGTTACTCACCCGTTCGCAACTCATCCAGAGAAGCAAGCTCCTCCTTCA
GCGTTCTACTTGCCATGTATTAGGCACGCCGCCAGCGTTCGTCCTGAGCCAGGATCAAACTCT
>520
CTGCTGCCGCCCGTAGGAGTCTGGGCCGTGTCTCAGTCCCAGTGTGGCCGATCACCCTCTCAGGTCGGCTATG
TATCGTCGCCTTGGTGAGCCGTTACCTCACCAACTAGCTAATACAACGCAGGTCCATCTGGTAGTGATGCAAT
TTGCACCTTTTAATTGACTATCATGCAATAATCAATTTTATGCGGTATTAGCCTATCGTTTCCAATAGTTACT
CCCCCGCTACCAGGCAGGTTACCTACGCGTTACTCACCCGTTCGCAACTCATCCAGAGAAGCAAGCTCCTCCT
TCAGCGTTCTACTTGCATGTATTACG
>521
CTGCTGCCGCCCGTAGGAGTCTGGGCCGTGTCTCAGTCCCAGTGTGGCCGATCACCCTCTCAGGTCGGCTATG
CATCGTCGCTTGGCAGGCCTTTACCCCACCAACTAGCTAATGCACCGCAAAGCCATCTCATAGTGTTAGCAAA
ACCAACTTTTAAACATCAACCATGCAGTTAATGCTGTTTATCCGGTATTTAGCTATCGTTTTCCAATAGTTTG
TCCCAGTCTATGAGGCAGGTTTTCTTTTACGTGTTTACTCACCCGTTTCGCCGCTAAGTTGTCGTCGACAGCG
AACCAGCAA
>522
CTGCTGCCGCCCGTAGGAGTCTGGGCCGTGTCTCAGTCCCAGTGTGGCCGATCACCCTCTCAGGTCGGCTATG
TATCGTCGCCTTGGTGAGCCCTTACCCCACCAACTAGCTAATACAACGCAGGTCCATCTGGTAGTGATGCAAT
TGCACCTTTCAAACAGTTGTCATGCAACAACTGCTATTACGCGGTATTAGCTATCGTTTCCAATAGTTATCCC
CCGCTACCAGGCAGGTTACCTACGCGTTACTCACCCGTTCGCAACTCATCCAGAGAAGCAAGCTCCTCCTCAG
CGTTCTACTTGCATGTATTAGGCACGCCGCCAGCGTTCGTCCTGAGCCAGGATCAAACTCT
>523
CTGCTGCCGCCCGTAGGAGTCTGGGCCGTGTCTCAGTCCCAGTGTGGCCGATCACCCTCTCAGGTCGGCTATG
TATCGTCGCCTTGGTGAGCCCTTACCCCACCAACTAGCTAATACAACGCAGGTCCATCTGGTAGTGATGCAAT
TGCACCCTTCAAACAGTTGTCATGCAACAACTGCTATTATGCGGTATTAGCTATCGTTTCCAATAGTTATCCC
CCGCTACCAGGCAGGTTACCTACGCGTACTCACCCGTTCGCAACTCATCCGGAGAAGCAAGCTCCTCCTCAGC
GTTCTACTTGCATGTATTAGGCACGCCGCCAGCGTTCGTCCTGAGCCAGGATCAAACTCT
>524
CTGCTGCCGCCCGTAGGAGTCTGGGCCGTGTCTCAGTCCCAGTGTGGCCGATCACCCTCTCAGGTCGGCTATG
TATCGTCGCCTTGGTGCAGCCTTACCCACCAACTAGCTAATACAACGCAGGTCCATCTGGTAGTGATGCAATT
GCACCTTTTCAACAGTTGTCATGCAACAACTGCTAATTTATGCGGTATTAGCTATCGTTTCCAATAGTTATCC
CCCGCTACCAGGCAGGTTACCTACGCGTTACTCACCCGTTCGCAACTCATCCAGAGAAGCAAGCTCCTCCTTC
AGCGTTCTACTTGCATGTATTAGGCACGCCGCCAGCGTTCGTCCTGAGCCAGGATCAAACTCT
>525
CTGCTGCCGCCCGTAGGAGTCTGGACCGTGTCTCAGTTCCAGTGTGGCCGATCACCCTCTCAGGTCGGCTACG
TATCGTTGCCTTGGTAAGCCGTTACCTTACCAACTAGCTAATACGGCGCAGGTCCATCTATAAGTGATAGCAA
AGCCATCTTTCACTATCGAACCATGCGGTTCGAAATATTATCCGGTATTAGCTCCGGTTTTCCCGAAGTTATC
CCAGTCTTATAGGTAGGTTTTACCTACGTGTTACTCACCCGTCCGCCGCTAACATCAAAGGAGCAAGCTCCTC
GTCTGTTCGCTCGACTTGCATGTATAGGCACGCCGTCCAGCGTTCATCCTGAGCCAGGATCAAACTCT
>526
CTGCTGCCGCCCGTAGGAGTCTGGGCCGTGTCTCAGTCCCAGTGTGGCCGATCACCCTCTCAGGTCGGCTATG
TATCGTCGCCTTGGTGAGCCGTTACCCCACCAACTAGCTAATACAACGCAGGTCCATCTGGTAGTGGTAGACA
TTGCACCTTTTAAGCAAATGTCATGCAACATCTACTGTTATGCGGTATTAGCTATCGTTTCCAATAGTTATCC
CCCGCTACCAGGCAGGTTACCTACGCGTTACTCACCCGTTCGCAACTCATCCGAGAAGACAGCTACGTCCTTC
CAGCCTCATGCT
>527
CTGCTGTCGCCCGTAGGAGTCTGGGCCGTGTCTCAGTCCCAGTGTGGCCGATCACCCTCTCAGGTCGGCTATG
TATCGTCGCCTTGGTGAGCCGTTACCCCACCAACTAGCTAATACAACGCAGGTCCATCTGGTAGTGGTGCAAT
TGCACCTTTTAAGCAAATGTCATGCAACATCTACTCTTATGCGGTATTAGCTATCGTTTTCCAATAGTTTATC
CCCCCGCTACCAGGCAGGTTACCTACGCGTTACTCACCCGTTCGCAACTCATCCGGAGAAGCAAGCTCCTCCT
CAGCGTTCTACTTGCATGTATTTAGGCACGCCGCCAGCGTTCGTCCTGAGCCAGGATCAAACTCT
>528
CTGCTGCCGCCCGTAGGAGTCTGGGCCGTGTCTCAGTCCCAGTGTGGCCGATCACCCTCTCAGGTCGGCTATG
TATCGTCGCCTTGGTGAGCCGTTACCTCACCAACTAGCTAATACAACGCAGGTCCATCTGGTAGTGATGCAAT

TGCACCTTTTAATTGACTATCATGCAATAATCAATTTTATGCGGTATTAGCCTATCGTTTTCCAATAGTTATC
CCCCGCTACCAGGCAGGTTACCTACGCGTTACTCACCCGTTCGCAACTCATCCAGAGAAGCAAGCTCCTCCTT
CAGCGTTCTACTTGCATGTATTAGGCACGCCGCCAGCGTTCGTCCTGAGCCAGGATCAAACTCT
>529
CTGCTGCCGCCCCGTAGGAGTCTGGGCCGTGTCTCAGTCCCAGTGTGGCCGATCACCCTCTCAGGTCGGCTAT
GTATCGTCGCCTTGGTGAGCCCTTACCCCACCAACTAGCTAATACAACGCAGGTCCATCTGGTAGTGATGCAA
TTGCACCTTTCAAACAGTTGTCATGCAACAACTGCTATTATGCGGTATTAGCTATCGTTTTCCAATAGTTATC
CCCCGCTACCAGGCAGGTTACCTACGCGTTACTCACCCGTTCGCAACTCATCCGGAGAAGCAAGCTCCTCCTT
CAGCGTTCTACTTGCATGTATTAGGCGCGCCGCCAGCGTTCGTCCTGAGCCAGGATCAAACTCT
>530
CTGCTGCCGCCCGTAGGAGTCTGGGCCGTGTCTCAGTCCCAGTGTGGCCGATCACCCTCTCAGGTCGGCTATG
TATCGTCGCCTTGGTGAGCCCTTACCCCACCAACTAGCTAATACAACGCAGGTCCATCTGGTAGTGATGCAAT
TGCACCTTTCAAACAGTTGTCATGCAACAACTGCTATTATGCGGTATTAGCTATCGTTTCCAATAGTTATCCC
CCGCTACCAGGCAGGTTACCTACGCGTTACTCACCCGTTCGCAACTCATCCAGAGAAGCAAGCTCCTCCTTCA
GCATTCTACTTGCATGTATTAGGCACGCCGCCAGCGTTCGTCCTGAGCCAGGATCAAACTCT
>531
CTGCTGCCGCCCGTAGGAGTCTGGGCCGTGTCTCAGTCCCAGTGTGGCCGGTCGCCCTCTCAGGCCGGCTACC
CGTCATCGCCTTGGTGAGCCATTACCTCACCAACAAACTGATAGGCCGCGAGTCCATCCCCCACCGAAAAACT
TTCCACAACACACCATGCGGTGCGAAGTCATATCCGGTATTACGCCCCGATTTCCCGAGGTTATCCCGAAGGA
GGGGGGCAGTTACTCACGTGTTACTCACCCGTTCGCCACTAATCGGACAGGGCAAGCCCTGTCGTCATCGTTC
GACTTGCATGTGTTAAGCACGCCGCCAGCGTTCGTCCTGAGCCAGGATCAAACTCT
>532
CTGCTGCCGCCCGTAGGAGTCTGGGCCGTGTCTCAGTCCCAGTGTGGCCGAACACCCTCTACCTGTCGAGCGT
ATGTATCGTCGCCTTGGTGAGCCCTTACCCCACCAACTAGCTAATACAACGCAGGTCCATCTGGTAGTGATAC
AATTGCACCTTTCAAACAGTTGTCATGCAACAACTGCTATTATGCGGTATTTAGCTATCGTTTCCAATAGTTA
TCCCCCGCTACCAGGCAGGTTACCTACGCGTTACTCACCCGTTCGCAACTCATCCAGAGAAGCAAGCCTCCTC
CTTCAGCGTTCTACTTGCCATGTATTTAGGCACGCCGCCAGCGTCGTCCTGAGCCAGGATCAAACTCT
>533
CTGCTGCCGCCCGTAGGAGTCTGGGCCGTGTCTCAGTCCCAGTGTGGCCGATCACCCTCTCAGGTCGGCTATG
TATCGTCGCCTTGGTGAGCCGTTACCCCACCAACTAGCTAATACAACGCAGGTCCATCTGGTAGTGGTGCAAT
TGCACCTTTTAAGCAAATGTCATGCAACATCTACTCTTATGCGGTATTAGCTATCGTTTTCCAATAGTTATCC
CCCGCTACCAGGCAGGTTACCTACGCGTTACTCACCCGTTCGCAACTCATCCGGAGAAGCAAGCTCCTCCTTC
AGCGTTCTACTTGCATGTATTAGGCACGCCGCCAGCGTTCGTCCTGAGCCAGGATCAAACTCT
>534
CTGCTGCCGCCCGTAGGAGTCTGGGCCGTGTCTCAGTCCCAGTGTGGCCGATCACCCTCTCAGGTCGGCTATG
CATCGTCGCCTTGGTAGGCCTTTACCCCACCAACTAGCTAATGCACCGCAAAGCCATCTCATAGTGTTAGCAA
AACCAACTTTTAAACATCAACCATGCAGTTAATGCTGTTATCCGGTATTAGCTATCGTTTCCAATAGTTGTCC
CAGTCTATGAGGCAGGTTCTTTACGTGTTACTCACCCGTTCGCCGCTAAGTTGTTCCGTCGACAGACATCCAA
AAGCTAACGTCTCGC
>535
CTGCTGCCGCCCGTAGGAGTCTGGGCCGTGTCTCAGTCCCAGTGTGGCCGATCACCCTCTCAGGTCGGCTATG
CATCGTCGCCTTGGTAGGCCTTTACCCCACCAACTAGCTAATGCACCGCAAAGCCATCTCATAGTGTTAGCAA
AACCAACTTTTAAACATCAACCATGCAGTTAATGCTGTTATCCGGTATTAGCTATCGTTTTTCCAATAGTTTT
GTCCCAGTCTATGAGGCAGGTTCTTTACGTGTTACTCACCCGTTCGCCGCTAAGTTTCGTTCGTCGACAGACA
CCAAAACTACGACTCGCTGACATTGC
>536
CTGCTGCCGCCCGTAGGAGGCTGGGCCGTGTCTCAGTCCCAGTGTGGCCGATCACCCTCTCAGGTCGGCTATG
TATCGTCGCCTTGGTGAGCCGTTACCCCACCAACTAGCTAATACAACGCAGGTCCATCTGGTAGTGGTGCAAT
TGCACCTTTTAAGCAAATGTCATGCAACATCTACTCTTATGCGGTATTAGCTATCGTTTTCCAATAGTTATCC
CCCGCTACCAGGCAGGTTACCTACGCGTACTCACCCGTTCGCAACTCATCCGGAGAAGCAAGCTCCTCCTTCA
GCGTTCTACTTGCATGTATTAGGCACGCCGCCAGCGTTCGTCCTGAGCCAGGATCAAACTCT
>537
CTGCTGCCGCCCGTAGGAGTCTGGGCCGTGTCTCAGTCCCAGTGTGGCCGATCACCCTCTCAGGTCGGCTATG
TATCGTCGCCTTGGTGAGCCCTTACCCCACCAACTAGCTAATACAACGCAGGTCCATCTGGTAGTGATGCAAT

TGCACCTTTCAAACAGTTGTCATGCAACAACTGCTATTATGCGGTATTTAGCTATCGTTTCCAATAGTTATCC
CCCGCTACCAGGCAGGTTACCTACGCGTTACTCACCCGTTCGCAACTCATCCGGAGAAGCAAGCTCCTCCTTC
AGCGTTCTACTTGCATGTATTTAGGCACGCCGCCAGCGTTCGTCCTGAGCCAGGATCAAACTCT
>538
CTGCTGCCGCCCGTAGGAGTCTGGGCCGTGTCTCAGTCCCAGTGTGGCCGATCACCCTCTCAGGTCGGCTATG
TATCGTCGCCTTGGTGAGCCGTTACCTCACCAACTAGCTAATACAACGCAGGTCCATCTGGTAGTGATGCAAT
TGCACCTTTTAATTGACTATCATGCAATAATCAATTTTATGCGGTATTAGCCTATCGTTTCCAATAGTTACTC
CCCCGCTACCAGGCAGGTTACCTACGCGTTACTCACCCGTTCGCAACTCATCCAGAGAAGCAAGCTCCTCCTT
CAGCGTTCTACTTGCATGTATTAGGCACGCCGCCAGCGTTCGTCCTGAGCCAGGATCAAACTCT
>539
CTGCTGCCGCCCGTAGGAGTCTGGGCCGTGTCTCAGTCCCAGTGTGGCCGATCACCCTCTCAGGTCGGCTATG
CATCGTCGCCTTGGTAGGCCTTTTACCCCACCAACTAGCTAATGCACCGCAAAGCCATCTCATAGTGTTAGCA
AAACCAACTTTTAAACATCAACCATGCAGTTTAATGCTGTTATCCGGTATTAGCTATCGTTTCCAATAGTTGT
CCCAGTCTATGAGGCAGGTTCTTTACGTGTTACTCACCCGTTCGCCGCTAAGTTGCGTCGCAGCAACCAGAAA
CTACGCAA
>540
CTGCTGCCGCCCGTAGGAGTCTGGGCCGTGTCTCAGTCCCAGTGTGGCCGATCACCCTCTCAGGTCGGCTATG
TATCGTCGCCTTGGTGAGCCGTTACCTCACCAACTAGCTAATACAACGCAGGTCCATCTGGTAGTGATGCAAT
TTGCACCTTTAATTGACTATCATGCAATAATCAATTTTATGCCGGTATTAGCCTATCGTTTCCAATAGTTACT
CCCCCGCTACCAGGCAGGTTACCTACGCGTACTCACCCGTTCGCAACTCATCCAGAGAAGCAAGCTCCTCCTT
CAGCGTTCTACTTGCATGTATTAGGCACGCCGCCAGCGTTCGTCCTGAGCCAGGATCAAACTCT
>541
CTGCTGCCGCCCGTAGGAGTCTGGGCCGTGTCTCAGTCCCAGTGTGGCCGGTCGCCCTCTCAGGCCGGCTACC
CGTCATCGCCTTGGTGAGCCATTACCTCACCAACAAACTGATAGGCCGCGAGTCCATCCCCCACCGAAAACTT
CCACAACACACCATGCGGTGCCGAAGTCATATCCGGTATTACGCCCCGATTTCCCGAGGTTATCCCGAAGAGG
GGGGCAGGTTACTCACGTGTTACTCACCCGTTCGCCACTAATCGGACAGGGCAAGCCCTGTCGTCATCGTTCG
ACTTGCATGTGTTAAGCACGCCGCCAGCGTTCGTCCTGAGCCAGGATCAAACTCT
>542
CTGCTGCCGCCCGTAGGAGTCTGGGCCGTATCTCAGTCCCAATGTGGCCGTCCACCCTCTCAGGCCGGCTACC
CGTCGCCGCCTTGGTAGGCCATTACCCCACCAACAAGCTGATAGGCCGCGAGCTCATCCTACACCGAAAAAAC
TTCCAACCATCACACTAAAAATGGCTCCTATCCGGTATTAGACCCAGCTTTCCCACGGCTTATCCCGAAGTGC
AGGGCAGATCACCCACGTGTTACTCACCCGTTCGCCACTCGAGTACCCTGCAAGCAGGGCCTTTCCGTTCGAC
TTGCAGTGTTAAGCACGCCGCCAGCGTTCGTCCTGAGCCAGGATCAAACTCT
>543
CTGCTGCCGCCCGTAGGAGTCTGGGCCGTGTCTCAGTCCCAGTGTGGCCGATCACCCTCTCAGGTCGGCTATG
TATCGTCGCCTTGGTGAGCCGTTACCCCACCAACTAGCTAATACAACGCAGGTCCATCTGGTAGTGGTGCAAT
TGCACCTTTTAAGCAAATGTCATGCAACATCTACTCTTATGCGGTATTAGCTATCGTTTCCAATAGTTATCCC
CCGCTACCAGGCAGGTTACCTACGCGTACTCACCCGTTCGCAACTCATCCGGAGAAGCAAGCTCCTCCTTCAG
CGTTCTACTTGCATGTATTAGGCACGCCGCCAGCGTTCGTCCTGAGCCAGAATCAAACTCT
>544
CTGCTGCCGCCCGTAGGAGTCTGGGCCGTGTCTCAGTCCCAGTGTGGCCGATCACCCTCTCAGGTCGGCTATG
TATCGTCGCCTTGGTGAGCCCTTACCCCACCAACTAGCTAATACAACGCAGGTCCATCTGGTAGTGACGCACA
TTGCACCTTTCAAACAGTTGTCATGCAACAACTGCTATTATGCGGTATTAGCTATCGTTTCCAATAGTTATCC
CCCGCTACCAGGCAGGTTACCTACGCGTTACTCACCCGTTCGCAACTCATCCAGAGAAGCAAGCTCCTCCTTC
AGCGTTCTACTTGCATGTATTAGGCACGCCGCCAGCGTTCGTCCTGAGCCAGGATCAAACTCT
>545
CTGCTGCCGCCCGTAGGAGTCTGGGCCGTGTCTCAGTCCCAGTGTGGCCGATCACCCTCTCAGGTCGGCTATG
TATCGTCGCCTTGGTGAGCCCTTACCCCACCAACTAGCTAATACAACGCAGGTCCATCTGGTAGTGATGCAAT
TGCACCTTTCAAACAGTTGTCATGCAACAACTGCTATTATGCGGTATTAGCTATCGTTTCCAATAGTTATCCC
CCGCTACCAGGCAGGTTACCTACGCGTTACTCACCCGTTCGCAACTCATCCAGAGAAGCAAGCTCCTCCTTCA
GCGTTCTACTTGCATGTATTAGGCACGCCGCCAGCGTTCGTCCTGAGCCAGGATCAAACTCT
>546
CTGCTGCCGCCCGTAGGAGTCTGGACCGTGTCGCAGTTCCAGTGTGGGGGTTATCCCTCTCGGGACCCCTAAC
CGTCATAGCCTTGGTAAGCCGTTACCTTACCAACTAGCTGATGGTGCCCAGGCCTCACTTTAAGCGTCTTGCG

ACTTTAGCATTACTGCCACATGGAGAATTATCCTACATTTCTATAGGTTATGCTCCACTTAAAGAAAGTTCCT
AGGTATTACTCACCCGTTCGCCGTGGGTCTAAACCCACTCGACTTGCATGTCTTAGGCACGCCGCCAGCGTTC
ATCCTGAGCCAGGATCAAACTCT
>547
CTGCTGCCGCCCGTAGGAGTCTGGGCCGTGTCTCAGTCCCAGTGTGGCCGATCACCCTCTCAGGTCGGCTATG
TATCGTCGCCTTGGTGAGCCGTTACCCCACCAACTAGCTAATACAACGCAGGTCCATCTGGTAGTGATGACAA
TTGCATCTTTTAAGTCAAATGTCATGCAACATCTACTATTATGCGGTATTAGCTATCGTTTTCCAATAGTTAT
CCCCCGCTACCAGGCAGGTTACCTACGCGTTACTCACCCGTTCGCAACTCATCCAGAGAAGCAAGCTCCCTCC
TCAGCGTTCTACTTGCATGTATTAGGCATGCCGCCAGCGTTCGTCCTGAGCCAGGATCAAACTCT
>548
CTGCTGCCGCCCGTAGGAGTCTGGGCCGTGTCTCAGTCCCAGTGTGGCCGATCACCCTCTCAGGTCGGCTATG
TATCGTCGCCTTGGTGAGCCGTTACCCCACCAACTAGCTAATACAACGCAGGTCCATCTGGTAGTGGTGCAAT
TGCACCTTTTAAGCAAATGTCATGCAACATCTACTCTTATGCGGTATTAGCTATCGTTTTCCAATAGTTTATC
CCCCGCTACCAGGCAGGTTACCTACGCGTTACTCACCCGTTCGCAACTCATCCGGAGAAGCAAGCTCCTCCTT
CAGCGTTCTACTTGCATGTATTAGGCACGCCGCCAGCGTTCGTCCTGAGCCAGGATCAAACTCT
>549
CTGCTGCCGCCCGTAGGAGTCTGGGCCGTGTCTCAGTCCCAGTGTGGCCGATCACCCTCTCAGGTCGGCTATG
CATCGTCGCCTTGGTGAGCCGTTACCTCACCAACTAGCTAATACAACGCAGGTCCATCTGGTAGTGATGCAAT
TGCACCTTTTAATTGACTATCATGCAATAATCAATTTTATGCGGTATTAGCTATCGTTTCCAATAGTTATCCC
CCCGCTACCAGGCAGGTTACCTACGCGTTACTCACCCGTTCGCAACTCATCCAGAGAAGCAAGCTCCTCCTTC
AACGTTCTACTTGCATGTATTTAGGCACGCCGCCAGCGTTCGTCCTGAGCCAGGATCAAACTCT
>550
CTGCTGCCGCCCGTAGGAGTCTGGGCCGTGTCTCAGTCCCAGTGTGGCTGATCATCCTCTCAGACCAGCTATG
GATCGTCGCCTTGGTAGGCCTTTACCCCACCAACTAGCTAATCCAACGCGGGCTCATCCATCTCCGATAAATC
TTTCTCCCGAAGGACGTATACGGTATTAGCACACGTTTCCATGCGTTATTCCGTAGGGATGGGTAGATTCCCA
CGCGTTACTCACCCGTCTGCCGCTCCCCTTGCGGGGCGCTCGACTTGCATGTGTTAAGCCTGCCGCCAGCGTT
CGTTCTGAGCCAGGATCAAACTCT
>551
CTGCTGCCGCCCGTAGGAGTCTGGAGCCGTGTCTCAGTCCCAGTGTGGCCGATCACCCTCTCAGGTCGGCTAT
GTATCGTCGCCTTGGTGAGCCGTTACCCCACCAACTAGCTAATACAACGCAGGTCCATCTGGTAGTGGTGCAA
TTGCACCTTTTAAGCAAATGTCATGCAACATCTACTCTTATGCGGTATTAGCTATCGTTTCCAATAGTTATCC
CCCGCTACCAGGCAGGTTACCTACGCGTTACTCACCCGTTCGCAACTCATCCGGAGAAGCAAGCTCCTCCTTC
AGCGTTCTACTTGCATGTATTAGGCACGCCGCCAGCGTTCGTCCTGAGCCAGGATCAAACTCT
>552
CTGCTGCCGCCCGTAGGAGTCTGGGCCGTGTCTCAGTCCCAGTGTGGCCGATCACCCTCTCAGGTCGGCTATG
TATCGTTGCCTTGGTGAGCCGTTACCTCACCAACTAGCTAATACAACGCAGGTCCATCTGGTAGTGATGCAAT
TGCACCTTTTAAGCAAATGTCATGCAACATCTACTATTGTGCGGTATTACGCTATCGTTTTCCAATAGTTATC
CCCCGCTACCAGGCAGGTTACCTACGCGTTACTCACCCGTTCGCAACTCATCCGGAGAAGCAAGCTCCTCCTT
CAGCGTTCTACTTGCATGTATTAGGCACGCCGCCAGCGTTCGTCCTGAGCCAGGATCAAACTCT
>553
CTGCTGCCGCCCGTAGGAGTCTGGGCCGTGTCTCAGTCCCAGTGTGGCCGATCACCCTCTCAGGTCGGCTATG
CATCGTCGCCTTGGTAGGCCTTTACCCCACCAACTAGCTAATGCACCGCAAAGCCATCTCATAGTGTTAGCAA
AACCAACTTTTAAACATCAACCATGCAGTTAATGCTGTTATCCGGTATTAGCTATCGTTTCCAATAGTTGTCC
CAGTCTATGAGGCAGGTTCTTTACGTGTTACTCACCCGTTCGCCGCTAAGTTCGTCGTCGCAGGCAACCAGTA
AACAACGTCAACTTCGACTTGCATAGTTATA
>554
CTGCTGCCGCCCGTAGGAGTCTGGGCCGTGTCTCAGTCCCAGTGTGGCCGATCACCCTCTCAGGTCGGCTATG
TATCGTCGCCTTGGTGAGCCGTTACCCCACCAACTAGCTAATACAACGCAGGTCCATCTGGTAGTGGTGCAAT
TGCACCTTTTTAAGCAAATGTCATGCAACATCTACTCTTTATGCGGTATTTAGCTGTCGTTTTCCAATAGTTT
ATCCCCCGCTACCAGGCAGGTTACCTACGCGTTACTCACCCGTTTCGCAACTCATCCGGAGAAGCAAGCTCCT
CCTCAGCGTTCTACTTGCATGTATTTAGGCACGCCGCCAGCGTTCGTCCTGAGCCAGGATCAAACTCT
>555
CTGCTGCCGCCCGTAGGAGTCTGGGCCGTGTCTCAGTCCCAGTGTGGCCGATCACCCTCTCAGGTCGGCTATG
TATCGTCGCCTTGGTGAGCCCTTACCCCACCAACTAGCTAATACAACGCAGGTCCATCTGGTAGTGATGCAAT

TGCACCTTTTCAAACAGTTTGTCATGCAACAACTGCTATTTATGCGGTATTTAGCTATCGTTTTTCCAATAGT
TTATCCCCCCCGCTACCAGGCAGGTTACCTACGCGTTACTCACCCGTTTCGAACTCATCCAGAGAAGCAAGCT
CCTCCTTTCAGC
>556
CTGCTGCCGCCCGTAGGAGTCTGGGCCGTGTCTCAGTCCCAGTGTGGCCGATCACCCTCTCAGGTCGGCTATG
TATCGTCGCCTTGGTGAGCCCTTACCCCACCAACTAGCTAATACAACGCAGGTCCATCTGGTAGTGATGCAAT
TGCACCTTTTCAAACAGTTTGTCATGCAACAACTGCTATTTATGCGGTTATTTAGCTATCGTTTTCCAATTAG
CTTATCCCCCCGCTACCAGGCAGGTTACCTTACGCGTTACTCACCCGTTCGCAACTCATCCAGAGAAGCAAGC
TCCTCCTTTCAGCGTTCTACTTGCATTGTATTTAGGCACGCCGCCAGCGTTCGTCCTGAGCCAGGATCAAACC
TCTT
>557
CTGCTGCCGCCCGTAGGAGTCTGGGCCGTGTCTCAGTCCCAGTGTGGCCGATCACCCTCTCAGGTCGGCTATG
TATCGTCGCCTTGGTGAGCCCTGTCACCCACCAACTACGAATACAACGCAGGTCCATCTGGTAGTGGTGCAAC
ATTGCACCTTTCAAACAGTTGTCATGCAACAACTGCTATTATGCGGTATTAGCTATCGTTTTCCAATAGTTAT
CCCCCGCTACCAGGCAGGTACCTACGCGTTACTCACCCGTTCGCAACTCATCCAGAAGAGCAAGCTCCTCCTC
AGCGTTCTACTGCATGTATTAGGCACGCCGCCAGCGTCGTCCTGAGCCAGGATCAAACTCT
>558
CTGCTGCCGCTCGTAGGAGTCTGGGCCGTGTCTCAGTCCCAGTGTGGCCGATCACCCTCTCAGGTCGGCTATG
TATCGTCGCCTTGGTGAGCCGTTACCCCACCAACTAGCTAATACAACGCAGGTCCATCTGGTAGTGGTGCAAT
TGCACCTTTTAAGCAAATGTCATGCAACATCTACTCTTATGCGGTATCAGCTATCGTTTTCCAATAGTTATCC
CCCGCTACCAGGCAGGTTACCTACGCGTTACTCACCCGTTCGCAACTCATCCAGAGAAGCAAGCTCCTCCTTC
AGCGTTCTACTTGCATGTATTAGGCACGCCGCCAGCGTTCGTCCTGAGCCAGGATCAAACCTCT
>559
CTGCTGCCGCCCGTAGGAGTCTGGGCCGTGTCTCAGTCCCAGTGTGGCCGATCACCCTCTCAGGTCGGCTATG
TATCGTCGCCTTGGTGAGCCGTTACCTCACCAACTAGCTAATACAACGCAGGTCCATCTGGTAGTGATGCAAT
TTGCACCTTTTAATTGACTATCATGCAATAATCAATTTTATGCGGTATTAGCCTATCGTTTCCAATAGTTATC
CCCCGCTACCAGGCAGGTTACCTACGCGTTACTCACCCGTTCGCAACTCATCCAGAGAAGCAAGCTCCTCCTC
AGCGTTCTACTTGCATGTATTAGGCACGCCGCCAGCGTCGTCCTGAGCCAGGATCAAACTCT
>560
CTGCTGCCGCCCGTAGGAGTCTGGGCCGTGTCTCAGTCCCAGTGTGGCCGATCACCCTCTCAGGTCGGCTATG
CATCGTCGCCTTGGTAGGCCTTTACCCCACCAACTAGCTAATGCACCGCAAAGCCATCTCATAGTGTTAGCAA
AACCAACTTTAAACATCAACCATGCAGTTAATGCTGTTATCCGGTATTAGCTATCGTTTTCCAATAGTTGTCC
CAGTCTATGAGGCAGGTTCTTTACGTGTTACTCACCCGTTCGCCGCTAACGTTTGTCGTGACAGACACACAGA
ACTCGCTGACATTCGT
>561
CTGCTGCCGCCCGTAGGAGTCTGGGCCGTGTCTCAGTCCCAGTGTGGCCGGTCGCCCTCTCAGGCCGGCTACC
CGTCATCGCCTTGGTGAGCCATTACCTCACCAACAAACTGATAGGCCGCGAGTCCATCCCCCACCGAAAAACT
TCCACAACACACCATGCGGTGCGAAGTCATATCCGGTATTACGCCCCGATTTCCCGAGGTTATCCCGAAGAGG
GGGGCAGGTTACGTCACGTGTTACTCACCCGTTCGCCACTAATCGGACAGGGCAAGCCCTGTCGTCATCGTTC
GACTTGCATGTGTTAAGCACGCCGCCAGCGTCCGTCCTGAGCCAGGATCAAACTCT
>562
CTGCTGCCGCCCGTAGGAGTCTGGGCCGTGTCTCAGTCCCAGTGTGGCCGATCACCCTCTCAGGTCGGCTATG
TATCGTCGCCTTGGTGAGCCCTTACCCCACCAACTAGCTAATACAACGCAGGTCCATCTGGTAGTGATGCAAT
TGCACCTTTCAAACAGTTGTCATGCAACAACTGCTATTATGCGGTATTAGCTATCGTTTCCAATAGTTATCCC
CCGCTACCAGGCAGGTTACCTACGCGTTACTCACCCGTTCGCAACTCATCCACGAGAAGCAAAGCCTCCTCCT
TCAGCGTTCTACTTGCCATGTATTAGGCACGCCGCCAGCGTTCGTCCTGAGCCAGGATCAAACTCT
>563
CTGCTGCCGCCCGTAGGAGTCTGGGCCGTGTCTCAGTCCCAGTGTGGCCGGTCGCCCTCTCAGGCCGGCTACC
CGTCATCGTCTTGGTGAGCCATTACCTCACCAACAAACTGATAGGCCGCGAGTCCATCCCCCACCGAATCAAT
CTTCCAACCCCCGCCATGCGACGAGGATTCATATCCGGTATTAGCCACGATTTCCCGAGGTTATCCCGAAGTG
GAGGGCAGGTTACTCACGTGTTACTCACCCGTTCGCCACTAATCAGACCGTGCAAGCACGGTCGTCATCGTTC
GACTTGCATGTGTTAAGCACGCCGCCAGCGTCGTCCTGAGCCAGGATCAAACTCT
>564

CTGCTGCCGCCCGTAGGAGTCTGGGCCGTGTCTCAGTCCCAGTGTGGCCGATCACCCTCTCAGGTCGGCTATG
TATCGTCGCCTTGGTGAGCCGTTACCCCACCAACTAGCTAATACAACGCAGGTCCATCTGGTAGTGGTGCAAT
TGCACCTTTTAAGCAAATGTCATGCAACATCTACTCTTATGCGGTATTAGCTATCGTTTTCCAATAGTTATCC
CCCGCTACCAGGCAGGTTACCTACGCGTACTCACCCGTTCGCAACTCATCCGGAGAAGCAAGCTCCTCCTCAG
CGTTCTACTTGCATGTATTTAGGCACGCCGCCAGCGTTCGTCCTGAGCCAGGATCAAACTCT
>565
CTGCTGCCGCCCGTAGGAGTCTGGGCCGTGTCTCAGTCCCAGTGTGGCCGATCACCCTCTCAGGTCGGCTATG
TATCGTCGCCTTGGTGAGCCCTTACCCCACCAACTAGCTAATACAACGCAGGTCCATCTGGTAGTGATGCAAT
TGCACCTTTCAAACAGTTGTCATGCAACAACTGCTATTATGCGGTATTTAGCTATCGTTTTTCCAATAGTTAT
CCCCCGCTACCAGGCAGGTTACCTACCGCGTTACTCACCCGTTCGCAACTCATCCAGAGAAGCAAGCTCCTCC
TTCAGCGTTCTACTTGCATGTATTAGGCACGCCGCCAGCGTTCGTCCTGAGCCAGGATAACCTCT
>566
CTGCTGCCGCCCGTAGGAGTCTGGACCGTGATCTCAGTTCCAGTGTGGCCCGATCACCCTCTCAGGTCGGCTA
CGTACTCGATTGCCTTGGGTAAGCCTGGTTACCTTACCAGCTAGCTAATACGGCGCAGGTCCATCTATAAGTG
ATAGCAAAGCCATCTTTCACTATCGAACCATGCAGTTCGAAATATTATCCGGTATTAGCTCCGGTTTTCCCGA
AGTTATCCCAGTCTTATAGGTAGGTTTTACCTACGTGTACTCACCCGTCCGCCGCTAACGTCAAGGAGCAAGC
TCCTCATCTGTCGCTCGACTCGCATGTATTAGGCACGCCGCCAGCGTTCATCCTGAGCCAGGATCAAACTCT
>567
CTGCTGCCGCCCGTAGGAGTCTGGGCCGTGTCTCAGTCCCAGTGTGGCCGATCACCCTCTCAGGTCGGCTATG
CATCGTCGCCTTGGTGAGCCGTTACCCCACCAACTAGCTAATACAACGCAGGTCCATCTGGTAGTGGTGCAAT
TGCACCTTTTAAGCAAATGTCATGCAACATCTACTCTTATGCGGTATTAGCTATCGTTTTCCAATAGTTATCC
CCCGCTACCAGGCAGGTTACCTACGCGTTACTCACCCGTTCGCAACTCATCCAGAGAAGCAAGCTCCTCCTTC
AGCGTTCTACTTGCATGTATTAGGCACGCCGCCAGCGTTCGTCCTGAGCCAGGATCAAACTCT
>568
CTGCTGCCGCCCGTAGGAGTCTGGGCCGTGTCTCAGTCCCAGTGTGGCCGATCACCCTCTCAGGTCGGCTATG
TATCGTCGCCTTGGTGAGCCGTTACCTCACCAACTAGCTAATACAACGCAGGTCCATCTGGTAGTGATGCAAT
TGCACCTTTTAATTGACTATCATGCAATAATCAATTTTATGCGGTATTAGCTATCGTTTCCAATAGTTATCCC
CCGCTACCAGGCAGGTTACCTACGCGTTACTCACCCGTTCGCAACTCGTCCAGAGAAGCGAGCTCCTCCTCAG
CGTTCTACTTGCATGTATTAGGCACGCCGCCAGCGTCGTCCTGAGCCAGGATCAAACTCT
>569
CTGCTGCCGCCCGTAGGAGTCTGGGCCGTGTCTCAGTCCCAGTGTGGCCGATCACCCTCTCAGGTCGGCTATG
TATCGTCGCCTTGGTGAGCCCTTACCCCACCAACTAGCTAATACAACGCAGGTCCATCTGGTAGTGATGCAAT
TGCACCTTTCAAACAGTTGTCATGCAACAACTGCTATTATGCGGTATTAGCTATCGTTTTCCAATAGTTATCC
CCCGCTACCAGGCAGGTTACCTACGCGTTACTCACCCGTTCGCAGCTCATCCAGAGAAGCAAGCCTCCTCCTT
CAGCGTTCTACTTGCATGTATTAGGCACGCCGCCAGCGTTCGTCCTGAGCCAGGATCAAACTCT
>570
CTGCTGCCGCCCGTAGGAGTCTGGGCCGTGTCTCAGTCCCAGTGTGGCCGATCACCCTCTCAGGTCGGCTATG
TATCGTCGCCTTGGTGAGCCCTTACCCCACCAACTAGCTAATACAACGCAGGTCCATCTGGTAGTGATGCAAT
TGCACCTTTCAAACAGTTGTCATGCAACAACTGCTATTATGCGGTATTAGCTATCGTTTCCAATAGTTATCCC
CCGCTACCAGGCAGGTCACCTACGCGTTACTCACCCGTTCGCAACTCATCCAGAGAAGCAAGCTCCTCCTTCA
GCGTTCTACTTGCATGTATTAGGCACGCCGCCAGCGTTCGTCCTGAGCCAGGATCAAACTCT
>571
CTGCTGCCGCCCGTAGGAGTCTGGGCCGTGTCTCAGTCCCAGTGTGGCCGATCACCCTCTCAGGTCGGCTATG
TATCGTCGCCTTGGTGAGCCGTTACCCCCACCAACTAGCTAATACAACGCAGGTCCATCTGGTAGTGATGCAA
TTGCACCTTTTAAGCAAATGTCATGCAACATCTACTCTTATGCGGTATTTAGCTATCGTTTTCCAATAGTTTA
TCCCCCGCTACCAGGCAGGTTACCTACGCCGTTACTCACCCGTTCGCAACTCATCCGGAGAAGCAAGCTCCTC
CTTCAAGCGTCCTACTTGCATGTATTTAGGCACGCCGCCAGCGTTCGTCCTGAGCCAGGATCAAACTCT
>572
CTGCTGCCGCCCGTAGGAGTCTGGGCCGTGTCTCAGTCCCAGTGTGGCCGATCACCCTCTCAGGTCGGCTATG
CATCGTCGCCTTGGTAGGCCTTTACCCCCCACCAACTAGCTAATGCACCGCAAAGCCATCTCATAGTGTTAGC
AAAACCAACTTTAAACATCAACCATGCAGTTAATGCTGTTTTACCGGTATTTAGCTATCGTTTTTCCCAATTA
GTTTGTTCCCCCAGTCTTATTGAGGCAGGTTTCTTTACGTGTTTACTCACCCCCCGTTTCGCCCGCTAAGTTT
GTTTGCGCAGCAACCAGAAACTA
>573

CTGCTGCCGCCCGTAGGAGTTCTGGGCCGTGTCTCAGTCCCAGTGTGGCCGATCACCCTCTCAGGTCGGCTAT
GTATCGTCGCCTTGGTGAGCCCTTACCCCACCAACTAGCTAATACAACGCAGGTCCATCTGGTAGTGATGACA
ATTTGCACCTTTCAAACAGTTGTCATGCAACAACTGCTATTTATGCGGTTATTTAGCTATCGTTTTCCAATAG
TTTATCCCCCCGCTACCAGGCAGGTTACCTACGCGTTACTCACCCGTTCGCAACTCATCCAGAGAAGCAAGCT
CCTCCTTTCAGCGTTCTACTTGCATTGTATTTAGGCACGCCGCCAGCGTTCGTCCTGAGCCAGGATCAAACTC
T
>574
CTGCTGCCGCCCGTAGGAGTCTGGGCCGTGTCTCAGTCCCAGTGTGGCCGATCACCCTCTCAGGTCGGCTATG
TATCGTCGCCTTGGTGAGCCCTTACCCCACCAACTAGCTAATACAACGCAGGTCCATCTGGTAGTGATGCAAT
TGCACCTTTCAAACAGTTGTCATGCAACAACTGCTATTATGCGGTATTAGCTATCGTTTCCAATAGTTATCCC
CCGCTACCAGGCAGGTTACCTACGCCGTTACTCACCCGTTCGCAACTCATCCAGAGAAGCAAGCTCCTCCTTC
AGCGTTCTACTTGCATGTATTAGGCACGCCGCCAGCGTTCGTCCTGAGCCAGGATCAAACTCT
>575
CTGCTGCCGCCCGTAGGAGTCTGGGCCGTGTCTCAGTCCCAGTGTGGCCGATCACCCTCTCAGGTCGGCTATG
TATCGTCGCCTTGGTGAGCCCTTACCCCACCAACTAGCTAATACAACGCAGGTCCATCTGGTAGTGATGCAAT
TGCACCTTTCAAACAGTTGTCATGCAACAACTGCTATTATGCGGTATTAGCTATCGTTTCCAATAGTTATCCC
CCGCTACCAGGCAGGTTACCTACGCGTTACTCACCCGTTCGCAACTCATCCGGAGAAGCAAGCTCCTCCTCAG
CGTTCTACTTGCATGTATTAGGCACGCCGCCAGCGTTCGTCCTGAGCCAGGGATCAAACTCT
>576
CTGCTGCCGCCCGTAGGAGTCTGGGCCGTGTCTCAGTCCCAGTGTGGCCGATCACCCTCTCAGGTCGGCTATG
TATCGTCGCCTTGGTGAGCCCTTACCCCACCAACTAGCTAATACAACGCAGGTCCATCTGGTAGTGATGCAAT
TGCACCTTTCAAACAGTTGTCATGCAACAACTGCTATTATGCGGCATTAGCTATCGTTTCCAATAGTTATCCC
CCGCTACCAGGCAGGTTACCTACGCGTTACTCACCCGTTCGCAACTCATCCAGAGAAGCAAGCTCCTCCTTCA
GCGTTCTACTTGCATGTATTAGGCACGCCGCCAGCGTTCGTCCTGAGCCAGGATCAAACTCT
>577
CTGCTGCCGCCCGTAGGAGTCTGGACCGTGTCTCAGTTCCAGTGTGGCTGATCACCCTCTCAGGTCGGCTATG
CATCGTTGCCTTGGTAAGCCGTTACCTTACCAACGAGCTAATGCAGCGCGGATCCATCTATAAGTGACAGCAA
GACCGTCTTTTACTTTTGAACCATGCGGTTCAAAATATTATCCGGTATTAGCTCCGGTTTTCCCGAAGTTATC
CCAGTCTTTATAGGTAGGTTATCCACGTGTTACTCACCCGTCCGCCGCTAACATCAGAGAAGCAAGCTTCTCG
TCCGTTCGCTCGACTTGCATGTATTAGGCACGCCGCCAGCGTTCATCCTGAGCCAGGATCAAACTCT
>578
CTGCTGCCGCCCGTAGGAGTCTGGGCCGTGTCTCAGTCCCAGTGTGGCCGATCACCCTCTCAGGTCGGCTATG
TATCGTCGCCTTGGTGAGCCCTTACCCCACCAACTAGCTAATACAACGCAGGTCCATCTGGTAGTGATGCAAT
TGCACCTTTCAAACAGTTGTCATGCAACAACTGCTATTTATGCGGTATTTAGCTATCGTTTTCCAATAGTTTA
TCCCCCCGCTACCAGGCAGGTTACCTACGCGTTACTCACCCGTTCGCAACTCATCCAGAGAGCAAGCTCCTCC
TTTCAGCGTTCTACTTGCCATTGTATTTAGGCACGCCGCCAGCGTCGTCCTGAGCCAGGATCAAACTCT
>579
CTGCTGCCGCCCGTAGGAGTCTGGGCCGTGTCTCAGTCCCAGTGTGGTCGATCACCCTCTCAGGTCGGCTATG
TATCGTCGCCTTGGTGAGCCGTTACCTCACCAACTAGCTAATACAACGCAGGTCCATCTGGTAGTGATGCAAT
TGCACCTTTTAAGCAAATGTCATGCAACATCTACTATTTATGCGGTATTTACGCTATCGTTTTTCCAATAGTT
TATCCCCCCCGCTACCAGGCAGGTTTACCTACGCGTTACTCACCCGTTTCGCAACTCATCCAGAGAAGCAAGC
TCCTCCTTTCAGCGTTCTACTTGCATTGTATTTAGGCACGCCGCCAGCGTTCGTCCTGAGCCAGGATCAAACT
CT
>580
CTGCTGCCGCCCGTAGGAGTCTGGGCCGTGTCTCAGTCCCAGTGTGGCCGATCGCCCTCTCAGGTCGGCTATG
TATCGTCGCCTTGGTGAGCCCTTACCCCACCAACTAGCTAATACAACGCAGGTCCATCTGGTAGTGATGCAAT
TGCACCTTTCAAACAGTTGTCATGCAACAACTGCTATTATGCGGTATTTAGCTATCGTTTTCCAATAGTTTAT
CCCCCGCTACCAGGCAGGTTACCTACGCGTTACTCACCCGTTCGCAACTCATCCAGAGAAGCAAGCTCCTCCT
TCAGCGTTCTACTTGCATGTATTAGGCACGCCGCCAGCGTCGTCCTGAGCCAGGATCAAACTCT
>581
CTGCTGCCGCCCGTAGGAGTCTGGGCCGTGTCTCAGTCCCAGTGTGGCCGATCACCCTCTCAGGTCGGCTACG
CATCGTTGCCTTGGTGGGCCGTTACCCCGCCAACCAGCTAATGCGCCGCAGGCCCATCCATCAGCGACAGCCG
AAACCGTCTTTCAACTCCCCACCCCGCGGTGGGGAGGATTATCCGGTATTAGCCACGGTTTTCCCGCAGTTAT

CCCCGGCTGATGGGCAGGTTGCCTACGTGTTACTCACCCGTCCGCCACTCGATCAGAGGGAGCAAGCTCCCTC
CTCTCGCGTTCGACTGCATGTATAGGCACGCCGCCAGCGTCATCCTGAGCCAGGATCAACTCT
>582
CTGCTGCCGCCCGTAGGAGTCTGGGCCGTGTCTCAGTCCCAGTGTGGCCGATCACCCTCTCAGGTCGGCTATG
TATCGTCGCCTTGGTGAGCCCTTACCCCACCAACTAGCTAATACAACGCAGGTCCATCTGGTAGTGATGCAAT
TGCACCTTTCAAACAGTTGTCATGCAACAACTGCTATTATGCGGTATTAGCTATCGTTTTCCAATAGTTATCC
CCCGCTACCAGGCAGGTTACCTACGCGTTACTCACCCGTTCGCAACTCATCCGGAGAAGCAAGCTCCTCCTCA
GCGTCTACTTGCATGTATTTAGGCACGCCGCCAGCGTTCGTCCTGAGCCAGGATCAAACTCT
>583
CTGCTGCCGCCCGTAGGAGTCTGGGCCGTGTCTCAGTCCCAGTGTGGCCGATCACCCTCTCAGGCCGGCTACC
CGTCGTCGCCTTGGTGAGCCATTACCTCACCAACAAGCTGATAGGCCGTGAGCCCATCCAAGACCAGTAAACC
CTTTCCACACACCACCATGCGGTGACATGTAGCATCCAGTATTAGACCCAGTTTTCCCGGGCTTATCCCAGAG
TCAAGGGCAGGTTACTCACGTATTACTCACCCGTTCGCCACTCATCCACACCAGCAAGCTGGTGCTTTCAGCG
TCGACTTGCATGTGTTAAGCACGCCGCCAGCGTTCGTCCTGAGCCAGGATCAAACTCT
>584
CTGCTGCCGCCCGTAGGAGTCTGGGCCGTGTCTCAGTCCCAGTGTGGCCGATCACCCTCTCAGGTCGGCTATG
TATCGTCGCCTTGGTGAGCCGTTACCCCACCAACTAGCTAATACAACGCAGGTCCATCTGGTAGTGGTGCAAT
TGCACCTTTTAAGCAAATGTCATGCAACATCTACTCTTATGCGGTATTAGCTATCGTTTTCCAATAGTTTATC
CCCCGCTACCAGGCAGGTTACCTACGCGTTACTCACCCGTTCGCAACTCATCCGGAGAAGCAAGCTCCTCCTC
AGCGTTCTACTGCATGTATTTAGGCACGCCGCCAGCGTTCGTCCTA
>585
CTGCTGCCGCCCGTAGGAGTCTGGGCCGTGTCTCAGTCCCAGTGTGGCCGATTACCTTCTTAGGTCGGCTATG
TATCGTCGCCTTGGTGAGCCCTTACCCCACCAACTAGCTAATACAACGCAGGTCCATCTGGTAGTGATGCAAT
TGCACCTTTCAAACAGTTGTCATGACAACAACTGCTATTATGCGGTATTAGCTATCGTTTCCAATAGTTATCC
CCCGCTACCAGGCAGGTTACCTACGCGTTACTCACCCGTTTCGCAACTCATCCAGAGAAGCAAGCTCCTCCTT
TCAGCGTTCTACTTGCATGTATTTAGGCACGCCGCCAGCGTTCGTCCTGAGCCAGGATCAAACTCT
>586
CTGCTGCCGCCCGTAGGAGTCTGGGCCGTGTCTCAGTCCCAGTGTGGCCGATCACCCTCTCAGGTCGGCTATG
TATCGTCGCCTTGGTGAGCCGTTACCTCACCAACTAGCTAATACAACGCAGGTCCATCTGGTAGTGATGCAAT
TGCACCTTTTAATTGACTATCATGCAATAATCAATTTTATGCGGTATTAGCCTATCGTTTTCCAATAGTTACT
CCCCCGCTACCAGGCAGGTTACCTACGCGTTACTCACCCGTTCGCAACTCATCCAGAGAAGCAAGCTCCTCCT
TCAGCGTTCTACTTGCATGTATTAGGCACGCCGCCAGCGTTCGTCCTGAGCCAGGATCAAACTCT
>587
CTGCTCCGCCCGTAGGAGTCTGGGCCGTGTCTCAGTCCCAGTGTGGCCGATCACCCTCTCAGGTCGGCTATGT
ATCGTCGCCTTGGTGAGCCGTTACCCCACCAACTAGCTAATACAACGCAGGTCCATCTGGTAGTGGTGCAATT
GCAACCTTTTAAGCAAATGTCATGCAACATCTACTCTTAATGCCGGTATTAGCCTATCGTTTTCCAATAGTTA
TCCCCCGCTACCAGGCAGGTACCTACGCGTTACTCACCCGTTCGCAACTCATCCGGAGAAGCAAGCTCCTCCT
CAGCGTTCTACTGCATGTATTAGGCACGCCGCCAGCGTTCGTCCTGAGCCAGGATCAAACTCT
>588
CTGCTGCCGCCCGTAGGAGTCTGGGCCGTGTCTCAGTCCCAGTGTGGCCGATCACCCTCTCAGGTCGGCTATG
TATCGTCGCCTTGGTGAGCCGTTACCTCACCAACTAGCTAATACAACGCAGGTCCATCTGGTAGTGATGCAAT
TGCACCTTTTAATTGACTATCATGCAATAATCAATTTTATGCGGTATTAGCCTATCGTTTCCAATAGTTATCC
CCCGCTACCAGGCAGGTTACCTACGCGTACTCACCCGTTCGCAACTCATCCAGAGAACGCAAGCTCCTCCTTC
AGCGTTCTACTGCCATGTATTAGGCACGCCGCCAGCGTTCGTCCTGAGCCAGGCATCA
>589
CTGCTGCCGCCCGTAGGAGTCTGGGCCGTGTCTCAGTCCCAGTGTGGCCGATCACCCTCTCAGGTCGGCTATG
TATCGTCGCCTTGGTGAGCCGTTACCTCACCAACTAGCTAATACAACGCAGGTCCATCTGGTAGTGATGCAAT
TGCACCTTTTAAGTAAATGTCATGCAACATCTACTCTTATGCGGTATTAGCCTATCGTTTCCAATAGTTATCC
CCCGCTACCAGGCAGGTTACCTACGCGTTACTCACCCGTTCGCAACTCATCCAGAGAAGCAAGCTCCTCCTTC
AGCGTTCTACTGCATGTATTAGGCACGCCGCCAGCGTTCGTCCTGAGCCAGGATCAAACTCT
>590
CTGCTGCCGCCCGTAGGAGTCTGGGCCGTGTCTCAGTCCCAGTGTGGCCGATCACCCTCTCAGGTCGGCTATG
TATCGTCGCCTTGGTGAGCCGTTACCCCACCAACTAGCTAATACAACGCAGGTCCATCTGGTAGTGGTGCAAT
TGCACCTTTTAAGCAAATGTCATGCAACATCTACTCTTATGCGGTATTAGCTATCGTTTCCAATAGTTATCCC

CCGCTACCAGGCAGGTTACCTACGCGTACTCACCCGTTCGCAACTCATCCGGAGAAGCAAGCTCCTCCTTCAG
CGTTCTACTGCATGTATTAGGCACGCCGCCAGCGTTCGTCCTGAGCCAGGATCAAACTCT
>591
CTGCTGCCGCCCGTAGGAGTCTGGGCCGTGTCTCAGTCCCAGTGTGGCCGATCACCCTCTCAGGTCGGCTATG
TATCGTCGCCTTGGTGAGCCCTTACCCCACCAACTAGCTAATACAACGCAGGTCCATCTGGTAGTGATGCAAT
TGCACCTTTCAAACAGTTGTCATGCAACAACTGCTATTATGCGGTATTAGCTATCGTTTCCAATAGTTATCCC
CCGCTACCAGGCAGGTTACCTACGCGTTACTCACCCGTTCGCAACTCATCCAGAGAAGCAAGCTCCTCCTTCA
GCGTTCTACCTTGCATGTATTAGGCACGCCGCCAGCGTTCGTCC
>592
CTGCTGCCGCCCGTAGGAGTCTGGGCCGTGTCTCAGTCCCAGTGTGGCCGATCACCCTCTCAGGTCGGCTATG
TATCGTCGCCTTGGTGAGCCGTTACCTCACCAACTAGCTAATACAACGCAGGTCCATCTGGTAGTGATGCAAT
TGCACCTTTTAATTGACTATCATGCAATAATCAATTTTATGCGGTATTAGCTATCGTTTCCAATAGTTATCCC
CCGCTACCAGGCAGGTTACCTACGCGTTACTCACCCGTTCGCAACTCATCCAGAGAAGCAAGCTCCTCCTTCA
GCGTTCTACTTGCATGTATTAGGCACGCCGCCAGCGTTCGTCCTGAGCCAGGATCAAACTCT
>593
CTGCTGCCGCCCGTAGGAGTCTGGGCCGTGTCTCAGTCCCAGTGTGGCCGATCACCCTCTCAGGTCGGCTATG
TATCGTCGCCTTGGTGAGCCGTTACCCCACCAACTAGCTAATACAACGCAGGTCCATCTGGTAGTGGTGCAAT
TGCACCTTTTAAGCAAATGTCATGCAACATCTACTCTTATGCGGTATTAGCTATCGTTTCCAATAGTTATCCC
CCGCTACCAGGCAGGTTACCTACGCGTACTCACCCGTTCGCAACTCCATCCGGAGAAGCAAGCTCCTCCTCAG
CGTTCTACTTGCATGTATTAGGCACGCCGCCAGCGTTCGTCCTGAGCCAAGGATCAAACTCT
>594
CTGCTGCCGCCCGTAGGAGTCTGGGCCGTGTCTCAGTCCCAGTGTGGCCGGTCGCCCTCTCAGGCCGGCTACC
CGTCATCGCCTTGGTGAGCCATTACCTCACCAACAAACTGATAGGCCGCGAGTCCATCCCCCACCGAAAAACT
TTCCACAACACACCATGCGGTGCGAAGTCATATCCGGTATTACGCCCCGATTTCCCGAGGTTATCCCGAAGA
GGGGGGCAGGTTACGTCACGTGTTACTCACCCGTTCGCCACTAATCGGACAGGGCAAGCCCTGTTCATCATCG
TTTCGACTTGTTATGTTGTTAAGCACGCCGCCAGCGTTCGTCCTGAGCCAGGATCAAACTCTT
>595
CTGCTGCCGCCCGTAGGAGTCTGGGCCGTGTCTCAGTCCCAGTGTGGCCGATCACCCTCTCAGGTCGGCTATG
TATCGTCGCCTTGGTGAGCCCTTACCCCACCAACTAGCTAATACAACGCAGGTCCATCTGGTAGTGATGCAAT
TGCACCTTTCAAACAGTTGTCATGCAACAACTGCTATTATGCGGTATTAGCTATCGTTTCCAATAGTTATCCC
CCGCTACCAGGCAGGTTACCTACGCGTTACTCACCCGTTCGCAACTCATCCAGAGAAGCAAGCTCCTCCTTCA
GCGTTCTACTTGCCATGTATTACGGCACGCCGCCAGCGTTCGTCCTGAGCCAGGATCAAACTCT
>596
CTGCTGCCGCCCGTAGGGGTCTGGGCCGTGTCTCAGTCCCAGTGTGGCCGATCACCCTCTCAGGTCGGCTATG
TATCGTCGCCTTGGTGAGCCGTTACCTCACCAACTAGCTAATACAACGCAGGTCCATCTGGTAGTGATGCAAT
TGCACCTTTTAATTGACTATCATGCAATAATCAATTTTATGCGGTATTAGCTATCGTTTCCAATAGTTATCCC
CCGCTACCAGGCAGGTTACCTACGCGTACTCACCCGTTCGCAACTCATCCAGAGAAGCAAGCTCCTCCTTCAG
CGTTCTACTGCATGTATTAGGCACGCCGCCAGCGTCGTCCTGAGCCAGGATCAAACTCT
>597
CTGCTGCCGCCCGTAGGAGTCTGGGCCGTGTCTCAATCCCAGTGTGGCCGATCACCCTCTCAGGTCGGCTATG
TATCGTTGCCTTGGTGAGCCGTTACCTCACCAACTAGCTAATACAACGCAGGTCCATCTGGTAGTGGTGCAAT
TTGCACCTTTTAAGCAGCTATCATGCGATATCTACTGTTATGCGGTATTACGCTATCGTTTCCAATAGTTATC
CCCCGCTACCAGGCAGGTTACCTACGCGTTACTCACCCGTTCGCAACTCATCCGGAAAGAGCAAGCTCCTTCC
TCAGCGTTCTACTTGCATGTATTAGGCACGCCGCCAGCGTTCGTCCTGAGCCAGGATCAAACTCT
>598
CTGCTGCCGCCCGTAGGAGTCTGGGCCGTGTCTCAGTCCCAGTGTGGCCGATCACCCTCTCAGGTCGGCTATG
TATCGTCGCCTTGGTGAGCCCTTACCCCACCAACTAGCTAATACAACGCAGGTCCATCTGGTAGTGATGCAAT
TGCACCTTTCAAACAGTTGTCATGCAACAACTGCTATTATGCGGTATTAGCCATCGTTTCCAATAGTTATCCC
CCGCTACCAGGCAGGTTACCTACGCGTTACTCACCCGTTCGCAACTCATCCAGAGAAGCAAGCTCCTCCTTCA
GCGTTCTACTTGCATGTATTAGGCACGCCGCCAGCGTTCGTCCTGAGCCAGGATCAAACTCT
>599
CTGCTGCCGCCCGTAGGAGTCTGGGCCGTGTCTCAGTCCCAGTGTGGCCGATCACCCTCTCAGGTCGGCTATG
TATCGTCGCCTTGGTGAGCCGTTACCTCACCAACTAGCTAATACAACGCAGGTCCATCTGGTAGTGATGCAAT
TTGCACCTTTTAATTGACTATCATGCAATAATCAATTTTATGCGGTATTAGCCTATCGTTTTCCAATAGTTTA

TCCCCCGCTACCAGGCAGGTTACCTACGCGTTACCCACCCGTTCGCAACTCATCCAGAGAAGCAAGCTCCTCC
TTCAGCGTTCTACTTGCATGTATTAGGCACGCCGCCAGCGTTCGTCCTGAGCCAGGATCAAACTCT
>600
CTGCTGCCGCCCGTAGGAGTCTGGGCCGTGTCTCAGTCCCAGTGTGGCCGATCACCCTCTCAGGTCGGCTATG
TATCGTCGCCTTGGTGAGCCCTTACCCCACCAACTAGCTAATACAACGCAGGTCCATCTGGTAGTGATGCAAT
TGCACCTTTCAAACAGTTGTCATGCAACAACTGCTATTATGCGGTATTAGCTATCGTTTCCAATAGTTATCCC
CCGCTACCAGGCAGGTTACCTACGCGTTACTCACCCGTTCGCAACTCATCCAGAGAAGCAAGCTCCTCCTCAG
CGTTCTACTTGCATGTATTAGGCACGCCGCCAGCGTCGTCCTGAGCCAGGATCAAACTCT
>601
CTGCTGCCGCCCGTAGGAGTCTGGGCCGTGTCTCAGTCCCAGTGTGGCCGATCACCCTCTCAGGTCGGCTATG
TATCGTCGCCTTGGTGAGCCGTTACCTCACCAACTAGCTAATACAACGCAGGTCCATCTGGTAGTGGTGCAAT
TTGCACCTTTCAAGCAGCTATCATGCGATATCTACTGTTATGCGGTATTAGCTATCGTTTCCAATAGTTATCC
CCCGCTACCAGGCAGGTTACCTACGCGTACTCACCCGTTCGCAACTCATCCGGAAAGAGCGAGCTCCTTCCTT
CAGCGTTCTACTTGCATGTATTAGGCACGCCGCCAGCGTTCGTCCTGAGCCAGGATCAAACTCT
>602
CTGCTGCCGCCCGTAGGAGTCTGGGCCGTGTCTCAGTCCCAGTGTGGCCGATCACCCTCTCAGGTCGGCTATG
TATCGTCGCCTTGGTGAGCCGTTACCTCACCAACTAGCTAATACAACGCAGGTCCATCTGGTAGTGATGCAAT
TTGCACCTTTTAATTGACTATCATGCAATAATCAATTTTATGCGGTATTAGCCTATCGTTTTCCAATAGTTAC
TCCCCCGCTACCAGGCAGGTTACCTACGCGTTACTCACCCGTTCGCAACTCATCCAGAGAAGCAAGCCTCCTC
CTTCAGCGTTCTACTTGCATGT
>603
TGCTGCCGCCCGTAGGAGTCTGGGCCGTGTCTCAGTCCCAGTGTGGCCGATCACCCTCTCAGGTCGGCTATGT
ATCGTCGCCTTGGTGAGCCGTTACCTCACCAACTAGCTAATACAACGCAGGTCCATCTGGTAGTGATGCAATT
GCACCTTTTAATTGACTATCATGCAATAATCAACTTTTATGCGGTATTAGCTATCGTTTCCAATAGTTATCCC
CCGCTACCAGGCAGGTTACCTACGCGTTACTCACCCGTTCGCAACTCATCCAGAGAAGCAAGCCTCCTCCTTC
AGCGTTCTACTTGCATGTATTAGGCACGCCGCCAGCGTTCGTCCTGAGCCAGGATCAAACTCT
>604
CTGCTGCCGCCCGTAGGAGTCTGGACCGTGTCTCAGTTCCAGTGTGGCCGATCACCCTCTCAGGTCGGCTACG
TATCGTTGCCTTGGTAAGCCGTTACCTTACCAACTAGCTAATACGGCGCAGGTCCATCTATAAGTGATAGCAA
AGCCATCTTTCACTATCGAACCATGCGGTTCGAAATATTATCCGGTATTAGCTCCGGTTTTTCCCGAAGTTAT
CCCAGTCTTATAGGTAGGTTACCTACGTGTTACTCACCCGTCCGCCGCTAACGTCGAAGGAGCAAGCTCCTCG
TCTGTTCGCTCGACTTGCATGTATTAGGCACGCCGCCAGCGTTCATCCTGAGCCCAGGATCAAACCTCTATCC
A
>605
CTGCTGCCGCCCGTAGGAGTCTGGGCCGTGTCTCAGTCCCAGTGTGGCCGATCACCCTCTCAGGTCGGCTATG
TATCGTCGCCTTGGTGAGCCCTTACCCCACCAACTAGCTAATACAACGCAGGTCCATCTGGTAGTGATGCAAT
TGCACCTTTCAAACAGTTGTCATGCAACAACTGCTATTATGCGGTATTAGCTATCGTTTCCAATAGTTATCCC
CCGCTACCAGGCAGGTTACCTACGCGTTACTCACCCGTTCGCAACTCATCCAGAGAAGCAAGCTCCTCCTTCA
GCGTTCTACTTGCATGTATTAGGCACGCCGCCAGCGTTCGTCCTGAGCCAGGATCAAACTCT
>606
CTGCTGCCGCCCGTAGGAGTCTGGGCCGTGTCTCAGTCCCAGTGTGGCCGATCACCCTCTCAGGTCGGCTATG
TATCGTCGCCTTGGTGAGCCGTTACCCCACCAACTAGCTAATACAACGCAGGTCCATCTGGTAGTGATGCAAT
TGCACCTTTTAAGTAAATGTCATGCAACATCTACTCTTATGCGGTATTAGCTATCGTTTTCCAATAGTTATCC
CCCGCTACCAGGCAGGTTACCTACGCGTTACTCACCCGTTCGCAACTCATCCAGAAGAGCAAGCTCCTCCTCA
GCGTTCTACTGCATGTATTAGGCACGCCGCCAGCGTCGTCCTGAGCCAGGATCAAACTCT
>607
CTGCTGCCGCCCGTAGGAGTCTGGGCCGTGTCTCAGTCCCAGTGTGGCCGACCACCCTCTCAGGTCGGCTATG
TATCGTCGCCTTGGTGAGCCGTTACCTCACCAACTAGCTAATACAACGCAGGTCCATCTGGTAGTGATGCAAT
TTGCACCTTTAATTGACTATCATGCAATAATCAATTTTATGCGGTATTAGCCTATCGTTTCCAATAGTTACTC
CCCCGCTACCAGGCAGGTTACCTACGCGTTACTCACCCGTTCGCAACTCATCCAGAGAAGCAAGCTCCTCCTT
CAGCGTTCTACTTGCATGTATTAGGCACGCCGCCAGCGTTCGTCCTGAGCCAGGATCAAACTCT
>608
CTGCTGCCGCCCGTAGGAGTCTGGGCCGTGTCTCAGTCCCAGTGTGGCCGATCACCCTCTCAGGTCGGCTATG
TATCGTCGCCTTGGTGAGCCCTTACCCCACCAACTAGCTAATACAACGCAGGTCCATCTGGTAGTGATGCAAT

TGCACCTTTCAAACAGTTGTCATGCAACAACTGCTATTATGCGGTATTAGCTATCGTTTCCAATAGTTATCCC
CCGCTACCAGGCAGGTTACCTACCGCGTTACTCACCCGTTCGCAACTCATCCGGAGAAGCAAGCTCCTCCTTC
AGCGTTCTACTTGCCATGTATTAGGCACGCCGCCAGCGTTCGTCCTGAGCCAGGATCAAACTCT
>609
CTGCTGCCGCCCGTAGGAGTCTGGGCCGTGTCTCAGTCCCAGTGTGGCTGGTCGCCCTCTCAGACCAGCTACA
GATCGCAGGCTTGGTAGGCTTTTACCCCACCAACTACCTAATCTGCCATCGGCCGCTCCAATCGCGCGAGGTC
TTGCGATCCCCCGCTTTCACCCTCAGGTCGTATGCGGTATTAGCGTAGCTTTTCGCTACGTTATCCCCCCCAC
GACTGGGCACGTTCCGATGTATTACTCACCCGTTCGCCACTCTCAAGGGTTGCCCCTCTACCGTTCGACTTGC
ATGTGTAAGGCATACCGCCAGCGTTCAATCTGAGCCAGGATCAAACTCT
>610
CTGCTGCCGCCCGTAGGAGTCTGGGCCGTGTCTCAGTCCCAGTGTGGCCGATCACCCTCTCAGGTCGGCTATG
TATCGTTGCCTTGGTGAGCCGTTACCTCACCAACTAGCTAATACAACGCAGGTCCATCTGGCAGTGATGCAAT
TGCACCTTTTAAGCAAATGTCATGCAACATCTACTATTTATGCGGTATTTACGCTATCGTTTTCCAATAGCTA
TCCCCCGCTACCAGGCAGGTTACCTACGCGTTACTCACCCGTTTCGCAACTCATCCGCTCGGTGCAAGCACCA
AGCTTTCAGCGTTCTACTTGCATGTATTTAGGCACGCCGCCAGCGTTCGTCCTGAGCCAGGATCAAACTT
>611
CTGCTGCCGCCCGTAGGAGTCTGGGCCGTGTCTCAGTCCCAGTGTGGCCGATCACCCTCTCAGGTCGGCTATG
CATCGTCGCCTTGGTAGGCCTTTACCCCACCAACTAGCTAATGCACCGCAAAGCCATCTCATAGTGTTAGCAA
AACCAACTTTTAAACATCAACCATGCAGTTAATGCTGTTATCCGGTATTAGCTATCGTTTCCAATAGTTGTCC
CAGTCTATGAGGCAGGTTCTTTACGTGTTACTCACCCGTTCGCCGCTAAGTTTCGTTCGCGACACGAACACCA
AAACTACGCTTCGCTGCATTGAGAGCAGCGCCGCC
>612
CTGCTGCCGCCCGTAGGAGTCTGGGCCGTGTCTCAGTCCCAGTGTGGCCGATCACCCTCTCAGGTCGGCTATG
CATCGTCGCCTTGGTAGGCCTTTACCCCACCAACTAGCTAATACAACGCAGGTCCATCTGGTAGTGATGCAAT
TGCATCTTTTAAGCAAATGTCATGCAACATCTACTCTTATGCGGTATTAGCTATCGTTTTCCAATAGTTTATC
CCCCGCTACCAGGCAGGTTACCTACGCGTTACTCACCCGTTCGCAACTCATCCGGAGAAGCAAGCTCCTCCTT
CAGCGTTCTACTTGCATGTATTTAGGCACGCCGCCAGCGTTCGTCCTGAGCCAGGATCAAACTCT
>613
CTGCTGCCGCCCGTAGGAGTCTGGGCCGTGTCTCAGTCCCAGTGTGGCCGATCACCCTCTCAGGTCGGCTATG
TATCGTCGCCTTGGTGAGCCGTTACCCCACCAACTAGCTAATACAACGCAGGTCCATCTGGTAGTGGTGCAAT
TGCACCTTTTTAAGCAAATGTCATGCAACATCTACTCTTTATGCGGTATTTAGCATCGTTTTCCAATAGTTTA
TCCCCCCCGCTACCAGGCAGGTTACCTACGCGTACTCACCCGTTTCGCAACTCATCCGGAGAAGCAAGCTCCT
CCTCAGCGTTCTACTTGCATGTATTTAGGCACGCCGCCAGCGTTCGTCCTGAGCCAGGATCAAACTCT
>614
CTGCTGCCGCCCGTAGGAGTCTGGGCCGTGTCTCAGTCCCAGTGTGGCCGATCACCCTCTCAGGTCGGCTATG
TATCGTCGCCTTGGTGAGCCCTTACCCCACCAACTAGCTAATACAACGCAGGTCCATCTGGTAGTGATGCAAT
TGCACCTTTCAAACAGTTGTCATGCAACAACTGCTATTATGCGGTATTAGCTATCGTTTCCAATAGTTATCCC
CCGCTACCAGGCAGGTTACCTACGCGTTACTCACCCGTTCGCAACTCATCCAGAGAAGCAAGCTCCTCCTTCA
GCGTTCTACTTGCATGTATTAGGCACGCCGCCAGCGTTCGTCCTGAGCCAGGATCAAACTCT
>615
CTGCTGCCGCCCGTAGGAGTCTGGGCCGTGTCTCAGTCCCAGTGTGGCCGATCACCCTCTCAGGTCGGCTATG
TATCGTCGCCTTGGTGAGCCGTTACCTCACCAACTAGCTAATACAACGCAGGTCCATCTGGTAGTGATGCAAT
TGCACCTTTTAATTGACTATCATGCAATAATCAATTTTATGCGGTATTAGCCTATCGTTTCCAATAGTTATCC
CCCGCTACCAGGCAGGTTACCTACGCGTTACTCACCCGTTCGCAACTCATCCAGAGAAGCAAGCTCCTCCTCA
GCGTTCTACTTGCCATGTATTAGGCACGCCGCCAGCGTTCGTCCTGAGCCAGGATCAAACCTC
>616
CTGCTGCCGCCCGTAGGAGTCTGGGCCGTGTCTCAGTCCCAGTGTGGCCGATCACCCTCTCAGGTCGGCTATG
TATCGTTGCCTTGGTGAGCCGTTACCTCACCAACTAGCTAATACAACGCAGGTCCATCTGGTAGTGGTGCAAT
TGCACCTTTCAAGCAGCTATCATGCGATATCTACTGTTATGCGGTATTACGCTATCGTTTCCAATAGTTATCC
CCCGCTACCAGGCAGGTTACCTACGCGTACTCACCCGTTCGCAACTCATCCGGAAAGAGCAAGCTCCTCCTCA
GCGTTCTACTGCATGTATTAGGCACGCCGCCAGCGTTCGTCCTGAGCCAGGATCAAACTCT
>617
CTGCTGCCGCCCGTAGGAGTCTGGGCCGTGTCTCAGTCCCAGTGTGGCCGATCACCCTCTCAGGTCGGCTATG
TATCGTCGCCTTGGTGAGCCGTTACCCCACCAACTAGCTAATACAACGCAGGTCCATCTGGTAGTGGTGCAAT

TGCACCCTTTAAGCAAATGTCATGCAACATCTACTCTTATGCGGTATTAGCTATCGTTTCCAATAGTTATCCC
CCGCTACCAGGCAGGTTACCTACGCGTTACTCACCCGTTCGCAACTCATCCGGAGAAGCAAGCTCCTCCTTCA
GCGTTCTACTTGCATGTATTAGGCACGCCGCCAGCGTTCGTCCTGAGCCAGGATCAAACTCT
>618
CTGCTGCCGCCCGTAGGAGTCTGGGCCGTGTCTCAGTCCCAGTGTGGCCGATCACCCTCTCAGGTCGGCTATG
TATCGTCGCCTTGGTGAGCCGTTACCTCACCAACTAGCTAATACAACGCAGGTCCATCTGGTAGTGATGCAAT
TGCACCTTTTAATCGACTATCATGCAATAATCAATTTTATGCGGTATTTAGCTATCGTTTTCCAATAGTTACT
CCCCCCCGCTACCAGGCAGGTTACCTACGCGTTACTCACCCGTTCGCAACTCATCCAGAGAAGCAAGCTCCTC
CTTCAGCGTTCTACTTGCATGTATTAGGCACGCCGCCAGCGTTCGTCCTGAGCCAGGATCAAACTCT
>619
CTGCTGCCGCCCGTAGGAGTCTGGGCCGTGTCTCAGTCCCAGTGTGGCCGATCACCCTCTCAGGTCGGCTACG
CATCGTTGCCTTGGTGGGCCGTTACCCCGCCAACCAGCTAATGCGCCGCAGGCCCATCCATCAGCGACAGCCG
AAACCGTCTTTCAACTCCCCACCCCGCGGTGGAGAGGATTATCCGGTATTAGCCACGGTTTTCCCGCAGTTAT
CCCCGGCTGATGGGCAGGTTTGCCTACGTGTTTACTCACCCGTCCGCCACTCGATCAGAGGGAGCAAGCTCCC
TCCTCTCGCGTCGACTTGCATGTATTAGGCACGCCGCCAGCGTCATCCTGAGCCAGGATCGAAACTCT
>620
CTGCTGCCGCCCGTAGGAGTCTGGGCCGTGTCTCAGTCCCAGTGTGGCTGATCACCCTCTCAGGTCGGCTATG
TATCGTCGCCTTGGTGAGCCGTTACCCCACCAACTAGCTAATACAACGCAGGTCCATCTGGTAGTGATGCAAT
TGCACCTTTTAAGTAAATGTCATGCAACATCTACTCTTATGCGGTATTAGCTATCGTTTCCAATAGTTATCCC
CCGCTACCAGGCAGGTACCTACGCGTACTCACCCGTTCGCAACTCATCCAGAAGAGCAAAGCTCCTCCTTCAG
CGTTCTACTTGCCATGT
>621
CTGCTGCCGCCCGTAGGAGTCTGGGCCGTGTCTCAGTCCCAGTGTGGCCGATCACCCTCTCAGGTCGGCTATG
TATCGTCGCCTTGGTGAGCCGTTACCTCACCAACTAGCTAATACAACGCAGGTCCATCTGGTAGTGATGCAAT
TGCACCTTTTAATTGACTATCATGCAATAATCAATTTTATGCGGTATTAGCTATCGTTTTCCAATAGTTATCC
CCCCCCGCTACCAGGCAGGTTACCTACGCGTTACTCACCCGTTCGCAACTCATCCAGAGAAGCAAGCTCCTCC
TCAGCGTCTACTTGCAGTATTAGGCACGCCGCCAGCGTCGTCCTG
>622
CTGCTGCCGCCCGTAGGAGTCTGGGCCGTGTCTCAGTCCCAGTGTGGCCGATCACCCTCTCAGGTCGGCTATG
TATCGTCGCCTTGGTGAGCCGTTACCCCACCAACTAGCTAATACAACGCAGGTCCATCTGGTAGTGGTGCAAT
TGCACCTTTTAAGCAAATGTCATGCAACATCTACTCTTATGCGGTATTAGCTATCGTTTCCAATAGTTATCCC
CCGCTACCAGGCAGGTTACCTACGCGTACTCACCCGTTCGCAACTCATCCGGAGAAGCAAGCTCCTCCTTCAG
CGTTCTACTTGCATGTATTAGGCACGCCGCCAGCGTTCGTCCTGAGCCAGGATCAAACTCT
>623
CTGCTGCCGCCCGTAGGAGTCTGGAGCCGTGTCTCAGTCCCAGTGTGGCCGATCACCCTCTCAGGTCGGCTAT
GCATCGTCGCCTTGGTAGGCCTTTTACCCCACCAACTAGCTAATGCACCGCAAAGCCATCTCATAGTGTTAGC
AAAACCAACTTTCAAACATCAACCATGCAGTTAATGCTATTTATCCGGTATTTAGCTTATTCGTTTTCCAATA
GTTTGTCCCAGTCTTATTGAGGCAGGTTTCTTACGTGTTACTCACCCGTTTCGCCGCTAAGTTGTCCGTCGAC
AGCGAACCAGCAACTAACTCG
>624
CTGCTGCCGCCCGTAGGAGTCTGGACCGTGTCTCAGTTCCAGTGTGGCCGATCACCCTCTCAGGTCGGCTATG
CATCGTTGCCTTGGTAAGCCGTTACCTTACCAACTAGCTAATGCAGCGCGGATCCATCTATAAGTGACAGCAA
GACCGTCTTTTACTTTGAACCATGCGGTTTCAAAATATTTATCCGGTTATTTAGCTTCCGGTTTTTCCCGAAG
TTATCCCAGTCTTTATAGGTAGGTTTATCCACGTGTTTACTCACCCGTCCGCCGCTAACATCAGAGAAGCAAG
CTTTCTCGTCCGTTCGCTCGACTTGCATTGTATTTAGGCACGCCGCCAGCGTTCATCCTGAGCCAGGATCAAA
CTCT
>625
CTGCTGCCGCCCGTAGGAGTCTGGGCCGTGTCTCAGTCCCAGTGTGGCCGATCACCCTCTCAGGTCGGCTATG
CATCGTCGCCTTGGTGGGCCGTTACCCCACCAACTAGCTAATGCACCGCAAGGCCATCCACGGGCGACGCCGG
AGCGCCTTTTCATCAGCGGACCATGCGGTCCGATGACACATCCGGTATTTAGCCCCCCCCGATTTTTCTCGTT
GGTTTATCCCCCCAGACCTATGGGCAGGTTTTTCTTTTACGTGTTTACTCACCCCCGTCCGCCGCTCATTTCC
ACCCGTCGTCCCCCCCCGAAGGGTTTCAGACGGCTTTCCTGCGCTCGACTTGCATGTATTAGGCACGCCGCCA
GCGTCGTCCTGAGCCAGGATCAAACTCT
>626

CTGCTGCCGCCCGTAGGAGTCTGGGCCGTGTCTCAGTCCCAGTGTGGCCGGTCACCCTCTCAGGCCGGCTACC
CGTCGTCGCCTTGGTGAGCCATTACCTCACCAACAAGCTGATAGGCCGTGAGCCCATCCAAGACCAGTAAACC
CTTTCCACACACCACCATGCGGTGACATGTAGCATCCAGTATTAGACCCAGTTTCCCGGGCTTATCCCAGAGT
CAAGGGCAGGTTACTCACGTATTACTCACCCGTTCGCCACTCATCCACACCAGCAAGCTGGTGCTTCAGCGTT
CGACTTGCATGTGTTAAGCACGCCGCCAGCGTTCGTCCTGAGCCAGGATCAAACTCT
>627
CTGCTGCCGCCCGTAGGAGTCTGGGCCGTGTCTCAGTCCCAGTGTGGCCGATCACCCTCTCAGGTCGGCTATG
TATCGTCGCCTTGGTGAGCCCTTACCCCACCAACTAGCTAATACAACGCAGGTCCATCTGGTAGTGATGCAAT
TGCACCTTTCAAACAGTTGTCATGCAACAACTGCTATTATGCGGTATTTAGCTATCGTTTTCCAATAGTTATC
CCCCCGCTACCAGGCAGGTTACCTACGCGTTACTCACCCGTTCGCAACTCATCCAGAGAAGCAAGCTCCTCCT
TCAGCGTTCTACTTGCATGTATTAGGCACGCCGCCAGCGTTCGTCCTGAGCCAGGATCAAACTCT
>628
CTGCTGCCGCCGTAGGAGTCTGGGCCGTGTCTCAGTCCCAGTGTGGCCGATCACCCTCTCAGGTCGGCTATGT
ATCGTCGCCTTGGTGAGCCCTTACCCCACCAACTAGCTAATACAACGCAGGTCCATCTGGTAGTGATGCAATT
GCACCTTTCAAACAGTTGTCATGCAACAACTGCTATTATGCGGTATTAGCTATCGTTTCCAATAGTTATCCCC
CGCTACCAGGCAGGTTACCTACGCGTTACTCACCCGTTCGCAACTCATCCGGAGAAGCAAGCTCCTCCTTCAG
CGTTCTACTTGCATGTATTAGGCACGCCGCCAGCGTTCGTCCTGAGCCAGGATCAAACTCT
>629
CTGCTGCCGCCCGTAGGAGTCTGGGCCGTGTCTCAGTCCCAGTGTGGCCGATCACCCTCTCAGGTCGGCTATG
TATCGTCGCCTTGGTGAGCCGTTACCCCACCAACTAGCTAATACAACGCAGGTCCATCTGGTAGTGGTGCAAT
TGCACCTTTTAAGCAAATGTCATGCAACATCTACTCTTATGCGGTATTAGCTATCGTTTCCAATAGTTATCCC
CCGCTACCAGGCAGGTTACCTACGCGTTACTCACCCGTTCGCAACTCATCCGGAGAAGCAAGCTCCTCCTTCA
GCGTTCTACTTGCATGTATTAGGCACGCCGCCAGCGTTCGTCCTGAGCCAGGATCAAACTCT
>630
CTGCTGCCGCCCGTAGGAGTCTGGGCCGTGTCTCAGTCCCAGTGTGGCCGATCACCCTCTCAGGTCGGCTATG
TATCGTCGCCTTGGTGAGCCCTTACCCCACCAACTAGCTAATACAACGCAGGTCCATCTGGTAGTGATGCAAT
TGCACCTTTCAAACAGTTGTCATGCAACAACTGCTATTATGCGGTATTTAGCTATCGTTTTCCAATAGTTATC
CCCCGCTACCAGGCAGGTTACCTACGCGTTACTCACCCGTTCGCAACTCATCCGGAGAAGCAAGCTCCTCCTC
AGCGTCTACTTGCATGTATTTAGGCACGCCGCCAGCGTCGTCCTGAGCCAGGATCAAACTCT
>631
CTGCTGCCGCCCGTAGGAGTCTGGGCCGTGTCTCAGTCCCAGTGTGGCCGATCACCCTCTCAGGTCGGCTATG
TATCGTCGCCTTGGTGAGCCCTTACCCCACCAACTAGCTAATACAACGCAGGTCCATCTGGTAGTGATGCAAT
TGCACCTTTCAAACAGTTGTCATGCAACAACTGCTATTATGCGGTATTTAGCTATCGTTTTCCAATAGTTTAT
CCCCCCGCTACCAGGCAGGTTACCTACGCGTTACTCACCCGTTCGCAACTCATCCGGAGAAGCAAGCTCCTCC
CTCAGCGTTCTACTTGCCATGTATTTAGGCACGCCGCCAGCGTTCGTCCTGAGCCAGGATCAAACTCT
>632
CTGCTGCCGCCCGTAGGAGTCTGGGCCGTGTCTCAGTCCCAGTGTGGCCGATCACCCTCTCAGGTCGGCTATG
TATCGTCGCCTTGGTGAGCCGTTACCTCACCAACTAGCTAATACAACGCAGGTCCATCTGGTAGTGATGCAAT
TTGCACCTTTTAATTGACTATCATGCAATAATCAATTTTATGCGGTATTAGCCTATCGTTTCCAATAGTTACT
CCCCCGCTACCAGGCAGGTTACCTACGCGTTACTCACCCGTTCGCAACTCATCCAGAGAAGCAAGCTCCTCCT
TCAGCGTTCTACTTGCATGTATTAGGCACGCCGCCAGCGTTCGTCCTGAGCCAGGATCAAACTCT
>633
CTGCTGCCGCCCGTAGGAGTCTGGGCCGTGTCTCAGTCCCAGTGTGGCCGATCACCCTCTCAGGTCGGCTATG
TATCGTCGCCTTGGTGAGCCCTTACCCCACCAACTAGCTAATACAACGCAGGTCCATCTGGTAGTGATGCAAT
TGCACCTTTTAAGCAAATGTCATGCAACATCTACTATTATGCGGTATTAGCTATCGTTTCCAATAGTTATCCC
CCGCTACCAGGCAGGTTACCTACGCGTACTCACCCGTTCGAACTCATCCGCTCGGTGCAAGCACCAAGCTTCA
GCGTTCTACTTGCATGTATTACGGCACGCCGCCAGCGTCGTCCTGAG
>634
CTGCTGCCGCCCGTAGGAGTCTGGGCCGTGTCTCAGTCCCAGTGTGGCCGATCACCCTCTCAGGTCGGCTATG
CATCGTCGCCTTGGTAGGCCTTTACCCCACCAACTAGCTAATGCACCGCAAAGCCATCTCATAGTGTTAGCAA
AACCAACTTTTAAACATCAACCATGCAGTTAATGCTGTTATCCGGTATTAGCTATCGTTTCCAATAGTTTGTT
CCAGTCTATGAGGCAGGTTCTTTACGTGTTACTCACCCGTTCGCCGCTAAGTTTCTGTGGCGCAAGCACCAAG
AAAACTTCGCTCGACTTGCATGTATTAGGCACGCCGCCAGCGTCTCGCTCGTCGAACCAGATATCCAAGCCTC
GACATGCCTAGTAGACC

>635
CTGCTGCCGCCCGTAGGAGTCTGGGCCGTGTCTCAGTCCCAGTGTGGCCGATCACCCTCTCAGGTCGGCTATG
TATCGTCGCCTTGGTGAGCCGTTACCTCACCAACTAGCTAATACAACGCAGGTCCATCTGGTAGTGATGCAAT
TTGCACCTTTTAATTGACTATCATGCAATAATCAATTTTATGCGGTATTTAGCCTATCGTTTTCCAATAGTTT
ACTCCCCCGCTACCAGGCAGGTTACCTACGCGTTACTCACCCGTTTCGCAACTCATCCAGAGAAGCAAGCTCC
TCCTTTCAGCGTTCTACTTGCATGTATTTAGGCACGCCGCCAGCGTTCGTCCTGAGCCAGGATCAAACTCT
>636
CTGCTGCCGCCCGTAGGAGTCTGGGCCGTGTCTCAGTCCCAGTGTGGCCGGTCACCCTCTCAGGCCGGCCTAC
CCGTCGTCGCCTTGGTGAGCCATTACCTCACCAACAAGCTGATAGGCCGTGAGCCCATCCAAGACCAGTAAAC
CCTTTTCCACACACCACCATGCGGTGACATGTAGCATCCAGTATTTAGACCCAGTTTTCCCGGGCTTTATCCC
AGAGCAAGGGCAGGTTACTCACGTATTTACTCACCCGTTTCGCCACTCATCCACACCAGCAAGCTGGTGCTTT
CAGCGTTCGACTTGCATGTGTAAGCACGCCGCCAGCGTTCGTCCTGAGCCAGGACAAACTCT
>637
CTGCTGCCGCCCGTAGGAGTCTGGGCCGTGTCTCAGTCCCAGTGTGGCCGATCACCCTCTCAGGTCGGCTATG
TATCGTCGCCTTGGTGAGCCGTTACCCCACCAACTAGCTAATACAACGCAGGTCCATCTGGTAGTGGTGCAAT
TGCACCTTTTTAAGCAAATGTCATGCAACATCTACTCTTTATGCGGTATTTAGCTATCGTTTTTTCCAATAGT
TTATCCCCCCCGCTACCAGGCAGGTTACCTACGCGTTACTCACCCGTTTCGCAACTCATCCGGAGAAGCAAGC
TCCTCCTTTCAGCGTTCTACTTGCATGTATTTAGGCACGCCGCCAGCGTTCGTCCTGAGCCAGGATCAAACTC
T
>638
CTGCTGCCGCCCGTAGGAGTCTGGGCCGTGTCTCAGTCCCAGTGTGGCCGACCACCCTCTCAGGTCGGCTATG
TATCGTCGCCTTGGTGAGCCGTTACCCCACCAACTAGCTAATACAACGCAGGTCCATCTGGTAGTGGTGCAAT
TGCACCTTTTAAGCAAATGTCATGCAACATCTACTCTTATGCGGTATTAGCTATCGTTTCCAATAGTTATCCC
CCGCTACCAGGCAGGTTACCTACGCGTTACTCACCCGTTCGCAACTCATCCGGAGAAGCAAGCTCCTCCTTCA
GCGTTCTACTTGCATGTATTAGGCACGCCGCCAGCGTTCGTCCTGAGCCAGGATCAAACTCT
>639
CTGCTGCCGCCCGTAGGAGTCTGGGCCGTGTCTCAGTCCCAGTGTGGCCGATCACCCTCTCAGGTCGGCTATG
TATCGTCGCCTTGGTGAGCCCTTACCCCACCAACTAGCTAATACAACGCAGGTCCATCTGGTAGTGATGCAAT
TGCACCTTTCAAACAGTTGTCATGCAACAACTGCTATTATGCGGTATTAGCTATCGTTTTCCAATAGTTATCC
CCCGCTACCAGGCAGGTTACCTACGCGTTACTCACCCGTTCGCAACTCATCCGGAGAAGCAAGCTACCTCCTC
CAGCGTGTACTTGCATGTATTAGGCACGCCGCCAGCGTTCGTCCTGAGCCAGGATCAAACTCT
>640
CTGCTGCCGCCCGTAGGAGTCTGGGCCGTGTCTCAGTCCCAGTGTGGCCGATCACCCTCTCAGGTCGGCTATG
TATCGTCGCCTTGGTGAGCCGTTACCCCACCAACTAGCTAATACAACGCAGGTCCATCTGGTAGTGGTGCAAT
TGCACCTTTTAAGCAAATGTCATGCAACATCTACTCTTATGCGGTATTAGCTATCGTTTTCCAATAGTTTATC
CCCCCGCTACCAGGCAGGTTACCTACGCGTACTCACCCGTTCGCAACTCATCCGGAGAAGCAAGCTCCTCCTT
CAGCGTTCTACTTGCATGTATTAGGCACGCCGCCAGCGTTCGTCCTGAGCCAGGATCAAACTCT
>641
CTGCTGCCGCCCGTAGGAGTCTGGGCCGTGTCTCAGTCCCAGTGTGGCCGATCACCCTCTCAGGTCGGCTATG
TATCGTCGCCTTGGTGAGCCGTTACCTCACCAACTAGCTAATACAACGCAGGTCCATCTGGTAGTGATGCAAT
TGCACCTTTTAATTGACTATCATGCAATAATCAATTTTATGCGGTATTAGCCTATCGTTTTCCAATAGTTTAC
TCCCCCCCGCTACCAGGCAGGTTACCTACGCGTTACTCACCCGTTCGCAACTCATCCAGAGAAGCAAGCTCCT
CCTTCAGCGTTCTACTGCATGTATTTAGGCACGCCGCCAGCGTCGTCCTGAGCCAGGATCAAACTCT
>642
CTGCTGCCGCCCGTAGGAGTCTGGGCCGTGTCTCAGTCCCAGTGTGGCCGATCACCCTCTCAGGTCGGCTATG
TATCGTCGCCTTGGTGAGCCCTTACCCCACCAACTAGCTAATACAACGCAGGTCCATCTGGTAGTGGTGCAAT
TGCACCTTTTAAGCAAATGTCATGCAACATCTACTCTTATGCGGTATTTATCATCGTTTTCCAATAGTTTATC
CCCCCCGCTACCAGGCAGGTTACCTACGCGTTACTCACCCGTTCGCAACTCATCCGGAGAAGCAAGCTCCTCC
TCAGCGTTCTACTTGCATGTATTTAGGCACGCCGCCAGCGTTCGTCCTGAGCCAGGATCAAACTCT
>643
CTGCTGCCGCCCGTAGGAGTCTGGGCCGTGTCTCAGTCCCAGTGTGGCCGATCACCCTCTCAGGTCGGCTATG
TATCGTCGCCTTGGTGAGCCCTTACCCCACCAACTAGCTAATACAACGCAGGTCCATCTGGTAGTGATGCAAT
TGCACCTTTCAAACAGTTGTCATGCAACAACTGCTATTATGCGGTATTAGCTATCGTTTTCCAATAGTTACTC

CCCGCTACCAGGCAGGTTACCTACGCGTTACTCACCCGTTCGCAACTCATCCGGAGAAGCAAGCTCCTCCTCA
GCGTTCTACTTGCCATGTATTTAGGCACGCCGCCAGCGTTCGTCCTGAGCCAGGATCAAACTCT
>644
CTGCTGCCGCCCGTAGGAGTCTGGGCCGTGTCTCAGTCCCAGTGTGGCCGATCACCCTCTCAGGTCGGCTATG
TATCGTCGCCTTGGTGAGCCGTTACCTCACCAACTAGCTAATACAACGCAGGTCCATCTGGTAGTGATGCAAT
TGCACCTTTTAATTGACTATCATGCAATAATCAATTTTATGCGGTATTAGCCTATCGTTTTCCAATAGTTACT
CCCCCGCTACCAGGCAGGTTACCTACGCGTTACTCACCCGTTCGCAACTCATCCAGAGAAGCAAGCTCCTCCT
CAGCGTTCTACTGCATGTATTAGGCACGCCGCCAGCGTTCGTCCTGAGCCAGGATCAAACTCT
>645
CTGCTGCCGCCCGTAGGAGTCTGGGCCGTGTCTCAGTCCCAGTGTGGCCGATCACCCTCTCAGGTCGGCTATG
TATCGTCGCCTTGGTGAGCCCTTACCCCACCAACTAGCTAATACAACGCAGGTCCATCCGGTAGTGATGCAAT
TGCACCTTTCAAACAGTTGTCATGCAACAACTGCTATTATGCGGTATTAGCTATCGTTTTCCAATAGTTATCC
CCCGCTACCAGGCAGGTTACCTACCGCGTTACTCACCCGTTCGCAACTCATCCGGAGAAGCAAGCTCCTCCTT
CAGCGTTCTACTTGCATGTATTAGGCACGCCGCCAGCGTTCGTCCTGAGCCAGGATCAAACTCT
>646
CTGCTGCCGCCCGTAGGAGTCTGGGCCGTGTCTCAGTCCCAGTGTGGCCGATCACCCTCTCAGGTCGGCTATG
TATCGTCGCCTTGGTGAGCCGTTACCCCACCAACTAGCTAATACAACGCAGGTCCATCTGGTAGTGGTGCAAT
TGCACCTTTTAAGCAAATGTCATGCAACATCTACTCTTATGCGGTATTAGCTATCGTTTCCAATAGTTATCCC
CCGCTACCAGGCAGGTTACCTACGCGTTACTCACCCGTTCGCAACTCATCCGGAGAAGCAAGCTCCTCCTCAG
CGTTCTACTTGCATGTATTAGGCACGCCGCCAGCGTTCGTCCTGAGCCAGGATCAAACTCT
>647
CTGCTGCCGCCCGTAGGAGTCTGGGCCGTGTCTCAGTCCCAGTGTGGCCGATCACCCTCTCAGGTCGGCTACG
CATCGTGGCCTTGGTGAGCCGTTACCTCACCAACTAGCTAATGCGCCGCGGGCCCATCCTGTAGTGACAGCCG
AAGCCGCCTTTCAACATCGGACCATGAGGTCCGAAGGATTATTCGGTATTAGCCCCGGTTTCCCGGAGTTATC
CCAATCTACAGGGCAGGTTGCCCACGTGTTACTCACCCGTCCGCCGCTGAATCTAGAGGAGCAAGCTCCTCTG
TCATCCGCTCGACTGCATGTATTAGGCACGCCGCCAGCGTCGTCCTGAGCCAGGATCAAACTCT
>648
CTGCTGCCGCCCGTAGGAGTCTGGGCCGTGTCTCAGTCCCAGTGTGGCCGATCACCCTCTCAGGTCGGCTATG
TATCGTCGCCTTGGTGAGCCGTTACCTCACCAACTAGCTAATACAACGCAGGTCCATCTGGTAGTGATGCAAT
TGCACCTTTTAATTGACTATCATGCAATAATCAATTTTATGCGGTATTAGCCTATCGTTTCCAATAGTTACTC
CCCCGCTACCAGGCAGGTTACCTACGCGTTACTCACCCGTTCGCAACTCATCCAGAGAAGCAAGCTCCTCCTC
AGCGTTCTACTTGCATGTATTACGGCAGCCGCCAGCGTCGGTC
>649
CTGCTGCCGCCCGTAGGAGTCTGGGCCGTGTCTCAGTCCCAGTGTGGCCGATCACCCTCTCAGGTCGGCTATG
TATCGTCGCCTTGGTGAGCCGTTACCCCACCAACTAGCTAATACAACGCAGGTCCATCTGGTAGTGGTGCAAT
TGCACCTTTCAAACAGCTGTCATGCAACAACTGCTATTATGCGGTATTAGCTATCGTTTCCAATAGTCATCCC
CCGCTACCAGGCAGGTTACCTACGCGTACTCACCCGTTCGCAACTCATCCAGAGAAGCAAGCTCCTCCTCAGC
GTTCTACTTGCATGTATTAGGCACGCCGCCAGCGTTCGTCCTGAGCCAGGATCAAACTCT
>650
CTGCTGCCGCCCGTAGGAGTCTGGGCCGTGTCTCAGTCCCAGTGTGGCCGATCACCCTCTCAGGTCGGCTATG
TATCGTCGCCTTGGTGAGCCGTTACCTCACCAACTAGCTAATACAACGCAGGTCCATCTGGTAGTGATGCAAT
TGCACCTTTTAATTGACTATCATGCAATAATCAATTTTATGCGGTATTAGCCTATCGTTTCCAATAGTTATCC
CCCGCTACCAGGCAGGTTACCTACGCGTTACTCACCCGTTCGCAACTCATCCAGAGAAGCAAGCTCCTCCTTC
AGCGTTCTACTTGCATGTATTAGGCACGCCGCCAGCGTTCGTCCTGAGCCAGGATCAAACTCT
>651
CTGCTGCCGCCCGTAGGAGTCTGGGCCGTGTCTCAGTCCCAGTGTGGCCGATCACCCTCTCAGGTCGGCTATG
TATCGTCGCCTTGGTGAGCCGTTACCTCACCAACTAGCTAATACAACGCAGGTCCATCTGGTAGTGATGCAAT
TGCACCTTTTAATTGACTATCATGCAATAATCAATTTTATGCGGTATTAGCTATCGTTTCCAATAGTTATCCC
CCGCTACCAGGCAGGTTACCTACGCGTTACTCACCCGTTCGCAACTCATCCAGAGAAGCAAGCTCCTCCTCAG
CGTTCTACTGCATGTATTAGGCACGCCGCCAGCGTTCGTCCTGAGCCAGGGATCAAACTCT
>652
CTGCTGCCGCCCGTAGGAGTCTGGGCCGTGTCTCAGTCCCAGTGTGGCCGATCACCCTCTCAGGTCGGCTATG
TATCGTCGCCTTGGTGAGCCGTTACCTCACCAACTAGCTAATACAACGCAGGTCCATCTGGTAGTGATGCAAT
TGCACCTTTTAATTGACTATCATGCAATAATCAATTTTATGCGGTATTAGCTATCGTTTTCCAATAGTTATCC

CCCGCTACCAGGCAGGTTACCTACGCGTTACTCACCCGTTCGCAACTCATCCAGAGAAGCAAGCTCCCCCTTC
AGCGTTCTACTTGCATGTATTTAGGCACGCCGCCAGCGTTCGTCCTGAGCCAGGATCAAACTCT
>653
CTGCTGCCGCCCGTAGGAGTCTGGGCCGTGTCTCAGTCCCAGTGTGGCCGATCACCCTCTCAGGTCGGCTATG
TATCGTCGCCTTGGTGAGCCCTTACCCCACCAACTAGCTAATACAACGCAGGTCCATCTGGTAGTGATGCAAT
TGCACCTTTCAAACAGTTGTCATGCAACAACTGCTATTATGCGGTATTAGCTATCGTTTCCAATAGTTATCCC
CCGCTACCAGGCAGGTTACCTACCGCGTTACTCACCCGTTCGCAACTCATCCAGAGAAGCAAGCTCCTCCTTC
AGCGTTCTACTTGCCATGTATTAGGCACGCCGCCAGCGTTCGTCCTGAGCCAGGC
>654
CTGCTGCCGCCCTAGGAGTCTGGGCCGTGTCTCAGTCCCAGTGTGGCCGATCACCCTCTCAGGTCGGCTATGC
ATCGTCGCCTTGGTAGGCCTTTACCCCCACCAACTAGCTAATGCACCGCAAAGCCATCTCATAGTGTTAGCAA
AACCAACTTTTAAACATCAACCATGCAGTTAATGCTGTTTATCCGGTATTTAGCTATCGTTTTCCAATAGTTT
GTCCCCCAGTCTATGAGGCAGGTTTCTTTACGTGTTTACTCACCCCCGTTTCGCCGCTAAGTTTTCGTTCGGC
ACACGAAACT
>655
CTGCTGCCGCCCGTAGGAGTCTGGGCCGTGTCTCAGTCCCAGTGTGGCCGATCACCCTCTCAGGTCGGCTATG
TATCGTCGCCTTGGTGAGCCGTTACCTCACCAACTAGCTAATACAACGCAGGTCCATCTGGTAGTGATGCAAT
TTGCACCTTTTAATTGACTATCATGCAATAATCAATTTTATGCGGTATTAGCCTATCGTTTCCAATAGTTACT
CCCCCGCTACCAGGCAGGTTACCTACGCGTTACTCACCCGTTCGCAACTCATCCAGAGAAGCAAGCTCCTCCT
TCAGCGTTCTACTTGCATGTATTAGGCACGCCGCCAGCGTTCGTCCTGAGCCAGGATCAAACTCT
>656
CTGCTGCCGCCCGTAGGAGTCTGGGCCGTGTCTCAGTCCCAGTGTGGCCGATCACCCTCTCAGGTCGGCTATG
CATCGTCGCCTTGGTAGGCCTTTACCCCACCAACTAGCTAATGCACCGCAAAGCCATCTCATAGTGTTAGCAA
AACCAACTTTTAAACATCAACCATGCAGTTAATGCTGTTTATCCGGTATTTTAGCTATTCGTTTTTCCAATTA
GTTTGTCCCAGTCTTATTGAGGCAGGTTTTCTTTACGTGTTTACTCACCCGTTTCGCCGCTAAGTTTTCGTTC
GCGACACAAACATCCAAAG
>657
CTGCTGCCGCCCGTAGGAGTCTGGGCCGTGTCTCAGTCCCAGTGTGGCCGATCACCCTCTCAGGTCGGCTATG
TATCGTCGCCTTGGTGAGCCCTTACCCCACCAACTAGCTAATACAACGCAGGTCCATCTGGTAGTGATGCAAT
TGCACCTTTCAAACAGTTGTCATGCAACAACTGCTATTATGCGGTATTTAGCTATCGTTTTTCCAATAGTTTA
TCCCCCGCTACCAGGCAGGTTACCTTACGCGTTACTCACCCGTTCGCAACTCATCCGGAGAAGCAAGCTCCTC
CTTCAGCGTTCTACTTGCATGTATTTAGGCACGCCGCCAGCGTTCGTTCCTGAGCCAGGATCAAACTCT
>658
CTGCTGCCGCCCGTAGGAGTCTGGGCCGTGTCTCAGTCCCAGTGTGGCCGATCACCCTCTCAGGTCGGCTATG
CATCGTCGCCTTGGTAGGCCTTTACCCCACCAACTAGCTAGTAGTACCACGCAAAGCCATCTCATAGTGCTAG
CAAAACCAACTTTTAAACATCAACCATGCAGTTAATGCTGTTTATCCGGTATTTAGCTTATTCGTTTTCCAAT
TAGTTTTGTCCCAGTCTTATTGAGGCAGGTTTTCTTTACGTGTTTACTCACCCGTTTCGCCGCTAAGTTTTCG
TTCGCGAGCACGAAACAT
>659
CTGCTGCCGCCCGTAGGAGTCTGGGCCGTGTCTCAGTCCCAGTGTGGCCGATCACCCTCTCAGGTCGGCTATG
TATCGTCGCCTTGGTGAGCCCTTACCCCACCAACTAGCTAATACAACGCAGGTCCATCTGGTAGTGATGCAAT
TGCACCTTTCAAACAGTTGTCATGCAACAACTGCTATTTATGCGGTATTTAGCTATCGTTTTTCCAATAGTTT
ATCCCCCGCTACCAGGCAGGTTACCTACGCGTTACTCACCCGTTTCGCAACTCATCCAGAGAAGCAAGCTCCT
CCTTTCAGCGTTCTACTTGCCATTGTATTAGGCACGCCGCCAGCGTTCGTCCTGAGCCAGGATCAAACTCT
>660
CTGCTGCCGCCCGTAGGAGTCTGGGCCGTGTCTCAGTCCCAGTGTGGCCGATCACCCTCTCAGGTCGGCTATG
TATCGTCGCCTTGGTGAGCCCTTACCCCACCAACTAGCTAATACAACGCAGGTCCATCTGGTAGTGATGCAAT
TGCACCTTTCAAACAGTTGTCATGCAACAACTGCTATTATGCGGTATTAGCTATCGTTTTCCAATAGTTATCC
CCCGCTACCAGGCAGGTTACCTACGCGTTACTCACCCGTTCGCAACTCATCCAGAGAAGCAAGCTCCTCCTTC
AGCGTTCTACTTGCATGTATTAGGCACGCCGCCAGCGTCGTCCTGAGCCAGGATCAAACTCT
>661
CTGCTGCCGCCCGTAGGAGTCTGGGCCGTGTCTCAGTCCCAGTGTGGCCGATCACCCTCTCAGGTCGGCTATG
TATCGTCGCCTTGGTGAGCCCTTACCCCACCAACTAGCTAATACAACGCAGGTCCATCTGGTAGTGATGCAAT
TGCACCTTTCAAACAGTTGTCATGCAACAACTGCTATTATGCGGTATTTAGCTATCGTTTTCCAATAGTTTAT

CCCCCCGCTACCAGGCAGGTTACCTACGCGTTACTCACCCGTTCGCAACTCATCCAGAGAAGCAAGCTCCTCC
TTTCAGCGTTCTACTTGCCATGTATTTAGGCACGCCGCCAGCGTTCGTCCTGAGCCAGGATCAAACTCT
>662
CTGCTGCCGCCCGTAGGAGTCTGGGCCGTGTCTCAGTCCCAGTGTGGCCGATCACCCTCTCAGGTCGGCTATG
TATCGTCGCCTTGGTGAGCCCTTACCCCACCAACTAGCTAATACAACGCAGGTCCATCTGGCAGTGATGCAAT
TGCACCTTTCAAACAGTTGTCATGCAACAACTGCTATTATGCGGTATTAGCTATCGTTTCCAATAGTTATCCC
CCGCTACCAGGCAGGTTACCTACGCGTTACTCACCCGTTCGCAACTCATCCAGAGAAGCAAGCTCCTCCTTCA
GCGTTCTACTTGCATGTATTAGGCACGCCGCCAGCGTTCGTCCTGAGCCAGGATCAAACTCT
>663
CTGCTGCCGCCCGTAGGAGTCTGGGCCGTGTCTCAGTCCCAGTGTGGCCGATCACCCTCTCAGGTCGGCTATG
TATCGTCGCCTTGGTGAGCCGTTACCTCACCAACTAGCTAATACAACGCAGGTCCATCTGGTAGTGATGCAAC
TGCACCTTTTAATTGACTATCATGCAATAATCAATTTTATGCGGTATTTAGCCTATCGTTTTCCAATAGTTTA
CTCCCCCCCGCTACCAGGCAGGTTACCTACGCGTTACTCACCCGTTTCGCAACTCATCCAGAGAAGCAAGCTC
CTCCTTTCAGCGTTCTACTTGCATGTATTTAGGCACGCCGCCAGCGTTCGTCCTGAGCCAGGATCAAACTCT
>664
CTGCTGCCGCCCGTAGGAGTCTGGGCCGTGTCTCAGTTCCCAGTTGTGGCCGGTCGCCCTCTCAGGCCGGCTA
CCCGTCATCGCCTTGGTGAGCCATTACCTCACCAACAAACTGATAGGCCGCGAGTCCATCCCCCACCGAAAAA
CTTTCCACAACACACCATGCGGTGCGAAGTCATATCCGGTATTTACGCCCCGATTTTTCCCGAAGGTTATCCC
GAAGAGGGGGGCAGTTACGTCACGTGTTACTCACCCGTTTCGCCACTAATCGGACAGGGCAAGCCCTGTCGTC
ATCGTTTCGACTTGCATGTGTTTAAGCACGCCGCCAGCGTTCGTCCTGAGCCAGGATCAAACTCTTA
>665
CTGCTGCCGCCCGTAGGAGTCTGGGCCGTGTCTCAGTCCCAATGTGGCCGATCACCCTCTCAGGTCGGCTATG
TATCGTCGCCTTGGTGAGCCCTTACCCCACCAACTAGCTAATACAACGCAGGTCCATCTGGTAGTGATGCAAT
TGCACCTTTCAAACAGTTGTCATGCAACAACTGCTATTTATGCGGTATTTAGCTATCGTTTTCCAATAGTTTA
TCCCCCGCTACCAGGCAGGTTACCTACGCGTTACTCACCCGTTCGCAACTCATCCAGAGAAGCAAGCTCCTCC
TTCAGCGTTCTACTTGCATTGTATAGGCACGCCGCCAGCGTCGTCCTGAGCCAGGATCAAACTCT
>666
CTGCTGCCGCCCGTAGGAGTCTGGGCCGTGTCTCAGTCCCAGTGTGGCCGATCACCCTCTCAGGTCGGCTATG
CATCGTCGCCTTGGTAGGCCTTTACCCCACCAACTAGCTAATGCACCGCAAAGCCATCTCATAGTGTTAGCAA
AACCAACTTTTAAACATCAACCATGCAGTTAATGCTGTTATCCGGTATTTAGCTATCGTTTTTCCAATAGTTT
GTCCCAGTCTATGAGGCAGGTTCTTTACGTGTTACTCACCCGTTTCGCCGCTAAGTTTCGTCGCGACACGAAA
CACCAAAA
>667
CTGCTGCCGCCCGTAGGAGTCTGGGCCGTGTCTCAGTCCCAGTGTGGCCGATCACCCCCTCAGGTCGGCTATG
TATCGTCGCCTTGGTGAGCCCTTACCCCACCAACTAGCTAATACAACGCAGGTCCATCTGGTAGTGATGCAAT
TGCACCTTTCAAACAGTTGTCATGCAACAACTGCTATTATGCGGTATTAGCTATCGTTTCCAATAGTTATCCC
CCGCTACCAGGCAGGTTACCTACGCGTTACTCACCCGTTCGCAACTCATCCAGAGAAGCAAGCTCCTCCTTCA
GCGTTCTACTTGCATGTATTAGGCACGCCGCCAGCGTTCGTCCTGAGCCAGGATCAAACTCT
>668
CTGCTGCCGCCCGTAGGAGTCTGGGCCGTGTCTCAGTCCCAGTGTGGCCGATCACCCTCTCAGGTCGGCTATG
TATCGTCGCCTTGGTGAGCCGTTACCCCACCAACTAGCTAATACAACGCAGGTCCATCTGGTAGTGATGCAAT
TGCACCTTTTAAGTAAATGTCATGCAACATCCACTCTTATGCGGTATTAGCTATCGTTTCCAATAGTTATCCC
CCGCTACCAGGCAGGTTACCTACGCGTTACTCACCCGTTCGCAACTCATCCAGAAGAGCAAGCTCCTCCTTCA
GCGTTCTACTTGCATGTATTAGGCACGCCGCCAGCGTTCGTCCTGAGCCAGGATCAAACTCT
>669
CTGCTGCCGCCCGTAGGAGTCTGGGCCGTGTCTCAGTCCCAGTGTGGCCGATCACCCTCTCAGGTCGGCTATG
TATCGTCGCCTTGGTGAGCCCTTACCCCACCAACTAGCTAATACAACGCAGGTCCATCTGGTAGTGATGCAAT
TGCACCTTTCAAACAGTTGCATGCAACAACTGCTATTATGCGGTATTAGCTATCGTTTCCAATAGTTATCCCC
CGCTACCAGGCAGGTTACCTACCGCGTTACTCACCCGTTCGCAACTCATCCAGAGAAGCAAGCTCCTCCTTCA
GCGTTCTACTTGCATGTATTAGGCACGCCGCCAGCGTTCGTCCTGAGCCAAGGATCAAACTCT
>670
CTGCTGCCGCCCGTAGGAGTCTGGGCCGTGTCTCAGTCCCAGTGTGGCCGATCACCCTCTCAGGTCGGCTATG
CATCGTCGCCTTGGTAGGCCTTTACCCCACCAACTAGCTAATGCACCGCAAAGCCATCTCATAGTGTTAGCAA
AACCAACTTTTAAACATCAACCATGCAGTTAATGCTGTTTATCCGGTATTTTAGCTATCGTTTTTCCAATAGT

TTGTCCCAGTCTATGAGGCAGGTTTCTTTACGTGTTTACTCACCCCGTTTCGCCGCTAAGTTGTCGTCGCAGC
GACCAAGCAAC
>671
CTGCTGCCGCCCGTAGGAGTCTGGGCCGTGTCTCAGTCCCAGTGTGGCCGATCACCCTCTCAGGTCGGCTATG
TATCGTCGCCTTGGTGAGCCGTTACCCCACCAACTAGCTAATACAACGCAGGTCCATCTGGTAGTGGTGCAAT
TGCACCTTTTAAGCAAATGTCATGCAACATCTACTCTTATGCGGTATTAGCTATCGTTTTCCAATAGTTATCC
CCCGCTACCAGGCAGGTTACCTACGCGTACTCACCCGTTCGCAACTCATCCGGAGAAGCAAGCTCCTCCTTCA
GCGTTCTACTTGCATGTATTAGGCACGCCGCCAGCGTTCGTCCTGAGCCAGGATCAAACTCT
>672
CTGCTGCCGCCCGTAGGAGTCTGGGCCGTGTCTCAGTCCCAGTGTGGCCGATCACCCTCTCAGGTCGGCTATG
TATCGTCGCCTTGGTGAGCCGTTACCTCACCAACTAGCTAATACAACGCAGGTCCATCTGGTAGTGATGCAAT
TTGCACCTTTTAATTGACTATCATACAATAATCAATTTTATGCGGTATTAGCCTATCGTTTTCCAATAGTTAT
CCCCCCGCTACCAGGCAGGTTACCTACGCGTTACTCACCCGTTCGCAACTCATCCAGAGAAGCAAGCTCCTCC
TTCAGCGTTCTACTTGCATGTATTAGGCACGCCGCCAGCGTTCGTCCTGAGCCAGGATCAAACTCT
>673
CTGCTGCCGCCCGTAGGAGTCTGGGCCGTGTCTCAGTCCCAGTGTGGCCGATCACCCTCTCAGGTCGGCTATG
TATCGTCGCCTTGGTGAGCCGTTACCCCACCAACTAGCTAATACAACGCAGGTCCATCTGGTAGTGGTGCAAT
TGCACCTTTTAAGCAAATGTCATGCAACATCTACTCTTATGCGGTATTAGCTATCGTTTCCAATAGTTATCCC
CCGCTACCAGGCAGGTTACCTACGCGTTACTCACCCGTTCGCAACTCATCCGGAGAAGCAAGCTCCTCCTTCA
GCGTTCTACTTGCATGTATTAGGCACGCCGCCAGCGTTCGTCCTGAGCCAGGATCAAACTCT
>674
CTGCTGCCGCCCGTAGGAGTCTGGACCGTGTCTCAGTTCCAGTGTGGCCGATCACCCTCTCAGGTCGGCTACG
TATCGTTGCCTTGGTAAGCCGTTACCTTACCAACTAGCTAATACGGCGCGGGTCCATCTATAAGTGATAGCAA
AACCATCTTTTCACTTTAGAACCATGCGGTTTCTAAATGTTATCCGGCATTAGCCCCGGTTTTTCCCGGAGTT
ATTTCCAGTCTTATTAGGTAGGTTTTACCCACGTGTTTACTCACCCGTCCGCCGCTAACGTCAAAGGAGCAAG
CTCCTTATCTGTTTCGCTCGACTTGCATGTATTAGGCACGCCGCCAGCGTTCATCCTGAGCCAGGATCAAACT
CT
>675
CTGCTGCCGCCCGTAGGAGTCTGGACCGTGTCTCAGTTCCAGTGTGGCTGATCATCCTCTCAGACCAGCTACG
GATCGTCGCCTTTGGTGGGCCTTTACCCCGCCAACTAGCTAATCCGACATCGGCTCATCTATCTGCGCGAAGC
CCGAAGGTCCTCCGCTTTCACCCGTAGGTCGTATGCGGTATTAGCGTAAGTTTCCCTACGTTATCCCCCACAA
ATAGGCAGATTCCGATGTATTCCTCACCCGTCCGCCACTCGCCACCCGAGGAGCAAGCTCCTCTGTGCTGCCG
TTCGACTGCATGTGTTAGGCCTGCCGCCAGCGTTCACTCTGAGCCAGGATCAAACTCT
>676
CTGCTGCCGCCCGTAGGAGTCTGGGCCGTGTCTCAGTCCCAGTGTGGCCGATCACCCTCTCAGGTCGGCTATG
TATCGTCGCCTTGGTGAGCCGTTACCTCACCAACTAGCTAATACAACGCAGGTCCATCTGGTAGTGATGCAAT
TGCACCTTTTAATTGACTATCATGCAATAATCAATTTTATGCGGTATTAGCCTATCGTTTCCAATAGTTATCC
CCCGCTACCAGGCAGGTTACCTACGCGTTACTCACCCGTTCGCAACTCATCCAGAAGAGCAAGCTCCTCCTCA
GCGTTCTACTTGCATGTATTAGGCACGCCGCCAGCGTTCGTCCTGAGCCAGGATCAAACTCT
>677
CTGCTGCCGCCCGTAGGAGTCTGGACCGTGTCTCAGTTCCAGTGTGGCCGATCACCCTCTCAGGTCGGCTATG
CATCGTTGCCTTGGTAAGCCGTTACCTTACCAACTAGCTAATGCAGCGCGGATCCATCTATAAGTGACAGCAA
GACCGTCTTTCACTTTTGAACCATGCGGTTTCAAAATATTATCCGGATATTTAGCTCCGGTTTTTCCCGAAGT
ATCCCCAGTCTTTATAGGTAGGTTTATCCACGTGTTACTCACCCGTCCGCCGCTAACATCAGAGAAGCAAGCT
TCTCATCCGTTCGCTCGACTTGCATGTATTAGGCACGCCGCCAGCGTTCATCCTGAGCCAGGATCAAACTCT
>678
CTGCTGCCGCCCGTAGGAGTCTGGGCCGTGTCTCAGTCCCAGTGTGGCCGATCACCCTCTCAGGTCGGCTATG
TATCGTCGCCTTGGTGAGCCGTTACCCCACCAACTAGCTAATACAACGCAGGTCCATCTGGTAGTGGTGCAAT
TGCACCTTTTAAGCAAATGTCATGCAACATCTACTCTTATGCGGTATTAGCTATCGTTTCCAATAGTTATCCC
CCGCTACCAGGCAGGTTACCTACGCGTTACTCACCCGTTCGCAACTCGTCCGGAGAAGCAAGCTCCTCCTTCA
GCGTTCTACTTGCATGTATTAGGCACGCCGCCAGCGTTCGTCCTGAGCCAGGATCAAACTCT
>679
CTGCTGCCGCCCGTAGGAGTCTGGGCCGTGTCTCAGTCCCAGTGTGGCCGATCACCCTCTCAGGTCGGCTATG
TATCGTCGCCTTGGTGAGCCGTTACCTCACCAACTAGCTAATACAACGCAGGTCCATCTGGTAGTGATGCAAT

TGCACCTTTTAATTGACTATCATGCAATAATCAATTTTATGCGGTATTAGCCTATCGTTTCCAATAGTTATCC
CCCGCTACCAGGCAGGTTACCTACGCGTTACTCACCCGTTCGCAACTCATCCAGAGAAGCAAGCTCCTCCTCA
GCGTTCTACTTGCATGTATTACGGCACGCCGCCAGCGTTCGTCCTGAGCCAGGATCAAACTCT
>680
CTGCTGCCGCCCGTAGGAGTCTGGGCCGTGTCTCAGTCCCAGCGTGGCCGATCACCCTCTCAGGTCGGCTATG
TATCGTCGCCTTGGTGAGCCGTTACCTCACCAACTAGCTAATACAACGCAGGTCCATCTGGTAGTGATGCAAT
TGCACCTTTTAATTGACTATCATGCAATAATCAATTTTATGCGGTATTAGCCTATCGTTTCCAATAGTTATCC
CCCGCTACCAGGCAGGTTACCTACGCGTTACTCACCCGTTCGCAACTCATCCAGAGAAGCAAGCTCCTCCTCA
GCGTTCTACTTGCATGTATTAGGCACGCCGCCAGCGTCGTCCTGAGCCAGGATCAAACTCT
>681
CTGCTGCCGCCCGTAGGAGTCTGGGCCGTGTCTCAGTCCCAGTGTGGCCGATCACCCTCTCAGGTCGGCTATG
TATCGTCGCCTTGGTGAGCCGTTACCCCACCAACTAGCTAATACAACGCAGGTCCATCTGGTAGTGGTGCAAT
TGCACCTTTTAAGCAAATGTCATGCAACATCTACTCTTATGCGGTATTAGCTATCGTTTCCAATAGTTATCCC
CCGCTACCAGGCAGGTTACCTACGCGTACTCACCCGTTCGCAACTCATCCGGAGAAGCAAGCTCCTCCTTCAG
CGTTCTACTTGCATGTATTAGGCACGCCGCCAGCGTTCGTCCTGAGCCAGGATCAAACTCT
>682
CTGCTGCCGCCCGTAGGAGTCTGGGCCGTGTCTCAGTCCCAGTGTGGCCGATCACCCTCTCAGGTCGGCTATG
TATCGTCGCCTTGGAGAGCCGTTACCCCACCAACTAGCTAATACAACGCAGGTCCATCTGGTAGTGGTGCAAT
TGCACCTTTTAAGCAAATGTCATGCAACATCTACTCTTATGCGGTATTAGCTATCGTTTTCCAATAGTTATCC
CCCGCTACCAGGCAGGTTACCTACGCGTTACTCACCCGTTCGCAACTCATCCGGAGAAGCAAGCTCCTCCTTC
AGCGTTCTACTTGCATGTATTAGGCACGCCGCCAGCGTTCGTCCTGAGCCAGGATCAAACTCT
>683
CTGCTGCCGCCCGTAGGAGTCTGGGCCGTGTCTCAGTCCCAGTGTGGCCGATCACCCTCTCAGGTCGGCTATG
TATCGTCGCCTTGGTGAGCCGTTACCCCACCAACTAGCTAATACAACGCAGGTCCATCTGGTAGTGGTGCAAT
TGCACCTTTTAAGCAAATGTCATGCAACATCTACTCTTATGCGGTATTAGCTATCGTTTCCAATAGTTATCCC
CCGCTACCAGGCAGGTTACCTACGCGTACTCACCCGTTCGCAACTCATCCGGAGAAGCAAGCTCCTCCTCAGC
GTCTACTTGCCATGTATTAGGCACGCCGCCAGCGTTCGTCCTGAGCCAGGATCAAACTCT
>684
CTGCTGCCGCCCGTAGGAGTCTGGGCCGTGTCTCAGTCCCAGTGTGGCCGATCACCCTCTCAGGTCGGCTATG
TATCGTCGCCTTGGTGAGCCCTTACCCCACCAACTAGCTAATACAACGCAGGTCCATCTGGTAGTGATGCAAT
TGCACCTTTCAAACAGTTGTCATGCAACAACTGCTATTACGCGGTATTAGCTATCGTTTTCCAATAGTTATCC
CCCGCTACCAGGCAGGTTACCTACGCGTTACTCACCCGTTCGCAACTCATCCGGAGAAGCAAGCTCCTCCTTC
AGCGTTCTACTTGCATGTATTTAGGCACGCCGCCAGCGTTCGTCCTGAGCCAGGATCAAACTCT
>685
CTGCTGCCGCCCGTAGGAGTCTGGGCCGTGTCTCAGTCCCAGTGTGGCCGATCACCCTCTCAGGTCGGCTATG
TATCGTCGCCTTGGTGAGCCGTTACCTCACCAACTAGCTAATACAACGCAGGTCCATCTGGTAGTGATGCAAT
TTGCACCTTTTAATTGACTATCATGCAATAATCAATTTTATGCGGTATTAGCTATCGTTTCCAATAGTTATCC
CCCGCTACCAGGCAGGTTACCTACGCGTTACTCACCCGTTCGCAACTCATCCAGAGAAGCAAGCTCCTCCTTC
AGCGTTCTACTTGCATGTATTAGGCACGCCGCCAGCGTTCGTCCTGAGCCAGGATCAAACTCT
>686
CTGCTGCCGCCCGTAGGAGTCTGGGCCGTGTCTCAGTCCCAGTGTGGCCGATCACCCTCTCAGGTCGGCTATG
CATCGTCGCCTTGGTAGGCCTTTACCCCACCAACTAGCTAATGCACCGCAAAGCCATCTCATAGTGTTAGCAA
AACCAACTTTCAAACATCAACCATGCAGTTAATGCTATTATCCGGTATTAGCTATCGTTTCCAATAGTTGTCC
CAGTCTATGAGGCAGGTTCTTTACGTGTTACTCTACCCGTTCGCCGCTAAGTTGTCGTCGCAGCGAACCACAA
CTAACTCGAACTCGC
>687
CTGCTGCCGCCCGTAGGAGTCTGGGCCGTGTCTCAGTCCCAGTGTGGCCGATCACCCTCTCAGGTCGGCTATG
TATCGTCGCCTTGGTGAGCCCTTACCCCACCAACTAGCTAATACAACGCAGGTCCATCTGGTAGTGATGCAAT
TGCACCTTTCAAACAGTTGTCATGCAACAACTGCTATTTATGCGGTATTTAGCTATCGTTTTCCAATAGTTTA
TCCCCCCGCTACCAGGCAGGTTACCTACGCGTTACTCACCCGTTCGCAACTCATCCAGAGAAGCAAGCTCCTC
CTTTCAGCGTTCTACTTGCATTGTATTTAGGCACGCCGCCAGCGTCGTCCTGAGCCAGGATCAAACTCT
>688
CTGCTGCCGCCCGTAGGAGTCTGGGCCGTGTCTCAGTCCCAGTGTGGCCGATCACCCTCTCAGGTCGGCTATG
TATCGTCGCCTTGGTGAGCCCTTACCCCACCAACTAGCTAATACAACGCAGGTCCATCTGGTAGTGATGCAAT

TGCACCTTTCAAACAGTTGTCATGCAACAACTGCTATTATGCGGTATTAGCTATCGTTTCCAATAGTTATCCC
CCGCTACCAGGCAGGTTACCTACGCGTTACTCACCCGTTCGCAACTCATCCGGAGAAGCAAGCTCCTCCTTCA
GCGTTCTACTTGCCATGTATTAGGCACGCCGCCAGCGTTCGTCCTGAGCCAGGATCAAACTCT
>689
CTGCTGCCGCGTAGGAGTCTGGGCCGTGTCTCAGTCCCAGTGTGGCCGATCACCCTCTCAGGTCGGCTATGTA
TCGTCGCCTTGGTGAGCCGTTACCCCCCACCAACTAGCTAATACAACGCAGGTCCATCTGGTAGTGGTGCAAT
TGCACCTTTTAAGCAAATGTCATGCAACATCTACTCTTATGCGGTATTTAGCTATCGTTTTCCAATAGTTTAT
CCCCCCCGCTACCAGGCAGGTTACCTACGCGTACTCACCCGTTCGCAACTCATCCGGAGAAGCAAGCTCCTCC
TTCAGCGTTCTACTGCATGTATTTAGGCACGCCGCCAGCGTTCACCTGAGCCAGGATCAAACTCT
>690
CTGCTGCCGCCCGTAGGAGTCTGGGCCGTGTCTCAGTCCCAGTGTGGCCGATCACCCTCTCAGGTCGGCTATG
TATCGTCGCCTTGGTGAGCCCTTACCCCCCACCAACTAGCTAATACAACGCAGGTCCATCTGGTAGTGATGCA
ATTGCACCTTTCAAACAGTTGTCATGCAACAACTGCTATTATGCGGTATTTAGCTATCGTTTTTCCAATAGTT
TATCCCCCCCCGCTACCAGGCAGGTTACCTACGCGTTACTCACCCGTTCGCAACTCATCCGGAGAAGCAAGCT
CCTCCTCAGCGTTCTACTGCATGTATTTAGGCACGCCGCCAGCGTTCGTCCTGAGCCAGGATCAAACTCT
>691
CTGCTGCCGCCCGTAGGAGTCTGGGCCGTGTCTCAGTCCCAGTGTGGCCGATCACCCTCTCAGGTCGGCTATG
TATCGTCGCCTTGGTGAGCCGTTACCTCATCAACTAGCTAATACAACGCAGGTCCATCTGGTAGTGATGCAAT
TTGCACCTTTTAATTGACTATCATGCAATAATCAATTTTATGCGGTATTAGCCTATCGTTTCCAATAGTTATC
CCCCGCTACCAGGCAGGTTACCTACGCGTTACTCACCCGTTCGCAACTCATCCAGAGAAGCAAGCTCCTCCTT
CAGCGTTCTACTTGCATGTATTAGGCACGCCGCCAGCGTTCGTCCTGAGCCAGGATCAAACTCT
>692
CTGCTGCCGCCCGTAGGAGTCTGGGCCGTGTCTCAGTCCCAGTGTGGCCGATCACCCTCTCAGGTCGGCTATG
TATCGTCGCCCTGGTGAGCCGTTACCTCACCAACTAGCTAATACAACGCAGGTCCATCTGGTAGTGATGCAAT
TTGCACCTTTTAATTGACTATCATGCAATAATCAATTTTATGCGGTATTAGCCTATCGTTTCCAATAGTTATC
CCCCGCTACCAGGCAGGTTACCTACGCGTTACTCACCCGTTCGCAACTCATCCAGAGAAGCAAGCTCCTCCTT
CAGCGTTCTACTTGCATGTATTAGGCACGCCGCCAGCGTTCGTCCTGAGCCAGGATCAAACTCT
>693
CTGCTGCCGCCCGTAGGAGTCTGGGCCGTGTCTCAGTCCCAGTGTGGCCGATCACCCTCTCAGGTCGGCTATG
TATCGTCGCCTAGGTGAGCCGTTACCTCACCTACTAGCTAATACAACGCAGGTCCATCTTGTAGTGGAGCAAT
TGCCCCTTTCAAATAAATGACATGTGTCATTCATTGTTATGCGGTATTAGCTATCCGTTCCAATAGTTATCCC
CCGCTACAAGGCAGGTTACCTACGCGTTACTCACCCGTTCGCAACTCATCCAAGAAGAGCAAGCTCCTCTCTT
CAGCGTTCTACTTGCATGTATTAGGCACGCCGCCAGCGTTCGTCCTGAGCCAGGATCAAACTCT
>694
CCTGCCGCCCGTAGGAGTCTGGGCCGTGTCTCAGTCCCAGTGTGGCCGATCACCCTCTCAGGTCGGCTATGTA
TCGTCGCCTTGGTGAGCCGTTACCTCACCAACTAGCTAATACAACGCAGGTCCATCTGGTAGTGATGCAATTG
CACCTTTTAATTGACTATCATGCAATAATCAATTTTATGCGGTATTTAGCTATTCGTTTTCCAATAGTTTATC
CCCCCGCTACCAGGCAGGTTTACCTACGCGTTACTCACCCGTTTCGCAACTCATCCAGAGAAGCAAGCTCCTC
CTTTCAGCGTTCTACTGCATTGTATTTAGGCACGCCGCCAGCGTTCGCCTGAGCCAGGATCAAACTCTT
>695
CTGCTGCCGCCCGTAGGAGTCTGGGCCGTGTCTCAGTCCCAGTGTGGCCGATCACCCTCTCAGGTCGGCTATG
TATCGTCGCCTTGGTGAGCCCTTATCCCACCCAACTACGCTAATACAACGCAGGTCCATCTGGTAGTGGTGCA
ATTGCACCTTTTAAGCAAATGTCATGCGACACTCTACTCTTATGCGGTATTAGCTATCGTTTTCCAATAGTTT
ATCCCCCCGCTACCAGGCAGGTACCTAGCGTACTCACCCGTTCGCAACCTCATCCGGAGAAGCAAGCTCCTCC
TCAGCGTTCTACTGCATGTATTTAGGCACGCCGCCAGCGTCGTCCTGAGCCAGGATCAAACTCT
>696
CTGCTGCCGCCCGTAGGAGTCTGGGCCGTGTCTCAGTCCCAGTGTGGCCGATCACCCTCTCAGGTCGGCTATG
TATCGTCGCCTTGGTGAGCCCTTACCCCACCAACTAGCTAATACAACGCAGGTCCATCTGGTAGTGATGCAAT
TGCACCTTTCAAACAGTTGTCATGCAACAACTGCTATTATGCGGTATTAGCTATCGTTTCCAATAGTTATCCC
CCGCTACCAGGCAGGTTACCTACCGCGTACTCACCCGTTCGCAACTCATCCGGAGAAGCAAGCTCCTCCTCAG
CGTTCTACTTGCATGTATTAGGCACGCCGCCAGCGTTCGTCCTGAGCCAGGATCAAACTCT
>697
CTGCTGCCGCCCGTAGGAGTCTGGACCGTGTCTCAGTTCCAGTGTGGCCGATCACCCTCTCAGGTCGGCTATG
CATCGTTGCCTTGGTAAGCCGTTACCTTACCAACTAGCTAATGCAGCGCGGATCCATCTATAAGTGACAGCAA

GACCGTCTTTCACTTTTGAACCAGCGGTTCAAAATATTATCCGGATATTAGCTCCGGTTTCCCGAAGTTATCC
CAGTCTTATAGGTAGGTTATCCACGTGTTACTCACCCGTCCGCCGCTAACATCAGAGGAGCAAGCTCCTCGTC
TGTCGCTCGACTTGCATGTATTAGGCACGCCGCCAGCGTTCATCCGTGAGCCAGGATCAAACTCT
>698
CTGCTGCCGCCCGTAGGAGTCTGGGCCGTGTCTCAGTCCCAGTGTGGCCGGTCGCCCTCTCAGGCCGGCTACC
CGTCATCGTCTTGGTGAGCCATCACCTCACCAACAAACTGATAGGCCGCGAGTCCATCCCCCACCGATAAATC
TTTCCAACACCCACCATGCGATGAGTGCTCATATCCGGTATTAGCCACGATTTCCCGAGGTTATCCCGAAGTG
GAGGGCAGGTTACTCACGTGTTACTCACCCGTTCGCCACTAATCAGGCGGTGCAAGCACCACCGTCATCGTTC
GACTTGCATGTGTTAAGCACGCCGCCAGCGTCGTCCTGAGCCAGGATCAAACTCT
>699
CTGCTGCCGCCCGTAGGAGTCTGGGCCGTGTCTCAGTCCCAGTGTGGCCGGTCGCCCTCTCAGGCCGGCTACC
CGTCATCGCCTTGGTGAGCCATTAGCTCACCAACAAACTGATAGGCCGCGAGTCCATCCCCCACCGAAAAACT
TTCCACAACACACCATGCGGTGCGAAGTCATATCCGGTATTAGCCCCGATTTCCCGAGGTTATCCCGAAGAGG
GGGGCAGGTTACTCACGTGCTACTCACCCGTTCGCCACTAATCGGACAGGGCAAGCCCTGTCGTCATCGTTCG
ACTTGCATGTGTTAAGCACGCCGCCAGCGTTCGTCCTGAGCCAGGATCAAACTCCT
>700
CTGCTGCCGCCCGTAGGAGTCTGGGCCGTGTCTCAGTCCCAGTGTGGCCGATCACCCTCTCAGGTCGGCTATG
TATCGTCGCCTTGGTGAGCCGTTACCTCACCAACTAGCTAATACAACGCAGGTCCATCTGGTAGTGATGCAAT
TGCACCTTTTAAGCAAATGTCATGCAACATCTACTATTATGCGGTATTACGCTATCGTTTCCAATAGTTATCC
CCCGCTACCAGGCAGGTACCTACGCGTACTCACCCGTTCGCAACTCATCCGCTCGGTGCAAGCACCAAGCCTT
CAGCGTTCTACTTGCATGTATTACGGCACGCCGCCAGCGATTCG
>701
CTGCTGCCGCCCGTAGGAGTCTGGGCCGTGTCTCAGTCCCAGCTGTGGCCGATCACCCTCTCAGGTCGGCTAT
GCATCGTCGCCTTGGTAGGCCTTTACCCCACCAACTAGCTAATGCACCGCAAAGCCATCTCATAGTGTTAGCA
AAACCAACTTTTAAACATCAACCATGCAGTTAATGCTGTTATCCGGTATTAGCCTATCGTTTCCAATAGTTGT
CCCAGTCTATGAGGCAGGTTCCTTTACGTGTTACTCACCCGTTCGCCGCTAAGTGTTCGTCGCAGCACCAAAC
TACGACTCGACTG
>702
CTGCTGCCGCCCGTAGGAGTCTGGGCCGTGTCTCAGTCCCAGTGTGGCCGATCACCCTCTCAGGTCGGCTATG
CATCGTCGCCTTGGTAGGCCTTTACCCCCCACCAACTAGCTAATGCACCGCAAAGCCATCTCATAGTGTTAGC
AAAACCAACTTTTAAACATCAACCATGCAGTTAATGCTGTTTATCCGGTATTTAGCTATTCGTTTTCCCCAAT
AGTTTGTCCCCCCAGTCTATTGAGGCAGGTTTCTTTACGTTGTTACTCACCCCCCCGTTTCGCCCGCTAAGTT
TCGTTGCGAGCACA
>703
CTGCTGCCGCCCGTAGGAGTCTGGGCCGTGTCTCAGTCCCAGTGTGGCCGATCACCCTCTCAGGCCGGCTACC
CGTCGTCGCCTGGCAGGCCCTTACCCCACCATCCAGCTGGTAGGCCGCGGGCTCATCCCATACCGCCGGAACT
TTCCACACCACCCCATCGCGAGGCGATGTCCTATCCGGTATTAGACCCGGTTTTTCCCAGGCTTTATCCCCGA
GTACAGGGCAGATTACCCACGTGTTACTCACCCGTTCGCCACTCATCCACCCCTCCGAAAAGGGGCTTCAGCG
TTCGACTTGCATGTGTTTAAGCACGCCGCCAGCGTTCGTCCTGAGCCAGGATCAAACTCT
>704
CTGCTGCCGCCCGTAGGAGTCTGGGCCGTGTCTCAGTCCCAGTGTGGCCGATCACCCTCTCAGGTCGGCTATG
TATCGTCGCCTTGGTGAGCCCTTACCCCACCAACTAGCTAATACAACGCAGGTCCATCTGGTAGTGATGCAAT
TGCACCTTTCAAACAGTTGTCATGCAACAACTGCTATTATGCGGTATTAGCTATCGTTTTCCAATAGTTATCC
CCCGCTACCAGGCAGGTTACCTACGCGTACTCACCCGTTCGCAACTCATCCGGAGAAGCAAGCTCCTCCTTCA
GCGTTCTACTTGCATGTATTAGGCACGCCGCCAGCGTTCGTCCTGAGCCAGGATCAAACTCT
>705
CTGCTGCCGCCCGTAGGAGTCTGGGCCGTGTCTCAGTCCCAGTGTGGCCGATCACCCTCTCAGGTCGGCTATG
TATCGTCGCCTTGGTGAGCCGTTACCCCACCAACTAGCTAATACAACGCAGGTCCATCTGGTAGTGATGCAAT
TGCACCTTTTAAGTAAATGTCATGCAACATCTATTCTTATGCGGTATTAGCTATCGTTTCCAATAGTTATCCC
CCGCTACCAGGCAGGTTACCTACGCGTTACTCACCCGTTCGCAACTCATCCAGAAGAGCAAGCCTCCTCCTTC
AGCGTTCTACTTGCATGTATTAGGCACGCCGCCAGCGTTCGTCCTGAGCCAGGATCAAACTCT
>706
CTGCTGCCGCCCGTAGGAGTCTGGGCCGTGTCTCAGTCCCAGTGTGGCCGATCACCCTCTCAGGTCGGCTATG
TATCGTCGCCTTGGTGAGCCGTTACCCCACCAACTAGCTAATACAACGCAGGTCCATCTGGTAGTGATGCAAT

TGCACCTTTTTAAGTAAACTGTCATGCAACATCTACTCTTATGCGGTATTTAGCATTTGTTTTTCCAATAGTT
ATCCCCCCGCTACCAGGCAGGTTACCTACGCGTTACTCACCCGTTTCGCAACTCATCCAGAAGAGCAAGCTCC
TCCTCAGCGTTCTACTGCATGTATTTAGGCACGCCGCCAGCGTCGTCCTGAGCCAGGATCAAACTCT
>707
CTGCTGCCGCCCGTAGGAGTCTGGACCGTGTCTCAGTTCCAGTGTGGCCGATCACCCTCTCAGGTCGGCTACG
TATCGTTGCCTTGGTAAGCCGTTACCTTACCAACTAGCTAATACGGCGCAGGTCCATCTATAAGTGATAGCAA
AGCCATCTTTCACTATCGAACCATGCGGTTCGAAATATTATCCGGTATTAGCTCCGGTTTCCCGAAGTTATCC
CAGTCTTATAGGTAGGTTACCTACGTGTTACTCACCCGTCCGCCGCTAACATCAAAGGAGCAAGCTCCTCGTC
TGTTCGCTCGACTTGCATGTATTAGGCACGCCGCCAGCGTTCATCCTGAGCCAGGATCAAACTCT
>708
CTGCTGCCGCCCGTAGGAGTCTGGGCCGTGTCTCAGTCCCAGTGTGGCCGATCACCCTCTCAGGTCGGCTATG
TATCGTCGCCTTGGTGAGCCGTTACCTCACCAACTAGCTAATACAACGCAGGTCCATCTGGTAGTGATGCAAT
CGCACCTTTTAATTGACTATCATGCAATAATCAATTTTATGCGGTATTAGCCTATCGTTTCCAATAGTTATCC
CCCGCTACCAGGCAGGTTACCTACGCGTACTCACCCGTTCGCAACTCATCCGGAGAAGCAAGCTCCTCCTTCA
GCGTTCTACTTGCATGTATTAGGCACGCCGCCAGCGTTCGTCCTGAGCCAGGATCAAACTCT
>709
CTGCTGCCGCCCGTAGGAGTCTGGGCCGTGTCTCAGTCCCAGTGTGGCCGATCACCCTCTCAGGTCGGCTATG
TATCGTCGCCTTGGTGAGCCCTTACCCCACCAACTAGCTAATACAACGCAGGTCCATCTGGTAGTGATGCAAT
TGCACCTTTCAAACAGTTGTCATGCAACAACTGCTATTATGCGGTATTAGCTATCGTTTCCAATAGTTATCCC
CCGCTACCAGGCAGGTTACCTACCGCGTTACTCACCCGTTCGCAACTCATCCAGAGAAGCAAGCTCCTCCTTC
AGCGTTCTACTTGCATGTATTAGGCACGCCGCCAGCGTTCGTCCTGAGCCAGGATCAAACTCT
>710
CTGCTGCCGCCCGTAGGAGTTTGGGCCGTGTCTCAGTCCCAATGTGGCTGATCATCCTCTCAGACCAGCTACT
GATCGTCGCCTTGGTGAGCCTTTACCTCACCAACTAGCTAATCAGACGCGGGCCGCTCTAAAGGCGATAAATC
TTTCCCCCGAAGGGCACATTCGGTATTAGCACAAGTTTTCCCTGAGTTATTCCGAACCTAAAGGCACGTTCCC
ACGTGTTACTCACCCGTTCGCCACTAGTCGGACAGGGCAAGCCCTGTCGTCATCGTTCGACTTGCATGTGTTA
AGCACGCCGCCAGCGTTCGTCCTGAGCCAGGATCAAACTCT
>711
CTGCTGCCGCCCGTAGGAGTCTGGGCCGTGTCTCAGTCCCAGTGTGGCCGATCACCCTCTCAGGTCGGCTATG
CATCGTCGCCTTGGTAGGCCTTTACCCCACCAACTAGCTAATGCACCGCAAAGCCATCTCATAGTGTTAGCAA
AACCAACTTTCAAACGTCAACCATGCAGTTAATGCTGTTATCCGGTATTAGCTATCGTTTCCAATAGTTGTCC
CAGTCTATGAGGCAGGTTCTTTACGTGTTACTCACCCGTTCGCCGCTAAGTTTTCTCAGCGCAAGCACCAGAA
AACTTCGACTCGACTGCATGTATTAGGCAGCCGTCCGCGTCGCCGAACCAGGATAAC
>712
CTGCTGCCGCCCGTAGGAGTCTGGGCCGTGTCTCAGTCCCAGTGTGGCCGATCACCCTCTCAGGTCGGCTATG
TATCGTCGCCTTGGTGAGCCGTTACCCCACCAACTAGCTAATACAACGCAGGTCCATCTGGTAGTGGTGCAAT
TGCACCTTTTAAGCAAATGTCATGCAACATCTACTCTTATGCGGTATTTAGCTATCGTTTTCCAATAGTTTAT
CCCCCCCGCTACCAGGCAGGTTACCTACGCGTTACTCACCCGTTCGCAACTCATCCGGAGAAGCAAGCTCCTC
CTCAGCGTCTACTTGCATGTATTTTAGGCACGCCGCCAGCGTTCGCCTGAGCCAGGATCAAACTCT
>713
CTGCTGCCGCCCGTAGGAGTCTGGGCCGTGTCTCAGTCCCAGTGTGGCCGATCACCCTCTCAGGTCGGCTATG
TATCGTCGCCTTGGTGAGCCGTTACCCCACCAACTAGCTAATACAACGCAGGTCCATCTGGTAGTGATGCAAT
TGCACCTTTTAAGTAAATGTCATGCAACATCTACTCTTATGCGGTATTAGCTATCGTTTCCAATAGTTATCCC
CCGCTACCAGGCAGGTTACCTACGCGTTACTCACCCGTTCGCAACTCATCCAGAAGAGCAAGCTCCTCCTCAG
CGTTCTACTTGCATGTATTAGGCACGCCGCCAGCGTCGTCCTGAGCCAGGATCAAACTCT
>714
CTGCTGCCGCCCGTAGGAGTCTGGGCCGTGTCTCAGTCCCAGTGTGGCCGATCACCCTCTCAGGTCGGCTATG
TATCGTTGCCTTGGTGAGCCGTTACCTCACCAACTAGCTAATACAACGCAGGTCCATCTGGTAGTGGTGCAAT
TGCACCTTTTAAGCAGCTACCATGCGATATCTACTGTTATGCGGTATTAGCTATCGTTTCCAATAGTTATCCC
CCGCTACCAGGCAGGTTACCTACGCGTTACTCACCCGTTCGCAACTCATCCGGAAAGAGCAAGCTCCTTCCTT
CAGCGTTCTACTTGCATGTATTAGGCACGCCGCCAGCGTTCGTCCTGAGCCAGGATCAAACTCT
>715
CTGCGCCGCCCGTAGGAGTCTGGGCCGTGTCTCAGTCCCAGTGTGGCCGGTCACCCTCTCAGGTCGGCTATGT
ATCGTCGCCTTGGTGAGCCGTTACCCCACCAACTAGCTAATACAACGCAGGTCCATCTGGTAGTGGTGCAATT

GCACCTTTTAAGCAAATGTCATGCAACATCTACTCTTATGCGGTATTAGCTATCGTTTTTCCAATAGTTTATC
CCCCCCGCTACCAGGCAGGTTACCTACGCGTTACTCACCCGTTCGCAACTCATCCGGAGAAGCAAGCTCCTCC
TCAGCGTTCTACTGCATGTATTTAGGCACGCCGCCAGCGTTCGTCCTGAGCCAGGATCAAACTCT
>716
CTGCTGCCGCCCGTAGGAGTCTGGGCCGTGTCTCAGTCCCAGTGTGGCCGATCACCCTCTCAGGTCGGCTATG
TATCGTCGCCTTGGTGAGCCGTTACCTCACCAACTAGCTAATACAACGCAGGTCCATCTGGTAGTGATGCAAT
TGCACCTTTTAATTGACTATCATGCAATAATCAATTTTATGCGGTATTAGCCTATCGTTTCCAATAGTTATCC
CCCGCTACCAGGCAGGTTACCTACGCGTTACTCACCCGTTCGCAACTCATCCAGAGAAGCAAGCTCCTCCTTC
AGCGTTCTACTTGCCATGTATTAGGCACGCCGCCAGCGTTCGTCCTGAGCCAGGATCAAACTCT
>717
CTGCTGCCGCCCGTAGGAGTCTGGGCCGTGTCTCAGTCCCAGTGTGGCCGATCACCCTCTCAGGTCGGCTATG
CATCGTCGCCTTGGTAGGCCTTTACCCCACCAACTAGCTAATGCACCGCAAAGCCATCTCATAGTGTTAGCAA
AACCAACTTTAAACATCAACCATGCAGTTAATGCTGTTATCCGGTATTAGCTATCGTTTCCAATAGTTATCCC
CCGCCACCAGGCAGGTTACCTACGCCGTACTCACCCGTTCGCAACTCATCCAGAGAAGCAAGCTCCTCCTTCA
GCGTTCTACTTGCATGTATTAGGCACGCCGCCAGCGTCGTCCTGAGCCAGGATCAACTCT
>718
CTGCTGCCGCCCGTAGGAGTCTGGGCCGTGTCTCAGTCCCAGTGTGGCCGATCACCCTCTCAGGTCGGCTATG
CATCGTCGCCTTGGTAGGCCTTTACCCCACCAACTAGCTAATGCACCGCAAAGCCATCTCATAGTGTTAGCAA
AACCAACTTTTAAACATCAACCATGCAGTTAATGCTGTTATCCGGTATTAGCTATCGTTTCCAATAGTTGTCC
CAGTCTATGAGGCAGGTTCTTTACGTGTTACTCACCCGTTCGCCGCTAATGTCTCGCGTACAGCACAGTAAAC
TCGCATCAGTACGTAT
>719
CTGCTGCCGCCCGTAGGAGTCTGGGCCGTGTCTCAGTCCCAGTGTGGCCGATCACCCTCTCAGGTCGGCTATG
TATCGTCGCCTTGGTGAGCCGTTACCTCACCAACTAGCTAATACAACGCAGGTCCATCTGGTAGTGATGCAAT
TGCACCTTTTAATTGACTATCATGCAATAATCAATTTTATGCGGTATTAGCCTATCGTTTCCAATAGTTATCC
CCCGCTACCAGGCAGGTTACCTACGCGTTACTCACCCGTTCGCAACTCATCCAGAGAAGCAAGCTCCTCCTCA
GCGTTCTACTTGCATGTATTAGGCACGCCGCCAGCGTTCGTCCTGAGCCAGGATCAAACTCT
>720
CTGCTGCCGCCCGTAGGAGTCTGGGCCGTGTCTCAGTCCCAGTGTGGCCGATCACCCTCTCAGGTCGGCTATG
TATCGTCGCCTTGGTGAGCCCTTACCCCACCAACTAGCTAATACAACGCAGGTCCATCTGGTAGTGATGCAAT
TTGCACCTTTCAAACAGTTGTCATGCAACAACTGCTATTATGCGGTATTTAGCTATCGTTTTCCAATAGTTAT
CCCCCGCTACCAGGCAGGTTACCTACGCGTTACTCACCCGTTCGCAACTCATCCAGAGAAGCAAGCTCCTCCT
TCAGCGTTCTACTTGCCATGTATTTAGGCACGCCGCCAGCGTTCGTCCGTGACGCCA
>721
CTGCTGCCGCCCGTAGGAGTCTGGGCCGTGCCTCAGTCCCAGTGTGGCCGATCACCCTCTCAGGTCGGCTATG
CATCGTCGCCTTGGTAGGCCTTTACCCCACCAACTAGCTAATGCACCGCAAAGCCATCTCATAGTGTTAGCAA
AACCAACTTTTAAACATCAACCATGCAGTTAATGCTGTTTATCCGGTATTTAGCTATCGTTTTCCAATAGTTT
GTCCCCCAGTCTATGAGGCAGGTTTTCTTTACGTGTTTACTCACCCGTTTCGCCGCTAAGTTTCGTTCGCGAC
ACGAAACATCC
>722
CTGCTGCCGCCCGTAGGAGTCTGGGCCGTGTCTCAGTCCCAGTGTGGCCGATCACCCTCTCAGGTCGGCTATG
TATCGTCGCCTTGGTGAGCCGTTACCCCACCAACTAGCTAATACAACGCAGGTCCATCTGGTAGTGGTGCAAT
TGCACCTTTCAAACAGCTGTCATGCAACAACTGCTATTATGCGGTATTAGCTATCGTTTTCCAATAGTTATCC
CCCGCTACCAGGCAGGTTACCTACGCGTTACTCACCCGTTCGCAACTCATCCAGAGAAGCAAGCTCCTCCTCA
GCGTTCTACTTGCATGTATTAGGCACGCCGCCAGCGTTCGTCCTGAGCCAGGATCAAACTCT
>723
CTGCTGCCGCCCGTAGGAGTCTGGGCCGTGTCTCAGTCCCAGTGTGGCCGATCACCCTCTCAGGTCGGCTATG
CATCGTCGCCTTGGTAGGCCTTTACCCCACCAACTAGCTAATGCACCGCAAAGCCATCTCATAGTGTTAGCAA
AACCAACTTTCAAACATCAACCATGCAGTTAATGCTGTTTATCCGGTATTTAGCTGTCGTTTTTCCAATAGTT
TGTCCCAGTCTATGAGGCAGGTTTCTTTACGTGTTTACTCACCCGTTTCGCCGCTAAGTTGTCGTCGCAGCAA
CCAGAAACTAACGTC
>724
CTGCTGCCGCCCGTAGGAGTCTGGGCCGTGTCTCAGTCCCAGTGTGGCCGATCACCCTCTCAGGTCGGCTATG
TATCGTCGCCTTGGTGAGCCGTTACCCCACCAACTAGCTAATACAACGCAGGTCCATCTGGTAGTGGTGCAAT

TGCACCTTTTAAGCAAATGTCATGCAACATCTACTCTTATGCGGTATTAGCTATCGTTTCCAATAGTTATCCC
CCGCTACCAGGCAGGTTACCTACGCGTTACTCACCCGTTCGCAACTCATCCGGAGAAGCAAGCTCCTCCTTCA
GCGTTCTACTTGCATGTATTAGGCACGCCGCCAGCGTTCGTCCTGAGCCAGGATCAAACTCT
>725
CTGCTGCCGCCCGTAGGAGTCTGGGCCGTGTCTCAGTCCCAGTGTGGCCGATCACCCTCTCAGGTCGGCTATG
TATCGTCGCCTTGGTGAGCCGTTACCTCACCAACTAGCTAATACAACGCAGGTCCATCTGGTAGTGATGCAAT
TGCACCTTTTTAATTGACTATCATGCAATAATCAATTTTATTGCGGTATTTTAGCCTATCGTTTTTCCAATAG
TTTACTCCCCCCCCGCTACCAGGCAGGTTTACCTACGCGTTACTTCACCCCCGTTTCGAACTCATCCAGAGAA
GCAAGCTCCTCCTTTCAGCGTTCTACTTGCATTGTATTTAGGCACGCCGCCAGCGTCGTCCTGAGCCAGGATC
AAACTCTT
>726
CTGCTGCCGCCCGTAGGAGTCTGGGCCGTGTCTCAGTCCCAGTGTGGCCGATCACCCTCTCAGGTCGGCTATG
TATCGTCGCCTTGGTGAGCCGTTACCTCACCAACTAGCTAATACAACGCAGGTCCATCTGGTAGTGATGCAAT
TGCACCTTTTCAAACAGTTGTCATGCAACAACTGCTTATTTATGCGGTTATTTAGCTTATTCGTTTTCCAATA
GTTTATTCCCCCCCGCTACCAGGCAGGTTTACCTACGCGTTACTCACCCCCCGTTTCGAACTCATCCAGAGAA
GCAAGCTCCTCCTTTCAGCGTTCTACTGCATTGTATTTAGGCACGCCGCCCAGCGTTCGTCCCCTGAGCCAGG
ATCAAACTCTTATCACCCCGACTGCCCCCCCATTAGAGAGGCTGAGACTGCCAAGGCACACAGGGGATAGG
>727
CTGCTGCCGCCCCGTAGGAGTCTGGGCCGTGATCTCAGTCCCAGCTGTGGCCCGATCAGCCCTCTCAGGTCGG
CTATCGTAATCGTCGAGCCTTGGGTGAGCCCTTACCCCACCAACTAGCTAATACAACGCAGGTCCATCTGGTA
GTGATGCAATTGCACCTTTCAAACAGTTGTCATGCAACAACTGCTATTATGCGGTATTTAGCTATCGTTTTCC
AATAGTTTATCCCCCCGCTACCAGGCAGGTTACCTACGCGTTACTCACCCGTTCGCAACTCATCCAGAGAAGC
AAGCTCCTCCTTCAGCGTTCTACTTGCATTGTATTAGGCACGCCGCCAGCGTCGTCCTGAGCCAAGATCAAAC
TCT
>728
CTGCTGCCGCCCGTAGGAGTCTGGGCCGTGTCTCAGTCCCAGTGTGGCCGATCACCCTCTCAGGTCGGCTATG
TATCGTCGCCTTGGTGAGCCGTTACCCCACCAACTAGCTAATACAACGCAGGTCCATCTGGTAGTGGTGCAAT
TGCACCTTTTAAGCAAATGTCATGCAACATCTACTCTTATGCGGTATTTAGCTATCGTTTTCCAATAGTTTAT
CCCCCGCTACCAGGCAGGTTACCTACGCGTTACTCACCCGTTCGCAACTCATCCGGAGAAGCAAGCTCCTCCT
TCAGCGTTCTACTTGCATGTATTTAGGCACGCCGCCAGCGTTCGTCCTGAGCCAGGATCAAACTCT
>729
CTGCTGCCGCCCGTAGGAGTCTGGGCCGTGTCTCAGTCCCAGTGTGGCCGATCACCCTCTCAGGTCGGCTATG
TATCGTCGCCTTGGTGAGCCGTTACCCCACCAACTAGCTAATACAACGCAGGTCCATCTGGTAGTGGTGCAAT
TGCACCTTTTAAGCAAATGTCATGCAACATCTACTCTTATGCGGTATTAGCTATCGTTTTCCAATAGTTTATC
CCCCCGCTACCAGGCAGGTTACCTACGCGTTACTCACCCGTTCGCAACTCATCCGGAGAAGCAAGCTCCTCCT
TCAGCGTTCTACTTGCATGTATTTAGGCACGCCGCCAGCGTTCGTCCTGAGCCAGGATCAAACTCT
>730
CTGCTGCCGCCCGTAGGAGTCTGGGCCGTGTCTCAGTCCCAGTGTGGCCGATCACCCTCTCAGGTCGGCTATG
TATCGTCGCCTTGGTGAGCCCTTACCCCACCAACTAGCTAATACAACGCAGGTCCATCTGGTAGTGATGCAAT
TGCACCTTTCAAACAGTTGTCATGCAACAACTGCTATTATGCGGTATTAGCTATCGTTTTCCAATAGTTATCC
CCCGCTACCAGGCAGGTTACCTACGCGTTACTCACCCGTTCGCAACTCATCCAGAGAAGCAAGCTCCTCCTTC
AGCGTTCTACTTGCATGTATTAGGCACGCCGCCAGCGTTCGTCCTGAGCCAGGATCAAACTCT
>731
CTGCTGCCGCCCGTAGGAGTCTGGGCCGTGTCTCAGTCCCAGTGTGGCCGATCACCCTCTCAGGTCGGCTATG
TATCGTCGCCTTGGTGAGCCCTTACCCCACCAACTAGCTAATACAACGCAGGTCCATCTGGTAGTGATGCAAT
TGCACCTTTCAAACAGTTGTCATGCAACAACTGCTATTTATGCGGTACTAGCTATCGTTTTCCAATAGTTTAT
CCCCCGCTACCAGGCAGGTTACCTACGCCGTACTCACCCGTTCGCAACTCATCCGGAGAAGCAAGCTCCTCCT
TCAGCGTTCTACTTGCATGTATTTAGGCACGCCGCCAGCGTTCGTCCTGAGCCAGGATCAAACTCT
>732
CTGCTGCCGCCCGTAGGAGTCTGGACCGTGTCTCAGTTCCAGTGTGGCCGATCACCCTCTCAGGTCGGCTACG
TATCGTTGCCTTGGTAAGCCGTTACCTTACCAACTAGCTAATACGGCGCAGGTCCATCTATAAGTGATAGCAA
AGCCATCTTTCACTATCGAACCATGCGGTTCGAAATATTATCCGGTATTAGCTCCGGTTTTTCCCGAAGTTAT
CCCAGTCTTATACGGTAGGTTACCTACGTGTTACTCACCCGTCCGCCGCTAACGTCGAAGGAGCAAGCTCCTC
GTCTGTTCGCTCGACTTGCATGTATAGGCACGCCGTCCAGCGTCATCCTGAGCCAGGATCAAACTCT

>733
CTGCTGCCGCCCGTAGGAGTCTGGGCCGTGTCTCAGTCCCAGTGTGGCCGATCCACCCCCTCAGGTCGGCTAT
GTATCGTCGCCTTGGTGAGCCGTTACCCCACCAACTAGCTAATACAACGCAGGTCCATCTGGTAGTGGTGCAA
TTGCACCTTTTAAGCAAATGTCATGCAACATCTACTCTTATGCGGTATTAGCTATCGTTTTCCAATAGTTATC
CCCCGCTACCAGGCAGGTTACCTACGCGTTACTCACCCGTTCGCAACTCATCCGGAGAAGCAAGCTCCTCCTT
CAGCGTTCTACTTGCATGTATTTAGGCACGCCGCCAGCGTTCGTCCTGAGCCAGGATCAAACTCT
>734
CTGCTGCCGCCCGTAGGAGTCTGGGCCGTGTCTCAGTCCCAGTGTGGCCGATCACCCTCTCAGGTCGGCTATG
TATCGTCGCCTTGGTGAGCCCTTACCCCACCAACTAGCTAATACGAACGCAGGTCCATCTGGTAGTGATGCAA
TTGCACCTTTTATACACTGATCATGCAATAACTCAATTTATGCGGTATTAGCTATCGTTTCCAATAGTTATCC
CCCGCTACCAGGCAGGTTACCTACGCGTTACTCACCCGTTCGCAACTCATCCAGAGAAGCAAGCTCTCCTTCA
GCGTTCTACTTGCAGTATTAGGCACGCCGCCAGCGTCGTCCTGAGCCAGGATCAAACTCT
>735
CTGCTGCCGCCCGTAGGAGTCTGGGCCGTGTCTCAGTCCCAGTGTGGCCGATCACCCTCTCAGGTCGGCTATG
TATCGTCGCCTTGGTGAGTCGTTACCTCACCAACTAGCTAATACAACGCAGGTCCATCTGGTAGTGATGCAAT
TGCACCTTTTAATTGACTATCATGCAATAATCAATTTTATGCGGTATTAGCTATCGTTTCCAATAGTTATCCC
CCGCTACCAGGCAGGTTACCTACGCGTTACTCACCCGTTCGCAACTCATCCAGAGAAGCAAGCTCCTCCTTCA
GCGTTCTACTTGCATGTATTAGGCACGCCGCCAGCGTTCGTCCTGAGCCAGGATCAAACTCT
>736
CTGCTGCCGCCCGTAGGAGTCTGGACCGTGTCTCAGTTCCAGTGTGTCTGATCGTCCTCTCAAACCAGATACT
GATCGTCGCCTTGGTAAGCCATTACCTTACCAACAAGCTAATCAGCCGCGAGCTTTTCTTTAGGCGGAAAAAT
CCGTTTCACTCTAGAGCATATGGGGTATTGAGCAATCGTTTCCAATTGTTGTCCCCCTCCTAAAGGTAAATTC
TCACGTGTTACTCACCCGTCCGCCACTTGAGTTAAAAACTCTCGTACGACTTGCATGTGTTAGACATACCGCC
AGCCGGTTCATCCTGAAGCCAGGATCAACCGTCTATC
>737
CTGCTGCCGCCCGTAGGAGTCTGGGCCGTGTCTCAGTCCCAGTGTGGCCGATCACCCTCTCAGGTCGGCTATG
TATCGTCGCCTTGGTGAGCCGTTACCCCACCAACTAGCTAATACAACGCAGGTCCATCTGGTAGTGGTGCAAT
TGCACCTTTTAAGCAAATGTCATGCAACATCTACTCTTATGCGGTATTAGCTATCGTTTCCAATAGTTATCCC
CCGCTACCAGGCAGGTTACCTACGCGTACTCACCCGTTCGCAACTCATCCGGAGAGGCAAGCTCCTCCTTCAG
CGTTCTACTTGCATGTATTAGGCACGCCGCCAGCGTTCGTCCTGAGCCAGGATCGAAACTCT
>738
CTGCTGCCGCCCGTAGGAGTCTGGACCGTGTCTCAGTTCCAGTGTGGCCGATCACCCTCTCAGGTCGGCTACG
TATCGTTGCCTTGGTAAGCCGTTACCTTACCAACTAGCTAATACGGCGCAGGTCCATCTATAAGTGATAGCAA
AGCCATCTTTCACTATCGAACCATGCGGTTCGAAATATTATCCGGTATTAGCTCCGGTTTTCCCGAAGTTATC
CCAGTCTTATACGGTAGGTTACCTACGTGTTACTCACCCGTCCGCCGCTAACGTCAAAGGAGCAAGCCTCCTC
GTCTGTTCGCTCGACTTGCATGTATTAGGCACGCCGCCAGCGTTCATCCTGAGCC
>739
CTGCTGCCGCCCGTAGGAGTCTGGGCCGTGTCTCAGTCCCAGTGTGGCCGATCACCCTCTCAGGTCGGCTATG
TATCGTCGCCTTGGTGAGCCGTTACCTCACCAACCAGCTAATACAACGCAGGTCCATCTGGTAGTGATGCAAT
TGCACCTTTTAATTGACTATCATGCAATAATCAATTTTATGCGGTATTAGCTATCGTTTCCAATAGTTATCCC
CCGCTACCAGGCAGGTTACCTACGCGTTACTCACCCGTTCGCAACTCATCCAGAGAAGCAAGCTCCTCCTCAG
CGTTCTACTGCATGTATTAGGCACGCCGCCAGCGTCGTCCTGAGCCAGGATCAACTCT
>740
CTGCTGCCGCCCGTAGGAGTCTGGGCCGTGTCTCAGTCCCAGTGTGGCCGATCACCCTCTCAGGTCGGCTATG
TATCGTCGCCTTGGTGAGCCTTTACCTCACCAACTAGCTAATACAACGCAGGTCCATCTCACAGTGAAGCAAT
TGCTCCTTTTAAATAACTAACATGTGTTAATTACTCTTATGCGGTATTAGCTATCGTTTTCCAATAGTTATCC
CCCGCTATGAGGCAGGTTACCTACGCGTCACTCACCCGTTCGCAACTCATCAGTCTAGTGTAAACACCAAACA
TCAGCGTTCTACTTGCATGTATTAGGCACGCCGCCAGCGTTCGTCCTGAGCCAGGATCAAACTCT
>741
CTGCTGCCGCCCGTAGGAGTCTGGGCCGTGTCTCAGTCCCAGTGTGGCCGATCACCCTCTCAGGTCGGCTATG
TATCGTCGCCTTGGTGAGCCCTTACCCCACCAACTAGCTAATACAACGCAGGTCCATCTGGTAGTGATGCAAT
TGCACCTTTCAAACAGTTGTCATGCAACAACTGCTATTATGCGGTATTAGCTATCGTTTCCAATAGTTATCCC
CCGCTACCAGGCAGGTTACCTACGCGTACTCACCCGTTCGCAACTCATCCGGAGAAGCAAGCTCCTCCTTCAG
CGTCTACTTGCCATGTATTAGGCACGCCGCCAGCGTTCGTCCTGAGCCAGGATCAAACTCT

>742
CTGCTGCCGCCCGTAGGAGTCTGGACCGTGTCTCAGTTCCAGTGTGGCCGATCACCCTCTCAGGTCGGCTACG
TATCGTTGCCTTGGTAAGCCGTTACCTTACCAACTAGCTAATACGGCGCAGGTCCATCTATAAGTGATAGCAG
ATCCATCTTTCACTATCGAACCATGCGGTTCGAAATATTATCCGGTATTAGCTCCGGTTTCCCGAAGTTATCC
CAGTCTTATAGGTAGGTTACCCACGTGTTACTCACCCGTCCGCCGCTAACGTCAAAGGAGCAAGCTCCTCGTC
TGTTCGCTCGACTTGCATGTATTAGGCACGCCGCCAGCGTTCATCCTGAGCCAGAATCAAACTCT
>743
CTGCTGCCGCCCGTAGGAGTCTGGGCCGTGTCTCAGTCCCAGTGTGGCCGATCACCCTCTCAGGTCGGCTATG
TATCGTCGCCTTGGTGAGCCGTTACCTCACCAACTAGCTAATACAACGCAGGTCCATCTGGTAGTGATGCAAT
TGCACCTTTTAATTGACTATCATGCAATAATCAATTTTATGCGGTATTTAGCCTATCGTTTTCCAATAGTTTA
TCCCCCCCGCTACCAGGCAGGTTACCTACGCGTTACTCACCCGTTTCGCAACTCATCCAGAGAAGCAAGCTCC
TCCTTTCAGCGTTCTACTTGCATGTATTTAGGCACGCCGCCAGCGTCGTCCTGAGCCAGGATCAAACTCT
>744
CTGCTGCCGCCCGTAGGAGTCTGGGCCGTGTCTCAGTCCCAGTGTGGCCGATCACCCTCTCAGGTCGGCTATG
TATCGTCGCCTTGGTGAGCCCTTACCCCACCAACTAGCTAATACAACGCAGGTCCATCTGGTAGTGATGCAAT
TGCACCTTTCAAACAGTTGTCATGCAACAACTGCTATTATGCGGTATTAGCTATCGTTTCCAATAGTTATCCC
CCGCTACCAGGCAGGTTACCTACGCGTTACTCACCCGTTCGCAACTCATCCAGAGAAGCAAGCTCCTCCTTCA
GCGTTCTACTTGCATGTATTAGGCACGCCGCCAGCGTCGTCCTGAGCCAGGATCAAACTCT
>745
CTGCTGCCGCCCGTAGGAGTCTGGACCGTGTCTCAGTTCCAGTGTGGCCGATCACCCTCTCAGGTCGGCTACG
CATCGTTGCCTTGGTAAGCCGTTACCTTACCAACTAGCTAATGCGGCGCGGATCCATCTATAAGTGACAGCAA
AACCGTCTTTCACTATGAACCATGCGGTTCAATATATTATCCGGTATTAGCTCCGGTTTCCCGAAGTTATCCC
AGTCTTATAGGTAGGTTATCCACGTGTTACTCACCCGTCCGCCGCTAACGTCAGAGGAGCAAGCTCCTCGTCT
GTTCGCTCGACTTGCATGTATTAGGCACGCCGCCAGCGTTCATCCTGAGCCAGGATCAACTCT
>746
CTGCTGCCGCCCGTAGGAGTCTGGGCCGTGTCTCAGTCCCAGTGTGGCCGATCACCCTCTCAGGTCGGCTATG
TATCGTCGCCTTGGTGAGCCCTTACCCCACCAACTAGCTAATACAACGCAGGTCCATCTGGTAGTGATGCAAT
TGCACCTTTCAAACAGTTGTCATGCAACAACTGCTATTATGCGGTATTAGCTATCGTTTCCAATAGTTATCCC
CCGCTACCAGGCAGGTTACCTACCGCGTTACTCACCCGTTCGCAACTCATCCGGAGAAGCAAGCTCCTCCTCA
GCGTTCTACTTGCCATGTATTAGGCACGCCGCCAGCGTTCGTCCTGAGCCAGGATCAAACTCT
>747
CTGCTGCCGCCCGTAGGAGTCTGGGCCGTGTCTCAGTCCCAGTGTGGCCGATCACCCTCTCAGGTCGGCTATG
TATCGTCGCCTTGGTGAGCCGTTACCTCACCAACTAGCTAATACAACGCAGGTCCATCTGGTAGTGATGCAAT
TGCACCTTTTAATTGACTATCATGCAATAATCAATTTATGCGGTATTAGCCTATCGTTTTCCAATAGTTATCC
CCCCCGCTACCAGGCAGGTTACCTACGCGTTACTCACCCGTTCGCAACTCATCCAGAGAAGCAAGCTCCTCCT
CAGCGTTCTACTGCATGTATTAGGCACGCCGCCAGGCG
>748
CTGCTGCCGCCCGTAGGAGTCTGGGGCCGTGCCTCAGTCCCAGTGTGGCCGATCACCCTCTCAGGTCGGCTAT
GTATCGTCGCCTTGGTGAGCCGTTACCCCACCAACTAGCTAATACAACGCAGGTCCATCTGGTAGTGATGCAA
TTGCACCTTTTAAGTAAATGTCATGCAACATCTACTCTTATGCGGTATTAGCTATCGTTTCCAATAGTTATCC
CCCGCTACCAGGCAGGTTACCTACGCGTACTCACCCGTTCGCAACTCATCCAGAAGAGCAAGCTCCTCCTCAG
CGTCTACTGCATGTATTAGGCACGCCGCCAGCGTCGTCCTGAGCCAGGATCGAAACTCT
>749
CTGCTGCCGCCCGTAGGAGTCTGGGCCGTGTCTCAGTCCCAGTGTGGCCGATCACCCTCTCAGGTCGGCTATG
TATCGTCGCCTTGGTGAGCCGTTACCCCACCAACTAGCTAATACAACGCAGGTCCATCTGGTAGTGGTGCAAT
TGCACCCTTTAAGCAAATGTCATGCAACATCTACTCTTATGCGGTATTAGCTATCGTTTCCAATAGTTATCCC
CCGCTACCAGGCAGGTTACCTACGCGTACTCACCCGTTCGCAACTCATCCGGAGAAGCAAGCTCCTCCTCAGC
GTCTACTTGCATGTATTAGGCACGCCGCCAGCGTCGTCCTGAGCCAGGATCAACTCT
>750
CTGCTGCCGCCCGTAGGAGTCTGGGCCGTGTCTCAGTCCCAGTGTGGCCGATCACCCTCTCAGGTCGGCTATG
CATCGTCGCCTTGGTAGGCCTTTACCCCACCAACTAGCTAATGCACCGCAAAGCCATCTCATAGTGTTAGCAA
AACCAACTTTAAACATCAACCATGCAGTTAATGCTGTTATCCGGTATTAGCTATCGTTTCCAATAGTTGTCCC
AGTCTATGAGGCAGGTTCTTTACGTGTTACTCACCCGTTCGCCGCTAATGTTCGTCGCAGCACCAAAGCTAAC
CTAACTCGC

>751
CTGCTGCCGCCCGTAGGAGTCTGGGCCGTGTCTCAGTCCCAGTGTGGCCGATCACCCTCTCAGGTCGGCTATG
TATCGTCGCCTTGGTGAGCCCTTACCCCACCAACTAGCTAATACAACGCAGGTCCATCTGGTAGTGATGCAAT
TGCACCTTTCAAACAGTTGTCATGCAACAACTGCTATTATGCGGTATTTAGCTATCGTTTTCCAATAGTTATC
CCCCCGCTACCAGGCAGGTTACCTACGCGTTACTCACCCGTTCGCAACTCATCCGGAGAAGCAAGCTCCTCCT
CAGCGTTCTACTTGCCATGTATTTAGGCACGCCGCCAGCGTTCGTCCTGAGCCAGGATCAAACTCT
>752
CTGCTGCCGCCCGTAGGAGTCTGGGCCGTGTCTCAGTCCCAGTGTGGCCGATCACCCTCTCAGGTCGGCTATG
TATCGTCGCCTTGGTGAGCCGTTACCTCACCAACTAGCTAATACAACGCAGGTCCATCTGGTAGTGATGCAAT
TGCACCTTTTAATTGACTATCATGCAATAATCAATTTTATGCGGTATTTAGCCTATCGTTTTCCAATAGTTTA
CTCCCCCCCGCTACCAGGCAGGTTTACCTACGCGTTACTCACCCGTTTCGCAACTCATCCAGAGAAGCAAGCT
CCTCCTTTCAGCGTTCTACTTGCATGTATTTAGGCACGCCGCCAGCGTTCGTCCTGAGCCAGGATCAAACTCT
T
>753
CTGCTGCCGCCCGTAGGAGTCTGGGCCGTGTCTCAGTCCCAGTGTGGCCGATCACCCTCTCAGGTCGGCTATG
TATCGTCGCCTTGGTGAGCCCTTACCCCACCAACTAGCTAATACAACGCAGGTCCATCTGGTAGTGATGCAAT
TGCACCTTTCAAACAGTTGTCATGCAACAACTGCTATTATGCGGTATTAGCTATCGTTTTCCAATAGTTATCC
CCCGCTACCAGGCAGGTTACCTACGCGTTACTCACCCGTTCGCAACTCATCCAGAGAAGCAAGCTCCTCCTTC
AGCGTTCGACTTGCATGTATTAGGCACGCCGCCAGCGTCGTCCTGAGCCAGGATCAAACTCT
>754
CTGCTGCCGCCCGTAGGAGTCTGGGCCGTGTCTCAGTCCCAGTGTGGCCGATCACCCTCTCAGGTCGGCTATG
TATCGTCGCCTTGGTGAGCCGTTACCCCACCAACTAGCTAATACAACGCAGGTCCATCTGGTAGTGGTGCAAT
TGCACCTTTTAAGCAAATGTCATGCAACATCTACTCTTATGCGGTATTAGCTATCGTTTCCAATAGTTATCCC
CCGCTACCAGGCAGGTTACCTACGCGTTACTCACCCGTTCGCAACTCATCCGGAGAAGCAAGCTCCTCCTTCA
GCGTTCTACTTGCATGTATTTAGGCACGCCGCCAGCGTTCGTCCTGAGCCAGGATCAAACTCT
>755
CTGCTGCCGCCCGTAGGAGTCTGGACCGTGTCTCAGTTCCAGTGTGGCCGATCACCCTCTCAGGTCGGCTACG
TATCGTTGCCTTGGTAAGCCGTTACCTTACCAACTAGCTAATACGGCGCAGGTCCATCTATAAGTGATAGCAA
AGCCATCTTTCACTATCGAACCATGCGGTTCGAAATATTATCCGGTATTAGCTCCGGTTTTTCCCGAAGTTAT
CCCAGTCTTATAGGTAGGTTTACCTACGTGTTACTCACCCGTCCGCCGCTAACGTCAAAGGAGCAAGCTCCTC
GTCTGTTCGCTCGACTTGCATGTATTAGGCACGCCGCCAGCGTTCATCCTGAGCCAGGATCAAACTCT
>756
CTGCTGCCGCCCGTAGGAGTCTGGGCCGTGTCTCAGTCCCAGTGTGGCCGATCACCCTCTCAGGTCGGCTATG
TATCGTCGCCTTGGTGAGCCGTTACCTCACCAACTAGCTAATACAACGCAGGTCCATCTGGTAGTGATGCAAT
TTGCACCTTTTAATTGACTATCATGCAATAATCAATTTTATGCGGTATTAGCCTATCGTTTCCAATAGTTACT
CCCCCGCTACCAGGCAGGTTACCTACGCGTTACTCACCCGTTCGCAACTCATCCAGAGAAGCAAGCTCCTCCT
TCAGCGTTCTACTTGCATGTATTAGGCACGCCGCCAGCGTTCGTCCTGAGCCAGGATCAAACTCT
>757
CTGCTGCCGCCCGTAGGAGTCTGGGCCGTGTCCCAGTCCCAGTGTGGCCGGTCGCCCTCTCAGGCCGGCTACC
CGTCATCGCCTTGGTGAGCCATTACCTCGCCAACAAACTGATAGGCCGCGAGTCCATCCCCCACCGAAAAACT
TTCCACAACACACCATGCGGTGTGAAGTCATATCCGGTATTAGCCCCGATTTCCCGAGGTTATCCCGAAGAGG
GGGGCAGGTTACTCACGCGTTACTCACCCGTTCGCCACTAATCGGACAGGGCAAGCCCTGTCGTCATCGTTCG
ACTTGCATGTGTTAAGCACGCCGCCAGCGCTCGTCCTGAGCCAGGATCAAACTCT
>758
CTGCTGCCGCCCGTAGGAGTCTGGGCCGTGTCTCAGTCCCAGTGTGGCCGATCACCCTCTCAGGTCGGCTATG
TATCGTCGCCTTGGTGAGCCGTTACCTCACCAACTAGCTAATACAACGCAGGTCCATCTGGTAGTGATGCAAT
TGCACCTTTTAATTGACTATCATGCAATAATCAATTTTATGCGGTATTAGCCTATCGTTTCCAATAGTTATCC
CCCGCTACCAGGCAGGTTACCTACGCGTTACTCACCCGTTCGCAACTCATCCAGAGAAGCAAGCTCCTCCTTC
AGCGTTCTACTTGCATGTATTAGGCACGCCGCCAGCGTTCGTCCTGAGCCAGGATCAAACTCT
>759
CTGCTGCCGCCCGTAGGAGTCTGGGCCGTGTCTCAGTCCCAGTGTGGCCGATCACCCTCTCAGGTCGGCTATG
TATCGTCGCCTTGGTGAGCCCTTACCCCACCAACTAGCTAATACAACGCAGGTCCATCTGGTAGTGATGCAAT
TGCACCTTTCAAACAGTTGTCATGCAACAACTGCTATTATGCGGTATTAGCTATCGTTTTCCAATAGTTATCC

CCCGCTACCAGGCAGGTTACCTACCGCGTTACTCACCCGTTCGCAACTCATCCAGAGAAGCAAGCTCCTCCTT
CAGCGTTCTACTTGCATGTATTAGGCACGCCGCCAGCGTTCGTCCTGAGCCAGGATCAAACTCT
>760
CTGCTGCCGCCCGTAGGAGTCTGGGCCGTGTCTCAGTCCCAGTGTGGCCGATCACCCTCTCAGGTCGGCTATG
TATCGTCGCCTTGGTGAGCCGTTACCTCACCAACTAGCTAATACAACGCAGGTCCATCTGGTAGTGATGCAAT
TGCACCTTTTTAATTGACTATCATGCAATAATCAATTTTATGCGGTATTTTAGCCTATCGTTTTTCCAATAGT
TTATCCCCCCCGCTACCAGGCAGGTTTACCTACGCGTTACTCACCCGTTTCGCAACTCATCCAGAGAAGCAAG
CTCCTCCTTTCAGCGTTCTACTTGCATGTATTTAGGCACGCCGCCAGCGTCGTCCTGAGCCAGGATCAAACTC
T
>761
CTGCTGCCGCCCGTAGGAGTCTGGGCCGTGTCTCAGTCCCAGTGTGGCCGATCACCCTCTCAGGTCGGCTATG
TATCGTCGCCTTGGTGAGCCGTTACCCCACCAACTAGCTAATACAACGCAGGTCCATCTGGTAGTGGTGCAAT
TGCACCTTTTTAAGCAAATGTCATGCAACATCTACTCTTTATGCGGTATTTAGCATCGTTTTTCCAATAGTTT
TATCCCCCCCGCTACCAGGCAGGTTACCTACGCGTTACTCACCCGTTCGCAACTCATCCGGAGAAGCAAGCTC
CTCCTCAGCGTTCTACTTGCATGTATTTTAGGCACGCCGCCAGCGTTCGCCTGAGCCAGGATCAAACTCT
>762
CTGCTGCCGCCCGTAGGAGTCTGGGCCGTGTCTCAGTCCCAGTGTGGCCGATCACCCTCTCAGGTCGGCTATG
TATCGTCGCCTTGGTGAGCCGTTACCCCACCAACTAGCTAATACAACGCAGGTCCATCTGGTAGTGATGCAAT
TGCACCTTTTAATTGACTATCATGCAATAATCAATTTTATGCGGTATTAGCTATCGTTTTCCAATAGTTATCC
CCCGCTACCAGGCAGGTTACCTACGCGTTACTCACCCGTTCGCAACTCATCCAGAGAAGCAAGCTCCTCCTTC
AGCGTTCTACTTGCATGTATTAGGCACGCCGCCAGCGTCGTCCTGAGCCAGGATCAAACTCT
>763
CTGCTGCCGCCCGTAGGAGTCTGGGCCGTGTCTCAGTCCCAGTGTGGCCGATCACCCTCTCAGGTCGGCTACG
TATCGTCGCCTTGGTAAGCCGTTACCTTACCAACTAGCTAATACGGCGCGGTCCATCTATAAGTGATAGCAAA
CCATCTTTCACTATCGAACCATGCGGTTCGAAATATTATCCGGTATTAGCTCCGGTTTTTCCCGAAGTTATCC
CAGTCTTATAGGTAGGTTTTACCCACGTGTTTACTCACCCGTCCGCCGCTAACGTCAAAGGAGCAAGCTCCTC
GTCTGTTCGCTCGACTTGCATGTATAGGCACGCCGCCAGCGTCATCCTGAGCCAGGATCAAACTCT
>764
CTGCTGCCGCCCGTAGGAGTCTGGGCCGTGTCTCAGTCCCAGTGTGGCCGATCACCCTCTCAGGTCGGCTATG
TATCGTCGCCTTGGTGAGCCGTTACCTCACCAACTAGCTAATACAACGCAGGTCCATCTGGTAGTGATGCAAT
TGCACCTTTTAATTGACTATCATGCAATAATCAATTTTATGCGGTATTAGCCTATCGTTTCCAATAGTTATCC
CCCGCTACCAGGCAGGTACCTACGCGTTACTCACCCGTTCGCAACTCATCCAGAGAAGCAAGCTCCTCCTTCA
GCGTTCTACTGCATGTATTACGGCACGCCGCCAGCGTTCGTCCTGAGCCAGGATCAAACTCT
>765
CTGCTGCCGCCCGTAGGAGTCTGGACCGTGTCTCAGTTCCAGTGTGGCCGATCACCCTCTCAGGTCGGCTACG
TATCGTTGCCTTGGTAAGCCGTTACCTTACCAACTAGCTAATACGGCGCAGGTCCGTCTATAAGTGATAGCAA
AGCCATCTTTCACTATCGAACCATGCAGTTCGAAATATTATCCGGTATTAGCTCCGGTTTTTCCCGAAGTTAT
CCCAGTCTTATAGGTAGGTTTTACCTACGTGTTTACTCACCCGTCCGCCGCTAACGTCAAAGGAGCAAGCTCT
CATCCTCGTC
>766
CTGCTGCCGCCCGTAGGAGTCTGGGCCGTGTCTCAGTCCCAGTGTGGCCGATCACCCTCTCAGGTCGGCTATG
CATCGTCGCCTTGGTAGGCCTTTACCCCACCAACTAGCTAATGCACCGCAAAGCCATCTCATAGTGTTAGCAA
AACCAACTTTTAAACATCAACCATGCAGTTAATGCTGTTATCCGGTATTAGCTATCGTTTTCCAATAGCTTGT
CCCAGTCTATGAGGCAGGTTTCTTTACGTGTTACTCACCCGTTTCGCCGCTAAGTTGTTCGTCGCAGCACCAA
AGCTAACCTAACTCGACTCGGC
>767
CTGCTGCCGCCCGTAGGAGTCTGGGCCGTGTCTCAGTCCCAGTGTGGCCGATCACCCTCTCAGGTCGGCTATG
TATCGTCGCCTTGGTGAGCCGTTACCCCACCAACTAGCTAATACAACGCAGGTCCATCTGGTAGTGGTGCAAT
TGCACCTTTTAAGCAAATGTCATGCAACATCTACTCTTATGCGGTATTAGCTATCGTTTCCAATAGTTATCCC
CCGCTACCAGGCAGGTTACCTACGCGTTACTCACCCGTTCGCAACTCATCCGGAGAAGCAAGCTCCTCCTCAG
CGTTCTACTTGCATGTATTAGGCACGCCGCCAGCGTCGTCCTGAGCCAGGATCAAACTCT
>768
CTGCTGCCGCCCGTAGGAGTCTGGGCCGTGTCTCAGTCCCAGTGTGGCCGATCACCCTCTCAGGTCGGCTATG
TATCGTCGCCTTGGTGAGCCGTTACCCCACCAACTAGCTAATACAACGCAGGTCCATCTGGTAGTGGTGCAAT

TGCACCTTTTAAGCAAATGTCATGCAACATCTACTCTTATGCGGTATTAGCTATCGTTTCCAATAGTTATCCC
CCGCTACCAGGCAGGTTACCTACGCGTACTCACCCGTTCGCAACTCATCCGGAGAAGCAAGCTCCTCCTCAGC
GTTCTACTTGCATGTATTAGGCACGCCGCCAGCGTTCGTCCTGAGCCAGGATCAAACTCT
>769
CTGCTGCCGCCCGTACGGAGTCTGGGCCGTGTCTCAGTCCCAGTGTGGCCGATCACCCTCTCAGGTCGGCTAT
GTATCGTCGCCTTGGTGAGCCGTTACCCCACCAACTAGCTAATACAACGCAGGTCCATCTGGTAGTGGTGCAA
TTGCACCTTTTAAGCAAACGTCATGCAACATCTACTCTTATGCGGTATTAGCTATCGTTTCCAATAGTATCCC
CCGCTACCAGGCAGGTTACCTACGCGTACTCACCCGTTCGCAACTCATCCGGAGAAGCAAGCTCCTCCTCAGC
GTTCTACTGCATGTATTAGGCACGCCGCCAGCGTTCGTCCTGAGCCAGGATCAAACTCT
>770
CTGCTGCCGCCCGTAGGAGTCTGGGCCGTGTCTCAGTCCCAGTGTGGCCGATCACCCTCTCAGGTCGGCTATG
TATCGTCGCCTTGGTGAGCCGTTACCTCACCAACTAGCTAATACAACGCAGGTCCATCTGGTAGTGATGCAAT
TGCACCTTTTAATTGACTATCATGCAATAATCAATTTTATGCGGTATTAGCTATCGTTTTCCAATAGTTATCC
CCCGCTACCAGGCAGGTTACCTACGCGTTACTCACCCGTTCGCAACTCATCCAGAGAAGCAAGCTCCTCCTCA
GCGTTCTACTTGCATGTATTAGGCACGCCGCCAGCGTTCGTCCTGAGCCAGGATCAAACTCT
>771
CTGCTGCCGCCCGTAGGAGTCTGGGCCGTGTCTCAGTCCCAGTGTGGCCGATCACCCTCTCAGGTCGGCTATG
CATCGTCGCCTTGGTAGGCCTTTACCCCACCAACTAGCTAATGCACCGCAAAGCCATCTCATAGTGTTAGCAA
AACCAACTTTTAAACATCAACCATGCAGTTAATGCTGTTATCCGGTATTTTAGCTATCGTTTTTCCCAATAGT
TTGTCCCAGTCTATGAGGCAGGTTTCTTTACGTGTTACTCACCCGTTTCGCCGCTAAGTTTCGTTCGCGACAG
ACACCAAAACTACGTACTCGCTGACATTGCGTAGGACTCAGCCGGC
>772
CTGCTGCCGCCCGTAGGAGTCTGGACCGTGTCTCAGTTCCAGTGTGTCTGATCGTCCTCTCAAACCAGATACT
GATCGTCGCCTTGGTAAGCCATTACCTTACCAACAAGCTAATCAGCCGCGAGCTTTTCTTTAGGCGGAAAAAT
CCGTTTCACTCTAGAGCATATGGGGTATTTGAGCAATCGTTTTCCAATTTGTTTTGTCCCCCTCCTAAAGGTA
AATTTCTCACGTGTTTACTCACCCGTCCGCCACTTGAGTTAAAAACTCTCGTACGACTTGCATGTGTTTAGGC
ATACCGCCAGCGTTCATCCTGAGCCAAGGATCAAACTCT
>773
CTGCTGCCGCCCGTAGGAGTCTGGGGCCGTGTCTCAGTCCCAGTGTGGCCGATCACCCCTCTCAGGTCGGCTA
TGTATCGTCGCCTTGGTGAGCCCTTACCCCACCAACTAGCTAATACAACGCAGGTCCATCTGGTAGTGATGCA
ATTGCACCTTTCAAACAGTTGTCATGCAACAACTGCTATTATGCGGTATTAGCTATCGTTTCCAATAGTTATC
CCCCGCTACCAGGCAGGTTACCTACGCGTACTCACCCGTTCGCAACTCATCCAGAGAAGCAAGCTCCTCCTCA
GCGTTCTACTTGCATGTATTAGGCACGCCGCCAGCGTTCGTCCTGAGCCAGGATCAAACTCT
>774
CTGCTGCCGCCCGTAGGAGTCTGGGCCGTGTCTCAGTCCCAGTGTGGCCGATCACCCTCTCAGGTCGGCTATG
TATCGTCGCCTTGGTGAGCCCTTACCCCACCAACTAGCTAATACAACGCAGGTCCATCTGGTAGTGATGCAAT
TGCACCTTTCAAACAGTTGTCATGCAACAACTGCTATTATGCGGTATTAGCTATCGTTTCCAATAGTTATCCC
CCGCTACCAGGCAGGTTACCTACGCGTTACTCACCCGTTCGCAACTCATCCGGAGAAGCAAGCTCCTCCTTCA
GCGTTCTACTTGCATGTATTAGGCACGCCGCCAGCGTTCGTCCTGAGCCAGGATCAAACTCT
>775
CTGCTGCCGCCCGTAGGAGTCTGGGCCGTGTCTCAGTCCCAGTGTGGCCGATCACCCTCTCAGGTCGGCTATG
TATCGTCGCCTTGGTGAGCCGTTACCCCACCAACTAGCTAATACAACGCAGGTCCATCTGGTAGTGGTGCAAT
TGCACCTTTCAAACAGCTGTCATGCAACAACTGCTATTATGCGGTATTAGCTATCGTTTTCCAATAGTTATCC
CCCGCTACCAGGCAGGTTACCTACGCGTTACTCACCCGTTCGCAACTCATCCGGAGAAGCAAGCTCCTCCTCA
GCGTTCTACTGCATGTATTTAGGCACGCCGCCAGCGTCGTCCTGAGCCAGGATCAAACTCT
>776
CTGCTGCCGCCCCGTAGGAGTCTGGGCCGTGTCTCAGTCCCAGTGTGGCCCGATCACCCTCTCAGGTCGGCTA
TCGTAATCGTCGAGCCTTGGGTGAGCCGTTACCCCACCAACTAGCTAATACAACGCAGGTCCATCTGGTAGTG
GTGCAATTGCACCTTTCAAACAGCTGTCATGCAACAACTGCTATTATGCGGTATTAGCTATCGTTTCCAATAG
TTATCCCCCGCTACCAGGCAGGTTACCTACGCGTACTCACCCGTTCGCAACTCATCCAGAGAAGCAAGCTCCT
CCTCAGCGTTCTACTTGCATGTATTAGGCACGCCGCCAGCGTCGTCCTGAGCCAGGATCAAACTCT
>777
CTGCTGCCGCCCGTAGGAGTCTGGGCCGTGTCTCAGTCCCAGTGTGGCCGATCACCCTCTCAGGTCGGCTATG
CATCGTCGCCTTGGTAGGCCTTTACCCCACCAACTAGCTAATGCACCGCAAAGCCATCTCATAGTGTTAGCAA

AACCAACTTTTAAACATCAACCATGCAGTTAATGCTGTTTATCCGGTATTAGCTATCGTTTTTCCAATAGTTT
TGTCCCAGTCTATGAGGCAGGTTTCTTTACGTGTTACTCACCCGTTCGCCGCTAAGTTTCGTTCGTCGACAGA
CACCAAAACTCGACTCGCTGACATTGCGTAG
>778
CTGCTGCCGCCCGTAGGAGTCTGGGCCGTGTCTCAGTCCCAGTGTGGCCGATCACCCTCTCAGGTCGGCTATG
TATCGTCGCCTTGGTGAGCCGTTACCCCACCAACTAGCTAATACAACGCAGGTCCATCTGGTAGTGATGCAAT
TGCACCTTTTAAGTAAATGTCATGCAACATCTACTCTTATGCGGTATTTAGCTATCGTTTTTCCAATAGTTTG
TCCCCCCCGCTACCAGGCAGGTTACCTACGCGTTACTCACCCGTTCGCAACTCATCCAGAGAAGCAAGCTCCT
CCTTCAGCGTTCTACTTGCATGTATTTAGGCACGCCGCCAGCGTCGTCCTGAGCCAGGATCAAACTCT
>779
CTGCTGCCGCCCGTAGGAGTCTGGGCCGTGTCTCAGTCCCAGTGTGGCCGATCACCCTCTCAGGTCGGCTATG
TATCGTCGCCTTGGTGAGCCGTTACCTCACCAACTAGCTAATACAACGCAGGTCCATCTGGTAGTGATGCAAT
TGCACCTTTTTAATTTGACTATCATGCAATAATCAATTTTTATTGCGGGTATTTTAGCTATCGTTTTTTCCAA
TAGTTTTATCCCCCCCGCTACCAGGCAGGTTTACCTACGCGTTACTCACCCCCGTTTCGAACTCATCCAGAGA
AGCAAGCTCCTCCTTTCAGCGTTCTACTTGCATTGTATTTAGGC
>780
CTGCTGCCGCCCGTAGGAGTCTGGGCCGTGTCTCAGTCCCAGTGTGGCCGATCACCCTCTCAGGTCGGCTATG
TATCGTCGCCTTGGTGAGCCGTTACCTCACCAACTAGCTAATACAACGCAGGTCCATCTGGTAGTGATGCAAT
TGCACCTTTTAATTGACTATCATGCAATAATCAATTTTATGCGGTATTAGCTATCGTTTCCAATAGTTATCCC
CCGCTACCAGGCAGGTTACCTACGCGTTACTCACCCGTTCGCAACTCATCCAGAGAAGCAAGCTCCTCCTCAG
CGTTCTACTTGCATGTATTAGGCACGCCGCCAGCGTTCGTCCTGAGCCAGGATCAAACTCT
>781
CTGCTGCCGCCCGTAGGAGTCTGGGCCGTGTCTCAGTCCCAGTGTGGCCGATCACCCTCTCAGGTCGGCTACG
TATCGTTGCCTTGGTGAGCCGTTACCTCACCAACTAGCTAATACAACGCAGGTCCATCTGGTAGTGATGCAAT
TGCACCTTTTAAGCAAATGTCATGCAACGTCTACTATTATGCGGTATTAGCTATCGTTTCCAATAGTTATCCC
CCGCTACCAGGCAGGTTACCTACGCGTACTCACCCGTTCGCAACTCATCCGCTCGGTGCAAGCACCAAGCTTC
AGCGTTCTACTTGCATGTATTAGGCACGCCGCCAGCGTTCGTCCTGAGCCAGGATCAAACTCT
>782
CTGCTGCCGCCCGTAGGAGTCTGGGCCGTGTCTCAGTCCCAGTGTGGCCGATCACCCTCTCAGGTCGGCTATG
TATCGTCGCCTTGGTGAGCCGTTACCTCACCAACTAGCTAATACAACGCAGGTCCATCTGGTAGTGATGCAAT
TTGCACCTTTTAATTGACTATCATGCAATAATCAATTTATGCGGTATTAGCTATCGTTTTCCAATAGTTATCC
CCCGCTACCAGGCAGGTACCTACGCGTACTCACCCGTTCGCAACTCATCCAGAGAAGCAAGCTCCTCCTTCAG
CGTTCTACTTGACATGTCATTA
>783
CTGCTGCCGCCCGTAGGAGTCTGGGCCGTGTCTCAGTCCCAGTGTGGCCGATCACCCTCTCAGGTCGGCTATG
TATCGTCGCCTTGGTGAGCCGTTACCCCACCAACTAGCTAATACAACGCAGGTCCATCTGGTAGTGGTGCAAT
TGCACCTTTTAAGCAAATGTCATGCAACATCTACTCTTATGCGGTATTAGCTATCGTTTCCAATAGTTATCCC
CCGCTACCAGGCAGGTACCTACGCGTACTCACCCGTTCGCAACTCATCCGGAGAAGCAAGCTCCTCCTCAGCG
TCTACTGCATGTATTAGGCACGCCGCCAGCGTCGTCCTGAGCCAGGATCAAACTCT
>784
CTGCTGCCGCCCGTAGGAGTCTGGGCCGTGTCTCAGTCCCAGTGTGGCCGATCACCCTCTCAGGTCGGCTATG
TATCGTCGCCTTGGTGAGCCGTTACCTCACCAACTAGCTAATACAACGCAGGTCCATCTGGTAGTGATGCAAT
TGCACCTTTTAATTGACTATCATGCAATAATCAATTTTATGCGGTATTAGCTATCGTTTTTCCAATAGTTATC
CCCCCCGCTACCAGGCAGGTTACCTACGCGTACTCACCCGTTCGCAACTCATCCAGAGAAGCAAGCTCCTCCT
CAGCGTTCTACTTGCATGTATTAGGCACGCCGCCAGCGTCGTCCTGAGCCAGGATCAAACTCT
>785
CTGCTGCCGCCCGTAGGAGTCTGGGCCGTGTCTCAGTCCCAGTGTGGCCGATCACCCTCTCAGGTCGGCTATG
TATCGTCGCCTTGGTGAGCCGTTACCTCACCAACTAGCTAATACAACGCAGGTCCATCTGGTAGTGATGCAAT
TTGCACCTTTAATTGACTATCATGCAATAATCAATTTTATGCGGTATTAGCCTATCGTTTCCAATAGTTACTC
CCCCGCTACCAGGCAGGTACCTACGCGTACTCACCCGTTCGCAACTCATCCAGAGAACGCAAGCTCCTCCTTC
AGTCGTTC
>786
CTGCTGCCGCCCGTAGGAGTCTGGGCCGTGTCTCAGTCCCAGTGTGGCCGATCACCCTCTCAGGTCGGCTATG
CATCGTCGCCTTGGTAGGCCTTTACCCCACCAACTAGCTAATGCACCGCAAAGCCATCTCATAGTGTTAGCAA

AACCAACTTTTAAACATCAACCATGCAGTTAATGCTGTTTATCCGGTATTTAGCTATCGTTTTCCAATAGTTT
GTCCCAGTCTATGAGGCAGGTTTCTTTACGTGTTTACTCACCCGTTTCGCCGCTAAGTTGTCGTCGCAGCGAA
CCACAACTAACGTCGAACTC
>787
CTGCTGCCGCCCGTAGGAGTCTGGGCCGTGTCTCAGTCCCAGTGTGGCCGATCACCCTCTCAGGTCGGCTATG
CATCGTCGCCTTGGTAGGCCTTTACCCCACCAACTAGCTAATGCACCGCAAAGCCATCTCATAGTGTTAGCAA
AACCAACTTTTAAACATCAACCATGCAGTTAATGCTGTTATCCGGTATTAGCTATCGTTTTCCAATAGTGTCC
CAGTCCATGAGGCAGGTTTCTTTACGTGTTACTCACCCGTTCGCCGCTAAGTTTCCGGTGCAAGCACCAAAAA
ACTTCGCTCGACTTGCATGATATTA
>788
CTGCTGCCGCCCGTAGGAGTCTGGCCGTGTCTCAGTCCCAGTGTGGCCGATCACCCTCTCAGGTCGGCTATGT
ATCGTCGCCTTGGTGAGCCGTTACCCCACCAACTAGCTAATACAACGCAGGTCCATCTGGTAGTGATGCAATT
GCACCTTTCAAACAGTTGTCATGCAACAACTGCTATTATGCGGTATTAGCTATCGTTTTCCAATAGTTATCCC
CCGCTACCAGGCAGGTTACCTACCGCGTTACTCACCCGTTCGCAACTCATCCGGAGAAGCAAGCTCCTCCTCA
GCGTTCTACTGCATGTATTAGGCACGCCGCCAGCGTTCGTCCTGACGCCAGGATCGAAACTCT
>789
CTGCTGCCGCCCGTAGGAGTCTGGGCCGTGTCTCAGTCCCAGTGTGGCCGATCACCCTCTCAGGTCGGCTATG
TATCGTCGCCTTGGTGAGCCCTGTCCACCCACCTAACTAGAATACAACGCAGGTCCATCTGGTAGTGATGCAA
TTGCACCTTTCAAACAGTTGTCATGCAACAACTGCTATTATGCGGTATTAGCTATCGTTTCCAATAGTTATCC
CCCGCTACCAGGCAGGTTACCTACCGCGTTACTCACCCGTTCGCAACTCATCCAGAGAAGCAAGCTCCTCCTT
CAGCGTTCTACTTGCCATGTATTAGGCACGCCGCCAGCGTTCGTCCTGAGCCAGGATCGAAACTCT
>790
CTGCTGCCGCCCGTAGGAGTCTGGACCGTGTCTCAGTTCCAGTGTGGCCGATCACCCTCTCAGGTCGGCTACG
TATCGTTGCCTTGGGTAAGCCGTTACCTTACCAACTAGCTAATACGGCGCAGGTCCATCTATAAGTGATAGCA
AAGCCATCTTTCACTATCGAACCATGCGGTTCGAAATATTATCCGGTATTAGCTCCGGTTTTTCCCGAAGTTA
TCCCAGTCTTATAGGTAGGTTTACCTACGTGTTACTCACCCGTCCGCCGCTAACGTCAAAGGAGCAAGCTCCT
CGTCTGTTCGCTCGACTTGCATGTATTAGGCACGCCGCCAGCGTTCATCCTGAGCCAGGATCAAACTCT
>791
CTGCTGCCGCCCGTAGGAGTCTGGGCCGTGTCTCAGTCCCAGTGTGGCTGATCGTCCTCTCAGACCAGCTACA
GATCGTCGGCTTGGTAAGCTTTTATCCCACCAACTACCTAATCTGCCATCGGCCGCTCTAGTAGCACAAGGTC
TTGCGATCCCCTGCTTTTCATCCGTAGATCTCATGCGGTATTTAGCCACTCTTTTCGAGTAGTTTATCCCCCC
CCACTACTAGGCACGTTCCGATGTATTTACTCACCCCCCGTTTCGCCACTCGTCAGCATCCGAAGACCTGTTT
TACCGTTTCGACTTGCATGTGTTAAGGCATGCCGCCAGCGTCAATCTGAGCCAGGATCAAACTCT
>792
CTGCTGCCGCCCGTAGGAGTCTGGGCCGTGTCTCAGTCCCAGTGTGGCCGATCACCCTCTCAGGTCGGCTATG
CATCGTCGCCTTGGTAGGCCTTTACCCCACCAACTAGCTAATGCACCGCAAAGCCATCTCATAGTGTTAGCAA
AACCAACTTTTAAACATCAACCATGCAGTTAATGCTGTTTATCCGGTATTTTAGCTATCGTTTTTCCAATAGT
TTGTCCCAGTCTATGAGGCAGGTTTCTTTACGTGTTTACTCACCCGTTTCGCCGCTAAGTTTCGTCGCGACAC
GACATCCAA
>793
CTGCTGCCGCCCGTAGGAGTCTGGGCCGTGTCTCAGTCCCAGTGTGGCCGATCACCCTCTCAGGTCGGCTATG
TATCGTCGCCTTGGTGAGCCGTTACCCCACCAACTAGCTAATACAACGCAGGTCCATCTGGTAGTGGTGCAAT
TGCACCTTTTAAGCAAATGTCATGCAACATCTACTCTTATGTGGTATTTAGCTATCGTTTTCCAATAGTTTAT
CCCCCCCGCTACCAGGCAGGTTACCTACGCGTTACTCACCCGTTTCGCAACTCATCCGGAGAAGCAAGCTCCT
CCTTCAGCGTTCTACTGCCATGTATTTAGGCACGCCGCCAGCGTTCGTCCTGAGCCAGGATCAAACTCT
>794
CTGCTGCCGCCCGTAGGAGTCTGGGCCGTGTCTCAGTCCCAGTGTGGCCGGTCGCCCTCTCAGGCCGGCTACC
CGTCATCGTCTTGTGAGCCATCACCTCACCAACAAACTGATAGGCCGCGAGTCCATCCCCCACCGATAATCTT
CCAACACCCACCATGCGATGAGTGCTCATATCCGGTATTACGCCACGATTTTCCCGAGGTTATCCCGAAGTGG
AGGGCAGGTTACTCACGTGTTACTCACCCGTTTCGCCACTAATCAGGCGGTGCGAGCACCACCGTCATCGTTT
CGACTTGCCATGTGTTTAAGCACGCCGCCAGCGTTC
>795
CTGCTGCCGCCCGTAGGAGTCTGGGCCGTGTCTCAGTCCCAGTGTGGCCGATCACCCTCTCAGGTCGGCTATG
TATCGTCGCCTTGGTGAGCCGTTACCCCACCAACTAGCTAATACAACGCAGGTCCATCTGGTAGTGGTGCAAT

TGCACCTTTTAAGCAAATGTCATGCAACATCTACTCTTATGCGGTATTAGCTATCGTTTTCCAATAGTTATCC
CCCGCTACCAGGCAGGTTACCTACGCGTTACTCACCCGTTCGCAACTCATCCGGAGAAGCAAGCTCCTCCTTC
AGCGTTCTACTTGCATGTATTTAGGCACGCCGCCAGCGTTCGTCCTGAGCCAGGATCAAACTCT
>796
CTGCTGCCGCCCGTAGGAGTCTGGGCCGTGTCTCAGTCCCAGTGTGGCCGATCACCCTCTCAGGTCGGCTATG
TATCGTCGCCTTGGTGAGCCCTTACCCCACCAACTAGCTAATACAACGCAGGTCCATCTGGTAGTGATGCAAT
TGCACCTTTCAAACAGTTGTCATGCAACAACTGCTATTATGCGGTATTAGCTATCGTTTTCCAATAGTTATCC
CCCGCTACCAGGCAGGTTACCTACCGCGTTACTCACCCGTTCGCAACTCATCCGGAGAAGCAAGCTCCTCCTT
CAGCGTTCTACTGCATGTATTAGGCACGCCGCCAGCGTTCGTCCTGAGCCAGGATCAAACTCT
>797
CTGCTGCCGCCCGTAGGAGTCTGGGCCGTGTCTCAGTCCCAGTGTGGCCGATCACCCTCTCAGGTCGGCTATG
TATCGTCGCCTTGGTGAGCCGTTACCCCACCAACTAGCTAATACAACGCAGGTCCATCTGGTAGTGGTGCAAT
TGCACCTTTTAAGCAAATGTCATGCAACATCTACTCTTATGCGGTATTAGCTATCGTTTCCAATAGTTATCCC
CCGCTACCAGGCAGGTTACCTACGCGTTACTCACCCGTTCGCAACTCATCCAGAGAAGCAAGCTCCTCCTTCA
GCGTTCTACTGCATGTATTAGGCACGCCGCCAGCGTTCGTCCTGAGCCAGGATCAAACTCT
>798
CTGCTGCCGCCCGTAGGAGTCTGGGCCGTGTCTCAGTCCCAGTGTGGCCGACCACCCTCTCAGGTCGGCTATG
TATCGTCGCCTTGGTGAGCCCTTACCCCACCAACTAGCTAATACAACGCAGGTCCATCTGGTAGTGATGCAAT
TTGCACCTTTCAAACAGTTGTCATGCAACAACTGCTATTATGCGGTATTTAGCTATCGTTTTCCAATAGTTTA
TCCCCCCGCTACCAGGCAGGTTACCTACGCGTTACTCACCCGTTCGCAACTCATCCGGAGAAGCAAGCTCCTC
CTCAGCGTTCTACTTGCCATGTATTTAGGCACGCCGCCAGCGTTCGTCCTGAGCCAGGATCAAACTCT
>799
CTGCTGCCGCCCGTAGGAGTCTGGGCCGTGTCTCAGTCCCAGTGTGGCCGATCACCCTCTCAGGTCGGCTATG
TATCGTTGCCTTGGTGAGCCGTTACCTCACCAACTAGCTAATACAACGCAGGTCCATCTGGTAGTGGTGCAAT
TGCACCTTTCAAGCAGCTATCATGCGATATCTACTGTTATGCGGTATTACGCTATCGTTTTTCCAATAGTTAT
CCCCCCCGCTACCAGGCAGGTTACCTACGCGTTACTCACCCGTTCGCAACTCATCCGGAAAGAGCAAGCTCCT
TCCTCAGCGTTCTACTTGCATGTATTTAGGCACGCCGCCAGCGTTCGTCCTGAGCCAGGATCAAACTCT
>800
CTGCTGCCGCCCGTAGGAGTCTGGGCCGTGTCTCAGTCCCAGTGTGGCCGATCACCCTCTCAGGTCGGCTATG
TATCGTCGCCTTGGTGAGCCCTTACCCCACCAACTAGCTAATACAACGCAGGTCCATCTGGTAGTGATGCAAT
TGCACCTTTCAAACAGTTGTCATGCAACAACTGCTATTATGCGGTATTTAGCTATCGTTTTCCAATAGTTATC
CCCCGCTACCAGGCAGGTTACCTACGCGTTACTCACCCGTTCGCAACTCATCCAGAGAAGCAAGCTCCTCCTT
CAGCGTTCTACTTGCCATGTATTAGGCACGCCGCCAGCGTCGTCCTGAGCCAGGATCAAACTCT
>801
CTGCTGCCGCCCGTAGGAGTCTGGGCCGTGTCTCAGTCCCAGTGTGGCCGATCACCCTCTCAGGTCGGCTATG
TATCGTCGCCTTGGTGAGCCCTTACCCCACCAACTAGCTAATACAACGCAGGTCCATCTGGTAGTGATGCAAT
TGCACCTTTTCAAACAGTTGTCATGCAACAACTGCTATTATGCGGTATTTAGCTATCGTTTTCCAATAGTTTA
TCCCCCCGCTACCAGGCAGGTTACCTACGCGTTACTCACCCGTCGAACTCATCCAGAGAAGCAAGCTCCTCCT
CAGCGTCTACTTGCATGTATTTAGGCACGCCGCCAGCGTCGTCCTGAGCCAGGATCAAACTCT
>802
CTGCTGCCGCCCGTAGGAGTCTGGACCGTGTCTCAGTTCCAGTGTGGCCGATCACCCTCTCAGGTCGGCTATG
CATCGTTGCCTTGGTAAGCCGTTACCTTACCAACTAGCTAATGCAGCGCGGATCCATCTATAAGTGACAGCAA
GACCGTCTTTCACTTTGAACCATGCGGTTCAAAATATTATCCGGATATTAGCTCCGGTTTTTCCCGAAGTATC
CCCAGTCTTATAGGTAGGTTATCCACGTGTTACTCGCCCGTCCGCCGCTAACATCAGAGAAGCAAGCTTCTCA
TCCGTTCGCTCGACTTGCATGTATTAGGCACGCCGCCAGCGTTCATCCTGAGCCAGGATCAAACTCT
>803
CTGCTGCCGCCCGTAGGAGTCTGGGCCGTGTCTCAGTCCCAGTGTGGCCGGTCACCCTCTCAGGCCGGCTACC
CGTCGTCGCCTTGGTGAGCCATTACCTCACCAACAAGCTGATAGGCCGTGAGCCCATCCAAGACCAGTAAACC
CTTTTCCACACACCACCATGCGGTGACATGTAGCATCCAGTATTAGACCCAGTTTTTCCCGGGCTTATCCCAG
AGTCAAGGGCAGGTTACTCACGTATTACTCACCCGTTCGCCACTCATCCACACCAGCAAGCTGGTGCTTTCAG
CGTTCGACTTGCATGTGTTAAGCACGCCGCCAGCGTTCGTCCTGAGCCAGGATCAAACTCT
>804
CTGCTGCCGCCCGTAGGAGTCTGGACCGTGTCTCAGTTCCAGTGTGGCTGGTCATCCTCTCAGACCAGCTAGA
GACCGCAGGCTTGGTAGGCCTTTACCCCACCAACTACCTAATCCCACTTGGGCTCATCTCATGGCATGCGGCC

TTGCGGTCCCGCACTTCGTCTCTCGACACTACGCGGTATTAGCTACAGTTTCCCGTAGTTATCCCCCTCCATA
AGCCAGATTCCCAAGCATTACTCACCCGTCCGCCACTCGTCAGCAAGAAAGCCAAGCTTCCTCCTGCTACCGT
TCGACTTGCATGTGTTAAGCCTGCCGCCAGCGTTCAATCTGAGCCAGGATCCAAACTCT
>805
CTGCTGCCGCCCGTAGGAGTCTGGGCCGTGTCTCAGTCCCAGTGTGGCCGATCACCCTCTCAGGTCGGCTATG
TATCGTCGCCTTGGTGAGCCGTTACCCCACCAACTAGCTAATACAACGCAGGTCCATCTGGTAGTGATGCAAT
TGCACCTTTTTAAGTAAATGTCATGCAACATCTACTCTTATGCGGTATTAGCTATCGTTTTCCAATAGTTATC
CCCCGCTACCAGGCAGGTTACCTACGCGTTACTCACCCGTTCGCAACTCATCCAGAAGAGCAAGCTCCTCCTC
AGCGTTCTACTTGCATGTATTAGGCACGCCGCCAGCGTCGTCCTGAGCCAGGATCAACTCT
>806
CTGCTGCCGCCCGTAGGAGTCTGGGCCGTGTCTCAGTCCCAGTGTGGCCGATCACCCTCTCAGGTCGGCTATG
TATCGTCGCCTTGGTGAGCCGTTACCCCACCAACTAGCTAATACAACGCAGGTCCATCTGGTAGTGGTGCAAT
TGCACCTTTTAAGCAAATGTCATGCAACATCTACTCTTATGCGGTATTAGCTATCGTTTCCAATAGTTATCCC
CCGCTACCAGGCAGGTTACCTACGCGTTACTCACCCGTTCGCAACTCATCCGGAGAAGCAAGCTCCTCCTTCA
GCGTTCTACTTGCATGTATTAGGCACGCCGCCAGCGTTCGTCCTGAGCCAGGATCAAACTCT
>807
CTGCTGCCGCCCGTAGGAGTCTGGGCCGTGTCTCAGTCCCAGTGTGGCCGATCACCCTCTCAGGTCGGCTATG
TATCGTCGCCTTGGTGAGCCGTTACCCCACCAACTAGCTAATACAACGCAGGTCCATCTGGTAGTGGTGCAAT
TGCACCTTTTAAGCAAATGTCATGCAACATCTACTCTTATGCGGTATTAGCTATCGTTTCCAATAGTTATCCC
CCGCTACCAGGCAGGTTACCTACGCGTACTCACCCGTTCGCAACTCATCCGGAGAAGCAAGCTCCTCCTCAGC
GTTCTACTTGCATGTATTAGGCACGCCGCCAGCGTTCGTCCTGAGCCAGGATCAAACTCT
>808
CTGCTGCCGCCCGTAGGAGTCTGGGCCGTGTCTCAGTCCCAGTGTGGCCGATCACCCTCTCAGGTCGGCTATG
TATCGTCGCCTTGGTGAGCCGTTACCCCACCAACTAGCTAATACAACGCAGGTCCATCTGGTAGTGGTGCAAT
TGCACCTTTTAAGCAAATGTCATGCAACATCTACTCTTATGCGGTATTAGCTATCGTTTCCAATAGTTATCCC
CCGCTACCAGGCAGGTTACCTACGCGTTACTCACCCGTTCGCAACTCATCCGGAGAAGCAAGCTCCTCCTTCA
GCGTTCTACTTGCATGTATTAGGCACGCCGCCAGCGTTCGTCCTGAGCCAGGATCAAACTCT
>809
CTGCTGCCGCTCGTAGGAGTCTGGGCCGTATCTCAGTCCCAATGTGGCCGTCCACCCTCTCAGGCCGGCTACC
CGTCGCCGCCTTGGTAGGCCATTACCCCACCAACAAGCTGATAGGCCGCGAGCTCATCCTACACCGAAAAAAC
TTCCAATCATCACACTAAAAATGGCTCCTATCCGGTATTAGACCCAGTTTCCCAGGCTTATCCCGAAGTGCAG
GGCAGATCACCCACGTGTTACTCACCCGTTCGCCACTCGAGTACCCTGCAAGCAGGGCCTTTCCGTTCGACTT
GCATGTGTTAACGCACGCCGCCAGCGTTCGTCCTGAGCCAGGATCAAACTCT
>810
CTGCTGCCGCCCGTAGGAGTCTGGGCCGTGTCTCAGTCCCAGTGTGGCCGATCACCCTCTCAGGTCGGCTATG
TATCGTCGCCTTGGTGAGCCGTTACCTCACCAACTAGCTAATACAACGCAGGTCCATCTGGTAGTGATGCAAT
TGCACCTTTTAATTGACTATCATGCAATAATCAATTTTATGCGGTATTAGCCTATCGTTTCCAATAGTTATCC
CCCGCTACCAGGCAGGTTACCTACGCGTTACTCACCCGTTCGCAACTCATCCAGAGAAGCAAGCTCCTCCTTC
AGCGTTCTACTTGCATGTATTAGGCACGCCGCCAGCGTTCGTCCTGAGCCAGGATCAAACTCT
>811
CTGCTGCCGCCCGTAGGAGTCTGGGCCGTGTCTCAGTCCCAGTGTGGCCGGTCGCCCTCTCAGGCCGGCTACC
CGTCATCGCCTTGGTGAGCCATTACCTCACCAACAAACTGATACGGCCGCGAGTCCATCCCCCACCGAAAAAC
TTTCCACAACACACCATGCGGCTGCGAAGTCATATCCCGGTATTTAGCCCCGATTTCCCGAGGTTATCCCGA
AGAGGGGGCAGGTTACTCACGTGTTACTCACCCGTTCGCCACTAATCGACAAGGGCAAGCCCTGTCGTCATCG
TTTCGACTTGCATGTGTTTAAGCAGCGCCGCCAGTCGTCC
>812
CTGCTGCCGCCCGTAGGAGTCTGGGCCGTGTCTCAGTCCCAGTGTGGCCGATCACCCTCTCAGGTCGGCTATG
CATCGTCGCCTTGGTAGGCCTTTACCCCCCCCAACTAGCTAATGCACCGCAAAGCCCATCTCATAGTGTTACG
CAAAACCAACCTTTTAAACATCAACCATGCAGTTAATGCTGTTATCCGGTATTAGCTATCGTTTCCAATAGTT
GTCCCAGTCTATGAGGCAGGTTCTTTACGTGTTACTCACCCGTTCGCCGCTAGTCGTCGCGACACGAACACCA
AACTTCGCTTCGCTGCAT
>813
CTGCTGCCGCCCGTAGGAGTCTGGGCCGTGTCTCAGTCCCAGTGTGGCCGATCACCCTCTCAGGTCGGCTATG
CATCGTCGCCTTGGTAGGCCTTTACCCCACCAACTAGCTAATACAACGCAGGTCCATCTGGTAGTGATGCAAT

TGCACCTTTCAAACAGTTGTCATGCAACAACTGCTATTATGCGGTATTAGCTATCGTTCCACATAGTTTATCC
CCCGCCTACCAGGCAGGTTACCTACGCGTTACTCACCCGTTCGCAACTCATCCAGAGAAGCAAGCTCCTCCTT
CAGCGTTCTACTTGCATGTATTAGGCACGCCGCCAGCGTTCGTCCTGAGCCAGGATCAAACTCT
>814
CTGCTGCCGCCCGTAGGAGTCTGGGCCGTGTCTCAGTCCCAGTGTGGCCGATTACTTTTTAGGTTGGCTATG
TATCGTCGCCTTGGTGAGCCCTTACCCCACCAACTAGCTAATACAACGCAGGTCCATCTGGTAGTGATGCAAT
TGCACCTTTCAAACAGTTGTCATGACAACAACTGCTATTATGCGGTATTACGCTATCGTTTTCCAATAGTTTA
TCCCCGCCTACCAGGCAGGTTTACCTACGCGTTACTCACCCGTTTCGCAACTCATCCAGAGAAGCAAGCTCCT
CCTTTCAGCGTTCTACTTGCATTGTATTTAGGCACGCCGCCAGCGTTCGTCCTGAGCCAGGATCGAAACTCT
>815
CTGCTGCCGCCCGTAGGAGTCTAGGCCGTGTCTCAGTCCCAGTGTGGCCAATCACCCTCTCAGGTCGGCTATG
TATCGTCGCCTTGGTGAGCCGTTACCCCACCAACTAGCTAATACAACGCAGGTCCATCTGGTAGTGGTGCAAT
TGCACCTTTTAAGCAAATGTCATGCAACATCTACTCTTATGCGGTATTTAGCTATCGTTTTCCAATAGTTTAT
CCCCCCGCTACCAGGCAGGTTACCTACGCGTTACTCACCCGTTTCGCAACTCATCCGGAGAAGCAAGCTCCTC
CTTCAGCGTTCTACTGCATGTATTTAGGCACGCCGCCAGCGTTCGTCCTGAGCCAGGATCAAACTCT
>816
CTGCTGCCGCCCGTAGGAGTCTGGGCCGTGTCTCAGTCCCAGTGTGGCCGATCACCCTCTCAGGTCGGCTATG
TATCGTCGCCTTGGTGAGCCGTTACCCCACCAACTAGCTAATACAACGCAGGTCCATCTGGTAGTGATGCAAT
TGCACCTTTTAAGCAAATGTCATGCAACATCTACTATTATGCGGTATTAGCTATCGTTTCCAATAGTTATCCC
CCGCTACCAGGCAGGTTACCTACGCGTTACTCACCCGTTCGCAACTCATCCAGAGAAGCAAGCTCCTCCTTCA
GCGTTCTACTTGCATGTATTAGGCACGCCGCCAGCGT
>817
CTGCTGCCGCCCTAGGAGTCTGGGCCGTGTCTCAGTCCCAGTGTGGCCGATCACCCTCTCAGGTCGGCTATGT
ATCGTCGCCTTGGTGAGCCGTTACCTCACCAACTAGCTAATACAACGCAGGTCCATCTGGTAGTGATGCAATT
TGCACCTTTTAATTGACTATCATGCAATAATCAATTTTATGCGGTATTAGCCTATCGTTTCCAATAGTTATCC
CCCGCTACCAGGCAGGTTACCTACGCGTTACTCACCCGTTCGCAACTCATCCAGAGAAGCAAGCTCCTCCTTC
AGCGTTCTACTTGCATGTATTAGGCACGCCGCCAGCGTTCGTCCTGAGCCAGGATCAAACTCT
>818
CTGCTGCCGCCCGTAGGAGTCTGGGCCGTGTCTCAGTCCCAGTGTGGCCGATCACCCTCTCAGGTCGGCTATG
TATCGTCGCCTTGGTGAGCCCTTACCCCACCAACTAGCTAATACAACGCAGGTCCATCTGGTAGTGATGCAAT
TGCACCTTTCAAACAGTTGTCATGCAACAACTGCTATTTATGCGGTTATTTAGCTATCGTTTTTCCAATAGT
TTATTCCCCCCGCTACCAGGCAGGTTTACCTACGCGTTACTCACCCGTTCGCAACTCATCCGGAGAAGCAAGC
TCCTCCTTCAGCGTCTACTTGCATGTTATTTAGGCACGCCGCCAGCGTTCGTTCCTGAGCCAGGGATCAAACT
CT
>819
CTGCTGCCGCCCGTAGGAGTCTGGGCCGTGTCTCAGTCCCAGTGTGGCCGATCACCCTCTCAGGTCGGCTATG
TATCGTCGCCTTGGTGAGCCGTTACCCCACCAACTAGCTAATACAACGCAGGTCCATCTGGTAGTGGTGCAAT
TGCACCTTTTAAGCAAATGTCATGCAACATCTACTCTTATGCGGTATTAGCTATCGTTTCCAATAGTTATCCC
CCGCTACCAGGCAGGTTACCTACGCGTACTCACCCGTTCGCAACTCATCCGGAGAAGCAAGCTCCTCCTCAGC
GTCTACTTGCATGTATTAGGCACGCCGCCAGCGTCGTCCTGAGCCAGGATCAAACTCT
>820
CTGCTGCCGCCCGTAGGAGTCTGGGCCGTGTCTCAGTCCCAGTGTGGCCGATCACCCTCTCAGGTCGGCTATG
TATCGTCGCCTTGGTGAGCCGTTACCCCACCAACTAGCTAATACAACGCAGGTCCATCTGGTAGTGATGCAAT
TGCACCTTTTAAGTAAATGTCATGCAACATCTACTCTTATGCGGTATTAGCTATCGTTTCCAATAGTTATCCC
CCGCTACCAGGCAGGTTACCTACGCGTTACTCACCCGTTCGCAACTCATCCAGAGAAGCAAGCTCCTCCTTCA
GCGTTCTACTTGCCATGTATTAGGCACGCCGCCAGCGTTCGTCCTGAGCCAGGATCAAACTCT
>821
CTGCTGCCGCCCGTAGGAGTCTGGGCCGTGTCTCAGTCCCAGTGTGGCCGATCACCCTCTCAGGTCGGCTATG
TATCGTCGCCTTGGTGAGCCGTTACCCCACCAACTAGCTAATACAACGCAGGTCCATCTGGTAGTGGTGCAAT
TGCACCTTTTAAGCAAATGTCATGCAACATCTACTTCTTATGCGGTTATTTAGCATCGTTTTCCAATAGTTTA
TCCCCCCGCTACCAGGCAGGTTACCTTACGCGTTACTCACCCGTTCGCAACTCATCCGGAGAAGCAAGCTCCT
CCTCAGCGTTCTACTTGCATTGTATTTAGGCACGCCGCCAGCGTTCGTCCTGAGCCAGGATCAAACTCTT
>822

CTGCTGCCGCCCGTAGGAGTCTGGGCCGTGTCTCAGTCCCAGTGTGGCCGATCACCCTCTCAGGTCGGCTATG
TATCGTCGCCTTGGTGAGCCCTTACCCCACCAACTAGCTAATACAACGCAGGTCCATCTGGTAGTGATGCAAT
TGCACCTTTCAAACAGTTGTCATGCAACAACTGCTATTATGCGGTATTAGCTATCGTTTTCCAATAGTTATCC
CCCGCTACCAGGCAGGTTACCTACGCGTACTCACCCGTTCGCAACTCATCCGAGAAGCAGCTACCTCCTCCAG
CTCGATGTACGTTAGTCCTAC
>823
CTGCTGCCGCCCGTAGGAGTCTGGGCCGTGTCTCAGTCCCAGTGTGGCCGATCACCCTCTCAGGTCGGCTATG
TATCGTCGCCTTGGTGAGCCGTTACCTCACCAACTAGCTAATACAACGCAGGTCCATCTGGTAGTGATGCAAT
TGCACCTTTTAATTGACTATCATGCAATAATCAATTTTATGCGGTATTAGCTATCGTTTCCAATAGTTATCCC
CCGCTACCAGGCAGGTTACCTACGCGTTACTCACCCGTTCGCAACTCATCCAGAGAAGCAAGCTCCTCCTTCA
GCGTTCTACTTGCATGTATTAGGCACGCCGCCAGCGTTCGTCCTGAGCCAGGATCAAACTCT
>824
CTGCTGCCGCCCGTAGGAGTCTGGGCCGTGTCTCAGTCCCAGTGTGGCCGATCACCCTCTCAGGTCGGCTATG
TATCGTCGCCTTGGTGAGCCGTTACCCCACCAACTAGCTAATACAACGCAGGTCCATCTGGTAGTGGTGCAAT
TGCACCTTTTAAGCAAATGTCATGCAACATCTACTCTTTATGCGGTATTTAGCTATCGTTTTTCCAATAGTTT
ATCCCCCCCGCTACCAGGCAGGTTACCTACGCGTTACTCACCCGTTCGCAACTCATCCGGAGAAGCAAGCTCC
TCCTCAGCGTTCTACTTGCATGTATTTAGGCACGCCGCCAGCGTTCGTCCTGAGCCAGGGATCAAACTCT
>825
CTGCTGCCGCCCGTAGGAGTCTGGGCCGTGTCTCAGTCCCAGTGTGGCCGATCACCCTCTCAGGTCGGCTATG
TATCGTCGCCTTGGTGAGCCGTTACCCCACCAACTAGCTAATACAACGCAGGTCCATCTGGTAGTGGTGCAAT
TGCACCTTTTAAGCAAATGTCATGCAACATCTACTCTTATGCGGTATTTAGCTATCGTTTTCCAATAGTTTAT
CCCCCCGCTACCAGGCAGGTTACCTACGCGTACTCACCCGTTCGCAACTCATCCGGAGAAGCAAGCTCCTCCT
CAGCGTTCTACTTGCATGTATTTAGGCACGCCGCCAGCGTTCGTCCTGAGCCAGGATCAAACTCT
>826
CTGCTGCCGCCCGTAGGAGTCTGGACCGTGTCTCAGTTCCAGTGTGGCCGATCACCCTCTCAGGTCGGCTACG
TATCGTTGCCTTGGTAAGCAGTTACCTTACCAACTAGCTAATACGGCGCAGGTCCATCTATAAGTGATAGCAG
AGCCATCTTTCACTATCGAACCATGCGGTTCGAAATATTATCCGGTATTAGCTCCGGTTTTTCCCGAAGTATC
CCAGTCTTATAGGTAGGTTACCCACGTGTACTCACCCGTCCGCCGCTAACGTCAAAGGAGCAAGCTCCTCGCT
GTCGCTCGACTTGCATGTATTTAGGCACGCCGCCAGCGTCACC
>827
CTGCTGCCGCCCGTAGGAGTCTGGGCCGTGTCTCAGTCCCAGTGTGGCCGATCACCCTCTCAGGTCGGCTATG
TATCGTCGCCTTGGTGAGCCGTTACCCCACCAACTAGCTAATACAACGCAGGTCCATCTGGTAGTGGTGCAAT
TGCACCTTTTAAGCAAATGTCATGCAACATCTACTCTTATGCGGTATTAGCTATCGTTTCCAATAGTTATCCC
CCGCTACCAGGCAGGTTACCTACGCGCTACTCACCCGTTCGCAACTCATCCGGAGAAGCAAGCTCCTCCTCAG
CGTCTACTTGCATGTATTAGGCACGCCGCCAGCGTTCGTCCTGAGCCAGGATCAAACTCT
>828
CTGCTGCCGCCCGTAGGAGTCTGGGCCGTGTCTCAGTCCCAGTGTGGCCGATCACCCTCTCAGGTCGGCTATG
TATCGTCGCCTTGGTGAGCCGTTACCCCACCAACTAGCTAATACAACGCAGGTCCATCTGGTAGTGGTGCAAT
TGCACCTTTTAAGCAAATGTCATGCAACATCTACTCTTATGCGGTATTAGCTATCGTTTCCAATAGTTATCCC
CCGCTACCAGGCAGGTTACCTACGCGTACTCACCCGTTCGCAACTCATCCGGAGAAGCAAGCTCCTCCTTCAG
CGTTCTACTGCATGTATTAGGCACGCCGCCAGCGTTCGTCCTGAGCCAGGATCAAACTCT
>829
CTGCTGCCGCCCGTAGGAGTCTGGGCCGTGTCTCAGTCCCAGTGTGGCCGATCACCCTCTCAGGTCGGCTATG
CATCGTCGCCTTGGTAGGCCTTTACCCCACCAACTAGCTAATGCACCGCAAAGCCATCTCATAGTGTTAGCAA
AACCAACTTTTAAACATCAACCATGCAGTTAATGCTGTTATCCGGTATTAGCTATCGTTTTCCAATAGTTGTC
CCAGTCTATGAGGCAGGTTCCTTTACGTGTTACTCACCCGTTCGCCGCTAAGTTCTTCGTCGCAGACACAAAA
CTAACGACTCGCGACTTGCAGC
>830
CTGCTGCCGCCCGTAGGAGTCTGGGCCGTGTCTCAGTCCCAGTGTGGCCGATCACCCTCTCAGGTCGGCTATG
TATCGTCGCCTTGGTGAGCCGTTACCCCACCAACTAGCTAATACAACGCAGGTCCATCTGGTAGTGGTGCAAT
TGCACCTTTTTAAGCAAATGTCATGCAACATCTACTCTTTATGCGGTATTTAGCTATCGTTTTTCCAATAGTT
TATCCCCCCCGCTACCAGGCAGGTTACCTAGCGTACTCACCCGTTCGCAACTCATCCGGAGAAGCAAGCTCCT
CCTCAGCGTTCTACTGCATGTATTTAGGCACGCCGCCAGCGTTCGTCCCGAGCCAGGATCAAACTCT
>831

CTGCTGCCGCCCGTAGGAGTCTGGGCCGTGTCTCAGTCCCAGTGTGGCCGATCACCCTCTCAGGTCGGCTATG
TATCGTCGCCTTGGTGAGCCGTTACCCCACCAACTAGCTAATACAACGCAGGTCCATCTGGTAGTGGTGCAAT
TGCACCTTTTTAAGCAAATGTCATGCAACATCTACTCTTATGCGGTATTTAGCTATCGTTTTCCAATAGTTTA
TCCCCCCGCTACCAGGCAGGTACCTACGCGTACTCACCCGTTCGCAACTCATCCGGAGAAGCAAGCTCCTCCT
CAGCGTTCTACTGCATGTATTTAGGCACGCCGCCAGCGTTCGTCCTGAGCCAGGATCAAACTCT
>832
CTGCTGCCGCCCGTAGGAGTCTGGGCCGTGTCTCAGTCCCAGTGTGGCCGATCACCCTCTCAGGTCGGCTATG
TATCGTCGCCTTGGTGAGCCCTTACCCCACCAACTAGCTAATACAACGCAGGTCCATCTGGTAGTGATGCAAT
TGCACCTTTCAAACAGTTGTCATGCAACAACTGCTATTATGCGGTATTAGCTATCGTTTCCAATAGTTATCCC
CCGCTACCAGGCAGGTTACCTACCGCGTACTCACCCGTTCGCAACCCATCCAGAGAACGCAAGCTCCCTCCTC
AAGCGTTCTACTTGCCATGTATTAGGCACGCCGCCA
>833
CTGCTGCCGCCCGTAGGAGTCTGGGCCGCGTCTCAGTCCCAGTGTGGCTGATCATCCTCTCAAACCAGCTATG
GATCGTCGGCTTGGTAGGCCATTACCCCACCAACTACCTAATCCAACGCGGGCTAATCCTTCCCCGATAAATC
TTTCCCCCAAAGGGCGTATGCGGTATTACTCTCAGTTTCCCGAGGCTATTCCGCAGAGAAGGGCATATTCCCA
CGCGTTACTCACCCGTCCGCCGCTAACCCTGAAGGGTCCGCTCGACTTGCATGTGTTAGGCCTGCCGCCAGCG
TTCGTTCTGAGCCAGGATCAAACTCT
>834
CTGCTGCCGCCCGTAGGAGTCTGGGCCGTGTCTCAGTCCCAGTGTGGCCGATCACCCTCTCAGGTCGGCTATG
CATCGTCGCCTTGGTAGGCCTTTACCCCCCACCAACTAGCTAATGCACCGCAAAGCCATCTCATAGTGTTAGC
AAAACCAACTTTAAACATCAACCATGCAGTTAATGCTGTTTATCCGGTATTTTAGCTATTCGTTTTCCAATAG
TTTGTCCCAGTCTATGAGGCAGGTTTTCTTTACGTGTTTACTCACCCGTTTCGCCGCTAAGTTTCGTTCGCGA
CACAAACATCCA
>835
CTGCTGCCGCCCGTAGGAGTCTGGGCCGTGTCTCAGTCCCAGTGTGGCCGATCACCCTCTCAGGTCGGCTATG
TATCGTCGCCTTGGTGAGCCGTTACCCCACCAACTAGCTAATACAACGCAGGTCCATCTGGTAGTGGTGCAAT
TGCACCTTTTAAGCAAATGTCATGCAACATCTACTCTTATGCGGTATTAGCTATCGTTTCCAATAGTTATCCC
CCGCTACCAGGCAGGTTACCTACGCGTACTCACCCGTTCGCAACTCATCCGGAGAAGCAAGCTCCTCCTTCAG
CGTTCTACTTGCATGTATTAGGCACGCCGCCAGCGTTCGTCCTGAGCCAGGATCAAACTCT
>836
CTGCTGCCGCCCGTAGGAGTCTGGGCCGTGTCTCAGTCCCAGTGTGGCCGATCACCCCCTCAGGTCGGCTATG
TATCGTCGCCTTGGTGAGCCCTTACCCCACCAACTAGCTAATACAACGCAGGTCCATCTGGTAGTGATGCAAT
TGCACCTTTCAAACAGTTGTCATGCAACAACTGCTATTATGCGGTATTTAGCTATCGTTTCCAATAGTTATCC
CCCGCTACCAGGCAGGTTACCTACGCGTTACTCACCCGTTCGCAACTCATCCAGAGAAGCAAGCTCCTCCTTC
AGCGTTCTACTTGCATGTATTAGGCACGCCGCCAGCGTTCGTCCTGAGCCAGGATCAAACTCT
>837
CTGCTGCCGCCCGTAGGAGTCTGGGCCGTGTCTCAGTCCCGGTGTGGCCGATCACCCTCTCAGGTCGGCTATG
TATCGTCGCCTTGGTGAGCCGTTACCCCACCAACTAGCTAATACAACGCAGGTCCATCTGGTAGTGGTGCAAT
TGCACCTTTTAAGCAAATGTCATGCAACATCTACTCTTATGCGGTATTAGCTATCGTTTCCAATAGTTATCCC
CCGCTACCAGGCAGGTTACCTACGCGTACTCACCCGTTCGCAACTCATCCGGAGAAGCAAGCTCCTCCTTCAG
CGTTCTACTTGCATGTATTAGGCACGCCGCCAGCGTTCGTCCTGAGCCAGGATCAAACTCT
>838
CTGCTGCCGCCCGTAGGAGTCTGGACCGTGTCTCAGTTCCAGTGTGGCCGATCACCCTCTCAGGTCGGCTACG
TATCGTTGCCTTGGTAAGCAGTTACCTTACCAACTAGCTAATACGGCGCAGGTCCATCTATAAGTGATAGCAG
AGCCATCTTCACTATCGAACCATGCGGTTCGAAATATTATCCGGTATTAGCTCCGGTTTCCCGAAGTTATCCC
AGTCTTATAGGTAGGTTACCCACGTGTTACTCACCCGTCCGCCGCTAACATCAAAGGAGACAAGCTCCTCGTC
TGTTCGCTCGACTTGCATGTATTAGGCACGCCGCCAGCGTTCATCCTGAGCCAGGATCAAACTCT
>839
CTGCTGCCGCCCGTAGGAGTCTGGGCCGTGTCTCAGTCCCAGTGTGGCCGATCACCCTCTCAGGTCGGCTATG
TATCGTCGCCTTGGTGAGCCCTTACCCCACCAACTAGCTAATACAACACAGGTCCATCTGGTAGTGATGCAAT
TGCACCTTTCAAACAGTTGTCATGCAACAACTGCTATTATGCGGTATTAGCTATCGTTTCCAATAGTTATCCC
CCGCTACCAGGCAGGTTACCTACGCCGTTACTCACCCGTTCGCAACTCATCCAGAGAAGCAAGCTCCTCCTTC
AGCGTTCTACTTGCCTGTATTAGGCACGCCGCCAGCGTTCGTCCTGACGCCAGGCATC
>840

CTGCTGCCGCCCGTAGGAGTCTGGGCCGTGTCTCAGTCCCAGTGTGGCCGATCACCCTCTCAGGTCGGCTATG
TATCGTCGCCTTGGTGAGCCCTTACCCCACCAACTAGCTAATACAACGCAGGTCCATCTGGTAGTGATGCAAT
TGCACCTTTCAAACAGTTGTCATGCAACAACTGCTATTATGCGGTATTAGCTATCGTTTCCAATAGTTATCCC
CCGCTACCAGGCAGGTTACCTACGCCGTTACTCACCCGTTCGCAACTCATCCACGAGAAGCAAGCCTCCTCCT
TCAGCGTTCTACTTGCCATGTATTAGGCACGCCGCCAGCGTCGT
>841
CTGCTGCCGCCCGTAGGAGTCTGGGCCGTGTCTCAGTCCCAGTGTGGCCGATCACCCTCTCAGGTCGGCTATG
TATCGTCGCCTTGGTGAGCCGTTACCCCACCAACTAGCTAATACAACGCAGGTCCATCTGGTAGTGATGCAAT
TGCACCTTTTAAGTAAATGTCGTGCAACATCTACTCTTATGCGGTATTAGCTATCGTTTTCCAATAGTTATCC
CCCGCTACCAGGCAGGTTACCTACGCGTTACTCACCCGTTCGCAACTCATCCAGAAGAGCAAGCTCCTCCTTC
AGCGTTCTACTTGCATGTATTAGGCACGCCGCCAGCGTTCGTCCTGAGCCAGGATCAAACTCT
>842
CTGCTGCCGCCCGTAGGAGTCTGGGCCGTGTCTCAGTCCCAGCTGTGGCCGATCACCCTCTCAGGTCGGCTAT
GTATCGTCGCCTTGGTGAGCCCTTACCCCACCAACTAGCTAATACAACGCAGGTCCATCTGGTAGTGATGCAA
TTGCACCTTTCAAACAGTTGTCATGCAACAACTGCTATTATGCGGTATTAGCTATCGTTTCCAATAGTTATCC
CCCGCTACCAGGCAGGTTACCTACGCGTTACTCACCCGTTCGCAACTCATCCAGAGAAGCAAGCTCCTCCTCA
GCGTTCTACTTGCCATGTATTAGGCACGCCGCCAGCGTCGTCCTGAGCCAGGATCAACTCT
>843
CTGCTGCCGCCCGTAGGAGTCTGGGCCGTGTCTCAGTCCCAGTGTGGCCGATCACCCCTCTCAGGTCGGCTAT
GCATCGTCGCCTTGGTAGGCCTTTACCCCACCAACTAGCTAATGCACCGCAAAGCCATCTCATAGTGTTAGCA
AAACCAACTTTTAAACATCAACCATGCAGTTAATGCTGTTATCCGGTATTAGCTATCGTTTTCCAATAGTTTG
TCCCAGTCTATGAGGCAGGTTTCTTTACGTGTTTACTCACCCGTTTCGCCGCTAAGTTGTCGTCGACAGCGAA
CCAGCAACTAACTCGAACCTCGGCA
>844
CTGCTGCCGCCCGTAGGAGTCTGGGCCGTGTCTCAGTCCCAGTGTGGCCGATCACCCTCTCAGGTCGGCTATG
CATCGTCGCCTTGGTGAGCCGTTACCCCACCAACTAGCTAATACAACGCAGGTCCATCTGGTAGTGATGCAAT
TGCACCTTTTAATTGACTATCATGCAATAATCAATTTTATGCGGTATTAGCTATCGTTTTCCAATAGTTATCC
CCCCGCTACCAGGCAGGTTACCTACGCGTTACTCACCCGTTCGCAACTCATCCAGAGAAGCAAGCTCCTCCTC
AGCGTTCTACTTGCATGTATTTAGGCACGCCGCCAGCGTTCGT
>845
CTGCTGCCGCCCGTAGGAGTCTGGGCCGTGTCCCAGTCCCAGTGTGGCCGATCACCCTCTCAGGTCGGCTATG
TATCGTCGCCTTGGTGAGCCGTTACCCCACCAACTAGCTAATACAACGCAGGTCCATCTGGTAGTGGTGCAAT
TGCACCTTTTAAGCAAATGTCATGCAACATCTACTCTTATGCGGTATTAGCTATCGTTTTCCAATAGTTATCC
CCCCGCTACCAGGCAGGTTTACCTACGCGTTACTCACCCGTTTCGCAACTCATCCGGAGAAGCAAGCTCCTCC
TTCAGCGTTCTACTTGCATGTATTTAGGCACGCCGCCAGCGTTCGTCCTGAGCCAGGATCAAACTCT
>846
CTGCTGCCGCCCGTAGGAGTCTGGGCCGTGTCTCAGTCCCAGTGTGGCCGATCACCCTCTCAGGTCGGCTATG
TATCGTCGCCTTGGTGAGCCCTTACCCCACCAACTAGCTAATACAACGCAGGTCCATCTGGTAGTGATGCAAT
TGCACCTTTCAAACAGTTGTCATGCAACAACTGCTATTATGCGGTATTAGCTATCGTTTCCAATAGTTATCCC
CCGCTACCAGGCAGGTTACCTACGCGTTACTCACCCGTTCGCAACTCATCCAGAGAAGCAAGCTCCTCCTTCA
GCGTTCTACTTGCATGTATTAGGCACGCCGCCAGCGTTCGTCCTGAGCCAGGATCAAACTCT
>847
CTGCTGCCGCCCGTAGGAGTCTGGGCCGTGTCTCAGTCCCAGTGTGGCCGATCACCCTCTCAGGTCGGCTATG
TATCGTCGCCTTGGTGAGCCGTTACCTCACCAACTAGCTAATACAACGCAGGTCCATCTGGTAGTGATGCAAT
TGCACCTTTTAATTGACTATCATGCAATAATCAATTTTACGCGGTATTAGCTATCGTTTCCAATAGTTATCCC
CCGCTACCAGGCAGGTTACCTACGCGTTACTCACCCGTTCGCAACTCATCCGGAGAAGCAAGCTCCTCCTTCA
GCGTTCTACTGCATGTATTAGGCACGCCGCCAGCGTTCGTCCTGAGCCAGGATCAAACTCT
>848
CTGCTGCCGCCCGTAGGAGTCTGGGCCGTGTCCCAGTCCCAGTGTGGCCGGTCGCCCTCTCAGGCCGGCTACC
CGTCATCGCCTTGGTGAGCCATTACCTCACCAACAAACTGATAGGCCGCGAGTCCATCCCCCACCGAAAAACT
TTCCACAACACACCATGCGGTGCGAAGTCATATCCGGTATTAGCCCCGATTTTCCCGAGGTTTATCCCGAAGA
GGGGGGCAGGTTACTCACGTGTTACTCACCCGTTCGCCACTAATCGGACAGGGCAAGCCCTGTCGTCATCGTT
CGACTTTGCATGTGTTTAAGCACGCCGCCAGCGTTCGCCTGAGCCAGGATCAAACTCT
>849

CTGCTGCCGCCCGTAGGAGTCTGGACCGTGTCTCAGTTCCAGTGTGGCCGATCACCCTCTCAGGTCGGCTACG
TATCGTTGCCTTGGTAAGCCGTTACCTTACCAACTAGCCAATACGGCGCAGGTCCATCTATAAGTGACAGCAA
AGCCATCTTTCACTATCGAACCATGCGGTTCGAAATATTATCCGGTATTAGCTCCGGTTTTTCCCGAAGTTAT
CCCAGTCTTATAGGTAGGTTTTACCTACGTGTTTACTCACCCGTCCGCCACTAACATCAAAGGAGCAAGCTCC
TCGTCTGTTTCGCTCGACTGCATGTATAGGCACGCCGTCCGCGTTCGCCTAGGC
>850
CTGCTGCCGCCCGTAGGAGTCTGGGCCGTGTCTCAGTCCCAGTGTGGCCGGTCACCCTCTCAGGCCGGCTACC
CGTCGTCGCCTTGGTAGGCCATTACCCCACCAACAAGCTGATAGGCCGCGAGTCCATCCACCACCGATAAATC
TTTCCACCCCATACCCATGCGAGTTGGAGTCATATCCAGTATTAGCACCGGTTTTTCCCGGAGTTATCCCAGA
GTGGTGGGCAGGTTACTCACGTGTTACTCACCCGTTCGCCACTAATCCACCCAGCAAGCTGGGCTTCATCGTT
CGACTTGCATGTGTTAAGCACGCCGCCAGCGTTCGTCCTGAGCCAGGATCAAACTCT
>851
CTGCTGCCGCCCGTAGGAGTCTGGGCCGTGTCTCAGTCCCAGTGTGGCCGATCACCCTCTCAGGTCGGCTATG
TATCGTCGCCTTGGTGAGCCCTTACCCCACCAACTAGCTAATACAACGCAGGTCCATCTGGTAGTGATGCAAT
TGCACCTTTCAAACAGTTGTCATGCAACAACTGCTATTATGCGGTATTAGCTATCGTTTCCAATAGTTATCCC
CCGCTACCAGGCAGGTTACCTACCGCGTTACTCACCCGTTCGCAACTCATCCGGAGAAGCAAGCTCCTCCTCA
GCGTTCTACTTGCATGTATTAGGCACGCCGCCAGCGTTCGTCCTGAGCCAGGATCAAACTCT
>852
CTGCTGCCGCCCGTAGGAGTCTGGGCCGTGTCTCAGTCCCAGTGTGGCCGATCACCCTCTCAGGTCGGCTATG
CATCGTCGCCTTGGTAGGCCTTTACCCCACCAACTAGCTAATGCACCGCAAAGCCATCTCATAGTGTTAGCAA
AACCAACTTTTAAACATCAACCATGCAGTTAATGCTGTTATCCGGTATCAGCTATCGTTTTTCCCAATAGTTG
TCCCAGTCTATGAGGCAGGTTTTCTTTACGTGTTTACTCACCCGTTTCGCCGCTAAGTTGTTCGTCGCAGCAC
CAAAACTAACGTACTCGCTGACATTGCAGAGGTATTCAGCC
>853
CTGCTGCCGCCCGTAGGAGTCTGGGCCGTGTCTCAGTCCCAGTGTGGCCGATCACCCTCTCAGGTCGGCTATG
TATCGTCGCCTTGGTGAGCCGTTACCCCACCAACTAGCTAATACAACGCAGGTCCATCTGGTAGTGGTGCAAT
TGCACCTTTCAAACAGCTGTCATGCAACAACTGCTATTATGCGGTATTAGCTATCGTTTCCAATAGTTATCCC
CCGCTACCAGGCAGGTTACCTACGCGTACTCACCCGTTCGCAACTCATCCAGAGAAGCAAGCTCCTCCTTCAG
CGTTCTACTTGCATGTATTAGGCACGCCGCCAGCGTCGTCCTGAGCCAGGATCAACTCT
>854
CTGCTGCCGCCCGTAGGAGTCTGGGCCGTGTCTCAGTCCCAGTGTGGCCGATCACCCTCTCAGGTCGGCTATG
CATCGTCGCCTTGGTAGGCCTTTACCCCACCAACTAGCTAATGCACCGCAAAGCCATCTCATAGTGTTAGCAA
AACCAACTTTTAAACATCAACCATGCAGTTAATGCTGTTATCCGGTATTAGCTATCGTTTCCAATAGTTGTCC
CAGTCTATGAGGCAGGTTCCTTTACGTGTTACTCACCCGTTCGCCGCTAAGTTGTTCGTCGCAGCACCAAACT
ACTACTCGCTGA
>855
CTGCTGCCGCCCGTAGGAGTCTGGACCGTGTCTCAGTTCCAGTGTGGCCGATCACCCTCTCAGGTCGGCTACG
TATCGTTGCCTTGGTAAGCCGTTACCTTACCAACTAGCTAATACGGCGCAGGTCCATCTATAAGTGATAGCAA
AGCCATCTTTCACTATCGAACCATGCGGTTCGAAATATTATCCGGTATTAGCTCCGGTTTCCCGAAGTTATCC
CAGTCTTATACGGTAGGTTACCTACGTGTTACTCACCCGTCCGCCGCTAACGTCGAAGGAGCAAGCTCCTCGT
CTGTTCGCTCGACTTGCATGTATTAGGCACGCCGCCAGCGTTCATCCTGAGCCAGGCATCAACTCT
>856
CTGCTGCCGCCCGTAGGAGTCTGGGCCGTGTCTCAGTCCCAGCGTGGCCGATCACCCTCTCAGGTCGGCTATG
TATCGTCGCCTTGGTGAGCCGTTACCCCACCAACTAGCTAATACAACGCAGGTCCATCTGGTAGTGGTGCAAT
TGCACCTTTTAAGCAAATGTCATGCAACATCTACTCTTATGCGGTATTAGCTATCGTTTCCAATAGTTATCCC
CCGCTACCAGGCAGGTTACCTACGCGTACTCACCCGTTCGCAACTCATCCGGAGAAGCAAGCTCCTCCTTCAG
CGTTCTACTTGCATGTATTAGGCGCGCCGCCAGCGTTCGTCCTGAGCCAGGATCAAACTCT
>857
CTGCTGCCGCCCGTAGGAGTCTGGGCCGTGTCTCAGTCCCAGTGTGGCCGATCACCCTCTCAGGTCGGCTATG
CATCGTCGCCTTGGTAGGCCTTTACCCCACCAACTAGCTAATGCACCGCAAAGCCATCTCATAGTGTTAGCAA
AACCAACTTTTAAACATCAACCATGCAGTTAATGCTGTTATCCGGTATTAGCTATCGTTTCCAATAGTTGTCC
CAGTCTATGAGGCAGGTTCTTTACGTGTTACTCACCCGTTCGCCGCTAAGTCTTCGTCGCAGCACCAAAACTA
CGAACTCGCTGACTTGCAGAGTATTCAGCGCCGCCAGCGGTTCGTCCTGAGCCACGG
>858

CTGCTGCCGCCCGTAGGAGTCTGGGCCGTGTCTCAGTCCCAGTGTGGCCGATCACCCTCTCAGGTCGGCTATG
TATCGTCGCCTTGGTGAGCCGTTACCTCACCAACTAGCTAATACAACGCAGGTCCATCTGGTAGTGATGCAAT
TGCACCTTTAATTGACTATCATGCAATAATCAATTTATGCGGTATTAGCCTATCGTTTCCAATAGTTATCCCC
CGCTACCAGGCAGGTTACCTACGCGTACTCACCCGTTCGCAACTCATCCAGAGAAGCAAGCTCCTCCTTCAGC
GTTCTACTGCCATGTATTAGGCACGCCGCCAGCGTCGTCCTGAGCCAGGATCAATCT
>859
CTGCTGCCGCCCGTAGGAGTCTGGGCCGTGTCTCAGTCCCAGTGTGGCCGATCACCCTCTCAGGTCGGCTATG
TATCGTCGCCTTGGTGAGCCCTTACCCCACCAACTAGCTAATACAACGCAGGTCCATCTGGTAGTGATGCAAT
TGCACCTTTCAAACAGTTGTCATGCAACAACTGCTATTATGCGGTATTAGCTATCGTTTCCAATAGTTATCCC
CCGCTACCAGGCAGGTTACCTACGCGTACTCACCCGTTCGCAACTCATCCAGAGAAGCAAGCTCCTCCTTCAG
CGTTCTACTTGCATGTATTAGGCACGCCGCCAGCGTTCGTCCTGAGCCAGGATCAAACTCT
>860
CTGCTGCCGCCCGTAGGAGTCTGGGCCGTGTCTCAGTCCCAGTGTGGCCGATCACCCTCTCAGGTCGGCTATG
TATCGTCGCCTTGGTGAGCCGTTACCCCACCAACTAGCTAATACAACGCAGGTCCATCTGGTAGTGGTGCAAT
TGCACCTTTTAAGCAAATGTCATGCAACATCTACTCTTATGCGGTATTAGCTATCGTTTCCAATAGTTATCCC
CCGCTACCAGGCAGGTTACCTACGCGTACTCACCTGTTCGCAACTCATCCGGAGAAGCAAGCTCCTCCTCAGC
GTTCTACTTGCATGTATTTAGGCACGCCGCCAGCGTTCGTCCTGAGCCAGGATCAAACTCT
>861
CTGCTGCCGCCCGTAGGGGTCTGGGCCGTGTCTCAGTCCCAGTGTGGCCGATCACCCTCTCAGGTCGGCTATG
TATCGTCGCCTTGGTGAGCCGTTACCCCACCAACTACGCTAATACAACGCAGGTCCATCTGGTAGTGGTGCAA
TTGCACCTTTTAAGCAAATGTCATGCAACATCTACTCTTATGCGGTATTAGCTATCGTTTCCAATAGTTATCC
CCCGCTACCAGGCAGGTTACCTACGCGTACTCACCCGTTCGCAACTCATCCGGAGAAGCAAGCTCCTCCTTCA
GCGTTCTACTTGCATGTATTAGGCACGCCGCCAGCGTTCGTCCTGAGCCAGGATCAAACTCT
>862
CTGCTGCCGCCCGTAGGAGTCTGGGCCGCGTCTCAGTCCCAGTGTGGCCGATCACCCTCTCAGGTCGGCTATG
TATCGTCGCCTTGGTGAGCCGTTACCTCACCAACTAGCTAATACAACGCAGGTCCATCTGGTAGTGATGCAAT
TGCACCTTTTAATTGACTATCATGCAATAATCAATTTTATGCGGTATTAGCCTATCGTTTCCAATAGTTACTC
CCCCGCTACCAAGCAGGTTACCTACGCGTTACTCACCCGTTCGCAACTCATCCAGAGAAGCAAGCTCCTCCTT
CAGCGTTCTACTTGCATGTATTAGGCACGCCGCCAGCGTTCGTCCTGAGCCAGGATCAAACTCT
>863
CTGCTGCCGCCCGTAGGAGTCTGGACCGTGTCTCAGTTCCAGTGTGGCCGATCACCCTCTCAGGTCGGCTACG
TATCGTTGCCTTGGTAAGCCGTTACCTTACCAACTAGCTAATACGGCGCAGGTCCATCTATAAGTGATAGCAA
AGCCATCTTTCACTATCGAACCATGCGGTTCGAAATATTATCCGGTATTAGCTCCGGTTTTTCCCGAAGTTAT
CCCAGTCTTATAGGTAGGTTTTACCTACGTGTTACTCACCCGTCCGCCGCTAACGTCAAAGGAGCAAGCTCCT
CGTCTGTTCGCTCGACTTGCATGTATTAGGCACGGCCGCCAGGCGTTCATCCTGAGCCCAGGATCAAAACT
>864
CTGCTGCCGCCCGTAGGAGTCTGGGCCGTGTCTCAGTCCCAGTGTGGCCGATCACCCTCTCAGGTCGGCTATG
TATCGTCGCCTTGGTGAGCCCTTACCCCACCAACTAGCTAATACAACGCAGGTCCATCTGGTAGTGGTGCAAT
TGCACCTTTTAAGCAAATGTCATGCAACATCTACTCTTATGCGGTATTAGCTATCGTTTCCAATAGTTATCCC
CCGCTACCAGGCAGGTTACCTACGCGTACTCACCCGTTCGCAACTCATCCGGAGAAGCAAGCTCCTCCTTCAG
CGTTCTACTTGCATGTATTAGGCACGCCGCCAGCGTTCGTCCTGAGCCAGGATCAAACTCT
>865
CTGCTGCCGTCCGTAGGAGTCTGGGCCGTGTCTCAGTCCCAGTGTGGCCGATCACCCTCTCAGGTCGGCTATG
TATCGTCGCCTTGGTGAGCCGTTACCTCACCAACTAGCTAATACAACGCAGGTCCATCTGGTAGTGATGCAAT
TGCACCTTTTAATTGACTATCATGCAATAATCAATTTTATGCCGGTATTAGCCTATCGTTTCCAATAGTTACT
CCCCCCGCTACCAGGCAGGTTACCTACGCGTACTCACCCGTTCGCAACTCATCCAGAGAAGCAAGTTCCTCCT
CAGCGTTCTACTTCGCATGTATTGGGCACGCCGCCAGCGTCGTCCTGA
>866
CTGCTGCCGCCCGTAGGAGTCTGGGCCGTGTCTCAGTCCCAGTGTGGCCGATCACCCTCTCAGGCCGGCTATG
TATCGTCGCCTTGGTGAGCCCTTACCCCACCAACTAGCTAATACAACGCAGGTCCATCTGGTAGTGATGCAAT
TGCACCTTTCAAACAGTTGTCATGCAACAACTGCTATTATGCGGTATTAGCTATCGTTTTCCAATAGTTATCC
CCCGCTACCAGGCAGGTTACCTACGCGTTACTCACCCGTTCGCAACTCATCCGGAGAAGCAAGCTCCTCCTTC
AGCGTTCTACTTGCATGTATTAGGCACGCCGCCAGCGTTCGTCCTGAGCCAGGATCAAACTCT
>867

CTGCTGCCGCCCGTAGGAGTCTGGGCCGTGTCTCAGTCCCAGTGTGGCCGATCACCCTCTCAGGTCGGCTATG
CATCGTCGCCTTGGTAGGCCTTTACCCCCCACCAACTAGCTAATGCACCGCAAAGCCATCTCATAGTGTTAGC
AAAACCAACTTTTAAACATCAACCATGCAGTTAATGCTGTTTATCCGGTATTTTAGCTATTCGTTTTTCCAAT
AGTTTTGTCCCCCAGTCTTATGAGGCAGGTTTTCTTTACGTGTTTACTCACCCCCCGTTTTCGCCGCTAAGTT
TTCGTTCGGC
>868
CTGCTGCCGCCCGTAGGAGTCTGGGCCGTGTCTCAGTCCCAGTGTGGCCGATCACCCTCTCAGGTCGGCTATG
TATCGTCGCCTTGGTGAGCCCTTACCCCACCAACTAGCTAATACAACGCAGGTCCATCTGGTAGTGATGCAAT
TGCACCTTTCAAACAGTCGTCATGCAACAACTGCTATTATGCGGTATTAGCTATCGTTTTCCAATAGTTATCC
CCCGCTACCAGGCAGGTTACCTACCGTGTTACTCACCCGTCCGCCGCTAACGTCAAAGGAGCAAGCTCCCCGT
CTGTTCGCTCGACTTCGCATGTATTAGGCACGCCGCCAGCGTTCATCCTGAGCCAGGATCAAACTCT
>869
CTGCTGCCGCCCGTAGGAGTCTGGGCCGTGTCTCAGTCCCAGTGTGGCCGATCACCCTCTCAGGTCGGCTATG
TATCGTCGCCTTGGTAAGCCGTTACCTCACCAACTAGCTAATACAACGCAGGTCCATCTGGTAGTGATGCAAT
TGCACCTTTTAATTGACTATCATGCAATAATCAATTTTATGCGGTATTAGCTATCGTTTCCAATAGTTATCCC
CCGCTACCAGGCAGGTTACCTACGCGTACTCACCCGTTCGCAACTCATCCAGAGAAGCAAGCCTCCTCCTTCA
GCGTTCTACTTGCCATGTATTAGGCACGCCGCCAGCGTCGTCCTGAGCCAGGATCAACTCT
>870
CTGCTGCCGCCCGTAGGAGTCTGGGCCGTGTCTCAGTCCCAGTGTGGCCGATCACCCTCTCAGGTCGGCTATG
TATCGTCGCCTTGGTGAGCCGTTACCCCACCAACTAGCTAATACAACGCAGGTCCATCTGGTAGTGGTGCAAT
TGCACCTTTTAAGCAAATGTCATGCAACATCTACTCTTATGCGGTATTAGCTATCGTTTCCAATAGTTATCCC
CCGCTACCAGGCAGGTTACCTACGCGTACTCACCCGTTCGCAACTCATCCGGAGAAGCAAGCTCCTCCTTCAG
CGTTCTACTTGCCATGTATTAGGCACACCGCCAGCGTTCGTCCTGAGCCAGGATCAAACTCT
>871
CTGCTGCCGCCCGTAGGAGTCTGGGCCGTGTCTCAGTCCCAGTGTGGCCGATCACCCTCTCAGGTCGGCTATG
TATCGTCGCCTTGGTGAGCCCTTACCCCACCAACTAGCTAATACAACGCAGGTCCATCTGGTAGTGATGCAAT
TGCACCTTTCAAACAGTTGTCATGCAACAACTGCTATTATGCGGTATTAGCTATCGTTTCCAATAGTTATCCC
CCGCTACCAGGCAGGTTACCTACCGCGTTACTCACCCGCTCGCAACTCATCCAGAGAAGCAAGCTCCTCCTCA
GCGTTCTACTTGCATGTATAGGCACGCCGCCAGCGTTCGCCGAGCCAGGATCAAACTCT
>872
CTGCTGCCGCCCGTAGGAGTCTGGGCCGTGTCTCAGTCCCAGTGTGGCCGATCACCCTCTCAGGTCGGCTATG
TATCGTCGCCTTGGTGAGCCGTTACCCCACCAACTAGCTAATACAACGCAGGTCCATCTGGTAGTGATGCAAT
TGCACCTTTTAAGTAAATGTCATGCAACATCTACTCTTATGCGGTATTAGCTATCGTTTCCAATAGTTATCCC
CCGCTACCAGGCAGGTTACCTACGCGTTACTCACCCGTTCGCAACTCATCCAGAAGAGCAAGCTCCTCCTTCA
GCGTTCTACTTGCACGTATTAGGCACGCCGCCAGCGTTCGTCCTGAGCCAGGATCAAACTCT
>873
CTGCTGCCGCCCGTAGGAGTCTGGGCCGTGTCTCAGTCCCAGTGTGGCCGATCACCCTCTCAGGTCGGCTATG
TATCGTCGCCTTGGTGAGCCGTTACCTCACCAACTAGCTAATACAACGCAGGTCCATCTGGTAGTGATGCAAT
TGCACCTTTTAATTGACTATCATGCAATAATCAATTTTATGCGGTATTAGCTATCGTTTCCAATAGTTATCCC
CCGCTACCAGGCAGGTTACCTACGCGTTACTCACCCGTTCGCAACCCATCCAGAGAAGCAAGCTCCTCCTTCA
GCGTTCTACTTGCATGTATTAGGCACGCCGCCAGCGTTCGTCCTGAGCCAGGATCAAACTCT
>874
CTGCTGCCGCCCGTAGGAGTCTGGGCCGTGTCTCAGTCCCAGTGTGGCCGATCACCCTCTCAGGTCGGCTATG
TATCGTCGCCTTGGTGAGCCGTTACCTCACCAACTAGCTAATACAACGCAGGTCCATCTGGTAGTGATGCAAT
TGCACCTTTTAATTGACTATCATGCAATAATCAATTTTATGCGGTATTAGCTATCGTTTTCCAATAGTTATCC
CCCGCTACCAGGCAGGTTACCTACGCGTTACTCACCCGTTCGCAACTCATCCAGAGAAGCAAGCTCCTCCTTC
AGCGTTCTACTTGCATGTATTAGGCACGCCGCCAGCGTTCGTCCTGAGCCAGGATCAAACTCT
>875
CTGCTGCCGCCCGTAGGAGTCTGGGCCGTGTCTCAGTCCCAGTGTGGCCGATCACCCTCTCAGGTCGGCTATG
CATCGTCGCCTTGGTAGGCCTTTACCCCACCAACTAGCTAATGCACCGCAAAGCCATCTCATAGTGTTAGCAA
AACCAACTTTTAAACATCAACCATGCAGTTAATGCTGTTATCCGGTATTAGCTATCGTTTCCAATAGTTGTCC
CAGTCTATGAGGCAGGTTCTTTACGTGTTACTCACCCGTTCGCCGCTAAGTTGTTCCGTCGACAGCACCAAAC
TAACACTCGCTGACTTGCA
>876

CTGCTGCCGCCCGTAGGAGTCTGGGCCGTGTCTCAGTCCCAGTGTGGCCGATCACCCTCTCAGGTCGGCTATG
CATCGTCGCCTTGGTAGGCCTTTACCCCACCAACTAGCTAATGCACCGCAAAGCCATCTCATAGTGTTAGCAA
AACCAACTTTTAAACATCAACCATGCAGTTAATGCTGTTATCCGGTATTAGCTATCGTTTCCAATAGTTGTCC
CAGTCTATGAGGCAGGTTCTTTACGTGTTACTCACCCGTTCGCCGCTAAGTTGTTCGTCGACAGCACCAAACT
AACACTCGCTGA
>877
CTGCTGCCGCCCGTAGGAGTCTGGGCCGTGTCTCAGTTCCAGTGTGGCCGATCACCCTCTCAGGTCGGCTATG
TATCGTCGCCTTGGTGAGCCGTTACCTCACCAACTAGCTAATACAACGCAGGTCCATCTGGTAGTGATGCAAT
TGCACCTTTTAATTGACTATCATGCAATAATCAATTTTATGCGGTATTAGCCTATCGTTTCCAATAGTTATCC
CCCGCTACCAGGCAGGTTACCTACGCGTTACTCACCCGTTCGCAACTCATCCAGAGAAGCAAGCTCCTCCCTT
CAGCGTTCTACTTGCC
>878
CTGCTGCCGCCCGTAGGAGTCTGGGCCGTGTCTCAGTCCCAGTGTGGCCGATCACCCTCTCAGGTCGGCTATG
TATCGTCGCCTTGGTGAGCCGTTACCCCACCAACTAGCTAATACAACGCAGGTCCATCTGGTAGTGGTGCAAT
TGCACCTTTTAAGCAAATGTCATGCAACATCTACTCTTATGCGGTATTAGCTATCGTTTCCAATAGTTATCCC
CCGCTACCAGGCAGGTTACCTACGCGTACTCACCCGTTCGCAACTCATCCGGAGGAGCAAGCTCCTCCTCAGC
GTTCTACTTGCATGTATTAGGCACGCCGCCAGCGTTCGCCCTGAGCCAGGATCAAACTCT
>879
CTGCTGCCGCCCGTAGGAGTCTGGGCCGTGTCTCAGTCCCAGTGTGGCCGATCACCCTCTCAGGCCGGCTATG
TATCGTCGCCTTGGTGAGCCCTTACCCCACCAACTAGCTAATACAACGCAGGTCCATCTGGTAGTGATGCAAT
TGCACCTTTCAAACAGTTGTCATGCAACAACTGCTATTTATGCGGTATTTAGCTATCGTTTTCCAATAGTTTA
TCCCCCCGCTACCAGGCAGGTTACCTACGCGTTACTCACCCGTTCGCAACTCATCCGGAGAAGCAAGCTCCTC
CTTCAGCGTTCTACTTGCATGTATTTAGGCACGCCGCCAGCGTTCGTCCTGAGCCAGGATCAAACTCT
>880
CTGCTGCCGCCCGTAGGAGTCTGGGCCGTGTCTCAGTCCCAGTGTGGCCGATCACCCTCTCAGGTCGGCTATG
TATCGTCGCCTTGGTGAGCCGTTACCCCACCAACTAGCTAATACAACGCAGGTCCATCTGGTAGTGGTGCAAT
TGCACCTTTTAAGCAAATGTCATGCAACATCTACTCTTATGCGGTATTAGCTATCGTTTCCAATAGTTATCCC
CCGCTACCAGGCAGGTTACCTACGCGTACTCACCCGTTCGCAACTCATCCGGAGAAGCAAGCTCCTCCTTCAG
CGTTCTACTTGCATGTATTAGGCACGCCGCCAGCGTTCGTCCTGAGCCAAGATCAAACTCT
>881
CTGCTGCCGCCCGTAGGAGTCTGGGCCGTGTCTCAGTCCCAGTGTGGCCGATCACCCTCTCAGGTCGGCTATG
TATCGTCGCCTTGGTGAGCCGTTACCCCCCACCAACTAGCTAATACAACGCAGGTCCATCTGGTAGTGGTGCA
ATTGCACCTTTAAGCAAATGTCATGCAACATCTACTCTTATGCGGTACTAGCTATCGTTTTCCAATAGTTTAT
CCCCCCGCTACCAGGCAGGTTACCTACGCGTACTCACCCGTTCGCAACTCATCCGGAGAAGCAAGCTCCTCCT
TCAGCGTTCTACTTGCATGTATTTAGGCACGCCGCCAGCGTTCGTCCTGAGCCAGGATCAAACTCT
>882
CTGCTGCCGCCCGTAGGAGTCTGGGCCGTGTCTCAGTCCCAGTGTGGCCGATCACCCTCTCAGGTCGGCTATG
TATCGTCGCCTTGGTGAGCCGTTACCCCACCAACTAGCTAATACAACGCAGGTCCATCTGGTAGTGGTGCAAT
TGCACCTTTTAAGCAAATGTCATGCAACATCTACTCTTATGCGGTATTAGCTATCGTTTTCCAATAGTTATCC
CCCCGCTACCAGGCAGGTTACCTACGCGTACTCACCCGTTCGCAACTCATCCAGAGAAGCAAGCTCCTCCTTC
AGCGTCTACTTGCATGTATTAGGCACGCCGCCAGCGTCGTCCTGAGCCAGGATCAAACTCT
>883
CTGCTGCCGCCCGTAGGAGTCTGGGCCGTGTCTCAGTCCCAGTGTGGCCGATCACCCTCTCAGGTCGGCTATG
TATCGTCGCCTTGGTGAGCCGTTACCTCACCAACTAGCTAATACAACGCAGGTCCATCTGGTAGTGATGCAAT
TTGCACCTTTTAATTGACTATCATGCAATAATCAATTTTATGCGGTATTAGCCTATCGTTTCCAATAGTTATC
CCCCGCTACCAGGCAGGTTACCTACGCGTTACTCACCCGTTCGCAACTCATCCAGAGAAGCAAGCTCCTCCTT
CAGCGTTCTACTTGCATGTATTAGGCACGCCGCCAGCGTTCGTCCTGAGCCAGGCATC
>884
CTGCTGCCGCCCGTAGGAGTCTGGACCGTGTCTCAATTCCAGTGTGGCCGATCACCCTCTCAGGTCGGCTATG
TATCGTCGCCTTGGTGAGCCGTTACCCCACCAACTAGCTAATACAACGCAGGTCCATCTGGTAGTGGTGCAAT
TGCACCTTTTAAGCAAATGTCATGCAACATCTACTCTTATGCGGTATTAGCTATCGTTTCCAATAGTTATCCC
CCGCTACCAGGCAGGTTACCTACGCGTACTCACCCGTTCGCAACTCATCCGGAGAAGCAAGCTCCTCCTCAGC
GTCTACTTGCATGTATTAGGCACGCCGCCAGCGTTCGTCCTGAGCCAGGATCAAACTCT
>885

CTGCTGCCGCCCGTAGGAGTCTGGGCCGTGTCTCAGTCCCAGTGTGGCCGATCACCCTCTCAGGTCGGCTATG
CATCGTCGCCTTGGTAGGCCTTTACCCCCCACCAACTAGCTAATGCACCGCAAAGCCATCTCATAGTGTTAGC
AAAACCAACTTTTAAACATCAACCATGCAGTTAATAGCTGTTTATCCGGTATTTAGCTATCGTTTTTCCAATA
GTTTTGTCCCAGTCTTATGAGGCAGGTTTCCTTTACGTGTTTACTCACCCCCGTTTTCGCCGCTAAGTTTCGT
TCGCGACACGAACATCCAAAAC
>886
CTGCTGCCGCCCGTAGGAGTCTGGGCCGTGTCTCAGTCCCAGTGTGGCCGATCACCCTCTCAGGTCGGCTATG
TATCGTCGCCTTGGTGAGCCGTTACCTCACCAACTAGCTAATACAACGCAGGTCCATCTGGTAGTGATGCAAT
TTGCACCTTTTAATTGACTATCATGCAATAATCAACTTTTATGCGGTATTAGCTATCGTTTCCAATAGTTATC
CCCCGCTACCAGGCAGGTTACCTACGCGTTACTCACCCGTTCGCAACTCATCCAGAGAAGCAAGCTCCTCCTT
CAGCGTTCTACTTGCATGTATTAGGCACGCCGCCAGCGTTCGTCCTGAGCCAGGATCAAACTCT
>887
CTGCTGCCGCCCGTACGGAGTCTGGACCGTGTCTCAGTTCCAGTGTGGCCGATCACCCTCTCAGGTCGGCTAC
GCATCGTTGCCTTGGTAAGCCGTTACCTTACCAGCTAGCTAATGCGGCGCGGATCCATCTATAAGTGACAGCA
AAACCGTCTTTCACTATGAACCATGCGGTTCAATATATTATCCGGTATTAGCTCCGGTTCCCGAAGTTATCCC
AGTCTTATGGGTAGGTTATCCACGTGTTACTCACCCGTCCGCCGCTAACGTCAGAGGAGCAAGCTCCTCGTCT
GTCGCTCGACTTGCATGTATTAGGCACGCCGCCAGCGTCATCCTGAGCCAGGATCAAACTCT
>888
CTGCTGCCGCCCGTAGGAGTCTGGGCCGTGTCTCAGTCCCAGTGTGGCCGATCACCCTCTCAGGTCGGCTATG
TATCGTCGCCTTGGTGAGCCGTTACCTCACCAACTAGCTAATACAACGCAGGTCCATCTGGTAGTGATGCAAT
TGCACCTTTTAATTGACTATCATGCAATAATCAATTTTATGCGGTATTAGCCTATCGTTTCCAATAGTTATCC
CCCGCTACCAGGCAGGTTACCTACGCGTTACTCACCCGTTCGCAACTCATCCAGAGAAGCAAGCTCCTCCTCA
GCGTTCTACTTGCATGTATTAGGCACGCCGCCAGCGTTCGTCCTGAGCCAGGATCAAACTCT
>889
CTGCTGCCGCCCGTAGGAGTCTGGGCCGTGTCTCAGTCCCAGTGTGGCCGATCACCCTCTCAGGTCGGCTATG
TATCGTCGCCTTGGTGAGCCGTTACCCCACCAACTAGCTAATACAACGCAGGTCCATCTGGTAGTGGTGCAAT
TGCACCTTTTAAGCAAATGTCATGCAACATCTACTCTTATGCGGTATTAGCTATCGTTTTCCAATAGTTATCC
CCCGCTACCAGGCAGGTTACCTACGCGTACTCACCCGTTCGCAACCCATCCGGAGAAGCAAGCTCCTCCTTCA
GCGTTCTACTTGCATGTATTAGGCACGCCGCCAGCGTTCGTCCTGAGCCGGAGATCAAACTCT
>890
CTGCTGCCGCCCGTAGGAGTCTGGGCCGTGTCTCAGTCCCAGTGTGGCCGATCACCCTCTCAGGTCGGCTATG
TATCGTCGCCTTGGTGAGCCGTTACCCCACCAACTAGCTAATACAACGCAGGTCCATCTGGTAGTGGTGCAAT
TGCACCTTTTAAGCAAATGTCATGCAACATCTACTCTTATGCGGTATTAGCTATCGTTTCCAATAGTTATCCC
CCGCTACCAGGCAGGTTACCTACGCGTACTCACCCGTTCGCAACTCATCCGGAGAAGCAAGCTCCTCCTCAGC
GTTCTACTTGCATGTATTAGGCACGCCGCCAGCGTTCGTCCTGAGCCAGGATCAAACTCT
>891
CTGCTGCCGCCCGTAGGAGTCTGGGCCGTGTCTCAGTCCCAGTGTGGCCGATCACCCTCTCAGGTCGGCTATG
TATCGTCGCCTTGGTGAGCCGTTACCCCACCAACTAGCTAATACAACGCAGGTCCATCTGATAGTGGTGCAAT
TGCACCTTTTAAGCAAATGTCATGCAACATCTACTCTTATGCGGTATTAGCTATCGTTTCCAATAGTTATCCC
CCGCTACCAGGCAGGTTACCTACGCGTACTCACCCGTTCGCAACTCATCCGGAGAAGCAAGCTCCTCCTCAGC
GTTTTACTGCATGTATTAGGCACGCCGCCAGCGTTCGTCCTGAGCCAGGATCAAACTCT
>892
CTGCTGCCGCCCGTAGGAGTCTGGGCCGTGTCTCAGTCCCAGTGTGGCCAATCACCCTCTCAGGTCGGCTATG
TATCGTCGCCTTGGTGAGCCGTTACCCCACCAACTAGCTGATACAACGCAGGTCCATCTGGTAGTGGTGCAAT
TGCACCTTTTAAGCAAATGTCATGCAACATCTACTCTTATGCGGTATTAGCTATCGTTTCCAATAGTTATCCC
CCGCTACCAGGCAGGTTACCTACGCGTACTCACCCGTTCGCAACTCATCCGGAGAAGCAAGCTCCTCCTCAGC
GTTCTACTTGCATGTATTAGGCACGCCGCAGCGTCGTCCTGAGCCAGGCACGAAACATCT
>893
CTGCTGCCGCCCGTAGGAGTCTGGGCCGTGTCTCAGTCCCAGTGTGGCCGATCACCCTCTCAGGTCGGCTATG
TATCGTCGCCTTGGTGAGCCCTTACCCCACCAACTAGCTAATACAACGCAGGTCCATCTGGTAGTGATGACAA
TTGCACCTTTCAAACAGTTGTCATGCAACAACTGCTATTATGCGGTATTAGCTATCGTTTCCAATAGTTATCC
CCCGCTACCAGGCAGGTTACCTACGCGTTACTCACCCGTTCGCAACTCATCCGAGAGACAGCACGCCTCCAGC
CTCATGTCGTCGTCCT
>894

CTGCTGCCGCCCGTACGGAGTCTGGGCCGTGTCTCAGTCCCAGTGTGGCTGATCATCCTCTCGGACCAGCTAC
TGATCATCGCCTTGGTAAGCTATTGCCTCACCAACTAGCTAATCAGACGCGAGCCCCTCCTCGGGCGGATTCC
TCCTTTTGCTCCTCAGCCTACGGGGTATTAGCAGCCGTTTTCAGCTGTTGTTCCCCTCCCAAGGGCAGGTTCT
TACGCGTTACTCACCCGTCCGCCACTGGAACACCACTTCCCGTCCGACTTGCATGTGTTAAGCATGCCGCCAG
CGTTCAGTCCGAGC
>895
CTGCTGCCGCCCGTAGGAGTCTGGGCCGTGTCTCAGTCCCAGTGTGGCCGATCACCCTCTCAGGTCGGCTATG
TATCGTCGCCTTGGTGAGCCCTTACCCCACCAACTAGCTAATACAACGCAGGTCCATCTGGTAGTGATGCAAT
TGCACCTTTCAAACAGTTGTCATGCAACAACTGCTATTATGCGGTATTAGCTATCGTTTCCAATAGTTATCCC
CCGCTACCAGGCAGGTTACCTACCGCGTACTCACCCGTTCGCAACTCATCCGGAGAAGCAAGCTCCTCCTCAG
CGTTCTACTTGCATGTATTAGGCACGCCGCCAGCGTCGTCCTGACGCCAGGCATCGAA
>896
CTGCTGCCGCCCGTAGGAGTCTGGGCCGTGTCTCAGTCCCAGTGTGGCCGATCACCCTCTCAGGTCGGCTATG
TATCGTCGCCTTGGTGAGCCGTTACCCCACCAACTAGCTAATACAACGCAGGTCCATCTGGTAGTGATGCAAT
TGCACCTTTTAAGTAAATGTCATGCAACATCTACTCTTATGCGGTATTAGCTATCGTTTCCAATAGTTATCCC
CCGCTACCAGGCAGGTTACCTACGCGTACTCACCCGTTCGCAACTCATCCAGAAGAGCAAGCTCCTCCTTCAG
CGTTCTACTTGCATGTATTAGGCACGCCGCCAGCGTCGTCCTGAGCCAGGATCAAACTCT
>897
CTGCTGCCGCCCGTAGGAGTCTGGGCCGTGTCTCAGTCCCAGTGTGGCCGATCACCCTCTCAGGTCGGCTATG
TATCGTCGCCTTGGTGAGCCGTTACCCCCACCAACTAGCTAATACAACGCAGGTCCATCTGGTAGTGATGCAA
TGCATCTTTTAAGCAAATGTCATGCAACATCTACTATTATGCGGTATTAGCTATCGTTTCCAATAGTTATCCC
CCGCTACCAGGCAGGTTACCTACGCGTACTCACCCGTTCGCAACTCATCCAGAGAAGCAAGCTCCTCCTTCAG
CGTTCTACTGCATGTATTAGGCACGCCGCCAGCGTCGTCCTGAGCCAGGATCAAACTCT
>898
CTGCTGCCGCCCGTAGGAGTCTGGGCCGTGTCTCAGTCCCAGTGTGGCCGATCACCCTCTCAGGTCGGCTATG
TATCGTCGCCTTGGTGAGCCGTTACCCCACCAACTAGCTAATACAACGCAGGTCCATCTGGTAGTGGTGCAAT
TGCACCTTTTAAGCAAATGTCATGCAACATCTACTCTTATGCGGTATTAGCTATCGTTTCCAATAGTTATCCC
CCGCTACCAGGCAGGTTACCTACGCGTACTCACCCGTTCGCAACTCATCCGGAGAAGCAAGCTCCTCCTTCAG
CGTTCTACTTGCATGTATTAGGCACGCCGCCAGCGTTCGTCCTGAGCCAGGATCAAACTCT
>899
CTGCTGCCGCCCGTAGGAGTCTGGGCCGTGTCTCAGTCCCAGTGTGGCCGATCACCCTCTCAGGTCGGCTATG
TATCGTCGCCTTGGTGAGCCGTTACCTCACCAACTAGCTAATACAACGCAGGTCCATCTGGTAGTGATGCAAT
TTGCACCTTTTAATTGACTATCATGCAATAATCAATTTTATGCGGTATTAGCCTATCGTTTTCCAATAGTTAT
CCCCCCGCTACCAGGCAGGTTACCTACGCGTTACTCACCCGTTCGCAACTCATCCAGAGAAGCAAGCTCCTCC
TTCAGCGTTCTACTTGCATGTATTAGGCACGCCGCCAGCGTTCGTCCTGAGCCAGGATCAAACTCT
>900
CTGCTGCCGCCCGTAGGAGTCTGGGCCGTGTCTCAGTCCCAGTGTGGCCGATCACCCTCTCAGGTCGGCTATG
TATCGTCGCCTTGGTGAGCCGTTACCTCACCAACTAGCTAATACAACGCAGGTCCATCTGGTAGTGATGCAAT
TTGCACCTTTTAATTGACTATCATGCAATAATCAATTTTATGCGGTATTAGCCTATCGTTTCCAATAGTTACT
CCCCCGCTACCAGGCAGGTTACCTACGCGTTACTCACCCGTTCGCAACTCATCCAGAGAAGCAAGCTCCTCCT
TCAGCGTTCTACTTGCATGTATTAGGCACGCCGCCAGCGTTCGTCCTGAGCCAGGATCAAACTCT
>901
CTGCTGCCGCCCGTAGGAGTCTGGGCCGTGTCTCAGTCCCAGTGTGGCCCGATCACCCTCTCAGGTCGGCTAT
GTATCGTCGCCTTGGTGAGCCCTTACCCCACCAACTAGCTAATACAACGCAGGTCCATCTGGTAGTGATGCAA
TTGCACCTTTCAAACAGTTGTCATGCAACAACTGCTATTATGCGGTATTAGCTATCGTTTCCAATAGTTATCC
CCCGCTACCAGGCAGGTTACCTACCGCGTACTCACCCGTTCGCAACTCATCCGGAGAAGCAGCTCCTCCTTCA
GCGTCTACTTGCATGTATTAGGCACGCCGCCAGCGTTCGTCCTGACGCCAGGATCAAACTCT
>902
CTGCTGCCGCCCGTAGGAGTCTGGACCGTGTCTCAGTTCCAGTGTGGCCGATCACCCTCTCAGGTCGGCTACG
TATCGTTGCCTTGGTAAGCCGTTACCTTACCAACTAGCTAATACGGCGCAGGTCCATCTATAAGTGATAGCAA
AGCCATCTTTCACTATCGAACCATGCAGTTCGAAATATTATCCGGTATTAGCTCCGGTTTCCCGAAGTTATCC
CAGTCTTATACGGTAGGTTTACCTACGTGTTACTCACCCGTCCGCCGCTAACGTCAAAGGCAGCAAGCTCCTC
ATCTGTTCGCTCGACTTGCATGTATTAGGCACGCCGCCAGCGTCATCCTGAGCCAGGATCAACTCT
>903

CTGCTGCCGCCCGTAGGAGTCTGGGCCGTGTCTCAGTCCCAGTGTGGCCGATCACCCTCTCAGGTCGGCTATG
TATCGTCGCCTTGGTGAGCCCTATCCACCCAACTACGACTAATACAACGCAGGTCTATCTGGTAGTGATGCAA
TTGCACCTTTCAAACAGTTGTCATGCAACAACTGCTATTATGCGGTATTAGCTATCGTTTTCCAATAGTTATC
CCCCGCTACCAGGCAGGTTACCTACCGCGTTACTCACCCGTTCGCAACTCATCCAGAGAAGCAAGCTCCTCCT
TCAGCGTTCTACTTGCCATGTATTAGGCACGCCGCCAGCGTTCGTCCTGACCAGGATCAACTCT
>904
CTGCTGCCGCCCGTAGGAGTCTGGGCCGTGTCTCAGTCCCAGTGTGGCCGATCACCCTCTCAGGTCGGCTATG
TATCGTCGCCTTGGTGAGCCCTTACCCCACCAACTAGCTAATACAACGCAGGTCCATCTGGTAGTGATGCAAT
TGCACCTTTTAATTGACTATCATGCAATAATCAATTTTATGCGGTATTAGCTATCGTTTTCCAATAGTTATCC
CCCGCTACCAGGCAGGTTACCTACGCGTTACTCACCCGTTCGCAACTCATCCAGAGAAGCAAGCTCCTCCTTC
AGCGTTCTACTTGCATGTATTAGGCACGCCGCCAGCGTTCGTCCTGAGCCAGGATCAAACTCT
>905
CTGCTGCCGCCCGTAGGAGTCTGGGCCGTGTCTCAGTCCCAGTGTGGCCGATCACCCTCTCAGGTCGGCTATG
TATCGTCGCCTTGGTGAGCCGTTACCCCCACCAACTAGCTAATACAACGCAGGTCCATCTGGTAGTGGTGCAA
TTGCACCTTTTAAGCAAATGTCATGCAACATCTACTCTTTATGCGGTATTTAGCTATCGTTTTCCAATAGTTT
ATCCCCCCCGCTACCAGGCAGGTTACCTACGCGTTACTCACCCGTTCGCAACTCATCCGGAGAAGCAAGCTCC
TCCTCAGCGTTCTACTTGCATGTATTTAGGCACGCCGCCAGCGTTCGTCCTGAGCCAGGATCAAACTCT
>906
CTGCTGCCGCCCGTAGGAGTCTGGGCCGTGTCTCAGTCCCAGTGTGGCCGATCACCCTCTCAGGTCGGCTATG
TATCGTTGCCTTGGTGAGCCGTTACCTCACCAACTAGCTAATACAACGCAGGTCCATCTGGTAGTGGCGCAAT
TGCACCTTTCAAGCAGCTATCATGCGATATCTACTGTTATGCGGTATTAGCTATCGTTTCCAATAGTTATCCC
CCGCTACCAGGCAGGTTACCTACGCGTACTCACCCGTTCGCAACTCATCCGGAAAGAGCAAGCTCCTTCCTCA
GCGTTCTACTTGCATGTATTAGGCACGCCGCCAGCGTTCGTCCTGAGCCAGGATCAAACTCT
>907
CTGCTGCCGCCCGTAGGAGTCTGGGCCGTGTCTCAGTCCCAGTGTGGCCGATCACCCTCTCAGGTCGGCTATG
TATCGTCGCCTTGGTGAGCCGTTACCTCACCAACTAGCTAATACAACGCAGGTCCATCTGGTAGTGATGCAAT
TGCACCTTTTAATTGACTATCATGCAATAATCAATTTTATGCGGTATTAGCCTATCGTTTTCCAATAGTTATC
CCCCGCTACCAGGCAGGTTACCTACGCGTTACTCACCCGTTCGCAACTCATCCAGAGAAGCAAGCTCCTCCTT
CAGCGTTCTACTTGCATGTATTAGGCACGCCGCCAGCGTTCGTCCTGAGCCAGGATCAAACTCT
>908
CTGCTGCCGCCCGTAGGAGTCTGGGCCGTGTCTCAGTCCCAGTGTGGCCGATCACCCTCTCAGGTCGGCTATG
TATCGTCGCCTTGGTGAGCCCTTACCCCACCAACTAGCTAATACAACGCAGGTCCATCTGGTAGTGATGCAAT
TGCACCTTTCAAACAGTTGTCATGCAACAACTGCTATTATGCGGTATTAGCTATCGTTTCCAATAGTTATCCC
CCGCTACCAGGCAGGTTACCTACCGCGTTACTCACCCGTTCGCAACTCATCCAGAGAAGCAAGCTCCTCCTTC
CAGCGTTCTACTTGCCATGTATTAGGCACGCCGCCAGCGTTCGTCCTGACGC
>909
CTGCTGCCGCCCGTAGGAGTCTGGGCCGTGTCTCAGTCCCAGCTGTGGCCGATCACCCTCTCAGGTCGGCTAT
GCATCGTCGCCTTGGTAGGCCTTTACCCCACCAACTAGCTAATGCACCGCAAAGCCATCTCATAGTGTTAGCA
AAACCAACTTTTAAACATCAACCATGCAGTTAATGCTGTTATCCGGTATTAGCCTATCGTTTCCAATAGTTGT
CCCAGTCTATGAGGCAGGTTCTTTACGTGTTACTCACCCGTTCGCCGCTAAGTTGTTCGTCGACAGACACCAA
ACTACGACTCGCTGACATTG
>910
CTGCTGCCGCCCGTAGGAGTATGGCCCGTGTCGCAGTGCCATTCTTGGGGGTCGCGCTCTCACGCCCCCTATC
CGTCATAGCCTTGGTGAGCCATTACCTCACCAACTAGCTGATAGACCGCAGACCATTCACAAAGCGGCTTGCG
CCTTTACCCTTGCGGGATCATGGGAAATTACCCCGTCTTTCGACGAGCTGTGCCCCACTTTGCGGTCATGTAT
CTACGTGTTACTAAGCCGTTCGCCGGTGACCTTGCGGTCCCCCCCTTTTGACTTGCTTTGCCGGACTCACCAT
TTTTCCAGCGTTTCACCCTGAGCCAGGATCAAACTCT
>911
CTGCTGCCGCCCGTAGGAGTCTGGGCCGTGTCTCAGTCCCAGTGTGGCCGATCACCCTTTATCGAGCGGCCTA
TGTATCGTCGCCTTGGTGAGCCGTTACCTCACCAACTAGCTAATACAACGCAGGTCCATCTGGTAGTGATGCA
ATTGCACCTTTTAATTGACTATCATGCAATAATCAATTTATGCGGTATTAGCCTATCGTTTCCAATAGTTATC
CCCCGCTACCAGGCAGGTTACCTACGCGTTACTCACCCGTTCGCAACTCATCCAGAGAAGCAAGCTCCTCCTT
CAGCGTTCTACTTGCATGTATTAGGCACGCCGCCAGCGTTCGTCCTGAGCCAGGATCAAACTCT
>912

CTGCTGCCGCCCGTAGGAGTCTGGGCCGTGTCTCAGTCCCAGTGTGGCCGATCACCCTCTCAGGTCGGCTATG
TATCGTCGCCTTGGTGAGCCGTTACCCCACCAACTAGCTAATACAACGCAGGTCCATCTGGTAGTGGTGCAAT
TGCACCTTTTAAGCAAATGTCATGCAACATCTACTCTTATGCGGTATTAGCTATCGTTTCCAATAGTTATCCC
CCGCTACCAGGCAGGTTACCTACGCGTACTCACCCGTTCGCAACTCATCCGGAGAAGCAAGCTCCTCCTTCAG
CGTTCTACTTGCATGTATTAGGCACGCCGCCAGCGTTCGTCCTGAGCCAGGATCAAACTCT
>913
CTGCTGCCGCCCGTAGGAGTCTGGGCCGTGTCTCAGTCCCAGTGTGGCCGATCACCCTCTCAGGTCGGCCATG
TATCGTCGCCTTGGTGAGCCGTTACCCCACCAACTAGCTAATACAACGCAGGTCCATCTGGTAGTGATGCAAT
TGCATCTTTAGTACAAATGTCATGCAACATCTACTATTATGCGGTATTAGCTATCGTTTCCAATAGTTATCCC
CCGCTACCAGGCAGGTTACCTACGCGTACTCACCCGTTCGCAACTCATCCGGAGAAGCAAGCTCCTCCTTCAG
CGTTCTACTTGCATGTATTAGGCACGCCGCCAGCGTTCGTCCTGAGCCAGGATCAAACTCT
>914
CTGCTGCCGCCCGTAGGAGTCTGGGCCGTGTCTCAGTCCCAGTGTGGCCGATCACCCTCTCAGGTCGGCTATG
CATCGTCGCCTTGGTAGGCCTTTACCCCACCAACTAGCTAATGCACCGCAAAGCCATCTCATAGTGTTAGCAA
AACCAACTTTTAAACATCAACCATGCAGTTAATGCTGTTATCCGGTATTAGCTATCGTTTCCAATAGTTGTCC
CAGTCTATGAGGCAGGTTCTTTACGTGTTACTCACCCGTTCGCCGCTAAGTTGTTCGTCGCAGCACCAAAGCT
AACCTAACTCGCTGGCTTGCAATAGTATAGGCGGCCC
>915
CTGCTGCCGCCCGTAGGAGTCTGGGCCGTGTCTCAGTCCCAGTGTGGCCGATCACCCTCTCAGGTCGGCTATG
TATCGTCGCCTTGGTGAGCCGTTACCTCACCAACTAGCTAATACAACGCAGGTCCATCTGGTAGTGATGCAAT
TGCACCTTTTAATTGACTATCATGCAATAATCAATTTTATGCGGTATTAGCCTATCGTTTTCCAATAGTTATC
CCCCGCTACCAGGCAGGTTACCTACGCGTACTCACCCGTTCGCAACTCATCCAGAGAAGCAAGCTCCTCCTCA
GCGTCTACTTGCATGTATTAGGCACGCCGCCAGCGTCGTCCTGAGCCAGGATCAAACTCT
>916
CTGCTGCCGCCCGTAGGAGTCTGGGCCGTGTCTCAGTCCCAGTGTGGCCGGTCACCCTCTCAGGCCGGCTACC
CGTCGTCGCCTTGGTGAGCCGTTACCTCACCAACAAGCTGATAGGCCGCGAGTCCATCCCAGACCGAAAAATC
TTTCCAAACGTCGACCATGCGGTCACGTCTCATATCCGGTATTAGACGCCGTTTTCCAGCGCTTATCCCAGAG
TCAGGGGCAGGTTACTCACGTGTTACTCACCCGTTCGCCACTGATCCACCAGAGCAAGCTCCAGCTTCATCGT
TCGACTTGCATGTGTTAAGCACGCCGCCAGCGTCATCCTGAGCCAGGATCAAACTCT
>917
CTGCTGCCGCCCGTAGGAGTCTGGGCCGTGTCTCAGTCCCAGTGTGGCCGATCACCCTCTCAGGTCGGCTATG
TATCGTCGCCTTGGTGAGCCGTTACCTCACCAACTAGCTAATACAACGCAGGTCCATCTGGTAGTGATGCAAT
TGCACCTTTTAATTGACTATCATGCAATAATCAATTTTATGCGGTATTAGCCTATCGTTTTCCAATAGTTATC
CCCCCCGCTACCAGGCAGGTTACCTACGCGTACTCACCCGTTCGCAACTCATCCAGAGAAGCAAGCTCCTCCT
TCAGCGTTCTACTTGCATGTATTAGGCACGCCGCCAGCGTCGTCCTGAGCCAGGCATCGAATCC
>918
CTGCTGCCGCCCGTAGGAGTCTGGGCCGTGTCTCAGTCCCAGTGTGGCCGATCACCCTCTCAGGTCGGCTATG
TATCGTCGCCTTGGTGAGCCGTTACCCCACCAACTAGCTAATACAACGCAGGTCCATCTGGTAGTGGTGCAAT
TGCACCTTTTTAAGCAAATGTCATGCAACATCTACTCTTATGCGGTATTAGCTATCGTTTTCCAATAGTTTAT
CCCCCCCGCTACCAGGCAGGTTACCTAGCGTACTCACCCGTTCGCAACTCATCCGGAGAAGCAAGCTCCTCCT
CAGCGTCTACTTGCATGTATTTAGGCACGCCGCCAGCGTCGTCCTGAGCCAGGATCGAAACTCT
>919
CTGCTGCCGCCCGTAGGAGTCTGGGCCGTGTCTCAGTCCCAGTGTGGCCGATCACCCTCTCAGGTCGGCTATG
TATCGTCGCCTTGGTGAGCCCTTACCCCACCAACTAGCTAATACAACGCAGGTCCATCTGGTAGTGATGCAAT
TTGCACCTTTCAAACAGTTGTCATGCAACAACTGCTATTTATGCGGTATTTAGCTATCGTTTTCCAATAGTTT
ATCCCCCCGCTACCAGGCAGGTTACCTACGCGTTACTCACCCGTTCGCAACTCATCCAGAGAAGCAAGCTCCT
CCTTTCAGCGTTCTACTTGCCATTGTATTAGGCACGCCGCCAGCATTCGTCCTGAGCCAGGATCAAACTCT
>920
CTGCTGCCGCCGTAGGAGTCTGGGCCGTGTCTCAGTCCCAGTGTGGCTGATCATCCTCTCAAACCAGCTATGG
ATCGTCGGCTTGGTAGGCCATTACCCCACCAACTACCTAATCCAACGCGGGCTAATCCTTCCCCGATAAATCT
TTCCCCCAAAGGGCGTATGCGGTATTACTCTCAGTTTCCCGAGGCTATTCCGCAGAGAAGGGCATATTCCCAC
GCGTTACTCACCCGTCCGCCGCTAACCCCGAAGGGTCCGCTCGACTTGCATGTGTTAGGCCTGCCGCCAGCGT
TCGTTCTGAGCCAGGATCAAACTCT
>921

CTGCTGCCGCCCGTAGGAGTCTGGGCCGTGTCTCAGTCCCAGTGTGGCCGATCACCCTCTCAGGTCGGCTATG
TATCGTCGCCTTGGTGAGCCGTTACCCCACCAACTAGCTAATACAACGCAGGTCCATCTGGTAGTGGTGCAAT
TGCGCCTTTTTAAGCAAATGTCATGCAACATCTACTCTTTATGCGGTATTTAGCTATCGTTTTCCAATAGTTT
ATCCCCCCGCTACCAGGCAGGTTACCTACGCGTTACTCACCCGTTCGCAACTCATCCGGAGAAGCAAGCTCCT
CCTTCAGCGTCTACTTGCATGTATTTAGGCACGCCGCCAGCGTTCGTCCTGAGCCAGGATCAAACTCT
>922
CTGCTGCCGCCCGTAGGAGTCTGGGCCGTGTCTCAGTCCCAGTGTGGCCGATCACCCTCTCAGGTCGGCTATG
TATCGTCGCCTTGGTGAGCCGTTACCCCACCAACTAGCTAATACAACGCAGGTCCATCTGGTAGTGATGCAAT
TGCACCTTTCAAACAGTTGTCATGCAACAACTGCTATTATGCGGTATTAGCTATCGTTTCCAATAGTTATCCC
CCGCTACCAGGCAGGTTACCTACCGCGTACTCACCCGTTCGCAACTCATCCGGAGAAGCAAGCTCCTCCTCAG
CGTTCTACTTGCCATGTATTAGGCACGCCGCCAGCGTTCGTCCTGAGCCAGGATCAACTCT
>923
CTGCTGCCGCCCGTAGGAGTCTGGGCCGTGTCTCAGTCCCAGTGTGGCCGATCACCCTCTCAGGTCGGCTATG
TATCGTCGCCTTGGTGAGCCGTTACCTCACCAACTAGCTAATACAACGCAGGTCCATCTGGTAGTGATGCAAT
TTGCACCTTTTAATTGACTATCATGCAATAATCAATTTATGCGGTATTTAGCCTATCGTTTTCCAATAGTTTA
TCCCCCCCGCTACCAGGCAGGTTTACCTACGCGTTACTCACCCGTTTCGCAACTCATCCAGAGAAGCAAGCTC
CTCCTTCAGCGTTCTACTGCATGTATTTAGGCACGCCGCCAGCGTTCGTCCTGAGCCAGGATCAAACTCTT
>924
CTGCTGCCGCCCGTAGGAGTCTGGGCCGTGTCTCAGTCCCAGTGTGGCCGATCACCCTCTCAGGTCGGCTATG
CATCGTCGCCTTGGTAGGCCTTTACCCCACCAACTAGCTAATGCACCGCAAAGCCATCTCATAGTGTTAGCAA
AACCAACTTTTAAACATCAACCATGCAGTTAATGCTGTTATCCGGTATTTAGCTATCGTTTTCCAATAGTTTG
TCCCAGTCTATGAGGCAGGTTTCTTTACGTGTTTACTCACCCGTTTCGCCGCTAAGTTTGTTCCGCGACACGA
ACACCAAAACTACGTCT
>925
CTGCTGCCGCCCGTAGGAGTCTGGACCGTGTCTCAGTTCCAGTGTGGCCGATCACCCTCTCAGGTCGGCTACG
TATCGTTGCCTTGGTAAGCCGTTACCTTACCAACTAGCTAATACGGCGCAGGTCCATCTATAAGTGATAGCAA
AGCCATCTTTCACTATCGAACCATGCGGTTCGAAATATTATCCGGTATAGCTCCGGTTTCCCGAAGTTATCCC
AGTCTTATAGGTAGGTTATCCACGTGTTACTCACCCGTCCGCCGCTAACGTCAGAGGAGCAAGCTCCTCGTCT
GTTCGCTCGACTTGCATGTATTAGGCACGCCGCCAGCGTTCATCCTGAGCCAGGATCAAACTCT
>926
CTGCTGCCGCCCGTAGGAGTCTGGGCCGTGTCTCAGTCCCAGTGTGGCTGATCGTCCTCTCAGACCAGCTACA
GATCGTCGGCTTGGTAAGCTTTTATCCCACCAACTACCTAATCTGCCATCGGCCGCTCTAGTAGCACAAGGTC
TTGCGATCCCCTGCTTTCATCCGTAGATCTCATGCGGTATTAGCCACTCTTTCGAGTAGTTATCCCCCACTAC
TAGGCACGTTCCGATGTATTACTCACCCGTTCGCCACTCGTCAGCATCCGAAGACCTGTTACCGTTCGACTTG
CATGTGTAAGGCATGCCGCCAGCGTTCAATCTGAGCCAGGATCAAACTCT
>927
CTGCTGCCGCCCGTAGGAGTCTGGGCCGTGTCTCAGTCCCAGTGTGGCCGATCACCCTCTCAGGTCGGCTATG
TATCGTCGCCTTGGTGAGCCGTTACCCCACCAACTAGCTAATACAACGCAGGTCCATCTGGTAGTGGTGCAAT
TGCACCTTTTAAGCAAATGTCATGCAACATCTACTCTTATGCGGTATTAGCTATCGTTTCCAATAGTTATCCC
CCGCTACCAGGCAGGTTACCTACGCGTTACTCACCCGTTCGCAACTCATCCAGAGAAGCAAGCTCCTCCTTCA
GCGTTCTACTTGCATGTATTAGGCACGCCGCCAGCGTTCGTCCTGAGCCAGGATCAAACTCT
>928
CTGCTGCCGCCCTAGGAGTCTGGGCCGTGTCTCAGTCCCAGTGTGGCCGATCACCCTCTCAGGTCGGCTATGT
ATCGTTGCCTTGGTGAGCCGTTACCTCACCAACTAGCTAATACAACGCAGGTCCATCTGGTAGTGGTGCAATT
GCACCTTTTAAGCAGCTATCATGCGATATCTACTGTTATGCGGTATTACGCTATCGTTTTCCAATAGTTATCC
CCCCGCTACCAGGCAGGTTACCTACGCGTTACTCACCCGTTCGCAACTCATCCGGAAAGAGCAAGCTCCTTCC
TTCAGCGTTCTACTGCATGTATTAGGCACGCCGCCAGCGTTCGTCCTGAGCCAGGATCAAACTCT
>929
CTGCTGCCGCCCGTAGGAGTCTGGGCCGTGTCTCAGTCCCAGTGTGGCCGATCACCCTCTCAGGTCGACTATG
TATCGTCGCCTTGGTGAGCCATTACCTCACCAACTAGCTAATACAACGCAGGTCCATCTGGTAGTGATGCAAT
TGCACCTTTTAATTGACTATCATGCAATAATCAATTTTATGCGGTATTAGCTATCGTTTCCAATAGTTATCCC
CCGCTACCAGGCAGGTTACCTACGCGTTACTCACCCGTTCGCAACTCATCCAGAGAAGCAAGCTCCTCCTTCA
GCGTTCTACTGCATGTATTAGGCACGCCGCCAGCGTCGTCCTGAGCAGGATCAAACTCT
>930

CTGCTGCCGCCCGTAGGAGTCTGGGCCGTGTCTCAGTCCCAGTGTGGCCGATCACCCTCTCAGGTCGGCTATG
TATCGTCGCCTTGGTGAGCCGTTACCCCACCAACTAGCTAATACAACGCAGGTCCATCTGGTAGTGGTGCAAT
TGCACCTTTTAAGCAAATGTCATGCAACATCTACTCTTATGCGGTATTAGCTATCGTTTCCAATAGTTATCCC
CCGCTACCAGGCAGGTTACCTACGCGTTACTCACCCGTTCGCAACTCATCCGGAGAAGCAAGCTCCTCCTCAG
CGTTCTACTTGCATGTATTAGGCACGCCGCCAGCGTTCGTCCTGAGCCAGGATCAAACTCT
>931
CTGCTGCCGCCCGTAGGAGTCTGGGCCGTGTCTCAGTCCCAGTGTGGCCGATCACCCTCTCAGGTCGGCTATG
TATCGTCGCCTTGGTGAGCCGTTACCTCACCAACTAGCTAATACAACGCAGGTCCATCTGGTAGTGATGCAAT
TGCACCTTTTAATTGACTATCATGCAATAATCAATTTTAGTGCGGTATTAGCCTATCGTTTTCCAATAGTTAT
CCCCCCGCTACCAGGCAGGTTACCTACGCGTTACTCACCCGTTCGCAACTCATCCAGAGAAGCAAGCTCCTCC
TCAGCGTTCTACTTGCATGTATTAGGCACGCCGCCAGCGTTCGTCCCTGAGCCAGGATCAAACTCT
>932
CTGCTGCCGCCCGTAGGAGTCTGGGCCGTGTCTCAGTCCCAGTGTGGCCGGTCGCCCCCTCAGGCCGGCTACC
CGTCATCGCCTTGGTGAGCCATTACCTCACCAACTAGCTAATACAACGCAGGTCCATCTGGTAGTGGTGCAAT
TGCACCTTTTAAGCAAATGTCAATGCAACATCTACTCTTATGCGGTATTAGCTATCGTTTCCAATAGTTATCC
CCCGCTACCAGGCAGGTTACCTACGCGTTACTCACCCGTTCGCAACTCATCCAGAGAAGCAAGCTCCTCCTTC
AGCGTTCTACTTGCATGTATTAGGCACGCCGCCAGCGTCGTCCTGAGCCAGGATCAAACTCT
>933
CTGCTGCCGCCCGTAGGAGTCTGGGCCGTGTCTCAGTCCCAGTGTGGCCGATCACCCTCTCAGGTCGGCTATG
TATCGTCGCCTTGGTGAGCCGTTACCTCACCAACTAGCTAATACAACGCAGGTCCATCTGGTAGTGATGCAAT
TGCACCTTTTAATTGACTATCATGCAATAATCAATTTTATGCGGTATTAGCTATCGTTTTCCAATAGTTATCC
CCCCGCTACCAGGCAGGTTACCTACGCGTTACTCACCCGTTCGCAACTCATCCAGAGAAGCAAGCTCCTCCTT
CAGCGTTCTACTTGCATGTATTAGGCACGCCGCCAGCGTTCGTCCTGAGCCAGGATCAAACTCT
>934
CTGCTGCCGCCCGTAGGAGTCTGGGCCGTGTCTCAGTCCCAGTGTGGCCGATCACCCTCTCAGGTCGGCTATG
TATCGTCGCCTTGGTGAGCCCTTACCCCACCAACTAGCTAATACAACGCAGGTCCATCTGGTAGTGATGCAAT
TGCACCTTTCAAACAGTTGTCATGCAACAACTGCTATTATGCGGTATTTAGCTATCGTTTTTCCAATAGTTTA
TCCCCCGCTACCAGGCAGGTTACCTACCGCGTTACCTCACCCGTTCGCAACTCATCCAGAGAAGCAAGCTCCT
CCTTCAGCGTTCTACTTGCATGTATTAGGCACGCCGCCAGCGTCGTCCTGAGCCAGGATCAAACTCT
>935
CTGCTGCCGCCCGTAGGAGTCTGGGCCGTGTCTCAGTCCCAGTGTGGCCGATCACCCTCTCAGGTCGGCTATG
TATCGTCGCCTTGGTGAGCCGTTACCCCACCAACTAGCTAATACAACGCAGGTCCATCTGGTAGTGATGCAAT
TGCACCTTTTAAGTAAATGTCATGCAACATCTACTCTTATGCGGTATTAGCTATCGTTTCCAATAGTTATCCC
CCGCTACCAGGCAGGTTACCTACGCGTTACTCACCCGTTCGCAACTCATCCAGAAGAGCAAGCTCCTCCTTCA
GCGTTCTACTGCATGTATTAGGCACGCCGCCAGCGTCGTCCTGAGCCAGGATCAAACTCT
>936
CTGCTGCCGCCCGTAGGAGTCTGGGCCGTGTCTCAGTCCCAGTGTGGCCGATCACCCTCTCAGGTCGGCTATG
TATCGTCGCCTTGGTGAGCCGTTACCCCACCAACTAGCTAATACAACGCAGGTCCATCTGGTAGTGGTGCAAT
TGCACCTTTTAAGCAAATGTCATGCAACATCTACTCTTATGCGGTATTAGCTATCGTTTCCAATAGTTATCCC
CCGCTACCAGGCAGGTTACCTACGCGTACTCACCCGTTCGCAACTCATCCGGAGAAGCAAGCTCCTCCTCAGC
GTCTACTTGCATGTATTAGGCACGCCGCCAGCGTTCGTCCTGAGCCAGGATCAAACTCT
>937
CTGCTGCCGCCCGTAGGAGTCTGGGCCGTGTCTCAGTCCCAGTGTGGCCGATCACCCTCTCAGGTCGGCTATG
TATCGTCGCCTTGGTGAGCCCTTACCCCACCAACTAGCTAATACAACGCAGGTCCATCTGGTAGTGATGCAAT
TGCACCTTTCAAACAGTTGTCATGCAACAACTGCTATTATGCGGTATTAGCTATCGTTTCCAATAGTTATCCC
CCGCTACCAGGCAGGTTACCTACCGCGTTACTCACCCGTTCGCAACTCATCCAGAGAAGCAAGCTCCTCCTTC
AGCGTTCTACTTGCATGTATTAGGCACGCCGCCAGCGTCGTCCCGAGCCAGGATCAAACTCT
>938
CTGCTGCCGCCCGTAGGAGTCTGGGCCGTGTCTCAGTCCCAGTGTGGCCGATCACCCTCTCAGGTCGGCTATG
TATCGTCGCCTTGGTGAGCCGTTACCTCACCAACTAGCTAATACAACGCAGGTCCATCTGGTAGTGATGCAAT
TGCACCTTTTAATTGACTATCATGCAATAATCAATTTTATGCGGTATTTAGCTATCGTTTTCCAATAGTTTAT
CCCCCCGCTACCAGGCAGGTTACCTACGCGTTACTCACCCGTTCGCAACTCATCCAGAGAAGCAAGCTCCTCC
TTCAGCGTTCTACTTGCATGTATTTAGGCACGCCGCCAGCGTTCGTCCTGAGCCAGGATCAAACTCT
>939

CTGCTGCCGCCCGTAGGAGTCTGGGCCGTGTCTCAGTCCCAGTGTGGCCGATCACCCTCTCAGGTCGGCTATG
TATCGTCGCCTTGGTGAGCCGTTACCTCACCAACTAGCTAATACAACGCAGGTCCATCTGGTAGTGATGCAAT
TGCACCTTTTAATTGACTATCATGCAATAATCAATTTTATGCGGTATTAGCTATCGTTTCCAATAGTTATCCC
CCGCTACCAGGCAGGTTACCTACGCGTTACTCACCCGTTCGCAACTCATCCAGAGAAGCAAGCTCCTCCTTCA
GCGTTCTACTTGCATGTATTAGGCACGCCGCCAGCGTTCGTCCTGAGCCAGGATCAAACTCT
>940
CTGCTGCCGCCCGTAGGAGCCTGGGCCGTGTCTCAGTCCCAGTGTGGCCGATCACCCTCTCAGGTCGGCTATG
TATCGTTGCCTTGGTGAGCCGTTACCTCACCAACTAGCTAATACAACGCAGGTCCATCTGGTAGTGATGCAAT
TTGCACCTTTTAAGCAAATGTCATGCAACATCTACTATTATGCGGTATTACGCTATCGTTTCCAATAGTTATC
CCCCGCTACCAGGCAGGTTACCTACGCGTTACTCACCCGTTCGCAACTCATCCAGAGAAGCAAGCTCCTCCTT
CAGCGTTCTACTGCATGTATTACGGCAGCCGCCAGCGTCGGTCCTGAGCCCAGGATCAAACTCT
>941
CTGCTGCCGCCCGTAGGAGTCTGGGCCGTGTCTCAGTCCCAGTGTGGCCGATCACCCTCTCAGGTCGGCTATG
CATCGTCGCCTTGGTAGGCCTTTACCCCACCAACTAGCTAATGCACCGCAAAGCCATCTCATAGTGTTAGCAA
AACCAACTTTAAACATCAACCATGCAGTTAATGCTGTTATCCGGTATTAGCTATCGTTTTTCCAATAGTTTTG
TCCCAGTCTATGAGGCAGGTTTTCTTTACGTGTTTTACTCACCCGTTTTTCGCCGCTAAGTTGTTCCGCGCAG
ACACCAAAAGCTAACCTAACGTCGCTGG
>942
CTGCTGCCGCCCGTAGGAGTCTGGACCGTGTCTCAGTTCCAGTGTAGCCGATCACCCTCTCAGGTCGGCTACG
TATCGTTGCCTTGGTAAGCCGTTACCTTACCAACTAGCTAATACGGCGCAGGTCCATCTATAAGTGATAGCAA
AGCCATCTTTCACTATCGAACCATGCGGTTCGAAATATTATCCGGTATTAGCTCCGGTTTTTCCCGAAGTATC
CCAGTCTTATACGGTAGGTTTTACCTACGTGTACTCACCGTCCGCGCTAACATCAAGGAGCAAGCTCCTCGCT
GTTCGCTCGACTTGCATGTATAGGCACGCCGCCAGCGTCATCCTGAGCCAGGATCAAACTCT
>943
CTGCTGCCGCCCGTAGGAGTCTGGGCCGTGTCTCAGTCCCAGCTGTGGCCGATCACCCTCTCAGGTCGGCTAT
GCATCGTCGCCTTGGTAGGCCTTTACCCCACCAACTAGCTAATGCACCGCAAAGCCATCTCATACGTGTCAGC
AAAACCAACTTTTAAACATCAACCATGCAGTTAATCGCTGTTATCCGGTATTAGCTATCGTTTCCAATAGTTG
TCCCAGTCTATGAGGCAGGTTCTTTACGTGTTACTCACCCGTTCGCCGCTAAGTGTTCGTCGACAGCACCAAA
TAACACTCGCGACTTGCAGAGGTATCAGC
>944
CTGCTGCCGCCCGTAGGAGTCTGGGCCGTGTCTCAGTCCCAGTGTGGCCGATCACCCTCTCAGGTCGGCTATG
TATCGTCGCCTTGGTGAGCCGTTACCCCACCAACTAGCTAATACAACGCAGGTCCATCTGGTAGTGGTGCAAT
TGCACCTTTTAAGCAAATGTCATGCAACATCTACTCTTATGCGGTATTAGCTATCGTTTCCAATAGTTATCCC
CCGCTACCAGGCAGGTTACCTACGCGTACTCACCCGTTCGCAACTCATCCGGAGAAGCAAGCTCCTCCTCAGC
GTTCTACTTGCATGTATTAGGCACGCCGCCAGCGTTCGTCCTGAGCCAGGATCAAACTCT
>945
CTGCTGCCGCCCGTAGGAGTCTGGGCCGTGTCTCAGTCCCAGTGTGGCCGATCACCCTCTCAGGTCGGCTATG
TATCGTCGCCTTGGTGAGCCGTTACCTCACCAACTAGCTAATACAACGCAGGTCCATCTGGTAGTGATGCAAT
TGCACCTTTTAATTGACTATCATGCAATAATCAATTTTATGCGGTATTAGCTATCGTTTTCCAATAGTTATCC
CCCCCGCTACCAGGCAGGTTACCTACGCGTACTCACCCGTTCGCAACTCATCCAGAGAAGCAAGCTCCTCCTC
AGCGTTCTACTTGCATGTATTAGGCACGCCGCCAGCGTCGTCCTGAGCCAGGATCAAACTCT
>946
CTGCTGCCGCCCGTAGGAGTCTGGGCCGTGTCTCAGTCCCAGTGTGGCCGATCACCCTCTCAGGTCGGCTATG
TATCGTCGCCTTGGTGAGCCGTTACCCCACCAACTAGCTAATACAACGCAGGTCCATCTGGTAGTGATGCAAT
TGCACCTTTTAAATAAATGTCATGCAACATCTACTCTTATGCGGTATTAGCTATCGTTTTCCAATAGTTATCC
CCCGCTACCAGGCAGGTTACCTACGCGTTACTCACCCGTTCGCAACTCATCCAGAAGAGCAAGCTCCTCCTCA
GCGTTCTACTTGCATGTATTAGGCACGCCGCCAGCGTTCGTCCTGAGCCAGGCATCG
>947
CTGCTGCCGCCCGTAGGAGTCTGGGCCGTGTCTCAGTCCCAGTGTGGCCGATCACCCTCTCAGGTCGGCTATG
TATCGTCGCCTTCGGTGAGCCGTTACCCCACCAACTAGCTAATACAACGCAGGTCCATCTGGCAGTGGTGCAA
TTGCACCTTTTAAGCAAATGTCATGCAACATCTACTCTTATGCGGTATTTAGCTATCGTTTTCCAATAGTTTA
TCCCCCCCGCTACCAGGCAGGTTACCTACGCGTTACTCACCCGTTCGCAACTCATCCGGAGAAGCAAGCTCCT
CCTTCAGCGTTCTACTTGCATGTATTTAGGCACGCCGCCAGCGTTCGTCCTGAGCCAGGATCAAACTCT
>948

CTGCTGCCGCCCGTAGGAGTCTGGGCCGTGTCTCAGTCCCAGTGTGGCCGATCACCCTCTCAGGTCGGCTATG
CATCGTCGCCTTGGTAGGCCTTTACCCCACCAACTAGCTAATGCACCGCAAAGCCATCTCATAGTGTTAGCA
AAACCAACTTTTAAACATCAACCATGCAGTTAATGCTGTTTATCCGGTATTTAGCTATCGTTTTTCCAATAGT
TTTGTCCCAGTCTATGAGGCAGGTTTTCTTTACGTGTTACTCACCCGTTTCGCCGCTAAGTTTCGTCGTCGAC
AGAACACCAAAACTACGACTCGCTGACATTGCG
>949
CTGCTGCCGCCCGTAGGAGTCTGGGCCGTGATCTCAGTCCCAGTGTGGCCCGATCACCCTCTCAGGTCGGCTA
TGTAAGTCGTCGCCTTGGGTGAGCCGTTACCTCACCAACTAGCTAATACAACGCAGGTCCATCTGGTAGTGAT
GCAATTGCACCTTTTAATTGACTATCATGCAATAATCAATTTTATGCGGTATTAGCCTATCGTTTCCAATAGT
TATCCCCCGCTACCAGGCAGGTTACCTACGCGTACTCACCCGTTCGCAACTCATCCAGAGAAGCAAGCTCCTC
CTTCAGCGTTCTACTTGCATGTATTAGGCACGCCGCCAGCGTTCGTCCTGAGCCAGGATCAAACTCT
>950
CTGCTGCCGCCCGTAGGAGTCTGGGCCGTGTCTCAGTCCCAGTGTGGCCGATCACCCTCTCAGGTCGGCTATG
TATCGTCGCCTTGGTGAGCCGTTACCCCACCAACTAGCTAATACAACGCAGGTCCATCTGGTAGTGGTGCAAT
TGCACCTTTTAAGCAAATGTCATGCAACATCTACTCTTATGCGGTATTAGCTATCGTTTCCAATAGTTATCCC
CCGCTACCAGGCAGGTTACCTACGCGTTACTCACCCGTTCGCAACTCATCCGGAGAAGCAGCTCCTCCTCAGC
GTTCTACTTGCATGTATTAGGCACGCCGCCAGCGTTCGTCCTGAGCCAGGATCAAACTCT
>951
CTGCTGCCGCCCGTAGGAGTCTGGACCGTGTCTCAGTTCCAGTGTGGCCGATCACCCTCTCAGGTCGGCTACG
TATCGTTGCCTTGGTAAGCCGTTACCTTACCAACTAGCTAATACGGCGCAGGTCCATCTATAAGTGATAGCAA
AGCCATCTTTCACTATCGAACCATGCAGTTCGAAATATTATCCGGTATTAGCTCCGGTTTCCCGAAGTTATCC
CAGTCTTATACGGTAGGTTACCTACGTGTTACTCACCCGTCCGCCGCTAACGTTAAAGGAGCAAGCTCCTCAT
CTGTTCGCTCGACTTGCATGTATTAGGCCACGCCGCCAGCGTTCATCCTGAGCCAGGATCAAA
>952
CTGCTGCCGCCCGTAGGAGTCTGGGCCGTGTCTCAGTCCCAGTGTGGCCGATCACCCTCTCAGGTCGGCTATG
TATCGTCGCCTTGGTGAGCCCTTACCCCACCAACTAGCTAATACAACGCAGGTCCATCTGGTAGTGATGCAAT
TGTACCTTTCAAACAGTTGTCATGCAACAACTGCTATTATGCGGTATTAGCTATCGTTTCCAATAGTTATCCC
CCGCTACCAGGCAGGTTACCTACCGCGTTACTCACCCGTTCGCAACTCATCCAGAGAAGCAAGCTCCTCCTTC
AGCGTTCTACTTGCCATGTATTAGGCACGCCGCCAGCGTTCGTCCTGAGCCAGGATCAAACCTCT
>953
CTGCTGCCGCCCGTAGGAGTCTGGGCCGTGTCTCAGTCCCAGTGTGGCCGATCACCCTCTCAGGTCGGCTATG
TATCGTCGCCTTGGTGAGCCGTTACCTCACCAACTAGCTAATACAACGCAGGTCCATCTGGTAGTGATGCAAT
TGCACCTTTTAATTGACTATCATGCAATAATCAATTTTATGCGGTATTAGCCTATCGTTTTCCAATAGTTATC
CCCCGCTACCAGGCAGGTTACCTACGCGTTACTCACCCGTTCGCAACTCATCCAGAGAAGCAAGCTCCTCCTC
AGCGTTCTACTTGCATGTATTAGGCACGCCGCCAGCGTTCGTCCTGAGCCAGGATCAAACTCT
>954
CTGCTGCCGCCCGTAGGAGTCTGGGCCGTGTCTCAGTCCCAGTGTGGCCGATCACCCTCTCAGGTCGGCTATG
TATCGTCGCCTTGGTGAGCCGTTACCCCACCAACTAGCTAATACAACGCAGGTCCATCTGGTAGTGGTGCAAT
TGCACCTTTTAAGCAAATGTCATGCAACATCTACTCTTATGCGGTATTAGCTATCGTTTCCAATAGTTATCCC
CCGCTACCAGGCAGGTTACCTACGCGTTACTCACCCGTTCGCAACTCATCCGGAGAAGCAAGCTCCTCCTCAG
CGTTCTACTTGCCATGTATTAGGCACGCCGCCAGCGTTCGTCCTGAGCCAGGATCAAACTCT
>955
CTGCTGCCGCCCGTAGGAGTCTGGGCCGTGTCTCAGTCCCAGTGTGGCCGATCACCCTCTCAGGTCGGCTATG
TATCGTCGCCTTGGTGAGCCGTTACCCCACCAACTAGCTAATACAACGCAGGTCCATCTGGTAGTGATGCAAT
TGCACCTTTTTAAGTAAATGTCATGCAACATCTACTCTTATGCGGTATTAGCTATCGTTTCCAATAGTTATCC
CCCGCTACCAGGCAGGTTACCTACGCGTTACTCACCCGTTCGCAACTCATCCAGAAGAGCAAGCTCCTCCTCA
GCGTTCTACTGCATGTATTAGGCACGCCGCCAGCGTCGTCCTGAGCCAGGATCAAACTCT
>956
CTGCTGCCGCCCGTAGGAGTCTGGGCCGTGTCTCAGTCCCAGTGTGGCCGATCACCCTCTCAGGTCGGCTATG
TATCGTCGCCTTGGTGAGCCGTTACCTCACCAACTAGCTAATACAACGCAGGTCCATCTGGTAGTGATGCAAT
TGCACCTTTTAAGCAAATGTCATGCAACATCTACTATTATGCGGTATTACGCTATCGTTTCCAATAGTTATCC
CCCGCTACCAGGCAGGTTACCTACGCGTACTCACCCGTTCGCAACTCATCCGCTCGGTGCAAGCACCAAGCTT
CAGCGTTCTACTTGCATGTATTACGGCACGCCGCCAGCGTTCGTCCTGAGCCAGGATCAAACTCT
>957

CTGCTGCCGCCCGTAGGAGTCTGGGCCGTGTCTCAGTCCCAGTGTGGCCGATCACCCTCTCAGGTCGGCTATG
TATCGTCGCCTTGGTGAGCCGTTACCCCACCAACTAGCTAATACAACGCAGGTCCATCTGGTAGTGGTGCAAT
TGCACCTTTTAAGCAAATGTCATGCAACATCTACTCTTATGCGGTATTAGCTATCGTTTCCAATAGTTATCCC
CCGCTACCAGGCAGGTTACCTACGCGTACTCACCCGTTCGCAACTCATCCGGAGAAGCAAGCTCCTCCTCAGC
GTTCTACTTGCATGTATTAGGCACGCCGCCAGCGTCGTCCTGAGCCAGGATCAACTCT
>958
CTGCTGCCGCCCGTAGGAGTCTGGGCCGTGTCTTAGTCCCAGTGTGGCCGATCACCCTCTCAGGTCGGCTATG
TATCGTCGCCTTGGTGAGCCGTTACCCCACCAACTAGCTAATACAACGCAGGTCCATCTGGTAGTGATGCAAT
TGCACCTTTTTAATTGACTATCATGCAATAATCAATTTTTATGCGGTATTAGCTATCGTTTTCCAATAGTTTA
TCCCCCCCGCTACCAGGCAGGTTACCTACGCGTACTCACCCGTTCGCAACTCATCCAGAGAAGCAAGCTCCTC
CTCAGCGTTCTACTGCATGTATTAGGCACGCCGCCAGCGTCGTCCTGAGCCAGGATCAAACTCGT
>959
CTGCTGCCGCCCGTAGGAGTCTGGGCCGTGTCTCAGTCCCAGTGTGGCCGATCACCCTCTCAGGTCGGCTATG
TATCGTCGCCTTGGTGAGCCGTTACCCCACCAACTAGCTAATACAACGCAGGTCCATCTGGTAGTGGTGCAAT
TGCACCTTTTAAGCAAATGTCATGCAACATCTACTCTTATGCGGTATTAGCTATCGTTTCCAATAGTTATCCC
CCGCTACCAGGCAGGTTACCTACGCGTACTCACCCGTTCGCAACTCATCCGGAGAAGCAAGCTCCTCCTCAGC
GTCTACTTGCATGTATTAGGCACGCCGCCAGCGTTCGTCCTGAGCCAGGATCAAACTCT
>960
CTGCTGCCGCCCGTAGGAGTCTGGGCCGTGTCTCAGTCCCAGTGTGGCCGATCACCCTCTCAGTCGGCTATGT
ATCGTCGCCTTGGTGAGCCGTTACCCCACCAACTAGCTAATACAACGCAGGTCCATCTGGTAGTGGTGCAATT
GCACCTTTTAAGCAAATGTCATGCAACATCTACTCTTATGCGGTATTAGCTATCGTTTCCAATAGTTATCCCC
CGCTACCAGGCAGGTTACCTACGCGTACTCACCCGTTCGCAACTCATCCGGAGAAGCAAGCTCCTCCTCAGCG
TTCTACTGCATGTATTAGGCACGCCGCCAGCGTCGTCCTGAGCCAGGATCAAACTCT
>961
CTGCTGCCGCCCGTAGGAGTCTGGGCCGCGTCTCAGTCCCAGTGTGGCCGATCACCCTCTCAGGTCGGCTATG
TATCGTCGCCTTGGTGAGCCGTTACCTCACCAACTAGCTAATACAACGCAGGTCCATCTGGTAGTGATGCAAT
TGCACCTTTAATTGACTATCATGCAATAATCAATTTTATGCGGTATTAGCCTATCGTTTCCAATAGTTATCCC
CCGCTACCAGGCAGGTTACCTACGCGTACTCACCCGTTCGCAACTCATCCAGAGAAGCAAGCTCCTCCTCAGC
GTTCTACTTGCATGTATTACGGCAGCCGCCAGCGATTCGGTCCCACT
>962
CTGCTGCCGCCCGTAGGAGTCTGGGCCGTGTCTCAGTCCCAGTGTGGCCGATCACCCTCTCAGGTCGGCTATG
TATCGTCGCCTTGGTGAGCCGTTACCTCACCAACTAGCTAATACAACGCAGGTCCATCTGGTAGTGATGCAAT
TGCACCTTTTAATTGACTATCATGCAATAATCAATTTTATGCGGTATTAGCTATCGTTTCCAATAGTTATCCC
CCCGCTACCAGGCAGGTTACCTACGCGTACTCACCCGTTCGCAACTCATCCAGAGAAGCAAGCTCCTCCTTCA
GCGTTCTACTTGCATGTATTAGGCACGCCGCCAGCGTTCGTCCTGAGCAGGCATCGA
>963
CTGCTGCCGCCCGTAGGAGTCTGGGCCGTGTCTCAGTCCCAGTGTGGCCGATCACCCTCTCAGGTCGGCTATG
TATCGTCGCCTTGGTGAGCCGTTACCCCACCAACTAGCTAATACAACGCAGGTCCATCTGGTAGTGATGCAAT
TGCACCTTTTAAGTAAATGATCATGCAACATCTACTCTTATGCGGTATTAGCTATCGTTTCCAACAGTTATCC
CCCGCTACCAGGCAGGTTACCTACGCGTTACTCACCCGTTCGCAACTCATCCAGAAGAGCAAGCTCCTCCTTC
AGCGTTCTACTGCATGTATTAGGCACGCCGCCAGCGTCGTCCTGAGCCAGGATCAAACTCT
>964
CTGCTGCCGCCCGTAGGAGTCTGGGCCGTGTCTCAGTCCCAGTGTGGCCGATCACCCTCTCAGGTCGGCTATG
TATCGTCGCCTTGGTGAGCCGTTACCTCACCAACTAGCTAATACAACGCAGGTCCATCTGGTAGTGATGCAAT
TTGCACCTTTTAATTGACTATCATGCAATAGTCAATTTTATGCGGTATTTAGCTATCGTTTTCCAATAGTTAT
CCCCCGCTACCAGGCAGGTTTACCTACGCGTTACTCACCCGTTTCGCAACTCATCCAGAGAAGCAAGCTCCCC
CTTCAGCGTTCTACTTGCATGTATTTAGGCACGCCGCCAGCGTTCGTCCTGAGCCAGGATCAAACTCT
>965
CTGCTGCCGCCCGTAGGAGTCTGGGCCGTGTCTCAGTCCCAGTGCGGCCGATCACCCTCTCAGGTCGGCTATG
TATCGTCGCCTTGGTGAGCCGTTACCCCACCAACTAGCTAATACAACGCAGGTCCATCTGGTAGTGGTGCAAT
TGCACCTTTTAAGCAAATGTCATGCAACATCTACTCTTATGCGGTATTAGCTATCGTTTCCAATAGTTATCCC
CCGCTACCAGGCAGGTTACCTACGCGTTACTCACCCGTTCGCAACTCATCCGGAGAAGCAAGCTCCTCCTTCA
GCGTTTACTTGCATGTATTAGGCACGCCGCCAGCGTTCGTCCTGAGCCAGGATCAAACTCT
>966

CTGCTGCCGCCCGTAGGAGTCTGGGCCGTGTCTCAGTCCCAGTGTGGCCGATCACCCTCTCAGGTCGGCTATG
TATCGTCGCCTTGGTGAGCCGTTACCTCACCAACTAGCTAATACAACGCAGGTCCATCTGGTAGTGATGCAAT
TGCACCTTTTAATTGACTATCATGCAATAATCAATTTTATGCGGTATTAGCCTATCGTTTCCAATAGTTATCC
CCCGCTACCAGGCAGGTTACCTACGCGTTACTCACCCGTTCGCAACTCATCCAGAGAAGCAAGCTCCTCCTTC
AGCGTTCTACTTGCCATGTATTACGGCAGCCGCCAGCGTTCGGTCCCTG
>967
CTGCTGCCGCCCGTAGGAGTCTGGGCCGTGTCTCAGTCCCAGTGTGGCCGATCACCCTCTCAGGTCGGCTATG
CATCGTCGCCTTGGTAGGCCTTTACCCCACCAACTAGCTAATGCACCGCAAAGCCATCTCATAGTGTTAGCAA
AACCAACTTTTAAACATCAACCATGCAGTTAATCGCTGTTATCCGGTATTAGCTATCGTTTCCAATAGTTGTC
CCAGTCTATGAGGCAGGTTCTTTACGTGTTACTCACCCGTTCGCCGCTAAGTTGTCGTCGACAGACACCAAAC
TACGCTCGCTGACATTCAGAGGTCTCAG
>968
CTGCTGCCGCCCGTAGGAGTCTGGACCGTGTCTCAGTTCCAGTGTGGCCGATCACCCTCTCAGGTCGGCTACG
TATCGTTGCCTTGGTAAGCCGTTACCTTACCAACTAGCTAATACGGCGCAGGTCCATCTATAAGTGATAGCAA
AGCCATCTTTCACTATCGAACCATGCGGTTCGAAATATTAATCCGGTATTAGCTCCGGTTTTTTCCCGAAGTT
ATCCCAGTCTTATACGGTAGGTTTTTACCACGTGTTTACTCACCCGTCCGCCGCTAACGTCGAAGGAGCAAGC
TCCTCGCTGTTCGCTCGACTTGCATGTATTAGGCACGCCGCCAGCGTCATCTGAGCCCAGGATCAAACTCCTA
TCACCGACCT
>969
CTGCTGCCGCCCGTAGGAGTCTGGGCCGTGTCTCAGTCCCAGTGTGGCCGATCACCCTCTCAGGTCGGCTATG
TATCGTCGCCTTGGTGAGCCGTTACCTCACCAACTAGCTAATACAACGCAGGTCCATCTGGTAGTGATGCAAT
TGCACCTTTTAATTGACTATCATGCAATAATCAATTTTATGCGGTATTAGCCTATCGTTTCCAATAGTTATCC
CCCGCTACCAGGCAGGTTACCTACGCGTTACTCACCCGTTCGCAACTCATCCAGAGAAGCAAGCTCCTCCTTC
AGCGTTCTACTTGCATGTATTAGGCACGCCGCCAGCGTTCGTCCTGAGCCAGGATCAAACTCT
>970
CTGCTGCCGCCCGTAGGAGTCTGGGCCGTGTCTCAGTCCCAGTGTGGCCGATCACCCTCTCAGGTCGGCTATG
TATCGTCGCCTTGGTGAGCCGTTACCCCACCAACTAGCTAATACAACGCAGGTCCATCTGGTAGTGGTGCAAT
TGCACCTTTTTAAGCAAATGTCATGCAACATCTACTCTTATGCGGTATTAGCTATCGTTTTCCAATAGTTTAT
CCCCCCCGCTACCAGGCAGGTACCTACGCGTACTCACCGTTCGCAACTCATCCGGAGAAGCAAGCTCCTCCTC
AGCGTCTACTGCATGTATTTAGGCACGCGCCAGCG
>971
CTGCTGCCGCCCGTAGGAGTCTGGACCGTGTCTCAGTTCCAGTGTAGCCGATCACCCTCTCAGGTCGGCTATG
CATCGTTGCCTTGGTAAGCCGTTACCTTACCAACTAGCTAATGCAGCGCGGATCCATCTATAAGTGACAGCAA
GACCGTCTTTCTACTTTGAACCAGCGGTTCAAAATATTATCCGGATATTAGCTCCGGTTTCCCGAAGTTATCC
CCAGTCTTATAGGTAGGTTATCCACGTGTTACTCACCCGTCCGCCGCTAACACTCAGAGAAGCAAGCTCTCGT
CCGTTCGCTCGTACTGCATGTATTAGGCACGCCGCCAGCGTCATCCTGAGCCAGGATCAACTCT
>972
CTGCTGCCGCCCGTAGGAGTCTGGGCCGTGTCTCAGTCCCAGTGTGGCCGATCACCCTCTCAGGTCGGCTATG
TATCGTCGCCTTGGTGAGCCGTTACCTCACCAACTAGCTAATACAACGCAGGTCCATCTGGTAGTGATGCAAT
TGCACCTTTAATTGACTATCATGCAATAATCAATTTATGCGGTATTAGCCTATCGTTTCCAATAGTTATCCCC
CGCTACCAGGCAGGTTACCTACGCGTACTCACCCGTTCGCAACTCATCCAGAGAAGCAAGCTCCACCTTCAGC
GTTCTACTTGCATGTATTACGGCAGCCGCCGACGTCGTCCCGTGAGCCCAAG
>973
CTGCTGCCGCCCGTAGGAGTCTGGGCCGTGTCTCAGTCCCAGTGTGGCCGATCACCCTCTCAGGTCGGCTATG
TATCGTTGCCTTGGTGAGCCGTTACCTCACCAACTAGCTAATACAACGCAGGTCCATCTGGTAGTGATGCAAT
TGCACCTTTTAATGACAATGTCATGCAACATCTACTATTATGCGGTATTACGCTATCGTTTCCAATAGTTATC
CCCCGCTACCAGGCAGGTTACCTACGCGTACTCACCCGTTCGCAACTCATCCGCTCGGTAGCAAGCACCAAGC
TTCAGCGTTCTATCGTTGC
>974
CTGCTGCCGCCCGTAGGAGTCTGGGCCGTGTCTCAGTCCCAGTGTGGCCGATCACCCTCTCAGGTCGGCTATG
TATCGTCGCCTTGGTGAGCCGTTACCTCACCAACTAGCTAATACAACGCAGGTCCATCTGGTAGTGATGCAAT
TGCACCTTTTAATTGACTATCATGCAATAATCAATTTATGCGGTATTAGCTATCGTTTCCAATAGTTATCCCC
CGCTACCAGGCAGGTTACCTACGCGTACTCACCCGTTCGCAACTCATCCAGAGAAGCAAGCTCCTCCCTCAGC
GTTCTACTTGCATGTATTACGGCAGCCGCCACGTTCGT

>975
CTGCTGCCGCCCGTAGGAGTCTGGGCCGTGTCTCAGTCCCAGTGTGGCCGATCACCCTCTCAGGTCGGCTATG
TATCGTCGCCTTGGTGAGCCGTTACCTCACCAACTAGCTAATACAACGCAGGTCCATCTGGTAGTGATGCAAT
TGCACCTTTTAATTGACTATCATGCAATAATCAATTTTATGCGGTATTAGCTATCGTTTCCAATAGTTATCCC
CCGCTACCAGGCAGGTTACCTACGCGTTACTCACCCGTTCGCAACTCATCCAGAGAAGCAAGCTCCTCCTTCA
GCGTTCTACTTGCATGTATTAGGCACGCCCGCCAGCGTTCGTCCCTGAGCCAGGATCAAACTCT
>976
CTGCTGCCGCCCGTAGGAGTCTGGACCGTGTCTCAGTTCCAGTGTGGCCGATCACCCTCTCAGGTCGGCTACG
TATCGTTGCCTTGGTAAGCCGTTACCTTACCAACTAGCTAATACGGCGCAGGTCCATCTATAAGTGATAGCAA
AGCCATCTTTCACTATCGAACCATGCAGTTCGAAATATTATCCGGTATTAGCTCCGGTTTTTCCCGAAGTTAT
CCCAGTCTTATAGGTAGGTTTTACCTACGTGTTACTCACCCGTCCGCCGCTAACGTCAAAGGAGCAAGCTCCT
CATCTGTCGCTCGACTTGCATGTATTAGGCACGCCGCCAGCGTCATCCTGAGCCAGGATCAAACTCT
>977
CTGCTGCCGCCGTAGGAGTCTGGGCCGTGTCTCAGTCCCAGTGTGGCCGATCACCCTCTCAGGTCGGCTATGT
ATCGTCGCCTTGGTGAGCCGTTACCCCCCACCAACTAGCTAATACAACGCAGGTCCATCTGGTAGTGGTGCAA
TTGCACCTTTTAAGCAAATGTCATGCAACATCTACTCTTATGCGGTATTAGCTATCGTTTTCCAATAGTTATC
CCCCCGCTACCAGGCAGGTTACCTACGCGTACTCACCCGTTCGCAACTCATCCGGAGAAGCAAGCTCCTCCTC
AGCGTTCTACTTGCATGTATTTAGGCACGCCGCCAGCGTTCGTCCTGAGCCAGGATCAAACTCT
>978
CTGCTGCCGCCCGTAGGAGTCTGGGCCGTGTCTCAGTCCCAGTGTGGCCGATCACCCTCTCAGGTCGGCTATG
TATCGTCGCCTTGGTGAGCCGTTACCCCACCAACTAGCTAATACAACGCAGGTCCATCTGGTAGTGGTGCAAT
TGCACCTTTTAAGCAAATGTCATGCAACATCTACTCTTATGCGGTATTAGCTATCGTTTCCAATAGTTATCCC
CCCGCTACCAGGCAGGTTACCTACGCGTACTCACCCGTTCGCAACTCATCCGGAGAAGCAAGCTCCTCCTCAG
CGTCTACTTGCATGTATTAGGCACGCCGCCAGCGTTCGTCCTGAGCCAGGATCAAACTCT
>979
CTGCTGCCGCCCGTAGGAGTCTGGGACCGTGTCTCAGTTCCAGCTGTGGCCGATCACCCTCTCAGGTCGGCTA
TGTATCGTCGCCTTGGTGAGCCGTTACCTCACCAACTAGCTAATACAACGCAGGCCCATCTGGTAGTGATGCA
ATTTGCACCTTTTAATTGACTATCATGCAATAATCAATTTTATGCGGTATTAGCTATCGTTTCCAATAGTTAT
CCCCCGCTACCAGGCAGGTTACCTACGCGTTACTCACCCGTTCGCAACTCATCCAGAGAAGCAAGCTCCTCCT
TCAGCGTTCTACTTGCATGTATTAGGCACGCCGCCAGCGTTCGTCCTGAGCCAGGATCAAACTCT
>980
CTGCTGCCGCCCGTAGGAGTCTGGGCCGTGTCTCAGTCCCAGTGTGGCCGATCACCCTCTCAGGTCGGCTATG
TATCGTCGCCTTGGTGAGCCGTTACCCCACCAACTAGCTAATACAACGCAGGTCCATCTGGTAGTGGTGCAAT
TGCACCTTTTAAGCAAATGTCATGCAACATCTACTCTTATGCGGTATTTAGCTATCGTTTTCCAATAGTTTAT
CCCCCCCGCTACCAGGCAGGTTACCTACGCGTACTCACCCGTTTCGCAACTCATCCGGAGAAGCAAGCTCCTC
CTCAGCGTCTACTTGCATGTATTTAGGCACGCCGCCAGCGTTCGTCCTGAGCCAGGATCAAACTCT
>981
CTGCTGCCGCCCGTAGGAGTCTGGGCCGTGTCTCAGTCCCAGTGTGGCCGATCACCCTCTCAGGTCGGCTATG
TATCGTCGCCTTGGTGAGCCGTTACCTCACCAACTAGCTAATACAACGCAGGTCCATCTGGTAGTGATGCAAT
TTGCACCTTTTAATTGACTATCATGCAATAATCAATTTTATGCGGTATTAGCCTATCGTTTCCAATAGCTATC
CCCCGCTACCAGGCAGGTTACCTACGCGTTACTCACCCGTTCGCAACTCATCCAGAGAAGCAAGCTCCTCCTC
AGCGTTCTACTGCATGTATTAGGCACGCCGCCAGCGTTCGTCCTGAGCCAGGATCAAACTCT
>982
CTGCTGCCGCCCGTAGGAGTCTGGGCCGTGTCTCAGTCCCAGTGTGGCCGATCACCCTCTCAGGTCGGCTATG
TATCGTCGCCTTGGTGAGCCGTTACCCCACCAACTAGCTAATACAACGCAGGTCCATCTGGTAGTGATGCAAT
GCATCTTTTAAGTCAAATGTCATGCAACATCTACTATTATGCGGTATTAGCTATCGTTTTCCAATAGTTATCC
CCCCGCTACCAGGCAGGTTACCTACGCGTTACTCACCCGTTCGCAACTCATCCAGAGAAGCAAGCTCCTCCTC
AGCGTTCTACTTGCATGTATTAGGCACGCCGCCAGCGTCGTCCTGAGCCAGGATCAAACTCT
>983
CTGCTGCCGCCCGTAGGAGTCTGGGCCGTGTCTCAGTCCCAGTGTGGCCGATCACCCTCTCAGGTCGGCTATG
TATCGTCGCCTTGGTGAGCCGTTACCCCACCAACTAGCTAATACAACGCAGGTCCATCTGGTAGTGGTGCAAT
TGCACCTTTTAAGCAAATGTCATGCAACATCTACTCTTATGCGGTATTAGCTATCGTTTCCAATAGTTATCCC
CCGCTACCAGGCAGGTTACCTACGCGTACTCACCCGTTCGCAACTCATCCGGAGAAGCAAGCTCCTCCTTCAG
CGTTCTACTTGCATGTATTAGGCACGCCGCCAGCGTTCGTCCTGAGCCAGGATCAAACTCT

>984
CTGCTGCCGCCCGTAGGAGTCTGGGCCGTGTCTCAGTCCCAGTGTGGCCGATCACCCTCCTCGAGTCGGCTAT
GTATCGTCGCCTTGGTGAGCCGTTACCCCACCAACTAGCTAATACAACGCAGGTCCATCTGGTAGTGATGACA
ATTGCACCTTTTTAAGTAATGATCGATGCAACATCTACTCTTATGCGGTATTTAGCTATCGTTTTCCAATAGT
TTATCCCCCCCGCTACCAGGCAGGTTACCTACGCGTTACTCACCCGTTCGCAACTCATCCAGAAGAGCAAGCT
CCTCCTCAGCGTTCACTTGCATGTATTTAGGCACGCCGCCAGCGTCGTCCTGAGCAGGATCAACTCT
>985
CTGCTGCCGCCCGTAGGAGTCTGGGCCGTGTCTCAGTCCCAGTGTGGCCGATCACCCTCTCAGGTCGGCTATG
TATCGTCGCCTTGGTGAGCCCTTACCCCACCAACTAGCTAATACAACGCAGGTCCATCTGGTAGTGATGCAAT
TGCACCTTTCAAACAGTTGTCATGCAACAACTGCTATTATGCGGTATTAGCTATCGTTTTCCAATAGTTATCC
CCCGCTACCAGGCAGGTTACCTACCGCGTTACTCACCCGTTCGCAACTCATCCAGAGAAGCAAGCTCCTCCTT
CAGCGTTCTACTTGCCATGTATTAGGCACGCCGCCAGCGTTCGTCCTGAGCCAGGATCAAACTCT
>986
CTGCTGCCGCCCGTAGGAGTCTGGGCCGTGTCTCAGTCCCAGTGTGGCCGATCACCCTCTCAGGTCGGCTGTG
TATCGTCGCCTTGGTGAGCCGTTACCCCACCAACTAGCTAATACAACGCAGGTCCATCTGGTAGTGATGCAAT
TGCACCTTTTAAGTAAATGTCATGCAACATCTACTCTTATGCGGTATTAGCTATCGTTTCCAATAGTTATCCC
CCGCTACCAGGCAGGTTACCTACGCGTACTCACCCGTTCGCAACTCATCCAGAAGAGCAAGCTCCTCCTCAGC
GTTCTACTTGCATGTATTAGGCACGCCGCCAGCGTCGTCCTGAGCCAGGATCAAACTCT
>987
CTGCTGCCGCCCGTAGGAGTCTGGGCCGTGTCTCAGTCCCAGTGTGGCCGATCACCCTCTCAGGTCGGCTATG
TATCGTCGCCTTGGTGAGCCGTTACCCCACCAACTAGCTAATACAACGCAGGTCCATCTGGTAGTGGTGCAAT
TGCACCTTTTTAAGCAAATGTCATGCAACATCTACTCTTATGCGGTATTAGCTATCGTTTTCCAATAGTTTAT
CCCCCCCGCTACCAGGCAGGTTACCTAGCGTACTCACCCGTTCGCAACTCATCCGGAGAAGCAAGCTCCTCCT
CAGCGTTCTACTGCATGTATTTAGGCACGCCGCCAGCGTCGTCCTGAGCCAGGATCAAACTCT
>988
CTGCTGCCGCCCGTAGGAGTCTGGGCCGTGTCTCAGTCCCAGTGTGGCCGATCACCCTCTCAGGTCGGCTATG
CATCGTCGCCTTGGTAGGCCTTTACCCCACCAACTAGCTAATGCACCGCAAAGCCATCTCATAGTGTTAGCAA
AACCAACTTTTAAACATCAACCATGCAGTTAATGCTGTTATCCGGTATTAGCTATCGTTTTTCCAATAGTTGT
CCCAGTCTATGAGGCAGGTTCTTTACGTGTTACTCACCCGTTCGCCGCTAAGTGTTCGTCGCAGACACCAAAA
GCTAACCTAAACTCGCGGCTTGCATGTATAGGCAC
>989
CTGCTGCCGCCCGTAGGAGTCTGGGCCGTGTCTCAGTCCCAGTGTGGCCGATCACCCTCTCAGGTCGGCTATG
TATCGTCGCCTTGGTGAGCCGTTACCTCACCAACTAGCTAATACAACGCAGGTCCATCTGGTAGTGATGCAAT
TGCACCTTTTAATTGACTATCATGCAATAATCAATTTTATGCGGTATTAGCTATCGTTTTCCAATAGTTATCC
CCCGCTACCAGGCAGGTTACCTACGCGTACTCACCCGTTCGCAACTCATCCAGAGAAGCAAGCTCCTCCTCAG
CGTTCTACTTGCATGTATTAGGCACGCCGCCAGCGTTCGTCCTGAGCCAGGATCAAACTCT
>990
CTGCTGCCGCCCGTAGGAGTCTGGGCCGTGTCTCAGTCCCAGTGTGGCCGATCACCCTCTCAGGTCGGCTACG
CATCGTTGCCTTGGTGGGCCGTTACCCCGCCAACCAGCTAATGCGCCGCAGGCCCATCCATCAGCGACAGCCG
AAACCGTCTTTCAACTCCCCACCCCGCGGTGGAGAGGATTATCCGGTATTAGCCACGGTTTCCCGCAGTTATC
CCCGGCTGATGGGCAGGTTGCCTACGTGTTACTCACCCGTCCGCCACTCGATCAGAGGGAGCAAGCTCCCTCC
TCTCGCGTCGACTTGCATGTATTAGGCACGCCGCCAGCGTCATCCTGAGCCAGGCATCAAATCACCT
>991
CTGCTGCCGCCCGTAGGAGTCTGGGCCGTGTCTCAGTCCCAGTGTGGCTGATCATCCTCTCAGACCAGCTACT
GATCGTCGCCTTGGTGAGCCATTACCTCACCAACTAGCTAATCAGACGCGGGCCGATCTCTCTGCGATAAATC
TTTCCCCAAAAGGGCGTATCCGGTATTACCTTTCGTTTCCAAAAAGCTATTCCGAACAAAGAGGTACGTTCCC
ACGTGTTACTCACCCGTCTGCCACTCACCCAGAAGGATGCGTTCGACTGCATGTGTTAAGCCTGCCGCCAGCG
TTCGCTCTGAGCCAGGATCAAACTCT
>992
CTGCTGCCGCCCGTAGGAGTCTGGGCCGTGTCTCAGTCCCAGTGTGGCCGATCACCCTCTCAGGTCGGCTATG
TATCGTCGCCTTGGTGAGCCGTTACCCCACCAACTAGCTAATACAACGCAGGTCCATCTGGTAGTGGTGCAAT
TGCACCCTTTAAGCAAATGTCATGCAACATCTACTCTTATGCGGTATTAGCTATCGTTTCCAATAGTTATCCC
CCGCTACCAGGCAGGTTACCTACGCGTACTCACCCGTTCGCAACTCATCCGGAGAAGCAAGCTCCTCCTCAGC
GTTCTACTGCATGTATTAGGCACGCCGCCAGCGTTCGTCCTGAGCCAGGATCAAACTCT

>993
CTGCTGCCGCCCGTAGGAGTCTGGGCCGTGTCTCAGTCCCAGTGTGGCCGATCACCCTCTCAGGTCGGCTATG
TATCGTCGCCTTGGTGAGCCCTTACCCCACCAACTAGCTAATACAACGCAGGTCCATCTGGTAGTGATGCAAT
TGCACCTTTCAAACAGTTGTCATGCAACAACTGCTATTATGCGGTATTGGCTATCGTTTCCAATAGTTATCCC
CCGCTACCAGGCAGGTTACCTACCGCGTTACTCACCCGTTCGCAACTCATCCACGAGAAGCAAAGCCTCCTCC
TTCCAGCGTTCTACTTGCCATGTATTAGGCACGCCGCCAGCGTCGCCCTGAGCCAGGCATCGA
>994
CTGCTGCCGCCCGTAGGAGTCTGGGCCGTGTCTCAGTCCCAGTGTGGCCGATCACCCTCTCAGGTCGGCTATG
TATCGTCGCCTTGGTGAGCCCTTACCCCACCAACTAGCTAATACAACGCAGGTCCATCTGGTAGTGATGCAAT
TGCACCTTTCAAACAGTTGTCATGCAACAACTGCTATTATGCGGTATTTAGCTATCGTTTTTCCAATAGTTTA
TCCCCCGCTACCAGGCAGGTTACCTACCGCGTACTCACCCGTTCGCAACTCATCCGGAGAAGCAAGCTCCTCC
TCAGCGTTCTACTTGCATGTATTAGGCACGCCGCCAGCGTTCGTCCTGAGCCAGGATCAAACTCT
>995
CTGCTGCCGCCCGTAGGAGTCTGGGCCGTGTCTCAGTCCCAGTGTGGCCGATCACCCTCTCAGGTCGGCTATG
TATCGTCGCCTTGGTGAGCCCTTACCCCACCAACTAGCTAATACAACGCAGGTCCATCTGGTAGTGATGCAAT
TGCACCTTTCAAACAGTTGTCATGCAACAACTGCTATTATGCGGTATTTAGCTATCGTTTTTCCAATAGTTTA
TCCCCCGCTACCAGGCAGGTTACCTACCGCGTTACCTCACCCGTTCGCAACTCATCCAGAGAAGCAAGCTCCT
CCTTCAGCGTTCTACTTGCC
>996
CTGCTGCCGCCCGTAGGAGTCTGGGCCGTGTCTCAGTCCCAGTGTGGCCGATCACCCTCTCAGGTCGGCTATG
TATCGTCGCCTTGGTGAGCCGTTACCTCACCAACTAGCTAATACAACGCAGGTCCATCTGGTAGTGATGCAAT
TGCACCTTTTAATTGACTATCATGCAATAATCAATTTTATGCGGTATTAGCTATCGTTTTCCAATAGTTATCC
CCCGCTACCAGGCAGGTTACCTACGCGTTACTCACCCGTTCGCAACTCATCCAGAGAAGCAAGCTCCTCCTTC
AGCGTTCTACTTGCCATGTATTAGGCACGCCGCCAGCGTCGTCCTGAGCCAGGATCAAACTCT
>997
CTGCTGCCGCCCGTAGGAGTCTGGGCCGTGTCTCAGTCCCAGTGTGGCCGATCACCCTCTCAGGTCGGCTATG
TATCGTCGCCTTGGTGAGCCGTTACCTCACCAACTAGCTAATACAACGCAGGTCCATCTGGTAGTGATGCAAT
TGCACCTTTTAATTGACTATCATGCAATAATCAATTTTATGCGGTATTAGCTATCGTTTCCAATAGTTATCCC
CCGCTACCAGGCAGGTTACCTACGCGTACTCACCCGTTCGCAACTCATCCAGAGAAGCAAGCTCCTCCTTCAG
CGTTCTACTTGCATGTATTAGGCACGCCGCCAGCGTTCGTCCTGAGCCAGGATCAAACTCT
>998
CTGCTGCCGCCCGTAGGAGTCTGGGCCGTGTCTCAGTCCCAGTGTGGCCGATCACCCTCTCAGGTCGGCTATG
TATCGTTGCCTTGGTGAGCCGTTACCTCACCAACTAGCTAATACAACGCAGGTCCATCTGGTAGTGATGCAAT
TGCACCTTTTAAGCAAATGTCATGCAACATCTACTATTATGCGGTATTAGCTATCGTTTCCAATAGTTATCCC
CCGCTACCAGGCAGGTTACCTACGCGTACTCACCCGTTCGCAACTCATCCGCTCGGTGCAAGCACCAAGCTTC
AGCGTTCTACTTGCATGTATTAGGCACGCCGCCAGCGTTCGTCCTGAGCCAGGATCAAACTCT
>999
CTGCTGCCGCCCGTAGGAGTCTGGGCCGTGTCTCAGTCCCAGTGTGGCCGATCACCCTCTCAGGTCGGCTATG
CATCGTCGCCTTGGTAGGCCTTTACCCCACCAACTAGCTAATGCACCGCAAAGCCATCTCATAGTGTTAGCAA
AACCAACTTTTAAACATCAACCATGCAGTTAATGCTGTTATCCGGTATTAGCCTATCGTTTTCCAATAGTTTG
TCCCAGTCTATGAGGCAGGTTCTTTACGTGTTACTCACCCGTTCGCCGCTAAGTTGTTCCGTCGACAGACACC
AAAACTACGTACTCGCCG
>1000
CTGCTGCCGCCCGTAGGAGTCTGGGCCGTGTCTCAGTCCCAGTGTGGCCGATCACCCTCTCAGGTCGGCTATG
TATCGTCGCCTTGGTGAGCCGTTACCTCACCAACTAGCTAATACAACGCAGGTCCATCTGGTAGTGGTGCAAT
TGCACCTTTTAAGCAAATGTCATGCAACATCTACTCTTATGCGGTATTAGCTATCGTTTCCAATAGTTATCCC
CCGCTACCAGGCAGGTTACCTACGCGTACTCACCCGTTCGCAACTCATCCGGAGAAGCAAGCTCCTCCTCAGC
GTTCTACTTGCATGTATTAGGCACGCCGCCAGCGTTCGTCCTGAGCCAGGATCAAACTCT

Additional file 3_B_P10

>1
CTGCTGCCGCCCGTAGGAGTCTGGTCCGTGTCTCAGTACCAGTGTGGGGGATCACCCTCTCAGGCCCCCTAAA
GATCGTCGCCTTGGTGAGCCGTTACCTCACCAACTAGCTAATCTTGCGCGTGCCCATCTCTATCCACCGTAGT
TTTCAATATCACCCGATGCCGAATAATATATTATGGGGTATTAATCTTCTCGAAAGGCTATCCCCTGATAAAG
GCAGGTTGCACACGTGTTCCGCACCCGTACGCCGCTCTCAATTACCCGAAAGTAATCTACCGCTCGGCTTGCA
TGTGTTAGGCCTCCCGCTAGCGTTCATCCTGAGCCAGGATCAAACTCT
>2
CTGCTGCCGCCCGTAGGAGTCTGGTCCGTGTCTCAGTACCAGTGTGGGGGATCACCCTCTCAGGCCCCCTAAA
GATCGTCGCCTGGTGAGCCGTTACCTCACCAACTAGCTAATCTTGCGCGTGCCCATCTCTATCCACCGTAGTT
TTCAATATCACCCGATGCCGAATAATATATTATGGGGTATTAATCTTCTTCGAAAGGCTATCCCCTGATAAAG
GCAGGTTGCACACGTGTTCCGCACCCGTACGCCGCTCTCAATTACCCGAAAGTAATCTACCGCTCGGCTTGCA
TGTGTTAGGCCTCCCGCTAGCGTTCATCCTGAGCCAGGATCAAACTCT
>3
CTGCTGCCGCCCGTAGGAGTCTGGTCCGTGTCTCAGTACCAGTGTGGGGGATCACCCTCTCAGGCCCCCTAAA
GATCGTCGCCTGTGAGCCGTTACCTCACCAACTAGCCTAATCTTGCGCGTGCCCATCTCTATCCACCGTAGTT
TTCAATATCACCCGATGCCGAATAATATATTATGGGGTATTAATCTTCTCGAAGGCTATCCCCTGATAAGGCA
GGTTGCACACGTGTCCGCACCCGTACGCCGCTCTCAATTTACCCGAAAGTAATCTACCGCTCCGGCTTGCATG
TGTTAGGCCTCCCGCTAGCGTCATCCTGAGCCAG
>4
CTGCTGCCGCCCGTAGGAGTCTGGTCCGTGTCTCAGTACCAGTGTGGGGGATCACCCTCTCAGGCCCCCTAAA
GATCGTCGCCTTGGTGAGCCGTTACCTCACCAACTAGCTAATCTTGCGCGTGCCCATCTCTATCCACCGTAGT
TTTCAATTATCACCCGATGCCGAATAATATATTATGGGGTATTAATCTTCTTCGAAAGGCTTATCCCCCTGAT
AAAGGCAGGTTGCACACGTGTTCCGCACCCGTACGCCGCTCTCAATTACCCGAAAGTAATCTACCGCTCGGCT
TGCATGTGTTAGGCCTCCCGCTAGCGTCTCCTGAGCAGGATCAACTCGT
>5
CTGCTGCCGCCCGTAGGAGTCTGGTCCGTGTCTCAGTACCAGTGTGGGGGATCACCCTCTCAGGTCCCCTAAA
GATCGTCGCCTGTGAGCCGTTACCTCACCAACTAGCTAATCTTGCGCGTGCCCATCTCTATCCACCGTAGTTT
TCAATATCACCCGATGCCGAATAATATATTATGGGGTATTAATCTTCTTCGAAAGGCTATCCCCTGATAAAGG
CAGGTTGCACACGTATTCCGCACCCGTACGCCGCTCTCAATTACCCGAAAGTAATCTACCGCTCGGCTTGCAT
GTGTTAGGCCTCCCGCTAGCGTTCATCCTGACGCAGGATCAACTCT
>6
CTGCTGCCGCCCGTAGGAGTCTGGACCGTGTCTCAGTTCCAGTGTGACTGATCATCCTCTCAGACCAGTACGG
ATCGTCGCCTTGGTAGGCCTTTACCCCACCAACTAGCTAATCCGACCTAGGCTCATCTGATAGCGTGAGGTCC
GAAGATCCCCCACTCTCCCTCAGGACGTATGCGGTATTAGCGCCCGTTTCCGGACGATCCCCACTACCAGGCA
GATTCCTAGGCATTACTCACCCGTCCGCCGCTGAATCCAGGAGCAAGCTCCCTCATCCGCTCGACTGCATGTG
TTAGGCCTGCCGCCAGCGTCAATCTGAGCCAGGATCAAACTCT
>7
CTGCTGCCGCCCGTAGGAGTCTGGTCCGTGTCTCAGTACCAGTGTGGGGGATCACCCTCTCAGGCCCCCTAAA
GATCGTCGCCTTGGTGAGCCGTTACCTCACCAACTAGCTAATCTTGCGCGTGCCCATCTCTATCCACCGTAGT
TTCAATATCACCCGATGCCGAATAATATATTATGGGGTATTAATCTTCTTCGAAAGGCTATCCCCCTGATAAA
GGCAGGTTGCACACGTGTTCCGCACCCGTACGCCGCTCTCAATACCCGAAAGTAATCTACCGCTCGGCTTGCA
TGTGTTAGGCCTCCCGCTAGCGTTCTCCTGAGCCAGGATCCAACTC
>8
CTGCTGCCGCCCGTAGGAGTCTGGTCCGTGTCTCAGTACCAGTGTGGGGGATCACCCTCTCAGGCCCCCTAAA
GATCGTCGCCTTGGTGAGCCGTTACCTCACCAACTAGCTAATCTTGCGCGTGCCCATCTCTATCCACCGTAGT
TTTCAATATCACCCGATGCCGAATAATATATTATGGGGTATTAATCTTCTCGAAAGGCTATCCCCCTGAATAA
AGGCAGGTTGCACACGTGTTCCGCACCCGTACGCCGCTCTCAATTACCCGAAAGTAATCTACCGCTCGGCTTG
CATGTGTTAGGCCTCCCGCTAGCGTTCATCCTGAGCCAGGATCAAACTCT
>9
CTGCTGCCGCCCGTAGGAGTCTGGTCCGTGTCTCAGTACCAGTGTGGGGGATCACCCTCTCAGGCCCCCTAAA
GATCGTCGCCTTGGTGAGCCGTTACCTCACCAACTAGCTAATCTTGCGCGTGCCCATCTCTATCCACCGTAGT
TTTCAATATCACCCGATGCCGAATAATATATTATGGGGTATTAATCTTCTTCGAAAGGCTATCCCCCTGATAA

AGGCAGGTTGCACACGTGTTCCGCACCCGTACGCCGCTCTCAATTACCCGAAAGTAATCTACCGCTCGGCTTG
CATGTGTTAGGCCTCCCGCTAGCGTTCATCCTGAGCCAGGATCAAACTCT
>10
CTGCTGCCGCCCGTAGGAGTCTGGTCCGTGTCTCAGTACCAGTGTGGGGGATCACCCTCTCAGGCCCCCTAAA
GATCGTCGCCTTGGTGAGCCGTTACCTCACCAACTAGCTAATCTTGCGCGTGCCCATCTCTATCCACCGTAGT
TTTCAATATCACCCGATGCCGAATAATATATTATGGGGTATTAATCTTCTTCGAAAGGCTATCCCCCTGATAA
AGGCAGGTTGCACACGTGTTCCGCACCCGTGCGCCGCTCTCAATTACCCGAAAGTAATCTACCGCTCGGCTTG
CATGTGTTAGGCCTCCCGCTAGCGTTCATCCTGAGCCAGGATCAAACTCT
>11
CTGCTGCCGCCCGTAGGAGTCTGGTCCGTGTCTCAGTACCAGTGTGGGGGATCACCCTCTCAGGCCCCCTAAA
GATCGTCGCCTTGGTGAGCCGTTACCTCACCAACTCAGCTAATCTTGCGCGTGCCCATCTCTATCCACCGTAG
TTTTCAATATCACCCGATGCCGAATAATATATTATGGGGTATTAATCTTCTTCGAAATGGCTATCCCCCTGAT
TAAAGGCAGGTTGCACACGTGTTCCGCACCCGTACGCCGCTCTCAATTACCCGAAAGTATCTACCGCTCGGCT
TGCATGTGTTAGGCCTCCCGCTAGCCGTTCATCCTGAGCCAGGATCAAACTCT
>12
CTGCTGCCGCCCGTAGGAGTCTGGTCCGTGTCTCAGTACCAGTGTGGGGGATCACCCTCTCAGGCCCCCTAAA
GATCGTCGCCTTGGTGAGCCGTTACCTCACCAACTAGCTAATCTTGCGCGTGCCCATCTCTATCCACCGTAGT
TTTCAATATCACCCGATGCCGAATAATATATTATGGGGTATTAATCTTCTTCGAAAGGCTATCCCCCTGATAA
AGGCAGGTTGCACACGTGTTCCGCACCCGTACGCCGCTCTCAATTACCCGAAAGTAATCTACCGCTCGGCTTG
CATGTGTTAGGCCTCCCGCTAGCGTTCATCCTGAGCCAGGATCAAACTCT
>13
CTGCTGCCGCCCGTAGGAGTCTGGTCCGTGTCTCAGTACCAGTGTGGGGGATCACCCTCTCAGGCCCCCTAAA
GATCGTCGCCTTGGTGAGCCGTTACCTCACCAACTAGCTAATCTTGCGCGTGCCCATCTCTATCCACCGTAGT
TTTCAATATCACCCGATGCCGAATAATATATTATGGGGTATTAATCTTCTTCGAAAGGCTATCCCCCTGATAA
AGGCAGGTTGCACACGTGTTCCGCACCCGTACGCCGCTCTCAATTACCCGAAAGTAATCTACCGCTCGGCTTG
CATGTGTTAGGCCTCCCGCTAGCGTTCATCCTGAGCCAGGATCAAACTCT
>14
CTGCTGCCGCCCGTAGGAGTCTGGTCCGTGTCTCAGTACCAGTGTGGGGGATCACCCTCTCAGGCCCCCTAAA
GATCGTCGCCTTGGTGAGCCGTTACCTCACCAACTAGCTAATCTTGCGCGTGCCCATCTCTATCCACCGTAGT
TTTCAATATCACCCGATGCCGAATAATATATTATGGGGTATTAATCTTCTTCGAAAGGCTATCCCCCTGATAA
AGGCAGGTTGCACACGTGTTCCGCACCCGTACGCCGCTCTCAATTACCCGAAAGTAATCTACCGCTCGGCTTG
CATGTGTTAGGCCTCCCGCTAGCGTTCATCCTGAGCCAGGATCAAACTCT
>15
CTGCTGCCGCCCGTAGGAGTCTGGTCCGTGTCTCAGTACCAGTGTGGGGGATCACCCTCTCAGGCCCCCTAAA
GATCGTCGCCCTGGTGAGCCGTTACCTCACCAACTAGCTAATCTTGCGCGTGCCCATCTCTATCCACCGTAGT
TTTCAATATCACCCGATGCCGAATAATATATTATGGGGTATTAATCTTCTCGAAAGCTATCCCCCTGATAAGG
CAGGTTGCACACGTGTTCCGCACCCGTACGCCGCTCTCAATTACCCGAAAGTAATCTACCTCTCGGCTTGCAT
GTGTTAGGCCTCCCGCTAGCGTCTCCTGAGC
>16
CTGCTGCCGCCCGTAGGAGTCTGGTCCGTGTCTCAGTACCAGTGTGGGGGATCACCCTCTCAGGCCCCCTAAA
GATCGTCGCCTTGGTGAGCCGTTACCTCACCAACTAGCTAATCTTGCGCGTGCCCATCTCTATCCACCGTAGT
TTCAATATCACCCGATGCCGAATAATATATTATGGGGTATTAATCTTCTCGAAAGCTATCCCCTGATAAGGCA
GGTTGCACACGTGTTCCGCACCCGTACGCCGCTCTCAATTACCCGAAAGTAATCTACCGCTCGGCTTGCATGT
GTTAGGCCTCCCGCTAGCGTTCATCCTGA
>17
CTGCTGCCGCCCGTAGGAGTCTGGTCCGTGTCTCAGTACCAGTGTGGGGGATCACCCTCTCAGGCCCCCTAAA
GATCGTCGCCTTGGTGAGCCGTTACCTCACCAACTAGCTAATCTTGCGCGTGCCCATCTCTATCCACCGTAGT
TTTCAATATCACCCGATGCCGAATAATATATTATGGGGTATTAATCTTCTTCGAAAGCTATCCCCCTGATAAA
GGCAGGTTGCACACGTGTTCCGCACCCGTACGCCGCTCTCAATTACCCGAAAGTAATCTACCGCTCGGCTTGC
ATGTGTTAGGCCTCCCGCTAGCGTTCTCCTGAGCAGGATCAACTCGT
>18
CTGCTGCCGCCCGTAGGAGTCTGGTCCGTGTCTCAGTACCAGTGTGGGGGATCACCCTCTCAGGCCCCCTAAA
GATCGTCGCCTTGGTGAGCCGTTACCTCACCAACTAGCTAATCTTGCGCGTGCCCATCTCTATCCACCGTAGT
TTTCAATATCACCCGATGCCGAATAATATATTATGGGGTATTAATCTTCTTCGAAAGGCTATCCCCCTGATAA

AGGCAGGTTGCACACGTGTTCCGCACCCGTACGCCGCCCTCAATTACCCGAAAGTAATCTACCGCTCGGCTTG
CATGTGTTAGGCCTCCCGCTAGCGTTCATCCTGAGCCAGGATCAAACTCT
>19
CTGCTGCCGCCCGTAGGAGTCTGGTCCGTGTCTCAGTACCAGTGTGGGGGGATCACCCTCTCCAGGCCCCTAA
AGATCGTCGCCTTGGTGAGCCGTTACCTCACCAACTAGCTAATCTTGCGCGTGCCCCATCTCTATCCACCGTA
GTTTCAATATCACCCGATGCCGAATAATATATTATGGGGTATTAATCTTCTCGAAAGGCTATCCCCCTGATAA
AGGCAGGTTGCACACGTGTTCCGCACCCGTACGCCGCTCTCAATTACCCGAAAGTAATCTACCGCTCGGCTTG
CATGTGTTAGGCCTCCCGCTAGCGTTCTCCTGAGCAGGATCAACTCGT
>20
CTGCTGCCGCCCGTAGGAGTCTGGTCCGTGTCTCAGTACCAGTGTGGGGGATCACCCTCTCAGGCCCCCTAAA
GATCGTCGCCTTGGTGAGCCGTTACCTCACCAACTAGCTAATCTTGCGCGTGCCCATCTCTATCCACCGTAGT
TTTCAATATCACCCGATGCCGAATAATATATTATGGGGTATTAATTCTTCTTCGAAAGGCTATCCCCCTGATA
AAGGCAGGTTGCACACGTGTTCCGCACCCGTACGCCGCTCTCAATTACCCGAAAGTAATCTACCGCTCGGCTT
GCATGTGTTAGGCCTCCCGCTAGCGTTCATCCTGAGCCAGGATCAAACTCT
>21
CTGCTGCCGCCCGTAGGAGTCTGGTCCGTGCCTCAGTACCAGTGTGGGGGATCACCCTCTCAGGCCCCCTAAA
GATCGTCGCCTTGGTGAGCCGTTACCTCACCAACTAGCTAATCTTGCGCGTGCCCACCTCTATCCACCGTAGT
TTTTCAATATCACCCGATGCCGAATAATATATTATGGGGGTATTAATCTTCTTCGAAAGGCTATCCCCCCTGA
TAAAGGCAGGTTGCACACGTGTTCCGCACCCGTACGCCGCTCTCAATTCCCGAAAGTAATCTACCGCTCGGCT
TGCATGTGTTAGGCCTCCCGCTAGCGTTCTCCTGAGCCAGGATCAAACTCT
>22
CTGCTGCCGCCCGTAGGAGTCTGGTCCGTGTCTCAGTACCAGTGTGGGGGATCACCCTCTCAGGCCCCCTAAA
GATCGTCGCCTGGTGAGCCGTTACCTCACCAACTAGCTAATCTTGCGCGTGCCCATCTCTATCCACCGTAGTT
TTCAATATCACCCGATGCCGAATAATATATTATGGGGGTATTAATTCTTCTTCGAAAGGCTATCCCCCTGATA
AAGGCAGGTTGCACACGTGTTCCGCACCCGTACGCCGCTCTCAATTACCCGAAAGTAATCTACCGCTCGGCTT
GCATGTGTTAGGCCTCCCGCTAGCGTTCATCCTGAGCCAGGATCAACTCT
>23
CTGCTGCCGCCCGTAGGAGTCTGGTCCGTGTCTCAGTACCAGTGTGGGGGATCACCCTCTCAGGCCCCCTAAA
GATCGTCGCCTGTGAGCCCGTTACCTCACCCAACTAGCTAATCTTGCGCGTGCCCATCTCTATCCACCGTAGT
TTTCAATATCACCCGATGCCGAATAATTATATTATGGGGTATTAATTCTTCTTCGAAAGGCTATCCCCCTGAT
AAGGCAGGTTGCACACGTGTTCCGCACCCGTACGCCGCTCTCAATTACCCCGAAAGTAATCTACCGCTCGGCT
TGCATGTGTTAGGCCTCCCGCTAGCGTTCTCCTGAGC
>24
CTGCTGCCCGCCCGTAGGAGTCTGGTCCCGTGTCTCAGTACCAGTGTGGGGGGATCACCCTCTCAGGCCCCCC
TAAAGATCGTCGCCTTGGTGAGCCCGTTACCTCACCCAACTAGCTAATCTTGCGCGTGCCCCATCTCTATCCA
CCGTAGTTTCAATATCACCCGATGCCGAATAATATATTATGGGGTATTAATCTTCTTCGAAGCTATCCCCCTG
ATAAAGGCAGGTTGCACACGTGTTCCGCACCCGTACGCCGCTCTCAATTACCCCGAAAGTAATCTACCGCTCG
GCTTGCATGTGTTAGGCCTCCCGCTAGCGTTCATCCTGAGCCAGGATCAAACTCT
>25
CTGCTGCCGCCCGTAGGAGTCTGGTCCGTGTCTCAGTACCAGTGTGGGGGATCACCCTCTCAGGCCCCCTAAA
GATCGTCGCCTTGTGAGCCGTTACCTCACCAACTAGCTAATCTTGCGCGTGCCCATCTCTATCCACCGTAGTT
TTCAATATCACCCGATGCCGAATAATATATTATGGGGTATTAATCTTCTTCGAAAGGCTATCCCCCTGATAAA
GGCAGGTTGCACACGTGTTCCGCACCCGTACGCCGCTCTCAATTACCCGAAAGTAATCTACCGCTCGGCTTGC
ATGTGTTAGGCCTCCCGCTAGCGTCATCCTGAGCCAGGATCAAACTCT
>26
CTGCTGCCGCCCGTAGGAGTCTGGTCCGTGTCTCAGTACCAGTGTGGGGGATCACCCTCTCAGGCCCCCTAAA
GATCGTCGCCTTGGTGAGCCGTTACCTCACCAACTAGCTAATCTTGCGCGTGCCCATCTCTATCCACCGTAGT
TTTCAATATCACCCGATGCCGAATAATATATTATGGGGTATTAATCTTCTTCGAAAGGCTATCCCCCTGATTA
AAGGCAGGTTGCACACGTGTTCCGCACCCGTACGCCGCTCTCAATTACCCGAAAGTAATCTACCGCTCGGCTT
GCATGTGTTAGGCCTCCCGCTAGCGTTCATCCTGAGCCAGGATCAACTCT
>27
CTGCTGCCGCCCGTAGGAGTCTGGTCCGTGTCTCAGTACCAGTGTGGGGGATCACCCTCTCAGGCCCCCTAAA
GATCGTCGCCTTGGTGAGCCGTTACCTCACCAACTAGCTAATCTTGCGCGTGCCCATTCTCTATCCACCGTAG
TTTCAATATCACCCGATGCCGAATAATTATATTATGGGGTATTAATCTTCTTCGAAAGGCTATCCCCCTGATT

AAAGGCAGGTTGCACACGTGTTCCGCACCCGTACGCCGCTCTCAATTACCCGAAAGTAATCTACCGCTCGGCT
TGCATGTGTTAGGCCTCCCGCTAGCGTTCTCC
>28
CTGCTGCTCGCCCGTAGGGGTCTGGTCCGTGTCTCAGTACCAGTGTGGGGGATCACCCTCTCAGGCCCCCTAA
AGATCGTCGCCTGGTGAGCCGTTACCTCACCAACTAGCTAATCTTGCGCGTGCCCATCTCTATCCACCGTAGT
TTTCAATATCACCCGATGCCGAATAATATATTATGGGGTATTAATCTTCTCGAAAGGCTATCCCCCTGATAAG
GCAGGTTGCACACGTGTTCCGCACCCGTACGCCGCTCTCAATTACCCGAAAGTAATCTACCGCTCGGCTTGCA
TGTGTTAGGCCTCCCGCTAGCGTCTCCTGAGCCAGGATCAAAC
>29
CTGCTGCCGCCCGTAGGAGTCTGGTCCGTGTCTCAGTACCAGTGTGGGGGATCACCCTCTCAGGCCCCCTAAA
GATCGTCGCCTTGGTGAGCCGTTACCTCACCAACTAGCTAATCTTGCGCGTGCCCATCTCTATCCACCGTAGT
TTTCAATATCACCCGATGCCGAATAATTATATTATGGGGTATTAATCTTCTTCTGAAAGGCTATCCCCCTGAT
AAAGGCAGGTTGCACACGTGTTCCGCACCCGTACGCCGCTCTCAATTACCCGAAAGTAATCTACCGCTCGGCT
TGCATGTGTTAGGCCTCCCGCTAGCGTTCTCCTGAGC
>30
CTGCTTGCCCGAGTAGGGAGCTGTCCTGTCTCAGTACCCAGTGTGGGGGGGATCACCCCTCTCAGGCCCCCTA
AAGATCGTCGCCTTGGTGAGCCGTTACCTCACCAACTAGCTAATCTTGCGCGTGCCCATCTCTATCCACCGTA
GTTTTCAATATCACCCGATGCCGAATAATATATTATGGGGTATTAATCTTCTTCGAAAGCTATCCCCCTGATA
AAGGCAGGTTGCACACGTGTTCCGCACCCGTACGCCGCTCTCAATTACCCGAAAGTAATCTACCGCTCGGCTT
GCATGTGTTAGGCCTCCCGCTAGCGTTCATCCTGAGCCAGGATCAACTCT
>31
CTGCTGCCGCCCGTAGGAGTCTGGTCCGTGTCTCAGTACCAGTGTGGGGGATCACCCTCTCAGGCCCCCTAAA
GATCGTCGCCTTGGTGAGCCGTTACCTCACCAACTAGCTAATCTTGCGCGTGCCCATCTCTATCCACCGTAGT
TTTCAATATCACCCGATGCCGAATAATATATTATGGGGTATTAATCTTCTTCGAAAGGCTATCCCCCTGATAA
AGGCAGGTTGCACACGTGTTCCGCACCCGTACGCCGCTCTCAATTACCCGAAAGTAATCTACCGCTCGGCTTG
CATGTGTTAGGCCTCCCGCTAGCGTTCTCCTGAGCAGGATCAACTCT
>32
CTGCTGCCGCCCGTAGGAGTCTGGACCGTGTCTCAGTTCCAGTGTGACTGATCATCCTCTCAGACCAGTACGG
ATCGTCGCCTTGGTAGGCCTTTACCCCACCAACTAGCTAATCCGACCTAGGCTCATCTGATAGCGTGAGGTCC
GAAGTCCCCCACTTCTCCCTCAGGACGTATGCGGTATTAGCGCCCGTTTCCGGACGTATCCCCCACTACCAGG
CAGATTCCTAGGCATTACTCACCCGTCCGCCGCTGAATCCAGGAGCAAGCCCCCTTCATCCGCTCGACTGCAT
GTGTTTAGGCTGCCGCCAGCGTCAATCTGAGCCAGGATCAAACTCT
>33
CTGCTGCCGCCCGTAGGAGTCTGGTCCGTGTCTCAGTACCAGTGTGGGGGATCACCCTCTCAGGCCCCCTAAA
GATCGTCGCCTTGGTGAGCCGTTACCTCACCAACTAGCTAATCTTGCGCGTGCCCATCTCTATCCACCGTAGT
TTTCAATATCACCCGATGCCGAATAATATATTATGGGGTATTAATCTTCTTCGAAAGGCTATCCCCCTGATAA
AGGCAGGTTGCACACGTGTTCCGCACCCGTACGCCGCTCTCAATTACCCGAAAGTAATCTACCGCTCGGCTTG
CATGTGTTAGGCCTCCCGCTAGCGTTCATCCTGAGCCAGGATCAAACTCT
>34
CTGCTGCCGCCCGTAGGAGTCTGGTCCGTGTCTCAGTACCAGTGTGGGGGATCACCCTCTCAGGCCCCCTAAA
GATCGTCGCCTTGGTGAGCCGTTACCTCACCAACTAGCTAATCTTGCGCGTGCCCATCTCTATCCACCGTAGT
TTTCAATATCACCCGATGCCGAATAATATATTATGGGGTATTAATCTTCTTCGAAAGGCTATCCCCCTGATAA
AGGCAGGTTGCACACGTGTTCCGCACCCGTACGCCGCTCTCAATTACCCGAAAGTAATCTACCGCTCGGCTTG
CATGTGTTAGGCCTCCCGCTAGCGTTCTCCTGAGCAGGTCAAACTCT
>35
CTGCTGCCGCCCGTAGGAGTCTGGTCCGTGTCTCAGTACCAGTGTGGGGGATCACCCTCTCAGGCCCCCTAAA
GATCGTCGCCTTGGTGAGCCGTTACCTCACCAACTAGCTAATCTTGCGCGTGCCCATCTCTATCCACCGTAGT
TTTCAATATCACCCGATGCCGAATAATATATTATGGGGTATTAATCTTCTTCGAAAGGCTATCCCCCTGATAA
AGGCAGGTTGCGCACGTGTTCCGCACCCGTACGCCGCTCTCAATTACCCGAAAGTAATCTACCGCTCGGCTTG
CATGTGTTAGGCCTCCCGCTAGCGTTCATCCTGAGCCAGGATCAACTCT
>36
CTGCTGCCGCCCGTAGGAGTCTGGACCGTGTCTCAGTTCCAGTGTGACTGATCATCCTCTCAGACCAGCTACG
GATCGTCGCCTGGTAGGCCTTTACCCCACCAACTAGCTAATCCGACCTAGGCTCATCTGATAGCGTGAGGTCC
GAAGTCCCCCACTTCTCCCTCAGGACGTATGCGGTATTAGCGCCCGTTTCCGGACGATCCCCCACTACCAGGC

AGATTCCTAGGCATTACTCACCCGTCCGCCGCTGAATCCAGGAGCAAGCCCCCTTCATCCGCTCGACTGCATG
TGTTAGGCCTGCCGCCAGCGTCAATCTGAGCCAGGATCAAACTCT
>37
CTGCTGCCGCCCGTAGGAGTCTGGTCCGTGTCTCAGTACCAGTGTGGGGGATCACCCTCTCAGGCCCCCTAAA
GATCGTCGCCTTGGTGAGCCGTTACCTCACCAACTAGCTAATCTTGCGCGTGCCCATCTCTATCCACCGTAGT
TTTCAATATCACCCGATGCCGAATAATATATTATGGGGTATTAATCTTCTTCGAAAGGCTATCCCCCTGATAA
AGGCAGGTTGCACACGTGTTCCGCACCCGTACGCCGCTCTCAATTACCCGAAAGTAATCTACCGCTCGGCTTG
CATGTGTTAGGCCTCCCGCTAGCGTTCTCCTGAGCAGGATCAACTCT
>38
CTGCTGCCGCCCGTAGGAGTCTGGTCCGTGTCTCAGTACCAGTGTGGGGGATCACCCTCTCAGGCCCCCTAAA
GATCGTCGCCTTGGTGAGCCGTTACCTCACCAACTAGCTAATCTTGCGCGTGCCCATCTCTATCCACCGTAGT
TTTCAATATCACCCGATGCCGAATAATATATTATGGGGTATTAATCTTCTTCGAAAGGCTATCCCCCTGATAA
AGGCAGGTTGCACACGTGTTCCGCACCCGTACGCCGCTCTCAATTACCCGAAAGTAATCTACCGCTCGGCTTG
CATGTGTTAGGCCTCCCGCTAGCGTTCATCCTGAGCCAGGATCAAACTC
>39
CTGCTGCCGCCCGTAGGAGTCTGGTCCGTGTCTCAGTACCAGTGTGGGGGATCACCCTCTCAGGCCCCCTAAG
ATCGTCGCCTGTGAGCCGTTACCTCACCAACTAGCTAATCTTGCGCGTGCCCATCTCTATCCACCGTAGTTTT
CAATATCACCCGATGCCGAATAATATATTATGGGGTATTAATCTTCCTTCGAAGAGCTATCCCCTGATAAGGC
AGGTTGCACACGTGTTCCGCACCCGTACGCCGCTCTCAATTACCCGAAAGTAATCTACCGCTCGGCTTGCATG
TGTTAGGCCTCCCGCTAGCGTCTCCT
>40
CTGCTGCCGCCCGTAGGAGTCTGGTCCGTGTCTCAGTACCAGTGTGGGGGATCACCCTCTCAGGCCCCCTAAA
GATCGTCGCCTTGGTGAGCCGTTACCTCACCAACTAGCTAATCTTGCGCGTGCCCATCTCTATCCACCGTAGT
TTTCAATATCACCCGATGCCGAATAATATATTATGGGGTATTAATCTTCTTCGAAAGGCTATCCCCCTGATAA
GGCAGGTTGCACACGTGTTCCGCACCCGTACGCCGCTCTCAATTACCCGAAAGTAATCTACCGCTCGGCTTGC
ATGTGTTAGGCCTCCCGCTAGCGTTCATCCTGAGCCAGGATCAAACTCT
>41
CTGCTGCCGCCCGTAGGAGTCTGGTCCGTGTCTCAGTACCAGTGTGGGGGATCACCCTCTCAGGCCCCCTAAA
GATCGTCGCCTTGTGAGCCGTTACCTCACCAACTAGCTAATCTTGCGCGTGCCCATCTCTATCCACCGTAGTT
TTCAATATCACCCGATGCCGAATAATATATTATGGGGTATTAATCTTCTTCGAAAGGCTATCCCCCTGATTAA
AGGCAGGTTGCACACGTGTTCCGCACCCGTACGCCGCTCTCAATTACCCGAAAGTAATCTACCGCTCGGCTTG
CACTGTGTTAGGCCTCCCGCTAGCGTTCTCCT
>42
CTGCTGCCGCCCGTAGGAGTCTGGTCCGTGTCTCAGTACCAGTGTGGGGGATCACCCTCTCAGGCCCCCTAAAG
ATCGTCGCCTGGTGAGCCGTTACCTCACCAACTAGCTAATCTTGCGCGTGCCCATCTCTATCCACCGTAGTTT
CAATATCACCCGATGCCGAATAATATATTATGGGGTATTAATCTTCTCGAAAGGCTATCCCCTGATAAAGGCA
GGTTGCACACGTGTTCCGCACCCGTACGCCGCTCTCAATTACCCGAAAGTAATCTACCGCTCGGCTTGCATGT
GTTAGGCCTCCCGCTAGCGTTCATCCTGAGCCAGGATCAACTCT
>43
CTGCTGCCGCCCGTAGGAGTCTGGTCCGTGTCTCAGTACCAGTGTGGGGGGATCACCCTCTCAGGCCCCCTAA
AGATCGTCGCCTTGGTGAGCCGTTACCTCACCAACTAGCTAATCTTGCGCGTGCCCATCTCTATCCACCGTAG
TTTTCAATATCACCCGATGCCGAATAATTATATTATGGGGTATTAATCTTCTTCGAAAGGCTATCCCCCTGAT
AAAGGCAGGTTGCACACGTGTTCCGCACCCGTACGCCGCTCTCAATTACCCGAAAGTAATCTACCGCTCGGCT
TGCATGTGTTAGGCCTCCCGCTAGCGTTCTCCTGAGCCAGGATCAACT
>44
CTGCTGCCGCCCGTAGGAGTCTGGTCCGTGTCTCAGTACCAGTGTGGGGGATCACCCTCTCAGGCCCCCTAAA
GATCGTCGCCTTGTGAGCCGTTACCTCACCAACTAGCTAATCTTGCGCGTGCCCATCTCTATCCACCGTAGTT
TTCAATATCACCCGATGCCGAATAATATATTATGGGGTATTAATCTTCTCGAAAGGCTATCCCCTGATAAAGG
CAGGTTGCACACGTGTTCCGCACCCGTACGCCGCTCTCAATTACCCGAAAGTAATCTACCGCTCGGCTTGCAT
GTGTTAGGCCTCCCGCTAGCGTCATCCTGAGCCAGGATCAACTCT
>45
CTGCTGCCGCCCGTAGGAGTCTGGTCCGTGTCTCAGTACCAGTGTGGGGGGATCACCCCTCTCAGGCCCCCCT
AAAGATCGTCGCCTTGGTGAGCCGTTACCTCACCCAACTAGCTAATCTTGCGCGTGCCCATCTCTATCCACCG
TAGTTTCAATATCACCCGATGCCGAATAATATATTATTGGGGTATTTAATCTTCTCGAAAGGCTATCCCCCTG

ATTAAAGGCAGGTTGCACACGTGTTCCGCACCCGTACGCCGCTCTCAATTACCCCGAAAGTAATCTACCGCTC
GGCTTGCATGTGTTAGGCCTCCCGCTAGCGTTCTTCCTGAGCAGGTCCA
>46
CTGCTGCCGCCCGTAGGAGTCTGGTCCGTGTCTCAGTACCAGTGTGGGGGATCACCCTCTCAGGCCCCCTAAA
GATCGTCGCCTTGTGAGCCGTTACCTCACCAACTAGCTAATCTTGCGCGTGCCCATCTCTATCCACCGTAGTT
TTCAATATCACCCGATGCCGAATAATATTATTATGGGGTATTAATCTTCTTCGAAAGGCTATCCCCCTGATAA
AGGCAGGTTGCACACGTGTTCCGCACCCGTACGCCGCTCTCAATTACCCGAAAGTAATCTACCGCTCGGCTTG
CATGTGTTAGGCCTCCCGCTAGCGTTCATTCCTGAGCCAGGATCAAACTCCT
>47
CTGCTGCCGCCCGTAGGAGTCTGGTCCGTGTCTCAGTACCAGTGTGGGGGGTCACCCTCTCAGGCCCCCTAAA
GATCGTCGCCTTGGTGAGCCGTTACCTCACCAACTAGCTAATCTTGCGCGTGCCCATCTCTATCCACCGTAGT
TTTCAATATCACCCGATGCCGAATAATATATTATGGGGTATTAATCTTCTTCGAAAGGCTATCCCCCTGATAA
AGGCAGGTTGCACACGTGTTCCGCACCCGTACGCCGCTCTCAATTACCCGAAAGTAATCTACCGCTCGGCTTG
CATGTGTTAGGCCTCCCGCTAGCGCTCATCCTGAGCCAGGATCAAACTCT
>48
CTGCTGCCGCCCGTAGGAGTCTGGTCCGTGTCCCAGTACCAGTGTGGGGGATCACCCTCTCAGGCCCCCTAAA
GATCGTCGCCTTGGTGAGCCGTTACCTCACCAACTAGCTAATCTTGCGCGTGCCCATCTCTATCCACCGTAGT
TTTCAATATCACCCGATGCCGAATAATATATTATGGGGTATTAATCTTCCTTCGAAAGCTATCCCCCTGATAA
AGGCAGGTTGCACACGTGTTCCGCACCCGTACGCCGCTCTCAATTACCCGAAAGTAATCTACCGCTCGGCTTG
CATGTGTTAGGCCTCCCGCTAGCGTTCATCCTGAGCCAGGATCAAACTCT
>49
CTGCTGCCGCCCGTAGGAGTCTGGTCCGTGTCTCAGTACCAGTGTGGGGGATCACCCTCTCAGGCCCCCTAAA
GATCGTCGCCTTGGTGAGCCGTTACCTCACCAACTAGCTAATCTTGCGCGTGCCCATCTCTATCCACCGTAGT
TTTCAATATCACCCGATGCCGAATAATATATTATGGGGTATTAATCTTCTTCGAAAGGCTATCCCCCTGATAA
AGGCAGGTTGCACACGTGTTCCGCACCCGTACGCCGCTCTCAATTACCCGAAAGTAATCTACCGCTCGGCTTG
CATGTGTTAGGCCTCCCGCTAGCGTTCTCCTGAG
>50
CTGCTGCCGCCCGTAGGAGTCTGGTCCGTGTCTCAGTACCAGTGTGGGGGATCACCCTCTCAGGCCCCCTAAA
GATCGTCGCCTTGGTGAGCCGTTACCTCACCAACTAGCTAATCTTGCGCGTGCCCATCTCTATCCACCGTAGT
TTTCAATATCACCCGATGCCGAATAATATATTATGGGGTATTAATCTTCTCGAAAGGCTATCCCCTGATAAAG
GCAGGTTGCACACGTGTTCCGCACCCGTACGCCGCTCTCAATTTACCCGAAAGTAATCTACCGCTCGGCTTGC
AATGTGTTAGGCCTCCCGCTAGCGTTCTCCTGAGCAGG
>51
CTGCTGCCGCCCGTAGGAGTCTGGTCCGTGTCTCAGTACCAGTGTGGGGGATCACCCTCTCAGGCCCCCTAAA
GATCGTCGCCTGGTGAGCCGTTACCTCACCAACTAGCTAATCTTGCGCGTGCCCATCTCTATCCACCGTAGTT
TTCAATATCACCCGATGCCGAATAATATATTATGGGGTATTAATCTTCTTCGAAAGGCTATCCCCTGATAAGG
CAGGTTGCACACGTGTTCCGCACCCGTACGCCGCTCTCAATTACCCGAAAGTAATCTACCGCTCCGGCTTGCA
TGTGTTAGGCCTCCCGCTAGCGTTCATCCTGAGCCAGGATCAAACTCT
>52
CTGCTGCCGCCCGTAGGAGTCTGGTCCGTGTCTCAGTACCAGTGTGGGGGATCACCCTCTCAGGCCCCCTAAA
GATCGTCGCCTTGGTGAGCCGTTACCTCACCAACTAGCTAATCTTGCGCGTGCCCATCTCTATCCACCGTAGT
TTTCAATATCACCCGATGCCGAATAATATATTATGGGGTATTAATTCTTCTTCGAAAGGCTATCCCCCTGATA
AAGGCAGGTTGCACACGTGTTCCGCACCCGTACGCCGCTCTCAATTACCCGAAAGTAATCTACCGCTCGGCTT
GCATGTGTTAGGCCTCCCGCTAGCGTTCATCCTGAGCCAGGATCAAACTCT
>53
CTGCTGCCGCCCGTAGGAGTCTGGTCCGTGTCTCAGTACCAGTGTGGGGGATCACCCTCTCAGGCCCCCTAAA
GATCGTCGCCTTGGTGAGCCGTTACCTCACCAACTAGCTAATCTTGCGCGTGCCCATCTCTATCCACCGTAGT
TTTCAATATCACCCGATGCCGAATAATATATTATGGGGTATTAATTCTTCTTCGAAAGGCTATCCCCCTGATA
AAGGCAGGTTGCACACGTGTTCCGCACCCGTACGCCGCTCTCAATTACCCGAAAGTAATCTACCGCTCGGCTT
GCATGTGTTAGGCCTCCCGCTAGCGTTCATCCTGAGCCAGGATCAACTCT
>54
CTGCTGCCGCCCGTAGGAGTCTGGTCCGTGTCTCAGTACCAGTGTGGGGGGATCACCCTCTCAGGCCCCCTAA
AGATCGTCGCCTTGGTGAGCCGTTACCTCACCAACTAGCTAATCTTGCGCGTGCCCATTCTCTATCCACCGTA
GTTTCAATATCACCCGATGCCGAATAATTATATTATGGGGTATTAATCTTCTTCGAAAGGCTATCCCCCTGAT

AAAGGCAGGTTGCACACGTGTTCCGCACCCGTACGCCGCTCTCAATTACCCGAAAGTAATCTACCGCTCGGCT
TGCATGTGTTAGGCCTCCCGCTAGCGTTCATCCTGAGCCAGGATCAAACTCT
>55
CTGCTGCCGCCCGTAGGAGTCTGGTCCGTGTCTCAGTACCAGTGTGGGGGATCACCCTCTCAGGCCCCCTAAA
GATCGTCGCCTTGGTGAGCCGTTACCTCACCAACTAGCTAATCTTGCGCGTGCCCATCTCTATCCACCGTAGT
TTTCAATATCACCCGATGCCGAATAATATATTATGGGGTATTAATCTTCTTCGAAAGGCTATCCCCCTGATAA
AGGCAGGTGCACACGTGTTCCGCACCCGTACGCCGCTCTCAATTACCCGAAAGTAATCTACCGCTCGGCTTGC
ATGTGTTAGGCCTCCCGCTAGCGTTCATCCTGAGCCAGGATCAACTCT
>56
CTGCTGCCGCCCGTAGGAGTCTGGTCCGTGTCTCAGTACCAGTGTGGGGGACCACCCTCTCAGGCCCCCTAAA
GATCGTCGCCTGGTGAGCCGTTACCTCACCAACTAGCTAATCTTGCGCGTGACCATCTCTATCCACCGTAGTT
TTCAATATCACCCGATGCCGAATAATATATTATGGGGTATTAATCTTCTTCGAAAGGCTATCCCCTGATAAAG
GCAGGTTGCACACGTGTTCCGCACCCGTACGCCGCTCTCAATTACCCGAAAGTAATCTACCGCTCGGCTTGCA
TGTGTTAGGCCTCCCGCTAGCGTTCATCCTGAGCCAGGATCAAACTCT
>57
CTGCTGCCGCCCGTAGGAGTCTGGTCCGTGTCTCAGTACCAGTGTGGGGGATCACCCTCTCCAGGCCCCCTAA
AGATCGTCGCCTTGGTGAGCCGTTACCTCACCAACTAGCTAATCTTGCGCGTGCCCATCTCTATCCACCGTAG
TTTTCAATATCACCCGATGCCGAATAATATATTATGGGGTATTAATCTTCTTCGAAAGGCTATCCCCCTGATA
AAGGCAGGTTGCACACGTGTTCCGCACCCGTACGCCGCTCTCAATTACCCGAAAGTAATCTACCGCTCGGCTT
GCATGTGTTAGGCCTCCCGCTAGCGTTCTCCTGAGCAGGTCAAACTCGT
>58
CTGCTGCCGCCCGTAGGAGTCTGGTCCGTGTCTCAGTACCAGTGTGGGGGATCACCCTCTCAGGCCCCCTAAA
AGATCGTCGCCTTGGTGAGCCGTTACCTCACCAACTAGCTAATCTTGCGCGTGCCCATCTCTATCCACCGTAG
TTTTCAATATCACCCGATGCCGAATAATATATTATGGGGTATTAATCTTCTTCGAAAGGCTATCCCCCTGATA
AAGGCAGGTTGCACACGTGTTCCGCACCCGTACGCCGCTCTCAATTACCCGAAAGTAATCTACCGCTCGGCTT
GCATGTGTTAGGCCTCCCGCTAGCGTTCTCCTGAGCCAGGATCAACTCT
>59
CTGCTGCCGCCCGTAGGAGTCTGGTCCGTGTCTCAGTACCAGTGTGGGGGATCACCCTCTCAGGCCCCCTAAA
GATCGTCGCCTGGTGAGCCGTTACCTCACCAACTAGCTAATCTTGCGCGTGCCCATCTCTATCCACCGTAGTT
TTCAATATCACCCGATGCCGAATAATATATTATGGGGTATTAATCTTCTTCGAAAGGCTATCCCCCTGATAAA
GGCAGGTTGCACACGTGTTCCGCACCCGTACGCCGCTCTCAATTACCCGAAAGTAATCTACCGCTCGGCTTGC
ATGTGTTAGGCCTCCCGCTAGCGTTCATCCTGAGCCAGGATCAACTCT
>60
CTGCTGCCGCCCCGTAGGAGTCTGGTCCCGTGTCTCAGTACCAGTGTGGGGGATCACCCCTCTCAGGCCCCCT
AAAGATCGTCGCCTTGGTGAGCCGTTACCTCACCCAACTAGCTAATCTTGCGCGTGCCCCATCTCTATCCACC
GTAGTTTTCAATATCACCCGATGCCGAATAATATATTATGGGGTATTAATCTTCTTCGAAAGGCTATCCCCTG
ATAAAGGCAGGTTGCACACGTGTTCCGCACCCGTACGCCGCTCTCAATTACCCCGAAAGTAATCTACCGCTCG
GCTTGCATGTGTTAGGCCTCCCGCTAGCGTTCTCCTGAGCAGGATCCAA
>61
CTGCTGCCGCCCGTAGGAGTCTGGTCCGTGTCTCAGTACCAGTGTGGGGGATCACCCTCTCAGGCCCCTAAGA
TCGTCGCCTTGTGAGCCGTTACCTCACCAACTAGCTAATCTTGCGCGTGCCCATCTCTATCCACCGTAGTTTT
CAATATCACCCGATGCCGAATAATATATTATGGGGTATTAATCTTCTTCGAAAGGCTATCCCCTGATAAAGGC
AGGTTGCACACGTGTTCCGCACCCGTACGCCGCTCTCAATTACCCGAAAGTAATCTACCGCTCCGGCTTGCAT
GTGTTAGGCCTCCCGCTAGCGTTCATCCTGAGCCAGGATCAAACTCT
>62
CTGCTGCCGCCCGTAGGAGTCTGGTCCGTGTCTCAGTACCAGTGTGGGGGATCACCCTCTCAGGCCCCCTAAA
GATCGTCGCCTTGGTGAGCCGTTACCTCACCAACTAGCTAATCTTGCGCGTGCCCATCTCTATCCACCGTAGT
TTTCAATATCACCCGATGCCGAATAATATATTATGGGGTATTAATCTTCTCTGAAAGGCTATCCCCCTGATAA
GGCAGGTTGCACACGTGTTCCGCACCCGTACGCCGCTCTCAATTTACCCGAAAGTAATCTACCGCTCCGGCTT
GCATGTGTTAGGCCTCCCGCTAGCGTTCATCCTGAGCCAGGATCAAACTCT
>63
CTGCTGCCGCCCGTAGGAGTCTGGTCCCGTGTCTCAGTACCAGTGTGGGGATCACCCTCTCAGGCCCCCTAAA
GATCGTCGCCTTGGTGAGCCGTTACCTCACCCAACTAGCTAATCTTGCGCGTGCCCCATCTCTATCCACCGTA
GTTTTCAATATCACCCGATGCCCGAATAATATATTATGGGGTATTAATCTTCTTCGAAAGGCTATCCCCTGAT

AAAGGCAGGTTGCACACGTGTTCCCGCACCCGTACGCCGCTCTCAATTACCCCGAAAGTAATCTACCGCTCGG
CTTGCATGTGTTAGGCCCTCCGCTAGCGTTCATTCCTGAGCCAGGATCAAACTCT
>64
CTGCTGCCGCCCGTAGGAGTCTGGTCCGTGTCTCAGTACCAGTGTGGGGGATCACCCTCTCAGGCCCCCTAAA
GATCGTCGCCTTGGTGAGCCGTTACCTCACCAACTAGCTAATCTTGCGCGTGCCCATCTCTATCCACCGTAGT
TTTCAATATCACCCGATGCCGAATAATATATTATGGGGTATTAATCTTCTTCGAAAGGCTATCCCCCTGATAA
AGGCAGGTTGCACACGTGTTCCGCACCCGTACGCCGCTCTCAATTACCCGAAAGTAATCTACCGCTCGGCTTG
CATGTGTTAGGCCTCCCGCTAGCGTTCTCCTGAGCAGGATCAACTCT
>65
CTGCTGCCGCCCGTAGGAGTCTGGTCCGTGTCTCAGTACCAGTGTGGGGGATCACCCTCTCAGGCCCCCTAAA
GATCGTCGCCTTGTGAGCCGTTACCTCACCAACTAGCTAATCTTGCGCGTGCCCATCTCTATCCACCGTAGTT
TTCAATATCACCCGATGCCGAATAATATATTATGGGGTATTAATCTTCTCGAAAGGCTATCCCCTGATAAAGG
CAGGTTGCACACGTGTTCCGCACCCGTACGCCGCTCTCAATTCCCGAAAGTAATCTACCGCCATCGGCTTGCA
TGTGTTAGGCCTCCCGCTAGCGTCTC
>66
CTGCTGCCGCCCCGTAGGAGTCTGGTCCGTGTCTCAGTACCAGTGTGGGGGATCACCCTCTCAGGCCCCCCTA
AAGATCGTCGCCTGTGAGCCCGTTACCTCACCCAACTAGCTAATCTTGCGCGTGCCCATCTCTATCCACCGTA
GTTTTCAATATCACCCGATGCCGAATAATATATTATGGGGTATTAATCTTCTTCGAAAGGCTATCCCCTGATA
AAGGCAGGTTGCACACGTGTTCCCGCACCCGTACGCCGCTCTCAATTACCCCGAAAGTAATCTACCGCTCGGC
TTGCATGTGTTAGGCCTCCCGCTAGCGTTCTCC
>67
CTGCTGCCGCCCGTAGGAGTCTGGTCCGTGCCTCAGTACCAGTGTGGGGGATCACCCTCTCAGGCCCCCTAAA
GATCGTCGCCTGGTGAGCCGTTACCTCACCAACTAGCTAATCTTGCGCGTGCCCATCTCTATCCACCGTAGTT
TTCAATATCAACCCGATGCCGAATAATATAATTATGGGGTATTAATCTTCTCGAAAGGCTATCCCCCTGATAA
AGGCAGGTTGCACACGTGTTCCGCACCCGTACGCCGCTCTCAATTACCCGAAAGTAATCTACCGCTCGGCTTG
CATGTGTTAGGCCTCCCGCTAGCGTTC
>68
CTGCTGCCGCCCGTAGGAGTCTGGTCCGTGTCTCAGTACCAGTGTGGGGGATCACCCTCTCAGGCCCCCTAAA
GATCGTCGCCTTGGTGAGCCGTTACCTCACCAACTAGCTAATCTTGCGCGCGCCCATCTCTATCCACCGTAGT
TTTCAATATCACCCGATGCCGAATAGTATATTATGGGGTATTAATCTTCTCTGAAAGGCTATCCCCCTGATAA
AGGCAGGTTGCACACGTGTTCCGCACCCGTACGCCGCTCTCAATTACCCGAAAGTAATCTACCGCTCGGCTTG
CATGTGTTAGGCCTCCCGCTAGCGTCATCCTGAGCCAGGATCAAACTCT
>69
CTGCTGCCGCCCGTAGGAGTCTGGTCCGTGTCTCAGTACCAGTGTGGGGGATCACCCTCTCAGGCCCCCTAAA
GATCGTCGCCTTGGTGAGCCGTTACCTCACCAACTAGCCTAATCTTGCGCGTGCCCATCTCTATCCACCGTAG
TTTTCAATATCACCCGATGCCGAATAATATATTATGGGGTATTAATCTTCTTCGAAAGGCTATCCCCCTGATA
AAGGCAGGTTGCACACGTGTTCCGCACCCGTACGCCGCTCTCAATTACCCGAAAGTAATCTACCGCTCCGGCT
TGCATGTGTTAGGCCTCCCGCTAGCGTTCATCCTGAGCCAGGATCAAACTCT
>70
CTGCTGCCGCCCGTAGGAGTCTGGTCCGTGTCTCAGTACCAGTGTGGGGATCACCCTCTCAGGCCCCCTAAAG
ATCGTCGCCTTGGTGAGCCGTTACCTCACCAACTAGCTAATCTTGCGCGTGCCCATCTCCATCCACCGTAGTT
TTCAATATCACCCGATGCCGAATAATATATTATGGGGTATTAATCTTCTTCGAAAGGCTATCCCCCTGATAAA
GGCAGGTTGCACACGTGTTCCGCACCCGTACGCCGCTCTCAATTCCCGAAAGTAATCTACCGCTCGGCTTGCA
TGTGTTAGGCCTCCCGCTAGCGTTCTCCTGAGCCAGGATCAACTCT
>71
CTGCTGCCGCCCGTAGGAGTCTGGTCCGTGTCTCAGTACCAGTGTGGGGGATCACCCTCTCAGGCCCCCTAAA
GATCGTCGCCTTGGTGAGCCGTTACCTCACCAACTAGCTAATCTTGCGCGTGCCCATCTCTATCCACCGTAGT
TTTCAATATCACCCGATGCCGAATAATATATTATGGGGTATTAATCTTCTTCGAAAGGCTATCCCCCTGATAA
AGGCAGGTTGCACACGTGTTCCGCACCCGTACGCCGCTCTCAATTACCCGAAAGTAATCTACCGCTCCGGCTT
GCATGTGTTAGGCCTCCCGCTAGCGTTCTCCTGAGCAGGATCAACTCT
>72
CTGCTGCCGCCCGTAGGAGTCTGGTCCGTGTCTCAGTACCAGTGTGGGGGGGATCACCCTCTCAGGCCCCCTA
AAGATCGTCGCCTTGGTGAGCCGTTACCTCACCAACTAGCTAATCTTGCGCGTGCCCATCTCTATCCACCGTA
GTTTTCAATATCACCCGATGCCGAATAATATATTATGGGGTATTAATCTTCTTCGAAAGGCTATCCCCCTGAT

AAAGGCAGGTTGCACACGTGTTCCGCACCCGTACGCCGCTCTCAATTACCCGAAAGTAATCTACCGCTCGGCT
TGCATGTGTTAGGCCTCCCGCTAGCGTTCATCCTGAGCCAGGATCAAACTCT
>73
CTGCTGCCGCCCGTAGGAGTCTGGTCCGTGTCTCAGTACCAGTGTGGGGGATCACCCTCTCAGGCCCCCTAAA
GATCGTCGCCTTGGTGAGCCGTTACCTCACCAACTAGCTAATCTTGCGCGTGCCCATCTCTATCCACCGTAGT
TTTCAATATCACCCGATGCCGAATAATATATTATGGGGTATTAATCTTCTTCGAAAGGCTATCCCCCTGATAA
AGGCAGGTTGCACACGTGTTCCGCACCCGTACGCCGCTCTCAATTACCCGAAAGTAATCTACCGCTCGGCTTG
CATGTGTTAGGCCTCCCGCTAGCGTTCATCCTGAGCCAGGATCAAACTCT
>74
CTGCTGCCGCCCGTAGGAGTCTGGTCCGTGTCTCAGTACCAGTGTGGGGGATCACCCTCTCAGGCCCCCTAAG
GATCGTCGCCTTGGTGAGCCGTTACCTCACCAACTAGCTAATCTTGCGCGTGCCCATCTCTATCCACCGTAGT
TTTCAATATCACCCGATGCCGAATAATATATTATGGGGTATTAATCTTCTCGAAAGGCTATCCCCCTGATAAA
GGCAGGTTGCACACGTGTTCCGCACCCGTACGCCGCTCTCAATTACCCGAAAGTAATCTACCGCTCGGCTTGC
ATGTGTTAGGCCTCCCGCTAGCGTCATCCTGAGCCAGGATCAAACTCT
>75
CTGCTGCCGCCCGTAGGAGTCTGGTCCGTGTCTCAGTACCAGTGTGGGGGATCACCCTCTCAGGCCCCCTAAA
GATCGTCGCCTGTGAGCCGTTACCTCACCAACTAGCTAATCTTGCGCGTGCCCATCTCTATCCACCGTAGTTT
TCAATATCACCCGATGCCGAATAATATATTATGGGGTATTAATCTTCTTCGAAAGGCTATCCCCCTGATAAAG
GCAGGTTGCACACGTGTTCCGCACCCGTACGCCGCTCTCAATTACCCGAAAGTAATCTACCGCTCGGCTTGCA
TGTGTTAGGCCTCCCGCTAGCGTTCTCCTGAGCCAGGATCA
>76
CTGCTGCCGCCCGTAGGAGTCTGGACCGTGTCTCAGTTCCAGTGTGACTGATCATCCTCTCAGACCAGTACGG
ATCGTCGCCTGGTAGGCCTTTACCCCACCAACTAGCTAATCCGACCTAGGCTCATCTGATAGCGTGAGGTCCG
AAGTCCCCCACTTCTCCCTCAGGACGTATGCGGTATTAGCGCCCGTTTCCGGACGATCCCCCACTACCAGGCA
GATTCCTAGGCATTACTCACCCGTCCGCCGCTGAATCCAGGAGCAAGCTCCCTTCATCCGCTCGACTGCATGT
GTTAGGCCTGCCGCCAGCGTCAATCTGAGCCAGGATCAAACTCT
>77
CTGCTGCCGCCCGTAGGAGTCTGGACCGTGTCTCAGTTCCAGTGTGACTGATCATCCTCTCAGACCAGTACGG
ATCGTCGCCTTGGTAGGCCTTTACCCCACCAACTAGCTAATCCGACCTAGGCTCATCTGATAGCGTGAGGTCC
GAAGATCCCCCACTTCTCCCTCAGGACGTATGCGGTATTAGCGCCCGTTTCCGGACGTATCCCCACTACCAGG
CAGATTCCCTAGGCATTACTCACCCGTCCGCCGCTGAATCCAGGAGCAAGCCCTCCTACTCCGCTCGACTGCA
TGTGTTAGGCCTGCCGCCAGCGTTCAATCTGAGCCAGGATCAAACTCT
>78
CTGCTGCCGCCCGTAGGAGTCTGGTCCGTGTCTCAGTACCAGTGTGGGGGATCACCCTCTCAGGCCCCCCTAA
AGATCGTCGCCTTGGTGAGCCCGTTACCTCACCCAACTAGCTTAATCTTGCGCGTGCCCCATCTCTATCCACC
GTAGTTTTCAATATCACCCGATGCCGAATAATATATTATGGGGTATTAATCTTCTTCGAAAGGCTATCCCCCT
GATAAAGGCAGGTTGCACACGTGTTCCGCACCCGTACGCCGCTCTCAATTACCCCGAAAGTAATCTACCGCTC
GGCTTGCATGTGTTAGGCCTCCCGCTAGCGTTCATCCTGAGCCAGGATCAAACTCT
>79
CTGCTGCCGCCCGTAGGAGTCTGGTCCGTGTCTCAGTACCAGTGTGGGGGATCACCCTCTCAGGCCCCCCTAA
AGATCGTCGCCTTGGTGAGCCGTTACCTCACCAACTAGCTAATCTTGCGCGTGCCCCATCTCTATCCACCGTA
GTTTTCAATATCACCCGATGCCGAATAATATATTATGGGGTATTAATCTTCTTCGAAAGGCTATCCCCCTGAT
AAAGGCAGGTTGCACACGTGTTCCGCACCCGTACGCCGCTCTCAATTACCCCGAAAGTAATCTACCGCTCGGC
TTGCATGTGTTAGGCCTCCCGCTAGCGTTCATTCCTGAGCCAGGATCAAACTCT
>80
CTGCTGCCGCCCGTAGGAGTCTGGCTCGTGTCTCAGTACCAGTGTGGGGGATCACCCTCTCAGGCCCCCTAAA
GATTGTCGCCTTGGTGAGCCGTTACCTCACCAACTAGCTAATCTTGCGCGTGCCCATCTCTATCCACCGTAGT
TTTCAACATCACCCGATGCCGAATAATATATTATGGGGTATTAATCTTCTTCGAAAGGCTATCCCCCTGATAA
AGGCAGGTTGCACACGTGTTCCGCACCCGTACGCCGCTCTCAATTACCCGAAAGTAATCTACCGCTCGGCTTG
CATGTGTTAGGCCTCCCGCTAGCGTTCATCCTGAGCCAGGATCAAACTCT
>81
CTGCTGCCGCCCGTAGGAGTCTGGACCGTGTCTCAGTTCCAGTGTGACTGATCATCCTCTCAGACCAGTACGG
ATCGTCGCCTTGGTAGGCCTTTACCCCACCAACTAGCTAATCCGACCTAGGCTCATCTGATAGCGTGAGGTCC
GAAGATCCCCCACTTCTCCCTCAGGACGTATGCGGTATTAGCGCGTTTCCGGGCGTATCCCCCACTACCAGGC

AGATTCCTAGGCATTACTCACTCGTCCGCCGCTGAATCCAGGAGCAAGCCCCCTTCATCCGCTCGACTGCATG
TGTTAGGCCTGCCGCCAGCGTCAATCTGAGCCAGGATCAAACTCT
>82
CTGCTGCCGCCCGTAGGAGTCTGGTCCGTGTCTCAGTACCAGTGTGGGGGATCACCCTCTCAGGCCCCCTAAA
GATCGTCGCCTTGGTGAGCCGTTACCTCACCAACTAGCTAATCTTGCGCGTGCCCATCTCTATCCACCGTAGT
TTTCAATATCACCCGATGCCGAATAATATATTATGGGGTATTAATCTTCTTCGAAAGGCTATCCCCCTGATAA
AGGCAGGTTGCACACGTGTTCCGCACCCGTACGCCGCTCTCAATTACCCGAAAGTAATCTACCGCTCGGCTTG
CATGTGTTAGGCCTCCCGCTAGCGTCTCCTGAGCAGGATCAA
>83
CTGCTGCCGCCCGTAGGAGTCTGGTCCGTGTCTCAGTACCAGTGTGGGGGATCACCCTCTCAGGCCCCCTAAA
GATCGTCGCCTTGGTGAGCCGTTACCTCACCAACTAGCTAATCTTGCGCGTGCCCATCTCTATCCACCGTAGT
TTTCAATATCACCCGATGCCGAATAATATATTATGGGGTATTAATCTTCTCGAAAGGCTATCCCCTGATAAAG
GCAGGTTGCACACGTGTTCCGCACCCGTACGCCGCTCTCAATTACCCGAAAGTAATCTACCGCTCGGCTTGCA
TGTGTCAGGCCTCCCGCTAGCGTTCATCCTGAGCCAAGGATCAAACTCT
>84
CTGCTGCCGCCCGTAGGAGTCTGGTCCGTGTCTCAGTACCAGTGTGGGGGATCACCCTCTCAGGCCCCCTAAA
GATCGTCGCCTTGGTGAGCCGTTACCTCACCAACTAGCTAATCTTGCGCGTGCCCATCTCTATCCACCGTAGT
TTTCAATATCACCCGATGCCGAATAATATATTATGGGGTATTAATCTTCCTTCGGAAGGCTATCCCCCTGATA
AAGGCAGGTTGCACACGTGTTCCGCACCCGTACGCCGCTCTCAATTACCCGAAAGTAATCTACCGCTCGGCTT
GCATGTGTTAGGCCTCCCGCTAGCGTTCATCCTGAGCCAGGATCAAACTCT
>85
CTGCTGCCGCCCGTAGGAGTCTGGTCCGTGTCTCAGTACCAGTGTGGGGATCACCCTCTCAGGCCCCCTAAAG
ATCGTCGCCTTGGTGAGCCGTTACCTCACCAACTAGCTAATCTTGCGCGTGCCCATCTCTATCCACCGTAGTT
TCAATATCACCCGATGCCGAATAATATATTATGGGGTATTAATCTTCTTCGAAAGGCTATCCCCCTGATAAAG
GCAGGTTGCACACGTGTTCCGCACCCGTACGCCGCTCTCAATTACCCGAAAGTAATCTACCGCTCGGCTTGCA
TGTGTTAGGCCTCCCGCTAGCGTTCATCCTGAGCCAGGATCAAACTCT
>86
CTGCTGCCGCCCGTAGGAGTCTGGTCCGTGTCTCAGTACCAGTGTGGGGGATCACCCTCTCAGGCCCCCTAAA
CGATCGTCGCCTTGGTGAGCCGTTTACCTCACCAACTAGCTAATCTTGCGCGTGCCCATCTCTATCCACCGTA
GTTTTCAATATCAACCCGATGCCGAATAATATATTATGGGGTATTAATCTTCTTCGAAAGGCTATCCCCCTGA
TAAGGCAGGTTGCACACGTGTTCCGCACCCGTACGCCGCTCTCAATTACCCGAAAGTAATCTACCGCTCGGCT
TGCATGTGTTACGGCCTCCCGCTAGGCCGTTCA
>87
CTGCTGCCGCCCGTAGGAGTCTGGACCGTGTCTCAGTTCCAGTGTGGCCGATCACCCTCTCAGGTCGGCTATG
CATCGTTGCCTTGGTAAGCCGTTACCTTACCAACTAGCTAATGCAGCGCGGATCCATCTATAAGTGACAGCAA
GACCGTCTTCACTAACCATGCGGTTCAAAATATTATCCGGTATTAGCTCCGGTTTCCCGAAGTTATCCCAGTC
TTATAGGTAGGTTATCCACGTGTTACTCACCCGTCCGCCGCTAACATCAGAGAAGCAAGCTTCTCGTCCGTTC
GCTCGACTGCATGTATTAGGCACGCCGCCAGCGTTCATCCTGAGCCAGGATCAACTCT
>88
CTGCTGCCGCCCGTAGGAGTCTGGACCGTGTCTCAGTTCCAGTGTGACTGATCATCCTCTCAGACCAGTACGG
ATCGTCGCCTTGGTAGGCCTTTACCCCACCAACTAGCTAATCCGACCTAGGCTCATCTGATAGCGTGAGGTCC
GAAGTCCCCACTTCTCCCTCAGGACGTATGCGGTATTAGCGCCCGTTTCCGGACGTATCCCCATTACCAGGCA
GATTCCTAGGCATTACTCACCCGTCCGCCGCTGAATCCAGGAGCAAGCCCCCTTCATCCGCTCGACTGCATGT
GTTAGGCCTGCCGCCAGCGTCAATCTGAGCCAGGATCAAACTCT
>89
CTGCTGCCGCCCGTAGGAGTCTGGTCCGTGTCTCAGTACCAGTGTGGGGGATCACCCTCTCAGGCCCCCTAAA
GATCGTCGCCTTGGTGAGCCGTTACCTCACCAACTAGCTAATCTTGCGCGTGCCCATCTCTATCCACCGTAGT
TTTCAATATCACCCGATGCCGAATAATATATTATGGGGTATTAATCTTCTTCGAAAGGCTATCCCCCTGATAA
AGGCAGGTTGCACACGTGTTCCGCACCCGTACGCCGCTCTCAATTACCCGAAAGTAATCTACCGCTCGGCTTG
CATGTGTTAGGCCTCCCGCTAGCGTTCATCCTGAGCCAGGATCAAACTCT
>90
CTGCTGCCGCCCGTAGGAGTCTGGTCCGTGTCTCAGTACCAGTGTGGGGGATCACCCTCTCAGGCCCCCTAAA
GATCGTCGCCTTGGTGAGCCGTTACCTCACCAACTAGCTAATCTTGCGCGTGCCCATCTCTATCCACCGTAGT
TTTCAATATCACCCGATGCCGAATAATATATTATGGGGGTATTAATCTTCTTCGAAAGGCTATCCCCCTGATA

AGGCAGGTTGCACACGTGTTCCGCACCCGTACGCCGCTCTCAATTACCCGAAAGTAATCTACCGCTCGGCTTG
CATGTGTTAGGCCTCCCGCTAGCGTTCATCCTGAGCCAGGATCAACTCT
>91
CTGCTGCCGCCCGTAGGGGTCTGGTCCGTGTCTCAGTACCAGTGTGGGGGATCACCCTCTCAGGCCCCCTAAA
GATCGTCGCCTTGGTGAGCCGTTACCTCACCAACTAGCTAATCTTGCGCGTGCCCATCTCTATCCACCGTAGT
TTTCAATATCACCCGATGCCGAATAATATATTATGGGGTATTAATCTTCTTCGAAAGGCTATCCCCCTGATAA
AGGCAGGTTGCACACGTGTTCCGCACCCGTACGCCGCTCTCAATTACCCGAAAGTAATCTACCGCTCGGCTTG
CATGTGTTAGGCCTCCCGCTAGCGTTCTCCTGAGCCAGGATCAACTCT
>92
CTGCTGCCGCCCGTAGGAGTCTGGTCCGTGTCTCAGTACCAGTGTGGGGGATCACCCTCTCAGGCCCCCTAAA
GATCGTCGCCTGGTGAGCCGTTACCTCACCAACTAGCTAATCTTGCGCGTGCCCATCTCTATCCACCGTAGTT
TTCAATATCACCCGATGCCGAATAATATATTATGGGGTATTAATCTTCTTCGAAAGGCTATCCCCCTGATAAA
GGCAGGTTGCACACGTGTTCCGCACCCGTACGCCGCTCTCAATTACCCGAAAGTAATCTACCGCTCGGCTTGC
ATGTGTTAGGCCTCCCGCTAGCGTTCATCCTGAGCCAGGATCAAACTCT
>93
CTGCTGCCGCCCGTAGGAGTCTGGTCCGTGTCTCAGTACCAGTGTGGGGGATCACCCTCTCAGGCCCCCTAAA
GATCGTCGCCTGGCGAGCCGTTACCTCACCAACTAGCTAATCTTGCGCGTGCCCATCTCTATCCACCGTAGTT
TTCAATATCACCCGATGCCGAATAATATATTATGGGGTATTAATCTTCTTCGAAAGGCTATCCCCCTGATAAA
GGCAGGTTGCCACACGTGTTCCGCACCCGTACGCCGCTCTCAATTACCCGAAAGTAATCTACCGCATCGGCTT
GCATGTGTTAGGCCTCCCGCTAGCGTTCTCCTGAGCAGGATCAAC
>94
CTGCTGCCGCCCGTAGGAGTCTGGTCCGTGTCTCAGTACCAGTGTGGGGGATCACCCTCTCAGGCCCCCTAAA
GATCGTCGCCTTGGTGAGCCGTTACCTCACCAACTAGCTAATCTTGCGCGTGCCCCATCTCTATCCACCGTAG
TTTTCAATATCACCCGATGCCGAATAATATATTATGGGGTATTAATTCTTCTTCGAAAGGCTATCCCCTGATA
AAGGCAGGTTGCACACGTGTTCCGCACCCGTACGCCGCTCTCAATTCCCCGAAAGTAATCTACCGCCATCGGC
TTGCACTGTGTTAGGCCTCCCGCTAGCGTTCTCCTGAGCAGGA
>95
CTGCTGCCGCCCGTAGGAGTCTGGTCCGTGTCTCAGTACCAGTGTGGGGATCACCCTCTCAGGCCCCCTAAGA
TCGTCGCCTGTGAGCCGTTACCTCACCAACTAGCTAATCTTGCGCGTGCCCATCTCTATCCACCGTAGTTTTC
AATATCACCCGATGCCGAATAATATATTATGGGGTATTAATCTTCTCTGAAAGGCTATCCCCCTGATAAAGGC
AGGTTGCACACGTGTTCCGCACCCGTACGCCGCTCTCAATTACCCGAAAGTAATCTACCGCTCCGGCTTGCAT
GTGTTAGGCCTCCCGCTAGCGTTCATCCTGAGCCAGGCATCAAACTCT
>96
CTGCTGCCGCCCGTAGGAGTCTGGACCGTGTCTCAGTTCCAGTGTGACTGATCATCCTCTCAGACCAGTACGG
ATCGTCGCCTGGTAGGCCTTTACCCCACCAACTAGCTAATCCGACCTAGGCTCATCTGATAGCGTGAGGTCCG
AAGTCCCCCACTTCTCCCTCAGGACGTATGCGGTATTAGCGCCCGTTTCCGGACGTATCCCCACTACCAGGCA
GATTCCTAGGCATTACTCACCCGTCCGCCGCTGAATCCAGGAGCAAGCTCCCTTCATCCGCTCGACTGCATGT
GTTAGGCCTGCCGCCAGCGTCAATCTGAGCCAGGATCAAACTCT
>97
CTGCTGCCGCCCGTAGGAGTCTGGTCCGTGTCTCAGTACCAGTGTGGGGGATCACCCTCTCAGGCCCCCTAAAG
ATCGTCGCCTTGGTGAGCCGTTACCTCACCAACTAGCTAATCTTGCGCGTGCCCATCTCTATCCACCGTAGTT
TTCAATATCACCCGATGCCGAATAATATATTATGGGGTATTAATCTTCTTCGAAAGGCTATCCCCTGATAAGG
CAGGTTGCACACGTGTTCCGCACCCGTACGCCGCTCTCAATTACCCCGAAAGTAATCTACCGCTCGGCTTGCA
TGTGTTAGGCCTCCCGCTAGCGTTCTCCTGAGCAG
>98
CTGCTGCCGCCCGTAGGAGTCTGGTCCGTGTCTCAGTACCAGTGTGGGGGATCACCCTCTCAGGCCCCCTAAA
GATCGTCGCCTTGTGAGCCGTTACCTCACCAACTAGCTAATCTTGCGCGTGCCCATCTCTATCCACCGTAGTT
TTCAATATCACCCGATGCCGAATAATATATTATGGGGTATTAATCTTCTTCGAAAGGCTATCCCCTGATAAAG
GCAGGTTGCACACGTGTTCCGCACCCGTACGCCGCTCCATATCCCGAAAGTAATCTACCGCTCGGCTTGCATG
TGTTAGGCCTCCCGCTAGCGTTCTCTGAGCCAG
>99
CTGCTGCCGCCCGTAGGAGTCTGGTCCGTGTCTCAGTACCAGTGTGGGGGGATCACCCTCTCAGGCCCCCCTA
AAGATCGTCGCCTTGGTGAGCCCGTTACCTCACCCAACTAGCTAATCTTGCGCGTGCCCCATCTCTATCCCAC
CGTAGTTTTCAATATCACCCGATGCCGAATAATATATTATGGGGTATTTAATCTTCTTCGAAAGGCTATCCCC

CTGATTAAAGGCAGGTTGCACACGTGTTCCGCACCCGTACGCCGCTCTCAATTACCCCGAAAGTAATCTACCG
CTCGGCTTGCATGTGTTAGGCCTCCCGCTAGCGTTCTTCCTGAGCCAGGATCCA
>100
CTGCTGCCGCCCGTAGGAGTCTGGACCGTGTCTCAGTTCCAGTGTGGCCGATCACCCTCTCAGGTCGGCTATG
CATCGTTGCCTTGGTAAGCCGTTACCTTACCAACTAGCTAATGCAGCGCGGATCCATCTATAAGTGACAGCAA
GACCGTCTTTCACTAACCATGCGGTTCAAAATATTATCCGGTATTAGCTCCGGTTTCCCGAAGTTATCCCAGT
CTTATAGGTAGGTTATCCACGTGTTACTCACCCGTCCGCCGCTAACACTCCAGAGAAGCAAGCTTCTCGTCCG
TTCGCTCGACTGCATGTATTAGGCACGCCGCCAGCGTTCTCCTGAGCCAGGATCAAACTCT
>101
CTGCTGCCGCCCGTAGGAGTCTGGTCCGTGTCTCAGTACCAGTGTGGGGGGATCACCCTCTCAGGCCCCCTAA
AGATCGTCGCCTTGGTGAGCCGTTACCTCACCAACTAGCTAATCTTGCGCGTGCCCATCTCTATCCACCGTAG
TTTTCAATATCACCCGATGCCGAATAATATATTATGGGGTATTAATCTTCTTCGAAAGGCTATCCCCCTGATA
AAGGCAGGTTGCACACGTGTTCCGCACCCGTACGCCGCTCTCAATTACCCGAAAGTAATCTACCGCTCGGCTT
GCATGTGTTAGGCCTCCCGCTAGCGTTCTCCTGAGCCAGG
>102
CTGCTGCCGCCCGTAGGAGTCTGGTCCGTGTCTCAGTACCAGTGTGGGGGATCACCCTCTCAGGCCCCCTAAA
GATCGTCGCCTTGGTGAGCCGTTACCTCACCAACTAGCTAATCTTGCGCGTGCCCATCTCTATCCACCGTAGT
TTTCAATATCACCCGATGCCGAATAATATATTATGGGGTATTAATCTTCTTCGAAAGGCTATCCCCCTGATAA
AGGCAGGTTGCACACGTGTTCCGCACCCGTACGCCGCTCTCAATTACCCGAAAGTAATCTACCGCTCGGCTTG
CATGTGTTAGGCCTCCCGCTAGCGTTCATCCTGAGCCAGGATCAACTCT
>103
CTGCTGCCGCCCGTAGGAGTCTGGTCCGTGTCTCAGTACCAGTGTGGGGGATCACCCTCTCAGGCCCCCTAAA
GATCGTCGCCTTGGTGAGCCGTTACCTCACCAACTAGCTAATCTTGCGCGTGCCCATCTCTATCCACCGTAGT
TTTCAATATCACCCGATGCCGAATAATATATTATGGGGTATTAATCTTCTTCGAAAGGCTATCCCCCTGATAA
AGGCAGGTTGCACACGTGTTCCGCACCCGTACGCCGCTCTCAATTACCCGAAAGTAATCTACCGCTCGGCTTG
CATGTGTTAGGCCTCCCGCTAGCGTTCATCCTGAGCCAGGATCAAACTCT
>104
CTGCTGCCGCCCGTAGGAGTCTGGTCCGTGTCTCAGTACCAGTGTGGGGGATCACCCTCTCAGGCCCCCTAAA
GATCGTCGCCTTGGTGAGCCGTTACCTCACCAACTAGCTAATCTTGCGCGTGCCCATCTCTATCCACCGTAGT
TTTCAATATCACCCGATGCCGAATAATATATTATGGGGGTATTAATCTTCTTCGAAAGGCTATCCCCCTGATA
AAGGCAGGTTGCACACGTGTTCCGCACCCGTACGCCGCTCTCAATTACCCGAAAGTAATCTACCGCTCGGCTT
GCATGTGTTAGGCCTCCCGCTAGCGTTCTCCTGAGC
>105
CTGCTGCCGCCCGTAGGAGTCTGGTCCGTGTCTCAGTACCAGTGTGGGGGATCACCCTCTCAGGCCCCCTAAA
GATCGTCGCCTTGGTGAGCCGTTACCTCACCAACTAGCTAATCTTGCGCGTGCCCATCTCTATCCACCGTAGT
TTTCAATATCACCCGATGCCGAATAATATATTATGGGGTATTAATCTTCTTCGAAAGGCTATCCCCCTGATAA
AGGCAGGTTGCACACGTGTTCCGCACCCGTACGCCGCTCTCAATTACCCGAAAGTAATCTACCGCTCGGCTTG
CATGTGTTAGGCCTCCCGCTAGCGTTCATCCTGAGCCAGGATCAAACTCT
>106
CTGCTGCCGCCCGTAGGAGTCTGGTCCGTGTCTCAGTACCAGTGTGGGGGATCACCCTCTCAGGCCCCCTAAA
GATCGTCGCCTTGGTGAGCCGTTACCTCACCAACTAGCTAATCTTGCGCGTGCCCATCTCTATCCACCGTAGT
TTTCAATATCACCCGATGCCGAATAATATATTATGGGGTATTAATCTTCTTCGAAAGGCTATCCCCCTGATAA
AGGCAGGTTGCACACGTGTTCCGCACCCGTACGCCGCTCTCAATTACCCGAAAGTAATCTACCGCTCCGGCTT
GCATGTGTTAGGCCTCCCGCTAGCGTTCATCCTGAGCCAGGATCAACTCT
>107
CTGCTGCCGCCCGTAGGAGTCTGGACCGTGTCTCAGTTCCAGTGTGACTGATCATCCTCTCAGACCAGTACGG
ATCGTCGCCTTGGTAGGCCTTTACCCCACCAACTAGCTAATCCGACCTAGGCTCATCTGATAGCGTGAGGTCC
GAAGATCCCCCACTTCTCCCTCAGGACGTATGCGGTATTAGCGCCCGTTTCCGGACGTATCCCCACTACCAGG
CAGATTCCTAGGCATTACTCACCCGTCCGCCGCTGAATCCAGGAGCAAGCTCCCTCATCCGCTCGACTGCCAT
GTGTTAGGCCTGCCGCCAGCGTCAATCTGAGCCAGGATCAAACTCT
>108
CTGCTGCCGCCCGTAGGAGTCTGGTCCGTGTCTCAGTACCAGTGTGGGGGATCACCCTCTCAGGCCCCCTAAG
ATCGTCGCCTTGGTGAGCCGTTACCTCACCAACTAGCTAATCTTGCGCGTGCCCATCTCTATCCACCGTAGTT
TTCAATATCACCCGATGCCGAATAATATATTATGGGGTATTAATCTTCTTCGAAAGGCTATCCCCCTGATAAA

GGCAGGTTGCCACACGTGTTCCGCACCCGTACGCCGCTCTCAATTACCCGAAAGTAATCTACCGCTCGGCTTG
CATGTGTTAGGCCTCCCGCTAGCGTTCTCCTGAGCAGGATCAACTCT
>109
CTGCTGCCGCCCGTAGGAGTCTGGTCCGTGTCTCAGTACCAGTGTGGGGGATCACCCTCTCAGGCCCCCTAAA
GATCGTCGCCTTGGTGAGCCGTTACCTCACCAACTAGCTAATCTTGCGCGTGCCCATCTCTATCCACCGTAGT
TTTCAATATCACCCGATGCCGAATAATATATTATGGGGTATTAATCTTCTTCGAAAGGCTATCCCCCTGATAA
AGGCAGGTTGCACACGTGTTCCGCACCCGTACGCCGCTCTCAATTACCCGAAAGTAATCTACCACTCGTCGTT
GCATGTGTTAGGCCTCCCGCTAGCGTTCATCCTGAGCCAGGATCAACTCT
>110
CTGCTGCCGCCCGTAGGAGTCTGGTCCGTGTCTCAGTACCAGTGTGGGGGATCACCCTCTCAGGCCCCCTAAA
GATCGTCGCCTGGTGAGCCGTTACCTCACCAACTAGCTAATCTTGCGCGTGCCCATCTCTATCCACCGTAGTT
TTCAATATCACCCGATGCCGAATAATATATTATGGGGTATTAATCTTCCCTTCGAAAGGCTATCCCCCTGATA
AAGGCAGGTTGCACACGTGTTCCGCACCCGTACGCCGCTCTCAATTACCCGAAAGTAATCTACCGCTCGGCTT
GCATGTGTTAGGCCTCCCGCTAGCGTTCATCCTGAGCCAGGATCAAACTCT
>111
CTGCTGCCGCCCGTAGGAGTCTGGTCCGTGTCTCAGTACCAGTGTGGGGGATCACCCTCTCAGGCCCCCTAAA
GATCGTCGCCTTGTGAGCCGTTACCTCACCAACTAGCTAATCTTGCGCGTGCCCATCTCTATCCACCGTAGTT
TTCAATATCACCCGATGCCGAATAATATATTATGGGGTATTAATCTTCTTCGAAAGCTATCCCCCTGATAAGG
CAGGTTGCACACGTGTTCCGCACCCGTACGCCGCTCTCAATTACCCGAAAGTAATCTACCGCTCGGCTTGCAT
GTGTTAGGCCTCCCGCTAGCGTTCTCCTGAGCAGGATCAACTCGT
>112
CTGCTGCCGCCCGTAGGAGTCTGGACTCGTGTCTCAGTTCCAGTGTGACCTGATCATCCTCTCAGACCAGTAC
GGATCGTCGCCTTGGTAGGCCTTTACCCCACCAACTAGCTAATCCGACCTAGGCTCATCTGATAGCGTGAGGT
CCGAAGTCCCCCACTTCTCCCTCAGGACGTATGCGGTATTAGCGCCCGTTTCCGGACGATCCCCACTACCAGG
CAGATTCCCTAGGCATTACTCACCCGTCCGCCGCTGAATCCAGGAGCAAGCCCTCCCTATCCGCTCGACCTGC
ATGTGTTAGGCCTGCCGCCAGCGTCAATCTGAGCCAGGATCAAACTCT
>113
CTGCTGCCGCCCGTAGGAGTCTGGTCCGTGTCTCAGTACCAGTGTGGGGGATCACCCTCTCAGGCCCCCTAAA
GATCGTCGCCTTGGTGAGCCGTTACCTCACCAACTAGCTAATCTTGCGCGTGCCCATCTCTATCCACCGTAGT
TTTCAATATCACCCGATGCCGAATAATATATTATGGGGTATTAATCTTCTTCTGAAAGGCTATCCCCCTGATA
AAGGCAGGTTGCACACGTGTTCCGCACCCGTACGCCGCTCTCAATTACCCGAAAGTAATCTACCGCTCCGGCT
TGCATGTGTTAGGCCTCCCGCTAGCGTTCTCCGTGAGCCAGG
>114
CTGCTGCCGCCCGTAGGAGTCTGGTCCGTGTCTCAGTACCAGTGTGGGGGATCACCCTCTCAGGCCCCCTAAA
GATCGTCGCCTTGGTGAGCCGTTACCTCACCAACTAGCTAATCTTGCGCGTGCCCATCTCTATCCACCGTAGT
TTTCAATATCACCCGATGCCGAATAATATATTATGGGGTATTAATCTTCTTCGAAAGGCTATCCCCCTGATAA
AGGCAGGTTGCACACGTGTTCCGCACCCGTACGCCGCTCTCAATTACCCGAAAGTAATCTACCGCTCCGGCTT
GCATGTGTTAGGCCTCCCGCTAGCGTTCTCCTGAGCAGGATCAACTCT
>115
CTGCTGCCGCCCGTAGGAGTCTGGTCCGTGTCTCAGTACCAGTGTGGGGGATCACCCTCTCAGGCCCCCTAAA
GATCGTCGCCTTGGTGAGCCGTTACCTCACCAACTAGCTAATCTTGCGCGTGCCCATCTCTATCCACCGTAGT
TTTCAATATCACCCGATGCCGAATAATATATTATGGGGTATTAATCTTCTTCGAAAGGCTATCCCCTGATAAA
GGCAGGTTGCACACGTGTTCCGCACCCGTACGCCGCTCTCAATTACCCGAAAGTAATCTACCGCTCGGCTTGC
ATGTGTTAGGCCTCCCGCTAGCGTTCATCCTGAGCCAGGATCAAACTCT
>116
CTGCTGCCGCCCGTAGGAGTCTGGTCCGTGTCTCAGTACCAGTGTGGGGGATCACCCTCTCAGGCCCCCTAAA
GATCGTCGCCTGGTGAGCCGTTACCTCACCAACTAGCTAATCTTGCGCGTGCCCATCTCTATCCACCGTAGTT
TTCAATATCACCCGATGCCGAATAATATATTATGGGGTATTAATCTTCTTCGAAAGGCTATCCCCTGATAAAG
GCAGGTTAGCACACGTGTTCCGCACCCGTACGCCGCTCTCAATTACCCGAAAGTAATCTACCGCTCGGCTTGC
ATGTGTTAGGCCTCCCGCTAGCGTTCATCCTGAGCCAGGATCAAACTCT
>117
CTGCTGCCGCCCGTAGGAGTCTGGTCCGTGTCTCAGTACCAGTGTGGGGGATCACCCTCTCAGGCCCCCTAAA
GATCGTCGCCTTGGTGAGCCGCTACCTCACCAACTAGCTAATCTTGCGCGTGCCCATCTCTATCCACCGTAGT
TTTCAATATCACCCGATGCCGAATAATATATTATGGGGTATTAATCTTCTTCGAAAGGCTATCCCCCTGATAA

AGGCAGGTTGCACACGTGTTCCGCACCCGTACGCCGCTCTCAATTACCCGAAAGTAATCTACCGCTCGGCTTG
CATGTGTTAGGCCTCCCGCTAGCGTTCATCCTGAGCCAGGATCAAACTCT
>118
CTGCTGCCGCCCGTAGGAGTCTGGTCCGTGTCTCAGTACCAGTGTGGGGGATCACCCTCTCAGGCCCCCTAAA
GATCGTCGCCTTGGTGAGCCGTTACCTCACCAACTAGCTAATCTTGCGCGTGCCCATCTTTATCCACCGTAGT
TTTTCAATATCACCCGATGCCGAATAATATATTATGGGGTATTAATCTTCTTCGAAAGGCTATCCCCCTGATA
AAGGCAGGTTGCACACGTGTTCCGCACCCGTACGCCGCTCTCAATTACCCGAAAGTAATCTACCGCTCGGCTT
GCATGTGTTAGGCCTCCCGCTAGCGTTCTCCTGAGCCAGGATCAAACTCT
>119
CTGCTGCCGCCCGTAGGAGTCTGGTCCGTGTCTCAGTACCAGTGTGGGGGATCACCCTCTCAGGCCCCCTAAA
GATCGTCGCCTTGGTGAGCCGTTACCTCACCAACTAGCTAATCTTGCGCGTGCCCATCTCTATCCACCGTAGT
TTTCAATATCACCCGATGCCGAATAATATATTATGGGGTATTAATCTTCTTCGAAAGGCTATCCCCCTGATAA
AGGCAGGTTGTCACACGTGTTCCGCACCCGTACGCCGCTCTCAATTACCCGAAAGTAATCTACCGCTCGGCTT
GCATGTGTTAGGCCTCCCGCTAGCGTTCATCCTGAGCCAGGATCAAACTCT
>120
CTGCTGCCGCCCGTAGGAGTCTGGTCCGTGTCTCAGTACCAGTGTGGGGGATCACCCTCTCAGGCCCCCTAAA
GATCGTCGCCTTGGTGAGCCGTTACCTCACCAACTAGCTAATCTTGCGCGTGCCCATCTCTATCCACCGTAGT
TTTCAATATCACCCGATGCCGAATAATATATTATGGGGTATTAATCTTCTTCGAAAGGCTATCCCCCTGATAA
AGGCAGGTTGCACACGTGTTCCGCACCCGTACGCCGCTCTCAATTACCCGAAAGTAATCTACCGCTCGGCTTG
CATGTGTTAGGCCTCCCGCTAGCGTTCTCCTGAGCAGGATCC
>121
CTGCTGCCGCCCGTAGGAGTCTGGACCGTGTCTCAGTTCCAGTGTGACTGATCATCCTCTCAGACCAGTTACG
GATCGTCGCCTTGGTAGGCCTTTACCCCACCAACTAGCTAATCCGACCTAGGCTCATCTGATAGCGTGAGGTC
CGAAGATCCCCCACTTCTCCCTCAGGACGTATGCGGTATTAGCGCCCGTTTCCGGACGTATCCCCACTACCAG
GCAGATTCCTAGGCATTACTCACCCGTCCGCCGCTGAATCCAGGAGCAAGCCCCCTTCATCCGCTCGACTGCA
TGTGTTAGGCCTGCCGCCAGCGTCAATCTGAGCCAGGATCAACTCT
>122
CTGCTGCCGCCCGTAGGAGTCTGGTCCGTGTCTCAGTACCAGTGTGGGGGATCACCCTCTCAGGCCCCCTAAA
GATCGTCGCCTGGTGAGCCGTTACCTCACCAACTAGCTAATCTTGCGCGTGCCCATCTCTATCCACCGTAGTT
TTCAATATCACCCGATGCCGAATAATATATTATGGGGTATTAATCTTCTTCGAAAGGCTATCCCCCTGATAAA
GGCAGGTTGCACACGTGTTCCGCACCCGTACGCCGCTCTCAATTACCCGAAAGTAATCTACCGCTCGGCTTGC
ATGTGTTAGGCCTCCCGCTAGTGTTCATCCTGAGCCAGGATCAACTCT
>123
CTGCTGCCGCCCGTAGGGAGTCTGGACCGTGTCTCAGTTCCAGTGTGACTGATCATCCTCTCAGACCAGTACG
GATCGTCGCCTGGTAGGCCTTACCCCACCAACTAGCTAATCCGACCTAGGCTCATCTGATAGCGTGAGGTCCG
AAGATCCCCCACTTCTCCCTCAGGACGTATGCGGTATTAGCGCCCGTTTCCGGACGATCCCCACTACCAGGCA
GATTCCTAGGCATTACTCACCCGTCCGCCGCTGAATCCAGGAGCAAGCTCCCTTCATCCGCTCGACTGCATGT
GTTAGGCCTGCCGCCAGCGTCAATCTGAGCCAGGATCAAACTCT
>124
CTGCTGCCGCCCGTAGGAGTCTGGTCCGTGTCTCAGTACCAGTGTGGGGGATCACCCTCTCAGGCCCCCTAAA
GATCGTCGCCTTGGTGAGCCGTTACCTCACCAACTAGCTAATCTTGCGCGTGCCCATCTCTATTCCACCGTAG
TTTCAATATCACCCGATGCCGAATAATATATTATGGGGTATTAATCTTCTTCGAAAGGCTATCCCCTGATAAA
GGCAGGTTGCACACGTGTTCCGCACCCGTACGCCGCTCTCAATTACCCGAAAGTAATCTACCGCTCGGCTTGC
ATGTGTTAGGCCTCCCGCTAGCGTCTCCTGAGCA
>125
TGCTGCCGCCCGTAGGAGTCTGGTCCGTGTCTCAGTGCCAGTGTGGGGGATCACCCTCTCAGGCCCCCTAAGG
ATCGTCGCCTTGGTGAGCCGTTACCTCACCAACTAGCTAATCTTGCGCGTGCCCATCTCTATCCACCGTAGTT
TTCAATATCACCCGATGCCGAATAATATATTATGGGGTATTAATCTTCTTCGAAAGGCTATCCCCCTGATAAA
GGCAGGTTGCACACGTGTTCCGCACCCGTACGCCGCTCTCAATTACCCGAAAGTAATCTACCGCTCGGCTTGC
ATGTGTTAGGCCTCCCGCTAGCGTTCTCCTGAGCCAGGATCAAACTCT
>126
CTGCTGCCGCCCGTAGGAGTCTGGTCCGTGTCTCAGTACCAGTGTGGGGGATCACCCTCTCAGGCCCCCTAAA
GATCGTCGCCTTGGTGAGCCGTTACCTCACCAACTAGCTAATCTTGCGCGTGCCCATCTCTATCCACCGTAGT
TTTCAATATCACCCGATGCCGAATAATATATTATGGGGGTATTAATCTTCTTCGAAAGGCTATCCCCCTGATA

AAGGCAGGTTGCACACGTGTTCCGCACCCGTACGCCGCTCTCAATTACCCGAAAGTAATCTACCGCTCGGCTT
GCATGTGTTAGGCCTCCCGCTAGCGTTCATCCTGAGCCAGGATCAACTCT
>127
CTGCTGCCGCCCGTAGGAGTCTGGTCCGTGTCTCAGTACCAGTGTGGGGGATCACCCTCTCAGGCCCCCTAAA
GATCGTCGCCTTGGTGAGCCGTTACCTCACCAACTAGCTAATCTTGCGCGTGCCCATCTCTATCCACCGTAGT
TTTCAATATCACCCGATGCCGAATAATATATTATGGGGTATTAATCTTCTTCGAAAGGCTATCCCCCTGATAA
AGGCAGGTTGCACACGTGTTCCGCACCCGTACGCCGCTCTCAATTACCCGAAAGTAATCTACCGCTCGGCTTG
CATGTGTTAGGCCTCCCGCTAGCGTTCATCCTGAGCCAGGATCAAACTCT
>128
CTGCTGCCGCCCGTAGGAGTCTGGTCCGTGTCTCAGTACCAGTGTGGGGGATCACCCTCTCAGGCCCCCTAAA
GATCGTCGCCTTGTGAGCCGTTACCTCACCAACTAGCTAATCTTGCGCGTGCCCATCTCTATCCACCGTAGTT
TTCAATATCACCCGATGCCGAATAATATATTATGGGGTATTAATCTTCTCGAAAGCTATCCCCCTGATAAAGG
CAGGTTGCACACGTGTTCCGCACCCGTACGCCGCTCTCAATTACCCGAAAGTAATCTACCGCTCGGCTTGCAT
GTGTTAGGCCTCCCGCTAGCGTCATCCT
>129
CTGCTGCCGCCCGTAGGAGTCTGGTCCGTGTCTCAGTACCAGTGTGGGGGATCACCCTCTCAGGCCCCCTAAA
GATCGTCGCCTTGGTGAGCCGTTACCTCACCAACTAGCTAATCTTGCGCGTGCCCATCTCTATCCACCGTAGT
TTTCAATATCACCCGATGCCGAATAATATATTATGGGGTATTAATCTTCTTCGAAAGGCTATCCCCCTGATAA
AGGCAGGTTGCACACGTGTTCCGCACCCGTACGCCGCTCTCAATTACCCGAAAGTAATCTACCGCTCGGCTTG
CATGTGTTAGGCCTCCCGCTAGCGTTCTCCTGAGCAGGATCAACTCT
>130
CTGCTGCCGCCCGTAGGAGTCTGGTCCGTGTCTCAGTACCAGTGTGGGGGATCACCCTCTCAGGCCCCCTAAA
GATCGTCGCCTTGGTGAGCCGTTACCTCACCAACTAGCTAATCTTGCGCGTGCCCATCTCTATCCACCGTAGT
TTTCAATATCACCCGATGCCGAATAATATATTATGGGGTATTAATCTTCTTCGAAAGCTATCCCCCTGATAAA
GGCAGGTTGCACACGTGTTCCGCACCCGTACGCCGCTCTCAATTACCCGAAAGTAATCTACCGCTCGGCTTGC
ATGTGTTAGGCCTCCCGCTAGCGTTCATCCTGAG
>131
CTGCTGCCGCCCGTAGGAGTCTGGTCCGTGTCTCAGTACCAGTGTGGGGGATCACCCTCTCAGGCCCCCTAAA
GATCGTCGCCTTGGTGAGCCGTTACCTCACCAACTAGCTAATCTTGCGCGTGCCCATCTCTATCCACCGTAGT
TTTCAATATCACCCGATGCCGAATAATATATTATGGGGTATTAATCTTCTTCGAAAGGCTATCCCCTGATAAA
GGCAGGTTGCACACGTGTTCCGCACCCGTACGCCGCTCTCAATTACCCGAAAGTAATCTACCGCTCGGCTTGC
ATGTGTTAGGCCTCCCGCTAGCGTTCTCCTGAGCAGGATCAA
>132
CTGCTGCCGCCCGTAGGAGTCTGGTCCGTGTCTCAGTACCAGTGTGGGGGATCACCCTCTCAGGCCCCCTAAA
GATCGTCGCCTGGTGAGCCGTTACCTCACCAACTAGCTAATCTTGCGCGTGCCCATCTCTATCCACCGTAGTT
TTCAATATCACCCGATGCCGAATAATATATTATGGGGTATTAATCTTCTTCGAAAGGCTATCCCCCTGATAAA
GGCAGGTTAGCACACGTGTTCCGCACCCGTACGCCGCTCTCAATTACCCGAAAGTAATCTACCGCTCGGCTTG
CATGTGTTAGGCCTCCCGCTAGCGTTCATCCTGAGCC
>133
CTGCTGCCGCCCGTAGGAGTCTGGTCCGTGTCTCAGTACCAGTGTGGGGGATCACCCTCTCAGGCCCCCTAAA
GATCGTCGCCTGGTGAGCCGTTACCTCACCAACTAGCTAATCTTGCGCGTGCCCATCTCTATCCACCGTAGTT
TTCAATATCACCCGATGCCGAATAATATATTATGGGGTATTAATCTTCTTCGAAAGGCTATCCCCCTGATAAA
GGCAGGTTGCACACGTGTTCCGCACCCGTACGCCGCTCTCAATTACCCGAAAGTAATCTACCGCTCGGCTTGC
ATGTGTTAGGCCTCCCGCTAGCGTTCTCCTGAGC
>134
CTGCTGCCGCCCGTAGGAGTCTGGTCCGTGTCTCAGTACCAGTGTGGGGGATCACCCTCTCAGGCCCCCTAAA
GATCGTCGCCTTGGTGAGCCGTTACCCCACCAACTAGCTAATCTTGCGCGTGCCCATCTCTATCCACCGTAGT
TTTCAATATCACCCGATGCCGAATAATATATTATGGGGTATTAATCTTCTTCGAAAGGCTATCCCCCTGATAA
AGGCAGGTTGCACACGTGTTCCGCACCCGTACGCCGCTCTCAATTACCCGAAAGTAATCTACCGCTCGGCTTG
CATGTGTTAGGCCTCCCGCTAGCGTTCTCCTGAGCCAGGATCAACTCT
>135
CTGCTGCCGCCCGTAGGAGTCTGGTCCGTGTCTCAGTACCAGTGTGGGGGATCACCCTCTCAGGCCCCCCTAA
AGATCGTCGCCTTGGTGAGCCGTTACCTCACCAACTAGCTAATCTTGCGCGTGCCCCATCTCTATCCACCGTA
GTTTTCAATATCACCCGATGCCGAATAATATATTATGGGGTATTAATCTTCTTCGAAAGGCTATCCCCCTGAT

AAAGGCAGGTTGCACACGTGTTCCGCACCCGTACGCCGCTCTCAATTACCCGAAAGTAATCTACCGCTCGGCT
TGCATGTGTTAGGCCTCCCGCTAGCGTTCATCCTGAGCCAGGATCAAACTCT
>136
CTGCTGCCCGCCCGTAGGAGCCTGGTCCCGTGTCTCAGTACCAGTGTGGGGGATCACCCCTCTCAGGCCCCCC
CTAAAGATCGTCGCCTTGGTGAGCCCGTTACCTCACCCAACTAGCTAATCTTGCGCGTGCCCCATCTCTATCC
ACCGTAGTTTTCAATATCACCCGATGCCCGAATAATATATTATGGGGTATTAATCTTCTTCGAAAGGCTATCC
CCCTGATAAAGGCAGGTTGCACACGTGTTCCCGCACCCGTACGCCGCTCTCAATTACCCCGAAAGTAATCTAC
CGCTCGGCTTGCATGTGTTAGGCCTCCCGCTAGCGTTCATCCTGAGCCAGGATCAAACTCT
>137
CTGCTGCCGCCCGTAGGAGTCTGGTCCGTGTCTCAGTACCAGTGTGGGGGATCACCCTCTCAGGCCCCCTAAA
GATCGTCGCCTTGGTGAGCCGTTACCTCACCAACTAGCTAATCTTGCGCGTGCCCATCTCTATCCACCGTAGT
TTTCAATATCACCCGATGCCGAATAATATATTATGGGGTATTAATCTTCTTCGAAAGGCTATCCCCTGATAAA
GGCAGGTTGCACACGTGTTCCGCACCCGTACGCCGCTCTCAATTACCCGAAAGTAATCTACCGCTCGGCTTGC
ATGTGTTAGGCCTCCCGCTAGCGTTCATCCTGAGCCAGGATCAAACTCT
>138
CTGCTGCCGCCCGTAGGAGTCTGGTCCGTGTCTCAGTACCAGTGTGGGGGATCACCCTCTCCAGGCCCCCTAA
AGATCGTCGCCTTGGTGAGCCGTTTACCTCACCAACTAGCTAATCTTGCGCGTGCCCATCTCTATCCACCGTA
GTTTTCAATATCACCCGATGCCGAATAATATATTATGGGGTATTAATCTTCTTCGAAAGGCTATCCCCCTGAT
AAAGGCAGGTTGCACACGTGTTCCGCACCCGTACGCCGCTCTCAATTACCCGAAAGTAATCTACCGCTCGGCT
TGCATGTGTTAGGCCTCCCGCTAGCGTTCTCCTGAGCAGGATCAACTCT
>139
TTGCTGCCGCCCGTAGGAGTCTGGTCCGTGTCTCAGTACCAGTGTGGGGGATCACCCTCTCAGGCCCCCTAAA
GATCGTCGCCTTGGTGAGCCGTTACCTCACCAACTAGCTAATCTTGCGCGTGCCCATCTCTATCCACCGTAGT
TTTCAATATCACCCGATGCCGAATAATATATTATGGGGTATTAATCTTCTTCGAAAGGCTATCCCCCTGATAA
AGGCAGGTTGCACACATGTTCCGCACCCGTACGCCGCTCTCAATTACCCGAAAGTAATCTACCGCTCGGCTTG
CATGTGTTAGGCCTCCCGCTAGCGTTCTCCTGAGCCAGGATCAAACTCT
>140
CTGCTGCCGCCCGTAGGAGCTGGTCCGTGTCTCAGTACCAGTGTGGGGGATCACCCTCTCAGGCCCCCTAAAG
ATCGTCGCCTTGGTGAGCCGTTACCTCACCAACTAGCTAATCTTGCGCGTGCCCATCTCTATCCACCGTAGTT
TCAATATCACCCGATGCCGAATAATATATTATGGGGTATTAATCTTCTTCGAAAGGCTATCCCCCTGATAAAG
GCAGGTTGCACACGTGTTCCGCACCCGTACGCCGCTCTCAATTACCCGAAAGTAATCTACCGCTCGGCTTGCA
TGTGTTAGGCCTCCCGCTAGCGTTCATCCTGAGCCAGGATCAAACTCT
>141
CTGCTGCCGCCCGTAGGAGTCTGGTCCGTGTCTCAGTACCAGTGTGGGGGATCACCCTCTCAGGCCCCCTAAA
GATCGTCGCCTTGGTGAGCCGTTACCTCACCAACTAGCTAATCTTGCGCGTGCCCATTCTCTATCCACCGTAG
TTTTCAATATCACCCGATGCCGAATAATATATTATGGGGTATTAATTCTTCTTCGAAAGGCTATCCCCTGATA
AAGGCAGGTTGCACACGTGTTCCGCACCCGTACGCCGCTCTCAATTACCCGAAAGTAATCTACCGCTCGGCTT
GCATGTGTTAGGCCTCCCGCTAGCGTTCTCCTGAGCAGGATTCAA
>142
CTGCTGCCGCCCGTAGGAGTCTGGTCCGTGTCTCAGTACCAGTGTGGGGGATCACCCTCTCAGGCCCCCTAAA
GATCGTCGCCTTGGTGAGCCGTTACCTCACCAACTAGCTAATCTTGCGCGTGCCCATCTCTTATCCCACCGTA
GTTTCAATATTCACCCGATTGCCCGAATAATATATTATGGGGTATTAATCTTCTTCGAAAGGCTTATTCCCCC
TGATAAAGGCAGGTTGCACACGTGTTCCGCACCCGTACGCCGCTCTCAATTTACCCGAAAGTAATCTACCGCT
TCGGCTTGCATGTGTTAGGCCTTCCCCGCTAGCGTTCATCCTGAGCCAGGATCAAACTCTT
>143
CTGCTGCCGCCCCGTAGGAGTCTGGTTCCCGTGTCTTCAGTACCAGTGTGGGGGATCACCCTCTCAGGCCCCC
TAAAGATCGTCGCCTTGGTGAGCCGTTACCCTCACCAACTAGCTTAATCTTGCGCGTGCCCATTCTCTATTCC
CACCCGTAGTTTTCAATATTCACCCCGATGCCCGAATAATTATATTTATTGGGGTATTTTAATTCTTTCTTCG
AAAGGCTATCCCCTGATTAAAGGCAGGTTTGCACACGTGTTTCCCGCACCCGTTACGCCGCTCTCAATTTACC
CCGAAAGTAATCTACCGCTCGGCTTGCATGTGTTTAGGCCTTCCCCGCTTAGCCGTTCATTCCTTGAGCCCAG
GATTCAAACTCT
>144
CTGCTGCCGCCCGTAGGAGTCTGGTCCCGTGTCTCAGTACCAGTGTGGGGGATCACCCTCTCAGGCCCCCCTA
AAGATCGTCGCCTTGGTGAGCCCGTTACCTCACCCAACTAGCTAATCTTGCGCGTGCCCCATCTCTATCCCAC

CGTAGTTTTCAATATCACCCGATGCCGAATAATATATTTATGGGGTATTAATCTTTCTTCGAAAGGCTATCCC
CCTGATAAAGGCAGGTTGCACACGTGTTCCCGCACCCGTACGCCGCTCTCAATTACCCCGAAAGTAATCTACC
GCTCGGCTTGCATGTGTTAGGCCCTTCCCGCTAGCGTTCTCCTGAGCAGGATCAA
>145
TGCTGCCGCCCGTAGGAGTCTGGTCCGTGTCTCAGTACCAGTGTGGGGGATCACCCTCTCAGGCCCCCTAAAG
ATCGTCGCCTGTGAGCCGTTACCTCACCAACTAGCTAATCTTGCGCGTGCCCATCTCTATCCACCGTAGTTTT
CAATATCACCCGATGCCGAATAATATATTATGGGGTATTAATCTTCTTCGAAAGGCTATCCCCCTGATAAAGG
CAGGTTGCACACGTGTTCCGCACCCGTACGCCGCTCTCAATTACCCGAAAGTAATCTACCGCTCGGCTTGCAT
GTGTTAGGCCTCCCGCTAGCGTTCTCCTGAGCCAGGATCAA
>146
CTGCTGCCGCCCGTAGGAGTCTGGTCCGTGTCTCAGTACCAGTGTGGGGGATCACCCTCTCAGGCCCCCTAAA
GATCGTCGCCTTGGTGAGCCGTTACCTCACCAACTAGCTAATCTTGCGCGTGCCCATCTCTATCCACCGTAGT
TTTCAACATCACCCGATGCCGAATAATATATTATGGGGTATTAATCTTCTTCGAAAGGCTATCCCCCTGATAA
AGGCAGGTTGCACACGTGTTCCGCACCCGTACGCCGCTCTCAATTACCCGAAGTAATCTACCGCATCGGCTTG
CATGTGTTAGGCCTCCCGCTAGCGTTCTCCTGAGCAGGATCAAACTCG
>147
CTGCTGCCGCCCGTAGGAGTCTGGTCCGTGTCTCAGTACCAGTGTGGGGGATCACCCTCTCAGGCCCCCTAAA
GATCGTCGCCTTGGTGAGCCGTTACCTCACCAACTAGCTAATCTTGCGCGTGCCCATCTCTATCCACCGTAGT
TTTCAATATCACCCGATGCCGAATAATATATTATGGGGGTATTAATCTTCTTCGAAAGGCTATCCCCCTGATA
AAGGCAGGTTGCACACGTGTTCCGCACCCGTACGCCGCTCTCAATTACCCGAAAGTAATCTACCGCTCGGCTT
GCATGTGTTAGGCCTCCCGCTAGCGTTCTCCTGAGCCAGGATCA
>148
CTGCTGCCGCCCCGTAGGAGTCTGGTCCCGTGTCTCAGTACCAGTGTGGGGGATCACCCTCTCAGGCCCCCCT
AAAGATCGTCGCCTTGGTGAGCCCGTTACCTCACCCAACTAGCTAATCTTGCGCGTGCCCATCTCTATCCACC
GTAGTTTTCAATATCACCCGATGCCGAATAATATATTATGGGGGTATTAATCTTCTTCGAAAGGCTATCCCCC
TGATAAAGGCAGGTTGCACACGTGTTCCGCACCCGTACGCCGCTCTCAATTACCCGAAAGTAATCTACCGCTC
GGCTTGCATGTGTTAGGCCTCCCGCTAGCGTTCTCCTGAGCAGGATCAACTCT
>149
CTGCTGCCGCCCGTAGGAGTCTGGTCCGTGTCTCAGTACCAGTGTGGGGGATCACCCTCTCAGGCCCCCTAAA
GATCGTCGCCTGTGAGCCGTTACCTCACCAACTAGCTAATCTTGCGCGTGCCCATCTCTATCCACCGTAGTTT
TCAATATCACCCGATGCCGAATAATATATTATGGGGTATTAATCTTCTTCGAAAGGCTATCCCCTGATAAAGG
CAGGTTGCACACGTGTTCCGCACCCGTACGCCGCTCTCAATTACCCGAAAGTAATCTACCGCTCGGCTTGCAT
GTGTTAGGCCTCCCGC
>150
CTGCTGCCGCCCCGTAGGAGTCTGGTCCCGTGTCTCAGTACCAGTGTGGGGGATCACCCTCTCAGGCCCCCCT
AAAGATCGTCGCCTTGGTGAGCCGTTACCTCACCCAACTAGCTAATCTTGCGCGTGCCCCATCTCTATCCACC
GTAGTTTTCAATATCACCCGATGCCGAATAATATATTATGGGGTATTAATTCTTCTTCGAAAGGCTATCCCCC
TGATAAAGGCAGGTTGCACACGTGTCCCGCACCCGTACGCCGCTCTCAATTACCCCGAAAGTAATCTACCGCT
CGGCTTGCATGTGTTAGGCCTCCCGCTAGCGTTCTTCCTGAGCA
>151
CTGCTGCCGCCCGTAGGAGTCTGGTCCGTGTCTCAGTACCAGTGTGGGGGATCACCCTCTCAGGCCCCCTAAA
GATCGTCGCCTTGGTGAGCCGTTACCTCACCAACTAGCTAGTATCTGCGCGTGCCCATCTCTATCCACCGTAG
TTTTCAATATCACCCGATGCCGAATAATATATTATGGGGTATTAATCTTCTTCGAAAGGCTATCCCCCTGATA
AAGGCAGGTTGCACACGTGTTCCGCACCCGTACGCCGCTCTCAATTACCCGAAAGTAATCTACCGCTCGGCTT
GCATGTGTTAGGCCTCCCGCTAGCGTTCATCCTGAGCCAGGATCAAACTCT
>152
CTGCTGCCGCCCGTAGGAGTCTGGTCCGTGTCTCAGTACCAGTGTGGGGGATCACCCTCTCAGGCCCCCTAAA
GATCGTCGCCTTGGTGAGCCGTTACCTCACCAACTAGCTAATCTTGCGCGTGCCCATCTCTATCCACCGTAGT
TTTCAATATCACCCGATGCCGAATAATATATTATGGGGTATTAATCTTCTTCGAAAGGCTATCCCCCTGATAA
AGGCAGGTTGCACACGTGTTCCGCACCGTACGCCGCTCTCAATTACCCGAAAGTAATCTACCGCTCGGCTTGC
ATGTGTTAGGCCTCCCGCTAGCGTTCTCCTGAGCAGGATCAACTCGT
>153
CTGCTGCCGCCCGTAGGAGTCTGGTCCGTGTCTCAGTACCAGTGTGGGGGATCACCCTCTCAGGCCCCCTAAA
GATCGTCGCCTTGGTGAGCCGTTACCTCACCAACTAGCTAATCTTGCGCGTGCCCATCTCTATCCACCGTAGT

TTTCAATATCACCCGATGCCGAATAATATATTATGGGGTATTAATCTTCTCGAAGCTATCCCCCTGATAAAGG
CAGGTTGCACACGTGTTCCGCACCCGTACGCCGCTCTCAATTTACCCGAAAGTAATCTACCGCATCGGCTTGC
ATGTGTTAGGCCTCCCGCTAGCGTTCTCCTGAGCCAGGATCAAACTCCGT
>154
CTGCTGCCGCCCGTAGGAGTCTGGTCCGTGTCTCAGTACCAGTGTGGGGGATCACCCTCTCAGGCCCCCTAAA
GATCGTCGCCTTGGTGAGCCGTTACCTCACCAACTAGCTAATCTTGCGCGTGCCCATCTCTATCCACCGTAGT
TTTCAATATCACCCGATGCCGAATAATATATTATGGGGTATTAATCTTCTTCGAAAGGCTATCCCCCTGATAA
AGGCAGGTTGCACACGTGTTCCGCACCCGTACGCCGCTCTCAATTACCCGAAAGTAATCTACCGCTCGGCTTG
CATGTGTTAGGCCTCCCGCTAGCGTTCATCCTGAGCCAGGATCAAACTCT
>155
CTGCTGCCGCCCGTAGGAGTCTGGTCCGTGTCCTCAGTACCAGTGTGGGGGATCACCCTCTCAGGCCCCCTAA
AGGTCGTCGCCTGGTGAGCCGTTACCTCACCAACTAGCTAATCTTGCGCGTGCCCATCTCTATCCACCGTAGT
TTTCAATATCACCCGATGCCGAATAATATATTATGGGGTATTAATCTTCTCGAAAGGCTATCCCCTGATAAAG
GCAGGTTGCACACGTGTTCCGCACCCGTACGCCGCTCTCAATTACCCGAAAGTAATCTACCGCTCGGCTTGCA
TGTGTTAGGCCTCCCGCTAGCGTTCTCCTGAGCCAGGATCAACTCT
>156
CTGCTGCCGCCCGTAGGAGTCTGGTCCGTGTCTCAGTACCAGTGTGGGGGATCACCCTCTCAGGCCCCCTAAA
GATCGTCGCCTTGGTGAGCCGTTACCTCACCAACTAGCTAATCTTGCGCGTGCCCATCTCTATCCACCGTAGT
TTTCAATATCACCCGATGCCGAATAATATATTATGGGGTATTTAATCTTCTTCGAAAGGCTATCCCCCTGATA
AAGGCAGGTTGCACACGTGTTCCGCACCCGTTACGCCGCTCTCAATTACCCGAAAGTAATCTACCGCTCGGCT
TGCATGTGTTAGGCCTCCCGCTAGCGTTCATCCTGAGCCAGGATCAAACTCT
>157
CTGCTGCCGCCCGTAGGAGTCTGGTCCGTGTCTCAGTACCAGTGTGGGGGATCACCCTCTCAGGCCCCCTAAA
GATCGTCGCCTTGGTGAGCCGTTACCTCACCAACTAGCTAATCTTGCGCGTGCCCATCTCTATCCACCGTAGT
TTTCAATAACACCCGATGCCGAATAATATATTATGGGGTATTAATCTTCTTCGAAAGGCTATCCCCTGATAAA
GGCAGGTTGCACACGTGTTCCGCACCCGTACGCCGCTCTCAATTACCCGAAAGTAATCTACCGCTCGGCTTGC
ATGTGTTAGGCCTCCCGCTAGCGTCTCTGAGCAGG
>158
CTGCTGCGCCCGTAGGAGTCTGGTCCGTGTCTCAGTACCAGTGTGGGGGATCACCCTCTCAGGCCCCCTAAAG
ATCGTCGCCTTGGTGAGCCGTTACCTCACCAACTAGCTAATCTTGCGCGTGCCCATCTCTATCCACCGTAGTT
TTCAATATCACCCGATGCCGAATAATATATTATGGGGTATTAATCTTCTTCGAAAGGCTATCCCCCTGATAAA
GGCAGGTTGCACACGTGTTCCGCACCCGTACGCCGCTCTCAATTACCCGAAAGTAATCTACCGCTCCGGCTTG
CATGTGTTAGGCCTCCCGCTAGCGTTCATCCTGAGCCA
>159
CTGCTGCCGCCCGTAGGAGTCTGGTCCGTGTCTCAGTACCAGTGTGGGGGATCACCCTCTCAGGCCCCCTAAA
GATCGTCGCCTTGGTGAGCCGTTACCTCACCAACTAGCTAATCTTGCGCGTGCCCATCTCTATCCACCGTAGT
TTTCAATATCACCCGATGCCGAATAATATATTATGGGGTATTAATCTTCTTCGAAAGGCTATCCCCTGTAAAG
GCAGGTTGCACACGTGTTCCGCACCCGTACGCCGCTCTCAATTACCCGAAAGTAATCTACCGCTCGGCTTGCA
TGTGTTAGGCCTCCCGCTAGCGTTCATCCTGAGCCAGGATCAAACTCT
>160
CTGCTGCCGCCCGAGGAGTCTGGTCCGTGTCTCAGTACCAGTGTGGGGGATCACCCTCTCAGGCCCCCTAAAG
ATCGTCGCCTTGGTGAGCCGTTACCTCACCAACTAGCTAATCTTGCGCGTGCCCATCTCTATCCACCGTAGTT
TTCAATATCACCCGATGCCGAATAATATATTATGGGGTATTAATCTTCTTCGAAAGGCTATCCCCTGATAAAG
GCAGGTTGCACACGTGTTCCGCACCCGTACGCCGCTCTCAATTACCCGAAAGTAATCTACCGCTCGGCTTGCA
TGTGTTAGGCCTCCCGCTAGCGTTCATCCTGAGCCAGGATCAAACTCT
>161
CTGCTGCCGCCCGTAGGAGTCTGGTCCGTGTTCTCAGTACCAGTGTGGGGATCACCCTCTCAGGCCCCTAAAG
ATCGTCGCCTTGGTGAGCCGTTACCTCACCAACTAGCTAATCTTTGCGCGTGCCCATCTCTATCCACCGTAGT
TTTTTCAATATCACCCGATGCCGAATAATATATTATGGGGTATTTAATCTTCTTCGAAAGGCTTATCCCCTGA
TAAAGGCAGGTTGCACACGTGTTCCGCACCCGTACGCCGCTCTCAATTTACCCGAAAGTAATCTACCGCTCGG
CTTGCATGTGTTAGGCCTCCCCGCTAGCGTTCATCCTGAGCCAGGATCAAACTCT
>162
CTGCTGCCGCCCGTAGGAGTCTGGTCCGTGTCTCAGTACCAGTGTGGGGATCACCCTCTCAGGCCCCTAAAGA
TCGTCGCCTTGGTGAGCCGTTACCTCACCAACTAGCTTAATCTTGCGCGTGCCCATCTCTATCCACCGTAGTT

TTCAATATCACCCGATGCCGAATAATATATTATGGGGTATTTAATCTTCTTCGAAGGCTATCCCCTGATAAGG
CAGGTTGCACACGTGTTCCGCACCCGTACGCCGCTCTCAATTACCCGAAAGTAATCTACCGCTCGGCTTGCAT
GTGTTAGGCCTCCCGCTAGCGTTCATTCCTGAGCCAGGATCAACTCT
>163
CTGCTGCCGCCCCGTAGGAGTCTGGACCCGTGTCTCAGTTCCAGTGTGGCCCGATCACCCTCTCAGGTCGGCT
ATGCATCGTTGCCTTGGTAAGCCGTTACCTTACCCAACTAGCTAATGCAGCGCGGATCCATCTATAAGTGACA
GCAAGACCGTCTTCACTAACCATGCGGTTCAAAATATTATCCGGTATTAGCTCCGGTTTCCCGAAGTATCCCA
GTCTTATAGGTAGGTTATCCACGTGTTACTCACCCGTCCGCCGCTAACATCAGAGAAGCAAGCTTCTCATCCG
TTCGCTCGACTGCATGTATTAGGCACACCGCCAGCGTTCATCCT
>164
CTGCTGCCGCCCGTAGGAGTCTGGTCCGTGTCTCAGTACCAGTGTGGGGGATCACCCTCTCAGGCCCCCTAAA
GATCGTCGCCTGGTGAGCCGTTACCTCACCAACTAGCTAATCTTGCGCGTGCCCATCTCTATCCACCGTAGTT
TTCAATATCACCCGATGCCGAATAATATATTATGGGGTATTAATCTTCTTCGAAAGGCTATTCCCCTGATAAA
GGCAGGTTGCACACGTGTTCCGCACCCGTACGCCGCTCTCAATTACCCGAAAGTAATCTACCGCTCGGCTTGC
ATGTGTTAGGCCTCCCGCTAGCGTTCATCCTGAGCCAGGATCAAACTCT
>165
CTGCTGCCGCCCGTAGGAGTCTGGTCCGTGTCTCAGTACCAGTGTGGGGGATCACCCTCTCAGGCCCCCTAAA
GATCGTCGCCTTGGTGAGCCGTTACCTCACCAACTAGCTAATCTTGCGCGTGCCCATCTCTATCCACCGTAGT
TTTCAATATCACCCGATGCCGAATAATATATTATGGGGTATTAATCTTCTTCGAAAGGCTATCCCCCTGATAA
AGGCAGGTTGCACACGTGTTCCGCACCCGTACGCCGCTCTCAATTACCCGAAAGTAATCTACCGCTCGGCTTG
CATGTGTTAGGCCTCCCGCTAGCGTTCATCCTGAGCCAGGATCAAACTCT
>166
CTGCTGCCGCCCGTAGGAGTCTGGTCCGTGTCTCAGTACCAGTGTGGGGGATCACCCTCTCAGGCCCCCTAAA
GATCGTCGCCTGGTGAGCCGTTACCTCACCAACTAGCTAATCTTGCGCGTGCCCATCTCTATCCACCGTAGTT
TTCAATATCACCCGATGCCGAATAATATATTATGGGGTATTAATCTTCTTCGAAAGCTATCCCCTGATAAAGG
CAGGTTGCACACGTGTTCCGCACCCGTACGCCGCTCTCAATTACCCGAAAGTAATCTACCGCTCGGCTTGCAT
GTGTTAGGCCTCCCGCTAGCGTTCTCCTGAGCAGGA
>167
CTGCTGCCGCCCGTAGGAGTCTGGTCCGTGTCTCAGTACCAGTGTGGGGGATCACCCTCTCAGGCCCCCTAAA
GATCGTCGCCTGGTGAGCCGTTACCTCACCAACTAGCTAATCTTGCGCGTGCCCATCTCTATCCACCGTAGTT
TTCAATATCACCCGATGCCGAATAATATATTATTGGGGTATTAATCTTCTTCGAAAGGCTATCCCCCTGATAA
AGGCAGGTTGCACACGTGTTCCGCACCCGTACGCCGCTCTCAATTACCCGAAAGTAATCTACCGCTCGGCTTG
CATGTGTTAGGCCTCCCGCTAGCGTTCATTCCTGAGCCAGGATCAAACTCT
>168
CTACTGCCGCCCGTAGGAGTCTGGTCCGTGTCTCAGTACCAGTGTGGGGGATCACCCTCTCAGGCCCCCTAAA
GATCGTCGCCTTGGTGAGCCGTTACCTCACCAACTAGCTAATCTTGCGCGTGCCCATCTCTATCCACCGTAGT
TTTCAATATCACCCGATGCCGAATAATATATTATGGGGTATTAATCTTCTTCGAAAGGCTATCCCCTGATAAA
GGCAGGTTGCACACGTGTTCCGCACCCGTACGCCGCTCTCAATTACCCGAAAGTAATCTACCGCTCGGCTTGC
ATGTGTTAGGCCTCCCGCTAGCGTTCATCCTGAGCCAGGATCAAACTCT
>169
CTGCTGCCGCCCGTAGGAGTCTGGTCCGTGTCTCAGTACCAGTGTGGGGGATCACCCTCTCAGGCCCCCTAAA
GATCGTCGCCTTGGTGAGCCGTTACCTCACCAACTAGCTAATCTTGCGCGTGCCCATCTCTATCCACCGTAGT
TTTCAATATCACCCGATGCCGAATAATATATTATGGGGTATTTAATTCTTCTTCGAAAGGCTATCCCCTGATA
AAGGCAGGTTGCACACGTGTTCCGCACCCGTACGCCGCTCTCAATTACCCGAAAGTAATCTACCGCTCGGCTT
GCATGTGTTAGGCCTCCCGCTAGCGTTCATTCCTGAGCCAGGATCAAACTCT
>170
CTGCTGCCGCCCCGTAGGAGTCTGGTCCCGTGTCTCAGTACCAGTGTGGGGGATCACCCTCTCAGGCCCCCTA
AAGATCGTCGCCTTGGTGAGCCGTTACCTCACCCAACTAGCTAATCTTGCGCGTGCCCATCTCTATCCACCGT
AGTTTTCAATATCACCCGATGCCGAATAATATATTATGGGGTATTAATCTTTCTTCGAAAGGCTATCCCCCTG
ATAAAGGCAGGTTGCACACGTGTTCCGCACCCGTACGCCGCTCTCAATTACCCCGAAAGTAATCTCCGCTCGG
CTTGCATGTGTTAGGCCTCCCGCTAGCGTTCTCCTGAGCAGGATTCAAC
>171
CTGCTGCCGCCCGTAGGAGTCTGGTCCGTGTCTCAGTACCAGTGTGGGGGATCACCCTCTCAGGCCCCCTAAA
GATCGTCGCCTTGGTGAGCCGTTACCTCACCAACTAGCTAATCTTGCGCGTGCCCATCTCTATCCACCGTAGT

TTTCAATATCACCCGATGCCGAATAATATATTATGGGGTATTAATCTTCTTCGAAAGGCTATCCCCCTGATAA
AGGCAGGTTGCACACGTGTTCCGCACCCGTACGCCGCTCTCAATTACCCGAAAGTAATCTACCGCTCGGCTTG
CATGTGTTAGGCCTCCCGC
>172
CTGCTGCCGCCCGTAGGAGTCTGGTCCGTGTCTCAGTACCAGTGTGGGGGATCACCCTCTCAGGCCCCTAAAG
ATCGTCGCCTGTGAGCCGTTTACCTCACCAACTAGCTAATCTTGCGCGTGCCCATCTCTATCCACCGTAGTTT
TCAATATCACCCGATGCCGAATAATATATTGTGGGGTATTAATCTTCCTCTCGAAAGGCTATCCCCCTGATAA
AGGCAGGTTGCACACGTGTTCCGCACCCGTACGCCGCTCTCAATTACCCGAAAGTAATCTACCGCTCGGCTTG
CATGTGTTAGGCCTCCCGCTAGCGTTCATCCTGAGCCAGGATCAAACTCT
>173
CTGCTGCCGCCCGTAGGAGTCTGGTCCGTGTCTCAGTACCAGTGTGGGGGATCACCCTCTCAGGCCCCCTAAA
GATCGTCGCCTGGTGAGCCGTTACCTCACCAACTAGCTAATCTTGCGCGTGCCCATCTCTATCCACCGTAGTT
TTCAATATCACCCGATGCCGAATAATATATTATGGGGTATTAATCTTCTTCGAAAGGCTATCCCCCTGATAAA
GGCAGGTTGCACACGTGTTCCGCACCCGTACGCCGCTCTCAATTACCCGAAAGTAATCTACCGCTCGGCTTGC
ATGTGTTAGGCCTCCCGCTAGCGTTCTCCTGAGCAGGATCAACTCT
>174
CTGCTGCCGCCCGTAGGAGTCTGGTCCGTGTCTCAGTACCAGTGTGGGGGATCACCCTCTCAGGCCCCCTAAA
GATCGTCGCCTTGGTGAGCCGTTACCTCACCAACTAGCTAATCTTGCGCGTGCCCATCTCTATCCACCGTAGT
TTTCAATATCACCCGATGCCGAATAATATATTATGGGGTATTAATCTTCTTCGAAAGGCTATCCCCCTGATAA
AGGCAGGTTGCACACGTGTTCCGCACCCGTACGCCGCTCTCAATTACCCGAAAGTAATCTACCGCTCGGCTTG
CATGTGTTAGGCCTCCCGCTAGCGTTCTCCTGAGCAGGATCAACTCGT
>175
TTGCTGCCGCCCGTAGGAGTCTGGTCCGTGTCTCAGTACCAGTGTGGGGGATCACCCTCTCAGGCCCCCCTAA
AAGATCGTCGCCTTGTGAGCCGTTACCTCACCAACTAGCTAATCTTGCGCGTGCCCATCTCTATCCACCGTAG
TTTTCAATATCACCCGATGCCGAATAATATATTATGGGGTATTAATCTTCTTCGAAAGCTATCCCCCTGATAA
AGGCAGGTTGCACACGTGTTCCGCACCCGTACGCCGCTCTCAATTACCCGAAAGTAATCTACCGCTCGGCTTG
CATGTGTTAGGCCTCCCGCTAGCGTTCATCCTGAGCCAGGATCAAACTCT
>176
CTGCTGCCGCCCGTAGGAGTCTGGTCCGTGTCTCAGTACCAGTGTGGGGGATCACCCTCTCAGGCCCCCTAAA
GATCGTCGCCTTGGTGAGCCGTTACCTCACCAACTAGCTAATCTTGCGCGTGCCCATCTCTATCCACCGTAGT
TTTCAATATCACCCGATGCCGAATAATATATTATGGGGTATTAATCTTCTTCGAAAGGCTATCCCCCTGATAA
AGGCAGGTTGCACACGTGTTCCGCACCCGTACGCCGCTCTCAATTACCCGAAAGTAATCTACCGCTCGGCTTG
CATGTGTTAGGCCTCCCGCTAGCGTTCATCCTGAGCCAGGATCAAACTCT
>177
CTGCTGCCGCCCGTAGGAGTCTGGTCCGTGTCTCAGTACCAGTGTGGGGGATCACCCTCTCAGGCCCCCTAAA
GATCGTCGCCTTGGTGAGCCGTTACCTCACCAACTAGCTAATCTTGCGCGTGCCCATCTCTATCCACCGTAGT
TTTCAATATCACCCGATGCCGAATAATATATTATGGGGTATTAATCTTCTTCGAAAGGCTATCCCCCTGATAA
AGGCAGGTTGCACACGTGTTCCGCACCCGTACGCCGCTCTCAATTACCCCGAAAGTAATCTACCGCTCGGCTT
GCATGTGTTAGGCCTCCCGCTAGCGTTCATCCTGAGCCA
>178
CTGCTGCCGCCCGTAGGAGTCTGGTCCGTGTCTCAGTACCAGTGTGGGGGATCACCCTCTCAGGCCCCCTAAA
GATCGTCGCCTTGGTGAGCCGTTACCTCACCAACTAGCTAATCTTGCGCGTGCCCATCTCTATCCACCGTAGT
TTTCAATATCACCCGATGCCGAATAATATATTATGGGGTATTAATCTTCTTCGAAAGGCTATCCCCCTGATAA
AGGCAGGTTGCACACGTGTTCCGCACCCGTACGCCGCTCTCAATTACCCGAAAGTAATCTACCGCTCGGCTTG
CATGTGTTAGGCCTCCCGCTAGCGTTCATCCTGAGCCAGGATCAAACTCT
>179
CTGCTGCCGCCCGTAGGAGTCTGGTCCGTGTCTCAGTACCAGTGTGGGGGATCACCCTCTCAGGCCCCCTAAA
GATCGTCGCCTTGGTGAGCCGTTACCTCACCAACTAGCTAATCTTGCGCGTGCCCATCTCTATCCACCGTAGT
TTTCAATATCACCCGATGCCGAATAATATATTATGGGGTATTGATCCTCTTCGAAGGCTATCCCCTGATAAAG
GCAGGTTGCACACGTGTTCCGCACCCGTACGCCGCTCTCAATTACCCGAAAGTAATCTACCGCTCGGCTTGCA
TGTGTTAGGCCTCCCGCTAGCGTTCATCCTGAGCCAGGATCAAACTCT
>180
CTGCTGCCGCCCGTAGGAGTCTGGTCCGTGTCTCAGTACCAGTGTGGGGGATCACCCTCTCAGGCCCCCTAAA
GATCGTCGCCTTGGTGAGCCGTTACCTCACCAACTAGCTAATCTTGCGCGTGCCCATCTCTATCCACCGTAGT

TTTCGATATCACCCGATGCCGAATAATATATTATGGGGTATTAATCTTCTCGAAGCTATCCCCTGATAAAGGC
AGGTTGCACACGTGTTCCGCACCCGTACGCCGCTCTCAATTACCCGAAAGTAATCTACCGCTCGGCTTGCATG
TGTTAGGCCTCCCGCTAGCGTCATCCTG
>181
CTGCTGCCGCCCGTAGGAGTCTGGTCCGTGTCTCAGTACCAGTGTGGGGATCACCCTCTCAGGCCCCTAAAGA
TCGTCGCCTTGGTGAGCCGTTACCTCACCAACTAGCTAATCTTGCGCGTGCCCATCTCTATCCACCGTAGTTT
TCAATACTCCATCCGACCGAATAATATATTATGGGGTATTAATCTTCTTCGAAAGGCTATCCCCTGATAAAGG
CAGGTTGCACACGTGTTCCGCACCCGTACGCCGCTCTCAATTACCCGAAAGTAATCTACCGCTCGGCTTGCAT
GTGTTAGGCCTCCCGCTAGCGTTCATCCTGAGCCAGGATCAAACTCT
>182
CTGCTGCCGCCCGTAGGAGTCTGGTCCGTGTCTCAGTACCAGTGTGGGGGATCACCCTCTCAGGCCCCCTAAA
GATCGTCGCCTTGGTGAGCCGTTACCTCACCAACTAGCTAATCTTGCGCGTGCCCATCTCTATCCACCGTAGT
TTTCAATATCACCCGATGCCGAATAATATATTATGGGGTATTAATCTTCTTCGAAAGGCTATCCCCCTGATAA
AGGCAGGTTGCACACGTGTTCCGCACCCGTACGCCGCTCTCAATTACCCGAAAGTAATCTACCGCTCGGCTTG
CATGTGTTAGGCCTCCCGCTAGCGTTCATCCCGAGCCAGGATCAACTCT
>183
CTGCTGCCGCCCGTAGGAGTCTGGTCCGTGTCTCAGTACCAGTGTGGGGGATCACCCTCTCAGGCCCCCTTAA
AGATCGTCGCCTTGGTGAGCCGTTACCTCACCAACTAGCTAATCTTGCGCGTGCCCATCTCTATCCACCGTAG
TTTTTCAATATCACCCGATGCCGAATAATATATTTATGGGGTATTTAATTCTTTCCTCTCGAAAGGCTATCCC
CCTGATAAAGGCAGGTTGCACACGTGTTCCGCACCCGTACGCCGCTCTCAATTACCCGAAAGTATCTACCGCT
CGGCTTGCATGTGTTAGGCCTCCCGCTAGCGTTCATTCCTGAGCCAGGATCAAACTCT
>184
CTGCTGCCGCCCGTAGGAGTCTGGTCCGTGTCTCAGTACCAGTGTGGGGGATCACCCTCTCAGGCCCCCTAAA
GATCGTCGCCTTGGTGAGCCGTTACCTCACCAACTAGCTTAATCTTGCGCGTGCCCATCTCTATCCACCGTAG
TTTTCAATATCACCCGATGCCGAATAATATATTATGGGGTATTTAATTCTTTCTTCGAAAGGCTATCCCCCTG
ATTAAAGGCAGGTTTGCACACGTGTTCCGCACCCGTACGCCGCTCTCAATTACCCGAAAGTAATCTACCGCTC
GGCTTGCATGTGTTAGGCCTCCCGCTAGCGTTCTTCCTGAG
>185
CTGCTGCCGCCGTAGGAGTCTGGTCCCGTGTCTCAGTACCAGTGTGGGGGATCACCCTCTCAGGCCCCCTAAA
GATCGTCGCCTTGGTGAGCCGTTACCTCACCCAACTAGCTAATCTTGCGCGTGCCCCATCTCTATCCCACCGT
AGTTTTTCAATATCACCCGATGCCGAATAATATATTTATGGGGTATTTAATCTTTTCTTCGAAAGGCTATCCC
CCTGATAAAGGCAGGTTGCACACGTGTTCCCGCACCCGTACGCCGCTCTCAATTTACCCCGAAAGTAATCTAC
CGCTCGGCTTGCAGTGTTAGGCCCTTCCCGCTAGCGTTCTCCTGAGCCAGGATCAACTCT
>186
CTGCTGCCGCCCGTAGGAGTCTGGTCCGTGTCTCAGTACCAGTGTGGGGGATCACCCTCTCAGGCCCCCTAAA
GATCGTCGCCTTGTGAGCCGTTACCTCACCAACTAGCTAATTCTTGCGCGTGCCCATCTCTATCCACCGTAGT
TTTTCAATTATCACCCGATGCCGAATAATATATTTATTGGGGTATTTAATCTTTCTTCGAAAGGCTATCCCCC
TGATAAAGGCAGGTTTGCACACGTGTTCCGCACCCGTACGCCGCTCTTCAATTTACCCGAAAGTAATCTACCG
CTCGGCTTGCATGTGTTAGGCCTTCCCGCTTAGCGTTCTCCTGAGCAGGATCCAACTCGT
>187
CTGCTGCCGCCCGTAGGAGTCTGGTCCGTGTCTCAGTACCAGTGTGGGGGATCACCCTCTCAGGCCCCCTAAA
GATCGTCGCCTTGGTGAGCCGTTACCTCACCAACTAGCTAATCTTGCGCGTGCCCATCTCTATCCACCGTAGT
TTCAATATCACCCGATGCCGAATAATATATTATGGGGTATTAATCTTCTTCGAAAGCTATCCCCCTGATAAAG
GCAGGTTGCACACGTGTTCCGCACCCGTACGCCGCTCTCAATTACCCGAAAGTAATCTACCGCTCGGCTTGCA
TGTGTTAGGCCTCCCGCTAGCGTTCATCCTGAGCCAGGATCAAACTCT
>188
CTGCTGCCGCCCGTAGGAGTCTGGTCCGTGTCTCAGTACCAGTGTGGGGGATCACCCTCTCAGGCCCCCTAAA
GATCGTCGCCTTGGTGAGCCGTTACCTCACCAACTAGCTAATCTTGCGCGTGCCCATCTCTATCCACCGTAGT
TTTCAATATCACCCGATGCCGAATAATATATTATGGGGTGTTAATCTTCTTCGAAAGGCTATCCCCTGATAAA
GGCAGGTTGCACACGTGTTCCGCACCCGTACGCCGCTCTCAATTACCCGAAAGTAATCTACCGCTCGGCTTGC
ATGTGTTAGGCCTCCCGCTAGCGTTCATCTGAGCCAG
>189
CTGCTGCCGCCCGTAGGAGTCTGGTCCGTGTCTCAGTACCAGTGTGGGGGATCACCCTCTCAGGCCCCCTAAA
GATCGTCGCCTTGTGAGCCGTTACCTCACCAACTAGCTAATCTTGCGCGTGCCCATCTCTATCCACCGTAGTT

TTCAATATCACCCGATGCCGAATAATATATTATGGGGTATTAATCTTCTTCGAAAGGCTATCCCCCTGATAAA
GGCAGGTTGCACACGTGTTCCGCACCCGTACGCCGCTCTCAATTACCCGAAAGTAATCTACCGCTCGGCTTGC
ATGTGTTAGGCCTCCCGCTAGCGTTCATCCTGAGCCAGGATCAACTCT
>190
CTGCTGCCGCCCGTAGGAGTCTGGTCCGTGTCTCAGTACCAGTGTGGGGGATCACCCTCTCAGGCCCCCTAAA
GATCGTCGCCTGTGAGCCGTTACCTCACCAACTAGCTAATCTTGCGCGTGCCCATCTCTATCCACCGTAGTTT
TCAATATCACCCGATGCCGAATAATATATTATGGGGTATTAATCTTCTCGAAAGGCTATCCCCCTGATAAAGG
CAGGTTGCACACGTGTTCCGCACCCGTACGCCGCTCTCAATTACCCGAAAGTAATCTACCGCTCGGCTTGCAT
GTGTTAGGCCTCCCGCTAGCGTTCATCCTGAGCCAGGATCAAACTCT
>191
CTGCTGCCGCCCGTAGGAGTCTGGTCCGTGTCTCAGTACCAGTGTGGGGGATCACCCTCTCAGGCCCCCTAAA
GATCGTCGCCTTGGTGAGCCGTTACCTCACCAACTAGCTAATCTTGCGCGTGCCCATCTCTATCCACCGTAGT
TTTCAATATCACCCGATGCCGAATAATATATTATGGGGTATTAATCTTCTTCGAAGGCTATCCCCTGATAAAG
GCAGGTTGCACACGTGTTCCGCACCCGTACGCCGCTCTCAATTACCCGAAAGTAATCTACCGCTCGGCTTGCA
TGTGTTAGGCCTCCCGCTAGCGTTCATCCGTGAGC
>192
CTGCTGCCGCCCGTAGGAGTCTGGTCCGTGTCTCAGTACCAGTGTGGGGGATCACCCTCTCAGGCCCCCTAAA
GATCGTCGCCTTGGTGAGCCGTTACCTCACCAACTAGCTAATCTTGCGCGTGCCCATCTCTATCCACCGTAGT
TTTCAATATCACCCGATGCCGAATAATATATTATGGGGTATTAATCTTCTTCGAAAGGCTATCCCCCTGATAA
AGGCAGGTTGCACACGTGTTCCGCACCCGTACGCCGCTCTCAATTACCCGAAAGTAATCTACCGCTCGGCTTG
CATGTGTTAGGCCTCCCGCTAGCGTTCATCCTGAGCCAGGATCAAACTCT
>193
CTGCTGCCGCCCGTAGGAGTCTGGTCCGTGTCTCAGTACCAGTGTGGGGGATCACCCTCTCAGGCCCCCTAAA
AGATCGTCGCCTTGGTGAGCCGTTACCTCACCAACTAGCTAATCTTGCGCGTGCCCATCTCTATCCACCGTAG
TTTTCAATATCACCCGATGCCGAATAATATATTATGGGGTATTAATCTTCTTCGAAGGCTATCCCCCTGGTAA
AGGCAGGTTGCACACGTGTTCCGCACCCGTACGCCGCTCTCAATTACCCGAAAGTAATCTACCGCTCGGCTTG
CATGTGTTAGGCCTCCCGCTAGCGTCTCCTGAGCAGGATCAAACTCT
>194
CTGCTGCCGCCCGTAGGAGTCTGGTCCGTGTCTCAGTACCAGTGTGGGGGATCACCCTCTCAGGCCCCCTAAA
GATCGTCGCCTTGGTGAGCCGTTACCTCACCAACTAGCTAATCTTGCGCGTGCCCATCTCTATCCACCGTAGT
TTTCAATATCACCCGATGCCGAATAATATATTATGGGGTATTAATCTTCTTCGAAAGGCTATCCCCCTGATAA
AGGCAGGTTGCACACGTGTTCCGCACCCGTACGCCGCTCTCAATTACCCGAAAGTAATCTACCGCTCGGCTTG
CATGTGTTAGGCCTCCCGCTAGCGTTCATCCTGAGCCAGGATCAACTCT
>195
CTGCTGCCGCCCGTAGGAGTCTGGTCCGTGCCTCAGTACCAGTGTGGGGGATCACCCTCTCAGGCCCCCTAAA
GATCGTCGCCTTGGTGAGCCGTTACCTCACCAACTAGCTAATCTTGCGCGTGCCCATCTCTATCCACCGTAGT
TTTCAATATCACCCGATGCCGAATAATATATTATGGGGTATTAATCTTCTTCGAAAGGCTATCCCCTGATAAA
GGCAGGTTGCACACGTGTTCCGCACCCGTACGCCGCTCTCAATTACCCGAAAGTAATCTACCGCTCGGCTTGC
ATGTGTTAGGCCTCCCGCTAGCGTTCTCTGAGCCAGG
>196
CTGCTGCCGCCCGTAGGAGTCTGGACCGTGTCTCAGTTCCAGTGTGACTGATCATCCTCTCAGACCAGTTACG
GATCGTCGCCTTGGTAGGCCTTTTACCCCACCAACTAGCTAATCCGACCTAGGCTCATCTGATAGCGTGAGGT
CCGAAGTCCCCCACTTCTCCCCCAGGACGTATGCGGTATTAACGCCCGTTTCCGGACGTATCCCCCCACTACC
AGGCAGATTCCTAGGCATTACTCACCCGTCCGCCGCTGAATCCAGGAGCAAGCTCCCTTCATCCGCTCGACTG
CATGTGTTAGGCCTGCCGCCAGCGTTCAATCTGAGCCAGGATCAAACTCT
>197
CTGCTGCCGCCCGTAGGAGTCTGGTCCGTGTCTCAGTACCAGTGTGGGGGATCACCCTCTCAGGCCCCCTAAA
GATCGTCGCCTTGGTGAGCCGTTACCTCACCAACTAGCTAATCTTGCGCGTGCCCATCTCTATCCACCGTAGT
TTTCAATATCACCCGATGCCGAATAATATATTATGGGGTATTAATCTTCTTCGAAAGGCTATCCCCCTGATAA
AGGCAGGTTGCACACGTGTTCCGCACCCGTACGCCGCTCTCAATTACCCGAAAGTAATCTACCGCTCGGCTTG
CATGTGTTAGGCCTCCCGCTAGCGTTCTCCTGAGCAGGATCAACTCGT
>198
CTGCTGCCGCCCGTAGGAGTCTGGTCCGTGTCTCAGTACCAGTGTGGGGGATCACCCTCTCAGGCCCCCTAAA
GATCGTCGCCTTGGTGAGCCGTTACCTCACCAACTAGCTAATCTTGCGCGTGCCCATCTCTATCCACCGTAGT

TTTCAATATCACCCGATGCCGAATAATATATTATGGGGTATTAATCTTCTTCGAAAGGCTATCCCCCTGATAA
AGGCAGGTTGCCACACGTGTTCCGCACCCGTACGCCGCTCTCAATTACCCGAAAGTAATCTACCGCTCCGGCT
TGCATGTGTTAGGCCTCCCGCTAGCGTTCTCCTGAGCCAGGTCAACTCGT
>199
CTGCTGCCGCCCGTAGGAGTCTGGTCCGTGTCTCAGTACCAGTGTGGGGGATCACCCTCTCAGGCCCCCTAAA
GATCGTCGCCTGGTGAGCCGTTACCTCACCAACTAGCTAATCTTGCGCGTGCCCATCTCTATCCACCGTAGTT
TTCAATATCACCCGATGCCGAATAATATATTATGGGGTATTAATCTTCTTCGAAAGGCTATCCCCTGATAAAG
GCAGGTTGCACACGTGTTCCGCACCCGTACGCCGCTCTCAATTACCCGAAAGTAATCTACCGCTCGGCTTGCA
TGTGTTAGGCCTCCCGCTAGCGTTCATCCTGAGCCAGGATCAACTCT
>200
CTGCTGCCGCCCGTAGGAGTCTGGACCGTGTCTCAGTTCCAGTGTGACTGATCATCCTCTCAGACCAGTACGG
ATCGTCGCCTTGGTAGGCCTTTACCCCACCAACTAGCTAATCCGACCTAGGCTCATCTGATAGCGTGAGGTCC
GAAGTCCCCCACTTCTCCCTCAGGACGTATGCGGTATTAGCGCCCGTTTCCGGACGATCCCCACTACCAGGCA
GATTCCCTAGGCATTACTCACCCGTCCGCCGCTGAATCCAGGAGCAAGCTCCCTCATCCGCTCGACTGCATGT
GTTAGGCCTGCCGCCAGCGTCAATCTGAGCCAGGATCAAACTCT
>201
CTGCTGCCGCCCGTAGGAGTCTGGTCCGTGTCTCAGTACCAGTGTGGGGGATCACCCTCTCAGGCCCCCTAAA
GATCGTCGCCTTGGTGAGCCGTTACCTCACCAACTAGCTAATCTTGCGCGTGCCCATCTCTATCCACCGTAGT
TTTCAATATCACCCGATGCCGAATAATATATTATGGGGTATTAATCTTCTTCGAAAGGCTATCCCCCTGATAA
AGGCAGGTTGCACACGTGTTCCGCACCCGTACGCCGCTCTCAATTACCCGAAAGTAATCTACCGCTCGGCTTG
CATGTGTTAGGCCTCCCGCTAGCGTTCGTCCTGAGCCAGGATCAAACTCT
>202
CTGCTGCCGCCCGTAGGAGTCTGGTCCGTGTCTCAGTACCAGTGTGGGGGATCACCCTCTCAGGCCCCCTAAA
GATCGTCGCCTTGGTGAGCCGTTACCTCACCAACTAGCTAATCTTGCGCGTGCCCATCTCTATCCACCGTAGT
TTTCAATATCACCCGATGCCGAATAATATATTATGGGGTATTAATCTTCTTCGAAAGGCTATCCCCCTGATAA
AGGCAGGTTGCACACGTGTTCCGCACCCGTACGCCGCTCTCAATTACCCGAAAGTAATCTACCGCTCGGCTGC
ATGTGTTAGGCCTCCCGCTAGCATTCATCTGAGCCAGGATCAAACTCT
>203
CTGCTGCCGCCCGTAGGAGTCTGGTCCGTGTCTCAGTACCAGTGTGGGGGATCACCCTCTCAGGCCCCCTAAA
GATCGTCGCCTTGGTGAGCCGTTACCTCACCAACTAGCTAATCTTGCGCGTGCCCATCTCTATCCACCGTAGT
TTCAATATCACCCGATGCCGAATAATATATTATGGGGTATTAATCTTCTTCGAAAGGCTATCCCCCTGATAAA
GGCAGGTTGCACACGTGTTCCGCACCCGTACGCCGCTCTCAATTACCCGAAAGTAATCTACCGCTCGGCTTGC
ATGTGTTAGGCCTCCCGCTAGCGTTCTCCTGAGCAGGATCAA
>204
CTGCTGCCGCCCGTAGGAGTCTGGTCCGTGTCTCAGTACCAGTGTGGGGGATCACCCTCTCAGGCCCCCTAAA
GATCGTCGCCTTGGTGAGCCGTTACCTCACCAACTAGCTAATCTTGCGCGTGCCCATCTCTATCCACCGTAGT
TTTCAATATCACCCGATGCCGAATAATATATTATGGGGTATTAATCTTCCTTCGAAAGGCTATCCCCCTGATA
AAGGCAGGTTGCACACGTGTTCCGCACCCGTACGCCGCTCTCAATTACCCGAAAGTAATCTACCGCTCGGCTT
GCATGTGTTAGGCCTCCCGCTAGCGTTCTCCTGAGCAGGATCAACTCG
>205
CTGCTGCCGCCCGTAGGAGTCTGGTCCGTGTCTCAGTACCAGTGTGGGGGATCACCCTCTCAGGCCCCCTAAA
GATCGTCGCCTTGGTGAGCCGTTACCTCACCAACTAGCTAATCTTGCGCGTGCCCATCTCTATCCACCGTAGT
TTTCAATATCACCCGATGCCGAATAATATATTATGGGGTATTAATCTTCTCGAAAGGCTATCCCCCTGATAAA
GGCAGGTTGCACACGTGTTCCGCACCCGTACGCCGCTCTCAATTACCCGAAAGTAATCTACCGCTCGGCTTGC
ATGTGTTAGGCCTCCCGCTAGCGTCATCTGAGCCAGGATCAACTCT
>206
CTGCTGCCGCCCTAGGAGCTGGTCCGTGTCTCAGTACCAGTGTGGGGGATCACCCTCTCAGGCCCCCTAAAGA
TCGTCGCCTTGGTGAGCCGTTACCTCACCAACTAGCTAATCTTGCGCGTGCCCATCTCTATCCACCGTAGTTT
TCAATATCACCCGATGCCGAATAATATATTATGGGGTATTAATCTTCTTCGAAAGGCTATCCCCTGATAAAGG
CAGGTTGCACACGTGTTCCGCACCGTACGCCGCTCTCAATTACCCGAAAGTAATCTACCGCTCGGCTTGCATG
TGTTAGGCCTCCGCTAGCGTCTCTGAGCAGTCAACTC
>207
CTGCTGCCGCCCGTAGGAGTCTGGTCCGTGTCTCAGTACCAGTGTGGGGATCACCCTCTCAGGCCCCTAAAGA
TCGTCGCCTTGGTGAGCCGTTACCTCACCAACTAGCTAATCTTGCGCGTGCCCATCTCTATCCACCGTAGTTT

TCAATATCACCCGATGCCGAATAATATATTATGGGGTATTAAATCTTCTCGAAAGGCTATCCCCCTGATAAAG
GCAGGTTGCACACGTGTTCCGCACCGTACGCCGCTCTCAATTACCCGAAAGTAATCTACCGCTCGGCTGCATG
TGTTAGGCCTCCCGCTAGCGTTCATCCTGAGCCAGGAT
>208
CTGCTGCCGCCCGTAGGAGTCTGGTCCGTGTCTCAGTACCAGTGTGGGGGATCACCCTCTCAGGCCCCCTAAA
GATCGTCGCCTTGGTGAGCCGTTACCTCACCAACTAGCTAATCTTGCGCGTGCCCATCTCTATCCACCGTAGT
TTCAATATCACCCGATGCCGAATAATATATTATGGGGTATTAATCTTCTTCGAAAGCTATCCCCTGATAAAGG
CAGGTTGCACACGTGTTCCGCACCCGTACGCCGCTCTCAATTACCCGAAAGTAATCTACCGCATCGGCTTGCA
TGTGTTAGGCCTCCCGCTAGCGTCTCCTGAG
>209
CTGCTGCCGCCCGTAGGAGTCTGGTCCGTGTCTCAGTACCAGTGTGGGGGATCACCCTCTCAGGCCCCCTAAA
GATCGTCGCCTTGGTGAGCCGTTACCTCACCAACTAGCTAATCTTGCGCGTGCCCATCTCTATCCACCGTAGT
TTTCAATATCACCCGATGCCGAATAATATATTATGGGGTATTAATCTTCTTCGAAAGGCTATCCCCCTGATAA
AGGCAGGTTGCACACGTGTTTCGCACCCGTACGCCGCTCTCAATTACCCGAAAGTAATCTACCGCTCGGCTTG
CATGTGTTAGGCCTCCCGCTAGCGTTCTCCTCGAGCAGGATCAACTCT
>210
CTGCTGCCGCCCGTAGGAGTCTGGACCGTGTCTCAGTTCCAGTGTGGCCGATCACCCTCTCAGGTCGGCTATG
CATCGTTGCCTTGGTAAGCCGTTACCTTACCAACTAGCTAATGCAGCGCGGATCCATCTATAAGTGACAGCAA
GACCGTCTTCACTAACCATGCGGTTCAAAATATTATCCGGTATTAGCTCCGGTTTCCCGAAGTTATCCCAGTC
TTATAGGTAGGTTATCCACGTGTTACTCACCCGTCCGCCGCTAACATCAGAGAAGCAAGCTTCTCGTCCGTTC
GCTCGACTGCATGTATTAGGCACGCGCCAGCGTCATCCGTGAGCCAAGGATAACTCT
>211
CTGCTGCCGCCCGTAGGAGTCTGGTCCGTGTCTCAGTACCAGTGTGGGGGATCACCCTCTCAGGCCCCCTAAA
GATCGTCGCCTTGGTGAGCCGTTACCTCACCAACTAGCTAATCTTGCGCGTGCCCCATCTCTATCCACCGTAG
TTTTCAATATCACCCGATGCCGAATAATATATTTATGGGGTATTTAATTCTTTCTTCGAAAGGCTATCCCCCT
GATAAAGGCAGGTTTGCACACGTGTTCCGCACCCGTACGCCGCTCTCAATTACCCCGAAAGTAATCTACCGCT
CGGCTTGCATGTGTTAGGCCTCCCGCTAGCGTTCTTCCTGAGCAGGATCAACTCGT
>212
CTGCTGCCGCCCGTAGGAGTCTGGTCCCGTGTCTCAGTACCAGTGTGGGGGATTCACCCTCTCAGGCCCCCCT
AAAGATCGTCGCCTTGGTGAGCCCGTTACCTCACCCAACTAGCTAATCTTGCGCGTGCCCCATCTCTTATCCC
ACCGTAGTTTTCAATATCACCCGATTGCCCGAATAATATATTTATGGGGTATTTAATCTTTCTTCGAAAGGCT
ATCCCCCTGATAAAGGCAGGTTGCACACGTGTTCCCGCACCCGTACGCCGCTCTCAATTACCCCGAAAGTAAT
CTACCGCTCGGCTGCATGTGTTTAGGCCCTTCCCGCTAGCGTTCTCCTGAGCAGGATCAACTCGT
>213
CTGCTGCCGCCCGTAGGAGTCTGGTCCGTGTCTCAGTACCAGTGTGGGGATCACCCTCTCAGGCCCCCTAAAG
ATCGTCGCCTTGGTGAGCCGTTACCTCACCAACTAGCTTAATCTTGCGCGTGCCCATCTCTATTCCACCGTAG
TTTTCAATTATCACCCGATGCCGAATAATATATTTATGGGGTATTTAATCTTTCTTCGAAAGGCTATCCCCTG
ATAAAGGCAGGTTTGCACACGTGTTCCGCACCCGTACGCCGCTCTCAATTTACCCCGAAAGTAATCTACCGCT
CGGCTTGCATGTGTTTAGGCCTCCCGCTAGCGTTCATCCTTGAGCC
>214
CTGCTGCCGCCCGTAGGAGTCTGGTCCGTATCTCAGTACCAGTGTGGGGGATCACCCTCTCAGGCCCCCTAAA
GATCGTCGCCTTGGTGAGCCGTTACCTCACCAACTAGCTAATTCTTGCGCGTGCCCATCTCTTATCCACCGTA
GTTTTTCAATATCACCCGATGCCGAATAATATATTATGGGGTATTTAATCTTTCTTCGAAAGGCTATCCCCCT
GATTAAAGGCAGGTTGCACACGTGTTCCGCACCCGTACGCCGCTCTCAATTACCCGAAAGTAATCTACCGCTC
GGCTTGCATGTGTTAGGCCTCCCGCTAGCGTTCATCCTGAGCCAGGATCAAACTCT
>215
CTGCTGCCGCCCGTAGGAGTCTGGTTCCGTGTCTCAGTACCAGTGTGGGGGATCACCCTCTCAGGCCCCCTAA
AGATCGTCGCCTTGTGAGCCGTTACCTCACCAACTAGCTAATCTTGCGCGTGCCCATCTCTTATCCACCGTAG
TTTCAATTATCACCCGATGCCGAATTAATATATTATGGGGTATTTAATCTTTCTCGAAAGGCTATCCCCTGAT
AAAGGCAGGTTTGCACACGTGTTCCGCACCCGTACGCCGCTTCTCAATTTACCCCGAAAGTAATCTACCGCTC
GGCTTGCATGTGTTAGGCCTT
>216
CTGCTGCCGCCCGTAGGAGTCTGGTCCGTGTCTCAGTACCAGTGTGGGGGATCACCCTCTCAGGCCCCTAAGA
TCGTCGCCTTGGTGAGCCGTTACCTCACCAACTAGCTAATTCTTGCGCGTGCCCATCTCTATCCACCGTAGTT

TTTCAATATCACCCGATGCCGAATAATATATTTATGGGGTATTTAATTCTTCTCGAAAGGCTATCCCCCTGAT
AAAGGCAGGTTGCACACGTGTTCCGCACCCGTACGCCGCTCTCAATTACCCGAAAGTAATCTACCGCTCCGGC
TTGCATGTGTTTAGGCCTCCCGCTAGCGTTCATCCTGAGCCAG
>217
CTGCTGCCGCCCGTAGGAGTCTGGTCCGTGTCTCAGTACCAGTGTGGGGGATCACCCTCTCAGGCCCCCTAAA
GATCGTCGCCTTGGTGAGCCGTTACCTCACCAACTAGCTAATCTTGCGCGTGCCCATCTCTTATCCACCGTAG
TTTCAATATCACCCGATGCCGAATAATATATTTATTGGGGTATTTAATCTTTCTTCGAAAGGCTATCCCCCTG
ATAAAGGCAGGTTGCACACGTGTTCCGCACCCGTACGCCGCTCTCAATTACCCGAAAGTAATCTACCGCTCGG
CTTGCATGTGTTAGGCCTTCCCGCTAGCGTTCTCCTGAGCAGGATCAACTCG
>218
CTGCTGCCGCCCGTAGGAGTCTGGTCCGTGTCTCAGTACCAGTGTGGGGGATCACCCTCTCAGGCCCCCTAAAG
ATCGTCGCCTTGTGAGCCGTTACCTCACCAACTAGCTAATCTTGCGCGTGCCCATCTCTATCCACCGTAGTTT
TCAATACCACCCGATGCCGAATAATATATTATGGGGTATTAATCTTCTTCGAAGGCTATCCCCCTGATAAAGG
CAGGTTGCACACGTGTTCCGCACCCGTACGCCGCTCTCAATTACCCGAAAGTAATCTACCGCTCGGCTTGCAT
GTGTTAGGCCTCCCGCTAGCGTTCTCCTGAGCAGGATCAACT
>219
CTGCTGCCGCCCGTAGGAGTCTGGTCCGTGTCTCAGTACCAGTGTGGGGGATCACCCTCTCAGGCCCCCCCTA
AAGATCGTCGCCTGTGAGCCGTTACCTCACCAACTAGCTAATCTCGCGCGTGCCCATCTCTATCCACCGTAGT
TTTCAATATCACCCGATGCCGAATAATATATTATGGGGTATTAATCTTCTTCGAAAGGCTATCCCCCTGATAA
AGGCAGGTTGCACACGTGTTCCGCACCCGTACGCCGCTCTCAATTACCCCGAAAGTAATCTACCGCTCGGCTT
GCATGTGTTAGGCCTCCCGCTAGCGTTCATCCTGAGCCAGGATCAACTCT
>220
CTGCTGCCGCCCGTAGGAGTCTGGTCCGTGTCTCAGTACCAGTGTGGGGGATCACCCTCTCAGGCCCCCTAAA
GATCGTCGCCTTGGTGAGCCGTTACCTCACCAACTAGCTAATCTTGCGCGTGCCCATCTCTATCCACCGTAGT
TTTCAATATCACCCGATGCCGAATAATATATTATGGGGTATTAATCTTCTTCGAAAGGCTATCCCCCTGATAA
AGGCAGGTTGCACACGTGTTCCGCACCCGTACGCCGCTCTCAATTACCCGAAAGTAATCTACCGCTCGGCTTG
CATGTGTTAGGCCTCCCGCTAGCGTTCATCCTGAGCCAGGATCAAACTCT
>221
CTGCTGCCGCCCGTAGGAGTCTGGTCCGTGTCTCAGTACCAGTGTGGGGGATCACCCTCTCAGGCCCCCTAAA
GATCGTCGCCTGGTGAGCCGTTACCTCACCAACTAGCTAATCTTGCGCGTGCCCATCTCTGTCCACCGTAGTT
TTCAATATCACCCGATGCCGAATAATATATTATGGGGTATTAATCTTCTTCGAAAGGCTATCCCCCTGATAAA
GGCAGGTTGCACACGTGTTCCGCACCCGTACGCCGCTCTCAATTACCCGAAAGTAATCTACCGCTCGGCTTGC
ATGTGTTAGGCCTCCCGCTAGCGTTCATCCTGAGCCAGGATCAAACTCT
>222
CTGCTGCCGCCCGTAGGAGTCTGGTCCGTGTCTCAGTACCAGTGTGGGGGATCACCCTCTCAGGCCCCCTAAA
GATCGTCGCCTTGGTGAGCCGTTACCTCACCAACTAGCTAATCTTGCGCGTGCCCATCTCTATCCACCGTAGT
TTTCAATATCACCCGATGCCGAATAATATATTATGGGGTATTAATCTTCTTCGAAAGGCTATCCCCCTGATTA
AAGGCAGGTTGCACACGTGTTCCGCACCCGTACGCCGCTCTCAATTACCCGAAAGTAATCTACCGCTCGGCTT
GCATGTGTTAGGCCTCCCGCTAGCGTTCATCCTGAGCCAGGATCAAACTCT
>223
CTGCTGCCGCCCGTAGGAGTCTGGTCCGTGTCTCAGTACCAGTGTGGGGGATCACCCTCTCAGGCCCCCTAAA
GATCGTCGCCTTGGTGAGCCGTTACCTCACCAACTAGCTTAATCTTGCGCGTGCCCATCTCTATCCACCGTAG
TTTTCAATATCACCCGATGCCGAATAATATATTATGGGGTATTAATCTTCTCGAAAGGCTATCCCCTGATAAA
GGCAGGTTGCACACGTGTTCCGCACCCGTACGCCGCTCTCAATTACCCGAAAGTAATCTACCGCTCGGCTTGC
ATGTGTTAGGCCTCCCGCTAGCGTTCTCCTGAGCAGGATCCA
>224
CTGCTGCCGCCCGTAGGAGTCTGGTCCGTGTCTCAGTACCAGTGTGGGGGATCACCCTCTCAGGCCCCCTAAA
GATCGTCGCCTTGGTGAGCCGTTACCTCACCAACTAGCTAATCTTGCGCGTGCCCATCTCTATCCACCGTAGT
TTTCAATATCACCCGATGCCGAATAATATATTATGGGGTATTAATCTTCTTCGAAAGGCTATCCCCCTGATAA
AGGCAGGTTGCACACGTGTTCCGCACCCGCTCTCAATTACCCGAAAGTAATCTACCGCTCGGCTTG
CATGTGTTAGGCCTCCCGCTAGCGTTCTCCTGA
>225
CTGCTGCCGCCCGTAGGAGTCTGGTCCGTGTCTCAGTACCAGTGTGGGGGATCACCCTCTCAGGCCCCCTAAA
GATCGTCGCCTTGGTGAGCCGTTACCTCACCAACTAGCTAATCTTGCGCGTGCCCATCTCTATCCACCGTAGT

TTTCAATATCACCGATGCCGAATAATATATTATGGGGTATTAATCTTCTTCGAAAGGCTATCCCCCTGATAAA
GGCAGGTTGCACACGTGTTCCGCACCCGTACGCCGCTCTCAATTACCCGAAAGTAATCTACCGCTCGGCTTGC
ATGTGTTAGGCCTCCCGCTAGCGTTCATCCTGAGCCAGGATCAAACTCT
>226
CTGCTGCCGCCCGTAGGAGTCTGGTCCGTGTCTCAGTACCAGTGTGGGGGATCACCCTCTCAGGCCCCCTAAA
GATCGTCGCCTTGGTGAGCCGTTACCTCACCAACTAGCTAATCTTGCGCGTGCCCATCTCTATCCACCGTAGT
TTTCAATATCACCCGATGCCGAATAATATATTATGGGGTATTAATCTTCTTCGAAAGGCTATCCCCCTGATAA
AGGCAGGTTGCACACGTGTTCCGCACCCGTACGCCGCTCTCAATTACCCGAAAGTAATCTACCGCTCGGCTTG
CATGTGTTAGGCCTCCCGCTAGCGTCTCCTGAG
>227
CTGCTGCCGCCCGTAGGAGTCTGGTCCGTGTCTCAGTACCAGTGTGGGGGATCACCCTCTCAGGCCCCCTAAA
CGATCGTCGCCTTGGTGAGCCGTTACCTCACCAACTAGCTAATCTTGCGCGTGCCCATCTCTATCCACCGTAG
TTTTCAATATCACCCGATGCCGAATAATATATTATGGGGTATTAATCTTCTTCGAAAGGCTATCCCCCTGATA
AAGGCAGGTTGCACACGTGTTCCGCACCCGTACGCCGCTCTCAATTACCCGAAAGTAATCTACCGCTCCGGCT
TGCATGTGTTAGGCCTCCCGCTAGCGTTCATCC
>228
CTGCTGCCGCCCGTAGGAGTCTGGTCCGTGTCTCAGTACCAGTGTGGGGGATCACCCTCTCAGGCCCCCTAAA
GATCGTCGCCTGGTGAGCCGTTACCTCACCAACTAGCTAATCTTGCGCGTGCCCATCTCTATCCACCGTAGTT
TTCAATATCACCCGATGCCGAATAATATATTATGGGGTATTAATCTTCTTCGAAAGGCTATCCCCCTGATAAA
GGCAGGTTGCACACGTGTTCCGCACCCGTACGCCGCTCTCAATTACCCGAAAGTAATCTACCGCTCGGCTTGC
ATGTGTTAGGCCTCCCGCCTAGCGTCATCCTGAGCCAGGATCAAACTCT
>229
CTGCTGCCGCCCGTAGGAGTCTGGTCCGTGTCTCAGTACCAGTGTGGGGGATCACCCTCTCAGGCCCCCTAAA
GATCGTCGCCTTGGTGAGCCGTTACCTCACCAACTAGCTAATCTTGCGCGTGCCCATCTCTATCCACCGTAGT
TTTCAATATCACCCGATGCCGAATAATATATTATGGGGTATTAATCTTCTTCGAAAGGCTATCCCCCTGATAA
AGGCAGGTTGCACACGTGTTCCGCACCCGTACGCCGCTCTCAATTACCCGAAAGTAATCTACCGCTCGGCTTG
CATGTGTTAGGCCTCCCGCTAGCGTTCATCCTGAGCCAGGATCAACTCT
>230
CTGCTGCCGCCCGTAGGAGTCTGGTCCGTGTCTCAGTACCAGTGTGGGGGATCACCCTCTCAGGCCCCCTAAA
GATCGTCGCCTGTGAGCCGTTACCTCACCAACTAGCTAATCTTGCGCGTGCCCATCTCTATCCACCGTAGTTT
TCAATATCACCCGATGCCGAATAATATATTATGGGGTATTAATCTTCTTCGAAAGGCTATCCCCCTGATAAAG
GCAGGTTGCACACGTGTTCCGCACCCGTACGCCGCTCTCAATTACCCGAAAGTAATCTACCGCTCGGCTTGCA
TGTGTTAGGCCTCCCGCTAGCGTTCTCCTGAGCAGGATCAACTCCGT
>231
CTGCTGCCGCCCGTAGGAGTCTGGACCGTGTCTCAGTTCCAGTGTGACTGATCATCCTCTCAGACCAGTACGG
ATCGTCGCCTGGTAGGCCTTTACCCCACCAACTAGCTAATCCGACCTAGGCTCATCTGATAGCGTGAGGTCCG
AAGATCCCCCACTCTCCCTCAGGACGTATGCGGTATTAGCGCCCGTTTCCGGACGTATCCCCACTACCAGGCA
GATTCCTAGGCATTACTCACCCGTCCGCCGCTGAATCCAGGAGCAAGCTCCCTCATCCGCTCGACTGCATGTG
TTAGGCCTGCCGCCAGCGTCAATCTGAGCCAGGATCAAACTCT
>232
CTGCTGCCGCCCGTAGGAGTCTGGTCCGTGTCTCAGTACCAGTGTGGGGGATCACCCTCTCAGGCCCCCTAAA
GATCGTCGCCTTGTGAGCCGTTACCTCACCAACTAGCTAATCTTGCGCGTGCCCATCTCTATCCACCGTAGTT
TTCAATATCACCCGATGCCGAATAATATATTATGGGGTATTAATCTTCTTCGAAAGGCTATCCCCCTGATAAA
GGCAGGTTGCACACGTGTTCCGCACCCGTACGCCGCTCTCAATTACCCGAAAGTAATCTACCGCTCCGGCTTG
CATGTGTTAGGCCTCCCGCTAGCGTCTCCTGAGCAGGATCAACTCT
>233
CTGCTGCCGCCCGTAGGAGTCTGGTCCGTGTCTCAGTACCAGTGTGGGGGATCACCCTCTCAGGCCCCCTAAA
GATCGTCGCCTTGGTGAGCCGTTACCTCACCAACTAGCTAATCTTGCGCGTGCCCATCTCTATCCACCGTAGT
TTTCAATATCACCCGATGCCGAATAATATATTATGGGGTATTAATCTTCTTCGAAAGGCTATCCCCCTGATAA
AGGCAGGTTGCACACGTGTTCCGCACCCGTACGCCGCTCTCAATTACCCGAAAGTAATCTACCGCTCGGCTTG
CATGTGTTAGGCCTCCCGCTAGCGTTCTCCTGAGCCAGGAT
>234
CTGCTGCCGCCCGTAGGAGTCTGGTCCGTGTCTCAGTACCAGTGTGGGGGATCACCCTCTCAGGCCCCCTAAA
GATCGTCGCCTTGGTGAGCCGTTACCTCACCAACTAGCTAATCTTGCACGTGCCCATCTCTATCCACCGTAGT

TTTCAATATCACCCGATGCCGAATAATATATTATGGGGTATTAATCTTCTTCGAAAGGCTATCCCCCTGATAA
AGGCAGGTTGCACACGTGTTCCGCACCCGTACGCCGCTCTCAATTACCCGAAAGTAATCTACCGCTCGGCTTG
CATGTGTTAGGCCTCCCGCTAGCGTTCATCCTGAGCCAGGATCAACTCT
>235
CTGCTGCCGCCCGTAGGAGTCTGGTCCGTGTCTCAGTACCAGTGTGGGGGATCACCCTCTCAGGCCCCCTAAA
GATCGTCGCCTTGGTGAGCCGTTACCTCACCAACTAGCTAATCTTGCGCGTGCCCATCTCTATCCACCGTAGT
TTTCAATATCACCCGATGCCGAATAATATATTATGGGGTATTAATCTTCTTCGAAAGGCTATCCCCCCGATAA
AGGCAGGTTGCACACGTGTTTCCGCACCCGTACGCCGCTCTCAATTACCCGAAAGTAATCTACCGCTCGGCTT
GCATGTGTTAGGCCTCCCGCTAGCGTTCATCCTGAGCCAGGATCAAACTCT
>236
CTGCTGCCGCCCGTAGGAGTCTGGTCCGTGTCTCAGTACCAGTGTGGGGGATCACCCTCTCAGGCCCCCTAAA
GATCGTCGCCTTGGTGAGCCGTTACCTCACCAACTAGCTAATCTTGCGCGTGCCCATCTCTATCCACCGTAGT
TTTCAATATCACCCGATGCCGAATAATATATTATGGGGTATTAATCTTCTTCGAAAGGCTATCCCCCTGATAA
AGGCAGGTTGCACACGTGTTCCGCACCCGTACGCCGCTCTCAATTTACCCGAAAGTAATCTACCGCTCCGGCT
TGCATGTGTTAGGCCCCTCGCTAGCGTTCATCCTGAGCCAGGATCAAACTCT
>237
CTGCTGCCGCCCGTAGGAGTCTGGTCCGTGTCTCAGTACCAGTGTGGGGGATCACCCTCTCAGGCCCCCTAAA
AGATCGTCGCCTTGGTGAGCCGTTACCTCACCAACTAGCTAATCTTGCGCGTGCCCATCTCTATCCACCGTAG
TTTTCAATATCACCCGATGCCGAATAATATATTATGGGGGTATTAATCTTCTTCGAAAGGCTATCCCCCTGAT
AAAGGCAGGTTGCACACGTGTTCCGCACCCGTACGCCGCTCTCAATTACCCGAAAGTAATCTACCGCTCGGCT
TGCATGTGTTAGGCCTCCCGCTAGCGTTCTCCTGAGCAGGATCCAACT
>238
CTGCTGCCGCCCGTAGGAGTCTGGTCCGTGTCTCAGTACCAGTGTGGGGGATCACCCTCTCAGGCCCCCTAAA
GATCGTCGCCTTGGTGAGCCGTTTACCTCACCAACTAGCTAATCTTGCGCGTGCCCATCTCTATCCACCGTAG
TTTTCAATATCACCCGATGCCGAATAATATATTATGGGGTATTAATCTTCTTCGAAAGGCTATCCCCCTGATA
AAGGCAGGTTGCACACGTGTTCCGCACCCGTACGCCGCTCTCAATTACCCGAAAGTAATCTACCGCTCCGGCT
TGCATGTGTTAGGCCTCCCGCTAGCGTTCATCCTGAGCCAGGATCAACTCT
>239
CTGCTGCCGCCCGTAGGAGTCTGGTCCGTGTCTCAGTACCAGTGTGGGGGATCACCCTCTCAGGCCCCCTAAA
AGATCGTCGCCTTGGTGAGCCGTTTACCTCACCAACTAGCTAATCTTGCGCGTGCCCATCTCTATCCACCGTA
GTTTTCAATATCACCCGATGCCGAATAATATATTATGGGGTATTAATCTTCTTCGAAAGGCTATCCCCCTGAT
AAAGGCAGGTTGCACACGTGTTCCGCACCCGTACGCCGCTCTCAATTACCCGAAAGTAATCTACCGCTCGGCT
TGCATGTGTTAGGCCTCCCGCTAGCGTTCATCCTGAGCCAGGATCAAACTCT
>240
CTGCTGCCCGCCCGTAGGAGTCTGGTCCCGTGTCTCAGTACCAGTGTGGGGGATCACCCCTCTCAGGCCCCCC
TAAAGATCGTCGCCTTGGTGAGCCCGTTACCTCACCCAACTAGCTAATCTTGCGCGTGCCCCATCTCTATCCA
CCGTAGTTTTCAATATCACCCGATGCCCGAATAATATATTATGGGGTATTAATCTTCTTCGAAAGGCTATCCC
CTGATAAAGGCAGGTTGCACACGTGTTCCGCACCCGTACGCCGCTCTCAATTACCCCGAAAGTAATCTACCGC
TCGGCTTGCATGTGTTAGGCCTCCCGCTAGCGTTCTCCTGAG
>241
CTGCTGCCGCCCGTAGGAGTCTGGTCCGTGTCTCAGTACCAGTGTGGGGGATCACCCTCTCAGGCCCCCTAAA
GATCGTCGCCTTGGTGAGCCGTTACCTCACCAACTAGCTAATCTTGCGCGTGCCCATCTCTATCCACCGTAGT
TTTCAATATCACCCGATGCCGAATAATATATTATGGGGTATTAATCTTCTTCGAAAGGCTATCCCCCTGATAA
AGGCAGGTTGCACACGTGTTCCGCACCCGTACGCCGCTCTCAATTACCCGAAAGTAATCTACCGCTCGGCTTG
CATGTGTTAGGCCTCCCGCTAGCGTTCTCCTCGAGCAGGAT
>242
CTGCTGCCGCCCGTAGGAGTCTGGTCCGTGTCTCAGTACCAGTCGTGGGGGATCACCCTCTCAGGCCCCCTAA
AGATCGTCGCCTTGGTGAGCCGTTACCTCACCAACTAGCTAATCTTGCGCGTGCCCATCTCTATCCACCGTAG
TTTTCAATATCACCCGATGCCGAATAATATATTATGGGGTATTAATCTTCTTCGAAAGGCTATCCCCCTGATA
AAGGCAGGTTGCACACGTGTTCCGCACCCGTACGCCGCTCTCAATTACCCGAAAGTAATCTACCGCTCGGCTT
GCATGTGTTAGGCCTCCCGCTAGCGTTCTCCTGAGCAGGATCAACTC
>243
CTGCTGCCGCCCGTAGGAGTCTGGTCCGTGTCTCAGTACCAGTGTGGGGGATCACCCTCTCAGGCCCCCTAAA
GATCGTCGCCTTGGTGAGCCGTTACCTCACCAACTAGCTAATCTTGCGCGTGCCCATCTCTATCCACCGTAGT

TTTCAATATCACCCGATGCCGAATAATATATTATGGGGTATTAATCTTCTTCGAAAGGCTATCCCCCTGATAA
AGGCAGGTTGCACACGTGTTCCGCACCCGTACGCCGCTCTCAATTACCCGAAAGTAATCTACCGCTCGGCTTG
CATGTGTTAGGCCTCCCGCTAGCGTTCTCCTGAGCAGGATCCAACTCT
>244
CTGCTGCCGCCCCGTAGGAGTCTGGTCCGTGTCTCAGTACCAGTGTGGGGGATCACCCTCTCAGGCCCCCTAA
AGATCGTCGCCTTGGTGAGCCGTTACCTCACCAACTAGCTAATCTTGCGCGTGCCCATCTCTATCCACCGTAG
TTTTCAATATCACCCGATGCCGAATAATATATTATGGGGTATTAATCTTCTTCGAAAGGCTATCCCCCTGATA
AAGGCAGGTTGCACACGTGTTCCGCACCCGTACGCCGCTCTCAATTACCCGAAAGTAATCTACCGCTCGGCTT
GCATGTGTTAGGCCTCCCGCTAGCGTTCATCCTGAGCCAGGATCAAACTCT
>245
CTGCTGCCGCCCGTAGGAGTCTGGACCGTGTCTCAGTTCCAGTGTGACTGATCATCCTCTCAGACCAGTACGG
ATCGTCGCCTTGGTAGGCCTTTACCCCACCAACTAGCTAATCCGACCTAGGCTCATCTGATAGCGTGAGGTCC
GAAGATCCCCCACTTCTCCCTCAGGACGTATGCGGTATTAGCGCCCGTTTCCGGACGATCCCCACTACCAGGC
AGATTCCTAGGCATTACTCACCCGTCCGCCGCTGAATCCAGGAGCAAGCTCCCCTTCATCCGCTCGACTGCAT
GTGTTAGGCCTGCCGCCAGCGTCAATCTGAGCCCAGGATCAAACTCT
>246
CTGCTGCCGCCCGTAGGAGTCTGGTCCGTGTCTCAGTACCAGTGTGGGGGATCACCCTCTCAGGCCCCCTAAA
GATCGTCGCCTTGGTGAGCCGTTACCTCACCAACTAGCTAATCTTGCGCGTGCCCATCTCTATCCACCGTAGT
TTCAATATCACCCGATGCCGAATAATATATTATGGGGTATTAATCTTCTTCGAAAGGCTATCCCCCTGATAAA
GGCAGGTTGCACACGTGTTCCGCACCCGTACGCCGCTCTCAATTACCCGAAAGTAATCTACCGCTCGGCTTGC
ATGTGTTAGGCCCTCCGCTAGCGTTCATCCTGAGCCAGGATCAAACTCT
>247
CTGCTGCCGCCCGTAGGAGTCTGGTCCGTGTCTCAGTACCAGTGTGGGGGATCACCCTCTCAGGCCCCCTAAA
GATCGTCGCCTTGGTGAGCCGTTACCTCACCAACTAGCTAATCTTGCGCGTGCCCATCTCTATCCACCGTAGT
TTTCAATATCACCCGATGCCGAATAATATATTATGGGGTATTAATCTTCTTCGAAAGCTATCCCCCTGATAAA
GGCAGGTTGCACACGTGTTCCGCACCCGTACGCCGCTCTCAATTACCCGAAAGTAATCTACCGCTCGGCTTGC
ATGTGTTAGGCCTCCCGCTAGCGTTCTCCTGAGCAGGATTCAACT
>248
CTGCTGCCGCCCGTAGGAGTCTGGTTCCGTGTCTCAGTACCAGTGTGGGGGATCACCCTCTCAGGCCCCCTAA
AGATCGTCGCCTTGGTGAGCCGTTACCTCACCAACTAGCTAATCTTGCGCGTGCCCATCTCTATCCACCGTAG
TTTCAATATCACCCGATGCCGAATAATATATTATGGGGTATTTAATCTTTCTTCGAAAGGCTATCCCCCTGAT
AAAGGCAGGTTTGCACACGTGTTCCGCACCCGTACGCCGCTCTCAATTACCCGAAAGTAATCTACCGCTCGGC
TTGCATGTGTTAGGCCTCCCGCTAGCGTTCATCCTGAGCCAGGATCAAACTCT
>249
CTGCTGCCGCCCGTAGGAGTCTGGTCCGTGTCTCAGTACCAGTGTGGGGGATCACCCTCTCAGGCCCCCTAAA
GATCGTCGCCTTGGTGAGCCGTTACCTCACCAACTAGCTAATCTTGCGCGTGCCCATCTCTATCCACCGTAGT
TTTCAATATCACCCGATGCCGAATAATATATTATGGGGTATTAATCTTCTTCGAAAGGCTATCCCCTGATAAG
GCAGGTTGCACACGTGTTCCGCACCCGTACGCCGCTCTCAATTACCCGAAAGTAATCTACCGCTCGGCTTGCA
TGTGTTAGGCCTCCCGCTAGCGTTCATTCCGT
>250
CTGCTGCCGCCCGTAGGAGTCTGGTCCGTGTCTCAGTACCAGTGTGGGGGATCACCCTCTCAGGCCCCCTAAA
GATCGTCGCCTTGGTGAGCCGTTACCTCACCAACTAGCTAATCTTGCGCGTGCCCATCTCTATCCACCGTAGT
TTCAATATCACCCGATGCCGAATAATATATTATGGGGTATTAATCTTCTTCGAAAGCTATCCCCTGATAAAGG
CAGGTTGCACACGTGTTCCCGCACCGTACGCCGCTCTCAATTACCCGAAAGTAATCTACCGCTCCGGCTTGCA
TGTGTTAGGCCTCCCGCTAGCGTTCATCCTGAGCCAGGATAAACTCT
>251
CTGCTGCCGCCCGTAGGAGTCTGGTCCGTGTCTCAGTACCAGTGTGGGGGATCACCCTCTCAGGCCCCCTAAA
GATCGTCGCCTTGGTGAGCCGTTACCTCACCAACTAGCTAATCTTGCGCGTGCCCATCTCTATCCACCGTAGT
TTCAATATCACCCGATGCCGAATAATATATTATTGGGGTATTAATCTTCTTCGAAAGGCTATCCCCCTGATAA
AGGCAGGTTGCACACGTGTTCCGCACCCGTACGCCGCTCTCAATTACCCGAAAGTAATCTACCGCTCCGGCTT
GCATGTGTTAGGCCTCCCGCTAGCGTTCATCCTGAGCCAGGATCAACTCT
>252
CTGCTGCCGCCCGTAGGAGTCTGGTCCGTGTCTCAGTACCAGTGTGGGGGATCACCCTCTCAGGCCCCCTAAA
GATCGTCGCCTTGGTGAGCCGTTACCTCACCAACTAGCTAATCTTGCGCGTGCCCATCTCTATTCCACCGTAG

TTTCAATATCACCCGATGCCGAATAATATATTTATGGGGTATTAATCTTCTTCGAAAGGCTATCCCCCTGATA
AAGGCAGGTTGCACACGTGTTCCGCACCCGTACGCCGCTCTCAATTACCCGAAAGTAATCTACCGCTCGGCTT
GCATGTGTTAGGCCTCCCGCTAGCGTTCATCCTGAGCCAAGGATCAAACTCT
>253
CTGCTGCCGCCCGTAGGAGTCTGGTCCGTGTCTCAGTACCAGTGTGGGGGATCACCCTCTCAGGCCCCCTAAA
GATCGTCGCCTTGGTGAGCCGTTACCTCACCAACTAGCTAATCTTGCGCGTGCCCATCTCTATCCACCGTAGT
TTTCAATATCACCCGATGCCGAATAATATATTATTGGGGTATTTAATCTTCTTCGAAAGGCTATCCCCTGATA
AGGCAGGTTGCACACGTGTTCCGCACCCGTACGCCGCTCTCAATTACCCGAAAGTAATCTACCGCTCGGCTTG
CATGTGTTAGGCCTCCCGCTAGCGTTCATTCCTGAGCCAGGATCAACTCT
>254
CTGCTGCCGCCCGTAGGAGTCTGGTCCGTGTCTCAGTACCAGTGTGGGGGATCACCCTCTCAGGCCCCCTAAA
GATCGTCGCCTTGGTGAGCCGTTACCTCACCAACTAGCTAATCTTGCGCGTGCCCATCTCTATCCACCGTAGT
TTTCAATATCACCCGATGCCGAATAATATATTATGGGGTATTAATCTTTCTCGAAAGGCTATCCCCTGATAAA
GGCAGGTTGCACACGTGTTCCGCACCCGTACGCCGCTCTCAATTACCCGAAAGTAATCTACCGCTCGGCTTGC
ATGTGTTAGGCCTCCCGCTAGCGTTCATCCTGAGCCAGGA
>255
CTGCTGCCGCCCGTAGGAGTCTGGTCCGTGTCTCAGTACCAGTGTGGGGATCACCCTCTCAGGCCCCCTAAAG
ATCGTCGCCTTGGTGAGCCGTTACCTCACCAACTAGCTAATCTTGCGCGTGCCCATCTCTATCCACCGTAGTT
TCAATATCACCCGATGCCGAATAATATATTATGGGGTATTAATCTTCCTCTCGAAAGGCTATCCCCCTGATAA
AGGCAGGTTGCACACGTGTTCCGCACCCGTACGCCGCTCTCAATTACCCGAAAGTAATCTACCGCTCGGCTTG
CATGTGTTAGGCCTCCCGCTAGCGTCATCCTGAGCCAGGATCAAACTCT
>256
CTGCTGCCGCCCGTAGGAGTCTGGTCCCGTGTCTCAGTACCAGTGTGGGGGATCACCCTCTCAGGCCCCCTAA
AAGATCGTCGCCTTGGTGAGCCGTTACCTCACCCAACTAGCTAATCTTGCGCGTGCCCCATCTTCTATCCACC
GTAGTTTTTCAATATCACCCGATGCCCGAATAATATATTTATGGGGTATTTAATTCTTTCTTCGAAAGGCTAT
CCCCCTGATAAAGGCAGGTTGCACACGTGTTCCGCACCCGTACGCCGCTCTCAATTACCCCGAAAGTAATCTA
CCGCTCGGCTTGCATGTGTTAGGCCTTCCCGCTAGCGTTCTCCTGAGCAGGATCAACTCGT
>257
CTGCTGCCGCCCGTAGGAGTCTGGTCCGTGTCTCAGTACCAGTGTGGGGGATCACCCTCTCAGGCCCCCTAAA
GATCGTCGCCTTGGTGAGCCGTTACCCCACCAACATCAGCTAATCTTGCGCGTGCCCCATCTCTATCCACCGT
AGTTTTTCAATATTCACCCGATGCCGAATAATATATTATGGGGTATTAATCTTCTTCGAAAGGCTATCCCCCT
GATAAAGGCAGGTTTGCACACGTGTTCCGCACCCGTACGCCGCTCTCAATTACCCCGAAAGTAATCTACCGCT
CGGCTTGCATGTGTTAGGCCTTCCCGCTAGCGTTCTCCT
>258
CTGCTGCCGCCCGTAGGAGTCTGGACCGTGTCTCAGTTCCAGTGTGACTGATCATCCTCTCAGACCAGTACGG
ATCGTCGCCTTGGTAGGCCCTTTACCCCCACCAACTAGCTAATCCGACCTAGGCTCATCTGATAGCGTGAGGT
CCCGAAGATCCCCACTTCTCCCCTCAGGACGTATGCGGTATTAGCGCCCGTTTCCGGACGTATCCCCACTACC
CAGGCAGATTCCTAGGCATTACTCACCCGTCCGCCGCTGAATCCAGGAGCAAGCTCCCCTCATCCGCTCGACT
GCATGTGTTAGGCCTGCCGCCAGCGTCAATTCTGAGCCAGGATCAAACTCT
>259
CTGCTGCCGCCCGTAGGAGTCTGGACCGTGTCTCAGTTCCAGTGTGACTGATCATCCTCTCAGACCAGTACGG
ATCGTCGCCTTGGTAGGCCTTTACCCCACCAACTAGCTAATCCGACCTAGGCTCATCTGATAGCGTGAGGTCC
GAAGTCCCCCACTTCTCCCTCAGGACGTATGCGGTATTAGCGCCCGTTTCCGGACGATCCCCCACTACCAGGC
AGATTCCTAGGCATTACTCACCCGTCCGCCGCTGAATCCAGGAGCAAGCTCCCTTCATCCGCTCGACTGCATG
TGTTAGGCCTGCCGCCAGCGTTCAATCTGAGCCAGGATCAAACTCT
>260
CTGCTGCCGCCCGTAGGAGTCTGGTCCGTGTCTCAGTACCAGTGTGGGGGATCACCCTCTCAGGCCCCCTAAA
GATCGTCGCCTTGGTGAGCCGTTACCTCACCAACTAGCTAATCTTGCGCGTGCCCATCTCTATCCACCGTAGT
TTTCAATATCACCCGATGCCGAATAATATATTATGGGGTATTAATCTTCTTCGAAAGGCTATCCCCCTGATAA
AGGCAGGTTGCACACGTGTTCCGCACCCGTACGCCGCTCTCAATTTACCCGAAAGTAATCTACCGCTCGGCTT
GCATGTGTTAGGCCTCCCGCTAGCGTTCATCCTGAGCCAGGATCAACTCT
>261
CTGCTGCCGCCCGTAGGAGTCTGGTCCGTGTCTCAGTACCAGTGTGGGGGATCACCCTCTCAGGCCCCCTAAA
GATCGTCGCCTTGGTGAGCCGTTACCTCACCAACTAGCTAATCTTGCGCGTGCCCATCTCTATCCACCGTAGT

TTTCAATATCACCCGATGCCGAATAATATATTATGGGGTATTAATCTTCTTCGAAAGGCTATCCCCCTGATAA
AGGCAGGTTGCACACGTGTTCCGCACCCGTACGCCGCTCTCAATTACCCGAAAGTAATCTACCGCTCGGCTTG
CATGTGTTAGGCCTCCCGCTAGCGTTCTCCTGAGCAGGATCAACTCGT
>262
CTGCTGCCGCCCGTAGGAGTCTGGTCCGTGTCTCAGTACCAGTGTGGGGGATCACCCTCTCAGGCCCCCTAAA
GATCGTCGCCTGGTGAGCCGTTACCTCACCAACTAGCTAATCTTGCGCGTGCCCATCTCTATCCACCGTAGTT
TTCAATATCACCCGATGCCGAATAATATATTATGGGGTATTAATCTTCTTCGAAAGGCTATCCCCCTGATAAA
GGCAGGTTAGCACACGTGTTCCGCACCCGTACGCCGCTCTCAATTACCCGAAAGTAATCTGCCGCTCGGCTTG
CATGTGTTAGGCCTCCCGCTAGCGTTCTCCTGAGC
>263
CTGCTGCCGCCCGTAGGAGTCTGGTCCGTGTCTCAGTACCAGTGTGGGGGATCACCCTCTCAGGCCCCCTAAA
GATCGTCGCCTTGGTGAGCCGTTACCTCACCAACTAGCTAATCTTGCGCGTGCCCATCTCTATCCACCGTAGT
TTTCAATATCACCCGATGCCGAATAATATATTATGGGGTATTAATCTTCTTCGAAAGGCTATCCCCCTGATAA
AGGCAGGTTGCACACGTGTTCCGCACCCGTACGCCGCTCTCAATTACCCGAAAGTAATCTACCGCATCGGCTT
GCATGTGTTAGGCCTCCCGCTAGCGTTCTCCTGAGCAGGATCAAACTCGT
>264
CTGCTGCCGCCCGTAGGAGTCTGGTCCGTGTCTCAGTACCAGTGTGGGGGATCACCCTCTCAGGCCCCCCTAA
AAGATCGTCGCCTGGTGAGCCGTTTACCTCACCAACTAGCTAATCTTGCGCGTGCCCATCTCTATCCACCGTA
GTTTTCAATATCACCCGATGCCGAATAATATATTATGGGGTATTAATCTTCTTCGAAAGGCTATCCCCCTGAT
AAAGGCAGGTTGCACACGTGTTCCGCACCCGTACGCCGCTCTCAATTTACCCGAAAGTAATCTACCGCTCGGC
GCATGTGTTTGAGGCCTCCCGCTAGCGTTCTCCTGAGCAG
>265
CTGCTGCCGCCCGTAGGAGTCTGGTCCGTGTCTCAGTACCAGTGTGGGGGATCACCCTCTCAGGCCCCCTAAA
GATCGTCGCCTTGGTGAGCCGTTTACCTCACCAACTAGCTAATCTTGCGCGTGCCCATCTCTATCCACCGTAG
TTTTCAATATCACCCGATGCCGAATAATATATTATGGGGTATTAATCTTCTTCGAAAGGCTATCCCCCTGATA
AAGGCAGGTTGCACACGTGTTCCGCACCCGTACGCCGCTCTCAATTACCCGAAAGTAATCTACCGCTCGGCTT
GCATGTGTTAGGCCTCCCGCTAGCGTCTCCTGAG
>266
CTGCTGCCGCCCGTAGGAGTCTGGTCCGTGTCTCAGTACCAGTGTGGGGGATCACCCTCTCAGGCCCCCTAAA
GATCGTCGCCTTGTGAGCCGTTACCTCACCAACTAGCTAATCTTGCGCGTGCCCATCTCTATCCACCGTAGTT
TTCAATATCACCCGATGCCGAATAATATATTATGGGGTATTAATCTTCCTCTCGAAAGGCTATCCCCCTGATA
AAGGCAGGTTGCACACGTGTTCCGCACCCGTACGCCGCTCTCAATTACCCGAAAGTAATCTACCGCTCGGCTT
GCATGTGTTAGGCCTCCCGCTAGCGTTCATCCTGAGCCAGGATCAAACTCT
>267
CTGCTGCCGCCCGTAGGAGTCTGGTCCGTGTCTCAGTACCAGTGTGGGGGATCACCCTCTCAGGCCCCCTAAA
GATCGTCGCCTTGGTGAGCCGTTACCTCACCAACTAGCTAATCTTGCGCGTGCCCATCTCTATCCACCGTAGT
TTTCAATATCACCCGATGCCGAATAATATATTATGGGGTATTAATCTTCTTCGAAAGGCTATCCCCCTGATAA
AGGCAGGTTGCACACGTGTTCCGCACCCGTACGCCGCTCTCAATTACCCGAAAGTAATCTACCGCTCGGCTTG
CATGTGTTAGGCCTCCCGCTAGCGTTCATCCTAGCAGGATCAACTCT
>268
CTGCTGCCGCCCGTAGGAGTCTGGTCCGTGTCTCAGTACCAGTGTGGGGGATCACCCTCTCCAGGCCCCCTAA
AGATCGTCGCCTTGGTGAGCCGTTACCTCACCAACTAGCTAATCTTGCGCGTGCCCATCTCTATCCACCGTAG
TTTTCAATATCACCCGATGCCGAATAATATATTATGGGGGTATTAATCTTCTTCGAAAGGCTATCCCCCTGAT
AAAGGCAGGTTGCACACGTGTTCCGCACCCGTACGCCGCTCTCAATTACCCGAAAGTAATCTACCGCTCGGCT
TGCATGTGTTAGGCCTCCCGCTAGCGTTCTCCTGAGCAGGATCAACTCGT
>269
CTGCTGCCGCCCGTAGGAGTCTGGTCCGTGTCTCAGTACCAGTGTGGGGGATCACCCTCTCAGGCCCCCTAAA
GATCGTCGCCTTGGTGAGCCGTTACCTCACCAACTAGCTAATCTTGCGCGTGCCCATCTCTATCCACCGTAGT
TTTCAATATCACCCGATGCCGAATAATATATTATGGGGGTATTAATCTTCTTCGAAAGGCTATCCCCCTGATA
AAGGCAGGTTGCACACGTGTTCCGCACCCGTACGCCGCTCTCAATTACCCGAAAGTAATCTACCGCTCGGCTT
GCATGTGTTAGGCCTCCCGCTAGCGTTCTCCTGAGCAGGATCAAC
>270
CTGCTGCCGCCCGTAGGAGTCTGGACCGTGTCTCAGTTCCAGTGTGACTGATCATCCTCTTAGACCAGTACGG
ATCGTCGCCTTGGTAGGCCTTACCCCACCAACTAGCTAATCCGACCTAGGCTCATCTGATAGCGTGAGGTCCG

AAGATCCCCCACTTCTCCCTCAGGACGCATGCGGTATTAGCGCCCGTTTCCGGACGATCCCCACTACCAGGCA
GATTCCTAGGCATTACTCACCCGTCCGCCGCTGAATCCAGGAGCAAGCTCCCTCATCCGCTCGACTGCATGTG
TTAGGCCTCCCGCTAGCGTTCTCCTGAGCCAGGATCAACTCT
>271
CTGCTGCCGCCCGTAGGAGTCTGGTCCGTGTCTCAGTACCAGTGTGGGGGATCACCCTCTCAGGCCCCCTAAA
GATCGTCGCCTTGGTGAGCCGTTACCTCACCAACTAGCTAATCTTGCGCGTGCCCATCTCTATCCACCGTAGT
TTTCAATATCACCCGATGCCGAATAATATATTATGGGGTATTAATCTTCTTCGAAAGGCTATCCCCCTGATAA
AGGCAGGTTGCACACGTGTTCCGCACCCGTACACCGCTCTCAATTACCCGAAAGTAATCTACCGCTCGGCTTG
CATGTGTTAGGCCTCCCGCTAGCGTTCATCCTGAGCCAGGATCAAACTCT
>272
CTGCTGCCGCCCGTAGGAGTCTGGTCCGTGTCTCAGTACCAGTGTGGGGGATCACCCTCTCAGGCCCCCTAAA
GATCGTCGCCTTGTGAGCCGTTACCTCACCAACTAGCTAATCTTGCGCGTGCCCATCTCTATCCACCGTAGTT
TTCAATATCACCCGATGCCGAATAATATATTATGGGGGTATTAATCTTCTTCGAAAGGCTATCCCCCTGATAA
AGGCAGGTTGCACACGTGTTCCGCACCCGTACGCCGCTCTCAATTACCCGAAAGTAATCTACCGCTCCGGCTT
GCATGTGTTAGGCCTCCCGCTAGCGTTCATCCTGAGCAGGATCAACTCT
>273
CTGCTGCCGCCCGTAGGAGTCTGGTCCGTGTCTCAGTACCAGTGTGGGGGATCACCCTCTCAGGCCCCCTAAA
GATCGTCGCCTTGGTGAGCCGTTACCTCACCAACTAGCTAATCTTGCGCGTGCCCATCTCTATCCACCGTAGT
TTTCAATATCAACCCGATGCCGAATAATATATTATGGGGGTATTAATCTTCTCGAAAGGCTATCCCCTGATAA
AGGCAGGTTGCACACGTGTTCCGCACCCGTACGCCGCTCTCAATTACCCGAAAGTAATCTACCGCTCGGCTTG
CATGTGTTAGGCCTCCCGCTAGCGTCTCCTGAGCCAGGATCAACTCGT
>274
CTGCTGCCGCCCGTAGGAGTCTGGTCCGTGTCTCAGTACCAGTGTGGGGGATCACCCTCTCAGGCCCCCTAAA
GATCGTCGCCTTGGTGAGCCGTTACCTCACCAACTAGCTAATCTTGCGCGTGCCCATCTCTATCCACCGTAGT
TTTCAATATCACCCGATGCCGAATAATATATTATGGGGTATTAATCTTCTTCGAAAGGCTATCCCCCTGATAA
AGGCAGGTTGCACACGTGTTCCGCACCCGTACGCCGCTCTCAATTACCCGAAAGTAATCTACCGCTCGGCTTG
CATGTGTTAGGCCTCCCGCTAGCGTTCATCCTGAGCCAGGATCAAACTCT
>275
CTGCTGCCGCCCGTAGGAGTCTGGTCCGTGTCTCAGTACCAGTGTGGGGGATCACCCTCTCAGGCCCCCTAAA
GATCGTCGCCTTGGTGAGCCGTTACCCTCAACCAACTAGCTAATCTTGCGCGTGCCCATCTCTATCCACCGTA
GTTTTCAATATCACCCGATGCCGAATAATATATTATGGGGTATTAATCTTCTTCGAAAGGCTATCCCCCTGAT
AAAGGCAGGTTGCACACGTGTTCCGCACCCGTACGCCGCTCTCAATTACCCGAAAGTAATCTACCGCTCGGCT
TGCATGTGTTAGGCCTCCCGCTAGCGTCTCCTGAGCAGGATCAACTCGT
>276
CTGCTGCCGCCCGTAGGAGTCTGGTCCGTGTCTCAGTACCAGTGTGGGGGATCACCCTCTCAGGCCCCCTAAA
GATCGTCGCCTTGGTGAGCCGTTACCTCACCAACTAGCTAATCTTGCGCGTGCCCATCTCTATCCACCGTAGT
TTTCAATATCACCCGATGCCGAATAATATATTATGGGGTATTAATCTTCTTCGAAAGGCTATCCCCCTGATAA
AGGCAGGTTGCACACGTGTTCCGCACCCGTACGCCGCTCTCAATTACCCGAAAGTAATCTACCGCTCGGCTTG
CATGTGTTAGGCCTCCCGCTAGCGTCATCCTGAGCCAGGATCAAACTCT
>277
CTGCTGCCGCCCGTAGGAGTCTGGTCCGTGTCTCAGTACCAGTGTGGGGGATCACCCTCTCAGGCCCCCTAAA
GATCGTCGCCTTGGTGAGCCGTTACCTCACCAACTAGCTAATCTTGCGCGTGCCCATCTCTATCCACCGTAGT
TTTCAATATCACCCGATGCCGAATAATATATTATGGGGTATTAATCTTCTTCGAAAGGCTATCCCCCTGATAA
AGGCAGGTTGCACACGTGTTCCGCACCCGTACGCCGCTCTCAATTACCCGAAAGTAATCTACCGCTCGGCTTG
CATGTGTTAGGCCTCCCGCTAGCGTTCATCCTGAGCCAGGATCAAACTCT
>278
CTGCTGCCGCCCGTAGGAGTCTGGTCCGTGTCTCAGTACCAGTGTGGGGGATCACCCTCTCAGGCCCCCTAAA
GATCGTCGCCTTGGTGAGCCGTTACCTCACCAACTAGCTAATCTTGCGCGTGCCCATCTCTATCCACCGTAGT
TTTCAATATCACCCGATGCCGAATAATATATTATGGGGTATTAATCTTCTTCGAAAGGCTATCCCCCTGATAA
AGGCAGGTTGCACACGTGTTCCGCACCCGTACGCCGCTCTCAATTACCCGAAAGTAATCTACCGCTCGGCTTG
CATGTGTTAGGCCTCCCGCTAGCGTTCATCCTGAGCCAGGATCAAACTCT
>279
CTGCTGCCGCCCGTAGGAGTCTGGTCCGTGTCTCAGTACCAGTGTGGGGGATCACCCTCTCAGGCCCCCTAAA
GATCGTCGCCTTGGTGAGCCGTTACCTCACCAACTAGCTAATCTTGCGCGTGCCCATCTCTATCCACCGTAGT

TTTCAATATCACCCGATGCCGAATAATATATTATGGGGTATTAATCTTCTTCGAAAGGCTATCCCCCTGATAA
AGGCAGGTTGCACACGTGTTCCGCACCCGTACGCCGCTCTCAATTACCCGAAAGTAATCTACCGCTCGGCTTG
CATGTGTTAGGCCTCCCGCTAGCGTTCATCCTGAGCCAGGATCAAACTCT
>280
CTGCTGCCGCCCGTAGGAGTCTGGTCCGTGTCTCAGTACCAGTGTGGGGGATCACCCTCTCAGGCCCCCTAAA
GATCGTCGCCTTGGTGAGCCGTTACCTCACCAACTAGCTAATCTTGCGCGTGCCCATCTCTATCCACCGTAGT
TTTCAATATCACCCGATGCCGAATAATATATTATGGGGTATTAATCTTCTTCTGAAAGGCTATCCCCTGATAA
AGGCAGGTTGCACACGTGTTCCGCACCCGTACGCCGCTCTCAATTACCCGAAAGTAATCTACCGCTCCGGCTT
GCATGTGTTAGGCCTCCCGCTAGCGTTCATCCTGAGCCAGGATCAAACTCT
>281
CTGCTGCCGCCCGTAGGAGTCTGGTCCGTGTCTCAGTACCAGTGTGGGGGATCACCCTCTCAGGCCCCCTAAA
GATCGTCGCCTTGGTGAGCCGTTACCTCACCAACTAGCTAATCTTGCGCGTGCCCATCTCTATCCACCGTAGT
TTTCAATATCACCCGATGCCGAATAATATATTATGGGGTATTAATCTTCTTCGAAAGGCTATCCCCCTGATAA
AGGCAGGTTGCACACGTGTTCCGCACCCGTACGCCGCTCTCAATTACCCGAAAGTAATCTACCGCTCGGCTTG
CATGTGTTAGGCCTCCCGCTAGCGTTCTCCTGAGCAGGATCAAC
>282
CTGCTGCCGCCCGTAGGAGTCTGGTCCGTGTCTCAGTACCAGTGTGGGGGATCACCCTCTCAGGCCCCCTAAA
GATCGTCGCCTGGTGAGCCGTTACCTCACCAACTAGCTAATCTTGCGCGTGCCCATCTCTATCCACCGTAGTT
TTCAATATCACCCGATGCCGAATAATATATTATGGGGTATTAATCTTCTTCGAAAGGCTATCCCCCTGATAAA
GGCAGGTTGCACACGTGTTCCGCACCCGTACGCCGCTCTCAATTACCCGAAAGTAATCTACCGCTCGGCTTGC
ATGTGTTAGGCCTCCCGCTAGCGTTCATCCTGAGCCAGGATCAAACTCT
>283
CTGCTGCCGCCCGTAGGAGTCTGGTCCGTGCCTCAGTACCAGTGTGGGGGATCACCCTCTCAGGCCCCCTAAA
GATCGTCGCCTTGGTGAGCCGTTACCTCACCAACTAGCTAATCTTGCGCGTGCCCATCTCTATCCACCGTAGT
TTTCAATATCACCCGATGCCGAATAATATATTATGGGGCATTAATCTTCTTCGAAAGGCTATCCCCCTGATAA
AGGCAGGTTGCACACGTGTTCCGCACCCGTACGCCGCTCTCAATTACCCGAAAGTAATCTACCGCTCGGCTTG
CATGTGTTAGGCCTCCCGCTAGCGTTCTCCTGAGCCAGGATCAACTCT
>284
CTGCTGCCGCCCGTAGGAGTCTGGTCCGTGTCTCAGTACCAGTGTGGGGGATCACCCTCTCAGGCCCCCTAAA
GATCGTCGCCTTGGTGAGCCGTTACCTCACCAACTAGCTAATCTTGCGCGTGCCCATCTCTATCCACCGTAGT
TTTCAATATCACCCGATGCCGAATAATATATTATGGGGTATTAATCTTCTTCGAAAGGCTATCCCCCTGATAA
AGGCAGGTTGCACACGTGTTCCGCACCCGTACGCCGCTCTCAATTTACCCGAAAGTAATCTACCGCTCGGCTT
GCATGTGTTAGGCCTCCCGCTAGCGTTCATCCTGAGCCAGGATCAACTCT
>285
CTGCTGCCGCCCGTAGGAGTCTGGTCCGTGTCTCAGTACCAGTGTGGGGGATCACCCTCTCAGGCCCCCTAAA
GATCGTCGCCTTGGTGAGCCGTTACCTCACCAACTAGCTAATCTTGCGCGTGCCCATCTCTATCCACCGTAGT
TTTCAATATCACCCGATGCCGAATAATATATTATGGGGTATTAATCTTCTTCGAAAGGCTATCCCCCTGATAA
AGGCAGGTTGCACACGTGTTCCGCACCCGTACGCCGCTCTCAATTACCCGAAAGTAATCTACCGCTCGGCTTG
CATGTGTTAGGCCTCCCGCTAGCGTTCATCCTGAGCCAGGATCAAACTCT
>286
CTGCTGCCGCCCGTAGGAGTCTGGTCCGTGTCTCAGTACCAGTGTGGGGGATCACCCTCTCAGGCCCCCTAAA
CGATCGTCGCCTTGGTGAGCCGTTACCTCACCAACTAGCTAATCTTGCGCGTGCCCATCTCTATCCACCGTAG
TTTCAATATCACCCGATGCCGAATAATATATTATGGGGTATTAATCTTCTCTGAAAGCTATCCCCCTGATAAA
GGCAGGTTGCACACGTGTTCCGCACCCGTACGCCGCTCTCAATTACCCGAAAGTAATCTACCGCTCGGCTTGC
ATGTGTTAGGCCTCCCGCTAGCGTTCTCCTGAGCAGGATCAACTCGT
>287
CTGCTGCCGCCCGTAGGAGTCTGGTCCGTGTCTCAGTACCAGTGTGGGGGATCACCCTCTCAGGCCCCCTAAA
GATCGTCGCCTTGGTGAGCCGTTACCTCACCAACTAGCTAATCTTGCGCGTGCCCATCTCTATCCACCGTAGT
TTTCAATATCACCCGATGCCGAATAATATATTATGGGGTATTAATCTTCTCGAAAGGCTATCCCCTGATAAAG
GCAGGTTGCACACGTGTTCCGCACCCGTACGCCGCTCTCAATTACCCGAAAGTAATCTACCGCTCGGCTTGCA
TGTGTTAGGCCTCCCGCTAGCGTTCATCCTGAGCCAGGAT
>288
CTGCTGCCGCCCGTAGGAGTCTGGTCCGTGTCTCAGTACCAGTGTGGGGGATCACCCTCTCAGGCCCCCTAAA
GATCGTCGCCTTGGTGAGCCGTTACCTCACCAACTAGCTAATCTTGCGCGTGCCCATCTCTATCCACCGTAGT

TTTCAATATCACCCGATGCCGAATAATATATTATGGGGTATTAATCTTCCTTCGAAATGCTATCCCCCTGATA
AAGGCAGGTTGCACACGTGTTCCGCACCCGTACGCCGCTCTCAATTACCCGAAAGTAATCTACCGCTCGGCTT
GCATGTGTTAGGCCTCCCGCTAGCGTTCATCCTGAGCCAGGATCAACTCT
>289
CTGCTGCCGCCCGTAGGGGTCTGGACCGTGTCTCAGTTCCAGTGTGACTGATCATCCTCTCAGACCAGTTACG
GATCGTCGCCTTGGTAGGCCTTTACCCACCAACTAGCTAATCCGACCTAGGCTCATCTGATAGCGTGAGGTCC
GAAGATTCCCCACTTCTCCCTCAGGACGTATGCGGTATTAGCGCCCGTTTCCGGACGTATTCCCCACTACCAG
GCAGATTCCTAGGCATTTACTCACCCGTTCCGCCGCTGAATCCAGGAGCAAGCTCCCTTCATCCGCTCGACTG
CATGTGTTAGGCCTTGCCGCCAGCGTTCAATCTGAGCCAGGATCAAACTCT
>290
CTGCTGCCGCCCGTAGGAGTCTGGTCCGTGTCTCAGTACCAGTGTGGGGATCACCCTCTCAGGCCCCTTAAAG
ATCGTCGCCTTGGTGAGCCGTTACCTCACCAACTAGCTAATCTTGCGCGTGCCCATCTCTATCCACCGTAGTT
TCAATATCACCCGATGCCGAATAATATATTATGGGGTATTAATTCTTCTTCGAAAGGCTATCCCCTGATAAAG
GCAGGTTGCACACGTGTTCCGCACCCGTACGCCGCTCTCAATTACCCCGAAAGTAATCTACCGCTCGGCTTGC
ATGTGCTTAGGCCCTCCCGCTAGCGTTCATCCTGAGCCAGGATCAACTCT
>291
CTGCTGCCGCCCGTAGGAGTCTGGTTCCGTGTCTCAGTACCAGTGTGGGGGATCACCCTCTCAGGCCCCTAAA
GATCGTCGCCTTGGTGAGCCGTTACCTCACCAACTAGCTAATCTTGCGCGTGCCCATCTCTATCCACCGTAGT
TTTCAATATCACCCGATGCCGAATAATATATTATGGGGTATTAATCTTCTCGAAAGGCTATCCCCCTGATAAG
GCAGGTTGCACACGTGTTCCGCACCCGTACGCCGCTCTCAATTACCCGAAAGTAATCTACCGCTCCGGCTTGC
ATGTGTTAGGCCTCCCGCTAGCG
>292
CTGCTGCCGCCCGTAGGAGTCTGGTCCGTGTCTCAGTACCAGTGTGGGGGATCACCCTCTCAGGCCCCTAAAG
ATCGTCGCCTTGGTGAGCCGTTACCTCACCAACTAGCTAATCTTGCGCGTGCCCATCTCTATCCACCGTAGTT
TTCAATATCACCCGATGCCGAATAATATATTATGGGGTATTAATCTTCTTCGAAAGGCTATCCCCTGATAAAG
GCAGGTTGCACACGTGTTCCGCACCCGTACGCCGCTCTCAATTACCCGAAAGTAATCTACCGCTCGGCTTGCA
TGTGTTAGGCCTCCCGCTAGCGTTCATCCTGAGCCAGGATCAAACTCT
>293
CTGCTGCCGCCCCGTAGGAGTCTGGTCCGTGTCCCAGTACCAGTGTGGGGGATCACCCTCTCAGGCCCCCTAA
AGATCGTCGCCTTGGTGAGCCGTTACCTCACCAACTAGCTAATCTTGCGCGTGCCCATCTCTATCCACCGTAG
TTTTCAATATCACCCGATGCCGAATAATATATTATGGGGTATTAATCTTCTTCTGAAAGGCTATCCCCCTGAT
AAAGGCAGGTTGCACACGTGTTCCGCACCCGTACGCCGCTCTCAATTACCCGAAAGTAATCTACCGCTCGGCT
TGCATGTGTTAGGCCTCCCGCTAGCGTTCATCCTGAGCCAGGATCAAACTCT
>294
CTGCTGCTGCCCGTAGGAGTCTGGTCCGTGTCTCAGTACCAGTGTGGGGGATCACCCTCTCAGGCCCCCTAAA
GATCGTCGCCTTGGTGAGCCGTTACCTCACCAACTAGCTAATCTTGCGCGTGCCCATCTCTATCCACCGTAGT
TTTCAATATCACCCGATGCCGAATAATATATTTATGGGGTATTTAATCTTCTTCGAAAGGCTATCCCCCTGAT
AAAGGCAGGTTGCACACGTGTTCCGCACCCGTACGCCGCTCTCAATTACCCGAAAGTAATCTACCGCTCGGCT
TGCATGTGTTAGGCCTCCCGCTAGCGTTCTCCTGAGCAGGATCCAACTCGT
>295
CTGCTGCCGCCCGTAGGAGTCTGGTTCCGTGTCTCAGTACCAGTGTGGGGGATCACCCTCTCAGGCCCCCTAA
AGATCGTCGCCTTGGTGAGCCGTTACCTCACCAACTAGCTAATTCTTGCGCGTGCCCATCTCTATCCACCGTA
GTTTTTCAATATCACCCGATGCCGAATAATATATTTATTGGGGTATTTAATCTTTCTTCGAAAGGCTATCCCC
CTGATAAAGGCAGGTTTGCACACGTGTTCCGCACCCGTACGCCGCTCTCAATTACCCGAAAGTAATCTACCGC
TCGGCTTGCATGTGTTAGGCCTTCCCGCTAGCGTTCTTCCTGAGCAGGATC
>296
CTGCTGCCGCCCGTAGGAGTCTGGTTCCGTGTCTCAGTACCAGTGTGGGGGATCACCCTCTCAGGCCCCCTAA
AGATCGTCGCCTTGGTGAGCCGTTACCTCACCAACTAGCTAATCTTGCGCGTGCCCATCTCTTATCCACCGTA
GTTTTTCAATACCACCCGATGCCGAATAATTATATTTATGGGGTATTAATCTTTCCTCGAAAGCTATCCCCCT
GATAAAGGCAGGTTTGCACACGTGTTCCGCACCCGTACGCCGCTCTCAATTACCCGAAAGTAATCTACCGCTC
GGCTTGCATGTGTTTAGGCCTCCCGCTAGCGTTCATTCC
>297
CTGCTGCCGCCCGTAGGAGTCTGGTCCGTGTCTCAGTACCAGTGTGGGGGATCACCCTCTCAGGCCCCCTAAA
GATCGTCGCCTTGGTGAGCCGTTACCTCACCAACTAGCTAATCTTGCGCGTGCCCATCTCTATCCACCGTAGT

TTTCAATATCACCCGATGCCGAATAATATATTATGGGGTATTTAATCTTTCTTCGAAAGGCTATCCCCCTGAT
AAAGGCAGGTTTGCACACGTGTTCCGCACCCGTACGCCGCTCTCAATTACCCGAAAGTAATCTACCGCTCGGC
TTGCATGTGTTAGGCCTCCCGCTAGCGTTCATCCTGAGCC
>298
CTGCTGCCGCCCGTAGGAGTCTGGTCCGTGTCTCAGTACCAGTGTGGGGGATCACCCTCTCAGGCCCCTAAAG
ATCGTCGCCTTGTGAGCCGTTACCTCACCAACTAGCTAATCTTGCGCGTGCCCATCTCTATCCACCGTAGTTT
TCAATATCACCCGATGCCGAATAATATATTATGGGGTATTAATCTTCTTCGAAAGGCTATCCCCTGATAAAGG
CAGGTTGCACACGTGTTCCGCACCCGTACGCCGCTCTCAATTACCCGAAAGTAATCTACCGCTCGGCTTGCAT
GTGTTAGGCCTCCCGCTAGCGTTCATC
>299
CTGCTGCCGCCCGTAGGAGTCTGGTCCGTGTCTCAGTACCAGTGTGGGGGATCACCCTCTCAGGCCCCCTAAA
GATCGTCGCCTTGGTGAGCCGTTACCTCACCAACTAGCTAATCTTGCGCGTGCCCATCTCTATCCACCGTAGT
TTTCAATATCACCCGATGCCGAATAATATATTATGGGGTATTAATCTTCTTCGAAAGGCTATCCCCCTGATAA
GGCAGGTTGCACACGTGTTCCGCACCCGTACGCCGCTCTCAATTACCCGAAAGTAATCTACCGCTCGGCTTGC
ATGTGTTAGGCCTCCCGCTAGCGTTCATCCTGAGCCAGGATCAACTCT
>300
CTGCTGCCGCCCGTAGGAGTCTGGTCCGTGTCTCAGTACCAGTGTGGGGGATCACCCTCTCAGGCCCCCTAAA
GATCGTCGCCTTGGTGAGCCGTTACCTCACCAACTAGCTAATCTTGCGCGTGCCCATCTCTATCCACCGTAGT
TTCAATATCACCCGATGCCGAATAATATATTATTGGGGTATTAATTCTTCTTCGAAGGCTATCCCCCTGACAA
GGCAGGTTGCACACGTGTTCCGCACCCGTACGCCGCTCTCAATTACCCGAAAGTAATCTACCGCTCGGCTTGC
ATGTGTTAGGCCTCCCGCTAGCGTTCTCCTGAGCCAGGATCAACTCT
>301
CTGCTGCCGCCCGTAGGAGTCTGGTCCCGTGTCTCAGTACCAGTGTGGGGGATCACCCTCTCAGGCCCCCTAA
AGATCGTCGCCTGTGAGCCGTTACCTCACCAACTAGCTAATCTTGCGCGTGCCCATCTCTATCCACCGTAGTT
TTCAATATCACCCGATGCCGAATAATATATTATGGGGTATTAATCTTCTTCGAAAGGCTATCCCCCTGATAAA
GGCAGGTTGCACACGTGTTCCGCACCCGTACGCCGCTCTCAATTACCCGAAAGTAATCTACCGCTCGGCTTGC
ATGTGTTAGGCCTCCCGCTAGCGTTCTCCTGAGCAGGATCAACTC
>302
CTGCTGCCGCCCCGTAGGAGTCTGGTCCCGTGTCTCAGTACCAGTGTGGGGGATCACCCTCTCAGGCCCCCCT
AAAGATCGTCGCCTTGGTGAGCCCGTTACCTCACCCAACTAGCTAATCTTGCGCGTGCCCCATCTCTATCCAC
CGTAGTTTTCAATATCACCCGATGCCCGAATAATATATTATGGGGGTATTTAATTCTTCTTCGAAAGGCTATC
CCCCTGATAAAGGCAGGTTGCACACGTGTTCCGCACCCGTACGCCGCTCTCAATTACCCCGAAAGTAATCTAC
CGCTCGGCTTGCATGTGTTAGGCCTCCCGCTAGCGTTCATCCTGAGCCAGGATCAAACTCT
>303
CTGCTGCCGCCCGTAGGAGTCTGGTCCCGTGTCTCAGTACCAGTGTGGGGGATCACCCTCTCAGGCCCCCTAA
AGATCGTCGCCTTGGTGAGCCGTCACCTCACCAACTAGCTGATTCTTGCGCGTGCCCATCTCTATTCCACCGT
AGTTTCAATATCACCCGATGCCGAATAATATATTATGGGGTATTAATCTTCTTCGAAAGGCTATCCCCCTGAT
AAAGGCAGGTTGCACACGTGTTCCGCACCCGTACGCCGCTCTCAATTTACCCGAAAGTAATCTACCGCTCCGG
CTTGCATGTGTTAGGCCTCCCGCTAGCGTTCTCCTGAGCAGGTCCAAACTCGT
>304
CTGCTGCCGCCCGTAGGAGTCTGGTCCGTGTCCCAGTACCAGTGTGGGGGATCACCCTCTCAGGCCCCCTAAA
GATCGTCGCCTTGGTGAGCCGTTACCTCACCAACTAGCTAATCTTGCGCGTGCCCATCTCTATCCACCGTAGT
TTTCAATATCACCCGATGCCGAATAATATATTATGGGGTATTAATCTTCTCGAAAGGCTATCCCCCTGATAAA
GGCAGGTTGCACACGTGTTCCGCACCCGTACGCCGCTCTCAATTACCCGAAAGTAATCTACCGCTCGGCTTGC
ATGTGTTAGGCCTCCCGCTAGCGTTCTCCTGAGCCAGGATCAAACTCT
>305
CTGCTGCCGCCCGTAGGAGTCTGGTCCGTGTCTCAGTACCAGTGTGGGGGATCACCCTCTCAGGCCCCCTAAA
AGATCGTCGCCTTGGTGAGCCGTTACCTCACCAACTAGCTAATCTTGCGCGTGCCCATCTCTATCCACCGTAG
TTTTCAATATCACCCGATGCCGAATAATATATTATGGGGGTATTAATCTTCTTCGAAAGGCTATCCCCCTGAT
AAAGGCAGGTTGCACACGTGTTCCGCACCCGTACGCCGCTCTCAATTACCCGAAAGTAATCTACCGCTCGGCT
TGCATGTGTTAGGCCTCCCGCTAGCGTTCATTCCTGAGCCAGGATCAAACTCT
>306
CTGCTGCCGCCCGTAGGAGTCTGGTCCGTGTCTCAGTACCAGTGTGGGGGATCACCCTCTCAGGCCCCCTAAA
GATCGTCGCCCTGGTGAGCCGTTACCTCACCAACTAGCTAATCTTGCGCGTGCCCATCTCTATCCACCGTAGT

TTTCAATATCACCCGATGCCGAATAATATATTATGGGGTATTAATCTTCTTCGAAAGCTATCCCCTGATAAAG
GCAGGTTGCACACGTGTTCCGCACCCGTACGCCGCTCTCAATTACCCGAAAGTAATCTACCGCTCGGCTTGCA
TGTGTTAGGCCTCCCGCTAGCGTCATCTGAGCCAG
>307
CTGCTGCCGCCCGTAGGAGTCTGGTCCGTGTCTCAGTACCAGTGTGGGGGATCACCCTCTCAGGCCCCCTAAA
GATCGTCGCCTTGGTGAGCCGTTACCTCACCAACTAGCTAATCTTGCGCGTGCCCATCTCTATCCACCGTAGT
TTCAATATCACCCGATGCCGAATAATATATTATGGGGTATTAATCTTCTTCGAAAGGCTATCCCCTGATAAAG
CAGGTTGCACACGTGTTCCGCACCCGTACGCCGCTCTCAATTACCCGAAAGTAATCTACCGCTCGGCTTGCAT
GTGTTAGGCCTCCCGCTAGCGTTCATCCTGAGCCAGGATCAAACTCT
>308
CTGCTGCCGCCCCGTAGGAGTCTGGTCCCGTGTCTCAGTACCAGTGTGGGGGGATCACCCCTCTCAGGCCCCC
CTAAAGATCGTCGCCTTGGTGAGCCGTTACCTCACCCAACTAGCTAATCTTGCGCGTGCCCCATCTCTATTCC
CACCGTAGTTTTCAATATCACCCGATGCCGAATAATATATTATGGGGTATTAATCTTCTTCGAAAGGCTATCC
CCCTGATAAAGGCAGGTTGCACACGTGTTCCCGCACCCGTACGCCGCTCTCAATTACCCCGAAAGTAATCTAC
CGCTCGGCTTGCATGTGTTAGGCCTCCCGCTAGCGTTCATCCTGAGCCAGGATCAAACTCT
>309
CTGCTGCCGCCCGTAGGAGTCTGGTCCGTGTCTCAGTACCAGTGTGGGGGATCACCCTCTCAGGCCCCCTAAA
GATCGTCGCCTGGTGAGCCGTTACCTCACCAACTAGCTAATCTTGCGCGTGCCCATCTCTATCCACCGTAGTT
TTCAATATCACCCGATGCCGAATAATATATTATGGGGTATTAATCTTCTTCGAAAGGCTATCCCCTGATAAAG
GCAGGTTGCACACGTGTTCCGCACCCGTACGCCGCTCTCAATTACCCGAAAGTAATCTACCGCTCGGCTTGCA
TGTGTTAGGCCTCCCGCTAGCGTTCTCCTGAGCAGGATCAACTCGT
>310
CTGCTGCCGCCCGTAGGAGTCTGGTCCGTGTCTCAGTACCAGTGTGGGGGATCACCCTCTCAGGCCCCCTAAA
GATCGTCGCCTTGGTGAGCCGTTACCTCACCAACTAGCTAATCTTGCGCGTGCCCATCTCTATCCACCGTAGT
TTTCAATATCACCCGATGCCGAATAATATATTATGGGGTATTAATCTTCTTCGAAAGGCTATCCCCTGATAAA
GGCAGGTTGCACACGTGTTCCGCACCCGTACGCCGCTCTCAATTACCCGAAAGTAATCTACCGCTCGGCTTGC
ATGTGTTAGGCCTCCCGCTAGCGTTCATCCTGAGCCAGGATCAAACTCT
>311
CTGCTGCCGCCCGTAGGAGTCTGGTCCGTGTCTCAGTACCAGTGTGGGGGATCACCCTCTCAGGCCCCCTAAA
GATCGTCGCCTTGGTGAGCCGTTACCTCACCAACTAGCTAATCTTGCGCGTGCCCATCTCTATCCACCGTAGT
TTTCAATATCACCCGATGCCGAATAATATATTATGGGGTATTAATCTTCTTCGAAGGCTATCCCCTGATAAAG
GCAGGTTGCACACGTGTTCCGCACCCGTACGCCGCTCTCAATTACCCGAAAGTAATCTACCGCTCGGCTTGCA
TGTGTTAGGCCTCCCGCTAGCGTTCTCCTGAGCAGGATCCA
>312
CTGCTGCCGCCCCGTAGGAGTCTGGTCCGTGTCTCAGTACCAGTGTGGGGGATCACCCCTCTCAGGCCCCCTA
AAGATCGTCGCCTTGGTGAGCCGTTACCTCACCAACTAGCTAATCTTGCGCGTGCCCATCTCTATCCACCGTA
GTTTCCAATATCACCCGATGCCGAATAATATATTATGGGGTATTAATCTTCTTCGAAAGGCTATCCCCCTGAT
AAAGGCAGGTTGCACACGTGTTCCGCACCCGTACGCCGCTCTCAATTACCCCGAAAGTAATCTACCGCTCGGC
TTGCATGTGTTAGGCCTCCCGCTAGCGTTCTCCTGAGCAGGATCAACTCT
>313
CTGCTGCCGCCCGTAGGAGTCTGGTCCGTGTCTCAGTACCAGTGTGGGGGATCACCCTCTCAGGCCCCCTAAA
GATCGTCGCCTTGGTGAGCCGTTACCTCACCAACTAGCTAATCTTGCGCGTGCCCATCTCTATCCACCGTAGT
TTCAATATCACCCGATGCCGAATAATATATTATGGGGTATTAATCTTCTCGAAAGGCTATCCCCCTGATAAAG
GCAGGTTGCACACGTGTTCCGCACCCGTACGCCGCTCTCAATTACCCGAAAGTAATCTACCGCTCCGGCTTGC
ATGTGTTAGGCCTCCCGCTAGCGTTCATCCTGAGCCAGGATCAACTCT
>314
CTGCTGCCGCCCGTAGGAGTCTGGTCCGTGTCTCAGTACCAGTGTGGGGGATCACCCTCTCAGGCCCCCTAAA
GATCGTCGCCTTGGTGAGCCGTTACCTCACCAACTAGCTAATCTTGCGCGTGCCCATCTCTATCCACCGTAGT
TTTCAATATCACCCGATGCCGAATAATATATTATGGGGTATTAATCTTCTTCGAAAGGCTATCCCCCTGATAA
AGGCAGGTTGCACACGTGTTCCGCACCCGTACGCCGCTCTCAATTACCCGAAAGTAATCTACCGCTCGGCTTG
CATGTGTTAGGCCTCCCGCTAGCGTTCATCCTGAGCCAGGATCAAACTCT
>315
CTGCTGCCGCCCGTAGGAGTCTGGTCCGTGTCTCAGTACCAGTGTGGGGGATCACCCTCTCAGGCCCCCTAAA
GATCGTCGCCTCGGTGAGCCGTTACCTCACCAACTAGCTAATCTTGCGCGTGCCCATCTCTATCCACCGTAGT

TTTCAATATCACCCGATGCCGAATAATATATTATGGGGTATTAATCTTCTTCTGAAAGGCTATCCCCCTGATA
AAGGCAGGTTGCACACGTGTTCCGCACCCGTACGCCGCTCTCAATTACCCGAAAGTAATCTACCGCTCCGGCT
TGCATGTGTTAGGCCTCCCGCTAGCGTTCATCCTGAGCCAGGATCAAACTCT
>316
CTGCTGCCGCCCGTAGGAGTCTGGTCCGTGTCTCAGTACCAGTGTGGGGGATCACCCTCTCAGGCCCCCTAAA
GATCGTCGCCTTGGTGAGCCGTTACCTCACCAACTAGCTAATCTTGCGCGTGCCCATCTCTATCCACCGTAGT
TTTCAATATCACCCGATGCCGAATAATATATTATGGGGTATTAATCTTCTTCGAAAGGCTATCCCCCTGATAA
AGGCAGGTTGCACACGTGTTCCGCACCCGTACGCCGCTCTCAATTACCCGGAAGTAATCTACCGCTCGGCTTG
CATGTGTTAGGCCTCCCGCTAGCGTTCATCCTGAGCCAGGATCAAACTCT
>317
CTGCTGCCGCCCGTAGGAGTCTGGTCCGTGTCTCAGTACCAGTGTGGGGGATCACCCTCTCAGGCCCCCTAAA
GATCGTCGCCTTGGTGAGCCGTTACCTCACCAACTAGCTAATCTTGCGCGTGCCCATCTCTATCCACCGTAGT
TTCAATATCACCCGATGCCGAATAATATATTATGGGGTATTAATCTTCTCGAAAGGCTATCCCCCTGATAAAG
GCAGGTTGCACACGTGTTCCGCACCCGTACGCCGCTCTCAATTACCCGAAAGTAATCTACCGCTCGGCTTGCA
TGTGTTAGGCCTCCCGCTAGCGTTCTCCTGAGCAGGATCAACTCGT
>318
CTGCTGCCGCCCGTAGGAGTCTGGTCCGTGTCTCAGTACCAGTGTGGGGGATCACCCTCTCAGGCCCCCTAAA
GATCGTCGCCTTGGTGAGCCGTTACCTCACCAACTAGCTAATCTTGCGCGTGCCCATCTCTATCCACCGTAGT
TTTCAATATCACCCGATGCCGAATAATATATTATTGGGGTATTTAATCTTCTTCGAAAGGCTATCCCCCTGAT
AAAGGCAGGTTGCACACGTGTTCCGCACCCGTACGCCGCTCTCAATTACCCGAAAGTAATCTACCGCTCGGCT
TGCATGTGTTAGGCCTCCCGCTAGCGTTCTCCTGAGCA
>319
CTGCTGCCGCCCGTAGGAGTCTGGACCGTGTCTCAGTTCCAGTGTGACTGATCATCCTCTCAGACCAGTTACG
GATCGTCGCCTTGGTAGGCCTTTACCCCACCAACTAGCTAATCCGACCTAGGCTCATCTGATAGCGTGAGGTC
CGAAGATCCCCACTTCTCCCTCAGGACGTATGCGGTATTAGCGCCCGTTTCCGGACGTATCCCCACTACCAGG
CAGATTCCTAGGCATTACTCACCCGTCCGCCGCTGAATCCAGGAGCAAGCCCCCTTCATCCGCTCGACTGCAT
GTGTTAGGCCTGCCGCCAGCGTTCAATCTGAGCCAGGATCAAACTCT
>320
CTGCTGCCGCCCGTAGGAGTCTGGTCCGTGTCTCAGTACCAGTGTGGGGGATCACCCTCTCAGGCCCCCTAAA
GATCGTCGCCTTGGTGAGCCGTTACCTCACCAACTAGCTAATCTTGCGCGTGCCCATCTCTATCCACCGTAGT
TTTCAATATCACCCGATGCCGAATAATTATATTATGGGGTATTTAATTCTTTCTTCGAAAGGCTATCCCCCTG
ATAAAGGCAGGTTGCACACGTGTTCCGCACCCGTACGCCGCTCTCAATTACCCCGAAAGTAATCTACCGCTCG
GCTTGCATGTGTTAGGCCTCCCGCTAGCGTTCATTCCTGAGCCAGGATCAACT
>321
CTGCTGCCGCCCGTAGGAGTCTGGTCCGTGTCTCAGTACCAGTGTGGGGGGATCACCCTCTCAGGCCCCCTAA
AGATCGTCGCCTTGGTGAGCCGTTACCTCACCAACTAGCTTAATCTTGCGCGTGCCCCATCTCTATCCACCGT
AGTTTTCAATATCACCCGATGCCGAATAATATATTTATTGGGGTATTTAATTCTTCTTCGAAAGGCTATCCCC
CTGATAAAGGCAGGTTTGCACACGTGTTCCGCACCCGTACGCCGCTCTCAATTACCCCGAAAGTAATCTACCG
CTCGGCTTGCATGTGTTAGGCCTCCCGCTAGCGTTCATCCTGAGCCAGGATCAAACTCT
>322
CTGCTGCCGCCCGTAGGAGTCTGGTCCGTGTCTCAGTACCAGTGTGGGGGATCACCCTCTCAGGCCCCCTAAA
GATCGTCGCCTTGGTGAGCCGTTACCTCACCAACTAGCTTAATCTTGCGCGTGCCCATCTCTATCCACCGTAG
TTTTTCAATATCACCCGATGCCGAATAATATATTTATTGGGGTATTTAATCTTCTTCGAAAGGCTATCCCCCT
GATTAAAGGCAGGTTGCACACGTGTTCCGCACCCGTACGCCGCTCTTCAATTACCCCGAAAGTAATCTACCGC
TCGGCTTGCATGTGTTTAGGCCTCCCGCTAGCGTTCATCCTGAGCCAGGATCAAACTCT
>323
CTGCTGCCGCCCGTAGGAGTCTGGACCGTGTCTCAGTTCCAGTGTGACTGATCATCCTCTCAGACCAGTACGG
ATCGTCGCCTTGGTAGGCCTTTACCCCACCAACTAGCTAATCCGACCTAGGCTCATCTGATAGCGTGAGGTCC
GAAGTCCCCCACTTCTCCCTCAGGACGTATGCGGTATTAGCGCCCGTTTCCGGACGATCCCCACTACCAGGCA
GATTCCTAGGCATTACTCACCCGTCCGCCGCTGAATCCAGGAGCAAGCTCCCTTCATCCGCTCGACTGCATGT
GTTAGGCCTGCCGCCAGCGTTCAATCTGAGCCAGGATCAAACTCT
>324
CTGCTGCCGCCCGTAGGAGTCTGGACCGTGTCTCAGTTCCAGTGTGACTGATCATCCTCTTCAGACCAGTTAC
GGATCGTCGCCTTGGTAGGCCTTTACCCCACCAACTAGCTAATCCGACCTAGGCTCATCTGATAGCGTGAGGT

CCGAAGATTCCCCCACTTCTCCCTCAGGACGTATGCGGTATTAGCGCCCGTTTCCGGACGTATCCCCACTACC
AGGCAGATTTCCTAGGCATTACTCACCCGTCCGCCGCTGAATCCAGGAGCAAGCTCCCTTCATCCGCTCGACT
GCATGTGTTAGGCCTGCCGCCAGCGTTCAATCTTGAGCCAGGATCAAACTCT
>325
CTGCTGCCGCCCGTAGGAGTCTGGTCCGTGTCTCAGTACCAGTGTGGGGGATCACCCTCTCAGGCCCCCTAAA
GATCGTCGCCTTGGTGAGCCGTTACCTCACCAACTAGCTAATCTTGCGCGTGCCCATCTCTTATCCACCGTAG
TTTTTCAATATCACCCGATGCCGAATAATATATTATGGGGTATTTAATCTTCTTCGAAAGGCTATCCCCTGAT
AAAGGCAGGTTGCACACGTGTTCCGCACCCGTACGCCGCTCTCAATTACCCGAAAGTAATCTACCGCTCGGCT
TGCATGTGTTAGGCCTCCCGCTAGCGTTCATCCTGAGC
>326
CTGCTGCCGCCCGTAGGAGTCTGGTCCGTGTCTCAGTACCAGTGTGGGGGATCACCCTCTCAGGCCCCCTTAA
AGATCGTCGCCTTGGTGAGCCGTTACCTCACCAACTAGCTAATCTTGCGCGTGCCCATCTTCTATCCACCGTA
GTTTTTCAATATCACCCGATGCCGAATAATATATTTATGGGGTATTTAATCTTCTTCGAAAGGCTATCCCCCT
GATAAAGGCAGGTTTGCACACGTGTTCCGCACCCGTACGCCGCTCTCAATTACCCGAAAGTAATCTACCGCCA
TCGGCTTTGCA
>327
CTGCTGCCGCCCGTAGGAGTCTGGTTCCGTGTCTCAGTACCAGTGTGGGGGATCACCCTCTCAGGCCCCCTAA
AGATCGTCGCCTTGGTGAGCCGTTACCTCCACCAACTAGCTAATCTTGCGCGTGCCCATCTCTATCCACCATA
GTTTTCAATATCACCCGATGCCGAATAATATATTTATGGGGTATTTAATTCTTTCTTCGAAAGGCTATCCCCC
TGATAAAGGCAGGTTGCACACGTGTTCCGCACCCGTACGCCGCTCTCAATTTACCCGAAAGTAATCTACCGCT
CGGCTTGCATGTGTTAGGCCTCCCGCTAGCGTTCATTCCTT
>328
CTGCTGCCGCCCGTAGGAGTCTGGTCCGTGTCTCAGTACCAGTGTGGGGGATCACCCTCTCAGGCCCCCTAAA
GATCGTCGCCTTGGTGAGCCGTTACCTCACCAACTAGCTAATCTTGCGCGTGCCCATCTCTATCCACCGTAGT
TTCAATATCACCCGATGCCGAATAATATATTATGGGGTATTAATCTTCTTCGAAAGGCTATCCCCCTGATAAA
GGCAGGTTGCACACGTGTTCCGCACCCGTACGCCGCTCTCAATTACCCGAAAGTAATCTACCGCTCGGCTTGC
ATGTGTTAGGCCTCCCGCTAGCGTTCATCCTGAGCCAGGATCAACTCT
>329
CTGCTGCCGCCCGTAGGAGTCTGGTCCGTGTCTCAGTACCAGTGTGGGGGATCACCCTCTCAGGCCCCCTAAA
GATCGTCGCCTTGGTGAGCCGTTACCTCACCAACTAGCTAATCTTGCGCGTGCCCATCTCTATCCACCGTAGT
TTCAATATCACCCGATGCCGAATAATATATTATGGGGTATTAATCTTCTTCGAAAGGCTATCCCCCTGATAAA
GGCAGGTTGCACACGTGTTCCGCACCCGTACGCCGCTCTCAATTACCCGAAAGTAATCTACCGCTCGGCTTGC
ATGTGTTAGGCCTCCCGCTAGCGTTCTCCTGAGCAGGATCAACTCGT
>330
CTGCTGCCGCCCGTAGGAGTCTGGTCCGTGTCTCAGTACCAGTGTGGGGGATCACCCTCTCAGGCCCCCTAAA
GATCGTCGCCTTGGTGAGCCGTTACCTCACCAACTAGCTAATCTTGCGCGTGCCCATCTCTATCCACCGTTAG
TTTCAATATCACCCGATGCCGAATAATATATTTATGGGGTATTTAATCTTTCTCGAAAGGCTATCCCCCTGAT
AAAGGCAGGTTTGCACACGTGTTCCGCACCCGTACGCCGCTCTCAATTACCCGAAAGTAATCTACCGCTCGGC
TTGCATGTGTTAGGCCTTCCCGCTAGCGTTCTCCTGAGCAG
>331
CTGCTGCCCGCCCCGTAGGAGTCTGGTCCCGTGTTCTCAGTACCCAGTGTGGGGGGATCACCCCTCTCAGGCC
CCCCCTAAAGATCGTCGCCTTGGTGAGCCCGTTTACCTCACCCAACTAGCTTAATCTTTGCGCGTGCCCCATC
TCTATTCCCACCCGTAGTTTTTCAATATCACCCGATGCCCGAATAATTATATTTATTGGGGTATTTTAATTCT
TTCTTCGAAAGGCTATCCCCCTGATTAAAGGCAGGTTTGCACACGTGTTCCCGCACCCGTTACGCCGCTCTCA
ATTTACCCCGAAAGTAATCTACCGCTCGGCTTGCATGTGTTTAGGCCCTTCCCC
>332
CTGCTGCCGCCCGTAGGAGTCTGGTCCGTGTCTCAGTACCAGTGTGGGGGATCACCCTCTCAGGCCCCCTAAA
GATCGTCGCCTTGTGAGCCGTTACCTCACCAACTAGCTAATCTTGCGCGTGCCCCATCTCTTATCCACCGTAG
TTTTTCAATATCACCCGATGCCGAATAATATATTTATGGGGTATTTAATCTTCTTCGAAAGGCTTATTCCCCC
TGATAAAGGCAGGTTGCACACGTGTTCCGCACCCGTACGCCGCTCTCAATTTACCCCGAAAGTAATCTACCGC
TCGGCTTGCATGTGTTTAGGCCTCCCGCTAGCGTTCATCCTGAGCCAGGATCAACTCT
>333
CTGCTGCCGCCCGTAGGAGTCTGGTCCGTGTCTCAGTACCAGTGTGGGGGATCACCCTCTCAGGCCCCCTAAA
GATCGTCGCCTTGGTGAGCCGTTACCTCACCAACTAGCTAATCTTGCGCGTGCCCATCTCTTATCCACCGTAG

TTTTTCAATATCACCCGATGCCGAATAATATATTTATGGGGTATTTAATCTTCTTCGAAAGGCTATCCCCCTG
ATAAAGGCAGGTTGCACACGTGTTCCGCACCGTACGCCGCTCTCAATTACCCGAAAGTAATCTACCGCTCCGG
CTTGCATGTGTTTAGGCCTTCCCGCTAGCGTTCATCCTGAGCCAGGATCAAACTCTT
>334
CTGCTGCCGCCCGTAGGAGTCTGGCTCGTGTCTCAGTTTCCAGTGTGACTGATCATCCTCTCAGACCAGTACG
GATCGTCGCCTTGGTAGGCCTTACCCCACCAACTAGCTAATCCGACCTAGGCTCATCTGATAGCGTGAGGTCC
GAAGTCCCCCACTTCTCCCCTCAGGACGTATGCGGTATTAGCGCCCGTTTCCGACGATCCCCACTACCAGGCA
GATCCCTAGGCATTACTCACCCGTCCGCCGCTGAATCCAGGAGCAAGCTCCCTTCATCCGCTCGACTGCATTG
TGTTAGGCCTGCCGCCAGCGTCAATCTGAGCCAGGATCAAACTCT
>335
CTGCTGCCGCCCGTAGGAGTCTGGTCCGTGTCTCAGTACCAGTGTGGGGGATCACCCTCTCAGGCCCCCTAAA
GATCGTCGCCTTGGTGAGCCGTTACCTCACCAACTAGCTAATCTTGCGCGTGCCCATCTCTATCCACCGTAGT
TTTCAATATCACCCGATGCCGAATAATATATTATGGGGTATTAATCTTCTTCGAAAGGCTATCCCCCTGATAA
AGGCAGGTTGCACACGTGTTCCGCACCCGTACGCCGCTCTCAATTACCCGAAAGTAATCTACCGCTCGGCTTG
CATGTGTTAGGCCTCCCGCTAGCGTCATCCTGAGCCAGGATCAAACTCT
>336
CTGCTGCCGCCCCGTAGGAGTCTGGTCCCGTGTCTCAGTACCAGTGTGGGGGATCACCCTCTCAGGCCCCCTA
AAGATCGTCGCCTTGGTGAGCCGTTACCTCACCAACTAGCTAATCTTGCGCGTGCCCATCTCTATCCACCGTA
GTTTTCAATATCACCCGATGCCGAATAATATATTATGGGGTATTAATCTTCTTCGAAAGGCTTATCCCCCTGA
TAAAGGCAGGTTGCACACGTGTTCCGCACCCGTACGCCGCTCTCAATTACCCGAAAGTAATCTACCGCTCGGC
TTGCATGTGTTAGGCCTCCCGCTAGCGTCATCT
>337
CTGCTGCTCGCCCGTAGGAGTCTGGTCCGTGTCTCAGTACCAGTGTGGGGGATCACCCTCTCAGGCCCCCTAA
AGATCGTCGCCTTGGTGAGCCGTTACCTCGCAACCATAGCTAATCTTGCGCGTGCCCATCTCTATCCACCGTA
GTTTTCAATATCACCCGATGCCGAATAATATATTATGGGGTATTAATCTTCTTCGAAAGGCTATCCCCCTGAT
AAAGGCAGGTTGCACACTGTTCCGCACCCGTACGCCGCTCCTCAATTACCCGAAAGTAATCTACCGCTCGGCT
GCATGTGTTAGGCCTCCCGCTAGCGTTCATCTGAGCCA
>338
CTGCTGCCGCCCGTAGGAGTCTGGTCCGTGTCTCAGTACCAGTGTGGGGGATCACCCTCTCAGGCCCCCTAAA
GATCGTCGCCTTGGTGAGCCGTTACCTCACCAACTAGCTAATCTTGCGCGTGCCCATCTCTATCCACCGTAGT
TTTCAATATCACCCGATGCCGAATAATATATTATGGGGTATTAATCTTCTTCGAAAGGCTATCCCCCTGATAA
AGGCAGGTTGCACACGTGTTCCGCACCCGTACGCCGCTCTCAATTACCCGAAAGTAATCTACCGCTCGACTGC
ATGTGTTAGGCCTCCCGCTAGCGTTCTCCTGAGCAGGTCAACTCGT
>339
CTGCTGCCGCCCGTAGGAGTCTGGTCCGTGTCTCAGTACCAGTGTGGGGGATCACCCTCTCAGGCCCCCTAAA
GATCGTCGCCTTGGTGAGCCGTTACCTCACCAACTAGCTAATCTTGCGCGTGCCCATCTCTATCCACCGTAGT
TTTCAATATCACCCGATGCCGAATAATATATTATGGGGTATTAATCTTCTCGAAAGGCTATCCCCTGATAAAG
GCAGGTTGCACACGTGTTCCGCACCCGTACGCCGCTCTCAATTACCCGAAAGTAATCTACCGCTCGGCTTGCA
TGTGTTAGGCCTCCCGCTAGCGTTCATCCTGAGCCAGGATCAAACTCT
>340
CTGCTGCCGCCCGTAGGAGTCTGGTCCGTGTCTCAGTACCAGTGTGGGGGATCACCCTCTCAGGCCCCCTAAA
GATCGTCGCCTTGGTGAGCCGTTACCTCACCAACTAGCTAATCTTGCGCGTGCCCATCTCTATCCACCGTAGT
TTTCAATATCACCCGATGCCGAATAATATATTATGGGGTATTAATCTTCTTCGAAAGGCTATCCCCCTGATAA
AGGCAGGTTGCACACGTGTTCCGCACCCGTACGCCGCTCTCAATTACCCGAAAGTAATCTACCGCTCCGGCTT
GCATGTGTTAGGCCTCCCGCTAGCGTCTCCTGAGCAGGATCAACTCGT
>341
CTGCTGCCGCCTAGGAGTCTGGTCCGTGTCTCAGTACCAGTGTGGGGGATCACCCTCTCAGGCCCCCTAAAGA
TCGTCGCCTTGGTGAGCCGTTACCTCACCAACTAGCTAATCTTGCGCGTGCCCATCTCTATCCACCGTAGTTT
TCAATATCACCCGATACCGAATAATATATTATGGGGTATTAATCTTCTTCTGAAAGGCTATCCCCCTGATAAA
GGCAGGTTGCACACGTGTTCCGCACCCGTACGCCGCTCTCAATTACCCGAAAGTAATCTACCGCTCGGCTTGC
ATGTGTTAGGCCTCCCGCTAGCGTTCATCCTGAGCCAGGATCAAACTCT
>342
CTGCTGCCGCCCGTAGGAGTCTGGTCCGTGTCTCAGTACCAGTGTGGGGGATCACCCTCTCAGGCCCCCTAAA
GATCGTCGCCTTGGTGAGCCGTTACCTCACCAACTAGCTAATCTTGCGCGTGCCCATCTCTATCCACCGTAGT

TTTCAATATCACCCGATGCGAATAATATATTATGGGGTATTAATCTTCTTCGAAAGGCTATCCCCCTGATAAA
GGCAGGTTGCACACGTGTTCCGCACCCGTACGCCGCTCTCAATTACCCGAAAGTAATCTACCGCTCGGCTTGC
ATGTGTTAGGCCTCCGCTAGCGTTCATCCTGAGCCAGGATCAAACTCT
>343
CTGCTGCCGCCCGTAGGAGTCTGGACCGTGTCTCAGTTCCAGTGTGGCCGATCACCCTCTCAGGTCGGCTATG
CATCGTTGCCTTGGTAAGCCGTTACCTTACCAACTAGCTAATGCAGCGCGGATCCATCTATAAGTGACAGCAA
GACCGTCTTTCACTTGGACCATGCGATTCAAAATATTATCCGGTATTAGCTCCGGTTTCCCGAAGTATCCCAG
TCTTATAGGTAGGTTATCCACGTGTTACTCACCCGTCCGCCGCTAACATCAGAGAAGCAAGCTTCTCGTCCGT
TCGCTCGACTGCATGTATTAGGCACGCCGCCAGCGTTCTCCTGAGCCAGGATCAACTCT
>344
CTGCTGCCGCCCGTAGGAGTCTGGTCCATGTCTCAGTACCAGTGTGGGGGATCACCCTCTCAGGCCCCCTAAA
GATCGTCGCCTTGGTGAGCCGTTACCTCACCAACTAGCTAATCTTGCGCGTGCCCATCTCTATCCACCGTAGT
TTTCAATATCACCCGATGCCGAATAATATATTATGGGGTATTAATCTTCTTCGAAAGGCTATCCCCCTGATAA
AGGCAGGTTAGCACACGTGTTCCGCACCCGTACGCCGCTCTCAATTACCCGAAAGTAATCTACCGCTCGGCTT
GCATGTGTTAGGCCTCCCGCTAGCGTTCTCCTGAGCAGGAT
>345
TGCTGCCGCCCGTAGGAGTCTGGTCCGTGTCTCAGTACCAGTGTGGGGGATCACCCTCTCAGGCCCCCTAAAG
ATCGTCGCCTTGTGAGCCGTTACCTCACCAACTAGCTAATCTTGCGCGTGCCCATCTCTATCCACCGTAGTTT
TCAATATCACCCGATGCCGAATAATATATTATGGGGTATTAATCTTCTTCGAAAGGCTATCCCCCTGATAAGG
CAGGTTGCACACGTGTTCCGCACCCGTACGCCGCTCTCAATTACCCGAAAGTAATCTACCGCTCGGCTTGCAT
GTGTTAGGCCTCCCGCTAGCGTTCTCCTGAGCCAGGATCAAACTCT
>346
CTGCTGCCGCCCGTAGGAGTCTGGTCCGTGTCTCAGTACCAGTGTGGGGGATCACCCTCTCAGGCCCCCTAAA
GATCGTCGCCTTGGTGAGCCGTTACCTCACCAACTAGCTAATCTTGCGCGTGCCCATCTCTATCCACCGTAGT
TTTCAATATCACCCGATGCCGAATAATATATTATGGGGTATTAATCTTCTTCGAAAGGCTATCCCCCTGATAA
AGGCAGGTTGCACACGTGCTCCGCACCCGTACGCCGCTCTCAATTACCCGAAAGTAATCTACCGCTCGGCTTG
CATGTGTTAGGCCTCCCGCTAGCGTTCATCCTGAGCCAGGATCAGACTCT
>347
CTGCTGCCGCCCGTAGGAGTCTGGTCCGTGTCTCAGTGCCAGTGTGGGGGATCACCCTCTCAGGCCCCCTAAA
GATCGTCGCCTTGGTGAGCCGTTACCTCACCAACTAGCTAATCTTGCGCGTGCCCATCTCTATCCACCGTAGT
TTTCAATATCACCCGATGCCGAATAATATATTATGGGGTATTAATCTTCTCGAAAGGCTATCCCCCTGATAAA
GGCAGGTTGCACACGTGTTCCGCACCCGTACGCCGCTCTCAATTACCCGAAAGTAATCTACCGCTCGGCTTGC
ATGTGTTAGGCCTCCCGCTAGCGTTCATCCTGAGCCAG
>348
CTGCTGCCGCCCGTAGGAGTCTGGTCCGTGTCTCAGTACCAGTGTGGGGGATCACCCTCTCAGGCCCCCTAAA
GATCGTCGCCTTGGTGAGCCGTTACCTCACCAACTAGCTAATCTTGCGCGTGCCCATCTCTATCCACCGTAGT
TTTCAATATCACCCGATGCCGAATAATATATTATGGGGTATTAATCTTCTCGAAAGGCTATCCCCCTGATAAA
GGCAGGTTGCACACGTGTTCCGCACCCGTACGCCGCTCTCAATTACCCGAAAGTAATCTACCGCTCGGCTTGC
ATGTGTTAGGCCTCCCGCTAGCGTTCTCCTGAGCAGGTCAACTCT
>349
CTGCTGCCGCCCGTAGGAGTCTGGTCCGTGTCTCAGTACCAGTGTGGGGGATCACCCTCTCAGGCCCCCTAAA
GATCGTCGCCTTGGTGAGCCGTTACCTCACCAACTAGCTAATCCTGCGCGTGCCCATCTCTATCCACCGTAGT
TTTCAATATCACCCGATGCCGAATAATATATTATGGGGTATTAATCTTCTTCGAAAGGCTATCCCCCTGATAA
AGGCAGGTTGCACACGTGTTCCGCACCCGTACGCCGCTCTCAATTACCCGAAAGTAATCTACCGCTCGGCTTG
CATGTGTTAGGCCTCCCGCTAGCGTTCATCCTGAGCCAGGATCAACTCT
>350
CTGCTGCCGCCCGTAGGAGTCTGGTCCGTGTCTCAGTACCAGTGTGGGGGATCACCCTCTCAGGCCCCCTAAA
GATCGTCGCCTTGGTGAGCCGTTACCTCACCAACTAGCTAATCTTGCGCGTGCCCATCTCTATCCACCGTAGT
TTTCAATATCACCCGATGCCGAATAATATATTATGGGGTATTAATCTTCTTCGAAAGGCTATCCCCCTGATAA
AGGCAGGTTGCACACGTGTTCCGCACCCGTACGCCGCTCTCAATTACCCGAAAGTAATCTACCGCTCGGCTTG
CATGTGTTAGGCCTCCCGCTAGCGTTCATCCTCG
>351
CTGCTGCCGCCCGTAGGAGTCTGGTCCGTGTCTCAGTACCAGTGTGGGGATCACCCTCTCAGGCCCCTTAAAG
ATCGTCGCCTTGGTGAGCCGTTACCTCACCAACTAGCTAATCTTGCGCGTGCCCATCTCTATCCACCGTAGTT

TTCAATATCACCCGATGCCGAATAATATATTATGGGGTATTAATCTTCTTCGAAAGGCTATCCCCTGATAAAG
GCAGGTTGCACACGTGTTCCGCACCCGTACGCCGCTCTCAATTACCCGAAAGTAATCTACCGCTCGGCTTGCA
TGTGTTAGGCCTCCCGCTAGCGTTCATCCTGAGCCAGGATCAAACTCT
>352
CTGCTGCCGCCCGTAGGAGTCTGGACCGTGTCTCAATTCCAGTGTGACTGATCATCCTCTCAGACCAGTACGG
ATCGTCGCCTTGGTAGGCCTTTACCCCACCAACTAGCTAATCCGACCTAGGCTCATCTGATAGCGTGAGGTCC
GAAGATCCCCCACTTCTCCCTCAGGACGTATGCGGTATTAGCGCCCGTTTCCGGACGTATCCCCACTACCAGG
CAGATTCCTCAGGCATTACTCACCCGTCCGCCGCTGAATCCAGGAGCAAGCTCCCTTCATCCGCTCGACTGCA
TGTGTTAGGCCTGCCGCCAGCGTCAATCTGAGCCAGGATCAAACTCT
>353
CTGCTGCCGCCCGTAGGAGTCTGGTCCGTGTCTCAGTACCAGTGTGGGGGATCACCCTCTCAGGCCCCCTAAA
GATCGTCGCCTTGGTGAGCCGTTACCTCACCAACTAGCTAATCTTGCGCGTGCCCATCTCTATCCACCGTAGT
TTTCAATATCACCCGATGCCGAATAATATATTATGGGGTATTAATCTTCTTCGAAAGGCTATCCCCTGATAAA
GGCAGGTTGCACACGTGTTCCGCACCCGTACGCCGCTCTCAATTACCCGAAAGTAATCTACCGCTCCGGCTTG
CATGTGTTAGGCCTCCCGCTAGCGTTCATCCTGAGC
>354
CTGCTGCCGCCCGTAGGAGTCTGGTCCGTGTCTCAGTACCAGTGTGGGGGATCACCCTCTCAGGCCCCCTAAA
GATCGTCGCCTTGGTGAGCCGTTACCTCACCAACTAGCTAATCTTGCGCGTGCCCATCTCTATCCACCGTAGT
TTTCAATATCACCCGATGCCGAATAATATATTATGGGGTATTAATCTTCTTCGAAAGGCTATCCCCCTGATAA
AGGCAGGTTGCACACGTGTTCCGCACCCGTACGCCGCTCTCAATTACCCGAAAGTAATCTACCGCTCGGCTTG
CATGTGTTAGGCCCCCGCTAGCGTT
>355
CTGCTGCCGCCCGTAGGAGTCTGGTCCGTGTCTCAGTACCAGTGTGGGGGATCACCCTCTCAGGCCCCCTAAA
GATCGTCGCCTTGGTGAGCCGTTACCTCACCAACTAGCTTAATCTTGCGCGTGCCCATCTCTATCCACCGTAG
TTTTTCAATATCACCCGATGCCGAATAATATATTTATGGGGTATTTAATTCTTTCTTCGAAAGGCTATCCCCC
TGATTAAAGGCAGGTTGCACACGTGTTCCGCACCCGTACGCCGCTCTCAATTACCCGAAAGTAATCTACCGCT
CGGCTTGCATGT
>356
CTGCTGCCGCCCGTAGGAGTCTGGTCCGTGTCTCAGTACCAGTGTGGGGGATCACCCTCTCAGGCCCCCTAAA
GATCGTCGCCTTGGTGAGCCGTTACCTCACCAACTAGCTAATCTTGCGCGTGCCCATCTCTATCCACCGTAGT
TTTTCAATATCACCCGATGCCGAATAATATATTTATGGGGTATTTAATCTTTCTTCGAAAGGCTATCCCCCTG
ATAAAGGCAGGTTGCACACGTGTTCCGCACCCGTACGCCGCTCTCAATTACCCGAAAGTAATCTACCGCTCCG
GCTTGCATGTGTTAGGCCTCCCGCTAGCGTTCATTCCTTG
>357
CTGCTGCCGCCCGTAGGAGTCTGGTCCGTGTCTCAGTACCAGTGTGGGGGATCACCCTCTCAGGCCCCCTAAA
GATCGTCGCCTTGGTGAGCCGTTACCTCACCAACTAGCTAATCTTGCGCGTGCCCATCTCTATCCACCGTAGT
TTTCAATATCACCCGATGCCGAATAATATATTATGGGGTATTTAATCTTTCTTCGAAAGGCTATCCCCCTGAT
AAAGGCAGGTTGCACACGTGTTCCGCACCCGTACGCCGCTCTCAATTACCCGAAAGTAATCTACCGCTCGGCT
TGCATGTGTTAGGCCTCCCGCTAGCGTTCATCCTGAGCCAGGATCAACTCT
>358
CTGCTGCCGCCCGTAGGAGTCTGGTCCGTGTCTCAGTACCAGTGTGGGGGATCACCCTCTCAGGCCCCCTAAA
GATCGTCGCCTTGGTGAGCCGTTACCTCACCAACTAGCTAATCTTGCGCGTGCCCATCTCTATCCACCGTAGT
TTTCAATATCACCCGATGCCGAATAATATATTATGGGGTATTAATCTTCTTCGAAAGGCTATCCCCCTGATAA
AGGCAGGTTGCACACGTGTTCCGCACCCGTACGCCGCTCTCAATTACCCGAAAGTAATCTACCGCTCCGGCTT
GCATGTGTTAGGCCTCCCGCTAGCGTTCATCCGTGCGCAGGATCAAACTCT
>359
CTGCTGCCGCCCGTAGGAGTCTGGTCCGTGTCTCAGTACCAGTGTGGGGGATCACCCTCTCAGGCCCCCTAAA
GATCGTCGCCTTGGTGAGCCGTTACCTCACCAACTAGCTAATCTTGCGCGTGCCCATCTCTATTCCACCGTAG
TTTTCAATATCACCCGATGCCGAATAATATATTATTGGGGTATTTAATCTTCTTCGAAAGGCTATCCCCTGAT
AAAGGCAGGTTGCACACGTGTTCCGCACCCGTACGCCGCTCTCAATTACCCGAAAGTAATCTACCGCTCGGCT
TGCATGTGTTAGGCCTCCCGCTAGCGTTCTCCTGAG
>360
CTGCTGCCGCCCGTAGGAGTCTGGACTCGTGTCTCAGTTCCAGTGTGACTGATCATCCTCTCAGACCAGTACG
GATCGTCGCCTTGGTAGGCTTTACCCCACCAACTAGCTAATCCGACCTAGGCTCATCTGATAGCGTGAGGTCC

GAAGATCCCCACTTCTCCTCAGGACGTATGCGGTATTAGCGCCCGTTCCGGACGTATCCCCACTACCAGGCAG
ATTTCCTAGGCATTACTCACCCGTCCGCCGCTGAATCCAGGAGCAAGCTCCCTCATCCGCTCGACTTGCATGT
GTTAGGCCTGCCGCCAGCGTCAATCTGAGCCAGGATCAAACTCT
>361
CTGCTGCCGCCCGTAGGAGTCTGGTTCCGTGTCTCAGTACCAGTGTGGGGGATCACCCTCTCAGGCCCCCTAA
AGATCGTCGCCTTGGTGAGCCGTTACTTCACCAACTAGCTAATCTTGCGCGTGCCCATCTTCTATCCACCGTA
GTTTTTCAATATCACCCGATGCCGAATAATATATTTATGGGGTATTTAATTCTTTCTTCGAAAGGCTATCCCC
CTGATAAAGGCAGGTTTGCACACGTGTTCCGCACCCGTACGCCGCTCTCAATTACCCGAAAGTAATCTACCGC
TCGGCTTGCATGTGTTAGGCCTCCCGCTAGCGTTCATTCCTGAGCCAGGATCAACTCT
>362
CTGCTGCCGCCCGTAGGAGTCTGGTCCGTGTCTCAGTACCAGTGTGGGGGATCACCCTCTCAGGCCCCCTAAA
GATCGTCGCCTTGGTGAGCCGTTACCTCACCAACTAGCTAATCTTGCGCGTGCCCCATCTCTATCCACCGTAG
TTTTTCAATATCACCCGATGCCGAATAATATATTTATGGGGTATTTAGTCTTTCCTTCGAAAGGCTATCCCCC
TGATAAAGGCAGGTTTGCACACGTGTTCCGCACCCGTACGCCGCTCTTCAATTACCCCGAAAGTAATCTACCG
CTCGGCTTGCATGTGTTTAGGCCTCCCGCTAGCGTTCATCCTGAGCCAGGATCAAACTCTT
>363
CTGCTGCCGCCCGTAGGAGTCTGGTCCGTGTCTCAGTACCAGTGTGGGGGATCACCCTCTCAGGCCCCCTAAA
GATCGTCGCCTTGGTGAGCCGTTACCTCACCAACTAGCTAATCTTGCGCGTGCCCATCTCTATCCACCGTAGT
TTTCAATATCACCCGATGCCGAATAATATATTATGGGGTATTAATCTTCTTCGAAAGGCTATCCCCCTGATAA
AGGCAGGTTGCACACGTGTTCCGCACCCGTACGCCGCTCTCAATTACCCGAAAGTAATCTACCGCTCGGCTTG
CATGTGTTAGGCCTCCCGCTAGCGTTCATCCTGAGCCAGGATCAACTCT
>364
CTGCTGCCGCCCGTAGGAGTCTGGTCCGTGTCTCAGTACCAGTGTGGGGGATCACCCTCTCCAGGCCCCCTAA
AGATCGTCGCCTTGGTGAGCCGTTACCTCACCAACTAGCTAATCTTGCGCGTGCCCATCTCTATCCACCGTAG
TTTTCAATATCACCCGATGCCGAATAATATATTATGGGGTATTAATCTTCTCGAAAGGCTATCCCCTGATAAA
GGCAGGTTGCACACGTGTTCCGCACCCGTACGCCGCTCTCAATTACCCGAAAGTAATCTACCGCTCGGCTTGC
ATGTGTTAGGCCTCCCGCTAGCGTTCTCCTGAGCAGGATCAAACTCGT
>365
CTGCTGCCGCCCGTAGGAGTCTGGTCCGTGTCTCAGTACCAGTGTGGGGGATCACCCTCTCAGGCCCCCTAAA
GATCGTCGCCTTGGTGAGCCGTTACCTCACCAACTAGCTAATCTTGCGCGTGCCCATCTCTATCCACCGTAGT
TTTCAATATCACCCGATGCCGAATAATATATTATGGGGTATTAATCTTCTTCGAAAGGCTATCCCCCTGATAA
AGGCAGGTTGCACACGTGTTCCGCACCCGTACGCCGCTCTCAATTACCCGAAAGTAATCTACCGCTCGGCTTG
CATGTGTTAGGCCTCCCGCTAGCGTCATCCTGAGCCAGGATCAAACTCT
>366
CTGCTGCCGCCCGTAGGAGTCTGGTCCGTGTCTCAGTACCAGTGTGGGGGATCACCCTCTCAGGCCCCCTAAA
GATCGTCGCCTTGGTGAGCCGTTACCTCACCAACTAGCTAATCTTGCGCGTGCCCATCTCTATCCACCGTAGT
TTTCAATATCACCCGATGCCGAATAATATATTATGGGGTATTAATCTTCTCGAAGCTATCCCCTGAATAAAGG
CAGGTTGCACACGTGTTCCGCACCCGTACGCCGCTCTCAATTACCCGAAAGTAATCTACGCTCCGGCTTGCAT
GTGTTAGGCCTCCCGCTAGCG
>367
CTGCTGCCGCCCGTAGGAGTCTGGTCCGTGTCTCAGTACCAGTGTGGGGGATCACCCTCTCAGGCCCCCTAAA
GATCGTCGCCTTGGTGAGCCGTTACCTCACCAACTAGCTAATCTTGCGCGTGCCCATCTCTATCCACCGTAGT
TTTCAATATCACCCGATGCCGAATAATATATTATGGGGTATTAATCTTCTTCGAAAGGCTATCCCCTGATAAA
GGCAGGTTGCACACGTGTTCCGCACCCGTACGCCGCTCTCAATTACCCGAAAGTAATCTACCGCTCGGCTTGC
ATGTGTTAGGCCTCCCGCTAGCGTTCTCCTGAGCAGGATCAACTCT
>368
CTGCTGCCGCCCGTAGGAGTCTGGTCCGTGTCTCAGTACCAGTGTGGGGGATCACCCTCTCAGGCCCCCTAAA
GATCGTCGCCTTGGTGAGCCGTTACCTCACCAACTAGCTAATCTTGCGCGTGCCCATCTCTATCCACCGTAGT
TTTCAATATCACCCGATGCCGAATAATATATTATGGGGTATTAATCTTCTTCGAAAGGCTATCCCCTGATAAA
GGCAGGTTGCACACGTGTTCCGCACCCGTACGCCGCTCTCAATTACCCGAAAGTAATCTACCGCTCCGGCTGC
ATGTGTTAGGCCTCCCGCAGCGTTCTCCTGAGCCAGGATCA
>369
CTGCTGCCGCCCGTAGGAGTCTGGTCCGTGTCTCAGTACCAGTGTGGGGGATCACCCTCTCAGGCCCCCTAAA
GATCGTCGCCTTGGTGAGCCGTTACCTCACCAACTAGCTAATCTTGCGCGTGCCCATCTCTATCCACCGTAGT

TTTCAATATCACCCGATGCCGAATAATATATTATGGGGTATTAATCTTCTTCGAAAGGCTATCCCCCTGATAA
AGGCAGGTTGCACACGTGTTCCGCACCCGTACGCCGCTCTCAATTACCCGAAAGTAATCTACCGCTCCGGCTT
GCATGTGTTAGGCCTCCCGCTAGCGTTCTCCTGAGCAGG
>370
CTGCTGCCGCCCGTAGGAGTCTGGTCCGTGTCTCAGTACCAGTGTGGGGGATCACCCTCTCAGGCCCCCTAAA
GATCGTCGCCTTGGTGAGCCGTTACCTCACCAACTAGCTAATCTTGCGCGTGCCCATCTCTATCCACCGTAGT
TTTCAATATCACCCGATGCCGAATAATATATTATGGGGTATTAATCTTCTTCGAAAGGCTATCCCCCTGATAA
AGGCAGGTTGCACACGTGTTCCGCACCCGTACGCCGCTCTCAATTACCCGAAAGTAATCTACCGCTCGGCTTG
CATGTGTTAGGCCTCCCGCTAGCGTTCATCCTGAGCCAGGATCAAACTCT
>371
CTGCTGCCGCCCGTAGGAGTCTGGTCCGTGTCTCAGTACCAGTGTGGGGGATCACCCTCTCAGGCCCCCTAAA
GATCGTCGCCTTGGTGAGCCGTTACCTCACCAACTAGCTAATCTTGCGCGTGCCCATCTCTATCCACCGTAGT
TTTCAATATCACCCGATGCCGAATAATATATTATGGGGTATTAATCTTCTTCGAAAGGCTATCCCCCTGATAA
AGGCAGGTTGCACACGTGTTCCGCACCCGTACGCCGCTCTCAATTACCCGAAAGTAATCTACCGCTCGGCTTG
CATGTGTTAGGCCTCCCGCTAGCGTTCTCCTGAGCCAGGAT
>372
CTGCTGCCGCCCGTAGGAGTCTGGTCCGTGTCTCAGTACCAGTGTGGGGGATCACCCTCTCAGGCCCCCTAAA
GATCGTCGCCTTGGTGAGCCGTTACCTCACCAACTAGCTAATCTTGCGCGTGCCCATCTCTATCCACCGTAGT
TTTCAATATCACCCGATGCCGAATAATATATTATGGGGTATTAATCTTCTTCGAAAGGCTATCCCCCTGATAA
AGGCAGGTTGCACACGTGTTCCGCACCCGTACGCCGCTCTCAATTACCCGAAAGTAATCTACCGCTCGGCTTG
CATGTGTTAGGCCTCCCGCTAGCGTCATCCTGAGCCAGGAT
>373
CTGCTGCCGCCCGTAGGAGTCTGGTCCGTGTCTCAGTACCAGTGTGGGGGATCACCCTCTCAGGCCCCCTAAA
GATCGTCGCCTTGGTGAGCCGTTACCTCACCAACTAGCTAATCTTGCGCGTGCCCATCTCTATCCACCGTAGT
TTTCAATATCACCCGATGCCGAATAATATATTATGGGGTATTAATCTTCTTCGAAAGGCTATCCCCTGATAAA
GGCAGGTTGCACACGTGTTCCGCACCCGTACGCCGCTCTCAATTACCCGAAAGTAATCTACCGCTCGGCTTGC
ATGTGTTAGGCCTCCCGCTAGCGTTCATCCCGAGCCAGGATCAACTCCT
>374
CTGCTGCCGCCCGTAGGAGTCTGGTCCGTGTCTCAGTACCAGTGTGGGGGATCACCCTCTCAGGCCCCCTAAA
GATCGTCGCCTTGGTGAGCCGTTACCTCACCAACTAGCTAATCTTGCGCGTGCCCATCTCTATCCACCGTAGT
TTTCAATATCACCCGATGCCGAATAATATATTATGGGGGTATTAATCTTCTTCGAAAGGCTATCCCCCTGATA
AAGGCAGGTTGCACACGTGTTCCGCACCCGTACGCCGCTCTCAATTACCCGAAAGTAATCTACCGCTCGGCTT
GCATGTGTTAGGCCTCCCGCTAGCGTTCTCTGAGC
>375
CTGCTGCCGCCCCGTAGGAGTCTGGTCCGTGTCTCAGTACCCAGTGTGGGGATCACCCTCTCAGGCCCCCCTA
AAGATCGTCGCCTTGGTGAGCCGTTACCTCACCAACTAGCTAATCTTGCGCGTGCCCCATCTCTATCCCACCG
TAGTTTTCAATATCACCCGATGCCGAATAATATATTATGGGGTATTTAATCTTTCCTTTCGAAAGGGCTATCC
CCCTGATAAAAGGGCAGGTTGCACACGTGTCCCGCACCCGTACGCCGCTCTCAACTACCCGGAAAAGTAATCT
ACCCGCTCGGCTTGCATGTGTTAGGCCTCCCGCTAGCGTTCTCCTGAGCCAGGATCAAACTCT
>376
CTGCTGCCGCCCGTAGGAGTCTGGTCCGTGTCTCAGTACCAGTGTGGGGGATCACCCTCTCAGGCCCCCTAAA
GATCGTCGCCTTGGTGAGCCGTTACCTCACCAACTAGCTAATCTTGCGCGTGCCCATCTCTATCCACCGTAGT
TTTCAATATCACCCGATGCCGAATAATATATTATGGGGGTATTTAATCTTCTTCGAAAGGCTATCCCCCTGAT
AAAGGCAGGTTGCACACGTGTTCCGCACCCGTACGCCGCTCTCAATTACCCGAAAGTAATCTACCGCTCGGCT
TGCATGTGTTAGGCCTCCCGCTAGCGTTCATTCCTGAGCCAGGATCAAACTCT
>377
CTGCTGCCGCCCGTAGGAGTCTGGTCCGTGTCTCAGTACCAGTGTGGGGGATCACCCTCTCAGGCCCCCCTAA
AAGATCGTCGCCTTGGTGAGCCGTTACCTCACCAACTAGCTAATCTTGCGCGTGCCCATCTCTATCCACCGTA
GTTTTCAATATCACCCGATGCCGAATAATATATTATGGGGGTATTAATCTTCTTCGAAAGGCTATCCCCCTGA
TAAAGGCAGGTTGCACACGTGTTCCGCACCCGTACGCCGCTCTCAATTACCCGAAAGTAATCTACCGCTCGGC
TTGCATGTGTTAGGCCTCCCGCTAGCGTTCATCCTGAGCCAGGATCAAACTCT
>378
CTGCTGCCGCCCGTAGGAGTCTGGTCCGTGTCTCAGTACCAGTGTGGGGGATCACCCTCTCAGGCCCCCTAAA
GATCGTCGCCTTGGTGAGCCGTTACCTCACCAACTAGCTAATCTTGCGCGTGCCCATCTCTATCCACCGTAGT

TTTCAATATCACCCGATGCCGAATAATATATTATGGGGTATTAATCTTCTTCGAAAGGCTATCCCCTGATAAA
GGCAGGTTGCACACGTGTTCCGCACCCGTACGCCGCTCTCAATTACCCGAAAGTAATCTACCGCTCGGCTTGC
ATGTGTTAGGCCTCCCGCTAGCGTCTCCTG
>379
CTGCTGCCGCCCGTAGGAGTCTGGTCCGTGTCTCAGTACCAGTGTGGGGGATCACCCTCTCAGGCCCCCTAAA
GATCGTCGCCTTGGTGAGCCGTTACCTCACCAACTAGCTAATCTTGCGCGTGCCCATCTCTATCCACCGTAGT
TTTCAATATCACCCGATGCCGAATAATATATTATGGGGTATTAATCTTCTTCGAAAGGCTATCCCCCTGATAA
AGGCAGGTTGCACACGTGTTCCGCACCCGTACGCCGCTCTCAATTACCCGAAAGTAATCTACCGCTCGGCTTG
CATGTGTTAGGCCTTCCGCTAGCGTTCATCCTGAGCCAGGATCAAACTCT
>380
CTGCTGCCGCCCGTAGGAGTCTGGTCCGTGTCTCAGTACCAGTGTGGGGATCACCCTCTCAGGCCCCCTAAAG
ATCGTCGCCTTGGTGAGCCGTTACCTCACCAACTAGCTAATCTTGCGCGTGCCCATCTCTATCCACCGTAGTT
TTCAATATCACCCGATGCCGAATAATATATTATGGGGTATTAATCTTCTTCGAAAGGCTATCCCCCTGATAAA
GGCAGGTTGCCACACGTGTTCCGCACCCGTACGCCGCTCTCAATTACCCGAAAGTAATCTACCGCTCGGCTTG
CATGTGTTAGGCCTCCCGCTAGCGTTCATCCTGAGCCAGGATCAAACTC
>381
CTGCTGCCGCCCGTAGGAGTCTGGTCCGTGTCTCAGTACCAGTGTGGGGGATCACCCTCTCAGGCCCCCTAAA
GATCGTCGCCTTGGTGAGCCGTTACCTCACCAACTAGCTAATCTTGCGCGTGCCCATCTCTATTCCACCGTAG
TTTTCAATATCACCCGATGCCGAATAATATATTATGGGGTATTAATCTTCTTCGAAAGGCTATCCCCCTGATA
AAGGCAGGTTGCACACGTGTTCCGCACCCGTACGCCGCTCTCAATTACCCGAAAGTAATCTACCGCTCGGCTT
GCATGTGTTAGGCCTCCCGCTAGCGTTCTCCTGAGCAGGATCAACTCT
>382
CTGCTGCCGCCCGTAGGAGTCTGGTCCGTGTCTCAGTACCAGTGTGGGGGATCACCCTCTCAGGCCCCCTAAA
GATCGTCGCCCTTGGTGAGCCGTTACCTCACCAACTAGCTAATCTTGCGCGTGCCCATCTCTATCCACCGTAG
TTTTCAATATCACCCGATGCCGAATAATATATTATGGGGTATTAATTCTTCTTCGAAAGGCTATCCCCCTGAT
AAAGGCAGGTTGCACACGTGTTCCGCACCCGTACGCCGCTCTCAATTACCCGAAAGTAATCTACCGCTCGGCT
TGCATGTGTTAGGCCTCCCGCTAGCGTTCATCCTGAGCCAGGATCAAACTCT
>383
CTGCTGCCGCCCGTAGGAGTCTGGTCCGTGTCTCAGTACCAGTGTGGGGGATCACCCTCTCAGGCCCCCTAAA
GATCGTCGCCTTGGTGAGCCGTTACCTCACCAACTAGCTAATCTTGCGCGTGCCCATCTCTATCCACCGTAGT
TTTCAATATCACCCGATGCCGAATAATATATTATGGGGTATTAATCTTCTTCGAAAGGCTATCCCCCTGATAA
AGGCAGGTTGCACACGTGTTCCGCACCCGTACGCCGCTCTCAATTACCCGAAAGTAATCTACCGCTCGGCTTG
CATGTGTTAGGCCTCCCGCTAGCGTTCATCCTGAGCCAGGATCAACTCT
>384
CTGCTGCCGCCCGTAGGAGTCTGGTCCGTGTCTCAGTACCAGTGTGGGGGATCACCCTCTCAGGCCCCCTAAA
GATCGTCGCCTTGGTGAGCCGTTACCTCACCAACTAGCTAATCTTGCGCGTGCCCATCTCTATCCACCGTAGT
TTTCAATATCACCCGATGCCGAATAATATATTATGGGGTATTAATCTTCTTCGAAAGGCTATCCCCCTGATAA
AGGCAGGTTGCACACGTGTTCCGCACCCGTACGCCGCTCTCAATTACCCGAAAGTAATCTACCGCTCGGCTTG
CATGTGTTAGGCCTCCCGCTAGCGTTCATCCTGAGCCAGGATCAACTCT
>385
CTGCTGCCGCCCGTAGGAGTCTGGTCCGTGTCTCAGTACCAGTGTGGGGGATCACCCTCTCAGGCCCCCTAAA
GATCGTCGCCTTGGTGAGCCGTTACCTCACCAACTAGCTAATCTTGCGCGTGCCCATCTCTATCCACCGTAGT
TTTCAATATCACCCGATGCCGAATAATATATTATGGGGTATTAATCTTCTTCGAAAGCTATCCCCTGATAAAG
GCAGGTTGCACACGTGTTCCGCACCCGTACGCCGCTCTCAATTACCCGAAAGTAATCTACCGCTCGGCTTGCA
TGTGTTAGGCCTCCCGCTAGCGTTCATCCGTGAG
>386
CTGCTGCCGCCCGTAGGAGTCTGGTCCGTGTCTCAGTACCAGTGTGGGGGATCACCCTCTCAGGCCCCCTAAA
GATCGTCGCCTTGGTGAGCCGTTACCTCACCAACTAGCTAATCTTGCGCGTGCCCATCTCTATTCCACCGTAG
TTTTCAATATCACCCGATGCCGAATAATATATTTATGGGGTATTTAATTCTTTCTTCGAAAGGCTATCCCCCT
GATAAAGGCAGGTTGCACACGTGTTCCGCACCCGTACGCCGCTCTCAATTACCCGAAAGTAATCTACCGCTCG
GCTTGCATGTGTTAGGCCTCCCGCTAGCGTTCATTCCTGAGCCAGGATCAAACTCT
>387
CTGCTGCCGCCCGTAGGAGTCTGGTCCGTGTCTCAGTACCAGTGTGGGGGATCACCCTCTCAGGCCCCCTAAA
GATCGTCGCCTTGGTGAGCCGTTACCTCACCAACTAGCTAATCTTGCGCGTGCCCATCTCTATCCACCGTAGT

TTTCAATATCACCCGATGCCGAATAATATATTATGGGGTATTAATCTTCTTCGAAAGGCTATCCCCTGATAAA
GGCAGGTTGCACACGTGTTCCGCACCCGTACGCCGCTCTCAATTACCCGAAAGTAATCTACCGCTCGGCTTGC
ATGTGTTAGGCCTCCCGCTAGCGTTCATCCTGAGCCAGGATCAAACTCT
>388
CTGCTGCCGCCCGTAGGAGTCTGGACCGTGTCTCAGTTCCAGTGTGACTGATCATCCTCTCAGACCAGTTACG
GATCGTCGCCTTGGTAGGCCTTTACCCCACCAACTAGCTAATCCGACCTAGGCTCATCTGATAGCGTGAGGTC
CGAAGATCCCCCACTTCTCCCTCAGGACGTATGCGGTATTAGCGCCCGTTTCCGGACGTATCCCCACTACCAG
GCAGATTCCTCAGGCATTACTCACCCGTCCGCCGCTGAATCCAGGAGCCAAGCTCCCTTCATCCGCTCGACTT
GCATGTGTTAGGCCTGCCGCCAGCGTTCAATCTGAGCCAGGATCAAACTCT
>389
CTGCTGCCGCCCGTAGGAGTCTGGTCCGTGTCTCAGTACCAGTGTGGGGGATCACCCTCTCAGGCCCCCTAAA
GATCGTCGCCTTGGTGAGCCGTTACCTCACCAACTAGCTAATCTTGCGCGTGCCCATCTCTATCCACCGTAGT
TTTCAATATCACCCGATGCCGAATAATATATTATGGGGTATTAATCTTCTTCGAAAGGCTATCCCCTGATAAA
GGCAGGTTGCACACGTGTTCCGCACCCGTACGCCGCTCTCAATTACCCGAAAGTAATCTACCGCATCGGCTTG
CATGTGTTAGGCCTCCCGCTAGCGTTCTCCTGAGCAGGATCAACTCGT
>390
CTGCTGCCGCCCGTAGGAGTCTGGTCCGTGTCTCAGTACCAGTGTGGGGGATCACCCTCTCAGGCCCCCTAAA
GATCGTCGCCTTGGTGAGCCGTTACCTCACCAACTAGCTAATCTTGCGCGTGCCCATCTCTATCCACCGTAGT
TTCAATATCACCCGATGCCGAATAATATATTATGGGGTATTTAATCTTCTCGAAAGGCTATCCCCTGATAAAG
GCAGGTTAGCACACGTGTTCCGCACCCGTACGCCGCTCTCAATTACCCGAAAGTAATCTACCGCTCGGCTTGC
ATGTGTTAGGCCTCCCGCTAG
>391
CTGCTGCCGCCCGTAGGAGTCTGGTCCGTGTCTCAGTACCAGTGTGGGGGATCACCCTCTCAGGCCCCCTAAA
GATCGTCGCCTTGGTGAGCCGTTACCTCACCAACTAGCTAATCTTGCGCGTGCCCATCTCTATCCACCGTAGT
TTTCAATATCACCCGATGCCGAATAATATATTATGGGGTATTAATCTTCTTCGAAAGGCTATCCCCCTGATAA
AGGCAGGTTGCACACGTGTTCCGCACCCGTACGCCGCTCTCAATTACCCGAAAGTAATCTACCGCTCCGGCTT
GCATGTGTTAGGCCTCCCGCTAGCGTTCATCCTGAGCCAGGATCAAACTCT
>392
CTGCTGCCGCCCGTAGGAGTCTGGTCCGTGTCTCAGTACCAGTGTGGGGGATCACCCTCTCAGGCCCCCTAAA
GATCGTCGCCTTGGTGAGCCGTTACCTCACCAACTAGCTAATCTTGCGCGTGCCCATCTCTATCCACCGTAGT
TTCAATATCACCCGATGCCGAATAATATATTATGGGGTATTAATCTTCTTCGAAAGGCTATCCCCCTGATAAA
GGCAGGTTGCACACGTGTTCCGCACCCGTACGCCGCTCTCAATTTACCCGAAAGTAATCTACCGCTCGGCTTG
CATGTGTTAGGCCTCCCGCTAGCGTCATCCTGAGCCAG
>393
CTGCTGCCGCCCGTAGGAGTCTGGTCCGTGTCTCAGTACCAGTGTGGGGGATCACCCTCTCAGGCCCCCTAAA
GATCGTCGCCTTGGTGAGCCGTTACCTCACCAACTAGCTAATCTTGCGCGTGCCCATCTCTATCCACCGTAGT
TTTCAATATCACCCGATGCCGAATAATATATTATGGGGTATTTAATCTTTCTTCGAAAGGCTATCCCCCTGAT
AAAGGCAGGTTGCACACGTGTTCCGCACCCGTACGCCGCTCTCAATTACCCGAAAGTAATCTACCGCTCGGCT
TGCATGTGTTTAGGCCTCCCGCTAGCGTTCATCCTGAGCCAGGATCAACTCT
>394
TGCTGCCGCCCGTAGGAGTCTGGTCCGTGTCTCAGTACCAGTGTGGGGGATCACCCTCTCAGGCCCCCTAAAG
ATCGTCGCCTTGGTGAGCCGTTACCTCACCAACTAGCTAATCTTGCGCGTGCCCATCTCTATCCACCGTAGTT
TTTCAATATCACCCGATGCCGAATAATATATTTATGGGGTATTTAATCTTTCTTCGAAAGGCTATCCCCCTGA
TAAAGGCAGGTTTGCACACGTGTTCCGCACCCGTACGCCGCTCTCAATTACCCGAAAGTAATCTACCGCTCGG
CTTGCATGTGTTAGGCCTCCCGCTAGCGTTCTCCTGAGCCAGGATCAACTCT
>395
CTGCTGCCGCCCGTAGGAGTCTGGTTCCGTGTCTCAGTACCAGTGTGGGGGATCACCCTCTCAGGCCCCCTAA
AGATCGTCGCCTTGGTGAGCCGTTACCTCACCAACTAGCTAATCTTGCGCGTGCCCATCTCTATCCACCGTAG
TTTTTCAATATCACCCGATGCCGAATAATATATTATGGGGTATTAATCTTCTTCGAAAGGCTATCCCCTGATT
AAAGGCAGGTTTGCACACGTGTTCCGCACCCGTACGCCGCTCCATTATCCCGAAAGTAATTCTACCGCTCGGC
TGCATGTGTTTAGGCCTTCCCGCTAGCGTTCATCCTGAGCCAGGATCAAACTCT
>396
CTGCTGCCGCCCGTAGGAGTCTGGTCCGTGTCTCAGTACCAGTGTGGGGATCACCCTCTCAGGCCCCCTAAAG
ATCGTCGCCTTGGTGAGCCGTTACCTCACCAACTAGCTAATCTTGCGCGTGCCCATCTCTTATCCACCGTAGT

TTTTCAATATCACCCGATGCCGAATAATATATTATGGGGTATTTTAATCTTTTCTTCGAAAGGCTATCCCCCT
GATAAAGGCAGGTTGCACACGTGTTCCGCACCCGTACGCCGCTCTTCAATTTACCCGAAAGTAATCTACCGCT
CGGCTTGCATGTGTTTAGGCCTCCCGCTAGCGTTCTCCTGAGCCAG
>397
CTGCTGCCGCCCGTAGGAGTCTGGTCCGTGTCTCAGTACCAGTGTGGGGGATCACCCTCTCAGGCCCCCTAAA
GATCGTCGCCTTGGTGAGCCGTTACCTCACCAACTAGCTTAATCTTGCGCGTGCCCATCTCTATCCACCGTAG
TTTTCAATATCACCCGATGCCGAATAATATATTTATTGGGGTATTTAATCTTCTTCGAAAGGCTATCCCCCTG
ATAAAGGCAGGTTTGCACACGTGTTCCGCACCCGTACGCCGCTCTCAATTACCCGAAAGTAATCTACCGCTCG
GCTTGCATGTGTTTAGGCCTTCCCGCTAGCGTTCATCCTGAGCCAGGATCAAACTCT
>398
CTGCTGCCGCCCGTAGGAGTCTGGTCCGTGTCTCAGTACCAGTGTGGGGGATCACCCTCTCAGGCCCCCTAAA
GATCGTCGCCTTGGTGAGCCGTTACCTCACCAACTAGCTAATCTTGCGCGTGCCCATCTCTATCCACCGTAGT
TTTCAATATCACCCGATGCCGAATAATATATTATGGGGTATTAATCTTCTTCGAAAGGCTATCCCCCTGAATA
AAGGCAGGTTGCCACACGTGTTCCGCACCCGTACGCCGCCCTCAATTACCCGAAAGTAATCTACCGCTCGGCT
TGCATGTGTTAGGCCTCCCGCTAGCGTTCTCCTGAGCAGATT
>399
CTGCTGCCGCCCGTAGGAGTCTGGTTCCGTGTCTCAGTACCAGTGTGGGGGATCACCCTCTCAGGCCCCCTAA
AGATCGTCGCCTTGGTGAGCCGTTACCTCACCAACTAGCTAATCTTGCGCGTGCCCATCTCTATCCACCGTAG
TTTTCAATATCACCCGATGCCGAATATATATTATGGGGTATTAATCTTCTTCTGAAAGGCTATCCCCCTGATA
AAGGCAGGTTGCACACGTGTTCCGCACCCGTACGCCGCTCTCAATTTACCCGAAAGTAATCTACGCTCGGCTT
GCATGTGTTAGGCCTCCCGCTAGCGTTCATCCTGAGCCAGGATCAAACTCT
>400
CTGCTGCCGCCCGTAGGAGTCTGGACCCGTGTCTCAGTTCCAGTGTGACTGATCATCCTCTCAGACCCAGTAC
GGATCGTCGCCTTGGTAGGCCCTTTTTACCCCCACCAACTAGCTAATCCGACCTAGGCTCATCTGATAGCGTG
AGGTCCGAAGATCCCCCACTTCTCCCCTCAGGACGTATGCGGTATTAGCGCCCCGTTTCCGGACGATCCCCAC
TACCAGGCAGATTCCTAGGCATTACTCACCCGTCCGCCGCTGAATCCAGGAGCAAGCTCCCTTCATCCGCTCG
ACTGCATGTGTTAGGCCTGCCGCCAGCGTTCAATCTGAGCCCAGGATCAACTCT
>401
CTGCTGCCGCCCGTAGGAGTCTGGTCCGTGTCTCAGTACCAGTGTGGGGGATCACCCTCTCAGGCCCCCTAAA
GATCGTCGCCTTGTGAGCCGTTACCTCACCAACTAGCTAATCTTGCGCGTGCCCATCTCTATTCCACCGTAGT
TTTCAATATCACCCGATGCCGAATAATATATTATGGGGTATTAATCTTCTTCGAAAGGCTATCCCCCTGATAA
AGGCAGGTTGCACACGTGTTCCGCACCCGTACGCCGCTCTCAATTACCCGAAAGTAATCTACCGCTCGGCTTG
CATGTGTTAGGCCTCCCGCTAGCGTTCTCCTGAGCAGGATCAACTCT
>402
CTGCTGCCGCCCGTAGGAGTCTGGTCCGTGTCTCAGTACCAGTGTGGGGGATCACCCTCTCAGGCCCCCTAAA
GATCGTCGCCTTGGTGAGCCGTTACCTCACCAACTAGCTAATCTTGCGCGTGCCCATCTCTATCCACCGTAGT
TTTCAATATCACCCGATGCCGAATAATATATTATGGGGTATTAATCTTCTTCGAAAGGCTATCCCCCTGATAA
AGGCAGGTTGCACACGTGTTCCGCACCCGTACGCCGCTCTCAATTACCCGAAAGTAATCTACCGCTCGGCTTG
CATGTGTTAGGCCTCCCGCTAGCGTTCATCTGAGCCAGGATCAACTCT
>403
CTGCTGCCGCCCGTAGGAGTCTGGTCCGTGTCTCAGTACCAGTGTGGGGGATCACCCTCTCAGGCCCCCTAAA
GATCGTCGCCTTGGTGAGCCGTTACCTCACCAACTAGCTAATCTTGCGCGTGCCCATCTCTATCCACCGTAGT
TTTCAATATCACCCGATGCCGAATAATATAATTATGGGGTATTAATCTTCTCGAAAGGCTATCCCCTGATAAA
GGCAGGTTGCACACGTGTTCCGCACCCGTACGCCGCTCTCAATTACCCGAAAGTAATCTACCGCTCGGCTTGC
ATGTGTTAGGCCTCCCGCTAGCGTTCATCCTGAG
>404
CTGCTGCCGCCCGTAGGAGTCTGGTCCGTGTCTCAGTACCAGTGTGGGGGATCACCCTCTCAGGCCCCCTAAA
GATCGTCGCCTTGGTGAGCCGTTACCTCACCAACTAGCTAATCTTGCGCGTGCCCATCTCTATCCACCGTAGT
TTTCAATATCACCCGATGCCGAATAATATATTATGGGGTATTAATCTTCTTCGAAAGGCTATCCCCCTGATAA
AGGCAGGTTGCACACGTGTTCCGCACCCGTACGCCGCTCTCAATTACCCGAAAGTAATCTACCGCTCGGCTTG
CATGTGTTAGGCCTCCCGCTAGCGTTCATCCTGAGCCAGGATCAAACTCT
>405
CTGCTGCCGCCCGTAGGAGTCTGGTCCGTGTCTCAGTACCAGTGTGGGGGATCACCCTCTCAGGCCCCCTAAA
GATCGTCGCCTTGGTGAGCCGTTACCTCACCAACTAGCTAATCTTGCGCGTGCCCATCTCTATCCACCGTAGT

TTTCAATACACCGATCGCCGAATAATATATTATGGGGTATTAATCTTCTTCGAAAGGCTATCCCCTGATAAAG
GCAGGTTGCCACACGTGTTCCGCACCCGTACGCCGCTCTCAATTACCCGAAAGTAATCTACCGCTCGGCTTGC
ATGTGTTAGGCCTCCCGCTAGCGTTCTCCTGAGCAGGATC
>406
CTGCTGCCGCCCGTAGGAGTCTGGTCCGTGTCTCAGTACCAGTGTGGGGGATCACCCTCTCAGGCCCCCTAAA
GATCGTCGCCTTGGTGAGCCGTTACCTCACCAACTAGCTAATCTTGCGCGTGCCCATCTCTATCCACCGTAGT
TTCAATATCACCCGATGCCGAATAATATATTATGGGGTATTAATCTTCTTCGAAAGGCTATCCCCTGATAAAG
GCAGGTTGCACACGTGTTCCGCACCCGTACGCCGCTCTCAATTACCCGAAAGTAATCTACCGCTCGGCTTGCA
TGTGTTAGGCCTCCCGCTAGCGTTCTCCTGAGCAGGATCAACTCT
>407
CTGCTGCCGCCCGTAGGAGTCTGGTCCGTGTCTCAGTACCAGTGTGGGGGATCACCCTCTCAGGCCCCCTAAA
GATCGTCGCCTTGGTGAGCCGTTACCTCACCAACTAGCTAATCTTGCGCGTGCCCATCTCTATCCACCGTAGT
TTTCAATATCACCCGATGCCGAATAATATATTATGGGGTATTAATCTTCTCGAAAGGCTATCCCCCTGATAAA
GGCAGGTTGCACACGTGTTCCGCACCCGTACGCCGCTCTCGATTACCCGAAAGTAATCTACCGCTCCGGCTTG
CATGTGTT
>408
CTGCTGCCGCCCGTAGGAGTCTGGTCCGTGTCTCAGTACCAGTGTGGGGGATCACCCTCTCAGGCCCCCTAAA
GATCGTCGCCTTGGTGAGCCGTTACCTCACCAACTAGCTAATCTTGCGCGTGCCCATCTCTATCCACCGTAGT
TTTCAATATCACCCGATGCCGAATAATATATTATGGGGTATTAATCTTCTTCGAAAGCTATCCCCTGATAAAG
GCAGGTTGCACACGTGTTCCGCACCCGTACGCCGCTCTCAATTACCCGAAAGTAATCTACCGCTCGGCTTGCA
TGTGTTAGACCTCCCGCTAGCGTTCATCCTGAGCCAGGATCAACTCT
>409
CTGCTGCCGCCCGTAGGAGTCTGGACCGTGTCTCAGTTCCAGTGTGACTGATCATCCTCTCAGACCAGTACGG
ATCGTCGCCTTGGTAGGCCTTTACCCCCACCAACTAGCTAATCCGACCTAGGCTCATCTGATAGCGTGAGGTC
CGAAGTCCCCCACTTCTCCCTCAGGACGTATGCGGTATTAGCGCCCGTTTCCGGACGTATCCCCACTACCAGG
CAGATTCCTAGGCATTACTCACCCGTCCGCCGCTGAATCCAGGAGCAAGCTCCCCTTCATCCGCTCGACTGCA
TTGTGTTAGGCCTGCCGCCAGCGTCAATCTGAGCCAGGATCAAACTCT
>410
CTGCTGCCGCCCGTAGGAGTCTGGTCCGTGTCTCAGTACCAGTGTGGGGGATCACCCTCTCAGGCCCCCTAAA
GATCGTCGCCTTGGTGAGCCGTTACCTCACCAACTAGCTAATCTTGCGCGTGCCCATCTCTATCCACCGTAGT
TTTCAATATCACCCGATGCCGAATAATATATTATGGGGTATTAATCTTCTTCGAAAGGCTATCCCCTGATAAA
GGCAGGTTGCACACGTGTTCCGCACCCGTACGCCGCTCTCAATTACCCGAAAGTAATCTACCGCTCCGGCTTG
CATGTGTTAGGCCTCCCGCTAGCGTTCTCCTGAGCAGGATCAACTCGT
>411
CTGCTGCCGCCCGTAGGAGTCTGGTCCGTGTCTCAGTACCAGTGTGGGGGATCACCCCCTCAGGCCCCCTAAA
GATCGTCGCCTTGGTGAGCCGTTACCTCACCAACTAGCTAATCTTGCGCGTGCCCATCTCTATCCACCGTAGT
TTTCAATATCACCCGATGCCGAATAATATATTATGGGGTATTAATCTTCTCGAAAGGCTATCCCCCTGATAAA
GGCAGGTTGCACACGTGTTCCGCACCCGTACGCCGCTCTCAATTACCCGAAAGTAATCTACCGCTCCGGCTTG
CATGTGTTAGGCCTCCCGCTAGCGTTCATCCTGAGCCAGGATCAAACTCT
>412
CTGCTGCCGCCCGTAGGAGTCTGGTCCGTGTCTCAGTACCAGTGTGGGGGATCACCCTCTCAGGCCCCCTAAA
GATCGTCGCCTTGGTGAGCCGTTACCTCACCAACTAGCTAATCTTGCGCGTGCCCATCTCTATCCACCGTAGT
TTTCAATATCACCCGATGCCGAATAATATATTATGGGGTATTAATCTTCTTCGAAAGGCTATCCCCCTGATAA
AGGCAGGTTGCACACGTGTTCCGCACCCGTACGCCGCTCTCAATTACCCGAAAGTAATCTACCGCTCGGCTTG
CATGTGTTAGGCCTCCCGCTAGCGTTCATCCTGAGCCAGGATCAA
>413
CTGCTGCCGCCCGTAGGAGTCTGGTCCGTGTCTCAGTACCAGTGTGGGGGATCACCCTCTCAGGCCCCCTAAA
GATCGTCGCCTGGTGAGCCGTTACCTCACCAACTAGCTAATCTTGCGCGTGCCCATCTCTATCCACCGTAGTT
TTCAATATCACCCGATGCCGAATAATATATTATGGGGTATTAATCTTCTTCTGAAAGGCTATCCCCCTGATAA
AGGCAGGTTGCACACGTGTTCCGCACCCGTACGCCGCTCTCAATTACCCGAAAGTAATCTACCGCTCGGCTTG
CATGTGTTAGGCCTCCCGCTAGCGTTCATCCTGAGCCAGGATCAAACTCT
>414
CTGCTGCCGCCCGTAGGAGTCTGGTCCGTGTCTCAGTACCAGTGTGGGGGATCACCCTCTCAGGCCCCCTAAA
GATCGTCGCCTTGGTGAGCCGTTACCTCACCAACTAGCTAATCTTGCGCGTGCCCACTCTCTATCCACCGTAG

TTTTCAATATCACCCGATGCCGAATAATATATTATGGGGTATTAATCTTCTTCGAAAGGCTATCCCCCTGATA
AAGGCAGGTTGCACACGTGTTCCGCACCCGTACGCCGCTCTCAATTACCCGAAAGTAATCTACCGCTCGGCTT
GCATGTGTTAGGCCTCCCGCTAGCGTTCTCCTG
>415
CTGCTGCCGCCCGTAGGAGTCTGGTCCGTGTCTCAGTACCAGTGTGGGGGATCACCCTCTCAGGCCCCCTAAA
GATCGTCGCCTTGGTGAGCCGTTACCTCACCAACTAGCTAATCTTGCGCGTGCCCATCTCTATCCACCGTAGT
TTTCAATATCACCCGATGCCGAATAATATATTATGGGGTATTAATCTTCTCGAAAGCTATCCCCTGATAAAGG
CAGGTTGCACACGTGTTCCGCACCCGTACGCCGCTCTCAATTACCCGAAAGTAATCTACCGCTCCGGCTTGCA
TGTGTTAGGCCTCCCGCTAGCGTTCATCCTGAGCCAGGATCAACTCT
>416
CTGCTGCCGCCCGTAGGAGTCTGGTCCGTGTCTCAGTACCAGTGTGGGGGGATCACCCTCTCAGGCCCCCTAA
AGATCGTCGCCTTGGTGAGCCGTTACCTCACCAACTAGCTAATCTTGCGCGTGCCCATCTCTATCCACCGTAG
TTTTCAATATCACCCGATGCCGAATAATATATTATGGGGTATTAATCTTCTTCGAAAGGCTATCCCCCTGATA
AAGGCAGGTTGCACACGTGTTCCGCACCCGTACGCCGCTCTCAATTACCCGAAAGTAATCTACCGCTCGGCTT
GCATGTGTTAGGCCTCCCGCTAGCGTTCATCCTGAGCCAGGATCAACTCT
>417
CTGCTGCCGCCCGTAGGAGTCTGGTCCGTGTCTCAGTACCAGTGTGGGGGATCACCCTCTCAGGCCCCCTAAA
GATCGTCGCCTTGGTGAGCCGTTACCTCACCAACTAGCTAATCTTGCGCGTGCCCATCTCTATCCACCGTAGT
TTTCAATATCACCCGATGCCGAATAATATATTATGGGGTATTTAATCTTCTTCGAAAGGCTATCCCCCTGATA
AAGGCAGGTTGCACACGTGTTCCGCACCCGTACGCCGCTCTCAATTACCCGAAAGTAATCTACCGCTCCGGCT
TGCATGTGTTAGGCCTCCCGCTAGCGTTCATTCCTCGA
>418
CTGCTGCCGCCCGTAGGAGTCTGGTCCGTGTCTCAGTACCAGTGTGGGGATCACCCTCTCAGGCCCCCTAAAG
ATCGTCGCCTTGGTGAGCCGTTACCTCACCAACTAGCTAATCTTGCGCGTGCCCATCTCTATCCACCGTAGTT
TTCAATATCACCCGATGCCGAATAATATATTATGGGGTATTAATCTTCTTCGAAAGGCTATCCCCCTGATAAA
GGCAGGTTGCACACGTGTTCCGCACCCGTACGCCGCTCTCAATTACCCGAAAGTAATCTACCGCTCGGCTTGC
ATGTGTTAGGCCTCCCGCTAGCGTCATCCTGAGCCAGGATCAACTCT
>419
CTGCTGCCGCCCGTAGGAGTCTGGTCCGTGTCTCAGTACCAGTGTGGGGGATCACCCTCTCAGGCCCCCTAAA
GATCGTCGCCTTGGTGAGCCGTTACCTCACCAACTAGCTAATCTTGCGCGTGCCCATCTCTATCCACCGTAGT
TTTCAATATCACCCGATGCCGAATAATATATTATGGGGTATTAATCTTCTTCGAAAGGCTATCCCCCTGATAA
AGGCAGGTTGCACACGTGTTCCGCACCCGTACGCCGCTCTCAATTACCCGAAAGTAACTCTACCGCTCGGCTT
GCATGTGTTAGGCCTCCCGCTAGCGTTCTCCTGAGCAGGATCAACTCT
>420
CTGCTGCCGCCCGTAGGAGTCTGGACCGTGTCTCAGTTCCAGTGTGACTGATCATCCTCTCAGACCAGTACGG
ATCGTCGCCTTGGTAGGCCTTTACCCCACCAACTAGCTAATCCGAACCTAGGCTCATCTGATAGCGTGAGGTC
CGAAGTCCCCCACTTCTCCCTCAGGACGTATGCGGTATTAGCGCCCGTTTCCGGACGTATCCCCACTACCAGG
CAGATTCCTAGGCATTACTCACCCGTCCGCCGCTGAATCCAGGAGCAAGCTCCCTCTCCGACTCGACTGCCAT
GTGTTAGGCCT
>421
CTGCTGCCGCCCGTAGGAGTCTGGTCCGTGTCTCAGTACCAGTGTGGGGGATCACCCCCTCAGGCCCCCTAAA
GATCGTCGCCTTGGTGAGCCGTTACCTCACCAACTAGCTAATCTTGCGCGTGCCCATCTCTATCCACCGTAGT
TTTCAATATCACCCGATGCCGAATAATATATTATGGGGTATTAATCTTCTTCGAAAGGCTATCCCCCTGATAA
AGGCAGGTTGCACACGTGTTCCGCACCCGTACGCCGCTCTCAATTACCCGAAAGTAATCTACCGCTCGGCTTG
CATGTGTTAGGCCTCCCGCTAGCGTTCATCCTGAGCCAGGATCAAACTCT
>422
CTGCTGCCGCCCGTAGGAGTCTGGACCGTGTCTCAGTTCCAGTGTGACTGATCATTCCTCTCAGACCAGTACG
GATCGTCGCCTTGGTAGGCCTTTACCCCACCAACTAGCTAATCCGACCTAGGCTCATCTGATAGCGTGAGGTC
CGAAGTCCCCCACTTCTCCCTCAGGACGTATGCGGTATTAGCGCCCGTTTCCGGACGATCCCCACTACCAGGC
AGATTCCTAGGCATTACTCACCCGTCCGCCGCTGAATCCAGGAGCAAGCTCCCTTCATCCGCTCGACTGCATG
TGTTAGGCCTGCCGCCAGCGTTCAATCTGAGCCAGGATCAAACTCT
>423
CTGCTGCCGCCCGTAGGAGTCTGGTCCGTGTCTCAGTACCAGTGTGGGGGATCACCCTCTCAGGCCCCCTAAA
GATCGTCGCCTTGGTGAGCCGTTACCTCACCAACTAGCTAAACTTGCGCGTGCCCATCTCTATCCACCGTAGT

TTTCAATATCACCCGATGCCGAATAATATATTATGGGGTATTTAATCTTCTTCGAAAGGCTATCCCCTGATAA
AGGCAGGTTGCACACGTGTTCCGCACCGTACGCCGCTCTCAATTACCCGAAAGTAATCTACCGCTCGGCTTGC
ATGTGTTAGGCCTCCCGC
>424
CTGCTGCCGCCCGTAGGAGTCTGGTCCGTGTCTCAGTACCAGTGTGGGGGATCACCCTCTCAGGCCCCCTAAA
GATCGTCGCCTTGGTGAGCCGTTACCTCACCAACTAGCTAATCTTGCGCGTGCCCATCTCTATCCACCGTAGT
TTTCAATATCACCCGATGCCGAATAATTATATTATGGGGTATTTAATTCTTCTCGAAAGGCTATCCCCTGATA
AAGGCAGGTTGCACACGTGTTCCGCACCCGTACGCCGCTCTCAATTACCCGAAAGTAATCTACCGCTCGGCTT
GCATGTGTTAGGCCTCCCGCTAGCGTTCTTCCTGAGCAG
>425
CTGCTGCCGCCCGTAGGAGTCTGGACCGTGTCTCAGTCCCAGTGTGACTGATATCCTCTCAGACCAGTACGGA
TCGTCGCCTTGGTAGGCCTTTACCCCACCAACTAGCTAATCCGACCTAGGCTCATCTGATAGCGTGAGGTCCG
AAGTCCCCCACTTCTCCCTCAGGACGTATGCGGTATTAGCGCCCGTTTCCGGACGTATCCCCACTACCAGGCA
GATTCCTAGGCATTACTCACCCGTCCGCCGCTGAATCCAGGAGCAAGCCCCCTCATCCGCTCGACTGCATGTG
TTAGGCCTGCCGCCAGCGTTCAATCTGAGCCAGGATCAAACTCT
>426
CTGCTGCCGCCCGTAGGAGTCTGGACCGTGTCTCAGTTCCAGTGTGACTGGTCATCCTCTCAGACCAGTACGG
ATCGTCGCCTTGGTAGGCCTTTACCCCACCAACTAGCTAATCCGACCTAGGCTCATCTGATAGCGTGAGGTCC
GAAGTCCCCCACTTCTCCCTCAGGACGTATGCGGTATTAGCGCCCGTTTCCGGACGTATCCCCGCACTACCAG
GCAGATTCCTAGGCATTACTCACCCGTCCGCCGCTGAATCCAGGAGCAAGCTCCCTTCATCCGCTCGACTGCA
TGTGTTAGGCCTGCCGCCAGCGTCAATCTGAGCCAGGATCAAACTCT
>427
CTGCTGCCGCCCGTAGGAGTCTGGTCCGTGTCTCAGTACCAGTGTGGGGGATCACCCTCTCAGGCCCCCTAAA
GATCGTCGCCTTGGTGAGCCGTTACCTCACCAACTAGCTAATCTTGCGCGTGCCCATCTCTATCCACCGTAGT
TTCAATATCACCCGATGCCGAATAATATATTATGGGGTATTTAATCTTCTTCGAAAGGCTATCCCCCTGATAA
AGGCAGGTTGCACACGTGTTCCGCACCCGTACGCCGCTCTCAATTACCCGAAAGTAATCTACCGCTCGGCTTG
CATGTGTTAGGCCTCCCGCTAGCGTTCATCCTGAGCCAGGATCAACTCT
>428
CTGCTGCCGCCCGTAGGGGTCTGGACCGTGTCTCAGTTCCAGTGTGACTGATCATCCTCTCAGACCAGTTACG
GATCGTCGCCTTGGTAGGCCTTTACCCCACCAACTAGCTAATCCGACCTAGGCTCATCTGATAGCGTGAGGTC
CGAAGATCCCCCACTTCTCCCTCAGGACGTATGCGGTATTAGCGCCCGTTTCCGGACGTATCCCCCACTACCA
GGCAGATTCCTAGGCATTACTCACCCGTCCGCCGCTGAATCCAGGAGCAAGCTCCCTTCATCGCTCGACTGCA
TGTGTTAGGCCTGCCGCCAGCTGATTCATCGCTGAGCCAAGGCATCAACATCT
>429
CTGCTGCCGCCCGTAGGAGTCTGGACCGTGTCTCAGTTCCAGTGTGACTGATCATCCTCTCAGACCAGTTACG
GATCGTCGCCTTGGTAGGCCTTTACCCCACCAACTAGCTAATCCGACCTAGGCTCATCTGATAGCGTGAGGTC
CGAAGATCCCCCACTTCTCCCTCAGGACGTATGCGGTATTAGCGCCCGTTTCCGGACGTATCCCCACTACCAG
GCAGATTCCCTAGGCATTACTCACCCGTCCGCCGCTGAATCCAGGAGCAAGCCCTCCTACTCCGCTCGACTGC
ATGTGTTAGGCCTGCCGCCAGCGTTCAATCTGAGCCAGGATCAACATCT
>430
CTGCTGCCGCCCGTAGGAGTCTGGTCCGTGTCTCAGTACCAGTGTGGGGGATCACCCTCTCAGGCCCCCTAAA
GATCGTCGCCTTGGTGAGCCGTTACCTCACCAACTAGCTAATCTTGCGCGTGCCCATCTCTATCCACCGTAGT
TTTTCAATATCACCCGATGCCGAATAATATATTATGGGGTATTTAATTCTTTCTTCGAAAGGCTATCCCCCTG
ATAAAGGCAGGTTTGCACACGTGTTCCGCACCCGTACGCCGCTCTCAATTTACCCGAAAGTAATCTACCGCTC
GGCTTGCATGTGTTTAGGCCTTCCCGCTAGCGTTCATCCTGAGCCAGGATCAACTCTT
>431
CTGCTGCCGCCCGTAGGAGTCTGGTCCGTGTCTCAGTACCAGTGTGGGGATCACCCTCTCAGGCCCCCTAAAG
ATCGTCGCCTTGGTGAGCCGTTACCTCACCAACTAGCTAATCTTGCGCGTGCCCATCTCTATCCACCGTAGTT
TTCAATATCACCCGATGCCGAATAATATATTATGGGGTATTAATCTTCTTCGAAAGGCTATCCCCTGATAAAG
GCAGGTTGCACACGTGTTCCGCACCCGTACGCCGCTCTCAATTACCCGAAACGTAATCTACCGCTACGGCTTG
ACATGTGTTA
>432
TCTGCTGCCGCCCGTAGGAGTCTGGTTCCGTGTCTCAGTACCAGTGTGGGGGATCACCCTCTCAGGCCCCCTA
AAGATCGTTCGCCTTGGTGAGCCGTTACCTCACCAACTAGCTAATCTTGCGCGTGCCCATCTCTATCCACCGT

AGTTTTCAATTATCACCCGATGCCGAATAATATATTTATGGGGTATTTAATCTTTCTTCGAAAGGCTATCCCC
CTTGATAAAGGCAGGTTTGCACACGTGTTCCGCACCCGTACGCCGCTCTCAATTACCCGAAAGTAATCTACCG
CTCCGGCTTGCATGTGTTAGGCCTTCCCGCTAGCGTCTCCTGA
>433
CTGCTGCCGCCCGTAGGAGTCTGGTCCGTGTCTCAGTACCAGTGTGGGGGATCACCCTCTCAGGCCCCCTAAA
GATCGTCGCCTGTGAGCCGTTACCTCACCAACTAGCTAATCTTGCGCGTGCCCATCTCTATCCACCGTAGTTT
TCAATATCACCCGATGCCGAATAATATATTATGGGGTATTAATCTTCTTCTGAAAGGCTATCCCCTGATAAAG
GCAGGTTGCACACGTGTCCGCACCCGTACGCCGCTCTCAATTACCCGAAAGTAATCTACCGCTCGGCTTGCAT
GTGTTAGGCCTCCCGCTAGCGTCATCTGAGCCAGGATCAAACTCT
>434
CTGCTGCCGCCCGTAGGAGTCTGGTCCGTGTCTCAGTACCAGTGTGGGGGATCACCCTCTCAGGCCCCCTAAA
GATCGTCGCCTGTGAGCCGTTACCTCACCAACTAGCTAATCTTGCGCGTGCCCATCTCTATCCACCGTAGTTT
TCAATATCACCCGATGCCGAATAATATATTATGGGGTATTAATCTTCTTCGAAAGGCTATCCCCCTGATAAAG
GCAGGTTGCACACGTGTTCCGCACCCGTACGCCGCTCTCAATTACCCGAAAGTAATCTACCGCTCCGGCTTGC
ATGTGTTAGGCCTCCCGCTAGCGTTCATCCTGAGCCAGGATCAACTCT
>435
CTGCTGCCGCCCGTAGGAGTCTGGTCCCGTGTCTCAGTACCAGTGTGGGGGATCACCCTCTCAGGCCCCCTAA
AGATCGTCGCCTGGTGAGCCCGTTACCTCACCCAACTAGCTAATCTTGCGCGTGCCCATCTCTATCCACCGTA
GTTTTCAATATCACCCGATGCCGAATAATATATTATTGGGGTATTTAATCTTCTTCGAAAGGCTATCCCCCTG
ATAAAGGCAGGTTGCACACGTGTTCCGCACCCGTACGCCGCTCTCAATTACCCCGAAAGTAATCTACCGCTCG
GCTTGCATGTGTTAGGCCTCCCGCTAGCGTTCTCCTGAGCAGGATCAACTCGT
>436
CTGCTGCTCGCCCGTAGGAGTCTGGTCCCGTGTCTCAGTACCAGTGTGGGGGATCACCCTCTCAGGCCCCCCT
AAAGATCGTCGCCTGGTGAGCCGTTACCTCACCAACTAGCTAATCTTGCGCGTGCCCATCTCTATCCACCGTA
GTTTTCAATATCACCCGATGCCGAATAATATATTATGGGGTATTAATCTTCTTCGAAAGGCTATCCCCTGATA
AAGGCAGGTTGCACACGTGTTCCGCACCCGTACGCCGCTCTCAATTTACCCCGAAAGTAATCTACCGCTCGGC
TTGCATGTGTTAGGCCTCCCGCTAGCGCTCTCTGCAGCACG
>437
CTGCTGCCGCCGTAGGAGTCTGGACCGTGTCTCAGTTCCAGTGTGACTGATCATCCTCTCAGACCAGTACGGA
TCGTCGCCTTGGTAGGCCTTACCCCACCAACTAGCTAATCCGACCTAGGCTCATCTGATAGCGTGAGGTCCGA
AGATCCCCCACTTCTCCCTCAGGACGTATGCGGTATTAGCGCCCGTTTCCGGACGTATCCCCCACTACCAGGC
AGATTCCTAGGCATTACTCACCCGTCCGCCGCTGAATCCAGGAGCAAGCTCCCCTCATCCGCTCGACTGCATG
TGTTAGGCCTGCCGCCAGCGTTCAATCTGAGCCAGGATCAAACTCT
>438
CTGCTGCCGCCCGTAGGAGTCTGGTCCGTGTCTCAGTACCAGTGTGGGGGATCACCCTCTCAGGCCCCCTAAA
GATCGTCGCCTTGGTGAGCCGTTACCTCACCAACTAGCTAACCTTGCGCGTGCCCATCTCTATCCACCGTAGT
TTTCAATATCACCCGATGCCGAATAATATATTATGGGGTATTAATCTTCTTCGAAAGGCTATCCCCCTGATAA
AGGCAGGTTGCACACGTGTTCCGCACCCGTACGCCGCTCTCAATTACCCGAAAGTAATCTACCGCTCGGCTTG
CATGTGTTAGGCCTCCGCTAGCGTCTCCTGAGC
>439
CTGCTGCCGCCCGTAGGAGTCTGGTCCGTGTCTCAGTACCAGTGTGGGGGATCACCCTCTCAGGCCCCCTAAA
GATCGTCGCCTTGTGAGCCGTTACCTCACCAACTAGCTAATCTTGCGCGTGCCCATCTCTATCCACCGTAGTT
TTCAATATCACCCGATGCCGAATAATATATTATGGGGTATTAATCTTCTTCGAAAGGCTATCCCCTGATAAAG
GCAGGTTGCACACGTGTTCCGCACCCGTACGCCGCTCTCAATTACCCGAAAGTAATCTACCGCTCCGGCTTGC
ATGTGTTAGGCCTCCCGCTAGCGTTCTCCTGAGCAGG
>440
CTGCTGCCGCCCGTAGGAGTCTGGTCCGTGTCTCAGTACCAGTGTGGGGGATCACCCTCTCAGGCCCCCTAAA
GATCGTCGCCTTGGTGAGCCGTTACCTCACCAACTAGCTAATCTTGCGCGTGCCCATCTCTATCCACCGTAGT
TTTCAATATCACCCGATGCCGAATAATATATTATGGGGTATTAATCTTCTTCGAAAGGCTATCCCCCTGATAA
AGGCAGGTTGCACACGTGTTCCGCACCCGTACGCCGCTCTCAATTACCCGAAAGTAATCTACCGCTCCGGCTT
GCATGTGTTAGGCCTCCCGCTAGCGTTCTCCTGAGC
>441
CTGCTGCCGCCCGTAGGAGTCTGGTCCGTGTCTCAGTACCAGTGTGGGGGATCACCCTCTCAGGCCCCCTAAA
GATCGTCGCCTTGGTGAGCCGTTACCTCACCAACTAGCTAATCTTGCGCGTGCCCATCTCTATCCACCGTAGT

TTTCAATATCACCCGATGCCGAATAATATATTATGGGGTATTAATCTTCTCGAAAGGCTATCCCCCTGATAAA
GGCAGGTTGCACACGTGTTCCGCACCCGTACGCCGCTCTCAATTACCCGAAAGTAATCTACCGCTCGGCTTGC
ATGTGTTAGGCCTCCCGCTAGCGTTCTCCTGAGCAGG
>442
CTGCTGCCGCCCGTAGGAGTCTGGTCCGTGTCTCAGTACCAGTGTGGGGGATCACCCTCTCAGGCCCCCTAAA
GATCGTCGCCTTGGTGAGCCGTTACCTCACCAACTAGCTAATCTTGCGCGTGCCCATCTCTATCCACCGTAGT
TTTCAATATCACCCGATGCCGAATAATATATTATGGGGTATTAATCTTCTTCGAAAGGCTATCCCCCTGATAA
AGGCAGGTTGCACACGTGTTCCGCACCCGTACGCCGCTCTCAATTACCCGAAAGTAATCTACCGCTCGGCTTG
CATGTGTTAGGCCTCCCGCTAGCGTTCATCCTGAGCCAGGATCAACTCT
>443
CTGCTGCCGCCCGTAGGAGTCTGGTCCGTGTCTCAGTACCAGTGTGGGGGATCACCCTCTCAGGCCCCCTAAA
GATCGTCGCCTTGGTGAGCCGTTACCTCACCAACTAGCTAATCTTGCGCGTGCCCATCTCTATCCACCGTAGT
TTTCAATATCACCCGATGCCGAATAATATAATTATGGGGTATTAATCTTCTCTGAAAGGCTATCCCCTGATAA
AGGCAGGTTGCACACGTGTTCCGCACCCGTACGCCGCTCTCAATTACCCGAAAGTAATCTACCGCTCCGGCTT
GCATGTGTTAGGCCTCCCGCTAGCGTTCATCCTGAGCCAGGATCAAACTCT
>444
CTGCTGCCGCCCGTAGGAGTCTGGTCCGTGTCTCAGTACCAGTGTGGGGGATCACCCTCTCAGGCCCCCTAAA
GATCGTCGCCTTGGTGAGCCGTTACCTCACCAACTAGCTAATCTTGCGCGTGCCCATCTCTATCCACCGTAGT
TTTCAATATCACCCGATGCCGAATAATATATTATGGGGTATTAATCTTCTTCGAAAGGCTATCCCCCTGATAA
AGGCAGGTTGCACACGTGTTCCGCACCCGTACGCCGCTCTCAATTACCCGAAAGTAATCACCGCTCGGCTTGC
ATGTGTTAGGCCTCCCGCTAGCGTTCTCCTGAGCAGGATCAAC
>445
CTGCTGCCGCCCGTAGGAGTCTGGTCCGTGTCTCAGTACCAGTGTGGGGGATCACCCTCTCAGGCCCCCTAAA
GATCGTCGCCTTGGTGAGCCGTTACCTCACCAACTAGCTAATCTTGCGCGTGCCCATCTCTATCCACCGTAGT
TTTCAATATCACCCGATGCCGAATAATATATTATGGGGTATTAATCTTCTTCGAAAGGCTATCCCCCTGATAA
AGGCAGGTTGCACACGTGTTCCGCACCCGTACGCCGCTCTCAATTACCCGAAAGTAATCTACCGCTCGGCTTG
CATGTGTTAGGCCTCCCGCTAGCGTTCATCCTGAGCCAGGATCAAACTCT
>446
CTGCTGCCGCCCCGTAGGAGTCTGGTCCCGTGTCTCAGTACCAGTGTGGGGGATCACCCTCTCAGGCCCCCCC
TAAAGATCGTCGCCTTGGTGAGCCCGTTTACCTCACCCAACTAGCTTAATCTTGCGCGTGCCCCATCTCTATC
CACCTAGTTTTCAATATCACCCGATGCCGAATAATATATTATGGGGTATTAATCTTCTTCGAAAGGCTATCCC
CTGATAAAGGCAGGTTGCACACGTGTTCCCGCACCCGTACGCCGCTCTCAATTACCCCGAAAGTAATCTACCG
CTCGGCTTGCATGTGTTAGGCCTCCCGCTAGCGTTCTCCTGAGCAGGA
>447
CTGCTGCCGCCCGTAGGAGTCTGGACCGTGTCTCAGTTCCAGTGTGACTGATCATCCTCTCAGACCAGTACGG
ATCGTCGCCTTGGTAGGCCTTTACCCCACCAACTAGCTAATCCGACCTAGGCTCATCTGATAGCGTGAGGTCC
GAAGTCCCCCACTTCTCCCTCAGGACGTATGCGGTATTAGCGCCCGTTTCCGGACGATCCCCACTACCAGGCA
GATTCCTAGGCATTACTCACCCGTCCGCCGCTGAATCCAGGAGCAAGCTCCCTTCATCCGCTCGACTGCATGT
GTTAGGCCTGCCGCCAGCGTTCAATCTGAGCCAGGATCAAACTCT
>448
CTGCTGCCGCCCGTAGGAGTCTGGTCCGTGTCTCAGTACCAGTGTGGGGGATCACCCTCTCAGGCCCCCTAAA
GATCGTCGCCTTGGTGAGCCGCTACCTCACCAACTAGCTAATCTTGCGCGTGCCCATCTCTATCCACCGTAGT
TTTCAATATCACCCGATGCCGAATAATATATTATGGGGTATTAATCTTCTCGAAAGCTATCCCCTGATAAAGG
CAGGTTGCACACGTGTTCCGCACCCGTACGCCGCTCTCAATTTACCCGAAAGTAATCTACCGCTCGGCTTGCA
TGTGTTAGGCCTCCCGCTAGCGTTCTCCTGAGCAGGATCAACTCGT
>449
CTGCTGCCGCCCGTAGGAGTCTGGTCCGTGTCTCAGTACCAGTGTGGGGGATCACCCTCTCAGGCCCCCTAAA
GATCGTCGCCTTGGTGAGCCGTTACCTCACCAACTAGCTAATCTTGCGCGTGCCCATCTCTATCCACCGTAGT
TTTCAATATCACCCGATGCCGAATAATATATTATGGGGTATTAATCTTCTCTGAAAGCTATCCCCTGATAAAG
GCAGGTTGCACACGTGTTCCGCACCCGTACGCCGCTCTCAATTACCCGAAAGTAATCTACCGCTCGGCTTGCA
TGTGTTAGGCCTCCCGCTAGCGTTCATCCTGAGCCAGGATCAAACTCT
>450
CTGCTGCCGCCCGTAGGAGTCTGGACCGTGTCTCAGTTCCAGTGTGACTGATCATCCTCTCAGACCAGTACGG
ATCGTCGCCTTGGTAGGCCTTTACCCCACCAACTAGCTAATCCGACCTAGGCTCATCTGATAGCGTGAGGTCC

GAAGTCCCCCACTTCTCCCTCAGGACGTATGCGGTATTAGCGCCCGTTTCCGGACGTATCCCCCACTACCAGG
CAGATTCCTAGGCATTACTCACCCGTCCGCCGCTGAATCCAGGAGCAAGCCCCCTTCATCCGCTCGACTGCAT
GTGTTAGGCCTGCCGCCAGCGTTCAATCTGAGCCAGGATCAAACTCT
>451
CTGCTGCCGCCCGTAGGAGTCTGGTCCGTGTCTCAGTACCAGTGTGGGGGATCACCCTCTCAGGCCCCCTAAA
GATCGTCGCCTTGGTGAGCCGTTACCTCACCAACTAGCTAATCTTGCGCGTGCCCATCTCTATCCACCGTAGT
TTTCAATATCACCCGATGCCGAATAATATATTATGGGGTATTAATCTTCTTCGAAAGGCTATCCCCCTGATAA
AGGCAGGTTGCACACGTGTTCCGCACCCGTACGCCGCTCTCAATTACCCGAAAGTAATCTACCGCTCGGCTGC
ATGTGTTAGGCCTCCCGCTAGCGTTCTCCTGAGCAGGATCC
>452
CTGCTGCCGCCCGTAGGAGTCTGGTCCGTGTCTCAGTACCAGTGTGGGGGATCACCCTCTCAGGCCCCCTAAA
GATCGTCGCCTTGGTGAGCCGTTACCTCACCAACTAGCTAATCTTGCGCGTGCCCATCTCTATCCACCGTAGT
TTTCAATATCACCCGATGCCGAATAATATATTATGGGGTATTAATCTTCTTCGAAAGGCTATCCCCCTGATAA
AGGCAGGTTGCACACGTGTTCCGCACCCGTACGCCGCTCTCAATTACCCGAAAGTAATCTACCGCTCGGCTTG
CATGTGTTAGGCCTCCCGCTAGCGTTCATCCTGAGCCAGGATCAAACTCT
>453
CTGCTGCCGCCCGTAGGAGTCTGGTCCGTGTCTCAGTACCAGTGTGGGGGATCACCCTCTCAGGCCCCCTAAA
GATCGTCGCCTTGGTGAGCCGTTACCTCACCAACTAGCTAATCTTGCGCGTGCCCATCTCTATCCACCGTAGT
TTTCAATATCAACCCGATGCCGAATAATATATTATGGGGTATTAATCTTCTTCGAAAGGCTATCCCCCTGATA
AAGGCAGGTTGCACACGTGTTCCGCACCCGTACGCCGCTCTCAATTACCCGAAAGTAATCTACCGCTCGGCTT
GCATGTGTTAGGCCTCCCGCTAGCGTTCATCCTGAGCCAGGATCAAACTCT
>454
CTGCTGCCGCCCGTAGGAGTCTGGACCGTGTCTCAGTTCCAGTGTGACTGATCATCCTCTCAGACCAGTTACG
GATCGTCGCCTTGGTAGGCCTTTACCCCACCAACTAGCTAATCCGACCTAGGCTCATCTGATAGCGTGAGGTC
CGAAGTCCCCCACTTCTCCCTCAGGACGTATGCGGTATTAGCGCCCGTTTCCGGACGTATCCCCACTACCAGG
CAGACTCCTAGGCATTACTCACCCGTCCGCCGCTGAATCCAGGAGCAAGCCCTCCTACTCCGCTCGACTGCAT
GTGTTAGGCCTGCCGCCAGCGTTCAATCTGAGCCAGGATCAAACTCT
>455
CTGCTGCCGCCCGTAGGAGTCTGGTCCGTGTCTCAGTACCAGTGTGGGGGATCACCCTCTCAGGCCCCCTAAA
GATCGTCGCCTTGGTGAGCCGTTACCTCACCACATAGCTAATCTTGCGCGTGCCCATCTCTATCCACCGTAGT
TTTCAATATCACCCGATGCCGAATAATATATTATGGGGTATTAATCTTCTTCGAAAGCTATCCCCCTGATAAA
GGCAGGTTGCACACGTGTTCCGCACCCGTACGCCGCTCTCAATTACCCGAAAGTAATCTACCGCTCGCTTGCA
TGTGTTAGGCCTCCCGCTAGCGTTCTCCTGAGCAGGATCAACTCGT
>456
CTGCTGCCGCCCATAGGAGTCTGGTCCGTGTCTCAGTACCAGTGTGGGGGATCACCCTCTCAGGCCCCCTAAA
GATCGTCGCCTTGGTGAGCCGTTACCTCACCAACTAGCTAATCTTGCGCGCCAGCCTATCTCATCCACCGTAG
TTTTCAATATCACCCGATGCCGAATAATATATTATGGGGTATTAATCTTCTTCGAAAGGCTATCCCCCTGATA
AAGGCAGGTTGCACACGTGTTCCGCACCCGTACGCCGCTCTCAATTACCCGAAAGTAATCTACCGCTCGGCTT
GCATGTGTTAGGCCTCCCGCTAGCGTTCATCCTGAGCCAGGATCAAACTCT
>457
CTGCTGCCGCCCGTAGGAGTCTGGTCCGTGTCTCAGTACCAGTGTGGGGGATCACCCTCTCAGGCCCCCTAAA
GATCGTCGCCTTGGTGAGCCGTTACCTCACCAACTAGCTAATCTTGCGCGTGCCCATCTCTATCCACCGTAGT
TTTCAATATCACCCGATGCCGAATAATATATTATGGGGTATTAATCTTCTTCGAAAGGCTATCCCCCTGATAA
AGGCAGGTTGCACACGTGTTCCGCACCCGTACGCCGCTCTCAATTACCCGAAAGTAATCTACCGCTCGGCTTG
CATGTGTTAGGCCTCCCGCTAGCGTTCATCCTGAGCAGGATCAAACTCT
>458
CTGCTGCCGCCCGTAGGAGTCTGGTCCGTGTCTCAGTACCAGTGTGGGGGATCACCCTCTCAGGCCCCCTAAA
GATCGTCGCCTTGGTGAGCCGTTACCTCACCAACTAGCTAATCTTGCGCGTGCCCATCTCTATCCACCGTAGT
TTTCAATATCACCCGATGCCGAATAATATATTTATGGGGTATTTAATTCTTTCTTCGAAAGGCTATCCCCCTG
ATAAAGGCAGGTTGCACACGTGTTCCGCACCCGTACGCCGCTCTCAATTACCCGAAAGTAATCTACCGCTCGG
CTTGCATGTGTTAGGCCTCCCGCTAGCGTTCATCCTGAGCCAGGATCAACTCT
>459
CTGCTGCCGCCCGTAGGAGTCTGGTCCGTGTCTCAGTACCAGTGTGGGGGATCACCCTCTCAGGCCCCCTAAG
ATCGTCGCCTTGTGAGCCGTTACCTCACCAACTAGCTAATCTTGCGCGTGCCCATCTTCTATCCACCGTAGTT

TTCAATATCACCCGATGCCGAATAATATATTATGGGGTATTAATCTTCTTCGAAAGGCTATCCCCCTGATAAA
GGCAGGTTGCACACGTGTTCCGCACCCGTACGCCGCTCTCAATTACCCGAAAGTAATCTACCGCTCCGGCTTG
CATGTGTTAGGCCTCCCGCTAGCGTTCTCCTGAGCAGGATCAACTCT
>460
CTGCTGCCGCCCGTAGGAGTCTGGTCCGTGTCTCAGTACCAGTGTGGGGGATCACCCTCTCAGGCCCCCTAAA
GGTCGTCGCCTTGGTGAGCCGTTACCTCACCAACTAGCTAATCTTGCGCGTGCCCATCTCTATCCACCGTAGT
TTTCAATATCACCCGATGCCGAATAATATATTATGGGGTATTAATCTTCTTCTGAAAGGCTATCCCCCTGATA
AAGGCAGGTTGCACACGTGTTCCGCACCCGTACGCCGCTCTCAATTACCCGAAAGTAATCTACCGCTCGGCTT
GCATGTGTTAGGCCTCCCGCTAGCGTTCTCCTGAGCAGGATCAAACTCT
>461
CTGCTGCCGCCCGTAGGAGTCTGGTCCGTGTTTCAGTACCAGTGTGGGGGATCACCCTCTCAGGCCCCCTAAA
GATCGTCGCCTTGGTGAGCCGTTACCTCACCAACTAGCTAATCTTGCGCGTGCCCATCTCTATCCACCGTAGT
TTTCAATATCACCCGATGCCGAATAATATATTATGGGGTATTAATCTTCTTCGAAAGGCTATCCCCCTGATAA
AGGCAGGTTGCACACGTGTTCCGCACCCGTACGCCGCTCTCAATTACCCGAAAGTAATCTACCGCTCGGCTTG
CATGTGTTAGGCCTCCCGCTAGCGTTCATCCTGAGCCAGGATCAAACTCT
>462
CTGCTGCCGCCCGTAGGAGTCTGGTCCGTGTCTCAGTACCAGTGTGGGGGATCACCCTCTCAGGCCCCCTAAA
GATCGTCGCCTTGGTGAGCCGTTACCTCACCAACTAGCTAATCTTGCGCGTGCCCATCTCTATCCACCGTAGT
TTCAATATCACCCGATGCCGAATAATATATTATGGGGTATTTAATTCTTTCTTCGAAAGGCTATCCCCCTGAT
AAAGGCAGGTTGCACACGTGTTCCGCACCCGTACGCCGCTCTCAATTACCCGAAAGTAATCTACCGCTCGGCT
TGCATGTGTTAGGCCTCCCGCTAGCGTTCTCCTGAGCAGGATCCAACTCGT
>463
CTGCTGCCGCCCGTAGGAGTCTGGTCCGTGTCTCAGTACCAGTGTGGGGATCACCCTCTCAGGCCCCCTAAAG
ATCGTCGCCTTGGTGAGCCGTTACCTCACCAACTAGCTAATCTTGCGCGTGCCCATCTCTTATCCACCGTAGT
TTTTCAATATCACCCGATGCCGAATAATTATATTATTGGGGTATTTAATTCTTTCTTCGAAAGGCTATCCCCC
TGATAAAGGCAGGTTTGCACACGTGTTCCGCACCCGTACGCCGCTCTTCAATTTACCCGAAAGTAATCTACCG
CATCGGCTTGCACGTGTTTAGGCCTCCCG
>464
CTGCTGCCGCCCGTAGGAGTCTGGTCCGTGTCTCAGTACCAGTGTGGGGGATCACCCTCTCAGGCCCCCTAAA
GATCGTCGCCTTGGTGAGCCGTTACCTCACCAACTAGCTAATCTTGCGCGTGCCCATCTCTATCCACCGTAGT
TTTCAATATCACCCGATGCCGAATAATATATTATGGGGTATTTAATCTTCTTCGAAAGGCTATCCCCCTGATA
AAGGCAGGTTGCACACGTGTTCCGCACCCGTACGCCGCTCTCAATTACCCGAAAGTAATCTACCGCTCGGCTT
GCATGTGTTAGGCCTCCCGCTAGCGTTCATCCTGAGCCAGGATCAAACTCT
>465
CTGCTGCCGCCCGTAGGAGTCTGGTCCGTGTCTCAGTACCAGTGTGGGGGATCACCCTCTCAGGCCCCCTAAA
GATCGTCGCCTTGGTGAGCCGTTACCTCACCAACTAGCTAATCTTGCGCGTGCCCATCTCTATCCACCGTAGT
TTTCAATATCACCCGATGCCGAATAATATATTTATGGGGTATTTAATCTTTCTTCGAAAGGCTATCCCCTGAT
AAAGGCAGGTTGCGCACGTGTTCCGCACCCGTTACGCCGCTCTCAATTACCCGAAAGTAATCTATCGCTCGGC
TTGCATGTGTTAGGCCTCCCGCTAGCGTTCTCCTGAGCAGG
>466
CTGCTGCCGCCCGTAGGAGTCTGGTCCGTGTCTCAGTACCAGTGTGGGGGATCACCCTCTCAGGCCCCCTAAA
GATCGTCGCCTTGGTGAGCCGTTACCTCACCAACTAGCTAATCTTGCGCGTGCCCATCTCTATCCACCGTAGT
TTTCAATATCACCCGATGCCGAATAATATATTATGGGGTATTAATCTTCTTCGAAAGGCTATCCCCCTGATAA
AGGCAGGTTGCACACGTGTTCCGCACCCGTACGCCGCTCTCAATTACCCGAAAGTAATCTACCGCTCGGCTTG
CATGTGTTAGGCCTCCCGCTAGCGTTCTCCTGAGCAGGATCAACTCGT
>467
TTGCTGCCGCCCGTAGGAGTCTGGTCCGTGTCTCAGTACCAGTGTGGGGGATCACCCTCTCAGGCCCCCTAAA
GATCGTCGCCTTGGTGAGCCGTTACCTCACCAACTAGCTAATCTTGCGCGTGCCCATCTCTATCCACCGTAGT
TTTCAATATCACCCGATGCCGAATAATATATTATGGGGTATTTAATCTTTCTTCGAAAGGCTATCCCCCTGAT
AAAGGCAGGTTGCACACGTGTTCCGCACCCGTACGCCGCTCTCAATTACCCGAAAGTAATCTACCGCTCGGCT
TGCATGTGTTAGGCCTCCCGCTAGCGTTCTCCTGAGCCAGGATCAAACTCT
>468
CTGCTGCCGCCCGTAGGAGTCTGGTCCGTGTCTCAGTACCAGTGTGGGGGATCACCCTCTCAGGCCCCCTAAA
GATCGTCGCCTTGGTGAGCCGTTACCTCACCAACTAGCTAATCTTGCGCGTGCCCATCTCTATCCACCGTAGT

TTTCAATATCACCCGATGCCGAATAATATATTATGGGGTATTAATCTTCTTCTGAAAGGCTATCCCCCTGATA
AAGGCAGGTTGCACACGTGTTCCGCACCCGTACGCCGCTCTCAATTACCCGAAAGTAATCTACCGCTCGGCTT
GCATGTGTTAGGCCTCCCGCTAGCGTTCTCCTCGAGCAGGATCCAACTCT
>469
CTGCTGCCGCCCGTAGGAGTCTGGTCCGTGTCTCAGTACCAGTGTGGGGATCACCCTCTCAGGCCCCCTAAAG
ATCGTCGCCTTGGTGAGCCGTTACCTCACCAACTAGCCTAATCTTGCGCGTGCCCATCTCTATCCACCGTAGT
TTCAATATCACCCGATGCCGAATAATATATTATGGGGTATTAATCTTCTTCGAAAGGCTATCCCCCTGATAAA
GGCAGGTTGCACACGTGTTCCGCACCCGTACGCCGCTCTCAATTACCCGAAAGTAATCTACCGCTCGGCTTGC
ATGTGTTAGGCCTCCCGCTAGCGTTCATCCTGAGCCAGGATCAACTCT
>470
CTGCTGCCGCCCGTAGGAGTCTGGTCCGTGTCTCAGTACCAGTGTGGGGGATCACCCTCCCAGGCCCCCTAAAG
ATCGTCGCCTTGGTGAGCCGTTACCTCACCAACTAGCTAATCTTGCGCGTGCCCATCTCTATCCACCGTAGTT
TTCAATATCACCCGATGCCGAATAATATATTATGGGGTATTAATCTTCTTCGAAAGGCTATCCCCCTGATAAA
GGCAGGTTGCGACGCGTGTCCGCACCCGTACGCCGCTCCCAATTACCCGAAAGTAATCTACCGCTCGGCTTGC
ATGTGTTAGGCCTCCCGCTAGCGTTCATCCTGAGCCAGGATCAAACTCT
>471
CTGCTGCCGCCCGTAGGAGTCTGGTCCGTGTCTCAGTACCAGTGTGGGGGATCACCCTCTCAGGCCCCCTAAA
GATCGTCGCCTTGGTGAGCCGTTACCTCACCAACTAGCTAATCTTGCGCGTGCCCATCTCTTATCCACCGTAG
TTTTTTCAATATCACCCGATGCCGAATAATATATTATGGGGTATTTAATTCTTTCTTCGAAAGGCTATCCCCC
TGATAAAGGCAGGTTTGCACACGTGTTCCGCACCCGTACGCCGCTCTCAATTTACCCGAAAGTAATCTACCGC
TCGGCTGCATGTGTTTAGGCCTCCCGCTAGCGTTCTCCTGAG
>472
CTGCTGCCGCCCGTAGGAGTCTGGTCCGTGTCTCAGTACCAGTGTGGGGGATCACCCTCTCAGGCCCCCTAAA
GATCGTCGCCTTGGTGAGCCGTTACCTCACCAACTAGCTAATCTTGCGCGTGCCCATCTCTATCCACCGTAGT
TTTCAATATCACCCGATGCCGAATAATATATTATGGGGTATTAATCTTCTTCTGAAAGGCTATCCCCCTGATA
AAGGCAGGTTGCACACGTGTTCCGCACCCGTACGCCGCTCTCAATTACCCGAAAGTAATCTACCGCTCGGCTT
GCATGTGTTAGGCCTCCCGCTAGCGTTCATCCTGAGCCAGGATCAAACTCT
>473
CTGCTGCCGCCCGTAGGAGTCTGGTCCGTGTCTCAGTACCAGTGGTGGGGATCACCCTCTCCAGGCCCCTAAA
GATCGTCGCCTTGGTGAGCCGTTTACCTCACCAACTAGCTAATCTTGCGCGTGCCCATCTCTTATCCACCGTA
GTTTTTCAATATCACCCGATGCCGAATAATATATTTATGGGGTATTTAATCTTCTTCGAAAGGCTTATCCCCC
TGATAAGGGCAGGTTGCACACGTGTTCCGCACCCGTACGCCGCTCTCAATTTACCCCGAAAGTAATCTACCGC
TCGGCTTGCATGTGTTAGGCCTCCCGCTAGCGTTCTCCTGAGCAGGTTCAAACTCGT
>474
CTGCTGCCGCCCGTAGGAGTCTGGTCCGTGTCTCAGTACCAGTGTGGGGGATCACCCTCTCAGGCCCCCTAAA
GATCGTTCGCCTTGGTGAGCCGTTACCTCACCAACTAGCTAATCTTGCGCGTGCCCATCTTCTATCCACCGTA
GTTTCAATTATCACCCGATGCCGAATAATATATTTATGGGGTATTTAATTCTTTCTTCGAAAGGCTATCCCCC
TGATAAAGGCAGGTTTGCACACGTGTTCCGCACCCGTACGCCGCTCTCAATTTACCCTAAAGTAATCTACCGC
TCGGCTTTGCA
>475
CTGCTGCCGCCCGTAGGAGTCTGGTCCGTGTCTCAGTACCAGTGTGGGGGATCACCCTTCTCAGGCCCCCTAA
AGATCGTCGCCTTGGTGAGCCGTTACCTCACCAACTAGCTTAATCTTGCGCGTGCCCATCTCTATTCCACCGT
AGTTTTCAATATCACCCGATGCCGAATAATATATTATTGGGGTATTTAATTCTTCTTCGAAAGCTATCCCCTG
ATAAAGGCAGGTTGCACACGTGTTCCGCACCCGTACGCCGCTCTCAATTTACCCGAAAGTAATCTACCGCTCG
GCTTGCATGTGTTTAGGCCTCCCGCTAGCGTTCTCCTGAGC
>476
CTGCTGCCGCCCGTAGGAGTCTGGACCGTGTCTCAGTTCCAGTGTGACTTGATCATCCTCTCAGACCAGTTAC
GGATCGTCGCCTTGGTAGGCCTTTACCCCACCAACTAGCTAATCCGACCTAGGCTCATCTGATAGCGTGAGGT
CCGAAGATCCCCACTTCTCCCTCAGGACGTATGCGGTATTTAGCGCCCGTTTCCGGACGTATCCCCACTACCA
GGCAGATTCCTAGGCATTACTCACCCGTCCGCCGCTGAATCCAGGAGCAAGCCCCCTTCATCCGCTCGACTGC
ATGTGTTAGGCCTGCCGCCAGCGTCAATCTGAGCCAGGATCAAACTCT
>477
CTGCTGCCGCCCGTAGGAGTCTGGTCCGTGTCTCAGTACCAGTGTGGGGGATCACCCTCTCAGGCCCCCTAAA
GATCGTCGCCTTGGTGAGCCGTTACCTCACCAACTAGCTAATCTTGCGCGTGCCCATCTCTATCCACCGTAGT

TTTCAATATCACCCGATGCCGAATAATATATTATGGGGTATTAATCTTCTTCGAAAGGCTATCCCCCTGATAA
AGGCAGGTTGCACACGTGTTCCGCACCCGTACGCCGCTCTCAATTACCCGAAAGTAATCTACCGCTCGGCTTG
CATGTGTTAGGCCTCCCGCTAGCGTTCATCCTGAGCCAGGATCAAACTCT
>478
CTGCTGCCGCCCGTAGGAGTCTGGTCCGTGTCTCAGTACCAGTGTGGGGGATCACCCTCTCAGGCCCCCTAAA
GATCGTCGCCTTGGTGAGCCGTTACCTCACCAACTAGCTAATCTTGCGCGTGCCCATCTCTATCCACCGTAGT
TTTCAATATCACCCGATGCCGAATAATATATTATGGGGGTATTAATCTTCTTCGAAAGGCTATCCCCCTGATA
AAGGCAGGTTGCACACGTGTTCCGCACCCGTACGCCGCTCTCAATTACCCGAAAGTAATCTACCGCTCGGCTT
GCATGTGTTAGGCCTCCCGCTAGCGTTCATCCTGAGCCAGGATCAAACTCT
>479
CTGCTGCCGCCCGTAGGAGTCTGGTCCGTGTCTCAGTACCAGTGTGGGGGATCACCCTCTCAGGCCCCCTAAA
GATCGTCGCCTTGGTGAGCCGTTACCTCACCAACTAGCTAATCTTGCGCGTGCCCATTCTCTATCCACCGTAG
TTTTCAATATCACCCGATGCCGAATAATATATTATGGGGTATTAATCTTCTTCGAAAGGCTATCCCCCTGATA
AAGGCAGGTTGCACACGTGTTCCGCACCCGTACGCCGCTCTCAATTACCCGAAAGTAATCTACCGCTCGGCTT
GCATGTGTTAGGCCTCCCGCTAGCGTTCATCCTGAGCCAGGATCAACTCT
>480
CTGCTGCCGCCCGTAGGAGTCTGGTCCGTGTCTCAGTACCAGTGTGGGGGATCACCCTCTCAGGCCCCCTAAA
GATCGTCGCCTTGGTGAGCCGTTACCTCACCAACTAGCTAATCTTGCGCGTGCCCATCTCTATCCACCGTAGT
TTCAATATCACCCGATGCCGAATAATATATTATGGGGTATTTAATCTTCTTCGAAAGGCTATCCCCTGATTAA
AGGCAGGTCGCACACGTGTTCCGCACCCGTACGCCGCTCTCAATTACCCGAAAGTAATCTACCGCTCGGCTTG
CATGTGTTAGGCCTCCCGCTAGCGTTCATCCTGAGCCAGGATCAAACTCT
>481
CTGCTGCCGCCCGTAGGAGTCTGGTCCGTGTCTCAGTACCAGTGTGGGGGATCACCCTCTCAGGCCCCCCTAA
AGATCGTCGCCTTGGTGAGCCGTTACCTCACCAACTAGCTAATCTTGCGCGTGCCCATCTCTATCCACCGTAG
TTTCAATATCACCCGATGCCGAATAATATATTATGGGGTATTAATCTTCTTCGAAAGGCTATCCCCTGATAAA
GGCAGGTTGCACACGTGTTCCGCACCCGTACGCCGCTCTCAATTACCCGAAAGTAATCTACCGCTCGGCTTGC
ATGTGTTAGGCCTCCCGCTAGCGTTCATCCTGAGCCAGGATCAACTCT
>482
CTGCTGCCGCCCGTAGGAGTCTGGTCCGTGTCTCAGTACCAGTGTGGGGGATCACCCTCTCAGGCCCCCTAAA
GATCGTCGCCTTGGTGAGCCGTTACCTCACCAACTAGCTAATCTTGCGCGTGCCCATCTCTATCCACCGTAGT
TTTCAATATCACCCGATGCCGAATAATATATTATGGGGTATTAATCTTCTTCGAAAGGCTATCCCCTGATAAA
GGCAGGTTGCACACGTGTTCCGCACCCGTACGCCGCTCTCAATTACCCGAAAGTAATCTACCGCTCGGCTTGC
ATGTGTTAGGCCTCCCGCTAGCGTTCATCCTGAGCCAGGATCAAACTCT
>483
CTGCTGCCGCCCCGTAGGAGTCTGGTCCGTGTCTCAGTACCAGTGTGGGGGGATCACCCTCTCAGGCCCCTAA
AGATCGTCGCCTTGGTAAGCCGTTACCTCACCAACTAGCTAATCTTGCGCGTGCCCCATCTCTTATCCACCGT
AGTTTTTCAATATCACCCGATGCCGAATAATATATTTATGGGGTATTTAATCTTCTTCGAAAGGCTATCCCCC
TGATAAAGGCAGGTTTGCACACGTGTTCCGCACCCGTACGCCGCTCTCAATTACCCGAAAGTAATCTACCGCT
CGGCTTGCATGTGTTAGGCCTCCCGCTAGCGTTCATCCTGAGCCAGGATCAAACTCT
>484
CTGCTGCCGCCCGTAGGAGTCTGGACCGTGTCTCAGTTCCAGTGTGACTGATCATCCTCTCAGACCAGTACGG
ATCGTCGCCTTGGTAGGCCTTTACCCCACCAACTAGCTAATCCGACCTAGGCTCATCTGATAGCGTGAGGTCC
GAAGTCCCCCACTTCTCCCTCAGGACGTATGCGGTATTAGCGCCCGTTTCCGGACGATCCCCACTACCAGGCA
GATTCCTAGGCATTACTCACCCGTCCGCCGCTGAATCCAGGAGCAAGCTCCCTTCATCCGCTCGACTGCATGT
GTTAGGCCTGCCGCCAGCGTCAATCTGAGCCAGGATCAAACTCT
>485
CTGCTGCCGCCCGTAGGAGTCTGGTCCGTGTCTCAGTACCAGGTGTGGGGGATCACCCTCTCCAGGCCCCCTA
AAGATCGTCGCCTTGGTGAGCCGTTACCTCACCAACTAGCTAATCTTGCGCGTGCCCATCTCTATCCACCGTA
GTTTTCAATATCACCCGATGCCGAATAATATATTATGGGGTATTAATCTTCTTCGAAAGGCTATCCCCCTGAT
AAAGGCAGGTTGCACACGTGTTCCGCACCCGTACGCCGCTCTCAATTACCCGAAAGTAATCTACCGCTCGGCT
TGCATGTGTTAGGCCTCCCGCTAGCGTTCATCCTGAGCCAGGATCAACTCT
>486
CTGCTGCCGCCCGTAGGAGTCTGGTCCGTGTCTCAGTACCAGTGTGGGGGATCACCCTCTCAGGCCCCCTAAA
GATCGTCGCCTTGGTGAGCCGTTACCTCACCAACTAGCTAATCTTGCGCGTGCCCATCTCTATCCACCGTAGT

TTTCAATATCACCCGATGCCGAATAATATATTATGGGGTATTAATCTTCTCGAAAGGCTATCCCCCTGATAAA
GGCAGGTTGCACACGTGTTCCGCACCCGTACGCCGCTCTCAATTACCCGAAAGTAATCTACCGCTCCGGCTTG
CATGTGTTAGGCCTCCCGCTAGCGTCATCTGAGCCAGGATCAAACTCT
>487
CTGCTGCCGCCCGTAGGAGTCTGGACCGTGTCTCAGTTCCAGTGTGACTGATCATCCTCTCAGACCCAGTACG
GATCGTCGCCTTGGTAGGCCCTTTACCCCCACCAACTAGCTAATCCGACCTAGGCTCATCTGATAGCGTGAGG
TCCGAAGTCCCCCCACTTCTCCCCTCAGGACGTATGCGGTATTGCGCCCGTTTCCGGACGATCCCCCACTACC
CAGGCAGATTCCTAGGCATTACTCACCCGTCCGCCGCTGAATCCAGGAGCAAGCCCCCTTCATCCGCTCGACT
GCATGTGTTAGGCCTGCCGCCAGCGTCAATCTGAGCCAGGATCAAACTCT
>488
CTGCTGCCGCCCGTAGGAGTCTGGTCCGTGTCTCAGTACCAGTGTGGGGGATCACCCTCTCAGGCCCCCTAAA
GATCGTCGCCTTGGTGAGCCGTTACCTCACCAACTAGCTAATCTTGCGCGTGCCCATCTCTATCCACCGTAGT
TTTCAATATCACCCGATGCCGAATAATATATTATGGGGTATTAATCTTCTTCGAAAGGCTATCCCCCTGATAA
AGGCAGGTTGCACACGTGTTCCGCACCCGTACGCCGCTCTCAATTACCCGAAAGTAATCTACCGCTCGGCTTG
CATGTGTTAGGCCTCCCGCTAGCGTTCTCCTGAGCAGGATCAACTCGT
>489
CTGCTGCCGCCCGTAGGAGTCTGGTCCCGTGTCTCAGTACCAGTGTGGGGGATCACCCTCTCAGGCCCCCTAA
AAGATCGTCGCCTGGTGAGCCGTTACCTCACCAACTAGCTAATCTTGCGCGTGCCCATCTCTATCCACCGTAG
TTTTCAATATCACCCGATGCCGAATAATATATTATGGGGTATTAATCTTCTCGAAAGCTATCCCCCTGATAAA
GGCAGGTTGCACACGTGTTCCGCACCCTACGCCGCTCTCAATTACCCGAAAGAATCTACCGCTCGGCTTGCAT
GTGTTAGGCCTCCCGCTAGCGTCTCCTGAGCAGGATCAACTCGT
>490
CTGCTGCTGCCCGTAGGAGTCTGGTCCGTGTCTCAGTACCAGTGTGGGGGATCACCCTCTCAGGCCCCCTAAA
GATCGTCGCCTTGGTGAGCCGTTACCTCACCAACTAGCTAATCTTGCGCGTGCCCATCTCTATCCACCGTAGT
TTTCAATATCACCCGATGCCGAATAACATATTATGGGGTATTAATCTTCTTCGAAAGGCTATCCCCCTGATAA
AGGCAGGTTGCACACGTGTTCCGCACCCGTACGCCGCTCTCAATTACCCGAAAGTAATCTACCGCTCGGCTTG
CATGTGTTAGGCCTCCCGCTAGCGTTCATCCTGAGCCAGGATCAAACTCT
>491
CGCTGCCGCCCGTAGGAGTCTGGTCCGTGTCTCAGTACCAGTGTGGGGGATCACCCTCTCAGGCCCCCTAAAG
ATCGTCGCCTTGGTGAGCCGTTACCTCACCAACTAGCTAATCTTGCGCGTGCCCATCTCTATCCACCGTAGTT
TTCAATATCACCCGATGCCGAATAATATATTATGGGGTATTAATCTTCTTCGAAAGGCTATCCCCCTGATAAA
GGCAGGTTGCACACGTGTTCCGCACCCGTACGCCGCTCTCAATTACCCGAAAGTAATCTACCGCTCGGCTTGC
ATGTGTTAGGCCTCCCGCTAGCGTTCATCCTGAGCCAGGATCAAACTCT
>492
CTGCTGCCGCCCGTAGGAGTCTGGTCCGTGTCTCAGTACCAGTGTGGGGGATCACCCTCTCAGGCCCCCTAAA
GATCGTCGCCTTGGTGAGCCGCTACCTCACCAACTAGCTAATCTTGCGCGTGCCCATCTCTATCCACCGTAGT
TTTCAATATCACCCGATGCCGAATAATATATTATGGGGTATTAATCTTCTTCGAAAGGCTATCCCCTGATAAA
GGCAGGTTGCACACGTGTTCCGCACCCGTACGCCGCTCTCAATTACCCGAAAGTAATCTACCGCTCGGCTTGC
ATGTGTTAGGCCTCCCGCTAGCGTTCATCCTGAGCCAGGATCAAACTCT
>493
CTGCTGCCGCCCGTAGGAGTCTGGTCCGTGTCTCAGTACCAGTGTGGGGGATCACCCTCTCAGGCCCCCTAAA
GATCGTCGCCTTGGTGAGCCGTTACCTCACCAACTAGCTAATCTTGCGCGTGCCCATCTCTATCCACCGTAGT
TTTCAATATCACCCGATGCCGAATAATATATTATGGGGTATTAATCTTCTTCGAAAGGCTATCCCCCTGATAA
AGGCAGGTTGCACACGTGTTCCGCACCCGTACGCCGCTCTCAATTACCCGAAAGTAATCTACCGCTCGGCTTG
CATGTGTTAGGCCTCCCGCTAGCGTTCA
>494
CTGCTGCCGCCCGTAGGAGTCTGGTCCGTGTCTCAGTACCAGTGTGGGGGATCACCCTCTCAGGCCCCCTAAA
GATCGTCGCCTTGGTGAGCCGTTACCTCACCAACTAGCTAATCTTGCGCGTGCCCATCTCTATCCACCGTAGT
TTTCAATATCACCCGATGCCGAATAATATATTATGGGGTATTAATCTTCTTCGAAAGGCTATCCCCCTGATAA
AGGCAGGTTGCACACGTGTTCCGCACCCGTACGCCGCTCTCAATTACCCGAAAGTAATCTACCGCTCGGCTTG
CATGTGTTAGGCCTCCCGCTAGCGTTCATCCTGAGCCAGGATCAACTCT
>495
CTGCTGCCGCCCGTAGGAGTCTGGTCCGTGTCTCAGTACCAGTGTGGGGGATCACCCTCTCAGGCCCCCTAAA
GATCGTCGCCTTGGTGAGCCGTTACCTCACCAACTAGCTAATCTTGCGCGTGCCCATCTCTATCCACCGTAGT

TTTCAATATCACCCGATGCCGAATAATATATTATGGGGTATTAATCTTCTTCGAAAGGCTATCCCCCTGATAA
AGGCAGGTTGCACACGTGTTCCGCACCCGTACGCCGCTCTCAATTACCCGAAAGTAATCTACCGCTCGGCTTG
CATGTGTTAGGCCTCCCGCTAGCGTTCTCCTGAGCAGGATCCAACTCGT
>496
CTGCTGCCGCCCGTAGGAGTCTGGTCCGTGTCTCAGTACCAGTGTGGGGGATCACCCTCTCAGGCCCCCTAAA
GATCGTCGCCTTGGTGAGCCGTTACCTCACCAACTAGCTAATCTTGCGCGTGCCCATCTCTATCCACCGTAGT
TTTCAATATCACCCGATGCCGAATAATATATTATGGGGTATTAATCTTCTTCGAAAGGCTATCCCCCTGATAA
AGGCAGGTTGCACACGTGTTCCGCACCCGTACGCCGCTCTCAATTACCCGAAAGTAATCTACCGCTCGGCTTG
CATGTGTTAGGCCTCCCGCTAGCGTTCTCCTGAGCAGGATCAACTCGT
>497
CTGCTGCCGCCCGTAGGAGTCTGGTCCGTGTCTCAGTACCAGTGTGGGGGATCACCCTCTCAGGCCCCCTAAA
GATCGTCGCCTTGGTGAGCCGTTACCTCACCAACTAGCTAATCTTGCGCGTGCCCATCTCTATCCACCGTAGT
TTTCAATATCACCCGATGCCGAATAATATATTATGGGGTATTAATCTTCTTCGAAAGGCTATCCCCCTGATAA
AGGCAGGTTGCACACGTGTTCCGCACCCGTACGCCGCTCTCAATTACCCGAAAGTAATCTACCGCTCGGCTTG
CATGTGTTAGGCCTCCCGCTAGCGTTCATCCTGAGCCAGGATCAAACTCT
>498
CTGCTGCCGCCCGTAGGAGTCTGGTCCGTGTCTCAGTACCAGTGTGGGGGATCACCCTCTCAGGCCCCCTAAA
AGATCGTCGCCTTGGTGAGCCGTTTACCTCACCAACTAGCTAATCTTGCGCGTGCCCATCTCTATCCACCGTA
GTTTTCAATATCACCCGATGCCGAATAATATATTATGGGGTATTAATCTTCTTCGAAAGGCTATCCCCCTGAT
AAAGGCAGGTTGCACACGTGTTCCGCACCCGTACGCCGCTCTCAATTACCCGAAAGTAATCTACCGCTCGGCT
TGCATGTGTTAGGCCTCCCGCTAGCGTTCATCCTGAGCCAGGATCAAACTCT
>499
CTGCTGCCGCCCGTAGGAGTCTGGTCCGTGTCTCAGTACCAGTGTGGGGGATCACCCTCTCAGGCCCCCTAAA
GATCGTCGCCTTGGTGAGCCGTTACCTCACCAACTAGCTAATCTTGCGCGTGCCCATCTCTATCCACCGTAGT
TTTCAATATCACCCGATGCCGAATAATATATTATGGGGTATTAATCTTCTTCGAAAGGCTATCCCCCTGATAA
AGGCAGGTTGCACACGTGTTCCGCACCCGTACGCCGCTCTCAATTACCCGAAAGTAATCTACCGCTCCGGCTT
GCATGTGTTAGGCCTCCCGCTAGCGTTCATCCTGAGCCAGGATCAACTCT
>500
CTGCTGCGCCCGTAGGAGTCTGGTCCGTGTCTCAGTACCAGTGTGGGGGATCACCCTCTCAGGCCCCCTAAAG
ATCGTCGCCTTGGTGAGCCGTTACCTCACCAACTAGCTAATCTTGCGCGTGCCCATCTCTATCCACCGTAGTT
TTCAATATCACCCGATGCCGAATAATATATTATGGGGTATTAATCTTCTCGAAAGGCTATCCCCTGATAAAGG
CAGGTTGCACACGTGTTCCGCACCCGTACGCCGCTCTCAATTACCCGAAAGTAATCTACCGCTCGGCTTGCAT
GTGTTAGGCCTCCCGCTAGCGTTCATCCTGAGCCAGGATCAAACTCT
>501
CTGCTGCCGCCCGTAGGAGTCTGGTCCGTGTCTCAGTACCAGTGTGGGGGATCACCCTCTCAGGCCCCCTAAA
GATCGTCGCCTTGGTGAGCCGTTACCTCACCAACTAGCTAATCTTGCGCGTGCCCATCTCTATCCACCGTAGT
TTTCAATATCACCCGATGCCGAATAATATATTATGGGGTATTAATCTTCTTCGAAAGGCTATCCCCCTGATAA
AGGCAGGTTGCACACGTGTTCCGCACCCGTACGCCGCTCTCAATTACCCGAAAGTAATCTACCGCTCGGCTTG
CATGTGTTAGGCCTCCCGCTAGCGTTCATCCTGAGCCAGGATCAACTCTT
>502
CTGCTGCCGCCCGTAGGAGTCTGGTCCGTGTCTCAGTACCAGTGTGGGGGATCACCCTCTCAGGCCCCCTAAA
GATCGTCGCCTTGGTGAGCCGTTACCTCACCAACTAGCTAATCTTGCGCGTGCCCATCTCTATCCACCGTAGT
TTTCAATATCACCCGATGCCGAATAATATATTATGGGGTATTAATCTTCTTCGAAAGGCTATCCCCCTGATAA
AGGCAGGTTGCACACGTGTTCCGCACCCGTACGCCGCTCTCAATTACCCGAAAGTAATCTACCGCTCGGCTTG
CATGTGTTAGGCCTCCCGCTAGCGTTCTCCTGAGCAGGATCAA
>503
CTGCTGCCGCCCGTAGGAGTCTGGTCCGTGTCTCAGTACCAGTGTGGGGGATCACCCTCTCAGGCCCCCTAAA
GATCGTCGCCTTGGTGAGCCGTTACCTCACCAACTAGCTAATCTTGCGCGTGCCCATCTCTATCCACCGTAGT
TTTCAATATCACCCGATGCCGAATAATATATTATGGGGTATTAATCTTCTTCGAAAGGCTATCCCCCTGAATA
AAGGCAGGTTGCACACGTGTTCCGCACCCGTACGCCGCTCTCAATTACCCGAAAGTAATCTACCGCTCGGCTT
GCATGTGTTAGGCCTCCCGCTAGCGTTCATCCTGAGCCAGGATCAAACTCT
>504
CTGCTGCCGCCCGTAGGAGTCTGGTCCGTGTCTCAGTACCAGTGTGGGGGATCACCCTCTCAGGCCCCCTAAA
GATCGTCGCCTTGGTGAGCCGTTACCTCACCAACTAGCTAATCTTGCGCGTGCCCATCTCTATCCACCGTAGT

TTTCAATATCACCCGATGCCGAATAATATATTATGGGGTATTAATCTTCTTCGAAAGGCTATCCCCTGATAAA
GGCAGGTTGCACACGTGTTCCGCACCCGTACGCCGCTCTCAATTACCCGAAAGTAATCTACCGCTCGGCTTGC
ATGTGTTAGGCCTCCCGCTAGCGTTCATCCTGAGCCAGGATCAACTCT
>505
CTGCTGCCGCCCGTAGGAGTCTGGTCCGTGTCTCAGTACCAGTGTGGGGGGGATCACCCTCTCAGGCCCCCCT
AAAGATCGTCGCCTTGGTGAGCCGTTACCTCACCAACTAGCTAATCTTGCGCGTGCCCATCTCTATCCACCGT
AGTTTTCAATATCACCCGATGCCGAATAATATATTATGGGGTATTAATCTTTCTCGAAAGGCTATCCCCCTGA
TAAAGGCAGGTTGCACACGTGTTCCGCACCCGTACGCCGCTCTCAATTACCCGAAAGTAATCTACCGCTCGGC
TTGCATGTGTTAGGCCTCCCGCTAGCGTTCTCCTGAGCAGGATCAACTCGT
>506
CTGCTGCCGCCCGTAGGAGTCTGGACCGTGTCTCAGTTCCAGTGTGACTGATCATCCTCTCAGACCAGTACGG
ATCGTCGCCTTGGTAGGCCTTTACCCCACCAACTAGCTAATCCGACCTAGGCTCATCTGATAGCGTGAGGTCC
GAAGTCCCCCACTTCTCCCTCAGGGCGTATGCGGTATTAGCGCCCGTTTCCGGACGTATCCCCACTACCAGGC
AGATTCCTAGGCATTACTCACCCGTCCGCCGCTGAATCCAGGAGCAAGCTCCCTTCATCCGCTCGACTGCATG
TGTTAGGCCTGCCGCCAGCGTCAATCTGAGCCAGGATCAAACTCT
>507
CTGCTGCCGCCCGTAGGAGTCTGGTCCGTGTCTCAGTACCAGTGTGGGGGATCACCCTCTCAGGCCCCCTAAA
GATCGTCGCCTGTGAGCCGTTACCTCACCAACTAGCTAATCTTGCGCGTGCCCATCTCTATCCACCGTAGTTT
TCAATATCACCCGATGCCGAATAATATATTATGGGGTATTAATCTTCTTCGAAAGGCTATCCCCCTGATAAAG
GCAGGTTGCACACGTGTTCCGCACCCGTACGCCGCTCTCAATTACCCGAAAGTAATCTACCGCTCCGGCTTGC
ATGTGTTAGGCCTCCCGCTAGCGTTCATCCTGAGCCAGGATCAAACTCT
>508
CTGCTGCCGCCCGTAGGAGTCTGGACCGTGTCTCAGTTCCAGTGTGACTGATCATCCTCTCAGACCAGTTACG
GATCGTCGCCTTGGTAGGCCTTTACCCCACCAACTAGCTAATCCGACCTAGGCTCATCTGATAGCGTGAGGTC
CGAAGATCCCCCACTTCTCCCTCAGGTCGTATGCGGTATTAGCGCCCGTTTCCGGACGATCCCCACTACCAGG
CAGATTCCTAGGCATTACTCACCCGTCCGCCGCTGAATCCAGGAGCAAGCTCCCTTCATCCGCTCGACTGCAT
GTGTTAGGCCTGCCGCCAGCGTCAATCTGAGCCAGGATCAAACTCT
>509
CTGCTGCCGCCCGTAGGAGTCTGGTCCGTGTCTCAGTACCAGTGTGGGGGATCACCCTCTCAGGCCCCCTAAA
GATCGTCGCCTTGGTGAGCCGTTACCTCACCAACTAGCTAATCTTGCGCGTGCCCATCTCTATCCACCGTAGT
TTTCAATATCACCCGATGCCGAATAATATATTATGGGGTATTAATCTTCTTCGAAAGGCTATCCCCCTGATAA
AGGCAGGTTGCACACGTGTTCCGCACCCGTACGCCGCTCTCAATTACCCGAAAGTAATCTACCGCGTCGCTTG
CATGTGTTAGGCCTCCCGCTAGCGTTCATCCTGAGCCAGGATCAAACTCT
>510
CTGCTGCCGCCCGTAGGAGTCTGGTCCGTGTCTCAGTACCAGTGTGGGGGATCACCCTCTCAGGCCCCCTAAA
AGATCGTCGCCTTGGTGAGCCGTTACCTCACCAACTAGCTAATCTTGCGCGTGCCCATCTCTATCCACCGTAG
TTTTCAATATCACCCGATGCCGAATAATATATTATGGGGTATTAATCTTCTTCGAAAGGCTATCCCCCTGATA
AAGGCAGGTTGCACACGTGTTCCGCACCCGTACGCCGCTCTCAATTACCCGAAAGTAATCTACCGCTCGGCTT
GCATGTGTTAGGCCTCCCGCTAGCGTTCTCCTGAGCAGGTCAAACTCGT
>511
CTGCTGCCGCCCGTAGGAGTCTGGTCCGTGTCTCAGTACCAGTGTGGGGGATCACCCTCTCAGGCCCCCTAAA
GATCGTCGCCTTGGTGAGCCGTTACCTCACCAACTAGCTAATCTTGCGCGTGCCCATCTCTATCCACCGTAGT
TTTCAATATCACCCGATGCCGAATAATATATTATGGGGTATTAATCTTCTTCGAAAGGCTATCCCCCTGATAA
AGGCAGGTTGCACACGTGTTCCGCACCCGTACGCCGCTCTCAATTACCCGAAAGTAATCTACCGCTCGGCTTG
CATGTGTTAGGCCTCCCGCTAGCGTTCATCCTGAGCCAGGATCAAACTCT
>512
CTGCTGCCGCCCGTAGGAGTCTGGTCCGTGTCTCAGTACCAGTGTGGGGGGATCACCCTCTCAGGCCCCCTAA
AGATCGTCGCCTTGGTGAGCCGTTACCTCACCAACTAGCTAATCTTGCGCGTGCCCATCTCTATCCACCGTAG
TTTTCAATATCACCCGATGCCGAATAATATATTATGGGGTATTTAATCTTCTTCGAAAGGCTATCCCCTGATA
AAGGCAGGTTGCACACGTGTTCCGCACCCGTACGCCGCTCTCAATTACCCGAAAGTAATCTACCGCTCGGCTT
GCATGTGTTAGGCCTCCCGCTAGCGTTCTCCTGAGCA
>513
CTGCTGCCGCCCGTAGGAGTCTGGTCCGTGTCTCAGTACCAGTGTGGGGGATCACCCTCTCAGGCCCCCTAAA
GATCGTCGCCTTGGTGAGCCGTTACCTCACCAACTAGCTAATCTTGCGCGTGCCCATCTCTATCCACCGTAGT

TTTCAATATCACCCGATGCCGAATAATATATTATGGGGTATTAATTCTTCTTCGAAAGGCTATCCCCCTGATA
AAGGCAGGTTGCACACGTGTTCCGCACCCGTACGCCGCTCTCAATTACCCGAAAGTAATCTACCGCTCGGCTT
GCATGTGTTAGGCCTCCCGCTAGCGTTCTTCCTGAGCAGGATCCAACTCGT
>514
CTGCTGCCGCCCGTAGGAGTCTGGTCCGTGTCTCAGTACCAGTGTGGGGGATCACCCTCTCAGGCCCCCTAAA
GATCGTCGCCTTGGTGAGCCGTTACCTCACCAACTAGCTAATCTTGCGCGTGCCCATCTCTATCCACCGTAGT
TTTCAATATCACCCGATGCCGAATAATATATTATGGGGTATTAATCTTCCTCTCGAAAGGCTATCCCCCTGAT
AAAGGCAGGTTGCACACGTGTTCCGCACCCGTACGCCGCTCTCAATTACCCGAAAGTAATCTACCGCTCGGCT
TGCATGTGTTAGGCCTCCCGCTAGCGTTCATCCTGAGCCAGGATCAAACTCT
>515
CTGCTGCCGCCCGTAGGAGTCTGGTCCGTGTCTCAGTACCAGTGTGGGGGATCACCCTCTCAGGCCCCCTAAA
GATCGTCGCCTTGGTGAGCCGTTACCTCACCAACTAGCTAATCTTGCGCGTGCCCATCTCTATCCACCGTAGT
TTTCAATATCACCCGATGCCGAATAATATATTATGGGGTATTAATCTTCTTCGAAAGGCTATCCCCCTGATAA
AGGCAGGTTGCACACGTGTTCCGCACCCGTACGCCGCTCTCAATTACCCGAAAGTAATCTACCGCTCGGCTTG
CATGTGTTAGGCCTCCCGCTAGCGTTCTCCTGAGCAGGATCAACTCGT
>516
CTGCTGCCGCCCGTAGGAGTCTGGTCCGTGTCTCAGTACCAGTGTGGGGGATCACCCTCTCAGGCCCCCTAAA
GATCGTCGCCTTGGTGAGCCGTTACCTCACCAACTAGCTAATCTTGCGCGTGCCCATCTCTATCCACCGTAGT
TTTCAATATCACCCGATGCCGAATAATATATTATGGGGTATTAATCTTCTTCGAAGGCTATCCCCCTGATAAA
GGCAGGTTGCACACGTGTTCCGCACCCGTACGCCGCTCTCAATTACCCGAAAGTAATCTACCGCTCGGCTTGC
ATGTGTTAGGCCTCCCGCTAGCGTTCATCCTGAGCCAGGATCAAACTCT
>517
CTGCTGCCGCCCGTAGGAGTCTGGTCCGTGTCTCAGTACCAGTGTGGGGGATCACCTCCCAGGCCCCCTAAAG
ATCGTCGCCTTGGTGAGCCGTTACCTCACCAACTAGCTAATCTTGCGCGTGCCCATCTCTATCCACCGTAGTT
TTCAATATCACCCGATGCCGAATAATATATTATGGGGTATTAATCTTCTCGAAGGCTATCCCCTGATAAAGGC
AGGTTGCACACGTGTTCCGCACCCGTACGCCGCTCTCAATTACCCGAAAGTAATCTACCGCTCCGGCTTGCAT
GTGTTAGGCC
>518
CTGCTGCCGCCCGTAGGAGTCTGGTCCGTGTCTCAGTACCAGTGTGGGGGATCACCCTCTCAGGCCCCCTAAA
GATCGTCGCCTTGGTGAGCCGTTACCTCACCAACTAGCTAATCTTGCGCGTGCCCATCTCTATCCACCGTAGT
TTTCAATATCACCCGATGCCGAATAATATATTATGGGGGTATTAATCTTCTTCGAAAGGCTATCCCCCTGATA
AAGGCAGGTTGCACACGTGTTCCGCACCCGTACGCCGCTCTCAATTACCCGAAAGTAATCTACCGCTCGGCTT
GCATGTGTTAGGCCTCCCGCTAGCGTTCTCCTGAGCAGGATCAACTCT
>519
CTGCTGCCGCCCGTAGGAGTCTGGTCCGTGTCTCAGTACCAGTGTGGGGGATCACCCTCTCAGGCCCCCTAAA
GATCGTCGCCTTGGTGAGCCGTTACCTCACCAACTAGCTAATCTTGCGCGTGCCCATCTCTATCCACCGTAGT
TTTCAATATCACCCGATGCCGAATAATATATTATGGGGTATTAATCTTCTTCGAAAGGCTATCCCCTGATAAA
GGCAGGTTGCACACGTGTTCCGCACCCGTACGCCGCTCTCAATTACCCGAAAGTAATCTACCGCTCCGGCTTG
CATGTGTTAGGCCTCCCGCTAGCGTTCATCCTGAGCCAGGATCAAACTCT
>520
CTGCTGCCGCCCGTAGGAGTCTGGTCCGTGTCTCAGTACCAGTGTGGGGGATCACCCTCTCAGGCCCCCTAAA
GATCGTCGCCTTGGTGAGCCGTTACCTCACCAACTAGCTAATCTTGCGCGTGCCCATCTCTATCCACCGTAGT
TTTCAATATCACCCGATGCCGAATAATATATTTATGGGGTATTTAATTCTTTCTTCGAAAGGCTATCCCCCTG
ATAAAGGCAGGTTGCACACGTGTTCCGCACCCGTACGCCGCTCTCAATTACCCGAAAGTAATCTACCGCTCCG
GCTTGCATGTGTTAGGCCTCCCGCTAGCGTTCTCCTGAGCAGGATCAACTCGT
>521
CTGCTGCCGCCCGTAGGAGTCTGGTCCGTGTCTCAGTACCAGTGTGGGGGATCACCCTCTCAGGCCCCCTAAA
GATCGTCGCCTTGGTGAGCCGTTACCTCACCAACTAGCTAATCTTGCGCGTGCCCATCTCTATCCACCGTAGT
TTTCAATATCACCCGATGCCGAATAATATATTTATGGGGTATTTAATCTTTCTTCGAAAGGCTATCCCCCTGA
TAAAGGCAGGTTGCACACGTGTTCCGCACCCGTACGCCGCTCTCAATTACCCGAAAGTAATCTACCGCTCCGG
CTTGCATGTGTTAGGCCTCCCGCTAGCGTTCATCCTGAGCCAGGATCAACTCT
>522
CTGCTGCCGCCCGTAGGAGTCTGGTCCGTGTCTCAGTACCAGTGTGGGGGATCACCCTCTCAGGCCCCCTAAA
GATCGTCGCCTTGGTGAGCCGTTACCTCACCAACTAGCTAATCTTGCGCGTGCCCATCTCTATCCACCGTAGT

TTTCAATATCACCCGATGCCGAATAATATATTATGGGGTATTAATCTTCTTCGAAAGGCTATCCCCCTGATAA
AGGCAGGTTGCACACGTGTTCCGCACCCGTACGCCGCTCTCAATTACCCGAAAGTAATCTACCGCTCGGCTTG
CATGTGTTAGGCCTCCCGCTAGCGTTCATCCTGAGCCAGGATCAACTCT
>523
CTGCTGCCGCCCGTAGGAGTCTGGTCCGTGTCTCAGTACCAGTGTGGGGGATCACCCTCTCAGGCCCCCTAAA
GATCGTCGCCTTGGTGAGCCGTTACCTCACCAACTAGCTAATCTTGCGCGTGCCCATCTCTATCCACCGTAGT
TTTCAATATCACCCGATGCCGAATAATATATTATGGGGTATTAATCTTCTCGAAAGGCTATCCCCCTGATAAA
GGCAGGTTGCACACGTGTTCCGCACCCGTACGCCGCTCTCAATTACCCGAAAGTAATCTACCGCTCGGCTTGC
ATGTGTTAGGCCTCCCGCTAGCGTTCTCCTGAGCAGGATCAACTCCGT
>524
CTTGCTGCCGCCCCGTAGGAGTCTGGTCCGTGTCTCAGTACCAGTGTGGGGGGATCACCCTCTCAGGCCCCCT
AAAGATCGTTCGCCTGGTGAGCCGTTTACCTCACCCAACTAGCTTAATCTTGCGCGTGCCCCATCTTCTTATC
CACCGTAGTTTTTCAATTATCACCCGATGCCGAATAATATATTATGGGGTATTTAATCTTTCTCGAAGGCTAT
CCCCTGAATAAAGGCAGGTTTGCACACGTGTTCCGCACCCGTACGCCGCTCTTCAATTTACCCCGAAAGTAAT
CACCGCTCCGGCTTGCATGTGTTAGGCCTTCCCGCTAGCGTTCATC
>525
CTGCTGCCGCCCGTAGGAGTCTGGTCCGTGTTCTCGGTACCAGTGTGGGGGATCACCCTCTCAGGCCCCCTAA
AGATCGTCGCCTTGGTGAGCCGTTACCTCACCAACTAGCTAATCTTGCGCGTGCCCATCTCTTATCCACCGTA
GTTTTTCAATATCACCCGATGCCGAATAATATATTATGGGGTATTTAATCTTCTTCGAAAGGCTATCCCCCTG
ATAAAGGCAGGTTTGCACACGTGTTCCGCACCCGTACGCCGCTCTCAATTTACCCGAAAGTAATCTACCGCTC
GGCTTGCATGTGTTTAGGCCTTCCCGCTAGCGTTCTCCTGAGCCAGGATCAACTC
>526
CTGCTGCCGCCCGTAGGAGTCTGGTCCGTGTCTCAGTACCAGTGTGGGGGATCACCCTCTCAGGCCCCCTAAA
AGATCGTCGCCTTGGTGAGCCGTTTACCTCACCAACTAGCTAATCTTGCGCGTGCCCATCTCTATCCACCGTA
GTTTCAATATCACCCGATGCCGAATAATATATTATGGGGTATTAATCTTCTTCGAAAGCTATCCCCCTGATAA
AGGCAGGTTGCACACGTGTTCCGCACCCGTACGCCGCTCTCAATTACCCGAAAGTAATCTACCGCTCGGCTTG
CATGTGTTAGGCCTCCCGCTAGCGTTCTCCTGAGCAGGATCAAACT
>527
CTGCTGCCGCCCGTAGGAGTCTGGTCCGTGTCTCAGTACCAGTGTGGGGGATCACCCTCTCAGGCCCCCTAAA
GATCGTCGCCTTGGTGAGCCGTTACCTCACCAACTAGCTAATCTTGCGCGTGCCCATCTCTATCCACCGTAGT
TTTCAATATCACCCGATGCCGAATAATATATTATGGGGTATTAATCTTCTCGAAAGGCTATCCCCCTGATAAA
GGCAGGTTGCACACGTGTTCCGCACCCGTACTCCGCTCTCAATTACCCGAAAGTAATCTACCGCTCCGGCTTG
CATGTGTTAGGCCTCCCGCTAGCGTTCATCCTGAGCCAGGATCAACTCT
>528
CTGCTGCCGCCCGTAGGAGTCTGGTCCGTGTCTCAGTACCAGTGTGGGGATCACCCTCTCAGGCCCCCTAAAG
ATCGTCGCCTTGGTGAGCCGTTACCTCACCAACTAGCTAATCTTGCGCGTGCCCATCTCTATCCACCGTAGTT
TTCAATATCACCCGATGCCGAATAATATATTATGGGGTATTAATCTTCTTCGAAAGGCTATCCCCCTGATAAG
GCAGGTTGCACACGTGTTCCGCACCCGTACGCCGCTCTCAATTACCCGAAAGTAATCTACCGCTCCGGCTTGC
ATGTGTTAGGCCTCCCGCTAGCGTTCTCCTGAGCAGGATCA
>529
CTGCTGCCGCCCGTAGGAGTCTGGTCCGTGTCTCAGTACCAGTGTGGGGGATCACCCTCTCAGGCCCCCTAAA
GATCGTCGCCTTGGTGAGCCGTTACCTCACCAACTAGCTAATCTTGCGCGTGCCCATCTCTATCCACCGTAGT
TTTCAATATCACCCGATGCCGAATAATATATTATGGGGTATTAATCTTCTTCTAAAGCTATCCCCCTGATAAA
GGCAGGTTGCACACGTGTTCCGCACCCGTACGCTGCTCTCAATTACCCGAAAGTAATCTACCGCTCGGCTTGC
ATGTGTTAGGCCTCCCGCTAGCGTTCATCTGAGCCAGGATCAAACTCT
>530
CTGCTGCCGCCCGTAGGAGTCTGGTCCGTGTCTCAGTACCAGTGTGGGGGATCACCCTCTCAGGCCCCCTAAA
GATCGTCGCCTTGGTGAGCCGTTACCTCACCAACTAGCTAATCTTGCGCGTGCCCATCTCTATCCACCGTAGT
TTTCAATATCACCCGATGCCGAATAATATATTATGGGGTATTAATCTTCTTCGAAAGGCTATCCCCCTGATAA
AGGCAGGTTGCACACGTGTTCCGCACCCGTACGCCGCTCTCAATTACCCGAAAGTAATCTACCGCTCGGCTTG
CATGTGTTAGGCCTCCCGCTAGCGTTCATCCTGAGCCAGGATCAAACTCT
>531
CTGCTGCCGCCCGTAGGAGTCTGGTCCGTGTCTCAGTACCAGTGTGGGGGATCACCCTCTCAGGCCCCCTAAA
GATCGTCGCCTTGGTGAGCCGTTACCTCACCAACTAGCTAATCTTGCGCGTGCCCATCTCTATCCACCGTAGT

TTTCAATATCACCCGATGCCGAATAATATATTATGGGGTATTAATCTTCTTCGAAAGGCTATCCCCCTGATAA
AGGCAGGTTGCACACGTGTTCCGCACCCGTACGCCGCTCTCAATTACCCGAAGGTAATCTACCGCTCGGCTTG
CATGTGTTAGGCCTCCCGCTAGCGTTCTCCTGAGCAGGATCAACTCGT
>532
CTGCTGCCGCCCGTAGGAGTCTGGTCCGTGTCTCAGTACCAGTGTGGGGGATCACCCTCTCAGGCCCCCTAAA
GATCGTCGCCTTGGTGAGCCGTTACCTCACCAACTAGCTAATCTTGCGCGTGCCCATCTCTATTCCACCGTAG
TTTCAATATCACCCGATGCCGAATAATATATTATGGGGTATTAATCTTCTTCGAAGGCTATCCCCCTGATAAA
GGCAGGTTGCACACGTGTTCCGCACCCGTACGCCGCTCTCAATTACCCGAAAGTAATCTACCGCTCGGCTTGC
ATGTGTTAGGCCTCCCGCTAGCGTTCATCCTGAGCCAGGATCAAACTCT
>533
CTGCTGCCGCCCGTAGGAGTCTGGTCCGTGTCTCAGTACCAGTGTGGGGGATCACCCTCTCAGGCCCCCTAAA
GATCGTCGCCTTGGTGAGCCGTTACCTCACCAACTAGCTAATCTTGCGCGTGCCCATTCTCTATTCACCGTAG
TTTCAATATCACCCGATGCCGAATAATATATTATGGGGTATTAATCTTCTTCGAAAGGCTATCCCCCTGATAA
AGGCAGGTTGCACACGTGTTCCGCACCCGTACGCCGCTCTCAATTACCCGAAAGTAATCTACCGCTCGGCTTG
CATTGTGTTAGGCCTCCCGCTAGCGTTCTCCTGAGCAGGATC
>534
CTGCTGCCGCCCGTAGGAGTCTGGTCCGTGTCTCAGTACCAGTGTGGGGGATCACCCTCTCAGGCCCCCTAAA
GATCGTCGCCTGGTGAGCCGTTACCTCACCAACTAGCTAATCTTGCGCGTGCCCATCTCTATCCACCGTAGTT
TTCAATATCACCCGATGCCGAATAATATATTATGGGGTATTAATCTTCTTCGAAAGGCTATCCCCCTGATAAA
GGCAGGTTGCACACGTGTTCCGCACCCGTACGCCGCTCTCAATTACCCGAAAGTAATCTACCGCTCGGCTTGC
ATGTGTTAGGCCTCCCGCTAGCGTCTCCTGAGCA
>535
CTGCTGCCGCCCGTAGGAGTCTGGTCCGTGTCTCAGTACCAGTGTGGGGGATCACCCTCTCAGGCCCCCTAAA
GATCGTCGCCTTGGTGAGCCGTTACCTCACCAACTAGCTAATCTTGCGCGTGCCCATCTCTATCCACCGTAGT
TTTCAATATCAACCCGATGCCGAATAATATATTATGGGGTATTAATCTTCTTCGAAAGGCTATCCCCCTGATA
AAGGCAGGTTGCACACGTGTTCCGCACCCGTACGCCGCTCTCAATTACCCGAAAGTAATCTACCGCTCGGCTT
GCATGTGTTAGGCCTCCCGCTAGC
>536
CTGCTGCCGCCCGTAGGAGTCTGGTCCGTGTCTCAGTACCAGTGTGGGGGATCACCCTCTCAGGCCCCCTAAA
GATCGTCGCCTTGGTGAGCCGTTACCTCACCAACTAGCTAATCTTGCGCGTGCCCATCTCTATCCACCGTAGT
TTTCAATATCAACCCGATGCCGAATAATATATTATGGGGTATTAATCTTCTTCGAAAGGCTATCCCCCTGATA
AAGGCAGGTTGCACACGTGTTCCGCACCCGTACGCCGCTCTCAATTACCCGAAAGTAATCTACCGCTCGGCTT
GCATGTGTTAGGCCTCCCGCTAGCGTCTCCTGAGCAGGATCAACTCGT
>537
CTGCTGCCGCCCGTAGGAGTCTGGTCCGTGTCTCAGTACCAGTGTGGGGGATCACCCTCTCAGGCCCCCTAAA
GATCGTCGCCTGGTGAGCCGTTACCTCACCAACTAGCTAATCTTGCGCGTGCCCATCTCTATCCACCGTAGTT
TTCAATATCACCCGATGCCGAATAATATATTATGGGGTATTAATCTTCTTCGAAAGGCTATCCCCCTGATAAA
GGCAGGTTGCACACGTGTTCCGCACCCGTACGCCGCTCTCAATTACCCGAAAGTAATCTACCGCTCGGCTTGC
ATGTGTTAGGCCTCCCGCTAGCGTTCTTCCTGAGCAG
>538
CTGCTGCCGCCCTAGGAGTCTGGTCCGTGTCTCAGTACCAGTGTGGGGGATCACCCTCTCAGGCCCCCTAAAG
ATCGTCGCCTGTGAGCCGTTACCTCACCAACTAGCTAATCTTGCGCGTGCCCATCTCTATCCACCGTAGTTTC
AATATCACCCGATGCCGAATAATATATTATGGGGTATTAATCTTCTTCGAAAGCTATCCCCTGATAAAGGCAG
GTTGCACACGTGTTCCGCACCCGTACGCCGCTCTCAATTACCCGAAAGTAATCTACCGCTCGGCTTGCATGTG
TTAGGCCTCCCGCTAGCGTTCTCCTGAGCA
>539
CTGCTGCCGCCCGTAGGAGTCTGGTCCGTGTCTCAGTACCAGTGTGGGGGATCACCCTCTCAGGCCCCCTAAA
GATCGTCGCCTTGGTGAGCCGTTACCTCACCAACTAGCTAATCTTGCGCGTGCCCATCTCTATCCACCGTAGT
TTTCAATATCACCCGATGCCGAATAATATATTATGGGGTATTAATCTTCTTCGAAAGGCTATCCCCCTGATAA
AGGCAGGTTGCACACGTGTTCCGCACCCGTACGCCGCTCTCAATTACCCGAAAGTAATCTACCGCTCGGCTTG
CATGTGTTAGGCCTCCCGCTAGCGTTCATCCTGAGCCAGGATCAAACTCT
>540
CTGCTGCCACCCGTAGGAGTCTGGTCCGTGTCTCAGTACCAGTGTGGGGGATCACCCTCTCAGGCCCCCTAAA
GATCGTCGCCTTGGTGAGCCGTTACCTCACCAACTAGCTAATCTTGCGCGTGCCCATCTCTATCCACCGTAGT

TTTCAATATCACCCGATGCCGAATAATATATTATGGGGTATTAATCTTCTTCGAAAGGCTATCCCCCTGATAA
AGGCAGGTTGCACACGTGTTCCGCACCCGTACGCCGCTCTCAATTACCCGAAAGTAATCTACCGCTCGGCTTG
CATGTGTTAGGCCTCCCGCTAGCGTTCTCCTGAGCCAGGATCAAACTCT
>541
CTGCTGCCGCCCGTAGGAGTCTGGTCCGTGTCTCAGTACCAGTGTGGGGGATCACCCTCTCAGGCCCCCTAAA
GATCGTCGCCTTGGTGAGCCGTTACCTCACCAACTAGCTTAATCTTGCGCGTGCCCATCTCTATCCACCGTAG
TTTCAATATCACCCGATGCCGAATAATATATTATGGGGTATTAATCTTCTTCGAAAGGCTATCCCCCTGATAA
AGGCAGGTTGCACACGTGTTCCGCACCCGTACGCCGCTCTCAATTACCCGAAAGTAATCTACCGCTCGGCTTG
CATGTGTTAGGCCTCCCGCTAGCGTTCATCCTGAGCAGGATCAACTCCT
>542
CTGCTGCCGCCCGTAGGGGTCTGGTCCGTGTCTCAGTACCAGTGTGGGGGATCACCCTCTCAGGCCCCCTAAA
GATCGTCGCCTTGGTGAGCCGTTACCTCACCAACTAGCTAATCTTGCGTGTGCCCATCTCTATCCACCGTAGT
TTTCAATATCACCCGATGCCGAATAATATATTATGGGGTATTAATCTTCTTCGAAAGGCTATCCCCCTGATTA
AAGGCAGGTTGCACACGTGTTCCGCACCCGTACGCCGCTCTCAATTACCCGAAAGTAATCTACCGCTCGGCTT
GCATGTGTTAGGCCTCCCGCTAGCGTTCTCCTGAGCCAGGATCAAACTCT
>543
CTGCTGCCGCCCGTAGGAGTCTGGTCCGTGTCTCAGTACCAGTGTGGGGGATCACCCTCTCAGGCCCCCTAAA
GATCGTCGCCTTGGTGAGCCGTTACCTCACCAACTAGCTAATCTTGCGCGTGCCCATCTCTATCCACCGTAGT
TTTCAATATCACCCGATGCCGAATAATATATTATGGGGTATTAATCTTCTCGAAAGGCTATCCCCTGATAAAG
GCAGGTTGCACACGTGTTCCGCACCCGTACGCCGCTCTCAATTACCCGAAAGTAATCTACCGCTCGGCTTGCA
TGTGTTAGGCCTCCCGCTAGCGTTCATCTGAGCCAGGATCAACTCT
>544
CTGCTGCCGCCCGTAGGAGTCTGGTCCGTGTCTCAGTACCAGTGTGGGGGATCACCCTCTCAGGCCCCCTAAA
GATCGTCGCCTTGGTGAGCCGTTACCCCACCAACTAGCTAATCTTGCGTGTGCCCATCTCTATCCACCGTAGT
TTTCAATATCACCCGATGCCGAATAATATATTATGGGGTATTTAATCTTCTTCGAAGGCTATCCCCTGATAAA
GGCAGGTTGCACACGTGTTCCGCACCCGTACGCCGCTCTCAATTACCCGAAAGTAATCTACCGCTCGGCTTGC
ATGTGTTAGGCCTCCCGCTAGCGTTCTCCTGAGCCAG
>545
CTGCTGCCGCCCGTAGGAGTCTGGTCCGTGTCTCAGTACCAGTGTGGGGGATCACCCTCTCAGGCCCCCTAAA
GATCGTCGCCTGGTGAGCCGTTACCTCACCAACTAGCTAATCTTGCGCGTGCCCATCTCTATCCACCGTAGTT
TTCAATATCACCCGATGCCGAATAATATATTATGGGGTATTAATCTTCTCGAAGGCTTATCCCCCTGATAAAG
GCAGGTTGCACACGTGTTCCGCACCCGTACGCCGCTCTCAATTACCCGAAAGTAATCTACCGCTCGGCTTGCA
TGTGTTAGGCCTCCCGCTAGCGTCTCCTGAGCAGGATCA
>546
CTGCTGCCGCCCGTAGGAGTCTGGTCCGTGTCTCAGTACCAGTGTGGGGGATCACCCTCTCAGGCCCCCTAAA
GATCGTCGCCTGTGAGCCGTTACCTCACCAACTAGCTAATCTTGCGCGTGCCCATCTCTATCCACCGTAGTTT
TCAATATCAACCCGATGCCGAATAATATATTATGGGGTATTAATCTTCTCGAAAGCTATCCCCTGATAAAGGC
AGGTTGCACACGTGTTCCGCACCCGTACGCCGCTCTCAATTACCCGAAAGTAATCTACCGCTCGGGCTTGCAT
GTGTTAGGCCTCCCGCTAGCGTCTCCTGAG
>547
CTGCTGCCGCCCGTAGGAGTCTGGTCCGTGTCTCAGTACCAGTGTGGGGGATCACCCTCTCAGGCCCCCTAAA
GATCGTCGCCTTGGTGAGCCGTTACCTCACCAACTAGCTAATCTTGCGCGTGCCCATCTCTATCCACCGTAGT
TTTCAATATCACCCGATGCCGAATAATATATTATGGGGTATTAATCTTCTTCGAAAGGCTATCCCCCTGATAA
AGGCAGGTTGCACACGTGTTCCGCACCCGTACGCCGCTCTCAATTACCCGAAAGTAATCTACCGCTCGGCTTG
CATGTGTTAGGCCTCCCGCTAGCGTTCTCCTGAGCAGGATCAACTCGT
>548
CTGCTGCCGCCCGTAGGAGTCTGGTCCGTGTCTCAGTACCAGTGTGGGGGATCACCCTCTCAGGCCCCCTAAA
AGATCGTCGCCTTGGTGAGCCGTTACCTCACCAACTAGCTAATCTTGCGCGTGCCCATCTCTATCCACCGTAG
TTTTCAATATCACCCGATGCCGAATAATATATTATGGGGGTATTAATCTTCTTCGAAAGGCTATCCCCCTGAT
AAAGGCAGGTTGCACACGTGTTCCGCACCCGTACGCCGCTCTCAATTACCCGAAAGTAATCTACCGCTCGGCT
TGCATGTGTTAGGCCTCCCGCTAGCGTTCATCCTGAGCCAGGATCAAACTCT
>549
CTGCTGCCGCCCGTAGGAGTCTGGACCGTGTCTCAGTTCCAGTGTGACTGATCATCCTCTCAGACCAGTACGG
ATCGTCGCCTTGGTAGGCCTTTACCCCACCAACTAGCTAATCCGACCTAGGCTCATCTGATAGCGTGAGGTCC

GAAGATCCCCCACTTCTCCCTCAGGACGTATGCGGTATTAGCGCCCGTTTCCGGACGATCCCCACTACCAGGC
AGATTCCTAGGCATTACTCACCCGTCCGCCGCTGAATCCAGGAGCAAGCCTCCTCTCCGCTCGACTGCCATGT
GTTAGGCCTGCCGCCAGCGTTCAATCTGAGCCAGGATCAAACTCT
>550
CTGCTGCCGCCCGTAGGAGTCTGGTCCGTGTCTCAGTACCAGTGTGGGGGATCACCCTCTCAGGCCCCCTAAA
GATCGTCGCCTGGTGAGCCGTTACCTCACCAACTAGCTAATCTTGCGCGTGCCCATCTCTATCCACCGTAGTT
TTCAATATCACCCGATGCCGAATAATATATTATGGGGTATTAATCTTCTTCGAAAGGCTATCCCCTGAATAAA
GGCAGGTTGCACACGTGTTCCGCACCCGTACGCCGTTCTCAATTACCCGAAAGTAATCTACCGCTCGGCTTGC
ATGTGTTAGGCCTCCCGCTAGCGTTCATCCTGAGCCAGGATCAAACTCT
>551
CTGCTGCCGCCCGTAGGAGTCTGGTCCGTGTCTCAGTACCAGTGTGGGGGATCACCCTCTCAGGCCCCCTAAA
GATCGTCGCCTTGGTGAGCCGTTACTCCACCAACTAGCTAATCTTGCGCGTGCCCATCTCTATCCACCGTAGT
TTTCAATATCACCCGATGCCGAATAATATATTATGGGGTATTAATCTTCTTCGAAAGGCTATCCCCCTGATAA
AGGCAGGTTGCACACGTGTTCCGCACCCGTACGCCGCTCTCAATTACCCGAAAGTAATCTACCGCTCGGCTTG
CATGTGTTAGGCCTCCCGCTAGCGTTCATCCTGA
>552
CTGCTGCCGCCCGTAGGAGTCTGGTCCGTGTCTCAGTACCAGTGTGGGGGATCACCCTCTCAGGCCCCCTAAA
GATCGTCGCCTTGGTGAGCCGTTACCTCACCAACTAGCTAATCTTGCGCGTGCCCATCTCTATCCACCGTAGT
TTTCAATATCACCCGATGCCGAATAATATATTATGGGGTATTAATCTTCTTCGAAAGGCTATCCCCCTGATAA
AGGCAGGTTGCACACGTGTTCCGCACCCGTACGCCGCTCTCAATTACCCGAAAGTAATCTACCGCTCGGCTTG
CATGTGTTAGGCCTCCCGCTAGCGTTCTCCTGAG
>553
CTGCTGCCGCCCGTAGGAGTCTGGTCCGTGTCTCAGTACCAGTGTGGGGGATCACCCTCTCAGGCCCCCTAAA
GATCGTCGCCTGGTGAGCCGTTACCTCACCAACTAGCTAATCTTGCGCGTGCCCATCTCTATCCACCGTAGTT
TTCAATATCACCCGATGCCGAATAATATATTATGGGGTATTAATCTTCTTCGAAAGGCTATCCCCCTGATAAA
GGCAGGTTGCACACGTGTTCCGCACCCGTACGCCGCTCTCAATTACCCGAAAGTAATCTACCGCTCGGCTTGC
ATGTGTTAGGCCTCCCGCTAGCGTTCATCCTGAGCCAGGGATCAAACTCT
>554
CTGCTGCCGCCCGTAGGAGTCTGGCTCGTGTCTCAGTACCAGTGTGGGGGATCACCCTCTCAGGCCCCCTAAA
GATCGTCGCCTTGGTGAGCCGTTACCTCACCAACTAGCTAATCTTGCGCGTGCCCATCTCTATCCATCGTAGT
TTTCAATATCACCCGATGCCGAATAATATATTATGGGGTATTAATCTTCTTCGAAAGGCTATCCCCCTGATAA
AGGCAGGTTGCACACGTGTTCCGCACCCGTACGCCGCTCTCAATTACCCGAAAGTAATCTACCGCTCGGCTTG
CATGTGTTAGGCCTCCCGCTAGCGTTCATTCCTGAGCCAGGATCAAACTCT
>555
CTGCTGCCGCCCGTAGGAGTCTGGTCCGTGTCTCAGTACCAGTGTGGGGGATCACCCTCTCAGGCCCCCTAAA
GATCGTCGCCTTGGTGAGCCGTTACCTCACCAACTAGCTAATCTTGCGCGTGCCCATCTCTATCCACCGTAGT
TTTCAATATCACCCGATGCCGAATAATATATTATGGGGTATTAATCTTCTTCGAAAGGCTATCCCCCTGATAA
AGGCAGGTTGCACACGTGTTCCGCACCCGTACGCCGCTCTCAATTACCCGAAAGTAATCTACCGCTCGGCTTG
CATGTGTTAGGCCTCCCGCTAGCGTTCATCCTGAGCCAGGATCAAACTCT
>556
CTGCTGCCGCCCGTAGGAGTCTGGTCCGTGTCTCAGTACCAGTGTGGGGGATCACCCTCTCAGGCCCCCTAAA
GATCGTCGCCTTGGTGAGCCGTTACCTCACCAACTAGCTAATCTTGCGCGTGCCCATCTCTATCCACCGTAGT
TTTCAATATCACCCGATGCCGAATAATATATTATGGGGTATTTAATCTTCTTCGAAAGGCTATCCCCCTGATA
AAGGCAGGTTGCACACGTGTTCCGCACCCGTACGCCGCTCTCAATTACCCGAAAGTAATCTACCGCTCGGCTT
GCATGTGTTAGGCCTCCCGCTAGCGTTCTCCTGAGCAGGATCAACTCGT
>557
CTGCTGCCGCCCGTAGGAGTCTGGACCGTGTCTCAGTTCCAGTGTGACTGATCATCCTCTCAGACCAGTACGG
ATCGTCGCCTTGGTAGGCCTTTACCCCACCAACTAGCTAATCCGACCTAGGCTCATCTGATAGCGTGAGGTCC
GAAGATCCCCCACTTCTCCCTCAGGACGTATGCGGTATTAGCGCCCGTTTCCGGACGTATCCCCACTACCAGG
CAGATTCCTAGGCATTACTCACCCGTCCGCCGCTGAATCCAGGAGCAAGCTCCCTTCATCCGCTCGACTGCCA
TGTGTTAGGCCTGCCGCCAGCGTCAATCTGAGCCAGGATCAAACTCT
>558
CTGCTGCCGCCCGTAGGAGTCTGGTCCGTGTCTCAGTACCAGTGTGGGGGATCACCCTCTCAGGCCCCCTAAA
GATCGTCGCCTTGGTGAGCCGTTACCTCACCAACTAGCTAATCTTGCGCGTGCCCATCTCTATCCACCGTAGT

TTTCAATATCACCCGATGCCGAATAATATATTATGGGGTATTAATCTTCTTCGAAAGGCTATCCCCCTGATAA
AGGCAGGTTGCACACGTGTTCCGCACCCGTACGCCGCTCTCAATTACCCGAAAGTAATCTACCGCTCGGCTTG
CATGTGTTAGGCCTCCCGCTAGCGTTCTCCTGAGCAGGATCC
>559
CTGCTGCCGCCCGTAGGAGTCTGGTCCGTGTCTCAGTACCAGTGTGGGGGATCACCCTCTCAGGCCCCCTAAA
GATCGTCGCCTTGGTGAGCCGTTACCTCACCAACTAGCTAATCTTGCGCGTGCCCATCTCTATCCACCGTAGT
TTTCAATATCACCCGATGCCGAATAATATATTATGGGGTATTAATCTTCTTCGAAAGGCTATCCCCCTGATAA
AGGCAGGTTGCACACGTGTTCCGCACCCGTACGCCGCTCTCAATTACCCGAAAGTAATCTACCGCTCGGCTTG
CATGTGTTAGGCCTCCCGCTAGCGTTCATCCTGAGCCAGGATCAACTCT
>560
CTGCTGCCGCCCGTAGGAGTCTGGTCCGTGTCTCAGTACCAGTGTGGGGGATCACCCTCTCAGGCCCCCTAAA
GATCGTCGCCTTGGTGAGCCGTTACCTCACCAACTAGCTAATCTTGCGCGTGCCCATCTCTATCCACCGTAGT
TTTCAATATCACCCGATGCCGAATAATATATTATGGGGTATTAATCTTCTTCGAAAGGCTATCCCCCTGATAA
AGGCAGGTTGCACACGTGTTCCGCACCCGTACGCCGCTCTCAATTACCCGAAAGTAATCTACCGCTCGGCTTG
CATGTGTTAGGCCTCCCGCTAGCGTTCATCCTGAGCCAGGATCAAACTCT
>561
CTGCTGCCGCCCGTAGGAGTCTGGTCCGTGTCTCAGTACCAGTGTGGGGGATCACCCTCTCAGGCCCCCTAAA
GATCGTCGCCTTGGTGAGCCGTTACCTCACCAACTAGCTAATCTTGCGCGTGCCCATCTCTATCCACCGTAGT
TTTCAATATCACCCGATGCCGAATAATATATTATGGGGTATTAATCTTCTTCGAAAGGCTATCCCCCTGATAA
AGGCAGGTTGCACACGTGTTCCGCACCCGTACGCCGCTCTCAATTACCCGAAAGTATCTACCGCTCGGCTTGC
ATGTGTTAGGCCTCCCGCTAGCGTTCATCCTGAGCCAGGATCAACTCCT
>562
CTGCTGCCGCCCGTAGGAGTCTGGTCCGTGTCTCAGTACCAGTGTGGGGGATCACCCTCTCAGGCCCCCTAAA
GATCGTCGCCTTGGTGAGCCGTTACCTCACCAACTAGCTAATCTTGCGCGTGCCCATCTCTATCCACCGTAGT
TTTCAATATCACCCGATGCCGAATAATATATTATGGGGTATTAATCTTCTTCGAAAGGCTATCCCCTGATAAA
GGCAGGTTGCACACGTGTTCCGCACCCGTACGCCGCTCTCAATTACCCGAAAGTAATCTACCGCTCGGCTTGC
ATGTGTTAGGCCTCCCGCTAGCGTTCATCCTGAGCCAGGATCAAACTCT
>563
CTGCTGCCGCCCGTAGGAGTCTGGTCCGTGTCTCAGTACCAGTGTGGGGGATCACCCTCTCAGGCCCCCTAAA
GATCGTCGCCTTGGTGAGCCGTTACCTCACCAACTAGCTAATCTTGCGCGTGCCCATCTCTATCCACCGTAGT
TTTCAATATCACCCGATGCCGAATAATATATTATGGGGTATTAATCTTCTTCGAAAGGCTATCCCCCTGATAA
AGGCAGGTTGCACACGTGTTCCGCACCCGTACGCCGCTCTCAATTACCCGAAAGTAATCTACCGCTCGGCTTG
CATGTGTTAGGCCTCCCGCTAGCGTTCTCCTGAGCAGGATCAACTC
>564
CTGCTGCCGCCCGTAGGAGTCTGGTCCGTGTCTCAGTACCAGTGTGGGGGATCACCCTCTCAGGCCCCCTAAA
GATCGTCGCCTTGGTGAGCCGTTACCTCACCAACTAGCTAATCTTGCGCGTGCCCATCTCTATCCACCGTAGT
TTTCAATATCACCCGATGCCGAATAATATATTATGGGGTATTAATCTTCTTCGAAAGGCTATCCCCCTGATAA
AGGCAGGTTGCACACGTGTTCCGCACCCGTACGCCGCTCTCAATTACCCGAAAGTAATCTACCGCTCGGCTTG
CATGTGTTAGGCCTCCCGCTAGCGTTCATCCTGAGCCAGGATCAAACTCT
>565
CTGCCGCCGCCCGTAGGAGTCTGGTCCGTGTCTCAGTACCAGTGTGGGGATCACCCTCTCAGGCCCCCTAAAG
ATCGTCGCCTTGGTGAGCCGTTACCTCACCAACTAGCTAATCTTGCGCGTGCCCATCTCTATCCACCGTAGTT
TTCAATATCACCCGATGCCGAATAATATATTATGGGGTATTAATCTTCTCTGAAAGGCTTATCCCCTGATAAA
GGCAGGTTGCACACGTGTTCCGCACCCGTACGCCGCTCTCAATTACCCGAAAGTAATCTACCGCTCGGCTTGC
ATGTGTTAGGCCTCCCGCTAGCGTTCTCCTGAGCAGGATCAACTC
>566
CTGCTGCCGCCCGTAGGAGTCTGGTCCGTGTCTCAGTACCAGTGTGGGGATCACCCTCTCAGGCCCCCTAAAG
ATCGTCGCCTTGGTGAGCCGTTACCTCACCAACTAGCTAATCTTGCGCGTGCCCATCTCTATCCACCGTAGTT
TTCAATATCACCCGATGCCGAATAATATATTATGGGGTATTAATCTTCTTCGAAAGGCTATCCCCCTGATAAA
GGCAGGTTGCACACGTGTTCCGCACCCGTACGCCGCTCTCAATTACCCGAAAGTAATCTACCGCTCGGCTTGC
ATGTGTTAGGCCTCCCGCTAGCGTTCATTCCTGAGCCAGGATCAAACTCT
>567
CTGCTGCCGCCCGTAGGAGTCTGGTCCGTGTCTCAGTACCAGTGTGGGGGATCACCCTCTCAGGCCCCCTAAA
GATCGTCGCCTTGGTGAGCCGTTACCTCACCAACTAGCTAATCTTGCGCGTGCCCATCTCTATCCACCGTAGT

TTTCAATATCACCCGATGCCGAATAATATATTATGGGGTATTAATCTTCTTCGAAAGGCTATCCCCCTGATAA
AGGCAGGTTGCACACGTGTTCCGCACCCGTACGCCGCTCTCAATTACCCGAAAGTAATCTACCGCTCGGCTTG
CATGTGTTAGGCCTCCCGCTAGCGTTCATCCTGAGCCAGGATCAAACTCT
>568
CTGCTGCCGCCCGTAGGAGTCTGGACCGTGTCTCAGTTCCAGTGTGACTGATCATCCTCTCAGACCAGTACGG
ATCGTCGCCTTGGTAGGCCTTTTACCCCACCAACTAGCTAATCCGACCTAGGCTCATCTGATAGCGTGAGGT
CCGAAGTCCCCACTTCTCCCTCAGGACGTATGCGGTATTAGCGCCCCGTTTCCGGACGTATCCCCACTACCAG
GCAGATTCCTAGGCATTACTCACCCGTCCGCCGCTGAATCCAGGAGCAAGCTCCCTCATCCGCTCGACTGCCA
TGTGTTAGGCCTGCCGCAGCGTCAATCTGAGCCAGGATCAAACTCT
>569
CTGCTGCCGCCCGTAGGAGTCTGGTCCGTGTCTCAGTACCAGTGTGGGGATCACCCTCTCAGGCCCCCTAAAG
ATCGTCGCCTTGGTGAGCCGTTGCCTCACCAACTAGCTAATCTTGCGCGTGCCCATCTCTATCCACCGTAGTT
TCAATACCACCGATGCCGAATAATATATTATGGGGTATTAATCTTCTTCGAAAGGCTATCCCCTGATAAAGGC
AGGTTGCACACGTGTTCCGCACCCGTACGCCGCTCTCAATTACCCGAAAGTAATCTACCGCTCGGCTGCATGT
GTTAGGCCTCCCGCTAGCGTCTCCTGAGCAGAT
>570
CTGCTGCCGCCCGTAGGAGTCTGGTCCGTGTCTCAGTACCAGTGTGGGGGATCACCCTCTCAGGCCCCCTAAA
GATCGTCGCCTTGGTGAGCCGTTACCTCACCAACTAGCTAATCTTGCGCGTGCCCATCTCTATCCACCGTAGT
TTTCAATATCACCCGATGCCGAATAATATATTATGGGGTATTAATCTTCTTCGAAAGGCTATCCCCTGATAAA
GGCAGGTTGCACACGTGTTCCGCACCCGTACGCCGCTCTCAATTACCCCGAAAGTAATCTACCGCTCCGGCTT
GCATGTGTTAGGCCTCCCGCTAGCGTTCTCCTGAGCAGGATCAACTCG
>571
CTGCTGCCGCCCGTAGGAGTCTGGTCCGTGTCTCAGTACCAGTGTGGGGGATCACCCTCTCAGGCCCCCTAAA
GATCGTCGCCTTGGTGAGCCGTTACCTCACCAACTAGCTAATCTTGCGCGTGCCCATCTCTATCCACCGTAGT
TTTCAATATCACCCGATGCCGAATAATATATTATGGGGTATTAATCTTCTTCGAAAGGCTATCCCCCTGATAA
AGGCGGTTGCACACGTGTTCCGCACCCGTACGCCGCTCTCAATTACCCGAAAGTAATCTACCGCTCGGCTTGC
ATGTGTTAGGCCTCCCGCTAGCGTTCTCCTGAGCCAGGATCAAACTCT
>572
CTGCTGCCGCCCGTAGGAGTCTGGTCCGTGTCTCAGTACCAGTGTGGGGGATCACCCTCTCAGGCCCCCTAAA
GATCGTCGCCTGGTGAGCCGTTACCTCACCAACTAGCTAATCTTGCGCGTGCCCATCTCTATCCACCGTAGTT
TTCAATATCACCCGATGCCGAATAATATATTATGGGGTATTAATCTTCTTCGAAAGGCTATCCCCCTGATAAA
GGCAGGTTGCACACGTGTTCCGCACCCGTACGCCGCTCTCAATTACCCGAAAGTAATCTACCGCTCGGCTTGC
ATGTGTTAGGCCTCCCGCTAGCGTTCATCCTGAGCCAGGATCAAACTCT
>573
CTGCTGCCGCCCGTAGGAGTCTGGTCCGTGTCTCAGTACCAGTGTGGGGATCACCTCTCAGGCCCCTAAAGAT
CGTCGCCTTGGTGAGCCGTTATCTCACCAACTAGCTAATCTTGCGCGTGCCCATCTCTATCCACCGTAGTTTC
AATATCACCCGATGCCGAATAATATATTATGGGGTATTAATCTTCTCGAAAGGCTATCCCCTGATAAAGGCAG
GTTGCACACGTGTTCCGCACCCGTACGCCGCTCTCAATTACCCGAAAGTAATCTAACCGCTCCGGCTTGCATG
TGTTAGGCCTCCCGCTAAGCGT
>574
CTGCTGCCGCCCGTAGGAGTCTGAGTCCGTGTCTCAGTACCAGTGTGGGGGATCACCCTCTCAGGCCCCCTAA
AGATCGTCGCCTTGGTGAGCCGTTACCTCACCAACTAGCTAATCTTGCGCGTGCCCATCTCTATCCACCGTAG
TTTTCAATATCACCCGATGCCGGAATAATATATTATGGGGTATTAATCTTCTTCGAAAGGCTATCCCCCTGAT
AAAGGCAGGTTGCACACGTGTTCCGCACCCGTACGCCGCTCTCAATTACCCGAAAGTAATCTACCGCTCCGGC
TTGCATGTGTTAGGCCTCCCGCTAGCGTTCATCCTGAGCCAGGATCAACTCT
>575
CTGCTGCCGCCCGTAGGAGTCTGGTCCGTGTCTCAGTACCAGTGTGGGGGATCACCCTCTCAGGCCCCCTAAAG
ATCGTCGCCTTGGTGAGCCGTTACCTCACCAACTAGCTAATCTTGCGCGTGCCCATCTCTATCCACCGTAGTT
TTTCAATATCACCCGATGCCGAATAATATATTATGGGGTATTTAATTCTTCTTCGAAAGCTATCCCCTGATAA
AGGCAGGTTGCACACGTGTTCCGCACCCGTTACGCCGCTCTCAATTACCCGAAAGTAATCTACCGCTCGGCTT
GCATGTGTTAGGCCTTCCCGCTAGCGTTCATCTGAGCCAGGATCAAACTCT
>576
CTGCTGCCGCCCGTAGGAGTCTGGTCCGTGTCTCAGTACCAGTGTGGGGGATCACCCTCTCAGGCCCCCTAAA
GATCGTCGCCTTGGTGAGCCGTTACCTCACCAACTAGCTAATCTTGCGCGTGCCCATCTCTATCCACCGTAGT

TTTCAATATCACCCGATGCCGAATAATATATTATGGGGTATTAATCTTCTTCGAAAGGCTATCCCCTGATAAA
GGCAGGTTGCACACGTGTTCCGCACCCGTACGCCGCTCTCAATTACCCGAAAGTAATCTACCGCTCCGGCTTG
CATGTGTTAGGCCTCCCGCTAGCGTTCTCCTGACAGGAT
>577
CTTGCTGCCGCCCGTAGGAGTCTGGTTCCGTGTCTCAGTACCAGTGTGGGGGATCACCTTCTCAGGCCCCCTA
AAGATCGTCGCCTTGGTGAGCCGTTACCTCACCAACTAGCTAATCTTGCGCGTGCCCATCTCTATCCACCGTT
AGTTTTTCAATATCACCCGATGCCGAATAATATATTTATGGGGTATTAATTCTTTCTTCGAAAGGCTATCCCC
CTGATTAAAGGCAGGTTGCACACGTGTTCCGCACCCGTACGCCGCTCTCAATTACCCGAAAGTAATTCTACCG
CTCCGGCTTGCATGTGTTAGGCCTTCCCGCTAGCGTTCTCCTGAGCAGGATCA
>578
CTGCTGCCGCCCGTAGGAGTCTGGTTCCGTGTCTCAGTACCAGTGTGGGGGATCACCCTCTCAGGCCCCCTAA
AGATCGTCGCCTTGGTGAGCCGTTACCTCACCAACTAGCTAATCTTGCGCGTGCCCATCTCTATCCACCGTAG
TTTTTCAATATCACCCGATGCCGAATAATATATTATTGGGGTATTTAATTCTTTCTCGAAAGGCTATCCCCCT
GATAAAGGCAGGTTTGCACACGTGTTCCGCACCCGTACGCCGCTCTCAATTACCCGAAAGTAATCTACCGCTC
CGGCTTGCATGTGTTAGGCCTCCCGCTAGCGTTCTCCTGAGCAGGATCAA
>579
CTGCTGCCGCCCGTAGGAGTCTGGTCCGTGTCTCAGTACCAGTGTGGGGGATCACCCTCTCAGGCCCCCTAAA
GATCGTCGCCTTGGTGAGCCGTTACCTCACCAACTAGCTAATCTTGCGCGTGCCCATCTCTATCCACCGTAGT
TTTCAATATCACCCGATGCCGAATAATATATTATGGGGTATTTAATCTTTCTTCGAAAGGCCTATCCCCCTGA
TAAAGGCAGGTTGTCACACGTGTTCCGCACCCGTACGCCGCTCTCAATTACCCGAAAGTAATCTACCGCTCGG
CTGCATGTGTTTAGGCCTTCCCGCTAGCGTTCATCCTGAGCCAGGATCAAACTCT
>580
CTGCTGCCGCCCGTAGGAGTCTGGTCCGTGTCTCAGTACCAGTGTGGGGGATCACCCTCTCAGGCCCCCTAAA
GATCGTCGCCTTGGTGAGCCGTTACCTCACCAACTAGCTTAATCTTGCGCGTGCCCATCTCTATCCACCGTAG
TTTTCAATTATCACCCGATGCCGAATAATATATTTATGGGGTATTAATCTTCCTTCGAAAGGCTTATCCCCCT
GATAAAGGCAGGTTGCACACGTGTTCCGCACCCGTACGCCGCTCTCAATTTACCCGAAAGTAATCTACCGCTC
GGCTTGCATGTGTTAGGCCTTCCCGCTTAGCGTTCACCTG
>581
CTGCTGCCGCCCGTAGGAGTCTGGTCCGTGTCTCAGTACCAGTGTGGGGGATCACCCTCTCAGGCCCCCTAAA
GATCGTCGCCTTGGTGAGCCGTTACCTCACCAACTAGCTAATCTTGCGCGTGCCCATCTCTATCCACCGTAGT
TTTCAATATCACCCGATGCCGAATAATATATTATGGGGTATTAATCTTCTTCGAAAGGCTATCCCCCTGATAA
AGGCAGGTTGCACACGTGTTCCGCACCCGTACGCCGCTCTCAATTACCCGAAAGTAATCTACCGCTCCGGCTT
GCATGTGTTAGGCCTCCCGCTAGCGTTCTCCTGAGCAGGATCAACTC
>582
CTGCTGCCGCCCGTAGGAGTCTGGTCCGTGTCTCAGTACCAGTGTGGGGGATCACCCTCTCAGGCCCCCTAAA
GATCGTCGCCTTGGTGAGCCGTTACCTCACCAACTAGCTAATCTTGCGCGTGCCCATCTCTATCCACCGTAGT
TTTCAATATCACCCGATGCCGAATAATATATTATGGGGTATTAATCTTCCTTCGAAAGGCTACCCCCTGATAA
AGGCAGGTTGCACACGTGTTCCGCACCCGTACGCCGCTCTCAATTACCCGAAAGTAATCTACCGCTCGGCTTG
CATGTGTTAGGCCTCCCGCTAGCGTTCTCCTGAGCAGGATCAACTCT
>583
CTGCTGCCGCCCGTAGGAGTCTGGACCGTGTCTCAGTTCCAGTGTGACTGATCATCCTCTCAGACCAGTACGG
ATCGTCGCCTTGGTAGGCCTTTACCCCACCAACTAGCTAATCCGACCTAGGCTCATCTGATAGCGTGAGGTCC
GAAGTCCCCCACTTCTCCCTCAGGACGTATGCGGTATTAGCGCCCGTTTCCGGACGATCCCCACTACCAGGCA
GATTCCCTAGGCATTACTCACCCGTCCGCCGCTGAATCCAGGAGCAAGCCCCCTTCATCCGCTCGACTGCATG
TGTTAGGCCTGCCGCCAGCGTCAATCTGAGCCAGGATCAAACTCT
>584
CTGCTGCCGCCCGTAGGAGTCTGGTCCGTGTCTCAGTACCAGTGTGGGGGATCACCCTCTCAGGCCCCCTAAA
GATCGTCGCCTTGGTGAGCCGTTACCTCACCAACTAGCTAATCTTGCGCGTGCCCATCTCTATCCACCGTAGT
TTTCAATATCACCCGATGCCGAATAATATATTATGGGGTATTAATCTTCTTCGAAAGGCTATCCCCCTGATAA
AGGCAGGTTGCACACGTGTTCCGCACCCGTACGCCGCTCTCAATTACCCGAAAGTAATCTACCGCTCGGCTTG
CATGTGTTAGGCCTCCCGCTAGCGTTCTCCTGAGCAGGTCAAACTCT
>585
CTGCTGCCGCCCGTAGGAGTCTGGTCCGTGTCTCAGTACCAGTGTGGGGGATCACCCTCTCAGGCCCCCTAAA
GATCGTCGCCTTGGTGAGCCGTTACCTCACCAACTAGCTAATCTTGCGCGTGCCCATCTCTATCCACCGTAGT

TTTCAATATCACCCGATGCCGAATAATATATTATGGGGTATTAATCTTCTTCGAAAGGCTATCCCCCTGATAA
AGGCAGGTTGCACACGTGTTCCGCACCCGTACGCCGCTCTCAATTACCCGAAAGTAATCTACCGCTCGGCTTG
CATGTGTTAGGCCTCCCGCTAGCGTTCATCCTGAGCCAGGATCAAACTCT
>586
CTGCTGCCGCCCGTAGGAGTCTGGTCCGTGTCTCAGTACCAGTGTGGGGGATCACCCTCTCAGGCCCCCTAAA
GATCGTCGCCTTGGTGAGCCGTTACCTCACCAACTAGCTAATCTTGCGCGTGCCCATCTCTATCCACCGTAGT
TTTCAATATCACCCGATGCCGAATAATATATTATGGGGTATTAATCTTCTTCGAAAGGCTATCCCCCTGATAA
AGGCAGGTTGCACACGTGTTCCGCACCCGTACGCCGCTCTCAATTACCCGAAAGTAATCTACCGCTCGGCTTG
CATGTGTTAGGCCTCCCGCTAGCGTTCATCCTGAGCAGGATCAAACTCT
>587
CTGCTGCCGCCCGTAGGAGTCTGGTCCGTGTCTCAGTACCAGTGTGGGGGATCACCCTCTCAGGCCCCCTAAA
GATCGTCGCCTTGGTGAGCCGTTACCTCACCAACTAGCTAATCTTGCGCGTGCCCATCTCTATCCACCGTAGT
TTTCAATATCACCCGATGCCGAATAATATATTATGGGGTATTAATCTTCTTCGAAAGGCTATCCCCCTGATAA
AGGCAGGTTGCACACGTGTTCCGCACCCGTACGCCACTCTCAATTACCCGAAAGTAATCTACCGCTCGGCTTG
CATGTGTTAGGCCTCCCGCTAGCGTTCTCCTGAGCAGGATCAACTCGT
>588
CTGCTGCCGCCCGTAGGAGTCTGGTCCGTGTCTCAGTACCAGTGTGGGGGATCACCCTCTCAGGCCCCCTAAA
GATCGTCGCCTTGGTGAGCCGTTACCTCACCAACTAGCTAATCTTGCGCGTGCCCATCTCTATCCACCGTAGT
TTTCAATATCACCCGATGCCGAATAATATATTATGGGGTATTAATCTTCTTCGAAAGGCTATCCCCCTGATAA
AGGCAGGTTGCACACGTGTTCCGCACCCGTACGCCGCTCTCAATTACCCGAAAGTAATCTACCGCTCGCTTGC
ATGTGTTAGGCCTCCCGCTAGCGTTCTCCTGAGCAGGATCCAACTCGT
>589
CTGCCGCCGCCCGTAGGAGTCTGGTCCGTGTCTCAGTACCAGTGTGGGGGATCACCCTCTCAGGCCCCCTAAA
GATCGTCGCCTGGTGAGCCGTTACCTCACCAACTAGCTAATCTTGCGCGTGCCCATCTCTATCCACCGTAGTT
TTCAATATCACCCGATGCCGAATAATATATTATGGGGTATTAATCTTCTTCGAAAGCTATCCCCTGATAAAGG
CAGGTTGCACACGTGTTCCGCACCCGTACGCCGCTCTCAATTACCCGAAAGTAATCTACCGCTCGGCTTGCGT
GTGTTAGGCCTCCCGCTAGCGTTCTCCTGAGCAGGATCAACTCT
>590
CTGCTGCCGCCCGTAGGAGTCTGGTTCCGTGTCTCAGTACCAGTGTGGGGGATCACCCTCTCAGGCCCCCTAA
AGATCGTCGCCTTGGTGAGCCGTTACCTCACCAACTAGCTAATCTTGCGCGTGCCCATCTCTATCCACCGTAG
TTTTCAATATCACCCGATGCCGAATAATATATTATGGGGTATTAATCTTCCTTCGAAAGGCTATCCCCTGATA
AAGGCAGGTTGCACACGTGTTCCGCACCCGTACGCCGCTCTCAATTACCCGAAAGTAATCTACCGCTCGGCTT
GCATGTGTTAGGCCTCCCGCTAGCGTTCTCCTGAGCAGGATCCAACTCGT
>591
CTGCTGCCGCCCGTAGGAGTCTGGTCCGTGTCTCAGTACCAGTGTGGGGGATCACCCTCTCAGGCCCCCTAAA
GATCGTCGCCTTGGTGAGCCGTTACCTCACCAACTAGCTAATCTTGCGCGTGCCCATCTCTATCCACCGTAGT
TTTCAATATCACCCGATGCCGAATAATATATTATGGGGTATTAATCTTCTTCGAAAGGCTATCCCCCTGATAA
AGGCAGGTTGCACACGTGTTCCGCACCCGTACGCCGCTCTCAATTACCCGAAAGTAATCTACCGCTCGGCTTG
CATGTGTTAGGCCTCCCGCTAGCGTTCTCCTGAGCAGGATCAACTCT
>592
CTGCTGCCGCCCGTAGGAGTCTGGACCGTGTCTCAGTTCCAGTGTGACTGATCATCCTCTCAGACCAGTACGG
ATCGTCGCCTTGGTAGGCCTTTACCCCACCAACTAGCTAATCCGACCTAGGCTCATCTGATAGCGTGAGGTCC
GAAGTCCCCCACTTCTCCCTCAGGACGTATGCGGTATTAGCGCCCGTTTCCGGACGATCCCCACTACCAGGCA
GATTCCTAGGCATTACTCACCCGTCCGCCGCTGAATCCAGGAGCAAGCTCCCTTCATCCGCTCGACTGCATGT
GTTAGGCCTGCCGCCAGCGTCAATCTGAGCCAGGATCAAACTCT
>593
CTGCTGCCGCCCGTAGGAGTCTGGTCCGTGTCTCAGTACCAGTGTGGGGGATCACCCTCTCAGGCCCCCTAAA
GATCGTCGCCTTGGTGAGCCGTTACCTCACCAACTAGCTAATCTTGCGCGTGCCCATCTCTATCCACCGTAGT
TTTCAATATCACCCGATGCCGAATAATCTATTATGGGGTATTAATCTTCTTCGAAAGGCTATCCCCTGATAAA
GGCAGGTTGCACACGTGTTCCGCACCCGTACGCCGCTCTCAATTACCCGAAAGTAATCTACCGCTCGGCTTGC
ATGTGTTAGGCCTCCCGCTAGCGTTCATCTGAGCCAGGATCAAACTCT
>594
CTGCTGCCGCCCGTAGGAGTCTGGTCCGTGTCTCAGTACCAGTGTGGGGGATCACCCTCTCAGGCCCCCTAAA
GATCGTCGCCTTGGTGAGCCGTTACCTCACCAACTAGCTAATCTTGCGCGTGCCCATCTCTATCCACCGTAGT

TTTCAATATCACCCGATGCCGAATAATATATTATGGGGTATTAATCTTCTTCGAAAGGCTATCCCCCTGATAA
AGGCAGGTTGCACACGTGTTCCGCACCCGTACGCCGCTCTCAATTACCCGAAAGTAATCTACCGCTCGGCTTG
CATGTGTTAGGCCTCCCGCTAGCGTTCATCCTGAGCCAGGATCAAACTCT
>595
CTGCTGCCGCCCGTAGGAGTCTGGTCCGTGTCTCAGTACCAGTGTGGGGGATCACCCTCTCAGGCCCCCTAAA
GATCGTCGCCTTGGTGAGCCGTTACCTCACCAACTAGCTAATCTTGCGCGTGCCCATCTCTATCCACCGTAGT
TTTCAATATCACCCGATGCCGAATAATATATTATGGGGTATTAATCTTCTTCGAAAGGCTATCCCCCTGATAA
AGGCAGGTTGCACACGTGTTCCGCACCCGTACGCCGCTCTCAATTACCCGAAAGTAATCTACCGCTCCGGCTT
GCATGTGTTAGGCCTCCCGCTAGCGTTCTCCTGAGCAGGAT
>596
CTGCTGCCGCCCGTAGGCGTCTGGACCGTGTCTCAGTTCCAGTGTGGGGGATCACCCTCTCAGGCCCCCTAAA
GATCGTCGCCTTGGTGAGCCGTTACCTCACCAACTAGCTAATCTTGCGCGTGCCCATCTCTATCCACCGTAGT
TTTCAATATCACCCGATGCCGAATAATATATTATGGGGTATTAATCTTCTCGAAAGCTATCCCCCTGATAAAG
GCAGGTTGCACACGTGTTCCGCACCCGTACGCCGCTCTCAATTACCCGAAAGTAATCTACCGCTCGGCTTGCA
TGTGTTAGGCCTCCCGCTAGCGTTCTCCT
>597
CTGCTGCCGCCCGTAGGAGTCTGGTCCGTGTCTCAGTACCAGTGTGGGGGATCACCCTCTCAGGCCCCCTAAA
GATCGTCGCCTTGGTGAGCCGTTACCTCACCAACTAGCTAATCTTGCGCGTGCCCATCTCTATCCACCGTAGT
TTTCAATATCACCCGATGCCGAATAATATATTATGGGGTATTAATCTTCTTCGAAAGGCTATCCCCCTGATAA
AGGCAGGTTGCACACGTGTTCCGCACCCGTACGCCGCTCTCAATTACCCGAAAGTAATCTACCGCTCGGCTTG
CATGTGTTAGGCCTCCCGCTAGCGTTCTCCTGAGCAGGATCAACTCGT
>598
CTGCTGCCGCCCGTAGGAGTCTGGTCCGTGTCTCAGTACCAGTGTGGGGGATCACCCTCTCAGGCCCCCTAAA
GATCGTCGCCTTGGTGAGCCGTTACCTCACCAACTAGCTAATCTTGCGCGTGCCCATCTCTATCCGCCGTAGT
TTTCAATATCACCCGATGCCGAATAATATATTATGGGGTATTAATCTTCTTCGAAAGGCTATCCCCCTGATAA
AGGCAGGTTGCACACGTGTTCCGCACCCGTACGCCGCTCTCAATTACCCGAAAGTAATCTACCGCTCGGCTTG
CATGTGTTAGGCCTCCCGCTAGCGTTCATCCTGAGCCAGGATCAAACTCT
>599
CTGCTGCCGCCCGTAGGAGTCTGGTCCGTGTCTCAGTACCAGTGTGGGGGATCACCCTCTCAGGCCCCCTAAA
GATCGTCGCCTGGTGAGCCGTTACCTCACCAACTAGCTAATCTTGCGCGTGCCCATCTCTATCCACCGTAGTT
TTCAATATCACCCGATGCCGAATAATATATTATGGGGTATTAATCTTCTTCGAAAGGCTATCCCCTGATAAAG
GCAGGTTGCACACGTGTTCCGCACCCGTACGCCGCTCTCAATTACCCGAAAGTAATCTACCGCTCGGCTTGCA
TGTGTTAGGCCTCCCGCTAGCGTTCATCCTGA
>600
CTGCTGCCGCCCGTAGGAGTCTGGTCCGTGTCTCAGTACCAGTGTGGGGGATCACCCTCTCAGGCCCCCTAAA
GATCGTCGCCTGGTGAGCCGTTACCTCACCAACTAGCTAATCTTGCGCGTGCCCATCTCTATCCACCGTAGTT
TTCAATATCACCCGATGCCGAATAATATATTATGGGGTATTAATCTTCTCGAAAGGCTATCCCCCTGATAAAG
GCAGGTTGCACACGTGTTCCGCACCCGTACGCGCTCTCAATTACCCGAAAGTAATCTACCGCTCGGCTGCATG
TGTTAGGCCTCCCG
>601
CTGCTGCCGCCCGTAGGAGTCTGGTCCGTGTCTCAGTACCAGTGTGGGGGATCACCCTCTCAGGCCCCCTAAA
GATCGTCGCCTTGGTGAGCCGTTACCTCACCAACTAGCTAATCTTGCGCGTGCCCATCTCTATCCACCGTAGT
TTTCAATATCACCCGATGCCGAATAGTATATTATGGGGTATTAATCTTCTTCGAAAGGCTATCCCCCTGATAA
AGGCAGGTTGCACACGTGTTCCGCACCCGTACGCCGCTCTCAATTACCCGAAAGTAATCTACCGCTCCGGCTT
GCATGTGTTAGGCCTCCCGCTAGCGTTCATCCTGAGCCAGGATCAAACTCT
>602
CTGCTGCCGCCCGTAGGAGTCTGGTCCGTGTCTCAGTACCAGTGTGGGGGATCACCCTCTCAGGCCCCCTAAA
GATCGTCGCCTTGGTGAGCCGTTACCTCACCAACTAGCTAATCTTGCGCGTGCCCATCTCTATCCACCGTAGT
TTTCAATATCACCCGATGCCGAATAATATATTATGGGGTATTAATCTTCTTCGAAAGGCTATCCCCCTGATAA
AGGCAGGTTGCACACGTGTTCCGCACCCGTACGCCGCTCTCAATTACCCGAAAGTAATCTACCGCTCGGCTTG
CATGTGTTAGGCCTCCCGCTAGCGTTCATCCTGAGCCAGGATCAAACTCT
>603
CTGCTGCCGCCCGTAGGAGTCTGGTCCGTGTCTCAGTACCAGTGTGGGGGATCACCCTCTCAGGCCCCCTAAA
GATCGTCGCCTTGGTGAGCCGTTACCTCACCAACTAGCTAATCTTGCGCGTGCCCATCTCTATCCACCGTAGT

TTTCAATATCACCCGATGCCGAATAATATATTATGGGGGTATTAATCTTCTCGAAAGGCTATCCCCCTGATAA
AGGCAGGTTGCACACGTGTTCCGCACCCGTACGCCGCTCTCAATTACCCGAAAGTAATCTACCGCTCGGCTTG
CATGTGTTAGGCCTCCCGCTAGCGTTCTCCTGAGCAGGTCAAACTCGT
>604
CTGCTGCCGCCCGTAGGAGTCTGGTCCGTGTCTCAGTACCAGTGTGGGGGATCACCCTCTCAGGCCCCCTAAA
GATCGTCGCCTTGGTGAGCCGTTACCTCACCAACTAGCTAATCTTGCGCGTGCCCATCTCTATCCACCGTAGT
TTTCAATATCACCCGATGCCGAATAATATATTATGGGGTATTAATCTTCTTCGAAGGCTTATCCCCTGATAAA
GGCAGGTTGCACACGTGTTCCGCACCCGTACGCCGCTCTCAATTACCCGAAAGTAATCTACCGCTCGGCTTGC
ATGTGTTAGGCCTCCCGCTAGCGTTCATCCGTGAGCCAGGATCAACTCT
>605
CTGCTGCCGCCCGTAGGAGTCTGGTCCGTGTCTCAGTACCAGTGTGGGGGATCACCCTCTCAGGCCCCCTAAA
GATCGTCGCCTGGTGAGCCGTTACCTCACCAACTAGCTAATCTTGCGCGTGCCCATCTCTATCCACCGTAGTT
TTCAATATCACCCGATGCCGAATAATATATTATGGGGTATTAATCTTCTTCGAAAGCTATCCCCTGATAAAGG
CAGGTTGCACACGTGTTCCGCACCCGTACGCCGCTCTCAATTACCCGAAAGTAATCTACCGCTCGGCTTGCAT
GTGTTAGGCCTCCCGCTAGCGTTCTCCTGAGCAGGATCA
>606
CTGCTGCCGCCCGTAGGAGTCTGGTCCGTGTCTCAATACCAGTGTGGGGGATCACCCTCTCAGGCCCCCTAAA
GATCGTCGCCTTGGTGAGCCGTTACCTCACCAACTAGCTAATCTTGCGCGTGCCCATCTCTATCCACCGTAGT
TTTCAATATCACCCGATGCCGAATAATATATTATGGGGTATTAATCTTCTTCGAAAGGCTATCCCCCTGATAA
AGGCAGGTTGCACACGTGTTCCGCACCCGTACGCCGCTCTCAATTACCCGAAAGTAATCTACCGCTCCGGCTT
GCATGTGTTAGGCCTCCCGCTAGCGTTCATCCTGAGCCAGGATCAAACTCT
>607
CTGCTGCCGCCCGTAGGAGTCTGGTCCGTGTCTCAGTACCAGTGTGGGGGATCACCCTCTCAGGCCCCCTAAA
GATCGTCGCCTTGGTGAGCCGTTACCTCACCAACTAGCTAATCTTGCGCGTGCCCATCTCTATCCACCGTAGT
TTTCAATATCACCCGATGCCGAATAATATATTATGGGGTATTAATCTTCTTCGAAAGGCTATCCCCCTGATAA
AGGCAGGTTGCACACGTGTTCCGCACCCGTACGCCGCTCTCAATTACCCGAAAGTAATCTACCGCTCGGCTTG
CATGTGTTAGGCCTCCCGCTAGCGTTCATCCTGAGCC
>608
CTGCTGCCGCCCGTAGGAGTCTGGTCCGTGTCTCAGTACCAGTGTGGGGGATCACCCTCTCAGGCCCCCTAAA
GATCGTCGCCTTGGTGAGCCGTTACCTCACCAACTAGCTAATCTTGCGCGTGCCCATCTCTATCCACCGTAGT
TTTCAATATCACCCGATGCCGAATAATATATTATGGGGTATTAATCTTCTTCGAAAGGCTATCCCCTGATAAA
GGCAGGTTGCACACGTGTTCCGCACCCGTACGCCGCTCTCAATTACCCGAAAGTAATCTACCGCTCGGCTTGC
ATGTGTTAGGCCTCCCGCTAGCGTTCATCCTGAG
>609
CTGCTGCCGCCCGTAGGAGTCTGGTCCGCGTCTCAGTACCAGTGTGGGGGATCACCCTCTCAGGCCCCCTAAA
GATCGTCGCCTTGGTGAGCCGTTACCTCACCAACTAGCTAATCTTGCGCGTGCCCATCTCTATCCACCGTAGT
TTTCAATATCACCCGATGCCGAATAATATATTATGGGGTATTAATCTTCCTTCGAAAGCTATCCCCCTGATAA
AGGCAGGTTGCACACGTGTTCCGCACCCGTACGCCGCTCTCAATTACCCGAAAGTAATCTACCGCTCGGCTTG
CATGTGTTAGGCCTCCCGCTAGCGTTCTCCGTGAGCAAGG
>610
CTGCTGCCGCCCGTAGGAGTCTGGTCCGTGTCTCAGTACCAGTGTGGGGGATCACCCTCTCAGGCCCCCTAAA
GATCGTCGCCTTGGTGAGCCGTTACCTCACCAACTAGCTAATCTTGCGCGTGCCCATCTCTATCCACCGTAGT
TTTCAATATCACCCGATGCCGAATAATATATTATGGGGTATTAATCTTCTTCGAAAGGCTATCCCCCTGATAA
AGGCAGGTTGCACACGTGTTCCGCACCCGTACGCCGCTCTCAATTACCCGAAAGTAATCTACCGCTCGGCTTG
CATGTGTTAGGCCTCCCGCTAGCGTTCATCCTGAGCCAGGATCAAACTCT
>611
CTGCTGCCGCCCGTAGGAGTCTGGTCCGTGTCTCAGTACCAGTGTGGGGGATCACCCTCTCAGGCCCCCTAAA
GATCGTCGCCTTGGTGAGCCGTTACCTCACCAACTAGCTAATCTTGCGCGTGCCCATCTCTATCCACCGTAGT
TTTCAATATCACCCGATGCCGAATAATATATTATGGGGTATTAATCTTCTTCGAAAGGCTATCCCCCTGATAA
AGGCAGGTTGCACACGTGTTCCGCACCCGTACGCCGCTCTCAATTACCCGAAAGTAATCTACCGCTCGGCTTG
CATGTGTTAGGCCTCCCGCTAGCGTTCATCCTGAGCCAGGATCAAACTCT
>612
CTGCTGCCGCCCGTAGGAGTCTGGTCCGTGTCTCAGTACCAGTGTGGGGGATCACCCTCTCAGGCCCCCTAAA
GATCGTCGCCTTGGTGAGCCGTTTGCCTCACCAACTAGCTAATCTTGCGCGTGCCCATCTCTATCCACCGTAG

TTTTCAATATCACCCGATGCCGAATAATATAATTATGGGGTATTAATCTTCTTCGAAAGGCTATCCCCCTGAT
AAAGGCAGGTTGCACACGTGTTCCGCACCCGTACGCCGCTCTCAATTACCCGAAAGTAATCTACCGCTCCGGC
TTGCATGTGTTAGGCCTCCCGCTAGCGTTCTCCTGAGCAAG
>613
CTGCTGCCGCCCGTAGGAGTCTGGTCCGTGTCTCAGTACCAGTGTGGGGGATCACCCTCTCAGGCCCCCTAAA
GATCGTCGCCTTGGTGAGCCGTTACCTCACCAACTAGCTAATCTTGCGCGTGCCCATCTCTATCCACCGTAGT
TTTCAATATCACCCGATGCCGAATAATATATTATGGGGTATTAATCTTCTTCGAAAGGCTATCCCCCTGATAA
AGGCAGGTTGCACACGTGTTCCGCACCCGTACGCCGCTCTCAATTACCCGAAAGTAATCTACCGCTCGGCTTG
CATGTGTTAGGCCTCCCGCTAGCGTTCATCCTGAGCCAGGATCAAACTCT
>614
CTGCTGCCGCCCGTAGGAGTCTGGTCCGTGTCTCAGTACCAGTGTGGGGGGATCACCCTCTCAGGCCCCCTAA
AGATCGTCGCCTTGGTGAGCCCGTTACCTCACCCAACTAGCTAATCTTGCGCGTGCCCCATCTCTATCCACCG
TAGTTTTCAATATCACCCGATGCCCGAATAATATATTTATTGGGGTATTTAATTCTTCTTCGAAAGGCTATCC
CCCTGATTAAAGGCAGGTTGCACACGTGTTCCCGCACCCGTACGCCGCTCTCAATTACCCCGAAAGTAATCTA
CCGCTCGGCTTGCATGTGTTAGGCCTCCCGCTAGCGTTCATCCTGAGCCAGGATCAAACTCT
>615
CTGCTGCCGCCCGTAGGAGTCTGGTCCGTGTCTCAGTACCAGTGTGGGGGATCACCCTCTCAGGCCCCCTAAA
GATCGTCGCCTTGGTGAGCCGTTACCTCACCAACTAGCTAATCTTGCGCGTGCCCATCTCTATCCACCGTAGT
TTTCAATATCACCCGATGCCGAATAATATATTATGGGGTATTAATCTTCTCGAAAGGCTATCCCCTGATAAAG
GCAGGTTGCACACGTGTTCCGCACCCGTACGCCGCTCTCAATTACCCGAAAGTAATCTACCGCATCGGCTTGC
ATGTGTTAGGCCTCCCGCTAGCGTTCTCCTGAGCAGGATCAACTCGT
>616
CTGCTGCCGCCCGTAGGAGTCTGGTCCGTGTCTCAGTACCAGTGTGGGGGATCACCCTCTCAGGCCCCCTAAA
GATCGTCGCCTTGGTGAGCCGTTACCTCACCAACTAGCTAATCTTGCGCGTGCCCATCTCTATCCACCGTAGT
TTTCAATATCACCCGATGCCGAATAATATATTATGGGGTATTAATCTTCTCGAAAGGCTTATCCCCCTGAATA
AAGGCAGGTTGCACACGTGTTCCGCACCCGTACGCCGCTCTCAATTACCCGAAAGTAATCTACCGCTCGGCTT
GCATGTGTTAGGCCTCCCGCTAGCGTCATCCTGA
>617
CTGCTGCCGCCCGTAGGAGTCTGGTCCCGTGTCTCAGTACCAGTGTGGGGGATCACCCTCTCAGGCCCCCCTA
AAGATCGTCGCCTTGGTGAGCCCGTTACCTCACCCAACTAGCTAATCTTGCGCGTGCCCCATCTCTATTCCCA
CCGTAGTTTCAATATCACCCGATGCCCGAATAATATATTATTGGGGTATTTAATCTTCTTCGAAAGGCTATC
CCCCTGATTAAAGGCGGTTGCACACGTGTTCCCGCACCCGTACGCCGCTCTCAATTACCCCGAAAGTAATCTA
CCGCTCGGCTTGCATGTGTTAGGCCCTCCCCGCTAGCGTTCTTCCTGAGCC
>618
CTGCTGCCGCCCGTAGGAGTCTGGTCCGTGTCTCAGTACCAGTGTGGGGGATCACCCTCTCAGGCCCCCTAAA
GATCGTCGCCTTGGTGAGCCGTTACCTCACCAACTAGCTAATCTTGCGCGTGCCCATCTCTATCCACCGTAGT
TTTCAATATCACCCGATGCCGAATAATATATTATGGGGTATTAATCTTCTTCGAAAGGCTATCCCCCTGATAA
AGGCAGGTTGCACACGTGTTCCGCACCCGTACGCCGCTCTCAATTACCCGAAAGTAATCTACCGCTCGGCTTG
CATGTGTTAGGCCTCCCGCTAGCGTTCATCCTGAGCCAGGATCAAACTCT
>619
CTGCTGCCGCCCGTAGGAGTCTGGTCCGTGTCTCAGTACCAGTGTGGGGGATCACCCTCTCAGGCCCCCTAAA
GATCGTCGCCTTGGTGAGCCGTTACCTCACCAACTAGCTAATCTTGCGCGTGCCCATCTCTATCCACCGTAGT
TTTCAATATCACCCGATGCCGAATAATATATTATGGGGTATTAATCTTCTTCGAAAGGCTATCCCCCTGATAA
GGAGCAGGTTGCACACGTGTTCCGCACCCGTACGCCGCTCTCAATTACCCGAAAGTAATCTACCGCTCGGCTT
GCATGTGTTAGGCCTCCCGCTAGCGTTCATCCTGAGCCAGGATCAAACTCT
>620
CTGCTGCCGCCCGTAGGAGTCTGGTCCGTGTCTCAGTACCAGTGTGGGGGATCACCCTCTCAGGCCCCCTAAA
GATCGTCGCCTTGGTGAGCCGTTACCTCACCAACTAGCTAATCTTGCGCGTGCCCATCTCTATCCACCGTAGT
TTTCAATATCACCCGATGCCGAATAATATATTATGGGGTATTAATCTTCTTCGAAAGGCTATCCCCCTGATAA
AGGCAGGTTGCACACGTGTTCCGCACCCGTACGCCGCTCTCAATTACCCGAAAGTAATCTACCGCTCGGCTTG
CATGTGTTAGGCCTCCCGCTAGCGTTCATCCTGAGCCAGGATCAAACTCT
>621
CTGCTGCCGCCCGTAGGAGTCTGGTCCGTGTCTCAGTACCAGTGTGGGGGATCACCCTCTCAGGCCCCCTAAA
GATCGTCGCCTTGGTGAGCCGTTACCTCACCAACTAGCTAATCTTGCGCGTGCCCATCTCTATCCACCGTAGT

TTTCAATATCACCCGATGCCGAATAATATATTATGGGGTATTAATCTTCTCGAAGGCTATCCCCTGATAAAGC
AGGTTGCACACGTGTTCCGCACCCGTACGCCGCTCTCAATTACCCGAAAGTAATCTACCGCTCGGCTTGCATG
TGTTAGGCCTCCCGCTAGCGTCATCCTGAGCCAGGATCAACTCGT
>622
CTGCTGCCGCCCGTAGGAGTCTGGTCCGTGTCTCAGTACCAGTGTGGGGGATCACCCTCTCAGGCCCCCTAAA
GATCGTCGCCTTGGTGAGCCGTTACCTCACCAACTAGCTAATCTTGCGCGTGCCCATCTCTATCCACCGTAGT
TTTCAATATCACCCGATGCCGAATAATATATTATGGGGTATTAATCTTCTTCGAAAGGCTATCCCCTGATAAA
GGCAGGTTGCACACGTGTTCCGCACCCGTACGCCGCTCTCAATTACCCGAAAGTAATCTACCGCTCGGCTTGC
ATGTGTTTAGGCCTCCCGCTAGCGTTCATCC
>623
CTGCTGCCGCCCGTAGGAGTCTGGTCCGTGTCTCAGTACCAGTGTGGGGGATCACCCTCTCAGGCCCCCTAAA
GATCGTCGCCTTGGTGAGCCGTTACCTCACCAACTAGCTAATCTTGCGCGTGCCCATCTCTATCCACCGTAGT
TTTCAATATCACCCGATGCCGAATAATATATTATGGGGTATTAATCTTCTTCGAAAGGCTATCCCCTGATAAA
GGCAGGTTGCACACGTGTTCCGCACCCGTACGCCGCTCTCAATTACCCGAAAGTAATCTACCGCTCGGCTTGC
ATGTGTTAGGCCTCCCGCTAGCGTTCTCCTGAGCAGGATCAACTCGT
>624
CTGCTGCCGCCCGTAGGAGTCTGGTCCGTGTCTCAGTACCAGTGTGGGGGATCACCCTCTCAGGCCCCCTAAA
GATCGTCGCCTTGGTGAGCCGTTACCTCACCAACTAGCTAATCTTGCGCGTGCCCATCTCTATCCACCGTAGT
TTTCAATATCACCCGATGCCGAATAATATATTATGGGGTATTAATCTTCTTCGAAAGGCTATCCCCTGATAAA
GGCAGGTTGCACACGTGTTCCGCACCCGTACGCCGCTCTCAATTACCCGAAAGTAATCTACCGCTCGGCTTGC
ATGTGTTAGGCCTCCCGCTAGCGTTCATCCTGAGCCAGGATCAAACTCT
>625
CTGCTGCCGCCCGTAGGAGTCTGGTCCGTGTCTCAGTACCAGTGTGGGGGATCACCCTCTCAGGCCCCCTAAA
GATCGTCGCCTTGGTGAGCCGTTACCTCACCAACTAGCTAATCTTGCGCGTGCCCATCTCTATCCACCGTAGT
TTTCAATATCACCCGATGCCGAATAATATATTATGGGGTATTAATCTTCTTCGAAAGGCTATCCCCCTGATAA
AGGCAGGTTGCACACGTGTTCCGCACCCGTACGCCGCTCTCAATTACCCGAAAGTAATCTACCGCTCGGCTTG
CATGTGTTAGGCCTCCCGCTAGCGTTCATCCCGAGCCAGGATCAACTCT
>626
CTGCTGCCGCCCGTAGGAGTCTGGTCCGTGTCTCAGTACCAGTGTGGGGGATCACCCTCTCCAGGCCCCTAAA
GATCGTCGCCTTGGTGAGCCGTTACCTCACCAGCTAGCTAATCTTGCGCGTGCCCATCTCTATCCACCGTAGT
TTTCAATATCACCCGATGCCGAATAATATATTATGGGGTATTAATCTTCTTCGAAAGGCTATCCCCTGATAAA
GGCAGGTTGCACACGTGTTCCGCACCCGTACGCCGCTCTCAATTACCCGAAAGTAATCTACCGCTCGGCTTGC
ATGTGTTAGGCCTCCCGCTAGCGTTCTCCTGAGCAGGATCAACTCGT
>627
CTGCTGCCGCCCGTAGGAGTCTGGTCCGTGTCTCAGTACCAGTGTGGGGGATCACCCTCTCAGGCCCCCTAAA
GATCGTCGCCTTGGTGAGCCGTTACCTCACCAACTAGCTAATCTTGCGCGTGCCCATCTCTATCCACCGTAGT
TTTCAATATCACCCGATGCCGAATAATATATTATGGGGTATTAATCTTCTTCGAAAGGCTATCCCCCTGATAA
AGGCAGGTTGCACACGTGTTCCGCACCCGTACGCCGCTCTCAATTACCCGAAAGTAATCTACCGCTCGGCTTG
CATGTGTTAGGCCTCCCGCTAGCGTTCTCCTGAGCAGGATCAACTCGT
>628
CTGCTGCCGCCCGTAGGAGTCTGGTCCGTGTCTCAGTACCAGTGTGGGGGATCACCCTCTCAGGCCCCCTAAA
GATCGTCGCCTTGGTGAGCCGTTACCTCACCAACTAGCTAATCTTGCGCGTGCCCATCTCTATCCACCGTAGT
TTTCAATATCACCCGATGCCGAATAATATATTATGGGGTATTAATCTTCTTCGAAAGGCTATCCCCCTGATAA
AGGCAGGTTGCACACGTGTTCCGCACCCGTACGCCGCTCTCAATTACCCGAAAGTAATCTACCGCTCGGCTTG
CATGTGTTAGGCCTCCCGCTAGCGTTCATCCTGAGCCAGGTCAAACTCT
>629
CTGCTGCCGCCCGTAGGAGTCTGGTCCGTGTCTCAGTACCAGTGTGGGGGATCACCCTCTCAGGCCCCCTAAA
GATCGTCGCCTTGGTGAGCCGTTACCTCACCAACTAGCTAATCTTGCGCGTGCCCATCTCTATCCACCGTAGT
TTTCAATATCACCCGATGCCGAATAATATATTATGGGGTATTAATCTTCTTCGAAAGCTATCCCCTGATAAAG
GCAGGTTGCACACGTGTTCCGCACCCGTACGCCGCTCTCAATTACCCGAAAGTAATCTACCGCTCCGGCTTGC
ATGTGTTAGGCCTCCCGCTAGCGTCTCTCGA
>630
CTGCTGCCGCCCCGTAGGGGTCTGGTCCGTGTCTCAGTACCAGTGTGGGGGATCACCCTCTCAGGCCCCCTAA
AGATCGTCGCCTTGGTGAGCCGTTACCTCACCAACTAGCTAATCTTGCGCGTGCCCATCTCTATCCACCGTAG

TTTTCAATATCACCCGATGCCGAATAATATATTATGGGGTATTAATCTTCTCGAAGGCTATCCCCCTGATAAA
GGCAGGTTGCACACGTGTTCCGCACCCGTACGCCGCTCTCAATTACCCGAAAGTAATCTACCGCTCGGCTTGC
ATGTGTTAGGCCTCCCGCTAGCGTTCTCCTGAGCCAGGATCAACTCT
>631
CTGCTGCCGCCCGTAGGAGTCTGGTCCGTGTCTCAGTACCAGTGTGGGGGATCACCCTCTCAGGCCCCCTAAA
GATCGTCGCCTTGGTGAGCCGTTACCTCACCAACTAGCTAATCTTGCGCGTGCCCATCTCTATCCACCGTAGT
TTTCAATATCACCCGATGCCGAATAATATATTATGGGGTATTTAATCTTTCTTCGAAAGGCTATCCCCCTGAT
AAAGGCAGGTTGCACACGTGTTCCGCACCCGTACGCCGCTCTCAATTACCCGAAAGTAATCTACCGCTCGGCT
TGCATGTGTTAGGCCTCCCGCTAGCGTTCATCCTGAGCCAGGATCAAACTCT
>632
CTGCTGCCGCCCGTAGGAGTCTGGTCCGTGTCTCAGTACCAGTGTGGGGGATCACCCTCTCAGGCCCCCTAAA
GATCGTCGCCTTGGTGAGCCGTTACCTCACCAACTAGCTAATCTTGCGCGTGCCCATCTCTATCCACCGTAGT
TTTCAATATCACCCGATGCCGAATAATATATTATGGGGTATTTAATCTTCTTCGAAAGGCTATCCCCTGATAA
AGGCAGGTTGCACACGTGTTCCGCACCCGTACGCCGCTCTCAATTACCCGAAAGTAATCTACCGCTCGGCTTG
CATGTGTTAGGCCTCCCGCTAGCGTTCTCCTGAGCAGGATCAACTCGT
>633
CTGCTGCCGCCCGTAGGAGTCTGGTCCGTGTCTCAGTACCAGTGTGGGGGATCACCCTCTCAGGCCCCCTAAA
GATCGTCGCCTTGGTGAGCCGTTACCTCACCAACTAGCTAATCTTGCGCGTGCCCATCTCTATCCACCGTAGT
TTCAATATCACCCGATGCCGAATAATATATTATGGGGTATTAATCTTCTTCGAAAGGCTATCCCCTGATAAAG
GCAGGTTGCACACGTGTTCCGCACCCGTACGCCGCTCTCAATTACCCGAAAGTAATCTACCGCTCGGCTTGCA
TGTGTTAGGCCTCCCGCTAGCGTTCATCCTGAGCCAGGATCAAACTCT
>634
CTGCTGCCGCCCGTAGGAGTCTGGTCCGTGTCTCAGTACCAGTGTGGGGGATCACCCTCTCAGGCCCCCTAAA
GATCGTCGCCTTGGTGAGCCGTTACCTCACCAACTAGCTAATCTTGCGCGTGCCCATCTCTATCCACCGTAGT
TTCAATATCACCCGATGCCGAATAATATATTATGGGGTATTAATCTTCTTCGAAAGGCTATCCCCTGATAAAG
GCAGGTTGCACACGTGTTCCGCACCGTACGCCGCTCTCAATTACCCGAAAGTAATCTACCGCTCCGGCTTGCA
TGTGTTAGGCCTCCCGCTAGCGTT
>635
CTGCTGCCGCCCGTAGGAGTCTGGTCCGTGTCTCAGTACCAGTGTGGGGGATCACCCTCTCAGGCCCCCTAAA
GATCGTCGCCTTGGTGAGCCGTTACCTCACCAACTAGCTAATCTTGCGCGTGCCCATCTCTATCCACCGTAGT
TTTCAATATCACCCGATGCCGAATAATATATTTATGGGGTATTTAATCTTTCTTCGAAAGGCTATCCCCTGAT
AAAGGCAGGTTGCACACGTGTTCCGCACCCGTACGCCGCTCTCAATTACCCGAAAGTAATCTACCGCTCCGGC
TTGCATGTGTTAGGCCTCCCGCTAGCGTTCTCCTGAGCAGGATCCAACTCT
>636
CTGCTGCCGCCCGTAGGAGTCTGGTCCGTGTCTCAGTACCAGTGTGGGGGATCACCCTCTCAGGCCCCCTAAA
GATCGTCGCCTTGGTGAGCCGTTACCTCACCAACTAGCTAATCTTGCGCGTGCCCATCTCTATCCACCGTAGT
TTTTCAATATCACCCGATGCCGAATAATATATTTATGGGGTATTTAATCTTTCTTCGAAAGGCTATCCCCCTG
ATAAAGGCAGGTTTGCACACGTGTTCCGCACCCGTACGCCGCTCCCAATTACCCGAAAGTAATCTACCGCTCG
GCTGCATGTGTTTAGGCCTTCCCGCTAGCGTTCATCCTGAGCCAGGATCAAACTCT
>637
CTGCTGCCGCCCGTAGGAGTCTGGTCCGTGTCTCAGTACCAGTGTGGGGGATCACCCTCTCAGGCCCCCTAAG
ATCGTCGCCTTGTGAGCCGTTACCTCACCAACTAGCTAATCTTGCGCGTGCCCATCTCTATCCACCGTAGTTT
TCAATATCACCCGATGCCGAATAATATATTATGGGGTATTTAATTCTTCTCGAAAGGCTATCCCCCTGATAAA
GGCAGGTTGCACACGTGTTCCGCACCCGTACGCCGCTCTCAATTACCCGAAAGTAATCTACCGCTCCGGCTTG
CATGTGTTAGGCCTCCCGCTAGCGTCATCCTGAG
>638
CTGCTGCCGCCCGTAGGAGTCTGGTCCGTGTCTCAGTACCAGTGTGGGGGATCACCCTCTCAGGCCCCCTAAA
GATCGTCGCCTTGGTGAGCCGTTACCTCACCAACTAGCTAATCTTGCGCGTGCCCATCTCTATCCACCGTAGT
TTTCAATATCACCCGATGCCGAATAATATATTATGGGGTATTAATCTTCTCTGAAAGGCTATCCCCCTGATAA
AGGCAGGTTGCACACGTGTTCCGCACCCGTACCGCCGCTCTCAATTACCCGAAAGTAATCTACCGCTCGGCTT
GCATGTGTTAGGCCTCCCGCTAGCGTTCATCCTGAGCCAGGATCAACTCT
>639
CTGCTGCCGCCCGTAGGAGTCTGGTCCGTGTCTCAGTACCAGTGTGGGGGATCACCCTCTCAGGCCCCCTAAA
GATCGTCGCCTTGGTGAGCCGTTACCTCACCAACTAGCTAATCTTGCGCGTGCCCATCTCTATCCACCGTAGT

TTTCAATATCACCCGATGCCGAATAATATATTATGGGGTATTAATCTTCTTCGAAAGGCTATCCCCCTGATAA
GGCAGGTTGCACACGTGTTCCGCACCCGTACGCCGCTCTCAATTACCCGAAAGTAATCTACCGCTCGGCTTGC
ATGTGTTAGGCCTCCCGCTAGCGTTCTCCTGAGC
>640
CTGCTGCCGCCCGTAGGAGTCTGGTCCGTGTCTCAGTACCAGTGTGGGGGATCACCCTCTCAGGCCCCCTAAA
GATCGTCGCCTGGTGAGCCGTTACCTCACCAACTAGCTAATCTTGCGCGTGCCCATCTCTATCCACCGTAGTT
TTCAATATCACCCGATGCCGAATAATATATTATGGGGTATTAATCTTCTCGAAAGGCTTATCCCCCTGAATAA
AGGCAGGTTGCAACACGTGTTCCGCACCCGTACGCCGCTCTCAATTACCCGAAAGTAATCTACCGCTCGGCTT
GCATGTGTTAGGCCTCCCGCTAGCGTTCATCC
>641
CTGCTGCCGCCCGTAGGAGTCTGGTCCGTGTCTCAGTACCAGTGTGGGGGATCACCCTCTCAGGCCCCCTAAA
GATCGTCGCCTTGGTGAGCCGTTACCTCACCAACTAGCTAATCTTGCGCGTGCCCATCTCTATCCACCGTAGT
TTTCAATATCACCCGATGCCGAATAATATATTATGGGGTATTAATCTTCTTCGAAAGGCTATCCCCCTGATAA
AGGCAGGTTGCACACGTGTTCCGCACCCGTACGCCGCTCTCAATTACCCGAAAGTAATCTACCGCTCGGCTTG
CATGTGTTAGGCCTCCCGCTAGCGTTCATCCTGAGCCAGGATCAAACTCT
>642
CTGCTGCCCGCCCCGTAGGAGTCTGGTCCCGTGTCTCAGTACCAGTGTGGGGGATCACCCTCTCAGGCCCCCC
TAAAGATCGTCGCCTTGGTGAGCCGTTACCTCACCCAACTAGCTAATCTTGCGCGTGCCCATCTCTATCCACC
GTAGTTTTCAATATCACCCGATGCCGAATAATATATTATGGGGTATTAATCTTCTTCGAAAGGCTATCCCCCT
GATAAAGGCAGGTTGCACACGTGTTCCGCACCCGTACGCCGCTCTCAATTACCCCGAAAGTAATCTACCGCTC
GGCTTGCATGTGTTAGGCCTCCCGCTAGCGTTCATCCTGAGCCAGGATCAAACTCT
>643
CTGCTGCCGCCCGTAGGAGTCTGGTCCGTGTCTCAGTACCAGTGTGGGGGATCACCCTCTCAGGCCCCCTAAA
GATCGTCGCCTTGGTGAGCCGTTTACCTCACCAACGCTGCTAATCTTGCGCGTGCCCATCTCTATCCACCGTA
GTTTTCAATATCACCCGATGCCGAATAATATATTATGGGGTATTAATCTTCTTCGAAAGGCTATCCCCCTGAT
AAAGGCAGGTTGCACACGTGTTCCGCACCCGTACGCCGCTCTCAATTACCCGAAAGTAATCTACCGCTCGGCT
TGCATGTGTTAGGCCTCCCGCTAGCGTTCATCCTGAGCCAGGATCAAACTCT
>644
CTGCTGCCGCCCGTAGGAGTCTGGTCCGTGTCTCAGTACCAGTGTGGGGGATCACCCTCTCAGGCCCCCTAAA
GATCGTCGCCTTGGTGAGCCGTTACCTCACCAACTAGCTAATCTTGCGCGTGCCCATCTCTATCCACCGTAGT
TTTCAATATCACCCGATGCCGAATAATATATTATGGGGTATTAATCTTCTTCGAAAGGCTATCCCCCTGATAA
AGGCAGGTTGCACACGTGTTCCGCACCCGTACGCCGCTCTCAATTACCCGAAAGTAATCTACCGCTCGGCTTG
CATGTGTTAGGCCTCCCGCTAGCGTTCTCCTGAGCAGGATCAACTCT
>645
CTGCTGCCGCCCGTAGGAGTCTGGTCCGTGTCTCAGTACCAGTGTGGGGGATCACCCTCTCAGGCCCCCTAAA
GATCGTCGCCTTGGTGAGCCGTTACCTCACCAACTAGCCAATCTTGCGCGTGCCCATCTCTATCCACCGTAGT
TTTCAATATCACCCGATGCCGAATAATATATTATGGGGTATTAATCTTCTTCGAAAGGCTATCCCCCTGATAA
AGGCAGGTTGCACACGTGTTCCGCACCCGTACGCCGCTCTCAATTACCCGAAAGTAATCTACCGCTCGGCTTG
CATGTGTTAGGCCTCCCGCTAGCGTTCATCCTGAGCCAGGATCAAACTCT
>646
CTGCTGCCGCCCGTAGGAGTCTGGTCCGTGTCTCAGTACCAGTGTGGGGGATCACCCTCTCAGGCCCCCTAAA
GATCGTCGCCTTGGTGAGCCGTTACCTCACCAACTAGCTAATCTTGCGCGTGCCCATCTCTATCCACCGTAGT
TTTCAATATCACCCGATGCCGAATAATATATTATGGGGTATTAATCTTCTTCGAAAGGCTATCCCCCTGATAA
AGGCAGGTTGCACACGTGTTCCGCACCCGTACGCCGCTCTCAATTACCCGAAAGTAATCTACCGCTCCGGCTT
GCATGTGTTAGGCCTCCCGCTAGCGTTCATCCTGAGCCAGGATCAAACTCT
>647
CTGCTGCCGCCCGTAGGAGTCTGGTCCGTGTCTCAGTACCAGTGTGGGGGATCACCCTCTCAGGCCCCCTAAA
GATCGTCGCCTGGTGAGCCGTTACCTCACCAACTAGCTAATCTTGCGCGTGCCCATCTCTATCCACCGTAGTT
TTCAATATCACCCGATGCCGAATAATATATTATGGGGTATTAATCTTCTTCGAAAGGCTATCCCCCTGATAAA
GGCAGGTTGCACACGTGTTCCGCACCCGTACGCCGCTCTCAATTACCCGAAAGTAATCTACCGCTCGGCTTGC
ATGTGTTAGGCCTCCCGCTAGCGTCTCCTGAGC
>648
CTGCTGCCGCCCGTAGGAGTCTGGTCCGTGTCTCAGTACCAGTGTGGGGGATCACCCTCTCAGGCCCCCTAAA
GATCGTCGCCTGGTGAGCCGTTACCTCACCAACTAGCTAATCTTGCGCGTGCCCATCTCTATCCACCGTAGTT

TTCAATATCACCCGATGCCGAATAATATATTATGGGGTATTAATCTTCTCGAAAGGCTATCCCCCTGATAAAG
GCAGGTTGCACACGTGTTCCGCACCCGTACGCCGCTCTCAATTACCCGAAAGTAATCTACCGCTCCGGCTTGC
ATGTGTTAGGCCTCCCGCTAGCGTTCATCCTGAGCCAGGATCAAACTCT
>649
CTGCTGCCGCCCGTAGGAGTCTGGTCCGTGTCTCAGTACCAGTGTGGGGGATCACCCTCTCAGGCCCCCTAAA
GATCGTCGCCTGTGAGCCGTTACCTCACCAACTAGCTAATCTTGCGCGTGCCCATCTCTATCCACCGTAGTTT
TCAATATCACCCGATGCCGAATAATATATTATGGGGTATTAATCTTCTCGAAAGGCTATCCCCTGATAAAGGC
AGGTTGCACACGTGTTCCGCACCCGTACGCGCTCTCAATTACCCGAAAGTAATCTACCGCTCGGCTTGCATGT
GTTAGGCCTCCCGCTAGCGT
>650
CTGCTGCCGCCCGTAGGAGTCTGGTCCGTGTCTCAGTACCAGTGTGGGGGATCACCCTCTCAGGCCCCCTAAA
GATCGTCGCCTTGGTGAGCCGTTACCTCACCAACTAGCTAATCTTGCGCGTGCCCATCTCTATCCACCGTAGT
TTTCAATATCACCCGATGCCGAATAATATATTATGGGGTATTAATCTTCTTCGAAAGGCTATCCCCTGATAAA
GGCAGGTTGCACACGTGTTCCGCACCCGTACGCCGCTCTCAATTACCCGAAAGTAATCTACCGCTCGGCTTGC
ATGTGTTAGGCTCCGCCTAGCGTTCATCTGAGCCAGGATCAAACTCT
>651
CTGCTGCCGCCCGTAGGAGTCTGGTCCGTGTCTCAGTACCAGTGTGGGGGATCACCCTCTCAGGCCCCCTAAA
GATCGTCGCCTTGGTGAGCCGTTACCTCACCAACTAGCTAATCTTGCGCGTGCCCATCTCTATCCACCGTAGT
TTTCAATATCACCCGATGCCGAATAATATATTATGGGGTATTAATCTTCTTCGAAAGGCTATCCCCCTGATAA
AGGCAGGTTGCACACGTGTTCCGCACCCGTACGCCGCTCTCAATTACCCGAAAGTAATCTACCGCTCGGCTTG
CATGTGTTATGCCTCCCGCTAGCGTTCATCCTGAGCCAGGATCAAACTCT
>652
CTGCTGCCGCCCGTAGGAGTCTGGTCCGTGTCTCAGTACCAGTGTGGGGGATCACCCTCTCAGGCCCCCTAAA
GATCGTCGCCTTGGTGAGCCGTTACCTCACCAACTAGCTAATCTTGCGCGTGCCCATCTCTATCCACCGTAGT
TTTCAATATCACCCGATGCCGAATAATATATTATGGGGTATTAATCTTCTTCGAAAGGCTATCCCCCTGATAA
AGGCAGGTTGCACACGTGTTCCGCACCCGTACGCCGCTCTCAATTACCCGAAAGTAATCTACCGCTCGGCTTG
CATGTGTTAGGCCTCCCGCTAGCGTTCATCCTGAGCCAGGATCAAACTCT
>653
CTGCTGCCGCCCGTAGGAGTCTGGTCCGTGTCTCAGTACCAGTGTGGGGGATCACCCTCTCAGGCCCCCTAAA
GATCGTCGCCTTGGTGAGCCGTTACCTCACCAACTAGCTAATCTTGCGCGTGCCCATCTCTATCCACCGTAGT
TTTCAATATCACCCGATGCCGAATAATATATTATGGGGTATTAATCTTCTTCGAAAGGCTATCCCCCTGATAA
AGGCAGGTTGCACACGTGTTCCGCACCCGTACGCCGCTCTCAATTACCCGAAAGTAATCTACCGCTCGGCTTG
CATGTGTTAGGCCTCCCGCTAGCGTTCATCCTGAGCCAGGATCAAACTCT
>654
CTGCTGCCGCCCGTAGGAGTCTGGTCCGTGTCTCAGTACCAGTGTGGGGGATCACCCTCTCAGGCCCCCTAAA
GATCGTCGCCTTGGTGAGCCGTTACCTCACCAACTAGCTAATCTTGCGCGTGCCCATCTCTATCCACCGTAGT
TTTCAATATCACCCGATGCCGAATAATATAATTATGGGGTATTAATCTTCTCGAAAGGCTATCCCCCTGATAA
AGGCAGGTTGCACACGTGTTCCGCACCCGTACGCCGCTCTCAATTACCCGAAAGTAATCTACCGCATCGGCTT
GCATGTGTTAGGCCTCCCGCTAGCGTTCTCCTGAGCAGGTCAAACTCT
>655
CTGCTGCCGCCCGTAGGAGTCTGGTCCGTGTCTCAGTACCAGTGTGGGGGATCACCCTCTCAGGCCCCCTAAA
GATCGTCGCCTTGGTGAGCCGTTACCTCACCAACTAGCTAATCTTGCGCGTGCCCATCTCTATCCACCGTAGT
TTTCAATATCACCCGATGCCGAATAATATATTATGGGGTATTAATCTTCTTCGAAAGGCTATCCCCCTGATAA
AGGCAGGTTGCACACGTGTTCCGCACCCGTACGCCGCTCTCAATTACCCGAAAGTAATCTACCGCTCGGCTTG
CATGTGTTAGGCCTCCCGCTAGCGTTCTCCTGAGCAGGATCCAACTCGT
>656
CTGCTGCCGCCCGTAGGAGTCTGGTCCGTGTCTCAGTACCAGTGTGGGGGATCACCCTCTCAGGCCCCCTAAA
GATCGTCGCCTTGGTGAGCCGTTACCTCACCAACTAGCTAATCTTGCGCGTGCCCATCTCTATCCACCGTAGT
TTTCAATATCACCCGATGCCGAATAATATATTATGGGGTATTAATCTTCTTCGAAAGGCTATCCCCCTGATAA
AGGCAGGTTGCACACGTGTTCCGCACCCGTACGCCGCTCTCAATTACCCGAAAGTAATCTACCGCTCCGGCTT
GCATGTGTTAGGCCTCCCGCTAGCGTTCATCCTGAGCCAGGATCAAACTCT
>657
CTGCTGCCGCCCGTAGGAGTCTGGTCCGTGTCTCAGTACCAGTGTGGGGGATCACCCTCTCAGGCCCCCTAAA
GATCGTCGCCTTGGTGAGCCGTTACCTCACCAACTAGCTAATCTTGCGCGTGCCCATCTCTATCCACCGTAGT

TTTCAATATCACCCGATGCCGAATAATATATTATGGGGTATTAATCTTCTCGAAAGGCTATCCCCTGATAAAG
GCAGGTTGCACACGTGTTCCGCACCCGTACGCCGCTCTCAATTACCCGAAAGTAATCTACCGCATCGGCTTGC
ATGTGTTAGGCCTCCCGCTAGCGTTCTCCTG
>658
CTGCTGCCGCCCGTAGGAGTCTGGTCCGTGTCTCAGTACCAGTGTGGGGGATCACCCTCTCAGGCCCCCTAAA
GATCGTCGCCTTGGTGAGCCGTTACCTCACCAACTAGCTAATCTTGCGCGTGCCCATCTCTATCCACCGTAGT
TTTCAATATCACCCGATGCCGAATAATATATTATGGGGTATTAATCTTCTTCGAAAGGCTATCCCCCTGATAA
AGGCAGGTTGCACACGTGTTCCGCACCCGTACGCCGCTCTCAATTACCCGAAAGTAATCTACCGCTCGGCTTG
CATGTGTTAGGCCTCCCGCTAGCGTTCATCCTGAGCCAGGATCAAACTCT
>659
CTGCTGCCGCCCGTAGGAGTCTGGTCCGTGTCTCAGTACCAGTGTGGGGGATCACCCTCTCAGGCCCCCTAAA
GATCGTCGCCTTGGTGAGCCGTTACCTCACCAACTAGCTAATCTTGCGCGTGCCCATCTCTATCCACCGTAGT
TTTCAATATCACCCGATGCCGAATAATATATTATGGGGTATTAATCTTCTTCGAAAGGCTATCCCCCTGATAA
AGGCAGGTTGCACACGTGTTCCGCACCCGTACGCCGCTCTCAATTACCCGAAAGTAATCTACCGCTCGGCTTG
CATGTGTTAGGCCTCCCGCTAGCGTTCATCCTGAGCCAGGATCAACTCT
>660
CTGCTGCCGCCCGTAGGAGTCTGGTCCGTGTCTCAGTACCAGTGTGGGGGATCACCCTCTCAGGCCCCCTAAA
GATCGTCGCCTTGGTGAGCCGTTACCTCACCAACTAGCTAATCTTGCGCGTGCCCATCTCTATCCACCGTAGT
TTTCAATATCACCCGATGCCGAATAATATATTATGGGGTATTAATCTTCTCGAAGCTATCCCCTGATAAAGGC
AGGTTGCACACGTGTTCCGCACCCGTACGCCGCTCTCAATTACCCGAAAGTAATCTACCGCTCCGGCTTGCAT
GTGTTAGGCCTCCCGCTAGCGTTCATCCTGAGCCAGGATCAAACTCT
>661
CTGCTGCCGCCCGTAGGAGTCTGGTCCGTGTCTCAGTACCAGTGTGGGGGATCACCCTCTCAGGCCCCCTAAA
GATCGTCGCCTTGGTGAGCCGTTACCTCACCAACTAGCTAATCTTGCGCGTGCCCATCTCTATCCACCGTAGT
TTTCAATATCACCCGATGCCGAATAATATATTATGGGGTATTAATCTTCTTCGAAAGGCTATCCCCCTGATAA
AGGCAGGTTGCACACGTGTTCCGCACCCGTACGCCGCTCTCAATTACCCGAAAGTAATCTACCGCTCGGCTTG
CATGTGTTAGGCCTCCCGCTAGCGTTCATCCTGAGCCAGGATCAAACTCT
>662
CTGCTGCCGCCCGTAGGAGTCTGGTCCGTGTCTCAGTACCAGTGTGGGGGATCACCCTCTCAGGCCCCCTAAA
GATCGTCGCCTTGGTGAGCCGTTACCTCACCAACTAGCTAATCTTGCGCGTGCCCATCTCTATTCCACCGTAG
TTTCAATATCACCCGATGCCGAATAATATATTATGGGGTATTAATCTTCTCGAAAGGCTATCCCCCTGATAAA
GGCAGGTTGCACACGTGTTCCGCACCCGTACGCCGCTCTCAATTACCCCGAAAGTAATCTACCGCTCGGCTTG
CATGTGTTAGGCCTCCCGCTAGCGTTCATCCTGAGCCAGGATCAAACTCT
>663
CTGCTGCCGCCCGTAGGAGTCTGGACCGTGTCTCAGTTCCAGTGTGACTGATCATCCTCTCAGACCAGTACGG
ATCGTCGCCTTGGTAGGCCTTTACCCCACCAACTAGCTAATCCGACCTAGGCTCATCTGATAGCGTGAGGTCC
GAAGATCCCCCACTTCTCCCTCAGGACGTATGCGGTATTAGCGCCCGTTTCCGGACGTATCCCCACTACCAGG
CAGATTCCCTAGGCATTACTCACCCGTCCGCCGCTGAATCCAGGAGCAAGCTCCCTTCATCCGCTCGACTGCA
TGTGTTAGGCCTGCCGCCAGCGTTCAATCTGAGCCAGGATCAAACTCT
>664
CTGCTGCCGCCCGTAGGAGTCTGGTCCGTGTCTCAGTACCAGTGTGGGGGATCACCCTCTCCAGGCCCCTAAA
GATCGTCGCCTTGGTGAGCCGTTACCTCACCACTAGCTAATCTTGCGCGTGCCCATCTCTATCCACCGTAGTT
TTCAATATCACCCGATGCCGAATAATATATTATGGGGTATTAATCTTCTTCGAAAGGCTATCCCCCTGATAAA
GGCAGGTTGCACACGTGTTCCGCACCCGTACGCCGCTCTCAATTACCCGAAAGTAATCTACCGCTCGGCTTGC
ATGTGTTAGGCCTCCCGCTAGCGTTCATCCTGAGCCAGGATCAACTCT
>665
CTGCTGCCGCCCGTAGGAGTCTGGTCCGTGTCTCAGTACCAGTGTGGGGGATCACCCTCTCAGGCCCCCTAAA
GATCGTCGCCTTGGTGAGCCGTTACCTCACCAACTAGCTAATCTTGCGCGTGCCCATCTCTATCCACCGTAGT
TTTCAATATCACCCGATGCCGAATAATATATTATGGGGTATTAATCTTCTTCGAAAGGCTATCCCCCTGATAA
AGGCAGGTTGCACACGTGTTCCGCACCCGTACGCCGCTCTCAATTACCCGAAAGTAATCTACCGCTCGGCTTG
CATGTGTTAGGCCTCCCGCTAGCGTTCATCCTGAGCAGGATCAACTCT
>666
CTGCTGCCGCCCGTAGGAGTCTGGTCCGTGTCTCAGTACCAGTGTGGGGGATCACCCTCTCAGGCCCCCTAAA
GATCGTCGCCTTGGTGAGCCGTTACCTCACCAACTAGCTAATCTTGCGCGTGCCCATCTCTATCCACCGTAGT

TTTCAATATCACCCGATGCCGAATAATATATTATGGGGTATTAATCTTCTTCGAAAGCTATCCCCCTGATAAA
GGCAGGTTGCCACACGTGTTCCGCACCCGTACCGCCGCTCTCAATTACCCGAAAGTAATCTACCGCTCGGCTT
GCATGTCGTTAGGCCTCCCGCTAGCGTTCTCCGTGA
>667
CTGCTGCCGCCCGTAGGAGTCTGGTCCGTGTCTCAGTACCAGTGTGGGGGATCACCCTCTCAGGCCCCCTAAA
GATCGTCGCCTTGGTGAGCCGTTACCTCACCAACTAGCTAATCTTGCGCGTGCCCATCTCTATCCACCGTAGT
TTTCAATATCACCCGATGCCGAATAATATATTATGGGGTATTAATCTTCTTCTCGAAAGGCTATCCCCCTGAT
AAAGGCAGGTTGCACACGTGTTCCGCACCCGTACGCCGCTCTCAATTACCCGAAAGTAATCTACCGCTCCGGC
TTGCATGTGTTAGGCCTCCCGCTAGCGTTCATCCTGAGCCAGGATCAACTCT
>668
CTGCTGCCGCCCGTAGGAGTCTGGTCCGTGTCTCAGTACCAGTGTGGGGGATCACCCTCTCAGGCCCCCTAAA
GATCGTCGCCTTGGTGAGCCGTTACCTCACCAACTAGCTAATCTTGCGCGTGCCCATCTCTATCCACCGTAGT
TTTCAATATCACCCGATGCCGAATAATATATTATGGGGGTATTAATCTTCTTCGAAAGGCTATCCCCCTGATA
AAGGCAGGTTGCACACGTGTTCCGCACCCGTACGCCGCTCTCAATTACCCGAAAGTAATCTACCGCTCCGGCT
TGCATGTGTTAGGCCTCCCGCTAGCGTTCATCCTGAGCCAGGATCAACTCT
>669
CTGCTGCCGCCCGTAGGAGTCTGGTCCGTGTCTCAGTACCAGTGTGGGGGATCACCCTCTCAGGCCCCCTAAA
GATCGTCGCCTTGGTGAGCCGTTACCTCACCAACTAGCTAATCTTGCGCGTGCCCATCTCTATCCACCGTAGT
TTCAATATCACCCGATGCCGAATAATATATTATGGGGGTATTAATCTTCTCGAAAGGCTATCCCCTGATAAAG
GCAGGTTGCACACGTGTTCCGCACCCGTACGCCGCTCTCAATTACCCGAAAGTAATCTACCGCTCGGCTTGCA
TGTGTTACGGCCTCCCGCTAGCTCGTC
>670
CTGCTGCCGCCCGTAGGAGTCTGGTCCGTGTCTCAGTACCAGTGTGGGGGATCACCCTCTCAGGCCCCCTAAA
GATCGTCGCCTTGGTGAGCCGTTACCTCACCAACTAGCTAATCTTGCGCGTGCCCATCTCTATCCACCGTAGT
TTTCAATATCACCCGATGCCGAATAATATATTATGGGGTATTAATCTTTCTTCGAAAGGCTATCCCCCTGATA
AAGGCAGGTTGCACACGTGTTCCGCACCCGTACGCCGCTCTCAATTACCCGAAAGTAATCTACCGCTCGGCTT
GCATGTGTTAGGCCTCCCGCTAGCGTTCTCCTGAGCAGGATCCAAACTCT
>671
CTGCTGCCGCCCGTAGGAGTCTGGTCCGTGTCTCAGTACCAGTGTGGGGGATCACCCTCTCAGGCCCCCTAAA
GATCGTCGCCTTGGTGAGCCGTTACCTCACCAACTAGCTTAATCTTGCGCGTGCCCATCTCTATCCACCGTAG
TTTTTCAATATCACCCGATGCCGAATAATATATTTATGGGGTATTTAATCTTTCTTCGAAAGGCTATCCCCCT
GATAAAGGCAGGTTGCACACGTGTTCCGCACCCGTACGCCGCTCTCAATTACCCGAAAGTAATCTACCGCTCG
GCTTGCATGTGTTAGGCCTTCCCGCTAGCGTTCTTCCTGAGCAGGATCCAACT
>672
CTGCTGCCGCCCGTAGGAGTCTGGTCCGTGTCTCAGTACCAGTGTGGGGGATCACCCTCTCAGGCCCCCTAAA
GATCGTCGCCTTGGTGAGCCGTTACCTCACCAGCTAGCTAATCTTGCGCGTGCCCATCTCTATCCACCGTAGT
TTTCAATATCACCCGATGCCGAATAATATATTATGGGGGTATTAATCTTCTTCGAAAGGCTATCCCCTGATAA
AGGCAGGTTGCACACGTGTTCCGCACCCGTACGCCGCTCTCAATTACCCGAAAGTAATCTACCGCTCGGCTTG
CATGTGTTAGGCCTCCCGCTAGCGTTCTCCTGAGCAGGATCAACTCGT
>673
CTGCTGCCGCCCGTAGGAGTCTGGTCCGTGTCTCAGTACCAGTGTGGGGGATCACCCTCTCAGGCCCCCTAAA
GATCGTCGCCTGGTGAGCCGTTACCTCACCAACTAGCTAATCTTGCGCGTGCCCATCTCTATCCACCGTAGTT
TTCAATATCACCCGATGCCGAATAATATATTATGGGGTATTAATCTTCTTCGAAAGGCTATCCCCCTGATAAA
GGCAGGTTGCACACGTGTTCCGCACCCGTACGCCGCTCTCAATTACCCGAAAGTAATCTACCGCTCGGCTTGC
ATGTGTTAGGCCTCCCGCTAGCGTTCATCCTGAGCCAGGATCAAACTCT
>674
CTGCTGCCGCCCGTAGGAGTCTGGTTCCGTGTCTCAGTACCAGTGTGGGGGATCACCCTCTCAGGCCCCCTAA
AGATCGTCGCCTTGGTGAGCCGTTACCTCACCAACTAGCTTAATCTTGCGCGTGCCCATCTCTATCCACCGTA
GTTTTTCAATATCACCCGATGCCGAATAATATATTATTGGGGTATTTAATCTTCTTCGAAAGGCTATCCCCTG
ATAAAGGCAGGTTGCACACGTGTTCCGCACCCGTACGCCGCTCTCAATTACCCGAAAGTAATCTACCGCTCGG
CTTGCATGTGTTTAGGCCTTCCCGCAGCGTTCTTCCTGAGC
>675
TCTGCTGCCGCCCGTAGGAGTCTGGTTCCGTGTCTCAGTACCAGTGTGGGGGATCACCCTCTCAGGCCCCCTA
AGATCGTCGCCTTGGTGAGCCGTTACCTCACCAACTAGCTTAATCTTGCGCGTGCCCATTCTCTATTCCACCG

TAGTTTTTCAATTATCACCCGATTGCCGAATAATATATTATTGGGGTATTTAATCTTCTTCGAAAGGCTATCC
CCCTGATTAAGGCAGGTTGCACACGTGTTCCGCACCCGTACGCCGCTCTCAATTTACCCGAAAGTAATCTACC
GCTCGGCTTGCATGTGTTTAGGCCTTCCCGCTAGCGTTCAT
>676
CTGCTGCCGCCCGTAGGAGTCTGGTCCGTATCTCAGTACCAGTGTGGGGGATCACCCTCTCAGGCCCCCTAAA
GATCGTCGCCTTGGTGAGCCGTTACCTCACCAACTAGCTAATCTTGCGCGTGCCCATCTCTATCCACCGTAGT
TTTCAATATCACCCGATGCCGAATAATTATATTATGGGGTATTAATCTTCTTCGAAAGGCTATCCCCCTGATA
AAGGCAGGTTGCACACGTGTTCCGCACCCGTACGCCGCTCTCAATTACCCGAAAGTAATCTACCGCTCGGCTT
GCATGTGTTAGGCCTCCCGCTAGCGTTCATCCTGAGCCAGGATCAAACTCT
>677
CTGCTGCCGCCCGTAGGAGTCTGGTCCGTGTCTCAGTACCAGTGTGGGGGATCACCCTCTCAGGCCCCCTAAA
GATCGTCGCCTGGTGAGCCGTTACCTCACCAACTAGCTAATCTTGCGCGTGCCCATCTCTATCCACCGTAGTT
TTCAATATCACCCGATGCCGAATAATATATTATGGGGTATTAATCTTCTTCGAAAGCTATCCCCCTGATAAAG
GCAGGTTGCACACGTGTTCCGCACCCGTACGCCGCTCTCAATTACCCGAAAGTAATCTACCGCTCGGCTTGCA
TGTGTTAGGCCTCCCGCTAGCGTTCTTCCTGAGCCAGGATT
>678
CTGCTGCCGCCCGTAGGAGTCTGGTCCGTGTCTCAGTACCAGTGTGGGGGATCACCCTCTCAGGCCCCCTAAA
GATCGTCGCCTTGGTGAGCCGTTACCTCACCAACTAGCTAATCTTGCGCGTGCCCATCTCTATTCCACCGTAG
TTTTCAATATCACCCGATGCCGAATAATATATTATGGGGTATTAATCTTCTTCGAAAGGCTATCCCCCTGATA
AGGCAGGTTGCACACGTGTTCCGCACCCGTACGCCGCTCTCAATTACCCGAAAGTAATCTACCGCTCGGCTTG
CATGTGTTAGGCCTCCCGCTAGCGTTCATCCTGAGCCAGGATCAAACTCT
>679
CTGCTGCCGCCCGTAGGAGTCTGGTCCGTGTCTCAGTACCAGTGTGGGGGATCACCCTCTCAGGCCCCCTAAA
GATCGTCGCCTTGGTGAGCCGTTACCTCACCAACTAGCTAATCTTGCGCGTGCCCATCTCTATCCACCGTAGT
TTTCAATATCACCCGATGCCGAATAATATATTATGGGGTATTAATCTTCTTCGAAAGGCTATCCCCCTGATAA
AGGCAGGTTGCACACGTGTTCCGCACCCGTACGCCGCTCTCAATTACCCGAAAGTAATCTACCGCTCGGCTTG
CATGTGTTAGGCTCCCGCTAGCGTCTCC
>680
CTGCTGCCGCCCGTAGGAGTCTGGTCCGTGTCTCAGTTCCAGTGTGACTGATCATCCTCTCAGACCAGTACGG
ATCGTCGCCTTGGTAGGCCTTTACCCCCACCAACTAGCTAATCCGACCTAGGCTCATCTGATAGCGTGAGGTC
CGAAGTCCCCCACTTCTCCCTCAGGACGTATGCGGTATTAGCGCCCGTTCCGGACGTATCCCCACTACCAGGC
AGATTCCTAGGCATTACTCACCCGTCCGCCGCTGAATCCAGGAGCAAGCTCCCTCATCCGCTCGACTGCATGT
GTTAGGCCTGCCGCCAGCGTCAATCTGAGCCAGGATCAAACTCT
>681
CTGCTGCCGCCCGTAGGAGTCTGGTCCGTGTCTCAGTACCAGTGTGGGGGATCACCCTCTCAGGCCCCCTAAA
GATCGTCGCCTTGGTGAGCCGTTACCTCACCAACTAGCTAATCTTGCGCGTGCCCATCTCTATCCACCGTAGT
TTTCAATATCACCCGATGCCGAATAATATATTATGGGGTATTAATCTTTCTTCGAAAGGCTATCCCCCTGATA
AAGGCAGGTTGCACACGTGTTCCGCACCCGTACGCCGCTCTCAATTACCCGAAAGTAATCTACCGCTCGGCTT
GCATGTGTTAGGCCTCCCGCTAGCGTTCATTCCTGAGCCAGGATCAAACTCT
>682
CTGCTGCCGCCCGTAGGAGTCTGGTCCGTGTCTCAGTACCAGTGTGGGGGATCACCCTCTCAGGCCCCCTAAA
GATCGTCGCCTGTGAGCCGTTACCTCACCAACTAGCTAATCTTGCGCGTGCCCACCTCTATCCACCGTAGTTT
CAATTATCACCCGATGCCGAATAATATATTATGGGGTATTAATCTTCTTCGAAAGGCTATCCCCCTGATAAAG
GCAGGTTGCACACGTGTTCCGCACCCGTACGCCGCTCTCAATTACCCGAAAGTAATCTACCGCTCGGCTTGCA
TGTGTTAGGCCTCCCGCTAGCGTTCATCCTGAGCCAGGATCAAACTCT
>683
CTGCTGCCGCCCGTAGGAGTCTGGTCCGTGTCTCAGTACCAGTGTGGGGGATCACCCTCTCAGGCCCCCTAAA
GATCGTCGCCTTGGTGAGCCGTTACCTCACCAACTAGCTAATCTTGCGCGTGCCCATCTCTATCCACCGTAGT
TTTCAATATCACCCGATGCCGAATAATATATTATGGGGTATTAATCTTCTTCGAAAGGCTATCCCCCTGATAA
AGGCAGGTTGCACACGTGTTCCGCACCCGTACGCCGCTCTCAATTACCCGAAAGTAATCTACCGCTCGGCTTG
CATGTGTTAGGCCTCCCGCTAGCGTTCATCCTGAGCCAGGATCAAACTCT
>684
CTGCTGCCGCCCGTAGGAGTCTGGTCCGTGTCTCAGTACCAGTGTGGGGATCACCCTCTCAGGCCCCCCTAAA
GATCGTCGCCTGGTGAGCCGTTACCTCACCAACTAGCTAATCTTGCGCGTGCCCATCTCTATCCACCGTAGTT

TTCAATTTCACCCGATGCCGAATAATATATTATGGGGTATTAATTCTTCTCGAAAGGCTATCCCCCTGATAAA
GGCAGGTTGGCACACGTGTTCCGCACCGTACGCCGCTCTCAATTACCCGAAAGTAATCTACCGCTCGGCTTGC
ATGTGTTAGGCCTCCCGCTAGCGTCTCCTGAGCAGGATCA
>685
CTGCTGCCGCCCGTAGGAGTCTGGTCCGTGTCTCAGTACCAGTGTGGGGGATCACCCTCTCAGGCCCCCTAAA
GATCGTCGCCTTGGTGAGCCGTTACCTCACCAACTAGCTAATCTTGCGCGTGCCCATCTCTATCCACCGTAGT
TTTCAATATCACCCGATGCCGAATAATATATTATGGGGTATTAATCTTCTCGAAGGCTATCCCCTGATAAAGG
CAGGTTGCACACGTGTTCCGCACCCGTACGCCGCTCTCAATTGCCCGAAAGTAATCTACCGCTCGGCTTGCAT
GTGTTAGGCCTCCCGCTAGCGTTCTCCTGAGCAGGTCAAA
>686
CTGCTGCCGCCCGTAGGAGTCTGGTCCGTGTCTCAGTACCAGTGTGGGGGATCTACCCTCTCAGGCCCCCTAA
AGATCGTCGCCTGGTGAGCCGTTACCTCACCAACTAGCTAATCTTGCGCGTGCCCATCTCTATCCACCGTAGT
TTTCAATATCACCCGATGCCGAATAATATATTATGGGGTATTAATCTTCTTCGAAAGCTATCCCCCTGATAAA
GGCAGGTTGCACACGTGTTCCGCACCCGTACGCCGCTCTCAATTACCCGAAAGTAATCTACCGCTCGGCTGCA
TGTGTTAGGCCTCCCGCTAGCGTTCATCCTGAGCCAGGATCAAACCTCT
>687
CTGCTGCCGCCCGTAGGAGTCTGGACCGTGTCTCAGTTCCAGTGTGACTGATCATCCTCTCAGACCAGTACGG
ATCGTCGCCTTGGTAGGCCTTACCCCACCAACTAGCTAATCCGACCTAGGCTCATCTGATAGCGTGAGGTCCG
AAGTCCCCCACTTCTCCCTCAGGACGTATGCGGTATTAGCGCCCGTTTCCGGACGTATCCCCCACTACCAGGC
AGATTCCTAGGCATTACTCACCCGTCCGCCGCTGAATCCAGGAGCAAGCTCCCTCATCCGCTCGACTGCCATG
TGTTAGGCCTGCCGCCAGCGTCAATCTGAGCCAGGATCAAACTCT
>688
CTGCTGCCGCCCGTAGGAGTCTGGTCCGTGTCTCAGTACCAGTGTGGGGGATCACCCTCTCAGGCCCCCTAAA
GATCGTCGCCTGGTGAGCCGTTACCTCACCAACTAGCTAATCTTGCGCGTGCCCATCTCTATCCACCGTAGTT
TTCAATATCACCCGATGCCGAATAATATATTATGGGGTATTAATCTTCTTCGAAAGGCTATCCCCCTGATAAG
AGCAGGTTGCACACGTGTTCCGCACCCGTACGCCGCTCTCAATTACCCGAAAGTAATCTACCGCTCGGCTTGC
ATGTGTTAGGCCTCCCGCTAGCGTTCTCCTGAGCAGTCCAAGCGT
>689
CTGCTGCCGCCCGTAGGAGTCTGGTCCGTGTCTCAGTACCAGTGTGGGGGATCACCCTCTCAGGCCCCCTAAA
GATCGTCGCCTGGTGAGCCGTTACCTCACCAACTAGCTAATCTTGCGCGTGCCCATCTCTATCCACCGTAGTT
TCAATATCACCCGATGCCGAATAATATAATTATGGGGTATTAATCTTCTCGAAAGGCTATCCCCTGATAAAGG
CAGGTTGCACACGTGTTCCGCACCGTACGCCGCTCTCAATTACCCGAAAGTAATCTACCGCTCGGCTTGCATG
TGTTAGGCCTCCCGCTAGCGTCT
>690
CTGCTGCCGCCCGTAGGAGTCTGGTCCGTGTCTCAGTACCAGTGTGGGGGATCACCCTCTCAGGCCCCCTAAA
GATCGTCGCCTTGGTGAGCCGTTACCTCACCAACTAGCTAATCTTGCGCGTGCCCACTCTCTATCCACCGTAG
TTTTCAATATCACCCGATGCCGAATAATATATTATGGGGTATTAATCTTCTTCGAAAGGCTATCCCCCTGATA
AGGCAGGTTAGCACACGTGTTCCGCACCCGTACGCCGCTCTCAATTACCCGAAAGTAATCTACCGCTCGGCTT
GCATGTGTTAGGCCTCCCGCTAGCGTTCTCCTGAGCAGGTCAACTCGT
>691
CTGCTGCCGCCCGTAGGAGTCTGGTCCGTGTCTCAGTACCAGTGTGGGGGATCACCCTCTCAGGCCCCCTAAA
GATCGTCGCCTTGGTGAGCCGTTACCTCACCAACTAGCTAATCTTGCGCGTGCCCATCTCTATCCACCGTAGT
TTTCAATATCACCCGATGCCGAATAATATATTATGGGGTATTAATCTTCTTCGAAAGGCTATCCCCCTGATAA
AGGCAGGTTGCACACGTGTTCCGCACCCGTACGCCGCTCTCAATTACCCGAAAGTAATCTACCGCTCGGCTTG
CATGTGTTAGGCCTCCCGCTAGCGTTCATCCTGAGCCAGGATCAAACTCT
>692
CTGCTGCGCCCGTAGGAGTCTGGTCCGTGTCTCAGTACCAGTGTGGGGGATCACCCTCTCAGGCCCCCTAAAG
ATCGTCGCCTTGGTGAGCCGTTACCTCACCAACTAGCTAATCTTGCGCGTGCCCATCTCTATCCACCGTAGTT
TTCAATATCACCCGATGCCGAATAATATATTATGGGGTATTAATCTTCTTCGAAAGGCTATCCCCCTGATAAA
GGCAGGTTGCACACGTGTTCCGCACCCGTACGCCGCTCTCAATTACCCGAAAGTAATCTACCGCTCGGCTTGC
ATGTGTTAGGCCTCCCGCTAGCGTTCATCCTGAGCCAGGATCAACTCT
>693
CTGCTGCCGCCCGTAGGAGTCTGGTCCGTGTCTCAGTACCAGTGTGGGGGATCACCCTCTCAGGCCCCCTAAA
GATCGTCGCCTTGGTGAGCCGTTACCTCACCAACTAGCTAATCTTGCGCGTGCCCATCTCTATCCACCGTAGT

TTTCAATATCACCCGATGCCGAATAATATATTATGGGGTATTAATCTTCCTTCGAGAGGCTATCCCCCTGATA
AAGGCAGGTTGCACACGTGTTCCGCACCCGTACGCCGCTCTCAATTACCCGAAAGTAATCTACCGCTCGGCTT
GCATGTGTTAGGCCTCCCGCTAGCGTTCTCCTGAGCAGGATCAAACTCT
>694
CTGCTGCCGCCCGTAGGAGTCTGGTCCGTGTCTCAGTACCAGTGTGGGGGATCACCCTCTCAGGCCCCCTAAA
GATCGTCGCCTTGGTGAGCCGTTACCTCACCAACTAGCTAATCTTGCGCGTGCCCATCTCTATCCACCGTAGT
TTTCAATATCACCCGATGCCGAATAATATATTATGGGGTATTAATCTTCTTCGAAAGGCTATCCCCCTGATAA
AGGCAGGTTGCACACGTGTTCCGCACCCGTACGCCGCTCTCAATTACCCGAAAGTAATCTACCGCTCGGCTTG
CATGTGTTAGGCCTCCCGCTAGCGTTCATCCTGAG
>695
CTGCTGCCGCCCGTAGGAGTCTGGTCCGTGTCTCAGTACCAGTGTGGGGGATCACCCTCTCAGGCCCCCTAAA
GATCGTCGCCTTGGTGAGCCGTTACCTCACCAACTAGCTAATCTTGCGCGTGCCCATCTCTATCCACCGTAGT
TTTCAATATCACCCGATGCCGAATAATATATTATGGGGTATTAATCTTCTTCGAAAGGCTATCCCCCTGATAA
AGGCAGGTTGCACACGTGTTCCGCACCCGTACGCCGCTCTCAATTACCCGAAAGTAATCTACCGCTCGGCTTG
CATGTGTTAGGCCTCCCGCTAGCGTTCTCCTGAGCAGGATCAACTCT
>696
CTGCTGCCGCCCGTAGGAGTCTGGTCCGTGTCTCAGTACCAGTGTGGGGGATCACCCTCTCAGGCCCCCTAAA
GATCGTCGCCTTGGTGAGCCGTTACCTCACCAACTAGCTAATCTTGCGCGTGCCCATCTCTATCCACCGTAGT
TTTCAATATCACCCGATGCCGAATAATATATTATGGGGTATTAATCTTCTTCGAAAGGCTATCCCCCTGATAA
AGGCAGGTTGCACACGTGTTCCGCACCCGTACGCCGCTCTCAATTACCCGAAAGTAATCTACCGCTCGGCTTG
CATGTGTTAGGCCTCCCGCTAGCGTTCTCCTGA
>697
CTGCTGCCGCCCGTAGGAGTCTGGTCCGTGTCTCAGTACCAGTGTGGGGGATCACCCTCTCAGGCCCCCTAAA
GATCGTCGCCTTGGTGAGCCGTTACCTCACCAACTAGCTAATCTTGCGCGTGCCCATCTCTATCCACCGTAGT
TTTCAATATCACCCGATGCCGAATAATATATTATGGGGTATTAATCTTCTCGAAAGGCTATCCCCCTGATAAA
GGCAGGTTGCACACGTGTTCCGCACCCGTACGCCGCTCTCAATTACCCGAAAGTAATCTACCGCTCGGCTTGC
ATGTGTTAGGCCTCCCGCTAGCGTCATCCTGAGCCAGGATCAAACTCT
>698
CTGCTGCCGCCCGTAGGAGTCTGGACCGTGTCTCAGTTCCAGTGTGACTGATCATCCTCTCAGACCAGTACGG
ATCGTCGCCTTGGTAGGCCTTTACCCCACCAACTAGCTAATCCGACCTAGGCTCATCTGATAGCGTGAGGTCC
GAAGATCCCCCACTTCTCCCTCAGGACGTATGCGGTATTAGCGCCCGTTTCCGGACGATCCCCACTACCAGGC
AGATTCCTAGGCATTACTCACCCGTCCGCCGCTGAATCCAGGAGCAAGCTCCCTTCATCCGCTCGACTGCATG
TGTTAGGCCTGCCGCCAGCGTTCAATCTGAGCCAGGATCAAACTCT
>699
CTGCTGCCGCCCGTAGGAGTCTGGACTCGTGTCTCGACGTCAGTGTGGGGGATCACCCTCTCAGGCCCCCTAA
AGATCGTCGCCTTGGTGAGCCGTTACCTCACCAACTAGCTAATCTTGCGCGTGCCCATCTCTATCCACCGTAG
TTTTCAATATCACCCGATGCCGAATAATATATTATGGGGTATTAATCTTCTTCGAAAGGCTATCCCCCTGATA
AAGGCAGGTTGCACACGTGTTCCGCACCCGTACGCCGCTCTCAATTACCCGAAAGTAATCTACCGCTCGGCTT
GCATGTGTTAGGCCTCCCGCTAGCGTTCATCCTGAGCCAGGATCAAACTCT
>700
CTGCTGCCGCCCCGTAGGAGTCTGGACCGTGTCTCAGTTCCAGTGTGACTGATCATCCTCTCAGACCCAGTTA
CGGATCGTCGCCTTGGTAGGCCCTTTTACCCCCACCAACTAGCTAATCCGACCTAGGCTCATCTGATAGCGTG
AGGTCCGAAGTCCCCCACTTCTCCCCTCAGGACGTATGCGGTATTAGCGCCGTTTCCGGACGTATCCCCACTA
CCCAGGCAGATTCCTAGGCATTACTCACCCGTCCCGCCGCTGAATCCAGGAGCAAGCCCCCTTCCATCCGCTC
GACTGCATTGTGTTAGGCCTGCCGCCAGCGTCAATCTGAGCCCAGGATCAAACTCT
>701
CTGCTGCCGCCCGTAGGAGTCTGGTCCGTGTCTCAGTACCAGTGTGGGGGATCACCCTCTCAGGCCCCCTAAA
GATCGTCGCCTTGGTGAGCCGTTACCTCACCAACTAGCTAATCTTGCGCGTGCCCATCTCTATCCACCGTAGT
TTTCAATATCACCCGATGCCGAATAATATATTATGGGGTATTAATCTTCTCGAAAGGCTATCCCCTGATAAAG
GCAGGTTGCACACGTGTTCCGCACCCGTACGCCGCTCTCAATTACCCGAAAGTAATCTACCGCTCGGCTTGCA
TGTGTTAGGCCTCCCGCTAGCGTTCATCCT
>702
CTGCTGCCGCCCGTAGGAGTCTGGTCCGTGTCTCAGTACCAGTGTGGGGGATCACCCTCTCAGGCCCCCTAAA
GATCGTCGCCTTGGTGAGCCGTTACCTCACCAACTAGCTAATCTTGCGCGTGCCCATCTCTATCCACCGTAGT

TTTCAATATCACCCGATGCCGAATAATATATTATGGGGTATTAATCTTCTTCGAAAGGCTATCCCCCTGATAA
AGGCAGGTTGCACACGTGTTCCGCACCCGTACGCCGCTCTCAATTACCCGAAAGTAATCTACCGCTCGGCTTG
CATGTGTTAGGCCTCCCGCTAGCGTTCATCCTGAGCCAGGATCAAACTCT
>703
CTGCTGCCGCCCGTAGGAGTCTGGTCCGTGTCTCAGTACCAGTGTGGGGGATCACCCTCTCAGGCCCCCTAAA
GATCGTCGCCTTGGTGAGCCGTTTACCTCACCAACTAGCTAATCTTGCGCGTGCCCATCTCTATCCACCGTAG
TTTTCAATATCACCCGATGCCGAATAATATATTATGGGGTATTAATCTTCTTCGAAAGGCTATCCCCCTGATA
AAGGCAGGTTGCACACGTGTTCCGCACCCGTACGCCGCTCTCAATTACCCGAAAGTAATCTACCGCTCGGCTT
GCATGTGTTAGGCCTCCCGCTAGCGTTCTCCTGAGCAGGATCAACT
>704
CTGCTGCCGCCCGTAGGAGTCTGGTCCGTGTCTCAGTACCAGTGTGGGGGGATCACCCTCTCAGGCCCCCTAA
AAGATCGTCGCCTTGGTGAGCCGTTACCTCACCAACTAGCTAATCTTGCGCGTGCCCATCTCTATCCACCGTA
GTTTTCAATATCACCCGATGCCGAATAATATATTATGGGGTATTAATCTTCTTCGAAAGGCTATCCCCCTGAT
AAAGGCAGGTTGCACACGTGTTCCGCACCCGTACGCCGCTCTCAATTACCCGAAAGTAATCTACCGCTCGGCT
TGCATGTGTTAGGCCTCCCGCTAGCGTTCTCCTCGAGCAGGA
>705
CTGCTGCCGCCCGTAGGAGTCTGGTCCGTGTCTCAGTACCAGTGTGGGGGATCACCCTCTCAGGCCCCCTAAA
GATCGTCGCCTTGGTGAGCCGTTACCTCACCAACTAGCTAATCTTGCGCGTGCCCATCTCTATCCACCGTAGT
TTTCAATATCACCCGATGCCGAATAATATATTATGGGGTATTAATCTTCTCGAAAGGCTATCCCCTGATAAGG
CAGGTTGCACACGTGTTCCGCACCCGTACGCCGCTCTCAATTACCCGAAAGTAATCTACCGCTCGGCTTGCAT
GTGTTAGGCTCCCGCCTAGCGTTC
>706
CTGCTGCCGCCCGTAGGAGTCTGGTCCGTGTCTCAGTACCAGTGTGGGGGATCACCCTCTCAGGCCCCCTAAA
GATCGTCGCCTTGGTGAGCCGTTACCTCACCAACTAGCTAATCTTGCGCGTGCCCATCTCTATCCACCGTAGT
TTTCAATATCACCCGATGCCGAATAATATATTATGGGGTATTAATCTTCTTCGAAAGGCTATCCCCCTGATAA
AGGCAGGTTGCACACGTGTTCCGCACCCGTACGCCGCTCTCAATTACCCGAAAGTAATCTACCGCTCGGCTTG
CATGTGTTAGGCCTCCCGCTAGCGTTCATCCTGAGCCAGGATCAAACTCT
>707
CTGCTGCCGCCCGTAGGAGTCTGGACCGTGTCTCAGTTCCAGTGTGACTGATCATCCTCTCAGACCAGTACGG
ATCGTCGCCTTGGTAGGCCTTACCCCACCAACTAGCTAATCCGAACCTAGGCTCATCTGATAGCGTGAGGTCC
GAAGATCCCCCACTTCTCCCTCAGGACGTATGCGGTATTAGCGCCCGTTTCCGGACGATCCCCACTACCAGGC
AGATTCCTAGGCATTACTCACCCGTCCGCCGCTGAATCCAGGAGCAAGCTCCCTCATCCGCTCGACTGCATGT
GTTAGGCCTGCCGCCAGCGTCAATCTGAGCCAGGATCAAACTCT
>708
CTGCTGCCGCCCGTAGGAGTCTGGTCCGTGTCTCAGTACCAGTGTGGGGGATCACCCTCTCAGGCCCCCTAAA
GATCGTCGCCTTGGTGAGCCGTTACCTCACCAACTAGCTAATCTTGCGCGTGCCCATCTCTATCCACCGTAGT
TTTCAATATCACCCGATGCCGAATAATATATTATGGGGTATTAATCTTCTTCGAAAGGCTATCCCCTGATAAA
GGCAGGTTGCACACGTGTTCCGCACCCGTACGCCGCTCTCAATTACCCGAAAGTAATCTACCGCTCGGCTTGC
ATGTGTTAGGCCTCCCGCTAGCGTTCATCCTGAGCCAGGATCAAACTCT
>709
CTGCTGCCGCCCGTAGGAGTCTGGTCCGTGTCTCAGTACCAGTGTGGGGGATCACCCTCTCAGGCCCCCTAAA
GATCGTCGCCTTGGTGAGCCGTTACCTCACCAACTAGCTAATCTTGCGCGTGCCCATCTCTATCCACCGTAGT
TTTCAATATCACCCGATGCCGAATAATATATTATGGGGTATTAATCTTCTCGAAAGGCTATCCCCCTGATAAA
GGCAGGTTGCACACGTGTTCCGCACCCGTACGCCGCTCTCAATTACCCGAAAGTAATCTACCGCTCGGCTTGC
ATGTGTTAGGCCTCCCGCTAGCGTTCTCCTGAGCAGGATCAACTCGT
>710
CTGCTGCCGCCCGTAGGAGTCTGGACCGTGTCTCAGTTCCAGTGTGACTGATCATCCTCTCAGACCAGTTACG
GATCGTCGCCTTGGTAGGCCTTACCCCACCAACTAGCTAATCCGACCTAGGCTCATCTGATAGCGTGAGGTCC
GAAGATCCCCCACTTCTCCCTCAGGACGTATGCGGTATTAGCGCCCGTTTCCGGACGTATCCCCACTACCAGG
CAGATTCCTAGGCATTACTCACCCGTCCGCCGCTGAATCCAGGAGCAAGCTCCCTTCATCCGCTCGACTTGCA
TGTGTTAGGCCTGCCGCCAGCGTCAATCTGAGCCAGGATCAAACTCT
>711
CTGCTGCCGCCCGTAGGAGTCTGGTCCGTGTCTCAGTACCAGTGTGGGGGATCACCCTCTCAGGCCCCCTAAA
GATCGTCGCCTTGGTGAGCCGTTACCTCACCAACTAGCTAATCTTGCGCGTGCCCATCTCTATCCACCGTAGT

TTCAATATCACCCGATGCCGAATAATATATTATGGGGTATTAATCTTCTTCGAAAGGCTATCCCCTGATAAAG
GCAGGTTGCACACGTGTTCCGCACCCGTACGCCGCTCTCAATTACCCGAAATAATCTACCGCTCGGCTTGCAT
GTGTTAGGCCTCCCGCTGAGCGTTCTCC
>712
CTGCTGCCGCCCGTAGGAGTCTGGTCCGTGTCTCAGTACCAGTGTGGGGGATCACCCTCTCAGGCCCCCTAAA
GATCGTCGCCTTGGTGAGCCGTTACCTCACCAACTAGCTAATCTTGCGCGTGCCCATCTCTATCCACCGTAGT
TTTCAATATCACCCGATGCCGAATAATATATTATGGGGTATTAATCTTCTTCGAAAGGCTATCCCCCTGATAA
AGGCAGGTTGCACACGTGTTCCGCACCCGTACGCCGCTCTCAATTACCCGAAAGTAATCTACCGCTCGGCTTG
CATGTGTTAGGCCTCCCGCTAGCGTTCATCCTGAGCCAGGATCAAACTCT
>713
CTGCTGCCGCCCGTAGGAGTCTGGACCGTGTCTCAGTTCCAGTGTGACTGATCATCCTCTCAGACCAGTACGG
ATCGTCGCCTTGGTAGGCCTTTACCCACCAACTAGCTAATCCGACCTAGGCTCATCTGATAGCGTGAGGTCCG
AAGATCCCCACTTCTCCCTCAGGACGTATGCGGTATTAGCGCCCGTTTCCGGACGTATCCCCACTACCAGGCA
GATTCCTAGGCATTACTCACCCGTCCGCCGCTGAATCCAGGAGCAAGCTCCCTTCATCCGCTCGACTGCATGT
GTTAGGCCTGCCGCCAGCGTTCAATCTGAGCCAGGATCAAACTCT
>714
CTGCTGCCGCCCGTAGGAGTCTGGTCCGTGTCTCAGTACCAGTGTGGGGGATCACCCTCTCAGGCCCCCTAAA
GATCGTCGCCTTGGTGAGCCGTTACCTCACCAACTAGCTAATCTTGCGCGTGCCCATCTCTATCCACCGTAGT
TTCAATATCACCCGATGCCGAATAATATATTATGGGGTATTAATCTTCTTCGAAAGGCTATCCCCCTGATAAA
GGCAGGTTGCACACGTGTTCCGCACCCGTACGCCGCTCTCAATTACCCGAAAGTAATCTACCGCTCCGGCTTG
CATGTGTTAGGCCTCCCGCTAGCGTCTCCTGAGCAGGATCAACTC
>715
CTGCTGCCGCCCGTAGGAGTCTGGTCCGTGTCTCAGTACCAGTGTGGGGGATCACCCTCTCAGGCCCCCTAAA
GATCGTCGCCTTGGTGAGCCGTTACCTCACCAACTAGCTTAATCTTGCGCGTGCCCATCTCTATCCACCGTAG
TTTCAATATCACCCGATGCCGAATTAATATATTATGGGGTATTTAATCTTCTTCGAAAGGCTATCCCCCTGAT
AAAGGCAGGTTGCACACGTGTTCCGCACCCGTACGCCGCTCTCAATTACCCGAAAGTAATCTACCGCTCGGCT
TGCATGTGTTAGGCCTCCCGCTAGCGTTCATCCTGAGCCAG
>716
CTGCTGCCGCCCGTAGGAGTCTGGTCCCGTGTCTCAGTACCAGTGTGGGGGATCACCCTCTCAGGCCCCCTAA
AGATCGTCGCCTTGGTGAGCCGTTTACCTCACCCAACTAGCTAATCTTGCGCGTGCCCATCTCTATCCACCGT
AGTTTTCAATATCACCCGATGCCGAATAATATATTATTGGGGTATTTAATTCTTCTTCGAAAGGCTATCCCCC
TGATAAAGGCAGGTTGCACACGTGTTCCGCACCCGTACGCCGCTCTCAATTACCCCGAAAGTAATCTACCGCT
CGGCTTGCATGTGTTAGGCCTCCCGCTAGCGTTCATTCCTGAGCCAGGATCAACTCT
>717
CTGCTGCCGCCCGTAGGAGTCTGGTCCGTGTCTCAGTACCAGTGTGGGGGATCACCCTCTCAGGCCCCCTAAA
GATCGTCGCCTGGTGAGCCGTTACCTCACCAACTAGCTAATCTTGCGCGTGCCCATCTCTATCCACCGTAGTT
TTCAATATCACCCGATGCCGAATAATATATTATGGGGTATTAATCTTCTCGAAAGGCTATCCCCTGATAAAGG
CAGGTTGCACACGTGTTCCGCACCCGTACGCCGCTCTCAATTACCCGAAAGTAATCTACCGCTCGGCTTGCAT
GTGTTAGGCCTCCCGCTAGCGTTCTCCTGAGC
>718
CTGCTGCCGCCCGTAGGAGTCTGGTCCGTGTCTCAGTACCAGTGTGGGGGATCACCCTCTCAGGCCCCCTAAA
GATCGTCGCCTTGGTGAGCCGTTACCTCACCAACTAGCTAATCTTGCGCGTGCCCATCTCTATCCACCGTAGT
TTTCAATATCACCCGATGCCGAATAATATATTATGGGGTATTAATCTTCTTCGAAAGGCTATCCCCCTGATAA
AGGCAGGTTGCACACGTGTTCCGCACCCGTACGCCGCTCTCAATTACCCGAAAGTAATCTACCGCTCGGCTTG
CATGTGTTAGGCCTCCCGCTAGCGTTCTCCTGAGCAGGATCAACTCGT
>719
CTGCTGCCGCCCGTAGGAGTCTGGACCGTGTCTCAGTTCCAGTGTGACTGATCATCCTCTCAGACCAGTACGG
ATCGTCGCCTTGGTAGGCCCTTTACCCCCACCAACTAGCTAATCCGACCTAGGCTCATCTGATAGCGTGAGGT
CCGAAGATCCCCCACTTCTCCCCTCAGGACGTATGCGGTATTAGCGCCCGTTTCCGGACGATCCCCACTACCA
GGCAGATTCCTAGGCATTACTCACCCGTCCGCCGCTGAATCCAGGAGCAAGCCCTCCTCTCCGCTCGACTGCA
TGTGTTAGGCCTGCCGCCAGCGTTCAATCTGAGCCAGGATCAAACTCT
>720
CTGCTGCCGCCATCGAGGTATGGTCCGTGTCTCAGTACCAGTGTGGGGGATCACCCTCTCAGGCCCCCTAAAG
ATCGTCGCCTTGGTGAGCCGTTACCTCACCAACTAGCTAATCTTGCGCGTGCCCATCTCTATCCACCGTAGTT

TTCAATATCACCCGATGCCGAATAATATATTATGGGGTATTAATCTTCTTCGAAAGGCTATCCCCCTGATAAA
GGCAGGTTGCACACGTGTTCCGCACCCGTACGCCGCTCTCAATTACCCGAAAGTAATCTACCGCTCGGCTTGC
ATGTGTTAGGCCTCCCGCTAGCGTTCTCCGTGAGCAGGATCCAACTCT
>721
CTGCTGCCGCCCGTAGGAGTCTGGTCCGTGTCTCAGTACCAGTGTGGGGGATCACCCTCTCAGGCCCCCTAAA
GATCGTCGCCTTGGTGAGCCGTTTACCTCACCAACTAGCTAATCTTGCGCGTGCCCATCTCTATCCACCGTAG
TTTTCAATATCACCCGATGCCGAATAATATATTATGGGGTATTAATCTTCTCGAAAGGCTATCCCCCTGATAA
AGGCAGGTTGCACACGTGTTCCGCACCCGTACGCCGCTCTCAATTACCCGAAAGTAATCTACCGCTCGGCTTG
CATGTGTTAGGCCTCCCGCTAGCGCTCATCCTGAGCCAGGATCAACTCT
>722
CTGCTGCCGCCCGTAGGAGTCTGGTCCGTGTCTCAGTACCAGTGTGGGGGATCACCCTCTCAGGCCCCCTAAA
GATCGTCGCCTTGGTGAGCCGTTACCTCACCAACTAGCTAATCTTGCGCGTGCCCATCTCTATCCACCGTAGT
TTTCAATATCACCCGATGCCGAATAATATATTATGGGGTATTAATCTTCTTCGAAAGGCTATCCCCCTGATAA
AGGCAGGTTGCACACGTGTTCCGCACCCGTACGCCGCTCTCAATTACCCGAAAGTAATCTACCGCTCGGCTTG
CATGTGTTAGGCCTCCCGCTAGCGTTCATCCTGAGCCAGGATCAAACTCT
>723
CTGCTGCCGCCCGTAGGAGTCTGGTCCGTGTCTCAGTACCAGTGTGGGGGATCACCCTCTCAGGCCCCCTAAA
AGATCGTCGCCTTGTGAGCCGTTACCTCACCAACTAGCTAATCTTGCGCGTGCCCATCTCTATCCACCGTAGT
TTTCAATATCACCCGATGCCGAATAATATATTATGGGGGTATTAATCTTCTTCGAAAGGCTATCCCCCTGATA
AAGGCAGGTTGCACACGTGTTCCGCACCCGTACGCCGCTCTCAATTACCCGAAAGTAATCTACCGCATCGGCT
TGCATGTGTTAGGCCTCCCGCTAGCGTTCTCCTGA
>724
CTGCTGCCGCCCGTAGGAGTCTGGTCCGTGTCTCAGTACCAGTGTGGGGGATCACCCTCTCAGGCCCCCTAAA
AGATCGTCGCCTTGGTGAGCCGTTACCTCACCAACTAGCTAATCTTGCGCGTGCCCATCTCTATCCACCGTAG
TTTTCAATATCACCCGATGCCGAATAATATAATTATGGGGTATTAATCTTCTTCGAAAGCTATCCCCCTGATA
AAGGCAGGTTGCACACGTGTTCCGCACCCGTACGCCGCTCTCAATTACCCGAAAGTAATCTACCGCTCGGCTT
GCATGTGTTAGGCCTCCCGCTAGCGTTCTCCTGAGCAGGATCC
>725
CTGCTGCCGCCCGTAGGAGTCTGGTCCGTGTCTCAGTACCAGTGTGGGGGATCACCCTCTCAGGCCCCCTAAA
GATCGTCGCCTTGGTGAGCCGTTACCTCACCAACTAGCTAATCTTGCGCGTGCCCATCTCTATCCACCGTAGT
TTTCAATATCACCCGATGCCGAATAATATATTATGGGGGTATTTAATCTTCTTCGAAAGGCTATCCCCCTGAT
AAAGGCAGGTTGCACACGTGTTCCGCACCCGTACGCCGCTCTCAATTACCCGAAAGTAATCTACCGCTCGGCT
TGCATGTGTTAGGCCTCCCGCTAGCGTTCATCCTGAGCCAGGATCAAACTCT
>726
CTGCTGCCGCCCGTAGGAGTCTGGTCCGTGTCTCAGTACCAGTGTGGGGGATCACCCTCTCAGGCCCCCTAAA
GATCGTCGCCTTGGTGAGCCGTTACCTCACCAACTAGCTAATCTTGCGCGTGCCCATCTCTATCCACCGTAGT
TTTCAATATCACCCGATGCCGAATAATATATTATGGGGTATTAATCTTCTTCGAAAGGCTATCCCCCTGATAA
AGGCAGGTTGCACACGTGTTCCGCACCCGTACGCCGCTCTCAATTACCCGAAAGTAATCTACCGCTCGGCTTG
CATGTGTTAGGCCTCCCGCTAGCGTTCTCCTGAGCAGGATCAACTC
>727
CTGCTGCCGCCCGTAGGAGTCTGGTCCGTGTCTCAGTACCAGTGTGGGGGATCACCCTCTCAGGCCCCCTAAA
GATCGTCGCCTTGGTGAGCCGTTACCTCACCAACTAGCTAATCTTGCGCGTGCCCATCTCTATCCACCGTAGT
TTTCAATATCACCCGATGCCGAATAATATATTATGGGGTATTAATCTTCTTCGAAAGGCTATCCCCCTGATAA
AGGCAGGTTGCACACGTGTTCCGCACCCGTACGCCGCTCTCAATTACCCGAAAGTAATCTACCGCTCGGCTTG
CATGTGTTAGGCCTCCCGCTAGCGTTCATCCTGAGCCAGGATCAAACTCT
>728
CTGCTGCCGCCCGTAGGAGTCTGGTCCGTGTCTCAGTACCAGTGTGGGGGATCACCCTCTCAGGCCCCCTAAA
GATCGTCGCCTTGTGAGCCGTTACCTCACCAACTAGCTAATCTTGCGCGTGCCCATCTCTATCCACCGTAGTT
TTCAATATCACCCGATGCCGAATAATATATTATGGGGTATTAATCTTCTCGAAAGGCTATCCCCCTGATAAAG
GCAGGTTGCACACGTGTTCCGCACCCGTACGCCGCTCTCAATTACCCGAAAGTATCTACCGCTCGGCTTGCAT
GTGTTAGGCCTCCCGCTAGCGTCATCCTGAGCCAGGATCAAACTCT
>729
CTGCTGCCGCCCGTAGGAGTCTGGACCGTGTCTCAGTTCCAGTGTGACTGATCATCCTCTCAGACCAGTACGG
ATCGTCGCCTTGGTAGGCCTTTACCCCACCAACTAGCTAATCCGACCTAGGCTCACTGATAGCGTGAGGTCCG

AAGTCCCCCACTTCTCCCTCAGGACGTATGCGGTATTAGCGCCCGTTTCCGGACGTATCCCCCACTACCAGGC
AGATTCCTAGGCATTACTCACCCGTCCGCCGCTGAATCCAGGAGCAAGCTCCCTTCATCCGCTCGACTGCATG
TGTTAGGCCTGCCGCCAGCGTCAATCTGAGCCAGGATCAAACTCT
>730
CTGCTGCCGCCCGTAGGAGTCTGGTCCGTGTCTCAGTACCAGTGTGGGGGATCACCCTCTCAGGCCCCCTAAA
GATCGTCGCCTTGGTGAGCCGTTACCTCACCAACTAGCTAATCTTGCGCGTGCCCATCTCTATCCACCGTAGT
TTTCAATATCACCCGATGCCGAATAATATATTATGGGGTATTAATCTTCTCGAAAGGCTATCCCCCTGATAAA
GGCAGGTTGCACACGTGTTCCGCACCCGTACGCCGCTCTCAATTACCCGAAAGTAATCTACCGCTCCGGCTTG
CATGTGTTAGGCCTCCCGCTAGCGTCTCCTGA
>731
CTGCTGCCGCCCGTAGGAGTCTGGACCGTGTCTCAGTTCCAGTGTGACTGATCATCCTCTCAGACCAGTACGG
ATCGTCGCCTTGGTAGGCCTTTACCCCACCAACTAGCTAATCCGACCTAGGCTCATCTGATAGCGTGAGGTCC
GAAGTCCCCCACTTCTCCCTCAGGACGTATGCGGTATTAGCGCCCGTTTCCGGACGTATCCCCACTACCAGGC
AGATTCCTAGGCATTACTCACCCGTCCGCCGCTGAATCCAGGAGCAAGCTCCCTCATCCGCTCGACTGCATGT
GTTAGGCCTGCCGCCAGCGTCAATCTGAGCCAGGGATCAAACTCT
>732
CTGCTGCCGCCCGTAGGAGTCTGGACCGTGTCTCAGTTCCAGTGTGACTGATCATCCTCTCAGACCAGTACGG
ATCGTCGCCTTGGTAGGCCTTTACCCCACCAACTAGCTAATCCGACCTAGGCTCATCTGATAGCGTGAGGTCC
GAAGTCCCCCACTTCTCCCTCAGGACGTATGCGGTATTAGCGCCCGTTTCCGGACGATCCCCCACTACCAGGC
AGATTCCTAGGCATTACTCACCCGTCCGCCGCTGAATCCAGGAGCAAGCTCCCTCATCCGCTCGACTGCATTG
TGTTAGGCCTGCCGCCAGCGTCAATCTGAGCCAGGATCAACTCT
>733
CTGCTGCCGCCCGTAGGAGTCTGGTCCGTGTCTCAGTACCAGTGTGGGGGATCACCCTCTCAGGCCCCCTAAA
GATCGTCGCCTTGGTGAGCCGTTACCTCACCAACTAGCTAATCTTGCGCGTGCCCATCTCTATCCACCGTAGT
TTTCAATATCACCCGATGCCGAATAATATATTATGGGGTATTAATCTTCTTCGAAAGGCTATCCCCCTGATAA
AGGCAGGTTGCACACGTGTTCCGCACCCGTACGCCGCTCTCAATTACCCGAAAGTAATCTACCGCTCGGCTTG
CATGTGTTAGGCCTCCCGCTAGCGTTCATCCTGAGCCAGGATCAAACTCT
>734
CTGCTGCCGCCCGTAGGAGTCTGGTCCGTGTCTCAGTACCAGTGTGGGGGATCACCCTCTCAGGCCCCCTAAA
GATCGTCGCCTTGGTGAGCCGTTACCTCACCAACTAGCTAATCTTGCGCGTGCCCATCTCTATCCACCGTAGT
TTTCAATATCACCCGATGCCGAATAATATATTATGGGGTATTAATCTTCTTCGAAAGGCTATCCCCCTGATAA
AGGCAGGTTGCACACGTGTTCCGCACCCGTACGCCGCTCTCAATTACCCGAAAGTAATCTACCGCTCGGCTTG
CATGTGTTAGGCCTCCCGCTAGCGTTCATCCTGAGCCAGGATCAAACTCT
>735
CTGCTGCCGCCCGTAGGAGTCTGGTCCGTGTCTCAGTACCAGTGTGGGGGATCGCCCTCTCAGGCCCCCTAAA
GATCGTCGCCTTGGTGAGCCGTTACCTCACCAACTAGCTAATCTTGCGCGTGCCCATCTCTATCCACCGTAGT
TTTCAATATCACCCGATGCCGAATAATATATTATGGGGTATTAATCTTCTTCGAAAGGCTATCCCCCTGATAA
AGGCAGGTTGCACACGTGTTCCGCACCCGTACGCCGCTCTCAATTACCCGAAAGTAATCTACCGCTCCGGCTT
GCATGTGTTAGGCCTCCCGCTAGCGTTCATCCTCGAGCCAGGATCAAACTCT
>736
CTGCTGCCGCCCGTAGGAGTCTGGTCCGTGTCTCAGTACCAGTGTGGGGGATCACCCTCTCAGGCCCCCTAAA
GATCGTCGCCTTGGTGAGCCGTTACCTCACCAACTAGCTAATCTTGCGCGTGCCCATCTCTATCCACCGTAGT
TTTCAATATCACCCGATGCCGAATAATATATTATGGGGTATTAATCTTCTTCGAAAGGCTATCCCCCTGATAA
AGGCAGGTTGCACACGTGTTCCGCACCCGTACGCCGCTCTCAATTACCCGAAAGTAATCTACCGCTCGGCTTG
CATGTGTTAGGCCTCCCGCTAGCGTTCATCCTGAGCCAGGATCAAACTCT
>737
CTGCTGCCGCCCGTAGGAGTCTGGTCCGTGTCTCAGTACCAGTGTGGGGGATCACCCTCTCAGGCCCCCTAAA
GATCGTCGCCTTGGTGAGCCGTTACCTCACATATAGCTAATCTTGCGCGTGCCCATCTCTATCCACCGTAGTT
TTCAATATCACCCGATGCCGAATAATATATTATGGGGTATTAATCTTCTTCGAAAGGCTATCCCCTGATAAAG
GCAGGTTGCACACGTGTTCCGCACCCGTACGCCGCTCTCAATTACCCGAAAGTAATCTACCGCTCGGCTTGCA
TGTGTTAGGCCTCCCGCTAGCGTTCTCCTGAGCAGGTCAACTCGT
>738
TGCTGCCGCCCGTAGGAGTCTGGACCGTGTCTCAGTTCCAGTGTGACTGATCATCCTCTCAGACCAGTACGGA
TCGTCGCCTTGGTAGGCCTTTACCCCACCAACTAGCTAATCCGACCTAGGCTCATCTGATAGCGTGAGGTCCG

AAGATCCCCCACTTCTCCCTCAGGACGTATGCGGTATTAGCGCCCGTTTCCGGACGATCCCCACTACCAGGCA
GATTCCTAGGCATTACTCACCCGTCCGCCGCTGAATCCAGGAGCAAGCTCCCCTCATCCGCTCGACTGCATGT
GTTAGGCCTGCCGCCAGCGTTCAATCTGAGCCAGGATCAAACTCT
>739
CTGCTGCCGCCCGTAGGAGTCTGGTCCGTGTCTCAGTACCAGTGTGGGGGATCACCCTCTCAGGCCCCCTAAA
GATCGTCGCCTTGGTGAGCCGTTACCTCACCAACTAGCTAATCTTGCGCGTGCCCATCTCTATCCACCGTAGT
TTTCAATATCACCCGATGCCGAATAATATATTATGGGGTATTAATCTTCTTCGAAAGGCTATCCCCTGATAAA
GGCAGGTTGCACACGTGTTCCGCACCCGTACGCCGCTCTCAATTACCCGAAAGTAATCTACCGCTCGGCTTGC
ATGTGTTAGGCCTCCCGCTAGCGTTCATCCTGAGCCAGGATCAAACTCT
>740
CTGCTGCCGCCCGTAGGAGTCTGGTCCGTGTCTCAGTACCAGTGTGGGGGATCACCCTCTCAGGCCCCCTAAA
GATCGTCGCCTTGGTGAGCCGTTACCTCACCAACTAGCTAATCTTGCGCGTGCCCATCTCTATCCACCGTAGT
TTTCAATATCACCCGATGCCGAATAATATATTATGGGGTATTAATCTTCTCGAAAGGCTATCCCCCTGATAAA
GGCAGGTTGCACACGTGTTCCGCACCCGTACGCCGCTCTCAATTACCCGAAAGTAATCTACCGCTCGGCTTGC
ATGTGTTAGGCCTCCCGCTAGCGTTCATCCTGAGCCAGGATCAAACTCT
>741
CTGCTGCCGCCCGTAGGAGTCTGGTCCGTGTCTCAGTACCAGTGTGGGGGATCACCCTCTCAGGCCCCCTAAA
GATCGTCGCCTTGGTGAGCCGTTACCTCACCAACTAGCTAATCTTGCGCGTGCCCATCTCTATCCACCGTAGT
TTTCAATATCACCCGATGCCGAATAATATATTATGGGGTATTAATCTTCTTCGAAAGGCTATCCCCCTGATAA
AGGCAGGTTGCACACGTGTTCCGCACCCGTACGCCGCTCTCAATTTACCCGAAAGTAATCTACCGCTCGGCTT
GCATGTGTTAGGCCTCCCGCTAGCGTTCTCCTGAGCAGGATCCAACTCGT
>742
CTGCTGCCGCCCGTAGGAGTCTGGTCCGTGTCTCAGTACCAGTGTGGGGGATCACCCTCTCAGGCCCCCTAAA
GATCGTCGCCTTGGTGAGCCGTTACCTCACCAACTAGCTAATCTTGCGCGTGCCCATCTCTATCCACCGTAGT
TTTCAATATCACCCGATGCCGAATAATATATTATGGGGTATTAATCTTCTTCGAAAGGCTATCCCCCTGATAA
AGGCAGGTTGCACACGTGTTCCGCACCCGTACGCCGCTCTCAATTACCCGAAAGTAATCTACCGCTCCGGCTT
GCATGTGTTAGGCCTCCCGCTAGCGTTCTCCTGACGCAGGATCAA
>743
CTGTGCCGCCCGTAGGAGTCTGGTCCGTGTCTCAGTACCAGTGTGGGGGGATCACCCTCTCAGGCCCCCTAAAG
ATCGTCGCCTTGGTGAGCCGTTACCTCACCAACTAGCTAATCTTGCGCGTGCCCCATCTCTTATCCCACCGTA
GTTTTTCAATATCACCCCGATGCCCGAATAATATATTTATGGGGTATTTAATCTTCTTCGAAAGGCTATCCCC
CTGATAAAGGCAGGTTGCACACGTGTTCCGCACCCGTACGCCGCTCTTCAATTACCCGAAAGTAATCTACCGC
TCGGCTTGCATGTGTTAGGCCTCCCGCTAGCGTTCTCCTGAGCCAGGATCA
>744
CTGCTGCCGCCCGTAGGAGTCTGGTCCGTGTCTCAGTACCAGTGTGGGGGATCACCCTCTCAGGCCCCCTAAA
GATCGTCGCCTTGGTGAGCCGTTACCTCACCAACTAGCTAATCTTGCGCGTGCCCATCTCTATCCACCGTAGT
TTTCAATATCACCCGATGCCGAATAATATATTATGGGGTATTAATCTTCTCGAAAGGCTATCCCCTGATAAAG
GCAGGTTGCACACGTGTTCCGCACCCGTACGCCGCTCTCAATTACCCGAAAGTAATCTACCGCTCGGCTTGCA
TGTGTTAGGCCTCCCGCTAGCGTTCTCCTGA
>745
CTGCTGCCGCCCGTAGGAGTCTGGTCCGTGTCTCAGTACCAGTGTGGGGGATCACCCTCTCAGGCCCCCTAAA
GATCGTCGCCTTGGTGAGCCGTTACCTCACCAACTAGCTAATCTTGCGCGTGCCCATCTCTATCCACCGTAGT
TTTCAATATCACCCGATGCCGAATAATATATTATGGGGTATTAATCTTCTTCGAAAGGCTATCCCCCTGATAA
AGGCAGGTTGCACACGTGTTCCGCACCCGTACGCCGCTCTCAATTACCCGAAAGTAATCTACCGCTCGGCTTG
CATGTGTTAGGCCTCCCGCTAGCGTTCATCCTGAGCCAGGATCAAACTCT
>746
CTGCTGCCGCCCGTAGGAGTCTGGTCCGTGTCTCAGTACCAGTGTGGGGGATCACCCTCTCAGGCCCCCTAAA
GATCGTCGCCTTGGTGAGCCGTTACCTCACCAACTAGCTAATCTTGCGCGTGCCCATCTCTATCCACCGTAGC
TTTCAATATCACCCGATGCCGAATAATATATTATGGGGTATTAATCTTTCTTCGAAAGGCTATCCCCCTGATA
AAGGCAGGTGTCACACGTGTTCCGCACCCGTACGCCGCTCTCAATTACCCGAAAGTAATCTACCGCTCGGCTT
GCATGTGTTAGGCCTCCCGCTAGCGTTCATCCTGAGCCAGGATCAAACTCT
>747
CTGCTGCCGCCCGTAGGAGTCTGGTCCGTGTCTCAGTACCAGTGTGGGGGATCACCCTCTCAGGCCCCCTAAA
GATCGTCGCCTTGGTGAGCCGTTACCTCACCAACTAGCTAATCTTGCGCGTGCCCATCTCTATCCACCGTAGT

TTTCAATATCACCCGATGCCGAATAATATATTATGGGGTATTAATCTTCTTCGAAAGGCTATCCCCCTGATAA
AGGCAGGTTGCACACGTGTTCCGCACCCGTACGCCGCTCTCAATTACCCGAAAGTAATCTACCGCTCGGCTTG
CATGTGTTAGGCCTCCCGCTAGCGTTCTCCTGAGCAGGATCCAACTCGT
>748
CTGCTGCCGCCCGTAGGAGTCTGGTCCGTGTCTCAGTACCAGTGTGGGGGATCACCCTCTCAGGCCCCCTAAA
GATCGTCGCCTTGGTGAGCCGTTACCTCACCAACTAGCTAATCTTGCGCGTGCCCATCTCTATCCACCGTAGT
TTTCAATATCACCCGATGCCGAATAATATATTATGGGGTATTAATCTTCTTCGAAGGCTATCCCCCTGATAAA
GGCAGGTTGCACACGTGTTCCGCACCCGTACGCCGCTCTCAATTACCCGAAAGTAATCTACCGCTCGGCTTGC
ATGCGTTAGGCCTCCCGCTAGCGTTCTCCTGAGCAGGTCCAAACTCGT
>749
CTGCTGCCGCCCGTAGGAGTCTGGTCCGTGTCTCAGTACCAGTGTGGGGGATCACCCTCTCAGGCCCCCTAAA
GATCGTCGCCTTGGTGAGCCGTTACCTCACCAACTAGCTAATCTTGCGTGTGCCCATCTCTATCCACCGTAGT
TTTCAATATCACCCGATGCCGAATAATATATTATGGGGTATTAATCTTCTTCGAAAGGCTATCCCCCTGATAA
AGGCAGGTTGCACACGTGTTCCGCACCCGTACGCCGCTCTCAATTACCCGAAAGTAATCTACCGCTCGGCTTG
CATGTGTTAGGCCTCCCGCTAGCGTTCATCCTGAGCCAGGATCAAACTCT
>750
CTGCTGCCGCCCGTAGGAGTCTGGTCCGTGTCTCAGTACCAGTGTGGGGGATCACCCTCTCAGGCCCCCTAAA
GATCGTCGCCTTGGTGAGCCGTTACCTCACCAACTAGCTAATCTTGCGCGTGCCCATCTCTATCCACCGTAGT
TTTCAATATCACCCGATGCCGAATAATATATTATGGGGTATTAATCTTCTTCGAAAGGCTATCCCCCTGATAA
AGGCAGGTTGCACACGTGTTCCGCACCCGTACGCCGCTCTCAATTACCCGAAAGTAATCTACCGCTCCGGCTT
GCATGTGTTAGGCCTCCCGCTAGCGTTCTCCTGAGCAGGATCCAACTCGT
>751
CTGCTGCCGCCCGTAGGAGTCTGGTCCGTGTCTCAGTACCAGTGTGGGGGATCACCCTCTCAGGCCCCCTAAA
GATCGTCGCCTTGGTGAGCCGTTACCTCACCAACTAGCTAATCTTGCGCGTGCCCATCTCTATCCACCGTAGT
TTTCAATATCACCCGATGCCGAATAATATATTATGGGGTATTAATCTTCTTCGAAGGCTATCCCCCTGATAAA
GGCAGGTTGCACACGTGTTCCGCACCCGTACGCCGCTCTCAATTACCCGAAAGTAATCTACCGCTCGGCTTGC
ATGTGTTAGGCCTCCCGCTAGCGTTCATCCTGAACCAGGATCAAACTCT
>752
CTGCTGCCGCCCGTAGGAGTCTGGACCGTGTCTCAGTTCCAGTGTGACTGATCATCCTCTCAGACCAGTACGG
ATCGTCGCCTTGGTAGGCCTTTACCCCACCAACTAGCTAATCCGACCTAGGCTCATCTGATAGCGTGAGGTCC
GAAGATCCCCCACTTCTCCCTCAGGACGTATGCGGTATTAGCGCCCGTTTCCGGACGTATCCCCACTACCAGG
CAGATTCCTAGGCATTACTCACCCGTCCGCCGCTGAATCCAGGAGCAAGCTCCCTTCATCCGCTCGACTGCCA
TGTGTTAGGCCTGCCGCCAAGCGTCAATCTGAGCCAGGATCAAACTCT
>753
CTGCTGCCGCCCGTAGGAGTCTGGTCCGTGTCTCAGTACCAGTGTGGGGGATCACCCTCTCAGGCCCCCTAAA
GATCGTCGCCTTGGTGAGCCGTTACCTCACCAACTAGCTAATCTTGCGCGTGCCCATCTCTATCCACCGTAGT
TTTCAATATCACCCGATGCCGAATAATATATTATGGGGTATTAATCTTCTTCGAAAGGCTATCCCCTGATAAA
GGCAGGTTGCACACGTGTTCCGCACCCGTACGCCGCTCTCAATTTACCCGAAAGTAATCTACCGCTCGGCTTG
CATGTGTTAGGCCTCCCGCTAGCGTTCTCCTGAGCAGG
>754
CTGCTGCCGCCCGTAGGAGTCTGGTCCGTGTCTCAGTACCAGTGTGGGGGATCACCCTCTCAGGCCCCCTAAA
GATCGTCGCCTTGGTGAGCCGTTACCTCACCAACTAGCTAATCTTGCGCGCGCCCATCTCTATCCACCGTAGT
TTTCAATATCACCCGATGCCGAATAATATATTATGGGGTATAATCTTCCCTTCGAAAGGCTATCCCCCTGATA
AAGGCAGGTTGCACACGTGTTCCGCACCCGTACGCCGCTCTCAATTACCCGAAAGTAATCTACCGCTCGGCTT
GCATGTGTTAGGCCTCCCGCTAGCGTTCATCCTGAGCCAGGATCAAACTCT
>755
CTGCTGCCGCCCGTAGGAGTCTGGTCCGTGTCTCAGTACCAGTGTGGGGGATCACCCTCTCAGGCCCCCTAAA
AGATCGTCGCCTTGGTGAGCCGTTACCTCACCAACTAGCTAATCTTGCGCGTGCCCATCTCTATCCACCGTAG
TTTCAATATCACCCGATGCCGAATAATATATTATGGGGTATTAATCTTCTTCGAAAGGCTATCCCCCTGATAA
AGGCAGGTTGCACACGTGTTCCGCACCCGTACGCCGCTCTCAATTACCCGAAAGTAATCTACCGCTCGGCTTG
CATGTGTTAGGCCTCCCGCTAGCGTTCATCCTGAGCCAGGATCAACTCT
>756
CTGCTGCCGCCCGTAGGAGTCTGGTCCGTGTCTCAGTACCAGTGTGGGGGATCACCCTCTCAGGCCCCCTAAA
GATCGTCGCCTTGGTGAGCCGTTACCTCACCAACTAGCTAATCTTGCGCGTGCCCATCTCTATCCACCGTAGT

TTCAATATCACCCGATGCCGAATAATATATTATGGGGTATTAATCTTCTTCGAAAGGCTATCCCCTGATAAAG
GCAGGTTGCACACGTGTTCCGCACCCGTACGCCGCTCTCAATTACCCGAAAGTAATCTACCGCTCGGCTTGCA
TGTGTTAGGCCTCCCGCTAGCGTTCTCCTGAGCAGATCAACT
>757
CTGCTGCCGCCCGTAGGAGTCTGGTCCGTGTCTCAGTACCAGTGTGGGGGATCACCCTCTCAGGCCCCCTAAA
GATCGTCGCCTTGGTGAGCCGTTACCTCACCAACTAGCTAATCTTGCGCGTGCCCATCTCTATCCACCGTAGT
TTTCAATATCACCCGATGCCGAATAATATATTATGGGGTATTAATCTTCTCGAAAGGCTATCCCCCTGATAAA
GGCAGGTTGCACACGTGTTCCGCACCCGTACGCCGCTCTCAATTACCCGAAAGTAATCTACCGCTCCGGCTTG
CATGTGTTAGGCCTCCCGCTAGCGTCATCCTGAGCAGGATCAACTCT
>758
CTGCTGCCGCCCGTAGGAGTCTGGTCCGTGTCTCAGTACCAGTGTGGGGGATCACCCTCTCAGGCCCCCTAAA
GATCGTCGCCTTGGTGAGCCGTTACCTCACCAACTAGCTAATCTTGCGCGTGCCCATCTCTATCCACCGTAGT
TTTCAATATCACCCGATGCCGAATAATATATTATGGGGTATTAATCTTCTTCGAAAGGCTATCCCCCTGATAA
AGGCAGGTTGCACACGTGTTCCGCACCCGTACGCCGCTCTCAATTACCCGAAAGTAATCTACCGCTCGGCTTG
CATGTGTTAGGCCTCCCGCTAGCGTTCATCCTGAGCCAGGATCAACTCT
>759
CTGCTGCCGCCCGTAGGAGTCTGGTTCCGTGTCTCAGTACCAGTGTGGGGGATCACCCTCTCAGGCCCCCTAA
AGATCGTCGCCTTGGTGAGCCGTTACCTCACCAACTAGCTAATCTTGCGCGTGCCCATCTCTTATCCACCGTA
GTTTTTCAATATCACCCGATTGCCGAATAATATATTTATGGGGTATTTAATCTTTCTTCGAAAGGCTATCCCC
CTGATAAAGGCAGGTTGCACACGTGTTCCGCACCCGTACGCCGCTCTCAATTACCCGAAAGTAATCTACCGCT
CGGCCTGCATGTGTTTAGGCCTTCCCGCTAGCGTTCATCCTGAGCCAGGATCAAACTCT
>760
CTGCTGCCGCCCGTAGGAGTCTGGACCGTGTCTCAGTTCCAGTGTGACTTGATCATTCCTCTCAGACCAGTTA
CGGATCGTCGCCTTGGTAGGCCTTTACCCCACCAACTAGCTAATCCGACCTAGGCTCATCTGATAGCGTGAGG
TCCGAAGATCCCCCACTTCTCCCTCAGGACGTATGCGGTATTAGCGCCCGTTTCCGGACGTATCCCCACTACC
AGGCAGATTTCCTGGCATTACTCACCCGTCCGCCGCTGAATCCAGGAGCAAGCCCCCTTCATCCGCTCGACTG
CATGTGTTAGGCCTGCCGCCAGCGTTCAATCTGAGCCAGGATCAAACTCT
>761
CTGCTGCCGCCCGTAGGAGTCTGGTCCGTGTCTCAGTACCAGTGTGGGGGATCACCCTCTCAGGCCCCCTAAA
GATCGTCGCCTTGGTGAGCCGTTACCTCACCAACTAGCTTAATCTGCGCGTGCCCATCTCTATCCACCGTAGT
TTTCAATATCACCCGATGCCGAATAATATATTATGGGGTATTTAATCTTTCTCGAAAGGCTATCCCCTGATAA
AGGCAGGTTGTCACACGTGTTCCGCACCCGTACGCCGCTCTCAATTACCCGAAAGTAATCTACCGCTCGGCTT
GCATGTGTTAGGCCTCCCGCTAGCGTTCATCC
>762
CTGCTGCCGCCCGTAGGAGTCTGGTCCGTGTCTCAGTACCAGTGTGGGGGGATCGCCCTCTCAGGCCCCCTAA
AGATCGTCGCCTTGGTGAGCCGTTACCTCACCAACTAGCTAATCTTGCGCGTGCCCATCTCTATCCACCGTAG
TTTTCAATATCACCCGATGCCGAATAATATATTATGGGGTATTTAATCTTCCTTTCGAAGGGCTATCCCCCTG
ATAAAGGCAGGTTGCACACGTGTTCCGCACCCGTACGCCGCTCTCAATTACCCGAAAGTAATCTACCGCTCGG
CTTGCATGTGTTAGGCCTCCCGCTAGCGTTCATCCTGAGCCAGGATCGAAACTCT
>763
CTGCTGCCGCCCGTAGGAGTCTGGTCCGTGTCTCAGTACCAGTGTGGGGGATCACCCTCTCAGGCCCCCTAAA
GATCGTCGCCTTGGTGAGCCGTTACCTCACCAACTAGCTAATCTTGCGCGTGCCCATCTCTATCCACCGTAGT
TTTCAATATCACCCGATGCCGAATAATATATTATGGGGTATTAATTCTTCTTCGAAGGCTATCCCCCTGATAA
AGGCAGGTTGCACACGTGTTCCGCACCCGTACGCCGCTCTCAATTACCCCGAAAGTAATCTACCGCTCGGCTT
GCATGTGTTAGGCCTCCCGCTAGCGTTCTCCTGAGC
>764
CTGCTGCCGCCCGTAGGAGTCTGGTCCGTGTCTCAGTACCAGTGTGGGGGATCACCCTCTCAGGCCCCCTAAA
GATCGTCGCCTTGGTGAGCCGTTACCTCACCAACTAGCTAATCTTGCGCGTGCCCATCTCTATCCACCGTAGT
TTTCAATATCACCCGATGCCGAATAATTATATTATGGGGTATTAATCTTCTTCGAAAGGCTATCCCCCTGATA
AAGGCAGGTTGCACACGTGTTCCGCACCCGTACGCCGCTCTCAATTACCCGAAAGTAATCTACCGCTCGGCTT
GCACGTGTTAGGCCTCCCGCTAGCGTTCTCCTGAGCAGGATCCAACTCG
>765
CTGCTGCCGCCCGTAGGAGTCTGGTCCGTGTCTCAGTACCAGTGTGGGGGATCACCCTCTCAGGCCCCCTAAA
GATCGTCGCCTTGGTGAGCCGTTACCTCACCAACTAGCTAATCTTGCGCGTGCCCATCTCTATCCACCGTAGT

TTTCAATATCACCCGATGCCGAATAATATATTATGGGGTATTAATCTTCTTCGAAAGGCTATCCCCCTGATAA
AGGCAGGTTGCACACGTGTTCCGCACCCGTACGCCGCTCTCAATTACCCGAAAGTAATCCACCGCTCGGCTTG
CATGTGTTAGGCCTCCCGCTAGCGTTCATCCTGAGCCAGGATCAAACTCT
>766
CTGCTGCCGCCCGTAGGAGTCTGGTCCGTGTCTCAGTACCAGTGTGGGGGATCACCCTCTCAGGCCCCCTAAA
GATCGTCGCCTTGGTGAGCCGTTACCTCACCAACTAGCTAATCTTGCGCGTGCCCATCTCTATCCACCGTAGT
TTTCAATATCACCCGATGCCGAATAATATATTATGGGGGTATTAATCTTCTTCGAAAGGCTATCCCCCTGATA
AAGGCAGGTTGCACACGTGTTCCGCACCCGTACGCCGCTCTCAATTACCCGAAAGTAATCTACCGCTCGGCTT
GCATGTGTTAGGCCTCCCGCTAGCGTTCTCCTGAGCAGGATCAACTCGT
>767
CTGCTGCCGCCTGTAGGAGTCTGGTCCGTGTCTCAGTACCAGTGTGGGGGATCACCCTCTCAGGCCCCCTAAA
GATCGTCGCCTTGGTGAGCCGTTACCTCACCAACTAGCTAATCTTGCGCGTGCCCATCTCTATCCACCGTAGT
TTTCAATATCACCCGATGCCGAATAATATATTATGGGGTATTAATCTTCTTCGAAAGGCTATCCCCCTGATAA
AGGCAGGTTGCACACGTGTTCCGCACCCGTACGCCGCTCTCAATTACCCGAAAGTAATCTACCGCTCGGCTTG
CATGTGTTAGGCCTCCCGCTAGCGTTCTCCTGAGCAAGG
>768
CTGCTGCCGCCCGTAGGAGTCTGGTCCGTGTCTCAGTACCAGTGTGGGGGATCACCCTCTCAGGCCCCCTAAA
GATCGTCGCCTTGGTGAGCCGTTACCTCACCAACTAGCTAATCTTGCGCGTGCCCATCTCTATCCACCGTAGT
TTTCAATATCACCCGATGCCGAATAATATATTATGGGGTATTAATCTTCTTCGAAAGGCTATCCCCCTGATAA
AGGCAGGTTGCACACGTGTTCCGCACCCGTACGCCGCTCTCAATTACCCGAAAGTAATCTACCGCTCGGCTTG
CATGTGTTAGGCCTCCCGCTAGCGTTCATCCTGAGCCAGGATCAAACTCT
>769
CTGCTGCCGCCCGTAGGAGTCTGGTCCGTGTCTCAGTACCAGTGTGGGGGATCACCCTCTCAGGCCCCCTAAA
AGATCGTCGCCTTGGTGAGCCGTTACCTCACCAACTAGCTAATCTTGCGCGTGCCCATCTCTATCCACCGTAG
TTTTCAATATCACCCGATGCCGAATAATATATTATGGGGTATTAATCTTCTTCGAAAGGCTATCCCCCTGATA
AAGGCAGGTTGCACACGTGTTCCGCACCCGTACGCCGCTCTCAATTACCCGAAAGTAATCTACCGCTCGGCTT
GCATGTGTTAGGCCTCCCGCTAGCGTTCATCCTGAGCCAGGATCAAACTCT
>770
CTGCTGCCGCCCGTAGGAGTCTGGTCCGTGTCTCAGTACCAGTGTGGGGGATCACCCTCTCAGGCCCCCTAAA
GATCGTCGCCTTGGTGAGCCGTTACCTCACCAACTAGCTAATCTTGCGCGTGCCCATCTCTATCCACCGTAGT
TTTCAATATCACCCGATGCCGAATAATATATTATGGGGGTATTAATCTTCTTCGAAAGGCTATCCCCCTGATA
AAGGCAGGTTGCACACGTGTTCCGCACCCGTACGCCGCTCTCAATTACCCGAAAGTAATCTACCGCTCGGCTT
GCATGTGTTAGGCCTCCCGCTAGCGTTCATCCTGAGCCAGGATCAAACTCT
>771
CTGCTGCCGCCCGTAGGAGTCTGGTCCGTGTCTCAGTACCAGTGTGGGGGATCACCCTCTCAGGCCCCCTAAA
GATCGTCGCCTTGGTGAGCCGTTACCTCACCAACTAGCTAATCTTGCGCGTGCCCATCTCTATCCACCGTAGT
TTTCAATATCACCCGATGCCGAATAATATATTATGGGGGTATTAATCTTCTTCGAAAGGCTATCCCCCTGATA
AAGGCAGGTTGCACACGTGTTCCGCACCCGTACGCCGCTCTCAATTACCCGAAAGTAATCTACCGCTCGGCTT
GCATGTGTTAGGCCTCCCGCTAGCGTTCATCCTGAGCCAGGATCAAACTCT
>772
CTGCTGCCGCCCGTAGGAGTCTGGACCGTGTCTCAGTTCCAGTGTGACTGATCATCCTCTCAGACCAGTACGG
ATCGTCGCCTTGGTAGGCCTTTACCCCACCAACTAGCTAATCCGACCTAGGCTCATCTGATAGCGTGAGGTC
CGAAGTCCCCCACTTCTCCCTCAGGACGTATGCGGTATTAGCGCCCGTTTCCGGACGTATCCCCCACTACCAG
GCAGATTCCTAGGCATTACTCACCCGTCCGCCGCTGAATCCAGGAGCAAGCCCCTCCATCCGCTCGACTGCAT
GTGTTAGGCCTGCCGCCAGCGTCAATCTGAGCCAGGATCAAACTCT
>773
CTGCTGCCGCCCGTAGGAGTCTGGTCCGTGTCTCAGTACCAGTGTGGGGGATCACCCTCTCAGGCCCCCTAAA
GATCGTCGCCTTGGTGAGCCGTTACCTCACCAACTAGCTAATCTTGCGCGTGCCCATCTCTATCCACCGTAGT
TTTCAATATCACCCGATGCCGAATAATATATTATGGGGTATTAATCTTCTCGAAAGGCTATCCCCCTGATAAA
GGCAGGTTGCACACGTGTTCCGCACCCGTACGCCGCTCTCAATTACCCGAAAGTAATCTACCGCTCGGCTTGC
ATGTGTTAGGCCTCCCGCTAGCGTTCTCCTGAGCAGGATCA
>774
CTGCTGCCGCCCGTAGGAGTCTGGTCCGTGTCTCAGTACCAGTGTGGGGGATCACCCTCTCAGGCCCCCTAAA
GATCGTCGCCTTGGTGAGCCGTTACCTCACCAACTAGCTAATCTTGCGCGTGCCCATCTCTATCCACCGTAGT

TTTCAATATCACCCGATGCCGAATAATATATTATGGGGTATTAATCTTCTTCGAAAGGCTATCCCCCTGATAA
AGGCAGGTTGCACACGTGTTCCGCACCCGTACGCCGCTCTCAATTACCCGAAAGTAATCTACCGCTCGGCTTG
CATGTGTTAGGCCTCCCGCTAGCGTTCTCCTGAGCAGGT
>775
CTGCTGCCGCCCGTAGGAGTCTGGTCCGTGTCTCAGTACCAGTGTGGGGGATCACCCTCTCAGGCCCCCTAAA
GATCGTCGCCTTGGTGAGCTGTTACCTCACCAACTAGCTAATCTTGCGCGTGCCCATCTCTATCCACCGTAGT
TTTCAATATCACCCGATGCCGAATAATATATTATGGGGTATTAATCTTCTCGAAAGGCTATCCCCCTGATAAA
GGCAGGTTGCACACGTGTTCCGCACCCGTACGCCGCTCTCAATTACCCGAAAGTAATCTACCGCTCGGCTTGC
ATGTGTTAGGCCTCCCGCTAGCGTTCATCCTGAGCCAGGATCAAACTCT
>776
CTCTGCTGCCGCCCGTAGGAGTCTGGTCCGTGTCTCAGTACCAGTGTGGGGGATCACCCTCTCAGGCCCCCTA
AAGATCGTCGCCTTGGTGAGCTGTTACCTCACCAACTAGCTAATCTTGCGCGTGCCCATCTCTATCCACCGTA
GTTTTCAATATCACCCGATGCCGAATAATATATTATGGGGTATTAATCTTCTTCGAAAGGCTATCCCCCTGAT
AAAGGCAGGTTGCACACGTGTTCCGCACCCGTACGCCGCTCTCAATTTACCCGAAAGTAATCTACCGCTCGGC
TTGCATGTGTTAGGCCTCCCGCTAGCGTTCTCCTGAGCAGGATCA
>777
CTGCTGCCGCCCGTAGGAGTCTGGTCCGTGTCTCAGTACCAGTGTGGGGGATCACCCTCTCAGGCCCCCTAAA
GATCGTCGCCTTGGTGAGCCGTTACCTCACCAACTAGCTAATCTTGCGCGTGCCCATCTCTATCCACCGTAGT
TTTCAATATCACCCGGTGCCGAATAATATATTATGGGGTATTAATCTTCTTCGAAAGGCTTATCCCCCTGATA
AAGGCAGGTTGCACACGTGTTCCGCACCCGTACGCCGCTCTCAATTACCCGAAAGTAATCTACCGCTCGGCTT
GCATGTGTTAGGCCTCCCGCTAGCGTTCATCCTGAGCCAGGATCAAACTCCT
>778
CTGCTGCCGCCCGTAGGAGTCTGGTCCGTGTCTCAGTACCAGTGTGGGGGATCACCCTCTCAGGCCCCCTAAA
GATCGTCGCCTTGGTGAGCCGTTACCTCACCAACTAGCTAATCTTGCGCGTGCCCATCTCTATCCACCGTAGT
TTCAATATCACCCGATGCCGAATAATATATTATGGGGTATTAATTCTTCTTCTGAAAGGCTATCCCCCTGATA
AAGGCAGGTTGCACACGTGTTCCGCACCCGTACGCCGCTCTCAATTACCCGAAAGTAATCTACCGCTCCGGCT
TGCATGTGTTAGGCCTCCCGCTAGCGTTCATCCTGAGCCAGGATCAAACTCT
>779
CTGCTGCCGCCCGTAGGAGTCTGGTCCGTGTCTCAGTACCAGTGTGGGGGATCACCCTCTCAGGCCCCCTAAA
GATCGTCGCCTTGGTGAGCCGTTACCTCACCAACTAGCTAATCTTGCGCGTGCCCATCTCTATCCACCGTAGT
TTTCAATATCACCCGATGCCGAATAATATATTATGGGGTATTAATCTTCTTCGAAAGGCTATCCCCTGATAAA
GGCAGGTTGCACACGTGTTCCGCACCCGTACGCCGCTCTCAATTACCCGAAAGTAATCTACCGCTCGGCTTGC
ATGTGTTAGGCCTCCCGCTAGCGTTCTCCTGAGCAGGATCAACTC
>780
CTGCTGCCGCCCGTAGGAGTCTGGTCCGTGTCTCAGTACCAGTGTGGGGGATCACCCTCTCAGGCCCCCTAAA
GATCGTCGCCTTGGTGAGCCTTACCTCACCAACTAGCTAATCTTGCGCGTGCCCATCTCTATCCACCGTAGTT
TTCAATATCACCCGATGCCGAATAATATATTATGGGGTATTAATCTTCTTCGAAAGGCTATCCCCTGATAAAG
GCAGGTTGCACACGTGTTCCGCACCCGTACGCCGCTCTCAATTACCCGAAAGTAATCTACCGCTCGGCTTGCA
TGTGTTAGGCCTCCCGCTAGCGTTCATCCTGAGCCAGGATCAAACTCT
>781
CTGCTGCCGCCCGTAGGAGTCTGGTCCGTGTCTCAGTACCAGTGTGGGGGATCACCCTCTCAGGCCCCCTAAA
GATCGTCGCCTTGGTGAGCCGTTACCTCACCAACTAGCTAATCTTGCGCGTGCCCATCTCTATCCACCGTAGT
TTTCAATATCACCCGATGCCGAATAATATATTATGGGGTATTAATCTTCTTCGAAAGGCTATCCCCCTGATAA
AGGCAGGTTGCACACGTGTTCCGCACCCGTACGCCGCTCTCAATTTACCCGAAAGTAATCTACCGCTCGGCTT
GCATGTGTTAGGCCTCCCGCTAGCGTTCTCCTGAGCAGGATCAA
>782
CTGCTGCCGCCCGTAGGAGTCTGGTCCGTGTCTCAGTACCAGTGTGGGGGATCACCCTCTCAGGCCCCCTAAA
AGATCGTCGCCTTGGTGAGCCGTTACCTCACCAACTAGCTAATCTTGCGCGTGCCCATCTCTATCCACCGTAG
TTTTCAATATCACCCGATGCCGAATAATATATTATGGGGTATTAATCTTCTTCGAAAGGCTATCCCCCTGATA
AAGGCAGGTTGCACACGTGTTCCGCACCCGTACGCCGCTCTCAATTACCCGAAAGTAATCTACCGCTCGGCTT
GCATGTGTTAGGCCTCCCGCTAGCGTTCATCCTGAGCCAGGATCAAACTCT
>783
CTGCTGCCGCCCGTAGGAGTCTGGTCCGTGTCTCAGTACCAGTGTGGGGGATCACCCTCTCAGGCCCCCTAAA
GATCGTCGCCTTGGTGAGCCGTTACCTCACCAACTAGCTTAATCTTGCGCGTGCCCATCTCTATCCACCGTAG

TTTTCAATATCACCCGATGCCGAATAATATATTATGGGGTATTAATCTTCTTCGAAAGGCTATCCCCCTGATT
AAAGGCAGGTTGCCACACGTGTTCCGCACCCGTACGCCGCTCTCAATTACCCGAAAGTAATCTACCGCTCGGC
TTGCATGTGTCGCTACGGCCTC
>784
CTGCTGCCGCCCGTAGGAGTCTGGTCCGTGTCTCAGTACCAGTGTGGGGGATCACCCTCTCAGGCCCCCTAAA
GATCGTCGCCTTGGTGAGCCGTTACCTCACCAACTAGCTAATCTTGCGCGTGCCCATCTCTATCCACCGTAGT
TTTCAATATCACCCGATGCCGAATAATATATTATGGGGTATTAATCTTCCTTCGAAAGGCTATCCCCCTGATA
AAGGCAGGTTGCACACGTGTTCCGCACCCGTACGCCGCTCTCAATTACCCGAAAGTAATCTACCGCTCGGCTT
GCATGTGTTAGGCCTCCCGCTAGCGTTCATCCTGAGCCAGGATCAAACTCT
>785
CTGCTGCCGCCCCGTAGGAGTCTGGACCCGTGTCTCAGTTCCAGTGTGACTGATCATCCTCTCAGACCAGTAC
GGATCGTCGCCTTGGTAGGCCTTTACCCACCAACTAGCTAATCCGAACCTAGGCTCATCTGATAGCGTGAGGT
CCGAAGTCCCCCACTCTCCCCTCAGGACGTATGCGGTATTAGCGCCCGTTTCCGGACGATCCCCACTACCAGG
CAGATTCCTAGGCATTACTCACCCGTCCGCCGCTGAATCCAGGAGCAAGCTCCCCTCATCGCTCGACTGCATG
TGTTAGGCCTGCCGCCAGCGTCATCTGAGCCAG
>786
CTGCTGCCGCCCGTAGGAGTCTGGACCGTGTCTCAGTTCCAGTGTGACTGATCATCCTCTCAGACCAGTACGG
ATCGTCGCCTTGGTAGGCCTTACCCCACCAACTAGCTAATCCGACCTAGGCTCATCTGATAGCGTGAGGTCCG
AAGTCCCCCACTTCTCCCTCAGGACGTATGCGGTATTAGCGCCCGTTTCCGGACGATCCCCACTACCAGGCAG
ATTCCTCAGGCATTACTCACCCGTCCGCCGCTGAATCCAGGAGCAAGCTCCCTCATCCGCTCGACTGCCATGT
GTTAGGCCTGCCGCCAGCGTCAATCTGAGCCAGGATCAAACTCT
>787
CTGCTGCCGCCCGTAGGAGTCTGGTCCGTGTCTCAGTACCAGTGTGGGGGATCACCCTCTCAGGCCCCCTAAA
GATCGTCGCCTTGGTGAGCCGTTACCTCACCAACTAGCTAATCTTGCGCGTGCCCATCTCTATCCACCGTAGT
TTTCAATATCACCCGATGCCGAATAATATATTATGGGGTATTAATCTTCTTCGAAAGGCTATCCCCCTGATAA
AGGCAGGTTGCACACGTGTTCCGCACCCGTACGCCGCTCTCAATTACCCGAAAGTAATCTACCGCTCGGCTTG
CATGTGTTAGGCCTCCCGCTAGCGTTCTCCTGAGCAGGATCAACTCGT
>788
CTGCTGCCGCCCGTAGGAGTCTGGTCCGTGTCTCAGTACCAGTGTGGGGGATCACCCTCTCAGGCCCCCTAAA
GATCGTCGCCTTGGTGAGCCGTTACCTCACCAACTAGCTAATCTTGCGCGTGCCCATCTCTATCCACCGTAGT
TTTCAATATCACCCGATGCCGAATAATATATTATGGGGTATTAATCTTCTTCGAAAGGCTATCCCCCTGATAA
AGGCAGGTTGCACACGTGTTCCGCACCCGTACGCCGCTCTCAATTACCCGAAAGTAATCTACCGCTCGGCCTG
CATGTGTTAGGCCTCCCGCTAGCGTTCATCCTGAGCCAGGATCAACTCT
>789
CTGCTGCCGCCCGTAGGAGTCTGGTCCGTGTCTCAGTACCAGTGTGGGGGATCACCCTCTCAGGCCCCCTAAA
GATCGTCGCCTTGGTGAGCCGTTACCTCACCAACTAGCTAATCTTGCGCGTGCCCATCTCTATCCACCGTAGT
TTCAATATCACCCGATGCCGAATAATATATTATGGGGTATTAATCTTCTTCGAAAGCTATCCCCTGATAAAGG
CAGGTTGCACACGTGTTCCGCACCCGTACGCCGCTCTCAATTACCCGAAAGTAATCTACCGCTCCGGCTTGCA
TGTGTTAGGCCTCCCGCTAGCGTT
>790
CTGCTGCCGCCCGTAGGAGTCTGGTCCGTGTCTCAGTACCAGTGTGGGGGATCACCCTCTCAGGCCCCCTAAA
GATCGTCGCCTTGGTGAGCCGTTACCTCACCAACTAGCTAATCTTGCAGTCGTGCCCATCTCTATCCACCGTA
GTTTTCAATATCACCCGATGCCGAATAATATAATTATGGGGTATTAATCTTCTTCGAAAGGCTATCCCCCTGA
TAAAGGCAGGTTGCACACGTGTTCCGCACCCGTACGCCGCTCTCAATTACCCGAAAGTAATCTACCGCTCGGC
TTGCATGTGTTAGGCCTCCCGCTAGCGTTCATCCTGAGCCAGGATCAAACTCT
>791
CTGCTGCCGCCCGTAGGAGTCTGGTCCGTGTCTCAGTACCAGTGTGGGGGATCACCCTCTCAGGCCCCCTAAA
GATCGTCGCCTTGGTGAGCCGTTACCTCACCAACTAGCTAATCTTGCGCGTGCCCATCTCTATCCACCGTAGT
TTTCAATATCACCCGATGCCGAATAATATATTATGGGGTATTAATCTTCCTTCGAAAGGCTATCCCCCTGATA
AAGGCAGGTTGCACACGTGTTCCGCACCCGTACGCCGCTTTCAATTACCCGAAAGTAATCTACCGCTCGGCTT
GCATGTGTTAGGCCTCCCGCTAGCGTTCATCCTGAGCCAGGATCAAACTCT
>792
CTGCTGCCGCCCGTAGGAGTCTGGTCCGTGTCTCAGTACCAGTGTGGGGGATCACCCTCTCAGGCCCCCTAAA
GATCGTCGCCTTGGTGAGCCGTTACCTCACCAACTAGCTAATCTTGCGCGTGCCCATCTCTATCCACCGTAGT

TTTCAATATCACCCGATGCCGAATAATATATTATGGGGTATTAATCTTCTTCGAAAGGCTATCCCCCTGATAA
AGGCAGGTTGCACACGTGTTCCGCACCCGTACGCCGCTCTCAATTACCCGAAAGTAATCTACCGCATCGGCTT
GCATGTGTTAGGCCTCCCGCTAGCGTTCTCCTGAGCAGGATCAACTCGT
>793
CTGCTGCCGCCCGTAGGAGTCTGGTCCGTGTCTCAGTACCAGTGTGGGGGATCACCCTCTCAGGCCCCCTAAA
GATCGTCGCCTTGGTGAGCCGTTACCTCACCAACTAGCTAATCTTGCGCGTGCCCATCTCTATCCACCGTAGT
TTTCAATATCACCCGATGCCGAATAATATATTATGGGGTATTAATCTTCTTCGAAAGGCTATCCCCTGATAAA
GGCAGGTTGCACACGTGTTCCGCACCCGTACGCCGCTCTCAATTACCCGAAAGTAATCTACCGCTCCGGCTTG
CATGTGTTAGGCCTCCCGCTAGCGTTCATCCTGAGCCAGGATCAAACTCT
>794
CTGCTGCCGCCCGTAGGAGTCTGGTCCGTGTCTCAGTACCAGTGTGGGGGATCACCCTCTCAGGCCCCCTAAA
GATCGTCGCCTTGGTGAGCCGTTACCTCACCAACTAGCTAATCTTGCGCGTGCCCATCTCTATCCACCGTAGT
TTTCAATATCACCCGATGCCGAATAATATATTATGGGGTATTAATCTTCTTCGAAAGGCTATCCCCCTGATAA
AGGCAGGTTGCACACGTGTTCCGCACCCGTACGCCGCTCTCAATTACCCGAAAGTAATCTACCGCTCAGCTTG
CATGTGTTAGGCCTCCCGCTAGCGTTCATCCTG
>795
CTGCTGCCGCCCGTAGGAGTCTGGTCCGTGTCTCAGTACCAGTGTGGGGATCACCCTCTCAGGCCCCCTAAAG
ATCGTCGCCTTGGTGAGCCGTTACCTCACCAACTAGCTAATCTTGCGCGTGCCCATCTCTATCCACCGTAGTT
TTCAATATCACCGATGCCGAATAATATATTATGGGGTATTAATCTTCTTCGAAAGGCTATCCCCCTGAATAAA
GGTCAGGTTGCAACACGTGTTCCGCACACGTACGCCGCTCTCAATTACCCGAAAGTAATCTACCGCTACGGCT
TGCATGTGTTAGGCCTCCCGCTAGCGTCATCCTGAGCCAGGATCAAACTCT
>796
CTGCTGCCGCCCGTAGGAGTCTGGTCCGTGTCTCAGTACCAGTGTGGGGGATCACCCTCTCAGGCCCCCTAAA
GATCGTCGCCTTGGTGAGCCGTTACCTCACCAACTAGCTAATCTTGCGCGTGCCCATCTCTATCCACCGTAGT
TTTCAATATCACCCGATGCCGAATAATATATTATGGGGTATTAATCTTCTCGAAAGGCTATCCCCTGATAAAG
GCAGGTTGCACACGTGTTCCGCACCCGTACGCCGCTCTCAATTACCCGAAAGTAATCTACCGCTCGGCTTGCA
TGTGTTAGGCCTCCCGCTAGCGTTCATCCTGAGCCAGGATCAAACTCT
>797
CTGCTGCCGCCCGTAGGAGTCTGGACCGTGTCTCAGTTCCAGTGTGACTGATCATCCTCTCAGACCAGTACGG
ATCGTCGCCTTGGTAGGCCTTTACCCCACCAACTAGCTAATCCGACCTAGGCTCATCTGATAGCGTGAGGTCC
GAAGATCCCCCACTTCTCCCTCGGGACGTATGCGGTATTAGCGCCCATTTCCGGACGTATCCCCCACTACCAG
GCAGATTCCCTAGGCATTACTCACCCGTCCGCCGCTGAATCCAGGAGCAAGCTCCCTTCATCCGCTCGACTGC
ATGTGTTAGGCCTGCCGCCAGCGTTCAATCTGAGCCAGGATCAACTCT
>798
CTGCTGCCGCCCGTAGGAGTCTGGACCGTGTCTCAGTTCCAGTGTGACTGATCATCCTCTCAGACCAGTACGG
ATCGTCGCCTTGGTAGGCCTTTACCCCACCAACTAGCTAATCCGACCTAGGCTCATCTGATAGCGTGAGGTCC
GAAGTCCCCCACTTCTCCCTCAGGACGTATGCGGTATTAGCGCCCGTTTCCGGACGTATCCCCGCACTACCCA
GGCAGATTCCTAGGCATTACTCACCCGTCCGCCGCTGAATCCAGGAGCAAGCTCCCTTCATCCGCTCGACTGC
ATGTGTTAGGCCTGCCGCCAGCGTTCAATCTGAGCCAGGATCAAACTCT
>799
CTGCTGCCGCCCGTAGGAGTCTGGTCCGTGTCTCAGTACCAGTGTGGGGGATCACCCTCTCAGGCCCCCTAAA
GATCGTCGCCTTGGTGAGCCGTTACCTCACCAACTAGCTAATCTTGCGCGTGCCCATCTCTATCCACCGTAGT
TTTCAATATCACCCGATGCCGAATAATATATTATGGGGTATTAATCTTCTTCGAAAGGCTATCCCCTGATAAA
GGCAGGTTGCACACGTGTTCCGCACCCGTACGCCGCTCTCAATTACCCGAAAGTAATCTACCGCTCGGCTTGC
ATGTGTTAGGCTCCCGCTAG
>800
CTGCTGCCGCCCGTAGGAGTCTGGTCCGTGTCTCAGTACCAGTGTGGGGGGATCACCCTCTCAGGCCCCCTAA
AGATCGTCGCCTTGGTGAGCCGTTACCTCACCAACTAGCTAATCTTGCGCGTGCCCCATCTCTATTCCACCGT
AGTTTTTCAATATCACCCGATGCCGAATAATATATTTATTGGGGTATTTAATTCTTTCTTCGAAAGGCTATCC
CCCTGATTAAAGGCAGGTTTGCACACGTGTTCCGCACCCGTACGCCGCTCTCAATTTACCCCGAAAGTAATCT
ACCGCTCGGCTTGCATGTGTTAGGCCTTCCCGCTAGCGTTCATCCTGAGCCAGGATCAAACTCT
>801
CTGCTGCCGCCCGTAGGAGTCTGGACCGTGTCTCAGTTCCAGTGTGACTGATCATCCTCTCAGACCAGTTACG
GATCGTCGCCTTGGTAGGCCTTTACCCCACCAACTAGCTAATCCGACCTAGGCTCATCTGATAGCGTGAGGTC

CGAAGATCCCCCACTTCTCCCTCAGGACGTATGCGGTATTAGCGCCCCGTTTCCGGACGTATCCCCACTACCA
GGCAGATTCCTAGGCATTACTCACCCGTCCGCCGCTGAATCCAGGAGCAAGCTCCCTTCATCCGCTCGACTGC
ATGTGTTAGGCCTGCCGCCAGCGTTCAATCTGAGCCAGGATCAAACTCT
>802
CTGCTGCCGCCCGTAGGAGTCTGGACTCGTGTCTCAGTTCCAGTGTGACTGATCATCCTCTCAGACCAGTACG
GATCGTCGCCTTGGTAGGCCTTTACCCCACCAACTAGCTAATCCGACCTAGGCTCATCTGATAGCGTGAGGTC
CGAAGATCCCCCACTTCTCCCTCAGGACGTATGCGGTATTAGCGCCCCGTTTCCGGACGATCCCCCACTACCA
GGCGATTCCTAGGCATTACTCACCCGTCCGCCGCTGAATCCAGGAGCAAGCTCCCTCATCGCTCGACTGCCAT
GTGTTAGGCCTGCCGCCAGCGTCATCCTGAGCCAGGATCAAACTCT
>803
CTGCTGCCGCCCGTAGGAGCTGGTCCGTGTCTCAGTACCAGTGTGGGGGATCACCCTCTCAGGCCCCCTAAAG
ATCGTCGCCTTGGTGAGCCGTTACCTCACCAACTAGCTAATCTTGCGCGTGCCCATCTCTATCCACCGTAGTT
TTCAATATCACCCGATGCCGAATAATATATTATGGGGTATTAATCTTTCTTCGAAAGGCTATCCCCCTGATAA
AAGGCAGGTTGCACACGTGTTCCGCACCCGTACGCCGCTCTCAATTACCCGAAAGTAATCTACCGCTCCGGCT
TGCATGTGTTAGGCCTCCCGCTAGCGTTCATTCCTGAGCCAGGATCAAACTCT
>804
CTGCTGCCGCCCGTAGGAGTCTGGTCCGTGTCTCAGTACCAGTGTGGGGGATCACCCTCTCAGGCCCCCTAAA
GATCGTCGCCTTGGTGAGCCGTTACCTCACCAACTAGCTAATCTTGCGCGTGCCCATCTCTATCCACCGTAGT
TTTCAATATCACCCGATGCCGAATAATATATTATGGGGTATTAATCTTCTTCGAAAGGCTATCCCCCTGATAA
AGGCAGGTTGCACACGTGTTCCGCACCCGTACGCCGCTCTCAATTACCCGAAAGTAATCTACCGCTCGGCTTG
CATGTGTTAGGCCTCCCGCTAGCGTTCATCCTGAGCCAGGATCAAACTCT
>805
CTGCTGCCGCCCGTAGGAGTCTGGTCCGTGTCTCAGTACCAGTGTGGGGGATCACCCTCTCAGGCCCCCTAAA
GATCGTCGCCTTGGTGAGCCGTTACCTCACCAACTAGCTAATCTTGCGCGTGCCCATCTCTATCCACCGTAGT
TTTCAATATCACCCGATGCCGAATAATATATTATGGGGTATTAATCTTCTTCGAAAGGCTATCCCCTGATAAA
GGCAGGTTGCACACGTGTTCCGCACCCGTACGCCGCTCTCAATTACCCGAAAGTAATCTACCGCTCGGCTTGC
ATGTGTTAGGCCTCCCGCTAGCGTTCATCCTGAGCCAGGATCAAACTCT
>806
CTGCTGCCGCCCGTAGGAGTCTGGTCCGTGTCTCAGTACCAGTGTGGGGGATCACCCTCTCAGGCCCCCTAAA
GATCGTCGCCTTGGTGAGCCGTTACCTCACCAACTAGCTAATCTTGCGCGTGCCCATCTCTATCCACCGTAGT
TTTCAATATCACCCGATGCCGAATAATATAATTATGGGGTATTAATCTTCTTCGAAGGCTATCCCCCTGAATA
AAGGCAGGTTGCACACGTGTTCCGCACCCGTACGCCGCTCTCAATTACCCGAAAGTAATCTACCGCTCGGCTT
GCATGTGTTAGGCCTCCCGCTAGCGTTCATCCTGAGCCAGGATCAAACTCT
>807
CTGCTGCCGCCCGTAGGAGTCTGGTCCGTGTCTCAGTACCAGTGTGGGGGATCACCCTCTCAGGCCCCCTAAA
GATCGTCGCCTTGGTGAGCCGTTACCTCACCAACTAGCTAATCTTGCGCGTGCCCATCTCTATCCACCGTAGT
TTTCAATATCACCCGATGCCGAATAATATATTATGGGGTATTAATCTTCTTCGAAAGGCTATCCCCTGATAAA
GGCAGGTTGCACACGTGTTCCGCACCCGTACGCCGCTCTCAATTACCCGAAAGTAGTCTACCGCTCGGCTTGC
ATGTGTTAGGCCTCCCGCTAGCGTTCTCCTCGAGCAAGGATCA
>808
CTGCTGCCGCCCGTAGGAGTCTGGACCGTGTCTCAGTTCCAGTGTGACTGATCATCCTCTCAGACCAGTACGG
ATCGTCGCCTTGGTAGGCCTTTACCCCACCAACTAGCTAATCCGACCTAGGCTCATCTGATAGCGTGAGGTCC
GAAGATCCCCCACTTCTCCCTCAGGACGTATGCGGTATTAGCGCCCGTTTCCGGACGTATCCCCACTACCAGG
CAGATTCCTAGGCATTACTCACCCGTCCGCCGCTGAATCCAGGAGCAAGCCTCCCTACTCCGCTCGACTGCAT
GTGTTAGGCCTGCCGCCAGCGTCAATCTGAGCCAGGATCAAACTCT
>809
CTGCTGCCGCCCGTAGGAGTCTGGTCCGTGTCTCAGTACCAGGTGTGGGGGATCACCCTCTCCAGGCCCCCTA
AAGATCGCCGCCTTGGTGAGCCGTTACCTCACCAACTAGCTAATCTTGCGCGTGCCCATCTCTATCCACCGTA
GTTTCAACATCACCCGATGCCGAATAATATATTATGGGGTATTAATCTTCCTTTCGAAGGGCTATCCCCCTGA
TAAAGGCAGGTTGCACACGTGTTCCGCACCCGTACGCCGCTCTCAATTACCCGAAAGTAATCTACCGCTCGGC
TTGCATGTGTTAGGCCTCCCGCTAGCGTTCATCCTGAGCCAGGATCAAACTCT
>810
CTGCTGCCGCCCGTAGGAGTCTGGTCCGTGTCTCAGTACCAGTGTGGGGGATCACCCTCTCAGGCCCCCTAAA
GATCGTCGCCTGGTGAGCCGTTACCTCACCAACTAGCTAATCTTGCGCGTGCCCATCTCTATCCACCGTAGTT

TTCAATATCACCCGATGCCGAATAATATATTATGGGGGTATTAATCTTCTCGAAAGGCTATCCCCTGATAAAG
GCAGGTTGCACACGTGTTCCGCACCCGTACGCCGCTCTCAATTACCCGAAAGTAATCTACCGCTCGGCTTGCA
TGTGTTAGGCCTCCCGCTAGCGTCATCTGAGCCAGGATCAACTCT
>811
CTGCTGCCGCCCGTAGGAGTCTGGTTCCGTGTCTCAGTACCAGTGTGGGGGATCACCCTCTCAGGCCCCCTAA
AGATCGTCGCCTTGGTGAGCCGTTACCTCACCAACTAGCTAATCTTGCGCGTGCCCATCTCTATCCACCGTAG
TTTTCAATATCACCCGATGCCGAATAATATATTATTGGGGTATTAATCTTCTTCGAAAGGCTATCCCCCTGAT
AAAGGCAGGTTGCACACGTGTTCCGCACCCGTACGCCGCTCTCAATTACCCGAAAGTAATCTACCGCTCGGCT
TGCATGTGTTAGGCCTCCCGCTAGCGTTCTCCTTGAGCAG
>812
CTGCTGCCGCCCGTAGGAGTCTGGACCGTGTCTCAGTTCCAGTGTGACTGATCATCTCTCAGACCAGTACGGA
TCGTCGCCTGGTAGGCCTTTACCCCACCAACTAGCTAATCCGACCCAGGCTCATCTGATAGCGTGAGGTCCGA
AGTCCCCCACTTCTCCCTCAGGACGTATGCGGTATTGCGCCCGTTTCCGGACGATCCCCCACTACCAGGCAGA
TTCCTAGGCATTACTCACCCGTCCGCCGCTGAATCCAGGAGCAAGCTCCCTTCATCCGCTCGACTGCATGTGT
TAGGCCTGCCGCCAGCGTCAATCTGAGCCAGGATCAAACTCT
>813
CTGCTGCCGCCCGTAGGAGTCTGGTCCGTGTCTCAGTACCAGTGTGGGGGATCACCCTCTCAGGCCCCCTAAA
GATCGTCGCCTTGGTGAGCCGTTACCTCACCAACTAGCTAATCTTGCGCGTGCCCATCTCTATCCACCGTAGT
TTTCAATATCACCCGATGCCGAATAATATATTATGGGGTATTAATCTTCTTCGAAAGGCTATCCCCCTGATAA
AGGCAGGTTGCACACGTGTTCCGCACCCGTACGCCGCTCTCAATTACCCGAAAGTAATCTACCGCTCGGCTTG
CATGTGTTAGGCCTCCCGCTAGCGTTCATCCTGAGCCAGGATCAACTCT
>814
CTGCTGCCGCCCGTAGGAGTCTGGTCCGTGTCTCAGTACCAGTGTGGGGGATCACCCTCTCAGGCCCCCTAAA
GATCGTCGCCTTGGTGAGCCGTTACCTCACCAACTAGCTAATCTTGCGCGTGCCCATCTCTATCCACCGTAGT
TTTCAATATCACCCGATGCCGAATAATATATTATGGGGGTATTAATCTTCTTCGAAAGGCTATCCCCCTGATA
AAGGCAGGTTGCACACGTGTTCCGCACCCGTACGCCGCTCTCAATTACCCGAAAGTAATCTACCGCTCGGCTT
GCATGTGTTAGGCCTCCCGCTAGCGTTCTCCTGAGCAGGATCAACTCT
>815
CTGCTGCCGCCCGTAGGAGTCTGGTCCGTGTCTCAGTACCAGTGTGGGGGATCACCCTCTCAGGCCCCCTAAA
GATCGTCGCCTTGGTGAGCCGTTACCTCACCAACTAGCTAATCTTGCGCGTGCCCATCTCTATCCACCGTAGT
TTTCAATATCACCCGATGCCGAATAATATATTATGGGGTATTAATCTTCTTCGAAAGGCTATCCCCCTGATAA
AGGCAGGTTGCACACGTGTTCCGCACCCGTACGCCGCTCTCAATTACCCGAAAGTAATCTACCGCTCGGCTTG
CATGTGTTAGGCCTCCCGCTAGCGTTCATCCTGAGCCAGGATCAAACTCT
>816
CTGCTGCCGCCCGTAGGAGTCTGGACCGTGTCTCAGTTCCAGTGTGACTGATCATCCTCTCAGACCAGTACGG
ATCGTCGCCTGGTAGGCCTTTACCCCCACCAACTAGCTAATCCGACCTAGGCTCATCTGATAGCGTGAGGTCC
GAAGATCCCCCACTTCTCCCTCAGGACGTATGCGGTATTAGCGCCCGTTTCCGGACGATCCCCACTACCAGGC
AGATTCCTAGGCATTACTCACCCGTCCGCCGCTGAATCCAGGAGCAAGCCTCCTCTCCGCTCGACTGCATGTG
TTAGGCCTGCCGCCAGCGTTCAATCTGAGCCAGGATCAAACTCT
>817
CTGCTGCCGCCCGTAGGAGTCTGGTCCGTGTCTCAGTACCAGTGTGGGGGATCACCCTCTCAGGCCCCCTAAA
GATCGTCGCCTGGTGAGCCGTTACCTCACCAACTAGCTAATCTTGCGCGTGCCCATCTCTATCCACCGTAGTT
TTCAATATCACCCGATGCCGAATAATATATTATGGGGTATTAATCTTCTTCGAAAGGCTATCCCCCTGATAAA
GGCAGGTTGCACACGTGTTCCGCACCCGTACGCCGCTCTCAATTACCCGAAAGTAATCTACCGCTCGGCTTGC
ATGTGTTAGGCCTCCCGCTAGCGTTCTCCTGAGCAGGATCAAC
>818
CTGCTGCCGCCCGTAGGAGTCTGGTCCGTGTCTCAGTACCAGTGTGGGGGATCACCCTCTCAGGCCCCCTAAA
GATCGTCGCCTGGTGAGCCGTTACCTCACCAACTAGCTAATCTTGCGCGTGCCCATCTCTATCCACCGTAGTT
TTCAATATCACCCGATGCCGAATAATATATTATGGGGTATTAATCTTCTCGAAAGGCTATCCCCTGATAAAGG
CAGGTTGCACACGTGTTCCGCACCCGTACGCCGCTCTCAATTACCCGAAAGTAATCTACCGCTCCGGCTTGCA
TGTGTTAGGCCTCCCGCTAGCGTCTCCTGAGCAGGATCAACTCGT
>819
CTGCTGCCGCTCGTAGGAGTCTGGTCCGTGTCTCAGTACCAGTGTGGGGGATCACCCTCTCAGGCCCCCTAAA
GATCGTCGCCTGGTGAGCCGTTACCTCACCAACTAGCTAATCTTGCGCGTGCCCATCTCTATCCACCGTAGTT

TTCAATATCACCCGATGCCGAATAATATATTATGGGGTATTAATCTTCTTCGAAAGGCTATCCCCCTGATAAA
GGCAGGTTGCACACGTGTTCCGCACCCGTACGCCGCTCTCAATTACCCGAAAGTAATCTACCGCTCGGCTTGC
ATGTGTTAGGCCTCCCGCTAGCGTTCATCCTGAGCCAGGATCAAACTCT
>820
CTGCTGCCGCCCGTAGGAGTCTGGTCCGTGTCTCAGTACCAGTGTGGGGGATCACCCTCTCAGGCCCCCTAAA
GATCGTCGCCTTGGTGAGCCGTTACCTCACCAACTAGCTAATCTTGCGCGTGCCCATCTCTATCCACCGTAGT
TTTCAATATCACCCGATGCCGAATAATATATTATGGGGTATTAATCTTCTTCGAAAGGCTATCCCCCTGATAA
AGGCAGGTTGCACACGTGTTCCGCACCCGTACGCCGCTCTCAATTACCCGAAAGTAATCTACCGCTCGGCTTG
CATGTGTTAGGCCTCCCGCTAGCGTTCATCCTGAGCCAGGATCAAACTCT
>821
CTGCTGCCGCCCGTAGGAGTCTGGTCCGTGTCTCAGTACCAGTGTGGGGGATCACCCTCTCAGGCCCCCTAAA
GATCGTCGCCTTGGTGAGCCGTTGCCTCACCAACTAGCTAATCTTGCGCGTGCCCATCTCTATCCACCGTAGT
TTTCAATATCGCCCGATGCCGAATAATATATTATGGGGTATTAATCTTCTTCGAAAGGCTATCCCCTGATAAA
GGCAGGTTGCACACGTGTTCCGCACCCGTACGCCGCTCTCAATTACCCGAAAGTAATCTACCGCTCGGCTTGC
ATGTGTTGACGCTC
>822
CTGCTGCCGCCCGTAGGAGTCTGGTCCGTGTCTCAGTACCAGTGTGGGGGATCACCCTCTCAGGCCCCCTAAA
GATCGTCGCCTTGGTGAGCCGTTACCTCACCAACTAGCTAATCTTGCGCGTGCCCATCTCTATCCACCGTAGT
TTTCAATATCACCCGATGCCGAATAATATATTATGGGGTATTAATCTTCTTCGAAAGGCTATCCCCCTGATAA
AGGCAGGTTGCACACGTGTTCCGCACCTGTACGCCGCTCTCAATTACCCGAAAGTAATCTACCGCTCGGCTTG
CATGTGTTAGGCCTCCCGCTAGCGTTCATCCTGAGCCAGGATCAAACTCT
>823
CTGCTGCCGCCCGTAGGAGTCTGGTCCGTGTCTCAGTACCAGTGTGGGGGATCACCCTCTCAGGCCCCCTAAA
GATCGTCGCCTTGGTGAGCCGTTACCTCACCAACTAGCTAATCTTGCGCGTGCCCATCTCTATCCACCGTAGT
TTTCAATATCACCCGATGCCGAATAATATATTATGGGGTATTAATCTTCTTCGAAAGGCTATCCCCCTGATAA
AGGCAGGTTGCACACGTGTTCCGCACCCGTACGCCGCTCTCAATTACCCGAAAGTAATCTACCGCTCGGCTTG
CATGTGTTAGGCCTCCCGCTAGCGTTCATCCTGAGCCAGGATCAAACTCT
>824
CTGCTGCCGCCCGTAGGAGTCTGGTCCGTGTCTCAGTACCAGTGTGGGGGATCACCCTCTCAGGCCCCCTAAA
GATCGTCGCCTTGGTGAGCCGTTACCTCACCAACTAGCTAATCTTGCGCGTGCCCATCTCTATCCACCGTAGT
TTTCAATATCACCCGATGCCGAATAATATATTATGGGGTATTAATCTTCTTCGAAAGGCTATCCCCCTGATAA
AGGCAGGTTGCACACGTGTTCCGCACCCGTACGCCGCTCTCAATTACCCGAAAGTAATCTACCGCTCGGCTTG
CATGTGTTAGGCCTCCCGCTAGCGTTCATCCTGAGCCAGGATCAACTCT
>825
CTGCTGCCGCCCGTAGGAGTCTGGTCCGTGTCTCAGTACCAGTGTGGGGGATCACCCTCTCAGGCCCCCTAAA
GATCGTCGCCTTGGTGAGCCGTTACCTCACCAACTAGCTAATCTTGCGCGTGCCCATCTCTATCCACCGTAGT
TTTCAATATCACCCGATGCCGAATAATATATTATGGGGTATTAATCTTCTTCGAAAGGCTATCCCCCTGATAA
AGGCAGGTTGCACACGTGTTCCGCACCCGTACGCCGCTCTCAATTACCCGAAAGTAATCTACCGCTCGGCTTG
CATGTGTTAGGCCTCCCGCTAGCGTTCATCCTGAGCCAGGATCAAACTCT
>826
CTGCTGCCGCCCGTAGGAGTCTGGTCCGTGTCTCAGTACCAGTGTGGGGGATCACCCTCTCAGGCCCCCTAAA
GATCGTCGCCTTGGTGAGCCGTTACCTCACCAACTAGCTAATCTTGCGCGTGCCCATCTCTATCCACCGTAGT
TTTCAATATCACCCGATGCCGAATAATATATTATGGGGTATTAATCTTCTTCGAAAGCTATCCCCTGATAAAG
GCAGGTTGCACACGTGTTCCGCACCCGTACGCCGCTCTCAATTACCCGAAAGTAATCTACCGCTCGGCTTGCA
TGTGTACGGCTCC
>827
CTGCTGCCGCCCGTAGGAGTCTGGTCCGTGTCTCAGTACCAGTGTGGGGGGATCACCCTCTCAGGCCCCCTAA
AAGATCGTCGCCTTGGTGAGCCGTTACCTCACCAACTAGCTAATCTTGCGCGTGCCCATCTCTATCCACCGTA
GTTTTCAATATCACCCGATGCCGAATAATATAATTATGGGGTATTAATCTTCTCGAAAGCTATCCCCTGATAA
AGGCAGGTTGCACACGTGTTCCGCACCCGTACGCCGCTCTCAATTACCCGAAAGTAATCTACCGCATCGGGCT
TGCATGTGTTAGGCCTCCCGCTAGCGTCATCCTGAGCCAGGATCAAACTCT
>828
CTGCTGCCGCCCCGTAGGAGTCTGGTCCCGTGTCTCAGTACCAGTGTGGGGGATCACCCTCTCAGGCCCCCCT
AAAGATCGTCGCCTTGGTGAGCCCGCTACCTCACCCAACTAGCTAATCTTGCGCGTGCCCCATCTCTATTCCC

ACCGTAGTTTTCAATATCACCCGATGCCCGAATAATATATTATGGGGTATTTAATTCTTCTTCGAAAGGCTAT
CCCCTGATTAAAGGCAGGTTGCACACGTGTTCCCGCACCCGTACGCCGCTCTCAATTACCCCGAAAGTAATCT
ACCGCTCGGCTTGCATGTGTTAGGCCTCCCGCTAGCGTTCATTCCTGAGCCAGGATCAAACTCT
>829
CTGCTGCCGCCCGTAGGAGTCTGGTCCGTGTCTCAGTACCAGTGTGGGGGATCACCCTCTCAGGCCCCCTAAA
GATCGTCGCCTTGGTGAGCCGTTACCTCACCAACTAGCTAATCTTGCGCGTGCCCATCTCTATCCACCGTAGT
TTTCAATATCACCCGATGCCGAATAATATATTATGGGGTATTAATCTTCTTCGAAAGGCTATCCCCCTGATAA
AGGCAGGTTGCACACGTGTTCCGCACCCGTACGCCGCTCTCAATTACCCGAAGAATCTACCGCTCGGCTGCAT
GTGTTAGGCCTCCCGCTAGCGTTCTCCTGAGCAGGATCA
>830
CTGCTGCCGCCCGTAGGAGTCTGGTCCGTGTCTCAGTACCAGTGTGGGGGATCACCCTCTCAGGCCCCCTAAA
GATCGTCGCCTTGGTGAGCCGTTACCTCACCAACTAGCTAATCTTGCGCGTGCCCATCTCTATCCACCGTAGT
TTTCAATATCACCCGATGCCGAATAATATATTATGGGGTATTAATCTTCTTCGAAAGGCTATCCCCCTGATAA
AGGCAGGTTGCACACGTGTTCCGCACCCGTACGCCGCTCTCAATTTACCCGAAAGTAATCTACCGCTCGGCTT
GCATGTGTTAGGCCTCCCGCTAGCGTTCATCCTGAGCC
>831
CTGCTGCCGCCCGTAGGAGTCTGGTCCGTGTCTCAGTACCAGTGTGGGGGATCACCCTCTCAGGCCCCCTAAA
GATCGTCGCCTTGGTGAGCCGTTACCTCACCAACTAGCTAATCTTGCGCGTGCCCATCTCTATCCACCGTAGT
TTTCAATATCACCCGATGCCGAATAATATATTATGGGGTATTAATCTTCTACGAAAGGCTATCCCCCTGATAA
AGGCAGGTTGCACACGTGTTCCGCACCCGTACGCCGCTCTCAATTACCCGAAAGTAATCTACCGCTCGGCTTG
CATGTGTTAGGCCTCCCGCTAGCGTTCATCCTGAGCCAGGATCAAACTCT
>832
CTGCTGCCGCCCGTAGGAGTCTGGTCCGTGTCTCAGTACCAGTGTGGGGGATCACCCTCTCAGGCCCCCTAAA
GATCGTCGCCTTGGTGAGCCGTTACCTCACCAACTAGCTAATCTTGCGCGTGCCCATCTCTATCCACCGTAGT
TTTCAATATCACCCGATGCCGAATAATATATTATGGGGTATTAATCTTCTTCGAAAGGCTATCCCCCTGATAA
AGGCAGGTTGCACACGTGTTCCGCACCCGTACGCCGCTCTCAATTACCCGAAAGTAATCTACCGCATCGGCTT
GCATGTGTTAGGCCTCCCGCTAGCGTTCTCCTGAGCCA
>833
CTGCTGCCGCCCGTAGGAGTCTGGTCCGTGTCTCAGTACCAGTGTGGGGGATCACCCTCTCAGGCCCCCTAAA
GATCGTCGCCTTGGTGAGCCGTTACCTCACCAACTAGCTAATCTTGCGCGTGCCCATCTCTATCCACCGTAGT
TTTCAATATCACCCGATGCCGAATAATATATTATGGGGGTATTAATCTTCTTCGAAAGGCTATCCCCCTGATA
AAGGCAGGTTGCACACGTGTTCCGCACCCGTACGCCGCTCTCAATTACCCGAAAGTAATCTACCGCTCGGCTT
GCATGTGTTAGGCCTCCCGCTAGCGTTCTCCTGAGCAGGATCAACTCGT
>834
CTGCTGCCGCCCGTAGGAGTCTGGTCCGTGTCTCAGTACCAGTGTGGGGGATCACCCTCTCAGGCCCCCTAAA
GATCGTCGCCTTGGTGAGCCGTTACCTCACCAACTAGCTAATCTTGCGCGTGCCCCATCTCTATTCCACCGTA
GTTTCAATATCACCCGATGCCGAATAATATATTATGGGGTATTAATCTTTCTTCGAAAGGCTATCCCCCTGAT
AAAGGCAGGTTGCACACGTGTTCCGCACCCGTACGCCGCTCTCAATTACCCCGAAAGTAATCTACCGCTCGGC
TTGCATGTGTTAGGCCTCCCGCTAGCGTTCATCCTGAGCCAGGATCAACTCT
>835
CTGCTGCCGCCCGTAGGAGTCTGGACCGTGTCTCAGTTCCAGTGTGACTGATCATCCTCTCAGACCAGTACGG
ATCGTCGCCTGGTAGGCCTTTACCCCACCAACTAGCTAATCCGAACCTAGGCTCATCTGATAGCGTGAGGTCC
GAAGATCCCCCACTTCTCCCTCAGGACGTATGCGGTATTAGCGCCCGTTTCCGGACGATCCCCACTACCAGGC
AGATTCCTAGGCATTACTCACCCGTCCGCCGCTGAATCCAGGAGCAAGCCCCCTTCATCCGCTCGACTGCAAT
GTGTTAGGCCTGCCGCCAGCGTCAATCTGAGCCAGGATCAAACTCT
>836
CTGCTGCCGCCCGTAGGAGTCTGGTCCGTGTCTCAGTACCAGTGTGGGGGATCACCCTCTCAGGCCCCCTAAA
GATCGTCGCCTTGGTGAGCCGTTACCTCACCAACTAGCTAATCTTGCGCGTGCCCATCTCTATCCACCGTAGT
TTTCAATATCACCCGATGCCGAATAATATATTATGGGGTATTAATCTTCTTCGAAAGGCTATCCCCTGATAAA
GGCAGGTTGCCACACGTGTTCCGCACCCGTACGCCGCTCTCAATTACCCGAAAGTAATCTACCGCTCGGCTTG
CATGTGTTAGGCCTCCCGCTAGCGTTCATCCTGAGCCAGGATCAAACTCT
>837
CTGCTGCCGCCCGTAGGAGTCTGGTCCGTGTCTCAGTACCAGTGTGGGGGATCACCCTCTCAGGCCCCCTAAA
GATCGTCGCCTTGGTGAGCCGTTACCTCACCAACTAGCTAATCTTGCGCGTGCCCATCTCTATCCACCGTAGT

TTTCAATATCACCCGATGCCGAATAATATATTATGGGGTATTAATCTTCTCGAAAGGCTATCCCCTGATAAAG
GCAGGTTGCACACGTGTTCCGCACCCGTACGCCGCTCTCAATTACCCGAAAGTAATCTACCGCTCGGCTTGCA
TGTGTTAGGCCTCCCGCTAGCGTTCATCC
>838
CTGCTGCCGCCCGTAGGAGTCTGGACCGTGTCTCAGTTCCAGTGTGACTGATCATCCTCTCAGACCAGTACGG
ATCGTCGCCTTGGTAGGCCTTTACCCCCACCAACTAGCTAATCCGACCTAGGCTCATCTGATAGCGTGAGGTC
CGAAGTCCCCCACTTCTCCCTCAGGACGTATGCGGTATTAGCGCCCGTTTCCGGACGATCCCCACTACCAGGC
AGATTCCTAGGCATTACTCACCCGTCCGCCGCTGGATCCAGGAGCAAGCTCCCTTCATCCGCTCGACTGCATG
TGTTAGGCCTGCCGCCAGCGTTCAATCTGAGCCAGGATCAAACTCT
>839
CTGCTGCCGCCCGTAGGAGTCTGGTCCGTATCTCAGTACCAGTGTGGGGGATCACCCTCTCAGGCCCCCTAAA
GATCGTCGCCTTGGTGAGCCGTTACCTCACCAACTAGCTAATCTTGCGCGTGCCCATCTCTATCCACCGTAGT
TTTCAATATCACCCGATGCCGAATAATATATTATGGGGTATTAATCTTCTTCGAAAGGCTATCCCCCTGATAA
AGGCAGGTTGCACACGTGTTCCGCACCCGTACGCCGCTCTCAATTACCCGAAAGTAATCTACCGCTCGGCTTG
CATGTGTTAGGCCTCCCGCTAGCGTTCATCCTGAGCCAGGATCAACTCT
>840
CTGCTGCCGCCCGTAGGAGTCTGGACCGTGTCTCAGTTCCAGTGTGACTGATCATCCTCTCAGACCAGTACGG
ATCGTCGCCTTGGTAGGCCTTTACCCCACCAACTAGCTAATCCGACCTAGGCTCATCTGATAGCGTGAGGTCC
GAAGTCCCCCACTTCTCCCTCAGGACGTATGCGGTATTAGCGCCCCGTTTCCGGACGATCCCCCACTACCAGG
CAGATTCCCTAGGCATTACTCACCCGTCCGCCGCTGAATCCAGGAGCAAGCCCCCTTCATCCGCTCGACTGCA
TGTGTTAGGCCTGCCGCCAGCGTTCAATCTGAGCCAGGATCAAACTCT
>841
CTGCTGCCGCCCGTAGGAGTCTGGTCCGTGTCTCAGTACCAGTGTGGGGGATCACCCTCTCAGGCCCCCTAAA
GATCGTCGCCTTGGTGAGCCGTTACCTCACCAACTAGCTAATCTTGCGCGTGCCCATCTCTATCCACCGTAGT
TTTCAATATCACCCGATGCCGAATAATATATTATGGGGTATTAATCTTCTTCGAAAGGCTATCCCCCTGATAA
AGGCAGGTTGCACACGTGTTCCGCACCCGTACGCCGCTCTCAATTACCCGAAAGTAATCTACCGCTCGGCTTG
CATGTGTTAGGCCTCCCGCTAGCGTTCATCCTGAGCCAGGATCAAACTCT
>842
CTGCTGCCGCCCGTAGGAGTCTGGTCCGTGTCTCAGTACCAGTGTGGGGGATCACCCTCTCAGGCCCCTAAAG
ATCGTCGCCTTGGTGAGCCGTTACCTCACCAACTAGCTAATCTTGCGCGTGCCCATCTCTATCCACCGTAGTT
TTCAATATCACCCGATGCCGAATAATATATTATGGGGTATTAATCTTCTTCGAAAGGCTATCCCCCTGATAAA
GGCAGGTTGCACACGTGTTCCGCACCCGTACGCCGCTCTCAATTACCCGAAAGTAATCTACCGCTCCGGCTTG
CATGTGTTAGGCCTCCCGCTAGCGTTCATCCTGAGCCAGGATCAACTCT
>843
CTGCTGCGCCCGTAGGAGTCTGGTCCGTGTCTCAGTACCAGTGTGGGGGATCACCCTCTCAGGCCCCCTAAAG
ATCGTCGCCTTGGTGAGCCGTTACCTCACCAACTAGCTAATCTTGCGCGTGCCCATCTCTATCCACCGTAGTT
TTCAATATCACCCGATGCCGAATAATATATTATGGGGTATTAATCTTCTTCGAAAGGCTATCCCCCTGATAAA
GGCAGGTTGCACACGTGTTCCGCACCCGTACGCCGCTCTCAATTACCCGAAAGTAATCTACCGCTCGGCTTGC
ATGTGTTAGGCCTCCCGCTAGCGTTCATCCTGAG
>844
CTGCTGCCGCCCGTAGGAGTCTGGTCCGTGTCTCAGTACCAGGTGTGGGGGATCACCCTCTCCAGGCCCCCTA
AAGATCGTCGCCTTGGTGAGCCGTTACCTCACCAACTAGCTAATCTTGCGCGTGCCCATCTCTATCCACCGTA
GTTTTCAATATCACCCGATGCCGAATAATATATTATGGGGTATTAATCTTCTTCGAAAGGCTATCCCCTGATA
AAGGCAGGTTGCACACGTGTTCCGCACCCGTACGCCGCTCTCAATTACCCGAAAGTAACTCTACCGCTCGGCT
TGCATGTGTTAGGCCTCCCGCTAGCGTTCATCCGTGACGCAGGATCAAACTCT
>845
CTGCTGCCGCCCGTAGGAGTCTGGTCCGTGTCTCAGTACCAGTGTGGGGGATCACCCTCTCAGGCCCCCTAAA
GATCGTCGCCTTGGTGAGCCGTTACCTCACCAACTAGCTAATCTTGCGCGTGCCCATCTCTATCCACCGTAGT
TTTCAATATCACCCGATGCCGAATAATATATTATGGGGTATTAATCTTCCTTCGAAGGGCTATCCCCCTGATA
AAGGCAGGTTGCACACGTGTTCCGCACCCGTACGCCGCTCTCAATTACCCGAAAGTAATCTACCGCTCGGCTT
GCATGTGTTAGGCCTCCCGCTAGCGTTCATCCTGAGCCAGGATCAAACTCT
>846
CTGCTGCCGCTCGTAGGAGTCTGGACCGTGTCTCAGTTCCAGTGTGACTGATCATCCTCTCAGACCAGTACGG
ATCGTCGCCTTGGTAGGCCTTTACCCCACCAACTAGCTAATCCGACCTAGGCTCATCTGATAGCGTGAGGTCC

GAAGATCCCCCACTTCTCCCTCAGGACGTATGCGGTATTGCGCCCGTTTCCGGACGTATCCCCCACTACCAGG
CAGATTCCTAGGCATTACTCACCCGTCCGCCGCTGAATCCAGGAGCAAGCCCCTTCATCCGCTCGACTGCATG
TGTTAGGCCTGCCGCCAGCGTCAATCTGAGCCAGGATCAACTCT
>847
CTGCTGCCGCCCGTAGGAGTCTGGTCCGTGTCTCAGTACCAGTGTGGGGGATCACCCTCTCAGGCCCCCTAAA
GATCGTCGCCTTGGTGAGCCGTTACCTCACCAACTAGCTAATCTTGCGCGTGCCCATCTCTATCCACCGTAGT
TTTCAATATCAACCCGATGCCGAATAATATATTATGGGGTATTAATCTTCTTCGAAAGCTATCCCCTGATAAA
GGCAGGTTGCACACGTGTTCCGCACCCGTACGCCGCTCTCAATTACCCGAAAGTAATCTACCGCTCGGCTTGC
ATGTGTTAGGCCTCCCGCTAGCGTTCTC
>848
CTGCTGCCGCCCGTAGGAGTCTGGTCCGTGTCTCAGTACCAGTGTGGGGGATCACCCTCTCAGGCCCCCTAAA
GATCGTCGCCTTGGTGAGCCGTTACCTCACCAACTAGCTAATCTTGCGCGTGCCCATCTCTATCCACCGTAGT
TTTCAATATCACCCGATGCCGAATAATATAATTATGGGGTATTAATCTTCTTCGAAAGGCTATCCCCCTGATA
AAGGCAGGTTGCACACGTGTTCCGCACCCGTACGCCGCTCTCAATTACCCGGAAAGTAACTACCGCTCGGCTT
GCATGTGTTAGGCCTCCCGCTA
>849
CTGCTGCCGCCCGTAGGAGTCTGGTCCGTGTCTCAGTACCAGTGTGGGGGATCACCCTCTCAGGCCCCCTAAA
GATCGTCGCCTTGGTGAGCCGTTACCTCACCAACTAGCTAATCTTGCGCGTGCCCATCTCTATCCACCGTAGT
TTTCAATATCACCCGATGCCGAATAATATATTATGGGGTATTAATCTTCTTCGAAAGGCTATCCCCCTGATAA
AGGCAGGTTGCACACGTGTTCCGCACCCGTACGCCGCTCTCAATTACCCGAAAGTAATCTACCGCTCGGCTTG
CATGTGTTAGGCCTCCCGCTAGCGTTCATCCTGAGCCAGGATCAAACTCT
>850
CTGCTGCCGCCCGTAGGAGTCTGGTCCGTGTCTCAGTACCAGTGTGGGGGATCACCCTCTCAGGCCCCCTAAA
GATCGTCGCCTTGGTGAGCCGTTACCTCACCAACTAGCTAATCTTGCGCGTGCCCATCTCTATCCACCGTAGT
TTTCAATATCACCCGATGCCGAATAATATATTATGGGGTATTAATCTTCTTCGAAAGGCTATCCCCCTGATAA
AGGCAGGTTGCACACGTGTTCCGCACCCGTACGCCGCTCTCAATTACCCGAAAGTAATCTACCGCTCGGCTTG
CATGTGTTAGGCCTCCCGCTAGCGTTCATCCTGAGCCAGGATCAAACTCT
>851
CTGCTGCCGCCCGTAGGAGTCTGGACCGTGTCTCAGTTCCAGTGTGACTGATCATCCCCTCAGACCAGTTACG
GATCGTCGCCTTGGTAGGCCTTTACCCACCAACCTAGCTAATCCGACCTAGGCTCATCTGATAGCGTGAGGTC
CGAAGATCCCCCACTTCTCCCTCAGGACGTATGCGGTATTAGCGCCCGTTTCCGGACGTATCCCCACTACCAG
GCAGATTCCTAGGCATTACTCACCCGTCCGCCGCTGAATCCAGGAGCAAGCTCCCTTCATCCGCTCGACTGCA
TGTGTTAGGCCTGCCGCCAGCGTTCAATCTGAGCCAGGATCAAACTCT
>852
CTGCTGCCGCCCGTAGGAGTCTGGTCCGTGTCTCAGTACCAGTGTGGGGGATCACCCTCTCAGGCCCCCTAAA
GATCGTCGCCTTGGTGAGCCGTTACCTCACCAACTAGCTAATCTTGCGCGTGCCCATCTCTATCCACCGTAGT
TTTCAATATCACCCGATGCCGAATAATATATTATGGGGTATTAATCTTCTTCGAAAGGCTATCCCCCTGATAA
AGGCAGGTTGCACACGTGTTCCGCACCCGTACGCCGCTCTCAATTTACCCGAAAGTAATCTACCGCTCGGCTT
GCATGTGTTAGGCCTCCCGCTAGCGTTCATCCTGAGCCAGGATCAAACTCT
>853
CTGCTGCCGCCCGTAGGAGTCTGGTCCGTGTCTCAGTACCAGTGTGGGGGATCACCCTCTCCAGGCCCCTAAA
GATCGTCGCCTTGGTGAGCCGTTACCTCACCAACTAGCTAATCTTGCGCGTGCCCATCTCTATCCACCGTAGT
TTTCAATATCACCCGATGCCGAATAATATATTATGGGGTATTAATCTTCTTCGAAAGGCTATCCCCCTGATAA
AGGCAGGTTGCACACGTGTTCCGCACCCGTACGCCGCTCTCAATTACCCGAAAGTAATCTACCGCTCGGCTTG
CATGTGTTAGGCCTCCCGCTAGCGTTCATCCTGAGCCAGGATCAAACTCT
>854
CTGCTGCCGCCCGTAGGAGTCTGGTCCGTGTCTCAGTACCAGTGTGGGGGATCACCCTCTCAGGCCCCCTAAA
GATCGTCGCCTTGGTGAGCCGTTACCTCACCAACTAGCTAATCTTGCGCGTGCCCATCTCTATCCACCGTAGT
TTTCAATATCACCCGATGCCGAATAATATATTATGGGGTATTAATCTTCTTCGAAAGGCTATCCCCCTGATAA
AGGCAGGTTGCACACGTGTTCCGCACCCGTACGCCGCTCTCAATTACCCGAAAGTAATCTACCGCTCGGCTTG
CATGTGTTAGGCCTCCCGCTAGCGTTCATCCTGAGCCAGGATCAAACTCT
>855
CTGCTGCCGCCCGTAGGAGTCTGGTCCGTGTCTCAGTACCAGTGTGGGGGATCACCCTCTCAGGCCCCCTAAA
GATCGTCGCCTTGGTGAGCCGTTACCTCACCAACTAGCTAATCTTGCGCGTGCCCATCTCTATCCACCGTAGT

TTTCAATATCACCCGATGCCGAATAATATATTATGGGGTATTAATCTTCTTCGAAAGGCTATCCCCTGATAAA
GGCAGGTTGCACACGTGTTCCGCACCCGTACGCCGCTCTCAATTACCCGAAAGTAATCTACCGCTCGGCTTGC
ATGTGTTAGGCCTCCCGCTAGCGTTCATC
>856
CTGCTGCCGCCCGTAGGAGTCTGGTCCGTGTCTCAGTACCAGTGTGGGGGATCACCCTCTCAGGCCCCCTAAA
GATCGTCGCCTGGTGAGCCGTTACCTCACCAACTAGCTAATCTTGCGCGTGCCCATCTCTATCCACCGTAGTT
TTCAATATCACCCGATGCCGAATAATATATTATGGGGTATTAATCTTCTTCGAAAGGCTATCCCCCTGATAAA
GGCAGGTTGCACACGTGTTCCGCACCCGTACGCCGCTCTCAATTACCCGAAAGTAATCTACCGCTCGGCTTGC
ATGTGTTAGGCCTCCCGCTAGCGTTCTCTGAGCAGTCAACTCGT
>857
CTGCTGCCGCCCGTAGGAGTCTGGTCCGTGTCTCAGTACCAGTGTGGGGGATCACCCTCTCAGGCCCCCTAAA
GATCGTCGCCTTGGTGAGCCGTTACCACACCAACTAGCTAATCTTGCGCGTGCCCATCTCTATCCACCGTAGT
TTTCAATATCACCCGATGCCGAATAATATATTATGGGGTATTAATCTTCTCGAAAGGCTATCCCCTGATAAAG
GCAGGTTGCACACGTGTTCCGCACCCGTACGCCGCTCTCAATTACCCGAAAGTAATCTACCGCTCGGCTTGCA
TGTGTTAGGCCTCCCGCTAGCGTTCATCCTGAGCCAGGA
>858
CTGCTGCCGCCCGTAGGAGTCTGGTCCGTGTCTCAGTACCAGTGTGGGGGATCACCCTCTCAGGCCCCCTAAA
GATCGTCGCCTTGGTGAGCCGTTACCTCACCAACTAGCTAATCTTGCGCGTGCCCATCTCTATCCACCGTAGT
TTTTCAATATCACCCGATGCCGAATAATATATTATTGGGGTATTTAATTCTTTCTTCGAAAGGCTATCCCCCT
GATAAAGGCAGGTTGCACACGTGTTCCGCACCCGTACGCCGCTCTCAATTACCCGAAAGTAATCTACCGCTCC
GGCTTGCATGTGTTTAGGCCTCCCGCTAGCGTTCTTCCTGAGCAGGATCA
>859
CTGCTGCCGCCCGTAGGAGTCTGGTCCGTGTCTCAGTACCAGTGTGGGGATCACCCTCTCAGGCCCCTAAAGA
TCGTCGCCTTGGTGAGCCGTTTACCTCACCAACTAGCTAATCTTGCGCGTGCCCATCTCTATCCACCGTAGTT
TTCAATATCACCCGATGCCGAATAATATATTATGGGGTATTAATCTTCTTCGAAAGGCTATCCCCCTGATAAA
GGCAGGTTGCACACGTGTTCCGCACCCGTACGCCGCTCTCAATTACCCGAAAGTAATCTACCGCTCGGCTTGC
ATGTGTTAGGCCTCCCGCTAGCGTTCATCCGTGAGCCAGGATCAAACTCT
>860
CTGCTGCCGCCCGTAGGAGTCTGGTCCGTGTCTCAGTACCAGTGTGGGGGATCACCCTCTCAGGCCCCCTAAA
GATCGTCGCCTTGGTGAGCCTTACCTCACCAACTAGCTAATCTTGCGCGTGCCCATCTCTATCCACCGTAGTT
TTCAATATCACCCGATGCCGAATAATATATTATGGGGTATTAATCTTCTTCGAAAGGCTATCCCCCTGATAAA
GGCAGGTTGCACACGTGTTCCGCACCCGTACGCCGCTCTCAATTTACCCGAAAGTAATCCACCGCTCCGGCTT
GCATGTGTTAGGCCTCCCGCTAGCGTTCATCC
>861
CTGCTGCCGCCCGTAGGAGTCTGGTCCGTGTCTCAGTACCAGTGTGGGGGATCACCCTCTCAGGCCCCCTAAG
ATCGTCGCCTGTGAGCCGTTACCTCACCAACTAGCTAATCTTGCGCGTGCCCATCTCTATCCACCGTAGTTTT
CAATATCACCCGATGCCGAATAATATATTATGGGGTATTAATCTTCTCGAAAGGCTATCCCCTGATAAGGCAG
GTTGCACACGTGTTCCGCACCCGTACGCCGCTCTCAATTACCCGAAAGTAATCTACCGCTCGGCTTGCATGTG
TTAGGCCTCCCGCTAGCGTTCATCCTGAGCCAGGATCAACTCT
>862
CTGCTGCCGCCCGTAGGAGTCTGGTCCGTGTCTCAGTACCAGTGTGGGGGATCACCCTCTCAGGCCCCCTAAA
GATCGTCGCCTTGGTGAGCCGTTGCCTCACCAACTAGCTAATCTTGCGCGTGCCCATCTCTATCCACCGTAGT
TTTCAATATCACCCGATGCCGAATAATATATTATGGGGTATTTAATCTTCTTCGAAAGGCTATCCCCCTGATA
AAGGCAGGTTGCACACGTGTTCCGCACCCGTACGCCGCTCTCAATTACCCGAAAGTAATCTACCGCTCGGCTT
GCATGTGTTAGGCCTCCCGCTAGCGTTCTTCCTGAGCAGGATCA
>863
CTGCTGCCGCCCGTAGGAGTCTGGACCGTGTCTCAGTTCCAGTGAGACTGATCATCCTCTCAGACCAGTACGG
ATCGTCGCCTTGGTAGGCCTTTACCCCACCAACTAGCTAATCCGACCTAGGCTCATCTGATAGCGTGAGGTCC
GAAGTCCCCCACTTCTCCCTCAGGACGTATGCGGTATTAGCGCCCGTTTCCGGACGTATCCCCCACTACCAGG
CAGATTCCTAGGCATTACTCACCCGTCCGCCGCTGAATCCAGGAGCAAGCTCCCTTCATCCGCTCGACTGCAT
GTGTTAGGCCTGCCGCCAGCGTTCTCCTGAGCCAGGATCAAACTCT
>864
CTGCTGCCGCCCGTAGGAGTCTGGTCCGTGTCTCAGTACCAGTGTGGGGGATCACCCTCTCAGGCCCCCTAAA
GATCGTCGCCTTGGTGAGCCGTTACCTCACCAACTAGCTAATCTTGCGCGTGCCCATCTCTATCCACCGTAGT

TTTCAATATCACCCGATGCCGAATAATATATTATGGGGTATTAATCTTCTTCGAAAGGCTATCCCCCTGATAA
AGGCAGGTTGCCACACGTGTTCCGCACCCGTACGCCGCTCTCAATTACCCGAAAGTAATCTACCGCTCGGCTT
GCATGTGTTAGGCCTCCCGCTAGCGTTCTCCGTGAGCAGGATCC
>865
CTGCTGCCGCCCGTAGGAGTCTGGTCCGTGTCTCAGTACCAGTGTGGGGGATCACCCTCTCAGGCCCCCTAAA
GATCGTCGCCTTGGTGAGCCGTTACCTCACCAACTAGCTAATCTTGCGCGTGCCCATCTCTATCCACCGTAGT
TTTCAATATCACCCGATGCCGAATAATATATTATGGGGTATTAATCTTCTTCGAAAGGCTATCCCCCTGATAA
AGGCAGGTTGCACACGTGTTCCGCACCCGTACGCCGCTCTCAATTACCCGAAAGTAATCTACCGCTCGGCTTG
CATGTGTTAGGCCTCCCGCTAGCGTTCATCCTGAGCCAGGATCAAACTCT
>866
CTGCTGCCGCCCGTAGGAGTCTGGTCCGTGTCTCAGTACCAGTGTGGGGGATCACCCTCTCAGGCCCCCTAAA
GATCGTCGCCTTGGTGAGCCGTTACCTCACCAACTAGCTAATCTTGCGCGTGCCCATCTCTATCCACCGTAGT
TTTCAATATCACCCGATGCCGAATAATATATTATGGGGTATTAATCTTCTCGAAAGGCTATCCCCCTGATAAA
GGCAGGTTGCACACGTGTTCCGCACCCGTACGCCGCTCTCAATTACCCGAAAGTAATCTACCGCTCGGCTTGC
ATGTGTTAGGCCTCCCGCTAGCGTTCACCCTGAGCCAGGATCAAACTCT
>867
CTGCTGCCGCCCGTAGGAGTCTGGTCCGTGTCTCAGTACCAGTGTGGGGGATCACCCTCTCAGGCCCCCTAAA
GATCGTCGCCTTGGTGAGCCGTTACCTCACCAACTAGCTAATCTTGCGCGTGCCCATTCTCTATCCACCGTAG
TTTTCAATATCACCCGATGCCGAATAATATATTATTGGGGGTATTAATCTTTCTTCGAAAGGCTATCCCCCTG
ATTAAAGGCAGGTTGCACACGTGTTCCGCACCCGTACGCCGCTCTCAATTACCCGAAAGTAATCTACCGCTCG
GCTTGCATGTGTTAGGCCTCCCGCTAGCGTTCATCCTGAGCCAGGATCAAACTCT
>868
CTGCTGCCGCCCGTAGGAGTCTGGTCCGTGTCTCAGTACCAGTGTGGGGGATCACCCTCTCAGGCCCCCTAAA
GATCGTCGCCTTGGTGAGCCGTTACCTCACCAACTAGCTAATCTTGCGCGTGCCCATCTCTATCCACCGTAGT
TTTCAATATCACCCGATGCCGAATAATATATTATGGGGTATTAATCTTCTTCGAAAGGCTATCCCCTGATAAA
GGCAGGTTGCACACGTGTTCCGCACCCGTACGCCGCTCTCAATTACCCGAAAGTAATCTACCGCTCGGCTTGC
ATGTGTTAGGCCTCCCGCTAGCGTTCTCCTGAGCAGTCAACTCGT
>869
CTGCTGCCGCCCGTAGGAGTCTGGTCCGTGTCTCAGTACCAGTGTGGGGGATCACCCTCTCAGGCCCCCTAAA
GATCGTCGCCTTGGTGAGCCGTTACCTCACCAACTAGCTAATCTTGCGCGTGCCCATCTCTATCCACCGTAGT
TTTCAATATCACCCGATGCCGAATAATATATTATGGGGTATTAATCTTCTTCGAAAGGCTATCCCCCTGATAA
AGGCAGGTTGCACACGTGTTCCGCACCCGTACGCCGCTCTCAATTACCCGAAAGTAATCTACCGCTCGGCTTG
CATGTGTTAGGCCTCCCGCTAGCGTTCATCCTGAGCCAGGATCAAACTCT
>870
CTGCTGCCGCCCGTAGGAGTCTGGTCCGTGTCTCAGTACCAGTGTGGGGGATCACCCTCTCAGGCCCCCTAAA
GATCGTCGCCTTGGTGAGCCGTTACCTCACCAACTAGCTAATCTTGCGCGTGCCCATTTCTATCCACCGTAGT
TTTCAATATCACCCGATGCCGAATAATATATTATGGGGTATTAATCTTCTTCGAAAGGCTATCCCCCTGATAA
AGGCAGGTTGCACACGTGTTCCGCACCCGTACGCCGCTCTCAATTACCCGAAAGTAATCTACCGCTCGGCTTG
CATGTGTTAGGCCTCCCGCTAGCGTTCTCCTGAGCCAGGATCAACTCT
>871
CTGCTGCCGCCCGTAGGAGTCTGGTCCGTGTCTCAGTACCAGTGTGGGGGATCACCCTCTCAGGCCCCCTAAA
GATCGTCGCCTTGGTGAGCCGTTACCTCACCAACTAGCTAATCTTGCGCGTGCCCATCTCTATCCACCGTAGT
TTTCAATATCACCCGATGCCGAATAATATATTATGGGGTATTAATCTTCTTCGAAAGGCTATCCCCCTGATAA
AGGCAGGTTGCACACGTGTTCCGCACCCGTACGCCGCTCCCAATTACCCGAAAGTAATCTACCGCTCGGCTTG
CATGTGTTAGGCCTCCCGCTAGCGTTCATCCTGAGCCAGGATCAAACTCT
>872
CTGCTGCCGCCCGTAGGAGTCTGGTCCGTGTCTCAGTACCAGTGTGGGGGATCACCCTCTCAGGCCCCCCTAA
AGATCGTCGCCTTGGTGAGCCGTTACCTCACCAACTAGCTAATCTTGCGCGTGCCCATCTCTATCCACCGTAG
TTTTCAATATCACCCGATGCCGAATAATATATTATGGGGTATTAATCTTCTTCGAAAGGCTATCCCCCTGATA
AAGGCAGGTTGCACACGTGTTCCGCACCCGTACGCCGCTCTCAATTACCCGAAAGTAATCTACCGCTCGGCTT
GCATGTGTTAGGCCTCCCGCTAGCGTTCATCCTGAGCCAGGATCAACTCT
>873
CTGCTGCCGCCCGTAGGAGTCTGGTCCGTGTCTCAGTACCAGTGTGGGGGATCACCCTCTCAGGCCCCCTAAA
GATCGTCGCCTTGGTGAGCCGTTACCTCACCAACTAGCTAATCTTGCGCGTGCCCATCTCTATCCACCGTAGT

TTCAATATCACCCGATGCCGAATAATATATTATGGGGTATTAATCTTCTTCGAAAGGCTATCCCCTGATAAGG
CAGGTTGCACACGTGTTCCGCACCCGTACGCCGCTCTCAATTACCCGAAAGTAATCTACCGCTCGGCTTGCAT
GTGTTAGGCCTCCCGCTAGCGTTCTCCTGAGCAGGTCAAACTCG
>874
CTGCTGCCGCCCGTAGGAGTCTGGTCCGTGTCTCAGTACCAGTGTGGGGGATCACCCTCTCAGGCCCCCTAAA
GATCGTCGCCTTGGTGAGCCGTTACCTCACCAACTAGCTAATCTTGCGCGTGCCCATCTCTATCCACCGTAGT
TTTCAATATCACCCGATGCCGAATAATATATTATGGGGTATTAATCTTCTTCGAAAGGCTATCCCCTGATAAA
GGCAGGTTGCACACGTGTTCCGCACCCGTACGCCGCTCTCAATTACCCGAAAGTAATCTACCGCATCGGCTTG
CACGTGTTAGGCCTCCCGCTAGCGTTCATCCTGAGCCAGGATCAAACTCT
>875
CTGCTGCCGCCCGTAGGAGTCTGGTCCGTGTCTCAGTACCAGTGTGGGGGATCACCCTCTCAGGCCCCCTAAA
GATCGTCGCCTTGGTGAGCCGTTACCTCACCAACTAGCTAATCTTGCGCGTGCCCATCTCTATCCACCGTAGT
TTTCAATATCACCCGATGCCGAATAATATATTATGGGGTATTAATCTTCTTCGAAAGGCTATCCCCCTGATAA
AGGCAGGTTGCACACGTGTTCCGCACCCGTACGCCGCTCTCAATTACCCGAAAGTAATCTACCGCTCGGCTTG
CATGTGTTAGGCCTCCCGCTAGCGTTCATCCTGAGCCAGGATCAAACTCT
>876
CTGCTGCCGCCCGTAGGAGTCTGGTCCGTGTCTCAGTACCAGTGTGGGGGATCACCCTCTCCAGGCCCCTAAA
GATCGTCGCCTTGGTGAGCCGTTACCTCACCAACTAGCTAATCTTGCGCGTGCCCATCTCTATCCACCGTAGT
TTTCAATATCACCCGATGCCGAATAATATATTATGGGGTATTAATCTTCTTCGAAAGGCTATCCCCTGATAAA
GGCAGGTTGCACACGTGTTCCGCACCCGTACGCCGCTCTCAATTACCCGAAAGTAATCTACCGCTCGGCTTGC
ATGTGTTAGGCCTCCCGCTAGCGTTCATCCTGAGCCAGGATCAACTCT
>877
CTGCTGCCGCCCGTAGGAGTCTGGTCCGTGTCTCAGTACCAGTGTGGGGGATCACCCTCTCAGGCCCCCTAAA
GATCGTCGCCTTGGTGAGCCGTTACCTCACCAACTAGCTAATCTTGCGCGTGCCCATCTCTATCCACCGTAGT
TTTCAATATCACCCGATGCCGAATAATATATTATGGGGTATTAATCTTCTTCGAAAGGCTATCCCCCTGATAA
AGGCAGGTTGCACACGTGTTCCGCACCCGTACGCCGCTCTCAATTACCCGAAAGTAATCTACCGCTCCGGCTT
GCATGTGTTAGGCCTCCCGCTAGCGTTCTCCTGAGCAGGATCAACTCGT
>878
CTGCTGCCGCCCGTAGGAGTCTGGTCCGTGTCTCAGTACCAGTGTGGGGGATCACCCTCTCAGGCCCCCTAAA
GATCGTCGCCTTGGTGAGCCGTTACCTCACCAACTAGCTAATCTTGCGCGTGCCCATCTCTATCCACCGTAGT
TTTCAATATCACCCGATGCCGAATAATATATTATGGGGTATTAATCTTCTTCGAAAGGCTATCCCCCTGATAA
AGGCAGGTTGCACACGTGTTCCGCACCCGTACGCCGCTCTCAATTACCCGAAAGTAATCTACCGCTCGGCTTG
CATGTGTTAGGCCTCCCGCTAGCGTTCATCCGTGAGCCAGGATCAACTCT
>879
CTGCTGCCGCCCGTAGGAGTCTGGTCCGTGTCTCAGTACCAGTGTGGGGGATCACCCTCTCAGGCCCCCTAAA
GATCGTCGCCTTGGTGAGCCGTTTACCTCACCAACTAGCTAATCTTGCGCGTGCCCATCTCTATCCACCGTAG
TTTTCAATATCACCCGATGCCGAATAATATATTATGGGGTATTAATCTTCTTCGAAAGGCTATCCCCCTGATA
AAGGCAGGTTGCACACGTGTTCCGCACCCGTACGCCGCTCTCAATTACCCGAAAGTAATCTACCGCTCGGCTT
GCATGTGTTAGGCCTCCCGCTAGCGTTCATCCTGAGCCAGGATCAACTCT
>880
CTGCTGCCGCCCGTAGGAGTCTGGTCCGTGTCTCAGTACCAGTGTGGGGGATCACCCTCTCAGGCCCCCTAAA
GATCGTCGCCTTGGTGAGCCGTTACCTCACCAACTAGCTAATCTTGCGCGTGCCCATCTCTATCCACCGTAGT
TTTCAATATCACCCGATGCCGAATAATATATTATGGGGTATTAATCTTCTTCGAAGGCTATCCCCCTGATAAA
GGCAGGTTGCACACGTGTTCCGCACCCGTACGCCGCTCTCAATTACCCGAAAGTAATCTACCGCTCGGCTTGC
ATGTGTTAGGCCTCCCGCTAGCGTTCTCCGTGAGCCAGGATCAACTCT
>881
CTGCTGCCGCCCGTAGGAGTCTGGTCCGTGTCTCAGTACCAGTGTGGGGGATCACCCTCTCAGGCCCCCTAAA
GATCGTCGCCTTGGTGAGCCGTTACCTCACCAACTAGCTAATCTTGCGCGTGCCCATCTCTATCCACCGTAGT
TTTCAATATCACCCGATGCCGAATAATATATTATGGGGTATTAATCTTCTTCGAAAGGCTATCCCCCTGATAA
AGGCAGGTTGCACACGTGTTCCGCACCCGTACGCCGCTCTCAATTACCCGAAAGTAATCTACCGCTCGGCTTG
CATGTGTTAGGCCTCCCGCTAGCGTTCTCCTCGAGCAGGATCA
>882
CTGCTGCCGCCCGTAGGAGTCTGGACCGTGTCTCAGTTCCAGTGTGACTGATCATCCTCTCAGACCAGTACGG
ATCGTCGCCTTGGTAGGCCTTTACCCCACCAACTAGCTAATCCGACCTAGGCTCATCTGATAGCGTGAGGTCC

GAAGATCCCCCACTTCTCCCTCAGGACGTATGCGGTATTAGCGCCCGTTTCCGGACGATCCCCACTACCAGGC
AGATTCCTAGGCATTACTCACCCGTCCGCCGCTAAATCCAGGAGCAAGCTCCCTTCATCCGCTCGACTGCATG
TGTTAGGCCTGCCGCCAGCGTCAATCTGAGCCAGGATCAAACTCT
>883
CTGCTGCCGCCCGTAGGAGTCTGGACCGTGTCTCAGTTCCAGTGTGACTGATCATCCTCTCAGACCAGTACGG
ATCGTCGCCTTGGTAGGCCTTTACCCCACCAACTAGCTAATCCGACCTAGGCTCATCTGATAGCGTGAGGTCC
GAAGTCCCCCACTTCTCCCTCAGGACGTATGCGGTATCAGCGTCCCGTTCCGGAGTCCTCCCCACTACCAGGC
CAGATTCCTAGGCATTACTCACCCGTCCGCCGCTGAATCCAGGAGCAAGCTCCCTTCATCCGCTCGACTGCCA
TGTGTTAGGCCTGCCGCCAGCGTCAATCTGAGCCAGGATCAAACTCT
>884
CTGCTGCCGCCCGTAGGAGTCTGGTCCGTGTCTCAGTACCAGTGTGGGGGATCACCCTCTCAGGCCCCCTAAA
GATCGTCGCCTTGGTGAGCCGTTACCTCACCAACTAGCTAATCTTGCGCGTGCCCATCTCTATCCACCGTAGT
TTTCAATATCACCCGATGCCGAATAATATATTATGGGGTATTAATCTTCTTCGAAAGGCTATCCCCCTGATAA
AGGCAGGTTGCACACGTGTTCCGCACCCGTACGCCGCTCTCAATTACCCGAAAGTAATCTACCGCTCGGCTTG
CATGTGTTAGGCCTCCCGCTAGCGTTCATCCGTGAGCCAGGATCAAACTCT
>885
CTGCTGCCGCCCGTAGGAGTCTGGTCCGTGTCTCAGTACCAGTGTGGGGGATCACCCTCTCAGGCCCCCTAAA
GATCGTCGCCTTGGTGAGCCGTTACCTCACCAACTAGCTAATCTTGCGCGTGCCCATCTCTATCCACCGTAGT
TTTCAATATCACCCGATGCCGAATAATATTATTATGGGGTATTAATCTTCTTCGAAAGGCTATCCCCCTGATA
AAGGCAGGTTGCCACACGTGTTCCGCACCCGTACGCCGCTCTCAATTACCCGAAAGTAATCTACCGCTCCGGC
TTGCATGTGTTAGGCCTCCCGCTAGCGTTCATCCTGAG
>886
CTGCTGCCGCCCGTAGGAGTCTGGTCCGTGTCTCAGTACCAGTGTGGGGGATCACCCTCTCAGGCCCCCTAAA
GACGTCGCCTGGTGAGCCGTTACCTCACCAACTAGCTAATCTTGCGCGTGCCCATCTCTATCCACCGTAGTTT
TCAATATCACCCGATGCCGAATAATATAATTATGGGGTATTAATCTTCTTCGAAAGGCTATCCCCTGATAAAG
GCAGGTTGCACACGTGTTCCGCACCCGTACGCCGCTCTCAATTACCCGAAAGTAATCTACCGCTCGGCTTGCA
TGTGTTAGGCCTCCCGCTAGCGTTCTCCTGAGCAGGATCAACTCT
>887
CTGCTGCCGCCCGTAGGAGTCTGGTCCGTGTCTCAGTACCAGTGTGGGGGATCACCCTCTCAGGCCCCCTAAA
GATCGTCGCCTTGGTGAGCCGTTACCTCACCAACTAGCTAATCTTGCGCGTGCCCATCTCTATCCACCGTAGT
TTTCAATATCACCCGATGCCGAATAATATATTATGGGGTATTAATCTTCTTCGAAAGGCTATCCCCTGATAAA
GGCAGGTTGCACACGTGTTCCGCACCCGTACGCCGCTCTCAATTACCCGAAAGTAATCTACCGCTCGGCTTGC
ATGTGTTAGGCCTCCCGCTAGCGTTCATCTGAG
>888
CTGCTGCCGCCCGTAGGAGTCTGGTCCGTGTCTCAGTACCAGTGTGGGGGATCACCCTCTCAGGCCCCCTAAA
GATCGTCGCCTTGGTGAGCCGTTACCTCACCAACTAGCTAATCTTGCGTGTGCCCATCTCTATCCACCGTAGT
TTTCAATATCACCCGATGCCGAATAATATATTATGGGGTATTAATCTTCTCGAAAGGCTATCCCCCTGATAAA
GGCAGGTTGCACACGTGTTCCGCACCCGTACGCCGCTCTCAATTACCCGAAAGTAATCTACCGCATCGGCTTG
CACTGTGTTAGGCTCCCGCTA
>889
CTGCTGCCGCCCGTAGGAGTCTGGTCCGTGTCTCAGTACCAGTGTGGGGGATCACCCTCTCAGGCCCCCTAAA
GATCGTCGCCTTGGTGAGCCGTTACCTCACCAACTAGCTAATCTTGCGCGTGCCCATCTCTATCCACCGTAGT
TTTCAATATCACCCGATGCCGAGTAATATATTATGGGGTATTAATCTTCTTCGAAAGGCTACCCCCTGATAAA
GGCAGGTTGCACACGTGTTCCGCACCCGTACGCCGCTCTCAATTTACCCGAAAGTAATCTACCGCTCGGCTTG
CATGTGTTAGGCCTCCCGCTAGCGTTCATCCTGAGCCAGGATCAACTCT
>890
CTGCTGCCGCCCGTAGGAGTCTGGTCCGTGTCTCAGTACCAGTGTGGGGGATCACCCTCTCAGGCCCCCTAAA
GATCGTCGCCTTGGTGAGCCGTTACCTCACCAACTAGCTAATCTTGCGCGTGCCCATCTCTATCCACCGTAGT
TTTCAATATCACCCGATGCCGAATAATATATTATGGGGTATTAATCTTCTTCGAAAGGCTATCCCCCTGATAA
AGGCAGGTTGCACACGTGTTCCGCACCCGTACGCCGCTCTCAATTACCCGAAAGTAATCTACCGCTCGGCTTG
CATGTGTTAGGCCTCCCGCTAGCGTTCATCCTGAGCCAGGATCAACTCT
>891
CTGCTGCCGCCCGTAGGAGTCTGGTCCGTGTCTCAGTACCAGTGTGGGGGATCACCCTCTCAGGCCCCCTAAA
GATCGTCGCCTTGGTGAGCCGTTACCTCACCAACTAGCTAATCTTGCGCGTGCCCATCTCTATCCACCGTAGT

TTTCAATATCACCCGATGCCGAATAATATATTATGGGGTATTAATCTTCTCGAAAGGCTTATCCCCCTGATAA
AGGCAGGTTGCACACGTGTTCCGCACCCGTACGCCGCTCTCAATTACCCGAAAGTAATCTACCGCTCCGGCTT
GCATGTGTTAGGCCTCCCGCTAGCGTTCATCGTGAGCCAGGATCAAACTCT
>892
CTGCTGCCGCCCGTAGGAGTCTGGTCCGTGTCTCAGTACCAGTGTGGGGGATCACCCTCTCAGGCCCCCTAAA
GATCGTCGCCTTGGTGAGCCGTTACCTCACCAACTAGCTAATCTTGCGCGTGCCCATCTCTATCCACCGTAGT
TTTCAATATCACCCGATGCCGAATAATATATTATTGGGGTATTAATCTTCTTCGAAGGCTATCCCCCTGATAA
AGGCAGGTTGCACACGTGTTCCGCACCCGTACGCCGCTCTCAATTACCCGAAAGTAATCTACCGCTCGGCTTG
CATGTGTTAGGCCTCCCGCTAGCGTTCATCCTGAGCCAGGATCAACTCT
>893
CTGCTGCCGCCCGTAGGAGTCTGGTCCGTGTCTCAGTACCAGTGTGGGGGATCACCCTCTCAGGCCCCCTAAA
GATCGTCGCCTTGGTGAGCCGTTACCTCACCAACTAGCTAATCTTGCGCGTGCCCATCTCTATCCACCGTAGT
TTTCAATATCACCCGATGCCGAATAATATATTATGGGGTATTAATCTTCTTCGAAGGCTATCCCCCTGATAAA
GGCAGGTTGCACACGTGTTCCGCACCCGTACGCCGCTCTCAATTACCCAAGAATATCACCGCATCGGCTTGCA
TGTGTTAGCCTCCCGCTAG
>894
CTGCTGCCGCCCGTAGGAGTCTGGTCCGTGTCTCAGTACCAGTGTGGGGGATCACCCTCTCAGGCCCCCTAAA
GATCGTCGCCTTGGTGAGCCGTTACCTCACCAACTAGCTAATCTTGCGCGTGCCCATCTCTATTCCACCGTAG
TTTTTCAATATCACCCGATGCCGAATAATATATTATTGGGGTATTTAATCTTCTTCGAAAGGCTATCCCCCTG
ATAAAGGCAGGTTTGCCACGCGTGTTCCGCACCCGTACGCCGCTCTCAATTACCCGAAAGTAATCTACCGCTC
CGGCTTGCATGTCGTTAGGCCTCCCGCTAGCGTTCATCCTGAGCCAGGATCAACTCT
>895
CTGCTGCCGCCCGTAGGAGTCTGGTCCGTGTCTCAGTACCAGTGTGGGGGATCACCCTCTCAGGCCCCCTAAA
GATCGTCGCCTTGGTGAGCCGTTACTCACCAACTAGCTAATCTTGCGCGTGCCCATCTCTATCCACCGTAGTT
TTCAATATCACCCGATGCCGAATAATATATTATGGGGTATTAATCTTCTTCGAAAGGCTATCCCCCTGATAAA
GGCAGGTTGCACACGTGTTCCGCACCCGTACGCCGCTCTCAATTACCCGAAAGTAATCTACCGCTCCGGCTTG
CATGTGTTAGGCCTCCCGCTAGCGTTCATCTCGAGCAGG
>896
CTGCTGCCGCCCGTAGGAGTCTGGACCGTGTCTCAGTTCCAGTGTGACTGATCATCCTCTCAGACCAGTTACG
GATCGTCGCCTTGGTAGGCCTTTACCCCACCAACTAGCTAATCCGACCTAGGCTCATCTGATAGCGTGAGGTC
CGAAGATCCCCCACTTTCTCCCTCAGGACGTATGCGGTATTAGCGCCCGTTTCCGGACGTATCCCCACTACCA
GGCAGATTCCTAGGCATTACTCACCCGTCCGCCGCTGAATCCAGGAGCAAGCTCCCTCATCCGCTCGACTTGC
ATGTGTTAGGCCTGCCGCCAGCGTTCAATCTGAGCCAGGATCAAACTCT
>897
CTGCTGCCGCCCGTAGGAGTCTGGTCCGTGTCTCAGTACCAGTGTGGGGGATCACCCTCTCAGGCCCCCTAAA
GATCGTCGCCTTGGTGAGCCGTTACCTCACCAACTAGCTAATCTTGCGCGTGCCCATCTCTATCCACCGTAGT
TTTCAATATCACCCGATGCCGAATAATATATTATGGGGTATTAATCCTCTTCGAAAGGCTATCCCCTGATAAA
GGCAGGTTGCACACGTGTTCCGCACCCGTACGCCGCTCTCAATTACCCGAAAGTAATCTACCGCTCGGCTTGC
ATGTGTTAGGCCTCCCGCTAGCGTCATCCTGAG
>898
CTGCTGCCGCCCGTAGGAGTCTGGTTCCGTGTCTCAGTACCAGTGTGGGGGATCACCCTCTCAGGCCCCCTAA
AGATCGTCGCCTTGGTGAGCCGTTACCTCACCAACTAGCTAATCTTGCGCGTGCCCATCTCTATTCCACCGTA
GTTTTCAATATCACCCGATGCCGAATAATATATTTATGGGGGTATTTAAGTCTTTCCTTCGAAAGGCTATCCC
CCTGATAAAGGCAGGTTTGCACACGTGTTCCGCACCCGTACGCCGCTCTCAATTACCCGAAAGTAATCTACCG
CTCGGCTTGCATGTGTTAGGCCTCCCGCTAGCGTTCATTCCTGAGCCAGGATCAAACTCT
>899
CTGCTGCCGCCCGTAGGAGTCTGGTCCGTGTCTCAGTACCAGTGTGGGGGATCACCCTCTCAGGCCCCCTAAA
GATCGTCGCCTTGGTGAGCCGTTACCTCACCAACTAGCTAATCCTGCGCGTGCCCATCTCTATCCACCGTAGT
TTCAATATCACCCGATGCCGAATAATATATTATGGGGTATTAATCTTCTTCGAAAGGCTATCCCCCTGATAAA
GGCAGGTTGCACACGTGTTCCGCACCCGTACGCCGCTCTCAATTACCCGAAAGTAATCACCGCTCGGCTTGCA
TGTGTTAGGCCTCCCGCTAGCGTTCATCCTGAGCCAGGATCAACTCT
>900
CTGCTGCCGCCCGTAGGAGTCTGGTCCGTGTCTCAGTACCAGTGTGGGGGATCACCCTCTCAGGCCCCCTAAA
GATCGTCGCCTGGTGAGCCGTTACCTCACCAACTAGCTAATCTTGCGCGTGCCCATCTCTATCCACCGTAGTT

TTCAATATCACCCGATGCCGAATAATATATTATGGGGTATTAATCTTCTCGAAAGCTATCCCCTGATAAAGGC
AGGTTGCACACGTGTTCCGCACCCGTACGCCGCTCTCAATTACCCGAAAGTAATCTACCGCTCGGCTTGCATG
TGTTAGGCCTCCCGCTAGCGTTCATC
>901
CTGCTGCCGCCCGTAGGAGTCTGGTCCGTGTCTCAGTACCAGTGTGGGGGATCACCCTCTCAGGCCCCCTAAA
GATCGTCGCCTTGGTGAGCCGTTACCTCACCAACTAGCTAATCTTGCGCGTGCCCATCTCTATCCACCGTAGT
TTTCAATATCACCCGATGCCGAATAATATATTATGGGGTATTAATCTTCTTCGAAAGGCTATCCCCTGATAAG
GCAGGTTGCACACGTGTTCCGCACCCGTACGCCGCTCTCAATTACCCGAAAGTAATCTACCGCTCGGCTTGCA
TGTGTTAGGCCTCCCGCTAGCGTTCTCCTGAGCAGGATCAAACTCT
>902
CTGCTGCCGCCCGTAGGAGTCTGGTCCGTGTCTCAGTACCAGTGTGGGGGATCACCCTCTCAGGCCCCCTAAA
GATCGTCGCCTTGGTGAGCCGTTACCTCACCAACTAGCTAATCTTGCGTGTGCCCATCTCTATCCACCGTAGT
TTTCAATATCACCCGATGCCGAATAATATATTATGGGGTATTAATCTTCTTCGAAAGGCTATCCCCCTGATAA
AGGCAGGTTGCACACGTGTTCCGCACCCGTACGCCGCTCTCAATTACCCGAAAGTAATCTACCGCTCGGCTTG
CATGTGTTAGGCCTCCCGCTAGCGTTCATCCTGAGCCAGGATCAAACTCT
>903
CTGCTGCCGCCCGTAGGAGTCTGGTCCGTGTCTCAGTACCAGTGTGGGGGATCACCCTCTCAGGCCCCCTAAA
GATCGTCGCCTTGGTGAGCCGTTACCTCACCAACTAGCTAATCTTGCGCGTGCCCATCTCTATCCACCGTAGT
TTTCAATATCACCCGATGCCGAATAATATATTATGGGGTATTAATCTTCTTCGAAAGGCTATCCCCCTGATAA
AGGCAGGTTGCACACGTGTTCCGCACCCGTACGCCGCTCTCAATTACCCGAAAGTAATCTACCGCTCGGCTTG
CATGTGTTAGGCCTCCCGCTAGCGTTCTCCTGAGCAGGATCAACTCGT
>904
CTGCTGCCGCCCGTAGGAGTCTGGTCCGTGTCTCAGTACCAGTGTGGGGGATCACCCTCTCAGGCCCCCTAAA
GATCGTCGCCTTGGTGAGCCGTTACCTCACCAACTAGCTAATCTTGCGCGTGCCCATCTCTATCCACCGTAGT
TTTCAATATCACCCGATGCCGAATAATATATTATGGGGTATTAATCTTCTTCGAAAGGCTATCCCCCTGATAA
AGGCAGGTTGCACACGTGTTCCGCACCCGTACGCCGCTCTCAATTACCCGAAAGTAATCTACCGCTCGGCTTG
CATGTGTTAGGCCTCCCGCTAGCGTTCATCCTGAG
>905
CTGCTGCCGCCCGTAGGAGTCTGGTCCGTGTCTCAGTACCAGTGTGGGGGATCACCCTCTCAGGCCCCCTAAA
AGATCGTCGCCTTGGTGAGCCGTTACCTCACCAACTAGCTAATCTTGCGCGTGCCCATCTCTATCCACCGTAG
TTTTCAATATCACCCGATGCCGAATAATATATTATGGGGTATTAATCTTCTCGAAAGGCTATCCCCTGATAAA
GGCAGGTTGCACACGTGTTCCGCACCCGTACGCCGCTCTCAATTACCCGAAAGTAATCTACCGCTCGGCTTGC
ATGTGTTAGGCCTCCCGCTAGCGTTCATCCTGAGCCAGGATCAAACTCT
>906
CTGCTGCCGCCCGTAGGAGTCTGGTCCGTGTCTCAGTACCAGTGTGGGGGGGTCACCCTCTCAGGCCCCCCTA
AAGATCGTCGCCTTGGTGAGCCCGTTACCTCACCAACTAGCTAATCTTGCGCGTGCCCATCTCTTATCCACCG
TAGTTTTCAATATCACCCGATGCCGAATAATATATTATGGGGTATTAATCTTCTTCGAAAGGCTATCCCCTGA
TTAAAGGCAGGTTGCACACGTGTTCCGCACCCGTACGCCGCTCTCAATTACCCCGAAAGTAATCTACCGCTCG
GCTTGCATGTGTTAGGCCTCCCGCTAGCGTTCTCCTGAGCAGGATCAACTCT
>907
CTGCTGCCGCCCGTAGGAGTCTGGTCCGTGTCTCAGTACCAGTGTGGGGGATCACCCTCTCAGGCCCCCTAAA
GATCGTCGCCTTGGTGAGCCGTTACCTCACCAACTAGCTAATCTTGCGCGTGCCCATCTCTATCCACCGTAGT
TTTCAATATCACCCGATGCCGAATAATATATTATGGGGTATTAATCTTCTTCGAAAGGCTATCCCCCTGATAA
AGGCAGGTTGCACACGTGCTCCGCACCCGTACGCCGCTCTCAATTACCCGAAAGTAATCTACCGCTCGGCTTG
CATGTCTTAGGCCTCCCGCTAGCGTTCATCCTGAGCCAGGATCAAACTCT
>908
CTGCTGCCGCCCGTAGGAGTCTGGACCGTGTCTCAGTTCCAGTGTGACTGATCATCCTCTCAGACCAGTTACG
GATCGTCGCCTTGGTAGGCCTTTACCCCACCAACTAGCTAATCCGACCTAGGCTCATCTGATAGCGTGAGGTC
CGAAGATCCCCCACTTCTCCCTCAGGACGTATGCGGTATTAGCGCCCGTTTCCGGACGTATCCCCACTACCAG
GCAGATTCCTAGGCATTACTCACCCGTCCGCCGCTGAATCCAGGAGCAAGCCCCTTCCATCCGCTCGACTGCA
TGTGTTAGGCCTGCCGCCAGCGTTCAATCTGAGCCAGGATCAAACTCT
>909
CTGCTGCCGCCCGTAGGAGTCTGGTCCGTGTCTCAGTACCAGTGTGGGGGATCACCCTCTCAGGCCCCCTAAA
GATCGTCGCCTTGGTGAGCCGTTACCTCACCAACTAGCTAATCTTGCGCGTGCCCATCTCTATCCACCGTAGT

TTTCAATATCACCCGATGCCGAATAATATATTATGGGGTATTAATCTTCTCGAAAGCTATCCCCTGATAAAGG
CAGGTTGCACACGTGTTCCGCACCCGTACGCCGCTCTCAATTACCCGAAAGTAATCTACCGCTCGGCTTGCAT
GTGTTAGGCCTCCCGCTAGCGTTCATCCTGAGCCAGGATCAAACTCT
>910
CTGCTGCCGCCCGTAGGAGTCTGGTCCGTGTCTCAGTACCAGTGTGGGGGATCACCCTCTCAGGCCCCCTAAA
GATCGTCGCCTTGGTGAGCCGTTACCTCACCAACTAGCTAATCTTGCGCGTGCCCATCTCTATCCACCGTAGT
TTTCAATATCACCCGATGCCGAATAATATATTATGGGGTATTAATCTTCTTCGAAAGGCTATCCCCCTGATAA
AGGCAGGTTGCACACGTGTTCCGCACCCGTACGCCGCTCTCAATTACCCGAAAGTAATCTACCGCTCGGCTTG
CATGTGTTAGGCCTCCCGCTAGCGTTCATCCTGAGCCAGGATCAAACTCT
>911
CTGCTGCCGCCCGTAGGAGTCTGGTCCGTGTCTCAGTACCAGTGTGGGGGATCACCCTCTCAGGCCCCCTAAA
GATCGTCGCCTTGGTGAGCCGTTACCTTACCAACTAGCTAATCTTGCGCGTGCCCATCTCTATCCACCGTAGT
TTTCAATATCACCCGATGCCGAATAATATATTATGGGGTATTAATCTTCTTCGAAAGGCTATCCCCCTGATAA
AGGCAGGTTGCACACGTGTTCCGCACCCGTACGCCGCTCTCAATTACCCGAAAGTAATCTACCGCTCGGCTTG
CATGTGTTAGGCCTCCCGCTAGCGTTCATCCTGAGCCAGGATCAAACTCT
>912
CTGCTGCCGCCCGTAGGAGTCTGGTCCGTGTCTCAGTACCAGTGTGGGGGATCACCCTCTCAGGCCCCCTAAA
GATCGTCGCCTTGGTGAGCCGTTACCTCACCAACTAGCTAATCTTGCGCGTGCCCATCTCTATCCACCGTAGT
TTTCAATATCACCCGATGCCGAATAATATATTATGGGGTATTAATCTTCTTCGAAAGGCTATCCCCCTGATAA
AGGCAGGTTGCACACGTGTTCCGCACCCGTACGCCGCTCTCAATTTACCCGAAAGTAATCTACCGCTCGGCTT
GCATGTGTTAGGCCTCCCGCTAGCGTTCATCCTGAGCCAGGATCAAACTCT
>913
CTGCTGCCGCCCGTAGGAGTCTGGTCCGTGTCTCAGTACCAGGTGTGGGGGATCACCCTCTCCAGGCCCCTAA
AGATCGTCGCCTTGGTGAGCCGTTTACCTCACCAACTAGCTAATCTTGCGCGTGCCCATCTCTATCCACCGTA
GTTTTCAATATCACCCGATGCCGAATAATATATTATGGGGTATTAATCTTCTTCGAAGCTATCCCCTGATAAA
GGCAGGTTGCACACGTGTTCCGCACCCGTACGCCGCTCTCAATTACCCGAAAGTAATCTACCGCTCGGCTTGC
ATGTGTTAGGCCTCCCGCTAGCGTTCATCCTGAGCCAGGATCAACTCT
>914
CTGCTGCCGCCCGTAGGAGTCTGGTCCGTGTCTCAGTACCAGTGTGGGGGATCACCCTCTCGCCCCCTAAACG
ATCGTCGCCTTGGTGAGCCGTTACCCCACCAACCTAGCTAATCTTGCGCGTGCCCATCTCTATCCACCGTAGT
TTTCAATATCACCCGATGCCGAATAATATATTATGGGGTATTAATCTTCTTCGAAAGGCTATCCCCCTGATAA
AGGCAGGTTGCACACGTGTTCCGCACCCGTACGCCGCTCTCAATTACCCGAAAGTAATCTACCGCATCGGCTT
GCATGTGTTAGGCCTCCCGCTAGCGTTCTCCTGAGCAGGATCAACTCT
>915
CTGCTGCCGCCCGTAGGAGTCTGGTCCGTGTCTCAGTACCAGGTGTGGGGGATCACCCTCTCCAGGCCCCCTT
AAAGATCGTCGCCTTGGTGAGCCGTTACCTCACCCAACTAGCTAATCTTGCGCGTGCCCATCTCTATCCACCG
TAGTTTTCAATATCACCCGATGCCGAATAATATATTATGGGGTATTTAATTCTTTCTTCGAAAGGCTATCCCC
CTGATAAAGGCAGGTTGCACACGTGTTCCGCACCCGTTACGCCGCTCTCAATTACCCCGAAAGTAATCTACCG
CTCGGCTTGCATGTGTTAGGCCTCCCGCTAGCGTTCTCCTGAGCAGGATCAACTCG
>916
CTGCTGCCGCCCGTAGGAGTCTGGACCGTGTCTCAGTTCCAGTGTGACTGATCATCCTCTCAGACCAGTACGG
ATCGTCGCCTTGGTAGGCCTTTACCCCACCAACTAGCTAATCCGACCTAGGCTCATCTGATAGCGTGAGGTCC
GAAGTCCCCCACTTCTCCCTCAGGACGTATGCGGTATTAGCGCCCGTTTCCGGACGTATCCCCACTACCAGGC
AGATTCCTAGGCATTACTCACCCGTCCGCCGCTGAATCCAGGAGCAAGCTCCCTCATCCGCTCGACTGCCATG
TGTTAGGCCTGCCGCCAGCGTCAATCTGAGCCAGGATCAAACTCT
>917
CTGCTGCCGCCCGTAGGAGTCTGGTCCGTGTCTCAGTACCAGTGTGGGGGATCACCCTCTCAGGCCCCCTAAA
GATCGTCGCCTTGGTGAGCCGTTACCTCACCAACTAGCTAATCTTGCGCGTGCCCATCTCTATCCACCGTAGT
TTTCAATATCACCCGATGCCGAATAATATATTATGGGGTATTAATCTTCTTCGAAAGGCTATCCCCCTGATAA
AGGCAGGTTGCACACGTGTTCCGCACCCGTACGCCGCTCTCAATTACCCGAAAGTAATCTACCGCTCGGCTTG
CATGTGTTAGGCCTCCCGCTAGCGTTCATCCTGAGCCAGGATCAAACTCT
>918
CTGCTGCCGCCGTAGGAGTCTGGTCCGTGTCTCAGTACCAGTGTGGGGGATCACCCTCTCAGGCCCCCTAAAG
ATCGTCGCCTTGGTGAGCCGTTACCTCACCAACTAGCTAATCTTGCGCGTGCCCATCTCTATCCACCGTAGTT

TTCAATATCACCCGATGCCGAATAATATATTATGGGGTATTAATCTTCTCGAAAGGCTTATCCCCCTGATAAA
GGCAGGTTGCACACGTGTTCCGCACCCGTACGCCGCTCTCAATTACCCGAAAGTAATCTACCGCTCGGCTTGC
ATGTGTTAGGCCTCCCGCTAGCGTCATCCTGAGCCAGGATAACTCT
>919
CTGCTGCCGCCCGTAGGAGTCTGGACCGTGTCTCAGTTCCAGTGTGACTGATCATCCTCTCAGACCAGTACGG
ATCGTCGCCTTGGTAGGCCTTTACCCCACCAACTAGCTAATCCGACCTAGGCTCATCTGATAGCGTGAGGTCC
GAAGTCCCCCACTTCTCCCTCAGGACGTATGCGGTATTAGCGCGTTTCCGGGCGTATCCCCACTACCAGGCAG
ATTCCTAGGCATTACTCACCCGTCCGCCGCTGAATCCAGGAGCAAGCTCCCTTCATCCGCTCGACTGCATGTG
TTAGGCCTGCCGCCAGCGTTCAATCTGAGCCAGGATCAAACTCT
>920
CTGCTGCCGCCCGTAGGAGTCTGGTCTGTGTCTCAGTACCAGTGTGGGGGATCACCCTCTCAGGCCCCCTAAA
GATCGTCGCCTTGGTGAGCCGTTACCTCACCAACTAGCTAATCTTGCGCGTGCCCATCTCTATCCACCGTAGT
TTTCAATATCACCCGATGCCGAATAATATATTATGGGGTATTAATCTTCTCGAAAGGCTATCCCCCTGATAAA
GGCAGGTTGCACACGTGTTCCGCACCCGTACGCCGCTCTCAATTACCCGAAAGTAATCTACCGCTCGGCTTGC
ATGTGTTAGGCCTCCCGCTAGCGTTCATCCTGAGCCAGGATCAAACTCT
>921
CTGCTGCCGCCCGTAGGAGTCTGGTCCGTGTCTCAGTACCAGTGTGGGGGATCACCCTCTCAGGCCCCCTAAA
GATCGTCGCCTTGGTGAGCCGTTACCTCACCAACTAGCTAATCTTGCGCGTGCCCATCTCTATCCACCGTAGT
TTTCAATATCACCCGATGCCGAATAATATATTATGGGGTATTAATCTTCTTCGAAAGGCTATCCCCCTGATAA
AGGCAGGTTGCACACGTGTTCCGCACCCGTACGCCGCTCTCAATTACCCGAAAGTAATCTACCGCTCGGCTTG
CATGTGTTAGGCCTCCCGCTAGCGTTCATCCTGAGCCAGGATCAAACTCT
>922
CTGCTGCCGCCCCGTAGGAGTCTGGTTCCCGTGTCTCAGTACCCAGTGTGGGGGGGGATCACCCCTCTCAGGC
CCCCTAAAAGATCGTCGCCTGGTGAGCCGTTACCTCACCAACTAACTAATCTTGCGCGTGCCCCATCTCTATC
CCACCGTAGTTTTCAATATCACCCGATGCCCGAATAATATATTTATGGGGTATTAATCTTTCTTCGAAAGGCT
ATCCCCCTGATAAAGGCAGGTTGCACACGTGTTCCCGCACCCGTACGCCGCTCTCAATTACCCCGAAAGTAAT
CTACCGCTCGGCTTGCATGTGTTAGGCCTCCCGCTA
>923
CTGCTGCCGCCCGTAGGAGTCTGGTCCGTGTCTCAGTACCAGTGTGGGGGATCACCCTCTCAGGCCCCCTAAA
AGATCGTCGCCTTGGTGAGCCGTTACCTCACCAACTAGCTAATCTTGCGCGTGCCCATCTCTATCCACCGTAG
TTTTCAATATCACCCGATGCCGAATAATATAATTATGGGGTATTAATCTTCTCGAAAGGCTATCCCCTGATAA
AGGCAGGTTGCACACGTGTTCCGCACCCGTACGCCGCTCTCAATTACCCGAAAGTAATCTACCGCTCGGCTTG
CATGTGTTAGGCCTCCCGCTAGCGTTCATCTGAGCCAGGATCAACTCT
>924
CTGCTGCCGCCCGTAGGAGTCTGGTCCGTGTCTCAGTACCAGTGTGGGGGATCACCCTCTCAGGCCCCCTAAA
GATCGTCGCCTTGGTGAGCCGTTTACCTCACCAACTAGCTAATCTTGCGCGTGCCCATCTCTATCCACCGTAG
TTTTCAATATCAACCCGATGCCGAATAATATATTATGGGGTATTAATCTTCTTCGAAAGGCTATCCCCCTGAT
AAAGGCAGGTTGCACACGTGTTCCGCACCCGTACGCCGCTCTCAATTACCCGAAAGTAATCTACCGCTCGGCT
TGCATGTGTTAGGCCTCCCGCTAGCGTTCATC
>925
CTGCTGCCGCCCGTAGGAGTCTGGTCCGTGTCTCAGTACCAGTGTGGGGGATCACCCTCTCAGGCCCCCTAAA
GATCGTCGCCTTGGTGAGCCGTTACCTCACCAACTAGCTAATCTTGCGCGTGCCCATCTCTATCCACCGTAGT
TTTCAATATCACCCGATGCCGAATAATATATTATGGGGTATTAATCTTCTCGAAAGGCTATCCCCCTGATAAA
GGCAGGTTGCACACGTGTTCCGCACCGTACGCCGCTCTCAATTACCCGAAAGTAATCTACCGCTCGGCTTGCA
TGTGTTAGGCCTCCCGCTAGCGTTCATCCTGAGCCAGGATCAACTCT
>926
CTGCTGCCGCCCGTAGGAGTCTGGTCCGTGTCTCATTACCAGTGTGGGGGATCACCCTCTCAGGCCCCCTAAA
GATCGTCGCCTTGGTGAGTCGTTACCTCACCAACTAGCTAATCTTGCGCGTGCCCATCTCTATCCACCGTAGT
TTTCAATATCACCCGATGCCGAATAATATATTATGGGGTATTAATCTTCTTCGAAAGGCTATCCCCCTGATAA
AGGCAGGTTGCACACGTGTTCCGCACCCGTACGCCGCTCTCAATTACCCGAAAGTAATCTACCGCTCGGCTTG
CATGTGTTAGGCCTCCCGCTAGCGTTCATCCTGAGCCAGGATCAACGATCCTC
>927
CTGCTGCCGCCCGTAGGAGTCTGGTCCGTGTCTCAGTACCAGTGTGGGGGATCACCCTCTCAGGCCCCCTAAA
GATCGTCGCCTTGGTGAGCCGTTACCTCACCAACTAGCTAATCTTGCGCGTGCCCATCTCTATCCACCGTAGT

TTTCAATATCACCCGATGCCGAATAATATATTATGGGGTATTAATCTTCTTCGAAAGGCTATCCCCCTGATAA
AGGCAGGTTGCACACGTGTTCCGCACCCGTACGCCGCTCTCAATTACCCGAAAGTAATCTACCGCTCCGGCTT
GCATGTGTTAGGCCTCCCGCTAGCGTTCATCCTGAGCCAGGATCAAACTCT
>928
CTGCTGCCGCCCGTAGGAGTCTGGTCCGTGTCTCAGTACCAGTGTGGGGGATCACCCTCTCAGGCCCCCTAAA
GATCGTCGCCTTGGTGAGCCGTTACCTCACCAACTAGCTAATCTTGCGCGTGCCCATCTCTATCCACCGTAGT
TTTCAATATCACCCGATGCCGAATAATATATTATGGGGTATTAATCTTCTCGAAAGGCTATCCCCCCGATAAA
GGCAGGTTGCACACGTGTTCCGCACCCGTACGCCGCTCTCAATTACCCGAAAGTAATCTACCGCTCGGCTTGC
ATGTGTTAGGCCTCCCGCTAGCGTTCTCCTGAGCCAAGGATCAAACTCT
>929
CTGCTGCCGCCCGTAGGAGTCTGGTCCGTGTCTCAGTACCAGTGTGGGGGATCACCCTCTCAGGCCCCCTAAA
GATCGTCGCCTTGGTGAGCCGTTACCTCACCAACTAGCTAATCTTGCGCGTGCCCATCTCTATCCACCGTAGT
TTTCAATATCACCCGATGCCGAATAATATATTATGGGGTATTAATCTTCTCGAAAGGCTATCCCCCTGATAAA
GGCAGGTTGCACACGTGTTCCGCACCCGTACGCCGCTCTCAATTACCCGAAAGTAATCTACCGCTCGGCTTGC
ATGTGTTAGGCCTCCCGCTAGCGTCATCCTGAGCCAGGATCAACTCT
>930
CTGCTGCCGCCCGTAGGAGTCTGGTCCGTGTCTCAGTACCAGTGTGGGGGATCACCCTCTCAGGCCCCCTAAA
GATCGTCGCCTTGGTGAGCCGTTACCTCACCAACTAGCTAATCTTGCGCGTGCCCATCTCTATCCACCGTAGT
TTTCAATATCACCCGATGCCGAATAATATATTATGGGGTATTAATCTTCTCGAAAGGCTATCCCCCTGATAAA
GGCAGGTTGCACACGTGTTCCGCACCCGTACGCCGCTCTCAATTACCCGAAAGTAATCTACCGCTCCGGCTTG
CATGTGTTAGGCCTCCCGCTAGCGTTCATCCTGAGCAGGATCAACTCT
>931
CTGCTGCCGCCCGTAGGAGTCTGGTCCGTGTCTCAGTACCAGTGTGGGGGATCACCCTCTCAGGCCCCCTAAA
GATCGTCGCCTTGGTGAGCCGTTACCTCACCAACTAGCTAATCTTGCGCGTGCCCATCTCTATCCACCGTAGT
TTTCAATATCACCCGATGCCGAATAATATATTATGGGGTATTAATCTTCTTCGAAAGGCTATCCCCTGATAAA
GGCAGGTTGCACACGTGTTCCGCACCCGTACGCCGCTCTCAATTACCCGAAAGTAATCTACCGCTCGGCTTGC
ATGTGTTAGGCCTCCCGCTAGCGTTCTCCGTGAGCAGGAT
>932
CTGCTGCCGCCCGTAGGAGTCTGGTCCGTGTCTCAGTACCAGTGTGGGGGATCACCCTCTCAGGCCCCCTAAA
GATCGTCGCCTTGGTGAGCCATTACCTCACCAACTAGCTAATCTTGCGCGTGCCCATCTCTATCCACCGTAGT
TTTCAATATCACCCGATGCCGAATAATATATTATGGGGTATTAATCTTCTCTGAAAGGCTATCCCCTGATAAA
GGCAGGTTGCACACGTGTTCCGCACCCGTACGCCGCTCTCAATTACCCGAAAGTAATCTACCGCTCGGCTTGC
ATGTGTTAGGCCTCCCGCTAGCGTTCATCCTGAGCAGGATCAACTCT
>933
CTGCTGCCGCCCGTAGGAGTCTGGTCCGTGTCTCAGTACCAGTGTGGGGGATCACCCTCTCAGGCCCCCTAAA
GATCGTCGCCTTGGTGAGCCGTTACCTCACCAACTAGCTAATCTTGCGCGTGCCCATCTCTATCCACCGTAGT
TTTCAATATCACCCGATGCCGAATAATATATTATGGGGTATTAATCTTCTTCGAAAGGCTATCCCCTGATAAA
GGCAGGTTGCACACGTGTTCCGCACCCGTACGCCGCTCTCAATTACCCGAAAGTAATCTACCGCTCCGGCTTG
CATGTGTTAGGCCTCCCGCTAGCGTTCATCCTGAGCCAGGATCAACTCT
>934
CTGCTGCCGCCCGTAGGAGTCTGGTCCGTGTCTCAGTACCAGTGTGGGGGATCACCCTCTCAGGCCCCCTAAA
GATCGTCGCCTTGGTGAGCCGTTACCTCACCAACTAGCTAATCTTGCGCGTGCCCATCTCTATCCACCGTAGT
TTTCAATATCACCCGATGCCGAATAATATATTATGGGGTATTAATCTTCTTCGAAAGGCTATCCCCCTGATAA
AGGCAGGTTGCACACGTGTTCCGCACCCGTACGCCGCTCTCAATTACCCGAAAGTAATCTACCGCTCGGCTTG
CATGTGTTAGGCCTCCCGCTAGCGTTCTCCTGAGCAG
>935
CTGCTGCCGCCCGTAGGAGTCTGGTCCGTGTCTCAGTACCAGTGTGGGGGATCACCCTCTCAGGCCCCCTAAA
GATCGTCGCCTTGGTGAGCCGTTACCTCACCAACTAGCTAATCTTGCGCGTGCCCATCTCTATCCACCGTAGT
TTTCAATATCACCCGATGCCGAATAATATATTATGGGGTATTAATCTTCTTCGAAAGGCTATCCCCCTGATAA
AGGCAGGTTGCACACGTGTTCCGCACCCGTACGCCGCTCTCAATTACCCGAAAGTAATCTACCGCTCGGCTTG
CATGTGTTAGGCCTCCCGCTAGCGTTCATCCTGAGCCAGGATCAACTCT
>936
CTGCTGCCGCCCGTAGGAGTCTGGTCCGTGTCTCAGTACCAGTGTGGGGGATCACCCTCTCAGGCCCCCTAAA
GATCGTCGCCTTGGTGAGCCGTTACCTCACCAACTAGCTAATCTTGCGCGTGCCCATCTCTATCCACCGTAGT

TTTCAATATCACCCGATGCCGAATAATATATTATGGGGTATTAATCTTCTCGAAAGGCTATCCCCCTGATAAA
GGCAGGTTGCACACGTGTTCCGCACCCGTACGCCGCTCTCAATTACCGAAAGTAATCTACCGCTCGGCTTGCA
TGTGTTAGGCCTCCCGC
>937
CTGCTGCCGCCCGTAGGAGTCTGGTCCGTGTCTCAGTACCAGTGTGGGGGATCACCCTCTCAGGCCCCCTAAA
GATCGTCGCCTTGGTGAGCCGTTACCTCACCAACTAGCTAATCTTGCGCGTGCCCATCTCTATCCACCGTAGT
TTTCAATATCACCCGATGCCGAATAATATATTATGGGGTATTAATCTTCTTCGAAAGGCTATCCCCCTGATAA
AGGCAGGTTGCACACGTGTTCCGCACCCGTACGCCGCTCTCAATTACCCGAAAGTAGTCTACCGCTCGGCTTG
CATGTGTTAGGCCTCCCGCTAGCGTTCATCCTGAGCCAGGATCAACTCT
>938
CTGCTGCCGCCCGTAGGAGTCTGGTCCGTGTCTCAGTACCAGTGTGGGGGATCACCCTCTCAGGCCCCCTAAA
GATCGTCGCCTTGGTGAGCCGTTACCTCACCAACTAGCTAATCTTGCGCGTGCCCATCTCTATCCACCGTAGT
TTTCAATATCACCCGATGCCGAATAATATATTATGGGGTATTAATCTTCTTCGAAAGGCTATCCCCCTGATAA
AGGCAGGTTGCACACGTGTTCCGCACCCGTACGCCGCTCTCAATTACCCGAAAGTAATCTACCGCTCGGCTTG
CATGTGTTAGGCCTCCCGCTAGCGTTCATCCTGAGCCAGGATCAACTCT
>939
CTGCTGCCGCCCGTAGGAGTCTGGTCCGTGTCTCAGTACCAGTGTGGGGGATCACCCTCTCAGGCCCCCTAAA
GATCGTCGCCTTGGTGAGCCGTTACCTCACCAACTAGCTAATCTTGCGCGTGCCCATCTCTATCCACCGTAGT
TTTCAATATCACCCGATGCCGAATAATATATTATGGGGTATTAATCTTCTTACGAAAGGCTATCCCCTGATAA
AGGCAGGTTGCACACGTGTTCCGCACCCGTACGCCGCTCTCAATTACCCGAAAGTAATCTACCGCATCGGCTT
GCATGTGTTAGGCCTCCCGCTAGCGTTCATCCCG
>940
CTGCTGCCGCCCGTAGGAGTCTGGTCCGTGTCTCAGTACCAGTGTGGGGGATCACCCTCTCAGGCCCCCTAAA
GATCGTCGCCTTGGTGAGCCGTTACCTCACCAACTAGCTAATCTTGCGCGTGCCCATCTCTATCCACCGTAGT
TTTCAATATCACCCGATGCCGAATAATATATTATGGGGTATTAATCTTCTTCGAAAGGCTATCCCCCTGATAA
AGGCAGGTTGCACACGTGTTCCGCACCCGTACGCCGCTCTCAATTACCCGAAAGTAATCTACCGCTCGGCTTG
CATGTGTTAGGCCTCCCGCTAGCGTTCATCCTGAGCCAGGATCAAACTCT
>941
CTGCTGCCGCCCGTAGGAGTCTGGTCCGTGTCTCAGTTACCAGTGTGGGGGATCACCCCTCTCAGGCCCCCCT
AAAGATCGTTCGCCTTGGTGAGCCGTTACCTCACCAACTAGCTAATCTTTGCGCGTGCCCCATCTTCTATCCC
ACCGTAGTTTTCAATTATCACCCCGATGCCCGAATAATATATTTATTGGGGTATTTAATCTTTCTTCGAAAGG
CTATCCCCTGATAAAGGCAGGTTTGCACACGTGTTCCCGCACCCGTACGCCGCTCTCAATTACCCGAAAGTAA
TCTACCGCTCGGCTTGCATGTGTTTAGGCCTTCCCCGCTTAG
>942
CTGCTGCCGCCCGTAGGAGTCTGGTCCGTGTCTCAGTACCAGTGTGGGGGATCACCCTCTCAGGCCCCCTAAA
GATCGTCGCCTTGGTGAGCCGTTACCTCACCAACTAGCTAATCTTGCGCGTGCCCATCTCTATCCACCGTAGT
TTTCAATATCACCCGATGCCGAATAATATATTATGGGGTATTAATCTTCTTCGAAAGGCTATCCCCCTGATAA
AGGCAGGTTGCACACGTGTTCCGCACCCGTACGCCGCTCTCAATTACCCGAAAGTAATCTACCGCTCGGCTTG
CATGTGTTAGGCCTCCCGCTAGCGTTCATCCTGAGCCAGGATCAACTCT
>943
CTGCTGCCGCCCGTAGGGGTCTGGTCCGTGTCTCAGTACCAGTGTGGGGGATCACCCTCTCAGGCCCCCTAAA
GATCGTCGCCTTGGTGAGCCGTTACCTCACCAACTAGCTAATCTTGCGCGTGCCCATCTCTATCCACCGTAGT
TTTCAATATCACCCGATGCCGAATAATATATTATGGGGGTATTAATCTTCTTCGAAAGGCTATCCCCCTGATA
AAGGCAGGTTGCACACGTGTTCCGCACCCGTACGCCGCTCTCAATTACCCGAAAGTAATCTACCGCTCGGCTT
GCATGTGTTAGGCCTCCCGCTAGCGTTCTCCTGAGCAGGATCAACTCT
>944
CTGCTGCCGCCCGTAGGAGTCTGGTCCGTGTCTCAGTACCAGTGTGGGGGATCACCCTCTCAGGCCCCCTAAA
GATCGTCGCCTTGGTGAGCCGTTACCTCACCAACTAGCTAATCTTGCGCGTGCCCATCTCTATCCACCGTAGT
TTCAATATCACCCGATGCCGAATAATATATTATGGGGTATTAATCTTCTTCGAAAGGCTATCCCCCTGATAAA
GGCAGGTTGCACACGTGTTCCGCACCCGTACGCCGCTCTCAATTACCCGAAAGTAATCTACCGCTCGGCTTGC
ATGTGTTAGGCCTCCCGCTAGCGTTCTCCTGAGCAGGATCAACTCT
>945
CTGCTGCCGCCCGTAGGAGTCTGGTCCGTGTCTCAGTACCAGTGTGGGGGATCACCCTCTCAGGCCCCCTAAA
GATCGTCGCCTTGGTGAGCCGTTACCTCACCAACTAGCTAATCTTGCGCGTGCCCATCTCTATCCACCGTAGT

TTTCAATATCACCCGATGCCGAATAATATATTATGGGGTATTAATCTTCTTCGAAAGGCTATCCCCCTGATAA
AGGCAGGTTGCACACGTGTTCCGCACCCGTACGCCGCTCTCAATTACCCGAAAGTAATCTACCGCTCGGCTTG
CATGTGTTAGGCCTCCCGCTAGCGTTCATCCTGAGCCAGGATCAAACTCT
>946
CTGCTGCCGCCCGTAGGAGTCTGGTCCGTGTCTCAGTACCAGTGTGGGGGATCACCCTCTCAGGCCCCCTAAA
GATCGTCGCCTTGGTGAGCCGTTACCTCACCAACTAGCTAATCTTGCGCGTGCCCATCTCTATCCACCGTAGT
TTTCAATATCACCCGATGCCGAATAATATATTATGGGGTATTAATCTTTCTTCGAAAGGCTATCCCCCTGATA
AAGGCAGGTTGCACACGTGTTCCGCACCCGTACGCCGCTCTCAATTACCCGAAAGTAATCTACCGCTCGGCTT
GCATGTGTTAGGCCCTCCGCTAGCGTTCATCCTGAGCCAGGATCAAACTC
>947
CTGCTGCCGCCCGTAGGAGTCTGGTCCGTGTCTCAGTACCAGTGTGGGGGATCACCCTCTCAGGCCCCCTAAA
GATCGTCGCCTTGGTGAGCCGTTACCTCACCAACTAGCTAATCTTGCGCGTGCCCATCTCTATCCACCGTAGT
TTTCAATATCACCCGATGCCGAATAATATATTATGGGGTATTAATCTTCTTCGAAAGGCTATCCCCCTGATAA
AGGCAGGTTGCACACGTGTTCCGCACCCGTACGCCGCTCTCAATTACCCGAAAGTAATCTACCGCTCGGCTTG
CATGTGTTAGGCCTCCCGCTAGCGTTCATCCTGAGCCAGGATCAAACTCT
>948
CTGCTGCCGCCCGTAGGAGTCTGGTCCGTGTCTCAGTACCAGTGTGGGGGATCACCCTCTCAGGCCCCCTAAA
GATCGTCGCCTTGGTGAGCCGTTACCTCACCAACTAGCTAATCTTGCGCGTGCCCATCTCTATCCACCGTAGT
TTTCAATATCACCCGATGCCGAATAATATATTATGGGGTATTAATCTTCTTCGAAAGGCTATCCCCCTGATAA
AGGCAGGTTGCACACGTGTTCCGCACCCGTACGCCGCTCTCAATTACCCGAAAGTAATCTACCGCTCGGCTTG
CATGTGTTAGGCCTCCCGCTAGCGTTCATCCTGAGCCAGGATCAAACTCT
>949
CTGCTGCCGCCGTAGGAGTCTGGTCCGTGTCTCAGTACCAGTGTGGGGGATCACCCTCTCAGGCCCCCTAAAG
ATCGTCGCCTTGGTGAGCCGTTACCTCACCAACTAGCTAATCTTGCGCGTGCCCATCTCTATCCACTGTAGTT
TTTCAATATCACCCGATGCCGAATAATATATTATGGGGTATTAATCTTCTTCGAAAGGCTATCCCCCTGATAA
AGGCAGGTTGCACACGTGTTCCGCACCCGTACGCCGCTCTCAATTACCCCGAAAGTAATCTACCGCTCGGCTT
GCATGTGTTAGGCCTCCCGCTAGCGTTCTCCTGAGCCAGGATCAAACTCT
>950
CTGCTGCCGCCCGTAGGAGTCTGGTCCGTGTCTCAGTACCAGTGTGGGGGATCACCCTCTCAGGCCCCCTAAA
GATCGTCGCCTTGGTGAGCCGTTACCTCACCAACTAGCTAATCTTGCGCGTGCCCATCTCTATCCACCGTAGT
TTTCAATATCACCCGATGCCGAATAATATATTATGGGGTATTAATCTTCCTTCCGAAAGGCTATCCCCCTGAT
AAAGGCCAGGTTGCACACGTGTTCCGCACCCGTACGCCGCTCTCAATTACCCGAAAGTAATCTACCGCTCGGC
TTGCATGTGTTAGGCCTCCCGCTAGCGTCTCCTGAGCAGGATCCAA
>951
CTGCTGCCGCCCGTAGGAGTCTGGTCCGTGTCTCAGTACCAGTGTGGGGGATCACCCTCTCAGGCCCCCTAAA
GATCGTCGCCTTGGTGAGCCGTTACCTCACCAACTAGCTAATCTTGCGCGTGCCCATCTCTATCCACCGTAGT
TTTCAATATCACCCGATGCCGAATAATATATTATGGGGTATTAATCTTCTTCGAAAGGCTATCCCCCTGATAA
AGGCAGGTTGCACACGTGTTCCGCACCCGTACGCCGCTCTCAATTACCCGAAAGTAATCTACCGCTCGGCTTG
CATGTGTTAGGCCTCCCGCTAGCGTTCATCCTGAGCCAGGATCAACTCT
>952
CTGCTGCCGCCCGTAGGAGTCTGGTCCGTGTCTCAGTACCAGTGTGGGGGATCACCCTCTCAGGCCCCCTAAA
GATCGTCGCCTTGGTGAGCCGTTACCTCACCAACTAGCTAATCTTGCGCGTGCCCATCTCTATCCACCGTAGT
TTTCAATATCACCCGATGCCGAATAATATATTATGGGGTATTAATCTTCTTCGAAAGGCTATCCCCCTGATAA
AGGCAGGTTGCACACGTGTTCCGCACCCGTACGCCGCTCTCAATTACCCGAAAGTAATCTACCGCTCGGCTTG
CATGTGTTAGGCCTCCCGCTAGCGTTCATCCTGAGCCAGGATCAAACTCT
>953
CTGCTGCCGCCCGTAGGAGTCTGGTCCGTGTCTCAGTACCAGTGTGGGGGATCACCCTCTCAGGCCCCCTAAA
GATCGTCGCCTTGGTGAGCCGTTACCTCACCAACTAGCTAATCTTGCGCGTGCCCATCTCTATCCACCGTAGT
TTTCAATATCACCCGATGCCGAATAATATATTATGGGGTATTAATCTTCTTCGAAAGGCTATCCCCCTGATAA
AGGCAGGTTGCACACGTGTTCCGCACCCGTGCGCGCTCTCAATTACCCGAAAGTAATCTACCGCTCGGCTTGC
ATGTGTTAGGCCTCCCGCTAGCGTTCATCCTGAGCCAGGATCAAACTCT
>954
CTGCTGCCGCCCGTAGGAGTCTGGTCCGTGTCTCAGTACCAGTGTGGGGGATCACCCTCTCAGGCCCCCTAAA
GATCGTCGCCTTGGTGAGCCGTTACCTCACCAACTAGCTAATCTTGCGCGTGCCCATCTCTATCCACCGTAGT

TTTCAATATCACCCGATGCCGAATAATATATTATGGGGTATTAATCTTCTTCGAAAGGCTATCCCCTGATAAA
GGCAGGTTGCACACGTGTTCCGCACCCGTACGCCGCTCTCAATTACCCGAAAGTAATCTACCGCTCGGCTTGC
ATGTGTTAGGCCTCCCGCTAGCGTTCATCCTGAGCCAGGATCAACTCT
>955
CTGCTGCCGCCCGTAGGAGTCTGGTCCGTGTCTCAGTACCAGTGTGGGGGATCACCCTCTCAGGCCCCCTAAA
GATCGTCGCCTTGGTGAGCCGTTACCTCACCAACTAGCTAATCTTGCGCGTGCCCATCTCTATCCACCGTAGT
TTTCAATATCACCCGATGCCGAATAATATATTATGGGGTATTAATCTTCTTCGAAAGGCTATCCCCTGATAAA
GGCAGGTTGCACACGTGTTCCGCACCCGTACGCCGCTCTCAATTACCCGAAAGTAATCTACCGCTCGGCTTGC
ATGTGTTAGGCCTCCCGCTAGCGTTCATCCTGAGCCAGGATCAAACTCT
>956
CTGCTGCCGCCCGTAGGAGTCTGGTCCGTGTCTCAGTACCAGTGTGGGGGATCACCCTCTCAGGCCCCCTAAA
GATCGTCGCCTTGGTGAGCCGTTACCTCACCAACTAGCTAATCTTGCGCGTGCCCATCTCTATCCACCGTAGT
TTTCAATATCACCCGATGCCGAATAATATATTATGGGGTATTAATCTTCTTCGAAAGGCTATCCCCTGATAAA
GGCAGGTTGCACACGTGTTCCGCACCCGTACGCCGCTCTCAATTACCCGAAAGTAATCTACCGCTCGGCTTGC
ATGTGTTAGGCCTCCCGCTAGCGTTCATCCTGAGCCAGGATCAACTCT
>957
CTGCTGCCGCCCGTAGGAGTCTGGTCCGTGTCTCAGTACCAGTGTGGGGGATCACCCTCTCAGGCCCCCTAAA
GATCGTCGCCTTGTGAGCCATTACCTCACCAACTAGCTAATCTTGCGCGTGCCCATCTCTATCCACCGTAGTT
TTCAATATCACCCGATGCCGAATAATATATTATGGGGTATTAATCTTCTTCGAAGGCTATCCCCCTGATAAAG
GCAGGTTGCACACGTGTTCCGCACCCGTACGCCGCTCTCAATTACCCGAAAGTAATCTACCGCTCGGCTTGCA
TGTGTTAGGCCTCCCGCTAGCGTTCATCCTGAGCCAGGATCAACTCT
>958
CTGCTGCCGCCCGTAGGAGTCTGGACCGTGTCTCAGTTCCAGTGTGACTTGATCATCCTCTCAGACCCAGTAC
GGATCGTCGCCTTGGTAGGCCCTTTACCCCCACCAACTAGCTAATCCGACCTAGGCTCATCTGATAGCGTGAG
GTCCGAAGATCCCCCACTTCTCCCCTCAGGACGTATGCGGTATTAGCGCCCGTTTCCGGACGTATCCCCACTA
CCAGGCAGATTCCTAGGCATTACTCACCCGTCCGCCGCTGAATCCAGGAGCAAGCTCCCCTTCATCCGCTCGA
CTGCATGTGTTAGGCCTGCCGCCAGCGTCAATCTGAGCCAGGATCAAACTCT
>959
CTGCTGCCGCCCGTAGGAGTCTGGTCCGTGTCTCAGTACCAGTGTGGGGATCACCCTCTCAGGCCCCCTAAAG
ATCGTCGCCTTGGTGAGCCGTTACCTCACCAACTAGCTAATCTTGCGCGTGCCCATCTCTATCCACCGTAGTT
TTCAATATCACCCGATGCCGAATAATATATTATGGGGTATTAATCTTCTACTGAAGGCTATCCCCTGATAAAG
GCAGGTTGCACACGTGTTCCGCACCCGTACGCCGCTCTCAATTACCCGAAAGTAATCTACCGCTCGGCTTGCA
TGTGTTAGGCCTCCCGCTAGCGTTCATCCTGAGCCAGGATCAAACTCT
>960
CTGCTGCCGCCCGTAGGAGTCTGGTCCGTGTCTCAGTACCAGTGTGGGGGATCACCCTCTCAGGCCCCCTAAA
GATCGTCGCCTTGGTGAGCCGTTACCTCACCAACTAGCTAATCTTGCGCGTGCCCATCTCTATCCACCGTAGT
TTTCAATATCACCCGATGCCGAATAATATAATTATGGGGTATTAATCTTCTCGAAAGCTATCCCCTGATAAAG
GCAGGTTGCACACGTGTTCCGCACCCGTACGCCGCTCTCAATTACCCGAAAGTAATCTACCGCTCGGCTTGCA
ATGTGTTAGGCCTCCCGCTAGCGTCATCCTGAGCCAGGATCAAACTCT
>961
CTGCTGCCGCCCGTAGGAGTCTGGTCCGTGTCTCAGTACCAGTGTGGGGGATCACCCTCTCAGGCCCCCTAAA
GATCGTCGCCTTGGTGAGCCGTTACCTCACCAACTAGCTAATCTTGCGCGTGCCCATCTCTATCCACCGTAGT
TTTCAATATCACCCGATGCCGAATAATATATTATGGGGTATTAATCTTCTTCGAAAGGCTATCCCCCTGATAA
AGGCAGGTTGCACGCGTGTTCCGCACCCGTACGCCGCTCTCAATTACCCGAAAGTAATCTACCGCTCGGCTTG
CATGTGTTAGGCCTCCCGCTAGCGTCATCCTGAGCCAGGATCAAACTCT
>962
CTGCTGCCGCCCGTAGGAGTCTGGTCCGTGTCTCAGTACCAGTGTGGGGGATCACCCTCTCAGGCCCCCTAAA
GATCGTCGCCTTGGTGAGCCGTTACCTCACCAACTAGCTAATCTTGCGCGTGCCCATCTCTATCCACCGTAGT
TTTCAATATCACCCGATGCCGAATAATATATTATGGGGTATTAATCTTCTTCGAAAGGCTATCCCCCTGATAA
AGGCAGGTTGCACACGTGTTCCGCACCCGTACGCCGCTCTCAATTACCCGAAAGTAATCTACCGCTCGGCTTG
CATGTGTTAGGCCTCCCGCTAGCGTTCATCCTGAGCCAGGATCAAACTCT
>963
CTGCTGCCGCCCGTAGGAGTCTGGTCCGTGTCCCCAGTACCAGTGTGGGGGATCACCCTCTCAGGCCCCCCTA
AAGATCGTCGCCTTGGTGAGCCGTTACCTCACCAACTAGCTAATCTTGCGCGTGCCCCATCTCTATCCACCGT

AGTTTTCAATATCACCCCGATGCCGAATAATATTATTATGGGGTATTAATCTTCTTCGAAAGGCTATCCCCCT
GATAAAGGCAGGTTGCACACGTGTTCCGCACCCGTACGCCGCTCTCAATTACCCCGAAAGTAATCTACCGCTC
GGCTTGCATGTGTTAGGCCTCCCGCTAGCGTTCTCCTGAGCCAGGATCAAACTCT
>964
CTGCTGCCGCCCGTAGGAGTCTGGTCCGTGTCTCAGTACCAGTGTGGGGGATCACCCTCTCAGGCCCCCTAAA
GATCGTCGCCTTGGTGAGCCGTTACCTCACCAACTAGCTAATCTTGCGCGTGCCCATCTCTATCCACCGTAGT
TTTCAATATCACCCGATGCCGAATAATATATTATGGGGTATTAATCTTCTTCGAAAGGCTATCCCCTGATAAA
GGCAGGTTGCACACGTGTTCCGCACCCGTACGCCGCTCTCAATTACCCGAAAGTAATCTACCGCTCGGCTTGC
ATGTGTTAGGCCTCCCGCTAGCGTTCATCCTGAGCCAGGATCAAACTCT
>965
CTGCTGCCGCCCGTAGGAGTCTGGTCCGTGTCTCAGTACCAGTGTGGGGGATCACCCTCTCAGGCCCCCTAAA
GATCGTCGCCTTGGTGAGCCGTTACCTCACCAACTAGCTAATCTTGCGCGTGCCCATCTCTATCCACCGTAGT
TTTCAATATCACCCGATGCCGAATAATATATTATGGGGTATTAATCTTCTTCGAAAGGCTATCCCCCTGATAA
AGGCAGGTTGCACACGTGTTCCGCACCCGTACGCCGCTCTCAATTACCCGAAAGTAATCTACCGCTCGGCTTG
CATGTGTTAGGCCTCCCGCTAGCGTTCATCCGTGAGCCAGGATCAAACTCT
>966
CTGCTGCCGCCCGTAGGAGTCTGGTCCGTGTCTCAGTACCAGTGTGGGGGATCACCCTCTCAGGCCCCCTAAA
GATCGTCGCCTGGTGAGCCGTTACCTCACCAACTAGCTAATCTTGCGCGTGCCCATCTCTATCCACCGTAGTT
TTCAATATCACCCGATGCCGAATAATATATTATGGGGTATTAATCTTCTTCGAAAGGCTATCCCCCTGATAAA
GGCAGGTTGCACACGTGTTCCGCACCCGTACGCCGCTCTCAATTACCCGAAAGTAATCTACCGCTCGGCTTGC
ATGTGTTAGGCCTCCCGCTAGCGTTCATCCTGAGCCAGGATCAAACTCT
>967
CTGCTGCCGCCCGTAGGAGTCTGGTCCGTGTCTCAGTACCAGTGTGGGGGATCACCCTCTCAGGCCCCCTAAA
GATCGTCGCCTTGGTGAGCCGTTACCTCACCAACTAGCTAATCTTGCGCGTGCCCATCTCTATCCACCGTAGT
TTTCAATATCACCCGATGCCGAATAATATATTATGGGGTATTAATCTTCTTCGAAGGCTATCCCCCTGAATAA
AGGCAGGTTGCACACGTGTTCCGCACCCGTACGCCGCTCTCAATTACCCGAAAGTAATCTACCGCTCGGCTTG
CATGTGTTAGGCCTCCCGCTAGCGTTCATCCTGAGCCAGGATCAAACTCT
>968
CTGCTGCCGCCCGTAGGAGCTGGTCCGTGTCTCAGTACCAGTGTGGGGGATCACCCTCTCAGGCCCCCTAAAG
ATCGTCGCCTTGGTGAGCCGTTACCTCACCAACTAGCTAATCTTGCGCGTGCCCATCTCTATCCACCGTAGTT
TTCAATATCACCCGATGCCGAATAATATATTATGGGGTATTAATCTTCTTCGAAAGGCTATCCCCCTGATAAA
GGCAGGTTGCACACGTGTTCCGCACCCGTACGCCGCTCTCAATTACCCGAAAGTAATCTACCGCTCGGCTTGC
ATGTGTTAGGCCTCCCGCTAGCGTTCATCCTGAGCCAGGATCAACTCT
>969
CTGCTGCCGCCCGTAGGAGTCTGGTCCGTGTCTCAGTACCAGTGTGGGGGATCACCCTCTCAGGCCCCCTAAA
GATCGTCGCCTTGGTGAGCCGTTACCTCACCAACTAGCTAATCTTGCGCGTGCCCATTTCTATCCACCGTAGT
TTTCAATATCACCCGATGCCGAATAATATATTATGGGGTATTAATCTTCTTCGAAAGGCTATCCCCTGATAAA
GGCAGGTTGCACACGTGTTCCGCACCCGTACGCCGCTCTCAATTACCCGAAAGTAATCTACCGCTCGGCTTGC
ATGTGTTAGGCCTCCCGCTAGCGTTCATCCGTGAGCCAGGATCAAACTCT
>970
CTGCTGCCGCCCGTAGGAGTCTGGACCGTGTCTCAGTTCCAGTGTGACTGATCATCCTCTCAGACCAGTACGG
ATCGTCGCCTTGGTAGGCCTTTACCCCACCAACTAGCTAATCCGACCTAGGCTCATCTGATAGCGTGAGGTCC
GAAGATCCCCCACTTCTCCCTCAGGACGTATGCGGTATTAGCGCCCGTTTCCGGACGTATCCCCACTACCAGG
CAGATTCCTAGGCATTACTCACCCGTCCGCCGCTGAATCCAGGAGCAAGCTCCCTTCATCCGCTCGACTGCAT
GTGTTAGGCCTGCCGCCAGCGTCAATCTGAGCCAGGATCAAACTCT
>971
CTGCTGCCGCCTCGGAGGATCGGTCCGTGTCTCAGTACCAGTGTGGGGGATCACCCTCTCAGGCCCCCTAAAG
ATCGTCGCCTTGGTGAGCCGTTACCTCACCAACTAGCTAATCTTGCGCGTGCCCATCTCTATCCACCGTAGTT
TTCAATATCACCCGATGCCGAATAATATATTATGGGGTATTAATCTTCTTCGAAAGCTATCCCCTGATAAAGG
CAGGTTGCACACGTGTTCCGCACCCGTACGCCGCTCTCAATTACCCGAAAGTAATCTACCGCTCGGCTTGCAT
GTGTTAGGCCTCCCGCTAGCGTTCATCCTGAGCCAGGATCAACTCT
>972
CTGCTGCCGCCCGTAGGAGTCTGGTCCGTGTCTCAGTACCAGTGTGGGGGATCACCCTCTCAGGCCCCCTAAA
GATCGTCGCCTTGGTGAGCCGTTACCTCACCAACTAGCTAATCTTGCGCGTGCCCATCTCTATCCACCGTAGT

TTTCAATATCACCCGATGCCGAATAATATATTATGGGGTATTAATCTTCTTCGAAAGGCTATCCCCCTGATAA
AGGCAGGTTGCACACGTGTTCCGCACCCGTACGCCGCTCTCAATTACCCGAAAGTAATCTACCGCTCGGCTTG
CATGTGTTAGGCCTCCCGCTAGCGTTCATCCTGAGCCAGGATCAAACTCT
>973
CTGCTGCCGCCCGTAGGAGTCTGGTCCGTGTCTCAGTACCAGTGTGGGGGATCACCCTCTCAGGCCCCTAAAG
ATCGTCGCCTTGGTGAGCCGTTACCTCACCAACTAGCTAATCTTGCGCGTGCCCATCTCTATCCACCGTAGTT
TTCAATATCACCCGATGCCGAATAATATATTATGGGGTATTAATCTTCTTCGAAAGGCTATCCCCCTGATAAA
GGCAGGTTGCACACGTGTTCCGCACCCGTACGCCGCTCTCAATTACCCGAAAGTAATCTACGCTCGGCTTGCA
TGTGTTAGGCTCCCGCTAGCGTC
>974
CTGCTGCCGCCCGTAGGAGTCTGGTCCGTGTCTCAGTACCAGTGTGGGGGATCACCCTCTCAGGCCCCCTAAA
GATCGTCGCCTTGGTGAGCCGTTACCTCACCAACTAGCTAATCTTGCGCGTGCCCATCTCTATCCACCGTAGT
TTTCAATATCACCCGATGCCGAATAATATATTATGGGGTATTTAATCTTCTTCGAAAGGCTATCCCCCTGATA
AAGGCAGGTTGCACACGTGTTCCGCACCCGTACGCCGCTCTCAATTACCCGAAAGTAATCTACCGCTCGGCTT
GCATGTGTTAGGCCTCCCGCTAGCGTTCATCCTGAGCCAGGATCAAACTCT
>975
CTGCTGCCGCCCGTAGGAGTCTGGTCCGTGTCTCAGTACCAGTGTGGGGGATCACCCTCTCAGGCCCCCTAAA
GATCGTCGCCTTGGTGAGCCGTTACCTCACCAACTAGCTAATCTTGCGCGTGCCCATCTCTATCCACCGTAGT
TTTCAATATCACCCGATGCCGAATAATATATTATGGGGTATTAATCTTCTTCGAAAGGCTATCCCCCTGATAA
AGGCAGGTTGCACACGTGTTCCGCACCCGTACGCCGCTCTCAATTACCCGAAAGTAATCTACCGCTCGGCTTG
CATGTGTTAGGCCTCCCGCTAGCGTTCATCCTGAGCCAGGATCAAACTCT
>976
CTGCTGCCGCCCGTAGGAGTCTGGTCCGTGTCTCAGTACCAGTGTGGGGGATCACCCTCTCAGGCCCCCTAAA
GATCGTCGCCTTGGTGAGCCGTTACCTCACCATCTAGCTAATCTTGCGCGTGCCCATCTCTATCCACCGTAGT
TTTCAATATCACCCGATGCCGAATAATATATTATGGGGTATTAATCCTCTTCGAAAGGCTATCCCCCTGATAA
AGGCAGGTTGCACACGTGTTCCGCACCCGTACGCCGCTCTCAATTACCCGAAAGTAATCTACCGCTCGGCTTG
CATGTGTTAGGCCTCCCGCTAGCGTTCATCCTGAGCAGGATCAACTCT
>977
CTGCTGCCGCCCGTAGGAGTCTGGTCCGTGTCTCAGTACCAGTGTGGGGGATCACCCTCTCAGGCCCCCTAAA
GATCGTCGCCTTGGTGAGCCGTTACCTCACCAACTAGCTAATCTTGCGCGTGCCCATCTCTATCCACCGTAGT
TTTCAATATCACCCGATGCCGAATAATATAATTATGGGGTATTAATCTTCTTCGAAAGGCTATCCCCTGATAA
AGGCAGGTTGCACACGTGTTCCGCACCCGTACGCCGCTCTCAATTTACCCGAAAGTAATCTACCGCTCGGCTT
GCATGTGTTAGGCCTCCCGCTAGCGTTCTCCTGAGCAGGTCAAACTCG
>978
CTGCTGCCGCCCGTAGGAGTCTGGTCCGTGTCTCAGTACCAGTGTGGGGGATCACCCTCTCAGGCCCCCTAAA
GATCGTCGCCTTGGTGAGCCGTTACCTCACCAACTAGCTAATCTTGCGCGTGCCCATCTCTATCCACCGTAGT
TTTCAATATCACCCGATGCCGAATAATATATTATGGGGTATTAATCTTCTTCGAAAGGCTATCCCCCTGATAA
AGGCAGGTTGCACACGTGTTCCGCACCCGTACGCCGCTCTCAATTACCCGAAAGTAATCTACCGCTCGGCTTG
CATGTGTTAGGCCTCCCGCTAGCGTTCATCCTGAGCCAGGATCAAACTCT
>979
CTGCTGCCGCCCGTAGGAGTCTGGTCCGTGTCTCAGTACCAGTGTGGGGGATCACCCTCTCAGGCCCCCTAAA
GATCGTCGCCTTGGTGAGCCGTTACCTCACCAACTAGCTAATCTTGCGCGTGCCCATCTCTATCCACCGTAGT
TTTCAATATCACCCGATGCCGAATAATATATTATGGGGTATTAATCTTCTTCGAAAGGCTATCCCCCTGATAA
AGGCAGGTTGCACACGTGTTCCGCACCCGTACGCCGCTCTCAATTACCCGAAAGTAATCTACCGCTCGGCTTG
CATGTGTTAGGCCTCCCGCTAGCGTTCATCCTGAGCCAGAATCAAACTCT
>980
CTGCTGCCGCCCGTAGGAGTCTGGTCCGTGTCTCAGTACCAGTGTGGGGGATCACCCTCTCAGGCCCCCTAAA
GATCGTCGCCTTGGTGAGCCGTTACCTCACCAACTAGCTAATCTTGCGCGTGCCCATCTCTATCCACCGTAGT
TTTCAATATCACCCGATGCCGAATAATATATTATGGGGTATTAATCTTCTTCGAAAGGCTATCCCCCCGATAA
AGGCAGGTTGCACACGTGTTCCGCACCCGTACGCCGCTCTCAATTACCCGAAAGTAATCTACCGCTCGGCTTG
CATGTGTTAGGCCTCCCGCTAGCGTTCTCCTGAGCCAGGATCA
>981
CTGCTGCTGCCCGTAGGAGTCTGGTCCGTGTCTCAGTACCAGTGTGGGGGATCACCCTCTCAGGCCCCCTAAA
GATCGTCGCCTTGGTGAGCCGTTACCTCACCAACTAGCTAATCTTGCGCGTGCCCATCTCTATCCACCGTAGT

TTTCAATATCACCCGATGCCGAATAATATATTATGGGGTATTAATCTTCTTCGAAAGGCTATCCCCCTGATAA
AGGCAGGTTGCACACGTGTTCCGCACCCGTACGCCGCTCTCAATTTACCCGAAAGTAATCTACCGCTCGGCTT
GCATGTGTTAGGCCTCCCGCTAGCGTTCATCCTGAGCCAGGATCAACTCT
>982
CTGCTGCCGCCCGTAGGAGTCTGGTCCGTGTCTCAGTACCAGTGTGGGGGATCACCCTCTCAGGCCCCCTAAA
GATCGTCGCCTTGGTGAGCCGTTACCTCACCAACTAGCTAATCTTGCGCGTGCCCATCTCTATCCACCGTAGT
TTTCAATATCACCCGATGCCGAATAATATATTATGGGGTATTAATCTTCTTCGAAAGGCTATCCCCTGATAAA
GGCAGGTTGCACACGTGTTCCGCACCCGTACGCCGCTCTCAATTACCCGAAAGTAATCTACCGCTCGGCTTGC
ATGTGTTAGGCCTCCCGCTAGCGTTCTCCTGAGCAGATCAACTCGT
>983
CTGCTGCCGCCCGTAGGAGTCTGGTCCGTGTCTCAGTACCAGTGTGGGGGATCACCCTCTCAGGCCCCCTAAA
GATCGTCGCCTTGGTGAGCCGTTACCTCACCAACTAGCTAATCTTGCGCGTGCCCATCTCTATCCACCGTAGT
TTTCAATATCACCCGATGCCGAATAATATATTATGGGGTATTAATCTTCTTCGAAAGGCTATCCCCTGATAAA
GGCAGGTTGCACACGTGTTCCGCACCCGTACGCCGCTCTCAATTTACCCGAAAGTAATCTACCGCTCGGCTTG
CATGTGTTAGGCCTCCCGCTAGCGTTCTCCTGAGCAGGATCCAAGTC
>984
CTGCTGCCGCCCGTAGGAGTCTGGTCCGTGTCTCAGTACCAGTGTGGGGGATCACCCTCTCAGGCCCCCTAAA
GATCGTCGCCTTGGTGAGCCGTTACCTCACCAACTAGCTAATCTTGCGCGTGCCCATCTCTATCCACCGTAGT
TTTCAATATCACCCGATGCCGAATAATATATTATGGGGTATTAATCTTCTCGAAAGGCTATCCCCTGATAAAG
GCAGGTTGCACACGTGTTCCGCACCCGTACGCCGCTCTCAATTACCCGAAAGTAATCTACCGCTCCGGCTTGC
ATGTGTTAGGCCTCCCGCTAGCGTTCATCCTGAGCCAGGATCAAACTCT
>985
CTGCTGCCGCCCGGAGGAGTCTGGTCCGTATCTCAGTACCAGTGTGGGGGATCACCCTCTCAGGCCCCCTAAA
GATCGTCGCCTTGGTGAGCCGTTACCTCACCAACTAGCTAATCTTGCGCGTGCCCATCTCTATCCACCGTAGT
TTTCAATATCACCCGATGCCGAATAATATATTATGGGGTATTAATCTTCTTCGAAAGGCTATCCCCCTGATAA
AGGCAGGTTGCACACGTGTTCCGCACCCGTACGCCGCTCTCAATTACCCGAAAGTAATCTACCGCTCGGCTTG
CATGTGTTAGGCCTCCCGCTAGCGTTCATCCTGAGCCAGGATCAAACTCT
>986
CTGCTGCCGCCCGTAGGAGTCTGGTCCGTGTCTCAGTTCCAGTGTGACTGATCATCCTCTCAGACCAGTACGG
ATCGTCGCCTTGGTAGGCCTTACCCCACCAACTAGCTAATCCGACCTAGGCTCATCTGATAGCGTGAGGTCCG
AAGTCCCCCACTTCTCCCTCAGGACGTATGCGGTATTAGCGCCCGTTTCCGGACGTATCCCCACTACCAGGCA
GATCCTAGGCATTACTCACCCGTCCGCCGCTGAATCCAGGAGCAAGCTCCCTCATCCGCTCGACTGCCATGTG
TTAGGCCTGCCGCCAGCGTTCAATCTGAGCCAGGATCAAACTCT
>987
CTGCTGCCGCCCGTAGGAGTCTGGTCCGTGTCTCAGTACCAGTGTGGGGGATCACCCTCTCAGGCCCCCTAAA
GATCGTCGCCTTGGTGAGCCGTTACCTCACCAACTAGCTAATCTTGCGCGTGCCCATCTCTATCCACCGTAGT
TTCAATATCACCCGATGCCGAATAATATATTATGGGGTATTAATCTTCTTCGAAAGGCTATCCCCCTGATAAA
GGCAGGTTGCACACGTGTTCCGCACCCGTACGCCGCTCTCAATTACCCGAAAGTAATCTACCGCTCCGGCTTG
CATGTGTTAGGCCTCCCGCTAGCGTTCAATCTGAGCCAGGATCAAACTCT
>988
CTGCTGCCGCCCGTAGGAGTCTGGTCCGTGTCTCAGTACCAGTGTGGGGGATCACCCTCTCAGGCCCCCTAAA
GATCGTCGCCTTGGTGAGCCGTTACCTCACCAACTAGCTAATCTTGCGCGTGCCCATCTCTATCCACCGTAGT
TTTCAATATCACCCGATGCCGAATAATATAATTATGGGGTATTAATCTTCTTCGAAAGGCTATCCCCCTGATA
AAGGCAGGTTAGCACACGTGTTCCGCACCCGTACGCCGCTCTCAATTACCCGAAAGTAATCTACCGCTCGGCT
TGCATGTGTTAGGCCTCCCGCTAGCGTTCATCCTGAGCAGG
>989
CTGCTGCCGCCCGTAGGAGTCTGGTCCGTGTCTCAGTACCAGTGTGGGGGATCACCCTCTCAGGCCCCCTAAA
GATCGTTCGCCTGGTGAGCCGTTACCTCACCAACTAGCTAATCTTGCGCGTGCCCATCTCTATCCACCGTAGT
TTTTCAATATCACCCGATGCCGAATAATATATTTATTGGGGTATTTAATCTTTCTCGAAAGGCTATCCCCCTG
ATAAAGGCAGGTTTGCACACGTGTTCCGCACCCGTACGCCGCTCTCAATTACCCGAAAGTAATCTACCGCTCC
GGCTTGCATGTGTTTAGGCCTTCCCGCTAGCGTTCTC
>990
CTGCTGCTGCCCGTAGGAGTCTGGTCCGTGTCTCAGTACCAGTGTGGGGGGGGATCACCCTCTCAGGCCCCCT
AAAGATCGTCGCCTGGTGAGCCGTTACCTCACCAACTAGCTAATCTTGCGCGTGCCCATCTCTATCCACCGTA

GTTTTCAATATCACCCGATGCCCGAATAATATATTATGGGGGTATTAATCTTCTCGAAAGGCTATCCCCTGA
TAAAGGGCAGGGTTGCACACGTGTTCCGCACCCGTACGCCGCTCTCAATTACCCCGAAAGTAATCTACCGCTC
GGGCTGCATGTGTTAGGCCTCCCGCTAGCGTCATCCGTGAGCCAGGGATCAAACTCT
>991
CTGCTGCCGCCCGTAGGAGTCTGGTTCCGTGTCTCAGTACCAGTGTGGGGATCACCCTCTCAGGCCCCCTAAA
GATCGTCGCCTTGTGAGCCGTTACCTCACCAACTAGCTAATCTTGCGCGTGCCCATCTCTATCCACCGTAGTT
TTCAATATCACCCGATGCCGAATAATATATTATGGGGTATTAATCTTCTTCGAAAGGCTATCCCCCTGATTAA
AGGCAGGTTGCACACGTGTTCCGCACCCGTACGCCGCTCTCAATTACCCGAAAGTAATCTACCGCTCGGCTTG
CATGTGTTACGGCCTCCCGCTAGGCCTCGTTC
>992
CTGCTGCCGCCCGTAGGAGTCTGGACCGTGTCTCAGTTCCAGTGTGACTGATCATCCCCTCAGACCAGTACGG
ATCGTCGCCTTGGTAGGCCTTTTACCCACCAACCTAGCTAATCCGACCTAGGCTCATCTGATAGCGTGAGGTC
CGAAGTCCCCCACTTCTCCCTCAGGACGTATGCGGTATTAGCGCCCGTTTCCGGACGATCCCCCACTACCAGG
CAGATTCCTAGGCATTACTCACCCGTCCGCCGCTGAATCCAGGAGCAAGCTCCCTTCATCCGCTCGACTGCCA
TTGTGTTAGGCCTGCCGCCAGCGTCAATCTGAGCCAGGATCAACTCT
>993
CTGCTGCCGCCCGTAGGAGTCTGGTCCGTGTCTCAGTACCAGTGTGGGGGATCACCCTCTCAGGCCCCCTAAA
GATCGTCGCCTTGGTGAGCCGTTACCTCACCAACTAGCTAATCTTGCGCGTGCCCATCTCTATCCACCGTAGT
TTTCAATATCACCCGATGCCGAATAATATATTATGGGGTATTAATCTTCTTCGAAAGGCTATCCCCCTGATAA
AGGCAGGTTGCACACGTGTTCCGCACCCGTACGCCGCTCTCAATTACCCGAAAGTAATCTACCGCTCGGCTTG
CATGTGTTAGGCCTCCCGCTAGCGTTCTCCTGAGCCAGGATCCAACTCGT
>994
CTGCTGCCGCCCGTAGGAGTCTGGTCCGTGTCTCAGTACCAGTGTGGGGGATCACCCTCTCAGGCCCCCTAAA
GATCGTCGCCTTGGTGAGCCGTTACCTCACCAACTAGCTAATCTTGCGCGTGCCCATCTCTATCCACCGTAGT
TTTCAATATCACCCGATGCCGAATAATATATTATGGGGTATTAATCTTCTTCGAAAGGCTATCCCCCTGATAA
AGGCAGGTTGCACACGTGTTCCGCACCCGTACGCCGCTCTCAATTACCCGAAAGTAATCTACCGCTCGGCTTG
CATGTGTTAGGCCTCCCGCTAGCGTTCATCCTGAGCCAGGATCAAACTCT
>995
CTGCTACCGCCCGTAGGAGTCTGGTCCGTGTCTCAGTACCAGTGTGGGGGATCACCCTCTCAGGCCCCCTAAA
GATCGTCGCCTTGGTGAGCCGTTACCTCACCAACTAGCTAATCTTGCGCGTGCCCATCTCTACCCACCGTAGT
TTTCAATATCACCCGATGCCGAATAATATATTATGGGGTATTAATCTTCTTCGAAAGGCTATCCCCCTGATAA
AGGCAGGTTGCACACGTGTTCCGCACCCGTACGCCGCTCTCAATTACCCGAAAGTAATCTACCGCTCGGCTTG
CATGTGTTAGGCCTCCCGCTAGCGTTCATTCCTGAGCCAGGATCAAACTCT
>996
CTGCTGCCGCCCGTAGGAGTCTGGTCCGTGTCTCAGTACCAGTGTGGGGGATCACCCTCTCAGGCCCCCTAAA
GATCGTCGCCTTGGTGAGCCGTTACCTCACCAACTAGCTAATCTTGCGCGTGCCCATCTCTATCCACCGTAGT
TTTCAATATCACCCGATGCCGAATAATATAATTATGGGGTATTAATCTTCTCGAAAGGCTATCCCCCTGATAA
AGGCAGGTTGCACACGTGTTCCGCACCCGTACGCCGCTCTCAATTACCCGAAAGTAATCTACCGCATCGGCTT
GCATGTGTTAGGCCTCCCGCTAGCGTTCTCCTGAGCAGGATCAACTCT
>997
CTGCTGCCGCCCGTAGGAGTCTGGTCCGTGTCTCAGTACCAGTGTGGGGGATCACCCTCTCAGGCCCCCTAAA
GATCGTCGCCTTGGTGAGCCGTTACCTCACCAACTAGCTAATCTTGCGCGTGCCCATCTCTATCCACCGTAGT
TTTCAATATCACCCGATGCCGAATAATATATTATGGGGTATTAATCTTCTTCGAAAGGCTATCCCCCTGATAA
AGGCAGGTTGCACACGTGTTCCGCACCCGTACGCCGCTCTCAATTACCCGAAAGTAATCTACCGCTCGGCTTG
CATGTGTTAGGCCTCCCGCTAGCGTTCATCCTGAGCCAGGATCAAACTCT
>998
CTGCTGCCGCCCGTAGGAGTCTGGTCCGTGTCTCAGTACCAGTGTGGGGGATCACCCTCTCAGGCCCCCTAAA
GATCGTCGCCTTGGTGAGCCGTTACCTCACCAACTAGCTAATCTTGCGCGTGCCCATCTCTATCCACCGTAGT
TTTCAATATCACCCGATGCCGAATAATATATTATGGGGTATTAATCTTCTTCGAAAGGCTATCCCCCTGATAA
AGGCAGGTTGCACACGTGTTCCGCACCCGTACGCCGCTCCCAATTACCCGAAAGTAATCTACCGCTCGGCTTG
CATGTGTTAGGCCTCCCGCTAGCGTTCATCCCGAGCCAGGATCAAACTCT
>999
CTGCTGCCGCCCGTAGGAGTCTGGTCCGTGTCTCAGTACCAGTGTGGGGGATCACCCTCTCAGGCCCCCTAAA
GATCGTCGCCTGGTGAGCCGTTACCTCACCAACTAGCTAATCTTGCGCGTGCCCATCTCTATCCACCGTAGTT

TTCAATATCACCCGATGCCGAATAATATATTATGGGGTATTAATCTTCTCGAAAGCTATCCCCCTGATAAAGG
CAGGTTGCACACGTGTTCCGCACCCGTACGCCGCTCTCAATTACCCGAAAGTAATCTACCGCTCGGCTTGCAT
GTGTTAGGCCTCCCGCTAGCGTTCATCCTGAGCCAGGATCAAACTCT
>1000
CTGCTGCCGCCCGTAGGAGTCTGGTCCGTGTCTCAGTACCAGTGTGGGGGATCACCCTCTCAGGCCCCCTAAA
GATCGTCGCCTTGGTGAGCCGTTACCTCACCAACTAGCTAATCTTGCGCGTGCCCATCTCTATCCACCGTAGT
TTTCAATATCACCCGATGCCGAATAATATATTATGGGGTATTAATCTTCTTCGAAAGGCTATCCCCCTGATAA
AGGCAGGTTGCACACGTGTTCCGCACCCGTACGCCGCTCTCAATTACCCGAAAGTAATCTACCGCTCGGCTTG
CATGTGTTAGGCCTCCCGCTAGCGTTCATCCTGAG
>1001
CTGCTGCCGCCCGTAGGAGTCTGGACCGTGTCTCAGTTCCAGTGTGGCCGATCACCCTCTCAGGTCGGCTATG
CATCGTTGCCTTGGTAAGCCGTTACCTTACCAACTAGCTAATGCAGCGCGGGTCCATCTATAAGTGACAGCAA
GACCGTCTTTCACTAACCATGCGGTTCAAAATATTATCCGGTATTAGCTTCCGGTTTCCCGAAGTTATCCCAG
TCTTATAGGTAGGTTATCCACGTGTTACTCACCCGTCCGCCGCTAACATCAGAGAAGCAAGCTTCTCGTCCGT
TCGCTCGACTGCATGTATTAGGCACGCCGCCAGCGTCTCCTGAGCCAGGATCAACTCT
>1002
CTGCTGCCGCCCGTAGGAGTCTGGTCCGTGTCTCAGTACCAATGTGGGGGATCACCCTCTCAGGCCCCCTAAA
GATCGTCGCCTTGGTGAGCCGTTACCTCACCAACTAGCTAATCTTGCGCGTGCCCATCTCTATCCACCGTAGT
TTTCAATATCACCCGATGCCGAATAATATATTATGGGGTATTAATCTTCTTCGAAAGGCTATCCCCCTGATAA
AGGCAGGTTGCACACGTGTTCCGCACCCGTACGCCGCTCTCAATTACCCGAAAGTAATCTACCGCTCGGCTTG
CATGTGTTAGGCCTCCCGCTAGCGTTCATCCTGAGCCAGGATCAAACTCT
>1003
CTGCTGCCGCCCGTAGGAGTCTGGTCCGTGTCTCAGTACCAGTGTGGGGGATCACCCTCTCAGGCCCCCTAAA
GATCGTCGCCTTGGTGAGCCGTTACCTCACCAACTAGCTAATCTTGCGCGTGCCCATCTCTATCCACCGTAGT
TTTCAATATCACCCGATGCCGAATAATATATTATGGGGTATTAATCTTCCTTCGAAGAGCTATCCCCCTGATA
AAGGCAGGTTGCACACGTGTTCCGCACCCGTACGCCGCTCTCAATTACCCGAAAGTAATCTACCGCTCGGCTT
GCATGTGTTAGGCCTCCCGCTAGCGTCATCCTGAGCCAGGATCAAACTCT
>1004
CTGCTGCCGCCCGTAGGAGTCTGGTCCGTGTCTCAGTACCAGTGTGGGGGATCACCCTCTCAGGCCCCCTAAA
GATCGTCGCCTGGTGAGCCGTTACCTCACCAACTAGCTAATCTTGCGCGTGCCCATCTCTATCCACCGTAGTT
TTCAATATCACCCGATGCCGAATAATATATTATGGGGTATTAATCTTCTCGAAAGGCTATCCCCTGATAAAGG
CAGGTTGCACACGTGTTCCGCACCCGTACGCCGCTCTCAATTACCCGAAAGTAATCTACCGCTCGGCTTGCAT
GTGTTAGGCCTCCCGCTAGCGTTCATCCTGAGCCAGGATCAACTCT
>1005
CTGCTGCCGCCCGTAGGAGTCTGGTCCGTGTCTCAGTACCAGTGTGGGGGATCACCCTCTCAGGCCCCCTAAA
GATCGTCGCCTTGGTGAGCCGTTACCTCACCAACTAGCTAATCTTGCGCGTGCCCATCTCTATCCACCGTAGT
TTTCAATATCACCCGATGCCGAATAATATGTTATGGGGTATTAATCTTCTTCGAAAGGCTATCCCCCTGATAA
AGGCAGGTTGCGCACGTGTTCCGCACCCGTACGCCGCTCTCAATTACCCGAAAGTAATCTACCGCTCGGCTTG
CATGTGTTAGGCCTCCCGCTAGCGTTCATCCTGAGCCAGGATCAAACTCT
>1006
CTGCTGCCGCCCGTAGGAGTCTGGTCCGTGTCTCAGTACCAGTGTGGGGGATCACCCTCTCAGGCCCCCTAAA
GATCGTCGCCTTGGTGAGCCGTTACCTCACCAACTAGCTAACCTTGCGCGTGCCCATCTCTATCCACCGTAGT
TTTCAATATCACCCGATGCCGAATAATATATTATGGGGTATTAATCTTCTTCGAAAGGCTATCCCCTGATAAA
GGCAGGTTGCACACGTGTTCCGCACCCGTACGCCGCTCTCAATTACCCGAAAGTAATCTACCGCTCGGCTTGC
ATGTGTTAGGCCTCCCGCTAGCGTTCATCCTG
>1007
CTGCTGCCGCCCGTAGGAGTCTGGTCCGTGTCTCAGTACCAGTGTGGGGGATCACCCTCTCAGGCCCCCTAAA
GATCGTCGCCTTGGTGAGCCGTTACCTCACCAACTAGCTAATCTTGCGCGTGCCCATCTCTATCCACCGTAGT
TTTCAATATCACCCGATGCCGAATAATATATTATGGGGTATTAATCTTCTTCGAAAGGCTATCCCCCTGATAA
AGGCAGGTTGCACACGTGTTCCGCACCCGTACGCCGCTCTCAATTACCCGAAAGTAATCTACCGCTCGGCTTG
CATGTGTTAGGCCTCCCGCTAGCGTTCATCCTGAGCCAGGATCAACTCT
>1008
CTGCTGCCGCCCGTAGGAGTCTGGTCCGTGTCTCAGTACCAGTGTGGGGGATCACCCTCTCAGGCCCCCTAAA
GATCGTCGCCTTGGTGAGCCGTTACCTCACCAACTAGCTAATCTTGCGCGTGCCCATCTCTATCCACCGTAGT

TTTCAATATCACCCGATGCCGAATAATATATTATGGGGTATTAATCTTCTTCGAAAGGCTATCCCCCTGATAA
AGGCAGGTTGCACACGTGTTCCGCACCCGTACGCCGCTCTCAATTACCCGAAAGTAATCTACCGCTCCGGCTT
GCATGTGTTAGGCCTCCCGCTAGCGTTCTCC
>1009
CTGCTGCCGCCCGTAGGAGTCTGGTCCGTGTCTCAGTACCAGTGTGGGGGGATCACCCTCTCAGGCCCCCTAA
AGATCGTCGCCTTGGTGAGCCGTTACCTCACCAACTAGCTAATCTTGCGCGTGCCCCATCTCTATCCACCGTA
GTTTTCAATATCACCCGATGCCGAATAATATATTTATGGGGTATTAATCTTTCTTCGAAAGGCTATCCCCTGA
TAAAGGCAGGTTGCACACGTGTTCCGCACCCGTACGCCGCTCTCAATTACCCCGAAAGTAATCTACCGCTCGG
CTTGCATGTGTTAGGCCTCCCGCTAGCGTTCTCCTGAGCAGGATCAACTCT
>1010
CTGCTGCCGCCCGTAGGAGTCTGGTCCGTGTCTCAGTACCAGTGTGGGGGATCACCCTCTCAGGCCCCCTAAA
GATCGTCGCCTTGTGAGCCGTTACCTCACCAACTAGCTAATCTTGCGCGTGCCCATCTCTATTCCACCGTAGT
TTTCAATATCAACCCGATGCCGAATAATATATTATGGGGTATTAATCTTCTTCGAAAGGCTATCCCCTGATAA
AGGCAGGTTGCACACGTGTTCCGCACCCGTACGCCGCTCTCAATTACCCGAAAGTAATCTACCGCTCGGCTTG
CATGTGTTAGGCCTCCCGCTAGCGTTCTCCTGAGCAGGA
>1011
TGCTGCCGCCCGTAGGAGTCTGGACCGTGTCTCAGTTCCAGTGTGACTGATCATCCTCTCAGACCAGTACGGA
TCGTCGCCTTGGTAGGCCTTTACCCCACCAACTAGCTAATCCGACCTAGGCTCATCTGATAGCGTGAGGTCCG
AAGATCCCCCACTTCTCCCTCAGGACGTATGCGGTATTAGCGCCCGTTTCCGGACGTATCCCCACTACCAGGC
AGATTCCTAGGCATTACTCACCCGTCCGCCGCTGAATCCAGGAGCAAGCTCCCTCATCCGCTCGACTGCATGT
GTTAGGCCTGCCGCCAGCGTCAATCTGAGCCAGGATCAAACTCT
>1012
CTGCTGCCGCCCGTAGGAGTCTGGTCCGTGTCTCAGTACCAGTGTGGGGGATCACCCTCTCAGGCCCCCTAAA
GATCGTCGCCTTGGTGAGCCGTTACCTCACCAACTAGCTAGTCTGCGCGTGCCCATCTCTATCCACCGTAGTT
TTCAATATCACCCGATGCCGAATAATATATTATGGGGTATTAATCTTCTTCGAAAGGCTATCCCCCTGATAAA
GGCAGGTTGCACACGTGTTCCGCACCCGTACGCCGCTCTCAATTACCCGAAAGTAATCTACCGCTCGGCTTGC
ATGTGTTAGGCCTCCCGCTAGCGTTCATCCCGTGACGCAGGATCAAACTCCT
>1013
CTGCTGCCGCCCGTAGGAGTCTGGTCCGTGTCTCAGTACCAGTGTGGGGGATCACCCTCTCAGGCCCCCTAAA
GATCGTCGCCTTGGTGAGCCGTTACCTCACCAACTAGCTAATCTTGCGCGTGCCCATCTCTATCCACCGTAGT
TTCAATATCACCCGATGCCGAATAATATATTATGGGGTATTAATCTTCTTCGAAAGGCTATCCCCCTGATAAA
GGCAGGTTGCACACGTGTTCCGCACCCGTACGCCGCTCTCAATTACCCGAAAGTAATCTACCGCTCGGCTTGC
ATGTGTTAGGCCTCCCGCTAGCGTTCATCCTGAGCCAGGATCAACTCT
>1014
CTGCTGCCGCCCGTAGGAGTCTGGTCCGTGTCTCAGTACCAGTGTGGGGGATCACCCTCTCAGGCCCCCTAAA
GATCGTCGCCTTGGTGAGCCGTTACCTCACCAACTAGCTAATCTTGCGCGTGCCCATCTCTATCCACCGTAGT
TTTCAATATCACCCGATGCCGAATAATATATTATGGGGTATTAATCTTCTTCGAAAGGCTATCCCCCTGATAA
AGGCAGGTTGCACACGTGTTCCGCACCCGTACGCCGCTCTCAATTACCCGAAAGTAATCTACCGCTCGGCTTG
CATGTGTTAGGCCTCCCGCTAGCGTTCTCCTGAGCAGGATCAACTCGT
>1015
CTGCTGCCGCCCGTAGGAGTCTGGTCCGTGTCTCAGTACCAGTGTGGGGGATCACCCTCTCAGGCCCCCTAAG
ATCGTCGCCTTGGTGAGCCGTTACCTCACCAACTAGCTAATCTTGCGCGTGCCCATCTCTATCCACCGTAGTT
TTCAATATCACCCGATGCCGAATAATATATTATGGGGTATTAATCTTCTTCGAAAGGCTATCCCCTGATAAAG
GCAGGTTGCACACGTGTTCCGCACCCGTACGCCGCTCTCAATTTACCCGAAAGTAATCTACCGCTCGGCTTGC
ATGTGTTAGGCCTCCCGCTAGCGTTCATCCTGA
>1016
CTGCTGCCGCCCGTAGGAGTCTGGTCCGTGTCTCAGTACCAGTGTGGGGGATCACCCTCTCAGGCCCCCTAAA
GATCGTCGCCTTGGTGAGCCGTTACCTCACCAACTAGCTAATCTTGCGCGTGCCCATCTCTATCCACCGTAGT
TTTCAATATCACCCGATGCCGAATAATATATTATGGGGTATTAATCTTCTTCGAAAGGCTATCCCCCTGATAA
AGGCAGGTTGCACACGTGTTCCGCACCCGTACGCCGCTCTCAATTACCCGAAAGTAATCTACCGCTCGGCTTG
CATGTGTTAGGCCTCCCGCTAGCGTTCATCCTCGAGCCAGGATCAAACTCT
>1017
CTGCTGCCGCCCGTAGGAGTCTGGTCCGTGTCTCAGTACCAGTGTGGGGGATCACCCTCTCAGGCCCCCTAAA
GATCGTCGCCTTGGTGAGCCGTTACCTCACCAACTAGCTAATCTTGCGCGTGCCCATCTCTATCCACCGTAGT

TTTCAATATCACCCGATGCCGAATAATATAATTATGGGGTATTAATCTTCTCGAAAGGCTATCCCCTGATAAA
GGCAGGTTGCACACGTGTTCCGCACCCGTACGCCGCTCTCAATTACCCGAGAGTAATCTAACCGCTCGGCTTG
CATGTGTTAGGCCTCCCGCTAGCGTTCTCCT
>1018
CTGCTGCCGCCCGTAGGAGTCTGGTTCCGTGTCTCAGTACCAGTGTGGGGGATCACCCTCTCAGGCCCCCTA
AAGATCGTCGCCTTGGTGAGCCGTTACCTCACCAACTAGCTAATCTTGCGCGTGCCCATCTCTATCCACCGTA
GTTTTCAATATCACCCGATGCCGAATAATATATTATGGGGTATTTAATCTTTCTTCGAAAGGCTATCCCCTGA
TAAAGGCAGGTTGCACACGTGTTCCGCACCCGTACGCCGCTCTCAATTACCCGAAAGTAATCTACCGCTCGGC
TTGCATGTGTTAGGCCTCCCGCTAGCGTTCTCCTGAGCAGGATCAACTCGT
>1019
CTGCTGCCCCCGTAGGAGTCTGGTCCGTGTCTCAGTACCAGTGTGGGGGATCACCCTCTCAGGCCCCCTAAAG
ATCGTCGCCTTGGTGAGCCGTTACCTCACCAACTAGCTTAATCTTGCGCGTGCCCATCTCTATTCCACCGTAG
TTTTCAATATCACCCGATGCCGAATAATATATTATGGGGTATTTAATCTTCTTCGAAGGCTATCCCCCTGATT
AAAGGCAGGTTGCACACGTGTTCCGCACCCGTACGCCGCTCTCAATTACCCGAAAGTAACCTACCGCTCCGGC
TTGCATGTGTTAGGCCTCCCGCTAGCGTTCTCCTGAGCCAGGATCAACATCT
>1020
CTGCTGCCGCCCGTAGGAGTCTGGTCCGTGTCTCAGTACCAGTGTGGGGGATCACCCTCTCAGGCCCCCTAAA
GATCGTCGCCTTGGTGAGCCGTTACCTCACCAACTAGCTAATCTTGCGCGTGCCCATCTCTATCCACCGTAGT
TTTCAATATCACCCGATGCCGAATAATATATTATGGGGTATTAATCTTCTTCGAAAGGCTATCCCCCTGATAA
AGGCAGGTTGCACACGTGTTCCGCACCCGTACGCCGCTCTCAATTACCCGAAAGTAATCTACCGCTCCGGCTT
GCATGTGTTAGGCCTCCCGCTAGCGTTCATCCTG
>1021
CTGCTGCCGCCCGTAGGAGTCTGGTCCGTGTCTCAGTACCAGTGTGGGGGATCACCCTCTCAGGCCCCCTAAA
GATCGTCGCCTTGGTGAGCCGTTACCTCACCAACTAGCTAATCTTGCGCGTGCCCATCTCTATCCACCGTAGT
TTTCAATATCACCCGATGCCGAATAATATATTATGGGGTATTAATCTTCTCGAAAGGCTATCCCCCTGATAAA
GGCAGGTTGCACACGTGTTCCGCACCCGTACGCCGCTCTCAATTACCCGAAAGTAATCTACCGCTCGGCTTGC
ATGTGTTAGGCCTCCCGCTAGCGTTCATCCTGAGCCAGGATCAAACTCT
>1022
CTGCTGCCGCCCGTAGGAGTCTGGTCCGTGTCTCAGTACCAGTGTGGGGGATCACCCTCTCAGGCCCCCTAAA
GATCGTCGCCTTGGTGAGCCGTTACCTCACCAACTAGCTAATCTTGCGCGTGCCCATCTCTATCCACCGTAGT
TTTCAATATCACCCGATGCCGAATAATATATTATGGGGGTATTAATCTTCTTCGAAAGGCTATCCCCCTGATA
AAGGCAGGTTGCCACACGTGTTCCGCACCCGTACGCCGCTCTCAATTACCCGAAAGTAATCTACCGCTCGGCT
TGCATGTGTTAGGCCTCCCGCTAGCGTTCTCCTGAGCAGGATCAACTCGT
>1023
CTGCTGCCGCCCGTAGGAGTCTGGTCCGTGTCTCAGTACCAGTGTGGGGGATCACCCTCTCAGGCCCCCTAAA
GATCGTCGCCTTGGTGAGCCGTTACCTCACCAACTAGCTAATCTTGCGCGTGCCCATCTCTATCCACCGTAGT
TTTCAATATCACCCGATGCCGAATAATATATTATGGGGTATTAATCTTCTTCGAAAGGCTATCCCCTGATAAA
GGCAGGTTGCCACACGTGTTCCGCACCCGTACGCCGCTCTCAATTACCCGAAAGTAATCTACCGCTCCGGCTT
GCATGTGTTAGGCCTCCCGCTAGCGTTCATCCTGAGCCAGGATCAACTCT
>1024
CTGCTGCCGCCCGTAGGAGTCTGGTCCGTGTCTCAGTACCAGTGTGGGGGATCACCCTCTCAGGCCCCCTAAA
GATCGTCGCCTTGGTGAGCCGTTACCTCACCAACTAGCTAATCTTGCGCGTGCCCATCTCTATCCACCGTAGT
TTTCAATATCACCCGATGCCGAATAATATATTATGGGGTATTAATCTTCTTCGAAAGGCTATCCCCTGATAAA
GGCAGGTTGCACACGTGTTCCGCACCCGTACGCCGCTCTCAATTACCCGAAAGTAATCTACCGCTCGGCTTGC
ATGTGTTAGGCCTCCCGCTAGCGTTCATCCTGAGCCAGGATCAAACTCT
>1025
CTGCTGCCGCCCGTAGGAGTCTGGTCCGTGTCTCAGTACCAGTGTGGGGGATCACCCTCTCAGGCCCCCTAAA
GATCGTCGCCTTGGTGAGCCGTTACCTCACCAACTAGCTAATCTTGCGCGTGCCCATCTCTATCCACCGTAGT
TTTCAATATCACCCGATGCCGAATAATATATTATGGGGTATTAATCTTCTTCGAAAGGCTATCCCCTGATAAA
GGCAGGTTGCACACGTGTTCCGCACCCGTACGCCGCTCTCAATTACCCGAAAGTAATCTACCGCTCGGCTTGC
ATGTGTTAGGCCTCCCGCTAGCGTTCATCTGAGCCAGGATCAAACTCT
>1026
CTGCTGCCGCCCGTAGGAGTCTGGTCCGTGTCTCAGTACCAGTGTGGGGGATCACCCTCTCAGGCCCCCTAAA
GATCGTCGCCTTGGTGAGCCGTTACCTCACCAACTAGCTAATCTTGCGCGTGCCCATCTCTATCCACCGTAGT

TTTCAATATCACCCGATGCCGAATAATATATTATGGGGTATTAATCTTCTTCGAAAGGCTACCCCCCTGATAA
AGGCAGGTTGCACACGTGTTCCGCACCCGTACGCCGCTCTCAATTACCCGAAAGTAATCTACCGCTCGGCTTG
CATGTGTTAGGCCTCCCGCTAGCGTTCATCCTGAGCCAGGATCAAACTCT
>1027
CTGCTGCCGCCCGTAGGAGTCTGGTCCGTGTCTCAGTACCAGTGTGGGGGATCACCCTCTCAGGCCCCCTAAA
GATCGTCGCCTTGGTGAGCCGTTACCTCACCAACTAGCTAATCTTGCGCGTGCCCATCTCTATCCACCGTAGT
TTTCAATATCACCCGATGCCGAATAATATATTATGGGGTATTAATCTTCCTTCGAAAGGCTATCCCCTGATAA
AGGCAGGTTGCACACGTGTTCCGCACCCGTACGCCGCTCTCAATTACCCGAAAGTAATCTACCGCTCGGCTTG
CATGTGTTAGGCCTCCCGCTAGCGTTCTCCTGAGCAGGATCAACTCGT
>1028
CTGCTGCCGCCCGTAGGAGTCTGGTCCGTGTCTCAGTACCAGTGTGGGGGATCACCCTCTCAGGCCCCCTAAA
GATCGTCGCCTTGGTGAGCCGTTACCTCACCAACTAGCTAATCTTGCGCGTGCCCATCTCTATCCACCGTAGT
TTTCAATATCACCCGATGCCGAATAATATATTATGGGGTATTAATCTTCTTCGAAAGGCTATCCCCCTGATAA
AGGCAGGTTGCACACGTGTTCCGCACCCGTACGCCGCTCTCAATTACCCGAAAGTAATCTACCGCTCGGCTTG
CATGTGTTAGGCCTCCCGCTAGCGTTCATCCGTGAGCCAGGATCAAACTCT
>1029
CTGCTGCCGCCCGTAGGAGTCTGGTCCGTGTCTCAGTACCAGTGTGGGGGATCACCCTCTCAGGCCCCCTAAA
GATCGTCGCCTTGGTGAGCCGTTACCTCACCAACTAGCTAATCTTGCGCGTGCCCATCTCTATCCACCGTAGT
TTTCAATATCACCCGATGCCGAATAATATATTATGGGGTATTAATCTTCTTCGAAAGGCTATCCCCCTGATAA
AGGCAGGTTGCACACGTGTTCCGCACCCGTACGCCGCTCTCAATTACCCGAAAGTAATCTACCGCTCCGGCTT
GCATGTGTTAGGCCTCCCGCTAGCGTTCTCCGTGAGCAGGATCA
>1030
CTGCTGCCGCCCGTAGGAGTCTGGACCGTGTCTCAGTTCCAGTGTGACTGATCATCCTCTCAGACCAGTACGG
ATCGTCGCCTTGGTAGGCCTTTACCCCACCAACTAGCTAATCCGACCTAGGCTCATCTGATAGCGTGAGGTCC
GAAGTCCCCCACTTCTCCCTCAGGACGTATGCGGTATTAGCGCCCGTTTCCGGACGTATCCCCACTACCAGGC
AGATTCCTAGGCATTACTCACCCGTCCGCCGCTGAATCCAGGAGCAAGCTCCCTCATCCGCTCGACTTGCATG
TGTTAGGCCTGCCGCCAGCGTCAATCTGAGCCAGGATCAAACTCT
>1031
CTGCTGCCGCCCGTAGGAGTCTGGTCCGTGTCTCAGTACCAGTGTGGGGGATCACCCTCTCAGGCCCCCTAAA
GATCGTCGCCTGGTGAGCCGTTACCTCACCAACTAGCTAATCTTGCGCGTGCCCATCTCTATCCACCGTAGTT
TTCAATATCACCCGATGCCGAATAATATATTATGGGGTATTAATCTTCTTCGAAAGGCTATCCCCTGATAAAG
GCAGGTTGCACACGTGTTCCGCACCCGTACGCCGCTCTCAATTACCCGAAAGTAATCTACCGCTCGGCTTGCA
TGTGTTAGGCCTCCCGCTAGCGTTCATCCGTGAGC
>1032
CTGCTGCCGCCCGTAGGAGTCTGGTCCGTGTCTCAGTACCAGTGTGGGGGATCACCCTCTCAGGCCCCCTAAA
GATCGTCGCCTTGGTGAGCCGTTACCTCACCAACTAGCTAATCTTGCGCGTGCCCATCTCTATCCACCGTAGT
TTTCAATATCACCCGATGCCGAATAATATATTATGGGGTATTAATCTTCTTCGAAAGGCTATCCCCCTGATAA
AGGCAGGTTGCACACGTGTTCCGCACCCGTACGCCGCTCTCAATTACCCGAAAGTAATCTACCGCTCGGCTTG
CATGTGTTAGGCCTCCCGCTAGCGTTCATCCGTGAGCCAGGATCAAACTCT
>1033
CTGCTGCCGCCCGTAGGAGTCTGGTCCGTGTCTCAGTACCAGTGTGGGGGATCACCCTCTCAGGCCCCCTAAA
GATCGTCGCCTTGGTGAGCCGTTACCTCACCAACTAGCTAATCTTGCGCGTGCCCATCTCTATCCACCGTAGT
TTTCAATATCACCCGATGCCGAATAATATATTATGGGGTATTAATCTTCTTCGAAAGGCTATCCCCCTGATAA
AGGCAGGTTGCACACGTGTTCCGTACCCGTACGCCGCTCTCAATTACCCGAAAGTAATCTACCGCTCGGCTTG
CATGTGTTAGGCCTCCCGCTAGCGTTCATCCTGAGCCAGGATCAAACTCT
>1034
CTGCTGCCGCCCGTAGGAGTCTGGTCCGTGTCTCAGTACCAGTGTGGGGGATCACCCTCTCAGGCCCCCTAAA
GATCGTCGCCTTGGTGAGCCGTTACCTCACCAACTAGCTAATCTTGCGCGTGCCCATCTCTATCCACCGTAGT
TTCAATATCACCCGATGCCGAATAATATATTATGGGGTATTAATCTTCTCGAAAGGCTATCCCCTGATAAAGG
CAGGTTGCACACGTGTTCCGCACCCGTACGCCGCTCTCAATTACCCGAAAGTAATCTACCGCTCGGCTTGCAT
GTGTTAGGCCTCCCGCTAGCGTTCATCCTCGAGCCAGGATCAAACTCT
>1035
CTGCTGCCGCCCGTAGGAGTCTGGTCCGTGTCTCAGTACCAGTGTGGGGGATCACCCTCTCAGGCCCCCTAAA
GATCGTCGCCTTGGTGAGCCGTTACCTCACCAACTAGCTAATCTTGCGCGTGCCCATCTCTATCCACCGTAGT

TTTCAATATCACCCGATGCCGAATAATATATTATGGGGTATTAATCTTCTTCGAAAGGCTATCCCCCTGATAA
AGGCAGGTTGCACACGTGTTCCGCACCCGTACGCCGCTCTCAATTACCCGAAAGTAATCTACCGCTCCGGCTT
GCAATGTGTTAGGCCTCCCGCTAGCGTTCATCCTCGAGCCAGGATCAACTCT
>1036
CTGCTGCCGCCCGTAGGAGTCTGGTCCGTGTCTCAGTACCAGTGTGGGGGATCACCCTCTCAGGCCCCCTAAA
GATCGTCGCCTGGTGAGCCGTTACCTCACCAACTAGCTAATCTTGCGCGTGCCCCATCTCTATCCACCGTAGT
TTTCAATATCACCCGATGCCGAATAATATATTATGGGGTATTAATCTTCTCGAAAGGCTATCCCCCTGATAAA
GGCAGGTTGCACACGTGTTCCGCACCCGTACGCCGCTCTCAATTACCCGAAAGAATCTACCGCTCGGCTGCAT
GTGTTAGGCCTCCCGCAGCGTCTCC
>1037
CTGCTGCCGCCCGTAGGAGTCTGGTCCGTGTCTCAGTACCAGTGTGGGGGATCACCCTCTCAGGCCCCCTAAA
GATCGTCGCCTTGGTGAGCCGTTACCTCACCAACTAGCTAATCTTGCGCGTGCCCATCTCTATCCACCGTAGT
TTTCAATATCACCCGATGCCGAATAATATATTATGGGGTATTTAATCTTCTTCGAAAGGCTATCCCCCTGATA
AAGGCAGGTTGCACACGTGTTCCGCACCCGTACGCCGCTCTCAATTACCCGAAAGTAATCTACCGCTCGGCTT
GCATGTGTTAGGCCTCCCGCTAGCGTTCATCCTGAGCCAGGATCAAACTCT
>1038
CTGCTGCCGCCCGTAGGAGTCTGGTCCGTGTCTCAGTACCAGTGTGGGGGATCACCCTCTCAGGCCCCCTAAA
GATCGTCGCCTTGGTGAGCCGTTACCTCACCAACTAGCTAATCTTGCGCGTGCCCATCTCTATCCACCGTAGT
TTTCAATATCACCCGATGCCGAATAATATATTATGGGGTATTAATCTTCTTCGAAGGCTATCCCCCTGATAAA
GGCAGGTTGCACACGTGTTCCGCACCCGTACGCCGCTCTCAATTACCCGAAAGTAATCTACGCTCGGCTTGCA
TGTGTTAGGCCTCCCGCTAGCGTTCATCCTGAGCCAGGATCAAACTCT
>1039
CTTGCTGCCGCCCGTAGGAGTCTGGTTCCGTGTCTCAGTACCAGTGTGGGGATCACCCTCTCAGGCCCCCTAA
AGATCGTCGCCTTGGTGAGCCGTTACCTCACCAACTAGCTAATCTTGCGCGTGCCCATCTCTATCCACCGTAG
TTTTCAATATCACCCGATGCCGAATAATATATTTATGGGGTATTAATCTTCTTCGAAAGGCTATCCCCTGATA
AAGGCAGGTTGCACACGTGTTCCGCACCCGTACGCCGCTCTCAATTACCCGAAAGTAATCTACCGCTCGGCTT
GCATGTGTTAGGCCTCCCGCTAGCGTTCATCCTGAGCCAGGATCAACTCT
>1040
CTGCTGCCGCCCGTAGGAGTCTGGTCCGTGTCTCAGTACCAGTGTGGGGGATCACCCTCTCAGGCCCCTAAAC
GATCGTCGCCTTGGTGAGCCGTTACCTCACCAACTAGCTAATCTTGCGCGTGCCCATCTCTATCCACCGTAGT
TTTCAATATCACCCGATGCCGAATAATATATTATGGGGTATTTAATCTTCTTCGAAAGGCTATCCCCCTGATA
AAGGCAGGTTGCACACGTGTTCCGCACCCGTACGCCGCTCTCAATTACCCGAAAGTAATCTACCGCTCGGCTT
GCATGTGTTAGGCCTCCCGCTAGCGTTCTTCCTGAGCAGG
>1041
CTGCTGCCGCCCGTAGGAGTCTGGTCCGTGTCTCAGTACCAGTGTGGGGGATCACCCTCTCAGGCCCCCTAAA
GATCGTCGCCTTGGTGAGCCGTTACCTCACCAACTAGCTAATCTTGCGCGTGCCCATCTCTATCCACCGTAGT
TTTCAATATCACCCGATGCCGAATAATATATTATGGGTATTAATCTTCTCGAAAGGCTTATCCCCCTGATAAA
GGCAGGTTGCACACGTGTTCCGCACCCGTACGCCGCTCTCAATTACCCGAAAGTAATCTACCGCTCGGCTTGC
ATGTGTTAGGCCTCCGCTAG
>1042
CTGCTGCCGCCCGTAGGAGTCTGGTCCGTGTCTCAGTACCAGTGTGGGGGATCACCCTCTCAGGCCCCCTAAA
GATCGTCGCCTTGTGAGCCGTTTACCTCACCAACTAGCTAATCTTGCGCGTGCCCATCTCTATCCACCGTAGT
TTTCAATATCACCCGATGCCGAATAATATATTATGGGGTATTAATCTTCTTCGAAAGGCTATCCCCCTGATAA
AGGCAGGTTGCACACGTGTTCCGCACCCGTACGCCGCTCTCAATTACCCGAAAGTAATCTACCGCTCGGCTTG
CATGTGTTAGGCCTCCCGCTAGCGTTCATCCTGAGCCAGGATCAACTCT
>1043
CTGCTGCCGCCCGTAGGAGTCTGGTCCGTGTCTCAGTACCAGTGTGGGGGATCACCCTCTCAGGCCCCCTAAA
GATCGTCGCCTTGGTGAGCCGTTACCTCACCAACTAGCTAATCTTGCGCGTGCCCATCTCTATCCACCGTAGT
TTTCAATATCACCCGATGCCGAATAATATATTATGGGGTATTAATCTTCTTCGAAAGGCTATCCCCCTGATAA
AGGCAGGTTGCACACGTGTTCCGCACCCGTACGCCGCTCTCAATTACCCGAAAGTAATCTACCGCTCGGCTTG
CATGTGTTAGGCCTCCCGCTAGCGTTCATCCTGAGCCAGGATCAAACTCT
>1044
CTGCTGCCGCCCGTAGGAGTCTGGTCCGTGTCTCAGTACCAGTGTGGGGGATCACCCTCTCAGGCCCCCTAAA
GATCGTCGCCTTGGTGAGCCGTTACCTCACCAACTAGCTAATCTTGCGCGTGCCCATCTCTATCCACCGTAGT

TTTCAATATCACCCGATGCCGAATAATATATTATGGGGTATTAATCTTCTTCGAAAGGCTATCCCCCTGATAA
AGGCAGGTTGCACACGTGTTCCGCACCCGTACGCCGCTCTCAATTACCCGAAAGTAATCTACCGCTCGGCTTG
CATGTGTTAGGCCTCCCGCTAGCGTCATCCTGAGCCAGGATCAAACTCT
>1045
CTGCTGCCGCCCGTAGGAGTCTGGTCCGTGTCTCAGTACCAGTGTGGGGGATCACCCTCTCAGGCCCCCTAAA
GATCGTCGCCTTGGTGAGCCGTTACCTCACCAACTAGCTAATCTTGCGCGTGCCCATCTCTATCCACCGTAGT
TTTCAATATCACCCGATGCCGAATAATATATTATGGGGTATTAATCTTCTTCGAAAGGCTATCCCCCTGATAA
AGGCAGGTTGCACACGTGTTCCGCACCCGTACGCCGCTCTCAATTACCCGAAAGTAATCTACCGCTCGGCTTG
CATGTGTTAGGCCTCCCGCTAGCGTTCATCCTGAGCCAGGATCAAACTCT
>1046
CTGCTGCCGCCCGTAGGAGTCTGGTCCGTGTCTCAGTACCAGTGTGGGGGATCACCCTCTCAGGCCCCCTAAA
GATCGTCGCCTTGGTGAGCCGTTACCTCACCAACTAGCTAATCTTGCGCGTGCCCATCTCTATCCACCGTAGT
TTTCAATATCACCCGATGCCGAATAATATATTATGGGGTATTAATCTTCTTCGAAAGCTATCCCCCTGATAAA
GGCAGGTTGCACACGTGTTCCGCACCCGTACGCCGCTCTCAATTACCCGAAAGTAATCTACCGCTCGGCTTGC
ATGTGTTAGGCCTCCCGCTAGCGTTCATCCTGAGCCAGGATCAAACTCT
>1047
CTGCTGCCGCCCGTAGGAGTCTGGTCCGTGTCTCAGTACCAGTGTGGGGGATCACCCTCTCAGGCCCCCTAAA
GATCGTCGCCTTGGTGAGCCGTTACCTCACCAACTAGCTAATCTTGCGCGTGCCCATCTCTATCCACCGTAGT
TTTCAATATCACCCGATGCCGAATAATATATTATGGGGTATTAATCTTCCTTCGAAAGGCTATCCCCCTGATA
AAGGCAGGTTGCACACGTGTTCCGCACCCGTACGCCGCTCTCAATTACCCGAAAGTAATCTACCGCTCGGCTT
GCATGTGTTAGGCCTCCCGCTAGCGTTCATCCTGAGCCAGGATCAAACTCT
>1048
CTGCTGCCGCCCGTAGGAGTCTGGTTCCCGTGTCTCAGTACCAGTGTGGGGGGGATCACCCCTCTCAGGCCCC
CTAAAGATCGTCGCCTTGGTGAGCCGTTACCTCACCCAACTAGCTAATCTTGCGCGTGCCCCATCTCTATCCA
CCGTAGTTTTCAATATCACCCGATGCCCGAATAATATATTTATGGGGTATTAATCTTTCTTCGAAAGGCTATC
CCCCTGATAAAGGCAGGTTTGCACACGTGTTCCGCACCCCGTACGCCGCTCTCAATTTACCCGAAAGTAATCT
ACCGCTCGGCTTGCATGTGTTTAGGCCTTCCCCGCTAGCGTCATCCTGAGCCAGGATCAAACTCT
>1049
CTGCTGCCGCCCGTAGGAGTCTGGTCCGTGTCTCAGTACCAGTGTGGGGGATCACCCTCTCAGGCCCCCTAAA
GATCGTCGCCTTGGTGAGCCGTTACCTCACCAACTAGCTAATCTTGCGCGTGCCCATCTCTATTCCACCGTAG
TTTTCAATATCACCCGATGCCGAATAATATATTATGGGGTATTAATCTTCTTCGAAAGGCTATCCCCCTGATA
AAGGCAGGTTGCACACGTGTTCCGCACCCGTACGCCGCTCTCAATTACCCGAAAGTAATCTACCGCTCGGCTT
GCATGTGTTAGGCCTCCCGCTAGCGTTCATCCTGAGCCAGGATCAAACTCT
>1050
CTGCTGCCGCCCGTAGGAGTCTGGTCCGTGTCTCAGTACCAGTGTGGGGGATCACCCTCTCAGGCCCCCTAAA
GATCGTCGCCTTGGTGAGCCGTTACCTCACCAACTAGCTAATCTTGCGCGTGCCCATCTCTATCCACCGTAGT
TTTCAATATCACCCGATGCCGAATAATATATTATGGGGTATTAATCTTCTTCGAAAGGCTATCCCCTGATAAA
GGCAGGTTGCCACACGTGTTCCGCACCCGTACGCCGCTCTCAATTACCCGAAAGTAATCTACCGCTCGGCTTG
CATGTGTTAGGCCTCCCGCTAA
>1051
CTGCTGCCGCCCGTAGGAGTCTGGTCCGTGTCTCAGTACCAGTGTGGGGGATCACCCTCTCAGGCCCCCTAAA
GATCGTCGCCTTGGTGAGCCGTTACCTCACCAACTAGCTAATCTTGCGCGTGCCCATCTCTATCCACCGTAGT
TTTCAATATCACCCGATGCCGAATAATATATTATGGGGTATTAATCTTCTTCGAAAGGCTATCCCCCTGATAA
AGGCAGGTTGCACACGTGTTCCGCACCCGTACGCCGCTCTCAATTACCCGAAAGTAATCTACCGCTCCGGCTT
GCATGTGTTAGGCCTCCCGCTAGCGTTCATCCGTGAGCAGGATCAACTCT
>1052
CTGCTGCCGCCCGTAGGGGTCTGGTCCGTGTCTCAGTACCAGTGTGGGGGGTCACCCTCTCAGGCCCCCCTAA
AGATCGTCGCCTTGGTGAGCCGTTACCTCACCAACTAGCTAATCTTGCGCGTGCCCCATCTCTATCCACCGTA
GTTTTCAATATCACCCGATGCCGAATAATATATTATTGGGGTATTAATCTTCTTCGAAAGGCTATCCCCCTGA
TTAAAGGCAGGTTGCACACGTGTTCCGCACCCGTACGCCGCTCTCAATTACCCGAAAGTAATCTACCGCTCGG
CTTGCATGTGTTAGGCCTCCCGCTAGCGTTCATTCCTGAGCCAGGATCAAACTCT
>1053
CTGCTGCCGCCCGTAGGAGTCTGGACCGTGTCTCAGTTCCAGTGTGACTGATCATCCTCTCAGACCAGTACGG
ATCGTCGCCTTGGTAGGCCTTTACCCCACCAACTAGCTAATCCGACCTAGGCTCATCTGATAGCGTGAGGTCC

GAAGATCCCCCACTTCTCCCTCAGGACGTATGCGGTATTAGCGCCCGTTTCCGGACGTATCCCCACTACCAGG
CAGATTCCTAGGCATTACTCACCCGTCCGCCGCTGAATCCAGGAGCAAGCCCCCTTCATCCGCTCGACTGCAT
GTGTTAGGCCTGCCGCCAGCGTCAATCTGAGCCAGGATCAAACTCT
>1054
CTGCTGCCGCCCGTAGGAGTCTGGTCCGTGTCTCAGTACCAGTGTGGGGGATCACCCTCTCAGGCCCCCTAAA
GATCGTCGCCTTGGTGAGCCGTTACCTCACCAACTAGCTAATCTTGCGCGTGCCCATCTCTATCCACCGTAGT
TTTCAATATCACCCGATGCCGAATAATATATTATGGGGTATTAATCTTCTTCGAAAGGCTATCCCCCTGATAA
AGGCAGGTTGCACACGTGTTCCGCACCCGTACGCCGCTCTCAATTACCCGAAAGTAATCTACCGCTCGGCTTG
CATGTGTTAGGCCTCCCGCTAGCGTTCATCCTGAGCCAGGATCAACTCT
>1055
CTGCTGCCGCCCGTAGGAGTCTGGTCCGTGTCTCAGTACCAGTGTGGGGGATCACCCTCTCAGGCCCCCTAAA
GATCGTCGCCTTGGTGAGCCGTTACCTCACCAACTAGCTAATCTTGCGCGTGCCCATCTCTATCCACCGTAGT
TTTCAATATCACCCGATGCCGAATAATATATTATGGGGTATTAATCTTCTTCGAAAGGCTATCCCCTGATAAA
GGCAGGTTGCACACGTGTTCCGCACCCGTACGCCGCTCTCAATTACCCGAAAGTAATCTACCGCTCCGGCTTG
CATGTGTTAGGCCTCCCGCTAGCGTTCTCCTGAGCAGGATCAACTCGT
>1056
CTGCTGCCGCCCGTAGGAGTCTGGTCCGTGTCTCAGTACCAGTGTGGGGGATCACCCTCTCAGGCCCCCTAAA
GATCGTCGCCTTGGTGAGCCGTTACCTCACCAACTAGCTAATCTTGCGCGTGCCCATCTCTATCCACCGTAGT
TTTCAATATCACCCGATGCCGAATAATATATTATGGGGTATTAATCTTCTTCGAAAGGCTATCCCCCTGATAA
AGGCAGGTTGCACACGTGTTCCGCACCCGTACGCCGCTCTCAATTTACCCGAAAGTAATCCACCGCTCGGCTT
GCATGTGTTAGGCCTCCCGCTAGCGTTCATCCTGAGCCAGGATCAAACTCT
>1057
CTGCTGCCGCCCGTAGGAGTCTGGTCCGTGTCTCAGTACCAGTGTGGGGGATCACCCTCTCAGGCCCCCTAAA
GATCGTCGCCTTGGTGAGCCGTTCGCCTACCAACTAGCTAATCTTGCGCGTGCCCATCTCTATCCACCGTAGT
TTTCAATATCACCCGATGCCGAATAATATATTATGGGGTATTAATCTTCTTCGAAAGGCTATCCCCTGATAAA
GGCAGGTTGCACACGTGTTCCGCACCCGTACGCCGCTCTCAATTACCCGAAAGTAATCTACCGCTCGGCTTGC
ATGTGTTAGGCCTCCCGCTA
>1058
CTGCTGCCGCCCGTAGGAGTCTGGTCCGTGTCTCAGTACCAGTGTGGGGGATCACCCTCTCAGGCCCCCTAAA
GATCGTCGCCTTGGTGAGCCGTTACCTCACCAACTAGCTAATCTTGCGCGTGCCCATCTCTATCCACCGTAGT
TTTCAATATCACCCGATGCCGAATAATATATTATGGGGTATTAATCTTCTTCGAAAGGCTATCCCCCTGATAA
AGGCAGGTTGCACACGTGTTCCGCACCCGTACGCCGCTCTCAATTACCCGAAAGTAATCTACCGCTCGGCTTG
CATGTGTTAGGCCTCCCGCTAGCGTTCATCCTGAGCCAGGATCAAACTCT
>1059
CTGCTGCCGCCCGTAGGAGTCTGGTCCGTGTCTCAGTACCAGTGTGGGGGATCACCCTCTCAGGCCCCCTAAA
GATCGTCGCCTTGGTGAGCCGTTACCTCACCAACTAGCTAATCTTGCGCGTGCCCATCTCTATCCACCGTAGT
TTTCAATATCACCCGATGCCGAATAATATATTATGGGGTATTAATCTTCTTCGAAAGGCTATCCCCCTGATAA
AGGCAGGTTGCACACGTGTTCCGCACCCGTACGCCGCTCTCAATTACCCGAAAGTAATCTACCGCTCCGGCTT
GCATGTGTTAGGCCTCCCGCTAGCGTTCATCTGAGCCAGGATCAACTCT
>1060
CTGCTGCCGCCCGTAGGAGTCTGGACCGTGTCTCAGTTCCAGTGTGACTGATCATCCTCTCAGACCAGTACGG
ATCGTCGCCTTGGTAGGCCTTTACCCCACCAACTAGCTAATCCGACCTAGGCTCATCTGATAGCGTGAGGTCC
GAAGTCCCCCACTTCTCCCTCAGGACGTATGCGGTATTACGCGCCCGTTTCCGGACGATCCCCACTACCAGGC
AGATTCCCTAGGCATTACTCACCCGTCCGCCGCTGAATCCAGGAGCAAGCTCCCTCATCCGCTCGACTGCCAT
GTGTTAGGCCTGCCGCCAGCGTCAATCTGAGCCAGGATCAACTCT
>1061
CTGCTGCCGCCCGTAGGAGTCTGGTCCGTGTCCCAGTACCAGTGTGGGGGATCACCCTCTCAGGCCCCCCTAA
AGATCGTCGCCTTGGTGAGCCGTTACCTCACCAACTAGCTAATCTTGCGCGTGCCCATCTCTATCCACCGTAG
TTTTCAATATCACCCGATGCCGAATAATATATTATGGGGTATTAATCTTTCTTCGAAAGGCTATCCCCCTGAT
AAAGGCAGGTTGCACACGTGTTCCGCACCCGTGCGCCGCTCTCAATTACCCGAAAGTAATCTACCGCTCGGCT
TGCATGTGTTAGGCCTCCCGCTAGCGTTCATCCTGAGCCAGGATCAAACTCT
>1062
CTGCTGCCGCCCGTAGGAGTCTGGTCCGTGTCTCAGTACCAGTGTGGGGGATCACCCTCTCAGGCCCCCTAAA
GATCGTCGCCTTGGTGAGCCGTTACCTCACCAACTAGCTAATCTTGCGCGTGCCCATCTCTATCCACCGTAGT

TTTCAATATCACCCGATGCCGAATAATATATTATGGGGTATTAATCTTCCTTCCGAAAGGCTATCCCCCTGAT
AAAGGCAGGTTGCACACGTGTTCCGCACCCGTACGCCGCTCTCAATTACCCGAAAGTAATCGACCGCTCGGCT
TGCATGTGTTAGGCCTCCCGCTAGCGTTCATCCTGAGCCAGATCAAACTCT
>1063
CTGCTGCCGCCCGTAGGAGTCTGGTCCGTGTCTCAGTACCAGTGTGGGGGATCACCCTCTCAGGCCCCCTAAG
ATCGTCGCCTGGTGAGCCGTTACCTCACCAACTAGCTAATCTTGCGCGTGCCCATCTCTATCCACCGTAGTTT
TCAATATCACCCGATGCCGAATAATATATTATGGGGTATTAATCTTCTTCGAAAGGCTATCCCCCTGATAAAG
GCAGGTTGCGCACGTGTTCCGCACCCGTACGCCGCTCTCAATTTACCCGAAAGTAATCTACCGCTCGGCTTGC
ATGTGTTAGGCCTCCCGCTAGCGTTCTCC
>1064
CTGCTGCCGCCCGTAGGAGTCTGGTCCGTGTCTCAGTACCAGTGTGGGGGATCACCCTCTCAGGCCCCCTAAA
GATCGTCGCCTTGGTGAGCCGTTACCTCACCAACTAGCTAATCTTGCGCGTGCCCATCTCTATCCACCGTAGT
TTTCAATATCACCCGATGCCGAATAATATATTATGGGGTATTAATCTTCTTCGAAAGGCTATCCCCCTGATAA
AGGCAGGTTGCACACGTGTTCCGCACCCGTACGCCGCTCTCAATTACCCGAAAGTAATCTACCGCTCGGCTTG
CATGTGTTAGGCCTCCCGCTAGCGTTCATCCTGAGCCAGGATCAACTCT
>1065
CTGCTGCCGCCCGTAGGAGTCTGGTCCGTGTCTCAGTACCAGTGTGGGGGATCACCCTCTCAGGCCCCCTAAA
GATCGTCGCCTTGGTGAGCCGTTACCACACCAACTGCTAATCTTGCGCGTGCCCATCTCTATCCACCGTAGTT
TTCAATATCACCCGATGCCGAATAATATATTATGGGGTATTAATCTTCTTCGAAAGCTATCCCCTGATAAAGG
CAGGTTGCACACGTGTTCCGCACCCGTACGCCGCTCTCAATTACCCGAAAGTAATCTACCGCTCCGGCTTGCA
TGTGTTAGGCCTCCCGCTAGCGTTCTCCTGAGCAGGTCAAC
>1066
CTGCTGCCGCCCGTAGGAGTCTGGTCCGTGTCTCAGTACCAGTGTGGGGGATCACCCTCTCAGGCCCCCCTAA
AGATCGTCGCCTTGGTGAGCCGTTACCTCACCCAACTAGCTAATCTTGCGCGTGCCCCATCTCTATCCACCGT
AGTTTTCAATATCACCCGATGCCGAATAATATATTATTGGGGTATTAATCTTTCTTCGAAAGGCTATCCCCCT
GATAAAGGCAGGTTGCACACGTGTTCCGCACCCGTACGCCGCTCTCAATTACCCCGAAAGTAATCTACCGCTC
GGCTTGCATGTGTTAGGCCTCCCGCTAGCGTTCATCCTGAGCCAGGATCAACTCT
>1067
CTGCTGCCGCCCGTAGGAGTCTGGTCCGTGTCTCAGTACCAGTGTGGGGGATCACCCTCTCAGGCCCCCTAAA
GATCGTCGCCTTGGTGAGCCGTTACCTCACCAACTAGCTAATCTTGCGCGTGCCCATCTCTATCCACCGTAGT
TTTCAATATCACCCGATGCCGAATAATATATTATGGGGTATTAATCTTCTTCGAAAGGCTATCCCCCTGATAA
AGGCAGGTTGCACACGTGTTCCGCACCCGTACGCCGCTCTCAATTACCCGAAAGTAATCTACCGCTCGGCTTG
CATGTGTTAGGCCTCCCGCTAGCGTTCATCCGT
>1068
CTGCTGCCGCCCGTAGGAGTCTGGTCCGTGTCTCAGTACCAGTGTGGGGGATCACCCTCTCAGGCCCCCTAAA
GATCGTCGCCTTGGTGAGCCGTTACCTCACCAACTAGCTAATCTTGCGCGTGCCCATCTCTATCCACCGTAGT
TTTCAATATCACCCGATGCCGAATAATATATTATGGGGTATTAATCTTCTTCGAAAGGCTATCCCCTGATAAA
GGCAGGTTGCACACGTGTTCCGCACCCGTACGCCGCTCTCAATTACCCGAAAGTAATCTACCGCTCGGCTTGC
ATGTGTTAGGCCTCCCGCTAGCGTTCATCCTGAG
>1069
CTGCTGCCGCCCGTAGGAGTCTGGTCCGTGTCTCAGTACCAGTGTGGGGGATCACCCTCTCAGGCCCCCTAAA
GATCGTCGCCTTGGTGAGCCGTTACCTCACCAACTAGCTAATCTTGCGCGTGCCCATCTCTATCCACCGTAGT
TTTCAATATCACCCGATGCCGAATAATATATTATGGGGTATTAATCTTCTTCGAAAGGCTATCCCCTGATAAA
GGCAGGTTGCACACGTGTTCCGCACCCGTACGCCGCTCTCAATTACCCGAAAGTAATCTACCGCTCGGCTTGC
ATGTGTTAGGCCTCCCGCTAGCGTTCATCCTGAGCCAGGATCAAACTCT
>1070
CTGCTGCCGCCCGTAGGAGTCTGGTCCGTGTCTCAGTACCAGTGTGGGGGATCACCCTCTCAGGCCCCCTAAA
GATCGTCGCCTTGGTGAGCCGTTACCTCACCAACTAGCTAATCTTGCGCGTGCCCATCTCTATCCACCGTAGT
TTTCAATATCACCCGATGCCGAATAATATATTATGGGGTATTAATCTTCTTCTGAAAGGCTATCCCCTGATAA
AGGCAGGTTGCACACGTGTTCCGCACCCGTACGCCGCTCTCAATTTACCCGAAAGTAATCTACCGCTCGGCTT
GCATGTGTTAGGCCTCCCGCTAGCGTTCATCCTGAGCCAGGATCAAACTCT
>1071
CTGCTGCCGCCCGTAGGAGTCTGGTCCGTGTCTCAGTACCAGTGTGGGGGATCACCCTCTCAGGCCCCCTAAA
GATCGTCGCCTTGGTGAGCCGTTACCTCACCAACTAGCTAATCTTGCGCGTGCCCATCTCTATCCACCGTAGT

TTTCAATATCACCCGATGCCGAATAATATATTATGGGGTATTAATCTTCTTCGAAAGGCTATCCCCCTGATAA
GCAGGTTGCACACGTGTTCCGCACCCGTACGCCGCTCTCAATTACCCGAAAGTAATCTACCGCTCGGCTTGCA
TGTGTTAGGCCTCCCGCTAGCGTTCATCCTGAGCCAGGATCAAACTCT
>1072
CTGCTGCCGCCCGTAGGAGTCTGGTCCGTGTCTCAGTACCAGTGTGGGGGATCACCCTCTCAGGCCCCCTAAA
GATCGTCGCCTTGGTGAGCCGTTACCTCACCAACTAGCTAATCTTGCGCGTGCCCATCTCTATCCACCGTAGT
TTTCAATATCACCCGATGCCGAATAATATATTATGGGGTATTAATCTTCTTCGAAAGGCTATCCCCCTGATAA
AGGCAGGTTGCACACGTGTTCCGCACCCGTACGCCGCTCTCAATTACCCGAAAGTAATCTACCGCTCGGCTTG
CATGTGTTAGGCCTCCCGCTAGCGTTCATCCTGAGCCAGGATCAAACTCT
>1073
CTGCTGCCGCCCGTAGGAGTCTGGACCGTGTCTCAGTTCCAGTGTGACTGATCATCCTCTCAGACCAGTACGG
ATCGTCGCCTTGGTAGGCCTTTTACCCCACCAACTAGCTAATCCGACCTAGGCTCATCTGATAGCGTGAGGTC
CGAAGTCCCCCACTTCTCCCTCAGGACGTATGCGGTATTAGCGCCCCGTTTCCGGACGTATCCCCACTACCAG
GCAGATTCCTAGGCATTACTCACCCGTCCGCCGCTGAATCCAGGAGCAAGCCCCTTCACTCCGCCTCGACTGC
ATGTGTTAGGCCTGCCGCCAGCGTCAATCTGAGCCAGGATCAAACTCT
>1074
CTGCTGCCGCCCGTAGGAGTCTGGTCCGTGTCTCAGTACCAGTGTGGGGGATCACCCTCTCAGGCCCCCTAAA
GATCGTCGCCTTGGTGAGCCGTTACCTCACCAACTAGCTAATCTTGCGCGTGCCCATCTCTATCCACCGTAGT
TTTCAATATCACCCGATGCCGAATAATATATTATGGGGTATTAATCTTCTCGAAAGGCTATCCCCCTGATAAA
GGCAGGTTGCACACGTGTTCCGCACCCGTACGCCGCTCTCAATTACCCGAAAGTAATCTACCGCATCGGCTTG
CATGTGTTAGGCCTCCCGCTAGCGTTCATCCTGAGCCAGGATCAAACTCT
>1075
CTGCTGCCGCCCCGTAGGAGTCTGGACCGTGTCTCAGTTCCAGTGTGACTGATCATTCCTCTCAGACCAGTAC
GGATCGTCGCCTTGGTAGGCCTTTACCCCCACCAACTAGCTAATCCGACCTAGGCTCATCTGATAGCGTGAGG
TCCGAAGTCCCCACTTCCCCCTCAGGACGTATGCGGTATTAGCGCCCGTTTCCGGACGTATCCCCACTACCAG
GCAGATTCCTAGGCATTACTCACCCGTCCGCCGCTGAATCCAGGAGCAAGCTCCCTCATCCGCTCGACTGCAT
GTGTTAGGCCTGCCGCCAGCGTCAATCTGAGCCAGGATCAAACTCT
>1076
CTGCTGCCGCCCGTAGGAGTCTGGTCCGTGTCTCAGTACCAGTGTGGGGGATCACCCTCTCAGGCCCCCTAAA
GATCGTCGCCTTGGTGAGCCGTTACCTCACCAACTAGCTAATCTTGCGCGTGCCCATCTCTATCCACCGTAGT
TTTCAATATCACCCGATGCCGAATAATATATTATGGGGTATTAATCTTCTTCGAAGGCTATCCCCCTGATAAA
GGCAGGTTGCACACGTGTTCCGCACCCGTACGCCGCTCTCAATTACCCGAAAGTAATCTACCGCTCGGCTTGC
ATGTGTTAGGCCTCCCGCTAGCGTTCATCCTCGAGCCAGGATCAACTCT
>1077
CTGCTGCCGCCCGTAGGAGTCTGGTCCGTGTCTCAGTACCAGTGTGGGGGATCACCCTCTCAGGCCCCCTAAA
GATCGTCGCCTTGGTGAGCCGTTACCTCACCAACTAGCTAATCTTGCGCGTGCCCATCTCTATCCACCGTAGT
TTTCAATATCACCCGATGCCGAATAATATATTATGGGGTATTAATCTTCCTTCGAAGGGCTATCCCCCTGATA
AAGGCAGGTTGCACACGTGTTCCGCACCCGTACGCCGCTCTCAATTACCCGAAAGTAATCTACCGCTCGGCTT
GCATGTGTTAGGCCTCCCGCTAGCGTTCATCCTGAGCCAGGATCAAACTCT
>1078
CTGCTGCCGCCCGTAGGAGTCTGGTCCGTGTCTCAGTACCAGTGTGGGGGATCACCCTCTCAGGCCCCCTAAA
GATCGTCGCCTTGGTGAGCCGTTACCTCACCAACTAGCTAATCTTGCGCGTGCCCATCTCTATCCACCGTAGT
TTCAATATCACCCGATGCCGAATAATATATTATGGGGTATTAATCTTCTTCGAAAGCTATCCCCCTGATAAAG
GCAGGTTGCACACGTGTTCCGCACCCGTACGCCGCTCTCAATTACCCGAAAGTAATCTACCGCTCGGCTTGAC
ATGTGTTAGGCCTCCCGCTAGCGTTCATCCTG
>1079
CTGCTGCCGCCTGTAGGAGTCTGGTCCGTGTCTCAGTACCAGTGTGGGGGATCACCCTCTCAGGCCCCCTAAA
GATCGTCGCCTTGGTGAGCCGTTACCTCACCAACTAGCTAATCTTGCGCGTGCCCATCTCTATTCCACCGTAG
TTTTCAATATCACCCGATGCCGAATAATATATTATGGGGTATTAATCTTCTTCGAAAGGCTATCCCCCTGATA
AAGGCAGGTTGCACACGTGTTCCGCACCCGTACGCCGCTCTCAATTTACCCGAAAGTAATCTACCGCTCGGCT
TGCATGTGTTAGGCCTCCCGCTAGCGTTCTCCTGAGCAGGATCAACTC
>1080
CTGCTGCCGCCCGTAGGAGTCTGGTCCGTGTCTCAGTACCAGTGTGGGGGATCACCCTCTCAGGCCCCCTAAA
GATCGTCGCCTTGGTGAGCCGTTACCTCACCAACTAGCTAATCTTGCGCGTGCCCATCTCTATCCACCGTAGT

TTTCAATATCACCCGATGCCGAATAATATATTATGGGGTATTAATCTTCTTCGAAAGGCTATCCCCCTGATAA
GGCAGGTTGCACACGTGTTCCGCACCCGTACGCCGCTCTCAATTACCCGAAAGTAATCTACCGCTCGGCTTGC
ATGTGTTAGGCCTCCCGCTAGCGTTCTCCTGAGCAGGATCAACTCG
>1081
CTGCTGCCGCCCGTAGGAGTCTGGTCCGTGTCTCAGTACCAGTGTGGGGGATCACCCTCTCAGGCCCCCTAAA
GATCGTCGCCTTGGTGAGCCGTTACCTCACCAACTAGCTAATCTTGCGCGTGCCCATCTCCATCCACCGTAGT
TTTCAATATCACCCGATGCCGAATAATATATTATGGGGTATTAATCTTCTTCGAAAGGCTATCCCCCTGATAA
AGACAGGTTGCACACGTGTTCCGCACCCGTACGCCGCTCTCAATTACCCGAAAGTAATCTACCGCTCGGCTTG
CATGTGTTAGGCCTCCCGCTAGCGTTCATCCTGAGCCAGGATCAAACTCT
>1082
CTGCTGCCGCCCGTAGGAGTCTGGTCCGTGTCTCAGTACCAGTGTGGGGGATCACCCTCTCAGGCCCCCTAAA
GATCGTCGCCTTGGTGAGCCGTTACCTCACCAACTAGCTAATCTTGCGCGTGCCCATCTCTATCCACCGTAGT
TTTCAATATCACCCGATGCCGAATAATATATTATGGGGTATTTAATCTTCTTCGAAAGGCTATCCCCCTGATA
AAGGCAGGTTGCACACGTGTTCCGCACCCGTACGCCGCTCTCAATTACCCGAAAGTAATCTACCGCTCGGCTT
GCATGTGTTAGGCCTCCCGCTAGCGTTCATCCTGAGCCAGGATCAAACTCT
>1083
CTGCTGCCGCCCGTAGGAGTCTGGTCCGTGTCTCAGTACCAGTGTGGGGGATCACCCTCTCAGGCCCCCTAAA
GATCGTCGCCTTGGTGAGCCGTTACCTCACCAACTAGCTAATCTTGCGCGTGCCCATCTCTATCCACCGTAGT
TTTCAATATCACCCGATGCCGAATAATATATTATGGGGTATTAATCTTCTTCGAAAGGCTATCCCCTGATAAA
GGCAGGTTGCACACGTGTTCCGCACCCGTACGCCGCTCTCAATTACCCGAAAGTAACTCTACCGCTCGGCTTG
CATGTGTTAGGCCTCCCGCTAGCGTTCATCCTGAGCCAGGATCAAACTCT
>1084
CTGCTGCCGCCCGTAGGAGTCTGGTCCGTGTCTCAGTACCAGTGTGGGGGATCACCCTCTCAGGCCCCCTAAAG
ATCGTCGCCTTGGTGAGCCGTTACCTCACCAACTAGCTAATCTTGCGCGTGCCCATCTCTATCCACCGTAGTT
TTCAATATCACCCGATGCCGAATAATATATTATGGGGTATTAATCTTCTCGAAAGCTATCCCCTGATAAAGGC
AGGTTGCACACGTGTTCCGCACCCGTACGCCGCTCTCAATTTACCCGAAAGTAATCTACCGCTCGGCTTGCAT
GTGTTAGGCCTCCCGCTAGCGTTCATCCTGAGCCAGGATCAAACTCT
>1085
CTGCTGCCGCCCGTAGGAGTCTGGTCCGTGTCTCAGTACCAGTGTGGGGGATCACCCTCTCAGGCCCCCTAAA
GATCGTCGCCTTGGTGAGCCGTTACCTCACCAACTAGCTAATCTTGCGCGTGCCCATCTCTATCCACCGTAGT
TTTCAATATCACCCGATGCCGAATAATATATTATGGGGTATTAATCTTCTTCGAAAGGCTATCCCCTGATAAA
GGCAGGTTGCACACGTGTTCCGCACCCGTACGCCGCTCTCAATTACCCGAAAGTAATCTACCGCTCGGCTTGC
ATGTGTTAGGCCTCCCGCTAGCGTTCTCTGA
>1086
CTGCTGCCGCCCGTAGGAGTCTGGTCCGTGTCTCAGTACCAGTGTGGGGGATCACCCTCTCAGGCCCCCTAAA
GATCGTCGCCTTGGTGAGCCGTTACCTCACCAACTAGCTAATCTTGCGCGTGCCCATCTCTATCCACCGTAGT
TTTCAATATCACCCGATGCCGAATAATATATTATGGGGTATTAATCTTCTTCGAAAGGCTATCCCCCTGATAA
AGGCAGGTTGCACACGTGTTCCGCACCCGTACGCCGCTCTCAATTACCCGAAAGTAATCTACCGCTCGGCTTG
CATGTGTTAGGCCTCCCGCTAGCGTTCATCCTGAGCCAGGATCAAACTCT
>1087
CTGCTGCCGCCCGTAGGAGTCTGGTCCGTGTCTCAGTACCAGTGTGGGGGATCACCCTCTCAGGCCCCCTAAA
GATCGTCGCCTGGTGAGCCGTTACCTCACCAACTAGCTAATCTTGCGCGTGCCCATCTCTATCCACCGTAGTT
TTCAATATCACCCGATGCCGAATAATATATTATGGGGTATTAATCTTCTCGAAAGGCTATCCCCCTGATAAAG
GCAGGTTGCACACGTGTTCCGCACCCGTACGCCGCTCTCAATTACCCGAAAGTAATCTACCGCTCGGCTTGCA
TGTGTTAGGCCTCCCGCTAGCGTTCATC
>1088
CTGCTGCCGCCCGTAGGAGTCTGGTCCGTGTCTCAGTACCAGTGTGGGGGATCACCCTCTCAGGCCCCCTAAA
GATCGTCGCCTTGGTGAGCCGTTACCTCACCAACTAGCTAATCTTGCGCGTGCCCATCTCTATCCACCGTAGT
TTTCAATATCACCCGATGCCGAATAATATATTATGGGGTATTAATCTTCTTCGAAAGGCTATCCCCTGATAAA
GGCAGGTTGCACACGTGTTCCGCACCCGTACGCCGCTCTCAATTACCCGAAAGTAATCTACCGCTCGGCTTGC
ATGTGTTAGGCCTCCCGCTAGCGTTCTCCTGAGCAGGATCCAACT
>1089
CTGCTGCCGCCCGTAGGAGTCTGGTCCGTGTCTCAGTACCAGTGTGGGGGATCACCCTCTCAGGCCCCCTAAA
GATCGTCGCCTTGGTGAGCCGTTACCTCACCAACTAGCTAATCTTGCGCGTGCCCATCTCTATCCACCGTAGT

TTTCAATATCAACCCGATGCCGAATAATATATTATGGGGTATTAATCTTCTCGAAAGGCTATCCCCTGATAAA
GGCAGGTTGCACACGTGTTCCGCACCCGTACGCCGCTCTCAATTACCCGAAAGTAATCTACCGCTCGGCTTGC
ATGTGTTAGGCCTCCCGCTAGCGTTCATCCTGAGCCAGGATCAAACTCT
>1090
CTGCTGCCGCCCGTAGGAGTCTGGTCCGTGTCTCAGTACCAGTGTGGGGGATCACCCTCTCAGGCCCCCTAAA
GATCGTCGCCTTGGTGAGCCGTTACCTCACCAACTAGCTAATCTTGCGCGTGCCCATCTCTATCCACCGTAGT
TTTCAATATCACCCGATGCCGAATAATATATTATGGGGTATTAATCTTCTTCGAAAGGCTATCCCCCTGATAA
AGGCAGGTTGCACACGTGTTCCGCACCCGTACGCCGCTCTCAATTACCCGAAAGTAATCTACCGCTCGGCTTG
CATGTGTTAGGCCTCCCGCTAGCGTTCTCCTGAGCCAGGATCAAACTCT
>1091
CTGCTGCCGCCCGTAGGAGTCTGGTCCGTGTCTCAGTACCAGTGTGGGGGATCACCCTCTCAGGCCCCCTAAA
GATCGTCGCCTTGGTGAGCCGTTACCTCACCAACTAGCTAATCTTGCGCGTGCCCATCTCTATCCACCGTAGT
TTCAACATCACCCGATGCCGAATAATATATTATGGGGTATTAATCTTCTTCGAAAGGCTATCCCCCTGATAAA
GGCAGGTTGCACACGTGTTCCGCACCCGTACGCCGCTCTCAATTACCCGAAAGTAATCTACCGCTCGGCTTGC
ATGTGTTAGGCCTCCCGCTAGCGTTCTCCTGAGCAGGATCAAC
>1092
CTGCTGCCGCCCGTAGGAGTCTGGTCCCGTGTCTCAGTACCAGTGTGGGGGATCACCCCTCTCAGGCCCCCTA
AAGATCGTCGCCTTTGGTGAGCCGTTACCTCACCCAACTAGCTAATCTTGCGCGTGCCCCATTCTCTATCCCA
CCGTAGTTTTCAATATCACCCGATGCCGAATAATATATTATGGGGTATTTAATCTTTCTTCGAAAGGCTATC
CCCTGATAAAGGCAGGTTGCACACGTGTTCCGCACCCGTACGCCGCTCTCAATTACCCCGAAAGTAATCTACC
GCTCGGCTGCATGTGTTTAGGCCTCCCGCTAGCGTTCTTCCTGAGCAGGA
>1093
CTGCTGCCGCCGTAGGAGTCTGGTCCGTGTCTCAGTACCAGTGTGGGGGATCACCCTCTCAGGCCCCCTAAAG
ATCGTCGCCTTGGTGAGCCGTTACCTCACCAACTAGCTAATCTTGCGCGTGCCCATCTCTATCCACCGTAGTT
TTCAATATCACCCGATGCCGAATAATATATTATGGGGTATTAATCTTCTTCGAAAGGCTATCCCCCTGATAAA
GGCAGGTTGCACACGTGTTCCGCACCCGTACGCCGCTCTCAATTACCCGAAAGTAATCTACCGCATCGGCTTG
CATGTGTTAGGCCTCCCGCTAGCGTTCTCCTGAGCAGGATCCAAA
>1094
CTGCTGCCGCCCGTAGGAGTCTGGTCCGTGTCTCAGTACCAGTGTGGGGGATCACCCTCTCAGGCCCCCTAAA
GATCGTCGCCTGGTGAGCCGTTACCTCACCAACTAGCTAATCTTGCGCGTGCCCATCTCTATCCACCGTAGTT
TTCAATATCACCCGATGCCGAATAATATATTATGGGGTATTAATCTTCTTCGAAAGGCTATCCCCCTGATAAA
GGCAGGTTGCACACGTGTTCCGCACCCGTACGCCGCTCTCAATTACCCGAAAGTAATCTACCGCTCGGCTTGC
ATGTGTTAGGCCTCCCGCTAGCGTTCTCCT
>1095
CTGCTGCCGCCCGTAGGAGTCTGGTCCGTGTCTCAGTACCAGTGTGGGGGATCACCCTCTCAGGCCCCCTAAA
GATCGTCGCCTTGGTGAGCCGTTACCTCACCAACTAGCTAATCTTGCGCGTGCCCATCTCTATCCACCGTAGT
TTTCAATATCACCCGATGCCGAATAATATATTATGGGGTATTAATCTTCTCGAAAGGCTATCCCCTGATAAAG
GCAGGTTGCACACGTGTTCCGCACCCGTACGCCGCTCTCAATTACCCGAAAGTAATCTACCGCTCGGCTTGCA
TGTGTTAGGCCTCCCGCTAGCGTTCATCTGAGCCAGGATCAACTC
>1096
CTGCTGCCGCCCGTAGGAGTCTGGTCCGTGTCTCAGTACCAGTGTGGGGGATCACCCTCTCAGGCCCCCTAAA
GATCGTCGCCTTGGTGAGCCGTTACCTCACCAACTAGCTAATCTTGCGCGTGCCCATCTCTATCCACCGTAGT
TTTCAATATCACCCGATGCCGAATAATATATTATGGGGTATTAATCTTCTTCGAAAGGCTATCCCCCTGATAA
AGGCAGGTTGCACACGTGTTCCGCACCCGTACGCCGCTCTCAATTACCCGAAAGTAATCTACCGCTCCGGCTT
GCATGTGTTAGGCCTCCCGCTAGCGTTCTCCTGAGCAGGATCAACTC
>1097
CTGCTGCCGCCCGTAGGAGTCTGGTCCGTGTCTCAGTACCAGTGTGGGGGATCACCCTCTCAGGCCCCCTAAA
GATCGTCGCCTTGGTGAGCCGTTACCTCACCAACTAGCTAATCTTGCGCGTGCCCATCTCTATCCACCGTAGT
TTTCAATATCACCCGATGCCGAATAATATATTATGGGGTATTAATCTTCTTCGAAAGGCTATCCCCCTGATAA
AGGCAGGTTGCACACGTGTTCCGCACCCGTACGCCGCTCTCAATTACCCGAAAGTAATCTACCGCTCCGGCTT
GCATGTGTTAGGCCTCCCGCTAGCGTTCTCCTGAGCAGGATCAACT
>1098
CTGCTGCCGCCCGTAGGAGTCTGGTCCGTGTCTCAGTACCAGTGTGGGGGATCACCCTCTCAGGCCCCCTAAA
GATCGTCGCCTTGGTGAGCCGTTACCTCACCAACTAGCTAATCTTGCGCGTGCCCATCTCTATCCACCGTAGT

TTTCAATATCACCCGATGCCGAATAATATATTATGGGGTATTAATCTTCTTCGAAAGGCTATCCCCCTGATAA
AGGCAGGTTGCACACGTGTTCCGCACCCGTACGCCGCTCTCAATTACCCGAAAGTAATCTACCGCTCGGCTTG
CATGTGTTAGGCCTCCCGCTAGCGTTCATCCTGAGCCAGGATCAAACTCT
>1099
CTGCTGCCGCCCGTAGGAGTCTGGTCCGTGTCTCAGTACCAGTGTGGGGGATCACCCTCTCAGGCCCCCTAAA
GATCGTCGCCTTGGTGAGCCGTTACCTCACCAACTAGCTAATCTTGCGCGTGCCCATCTCTATCCACCGTAGT
TTTCAATATCACCCGATGCCGAATAATATATTATGGGGTATTAATCTTCTTCGAAAGGCTATCCCCCTGATAA
AGGCAGGTTGCACACGTGTTCCGCACCCGTACGCCGCTCTCAATTACCCGAAAGTAATCTACCGCTCCGGCTT
GCATGTGTTAGGCCTCCCGCTAGCGTTCATCCTGAGCCAGGATCAACTCT
>1100
CTGCTGCCGCCCGTAGGAGTCTGGTCCGTGTCTCAGTACCAGTGTGGGGGGATCACCCTCTCAGGCCCCCCTA
AAGATCGTCGCCTTGGTGAGCCGTTACCTCACCCAACTAGCTTAATCTTGCGCGTGCCCCATCTCTATCCACC
GTAGTTTTCAATATCACCCGATGCCGAATAATATATTATTGGGGTATTTAATCTTCTTCGAAAGGCTATCCCC
TGATAAAGGCAGGTTGCACACGTGTTCCGCACCCGTACGCCGCTCTCAATTACCCCGAAAGTAATCTACCGCT
CGGCTTGCATGTGTTAGGCCTCCCGCTAGCGTTCTTCCTGAGCAGGATCAACTCGT
>1101
CTGCTGCCGCCCGTAGGAGTCTGGTCCGTGTCTCAGTACCAGTGTGGGGGATCACCCTCTCAGGCCCCCTAAAG
ATCGTCGCCTTGGTGAGCCGTTACCTCACCAACTAGCTAATCTTGCGCGTGCCCATCTCTATCCACCGTAGTT
TTCAATATCACCCGATGCCGAATAATATATTATGGGGTATTAATCTTCTTCGAAAGGCTATCCCCTGATAAGG
CAGGTTGCACACGTGTTCCGCACCCGTACGCCGCTCTCAATTACCCGAAAGTAATCTACCGCTCCGGCTTGCA
TGTGTTAGGCCTCCCGCTAGCGTCATCCTGAGCCAGGATCAAACTCT
>1102
CTGCTGCCGCCCGTAGGAGTCTGGTCCGTGTCTCAGTACCAGTGTGGGGGATCACCCTCTCAGGCCCCCTAAA
GATCGTCGCCTTGGTGAGCCGTTACCTCACCAACTAGCTAATCTTGCGCGTGCCCATCTCTATCCACCGTAGT
TTTCAATATCACCCGATGCCGAATAATATATTATGGGGTATTAATCTTCTTCGAAAGGCTATCCCCCTGATAA
AGGCAGGTTGCACACGTGTTCCGCACCCGTACGCCGCTCTCAATTACCCGAAAGTAATCTACCGCTCCGGCTT
GCATGTGTTGACGGCTCCCGCTAGCGTTCTCTGAGCAGGATCAACATCCT
>1103
CTGCTGCCGCCCGTAGGAGTCTGGTCCGTGTCTCAGTACCAGTGTGGGGGATCACCCTCTCAGGCCCCCTAAA
GATCGTCGCCTTGGTGAGCCGTTACCTCACCAACTAGCTAATCTTGCGCGTGCCCATCTCTATCCACCGTAGT
TTTCAATATCACCCGATGCCGAATAATATATTATGGGGTATTAATCTTCTTCGAAAGGCTATCCCCTGATAAA
GGCAGGTTGCACACGTGTTCCGCACCCGTACGCCGCTCTCAATTACCCGAAAGTAATCTACCGCCCGGCTTGC
ATGTCGTTAGGCCTCCCGCTAGCGTTCATCCTGAGCCAGGATCAAACTCT
>1104
CTGCTGCCGCCCGTAGGAGTCTGGTCCGTGTCTCAGTACCAGTGTGGGGGATCACCCTCTCAGGCCCCCTAAA
GATCGTCGCCTTGGTGAGCCGTTACCTCACCAACTAGCTAATCTTGCGCGTGCCCATCTCTATCCACCGTAGT
TTTCAATATCACCCGATGCCGAATAATATATTATGGGGTATTAATCTTCTTCGAAAGGCTATCCCCTGATAAA
GGCAGGTTGCACACGTGTTCCGCACCCGTACGCCGCTCTCAATTTACCCGAAAGTAATCTACCGCTCGGCTTG
CATGTGTTAGGCCTCCCGCTAGCGTTCATCCTGAGCCAGGATC
>1105
CTGCTGCCGCCCGTAGGAGTCTGGTCCGTGTCTCAGTACCAGTGTGGGGGATCACCCTCTCAGGCCCCCTAAA
GATCGTCGCCTTGGTGAGCCGTTACCTCACCAACTAGCTAATCTTGCGCGTGCCCATCTCTATCCACCGTAGT
TTTCAATATCACCCGATGCCGAATAATATATTATGGGGTATTAATCTTCTTACGAAAGGCTATCCCCTGATAA
AGGCAGGTTGCACACGTGTTCCGCACCCGTACGCCGCTCTCAATTACCCGAAAGTAATCTACCGCATCGGCTT
GCACTGTGTTAGGCCTCCCGCTA
>1106
TTGCTGCCGCCCGTAGGAGTCTGGTCCGTGTCTCAGTACCAGTGTGGGGGATCACCCTCTCAGGCCCCCTAAA
GATCGTCGCCTTGGTGAGCCGTTACCTCACCAACTAGCTAATCTTGCGCGTGCCCATCTCTATCCACCGTAGT
TTTCAATATCACCCGATGCCGAATAATATATTATGGGGTATTAATCTTCTTCGAAAGGCTATCCCCCTGATAA
AGGCAGGTTGCACACGTGTTCCGCACCCGTACGCCGCTCTCAATTACCCGAAAGTAATCTACCGCTCGGCTTG
CATGTGTTAGGCCTCCCGCTAGCGTTCTCCT
>1107
CTGCTGCCGCCCGTAGGAGTCTGGTCCGTGTCTCAGTACCAGTGTGGGGGATCACCCTCTCAGGCCCCCTAAA
GATCGTCGCCTTGGTGAGCCGTTACCTCACCAACTAGCTAATCTTGCGCGTGCCCATCTCTATCCACCGTAGT

TTTCAATATCACCCGATGCCGAATAATATATTATGGGGTATTAATCTTCTTCGAAAGGCTATCCCCCTGATAA
AGGCAGGTTGCACACGTGCTCCGCACCCGTACGCCGCTCTCAATTACCCGAAAGTAATCTACCGCTCGGCTTG
CATGTGTTAGGCCTCCCGCTAGCGTTCATCCTGAGCCAGGATCAAACTCT
>1108
CTGCTGCCGCCCGTAGGAGTCTGGTCCGTGTCTCAGTACCAGTGTGGGGGATCACCCTCTCAGGCCCCCTAAA
GATCGTCGCCTTGGTGAGCCGTTACCTCACCAACTAGCTAATCCTGCGCGTGCCCATCTCTATCCACCGTAGT
TTTCAATATCACCCGATGCCGAATAATATATTATGGGGTATTAATCTTCTCGAAAGGCTATCCCCTGATAAAG
GCAGGTTGCACACGTGTTCCGCACCCGTACGCCGCTCTCAATTACCCGAAAGTAATCTACCGCTCGGCTTGCA
TGTGTTAGGCCTCCCGCTAGCGTTCATCCTGAGCCAGGATCAACTCT
>1109
CTGCTGCCGCCCGTAGGGGTCTGGTCCGTGTCTCAGTACCAGTGTGGGGGATCACCCTCTCAGGCCCCCTAAA
GATCGTCGCCTTGGTGAGCCGTTACCTCACCAACTAGCTAATCTTGCGCGTGCCCATCTCTATCCACCGTAGT
TTTCAATATCACCCGATGCCGAATAATATATTATGGGGTATTAATCTTCTTCTGAAAGGCTATCCCCCTGATA
AAGGCAGGTTGCACACGTGTTCCGCACCCGTACGCCGCTCTCAATTACCCGAAAGTAATCTACCGCTCGGCTT
GCATGTGTTAGGCCTCCCGCTAGCGTTCATCCTGAGCCAGGATCAAACTCT
>1110
CTGCTGCCGCCCGTAGGAGTCTGGTCCGTGTCTCAGTACCAGTGTGGGGGATCACCCTCTCAGGCCCCCTAAA
AGATCGTCGCCTTGGTGAGCCGTTACCTCACCAACTAGCTAATCTTGCGCGTGCCCATCTCTATCCACCGTAG
TTTTCAATATCACCCGATGCCGAATAATATATTATGGGGTATTTAATCTTTCTTCGAAAGGCTATCCCCCTGA
TAAAAGGCAGGTTACACACGTGTTCCGCACCCGTACGCCGCTCTCAATTACCCGAAAGTAATCTACCGCTCGG
CTTGCATGTGTTAGGCCTCCCGCTAGCGTTCTCCTGAGCAGGATCCAAACTCGT
>1111
CTGCTGCCGCCCGTAGGAGTCTGGTCCGTGTCTCAGTACCAGTGTGGGGGGATCACCCTCTCAGGCCCCCCTA
AAGATCGTCGCCTTGGTGAGCCGTTACCTCACCCAACTAGCTAATCTTGCGCGTGCCCCATCTCTATCCACCG
TAGTTTTCAATATCACCCGATGCCGAATAATATATTTATGGGGTATTTAATCTTTCTTCGAAAGGCTATCCCC
CTGATAAAGGCAGGTTGCACACGTGTTCCGCACCCGTACGCCGCTCTCAATTACCCCCGAAAGTAATCTACCG
CTCGGCTTGCATGTGTTAGGCCTCCCGCTAGCGTTCTTCCTGAGCAGGATCCAACT
>1112
CTGCTGCCGCCCGTAGGAGTCTGGTCCGTGTCTCAGTACCAGTGTGGGGGATCACCCTCTCAGGCCCCCTAAA
GATCGTCGCCTTGGTGAGCCGTTACCTCACCAACTAGCTAATCTTGCGCGTGCCCATCTCTATCCACCGTAGT
TTTCAATATCACCCGATGCCGAATAATATATTATGGGGTATTAATCTTCTTCGAAAGGCTATCCCCCTGATAA
AGGCAGGTTGCACACGTGTTCCGCACCCGTACGCCGCTCTCAATTACCCGAAAGTAATCTACCGCTCGGCTTG
CATGTGTTAGGCCTCCCGCTAGCGTTCATCCTGAGCCAAGATCAACTCT
>1113
CTGCTGCCGCCCGTAGGAGTCTGGTCCGTGTCTCAGTACCAGTGTGGGGGATCACCCTCTCAGGCCCCCTAAA
GATCGTCGCCTTGGTGAGCCGTTACCTCACCAACTAGCTAATCTTGCGCGTGCCCATCTCTATCCACCGTAGT
TTTCAATATCACCCGATGCCGAATAATATATTATGGGGTATTAATCTTCTTCGAAAGGCTATCCCCCTGATAA
AGGCAGGTTGCACACGTGTTCCGCACCCGTACGCCGCTCTCAATTACCCGAAAGTAATCTACCGCTCGGCTTG
CATGTGTTAGGCCTCCCGCTAGCGTTCATCCTGAGCCAGGATCAAACTCT
>1114
CTGCTGCCGCCCGTAGGAGTCTGGTCCGTGTCTCAGTACCAGTGTGGGGGATCACCCTCTCAGGCCCCCTAAA
GATCGTCGCCTTGGTGAGCCGTTACCTCACCAACTAGCTAATCTTGCGCGTGCCCATCTCTATCCACCGTAGT
TTTCAATATCACCCGATGCCGAATAATATATTATGGGGTATTAATCTTCTTCGAAAGGCTATCCCCCTGATAA
AGGCAGGTTGCACACGTGTTCCGCACCCGTACGCCGCTCTCAATTACCCGAAAGTAATCTACCGCTCGGCTTG
CATGTGTTAGGCCTCCCGCTAGCGTTCATCCTGAGCCAGGATCAAACTCT
>1115
CTGCTGCCGCCCGTAGGAGTCTGGTCCGTGTCTCAGTACCAGTGTGGGGGATCACCCTCTCAGGCCCCCTAAA
GATCGTCGCCTTGGTGAGCCGTTACCTCACCAACTAGCTAATCTTGCGCGTGCCCATCTCTATCCACCGTAGT
TTTCAATATCACCCGATGCCGAATAATATATTATGGGGTATTAATCTTCTTCGAAAGGCTATCCCCTGATAAA
GGCAGGTTGCACACGTGTTCCGCACCCGTACGCCGCTCTCAATTACCCGAAAGTAATCTACCGCTCGGCTTGC
ATGTGTTAGGCCTCCCGCTAGCGTTCATCCCGTGCGCAAGGCAT
>1116
CTGCTGCCGCCCGTAGGAGTCTGGACCGTGTCTCAGTTCCAGTGTGACTGATCATCCTCTCAGACCAGTACGG
ATCGTCGCCTTGGTAGGCCTTTACCCCACCAACTAGCTAATCCGACCTAGGCTCATCTGATAGCGTGAGGTCC

GAAGTCCCCCACTTCTCCCTCAGGACGTATGCGGTATTAGCGCCCGTTTCCGGACGTATCCCCACTACCAGGC
AGATTCCTAGGCATTACTCACCCGTCCGCCGCTGAATCCAGGAGCAAGCTCCCTCATCCGCTCGACTGCATGT
GTTAGGCCTGCCGCCAGCGTTCAATCTGAGCCAGGATCAACTCT
>1117
CTGCTGCCGCCCGTAGGAGTCTGGTCCGTGTCTCAGTACCAGTGTGGGGGATCACCCTCTCAGGCCCCCTAAA
GATCGTCGCCTTGGTGAGCCGTTACCTCACCAACTAGCTAATCTTGCGCGTGCCCATCTCTATCCACCGTAGT
TTTCAATATCACCCGATGCCGAATAATATATTATGGGGTATTAATCTTCTTCGAAAGGCTATCCCCCTGATAA
AGGCAGGTTGCACACGTGTTCCGCACCCGTACGCCGCTCTCAATTACCCGAAAGTAATCTACCGCTCGGCTTG
CATGTGTTAGGCCTCCCGCTAGCGTTCATCCTGAGCA
>1118
CTGCTGCCGCCCGTAGGAGTCTGGTCCGTGTCTCAGTACCAGTGTGGGGGATCACCCTCTCAGGCCCCCTAAA
GATCGTCGCCTTGGTGAGCCGTTACCTCACCAACTAGCTAATCTTGCGCGTGCCCATCTCTATCCACCGTAGT
TTTCAATATCACCCGATGCCGAATAATATATTATGGGGTATTAATCTTCTTCGAAAGGCTATCCCCTGATAAA
GGCAGGTTGCACACGTGTTCCGCACCCGTACGCCGCTCTCAATTACCCGAAAGTAATCTACCGCTCGGCTTGC
ATGTGTTAGGCCTCCCGCTAGCGTTCTCCTCGAGCAGG
>1119
CTGCTGCCGCCCGTAGGAGTCTGGTCCGTGTCTCAGTACCAGTGTGGGGATCACCCTCTCAGGCCCCTAAAGA
TCGTCGCCTTGGTGAGCCGTTACCTCACCAACTAGCTAATCTTGCGCGTGCCCATCTCTATCCACCGTAGTTT
TCAATATCACCCGATGCCGAATAATATATTATGGGGTATTAATCTTCTCGAAAGGCTATCCCCTGATAAGGCA
GGTTAGCACACGTCGTTCCGCACCGTACGCCGCTCTCAATTACCCGAAAGTAATCTACCGCTCCGGCTTGCAA
TGTGTTAGGCCTCCCGCTAGCGTTCATCCTG
>1120
CTGCTGCCGCCCGTAGGAGTCTGGTCCGTGTCTCAGTACCAGTGTGGGGGATCACCCTCTCAGGCCCCCTAAA
GATCGTCGCCTTGGTGAGCCGTTACCTCACCAACTAGCTAATCTTGCGCGTGCCCATCTCTATCCACCGTAGT
TTTCAATATCACCCGATGCCGAATAATATATTATGGGGTATTAATCTTCTTCGAAAGGCTATCCCCCTGATAA
AGGCAGGTTGCACACGTGTTCCGCACCCGTACGCCGCTCTCAATTACCCGAAAGTAATCTACCGCTCGGCTTG
CATGTGTTAGGCCTCCCGCTAGCGTTCATCCTCGAGC
>1121
CTGCTGCCGCCCGTAGGAGTCTGGTCCGTGTCTCAGTACCAGTGTGGGGGATCACCCTCTCAGGCCCCCTAAA
GATCGTCGCCTTGGTGAGCCGTTACCTCACCAACTAGCTAATCTTGCGCGTGCCCATCTCTATCCACCGTAGT
TTTCAATATCACCCGATGCCGAATAATATAATTATGGGGTATTAATCTTCTCGAAAGGCTATCCCCCTGATAA
AGGCAGGTTGCACACGTGTTCCGCACCCGTACGCCGCTCTCAATTACCCGAAAGTAATCTACCGCTCGGCTTG
CATGTGTTAGGCCTCCCGCTAGCGTTCATCCTGAGCCAGGATCAACTCT
>1122
CTGCTGCCGCCCGTAGGAGTCTGGTCCGTGTCTCAGTACCAGTGTGGGGGATCACCCTCTCAGGCCCCCTAAA
GATCGTCGCCTTGGTGAGCCGTTACCTCACCAACTAGCTAATCTTGCGCGTGCCCATCTCTATCCACCGTAGT
TTCAATATCACCCGATGCCGAATAATATATTATGGGGTATTAATCTTCTTCGAAAGGCTATCCCCCTGATAAA
GGCAGGTTGCACACGTGTTCCGCACCCGTACGCCGCTCTCAATTACCCGAAAGTAATCTACCGCTCGGCTTGC
ATGTGTTAGGCCTCCCGCTAGCGTTCTCCTCGAGCAGGATC
>1123
CTGCTGCCGCCGTAGGAGTCTGGTCCGTGTCTCAGTACCAGTGTGGGGGATCACCCTCTCAGGCCCCCTAAAG
ATCGTCGCCTTGGTGAGCCGTTACCTCACCAACTAGCTAATCTTGCGCGTGCCCATCTCTATCCACCGTAGTT
TTCAATATCACCCGATGCCGAATAATATATTATGGGGTATTAATCTTCTTCGAAAGGCTATCCCCCTGATAAA
GGCAGGTTGCACACGTGTTCCGCACCCGTACGCCGCTCTCAATTACCCGAAAGTAATCTACCGCTCCGGCTTG
CATGTGTTAGGCCTCCCGCTAGCGTTCTCCTCGAGCAGGA
>1124
CTGCTGCCGCCCGTAGGAGTCTGGTCCGTGTCTCAGTACCAGTGTGGGGGATCACCCTCTCAGGCCCCCTAAA
GATCGTCGCCTTGGTGAGCCGTTACCTCACCAACTAGCTAATCTTGCGCGTGCCCATCTCTATCCACCGTAGT
TTTCAATATCACCCGATGCCGAATAATATATTATGGGGTATTAATCTTCTCGAAAGGCTATCCCCTGATAAAG
GCAGGTTGCACACGTGTTCCGCACCCGTACGCCGCTCTCAATTACCCGAAAGTAATCTACCGCTCCGGCTTGC
ATGTGTTAGGCCTCCCGCTAGCGTTCATCCGTCGAGCCAGGATCAACTCT
>1125
CTGCTGCCGCCCGTAGGAGTCTGGTCCGTGTCTCAGTACCAGTGTGGGGGATCACCCTCTCAGGCCCCCTAAA
GATCGTCGCCTTGGTGAGCCGTTACCTCACCAACTAGCTAATCTTGCGCGTGCCCATCTCTATCCACCGTAGT

TTTCAATATCACCCGATGCCGAATAATATATTATGGGGGTATTAATCTTCTCGAAAGGCTATCCCCTGATAAA
GGCAGGTTGCACACGTGTTCCGCACCCGTACGCCGCTCTCAATTACCCGAAAGTAATCTACCGCTCCGGGCTT
GCATGTGTTAGGCCTCCCGCTAGCGTTCATCCGTGAGCCAGGATCAACTCT
>1126
CTGCTGCCGCCCGTAGGAGTCTGGTCCGTGTCTCAGTACCAGTGTGGGGGATCACCCTCTCAGGCCCCTAAAG
ATCGTCGCCTGGTGAGCCGTTACCTCACCAACTAGCTAATCTTGCGCGTGCCCATCTCTATCCACCGTAGTTT
TCAATATCACCCGATGCCGAATAATATATTATGGGGTATTAATCTTCTCGAAAGGCTATCCCCCTGATAAAGG
CAGGTTGCACACGTGTTCCGCACCCGTACGCCGCTCTCAATTACCCCGAAAGTGATCTACCGCTCGGCTTGCA
TGTGTTAGGCCTCCCGCTAGCGTTCATCCTC
>1127
CTGCTGCCGCCCGTAGGAGTCTGGTCCGTGTCTCAGTACCAGTGTGGGGGATCACCCTCTCAGGCCCCCTAAA
GATCGTCGCCTTGGTGAGCCGTTACCTCACCAACTAGCTAATCTTGCGCGTGCCCATCTCTATCCACCGTAGT
TTTCAATATCACCCGATGCCGAATAATATATTATGGGGTATTAATCTTCTTCGAAAGGCTATCCCCCTGATAA
AGGCAGGTTGCACACGTGTTCCGCACCCGTACGCCGCTCTCAATTACCCGAAAGTAATCTACCGCTCGGCTTG
CATGTGTTAGGCCTCCCGCTAGCGTTCATCCTGAGCCAGAATCAAACTCT
>1128
CTGCTGCCGCCCGTAGGAGTCTGGTCCGTGTCTCAGTACCAGTGTGGGGGATCACCCTCTCAGGCCCCCTAAAG
ATCGTCGCCTTGGTGAGCCGTTACCTCACCAACTAGCTAATCTTGCGCGTGCCCATCTCTATCCACCGTAGTT
TTCAATATCACCCGATGCCGAATAATATATTATGGGGTATTAATCTTCTTCGAAAGGCTATCCCCCTGATAAA
GGCAGGTTGCACACGTGTTCCGCACCCGTACGCCGCTCTCAATTACCCGAAAGTAATCTACCGCTCGGCTTGC
ATGTGTTAGGCCTCCCGCTAGCGTTCTCCTGAGCAGGATCAACTCGT
>1129
CTGCTGCCGCCCGTAGGAGTCTGGTCCGTGTCTCAGTACCAGTGTGGGGGATCACCCTCTCAGGCCCCCTAAA
GATCGTCGCCTTGGTGAGCCGTTACCTCACCAACTAGCTAATCTTGCGCGTGCCCACCTCTATCCACCGTAGT
TTTCAATATCACCCGATGCCGAATAATATATTATGGGGTATTAATTCTTCTTCGAAAGGCTATCCCCCTGATA
AAGGCAGGTTGCACACGTGTTCCGCACCCGTACGCCGCTCTCAATTACCCGAAAGTAATCTACCGCTCGGCTT
GCATGTGTTAGGCCTCCCGCTAGCGTTCATTCCTGAGCCAGGATCAAACTCT
>1130
CTGCTGCCGCCCGTAGGAGTCTGGTCCGTGTCTCAGTACCAGTGTGGGGGATCACCCTCTCAGGCCCCCTAAA
GATCGTCGCCTTGGTGAGCCGTTACCTCACCAACTAGCTAATCTTGCGCGTGCCCATCTCTATCCACCGTAGT
TTTCAATATCACCCGATGCCGAATAATATATTATGGGGTATTAATCTTCTTCGAAAGGCTATCCCCCTGATAA
AGGCAGGTTGCACACGTGTTCCGCACCCGTACGCCGCTCTCAATTACCCGAAAGTAATCTACCGCTCGGCTTG
CATGTGTTAGGCCTCCCGCTAGCGTTCATCCTGAGCCAGGATCAACTCT
>1131
CTGCTTGCCGCCCCGTAGGAGTCTGGACCCGTGTCTCAGTTCCAGTGTGACTGATCATCCTCTCAGACCCAGT
ACGGATCGTCGCCTTGGTAGGCCCTTTTACCCCCACCAACTAGCTAATCCGACCTAGGCTCATCTGATAGCGT
GAGGTCCGAAGTCCCCCACTTCTCCCCTCAGGACGTATGCGGTATTAGCGCCCGTTTCCGGACGTATCCCCCA
CTGCCCAGGCAGATTCCTAGGCATTACTCACCCGTCCGCCGCTGAATCCAGGAGCAAGCTCCCCTCATCGCTC
GACTGCATGTGTTAGGCCTGCCGCCAGCGTCAATCTG
>1132
CTGCTGCCGCCCGTAGGAGTCTGGTCCGTGTCTCAGTACCAGTGTGGGGGATCACCCTCTCAGGCCCCCTAAA
GATCGTCGCCTTGTGAGCCGTTACCTCACCAACTAGCTAATCTTGCGCGTGCCCATCTCTATCCACCGTAGTT
TTCAATATCACCCGATGCCGAATAATATATTATGGGGTATTAATCTTCTCGAAAGGCTTATCCCCCTGATAAA
GGCAGGTTGCACACGTGTTCCGCACCCGTACGCCGCTCTCAATTTACCCGAAAGTAATCTACCGCTCGGCTTG
CAATGTGTTAGGCCTCCCGCTAGCGTTCATCCTGAGCCAGGATCAACTCT
>1133
CTGCTGCCGCCCGTAGGAGTCTGGTCCGTGTCTCAGTACCAGTGTGGGGGATCACCCTCTCAGGCCCCCTAAA
AGATCGTCGCCTTGGTGAGCCGTTACCTCACCAACTAGCTAATCTTGCGCGTGCCCATCTCTATCCACCGTAG
TTTTCAATATCACCCGATGCCGAATAATATATTATGGGGTATTAATCTTCTTCGAAAGGCTATCCCCCTGATA
AAGGCAGGTTGCACACGTGTTCCGCACCCGTACGCCGCTCTCAATTACCCGAAAGTAATCTACCGCTCGGCTT
GCATGTGTTAGGCCTCCCGCTAGCGTTCATCCTGAGCCAGGATCAAACTCT
>1134
CTGCTGCCGCCCGTAGGAGTCTGGACCGTGTCTCAGTTCCAGTGTGACTGATCATCCTCTCAGACCAGTACGG
ATCGTCGCCTTGGTAGGCCTTTACCCCACCAACTAGCTAATCCGACCTAGGCTCATCTGATAGCGTGAGGTCC

GAAGATCCCCCACTTCTCCCTCAGGACGTATGCGGTATTAGCGCCCGTTTCCGGACGTATCCCCACTACCAGG
CAGATTCCTAGGCATTACTCACCCGTCCGCCGCTGAATCCAGGAGCAAGCTCCCTCATCCGCTCGACTGCATG
TGTTAGGCCTGCCGCCAGCGTCAATCTGAGCCAGGATCAAACTCT
>1135
CTGCTGCCGCCCGTAGGAGTCTGGTCCGTGTCTCAGTACCAGTGTGGGGGATCACCCTCTCAGGCCCCCTAAA
GATCGTCGCCTTGGTGAGCCGTTACCTCACCAACTAGCTAATCTTGCGCGTGCCCATCTCTATCCACCGTAGT
TTTCAATATCACCCGATGCCGAATAATATATTATGGGGTATTAATCTTCTTCGAAAGGCTATCCCCCTGATAA
AGGCAGGTTGCACACGTGCTCCGCACCCGTACGCCGCTCTCAATTACCCGAAAGTAATCTACCGCTCGGCTTG
CATGTGTTAGGCCTCCCGCTAGCGTTCATCCTGAGCCAGGATCAAACTCT
>1136
CTGCTGCCGCCCGTAGGAGTCTGGTCCGTGTCTCAGTACCAGTGTGGGGGATCACCCTCTCAGGCCCCCTAAA
GATCGTCGCCTTGGTGAGCCGTTACCTCACCAACTAGCTAATCTTGCGCGTGCCCATCTCTATCCACCGTAGT
TTTCAATATCACCCGATGCCGAATAATATATTATGGGGTATTAATCTTCTTCGAAAGGCTATCCCCCTGATAA
AGGCAGGTTGCACACGTGTTCCGCACCCGTACGCCGCTCTCAATTACCCGAAAGTAATCTACCGCTCGGCTTG
CATGTGTTAGGCCTCCCGCTAGCGTTCATCCTGAGCAGGATCAACTCT
>1137
CTGCTGCCGCCCGTAGGAGTCTGGTCCGTGTCTCAGTACCAGTGTGGGGGATCACCCTCTCAGGCCCCCTAAA
GATCGTCGCCTGGTGAGCCGTTACCTCACCAACTAGCTAATCTTGCGCGTGCCCATCTCTATCCACCGTAGTT
TTCAATATCACCCGATGCCGAATAATATATTATGGGGTATTAATCTTCTTCGAAAGGCTATCCCCCTGATAAA
GGCAGGTTGCACACGTGTTCCGCACCCGTACGCCGCTCTCAATTACCCGAAAGTAATCTACCGCTCGGCTTGC
ATGTGTTAGGCCTCCCGCTAGCGTTCATCCTGAGCAG
>1138
CTGCTGCCGCCCGTAGGAGTCTGGTCCGTGTCTCAGTACCAGTGTGGGGGATCACCCTCTCAGGCCCCCTAAA
GATCGTCGCCTTGGTGAGCCGTTACCTCACCAACTAGCTAATCTTGCGCGTGCCCATCTCTATCCACCGTAGT
TTTCAATATCACCCGATGCCGAATAATATAATTATGGGGTATTAATCTTCTTCGAAAGGCTATCCCCTGATAA
AGGCAGGTTGCACACGTGTTCCGCACCCGTACGCCGCTCTCAATTACCCGAAAGTAATCTACCGCTCGGCTTG
CATGTGTTAGGCCTCCCGCTAGCGTTCATCCTGAGCCAGGATCAACTCT
>1139
CTGCTGCCGCCCGTAGGAGTCTGGTCCGTGTCTCAGTACCAGTGTGGGGGATCACCCTCTCAGGCCCCCTAAA
GATCGTCGCCTTGGTGAGCCGTTACCTCACCAACTAGCTAATCTTGCGCGTGCCCATCTCTATCCACCGTAGT
TTTCAATATCACCCGATGCCGAATAATATATTATGGGGTATTAATCTTCTTCGAAAGGCTATCCCCCTGATAA
AGGCAGGTTGCACACGTGTTCCGCACCCGTACGCCGCTCTCAATTACCCGAAAGTAATCTACCGCTCGGCTTG
CATGTGTTAGGCCTCCCGCTAGCGTTCATCCTGAGCCAGGATCAAACTCT
>1140
CTGCTGCCGCCCGTAGGAGTCTGGTCCGTGTCTCAGTACCAGTGTGGGGGATCACCCTCTCAGGCCCCCTAAA
GATCGTCGCCTTGGTGAGCCGTTACCTCACCAACTAGCTAATCTTGCGCGTGCCCATCTCTATCCACCGTAGT
TTTCAATATCACCCGATGCCGAATAATATATTATGGGGTATTAATCTTCTTCGAAAGCTATCCCCCTGATAAA
GGCAGGTTGCACACGTGTTCCGCACCCGTACGCCGCTCTCAATTACCCGAAAGTAATCTACCGCTCCGGCTTG
CATGTGTTAGGCCTCCCGCTAGCGTTCTCCTGAGCAGGATCAACTCT
>1141
CTGCTGCCGCCTGTAGGAGTCTGGTCCGTGTCTCAGTACCAGTGTGGGGGATCACCCTCTCAGGCCCCCTAAA
GATCGTCGCCTTGGTGAGCCGTTACCCCACCAACTAGCTAATCTTGCGCGTGCCCATCTCTATCCACCGTAGT
TTTCAATATCACCCGATGCCGAATAATATATTATGGGGTATTAATCTTCTCGAAAGGCTATCCCCTGATAAAG
GCAGGTTGCACACGTGTTCCGCACCCGTACGCCGCTCTCAATTACCCGAAAGCAATCTACCGCTCGGCTTGCA
TGTGTTAGGCCTCCCGCTAGCGTTCATCCTGAGCCAGGATCAAACTCT
>1142
CTGCTGCCGCCCGTAGGAGTCTGATCCGTGTCTCAGTACCAGTGTGGGGGATCACCCTCTCAGGCCCCCTAAA
GATCGTCGCCTTGGTGAGCCGTTACCTCACCAACTAGCTAATCTTGCGCGTGCCCATCTCTATCCACCGTAGT
TTTCAATATCACCCGATGCCGAATAATATATTATGGGGTATTAATCTTCTTCGAAAGGCTATCCCCCTGATAA
AGGCAGGTTGCACACGTGTTCCGCACCCGTACGCCGCTCTCAATTACCCGAAAGTAATCTACCGCTCGGCTTG
CATGTGTTAGGCCTCCCGCTAGCGTTCATCCTGAGCCAGGATCAACTCT
>1143
CTGCTGCCGCCCGTAGGAGTCTGGTCCGTGTCTCAGTACCAGTGTGGGGGATCACCCTCTCAGGCCCCCTAAA
GATCGTCGCCTTGGTGAGCCGTTACCTCACCAACTAGCTAATCTTGCGCGTGCCCATCTCTATTCCACCGTAG

TTTCAATATCACCCGATGCCGAATAATTATATTATGGGGTATTTAATCTTCTTCGAAAGGCTATCCCCTGATA
AAGGCAGGTTGCACACGTGTTCCGCACCCGTACGCCGCTCTCAATTACCCGAAAGTAATCTACCGCTCGGCTT
GCATGTGTTAGGCCTCCCGCTAGCGTTCATCCTGAGCCAG
>1144
CTGCTGCCGCCCGTAGGAGTCTGGCCCCGTGTCTCAGTACCAGTGTGGGGGATCACCCTCTCAGGCCCCCTAA
AGATCGTCGCCTTGGTGAGCCGTTACCTCACCAACTAGCTAATCTTGCGCGTGCCCATCTCTATCCACCGTAG
TTTTCAATATCACCCGATGCCGAATAATATATTATGGGGTATTAATCTTCTTCGAAAGGCTATCCCCCTGATA
AAGGCAGGTTGCACACGTGTTCCGCACCCGTACGCCGCTCTCAATTACCCGAAAGTAATCTACCGCTCCGGCT
TGCATGTGTTAGGCCTCCCGCTAGCGTCCATTCCTGAGCCAGGATCAACTCT
>1145
CTGCTGCCGCCCGTAGGAGTCTGGTCCGTGTCTCAGTACCAGTGTGGGGGATCACCCTCTCAGGCCCCCTAAA
GATCGTCGCCTTGGTGAGCCGTTACCTCACCAACTAGCTAATCTTGCGCGTGCCCATCTCTATCCACCGTAGT
TTTCAATATCACCCGATGCCGAATAATATATTATGGGGTATTAATCTTCTTCGAAAGGCTATCCCCCTGATAA
AGGCAGGTTGCACACGTGTTCCGCACCCGTACGCCGCTCTCAATTACCCGAAAGTAATCTACCGCTCGGCTTG
CATGTGTTAGGCCTCCCGCTAGCGCTCATCCTGAGCCAGGATCAAACTCT
>1146
CTGCTGCCGCCCGTAGGAGTCTGGTCCGTGTCTCAGTACCAGTGTGGGGGATCACCCTCTCAGGCCCCCTAAA
GATCGTCGCCTTGGTGAGCCGTTACCTCACCAACTAGCTAATCTTGCGCGTGCCCATCTCTATCCACCGTAGT
TTTCAATATCACCCGATGCCGAATAATATAATTATGGGGTATTAATCTTCTTCGAAAGGCTATCCCCCTGATA
AAGGCAGGTTGCACACGTGTTCCGCACCCGTACGCCGCTCTCAATTACCCGAAAGTAATCTACCGCTCGGCTT
GCATGTGTTAGGCCTCCCGCTAGCGTTCTCCTGAGCAGGATCAACTCGT
>1147
CTGCTGCCGCCCGTAGGAGTCTGGTCCCGTGTCTCAGTACCAGTGTGGGGGATCACCCTCTCAGGCCCCCCTA
AAGATCGTCGCCTTGGTGAGCCGTTACCTCACCAACTAGCTTAATCTTGCGCGTGCCCCATCTCTATCCACCG
TAGTTTTCAATATCACCCGATGCCGAATAATATATTATGGGGTATTTAATCTTCTTCGAAAGGCTATCCCCCT
GATAAAGGCAGGTTGCACACGTGTTCCGCACCCGTACGCCGCTCTCAATTACCCCGAAAGTAATCTACCGCTC
GGCTTGCATGTGTTAGGCCTCCCGCTAGCGTTCATCCTGAGCCAGGATCAAACTCT
>1148
CTGCTGCCGCCCGTAGGAGTCTGGTCCGTGTCTCAGTACCAGTGTGGGGGATCACCCTCTCAGGCCCCCTAAA
GATCGTCGCCTTGGTGAGCCGTTACCTCACCAACTAGCTAATCTTGCGCGTGCCCATCTCTATCCACCGTAGT
TTTCAATATCACCCGATGCCGAATAATATATTATGGGGTATTAATCTTCTTCGAAAGGCTATCCCCCTGATAA
AGGCAGGTTGCACACGTGTTCCGCACCCGTACGCCGCTCTCAATTACCCGAAAGTAATCTACCGCTCGGCTTG
CATGTGTTAGGCCTCCCGCTAGCGTTCTCCTCGAGCAGGATCAACATCT
>1149
CTGCTGCCGCCCGTAGGAGTCTGGTCCGTGTCTCAGTACCAGTGTGGGGGATCACCCTCTCAGGCCCCCTAAA
GATCGTCGCCTTGGTGAGCCGTTACCTCACCAACTAGCTAATCTTGCGCGTGCCCATCTCTATCCACCGTAGT
TTTCAATATCACCCGATGCCGAATAATATATTATGGGGTATTAATCTTCTTCGAAAGGCTATCCCCCTGATAA
AGGCAGGTTGCACACGTGTTCCGCACCCGTACGCCGCTCTCAATTACCCGAAAGTAATCTACCGCTCCGGCTT
GCATGTGTTAGGCCTCCCGCTAGCGTTCTCCTGAGCAGGATCAACTCG
>1150
CTGCTGCCGCCGTAGGAGTCTGGACCGTGTCTCAGTTCCAGTGTGACTGATCATCCTCTCAGACCAGTACGGA
TCGTCGCCTGGTAGGCCTTTACCCCACCAACTAGCTAATCCGACCTAGGCTCATCTGATAGCGTGAGGTCCGA
AGATCCCCCACTTCTCCCTCAGGACGTATGCGGTATTAGCGCCCGTTTCCGGACGATCCCCACTACCAGGCCA
GATTCCTAGGCATTACTCACCCGTCCGCCGCTGAATCCAGGAGCAAGCCTCCTCTCCGCTCGACTGCATGTGT
TAGGCCTGCCGCCAGCGTCAATCTGAGCCAGGATCAAACTCT
>1151
CTGCTGCCGCCCGTAGGAGTCTGGTCCGTGTCTCAGTACCAGTGTGGGGGATCACCCTCTCAGGCCCCCTAAA
GATCGTCGCCTTGGTGAGCCGTTACCTCACCAACTAGCTAATCTTGCGCGTGCCCATCTCTATCCACCGTAGT
TTCAATATCACCCGATGCCGAATAATATATTATGGGGTATTAATCTTCTTCGAAAGGCTATCCCCTGATAAAG
GCAGGTTGCACACGTGTTCCGCACCCGTACGCCGCTCTCAATTACTCGAAAGTAACTCTACCGCTCGGCTTGC
ATGTGTTAGGCCTCCCGCTAGCGTTCTCCTGAGCAGGATCAACATCT
>1152
CTGCTGCCGCCCGTAGGAGTCTGGTCCGTGTCTCAGTACCAGTGTGGGGGATCACCCTCTCAGGCCCCCTAAA
GATCGTCGCCTGGTGAGCCGTTACCTCACCAACTAGCTAATCCGACCTAGGCTCATCTGATAGCGTGAGGTCC

GAAGATCCCCCACTTCTCCCTCAGGACGTATGCGGTATTGCGCCCGTTTCCGGACGATCCCCCACTACCAGGC
CAGATTCCTAGGCATTACTCACCCGTCCGCCGCTGAATCCAGGAGCAAGCTCCCTCATCGCTCGACTGCCATG
TGTTAGGCCTGCCGCCAGCGTCAATCTGAGCCAGGATCAACTCT
>1153
CTGCTGCCGCCCGTAGGAGTCTGGTCCGTGTCTCAGTACCAGTGTGGGGGATCACCCTCTCAGGCCCCCTAAA
GATCGTCGCCTTGGTGAGCCGTTACCTCACCAACTAGCTAATCTTGCGCGTGCCCATCTCTATCCACCGTAGT
TTTCAATATCACCCGATGCCGAATAATATATTATGGGGTATTAATCTTCTTCGAAAGGCTATCCCCCTGATAA
AGGCAGGTTGCACACGTATTCCGCACCCGTACGCCGCTCTCAATTACCCGAAAGTAATCTACCGCTCGGCTTG
CATGTGTTAGGCCTCCCGCTAGCGTTCATCCTGAGCCAGGATCAAACTCT
>1154
CTGCTGCCGCCCGTAGGAGTCTGGTCCGTGTCTCAGTACCAGTGTGGGGGATCACCCTCTCAGGCCCCCTAAA
GATCGTCGCCTTGGTGAGCCGTTACCTCACCAACTAGCTAATCTTGCGCGTGCCCATCTCTATCCACCGTAGT
TTTCAATATCACCGATGCCGAATAATATATTATGGGGGTATTAATCTTCTTCGAAAGGCTATCCCCCTGATAA
AGGCAGGTTGCACACGTGTTCCGCACCCGTACGCCGCTCTCAATTACCCGAAAGTAATCTACCGCTCGGCTTG
CATGTGTTAGGCCTCCCGCTAGCGTTCTCCTGAGCAGGATCCAACTC
>1155
CTGCTGCCGCCCGTAGGAGTCTGGTCCGTGTCTCAGTACCAGTGTGGGGGATCACCCTCTCAGGCCCCCTAAA
AGATCGTCGCCTTGGTGAGCCGTTACCTCACCAACTAGCTAATCTTGCGCGTGCCCATCTCTATCCACCGTAG
TTTTCAATATCACCCGATGCCGAATAATATATTATGGGGTATTAATCTTCTTCGAAAGGCTATCCCCCTGATA
AAAGGCAGGTTGCACACGTGTTCCGCACCCGTACGCCGCTCTCAATTACCCGAAAGTAATCTACCGCTCGGCT
TGCATGTGTTAGGCCTCCCGCTAGCGTTCATCTGAGCAGGATCAAACTCT
>1156
CTGCTGCCGCCCGTAGGAGTCTGGTCCGTGTCTCAGTACCAGTGTGGGGGATCACCCTCTCAGGCCCCCTAAA
GATCGTCGCCTTGGTGAGCCGTTACCTCACCAACTAGCTAATCTTGCGCGTGCCCATCTCTATCCACCGTAGT
TTTCAATATCACCCGATGCCGAATAATATATTATGGGGTATTAATCTTCTTCGAAAGGCTATCCCCCTGATAA
AGGCAGGTTGCACACGTGTTCCGCACCCGTACGCCGCTCTCAATTACCCGAAAGTAATCTACCGCTCGGCTTG
CATGTGTTAGGCCTCCCGCTAGCGTTCATCCTGAGCCAGGCATCAACTCT
>1157
CTGCTGCCGCCCGTAGGAGTCTGGTCCGTGTCTCAGTACCAGTGTGGGGGATCACCCTCTCAGGCCCCCTAAA
GATCGTCGCCTTGGTGAGCCGTTACCTCACCAACTAGCTAATCTTGCGCGTGCCCATCTCTATCCACCGTAGT
TTTCAATATCACCCGATGCCGAATAATATATTATGGGGTATTAATCTTCTTCGAAAGGCTATCCCCCTGATAA
GGCAGGTTGCACACGTGTTCCGCACCCGTACGCCGCTCTCAATTACCCGAAAGTAATCTACCGCTCGGCTTGC
ATGTGTTAGGCCTCCCGCTAGCGTTCTCCTGAG
>1158
CTGCTGCCGCCCGTAGGAGTCTGGTCCGTGTCTCAGTACCAGTGTGGGGGATCACCCTCTCAGGCCCCCTAAA
GATCGTCGCCTTGGTGAGCCGTTACCTCACCAACTAGCTAATCTTGCGCGTGCCCATCTCTATCCACCGTAGT
TTTCAATATCACCCGATGCCGAATAATATATTATGGGGTATTAATCTTCTTCGAAAGGCTATCCCCTGATAAA
GGCAGGTTGCACACGTGTTCCGCACCCGTACGCCGCTCTCAATTACCCGAAAGTAATCTACCGCTCGGCTTGC
ATGTGTTAGGCCTCCCGCTAGCGTTCATCCTGAGCCAGGATCAAACTCT
>1159
CTGCTGCCGCCCGTAGGAGTCTGGTCCGTGTCTCAGTACCAGTGTGGGGGATCACCCTCTCAGGCCCCCTAAA
GATCGTCGCCTTGGTGAGCCGTTACCTCACCAACTAGCTAATCTTGCGCGTGCCCATCTCTATCCACCGTAGT
TTTCAATATCACCCGATGCCGAATAATATATTATGGGGTATTAATCTTCTTCGAAAGGCTATCCCCTGATAAA
GGCAGGTTGCACACGTGTTCCGCACCCGTACGCCGCTCTCAATTACCCGAAAGTAATCTACCGCTCGGCTTGC
ATGTGTTAGGCCTCCCGCTAGCGTTCATCCGTGA
>1160
CTGCTGCCGCCCGTAGGAGTCTGGTCCGTGTCTCAGTACCAGTGTGGGGGATCACCCTCTCAGGCCCCCTAAA
GATCGTCGCCTTGGTGAGCCGTTACCTCACCAACTAGCTAATCTTGCGCGTGCCCATCTCTATCCACCGTAGT
TTTCAATATCACCCGATGCCGAATAATATATTATGGGGTATTAATCTTCTTCTGAAAGGCTATCCCCTATAAA
GGCAGGTTGCACACGTGTTCCGCACCCGTACGCCGCTCTCAATTACCCGAAAGTAATCTACCGCTCGGCCTGC
ATGTGTTAGGCCTCCCGCTAGCGTTCATCCTGAGCCAGGACAACTCT
>1161
CTGCTGCCGCCCGTAGGAGTCTGGTCCGTGTCTCAGTACCAGTGTGGGGGATCACCCTCTCAGGCCCCCTAAA
GATCGTCGCCTTGGTGAGCCGTTACCTCACCAACTAGCTAATCTTGCGCGTGCCCATCTCTATCCACCGTAGT

TTTCAATATCACCCGATGCCGAATAATATATTATGGGGTATTAATCTTCTTCGAAAGGCTATCCCCTGATAAA
GGCAGGTTGCACACGTGTTCCGCACCCGTACGCCGCTCTCAATTACCCGAAAGTAATCTACCGCTCGGCTTGC
ATGTGTTAGGCCTCCCGCTAGC
>1162
CTGCTGCCGCCCGTAGGAGTCTGGTCCGTGTCTCAGTACCAGTGTGGGGGATCACCCTCTCAGGCCCCCTAA
AGATCGTCGCCTTAGTGAGCCGTTACCTCACCAACTAGCTAATCTTGCGCGTGCCCATCTCTATCCACCGTAG
TTTTCAATATCACCCGATGCCGAATAATATATTATGGGGTATTAATCTTTCTCGAAAGGCTATCCCCTGATAA
AGGCAGGTTGCACACGTGTTCCGCACCCGCACGCGCTCTCAATTACCCGAAAGTAATCTACCGCTCGGCTTGC
ATGTGTTAGGCCTCCCGCTAGCGTTCATCCTGAGCCAGGATCAAACTCT
>1163
CTGCTGCCGCCCGTAGGAGTCTGGTCCGTGTCTCAGTACCAGTGTGGGGGATCACCCTCTCAGGCCCCCTAAA
GATCGTCGCCTTGGTGAGCCGTTACCTCACCAACTAGCTAATCTTGCGCGTGCCCATCTCTATCCACCGTAGT
TTTTCAATATCACCCGATGCCGAATAATATATTATGGGGTATTAATCTTCTCGAAAGGCTATCCCCCTGATAA
AGGCAGGTTGCACACGTGTTCCGCACCCGTACGCCGCTCTCAATTACCCGAAAGTAATCTACCGCTCGGCTTG
CATGTGTTAGGCCTCCCGCTAGCGTTCATCCTGAGCCAGGATCAACTCT
>1164
CTGCTGCCGCCCGTAGGAGTCTGGTCCGTGTCTCAGTACCAGTGTGGGGGATCACCCTCTCAGGCCCCCTAAA
GATCGTCGCCTTGGTGAGCCGTTACCTCACCAACTAGCTAATCTTGCGCGTGCCCATCTCTATCCACCGTAGT
TTTCAATATCACCCGATGCCGAATAATATATTATGGGGTATTAATCTTCTTCGAAAGGCTATCCCCCTGATAA
AGGCAGGTTGCACACGTGTTCCGCACCCGTACGCCGCTCTCAATTACCCGAAAGTAATCTACCGCATCGGCTT
GCATGTGTTAGGCCTCCCGCTAGCGTTCTCCGTGAGCAGG
>1165
CTGCTGCCGCCCGTAGGAGTCTGGTCCGTGTCTCAGTACCAGTGTGGGGGATCACCCTCTCAGGCCCCCTAAA
GATCGTCGCCTTGGTGAGCCGTTACCTCACCAACTAGCTAATCTTGCGCGTGCCCATCTCTATCCACCGTAGT
TTTCAATATCACCCGATGCCGAATAATATAATTATGGGGGTATTAATCTTCTTCGAAAGGCTATCCCCTGATA
AAGGCAGGTTGCACACGTGTTCCGCGCCGTACGCCGCTCTCAATTACCCGAAAGTAATCTACCGCTCGGCTTG
CATGTGTTAGGCCTCCCGCTAGCGTTCATCCTGAGCCAGGATCAAACTCT
>1166
CTGCTGCCGCCCGTAGGAGTCTGGACCGTGTCTCAGTTCCAGTGTGACTGATCATCCTCTCAGACCAGTTACG
GATCGTCGCCTTGGTAGGCCTTTACCCCACCAACTAGCTAATCCGACCTAGGCTCATCTGATAGCGTGAGGTC
CGAAGATCCCCCACTTCTCCCTCAGGACGTATGCGGTATTAGCGCCCGTTTCCGGACGTATCCCCACTACCAG
GCAGATTCCTAGGCATTACTCACCCGTCCGCCGCTGAATCCAGGAGCAAGCCTCCCTCATCCGCTCGACTGCA
TGTGTTAGGCCTGCCGCCAGCGTCATCTGAGCCAGGATCAAACTCT
>1167
CTGCTGCCGCCCGTAGGAGTCTGGACCGTGTCTCAGTTCCAGTGTGACTGATCATCCTCTCAGACCAGTACGG
ATCGTCGCCTGGTAGGCCTTTACCCCACCAACTAGCTAATCCGACCTAGGCTCATCTGATAGCGTGAGGTCCG
AAGATCCCCCACTCTCCCTCAGGACGTATGCGGTATTAGCGCCCGTTTCCGGACGATCCCCCACTACCAGGCA
GATCCCTAGGCATTACTCACCCGTCCGCCGCTGAATCCAGGAGCAAGCTCCCTCATCCGCTCGACTGCATGTG
TTAGGCTGCCGCCAGCGTCAATCTGAGCCAGGATCAACTCT
>1168
CTGCTGCCGCCCGTAGGAGTCTGGTCCGTGTCTCAGTACCAGTGTGGGGGATCACCCTCTCAGGCCCCCTAAA
GATCGTCGCCCTGGTGAGCCGTTACCTCACCAACTAGCTAATCTTGCGCGTGCCCATCTCTATCCACCGTAGT
TTTCAATATCACCCGATGCCGAATAATATATTATGGGGTATTAATCTTCTTCGAAAGGCTATCCCCCTGATAA
AGGCAGGTTGCCACACGTGTTCCGCACCCGTACGCCGCTCTCAATTACCCGAAAGTAATCTACCGCTCGGCTT
GCATGTGTTAGGCCTCCCGCTAGCGTTCATCCTGAGCCAGGATCAAACTCT
>1169
CTGCTGCCGCCCGTAGGAGTCTGGTCCGTGTCTCAGTACCAGTGTGGGGGATCACCCTCTCAGGCCCCCTAAA
GATCGTCGCCTTGGTGAGTCGTTACCTCACCAACTAGCTAATCTTGCGCGTGCCCATCTCTATCCACCGTAGT
TTTCAATATCACCCGATGCCGAATAATATATTATGGGGTATTAATCTTCTTCGAAAGGCTATCCCCCTGATAA
AGGCAGGTTGCACACGCGTTCCGCACCCGTACGCCGCTCTCAATTACCCGAAAGTAATCTACCGCTCGGCTTG
CATGTGTTAGGCCTCCCGCTAGCGTTCATCCTGAGCCAGG
>1170
CTGCTGCCGCCCGTAGGAGTCTGGTCCGTGTCTCAGTACCAGTGTGGGGGATCACCCTCTCAGGCCCCCTAAA
GATCGTCGCCTTGGTGAGCCGTTACCTCACCAACTAGCTAATCTTGCGCGTGCCCATCTCTATCCACCGTAGT

TTTCAATATCACCCGATGCCGAATAATATATTATGGGGGTATTAATCTTCTTCGAAAGGCTATCCCCCTGATA
AAGGCAGGTTGCACACGTGTTCCGCACCCGTACGCCACTCTCAATTACCCGAAAGTAATCTACCGCTCGGCTT
GCATGTGTTAGGCCTCCCGCTAGCGTTCATCCTGAGCCAGGATCAAACTCT
>1171
CTGCTGCCGCCCGTAGGAGTCTGGTCCGTGTCTCAGTACCAGTGTGGGGGATCACCCTCTCAGGCCCCCTAAA
GATCGTCGCCTTGGTGAGCCGTTACCTCACCAACTAGCTAATCTTGCGCGTGCCCATCTCTATCCACCGTAGT
TTTCAATATCACCCGATGCCGAATAATATATTATGGGGTATTAATCTTCTTCGAAAGGCTATCCCCTGATAAA
GGCAGGTTGCACACGTGTTCCGCACCCGTACGCCGCTCTCAATTACCCGAAAGTAATCTACCGCTCGGCTTGC
ATGTGTTAGGCCTCCCGCTAGCGTTCTCCTGAGCAGGATCCAAACTCGT
>1172
CTGCTGCCGCCCGTAGGAGTCTGGTCCGTGTCTCAGTACCAGTGTGGGGGATCACCCTCTCAGGCCCCCTAAA
AGATCGTCGCCTTGGTGAGCCGTTACCTCACCAACTAGCTAATCTTGCGCGTGCCCATCTCTATCCACCGTAG
TTTTCAACATCACCCGATGCCGAATAATATATTATGGGGTATTAATCTTCTTCGAAAGGCTATCCCCCTGATA
AAGGCAGGTTGCACACGTGTTCCGCACCCGTACGCCGCTCTCAATTACCCGAAAGTAATCTACCGCTCGGCTT
GCATGTGTTAGGCCTCCCGCTAGCGTTCATCCTGAGCCAGGATCAAACTCT
>1173
CTGCTGCCGCCCGTAGGAGTCTGGTCTGTCTCAGTACCAGTGTGGGGGATCACCCTCTCAGGCCCCCTAAAGA
TCGTCGCCTTGGTGAGCCGTTACCTCACCAACTAGCTAATCTTGCGCGTGCCCATCTCTATCCACCGTAGTTT
TCAATATCACCCGATGCCGAATAATATATTATGGGGTATTAATCTTCTTCGAAAGGCTATCCCCCTGATAAAG
GCAGGTTGCACACGTGTTCCGCACCCGTACGCCGCTCTCAATTACCCGAAAGTAATCTACCGCTCGGCTTGCA
TGTGTTAGGCCTCCCGCTAGCGTTCATCCTGAGCCAGGATCAAACTCT
>1174
CTGCTGCCGCCCGTAGGAGTCTGGTCCGTGTCTCAGTACCAGTGTGGGGGATCACCCTCTCAGGCCCCCTAAA
GATCGTCGCCTTGGTGAGCCGTTACCTCACCAACTAGCTAATCTTGCGCGTGCCCATCTCTATCCACCGTAGT
TTTCAATATCACCCGATGCCGAATAATATATTATGGGGTATTAATCTTCTTCGAAAGGCTATCCCCTGATAAA
GGCAGGTTGCACACGTGTTCCGCACCCGTTACGCCGCTCTCAATTACCCGAAAGTAATCTACCGCTCGGCTTG
CATGTGTTAGGCCTCCCGCTAGCGTTCATCCTGAGCCAGGATCAAACTCT
>1175
CTGCTGCCGCCCGTAGGAGTCTGGACCGTGTCTCAGTTCCAGTGTGACTGATCATCCTCTCAGACCAGTACGG
ATCGTCGCCTTGGTAGGCCTTTACCCCACCAACTAGCTAATCCGAACCTAGGCTCATCTGATAGCGTGAGGTC
CGAAGATCCCCCACTTCTCCCTCAGGACGTATGCGGTATTAGCGCCCCGTTTCCGGACGATCCCCACTACCAG
GCAGATTCCTAGGCATTACTCACCCGTCCGCCGCTGAATCCAGGAGCAAGCTCCCTCATCGCTCGACTGCATG
TGTTAGGCCTGCCGCCAGCGTTCAATCTGAGCCAGGATCAAACTCT
>1176
CTGCTGCCGCCCGTAGGAGTCTGGTCCGTGTCTCAGTACCAGTGTGGGGGATCACCCTCTCAGGCCCCCTAAA
GATCGTCGCCTTGGTGAGCCGTTACCTCACCAACTAGCTAATCTTGCGCGTGCCCATCTCTATCCACCGTAGT
TTTCAATATCACCCGATGCCGAATAATATATTATGGGGTATTAATCTTCTTCGAAAGGCTATCCCCCTGATAA
AGGCAGGTTGCACACGTGTTCCGCACCCGTACGCCGCTCTCAATTACCCGAAAGTAATCTACCGCTCGGCTTG
CATGTGTTAGGCCTCCCGCTAGCGTTCATCCTGAGCCAGGATCAAACTCT
>1177
CTGCTGCCGCCCGTAGGAGTCTGGTCCGTGTCTCAGTACCAGTGTGGGGGATCACCCTCTCAAGCCCCCTAAA
GATCGTCGCCTTGGTGAGCCGTTACCTCACCAACTAGCTAATCTTGCGCGTGCCCATCTCTATCCACCGTAGT
TTTCAATATCACCCGATGCCGAATAATATATTATGGGGTATTAATCTTCTTCGAAAGGCTATCCCCCTGATAA
AGGCAGGTTGCACACGTGTTCCGCACCCGTACGCCGCTCTCAATTACCCGAAAGTAATCTACCGCTCGGCTTG
CATGTGTTAGGCCTCCCGCTAGCGTTCATCCTGAGCCAGGATCAAACTCT
>1178
CTGCTGCCGCCCGTAGGAGTCTGGTCCGTGTCTCAGTACCAGTGTGGGGGATCACCCTCTCAGGCCCCCTAAA
GACCGTCGCCTTGGTGAGCCGTTACCTCACCAACTAGCTAATCTTGCGCGTGCCCATCTCTATCCACCGTAGT
TTTCAATATCACCCGATGCCGAATAATATATTATGGGGTATTAATCTTCTTCGAAAGGCTATCCCCCTGATAA
AGGCAGGTTGCACACGTGTTCCGCACCCGTACGCCGCTCTCAATTACCCGAAAGTAATCTACCGCTCGGCTTG
CATGTGTTAGGCCTCCCGCTAGCGTTCTCCTGAGCAGGATCAAACTCT
>1179
CTGCTGCCGCCCGTAGGAGTCTGGTCCGTGTCTCAGTACCAGTGTGGGGGATCACCCTCTCAGGCCCCCTAAA
GATCGTCGCCTTGGTGAGCCGTTACCTCACCAACTAGCTAATCTTGCGCGTGCCCATCTCTATCCACCGTAGT

TTCAATATCACCCGATGCCGAATAATATATTATGGGGTATTAATCTTTCTTCGAAAGGCTATCCCCCTGATAA
AGGCAGGTTGCACACGTGTTCCGCACCCGTACGCCGCTCTCAATTACCCGAAAGTAATCTACCGCTCGGCTTG
CATGTGTTAGGCCTCCCGCTAGCGTTCTCCTCGAGCAGGATCCAACTCT
>1180
CTGCTGCCGCCCGTAGGAGTCTGGTCCGTGTCTCAGTACCAGTGTGGGGGATCACCCTCTCAGGCCCCCTAAA
GATCGTCGCCTTGGTGAGCCGTTACCTCACCAACTAGCTAATCTTGCGCGTGCCCATCTCTATCCACCGTAGT
TTTCAATATCACCCGATGCCGAATAATATATTATGGGGTATTAATCTTCTTCGAAAGCTATCCCCTGATAAAG
GCAGGTTGCACACGTGTTCCGCACCCGTACGCCGCTCTCAATTACCCGAAAGTAATCTACCGCTCCGGCTTGC
ATGTGTTAGGCCTCCCGCTAGCGTTCTCCTGAGCAGGATCAACTCGT
>1181
CTGCTGCCGCCCGTAGGAGTCTGGTCCGTGTCTCAGTACCAGTGTGGGGGGATCACCCTCTCAGGCCCCCTAA
AGATCGTCGCCTTGGTGAGCCGTTACCTCACCAACTAGCTAATCTTGCGCGTGCCCATCTCTATCCACCGTAG
TTTTCAATATCACCCGATGCCGAATAATATATTATGGGGTATTAATCTTCTTCGAAAGGCTATCCCCCTGATA
AAGGCAGGTTGCACACGTGTTCCGCACCCGTACGCCGCTCTCAATTACCCGAAAGTAATCTACCGCTTGGCCT
TGCATGTGTTAGGCCTCCCGCTAGCGTTCATCCTGAGCCAGGATCAAACTCT
>1182
CTGCTGCCGCCCGTAGGAGTCTGGTCCGTGTCTCAGTACCAGTGTGGGGGATCACCCTCTCAGGCCCCCTAAA
GATCGTCGCCTTGGTGAGCCGTTACCTCACCGACTAGCTAATCTTGCGCGTGCCCATCTCTATCCACCGTAGT
TTTCAATATCACCCGATGCCGAATAATATATTATGGGGTATTAATCTTCTCGAAAGGCTATCCCCCTGATAAA
GGCAGGTTGCACACGTGTTCCGCACCCGTACGCCGCTCTCAATTACCCGAAAGTAATCTACCGCTCCGGCTTG
CATGTGTTACGG
>1183
CTGCTGCCGCCCGTAGGAGTCTGGTCCGTGTCTCAGTACCAGTGTGGGGGATCACCCTCTCAGGCCCCCTAAA
GATCGTCGCCTTGGTGAGCCGTTACCTCACCAACTAGCTAATCTTGCGCGTGCCCATCTCTATCCACCGTAGT
TTTCAATATCACCCGATGCCGAATAATATATTATGGGGTATTAATCTTCTTCGAAAGGCTATCCCCCTGATAA
AGGCAGGTTGCACACGTGTTCCGCACCCGTACGCCGCTCTCAATTACCCGAAAGTAATCTACCGCTCGGCTTG
CATGTGTTAGGCCTCCCGCTAGCGTTCTCCTGAGCAG
>1184
CTGCTGCCGCCCGTAGGAGTCTGGTCCGTGTCTCAGTACCAGTGTGGGGGATCACCCTCTCAGGCCCCCTAAA
GATCGTCGCCTTGGTGAGCCGTTACCTCACCAACTAGCTAATCTTGCGCGTGCCCATCTCTATCCACCGTAGT
TTTCAATATCACCCGATGCCGAATAATATATTATGGGGTATTAATCTTCTCGAAAGGCTACCCCCTGATAAAG
GCAGGTTGCACACGTGTTCCGCACCCGTACGCCGCTCTCAATTACCCGAAAGTAATCTACCGCTCGGCTTGCA
TGTGTTAGGCCTCCCGCTAGCGTTCTCCTGAGCAAGGTCC
>1185
CTGCTGCCGCCCGTAGGAGTCTGGTCCGTGTCTCAGTACCAGTGTGGGGGATCACCCTCTCAGGCCCCCTAAA
GATCGTCGCCTTGGTGAGCCGTTACCTCACCAACTAGCTTAATCTTGCGCGTGCCCATCTCTATTCCACCGTA
GTTTCAATATCACCCGATGCCGAATAATATATTATTGGGGTATTTAATCTTCTTCGAAAGGCTATCCCCTGAT
AAAGGCAGGTTGCACACGTGTTCCGCACCCGTACGCCGCTCTCAATTACCCGAAAGTAATCTACCGCTCGGCT
TGCACGTGTTAGGCCTCCCGCTAGCGTTCATCCTGA
>1186
CTGCTGCCGCCCGTAGGAGTCTGGACCGTGTCTCAGTTCCAGTGTGACTGATCATCCTCTCAGACCAGTACGG
ATCGTCGCCTTGGTAGGCCTTTACCCCACCAACTAGCTAATCCGACCTGGGCTCATCTGATAGCGTGAGGTCC
GAAGTCCCCCACTTCTCCCTCAGGACGTATGCGGTATTAGCGCCCGTTTCCGGACGATCCCCACTACCAGGCC
AGATTCCTAGGCATTACTCACCCGTCCGCCGCTGAATCCAGGAGCAAGCTCCCTCATCGCTCGACTGCATGTG
TTAGGCCTGCCGCCAGCGTCAATCTGAGCCAGGATCAAACTCT
>1187
CTGCTGCCGCCCGTAGGAGTCTGGTCCGTGTCTCAGTACCAGTGTGGGGGATCACCCTCTCAGGCCCCCTAAA
GATCGTCGCCTTGGTGAGCCGTTACCTCACCAACTAGCTAATCTTGCGCGTGCCCATCTCTATCCACCGTAGT
TTTCAATATCACCCGATGCCGAATAATATATTATGGGGTATTAATCTTCTCGAAAGGCTATCCCCTGATAAAG
GCAGGTTGCACACGTGTTCCGCACCCGTACGCCGCTCTCAATTACCCGAAAGTAATCTACCGCTCGGCTTGCA
TGTGTTAGGCCTCCCGCTAGCGTTCATCCTGACCAGGATCAAACTCT
>1188
CTGCTGCCGCCCCGTAGGAGTCTGGTCCGTGTCTCAGTACCAGTGTGGGGGATCACCCTCTCAGGCCCCCCCTA
AAGATCGTCGCCTTGGTGAGCCGTTACCTCACCCAACTAGCTAATCTTGCGCGTGCCCCATCTCTATTCCACC

GTAGTTTCAATATCACCCGATGCCCGAATAATATATTATTGGGGTATTTAATTCTTCTTCGAAAGGCTATCCC
CTGATTAAAGGCAGGTTGCACACGTGTTCCCGCACCCGTACGCCGCTCTCAATTACCCCGAAAGTAATCTACC
GCTCGGCTTGCATGTGTTAGGCCTTCCCGCTAGCGTTCATTCCTGAGCCAGGATCAAACTCT
>1189
CTGCTGCCGCCCGTAGGAGTCTGGTCCGTGTCTCAGTACCAGTGTGGGGGATCACCCTCTCAGGCCCCCTAAA
GATCGTCGCCTTGGTGAGCCGTTACCTCACCAACTAGCTAATCTTGCGCGTGCCCATCTCTATCCACCGTAGT
TTTCAATATCACCCGATGCCGAATAATATATTATGGGGTATTAATCTTCTTCGAAAGGCTATCCCCTGATAAA
GGCAGGTTGCACACGTGTTCCGCACCCGTACGCCGCTCTCAATTACCCGAAAGTAATCTACCGCTCGGCTTGC
ATGTGTTAGGCCTCCCGCTAGCGTTCATCCGTGAG
>1190
CTGCTGCCGCCCGTAGGAGTCTGGTCCGTGTCTCAGTACCAGTGTGGGGGATCACCCTCTCAGGCCCCCTAAA
GATCGTCGCCTTGGTGAGCCGTTACCTCACCAACTAGCTAATCTTGCGCGTGCCCATCTCTATCCACCGTAGT
TTTCAATATCACCCGATGCCGAATAATATATTATGGGGTATTAATCTTCTTCGAAAGGCTATCCCCCTGATAA
AGGCAGGTTGCACACGTGTTCCGCACCCGTACGCCGCTCTCAATTACCCGAAAGTAATCTACCGCTCGGCTTG
CATGTGTTAGGCCTCCCGCTAGCGTTCATCCTGAGCCAGGATCAAACTCT
>1191
CTGCTGCCGCCCGTAGGAGTCTGGTCCGTGTCTCAGTACCAGTGTGGGGGATCACCCTCTCAGGCCCCCTAAA
GATCGTCGCCTTGGTGAGCCGTTACCTCACCAACTAGCTAATCTTGCGCGTGCCCATCTCTATCCACCGTAGT
TTTCAATATCACCCGATGCCGAATAATATATTATGGGGTATTAATCTTCTTCGAAAGGCTATCCCCTGATAAA
GGCAGGTTGCACACGTGTTCCGCACCCGTACGCCGCTCTCAATTACCCGAAAGTAATCTACCGCTCGGCTTGC
ATGTGTTAGGCCTCCCGCT
>1192
CTGCTGCCGCCCGTAGGAGTCTGGTCCGTGTCTCAGTACCAGTGTGGGGGATCACCCTCTCAGGCCCCCTAAA
GATCGTCGCCTTGGTGAGCCGTTACCTCACCAACTAGCTAATCTTGCGCGTGCCCATCTCTATCCACCGTAGT
TTTCAATATCACCCGATGCCGAATAATATATTATGGGGTATTAATCTTCTTCGAAAGGCTATCCCCCTGATAA
AGGCAGGTTGCACACGTGTTCCGCACCCGTACGCCGCTCTCAATTACCCGAAAGTAATCTACCGCTCGGCTTG
CATGTGTTAGGCCTCCCGCTAGCGTTCATCCTGAGCCAGGATCAAACTCT
>1193
CTGCTGCCGCCCGTAGGAGTCTGGTCCGTGTCTCAGTACCAGTGTGGGGGATCACCCTCTCAGGCCCCCTAAA
GATCGTCGCCTTGGTGAGCCGTTACCTCACCAACTAGCTAATCTTGCGCGTGCCCATCTCTATCCACCGTAGT
TTTCAATATCACCCGATGCCGAATAATATATTATGGGGTATTAATCTTCTTCGAAAGGCTATCCCCCTGATAA
AGGCAGGTTGCACACGTGTTCCGCACCCGTACGCCGCTCTCAATTACCCGAAAGTAATCTACCGCTCGGCTTG
CATGTGTTAGGCCTCCCGCTAGCGTTCATCCTGAGCCAGGATCAAACTCT
>1194
CTGCTGCCGCCCGTAGGAGTCTGGTCCGTGTCTCAGTACCAGTGTGGGGGATCACCCTCTCAGGCCCCCTAAA
GATCGTCGCCTTGGTGAGCCGTTACCTCACCAACTAGCTAATCTTGCGCGTGCCCATCTCTATCCACCGTAGT
TTTCAATATCACCCGATGCCGAATAATATATTATGGGGTATTAATCTTCTTCGAAAGGCTATCCCCCTGATAA
AGGCAGGTTGCACACGTGTTCCGCACCCGTACGCCGCTCTCAATTACCCGAAAGTAATCTACCGCTCGGCTTG
CATGTGTTAGGCCTCCCGCTAGCGTTCTCCTGAGCAGGATCAA
>1195
CTGCTGCCGCCCGTAGGAGTCTGGTCCGTGTCTCAGTACCAGTGTGGGGGATCACCCTCTCAGGCCCCCTAAA
GATCGTCGCCTTGGTGAGCCGTTACCTCACCAACTAGCTAATCTTGCGCGTGCCCATCTCTATCCACCGTAGT
TTTCAATATCACCCGATGCCGAATAATATATTATGGGGTATTAAATCTTCTCGAAAGGCTTATCCCCCTGAAT
AAAGGCAGGTTGCACACGTGTTCCGCACCCGTACGCCGCTCTCAATTACCCGAAAGTAATCTACCGCTCGGCT
TGCATGTGTTAGGCCTCCCGCTAGCGTTCATCCTGAG
>1196
CTGCTGCCGCCCGTAGGAGTCTGGTCCGTGTCTCAGTACCAGTGTGGGGGATCACCCTCTCAGGCCCCCTAAA
GATCGTCGCCTTGGTGAGCCGTTACCTCACCAACTAGCTAATCTTGCGCGTGCCCATCTCTATCCACCGTAGT
TTTCAATATCACCCGATGCCGAATAATATATTATGGGGTATTAATCTTCTTCGAAAGGCTATCCCCCTGATAA
AGGCAGGTTGCACACGTGTTCCGCACCCGTACGCCGCTCTCAATTACCCGAAAGTAATCTACCGCTCGGCTTG
CATGTGTTAGGCCTCCCGCTAGCGTTCATCTGAGCCAGGATCAACTCT
>1197
CTGCTGCCGCCCGTAGGAGTCTGGACCGTGTCTCAGTTCCAGTGTGACTGATCATCCTCTCAGACCAGTACGG
ATCGTCGCCTTGGTAGGCCTTTACCCCACCAACTAGCTAATCCGACCTAGGCTCATCTGATAGCGTGAGGTCC

GAAGTCCCCCACTTCTCCCTCGGGACGTATGCGGTATTAGCGCCCGTTTCCGGACGATCCCCACTACCAGGCC
AGATTCCCTAGGCATTACTCACCCGTCCGCCGCTGAATCCAGGAGCAAGCTCCCTCATCCGCTCGACTGCCAT
GTGTTAGGCCTGCCGCCAGCGTTCAATCTGAGCCAGGATCAACTCT
>1198
CTGCTGCCGCCCGTAGGAGTCTGGTCCGTGTCTCAGTACCAGTGTGGGGGATCACCCTCTCAGGCCCCCTAAA
GATCGCCGCCTTGGTGAGCCGTTACCTCACCAACTAGCTAATCTTGCGCGTGCCCATCTCTATCCACCGTAGT
TTTCAATATCACCCGATGCCGAATAATATAATTATGGGGTATTAATCTTCTTCGAAAGGCTATCCCCTGATAA
AGGCAGGTTGCACACGTGTTCCGCACCCGTACGCCGCTCTCAATTACCCGAAAGTAATCTACCGCTCGGCTTG
CATGTGTTAGGCCTCCCGCTAGCGTTCATCCTGAGCCAGGATCAACTCT
>1199
CTGCTGCCGCCCGTAGGAGTCTGGTCCGTGTCTCAGTACCAGTGTGGGGGATCACCCTCTCAGGCCCCCTAAA
GATCGTCGCCTTGGTGAGCCGTTACCTCACCAACTAGCTAATCTTGCGCGTGCCCATCTCTATCCACCGTAGT
TTTCAATATCACCCGATGCCGAATAATATATTATGGGGTATTAATCTTCTTCGAAAGGCTATCCCCTGATAAA
GGCAGGTTGCACACGTGTTCCGCACCCGTACGCCGCTCTCAATTACCCGAAAGTAATCTACCGCTCGGCTTGC
ATGTGTTAGGCCTCCCGCTAGCGTTCATCCTGAGCCAGGATCAACTCT
>1200
CTGCTGCCGCCCGTAGGAGTCTGGTCCGTGTCTCAGTACCAGTGTGGGGGATCACCCTCTCAGGCCCCCTAAA
GATCGTCGCCTTGGTGAGCCGTTACCTCACCAACTAGCTAATCTTGCGCGTGCCCATCTCTATCCACCGTAGT
TTTCAATATCACCCGATGCCGAATAATATAATTATGGGGTATTAATCTTCTTCGAAAGGCTATCCCCTGATAA
AGGCAGGTTGCACACGTGTTCCGCACCCGTACGCCGCTCTCAATTACCCGAAAGTAATCTACCGCTCGGCTTG
CATGTGTTAGGCCTCCCGCTAGCGTTCATCCTCGAGCAGG
>1201
CTGCTGCCGCCCGTAGGAGTCTGGTCCGTGTCTCAGTACCAGTGTGGGGGATCACCCTCTCAGGCCCCCTAAA
GATCGTCGCCTTGGTGAGCCGTTACCTCACCAACTAGCTAATCTTGCGCGTGCCCATCTCTATCCACCGTAGT
TTTCAATATCACCCGATGCCGAATAATATATTATGGGGTATTAATCTTCTCGAAAGGCTATCCCCTGATAAAG
GCAGGTTGCACACGTGTTCCGCACCCGTACGCCGCTCTCAATTACCCCGAAAGTAATCTACCGCTCGGCTTGC
ATGTGTTAGGCTCCCG
>1202
CTGCTGCCGCCCGTAGGAGTCTGGTCCGTGTCTCAGTACCAGTGTGGGGGATCACCCTCTCAGGCCCCCTAAA
GATCGTCGCCTTGGTGAGCCGTTACCTCACCAACTAGCTAATCTTGTGCGTGCCCATCTCTATCCACCGTAGT
TTTCAATATCACCCGATGCCGAATAGTATATTATGGGGTATTAATCTTCTTCGAAAGGCTATCCCCCTGATAA
AGGCAGGTTGCACACGTGTTCCGCACCCGTACGCCGCTCTCAATTACCCGAAAGTAATCTACCGCTCCGGCTT
GCATGTGTTAGGCCTCCCGCTAGCGTTCAT
>1203
CTGCTGCCGCCCGTAGGAGTCTGGTCCGTGTCTCAGTACCAGTGTGGGGGATCACCCTCTCAGGCCCCCTAAA
GATCGTCGCCTTGGTGAGCCGTTACCTCACCAACTAGCTAATCTTGCGCGTGCCCATCTCTATCCACCGTAGT
TTTCAATATCACCCGATGCCGAATAATATATTATGGGGTATTAATCTTCTTCGAAAGGCTATCCCCTGATAAA
GGCAGGTTGCACACGTGTTCCGCACCCGTACGCCGCTCTCAATTACCCGAAAGTAATCTACCGCATCGGCTTG
CACTGTGTTAGGCCTCCC
>1204
CTGCTGCCGCCCGTAGGAGTCTGGTCCGTGTCTCAGTACCAGTGTGGGGGATCACCCTCTCAGGCCCCCTAAA
GATCGTCGCCTTGGTGAGCCGTTACCTCACCAACTAGCTAATCTTGCGCGTGCCCATCTCTATCCACCGTAGT
TTTCAATATCACCCGATGCCGAATAATATATTATGGGGTATTAATCTTCTTCGAAAGGCTATCCCCCTGATAA
AGGCAGGTTGCACACGTGTTCCGCACCCGTACGCCGCTCTCAATTACCCGAAAGTAATCTACCGCTCGGCTTG
CATGTGTTAGGCCTCCCGCTAGCGTTCTCCTCGAGCAGGATCAACATCT
>1205
CTGCTGCCGCCCGTAGGAGTCTGGTCCGTGTCTCAGTACCAGTGTGGGGGATCACCCTCTCAGGCCCCCTAAA
GATCGTCGCCTTGGTGAGCCGTTACCTCACCAACTAGCTAATCTTGCGCGTGCCCATCTCTATCCACCGTAGT
TTTTCAATATCACCCGATGCCGAATAATATATTATGGGGTATTTAATTCTTTCTCGAAAGGCTATCCCCCTGA
TAAAGGCAGGTTGCACACGTGTTCCGCACCCGTACGCCGCTCTCAATTACCCGAAAGTAATCTACCGCTCCGG
CTTGCATGTGTTAGGCCTCCCGCTAGCGTTCATTCCT
>1206
CTGCTGCCGCCCGTAGGAGTCTGGTCCGTGTCTCAGTACCAGTGTGGGGGATCACCCTCTCAGGCCCCCTAAA
GATCGTCGCCTTGTGAGCCGTTTACCTCACCAACTAGCTAATCTTGCGCGTGCCCATCTCTATCCACCGTAGT

TTTCAATATCACCCGATGCCGAATAATATATTATGGGGTATTAATCTTCTTCGAAAGGCTATCCCCCTGATAA
AGGCAGGTTGCACACGTGTTCCGCACCCGTACGCCGCTCTCAATTACCCGAAAGTAATCTACCGCTCGGCTTG
CATGTGTTTAGGCCTCCCGCTAGCGTTCTCCTGAGCAGGATCC
>1207
CTGCTGCCGCCCGTAGGAGTCTGGTCCGTGTCTCAGTACCAGTGTGGGGGATCACCCTCTCAGGCCCCCTAAA
GATCGTCGCCTTGGTGAGCCGTTACCTCACCAACTAGCTAATCTTGCGCGTGCCCATCTCTATCCACCGTAGT
TTTCGATATCACCCGATGCCGAATAATATATTATGGGGTATTAATCTTCTTCGAAAGCTATCCCCCTGATAAA
GGCAGGTTGCACACGTGTTCCGCACCCGTACGCCGCTCTCAATTACCCGAAAGTAATCTACCGCTCGGCTTGC
ATGTGTTAGGCCTCCCGCTAGCGTTCTCTGAGCA
>1208
CTGCTGCCGCCCGTAGGAGTCTGGTCCGTGTCTCAGTACCAGTGTGGGGGATCACCCTCTCAGGCCCCCTAAA
GATCGTCGCCTTGGTGAGCCGTTACCTCACCAACTAGCTAATCTTGCGCGTGCCCATCTCTATCCACCGTAGT
TTTCAATATCACCCGATGCCGAATAATATATTATGGGGTATTAATCTCTTCGAAAGGCTATCCCCTGATAAAG
GCAGGTTGCACACGTGTTCCGCACCCGTACGCCGCTCTCAATTACCCGAAAGTAATCTACCGCTCGGCTTGCA
TGTGTTAGGCCTCCTCGTAGCGTTCATCCTGAGCCAGGATCAAACTCT
>1209
CTGCTGCCGCCCGTAGGAGTCTGGACCGTGTCTCAGTTCCAGTGTGGCTGATCATCCTCTCAGACCAGTACGG
ATCGTCGCCTTGGTAGGCCTTTTACCCCACCAACTAGCTAATCCGACCTAGGCTCATCTGATAGCGTGAGGTC
CGAAGATCCCCCACTTCTCCCTCAGGACGTATGCGGTATTAGCGCCCGTTTCCGGACGTATCCCCACTACCAG
GCAGATTCCTAGGCATTACTCGCCCGTCCGCCGCTGAATCCAGGAGCAAGCCCCCTTCATCCGCTCGACTGCA
TGTGTTAGGCCTGCCGCCAGCGTCAATCTGAGCCAGGATCAAACTCT
>1210
CTGCTGCCGCCCGTAGGAGTCTGGTCCGTGTCTCAGTACCAGTGTGGGGGATCACCCTCTCAGGCCCCCTAAA
AGATCGTCGCCTGGTGAGCCGTTACCTCACCAACTAGCTAATCTTGCGCGTGCCCATCTCTATCCACCGTAGT
TTTCAATATCACCCGATGCCGAATAATATATTATGGGGTATTAATCTTCTCGAAAGCTATCCCCCTGATAAAG
GCAGGTTGCACACGTGTTCCGCACCCGTACGCCGCTCTCAATTACCCGAAAGTAATCTACCGCTCGGCTTGCA
TGTGTTAGGCCTCCCGCTAGCGTTCATCCTGAGCCAGGATCAAACTCT
>1211
CTGCTGCCGCCCGTAGGAGTCTGGTCCGTGTCTCAGTACCAGTGTGGGGGATCACCCTCTCAGGCCCCCTAAA
GATCGTCGCCTTGGTGAGCCGTTACCTCACCAACTAGCTAATCTTGCGCGTGCCCATCTCTATCCACCGTAGT
TTTCAATATCACCCGATGCCGAATAATATATTATGGGGTATTAATCTTCTCGAAAGGCTATCCCCCTGATAAA
GGCAGGTTGCACACGTGTTTCCGCACCCGTACGCCGCTCTCAATTACCCGAAAGTAATCTACCGCTCGGCTTG
CATGTGTTAGGCCTCCCGCTAGCGTTCATCCTGAGCCAGGATCAAACTCT
>1212
CTGCTGCCGCCCGTAGGAGTCTGGTCCGTGTCTCAGTACCAGTGTGGGGGATCACCCTCTCAGGCCCCCTAAA
GATCGTCGCCTTGGTGAGCCGTTACCTCACCAACTAGCTAATCTTGCGCGTGCCCATCTCTATCCACCGTAGT
TTTCAATATCACCCGATGCCGAATAATATATTATGGGGTATTAATCTTCTCGAAAGGCTATCCCCCTGATAAA
GGCAGGTTGCACACGTGTTCCGCACCCGTACGCCGCTCTCAATTACCCGAAAGTAATCTACCGCTCGGCTTGC
ATGTGTTAGGCCTCCCGCTAGCGTCATCT
>1213
CTGCTGCCGCCCGTAGGAGTCTGGTCCGTGTCTCAGTACCAGTGTGGGGGATCACCCTCTCAGGCCCCCTAAA
GATCGTCGCCTTGGTGAGCCGTTACCTCACCAACTAGCTAATCTTGCGCGTGCCCGTCTCTATCCACCGTAGT
TTTCAATATCACCCGATGCCGAATAATATATTATGGGGTATTAATCTTCTTCGAAAGGCTATCCCCCTGATAA
AGGCAGGTTAGCACACGTGTTCCGCACCCGTACGCCGCTCTCAATTACCCGAAAGTAATCTACCGCTCGGCTT
GCATGTGTTAGGCCTCCCGCTAGCGTTCATCCTGAGCCAGGATCAAACTCT
>1214
CTGCTGCCGCCCGTAGGAGTCTGGTCCCGTGTCTCAGTACCAGTGTGGGGGATCACCCTCTCAGGCCCCCCCT
AAAGATCGTCGCCTTGGTGAGCCGTTACCTCACCCAACTAGCTAATCTTGCGCGTGCCCCATCTCTATCCACC
GTAGTTTTCAATATCACCCGATGCCCGAATAATATATTATTGGGGTATTAATCTTCTTCGAAAGGCTATCCCC
CTGATAAAGGCAGGTTGCACACGTGTTCCGCACCCGTACGCCGCTCTCAATTACCCCCGAAAGTAATCTACCG
CTCGGCTTGCATGTGTTAGGCCTCCCGCTAGCGTTCATCCTGAGCCAGGATCAAACTCT
>1215
CTGCTGCCCCCGTAGGAGTCTGGTCCGTGTCTCAGTACCAGTGTGGGGGATCACCCTCTCAGGCCCCCTAAAG
ATCGTCGCCTTGGTGAGCCGTTACCTCACCAACTAGCTAATCTTGCGCGTGCCCATCTCTATCCACCGTAGTT

TTCAATATCACCCGATGCCGAATAATATATTATGGGGTATTAATCTTCTTCGAAAGGCTATCCCCCTGATAAA
GGCAGGTTGCACACGTGTTCCGCACCCGTACGCCGCTCTCAATTACCCGAAAGTAATCTACCGCTCGGCTTGC
ATGTGTTAGGCCTCCCGCTAGCGTTCATCCTGAGCAGGATCAAACTCT
>1216
CTGCTGCCGCCCGTAGGAGTCTGGTCCGTGTCTCAGTACCAGTGTGGGGGGATCACCCTCTCAGGCCCCCTAA
AGATCGTCGCCTTGGTGAGCCGTTACCTCACCAACTAGCTAATCTTGCGCGTGCCCATCTCTATCCACCGTAG
TTTTCAATATCACCCGATGCCGAATAATATATTATGGGGTATTAATCTTCTACGAAAGCTATCCCCCTGATAA
AGGCAGGTTGCACACGTGTTCCGCACCCGTACGCCGCTCTCAATTACCCGAAAGTAATCTACCGCTCGGCTTG
CATGTGTTAGGCCTCCCGCTAGCGTTCATCCGTGAGCCAGGATCAACATCT
>1217
CTGCTGCCGCCCGTAGGAGTCTGGTCCGTGTCTCAGTACCAGTGTGGGGGATCACCCTCTCAGGCCCCCTAAA
GATCGTCGCCTTGGTGAGCCGTTACCTCACCAACTAGCTAATCTTGCGCGTGCCCATCTCTATCCACCGTAGT
TTTCAATATCACCCGATGCCGAATAATATAATTATGGGGTATTAATCTTCTTCGAAAGGCTATCCCCCTGATA
AAGGCAGGTTGCACACGTGTTCCGCACCCGTACGCCGCTCTCAATTACCCGAAAGTAATCTACCGCTCGGCTT
GCATGTGTTAGGCCTCCCGCTAGCGTTCTCCTGAGCAGGATCCAAC
>1218
CTGCTGCCGCCCGTAGGAGTCTGGTCCGTGTCTCAGTACCAGTGTGGGGGATCACCCTCTCAGGCCCCCTAAA
GATCGTCGCCTTGGTGAGCCGTTACCTCACCAACTAGCTAATCTTGCGCGTGCCCATCTCTATCCACCGTAGT
TTTCAATATCACCCGATGCCGAATAATATATTATGGGGTATTAATCTTCTTCGAAAGGCTATCCCCCTGATAA
AGGCAGGTTGCACACGTGTTCCGCACCCGTACGCCGCTCTCAATTACCCGAAAGTAATCTACCGCTCCGGCTT
GCATGTGTTAGGCCTCCCGCTAGCGTTCTCCTGAGCAGGATCAACTCGT
>1219
CTGCTGCCGCCCGTAGGAGTCTGGTCCGCGTCTCAGTACCAGTGTGGGGGATCACCCTCTCAGGCCCCCTAAA
GATCGTCGCCTTGGTGAGCCGTTACCTCACCAACTAGCTAATCTTGCGCGTGCCCATCTCTATTCCACCGTAG
TTTCAATATCACCCGATGCCGAATAATATATTATGGGGTATTAATTCTTCTTCGAAAGGCTATCCCCCTGATA
AAGGCAGGTTGCACACGTGTTCCGCACCCGTACGCCGCTCTCAATTACCCGAAAGTAATCTACCGCTCCGGCT
TGCATGTGTTAGGCCTCCCGCTAGCGTTCTCCTGA
>1220
CTGCTGCCGCCCGTAGGAGTCTGGTCCGTGTCTCAGTACCAGTGTGGGGGATCACCCTCTCAGGCCCCCTAAA
GATCGTCGCCTGTGAGCCGTTACCTCACCAACTAGCTAATCTTGCGCGTGCCCATCTCTATCCACCGTAGTTT
TCAATATCACCCGATGCCGAATAATATATTATGGGGTATTAATCTTCCTTCGAGAGGCTATCCCCTGATAAAG
GCAGGTTGCACACGTGTTCCGCACCCGTACGCACTCTCAATTACCCGAAAGTAATCTACCGCTCGGCTTGCAT
GTGTTAGGCCC
>1221
CTGCTGCCGCCCGTAGGAGTCTGGTCCGTGTCTCAGTACCAGTGTGGGGGATCACCCTCTCAGGCCCCCTAAA
GATCGTCGCCTTGGTGAGCCGTTACCTCACCAACTAGCTAATCTTGCGCGTGCCCATCTCTATCCACCGTAGT
TTTCAATATCACCCGATGCCGAATAATATATTATGGGGTATTAATCTTCTTCGAAAGGCTATCCCCCTGATAA
AGGCAGGTTGCACACGTGTTCCGCACCCGTACGCCGCTCTCAATTACCCGAAAGTAATCTACCGCTCGGCTTG
CATGTGTTAGGCCTCCCGCTAGCGTTCATCCTCGAGCCAGGATCAAACTCT
>1222
CTGCTGCCGCCCGTAGGAGTCTGGTCCGTGTCTCAGTACCAGTGTGGGGGATCACCCTCTCAGGCCCCCTAAA
GATCGTCGCCTTGGTGAGCCGTTACCTCACCAACTAGCTAATCTTGCGCGTGCCCATCTCTATCCACCGTAGT
TTTCAATATCACCCGATGCCGAATAATATATTATGGGGTATTAATCTTCTCGAAGCTATCCCCTGATAAGGCA
GGTTGCACACGTGTTCCGCACCCGTACGCCGCTCTCAATTACCCGAAAGTAATCTACCGCTCCGGCTTGCATG
TGTTAGGCCTCCCGCTAGCGTTC
>1223
CTGCTGCCGCCCGTAGGAGTCTGGTCCGTGTCTCAGTACCAGTGTGGGGGATCACCCTCTCAGGCCCCCTAAA
GATCGTCGCCTTGGTGAGCCGTTACCTCACCAACTAGCTAATCTTGCGCGTGCCCATCTCTATCCACCGTAGT
TTTCAATATCACCCGATGCCGAATAATATATTATGGGGTATTAATCTTCTTCGAAAGGCTATCCCCCTGATAA
AGGCAGGTTGCACACGTGTTCCGCACCCGTACGCCGCTCTCAATTACCCGAAAGTATCTACCGCTCGGCTTGC
ATGTGTTAGGCCTCCCGCTAGCGTTCTCCTGAGCAGGATCAACTCGT
>1224
CTGCTGCCGCCCGTAGGAGTCTGGACCGTGTCTCAGTTCCAGTGTGACTGATCATCCTCTCAGACCAGTACGG
ATCGTCGCCTTGGTAGGCCTTTACCCCACCAACTAGCTAATCCGACCTAGGCTCATCTGATAGCGTGAGGTCC

GAAGTCCCCCACTTCTCCCTCAGGACGTATGCGGTATTAGCGCCCGTTTCCGGACGTATCCCCCACTACCAGG
CAGATTCCTAGGCATTACTCACCCGTCCGCCGCTGAATCCAGGAGCAAGCCCCCTTCATCCGCTCGACTGCAT
GTGTTAGGCCTGCCGCCAGCGTCAATCTGAGCCAGGATCAACTCT
>1225
CTGCTGCCGCCCGTAGGAGTCTGGTCCGTGTCTCAGTACCAGTGTGGGGGATCACCCTCTCAGGCCCCCTAAA
AGATCGTCGCCTTGGTGAGCCGTTACCTCACCAACTAGCTAATCTTGCGCGTGCCCATCTCTATCCACCGTAG
TTTTCAATATCACCCGATGCCGAATAATATAATTATGGGGTATTAATCTTCTCGAAAGGCTATCCCCTGATAA
AGGCAGGTTGCACACGTGTTCCGCACCCGTACGCCGCTCTCAATTACCCGAAAGTAATCTACCGCTCGGCTTG
CATGTGTTAGGCCTCCCGCTAGCGTCATCCTGAGCAGGATCAACTCT
>1226
CTGCTGCCGCCCGTAGGAGTCTGGTCCGTGTCTCAGTACCAGTGTGGGGGATCACCCTCTCAGGCCCCCTAAA
GATCGTCGCCTTGGTGAGCCGTTACCTCACCAACTAGCTAATCTTGCGCGTGCCCATCTCTATCCACCGTAGT
TTTCAATATCACCCGATGCCGAATAATATATTATGGGGTATTAATCTTCTTCGAAAGGCTATCCCCCTGATAA
AGGCAGGTTGCACACGTGTTCCGCACCCGTACGCCGCTCTCAATTACCCGAAAGTAATCTACCGCTCGGCTTG
CATGTGTTAGGCCTCCCGCTAGCGTTCTCCTGAGCAGGTCAAACTC
>1227
CTGCTGCCGCCCGTAGGAGTCTGGTCCGTGTCTCAGTACCAGTGTGGGGGATCACCCTCTCAGGCCCCCTAAA
GATCGTCGCCTTGGTGAGCCGTTACCTCACCAACTAGCTAATCTTGCGCGTGCCCATCTCTATCCACCGTAGT
TTTCAATATCACCCGATGCCGAATAATATATTATGGGGTATTAATCTTTCTTCGAAAGGCTATCCCCCTGATA
AAGGCAGGTTGCGCACGTGTTCCGCACCCGTACGCCGCTCTCAATTACCCGAAAGTAATCTACCGCTCGGCTT
GCATGTGTTAGGCCTCCCGCTAGCGTTCATCCTGAGCCAGGATCAACTCT
>1228
CTGCTGCCGCCCGTAGGAGTCTGGTCCGTGTCTCAGTACCAGTGTGGGGGATCACCCTCTCAGGCCCCCTAAA
GATCGTCGCCTTGGTGAGCCGTTACCTCACCAACTAGCTAATCTTGCGCGTGCCCATCTCTATCCACCGTAGT
TTTCAATATCACCCGATGCCGAATAATATATTATGGGGTATTAATCTTCTTCGAAAGGCTATCCCCCTGATAA
AGGCAGGTTGCACACGTGTTCCGCACCCGTACGCCGCTCTCAATTACCCGAAAGTAATCTACCGCTCGGCTTG
CATGTGTTAGGCCTCCCGCTAGCGTTCATCC
>1229
CTGCTGCCGCCCGTAGGAGTCTGGTCCGTGTCTCAGTACCAGTGTGGGGGATCACCCTCTCAGGCCCCCTAAA
GATCGTCGCCTTGGTGAGCCGTTACCTCACCAACTAGCTAATCTTGCGCGTGCCCATCTCTATCCACCGTAGT
TTTCAATATCACCCGATGCCGAATAATATATTATGGGGTATTAATCTTCTTCGAAAGGCTATCCCCCTGATAA
AGGCAGGTTGCACACGTGTTCCGCACCCGCACGCGCTCTCAATTACCCGAAAGTAATCTACCGCTCGGCTTGC
ATGTGTTAGGCCTCCCGCTAGCGTTCATCCTGAGCCAGGATCAAACTCT
>1230
CTGCTGCCGCCCGCAGGAGTCTGGTCCGTGTCTCAGTACCAGTGTGGGGGATCACCCTCTCAGGCCCCCTAAA
GATCGTCGCCTTGGTGAGCCGTTACCTCACCAACTAGCTAATCTTGCGCGTGCCCATCTCTATCCACCGTAGT
TTTCAATATCACCCGATGCCGAATAATATATTATGGGGTATTAATCTTCTTCGAAAGGCTATCCCCTGATAAA
GGCAGGTTGCACACGTGTTCCGCACCCGTACGCCGCTCTCAATTACCCGAAAGTAATCTACCGCTCGGCTTGC
ATGTGTTAGGCCTCCCGCTAGCGTTCATCCTGAGCCAGGATCAACTCT
>1231
CTGCTGCCGCCCGTAGGAGTCTGGTCCGTGTCTCAGTACCAGTGTGGGGGATCACCCTCTCAGCCCCCCTAAA
GATCGTCGCCTTGGTGAGCCGTTACCTCACCAACTAGCTAATCTTGCGCGTGCCCATCTCTATCCACCGTAGT
TTTCAATATCACCCGATGCCGAATAATATATTATGGGGTATTAATCTTCTTCTGAAAGGCTATCCCCCTGATA
AAGGCAGGTTGCACACGTGTTCCGCACCCGTACGCCGCTCTCAATTACCCGAAAGTAATCTACCGCTCGGCTT
GCATGTGTTAGGCCTCCCGCTAGCGTTCATCCTGAGCCAGGATCAACTCT
>1232
CTGCTGCCGCCGTAGGAGTCTGGTCCGTGTCTCAGTACCAGTGTGGGGGATCACCCTCTCAGGCCCCCTAAAG
ATCGTCGCCTTGGTGAGCCGTTACCTCACCAACTAGCTAATCTTGCGCGTGCCCATCTCTATCCACCGTAGTT
TTCAATATCACCCGATGCCGAATAATATATTATGGGGTATTAATCTTCTTCGAAAGGCTATCCCCCTGATAAA
GGCAGGTTGCACACGTGTTCCGCACCCGTACGCCGCTCTCAATTACCCGAAAGTAATCTACCGCTCGGCTTGC
ATGTGTTAGGCCTCCCGCTAGCGTTCTCCTGAGCAGGATCAACTCG
>1233
CTGCTGCCGCCCGTAGGAGTCTGGTCCGTGTCTCAGTACCAGTGTGGGGGATCACCCTCTCAGGCCCCCTAAA
GATCGTCGCCTTGGTGAGCCGTTACCTCACCAACTAGCTAATCTTGCGCGTGCCCATCTCTATCCACCGTAGT

TTTCAATATCACCCGATGCCGAATAATATATTATGGGGTATTAATCTTCTCGAAAGGCTATCCCCTGATAAAG
GCAGGTTGCACACGTGTTCCGCACCCGTACGCCGCTCTCAATTACCCGAAAGTAATCTACCGCTCGGCTTGCA
TGTGTTAGGCCTCCCGCTAGC
>1234
CTGCTGCCGCCCGTAGGAGTCTGGTCCGTGTCTCAGTACCAGTGTGGGGGATCACCCTCTCAGGCCCCCTAAA
GATCGTCGCCTTGGTGAGCCGTTACCTCACCAACTAGCTAATCTTGCGCGTGCCCATCTCTATCCACCGTAGT
TTTCAATATCACCCGATGCCGAATAATATATTATGGGGTATTAATCTTCTTCGAAAGGCTATCCCCTGATAAA
GGCAGGTTGCACACGTGTTCCGCACCCGTACGCCGCTCTCAATTACCCGAAAGTAATCTACCGCTCCGGCTTG
CATGTGTTAGGCCTCCCGCTAGCGTTCTCCTGAGCAGGATCAACTCGT
>1235
CTGCTGCCGCCCGTAGGAGTCTGGTCCGTGTCTCAGTACCAGTGTGGGGGATCACCCTCTCAGGCCCCCTAAA
GATCGTCGCCTTGGTGAGCCGTTACCTCACCAACTAGCTAATCTTGCGCGTGCCCATCTCTATCCACCGTAGT
TTTCAATATCACCCGATGCCGAATAATATATTATGGGGTATTAATCTTCTTCGAAAGGCTATCCCCCTGATAA
AGGCAGGTTGCACACGTGTTCCGCACCCGTACGCCGCTCTCAATTACCCGAAAGTAATCTACCGCTCGGCTTG
CATGTGTTAGGCCTCCCGCTAGCGTTCATCCTGAG
>1236
CTGCTGCCGCCCGTAGGAGTCTGGTCCGTGTCTCAGTACCAGTGTGGGGGATCACCCTCTCAGGCCCCCTAAA
GATCGTCGCCTTGGTGAGCCGTTACCTCACCAACTAGCTAATCTTGCGCGTGCCCATCTCTATCCACCGTAGT
TTTCAATATCACCCGATGCCGAATAATATATTATGGGGTATTAATCTTCTTCGAAAGGCTATCCCCCTGATAA
AGGCAGGTTGCACACGTGTTCCGCACCCGTACGCCGCTCTCAATTACCCGAAAGTAATCTACCGCATCGGCTT
CGGCACTGTGTTAGGCCTCCCGCTAGCGTTCATCCTGAGCCAGGATCAAACTCT
>1237
CTGCTGCCGCCCGTAGGAGTCTGGTCCGTGTCTCAGTACCAGTGTGGGGGATCACCCTCTCAGGCCCCCTAAA
GATCGTCGCCTTGGTGAGCCGTTACCTCACCAACTAGCTAATCTTGCGCGTGCCCATCTCTATCCACCGTAGT
TTTCAATATCACCCGATGCCGAATAATATATTATGGGGTATTAATCTTCTTCGAAAGGCTATCCCCCTGATAA
AGGCAGGTTGCACACGTGTTCCGCACCCGTACGCCGCTCTCAATTACCCGAAAGTAATCTACCGCTCCGGCTT
GCATGTGTTAGGCCTCCCGCTAGCGTTCATCCGTGAGCCAGGATCAACTCT
>1238
CTGCTGCCGCCCGTAGGAGTCTGGTCCGTGTCTCAGTACCAGTGTGGGGGATCACCCTCTCAGGCCCCCTAAA
GATCGTCGCCTTGGTGAGCCGTTACCTCACCAACTAGCTAATCTTGCGCGTGCCCATCTCTATCCACCGTAGT
TTTTCAATATCACCCAATGCCGAATAATATATTATGGGGTATTTAATCTTCTTCGAAAGGCTATCCCCCTGAT
AAAGGCAGGTTGCACACGTGTTCCGCACCCGTACGCCGCTCTCAATTACCCGAAAGTAATCTACCGCTCGGCT
TGCATGTGTTAGGCCTCCCGCTAGCGTTCTCCTGAGCAGGATCAAACTCGT
>1239
CTGCTGCCGCCCGTAGGAGTCTGGTCCGTGTCTCAGTACCAGTGTGGGGGATCACCCTCTCAGGCCCCCTAAA
AGATCGTCGCCTTGGTGAGCCGTTACCTCACCAACTAGCTAATCTTGCGCGTGCCCATCTCTATCCACCGTAG
TTTTCAATATCACCCGATGCCGAATAATATATTATGGGGTATTAATCTTCTACGAAAGCTATCCCCTGATAAA
GGCAGGTTGCACACGTGTTCCGCACCCGTACGCCGCTCTCAATTACCCGAAAGTAATCTACCGCTCGGCTTGC
ATGTGTTAGGCCTCCGCTAGCGTTCATCCTGA
>1240
CTGCTGCCGCCCGTAGGAGTCTGGTCCGTGTCTCAGTACCAGTGTGGGGGATCACCCTCTCAGGCCCCCTAAA
GATCGTCGCCTTGGTGAGCCGTTACCTCACCAACTAGCCTAATCTTGCGCGTGCCCATCTCTATTCCACCGTA
GTTTTCAATATCACCCGATGCCGAATAATATATTATGGGGTATTTAATCTTCTCGAAAGGCTATCCCCCTGAT
AAAGGCAGGTTGCACACGTGTTCCGCACCCGTACGCCGCTCTCAATTACCCGAAAGTAATCTACCGCTCGGCT
TGCATGTG
>1241
CTGCTGCCGCCCGTAGGAGTCTGGTCCGTGTCTCAGTACCAGTGTGGGGGATCACCCTCTCAGGCCCCCTAAA
GATCGTCGCCTTGGTGAGCCGTTACCTCACCAACTAGCTAATCTTGCGCGTGCCCATCTCTATCCACCGTAGT
TTTCAATATCACCCGATGCCGAATAATATATTATGGGGTATTAATCTTCTTCGAAAGGCTATCCCCCTGATAA
AGGCAGGTTGCACACGTGTTCCGCACCCGTACGCCGCTCTCAATTACCCGAAAGTAATCTACCGCTCGGCTTG
CATGTGTTAGGCCTCCCGCTAGCGTTCATCCTC
>1242
CTGCTGCCGCCCGTAGGAGTCTGGTCCGTGTCTCAGTACCAGTGTGGGGGATCACCCTCTCAGGCCCCCTAAA
GATCGTCGCCTTGGTGAGCCGTTACCTCACCAACTAGCTAATCTTGCGCGTGCCCATCTCTATCCACCGTAGT

TTTCAATATCACCCGATGCCGAATAATATATTATGGGGTATTAATCTTCTTCGAAAGCTATCCCCTGATAAAG
GCAGGTTGCACACGTGTTCCGCACCCGTACGCCGCTCTCAATTACCCGAAAGTAATCTACCGCTCCGGCTTGC
ATGTGTTAGGCCTCCCGCTAGCGTTCATCCTGAGCCAGG
>1243
CTGCTGCCGCCCGTAGGAGTCTGGTCCGTGTCTCAGTACCAGTGTGGGGGATCACCCTCTCAGGCCCCCTAAA
GATCGTCGCCTTGGTGAGCCGTTACCTCACCAACTAGCTAATCTTGCGCGTGCCCATCTCTATCCACCGTAGT
TTTCAATATCACCCGATGCCGAATAATATATTATGGGGTATTAATCTTCTTCGAAAGGCTATCCCCCTGATAA
AGGCAGGTTGCACACGTGTTCCGCACCCGTACGCCGCTCTCAATTACCCGAAAGTAATCTACCGCTCGGCTTG
CATGTGTTAGGCCTCCCGCTAGCGTTCTCCTCGAGCAA
>1244
CTGCTGCCGCCCGTAGGAGTCTGGTCCGTGTCTCAGTACCAGTGTGGGGGATCACCCTCTCAGGCCCCCTAAA
GATCGTCGCCTTGGTGAGCCGTTACCTCACCAACTAGCTAATCTTGCGCGTGCCCATCTCTATCCACCGTAGT
TTTCAATATCACCCGATGCCGAATAATATAATTATGGGGTATTAATCTTCTCGAAAGGCTATCCCCTGATAAA
GGCAGGTTGCACACGTGTTCCGCACCCGTACGCCGCTCTCAATTACCCGAAAGTAATCTACCGCTCGGCTTGC
ATGTGTTAGGCCTCCCGCTAGCGTTCATCCTGAGCAGGCATCAAACTCT
>1245
CTGCTGCCGCCCGTAGGAGTCTGGTCCGTGTCTCAGTACCAGTGTGGGGGATCACCCTCTCAGGCCCCCTAAA
GATCGTCGCCTTGGTGAGCCGTTACCTCACCAACTAGCTAATCTTGCGCGTGCCCATCTCTATCCACCGTAGT
TTTCAATATCACCCGATGCCGAATAATATATTATGGGGTATTAATCTTCTCGAAAGGCTATCCCCTGATAAAG
GCAGGTTGCACACGTGTTCCGCACCGTACGCCGCTCTCAATTACCCGAAAGTAATCTACCGCTCGGCTTGCAT
GTGTTAGGCCTCCCGCTAGCGTTCTCTCGAG
>1246
CTGCTGCCGCCCGTAGGAGTCTGGTCCGTGTCTCAGTACCAGTGTGGGGGATCACCCTCTCAGGCCCCCTAAA
GATCGTCGCCTTGGTGAGCCGTTACCTCACCAACTAGCTAATCTTGCGCGTGCCCATCTCTATCCACCGTAGT
TTTCAATATCACCCGATGCCGAATAATATATTATGGGGTATTAATCTTCTCGAAAGGCTATCCCCCTGATAAA
GGCAGGTTGCACACGTGTTCCGCACCCGTACGCCGCTCTCAATTACCCGAAAGTAATCTACCGCTCGGCTTGC
ATGTGTTAGGCCTCCCGCTAGCGTTCATCCTCG
>1247
CTGCTGCCGCCCGTAGGAGTCTGGTCCGTGTCTCAGTACCAGTGTGGGGGATCACCCTCTCAGGCCCCCTAAA
GATCGTCGCCTTGGTGAGCCGTTACCTCACCAACTAGCTAATCTTGCGCGTGCCCATCTCTATCCACCATAGT
TTCAATATCACCCGATGCCGAATAATATAATTATGGGGTATTAATCTTCTTCGAAAGGCTATCCCCTGATAAA
GGCAGGTTGCACACGTGTTCCGCACCCGTACGCCGCCCTCAATTACCCGAAAGGTAATCTACCGCTCGGCTTG
CAATGTGTTAGGCCTCCCGCTAGCGTTCATCCCGAGCCAGGATCAAACTCGT
>1248
CTGCTGCCGCCCGTAGGAGTCTGGTCCGTGTCTCAGTACCAGTGTGGGGGATCACCCTCTCAGGCCCCCTAAA
GATCGTCGCCTTGGTGAGCCGTTACCTCACCAACTAGCTAATCTTGCGCGTGCCCATCTCTATCCACCGTAGT
TTTTCAATATCACCCGATGCCGAATAATATATTATGGGGTATTTAATTCTTTCTCGAAAGGCTATCCCCCTGA
TAAAGGCAGGTTGCCACACGTGTTCCGCACCCGTACGCCGCTCTCAATTACCCGAAAGTAATCTACCGCTCGG
CTTGCATGTGTTAGGCCTCCCGCTAGCGTTCATCTCGAGCC
>1249
CTGCTGCCGCCCGTAGGAGTCTGGTCCGTGTCTCAGTACCAGTGTGGGGGATCACCCTCTCAGGCCCCCTAAA
GATCGTCGCCTTGGTGAGCCGTTACCTCACCAACTAGCTAATCTTGCGCGTGCCCATCTCTATCCACCGTAGT
TTCAATATCACCCGATGCCGAATAATATATTATGGGGTATTAATCTTCTTCGAAAGGCTATCCCCCTGATAAA
GGCAGGTTGCACACGTGTTCCGCACCCGTACGCCGCTCTCAATTACCCGAAAGTAATCTACCGCTCGGCTTGC
ATGTGTTAGGCCTCCCGCTAGCGTTCTCCTCGAGCAGGATCAACATCCT
>1250
CTGCTGCCGCCCGTAGGAGTCTGGTCCGTGTCTCAGTACCAGTGTGGGGGATCACCCTCTCAGGCCCCCTAAA
GATCGTCGCCTTGGTGAGCCGTTACCTCACCAACTAGCTAATCTTGCGCGTGCCCATCTCTATCCACCGTAGT
TTTTTCAATATCACCCGATGCCGAATAATATATTTATGGGGTATTTAATCTTTCTCGAAAGGCTATCCCCCTG
ATAAAGGCAGGTTGCACACGTGTTCCGCACCCGTACGCCGCTCTCAATTACCCGAAAGTAATCTACCGCTCGG
CTTGCATGTGTTAGGCCTCCCGCTAGCGTTCTCCTCGAGCAGGATCCAACATCT
>1251
CTGCTGCCGCCCGTAGGAGTCTGGTCCGTGTCTCAGTACCAGTGTGGGGGATCACCCTCTCAGGCCCCCTAAA
GATCGTCGCCTTGGTGAGCCGTTACCCACCACCATAGCTAATCTTGCGCGTGCCCATCTCTATCCACCGTAGT

TTTCAATATCACCCGATGCCGAATAATATATTATGGGGTATTAATCTTCTTCGAAAGGCTATCCCCTGATAAA
GGCAGGTTGCACACGTGTTCCGCACCCGTACGCCGCTCTCAATTACCCGAAAGTAATCTACCGCTCGGCTTGC
ATGTGTTAGGCCTCCCGCTAGCGTTCTCTGAGCCAGGATCAACTCT
>1252
CTGCTGCCGCCCGTAGGAGTCTGGTCCGTGTCTCAGTACCAGTGTGGGGGATCACCCTCTCAGGCCCCCTAAA
GATCGTCGCCTTGGTGAGCCGTTACCTCACCAACTAGCTAATCTTGCGCGTGCCCATCTCTATCCACCGTAGT
TTTCAATATCACCCGATGCCGAATAATATATTATGGGGTATTAATCTTCTTCGAAAGGCTATCCCCCTGATAA
AGGCAGGTTGCACACGTGTTCCGCACCCGTACGCCGCTCTCAATTACCCGAAAGTAATCTACCGCTCGGCTTG
CATGTGTTAGGCCTCCCGCTAGCGTTCATCCTGAGCCAGGATCAAACTCT
>1253
CTGCTGCCGCCCGTAGGAGTCTGGTCCGTGTCTCAGTACCAGTGTGGGGGATCACCCTCTCAGGCCCCCTAAA
GATCGTCGCCTTGGTGAGCCGTTACCTCACCAACTAGCTAATCTTGCGCGTGCCCATCTCTATCCACCGTAGT
TTTCAATATCACCCGATGCCGAATAATATATTATGGGGTATTTAATCTTCTTCGAAAGGCTATCCCCCTGATA
AAGGCAGGTTGCACACGTGTTCCGCACCCGTACGCCGCTCTCAATTACCCGAAAGTAATCTACCGCTCGGCTT
GCATGTGTTAGGCCTCCCGCTAGCGTTCATCCTGAGCCAGGATCAACTCT
>1254
CTGCTGCCGCCCGTAGGAGTCTGGTCTGTCTCAGTACCAGTGTGGGGGATCACCCTCTCAGGCCCCCTAAAGA
TCGTCGCCTTGGTGAGCCGTTACCTCACCAACTAGCTAATCTTGCGCGTGCCCATCTCTATCCACCGTAGTTT
TCAATATCACCCGATGCCGAATAATATATTATGGGGTATTAATCTTCTTCGAAAGGCTATCCCCCTGATAAAG
GCAGGTTGCACACGTGTTCCGCACCCGTACGCCGCTCTCAATTACCCGAAAGTAATCTACCGCTCGGCTTGCA
TGTGTTAGGCCTCCCGCTAGCGTTCATCCTGAGCCAGGATCAAACTCT
>1255
CTGCTGCCGCCCGTAGGAGTCTGGACCGTGTCTCAGTACCAGTGTGGGGGATCACCCTCTCAGGCCCCCTAAA
GATCGTCGCCTTGGTGAGCCGTTACCTCACCAACTAGCTAATCTTGCGCGTGCCCATCTCTATCCACCGTAGT
TTTCAATATCACCCGATGCCGAATAATATATTATGGGGTATTAATCTTCTTCGAAAGGCTATCCCCCTGATAA
AGGCAGGTTGCACACGTGTTCCGCACCCGTACGCCGCTCTCAATTACCCGAAAGTAATCTACCGCTCGGCTTG
CATGTGTTAGGCCTCCCGCTAGCGTTCATCCTGAGCCAGGATCAAACTCT
>1256
CTGCTGCCGCCCGTAGGAGTCTGGTCCGTGTCTCAGTACCAGTGTGGGGGATCACCCTCTCAGGCCCCCTAAA
GATCGTCGCCTTGGTGAGCCGTTACCTCACCAACTAGCTAATCTTGCGCGTGCCCATCTCTATCCACCGTAGT
TTTCAATATCACCCGATGCCGAATAATATAATTATGGGGTATTAATCTTCTTCGAAAGGCTATCCCCCTGATA
AAAGGCAGGTTGCACACGTGTTCCGCACCCGTACGCCGCTCTCAATTACCCGAAAGTAATCTACCGCTCGGCT
TGCATGTGTTAGGCCTCCCGCTAGCGTTCATCCTGAGCCAGGATCAAACTCT
>1257
CTGCTGCCGCCCGTAGGAGTCTGGTCCGTGTCTCAGTACCAGTGTGGGGGATCACCCTCTCAGGCCCCCTAAA
GATCGTCGCCTTGGTGAGCCGTTACCTCACCAACTAGCTAATCTTGCGCGTGCCCATCTCTATCCACCGTAGT
TTTCAATATCACCCGATGCCGAATAATATATTATGGGGTATTAATCTTCTTCGAAAGGCTATCCCCCTGATAA
AGGCAGGTTGCACACGTGTTCCGCACCCGTACGCCGCTCTCAATTACCCGAAAGTAATCTACCGCTCGGCTTG
CATGTGTTAGGCCTCCCGCTAGCGTTCTCCTGAGCAGGATCCAACTCGT
>1258
CTGCTGCCGCCCGTAGGAGTCTGGTCCGTGTCTCAGTACCAGTGTGGGGGATCACCCTCTCAGGCCCCCTAAA
GATCGTCGCCTTGGTGAGCCGTTACCTCACCAACTAGCTAATCTTGCGCGTGCCCATCTCTATCCACCGTAGT
TTTCAATATCACCCGATGCCGAATAATATATTATGGGGTATTAATCTTCTTCGAAAGGCTATCCCCCTGATAA
AGGCAGGTTGCACACGTGTTCCGCACCCGTACGCCGCTCTCAATTACCCGAAAGTAATCTACCGCTCGGCTTG
CATGTGTTAGGCCTCCCGCTAGCGTTCATCCTGAGCCAGGATCAAACTCT
>1259
CTGCTGCCGCCCGTAGGAGTCTGGTCCGTGTCTCAGTACCAGTGTGGGGGATCACCCTCTCAGGCCCCCTAAA
GATCGTCGCCTTGGTGAGCCGTTACCTCACCAACTAGCTAATCTTGCGCGTGCCCATCTCTATCCACCGTAGT
TTTCAATATCACCCGATGCCGAATAATATATTATGGGGTATTAATCTTCTTCGAAAGGCTATCCCCTGATAAA
GGCAGGTTGCACACGTGTTCCGCACCCGTACGCCGCTCTCAATTACCCGAAAGTAATCTACCGCTCGGCTTGC
ATATGTTAGGCCTCCCGCTAGCGTTCATCCTGAGCCAGGATCA
>1260
CTGCTGCCGCCCGTAGGAGTCTGGTCCGTGTCTCAGTACCAGTGTGGGGGATCACCCTCTCAGGCCCCCTAAA
GATCGTCGCCTTGGTGAGCCGTTACCTCACCAACTAGCTAATCTTGCGCGTGCCCATCTCTATCCACCGTAGT

TTTCAATATCACCCGATGCCGAATAATATATTATGGGGTATTAATCTTCTTCGAAAGGCTATCCCCCTGATAA
AGGCAGGTTGCACACGCGTCCGCACCCGTACGCCGCTCTCAATTACCCGAAAGTAATCTACCGCTCGGCTTGC
ATGTGTTAGGCCTCCCGCTAGCGTTCTCCTGAGCAGGATCCAACTCG
>1261
CTGCTGCCGCCCGTAGGAGTCTGGTCCGTGTCTCAGTACCAGTGTGGGGGATCACCCTCTCAGGCCCCCTAAA
GATCGTCGCCTTGGTGAGCCGTTACCTCACCAACTAGCTAATCTTGCGCGTGCCCATCTCTATCCACCGTAGT
TTTCAATATCACCCGATGCCGAATAATATATTATGGGGTATTAATCTTCTTCGAAAGGCTATCCCCCTGATAA
AGGCAGGTTGCACACGTGTTCCGCACCCGTACGCCGCTCTCAATTACCCGAAAGTAATCTACCGCATCGGCTC
GCACTGTGTTAGGCCTCCCGCTAGCGTTCTCCTCGAGCCAGGATCAAACTCT
>1262
CTGCTGCCGCCCGTAGGAGTCTGGTCCGTGTCTCAGTACCAGTGTGGGGGATCACCCTCTCAGGCCCCCTAAA
GATCGTCGCCTTGGTGAGCCGTTACCTCACCAACTAGCTAATCTTGCGCGTGCCCATCTCTATCCACCGTAGT
TTTCAATATCACCCGATGCCGAATAATATATTATGGGGTATTAATCTTCTTCGAAAGGCTATCCCCCTGATAA
AGGCAGGTTGCACACGTGTTCCGCACCCGTACGCCGCTCTCAATTACCCGAAAGTAATCTACCGCTCCGGCTT
GCATGTGTTAGGCCTCCCGCTAGCGTTCATCCTGAGCCAGGATCAAACTCT
>1263
CTGCTGCCGCCCGTAGGAGTCTGGTCCGTGTCTCAGTACCAGTGTGGGGGATCACCCTCTCAGGCCCCCTAAA
GATCGTCGCCTTGGTGAGCCGTTACCTCACCAACTAGCTAATCTTGCGCGTGCCCATCTCTATCCACCGTAGT
TTTCAATATCACCCGATGCCGAATAATATATTATGGGGTATTAATCTTCTTCGAAAGGCTATCCCCCTGATAA
AGGCAGGTTGCACACGTGTTCCGCACCCGTACGCCGCTCTCAATTACCCGAAAGTAATCTACCGCTCGGCTTG
CATGTGTTAGGCCTCCCGCTAGCGTTCATCCTGAGCCAGGATCAAACTCT
>1264
CTGCTGCCGCCCGTAGGAGTCTGGACCGTGTCTCAGTTCCAGTGTGGCCGATCACCCTCTCAGGTCGGCTATG
CATCGTTGCCTTGGTAAGCCGTTACCTTACCAACTAGCTGATGCAGCGCGGATCCATCTATAAGTGACAGCAA
GACCGTCTTCACTGAACCATGCGGTTCAAAATATTGTCCGGTATTAGCTCCGGTTTCCCGAAGTATCCCAGTC
TTATAGGTAGGTTATCCACGTGTTACTCACCCGTCCGCCGCTAACATCAGAGAAGCAAGCTTCTCATCCGTTC
GCTCGACTGCATGTATTAGGCACGCCGCCAGCGTTCATCTGAGCCAGGATCAACTCT
>1265
CTGCTGCCGCCCGTAGGAGTCTGGTCCGTGTCTCAGTACCAGTGTGGGGGATCACCCTCTCAGGCCCCCTAAA
GATCGTCGCCTTGGTGAGCCGTTACCTCACCAACTAGCTAATCTTGCGCGTGCCCATCTCTATCCACCGTAGT
TTTCAATATCACCCGATGCCGAATAATATATTATGGGGTATTTAATCTTCTTCGAAAGGCTATCCCCCTGATA
AAGGCAGGTTGCACACGTGTTCCGCACCCGTACGCCGCTCTCAATTACCCGAAAGTAATCTACCGCTCGGCTT
GCATGTGTTAGGCCTCCCGCTAGCGTTCATCCTGAGCCAGGATCAAACTCT
>1266
CTGCTGCCGCCCGTAGGAGTCTGGTCCGTGTCTCAGTACCAGTGTGGGGGATCACCCTCTCAGGCCCCCTAAA
GATCGTCGCCTTGGTGAGCCGTTACCTCACCAACTAGCTAATCTTGCGCGTGCCCATCTCTATCCACCGTAGT
TTTCAATATCACCCGATGCCGAATAATATATTATGGGGTATTAATCTTCTTCGAAAGCTATCCCCCTGATAAA
GGCAGGTTGCACACGTGTTCCGCACCCGTACGCCGCTCTCAATTACCCGAAAGTAATCTACCGCTCGGCTTGC
ATGTGTTAGGCCTCCCGCTAGCGTTCTCCTGAGCAGGATCAA
>1267
CTGCTGCCGCCCGTAGGAGTCTGGTCCGTGTCTCAGTACCAGTGTGGGGGATCACCCTCTCAGGCCCCCTAAA
GATCGTCGCCTTGGTGAGCCGTTACCTCACCAACTAGCTAATCTTGCGCGTGCCCATCTCTATCCACCGTAGT
TTTCAATATCACCCGATGCCGAATAATATATTATGGGGTATTAATCTTCTTCGAAAGGCTATCCCCCTGATAA
AGGCAGGTTGCACACGTGTTCCGCACCCGTACGCCGCTCTCAATTACCCGAAAGTAATCTACCGCTCGGCTTG
CATGTGTTAGGCCTCCCGCTAGCGTTCTCCTCGA
>1268
CTGCTGCCGCCCGTAGGAGTCTGGACCGTGTCTCAGTTCCAGTGTGACTGATCATCCTCTCAGACCAGTACGG
ATCGTCGCCTTGGTAGGCCTTTACCCCACCAACTAGCTAATCCGACCTAGGCTCATCTGATAGCGTGAGGTCC
GAAGATCCCCCACTTCTCCCTCAGGACGTATGCGGTATTAGCGCCCGTTTCCGGACGATCCCCACTACCAGGC
CAGATTCCTAGGCATTACTCACCCGTCCGCCGCTGAATCCAGGAGCAAGCTCCCTCATCCGCTCGACTGCATG
TGTTAGGCCTGCCGCCAGCGTCAATCTGAGCCAGGATCAAACTCT
>1269
CTGCTGCCGCCCGTAGGAGTCTGGTCCGTGTCTCAGTACCAGTGTGGGGGATCACCCTCTCAGGCCCCCTAAA
GATCGTCGCCTTGGTGAGCCGTTACCTCACCAACTAGCTAATCTTGCGCGTGCCCATCTCTATCCACCGTAGT

TTTCAATATCACCCGATGCCGAATAATATATTATGGGGTATTAATCTTCTCGAAAGGCTATCCCCTGATAAAG
GCAGGTTGCACACGTGTTCCGCACCCGTACGCCGCTCTCAATTACCCGAAAGTAATCTACCGCTCGGCTGCAT
GTGTTAGGCCTCCCGCTAGCGTTCATCTGAG
>1270
CTGCTGCCGCCCGTAGGAGTCTGGTCCGTGTCTCAGTACCAGTGTGGGGGATCACCCTCTCAGGCCCCCTAAA
GATCGTCGCCTTGGTGAGCCGTTACCTCACCAACTAGCTAATCTTGCGCGTGCCCATCTCTATCCACCGTAGT
TTTCAATATCACCCGATGCCGAATAATATATTATGGGGTATTAATCTTCTTCGAAAGGCTATCCCCTGATAAA
GGCAGGTTGCACACGTGTTCCGCACCCGTACGCCGCTCTCAATTACCCGAAAGTAATCTACCGCTCGGCTTGC
ATGTGTTAGGCCTCCCGCTAGCGTTCATCCTGAGC
>1271
CTGCTGCCGCCCGTAGGAGTCTGGACCGTGTCTCAGTTCCAGTGTGACTGATCATCCTCTCAGACCAGTTACG
GATCGTCGCCTTGGTAGGCCTTTACCCCACCAACTAGCTAATCCGACCTAGGCTCATCTGATAGCGTGAGGTC
CGAAGATCCCCCACTTCTCCCTCAGGACGTATGCGGTATTAGCGCCCGTTTCCGGACGTATCCCCACTACCAG
GCAGATTCCTAGGCATTACTCACCCGTCCGCCGCTGAATCCAGGAGCAAGCCCCCTTCATCCGCTCGACTGCA
TGTGTTAGGCCTGCCGCCAGCGTCAATCTGAGCCAGGATCAAACTCT
>1272
CTGCTGCCGCCCGTAGGAGTCTGGACCGTGTCTCAGTTCCAGTGTGACTGATCATCCTCTCAGACCAGTACGG
ATCGTCGCCTTGGTAGGCCTTTACCCCACCAACTAGCTAATCCGACCTAGGCTCATCTGATAGCGTGAGGTCC
GAAGATCCCCCACTTCTCCCTCAGGACGTATGCGGTATTAGCGCCCGTTTCCGGACGTATCCCCACTACCAGG
CAGGTTCCTAGGCATTACTCACCCGTCCGCCGCTGAATCCAGGAGCAAGCTCCCTCATCCGCTCGACTGCATG
TGTTAGGCCTGCCGCCAGCGTCAATCTGAGCCAGGATCAAACTCT
>1273
CTGCTGCCGCCCGTAGGAGTCTGGTCCGTGTCTCAGTACCAGTGTGGGGGATCACCCTCTCAGGCCCCCTAAA
GATCGTCGCCTTGGTGAGCCGTTACCTCACCAACTAGCTAATCTTGCGCGTGCCCATCTCTATCCACCGTAGT
TTTCAATATCACCCGATGCCGAATAATATATTATGGGGTATTAATCTTCTTCGAAAGGCTATCCCCCTGATAA
AGGCAGGTTGCACGCGTGTTCCGCACCCGTACGCCGCTCTCAATTACCCGAAAGTAATCTACCGCTCGGCTTG
CATGTGTTAGGCCTCCCGCTAGCGTTCATCCTGAGCCAGGATCAACTCT
>1274
CTGCTGCCGCCCGTAGGGGTCTGGTCCGTGTCTCAGTACCAGTGTGGGGGATCACCCTCTCAGGCCCCCTAAA
GATCGTCGCCTTGGTGAGCCGTTACCTCACCAACTAGCTAATCTTGCGCGTGCCCATCTCTATCCACCGTAGT
TTTCAATATCACCCGATGCCGAATAATATATTATGGGGTATTAATCTTCTTCTGAAAGGCTATCCCCTGATAA
AGGCAGGTTGCACACGTGTTCCGCACCCGTACGCCGCTCTCAATTACCCGAAAGTAATCTACCGCTCGGCTTG
CATGTGTTAGGCCTCCCGCTAGCGTTCATCCTGAGCCAGGATCAAACTCT
>1275
CTGCTGCCGCCCGTAGGAGTCTGGTCCGTGTCTCAGTACCAGTGTGGGGGATCACCCTCTCAGGCCCCCTAAA
GATCGTCGCCTTGGTGAGCCGTTACCTCACCAACTAGCTAATCTTGCGCGTGCCCACCTCTATCCACCGTAGT
TTTCAATATCACCCGATGCCGAATAATATATTATGGGGTATTAATCTTCTCGAAAGCTTATCCCCTGATAAAG
GCAGGTTGCACACGTGTTCCGCACCCGTACGCCGCTCTCAATTACCCGAAAGTAATCTACCGCATCGGTTGCA
TGTGTTAGGCCTCCCGCTAGCGTCTCTGAGC
>1276
CTGCTGCCGCCCGTAGGAGTCTGGACCGTGTCTCAGTTCCAGTGTGACTGATCATCCTCTCAGACCAGTACGG
ATCGTCGCCTTGGTAGGCCTTTACCCCACCAACTAGCTAATCCGACCTAGGCTCATCTGATAGCGTGAGGTCC
GAAGTCCCCACTTCTCCCTCAGGACGTATGCGGTATTAGCGCCCGTTTCCGGACGTATCCCCGCACTACCAGG
CAGATTCCTAGGCATTACTCACCCGTCCGCCGCTGAATCCAGGAGCAAGCTCCCTCATCGCTCGACTGCATGT
GTTAGGCCTGCCGCCAGCGTTCAATCTGAGCCAGGATCAAACTCT
>1277
CTGCTGCCGCCCGTAGGAGTCTGGTCCGTGTCTCAGTACCAGTGTGGGGGATCACCCTCTCAGGCCCCCTAAA
AGATCGTCGCCTTGGTGAGCCGTTACCTCACCAACTAGCTAATCTTGCGCGTGCCCATCTCTATCCACCGTAG
TTTTCAATATCACCCGATGCCGAATAATATATTATGGGGTATTAATCTTCTCGAAAGCTATCCCCTGATAAAG
GCAGGTTGCACACGTGTTCCGCACCCGTACGCCGCTCTCAATTACCCGAAAGTAATCTACCGCTCGGCTTGCA
TGTGTTAGGCCTCCCGCTAGCGTCTCTGAGCAGTCAACTCGT
>1278
CTGCTGCCGCCCGTAGGAGTCTGGTCCGTGTCTCAGTACCAGTGTGGGGGATCACCCTCTCAGGCCCCCTAAA
GATCGTCGCCTTGGTGAGCCGTTACCTCACCAACTAGCTAATCTTGCGCGTGCCCATCTCTATCCACCGTAGT

TTTCAATATCACCCGATGCCGAATAATATATTATGGGGTATTAATCTTCTTCGAAAGCTATCCCCTGATAAAG
GCAGGTTGCACACGTGTTCCGCACCCGTACGCCGCTCTCAATTACCCGAAAGTAATCTACCGCTCGGCTTGCA
TGTGTTAGGCCTCCCGCTAGCGTTCATCCTCGAGCCAGG
>1279
CTGCTGCCGCCCGTAGGAGTCTGGTCCGTGTCTCAGTACCAGTGTGGGGGATCACCCTCTCAGGCCCCCTAAA
GATCGTCGCCTTGGTGAGCCGTTACCTCACCAACTAGCTAATCTTGCGCGTGCCCATCTCTATCCACCGTAGT
TTTCAATATCACCCGATGCCGAATAATATATTATGGGGTATTAATCTTCTTCGAAAGGCTATCCCCCTGATAA
AGGCAGGTTGCACACGTGTTCCGCACCCGTACGCCGCTCTCAATTACCCGAAAGTAATCTACCGCTCCGGCTT
GCATGTGTTAGGCCTCCCGCTAGCGTTCATCGTCGAGCC
>1280
CTGCTGCCGCCCGTAGGAGTCTGGACCGTGTCTCAGTTCCAGTGTGACTGATCATCCTCTCAGACCAGTACGG
ATCGTCGCCTTGGTAGGCCTTTACCCCACCAACTAGCTAATCCGAACCTAGGCTCATCTGATAGCGTGAGGTC
CGAAGTCCCCCACTTCTCCCTCAGGACGTATGCGGTATTAGCGCCCGTTTCCGGACGTATCCCCACTACCAGG
CAGATTCCCTAGGCATTACTCACCCGTCCGCCGCTGAATCCAGGAGCAAGCCCCCTCATCCGCTCGACTGCAT
GTGTTAGGCCTGCCGCCAGCGTCAATCTGAGCCAGGATCAAACTCT
>1281
CTGCTGCCGCCCGTAGGAGTCTGGACCGTGTCTCAGTTCCAGTGTGACTGATCATCCTCTCAGACCAGTACGG
ATCGTCGCCTTGGTAGGCCTTTACCCCACCAACTAGCTAATCCGACCTAGGCTCATCTGATAGCGTGAGGTCC
GAAGATCCCCCACTTCTCCCTCAGGACGTATGCGGTATTAGCGCCCGTTTCCGGACGTATCCCCACTACCAGG
CAGATTCCTAGGCATTACTCACCCGTCCGCCGCTGAATCCAGGAGCAAGCTCCCTCATCCGCTCGACTGCATG
TGTTAGGCCTGCCGCCAGCGTCAATCTGAGCCAGGATCAAACTCT
>1282
CTGCTGCCGCCCGTAGGAGTCTGGTCCGTGTCTCAGTACCAGTGTGGGGGATCACCCTCTCAGGCCCCCTAAA
GATCGTCGCCTTGGTGAGCCGTTACCTCACCAACTAGCTAATCTTGCGCGTGCCCATCTCTATCCACCGTAGT
TTTCAATATCACCCGATGCCGAATAATATATTATGGGGTATTAATCTTCTTCGAAAGGCTATCCCCCTGATAA
AGGCAGGTTGCACACGTGTTCCGCACCCGTACGCCGCTCTCAATTACCCGAAAGTAATCTACCGCTCCGGCTT
GCATGTGTTAGGCCTCCCGCTAGCGTTCATCCGTCGAGCCAGG
>1283
CTGCTGCCGCCCGTAGGAGTCTGGACCGTGTCTCAGTTCCAGTGTGACTGATCATCTCTCAGACCAGTTACGG
ATCGTCGCCTTGGTAGGCCTTTACCCCACCAACTAGCTAATCTTGCGCGTGCCCATCTCTATCCACCGTAGTT
TTCAATATCACCCGATGCCGAATAATATATTATGGGGTATTAATCTTCTCGAAAGCTATCCCCTGATAAGGCA
GGTTGCACACGTGTTCCGCACCCGTACGCCGCTCTCAATCACCCGAAAGTAATCTACCGCTCGGCTTGCATGT
GTTAGGCCTCCCGCTAGCGTCATCCTGAGCCAGGATCAACTCT
>1284
CTGCTGCCGCCCGTAGGAGTCTGGTCCGTGTCTCAGTACCAGTGTGGGGGATCACCCTCTCAGGCCCCCTAAAG
ATCGTCGCCTTGGTGAGCCGTTACCTCACCAACTAGCTAATCTTGCGCGTGCCCATCTCTATCCACCGTAGTT
TCAATATCACCCGATGCCGAATAATATATTATGGGGTATTAATCTTCTTCGAAGGCTATCCCCTGAATAAAGG
CAGGTTGCACACGTGTTCCGCACCCGTACGCCGCTCTCAATTACCCGAAAGTAATCTACCGCTCGGCTTGCAT
GTGTTAGGCCTCCCGCTAGCGTTCATTCCTG
>1285
CTGCTGCCGCCCGTAGGAGTCTGGTCCGTGTCTCAGTACCAGTGTGGGGGATCACCCTCTCAGGCCCCCCTAA
AGATCGTCGCCTTGGTGGCCGTTACCTCACCAACTAGCTAATCTTGCGCGTGCCCCATCTCTATCCACCGTAG
TTTTCAATATCACCCGATGCCGAATAATATATTATGGGGTATTTAATCTTCTTCGAAAGGCTATCCCCCTGAT
AAAGGCAGGTTGCACACGTGTTCCGCACCCGTACGCCGCTCTCAATTACCCCGAAAGTAATCTACCGCTCGGC
TTGCATGTGTTAGGCCTCCCGCTAGCGTTCTCCTGAGC
>1286
CTGCTGCCGCCCGTAGGAGTCTGGTCCGTGTCTCAGTACCAGTGTGGGGGATCACCCTCTCAGGCCCCCTAAA
GATCGTCGCCTTGGTGAGCCGTTACCTCACCAACTAGCTAATCTTGCGCGTGCCCATCTCTATCCACCGTAGT
TTTCAATATCACCCGATGCCGAATAATATATTATGGGGTATTAATCTTCTTCGAAAGGCTATCCCCTGATAAA
GGCAGGTTGCACACGTGTTCCGCACCCGTACGCCGCTCTCAATTACCCGAAAGTAATCTACCGCTCGGCTTGC
ATGTGTTAGGCCTCCCGCTAGCGTTCATCCTGAGCCAGGATCAAACTCT
>1287
CTGCTGCCGCCCGTAGGAGTCTGGTCCGTGTCTCAGTACCAGTGTGGGGGATCACCCTCTCCAGGCCCCTAAA
GATCGTCGCCTTGGTGAGCCGTTACCTCACCAACTGGCTAATCTTGCGCGTGCCCATCTCTATCCACCGTAGT

TTTCAATATCACCCGATGCCGAATAATATATTATGGGGTATTAATTCTTCTTCGAAAGGCTATCCCCCTGATA
AAGGCAGGTTGCACACGTGTTCCGCACCCGTACGCCGCTCTCAATTACCCGAAAGTAATCTACCGCTCGGCTT
GCATGTGTTAGGCCTCCCGCTAGCGTTCATCCTGAGCCAGGATCAAACTCT
>1288
CTGCTGCCGCCCGTAGGAGTCTGGTCCGTGTCTCAGTACCAGTGTGGGGGATCACCCTCTCAGGCCCCCTAAA
GATCGTCGCCTTGGTGAGCCGTTACCTCACCAACTAGCTAATCTTGCGCGTGCCCATCTCTATCCACCGTAGT
TTTCAATATCACCCGATGCCGAATAATATATTATGGGGTATTAATCTTCTTCGAAAGGCTATCCCCCTGATAA
AGGCAGGTTGCACACGTGTTCCGCACCCGTACGCCGCTCTCAATTACCCGAAAGTAATCTACCGCTCCGGCTT
GCATGTGTTAGGCCTCCCGCTAGCGTTCATCCTGAGCCAGGATCAAACTCT
>1289
CTGCTGCCGCCCGTAGGAGTCTGGACCGTGTCTCAGTTCCAGTGTGACTGATCATCCTCTCAGACCAGTACGG
ATCGTCGCCTTGGTAGGCCTTTTACCCCACCAACTAGCTAATCCGACCTAGGCTCATCTGATAGCGTGAGGTC
CGAAGTCCCCCACTTCCCCCTCAGGACGTATGCGGTATTAGCGCCCGTTCCGGACGATCCCCACTACCAGGCA
GATTCCTAGGCATTACTCACCCGTCCGCCGCTGAATCCAGGAGCAAGCTCCCTTCATCCGCTCGACTGCATGT
GTTAGGCCTGCCGCCAGCGTCAATCTGAGCCAGGATCAAACATCT
>1290
CTGCTGCCGCCCGTAGGAGTCTGGTCTGTGTCTCAGTACCAGTGTGGGGGATCACCCTCTCAGGCCCCCTAAA
GATCGTCGCCTTGGTGAGCCGTTACCTCACCAACTAGCTAATCTTGCGCGTGCCCATCTCTATCCACCGTAGT
TTTCAATATCACCCGATGCCGAATAATATATTATGGGGTATTAATCTTCTTCGAAGGCTATCCCCCTGAATAA
AGGCAGGTTGCACACGTGTTCCGCACCCGTACGCCGCTCTCAATTACCCGAAAGTAATCTACCGCTCGGCTTG
CATGTGTTAGGCCTCCCGCTAGCGTTCATCCTGAGCCAGGATCAACTCT
>1291
CTGCTGCCGCCCGTAGGAGTCTGGTCCGTGTCTCAGTACCAGTGTGGGGGATCACCCTCTCAGGCCCCCTAAA
GATCGTCGCCTTGGTGAGCCGTTACCTCACCAACTAGCTAATCTTGCGCGTGCCCATCTCTATCCACCGTAGT
TTTCAATATCACCCGATGCCGAATAATATATTATGGGGTATTAATCTTCTCGAAAGGCTATCCCCTGATAAAG
GCAGGTTGCACACGTGTTCCGCACCCGTACGCCGCTCTCAATTACCCGAAAGTAATCTACCGCTCGGCTTGCA
TGTGTTAGGCCTCCCGCTAGCGTTCATCCTGAGCCA
>1292
CTGCTGCCGCCCGTAGGAGTCTGGACCGTGTCTCAGTTCCAGTGTGACTGATCATCCTCTCAGACCAGTACGG
ATCGTCGCCTTGGTAGGCCTTTACCCCACCAACTAGCTAATCCGACCTAGGCTCATCTGATAGCGTGAGGTCC
GAAGATCCCCCACTTCTCCCTCAGGACGTATGCGGTATTAGCGCCCGTTTCCGGACGTATCCCCACTACCAGG
CAGATTCCTAGGCATTACTCACCCGTCCGCCGCTGAATCCAGAGCAAGCTCCCTTCATCCGCTCGACTGCATG
TGTTAGGCCTGCCGCCAGCGTTCAATCTGAGCCAGGATCAAACTCT
>1293
CTGCTGCCGCCCGTAGGAGTCTGGTCCGTGTCTCAGTACCAGTGTGGGGGATCACCCTCTCAGGCCCCCTAAA
GATCGTCGCCTTGGTGAGCCGTTACCTCACCAACTAGCTAATCTTGCGCGTGCCCATCTCTATTCCACCGTAG
TTTTCAATATCACCCGATGCCGAATAATATATTTATGGGGTATTTAATTCTTCTCGAAAGGCTATCCCCTGAT
AAAGGCAGGTTGCACACGTGTTCCGCACCCGTACGCCGCTCTCAATTACCCGAAAGTAATCTACCGCTCCGGC
TTGCATGTGTTAGGCCTCCCGCTAGCGT
>1294
CTGCTGCCGCCCGTAGGAGTCTGGTCCGTGTCTCAGTACCAGTGTGGGGGATCACCCTCTCAGGCCCCCTAAA
GATCGTCGCCTTGGTGAGCCGTTACCTCACCAACTAGCTAATCTTGCGCGTGCCCATCTCTATCCACCGTAGT
TTTCAATATCACCCGATGCCGAATAATATATTATGGGGTATTAATCTTCTTCGAAAGGCTATCCCCCTGATAA
AGGCAGGTTGCACACGTGTTCCGCACCCGTACGCCGCTCTCAATTACCCGAAAGTAATCTACCGCTCGGCTTG
CATGTGTTAGGCCTCCCGCTAGCGTTCATCCTGAGCCAGGATCAACTCT
>1295
CTGCTGCCGCCCGTAGGAGTCTGGTCCGTGTCTCAGTACCAGTGTGGGGGATCACCCTCTCAGGCCCCCTAAA
GATCGTCGCCTTGGTGAGCCGTTACCTCACCAACTAGCTAATCTTGCGCGTGCCCATCTCTATCCACCGTAGT
TTTCAATATCACCCGATGCCGAATAATATATTATGGGGTATTAATCTTCCTTCGAAAGGCTATCCCCCTGATA
AAGGCAGGTTGCACACGTGTTCCGCACCCGTACGCCGCTCTCAATTACCCGAAAGTAATCTACCGCTCGGCTT
GCATGTGTTAGGCCTCCCGCTAGCGTTCATCCGTGACGCAGGATCAAACTCCT
>1296
CTGCTGCCGCCCGTAGGAGTCTGGTCCGTGTCTCAGTACCAGTGTGGGGGATCACCCTCTCAGGCCCCCTAAA
GATCGTCGCCTTGGTGAGCCGTTACCTCACCAACTAGCTAATCTTGCGCGTGCCCATCTCTATCCACCGTAGT

TTTCAATATCACCCGATGCCGAATAATATATTATGGGGTATTAATCTTCTTCGAAAGGCTATCCCCCTGATAA
AGGCAGGTTGCACACGTGTTCCGCACCCGTACGCCGCTCTCAATTACCCGAAAGTAATCTACCGCTCCGGCTT
GCATGTGTTAGGCCTCCCGCTAGCGTTCATCCTGAGCCAGATCAAACTCT
>1297
CTGCTGCCGCCTGTAGGAGTCTGGTCCGTGTCTCAGTACCAGTGTGGGGGATCACCCTCTCAGGCCCCCTAAA
GATCGTCGCCTTGGTGAGCCGTTACCTCACCAACTAGCTAATCTTGCGCGTGCCCATCTCTATCCACCGTAGT
TTTCAATATCACCCGATGCCGAATAATATATTATGGGGTATTAATCTTCTTCGAAAGGCTATCCCCCTGATAA
AGGCAGGTTGCACACGTGTTCCGCACCCGTACGCCGCTCTCAATTACCCGAAAGTAATCTACCGCTCGGCTTG
CATGTGTTAGGCCTCCCGCTAGCGTTCATCCTGAGCCAGGATCAACTCT
>1298
CTGCTGCCGCCCGTAGGAGTCTGGTCCGTGTCTCAGTACCAGTGTGGGGGATCACCCTCTCAGGCCCCCTAAA
GATCGTCGCCTTGGTGAGCCGTTACCTCACCAACTAGCTAATCTTGCGCGTGCCCATCTCTATCCACCGTAGT
TTTCAATATCACCCGATGCCGAATAATATAATTATGGGGTATTAATCTTCTCGAAGGCTATCCCCTGATAAGG
CAGGTTGCACACGTGTTCCGCACCCGTACGCCGCTCTCAATTACCCGAAAGTAATCTACCGCTCGGCTTGCAT
GTGTTAGGCCTCCCGCTAGCGTTCATCCTG
>1299
CTGCTGCCGCCCGTAGGAGTCTGGTCCGTGTCTCAGTACCAGTGTGGGGGATCACCCTCTCAGGCCCCCTAAA
GATCGTCGCCTTGGTGAGCCGTTACCTCACCAACTAGCTAATCTTGCGCGTGCCCATCTCTATCCACCGTAGT
TTTCAATATCACCCGATGCCGAATAATATATTATGGGGTATTAATCTTCTTCGAAAGGCTATCCCCCTGATAA
AGGCAGGTTGCACACGTGTTCCGCACCCGTACGCCGCTCTCAATTACCCGAAAGTAATCTACCGCTCGGCTTG
CATGTGTTAGGCCTCCCGCTAGCGTTCTCCTCGAGCAGGA
>1300
CTGCTGCCGCCCGTAGGAGTCTGGTCCGTGTCTCAGTACCAGTGTGGGGGATCACCCTCTCAGGCCCCCTAAA
GATCGTCGCCTTGGTGAGCCGTTACCTCACCAACTAGCTAATCTTGCGCGTGCCCATCTCTATCCACCGTAGT
TTTCAATATCACCCGATGCCGAATAATATAATTATGGGGTATTAATCTTCTCGAAAGGCTATCCCCTGATAAA
GGCAGGTTGCACACGTGTTCCGCACCCGTACGCCGCTCTCAATTACCCGAAAGTAATCTACCGCTCCGGCTTG
CATGTGTTAGGCCTCCCGCTAGCGTCATCCTGAGCAGGATCAACTCCT
>1301
CTGCTGCCGCCCGTAGGAGTCTGGTCCGTGTCTCAGTACCAGTGTGGGGGATCACCCTCTCAGGCCCCCTAAA
GATCGTCGCCTTGGTGAGCCGTTACCTCACCAACTAGCTAATCTTGCGCGTGCCCATCTCTATCCACCGTAGT
TTCAATATCACCCGATGCCGAATAATATATTATGGGGTATTAATCTTCTTCGAAAGCTATCCCCCTGATAAAG
GCAGGTTGCACACGTGTTCCGCACCCGTACGCCGCTCTCAATTACCCGAAAGTAATCTACCGCTCGGCTTGCA
TGTGTTAGGCCTCCCGCTAGCGTTCATCCTGAGCCAGGATCAACTCT
>1302
CTGCTGCCGCCCCGTAGGAGTCTGGTCCGTGTCTCAGTACCAGTGTGGGGGGATCACCCTCTCAGGCCCCCCT
AAAGATCGTCGCCTTGGTGAGCCGTTACCCCCACCAACTAGCTAATCTTGCGCGTGCCCCATCTCTATCCACC
GTAGTTTTTCAATATCACCCGATGCCGAATAATATATTTATGGGGTATTTAATCTTCTTCGAAAGGCTATCCC
CTGATAAAGGCAGGTTGCACACGTGTTCCGCACCCGTACGCCGCTCTCAATTACCCCGAAAGTAATCTACCGC
TCGGCTTGCATGTGTTAGGCCTCCCGCTAGCGTTCATCCTGAGCAGGATCAAACTCT
>1303
CTGCTGCCGCCCGTAGGAGTCTGGTCCGTGTCTCAGTACCAGTGTGGGGGGATCACCCCTCTCAGGCCCCCCT
AAAGATCGTCGCCTTGGTGAGCCGTTACCTCACCCAACTAGCTAATCTTGCGCGTGCCCCATCTCTATCCACC
GTAGTTTTCAATATCACCCGATGCCGAATAATATATTTATTGGGGTATTTAATCTTCTTCGAAAGGCTATCCC
CCTGATAAAGGCAGGTTGCACACGTGTTCCGCACCCGTACGCCGCTCTCAATTACCCCCGAAAGTAATCTACC
GCTCGGCTTGCATGTGTTTAGGCCTCCCGCTAGCGTTCTCCTCGAG
>1304
CTGCTGCCGCCCGTAGGAGTCTGGTCCGTGTCTCAGTACCAGTGTGGGGGATCACCCTCTCAGGCCCCCTAAA
GATCGTCGCCTTGGTGAGCCGTTACCTCACCAACTAGCTAATCTTGCGCGTGCCCATCTCTATCCACCGTAGT
TTTCAATATCACCCGATGCCGAATAATATATTATGGGGTATTAATCTTCTTCGAAAGGCTATCCCCTGATAAA
GGCAGGTTGCACACGTGTTCCGCACCCGTACGCCGCTCTCAATTACCCGAAAGTAATCTACCGCTCGGCTTGC
ATGTGTTAGGCCTCCCGCTAGCGTTCATCTGAGCCAGGATCAACTCT
>1305
CTGCTGCCGCCCGTAGGAGTCTGGTCCGTGTCTCAGTACCAGTGTGGGGGATCACCCTCTCAGGCCCCCTAAA
GATCGTCGCCTTGGTGAGCCGTTACCTCACCAACTAGCTAATCTTGCGCGTGCCCATCTCTATCCACCGTAGT

TTTCAATATCACCCGATGCCGAATAATATATTATGGGGTATTAATCTTCTTCGAAAGGCTATCCCCTGATAAA
GGCAGGTTGCACACGTGTTCCGCACCCGTACGCCGCTCTCAATTACCCGAAAGTAATCTACCGCTCGGCTTGC
ATGTGTTAGGCCTCCCGCTAGCGTTCATCCGTCGAGCCAGGATCAAACTCT
>1306
CTGCTGCCGCCCGTAGGAGTCTGGACCGTGTCTCAGTTCCAGTGTGACTGATCATCCTCTCAGACCAGTACGG
ATCGTCGCCTTGGTAGGCCTTTACCCCACCAACTAGCTAATCCGACCTAGGCTCATCTGATAGCGTGAGGTCC
GAAGTCCCCCACTTCTCCCTCAGGACGTATGCGGTATTAGCGCCCGTTTCCGGACGTATCCCCACTACCAGGC
AGATTCCTAGGCATTACTCACCCGTCCGCCGCTGAATCCAGGAGCAAGCTCCCTCATCCGCTCGACTGCATGT
GTTAGGCCTGCCGCCAGCGTTCAATCTGAGCCAGGATCAAACTCT
>1307
CTGCTGCCGCCCGTAGGAGTCTGGTCCGTGTCTCAGTACCAGTGTGGGGGATCACCCTCTCAGGCCCCCTAAA
GATCGTCGCCTTGGTGAGCCGTTACCTCACCAACTAGCTAATCTTGCGCGTGCCCACCTCTATCCACCGTAGT
TTTCAATATCACCCGATGCCGAGTAATATATTTATGGGGTATTTAATCTTTCTCGAAAGGCTTATCCCCCTGA
TAAAGGCAGGTTGCACACGTGTTCCGCACCCGTACGCCGCTCTCAATTACCCGAAAGTAATCTACCGCTCGGC
TTGCATGTGTTAGGCCTCCCGCTAGCGTTCTCCTGAGCAGGATCAACTCCGT
>1308
CTGCTGCCCGCCCCGTAGGAGTCTGGTCCGTGTCTCAGTACCAGTGTGGGGGATCACCCCTCTCAGGCCCCCT
AAAGATCGTCGCCTTGGTGAGCCCGTTACCTCACCAACTAGCCTTAATCTTGCGCGTGCCCCATCTCTATCCC
ACCCGTAGTTTTTTCAATATCACCCGATGCCCGAATAATATATTTATTGGGGTATTTAATTCTTCTCGAAAGG
CTATCCCCCTGATAAAGGCAGGTTTGCACACGTGTTCCCGCACCCGTACGCGCTCTCAATTACCCGAAAGTAA
TCTACCGCTCGGCTTGCATGTGTTTAGGCCTTCCCGCTAGCGTTCATCCTGAGCCAGGCATCGAAACTCT
>1309
CTGCTGCCGCCCGTAGGAGTCTGGTCCGTGTCTCAGTACCAGTGTGGGGGATCACCCTCTCAGGCCCCCTAAA
GATCGTCGCCTTGGTGAGCCGTTACCTCACCAACTAGCTAATCTTGCGCGTGCCCATCTCTATCCACCGTAGT
TTTCAATATCACCCGATGCCGAATAATATATTATGGGGTATTAATCTTCTTCGAAAGGCTATCCCCCTGATAA
AGGCAGGTTGCACACGTGTTCCGCACCCGTACGCCGCTCTCAATTACCCGAAAGTAATCTACCGCTCGGCTTG
CATGTGTTAGGCCTGCCGCCAGCGTCAATCGTGAGCCAGGATCAAACTCT
>1310
CTGCTGCCGCCCGTAGGAGTCTGGTCCGTGTCTCAGTACCAGTGTGGGGGATCACCCTCTCAGGCCCCCTAAA
GATCGTCGCCTTGGTGAGCCGTTACCTCACCAACTAGCTAATCTTGCGCGTGCCCATCTCTATCCACCGTAGT
TTCAATATCACCCGATGCCGAATAATATATTATGGGGTATTAATCTTCTCGAAAGGCTATCCCCCTGATAAAG
GCAGGTTGCACACGTGTTCCGCACCCGTACGCCGCTCTCAATTACCCGAAAGTAATCTACCGCTCGGCTTGCA
TGTGTTAGGCCTCCCGCTAGCGTTCATCCTCGAGCCAGGATCAAACTCT
>1311
CTGCTGCCGCCCGTAGGAGTCTGGACCGTGTCTCAGTTCCAGTGTGACTGATCATCCTCTCAGACCAGTACGG
ATCGTCGCCTTGGTAGGCCTTTACCCCACCAACTAGCTAATCCGACCTAGGCTCATCTGATAGCGTGAGGTCC
GAAGATCCCCCACTTCTCCCTCAGGACGTATGCGGTATTAGCGCCCGTTTCCGGACGATCCCCGCACTACCAG
GCAGATTCCTAGGCATTACTCACCCGTCCGCCGCTGAATCCAGGAGCAAGCTCCCTCACCGCTCGATGCATGT
GTTAGGCCTGCCGCCAGCGTCAATCTGAGCCAGGATCAAACTCT
>1312
CTGCTGCCGCCCGTAGGAGTCTGGTCCGTGTCTCAGTACCAGTGTGGGGGATCACCCTCTCAGGCCCCCTAAA
GATCGTCGCCTTGGTGAGCCGTTACCTCACCAACTAGCTAATCTTGCGCGTGCCCATCTCTATCCACCGTAGT
TTTCAATATCACCCGATGCCGAATAATATATTTATGGGGTATTTAATCTTTCTCGAAAGCTATCCCCTGATAA
AGGCAGGTTGCACACGTGTTCCGCACCCGTACGCCGCTCTCAATTACCCGAAAGTAATCTACCGCTCCGGCTT
AGCATGTG
>1313
CTGCTGCCGCCCGTAGGAGTCTGGTCCGTGTCTCAGTACCAGTGTGGGGGATCACCCTCTCAGGCCCCCTAAA
GATCGTCGCCTTGGTGAGCCGTTTACCTCACCAACTAGCTTAATCTTGCGCGTGCCCATCTCTATCCACCGTA
GTTTTCAATATCACCCGATGCCGAATAATATATTATTGGGGTATTAATCTTCTTCGAAAGGCTATCCCCCTGA
TAAAGGCAGGTTGCACACGTGTTCCGCACCCGTACGCCGCTCTCAATTACCCGAAAGTAATCTACCGCTCGGC
TTGCATGTGTTAGGCCTCCCGCTAGCGTTCATCCTGAGCCAGGATCAAACTCT
>1314
CTGCTGCCGCCCGTAGGAGTCTGGACCGTGTCTCAGTTCCAGTGTGACTGATCATCCTCTCAGACCAGTACGG
ATCGTCGCCTTGGTAGGCCTTTACCCCACCAACTAGCTAATCCGACCTAGGCTCATCTGATAGCGTGAGGTCC

GAAGTCCCCCACTTCTCCCTCAGGACGTATGCGGTATTAGCGCCCGTTTCCGGACGATCCCCACTACCAGGCA
GATTCCTAGGCATTACTCACCCGTCCGCCGCTGAATCCAGGAGCAAGCCCCCTTCATCCGCTCGACTGCATGT
GTTAGGCCTGCCGCCAGCGTCAATCTGAGCCAGGATCAAACTCT
>1315
CTGCTGCCGCCCGTAGGAGTCTGGTCCGTGTCTCAGTTCCAGTGTGACTGATCATCCTCTCAGACCAGTACGG
ATCGTCGCCTTGGTAGGCCTTTACCCCACCAACTAGCTAATCCGACCTAGGCTCATCTGATAGCGTGAGGTCC
GAAGTCCCCCACTTCTCCCTCAGGACGTATGCGGTATTAGCGCCCGTTTCCGGACGTATCCCCCACTACCAGG
CAGATTCCTAGGCATTACTCACCCGTCCGCCGCTGAATCCAGGAGCAAGCCCCCTTCATCCGCTCGACTGCAT
GTGTTAGGCCTGCCGCCAGCGTCAATCTGAGCCAGGATCAAACTCT
>1316
CTGCTGCCGCCCGTAGGAGTCTGGTCCGTGTCTCAGTACCAGTGTGGGGGATCACCCTCTCAGGCCCCCTAAA
GATCGTCGCCTTGGTGAGCCGTTACCTCACCAACTAGCTAATCTTGCGCGTGCCCATCTCTATCCACCGTAGT
TTTCAATATCACCCGATGCCGAATAATATATTATGGGGTATTAATCTTCTTCGAAAGGCTATCCCCCTGATAA
AGGCAGGTTGCACACGTGTTCCGCACCCGTACGCCGCTCTCAATTACCCGAAAGTAATCTACCGCTCGGCTTG
CATGTGTTAGGCCTCCCGCTAGCGTTCATCCTGAGCCAGGATCAAACTCT
>1317
CTGCTGCCGCCCGTAGGAGTCTGGTCCGTGTCTCAGTACCAGTGTGGGGGATCACCCTCTCAGGCCCCCTAAA
GATCGTCGCCTTGGTGAGCCGTTACCTCACCAACTAGCTAATCTTGCGCGTGCCCATCTCTATCCACCGTAGT
TTTCAATATCACCCGATGCCGAATAATATATTATGGGGTATTAATCTTCCTTCGAAAGGCTATCCCCCTGATA
AAGGCAGGTTGCCACACGTGTTCCGCACCCGTACGCCGCTCTCAATTACCCGAAAGTAATCTACCGCTCGGCT
TGCATGTGTTAGGCCTCCCGCTAGCGTTCATCCTGAGCCAGGATCAAACTCT
>1318
CTGCTGCCGCCCGTAGGAGTCTGGTCCGTGTCTCAGTACCAGTGTGGGGGATCACCCTCTCAGGCCCCCTAAA
GATCGTCGCCTTGGTGAGCCGTTACCTCACCAACTAGCTAATCTTGCGCGTGCCCATCTCTATCCACCGTAGT
TTTCAATATCACCCGATGCCGAATAATATATTATGGGGTATTAATCTTCTCGAAAGCTATCCCCCTGATAAAG
GCAGGTTGCAACACGTGTTCCGCACCCGTACGCCGCTCTCAATTACCCGAAAGTAATCTACCGCTCGGCTTGA
CATGTGTTAGGCCTCCCG
>1319
CTGCTGCCGCCCGTAGGAGTCTGGTCCGTGTCTCAGTACCAGTGTGGGGGATCACCCTCTCAGGCCCCCTAAA
GATCGTCGCCTTGGTGAGCCGTTACCTCACCAACTAGCTAATCTTGCGTGTGCCCATCTCTATCCACCGTAGT
TTTCAATATCACCCGATGCCGAATAATATATTATGGGGTATTAATCTTCTTCGAAAGGCTATCCCCTGATAAA
GGCAGGTTGCACACGTGTTCCGCACCCGTACGCCGCTCTCAATTACCCGAAAGTAATCTACCGCTCGGCTTGC
ATGTGTTAGGCCTCCCGCTAGCGTTCATCCTGAGCCAGGATCAAACTCT
>1320
CTGCTGCCGCCCGTAGGAGTCTGGACCGTGTCTCAGTTCCAGTGTGACTGATCATCCTCTCAGACCAGTACGG
ATCGTCGCCTTGGTAGGCCTTTACCCCACCAACTAGCTAATCCGACCTAGGCTCATCTGATAGCGTGAGGTCC
GAAGATCCCCCACTTCTCCCTCAGGACGTATGCGGTATTAGCGCCCGTTTCCGGACGTATCCCCCACTACCAG
GCAGATTCCTAGGCATTACTCACCCGTCCGCCGCTGAATCCAGGAGCAAGCCCCTCCATCCGCTCGACTGCAT
GTGTTAGGCCTGCCGCCAGCGTTCAATCTGAGCCAGGATCAAACTCT
>1321
CTGCTGCCGCCCGTAGGAGTCTGGTCCGTGTCTCAGTACCAGTGTGGGGGATCACCCTCTCAGGCCCCCTAAA
GATCGTCGCCTTGGTGAGCCGTTACCTCACCAACTAGCTAATCTTGCGCGTGCCCATCTCTATCCACCGTAGT
TTTCAATATCACCCGATGCCGAATAATATATTATGGGGTATTAATCTTCTTCGAAGGCTATCCCCTGATAAAG
GCAGGTTAGCACACGTGTTCCGCACCCGTACGCCGCTCTCAATTACCCGAAAGTAATCTACCGCTCGGCTTGC
ATGTGTTAGGCCTCCCGCTAGCGTTCATCCTGAGCCAGGATCAAACTCT
>1322
CTGCTGCCGCCCGTAGGAGTCTGGTCCGTGTCTCAGTACCAGTGTGGGGGATCACCCTCTCAGGCCCCCTAAA
GATCGTCGCCTTGGTGAGCCGTTACCTCACCAACTAGCTAATCTTGCGCGTGCCCATCTCTATCCACCGTAGT
TTTCAATATCACCCGATGCCGAATAATATATTATGGGGTATTTAATCTTCTTCGAAAGGCTATCCCCCTGATA
AAGGCAGGTTGCACACGTGTTCCGCACCCGTACGCCGCTCTCAATTACCCGAAAGTAATCTACCGCTCGGCTT
GCATGTGTTAGGCCTCCCGCTAGCGTTCATCCTGAGCCAGGATCAAACTCT
>1323
CTGCTGCCGCCCGTAGGAGTCTGGTCCGTGTCTCAGTACCAGGTGTGGGGGATCACCCTCTCAGGCCCCCTAA
AGATCGTCGCCTTGGTGAGCCGTTACCTCACCAACTAGCTAATCTTGCGCGTGCCCATCTCTATCCACCGTAG

TTTTCAATATCACCCGATGCCGAATAATATATTATGGGGTATTAAATCTTCTTCGAAAGGCTATCCCCCTGAT
AAAGGCAGGTTGCACACGTGTTCCGCACCCGTACGCCGCTCTCAATTACCCGAAAGTAATCTACCGCATCGGC
TTGCATGTGTTAGGCCTCCCGCTAGCGTTCATCCTGAGCCAGGATCAAACTCT
>1324
CTGCTGCCGCCCGTAGGAGTCTGGTCCGTGTCTCAGTACCAGTGTGGGGGATCACCCTCTCAGGCCCCCTAAA
GATCGTCGCCTTGGTGAGCCGTTACCTCACCAACTAGCTAATCTTGCGCGTGCCCATCTCTATCCACCGTAGT
TTTCAATATCACCCGATGCCGAATAATATATTATGGGGTATTAATCTTCTCGAAAGCTATCCCCCTGATAAAG
GCAGGTTGCACACGTGTTCCGCACCCGTACGCCGCTCTCAATTACCCGAAAGTAATCTACCGCATCGGCTTGC
ATGTGTTAGGCCTCCCGCTAGCGTTCTCCTGAGCAGGAT
>1325
CTGCTGCCGCCCGTAGGAGTCTGGTCCGTGTCTCAGTACCAGTGTGACTGATCATCCTCTCAGACCAGTACGG
ATCGTCGCCTTGGTAGGCCTTTACCCCACCAACTAGCTAATCCGACCTAGGCTCATCTGATAGCGTGAGGTCC
GAAGATCCCCCACTTCTCCCTCAGGACGTATGCGGTATTAGCGCCCGTTTCCGGACGTATCCCCACTACCAGG
CAGATTCCTAGGCATTACTCACCCGTCCGCCGCTGAATCCAGGAGCAAGCTCCCTTCATCCGCTCGACTGCAT
GTGTTAGGCCTGCCGCCAGCGTTCAATCTGAGCCAGGATCAAACTCT
>1326
CTGCTGCCGCCCGTAGGAGTCTGGTCCGTGTCTCAGTACCAGTGTGGGGGATCACCCTCTCAGGCCCCCTAAA
GATCGTCGCCTTGGTGAGCCGTTACCTCACCAACTAGCTAATCTTGCGCGTGCCCATCTCTATCCACCGTAGT
TTTCAATATCACCCGATGCCGAATAATATATTATGGGGTATTAATCTTCTTCGAAAGGCTATCCCCCTGATAA
AGGCAGGTTGCACACGTGTTCCGCACCCGTACGCCGCTCTCAATTACCCGAAAGTAATCTACCGCTCGGCTTG
CATGTGTTAGGCCTCCCGCTAGCGTTCTCCTGAGCAGGATCAACTCGT
>1327
CTGCTGCCGCCCGTAGGAGTCTGGTCCGTGTCTCAGTACCAGTGTGGGGGATCACCCTCTCAGGCCCCCTAAA
AGATCGTCGCCTTGGTGAGCCGTTACCTCACCAACTAGCTAATCTTGCGCGTGCCCATCTCTATCCACCGCAG
TTTTCAATATCACCCGATGCCGAATAATATATTATGGGGTATTAATCTTCTCGAAAGGCTATCCCCTGATAAA
GGCAGGTTGCACACGTGTTCCGCACCCGTACGCCGCTCTCAATTACCCGAAAGTAATCTACCGCTCGGCTTGC
ATGTGTTAGGCCTCCCGCTAGCGTTCATCCTGAGCAGGATCAACTCT
>1328
CTGCTGCCGCCCGTAGGAGTCTGGACCGTGTCTCAGTTCCAGTGTGACTGATCATCCTCTCAGACCAGTACGG
ATCGTCGCCTGTGGGCCTTACCCCACCAACTAGCTAATCCGACCTAGGCTCATCTGATAGCGTGAGGTCCGAA
GATCCCCCACTTCTCCCTCAGGACGTATGCGGTATTAGCGCCCGTTTCCGGACGTATCCCCACTACCAGCAGA
TTCCTAGGCATTACTCACCCGTCCGCCGCTGAATCCAGGAGCAAGCTCCCTCATCGCTCGACTGCCATGTGTT
AGGCCTGCCGCCAGCGTCAATCTGAGCCAGGATCAACTCT
>1329
CTGCTGCCGCCCGTAGGAGTCTGGTCCGTGTCTCAGTACCAGTGTGGGGGATCACCCTCTCAGGCCCCCTAAA
GATCGTCGCCTTGGTGAGCCGTTACCTCACCAACTAGCTAATCTTGCGCGTGCCCATCTCTATCCACCGTAGT
TTTCAATATCACCCGATGCCGAATAATATATTATGGGGTATTAATCGTTCTTCGAAAGGCTATCCCCCTGATA
AAGGCAGGTTGCACACGTGTTCCGCACCCGTACGCCGCTCTCAATTACCCGAAAGTAATCTACCGCTCGGCTT
GCATGTGTTAGGCCTCCCGCTAGCGTTCATCCTGAGCCAGGATCAAACTCT
>1330
CTGCTGCCGCCCGTAGGAGTCTGGTCCGTGTCTCAGTACCAGTGTGGGGGATCACCCTCTCAGGCCCCCTAAA
GATCGTCGCCTTGGTGAGCCGTTACCTCACCAACTAGCTAATCTTGCGCGTGCCCATCTCTATCCACCGTAGT
TTTCAATATCACCCGATGCCGAATAATATATTATGGGGTATTAATCTTCTTCGAAAGGCTATCCCCCTGATAA
AGGCAGGTTGCACACGTGTTCCGCACCCGTACGCCGCTCTCAATTACCCGAAAGTAATCTACCGCTCGGCTTG
CATGTGTTAGGCCTCCCGCTAGCGTTCATCCTGAGCCAGGATCAAACTCT
>1331
CTGCTGCCGCCCGTAGGAGTCTGGTCCGTGTCTCAGTACCAGTGTGGGGGATCACCCTCTCAGGCCCCCTAAA
GATCGTCGCCTTGGTGAGCCGTTACCTCACCAACTAGCTAATCTTGCGCGTGCCCATCTCTATCCACCGTAGT
TTTCAATATCACCCGATGCCGAATAATATATTATGGGGTATTAATCTTCTCGAAAGGCTATCCCCTGATAAAG
GCAGGTTGCACACGTGTTCCGCACCCGTACGCCGCTCTCAATTACCCGAAAGTAATCTACCGCTCGGCTTGCA
TGTGTTAGGCCTCCCGCTAGCGTTCATCC
>1332
CTGCTGCCGCCCGTAGGAGTCTGGTCCGTGTCTCAGTACCAGTGTGGGGGATCACCCTCTCAGGCCCCCTAAA
GATCGTCGCCTTGGTGAGCCGTTACCTCACCAACTAGCTAATCTTGCGCGTGCCCATCTCTATCCACCGTAGT

TTTCAATATCACCCGATGCCGAATAATATATTATGGGGTATTAATCTTCTTCGAAAGGCTATCCCCCTGATAA
AGGCAGGTTGCACACGTGTTCCGCACCCGTACGCCGCTCTCAATTACCCGAAAGTAATCTACCGCTCGGCTTG
CATGTGTTAGGCCTCCCGCTAGCGTTCATCCGTGAGCCAGGATCAACTCT
>1333
CTGCTGCCGCCCGTAGGAGTCTGGACCGTGTCTCAGTTCCAGTGTGACTGATCATCCTCTCAGACCAGTACGG
ATCGTCGCCTTGGTAGGCCTTTACCCCCACCAACTAGCTAATCCGACCTAGGCTCATCTGATAGCGTGAGGTC
CGAAGATCCCCCACTTCTCCCTCAGGACGTATGCGGTATTAGCGCCCGTTTCCGGACGTATCCCCACTACCAG
GCAGATTCCTAGGCATTACTCACCCGTCCGCCGCTGAATCCAGGAGCAAGCCCCCTTCATCCGCTCGACTGCA
TGTGTTAGGCCTGCCGCCAGCGTCAATCTGAGCCAGGATCAAACTCT
>1334
CTGCTGCCGCCCGTAGGAGTCTGGTCCGTGTCTCAGTACCAGTGTGGGGGGATCACCCTCTCAGGCCCCCTAA
AGATCGTCGCCTTGGTGAGCCGTTACCTCACCAACTAGCTAATCTTGCGCGTGCCCATCTCTATCCACCGTAG
TTTTCAATATCACCCGATGCCGAATAATATATTATGGGGTATTTAATCTTCTTCGAAAGGCTATCCCCTGATA
AAGGCAGGTTGCACACGTGTTCCGCACCCGTACGCCGCTCTCAATTACCCGAAAGTAATCTACCGCTCGGCTT
GCATGTGTTAGGCCTCCCGCTAGCGTTCATCCTGAGCCAGGATCAAACTCT
>1335
CTGCTGCCGCCCGTAGGAGTCTGGTCCGTGTCTCAGTACCAGTGTGGGGGATCACCCTCTCAGGCCCCCTAAA
GATCGTCGCCTTGGTGAGCCGTTACCTCACCAACTAGCTAATCTTGCGCGTGCCCATCTCTATCCACCGTAGT
TTTCAATATCACCCGATGCCGAATAATATATTATGGGGTATTAATCTTCTTCGAAAGGCTATCCCCTGATAAA
GGCAGGTTGCACACGTGTTCCGCACCCGTACGCCGCTCTCAATTACCCGAAAGTAATCTACCGCTCCGGCTTG
CATGTGTTAGGCCTCCCGCTAGCGTTCATCCTCGAGCCAGGATCAAACTCT
>1336
CTGCTGCCGCCCGTAGGAGTCTGGTCCGTGTCTCAGTACCAGTGTGGGGGATCACCCTCTCAGGCCCCCTAAA
GATCGTCGCCTTGGTGAGCCGTTACCTCACCAACTAGCTAATCTTGCGCGTGCCCATCTCTATCCACCGTAGT
TTTCAATATCACCCGATGCCGAATAATATATTATGGGGTACTAATCTTCTTCGAAAGGCTATCCCCCTGATAA
AGGCAGGTTGCACACGTGTTCCGCACCCGTACGCCGCTCTCAATTACCCGAAAGTAATCTACCGCTCCGGCTT
GCATGTGTTAGGCCTCCCGCTAGCGTTCATCCTCGAGCCAGGATCAACTCT
>1337
CTGCTGCCGCCCGTAGGAGTCTGGACCGTGTCTCAGTTCCAGTGTGACTGATCATCCTCTCAGACCAGTACGG
ATCGTCGCCTTGGTAGGCCTTACCCCACCAACTAGCTAATCCGACCTAGGCTCATCTGATAGCGTGAGGTCCG
AAGTCCCCCACTTCTCCCTCAGGACGTATGCGGTATTAGCGCCCGTTTCCGGACGTATCCCCACTACCAGGCA
GATTCCTAGGCATTACTCACCCGTCCGCCGCTGAATCCAGGAGCAAGCTCCCTCATCCGCTCGACTGCATGTG
TTAGGCCTGCCGCCAGCGTCAATCTGAGCCAGGATCAAACTCT
>1338
CTGCTGCCGCCCGTAGGAGTCTGGTCCGTGTCTCAGTACCAGTGTGGGGGATCACCCTCTCAGGCCCCCTAAA
GATCGTCGCCTTGGTGAGCCGTTACCTCACCAACTAGCTAATCTTGCGCGTGCCCATCTCTATCCACCGTAGT
TTTCAATATCACCCGATGCCGAATAATATATTATGGGGTATTAATCTTCTTCGAAAGGCTATCCCCTGATAAA
GGCAGGTTGCACACGTGTTCCGCACCCGTACGCCGCTCTCAATTACCCGAAAGTAATCTACCGCTCCGGCTTG
CATGTGTTAGGCCTCCCGCTAGCGTTCATCCTGAGCCAGGATCAACTCT
>1339
CTGCTGCCGCCCGTAGGAGTCTGGTCCGTGTCTCAGTACCAGTGTGGGGGATCACCCTCTCAGGCCCCCTAAA
GATCGTCGCCTTGGTGAGCCGTTACCTCACCAACTAGCTAATCTTGCGCGTGCCCATCTCTATCCACCGTAGT
TTTCAATATCACCCGATGCCGAATAATATATTATGGGGTATTAATCTTCTTCGAAAGCTATCCCCCTGATAAA
GGCAGGTTGCACACGTGTTCCGCACCCGTACGCCGCTCTCAATTACCCGAAAGTAATCTACCGCTCGGCTTGC
ATGTGTTAGGCCTCCCGCTAGCGTTCATCCTGAGCCAGGATCAACTCT
>1340
CTGCTGCCGCCCGTAGGGGTCTGGTCCGTGTCTCAGTACCAGTGTGGGGGATCACCCTCTCAGGCCCCCTAAA
GATCGTCGCCTTGGTGAGCCGTTACCTCACCAACTAGCTAATCTTGCGCGTGCCCATCTCTATCCACCGTAGT
TTTCAATATCACCCGATGCCGAATAATATATTATGGGGTATTAATCTTCTTCGAAGGCTATCCCCCTGATAAA
GGCAGGTTGCACACGTGTTCCGCACCCGTACGCCGCTCTCAATTACCCGAAAGTATCTACCGCTCGGCTTGCA
TGTGTTAGGCCTCCCGCTAGCGTTCTCCTGAGCAGGATCAAACTCGT
>1341
CTGCTGCCGCCCGTAGGAGTCTGGTCCGTGTCTCAGTACCAGTGTGGGGGATCACCCTCTCAGGCCCCCTAAA
GATCGTCGCCTTGGTGAGCCGTTACCTCACCAACTAGCTAATCTTGCGCGTGCCCATCTCTATCCACCGTAGT

TTTCAATATCACCCGATGCCGAATAATATATTATGGGGTATTAATCTTCTTCGAAAGGCTATCCCCCTGATAA
AGGCAGGTTGCACACGTGTTCCGCACCCGTACGCCGCTCTCAATTACCCGAAAGTAATCTACCGCTCCGGCTT
GCATGTGTTAGGCCTCCCGCTAGCGTTCATCCTCGAGCCAGGATCAACTCT
>1342
CTGCTGCCGCCCGTAGGAGTCTGGTCCGTGTCTCAGTACCAGTGTGGGGGATCACCCTCTCAGGCCCCCCAAA
GATCGTCGCCTTGGTGAGCCGTTACCTCACCAACTAGCTAATCTTGCGCGTGCCCATCTCTATCCACCGTAGT
TTTCAATATCACCCGATGCCGAATAATATATTATGGGGTATTAATCTTCTCTGAAAGGCTATCCCCTGATAAA
GGCAGGTTGCACACGTGTTCCGCACCCGTACGCCGCTCTCAATTACCCGAAAGTAATCTACCGCTCGGCTTGC
ATGTGTTAGGCCTCCCGCTAGCGTTCATCCTGAGCAGGATCAAACTCT
>1343
CTGCTGCCGCCCGTAGGAGTCTGGTCCGTGTCTCAGTACCAGTGTGGGGGATCACCCTCTCAGGCCCCCTAAA
GATCGTCGCCTTGGTGAGCCGTTACCTCACCAACTAGCTAATCTTGCGCGTGCCCATCTCTATCCACCGTAGT
TTTCAATATCACCCGATGCCGAATAATATATTATGGGGTATTAATCTTCTTCGAAAGGCTATCCCCTGATAAA
GGCAGGTTGCACACGTGTTCCGCACCCGTACGCCGCTCTCAATTACCCGAAAGTAATCTACCGCTCGGCTTGC
ATGTGTTAGGCCTCCCGCTAGCGTTCATCCGTGAGCCAGGATCAAACTCT
>1344
CTGCTGCCGCCCGTAGGAGTCTGGTCCGTGTCTCAGTACCAGTGTGGGGGATCACCCTCTCAGGCCCCCTAAA
GATCGTCGCCTTGGTGAGCCGTTACCTCACCAACTAGCTAATCTTGCGCGTGCCCATCTCTATCCACCGTAGT
TTTCAATATCACCCGATGCCGAATAATATATTATGGGGTATTAATCTTCTTCGAAAGGCTATCCCCCTGATAA
AGGCAGGTTGCACACGTGTTCCGCACCCGTACGCCGCTCTCAATTACCCGAAAGTAATCTACCGCTCGGCTTG
CATGTGTTAGGCCTCCCGCTAGCGTTCATCCTGAGCAGGATCAACTCT
>1345
CTGCTGCCGCCCGTAGGAGTCTGGTCCGTGTCTCAGTACCAGTGTGGGGGATCACCCTCTCAGGCCCCCTAAA
GATCGTCGCCTTGGTGAGCCGTTACCTCACCAACTAGCTAATCTTGCGCGTGCCCATCTCTATCCACCGTAGT
TTTCAATATCACCCGATGCCGAATAATATATTATGGGGGTATTAATCTTCTTCGAAAGGCTATCCCCCTGATA
AAGGCAGGTTGCACACGTGTTCCGCACCCGTACGCCGCTCTCAATTACCCGAAAGTAATCTACCGCTCGGCTT
GCATGTGTTAGGCCTCCCGCTAGCGTTCATCCTGAGCCAGGATCAAACTCT
>1346
CTGCTGCCGCCCGTAGGAGTCTGGTCCGTGTCTCAGTACCAGTGTGGGGGATCACCCTCTCAGGCCCCCTAAA
GATCGTCGCCTTGGTGAGCCGTTACCTCACCAACTAGCTAATCTTGCGCGTGCCCATCTCTATCCACCGTAGT
TTTCAATATCACCCGATGCCGAATAATATAATTATGGGGTATTAATCTTCTCGAAGGCTATCCCCTGATAAAG
GCAGGTTGCACACGTGTTCCGCACCCGTACGCCGCTCTCAATTACCCCGAAAGTAATCTACCGCTCGGCTTGC
ATGTGTTAGGCCTCCCGCTAGCGTTCATCCTGAGCCAGGATCAAACTCT
>1347
CTGCTGCCGCCCGTAGGAGTCTGGTCCGTGTCTCAGTACCAGTGTGGGGGATCACCCTCTCAGGCCCCCTAAA
GATCGTCGCCTTGGTGAGCCGTTACCTCACCAACTAGCTAATCTTGCGCGTGCCCATCTCTATCCACCGTAGT
TTTCAATATCACCCGATGCCGAATAATAATATTATGGGGTATTAATCTTCTCGAAAGGCTATCCCCTGATAAA
GGCAGGTTGCACACGTGTTCCGCACCCGTACGCCGCTCTCAATTACCCGAAAGTAATCTACCGCATCGGCTTG
CATGTGTTAGGCCTCCCGCTAGCGTTCATCCTGAGCAGGATCAAACTCT
>1348
CTGCTGCCGCCCGTAGGAGTCTGGTCCGTGTCTCAGTACCAGTGTGGGGGATCACCCTCTCAGGCCCCCTAAA
GATCGTCGCCTTGGTGAGCCGTTACCTCACCAACTAGCTAATCTTGTGCGTGCCCATCTCTATCCACCGTAGT
TTTCAATATCACCCGATGCCGAATAATATATTATGGGGTATTAATCTTCTTCGAAAGGCTATCCCCTGATAAA
GGCAGGTTGCACACGTGTTCCGCACCCGTACGCCGCTCTCAATTACCCGAAAGTAATCTACCGCTCGGCTTGC
ATGTGTTAGGCCTCCCGCTAGCGTTCATCTGAGCCAGGATCAACTCT
>1349
CTGCTGCCGCCCGTAGGAGTCTGGTCCGTGTCTCAGTACCAGTGTGGGGGATCACCCTCTCAGGCCCCCTAAA
GATCGTCGCCTTGGTGAGCCGTTACCTCACCAACTAGCTAATCTTGCGCGTGCCCATCTCTATCCACCGTAGT
TTTCAATATCACCCGATGCCGAATAATATATTATGGGGTATTAATCTTCTTCGAAAGGCTATCCCCCTGATAA
AGGCAGGTTAGCACGCGTTCCGCACCCGTACGCCGCTCTCAATTACCCGAAAGTAATCTACCGCTCCGGCTTG
CATGTGTTAGGCCTCCCGCTAGCGTTCTCCTCGAGCAGGATCCAATCATCCT
>1350
CTGCTGCCGCCCGTAGGAGTCTGGACCGTGTCTCAGTTCCAGTGTGACTGATCATCCTCTCAGACCAGTACGG
ATCGTCGCCTTGGTAGGCCTTTACCCCACCAACTAGCTAATCCGACCTAGGCTCATCTGATAGCGTGAGGTCC

GAAGATCCCCCACTTCTCCCTCAGGACGTATGCGGTATTGCGCCCGTTCCCGGACGATCCCCACTACCAGGCA
GATTCCCTAGGCATTACTCACCCGTCCGCCGCTGAATCCAGGAGCAAGCCCCCTTCATCCGCTCGACTGCCAT
GTGTTAGGCCTGCCGCCAGCGTCAATCTGAGCCAGGATCAAACTCT
>1351
CTGCTGCCGCCCGTAGGAGTCTGGTCCGTGTCTCAGTACCAGTGTGGGGGATCACCCTCTCAGGCCCCCTAAA
GATCGTCGCCTTGGTGAGCCGTTACCTCACCAACTAGCTAATCTTGCGCGTGCCCATCTCTATCCACCGTAGT
TTTTCAATATCACCCGATGCCGAATAATATATTATGGGGTATTTAATTCTTCTTCGAAAGGCTATCCCCCTGA
TAAAGGCAGGTTGCACACGTGTTCCGCACCCGTACGCCGCTCTCAATTACCCGAAAGTAATCTACCGCTCGGC
TTGCATGTGTTAGGCCTCCCGCTAGCGTTCATCCTCGAGCCAGGATCAACTCT
>1352
CTGCTGCCGCCCGTAGGAGTCTGGTCCGTGTCTCAGTACCAGTGTGGGGGATCACCCTCTCAGGCCCCCTAAA
GATCGTCGCCTTGGTGAGCCGTTACCTCACCAACTAGCTAATCTTGCGCGTGCCCATCTCTATCCACCGTAGT
TTTCAATATCACCCGATGCCGAATAATATATTATGGGGTATTAATCTTCTTCGAAAGGCTATCCCCCTGATAA
AGGCAGGTTGCACACGTGTTCCGCACCCGTACGCCGCTCTCAATTACCCGAAAGTAATCTACCGCTCGGCTTG
CATGTGTTAGGCCTCCCGCTAGCGTTCATCCTCGAGCCAGGATCAAACTCT
>1353
CTGCTGCCGCCCGTAGGAGTCTGGACCGTGTCTCAGTTCCAGTGTGACTGATCATCCTCTCAGACCAGTTACG
GATCGTCGCCTTGGTAGGCCTTTACCCCACCAACTAGCTAATCCGACCTAGGCTCATCTGATAGCGTGAGGTC
CGAAGATCCCCCACTTCTCCCTCAGGACGTATGCGGTATTAGCGCCCGTTTCCGGACGTATCCCCACTACCAG
GCAGATTCCTAGGCATTACTCACCCGTCCGCCGCTGAATCCAGGAGCAAGCTCCCTCATCCGCTCGACTGCCA
TGTGTTAGGCCTGCCGCCAGCGTCAATCTGAGCCAGGATCAAACTCT
>1354
CTGCTGCCGCCCGTAGGAGTCTGGTCCGTGTCTCAGTACCAGTGTGGGGGATCACCCTCTCAGGCCCCCTAAA
GATCGTCGCCTTGGTGAGCCGTTACCTCACCAACTAGCTAATCTTGCGCGTGCCCATCTCTATCCACCGTAGT
TTTCAATATCACCCGATGCCGAATAATATATTATGGGGTATTAATCTTCTTCTGAAAGCTATCCCCTGATAAG
GCAGGTTGCACACGTGTTCCGCACCCGTACGCCGCTCTCAATTACCCGAAAGTAATCTACCGCTCGGCTTGCA
TGTGTTAGGCCTCCCGCTAGCGTTCATCCTGAGCCAGGATCAACTCT
>1355
CTGCTGCCGCCCGTAGGAGTCTGGTCCGTGTCTCAGTACCAGTGTGGGGATCACCCTCTCAGGCCCCCTAAAG
ATCGTCGCCTTGGTGAGCCGTTACCTCACCAACTAGCTAATCCTGCGCGTGCCCATCTCTATCCACCGTAGTT
TTCAATATCACCCGATGCCGAATAATATATTATGGGGTATTAATCTTCTCGAAAGGCTTATCCCCCTGATAAA
GGCAGGTTGCACACGTGTTCCGCACCCGTACGCCGCTCTCAATTACCCGAAAGTAATCTACCGCTCGGCTTGC
ATGTGTTAGGCCTCCCGCTAGCGTTCTCCTGAGCAGG
>1356
CTGCTTGCCGCCCGTAGGAGTCTGGTCCGTGTCTCAGTACCAGTGTGGGGGATCACCCTCTCAGGCCCCCTAA
AGATCGTCGCCTTGGTGAGCCGTTACCTCACCAACTAGCTAATCTTGCGCGTGCCCATCTCTATCCACCGTAG
TTTTCAATATCACCCGATGCCGAATAATATATTATGGGGTATTAATCTTCTCGAAAGGCTATCCCCCTGATAA
AGGCAGGTTGCACACGTGTTCCGCACCCGTACGCCGCTCTCAATTACCCGAAAGTAATCTACCGCTCGGCATG
CATGTGTTAGGCCTCCCGCTAGCGTTCATCCTGAGCCAGGATCAAACTCT
>1357
CTGCTGCCGCCCGTAGGAGTCTGGTCCGTGTCTCAGTACCAGTGTGGGGGATCACCCTCTCAGGCCCCCTAAA
GATCGTCGCCTTGGTGAGCCGTTACCTCACCAACTAGCTAATCTTGCGCGTGCCCATCTCTATCCACCGTAGT
TTTCAATATCACCCGATGCCGAATAATATATTATTGGGGTATTTAATTCTTCTTCGAAAGGCTATCCCCCTGA
TAAAGGCAGGTTGCACACGTGTTCCGCACCCGTACGCCGCTCTCAATTACCCGAAAGTAATCTACCGCTCGGC
TTGCATGTGTTAGGCCTCCCGCTAGCGTTCATTCCTGAGCCAGGATCAAACTCT
>1358
CTGCTGCCGCCCGTAGGAGTCTGGTCCGTGTCTCAGTACCAGTGTGGGGGATCACCCTCTCAGGCCCCCTAAA
AGATCGTCGCCTTGGTGAGCCGTTACCTCACCAACTAGCTAATCTTGCGCGTGCCCATCTCTATCCACCGTAG
TTTTCAATATCACCCGATGCCGAATAATATATTATGGGGTATTAATCTTCTTCGAAAGGCTATCCCCCTGATA
AAGGCAGGTTGCACACGTGTTCCGCACCCGTACGCCGCTCTCAATTACCCGAAAGTAATCTACCGCTCGGCTT
GCATGTGTTAGGCCTCCCGCTAGCGTTCATCCTGAGCCAGGATCAAACTCT
>1359
CTGCTGCCGCCCGTAGGAGTCTGGACCGTGTCTCAGTTCCAGTGTGACTGATCATCCTCTCAGACCAGTTACG
GATCGTCGCCTTGGTAGGCCTTTACCCCACCAACTAGCTAATCCGACCTAGGCTCATCTGATAGCGTGAGGTC

CGAAGTCCCCCACTTCTCCCTCAGGACGTATGCGGTATTAGCGCCCGTTTCCGGACGATCCCCCACTACCAGG
CAGATTCCTAGGCATTACTCACCCGTCCGCCGCTGAATCCAGGAGCAAGCTCCCTTCATCCGCTCGACTGCAT
GTGTTAGGCCTGCCGCCAGCGTCAATCTGAGCCAGGATCAAACTCT
>1360
CTGCTGCCGCCCGTAGGAGTCTGGTCCGTGTCTCAGTACCAGTGTGGGGGATCACCCTCTCAGGCCCCCTAAA
GATCGTCGCCTTGGTGAGCCGTTACCTCACCAACTAGCTAATCTTGCGCGTGCCCCATCTCTATCCACCGTAG
TTTTCAATATCACCCGATGCCGAATAATATATTTATGGGGTATTAATTCTTCTTCGAAAGGCTATCCCCCTGA
TAAAGGCAGGTTGCACACGTGTTCCGCACCCGTACGCCGCTCTCAATTACCCGAAAGTAATCTACCGCTCGGC
TTGCATGTGTTAGGCCTCCCGCTAGCGTTCATCCTGAGCCAGGATCAAACTCT
>1361
CTGCTGCCGCCCGTAGGAGTCTGGTCCGTGTCTCAGTACCAGTGTGGGGGATCACCCTCTCAGGCCCCCTAAA
GATCGTCGCCTTGGTGAGCCGTTACCTCACCAACTAGCTAATCTTGCGCGTGCCCATCTCTATCCACCGTAGT
TTTCAATATCACCCGATGCCGAATAATATATTATGGGGTATTTAATCTTCTTCGAAAGGCTATCCCCCTGATT
AAAGGCAGGTTGCACACGTGTTCCGCACCCGTACGCCGCTCTCAATTACCCGAAAGTAATCTACCGCTCGGCT
TGCATGCGTTAGGCCTCCCGCTAGCGTCTCCTGAGCAGGATCAACTCGT
>1362
CTGCTGCCGCCCGTAGGAGTCTGGTCCGTGTCTCAGTACCAGTGTGGGGGATCACCCTCTCAGGCCCCCTAAA
GATCGTCGCCTTGGTGAGCCGTTACCTCACCAACTAGCTAATCTTGCGCGTGCCCATCTCTATCCACCGTAGT
TTTCAATATCACCCGATGCCGAATAATATTATTATGGGGTATAATCTTTCTTCGAAAGGCTATCCCCCTGATA
AAGGCAGGTTGCACACGTGTTCCGCACCCGTACGCCGCTCTCAATTACCCGAAAGTAATCTACCGCTCCGGCT
TGCATGTGTTAGGTTCTCCCGCTAGCGTTCTCCTGAGCCAGGATCAACTCT
>1363
CTGCTGCCGCCCGTAGGAGTCTGGTCCGTGTCTCAGTACCAGTGTGGGGGATCACCCTCTCAGGCCCCCTAAA
GATCGTCGCCTTGGTGAGCCGTTACCTCACCAACTAGCTAATCTTGCGCGTGCCCATCTCTATCCACCGTAGT
TTTCAATATCACCCGATGCCGAATAATATATTATGGGGGTATTAATCTTCCTTTCGGAAGGCTATCCCCCTGA
TAAAAGCAGGTTGCACACGTGTTCCGCACCCGTACGCCGCTCTCAATTACCCGAAAGTAATCTACCGCTCGGC
TTGCATGTGTTAGGCCTCCCGCTAGCGTTCATCCTGAGCCAGGATCAAACTCT
>1364
CTGCTGCCGCCCGTAGGAGTCTGGTCCGTGTCTCAGTACCAGTGTGGGGGATCACCCTCTCAGGCCCCCTAAA
GATCGTCGCCTTGGTGAGCCGTTACCTCACCAACTAGCTAATCTTGCGCGTGCCCATCTCTATCCACCGTAGT
TTTCAATATCACCCGATGCCGAATAATATATTATGGGGTATTAATCTTTCTTCGAAAGGCTATCCCCCTGATA
AAGGCAGGTTGCACACGTGTTCCGCACCCGTACGCCGCTCTCAACTACCCGAAAGTAATCTACCGCTCGGCTT
GCATGTGTTAGGCCTCCCGCTAGCGTTCATCCTCGAGCCAGGATCAAACTCT
>1365
CTGCTGCCGCCCGTAGGAGTCTGGTCCGTGTCTCAGTACCAGTGTGGGGGATCACCCTCTCAGGCCCCCTAAA
GATCGTCGCCTTGGTGAGCCGTTACCTCACCAACTAGCTAATCTTGCGCGTGCCCATCTCTATCCACCGTAGT
TTTCAATATCACCCGATGCCGAATAATATATTATGGGGTATTAATCTTCTTCGAAAGGCTATCCCCCTGATAA
AGGCAGGTTGCACACGTGTTCCGCACCCGTACGCCGCTCTCAATTACCCGAAAGTAATCTACCGCTCGGCTTG
CATGTGTTAGGCCTCCCGCTAGCGTTCATCCTGAGCCAGGATCAACTCT
>1366
CTGCTGCCGCCCGTAGGAGTCTGGTCCGTGTCTCAGTACCAGTGTGGGGGATCACCCTCTCAGGCCCCCTAAA
AGATCGTCGCCTTGGTGAGCCGTTACCTCACCAACTAGCTAATCTTGCGCGTGCCCATCTCTATCCACCGTAG
TTTTCAATATCACCCGATGCCGAATAATATATTATGGGGTATTAATCTTCTTCGAAAGGCTATCCCCTGATAA
AGGCAGGTTGCACACGTGTTCCGCACCCGTACGCCGCTCTCAATTACCCGAAAGTAATCTACCGCTCGGCTTG
CATGTGTTAGGCCTCCCGCTAGCGTTCATCCTGAGCAGGATCAACTCT
>1367
CTGCTGCCGCCCGTAGGAGTCTGGTCCGTGTCTCAGTACCAGTGTGGGGGATCACCCTCTCAGGCCCCCTAAA
GATCGTCGCCTTGGTGAGCCGTTACCTCACCAACTAGCTAATCTTGCGCGTGCCCATCTCTATCCACCGTAGT
TTTCAATATCACCCGATGCCGAATAATATATTATGGGGTATTAATCTTCTCGAAAGGCTATCCCCTGATAAAG
GCAGGTTGCACACGTGTTCCGCACCCGTACGCCGCTCTCAATTACCCGAAAGTAATCTACCGCTCCGGCTTGC
ATGTGTTAGGCCTCCCGCTAGCGTTCTCCGTGAGCAGG
>1368
CTGCTGCCGCCGTAGGAGTCTGGTCCGTGTCTCAGTACCAGTGTGGGGGATCACCCTCTCAGGCCCCCTAAAG
ATCGTCGCCTTGGTGAGCCGTTACCTCACCAACTAGCTAATCTTGCGCGTGCCCATCTCTATCCACCGTAGTT

TCAATATCACCCGATGCCGAATAATATATTATGGGGTATTAATCTTCTTCGAAAGGCTATCCCCCTGATAAAG
GCAGGTTGCACACGTGTTCCGCACCCGTACGCCGCTCTCAATTACCCGAAAGTAATCTACCGCTCGGCTTGCA
TGTGTTAGGCCTCCCGCTAGCGTTCATCCTGAGCCAGGATCAAACTCT
>1369
CTGCTGCCGCCCGTAGGAGTCTGGTCCGTGTCTCAGTACCAGTGTGGGGGATCACCCTCTCAGGCCCCCTAAA
GATCGTCGCCTTGGTGAGCCGTTACCTCACCAACTAGCTAATCTTGCGCGTGCCCATCTCTATCCACCGTAGT
TTTCAATATCACCCGATGCCGAATAATATAATTATGGGGTATTAATCTTCTCTGAAAGGCTATCCCCTATAAA
GGCAGGTTGCACACGTGTTCCGCACCCGTACGCGCTCTCAATTACCCGAAAGTAATCTACCGCTCGGCTTGCA
TGTGTTAGGCCTCCCGCTAGCGTTCATCCTGAGCCAGGATCAAACTCT
>1370
CTGCTGCCGCCCGTAGGAGTCTGGACCGTGTCTCAGTTCCAGTGTGACTGATCATCCTCTCAGACCAGTTACG
GATCGTCGCCTTGGTAGGCCTTTACCCCACCAACTAGCTAATCCGACCTAGGCTCATCTGATAGCGTGAGGTC
CGAAGATCCCCCACTTCTCCCTCAGGACGTATGCGGTATTAGCGCCCGTTTCCGGACGTATCCCCACTACCAG
GCAGATTCCTAGGCATTACTCACCCGTCCGCCGCTGAATCCAGGAGCAAGCTCCCTTCATCCGCTCGACTGCA
TGTGTTAGGCCTGCCGCCAGCGTTCAATCTGAGCCAGGATCAAACTCT
>1371
CTGCTGCCGCCCGTAGGAGTCTGGTCCGTGTCTCAGTACCAGTGTGGGGGATCACCCTCTCAGGCCCCCTAAA
GATCGTCGCCTTGGTGAGCCGTTACCTCACCAACTAGCTAATCTTGCGCGTGCCCATCTCTATCCACCGTAGT
TTTCAATATCACCCGATGCCGAATAATATATTATGGGGTATTAATCTTTCTTCTGAAAGGCTATCCCCCTGAT
AAAGGCAGGTTGCACACGTGTTCCGCACCCGTACGCCGCTCTCAATTACCCGAAAGTAATCTACCGCTCGGCT
TGCATGTGTTAGGCCTCCCGCTAGCGTTCATCCTGAGCCAGGATCAAACTCT
>1372
CTGCTGCCGCCCGTAGGAGTCTGGTCCGTGTCTCAGTACCAGTGTGGGGGATCACCCTCTCAGGCCCCCTAAA
GATCGTCGCCTTGGTGAGCCGTTACCTCACCAACTAGCTAATCTTGCGCGTGCCCATCTCTATCCACCGTAGT
TTTCAATATCACCCGATGCCGAATAATATATTATGGGGTATTAATCTTCTTCGAAAGGCTATCCCCCTGATAA
GGCAGGTTGCACACGTGTTCCGCACCCGTACGCCGCTCTCAATTACCCGAAAGTAATCTACCGCTCGGCTTGC
ATGTGTTAGGCCTCCCGCTAGCGTTCATCCTGAGCCAGGATCAACTCT
>1373
CTGCTGCCGCCCGTAGGAGTCTGGTCCGTGTCTCAGTACCAGTGTGGGGGATCACCCTCTCAGGCCCCCTAAA
GATCGTCGCCTTGGTGAGCCGTTACCTCACCAACTAGCTAATCTTGCGCGTGCCCATCTCTATCCACCGTAGT
TTTCAATATCACCCGATGCCGAATAATATATTATGGGGTATTAATCTTCTTCGAAAGGCTATCCCCTGATAAA
GGCAGGTTGCACACGTGTTCCGCACCCGTACGCCGCTCTCAATTACCCGAAAGTAATCTACCGCTCGGCTTGC
ATGTGTTAGGCCTCCCGCTAGCGTTCATCCTGAGCCAGGATCAAACTCT
>1374
CTGCTGCCGCCCGTAGGAGTCTGGTCCGTGTCTCAGTACCAGTGTGGGGGATCACCCTCTCAGGCCCCCTAAA
GATCGTCGCCTTGGTGAGCCGTTACCTCACCAACTAGCTAATCTTGCGCGTGCCCATCTCTATCCACCGTAGT
TTTCAATATCACCCGATGCCGAATAATATATTATGGGGTATTAATCTTCTTCGAAAGGCTATCCCCCTGATAA
AGGCAGGTTGCACACGTGTTCCGCACCCGTACGCCGCTCTCAATTACCCGAAAGTAATCTACCGCTCGGCTTG
CATGTGTTAGGCCTCCCGCTAGCGTTCATCCTGAGCCAGGTCAATCATCT
>1375
CTGCTGCCGCCCGTAGGAGTCTGGTCCGTGTCTCAGTACCAGTGTGGGGGATCACCCTCTCAGGCCCCCTAAA
GATCGTCGCCTTGGTGAGCCGTTACCTCACCAACTAGCTAATCTTGCGCGTGCCCATCTCTATCCACCGTAGT
TTTCAATATCACCCGATGCCGAATAATATATTATGGGGTATTAATCTTCTCGAAGGCTTATCCCCTGAATAAA
GGCAGGTTGCACACGTGTTCCGCACCCGTACGCCGCTCTCAATTACCCGAAAGTAATCTACCGCTCGGCTTGC
ATGTGTTAGGCCTCCCGCTAGCGTTCTCCTGAGCAGGTC
>1376
CTGCTGCCGCCCGTAGGAGTCTGGACCGTGTCTCAGTTCCAGTGTGACTGATCATCCTCTCAGACCAGTTACG
GATCGTCGCCTTGGTAGGCCTTTACCCCACCAACTAGCTAATCCGACCTAGGCTCATCTGATAGCGTGAGGTC
CGAAGATCCCCCACTTCTCCCTCAGGACGTATGCGGTATTAGCGCCCGTTTCCGGACGTATCCCCACTACCAG
GCAGATTCCTAGGCATTACTCACCCGTCCGCCGCTGAATCCAGGAGCAAGCCCCCTTCATCCGCTCGACTGCA
TGTGTTAGGCCTGCCGCCAGCGTCAATCTGAGCCGGGATCAAACTCT
>1377
CTGCTGCCGCCCGTAGGAGTCTGGTCCGTGTCTCAGTACCAGTGTGGGGGATCACCCTCTCAGGCCCCCTAAA
GATCGTCGCCTTGGTGAGCCGTTACCTCACCAACTAGCTAATCTTGCGCGTGCCCATCTCTATCCACCGTAGT

TTTCAATATCACCCGATGCCGAATAATATATTATGGGGTATTAATCTTCTCGAAAGCTATCCCCTGATAAAGG
CAGGTTGCACACGTGTTCCGCACCCGTACGCCGCTCTCAATTACCCGAAAGTAATCTACCGCTCGGCTTGCAT
GTGTTAGGCCTCCCGCTGAGCGTTCT
>1378
CTGCTGCCGCCCGTAGGAGTCTGGTCCGTGTCTCAGTACCAGGTGTGGGGGATCACCCTCTCCAGGCCCCCTA
AAGATCGTCGCCTTGGTGAGCCGTTACCTCACCAACTAGCTAATCTTGCGCGTGCCCATCTCTATCCACCGTA
GTTTTCAATATCACCCGATGCCGAATAATATATTATGGGGTATTAATCTTCTTCGAAAGGCTATCCCCTGATA
AAGGCAGGTTGCACACGTGTTCCGCACCCGTACGCCGCTCTCAATTACCCGAAAGTAATCTACCGCTCGGCTT
GCATGTGTTAGGCCTCCCGCTAGCGTTCATCCTGAGCCAGGATCAACTCT
>1379
CTGCTGCCGCCCGTAGGAGTCTGGTCCGTGTCTCAGTACCAGTGTGGGGGATCACCCTCTCAGGCCCCCTAAA
GATCGTCGCCTTGGTGAGCCGTTACCTCACCAACTAGCTAATCTTGCGCGTGCCCATCTCTATCCACCGTAGT
TTTCAATATCACCCGATGCCGAATAATATATTATGGGGGTATTAATCTTCTTCGAAAGGCTATCCCCCTGATA
AAGGCAGGTTGCACACGTGTTCCGCACCCGTACGCCGCTCTCAATTACCCGAAAGTAATCTACCGCTCGGCTT
GCATGTGTTAGGCCTCCCGCTAGCGTTCTCCTGAGCAGGATCAACTCG
>1380
CTGCTGCCGCCCGTAGGAGTCTGGTCCGTGTCTCAGTACCAGTGTGGGGGATCACCCTCTCAGGCCCCCTAAA
GATCGTCGCCTTGGTGAGCCGTTACCTCACCAACTAGCTAATCTTGCGCGTGCCCATCTCTATCCACCGTAGT
TTTCAATATCACCCGATGCCGAATAATATATTATGGGGTATTAATCTTCTTCGAAAGGCTATCCCCCTGATAA
AGGCAGGTTGCACACGTGTTCCGCACCCGTACGCCGCTCTCAATTACCCGAAAGTAATCTACCGCTCGGCTTG
CATGTGTTAGGCCTCCCGCTAGCGTTCATCCTGAGCCAGGATCAACTCT
>1381
CTGCTGCCGCCCGTAGGAGTCTGGTCCGTGTCTCAGTACCAGTGTGGGGGATCACCCTCTCAGGCCCCCTAAA
GATCGTCGCCTTGGTGAGCCGTTACCTCACCAACTAGCTAATCTTGCGCGTGCCCATCTCTATCCACCGTAGT
TTTCAATATCACCCGATGCCGAATAATATATTATGGGGTATTAATCTTCTTCGAAAGCTATCCCCTGATAAAG
GCAGGTTGCACACGTGTTCCGCACCCGTACGCCGCTCTCAATTACCCGAAAGTAATCTACCGCTCGGCTTGCA
TGTGTTAGGCCTCCCGCTAGCGTTCTCCTGAGCAGGATCAACTCT
>1382
CTGCTGCCGCCCGTAGGAGTCTGGACCGTGTCTCAGTTCCAGTGTGACTGATCATCCTCTCAGACCAGTTACG
GATCGTCGCCTTGGTAGGCCTTTACCCCACCAACTAGCTAATCCGACCTAGGCTCATCTGATAGCGTGAGGTC
CGAAGATCCCCCACTTCTCCCTCAGGACGTATGCGGTATTAGCGCCCGTTTCCGGACGTATCCCCACTACCAG
GCAGATTCCTAGGCATTACTCACCCGTCCGCCACTGAATCCAGGAGCAAGCTCCCTCATCCGCTCGACTGCAT
GTGTTAGGCCTGCCGCCAGCGTCAATCTGAGCCAGGATCAAACTCT
>1383
CTGCTGCCGCCCGTAGGAGTCTGGTCCGTGTCTCAGTACCAGTGTGGGGGATCACCCTCTCAGGCCCCCTAAA
GATCGTCGCCTTGGTGAGCCGTTACCTCACCAACTAGCTAATCTTGCGCGTGCCCATCTCTATCCACCGTAGT
TTTCAATATCACCCGATGCCGAATAATATATTATGGGGTATTAATCTTCTTCGAAAGGCTATCCCCTGATAAA
GGCAGGTTGCACACGTGTTCCGCACCCGTACGCCGCTCTCAATTACCCGAAAGTAATCTACCGCTCCGGCTTG
CATGTGTTAGGCCTCCCGCTAGCGTTCATCCTGAGCCAGGATCAAACTCT
>1384
CTGCTGCCGCCCGTAGGAGTCTGGTCCGTGTCTCAGTACCAGTGTGGGGGATCACCCTCTCAGGCCCCCTAAA
GATCGTCGCCTTGGTGAGCCGTTACCTCACCAACTAGCTAATCTTGCGCGTGCCCATCTCTATCCACCGTAGT
TTTCAATATCACCCGATGCCGAATAATATATTATGGGGTATTAATCTTCTTCGAAAGGCTATCCCCTGATAAA
GGCAGGTTGCACACGTGTTCCGCACCCGTACGCCGCTCTCAATTACCCGAAAGTAATCTACCGCTCGGCTTGC
ATGTGTTAGGCCTCCCGCTAGCGTTCATCCTGAGCCAGGATCAAACTCT
>1385
CTGCTGCCGCCCGTAGGAGTCTGGTCCGTGTCTCAGTACCAGTGTGGGGGATCACCCTCTCAGGCCCCCTAAA
GATCGTCGCCTTGGTGAGCCGTTACCTCACCAACTAGCTAATCTTGCGCGTGCCCATCTCTATCCACCGTAGT
TTTCAATATCACCCGATGCCGAATAATATATTATGGGGTATTAATCTTCTTCGAAAGGCTATCCCCCTGATAA
AGGCAGGTTGCACACGTGTTCCGCACCCGTACGCCGCTCTCAATTACCCGAAAGTAATCTACCGCTCGGCTTG
CATGTGTTAGGCCTCCCGCTAGCGTTCATCCTCGAGCCAGGATCAACTCT
>1386
CTGCTGCCGCCCGTAGGAGTCTGGTCCGTGTCTCAGTACCAGTGTGGGGGATCACCCTCTCAGGCCCCCTAAA
GATCGTCGCCCTGGTGAGCCGTTACCTCACCAACTAGCTAATCTTGCGCGTGCCCATCTCTATCCACCGTAGT

TTTCAATATCACCCGATGCCGAATAATATATTATGGGGTATTAATCTTCTTCGAAAGGCTATCCCCCTGATAA
AGGCAGGTTGCACACGTGTTCCGCACCCGTACGCCGCTCTCAATTACCCGAAAGTAATCTACCGCTCCGGCTT
GCATGTGTTAGGCCTCCCGCTAGCGTTCATCCTGAGCCAGGATCAAACTCT
>1387
CTGCTGCCGCCCGTAGGAGTCTGGTCCGTGTCTCAGTACCAGTGTGGGGGATCACCCTCTCAGGCCCCCTAAA
GATCGTCGCCTTGGTGAGCCGTTACCTCACCAACTAGCTAATCTTGCGCGTGCCCATCTCTATCCACCGTAGT
TTTCAATATCACCCGATGCCGAATAATATATTATGGGGTATTAATCTTCTCGAAAGGCTATCCCCTGATAAAG
GCAGGTTGCACACGTGTTCCGCACCCGTACGCCGCTCTCAATTTACCCGAAAGTAATCTACCGCTCCGGCTTG
CATGTGTTAGGCCTCCCGCTAGCGTTCATCCGTGAGCCAGGATCAAACTCT
>1388
CTGCTGCCGCCCGTAGGAGTCTGGTCCGTGTCTCAGTACCAGTGTGGGGGATCACCCTCTCAGGCCCCCTAAA
GATCGTCGCCTTGGTGAGCCGTACCTCACCAACTAGCTAATCTTGCGCGTGCCCATCTCTATCCACCGTAGCT
TTCAATATCACCCGATGCCGAATAATATATTATGGGGTATTAATCTTCTCGAAGCTATCCCTGATAAAGGCAG
GTTGCACACGTGTTCCGCACCCGTACGCCGCTCTCAATTACCCGAAAGTAATCTACCGCTCGGCTTGCATGTC
GTTAGGCCTCCCGCTAAGCGTCATC
>1389
CTGCTGCCGCCCGTAGGAGTCTGGTCCGTGTCTCAGTACCAGTGTGGGGGATCACCCTCTCAGGCCCCCTAAA
GATCGTCGCTTGGTGAGCCGTTACCTCACCAACTAGCTAATCTTGCGCGTGCCCATCTCTATCCACCGTAGTT
TTCAATATCACCCGATGCCGAATAATATATTATGGGGTATTAATCCTCTTCGAAAGGCTATCCCCTGATAAAG
GCAGGTTGCACACGTGTTCCGCACCCGTACGCCGCTCTCAATTACCCGAAAGTAATCTACCGCTCCGGCTTGC
ATGTGTTAGGCCTCCCGCTAGCGTTCTCCTGAGCAGGATCAAACTCGT
>1390
CTGCTGCCGCCCGTAGGAGTCTGGTCCGTGTCTCAGTACCAGTGTGGGGGATCACCCTCTCAGGCCCCCTAAA
GATCGTCGCCTTGGTGAGCCGTTACCTCACCAACTAGCTAATCTTGCGCGTGCCCATCTCTATCCACCGTAGT
TTTCAATATCACCCGATGCCGAATAATATATTATGGGGTATTAATCTTCTTCGAAAGGCTATCCCCCTGATAA
AGGCAGGTTGCACACGTGTTCCGCACCCGTACGCCGCTCTCAATTACCCGAAAGTAATCTACCGCTCGGCTTG
CATGTGTTAGGCCTCCCGCTAGCGTTCATCCTGAGCAGGATCAACTCT
>1391
CTGCTGCCGCCCGTAGGAGTCTGGTCCGTGTCTCAGTACCAGTGTGGGGGATCACCCTCTCAGGCCCCCTAAA
GATCGTCGCCTTGGTGAGCCGTTACCTCACCAACTAGCTAATCTTGCGCGTGCCCATCTCTATCCACCGTAGT
TTTCAATATCACCCGATGCCGAATAATATATTATGGGGTATTAATCTTCTTCGAAAGGCTATCCCCCTGATAA
AGGCAGGTTGCACACGTGTTCCGCACCCGTACGCCGCTCTCAATTACCCGAAAGTAATCTACCGCTCGGCTTG
CATGTGTTAGGCCTCCCGCTAGCGTTCATCCTCGAGCCAGGATCAAACTCT
>1392
CTGCTGCCGCCCGTAGGAGTCTGGTCCGTGTCTCAGTACCAGTGTGGGGGATCACCCTCTCAGGCCCCCTAAA
GATCGTCGCCTTGGTGAGCCGTTACCTCACCAACTAGCTAATCTTGCGCGTGCCCATCTCTATCCACCGTAGT
TTTCAATATCACCCGATGCCGAATAATATATTATGGGGTATTAATCTTCTTCGAAAGGCTATCCCCTGATAAA
GGCAGGTTGCACACGTGTTCCGCACCCGTACGCCGCTCTCAATTACCCGAAAGTAATCTACCGCTCGGCTTGC
ATGTGTTAGGCCTCCCGCTAGCGTT
>1393
CTGCTGCCGCCCGTAGGAGTCTGGTCCGTGTCTCAGTACCAGTGTGGGGGATCACCCTCTCAGGCCCCCTAAA
GATCGTCGCCTTGGTGAGCCGTTACCTCACCAACTAGCTAATCTTGCGCGTGCCCATCTCTATCCACCGTAGT
TTTCAATATCACCCGATGCCGAATAATATATTATGGGGTATTAATCTTCTTCGAAAGGCTATCCCCCTGATAA
AGGCAGGTTGCACACGTGTTCCGCACCCGTACGCCGCTCTCAATTACCCGAAAGTAATCTACCGCTCGGCTTG
CATGTGTTAGGCCTCCCGCTAGCGTTCATCCGTGAGCCAGGATCAACTCT
>1394
CTGCTGCCGCCCGTAGGAGTCTGGTCCGTGTCTCAGTACCAGTGTGGGGGATCACCCTCTCAGGCCCCCTAAA
GATCGTCGCCTTGGTGAGCCGTTACCTCACCAACTAGCTAATCTTGCGCGTGCCCATCTCTATCCACCGTAGT
TTTCAATATCACCCGATGCCGAATAATATATTATGGGGTATTAATCTTCTTCGAAAGGCTATCCCCCTGATAA
AGGCAGGTTGCACACGTGTTCCGCACCCGTACGCCGCTCTCAATTACCCGAAAGTAATCTACCGCTCGGCTTG
CATGTGTTAGGCCTCCCGCTAGCGTTCTCCTCGAGCAGGATCCAACATCT
>1395
CTGCTGCCGCCCGTAGGAGTCTGGTCCGTGTCTCAGTACCAGTGTGGGGGATCACCCTCTCAGGCCCCCTAAA
GATCGTCGCCTTGGTGAGCCGTTACCTCACCAACTAGCTAATCTTGCGCGTGCCCATCTCTATCCACCGTAGT

TTTCAATATCACCCGATGCCGAATAATATATTATGGGGTATTAATCTTCTCGAAAGGCTATCCCCTGATAAGG
CAGTATCACACGTGTTCCGCACCCGTACGCCGCTCTCAATTACCCGAAAGTAATCTACCGCTCGGCTTGCATG
TGTTAGGCCTCCCGCTAGCGTTCATCCTGAGCCAGGATCAACTCT
>1396
CTGCTGCCGCCTGTAGGAGTCTGGTCCGTGTCTCAGGTACCAGTGTGGGGGATCACCCTCTCAGGCCCCCTAA
AGATCGTCGCCTTGGTGAGCCGTTACCTCAACCAACTAGCTAATCTTGCGCGTGCCCATCTCTATCCACCGTA
GTTTTCAATATCACCCGATGCCGAATAATATATTATGGGGTATTAATCTTCTCGAAAGGCTATCCCCCTGATA
AAGGCAGGTTGCACACGTGTTCCGCACCCGTACGCCGCTCTCAATTACCCGAAAGTAATCTACCGCTCCGGCT
TAGCCATG
>1397
CTGCTGCTGCCCGTAGGAGTCTGGTCCGTGTCTCAGTACCAGTGTGGGGGATCACCCTCTCAGGCCCCCTAAA
GATCGTCGCCTTGGTGAGCCGTTACCTCACCAACTAGCTAATCTTGCGCGTGCCCATCTCTATCCACCGTAGT
TTTCAATATCACCCGATGCCGAATAATATATTATGGGGTATTAATCTTCTCGAAAGGCTATCCCCTGATAAAG
GCAGGTTGCACACGTGTTCCGCACCCGTACGCCGCTCTCAATTACCCGAAAGTAATCTACCGCTCGGCTTGCA
TGTGTTAGGCCTCCCGCTAGCGTCATCCTGAGCAGGATCAACTCT
>1398
CTGCTGCCGCCCGTAGGAGTCTGGTCCGTGTCTCAGTACCAGTGTGGGGGATCACCCTCTCAGGCCCCCTAAA
GATCGTCGCCTTGGTGAGCCGTTACCTCACCAACTAGCTAATCTTGCGCGTGCCCATCTCTATCCACCGTAGT
TTTCAATATCACCCGATGCCGAATAATATATTATGGGGTATTAATCTTCTTCGAAAGGCTATCCCCTGATAAA
GGCAGGTTGCACACGTGTTCCGCACCCGTACGCCGCTCTCAATTACCCGAAAGTAATCTACCGCTCCGGCTTG
CATGTGTTAGGCCTCCCGCTAGCGTTCTCTGAGCAGGATCAACTCGT
>1399
CTGCTGCCGCCCGTAGGAGTCTGGTCCGTGTCTCAGTACCAGTGTGGGGGATCACCCCCTCAGGCCCCCTAAA
GATCGTCGCCTTGGTGAGCCGTTACCTCACCAACTAGCTAATCTTGCGCGTGCCCATCTCTATCCACCGTAGT
TTTCAATATCACCCGATGCCGAATAATATATTATGGGGTATTAATCTTCTTCGAAAGGCTATCCCCCTGATAA
AGGCAGGTTGCACACGTGTTCCGCACCCGTACGCCGCTCTCAATTACCCGAAAGTAATCTACCGCTCGGCTTG
CATGTGTTAGGCCTCCCGCTAGCGTTCATCCTGAGCCAGGATCAAACTCT
>1400
CTGCTGCCGCCCGTAGGAGTCTGGTCCGTGTCTCAGTACCAGTGTGGGGGATCACCCTCTCAGGCCCCCTAAA
GATCGTCGCCTTGGTGAGCCGTTACCTCACCAACTAGCTAATCTTGCGCGTGCCCATCTCTATCCACCGTAGT
TTTCAATATCACCCGATGCCGAATAATATATTATGGGGTATTAATCTTCTTCGAAAGGCTATCCCCCTGATAA
AGGCAGGTTGCACACGTGTTCCGCACCCGTACGCCGCTCTCAATTACCCGAAAGTAATCTACCGCTCCGGCTT
GCATGTGTTAGGCCTCCCGCTAGCGTTCATCCGTGAGCCAGGCATCAAACTCT
>1401
CTGCTGCCGCCCGTAGGAGTCTGGACCGTGTCTCAGTTCCAGTGTGACTGATCATCCTCTCAGACCAGTACGG
ATCGTCGCCTTGGTAGGCCTTTTACCCCACCAACTAGCTAATCCGACCTAGGCTCATCTGATAGCGTGAGGTC
CGAAGATCCCCCACTTCTCCCTCAGGACGTATGCGGTATTAGCGCCCGTTTCCGGACGTATCCCCACTACCAG
GCAGATTCCTAGGCATTACTCACCCGTCCGCCGCTGAATCCAGGAGCAAGCTCCCTTCATCCGCTCGACTGCA
TGTGTTAGGCCTGCCGCCAGCGTTCAATCTGAGCCAGGATCAAACTCT
>1402
CTGCTGCCGCCCGTAGGAGTCTGGTCCGTGTCTCAGTACCAGTGTGGGGGATCACCCTCTCAGGCCCCCTAAA
GATCGTCGCCTTGGTGAGCCGTTACCTCACCAACTAGCTAATCTTGCGCGTGCCCATCTCTATCCACCGTAGT
TTTCAATATCACCCGATGCCGAATAATAATATTATGGGGTATTAATCTTCTCGAAGGCTATCCCCTGATAAAG
CAGGTTGCACACGTGTTCCGCACCCGTACGCCGCTCTCAATTACCCGAAAGTAATCTACCGCTCCGGCTTGCA
TGTGTTAGGCCTCCCGCTAGCGTTCATCCTGAGCAGGCATCGAAACTCT
>1403
CTGCTGCCGCCCGTAGGAGTCTGGTCCGTGTCTCAGTACCAGTGTGGGGGATCACCCTCTCAGGCCCCCTAAA
GATCGTCGCCTTGGTGAGCCGTTACCTCACCAACTAGCTAATCTTGCGCGTGCCCATCTCTATCCACCGTAGT
TTTCAATATCACCCGATGCCGAATAATATATTATGGGGTATTAATCTTCTTCGAAAGGCTATCCCCCTGATAA
AGGCAGGTTGCACACGTGTTCCGCACCCGTACGCCGCTCTCAATTACCCGAAAGTAATCTACCGCTCCGGCTT
GCATGTGTTAGGCCTCCCGCTAGCGTTCATCCTGAGCCAGGATCAACTCT
>1404
CTGCTGCCGCCCGTAGGAGTCTGGTCCGTGTCTCAGTACCAGTGTGGGGGATCACCCTCTCAGGCCCCCTAAA
GATCGTCGCCTTGGTGAGCCGTTACCTCACCAACTAGCTAATCTTGCGCGTGCCCATCTCTATCCACCGTAGT

TTTCAATATCACCCGATGCCGAATAATATATTATGGGGTATTAATCTTCTTCGAAAGGCTATCCCCTGATAAA
GGCAGGTTGCACACGTGTTCCGCACCCGTACGCCGCTCTCAATTACCCGAAAGTAATCTACCGCTCCGGCTTG
CATGTGTTAGGCCTCCCGCTAGCGTTCATCTCGAGCCAGGATCAACTCT
>1405
CTGCTGCCGCCCGTAGGAGTCTGGTCCGTGTCTCAGTACCAGTGTGGGGGATCACCCTCTCAGGCCCCCTAAA
GATCGTCGCCTTGGTGAGCCGTTACCTCACCAACTAGCTAATCTTGCGCGTGCCCATCTCTATCCACCGTAGT
TTTCAATATCACCCGATGCCGAATAATATATTATGGGGTATTAATCTTCTCGAAAGCTATCCCCTGATAAAGG
CAGGTTGCACACGTGTTCCGCACCCGTACGCCGCTCTCAATTACCCGGAAAGTAATCTACCGCTCCGGCTTGA
CATGTGTTAGGCCTCCCGCTAGCGTTCATCTCGAGCAGGATCAACTCCT
>1406
CTGCTGCCGCCCGTAGGAGTCTGGTCCGTGTCTCAGTACCAGTGTGGGGGATCACCCTCTCAGGCCCCCTAAA
GATCGTCGCCTTGGTGAGCCGTTACCTCACCAACTAGCTAATCTTGCGCGTGCCCATCTCTATCCACCGTAGT
TTTCAATATCACCCGATGCCGAATAATATATTATGGGGTATTAATCTTCTTCGAAAGGCTATCCCCCTGATAA
AGGCAGGTTGCACACGTGTTCCGCACCCGTACGCCGCTCTCAATTACCCGAAAGTAATCTACCGCTCGGCTTG
CATGTGTTAGGCCTCCCGCTAG
>1407
CTGCTGCCGCCCGTAGGAGTCTGGTCCGTGTCTCAGTACCAGTGTGGGGGATCACCCTCTCAGGCCCCCTAAA
GATCGTCGCCTTGGTGAGCCGTTACCTCACCAACTAGCTAATCTTGCGCGTGCCCATCTCTATCCACCGTAGT
TTTCAATATCACCCGATGCCGAATAATATATTATGGGGTATTAATCTTCTTCGAAAGGCTATCCCCCTGATAA
AGGCAGGTTGCACACGTGTCCCGCACCCGTACGCCGCTCTCAATTACCCGAAAGTAATCTACCGCTCGGCTTG
CATGTGTTAGGCCTCCCGCTAGCGTTCATCCTGAGCCAGGATCAAACTCT
>1408
CTGCTGCCGCCCGTAGGAGTCTGGACCGTGTCTCAGTTCCAGTGTGACTGATCATCCTCTCAGACCAGTACGG
ATCGTCGCCTTGGTAGGCCTTTGCCCCACCAACTAGCTAATCCGACCTAGGCTCATCTGATAGCGTGAGGTCC
GAAGATCCCCCACTTCTCCCTCAGGACGTATGCGGTATTAGCGCCCGTTTCCGGACGATCCCCCACTACCAGG
CAGATTCCTAGGCATTACTCACCCGTCCGCCGCTGAATCCAGGAGCAAGCCCCCTTCATCGCTCGACTGCATG
TGTTAGGCCTGCCGCCAGCGTCAATCTGAGCCAGGATCAAACTCT
>1409
CTGCTGCCGCCCGTAGGAGTCTGGTCCGTGTCTCAGTACCAGTGTGGGGGATCACCCTCTCAGGCCCCCTAAA
GATCGTCGCCTTGGTGAGCCGTTACCTCACCAACTAGCTAATCTTGCGTGTGCCCATCTCTATCCACCGTAGT
TTCAATATCACCCGATGCCGAATAATATATTATGGGGTATTAATTCTTCTTCGAAAGGCTATCCCCTGATAAA
GGCAGGTTGCACACGTGTTCCGCACCCGTACGCCGCTCTCAATTACCCGAAAGTAATCTACCGCTCGGCTTGC
ATGTGTTAGGCCTCCCGCTAGCGTTCATCCTGAGCCAGGATCAACTCT
>1410
CTGCTGCCGCCCGTAGGAGTCTGGTCCGTGTCTCAGTACCAGTGTGGGGGATCGCCCTCTCAGGCCCCCTAAA
GATCGTCGCCTTGGTGAGCCGTTACCTCACCAACTAGCTAATCTTGCGCGTGCCCATCTCTATCCACCGTAGT
TTTCAATATCACCCGATGCCGAATAATATATTATGGGGTATTTAATCTTTCTTCGAAAGGCTATCCCCCTGAT
AAAGGCAGGTTGCACACGTGTTCCGCACCCGTACGCCGCTCTCAATTACCCGAAAGTAATCTACCGCTCGGCT
TGCATGTGTTAGGCCTCCCGCTAGCGTTCATTCCTGAGCCAGGATCAAACTCT
>1411
CTGCTGCCGCCCGTAGGAGTCTGGTCCGTGTCTCAGTACCAGTGTGGGGGATCACCCTCTCAGGCCCCCTAAA
GATCGTCGCCTTGGTGAGCCGTTACCTCACCAACTAGCTAATCTTGCGCGTGCCCATCTCTATCCACCGTAGT
TTTCAATATCACCCGATGCCGAATAATATATTATTGGGGTATTTAATCTTCTTCGAAAGGCTATCCCCCTGAT
AAAGGCAGGTTGCACACGTGTTCCGCACCCGTACGCCGCTCTCAATTACCCGAAAGTAATCTACCGCTCGGCT
TGCATGTGTTAGGCCTCCCGCTAGCGTTCATCCTGAGCCAGGATCAAACTCT
>1412
CTGCTGCCGCCCGTAGGGGTCTGGTCCGTGTCTCAGTACCAGTGTGGGGGGATCACCCCCTCAGGCCCCCTAA
AAGATCGTCGCCTTGGTGAGCCGTTACCTCACCAACTAGCTAATCTTGCGCGTGCCCATCTCTATCCACCGTA
GTTTTCAATATCACCCGATGCCGAATAATATATTATGGGGTATTAATCTTCTTCGAAAGGCTATCCCCTGATA
AAGGCAGGTTGCACACGTGTTCCGCACCCGTACGCCGCTCTCAATTACCCGAAAGTAATCTACCGCTCGGCTT
GCATGTGTTAGGCCTCCCGCTAGCGTTCTCCTGAGCAGGATCAAACTCGT
>1413
CTGCTGCCGCCCGTAGGAGTCTGGACCGTGTCTCAGTTCCAGTGTGACTGATCATCCTCTCAGACCAGTACGG
ATCGTCGCCTGGTAGGCCTTTACCCCACCAACTAGCTAATCCGACCTAGGCTCATCTGATAGCGTGAGGTCCG

AAGTCCCCCACTTCTCCCTCAGGGCGTATGCGGTATTAGCGCCCGTTTCCGGACGTATCCCCGCACTACCCAG
GCAGATTCCCTAGGCATTACTCACCCGTCCGCCGCTGAATCCAGGAGCAAGCTCCCTCATCCGCTCGACTGCC
ATGTGTTAGGCCTGCCGCCAGCGTCAATCTGAGCCAGGATCAAACTCT
>1414
CTGCTGCCGCCCGTAGGAGTCTGGTCCGTGTCTCAGTACCAGTGTGGGGGATCACCCTCTCAGGCCCCCTAAA
GATCGTCGCCTTGGTGAGCCGTTACCTCACCAACTAGCTAATCTTGCGCGTGCCCATCTCTATCCACCGTAGT
TTTCAATATCACCCGATGCCGAATAATATATTATGGGGTATTAATCTTCTTCGAAAGGCTATCCCCCTGATAA
AGGCAGGTTGCACACGTGTTCCGCACCCGTACGCCGCTCTCAATTACCCGAAAGTAATCTACCGCTCGGCTTG
CATGTGTTAGGCCTCCCGCTAGCGTTCTCCTGAGCAGGATCAACTCGT
>1415
CTGCTGCCGCCCGTAGGAGTCTGGTCCGTGTCTCAGTACCAGTGTGGGGGATCACCCTCTCAGGCCCCCTAAA
GATCGTCGCCTTGGTGAGCCGTTACCTCACCAACTAGCTAATCTTGCGCGTGCCCATCTCTATCCACCGTAGT
TTTCAATATCACCCGATGCCGAATAATATATTATGGGGTATTAATCTTCTTCGAAAGGCTATCCCCTGATAAA
GGCAGGTTGCACACGTGTTCCGCACCCGTACGCCGCTCTCAATTACCCGAAAGTAATCTACCGCTCGGCTTGC
ATGTGTTAGGCCTCCCGCTAGCGTTCTCCTGAGCAGGTCAACT
>1416
CTGCTGCCGCCCGTAGGAGTCTGGTCCGTGTCTCAGTACCAGTGTGGGGGATCACCCTCTCAGGCCCCCTAAA
GATCGTCGCCTGGTGAGCCGTTACCTCACCAACTAGCTAATCTTGCGCGTGCCCATCTCTATCCACCGTAGTT
TTCAATATCACCCGATGCCGAATAATATATTATGGGGTATTAATCTTCTCGAAAGGCTATCCCCCTGATAAAG
GCAGGTTGCACACGTGTTCCGCACCCGTACGCCGCTCTCAATTACCCGAAAGTAATCTACCGCTCCGGCCTGC
ATGTGTTAGGCCTCCCGCTAGCGTTCATCCTGAGCCAGGATCAACTCT
>1417
CTGCTGCCGCCCGTAGGAGTCTGGTCCGTGTCTCAGTACCAGTGTGGGGGATCACCCTCTCAGGCCCCCTAAA
GATCGTCGCCTTGGTGAGCCGTTACCTCACCAACTAGCTAATCTTGCGCGTGCCCATCTCTATCCACCGTAGT
TTTCAATATCACCCGATGCCGAATAATATATTATGGGGTATTAATCTTCTCGAAAGGCTATCCCCTGATAAAG
GCAGGTTGCACACGTGTTCCGCACCCGTACGCCGCTCTCAATTACCCGAAAGTAATCTACCGCATCGGCTTGC
ATGTGTTAGGCCTCCCGCTAGCGTTCATCCTGAGCCAGGATCAACTCT
>1418
CTGCTGCCGCCCGTAGGAGTCTGGTCCGTGTCTCAGTACCAGTGTGGGGGATCACCCTCTCAGGCCCCCTAAA
GATCGTCGCCTGGTGAGCCGTTACCTCACCAACTAGCTAATCTTGCGCGTGCCCATCTCTATCCACCGTAGTT
TCAATATCACCCGATGCCGAATAATATATTATGGGGTATTAATCTTCTCGAAAGGCTTATCCCCCTGATAAAG
GCAGGTTGCCACACGTGTTCCGCACCCGTACGCCGCTCTCAATTACCCGAAAGTAATCTACCGCTCGGCTTGC
ATGTGTTAGGCCTCCCGCTAGCGTTCATC
>1419
CTGCTGCCGCCCGTAGGAGTCTGGTCCGTGTCTCAGTACCAGTGTGGGGGATCACCCTCTCAGGCCCCCTAAA
GATCGTCGCCTTGGTGAGCCGTTACCTCACCAACTAGCTAATCTTGCGCGTGCCCCATCTCTATCCACCGTAG
TTTTCAATATCACCCGATGCCGAATAATATATTTATTGGGGTATTTAATCTTCTTCGAAAGGCTATCCCCCTG
ATAAAGGCAGGTTGCACACGTGTTCCGCACCCGTACGCCGCTCTCAATTACCCCGAAAGTAATCTACCGCTCG
GCTTGCATGTGTTAGGCCTCCCGCTAGCGTTCATCCTGAGCCAGGATCAACTCT
>1420
CTGCTGCCGCCCGTAGGAGTCTGGTCCGTGTCTCAGTACCAGTGTGGGGGATCACCCTCTCAGGCCCCCTAAA
GATCGTCGCCTTGGTGAGCCGTTACCTCACCAACTAGCTAATCTTGCGCGTGCCCATCTCTATCCACCGTAGT
TTTCAATATCACCCGATGCCGAATAATATATTATGGGGTATTAATCTTCTTCGAAAGGCTATCCCCTGATAAA
GGCAGGTTGCACACGTGTTCCGCACCCGTACGCCGCTCTCAATTACCCGAAAGTAATCTACCGCTCCGGCTTG
CATGTGTTAGGCCTCCCGCTAGCGTTCTCCTGAGCAGGATCAACTCGT
>1421
CTGCTGCCGCCCGTAGGAGTCTGGTCCGTGTCTCAGTACCAGTGTGGGGGATCACCCTCTCAGGCCCCCTAAA
GATCGTCGCCTTGGTGAGCCGTTACCTCACCAACTAGCTAATCTTGCGCGTGCCCATCTCTATCCACCGTAGT
TTTCAATATCACCCGATGCCGAATAATATATTATGGGGTATTAATCTTCTTCGAAAGGCTATCCCCCTGATAA
AGGCAGGTTGCACACGTGTTCCGCACCCGTACGCCGCTCTCAATTACCCGAAAGTAATCTACCGCTCGGCTTG
CATGTGTTAGGCCTCCCGCTAGCGTTCATCCTGAGCCAGGATCAAACTCT
>1422
TGCTGCCGCCCGTAGGAGTCTGGTCCGTGTCTCAGTACCAGTGTGGGGGATCACCCTCTCAGGCCCCCTAAAG
ATCGTCGCCTTGGTGAGCCGTTACCTCACCAACTAGCTAATCTTGCGCGTGCCCATCTCTATCCACCGTAGTT

TTCAATATCACCCGATGCCGAATAATATATTATGGGGTATTAATCTTCTTCGAAAGGCTATCCCCCTGATAAA
GGCAGGTTGCACACGTGTTCCGCACCCGTACGCCGCTCTCAATTACCCGAAAGTAATCTACCGCTCGGCTTGC
ATGTGTTAGGCCTCCCGCTAGCGTTCATCCTGAGCCAGGATCAACTCT
>1423
CTGCTGCCGCCCGTAGGAGTCTGGTCCGTGTCTCAGTACCAGTGTGGGGGATCACCCTCTCAGGCCCCCTAAA
GATCGTCGCCTTGGTGAGCCGTTACCTCACCAACTAGCTAATCTTGCGCGTGCCCATCTCTATCCACCGTAGT
TTTCAATATCACCCGATGCCGAATAATATATTATGGGGTATTAATCTTCTCGAAAGGCTATCCCCTGATAAAG
GCAGGTTGCACACGTGTTCCGCACCCGTACGCCGCTCTCAATTACCCGAAAGTAATCTACCGCTCGGCTTGCA
TGTGTTAGGCCTCCCGCTAGCGTTCTCCTGAGCAGGATCAACTCG
>1424
CTGCTGCCGCCCGTAGGAGTCTGGTCCGTGTCTCAGTACCAGTGTGGGGGATCACCCTCTCCAGGCCCCCTAA
AGATCGTCGCCTTGGTGAGCCGTTACCTCACCAACTAGCTAATCTTGCGCGTGCCCATCTCTATCCACCGTAG
TTTTCAATATCACCCGATGCCGAATAATATATTATGGGGTATTAATCTTCTTCGAAAGGCTATCCCCCTGATA
AAGGCAGGTTGCACACGTGTTCCGCACCCGTACGCCGCTCTCAATTACCCGAAAGTAATCTACCGCTCGGCTT
GCATGTGTTAGGCCTCCCGCTAGCGTTCATCCTGAGCCAGGATCAACTCT
>1425
CTGCTGCCGCCCGTAGGAGTCTGGTCCGTGTCTCAGTACCAGTGTGGGGGATCACCCTCTCAGGCCCCCCTAA
AGATCGTCGCCTTGGTGAGCCGTTACCTCACCAACTAGCTAATCTTGCGCGTGCCCCATCTCTATCCACCGTA
GTTTTCAATATCACCCGATGCCGAATAATATATTATTGGGGTATTTAATCTTCTTCGAAAGGCTATCCCCCTG
ATAAAGGCAGGTTGCACACGTGTTCCGCACCCGTACGCCGCTCTCAATTACCCCGAAAGTAATCTACCGCTCG
GCTTGCATGTGTTAGGCCTCCCGCTAGCGTTCATTCCTCGAGCCAGGATCAAACTCT
>1426
CTGCTGCCGCCCGTAGGAGTCTGGTCCGTGTCTCAGTACCAGTGTGGGGGATCACCCTCTCAGGCCCCCTAAA
GATCGTCGCCTTGGTGAGCCGTTACCTCACCAACTAGCTAATCTTGCGCGTGCCCATCTCTATCCACCGTAGT
TTTCAATATCACCCGATGCCGAATAATATATTATGGGGTATTAATCTTCTTCGAAAGGCTATCCCCTGATAAA
GGCAGGTTGCACACGTGTTCCGCACCCGTACGCCGCTCTCAATTACCCGAAAGTAATCTACCGCTCGGCTTGC
ATGTGTTAGGCCTCCCGCTAGCGTTCATCCTGAGCCAGGATCAACTCT
>1427
CTGCTGCCGCCCGTAGGAGTCTGGACCGTGTCCCAGTTCCAGTGTGACTGATCATCCTCTCAGACCAGTTACG
GGTCGTCGCCTTGGTAGGCCTTTACCCCACCAACTAGCTAATCCGACCTAGGCTCATCTGATAGCGTGAGGTC
CGAAGATCCCCCACTTCTCCCTCAGGACGTATGCGGTATTAGCGCCCGTTTCCGGACGTATCCCCACTACCAG
GCAGATTCCTAGGCATTACTCACCCGTCCGCCGCTGAATCCAGGAGCAAGCTCCCTTCATCCGCTCGACTGCA
TGTGTTAGGCCTGCCGCCAGCGTTCAATCTGAGCCAGGATCAAACTCT
>1428
CTGCTGCCGCCCGTAGGAGTCTGGTCCGTGTCTCAGTACCAGTGTGGGGGATCACCCTCTCAGGCCCCCTAAA
GATCGTCGCCTTGGTGAGCCGTTACCTCACCAACTAGCTAATCTTGCGCGTGCCCATCTCTATCCACCGTAGT
TTTCAATATCACCCGATGCCGAATAATATATTATGGGGTATTAATCTCTTCGAAAGGCTATCCCCTGATAAAG
GCAGGTTGCACACGTGTTCCGCACCCGTACGCCGCTCTCAATTACCCGAAAGTAATCTACCGCTCCGGCTTGC
ATGTGTTAGGCCTCCCGCTAGCGTTCATCCTGAGCCAGGATCAAACTCT
>1429
CTGCTGCCGCCCGTAGGAGTCTGGACCGTGTCTCAGTTCCAGTGTGACTGATCATCCTCTCAGACCAGTACGG
ATCGTCGCCTTGGTAGGCCTTTACCCCACCAACTAGCTAATCCGACCTAGGCTCATCTGATAGCGTGAGGTCC
GAAGATCCCCCACTTCTCCCTCAGGACGTATGCGGTATTAGCGCCCGTTTCCGGACGTATCCCCACTACCAGG
CAGATTCCTAGGCATTACTCACCCGTCCGCCGCTGAATCCAGGAGCAAGCTCCCTCATCCGCTCGACTGCATG
TGTTAGGCCTGCCGCCAGCGTCAACCTGAGCCAGGATCAAACTCT
>1430
CTGCTGCCGCCCGTAGGAGTCTGGTCCGTGTCTCAGTACCAGTGTGGGGGATCACCCTCTCAGGCCCCCTAAA
GATCGTCGCCTTGGTGAGCCGTTACCTCACCAACTAGCTAATCTTGCGCGTGCCCATCTCTATCCACCGTAGT
TTTCAATATCACCCGATGCCGAATAATATATTATGGGGTATTAATCTTCTTCGAAAGGCTATCCCCTGATAAA
GGCAGGTTGCACACGTGTTCCGCACCCGTACGCCGCTCTCAATTACCCGAAAGTAATCTACCGCTCGGCTTGC
ATGTGTTAGGCCTCCCGCTAGCGTTCATCCTGAGCCAGGATCAACTCT
>1431
CTGCTGCCGCCCGTAGGAGTCTGGTCCGTGTCTCAGTACCAGTGTGGGGGATCACCCTCTCAGGCCCCCTAAA
GATCGTCGCCTTGGTGAGCCGTTACCTCACCAACTAGCTAATCTTGCGCGTGCCCATCTCTATCCACCGTAGT

TTTCAATATCACCCGATGCCGAATAATATATTATGGGGTATTAATCTTCTTCGAAAGCTATCCCCCTGATAAA
GGCAGGTTGCACACGTGTTCCGCACCCGTACGCCGCTCTCAATTACCCGAAAGTAATCTACCGCTCCGGCTTG
CATGTGTTAGGCCTCCCGCTAGCGTTCATCCTCGAGCCAGGATCAACTCT
>1432
CTGCTGCCGCCCGTAGGAGTCTGGTCCGTGTCTCAGTACCAGTGTGGGGGATCACCCTCTCAGGCCCCCTAAA
GATCGTCGCCTTGGTGAGCCGTTACCTCACCAACTAGCTAATCTTGCGCGTGCCCATCTCTATCCACCGTAGT
TTTCAATATCACCCGATGCCGAATAATATATTATGGGGTATTAATCTTCTTCGAAAGGCTATCCCCTGATAAA
GGCAGGTTGCACACGTGTTCCGCACCCGTACGCCGCTCTCAATTACCCGAAAGTAATCTACCGCTCCGGCTTG
CATGTGTTAGGCCTCCCGCTAGCGTTCATCCTGAGCCAGGATCAACTCT
>1433
CTGCTGCCGCCCGTAGGAGTCTGGTCCGTGTCTCAGTACCAGTGTGGGGGATCACCCTCTCAGGCCCCCTAAA
GATCGTCGCCTTGGTGAGCCGTTACCTCACCAACTAGCTAATCTTGCGCGTGCCCATCTCTATCCACCGTAGT
TTTCAATATCACCCGATGCCGAATAATATATTATGGGGTATTAATCCTCTTCGAAAGGCTATCCCCTGATAAA
GGCAGGTTGCACACGTGTTCCGCACCCGTACGCCGCTCTCAATTACCCGAAAGTAATCTACCGCTCCGGCTTG
CATGTGTTAGGCCTCCCGCTAGCGTTCATCCGTGAGCCAGGATCAAACTCT
>1434
CTGCTGCCGCCCGTAGGAGTCTGGTCCGTGTCTCAGTACCAGTGTGGGGGATCACCCTCTCAGGCCCCCTAAA
GATCGTCGCCTTGGTGAGCCGTTACCTCACCAACTAGCTAATCTTGCGCGTGCCCATCTCTATCCACCGTAGT
TTTTCAATATCACCCGATGCCGAATAATATATTATGGGGTATTTAATCTTTCTTCGAAAGGCTATCCCCCTGA
TAAAGGCAGGTTGCACACGTGTTCCGCACCCGTACGCCGCTCTCAATTACCCGAAAGTAATCTACCGCTCGGC
TTGCATGTGTTAGGCCTCCCGCTAGCGTTCTCCTCGAGCAGGATCCAACATCT
>1435
CTGCTGCCGCCCGTAGGAGTCTGGTCCGTGTCTCAGTACCAGTGTGGGGGATCACCCTCTCAGGCCCCCTAAA
GATCGTCGCCTTGGTGAGCCGTTACCTCACCAACTAGCTAATCTTGCGCGTGCCCATCTCTATCCACCGTAGT
TTTCAATATCACCCGATGCCGAATAATATATTATGGGGTATTAATCTTCTTCGAAAGGCTATCCCCCTGATAA
AGGCAGGTTGCACACGTGTTCCGCACCCGTACGCCGCTCTCAATTTACCCGAAAGAATCTACCGCTCCGGGCT
TGCATGTGTTAGGCCTCCCGCTAGCGTTCATCCTGAGCCAGGATCAACTCT
>1436
CTGCTGCCGCCCGTAGGAGTCTGGTCCGTGTCTCAGTACCAGTGTGGGGGATCACCCTCTCAGGCCCCCTAAA
GATCGTCGCCTTGGTGAGCCGTTACCTCACCAACTAGCTAATCTTGCGCGTGCCCATCTCTATCCACCGTAGT
TTTCAATATCACCCGATGCCGAATAATATATTATGGGGTATTAATCTTCTTCGAAAGGCTATCCCCCTGATAA
AGGCAGGTTGCACACGTGTTCCGCACCCGTACGCCGCTCTCAATTACCCGAAAGTAATCTACCGCTCGGCTTG
CATGTGTTAGGCCTCCCGCTAGCGTTCATCCGTCGAGCAGGCATCAACTCT
>1437
CTGCTGCCGCCCGTAGGAGTCTGGTCCGTGTCTCAGTACCAGTGTGGGGGATCACCCTCTCAGGCCCCCTAAA
GATCGTCGCCTTGGTGAGCCGTTACCTCACCAACTAGCTAATCTTGCGCGTGCCCATCTCTATCCACCGTAGT
TTTCAATATCACCCGATGCCGAATAATATATTATGGGGTATTAATCTTCTTCGAAAGCTATCCCCTGATAAAG
GCAGGTTGCACACGTGTTCCGCACCCGTACGCCGCTCTCAATTACCCGAAAGTAATCTACCGCTCCGGCTTGC
ATGTGTTAGGCCTCCCGCTAGCGTTCATCCGTCGAGCCAGGATCAAACTCT
>1438
CTGCTGCCGCCCGTAGGAGTCTGGTCCGTGTCTCAGTACCAGTGTGGGGGATCACCCTCTCAGGCCCCCTAAA
GATCGTCGCCTTGGTGAGCCGTTACCTCACCAACTAGCTAATCTTGCGCGTGCCCATCTCTATCCACCGTAGT
TTTCAATATCACCCGATGCCGAATAATATATTATGGGGTATTAATCTTCTTCGAAAGGCTATCCCCTGATAAA
GGCAGGTTGCACACGTGTTCCGCACCCGTACGCCGCTCTCAATTACCCGAAAGTAATCTACCGCTCCGGCTTG
CATGTGTTAGGCCTCCCGCTAGCGTTCATCCGTGAGCAGGCATCAACTCT
>1439
CTGCTGCCGCCCGTAGGAGTCTGGTCCGTGTCTCAGTACCAGTGTGGGGGATCACCCTCTCAGGCCCCCTAAA
GATCGTCGCCTTGGTGAGCCGTTACCTCACCAACTAGCTAATCTTGCGCGTGCCCATCTCTATCCACCGTAGT
TTTCAATATCACCCGATGCCGAATAATATATTATGGGGTATTAATCTTCTTCGAAAGGCTATCCCCCTGATAA
AGGCAGGTTGCACACGTGTTCCGCACCCGTACGCCGCTCTCAATTACCCGAAAGTAATCTACCGCTCCGGCTT
GCATGTGTTAGGCCTCCCGCTAGCGTTCATCCTGAGCCAGGATCAACTCT
>1440
CTGCTGCCGCCCGTAGGAGTCTGGTCCGTGTCTCAGTACCAGTGTGGGGGATCACCCTCTCAGGCCCCCTAAA
GATCGTCGCCTTGGTGAGCCGTTACCTCACCAACTAGCTAATCTTGCGCGTGCCCATCTCTATCCACCGTAGT

TTTCAATATCACCCGATGCCGAATAATATATTATGGGGTATTAATCTTCTTCGAAAGGCTATCCCCTGATAAA
GGCAGGTTGCACACGTGTTCCGCACCCGTACGCCGCTCTCAATTACCCGAAAGTAATCTACCGCTCGGCTTGC
ATGTGTTAGGCCTCCCGCTAGCGTTCATCCTGAGCAGGCATCAAACTCT
>1441
CTGCTGCCGCCCGTAGGAGTCTGGTCCGTGTCTCAGTACCAGTGTGGGGGATCACCCTCTCAGGCCCCCTAAA
GATCGTCGCCTTGGTGAGCCGTTACCTCACCAACTAGCTAATCTTGCGCGTGCCCATCTCTATCCACCGTAGT
TTTCAATATCACCCGATGCCGAATAATATATTATGGGGTATTAATCTTCTTCGAAAGGCTATCCCCTGATAAA
GGCAGGTTGCACACGTGTTCCGCACCCGTACGCCGCTCTCAATTACCCGAAAGTAATCTACCGCTCGGCTTGC
ATGTGTTAGGCCTCCCGCAGCGTTCTC
>1442
CTGCTGCCGCCCGTAGGAGTCTGGTCCGTGTCTCAGTACCAGTGTGGGGATCACCCTCTCAGGCCCCCTAAAG
ATCGTCGCCTTGGTGAGCCGTTACCTCACCAACTAGCTAATCTTGCGCGTGCCCATCTCTATCCACCGTAGTT
TCAATATCACCCGATGCCGAATAATATATTATGGGGTATTAATCTTCTCGAAAGGCTATCCCCCTGATAAAGG
CAGGTTGCACACGTGTTCCGCACCCGTACGCCGCTCTCAATTACCCGAAAGTAATCTACCGCTCCGGCTTGCA
TGTGTTAGGCCTCCCGCTAAGC
>1443
CTGCTGCCGCCCGTAGGAGTCTGGTCCGTGTCTCAGTACCAGTGTGGGGGATCACCCTCTCAGGCCCCCTAAA
GATCGTCGCCTTGGTGAGCCGTTACCTCACCAACTAGCTAATCTTGCGCGTGCCCATCTCTATCCACCGTAGT
TTTCAATATCACCCGATGCCGAATAATATATTATGGGGTATTAATCTTCTTCGAAAGGCTATCCCCCTGATAA
AAGGCAGGTTGCACACGTGTTCCGCACCCGTACGCCGCTCTCAATTACCCGAAAGTAATCTACCGCTCGGCTT
GCATGTGTTAGGCCTCCCGCTAGCGTTCATCCTCGAGCAGGCATCAAACTCT
>1444
CTGCTGCCGCCCGTAGGAGTCTGGTCCGTGTCTCAGTACCAGTGTGGGGGATCACCCTCTCAGGCCCCCTAAA
GATCGTCGCCTTGGTGAGCCGTTACCTCACCAACTAGCTAATCTTGCGCGTGCCCATCTCTATCCACCGTAGT
TTTCAATATCACCCGATGCCGAATAATATATTATGGGGTATTAATCTTCTCGAAAGCTATCCCCTGATAAAGG
CAGGTTGCACACGCGTCCGCACCCGTACGCCGCTCTCAATTACCCGAAAGAATCTACCGCTCGGCTTGCATGT
GTTAGGCCTCCGCTAGCGTCTCTCGAGCAGGATCCAACAT
>1445
CTGCTGCCGCCCGTAGGAGTCTGGTCCGTGTCTCAGTACCAGTGTGGGGGATCACCCTCTCAGGCCCCCTAAA
GATCGTCGCCTTGGTGAGCCGTTACCTCACCAACTAGCTAATCTTGCGCGTGCCCATCTCTATCCACCGTAGT
TTTCAATATCACCCGATGCCGAATAATATATTATGGGGTATTAATCTTCTTCGAAAGCTATCCCCCTGAATAA
AGGCAGGTTGCACACGTGTTCCGCACCCGTACGCCGCTCTCAATTACCCGAAAGTAATCTACCGCTCGGCTTG
CATGTGTTAGGCCTCCCGCTAGCGTTCATCCTGAGCCAGGATCAAACTCT
>1446
CTGCTGCCGCCCGTAGGAGTCTGGTCCGTGTCTCAGTACCAGTGTGGGGGATCACCCTCTCAGGCCCCCTAAA
GATCGTCGCCTTGGTGAGCCGTTACCTCACCAACTAGCTAATCTTGCGCGTGCCCATCTCTATCCACCGTAGT
TTTCAATATCACCCGATGCCGAATAATATATTATGGGGTATTAATCTTCTTCGAAAGGCTATCCCCCTGATAA
AGGCAGGTTGCACACGTGTTCCGCACCCGTACGCCGCTCTCAATTACCCGAAAGTAATCTACCGCTCGGCTTG
CATGTGTTAGGCCTCCCGCTAGCGTTCATCCTGAGCCAGGATCAAACTCT
>1447
CTGCTGCCGCCCGTAGGAGTCTGGTCCGTGTCTCAGTACCAGTGTGGGGGATCACCCTCTCAGGCCCCCTAAA
AGATCGTCGCCTTGGTGAGCCGTTACCTCACCAACTAGCTAATCTTGCGCGTGCCCATCTCTATCCACCGTAG
TTTTCAATATCACCCGATGCCGAATAATATATTATGGGGGTATTAATCTTCTCGAAAGGCTATCCCCTGATAA
AGGCAGGTTGCACACGTGTTCCGCACCCGTACGCCGTTCTCAATTACCCGAAAGTAATCTACCGCTCGGCTTG
CATGTGTTAGGCCTCCCGCTAGCGTTCATCCTGAGCCAGGATCAAACTCT
>1448
CTGCTGCCGCCCGTAGGAGTCTGGTCCGTGTCTCAGTACCAGTGTGGGGGATCACCCTCTCAGGCCCCCTAAA
GATCGTCGCCTTGGTGAGCCGTTACCTCACCAACTAGCTAATCTTGCGCGTGCCCATCTCTATCCACCGTAGT
TTTCAATATCACCCGATGCCGAATAATATATTATGGGGTATTAATCTTCTTCGAAGGCTATCCCCCTGATAAA
GGCAGGTTGCACACGTGTTCCGCACTCGTACGCCGCTCTCAATTACCCGAAAGTAATCTACCGCTCGGCTTGC
ATGTGTTAGGCCTCCCGCTAGCGTTCATCCTGAGCCAGGATCAAACTCT
>1449
CTGCTGCCGCCCGTAGGAGTCTGGTCCGTGTCTCAGTACCAGTGTGGGGGATCACCCTCTCAGGCCCCCTAAA
GATCGTCGCCTTGGTGAGCCGTTACCTCACCAACTAGCTAATCTTGCGCGTGCCCATCTCTATCCACCGTAGT

TTTCAATATCACCCGATGCCGAATAATATATTATGGGGTATTTAATCTTCTTCGAAAGGCTATCCCCCTGATA
AAGGCAGGTTGCACACGTGTTCCGCACCCGTACGCCGCTCTCAATTACCCGAAAGTAATCTACCGCTCGGCTT
GCAATGTGTTAGGCCTCCCGCTAGCGTTCATTCCTGAGCCAGGACAACTCT
>1450
CTGCTGCCGCCCGTAGGAGTCTGGTCCGTGTCTCAGTACCAGTGTGGGGGATCACCCTCTCAGGCCCCCTAAA
GATCGTCGCCTTGGTGAGCCGTTACCTCACCAACTAGCTAATCCTGCGCGTGCCCATCTCTATCCACCGTAGT
TTTCAATATCACCCGATGCCGAATAATATAATTATGGGGTATTAATCTTCTCGAAAGGCTATCCCCTGATAAA
GGCAGGTTGCACACGTGTTCCGCACCGTACGCCGCTCTCAATTACCCGAAAGTAATCTACCGCTCGGCTTGCA
TGTGTTAGGCCTCCCGCTAGCGT
>1451
CTGCTGCCGCCCGTAGGAGTCTGGTCCGTGTCTCAGTACCAGTGTGGGGGATCACCCTCTCAGGCCCCCTAAA
GATCGTCGCCTTGGTGAGCCGTTACCTCACCAACTAGCTAATCTTGCGCGTGCCCATCTTTATCCACCGTAGT
TTTCAATATCACCCGATGCCGAATAATATATTATGGGGTATTAATCTTCTTCGAAAGGCTATCCCCCTGATAA
AGGCAGGTTGCACACGTGTTCCGCACCCGTACGCCGCTCTCAATTACCCGAAAGTAATCTACCGCTCGGCTTG
CATGTGTTAGGCCTCCCGCTAGCGTTCATCCTGAGCCAGGATCAAACTCT
>1452
CTGCTGCCGCCCGTAGGGGTCTGGTCCGTGTCTCAGTACCAGTGTGGGGGATCACCCTCTCAGGCCCCCTAAA
GATCGTCGCCTTGGTGAGCCGTTACCTCACCAACTAGCTAATCTTGCGCGTGCCCATCTCTATCCACCGTAGT
TTTCAATATCACCCGATGCCGAATAATATATTATGGGGTATTAATCTTCTTCGAAAGGCTATCCCCCTGATAA
AGGCAGGTTGCACACGTGTTCCGCACCCGTACGCCGCTCTCAATTACCCGAAAGTAATCTACCGCTCGGCTTG
CATGTGTTAGGCCTCCCGCTAGCGTTCATCCTGAGCCAGGATCAAACTCT
>1453
CTGCTGCCGCCCGTAGGAGTCTGGTCCGTGTCTCAGTACAGTGTGGGGGATCACCCTCTCAGGCCCCCTAAAG
ATCGTCGCCTTGGTGAGCCGTTACCTCACCAACTAGCTAATCTTGCGCGTGCCCATCTCTATCCACCGTATTT
TCAATATCACCCGATGCCGAATAATATAATTATGGGGTATTAATCTTCTCGAAGGCTATCCCCCTGATAAAGG
CAGGTTGCACACGTGTTCCGCACCCGTACGCCGCTCTTCAATTACCCGAAAGAATCTACCGCACGGCTTGCAT
GTGTAGGCCTCCCGCTAGCGTTCTCCTGAGCAGGATCAACTCGT
>1454
CTGCTGCCGCCCGTAGGAGTCTGGTCCGTGTCTCAGTACCAGTGTGGGGGATCACCCTCTCAGGCCCCCTAAA
GATCGTCGCCTTGGTGAGCCGTTACCTCACCAACTAGCTAATCTTGCGCGTGCCCATCTCTATCCACCGTAGT
TTTCAATATCACCCGATGCCGAATAATATATTATGGGGTATTAATCTTCTTCGAAAGGCTATCCCCCTGATAA
AGGCAGGTTGCACACGTGTTCCGCACCCGTACGCCGCTCTCAATTACCCGAAAGTAATCTACCGCTCGGCTTG
CATGTGTTAGGCCTCCCGCTAGCGTTCATCCTGAGCCAGGATCAAACTCT
>1455
CTGCTGCCGCCCGTAGGAGTCTGGTCCGTGTCTCAGTACCAGTGTGGGGGATCACCCTCTCAGGCCCCCTAAA
GATCGTCGCCTTGGTGAGCCGTTACCTCACCAACTAGCTAATCTTGCGCGTGCCCATCTCTATCCACCGTAGT
TTTCAATATCAACCCGATGCCGAATAATATAATTATGGGGTATTAATCTTCTTCGAAAGGCTATCCCCCTGAT
AAAGGCAGGTTGCACACGTGTTCCGCACCCGTACGCCGCTCTCAATTTACCCGAAAGTAATCTACCGCTCGGC
TTGCATGTGTTAGGCCTCCCGCTAGCGTTCATCCTGAGCCAGGATCAAACTCT
>1456
CTGCTGCCGCCCGTAGGAGTCTGGTCCGTGTCTCAGTACCAGTGTGGGGGATCACCCTCTCAGGCCCCCTAAA
GATCGTCGCCTTGGTGAGCCGTCACCTCACCAACTAGCTTAATCTTGCGCGTGCCCATCTCTATCCACCGTAG
TTTTCAATATCACCCGATGCCGAATAATATAATTATGGGGGTATTAATCTTCTCGAAAGGCTATCCCCCTGAT
AAAGGCAGGTTGCACACGTGTTCCGCACCCGTACGCCGCTCTCAATTACCCGAAAGTAATCTACCGCTCGGCT
TGCATGTGTTAGGCCTCCCGCTAGCGTTCATCCTGAGCCAGGATCAACTCT
>1457
CTGCTGCCGCCCGTAGGAGTCTGGTCCGTGTCTCAGTACCAGTGTGGGGGATCACCCTCTCAGGCCCCCTAAA
GATCGTCGCCTTGGTGAGCCGTTACCTCACCAACTAGCTAATCTTGCGCGTGCCCATCTCTATCCACCGTAGT
TTTCAATATCACCCGATGCCGAATAATATATTATGGGGTATTAATCTTCTTCGAAAGGCTATCCCCTGATAAA
GGCAGGTTGTCACACGTGTTCCGCACCCGTACGCCGCTCTCAATTACCCGAAAGTAATCTACCGCTCGGCTTG
CATGTGTTAGGCCTCCCGCTAGCGTTCTCCTGAGCAGGATC
>1458
CTGCTGCCGCCTAGGAGTCTGGCTCGTGTCTCAGTACCAGTGTGGGGGATCACCCTCTCAGGCCCCCTAAAGA
TCGTCGCCTTGGTGAGCCGTTACCTCACCAACTAGCTAATCTTGCGCGTGCCCATCTCTATCCACCGTAGTTT

TCAATATCACCCGATGCCGAATAATATATTATGGGGTATTAATCTTCTCGAAAGCTATCCCCTGATAAAGGCA
GGTTGCACACGTGTTCCGCACCCGTACGCCGCTCTCAATTACCCGAAAGTAATCTACCGCTCCGGCTTGCATG
TGTTAGGCCTCCCGCTAGCGTTCATCCGTG
>1459
CTGCTGCCGCCCGTAGGAGTCTGGTCCGTGTCTCAGTACCAGTGTGGGGGATCACCCTCTCAGGCCCCCTAAA
GATCGTCGCCTTGGTGAGCCGTTACCTCACCAACTAGCTAATCTTGCGCGTGCCCATCTCTATCCACCGTAGT
TTTCAATATCACCCGATGCCGAATAATATATTATTGGGGTATTAATCTTCTTCGAAAGGCTATCCCCCTGATA
AAGGCAGGTTGCACACGTGTTCCGCACCCGTACGCCGCTCTCAATTACCCGAAAGTAATCTACCGCTCCGGCT
TGCATGTGTTAGGCCTCCCGCTAGCGTTCTCCTCGAGCAGGATCAACTCT
>1460
CTGCTGCCGCCCGTAGGAGTCTGGTCCGTGTCTCAGTACCAGTGTGGGGGATCACCCTCTCAGGCCCCCTAAA
GATCGTCGCCTTGGTGAGCCGTTACCTCACCAACTAGCTAATCTTGCGCGTGCCCATCTCTATCCACCGTAGT
TTTCAATATCACCCGATGCCGAATAATATATTATGGGGTATTAATCTTCTTCGAAAGGCTATCCCCCTGATAA
AGGCAGGTTGCACACGTGTTCCGCACCCGTACGCCGCTCTCAATTACCCGAAAGTAATCTACCGCTCGGCTTG
CATGTGTTAGGCCTCCCGCTAGCGTTCATCCTGAGCCAGGATCAAACTCT
>1461
CTGCTGCCGCCCCGTAGGAGTCTGGTCCCGTGTCTCAGTACCAGTGTGGGGGGATCACCCCTCTCAGGCCCCC
CTAAAGATCGTCGCCTTGGTGAGCCGTTACCTCACCCAACTAGCTAATCTTGCGCGTGCCCCATCTCTATCCA
CCGTAGTTTTTCAATATCACCCCGATGCCGAATAATATATTTATTGGGGTATTTAATCTTCTTCGAAAGGCT
ATCCCCCTGATTAAAGGCAGGTTGCACACGTGTTCCCGCACCCGTACGCCGCTCTCAATTTACCCCCGAAAGT
AATCTACCGCTCGGCTTGCATGTGTTTAGGCCTCCCGCTAGCGTTCATCCTGAGCCAGGATCAAACTCT
>1462
CTGCTGCCGCCCCGTAGGAGTCTGGTCCCGTGTCTCAGTACCAGTGTGGGATCACCCCTCTCGCCCCCTAAAC
GAATCGTTCGCCTTGGTGAGCCGTTACCTCACCCCAACTAAGCTAATCTTGCGCGTGCCCCATCTCTATCCAC
CGTAGTTTTCAATATCACCCGATGCCGAATAATATAATTTATGGGGTATTTAATTCTTTCTCTGAAAGGCTTA
TCCCCCTGATAAAGGCAGGTTGCACACGTGTTCCGCACCCGTACGCCGCTCTCAATTACCCGAAAGTAATCTA
CCGCTCCGGCTTGACA
>1463
CTGCTGCCGCCCGTAGGAGTCTGGTCCGTGTCTCAGTACCAGTGTGGGGGATCACCCTCTCAGGCCCCCTAAA
GATCGTCGCCTTGGTGAGCCGTTACCTCACCAACTAGCTAATCTTGCGCGTGCCCATCTCTATCCACCGTAGT
TTTCAATATCACCCGATGCCGAATAATATATTATGGGGTATTAATCTTCTTCGAAAGGCTATCCCCTGATAAA
GGCAGGTTGCACACGTGTTCCGCACCCGTACGCCGCTCTCAATTACCCGAAAGTAATCTACCGCTCGGCTTGA
CATGTGTTAGGCCTCCCGCTAGCGCTCATCCTGAGCCAGGATCAACTCT
>1464
CTGCTGCCGCCCGTAGGAGTCTGGTCCGTGTCTCAGTACCAGTGTGGGGGATCACCCTCTCAGGCCCCCTAAA
GATCGTCGCCTTGGTGAGCCGTTACCTCACCAACTAGCTAATCTTGCGCGTGCCCATCTCTATCCACCGTAGT
TTTCAATATCACCCGATGCCGAATAATATATTATGGGGTATTAATCTTCTTCGAAAGGCTATCCCCCTGATAA
AGGCAGGTTGCACACGTGTTCCGCACCCGTACGCCGCTCTCAATTACCCGAAAGTAATCTACCGCTCGGCTTG
CATGTGTTAGGCCTCCCGCTAGCGTTCATCCTGAGCCAGGATCAACTCT
>1465
CTGCTGCCGCCCGTAGGAGTCTGGTCCGTGTCTCAGTACCAGTGTGGGGGATCACCCTCTCAGGCCCCCTAAA
GATCGTCGCCTTGGTGAGCCGTTACCTCACCAACTAGCTAATCTTGCGCGTGCCCATCTCTATCCACCGTAGT
TTTCAATATCACCCGATGCCGAATAATATATTATGGGGTATTAATCTTCTCGAAAGGCTATCCCCTGATAAAG
GCAGGTTGCACACGTGTTCCGCACCCGTACGCCGCTCTCAATTACCCGAAAGTAATCTACCGCTCCGGCTTGC
ATGTGTTAGGCCACC
>1466
CTGCTGCCGCCCGTAGGAGTCTGGTCCGTGTCTCAGTACCAGTGTGGGGGATCACCCTCTCAGGCCCCCTAAA
GATCGTCGCCTTGGTGAGCCGTTACCTCACCAACTAGCTAATCTTGCGCGTGCCCATCTCTATCCACCGTAGT
TTTCAATATCACCCGATGCCGAATAATATATTATGGGGTATTAATCTTCTTCGAAAGGCTATCCCCTGATAAA
GGCAGGTTGCACACGTGTTCCGCACCCGTACGCCGCTCTCAATTACCCGAAAGTAATCTACCGCTCGGCTTGC
ATGTGTTAGGCCTCCCGCTAGCGTTCTCCTGAGCAGGATCAACTCG
>1467
CTGCTGCCGCCCGTAGGAGTCTGGTCCGTGTCTCAGTACCAGTGTGGGGGATCACCCTCTCAGGCCCCCTAAA
GATCGTCGCCTTGGTGAGCCGTTACCTCACCAACTAGCTAATCTCGCGCGTGCCCATCTCTATCCACCGTAGT

TTTCAATATCACCCGATGCCGAATAATATATTATGGGGTATTAATCTTCTCGAAAGGCTATCCCCCTGATAAA
GGCAGGTTGCACACGTGTTCCGCACCCGTACGCCGCTCTCAATTACCCGAAAGTAATCTACCGCTCCGGCTTG
CATGTGTTAGGCCTCCCGCTAGCGTTCATCCTGAGC
>1468
CTGCTGCCGCCCGTAGGAGTCTGGACCGTGTCTCAGTTCCAGTGTGACTGATCATCCTCTCAGACCAGTTACG
GATCGTCGCCTTGGTAGGCCTTTACCCCACCAACTAGCTAATCCGACCTAGGCTCATCTGATAGCGTGAGGTC
CGAAGATCCCCCACTTCTCCCTCAGGACGTATGCGGTATTAGCGCCCGTTTCCGGACGTATCCCCACTACCAG
GCAGATTCCTAGGCATTACTCACCCGTCCGCCGCTGAATCCAGGAGCAAGCTCCCTCATCCGCTCGACTTGCA
TGTGTTAGGCCTGCCGCCAGCGTCAATCTGAGCCAGGATCAAACTCT
>1469
CTGCTGCCGCCCGTAGGAGTCTGGTCCGTGTCTCAGTACCAGTGTGGGGGATCACCCTCTCAGGCCCCCTAAA
GATCGTCGCCTTGGTGAGCCGTTACCTCACCAACTAGCTAATCTTGCGCGTGCCCATCTCTATCCACCGTAGT
TTTCAGTATCACCCGATGCCGAATAATATATTATGGGGTATTAATCTTCTCTAAAGGCTATCCCCCTGATAAA
GGCGGTTGCACACGTGTTCCGCACCCGTACGCCGCTCTCAATTACCCGAAAGTAATCTACCGCTCGGCTTGCA
TGTGTTAGGCCTCCCGCTAGCGTTCTCCTGAGCAGGATCAACTCT
>1470
CTGCTGCCGCCCGTAGGAGTCTGGTCCGTGTCTCAGTACCAGTGTGGGGGGATCACCCTCTCAGGCCCCCTAA
AGATCGTCGCCTTGGTGAGCCGTTACCTCACCAACTAGCTAATCTTGCGCGTGCCCATCTCTATCCACCGTAG
TTTTCAATATCACCCGATGCCGAATAATATATTATGGGGTATTAATCTTTCTCGAAAGGCTATCCCCCTGATA
AGAGCAGGTTGCACACGTGTTCCGCACCCGTACGCCGCTCTCAATTACCCGAAAGTAATCTACCGCTCCGGCT
TGCATGTGTTAGGCCTCCCGCTAGCGTTCTCCTCGAGAAG
>1471
CTGCTGCCGCCCGTAGGAGTCTGGTCCGTGTCTCAGTACCAGTGTGGGGGATCACCCTCTCAGGCCCCCTAAA
GATCGTCGCCTTGGTGAGCCGTTACCTCACCAACTAGCTAATCTTGCGCGTACCCATCTCTATCCACCGTAGT
TTTCAATATCACCCGATGCCGAATAATATAATTATGGGGTATTAATCTTCTTCGAAAGGCTATCCCCTGATAA
AGGCAGGTTGCACACGTGTTCCGCACCCGTACGCCGCTCTCAATTACCCGAAAGTAATCTACCGCTCGGCTTG
CATGTGTTAGGCCTCCCGCTAGCGTTCTCCTCGAGCAG
>1472
CTGCTGCCGCCCGTAGGAGTCTGGTCCGTGTCTCAGTACCAGTGTGGGGGATCACCCTCTCAGGCCCCCTAAA
GATCGTCGCCTTGGTGAGCCGTTACCTCACCAACTAGCTAATCTTGCGCGTGCCCATCTCTATCCACCGTAGT
TTTCAATATCACCCGATGCCGAATAATATATTATGGGGTATTAATCTTCTTCGAAGGCTATCCCCTGATAAAG
GCAGGTTGCACACGTGTTCCGCACCCGTACGCCGCTCTCAATTACCCGAAAGTAATCTACCGCTCCGGCTTGC
ATGTGTTAGGCCTCCCGCTAGCGTTCATCCGTGAGCCAGGATCAAACTCT
>1473
TTGCTGCCGCCCGTAGGAGCCTGGTCCGTGTCTCAGTACCAGTGTGGGGGATCACCCTCTCAGGCCCCCTAAA
GATCGTCGCCTGGTGAGCCGTTACCTCACCAACTAGCTAATCTTGCGCGTGCCCATCTCTATCCACCGTAGTT
TTCAATATCACCCGATGCCGAATAATATATTATGGGGTATTAATCTTCCTTCGAGAGGCTATCCCCCTGATAA
AGGCAGGTTGCACACGTGTTCTGCACCCGTACGCCGCTCTCAATTACCCGAAAGTAATCTACCGCTCGGCTTG
CATGTGTTAGGCCTCCCGCTAGCGTTCATCCTGAGCCAGGATCAAACTCT
>1474
CTGCTGCCGCCCGTAGGAGTCTGGTCCGTGTCTCAGTACCAGTGTGGGGGATCACCCTCTCAGGCCCCCTAAA
GATCGTCGCCTTGGTGAGCCGTTACCTCACCAACTAGCTAATCTTGCGCGTGCCCATCTCTATCCACCGTAGT
TTTCAATATCACCCGATGCCGAATAATATATTATGGGGTATTAATCTTCTCGAAAGGCTATCCCCTGATAAAG
GCAGGTTGCACACGTGTTCCGCACCCGTACGCCGCTCTCAATTACCCGAAAGTAATCTACCGCTCGGCTTGCA
TGTGTTAGGCCTCCCGCTAGCGTTCATCTCGAGCCAGGATCAAACTCT
>1475
CTGCTGCCGCCCGTAGGAGTCTGGTCCGTGTCTCAGTACCAGTGTGGGGGATCACCCTCTCAGGCCCCCTAAA
GATCGTCGCCTTGGTGAGCCGTTACCTCACCAACTAGCTAATCTTGCGCGTGCCCATCTCTATCCACCGTAGT
TTTCAATATCACCCGATGCCGAATAATATATTATGGGGTATTAATCTTCCGTTCGAAAGGCTATCCCCCTGAT
AAAGGCAGGTTGCACACGTGTTCCGCACCCGTACGCCGCTCTCAATTACCCGAAAGTAATCTACCGCTCGGCT
TGCATGTGTTAGGCCTCCCGCTAGCGTTCATCCTGAGCCAGGATCAAACTCT
>1476
CTGCTGCCGCCCGTAGGAGTCTGGTCCGTGTCTCAGTACCAGTGTGGGGGATCACCCTCTCAGGCCCCCTAAA
GATCGTCGCCTTGGTGAGCCGTTACCTCACCAACTAGCTAATCTTGCGCGTGCCCATCTCTATCCACCGTAGT

TTTCAATATCACCCGATGCCGAATAATATATTATGGGGTATTAATCTTCTTCGAAAGCTATCCCCTGAATAAA
GGCAGGTTGCACACGTGTTCCGCACCCGTACGCCGCTCTCAATTACCCGAAAGTAATCTACCGCTCGGCTTGC
ATGTGTTAGGCCTCCCGCTAGCGTTCATCTGAGCCAGGATCAACTCT
>1477
CTGCTGCCGCCCGTAGGAGTCTGGTCCGTGTCTCAGTACCAGTGTGGGGGATCACCCTCTCAGGCCCCCTAAA
GATCGTCGCCTTGGTGAGCCGTTACCTCACCAACTAGCTAATCTTGCGCGTGCCCGTCTCTATCCACCGTAGT
TTTCAATATCACCCGATGCCGAATAATATATTATGGGGTATTAATCTTCTTCGAAAGGCTATCCCCCTGATAA
AGGCAGGTTGCACACGTGTTCCGCACCCGTACGCCGCTCTCAATTACCCGAAAGTAATCTACCGCTCGGCTTG
CATGTGTTAGGCCTCCCGCTAGCGTTCATCCTGA
>1478
CTGCTGCCGCCCGTAGGAGACTGGTCCGTGTCTCAGTACCAGTGTGGGGGATCACCCTCTCAGGCCCCCTAAA
GATCGTCGCCTTGGTGAGCCGTTACCTCACCAACTAGCTAATCTTGCGCGTGCCCATCTCTATCCACCGTAGT
TTTCAATATCACCCGATGCCGAATAATATATTATGGGGTATTAATCTTCTCGAAGCTATCCCCTGATAAAGGC
AGGTTGCACACGTGTTCCGCACCCGTACGCCGCTCTCAATTACCCGAAAGTAATCTACCGCTCGGCTTGCATG
TGTTAGGCCTCCCGCTAGCGTTCTCCTCGAGCAGGATCCAATATCTCACGCAACCTGACCTGCCGTAAGAGGC
AGGATAGGACCAGTCAC
>1479
CTGCTGCCGCCCGTAGGAGTCTGGTCCGTGTCTCAGTACCAGTGTGGGGGATCACCCTCTCAGGCCCCCTAAA
GATCGTCGCCTTGGTGAGCCGTTACCTCACCAACTAGCTAATCTTGCGCGTGCCCATCTCTATCCACCGTAGT
TTTCAATATCACCCGATGCCGAATAATATATTATGGGGTATTAATCTTCTTCGAAAGGCTATCCCCTGATAAA
GGCAGGTTGCACACGTGTTCCGCACCCGTACGCCGCTCTTAATTACCCGAAAGTAATCTACCGCTCCGGCTTG
CATGTGTTAGGCCTCCCGCTAGCGTTCTCCTCGAGCAGGATCCAACATCT
>1480
CTGCTGCCGCCCGTAGGAGTCTGGTCCGTGTCTCAGTACCAGTGTGGGGGATCACCCTCTCAGGCCCCCTAAA
GATCGTCGCCTTGGTGAGCCGTTACCTCACCAACTAGCTAATCTTGCGCGTGCCCATCTCTATCCACCGTAGT
TTTTCAATATCACCCGATGCCGAATAATATATTATGGGGTATTAATCTTCTTCGAAAGGCTATCCCCCTGATA
AAGGCAGGTTGCACACGTGTTCCGCACCCGTACGCCGCTCTCAATTACCCGAAAGTAATCTACCGCTCCGGCT
TGCATGTGTTAGGCCTCCCGCTAGCGTTCATCCGTCG
>1481
CTGCTGCCGCCCGTAGGAGTCTGGTCCGTGTCTCAGTACCAGTGTGGGGGATCACCCTCTCAGGCCCCCTAAA
GATCGTCGCCTTGGTGAGCCGTTACCTCACCAACTAGCTAATCTTGCGCGTGCCCATCTCTATCCACCGTAGT
TTTCAATATCACCCGATGCCGAATAATATATTATTGGGGTATTAATCTTTCTTCGAAAGGCTATCCCCCTGAT
AAAGGCAGGTTGCACACGTGTTCCGCACCCGTACGCCGCTCTCAATTACCCGAAAGTAATCTACCGCTCGGCT
TGCATGTGTTAGGCCTCCCGCTAGCGTTCATTCCGTGAGCCAGGCAA
>1482
CTGCTGCCGCCCGTAGGAGTCTGGACCGTGTCTCAGTTCCAGTGTGACTGATCATCCTCTCAGACCAGTACGG
ATCGTCGCCTTGGTAGGCCTTTTACCCCACCAACTAGCTAATCCGACCTAGGCTCATCTGATAGCGTGAGGTC
CGAAGTCCCCCACTTCTCCCTCAGGACGTATGCGGTATTAGCGCCCCGTTTCCGGACGTATCCCCCACTACCA
GGCAGATTCCTAGGCATTACTCACCCGTCCGCCGCTGAATCCAGGAGCAAGCTCCCTTCATCCGCTCGACTGC
ATGTGTTAGGCCTGCCGCCAGCGTTCAATCTGAGCCAGGATCAAACTCT
>1483
CTGCTGCCGCCCGTAGGAGTCTGGTCCGTGTCTCAGTACCAGTGTGGGGGATCACCCTCTCAGGCCCCCTAAA
GATCGTCGCCTTGGTGAGCCGTTACCTCACCAACTAGCTAATCTTGCGCGTGCCCATCTCTATCCACCGTAGT
TTTCAATATCACCCGATGCCGAATAATATATTATGGGGTATTTAATCTTTCTTCGAAAGGCTATCCCCCTGAT
AAAGGCAGGTTGCACACGTGTTCCGCACCCGTACGCCGCTCTCAATTACCCGAAAGTAATCTACCGCTCGGCT
TGCATGTGTTAGGCCTCCCGCTAGCGTTCATCCTGAGCCAGGATCAAACTCT
>1484
CTGCTGCCGCCCGTAGGAGTCTGGACCGTGTCTCAGTTCCAGTGTGACTGATCATCCTCTCAGACCAGTACGG
ATCGTCGCCTTGGTAGGCCTTTACCCCACCAACTAGCTAATCCGACCTAGGCTCATCTGATAGCGTGAGGTCC
GAAGATCCCCCACTTCTCCCTCAGGACGTATGCGGTATTAGCGCCCGTTTCCGGACGTATCCCCACTACCAGG
CAGATTCCTAGGCATTACTCACCCGTCCGCCGCTGAATCCAGGAGCAAGCCTCCTACATCCGCTCGACTGCAT
GTGTTAGGCCTGCCGCCAAGCGCATCATGAGCAGGC
>1485

CTGCTGCCGCCCGTAGGAGTCTGGTCCGTGACTCAGTACCAGTGTGGGGGATCACCCTCTCAGGCCCCCTAAA
GATCGTCGCCTTGGTGAGCCGTTACCTCACCAACTAGCTAATCTTGCGCGTGCCCATCTCTATCCACCGTAGT
TTTCAATATCACCCGATGCCGAATAATATATTATGGGGTATTAATCTTCTTCGAAAGGCTATCCCCTGATAAA
GGCAGGTTGCACACGTGTTCCGCACCCGTACGCCGCTCTCAATTACCCGAAAGTAATCTACCGCTCGGCTTGC
ATGTGTTAGGCCTCCCGCTAGCGTTCATCCTGAGCCAGGCATCAAACTC
>1486
CTGCTGCCGCCCGTAGGAGTCTGGACCGTGTCTCAGTTCCAGTGTGACTGATCATCCTCTCAGACCAGTACGG
ATCGTCGCCTTGGTAGGCCTTTACCCCCACCAACTAGCTAATCCGACCTAGGCTCATCTGATAGCGTGAGGTC
CGAAGATCCCCCACTTCTCCCTCAGGACGTATGCGGTATTAGCGCCCGTTTCCGGACGTATCCCCACTACCAG
GCAGATTCCTAGGCATTACTCACCCGTCCGCCGCTGAATCCAGGAGCAAGCCCCCTTCATCCGCTCGACTGCA
TGTGTTAGGCCTGCCGCCAGCGTTCATCTGAGCCAGGATCAAACTCT
>1487
CTGCTGCCGCCCGTAGGAGTCTGGTCCGTGTCTCAGTACCAGTGTGGGGGATCACCCTCTCAGGCCCCCTAAA
GATCGTCGCCTTGGTGAGCCGTTACCTCACCAACTAGCTAATCTTGCGCGTGCCCATCTCTATCCACCGTAGC
TTTCAATATCACCCGATGCCGAATAATATATTATGGGGTATTAATCTTCTCGAAAGGCTATCCCCCTGATAAA
GGCAGGTTGCACACGTGTACCGCACCCGTACGCCGCTCTCAATTACCCGAAAGTAATCTACCGCTCCGGCTTA
GCATGTGTTAGGCCTCCCGCTAGCGTTCTCCTGAGCCAAGGATCAAACTCT
>1488
CTGCTGCCGCCCCGTAGGAGTCTGGTCCGTGTCTCAGTACCAGTGTGGGGGATCACCCTCTCAGGCCCCCCTA
AAGATCGTCGCCTTGGTGAGCCGTTACCTCACCCAACTAGCTAATCTTGCGCGTGCCCCATCTCTATTCCACC
GTAGTTTCAATATCACCCGATGCCGAATAATATATTTATGGGGTATTAATCTTCTTCGAAAGGCTATCCCCCT
GATAAAGGCAGGTTGCACACGTGTTCCCGCACCCGTACGCCGCTCTCAATTACCCCGAAAGTAATCTACCGCT
CGGCTTGCATGTGTTAGGCCTCCCGCTAGCGTTCATTCCTGAGCCAGGATCAAACTCT
>1489
CTGCTGCCGCCCGTAGGAGTCTGGTCCGTGTCTCAGTACCAGTGTGGGGGATCACCCTCTCAGGCCCCCTAAA
GATCGTCGCCTTGGTGAGCCGTTACCTCACCAACTAGCTAATCTTGCGCGTGCCCATCTCTATCCACCGTAGT
TTTCAATATCACCCGATGCCGAATAATATATTATGGGGTATTAATCTTCTTCGAAAGGCTATCCCCCTGATAA
AGGCAGGTTGCACACGTGTTCCGCACCCGTACGCCGCTCTCAATTACCCGAAAGTAATCTACCGCTCGGCTTG
CATGTGTTAGGCCTCCCGCTAGCGTTCATCCTGAGCCAGGATCAACTCT
>1490
CTGCTGCCGCCCGTAGGAGTCTGGTCCGTGTCTCAGTACCAGTGTGGGGGATCACCCTCTCAGGCCCCCTAAA
GATCGTCGCCTTGGTGAGCCGTTACCTCACCAACTAGCTAATCTTGCGCGTGCCCATCTCTATCCACCGTAGT
TTTCAATATCACCCGATGCCGAATAATATATTATGGGGTATTAATCTTCTTCGAAAGGCTATCCCCTGATAAA
GGCAGGTTGCACACGTGTTCCGCACCCGTACGCCGCTCTCAATTACCCGAAAGTAATCTACCGCTCGGCTTGC
ATGTGTTAGGCCTCCCGCTAGCGTTCATCCTGAGCCAGGATCAACTCT
>1491
CTGCTGCCGCCCGTAGGAGTCTGGTCCGTGTCTCAGTACCAGTGTGGGGGATCGCCCTCTCAGGCCCCCTAAA
GATCGTCGCCTTGGTGAGCCGTTACCTCACCAACTAGCTAATCTTGCGCGTGCCCATCTCTATCCACCGTAGT
TTTCAATATCACCCGATGCCGAATAATATATTATGGGGTATTAATCTTCTTCGAAGGCTATCCCCCTGATAAA
GGCAGGTTGCACACGTGTTCCGCACCCGTACGCCGCTCTCAATTACCCGAAAGTAATCTACCGCATCGGCTTG
CACTGTGTTAG
>1492
CTGCTGCCGCCCGTAGGAGTCTGGTCCGTGTCTCAGTACCAGTGTGGGGGATCACCCTCTCAGGCCCCCTAAA
GATCGTCGCCTTGGTGAGCCGTTACCTCACCAACTAGCTAATCTTGCGCGTGCCCATCTCTATCCACCGTAGT
TTTCAATATCACCCGATGCCGAATAATATATTATGGGGTATTAATCTTCTCGAAAGGCTATCCCCCTGATAAA
GGCAGGTTGCACACGTGTTCCGCACCCGTACGCCGCTCTCAATTACCCGAAAGTAATCTACCGCTCGGCTTGC
ATGTGTTAGGCCTCCCGCTAGCGTTCATCCTGAGCCAGGATCAAACTCT
>1493
CTGCTGCCGCCCGTAGGAGTCTGGTCCGTGTCTCAGTACCAGTGTGGGGGATCACCCTCTCAGGCCCCCTAAA
GATCGTCGCCTTGGTGAGCCGTTACCTCACCAACTAGCTAATCTTGCGCGTGCCCATCTCTATCCACCGTAGT
TTTCAATATCACCCGATGCCGAATAATATATTATGGGGTATTAATCTTCTTCGAAAGGCTATCCCCCTGATAA
AGGCAGGTTGCACACGTGTTCCGCACCCGTACGCCGCTCTCAATTACCCGAAAGTAATCTACCGCTCGGCTTG
CATGTGTTAGGCCTCCCGCTAGCGTTCATCCTGAGCAGGATCAAACTCT
>1494

CTGCTGCCGCCCGTAGGAGTCTGGTCCGTGTCTCAGTACCAGTGTGGGGGATCACCCTCTCAGGCCCCCTAAA
GATCGTCGCCTTGGTGAGCCGTTACCTCACCAACTAGCTAATCTTGCGCGTGCCCATCTCTATCCACCGTAGT
TTTCAATATCACCCGATGCCGAATAATATATTATGGGGTATTAATCTTCTTCGAAAGGCTTATCCCCCTGATA
AAAGGCAGGTTGCACACGTGTTCCGCACCCGTACGCCGCTCTCAATTACCCGAAAGTAATCTACCGCTCGGCT
TGCATGTGTTAGGCCTCCCGCTAGCGTTCATCCTGAGCCAGGATCAAACTCT
>1495
CTGCTGCCGCCCGTAGGAGTCTGGTCCGTGTCTCAGTACCAGTGTGGGGGATCACCCTCTCAGGCCCCCTAAA
AGATCGTCGCCTTGGTGAGCCGTTACCTCACCAACTAGCTAATCTTGCGCGTGCCCATCTCTATCCACCGTAG
TTTTCAATATCACCCGATGCCGAATAATATATTATGGGGTATTTAATCTTCTTCGAAAGGCTATCCCCCTGAT
AAAGGCAGGTTGCACACGTGTTCCGCACCCGTACGCCGCTCTCAATTACCCGAAAGTAATCTACCGCTCGGCT
TGCATGTGTTAGGCCTCCCGCTAGCGTTCATCCTGAGCCAGGATCAACTCT
>1496
CTGCTGCCGCCCGTAGGAGTCTGGACCGTGTCTCAGTTCCAGTGTGACTGATCATCCTCTCAGACCAGTACGG
ATCGTCGCCTTGGTAGGCCTTTACCCCACCAACTAGCTAATCCGACCTAGGCTCATCTGATAGCGTGAGGTCC
GAAGATCCCCCACTTCTCCCTCAGGACGTATGCGGTATTAGCGCCCGTTTCCGGACGTATCCCCCACTACCAG
GCAGATTCCTAGGCATTACTCACCCGTCCGCCGCTGAATCCAGGAGCAAGCCCCCTTCATCCGCTCGACTGCA
TGTGTTAGGCCTGCCGCCAGCGTCAATCTGAGCCAGGATCAAACTCT
>1497
CTGCTGCCGCCCGTAGGAGTCTGGTCCGTGTCTCAGTACCAGTGTGGGGGATCACCCTCTCAGGCCCCCTAAA
GATCGTCGCCTTGGTGAGCCGTTACCTCACCAACTAGCTAATCTTGCGCGTGCCCATCTCTATCCACCGTAGT
TTTCAATATCACCCGATGCCGAATAATATATTATGGGGTATTAATCTTCTTCGAAAGGCTATCCCCCTGATAA
AGGCAGGTTGCACACGTGTTCCGCACCCGTACGCCGCTCTCAATTTACCCGAAAGTAATCTACCGCTCGGCTT
GCATGTGTTAGGCCTCCCGCTAGCGTTCATCCTCGA
>1498
CTGCTGCCGCCCGTAGGAGTCTGGTCCGTGTCTCAGTACCAGTGTGGGGATCACCCTCTCAGGCCCCCCTAAA
GACTCGTCGCCTTGGTGAGCCATTACCTCACCAACTAGCTAATCTTGCGCGTGCCCATCTCTATCCACCGTAG
TTTTCAATATCACCCGATGCCGAATAATATATTATGGGGTATTTAATCTTCTCGAAGGCTATCCCCCTGATAA
AGGCAGGTTGCCACACGTGTTCCGCACCCGTACGCCGCTCTCAATTACCCGAAAGTAATCTACCGCTCGGCTT
GCATGTGTTAGGCCTCCGCTAGCGTTCATCCTGAGCCAGGATCAAACTCT
>1499
CTGCTGCCGCCCGTAGGAGTCTGGTCCGTGTCTCAGTACCAGTGTGGGGGATCACCCTCTCAGGCCCCCTAAA
GATCGTCGCCTTGGTGAGCCGTTACCTCACCAACTAGCTAATCTTGCGCGTGCCCATCTCTATCCACCGTAGT
TTTCAATATCACCCGATGCCGAATAATATATTATGGGGTATTAATCTTCTTCGAAAGGCTATCCCCCTGATAA
AGGCAGGTTGCACACGTGTTCCGCACCCGTACGCCGCTCTCAATTACCCGAAAGTAATCTACCGCTCCGGCTT
GCATGTGTTAGGCCTCCCGCTAGCGTTCATCCGTGA
>1500
CTGCTGCCGCCCGTAGGAGTCTGGTCCGTGTCTCAGTACCAGTGTGGGGGATCACCCTCTCAGGCCCCCTAAA
GATCGTCGCCTTGGTGAGCCGTTACCTCACCAACTAGCTAATCTTGCGCGTGCCCATCTCTATCCACCGTAGT
TTCAATATCACCCGATGCCGAATAATATATTATGGGGTATTAATCTTCTTCGAAAGGCTATCCCCCTGATAAA
GGCAGGTTGCACACGTGTTCCGCACCCGTACGCCGCTCTCAATTACCCGAAAGTAATCTACCGCTCGGCTTGC
ATGTGTTAGGCCTCCCGCTAGCGTCATCCTGAGCCAGGATCAAACTCT
>1501
TTGCTGCCGCCCGTAGGAGTCTGGTCCGTGTCTCAGTACCAGTGTGGGGGATCACCCTCTCAGGCCCCCTAAA
GATCGTCGCCTTGGTGAGCCGTTACCTCACCAACTAGCTAATCTTGCGCGTGCCCATCTCTATCCACCGTAGT
TTTCAATATCACCCGATGCCGAATAATATATTATGGGGTATTTAATCTTCTTCGAAAGGCTATCCCCCTGATA
AAGGCAGGTTGCACACGTGTTCCGCACCCGTACGCCGCTCTCAATTACCCGAAAGTAATCTACCGCTCGGCTT
GCATGTGTTAGGCCTCCCGCTAGCGTTCATCCTGAGCCAGGATCAAACTCT
>1502
CTGCTGCCGCCCGTAGGAGTCTGGTCCGTGTCTCAGTACCAGTGTGGGGGATCACCCTCTCAGGCCCCCTAAA
GATCGTCGCCTTGGTGAGCCGTTACCTCACCAACTAGCTAATCTTGCGCGTGCCCATCTCTATCCACCGTAGT
TTCAATATCACCCGATGCCGAATAATATATTATGGGGTATTAATCTTCTTCGAAAGGCTATCCCCCTGATAAA
GGCAGGTTGCACACGTGTTCCGCACCCGTACGCCGCTCTCAATTACCCGAAAGTAATCTACCGCTCCGGCTTG
CATGTGTTAGGCCTCCCGCTAGCGTTCATCCTGAGCCAGGATCAAACTCT
>1503

CTGCTGCCGCCCGTAGGAGTCTGGTCCGTGTCTCAGTACCAGTGTGGGGGATCACCCTCTCAGGCCCCCTAAA
GATCGTCGCCTTGGTGAGCCGTTACCTCACCAACTAGCTAATCTTGCGCGTGCCCATCTCTATCCACCGTAGT
TTTCAATATCACCCGATGCCGAATAATATATTATGGGGTATTAATCTTCTTCGAAAGGCTATCCCCCTGATAA
AGGCAGGTTGCACACGTGTTCCGCACCCGTACGCCGCTCTCAATTACCCGAAAGTAATCTACCGCTCCGGCTT
GCATGTGTTAGGCCTCCCGCTAGCGTTCATCCTGAGCCAGGATCAACTCT
>1504
CTGCTGCCGCCCGTAGGAGTCTGGTCCGTGTCTCAGTACCAGTGTGGGGGATCACCCTCTCAGGCCCCCTAAA
GATCGTCGCCTTGGTGAGCCGTTACCTCACCAACTAGCTAATCTTGCGCGTGCCCATCTCTATCCACCGTAGT
TTTCAATATCACCCGATGCCGAATAATATATTATGGGGTATTAATCTTCTTCGAAAGGCTATCCCCTGATAAA
GGCAGGTTGCACACGTGTTCCGCACCCGTACGCCGCTCTCAATTACCCGAAAGTAATCTACCGCTCCGGCTTG
CATGTGTTAGGCCTCCCGCTAGCGTTCATCCTCGAGCCAGGATCAACTCT
>1505
CTGCTGCCGCCCGTAGGAGTCTGGTCCGTGTCTCAGTACCAGTGTGGGGGATCACCCTCTCAGGCCCCCTAAA
GATCGTCGCCTTGGTGAGCCGTTACCTCACCAACTAGCTAATCTTGCGCGTGCCCATCTCTATCCACCGTAGT
TTTCAATATCACCCGATGCCGAATAATATATTATGGGGTATTAATCTTCCCTTCGAAAGGCTACCCCCCTGAA
TAAAGGCAGGTTGCACACGTGTTCCGCACCCGTACGCCGCTCTCAATTACCCGAAAGTAATCTACCGCTCGGC
TTGCATGTGTTAGGCCTCCCGCTAGCGTTCATCCTGAGCAGGATCAAACTCT
>1506
CTGCTGCCGCCCGTAGGAGTCTGGTCCGTGTCTCAGTACCAGTGTGGGGGATCACCCTCTCAGGCCCCCTAAA
GATCGTCGCCTTGGTGAGCCGTTACCTCACCAACTAGCTAATCTTGCGCGTGCCCATCTCTATCCACCGTAGT
TTTCAATATCACCCGATGCCGAATAATATATTATGGGGTATTAATCTTCTTCGAAAGGCTATCCCCTGATAAA
GGCAGGTTGCACACGTGTTCCGCACCCGTACGCCGCTCTCAATTACCCGAAAGTAATCTACCGCTCCGGCTTG
CATGTGTTAGGCCTCCCGCTAGCGTTCATCCGTCGAGCCAGGCATCAAACTCT
>1507
CTGCTGCCGCCCGTAGGAGTCTGGACCGTGTCTCAGCTCCAGTGTGACTGATCATCCTCTCAGACCAGTACGG
ATCGTCGCCTTGGTAGGCCTTTACCCCACCAACTAGCTAATCCGACCTAGGCTCATCTGATAGCGTGAGGTCC
GAAGATCCCCCACTTCTCCCTCAGGACGTATGCGGTATTAGCGCCCGTTTCCGGACGTATCCCCACTACCAGG
CAGATTCCTAGGCATTACTCACCCGTCCGCCGCTGAATCCAGGAGCAAGCTCCCTCATCCGCTCGACTGCATG
TGTTAGGCCTGCCGCCAGCGTTCAATCTGAGCCAGGATCAAACTCT
>1508
CTGCTGCCGCCCGTAGGAGTCTGGTCCGTGTCTCAGTACCAGTGTGGGGGGATCACCCTCTCAGGCCCCCTAA
AGATCGTCGCCTTGGTGAGCCGTTACCTCACCAACTAGCTAATCTTGCGCGTGCCCATCTCTATCCACCGTAG
TTTTTCAATATCACCCGATGCCGAATAATATATTATGGGGTATTTAATCTTCCTTTCGAAAAGACTATCCCCC
TGATAAAGGCAGGTTTGCACACGTGTTCCGCACCCGTACGCCGCTCTCAATTACCCGAAAGTAATCTACCGCT
CGGCTTGCATGTGTTAGGCCTCCCGCTAGCGTTCATCCTGAGCCAGGATCAAACTCT
>1509
CTGCTGCCGCCCGTAGGAGTCTGGTCCGTGTCTCAGTACCAGTGTGGGGGATCACCCTCTCAGGCCCCCTAAA
GATCGTCGCCTTGGTGAGCCGTTACCTCACCAACTAGCTAATCTTGCGCGTGCCCATCTCTATCCACCGTAGT
TTTCAATATCACCCGATGCCGAATAATATATTATGGGGTATTAATCTTCTCGAAAGCTATCCCCTGATAAAGG
CAGGTTGCACACGTGTTCCGCACCCGTACGCCGCTCTCAATTACCCCGAAAGTAATCTACCGCTCCGGCTTGC
ATGTGTTAGGCCTCCCGCTAGCGTTCTCCTCGAGCAGGATC
>1510
CTGCTGCCGCCCGTAGGAGTCTGGTCCGTGTCTCAGTACCAGTGTGGGGGATCACCCTCTCAGGCCCCCTAAA
GATCGTCGCCTTGGTGAGCCGTTACCTCACCAACTAGCTAATCTTGCGCGTGCCCATCTCTATCCACCGTAGT
TTTCAATATCACCCGATGCCGAATAATATATTATGGGGTATTAATCTTCTTCGAAAGGCTGTCCCCTGATAAA
GGCAGGTTGCACACGTGCTCCGCACCCGTACGCCGCTCTCAATTACCCGAAAGTAATCTACCGCTCGGCTTGC
ATGTGTTAGGCCTCCCGCTAGCGTTCATCCTGAGCCAGGATCAAACTCT
>1511
CTGCTGCCGCCCGTAGGAGTCTGGACCGTGTCTCAGTTCCAGTGTGACTGATCATCCTCTCAGACCAGTTACG
GATCGTCGCCTTGGTAGGCCTTTACCCCACCAACTAGCTAATCCGACCTAGGCTCATCTGATAGCGTGAGGTC
CGAAGATCCCCCACTTCTCCCTCAGGACGTATGCGGTATTAGCGCCCGTTTCCGGACGTATCCCCGCACTACC
AGGCAGATTCCTAGGCATTACTCACCCGTCCGCCGCTGAATCCAGGAGCAAGCCCCCTCATCCGCTCGACTGC
ATGTGTTAGGCCTGCCGCCAGCGTCAATCTGAGCCAGGATCAAACTCT
>1512

CTGCTGCCGCCCGTAGGAGTCTGGACCGTGTCTCAGTTCCAGTGTGACTGATCATCCTCTCAGACCAGTTACG
GATCGTCGCCTTGGTAGGCCTTTACCCCACCAACTAGCTAATCCGACCTAGGCTCATCTGATTAGCGTGAGGT
CCGAAGTCCCCACTTCTCCCTCAGGACGTATGCGGTATTAGCGCCCGTTTCCGGACGTATCCCCACTACCAGG
CAGATTTCCTAGGCATTACTCACCCGTCCGCCGCTGAATCCAGGAGCAAGCTCCCTTCATCCGCTCGACTTGC
ATGTGTTAGGCCTGCCGCCAGCGTCAATCTTGAGCCAGGATCAAACTCT
>1513
CTGCTGCCGCCCGTAGGAGTCTGGACCGTGTCTCAGTTCCAGTGTGGCCGATCACCCTCTCAGGTCGGCTATG
CATCGTTGCCCTGGTAAGCCGTTACCTTACCAACTAGCTAATGCAGCGCGGATCCATCTATAAGTGACAGCAA
GACCGTCTTCACTAACCATGCGGTTCAAAATATTATCCGGTATTAGCTCCGGTTTCCCGAAGTTATCCCAGTC
TTATAGGTAGGTTATCCACGTGTTACTCACCCGTCCGCCGCTAACACTCAGAGAAGCAAGCTTCTCGTCCGTG
CTCGACTGCATGTATTAGGCACGCCGCCAGCGTCATCTGAGCCAGGATCAACTCT
>1514
CTGCTGCCGCCCGTAGGAGTCTGGTCCGTGTCTCAGTACCAGTGTGGGGGATCACCCTCTCAGGCCCCCTAAA
GATCGTCGCCTTGGTGAGCCGTTACCTCACCAACTAGCTAATCTTGCGCGTGCCCATCTCTATCCACCGTAGT
TTCAATATCACCCGATGCCGAATAATATATTATGGGGTATTAATCTTCTTCGAAAGGCTATCCCCCTGATAA
AGGCAAGTTGCACACGTGTTCCGCACCCGTACGCCGCTCTCAATTACCCGAAGTAATCTACCGCTCGGCTTGC
ATGTGTTAGGCCTCCCGCTAGCGTTCTTCCTGAGCAGGATCCAAACTCT
>1515
CTGCTGCCGCCCGTAGGAGTCTGGACCGTGTCTCAGTTCCAGTGTGACTGATCATCCTCTCAGACCAGTACGG
ATCGTCGCCTTGGTAGGCCTTTACCCCACCAACTAGCTAATCCGACCTAGGCTCATCTGATAGCGTGAGGTCC
GAAGATCCCCCACTTCTCCCTCAGGACGTATGCGGTATTAGCGCCCGTTTCCGGACGTATCCCCACTACCAGG
CAGATTCCTAGGCATTACTCACCCGTCCGCCGCTGAATCCAGGAGCAAGCTCCCTTCATCCGCTCGACTGCAT
GTGTTAGGCCTGCCGCCAGCGTTCAATCTGAGCCAGGATCAAACTCT
>1516
CTGCTGCCGCCCGTAGGAGTCTGGTCCGTGTCTCAGTACCAGTGTGGGGGATCACCCTCTCAGGCCCCCTAAA
GATCGTCGCCTTGGTGAGCCGTTACCTCACCAACTAGCTAATCTTGCGCGTGCCCATCTCTATCCACCGTAGT
TTTCAATATCACCCGATGCCGAATAATATATTATGGGGTATTAATCTTCTTCGAAAGGCTATCCCCCTGATAA
AGGCAGGTTGCACACGTGTTCCGCACCCGTACGCCGCTCTCAATTACCCGAAAGTAATCTACCGCATCGGCTT
GCATGTGTTAGGCCTCCCGCTAGCGTTCTCCTGAGCAGGATCAACTC
>1517
CTGCTGCCGCCCGTAGGAGTCTGGTCCGTGTCTCAGTACCAGTGTGGGGGATCACCCTCTCAGGCCCCCTAAA
GATCGTCGCCTTGGTGAGCCGTTACCTCACCAACTAGCTAATCTTGCGCGTGCCCATCTCTATCCACCGTAGT
TTTCAATATCACCCGATGCCGAATAATATATTATGGGGTATTAATCTTCTTCGAAAGGCTTATCCCCCTGATA
AAGGCAGGTTGCACACGTGTTCCGCACCCGTACGCCGCTCTCAATTACCCGAAAGTAATCTACCGCTCGGCTT
GCATGTGTTAGGCCTCCCGCTAGCGTTCATCCTGAGCCAGGATCAAACTCT
>1518
CTGCTGCCGCCCGTAGGAGTCTGGTCCGTGTCTCAGTACCAGTGTGGGGGATCACCCTCTCAGGCCCCCTTAA
AGATCGTCGCCTTGGTGAGCCGTTACCTCACCCAACTAGCTTAATCTTGCGCGTGCCCCATCTCTATCCACCG
TAGTTTTTCAATATCACCCGATGCCGAATAATATATTTATTGGGGTATTTAATTCTTCCTTCGAAAGGCTATC
CCCTGATTAAAGGCAGGTTGCCACACGTGTTCCCGCACCCGTACGCCGCTCTCAATTACCCCGAAAGTAATCT
ACCGCTCGGCTTGCATGTGTTAGGCCTTCCCCGCTAGC
>1519
CTGCTGCCGCCCGTAGGAGTCTGGTCCGTGTCTCAGTACCAGTGTGACTGATCATCCTCTCAGACCAGTACGG
ATCGTCGCCTTGGTAGGCCTTTACCCCACCAACTAGCTAATCCGACCTAGGCTCATCTGATAGCGTGAGGTCC
GAAGATCCCCCACTTCTCCCTCAGGACGTATGCGGTATTAGCGCCCGTTTCCGGACGTATCCCCACTACCAGG
CAGATTCCTAGGCATTACTCACCCGTCCGCCGCTGAATCCAGGAGCAAGCTCCCTTCATCCGCTCGACTGCAT
GTGTTAGGCCTGCCGCCAGCGTCAATCTGAGCCAGGATCAAACTCT
>1520
CTGCTGCCGCCCGTAGGAGTCTGGTCCGTGTCTCAGTACCAGTGTGGGGGATCACCCTCTCAGGCCCCCTAAA
GATCGTCGCCTTGGTGAGCCGTTACCTCACCAACTAGCTAATCTTGCGCGTGCCCATCTCTATCCACCGTAGT
TTTCAATATCACCCGATGCCGAATAATATATTATGGGGTATTAATCTTCTTCGAAAGGCTATCCCCCTGATAA
AGGCAGGTTGCACACGTGTTCCGCACCCGTACGCCGCTCTCAATTACCCGAAAGTAATCTACCGCTCGGCTTG
CATGTGTTAGGCCTCCCGCTAGCGTTCATCCTGAGCCAGGATCAAACTCT
>1521

CTGCTGCCGCCCGTAGGAGTCTGGTCCGTGTCTCAGTACCAGTGTGGGGGATCACCCTCTCAGGCCCCCTAAA
GATCGTCGCCTTGGTGAGCCGTTACCTCACCAACTAGCTAATCTTGCGCGTGCCCATCTCTATCCACCGTAGT
TTTCAATATCACCCGATGCCGAATAATATATTATGGGGTATTAATCTTCTTCGAAAGGCTATCCCCCTGATAA
AAGGCAGGTTGCACACGTGTTCCGCACCCGTACGCCGCTCTCAATTACCCGAAAGTAATCTACCGCTCGGCTT
GCATGTGTTAGGCCTCCCGCTAGCGTTCATCCTGAGCCAGGATCAAACTCT
>1522
CTGCTGCCGCCCCGTAGGAGTCTGGTCCCGTGTCTCAGTACCAGTGTGGGGGATCACCCTCTCAGGCCCCCTA
AAGATCGTCGCCTTGGTGAGCCGTTACCTCACCCAACTAGCTTATTCTTGCGCGTGCCCCATCTCTATCCACC
GTAGTTTTCAATATCACCCGATGCCGAATAATATATTTATGGGAGTTAATTATCTTCCTCGAAGGCTATCCCC
CTGATAAAGGCAGGTTGCACACGTGTTCCGCACCCGTACGCCGCTCTCAATTACCCCGAAAGTAATCTACCGC
TCGGCTTGCATGTGTTAGGCCTCCCGCTAGCGTTCATCCTGAGCCAGGATCAAACTCT
>1523
CTGCTGCCGCCCGTAGGAGTCTGGTCCGTGTCTCAGTACCAGTGTGGGGGATCACCCTCTCAGGCCCCCTAAA
GATCGTCGCCTTGGTGAGCCGTTACCTCACCAACTAGCTAATCTTGCGCGTGCCCATCTCTATCCACCGTAGT
TTTCAATATCACCCGATGCCGAATAATATAATTATGGGGTATTAATCTTCTTCGAAAGGCTATCCCCCTGATA
AAGGCAGGTTGCACACGTGTTCCGCACCCGTACGCCGCTCTCAATTACCCGAAAGTAATCTACCGCTCGGCTT
GCATGTGTTAGGCCTCCCGCTAGCGTTCATCCTGAGCCAGGATCAAACTCT
>1524
CTGCTGCCGCCCGTAGGAGTCTGGACCGTGTCTCAGTTCCAGTGTGACTGATCATCCTCTCAGACCAGTTACG
GATCGTCGCCTTGGTAGGCCTTTACCCCACCAACTAGCTAATCCGACCTAGGCTCATCTGATAGCGTGAGGTC
CGAAGATCCCCCACTTCTCCCTCAGGACGTATGCGGTATTAGCGCCCGTTTCCGGACGTATCCCCACTACCAG
GCAGATTCCCTAGGCATTACTCACCCGTCCGCCGCTGAATCCAGGAGCAAGCTCCCTCATCCGCTCGACTGCA
TGTGTTAGGCCTGCCGCCAGCGTTCAATCTGAGCCAGGATCAAACTCT
>1525
CTGCTGCCGCCCGTAGGAGTCTGGTCCGTGTCTCAGTACCAGTGTGGGGGATCACCCTCTCAGGCCCCCTAAA
GATCGTCGCCTTGGTGAGCCGTTACCTCACCAACTAGCTAATCTTGCGCGTGCCCATCTCTATCCACCGTAGT
TTTCAATATCACCCGATGCCGAATAATATATTATGGGGTATTAATCTTCTTCGAAAGGCTATCCCCCTGATAA
AGGCAGGTTGCACACGTGTTCCGCACCCGTACGCCGCTCTCAATTACCCGAAAGTAATCTACCGCTCGGCTTG
CATGTGTTAGGCCTCCCGCTAGCGTTCTCCTGAGCAGGATCAACTCT
>1526
CTGCTGCCGCCCGTAGGAGTCTGGTCCGTGTCTCAGTACCAGTGTGGGGGATCACCCTCTCAGGCCCCCTAAA
GATCGTCGCCTTGGTGAGCCGTTACCTCACCAACTAGCTAATCTTGCGCGTGCCCATCTCAATCCACCGTAGT
TTTCAACTATCACCGATGCCGAATAATATATTATGGGGTATTAATCTTCTTCGAAAGCTATCCCCCTGAATAA
AGGCAGGTTGCACACGTGTTCCGCACCCGTACGCCGCTCTCAATTACCCGAAAGTAATCTACCGCTCGGCTTG
CATGTGTTAGGCCTCCCGCTAGCGTTCTC
>1527
CTGCTGCCGCCCGTAGGAGTCTGGTCCGTGTCTCAGTACCAGTGTGGGGGATCACCCTCTCAGGCCCCCTAAA
GATCGTCGCCTTGGTGAGCCGTTACCTCGCCAACTAGCTAATCTTGCGCGTGCCCATCTCTATCCACCGTAGT
TTTCAATATCACCCGATGCCGAATAATATATTATGGGGTATTAATCTTCTTCGAAAGGCTATCCCCTGATAAA
GGCAGGTTGCACACGTGTTCCGCACCCGTACGCCGCTCTCAATTACCCGAAAGTAATCTACCGCTCGGCTTGC
ATGTGTTAGGCCTCCCGCTAGCGTTCATCCTGAGCCAGGATCAAACTCT
>1528
CTGCTGCCGCCCGTAGGAGTCTGGTCCGTGTCTCAGTACCAGTGTGGGGGATCACCCTCTCAGGCCCCCTAAA
GATCGTCGCCTTGGTGAGCCGTTACCTCACCAACTAGCTAATCTTGCGCGTGCCCATCTCTATCCACCGTAGT
TTTCAATATCACCCGATGCCGAATAATATATTATGGGGTATTAATCTTCTTCGAAAGGCTATCCCCTGATAAA
GGCAGGTTGCACACGTGTTCCGCACCCGTACGCCGCTCTCAATTACCCGAAAGTAATCTACCGCTCGGCTTGC
ATGTGTTAGGCCTCCCGCTAGCGTTCTCCTGAGCAGGATCAACTCGT
>1529
CTGCTGCCGCCCGTAGGAGTCTGGTCCGTGTCTCAGTACCAGTGTGGGGGATCACCCTCTCAGGCCCCCTAAA
GATCGTCGCCTTGGTGAGCCGTTACCTCACCAACTAGCTAATCTTGCGCGTGCCCATCTCTATCCACCGTAGT
TTTCAATATCACCCGATGCCGAATAATATATTATGGGGTATTAATCTTCTTCGGAAGGCTATCCCCTGATAAA
GGCAGGTTGCACACGTGTTCCGCACCCGTACGCCGCTCTCAATTACCCGAAAGTAATCTACCGCTCGGCTTGC
ATGTGTTAGGCCTCCCGCTAGCGTTCATCCTGAGCCAGGATCAAACTCT
>1530

CTGCTGCCGCCCGTAGGAGTCTGGTCCGTGTCTCAGTACCAGTGTGGGGGATCACCCTCTCAGGCCCCCTAAA
GATCGTCGCCTTGGTGAGCCGTTACCTCACCAACTAGCTAATCTTGCGCGTGCCCATCTCTATCCACCGTAGT
TTTCAATATCACCCGATGCCGAATAATATATTATGGGGTATTTAATCTTCCTCTCGAAAGGCTATCCCCCTGA
TAAAGGCAGGTTTGCACACGTGTTCCGCACCCGTACGCCGCTCTCAATTACCCGAAAGTAATCTACCGCTCGG
CTTGCATGTGTTAGGCCTCCCGCTAGCGTTCATCCTGAGCCAGGATCAAACTCT
>1531
CTGCTGCCGCCCGTAGGAGTCTGGTCCGTGTCTCAGTACCAGTGTGGGGGATCACCCTCTCAGGCCCCCTAAA
GATCGTCGCCTTGGTGAGCCGTTACCCCACCAACTAGCTAATCTTGCGCGTGCCCATCTCTATCCACCGTAGT
TTTCAATATCACCCGATGCCGAATAATATATTATGGGGTATTTAATCTTCTCGAAAGGCTATCCCCCTGATAA
AGGCAGGTTGCACACGTGTTCCGCACCCGTACGCCGCTCTCAATTACCCGAAAGTAATCTACCGCTCGGCTTG
CATGTGTTAGGCCTCCCGCTAGCGTTCTCCTGAGCAGGATCAACTCT
>1532
CTGCTGCCGCCCGTAGGAGTCTGGTCCGTGTCTCAGTACCAGTGTGGGGGATCACCCTCTCAGGCCCCCTAAA
GATCGTCGCCTTGGTGAGCCGTTACCTCACCAACTAGCTAATCTTGCGCGTGCCCATCTCTATCCACCGTAGT
TTTCAATATCACCCGATGCCGAATAATATATTATGGGGTATTAATCTTCTTCTGAAAGGCTATCCCCTGATAA
AGGCAGGTTGCACACGTGTTCCGCACCCGTACGCCGCTCTCAATTTACCCCGAAAGTAATCTACCGCTCGGCT
TGCAATGTGTTAGGCCTCCCGCTAGCGTTCATCCTGAGCCAGGATCAAACTCT
>1533
CTGCTGCCGCCCGTAGGAGTCTGGTCCGTGTCTCAGTACCAGTGTGGGGGATCACCCTCTCAGGCCCCCTAAA
GATCGTCGCCTTGGTGAGCCGTTACCTCACCAACTAGCTAATCTTGCGCGTGCCCATCTCTATCCACCGTAGT
TTTCAATATCACCCGATGCCGGATAATATATTATGGGGTATTAATCTTCTTCGAAAGCTATCCCCCTGATAAA
GGCAGGTTGCACACGTGTTCCGCACCCGTACGCCGCTCTCAATTACCCGAAAGTAATCTACCGCTCGGCTTGC
GTGTGTTAGGCCTCCCGCTAGCGTTCATCCTCGAGCCAGGATCAACTCT
>1534
CTGCTGCCGCCCGTAGGAGTCTGGTCCGTGTCTCAGTACCAGTGTGGGGGATCACCCTCTCAGGCCCCTAAAG
ATCGTCGCCTTGTGAGCCGTTACCTCACCAACTAGCTAATCTTGCGCGTGCCCATCTCTATCCACCGTAGTTT
CAATATCACCCGATGCCGAATAATATATTATGGGGTATTAATCTTCCTTCGAAAGGCTATCCCCTGATAAAGG
CAGGTTGCACACGTGTTCCGCACCCGTACGCCGCTCTCAATTACCCGAAAGTAATCTACCGCTCGGCTGCATG
TGTTAGGCCTCCCGCTAGCGTCTCCTGAG
>1535
CTGCTGCCGCCCGTAGGAGTCTGGTCCGTGTCTCAGTACCAGTGTGGGGGATCACCCTCTCAGGCCCCCTAAA
GATCGTCGCCTTGGTGAGCCGTTACCTCACCAACTAGCTAATCTTGCGCGTGCCCATCTCTATCCACCGTAGT
TTTCAATATCACCCGATGCCGAATAATATATTATGGGGTATTAATCTTCTCGAAAGGCTATCCCCTGATAAAG
GCAGGTTGCACACGTGTTCCGCACCCGTACGCCGCTCTCAATTACCCGAAAGTAATCTACCGCTCGGCTTAGC
ATGTGTTACGGCC
>1536
CTGCTGCCGCCCGTAGGAGTCTGGTCCGTGTCTCAGTACCAGTGTGGGGGATCACCCTCTCAGGCCCCCTAAA
GATCGTCGCCTTGGTGAGCCGTTACCTCACCAACTAGCTAATCTTGCGCGTGCCCATCTCTATCCACCGTAGT
TTTCAATATCACCCGATGCCGAATAATATATTATGGGGTATTAATCTTCTTCGAAAGGCTATCCCCTGATAAA
GGCAGGTTGCACACGTGTTCCGCACCCGTACGCCGCTCTCAATTACCCGAAAGTAATCTACCGCTCCGGCTTG
CATGTGTTAGGCCTCCCGCTAGCGTTCATCCTGAGCCAGGATCAACTCT
>1537
CTGCTGCCGCCCGTAGGAGTCTGGTCCGTGTCTCAGTACCAGTGTGGGGGATCACCCTCTCAGGCCCCCTAAA
GATCGTCGCCTTGGTGAGCCGTTACCTCACCAACTAGCTAATCTTGCGCGTGCCCATCTCTATCCACCGTAGT
TTTTCAATATCACCCGATGCCGAATAATATATTATGGGGTATTAATCTTCTTCGAAAGGCTATCCCCCTGATA
AAGGCAGGTTGCACACGTGTTCCGCACCCGTATGCCGCTCTCAATTACCCGAAAGTAATCTACCGCTCGGCTT
GCATGTGTTAGGCCTCCCGCTAGCGTTCATCCTGAGCCAGGATCAAACTCT
>1538
CTGCTGCCGCCCGTAGGAGTCTGGTCCGTGTCTCAGTACCAGTGTGGGGGATCACCCTCTCAGGCCCCCTAAA
GATCGTCGCCTTGGTGAGCCGTTACCTCACCAACTAGCTTAATCTTGCGCGTGCCCATCTCTATCCACCGTAG
TTTTTTCAATATCACCCGATGCCGAATAATATATTTATTGGGGTATTTAATTCTTTCTCGAAAGGCTATCCCC
CTGATAAAGGCAGGTTTGCACACGTGTTCCGCACCCGTACGCCGCTCTCAATTACCCGAAAGTAATCTACCGC
TCCGGCTTGCATGTGTTAGGCCTTCCCGCTAGCGTTCATTCCGTCGAGCCAGGC
>1539

CTGCTGCCGCCCGTAGGAGTCTGGTCCGTGTCTCAGTACCAGTGTGGGGGATCACCCTCTCAGGCCCCCTAAA
GATCGTCGCCTTGGTGAGCCGTTACCTCACCAACTAGCTAATCTTGCGCGTGCCCATCTCTATCCACCGTAGT
TTTCAATATCACCCGATGCCGAATAATATATTATGGGGTATTAATCTTTCTCGAAAGCTATCCCCTGATAAAC
GGCAGGTTGCACACGTGTTCCGCACCCGTACGCCGCTCTCAATTACCCGAAAGTAATCTACCGCTCCGGCTTG
CATGTGTTAGGCCTCCCGCTAGCGTTCTCCGTCGAGCAGGCATCAATCATCCT
>1540
CTGCTGCCGCCCGTAGGAGTCTGGTCCGTGTCTCAGTACCAGTGTGGGGGATCACCCTCTCAGGCCCCCTAAA
GATCGTCGCCTTGGTGAGCCGTTACCTCACCAACTAGCTAATCTTGCGCGTGCCCATCTCTATCCACCGTAGT
TTTCAATATCACCCGATGCCGAATAATATATTATGGGGTATTAATCTTCTCGAAAGGCTATCCCCTGATAAGG
CAGGTTGCACACGTGTTCCGCACCCGTACGCCGCTCTCAATTACCCGAAACGTAATCTACCGCTACGGCTTGA
CATGTGTT
>1541
CTGCTGCCGCCCGTAGGAGTCTGGTCCGTGTCTCAGTACCAGTGTGGGGGATCACCCTCTCAGGCCCCCTAAA
GATCGTCGCCTTGGTGAGCCGTTACCTCACCAACTAGCTAATCTTGCGCGTGCCCATCTCTATCCACCGTAGT
TTCAATATCACCCGATGCCGAATAATATATTATGGGGTATTAATCTTCTCGAAGCTATCCCCTGATAAAGGCA
GGTTGCACACGTGTTCCGCACCCGTACGCCGCTCTCAATTACCCGAAAGTAATCTACCGCTCCGGCTTGCATG
TGTTAGGCCTCCCGCTAGCGTTCTCCGTCGAGAAGGCTCAATCATCCGT
>1542
CTGCTGCCGCCCGTAGGAGTCTGGTCCGTGTCTCAGTACCAGTGTGGGGGATCACCCTCTCAGGCCCCCTAAA
GATCGTCGCCTTGGTGAGCCGTTACCTCACCAACTAGCTAATCTTGCGCGTGCCCATCTCTATCCACCGTAGT
TTTCAATATCACCCGATGCCGAATAATATATTATGGGGTATTTAATCTTCTTCGAAAGGCTATCCCCCTGATA
AAGGCAGGTTGCACACGTGTTCCGCACCCGTACGCCGCTCTCAATTACCCGAAAGTAATCTACCGCTCGGCTT
GCATGTGTTAGGCCTCCCGCTAGCGTCCATCCTGAGCCAGGATCAAACTCT
>1543
CTGCTGCCGCCCGTAGGAGTCTGGTCCGTGTCTCAGTACCAGTGTGGGGGATCGCCCTCTCAGGCCCCCTAAA
GATCGTCGCCTTGGTGAGCCGTTACCTCACCAACTAGCTAATCTTGCGCGTGCCCATCTCTATCCACCGTAGT
TTTCAATATCACCCGATGCCGAATAATATATTATGGGGTATTAATCTTTCTTCGAAGGCTATCCCCCTGATAA
AGGCAGGTTGCACACGTGTTCCGCACCCGTACGCCGCTCTCAATTACCCCGAAAGTAATCTACCGCTCGGCTT
GCATGTGTTAGGCCTCCCGCTAGCGTTCATCCTGAGCCAGGATCAAACTCT
>1544
CTGCTGCCGCCCGTAGGAGTCTGGTCCGTGTCTCAGTACCAGTGTGGGGGATCACCCTCTCAGGCCCCCTAAA
GATCGTCGCCTTGGTGAGCCGTTACCTCACCAACTAGCTAATCTTGCGCGTGCCCATCTCTATCCACCGTAGT
TTTCAATATCACCCGATGCCGAATAATATATTATGGGGGTATTAATCTTCTTCGAAAGGCTATCCCCCTGATA
AAGGCAGGTTGCACACGTGTTCCGCACCCGTACGCCGCTCTCAATTACCCGAAAGTAATCTACCGCTCGGCTT
GCATGTGTTAGGCCTCCCGCTAGCGTTCATCCTGAGCCAGGATCAACTCT
>1545
CTGCTGCCGCCCGTAGGAGTCTGGTCCGTGTCTCAGTACCAGTGTGGGGGATCACCCTCTCAGGCCCCCTAAA
GATCGTCGCCTTGGTGAGCCGTTACCTCACCAACTAGCTAATCTTGCGCGTGCCCATCTCTATCCACCGTAGT
TTTCAATATCACCCGATGCCGAATAATATATTATGGGGTATTAATTCTTCTTCGAAAGGCTATCCCCCTGATA
AAGGCAGGTTGCACACGTGTTCCGCACCCGTACGCCGCTCTCAATTACCCGAAAGTAATCTACCGCTCGGCTT
GCATGTGTTAGGCCTCCCGCTAGCGTTCATCCTGAGCCAGGATCAACTCT
>1546
CTGCTGCCGCCCGTAGGAGTCTGGTCCGTGTCTCAGTACCAGTGTGGGGGATCACCCTCTCAGGCCCCCCTAA
AGATCGTCGCCTTGGTGAGCCGTTACCTCACCAACTAGCTAATCTTGCGCGTGCCCCATCTCTATCCACCGTA
GTTTTCAATATCACCCGATGCCGAATAATATATTATGGGGTATTTAATTCTTTCTTCGAAAGGCTATCCCCCT
GATAAAAGGCAGGTTGCACACGTGTTCCGCACCCGTACGCCGCTCTCAATTACCCCGAAAGTAATCTACCGCT
CGGCTTGCATGTGTTAGGCCTCCCGCTAGCGTTCATTCCTGAGCCAGGATCAAACTCT
>1547
CTGCTGCCGCCCGTAGGAGTCTGGACCGTGTCTCAGTTCCAGTGTGACTGATCATCCTCTCAGACCAGTTACG
GATCGTCGCCTTGGTAGGCCTTTTACCCCACCAACTAGCTAATCCGACCTAGGCTCATCTGATAGCGTGAGGT
CCGAAGATCCCCCACTTCTCCCTCAGGACGTATGCGGTATTAGCGCCCCGTTTCCGGACGTATCCCCACTACC
AGGCAGATTCCTAGGCATTACTCACCCGTCCGCCGCTGAATCCAGGAGCAAGCCCCCTTCATCCGCTCGACTG
CATGTGTTAGGCCTGCCGCCAGCGTCAATCTGAGCCAGGATCAAACTCT
>1548

CTGCTGCCGCCCGTAGGAGTCTGGTCCGTGTCTCAGTACCAGTGTGGGGGATCACCCTCTCAGGCCCCCTAAA
GATCGTCGCCTTGGTGAGCCGTTACCTCACCAACTAGCTAATCTTGCGCGTGCCCATCTCTATCCACCGTAGT
TTTCAATATCACCCGATGCCGAATAATATATTATGGGGTATTAATCTTCCCTTCGAAAGGCTATCCCCCTGAT
AAAGGCAGGTTGCACACGTGTTCCGCACCCGTACGCCGCTCTCAATTACCCGAAAGTAATCTACCGCTCGGTT
TGCATGTGTTAGGCCTCCCGCTAGCGTTCATCCTGAGCCAGGATCAAACTCT
>1549
CTGCTGCCGCCCGTAGGAGTCTGGTCCGTGTCTCAGTACCAGTGTGGGGGATCACCCTCTCAGGCCCCCTAAA
GATCGTCGCCTTGGTGAGCCGTTACCTCACCAACTAGCTAATCTTGCGCGTGCCCATCTCTATCCACCGTAGT
TTTCAATATCACCCGATGCCGAATAATATATTATGGGGTATTTAATCTTTCTTCGAAAGGCTATCCCCCTGAT
AAAGGCAGGTTGCACACGTGTTCCGCACCCGTACGCCGCTCTCAATTACCCGAAAGTAATCTACCGCTCGGCT
TGCATGTGTTAGGCCTCCCGCTAGCGTTCTCCTGAGCAGGATCAACTCGT
>1550
CTGCTGCCGCCCGTAGGAGTCTGGTCCGTGTCTCAGTACCAGTGTGGGGGATCACCCTCTCAGGCCCCCTAAA
GATCGTCGCCTTGGTGAGCCGTTACCTCACCAACTAGCTAATCTTGCGCGTGCCCATCTCTATCCACCGTAGT
TTTCAATATCACCCGATGCCGAATAATATATTATGGGGTATTAATCTTCTTCGAAAGGCTATCCCCCTGATAA
AGGCGGGTTGCACACGTGTTCCGCACCCGTACGCCGCTCTCAATTACCCGAAAGTAATCTACCGCTCGGCTTG
CATGTGTTAGGCCTCCCGCTAGCGTTCATCCTGAGCCAGGATCAAACTCT
>1551
CTGCTGCCGCCCGTAGGAGTCTGGTCCGTGTCTCAGTACCAGTGTGGGGGATCACCCTCTCAGGCCCCCTAAA
AGATCGTCGCCTTGGTGAGCCGTTACCTCACCAACTAGCTAATCTTGCGCGTGCCCATCTCTATCCACCGTAG
TTTTCAATATCACCCGATGCCGAATAATATATTATGGGGTATTAATCTTCTTCGAAAGGCTATCCCCCTGATA
AAGGCAGGTTGCACACGTGTTCCGCACCCGTACGCCGCTCTCAATTACCCGAAAGTAATCTACCGCTCGGCTT
GCATGTGTTAGGCCTCCCGCTAGCGTTCACCTGAGCCAGGATCAAACTCT
>1552
CTGCTGCTGCCCGTAGGAGTCTGGTCCGTGTCTCAGTACCAGTGTGGGGGATCACCCTCTCAGGCCCCCTAAA
GATCGTCGCCTGGTGAGCCGTTACCTCACCAACTAGCTAATCTTGCGCGTGCCCATCTCTATCCACCGTAGTT
TTCAATATCACCCGATGCCGAATAATATATTATGGGGTATTAATCTTCTTCGAAAGGCTATCCCCCTGATAAA
GGCAGGTTGCACACGTGTTCCGCACCCGTACGCCGCTCTCAATTACCCGAAAGTAATCTACCGCTCGGCTTGC
ATGTGTTAGGCCTCCCGCTAGCGTTCATCCTGAGCCAGGATCAACTCT
>1553
CTGCTGCCGCCCGTAGGAGTCTGGTCCGTGACTCAGTACCAGTGTGGGGGATCACCCTCTCAGGCCCCCTAAA
GATCGTCGCCTTGGTGAGCCGTTACCTCACCAACTAGCTAATCTTGCGCGTGCCCATCTCTATCCACCGTAGT
TTTCAATATCACCCGATGCCGAATAATATATTATGGGGTATTAATCTTCTTCGAAAGGCTATCCCCCTGATAA
AGGCAGGTTGCACACGTGTTCCGCACCCGTACGCCGCTCTCAATTACCCGAAAGTAATCTACCGCTCGGCTGC
ATGTGTTAGGCCTCCCGCTAGCGTCTCCTGAGC
>1554
CTGCTGCCGCCCGTAGGAGTCTGGTCCGTGTCTCAGTACCAGTGTGGGGGATCACCCTCTCAGGCCCCCTAAA
GATCGTCGCCTTGGTGAGCCGTTACCTCACCAACTAGCTAATCTTGCGCGTGCCCATCTCTATCCACCGTAGT
TTTCAATATCACCCGATGCCGAATAATATATTATGGGGTATTAATCTTCTTCGAAAGGCTATCCCCCTGATAA
AGGCAGGTTGCACACGTGTTCCGCACCCGTACGCCGCTCTCAATTACCCGAAAGTAATCTACCGCTCGGCTTG
CATGTGTTAGGCCTCCCGCTAGCGTTCATCCTGAGCCAGGATCAAACTCT
>1555
CTGCTGCCGCCCGTAGGAGTCTGGTCCGTGTCTCAGTACCAGTGTGGGGGATCACCCTCTCAGGCCCCCTAAA
GATCGCCGCCTTGGTGAGCCGTTACCTCACCAACTAGCTAATCTTGCGCGTGCCCATCTCTATCCACCGTAGT
TTTCAATATCACCCGATGCCGAATAATATATTATGGGGTATTAATCTTCTTCGAAAGCTATCCCCTGATAAAG
GCAGGTTGCACACGTGTTCCGCACCCGTACGCCGCTCTCAATTACCCGAAAGTAATCTACCGCTCGGCTTGCA
TGTGTTAGGCCTCCCGCTAGCGTTCATCTGAGCCAGGATCAAACTCGT
>1556
CTGCTGCCGCCCGTAGGAGTCTGGTCCGTGTCTCAGTACCAGTGTGGGGGATCACCCTCTCAGGCCCCCTAAA
GATCGTCGCCTTGGTGAGCCGTTACCTCACCAACTAGCTAATCTTGCGCGTGCCCATCTCTATCCACCGTAGT
TTCAATATCACCCGATGCCGAATAATATATTATGGGGTATTAATTCTTCTTCGAAGGCTATCCCCCTGATAAA
GGCAGGTTGCACACGTGTTCCGCACCCGTACGCCGCTCTCAATTACCCGAAAGTAATCTACCGCTCGGCTTGC
ATGTGTTAGGCCTCCCGCTAGCGTTCATCCTGAGCCAGGATCAAACTCT
>1557

CTGCTGCCGCCCCGTAGGAGTCTGGTCCGTGTCTCAGTACCAGTGTGGGGGATCACCCTCTCAGGCCCCCTAA
AGATCGTCGCCTTGGTGAGCCGTTACCTCACCAACTAGCTAATCTTGCGCGTGCCCATCTCTATCCACCGTAG
TTTCAATATCACCCGATGCCGAATAATATATTATGGGGTATTAATCTTCTTCGAAAGGCTATCCCCCTGATAA
AGGCAGGTTGCACACGTGTTCCGCACCCGTACGCCGCTCTCAATTACCCCGAAAGTAATCTACCGCTCGGCTT
GCACTGTGTTACGGCTCCCGCTAGGCT
>1558
CTGCTGCCGCCCGTAGGAGTCTGGTCCGTGTCTCAGTACCAGTGTGGGGGATCACCCTCTCAGGCCCCCTAAA
GATCGTCGCCTTGGTGAGCCGTTACCTCACCAACTAGCTAATCTTGCGCGTGCCCATCTCTATCCACCGTAGT
TTTCAATATCACCCGATGCCGAATAATATATTATGGGGTATTAATCTTCTCGAAAGGCTATCCCCCTGATAAA
GGCAGGTTGCACACGTGTTCCGCACCCGTACGCCGCTCTCAATTACCCGAAAGTAATCTACCGCTCCGGCTTG
CATGTGTTAGGCCTCCCGCTAGCGTTCATC
>1559
CTGCTGCCGCCCGTAGGAGTCTGGTCCGTGTCTCAGTACCAGTGTGGGGGATCACCCTCTCAGGCCCCCTAAA
GATCGTCGCCTTGGTGAGCCGTTACCTCACCAACTAGCTAATCTTGCGCGTGCCCATCTCTATCCACCGTAGT
TTTCAATATCACCCGATGCCGAATAATATATTATGGGGTATTAATCTTCTTCGAAAGGCTATCCCCCTGATAA
AGGCAGGTTGCACACGTGTTCCGCACCCGTACGCCGCTCTCAATTACCCGAAAGTAATCTACCGCTCCGGCTT
GCATGTGTTAGGCCTCCCGCTAGCTGTTCAT
>1560
CTGCTGCCGCCCGTAGGAGTCTGGTCCGTGTCTCAGTACCAGTGTGGGGGATCACCCTCTCAGGCCCCCTAAA
GATCGTCGCCTTGGTGAGCCGTTACCTCACCAACTAGCTAATCTTGCGCGTGCCCATCTCTATCCACCGTAGT
TTCAATATCACCCGATGCCGAATAATATATTATGGGGTATTAATCTTCTCGAAAGCTATCCCCCTGATAAAGG
CAGGTTGCACACGTGTTCCGCACCCGTACGCCGCTCTCAATTACCCGAAAGTAATCTACCGCTCGGCTTGCAT
GCGTTAGGCCTCCCGCTAGCGTTCATCCGTG
>1561
CTGCTGCCGCCCGTAGGAGTCTGGTCCGTGTCTCAGTACCAGTGTGGGGGATCACCCTCTCAGGCCCCCTAAA
GATCGTCGCCTTGGTGAGCCGTTACCTCACCAACTAGCTAATCTTGCGCGTGCCCATCTCTATCCACCGTAGT
TTTCAATATCACCCGATGCCGAATAATATATTATGGGGTATTAATCTTCTCGAAGGCTATCCCCTGATAAAGG
CAGGTTGCACACGTGTTCCGCACCCGTACGCCGCTCTCAATTACCCGAAAGTAATCTACCGCTCCGGCTTGCA
TGTGTTAGGCCTCCCGCTAGCGTTCTCCTCGAGCAGGATCAACTCT
>1562
CTGCTGCCGCCCGTAGGAGTCTGGTCCGTGTCTCAGTACCAGTGTGGGGGATCACCCTCTCAGGCCCCCTAAA
GATCGTCGCCTTGGTGAGCCGTTACCTCACCAACTAGCTAATCTTGCGCGTGCCCATCTCTATCCACCGTAGT
TTTCAATATCACCCGATGCCGAATAATATATTATGGGGTATTAATCTTCTTCGAAAGCTATCCCCTGATAAAG
GCAGGTTGCACACGTGTTCCGCACCCGTACGCCGCTCTCAATTACCCGAAAGTAATCTACCGCTCGGCTTGCA
TGTGTTAGGCCTCCCGCTAGCGTTCTCCTCGAGCAGGATCAATCATCT
>1563
CTGCTGCCGCCCGTAGGAGTCTGGTCCGTGTCTCAGTACCAGTGTGGGGGATCACCCTCTCAGGCCCCCTAAA
GATCGTCGCCTTGGTGAGCCGTTACCTCACCAACTAGCTAATCTTGCGCGTGCCCATCTCTATCCACCGTAGT
TTTCAATATCACCCGATGCCGAATAATATATTATGGGGTATTAATCTTCTTCGAAAGGCTATCCCCCTGATAA
AGGCAGGTTGCACACGTGTTCCGCACCCGTACGCCGCTCTCAATTACCCGAAAGTAATCTACCGCTCCGGCTT
GCATGTGTTAGGCCTCCCGCTAGCGTTCATCCTCGAGCCAGGATCAAACTCT
>1564
CTGCTGCCGCCCGTAGGAGTCTGGTCCGTGTCTCAGTACCAGTGTGGGGGATCACCCTCTCAGGCCCCCTAAA
GATCGTCGCCTTGGTGAGCCGTTACCTCACCAACTAGCTAATCTTGCGCGTGCCCATCTCTATCCACCGTAGT
TTTCAATATCACCCGATGCCGAATAATATATTATGGGGTATTAATCTTCTTCGAAGCTATCCCCCTGAATAAA
GGCAGGTTGCACACGTGTTCCGCACCCGTACGCCGCTCTCAATTACCCGAAAGTAATCTACCGCTCGGCTTGC
ATGTGTTAGGCCTCCCGCTAGCGTTCATCCTGAGCCAGGATCAAACTCCGT
>1565
CTGCTGCCGCCCGTAGGAGTCTGGTCCGTGTCTCAGTACCAGTGTGGGGGATCACCCTCTCAGGCCCCCTAAA
GATCGTCGCCTTGGTGAGCCGTTACCTCACCAACTAGCTAATCTTGCGCGTGCCCATCTCTATCCACCGTAGT
TTTCAATATCACCCGATGCCGAATAATATATTATGGGGTATTAATCTTCTTCGAAAGGCTATCCCCTGATAAA
GGCAGGTTGCACACGTGGTTCCGCACCCGTACGCCGCTCTCAATTACCCGAAAGTAATCTACCGCTCCGGCTT
GCATGTGTTAGGCCTCCCGCTAGCGTTCATCC
>1566

CTGCTGCCGCCCGTAGGAGTCTGGTCCGTGTCTCAGTACCAGTGTGGGGGATCACCCTCTCCAGGCCCCCTAA
AGATCGTCGCCTTGGTGAGCCGTTACCTCACCAACTAGCTAATCTTGCGCGTGCCCATCTCTATCCACCGTAG
TTTTCAATATCACCCGATGCCGAATAATATATTATGGGGTATTAATCTTCTTCGAAAGGCTATCCCCCTGATA
AAGGCAGGTTGCACACGTGTTCCGCACCCGTACGCCGCTCTCAATTACCCGAAAGTAATCTACCGCTCGGCTT
GCATGTGTTAGGCCTCCCGCTAGCGTTCATCCTCGAGCCAGGATCAACTCT
>1567
CTGCTGCCGCCCGTAGGAGTCTGGTCCGTGTCTCAGTACCAGTGTGGGGGATCACCCTCTCAGGCCCCCTAAA
GATCGTCGCCTTGGTGAGCCGTTACCTCACCAACTAGCTAATCTTGCGCGTGCCCATCTCTATCCACCGTAGT
TTTCAATATCACCCGATGCCGAATAATATATTATGGGGTATTAATCTTCTTCGAAAGCTATCCCCCTGATAAA
GGCAGGTTGCACACGTGTTCCGCACCCGTACGCCGCTCTCAATTACCCGAAAGTAATCTACCGCTCCGGCTTG
CATGTGTTAGGCCTCCCGCTAGCGTTCATCCGTGAGCCAGGATCAACTCT
>1568
CTGCTGCCGCCCGTAGGAGTCTGGTCCGTGTCTCAGTACCAGTGTGGGGGATCACCCTCTCAGGCCCCCTAAA
GATCGTCGCCTTGGTGAGCCGTTACCTCACCAACTAGCTAATCTTGCGCGTGCCCATCTCTATCCACCGTAGT
TTTCAATATCACCCGATGCCGAATAATATATTATGGGGTATTTAATCTTTCTTCGAAAGGCTATCCCCCTGAT
AAAGGCAGGTTGCACACGTGTTCCGCACCCGTACGCCGCTCTCAATTACCCGAAAGTAATCTACCGCTCGGCT
TGCATGTGTTAGGCCTCCCGCTAGCGTTCTCCTCGAGCAGGATCAACATCT
>1569
CTGCTGCCGCCCGTAGGAGTCTGGTCCGTGTCTCAGTACCAGTGTGGGGGATCACCCTCTCAGGCCCCCTAAA
GATCGTCGCCTTGGTGAGCCGTTACCTCACCAACTAGCTAATCTTGCGCGTGCCCATCTCTATCCACCGTAGT
TTTCAATATCACCCGATGCCGAATAATATATTATGGGGTATTAATCTTCTTCGAAGGCTATCCCCTGATAAAG
GCAGGTTGCACACGTGTTCCGCACCCGTACGCCGCTCTCAATTACCCGAAAGTAATCTACCGCTCCGGCTTGC
ATGTGTTAGGCCTCCCGCTAGCGTTCTCCTCGAGCAGGA
>1570
CTGCTGCCGCCCGTAGGAGTCTGGTCCGTGTCTCAGTACCAGTGTGGGGGATCACCCTCTCAGGCCCCCTAAA
GATCGTCGCCTTGGTGAGCCGTTACCTCACCAACTAGCTAATCTTGCGCGTGCCCATCTCTATCCACCGTAGT
TTCCAATATCACCCGATGCCGAATAATATATTATGGGGTATTAATCTTCTCGAAAGGCTATCCCCTGATAAAG
GCAGGTTGCACACGTGTTCCGCACCCGTACGCCGCTCTCAATTACCCGAAAGTAATCTACCGCTCCGGCTTGC
ATGTGTTAGGCCTCCCGCTAGCGTTCTCCGCTC
>1571
CTGCTGCCGCCCGTAGGAGTCTGGTCCGTGTCTCAGTACCAGTGTGGGGGATCACCCTCTCAGGCCCCCTAAA
GATCGTCGCCTTGGTGAGCCGTTACCTCACCAACTAGCTAATCTTGCGCGTGCCCATCTCTATCCACCGTAGT
TTTCAATATCACCCGATGCCGAATAATATATTATGGGGTATTAATCTTCTTCGAAGGCTATCCCCTGATAAAG
GCAGGTTGCACACGTGTTCCGCACCCGTACGCCGCTCTCAATTACCCGAAAGTAATCTACCGCTCGGCTTAGC
ATGTGTTACGGCC
>1572
CTGCTGCCGCCCGTAGGAGTCTGGTCCGTGTCTCAGTACCAGTGTGGGGGATCACCCTCTCAGGCCCCCTAAA
GATCGTCGCCTTGGTGAGCCGTTACCTCACCAACTAGCTAATCTTGCGCGTGCCCATCTCTATCCACCGTAGT
TTTCAATATCACCCGATGCCGAATAATATATTATGGGGTATTAATCTTCCCTTCGAAAGGCTATCCCCCTGAT
AAAGGCAGGTTGCACACGTGTTCCGCACCCGTACGCCGCTCTCAATTACCCGAAAGTAATCTACCGCTCGGCT
TGCATGTGTTAGGCCTCCCGCTAGCGTTCATCCTCGAGCCAGGATCAAACTCT
>1573
CTGCTGCCGCCCGTAGGAGTCTGGTCCGTGTCTCAGTACCAGTGTGGGGGATCACCCTCTCAGGCCCCCTAAA
GATCGTCGCCTTGGTGAGCCGTTACCTCACCAACTAGCTAATCTTGCGCGTGCCCATCTCTATCCACCGTAGT
TTTCAATATCACCCGATGCCGAATAATATATTATGGGGTATTAATCTTCTTCGAAAGGCTTATCCCCCTGATA
AAGGCAGGTTGCACACGTGTTCCGCACCCGTACGCCGCTCTCAATTACCCGAAAGTAATCTACCGCTCGGCTT
GCATGTGTTAGGCCTCCGCTAG
>1574
CTGCTGCCGCCCGTAGGAGTCTGGTCCGTGTCTCAGTACCAGTGTGGGGGATCACCCTCTCAGGCCCCTAAAG
ATCGTCGCCTTGGTGAGCCGTTACCTCACCAACTAGCTAATCTTGCGCGTGCCCATCTCTATCCACCGTAGTT
TTCAATATCACCCGATGCCGAATAATATATTATGGGGTATTAATCTTCTTCGAAAGGCTATCCCCTGATAAAG
GCAGGTTGCACACGTGTTCCGCACCCGTACGCCGCTCTCAATTACCCGAAAGTAATCTACCGCTCGGCTTGAC
ATGTGTTAGGCCTCCCGCTAGCGTCCATCCTGAGCCAGGATCAAACTCT
>1575

CTGCTGCCGCCCGTAGGAGTCTGGTCCGTGTCTCAGTACCAGTGTGGGGGATCACCCTCTCAGGCCCCCTAAA
GATCGTCGCCTTGGTGAGCCGTTACCTCACCAACTAGCTAATCTTGCGCGTGCCCATCTCTATCCACCGTAGT
TTTCAATATCACCCGATGCCGAATAATATATTATTGGGGTATTAATCTTCTTCGAAGGCTTATCCCCCTGATA
AAGGCAGGTTGCACACGTGTTCCGCACCCGTACGCCGCTCTCAATTACCCGAAAGTAATCTACCGCTCGGCTT
GCATGTGTTAGGCCTCCCGCTAGCGTTCATCCTGAGCCAG
>1576
CTGCTGCCGCCCGTAGGAGTCTGGTCCGTGTCTCAGTACCAGTGTGGGGGATCACCCTCTCAGGCCCCCTAAA
GATCGTCGCCTTGGTGGGCCGTTACCTCACCAACTAGCTAATCTTGCGCGTGCCCATCTCTATCCACCGTAGT
TTTCAATATCACCCGATGCCGAATAATATATTATGGGGTATTAATCTTCTTCGAAAGGCTATCCCCCTGATAA
AGGCAGGTTAGCACACGTGTTCCGCACCCGTACGCCGCTCTCAATTACCCGAAAGTAATCTACCGCTCGGCTT
GCATGTGTTAGGCCTCCCGCTAGCGTTCTCCTGAACC
>1577
CTGCTGCCGCCCGTAGGAGTCTGGTCCGTGTCTCAGTACCAGTGTGGGGGATCACCCTCTCAGGCCCCCTAAA
GATCGTCGCCTTGGTGAGCCGTTACCTCACCAGCTAGCTAATCTTGCGCGTGCCCCATCTCTATCCACCGTAG
TTTTCAATATCACCCGATGCCGAATAATATATTATGGGGGTATTTAATCTTCTTCGAAAGGCTATCCCCCTGA
TAAAGGCAGGTTGCACACGTGTTCCGCACCCGTACGCCGCTCTCAATTACCCGAAAGTAATCTACCGCTCGGC
TTGCATGTGTTAGGCCTCCCGCTAGCGCTCATCCTGAGCCAGAATCAAACTCT
>1578
CTGCTGCCGCCCGTAGGAGTCTGGTCCGTGTCTCAGTACCAGTGTGGGGGATCACCCTCTCAGGCCCCCTAAA
AGATCGTCGCCTTGGTGAGCCGTTACCTCACCAACTAGCTAATCTTGCGCGTGCCCATCTCTATCCACCGTTA
GTTTTCAATATCACCCGATGCCGAATAATATATTTATGGGGTATTTAATTCTTCTTCGAAAGGCTATCCCCTG
ATAAAGGCAGGTTGCACACGTGTTCCGCACCCGTACGCCGCTCTCAATTACCCGAAAGTAATCTACCGCTCGG
CTTGCATGTGTTAGGCCTCCCGCTAGCGTTCTCCTGAGCA
>1579
CTGCTGCCGCCCGTAGGAGTCTGGTCCGTGTCTCAGTACCAGTGTGGGGGATCACCCTCTCAGGCCCCCTAAA
GATCGTCGCCTTGTGAGCCGTTACCTCACCAACTAGCTAATCTTGCGCGTGCCCATCTCTATCCACCGTAGTT
TTCAATATCACCCGATGCCGAATAATATATTATGGGGTATTAATCTTCTCGAAAGGCTATCCCCTGATAAAGG
CAGGTTGCACACGTGTTCCGCACCCGTACGCCGCTCTCAATTACCCGAAAGTAATCTACCGCTCGGCTTGCAT
GTGTTAGGCCTCCCGCTAGCGTTCATCCTGAGCAGGATCAACTCT
>1580
CTGCTGCCGCCCGTAGGAGTCTGGTCCGTGTCTCAGTACCAGTGTGGGGGATCACCCTCTCAGGCCCCCTAAA
GATCGTCGCCTTGGTGAGCCGTTACCTCACCAACTAGCTAATCTTGCGCGTGCCCATCTCTATCCACCGTAGT
TTTCAATATCACCCGATGCCGAATAATATATTATGGGGTATTTAATCTTCTTCGAAAGGCTATCCCCCTGATA
AAGGCAGGTTGCACACGTGTTCCGCACCCGTACGCCGCTCTCAATTACCCGAAAGTAATCTACCGCTCGGCTT
GCATGTGTTAGGCCTCCCGCTAGCGTTCATCCTGAGCCAGGATCAAACTCT
>1581
CTGCTGCCGCCCGTAGGAGTCTGGTCCGTGTCTCAGTACCAGTGTGGGGGATCACCCTCTCAGGCCCCCTAAA
GATCGTCGCCTTGGTGAGCCGTTACCTCACCAACTAGCTAATCTTGCGCGTGCCCATCTCTATCCACGTAGTT
TCAATATCACCCGATGCCGAATAATATATTATGGGGTATTAATCTTCTCGAAAGGCTATCCCCCTGATAAAGG
CAGGTTGCACACGTGTTCCGCACCCGTACGCCGCTCTCAATTACCCGAAAGTAATCTACCGCTCGGCTTGCAT
GTGTTAGGCCTCCCGCTAGCGTTCATCTGAGCCAGGATCAAACTCT
>1582
CTGCTGCCGCCCGTAGGAGTCTGGTCCGTGTCTCAGTACCAGTGTGGGGGATCACCCTCTCAGCCCCCTAAAG
ATCGTCGCCTTGGTGAGCCGTTACCTCACCAACTAGCTAATCTTGCGCGTGCCCATCTCTATCCACCGTAGTT
TTCAATATCACCCGATGCCGAATAATATATTATGGGGTATTAATCTTCTTCGAAAGGCTATCCCCTGATAAAG
GCAGGTTGCACACGTGTTCCGCACCCGTACGCCGCTCTCAATTACCCGAAAGTAATCTACCGCTCGGCTTGCA
TGTGTTAGGCCTCCCGCTAGCGTTCATCCTGAGCCAGGA
>1583
CTGCTGCCGCCCGTAGGAGTCTGGTCCGTGTCTCAGTACCAGTGTGGGGGATCACCCTCTCAGGCCCCCTAAA
GATCGTCGCCTGGTGAGCCGTTACCTCACCAACTAGCTAATCTTGCGCGTGCCCATCTCTATCCACCGTAGTT
TTCAATATCACCCGATGCCGAATAATATATTATGGGGTATTAATCTTCTTCGAAAGGCTATCCCCCTGATAAA
GGCAGGTTGCACGCGTTGTCCGCACCCGTACGCCGCTCTCAATTACCCGAAAGTAATCTACCGCTCGGCTTGC
ATGTGTTAGGCCTCCCGCTAGCGTTCATCCTGAGCCAGGATCAAACTCT
>1584

CTGCTGCCGCCCGTAGGAGTCTGGTCCGTGTCTCAGTACCAGTGTGGGGGATCACCCTCTCAGGCCCCCTAAA
GATCGTCGCCTTGGTGAGCCGTTACCTCACCAACTAGCTAATCTTGCGCGTGCCCATCTCTATCCACCGTAGT
TTTCAATATCACCCGATGCCGAATAATATATTATGGGGTATTAATCTTCTTCGAAAGCTATCCCCCTGATAAA
GGCAGGTTGCACACGTGTTCCGCACCCGTACGCCGCTCTCAATTACCCGAAAGTAATCTACCGCTCGGCTTGC
ATGTGTTAGGCCTCCCGCTAGCGTTCATCCTGAGC
>1585
CTGCTGCCGCCCGTAGGAGTCTGGACCGTGTCTCAGTTCCAGTGTGACTGATCATCCTCTCAGACCAGTACGG
ATCGTCGCCTTGGTAGGCCTTTACCCCACCAACTAGCTAATCCGACCTAGGCTCATCTGATAGCGTGAGGTCC
GAAGATCCCCCACTTCTCCCTCAGGACGTATGCGGTATTAGCGCCCGTTTCCGGACGTATCCCCACTACCAGG
CAGATTCCTAGGCATTACTCACCCGTCCGCCGCTGAATCCAGGAGCAAGCTCCCTCATCCGCTCGACTGCATG
TGTTAGGCCTGCCGCCAGCGTTCAATCTGAGCCAGGATCAAACTCT
>1586
CTGCTGCCGCCCGTAGGAGTCTGGTCCGTGTCTCAGTACCAGTGTGGGGGATCACCCTCTCAGGCCCCCTAAA
GATCGTCGCCTTGGTGAGCCGTTACCTCACCAACTAGCTAATCTTGCGCGTGCCCATCTCTATCCACCGTAGT
TTTCAATATCAACCCGATGCCGAATAATATATTATGGGGTATTAATCTTCTCGAAAGGCTATCCCCTGATAAA
GGCAGGTTGCACACGTGTTCCGCACCCGTACGCCGCTCTCAATTACCCGAAAGTAATCTACCGCTCGGCTTGC
ATGTGTTAGGCCTCCCGCTAGCGTTCATC
>1587
CTGCTGCCGCCCGTAGGAGTCTGGTCCGTGTCTCAGTACCAGTGTGGGGGATCACCCTCTCAGGCCCCCTAAA
GATCGTCGCCTGGTGAGCCGTTACCTCACCAACTAGCTAATCTTGCGCGTGCCCATCTCTATCCACCGTAGTT
TTCAATATCACCCGATGCCGAATAATATATTATGGGGTATTAATCTTCTTCGAAAGGCTATCCCCTGATAAAG
GCAGGTTGCACACGTGTTCCGCACCCGTACGCCGCTCTCAATTACCCGAAAGTAATCTACCGCTCGGCTTGCA
TGTGTTAGGCCTCCCGCTAGCGTTCATCCTGAG
>1588
CTGCTGCCGCCCGTAGGAGTCTGGTCCGTGTCTCAGTACCAGTGTGGGGGATCACCCTCTCAGGCCCCCTAAA
GATCGTCGCCTTGGTGAGCCGTTACCTCACCAACTAGCTAATCTTGCGCGTGCCCATCTCTATCCACCGTAGT
TTTCAATATCACCCGATGCCGAATAATATATTATGGGGTATTAATCTTCTCTGAAAGCTATCCCCCTGATAAA
GGCAGGTTGCACACGTGTTCCGCACCCGTACGCCGCTCTCAATTACCCGAAAGTAATCTACCGCTCCGGCTTG
CATGTGTTAGGCCTCCCGCTAGCGTTCATCCTGAGCCAGGATCAAACTCT
>1589
CTGCTGCCGCCCGTAGGAGTCTGGTCCGTGTCTCAGTACCAGTGTGGGGGATCACCCTCTCAGGCCCCCTAAA
GATCGTCGCCTTGGTGAGCCGTTACCTCACCAACTAGCTAATCTTGCGCGTGCCCATCTCTATCCACCGTAGT
TTTCAATATCACCCGATGCCGAATAATATATTATGGGGTATTAATCTTCTTCGAAAGGCTATCCCCCTGATAA
AGGCAGGTTGCACACGTGTTCCGCACCCGTACACCGCTCTCAATTACCCGAAAGTAGTCTACCGCTCGGCTTG
CATGTGTTAGGCCTCCCGCTAGCGTTCATCCTGAGCCAGGATCAAACTCT
>1590
CTGCTGCCGCCCGTAGGAGTCTGGTCCGTGTCTCAGTACCAGTGTGGGGGATCACCCTCTCAGGCCCCCTAAA
GATCGTCGCCTTGGTGAGCCGTTACCTCACCAACTAGCTAATCTTGCGCGTGCCCATCTCTATCCACCGTAGT
TTTCAATATCACCCGATGCCGAATAATATATTATGGGGTATTAATCTTCTTCGAAAGCTATCCCCTGATAAAG
GCAGGTTGCACACGTGTTCCGCACCCGTACGCCGCTCTCAATTACCCGAAAGTAATCTACCGCTCGGCTTGCA
TGTGTTAGGCCTCCCGCTAGCGTTCATCCTGAGCCAGGATCAAACTCT
>1591
CTGCTGCCGCCCGTAGGAGTCTGGTCCGTGTCTCAGTACCAGTGTGGGGGATCACCCTCTCAGGCCCCCTAAA
GATCGTCGCCTTGGTGAGCCGTTACCTCACCAACTAGCTAATCTTGCGCGTGCCCATCTCTATCCACCGTAGT
TTTCAATATCACCCGATGCCGAATAATATATTATGGGGTATTAAATCTTCTCGAAAGGCTTATCCCCCTGATA
AAGGCAGGTTGCACACGTGTTCCGCACCCGTACGCCGCTCTCAATTACCCGAAAGTAATCTACCGCTCGGCTT
GCATGTGTTAGGCCTCCCGCTAGCGTTCATCCTGAGCCAGGCATCGAACTCT
>1592
CTGCTGCCGCCCGTAGGAGTCTGGTCCGTGTCTCAGTACCAGTGTGGGGGATCACCCTCTCAGGCCCCCTAAA
GATCGTCGCCTTGGTGAGCCGTTACCTCACCAACTAGCTAATCTTGCGCGTGCCCATCTCTATCCACCGTAGT
TTTCAATATCCACCGATGCCGAATAATATATTATGGGGTATTAATCTTCTCGAAAGGCTATCCCCTGATAAGG
CAGGTTAGCACACGTCGTTCCGCACCGTACGCCGCTCTCAATTACCCGAAAGTAATCACCGCTCCGGCTTGCA
TGTGTTAGGCCTCCCGCTAGCGTCATCCTGAGCAGGATAACTCCT
>1593

CTGCTGCCGCCCGTAGGAGTCTGGTCCGTGTCTCAGTACCAGTGTGGGGGATCACCCTCTCAGGCCCCCTAAA
GATCGTCGCCTTGGTGAGCCGTTACCTCACCAACTAGCTAATCTTGCGCGTGCCCATCTCTATCCACCGTAGT
TTTCAATATCACCCGATGCCGAATAATATATTATGGGGTATTAATCTTCTCGAAAGGCTATCCCCTGATAAAG
GCAGGTTGCACACGTGTTCCGCACCCGTACGCCGCTCTCAATTACCCGAAAGTAATCTACCGCTCCGGCTTGC
ATGTGTTAGGCCTCCCGCTAGCGTTCAT
>1594
CTGCTGCCGCCCGTAGGAGTCTGGTCCGTGTCTCAGTACCAGTGTGGGGGATCACCCTCTCAGGCCCCCTAAA
GATCGTCGCCTTGGTGAGCCGTTACCTCACCAACTAGCTAATCTTGCGCGTGCCCATCTCTATCCACCGTAGT
TTTCAATATCACCCGATGCCGAATAATATATTATGGGGTATTAATCTTCTTCGAAAGGCTATCCCCCTGATAA
AGGCAGGTTGCACACGTGTTCCGCACCCGTGCGCCGCTCTCAATTACCCGAAAGTAATCTACCGCTCGGCTTG
CATGTGTTAGGCCTCCCGCTAGCGTTCATCCTGAGCCAGGATCAAACTCT
>1595
CTGCTGCCGCCCGTAGGAGTCTGGTCCGTGTCTCAGTACCAGTGTGGGGGATCACCCTCTCAGGCCCCCTAAA
GATCGTCGCCTTGGTGAGCCGTTACCTCACCAACTAGCTAATCTTGCGCGTGCCCATCTCTATCCACCGTAGT
TTCAATATCACCCGATGCCGAATAATATATTATGGGGTATTAATCTTCTCTCGAAAGGCTATCCCCTATAAGG
CAGGTTGCACACGTGTTCCGCACCCGTACGCCGCTCTCAATTACCCGAAAGTAATCTACCGCTCCGGCTTGCA
TGTGTTAGGCCTCCCGCTAGCGTTCATCCTGAGCCAGGATCAACTCT
>1596
CTGCTGCCGCCCGTAGGAGTCTGGTCCGTGTCTCAGTACCAGTGTGGGGGATCACCCTCTCAGGCCCCCTAAA
GATCGTCGCCTTGGTGAGCCGTTACCTCACCAACTAGCTAATCTTGCGCGTGCCCATCTCTATCCACCGTAGT
TTTCAATATCACCCGATGCCGAATAATATATTATGGGGTATTAATCTTCTTCGAAAGCTATCCCCTGATAAAG
GCAGGTTGCACACGTGTTCCGCACCCGTACGCCGCTCTCAATTTACCCGAAAGTAATCTACCGCTCCGGCTTG
CATGTGTTAGGCCTCCCGCTAGCGTTCATCGTC
>1597
CTGCTGCCGCCCGTAGGAGTCTGGTCCGTGTCTCAGTACCAGTGTGGGGGATCACCCTCTCAGGCCCCCTAAA
GATCGTCGCCTTGGTGAGCCGTTACCTCACCAACTAGCTAATCTTGCGCGTGCCCATCTCTATCCACCGTAGT
TTTCAATATCACCCGATGCCGAATAATATATTATGGGGTATTAATCTTCTCTAAAGGCTATCCCCTGATAAAG
GCAGGTTGCACACGTGTTCCGCACCCGTACGCCGCTCTCAATTACCCGAAAGTAATCTACCGCTCCGGCTTGC
ATGTGTTAGGCCTTCC
>1598
CTGCTGCCGCCCGTAGGAGTCTGGTCCGTGTCTCAGTACCAGTGTGGGGGATCACCCTCTCAGGCCCCCTAAA
GATCGTCGCCTTGGTGAGCCGTTACCTCACCAACTAGCTAATCTTGCGCGTGCCCATCTCTATCCACCGTAGT
TTTCAATATCACCCGATGCCGAATAATATATTATGGGGTATTAATCTTCTCGAAAGCTATCCCCTGATAAAGG
CAGGTTGCACACGTGTTCCGCACCCGTACGCCGCTCTCAATTACCCGAAAGTAATCTACCGCTCCGGCTTGCA
TGTGTTAGGCCTCCCGCTAGCGTTCATCCTGAGCAGGATCAACTCT
>1599
CTGCTGCCGCCCGTAGGAGTCTGGTCCGTGTCTCAGTACCAGTGTGGGGGATCACCCTCTCAGGCCCCCTAAA
GATCGTCGCCTTGGTGAGCCGTTACCTCACCAACTAGCTAATCTTGCGCGTGCCCATCTCTATCCACCGTAGT
TTTCAATATCACCCGATGCCGAATAATATATTATGGGGTATTAATCTTCTTCGAAAGCTATCCCCCTGATAAA
GGCAGGTTGCACACGTGTTCCGCACCCGTACGCCGCTCTCAATTACCCGAAAGTAATCTACCGCTCGGCTTGC
ATGTGTTAGGCCTCCCGCTAGCGTTCTCCGTGAGCAAGGCATCCAATCATGCCT
>1600
CTGCTGCCGCCCGTAGGAGTCTGGTCCGTGTCTCAGTACCAGTGTGGGGGATCACCCTCTCAGGCCCCCTAAA
GATCGTCGCCTTGGTGAGCCGTTACCTCACCAACTAGCTAATCTTGCGCGTGCCCATCTCTATCCACCGTAGT
TTTCAATATCACCCGATGCCGAATAATATATTATGGGGTATTAATCTTCTCGAAGGCTATCCCCTGATAAAGC
AGGTTGCACACGTGTTCCGCACCCGTACGCCGCTCTCAATTACCCGAAAGTAATCTACCGCTCCGGCTTGACA
TGTGTTAGGCCTCCGCTAGCGTTCTCC
>1601
CTGCTGCCGCCCGTAGGAGTCTGGTCCGTGTCTCAGTACCAGTGTGGGGGATCACCCTCTCAGGCCCCCTAAA
GATCGTCGCCTTGGTGAGCCGTCACCTCACCAACTAGCTAATCTTGCGCGTGCCCATCTCTATCCACCGTAGT
TTTCAATATCACCCGATGCCGAATAATATATTATGGGGTATTAATCTTCTCGAAGGCTATCCCCTGAATAAAG
GCAGGTTGCACACGTGTTCCGCACCCGTACGCCGCTCTCAATTACCCGAAAGTAATCTACCGCTCCGGCTTGC
ATGTGTTAGGCCTCCCGCTAGCGTTCTACCTCGAGCAGGATCAATCATCT
>1602

CTGCTGCCGCCCGTAGGAGTCTGGACCGTGTCTCAGTTCCAGTGTGGCTGATCATCCTCTCAGACCAGCTACG
GATCGTCGCCTTTGGTGGGCCTTTACCCCGCCAACTAGCTAATCCGACATCGGCTCATTCAATCGCGCAAGGT
CCGAAGATCCCCTGCTTCACCCGTAGGTCGTATGCGGTATTAGCGTAAGTTTCCCTACGTTATCCCCCACGAA
AAGTAGATTCCGATGTATTCCTCACCCGTCCGCCACTCTCCACCCGTAAGAGCAAGCTCTTACTGTGCTGCCG
TTCGACTGCATGTGTTAGGCTACCGCCAGCGTCACTCTGAGCCAGGATCAAACTCT
>1603
CTGCTGCCGCCCGTAGGAGTCTGGTCCGTGTCTCAGTACCAGTGTGGGGGATCACCCTCTCAGGCCCCCTAAA
GATCGTCGCCTTGGTGAGCCGTTACCTCACCAACTAGCTAATCTTGCGCGTGCCCATCTCTATCCACCGTAGT
TTTCAATATCACCCGATGCCGAATAATATATTATGGGGTATTAATCTTCTCGAAGCTATCCCCTGAATAAAGG
CAGGTTGCACACGTGTTCCGCACCGTACGCCGCTCCTCAATTTACCCCGAAACGTAATCTACCGCATACGGCT
TCGGCCATGG
>1604
CTGCTGCCGCCCGTAGGAGTCTGGACTCGTGTCTCAGTTCCAGTGTGACTGATCATCCTCTCAGACCAGTACG
GATCGTCGCCTTGGTAGGCCTTTACCCCACCAACTAGCTAATCCGACCTAGGCTCATCTGATAGCGTGAGGTC
CGAAGTCCCCCACTTCTCCCTCAGGACGTATGCGGTATTAGCGCCCGTTTCCGGACGTATCCCCACTACCAGG
CAGATTCCTAGGCATTACTCACCCGTCCGCCGCTGAATCCAGGAGCAAGCCCTCCATCGCTCGACTGCCATGT
GTTAGGCCTGCCGCCAGCGTCAATCTGAGCCAGGATCAACATCT
>1605
CTGCTGCCGCCCGTAGGAGTCTGGTCCGTGTCTCAGTTCCAGTGTGACTGATCATCCTCTCAGACCAGTTACG
GATCGTCGCCTTGGTAGGCCTTTACCCCACCAACTAGCTAATCCGACCTAGGCTCATCTGATAGCGTGAGGTC
CGAAGATCCCCACTTCTCCCTCAGGACGTATGCGGTATTAGCGCCCGTTCCGGACGTATCCCACTACCAGCAG
ATTCCTCAGGCATTACTCACCCGTCCGCGCTGAATCCAGGAGCAAGCCCCCTTCCATCGCTCGACTGCATGTG
TTAGGCCTGCCGCCAGCGTCATCTGAGCCAGGATCAAACTCT
>1606
CTGCTGCCGCCCGTAGGAGTCTGGTCCGTGTCTCAGTACCAGTGTGGGGGATCACCCTCTCAGGCCCCCTAAA
GATCGTCGCCTGGTGAGCCGTTACCTCACCAACTAGCTAATCTTGCGCGTGCCCATCTCTATCCACCGTAGTT
TTCAATATCACCCGATGCCGAATAATATATTATGGGGTATTAATCTTCTTCGAAAGCTATCCCCTGATAAAGG
CAGGTTGCACACGTGTTCCGCACCCGTACGCCGCTCTCAATTACCCGAAAGTAATCTACCGCTCGGCTTGCAT
GTGTTAGGCCTCCCGCTAGCGTTCTCCTGAGCAGGATCAACTCGT
>1607
CTGCTGCCGCCCCGTAGGAGTCTGGTCCCGTGTCTCAGTACCAGTGTGGGGGATCACCCCTCTCAGGCCCCCC
TAAAGATCGTCGCCTTGGTGAGCCGTTACCTCACCCAACTAGCTAATCTTGCGCGTGCCCCATCTCTATCCCA
CCGTAGTTTTTCAATTATCACCCGATGCCCGAATAATATATTATTGGGGTATTTAATCTTCTTCGAAGGCTAT
CCCCTGATTAAAGGCAGGTTGCACACGTGTTCCCGCACCCGTACGCCGCTCTCAATTACCCCGAAAGTAATCT
ACCGCTCGGCTTGCATGTGTTAGGCCTTCCCCGCTAGCGTTCTTCCTGAGCAGGTCCAAAC
>1608
CTGCTGCCGCCCGTAGGAGTCTGGTCCGTGTCTCAGTACCAGTGTGGGGGATCACCCTCTCAGGCCCCCTAAA
GATCGTCGCCTTGGTGAGCCGTTACCTCACCAACTAGCTAATCTTGCGCGTGCCCATCTCTATCCACCGTAGT
TTTCAATATCACCCGATGCCGAATAATATATTATGGGGTATTAATCTTCTTCGAAAGGCTATCCCCCTGATAA
AGGCAGGTTGCACACGTGTTCCGCACCCGTACGCCGCTCTCAATTACCCGAAAGTAATCTACCGCTCGGCTTG
CATGTGTTAGGCCTCCCGCTAGCGTTCATCCTGAGCCAGGATCAAACTCT
>1609
CTGCTGCCGCCCGTAGGAGTCTGGTCCGTGTCTCAGTACCAGTGTGGGGGATCACCCTCTCAGGCCCCCTAAA
GATCGTCGCCTTGGTGAGCCGTTACCTCACCAACTAGCTAATCTTGCGCGTGCCCATCTCTATCCACCGTAGT
TTTCAATATCACCCGATGCCGAATAATATATTATGGGGTATTAATCTTCTCGAAAGGCTTATCCCCCTGAATA
AAGGCAGGTTGCACACGTGTTCCGCACCCGTACGCCGCTCTCAATTACCCGAAAGTAATCTACCGCTCGGCTT
GCATGTGTTAGGCCTCCCGCTAGCGTTCATCCTGAGCCAGGATCAAACTCT
>1610
CTGCTGCCGCCCGTAGGAGTCTGGTCCGTGTCTCAGTACCAGTGTGGGGGATCACCCTCTCAGGCCCCCTAAA
GATCGTCGCCTTGGTGAGCCGTTACCTCACCAACTAGCTAATCTTGCGCGTGCCCATCTCTATCCACCGTAGT
TTCAATATCACCCGATGCCGAATAATATATTATGGGGTATTAATCTTCTTCGAAAGGCTATCCCCCTGATAAA
GGCAGGTTGCACACGTGTTCCGCACCCGTACGCCGCTCTCAATTACCCGAAAGTAATCTACCGCTCGGCTTGC
ATGTGTTAGGCCTCCCGCTAGCGTTCATCCTGAGCCAGGATCAAACTCT
>1611

CTGCTGCCGCCCGTAGGAGTCTGGTCCGTGTCTCAGTACCAGTGTGGGGGATCACCCTCTCAGGCCCCCTAAA
GATCGTCGCCTTGGTGAGCCGTTACCTCACCAACTAGCTAATCTTGCGCGTGCCCATCTCTATCCACCGTAGT
TTTCAATATCACCCGATGCCGAATAATATATTATGGGGTATTTAATCTTCTTCGAAAGGCTATCCCCTGATAA
AGGCAGGTTGCACACGTGTTCCGCACCCGTACGCCGCTCTCAATTACCCGAAAGTAATCTACCGCTCGGCTTG
CATGTGTTAGGCCTCCCGCTAGCGTTCATCCTGAGCCAGGATCAAACTCT
>1612
CTGCTGCCGCCCGTAGGAGTCTGGTCCGTGTCTCAGTACCAGTGTGGGGGATCACCCTCTCAGGCCCCCTAAA
GATCGTCGCCTTGGTGAGCCGTTACCTCACCAACTAGCTAATCTTGCGCGTGCCCATCTCTATCCACCGTAGT
TTTCAATATCACCCGATGCCGAATAATATATTATGGGGTATTAATCTTCTTCGAAAGGCTATCCCCCTGATAA
AGGCAGGTTGCACACGTGTTCCGCACCCGTACGCCGCTCTCAATTACCCGAAAGTAATCTACCGCTCCGGCTT
GCATGTGTTAGGCCTCCCGCTAGCGTTCATCCTGAGCCAGGATCAAACTCT
>1613
CTGCTGCCGCCCGTAGGAGTCTGGTCCGTGTCTCAGTACCAGTGTGGGGGACCACCCTCTCAGGCCCCCTAAA
GATCGTCGCCTTGGTGAGCCGTTACCTCACCAACTAGCTAATCTTGCGCGTGCCCATCTCTATCCACCGTAGT
TTTCAATATCACCCGATGCCGAATAATATATTATGGGGTATTAATCTTCTTCGAAAGGCTATCCCCCTGATAA
AGGCAGGTTGCACACGTGTTCCGCACCCGTACGCCGCTCTCAATTACCCGAAAGTAATCTACCGCTCGGCTTG
CATGTGTTAGGCCTCCCGCTAGCGTTCATCCTGAGCCAGGATCAAACTCT
>1614
CTGCTGCCGCCCGTAGGAGTCTGGTCCGTGTCTCAGTACCAGTGTGGGGGATCACCCTCTCAGGCCCCCTAAA
GATCGTCGCCTTGGTGAGCCGTTACCTCACCAACTAGCTAATCTTGCGCGTGCCCATCTCTATCCACCGTAGT
TTTCAATATCACCCGATGCCGAATAATATATTATGGGGTATTAATCTTCTTCGAAAGGCTATCCCCCTGATAA
AGGCAGGTTGCACACGTGTTCCGCACCCGTACGCCGCTCTCAATTACCCGAAAGTAATCTACCGCTCGGCTTG
CATGTGTTAGGCCTCCCGCTAGCGTTCATCC
>1615
CTGCTGCCGCCCGTAGGAGTCTGGTCCGTGTCTCAGTACCAGTGTGGGGGATCACCCTCTCAGGCCCCCTAAA
AGATCGTCGCCTTGGTGAGCCGTTACCTCACCAACTAGCTAATCTTGCGCGTGCCCATCTCTATCCACCGTAG
TTTTCAATATCACCCGATGCCGAATAATATATTTATGGGGTATTAATCTTCTTCGAAAGGCTATCCCCCTGAT
AAAGGCAGGTTGCACACGTGTTCCGCACCCGTACGCCGCTCTCAATTACCCGAAAGTAATCTACCGCTCGGCT
TGCATGTGTTAGGCCTCCCGCTAGCGTTCATCCTGAGCCAGGATCAAACTCT
>1616
CTGCTGCCGCCCGTAGGAGTCTGGACCGTGTCTCAGTTCCAGTGTGACTGATCATCCTCTCAGACCAGTACGG
ATCGTCGCCTTGGTAGGCCTTTACCCCACCAACTAGCTAATCCGACCTAGGCTCATCTGATAGCGTGAGGTCC
GAAGATCCCCCACTTCTCCCTCAGGACGTATGCGGTATTAGCGCCCGTTTCCGGACGTATCCCCACTACCAGG
CAGATTCCTAGGCATTACTCACCCGTCCGCCGCTGAATCCAGGAGCAAGCTCCCTTCATCCGCTCGACTGCAT
GTGTTAGGCCTGCCGCCAGCGTTCAATCTGAGCCAGGATCAAACTCT
>1617
CTGCTGCCGCCCGTAGGAGTCTGGACCGTGTCTCAGTTCCAGTGTGACTGATCATCCTCTCAGACCAGTACGG
ATCGTCGCCTTGGTAGGCCTTTACCCCACCAACTAGCTAATCCGACCTAGGCTCATCTGATAGCGTGAGGTCC
GAAGATCCCCCACTTCTCCCTCAGGACGTATGCGGTATTAGCGCCCGTTTCCGGACGATCCCCACTACCAGGC
AGATTCCTAGGCATTACTCACCCGTCCGCCGCTGAATCCAGGAGCAAGCTCCCTTCAATCCGCTCGACTGCAT
GTGTTGGGCCTGCCGCCAGCGTTCAATCTGAGCCAGGATCAAACTCT
>1618
CTGCTGCCGCCCGTAGGAGTCTGGTCCGTGTCTCAGTACCGGTGTGGGGGATCACCCTCTCAGGCCCCCTAAA
GATCGTCGCCTTGGTGAGCCGTTACCTCACCAACTAGCTAATCTTGCGCGTGCCCATCTCTATCCACCGTAGT
TTTCAATATCACCCGATGCCGAATAATATATTATGGGGTATTTAATCTTCTTCGAAAGGCTATCCCCCTGATA
AAGGCAGGTTGCACACGTGTTCCGCACCCGTACGCCGCTCTCAATTACCCGAAAGTAATCTACCGCTCGGCTT
GCATGTGTTAGGCCTCCCGCTAGCGTTCTCCTGAGCAGGATCCAACTCGT
>1619
CTGCTGCCGCCCGTAGGAGTCTGGTCCGTGTCTCAGTACCAGTGTGGGGGATCACCCTCTCAGGCCCCCTAAA
GATCGTCGCCTTGGTGAGCCGTTACCTCACCAACTAGCTAATCTTGCGCGTGCCCATCTCTATCCACCGTAGT
TTTCAATATCACCCGATGCCGAATAATATATTATGGGGTATTAATCTTCTTCGAAAGCTATCCCCCTGATAAA
GGCAGGTTGCACACGTGTTCCGCACCCGTACGCCGCTCTCAATTACCCGAAAGTAATCTACCGCTCCGGCTTG
CATGTGTTAGGCCTCCCGCTAGCGTTCATCCTGAGCCAGGATCAAACTCT
>1620

CTGCTGCCGCCCGTAGGAGTCTGGTCCGTGTCTCAGTACCAGTGTGGGGGATCACCCTCTCAGGCCCCCTAAA
GATCGTCGCCTTGGTGAGCCGTTACCTCACCAACTAGCTAATCTTGCGCGTGCCCATCTCTATCCACCGTAGT
TTTCAATATCACCCGATGCCGAATAATATATTATGGGGTATTAATCTTCTTCGAAAGGCTATCCCCCTGATAA
AGGCAGGTTGCACACGTGTTCCGCACCCGTACGCCGCTCTCAATTACCCGAAAGTAATCTACCGCTCGGCTTG
CATGTGTTAGGCCTCCCGCTAGCGTTCATCCTCGA
>1621
CTGCTGCCGCCCGTAGGAGTCTGGTCCGTGTCTCAGTACCAGTGTGGGGGATCACCCTCTCAGGCCCCCTAAA
GATCGTCGCCTTGGTGAGCCGTTACCTCACCAACTAGCTAATCTTGCGCGTGCCCATCTCTATCCACCGTAGT
TTTCAATATCACCCGATGCCGAATAATATAATTATTGGGGTATTAATCTTCTTCGAAAGGCTATCCCCCTGAT
AAAGGCAGGTTGCACACGTGTTCCGCACCCGTACGCCGCTCTCAATTACCCGAAAGTAATCTACCGCTCGGCT
TGCATGTGTTAGGCCTCCCGCTAGCGTTCATCCTCGAGCCAGGATCAAACTCT
>1622
CTGCTGCCGCCCGTAGGAGTCTGGTCCGTGTCTCAGTACCAGTGTGGGGGATCACCCTCTCAGGCCCCCTAAA
GATCGTCGCCTTGGTGAGCCGTTACCTCACCAACTAGCTAATCTTGCGCGTGCCCATCTCTATCCACCGTAGT
TTTCAATATCACCCGATGCCGAATAATATATTATGGGGTATTAATCTTCTTCGAAAGGCTATCCCCCTGATAA
AGGCAGGTTGCACACGTGTTCCGCACCCGTACGCCGCTCTCAATTACCCGAAAGTAATCTACCGCTCCGGCTT
GCATGTGTTAGGCCTCCCGCTAGCGTTCATCCCGAGCCAGGATCAAACTCCT
>1623
CTGCTGCCGCCCGTAGGAGTCTGGTCCGTGTCTCAGTACCAGTGTGGGGGATCACCCTCTCAGGCCCCCTAAA
GATCGTCGCCTTGGTGAGCCGTTACCTCACCAACTAGCTAATCTTGCGCGTGCCCATCTCTATCCACCGTAGT
TTTCAATATCACCCGATGCCGAATAATATATTATGGGGTATTAATCTTCTTCGAAAGGCTATCCCCCTGATAA
AGGCAGGTTGCACACGTGTTCCGCACCCGTACGCCGCTCTCAATTACCCGAAAGTAATCTACCGCTCGGCTTG
CATGTGTTAGGCCTCCCGCTAGCGTTCATCCGTGAGCCAGGATCAAACTCT
>1624
CTGCTGCCGCCCGTAGGAGTCTGGTCCGTGTCTCAGTACCAGTGTGGGGGATCACCCTCTCAGGCCCCCTAAA
GATCGTCGCCTTGGTGAGCCGTTACCTCACCAACTAGCTAATCTTGCGCGTGCCCATCTCTATCCACCGTAGT
TTTCAATATCACCCGATGCCGAATAATATATTATGGGGTATTAATCTTCTTCGAAAGGCTATCCCCTGATAAA
GGCAGGTTGCACACGTGTTCCGCACCCGTACGCCGCTCTCAATTACCCGAAAGTAATCTACCGCTCGGCTTGC
ATGTGTTAGGCCTCCCGCTAGCGTTCATCCGTC
>1625
CTGCTGCCGCCCGTAGGAGTCTGGTCCGTGTCTCAGTACCAGTGTGGGGGATCACCCTCTCAGGCCCCCTAAA
GATCGTCGCCTTGGTGAGCCGTTACCTCACCAACTAGCTAATCTTGCGCGTGCCCATCTCTATCCACCGTAGT
TTTCAATATCACCCGATGCCGAATAATATATTATGGGGTATTAATCTTCTTCGAAAGGCTATCCCCCTGATAA
AGGCAGGTTGCACACGTGTTCCGCACCCGTACGCCGCTCTCAATTACCCGAAAGTAATCTACCGCTCCGGCTT
GCATGTGTTAGGCCTCCCGCTAGCGTTCATCCTCGAGCCAGGATCAACTCT
>1626
CTGCTGCCGCCCGTAGGAGTCTGGTCCGTGTCTCAGTACCAGTGTGGGGGATCACCCTCTCAGGCCCCCTAAA
GATCGTCGCCTTGGTGAGCCGTTACCTCACCAACTAGCTAATCTTGCGCGTGCCCATCTCTATCCACCGTAGT
TTTCAATATCACCCGATGCCGAATAATATATTATGGGGTATTAATCTTCTTCGAAAGCTATCCCCCTGATAAA
GGCAGGTTGCACACGTGTTCCGCACCCGTACGCCGCTCTCAATTACCCGAAAGTAATCTACCGCTCCGGCTTG
CATGTGTTAGGCCTCCCGCTAGCGTTCATCCTCGAGCCAGGATCAACTCT
>1627
CTGCTGCCGCCCGTAGGAGTCTGGTCCGTGTCTCAGTACCAGTGTGGGGGATCACCCTCTCAGGCCCCCTAAA
GATCGTCGCCTTGGTGAGCCGTTACCTCACCAACTAGCTTAATCTTGCGCGTGCCCATCTCTATTCCACCGTA
GTTTTTCAATATCACCCGATGCCGAATAATATATTATTGGGGTATTTAATTCTTCTTCGAAAGGCTATCCCCC
TGATAAAGGCAGGTTGCACACGTGTTCCGCACCCGTACGCCGCTCTCAATTACCCGAAAGTAATCTACCGCTC
GGCTGCATGTGTTTAGGCCTTCCCGCTAGCGTTCATTCCGTCGAGCCAGGATCAAACTCT
>1628
CTGCTGCCGCCCGTAGGAGTCTGGTCCGTGTCTCAGTACCAGTGTGGGGGATCACCCTCTCAGGCCCCCTAAA
GATCGTCGCCTTGGTGAGCCGTTACCTCACCAACTAGCTAATCTTGCGCGTGCCCATCTCTATCCACCGTAGT
TTTCAATATCACCCGATGCCGAATAATATATTATGGGGTATTAATCTTCTCGAAAGCTATCCCCTGATAAAGG
CAGGTTGCACACGTGTTCCGCACCCGTACGCCGCTCTCAATTACCCGAAAGTAATCTACCGCTCCGGCTTGCA
TGTGTTAGGCCTCCCGCTAGCGTTCATCCTCGAGCCAGGATCAAACTCT
>1629

CTGCTGCCGCCCGTAGGAGTCTGGTCCGTGTCTCAGTACCAGTGTGGGGGATCACCCTCTCAGGCCCCCTAAA
GATCGTCGCCTTGGTGAGCCGTTACCTCACCAACTAGCTAATCTTGCGCGTGCCCATCTCTATCCACCGTAGT
TTTCAATATCACCCGATGCCGAATAATATAATTATGGGGTATTAATCTTCTCTGAAAGCTATCCCCTGATAAA
GGCAGGTTGCACACGTGTTCCGCACCCGTACGCCGCTCTCAATTACCCGAAAGTAATCTACCGCTCGGCTTGC
ATGTGTTAGGCCTCCCGCTAGCGTTCATCCTGAGCCAGGATCAACTCT
>1630
CTGCTGCCGCCCGTAGGAGTCTGGTCCGTGTCTCAGTACCAGTGTGGGGGGTCACCCTCTCAGGCCCCCTAAA
GATCGTCGCCTTGGTGAGCCGTTACCTCACCAACTAGCTAATCTTGCGCGTGCCCATCTCTATCCACCGTAGT
TTTCAATATCACCCGATGCCGAATAATATATTATGGGGTATTAATCTTCTCTGAAAGGCTATCCCCTGATCAA
AGGCGGTTAGGCACACGTGTTCCGCACCCGTACGCCGCTCTCAATTACCCGAAAGTAATCTACCGCTCGGCTT
GCATGTGTTAGGCCTCCCGCTAGCGTTCTCCTCGAGCAGGATCCAAC
>1631
CTGCTGCCGCCCGTAGGAGTCTGGTCCGTGTCTCAGTACCAGTGTGGGGGATCACCCTCTCAGGCCCCCCTAA
AGATCGTCGCCTTGGTGAGCCGTTACCTCACCAACTAACTAATCTTGCGCGTGCCCATCTCTATCCACCGTAG
TTTTTCAATATCACCCGATGCCGAATAATATATTTATGGGGTATTTAATCTTTCCTTTCGAAGGGCTATCCCC
CTGATTAAAGGCAGGTTGCACACGTGTTCCGCACCCGTACGCCGCTCTCAATTACCCGAAAGTAATCTACCGC
TCGGCTTGCATGTGTTAGGCCTCCCGCTAGCGTTCATCCTGAGCCAGGATCAAACTCT
>1632
CTGCTGCCGCCCGTAGGAGTCTGGTCCGTGTCTCAGTACCAGTGTGGGGGATCACCCTCTCAGGCCCCCTAAA
GATCGTCGCCTTGGTGAGCCGTTACCTCACCAACTAGCTAATCTTGCGCGTGCCCATCTCTATCCACCGTAGT
TTTCAATATCACCCGATGCCGAATAATATATTATGGGGTATTAATCTTCTCGAAAGGCTATCCCCTGATAAAG
GCAGGTTGCACACGTGTTCCGCACCCGTACGCCGCTCTCAATTACCCGAAAGTAATCTACCGCTCGGCTTGCA
TGTGTTAGGCCTCCCGCTAGCGTTCTCCTCGAGCAGGATCCAA
>1633
CTGCTGCCGCCCGTAGGAGTCTGGTCCGTGTCTCAGTACCAGTGTGGGGGATCACCCTCTCAGGCCCCCTAAA
GATCGTCGCCTTGGTGAGCCGTTACCTCACCAACTAGCTAATCTTGCGCGTGCCCATCTCTATCCACCGTAGT
TTTCAATATCACCCGATGCCGAATAATATATTATGGGGTATTTAATCTTCTTCGAAAGGCTATCCCCCTGATAA
AGGCAGGTTGCACACGTGTTCCGCACCCGTACGCCGCTCTCAATTACCCGAAAGTAATCTACCGCTCGGCTTG
CATGTGTTAGGCCTCCCGCTAGCGTTCATCCTGAGCCAGGATCAACTCT
>1634
CTGCTGCCGCCCGTAGGAGTCTGGACCGTGTCTCAGTTCCAGTGTGACTGATCATCCTCTCAGACCAGTTACG
GATCGTCGCCTTGGTAGGCCTTTACCCCACCAACTAGCTAATCCGACCTAGGCTCATCTGATAGCGTGAGGTC
CGAAGTCCCCCACTTCTCCCTCAGGACGTATGCGGTATTAGCGCCCGTTTCCGGACGTATCCCCACTACCAGG
CAGATTCCTAGGCATTACTCACCCGTCCGCCGCTGAATCCAGGAGCAAGCTCCCTCATCCGCTCGACTGCCAT
GAGTTAGGCCTGCCGCCAGCGTTCAATCTGAGCCAGGATCAACTCT
>1635
CTGCTGCCGCCCGTAGGAGTCTGGTCCGTGTCTCAGTACCAGTGTGGGGGATCACCCTCCCAGGCCCCCTAAA
GATCGTCGCCTGGTGAGCCGTTACCTCACCAACTAGCTAATCTTGCGCGTGCCCATCTCTATCCACCGTAGTT
TTCAATATCACCCGATGCCGAATAATATATTATGGGGTATTTAATCTTTCTCTGAAAGGCTATCCCCTGATAA
AGGCAGGTTGCACACGTGTTCCGCACCCGTACGCCGCTCTCAATTACCCGAAAGTAATCTACCGCTCCGGCTT
GCATGTGTTAGGCCTCCCGCTAGCGTTCATCCTGAGCCAGGATCAAACTCT
>1636
CTGCTGCCGCCCGTAGGAGTCTGGTCCGTGTCTCAGTACCAGTGTGGGGGATCACCCTCTCAGGCCCCCTAAA
GATCGTCGCCTTGGTGAGCCGTTACCTCACCAACTAGCTAATCTTGCGCGTGCCCATCTCTATCCACCGTAGT
TTTCAATATCACCCGATGCCGAATAATATATTATGGGGTATTAATCTTCTTCGAAAGGCTATCCCCCTGATAA
AGGCAGGTTGCACACGTGTTCCGCACCCGTACGCCGCTCTCAATTACCCGAAAGTAATCTACCGCTCCGGCTT
GCATGTGTTAGGCCTCCCGCTAGCGTTCTCCTCGAGCAGGATCCAATCATCCT
>1637
CTGCTGCCGCCCGTAGGAGTCTGGTCCGTGTCTCAGTACCAGTGTGGGGGATCACCCTCTCAGGCCCCCTAAA
GATCGTCGCCTTGGTGAGCCGTTACCTCACCAACTAGCTAATCTTGCGCGTGCCCATCTCTATCCACCGTAGT
TTTCAATATCACCCGATGCCGAATAATATATTATGGGGTATTAATCTTCTTCGAAAGGCTATCCCCCTGATAA
AGGCAGGTTGCACACGTGTTCCGCACCCGTACGCCGCTCTCAATTACCCGAAAGTAATCTACCGCTCCGGCTG
CATGTGTTAGGCCTCCCGCTAGCGTTCATCCGTCGCAGGATCAAACTCCT
>1638

CTGCTGCCGCCCGTAGGAGTCTGGTCCGTGTCTCAGTACCAGTGTGGGGGATCACCCTCTCAGGCCCCCTAAA
GATCGTCGCCTTGGTGAGCCGTTACCTCACCAACTAGCTAATCTTGCGCGTGCCCATCTCTATCCACCGTAGT
TTTCAATATCACCCGATGCCGAATAATATATTATGGGGTATTAATCTTCCTTCGAGAGGCTATCCCCCTGATA
AGGCAGGTTGCACACGTGTTCCGCACCCGTACGCCGCTCTCAATTACCCGAAAGTAATCTACCGCTCGGCTTG
CATGTGTTAGGCCTCCCGCTAGCGTTCATCCTGAGCAGGCATCAACTCT
>1639
CTGCTGCCGCCCGTAGGAGTCTGGTCCGTGTCTCAGTACCAGTGTGGGGGATCACCCTCTCAGGCCCCCTAAA
GATCGTCGCCTTGGTGAGCCGTTACCTCACCAACTAGCTAATCTTGCGCGTGCCCATCTCTATCCACCGTAGT
TTTCAATATCACCCGATGCCGAATAATATATTATGGGGTATTAATCTTCTTCGAAAGGCTATCCCCCTGATAA
AGGCAGGTTGCACACGTGTTCCGCACCCGTACGCCGCTCTCAATTACCCGAAAGTAATCTACCGCTCGGCTTG
CATGTGTTAGGCCTCCCGCTAGCGTTCATCCTGAGCCAGGATCAAACTCT
>1640
CTGCTGCCGCCCGTAGGAGTCTGGTCCGTGTCTCAGTACCAGTGTGGGGGATCACCCTCTCAGGCCCCCTAAA
GATCGTCGCCTTGGTGAGCCGTTACCTCACCAACTAGCTAATCTTGCGCGTGCCCATCTCTATCCACCGTAGT
TTTCAATATCACCCGATGCCGAATAATATATTATGGGGTATTAATCTTCTTCGAAAGCTATCCCCTGATAAAG
GCAGGTTGCACACGTGTTCCGCACCCGTACGCCGCTCTCAATTTACCCGAAAGTAATCTACCGCTCGGCTTGC
ATGTGTTAGGCCTCCCGCTAGCGTTCATCCTGAGCCAGGATCAACTCT
>1641
CTGCTGCCGCCCGTAGGAGTCTGGTCCGTGTCTCAGTACCAGTGTGGGGGATCACCCTCTCAGGCCCCCTAAA
GATCGTCGCCTTGGTGAGCCGTTACCTCACCAACTAGCTAATCTTGCGCGTGCCCATCTCTATCCACCGTAGT
TTCAATATCACCCGATGCCGAATAATATATTTATGGGGTATTTAATCTTCTTCGAAAGGCTATCCCCCTGATA
AAGGCAGGTTGCACACGTGTTCCGCACCCGTACGCCGCTCTCAATTACCCGAAAGTAATCTACCGCTCGGCTT
GCATGTGTTAGGCCTCCCGCTAGCGTTCATCCTGAGCCAGGATCAACTCT
>1642
CTGCTGCCGCCCGTAGGAGTCTGGTCCGTGTCTCAGTACCAGTGTGGGGGATCACCCTCTCAGGCCCCCTAAA
GATCGTCGCCTTGGTGAGCCGTTACCTCACCAACTAGCTAATCTTGCGCGTGCCCATCTCTATCCACCGTAGT
TTTCAATATCACCCGATGCCGAATAATATATTATGGGGTATTAATCTTCTTCGAAAGGCTATCCCCCTGATAA
AGGCAGGTTGCACACGTGTTCCGCACCCGTACGCCGCTCTCAATTACCCGAAAGTAATCTACCGCTCGGCTGC
ATGTGTTAGGCCTCCCGCTAGCGTTCATCCTGAGCCAGGATCAAACTCT
>1643
CTGCTGCCGCCCGTAGGAGTCTGGTCCGTGTCTCAGTACCAGTGTGGGGGATCACCCCTCTCAGGCCCCCCTA
AAGATCGTCGCCTTGGTGAGCCGTTACCTCACCAACTAGCTAATCTTGCGCGTGCCCCATCTCTATCCACCGT
AGTTTTTCAATATCACCCGATGCCGAATAATATATTTATTGGGGTATTTAATTCTTCTTCGAAAGGCTATCCC
CCTGATTAAAGGCAGGTTGCACACGTGTTCCGCACCCGTTACGCCGCTCTCAATTACCCCGAAAGTAATCTAC
CGCTCGGCTTGCATGT
>1644
CTGCTGCCGCCCGTAGGAGTCTGGTCCGTGTCTCAGTACCAGTGTGGGGGATCACCCTCTCAGGCCCCCTAAA
GATCGTCGCCTTGGTGAGCCGTTACCTCACCAACTAGCTAATCTTGCGCGTGCCCATCTCTATCCACCGTAGT
TTTCAATATCACCCGATGCCGAATAATATATTATGGGGTATTAATCTTCCTTCGAAGGAGCTATCCCCTGATA
AAGGCAGGTTGCACACGTGTTCCGCACCCGTACGCCGCTCTCAATTACCCGAAAGTAATCTACCGCTCGGCTT
GCATGTGTTAGGCCTCCCGCTAGCGTTCATCCTGAGCCAGGATCAAACTCT
>1645
CTGCTGCCGCCCGTAGGAGTCTGGACCGTGTCTCAGTTCCAGTGTGACTGATCATCCTCTCAGACCAGTACGG
ATCGTCGCCTTGGTAGGCCTTTACCCCCACCAACTAGCTAATCCGACCTAGGCTCATCTGATAGCGTGAGGTC
CGAAGATCCCCCACTTCTCCCTCAGGACGTATGCGGTATTAGCGCCCGTTTCCGGACGATCCCCACTACCAGG
CAGATTCCTAGGCATTACTCACCCGTCCGCCGCTGAATCCAGGAGCAAGCTCCCTTCATCCGCTCGACTGCAT
GTGTTAGGCCTGCCGCCAGCGTCAATCTGAGCCAGGATCAAACTCT
>1646
CTGCTGCCGCCCTAGGAGTCTGGACCGTGTCTCAGTTCCAGTGTGACTGATCATCCTCTCAGACCAGTTACGG
ATCGTCGCCTTGGTAGGCCTTTACCCCACCAACTAGCTAATCCGACCTAGGCTCATCTGATAGCGTGAGGTCC
GAAGTCCCCCACTTCTCCCTCAGGGACGTATGCGGTATTAGCGCCCGTTTCCGGACGTATCCCCACTACCAGG
CAGATTCCCTAGGCATTACTCACCCGTCCGCCGCTGAATCCAGGAGCAAGCTCCCTCATCCGCTCGACTGCAT
GTGTTAGGCCTGCCGCCAGCGTTCAATCTGAGCCAGGATCAAACTCT
>1647

CTGCTGCCGCCCGTAGGAGTCTGGTCCGTGTCTCAGTACCAGTGTGGGGGATCACCCTCTCAGGCCCCCTAAA
GATCGTCGCCTTGGTGAGCCGTTACCTCACCAACTAGCTAATCTTGCGCGTGCCCATCTCTATCCACCGTAGT
TTTCAATATCACCCGATGCCGAATAATATATTATGGGGTATTAATCTTCTTCGAAAGGCTATCCCCCTGATAA
AGGCAGGTTGCACACGTGTTCCGCACCCGTACGCCGCTCTCAATTACCCGAAAGTAATCTACCGCTCGGCTTG
CATGTGTTAGGCCTCCCGCTAGCGTTCATCCTGAGCCAGGATCAAACTCT
>1648
CTGCTGCCGCCCGTAGGAGTCTGGTCCGTGTCTCAGTACCAGTGTGGGGGATCACCCTCTCAGGCCCCCTAAA
GATCGTCGCCTTGGTGAGCCGTTACCTCACCAACTAGCTAATCTTGCGCGTGCCCATCTCTATCCACCGTAGT
TTTCAATATCACCCGATGCCGAATAATATATTATGGGGTATTAATCTTCTTCGAAAGGCTATCCCCCCGATAA
AGGCAGGTTGCACACGTGTTCCGCACCCGTACGCCGCTCTCAATTACCCGAAAGTAATCTACCGCTCGGCTTG
CATGTGTTAGGCCTCCCGCTAGCGTTCATCCTGAGCCAGGATCAAACTCT
>1649
CTGCTGCCGCCCGTAGGAGTCTGGTCCGTGTCTCAGTACCAGTGTGGGGGATCACCCTCTCAGGCCCCCTAAA
GATCGTCGCCTTGGTGAGCCGTTACCTCACCAACTAGCTAATCTTGCGCGTGCCCATCTCTATCCACCGTAGT
TTTCAATATCACCCGATGCCGAATAATATATTATGGGGTATTAATCTTCTTCGAAAGGCTATCCCCCTGATAA
AGGCGGTTGCACACGTGTTCCGCACCCGTACGCCGCTCTCAATTACCCGAAAGTAATCTACCGCTCGGCTTGC
ATGTGTTAGGCCTCCCGCTAGCGTCTCCTGAGCCAGGATCAAACTCGT
>1650
CTGCTGCCGCCCGTAGGAGTCTGGTCCGTGTCTCAGTACCAGTGTGGGGGATCACCCTCTCAGGCCCCCTAAAG
ATCGTCGCCTGTGAGCCGTTACCTCACCAACTAGCTAATCTTGCGCGTGCCCATCTCTATCCACCGTAGTTTT
CAATATCACCCGATGCCGAATAATATATTATGGGGTATTAATCTTCTCGAAAGGCTATCCCCTGATAAGGCAG
GTTGCACACGTGTTCCGCACCCGTACGCGCTCTCAATTACCCGAAAGAATCTACCGCTCGGCTTGCATGTGTT
ACGGCCTCCCGCTAGGCCT
>1651
CTGCTGCCGCCCGTAGGAGTCTGGTCCGTGTCTCAGTACCAGTGTGGGGGATCACCCTCTCAGGCCCCCTAAA
GATCGTCGCCTTGGTGAGCCGTTACCTCACCAACTAGCTAATCTTGCGCGTGCCCATCTCTATCCACCGTAGT
TTTCAATATCACCCGATGCCGAATAATATATTATGGGGTATTAATCTTCTTCGAAAGGCTATCCCCTGATAAA
GGCAGGTTGCACACGTGTTCCGCACCCGTACGCCGCTCTCAATTACCCGAAAGTAATCTACCGCTCGGCTGCA
TGTGTTAGGCCTCCCGCTAGCGTTCTCCTGAGCAGGATCAACTC
>1652
CTGCTGCCGCCCGTAGGAGTCTGGTCCGTGTCTCAGTACCAGTGTGGGGGATCACCCTCTCAGGCCCCCTAAA
AGATCGTCGCCTTGGTGAGCCGTTACCTCACCAACTAGCTAATCTTGCGCGTGCCCATCTCTATCCACCGTAG
TTTTCAATATCACCCGATGCCGAATAATATATTATGGGGTATTAATCTTCTTCGAAAGGCTATCCCCCTGATA
AAGGCAGGTTGCACACGTGTTCCGCACCCGTACGCCGCTCTCAATTACCCGAAAGTAATCTACCGCTCGGCTT
GCATGTGTTAGGCCTCCCGCTAGCGTTCTCCTGAGCAGGATCAAC
>1653
CTGCTGCCGCCCGTAGGAGTCTGGTCCGTGTCTCAGTACCAGTGTGGGGGATCACCCTCTCAGGCCCCCTAAA
GATCGTCGCCTTGGTGAGCCGTTACCTCACCAACTAGCTAATCTTGCGCGTGCCCATCTCTATCCACCGTAGT
TTTCAATATCACCCGATGCCGAATAATATAATTATGGGGTATTAATTCTTCTTCGAAAGGCTATCCCCCTGAT
AAAGGCAGGTTGCACACGTGTTCCGCACCCGTACGCCGCTCTCAATTACCCGAAAGTAATCTACCGCTCGGCT
TGCATGTGTTAGGCCTCCCGCTAGCGTTCATCCTGAGCCAGAATCAAACTCT
>1654
CTGCTGCCGCCCGTAGGAGTCTGGTCCGTGTCTCAGTACCAGTGTGGGGGATCACCCTCTCAGGCCCCCTAAA
GATCGTCGCCTTGGTGAGCCGTTACCTCACCAACTAGCTAATCTTGCGCGTGCCCATCTCTATCCACCGTAGT
TTTCAATATCACCCGATGCCGAATAATATATTATGGGGTATTAATCTTCTTCGAAAGGCTATCCCCCTGATAA
AGGCAGGTTGCACACGTGTTCCGCACCCGTACGCCGCTCTCAATTACCCGAAAGTAATCTACCGCTCGGCTTG
CATGTGTTAGGCCTCCCGCTAGCGTTCATCCTGAGCCAGGATCAACTCT
>1655
CTGCTGCCGCCCGTAGGAGTCTGGTCCGTGTCTCAGTACCAGTGTGGGGGATCACCCTCTCAGGCCCCCTAAA
GATCGTCGCCTTGGTGAGCCGCTACCTCACCAACTAGCTAATCTTGCGCGTGCCCATCTCTATCCACCGTAGT
TTTCAATATCACCCGATGCCGAATAATATATTATGGGGTATTAATCTTCTTCGAAAGGCTATCCCCTGATAAA
GGCAGGTTGCACACGTGTTCCGCACCCGTACGCCGCTCTCAATTACCCGAAAGTAATCTACCGCTCGGCTTGC
ATGTGTTAGGCCTCCCGCTAGCGTTCATCCGTCGAGC
>1656

CTGCTGCCGCCCGTAGGAGTCTGGTCCGTGTCTCAGTACCAGTGTGGGGGATCACCCTCTCAGGCCCCCTAAA
GATCGTCGCCTTGGTGAGCCGTTACCTCACCAACTAGCTAATCTTGCGCGTGCCCATCTCTATCCACCGTAGT
TTTCAATATCACCCGATGCCGAATAATATATTATGGGGTATTAATCTTCTTCGAAAGGCTATCCCCCTGATAA
AGGCAGGTTGCACACGTGTTCCGCACCCGTACGCCGCTCTCAATTACCCGAAAGTAATCTACCGCTCGGCTTG
CATGTGTTAGGCCTCCCGCTAGCGTTCATCCTGAGCCAGGATCAACTCT
>1657
CTGCTGCCGCCCGTAGGAGTCTGGACCGTGTCTCAGTTCCAGTGTGAGCTGATCGTCCTCTCAGACCAGTTAC
GGATCGTCGCCTTGGTAGGCCTTTACCCCACCAACTAGCTAATCCGACCTAGGCTCATCTGATAGCGTGAGGT
CCGAAGTCCCCCACTTCTCCCTCAGGACGTATGCGGTATTAGCGCCCGTTTCCGGACGTATCCCCCACTACCAG
GCAGATTCCTAGGCATTACTCACCCGTCCGCCGCTGAATCCAGGAGCAAGCTCCCTCATCCGCTCGACTGCAT
GTGTTAGGCCTGCCGCCAGCGTCAACCTGAGCCAGGATCAAACTCT
>1658
CTGCTGCCGCCCGTAGGAGTCTGGTCCGTGTCTCAGTACCAGTGTGGGGGATCACCCTCTCAGGCCCCCTAAA
GATCGTCGCCTTGGTGAGCCGTTACCTCACCAACTAGCTAATCTTGCGCGTGCCCATCTCTATCCACCGTAGT
TTTCAATATCGCCCGATGCCGAATAATATATTATGGGGTATTAATCTTCTTCGAAAGGCTATCCCCCTGATAA
AGGCAGGTTAGCACGTGTTCCGCACCCGTACGCCGCTCTCAATTACCCGAAAGTAATCTACCGCTCGGCTTGC
ATGTGTTAGGCCTCCCGCTAGCGTTCATCCTGAGCCAGGATCAACTCT
>1659
CTGCTGCCGCCCGTAGGAGTCTGGTCCGTGTCTCAGTACCAGTGTGGGGGATCACCCTCTCAGGCCCCCTAAA
GATCGTCGCCTTGGTGAGCCGTTACCTCACCAACTAGCTAATCTTGCGCGTGCCCATCTCTATCCACCGTAGT
TTTCAATATCACCCGATGCCGAATAATATATTATGGGGTATTAATCTTCTTCGAAGGCTATCCCCCTGATAAA
GGCAGGTTGCACACGTGTTCCGCACCCGTACGCCGCTCTCAATTACCCGAAAGTAATCTACCGCTCGGCTTGC
ATGTGTTAGGCCTCCCGCTAGCGTTCATCCTGAGCCAGGATCAACTCT
>1660
CTGCTGCCGCCCGTAGGAGTCTGGTCCGTGTCTCAGTACCAGTGTGGGGGATCACCCTCTCAGGCCCCCTAAA
GATCGTCGCCTTGGTGAGCCGTTACCTCACCAACTAGCTAATCTTGCGCGTGCCCATCTCTATCCACCGTAGT
TTTCAATATCACCCGATGCCGAATAATATATTATGGGGTATTAATCTTCTCGAAAGGCTATCCCCTGATAAAG
GCAGGTTGCACACGTGTTCCGCACCCGTACGCCGCTCTCAATTACCCGAAAGTAATCTACCGCTCGGCTTGCA
TGTGTTAGGCCTCCCGCTAGCGTTCTCCGTCGAGCAGG
>1661
CTGCTGCCGCCCCGTAGGAGTCTGGTCCGTGTCTCAGTACCAGTGTGGGGGATCACCCTCTCAGGCCCCCCTA
AAGATCGTCGCCTTGGTGAGCCGTTACCTCACCAACTAGCTAATCTTGCGCGTGCCCCATCTCTATCCACCGT
AGTTTTTCAATATCACCCGATGCCGAATAATATATTATGGGGTATTAATTCTTTCTCGAAAGGCTATCCCCTA
ATAAACGGCAGGTTGCACACGTGTTCCGCACCCGTACGCCGCTCTCAATTACCCCGAAAGTAATCTACCGCAT
CGGCTTGCACTGTCGTTTAGGCC
>1662
CTGCTGCCGCCCGTAGGAGTCTGGTCCGTGTCTCAGTACCAGTGTGGGGGATCACCCTCTCAGGCCCCCTAAA
GATCGTCGCCTTGGTGAGCCGTTACCTCACCAACTAGCTAATCTTGCGCGTGCCCATCTCTATCCACCGTAGT
TTTCAATATCACCCGATGCCGAATAATATATTATGGGGTATTAATCTTCTTCGAAAGGCTATCCCCTGATAAA
GGCAGGTTGCACACGTGTTCCGCACCCGTACGCCGCTCTCAATTACCCGAAAGTAATCTACCGCTCGGCTTGC
ATGTGTTAGGCCTCCCGCTAGCGTTCATCTGAGCCAGGATCAACTCT
>1663
CTGCTGCCGCCCGTAGGAGTCTGGTCCGTGTCTCAGTACCAGTGTGGGGGATCACCCTCTCAGGCCCCCTAAA
GATCGTCGCCTTGGTGAGCCGTTACCTCACCAACTAGCTAATCTTGCGCGTGCCCATCTCTATCCACCGTAGT
TTTCAATATCACCCGATGCCGAATAATATATTATGGGGTATTAATCTTCTTCGAAAGCTATCCCCTGATAAAG
GCAGGTTGCACACGTGTTCCGCACCCGTACGCCGCTCTCAATTACCCGAAAGTAATCTACCGCTCGGCTTGCA
TGTGTTAGGCCTCCCGCTAGCGTTCATCCGTGAGCCAGGGATCAAACTCT
>1664
CTGCTGCCGCCCGTAGGAGTCTGGTCCGTGTCTCAGTACCAGTGTGGGGGATCACCCTCTCAGGCCCCCTAAA
GATCGTCGCCTTGGTGAGCCGTTACCTCACCAACTAGCTAATCTTGCGCGTGCCCATCTCTATCCACCGTAGT
TTTCAATATCACCCGATGCCGAATAATATATTATGGGGTATTAATCTTCTTCGAAAGCTATCCCCTGATAAAG
GCAGGTTGCACACGTGTTCCGCACCCGTACGCCGCTCTCAATTACCCGAAAGTAATCTACCGCTCCGGCTTAG
CATGTGTTCGGC
>1665

CTGCTGCCGCCCGTAGGAGTCTGGTCCGTGTCTCAGTACCAGTGTGGGGGATCACCCTCTCAGGCCCCCTAAA
GATCGTCGCCTTGGTGAGCCGTTACCTCACCAACTAGCTAATCTTGCGCGTGCCCATCTCTATCCACCGTAGT
TTTCAATATCACCCGATGCCGAATAATATATTATGGGGTATTAATCTTCTCGAAAGCTATCCCCTGATAAAGG
CAGGTTGCACACGTGTTCCGCACCCGTACGCCGCTCTCAATTACCCGAAAGTAATCTACCGCTCCGGCTTGCA
TGTGTTAGGCCTCCCGCTAGCGTTCTCCTCGAGCAGGATCA
>1666
CTGCTGCCGCCCGTAGGAGTCTGGTCCGTGTCTCAGTACCAGTGTGGGGGATCACCCTCTCAGGCCCCCTAAA
GATCGTCGCCTTGGTGAGCCGTTACCTCACCAACTAGCTAATCTTGCGCGTGCCCATCTCTATCCACCGTAGT
TTTCAATATCACCCGATGCCGAATAATATATTATGGGGTATTAATCTTCTCGAAAGGCTATCCCCTGATAAAG
GCAGGTTGCACACGTGTTCCGCACCCGTACGCCGCTCTCAATTACCCGAAAGTAATCTACCGCTCGGCTTGCA
TGTGTTAGGCCTCCCGCTAGCGTTCTCCTCGAGCAGGATCCAACATCT
>1667
CTGCTGCCGCCCGTAGGAGTCTGGTCCGTGTCTCAGTACCAGTGTGGGGGATCACCCTCTCAGGCCCCCTAAA
GATCGTCGCCTTGGTGAGCCGTTACCTCACCAACTAGCTAATCTTGCGCGTGCCCATCTCTATCCACCGTAGT
TTTTCAATATCGCCCGATGCCGAATAATATATTATGGGGTATTAATCTTCTCGAAGCTATCCCCTGATAAAGG
CAGGTTGCACACGTGTTCCGCACCCGTACGCCGCTCTCAATTACCCGAAAGTAATCTACCGCTCCGGCTTAGC
ATGTGTTCGGCTCCC
>1668
CTGCTGCCGCCCGTAGGAGTCTGGTCCGTGTCTCAGTACCAGTGTGGGGGATCACCCTCTCAGGCCCCCTAAA
GATCGTCGCCTTGGTGAGCCGTTACCTCACCAACTAGCTAATCTTGCGCGTGCCCATCTCTATCCACCGTAGT
TTCAATATCACCCGATGCCGAATAATATATTATGGGGTATTAATCTTCTTCGAAAGGCTATCCCCTGAATAAA
GGCAGGTTGCACACGTGTTCCGCACCCGTACGCCGCCCTCAATTACCCGAAAGTAATCTACCGCTCGGCTTGC
ATGTGTTAGGCCTCCCGCTAGCG
>1669
CTGCTGCCGCCCGTAGGAGTCTGGTCCGTGTCTCAGTACCAGTGTGGGGGATCACCCTCTCAGGCCCCCTAAA
GATCGTCGCCTTGGTGAGCCGTTACCTCACCAACTAGCTAATCTTGCGCGTGCCCATCTCTATCCACCGTAGT
TTTCAATATCACCCGATGCCGAATAATATATTATGGGGTATTAATCTTCTTCGAAAGGCTACCCCCCTGATAA
AGGCAGGTTGCACACGTGTTCCGCACCCGTACGCCGCTCTCAATTACCCGAAAGTAATCTACCGCTCGGCTTG
CATGTGTTAGGCCTCCCGCTAGCGTTCATCCTGAGCCAGGATCAACTCT
>1670
CTGCTGCCGCCCGTAGGAGTCTGGTCCGTGTCTCAGTACCAGTGTGGGGGATCACCCTCTCAGGCCCCCTAAA
GATCGTCGCCTTGGTGAGCCGTTACCTCACCAACTAGCTAATCTTGCGCGTGCCCATCTCTATCCACCGTAGT
TTTCAATATCACCCGATGCCGAATAATATATTATGGGGTATTAATCTTCTTCGAAAGGCTATCCCCCTGATAA
AGGCAGGTTGCACACGTGTTCCGCACCCGTACGCCGCTCTCAATTACCCGAAAGTAATCTACCGCTCGGCTTG
CATGTGTTAGGCCTCCCGCTAGCGTTCATCCTGAGCCAGGATCAACTCT
>1671
CTGCTGCCGCCCGTAGGAGTCTGGTCCGTGTCTCAGTACCAGTGTGGGGGATCACCCTCTCAGGCCCCCTAAA
GATCGTCGCCTTGGTGAGCCGTTACCTCACCAACTAGCTAATCTTGCGCGTGCCCATCTCTATCCACCGTAGT
TTTCAATATCACCCGATGCCGAATAATATATTATGGGGTATTTAATCTTCTTCGAAAGGCTATCCCCCTGATA
AAGGCAGGTTGCACACGTGTTCCGCACCCGTACGCCGCTCTCAATTACCCAAAGTAATCTACCGCTCGGCTTG
CATGTGTTAGGCCTCCCGCTAGCGTTCATCCTGAGCCAGGATCAAACTCT
>1672
CTGCTGCCGCCCGTAGGAGTCTGGTCTGTGCTCAGTACCAGTGTGGGGGATCACCCTCTCAGGCCCCCTAAAG
ATCGTCGCCTTGGTGAGCCGTTACCTCACCAACTAGCTAATCTTGCGCGTGCCCATCTCTATCCACCGTAGTT
TTCAATATCACCCGATGCCGAATAATATATTTATGGGGTATTAATCTTCTTCGAAAGCTATCCCCCTGATAAG
AGCAGGTTGCACACGTGTTCCGCACCCGTACGCCGCTCTCAATTACCCGAAAGTAATCTACCGCTCGGCTTGC
ATGTGTTAGGCCTCCCGCTAGCGTTCATCCTGAGCCAGATCAAACTCT
>1673
CTGCTGCCGCCCGTAGGAGTCTGGTCCGTGTCTCAGTACCAGTGTGGGGGATCACCCTCTCAGGCCCCCTAAA
GATCGTCGCCTGGTGAGCCGTTACCTCACCAACTAGCTAATCTTGCGCGTGCCCATCTCTATCCACCGTAGTT
TCAATATCACCCGATGCCGAATAATATATTATGGGGTATTAATCTTCTTCGAAAGGCTATCCCCTGATAAAGG
CAGGTTGCACACGTGTTCCGCACCCGTACGCCGCTCTCAATTACCCGAAAGTAATCTACCGCTCGGCTTGCAT
GTGTTAGGCCTCCCGCTAGCGTCATCCTGAGCCAGGATCAAACTCT
>1674

CTGCTGCCGCCCGTAGGAGTCTGGTCCGTGTCTCAGTACCAGTGTGGGGGATCACCCTCTCAGGCCCCCTAAA
GATCGTCGCCTTGGTGAGCCGTTACCTCACCAACTAGCTAATCTTGCGCGTGCCCATCTCTATCCACCGTAGT
TTTCAATATCACCCGATGCCGAATAATATATTATGGGGTATTAATCTTCTCGAAAGGCTATCCCCTGATAAAG
GCAGGTTGCACATGTGTTCCGCACCCGTACGCCGCTCTCAATTTACCCGAAAGTAATCTACGCTCGGCTTGCA
TGTGTTAGGCCTCCCGCTAGCGTCATCGTGAGCCAGGATCAAACTCT
>1675
CTGCTGCCGCCCGTAGGAGTCTGGTCCGTGTCTCAGTACCAGTGTGGGGGATCACCCTCTCAGGCCCCCTAAA
GATCGTCGCCTTGGTGAGCCGTTACCTCACCAACTAGCTAATCTTGCGCGTGCCCATCTCTATCCACCGTAGT
TTTCAATATCACCCGATGCCGAATAATATATTATGGGGTATTAATCTTCTTCGAAAGGCTATCCCCTGATAAA
GGCAGGTTGCACACGTGTTCCGCACCCGTACGCCGCTCTCAATTACCCGAAAGTAATCTACCGCTCGGCTTGC
ATGTGTTAGGCCTCCCGCTAGCGTTCATCCTGAGCCAGGA
>1676
CTGCTGCCGCCCGTAGGAGTCTGGTCCGTGTCTCAGTACCAGTGTGGGGGATCACCCTCTCAGGCCCCCTAAA
GATCGTCGCCTTGGTGAGCCGTTACCTCACCAACTAGCTAATCTTGCGCGTGCCCATCTCTATCCACCGTAGT
TTTCAATATCACCCGATGCCGAATAATATATTATGGGGTATTAATCTTCTTCGAAAGCTATCCCCCTGATAAA
GGCAGGTTGCACACGTGTTCCGCACCCGTACGCCGCTCTCAATTACCCGAAAGTAATCTACCGCTCGGCTTGC
ATGTGTTAGGCCTCCCGCTAGCGTTCTCCTGAGCAGGATCAACTCT
>1677
CTGCTGCCGCCCGTAGGAGTCTGGTCCGTGTCTCAGTACCAGTGTGGGGGATCACCCTCTCAGGCCCCCTAAA
GATCGTCGCCTTGGTGAGCCGTTACCTCACCAACTAGCTAATCTTGCGCGTGCCCATCTCTATCCACCGTAGT
TTTCAATATCACCCGATGCCGAATAATATATTATGGGGTATTAATTCTTCTTCGAAAGGCTATCCCCCTGATA
AAGGCAGGTTGCACACGTGTTCCGCACCCGTACGCCGCTCTCAATTACCCGAAAGTAATCTACCGCTCGGCTT
GCATGTGTTAGGCCTCCCGCTAGCGTTCATCCTGAGCCAGGATCAAACTCT
>1678
CTGCTGCCGCCCGTAGGAGTCTGGTCCGTGTCTCAGTACCAGTGTGGGGGATCACCCTCTCAGGCCCCCTAAA
GATCGTCGCCTTGGTGAGCCGTTACCTCACCAACTAGCTAATCTTGCGCGTGCCCATCTCTATCCACCGTAGT
TTTCAATATCACCCGATGCCGAATAGTATATTATGGGGTATTTAATTCTTCTTCGAAAGGCTATCCCCCTGAT
AAAGGCAGGTTGCACACGTGTTCCGCACCCGTACGCCGCTCTCAATTACCCGAAAGTAATCTACCGCTCCGGC
TTGCATGTGTTAGGCCTCCCGCTAGCGTATCTCCTGAGCACGAT
>1679
CTGCTGCCGCCCGTAGGAGTCTGGTCCGTGTCTCAGTACCAGTGTGGGGGATCACCCTCTCAGGCCCCCTAAA
GATCGTCGCCTTGGTGAGCCGTTACCTCACCAACTAGCTAATCTTGCGCGTGCCCATCTCTATCCACCGTAGT
TTTCAATATCACCCGATGCCGAATAATATATTATGGGGTATTAATCTTCTTCGAAAGGCTATCCCCCTGATAA
AGGCAGGTTGCACACGTGTTCCGCACCCGTACGCCGCTCTCAATTACCCGAAAGTAATCTACCGCTCGGCTTG
CATGTGTTAGGCCTCCCGCTAGCGTTCATCCTGAGCCAGGATCAAACTCT
>1680
CTGCTGCCGCCCGTAGGAGTCTGGTCCGTGTCCCAGTACCAGTGTGGGGGATCACCCTCTCAGGCCCCCTAAA
GATCGTCGCCTTGGTGAGCCGTTACCTCACCAACTAGCTAATCTTGCGCGTGCCCATCTCTATCCACCGTAGT
TTTCAATATCACCCGATGCCGAATAATATATTATGGGGTATTAATCTTCTTCGAAAGGCTATCCCCCTGATAA
AGGCAGGTTGCACACGTGTTCCGCACCCGTACGCCGCTCTCAATTACCCGAAAGTAATCTACCGCTCGGCTTG
CATGTGTTAGGCCTCCCGCTAGCGTTCATCCTGAGCCAGGATCAAACTCT
>1681
CTGCTGCCGCCCGTAGGAGTCTGGTCCGTGTCTCAGTACCAGTGTGGGGGATCACCCTCTCAGGCCCCCTAAA
GATCGTCGCCTTGGTGAGCCGTTACCTCACCAACTAGCTAATCTTGCGCGTGCCCATCTCTATCCACCGTAGT
TTTCAATATCACCCGATGCCGAATAATATATTATGGGGTATTAATCTTCTTCGAAAGGCTATCCCCCTGATAA
AGGCAGGTTGCACACGTGTTCCGCACCCGTACGCCGCTCTCAATTACCCGAAAGTAATCTACCGCTCGGCTTG
CATGTGTTAGGCCTCCCGCTAGCGTTCATCCTGAGCCAGGATCAACTCT
>1682
CTGCTGCCGCCCGTAGGAGTCTGGTCCGTGTCTCAGTACCAGTGTGGGGGATCACCCTCTCAGGCCCCCTAAA
GATCGTCGCCTTGGTGAGCCGTTACCTCACCAACTAGCTAATCTTGCGCGTGCCCATCTCTATCCACCGTAGT
TTTCAGTATCACCCGATGCCGAATAATATATTATGGGGTATTAATCTTCTCGAAAGGCTATCCCCCTGATAAA
GGCAGGTTGCACACGTGTTCCGCACCCGTACGCCGCTCTCAATTACCCGAAAGTAATCTACCGCTCGGCTTGC
ATGTGTTAGGCCTCCCGCTAGCGTTCATCCTGAGCCAGGATCAAACTCT
>1683

CTGCTGCCGCCCGTAGGAGTCTGGTCCGTGTCTCAGTACCAGTGTGGGGGATCACCCTCTCAGGCCCCCTTAA
AAGATCGTCGCCTTGGTGAGCCGTTACCTCACCAACTAGCTAATCTTGCGCGTGCCCATCTCTATCCACCGTA
GTTTCAATATCACCCGATGCCGAATAATATATTATGGGGTATTAATCTTCTCGAAAGGCTATCCCCTGATAAA
GGCAGGTTGCACACGTGTTCCGCACCCGTACGCCGCTCTCAATTACCCGAAAGTAATCTACCGCTCGGCTTGC
ATGTGTTAGGCCTCCCGCTAGCGTTCTCCTGAGCAGGATCAA
>1684
CTGCTGCCGCCCGTAGGAGTCTGGTCCGTGTCTCAGTACCAGTGTGGGGGATCACCCTCTCAGGCCCCCTAAA
GATCGTCGCCTTGGTGAGCCGTTACCTCACCAACTAGCTAATCTTGCGCGTGCCCATCTCTATCCACCGTAGT
TTTCAATATCACCCGATGCCGAATAATATATTTATGGGGTATTTAATTCTTCTTCGAAAGGCTATCCCCTGAT
AAGGGCAGGTTGCACACGTGTTCCGCACCCGTACGCCGCTCTCAATTACCCGAAAGTAATCTACCGCTCGGCT
TGCATGTGTTAGGCCTCCCGCTAGCGTTCTCCTGAGCAGGATCAACTCGT
>1685
CTGCTGCCGCCCGTAGGAGTCTGGACCGTGTCTCAGTTCCAGTGTGACTGATCATCCTCTCAGACCAGTTACG
GATCGTCGCCTTGGTAGGCCTTTACCCCACCAACTAGCTAATCCGACCTAGGCTCATCTGATAGCGTGAGGTC
CGAAGATCCCCCACTTCTCCCTCAGGACGTATGCGGTATTAGCGCCCGTTTCCGGACGTATCCCCACTACCAG
GCAGATTCCTAGGCATTACTCACCCGTCCGCCGCTGAATCCAGGAGCAAGCTCCCTTCATCCGCTCGACTGCA
TGTGTTAGGCCTGCCGCCAGCGTTCAATCTGAGCCAGGATCAAACTCT
>1686
CTGCTGCCGCCCGTAGGAGTCTGGTCCGTGTCTCAGTACCAGTGTGGGGGATCACCCTCTCAGGCCCCCTAAA
GATCGTCGCCTTGGTGAGCCGTTACCTCACCAACTAGCTAATCTTGCGCGTGCCCATCTCTATCCACCGTAGT
TTTCAATATCACCCGATGCCGAATAATATATTATGGGGTATTAATCTTCTTCGAAAGGCTATCCCCTGATAAA
GGCAGGTTGCACACGTGTTCCGCACCCGTACGCCGCTCTCAATTACCCGAAAGTAATCTACCGCTCGGCTTGC
ATGTGTTAGGCCTCCCGCTAGCGTTCATCCTGAGCCAGGATCAACTCT
>1687
CTGCTGCCGCCCGTAGGAGTCTGGTCCGTGTCTCAGTACCAGTGTGGGGGATCACCCTCTCAGGCCCCCTAAA
GATCGTCGCCTTGGTGAGCCGTTACCTCACCAACTAGCTAATCTTGCGCGTGCCCATCTCTATCCACCGTAGT
TTTCAATATCACCCGATGCCGAATAATATATTATGGGGTATTAATCTTCTCGAAAGGCTATCCCCTGATAAAG
GCAGGTTGCACACGTGTTCCGCACCCGTACGCCGCTCTCAATTACCCGAAAGTAATCTACCGCTCGGCTTGCA
TGTGTTAGGCCTCCCGCTAGCGTCATCCTGAGCCAGGATCAACTCT
>1688
CTGCTGCCGCCCGTAGGAGTCTGGTCCGTGTCTCAGTACCAGTGTGGGGGATCACCCTCTCAGGCCCCCTAAA
GATCGTCGCCTTGGTGAGCCGTTACCTCACCAACTAGCTAATCTTGCGCGTGCCCATCTCTATCCACCGTAGT
TTTCAATATCACCCGATGCCGAATAATATATTATGGGGTATTAATCTTCTTCGAAAGGCTATCCCCCTGATAA
AGGCAGGTTGCACACGTGTTCCGCACCCGTACGCCGCTCTCAATTACCCGAAAGTAATCTACCGCTCCGGCTT
GCATGTGTTAGGCCTCCCGCTAGCGTTCATCTCGAGCCAGGATCAACTCT
>1689
CTGCTGCCGCCCGTAGGAGTCTGGTCCGTGTCTCAGCACCAGTGTGGGGGATCACCCTCTCAGGCCCCCTAAA
GATCGTCGCCTTGGTGAGCCGTTACCTCACCAACTAGCTAATCTTGCGCGTGCCCATCTCTATCCACCGTAGT
TTTCAATATCACCCGATGCCGAATAATATATTATGGGGTATTAATCTTCTTCGAAAGGCTATCCCCCTGATAA
AGGCAGGTTGCACACGTGTTCCGCACCCGTACGCCGCTCTCAATTACCCGAAAGTAATCTACCGCTCCGGCTT
GCATGTGTTAGGCCTCCCGCTAGCGTTCTCCTGAGCAGATC
>1690
CTGCTGCCGCCCGTAGGAGTCTGGTCCGTGTCTCAGTACCAGTGTGGGGGATCACCCTCTCAGGCCCCCTAAA
GATCGTCGCCTTGGTGAGCCGTTACCTCACCAACTAGCTAATCTTGCGCGTGCCCATCTCTATCCACCGTAGT
TTTTCAATATCACCCGATGCCGAATAATATATTATGGGGTATTTAATCTTCCTTCGGAAGGCTATCCCCCTGA
TAAAGGCAGGTTTGCACACGTGTTCCGCACCCGTACGCCGCTCTCAATTACCCGAAAGTAATCTACCGCTCGG
CTTGCATGTGTTAGGCCTCCCGCTAGCGTTCATCCTGAGCCAGGATCAAACTCT
>1691
CTGCTGCCGCCCGTAGGAGTCTGGTCCGTGTCTCAGTACCAGTGTGGGGGATCACCCTCTCAGGCCCCCTAAA
GATCGTCGCCTTGGTGAGCCGTTACCTCACCAACTAGCTAATCTTGCGCGTGCCCATCTCTATCCACCGTAGT
TTTCAATATCACCCGATGCCGAATAATATATTATGGGGTATTAATCTTCTTCGAAAGCTATCCCCTGATAAAG
GCAGGTTGCACACGTGTTCCGCACCCGTACGCCGCTCTCAATTACCCGAAAGTAATCTACCGCTCGGCTTGCA
TGTGTTAGGCCTCCCGCTAGCGTTCTCCTCGAGCAGGAT
>1692

CTGCTGCCGCCCGTAGGAGTCTGGTCCGTGTCTCAGTACCAGTGTGGGGGATCACCCTCTCAGGCCCCCCTA
AAGATCGTCGCCTTGGTGAGCCGTTACCTCACCAACTAGCTAATCTTGCGCGTGCCCCATCTCTATCCACCGT
AGTTTTCAATATCACCCGATGCCGAATAATATATTATGGGGTATTTAATTCTTTCTTCGAAAGGCTATCCCCC
TGATAAAGGCAGGTTGCACACGTGTTCCGCACCCGTACGCCGCTCTCAATTACCCCGAAAGTAATCTACCGCT
CGGCTTGCATGTGTTAGGCCTCCCGCTAGCGTTCTCCTCGAGCAGGATCCAACATCT
>1693
CTGCTGCCGCCCGTAGGAGTCTGGTCCGTGTCTCAGTACCAGTGTGGGGGATCACCCTCTCAGGCCCCCTAAA
GATCGTCGCCTTGGTGAGCCGTTACCTCACCAACTAGCTAATCTTGCGCGTGCCCATCTCTATCCACCGTAGT
TTCAATATCACCCGATGCCGAATAATATATTATGGGGTATTAATCTTCTTCGAAAGCTATCCCCCTGATAAAG
GCAGGTTGCACACGTGTTCCGCACCCGTACGCCGCTCTCAATTACCCGAAAGTAATCTACCGCTCGGCTTGCA
TGTGTTAGGCCTCCCGCTAGCGTTCTCCTGAGC
>1694
CTGCTGCCGCCCGTAGGAGTCTGGTCCGTGTCTCAGTACCAGTGTGGGGGATCACCCTCTCAGGCCCCCTAAA
GATCGTCGCCTTGGTGAGCCGTTACCTCACCAACTAGCTAATCTTGCGCGTGCCCATCTCTATCCACCGTAGT
TTTCAATATCACCCGATGCCGAATAATATATTATGGGGTATTAATCTTCTTCGAAAGGCTATCCCCCTGATAA
AGGCAGGTTGCACACGTGTTCCGCACCCGTACGCCGCTCTCAATTACCCGAAAGTAATCTACCGCTCGGCTTG
CATGTGTTAGGCCTCCCGCTAGCGTCCATCCTGAGCCAGGATCAACTCT
>1695
CTGCTGCCGCCCGTAGGAGTCTGGTCCGTGTCTCAGTACCAGTGTGGGGGATCACCCTCTCAGGCCCCCTAAA
GATCGTCGCCTTGGTGAGCCGTTACCTCACCAACTAGCTAATCTTGCGCGTGCCCATCTCTATCCACCGTAGT
TTTCAATATCACCCGATGCCGAATAATATATTATGGGGTATTAATCTTCTTCGAAAGCTATCCCCCTGATAAA
GGCAGGTTGCACACGTGTTCCGCACCCGTACGCCGCTCTCAATTACCCGAAAGTAATCTACCGCTCCGGCTTG
CATGTGTTAGGCCTCCCGCTAGCGTTCATCCTGAGCCAGGATCAACTCT
>1696
CTGCTGCCGCCCGTAGGAGTCTGGTCCGTGTCTCAGTACCAGTGTGGGGGATCACCCTCTCAGGCCCCCTAAA
GATCGTCGCCTTGGTGAGCCGTTACCTCACCAACTAGCTAATCTTGCGCGTGCCCATCTCTATCCACCGTAGT
TTCAATATCACCCGATGCCGAATAATATATTATGGGGTATTAATCTTCTTCGAAGGCTATCCCCTGATAAAGG
CAGGTTGCACACGTGTTCCGCACCCGTACGCCGCTCTCAATTACCCGAAAGTAATCTACCGCTCCGGCTTGCA
TGTGTTAGGCCTCCCGCTAGCGTTCTCCTCGAGCAGGATCCAACATCT
>1697
CTGCTGCCGCCCGTAGGAGTCTGGTCCGTGTCTCAGTACCAGTGTGGGGGATCACCCTCTCAGGCCCCCTAAA
GATCGTCGCCTTGGTGAGCCGTTACCTCACCAACTAGCTAATCTTGCGCGTGCCCATCTCTATCCACCGTAGT
TTTCAATATCACCCGATGCCGAATAATATATTATGGGGTATTAATCTTCTCGAAGCTATCCCCTGAATAAAGG
CAGGTTGCACACGTGTTCCGCACCCGTACGCCGCTCTCAATTACCCGAAAGTAATCTACCGCTCGGCTTGCAT
GTGTTAGGCCTCCCGCTAGCGTTCTCC
>1698
CTGCTGCCGCCCGTAGGAGTCTGGACCGTGTCTCAGTTCCAGTGTGACTGATCATCCTCTCAGACCAGTTACG
GATCGTCGCCTTGGTAGGCCTTTACCCCACCAACTAGCTAATCCGACCTAGGCTCATCTGATAGCGTGAGGTC
CGAAGATCCCCCACTTCTCCCTCAGGACGTATGCGGTATTAGCGCCCGTTTCCGGACGTATCCCCACTACCAG
GCAGATTCCTAGGCATTACTCACCCGTCCGCCGCTGAATCCAGGAGCAAGCTCCCTCATCCGCTCGACTGCAT
GTGTTAGGCCTGCCGCCAGCGTCAATCTGAGCCAGGATCAAACTCT
>1699
CTGCTGCCGCCCGTAGGAGTCTGGTCCGTGTCTCAGTACCAGTGTGGGGGATCACCCTCTCAGGCCCCCTAAA
GATCGTCGCCTTGGTGAGCCGTTACCTCACCAACTAGCTAATCTTGCGCGTGCCCATCTCTATCCACCGTAGT
TTTCAATATCACCCGATGCCGAATAATATATTATGGGGTATTAATCTTCTCGAAAGCTATCCCCCTGATAAAG
GCAGGTTGCACACGTGTTCCGCACCCGTACGCCGCTCTCAATTACCCGAAAGTAATCTACCGCTCCGGCTTGC
ATGTGTTAGGCCTCCCGCTAGCGTTCATCCGTCGAGCCAGGCATCAAACTCT
>1700
CTGCTGCCGCCCGTAGGAGTCTGGTCCGTGTCTCAGTACCAGTGTGGGGGATCACCCTCTCAGGCCCCCTAAA
GATCGTCACCTTGTGAGCCGTTACCTCACCAACTAGCTAATCTTGCGCGTGCCCATCTCTATCCACCGTAGTT
TTCAATATCACCCGATGCCGAATAATATATTATGGGGTATTAATTCTTCTCGAAGCTATCCCCTGATAAAGGC
AGGTTGCACACGTGTTCCGCACCCGTACGCCGCTCTCAATTACCCGAAAGTAATCTACCGCATCGGCTTGCAT
GTGTTAGGCCTCCCGCTAGCGTTCTTC
>1701

CTGCTGCCGCCCGTAGGAGTCTGGTCCCGTGTCTCAGTACCAGTGTGGGGGATCACCCCTCTCAGGCCCCCTA
AAGATCGTCGCCTTGGTGAGCCGTTACCTCACCCAACTAGCTAATCTTGCGCGTGCCCCATCTCTATCCACCG
TAGTTTCAATATCACCCGATGCCGAATAATATATTATTGGGGTATTTAATTCTTCTTCGAAAGGCTATCCCCT
GATAAAGGCAGGTTGCACACGTGTTCCGCACCCGTACGCCGCTCTCAATTACCCCGAAAGTAATCTACCGCTC
GGCTTGCATGTGTTAGGCCTCCCGCTAGCGTTC
>1702
CTGCTGCCGCCCGTAGGAGTCTGGTCCGTGTCTCAGTACCAGTGTGGGGGATCACCCTCTCAGGCCCCCTAAA
GATCGTCGCCTTGGTGAGCCGTTACCTCACCAACTAGCTAATCTTGCGCGTGCCCATCTCTATCCACCGTAGT
TTCAATATCACCCGATGCCGAATAATATATTATTGGGGTATTTAATCTTCTTCGAAAGGCTATCCCCTGATAA
AGGCAGGTTGCACACGTGTTCCGCACCCGTACGCCGCTCTCAATTACCCGAAAGTAATCTACCGCTCGGCTTG
CATGTGTTAGGCCTCCCGCTAGCGTTCATCCTGAGCCAGGATCAAACTCT
>1703
CTGCTGCCGCCCGTAGGAGTCTGGTCCGTGTCTCAGTACCAGTGTGGGGGATCACCCTCTCAGGCCCCCTAAA
GATCGTCGCCTTGGTGAGCCGTTACCTCACCAACTAGCTAATCTTGCGCGTGCCCATCTCTATCCACCGTAGT
TTTCAATATCACCCGATGCCGAATAATATATTATGGGGTATTAATCTTCTTCGAAAGGCTATCCCCTGATAAA
GGCAGGTTGCACACGTGTTCCGCACCCGTACGCCGCTCTCAATTTACCCGAAAGTAATCTACCGCTCGGCTTG
CATGTGTTAGGCCTCCCGCTAGCGTTCATCTGAGCCAGGATCAAACTCT
>1704
CTGCTGCCGCCCGTAGGAGTCTGGTCCGTGTCTCAGTACCAGTGTGGGGGATCACCCTCTCAGGCCCCCTAAA
GATCGTCGCCTTGGTGAGCCGTTACCTCACCAACTAGCTAATCTTGCGCGTGCCCATCTCTATTCCACCGTAG
TTTTCAATATCACCCGATGCCGAATAATATATTATGGGGTATTTAATCTTCTTCGAAAGGCTATCCCCCTGAT
AAAGGCAGGTTGCACACGTGTTCCGCACCCGTACGCCGCTCTCAATTACCCCGAAAGTAATCTACCGCTCGGC
TTGCATGTGTTAGGCCTCCCGCTAGCGTTCTCCTGAGCAGGAT
>1705
CTGCTGCCGCCCCGTAGGAGTCTGGTTCCCGTGTCTCAGTACCAGTGTGGGGGATCACCCCTCTCAGGCCCCC
CTAAAGATCGTCGCCTTGGTGAGCCGTTACCTCACCCAACTAGCTAATCTTGCGCGTGCCCCATCTCTATCCA
CCGTAGTTTTTTCAATATCACCCGATGCCGAATAATATATTTATGGGGTATTTAATCTTTCTTCGAAAGGCTA
TCCCCCTGATTAAAGGCAGGTTGCACACGTGTTCCGCACCCGTACGCCGCTCCATTATCCCCGAAAGTAATCT
ACCGCTCGGCTTGCATGTGTTAGGCCTTCCCGCTAGCGTTCATTCCTCGAGCCAGGATCAAACTCT
>1706
CTGCTGCCGCCCGTAGGAGTCTGGTCCGTGTCTCAGTACCAGTGTGGGGGATCACCCTCTCAGGCCCCCTAAA
GATCGTCGCCTTGGTGAGCCGTTACCTCACCAACTAGCTAATCTTGCGCGTGCCCATCTCTATCCACCGTAGT
TTTCAATATCACCCGATGCCGAATAATATAATTATGGGGTATTAATCTTCTTCGAAAGGCTATCCCCCTGATA
AAGGCAGGTTGCACACGTGTTCCGCACCCGTACGCCGCTCTCAATTACCCGAAAGTAATCTACCGCTCGGCTT
GCATGTGTTAGGCCTCCCGCTAGCGTTCATCCTGAGCCAGGATCAAACTCT
>1707
CTGCTGCCGCCCGTAGGAGTCTGGTCCGTGTCTCAGTACCAGTGTGGGGGATCACCCTCTCAGGCCCCCTAAA
GATCGTCGCCTTGGTGAGCCGTTACCTCACCAACTAGCTAATCTTGCGCGTGCCCATCTCTATCCACCGTAGT
TTTCAATATCACCCGATGCCGAATAATATATTATGGGGTATTAATCTTCTTCGAAAGGCTATCCCCCTGATAA
AGGGCAGGTCGCACACGTGTTCCGCACCCGTACGCCGCTCTCAATTACCCGAAAGTAATCTACCGCTCGGCTT
GCATGTGTTAGGCCTCCCGCTAGCGTTCATCCTGAGCCAGGATCAACTCT
>1708
CTGCTGCCGCCCGTAGGAGTCTGGTCCGTGTCTCAGTACCAGTGTGGGGGATCACCCTCTCAGGCCCCCTAAA
GATCGTCGCCTTGGTGAGCCGTTACCTCACCAACTAGCTAATCTTGCGCGTGCCCATCTCTATCCACCGTAGT
TTTCAATATCACCCGATGCCGAATAATATATTATGGGGTATTAATCTTCTCGAAAGGCTATCCCCTGATAAAG
GCAGGTTGCACACGTGTTCCGCACCCGTACGCCGCTCTCAATTACCCGAAAGTAATCTACCGCTCGGCTTGCA
TGTGTTAGGCCTCCCGCTAGCGTCATCCTGAGCCAGGATCAAACTCT
>1709
CTGCTGCCGCCCGTAGGAGTCTGGTCCGTGTCTCAGTACCAGTGTGGGGGATCACCCTCTCAGGCCCCCTAAA
GATCGTCGCCTTGGTGAGCCGTTACCTCACCAACTAGCTAATCTTGCGCGTGCCCATCTCTATCCACCGTAGT
TTTCAATATCACCCGATGCCGAATAATATATTATGGGGTATTAATCTTCTTCGAAAGGCTATCCCCCTGATAA
AGGCAGGTTGCACACGTGTTCCGCACCCGTACGCCGCTCTCAATTACCCGAAAGTAATCTACCGCTCGGCTTG
CATGTGTTAGGCCTCCCGCTAGCGTTCATCCTGAGCCAGGATCAAACTCT
>1710

CTGCTGCCGCCCGTAGGAGTCTGGTCCGTGTCTCAGTACCAGTGTGGGGGATCACCCTCTCAGGCCCCCTAAA
GATCGTCGCCTTGGTGAGCCGTTACCTCACCAACTAGCTAATCTTGCGCGTGCCCATCTCTATCCACCGTAGT
TTTCAATATCACCCGATGCCGAATAATATATTATGGGGTATTAATCTTCTTCGAAAGGCTATCCCCCTGATAA
AGGCAGGTTGCACACGTGTTCCGCACCCGTACGCCGCTCTCAATTACCCGAAAGTAATCTACCGCTCGGCTTG
CATGTGTTAGGCCTCCCGCTAGCGTTCATCCTCGAGCCAGGATCAAACTCT
>1711
CTGCTGCCGCCCGTAGGAGTCTGGTCCGTGTCTCAGTACCAGTGTGGGGGATCACCCTCTCAGGCCCCCTAAA
GATCGCGCCTTGGTGAGCCGTTACCTCACCAACTAGCTAATCTTGCGCGTGCCCATCTCTATCCACCGTAGTT
TTCAATATCACCCGATGCCGAATAATATATTATGGGGGTATTAATCTTCTTCGAAAGGCTATCCCCCTGATAA
AGGCAGGTTGCACACGTGTTCCGCACCCGTACGCCGCTCTCAATTACCCGAAAGTAATCTACCGCTCGGCTTG
CATGTGTTAGGCCTCCCGCTAGCGTTCATCCTGAGCCAGGATCAAACTCT
>1712
CTGCTGCCGCCCGTAGGAGTCTGGTCCGTGTCTCAGTACCAGTGTGGGGGATCACCCTCTCAGGCCCCCTAAA
GATCGTCGCCTTGGTGAGCCGTTACCTCACCAACTAGCTAATCTTGCGCGTGCCCATCTCTATCCACCGTAGT
TTTCAATATCACCCGATGCCGAATAATATATTATGGGGTATTAATCTTCCCTTCGAAAGGCTATCCCCCTGAT
AAAGGCAGGTTGCACACGTGTTCCGCACCCGTACGCCGCTCTCAATTACCCGAAAGTAATCTACCGCTCGGCT
TGCATGTGTTAGGCCTCCCGCTAGCGTTCATCCTGAGCCAGGATCAAACTCT
>1713
CTGCTGCCGCCCGTAGGAGTCTGGTCCGTGTCTCAGTACCAGTGTGGGGGATCACCCTCTCAGGCCCCCTAAA
GATCGTCGCCTTGGTGAGCCGTTACCTCACCAACTAGCTAATCTTGCGCGTGCCCATCTCTATCCACCGTAGT
TTTCAATATCACCCGATGCCGAATAATATATTATGGGGTATTAATCTTCTTCGAAAGGCTATCCCCTGATAAA
GGCAGGTTGCACACGTGTTCCGCCCCGTACGCCGCTCTCAATTACCCGAAAGTAATCTACCGCTCGGCTTGCA
TGTGTTAGGCCTCCCGCTAGCGTTCATCCTGAGCAGGA
>1714
CTGCTGCCGCCCGTAGGAGTCTGGTCCGTGTCTCAGTACCAGTGTGGGGGATCACCCTCTCAGGCCCCCTAAA
GATCGTCGCCTTGGTGAGCCGTTACCTCACCAACTAGCTAATCTTGCGCGTGCCCATCTCTATCCACCGTAGT
TTTCAATATCACCCGATGCCGAATAATATATTATGGGGTATTAATCTTCTTCGAAAGGCTATCCCCTGATAAA
GGCAGGTTGCACACGTGTTCCGCACCCGTACGCCGCTCTCAATTACCCGAAAGTAATCTACCGCTCCGGCTTG
CATGTGTTAGGCCTCCCGCTAGCTGTTC
>1715
CTGCTGCCGCCCGTAGGAGTCTGGTCCGTGTCTCAGTACCAGTGTGGGGGATCACCCTCTCAGGCCCCCTAAA
GATCGTCGCCTTGGTGAGCCGTTACCTCACCAACTAGCTAATCTTGCGCGTGCCCATCTCTATCCACCGTAGT
TTTCAATATCACCCGATGCCGAATAATATATTATGGGGTATTAATCTTCTTCGAAAGGCTATCCCCTGATAAA
GGCAGGTTGCACACGTGTTCCGCACCCGTACGCCGCTCTCAATTTACCCGAAAGTAATCTACCGCTCCGGCTT
GCATGTGTTAGGCCTCCCGCTAGCGTTCATCCTGAGCCAGGATCAAACTCT
>1716
CTGCTGCCGCCCGTAGGAGTCTGGTCCGTGTCTCAGTACCAGTGTGGGGGATCACCCTCTCAGGCCCCCTAAA
GATCGTCGCCTTGGTGAGCCGTTACCTCACCAACTAGCTAATCTTGCGCGTGCCCATCTCTATCCACCGTAGC
TTTCAATATCACCCGATGCCGAATAATATATTATGGGGGTATTAATCTTCTCGAAAGGCTATCCCCTGATAAA
GGCAGGTTGCACACGTGTTCCGCACCCGTACGCCGCTCTCAATTACCCGAAAGTAATCTACCGCTCGGCTTGC
ATGTGTTAGGCCTCCCGCTAGCGTTCTCCGTG
>1717
CTGCTGCCGCCCGTAGGAGTCTGGTCCGTGTCTCAGTACCAGTGTGGGGGATCACCCTCTCAGGCCCCCTAAA
GATCGTCGCCTTGGTGAGCCGTTACCTCACCAACTAGCTAATCTTGCGCGTGCCCATCTCTATCCACCGTAGT
TTTCAATATCACCCGATGCCGAATAATATATTATGGGGTATTAATCTTCTTCGAAAGCTATCCCCCTGATAAA
GGCAGGTTGCACACGTGTTCCGCACCCGTACGCCGCTCTCAATTACCCGAAAGTAATCTACCGCTCGGCTTGC
ATGTGTTGGGCCTCCCGCTAGCGTTCTCCTCGAGCAGGATCAACATCT
>1718
CTGCTGCCGCCCGTAGGAGTCTGGTCCGTGTCTCAGTACCAGTGTGGGGGATCACCCTCTCAGGCCCCCCTAA
AGATCGTCGCCTTGGTGAGCCGTTACCTCACCAACTAGCTAATCTTGCGCGTGCCCATCTCTATCCACCGTAG
TTTTTCAATATCACCCGATGCCGAATAATATATTTATGGGGTATTTAATTCTTCTCGAAAGCTATCCCCCTGA
TAAAGGCAGGTTGCACACGTGTTCCGCACCCGTACGCCGCTCTCAATTACCCCGAAAGTAATCTACCGCTCGG
CTTGCATGTGTTAGGCCTTCCCGCTAGCGTTCTCCTCGAG
>1719

CTGCTGCCGCCCGTAGGAGTCTGGTCCGTGTCTCAGTACCAGTGTGGGGGATCACCCTCTCAGGCCCCCTAAA
GATCGTCGCCTTGGTGAGCCGTTACCTCACCAACTAGCTAATCTTGCGCGTGCCCATCTCTATCCACCGTAGT
TTTCAATATCACCCGATGCCGAATAATATATTATGGGGTATTAATCTTCTTCGAAAGGCTATCCCCCTGATAA
AGGCAGGTTGCACACGTGTTCCGCACCCGTACGCCGCTCTCAATTACCCGAAAGTAATCTACCGCTCGGCTTG
CATGTGTTAGGCCTCCCGCTAGCGTTCATCCTGAGCA
>1720
CTGCTGCCGCCCGTAGGAGTCTGGTCCGTGTCTCAGTACCAGTGTGGGGGATCACCCTCTCAGGCCCCCTAAA
GATCGTCGCCTTGGTGAGCCGTTACCTCACCAACTAGCTAATCTTGCGCGTGCCCATCTCTATCCACCGTAGT
TTTCAATATCACCCGATGCCGAATAATATATTATGGGGGTATTAATCTTCTTCGAAAGGCTATCCCCTGATAA
AGGCAGGTTGCACACGTGTTCCGCACCCGTACGCCGCTCTCAATTTACCCGAAAAGTAATCTACCGCTCCGGC
TTGCATGTGTTAGGCCTCCCGCTAGCGTTCTCCTCGAGCAGGAT
>1721
CTGCTGCCGCCCGTAGGAGTCTGGTCCGTGTCTCAGTACCAGTGTGGGGGATCACCCTCTCAGGCCCCCTAAA
GATCGTCGCCTTGGTGAGCCGTTACCTCACCAACTAGCTAATCTTGCGCGTGCCCATCTCTATCCACCGTAGT
TTTCAATATCACCCGATGCCGAATAATATATTATGGGGTATTAATCTTCTTCGAAAGGCTATCCCCTGATAAA
GGCAGGTTGCACACGTGTTCCGCACCCGTACGCCGCTCTCAATTACCCGAAAGTAATCTACCGCTCCGGCTGC
ATGTGTTAGGCCTCCCGCTAGCGTTCTCTCGAGCAGGATCAAC
>1722
CTGCTGCCGCCCGTAGGAGTCTGGTCCGTGTCTCAGTACCAGTGTGGGGGATCACCCTCTCAGGCCCCCTAAA
GATCGTCGCCTTGGTGAGCCGTTACCTCACCAACTAGCTAATCTTGCGCGTGCCCATCTCTATCCACCGTAGT
TTTCAATATCACCCGATGCCGAATAATATATTATGGGGTATTAATCTTCTTCGAAAGGCTATCCCCTGATAAA
GGCAGGTTGCACACGTGTTCCGCACCCGTACGCCGCTCTCAATTACCCGAAAGTAATCACCGCTCCGGCTTGC
ATGTGTTAGGCCTCCCGCTAGCGTTCATCTGAGCAGGCATCAAACTCCT
>1723
CTGCTGCCGCCCGTAGGGGTCTGGTCCGTGTCTCAGTACCAGTGTGGGGGATCACCCTCTCAGGCCCCCTAAA
GATCGTCGCCTTGGTGAGCCGTTACCTCACCAACTAGCTAATCTTGCGCGTGCCCATCTCTATCCACCGTAGT
TTTCAATATCACCCGATGCCGAATAATATATTATGGGGTATTAATCTTCTCGAAAGGCTTATCCCCTGATAAA
GGCAGGTTGCACACGTGTTCCGCACCCGTACGCCGCTCTCAATTACCCGAAAGTAATCTACCGCTCGGCTTGC
ATGTGTTAGGCCTCCCGCTAGCGTTCATCCTGAGCCAGGATCAACTCT
>1724
CTGCTGCCGCCCGTAGGAGTCTGGTCCGTGTCTCAGTACCAGTGTGGGGGATCACCCTCTCAGGCCCCCTAAA
GATCGTCGCCTTGGTGAGCCGTTACCTCACCAACTAGCTAATCTTGCGCGTGCCCATCTCTATCCACCGTAGT
TTTCAATATCACCCGATGCCGAATAATATATTATGGGGTATTAATCTTCTTCGAAAGGCTATCCCCTGATAAA
GGCAGGTTGCACACGTGTTCCGCACCCGTACGCCGCTCTCAATTACCCGAAAGTAATCTACCGCTCGGCTTGC
ATGTGTTAGGCCTCCCGCTAGCGTTCTCCTGAGCAGGATCAACTCGT
>1725
CTGCTGCCGCCCGTAGGAGTCTGGACCGTGTCTCAGTTCCAGTGTGACTGATCATCCTCTCAGACCAGTACGG
ATCGTCGCCTTGGTAGGCCTTTACCCCACCAACTAGCTAATCCGACCTAGGCTCATCTGATAGCGTGAGGTCC
GAAGTCCCCCACTTCTCCCTCAGGACGTATGCGGTATTAGCGCCCGTTTCCGGACGTATCCCCACTACCAGGC
AGATTCCTAGGCATTACTCACCCGTCCGCCGCTGAATCCAGGAGCAAGCTCCCTCATCCGCTCGACTGCATGT
GTTAGGCCTGCCGCCAGCGTCAATCTGAGCCAGGATCAACTCT
>1726
CTGCTGCCGCCCGTAGGAGTCTGGACCGTGTCTCAGTTCCAGTGTGACTGATCATCCTCTCAGACCAGTTACG
GATCGTCGCCTTGGTAGGCCTTTACCCCACCAACTAGCTAATCCGACCTAGGCTCATCTGATAGCGTGAGGTC
CGGAGTCCCCCACTTCTCCCTCAGGACGTATGCGGTATTAGCGCCCGTTTCCGGACGTATCCCCACTACCAGG
CCAGATTCCTAGGCATTACTCACCCGTCCGCCGCTGAATCCAGGAGCAAGCCCCCTCATCCGCTCGACTGCAT
GTGTTAGGCCTGCCGCCAGCGTCAATCTGAGCCAGGATCAAACTCT
>1727
CTGCTGCCGCCCGTAGGAGTCTGGTCCGTGTCTCAGTACCAGTGTGGGGGATCACCCTCTCAGGCCCCCTAAA
GATCGTCGCCTTGGTGAGCCGTTACCTCACCAACTAGCTAATCTTGCGCGTGCCCATCTCTATCCACCGTAGT
TTTCAATATCACCCGATGCCGAATAATATATTATGGGGTATTAATCTTCTTCGAAAGGCTATCCCCTGATAAA
GGCAGGTTGCACACGTGTTCCGCACCCGTACGCCGCTCTCAATTACCCGAAAGTAATCTACCGCTCCGGCTTG
CATGTGTTAGGCCTCCCGCTAGCGTTCTCCGTCGAGCAGGCATCAATCAT
>1728

CTGCTGCTGCCCGTAGGAGTCTGGTCCGTGTCTCAGTACCAGTGTGGGGGATCACCCTCTCAGGCCCCCTAAA
GATCGTCGCCTTGGTGAGCCGTTACCTCACCAACTAGCTAATCTTGCGCGTGCCCATCTCTATCCACCGTAGT
TTTCAATATCACCCGATGCCGAATAATATATTATGGGGTATTAATCTTCTCGAAAGCTATCCCCTGATAAAGG
CAGGTTGCACACGTGTTCCGCACCCGTACGCCGCTCTCAATTACCCGAAAGTAATCTACCGCTCGGCTTGCAT
GTGTTAGGCCTCCCGCTAGCGTTCATCCTCGAGCAGGCATCAACTCT
>1729
CTGCTGCCGCCCCGTAGGAGTCTGGTCCGTGTCTCAGTACCAGTGTGGGGGATCACCCTCTCAGGCCCCCCTA
AAGATCGTCGCCTTGGTGAGCCGTTACCTCACCAACTAGCTAATCTTGCGCGTGCCCATCTCTATCCACCGTA
GTTTTTCAATATCACCCGATGCCGAATAATATAATTATTGGGGTATTTAATCTTTCTCGAAAGGCTATCCCCT
GATAAAGGCAGGTTGCACACGTGTTCCGCACCCGTACGCCGCTCTCAATTACCCGAAAGTAATCTACCGCTCC
GGCTTGCATGTGTTAGGCCTCCCGCTAGCGTTCTCCGTCGAGCAGGCA
>1730
CTGCTGCCGCCCGTAGGAGTCTGGACCGTGTCTCAGTTCCAGTGTGACTGATCATCCTCTCAGACCAGTACGG
ATCGTCGCCTTGGTAGGCCTTTACCCCACCAACTAGCTAATCCGAACCTAGGCTCATCTGATAGCGTGAGGTC
CGAAGATCCCCCACTTCTCCCTCAGGACGTATGCGGTATTAGCGCCCGTTTCCGGACGTATCCCCACTACCAG
GCCAGATTCCTAGGCATTACTCACCCGTCCGCCGCTGAATCCAGGACGCAAGCTCCCTCATCGCTCGATCGCA
CTGGTCTTA
>1731
CTGCTGCCGCCCGTAGGAGTCTGGCTCCGTGATCTCAGTACCAGTGTGGGGGACTCACCCTCTCAGGCCCCCT
AAAGATCGTCGCCTTGGTGAGGCCGTTACCTCACCAACTAGCTATCTTGCGCGTGCCCATCTCTATCCACCGT
AGTTTTCAATATCACCCGATGCCGAATAATATATTATGGGGTATTAATCTTCTCGAAGCTATCCCCTGATAAA
GGCAGGTTGCACACGTGTTCCGCACCCGTACGCCGCTCTCAATTACCCGAAAGTAATCTACCGCTCCGGCTTG
CATGTGTTAGGCCTCCCGCTAGCGTTCATCCGTCGAGCCAGGCATCAATCTCT
>1732
CTGCTGCCGCCCCGTAGGAGTCTGGTCCCGTGTCTCAGTACCAGTGTGGGGGATCACCCTCTCAGGCCCCCCT
AAAGATCGTCGCCTTGGTGAGCCCGTTACCTCACCCAACTAGCTAATCTTGCGCGTGCCCCATCTCTATCCCA
CCGTAGTTTTCAATATCACCCGATGCCCGAATAATATATTATGGGGTATTAATCTTCTTCGAAAGGCTATCCC
CCTGATTAAAGGCAGGTTGCACACGTGTTCCCGCACCCGTACGCCGCTCTCAATTACCCCGAAAGTAATCTAC
CGCTCGGCTTGCATGTGTTAGGCCTCCCGCTAGCGTTCTTCCTGAGCAGGATTCAA
>1733
CTGCTGCCGCCCGTAGGAGTCTGGTCCCGTGTCTCAGTACCAGTGTGGGGGATCACCCCTCTCAGGCCCCCCC
TAAAGATCGTCGCCTTGGTGAGCCCGTTACCCTCACCCAACTAGCTAATCTTGCGCGTGCCCCATCTCTATCC
ACCGTAGTTTTCAATATCACCCGATGCCCGAATAATATATTATTGGGGTATTTAATTCTTCTTCGAAAGGCTT
ATCCCCCTGATTAAAGGCAGGTTGCACACGTGTTCCCGCACCCGTACGCCGCTCTCAATTACCCCGAAAGTAA
TCTACCGCTCGGCTTGCATGTGTTAGGCCCTTCCCGCTAGCGTTCTCCTGAGCA
>1734
CTGCTGCCGCCCGTAGGAGTCTGGTCCGTGTCTCAGTACCAGTGTGGGGGATCACCCTCTCAGGCCCCCTAAA
GATCGTCGCCTTGGTGAGCCGTTACCTCACCAACTAGCTAATCTTGCGCGTGCCCATCTCTATCCACCGTAGT
TTTCAATATCACCCGATGCCGAATAATATATTATGGGGTATTTAATCTTCTTCGAAAGGCTATCCCCCTGATA
AAGGCAGGTTGCACACGTGTTCCGCACCCGTACGCCGCTCTCAATTACCCGAAAGTAATCTACCGCTCGGCTT
GCATGTGTTAGGCCTCCCGCTAGCGTTCATCCTCGAGCCAGGATCAAACTCT
>1735
CTGCTGCCGCCCGTAGGAGTCTGGTCCGTGTCTCAGTACCAGTGTGGGGGGATCACCCTCTCAGGCCCCCTAA
AGATCGTCGCCTTGGTGAGCCGTTACCTCACCCAACTAGCTAATCTTGCGCGTGCCCATCTCTATCCACCGTA
GTTTTTCAATATCACCCGATGCCGAATAATATATTTATGGGGTATTTAATCTTCTTCGAAAGGCTATCCCCCT
GATTAAAGGCAGGTTGCACACGTGTTCCGCACCCGTACGCCGCTCTCAATTACCCCGAAAGTAATCTACCGCT
CGGCTTGCATGTGTTAGGCCTCCCGCTAGCGTTCATTCCTTGAG
>1736
CTGCTGCCGCCCGTAGGAGTCTGGTCCGTGTCTCAGTACCAGTGTGGGGGATCACCCTCTCAGGCCCCCTAAA
GATCGTCGCCTTGGTGAGCCGTTACCTCACCAACTAGCTAATCTTGCGCGTGCCCATCTCTATCCACCGTAGT
TTTCAATATCACCCGATGCCGAATAATATATTATGGGGTATTAATCTTCTTCGAAAGGCTATCCCCCTGATAA
AGGCAGGTTGCACACGTGTTCCGCACCCGTACGCCGCTCTCAATTACCCGAGAGTAATCTACCGCTCGGCTTG
CATGTGTTAGGCCTCCCGCTAGCGTTCATCCTCGAGCCAGGATCAACTCT
>1737

CTGCTGCCGCCCGTAGGAGTCTGGTCCGTGTCTCAGTACCAGTGTGGGGGATCACCCTCTCAGGCCCCCTAAA
GATCGTCGCCTTGGTGAGCCGTTACCTCACCAACTAGCTAATCTTGCGCGTGCCCATCTCTATCCACCGTAGT
TTTCAATATCACCCGATGCCGAATAATATATTATGGGGTATTTAATCTTCTTCGAAAGGCTATCCCCCTGATA
AAGGCAGGTTGCACACGTGTTCCGCACCCGTACGCCGCTCTCAATTACCCGAAAGTAATCTACCGCTCGGCTT
GCATGTGTTAGGCCTCCCGCTAGCGTTCATTCCTGAGCAGGAT
>1738
CTGCTGCCGCCCGTAGGAGTCTGGTCCGTGTCTCAGTACCAGTGTGGGGGATCACCCTCTCAGGCCCCCTAAA
GATCGTCGCCTTGGTGAGCCGTTACCTCACCAACTGACTAATCTTGCGCGTGCCCATCTCTATCCACCGTAGT
TTTCAATATCACCCGATGCCGAATAATATATTATGGGGTATTAATCTTCTTCGAAAGGCTATCCCCCTGATAA
AGGCAGGTTGCACACGTGTTCCGCACCCGTACGCCGCTCTCAATTACCCGAAAGTAATCTACCGCTCGGCTTG
CATGTGTTAGGCCTCCCGCTAGCGTTCATCCTGAGCCAGGATCAACTCT
>1739
CTGCTGCCGCCCGTAGGAGTCTGGTCCGTGTCTCAGTACCAGTGTGGGGGATCACCCTCTCAGGCCCCCTAAA
GATCGTCGCCTTGGTGAGCCGTTACCTCACCAACTAGCTAATCTTGCGCGTGCCCATCTCTATCCACCGTAGT
TTTCAATATCACCCGATGCCGAATAATATATTATGGGGTATTTAATTCTTCTTCGAAAGGCTATCCCCCTGAT
AAAGGCAGGTTGCACACGTGTTCCGCACCCGTACGCCGCTCTCAATTACCCGAAAGTAATCTACCGCTCGGCT
TGCATGTGTTAGGCCTCCCGCTAGCGTTCATCCTGAGCCAGGATCAACTCT
>1740
CTGCTGCCGCCCGTAGGAGTCTGGTCCGTGTCTCAGTACCAGTGTGGGGGATCACCCTCTCAGGCCCCCTAAA
GATCGTCGCCTTGGTGAGCCGTTACCTCACCAACTAGCTAATCTTGCGCGTGCCCATCTCTATCCACCGTAGT
TTTCAATATCACCCGATGCCGAATAATATATTATGGGGTATTAATCTTCCTTCGAAAGGCTATCCCCCTGATA
AAGGCAGGTTGCACACGTGTTCCGCACCCGTACGCCGCTCTCAATTACCCGAAAGTAATCTACCGCTCGGCTC
GCATGTGTTAGGCCTCCCGCTAGCGTTCATCCTGAGCCAGGATCAAACTCT
>1741
CTGCTGCCGCCCGTAGGAGTCTGGTCCGTGTCTCAGTACCAGTGTGGGGGATCACCCTCTCAGGCCCCCTAAA
GATCGTCGCCTTGGTGAGCCGTTACCTCACCAACTAGCTAATCTTGCGCGTGCCCATCTCTATCCACCGTAGT
TTTCAATATCACCCGATGCCGAATAATATATTATGGGGTATTAATCTTCTTCGAAAGGCTATCCCCCTGATAA
AGGCAGGTTGCACACGTGTTCCGCACCCGTACGCCGCTCTCAATTACCCGAAAGTAATCTACCGCTCGGCTTG
CATGTGTTAGGCCTCCCGCTAGCGTCATCCTGAGCAGGATCAACTCCT
>1742
CTGCTGCCGCCCGTAGGAGTCTGGTCCGTGTCTCAGTACCAGTGTGGGGGATCACCCTCTCAGGCCCCCTAAA
GATCGTCGCCTTGGTGAGCCGTTACCTCACCAACTAGCTAATCTTGCGCGTGCCCATCTCTATCCACCGTAGT
TTTCAATATCACCCGATGCCGAATAATATATTATGGGGTATTAATCTTCTTCGAAAGGCTATCCCCCTGATAA
AGGCAGGTTGCACACGTGTTCCGCACCCGTACGCCGCTCTCAATTACCCGAAAGTAATCTACCGCTCGGCTTG
CATGTGTTAGGCCTCCCGCTAGCGTTCATCCTGAGCCAGGATCAACTCT
>1743
CTGCTGCCGCCCGTAGGAGTCTGGTCCGTGTCTCAGTACCAGTGTGGGGGGATCACCCTCTCAGGCCCCCTAA
AGATCGTCGCCTTGGTGAGCCGTTACCTCACCAACTAGCTAATCTTGCGCGTGCCCATCTCTATCCACCGTAG
TTTTCAATATCACCCGATGCCGAATAATATATTTATGGGGTATTTAATCTTTCTTCGAAAGGCTATCCCCCTG
ATAAAGGCAGGTGCACACGTGTTCCGCACCCGTACGCCGCTCTCAATTACCCGAAAGTAATCTACCGCATCGG
CTTGCATGTGTTAGGCCTCCCGCTAGCGTTCATTCCTGAGCCAG
>1744
CTGCTGCCGCCCGTAGGAGTCTGGTCCGTGTCTCAGTACCAGTGTGGGGGATCACCCTCTCAGGCCCCCTAAA
GATCGTCGCCTTGGTGAGCCGTTACCTCACCAACTAGCTAATCTTGCGCGTGCCCATCTCTATCCACCGTAGT
TTTCAATATCACCCGATGCCGAATAATATATTATGGGGTATTAATCTTCTTCGAAAGGCTATCCCCCTGATAA
AGGCAGGTTGCACACGTGTTCCGCACCCGTACGCCGCTCTCAATTACCCGAAAGTAATCTACCGCTCGGCTTG
CATGTGTTAGGCCTCCCGCTAGCGTTCATCCTGAGCCAGGATCAAACTCT
>1745
CTGCTGCCGCCCGTAGGAGTCTGGTCCGTGTCTCAGTACCAGTGTGGGGGATCACCCTCTCAGGCCCCCTAAA
GATCGTCGCCTTGGTGAGCCGTTACCTCACCAACTAGCTAATCTTGCGCGTGCCCATCTCTATCCACCGTAGT
TTTCAATATCACCCGATGCCGAATAATATATTATGGGGTATTAATCTTCTTCGAAAGGCTATCCCCCTGATAA
AGGCAGGTTGCACACGTGTTCCGCACCCGTACGCCGCTCTCAATTACCCGAAAGTAATCTACCGCTCCGGCTT
GCATGTGTTAGGCCTCCCGCTAGCGTTCTCCTGAGCAGGTCAAACTC
>1746

CTGCTGCCGCCCGTAGGAGTCTGGTCCGTGTCTCAGTACCAGTGTGGGGGATCACCCTCTCAGGCCCCCTAAA
GATCGTCGCCTTGGTGAGCCGTTACCTCACCAACTAGCTAATCTTGCGCGTGCCCATCTCTATTCCACCGTAG
TTTCAATATCACCCGATGCCGAATAATATATTATGGGGTATTAATCTTCTTCGAAAGGCTATCCCCCTGATAA
AGGCAGGTTGCCACACGTGTTCCGCACCCGTACGCCGCTCTCAATTACCCGAAAGTAATCTACCGCTCGGCTT
GCATGTGTTAGGCCTCCCGCTAGCGTTCATCCTGAGCCAGGATCAACTCT
>1747
CTGCTGCCGCCCGTAGGAGTCTGGTCCGTGTCTCAGTACCAGTGTGGGGGATCACCCTCTCAGGCCCCCTAAA
GATCGTCGCCTTGGTGAGCCGTTACCTCACCAACTAGCCTAATCTTGCGCGTGCCCATCTCTATCCACCGTAG
TTTTCAATATCACCCGATGCCGAATAATATATTATGGGGTATTAATCTTCTTCGAAGGGCTATCCCCTGATAA
GGCAGGTTGCACACGTGTTCCGCACCTGTACGCCGCTCTCAATTACCCGAAAGTAATCTACCGCTCGGCTTGC
ATGTGTTAGGCCTCCCGCTAGCGTCATCCTGAGCCAGGATCAAACTCT
>1748
CTGCTGCCGCCCGTAGGAGTCTGGACCGTGTCTCAGTTCCAGTGTGACTGATCATCCTCTCAGACCAGTACGG
ATCGTCGCCTTGGTAGGCCTTTACCCCACCAACTAGCTAATCCGACCTAGGCTCATCTGATAGCGTGAGGTCC
GAAGATCCCCCACTTCTCCCTCAGGACGTATGCGGTATTAGCGCCCGTTTCCGGACGTATCCCCACTACCAGG
CAGATTCCTAGGCATTACTCACCCGTCCGCCGCTGAATCCAGGAGCAAGCTCCCTCATCCGCTCGACTGCCAT
GTGTTAGGCCTGCCGCCAGCGTTCAATCTGAGCCAAGGATCAACTCT
>1749
CTGCTGCCGCCCGTAGGAGTCTGGTCCGTGTCTCAGTACCAGTGTGGGGGATCACCCTCTCAGGCCCCCTAAA
GATCGTCGCCTTGGTGAGCCGTTACCTCACCAACTAGCTAATCTTGCGCGTGCCCATCTCTATCCACCGTAGT
TTTCAATATCACCCGATGCCGAATAATATATTATGGGGTATTAATCTTCTTCGAAAGGCTATCCCCTGATAAA
GGCAGGTTGCACACGTGTTCCGCACCCGTACGCCGCTCTCAATTACCCGAAAGTAATCTACCGCTCGGCTTGC
ATGTGTTAGGCCTCCCGCTAGCGTTCATCCTGAGCCAGGATCAACTCT
>1750
CTGCTGCCGCCCGTAGGAGTCTGGTCCGTGTCTCAGTACCAGTGTGGGGGATCACCCTCTCAGGCCCCCTAAA
GATCGTCGCCTTGGTGAGCCGTTACCTCACCAACTAGCTAATCTTGCGCGTGCCCATCTCTATCCACCGTAGT
TTTCAATATCACCCGATGCCGAATAATATATTATGGGGTATTAATCTTCTCGAAGCTATCCCCCTGATAAAGG
CAGGTTGCACACGTGTTCCGCACCCGTACGCCGCTCTCAATTACCCGAAAGTAATCACCGCTCCGGCTTGCAT
GTCGTTAGGCCTCCCGCTAGCGTTCATCCCGTAGCAGGCATCAAACTCCT
>1751
CTGCTGCCGCCCGTAGGAGTCTGGACCGTGTCTCAGTTCCAGTGTGACTGATCATCCTCTCAGACCAGTACGG
ATCGTCGCCTTGGTAGGCCTTTACCCCACCAACTAGCTAATCCGACCTAGGCTCATCTGATAGCGTGAGGTCC
GAAGATCCCCCACTTCTCCCTCAGGACGTATGCGGTATTAGCGCCCGTTTCCGGACGTATCCCCACTACCAGG
CAGATTCCTAGGCATTACTCACCCGTCCGCCGCTGAATCCAGGAGCAAGCTCCCTCATCCGCTCGACTGCATG
TGTTAGGCCTGCCGCCAGCGTCAATCTGAGCCAGGATCAACTCT
>1752
CTGCTGCCGCCCGTAGGAGTCTGGACCGTGTCTCAGTTCCAGTGTGACTGATCATCCTCTCAGACCAGTACGG
ATCGTCGCCTTGGTAGGCCTTTACCCCACCAACTAGCTAATCCGACCTAGGCTCATCTGATAGCGTGAGGTCC
GAAGATCCCCCACTTCTCCCTCAGGACGTATGCGGTATTAGCGCCCGTTTCCGGACGTATCCCCGCACTACCC
AGGCAGATTCCTAGGCATTACTCACCCGTCCGCCGCTGAATCCAGGAGCAAGCTCCCTCATCCGCTCGACTGC
CATGTGTTAGGCCTGCCGCCAGCGTTCAATCTGAGCCAGGATCAAACTCT
>1753
CTGCTGCCGCCCGTAGGAGTCTGGTCCGTGTCTCAGTACCAGTGTGGGGGATCACCCTCTCAGGCCCCCTAAA
GATCGTCGCCTTGGTGAGCCGTTACCTCACCAACTAGCTAATCTTGCGCGTGCCCATCTCTATCCACCGTAGT
TTTCAATATCACCCGATGCCGAATAATATATTATGGGGTATTAATCTTCTTCGAAGCTATCCCCTGATAAAGG
CAGGTTGCACACGTGTTCCGCACCCGTACGCCGCTCTCAATTACCCGAAAGTAATCTACCGCTCCGGCTTGCA
TGTGTTAGGCCTCCCGCTAGCGTTCATCCGTCGAGCCAGGCATCAATCT
>1754
CTGCTGCCGCCCGTAGGAGTCTGGACCGTGTCTCAGTTCCAGTGTGACTGATCATCCTCTCAGACCAGTTACG
GATCGTCGCCTTGGTAGGCCTTTACCCCACCAACTAGCTAATCCGACCTAGGCTCATCTGATAGCGTGAGGTC
CGAAGATCCCCCACTTCTCCCTCAGGACGTATGCGGTATTAGCGCCCGTTTCCGGACGTATCCCCGCACTACC
CAGGCAGATTCCTAGGCATTACTCACCCGTCCGCCGCTGAATCCAGGAGCAAGCTCCCTCATCCGCTCGACTG
CCATGTGTTAGGCCTGCCGCCAGCGTCAATCTGAGCCAGGATCAAACTCT
>1755

CTGCTGCCGCCCGTAGGAGTCTGGTCCGTGTCTCAGTACCAGTGTGGGGGATCACCCTCTCAGGCCCCCTAAA
GATCGTCGCCTTGGTGAGCCGTTACCTCACCAACTAGCTAATCTTGCGCGTGCCCATCTCTATCCACCGTAGT
TTTCAATATCACCCGATGCCGAATAATATATTATGGGGTATTAATCTTCTTCGAAAGGCTATCCCCTGATAAA
GGCAGGTTGCACACGTGTTCCGCACCCGTACGCCGCTCTCAATTACCCGAAAGTAATCTACCGCTCGGCTTGC
ATGTGTTAGGCCTCCCGCTAGCGTTCATCCTGAGCCAGGATCAAACT
>1756
CTGCTGCCGCCCGTAGGAGTCTGGTCCGTGTCTCAGTACCAGTGTGGGGGATCACCCTCTCAGGCCCCCTAAA
GATCGTCGCCTTGGTGAGCCGTTACCTCACCAACTAGCTAATCTTGCGCGTGCCCATCTCTATCCACCGTAGT
TTTCAATATCACCCGATGCCGAATAATATATTTATGGGGTATTTAATTCTTTCTTCGAAAGGCTATCCCCCTG
ATAAAGGCAGGTTGCACACGTGTTCCGCACCCGTACGCCGCTCTCAATTACCCGAAAGTAATCTACCGCTCGG
CTTGCATGTGTTAGGCCTCCCGCTAGCGTTCATCCTGAGCCAGGATCAACTCT
>1757
CTGCTGCCGCCCGTAGGAGTCTGGTCCGTGTCTCAGTACCAGTGTGGGGGATCACCCTCTCAGGCCCCCTAAA
GATCGTCGCCTTGGTGAGCCGTTACCTCACCAACTAGCTAATCTTGCGCGTGCCCATCTCTATCCACCGTAGT
TTTCAATATCACCCGATGCCGAATAATATATTATGGGGTATTAATCTTCTTCGAAAGGCTATCCCCCTGATAA
AGGCAGGTTGCACACGTGTTCCGCACCCGTACGCCGCTCTCAATTACCCGAAAGTAATCTACCGCTCGGCTTG
CATGTGTTAGGCCTCCCGCTAGCGTTCATCCTGAGCCAGGATCAACTCT
>1758
CTGCTGCCGCCCGTAGGAGTCTGGACCGTGTCTCAGTTCCAGTGTGACTGATCATCCTCTCAGACCAGTACGG
ATCGTCGCCTTGGTAGGCCTTTACCCCACCAACTAGCTAATCCGACCTAGGCTCATCTGATAGCGTGAGGTCC
GAAAGATCCCCCACTTCTCCCTCAGGACGTATGCGGTATTAGCGCCCGTTTCCGGACGATCCCCACTACCAGG
CAGATTCCTAGGCATTACTCACCCGTCCGCCGCTGAATCCAGGAGCAAGCTCCCTTCATCCGCTCGACTGCAT
GTGTTAGGCCTGCCGCCAGCGTCAATCTGAGCCAGGATCAACTCT
>1759
CTGCTGCCGCCCGTAGGAGTCTGGTCCGTGTCTCAGTACCAGTGTGGGGGATCACCCTCTCAGGCCCCCTAAA
GATCGTCGCCTTGGTGAGCCGTTACCTCACCAACTAGCTAATCTTGCGCGTGCCCATCTCTATCCACCGTAGT
TTTCAATATCACCCGATGCCGAATAATATATTATGGGGTATTAATCTTCTTCGAAAGGCTATCCCCCTGATAA
AGGCAGGTTGCACACGTGTTCCGCACCCGTACGCCGCTCTCAATTACCCGAAAGTAATCTACCGCTCGGCTTG
CATGTGTTAGGCCTCCCGCTAGCGTTCATCCTGAGCCAGGATCAAACTCT
>1760
CTGCTGCCGCCCGTAGGAGTCTGGTCCGTGTCTCAGTACCAGTGTGGGGGATCACCCTCTCAGGCCCCCTAAA
GATCGTCGCCTTGGTGAGCCGTTACCTCACCAACTAGCTAATCTTGCGCGTGCCCATCTCTATCCACCGTAGT
TTTCAATATCACCCGATGCCGAATAATATATTATGGGGGTATTAATCTTCTTCGAAAGGCTATCCCCCTGATA
AAGGCAGGTTGCACACGTGTTCCGCACCCGTACGCCGCTCTCAATTACCCGAAAGTAATCTACCGCTCGGCTT
GCATGTGTTAGGCCTCCCGCTGGCGTCATCCTGAGCCAGGATCAACTCT
>1761
CTGCTGCCGCCCGTAGGAGTCTGGTCCGTGTCTCAGTACCAGTGTGGGGGATCACCCTCTCAGGCCCCCTAAA
GATCGTCGCCTTGGTGAGCCGTTACCTCACCAACTAGCTAATCTTGCGCGTGCCCATCTCTATCCACCGTAGT
TTTCAATATCACCCGATGCCGAATAATATATTATGGGGTATTAATCTTCTCGAAAGGCTATCCCCTGATAAAG
GCAGGTTGCACACGTGTTCCGCACCCGTACGCCGCTCTCAATTACCCGAAAGTAATCTACCGCTCCGGCTTGC
ATGTGTTAGGCCTCCCGCTAGCGTTCATCCTGAGCAGGATCAACTCT
>1762
CTGCTGCCGCCCGTAGGAGTCTGGTCCGTGTCTCAGTACCAGTGTGGGGGATCACCCTCTCAGGCCCCCTAAA
GATCGTCGCCTTGGTGAGCCGTTACCTCACCAACTAGCTAATCTTGCGCGTGCCCATCTCTATTCCACCGTAG
TTTCAATATCACCCGATGCCGAATAATATATTATGGGGTATTAATCTTCTTCGAAAGGCTATCCCCCTGATAA
AGGCAGGTTGCACACGTGTTCCGCACCCGTACGCCGCTCTCAATTACCCGAAAGTAATCTACCGCTCCGGCTT
GCATGTGTTAGGCCTCCCGCTAGCGTTCATCCTGAGCCAGGATCAACTCT
>1763
CTGCTGCCGCCCGTAGGAGTCTGGTCCGTGTCTCAGTACCAGTGTGGGGGATCACCCTCTCAGGCCCCCTAAA
GATCGTCGCCTTGGTGAGTCGTTACCTCACCAACTAGCTAATCTTGCGCGTGCCCATCTCTATCCACCGTAGT
TTTCAATATCACCCGATGCCGAATAATATATTATGGGGTATTAATCTTCTTCGAAAGGCTATCCCCTGATAAA
GGCAGGTTGCACACGTGTTCCGCACCCGTACGCCGCTCTCAATTACCCGAAAGTAATCTACCGCTCGGCTTGC
ATGTGTTAGGCCTCCCGCTAGCGTTCATCCTGAGCCAGGATCAAACTCT
>1764

CTGCTGCCGCCCGTAGGAGTCTGGTCCGTGTCTCAGTACCAGTGTGGGGGATCACCCTCTCAGGCCCCCTAAA
GATCGTCGCCTTGGTGAGCCGTTACCTCACCAACTAGCTAATCTTGCGCGTGCCCATCTCTATCCACCGTAGT
TTTCAATATCACCCGATGCCGAATAATATATTATGGGGTATTAATCTTCTTCGAAAGGCTATCCCCTGATAAA
GGCAGGTTGCATACGTGTTCCGCACCCGTACGCCGCTCTCAATTACCCGAAAGTAATCTACCGCTCGGCTTGC
ATGTGTTAGGCCTCCCGCTAGCGTTCATCCTGAGCAGGATCAACTCT
>1765
CTGCTGCCGCCCGTAGGAGTCTGGTCCGTGTCTCAGTACCAGTGTGGGGGATCACCCTCTCAGGCCCCCTAAA
GATCGTCGCCTTGGTGAGCCGTTACCTCACCAACTAGCTAATCTTGCGCGTGCCCATCTCTATCCACCGTAGT
TTTCAATATCACCCGATGCCGAATAATATATTATGGGGTATTAATCTTCTTCGAAAGGCTATCCCCCTGATAA
AGGCAGGTTGCACACGTGTTCCGCACCCGTACGCCGCTCTCAATTACCCGAAAGTAATCTACCGCTCGGCTTG
CATGTGTTAGGCCTCCCGCTAGCGTTCATCCTGAGCCAGGATCAAACTCT
>1766
CTGCTGCCGCCCGTAGGAGTCTGGTCCGTGTCTCAGTACCAGTGTGGGGGATCACCCTCTCAGGCCCCCTAAA
GATCGTCGCCTTGGTGAGCCGTTACCCTCACCAACTAGCTAATCTTGCGCGTGCCCATCTCTATCCACCGTAG
TTTTCAATATCACCCGATGCCGAATAATATATTATGGGGTATTAATCTTCTTCGAAAGGCTATCCCCCTGATA
AAGGCAGGTTGCACACGTGTTCCGCACCCGTACGCCGCTCTCAATTACCCGAAAGTAATCTACCGCTCGGCTT
GCATGTGTTAGGCCTCCCGCTAGCGTTCATCCTGAGCCAGGATCAAACTCT
>1767
CTGCTGCCGCCCGTAGGAGTCTGGTCCGTGTCTCAGTACCAGTGTGGGGGATCACCCTCTCAGGCCCCCTAAA
GATCGTCGCCTTGGTGAGCCGTTACCTCACCAACTAGCTAATCTTGCGCGTGCCCATCTCTATCCACCGTAGT
TTTCAATATCACCCGATGCCGAATAATATATTATGGGGTATTTAATCTTTCTTCGAAAGGCTATCCCCCTGAT
AAAGGCAGGTTGCACACGTGTTCCGCACCCGTACGCCGCTCTCAATTACCCGAAAGTAATCTACCGCTCGGCT
TGCATGTGTTAGGCCTCCCGCTAGCGTTCATCCTGAGCCAGGATCAAACTCT
>1768
CTGCTGCCGCCCGTAGGAGTCTGGTCCGTGTCTCAGTACCAGTGTGGGGGATCACCCTCTCAGGCCCCCTAAA
GATCGTCGCCTTGGTGAGCCGTTACCTCACCAACTAGCTAATCTTGCGCGTGCCCATCTCTATCCACCGTAGT
TTTCAATATCACCCGATGCCGAATAATATATTATGGGGTATTAATCTTCTTCGAAAGGCTATCCCCTGATAAA
GGCAGGTTGCACACGTGTTCCGCACCCGTACGCCGCTCTCAATTACCCGAAAGTAATCTACCGCTCGGCTTGC
ATGTGTTAGGCCTCCCGCTAGCGTTCATCCTGAGCAGGATCAAACTCT
>1769
CTGCTGCCGCCCGTAGGAGTCTGGTCCGTGTCTCAGTACCAGTGTGGGGGATCACCCTCTCAGGCCCCCTAAA
GATCGTCGCCTTGGTGAGCCGTTACCTCACCAACTAGCTAATCTTGCGCGTGCCCATCTCTATCCACCGTAGT
TTTCAATATCACCCGATGCCGAATAATATATTATGGGGTATTAATCTTCTTCGAAAGCTATCCCCCTGATAAA
GGCAGGTTGCACACGTGTTCCGCACCCGTACGCCGCTCTCAATTACCCGAAAGTAATCTACCGCTCCGGCTTG
CATGTGTTAGGCCTCCCGCTAGCGTCTCCGTCGAGCAGGCATCAATCATGCCT
>1770
CTGCTGCGCCCGTAGGAGTCTGGTCCGTGTCTCAGTACCAGTGTGGGGGATCACCCTCTCAGGCCCCCTAAAG
ATCGTCGCCTTGGTGAGCCGTTACCTCACCAACTAGCTAATCTTGCGCGTGCCCATCTCTATCCACCGTAGTT
TTCAATATCACCCGATGCCGAATAATATATTATGGGGTATTAATCTTCTTCGAAAGGCTATCCCCTGATAAAG
GCAGGTTGCACACGTGTTCCGCACCCGTACGCCGCTCTCAATTACCCGAAAGTAATCTACCGCTCCGGCTTGC
ATGTGTTAGGCCTCCCGCTAGCGTTCTCCTCGAGCAGGATC
>1771
CTGCTGCCGCCCGTAGGAGTCTGGTCCGTGTCTCAGTACCAGTGTGGGGGATCACCCTCTCAGGCCCCCTAAA
GATCGTCGCCTTGGTGAGCCGTTACCTCACCAACTAGCTAATCTTGCGCGTGCCCATCTCTATCCACCGTAGT
TTCAATATCACCCGATGCCGAATAATATATTATGGGGTATTAATCTTCTCGAAGCTATCCCCTGATAAAGGCA
GGTTGCCACACGTGTTCCGCACCCGTACCGCCGCTCTCAATTACCCGAAAGTAATCACCGCTCCGGCTTGCAT
GTGTTACGGCCTCCCGCTAGGCCTCGTTCTAGCTCGTAGCAA
>1772
CTGCTGCCGCCCGTAGGAGTCTGGTCCGTGTCTCAGTACCAGTGTGGGGGATCACCCTCTCAGGCCCCCTAAA
GATCGTCGCCTTGGTGAGCCGTTACCTCACCAACTAGCTAATCTTGCGCGTGCCCATCTCTATCCACCGTAGT
TTCAATATCACCCGATGCCGAATAATATATTATGGGGTATTAATCTTCTCTGAAAGCTATCCCCTGATAAAGG
CAGGTTGTCACACGTGTTCCGCACCCGTACGCCGCTCTCAATTACCCGAAAGTAATCTACCGCTCCGGCTTGC
ATGTGTTAGGCCTCCCGCTAGCGTTCATCCTCGAGC
>1773

CTGCTGCCGCCCGTAGGAGTCTGGACCGTGTCTCAGTTCCAGTGTGACTGATCATCCTCTCAGACCAGTACGG
ATCGTCGCCTTGGTAGGCCTTTACCCCACCAACTAGCTAATCCGACCTAGGCTCATCTGATAGCGTGAGGTCC
GAAGTCCCCCACTTCTCCCTCAGGACGTATGCGGTATTAGCGCCCGTTTCCGGACGTATCCCGCACTACCCAG
GCAGACTTCCAGGCATTACTCACCCGTCCGCCGCTGAATCCAGGAGCAAGCCCCTCCACTCCGCTCGACTGCA
TGTGTTAGGCCTGCCGCCAGCGTCAATCGTGACGCCCAAGGCATCAAATCATCT
>1774
CTGCTGCCGCCCGTAGGAGTCTGGACCGTGTCTCAGTTCCAGTGTGACTGATCATCCTCTCAGACCAGTACGG
ATCGTCGCCTTGGTAGGCCTTTGCCCCACCAACTAGCTAATCCCGACCTAGGCTCATCTGATAGCGTGAGGTC
CGAAGATCCCCACTTCTCCCTCAGGACGTATGCGGTATTAGCGCCCGTTTCCGGACGTATCCCGCACTACCAG
GCAGATTCCCTAGGCATTACTCACCCGTCCGCCGCTGAATCCAGGAGCAAGCCCCCTCATCGCCTCGACTGCA
TGTGTTAGGCCTGCCGCCAGCGTCAATCTGAGCCAGGATCAACTCT
>1775
CTGCTGCCCGCCCCGTAGGAGTCTGGTCCGTGTCTCAGTACCAGTGTGGGGGATCACCCTCTCAGGCCCCCCT
AAAGATCGTCGCCTGGTGAGCCCGTTTACCCTCACCCAACTAGCTTAATCTTGCGCGTGCCCCATCTCTATTC
CCACCCGTAGTTTTCAATATCACCCGATGCCCGAATAATATATTATGGGGTATTTAATTCTTCTTCGAAAGGC
TATCCCCTGATAAAGGCAGGTTGCACACGTGTTCCCGCACCCGTACGCCGCTCTCAATTACCCCGAAAGTAAT
CTACCGCTCGGCTTGCATGTGTTAGGCCCTCCCGCTAGCGTTCTTCCTGAGCAGGATCAACTCT
>1776
CTGCTGCCGCCCGTAGGAGTCTGGTCCGTGTCTCAGTACCAGTGTGGGGGATCACCCTCTCAGGCCCCCTAAA
GATCGTCGCCTTGGTGAGCCGTTACCTCACCAACTAGCTAATCTTGCGCGTGCCCATCTCTATCCACCGTAGT
TTCAATATCACCCGATGCCGAATAATATATTATTGGGGTATTTAATCTTCCTTCGAGAGGCTATCCCCCTGAT
AAAGGCAGGTTGCACACGTGTTCCGCACCCGTACGCCGCTCTCAATTACCCCGAAAGTAATCTACCGCTCGGC
TTGCATGTGTTAGGCCTCCCGCTAGCGTTCTCCTGAGCAGGATCAACTCT
>1777
CTGCTGCCGCCCGTAGGAGTCTGGTCCGTGTCTCAGTACCAGTGTGGGGGATCACCCTCTCAGGCCCCCTAAA
GATCGTCGCCTTGGTGAGCCGTTACCTCACCAACTAGCTAATCTTGCGCGTGCCCATCTCTATCCACCGTAGT
TTTCAATATCACCCGATGCCGAATAATATATTATGGGGTATTAATCTTCTTCGAAAGGCTATCCCCCTGAATA
AAGGCAGGTTGCACACGTGTTCCGCACCCGTACGCCGCTCTCAATTTACCCGAAAGTAATCTACCGCTCGGCT
GCATGTGTTAGGCCTCCCGCTAGCGTTCATCCTCGAGCC
>1778
CTGCTGCCGCCCGTAGGAGTCTGGTCCCGTGTCTCAGTACCAGTGTGGGGGATCACCCTCTCAGGCCCCCTAA
AGATCGTCGCCTTGGTGAGCCGTACCTCACCCAACTAGCTAATCTTGCGCGTGCCCCATCTCTATTCCACCGT
AGTTTCAATATCACCCGATGCCGAATAATATATTATTGGGGTATTTAATCTTCTTCGAAAGGCTATCCCCCTG
ATAAAGGCAGGTTGCACACGTGTTCCGCACCCGTACGCCGCTCTCAATTACCCCCGAAAGTAATCTACCGCTC
GGCTTGCATGTGTTAGGCCTCCCGCTAGCGTTCTTCCTGAGCAGGATCCAAAC
>1779
CTGCTGCCGCCCGTAGGAGTCTGGTCCGTGTCTCAGTACCAGTGTGGGGGATCACCCTCTCAGGCCCCCTAAA
GATCGTCGCCTTGGTGAGCCGTTACCTCACCAACTAGCTAATCTTGCGCGTGCCCATCTCTATCCACCGTAGT
TTCAATATCACCCGATGCCGAATAATATATTATGGGGTATTAATCTTCTTCGAAAGGCTATCCCCCTGATAAA
GGCAGGTTGCACACGTGTTCCGCACCCGTACGCCGCTCTCAATTACCCGAAAGTAATCTACCGCTCGGCTTGC
ATGTGTTAGGCCTCCCGCTAGCGTTCATCCTGAGCCAGGATCAAACTCT
>1780
CTGCTGCCGCCCGTAGGAGTCTGGTCCGTGTCTCAGTACCAGTGTGGGGGATCACCCTCTCAGGCCCCCTAAA
GATCGTCGCCTTGGTGAGCCGTTACCTCACCAACTAGCTTAATCTTGCGCGTGCCCATCTCTATCCACCGTAG
TTTTCAATATCACCCGATGCCGAATAATATATTTATTGGGGTATTTAATCTTCTTCGAAAGGCTATCCCCCTG
ATAAAGGCAGGTTGCACACGTGTTCCGCACCCGTACGCCGCTCTCAATTACCCGAAAGTAATCTACCGCTCGG
CTTGCATGCGTTAGGCCTCCCGCTAGCGTTCATCCTGAGCCAGGATCAAACTCT
>1781
CTGCTGCCGCCCGTAGGAGTCTGGTCCGTGTCTCAGTACCAGTGTGGGGGATCACCCTCTCAGGCCCCCTAAA
GATCGTCGCCTTGGTGAGCCGTTACCTCACCAACTAGCTAATCTTGCGCGTGCCCATCTCTATCCACCGTAGT
TTTCAATATCACCCGATGCCGAATAATATATTATGGGGTATTAATCTTCTTCGAAAGGCTATCCCCTGATAAA
GGCAGGTTGCACACGTGTTCCGCACCCGTACGCCGCTCTCAATTACCCGAAAGTAATCTACCGCTCGGCTTGC
ATGTGTTAGGCCTCCCGCTAGCGTTCATCCTGAGCAGGATCAACTCT
>1782

CTGCTGCCGCCCGTAGGAGTCTGGTCCGTGTCTCAGTACCAGTGTGGGGGATCACCCTCTCAGGCCCCCTAAA
GATCGTCGCCTTGGTGAGCCGTTACCTCACCAACTAGCTAATCTTGCGCGTGCCCATCTCTATCCACCGTAGT
TTTCAATATCACCCGATGCCGAATAATATATTATGGGGTATTAATCTTCTTCGAAAGGCTATCCCCCTGATAA
AGGCAGGTTGCACACGTGTTCCGCACCCGTACGCCGCTCTCAATTACCCGAAAGTAATCTACCGCTCGGCTTG
CATGTGTTAGGCCTCCCGCTAGCGTTCATCCTGAGCCAGGATCAAACTCT
>1783
CTGCTGCCGCCCGTAGGAGTCTGGTCCGTGTCTCAGTACCAGTGTGGGGGATCACCCTCTCAGGCCCCCTAAA
GATCGTCGCCTTGGTGAGCCGTTACCTCACCAACTAGCTAATCTTGCGCGTGCCCATCTCTATCCACCGTAGT
TTTCAATATCACCCGATGCCGAATAATATATTATGGGGTATTAATCTTCTTCGAAGGCTATCCCCTGATAAAG
GCAGGTTGCACACGTGTTCCGCACCCGTACGCCGCTCTCAATTACCCGAAAGTAATCTACCGCTCGGCTTGCA
TGTGTTAGGCCTCCCGCTAGCGTTCATCCTGAGCCAGGATCAACTCT
>1784
CTGCTGCCGCCCGTAGGAGTCTGGTCCGTGTCTCAGTACCAGTGTGGGGGATCACCCTCTCAGGCCCCCTAAA
GATCGTCGCCTTGGTGAGCCGTTACCTCACCAACTAGCTAATCTTGCGCGTGCCCATCTCTATCCACCGTAGT
TTTTCAATATCACCCGATGCCGAATAATATATTATGGGGTACTAATCTTTCTCGAAAGGCTATCCCCTGATAA
AGGCAGGTTGCCACACGCGTTCCGCACCCGTACGCGCTCTCAATTTACCCGAAACGTAATCTACTCGCTACGG
CTTGATCATG
>1785
CTGCTGCCGCCCGTAGGAGTCTGGTCCGTGTCTCAGTACCAGTGTGGGGGATCACCCTCTCAGGCCCCCTAAA
GATCGTCGCCTTGGTGAGCCGTTACCTCACCAACTAGCTAATCTTGCGCGTGCCCATCTCTATCCACCGTAGT
TTTCAATATCACCCGATGCCGAATAATATATTATGGGGTATTAATCTTCTTCGAAAGGCTATCCCCTGATAAA
GGCAGGTTGCACACGTGTTCCGCACCCGTACGCCGCTCTCAATTACCCGAAAGTAATCTACCGCTCGGCTTGC
ATGTGTTAGGCCTCCCGCTAGCGTTCATCCTCGA
>1786
CTGCTGCCGCCCGTAGGAGTCTGGACCGTGTCTCAGTTCCAGTGTGACTGATCATCCTCTCAGACCAGTTACG
GATCGTCGCCTTGGTAGGCCTTTTACCCCCCACCAACTAGCTAATCCGACCTAGGCTCATCTGATAGCGTGAG
GTCCGAAGATCCCCACTTCTCCCTCAGGACGTATGCGGTATTAGCGCCCGTTTCCGGACGTATCCCCACTACC
AGGCAGATTCCTAGGCATTACTCACCCGTCCGCCGCTGAATCCAGGAGCAAGCCCCCTCATCCGCTCGACTGC
ATGTGTTAGGCCTGCCGCCAGCGTCAATCTGAGCCAGGATCAAACTCT
>1787
CTGCTGCCGCCCGTAGGAGTCTGGTCCGTGTCTCAGTACCAGTGTGGGGGGATCACCCTCTCAGGCCCCCCTA
AAGATCGTCGCCTTGGTGAGCCGTTACCTCACCAACTAGCTTAATCTTGCGCGTGCCCATCTCTATTCCGCCG
TAGTTTTTTCAATATCACCCGATGCCGAATAATATATTTATGGGGTATTTAATTCTTTCTCGAAAGGCTATCC
CCCTGATAAAGGCAGGTTGCACACGTGTTCCGCACCCGTACGCCGCTCTCAATTACCCCCGAAAGTAATCTAC
CGCTCGGCTTGCATGTGTTAGGCCTCCCGCTAGCGTTCATTCCGTCG
>1788
CTGCTGCCGCCCGTAGGAGTCTGGTCCGTGTCTCAGTACCAGTGTGGGGGATCACCCTCTCAGGCCCCCTAAA
GATCGTCGCCTTGGTGAGCCGTTACCTCACCAACTAGCTAATCTTGCGCGTGCCCATCTCTATCCACCGTAGT
TTTCAATATCACCCGATGCCGAATAATATATTATGGGGTATTAATCTTCTTCGAAAGCTATCCCCTGATAAAG
GCAGGTTGCACACGTGTTCCGCACCCGTACGCCGCTCTCAATTACCCGAAAGTAATCTACCGCTCGGCTTGCA
TGTGTTAGGCCTCCCGCTAGCGTTCATCCGTCGA
>1789
CTGCTGCCGCCCGTAGGAGTCTGGTCCGTGTCTCAGTACCAGTGTGGGGGATCACCCTCTCAGGCCCCCTAAA
GATCGTCGCCTTGGTGAGCCGTTACCTCACCAACTAGCTAATCTTGCGCGTGCCCATCTCTATCCACCGTAGT
TTTTCAATATCACCCGATGCCGAATAATATATTATGGGGTATTTAATCTTTCTCGAAAGCTATCCCCTGATAA
AGGCAGGTTGCACACGTGTTCCGCACCCGTACGCCGCTCTCAATTACCCGAAAGTAATCTACCGCTCCGGCTT
AGCATGTGTTCGGCCTTCCC
>1790
CTGCTGCCGCCCGTAGGAGTCTGGTCCGTGTCTCAGTACCAGTGTGGGGGATCACCCTCTCAGGCCCCCTAAA
GATCGTCGCCTTGGTGAGCCGTTACCTCACCAACTAGCTAATCTTGCGCGTGCCCATCTCTATCCACCGTAGT
TTTCAATATCACCCGATGCCGAATAATATATTATGGGGTATTAATCTTCTCGAAAGGCTATCCCCTGATAAAG
GCAGGTTGCACACGTGTTCCGCACCCGTACGCCGCTCTCAATTACCCGAAAGTAATCTACCGCTCCGGCTTGC
ATGTGTTAGGCCTCCCGCTAGCGTTCATCCTGAGCAGGATCAACTCT
>1791

CTGCTGCCGCCCGTAGGAGTCTGGTCCGTGTCTCAGTACCAGTGTGGGGGATCACCCTCTCAGGCCCCCTAAA
GATCGTCGCCTTGGTGAGCCGTTACCTCACCAACTAGCTAATCTTGCGCGTGCCCATCTCTATCCACCGTAGT
TTTCAATATCACCCGATGCCGAATAATATATTATGGGGTATTAATCTTCTTCGAAAGCTATCCCCTGATAAAG
GCAGGTTGCACACGTGTTCCGCACCCGTACGCCGCTCTCAATTACCCGAAAGTAATCTACCGCTCCGGCTTGC
ATGTCGTTAGGCCTCC
>1792
CTGCTGCCGCCCGTAGGAGTCTGGTCCGTGTCTCAGTACCAGTGTGGGGGATCACCCTCTCAGGCCCCCTAAA
GATCGTCGCCTTGGTGAGCCGTTACCTCACCAACTAGCTAATCTTGCGCGTGCCCATCTCTATCCACCGTAGT
TTTCAATATCACCCGATGCCGAATAATATATTATGGGGTATTAATCTTCCTTTCGAAGGGCTATCCCCCTGAT
AAAGGCAGGTTGCACACGTGTTCCGCACCCGTACGCCGCTCTCAATTACCCGAAAGTAATCTACCGCTCGGCT
TGCATGTGTTAGGCCTCCCGCTAGCGTTCATCCTCGAGCC
>1793
CTGCTGCCGCCCTAGGAGTCTGGACCGTGTCTCAGTTCCAGTGTGACTGATCATCCTCTCAGACCAGTTACGG
ATCGTCGCCTTGGTAGGCCTTTACCCCATCAACTAGCTAATCCCGACCTAGGCTCATCTGATAGCGTGAGGTC
CGAAGTCCCCCACTTCTCCCTCAGGACGTATGCGGTATTAGCGCCCGTTTCCGGACGTATCCCGCACTACCAG
CAGATTCCAGGCATTACTCACCCGTCCGCCGCTGAATCCAGGAGCAAGCTCCCTCATCGCTCGACTGCCATCG
TGTTAGGCTCGCCGCAGCGTTCATCGCTCGAGCCAAGGTAAACATCTATACGCAGCCCACGGAACTGCCCTTA
GAGAAGGTGAGACCATGCCCAAAGGGCACACAGGGGATAGG
>1794
CTGCTGCCGCCCGTAGGAGTCTGGTCCGTGTCTCAGTACCAGTGTGGGGGATCACCCTCTCAGGCCCCCTAAA
GATCGTCGCCTTGTGAGCCGTTACCTCACCAACTAGCTAATCTTGCGCGCGCCCATCTCTATCCACCGTAGTT
TTCAATATCACCCGATGCCGAATAATATATTATGGGGTATTAATCTTCTTCGAAAGCTATCCCCTGATAAAGG
CAGGTTGCACACGTGTTCCGCACCCGTACGCCGCTCTCAATTACCCGAAAGTAATCTACCGCTCCGGCTTGCA
TGTGTTAGGCCTCTCGCTAGCGTTCTCTGAGCCAGG
>1795
CTGCTGCCGCCCGTAGGAGTCTGGTCCGTGTCTCAGTACCAGTGTGGGGGATCACCCTCTCAGGCCCCCTAAA
GATCGTCGCCTTGGTGAGCCGTTACCTCACCAACTAGCTAATCTTGCGCGTGCCCATCTCTATCCACCGTAGT
TTCAATATCACCCGATGCCGAATAATATATTATGGGGTATTAATCTTCTTCGAAAGGCTATCCCCCTGATAAA
GGCAGGTTGCACACGTGTTCCGCACCCGTACGCCGCTCTCAATTTACCCGAAAGTAATCTACCGCTCGGCTTG
CATGTGTTAGGCCTCCCGCTAGCGTTCTCCTGAGCAGGATCAACT
>1796
CTGCTGCCGCCCGTAGGAGTCTGGTCCGTGTCTCAGTACCAGCGTGGGGGATCACCCTCTCAGGCCCCCTAAA
GATCGTCGCCTTGGTGAGCCGTTACCTCACCAACTAGCTAATCTTGCGCGTGCCCATCTCTATCCACCGTAGT
TTCAATATCACCCGATGCCGAATAATATATTTATTGGGGTATTTAATCTTCTTCGAAAGGCTATCCCCCTGAT
AAAGGCAGGTTGCACACGTGTTCCGCACCCGTACGCCGCTCTCAATTACCCGAAAGTAATCTACCGCTCGGCT
TGCATGTGTTAGGCCTCCCGCTAGCGTTCTCCTGAGCAGGATCCAA
>1797
CTGCTGCCGCCCGTAGGAGTCTGGTCCGTGTCTCAGTACCAGTGTGGGGGATCACCCTCTCAGGCCCCCTAAA
GATCGTCGCCTTGGTGAGCCGTTACCTCACCAACTAGCTAATCTTGCGCGTGCCCATCTCTATTCCACCGTAG
TTTTCAATATCACCCGATGCCGAATAATATATTATGGGGTATTTAATCTTCTTCGAAAGGCTATCCCCTGATA
AAGGCAGGTTGCGCACGTGTTCCGCACCCGTACGCCGCTCTCAATTACCCGAAAGTAATCTACCGCTCGGCTT
GCATGTGTTAGGCCTCCCGCTAGCGTTCTCCTGAGCAGGATCAACTCGT
>1798
CTGCTGCCGCCCGTAGGAGTCTGGTCCGTGTCTCAGTACCAGTGTGGGGGATCACCCTCTCAGGCCCCCTAAA
GATCGTCGCCTTGGTGAGCCGTTACCTCACCAACTAGCTAATCTTGCGCGTGCCCATCTCTATCCACCGTAGT
TTTCAATATCACCCGATGCCGAATAATATATTATGGGGTATTAATCTTCCTTCGAAGGGCTATCCCCCTGATA
AAGGCAGGTTGCACACGTGTTCCGCACCCGTACGCCGCTCTCAATTACCCGAAAGTAATCTACCGCTCGGCTT
GCATGTGTTAGGCCTCCCGCTAGCGTTCATCCTGAGCCAGGATCAAACTCT
>1799
CTGCTGCCCGCCCCGTAGGAGTCTGGTCCCGTGTCTCAGTACCAGTGTGGGGGATCACCCCTCTCAGGCCCCC
CTAAAGATCGTCGCCTTGGTGAGCCCGTTACCTCACCCAACTAGCTTAATCTTGCGCGTGCCCCATCTCTATC
CACCGTAGTTTCAATATTCACCCGATGCCCGAATAATATATTATTGGGGTATTTAATTCTTTCTTCGAAAGGC
TATCCCCCTGATTAAAGGCAGGTTGCACACGTGTTCCCGCACCCGTACGCCGCTCTCAATTTACCCGAAAGTA
ATCTACCGCTCCGGCTGCATGTGTTTAGGCCCTTCCCGCTAGCGTTCTCCTGAGCAGGATCAACTCGT

>1800
CTGCTGCCGCCCGTAGGAGTCTGGTCCGTGTCTCAGTACCAGTGTGGGGGATCACCCTCTCAGGCCCCCTAAA
GATCGTCGCCTGGTGAGCCGTTACCTCACCAACTAGCTAATCTTGCGCGTGCCCATCTCTATCCACCGTAGTT
TTCAATATCACCCGATGCCGAATAATATATTATGGGGTATTAATCTTCTTCGAAAGGCTATCCCCCTGATAAA
GGCAGGTTGCACACGTGTTCCGCACCCGTACGCCGCTCTCAATTACCCGAAAGTAATCTACCGCTCCGGCTTG
CATGTGTTAGGCCTCCCGCTAGCGTTCATCCTGAGCCAGGATCAAACTCT
>1801
CTGCTGCCGCCCGTAGGAGTCTGGTCCGTGTCTCAGTACCAGTGTGGGGGATCACCCTCTCAGGCCCCCTAAA
GGTCGTCGCCTGGTGAGCCGTTACCTCACCAACTAGCTAATCTTGCGCGTGCCCATCTCTATCCACCGTAGTT
TTCAATATCACCCGATGCCGAATAATATATTATGGGGTATTAATCTTCTTCGAAAGGCTATCCCCCTGATAAA
GGCAGGTTGCACACGTGTTCCGCACCCGTACGCCGCTCTCAATTACCCGAAAGTAATCTACCGCTCGGCTTGC
ATGTGTTAGGCCTCCCGCTAGCGTTCATCCTGAGCCAGGATCAAACTCT
>1802
CTGCTGCCGCCCGTAGGAGTCTGGTCCGTGTCTCAGTACCAGTGTGGGGGATCACCCTCTCAGGCCCCCTAAA
GATCGTCGCCTTGGTGAGCCGTTACCTCACCAACTAGCTAATCTTGCGCGTGCCCATCTCTATCCACCGTAGT
TTTCAATATCACCGATGCCGAATAATATATTATGGGGTATTAATCTTTCTCGAAGGCTATCCCCTGATAAAGG
CAGGTTGCACACGTGTTCCGCACCCGTACGCCGCTCTCAATTACCCGAAAGTAATCTACCGCTCGGCTTGCAT
GTGTTAGGCCTCCCGCTAGCGTTCATCCTGAGCCAGGATCAACTCT
>1803
CTGCTGCCGCCCGTAGGAGTCTGGTCCGTGTCTCAGTACCAGTGTGGGGGATCACCCTCTCAGGCCCCCTAAA
GATCGTCGCCTTGGTGAGCCGCTACCTCACCAACTAGCTAATCTTGCGCGTGCCCATCTCTATCCACCGTAGT
TTTCAATATCACCCGATGCCGAATAATATATTATGGGGTATTAATCTTCTTCGAAAGGCTATCCCCCTGATAA
AGGCAGGTTGCACACGTGTTCCGCACCCGTACGCCGCTCTCAATTACCCGAAAGTAATCTACCGCTCGGCTTG
CATGTGTTAGGCCTCCCGCTAGCGTTCATCCTGAGCCAGGATCAACTCT
>1804
CTGCTGCCGCCCGTAGGAGTCTGGTCCGTGTCTCAGTACCAGTGTGGGGGATCACCCTCTCAGGCCCCCTAAA
GATCGTCGCCTTGGTGAGCCGTTACCTCACCAACTAGCTAATCTTGCGCGTGCCCATCTCTATCCACCGTAGT
TTTCAATATCACCCGATGCCGAATAATATATTATGGGGTATTAATCTTCTCGAAAGGCTATCCCCTGATAAAG
GCAGGTTGCACACGTGTTCCGCACCCGTACGCCGCTCTCAATTACCCGAAAGTAATCTACCGCTCCGGCTTGC
ATGTGTTAGGCCTCCCGCTAGCGTTCATCCCGTAGCAGGCATCAAACTCCT
>1805
CTGCTGCCGCCCGTAGGAGTCTGGTCCGTGTCTCAGTACCAGTGTGGGGGATCACCCTCTCAGGCCCCCTAAA
GATCGTCGCCTTGGTGAGCCGTTACCTCACCAACTAGCTAATCTTGCGCGTGCCCATCTCTATCCACCGTAGT
TTTCAATATCACCCGATGCCGAATAATATATTATGGGGTATTAATCTTCTTCGAAAGGCTATCCCCCTGAATA
AAGGCAGGTTGCACACGTGTTCCGCACCCGTACGCCGCTCTCAATTACCCGAAAGTAATCTACCGCTCGGCTT
GCATGTGTTAGGCCTCCCGCTAGCGTTCATCCTGAGCCAGGATCAACTCT
>1806
CTGCTGCCGCCCGTAGGAGTCTGGTCCGTGTCTCAGTACCAGTGTGGGGGATCACCCTCTCAGGCCCCCTAAA
GATCGTCGCCTTGGTGAGCCGTTACCTCACCAACTAGCTAATCTTGCGCGTGCCCATCTCTATCCACCGTAGT
TTTCAATATCACCCGATGCCGAATAATATATTATGGGGTATTAATCTTCTTCGAAAGGCTATCCCCTGATAAA
AGGCAGGTTGCACACGTGTTCCGCACCCGTACGCCGCTCTCAATTACCCGAAAGTAATCTACCGCTCGGCTTG
CATGTGTTAGGCCTCCCGCTAGCGTTCATCCG
>1807
CTGCTGCCGCCCGTAGGAGTCTGGTCCGTGTCTCAGTACCAGTGTGGGGGATCACCCTCTCAGGCCCCCTAAA
GATCGTCGCCTTGGTGAGCCGTTACCTCACCAACTAGCTAATCTTGCGCGTGCCCATCTCTATCCACCGTAGT
TTTCAATATCAACCCGATGCCGAATAATATATTATGGGGTATTAAATCTTCTCGAAAGGCTATCCCCTGATAA
AGGCAGGTTGCACACGTGTTCCGCACCCGTACGCCGACTCTCAATTACCCGAAAGTAATCTACCGCTCGGCTT
GCATGTGTTAGGCCTCCCGCTAAGCGTCATCCTGAGCAGGACAAACTCT
>1808
CTGCTGCCGCCCGTAGGAGTCTGGTCCGTGCCTCAGTACCAGTGTGGGGGATCACCCTCTCAGGCCCCCTAAA
GATCGTCGCCTTGGTGAGCCGTTACCTCACCAACTAGCTAATCTTGCGCGTGCCCATCTCTATCCACCGTAGT
TTTCAATATCACCCGATGCCGAATAATATATTATGGGGTATTAATCTTCTTCGAAAGGCTATCCCCCTGATAA
AGGCAGGTTGCACACGTGTTCCGCACCCGTACGCCGCTCTCAATTACCCGAAAGTAATCTACCGCTCGGCTTG
CATGTGTTAGGCCTCCCGCTAGCGTTCATCCTGAGCCAGGATCAAACTCT

>1809
TGCTGCCGCCCGTAGGAGTCTGGTCCGTGTCTCAGTACCAGTGTGGGGGATCACCCTCTCAGGCCCCCTAAAG
ATCGTCGCCTTGGTGAGCCGTTACCTCACCAACTAGCTAACCTTGCGCGTGCCCATCTCTATCCACCGTAGTT
TTCAATATCACCCGATGCCGAATAATATATTATGGGGTATTAATCTTCTTCGAAAGGCTATCCCCCTGATAAA
GGCAGGTTGCACACGTGTTCCGCACCCGTACGCCGCTCTCAATTACCCGAAAGTAATCTACCGCTCGGCTTGC
ATGTGTTAGGCCTCCCGCTAGCGTTCATCCTGAGCCAGGATCAAACTCT
>1810
CTGCTGCCGCCCGTAGGAGTCTGGTCCGTGTCTCAGTACCAGTGTGGGGGATCACCCTCTCAGGCCCCCTAAA
GATCGTCGCCTTGGTGAGCCGTTACCTCACCAACTAGCTAATCTTGCGCGTGCCCATCTCTATCCACCGTAGT
TTTCAATATCACCCGATGCCGAATAATATATTATGGGGTATTAATCTTCTCGAAAGGCTATCCCCCTGATAAA
GGCAGGTTGCACACGTGTTCCGCACCCGTACGCCGCTCTCAATTACCCGAAAGTAATCTACCGCTCCGGCTTG
CATGTGTTAGGCCTCCCGCTAGCGTTCATCCTGAGCCAGGATCAACTCT
>1811
CTGCTGCCGCCCGTAGGAGTCTGGTCCGTGTCTCAGTACCAGTGTGGGGGATCACCCTCTCAGGCCCCCTAAA
GATCGTCGCCTTGGTGAGCCGTTACCTCACCAGCTAGCTAATCTTGCGCGTGCCCATCTCTATCCACCGTAGT
TTTCAATATCACCCGATGCCGAATAATATATTATGGGGTATTAATCTTCTTCGAAAGGCTATCCCCTGATAAA
GGCAGGTTGCACACGTGTTCCGCACCCGTACGCCGCTCTCAATTACCCGAAAGTAATCTACCGCTCCGGCTTG
CATGTGTTAGGCCTCCCGCTAGCGTTCATCCTGAGCCAGGATCAACTCT
>1812
CTGCTGCCGCCCGTAGGAGTCTGGTCCGTGTCTCAGTACCAGTGTGGGGGATCACCCTCTCAGGCCCCCTAAA
GATCGTCGCCTTGGTGAGCCGTTACCTCACCAACTAGCTAATCTTGCGCGTGCCCATCTCTATCCACCGTAGT
TTCAATATCACCCGATGCCGAATAATATATTATGGGGTATTAAATCTTCTCGAAAGGCTATCCCCTGATAAAG
GCAGGTTGCACACGTGTTCCGCACCCGTACGCCGCTCTCAATTACCCGAAAGTAATCTACCGCTCGGCTTGCA
TGTGTTAGGCCTCCCGCTAGCGTTC
>1813
CTGCTGCCGCCCGTAGGAGTCTGGACCGTGTCTCAGTTCCAGTGTGACTGATCATCCTCTCAGACCAGTACGG
ATCGTCGCCTTGGTAGGCCTTTACCCCACCAACTAGCTAATCCGACCTAGGCTCATCTGATAGCGTGAGGTCC
GAAGTCCCCACTTCTCCCTCAGGACGTATGCGGTATTAGCGCCCGTTTCCGGACGTATCCCCACTACCAGGCA
GATTCCTAGGCATTACTCACCCGTCCGCCGCTGAATCCAGGAGCAAGCTCCCTTCATCCGCTCGACTGCATGT
GTTAGGCCTGCCGCCAGCGTCAATCTGAGCCAGGATCAACTCT
>1814
CTGCTGCCGCCCGTAGGAGTCTGGACCGTGTCTCAGTTCCAGTGTGACTGATCATCCTCTCAGACCAGTACGG
ATCGTCGCCTTGGTAGGCCTTTACCCCACCAACTAGCTAATCCGACCTAGGCTCATCTGATAGCGTGAGGTCC
GAAGTCCCCCACTTCTCCCTCAGGACGTATGCGGTATTAGCGCCCGTTTCCGGACGTATCCCCACTACCAGGC
AGATTCCTAGGCATTACTCACCCGTCCGCCGCTGAATCCAGGAGCAAGCTCCCTCATCCGCTCGACTGCATGT
GTTAGGCCTGCCGCCAGCGTCAATCTGAGCCAGGATCAAACTCT
>1815
CTGCTGCCGCCCGTAGGAGTCTGGTCCGTGTCTCAGTACCAGTGTGGGGGATCACCCTCTCAGGCCCCCTAAA
GATCGTCGCCTTGGTGAGCCGTTACCTCACCAACTAGCTAATCTTGCGCGTGCCCATCTCTATCCACCGTAGT
TTTCAATATCACCCGATGCCGAATAATATATTATGGGGTATTAATCTTCCTTCGAAGGGCTATCCCCCTGATA
AAGGCAGGTTGCACACGTGTTCCGCACCCGTACGCCGCTCTCAATTACCCGAAAGTAATCTACCGCTCGGCTT
GCATGTGTTAGGCCTCCCGCTAGCGTTCATCCTGAGCCAGGATCAAACTCT
>1816
CTGCTGCCGCCCGTAGGAGTCTGGTCCGTGTCTCAGTACCAGTGTGGGGGATCACCCTCTCAGGCCCCCTAAA
GATCGTCGCCTTGGTGAGCCGTTACCTCACCAACTAGCTAATCTTGCGCGTGCCCATCTCTATCCACCGTAGT
TTTCAATATCACCCGATGCCGAATAATATATTATGGGGTATTAATCTTCTTCGAAAGCTATCCCCTGATAAAG
GCAGGTTGCACACGTGTTCCGCACCCGTACGCCGCTCTCAATTACCCGAAAGTAATCTACCGCTCCGGCTTGC
ATGTGTTAGGCCTCCCGCTAGCGTTCTCCGTCGAG
>1817
CTGCTGCCGCCCGTAGGAGTCTGGTCCGTGTCTCAGTACCAGTGTGGGGGATCACCCTCTCAGGCCCCCTAAA
GATCGTCGCCTTGGTGAGCCGTTACCTCACCAACTAGCTAATCTTGCGCGTGCCCATCTCTATCCACCGTAGT
TTTCAATATCACCCGATGCCGAATAATATATTATGGGGTATTAATCTTCCTTCGAAGAGCTATCCCCTGATAA
AGGCAGGTTGCACACGTGTTCCGCACCCGTACGCCGCTCTCAATTACCCGAAAGTAATCTACCGCTCGGCTTG
CATGTGTTAGGCCTCCCGCTAGCGTTCACCTGAGCCAGGATCAACTCT

>1818
CTGCTGCCGCCCGTAGGAGTCTGGTCCGTGTCTCAGTACCAGTGTGGGGGATCACCCTCTCAGGCCCCCTAAA
GATCGTCGCCTTGGTGAGCCGTTACCTCACCAACTAGCTAATCTTGCGCGTGCCCATCTCTATCCACCGTAGT
TTCAATATCACCCGATGCCGAATAATATATTATGGGGTATTAATCTTCCTTCGAAAGGCTATCCCCTGATAAA
GGCAGGTTGCACACGTGTTCCGCACCCGTACGCCGCTCTCAATTACCCGAAAGTAATCTACCGCTCGGCTTGC
ATGTGTTAGGCCTCCCGCTAGCGTCATCTGAGCCAGGATCAAACTCT
>1819
CTGCTGCCGCCCGTAGGAGTCTGGTCCGTGTCTCAGTACCAGTGTGGGGGATCACCCTCTCAGGCCCCCTAAA
GATCGTCGCCTTGGTGAGCCGTTACCTCACCAACTAGCTAATCTTGCGCGTGCCCATCTCTATCCACCGTAGT
TTTCAATATCACCCGATGCCGAATAATATTATTATGGGGTATTTAATCTTCTTCGAAAGGCTATCCCCTGATA
AAGGCAGGTTGCACACGTGTTCCGCACCCGTACGCCGCTCTCAATTTACCCGAAAGTAATCTACCGCTCGGCT
TGCATGTGTTAGGCCTCCCGCTAGCGTTCATTCCTGA
>1820
CTGCTGCCGCCCGTAGGAGTCTGGTCCGTGTCTCAGTACCAGTGTGGGGGATCACCCTCTCAGGCCCCCTAAA
GATCGTCGCCTTGTGAGCCGTTACCTCACCAACTAGCTAATCTTGCGCGTGCCCATCTCTATCCACCGTAGTT
TTCAATATCACCCGATGCCGAATAATATATTATGGGGTATTAATTCTTTCTCGAAAGGCTATCCCCCTGATAA
AGGCAGGTTGCACACGTGTTCCGCACCCGTACGCCGCTCTCAATTACCCGAAAGTAATCTACCGCTCCGGCTG
CATGTGTTAGGCCTCCCGCTAGCGTTCTCCGTCGAG
>1821
CTGCTGCCGCCCGTAGGAGTCTGGTCCGTGTCTCAGTACCAGTGTGGGGGATCACCCCTCTCAGGCCCCCCTA
AAGATCGTCGCCTTGGTGAGCCCGTTACCTCACCCAACTAGCTAATCTTGCGCGTGCCCCATCTCTATTCCCA
CCGTAGTTTCAATATCACCCCGATGCCCGAATAATATATTTATGGGGTATTTAATTCTTCTTCGAAAGGCTAT
CCCCCTGATAAAGGCAGGTTGCACACGTGTTCCCGCACCCGTACGCCGCTCTCAATTACCCCGAAAGTAATCT
ACCGCTCGGCTTGCATGTGTTAGGCCTCCCGCTAGCGTTCATCCTGA
>1822
CTGCTGCCGCCCCGTAGGAGTCTGGACCGTGTCTCAGTTCCAGTGTGACTGATCATCCTCTCAGACCAGTACG
GATCGTCGCCTTGGTAGGCCTTTACCCCCACCAACTAGCTAATCCGACCTAGGCTCATCTGATAGCGTGAGGT
CCGAAGATCCCCCACTTCTCCCCTCAGGACGTATGCGGTATTAGCGCCCGTTTCCGGACGTATCCCCACTACC
AGGCAGATTCCTAGGCATTACTCACCCGTCCGCCGCTGAATCCAGGAGCAAGCTCCCTCATCCGCTCGACTGC
ATGTGTTAGGCCTGCCGCCAGCGTCAATCTGAGCCAGGATCAAACTCT
>1823
CTGCTGCCGCCCGTAGGAGTCTGGACTCGTGTCTCAGTTCCAGTGTGACTGATCATCCTCTCAGACCAGTACG
GATCGTCGCCTTGGTAGGCCTTTTACCCCCACCAACTAGCTAATCCGACCTAGGCTCATCTGATAGCGTGAGG
TCCGAAGATCCCCCACTTCTCCCCTCAGGACGTATGCGGTATTAGCGCCCGTTTCCGGACGTATCCCCACTAC
CAGGCAGATTCCTAGGCATTACTCACCCGTCCGCCGCTGAATCCAGGAGCAAGCTCCCTCATCCGCTCGACTG
CATGTGTTAGGCCTGCCGCCAGCGTCAATCTGAGCCAGGATCAAACTCT
>1824
CTGCTGCCGCCCGTAGGAGTCTGGTCCGTGTCTCAGTACCAGTGTGGGGGATCACCCTCTCAGGCCCCCTAAA
GATCGTCGCCTTGTGAGCCGTTACCTCACCAACTAGCTAATCTTGCGCGTGCCCATCTCTATCCACCGTAGTT
TTCAATATCACCCGATGCCGAATAATATATTATGGGGTATTAATCTTCTTCGAAAGGCTATCCCCCTGATAAA
GGCAGGTTGCACACGTGTTCCGCACCCGTACGCCGCTCTCAATTACCCGAAAGTAATCTACCGCTCGGCTTGC
ATGTGTTAGGCCTCCCGCTAGCGTCATCCGT
>1825
CTGCTGCCGCCCGTAGGAGTCTGGTCCGTGTCTCAGTACCAGTGTGGGGGATCACCCTCTCAGGCCCCCTAAA
GATCGTCGCCTGGTGAGCCGTTACCTCACCAACTAGCTAATCTTGCGCGTGCCCATCTCTATCCACCGTAGTT
TTCAATATCACCCGATGCCGAATAATATATTATGGGGTATTAAATCTTCTCGAAGGCTATCCCCCTGAATAAA
GGCAGGTTGCACACGTGTTCCGCACCCGTACGCCGCTCTCAATTACCCGAAAGAATCTACGCTCCGGCTGCAA
TGTGTTAGGCCTCCCGCTAGCGTTCTACCTGAGCCAGGATCAACATCT
>1826
CTGCTGCCGCCCGTAGGAGTCTGGTCCGTGTCTCAGTACCAGTGTGGGGGATCACCCTCTCAGGCCCCCTAAA
GATCGTCGCCTTGGTGAGCCGTTACCTCACCAACTAGCTAATCTTGCGCGTGCCCATCTCTATCCACCGTAGT
TTTCAATATCACCCGATGCCGAATAATATATTATGGGGTATTAATCTTCTTCGAAAGGCTATCCCCCTGATAA
AGGCAGGTTGCACACGTGTTCCGCACCCGTACGCCGCTCTCAATTACCCGAAAGTAATCTACCGCTCGGCTTG
CATGTGTTAGGCCTCCCGCTAGCGTTCATCCTGAGCCAGGATCAACTCT

>1827
CTGCTGCCGCCCGTAGGAGTCTGGTCCGTGTCTCAGTACCAGTGTGGGGGATCACCCTCTCAGGCCCCCTAAA
AGATCGTCGCCTTGGTGAGCCGTTACCTCACCAACTAGCTAATCTTGCGCGTGCCCATCTCTATCCACCGTAG
TTTCAATATCACCCGATGCCGAATAATATATTATGGGGTATTAATCTCTTCGAAAGGCTATCCCCTGATAAAG
GCAGGTTGCACACGTGTTCCGCACCCGTACGCCGCTCTCAATTACCCGAAAGTAATCTACCGCTCGGCTTGCA
TGTGTTAGGCCTCCCGACTAGCGTTCATC
>1828
CTGCTGCCGCCCGTAGGAGTCTGGACCGTGTCTCAGTTCCAGTGTGACTGATCATCCTCTCAGACCAGTACGG
ATCGTCGCCTTGGTAGGCCTTTACCCCACCAACTAGCTAATCCGACCTAGGCTCATCTGATAGCGTGAGGTCC
GAAGATCCCCCACTTCTCCCTCAGGACGTATGCGGTATTAGCGCCCGTTTCCGGACGTATCCCCACTACCAGG
CAGATTCCTAGGCATTACTCACCCGTCCGCCGCTGAATCCAGGAGCAAGCTCCCTTCATCCGCTCGACTGCAT
GTGTTAGGCCTGCCGCCAGCGTTCAATCTGAGCCAGGATCAAACTCT
>1829
CTGCTGCCGCCCGTAGGAGTCTGGTCCGTGTCTCAGTACCAGTGTGGGGGATCACCCTCTCAGGCCCCCTAAA
GATCGTCGCCTTGGTGAGCCGTTACCTCACCAACTAGCTAATCTTGCGCGTGCCCATCTCTATCCACCGTAGT
TTTCAATATCACCCGATGCCGAATAATATATTATGGGGTATTAATCTTCTTCGAAAGGCTATCCCCCTGATAA
AGGCAGGTTGCACACGTGTTCCGCACCGTACGCCGCTCCCAATTACCCGAAAGTAATCTACCGCTCGGCTTGC
ATGTGTTAGGCCTCCCGCTAGCGTTCTCCTCGAGCAGGATCCAACTCT
>1830
CTGCTGCCGCCCGTAGGAGTCTGGTCCGTGTCTCAGTACCAGTGTGGGGGATCACCCTCTCAGGCCCCCTAAA
GATCGTCGCCTTGGTGGAGCCGTTACCTCACCAACTAGCTAATCTTGCGCGTGCCCATCTCTATCCACCGTAG
TTTTCAATATCACCCGATGCCGAATAATATATTATGGGGTATTAATCTTCTTCGAAAGGCTATCCCCTGATAA
AGCAGGTTGCACACGTGTTCCGCACCCGTACGCCGCTCTCAATTACCCGAAAGTAATCTACCGCTCGGCTTGC
ATGTGTTGGCGCTCCCGCTAGCCTCG
>1831
CTGCTGCCGCCCGTAGGAGTCTGGTCCGTGTCTCAGTACCAGTGTGGGGGATCACCCTCTCAGGCCCCCTAAA
GATCGTCGCCTTGGTGAGCCGTTACCTCACCAACTAGCTAATCTTGCGCGTGCCCATCTCTATCCACCGTAGT
TTTCAATATCACCCGATGCCGAATAATATATTATGGGGTATTAATCTTCTTCGAAAGGCTATCCCCCTGATAA
AGGCAGGTTGCACACGTGTTCCGCACCCGTACGCCGCTCTCAATTACCCGAAAGTAATCTACCGCTCGGCTTG
CATGTGTTAGGCCTCCCGCTAGCGTTCATCTGAGCCAGGATCAAACTCT
>1832
CTGCTGCCGCCCGTAGGGGTCTGGTCCGTGTCTCAGTACCAGTGTGGGGGATCACCCTCTCAGGCCCCCTAAA
GATCGTCGCCTTGGTGAGCCGTTACCTCACCAACTAGCTAATCTTGCGCGTGCCCATCTCTATCCACCGTAGT
TTTCAATATCACCCGATGCCGAATAATATATTGTGGGGGTATTAATCTTCTTCGAAAGGCTATCCCCCTGATA
AAGGCAGGTTGCACACGTGTTCCGCACCCGTACGCCGCTCTCAATTACCCGAAAGTAATCCACCGCATCGGCT
TGCATGTGTTAGGCCTCCCGCTAGCGTTCTCCTGAGCAGGATCAACTCT
>1833
CTGCTGCCGCCCGTAGGAGTCTGGTCCGTGTCTCAGTACCAGTGTGGGGGATCACCCTCTCAGGCCCCCTAAA
GATCGTCGCCTTGGTGAGCCGTTACCTCACCAACTAGCTAATCTTGCGCGTGCCCATCTCTATCCACCGTAGT
TTTCAATATCACCCGATGCCGAATAATATATTATGGGGTATTAAATCTTCTTCGAAAGGCTATCCCCTGATAA
AGGCAGGTTGCACACGTGTTCCGCACCCGTACGCCGCTCTCAATTACCCGAAAGTAATCTACCGCTCGGCTTG
CATGTGTTAGGCCTCCCGCTAGCGTTCATCCTGAGCCAGGATCAAACTCT
>1834
CTGCTGCCGCCCGTAGGAGTCTGGTCCGTGTCTCAGTACCAGTGTGGGGGATCACCCTCTCAGGCCCCCTAAA
GATCGTCGCCTTGGTGAGCCGTTACCTCACCAACTAGCTAATCTTGCGCGTGCCCATCTCTATCCACCGTAGT
TTTCAATATCACCCGATGCCGAATAATATAATTATGGGGTATTAATCTTCTTCGAAAGGCTATCCCCCTGATA
AGGAGCAGGTTGCACACGTGTTCCGCACCCGTACGCCGCTCTCAATTACCCGAAAGTAATCTACCGCTCGGCT
TGCATGTGTTAGGCCTCCCGCTAGCGTTCTCCTGAGCAGTCCAAAGCGT
>1835
CTGCTGCCGCCCGTAGGAGTCTGGTCCGTGTCTCAGTACCAGTGTGGGGGATCACCCTCTCAGGCCCCCTAAA
GATCGTCGCCTTGGTGAGCCGTTACCTCACCAACTAGCTAATCTTGCGCGTGCCCATCTCTATCCACCGTAGT
TTTCAATATCACCCGATGCCGAATAATATAATTATGGGGTATTAATCTTCTTCGAAAGGCTATCCCCCTGATA
AAGGCAGGTTGCACACGTGTTCCGCACCCGTACGCCGCTCTCAATTACCCGAAAGTAATCTACCGCTCGGCTT
GCATGTGTTAGGCCTCCCGCTAGCGTTCATCCTGAGCCAGGATCAAACTCT

>1836
CTGCTGCCGCCCGTAGGAGTCTGGTCCGTGTCTCAGTACCAGTGTGGGGGATCACCCTCTCAGGCCCCCTAAA
GATCGTCGCCTTGGTGAGCCGTTACCTCACCAACTAGCTAATCTTGCGCGTGCCCATCTCTATCCACCGTAGT
TTTCAATATCACCCGATGCCGAATAATATATTATGGGGTATTAATCTTCTTCGAAAGGCTATCCCCTGATAAA
GGCAGGTTGCACACGTGTTCCGCACCCGTACGCCGCTCTCAATTACCCGAAAGTAATCTACCGCTCGGCTTGC
ATGTGTTAGGCCTCCCGCTAGCGTTCTCTGAGC
>1837
CTGCTGCCGCCCGTAGGAGTCTGGTCCGTGTCTCAGTACCAGTGTGGGGGATCACCCTCTCAGGCCCCCTAAA
GATCGTCGCCTTGGTGAGCCGTTACCTCACCAACTAGCTAATCTTGCGCGTGCCCATCTCTATCCACCGTAGT
TTTCAATATCACCCGATGCCGAATAATATATTATGGGGTATTAATCTTCTTCGAAAGGCTATCCCCCTGATAA
AGGCAGGTTGCACACGTGTTCCGCACCCGTACGCCGCTCTCAATTACCCGAAAGTAATCTACCGCTCCGGCTT
GCATGTGTTAGGCCTCCCGCTAGCGTTCATCCTCGAG
>1838
CTGCTGCCGCCCGTAGGAGTCTGGTCCGTGTCTCAGTACCAGTGTGGGGGATCACCCTCTCAGGCCCCCTAAA
GATCGTCGCCTTGGTGAGCCGTTACCTCACCAACTAGCTAATCTTGCGCGTGCCCATCTCTATCCACCGTAGT
TTTCAATATCACCCGATGCCGAATAATATATTATGGGGTATTAATCTTCTCGAAAGGCTATCCCCCTGATAAA
GGCAGGTTGCACACGTGTTCCGCACCCGTACGCCGCTCTCAATTACCCGAAAGTAATCTACCGCTCGGCTTGC
ATGTGTTAGGCCTCCCGCTAGCGTTCTACCTCGAGCAGGATCAACATCGT
>1839
CTGCTGCCGCCCGTAGGAGTCTGGTCCGTGTCTCAGTACCAGTGTGGGGGGATCACCCTCTCAGGCCCCCTAA
AGATCGTCGCCTTGGTGAGCCGTTACCTCACCAACTAGCTAATCTTGCGCGTGCCCATCTCTATCCACCGTAG
TTTTCAATATCACCCGATGCCGAATAATATATTATGGGGTATTTAATCTTCTTCGAAAGCTATCCCCCCTGATA
AAGGCAGGTTGCACACGTGTTCCGCACCCGTACGCCGCTCTCAATTACCCGAAAGTAATCTACCGCTCCGGCT
TAGCATGTGTTCGGCCTTC
>1840
CTGCTGCCGCCCGTAGGAGTCTGGACCGTGTCTCAGTTCCAGTGTGACTGATCATTCCTCTCAGACCAGTACG
GATCGTCGCCTTGGTAGGCCTTACCCCCACCAACTAGCTAATCCGACCTAGGCTCATCTGATTAGCGTGAGGT
CCGAAGTCCCCCACTTCTCCCTCAGGACGTATGCGGTATTAGCGCCCGTTTCCGGACGATCCCCACTACCAGG
CAGATTCCTAGGCATTACTCACCCGTCCGCCGCTGAATCCAGGAGCAAGCCCCTTCATCCGCCTCGACTGCAT
TGTGTTAGGCCTGCCGCCAGCGTCAATCTGAGCCAGGATCAACTCT
>1841
CTGCTGCCGCCCGTAGGAGTCTGGTCCGTGTCTCAGTACCAGTGTGGGGGATCACCCTCTCAGGCCCCCTAAAG
ATCGTCGCCTGGTGAGCCGTTACCTCACCAACTAGCTAATCTTGCGCGTGCCCATCTCTATCCACCGTAGTTT
TCAATACCACCCGATGCCGAATAATATATTATGGGGTATCAATCTCCCTTCGAAAGGCTATCCCCTGATAAAG
GCAGGTTGCACACGTGTTCCGCACCCGTACGCCGCTCTCAATTACCCGAAAGTAATCTACCGCTCGGCTTGCA
TGTGTTAGGCCTCCCGCTAG
>1842
CTGCTGCCGCCCGTAGGAGTCTGGACCGTGTCTCAGTTCCAGTGTGACTGATCATCCTCTCAGACCAGTACGG
ATCGTCGCCTTGGTAGGCCTTTACCCCACCAACTAGCTAATCCGACCTAGGCTCATCTGATTAGCGTGAGGTC
CGAAGTCCCCCACTTCTCCCTCAGGACGTATGCGGTATTAGCGCCCGTTTCCGGACGTATCCCCATTACCAGG
CAGATTCCTAGGCATTACTCACCCGTCCGCCGCTGAATCCAGGAGCAAGCTCCCTCATCCGCTCGACTGCATG
TGTTAGGCCTGCCGCCAGCGTCAATCTGAGCCAGGATCAAACTCT
>1843
CTGCTGCCGCCCGTAGGAGTCTGGTCCGTGTCTCAGTACCAGTGTGGGGGATCACCCTCTCAGGCCCCCTAAA
GATCGTCGCCTTGGTGAGCCGTTACCTCACCAACTAGCTAATCTTGCGCGTGCCCATCTCTATCCACCGTAGT
TTTCAATATCACCCGATGCCGAATAATATATTATGGGGTATTAATCTTCTTCGAAAGGCTATCCCCTGATAAA
GGCAGGTTGCACACGTGTTCCGCACCCGTACGCCGCTCTCAATTACCCGAAAGTAATCTACCGCTCGGCTTGC
ATGTGTTAGGCCTCCCGCTAGCGTTCTCCTGAGCAG
>1844
CTGCTGCCGCCCGTAGGAGTCTGGTCCGTGTCTCAGTACCAGTGTGGGGGATCACCCTCTCAGGCCCCCTAAA
GATCGTCGCCTTGGTGAGCCGTTACCTCACCAACTAGCTAATCTTGCGCGTGCCCATCTCTATCCACCGTAGT
TTTCAATATCACCCGATGCCGAATAATATATTATGGGGGTATAATCTTCTTCGAAAGGCTATCCCCCTGATAA
AGGCAGGTTGCACACGTGTTCCGCACCCGTACGCCGCTCTCAATTTACCCGAAAGTAATCTACCGCTCCGGCT
TGCATGTGTTAGGCCTCCCGCTAGCGTTCATCCTGAGCCAGGATCAAACTCT

>1845
CTGCTGCCGCCCGTAGGAGTCTGGTCCGTGTCTCAGTACCAGTGTGGGGGATCACCCTCTCAGGCCCCCTAAA
GATCGTCGCCTTGGTGAGCCGTTACCTCACCAACTAGCTAATCTTGCGCGTGCCCATCTCTATCCACCGTAGT
TTCAATATCACCCGATGCCGAATAATATATTATGGGGTATTAATCTTCTTCGAAAGGCTATCCCCCTGATAAA
GGCAGGTTGCACACGTGTTCCGCACCCGTACGCCGCTCTCAATTACCCGAAAGTAATCTACCGCTCCGGCTTG
CATGTGTTAGGCCTCCCGCTAGCGTTCATCCTGAGCCAGGATCAACTCT
>1846
CTGCTGCCGCCCGTAGGAGTCTGGTCCGTGTCTCAGTACCAGTGTGGGGGATCACCCTCTCAGGCCCCCTAAA
GATCGTCGCCTTGGTGAGCCGTTACCTCACCAACTAGCTAATCTTGCGCGTGCCCATCTCTATCCACCGTAGT
TTTCAATATCACCCGATGCCGAATAATATATTATGGGGTATTAATCTTCTTCGAAAGGCTATCCCCCTGATAA
AGGCAGGTTGCACACGTGTTCCGCACCCGTACGCCGCTCTCAATTACCCGAAAGTAATCTACCGCTCGGCTTG
CATGTGTTAGGCCTCCCGCTAGCGTTCATCCTGAGCCAGGATCAAACTCT
>1847
CTGCTGCCGCCCGTAGGAGTCTGGACCGTGTCTCAGTTCCAGTGTGACTGATCATCCTCTCAGACCAGTTACG
GATCGTCGCCTTGGTAGGCCTTTACCCCACCAACTAGCTAATCCGACCTAGGCTCATCTGATAGCGTGAGGTC
CGAAGATCCCCCACTTCTCCCTCAGGACGTATGCGGTATTAGCGCCCGTTTCCGGACGTATCCCCACTACCAG
GCAGATTCCTAGGCATTACTCACCCGTCCGCCGCTGAATCCAGGAGCAAGCCCCCTTCATCCGCTCGACTGCA
TGTGTTAGGCCTGCCGCCAGCGTTCAATCTGAGCCAGGATCAAACTCT
>1848
CTGCTGCCGCCCGTAGGAGTCTGGTCCGTGTCTCAGTACCAGTGTGGGGGATCACCCTCTCAGGCCCCCTAAA
GATCGTCGCCTTGGTGAGCCGTTACCTCACCAACTAGCTAATCTTGCGCGTGCCCATCTCTATCCACCGTAGT
TTTCAATATCACCCGATGCCGAAATAATATATTATGGGGTATTAATCTTCTTCGAAAGGCTATCCCCTGATAA
AGGCAGGTTAGCAACACGTGTTCCGCACCCGTACGCCGCTCTCAATTTACCCGAAAAGTAATCTACCGCTCGG
CTTGCAATGTGTTAGGCCTCCCGCTAGCGTTCTCCTGAGCAAGGTCAAACTCGT
>1849
CTGCTGCCGCCCGTAGGAGTCTGGTCCGTGTCTCAGTACCAGTGTGGGGGATCACCCTCTCAGGCCCCCTAAA
GATCGTCGCCTTGGTGAGCCGTTACCTCACCAACTAGCTAATCTTGCGCGTGCCCATCTCTATCCACCGTAGT
TTTCAATATCACCCGATGCCGAATAATATATTATGGGGTATTAATCTTCTTCGAAAGGCTATCCCCTGATAAA
GGCAGGTTGCACACGTGTTCCGCACCCGTACGCCGCTCTCAATTACCCGAAAGTAATCTACCGCTCGGCTTGA
CATGTGTTAGGCCTCCCGCTAGCGTTCATCCTGAGCCAGGATCAAACTCT
>1850
CTGCTGCCGCCCGTAGGAGTCTGGTCCGTGTCTCAGTACCAGTGTGGGGGATCACCCTCTCAGGCCCCCTAAA
GATCGTCGCCTTGGTGAGCCGTTACCTCACCAACTAGCTAATCTTGCGCGTGCCCATCTCTATCCACCGTAGT
TTTCAATATCACCCGATGCCGAATAATATATTATGGGGTATTAATCTTCTCGAAAGGCTATCCCCTGATAAAG
GCAGGTTGCACACGTGTTCCGCACCCGTACGCCGCTCTCAATTACCCGAAAGTAATCTACCGCTCGGCTTGCA
TGTGTTAGGCCTCCCGCTAGCGTTCATCCTGAGCAGGATCAACTCCT
>1851
CTGCTGCCGCCCGTAGGAGTCTGGTCCGTGTCTCAGTACCAGTGTGGGGGATCACCCTCTCAGGCCCCCTAAA
GATCGTCGCCTTGGTGAGCCGTTACCTCACCAACTAGCTAATCTTGCGCGTGCCCATCTCTATCCACCGTAGT
TTTCAATATCACCCGATGCCGAATAATATATTATGGGGTATTAATCTTCTTCGAAAGGCTATCCCCCTGATAA
AGGCAGGTTGCACACGTGTTCCGCACCCGTACGCCGCTCTCAATTACCCGAAAGTAATCTACCGCTCGGCTTG
CATGTGTTAGGCCTCCCGCTAGCGTTCATCCTGAGCAGGATCAAACTCT
>1852
CTGCTGCCGCCCGTAGGAGTCTGGTTCCGTGTCTCAGTACCAGTGTGGGGGGATCACCCTCTCAGGCCCCCTA
AAGATCGTCGCCTTGGTGAGCCGTTACCTCACCAACTAGCTTAATCTTGCGCGTGCCCATCTCTATCCACCGT
AGTTTTTCAATATCACCCGATGCCGAATAATATATTTATGGGGTATTTAATCTTTCTACGAAAGGCTATCCCC
TGATAAAGGCAGGTTGCACACGTGTTCCGCACCCGTACGCCGCTCTCAATTACCCGAAAGTAATCTACCGCTC
GGCTTGCATGTGTTAGGCCTCCCGCTAGCGTTCTCCTGAGCAGGATCAACTC
>1853
CTGCTGCCGCCCGTAGGAGTCTGGTCCGTGTCTCAGTACCAGTGTGGGGGATCACCCTCTCAGGCCCCCTAAA
GATCGTCGCCTTGGTGAGCCGTTACCTCACCAACTAGCTAATCCTGCGCGTGCCCATCTCTATCCACCGTAGT
TTTCAATATCACCCGATGCCGAATAATATATTATGGGGTATTAATCTTCTTCGAAAGGCTATCCCCTGATAAA
GGCAGGTTGCACACGTGTTCCGCACCCGTACGCCGCTCTCAATTACCCGAAAGTAATCTACCGCTCGGCTTGC
ATGTGTTAGGCCTCCCGCTAGCGTTCATCCTGAGCCAGGATCAACTCT

>1854
CTGCTGCCGCCCGTAGGAGTCTGGTCCGTGTCTCAGTACCAGTGTGGGGGATCACCCTCTCAGGCCCCCTAAA
GATCGTCGCCTTGGTGAGCCGTTACCTCACCAACTAGCTAATCTTGCGCGTGCCCATCTCTATCCACCGTAGT
TTTCAATATCACCCGATGCCGAATAATATATTATGGGGTATTAATCTTCTTCGAAAGGCTATCCCCTGATAAA
GGCAGGTTGCACACGTGTTCCGCACCCGTACGCCGCTCTCAATTACCCGAAAGTAATCTACCGCTCGGCTTGC
ATGTGTTAGGCCTCCCGCTAGCGTTCATCCGTC
>1855
CTGCTGCCGCCCGTAGGAGTCTGGTCCGTGTCTCAGTACCAGTGTGGGGGATCACCCTCTCAGGCCCCCTAAA
GATCGTCGCCTTGGTGAGCCGTTACCTCACCAACTAGCTAATCTTGCGCGTGCCCATCTCTATCCACCGTAGT
TTTCAATATCACCCGATGCCGAATAATATATTATGGGGTATTAATCTTCTCGAAAGGCTATCCCCCTGATAAA
GGCAGGTTGCACACGTGTTCCGCACCCGTACGCCGCTCTCAATTACCCGAAAGTAATCTACCGCTCGGCTTAG
CATGTGTTACGGCCTTCCC
>1856
CTGCTGCCGCCGTAGGAGTCTGGACCGTGTCTCAGTTCCAGTGTGACTGATCATCCTCTCAGACCAGTACGGA
TCGTCGCCTTGGTAGGCCTTTACCCCACCAACTAGCTAATCCGACCTAGGCTCATCTGATAGCGTGAGGTCCG
AAGATCCCCCACTTCTCCCTCAGGACGTATGCGGTATTAGCGCCCGTTTCCGGACGTATCCCCACTACCAGGC
AGATTCCTAGGCATTACTCACCCGTCCGCCGCTGAATCCAGGAGCAAGCCCTCCACTCGCTCGACTGCCATGT
GTTAGGCCTGCCGCCAGCGTCAATCTGAGCCAGGATCAACTCT
>1857
CTGCTGCCGCCCGTAGGAGTCTGGTCCGTGTCTCAGTACCAGTGTGGGGGATCACCCTCTCAGGCCCCCTAAA
GATCGTCGCCTTGGTGAGCCGTTACCTCACCAACTAGCTAATCTTGCGCGTGCCCATCTCTATCCACCGTAGT
TTTCAATATCACCCGATGCCGAATAATATATTATGGGGTATTAATCTTCTTCGAAAGCTATCCCCTGATAAAG
GCAGGTTGCACACGTGTTCCGCACCCGTACGCCGCTCTCAATTACCCGAAAGTAATCTACCGCTCCGGCTTAG
CATGTGTTGACGGC
>1858
CTGCTGCCGCCCGTAGGAGTCTGGTCCGTGTCTCAGTACCAGTGTGGGGGATCACCCTCTCAGGCCCCCTAAA
GATCGTCGCCTTGGTGAGCCGTTACCTCACCAACTAGCTAATCTTGCGCGTGCCCATCTCTATCCACCGTAGT
TTTTCAATATCACCCGATGCCGAATAATATATTATGGGGTATTAATCTTCTCGAAGCTATCCCCTGATAAAGG
CAGGTTGCACACGTGTTCCGCACCCGTACGCCGCTCTCAATTACCCGAAAGTAATCTACCGCTCCGGCTTGCA
TGTGTTAGGCCTCCCGCTAGCGTTCTCCTCGAGCAGGATCCAAT
>1859
CTGCTGCCGCCCGTAGGAGTCTGGTCCGTGTCTCAGTACCAGTGTGGGGGATCACCCTCTCAGGCCCCCTAAA
GATCGTCGCCTTGGTGAGCCGTTACCTCACCAACTAGCTAATCTTGCGCGTGCCCATCTCTATCCACCGTAGT
TTCAATATCACCCGATGCCGAATAATATATTATGGGGTATTAATCTTCTTCGAAAGGCTATCCCCCTGATAAA
GGCAGGTTGCACACGTGTTCCGCACCCGTACGCCGCTCTCAATTACCCGAAAGTAATCTACCGCTCGGCTTGC
ATGTGTTAGGCCTCCCGCTAGCGTTCTCCTGAGCAGGATCAACTCT
>1860
CTGCTGCCGCCCGTAGGAGTCTGGTCCGTGTCTCAGTACCAGTGTGGGGGATCACCCTCTCAGGCCCCCTAAA
GATCGTCGCCTGGTGAGCCGTTACCTCACCAACTAGCTAATCTTGCGCGTGCCCATCTCTATCCACCGTAGTT
TTCAATATCACCCGATGCCGAATAATATATTATGGGGTATTAATCTTCTCGAAAGGCTATCCCCTGATAAAGG
CAGGTTGCACACGTGTTCCGCACCCGTACGCCGCTCTCAATTACCCGAAAGTAATCTACCGCTCGGCTTGCAT
GTGTTAGGCCTCCCGCTAGCGT
>1861
CTGCTGCCGCCCGTAGGAGTCTGGTCCCGTGTCTCAGTACCCAGTGTGGGGGGATCACCCCTCTCAGGCCCCC
CTAAAGATCGTCGCCTTGGTGAGCCCGTTACCTCACCCAACTAGCTTAATCTTGCGCGTGCCCCATCTCTATC
CCACCGTAGTTTTTCAATATCACCCGATGCCCGAATAATATATTTATGGGGTATTTAATCTTTCTCGAAAGGC
TATCCCCCTGATTAAAGGCAGGTTGCACACGTGTTCCCGCACCCGTACGCCGCTCTCAATTACCCGAAAGTAA
TCTACCGCTCGGCTGCATGTGTTTAGGCCCTTCCCGCTAGCCGTTCATC
>1862
CTGCTGCCGCCCCGTAGGAGTCTGGACCCGTGTCTCAGTTCCAGTGTGACTGATCATCCTCTCAGACCAGTAC
GGATCGTCGCCTTGGTAGGCCTTTACCCCCACCAACTAGCTAATCCGACCTAGGCTCATCTGATAGCGTGAGG
TCCGAAGTCCCCCACTTCTCCCCTCAGGACGTATGCGGTATTAGCGCCCGTTTCCGGACGTATCCCCACTACC
AGGCAGATTCCTAGGCATTACTCACCCGTCCGCCGCTGAATCCAGGAGCAAGCTCCCTCATCCGCTCGACTGC
ATGTGTTAGGCCTGCCGCCAGCGTCAATCTGAGCCAGGATCAAACTCT

>1863
CTGCTGCCGCCCGTAGGAGTCTGGACCGTGTCTCAGTTCCAGTGTGACTGATCATCCTCTCAGACCAGTACGG
ATCGTCGCCTTGGTAGGCCTTTACCCCACCAACTAGCTAATCCGACCTAGGCTCATCTGATAGCGTGAGGTCC
GAAGATCCCCCACTTCTCCCTCAGGACGTATGCGGTATTAGCGCCCGTTTCCGGACGTATCCCCACTACCAGG
CAGATTCCTAGGCATTACTCACCCGTCCGCCGCTGAATCCAGGAGCAAGCTCCCTTCATCGCTCGACTGCATT
GTGTTAGGCCTGCCGCCAGCGTCAATCTGAGCCAGAATCAAACTCT
>1864
CTGCTGCCGCCCGTAGGAGTCTGGTCCGTGTCTCAGTACCAGTGTGGGGGATCACCCTCTCAGGCCCCCCTAA
AGATCGTCGCCTTGGTGAGCCGTTACCTCACCCAACTAGCTAATCTTGCGCGTGCCCATCTCTATCCACCGTA
GTTTTCAATATCACCCGATGCCGAATAATATATTATGGGGTATTTAATTCTTCTCGAAAGGCTATCCCCTGAT
AAAGGCAGGTTTGCACACGTGCTCCGCACCCCGTACGCCGCTCTCAATTACCCGAAAGAACTCTACCGCTCGG
CTTGCATGTGTTAGGCCTCCCGCTAGCGTTCATCCTGAGCCAGGATCAAACTCT
>1865
CTGCTGCCGCCCGTAGGAGTCTGGTCCGTGTCTCAGTACCAGTGTGGGGGATCACCCTCTCAGGCCCCCTAAA
GATCGTCGCCTTGGTGAGCCGTTACCTCACCAACTAGCTAATCTTGCGCGTGCCCATCTCTATCCACCGTAGT
TTTCAATATCACCCGATGCCGAATAATATATTTATGGGGTATTTAATCTTTCTTCGAAAGGCTATCCCCCTGA
ATTAAAGGCAGGTTGCACACGTGTTCCGCACCCGTACGCCGCTCTCAATTACCCGAAAGTAATCTACCGCTCG
GCTTGCATGTGTTAGGCCTCCCGCTAGCGTTCTCTCGAGCAGGATCAACATCCT
>1866
CTGCTGCCGCCCGTAGGAGTCTGGTCCGTGTCTCAGTACCAGTGTGGGGGATCACCCTCTCAGGCCCCCTAAA
GATCGTCGCCTTGGTGAGCCGTTACCTCACCAACTAGCTAATCTTGCGCGTGCCCATCTCTATCCACCGTAGT
TTTCAATATCACCCGATGTCGAATAATATATTATGGGGGTATTAATCTTCTTCGAAAGGCTATCCCCCTGATA
AAGGCAGGTTGCACACGTGTTCCGCACCCGTACGCCGCTCTCAATTTACCCGAAAGTAATCTACCGCTCCGGC
TTGCATGTGTTAGGCCTCCCGCTAGCGTTCATCCTGAGCCAGGATCAACTCT
>1867
CTGCTGCCGCCCGTAGGAGTCTGGTCCGTGTCTCAGTACCAGTGTGGGGGATCACCCTCTCAGGCCCCCTAAA
GATCGTCGCCTTGGTGAGCCGTTACCTCACCAACTAGCTAATCTTGCGCGTGCCCATCTCTATCCACCGTAGT
TTTCAATATCACCCGATGCCGAATAATATATTATGGGGTATTAATCTTCTTCGAAAGGCTATCCCCCTGATAA
AGGCAGGTTGCACACGTGTTCCGCACCCGTACGCCGCTCTCAATTACCCGAAAGTAATCTACCGCTCGGCTTG
CATGTGTTAGGCCTCCCGCTAGCGTTCATCCGTG
>1868
CTGCTGCCGCCCGTAGGAGTCTGGTCCGTGTCTCAGTACCAGTGTGGGGGATCACCCTCTCAGGCCCCCTAAA
GATCGTCGCCTTGGTGAGCCGTTACCTCACCAACTAGCTAATCTTGCGCGTGCCCATCTCTATCCACCGTAGT
TTTCAATATCACCCGATGCCGAATAATATATTATGGGGTATTAATCTTCTTCGAAGGCTATCCCCTGGTAAAG
GCAGGTTGCACACGTGTTCCGCACCCGTACGCCGCTCTCAATTTACCCGAAAGTAATCTACCGCTCGGCTTGC
ATGTGTTAGGCCTCCCGCTAGCGTTCTCCTGAGCAGGATCAACTCT
>1869
CTGCTGCCGCCCGTAGGAGTCTGGTCCGTGTCTCAGTACCAGTGTGGGGGATCACCCTCTCAGGCCCCCTAAA
GATCGTCGCCTTGGTGAGCCGTTACCTCACCAACTAGCTAATCTTGCGCGTGCCCATCTCTATCCACCGTAGT
TTTCAATATCACCCGATGCCGAATAATATATTATGGGGGTATTAATCTTCTTCGAAAGGCTATCCCCCTGATA
AAGGCAGGTTGCACACGTGTTCCGCACCCGTACGCCGCTCTCAATTACCCGAAAGTAATCTACCGCTCGGCTT
GCATGTGTTAGGCCTCCCGCTAGCGTTCATCCTGAGCA
>1870
CTGCTGCCGCCCGTAGGAGTCTGGTCCGTGTCTCAGTACCAGTGTGGGGGATCACCCTCTCAGGCCCCCTAAA
GATCGTCGCCTTGGTGAGCCGTTACCTCACCAACTAGCTAATCTTGCGCGTGCCCATCTCTATCCACCGTAGT
TTTCAATATCACCCGATGCCGAATAATATATTATGGGGTATTAATCTTCCTCTCGAAAGGCTATCCCCCTGAT
AAAGGCAGGTTGCACACGTGTTCCGCACCCGTACGCCGCTCTCAATTACCCGAAAGTAATCTACCGCTCGGCT
TGCATGTGTTAGGCCTCCCGCTAGCGTTCATCCTGAGCCAGGATCAAACTCT
>1871
CTGCTGCCGCCCGTAGGAGTCTGGTCCGTGTCTCAGTACCAGTGTGGGGGATCACCCTCTCAGGCCCCCTAAA
GATCGTCGCCTTGGTGAGCCGTTACCTCACCAACTAGCTTAATCTTGCGCGTGCCCATCTCTATCCACCGTAG
TTTTTCAATATCACCCGATGCCGAATAATATATTTATGGGGTATTTAATTCTTTCTTCGAAAGGCTATCCCCC
TGATAAAAGGCAGGTTGCACACGTGTTCCGCACCCGTACGCCGCTCTCAATTACCCGAAAGTAATCTACCGCT
CGGCTTGCATGTGTTAGGCCTCCCGCTAGCGTTCATTCCTGAGCCAGGATCAAACTCT

>1872
CTGCTGCCGCCCGTAGGAGTCTGGTCCGTGTCTCAGTACCAGTGTGGGGGATCACCCTCTCAGGCCCCCTAAA
GATCGTCGCCTTGGTGAGCCGTTACCTCACCAACTAGCTAATCTTGCGCGTGCCCATCTCTATCCACCGTAGT
TTTCAATATCACCCGATGCCGAATAATATATTATGGGGTATTAATCTTCTTCGAAAGGCTATCCCCTGATAAA
GGCAGGTTGCACACGTGTTCCGCACCCGTACGCCGCTCTCAATTACCCGAAAGTAATCTACCGCTCGGCTTGC
ATGTGTTAGGCCTCCCGCTAGCGTTCATCCTGAGCAGGATCAAACTCGT
>1873
CTGCTGCCGCCCGTAGGAGTCTGGTCCGTGTCTCAGTACCAGTGTGGGGGATCACCCTCTCAGGCCCCCTAAA
GATCGTCGCCTTGGTGAGCCGTTACCTCACCAACTAGCTAATCTTGCGCGTGCCCATCTCTATCCACCGTAGT
TTTTCAATATCACCCGATGCCGAATAATATAATTATGGGGTATTTAATCTTTCTTCGAAAGGCTATCCCCCTG
ATAAAGGCAGGTTGCACACGTGTTCCGCACCCGTACGCCGCTCTCAATTACCCGAAAGTAATCTACCGCTCCG
GCTTGCATGTGTTAGGCC
>1874
CTGCTGCCGCCCGTAGGAGTCTGGTCCGTGTCTCAGTACCAGTGTGGGGGATCACCCTCTCAGGCCCCCTAAA
GATCGTCGCCTTGGTGAGCCGTTACCTCACCAACTAGCTAATCTTGCGCGTGCCCATCTCTATCCACCGTAGT
TTTCAATATCACCCGATGCCGAATAATATATTATGGGGTATTAATCTTCTTCGAAAGGCTATCCCCTGATAAA
GGCAGGTTGCACACGTGTTCCGCACCCGTACGCCGCTCTCAATTACCCGAAAGTAATCTACCGCTCCGGCTTG
CATGTGTTAGGCCTCCCGCTAGCGTTCATCCTGAGCA
>1875
CTGCTGCCGCCCGTAGGAGTCTGGTCCGTGTCTCAGTACCAGTGTGGGGGATCACCCTCTCAGGCCCCCTAAA
GATCGTCGCCTTGGTGAGCCGTTACCTCACCAACTAGCTAATCTTGCGCGTGCCCATCTCTATCCACCGTAGT
TTTCAATATCACCCGATGCCGAATAATATATTATGGGGTATTAATCTTCTCGAAAGGCTATCCCCTGATAAAG
GCAGGTTGCACACGTGTTCCGCACCCGTACGCCGCTCTCAATTACCCGAAAGTAATCTACCGCTCCGGCTTGC
ATGTGTTAGGCCTCCCGCTAGCGTTCATCCTGAGCCAGGATCAACTCT
>1876
CTGCTGCCGTCCGTAGGAGTCTGGACCGTGTCTCAGTTCCAGTGTGACTGATCATCCTCTCAGACCAGTACGG
ATCGTCGCCTGGTAGGCCTTACCCCACCAACTAGCTAATCCGAACCTAGGCTCATCTGATAGCGTGAGGTCCG
AAAGATCCCCCACTTCTCCCTCAGGACGTATGCGGTATTAGCGCCCGTTTCCGGACGATCCCCACTACCAGGC
AGATTCCTAGGCATTACTCACCCGTCCGCCGCTGAATCCAGGAGCAAGCCTCCCTCATCCGCTCGACTGCAAT
GTGTTAGGCCTGCCGCCAGCGTCAATCTGAGCCAGGATAAACTCT
>1877
CTGCTGCCGCCCGTAGGAGTCTGGTCCGTGTCTCAGTACCAGTGTGGGGGATCACCCTCTCAGGCCCCCTAAA
GATCGTCGCCTTGGTGAGCCGTTACCTCACCAACTAGCTAATCTTGCGCGTGCCCATCTCTATCCACCGTAGT
TTTCAATATCACCCGATGCGAATAATATATTATGGGGTATTAATCTTCTCGAAGGCTATCCCCTGATAAAGGC
AGGTTGCACACGTGTTCCGCACCCGTACGCCGCTCTCAATTACCCGAAAGTAATCTACCGCTCCGGCTTGCAT
GTGTTAGGCCTCCCGCTAGCGTTCATCC
>1878
CTGCTGCCGCCCGTAGGAGTCTGGTCCGTGTCTCAGTACCAGTGTGGGGGATCACCCTCTCAGGCCCCCTAAA
GATCGTCGCCTTGGTGAGCCGTTACCTCACCAACTAGCTAATCTTGCGCGTGCCCATCTCTATCCACCGTAGT
TTTTCAATATCACCCGATGCCGAATAATATAATTTATTGGGGTATTTAATTCTTTCTCGAAAGGCTATCCCCC
TGATAAAAGGCAGGTTGCACACGTGTTCCGCACCCGTACGCCGCTCTCAATTACCCGAAAGTAATCTACCGCT
CCGGCTTGCATGTGTTAGGCCTCCCGCTAGCGTTCTTCCGTCGAGC
>1879
CTGCTGCCGCCCGTAGGAGTCTGGTCCGTGTCTCAGTACCAGTGTGGGGGATCACCCTCTCAGGCCCCCTAAA
GATCGTCGCCTTGGTGAGCCGTTACCTCACCAACTAGCTAATCTTGCGCGTGCCCATCTCTATCCACCGTAGT
TTTCAATATCACCCGATGCCGAATAATATATTATGGGGTATTAATCTTCTCGAAAGGCTATCCCCTGATAAAG
GCGGTTGCACACGTGTTCCGCACCCGTACGCCGCTCTCAATTACCCGAAAGTAATCTACCGCTCGGCTTGCAT
GTGTTAGGCCTCCCGCTAGCGTTCTCCTGAGCAGGATCAACTCT
>1880
CTGCTGCCGCCCGTAGGAGTCTGGTCCGTGTCTCAGTACCAGTGTGGGGGATCACCCTCTCAGGCCCCCTAAA
GATCGTCGCCTTGGTGAGCCGTTACCTCACCAACTAGCTAATCTTGCGCGTGCCCATCTCTATCCACCGTAGT
TTTCAATATCACCCGATGCCGAATAATATATTATGGGGTATTAATCTTCTTCGAAAGGCTATCCCCCTGATAA
AGGCAGGTTGCACACGTGTTCCGCACCCGTACGCCGCTCTCAATTACCCCGAAAGTAATCTACCGCTCGGCTT
GCATGTGTTAGGCCTCCCGCTAGCGTCTCT

>1881
CTGCTGCCGCCCCGTAGGAGTCTGGTCCCGTGTCTCAGTACCAGTGTGGGGGATCACCCCTCTCAGGCCCCCC
TAAAGATCGTCGCCTTGGTGAGCCGTTACCTCACCCAACTAGCTAATCTTGCGCGTGCCCCATCTCTATCCCA
CCGTAGTTTTCAATATCACCCGATGCCCGAATAATTATATTATGGGGGTATTTAATTCTTCTTCGAAAGGCTA
TCCCCCTGATAAAGGCAGGTTGCACACGTGTTCCGCACCCGTACGCCGCTCTCAATTACCCCGAAAGTAATCT
ACCGCTCGGCTTGCATGTGTTAGGCCTCCCGCTAGCGTTCTCCTGAGCA
>1882
CTGCTGCCGCCCGTAGGAGTCTGGTCCGTGTCTCAGTACCAGTGTGGGGGATCACCCTCTCAGGCCCCCTAAA
GATCGTCGCCTTGGTGAGCCGTTACCTCACCACTAGCTAATCTTGCGCGTGCCCATCTCTATCCACCGTAGTT
TCAATATCACCCGATGCCGAATAATATATTATGGGGTATTAATCTTCTTCGAAGGCTATCCCCCTGATAAAGG
CAGGTTGCACACGTGTTCCGCACCCGTACGCCGCTCTCAATTACCCGAAAGTAATCTACCGCTCGGCTTGCAT
GTGTTAGGCCTCCCGCTAGCGTTCTCCTGAGCAGGATCAACTCGT
>1883
CTGCTGCCGCCCGTAGGAGTCTGGTCCGTGTCTCAGTACCAGTGTGGGGGATCACCCTCTCAGGCCCCCTAAA
GATCGTCGCCTTGGTGAGCCGTTACCTCACCAACTAGCTAATCTTGCGCGTGCCCATCTCTATTCCACCGTAG
TTTCAATATCACCCGATGCCGAATAATATAATTATGGGGTATTAATCTTCTCTGAAAGGCTATCCCCTGATAA
AGGCAGGTTGCACACGTGTTCCGCACCCGTACGCCGCTCTCAATTTACCCGAAAGTAATCTACCGCTCGGCTT
GCATGTGTTAGGCCTCCCGCTAGCGTTCATCCTGAGCCAGGATCAACTCT
>1884
CTGCTGCCGCCCGTAGGAGTCTGGACCGTGTCTCAGTTCCAGTGTGACTGATCATCCTCTCAGACCAGTACGG
ATCGTCGCCTTGGTAGGCCTTTTACCCCCACCAACTAGCTAATCCGACCTAGGCTCATCTGATAGCGTGAGGT
CCGAAGATCCCCCACTTCTCCCTCAGGACGTATGCGGTTATTAGCGCCCGTTTCCGGACGATCCCCACTACCA
GGCAGATTTCCTAGGCATTACTCACCCGTCCGCCGCTGAATCCAGGAGCAAGCCCCTCCATCCGCTCGACTGC
ATGTGTTAGGCCTGCCGCCAGCGTCAATCTGAGCCAGGATCAAACTCT
>1885
CTGCTGCCGCCCGTAGGAGTCTGGTCCGTGTCTCAGTACCAGTGTGGGGGATCACCCTCTCAGGCCCCCTAAA
GATCGTCGCCCTGGTGAGCCGTTACCTCACCAACTAGCTAATCTTGCGCGTGCCCATCTCTATCCACCGTAGT
TTTCAATATCACCCGATGCCGAATAATATATTATGGGGTATTTAATCTTCTCGAAGGCTATCCCCTGATAAAG
GCAGGTTGCACACGTGTTCCGCACCCGTACGCCGCTCTCAATTACCCGAAAGTAATCTACCGCTCGGCTTGCA
TGTGTTAGGCCTCCCGCTAGCGTTC
>1886
CTGCTGCCGCCCGTAGGAGTCTGGACTCGTGTCTCAGTTCCAGTGTGACTGATCATCCTCTCAGACCAGTACG
GATCGTCGCCTTGGTAGGCCTTTACCCCACCAACTAGCTAATCCGACCTAGGCTCATCTGATAGCGTGAGGTC
CGAAGTCCCCCACTTCTCCCCACGAGAGTATGCGGTATTAGCGCCCGTTTCCGGACGTATCCCCACTACCAGG
CAGATTCCCTAGGCATTACTCACCCGTCCGCCGCTGAATCCAGGAGCAAGCCCTCCATCCGCTCGACTGCATG
TGTTAGGCCTCGCCGCAGCGTTCATCTGAGCCAGGATCAAACTCT
>1887
CTGCTGCCGCCCGTAGGAGTCTGGACCGTGTCTCAGTTCCAGTGTGACTGATCATCCTCTCAGACCAGTACGG
ATCGTCGCCTTGGTAGGCCTTTACCCCACCAACTAGCTAATCCGACCTAGGCTCATCTGATAGCGTGAGGTCC
GAAGTCCCCCACTTCTCCCTCAGGACGTATGCGGTATTAGCGCCCGTTTCCGGACGTATCCCCACTACCAGGC
AGATTCCTAGGCATTACTCACCCGTCCGCCGCTGAATCCAGGAGCAAGCTCCCTCATCCGCTCGACTGCCATG
TGTTAGGCCTGCCGCCAGCGTCAATCTGAGCCAGGATCAAACTCT
>1888
CTGCTGCCGCCCGTAGGAGTCTGGTCCGTGTCTCAGTACCAGTGTGGGGGATCACCCTCTCAGGCCCCCTAAA
GATCGTCGCCTTGGTGAGCCGTTACCTCACCAACTAGCTAATCTTGCGCGTGCCCATCTCTATCCACCGTAGT
TTCAATATCACCCGATGCCGAATAATATATTATGGGGTATTAATCTTCTTCGAAAGGCTATCCCCTGATAAAG
GCAGGTTGCACACGTGTTCCGCACCCGTACGTCGCTCTCAATTACCCGAAAGTAATCTACCGCTCGGCTTGCA
TGTGTTAGGCCTCCCGCTAGCGTCATCCTGAGCCAGGATCAAACTCT
>1889
CTGCTGCCGCCCGTAGGAGTCTGGTCCGTGTCTCAGTACCAGTGTGGGGGATCACCCTCTCAGGCCCCCTAAA
GATCGTCGCCTTGGTGAGCCGTTACCTCACCAACTAGCTAATCTTGCGCGTGCCCATCTCTATCCACCGTAGT
TTTCAATATCACCCGATGCCGAATAATATATTATGGGGTATTAATCTTCTCGAAGGCTATCCCCTGAATAAAG
GCAGGTTGCCACACGTGTTCCGCACCCGTACGCCGCTCTCAATTACCCGAAAGTAATCTACCGCTCGGCTTGC
ATGTGTTAGGCCTCCCGCTAGCGTTCATCCTGAGCA

>1890
CTGCTGCCGCCCGTAGGAGTCTGGTCCGTGTCTCAGTACCAGTGTGGGGGATCACCCTCTCAGGCCCCCTAAA
GATCGTCGCCTTGGTGAGCCGTTACCTCACCAACTAGCTAATCTTGCGCGTGCCCATCTCTATCCACCGTAGT
TTTCAATATCACCCGATGCCGAATAATATATTATGGGGTATTAATCTTCTTCGAAAGGCTATCCCCCTGATAA
AGGCAGGTTGCACACGTGTTCCGCACCCGTACGCCGCTCTCAATTACCCGAAAGTAATCTACCGCTCCGGCTT
GCATGTGTTAGGCCTTCCGCTAGCGTTCTCCTGAGCAGGATCAACTCGT
>1891
CTGCTGCCGCCCGTAGGAGTCTGGTCCGTGTCTCAGTACCAGTGTGGGGGATCACCCTCTCAGGCCCCCTAAA
GATCGTCGCCTTGGTGAGCCGTTACCTCACCAACTAGCTAATCTTGCGCGTGCCCATCTCTATCCACCGTAGT
TTCAATATCACCCGATGCCGAATAATATATTATGGGGTATTAATCTTCTCTGAAAGGCTATCCCCTGATAAAG
GCAGGTTGCACACGTGTTCCGCACCCGTACGCCGCTCTCAATTACCCGAAAGTAATCTACCGCTCCGGCTTGC
ATGTGTTAGGCCTCCCGCTAGCGTTCATCCTGAGCCAGGATCAAACTCT
>1892
CTGCTGCCGCCCGTAGGAGTCTGGTCCGTGTCTCAGTACCAGTGTGGGGGATCACCCTCTCAGGCCCCCTAAA
GATCGTCGCCTTGGTGAGCCGTTACCTCACCAACTAGCTAATCTTGCGCGTGCCCATCTCTATCCACCGTAGT
TTTCAATATCACCCGATGCCGAATAATATATTATGGGGTATTAATCTTCTTCGAAAGGCTATCCCCCTGATAA
AGGCAGGTTGCACACGTGTTCCGCACCCGTACGCCGCTCTCAATTACCCGAAAGTAATCTACCGCTCCGGCTT
GCATGTGTTAGGCCTCCCGCTAGCGTTCATCCTGAGCCA
>1893
CTGCTGCCGCCCGTAGGAGTCTGGTCCGTGTCTCAGTACCAGTGTGGGGGATCGCCCTCTCAGGCCCCCTAAA
GATCGTCGCCTTGGTGAGCCGTTACCTCACCAACTAGCTAATCTTGCGCGTGCCCATCTCTATCCACCGTAGT
TTTCAATATCACCCGATGCCGAATAATATATTATGGGGTATTAATCTTCTTCGAAAGCTATCCCCTGATAAAG
GCAGGTTGCACACGTGTTCCGCACCCGTACGCCGCTCTCAATTACCCGAAAGTAATCTACCGCTCGGCTTGCA
TGTGTTAGGCCTCCCGCTAGCGTCATCCTGAGCCAGGATCAACTCT
>1894
CTGCTGCCGCCCGTAGGAGTCTGGTCCGTGTCTCAGTACCAGTGTGGGGGATCACCCTCTCAGGCCCCCTAAA
GATCGTCGCCTGGTGAGCCGTTACCTCACCAACTAGCTAATCTTGCGCGTGCCCATCTCTATCCACCGTAGTT
TTCAATATCACCCGATGCCGAATAATATATTATGGGGTATTAATCTTCTTCGAAAGGCTATCCCCTGATAAAG
GCAGGTTGCACACGTGTTCCGCACCCGTACGCCGCTCTCAATTACCCGAAAGTAATCTACCGCTCGGCTTGCA
TGTGTTAGGCCTCCCGCTAGCGTTCATC
>1895
CTGCTGCCGCCCGTAGGAGTCTGGTCCGTGTCTCAGTACCAGTGTGGGGGATCACCCTCTCAGGCCCCCTAAA
GATCGTCGCCTTGGTGAGCCGTTACCTCACCAACTAGCTAATCTTGCGCGTGCCCATCTCTATCCACCGTAGT
TTTCAATATCACCCGATGCCGAATAATATATTATGGGGTATTAATCTTCTTCGAAAGGCTATCCCCCTGATAA
AAGGCAGGTTGCACACGTGTTCCGCACCCGTACGCCGCTCTCATGTACCCGAAAGTAATCTACCGCATCGGCT
TGCATGTGTTAGGCCTCC
>1896
CTGCTGCCGCCCCGTAGGAGTCTGGTCCCGTGTCTCAGTACCAGTGTGGGGGATCACCCCTCTCAGGCCCCCT
AAAGATCGTCGCCTTGGTGAGCCGTTACCTCACCCAACTAGCTTAATCTTGCGCGTGCCCCATCTCTATCCAC
CGTTAGTTTTTCAATATCACCCGATGCCGAATAATATATTTATGGGGTATTTAATCTTTCTCGAAAGGCTAT
CCCCTGATAAAGGCAGGTGCACACGTGTTCCCGCACCCGTACGCCGCTCTCAATTTACCCCCGAAAGTAATCT
ACCGCTCGGCTTGCATGTGTTAGGCCTTCCC
>1897
CTGCTGCCGCCCGTAGGAGTCTGGTCCGTGTCTCAGTACCAGTGTGGGGGATCACCCTCTCAGGCCCCCTAAA
GATCGTCGCCTTGGTGAGCCGTTACCTCACCAACTAGCTAATCTTGCGCGTGCCCATCTCTATCCACCGTAGT
TTTCAATATCACCCGATGCCGAATAATATATTATGGGGTATTAATCTTCTTCGAAAGGCTATCCCCTGATAAG
GCAGGTTGCACACGTGTTCCGCACCCGTACGCCGCTCTCAATTACCCGAAAGTAATCTACCGCTCGGCTTGCA
TGTGTTAGGCCTCCCGCTAGCGTTCATCCTGAGCCAGGATCAAACTCT
>1898
CTGCTGCCGCCCGTAGGAGTCTGGTCCGTGTCTCAGTACCAGTGTGGGGGATCACCCTCTCAGGCCCCCTAAA
GATCGTCGCCTTGGTGAGCCGTTACCTCACCAACTAGCTAATCTTGCGCGTGCCCATCTCTATCCACCGTAGT
TTTCAATATCACCCGATGCCGAATAATATATTATGGGGTATTAATCTTCTCGAAAGGCTATCCCCTGATAAAG
GCAGGTTGCACACGTGTTCCGCACCCGTACGCCGCTCTCAATTACCCGAAAGTAATCTACCGCTCCGGCTTGC
ATGCGTTAGGCCTCCCGCTAGCGTCATCCGTGAGCCAGGATCAAACTCT

>1899
CTGCTGCCGCCCGTAGGAGTCTGGTCCGTGTCTCAGTACCAGTGTGGGGGATCACCCTCTCAGGCCCCCTAAA
GATCGTCGCCTTGGTGAGCCGTTACCTCACCAACTAGCTAATCTTGCGCGTGCCCATCTCTATCCACCGTAGT
TTTCAATATCACCCGATGCCGAATAATATATTATGGGGTATTAATCTTCTACGAAAGGCTATCCCCCTGATAA
AGGCAGGTTGCACACGTGTTCCGCACCCGTACGCCGCTCTCAATTACCCGAAAGTAATCTACCGCTCGGCTTG
CATGTGTTAGGCCTCCCGCTAGCGTTCATCCTGAGCCAGGATCAAACTCT
>1900
CTGCTGCCGCCCGTAGGAGTCTGGTCCGTGTCTCAGTACCAGTGTGGGGGATCACCCTCTCAGGCCCCCTAAA
GATCGTCGCCTTGGTGAGCCGTTACCTCACCAACTAGCTAATCTTGCGCGTGCCCATCTCTATCCACCGTAGT
TTTCAATATCACCCGATGCCGAATAATATATTATGGGGTATTAATCTTCTTCGAAAGGCTATCCCCCTGATAA
AGGCAGGTTGCACACGTGTTCCGCACCCGTACGCCGCTCTCAATTACCCGAAAGTAATCTACCGCTCGGCTTG
CATGTGTTAGGCCTCCCGCTAGCGTTCATCCTGAGCCAGGATCAAACTCT
>1901
CTGCTGCCGCCCGTAGGAGTCTGGTCCGTGTCTCAGTACCAGTGTGGGGGATCACCCTCTCAGGCCCCCTAAA
GATCGTCGCCTTGGTGAGCCGTTACCTCACCAACTAGCTAATCTTGCGCGTGCCCATCTCTATCCACCGTAGT
TTTCAATATCACCCGATGCCGAATAATATATTATGGGGTATTAATCTTCTCGAAAGGCTATCCCCTGATAAGC
AGGTTGCACACGTGTTCCGCACCCGTACGCCGCTCTCAATTACCCGAAAGTAATCTACCGCTCCGGCTTGCAT
GTGTTAGGCCTCCCGCTAGCGTTCATCC
>1902
CTGCTGCCGCCCGTAGGAGTCTGGTCCGTGTCTCAGTACCAGTGTGGGGGATCACCCTCTCAGGCCCCCTAAA
GATCGTCGCCTTGGTGAGCCGTTACCTCACCAACTAGCTAATCTTGCGCGTGCCCATCTCTATCCACCGTAGT
TTTCAATATCACCCGATGCCGAATAATAATATTATGGGGTATTAATCTTCTCCGAAAGGCTGTCCCCTGATAA
AGCAGGTTGCACACGTGTTCCGCACCCGTACGCCGCTCTCAATTACCCGAAAGTAATCTACCGCTCGGCTTGC
ATGTGTTAGGCCTCCCGCTAGCGTTCTCTCGAGCAAGGATCCAATATCCT
>1903
CTGCTGCCGCCCGTAGGAGTCTGGTCCGTGTCTCAGTACCAGTGTGGGGGATCACCCTCTCAGGCCCCCCAAA
GATCGTTGCCTTGGTGAGCCGTTACCTCACCAACTAGCTAATCTTGCGCGTGCCCATCTCTATCCACCGTAGT
TTTCAATATCACCCGATGCCGAGTAATATATTATGGGGTATTAATCGTTCTTCTGAAAGGCTATCCCCTGATC
AAAGGCAGGTTAGGCCACACGTGTTCCGCACCCGTACGCCGCTCTCAATTACCCGAAAGTAATCTACCGCTCC
GGCTTGCATGTGTTAGGCCTCCCGCTAGCGTTCATCTCGAGCC
>1904
CTGCTGCCGCCCGTAGGAGTCTGGTCCGTGTCTCAGTACCAGTGTGGGGGATCACCCTCTCAGGCCCCCTAAA
GATCGTCGCCTTGGTGAGCCGTTACCTCACCAACTAGCTAATCTTGCGCGTGCCCATCTCTATCCACCGTAGT
TTCAATATCACCCGATGCCGAATAATATATTATGGGGTATTAATCTTCTCGAAAGGCTATCCCCTGATAAAGG
CAGGTTGCACACGTGTTCCGCACCCGTACGCCGCTCTCAATTACCCGAAAGTAATCTACCGCTCGGCTTGCAT
GTGTTAGGCCTCCCGCTAGCGTCTCCTGA
>1905
CTGCTGCCGCCCGTAGGAGTCTGGTCCGTGTCTCAGTACCAGTGTGGGGGATCACCCTCTCAGGCCCCCTAAA
GATCGTCGCCTTGGTGAGCCGTTACCTCACCAACTAGCTAATCTTGCGCGTGCCCATCTCTATCCACCGTAGT
TTTCAATATCACCCGATGCCGAATAATATATTATGGGGTATTAATCTTCTTCGAAAGCTATCCCCCTGATAAA
GGCAGGTTGCAACACGTGTTCCGCACCCGTACGCCGCTCTCAATTACCCGAAAGTAATCTACCGCTCGGCTTG
CATGTGTTAGGCCTCCCGCTAGCGTTCTCTGAG
>1906
CTGCTGCCGCCCGTAGGAGTCTGGTCCGTGTCTCAGTACCAGTGTGGGGGATCACCCTCTCAGGCCCCCTAAA
GATCGTCGCCTTGGTGAGCCGTTACCTCACCAACTAGCTAATCTTGCGCGTGCCCACCTCTATCCACCGTAGT
TTTCAATATCACCCGATGCCGAATAATATATTATGGGGTATTAATCTTCTTCGAAAGGCTATCCCCCTGATAA
AGGCAGGTTGCACACGTGTTCCGCACCCGTACGCCGCTCTCAATTACCCGAAAGTAATCTACCGCTCGGCTTG
CATGTGTTAGGCCTCCCGCTAGCGTTCATCCTGAGCAGGATCAACTCT
>1907
CTGCTGCCGCCCGTAGGAGTCTGGTCCGTGTCTCAGTACCAGTGTGGGGGATCACCCTCTCAGGCCCCCTAAA
GATCGTCGCCTTGGTGAGCCGTTACCTCACCAACTAGCTAATCTTGCGCGTGCCCATCTCTATCCACCGTAGT
TTTCAATATCACCCGATGCCGAATAATATATTATGGGGTATTAATCTTTCTTCGAAAGGCTATCCCCCTGATA
AAGGCAGGTTGCACACGTGTTCCGCACCCGTACGCCGCTCTCAATTACCCGAAAGTAATCTACCGCTCGGCTT
GCATGTGTTAGGCCTCCCGCTAGCGTTCTCCGTCGA

>1908
CTGCTGCCGCCCGTAGGAGTCTGGACCGTGCCTCAGCTCCAGTGTGACTTGATCATCCTCTCAGACCAGTACG
GATCGTCGCCTTGGTAGGCCTTTACCCCACCAACTAGCTAATCCGACCTAGGCTCATCTGATAGCGTGAGGTC
CGAAGTCCCCCACTTCTCCCTCAGGACGTATGCGGTATTAGCGCCCGTTTCCGGACGTATCCCCGCACTACCA
GGCAGATTCCCTAGGCATTACTCACCCGTCCGCCGCTGAATCCAGGAGCAAGCCCCCTTCATCCGCTCGACTG
CATGTGTTAGGCCTGCCGCCAGCGTCAATCTGAGCCAGGATCAAACTCT
>1909
CTGCTGCCGCCCGTAGGAGCTGGTCCGTGTCTCAGTACCAGTGTGGGGGATCACCCTCTCAGGCCCCCTAAAG
ATCGTCGCCTGGTGAGCCGTTACCTCACCAACTAGCTAATCTTGCGCGTGCCCATCTCTATCCACCGTAGTTT
TCAATATCACCCGATGCCGAATAATATATTATGGGGTATTAATCTTCTCGAAAGGCTATCCCCTGATAAAGGC
AGGTTGCACACGTGTTCCGCACCCGTACGCCGCTCTCAATTACCCGAAAGTAATCTACCGCTCGGCTTGCATG
TGTTGACGGCTCCCGCTAGGCCTCGTT
>1910
CTGCTGCCGCCCGTAGGAGTCTGGTCCGTGTCTCAGTACCAGTGTGGGGGATCACCCTCTCAGGCCCCCTAAA
GATCGTCGCCTGGTGAGCCGTTACCTCACCAACTAGCTAATCTTGCGCGTGCCCATCTCTATCCACCGTAGTT
TTCAATATCACCCGATGCCGAATAATATAATTATGGGGGTATTAATCTTCTCGAAAGGCTATCCCCTGATAAA
GGCAGGTTGCACACGTGTTCCGCACCCGTACGCCGCTCTCAATTACCCGAAAGAATCTACCGCTCGGCTTGCA
TGTGTTAGGCCTCCCGCTAGCGT
>1911
CTGCTGCCGCCCGTAGGAGTCTGGTCCGTGTCTCAGTACCAGTGTGGGGGATCACCCTCTCAGGCCCCCTAAA
GATCGTCGCCTTGGTGAGCCGTTACCTCACCAACTAGCTAATCTTGCGCGTGCCCATCTCTATCCACCGTAGT
TTTCAATATCACCCGATGCCGAATAATATATTATGGGGTATTAATCTTCTTCGAAAGGCTATCCCCCTGATAA
AGGCAGGTTGCACACGTGTTCCGCACCCGTACGCCGCTCTCAATTACCCGAAAGTAATCTACCGCTCCGGCTT
GCATGTGTTAGGCCTCCCGCTAGCGTTCATCTCGAGCCAGGATCAACTCT
>1912
CTGCTGCCGCCCGTAGGAGTCTGGTCCGTGTCTCAGTACCAGTGTGGGGGATCACCCTCTCAGGCCCCCTAAA
GATCGTCGCCTTGGTGAGCCGTTACCTCACCAACTAGCTAATCTTGCGCGCGCCCATCTCTATCCACCGTAGT
TTTCAATATCACCCGATGCCGAATAATATATTATGGGGTATTAATCTTCTTCGAAAGGCTATCCCCTGATAAA
GGCAGGTTGCACACGTGTTCCGCACCCGTACGCCGCTCTCAATTACCCGAAAGTAATCTACCGCTCGGCTTGC
ATGTGTTAGGCCTCCCGCTAGCGTTCATCCTCGAGCAGGCATCAAACTCT
>1913
CTGCTGCCGCCCGTAGGAGTCTGGTCCGTGTCTCAGTACCGGTGTGGGGGATCACCCTCTCAGGCCCCCTAAA
GATCGTCGCCTTGGTGAGCCGTTACCTCACCAACTAGCTAATCTTGCGCGTGCCCATCTCTATCCACCGTAGT
TTTCAATATCACCCGATGCCGAATAATATATTATGGGGTATTAATCTTCTTCGAAGGCTATCCCCCTGAATAA
AGGCAGGTTGCACACGTGTTCCGCACCCGTACGCCGCTCTCAATTACCCGAAAGTAATCTACCGCTCCGGCTT
GCATGTGTTAGGCCTCCCGCTAGCGTTCATCTGAGCCAGGATCAAACTCT
>1914
CTGCTGCCGCCCGTAGGAGTCTGGACCGTGTCTCAGTTCCAGTGTGACTGATCATCCTCTCAGACCAGTACGG
ATCGTCGCCTTGGTAGGCCTTTACCCCACCAACTAGCTAATCCGACCTAGGCTCATCTGATAGCGTGAGGTCC
GAAGATCCCCCACTTCTCCCTCAGGACGTATGCGGTATTAGCGCCCGTTTCCGGACGTATCCCCACTACCAGG
CAGATTCCTAGGCATTACTCACCCGTCCGCCGCTGAATCCAGGAGCAAGCTCCCTCATCCGCTCGACTGCCAT
GTGTTAGGCCTGCCGCCAGCGTTCAATCTGAGCCAGGATCAAACTCT
>1915
CTGCTGCCGCCCGTAGGAGTCTGGTCCGTGTCTCAGTACCAGTGTGGGGGATCACCCTCTCAGGCCCCCTAAA
GATCGTCGCCTTGGTGAGCCGTTACCCTCAACCAACTAGCTAATCTTGCGCGTGCCCATCTCTATCCACCGTA
GTTTTTCAATATCACCCGATGCCGAATAATATATTATGGGGTATTTAATCTTCTCGAAAGCTATCCCCTGATA
AAGGCAGGTTGCACACGTGTTCCGCACCCGTACGCCGCTCTCAATTACCCGAAAGTAATCTACCGCTCCGGCT
TGCATGTGTAGGCTCCCGCTAGCGTTCTCCTCGAGCAGGATCAAT
>1916
CTGCTGCCGCCCGTAGGAGTCTGGTCCGTGTCTCAGTACCAGTGTGGGGGATCACCCTCTCAGGCCCCCTAAA
GATCGTCGCCTTGGTGAGCCCGTTACCTCACCCAACTAGCTTAATCTTGCGCGTGCCCATCTCTATCCACCGT
AGTTTCAATATCACCCGATGCCGAATAATATATTATTGGGGTATTAATTCTTCTTCGAAAGGCTATCCCCCTG
ATAAAGGCAGGTTGCACACGTGTTCCGCACCCGTACGCCGCTCTCAATTACCCCGAAAGTAATCTACCGCTCG
GCTTGCATGTGTTAGGCCTCCCGCTAGCGTTCATCCTGAGCCA

>1917
CTGCTGCCGCCCGTAGGAGTCTGGTCCGTGTCTCAGTACCAGTGTGGGGGATCACCCTCTCAGGCCCCCTAAG
ATCGTCGCCTTGGTGAGCCGTTACCTCACCAACTAGCTAATCTTGCGCGTGCCCATCTCTATCCACCGTAGTT
TTCAATATCACCCGATGCCGAATAATATATTATGGGGTATTAATCTTCTCGAAGGCTATCCCCTGATAAGGCA
GGTTGCACACGTGTTCCGCACCCGTACGCCGCTCTCAATTACCCGAAAGTAATCTACCGCTCCGGCTTGCATG
TGTTAGGCCTCCCGCTAGCGT
>1918
CTGCTGCCGCCCGTAGGAGTCTGGTCCGTGTCTCAGTACCAGTGTGGGGGATCACCCTCTCAGGCCCCCTAAA
GATCGTCGCCTGGTGAGCCGTTACCTCACCAACTAGCTAATCTTGCGCGTGCCCATCTCTATCCACCGTAGTT
TCAATATCACCCGATGCCGAATAATATATTATGGGGTATTTAATCTTCTCGAAAGGCTATCCCCTGATAAAGG
CAGGTTGCACACGTGTTCCGCACCCGTACGCCGCTCTCAATTACCCGAAAGTAATCTACCGCTCCGGCTTGCA
TGTGTTAGGCCTCCCGCTAGCGTTCTCCGTGAGCAGGATCCAACATCT
>1919
CTGCTGCCGCCCGTAGGAGTCTGGTCCGTGTCTCAGTACCAGTGTGGGGGATCACCCTCTCAGGCCCCCTAAA
GATCGTCGCCTTGGTGAGCCGTTACCTCACCAACTAGCTAATCTTGCGCGTGCCCATCTCTATCCACCGTAGT
TTTCAATATCACCCGATGCCGAATAATATATTATGGGGTATTAATCTTCTTCGAAAGGCTATCCCCCTGATAA
AGGCAGGTTGCACACGTGTTCCGCACCCGTACGCCGCTCTCAATTACCCGAAAGTAATCTACCGCTCGGCTTG
CATGTGTTAGGCCTCCCGCTAGCGTTCATC
>1920
CTGCTGCCGCCCGTAGGAGTCTGGTCCGTGTCTCAGTACCAGTGTGGGGGATCACCCTCTCAGGCCCCCTAAA
GATCGTCGCCTTGGTGAGCCGTTACCTCACCAACTAGCTAATCTTGCGCGTGCCCATCTCTATCCACCGTAGT
TTTCAATATCACCCGATGCCGAATAATATAATTATGGGGTATTAATCTTCTCGAAAGGCTATCCCCCTGATAA
AGGCAGGTTGCACACGTGTTCCGCACCGTACGCCGCTCTCAATTACCCGAAAGTAATCTACCGCTCCGGCTTG
CACGTGTTAGGCCTCCCGCTAGCGTCATCCGTC
>1921
CTGCTGCCGCCCGTAGGAGTCTGGTCCGTGTCTCAGTACCAGTGTGGGGGATCACCCTCTCAGGCCCCCTAAA
GATCGTCGCCTGGTGAGCCGTTACCTCACCAACTAGCTAATCTTGCGCGTGCCCATCTCTATCCACCGTAGTT
TTCAATATCACCCGATGCCGAATAATATATTATGGGGTATTAATCTTCTTCGAAAGGCTATCCCCTGATAAAG
GCAGGTTGCACACGTGTTCCGCACCCGTACGCCGCTCTCAATTACCCGAAAGTAATCTACCGCTCGGCTTGCA
TGTGTTAGGCCTCCCGCTAGCGTTCATCCTGAGCCAGGATCAACTCT
>1922
CTGCTGCCGCCCGTAGGAGTCTGGTCCGTGTCTCAGTACCAGTGTGGGGGATCACCCTCTCAGGCCCCCTAAA
GATCGTCGCCTTGGTGAGCCGTTACCTCACCAACTAGCTAATCTTGCGCGTGCCCATCTCTATCCACCGTAGT
TTTCAATATCACCCGATGCCGAATAATATATTATGGGGTATTAATCTTCTCGAAGGCTATCCCCTGATAAAGG
CAGGTTGCACACGTGTTCCGCACCCGTACGCCGCTCTCAATTTACCCGAAAGTAGTCTACCGCTCGGCTTGCA
TGTGTTAGGCCTCCCG
>1923
CTGCTGCCGCCCGTAGGAGTCTGGTCCGTGTCTCAGTACCAGTGTGGGGGATCACCCTCTCAGGCCCCCTAAA
GATCGTCGCCTTGGTGAGCCGTTACCTCACCAACTAGCTAATCTTGCGCGTGCCCATCTCTATTCCACCGTAG
TTTCAATATCACCCGATGCCGAATAATATATTATGGGGTATTAATCTTTCTCGAAAGGCTATCCCCCTGATAA
AGGCAGGTTGCACACGTGTTCCGCACCCGTACGCCGCTCTCAATTACCCGAAAGTAATCTACCGCATCGGCTT
GCACTGTGTTAGGCTCCCGCCTAGCGTTCTCCTGAGCAGGATCCAACTC
>1924
CTGCTGCCGCCCGTAGGAGTCTGGTCCGTGTCTCAGTACCAGTGTGGGGGATCACCCTCTCAGGCCCCCTAAA
GATCGTCGCCTTGGTGAGCCGTTACCTCACCAACTAGCTAATCTTGCGCGTGCCCATCTCTATCCACCGTAGT
TTTCAATATCACCCGATGCCGAATAATATATTATGGGGTATTTAATCTTCTTCGAAAGGCTATCCCCTGATAA
AGGCAGGTTGCACACGTGTTCCGCACCCGTACGCCGCTCTCAATTACCCGAAAGTAATCTACCGCTCCGGCCT
GCATGTGTTAGGCCTCCCGCTAGCGTTCTCCGTCGAGCAGG
>1925
CTGCTGCCGCCCGTAGGAGTCTGGTCCGTGTCTCAGTACCAGTGTGGGGGATCACCCTCTCAGGCCCCCTAAA
GATCGTCGCCTTGGTGAGCCGTTACCTCACCAACTAGCTAATCTTGCGCGTGCCCATCTCTATCCACCGTAGT
TTTCAATATCACCCGATGCCGAATAATATATTATTGGGGTATTTAATCTTCTTCGAAAGGCTATCCCCCTGAT
AAAAGGCAGGTTGCACACGTGTTCCGCACCCGTACGCCGCTCTCAATTACCCGAAAGTAATCTACCGCTCGGC
TTGCATGTGTTAGGCCTCCCGCTAGCGTTCTCCTCGAGCAGGATC

>1926
CTGCTGCCGCCCGTAGGAGTCTGGTCCGTGTCTCAGTACCAGTGTGGGGGATCACCCTCTCAGGCCCCCTAAA
GATCGTCGCCTGGTGAGCCGTTACCTCACCAACTAGCTAATCTTGCGCGTGCCCATCTCTATCCACCGTAGTT
TCAACATCACCCGATGCCGAATAATATATTATGGGGTATTAATCTTCTTCGAAAGGCTATCCCCTGATAAAGG
CAGGTTGCACACGTGTTCCGCACCCGTACGCCGCTCTCAATTACCCGAAAGTAATCTACCGCTCCGGCTTGCA
TGTGTTAGGCCTCCCGCTAGCGTTCATCCTGAGCAGGCATAACTCT
>1927
CTGCTGCCGCCCGTAGGAGTCTGGACCGTGTCTCAGTTCCAGTGTGACTGATCATCCTCTCAGACCAGTTACG
GATCGTCGCCTTGGTAGGCCTTTACCCCACCAACTAGCTAATCCGACCTAGGCTCATCTGATAGCGTGAGGTC
CGAAGATCCCCCACTTCTCCCTCAGGACGTATGCGGTATTAGCGCCCGTTTCCGGACGTATCCCCACTACCAG
GCAGATTCCTAGGCATTACTCACCCGTCCGCCGCTGAATCCAGGAGCAAGCCCCCTTCATCCGCTCGACTGCA
TGTGTTAGGCCTGCCGCCAGCGTCAATCTGAGCCAGGATCAAACTCT
>1928
CTGCTGCCGCCGTAGGAGTCTGGTCCGTGTCTCAGTACCAGTGTGGGGGATCACCCTCTCAGGCCCCCTAAAG
ATCGTCGCCTTGGTGAGCCGTTACCTCACCAACTAGCTAATCTTGCGCGTGCCCATCTCTATCCACCGTAGTT
TTCAATATCACCCGATGCCGAATAATATATTATGGGGTATTAATCTTCTTCGAAAGGCTATCCCCCTGATAAA
GGCAGGTTGCACACGTGTTCCGCACCCGTACGCCGCTCTCAATTACCCGAAAGTAATCTACCGCTCGGCTTGC
ATGTGTTAGGCCTCCCGCTAGCGTTCATCCTGAG
>1929
CTGCTGCCGCCCGTAGGAGTCTGGTCCGTGTCTCAGTACCAGTGTGGGGGATCACCCTCTCAGGCCCCCTAAA
GATCGTCGCCTTGGTGAGCCGTTACCTCACCAACTAGCTAATCTTGCGCGTGCCCATCTCTATCCACCGTAGT
TTTCAATATCACCCGATGCCGAATAATATATTATGGGGTATTAATCTTCTTCGAAAGCTATCCCCTGATAAAG
GCAGGTTGCACACGTGTTCCGCACCCGTACGCCGCTCTCAATTACCCGAAAGTAATCTACCGCTCGGCTTGCA
TGTGTTAGGCCTCCCGCTAGCGTTCATCCTCGAGCCAGGATCAAACTCT
>1930
CTGCTGCCGCCCGTAGGAGTCTGGTCCGTGTCTCAGTACCAGTGTGGGGGATCACCCTCTCAGGCCCCCTAAA
GATCGTCGCCTTGGTGAGCCGTTACCTCACCAACTAGCTAATCTTGCGCGTGCCCATCTCTATCCACCGTAGT
TTTCAATATCACCCGATGCCGAATAATATATTATGGGGTATTTAATCTTTCTCGAAAGGCTATCCCCTGATAA
AGGCAGGTTGCACACGTGTTCCGCACCCGTACGCCGCTCTCAATTACCCGAAAGTAATCTACCGCTCGGCTTG
CATGTGTTAGGCCTCCCGCTAGCGTTCTCCTCGAGCAGGATCCAACATCT
>1931
CTGCTGCCGCCCGTAGGAGTCTGGTCCGTGTCTCAGTACCAGTGTGGGGGATCACCCTCTCAGGCCCCCTAAA
GATCGTCGCCTTGGTGAGCCGTTACCTCACCAACTAGCTAATCTTGCGCGTGCCCATCTCTATCCACCGTAGT
TTTCAATATCACCCGATGCCGAATAATATATTATGGGGTATTAATCTTCTCGAAAGCTATCCCCTGATAAAGG
CAGGTTGCACACGTGTTCCGCACCCGTACGCCGCTCTCAATTACCCGAAAGTAATCTACCGCTCCGGCTTGCA
TGTGTTAGGCCTCCCGCTAGCGTTCATCCGTGAGCAGGATCAACTCT
>1932
CTGCTGCCGCCCGTAGGAGTCTGGTCCGTGTCTCAGTACCAGTGTGGGGGATCACCCTCTCAGGCCCCCTAAA
GATCGTCGCCTTGGTGAGCCGTTACCTCACCAACTAGCTAATCTTGCGCGTGCCCATCTCTATCCACCGTAGT
TTCAATATCACCCGATGCCGAATAATATATTATGGGGTATTAATCTTCTCGAAAGGCTATCCCCTGATAAAGG
CAGGTTGCACACGTGTTCCGCACCCGTACGCCGCTCTCAATTACCCGAAAGTAATCTACCGCTCCGGCTTGCA
TGTGTTAGGCCTCCCGCTAGCGTTCATCCGTCGAGCAGGCATCAATCTCCT
>1933
CTGCTGCCGCCCGTAGGAGTCTGGTCCGTGTCTCAGTACCAGTGTGGGGGATCACCCTCTCAGGCCCCCTAAA
GATCGTCGCCTGTGAGCCGTTACCTCACCAACTAGCTAATCTTGCGCGTGCCCATCTCTATCCACCGTAGTTT
CAATATCACCCGATGCCGAATAATATATTATGGGGTATTAATCTTCTTCGAAAGGCTATCCCCCTGATAAAGG
CAGGTTGCACACGTGTTCCGCACCCGTACGCCGCTCTCAATTACCCGAAAGTAATCTACCGCTCCGGCTTGCA
TGTGTTAGGCCTCCCGCTAGCGTTCTCCTGAGCAGGATCAACTCGT
>1934
CTGCTGCCGCCCGTAGGAGTCTGGACCGTGTCTCAGTTCCAGTGTGACTGATCATCCTCTCAGACCCAGTACG
GATCGTCGCCTGGTAGGCCCTTTACCCCACCAACTAGCTAATCCGACCTAGGCTCATCTGATAGCGTGAGGTC
CGAAGTCCCCACTTCTCCCCTCAGGACGTATGCGGTATTGCGCCCGTTTCCGGACGATCCCCGCACTACCCAG
GCAGATTCCTAGGCATTACTCACCCGTCCGCCGCTGAATCCAGGAGCAAGCTCCCCTTCATCCGCTCGACTGC
ATGTGTTAGGCCTCCGCCGCAGCGTTCATCTGAGCCAGGATCAAACTCT

>1935
CTGCTGCCGCCCGTAGGAGTCTGGTCCGTGTCTCAGTACCAGTGTGGGGGATCACCCTCTCAGGCCCCCTAAA
GATCGTCGCCTTGGTGAGCCGTTACCTCACCAACTAGCTAATCTTGCGCGTGCCCATCTCTATCCACCGTAGT
TTCAATATCACCCGATGCCGAATAATATATTATGGGGTATTAATTCTTCTTCGAAAGGCTATCCCCCTGATAA
AGGCAGGTTGCACACGTGTTCCGCACCCGTACGCCGCTCTCAATTACCCGAAAGTAATCTACCGCTCGGCTTG
CATGTGTTAGGCCTCCCGCTAGCGTTCTCCTGAGCAGGATCAACTCGT
>1936
CTGCTTGCCGCCCCGTAGGAGTCTGGTCCCGTGTCTCAGTACCAGTGTGGGGGATCACCCTCTCAGGCCCCCT
AAAGATCGTCGCCTTGGTGAGCCCGTTACCCTCACCCAACTAGCTAATCTTGCGCGTGCCCCATCTCTATCCC
ACCCGTAGTTTTTTCAATATCACCCGATGCCCGAATAATATATTTATGGGGGTATTTAATTCTTTCTTCGAA
AGGCTATCCCCCTGATAAAGGCAGGTTGCACACGTGTTCCCGCACCCGTACGCCGCTCTCAATTACCCGAAAG
TAATCTACCGCTCGGCTTGCATTGTGTTTAGGCCTCCCGCTAGCGTTCTCCTGAGCAG
>1937
CTGCTGCCGCCCGTAGGAGTCTGGTCCGTGTCTCAGTACCAGTGTGGGGGATCACCCTCTCAGGCCCCCTAAA
GATCGTCGCCTTGGTGAGCCGTTACCTCACCAACTAGCTTAATCTTGCGCGTGCCCATCTCTATCCACCGTAG
TTTTCAATATCACCCGATGCCGAATAATATATTATTGGGGGTATTTAATTCTTCTTCGAAAGGCTATCCCCCT
GATAAAGGCAGGTTGCACACGTGTTCCGCACCCGTACGCCGCTCTCAATTACCCGAAAGTAATCTACCGCTCG
GCTTGCATGTGTTAGGCCTCCCGCTAGCGTTCTCCTGAGCAGGTCAACTCGT
>1938
CTGCTGCCGCCCGTAGGAGTCTGGTCCGTGTCTCAGTACCAGTGTGGGGGATCACCCTCTCAGGCCCCTAAAG
ATCGTCGCCTTGGTGAGCCGTTACCTCACCAACTAGCTAATCTTGCGCGTGCCCATCTCTATCCACCGTAGTT
TTCAATATCACCCGATGCCGAATAATATATTATGGGGTATTAATCTTCTTCGAAAGGCTATCCCCTGATAAAG
GCAGGTTGGCACACGTGTTCCGCACCCGTACGCCGCTCTCAATTACCCGAGAGTAATCTACCGCTCGGCTTGC
ATGTGTTAGGCCTCCCGCTAGCG
>1939
CTGCTGCCGCCCGTAGGAGTCTGGTCCGTGTCTCAGTACCAGTGTGGGGGATCACCCTCTCAGGCCCCCTAAA
AGATCGTCGCCTTGGTGAGCCGTTACCTCACCAACTAGCTAATCTTGCGCGTGCCCATCTCTATCCACCGTAG
TTTTCAATATCACCCGATGCCGAATAATATATTATGGGGTATTAATCTTCTTCGAAGAGCTATCCCCTGATAA
AGGCAGGTTGCACACGTGTTCCGCACCCGTACGCCGCTCTCAATTACCCGAAAGTAATCTACCGCTCGGCTTG
CATGTGTTAGGCCTCCCGCTAGCGTTCATCCTGAGCCAGGATCAAACTCT
>1940
CTGCTGCCGCCCGTAGGAGTCTGGTCCGTGTCTCAGTACCAGTGTGGGGGATCACCCTCTCAGGCCCCCTAAA
GATCGTCGCCTTGGTGAGCCGTTACCTCACCAACTAGCTAATCTTGCGCGTGCCCATCTCTATTCCACCGTAG
TTTTCAATATCACCCGATGCCGAATAATATATTTATGGGGTATTAATCTTCTTCGAAAGCTATCCCCTGATAA
AGGCAGGTTGCACACGTGTTCCGCACCCGTACGCCGCTCTCAATTACCCGAAAGTAATCTACCGCTCGGCTTG
CATGTGTTACGGCCTCCCGCTAGGCTC
>1941
CTGCTGCCGCCCGTAGGAGTCTGGTCCGTGTCTCAGTACCAGTGTGGGGGATCACCCTCTCAGGCCCCCTAAA
GATCGTCGCCTGGTGAGCCGTTACCTCACCAACTAGCTAATCCTGCGCGTGCCCATCTCTATCCACCGTAGTT
TTCAATATCACCCGATGCCGAATAATATATTATGGGGTATTAATCTTCTCGAAAGGCTATCCCCTGATAAAGG
CAGGTTGCACACGTGTTCCGCACCCGTACGCCGCTCTCAATTACCCGAAAGTAATCTACCGCTCCGGCTTGCA
TGTGTTAGGCCTCCCGCTAGCGTTCATCCTGAGCAGGATCAACTCT
>1942
CTGCTGCCGCCCGTAGGAGTCTGGTCCGTGTCTCAGTACCAGTGTGGGGGATCACCCTCTCAGGCCCCCTAAA
GATCGTCGCCTTGGTGAGCCGTTACCTCACCAACTAGCTAATCTTGCGCGTGCCCATCTCTATCCACCGTAGT
TTTCAATATCACCCGATGCCGAATAATATATTATGGGGTATTAATCTTCCTCGAAAGGCTATCCCCCTGATAA
AGGCAGGTTGCACACGTGTTCCGCACCCGTACGCCGCTCTCAATTACCCGAAAGTAATCTACCGCTCCGGCTT
GCACGTGTTAGGCCTCCCGC
>1943
CTGCTGCCGCCCGTAGGAGTCTGGTCCGTGTCTCAGTACCAGTGTGGGGGATCACCCTCTCAGGCCCCCTAAA
GATCGTCGCCTTGGTGAGCCGTTACCTCACCAACTAGCTAATCTTGCGCGTGCCCATCTCTATCCACCGTAGT
TTTCAATATCACCCGATGCCGAATAATATATTATGGGTATTAATCCTCTCGAAAGGCTATCCCCTGATAAAGG
CAGGTTGCACACGTGTTCCGCACCCGTACGCCGCTCTCAATTACCCGAAAGTAATCACCGCTCCGGCTTGCAT
GTGTTACGGCCTCCCGCTAGGCTCG

>1944
CTGCTGCCGCCCGTAGGAGTCTGGCTCGTGTCTCAGTACCAGTGTGGGGGATCACCCTCTCAGGCCCCCTAAA
GATCGTCGCCTTGGTGAGCCGTTACCTCACCAACTAGCTAATCTTGCGCGTGCCCATCTCTATCCACCGTAGT
TTTCAATATCACCCGATGCCGAATAATATATTATGGGGTATTAATCTTCTCGAAGGCTATCCCCCTGATAAAG
GCAGGTTGCCACACGTGTTCCGCACCCGTACGCCGCTCTCAATTACCCGAAAGTAATCTACCGCTCCGGCTTG
CATGTGTTAGGCCTCCCGCTAGCGTTCATCCTCGAGCCAGGATCAACTCT
>1945
CTGCTGCCGCCCGTAGGAGTCTGGTCCGTGTCTCAGTACCAGTGTGGGGGATCACCCTCTCAGGCCCCCTAAA
GATCGTCGCCTTGGTGAGCCGTTACCTCACCAACTAGCTAATCTTGCGCGTGCCCATCTCTATCCACCGTAGT
TTTCAATATCACCCGATGCCGAATAATATATTATGGGGTATTAATCTTCTCGAAAGGCTATCCCCCTGATAAA
GGCAGGTTGCCACACGTGTTCCGCACCCGTACGCCGCTCTCAATTACCCGAAAGTAATCTACCGCTCCGGCTC
GCATGTGTTAGGCCTCCCGCTAGCCG
>1946
CTGCTGCCGCCCGTAGGAGTCTGGTCCGTGTCTCAGTACCAGTGTGGGGGATCACCCTCTCAGGCCCCCTAAA
GATCGTCGCCTTGGTGAGCCGTTACCTCACCAACTAGCTAATCTTGCGCGTGCCCATCTCTATCCACCGTAGT
TTTCAATATCACCCGATGCCGAATAATAATATTATGGGGTATTAATCTTCTCGAAAGGCTATCCCCTGATAAG
GCAGGTTAGCACACGTGTTCCGCACCCGTACGCCGCTCTCAATTACCCGGAAAGTAATCTACCGCTCGGCTTG
CATGTGTTAGGCCTCCCGCTAGCGTTCTCCTCGAGCAGGA
>1947
CTGCTGCCGCCCGTAGGAGTCTGGTCCGTGTCTCAGTACCAGTGTGGGGGATCACCCTCTCAGGCCCCCTAAA
GATCGTCGCCTTGGTGAGCCGTTACCTCACCAACTAGCTAATCTTGCGCGTGCCCATCTCTATCCACCGTAGT
TTTCAATATCACCCGATGCCGAATAATATATTATGGGGTATTAATCTTCTTCGAAAGGCTATCCCCTGATAAA
GGCAGGTTGCACACGTGTTCCGCACCCGTACGCCGCTCTCAATTACCCGAAAGTAATCTACCGCTCGGCTTGC
ATGTGTTAGGCCTCCCGCTAGCGTTCTACCGTCGA
>1948
CTGCTGCCGCCCGTAGGAGTCTGGTCCGTGTCTCAGTACCAGTGTGGGGGATCACCCTCTCAGGCCCCCTAAA
GATCGTCGCCTTGGTGAGCCGTTACCTCACCAACTAGCTAATCTTGCGCGTGCCCATCTCTATCCACCGTAGT
TTCAATATCACCCGATGCCGAATAATATAATTATGGGGTATTTAATCTTCTTCGAAAGGCTATCCCCCTGATA
AAAGGCAGGTTGCACACGTGTTCCGCACCCGTACGCCGCTCTCAATTACCCGAAAGTAATCTACCGCTCGGCT
TGCATGTGTTAGGCCTCCCGCTAGCGTTCTCCTGAGCAGGTCAACT
>1949
CTGCTGCCGCCCGTAGGAGTCTGGACCCGTGTCTCAGTTCCAGTGTGACTGATCATCCTCTCAGACCCAGTAC
GGATCGTCGCCTTGGTAGGCCCTTTTACCCCCACCAACTAGCTAATCCGACCTAGGCTCATCTGATAGCGTGA
GGTCCGAAGTCCCCCACTTCTCCCCTCAGGACGTATGCGGTATTAGCGCCCGTTTCCGGACGTATCCCCACTA
CCCAGGCAGATTCCTAGGCATTACTCACCCGTCCGCCGCTGAATCCAGGAGCAAGCTCCCTTCATCCGCTCGA
CTGCATGTGTTAGGCCTGCCGCCAGCGTCAATCTGAGCCCAGGATCAAACTCT
>1950
CTGCTGCCGCCCGTAGGAGTCTGGTCCGTGTCTCAGTACCAGTGTGGGGGATCACCCTCTCAGGCCCCCTAAA
GATCGTCGCCTTGGTGAGCCGTTACCTCACCAACTAGCTAATCTTGCGCGTGCCCATCTCTATTCCACCGTAG
TTTCAATATCACCCGATGCCGAATAATATATTATTGGGGTATTTAATTCTTTCTTCGAAAGGCTATCCCCCTG
ATAAAGGCAGGTTGCACACGTGTTCCGCACCCGTACGCCGCTCTCAATTACCCGAAAGTAATCTACCGCTCGG
CTTGCATGTGTTAGGCCTCCCGCTAGCGTTCTTCCTCGAGC
>1951
CTGCTGCCGCCCGTAGGAGTCTGGTCCGTGTCTCAGTACCAGTGTGGGGGATCACCCTCTCAGGCCCCCTAAA
AGATCGTCGCCTTGGTGAGCCGTTACCTCACCAACTAGCTAATCTTGCGCGTGCCCATCTCTATCCACCGTAG
TTTTCAATATCACCCGATGCCGAATAATATATTATGGGGTATTAATCTTCTTCGAAAGCTATCCCCTGATAAA
GGCAGGTTGCACACGTGTTCCGCACCCGTACGCCGCTCTCAATTACCCGAAAGTAATCTACCGCTCCGGCTTG
CATGTGTTAGGCCTCCCGCTAGCGTTCATCTCGA
>1952
CTGCTGCCGCCCGTAGGAGTCTGGTTCCGTGTCTCAGTACCAGTGTGGGGGATCACCCTCTCAGGCCCCCTAA
AGATCGTCGCCTTGGTGAGCCGTTACCTCACCAACTAGCTAATCTTGCGCGTGCCCATCTCTATCCACCGTAG
TTTCAATATCACCCGATGCCGAATAATATATTATGGGGTATTAATCTTCCCTTCGAAAGGCTATCCCCCTGAT
AAAGGCAGGTTGCACACGTGTTCCGCACCCGTACGCCGCTCTCAATTACCCGAAAGTAATCTACCGCTCGGCT
TGCATGTGTTAGGCCTCCCGCTAGCGTTCATCCTGAGCCAGGATCAAACTCT

>1953
CTGCTGCCGCCCGTAGGAGTCTGGTCCGTGTCTCAGTACCAGTGTGGGGGACCACCCTCTCAGGCCCCCTAAA
GATCGTCGCCTTGGTGAGCCGTTACCTCACCAACTAGCTAATCTTGCGCGTGCCCATCTCTATCCACCGTAGT
TTTCAATATCACCCGATGCCGAATAATATATTATTGGGGTATTAATCTTCTTCGAAAGGCTATCCCCCTGATA
AAGGCAGGTTGCACACGTGTTCCGCACCCGTACGCCGCTCTCAATTACCCGAAAGTAATCTACCGCTCGGCTT
GCATGTGTTAGGCCTCCCGCTAGCGTCATCCTGAG
>1954
CTGCTGCCGCCCGTAGGAGTCTGGTCCGTGTCTCAGTACCAGTGTGGGGGATCACCCTCTCAGGCCCCCTAAA
GATCGTCGCCTTGGTGAGCCGTTACCTCACCAACTAGCTAATCTTGCGCGTGCCCATCTCTATCCACCGTAGT
TTCAATATCACCCGATGCCGAATAATATATTATGGGGTATTAATCTTCTCGAAAGGCTTATCCCCCTGATAAA
GGCAGGTTGCACACGTGTTCCGCACCCGTACGCCGCTCTCAATTACCCGAAAGTAATCTACCGCTCGGCTTGC
ATGTGTTAGGCCTCCCGCTAGCGTCTCC
>1955
CTGCTGCCGCCCGTAGGAGTCTGGTCCGTGTCTCAGTACCAGTGTGGGGGATCACCCTCTCAGGCCCCCTAAA
GATCGTCGCCTTGGTGAGCCGTTACCTCACCAACTAGCTAATCTTGCGCGTGCCCATCTCTATCCACCGTAGT
TTTCAATATCACCCGATGCCGAATAATATATTATGGGGTATTTAATCTTCTTCGAAGGCTATCCCCCTGATAA
AGGCAGGTTGCACACGTGTTCCGCACCCGTACGCCGCTCTCAATTACCCGAAAGTAATCTACCGCTCCGGCTT
GCATGTGTTAGGCCTCCCGCTAGCGTTCATCCGT
>1956
CTGCTGCCGCCCGTAGGAGTCTGGTCCGTGTCTCAGTACCAGTGTGGGGGATCACCCTCTCAGGCCCCCTAAA
GATCGTCGCCTTGGTGAGCCGTTACCTCACCAACTAGCTAATCTTGCGCGTGCCCATCTCTATCCACCGTAGT
TTTCAATATCACCCGATGCCGAATAATATATTTATGGGGTATTAATCTTCTTCTGAAAGGCTATCCCCCTGAT
AAAGGCAGGTTGCACACGTGTTCCGCACCCGTACGCCGCTCTCAATTTACCCGAAAGTAATCTACCGCTCCGG
CTTGCATGTGTTAGGCCTCCCGCTAGCGTCTCCTGAGCAGGATCAACTCT
>1957
CTGCTGCCGCCCGTAGGAGTCTGGTCCGTGTCTCAGTACCAGTGTGGGGGATCACCCTCTCAGGCCCCCTAAA
GATCGTCGCCTTGGTGAGCCGTTACCTCACCAACTAGCTAATCTTGCGCGTGCCCATCTCTATCCACCGTAGT
TTTCAATATCACCCGATGCCGAATAATATATTATGGGGTATTAATCTTCTCGAAGGCTATCCCCTGATGAAGG
CAGGTTGCACACGTGTTCCGCACCCGTACGCCGCTCTCAATTACCCGAAAGTAATCTACCGCATCGGCTTGCA
CTGTCGTTAGGCTCCCGCTAGCGTTCATCGCGAGC
>1958
CTGCTGCCGCCCGTAGGAGTCTGGTCCGTGTCTCAGTACCAGTGTGGGGGATCACCCTCTCAGGCCCCCTAAA
GATCGTCGCCTTGGTGAGCCGTTACCTCACCAACTAGCTAATCTTGCGCGTGCCCATCTCTATCCACCGTAGT
TTTCAATATCACCCGATGCCGAATAATATATTATGGGGTATTAATCTTCTTCGAAAGGCTATCCCCCTGATAA
AGGCAGGTTGCACACGTGTTCCGCACCCGTACGCCGCTCTCAATTACCCGAAAGTAATCTACCGCTCGGCTTG
CATGTGTTAGGCCTCCCGCTAGCGTTCATCCTCGAGCCAGGATCAAACTCT
>1959
CTGCTGCCGCCCGTAGGAGTCTGGTCCGTGTCTCAGTACCAGTGTGGGGGATCACCCTCTCAGGCCCCCTAAA
GATCGTCGCCTTGGTGAGCCGTTACCTCACCAACTAGCTAATCTTGCGCGTGCCCATCTCTATCCACCGTAGT
TTTCAATATCAACCCGATGCCGAATAATATATTATGGGGTATTAATCTTCTCGAAAGGCTATCCCCCTGATAA
AGGCAGGTTGCACACGTGTTCCGCACCCGTACGCCGCTCTCAATTACCCGAAAGTAATCTACCGCTCGGCTTG
TATGTGTTAGGCCCTCCCGCTAGCGTTCACCTGAGCAGGATC
>1960
CTGCTGCCGCCCGTAGGAGTCTGGACCGTGTCTCAGTTCCAGTGTGACTGATCATCCTCTCAGACCAGTTACG
GATCGTCGCCTTGGTAGGCCTTTACCCCACCAACTAGCTAATCCGACCTAGGCTCATCTGATAGCGTGAGGTC
CGAAGATCCCCCACTTCTCCCTCAGGACGTATGCGGTATTAGCGCCCGTTTCCGGACGTATCCCCACTACCAG
GCAGATTCCTAGGCATTACTCACCCGTCCGCCGCTGAATCCAGGAGCAAGCTCCCTTCATCCGCTCGACTGCA
TGTGTTAGGCCTGCCGCCAGCGTCAATCTGAGCCAGGATCAAACTCT
>1961
CTGCTGCCGCCCGTAGGAGTCTGGTCCGTGTCTCAGTACCAGTGTGGGGGATCACCCTCTCAGGCCCCCTAAA
GATCGTCGCCTTGGTGAGCCGTTACCTCACCAACTAGCTAATCTTGCGCGTGCCCATCTCTATCCACCGTAGT
TTTCAATATCACCCGATGCCGAATAATATATTATGGGGTATTAATCTTCTTCGAAAGGCTATCCCCTGATAAA
AGGCAGGTTGCACACGTGTTCCGCACCCGTACGCCGCTCTCAATTACCCGAAAGTAATCTACCGCATCGGCTT
GCATGTGTTAGGCCTCCCGCTAGCGTTCATCCTGAGCAGGATCAACTCT

>1962
CTGCTGCCGCCCGTAGGAGTCTGGTCCGTGTCTCAGTACCAGTGTGGGGGATCACCCTCTCAGGCCCCCTAAA
GATCGTCGCCTTGGTGAGCCGTTACCTCACCAACTAGCTAATCTTGCGCGTGCCCATCTCTATCCACCGTAGT
TTTCAATATCACCCGATGCCGAATAATATATTATGGGGTATTAATCTTCTTCGAAAGCTATCCCCTGATAAAG
GCAGGTTGCACACGTGTTCCGCACCCGTACGCCGCTCTCAATTACCCGAAAGTAATCTACCGCTCCGGCTTGC
ATGTGTTAGGCCTCCCGCTAGCGTTCATCTCGAGCCAGGATCAACTCT
>1963
CTGCTGCCGCCCGTAGGAGTCTGGTCCGTGTCTCAGTACCAGTGTGGGGGATCACCCTCTCAGGCCCCCTAAA
GATCGTCGCCTTGGTGAGCCGTTACCTCACCAACTAGCTAATCTTGCGCGTGCCCATCTCTATCCACCGTAGT
TTTCAATATCACCCGATGCCGAATAATATATTATGGGGTATTAATCTTCTCGAAAGCTATCCCCTGATAAAGG
CAGGTTGCACACGTGTTCCGCACCCGTACGCCGCTCTCAATTACCCGAAAGTAATCTACCGCATCGGCTTGCA
TGTGTTAGGCCTCCCGCTAGCGTTCATCCGTCGAGCCAGGATCAAACTCT
>1964
CTGCTGCCGCCCGTAGGAGTCTGGTCCGTGTCTCAGTACCAGTGTGGGGGATCACCCTCTCAGGCCCCCTAAA
GATCGTCGCCTTGGTGAGCCGTTACCTCACCAACTAGCTAATCTTGCGCGTGCCCATCTCTCATCCACCGTAG
TTTTCAATATCACCCGATGCCGAATAATATATTATGGGGGTATTAATCTTCTTCGAAAGGCTATCCCCCTGAT
AAAGGCAGGTTGCACACGTGTTCCGCACCCGTACGCCGCTCTCAATTACCCGAAAGTAATCTACCGCTCGGCT
TGCATGTGTTAGGCCTCCCGCTAGCGTTCTCCTGAGCAGGATCAACTCGT
>1965
CTGCTGCCGCCCGTAGGAGTCTGGTCCGTGTCTCAGTACCAGTGTGGGGGATCACCCTCTCAGGCCCCCTAAA
AGATCGTCGCCTTGGTGAGCCGTTACCTCACCAACTAGCTAATCTTGCGCGTGCCCATCTCTATCCACCGTAG
TTTTCAATATCACCCGATGCCGAATAATATATTATGGGGTATTAATCTTCTCGAAAGGCTATCCCCCTGATAA
AGGCAGGTTGCACACGTGTTCCGCACCCGTACGCCGCTCTCAATTACCCGAAAGTAATCTACCGCTCGGCTTG
CATGTGTTAGGCCTCCCGCTAGCGTTCATCCTGA
>1966
CTGCTGCCGCCCGTAGGAGTCTGGACCGTGTCTCAGTTCCAGTGTGACTGATCATCCTCTCAGACCAGTACGG
ATCGTCGCCTTGGTAGGCCTTTACCCCACCAACTAGCTAATCCGACCTAGGCTCATCTGATAGCGTGAGGTCC
GAAGTCCCCCACTTCTCCCTCAGGACGTATGCGGTATTAGCGCCCGTTTCCGGACGTATCCCCACTACCAGGC
AGATTCCTAGGCATTACTCACCCGTCCGCCGCTGAATCCAGGAGCAAGCCCCCTTCATCCGCTCGACTGCATG
TGTTAGGCCTGCCGCCAGCGTCAATCTGAGCCAGGATCAACATCT
>1967
CTGCTGCCGCCCGTAGGGAGTCTGGTCCGTGTCTCAGTACCAGGTGTGGGGGACTCACCCTCTCCAGGCCCCC
TAAAGATCGTCGCCTTGGTGAGCCGTTACCTCACCAACTAGCTAATCTTGCGCGTGCCCATCTCTATCCACCG
TAGTTTTCAATATCACCCGATGCCGAATAATATATTATGGGGTATTAATCTTCTCGAAAGCTATCCCCTGATA
AAGGCAGGTTGCACACGTGTTCCGCACCCGTACGCCGCTCTCAATTACCCGAAAGTAATCTACCGCCGATCGG
CTTGCATGTGTTAGGCCTCCCGCTAGCGTTCATCCTCGAGCCAGGATCAAACTCT
>1968
CTGTGCCGCCCGTAGGAGTCTGGTCCGTGTCTCAGTACCAGTGTGGGGGATCACCCTCTCAGGCCCCCTAAAG
ATCGTCGCCTTGGTGAGCCGTTACCTCACCAACTAGCTAATCTTGCGCGTGCCCATCTCTATCCACCGTAGTT
TTTCAATATCACCCGATGCCGAATAATATATTATGGGGTATTTAATCTTCTCGAAAGGCTATCCCCTGATAAA
GGCAGGTTGCACACGTGTTCCGCACCCGTACGCCGCTCTCAATTACCCGAAAGTAATCTACCGCTCCGGCTTG
CATGTGTTAGGCCTCCCGCTAGCGTTCTCCTGAGCAGGATCAACTCGT
>1969
CTGCTGCCGCCCGTAGGAGTCTGGTCCGTGTCTCAGTACCAGTGTGGGGGATCACCCTCTCAGGCCCCCTAAA
GATCGTCGCCTTGGTGAGCCGTTACCTCACCAACTAGCTAATCTTGCGCGTGCCCATCTCTATCCACCGTAGT
TTTCAATATCACCCGATGCCGAATAATATATTATGGGGTATTAATCTTCTCGAAAGGCTTATCCCCCTGATAA
AGGCCAGGTTGCACACGTGTTCCGCACCCGTACGCCGCTCTCAATTACCCGAAAGTAATCTACCGCTCCGGCT
TGCATGTGTTAGGCCTCCCGCTAGCGTTCATCCTGAGCCAGGCATCAAACTCT
>1970
CTGCTGCCGCCCGTAGGAGTCTGGTCCGTGTCTCAGTACCAGTGTGGGGGATCACCCTCTCCAGGCCCCTAAA
CGATCGTCGCCTTGGTGAGCCGCTACCTCACCAACTAGCTAATCTTGCGCGTGCCCATCTCTATCCACCGTAG
TTTTCAATATCACCCGATGCCGAATAATATATTATGGGGTATTAATCTTCTCGAAGCTATCCCCTGATAAAGG
CAGGTTGCACACGTGTTCCGCACCCGTACGCCGCTCTCAATTTACCCGAAAGTAATCTACCGCTCCGGCTTGA
CATGTGTTACGGC

>1971
CTGCTGCCGCCCGTAGGAGTCTGGTCCGTGTCTCAGTACCAGTGTGGGGGATCACCCTCTCAGGCCCCCTAAA
GATCGTCGCCTTGGTGAGCCGTTACCTCACCAACTAGCTAATCTTGCGCGTGCCCATCTCTATCCACCGTAGT
TTTCAATATCACCCGATGCCGAATAATATATTATGGGGTATTAATCTTCTCGAAAGGCTATCCCCTGATAAAC
GGCAGGTTGCACACGTGTTCCGCACCCGTACGCCGCTCTCAATTACCCGAAAGTAATCTACCGCTCCGGCTTG
CATGTGTTAGGCCTCCCGCTAGCGTTCATCCGCT
>1972
CTGCTGCCGCCCGTAGGAGTCTGGTCCGTGTCTCAGTACCAGTGTGGGGGATCACCCTCTCAGGCCCCCTAAA
GATCGTCGCCTTGGTGAGCCGTTACCTCACCAACTAGCTAATCTTGCGCGTGCCCATCTCTATCCACCGTAGT
TTTCAATATCACCCGATGCCGAATAATATATTATGGGGTATTAATCTTCTCGAAGGCTATCCCCTGATAAAGG
CAGGTTGCACACGTGTTCCGCACCCGTACGCCGCTCTCAATTACCCGAAAGTAATCTACCGCTCCGGCTTGCA
TGTGTTAGGCCTCCCGCTAGCGTTCTCGTCGAGCAAGGCATCAAT
>1973
CTGCTGCCGCCCCGTAGGAGTCTGGTCCGTGTCTCAGTACCAGTGTGGGGGATCACCCTCTCAGGCCCCCCTA
AAGATCGTCGCCTTGGTGAGCCCGTTACCTCACCCAACTAGCTAATCTTGCGCGTGCCCCATCTCTTATCCCA
CCGTAGTTTTTCAATATCACCCGATGCCGAATAATATATTTATGGGGGTATTTAATCTTTCTTCGAAAGGCT
ATTCCCCCTGATAAAGGCAGGTTTGCACACGTGTTCCCGCACCCGTACGCCGCTCTCAATTACCCCGAAAGTA
ATCTACCGCTCGGCTTGCATGTGTTTAGGCCTTCCCGCTAGCGTTCTCCTGAGCAGGA
>1974
CTGCTGCCGCCCGTAGGAGTCTGGTCCGTGTCTCAGTACCAGTGTGGGGGATCACCCTCTCAGGCCCCCTTAA
AGATCGTCGCCTTGGTGAGCCGTTACCTCACCAACTAGCTAATCTTGCGCGTGCCCATCTCTATCCACCGTAG
TTTTCAATATCACCCGATGCCGAATAATATATTATGGGGGTATTTAATTCTTTCTTCGAAAGGCTATCCCCCT
GATAAAGGCAGGTTGCACACGTGTTCCGCACCCGTACGCCGCTCTCAATTACCCCGAAAGTAATCTACCGCTC
GGCTTGCATGTGTTAGGCCTCCCGCTAGCGTTCTCCTGAGCAG
>1975
CTGCTGCCGCCCGTAGGAGTCTGGTCCGTGTCTCAGTACCAGTGTGGGGGATCACCCTCTCAGGCCCCCTAAA
GATCGTCGCCTGGTGAGCCGTTACCTCACCAACTAGCTAATCTTGCGCGTGCCCATCTCTATCCACCGTAGTT
TTCAATATCACCCGATGCCGAATAATATATTATGGGGTATTAATCTTCTCGAAAGGCTATCCCCTGATAAAGG
CAGGTTGCACACGTGTTCCGCACCGTACGCGCTCTCAATTACCCGAAAGTAATCTACCGCTCGGCTTGCATGT
GTTAGGCCTCCCGCTAGCGTCT
>1976
CTGCTGCCGCCCGTAGGAGTCTGGTCCGTGTCTCAGTACCAGTGTGGGGGATCACCCTCTCAGGCCCCCTAAA
GATCGTCGCCTTGGTGAGCCGTTACCTCACCAACTAGCTAATCTTGCGCGTGCCCATCTCTATCCACCGTAGT
TTTCAATATCACCCGATGCCGAATAATATATTATGGGGTATTAATCCTCTCTGAAAGGCTATCCCCTGATAAA
GGCAGGTTGCACACGTGTTCCGCACCCGTACGCGCTCTCAATTACCCGAAAGTAATCTACCGCTCGGCTTGCA
TGTGTTAGGCCTCCCGCTAGCGTTCTCCTGAGCAG
>1977
CTGCTGCCGCCCGTAGGAGTCTGGTCCGTGTCTCAGTACCAGTGTGGGGGATCACCCTCTCAGGCCCCCTAAA
GATCGTCGCCTTGGTGAGCCGTTACCTCACCAACTAGCTAATCTTGCGCGTGCCCATCTCTATCCACCGTAGT
TTTCAATATCACCCGATGCCGAATAATATATTATGGGGTATTAATCTTCCTTCGAAAGGCTATCCCCCTGATA
AAGGCAGGTTGCACATGTGTTCCGCACCCGTACGCCGCTCTCAATTACCCGAAAGTAATCTACCGCTCGGCTT
GCATGTGTTAGGCCTCCCGCTAGCGTTCTCCTCGAGCAGGATCAACATCT
>1978
CTGCTGCCGCCCGTAGGAGTCTGGTCCGTGTCTCAGTACCAGTGTGGGGGATCACCCTCTCAGGCCCCCTAAA
GATCGTCGCCTTGGTGAGCCGTTACCTCACCAACTAGCTAATCTTGCGCGTGCCCATCTCTATCCACCGTAGT
TTCAATATCACCCGATGCCGAATAATATATTATGGGGTATTAATCTTCTTCGAAAGGCTATCCCCCTGATAAA
GGCAGGTTGCACACGTGTTCCGCACCCGTACGCCGCTCTCAATTACCCGAAAGTAATCTACCGCTCGGCTTGC
ATGTGTTAGGCCTCCCGCTAGCGTTCATCTCG
>1979
CTGCTGCCGCCCGTAGGAGTCTGGTCCGTGTCTCAGTACCAGTGTGGGGGATCACCCTCTCAGGCCCCCTAAA
GATCGTCGCCTTGGTGAGCCGTTACCTCACCAACTAGCTAATCTTGCGCGTGCCCATCTCTATCCACCGTAGT
TTTCAATATCACCCGATGCCGAATAATATATTATGGGGTATTAATCTTCTTCGAAAGGCTATCCCCTGATAAA
GGCAGGTTGCACACGTGTTCCGCACCCGTACGCCGCCTCATATCCCGAAAGTAATCTACCGCTCGGCTTGCAT
GTGTTAGGCCTCCCGCTAGCGTTCTCCTGAGCAGGATCAACTCGT

>1980
CTGCTGCCGCCCGTAGGAGTCTGGTCCGTGTCTCAGTACCAGTGTGGGGGATCACCCTCTCAGGCCCCCTAAA
GATCGTCGCCTTGGTGAGCCGTTACCTCACCAACTAGCTTAATCTTGCGCGTGCCCATCTCTATCCACCGTAG
TTTCAATATCACCCGATGCCGAATAATATATTATGGGGTATTTAATCTTCTTCGAAAGGCTATCCCCCTGATA
AAGGCAGGTTGCACACGTGTTCCGCACCCGTACGCCGCTCTCAATTACCCGAAAGTAATCTACCGCTCCGGCT
TGCATGTGTTAGGCCTCCCGCTAGCGTTCATCCGTCGAG
>1981
CTGCTGCCGCCCGTAGGAGTCTGGTCCGTGTCTCAGTACCAGTGTGGGGGATCACCCTCTCAGGCCCCCTAAA
GATCGTCGCCTTGGTGAGCCGTTACCTCACCAACTAGCTAATCTTGCGCGTGCCCATCTCTATCCACCGTAGT
TTCAATATCACCCGATGCCGAATAATATATTATGGGGTATTAATCGTTCTTCGAAAGGCTATCCCCTGATAAA
GGCAGGTTGCACACGTGTTCCGCACCCGTACGCCGCTCTCAATTACCCGAAAGTAATCTACCGCTCCGGCTTA
GCATGTGTTCGGCC
>1982
CTGCTGCCGCCCGTAGGAGTCTGGTCCGTGTCTCAGTACCAGTGTGGGGGATCACCCTCTCAGGCCCCCTAAA
GATCGTCGCCTTGGTGAGCCGTTACCTCACCAACATGCTAAATCTTGCGCGTGCCCATCTCTATCCACCGTAG
TTTTTCAATATCACCCGATGCCGAATAATATATTATGGGGTATTTAATCTTCCTTCGAAGAGCTATCCCCTGA
TAAAGGCAGGTTGCACACGTGTTCCGCACCCGTACGCCGCTCTCAATTACCCGAAAGTAATCTACCGCTCGGC
TTGCCATGTGTTAGGCCTCCCGCTAGCGTTCATCCTGAGCCAGGCATCGAAACTCT
>1983
CTGCTGCCGCCCGTAGGAGTCTGGACCGTGTCTCAGTTCCAGTGTGACTGATCATCTCTCAGACCAGTACGGA
TCGTCGCCTTGGTAGGCCTTTACCCCACCAACTAGCTAATCCGACCTAGGCTCATCTGATAGCGTGAGGTCCG
AAGATCCCCCACTTCTCCCTCAGGACGTATGCGGTATTAGCGCCCGTTTCCGGACGTATCCCCACTACCAGGC
AGATCCTAGGCATTACTCACCCGTCCGCCGCTGAATCCAGGAGCAAGCTCCCTCATCGCTCGACTGCAATGTG
TTAGGCCTCCGCGCAG
>1984
CTGCTGCCGCCCGTAGGAGTCTGGACCGTGTCTCAGTTCCAGTGTGACTGATCATCCTCTCAGACCAGTACGG
ATCGTCGCCTTGGTAGGCCTTTACCCCACCAACTAGCTAATCCGACCTAGGCTCATCTGATAGCGTGAGGTCC
GAAGATCCCCCACTTCTCCCTCAGGACGTATGCGGTATTAGCGCCCGTTTCCGGACGTATCCCCGCACTACCC
AGGCAGATTCCAGGCATTACTCACCCGTCCGCCGCTGAATCCAGGAGCAAGCCCCCTCATCCGCTCGACTGCA
TGTGTTAGGCCTGCCGCCAGCGTCATCTGAGCCAGGATAACATCT
>1985
CTGCTGCCGCCCGTAGGAGTCTGGTCCGTGTCTCAGTACCAGTGTGGGGGATCACCCTCTCAGGCCCCCTAAA
GATCGTCGCCTTGGTGAGCCGTTACCTCACCAACTAGCTAATCTTGCGCGTGCCCATCTCTATCCACCGTAGT
TTTCAATATCACCCGATGCCGAATAATATATTATGGGGTATTTAATTCTTCTCGAAAGGCTATCCCCTGATAA
AGGCAGGTTGCACACGTGTTCCGCACCCGTACGCCGCTCTCAATTACCCGAAAGTAATCTACCGCTCGGCTTG
CATGTGTTAGGCCTCCCGCTAGCGTTCTTCCTGAGC
>1986
CTGCTGCCGCCCGTAGGAGTCTGGTCCGTGTCTCAGTACCAGTGTGGGGGATCACCCTCTCAGGCCCCCTAAA
GATCGTCGCCTTGGTGAGCCGTTACCTCACCAACTAGCTAATCTTGCGCGTGCCCATCTCTATCCACCGTAGT
TTTCAATATCACCCGATGCCGAATAATATATTATGGGGTATTAATCTTCTCGAAAGGCTATCCCCCTGATAAA
GGCAGGTTGCACACGTGTTCCGCACCCGTACGCCGCTCTCAATTACCCGAAAGTAATCTACCGCTCGGCTTGC
ATGTGTTAGGCCTCCCGCTAGCGTTCTCCTGAGCAGG
>1987
CTGCTGCCGCCCGTAGGAGTCTGGACCGTGTCTCAGTTCCAGTGTGACCGATCATCCTCTCAGACCAGTTACG
GATCGTCGCCTTGGTAGGCCTTTACCCCACCAACTAGCTAATCCGACCTAGGCTCATCTGATAGCGTGAGGTC
CGAAGTCCCCCACTTCTCCCTCAGGACGTATGCGGTATTAGCGCCCGTTTCCGGACGTATTCCCCGCACTACC
CAGGCAGATTCCCTAGGCATTACTCACCCGTCCGCCGCTGAATCCAGGAGCAAGCCCCCTCATCGCCTCGACT
GCCATGTGTTAGGCTGCCGCCAGCGTCATCTGAGCCAGGATCAACATCGT
>1988
CTGCTGCCGCCCGTAGGAGTCTGGTCCGTGTCTCAGTACCAGTGTGGGGGATCACCCTCTCAGGCCCCCTAAA
GATCGTCGCCTTGGTGAGCCGTTACCTCACCAACTAGCTAATCTTGCGCGTGCCCATCTCTATCCACCGTAGT
TTCAATATCACCCGATGCCGAATAATATATTATGGGGTATTAATCTTCTCGAAAGGCTATCCCCTGATAAAGG
CAGGTTGCACACGTGTTCCGCACCCGTACGCCGCTCTCAATTACCCGAAAGAATCTACCGCTCCGGCTGCATG
TGTTAGGCCTCCCGCTAGCGTCTCTGAGCAGGATCAACT

>1989
CTGCTGCCGCCCGTAGGAGTCTGGTCCGTGTCTCAGTACCAGTGTGGGGGATCACCCTCTCAGGCCCCCTAAA
GATCGTCGCCTTGGTGAGCCGTTACCTCACCAACTAGCTAATCTTGCGCGTGCCCATCTCTATCCACCGTAGT
TTCCAATATCACCCGATGCCGAATAATATATTATGGGGTATTAATCTTCTTCGAAAGGCTATCCCCCTGATAA
AGGCAGGTTGCACACGTGTTCCGCACCCGTACGCCGCTCTCAATTACCCGAAAGTAATCTACCGCTCCGGCTT
GCATGTGTTAGGCCTCCCGCTAGCGTTCATCCTCGAG
>1990
CTGCTGCCGCCCGTAGGAGTCTGGACCGTGTCTCAGTTCCAGTGTGACTGATCATCCTCTCAGACCAGTTACG
GATCGTCGCCTTGGTAGGCCTTTACCCCACCAACTAGCTAATCCGACCTAGGCTCATCTGATAGCGTGAGGTC
CGAAGATCCCCCACTTCTCCCTCAGGACGTATGCGGTATTAGCGCCCGTTTCCGGACGTATCCCCGCACTACC
AGGCAGATTCCTAGGCATTACTCACCCGTCCGCCGCTGAATCCAGGAGCAAGCCCTCCTATCCGCTCGACTGC
ATGTGTTAGGCCTGCCGCCAGCGTTCAATCTGAGCCAGGATCAACATCT
>1991
CTGCTGCCGCCCGTAGGAGTCGTGGTCCGTGTCTCAGTACCAGGTGTGGGGACTCACCCTCTCCAGGCCCCTA
AAGATCGTCGCCTTGGTGAGCCGTTACCTCACCAACTAGCTTAATCTTGCGCGTGCCCATCTCTATTCCACCG
TAGTTTTCAATATCACCCGATGCCGAATAATATATTTATTGGGGTATTTAATCTTCTCGAAGGCTATCCCCTG
ATAAAGGCAGGTTTGCACACGTGTTCCGCACCGTACGCCGCTCTCAATTACCCGAAAGTAATCACCGCTCCGG
CTTGCATGTGTTAGGCCTCCGCTAGCGTTCTCCGTCGAGC
>1992
CTGCTGCCGCCCGTAGGAGTCTGGTCCGTGTCTCAGTACCAGTGTGGGGGATCACCCTCTCAGGCCCCCTAAA
GATCGCCGCCTTGGTGAGCCGTTACCTCACCAACTAGCTAATCTTGCGCGTGCCCATCTCTATCCACCGTAGT
TTTCAATATCACCCGATGCCGAATAATATATTATGGGGTATTAATCTTCTTCGAAAGCTATCCCCTGATAAAG
GCAGGTTGCACACGTGTTCCGCACCCGTACGCCGCTCTCAATTACCCGAAAGTAATCTACCGCTCCGGCTTAG
CATGTGTTCGGC
>1993
CTGCTGCCGCCCGTAGGAGTCTGGTCCGTGTCTCAGTACCAGTGTGGGGGATCACCCTCTCAGGCCCCCTAAA
GATCGTCGCCTTGGTGAGCCGTTACCTCACCAACTAGCTAATCTTGCGCGTGCCCATCTCTATCCACCGTAGT
TTTCAATATCACCCGATGCCGAATAATATATTATGGGGTATTAATCTTCTCGAAGGCTATCCCCTGATAAAGG
CAGTTGCACACGTGTTCCGCACCCGTACGCCGCTCTCAATTACCCGAAAGTAATCTACCGCTCCGGCTTGCAT
GTGTTAGGCCTCCCGCTAGCGTCTCGCTCCGTGC
>1994
CTGCTGCCGCCCGTAGGAGTCTGGACCGTGTCTCAGTTCCAGTGTGACTGACCATCCTCTCAGACCAGTTACG
GATCGTCGCCTTGGTAGGCCTTTACCCCACCAACTAGCTTAATCCGACCTAGGCTCATCTGATAGCGTGAGGT
CCGAAGTCCCCCACTTCTCCCTCAGGACGTATGCGGTATTAGCGCCCGTTTCCGGACGTATCCCCGCACTACC
CAGGCAGATTCCAGGCATTTACTCACCCGTCCGCCGCTGAATCCAGGAGCCAAGCTCCCTCATCCGCTCGACT
GCCATGTGTTAGGCCTGCCGCCAGCGTCAATTCTGAGCCAGGATCAACTCTT
>1995
CTGCTGCCGCCCGTAGGAGTCTGGTCCGTGTCTCAGTACCAGTGTGGGGGATCACCCTCTCAGGCCCCCTAAA
GATCGTCGCCTTGGTGAGCCGTTACCTCACCAACTAGCTAATCTTGCGCGTGCCCATCTCTATCCACCGTAGT
TTTCAATATCACCCGATGCCGAATAATATATTATGGGGTATTAATCTTCTCGAAAGGCTATCCCCTGATAAAG
GCAGGTTGCACACGTGTTCCGCACCCGTACGCCGCTCTCAATTACCCGAAAGTAATCTACCGCTCCGGCTTGC
ATGTGTTAGGCCTCCCGCTAGCGTTCATCCTGAGCAGGCATCAACTCCT
>1996
CTGCTGCCGCCCGTAGGAGTCTGGTCCGTGTCTCAGTACCAGTGTGGGGGATCACCCTCTCAGGCCCCCTAAA
GATCGTCGCCTTGGTGAGCCGTTACCTCACCAACTAGCTAATCTTGCGCGTGCCCATCTCTATCCACCGTAGT
TTTCAATATCACCCGATGCCGAATAATATATTATGGGGTATTAATCTTCTCGAAAGGCTATCCCCCTGATAAA
GGCAGGTTGCACACGTGTTCCGCACCCGTACGCCGCTCTCAATTACCCGAAAGTAATCTACCGCTCGGCTTGC
ATGTGTTAGGCCTCCCGCTAGCGTTCTCCTCGAGCAGG
>1997
CTGCTGCCGCCCGTAGGAGTCTGGTCCGTGTCTCAGTACCAGTGTGGGGGATCACCCTCTCAGGCCCCCTAAA
GATCGTCGCCTTGGTGAGCCGTTACCTCACCAACTAGCTAATCTTGCGCGTGCCCATCTCTATCCACCGTAGT
TTTCAATATCACCCGATGCCGAATAGTATATTATGGGGTATTAATCTTCTGTGAAAGGCTATCCCCCTAATAA
AGGCAGGTTGCACACGTGTTCCGCACCCGTACGCCGCTCTCAATTACCCGAAAGTAATCTACCGTCTCCGGCT
TGCATGTGTTAGGCCTCCGCTAGCGTCTACTCGAGCAGG

>1998
CTGCTGCCGCCCGTAGGAGTCTGGTCCGTGTCTCAGTACCAGTGTGGGGGATCACCCTCTCAGGCCCCCTAAA
GATCGTCGCCTTGGTGAGCCGTTACCTCACCAACTAGCTAATCTTGCGCGTGCCCATCTCTATCCACCGTAGT
TTTTCAATATCACCCGATGCCGAATAATATATTATGGGGTATTTAATCTTTCTCGAAGGCTATCCCCTGATAA
AGGCAGGTTGCACACGTGTTCCGCACCCGTACGCCGCTCTCAATTACCCGAAACGTAATCTACCGCATACGGC
TTCGCCATGG
>1999
CTGCTGCCGCCCGTAGGAGTCTGGTCCGTGTCTCAGTACCAGTGTGGGGGATCACCCTCTCAGGCCCCCTAAA
GATCGTCGCCTTGGTGAGCCGTTACCTCACCAACTAGCTAATCTTGCGCGTGCCCATCTCTATTCCACCGTAG
TTTTTCAATATCACCCGATGCCGAATAATATATTATGGGGTATTTAATCTTTCTCGAAGGCTATCCCCCTGAT
AAAGGCAGGTTGCCAACACGTGTTCCGCACCCGTACGCCGCTCTCAATTACCCCGAAACGTAATCACCGTATA
CCGGCTTGACCGCTATTGGTCGTT
>2000
CTGCTGCCGCCCGTAGGAGTCTGGTCCGTGTCTCAGTACCAGTGTGGGGGATCACCCTCTCAGGCCCCCTAAA
GATCGTCGCCTTGGTGAGCCGTTACCTCACCAACTAGCTAATCTTGCGCGTGCCCATCTCTATCCACCGTAGT
TTTCAATATCACCCGATGCCGAATAATATATTATGGGGTATTAATCGTTCTTCGAAAGGCTATCCCCCTGATA
AAGGCAGGTTGCCACACGTGTTCCGCACCCGTACGCCGCTCTCAATTACCCGAAAGTAATCACCGCTCCGGCT
TGCATGTGTTAGGCCTCCGCTA

Additional file 3_B_P11

>1
CTGCTGCCGCCCGTAGGAGTCTGGACCGTGTCTCAGTTCCAGTGTGGCTGGTCATCCTCTCAGACCAGCTAGG
GATCGTCGCCTAGGTGAGCCGTTACCCCACCTACTAGCTAATCCCATCTGGGCACATCCGATGGCAAGAGGCC
CTAAGGTCCCCCTCTTGGTCTTGCGACGTATGCGGTATTAGCTACCGTTTCCAGTAGTTATCCCCCTCCATCG
GGCAGTTTCCCAGACATTACTCACCCGTCCGCCACTCGTCAGCAAAGCAGCAAGCTGCTTCCTGTTACCGTTC
GACTGCATGTGTTAGGCCTGCCGCCAGCGTCAATCTGAGCCAGGATCAACTCT
>2
CTGCTGCCGCCCGTAGGAGTCTGGACCGTGTCTCAGTTCCAGTGTGGCTGGTCATCCTCTCAGACCAGCTAGG
GATCGTCGCCTAGGTGAGCCGTTACCCCACCTACTAGCTAATCCCATCTGGGCACATCCGATGGCAAGAGGCC
CTAAGGTCCCCCTCTTGTCTTGCGACGTATGCGGTATTAGCTACCGTTTCCAGTAGTTATCCCCCTCCATCGG
GCAGTTTCCCAGACATTACTCACCCGTCCGCCACTCGTCAGCAAAGCAGCAAGCTGCTTCCTGTTACCGTTCG
ACTGCATGTGTTAGGCCTGCCGCCAGCGTCAATCTGAGCCAGGATCAAACTCT
>3
CTGCTGCCGCCCGTAGGAGTCTGGACCGTGTCTCAGTTCCAGTGTGGCTGGTCATCCTCTCAGACCAGCTAGG
GATCGTCGCCTAGGTGAGCCGTTACCCCACCTACTGGCTAATCCCATCTGGGCACATCCCGATGGCAAGAGGC
CCTAAGGTCCCCCTCTTGTCTGCGACGTATGCGGTATTAGCTACCGTTTCCAGTAGTTATCCCCCTCCATCAG
GCAGTTTCCCAGACATTACTCACCCGTCCGCCACTCGTCAGCAAAGCAGCAAGCTGCTTCTGTTACCGTTCGA
CTGCATGTGTTAGGCCTGCCGCCAGCGTCAATATGAGCCAGGATCAAACTCT
>4
CTGCTGCCGCCCGTAGGAGTCTGGACCGTGTCTCAGTTCCAGTGTGGCTGGTCATCCTCTCAGACCAGCTAGG
GATCGTCGCCTAGGTGAGCCGTTACCCCACCTACTAGCTAATCCCATCTGGGCACATCCGATGGCAAGAGGCC
CTAAGGTCCCCCTCTTGTCTGCGACGTATGCGGTATTAGCTACCGTTTCCAGTAGTTATCCCCCTCCATCAGG
CAGTTTCCCAGACATTACTCACCCGTCCGCCACTCGTCAGCAAAGCAGCAAGCTGCTTCTGTTACCGTTCGAC
TGCATGTGTTAGGCCTGCCGCCAGCGTCAATCTGAGCCAGGATCAAACTCT
>5
CTGCTGCCGCCCGTAGGAGTCTGGACCGTGTCTCAGTTCCAGTGTGGCTGGTCATCCTCTCAGACAGCTAGGG
ATCGTCGCCTAGGTGAGCCGTTACCCCACCTACTAGCTAATCCTATCTGGGCACATCCGATGGCAAGAGGCCC
TACGGTCCCCCTCTTGGTCTTGCGACGTATGCGGTATTAGCTACCGTTTCCAGTAGTTATCCCCCTCCATCAG
GCAGTTTCCCAGACATTACTCACCCGTCCGCCACTCGTCAGCAAAGCAGCAAGCTGCTTCCTGTTACCGTTCG
ACTGCATGTGTTAGGCCTGCCGCCAGCGTCAATCTGAGCCAGGATCAACTCT
>6
CTGCTGCCGCCCGTAGGAGTCTGGACCGTGTCTCAGTTCCAGTGTGGCTGGTCATCCTCTCAGACCAGCTAGG
GATCGTCGCCTAGGTGAGCCGTTACCCCACCTACTAGCTAATCCCATCTGGGCACATCCGATGGCAAGAGGCC
CTAAGGTCCCCCTCTTGTCTTGCGACGTATGCGGTATTAGCTACCGTTTCCAGTAGTTATCCCCCTCCATCGG
GCAGTTTCCCAGACATTACTCACCCGTCCGCCACTCGTCAGCAAAGCAGCAAGCTGCTTCCTGTTACCGTTCG
ACTGCATGTGTTAGGCCTGCCGCCAGCGTCAATCTGAGCCAGGATCAACTCT
>7
CTGCTGCCGCCCGTAGGAGTCCGACCGTGTCTCAGTTCCAGTGTGGCTGGTCATCCTCTCAGACCAGCTAGGG
ATCGTCGCCTAGGTGAGCCGTTACCCCACCTACTAGCTAATCCCATCTGGGCACATCCGATGGCAAGAGGCCC
TAAGGTCCCCCTCTTGTCTTGCGACGTATGCGGTATTAGCTACCGTTTCCAGTAGTTATCCCCCTCCATCGGG
CAGTTTCCCAGACATTACTCACCCGTCCGCCACTCGTCAGCAAAGCAGCAAGCTGCTTCCTGTTACCGTTCGA
CTGCATGTGTTAGGCCTGCCGCCAGCGTCAATCTGAGCCAGGATCAAACTCT
>8
CTGCTGCCGCCCGTAGGAGTCTGGACCGTGTCTCAGTTCCAGTGTGGCTGGTCATCCTCTCAGACCAGCTAGG
GATCGTCGCCTAGGTGAGCCGTTACCCCACCTACTAGCTAATCCCATCTGGGCACATCCGATGGCAAGAGGCC
CTAAGGTCCCCCTCTTTGTCTTGCGACGTATGCGGTATTAGCTACCGTTTCCAGTAGTTATCCCCCTCCATCA
GGCAGTTTCCCAGACATTACTCACCCGTCCGCCACTCGTCAGCAAAGCAGCAAGCTGCTTCTGTTACCGTTCG
ACTGCATGTGTTAGGCCTGCCGCCAGCGTTCAATCTGAAGCCAGGATCAAACTCT
>9
CTGCTGCCGCCCTAGGAGTCTGGACCGTGTCTCAGTTCCAGTGTGGCTGGTCATCCTCTCAGACCAGCTAGGG
ATCGTCGCCTAGGTGAGCCGTTACCCCACCTACTAGCTAATCCCATCTGGGCACATCCGATGGCAAGAGGCCC
TAAGGTCCCCCTCTTGGTCTGCGACGTATGCGGTATTAGCTACCGTTTCCAGTAGTTATCCCCCTCCATCAGG

CAGTTTCCCAGACATTACTCACCCGTCCGCCACTCGTCAGCAAAGCAGCAAGCTGCTTCCTGTTACCGTTCGA
CTGCATGTGTTAGGCCTGCCGCCAGCGTCAATCTGAGCCAGGATCAAACTCT
>10
CTGCTGCCGCCCGTAGGAGTCTGGACCGTGTCTCAGTTCCAGTGTGGCTGGTCATCCTCTCAGACAGCTAGGG
ATCGTCGCCTAGGTGAGCCGTTACCCCACCTACTAGCTAATCCCATCTGGGCACATCCGATGGCAAGAGGCCC
TAAGGTCCCCCTCTTGTCTGCGACGTATGCGGTATTAGCTACCGTTTCCAGTAGTTATCCCCTCCATCAGGCA
GTTTCCCAGACATTACTCACCGTCCGCCACTCGTCAGCAAACAGCAAGCTGCTTCCTGTTACCGTTCGAACTG
CATGTCGTTAGGCCTGCCGCCAGCGTTCAATCT
>11
CTGCTGCCGCCCGTAGGAGTCTGACCGTGTCTCAGTCCAGTGTGGCTGGTCATCCTCTCAGACCAGCTAGGGA
TCGTCGCCTAGGTGAGCCGTTTACCCCACCTACTAGCTAATCCCATCTGGCACATCCGATGGCAAGAGGCCCT
AAGGTCCCCCTCTTGTCTGCGACGTTATGCGGTATTAGCTACCGTTTCCAGTAGTTATCCCCTCCATCGGGCA
GTTTCCCAGACATTACTCACCCGTCCGCCACTCGTCAGCAAAGCAGCAAGCTGCTTCCTGTTACCGTTCGACT
GCATGTGTTAGGCCTGCCGCCAGCGTCAATCTGAGCCAGGATCAAACTCT
>12
CTGCTGCCGCCCGTAGGAGTCTGGACCGTGTCTCAGTTCCAGTGTGGCTGGTCATCCTCTCAGACCAGCTAGG
GATCGTCGCCTAGGTGAGCCGTTACCCCACCTACTAGCTAATCCCATCTGGGCACATCCGATGGCAAGAGGCC
CTAAGGTCCCCCTCTTGTCTGCGACGTATGCGGTATTAGCTACCGTTTCCAGTAGTTATCCCCCTCCATCAGG
CAGTTTCCCAGACATTACTCACCCGTCCGCCACTCGTCAGCAAAGCAGCAAGCTGCTTCTGTTACCGTTCGAC
TGCATGTGTTAGGCCTGCCGCCAGCATTCAATCTGAGCCAGGATCAAACTCT
>13
CTGCTGCCGCCCGTAGGAGTCTGGACCGTGTCTCAGTTCCAGTGTGGCTGGTCATCCTCTCAGACCAGCTAGG
GATCGTCGCCTAGGTGAGCCGTTACCCCACCTACTAGCTAATCCCATCTGGGCACATCCGATGGCAAGAGGCC
CTAAGGTCCCCCTCTTGTCTGCGACGTATGCGGTATTAGCTACCGTTTCCAGTAGTTATCCCCCTCCATCGGG
CAGTTTCCCAGACATTACTCACCCGTCCGCCACTCGTCAGCAAAGCAGCAAGCTGCTTCCTGTTACCGTTCGA
CTGCATGTGTTAGGCCTGCCGCCAGCGTCAATCTGAGCCAGGATCAAACTCT
>14
CTGCTGCCGCCCGTAGGAGTCTGGACCGTGTCTCAGTTCCAGTGTGGCTGGTCATCCTCTCAGACCAGCTAGG
GACCGTCGCCTAGGTGAGCCCGTTTACCCCCACCTACTAGCTAATCCCATCTGGGCACATCCCGATGGCAAGA
GGCCCCTAAGGTCCCCCTCTTGTCTGCGACGTTATGCGGTATTAGCTACCGTTTCCAGTAGTTATCCCCCTCC
ATCAGGCAGTTTCCCCAGACATTACTCACCCGTCCGCCACTCGTCAGCAAAGCAGCAAGCTGCTTTCCTGTTA
CCGTTCGACTGCATGTGTTAGGCCTGCCGCCAGCGTCAATCTGAGCCAGGATCAAACTCT
>15
CTGCTGCCGCCCGTAGGAGTCTGGACCGTGTCTCAGTTCCAGTGTGGCTGGTCATCCTCTCAGACCAGCTAGG
GATCGTCGCCTAGGTGAGCCCGTTACCCCACCTACTAGCTAATCCCATCTGGGCACATCCGATGGCAAGAGGC
CCCTAAGGTCCCCCCCCTCTTGTCTGCGACGTATGCGGTATTAGCTACCGTTTCCAGTAGTTATCCCCTCCAT
CGGGCAGTTTTCCCAGACATACTCACCCTCCGCCACTCGTCAGCAAAGCACAAGCCGCTTCCTGTTACCCGTT
CGACTTGCATGTGTTAGGCCTGCCCGCCAGCGTCAATCTGAGCCCAGGATCAAACTCT
>16
CTGCTGCCGCCCGTAGGAGTCTGGACCGTGTCTCAGTTCCAGTGTGGCTGGTCATCCTCTCAGACAGCTAGGG
ATCGTCGCCTAGGTGAGCCGTTACCCCACCTACTAGCTAATCCCATCTGGGCACATCCGATGGCAAGAGGCCC
TAAGGTCCCCCTCTTGTCTGCGACGTATGCGGTATTAGCTACCGTTTCCAGTAGTTATCCCCCTCCATCAGGC
AGTTTCCCAGACATTACTCACCCGTCCGCCACTCGTCAGCAAGCAGCAAGCTGCTTCCTGTTACCGTTCGACT
GCATGTGTTAGGCCTGCCGCCAGCGTCATCTGAGCCAGGATCAAACTCT
>17
CTGCTGCCGCCCGTAGGAGTCTGGACCGTGTCTCAGTTCCAGTGTGGCTGGTCATCCTCTCAGACCAGCTAGG
GATCGTCGCCTAGGTGAGCCGTTACCCCACCTACTAGCTAATCCCATCTGGGCACATCCGATGGCAAGAGGCC
CTAAGGTCCCCCTCTTGTCTTGCGACGTTATGCGGTATTAGCTACCGTTTCCAGTAGTTATCCCCCTCCATCG
GGCAGTTTCCCAGACATTACTCACCCGTCCGCCACTCTCAGCAAGCAGCAAGCTGCTTCCTGTTACCGTTCGA
CTGCATGTGTTAGGCCTGCCGCCAGCGTCAATCTGAGCCAGGATCAAACTCT
>18
CTGCTGCCGCCCGTAGGAGTTGGACCGTGTCTCAGTTCCAGTGTGGCTGGTCATCCTCTCAGACCAGCTAGGG
ATCGTCGCCTAGGTGAGCCGTTACCCCACCTACTAGCTAATCCCATCTGGGCACATCCGATGGCAAGAGGCCC
TAAGGTCCCCCTCTTGTCTGCGACGTATGCGGTATTAGCTACCGTTTCCAGTAGTTATCCCCCTCCATCGGGC

AGTTTCCCAGACATTACTCACCCGTCCGCCACTCGTCAGCAAAGCAGCAAGCTGCTTCCTGTTACCGTTCGAC
TGCATGTGTTAGGCCTGCCGCCAGCGTCAATCTGAGCCAGGATCAAACTCT
>19
CTGCTGCCGCCCGTAGGAGTCTGGACCGTGTCTCAGTTCCAGTGTGGCTGGTCATCCTCTCAGACCAGCTAGG
GATCGTCGCCTAGGTGAGCCGTTACCCCACCTACTAGCTAATCCCATCTGGGCACATCCGATGGCAAGAGGCC
CTAAGGTCCCCCTCTTTGGTCTTGCGACGTATGCGGTATTAGCTACCGTTTCCAGTAGTTATCCCCCTCCATC
AGGCAGTTTTCCCAGACATTACTCACCCGTCCGCCACTCGTCAGCAAAGCAGCAAGCTGCTTCTGTTACCGTT
CGACTGCATGTGTTAGGCCTGCCGCCAGCGTCAATCTGAGCCAGGATCAAACTCT
>20
CTGCTGCCGCCCGTAGGAGTCTGGACCGTGTCTCAGTTCCAGTGTGGCTGGTCATCCTCTCAGACCAGCTAGG
GATCGTCGCCTAGGTGAGCCGTTACCCCACCTACTAGCTAATCCCATCTGGGCACATCCGATGGCAAGAGGCC
CTAAGGTCCCCCTCTTTGTCTTGCGACGTATGCGGTATTAGCTACCGTTTCCAGTAGTTATCCCCCTCCATCG
GGCAGTTTTCCCAGACATTACTCACCCGTCCGCCACTCGTCAGCAAAGCAGCAAGCTGCTTCCTGTTACCGTT
CGACTGCATGTGTTAGGCCTGCCGCCAGCGTCAATCTGAGCCAGGATCAAACTCT
>21
CTGCTGCCGCCCGTAGGAGTCTGGACCGTGTCTCAGTTCCAGTGTGGCTGGTCATCCTCTCAGACCAGCTAGG
GATCGTCGCCTAGGTGAGCCGTTACCCCACCTACTAGCTAATCCCATCTGGGCACATCCGATGGCAAGAGGCC
CTAAGGTCCCCCTCTTTGGTCTTGCGACGTACGCGGTATTAGCTACCGTTTCCAGTAGTTATCCCCCTCCATC
AGGCAGTTTCCCAGACATTACTCACCCGTCCGCCACTCGTCAGCAAAGCAGCAAGCTGCTTCCTGTTACCGTT
CGACTGCATGTGTTAGGCCTGCCGCCAGCGTCAATCTGAGCCAGGATCAAACTCT
>22
CTGCTGCCGCCCGTAGGAGTCTGGACCGTGTCTCAGTTCCAGTGTGGCTGGTCATCCTCTCAGACAGCTAGGG
ATCGTCGCCTAGGTGAGCCGTTACCCCACCTACTAGCTAATCCCATCTGGGCACATCCGATGGCAAGAGGCCC
TAAGGTCCCCCTCTTTGTCTTGCGACGTATGCGGTATTAGCTACCGTTTCCAGTAGTTATCCCCCTCCATCAG
GCAGTTTCCCAGACATTACTCACCCGTCCGCCACTCGTCAGCAAAGCAGCAAGCTGCTTCCTGTTACCGTTCG
ACTGCATGTGTTAGGCCTGCCGCCAGCGTTCAATCTGAGCCAGGATCAAACTCT
>23
CTGCTGCCGCCCGTAGGAGTCTGGACCGTGTCTCAGTTCCAGTGTGGCTGGTCATCCTCTCAGACCAGCTAGG
GATCGTCGCCTAGGTGAGCCGTTACCCCACCTACTAGCTAATCCCATCTGGGCACATCCGATGGCAAGAGGCC
CTAAGGTCCCCCTCTTTGTCTGCGACGTATGCGGTATTAGCTACCGTTTCCAGTAGTTATCCCCCTCCATCGG
GCAGTTTTCCCAGACATTACTCACCCGTCCGCCACTCGTCAGCAAAGCAGCAAGCTGCTTCCTGTTACCGTTC
GACTGCATGTGTTAGGCCTGCCGCCAGCGTCAATCTGAGCCAGGATCAAACTCT
>24
CTGCTGCCGCCCGTAGGAGTCTGGACCGTGTCTCAGTTCCAGTGTGGCTGGTCATCCTCTCAGACCAGCTAGG
GATCGTCGCCTAGGTGAGCCGTTACCCCACCTACTAGCTAATCCCATCTGGGCACATCCGATGGCAAGAGGCC
CTAAGGTCCCCCTCTTGGTCTTGCGACGTATGCGGTATTAGCTACCGTTTCCAGTAGTTATCCCCCTCCATCA
GGCAGTTTCCCAGACATTACTCACCCGTCCGCCACTCGTCAGCAAACAGCAAGCTGCTTCCTGTTACCGTTCG
ACTGCATGTGTTAGGCCTGCCGCCAGCGTCAATCTGAGCCAGGATCAAACTCT
>25
CTGCTGCCGCCCGTAGGAGTCTGGACCGTGTCTCAGTTCCAGTGTGGCTGGTCATCCTCTCAGACCAGCTAGG
GATCGTCGCCTAGGTGAGCCGTTACCCCACCTACTAGCTAATCCCATCTGGGCACATCCGATGGCAAGAGGCC
CTAAGGTCCCCTCTTGTCTTGCGACGTATGCGGTATTAGCTACCGTTTCCAGTAGTTATCCCCCTCCATCAGG
CAGTTTTCCCAGACATTACTCACCCGTCCGCCACTCGTCAGCAAACAGCAAGCTGCTTCCTGTTACCGTTCGA
CTGCATGTGTTAGGCCTGCCGCCAGCGTCAATCTGAGCCAGGATCAAACTCT
>26
CTGCTGCCGCCCGTAGGAGTCTGGACCGTGTCTCAGTTCCAGTGTGGCTGGTCATCCTCTCAGACAGCTAGGG
ATCGTCGCCTAGGTGAGCCGTTACCCCACCTACTAGCTAATCCCATCTGGGCACATCCGATGGCAAGAGGCCC
TAAGGTCCCCCTCTTGTCTGCGACGTATGCGGTATTAGCTACCGTTTCCAGTAGTTATCCCCCTCCATCAGGC
AGTTTCCCAGACATACTCACCCGTCCGCCACTCGTCAGCAAAGCAGCAAGCTGCTTCTGTTACCGTTCGACTG
CATGTCGTTAGGCCTGCCGCCAGCGTCAATCTGAGCCAGGATCAACTCT
>27
CTGCTGCCGCCCGTAGGAGTCTGGACCGTGTCTCAGTTCCAGTGTGGCTGGTCATCCTCTCAGACCAGCTAGG
GATCGTCGCCTAGGTGAGCCGTTACCCCACCTACTAGCTAATCCCATCTGGGCACATCCGATGGCAAGAGGCC
CTAAGGTCCCCCTCTTGTCTGCGACGTATGCGGTATTAGCTACCGTTTCCAGTAGTTATCCCCCTCCATCGGG

CAGTTTTCCCAGACATTACTCACCCGTCCGCCACTCGTCAGCAAAGCAGCAAGCTGCTTCCTGTTACCGTTCG
ACTGCATGTGTTAGGCCTGCCGCCAGCGTCAATCTGAGCCAGGATCAAACTCT
>28
CTGCTGCCGCCCGTAGGAGTCTGGACCGTGTCTCAGTTCCAGTGTGGCTGGTCATCCTCTCAGACCAGCTAGG
GATCGTCGCCTAGGTGAGCCGTTACCCCACCTACTAGCTAATCCCATCTGGGCACATCCGATGGCAAGAGGCC
CTAAGGTCCCCCTCTTGTCTTGCGACGTATGCGGTATTAGCTACCGTTTCCAGTAGTTATCCCCCTCCATCAG
GCAGTTTCCCAGACATTACTCACCCGTCCGCCACTCGTCAGCAAACAGCAAGCTGCTTCCTGTTACCGTTCGA
CTGCATGTGTTAGGCCTGCCGCCAGCGTCAATCTGAGCCAGGATCAAACTCT
>29
CTGCTGCCGCCCGTAGGAGTCTGGACCGTGTCTCAGTTCCAGTGTGGCTGGTCATCCTCTCAGACCAGCTAGG
GATCGTCGCCTAGGTGAGCCGTTACCCCACCTACTAGCTAATCCCATCTGGGCACATCCGATGGCAAGAGGCC
CTAAGGTCCCCCTCTTGTCTTGCGACGTATGCGGTATTAGCTACCGTTTCCAGTAGTTATCCCCCTCCATCAG
GCAGTTTTCCCAGACATTACTCACCCGTCCGCCACTCTCAGCAAAGCAGCAAGCTGCTTCCTGTTACCGTTCG
ACTGCATGTGTTAGGCCTGCCGCCAGCGTCAATCTGAGCCAGGATCAAACTCT
>30
CTGCTGCCGCCCCTAGGAGTCTGGACCGTGTCTCAGTTCCAGTGTGGCTGGTCATCCTCTCAGACCAGCTAGG
GATCGTCGCCTAGGTGAGCCGTTACCCCACCTACTAGCTAATCCCATCTGGGCACATCCGATGGCAAGAGGCC
CTAAGGTCCCCCTCTTGTCTTGCGACGTATGCGGTATTAGCTACCGTTTCCAGTAGTTATCCCCCTCCATCAG
GCAGTTTCCCAGACATTACTCACCCGTCCGCCACTCGTCAGCAAAGCAGCAAGCTGCTTCTGTTACCGTTCGA
CTGCATGTGTTAGGCCTGCCGCCAGCGTTCAATCTGAGCCAGGATCAAACTCT
>31
CTGCTGCCGCCCGTAGGAGTCTGGACCGTGTCTCAGTTCCAGTGTGGCTGGTCATCCTCTCAGACCAGCTAGG
GATCGTCGCCTAGGTGAGCCGTTACCCCACCTACTAGCTAATCCCATCTGGGCACATCCGATGGCAAGAGGCC
CTAAGGTCCCCCTCTTTGTCTGCGACGTATGCGGTATTAGCTACCGTTTCCAGTAGTTATCCCCCTCCATCAG
CAGTTTCCCAGACATTGCTCACCCGTCCGCCACTCGTCAGCAAAGCAGCAAGCTGCTTCCTGTTACCGTTCGA
CTGCATGTGTTAGGCCTGCCGCCAGCGTCAATCTGAGCCAGGATCAAACTCT
>32
CTGCTGCCGCCCGTAGGAGTCTGGACCGTGTCTCAGTTCCAGTGTGGCTGGTCATCCTCTCAGACCAGCTAGG
GATCGTCGCCTAGGTGAGCCGTTACCCCACCTACTAGCTAATCCCATCTGGGCACATCCGATGGCAAGAGGCC
CTAGGGTCCCCCTCTTGTCTTGCGACGTATGCGGTATTAGCTACCGTTTCCAGTAGTTATCCCCCTCCCATCA
GGCAGTTTCCCAGACATACTCACCCGTCCGCCATTCGTCAGCAAAGCAGCAAGCTGCTTCCTGTTACCGTTCG
ACTGCATGTGTTAGGCCTGCCGCCAGCGTCAATCTGAGCCAGGATCAAACTCT
>33
CTGCTGCCGCCCGTAGGAGTCTGGACCGTGTCTCAGTTCCAGTGTGGCTGGTCATCCTCTCAGACCAGCTAGG
GATCGTCGCCTAGGTGAGCCGTTACCCCACCTACTAGCTAATCCCATCTGGGCACATCCGATGGCAAGAGGCC
CTAAGGTCCCCCTCTTGTCTTGCGACGTTATGCGGTATTAGCTACCGTTTCCAGTAGTTATCCCCCTCCATCA
GGCAGTTTCCCAGACATTACTCACCCGTCCGCCACTCGTCAGCAAAGCAGCAAGCTGCTTCCTGTTACCGTTC
GACTGCATGTGTTAGGCCTGCCGCCAGCGTCAATCTGAAGCCAGGATCAACTCT
>34
CTGCTGCCGCCCGTAGGAGTCTGGACCGTGTCTCAGTTCCAGTGTGGCTGGTCATCCTCTCAGACCAGCTAGG
GATCGTCGCCTAGGTGAGCCGTTACCCCACCTACTAGCTAATCCCATCTGGGCACATCCGATGGCAAGAGGCC
CTAAGGTCCCCCTCTTTGTCTTGCGACGTTATGCGGTATTAGCTACCGTTTCCAGTAGTTATCCCCCTCCATC
AGGCAGTTTTCCCCAGACATTACTCACCCGTCCGCCACTCGTCAGCAAAGCAGCAAGCTGCTTCCTGTTACCG
TTCGACTGCATGTGTTAGGCCTGCCGCCAGCGTCAATCTGAGCCAGGATCAAACTCT
>35
CTGCTGCCGCCCGTAGGAGTCTGGACCGTGTCTCAGTTCCAGTGTGGCTGGTCATCCTCTCAGACCAGCTAGG
GATCGTCGCCTAGGTGAGCCGTTTACCCCACCTACTAGCTAATCCCATCTGGGCACATCCGATGGCAAGAGGC
CCTAAGGTCCCCCTCTTGTCTTGCGACGTATGCGGTATTAGCTACCGTTTCCAGTAGTTATCCCCCTCCATCA
GGCAGTTTCCCAGACATTACTCACCCGTCCGCCACTCGTCAGCAAAGCAGCAAGCTGCTTCTGTTACCGTTCG
ACTGCATGTGTTAGGCCTGCCGCCAGCGTCAATCTGAGCCAGGATCAAACTAT
>36
CTGCTGCCGCCCGTAGGAGTCTGGACCGTGTCTCAGTTCCAGTGTGGCTGGTCATCCTCTCAGACCAGCTAGG
GATCGTCGCCGTAGGTAGCACTGTCCACCCTACTAGCTAATCCCATCTGGGCACATCCGATGGCAAGAGGCCC
TAAGGTCCCCCTCTTTGTCTTGCGACGTATGCGGTATTAGCTACCGTTTCCAGTAGTTATCCCCCTCCATCAG

GCAGTTTCCCAGACATTACTCACCCGTCCGCCACTCGTCAGCAAAGCAGCAAGCTGCTTCTGTTACCGTTCGA
CTGCATGTGTTAGGCCTGCCGCCAGCGTTCAATCTGAGCCAGGATCAAACTCT
>37
CTGCTGCCGCCCGTAGGAGTCTGGACCGTGTCTCAGTTCCAGTGTGGCTGGTCATCCTCTCAGACCAGCTAGG
GATCGTCGCCTAGGTGAGCCGTTACCCCACCTACTAGCTAATCCCATCTGGGCACATCCGATGGCAAGAGGCC
CTAAGGTCCCCCTCTTTGTCTGCGACGTATGCGGTATTAGCTACCGTTTCCAGTAGTTATCCCCCTCCATCGG
GCAGTTTCCCAGACATTACTCACCCGTCCGCCACTCGTCAGCAAAGCAGCAAGCTGCTTCCTGTTACCGTTCG
ACTGCATGTGTTAGGCCTGCCGCCAGCGTCAATCTGAGCCAGGATCAAACTCT
>38
CTGCTGCCGCCCGTAGGAGTCTGGACCGTGTCTCAGTTCCAGTGTGGCTGGTCATCCTCTCAGACCAGCTAGG
GATCGTCGCCTAGGTGAGCCGTTACCCCACCTACTAGCTAATCCCATCTGGGCACATCCGATGGCAAGAGGCC
CTAAGGTCCCCCTCTTTGTCTGCGACGTATGCGGTATTAGCTACCGTTTCCAGTAGTTATCCCCCTCCATCGG
GCAGTTTCCCAGACATTACTCACCCGTCCGCCACTCGTCAGCAAAGCAGCAAGCTGCTTCCTGTTACCGTTCG
ACTGCATGTGTTAGGCCTGCCGCCAGCGTCAATCTGAGCCAGGATCAAACTCT
>39
CTGCTGCCGCCCGTAGGAGTCTGGACCGTGTCTCAGTTCCAGTGTGGCTGGTCATCCTCTCAGACCAGCTAGG
GATCGTCGCCTAGGTGAGCCGTTTACCCCCACCTACTAGCTAATCCCATCTGGGCACATCCGATGGCAAGAGG
CCCTAAGGTCCCCCTCTTGTCTGCGACGTTATGCGGTATTAGCTACCGTTTCCAGTAGTTATCCCCCTCCATC
AGGCAGTTTTCCCAGACATTACTCACCCGTCCGCCACTCGTCAGCAAAGCAGCAAGCTGCTTCCTGTTACCGT
TCGACTGCATGTGTTAGGCCTGCCGCCAGCGTCAATCTGAGCCAGGATCAAACTCT
>40
CTGCTGCCGCCCGTAGGAGTCTGGACCGTGTCTCAGTTCCAGTGTGGCTGGTCATCCTCTCAGACCAGCTAGG
GATCGTCGCCTAGGTGAGCCGTTACCCCACCTACTAGCTAATCCCATCTGGGCACATCCGATGGCAAGAGGCC
CTAAGGTCCCCCTCTTGTCTGCGACGTATGCGGTATTAGCTACCGTTTCCAGTAGTTATCCCCCTCCATCGGG
CAGTTTCCCAGACATTACTCACCCGTCCGCCACTCGTCAGCAAGCAGCAAGCTGCTTCCTGTTACCGTTCCGA
CTGCATGTGTTAGGCCTGCCGCCAGCGTCAATCTGAGCCAGGATCAAACTCT
>41
CTGCTGCCGCCCGTAGGAGTCTGGACCGTGTCTCAGTTCCAGTGTGGCTGGTCATCCTCTCAGACCAGCTAGG
GATCGTCGCCTAGGTGAGCCGTTACCCCACCTACTAGCTAATCCCATCTGGGCACATCCGATGGCAAGAGGCC
CTAAGGTCCCCCTCTTGTCTTGCGACGTTATGCGGTATTAGCTACCGTTTCCAGTAGTTATCCCCTCCATCGG
GCAGTTTCCCAGACATTACTCACCCGTCCGCCACTCGTCAGCAAAGCAGCAAGCTGCTTCCTGTTACCGTTCG
ACTGCATGTGTTAGGCCTGCCGCCAGCGTCAATCTGAGCCAGGATCAACTCT
>42
CTGCTGCCGCCCGTAGGAGTCTGGACCGTGTCTCAGTTCCAGTGTGGCTGGTCATCCTCTCAGACCAGCTAGG
GATCGTCGCCTAGGTGAGCCGTTACCCCACCTACTAGCTAATCCCATCTGGGCACATCCGATGGCAAGAGGCC
CTAAGGTCCCCCTCTTGTCTTGCGACGTTATGCGGTATTAGCTACCGTTTCCAGTAGTTATCCCCCTCCATCG
GGCAGTTTCCCAGACATTACTCACCCGTCCGCCACTCGTCAGCAAAGCAGCAAGCTGCTTCCTGTTACCGTTC
GACTGCATGTGTTAGGCCTGCCGCCAGCGTTCAATCTGAGCCAGGATCAAACTCT
>43
CTGCTGCCGCCCGTAGGAGTCTGGACCGTGTCTCAGTTCCAGTGTGGCTGGTCATCCTCTCAGACCAGCCAGG
GATCGTCGCCTAGGTGAGCCGTTACCCCACCTACTAGCTAATCCCATCTGGGCACATCCCGATGGCAAGAGGC
CCTAAGGTCCCCCTCTTTGTCTTGCGACGTTATGCGGTATTAGCTACCGTTTCCAGTAGTTATCCCCCTCCAT
CAGGCAGTTTCCCAGACATTACTCACCCGTCCGCCACTCGTCAGCAAAGCAGCAAGCTGCTTCCTGTTACCGT
TCGACTGCATGTGTTAGGCCTGCCGCCAGCGTCAATCCGAGCCAGGATCAAACTCT
>44
CTGCTGCCGCCCGTAGGAGTCTGGACCGTGTCTCAGTTCCAGTGTGGCTGGTCATCCTCTCAGACCAGCTAGG
GATCGTCGCCTAGGTGAGCCGTTACCCCACCTACTAGCTAATCCCATCTGGGCACATCCGATGGCAAGAGGCC
CTAAGGTCCCCCTCTTGGTCTGCGACGTATGCGGTATTAGCTACCGTTTCCAGTAGTTATCCCCCTCCATGGG
CAGTTTCCCAGACATTACTCACCCGTCCGCCACTCGTCAGCAAAGCAGCAAGCTGCTTCCTGTTACCGTTCGA
CTGCATGTGTTAGGCCTGCCGCCAGCGTCAATCTGAGCCAGGAGCAAACTCT
>45
TGCTGCCGCCCGTAGGAGTCTGGACCGTGTCTCAGTCCAGTGTGGCTGGTCATCCTCTCAGACCAGCTAGGGA
TCGTCGCCTAGGTGAGCCGTTACCCCACCTACTAGCTAATCCCATCTGGGCACATCCGATGGCAAGAGGCCCT
AAGGTCCCCCTCTTGGTCTTGCGACGTTATGCGGTATTAGCTACCGTCTCCAGTAGTTATCCCCCTCCATCAG

GCAGTTTCCCAGACATTACTCACCCGTCCGCCACTCGTCAGCAAAGCAGCAAGCTGCTTCCTGTTACCGTTCG
ACTGCATGTGTTAGGCCTGCCGCCAGCGTCAATCTGAGCCAGGATCAAACTCT
>46
CTGCTGCCGCCCGTAGGAGTCTGGACCGTGTCTCAGTTCCAGTGTGGCTGGTCATCCTCTCAGACCAGCTAGG
GATCGTCGCCTAGGTGAGCCGTTACCCCACCTACTAGCTAATCCCATCTGGGCACATCCGATGGCAAGAGGCC
CTAAGGTCCCCCTCTTTGGTCTTGCGACGTCATGCGGTATTAGCTACCGTTTCCAGTAGTTATCCCCCTCCAT
CGGGCAGTTTTCCCAGACATTACTCACCCGTCCGCCACTCGTCAGCAAAGCAGCAAGCTGCTTCCTGTTACCG
TTCGACTGCATGTGTTAGGCCTGCCGCCAGCGTCAATCTGAGCCAGGATCAAACTCT
>47
CTGCTGCCGCCCGTAGGAGTCTGGACCGTGTCTCAGTTCCAGTGTGGCTGGTCATCCTCTCAGACCAGCTAGG
GATCGTCGCCTAGGTGAGCCGTTACCCCACCTACTAGCTAATCCCATCTGGGCACATCCGATGGCAAGAGGCC
CTAAGGTCCCCCTCTTTGGTCTGCGACGTATGCGGTATTAGCTACCGTTTCCAGTAGTTATCCCCCTCCATCG
GGCAGTTTCCCAGACATTACTCACCCGTCCGCCACTCGTCAGCAAAGCAGCAAGCTGCTTCCTGTTACCGTTC
GACTGCATGTGTTAGGCCTGCCGCCAGCGTCAATCTGAGCCAGGATCAAACTCT
>48
CTGCTGCCGCCCGTAGGAGTCTGGACCGTGTCTCAGTTCCAGTGTGGCTGGTCATCCTCTCAGACCAGCTAGG
GATCGTCGCCTAGGTGAGCCGTTACCCCACCTACTAGCTAATCCCATCTGGGCACATCCGATGGCAAGAGGCC
CTAAGGTCCCCCTCTTTGGTCTTGCGACGTATGCGGTATTAGCTACCGTTTCCAGTAGTTATCCCCCTCCATC
AGGCAGTTTTCCCCAGACATTACTCACCCGTCCGCCACTCGTCAGCAAACAGCAAGCTGCTTCCTGTTACCGT
TCGACTGCATGTGTTAGGCCTGCCGCCAGCGTCAATCTGAGCCAGGATCAAACTCT
>49
CTGCTGCCGCCCGTAGGAGTCTGGACCGTGTCTCAGTTCCAGTGTGGCTGGTCATCCTCTCAGACAGCTAGGG
ATCGTCGCCTAGGTGAGCCGTTACCCCACCTACTAGCTAATCCCATCTGGGCACATCCGATGGCAAGAGGCCC
TAAGGTCCCCCTCTTGTCTTGCGACGTATGCGGTATTAGCTACCGTTTCCAGTAGTTATCCCCCTCCATCAGG
CAGTTTCCCAGACATTACTCACCCGTCCGCCACTCGTCAGCAAAGCAGCAAGCTGCTTCCTGTTACCGTTCGA
CTGCATGTGTTAGGCCTGCCGCCAGCGTCAATCTGAGCCAGGATCAAACTCT
>50
CTGCTGCCGCCCGTAGGAGTCTGGACCGTGTCTCAGTTCCAGTGTGGCTGGTCATCCTCTCAGACCAGCTAGG
GATCGTCGCCTAGGTGAGCCGTTACCCCACCTACTAGCTAATCCCATCTGGGCACATCCGATGGCAAGAGGCC
CTAAGGTCCCCCTCTTTGTCTTGCGACGTATGCGGTATTAGCTACCGTTTCCAGTAGTTATCCCCTCCATCGG
GCAGTTTCCCAGACATTACTCACCCGTCCGCCACTCGTCAGCAAACAGCAAGCTGCTTCCTGTTACCGTTCGA
CTGCATGTGTTAGGCCTGCCGCCAGCGTCAATCTGAGCCAGGATCAAACTCT
>51
CTGCTGCCGCCCGTAGGAGTCTGGACCGTGTCTCAGTTCCAGTGTGGCTGGTCATCCTCTCAGACCAGCTAGG
GATCGTCGCCTAGGTGAGCCGTTACCCCACCTACTAGCTAATCCCATCTGGGCACATCCGATGGCAAGAGGCC
CTAAGGTCCCCCTCTTTGTCTGCGACGTTATGCGGTATTAGCTACCGTTTCCAGTAGTTATCCCCCTCCATCA
GGCAGTTTTCCCAGACATTACTCACCCGTCCGCCACTCGTCAGCAAAGCAGCAAGCTGCTTCCTGTTACCGTT
CGACTGCATGTGTTAGGCCTGCCGCCAGCGTCAATCTGAGCCAGGATCAAACTCT
>52
CTGCTGCCGCCCGTAGGAGTCTGGACCGTGTCTCAGTTCCAGTGTGGCTGGTCATCCTCTCAGACCAGCTAGG
GATCGTCGCCTAGGTGAGCCGTTACCCCACCTACTAGCTAATCCCATCTGGGCACATCCGATGGCAAGAGGCC
CTAAGGTCCCCCTCTTTGTCTTGCGACGTATGCGGTATTAGCTACCGTTTCCAGTAGTTATCCCCCTCCATCA
GGCAGTTTCCCAGACATTACTCACCCGTCCGCCACTCGTCAGCAAAGCAGCAAGCTGCTTCCTGTTACCGTTC
GACTGCATGTGTTAGGCCTGCCGCCAGCGTCAATCTGAGCCAGGATCAAACTCT
>53
CTGCTGCCGCCCGTAGGAGTCTGGACCGTGTCTCAGTTCCAGTGTGGCTGGTCATCCTCTCAGACCAGCTAGG
GATCGTCGCCTAGGTGAGCCGTTACCCCACCTACTAGCTAATCCCATCTGGGCACATCCGATGGCAAGAGGCC
CTAAGGTCCCCCTCTTTGGTCTTGCGACGTTATGCGGTATTAGCTACCGTTCCTCAGTAGTTATCCCCCTCCA
TCAGGCAGTTTCCCAGACATTACTCACCCGTCCGCCACTCGTCAGCAAAGCAGCAAGCTGCTTCCTATTACCG
TCCGACTGCATGTGTTAGGCCTGCCGCCAGCGTTCAATCTGAGCCAGGATCAAACTCT
>54
CTGCTGCCGCCCGTAGGAGTCTGGACCGTGTCTCAGTTCCAGTGTGGCTGGTCATCCTCTCAGACCAGCTAGG
GATCGTCGCCTAGGTGAGCCGTTACCCCACCTACTAGCTAATCCCATCTGGGCACATCCGATGGCAAGAGGCC
CTAAGGTCCCCCTCTTTGTCTGCGACGTTATGCGGTATTAGCTACCGTTTCCAGTAGTTATCCCCTCCATCGG

GCAGTTTCCCAGACATTACTCACCCGTCCGCCACTCGTCAGCAAAGCAGCAAGCTGCTTCCTGTTACCGTTCG
ACTGCATGTGTTAGGCCTGCCGCCAGCGTCAATCTGAGCCAGGATCAAACTCT
>55
CTGCTGCCGCCTGTAGGAGTCTGGACCGTGTCTCAGTTCCAGTGTGGCTGGTCATCCTCTCAGACCAGCTAGG
GATTGTCGCCTAGGTGAGCCGTTACCCCACCTACTAGCTAATCCCATCTGGGCACATCCGATGGCAAGAGGCC
CTAAGGTCCCCCTCTTGTCTTGCGACGTATGCGGTATTAGCTACCGTTTCCAGTAGTTATCCCCCTCCATCGG
GCAGTTTCCCAGACATTACTCACCCGTCCGCCACTCGTCAGCAAAGCAGCAAGCTGCTTCCTGTACCGTTCGA
CTGCATGTGTTAGGCCTGCCGCCAGCGTCAATCTGAGCCAGGATCAAACTCT
>56
CTGCTGCCGCCCGTAGGAGTCTGGACCGTGTCTCAGTTCCAGTGTGGCTGGTCATCCTCTCAGACCAGCTAGG
GATCGTCGCCTAGGTGAGCCGTTACCCCACCTACTAGCTAATCCCATCTGGGCACATCCGATGGCAAGAGGCT
CTAAGGTCCCCCTCTTTGGTCTTGCGACGTATGCGGTATTAGCTACCGTTTCCAGTAGTTATCCCCCTCCATC
AGGCAGTTTTCCCAGACATTACTCACCCATCCGCCACTCGTCAGCAAAGCAGCAAGCTGCTTCCTGTTACCGT
TCGACTTGCATGTGTTAGGCCTGCCGCCAGCGTTCAATCTGAGCCAGGATCAAACTCT
>57
CTGCTGCCGCCCGTAGGAGTCTGGACCGTGTCTCAGTTCCAGTGTGGCTGGTCATCCTCTCAGACCAGCTAGG
GATCGTCGCCTAGGTGAGCCGTTACCCCACCTACTAGCTAATCCCATCTGGGCACATCCGATGGCAAGAGGCC
CTAAGGTCCCCCTCTTTGTCTGCGACGTATGCGGTATTAGCTACCGTTTCCAGTAGTTATCCCCCTCCATCAG
GCAGTTTCCCAGACATTACTCACCCGTCCGCCACTCGTCAGCAAACAGCAAGCTGCTTCCTGTTACCGTTCGA
CTGCATGTGTTAGGCCTGCCGCCAGCGTTCAATCTGAGCCAGGATCAAACTCT
>58
CTGCTGCCGCCCGTAGGAGTCTGGACCGTGTCTCAGTTCCAGTGTGGCTGGTCATCCTCTCAGACCAGCTAGG
GATCGTCGCCTAGGTGAGCCGTTACCCCACCTACTAGCTAATCCCATCTGGGCACATCCGATGGCAAGAGGCC
CTAAGGTCCCCCTCTTTGTCTTGCGACGTTATGCGGTATTAGCTACCGTTTCCAGTAGTTATCCCCCTCCATC
GGGCAGTTTTCCCAGACATTACTCACCCGTCCGCCACTCGTCAGCAAAGCAGCAAGCTGCTTCCTGTTACCGT
TCGACTGCATGTGTTAGGCCTGCCGCCAGCGTCAATCTGAGCCAGGATCAAACTCT
>59
CTGCTGCCGCCCGTAGGAGTCTGGACCGTGTCTCAGTTCCAGTGTGGCTGGTCATCCTCTCAGACCAGCTAGG
GATCGTCGCCTAGGTGAGCCGTTACCCCACCTACTAGCTAATCCCATCTGGGCACATCCGATGGCAAGAGGCC
CTAAGGTCCCCCTCTTTGTCTTGCGACGTTATGCGGTATTAGCTACCGTTTCCAGTAGTTATCCCCCTCCATC
GGGCAGTTTTCCCAGACATTACTCACCCGTCCGCCACTCGTCAGCAAAGCAGCAAGCTGCTTCCTGTTACCGT
TCGACTGCATGTGTTAGGCCTGCCGCCAGCGTCAATCTGAGCCAGGATCAAACTCT
>60
CTGCTGCCGCCCGTAGGAGTCTGGACCATGTCTCAGTTCCAGTGTGGCTGGTCATCCTCTCAGACCAGCTAGG
GATCGTCGCCTAGGTGAGCCGTTACCCCACCTACTAGCTAATCCCATCTGGGCACATCCGATGGCAAGAGGCC
CTAAGGTCCCCCTCTTTGGTCTTGCGACGTATGCGGTATTAGCTACCGTTTCCAGTAGTTATCCCCCTCCATC
GGGCAGTTTCCCAGACATTACTCACCCGTCCGCCACTCGTCAGCAAAGCAGCAAGCTGCTTCCTGTTACCGTT
CGACTGCATGTGTTAGGCCTGCCGCCAGCGTCAATCTGAGCCAGGATCAAATCT
>61
CTGCTGCCGCCCGTAGGAGTCTGGACCGTGTCTCAGTTCCAGTGTGGCTGGTCATCCTCTCAGACCAGCTAGG
GATCGTCGCCTAGGTGAGCCGTTACCCCACCTACTATCTAATCCCATCTGGGCACATCCGATGGCAAGAGGCC
CTAAGGTCCCCCTCTTTGGTCTTGCGACGTTATGCGGTATTAGCTACCGTTTCCAGTAGTTATCCCCCTCCAT
CAGGCAGTTTTCCCAGACATTACTCACCCGTCCGCCACTCGTCAGCAAAGCAGCAAGCTGCTTCCTGTTACCG
TTCGACTGCATGTGTTAGGCCTGCCGCCAGCGTCAATCTGAGCCAGGATCAAACTCT
>62
CTGCTGCCGCCCGTAGGAGTCTGGACCGTGTCTCAGTTCCAGTGTGGCTGGTCATCCTCTCAGACCAGCTAGG
GATCGTCGCCTAGGTGAGCCGTTACCCCACCTACTAGCTAATCCCATCTGGGCACATCCGATGGCAAGAGGCC
CTAAGGTCCCCCTCTTTGTCTTGCGACGTATGCGGTATTAGCTACCGTTTCCAGTAGTTATCCCCCTCCATCA
GGCAGTTTTCCCAGACATTACTCACCCGTCCGCCACTCGTCAGCAAAGCAGCAAGCTGCTTCCTGTTACCGTT
CGACTGCATGTGTTAGGCCTGCCGCCAGCGTCAATCTGAGCCAGGATCAAACTCT
>63
CTGCTGCCGCCCGTAGGAGTCTGGACCGTGTCTCAGTTCCAGTGTGGCTGGTCATCCTCTCAGACCAGCTAGG
GATCGTCGCCTAGGTGAGCCGTTACCCCACCTACTAGCTAATCCCATCTGGGCACATCCGATGGCAAGAGGCC
CTAAGGTCCCCCTCTTGTCTGCGACGTATGCGGTATTAGCTACCGTTTCCAGTAGTTATCCCCCTCCATCGGG

CAGTTTCCCAGACATTACTCACCCGTCCGCCACTCGCAGCAAAGCAGCAAGCTGCTTCCTGTTACCGTTCGAC
TGCATGTGTTAGGCCTGCCGCCAGCGTCAATCTGAGCCAGGATCAAACTCT
>64
CTGCTGCCGCCCGTAGGAGTCTGGACCGTGTCTCAGTTCCAGTGTGGCTGGTCATCCTCTCAGACCAGCTAGG
GATCGTCGCCTAGGTGAGCCGTTACCCCACCTACTAGCTAATCCCATCTGGGCACATCCGATGGCAAGAGGCC
CTAAGGTCCCCCTCTTGTCTGCGACGTATGCGGTATTAGCTACCGTTTCCAGTAGTTATCCCCCTCCATCGGG
CAGTTTCCCAGACATTACTCACCCGTCCGCCACTCGTCAGCAAAGCAGCAAGCTGCTTCCTGTTACCGTTCGA
CTGCATGTGTTAGGCCTGCCGCCAGCGTCAATCTGAGCCAGGATCAAACTCT
>65
TGCTGCCCCCTAGGAGTCTGGACCGTGTCTCAGTTCCAGTGTGGCTGGTCATCCTCTCAGACCAGCTAGGGAT
CGTCGCCTAGGTGAGCCGTTACCCCACCTACTAGCTAATCCCATCTGGGCACATCCGATGGCAAGAGGCCCTA
AGGTCCCCCTCTTGTCTTGCGACGTTATGCGGTATTAGCTACCGTTTCCAGTAGTTATCCCCCTCCATCAGGC
AGTTTCCCAGACATTACTCACCCGTCCGCCACTCGTCAGCAAAGCAGCAAGCTGCTTCCTGTTACCGTTCGAC
TGCATGTGTTAGGCCTGCCGCCAGCGTTCAATCTGAGCCAGGATCAACTCT
>66
CTGCTGCCGCCCGTAGGAGTCTGGACCGTGTCTCAGTTCCAGTGTGGCTGGTCATCCTCTCAGACCAGCTAGG
GATCGTCGCCTAGGTGAGCCGTTACCCCACCTACTAGCTAATCCCATCTGGGCACATCCGATGGCAAGAGGCC
CTAAGGTCCCCCCTCTTTGGTCTTGCGACGTATGCGGTATTAGCTACCGTTTCCAGTAGCTATCCCCCTCCAT
CGGGCAGTTTCCCAGACATTACTCACCCGTCCGCCACTCGTCAGCAAAGCAGCAAGCTGCTTCCTGTTACCGT
TCGACTGCATGTGTTAGGCCTGCCGCCAGCGTCAATCTGAGCCAGGATCAAACTCT
>67
CTGCTGCCGCCCGTAGGAGTCTGGACCGTGTCTCAGTTCCAGTGTGGCTGGTCATCCTCTCAGACCAGCTAGG
GATCGTCGCCTAGGTGAGCCGTTACCCCACCTACTAGCTAACCCATCTGGGCACATCCGATGGCAAGAGGCCC
TAAGGTCCCCCTCTTGTCTGCGACGTATGCGGTATTAGCTACCGTTTCCAGTAGTTATCCCCCTCCATCGGGC
AGTTTCCCAGACATACTCACCCGTCCGCCACTCGTCAGCAAGCAGCGAGCTGCTTCCTGTTCCGTTCGACTGC
ATGTGTTAGGCCTGCCGCCAGCGTCAATCTGAGCCAGGATCAACTCT
>68
CTGCTGCCGCCCGTAGGAGTCTGGACCGTGTCTCAGTTCCAGTGTGGCTGGTCATCCTCTCAGACAGCTAGGG
ATCGTCGCCTAGGTGAGCCGTTACCCCACCTACTAGCTAATCCCATCTGGGCACATCCGATGGCAAGAGGCCC
TAAGGTCCCCCTCTTGGTCTGCGACGTATGCGGTACTAGCTACCGTTTCCAGTAGTTATCCCCCTCCATCAGG
CAGTTTCCCAGACATTACTCACCCGTCCGCCACTCGTCAGCAAAGCAGCAAGCTGCTTCCTGTTACCGTCGAC
TGCATGTGTTAGGCCTGCCGCCAGCGTCAATCTGAGCCAGGATCAAACTCT
>69
CTGCTGCCGCCCGTAGGAGTCTGGACCGTGTCTCAGTTCCAGTGTGGCTGGTCATCCTCTCAGACCAGCTAGG
GATCGTCGCCTAGGTGAGCCGTTACCCCACCTACTAGCTAATCCCATCTGGGCACATCCGATGGCAAGAGGCC
CTAAGGTCCCCCTCTTTGTCTTGCGACGTATGCGGTATTAGCTACCGTTTCCAGTAGTTATCCCCCTCCATCA
GGCAGTTTCCCAGACATTACTCACCCGTCCGCCACTCGTCAGCAAAGCAGCAAGCTGCTTTCTGTTACCGTTC
GACTGCATGTGTTAGGCCTGCCGCCAGCGTCAATCTGAGCCAGGATCAAACTCT
>70
CTGCTGCCGCCCCGTAGGGAGTCGTGACCGTGTCTCAGTTCCAGTGTGGCTGGTCATCCTCTCAGACCAGCTA
GGGATCGTCGCCTAGGTGAGCCGTTACCCCACCTACTAGCTAATCCCATCTGGGCACATCCGATGGCAAGAGG
CCCTAAGGTCCCCCTCTTGTCTGCGACGTATGCGGTATTAGCTACCGTTTCCAGTAGTTATCCCCCTCCATCG
GGCAGTTTCCCAGACATTACTCACCCGTCCGCCACTCGTCAGCAAAGCAGCAAGCTGCTTCCTGTTACCGTTC
GACTGCATGTGTTAGGCCTGCCGCCAGCGTCAATCTGAGCCAGGATCAAACTCT
>71
CTGCTGCCGCCCGTAGGAGTCTGGACCGTGTCTCAGTTCCAGTGTGGCTGGTCATCCTCTCAGACCAGCTAGG
GATCGTCGCCTAGGTGAGCCGTTACCCCACCTACTAGCTAATCCCATCTGGGCACATCCGATGGCAAGAGGCC
CTAAGGTCCCCCTCTTGTCTTGCGACGTATGCGGTATTAGCTACCGTTTCCAGTAGTTATCCCCCTCCATCAG
GCAGTTTCCCAGACATTACTCACCCGTCCGCCACTCGTCAGCAAAGCAGCAAGCTGCTTCTGTTACCGTTCGA
CTGCATGTGTTAGGCCTGCCGCCAGCGTCAATCTGAGCCAGGATCAAACTCT
>72
CTGCTGCCGCCCGTAGGAGTCTGGACCGTGTCCCAGTTCCAGTGTGGCTGGTCATCCTCTCAGACCAGCTAGG
GATCGTCGCCTAGGTGAGCCGTTACCCCACCTACTAGCTAATCCCATCTGGGCACATCCGATGGCAAGAGGCC
CTAAGGTCCCCCTCTTTGTCTTGCGACGTATGCGGTATTAGCTACCGTTTCCAGTAGTTATCCCCCTCCATCG

GGCAGTTTCCCAGACATTACTCACCCGTCCGCCACTCGTCAGCAAAGCAGCAAGCTGCTTCCTGTTACCGTTC
GACTGCATGTGTTAGGCCTGCCGCCAGCGTCAATCTGAGCCAGGATCAACTCT
>73
CTGCTGCCGCCCGTAGGAGTCTGGACCGTGTCTCAGTTCCAGTGTGGCTGGTCATCCTCTCAGACCAGCTAGG
GATCGTCGCCTAGGTGAGCCGTTACCCCACCTACTAGCTAATCCCATCTGGGCACATCCCGATGGCAAGAGGC
CCTAAGGTCCCCCTCTTGTCTTGCGACGTTATGCGGTATTAGCTACCGTTTCCAGTAGTTATCCCCTCCATCA
GGCAGTTTCCCAGACATTACTCACCCGTCCGCCACTCGTCAGCAAAGCAGCAAGCTGCTTCTGTTACCGTTCG
ACTGCATGTGTTAGGCCTGCCGCCA
>74
CTGCTGCCGCCCGTAGGAGTCTGGACCGTGTCTCAGTTCCAGTGTGGCTGGTCATCCTCTCAGACCAGCTAGG
GATCGTCGCCTAGGTGAGCCGTTACCCCACCTACTAGCTAATCCCATCTGGGCACATCCGATGGCAAGAGGCC
CTAAGGTCCCCCTCTTTGTCTTGCGACGTATGCGGTATTAGCTACCGTTTCCAGTAGTTATCCCCCTCCATCA
GGCAGTTTCCCAGACATTACTCACCCGTCCGCCACTCGTCAGCAAAGCAGCAAGCTGCTTCCTGTTACCGTTC
GACTGCATGTGTTAGGCCTGCCGCCAGCGTCAATCTGAGCCAGGATCAACTCT
>75
CTGCTGCGCCCGTAGGAGTCTGGACCGTGTCTCAGTTCCAGTGTGGCTGGTCATCCTCTCAGACCAGCTAGGG
ATCGTCGCCTAGGTGAGCCGTTACCCCACCTACTAGCTAATCCCATCTGGGCACATCCCGATGGCAAGAGGCC
CTAAGGTCCCCCTCTTTGTCTTGCGACGTATGCGGTATTAGCTACCGTTTCCAGTAGTTATCCCCCTCCATCA
GGCAGTTTTCCCAGACATTACTCACCCGTCCGCCACTCGTCAGCAAAGCAGCAAGCTGCTTCCTGTTACCGTT
CGACTGCATGTGTTAGGCCTGCCGCCAGCCGTTCAATCTGAGCCAGGATCAAACTCT
>76
CTGCTGCCGCCCGTAGGAGTCTGGACCGTGTCTCAGTTCCAGTGTGGCTGGTCATCCTCTCAGACCAGCTAGG
GATCGTCGCCTAGGTGAGCCGTTACCCCACCTACTAGCTAATCCCATCTGGGCACATCCGATGGCAAGAGGCC
CTAAGGTCCCCCTCTTTGTCTGCGACGTATGCGGTATTAGCTACCGTTTCCAGTAGTTATCCCCCTCCATCGG
GCAGTTTCCCAGACATTACTCACCCGTCCGCCACTCGTCAGCAAAGCAGCAAGCTGCTTCCTGTTACCGTTCG
ACTGCATGTGTTAGGCCTGCCGCCAGCGTCAATCTGAGCCAGGATCAAACTCT
>77
CTGCTGCCGCCCTAGGAGTCTGGACCGTGTCTCAGTTCCAGTGTGGCTGGTCATCCTCTCAGACCAGCTAGGG
ATCGTCGCCTAGGTGAGCCGTTACCCCACCTACTAGCTAATCCCATCTGGGCACATCCGATGGCAAGAGGCCC
TAAGGTCCCCCTCTTGTCTTGCGACGTATGCGGTATTAGCTACCGTTTCCAGTAGTTATCCCCCTCCATCAGG
CAGTTTTCCCAGACATTACTCACCCGTCCGCCACTCTCAGCAAGCAGCAAGCTGCTTCCTGTTACCGTTCGAC
TGCATGTGTTAGGCCTG
>78
CTGCTGCCGCCCCGTAGGAGCTGGACCGTGTCTCAGTTCCAGTGTGGCTGGTCATCCTCTCAGACCAGCTAGG
GATCGTCGCCTAGGTGAGCCGTTACCCCACCTACTAGCTAATCCCATCTGGGCACATCCGATGGCAAGAGGCC
CTAAGGTCCCCCTCTTGTCTTGCGACGTTATGCGGTATTAGCTACCGTTTCCAGTAGTTATCCCCCTCCATCA
GGCAGTTTCCCAGACATTACTCACCCGTCCGCCACTCGTCAGCAAAGCAGCAAGCTGCTTCCTGTACCGTTCG
ACTGCATGTGTTAGGCCTGCCGCCAGCGTTCAATCGAGCCAGGATCAACTCT
>79
CTGCTGCCGCCCCGTAGGAGTCTGACCGTGTCTCAGTCCAGTGTGGCTGGTCATCCTCTCAGACCAGCTAGGG
ATCGTCGCCTAGGTGAGCCGTTACCCCACCTACTAGCTAATCCCATCTGGGCACATCCGATGGCAAGAGGCCT
AAGGTCCCCCTCTTGTCTGCGACGTATGCGGTATTAGCTACCGTTTCCAGTAGTTATCCCCCTCCATCAGGCA
GTTTCCCAGACATTACTCACCCGTCCGCCACTCGTCAGCAAAGCAGCAAGCTGCTTCCTGTTACCGTTCGACT
GCATGTGTTAGGCCTGCCGCCAGCGTCAATCTGAGCCAGGATCAACTCT
>80
CTGCTGCCGCCCGTAGGAGTCTGGACCGTGTCTCAGTTCCAGTGTGGCTGGTCATCCTCTCAGACCAGCTAGG
GATCGTCGCCTAGGTGAGCCGTTACCCCACCTACTAGCTAATCCCATCTGGGCACATCCCGATGGCAAGAGGC
CCTAAGGTCCCCTCTTTGTCTTGCGACGTATGCGGTATTAGCTACCGTTTCCAGTAGTTATCCCCCTCCATCA
GGCAGTTTTCCCCAGACATTACTCACCCGTCCGCCACTCGTCAGCAAAGCAGCAAGCTGCTTCCTGTTACCGT
TCGACTGCATGTGTTAGGCCTGCCGCCAGCGTCAATCTGAGCCAGGATCAAACTCT
>81
CTGCTGCCGCCCGTACGGAGTCTGGACCGTGTCTCAGTTCCAGTGTGGCTGGTCATCCTCTCAGACCAGCTAG
GGATCGTCGCCTAGGTCGAGCCGTTACCCCACCTACTACGCTAATCCCATCTGGGCACATCCCGATGGCAAGA
GGCCCTAAGGTCCCCCTCTTGTCTGCGACGTATGCGGTATTCGCTACCGTTTCCAGTAGTTATCCCCCTCCAT

CGGGCAGTTTCCCAGACATTACTCACCCGTCCGCCACTCGTCAGCAAAGCAGCAAGCTGCTTCCTGTTACCGT
TCGACTGCATGTGTTAGGCCTGCCGCCAGCGTCAATCTGAACCAGGATCAAACTCT
>82
CTGCTGCCGCCCGTAGGAGTCTGGACCGTGTCTCAGTTCCAGTGTGGCTGGTCATCCTCTCCGACAGCTAGGG
ATCGTCGCCTAGGTGAGCCGCTACCCCACCTACTAGCTAATCCCATCTGGGCACATCCGATGGCAAGAGGCCC
TAAGGTCCCCCTCTTGGTCTGCGACGTATGCGGTATTAGCTACCGTTTCCAGTAGTTTATCCCCCTCCATCAG
CAGTTTCCCAGACATTACTCACCCGTCCGCCACTCGTCAGCAAAGCAGCAAGCTGCTTCTGTTACCGTTCGAC
TGCATGTGTTAGGCCTGCCGCCAGCGTTCAATCTGAAGCCAGGATCAAACTCT
>83
CTGCTGCCGCCCGTAGGAGTCTGGACCGTGTCTCAGTTCCAGTGTGGCTGGTCATCCTCTCAGACCAGCTAGG
GATCGTCGCCTAGGTGAGCCGTTACCCCACCTACTAGCTAATCCCATCTGGGCACATCCGATGGCAAGAGGCC
CTAAGGTCCCCCTCTTGTCTGCGACGTTATGCGGTATTAGCTACCGTTTCCAGTAGTTATCCCCCTCCATCAG
GCAGTTTCCCAGACATTACTCACCCGTCCGCCACTCGTCAGCAAAGCAGCAAGCTGCTTCCTGTTACCGTTCG
ACTGCATGTGTTAGGCCTGCCGCCAGCGTCAATCTGAGCCAGGATCAACTCT
>84
CTGCTGCCGCCCGTAGGAGTCTGGACCGTGTCTCAGTTCCAGTGTGGCTGGTCATCCTCTCAGACCAGCTAGG
GATCGTCGCCTAGGTGAGCCGTTACCCCACCTACTAGCTAATCCCATCTGGGCACATCCGATGGCAAGAGGCC
CTAAGGTCCCCCTCTTGGTCTTGCGACGTATGCGGTATTAGCTACCGTTTCCAGTAGTTATCCCCCTCCATCG
GGCAGTTTCCCAGACATTACTCACCCGTCCGCCACTCGTCAGCAAAGCAGCAAGCTGCTTCCTGTTACCGTTC
GACTGCATGTGTTAGGCCTGCCGCCAGCGTTCAATCTGAGCCAGGATCAAACTCT
>85
CTGCTGCCGCCCGTAGGAGTCTGGACCGTGTCTCAGTTCCAGTGTGGCTGGTCATCCTCTCAGACCAGCTAGG
GATCGTCGCCTAGGTGAGCCGTTACCCCACCTACTAGCTAATCCCATCTGGGCACATCCGATGGCAAGAGGCC
CTAAGGTCCCCCTCTTTGTCTGCGACGTATGCGGTATTAGCTACCGTTTCCAGTAGTTATCCCCCTCCATCAG
GCAGTTTCCCAGACATTACTCACCCGTCCGCCACTCGTCAGCAAACAGCAAGCTGCTTCCTGTTACCGTTCGA
CTGCATGTGTTAGGCCTGCCGCCAGCGTCAATCTGAGCCAGGATCAAACTCT
>86
CTGCTGCCGCCCGTAGGAGTCTGGACCGTGTCTCAGTTCCAGTGTGGCTGGTCATCCTCTCAGACCAGCTAGG
GATCGTCGCCTAGGTGAGCCGTTACCCCACCTACTAGCTAATCCCATCTGGGCACATCCGATGGCAAGAGGCC
CTAAGGTCCCCCTCTTGTCTTGCGACGTATGCGGTATTAGCTACCGTTTCCAGTAGTTATCCCCCTCCATCAG
GCAGTTTCCCAGACATTACTCACCCGTCCGCCACTCGTCAGCAAAGCAGCAAGCTGCTTCTGTTACCGTTCGA
CTGCATGTGTTAGGCCTGCCGCCAGCGTTCAATCTGAGCCAGGATCAAACTCT
>87
CTGCTGCCGCCCGTAGGAGTCTGGACCGTGTCTCAGTTCCAGTGTGGCTGGTCATCCTCTCAGACCAGCTAGG
GATCGTCGCCTAGGTGAGCCGTTACCCCACCTACCAGCTAATCCCATCTGGGCACATCCGATGGCAAGAGGCC
CTAAGGTCCCCCTCTTGTCTTGCGACGTATGCGGTATTAGCTACCGTTTCCAGTAGTTATCCCCCTCCATCGG
GCAGTTTCCCAGACATTACTCACCCGTCCGCCACTCGTCAGCAAAGCAGCAAGCTGCTTCCTGTTACCGTTCG
ACTGCATGTGTTAGGCCTGCCGCCAGCGTCAATCTGAGCCAGGATCAAACTCT
>88
CTGCTGCCGCCCGTAGGAGTCTGGACTGTGTCTCAGTTCCAGTGTGGCTGGTCATCCTCTCAGACCAGCTAGG
GATCGTCGCCTAGGTGAGCCGTTACCCCACCTACTAGCTAATCCCATCTGGGCACATCCGATGGCAAGAGGCC
CTAAGGTCCCCCTCTTGTCTTGCGACGTATGCGGTATTAGCTACCGTTTCCAGTAGTTATCCCCCTCCATCAG
GCAGTTTTCCCAGACATTACTCACCCGTCCGCCACTCGTCAGCAAAGCAGCAAGCTGCTTCTGTTACCGTTCG
ACTGCATGTGTTAGGCCTGCCGCCAGCGTCAATCTGAGCCAGGATCAAACTCT
>89
CTGCTGCCGCCCGTAGGAGTCTGGACCGTGTCTCAGTTCCAGTGTGGCTGGTCATCCTCTCAGACCAGCTAGG
GATCGTCGCCTAGGTGAGCCGTTACCCCACCTACTAGCTAATCCCATCTGGGCACATCCGATGGCAAGAGGCC
CTAAGGTCCCCCTCTTGTCTGCGACGTATGCGGTATTAGCTACCGTTTCCAGTAGTTATCCCCCTCCATCGGG
CAGTTTCCCAGACATTACTCACCCGTCCGCCACTCGTCAGCAAAGCAGCAAGCTGCTTCCTGTTACCGTTCGA
CTGCATGTGTTAGGCCTGCCGCCAGCGTCAATCTGAGCCAGGATCAAACTCT
>90
CTGCTGCCGCCCGTAGGAGTCTGGACCGTGTCTCAGTTCCAGTGTGGCTGGACATCCTCTCGGACCAGCTAGG
GATCGTCGCCTAGGTGAGCCGTTACCCCACCTACTAGCTAATCCCATCTGGGCACATCCGATGGCAAGAGGCC
CTAAGGTCCCCCTCTTTGTCTTGCGACGTATGCGGTATTAGCTACCGTTTCCAGTAGTTATCCCCCTCCATCA

GGCAGTTTTCCCCAGACATTACTCACCCGTCCGCCACTCGTCAGCAAAGCAGCAAGCTGCTTTCTGTTACCGT
TCGACTGCATGTGTTAGGCCTGCCGCCAGCGTCAATCTGAGCCAGGATCAAACTCT
>91
CTGCTGCCGCCCGTAGGAGTCTGGACCGTGTCTCAGTTCCAGTGTGGCTGGTCATCCTCTCAGACCAGCTAGG
GATCGTCGCCTAGGTGAGCCGTTACCCCACCTACTAGCTAATCCCATCTGGGCACATCCGATGGCAAGAGGCC
CTAGGGTCCCCCTCTTGTCTTGCGACGTTATGCGGTATTAGCTACCGTTTCCAGTAGTTATCCCCCTCCATCG
GGCAGTTTCCCAGACATTACTCACCCGTCCGCCACTCGTCAGCAAAGCAGCAAGCTGCTTCCTGTTACCGTTC
GACTGCATGTGTTAGGCCTGCCGCCAGCGTCAATCTGAGCCAGGATCAAACTCT
>92
CTGCTGCCGCCCTAGGAGTCTGGACCGTGTCTCAGTTCCAGTGTGGCTGGTCATCCTCTCAGACCAGCTAGGG
ATCGTCGCCTAGGTGAGCCGTTACCCCACCTACTAGCTAATCCCATCTGGGCACATCCGATGGCAAGAGGCCC
TAAGGTCCCCCTCTTGTCTGCGACGTATGCGGTATTAGCTACCGTTTCCAGTAGTTATCCCCCTCCATCGGGC
AGTTTTCCCAGACATTACTCACCCGTCCGCCACTCGTCAGCAAAGCAGCAAGCTGCTTCCTGTTACCGTTCGA
CTGCATGTGTTAGGCCTGCCGCCAGCGTCAATCTGAGCCAGGATCAAACTCT
>93
CTGCTGCCGCCCGTAGGAGTCTGGACCGTGTCTCAGTTCCAGTGTGGCTGGTCATCCTCTCAGACCAGCTAGG
GATCGTCGCCTAGGTGAGCCGTTGCCCCACCTACTAGCTAATCCCATCTGGGCACATCCGATGGCAAGAGGCC
CTAAGGTCCCCCTCTTGTCTGCGACGTATGCGGTATTAGCTACCGTTTCCAGTAGTTATCCCCCTCCATCAGG
CGGTTTCCCAGACATTACTCACCCGTCCGCCACTCGTCAGCAAACAGCAAGCTGCTTCCTGTTACCGTTCGAC
TGCATGTGTTAGGCCTGCCGCCAGCGTCAATCTGAGCCAGGATCAAACTCT
>94
CTGCTGCCGCCCGTAGGAGTCTGGACCGTGTCTCAGTTCCAGTGTGGCTGGTCATCCTCTCAGACAGCTAGGG
ATCGTCGCCTAGGTGAGCCGTTACCCCACCTACTAGCTAATCCCATCTGGGCACATCCGATGGCAAGAGGCCC
TAAGGTCCCCCTCTTGGTCTTGCGACGTATGCGGTATTAGCTACCGTTTCCAGTAGTTATCCCCTCCATCGGG
CAGTTTCCCAGACATTACTCACCCGTCCGCCACTCGTCAGCAAGCAGCAAGCTGCTTCCTGTTACCGTTCGAC
TGCATGTGTTAGGCCTGCCGCCAGCGTTCAATCTGAGCCAGGATCAAACTCT
>95
CTGCTGCCGCCCGTAGGAGTCTGGACCGTGTCTCAGTTCCAGTGTGGCTGGTCATCCTCTCAGACCAGCTAGG
GATCGTCGCCTAGGTGAGCCGTTACCCCACCTACTAGCTAATCCCATCTGGGCACATCCGATGGCAAGAGGCC
CTAAGGTCCCCCTCTTGTCTTGCGACGTATGCGGTATTACGTACCGTTTCCAGTAGTTATCCCCCTCCATCAG
GCAGTTTTCCCAGACATTACTCACCCGTCCGCCACTCGTCAGCAAACAGCAAGCTGCTTCCTGTTACCGTTCG
ACTGCATGTGTTAGGCCTGCCGCCAGCGTTCAATCTGAGCCAGGATCAAACTCT
>96
CTGCTGCCGCCCGTAGGAGTCTGGACCGTGTCTCAGTTCCAGTGTGGCTGGTCATCCTCTCAGACCAGCTAGG
GATCGTCGCCTAGGTGAGCCGTTACCCCACCTACTAGCTAATCCCATCTGGGCACATCCGATGGCAAGAGGCC
CTAAGGTCCCCCTCTTTGGTCTTGCGACGTTATGCGGTATTAGCTACCGTTTCCAGTAGTTTATCCCCCTCCA
TCAGGCAGTTTTCCCAGACATTACTCACCCGTCCGCCACTCGTCAGCAAACAGCAAGCTGCTTCCTGTTACCG
TTCGACTGCATGTGTTAGGCCTGCCGCCAGCGTCAATCTGAGCCAGGATCAAACTCT
>97
CTGCTGCCGCCCGTAGGAGTCTGGACCGTGTCTCAGTTCCAGTGTGGCTGGTCATCCTCTCAGACCAGCTAGG
GATCGTCGCCTAGGTGAGCCGTTACCCCACCTACTAGCTAATCCCATCTGGGCACATCCGATGGCAAGAGGCC
CTAAGGTCCCCCTCTTTGTCTTGCGACGTATGCGGTATTAGCTACCGTTTCCAGTAGTTATCCCCCTCCATCA
GGCAGTTTCCCAGACATTACTCACCCGTCCGCCACTCGTCAGCAAACAGCAAGCTGCTTCCTGTTACCGTTCG
ACTGCATGTGTTAGGCCTGCCGCCAGCGTTCAATCTGAGCCAGGATCAAACTCT
>98
CTGCTGCCGCCCGTAGGAGTCTGGACCGTGTCTCAGTTCCAGTGTGGCTGGTCATCCTCTCAGACCAGCTAGG
GATCGTCGCCTAGGTGAGCCGTTACCCCACCTACTAGCTAATCCCATCTGGGCACATCCGATGGCAAGAGGCC
CTAAGGTCCCCCTCTTGTCTGCGACGTATGCGGTATTAGCTACCGTTTCCAGTAGTTATCCCCCTCCATCAGG
CAGTTTCCCAGACATTACTCACCCGTCCGCCACTCGTCAGCAAACAGCAAGCTGCTTCCTGTTACCGTTCGAC
TGCATGTGTTAGGCCTGCCGCCAGCGTTCAATCTGAGCCAGGATCAAACTCT
>99
CTGCTGCCGCCCGTAGGAGTCTGGACCGTGTCTCAGTTCCAGTGTGGCTGGTCATCCTCTCAGACCAGCTAGG
GATCGTCGCCTAGGTGAGCCGTTACCCCACCTACTAGCTAATCCCATCTGGGCACATCCGATGGCAAGAGGCC
CTAAGGTCCCCCTCTTGTCTGCGACGTATGCGGTATTAGCTACCGTTTCCAGTAGTTATCCCCCTCCATCGGG

CAGTTTCCCAGACATTACTCACCCGTCCGCCACTCGTCAGCAAACACACAAGCTGCTTCCTGTTACCGTTCGA
CTGCATGTGTTAGGCCTGCCGCCAGCGTCAATCTGAGCCAGGATCAAACTCT
>100
CTGCTGCCGCCCGTAGGAGTCTGGACCGTGTCTCAGTTCCAGTGTGGCTGGTCATCCTCTCAGACCAGCTAGG
GATCGTCGCCTAGGTGAGCCGTTACCCCACCTACTAGCTAATCCCATCTGGGCACATCCGATGGCAAGAGGCC
CTAAGGTCCCCCTCTTTGTCTTGCGACGTATGCGGTATTAGCTACCGTTTCCAGTAGTTATCCCCCTCCATCA
GGCAGTTTCCCAGACATTACCCACCCGTCCGCCACTCGTCAGCAAAGCAGCAAGCTGCTTCCTGTTACCGTTC
GACTGCATGTGTTAGGCCTGCCGCCAGCGTCAATCTGAGCCAGGATCAAACTCT
>101
CTGCTGCCGCCCGTAGGAGTCTGGACCGTGTCTCAGTTCCAGTGTGGCTGGTCATCCTCTCAGACCAGCTAGG
GATCGTCGCCTAGGTGAGCCGTTACCCCACCTACTAGCTAATCCCATCTGGGCACATCCGATGGCAAGAGGCC
CTAAGGTCCCCCTCTTGTCTTGCGACGTATGCGGTATTAGCTACCGTTTCCAGTAGTTATCCCCCTCCATCAG
GCAGTTTCCCAGACATTACTCACCCGTCCGCCACTCGTCAGCAAAGCAGCAAGCTGCTTCCTGTTACCGTTCG
ACTGCATGTGTTAGGCCTGCCGCCAGCGTCAATCTGAGCCAGGATCAAACTCT
>102
CTGCTGCCGTCCGTAGGAGTCTGGACCGTGTCTCAGTTCCAGTGTGGCTGGTCATCCTCTCAGACCAGCTAGG
GATCGTCGCCTAGGTGAGCCGTTACCCCACCTACTAGCTAATCCCATCTGGGCACATCCGATGGCAAGAGGCC
CTAAGGTCCCCCTCTTGTCTGCGACGTATGCGGTATTAGCTACCGTTTCCAGTAGTTATCCCCCTCCATCAGG
CAGTTTCCCAGACATTACTCACCCGTCCGCCACTCGTCAGCAAACAGCAAGCTGCTTCCTGTTACCGTTCGAC
TGCATGTGTTAGGCCTGCCGCCAGCGTTCAATCTG
>103
CTGCTGCCGCCCGTAGGAGTCTGGACCGTGTCTCAGTTCCAGTGTGGCTGGTCATCCTCTCAGACCAGCTAGG
GATCGTCGCCTAGGTGAGCCGTTACCCCACCTACTAGCTAATCCCATCTGGGCACATCCGATGGCAAGAGGCC
CTAAGGTCCCCCTCTTTGTCTTGCGACGTATGCGGTATTAGCTACCGTTTCCAGTAGTTATCCCCTCCATCGG
GCAGTTTCCCAGACATTACTCACCCGTCCGCCACTCGTCAGCAAAGCAGCAAGCTGCTTCCTGTTACCGTTCG
ACTGCATGTGTTAGGCCTGCCGCCAGCGTCAATCTGAGCCAGGATCAAACTCT
>104
CTGCTGCCGCCCGTAGGAGTCTGGACCGTGTCTCAGTTCCAGTGTGGCTGGTCATCCTCTCAGACCAGCTGGG
GATCGTCGCCTAGGTGAGCCGTTACCCCACCTACTAGCTAATCCCATCTGGGCACACCCGATGGCAAGAGGCC
CTAAGGTCCCCCTCTTGTCTGCGACGTATGCGGTATTAGCTACCGTTTCCAGTAGTTATCCCCCTCCATCAGG
CAGTTTCCCAGACATACTCACCCGTCCGCCACTCGTCAGCAAACAGCAAGCTGCTTCCTGTTACCGTTCGACT
GCATGTGTTAGGCCTGCCGCCAGCGTCAATCTGAGCCAGGATCAAACTGT
>105
CTGCTGCCGCCCGTAGGAGTCTGGACCGTGTCTCAGTTCCAGTGTGGCTGGTCATCCTCTCAGACCAGCTAGG
GATCGTCGCCTAGGTGAGCCGTTACCCCACCTACTAGCTAATCCCATCTGGGCACATCCGATGGCAAGAGGCC
CTAAGGTCCCCCCCTTGTCTTGCGACGTATGCGGTATTAGCTACCGTTTCCAGTAGTTATCCCCCTCCATCAG
GCAGTTTCCCAGACATTACTCACCCGTCCGCCACTCGTCAGCAAAGCAGCAAGCTGCTTCTGTTACCGTTCGA
CTGCATGTGTTAGGCCTGCCGCCAGCGTTCAATCTGAGCCAGGATCAAACTCT
>106
CTGCTGCCGCCCGTAGGAGTCTGGACCGTGTCTCAGTTCCAGTGTGGCTGGTCATCCTCTCAGACCAGCTAGG
GATCGTCGCCTAGGTGAGCCGTTACCCCACCTACTAGCTAATCCCATCTGGGCACATCCGATGGCAAGAGGCC
CTAAGGTCCCCCTCTTTGTCTGCGACGTTATGCGGTATTAGCTACCGTTTCCAGTAGTTATCCCCCTCCATCA
GGCAGTTTCCCAGACATTACTCACCCGTCCGCCACTCGTCAGCAAAAGCAGCAAGCTGCTTCCTGTTACCGTT
CAACTGCATGTGTTAGGCCTGCCGCCAGCGTTCAATCTGAGCCAGGATCAAACTCT
>107
CTGCTGCCGCCCGTAGGAGTCTGGACCGTGTCTCAGTTCCAGTGTGGCTGGTCATCCTCTCAGACCAGCTAGG
GATCGTCGCCTAGGTGAGCCGTTACCCCACCTACTAGCTAATCCCATCTGGGCACATCCGATGGCAAGAGGCC
CTAAGGTCCCCCTCTTGTCTTGCGACGTATGCGGTATTAGCTACCGTTTCCAGTAGTTATCCCCCTCCATCAG
GCAGTTTCCCAGACATTACTCACCCGTCCGCCACTCGTCAGCAAAGCAGCAAGCTGCTTTCTGTTACCGTTCG
ACTGCATGTGTTAGGCCTGCCGCCAGCGTTCAATCTGAGCCAGGATCAAACTCT
>108
CTGCTGCCGCCCGTAGGAGTCTGGACCGTGTCTCAGTTCCAGTGTGGCTGGTCATCCTCTCAGACCAGCTAGG
GATCGTCGCCTAGGTGAGCCGTTACCCCCACCTACTAGCTAATCCCATCTGGGCACATCCGATGGCAAGAGGC
CCTAAGGTCCCCCTCTTGGTCTTGCGACGTATGCGGTATTAGCTACCGTTTCCAGCAGTTATCCCCCTCCATC

GGGCAGTTTCCCAGACATTACTCACCCGTCCGCCACTCGTCAGCAAAGCAGCAAGCTGCTTCCTGTTACCGTT
CGACTGCATGTGTTAGGCCTGCCGCCAGCGTTCAATCTGAGCCAGGATCAAACTCT
>109
CTGCTGCCGCCCGTAGGAGTCTGGACCGTGTCTCAGTTCCAGTGTGGCTGGTCATCCTCTCAGACCAGCTAGG
GATCGTCGCCTAGGTGAGCCGTTACCCCACCTACTAGCTAATCCCATCTGGGCACATCCGATGGCAAGAGGCC
CTAAGGTCCCCCTCTTGTCTTGCGACGTATGCGGTATTAGCTACCGTTTCCAGTAGTTATCCCCCTCCATCAG
GCAGTTTCCCAGACATACTCACCCGTCCGCCACTCGTCAGCAAACAGCAAGCTGCTTCCTGTTACCGTTCGAC
TGCATGTGTTAGGCCTGCCGCCAGCGTCAATCTGAGCCAGGATCAAACTCT
>110
CTGCTGCCGCCCGTAGGAGTCTGGACCGTGTCTCAGTTCCAGTGTGGCTGGTCATCCTCTCAGACCAGCTAGG
GATCGTCGCCTAGGTGAGCCGTTACCCCACCTACTAGCTAATCCCATCTGGGCACATCCGATGGCAAGAGGCC
CTAAGGTCCCCCTCTTGTCTTGCGACGTATGCGGTATTAGCTACCGTTTCCAGTAGTTATCCCCCTCCATCAG
GCAGTTTTCCCAGACATTACTCACCCGTCCGCCACTCGTCAGCAAAGCAGCAAGCTGCTTTCTGTTACCGTTC
GACTGCATGTGTTTAGGCCTGCCGCCAGCGTCAATCTGAGCCAGGATCAAACTCT
>111
CTGCTGCCGCCCGTAGGAGTCTGGACCGTGTCTCAGTTCCAGTGTGGCTGGTCATCCTCTCAGACCAGCTAGG
GATCGTCGCCTAGGTGAGCCGTTACCCCACCTACTAGCTAATCCCATCTGGGCACATCCGATGGCAAGAGGCC
CTAAGGTCCCCCTCTTGTCTTGCGACGTATGCGGTATTAGCTACCGTTTCCGGTAGTTATCCCCCTCCATCAG
GCAGTTTCCCAGACATTACTCACCCGTCCGCCACTCGTCAGCAAACAGCAAGCTGCTTCCTGTTACCGTTCGA
CTGCATGTGTTAGGCCTGCCGCCAGCGTCAATCTGAGCCAGGATCAACTCT
>112
CTGCTGCCGCCCGTAGGAGTCTGGACCGTGTCTCAGTTCCAGTGTGGCTGGTCATCCTCTCAGACAGCTAGGG
ATCGTCGCCTGGGTGAGCCGTTACCCCACCTACTAGCTAATCCCATCTGGGCACATCCGATGGCAAGAGGCCC
TAAGGTCCCCCTCTTGTCTGCGACGTATGCGGTATTAGCTACCGTTTCCAGTAGTTATCCCCCTCCATCGGGC
AGTTCCCAGACATACTCACCCGTCCGCCACTCTCAGCAAGCAGCAAGCTGCTTCTGTTACCGTTCGACTGCAT
GTGTTAGGCCTGCCGCCAGCGTCAATCGTGACGCCAAGGGATCAAACTCT
>113
CTGCTGCCGCCCGTAGGAGTCTGGACCGTGTCTCAGTTCCAGTGTGGCTGGTCATCCTCTCAGACCAGCTAGG
GATCGTCGCCTAGGTGAGCCGTTACCCCACCTACTAGCTAATCCCATCTGGGCACATCCGATGGCAAGAGGCC
CTAAGGTCCCCCTCTTTGGTCTTGCGACGTATGCGGTATTAGCTACCGTTTCCAGTAGTTATCCCCCTCCATC
AGGCAGTTTCCCAGACATTACTCACCCGTCCGCCACTCGTCAGCAAACAGCAAGCTGCTTCCTGTTACTGTTC
GACTGCATGTGTTAGGCCTGCCGCCAGCGTCAATCTGAGCCAGGATCAAACTCT
>114
CTGCTGCCGCCCCGTAGGAGTCTGGACCGTGTCTCAGTTCCAGTGTGGCTGGTCATCCTCTCAGACCAGCTAG
GGATCGTCGCCTAGGTGAGCCGTTACCCCACCTACTAGCTAATCCCATCTGGGCACATCCGATGGCAAGAGGC
CCTAAGGTCCCCCTCTTGGTCTGCGACGTATGCGGTATTAGCTACCGTTTCCAGTAGTTATCCCCCTCCATCG
GGCAGTTTCCCAGACATACTCACCCGTCCGCCACTCTCAGCAAAGCAGCAAGCTGCTTCCTGTTACCGTTCGA
CTTGCATGTGTTAGGCCTGCCGCCAGCGTCAATCTGAGCCAGGATCAACTCT
>115
CTGCTGCCGCCCGTAGGAGTCTGGACCGTGTCTCAGTTCCAGTGTGGCTGGTCATCCTCTCAGACCAGCTAGG
GATCGTCGCCTAGGTGAGCCGTTACCCCACCTACTAGCTAATCCCATCTGGGCACATCCGATGGCAAGAGGCC
CTAAGGTCCCCCTCTTCGTCTGCGACGTTATGCGGTATTAGCTACCGTTTCCAGTAGTTATCCCCCTCCATCG
GGCAGTTTCCCAGACATTACTCACCCTCCGCCACTCGTCAGCAAAGCAGCAAGCTGCTTCCTGTTACCGTTCG
ACTGCATGTGTTAGGCCTGCCGCCAGCGTCAATCTGAGCCAGGATCAAACTCT
>116
CTGCTGCCGCCCGTAGGAGTCTGGACCGTGTCTCAGTTCCAGTGTGGCTGGTCATCCTCTCAGACAGCTAGGG
ATCGTCGCCTAGGTGAGCCGTTACCCCACCTACTAGCTAATCCCATCTGGGCACATCCGATGGCAAGAGGCCC
TAAGGTCCCCCTCTTGTCTTGCGACGTATGCGGTATTAGCTACCGTTTCCAGTAGTTATCCCCCTCCATCGGG
CAGTTTCCCAGACATTACTCACCCGTCCGCCACTCGTCAGCAAAGCAGCAAGCTGCTTCCTGTTACCGTTCGA
CTTGCATGTGTTAGGCCTGCCGCCAGCGTCAATCTGAGCCAGGATCAAACTCT
>117
CTGCTGCCGCCCGTAGGAGTCTGGACCGTGTCTCAGTTCCAGTGTGGCTGGTCATCCTCTCAGACCAGCTAGG
GATCGTCGCCTAGGTGAGCCGTTACCCCACCTACTAGCTAATCCCATCTGGGCACATCCGATGGCAAGAGGCC
CTAAGGTCCCCCCTCTTTGGTCTTGCGACGTTATGCGGTATTAGCTACCGTTTCCAGTAGTTATCCCCCTCCA

TCGGGCAGTTTTCCCAGACATTACTCACCCGTCCGCCACTCGTCAGCAAAGCAGCAAGCTGCTTCCTGTTACC
GTTCGACTGCATGTGTTAGGCCTGCCGCCAGCGTTCAATCTGAGCCAGGATCAAACTCT
>118
CTGCTGCCGCCCGTAGGAGTCTGGACCGTGTCTCAGTTCCAGTGTGGCTGGTCATCCTCTCAGACCAGCTAGG
GATCGTCGCCTAGGTGAGCCGTTACCCCACCTACTAGCTAATCCCATCTGGGCACATCCGATGGCAAGAGGCC
CTAAGGTCCCCCTCTTTGTCTTGCGACGTTATGCGGTATTAGCTACCGTTTCCAGTAGTTATCCCCCTCCATC
AGGCAGTTTCCCAGACATTACTCACCCGTCCGCCACTCGTCAGCAAACAGCAAGCTGCTTCCTGTTACCGTTC
GACTGCATGTGTTAGGCCTGCCGCCAGCGTCAATCTGAGCCAGGATCAACTCT
>119
CTGCTGCCGCCCGTAGGAGTCTGGACCGTGTCTCAGTTCCAGTGTGGCTGGTCATCCTCTCAGACCAGCTAGG
GATCGTCGCCTAGGTGAGCCGTTACCCCACCTACTAGCTAATCCCATCTGGGCACATCCGATGGCAAGAGGCC
CTAAGGTCCCCCTCTTGGTCTTGCGACGTATGCGGTATTAGCTACCGTTTCCAGTAGTTATCCCCCTCCATCA
GGCAGTTTTCCCAGACATTACTCACCCGTCCGCCACTCGTCAGCAAAGCAGCAAGCTGCTTCCTGTTACCGTT
CGACTGCATGTGTTAGGCCTGCCGCCAGCGTCAATCTGAGCCAGGATCAAACTCT
>120
CTGCTGCCGCCCGTAGGAGTCTGGACCGTGTCTCAGTTCCAGTGTGGCTGGTCATCCTCTCAGACCAGCTAGG
GATCGTCGCCTAGGTGAGCCGTTACCCCACCTACTAGCTAATCCCATCTGGGCACATCCGATGGCAAGAGGCC
CTAAGGTCCCCCTCTTGTCTGCGACGTATGCGGTATTAGCTACCGTTTCCAGTAGTTATCCCCCTCCATCAGG
CAGTTTCCCAGACATTACTCACCCGTCCGCCACTCGTCAGCAAAGCAGCAAGCTGCTTCCTGTTACCGTTCGA
CTGCATGTGTTAGGCCTGCCGCCAGCGTCAATCTGAGCCAGGATCAAACTCT
>121
CTGCTGCCGCCCCGTAGGAGTCTGGACCGTGTCTCAGTTCCAGTGTGGCTGGTCATCCTCTCAGACCAGCTAG
GGATCGTCGCCTAGGTGAGCCGTTACCCCACCTACTAGCTAATCCCATCTGGGCACATCCGATGGCAAGAGGC
CCTAAGGTCCCCCTCTTTGTCTTGCGACGTTATGCGGTATTAGCTACCGTTTCCAGTAGTTATCCCCCTCCAT
CAGGCAGTTTCCCAGACATTACTCACCCGTCCGCCACTCGTCAGCAAACAGCAAGCTGCTTCCTGTTACCGTT
CGACTGCATGTGTTAGGCCTGCCGCCAGCGTTCAATCTGAGCCAGGATCAAACTCT
>122
CTGCTGCCGCCCGTAGGAGTCTGGACCGTGTCTCAGTTCCAGTGTGGCTGGTCATCCTCTCAGACAGCTAGGG
ATCGTCGCCTAGGTGAGCCGTTACCCCACCTACTAGCTAATCCCATCTGGGCACATCCGATGGCAAGAGGCCC
TAAGGTCCCCCTCTTTGTCTTGCGACGTATGCGGTATTAGCTACCGTTTCCAGTAGTTATCCCCCTCCATCAG
GCAGTTTCCCAGACATTACTCACCCGTCCGCCACTCGTCAGCAAAGCAGCAAGCTGCTTTCTGTTACCGTTCG
ACTGCATGTGTTAGGCCTGCCGCCAGCGTTCAATCTGAGCCAGGATCAAACTCT
>123
CTGCTGCCGCCCGTAGGAGTCTGGACCGTGTCTCAGTTCCAGTGTGGCTGGTCATCCTCTCAGACCAGCTAGG
GATCGTCGCCTAGGTGAGCCGTTACCCCACCTACTAGCTAATCCCATCTGGGCACATCCGATGGCAAGAGGCC
CTAAGGTCCCCCTCTTGTCTGCGACGTATGCGGTATTAGCTACCGTTTCCAGTAGTTATCCCCCTCCATCAGG
CAGTTTCCCAGACATTACTCACCCGTCCGCCACTCGTCAGCAAAGCAGCAAGCTGCTTCCTGTTACCGTTCGA
CTGCATGTGTTAGGCCTGCCGCCAGCGTCAATCTGAGCCAGGATCAAACTCT
>124
CTGCTGCCGCCCTAGGAGTCTGGACCGTGTCTCAGTTCCAGTGTGGCTGGTCATCCTCTCAGACCAGCTAGGG
ATCGTCGCCTAGGTGAGCCGTTACCCCACCTACTAGCTAATCCCATCTGGGCACATCCGATGGCAAGAGGCCC
TAAGGTCCCCCTCTTTGGTCTTGCGACGTTATGCGGTATTAGCTACCGTTTCCAGTAGTTATCCCCTCCATCG
GGCAGTTTCCCAGACATTACTCACCCGTCCGCCACTCGTCAGCAAAGCAGCAAGCTGCTTCCTGTTACCGTTC
GACTTGCATGTGTTAGGCCTGCCGCCAGCGTCAATCTGAGCCAGGATCAAACTCT
>125
CTGCTGCCGCCCGTAGGAGTCTGGACCGTGTCTCAGTTCCAGTGTGGCTGGTCATCCTCTCAGACAGCTAGGG
ATCGTCGCCTAGGTGAGCCGTTACCCCACCTATAGCTAATCCCATCTGGGCACATCCGATGGCAAGAGGCCCT
AAGGTCCCCCTCTTTGTCTTGCGACGTTATGCGGTATTAGCTACCGTTTCCAGTAGTTATCCCCCTCCATCAG
GCAGTTTCCCAGACATTACTCACCCGTCCGCCACTCGTCAGCAAACAGCAAGCTGCTTCCTGTTACCGTTCGA
CTGCATGTGTAGGCCTGCCGCCAG
>126
CTGCTGCCGCCCGTAGGAGTCTGGACCGTGTCTCAGTTCCAGTGTGGCTGGTCATCCTCTCAGACCAGCTAGG
GATCGTCGCCTAGGTGAGCCGTTACCCCACCTACTAGCTAATCCCATCTGGGCACATCCGATGGCAAGAGGCC
CTAAGGTCCCCCTCTTTGGTCTTGCGACGTATGCGGTATTAGCTACCGTTTCCAGTAGTTATCCCCCTTCCAT

CAGGCAGTTTCCCAGACATTACTCACCCGTCCGCCACTCGTCAGCAAGCAGCAAGCTGCTTCTGTTACCGTTC
GACTGCATGTGTTAGGCCTGCCGCCAGCGTTCAATCTGAGCCAGGATCAAACTCT
>127
CTGCTGCCGCCCGTAGGAGTCTGGACCGTGTCTCAGTTCCAGTGTGGCTGGTCATCCTCTCAGACCAGCTAGG
GATCGTCGCCTAGGTGAGCCGTTACCCCACCTACTAGCTAATCCCATCTGGGCACATCCGATGGCAAGAGGCC
CTAAGGTCCCCCTCTTGTCTGCGACGTATGCGGTATTAGCTACCGTTTCCAGTAGTTATCCCCCTCCATCGGG
CAGTTTCCCAGACATTACTCACCCGTCCGCCACTCGTCAGCAAAGCAGCAAGCTGCTTCCTGTTACCGTTCGA
CTGCATGTGTTAGGCCTGCCGCCAGCGTCAATCTGAGCCAGGATCAAACTCT
>128
CTGCTGCCGCCCGTAGGAGTCTGGACCGTGTCTCAGTTCCAGTGTGGCTGGTCATCCTCTCAGACCAGCTAGG
GATCGTCGCCTAGGTGAGCCGTTACCCCACCTACTGGCTAATCCCATCTGGGCACATCCGATGGCAAGAGGCC
CTAAGGTCCCCCTCTTGTCTTGCGACGTATGCGGTATTAGCTACCGTTTCCAGTAGTTATCCCCCTCCATCAG
GCAGTTTCCCAGACATTACTCACCCGTCCGCCACTCGTCAGCAAAGCAGCAAGCTGCTTCCTGTTACCGTTCG
ACTGCATGTGTTAGGCCTGCCGCCAGCGTCAATCTGAAGCCAGGATCAAACTCT
>129
CTGCTGCCGCCCGTAGGAGTCTGGACCGTGTCTCAGTTCCAGTGTGGCTGGTCATCCTCTCAGACCAGCTAGG
GATCGTCGCCTAGGTGAGCCGTTACCCCACCTACTAGCTAATCCCATCTGGGCACATCCGATGGCAAGAGGCC
CTAAGGTCCCCCTCTTTGGTCTTGCGACGTATGCGGTATTAGCTACCGTTTCCAGTAGTTATCCCCCTCCATC
AGGCAGTTTCCCAGACATTACTCACCCGTCCGCCACTCGTCAGCAAAGCAGCAAGCTGCTTCCTGTTACCGTT
CGACTGCATGTGTTAGGCCTGCCGCCAGCGTCAATCTGAGCCAGGATCAAACTCT
>130
CTGCTGCCGCCCGTAGGAGTCTGGACCGTGTCTCAGTTCCAGTGTGGCTGGTCATCCTCTCAGACCAGCTAGG
GATCGTCGCCTAGGTGAGCCGTTACCCCACCTACTAGCTAATCCCATCTGGGCACATCCGATGGCAAGAGGCC
CTAAGGTCCCCCTCTTTGTCTGCGACGTTATGCGGTATTAGCTACCGTTTCCAGTAGTTATCCCCCTCCATCG
GGCAGTTTCCCAGACATTACTCACCCGTCCGCCACTCGTCAGCAAAGCACAAGCTGCTTCCTGTTACCGTTCG
ACTGCATGTGTTAGGCCGCCGCCAGCGTCAATCTGAGCCAGGATCAAACTCT
>131
CTGCTGCGCCCGTAGGAGTCTGGACCGTGTCTCAGTTCCAGTGTGGCTGGTCATCCTCTCAGACCAGCTAGGG
ATCGTCGCCTAGGTGGCACTATCCACCCTACTAGCTAATCCCATCTGGGCACATCCGATGGCAAGAGGCCCTA
AGGTCCCCCTCTTGTCTGCGACGTATGCGGTATTAGCTACCGTTTCCAGTAGTTATCCCCCTCCATCAGGCAG
TTTTCCCAGACATTACTCACCCGTCCGCCACTCGTCAGCAAAGCAGCAAGCTGCTTCCTGTTACCGTTCGACT
GCATGTGTTAGGCCTGCCGCCAGCGTCAATCTGAGCCAGGATCAAACTCT
>132
CTGCTGCCGCCCGTAGGAGTCTGGACCGTGTCTCAGTTCCAGTGTGGCTGGTCATCCTCTCAGACAGCTAGGG
ATCGTCGCCTAGGTGAGCCGTTACCCCACCTACTAGCTAATCCCATCTGGGCACATCCGATGGCAAGAGGCCC
TAAGGTCCCCCTCTTGTCTGCGACGTATGCGGTATTAGCTACCGTTTCCAGTAGTTATCCCCCTCCATCAGGC
AGTTTCCCAGACATTACTCACCCGTCCGCCACTCGTCAGCAAAGCAGCAAGCTGCTTCCTGTTACCGTTCGAC
TGCATGTGTTAGGCCTGCCGCCAGCGTCAATCTGAGCCAGGATCAAACTCT
>133
CTGTTGCCGCCCGTAGGAGTCTGGACCGTGTCTCAGTTCCAGTGTGGCTGGTCATCCTCTCAGACCAGCTAGG
GATCGTCGCCTAGGTGAGCCGTTACCCCCACCTACTAGCTAATCCCATCTGGGCACATCCGATGGCAAGAGGC
CCTAAGGTCCCCCTCTTTGTCTGCGACGTATGCGGTATTAGCTACCGTTTCCAGTAGTTATCCCCCTCCATCA
GGCAGTTTTCCCAGGCATTACTCACCCGTCCGCCACTCGTCAGCAAAGCAGCAAGCTGCTTCTGTTACCGTTC
GACTGCATGTGTTAGGCCTGCCGCCAGCGTCAATCTGAGCCAGGATCAAACTCT
>134
CTGCTGCCGCCCGTAGGAGTCTGGACCGTGTCTCAGTTCCAGTGTGGCTGGTCATCCTCTCAGACCAGCTAGG
GATCGTCGCCTAGGTGAGCCGTTACCCCACCTACTAGCTAATCCCATCTGGGCACATCCGATGGCAAGAGGCC
CTAAGGTCCCCCTCTTGTCTGCGACGTATGCGGTATTAGCTACCGTTTCCAGTAGTTATCCCCCTCCATCGGG
CAGTTTCCCAGACATTACTCACCCGTCCGCCACTCTCAGCAAAGCAGCAAGCTGCTTCCTGTTACCGTTCGAC
TGCATGTGTTAGGCCTGCCGCCAGCGCATCATGAGCCAGGCATCGAATCAACTC
>135
CTGCTGCCGCCCGTAGGAGTCTGGACCGTGTCTCAGTTCCAGTGTGGCTGGTCATCCTCTCAGACCAGCTAGG
GATCGTCGCCTAGGTGAGCCGTTACCCCACCTACTAGCTAATCCCATCTGGGCACATCCGATGGCAAGAGGCC
CTAAGGTCCCCCTCTTGTCTTGCGACGTATGCGGTATTAGCTACCGTTTCCAGTAGTTATCCCCCTCCATCAG

GCAGTTTCCCAGACATTACTCACCCGTCCGCCACTCGTCAGCAAAGCAGCAAGCTGCTTCCTGTTACCGTTCG
ACTGCATGTGTTAGGCCTGCCGCCAGCCGTTCAATCTGAGCCAGGATCAAACTCT
>136
CTGCTGCCGCCCGTAGGAGTCTGGACCGTGTCTCAGTTCCAGTGTGGCTGGTCATCCTCTCAGACCAGCTAGG
GATCGTCGCCTAGGTGAGCCGTTACCCCACCTACTAGCTAATCCCATCTGGGCACATCCGATGGCAAGAGGCC
CTAAGGTCCCCCCTCTTTGTCTTGCGACGTATGCGGTATTAGCTACCGTTTCCAGTAGTTATCCCCCTCCATC
AGGCAGTTTCCCAGACATTACTCACCCGTCCGCCACTCGTCAGCAAAGCAGCAAGCTGCTTCCTGTTACCGTT
CGACTGCATGTGTTAGGCCTGCCGCCAGCGTTCAATCTGAGCCAGGATCAAACTCT
>137
CTGCTGCCGCCCGTAGGAGTCTGGACCGTGTCTCAGTTCCAGTGTGGCTGGTCATCCTCTCAGACCAGCTAGG
GATCGTCGCCTAGGTGAGCCGTTACCCCACCTACTAGCTAATCCCATCTGGGCACATCCGATGGCAAGAGGCC
CTAAGGTCCCCCTCTTGTCTGCGACGTATGCGGTATTAGCTACCGTTTCCAGTAGTTATCCCCCTCCATCGGG
CAGTTTCCCAGACATTACTCACCCGTCCGCCACTCGTCAGCAAAGCAGCCAAGCTGCTTCCTGTTACCGTTCG
ACTGCATGTGTTAGGCCTGCCGCCAGCGTCAATCTGAGCCAGGATCAAACTCT
>138
CTGCTGCCGCCCGTAGGAGTCTGGACCGTGTCTCAGTTCCAGTGTGGCTGGTCATCCTCTCAGACCAGCTAGG
GATCGTCGCCTAGGTGAGCCGTTACCCCACCTACTAGCTAATCCCATCTGGGCACATCCGATGGCAAGAGGCC
CTAAGGTCCCCCCTCTTGTCTTGCGACGTATGCGGTATTAGCTACCGTTTCCAGTAGTTATCCCCCTCCATCA
GGCAGTTTCCCCAGACATTACTCACCCGTCCGCCACTCGTCAGCAAAGCAGCAAGCTGCTTCCTGTTACCGTT
CGACTGCATGTGTTAGGCCTGCCGCCAGCGTCAATCTGAGCCAGGATCAAACTCT
>139
CTGCTGCCGCCCGTAGGAGTCTGGACCGTGTCTCAGTTCCAGCGTGGCTGGTCATCCTCTCAGACAGCTAGGG
ATCGTCGCCTAGGTGAGCCGTTACCCCACCTATAGCTAATCCCATCTGGGCACATCCGATGGCAAGAGGCCCT
AAGGTCCCCCTCTTGTCTGCGACGTATGCGGTATTAGCTACCGTTTCCAGTAGTTATCCCCTCCATCAGGCAG
TTTCCCAGACATACTCACCCGTCCGCCACTCGTCAGCAAACAGCAAGCTGCTTCCTGTTACCGTTCGACTGCA
TGTGTTAGGCCTGCCG
>140
CTGCTGCCGCCCGTAGGAGTCTGGACCGTGTCTCAGTTCCAGTGTGGCTGGTCATCCTCTCAGACCAGCTAGG
GATCGTCGCCTAGGTGAGCCGTTACCCCACCTACTAGCTAATCCCATCTGGGCACATCCGATGGCAAGAGGCC
CTAAGGTCCCCCTCTTTGGTCTTGCGACGTTATGCGGTATTAGCTACCGTTTCCAGTAGTTATCCCCCTCCAT
CAGGCAGTTTCCCAGACATTACTCACCCGTCCGCCACTCGTCAGCAAAGCAGCAAGCTGCTTCCTGTTACCGT
TCGACTGCATGTGTTAGGCCTGCCGCCAGCGTCAATCTGAGCCAGGATCAAACTCT
>141
CTGCTGTCGCCCGTAGGAGTCTGGACCGTGTCTCAGTTCCAGTGTGGCTGGTCATCCTCTCAGACCAGCTAGG
GATCGTCGCCTAGGTGAGCCGTTACCCCACCTACTAGCTAATCCCATCTGGGCACATCCGATGGCAAGAGGCC
CTAAGGTCCCCCTCTTTGTCTTGCGACGTATGCGGTATTAGCTACCGTTTCCAGTAGTTATCCCCCTCCATCA
GGCAGTTTTCCCAGACATTACTCACCCGTCCGCCACTCGTCAGCAAAGCAGCAAGCTGCTTCCTGTTACCGTT
CGACTGCATGTGTTAGGCCTGCCGCCAGCGTTCAATCTGAGCCAGGATCAAACTCT
>142
CTGCTGCCGCCCGTAGGAGTCTGGACCGTGTCTCAGTTCCAGTGTGGCTGGTCATCCTCTCAGACCAGCTAGG
GATCGTCGCCTAGGTGAGCCGTTACCCCACCTACTAGCTAATCCCATCTGGGCACATCCGATGGCAAGAGGCC
CTAAGGTCCCCCTCTTGTCTTGCGACGTATGCGGTATTAGCTACCGTTTCCAGTAGTTATCCCCCTCCATCAG
GCAGTTTCCCAGACATTACTCACCCGTCCGCCACTCGTCAGCAAAGCAGCAAGCTGCTTCCTGTTACCGTTCG
ACTGCATGTGTTAGGCCTGCCGCCAGCGTCAATCTGAGCCAGGATCAAACTCT
>143
CTGCTGCCGCCCGTAGGAGTCTGGACCGTGTCTCAGTTCCAGTGTGGCTGGTCATCCTCTCAGACCAGCTAGG
GATCGTCGCCTAGGTGAGCCGTTACCCCACCTACTAGCTAATCCCATCTGGGCACATCCGATGGCAAGAGGCC
CTAAGGTCCCCCTCTTTGTCTTGCGACGTATGCGGTATTAGCTACCGTTTCCAGTAGTTATCCCCCTCCATCG
GGCAGTTTCCCAGACATTACTCACCCGTCCGCCACTCGTCAGCAAAGCAGCAAGCTGCTTCCTGTTACCGTTC
GACTGCATGTGTTAGGCCTGCCGCCAGCGTCAATCTGAGCCAGGATCAAACTCT
>144
TGCTGCCGCCCCGTAGGAGTCTGGACCGTGTCTCAGTTCCAGTGTGGCTGGTCATCCTCTCAGACCAGCTAGG
GATCGTCGCCTAGGTGAGCCGTTACCCCACCTACTAGTTAATCCCATCTGGGCACATCCGATGGCAAGAGGCC
CTAAGGTTCCCCCTCTTTGGTCTTGCGACGTTATGCGGTATTAGCTACCCGTTTCCCAGTAGTTATCCCCTCC

ATCGGGCAGTTTCCCCAGACCATTACTCACCCCGTCCGCCCACTCGTCAGCAAAGCAGCAAGCTGCTTCCTGT
TACCGTTCGACTTGCATGTGTTAGGCCTGCCGCCCAGCGTTCAATCTGAGCCCAGGATCAAACTCTT
>145
CTGCTGCCGCCCGTAGGAGTCTGGACCGTGTCTCAGTTCCAGTGTGGCTGGTCATCCTCTCAGACCAGCTAGG
GATCGTCGCCTAGGTGAGCCGTTACCCCACCTACTAGCTAATCCCATCTGGGCACATCCCGATGGCAAGAGGC
CCTAAGGTCCCCCTCTTTGTCTTGCGACGTTATGCGGTATTAGCTACCGTTTCCAGTAGTTATCCCCCTCCAT
CAGGCAGTTTTCCCAGACATTACTCACCCGTCCGCCACTCGTCAGCAAAGCAGCAAGCTGCTTCTGTTACCGT
CGACTGCATGTGTTAGGCCTGCCGCCAGCGTTCAA
>146
CTGCTGCCGCCCGTAGGAGTCTGGACCGTGTCTCAGTTCCAGTGTGGCTGGTCATCCTCTCAGACCAGCTAGG
GATCGTCGCCTAGGTGAGCCGTTACCCCACCTACTAGCTAATCCCATCTGGGCACATCCGATGGCAAGAGGCC
CTAAGGTCCCCCTCTTTGTCTTGCGACGTTATGCGGTATTAGCTACCGTTTCCAGTAGTTATCCCCTCCATCA
GGCAGTTTCCCAGACATTACTCACCCGTCCGCCACTCGTCAGCAAAGCAGCAAGCTGCTTCCTGTTACCGTTC
GACTGCATGTGTTAGGCCTGCCGCCAGCGTTCAATCTGAGCCAGGATCAAACTCT
>147
CTGCTGCCGCCCGTAGGAGTCTGGACCGTGTCTCAGTTCCAGTGTGGCTGGTCATCCTCTCAGACCAGCTAGG
GATCGTCGCCTAGGTGGCCGTACCCACCCTACTAGCTAATCCCATCTGGGCACATCCGATGGCAAGAGGCCCT
AAGGTCCCCCTCTTTGGTCTCGCGACGTTATGCGGTATTAGCTACCGTTTCCAGTAGTTATCCCCCTCCATCA
GGCAGTTTTCCCAGACATTACTCACCCGTCCGCCACTCGTCAGCAAAGCAGCAAGCTGCTTTCTGTTACCGTT
CGACTGCATGTGTTAGGC
>148
CTGCTGCCGCCCGTAGGAGTCTGGACCGTGTCTCAGTTCCAGTGTGGCTGGTCATCCTCTCAGACCAGCTAGG
GATCGTCGCCTAGGTGAGCCGTTACCCCACCTACTAGCTAATCCCATCTGGGCACATCCCGATGGCAAGAGGC
CCTAAGGTCTCCCTCTTGGTCTTGCGACGTATGCGGTATTAGCTACCGTTTCCAGTAGTTATTCCCCCTCCAT
CGGGCAGTTTTCCCAGACATTACTCACCCGTCCGCCACTCGTCAGCAAACAGCAAGCTGCTTCCTGTTACCGT
TCGACTGCATTGTGTTAGGCCTGCCGCCAGCGTCAATCTGAGCCAGGATCAAACTCT
>149
CTGCTGCCGCCCGTAGGAGTCTGGACCGTGTCTCAGTTCCAGTGTGGCTGGTCATCCTCTCAGACCAGCTAGG
GATCGTCGCCTAGGTGAGCCGTTACCCCACCTACTAGCTAATCCCATCTGGGCACATCCGATGGCAAGAGGCC
CTAAGGTCCCCCTCTTTGTCTTGCGACGTTATGCGGTATTAGCTACCGTTTCCAGTAGTTATCCCCCTCCATC
AGGCAGTTTCCCAGACATTACTCACCCGTCCGCCACTCGTCAGCAAAGCAGCAAGCTGCTTCCTGTTACCGTT
CGACTGCATGTGTTAGGCCTGCCGCCAGCGTCAATCTGAGCCAGGATCAAACTCT
>150
CTGCTGCCGCCCGTAGGAGTCTGGACCGTGTCTCAGTTCCAGTGTGGCTGGTCATCCTCTCAGACCAGCTAGG
GATCGTCGCCTAGGTGAGCCGTTACCCCACCTACTGGCTAATCCCATCTGGGCACATCCGATGGCAAGAGGCC
CTAAGGTCCCCTCTTGTCTGCGACGTATGCGGTATTAGCTACCGTTTCCAGTAGTTATCCCCTCCATCAGGCA
GTTTTCCCAGACATTACTCACCCGTCCGCCACTCGTCAGCAAAGCAGCAAGCTGCTTCCTGTTACCGTTCGAA
CTGCATGTGTTAGGCCTGCCGCCAGCGTCAATCT
>151
CTGCTGCCGCCCGTAGGAGTCTGGACCGTGTCTCAGTTCCAGTGTGGCTGGTCATCCTCTCAGACCAGCTAGG
GATCGTCGCCTAGGTGAGCCGTTACCCCACCTACTAGCTAATCCCATCTGGGCACATCCGATGGCAAGAGGCC
CTAAGGTCCCCCTCTTTGTCTTGCGACGTTATGCGGTATTAGCTACCGTTCCAGTAGTTATCCCCCTCCATCA
GGCAGTTTCCCAGACATTACTCACCCGTCCGCCACTCGTCAGCAAAGCAGCAAGCTGCTTCCTGTTACCGTTC
GGCTTGCATGTGTTAGGCCTGCCGCCAGCGTTCAATCTGAGCCAGGATCAAACTCT
>152
CTGCTGCCGCCCGTAGGAGTCTGGACCGTGTCTCAGTTCCAGTGTGGCTGGTCATCCTCTCAGACCAGCTAGG
GATCGTCGCCTAGGTGAGCCGTTACCCCACCTACTAGCTAATCCCATCTGGGCACATCCGATGGCAAGAGGCC
CTAAGGTCCCCCTCTTTGGTCTTGCGACGTATGCGGTATTAGCTACCGTTTCCAGTAGTTATCCCCCTCCATC
AGGCAGTTTCCCAGACATTACTCACCCGTCCGCCACTCGTCAGCAAACAGCAAGCTGCTTCCTGTTACCGTTC
GACTGCATGTGTTAGGCCTGCCGCCAGCGTCAATCTGAGCCAGGATCAAACTCT
>153
CTGCTGCCGCCCGTAGGAGTCTGGACCGTGTCTCAGTTCCAGTGTGGCTGGTCATCCTCTCAGACCAGCTAGG
GATCGTCGCCTAGGTGAGCCGTTACCCCACCTACTAGCTAATCCCATCTGGGCACATCCGATGGCAAGAGGCC
CTAAGGTCCCCCTCTTGTCTGCGACGTATGCGGTATTAGCTACCGTTCCAGTAGTTATCCCCCTCCATCGGGC

AGTTTCCCAGACATTACTCACCCGTCCGCCACTCGTCAGCAAAGCAGCAAGCTGCTTCCTGTTACCGTTCGAC
TGCATGTGTTAGGCCTGCCGCCAGCGTCAATCTGAGCCAGGATCAAACTCT
>154
CTGCTGCCGCCCGTAGGAGTCTGGACCGTGTCTCAGTTCCAGTGTGGCTGGTCATCCTCTCAGACCAGCTAGG
GATCGTCGCCTAGGTGAGCCGTTACCCCACCTACTAGCTAATCCCATCTGGGCACGTCCGATGGCAAGAGGCC
CTAAGGTCCCCCTCTTTGGTCTTGCGACGTTATGCGGTATTAGCTACCGTTTCCAGTAGTTATCCCCCTCCAT
CAGGCAGTTTCCCAGACATTACTCACCCGTCCGCCACTCGTCAGCAAAGCAGCAAGCTGCTTCCTGTTACCGT
TCGACTGCATGTGTTAGGCCTGCCGCCAGCGTCAATCTGAGCCAGGATCAAACTCT
>155
CTGCTGCCGCCCGTAGGAGTCTGGACCGTGTCTCAGTTCCAGTGTGGCTGGTCATCCTCTCAGACCAGCTAGG
GATCGTCGCCTAGGTGAGCCGTTACCCCACCTACTAGCTAATCCCATCTGGGCACATCCGATGGCAAGAGGCC
CTAAGGTCCCCCTCTTGGTCCTGCGACGTATGCGGTATTAGCTACCGTTTCCAGTAGTTATCCCCCTCCATCA
GGCAGTTTTCCCAGACATTACTCACCCGTCCGCCACTCGTCAGCAAAGCAGCAAGCTGCTTCTGTTACCGTTC
GACTGCATGTGTTAGGCCTGCCGCCAGCGTCAATCTGAGCCAGGATCAAACTCT
>156
CTGCTGCCGCCCGTAGGAGTCTGGACCGTGTCTCAGTTCCAGTGTGGCTGGTCATCCTCTCAGACCAGCTAGG
GATCGTCGCCTAGGTGAGCCGTTACCCCACCTACTAGCTAATCCCATCTGGGCACATCCGATGGCAAGAGGCC
CTAAGGTCCCCCTCTTGTCTTGCGACGTATGCGGTATTAGCTACCGTTTCCAGTAGTTATCCCCCTCCATCGG
GCAGTTTCCCAGACATTACTCACCCGTCCGCCACTCGTCAGCAAGCAGCAAGCTGCTTCCTGTTACCGTTCGA
CTGCATGTGTTAGGCCTGCCGCCAGCGTCAATCTGAGCCAGGATCAAACTCT
>157
CTGCTGCCGCCCGTAGGAGTCTGGACCGTGTCTCAGTTCCAGTGTGGCTGGTCATCCTCTCAGACCAGCTAGG
GATCGTCGCCTAGGTGAGCCGTTACCCCACCTACTAGCTAATCCCATCTGGGCACATCCGATGGCAAGAGGCC
CTAAGGTCCCCCTCTTGTCTGCGACGTATGCGGTATTAGCTACCGTTTCCAGTAGTTATCCCCTCCATCGGGC
AGTTTCCCAGACATACTCACCCGTCCGCCACTCGTCAGCAAAGCAGCAAGCTGCTTCCTGTTACCGTTCGACT
GCATGTGTTAGGCCTGCCGCCAGCGTCAATCTGAGCCAGATCAAACTCT
>158
CTGCTGCCCCCGTAGGAGTCTGGACCGTGTCTCAGTTCCAGTGTGGCTGGTCATCCTCTCAGACCAGCTAGGG
ATCGTCGCCTAGGTGAGCCGTTACCCCACCTACTAGCTAATCCCATCTGGGCACATCCGATGGCAAGAGGCCC
TAAGGTCCCCCTCTTTGTCTTGCGACGTATGCGGTATTAGCTACCGTTTCCAGTAGTTATCCCCCTCCATCGG
GCAGTTTTCCCAGACATTACTCACCCGTCCGCCACTCGTCAGCAAAGCAGCAAGCTGCTTCCTGTTACCGTTC
GACTGCATGTGTTAGGCCTGCCGCCAGCGTCAATCTGAGCCAGGATCAAACTCT
>159
CTGCTGCCGCCCGTAGGAGTCTGGACCGTGTCTCAGTTCCAGTGTGGCTGGTCATCCTCTCAGACCAGCTAGG
GATCGTCGCCTAGGTGAGCCGTTACCCCACCTACTAGCTAATCCCATCTGGGCACATCCGATGGCAAGAGGCC
CTAAGGTCCCCCTCTTGTCTTGCGACGTTATGCGGTATTAGCTACCGTTTCCAGTAGTTATCCCCCTCCATCA
GGCAGTTTCCCAGACATTACTCACCCGTCCGCCACTCGTCAGCAAAGCAGCAAGCTGCTTTCCTGTTACCGTT
CGACTGCATGTGTTAGGCCTGCCGCCAGCGTTCAATCTGAGCCAGGATCAAACTCT
>160
CTGCTGCCGCCCGTAGGAGTCTGGACCGTGTCTCAGTTCCAGTGTGGCTGGTCATCCTCTCAGACCAGCTAGG
GATCGTCGCCTAGGTGAGCCGTTACCCCACCTACTAGCTAATCCCATCTGGGCACATCCGATGGCAAGAGGCC
CTAAGGTCCCCCTCTTTGGTCTTGCGACGTTATGCGGTATTAGCTACCGTTTCCAGTAGTTATCCCCCTCCAT
CAGGCAGTTTCCCAGACATTACTCACCCGTCCGCCACTCGTCAGCAAAGCAGCAAGCTGCTTCCTGTTACCGT
TCGACTGCATGTGTTAGGCCTGCCGCCAGCGTTCAATCTGAGCCAGGATCAAACTCT
>161
CTGCTGCCGCCCGTAGGAGTCTGGACCGTGTCTCAGTTCCAGTGTGGCTGGTCATCCTCTCAGACCAGCTAGT
GATCGTCGCCTAGGTGAGCCGTTACCCACCTACTAGCTAATCCCATCTGGGCACATCCGATGGCAAGAGGCCC
TAAGGTCCCCCTCTTTGTCTTGCGACGTTATGCGGTATTAGCTACCGTTTCCAGTAGTTATCCCCCTCATCAG
GCAGTTTCCCAGACATTACTCACCCGTCCGCCACTCGTCAGCAAAGCAGCAAGCTGCTTCCTGTTACCGTTCG
ACTGCATGTGTTAGGCCTGCCGCCAGCGTCAATCTGAGCCAGGATCAAACTCT
>162
CTGCTGCCGCCCGTAGGAGTCTGGACCGTGTCTCAGTTCCAGTGTGGCTGGTCATCCTCTCAGACCAGCTAGG
GATCGTCGCCTAGGTGAGCCGTTACCCCACCTACTAGCTAATCCCATCTGGGCACATCCGATGGCAAGAGGCC
CTAAGGTCCCCTCTTGTCTGCGACGTATGCGGTATTAGCTACCGTTTCCAGTAGTTATCCCCCTCCATCAGGC

AGTTTCCCAGACATTACTCACCCGTCCGCCACTCGTCAGCAAAGCAGCAAGCTGCTTCCTGTTACCGTTCGAC
TCGCATGTGTTAGGCCTGCCGCCAGCGTCAATCTGAGCCAGGATCAAACTG
>163
CTGCTGCCGCCCGTAGGAGTCTGGACCGTGTCTCAGTTCCAGTGTGGCTGGTCATCCTCTCAGACCAGCTAGG
GATCGTCGCCTAGGTGAGCCGTTACCCCACCTACTAGCTAATCCCATCTGGGCACATCCGATGGCAAGAGGCC
CTAAGGTCCCCCTCTTGTCTGCGACGTTATGCGGTATTAGCTACCGTTTCCAGTAGTTATCCCCTCCATCGGG
CAGTTTTCCCAGACATTACTCACCCGTCCGCCACTCTCAGCAAAGCAGCAAGCTGCTTCCTGTACCGTTCGAC
TGCATGTGTTAGGCCTGCCGCCAGCGTCAATCTGAGCC
>164
CTGCTGCCGCCCGTAGGAGTCTGGACCGTGTCTCAGTTCCAGTGTGGCTGGTCATCCTCTCAGACCAGCTAGG
GATCGTCGCCTAGGTGAGCCGTTACCCCACCTACTAGCTAATCCCATCTGGGCACATCCGATGGCAAGAGGCC
CTAAGGTCCCCCTCTTTGTCTTGCGACGTTATGCGGTATTAGCTACCGTTTCCAGTAGTTATCCCCCTCCATC
GGGCAGTTTCCCAGACATTACTCACCCGTCCGCCACTCGTCAGCAAAGCAGCAAGCTGCTTCCTGTTACCGTT
CGACTGCATGTGTTAGGCCTGCCGCCAGCGTTCAATCTGAGCCAGGATCAACTCT
>165
TGCTGCCGCCCGTAGGAGTCTGGACCGTGTCTCAGTTCCAGTGTGGCTGGTCACCCTCTCAGACCAGCTAGGG
ATCGTCGCCTAGGTGAGCTGTTACCCCACCTACTAGCTAATCCCATTCTGGGCACATCCGATGGCAAGAGGCC
CTAAGGTCCCCCTCTTTGGTCTTGCGACGTTATGCGGTATTAGCTACCGTTTCCAGTAGTTATCCCCTCCATC
GGGCAGTTTCCCAGACATTACTCACCCGTCCGCCACTCGTCAGCAAAGCAGCAAGCTGCTTCCTGTTTACCGT
TCGACTGCATGTGTTAGGCCTGCCGCCAGCGTCAATCTGAGCCAGGATCAAACTCT
>166
CTGCTGCCGCCCGTAGGAGTCTGGACCGTGTCTCAGTTCCAGTGTGGCTGGTCATCCTCTCAGACCAGCTAGG
GATCGTCGCCTAGGTGAGCCGTTACCCCACCTACTAGCTAATCCCATCTGGGCACATCCCGATGGCAAGAGGC
CCTAAGGTCCCCCTCTTGTCTTGCGACGTATGCGGTATTAGCTACCGTTCCAGTAGTTATCCCCCTCCATCAG
GCAGTTTCCCAGACATTACTCACCCGTCCGCCACTCGTCAGCAAGCAGCAAGCTGCTTCTGCTACCGTTCGAC
TGCAATGTGTTAGGCCTGCCGCCAGCGTCAATCTGAGCCAGGATCAAACTCT
>167
CTGCTGCCGCCCGTAGGAGTCTGGACCGTGTCTCAGTTCCAGTGTGGCTGGTCATCCTCTCAGACCAGCTAGG
GATCGTCGCCTAGGTGAGCCGTTACCCCACCTACTAGCTAATCCCATCTGGGCACATCCCGATGGCAAGAGGC
CCTAAGGTCCCCCTCTTTGTCTGCGACGTTATGCGGTATTAGCTACCGTTTCCAGTAGTTATCCCCCTCCATC
AGGCAGTTTCCCAGACATTACTCACCCGTCCGCCACTCGTCAGCAAAGCAGCAAGCTGCTTCCTGTTACCGTT
CGACTGCATGTGTTAGGCCTGCCGCCAGCGTCAATCTGAGCCAGGATCAAACTCT
>168
CTGCTGCCGCCCGTAGGAGTCTGGACCGTGTCTCAGTTCCAGTGTGGCTGGTTATCCTCTCAGACCAGCTAGG
GATCGTCGCCTAGGTGAGCCGTTACCCCACCTACTAGCTAATCCCATCTGGGCACATCCGATGGCAAGAGGCC
CTAAGGTCCCCCTCTTGTCTTGCGACGTATGCGGTATTAGCTACCGTTTCCAGTAGTTATCCCCCTCCATCAG
GCAGTTTCCCAGACACTACTCACCCGTCCGCCACTCGTCAGCAAAACAGCAAGCTGCTTCCTGTTACCGTTCG
ACTGCATGTGTTAGGCCTGCCGCCAGCGTTCAATCTGAGCCAGGATCAAACTCT
>169
CTGCTGCCGCCCGTAGGAGTCTGGACCGTGTCTCAGTTCCAGTGTGGCTGGTCATCCTCTCAGACCAGCTAGG
GATCGTCGCCTAGGTGAGCCGTTACCCCACCTACTAGCTAATCCCATCTGGGCACATCCGATGGCAAGAGGCC
CTAAGGTCCCCCTCTTTGTCTTGCGACGTATGCGGTATTAGCTACCGTTTCCAGTAGTTATCCCCCTCCATCA
GGCAGTTTCCCAGACATTACTCACCCGTCCGCCACTCGTCAGCAAACAGCAAGCTGCTTCCTGTTACCGTTCG
ACTGCATGTGTTAGGCCCGCCGCCAGCG
>170
CTGCTGCCGCCCGTAGGAGTCTGGACCGTGTCTCAGTTCCAGTGTGGCTGGTCATCCTCTCAGACCAGCTAGG
GATCGTCGCCTAGGTGAGCCGTTACCCCACCTACTAGCTAATCCCATCTGGGCACATCCGATGGCAAGAGGCC
CTAAGGTCCCCCTCTTGGTCTTGCGACGTTATGCGGTATTAGCTACCGTTCCAGTAGTTATTCCCCCTCCATC
GGGCAGTTTCCCAGACATTACTCACCCGTCCGCCACTCGTCAGCAAAGCAGCAGGCTGCTTTCCTGTTACCGT
CGACTGCATGTGTTAGGCCTGCCGCCAGCGTCAATCTGAGCCAAGATCAAACTCT
>171
CTGCTGCCGCCCGTAGGAGTCTGGACCGTGTCTCAGTTCCAGTGTGGCTGGTCATCCTCTCAGACCAGCTAGG
GATCGTCGCCTAGGTGAGCCGTTACCCCACCTACTAGCTAATCCCATCTGGGCACATCCCGATGGCAAGAGGC
CCTAAGGTCCCCCTCTTGTCTTGCGACGTATGCGGTATTAGCTACCGTTTCCAGTAGTTATCCCCCTCCATCA

GGCAGTTTCCCAGACATTACTCACCCGTCCGCCACTCGTCAGCAAAGCAGCAAGCTGCTTCTGTTACCGTTCG
ACTGCATGTGTTAGGCCTGCCGCCAGCGTCAATCTGAGCCAGGATCAAACTCT
>172
CTGCTGCCGCCCGTAGGAGTCTGGACCGTGTCTCAGTTCCAGTGTGGCTGGTCATCCTCTCAGACCAGCTAGG
GATCGTCGCCTAGGTGAGCCGTTACCCCACCTACTAGCTAATCCCATCTGGGCACATCCGATGGCAAGAGGCC
CTAAGGTCCCCCTCTTGTCTGCGACGTATGCGGTATTAGCTACCGTTCCAGTAGTTATCCCCCTCCATCAGGC
AGTTTCCCAGACATTACTCACCCGTCCGCCACTCGTCAGCAAACAGCAAGCTGCTTCCTGTTACCGTTCGACT
GCATGTGTTAGGCCTGCCGCCAGCGTCAGTCT
>173
CTGCTGCCGCCCTAGGAGTCTGGACCGTGTCTCAGTTCCAGTGTGGCTGGTCATCCTCTCAGACAGCTAGGGA
TCGTCGCCTAGGTGAGCCGTTACCCCACCTACTAGCTAATCCCATCTGGGCACATCCGATGGCAAGAGGCCCG
AAGGTCCCCCTCTTTGTCTTGCGACATTATGCGGTATTAGCTACCGTTTCCAGTAGTTATCCCCCTCCATCAG
GCAGTTTTCCCAGACATTACTCACCCGTCCGCCACTCGTCAGCGACAGCAAGCTGTTTCCTGTTACCGTTCGA
CTGCATGTGTTAGGCCTGCCGCCAGCGTCAATCTGAGCCAGGATCAAACTCT
>174
CTGCTGCCGCCCGTAGGAGTCTGGACCGTGTCTCAGTTCCAGTGTGGCTGGTCATCCTCTCAGACCAGCTAGG
GATCGTCGCCTAGGTGAGCCGTTACCCCACCTACTAGCTAATCCCATCTGGGCACATCCGATGGCAAGAGGCC
CTAAGGTCCCCCTCTTGTCTTGCGACGTATGCGGTATTAGCTACCGTTTCCAGTAGTTATCCCCCTCCATCAG
GCAGTTTCCCAGACATTACTCACCCGTCCGCCACTCGTCAGCAAAGCAGCAAGCTGCTTCCTGTTACCGTTCG
ACTGCATGTGTTAGGCCTGCCGCCAGCGTCAATCTGAGCCAGGATCAAACTCT
>175
CTGCTGCCGCCCGTAGGAGTCTGGACCGTGTCTCAGTTCCAGTGTGGCTGGTCATCCTCTCAGACCAGCTAGG
GATCGTCGCCTAGGTGAGCCGTTACCCCACCTACTAGCTAATCCCATCTGGGCACATCCGATGGCAGGAGGCC
CTAAGGTCCCCCTCTTGGTCTTGCGACGTTATGCGGTATTAGCTACCGTTTCCAGTAGTTATCCCCCTCCATC
AGGCAGTTTCCCAGACATTACTCACCCGTCCGCCACTCGTCAGCAAAGCAGCAAGCTGCTTCCTGTCACCGTT
CGACTGCATGTGTTAGGCCTGCCGCCAGCGTCAATCTGAGCCAGGATCAAACTCT
>176
CTGCTGCCGCCCGTAGGAGTCTGGACCGTGTCTCAGTTCCAGTGTGGCTGGTCATCCTCTCAGACCGGCTAGG
GATCGTCGCCTAGGTGAGCCGTTACCCCACCTACTAGCTAATCCCATCTGGGCACATCCGATGGCAAGAGGCC
CTAAGGTCCCCCTCTTGTCTTGCGACGTATGCGGTATTAGCTACCGTTTCCAGTAGTTATCCCCCTCCATCGG
GCAGTTTCCCAGACATTACTCACCCGTCCGCCACTCGTCAGCAAGCAGCAAGCTGCTTCCTGTTACCGTTCGA
CTGCATGTGTTAGGCCTGCCGCCAGCGTCAATCTGAGCCAGGATCAAACTCT
>177
CTGCTGCCGCCCGTAGGAGTCTGGACCGTGTCTCAGTTCCAGTGTGGCTGGTCATCCTCTCAGACCAGCTAGG
GATCGTCGCCTAGGTGAGCCGTTACCCCACCTACTAGCTAATCCCATCTGGGCACATCCGATGGCAAGAGGCC
CTAAGGTCCCCCTCTTTGTCTTGCGACGTATGCGGTATTAGCTACCGTTTCCAGTAGTTATCCCCCTCCATCA
GGCAGTTTCCCAGACATTACTCACCCGTCCGCCACTCGTCAGCAAACAGCAAGCTGCTTCCTGTTACCGTTCG
ACTGCATGTGTTAGGCCTGCCGCCAGCGTCAATCTGAGCCAGGATCAAACTCT
>178
CGCTGCCGCCCGTAGGAGTCTGGACCGTGTCTCAGTCCGGTGTGGCTGGTCATCCTCTCAGACCAGCTAGGGA
TCGTCGCCTAGGTGAGCCGTTACCCCACCTACTAGCTAATCCCATCTGGGCACATCCGATGGCAAGAGGCCCT
AAGGTCCCCTCTTTGGTCTTGCGACGTTATGCGGTATTAGCTACCGTTTCCAGTAGTTATCCCCCTCCATCAG
GCAGTTTTCCCAGACATTACTCACCCGTCCGCCACTCGTCAGCAAAGCAGCAAGCTGCTTCCTGTTACCGTTC
GACTGCATGTGTTAGGCCTGCCGCCAGCGTCAATCTGAGCCAGGATCAAACTCT
>179
TGCTGCCGCCCGTAGGAGTCTGGACCGTGTCTCAGTTCCAGTGTGGCTGGTCATCCTCTCAGACCAGCTAGGG
ATCGTCGCCTAGGTGAGCCGTTACCCCACCTACTAGCTAATCCCATCTGGGCACATCCGATGGCAAGAGGCCC
TAAGGTCCCCCTCTTTGTCTTGCGACGTTATGCGGTATTAGCTACCGTTTCCAGTAGTTATCCCCCTCCATCA
GGCAGTTTTCCCAGACATTACTCACCCGTCCGCCACTCGTCAGCAAAGCAGCAAGCTGCTTCTGTTACCGTTC
GACTGCATGTGTTAGGCCTGCCGCCAGCGTCAATCTGAGCCAGGATCAAACTCT
>180
CTGCTGCCGCCCGTAGGAGTCTGGACCGTGTCTCAGTTCCAGTGTGGCTGGTCATCCTCTCAGACCAGCTAGG
GATCGTCGCCTAGGTGAGCCGTTACCCCACCTACTAGCTAATCCCATCTGGGCACATCCCGATGGCAAGAGGC
CCTAAGGTCCCCCTCTTGTCTTGCGACGTTATGCGGTATTAGCTACCTCTCAGTAGTTTATCCCCCTCCATCG

GGCAGTTTTCCCAGACATTACTCACCCGTCCGCCACTCGTCAGCAAAGCAGCAAGCTGCTTCCTGTTACCGTT
CGACTGCATGTGTTAGGCCTGCCGCCAGCGTCAATCTGAGCCAGGATCAAACTCT
>181
CTGCTGCCGCCCGTAGGAGTCTGGACCGTGTCTCAGTTCCAGTGTGGCTGGTCATCCTCTCAGACCAGCTAGG
GATCGTCGCCTAGGTGAGCCGTTACCCCACCTACTAGCTAATCCCATCTGGGCACATCCGATGGCAAGAGGCC
CTAAGGTCCCCCTCTTGTCTGCGACGTATGCGGTATTAGCTACCGTTTCCAGTAGTTATCCCCTCCATCGGGC
AGTTTCCCAGACATTACTCACCCGTCCGCCACTCGTCAGCAAGCAGCAAGCTGCTTCCTGTTACCGTTCGACT
GCATGTGTTAGGCCTGCCGCCAGCGTCAATCTGAGCCAGGATCAAACTCT
>182
CTGCTGCCGCCCGTAGGAGTCTGGACCGTGTCTCAGTTCCAGTGTGGCTGGTCATCCTCTCAGACCAGCTAGG
GATCGTCGCCTAGGTGAGCCGTTACCCCACCTACTAGCTAATCCCATCTGGGCACATCCGATGGCAAGAGGCC
CTAAGGTCCCCCTCTTGTCTGCGACGTATGCGGTATTAGCTACCGTTTCCAGTAGTTATCCCCCTCCATCGGG
CAGTTTCCCAGACATTACTCACCCGTCCGCCACTCGTCAGCAAAGCAGCAAGCTGCTTCCTGTTACCGTTCGA
CTGCATGTGTTAGGCCTGCCGCCAGCGTCAATCTGAGCCAGGATCAAACTCT
>183
CTGCTGCCGCCCGTAGGAGTCTGGACCGTGTCTCAGTTCCAGTGTGGCTGGTCATCCTCTCAGACCAGCTAGG
GATCGTCGCCTAGGTGAGCCGTTACCCCACCTACTAGCTAATCCCATCTGGGCACATCCGATGGCAAGAGGCC
CTAAGGTCCCCCTCTTTGGTCTTGCGACGTATGCGGTATTAGCTACCGTTTCCAGTAGTTATCCCCCTCCATC
AGGCAGTTTTCCCAGACATTACTCACCCGTCCGCCACTCGTCAGCAAAGCAGCAAGCTGCTTCCTGTTACCGT
TCGACTGCATGTGTTAGGCCTGCCGCCAGCGTTCAATCTGAGCCAGGATCAAACTCT
>184
CTGCTGCCGCCCGTAGGAGTCTGGACCGTGTCTCAGTTCCAGTGTGGCTGGTCATCCTCTCAGACCAGCTAGG
GATCGTCGCCTAGGTGAGCCGTTACCCCACCTACTAGCTAATCCCATCTGGGCACATCCGATGGCAAGAGGCC
CTAAGGTCCCCCTCTTGTCTGCGACGTATGCGGTATTAGCTACCGTTTCCAGTAGTTATCCCCCTCCATCGGG
CAGTTTCCCAGACATTACTCACCCGTCCCGCCACTCGTCAGCAAAGCAGCAAGCTGCTTCCTGTTACCGTTCG
ACTGCATGTGTTAGGCCTGCCGCCGGCGTCAATCTGAGCCAGGATCAAACTCT
>185
CTGCTGCCGCCCGTAGGAGTCTGGACCGTGTCTCAGTTCCAGTGTGGCTGGTCATCCTCTCAGACCAGCTAGG
GATCGTCGCCTAGGTGAGCCGTTACCCCACCTACTAGCTAATCCCATCTGGGCACATCCGATGGCAAGAGGCC
CTAAGGTCCCCCTCTTGTCTTGCGACGTTATGCGGTATTAGCTACCGTTTCCAGTAGTTATCCCCCTCCATCG
GGCAGTTTCCCAGACATTACTCACCCGTCCGCCACTCGTCAGCAAAGCAGCAAGCTGCTTCCTGTTACCGTTC
GACTGCATGTGTTAGGCCTGCCGCCAGCGTCAATCTGAGCCAGGATCAAACTCT
>186
CTGCTGCCGCCCGTAGGAGTCTGGACCGTGTCTCAGTTCCAGTGTGGCTGGTCATCCTCTCAGACCAGCTAGG
GATCGTCGCCTAGGTGAGCCGTTACCCCACCTACTAGCTAATCCCATCTGGGCACATCCGATGGCAAGAGGCC
CTAAGGTCCCCCTCTTTGTCTGCGACGTATGCGGTATTAGCTACCGTTTCCAGTAGTTATCCCCCTCCATCAG
GCAGTTTCCCAGACATTACTCACCCGTCCGCCACTCGTCAGCAAAACAGCAAGCTGCTTCCTGTTACCGTTCG
ACTGCATGTGTTAGGCCTGCCGCCAGCGTCAATCTGAGCCAGGATCAAACTCT
>187
CTGCTGCCGCCCGTAGGAGTCTGGACCGTGTCTCAGTCCCAGTGTGGCTGGTCATCCCCTCAGACCAGCTAGG
GATCGTCGCCTAGGTGAGCCGTTACCCCACCTACTAGCTAATCCCATCTGGGCACATCCGATGGCAAGAGGCC
CTAAGGTCCCCCTCTTGTCTTGCGACGTATGCGGTATTAGCTACCGTTTCCAGTAGTTATCCCCTCCATCGGG
CAGTTTCCCAGACATTACTCACCCGTCCGCCACTCGTCAGCAAAGCAGCAAGCTGCTTCCTGTTACCGTTCGA
CTGCATGTGTTAGGCCTGCCGCCAGCGTCAATCTGAGCCAGGATCAAACTCT
>188
ATGCTGCCGCCCGTAGGAGTCTGGACCGTGTCTCAGTTCCAGTGTGGCTGGTCATCCTCTCAGACCAGCTAGG
GATCGTCGCCTAGGTGAGCCGTTACCCCACCTACTAGCTAATCCCATCTGGGCACATCCGATGGCAAGAGGCC
CTAAGGTCCCCCTCTTTGGTCTGCGACGTATGCGGTATTAGCTACCGTTTCCAGTAGTTATCCCCCTCCATCG
GGCAGTTTCCCAGACATTACTCACCCGTCCGCCACTCGTCAGCAAAGCAGCAAGCTGCTTCCTGTTACCGTTC
GACTGCATGTGTTAGGCCTGCCGCCAGCGTCAATCTGAGCCAGGATCAAACTCT
>189
CTGCTGCCGCCCGTAGGAGTCTGGACCGTGTCTCAGTTCCAGTGTGGCTGGTCATCCTCTCAGACCAGCTAGG
GATCGTCGCCTAGGTGAGCCGTTACCCCACCTACTAGCTAATCCCATCTGGGCACATCCGATGGCAAGAGGCC
CTAAGGTCCCCCTCTTTGGTCTGCGACGTATGCGGTATTAGCTACCGTTTCCAGTAGTTATCCCCCTCCATCG

GGCAGTTTCCCAGACATTACTCACCCGTCCGCCACTCGTCAGCAAAGCAGCAAGCTGCTTCCTGTTACCGTTC
GACTTGCATGTGTTAGGCCTGCCGCCAGCGTCAATCTGAGCCAGGATCAAACTCT
>190
CTGCTGCCGCCCGTAGGAGTCTGGACCGTGTCTCAGTTCCAGTGTGGCTGGTCATCCTCTCAGACCAGCTAGG
GATCGTCGCCTAGGTGAGCCGTTACCCCACCTACTAGCTAATCCCATCTGGGCACATCCGATGGCAAGAGGCC
CTGAGGTCCCCCTCTTTGTCTTGCGACGTATGCGGTATTAGCTACCGTTTCCAGTAGTTATCCCCCTCCATCA
GGCAGTTTCCCAGACATTACTCACCCGTCCGCCACTCGTCAGCAAAACAGCAAGCTGCTTCCTGTTACCGTTC
GACTGCATGTGTTAGGCCTGCCGCCAGCGTCAATCTGAGCCAGGATCAAACTCT
>191
CTGCTGCCGCCCGTAGGAGTCTGGACCGTGTCTCAGTTCCAGTGTGGCTGGTCATCCTCTCAGACCAGCTAGG
GATCGTCGCCTAGGTGAGCCGTTACCCCACCTACTAGCTAATCCCATCTGGGCACATCCGATGGCAAGAGGCC
CTAAGGTCCCCCTCTTGTCTTGCGACGTATGCGGTATTAGCTACCGTTTCCAGTAGTTATCCCCCTCCATCAG
GCAGTTTTCCCAGACATTACTCACCCGTCCGCCACTCGTCAGCAAAGCAGCAAGCTGCTTCCTGTTACCGTTC
GACTGCATGTGTTAGGCCTGCCGCCAGCGTCAATC
>192
CTGCTGCCGCCCGTAGGAGTCTGGACCGTGTCTCAGTTCCAGTGTGGCTGGTCATCCTCTCAGACCAGCTAGG
GATCGTCGCCTAGGTGAGCCGTTACCCCACCTACTAGCTAATCCCATCTGGGCACATCCGATGGCAAGAGGCC
CTAAGGTCCCCCTCTTGTCTTGCGACGTATGCGGTATTAGCTACCGTTTCCAGTAGTTATCCCCCTCCATCAG
GCAGTTTCCCAGACATTACTCACCCGTCCGCCACTCGTCAGCAAACAGCAAGCTGCTTCCTGTTACCGTTCGA
CTTGCATGTGTTAGGCCTGCCGCCAGCGTCAATCTGAGCCAGGATCAACTCT
>193
CTGCTGCCGCCCGTAGGAGTCTGGACCGTGTCTCAGTTCCAGTGTGGCTGGTCATCCTCTCAGACCAGCTAGG
GATCGTCGCCTAGGTGAGCCGTTACCCCACCTACTAGCTAATCCCATCTGGGCACATCCGATGGCAAGAGGCC
CTAAGGTCCCCCTCTTTGTCTGCGACGTTATGCGGTATTAGCTACCGTTTCCAGTAGTTATCCCCCTCCATCG
GGCAGTTTTCCCAGACATTACTCACCCGTCCGCCACTCGTCAGCAAAGCAGCAAGCTGCTTCCTGTTACCGTT
CGACTGCATGTGTTAGGCCTGCCGCCAGCGTCAATCTGAGCCAGGATCAAACTCT
>194
CTGCTGCCGCCCGTAGGAGTCTGGACCGTGTCTCAGTTCCAGTGTGGCTGGTCATCCTCTCAGACCAGCTAGG
GATCGTCGCCTAGGTGAGCCGTTACCCCACCTACTAGCTAATCCCATCTGGGCACATCCGATGGCAAGAGGCC
CGAAGGTCCCCCTCTTTGGTCTGCGACATTATGCGGTATTAGCTACCGTTTCCAGTAGTTATCCCCCTCCATC
AGCAGTTTTCCCAGACATTACTCACCCGTCCGCCACTCGTCAGCGACAGCAAGCTGTTTTCCTGTTACCGTTC
GACTGCATGTGTTAGGCCTGCCGCCAGCGTCAATCTGAGCCAGGATCAACTCT
>195
CTGCTGCCGCCCGTAGGAGTCTGGACCGTGTCTCAGTTCCAGTGTGGCTGGTCATCCTCTCAGACCAGCTAGG
GATCGTCGCCTAGGTGAGCCGTTACCCCACCTACTAGCTAATCCCATCTGGGCACATCCCGATGGCAAGAGGC
CCTAAGGTCCCCCTCTTTGTCTTGCGACGTTATGCGGTATTAGCTACCGTTTCCAGTAGTTATCCCCCTCCAT
CAGGCAGTTTCCCAGACATTACTCACCCGTCCGCCACTCGTCAGCAAAGCAGCAAGCTGCTTCCTGTTACCGT
TCGACTGCATGTGTTAGGCCTGCCGCCAGCGTCAATCTGAGCCAGGATCAAACTCT
>196
CTGCTGCCGCCCGTAGGAGTCTGGACCGTGTCTCAGTTCCAGTGTGGCTGGTCATCCCCTCAGACCAGCTAGG
GATCGTCGCCTAGGTGAGCCGTTACCCCACCTACTAGCTAATCCCATCTGGGCACATCCGATGGCAAGAGGCC
CTAAGGTCCCCCTCTTGGTCTTGCGACGTTATGCGGTATTAGCTACCGTTTCCAGTAGTTATCCCCTCCATCG
GGCAGTTTCCCAGACATTACTCACCCGTCCGCCACTCGTCAGCAAAGCAGCAAGCTGCTTCCTGTTTACCGTT
CGACTGCATGTGTTAGGCCTGCCGCCAGCGTTCAATCTGAGCCAGGATCAAACTCT
>197
CTGCTGCCGCCCGTAGGAGTCTGGACCGTGTCTCAGTTCCAGTGTGGCTGGTCATCCTCTCAGACCAGCTAGG
GATCGTCGCCTAGGTGAGCCGTTACCCCACCTACTAGCTAATCCCATCTGGGCACATCCCGATGGCAAGAGGC
CCTAAGGTCCCCCTCTTGTCTTGCGACGTATGCGGTATTAGCTACCGTTTCCAGTAGTTCTCCCCCTCCATCGG
GCAGTTTCCCAGACATTACTCACCCGTCCGCCACTCTCAGCAAGCAGCAAGCTGCTTCCTGTTACCGTTCGAC
TGCATGTGTAGCT
>198
CTGCTGCCGCCCGTAGGAGTCTGGACCGTGTCTCAGTTCCAGTGTGGCTGGTCATCCTCTCAGACCAGCTAGG
GATCGTCGCCTAGGTGAGCCGTTACCCCACCTACTAGCTAATCCCATCTGGGCACATCCCGATGGCAAGAGGC
CCTAAGGTCCCCCTCTTGTCTGCGACGTATGCGGTATTAGCTACCGTTTCCAGTAGTTATCCCCCTCCATCGG

GCAGTTTCCCAGACATTACTCACCCGTCCGCCACTCGTCAGCAAAGCAGCAAGCTGCTTCCTGTTACCCGTTC
GACTGCATGTGTTAGGCCTGCCGCCAGCGTCAATCTGAGCCAGGATCAACTCT
>199
CTGCTGCCGCCCGTAGGAGTCTGGACCGTGTCTCAGTTCCAGTGTGGCTGGTCATCCTCTCAGACCAGCTAGG
GATCGTCGCCTAGGTGAGCCGTTACCCCACCTACTAGCTAATCCCATCTGGGCACATCCGATGGCAAGAGGCC
CTAAGGTCCCCCTCTTTGTCTTGCGACGTATGCGGTATTAGCTACCGTTTCCAGTAGTTATCCCCCTCCATCA
GGCAGTTTTCCCAGACATTACTCACCCGTCCGCCACTCGTCAGCAAAGCAGCAAGCTGCTTCCTGTTACCGTT
CGACTGCATGTGTTAGGCCTGCCGCCAGCGTCAATCTGAGCCAGGATCAAACTCT
>200
CTGCTGCCGCCCGTAGGAGTCTGGACCGTGTCTCAGTTCCAGTGTGGCTGGTCATCCTCTCAGACCAGCTAGG
GATCGTCGCCTAGGTGAGCCGTTACCCCACCTACTAGCTAATCCCATCTGGGCACATCCGATGGCAAGAGGCC
CTAAGGTCCCCCTCTTGTCTGCGACGTATGCGGTATTAGCTACCGTTTCCAGTAGTTATCCCCCTCCATCAGG
CAGTTTCCCAGACATTACTCACCCGTCCGCCACTCGTCAGCAAAGCAGCAAGCTGCTTCCTGTTACCGTTCGA
CTGCATGTGTTAGGCCTGCCGCCAGCGTTCAATCTGAGCCAGGATCAAACTCT
>201
CTGCTGCCGCCCGTAGGAGTCTGGACCGTGTCTCAGTTCCAGTGTGGCTGGTCATCCTCTCAGACCAGCTAGG
GATCGTCGCCTAGGTGAGCCGTTACCCCACCTACTAGCTAATCCCATCTGGGCACATCCGATGGCAAGAGGCC
CTAAGGTCCCCCTCTTGTCTGCGACGTATGCGGTATTAGCTACCGTTTCCAGTAGTTATCCCCCTCCATCAGG
CAGTTTCCCAGACATTACTCACCCGTCCGCCACTCGTCAGCAAACAGCAAGCTGCTTCCTGTTACCGTTCGAC
TGCATGTGTTAGGCCTGCCGCCAGCGGTCAATCTGAGCCAGGATCAAACTCT
>202
CTGCTGCCGCCCGTAGGAGTCTGGACCGTGTCTCAGTTCCAGTGTGGCTGGTCATCCTCTCAGACCAGCTAGG
GATCGTCGCCTAGGTGAGCCGTTACCCCACCTACTAGCTAATCCCATCTGGGCACATCCGATGGCAAGAGGCC
CTAAGGTCCCCCTCTTGTCTTGCGACGTATGCGGTATTAGCTACCGTTTCCAGTAGTTATCCCCCTCCATCGG
GCAGTTTCCCAGACATTACTCACCCGTCCGCCACTCGTCAGCAAAGCAGCAAGCTGCTTCCTGTTACCGTTCG
ACTGCATGTGTTAGGCCTGCCGCCAGCGTCAATCTGAGCCAAGGATCAAACTCT
>203
CTGCTGCCGCCCGTAGGAGTCTGGACCGTGTCTCAGTTCCAGTGTGGCTGGTCATCCTCTCAGACCAGCTAGG
GATCGTCGCCTAGGTGAGCTGTTACCCCACCTACTAGCTAATCCCATCTGGGCACATCCCGATGGCAAGAGGC
CCTAAGGTCCCCCTCTTGTCTGCGACGTTATGCGGTATTAGCTACCGTTTCCAGTAGTTATCCCCCTCCATCG
GGCAGTTTCCCAGACATTACTCACCCGTCCGCCACTCGTCAGCAAAGCAGCAAGCTGCTTCCTGTACCGTTCG
ACTGCATGTGTTAGGCCTGCCGCCAGCGTCAATCTGAGCCAGGATCAAACTCT
>204
CTGCTGCCGCCCGTAGGAGTCTGGACCGTGTCTCAGTTCCAGTGTGGCTGGTCATCCTCTCAGACCAGCTAGG
GATCGTCGCCTAGGTGAGCCGTTACCCCACCTACTAGCTAATCCCATCTGGGCACATCCGATGGCAAGAGGCC
CTAAGGTCCCCCTCTTGTCTTGCGACGTATGCGGTATTAGCTACCGTTTCCAGTAGTTATCCCCCTCCATCAG
GCAGTTTCCCAGACATTACTCACCCGTCCGCCACTCGTCAGCAAAGCAGCAAGCTGCTTCCTGTTACCGTTCG
ACTTGCATGTGTTAGGCCTGCCGCCAGCGTCAATCTGAGCCAGGATCAAACTCT
>205
CTGCTGCCGCCCGTAGGAGTCTGGACCGTGTCTTAGTCTCAGTGTGGCTGGTCATCCTCTCAGACCAGCTAGG
GATCGTCGCCTAGGTGAGCCGTTACCCCACCTACTAGCTAATCCCATCTGGGCACATCCGATGGCAAGAGGCC
CTAAGGTCCCCCTCTTTGTCTTGCGACGTATGCGGTATTAGCTACCGTTTCCAGTAGTTATCCCCCTCCATCA
GGCAGTTTCCCAGACATTACTCACCCGTCCGCCACTCGTCAGCAAAGCAGCAAGCTGCTTCCTGTTACCGTTC
GACTGCATGTGTTAGGCCTGCCGCCAGCGTCAATCTGAGCCAGGATCAAACTCT
>206
CTGCTGCCGCCCGTAGGAGTCTGGACCGTGTCTCAGTTCCAGTGTGGCTGGTCATCCTCTCAGACCAGCTAGG
GATCGTCGCCTAGGTGAGCCGTTACCCCACCTACTAGCTAATCCCATCTGGGCACATCCGATGGCAAGAGGCC
CTAAGGTCCCCCTCTTTGGTCTTGCGACGTATGCGGTATTAGCTACCGTTTCCAGTAGTTATCCCCCTCCATC
GGGCAGTTTTCCCAGACATTACTCACCCGTCCGCCACTCGTCAGCAAAGCAGCAAGCTGCTTCCTGTTACCGT
TCGACTGCATGTGTTAGGCCTGCCGCCAGCGTCAATCTGAGCCAGGATCAAACTCT
>207
CTGCTGCCGCCCGTAGGAGTCTGGACCGTGTCTCAGTTCCAGTGTGGCTGGTCATCCTCTCAGACCAGCTAGG
GATCGTCGCCTAGGTGAGCCGTTACCCCACCTACTAGCTAATCCCATCTGGGCACATCCGATGGCAAGAGGCC
CTAAGGTCCCCCTCTTTGTCTTGCGACGTATGCGGTATTAGCTACCGTTTCCAGTGGTTATCCCCCTCCATCG

GGCAGTTTCCCAGACATTACTCACCCGTCCGCCACTCGTCAGCAAAGCAGCAAGCTGCTTCCTGTTACCGTTC
GACTGCATGTGTTAGGCCTGCCGCCAGCGTCAATCTGAGCCAGGATCAAACTCT
>208
CTGCTGCCGCCCGTAGGAGTCGTGACCGTGTCTCAGTTCCAGTGTGGCTGGTCATCCTCTCAGACCAGCTAGG
GATCGTCGCCTAGGTGAGCCGTTACCCCACCTACTAGCTAATCCCATCTGGGCACATCCGATGGCAAGAGGCC
CTAAGGTCCCCCTCTTTGTCTTGCGACGTATGCGGTATTAGCTACCGTTTCCAGTAGTTATCCCCCTCCATCA
GGCAGTTTCCCAGACATTACTCACCCGTCCGCCACTCGTCAGCAAGCAGCAAGCTGCTTCTGTTACCGTTCGA
CTGCATGTGCTAGGCCTGCCGCCAGCGTTCAATCTGAGCCAGGATCAAACTCT
>209
CTGCTGCCGCCCGTAGGAGTCTGGACCGTGTCTCAGTTCCAGTGTGGCTGGTCATCCTCTCAGACAGCTAGGG
ATCGTCGCCTAGGTGAGCCGTTACCCCACCTACTAGCTAATCCCATCTGGGCACATCCGATGGCAAGAGGCCC
TAAGGTCCCCCTCTTGTCTTGCGACGTATGCGGTATTAGCTACCGTTTCCAGTACGTTATCCCCCTCCATCGG
GCAGTTCCCAGACATACTCACCCGTCCGCCACTCTCAGCAAAGCAGCAAGCTGCTTCCTGTTACCGTTCGACT
GCATGTGTTAGGCCTGCCGCCAGCGTCAATCTGAG
>210
CTGCTGCCGCCCGAGGAGTCTGGACCGTGTCTCAGTTCCAGTGTGGCTGGTCATCCTCTCAGACCAGCTAGGG
ATCGTCGCCTAGGTGAGCCGTTACCCCACCTACTAGCTAATCCCATCTGGGCACATCCGATGGCAAGAGGCCC
TAAGGTCCCCCTCTTTGTCTTGCGACGTATGCGGTATTAGCTACCGTTTCCAGTAGTTATCCCCCTCCATCGG
GCAGTTTTCCCAGACATTACTCACCCGTCCGCCACTCGTCAGCAAAGCAGCAAGCTGCTTCCTGTTACCGTTC
GACTGCATGTGTTAGGCCTGCCGCCAGCGTCAATCTGAGCCAGGATCAAACTCT
>211
CTGCTGCCGCCCGTAGGAGTCTGGACCGTGTCTCAGTTCCAGTGTGGCTGGTCATCCTCTCAGACCAGCTAGG
GATCGTCGCCTAGGTGAGCCGTTACCCCACCTACTAGCTAATTCCCATCTGGGCACATCCGATGGCAAGAGGC
CCTAAGGTCCCCCTCTTTGTCTGCGACGTATGCGGTATTAGCTACCGTTTCCAGTAGTTATCCCCCTCCATCA
GGCAGTTTCCCAGACATACTCACCCGTCCGCCACTCGTCAGCAAAACAGCAAGCTGCTTCCTGTTACCGTTCG
ACTGCATGTGTTAGGCCTGCCGCCAGCGTCAATCTGAG
>212
CTGCTGCCGCCCGTAGGAGTCTGGACCGTGTCTCAGTTCCAGTGTGGCTGGTCATCCTCTCAGACCAGCTAGG
GATCGTCGCCTAGGTGAGCCGTTACCCCACCTACTAGCTAATCCCATCTGGGCACATCCGATGGCAAGAGGCC
CTAAGGTCCCCCTCTTGTCTTGCGACGTTATGCGGTATTAGCTACCGTTTCCAGTAGTTATCCCCCTCCCATC
AGGCAGTTTCCCAGACATTACTCACCCGTCCGCCACTCGTCAGCAAACAGCAAGCTGCTTCCTGTTACCGTTC
GACTGCATGTGTTAGGCCTGCCGCCAGCGTTCAACTCTGAGCCAGGATCAAACTCT
>213
CTGCTGCCGCCCGTAGGAGTCTGGACCGTGTCTCAGTTCCAGTGTGGCTGGTCATCCTCTCAGACCAGCTAGG
GATCGTCGCCTAGGTGAGCCGTTACCCCACCTACTAGCTAATCCCATCTGGGCACATCCGATGGCAAGAGGCC
CTAAGGTCCCCCTCTTTGGTCTCGCGACGTATGCGGTATTAGCTACCGTTTCCAGTAGTTATCCCCCTCCATC
GGGCAGTTTCCCAGACATTACTCACCCGTCCGCCACTCGTCAGCAAAGCAGCAAGCTGCTTCCTGTTACCGTT
CGACTGCATGTGTTAGGCCTGCCGCCAGCGTCAATCTGAGCCAGGATCAAACTCT
>214
TGCTGCCGCCCGTAGGAGTCTGGACCGTGTCTCAGTTCCAGTGTGGCTGGTCATCCTCTCAGACCAGCTAGGG
ATCGTCGCCTAGGTGAGCCGTTACCCCACCTACTAGCTAATCCCATCTGGGCACATCCGATGGCAAGAGGCCC
TAAGGTCCCCCTCTTTGTCTTGCGACGTTATGCGGTATTAGCTACCGTTTCCAGTAGTTATCCCCCTCCATCA
GGCAGTTTCCCAGACATTACTCACCCGTCCGCCACTCGTCAGCAAAGCAGCAAGCTGCTTCTGTTACCGTTCG
ACTGCATGTGTTAGGCCTGCCGCCAGCGTTCAATCTGAGCCAGGATCAAACTCT
>215
CTGCTGCCGCCCGTAGGAGTCTGGACCGTGTCTCAGTTCCAGTGTGGCTGGTCATCCTCTCAGACCAGCTAGG
GATCGTCGCCTAGGTGAGCCGTTACCCCACCTACTAGCTAATCCCATCTGGGCACATCCGATGGCAAGAGGCC
CTAAGGTCCCCCTCTTGTCTTGCGACGTATGCGGTATTAGCTACCGTTTCCAGTAGTTATCCCCCTCCATCAG
GCAGTTTTCCCCAGACATTACTCACCCGTCCGCCACTCGTCAGCAAAGCAGCAAGCTGCTTCTGTTACCGTTC
GACTGCATGTGTTAGGCCTGC
>216
CTGCTGCCGCCCGTAGGAGTCTGGACCGTGTCTCAGTTCCAGTGTGGCTGGTCATCCTCTCAGACCAGCTAGG
GATCGTCGCCTAGGTGAGCCCGTTTACCCCACCTACTAGCTAATCCCATCTGGGCACATCCGATGGCAAGAGG
CCCTAAGGTCCCCTCTTGGTCTTGCGACGTTATGCGGTATTAGCTACCGTTCCAGTAGTTATCCCCTCCATCA

GGCAGTTTCCCAGACATTACTCACCCGTCCGCCACTCGTCAGCAAAGCAGCAAGCTGCTTCTGTTACCGTTCG
ACTGCATGTGTTAGGCCTGCCGCCAGCGTCAATCTGAGCCAGGATCAACTCT
>217
CTGCTGCCGCCCGTAGGAGTCTGGACCGTGTCTCAGTTCCAGTGTGGCTGGTCATCCTCTCAGACCAACTAGG
GATCGTCGCCTAGGTGAGCCGTTACCCCACCTATTAGCTAATCCCATCTGGGCACATCCGATGGCAAGAGGCC
CTAAGGTCCCCCTCTTGTCTTGCGACGTTATGCGGTATTAGCTACCGTTTCCAGTAGTTATCCCCCTCCATCA
GGCAGTTTCCCAGACATTACTCACCCGTCCGCCACTCGTCAGCAAACAGCAAGCTGCTTCCTGTTACCGTTCG
ACTGCATGTGTTAGGCCTGCCGCCAGCGTCAATCTGAGCCAGGATCAAACTCT
>218
CTGCTGCCGCCCGTAGGAGTCTGGACCGTGTCTCAGTTCCAGTGTGGCTGGTCATCCTCTCAGACCAGCTAGG
GATCGTCGCCTAGGTGAGCCGTTACCCCACCTACTAGCTAATCCCATCTGGGCACATCCGATGGCAAGAGGCC
CTAAGGTCCCCCTCTTGTCTTGCGACGTTATGCGGTATTAGCTACCGTTTCCAGTAGTTATCCCCCTCCATCA
GGCAGTTTCCCAGACATTACTCACCCGTCCGCCACTCGTCAGCAAAGCAGCAAGCTGCTTTCTGTTACCGTTC
GACTGCATGTGTTAGGCCTGCCGCCAGCGTTCAATCTGAGCCAGGATCAAACTCT
>219
CTGCTGCCGCCCGTAGGAGTCTGGGCCGTGTCTCAGTTCCAGTGTGGCTGGTCATCCTCTCAGACCAGCTAGG
GATCGTCGCCTAGGTGAGCCGTTACCCCACCTACTAGCTAATCCCATCTGGGCACATCCGATGGCAAGAGGCC
CTAAGGTCCCCCTCTTTGTCTTGCGACGTTATGCGGTATTAGCTACCGTTTCCAGTAGTTATCCCCCTCCATC
AGCAGTCTCCCAGACATTACTCACCCGTCCGCCACTCTCAGCAAGCAGCAAGCTGCTTTCTGTTACCGTTCGA
CTGCATGTGTTAGGCCTGCCGCCAGCGTCGATCTGAGCCAGGATCAAACTCT
>220
CTGCTGCCGCCCGTAGGAGTCTGGACCGTGTCTCAGTTCCAGTGTGGCTGGTCATCCTCTCAGACCAGCTAGG
GATCGTCGCCTAGGTGAGCCGTTACCCCACCTACTAGCTAATCCCATCTGGGCACATCCGATGGCAAGAGGCC
CTAAGGTCCCCCTCTTTGGTCTTGCGACGTTATGCGGTATTAGCTACCGTTTCCAGTAGTTATCCCCCTCCAT
CAGGCAGTTTCCCAGACATTACTCACCCGTCCGCCACTCGTCAGCAAAGCAGCAAGCTGCTTCCTGTTACCGT
TCGACTGCATGTGTTAGGCCTGCCGCCAGCGTTCAATCTGAGCCAGGATCAAACTCT
>221
CTGCTGCCGCCCGTAGGAGTCTGGACCGTGTCTCAGTTCCAGTGTGGCTGGTCATCCTCTCAGACCAGCTAGG
GATCGTCGCCTAGGTGAGCCGTTACCCCACCTACTAGCTAATCCCATCTGGGCACATCCGATGGCAAGAGGCC
CTAAGGTCCCCCTCTTGTCTTGCGACGTTATGCGGTATTAGCTACCGTTTCCAGTAGTTATCCCCCTCCATCA
GGCAGTTTTCCCAGACATTACTCACCCGTCCGCCACTCGTCAGCAAACAGCAAGCTGCTTCCTGTTACCGTTC
GACTGCATGTGTTAGGCCTGCCGCCAGCGTCAATCTGAGCCAGGATCAAACTCT
>222
CTGCTGCCGCCCGTAGGAGTCTGGACCGTGTCTCAGTTCCAGTGTGGCTGGTCATCCTCTCAGACCAGCTAGG
GATCGTCGCCTAGGTGAGCCGTTACCCCACCTACTAGCTAATCCCATCTGGGCACATCCGATGGCAAGAGGCC
CTAAGGTCCCCCTCTTTGTCTTGCGACGTTATGCGGTATTAGCTACCGTTTCCAGTAGTTATCCCCCTCCATC
AGGCAGTTTCCCAGACATTACTCACCCGTCCGCCACTCGTCAGCAAAGCAGCAAGCTGCTTCCTGTTACCGTT
CGACTGCATGTGTTAGGCCTGCCGCCAACGTCAATCTGAGCCAGGATCAAACTCT
>223
CTGCTGCCGCCCGTAGGAGTCTGGACCGTGTCTCAGTTCCAGTGTGGCTGGTCATCCTCTCAGACCAGCTAGG
GATCGTCGCCTAGGTGAGCCGTTACCCCACCTACTAGCTAATCCCATCTGGGCACATCCGATGGCAAGAGGCC
CTAAGGTCCCCCTCTTTGTCTGCGACGTATGCGGTATTAGCTACCGTTTCCAGTAGTTATCCCCCTCCATCAG
GCAGTTTCCCAGACATTACTCACCCGTCCGCCACTCGTCAGCAAAGCAGCAAGCTGCTTCTGTTACCGTTCGA
CTGCATGTGTTAGGCCTGCCGCCAGCGTCAATCTGAGCCAGGATCAAACTCT
>224
CTGCTGCCGCCCGTAGGAGTCTGGACCGTGTCTCAGTTCCAGTGTGGCTGGTCATCCTCTCAGACCAGCTAGG
GATCGTCGCCTAGGTGAGCCGTTTACCCCACCTACTAGCTAATCCCATCTGGGCACATCCGATGGCAAGAGGC
CCTAAGGTCCCCCTCTTGTCTTGCGACGTTATGCGGTATTAGCTACCGTTTCCAGTAGTTATCCCCCTCCATC
AGGCAGTTTCCCAGACATTACTCACCCGTCCGCCACTCGTCAGCAAAGCAGCAAGCTGCTTCCTGTTACCGTT
CGACTGCATGTGTTAGGCCTGCCGCCAGCGTCAATCTGAGCCAGGATCAAACTCT
>225
CTGCTGCCGCCCGTAGGAGTCTGGACCGTGTCTCAGTTCCAGTGTGGCTGGTCATCCTCTCAGACCAGCTAGG
GATCGTCGCCTAGGTGAGCCGTTACCCCACCTACTAGCTAATCCCATCTGGGCACATCCGATGGCAAGAGGCC
CTAAGGTCCCCCTCTTTGTCTTGCGACGTTATGCGGTATTAGCTACCGTTTCCAGTAGTTATCCCCCTCCATC

AGGCAGTTTCCCAGACATTACTCACCCGTCCGCCACTCGTCAGCAAACAGCAAGCTGCTTCCTGTTACCGTTC
GACTGCATGTGTTAGGCCTGCCGCCAGCGTTCAATCTGAGCCAGGATCAAACTCT
>226
CTGCTGCCGCCCGTAGGAGTCTGGACCGTGTCTCAGTTCCAGTGTGGCTGGTCATCCTCTCAGACCAGCTAGG
GATCGTCGCCTAGGTGAGCCGTTACCCCACCTACTAGCTAATCCCATCTGGGCACATCCGATGGCAAGAGGCC
CTAAGGTCCCCTCTTGTCTTGCGACGTATGCGGTATTAGCTACCGTTTCCAGTAGTTATCCCCCTCCATCAGG
CAGTTTCCCAGACATTACTCACCCGTCCGCCACTCGTCAGCAAACAGCAAGCTGCTTCCTGTTACCGTTCGAC
TGCATGTGTTAGGCCTGCCGCCAGCGTTCAATCTGAGCCAGGATCAAACTCT
>227
CTGCTGCCGCCCGTAGGAGTCTGGACCGTGTCTCAGTTCCAGTGTGGCTGGTCATCCTCTCAGACCAGCTAGG
GATCGTCGCCTAGGTGAGCCGTTACCCCACCTACTAGCTAATCCCATCTGGGCACATCCCGATGGCAAGAGGC
CCTAAGGTCCCCCTCTTTGTCTTGCGACGTTATGCGGTATTAGCTACCGTTTCCAGTAGTTATCCCCCTCCAT
CAGGCAGTTTTCCCAGACATTACTCACCCGTCCGCCACTCGTCAGCAAAGCAGCAAGCTGCTTCCTGTTACCG
TTCGACTGCATGTGTTAGGCCTGCCGCCAGCGTTCAATCTGAGCCAGGATCAAACTCT
>228
CTGCTGCCGCCCGTAGGAGTCTGGACCGTGTCTCAGTTCCAGTGTGGCTGGTCATCCTCTCAGACCAGCTAGG
GATCGTCGCCTAGGTGAGCCGTTACCCCACCTACTAGCTAATCCCATCTGGGCACATCCCGATGGCAAGAGGC
CCTAAGGTCCCCCTCTTTGTCTGCGACGTATGCGGTATTAGCTACCGTTTCCAGTAGTTATCCCCCTCCATCG
GGCAGTTTCCCAGACATTACTCACCCGTCCACCACTCGTCAGCAAAGCAGCAAGCTGCTTCCTGTTACCGTTC
GACTGCATGTGTTAGGCCTGCCGCCAGCGTTCAATCTGAGCCAGGATCAAACTCT
>229
CTGCTGCCGCCCGTAGGAGTCTGGACCGTGTCTCAGTTCCAGTGTGGCTGGTCATCCTCTCAGACCAGCTAGG
GATCGTCGCCTAGGTGAGCCGTTACCCCACCTACTAGCTAATCCCATCTGGGCACATCCGATGGCAAGAGGCC
CTAAGGTCCCCCTCTTGGTCTGCGACGTATGCGGTATTAGCTACCGCTTCCAGTAGTTATCCCCCTCCATCGG
GCAGTTCCCAGACATACTCACCCGTCCGCCACTCGTCAGCAAGCAGCAAGCTGCTTCCTGTTACCGTTCGACT
TGCATGTGTTAGGCCTGCCGCCAGCGTCAATCTGAGCCAGGATCAAACTCT
>230
CTGCTGCCGCCCGTAGGAGTCTGGACCGTGTCTCAGTTTCCAGTGTGGCTGGTCATCCTCTCAGACCAGCTAG
GGATCGTCGCCTAGGTGAGCCGTTACCCCACCTACTAGCTAATCCCATCTGGGCACATCCCGATGGCAAGAGG
CCCTAAGGTCCCCCTCTTTGTCTTGCGACGTATGCGGTATTACTACCGTTTCCAGTAGTTATCCCCCTCCATC
AGGCAGTTTTCCCCAGACATTACTCACCCGTCCGCCACTCGTCAGCAAAACAGCAAGCTGCTTCCTGTTACCG
TTCGACTGCATGTGTTAGGCCTGCCGCCAGCGTTCAATCTGAGCCAGGATCAAACTCT
>231
CTGCTGCCGCCCGTAGGAGTCTGGACCGTGTCTCAGTTCCAGTGTGGCTGGTCATCCTCTCAGACCAGCTAGG
GATCGTCGCCTAGGTGAGCCGTTACCCCACCTACTAGCTAATCCCATCTGGGCACATCCGATGGCAAGAGGCC
CTAAGGTCCCCCTCTTTGTCTTGCGACGTATGCGGTATTAGCTACCGTTTCCAGTAGTTATCCCCCTCCATCA
GGCAGTTTCCCAGACATTACTCACCCGTCCGCCACTCGTCAGCAAAGCAGCAAGCTGCTTCCTGTTACCGTTC
GACTGCATGTGTTAGGCCTGCCGCCAGCGTCAATCTGAGCCAGGATCAAACTCT
>232
CTGCTGCCGCCCGTAGGAGTCTGGACCGTGTCTCAGTTCCAGTGTGGCTGGTCATCCTCTCAGACCAGCTAGG
GATCGTCGCCTAGGTGAGCCGTTACCCCACCTACTAGCTAATCCCATCTGGGCACATCCGATGGCAAGAGGCC
CTAAGGTCCCCCTCTTTGTCTTGCGACGTATGCGGTATTAGCTACCGTTTCCAGTAGTTATCCCCTCCATCGG
GCAGTTTTCCCAGACATACTCACCCTCCGCCACTCGTCAGCAAAGCAGCAAGCTGCTTCCTGTTACCGTTCGA
CTGCATGTGTTAGGCCTGCCGCCAGCGTCAATCTGAGCCAGGATCAAACTCT
>233
CTGCTGCCGCCCGTAGGAGTCTGGACCGTGTCTCAGTTCCAGTGTGGCTGGTCATCCTCTCAGACCAGCTAGG
GATCGTCGCCTAGGTGAGCCGTTACCCCACCTACTAGCTAATCCCATCTGGGCACATCCGATGGCAAGAGGCC
CTAAGGTCCCCCTCTTGTCTTGCGACGTATGCGGTATTAGCTACCGTTTCCAGTAGTTATCCCCCTCCATCAG
GCAGTTTCCCAGACATTACTCACCCGTCCGCCACTCGTCAGCAAACAGCAAGCTGCTTCCTGTTACCGTTCGA
CTGCATGTGTTAGGCCTGCCGCCAGCGTCAATCTGAGCCAGGATCAAACTCT
>234
CTGCTGCCGCCCGTAGGAGTCTGGACCGTGTCTCAGTTCCAGTGTGGCTGGTCATCCTCTCAGACCAGCTAGG
GATCGTCGCCTAGGTGAGCCGTTACCCCACCTACTAGCTAATCCCATCTGGGCACATCCGATGGCAAGAGGCC
CTAAGGTCCCCCTCTTGTCTGCGACGTATGCGGTATTAGCTACCGTTTCCAGTAGTTATCCCCCTCCATCAGG

CAGTTTCCCAGACATTACTCACCCGTCCGCCACTCGTCAGCAAAGCAGCAAGCTGCTTCCTGTTACCGTTCGA
CTGCATGTGTTTAGGCCTGCCGCCAGCGTCAATCTGAGCCAGGATCAACTCT
>235
CTGCTGCCGCCCGTAGGAGTCTGGACCGTGTCTCAGTTCCAGTGTGGCTGGTCATCCTCTCAGACCAGCTGAG
GATCGTCGCCTAGGTGAGCCGTTACCCCACCTTCTAGCTAATCCCATCTGGGCACATCCGATGGCAAGAGGC
CCTAAGGTCCCCCCTCTTTGTCTGCGACGTATGCGGTATTAGCTACCGTTTCCAGTAGTTATCCCCCTCCATC
GGGCAGTTTTCCCCAGACATTACTCACCCGTCCGCCACTCGTCAGCAAAGCAGCAAGCTGCTTCCTGTTACCG
TTCGACTGCATGTGTTAGGCCTGCCGCCAGCGTTCAATCTGAGCCAGGATCAAACTCT
>236
CTGCTGCCGCCCGTAGGAGTCTGGACCGTGTCTCAGTTCCAGTGTGGCTGGTCATCCTCTCAGACCAGCTAGG
GATCGTCGCCTAGGTGAGCCGTTACCCCACCTACTAGCTAATCCCATCTGGGCACATCCGATGGCAAGAGGCC
CTAAGGTCCCCCTCTTTGTCTTGCGACGTATGCGGTATTAGCTACCGTTTCCAGTAGTTATCCCCCTCCATCA
GGCAGTTTCCCAGACATTACTCACCCGTCCGCCACTCGTCAGCAAAGCAGCAAGCTGCTTCCTGTTACCGTTC
GACTGCATGTGTTAGGCCTGCCGCCAGCGTCAATCTGAGCCAGGATCAAACTCT
>237
CTGCTGCCGCCCGTAGGAGTCTGGACCGTGTCTCAGTTCCAGTGTGGCTGGTCATCCTCTCAGACCAGCTAGG
GATCGTCGCCTAGGTGAGCCGTTACCCCACCTACTAGCTAATCCCATCTGGGCACATCCGATGGCAAGAGGCC
CTAAGGTCCCCCTCTTTGTCTTGCGACGTATGCGGTATTAGCTACCGTTTCCAGTAGTTATCCCCCTCCATCA
GGCAGTTTCCCAGACATTACTCACCCGTCCGCCACTCGTCAGCAAAGCAGCAAGCTGCTTCCTGTTACCGTTC
GACTTGCATGTGTTAGGCCTGCCGCCAGCGTCAATCTGAGCCAGGATCAAACTCT
>238
CTGCTGCCGCCCGTAGGAGTCTGGACCGTGTCTCAGTTCCAGTGTGGCTGGTCATCCTCTCAGACAGCTAGGG
ATCGTCGCCTAGGTGAGCCGTTACCCCACCTACTAGCTAATCCCATCTGGGCACATCCGATGGCAAGAGGCCC
TAAGGTCCCCCTCTTTGTCTTGCGACGTATGCGGTATTAGCTACCGTTTCCAGTAGTTATCCCCCTCCATCGG
GCAGTTTCCCAGACATTACTCACCCGTCCGCCACTCGTCAGCAAAGCAGCAAGCTGCTTCCTGTTACCGTTCG
ACTGCATGTGTTAGGCCTGCCGCCAGCGTCAATCTGAGCCAGGATCAAACTCT
>239
CTGCTGCCGCCCGTAGGAGTCTGGACCGTGTCTCAGTTCCAGTGTGGCTGGTCATCCTCTCAGACCAGCTAGG
GATCGTCGCCTAGGTGAGCCGTTACCCCACCTACTAGCTAATCCCATCTGGGCACATCCGATGGCAAGAGGCC
CTAAGGTCCCCCTCTTTGTCTTGCGACGTATGCGGTATTAGCTACCGTTTCCAGTAGTTATCCCCCTCCATCA
GGCAGCTTCCCAGACATTACTCACCCGTCCGCCACTCGTCAGCAAAGCAGCAAGCTGCTTCCTGTTACCGTTC
GACTGCATGTGTTAGGCCTGCCGCCAGCGTCAATCTGAGCCAGGATCAAACTCT
>240
CTGCTGCCGCCCTAGGAGTCTGGACCGTGTCTCAGTTCCAGTGTGGCTGGTCATCCTCTCAGACCAGCTAGGG
ATCGTCGCCTAGGTGAGCCGTTACCCCACCTACTAGCTAATCCCATCTGGGCACATCCGATGGCAAGAGGCCC
TAAGGTCCCCCTCTTGTCTTGCGACGTATGCGGTATTAGCTACCGTTTCCAGTAGTTATCCCCCTCCATCGGG
CAGTTTCCCAGACATACTCACCCGTCCGCCACTCTCAGCAAAGCAGCAAGCTGCTTCCTGTTACCGTTCGACT
GCATGTGTTAGGCCTGCCGCCAGCGTCAATCTGAGCCAGGATCAACTCT
>241
CTGCTGCCGCCCGTAGGAGTCTGGACCGTGTCTCAGTTCCAGTGTGGCTGGTCATCCTCTCAGACCAGCTAGG
GATCGTCGCCTAGGTGAGCCGTTACCCCACCTACTAGCTAATCCCATCTGGGCACATCCGATGGCAAGAGGCC
CTAAGGTCCCCCTCTTGTCTTGCGACGTATGCGGTATTAGCTACCGTTTCCAGTAGTTATCCCCCTCCATCGG
GCAGTTTTCCCAGACATTACTCACCCGTCCGCCACTCGTCAGCAAAGCAGCAAGCTGCTTCCTGTTACCGTTC
GACTGCATGTGTTAGGCCTGCCGCCAGCGTCAATCTGAGCCAGGATCAAACTCT
>242
CTGCTGCCGCCCGTAGGAGTCTGGACCGTGTCTCAGTTCCAGTGTGGCTGGTCATCCTCTCAGACCAGCTAGG
GATCGTCGCCTAGGTGAGCCGTTACCCCACCTACTAGCTAATCCCATCTGGGCACATCCGATGGCAAGAGGCC
CTAAGGCCCCCCTCTTGTCTTGCGACGTATGCGGTATTAGCTACCGTTTCCAGTAGTTATCCCCCTCCATCGG
GCAGTTTTCCCAGACATTACTCACCCGTCCGCCACTCGTCAGCAGAGCAGCAAGCTGCTTCCTGTTACCGTTC
GACTGCATGTGTTAGGCCTGCCGCCAGCGTCAATCTGAGCCAGGATCAACTCT
>243
CTGCTGCCGCCCGTAGGAGTCTGGACCGTGTCTCAGTTCCAGTGTGGCTGGTCATCCTCTCAGACCAGCTAGG
GATCGTCGCCTAGGTGAGCCGTTACCCCACCTACTAGCTAATCCCATCTGGGCACATCCCGATGGCAAGAGGC
CCTAAGGTCCCCCTCTTGTCTTGCGACGTCTGCGGTATTAGCTACCGTTTCCAGTAGTTATCCCCCTCCCATC

AGGCAGTTTTCCCAGACATTACTCACCCGTCCGCCACTCGTCAGCAAAGCAGCAAGCTGCTTCTGTTACCGTT
CGACTGCATGTGTTAGGCCTGCCGCCAGCCGTTCAAT
>244
CTGCTGCCGCCCGTAGGAGTCTGGACCGTGTCTCAGTTCCAGTGTGGCTGGTCACTCCTCTCAGACGAGCTAG
GATCGTCGCGTAGGTGCAGCCGTTCACCCACTACTAGCTAATCCCATCTGGGCACATCCGATGGCAAGAGGCC
CTAAGGTCCCCTCTTGGTCTTGCGACGTATGCGGTATTAGCTACCGTTCCAGTAGTTATCCCCTCCATCAGGC
AGTTTCCCAGACATTACTCACCGTCCGCCACTCGTCAGCAAAGCAGCAAGCTGCTTCCTGTTACCGTTCGACT
GCATGTGTTAGGCCTGCCGCCAGCGTCAATCTGAGCCAGGATC
>245
CTGCTGCCGCCCGTAGGAGTCTGGACCGTGTCTCAGTTCCAGTGTGGCTGGTCATCCTCTCAGACCAGCTAGG
GATCGTCGCCTAGGTGAGCCGTTACCCCACCTACTAGCTAATCCCATCTGGGCACATCCGATGGCAAGAGGCC
CTAAGGTCCCCCTCTTTGTCTTGCGACGTTATGCGGTATTAGCTACCGTTTCCAGTAGTTATCCCCCTCCATC
GGGCAGTTTTCCCAGACATTACTCACCCGTCCGCCACTCGTCAGCAAACAGCAAGCTGCTTCCTGTTACCGTT
CGACTTGCATGTGTTAGGCCTGCCGCCAGCGTCAATCCGAGCCAGGATCAAACTCT
>246
CTGCTGCCGCCCGTAGGAGTCTGGACCGTGTCTCAGTTCCAGTGTGGCTGGTCATCCTCTCAGACCAGCTAGG
GATCGTCGCCTAGGTGAGCCGTTTACCCCACCTACTAGCTAATCCCATCTGGGCACATCCGATGGCAAGAGGC
CCTAAGGTCCCCCTCTTTGGTCTTGCGACGTTATGCGGTATTAGCTACCGTTTCCAGTAGTTATCCCCCTCCA
TCAGGCAGTTTCCCAGACATTACTCACCCGTCCGCCACTCGTCAGCAAAGCAGCAAGCTGCTTCCTGTTACCG
TTCGACTGCATGTGTTAGGCCTGCCGCCAGCGTCAATCTGAGCCAGGATCAAACTCT
>247
CTGCTGCCGCCCGTAGGAGTCTGGACCGTGTCTCAGTTCCAGTGTGGCTGGTCATCCTCTCAGACCAGCTAGG
GATCGTCGCCTAGGTGAGCCGTTACCCCACCTACTAGCTAATCCCATCTGGGCACATCCGATGGCAAGAGGCC
CTAAGGTCCCCCTCTTTGGTCTTGCGACGTTATGCGGTATTAGCTACCGTTTCCAGTAGTTATCCCCCTCCAT
CGGGCAGTTTCCCAGACATTACTCACCCGTCCGCCACTCGTCAGCAAAGCAGCAAGCTGCTTCCTGTTACCGT
TCGACTGCATGTGTTAGGCCTGCCGCCAGCGTTCAATCTGAGCCAGGATCAAACTCT
>248
CTGCTGCCGCCCGTAGGAGTCTGGACCGTGTCTCAGTTCCAGTGTGGCTGGTCATCCTCTCAGACCAGCTAGG
GATCGTCGCCTAGGTGAGCCGTTACCCCACCTACTAGCTAATCCCATCTGGGCACATCCGATGGCAAGAGGCC
CTAAGGTCCCCCTCTTTGTCTTGCGACGTTATGCGGTATTAGCTACCGTTTCCAGTAGTTATCCCCCTCCATC
AGGCAGTTTTCCCAGACATTACTCACCCGTCCGCCACTCGTCAGCAAAGCAGCAAGCTGCTTCCTGTTACCGT
TCGACTGCATGTGTTAGGCCTGCCGCCAGCGTCAATCTGAGCCAGGATCAAACTCT
>249
CTGCTGCCGCCCGTAGGAGTCTGGACCGTGTCTCAGTTCCAGTGTGGCTGGTCATCCTCTCAGACCAGCTAGG
GATCGTCGCCTAGGTCGAGCCGTTACCCCACCTACTACGCTAATCCCATCTGGGCACATCCCGATGGCAAGAG
GCCCTAAGGTCCCCCTCTTTGTCTTGCGACGTTATGCGGTATTAGCTACCGTTCCAGTAGTTATCCCCCTCCA
TCGGGCAGTTTCCCAGACATTACTCACCCGTCCGCCACTCGTCAGCAAAGCAGCAAGCTGCTTCCTGTTACCG
TTCGACTGCATGTGTTAGGCCTGCCGCCAGCGTCAATCTGAGCCAGGATCAAACTCT
>250
CTGCTGCCGCCCGTAGGAGTCTGGACCGTGTCTCAGTTCCAGTGTGGCTGGTCATCCTCTCAGACCAGCTAGG
GATCGTCGCCTAGGTGAGCCGTTACCCCACCTACTAGCTAATCCCATCTGGGCACATCCCGATGGCAAGAGGC
CCTAAGGTCCCCCTCTTGTCTTGCGACGTATGCGGTATTAGCTACCGTTTCCAGTAGTTATCCCCCTCCATCA
GGCAGTTTCCCAGACATTACTCACCGTCCGCCACTCGTCAGCAAAGCAGCAAGCTGCTTCCTGTTACCGTTCG
ACTGCATGTGTTAGGCCTGCCGCCAGCGTCAATC
>251
CTGCTGCCGCCCGTAGGAGTCTGGACCGTGTCTCAGTTCCAGTGTGGCTGGTCATCCTCTCAGACCAGCTAGG
GATCGTCGCCTAGGTGAGCCGTTACCCCACCTACTAGCTAATCCCATCTGGGCACATCCGATGGCAAGAGGCC
CTAAGGTCCCCCTCTTTGTCTTGCGACGTATGCGGTATTAGCTACCGTTTCCAGTAGTTATCCCCCTCCATCA
GGCAGTTTCCCAGACATTACTCACCCGTCCGCCACTCGTCAGCAAAACAGCAAGCTGCTTCCTGTTACCGTTC
GACTGCATGTGTTAGGCCTGCCGCCAGCGTCAATCTGAGCCAGGATCAAACTCT
>252
CTGCTGCCGCCCGTAGGAGTCTGGACCGTGTCTCAGTTCCAGTGTGGCTGGTCATCCTCTCAGACCAGCTAGG
GATCGTCGCCTAGGTGAGCCGTTACCCCACCTACTAGCTAATCCCATCTGGGCACATCCGATGGCAAGAGGCC
CTAAGGTCCCCCTCTTTGGTCTTGCGACGTATGCGGTATTAGCTACCGTTTCCAGTAGTTATCCCCCTCCATC

AGGCAGTTTCCCAAGACATTACTCACCCGTCCGCCACTCGTCAGCAAAGCAGCAAGCTGCTTCCTGTTACCGT
TCGACTGCATGTGTTAGGCCTGCCGCCAGCGTCAATCTGAGCCAGGATCAAACTCT
>253
CTGCTGCCGCCCGTAGGAGTCTGGACCGTGTCTCAGTTCCAGTGTGGCTGGTCATCCTCTCAGACCAGCTAGG
GATCGTCGCCTAGGTGAGCCGTTACCCCACCTACTAGCTAATCCCATCTGGGCACATCCGATGGCAAGAGGCC
CTAAGGTCCCCCTCTTTGTCTTGCGACGTATGCGGTATTAGCTACCGTTTCCAGTAGTTATCCCCCTCCATCA
GGCAGTTTCCCAGACATTACTCACCCGTCCGCCACTCGTCAGCAAAGCAGCAAGCTGCTTTCTGTTACCGTTC
GACTGCATGTGTTAGGCCTGCCGCCAGCGTTCAATCTGAGCCAGGATCAAACTCT
>254
CTGCTGCCGCCCGTAGGAGTCTGGACCGTGTCTCAGTTCCAGTGTGGCTGGTCATCCTCTCAGACCAGCTAGG
GATCGTCGCCTAGGTGAGCCGTTACCCCACCTACTAGCTAATCCCATCTGGGCACATCCGATGGCAAGAGGCC
CTAAGGTCCCCCTCTTTGTCTTGCGACGTTATGCGGTATTAGCTACCGTTTCCAGTAGTTATCCCCCTCCATC
AGGCAGTTTCCCAGACATTACTCACCCGTCCGCCACTCGTCAGCAAAGCAGCAAGCTGCTTCCTGTTACCGTT
CGACTGCATGTGTTAGGCCTGCCGCCAGCGTTCAATCTGAGCCAGGATCAAACTCT
>255
TGCTGCCGCCCGTAGGAGTCTGGACCGTGTCTCAGTTCCAGTGTGGCTGGTCATCCTCTCAGACCAGCTAGGG
ATCGTCGCCTAGGTGAGCCGTTACCCCACCTACTAGCTAATCCCATCTGGGCACATCCGATGGCAAGAGGCCC
TAAGGTCCCCCTCTTTGTCTTGCGACGTTATGCGGTATTAGCTACCGTTTCCAGTAGTTATCCCCCTCCATCG
GGCAGTTTTCCCAGACATTACTCACCCGTCCGCCACTCGTCAGCAAAGCAGCAAGCTGCTTCCTGTTACCGTT
CGACTGCATGTGTTAGGCCTGCCGCCAGCGTCAATCTGAGCCAGGATCAAACTCT
>256
CTGCTGCCGCCCGTAGGAGTCTGGACCGTGTCTCAGTTCCAGTGTGGCTGGTCATCCTCTCAGACCAGCTAGG
GATCGTCGCCTAGGTGAGCCGTTACCCCACCTACTAGCTAATCCCATCTGGGCACATCCCGATGGCAAGAGGC
CCTAAGGTCCCCCTCTTGTCTTGCGACGTATGCGGTATTCGCTACCGTTTCCAGTAGTTATCCCCCTCCATCG
GGCAGTTTCCCAGACATTACTCACCCGTCCGCCACTCGTCAGCAAACAGCAAGCTGCTTCCTGTTACCGTTCG
ACTGCATGTGTTAGGCCTGCCGCCAGCGTCAATCTGAGCCAGGATCAAACTCT
>257
CTGCTGCCGCCCGTAGGAGTCTGGACCGTGTCTCAGTTCCAGTGTGGCTGGTCATCCTCTCAGACCAGCTAGG
GATCGTCGCCTAGGTGAGCCGTTACCCCACCTACTAGCTAATCCCATCTGGGCACATCCCGATGGCAAGAGGC
CCTAAGGTCCCCCTCTTTGTCTTGCGACGTTATGCGGTATTAGCTACCGTTTCCAGTAGTTATCCCCCTCCAT
CAGGCAGTTTCCCAGACATTACTCACCCGTCCGCCACTCGTCAGCAAAACAGCAAGCTGCTTCCTGTTACCGT
TCGACTGCATGTGTTAGGCCTGCCGCCAGCGTCAATCTGAGCCAGGATCAAACTCT
>258
CTGCTGCCGCCCGTAGGAGTCTGGACCGTGTCTCAGTTCCAGTGTGGCTGGTCATCCTCTCAGACCAGCTAGG
GATCATCGCCTAGGTGAGCCGTTACCCCACCTACTAGCTAATCCCATCTGGGCACATCCGATGGCAAGAGGCC
CTAAGGTCCCCCTCTTGTCTTGCGACGTATGCGGTATTAGCTACCGTTTCCAGTAGTTATCCCCTCCCATCAG
GCAGTTTCCCAGACATTACTCACCCGTCCGCCACTCGTCAGCAAAGCAGCAAGCTGCTTCCTGTTACCGTTCG
ACTGCATGTCGTTAGGCCTGCCGCCAGCGTTCAATCTGAGCCAGGATCAAACTCT
>259
CTGCTGCCGCCCGTAGGAGTCTGGACCGTGTCTCAGTTCCAGTGTGGCTGGTCATCCTCTCAGACCAGCTAGG
GATCGTCGCCTAGGTGAGCCGTTACCCCACCTACTAGCTAATCCCATCTGGGCACATCCGATGGCAAGAGGCC
CTAAGGTCCCCCTCTTTGTCTTGCGACGTATGCGGTATTAGCTACCGTTTCCAGTAGTTATCCCCCTCCATCG
GGCAGTTTCCCAGACATTACTCACCCGTCCGCCACTCGTCAGCAAAGCAGCAAGCTGCTTCCTGTTACCGTTC
GACTGCATGTGTTAGGCCTGCCGCCAGCGTCAATCTGAGCCAGGATCAAACTCT
>260
CTGCTGCCGCCCTAGGAGTCTGGACCGTGTCTCAGTTCCAGTGTGGCTGGTCATCCTCTCAGGCAGCTAGGGA
TCGTCGCCTAGGTGAGCCGTTACCCCACCTACTAGCTAATCCCCATCTGGGCACATCCGATGGCAAGAGGCCC
TAAGGTCCCCCTCTTGTCTTGCGACGTATGCGGTATTAGCTACCGTTTCCAGTAGTTATCCCCCTCCATCAGG
CAGTTTCCCAGACATTACTCACCCGTCCGCCACTCGTCAGCAAACAGCAAGCTGCTTCCTGTTACCGTTCGAC
TGCATGTGGCTG
>261
CTGCTGCCGCCCGTAGGAGTCTGGACCGTGTCTCAGTTCCAGTGTGGCTGGTCATCCTCTCAGACCAGCTAGG
GATCGTCGCCTAGGTGAGCCGTTACCCCACCTACTAGCTAATCCCATCTGGGCACATCCGATGGCAAGAGGCC
CTAAGGTCCCCCTCTTTGTCTGCGACGTATGCGGTATTAGCTACCGTTTCCAGTAGTTATCCCCCTCCATCGG

GCAGTTCCCAGACATTACTCACCCGTCCGCCACTCGTCAGCAAAGCAGCAAGCTGCTTCCTGTTACCGTTCGA
CTGCATGTGTTAGGCCTGCCGCCAGCGTCAATCTGAGCCAGGATCAACTCT
>262
CTGCTGCCGCCCGTAGGAGTCTGGACCGTGTCTCAGTTCCAGTGTGGCTGGTCATCCTCTCAGACCAGCTAGG
GATCGTCGCCTAGGTGAGCCGTTACCCCACCTACTAGCTAATCCCATCTGGGCACATCCGATGGCAAGAGGCC
CTAAGGTCCCCCTCTTTGTCTTGCGACGTTATGCGGTATTAGCTACCGTTTCCAGTAGTTATCCCCCTCCATC
GGGCAGTTTTCCCAGACATTACTCACCCGTCCGCCACTCGTCAGCAAAGCAGCAAGCTGCTTCCTGTTACCGT
TCGACTGCATGTGTTAGGCCTGCCGCCAGCGTCAATCTGAGCCAGGATCAAACTCT
>263
CTGCTGCCGCCCGTAGGAGTCTGGACCGTGTCTCAGTTCCAGTGTGGCTGGTCATCCTCTCAGACCAGCTAGG
GATCGTCGCCTAGGTGAGCCGTTACCCCACCTACTAGCTAATCCCATCTGGGCACATCCGATGGCAAGAGGCC
CTAAGGTCCCCCTCTTGTCTGCGACGTATGCGGTATTAGCTACCGTTTCCAGTAGTTATCCCCTCCATCGGGC
AGTTTCCCAGACATACTCACCCGTCCGCCACTCGTCAGCAAAGCAGCAAGCTGCTTCCTGTTACCGTCGACTG
CATGTGTTAGGCCTGCCGCCAGCGTCAATCTGAGCCAGGATCAAACTCT
>264
CTGCTGCCGCCCGTAGGAGTCTGGACCGTGTCTCAGTTCCAGTGTGGCTGGTCATCCTCTCAGACCAGCTAGG
GATCGTCGCCTAGGTGAGCCGTTACCCCACCTACTAGCTAATCCCATCTGGGCACATCCGATGGCAAGAGGCC
CTAAGGTCCCCCTCTTTGTCTGCGACGTATGCGGTATTAGCTACCGTTTCCAGTAGTTATCCCCCTCCATCAG
GCAGTTTTCCCAGACATTACTCACCCGTCCGCCACTCGTCAGCAAAGCAGCAAGCTGCTTCCTGTTACCGTTC
GACTGCATGTGTTAGGCCTGCCGCCAGCGTCAATCTGAGCCAGGATCAAACTCT
>265
CTGCTGCCGCCCGTAGGAGTCTGGACCGTGTCTCAGTTCCAGTGTGGCTGGTCATCCTCTCAGACCAGCTAGG
GATCGTCGCCTAGGTGAGCCGTTACCCCACCTACTAGCTAATCCCATCTGGGCACATCCGATGGCAAGAGGCC
CTAAGGTCCCCCTCTTTGTCTTGCGACGTATGCGGTATTAGCTACCGTTTCCAGTAGTTATCCCCCTCCATCG
GGCAGTTTCCCAGACATTACTCACCCGTCCGCCACTCGTCAGCAAAGCAGCAAGCTGCTTCCTGTTACCGTTC
GACTGCATGTGTTAGGCCTGCCGCCAGCGTCAATCTGAGCCAGGATCAAACTCT
>266
CTGCTGCCGCCCGTAGGAGTCTGGACCGTGTCTCAGTTCCAGTGTGGCTGGTCATCCTCTCAGACCAGCTAGG
GATCGTCGCCTAGGTGAGCCGTTACCCCACCTACTAGCTAATCCCATCTGGGCACATCCGATGGCAAGAGGCC
CTAAGGTCCCCCTCTTGGTCTTGCGACGTTATGCGGTATTAGCTACCGTTTCCAGTAGTTATCCCCCTCCATC
AGGCAGTTTCCCAGACATTACTCACCCGTCCGCCACTCGTCAGCAAAAGCAGCAAGCTGCTTCCTGTTACCGT
TCGACTGCATGTGTTAGGCCTGCCGCCAGCGTCAATCTGAGCCAGGATCAAACTCT
>267
CTGCTGCCGCCCGTAGGAGTCTGGACCGTGTCTCAGTTCCAGTGTGGCTGGTCATCCTCTCAGACCAGCTAGT
GGATCGCGCTAGGTGAGCCGTTACCCCCACCTACTAGCTAATCCCATCTGGGCACATCCGATGGCAAGAGGCC
CTAAGGTCCCCCCTCTTTGTCTGCGACGTATGCGGTATTAGCTACCGTTTCCAGTAGTTATCCCCCTCCATCA
GGCAGTTTTCCCCAGACATTACTCACCCGTCCGCCACTCGTCAGCAAAGCAGCAAGCTGCTTCCTGTTACCGT
TCGACTGCATGTGTTAGGCCTGCCGCCAGCGTCAATCTGAGCCAGGATCAAACTCT
>268
CTGCTGCCGCCCGTAGGAGTCTGGACCGTGTCTCAGTTCCAGTGTGGCTGGTCATCCTCTCAGACCAGCTAGG
GATCGTCGCCTAGGTGAGCCGTTACCCCACCTACTAGCTAATCCCATCTGGGCACATCCGATGGCAAGAGGCC
CTAAGGTCCCCCTCTTGTCTTGCGACGTATGCGGTATTAGCTACCGTTTCCAGTAGTTATCCCCCTCCATCGG
GCAGTTTTCCCAGACATTACTCACCCGTCCGCCACTCGTCAGCAAAGCAGCAAGCTGCTTCCTGTTACCGTTC
GACTGCATGTGTTAGGCCTGCCGCCAGCGTCAATCTGAGCCAGGATCAAACTCT
>269
CTGCTGCCGCCCGTAGGAGTCTGGACCGTGTCTCAGTTCCAGTGTGGCTGGTCATCCTCTCAGACCAGCTAGG
GATCGTCGCCTAGGTGAGCCGTTACCCCACCTACTAGCTAGTCCCATCTGGGCACATCCGATGGCAAGAGGCC
CTAAGGTCCCCCTCTTGTCTTGCGACGTTATGCGGTATTAGCTACCGTTTCCAGTAGTTATCCCCCTCCATCA
GGCAGTTTCCCAGACATTACTCACCCGTCCGCCACTCGTCAGCAAAGCAGCAAGCTGCTTCTGTTACCGTTCG
ACTGCATGTGTTAGGCCTGCCGCCAGCGTCAATCTGAGCCAGGATCAAACTCT
>270
CTGCTGCCGCCCGTAGGAGTCTGGACCGTGTCTCAGTTCCAGTGTGGCTGGTCATCCTCTCAGACCAGCTAGG
GATCGTCGCCTAGGTGAGCCGTTACCCCACCTACTAGCTAATCCCATCTGGGCACATCCGATGGCAAGAGGCC
CTAAGGTCCCCCTCTTTGTCTTGCGACGTATGCGGTATTAGCTACCGTTTCCAGTAGTTATCCCCCTCCATCA

GGCAGTTTCCCAGACATTACTCACCCGTCCGCCACTCGTCAGCAAAGCAGCAAGCTGCTTCCTGTTACCGTTC
GACTGCATGTGTTAGGCCTGCCGCCAGCGTCAATCTGAGCCAGGATCAAACTCT
>271
CTGCTGCCGCCCGTAGGAGTCTGGACCGTGTCTCAGTTCCAGTGTGGCTGGTCATCCTCTCAGACCAGCTAGG
GATCGTCGCCTAGGTGAGCCGTTACCCCACCTACTAGCTAATCCCATCTGGGCACATCCGATGGCAAGAGGCC
CTAAGGTCCCCCTCTTTGTCTTGCGACGTTATGCGGTATTAGCTACCGTTTCCAGTAGTTATCCCCCTCCATC
AGGCAGTTTCCCAGACATTACTCACCCGTCCGCCACTCGTCAGCAAAGCAGCAAGCTGCTTCTGTTACCGTTC
GACTGCATGTGTTAGGCCTGCCGCCAGCGTCAATCTGAGCCAGGATCAAACTCT
>272
CTGCTGCCGCCCGTAGGAGTCTGGACCGTGTCTCAGTTCCAGTGTGGCTGGTCATCCTCTCAGACCAGTTAGG
GATCGTCGCCTAGGTGAGCCGTTACCCCACCTACTAGCTAATCCCATCTGGGCACATCCGATGGCAAGAGGCC
CTAAGGTCCCCCTCTTGTCTGCGACGTATGCGGTATTAGCTACCGTTTCCAGTAGTTATCCCCCTCCATCAGG
CAGTTTCCCAGACATTACTCACCCGTCCGCCACTCGTCAGCAAAGCAGCAAGCTGCTTCCTGTTACCGTTCGA
CTGCATGTGTTAGGCCTGCCGCCAGCGTCAATCTGAGCCAGGATCAAACTCT
>273
CTGCTGCCGTAGGAGTCTGGACCGTGTCTCAGTTCCAGTGTGGCTGGTCATCCTCTCAGACCAGCTAGGGATC
GTCGCCTAGGTGAGCCGTTACCCCACCTACTAGCTAATCCCATCTGGGCACATCCGATGGCAAGAGGCCCTAA
GGTCCCCCTCTTTGTCTGCGACGTATGCGGTATTAGCTACCGTTTCCAGTAGTTATCCCCCTCCATCAGGCAG
TTTTCCCAGACATTACTCACCCGTCCGCCACTCGTCAGCAAAGCAGCAAGCTGCTTCCTGTTACCGTTCGACT
TGCATGCGTTAGGCCTGCCGCCAGCGTCAATCTGAGCCAGGATCAAACTCT
>274
CTGCTGCCGCCCGTAGGAGTCTGGACCGTGTCTCAGTTCCAGTGTGGCTGGTCATCCTCTCAGACCAGCTAGG
GATCGTCGCCTAGGTGAGCCGTTACCCCACCTACTAGCTAATCCCATCTGGGCACATCCCGATGGCAAGAGGC
CCTAAGGTCCCCCTCTTGTCTTGCGACGTATGCGGTATTAGCTACCGTTTCCAGTAGTTATCCCCTCCATCAG
GCAGTTTCCCAGACATTACTCACCCGTCCGCCACTCGTCAGCAAAGCAGCAAGCTGCTTCCTGTTACCGTTCG
ACTGCATGTCGTTAGGCCTGCCGCCAGCGTTCAGTCTGAGCCAGGATCAAACTCT
>275
CTGCTGCCGCCCGTAGGAGTCTGGACCGTGTCTCAGTTCCAGTGTGGCTGGTCATCCTCTCAGACCAGCTAGG
GATCGTCGCCTAGGTGAGCCGTTACCCCACCTACTAGCTAATCCCATCTGGGCACATCCGATGGCAAGAGGCC
CTAAGGTCCCCCTCTTTGGTCTTGCGACGTATGCGGTATTAGCTACCGTTTCCAGTAGTTATCCCCCTCCATC
GGGCAGTTTTCCCAGACATACTCACCCGTCCGCCACTCGTCAGCAAAGCAGCAAGCTGCTTCCTGTTACCGTT
CGACTGCATGTGTTAGGCCTGCCGCCAGCGTCAATCTGAGCCAGGATCAAACTCT
>276
TGCTGCCGCCCGTAGGAGTCTGGACCGTGTCTCAGTTCCAGTGTGGCTGGTCATCCTCTCAGACCAGCTAGGG
ATCGTCGCCTAGGTGAGCCGTTACCCCACCTACTAGCTAATCCCATCTGGGCACATCCGATGGCAAGAGGCCC
TAAGGTCCCCCTCTTTGGTCTTGCGACGTATGCGGTATTAGCTACCGTTTCCAGTAGTTATCCCCCTCCATCG
GGCAGTTTCCCAGACATTACTCACCCGTCCGCCACTCGTCAGCAAAGCAGCAAGCTGCTTCCTGTTACCGTTC
GACTGCATGTGTTAGGCCTGCCGCCAGCGTCAATCTGAGCCAGGATCAAACTCT
>277
CTGCTGCCGCCCGTAGGAGTCTGGACCGTGTCTCAGTTCCAGTGTGGCTGGTCATCCTCTCAGACCAGCTAGG
GATCGTCGCCTAGGTGAGCCGTTACCCCACCTACTAGCTAATCCCATCTGGGCACATCCGATGGCAAGAGGCC
CTAAGGTCCCCCTCTTTGGTCTTGCGACGTATGCGGTATTAGCTACCGTTTCCAGTAGTTATCCCCCTCCATC
GGGCAGTTTCCCAGACATTACTCACCCGTCCGCCACTCGTCAGCAAAGCAGCAAGCTGCTTCCTGTTACCGTT
CGACTGCATGTGTTAGGCCTGCCGCCAGCGTCAATCTGAGCCAGGATCAAACTCT
>278
CTGCTGCCGCCCGTAGGAGTCTGGACCGTGTCTCAGTTCCAGTGTGGCTGGTCATCCTCTCAGACCAGCTAGG
GATCGTCGCCTAGGTGAGCCGTTACCCCACCTACTAGCTAATCCCATCTGGGCACATCCGATGGCAAGAGGCC
CTAAGGTCCCCCTCTTTGTCTTGCGACGTTATGCGGTATTAGCTACCGTTTCCAGTAGTTATCCCCCTCCATC
AGGCAGTTTCCCAGACATTACTCACCCGTCCGCCACTCGTCAGCAAAGCAGCAAGCTGCTTCCTGTTACCGTT
CGACTGCATGTGTTAGGCCTGCCGCCAGCGTCAATCTGAGCCAGGATCAAACTCT
>279
CTGCTGCCGCCCGTAGGAGTCTGGACCGTGTCTCAGTTCCAGTGTGGCTGGTCATCCTCTCAGACCAGCTAGG
GATCGTCGCCTAGGTGAGCCGTTACCCCACCTACTAGCTAATCCCATCTGGGCACATCCGATGGCAAGAGGCC
CTAAGGTCCCCCTCTTTGTCTTGCGACGTTATGCGGTATTAGCTACCGTTTCCAGTAGTTATCCCCCTCCATC

GGGCAGTTTCCCAGACATTACTCACCCGTCCGCCACTCGTCAGCAAAGCAGCAAGCTGCTTCCTGTTACCGTT
CGACTGCATGTGTTAGGCCTGCCGCCAGCGTTCAATCTGAGCCAGGATCAAACTCT
>280
CTGCTGCCGCCCGTAGGAGTCTGGACCGTGTCTCAGTTCCAGTGTGGCTGGTCATCCTCTCAGACCAGCTAGG
GATCGTCGCCTAGGTGAGCCGTTACCCCACCTACTAGCTAATCCCATCTGGGCACATCCGATGGCAAGAGGCC
CTAAGGTCCCCCTCTTGTCTTGCGACGTATGCGGTATTAGCTACCGTTTCCAGTACGTTATCCCCCTCCATCG
GGCAGTTTCCCAGACATTACTCACCCGTCCGCCACTCGTCAGCAAAGCAGCAAGCTGCTTCCTGTTACCGTTC
GACTGCATGTGTTAGGCCTGCCGCCAGCGTCAATCTGAGCCAGGATCAACTCT
>281
CTGCTGCCGCCCCGTAGGGGTCTGGACCGTGTCTCAGTTCCAGTGTGGCTGGTCATCCTCTCAGACCAGCTAG
GGATCGTCGCCTAGGTGAGCCGTTACCCCACCTACTAGCTAATCCCATCTGGGCACATCCGATGGCAAGAGGC
CCTAAGGTCCCCCTCTTGTCGTTCGCGACGTATGCGGTATTAGCTACCGTTTCCAGTAGTTTATCCCCTCCAT
CAGGCAGTTTCCCAGACATTACTCACCCGTCCGCCACTCGTCAGCAAAGCAGCAAGCTGCTTCCTGTTACCGT
TCGACTGCATGTGTTAGGCCTGCCGCCAGCGTCATCTGAGCCAGGATCAAACTCT
>282
TGCTGCCGCCCCGTAGGAGTCTGGACCGTGTCTCAGTCCAGTGTGGCTGGTCATCCTCTCAGACCAGCTAGGG
ATCGTCGCCTAGGTGAGCCGTTACCCCACCTACTAGCTAATCCCATCTGGGCACATCCGATGGCAAGAGGCTC
TAAGGTCCCCCTCTTGGTCTTGCGACGTTATGCGGTATTAGCTACCGTTTCCAGTAGTTATCCCCCTCCATCA
GGCAGTTTCCCAGACATTACTCACCCGTCCGCCACTCGTCAGCAAAGCAGCAAGCTGCTTCCTGTTACCGTTC
GACTGCATGTGTTAGGCCTGCCGCCAGCGTCAATCTGAGCCAGGATCAAACTCT
>283
CTGCTGCCGCCCGTAGGAGTCTGGACCGTGTCTCAGTTCCAGTGTGGCTGGTCATCCTCTCAGACCAGCTAGG
GATCGTCGCCTAGGTGAGCCGTTACCCACCTACTAGCTAATCCCATCTGGGCACATCCGATGGCAAGAGGCCC
TAAGGTCCCCCTCTTGGTCTTGCGACGTTATGCGGTATTAGCTACCGTTTCCAGTAGTTATCCCCTCCATCGG
GCAGTTTCCCAGACATTACTCACCCGTCCGCCACTCGTCAGCAAAGCAGCAAGCTGCTTCCTGTTACCGTTCG
ACTGCATGTGTTAGGCCTGCCGCCAGCGTCAATCTGAGCCAGGATCAACTCT
>284
CTGCTGCCGCCCGTAGGAGTCTGGACCGTGTCTCAGTTCCAGTGTGGCTGGTCATCCTCTCAGACCAGCTAGG
GATCGTCGCCTAGGTGAGCCGTTACCCCACCTACTAGCTAATCCCATCTGGGCACATCCGATGGCAAGAGGCC
CTAAGGTCCCCCTCTTGGTCTTGCGACGTTATGCGGTATTAGCTACCGTTTCCAGTAGTTATCCCCCTCCATC
AGGCAGTTTCCCAGACATTACTCACCCGTCCGCCACTCGTCAGCAAGGCAGCAAGCTGCTTCTGTTACCGTTC
GACTGCATGTGTTAGGCCTGCCGCCAGCGTTCAATCTGAGCCAGGATCAAACTCT
>285
CTGCTGCCGCCCGTAGGAGTCTGGACCGTGTCTCAGTTCCAGTGTGGCTGGTCATCCTCTCAGACCAGCTAGG
GATCGTCGCCTAGGTGAGCCGTTACCCCACCTACTAGCTAATCCCATCTGGGCACATCCGATGGCAAGAGGCC
CTAAGGTCCCCTCTTGTCTTGCGACGTATGCGGTATTAGCTACCGTTTCCAGTAGTTATCCCCCTCCATCGGG
CAGTTTCCCAGACATTACTCACCCGTCCGCCACTCGTCAGCAAAGCAGCAAGCTGCTTCCTGTTACCGTTCGA
CTGCATGTGTTAGGCCTGCCGCCAGCGTCAATCTGAGCCAGGATCAAACTCT
>286
CTGCTGCCGCCCGTAGGAGTCTGGACCGTGTCTCAGTTCCAGTGTGGCTGGTCATCCTCTCAGACCAGCTAGG
GATCGTCGCCTAGGTGAGCCGTTACCCCACCTACTAGCTAATCCCATCTGGGCACATCCGATGGCAAGAGGCC
CTAAGGTCCCCCTCTTTGTCTTGCGACGTTATGCGGTATTAGCTACCGTTTCCAGTAGTTATCCCCCTCCATC
GGGCAGTTTTCCCAGACATTACTCACCCGTCCGCCACTCGTCAGCAAAGCAGCAAGCTGCTTCCTGTTACCGT
TCGACTGCATGTGTTAGGCCTGCCGCCAGCGTCAATCTGAGCCAGGATCAAACTCT
>287
CTGCTGCCGCCCGTAGGAGTCTGGACCGTGTCTCAGTTCCAGTGTGGCTGGTCATCCTCTCAGACCAGCTAGG
GATCGTCGCCTAGGTGAGCCGTTACCCCACCTACTAGCTAATCCCATCTGGGCACATCCGATGGCAAGAGGCC
CTAAGGTCCCCCTCTTGTCTTGCGACGTTATGCGGTATTAGCTACCGTTTCCAGTAGTTATCCCCCTCCATCG
GGCAGTTTCCCAGACATTACTCACCCGTCCGCCACTCGTCAGCAAAGCAGCAAGCTGCTTCCTGTTACCGTTC
GACTGCATGTGTTAGGCCTGCCGCCAGCGTCAATCTGAGCCAGGATCAACATCT
>288
CTGCTGCCGCCCGTAAGAGTCTGGACCGTGTCTCAGTTCCAGTGTGGCTGGTCATCCTCTCAGACCAGCTAGG
GATCGTCGCCTAGGTGAGCCGTTACCCCACCTACTAGCTAATCCCATCTGGGCACATCCGATGGCAAGAGGCC
CTAAGGTCCCCCTCTTTGTCTTGCGACGTTATGCGGTATTAGCTACCGTTTCCAGTAGTTATCCCCCTCCATC

GGGCAGTTTCCCAGACATTACTCACCCGTCCGCCACTCGTCAGCAAAGCAGCAAGCTGCTTCCTGTTACCGTT
CGACTGCATGTGTTAGGCCTGCCGCCAGCGTTCAATCTGAGCCAGGATCAAACTCT
>289
CTGCTGCCGCCCGTAGGAGTCTGGACCGTGTCTCAGTTCCAGTGTGGCTGGTCATCCTCTCAGACCAGCTAGG
GATCGTCGCCTAGGTGAGCCGTTACCCCACCTACTAGCTAATCCCATCTGGGCACATCCGATGGCAAGAGGCC
CTAAGGTCCCCCTCTTGTCTGCGACGTATGCGGTATTAGCTACCGTTCCAGTAGTTATCCCCTCCATCGGGCA
GTTTCCCAGACATACTCACCCGTCCGCCACTCGTCAGCAAAGCAGCAAGCTGCTTCCTGTTACCGTTCGACTG
CATGTGTTAGGCCTGCCGCCAGCGTCAATCTGAGCCAGGATCAAACTCT
>290
CTGCTGCCGCCCGTAGGAGTCTGGACCGTGTCTCAGTTCCAGTGTGGCTGGTCATCCTCTCAGACCAGCTAGG
GATCGTCGCCTAGGTGAGCCGTTACCCCACCTACTAGCTAATCCCATCTGGGCACATCCGATGGCAAGAGGCC
CTAAGGTCCCCCTCTTGTCTTGCGACGTATGCGGTATTAGCTACCGTTTCCAGTAGTTATCCCCCTCCATCGG
CAGTTTCCCAGACATTACTCACCCGTCCGCCACTCGTCAGCAAGCAGCAAGCTGCTTCCTGTTACCTTCGACT
GCATGTGTTAGGCCTGCCGCCAGCGTCAATCTGAGCCAGGATCAAACTCT
>291
CTGCTGCCGCCCGTAGGAGTCTGGACCGTGTCTCAGTTCCAGTGTGGCTGGTCATCCTCTCAGACCAGCTAGG
GATCGTCGCCTAGGTGAGCCGTTACCCCACCTACTAGCTAATCCCATCTGGGCACATCCGATGGCAAGAGGCC
CTAAGGTCCCCCTCTTTGTCTTGCGACGTTATGCGGTATTAGCTACCGTTTCCAGTAGTTATCCCCCTCCATC
GGGCAGTTTTCCCAGACATTACTCACCCGTCCGCCACTCGTCAGCAAAGCAGCAAGCTGCTTCCTGTTACCGT
TCGACTGCATGTGTTAGGCCTGCCGCCAGCGTCAATCTGAGCCAGGATCAAACTCT
>292
CTGCTGCCGCCCGTAGGAGTCTGGACCGTGTCTCAGTTCCAGTGTGGCTGGTCATCCTCTCAGACCAGCTAGG
GATCGTCGCCTAGGTGAGCCGTTACCCCACCTACTAGCTAATCCCATCTGGGCACATCCGATGGCAAGAGGCC
CTAAGGTCCCCCTCTTTGTCTTGCGACGTTATGCGGTATTAGCTACCGTTTCCAGTAGTTATCCCCCTCCATC
GGGCAGTTTCCCAGACATTACTCACCCGTCCGCCACTCGTCAGCAAAGCAGCAAGCTGCTTCCTGTTACCGTT
CGACTGCATGTGTTAGGCCTGCCGCCAGCGTTCAATCTGAGCCAGGATCAAACTCT
>293
CTGCTGCCCCCGTAGGAGTCTGGACCGTGTCTCAGTTCCAGTGTGGCTGGTCATCCTCTCAGACCAGCTAGGG
ATCGTCGCCTAGGTGAGCCGTTACCCCACCTACTAGCTAATCCCATCTGGGCACATCCGATGGCAAGAGGCCC
TAAGGTCCCCCTCTTTGTCTTGCGACGTTATGCGGTATTAGCTACCGTTTCCAGTAGTTATCCCCCTCCATCG
GGCAGTTTCCCAGACATTACTCACCCGTCCGCCACTCGTCAGCAAAGCAGCAAGCTGCTTCCTGTTACCGTTC
GACTGCATGTGTTAGGCCTGCCGCCAGCGTCAATCTGAGCCAGGATCAAACTCT
>294
CTGCTGCCGCCCGTAGGAGTCTGGACCGTGTCTCAGTTCCAGTGTGGCTGGTCATCCTCTCAGACCAGCTAGG
GATCGTCGCCTAGGTGAGCCGTTACCCCACCTACTAGCTAATCCCATCTGGGCACATCCGATGGCAAGAGGCC
CTAAGGTCCCCCTCTTTGTCTTGCGACGTTATGCGGTATTAGCTACCGTTTCCAGTAGTTATCCCCCTCCATC
GGGCAGTTTCCCAGACATTACTCACCCGTCCGCCACTCGTCAGCAAAGCAGCAAGCTGCTTCCTGTTACCGTT
CGACTGCATGTGTTAGGCCTGCCGCCAGCGTCAATCTGAGCCAGGATCAAACTCT
>295
TGCTGCCGCCCGTAGGAGTCTGGACCGTGTCTCAGTTCCAGTGTGGCTGGTCATCCTCTCAGACAGCTAGGGA
TCGTCGCCTAGGTGAGCCGTTACCCCACCTACTAGCTAATCCCATCTGGGCACATCCGATGGCAAGAGGCCCT
AAGGTCCCCCTCTTTGTCTTGCGACGTTATGCGGTATTAGCTACCGTTTCCAGTAGTTATCCCCCTCCATCAG
GCAGTTTCCCAGATATTACTCACCCGTCCGCCACTCGTCAGCAAAGCAGCAAGCTGCTTTCTGTTACCGTTCG
ACTGCATGTGTTAGGCCTGCCGCCAGCGTCAATCTGAGCCAGGATCAAACTCT
>296
CTGCTGCCGCCCGTAGGAGTCTGGACCGTGTCTCAGTTCCAGTGTGGCTGGTCATCCTCTCAGACAGCTAGGG
ATCGTCGCCTAGGTGAGCCGTTACCCCACCTACTAGCTAATCCCATCTGGGCACATCCGATGGCAAGAGGCCC
TAAGGTCCCCCTCTTGTCTGCGACGTATGACGGTATTAGCTACCGTTTCCAGTAGTTATCCCCTCCATCGGGC
AGTTTCCCAGACATTACTCACCCGTCCGCCACTCGTCAGCAAAGCAGCAAGCTGCTTCCTGTTACCGTTCGAC
TGCATGTGTTAGGCCTGCCGCCAGCGTCAATCTGAGCCAAGGA
>297
CTGCTGCCGCCCGTAGGAGTCTGGACCGTGTCTCAGTTCCAGTGTGGCTGGTCATCCTCTCAGACCAGCTAGG
GATCGTCGCCTAGGTGAGCCGTTACCCCACCTACTAGCTAATCCCATCTGGGCACATCCCGATGGCAAGAGGC
CCTAAGGTCCCCCTCTTGTCTTGCGACGTTATGCGGTATTAGCTACCGTTTCCAGTAGTTATCCCCCTCCATC

GGGCAGTTTCCCAGACATTACTCACCCGTCCGCCACTCGTCAGCAAGCAGCAAGCTGCTTCCTGTTACCGTTC
GACTGCATGTGTTAGGCCTGCCGCCAGCGTCAATCTGAGCCAGGATCAACTCT
>298
CTGCTGCCGCCCGTAGGAGTCTGGACCGTGTCTCAGTTCCAGTGTGGCTGGTCATCCTCTCAGACCAGCTAGG
GATCGTCGCCTAGGTGAGCCGTTACCCCACCTACTAGCTAATCCCATCTGGGCACATCCGATGGCAAGAGGCC
CTAAGGTCCCCCTCTTTGTCTTGCGACGTATGCGGTATTAGCTACCGTTTCCAGTAGTTATCCCCCTCCATCA
GGCAGTTTCCCAGACATTACTCACCCGTCCGCCACTCGTCAGCAAACAGCAAGCTGCTTCCTGTTACCGTTCG
ACTGCATGTGTTAGGCCTGCCGCCAGCGTTCAATCTGAGCCAGGATCAAACTCT
>299
CTGCTGCCGCCCGTAGGAGTCTGGACCGTGTCTCAGTTCCAGTGTGGCTGGTCATCCTCTCAGACCAGCTAGG
GATCGTCGCCTAGGTGAGCCGTTACCCCACCTACTAGCTAATCCCATCTGGGCACATCCGATGGCAAGAGGCC
CTAAGGTCCCCCTCTTGTCTTGCGACGTATGCGGTATTAGCTACCGTTTCCAGTAGTTATCCCCCTCCATCAG
GCAGTTTCCCAGACATTACTCACCCGTCCGCCACCGTCAGCAAAGCAGCAAGCTGCTTCCTGTTACCGTTCGA
CTGCATGTGTTAGGCCTGCCGCCAGCGTTCAATCTGAGCCAGG
>300
CTGCTGCCGCCCGTAGGAGTCTGGACCGTGTCTCAGTTCCAGTGTGGCTGGTCATCCTCTCAGACCAGCTAGG
GATCGTCGCCTAGGTGAGCCGTTACCCCACCTACTAGCTAATCCCATCTGGGCACATCCGATGGCAAGAGGCC
CTAAGGTCCCCCTCTTTGTCTGCGACGTTATGCGGTATTAGCTACCGTTTCCAGTAGTTATCCCCCTCCATCG
GGCAGTTTCCCAGACATTACTCACCCGTCCGCCACTCGTCAGCAAAGCAGCAAGCTGCTTCCTGTTACCGTTC
GACTGCATGTGTTAGGCCTGCCGCCACGTCAATCTGAGCCAGGATCAAACTCT
>301
CTGCTGCCGCCCGTAGGAGTCTGGACCGTGTCTCAGTTCCAGTGTGGCTGGTCATCCTCTCAGACCAGCTAGG
GATCGTCGCCTAGGTGAGCCGTTACCCCACCTACTAGCTAATCCCATCTGGGCACATCCGATGGCAAGAGGCC
CTAAGGTCCCCCTCTTGTCTTGCGACGTATGCGGTATTAGCTACCGTTTCCAGTAGTTATCCCCCTCCCATCA
GGCAGTTTCCCAGACATTACTCACCCGTCCGCCACTCGTCAGCAAAGCAGCAAGCTGCTTCCTGTTACCGTTC
GACTGCATGTGTTAGGCCTGCCGCCAGCCGTTCAATCTGAAGCCAGGATCAAACTCT
>302
CTGCTGCCGCCCGTAGGAGTCTGGACCGTGTCTCAGTTCCAGTGTGGCTGGTCATCCTCTCAGACCAGCTAGG
GATCGTCGCCTAGGTGAGCCGTTACCCCACCTACTAGCTAATCCCATCTGGGCACATCCGATGGCAAGAGGCC
CTAAGGTCCCCCTCTTTGTCTTGCGACGTTATGCGGTATTAGCTACCGTTTCCAGTAGTTATCCCCCTCCATC
AGGCAGTTTCCCAGACATTACTCACCCGTCCGCCACTCGTCAGCAAAGCAGCAAGCTGCTTCTGTTACCGTTC
GACTGCATGTGTTAGGCCTGCCGCCAGCGTCAATCTGAGCCAGGATCAAACTCT
>303
CTGCTGCCGCCCGTAGGAGTCTGGACCGTGTCTCAGTTCCAGTGTGGCTGGTCATCCTCTCAGACCAGCTAGG
GATCGTCGCCTAGGTGAGCCGTTACCCCACCTACTAGCTAATCCCATCTGGGCACATCCGATGGCAAGAGGCC
CTAAGGTCCCCCTCTTTGGTCTTGCGACGTATGCGGTATTAGCTACCGTTTCCAGTAGTTATCCCCTCCATCG
GGCAGTTTCCCAGACATTACTCACCCGTCCGCCACTCGTCAGCAAAGCAGCAAGCTGCTTCCTGTTACCGTTC
GACTGCATGTGTTAGGCCTGCCGCCAGCGTCAATCTGAGCCAGGATCAAACTCT
>304
CTGCTGCCGCCCGTAGGAGTCTGGACCGTGTCTCAGTTCCAGTGTGGCTGGTCATCCTCTCAGACCAGCTAGG
GATCGTCGCCTAGGTGAGCCGTTACCCCACCTACTAGCTAATCCCATCTGGGCACATCCGATGGCAAGAGGCC
CTAAGGTCCCCCTCTTTGGTCTTGCGACGTTATGCGGTATTAGCTACCGTTTCCAGTAGTTATCCCCCTCCAT
CAGGCAGTTTCCCAGACATTACTCACCCGTCCGCCACTCGTCAGCAAAGCAGCAAGCTGCTTCTGTTACCGTT
CGACTGCATGTGTTAGGCCTGCCGCCAGCGTCAATCTGAGCCAGGATCAAACTCT
>305
CTGCTGCCGCCCGTAGGAGTCTGGACCGTGTCTCAGTTCCAGTGTGGCTGGTCATCCTCTCAGACCAGCTAGG
GATCGTCGCCTAGGTGAGCCGTTACCCCACCTACTAGCTAATCCCATCTGGGCACATCCGATGGCAAGAGGCC
CTAGGTCCCCCTCTTGTCTTGCGACGTATGCGGTATTAGCTACCGTTTCCAGTAGTTATCCCCTCCATCAGGC
AGTTTCCCAGACATTACTCACCCGTCCGCCACTCGTCAGCAAACAGCAAGCTGCTTCCTGTTACCGTTCGACT
GCATGTGTTAGGCCTGCCGCCAGCGTTCAATCTGAGCCAGGATCAAACTCT
>306
CTGCTGCCGCCCGTAGGAGTCTGGACCGTGTCTCAGTTCCAGTGTGGCTGGTCATCCTCTCAGACCAGCTAGG
GATCGTCGCCTAGGTGAGCCGTTACCCCACCTACTAGCTAATCCCATCTGGGCACATCCGATGGCAAGAGGCC
CTAAGGTCCCCCTCTTGTCTGCGACGTATGCGGTATTAGCTACCGTTTCCAGTAGTTATCCCCCTCCATCAGG

CAGTTTCCCAGACATTACTCACCCGTCCGCCACTCGTCAGCAAAGCAGCAAGCTGCTTCTGTTACCGTTCGAC
TGCATGTGTTAGGCCTGCCGCCAGCGTCAATCTGAGCCAGGATCAACTCT
>307
TGCTGCCGCCCGTAGGAGTCTGGACCGTGTCTCAGTTCCAGTGTGGCTGGTCATCCTCTCAGACCAGCTAGGG
ATCGTCGCCTAGGTGAGCCGTTACCCCACCTACTAGCTAATCCCATCTGGGCACATCCGATGGCAAGAGGCCC
TAAGGTCCCCCTCTTGTCTGCGACGTATGCGGTATTAGCTACCGTTTCCAGTAGTTATCCCCCTCCATCAGGC
AGTTTCCCAGACATTACTCACCCGTCCGCCACTCGTCAGCAAACAGCAAGCTGCTTCCTGTTACCGTTCGACT
GCATGTGTTAGGCCTGCCGCCAGCGTCAATCTGAGCCAGG
>308
CTGCTGCCGCCCGTAGGAGCCTGGACCGTGTCTCAGTTCCAGTGTGGCTGGTCATCCTCTCAGACCAGCTAGG
GATCGTCGCCTAGGTGAGCCGTTACCCCACCTACTAGCTAATCCCATCTGGGCACATCCCGATGGCAAGAGGC
CCTAAGGTCCCCCTCTTTGTCTTGCGACGTATGCGGTATTAGCTACCGTTTCCAGTAGTTATCCCCCTCCATC
AGGCAGTTTCCCAGACATTACTCACCCGTCCGCCACTCGTCAGCAAAGCAGCAAGCTGCTTCTGTTACCGTTC
GACTGCATGTGTTAGGCCTGCCGCCAGCGTTCAATCTGAGCCAGGATCAAACTCT
>309
CTGCTGCCGCCCGTAGGAGTCTGGACCGTGTCTCAGTTCCAGTGTGGCTGGTCATCCTCTCAGACCAGCTAGG
GATCGTCGCCTAGGTGAGCCGTTACCCCACCTACTAGCTAATCCCATCTGGGCACATCCCGATGGCAAGAGGC
CCTAAGGTCCCCCTCTTGTCTTGCGACGTATGCGGTATTAGCTACCGTTTCCAGTAGTTATCCCCCTCCATCG
GGCAGTTTCCCAGACATTACTCACCCGTCCGCCGCTCGTCAGCAAAGCAGCAAGCTGCTTCCTGTTACCGTTC
GACTGCGTGTGTTAGGCCTGCCGCCAGCGTTCAATCTGAGCCAGGATCAAACTCT
>310
CTGCTGCCGCCCGTAGGAGTCTGGACCGTGTCTCAGTTCCAGTGTGGCTGGTCATCCTCTCAGACCAGCTAGG
GATCGTCGCCTAGGTGAGCCGTTACCCCACCTACTAGCTAATCCCATCTGGGCACATCCGATGGCAAGAGGCC
CTAAGGTCCCCCTCTTTGTCTTGCGACGTATGCGGTATTAGCTACCGTTTCCAGTAGTTATCCCCCTCCATCG
GGCAGTTTCCCAGACATTACTCACCCGTCCGCCACTCGTCAGCAAAGCAGCAAGCTGCTTCCTGTTACCGTTC
GACTGCATGTGTTAGGCCTGCCGCCAGCGTCAATCTGAGCCAGGATCAAACTCT
>311
CTGCTGCCGCCCGTAGGAGTCTGGACCGTGTCTCAGTTCCAGTGTGGCTGGTCATCCTCTCAGACCAGCTAGG
GATCGTCGCCTAGGTGAGCCGTTACCCCACCTACTAGCTAATCCCATCTGGGCACATCCCGATGGCAAGAGGC
CCTAAGGTCCCCCTCTTGTCTTGCGACGTTATGCGGTATTAGCTACCGTTTCCAGTAGTTATCCCCCTCCATC
GGGCAGTTTCCCAGACATTACTCACCCTCCGCCACTTGTCAGCAAACAGCAAGCTGCTCCTGTTACCGTTCGA
CTGCATGTGTTAGGCCTG
>312
CTGCTGCCGCCCGTAGGAGTCTGGACCGTGTCTCAGTTCCGTGTGGCTGGTCATCCTCTCAGACCAGCTAGGG
ATCGTCGCCTAGGTGAGCCGTTACCCCACCTACTAGCTAATCCCATCTGGGCACATCCGATGGCAAGAGGCCC
TAAGGTCCCCCTCTTGTCTTGCGACGTATGCGGTATTAGCTACCGTTTCCAGTAGTTATCCCCCTCCATCGGG
GCAGTTTTCCCAGACATTACTCACCCGTCCGCCACTCGTCAGCAAAGCAGCAAGCTGCTTCCTGTTACCGTTC
GACTGCATGTGTCGCTAGGCCCTCGCCGCCAAG
>313
CTGCTGCCGCCCGTAGGAGTCTGGACCGTGTCTCAGTTCCAGTGTGGCTGGTCATCCTCCCAGACCAGCTAGG
GATCGTCGCCTAGGTGAGCCGTTACCCCACCTACTAGCTAATCCCATCTGGGCACATCCGATGGCAAGAGGCC
CTAAGGTCCCCCTCTTTGTCTTGCGACGTTATGCGGTATTAGCTACCGTTTCCAGTAGTTATCCCCCTCCATC
AGGCAGTTTTCCCAGACATTACTCACCCGTCCGCCACTCGTCAGCAAAGCAGCAAGCTGCTTCCTGTTACCGT
TCGACTGCATGTGTTAGGCCTGCCGCCAGCGTCAATCTGAGCCAGGATCAAACTCT
>314
CTGCTGCCGCCCGTAGGAGTCTGGACCGTGTCTCAGTTCCAGTGTGGCTGGTCATCCTCTCAGACCAGCTAGG
GATCGTCGCCTAGGTGAGCCGTTACCCCACCTACTAGCTAATCCCATCTGGGCACATCCGATGGCAAGAGGCC
CTAAGGTCCCCCTCTTGTCTGCGACGTATGCGGTATTAGCTACCGTTTCCAGTAGTTATCCCCCTCCATCAGG
CAGTTTCCCAGACATTACTCACCCGTCCGCCACTCGTCAGCAAACAGCAAGCTGCTTCCTGTTACCGTTCGAC
TGCATGTGTTAGGCCTGCCGCCAGCGTTCAATCTGAGCCAGGATCAAACTCT
>315
CTGCTGCCGCCCGTAGGAGTCTGGACCGTGTCTCAGTTCCAGTGTGGCTGGTCATCCTCTCAGACCAGCTAGG
GATCGTCGCCTAGGTGAGCCGTTACCCCACCTACTAGCTAATCCCATCTGGGCACATCCGATGGCAAGAGGCC
CTAAGGTCCCCCTCTTGTCTGCGACGTATGCGGTATTAGCTACCGTTCCAGTAGTTATCCCCTCCATCGGGCA

GTTCCCAGACATTACTCACCCGTCCGCCACTCGTCAGCAAAGCAGCAAGCTGCTTCCTGTTACCGTTCGACTG
CATGTGTTAGGCCTGCCGCCAGCGTCAATCTGAGCCAAGGATCAAACTCT
>316
CTGCTGCCGCCCGTAGGAGTCTGGACCGTGTCTCAGTTCCAGTGTGGCTGGTCATCCTCTCAGACCAGCTAGG
GATCGTCGCCTAGGTGAGCCGTTACCCCACCTACTAGCTAATCCCATCTGGGCACATCCCGATGGCAAGAGGC
CCTAAGGTCCCCCTCTTTGTCTTGCGACGTTATGCGGTATTAGCTACCGTTTCCAGTAGTTATCCCCCTCCAT
CGGGCAGTTTCCCAGACATTACTCACCCGTCCGCCACTCGTCAGCAAAGCAGCAAGCTGCTTCCTGTTACCGT
TCGACTGCATGTGTTAGGCCTGCCGCCAGCGTCAATCTGAGCCAGGATCAAACTCT
>317
CTGCTGCCGCCCGTAGGAGTCTGGACCGTGTCTCAGTTCCAGTGTGGCTGGTCATCCTCTCAGACCAGCTAGG
GATCGTCGCCTAGGTGAGCCGTTACCCCACCTACTAGCTAATCCCATCTGGGCACATCCGATGGCAAGAGGCC
CTAAGGTCCCCCTCTTGTCTTGCGACGTATGCGGTATTAGCTACCGTTTCCAGTAGTTATCCCCCTCCATCAG
GCAGTTTTCCCAGACATTACTCACCCGTCCGCCACTCGTCAGCAAAGCAGCAAGCTGCTTCCTGTTACCGTTC
GACTGCATGTCGTTAGGCCTGCCGCCAGCGTCAATCTGAGCCAGGATCAAACTGT
>318
CTGCTGCCGCCCGTAGGAGTCTGGACCGTGTCTCAGTTCCAGTGTGGCTGGTCATCCTCTCAGACCAGCTAGG
GATCGTCGCCTAGGTGAGCCGTTACCCCACCTACTAGCTAATCCCATCTGGGCACATCCGATGGCAAGAGGCC
CTAAGGTCCCCCTCTTTGTCTTGCGACGTTATGCGGTATTAGCTACCGTTTCCAGTAGTTATCCCCCTCCATC
AGGCAGTTTTCCCAGACATTACTCACCCGTCCGCCACTCGTCAGCAAACAGCAAGCTGCTTCTGTTACCGTTC
GACTGCATGTGTTAGGCCTGCCGCCAGCGTTCAATCTGAGCCAGGATCAAACTCT
>319
CTGCTGCCGCCCGTAGGAGTCTGGACCGTGTCTCAGTTCCAGTGTGGCTGGTCATCCTCTCAGACCAGCTAGG
GATCGTCGCCTAGGTGAGCCGTTACCCCACCTACTAGCTAATCCCATCTGGGCAGCATCCGATGGCAAGAGGC
CCTAAGGTCCCCCTCTTGTCTTGCGACGTATGCGGTATTAGCTACCGTTTCCAGTAGTTATCCCCCTCCCATC
AGGCAGTTTTCCCCAGACATTACTCACCCGTCCGCCACTCGTCAGCAAAACAGCAAGCTGCTTCCTGTTACCG
TTTCGACTGCATGTGTTTA
>320
TGCTGCCCCCGTAGGAGTCTGGACCGTGTCTCAGTTCCAGTGTGGCTGGTCATCCTCTCAGACCAGCTAGGGA
TCGTCGCCTAGGTGAGCCGTTACCCCACCTACTAGCTAATCCCATCTGGGCACATCCGATGGCAAGAGGCCCT
AAAGGTCCCCCCTCTTTGGTCTTGCGACGTTATGCGGTATTAGCTACCGTTTCCAGTAGTTATCCCCCTCCCA
TCAGGCAGTTTCCCAGACATTACTCACCCGTCCGCCACTCGTCAGCAAAGCAGCAAGCTGCTTCCTGTTACCG
TTCGACTGCATGTGTTAGCCTCGCCGCCAGCGTCAATCTGAGCCAGGATCAAACTCT
>321
CTGCTGCCGCCCGTAGGAGTCTGGACCGTGTCTCAGTTCCAGTGTGGCTGGTCATCCTCTCAGACCAGCTAGG
GATCGTCGCCTAGGTGAGCCGTTACCCCACCTACTAGCTAATCCCATCTGGGCACATCCGATGGCAAGAGGCC
CTAAGGTCCCCCTCTTGTCTTGCGACGTTATGCGGTATTAGCTACCGTTTCCAGTAGTTATCCCCCTCCATCA
GGCAGTTTCCCAGACATTACTCACCGTCCGCCACTCGTCAGCAAACAGCAAGCTGCTTCCTGTTACCGTTCGA
CTGCATGTGTTAGGCCTGCCGCCAGCGTTCAATCTGAGCCAGGATCAAACTGT
>322
CTGCTGCCGCCCGTAGGAGTCTGGACCGTGTCTCAGTTCCAGTGTGGCTGGTCATCCTCTCAGACCAGCTAGG
GATCGTCGCCTAGGTGAGCCGTTACCCCACCTACTAGCTAATCCCATCTGGGCACATCCGATGGCAGGAGGCC
CTAAGGTCCCCCTCTTGTCTTGCGACGTTATGCGGTATTAGCTACCGTTCCAGTAGTTATCCCCTCCATCAGG
CAGTTTCCCAGACATTACTCACCGTCCGCCACTCGTCAGCAAAGCAGCAAGCTGCTTTCTGTTACCGTTCGAC
TGCATGTGTTAGGCCTGACCGCCAGCGTCAATCTGAGCCAGGATCAAACTTAT
>323
CTGCTGCCGCCCGTAGGAGTCTGGACCGTGTCTCAGTTCCAGTGTGGCTGGTCATCCTCTCAGACCAGCTAGG
GATCGTCGCCTAGGTGAGCCGTTACCCCACCTACTAGCTAATCCCATCTGGGCACATCCGATGGCAAGAGGCC
CTAAGGTCCCCCTCTTTGTCTGCGACGTTATGCGGTATTAGCTACCGTTTCCAGTAGTTATCCCCCTCCATCA
GGCAGTTTCCCAGACATTACTCACCCGTCCGCCACTCGTCAGCAAAGCAGCAAGCTGCTTCCTGTTACCGTTC
GACTGCATGTGTTAGGCCTGCCGCCAGCGTTCAATCTGAGCCAGGATCAAACTCT
>324
CTGCTGCCGCCCGTAGGAGTCTGGACCGTGTCTCAGTTCCAGTGTGGCTGGTCATCCTCTCAGACCAGCTAGG
GATCGTCGCCTAGGTGAGCCGTTACCCCACCTACTAGCTAATCCCATCTGGGCACATCCGATGGCAAGAGGCC
CTAAGGTCCCCCTCTTGGTCTTGCGACGTTATGCGGTATTAGCTACCGTTTCCAGTAGTTATCCCCCTCCATC

AGGCAGTTTCCCAGACATTACTCACCCGTCCGCCACTCGTCAGCAAACAGCAAGCTGCTTCCTGTTACCGTTC
GACTGCATGTGTTAGGCCTGCCGCCAGCGTTCAATCTGAGCCAGGATCAAACTCT
>325
CTGCTGCCGCCCGTAGGAGTCTGGACCGTGTCTCAGTTCCAGTGTGGCTGGTCATCCTCTCAGACCAGCTAGG
GATCGTCGCCTAGGTGAGCCGTTACCCCACCTACTAGCTAATCCCATCTGGGCACATCCGATGGCAAGAGGCC
CTAAGGTCCCCCTCTTTGTCTTGCGACGTATGCGGTATTAGCTACCGTTTCCAGTAGTTACCCCCCTCCATCA
GGCAGTTTCCCAGACATTACTCACCCGTCCGCCACTCGTCAGCAAAGCAGCAAGCTGCTTCCTGTTACCGTTC
GACTGCATGTGTTAGGCCTGCCGCCAGCGTCAATCTGAGCCAGGATCAAACTCT
>326
CTGCTGCCGCCCGTAGGAGTCTGGACCGTGTCTCAGTTCCAGTGTGGCTGGTCATCCTCTCAGACCAGCTAGG
GATCGTCGCCTAGGTGAGCCGTTACCCCACCTACTAGCTAATCCCATCTGGGCACATCCGATGGCAAGAGGCC
CTAAGGTCCCCCTCTTTGTCTTGCGACGTTATGCGGTATTAGCTACCGTTTCCAGTAGTTATCCCCCTCCATC
AGGCAGTTTCCCAGACATTACTCACCCGTCCGCCACTCGTCAGCAAAGCAGCAAGCTGCTTCTGTTACCGTTC
GACTGCATGTGTTAGGCCTGCCGCCAGCGTTCAATCTGAGCCAGGATCAAACTCT
>327
TGCTGCCGCCCGTAGGAGTCTGGACCGTGTCTCAGTTCCAGTGTGGCTGGTCATCCTCTCAGACCAGCTAGGG
ATCGTCGCCTAGGTGAGCCGTTACCCCACCTACTAGCTAATCCCATCTGGGCACATCCGATGGCAAGAGGCCC
TAAGGTCCCCCTCTTGTCTTGCGACGTATGCGGCATTAGCTACCGTTTCCAGTAGTTATCCCCCTCCATCAGG
CAGTTTCCCAGACATTACTCACCCGTCCGCCACTCGTCAGCAAACAGCAAGCTGCTTCCTGTTACCGTTCGAC
TGCATGTGTTAGGCCTGCCGCCAGCGTTCAATCTGAGCCAGGATCAAACTCT
>328
CTGCTGCCGCCCGTAGGAGTCTGGACCGTGTCTCAGTTCCAGTGTGGCTGGTCATCCTCTCAGACCAGCTAGG
GATCGTCGCCTAGGTGAGCCGTTACCCCACCTACTAGCTAATCCCATCTGGGCACATCCGATGGCAAGAGGCC
CTAAGGTCCCCCTCTTGTCTGCGACGTTATGCGGTATTAGCTACCGTTTCCAGTAGTTATCCCCCTCCATCGG
GCAGTTTCCCAGACATTACTCACCCGTCCGCCACTCGTCAGCAAGCAGCAAGCTGCTTCCTGTTACCGTTCGA
CTGCATGTGTTAGGCCTGCCGCCAGCGTCAATCTGAGCCAGGATCAAACTCT
>329
CTGCTGCCGCCCGTAGGAGTCTGGACCGTGTCTCAGTTCCAGTGTGGCTGGTCATCCTCTCAGACCAGCTAGG
GATCGTCGCCTAGGTGAGCCGTTACCCCACCTACTAGCTAATCCCATCTGGGCACATCCGATGGCAAGAGGCC
CTAAGGTCCCCCTCTTGTCTGCGACGTATGCGGTATTAGCTACCGTTTCCAGTAGTTATCCCCCTCCATCAGG
CAGTTTCCCAGACATTACTCACCCGTCCGCCACTCGTCAGCAAACAGCAAGCTGCTTCCTGTTACCGTTCGAC
TGCATGTGTTAGGCCTGCCGCCAGCGTTCAATC
>330
CTGCTGCCGCCCGTAGGAGTCTGGACCGTGTCTCAGTTCCAGTGTGGCTGGTCATCCTCTCAGACCAGCTAGG
GATCGTCGCCTAGGTGAGCCGTTACCCCACCTACTAGCTAATCCCATCTGGGCACATCCGATGGCAAGAGGCC
CTAAGGTCCCCCTCTTTGTCTGCGACGTTATGCGGTATTAGCTACCGTTTCCAGTAGTTATCCCCCTCCATCG
GGCAGTTTTCCCAGACATTACTCACCCGTCCGCCACTCGTCAGCAAGCAGCAAGCTGCTTCCTGTTACCGTTC
GACTGCATGTGTTAGGCTGCCGCCAGCGTCAATCTGAGCCAGGATCAAACTCT
>331
CTGCTGCCGCCCGTAGGAGTCTGGACCGTGTCTCAGTTCCAGTGTGGCTGGTCATCCTCTCAGACCAGCTAGG
GATCGTCGCCTAGGTGAGCCGTTACCCCACCTACTAGCTAATCCCATCTGGGCACATCCCGATGGCAAGAGGC
CCTAAGGTCCCCCTCTTTGTCTTGCGACGTTATGCGGTATTAGCTACCGTTTCCAGTAGTTATCCCCCTCCAT
CAGGCAGTTTCCCAGACATTACTCACCCGTCCGCCACTCGTCAGCAAAGCAGCAAGCTGCTTCCTGTTACCGT
TCGACTGCATGTGTTAGGCCTGCCGCCAGCGTTCAATCTGAGCCAGGATCAAACTCT
>332
CTGCTGCCGCCCGTAGGAGTCTGGACCGTGTCTCAGTTCCAGTGTGGCTGGTCATCCTCTCAGACCAGCTAGG
GATCGTCGCCTAGGTGAGCCGTTACCCCACCTACTAGCTAATCCCATCTGGGCACATCCGATGGCAAGGGGGC
CCTAAGGTCCCCCTCTTTGGTCTTGCGACGTTATGCGGTATTAGCTACCGTTTCCAGTAGTTATCCCCCTCCA
TCGGGCAGTTTCCCAGACATTACTCACCCGTCCGCCACTCGTCAGCAAAGCAGCAAGCTGCTTCCTGTTACCG
TTCGACTGCATGTGTTAGGCCTGCCGCCAGCGTTCAATCTGAGCCAGGATCAAACTCT
>333
CTGCTGCCGCCCGTAGGAGTCTGGACCGTGTCTCAGTTCCAGTGTGGCTGGTCATCCTCTCAGACCAGCTAGG
GATCGTCGCCTAGGTGAGCCGTTACCCCACCTACTAGCTAATCCCATCTGGGCACATCCGATGGCAAGAGGCC
CTAAGGTCCCCCTCTTGTCTTGCGACGTATGCGGTATTAGCTACCGTTTCCAGTAGTTATCCCCCTCCATCGG

GCAGTTTCCCAGACATTACTCACCCGTCCGCCACTCGTCAGCAAGCAGCAAGCTGCTTCCTGTTACCGTTCGA
CTGCATGTGTTAGGCCTGCCGCCAGCGTCAATCTGAGCCAGGATCAACTCT
>334
CTGCTGCCGCCCGTAGGAGTCTGGACCGTGTCTCAGTTCCAGTGTGGCTGGTCATCCTCTCAGACCAGCTAGG
GATCGTCGCCTAGGTGAGCCGTTACCCCACCTACTAGCTAATCCCATCTGGGCACATCCGATGGCAAGAGGCC
CTAAGGTCCCCCTCTTTGGTCTTGCGACGTTATGCGGTATTAGCTACCGTTTCCAGTAGTTATCCCCCTCCAT
CAGGCAGTTTCCCAGACATTACTCACCCGTCCGCCACTCGTCAGCAAAGCAGCAAGCTGCTTCCTGTTACCGT
TCGACTGCATGTGTTAGGCCTGCCGCCAGCGTTCAATCTGAGCCAGGATCAAACTCT
>335
CTGCTGCCGCCCGTAGGAGTCTGGACCGTGTCTCAGTTCCAGTGTGGCTGGTCATCCTCTCAGACCAGCTAGG
GATCGTCGCCTAGGTGAGCCGTTACCCCACCTACTAGCTAATCCCATCTGGGCACATCCGATGGCAAGAGGCC
CTAAGGTCCCCCTCTTTGTCTTGCGACGTATGCGGTATTAGCTACCGTTTCCAGTAGTTATCCCCCTCCATCA
GGCAGCTTTCCCAGACATTACTCACCCGTCCGCCACTCGTCAGCAAAGCAGCAAGCTGCTTCTGTTACCGTTC
GACTGCATGTGTTAGGCCTGCCGCCAGCGTCAATCTGAGCCAGGATCAAACTCT
>336
CTGCTGCCGCCCGTAGGAGTCTGGACCGTGTCACAGTTCCAGTGTGGCTGGTCATCCTCTCAGACCAGCTAGG
GATCGTCGCCTAGGTGAGCCGTTACCCCACCTACTAGCTAATCCCATCTGGGCACATCCGATGGCAAGAGGCC
CTAAGGTCCCCCTCTTGTCTTGCGACGTATGCGGTATTAGCTACCGTTTCCAGTAGTTATCCCCCTCCATCAG
GCAGTTTCCCAGACATTACTCACCCGTCCGCCACTCGTCAGCAAAGCAGCAAGCTGCTTCCTGTTACCGTTCG
ACTGCATGTGTTAGGCCTGCCGCCAGCGTCAATCTGAGCCAGGATCAAACTCT
>337
CTGCTGCCGCCCTAGGAGTCTGGACCGTGTCTCAGTTCCAGTGTGGCTGGTCATCCTCCCAGACCAGCTAGGG
ATCGTCGCCTAGGTGAGCCGTTACCCCACCTACTAGCTAATCCCATCTGGGCACATCCGATGGCAAGAGGCCC
TAAGGTCCCCCTCTTGTCTTGCGACGTATGCGGTATTAGCTACCGTTTCCAGTAGTTATCCCCCTCCATCAGG
CAGTTTCCCAGACATACTCACCCGTCCGCCACTCGTCAGCAAAGCAGCAAGCTGCTTCCTGTTACCGTTCGAC
TGCATGTGTTAGGCCTGCCGCCAGCGTTCAATCTGAGCCAGGATCAAACTCT
>338
CTGCTGCCGCCCGTAGGAGTCTGGACCGTGTCTCAGTTCCAGTGTGGCTGGTCATCCTCTCAGACCAGCTAGG
GATCGTCGCCTAGGTGAGCCGTTACCCCACCTACTAGCTAATCCCATCTGGGCACATCCGATGGCAAGAGGCC
CTAAGGTCCCCCTCTTGTCTGCGACGTATGCGGTATTAGCTACCGTTTCCAGTAGTTATCCCCCTCCATCGGG
CAGTTTCCCAGACATACTCACCCGTCCGCCACTCGTCAGCAAGCAGCAAGCTGCTTCCTGTTACCGTTCGACT
GCATGTGTTAGGCCTGCCGCCAGCGTCAATCTGAGCCAGGATCAACTCT
>339
CTGCTGCCGCCCGTAGGAGTCTGGACCGTGTCTCAGTTCCAGTGTAGCTGGTCATCCTCTCAGACCAGCTAGG
GATCGTCGCCTAGGTGAGCCGTTACCCCACCTACTAGCTAATCCCATCTGGGCACATCCGATGGCAAGAGGCC
CTAAGGTCCCCCTCTTTGGTCTTGCGACGTGTGCGGTATTAGCTACCGTTTCCAGTAGTTATCCCCCTCCATC
GGGCAGTTTCCCAGACATTACTCACCCGTCCGCCACTCGTCAGCAAAGCAGCAAGCTGCTTCCTGTTACCGTT
CGACTGCATGTGTTAGGCCTGCCGCCAGCGTCAATCTGAGCCAGGATCAAACTCT
>340
TTGCTGCCGCCCGTAGGAGTCTGGACCGTGTCTCAGTTCCAGTGTGGCTGGTCATCCTCTCAGACAGCTAGGG
ATCGTCGCCTAGGTGAGCCGTTACCTCACCTACTAGCTAATCCCCATCTGGGCACATCCGATGGCAAGAGGCC
CTAAGGTCCCCCTCTTGTCTGCGACGTATGCGGTATTAGCTACCGTTTCCAGTAGTTATCCCCCTCCATCAGG
CAGTTTCCCAGACATTACTCACCCGTCCGCCACTCGTCAGCAAAGCAGCAAGCTGCTTCCTGTTACCGTTCGA
CTGCATGTGTTAGGCCTGCCGCCAGCGTCAATCTGAAGCCAGGATCAAACTTCT
>341
CTGCTGCCGCCCGTAGGAGTCTGGACCGTGTCTCAGTTCCAGTGTGGCTGGTCATCCTCTCAGACCAGCTAGG
GATCGTCGCCTAGGTGAGCCGTTACCCCACCTACTAGCTAATCCCATCTGGGCACATCCGATGGCAAGAGGCC
CTAAGGTCCCCCTCTTGTCTGCGACGTATGCGGTATTAGCTACCGTTTCCAGTAGTTATCCCCCTCCATCGGG
CAGTTTTCCCAGACATTACTCACCCGTCCGCCACTCGTCAGCAAAGCAGCAAGCTGCTTCCTGTTACCGTTCG
ACTGCATGTGTTAGGCACTCGC
>342
CTGCTGCCCCCGTAGGAGTCTGGACCGTGTCTCAGTTCCAGTGTGGCTGGTCATCCTCTCAGACCAGCTAGGG
ATCGTCGCCTAGGTGAGCCGTTACCCCACCTACTAGCTAATCCCATCTGGGCACATCCGATGGCAAGAGGCCC
TAAGGTCCCCCTCTTTGTCTGCGACGTTATGCGGTATTAGCTACCGTTTCCAGTAGTTATCCCCTCCATCGGG

CAGTTTCCCAGACATTACTCACCCGTCCGCCACTCTCAGCAAAGCAGCAAGCTGCTTCCTGTTACCGTTCGAC
TGCATGTGTTAGGCCTGCCGCCAGCGTCAATCTGAGCCAGGATCAAACTCT
>343
CTGCTGCCGCCCGTAGGAGTCTGGACCGTGTCTCAGCTCCAGTGTGGCTGGTCATCCTCTCAGACCAGCTAGG
GATCGTCGCCTAGGTGAGCCGTTACCCCACCTACTAGCTAATCCCATCTGGGCACATCCGATGGCAAGAGGCC
CTAAGGTCCCCCTCTTGTCTTGCGACGTATGCGGTATTAGCTACCGTTTCCAGTAGTTATCCCCCTCCATCGG
GCAGTTTCCCAGACATACTCACCCGTCCGCCACTCGTCAGCAAAGCAGCAAGCTGCTTCCTGTTACCGTTCGA
CTGCATGTGTTAGGCCTGCCGCCAGCGTCAATCTGAGCCAGGATCAAACTCT
>344
CTGCTGCCGCCCGTAGGAGTCTGGACCGTGTCTCAGTTCCAGTGTGGCTGGTCATCCTCTCAGACCAGCTAGG
GATCGTCGCCTAAGGTGAGCCGTTACCCCACCTACTAGCTAATCCCATCTGGGCACATCCGATGGCAAGAGGC
CCTAAGGTCCCCCTCTTTGGTCTTGCGACGTTATGCGGTATTAGCTACCGTTTCCAGTAGTTATCCCCCTCCA
TCAGGCAGTTTCCCAGACATTACTCACCCGTCCGCCACTCGTCAGCAAACAGCAAGCTGCTTCCTGTTACCGT
TCGACTGCATGTGTTAGGCCTGCCGCCAGCGTCAATCTGAGCCAGGATCAAACTCT
>345
CTGCTGCCGCCCGTAGGAGTCTGGACCGTGTCTCAGTTCCAGTGTGGCTGGTCATCCTCTCAGACCAGCTAGG
GATCGTCGCCTAGGTGAGCCGTTACCCCACCTACTAGCTAATCCCATCTGGGCACATCCGATGGCAAGAGGCC
CTAAGGTCCCCCTCTTGTCTGCGACGTATGCGGTATTAGCTACCGTTTCCAGTAGTTATCCCCCTCCATCAGG
CAGTTTCCCAGACATTACTCACCCGTCCGCCACTCGTCAGCAAACAGCAAGCTGCTTCCTGTTACCGTTCGAC
TGCATGTGTTAGGCCTGCCGCCAGCGTTCAATCTGAGCCAGGATCAAACTCT
>346
CTGCTGCCGCCCGTAGGAGTCTGGACCGTGTCTCAGTTCCAGTGTGGCTGGTCATCCTCTCAGACCAGCTAGG
GATCGTCGCCTAGGTGAGCCGTTACCCCACCTACTAGCTAATCCCATCTGGGCACATCCGATGGCAAGAGGCC
CTAAGGTCCCCCTCTTGTCTTGCGACGTTATGCGGTATTAGCTACCGTTTCCAGTAGTTATCCCCCTCCATCA
GGCAGTTTCCCAGACATTACTCACCCGTCCGCCACTCGTCAGCAAAGCAGCAAGCTGCTTCTGTTACCGTTCG
ACTGCATGTGTTAGGCCTGCCGCCAGCGTCAATCTGAGCCAGGATCAAACTCT
>347
CTGCTGCCGCCCGTAGGAGTCTGGACCGTGTCTCAGTTCCAGTGTGGCTGGTCATCCTCTCAGACCAGCTAGG
GATCGTCGCCTAGGTGAGCCGTTACCCCACCTACTAGCTAATCCCATCTGGGCACATCCGATGGCAAGAGGCC
CTAAGGTCCCCCTCTTTGTCTTGCGACGTTATGCGGTATTAGCTACCGTTTCCAGTAGTTATCCCCCTCCATC
AGGCAGTTTCCCAGACATTACTCACCCGTCCGCCACTCGTCAGCAAAGCAGCAAGCTGCTTTCTGTTACCGTT
CGACTGCATGTGTTAGGCCTGCCGCCAGCGTCAATCTGAGCCAGGATCAAACTCT
>348
CTGCTGCCGCCCGTAGGAGTCTGGACCGTGTCTCAGTTCCAGTGTGGCTGGTCATCCTCTCAGACCAGCTAGG
GATCGTCGCCTAGGTGAGCCGTTACCCCACCTACTAGCTAATCCCATCTGGGCACATCCGATGGCAAGAGGCC
CTAAGGTCCCCCTCTTTGGTCCTGCGACGTATGCGGTATTAGCTACCGTTTCCAGTAGTTATCCCCCTCCATC
AGGCAGTTTTCCCAGACATTACTCACCCGTCCGCCACTCGTCAGCAAAACAGCAAGCTGCTTCCTGTTACCGT
TCGACTGCATGTGTTAGGCCTGCCGCCAGCGTTCAATCTGAGCCAGGATCAAACTCT
>349
CTGCTGCCGCCCGTAGGAGTCTGGACCGTGTCTCAGTTCCAGTGTGGCTGGTCATCCTCTCAGACCAGCTAGG
GATCGTCGCCTAGGTGAGCCGTTACCCCACCTACTAGCTAATCCCATCTGGGCACATCCGATGGCAAGAGGCC
CTAAGGTCCCCCTCTTTGTCTTGCGACGTATGCGGTATTAGCTACCGTTTCCAGTAGTTATCCCCCTCCATCA
GGCAGTTTTCCCCAGACATTACTCACCCGTCCGCCACTCGTCAGCAAAACAGCAAGCTGCTTCCTGTTACCGT
TCGACTGCATGTGTTAGGCCTGCCGCCAGCGTCAATCTGAGCCAGGATCAAACTCT
>350
CTGCTGCCGCCCGTAGGAGTCTGGACCGTGTCTCAGTTCCAGTGTGGCTGGTCATCCTCTCAGACCAGCTAGG
GATCGTCGCCTAGGTGAGCCGTTACCCCACCTACTAGCTAATCCCATCTGGGCACATCCGATGGCAAGAGGCC
CTAAGGTCCCCCTCTTTGGTCTTGCGACGTTATGCGGTATTAGCTACCGTTTCCAGTAGTTATCCCCCTCCAT
CAGGCAGTTTTCCCAGACATTACTCACCCGTCCGCCACTCGTCAGCAAAGCAGCAAGCTGCTTTCTGTTACCG
TTCGACTGCATGTGTTAGGCCTGCCGCCAGCGTCAATCTGAGCCAGGATCAAACTCT
>351
CTGCTGCCGCCCGTAGGAGTCTGGACCGTGTCTCAGTTCCAGTGTGGCTGGTCATCCTCTCAGACCAGCTAGG
GATCGTCGCCTAGGTGAGCCGTTACCCCACCTACTAGCTAATCCCATCTGGGCACATCCGATGGCAAGAGGCC
CTAAGGTCCCCCTCTTGTCTGCGACGTATGCGGTATTAGCTACCGTTTCCAGTAGTTATCCCCCTCCATCGGG

CAGTTTCCCAGACATTACTCACCCGTCCGCCACTCGTCAGCAAAGCAGCAAGCTGCTTCCTGTTACCGTTCGA
CTGCATGTGTTAGGCCTGCCGCCAGCGTCAATCTGAGCCAGGATCAAACTCT
>352
CTGCTGCCGCCCGTAGGAGTCTGGACCGTGTCTCAGTTCCAGTGTGGCTGGTCATCCTCTCAGACCAGCTAGG
GATCGTCGCCTAGGTGAGCCGTTACCCCACCTACTAGCTAATCCCATCTGGGCACATCCGATGGCAAGAGGCC
CTAAGGTCCCCCTCTTTGGTCTTGCGACGTTATGCGGTATTAGCTACCGTTTCCAGTAGTTATCCCCCTCCAT
CAGGCAGTTTCCCAGACATTACTCACCCGTCCGCCACTCGTCAGCAAAGCAGCAAGCTGCTTTCTGTTACCGC
TCGACTGCATGTGTTAGGCCTGCCGCCAGCGTTCAATCTGAGCCAGGATCAAACTCT
>353
CTGCTGCCGCCCGTAGGAGTCTGGACCGTGTCTCAGTTCCAGTGTGGCTGGTCATCCTCTCAGACCAGCTAGG
GATCGTCGCCTAGGTGAGCCGTTACCCACCTACTAGCTAATCCCATCTGGGCACATCCGATGGCAAGAGGCCC
TAAGGTCCCCCTCTTGTCTGCGACGTATGCGGTATTAGCTACCGTTTCCAGTAGTTATCCCCTCCATCAGGCA
GTTTCCCAGACATTACTCACCCGTCCGCCACTCGTCAGCAAAGCAGCAAGCTGCTTCCTGTTACCGTCGACTT
GCATGTGTTAGGCCTGCCGCCAGCGTCAATCTGAGCCAGGATCAACTCT
>354
CTGCTGCCGCCCGTAGGAGTCTGGACCGTGTCTCAGTTTCCAGTGTGGCTGGTCATCCTCTCAGACCAGCTAG
GGATCGTCGCCTAGGTGAGCCGTTTACCCCACCTACTAGCTAATCCCATCTGGGCACATCCGATGGCAAGAGG
CCCTAAGGTCCCCCTCTTTGTCTTGCGACGTTATGCGGTATTAGCTACCGTTTCCAGTAGTTATTCCCCCTCC
ATCGGGCAGTTTTCCCAGACATTACTCACCCGTCCGCCACTCGTCAGCAAAGCAGCAAGCTGCTTCCTGTTAC
CGTTCGACTGCATGTGTTAGGCCTGCCGCCAGCGTCAATCTGAGCCAGGATCAAACTCT
>355
CTGCTGCCGCCCGTAGGAGTCTGGACCGTGTCTCAGTTCCAGTGTGGCTGGTCATCCTCTCAGACCAGCTAGG
GATCGTCGCCTAGGTGAGCCGTTACCCCACCTACTAGCTAATCCCATCTGGGCACATCCGATGGCAAGAGGCC
CTAAGGTCCCCCTCTTGTCTTGCGACGTATGCGGTATTAGCTACCGTTTCCAGTAGTTATCCCCCTCCATCGG
GCAGTTCCCAGACATTACTCACCCGTCCGCCACTCGTCAGCAAGCAGCAAGCTGCTTCCTGTTACCGTTCGAC
TGCATGTGTTAGGCCTGCCGCCAGCGTCAATCTGAGCCAGGATCAACTCT
>356
CTGCTGCCGCCCGTAGGAGTCTGGACCGTGTCTCAGTTCCAGTGTGGCTGGTCATCCTCTCAGACCGAGCTAG
GGATCGTCGCCGTAGGTGCAGCCTGTTACCCCACCTACTAGCTAATCCCATCTGGGCACATCCGATGGCAAGA
GGCCCTAAGGTCCCCCTCTTGTCTTGCGACGTATGCGGTATTAGCTACCGTTTCCAGTAGTTATCCCCCTCCA
TCAGGCAGTTTCCCAGACATTACTCACCCGTCCGCCACTCGTCAGCAAAGCAGCAAGCTGCTTCCTGTTACCG
TTCGACTGCATGTGTTAGGCCTGCCGCCAGCGTCAATCTGAGCCAGGATCAAACTCT
>357
CTGCTGCCGCCCGTAGGAGTCTGGACCGTGTCTCAGTTCCAGTGTGGCTGGTCATCCTCTCAGACCAGCTAGG
GATCGTCGCCTAGGTGAGCCGTTACCCCACCTACTAGCTAATCCCATCTGGGCACATCCGATGGCAAGAGGCC
CTAAGGTCCCCCTCTTTGTCTTGCGACGTTATGCGGTATTAGCTACCGTTTCCAGTAGTTATCCCCCTCCATC
GGGCAGTTTCCCAGACATTACTCGCCCGTCCGCCACTCGTCAGCAAAGCAGCAAGCTGCTTCCTGTTACCGTT
CGACTGCATGTGTTAGGCCTGCCGCCAGCGTCAATCTGAGCCAGGATCAAACTCT
>358
CTGCTGCCGCCCGTAGGAGTCTGGACCGTGTCTCAGTTCCAGTGTGGCTGGTCATCCTCTCAGACCAGCTAGG
GATCGTCGCCTAGGTGAGCCGTTACCCCACCTACTAGCTAATCCCATCTGGGCACATCCGATGGCAAGAGGCC
CTAAGGTCCCCCTCTTGTCTTGCGACGTATGCGGTATTAGCTACCGTTTCCAGTAGTTATCCCCCTCCATCAG
GCAGTTTCCCAGACATTACTCACCCGTCCGCCACTCGTCAGCAAAGCAGCAAGCTGCTTCCTGTTACCGTTCG
ACTGCATGTGTTAGGCCTGCCGCCAGCGTTCAATCTGAGCCAGGATCAAACTCT
>359
CTGCTGCCGCCCGTAGGAGTCTGGACCGTGTCTCAGTTCCAGTGTGGCTGGTCATCCTCTCAGACCAGCTAGG
GATCGTCGCCTAGGTGAGCCGTTACCCCACCTACTAGCTAATCCCATCTGGGCACATCCGATGGCAAGAGGCC
CTAAGGTCCCCCTCTTGTCTTGCGACGTTATGCGGTATTAGCTACCGTTCCAGTAGTTATCCCCCTCCATCAG
GCAGTTTCCCAGACATTACTCACCCGTCCGCCACTCGTCAGCAAAGCAGCAAGCTGCTTCTGTTACCGTTCGA
CTGCATGTGTTAGGCCTGCCGCCAGCGTTCAATCTGAGCCAGGATCAAACTCT
>360
CTGCTGCCGCCCGTAGGAGTCTGGACCGTGTCTCAGTTCCAGTGTGGCTGGTCATCCTCTCAGACCAGCTAGG
GATCGTCGCCTAGGTGAGCCGTTACCCCACCTACTAGCTAATCCCATCTGGGCACATCCGATGGCAAGAGGCC
CTAAGGTCCCCCTCTTTGTCTTGCGACGTTATGCGGTATTAGCTACCGTTTCCAGTAGTTATCCCCCTCCATC

GGGCAGTTTTCCCAGACATTACTCACCCGTCCGCCACTCGTCAGCAAAGCAGCAAGCTGCTTCCTGTTACCGT
TCGACTGCATGTGTTAGGCCTGCCGCCAGCGTCAATCTGAGCCAGGATCAAACTCT
>361
TGCTGCCGCCCGTAGGAGTCTGGACCGTGTCTCAGTTCCAGTGTGGCTGGTCATCCTCTCAGACCAGCTAGGG
ATCGTCGCCTAGGTGAGCCGTTACCCCACCTACTAGCTAATCCCATCTGGGCACATCCGATGGCAAGAGGCCC
GAAGGTCCCCCTCTTGGTCTTGCGACGTATGCGGTATTAGCTACCGTTTCCAGTAGTTATCCCCTCCATCGGG
CAGTTTCCCAGACATTACTCACCCGTCCGCCACTCGTCAGCAAAGCAGCAAGCTGCTTCCTGTTACCGTTCGA
CTGCATGTGTTAGGCCTGCCGCCAGCGTCAATCTGAGCCAGGATCAAACTCT
>362
CTGCTGCCGCCCGTAGGAGTCTGGACCGTGTCTCAGTTCCAGTGTGGCTGGTCATCCTCTCAGACCAGCTAGG
GATCGTCGCCTAGGTGAGCCGTTACCCCACCTACTAGCTAATCCCATCTGGGCACATCCGATGGCAAGAGGCC
CTAAGGTCCCCCTCTTGTCTTGCGACGTATGCGGTATTAGCTACCGTTTCCAGTAGTTATCCCCCTCCATCAG
GCAGTTTCCCAGACATTACTCACCCGTCCGCCACTCGTCAGCAAACAGCAAGCTGCTTCCTGTTACCGTTCGA
CTGCATGTGTTAGGCCTGCCGCCAGCGCTACACTCG
>363
CTGCTGCCGCCCGTAGGAGTCTGGACCGTGTCCCAGTTCCAGTGTGGCTGGTCATCCTTTCAGACCAGCTAGG
GATCGTCGCCTAGGTGAGCCGTTACCCCACCTACTAGCTAATCCCATCTGGGCACATCCGATGGCAAGAGGCC
CTAAGGTCCCCCTCTTTGTCTTGCGACGTATGCGGTATTAGCTACCGTTTCCAGTAGTTATCCCCCTCCATCA
GGCAGTTTCCCAGACATTACTCACCCGTCCGCCACTCGTCAGCAAAGCAGCAAGCTGCTTCCTGTTACCGTTC
GACTGCATGTGTTAGGCCTGCCGCCAGCGTTCAATCTGAGCCAGGATCAAACTCT
>364
CTGCTGCCGCCCGTAGGAGTCTGGACCGTGTCTCAGTTCCAGTGTGGCTGGTCATCCTCTCAGACCAGCTAGG
GATCGTCGCCTAGGTGAGCCGTTACCCCACCTACTAGCTAATCCCATCTGGGCACATCCGATGGCAAGAGGCC
CTAAGGTCCCCCTCTTGTCTTGCGACGTTATGCGGTATTAGCTACCGTTTCCAGTAGTTATCCCCCTCCATCG
GGCAGTTTCCCAGACATTACTCACCCGTCCGCCACTCGTCAGCAAAGCAGCAAGCTGCTTCCTGTTACCGTTC
GACTGCATGTGTTAGGCCTGCCGCCAGCGTCAATCTGAGCCAGGATCAAACTCT
>365
CTGCTGCCGCCCGTAGGAGTCTGGACCGTGTCTCAGTTCCAGTGTGGCTGGTCATCCTCTCAGACAGCTAGGG
ATCGTCGCCTAGGTGAGCCGTTACCCCACCTACTAGCTAATCCCATCTGGGCACATCCGATGGCAAGAGGCCC
TAAGGTCCCCCTCTTGTCTGCGACGTATGCGGTATTAGCTACCGTTCCAGTAGTTATCCCCCTCCATCAGGCA
GTTTCCCAGACATTACTCACCCGTCCGCCACTCGTCAGCAAGCAGCAAGCTGCTTCCTGTTACCGTCGACTGC
ATGTGTTAGGCCTGC
>366
CTGCTGCCGCCCGTAGGAGTCTGGACCGTGTCTCAGTTCCAGTGTGGCTGGTCATCCTCTCAGACCAGCTAGG
GATCGTCGCCTAGGTGAGCCGTTACCCCACCTACTAGCTAATCCCATCTGGGCACATCCGATGGCAAGAGGCC
CTAAGGTCCCCCTCTTTGGTCTTGCGACGTTATGCGGTATTAGCTACCGTTTCCAGTAGTTATCCCCCTCCAT
CAGGCAGTTTCCCAGACATTACTCACCCGTCCGCCACTCGTCAGCAAACAGCAAGCTGCTTCCTGTTACCGTT
CGACTGCATGTGTTAGGCCTGCCGCCAGCGTTCAATCTGAGCCAGGATCAAACTCT
>367
CTGCTGCCGCCCGTAGGAGTCTGGACCGTGTCTCAGTTCCAGTGTGGCTGGTCATCCTCTCAGACCAGCTAGG
GATCGTCGCCTAGGTGAGCCGTTACCCCACCTACTAGCTAATCCCATCTGGGCACATCCGATGGCAAGAGGCC
CTAAGGTCCCCCTCTTTGTCTGCGACGTATGCGGTATTAGCTACCGTTTCCAGTAGTTATCCCCCTCCATCGG
GCAGTTTCCCAGACATTACTCACCCGTCCGCCACTCTCAGCAAGCAGCAAGCTGCTTCCTGTTACCGTTCGAC
TGCATGTGTTAGGCCTGCCGCCAGCGTCAATCTGAGCCAGGATCAAACTCT
>368
CTGCTGCCCCGTCCAGAGGTCTGGACCGTGTCTCAGTTCCAGTGTGGCTGGTCATCCTCTCAGACCAGCTAGG
GATCGTCGCCTAGGTGAGCCGTTACCCCACCTACTAGCTAATCCCATCTGGGCACATCCGATGGCAAGAGGCC
CTAAGGCCCCCTCTTGTCTGCGACGTATGCGGTATTAGCTACCGTTTCCAGTAGTTATCCCCCTCATCGGGCA
GTTTCCCAGACATTACTCACCCGTCCGCCACTCGTCAGCAAAGCAGCAAGCTGCTTCCTGTTACCGTTCGACT
GCACGTGTTAGGCCTGCCGCCAGCGTCAATCTGAGCCGAGATCAAACTCT
>369
CTGCTGCCGCCCGTAGGAGTCTGGACCGTGTCTCAGTTCCAGTGTGGCTGGTCATCCTCTCAGACCAGCTAGG
GATCGTCGCCTAGGTGAGCCGTTTACCCCACCTACTAGCTAATCCCATCTGGGCACATCCCGATGGCAAGAGG
CCCTAAGGTCCCCCTCTTTGTCTGCGACGTATGCGGTATTAGCTACCGTTTTCCAGTAGTTATCCCCCTCCAT

CAGGCAGTTTCCCAGACATTACTCACCCGTCCGCCACTCGTCAGCAAGCAGCAAGCTGCTTCCTGTTACCGTT
CGACTGCATGTGTTAGGCCTGCCGCCAGCGTCAATCTAGCCAGGA
>370
CTGCTGCCGCCCGTAGGAGTCTGGACCGTGTCTCAGTTCCAGTGTGGCTGGTCATCCTCTCAGACCAGCTAGG
GATCGTCGCCTAGGTGAGCCGTTACCCCACCTACTAGCTAATCCCATCTGGGCACATCCGATGGCAAGAGGCC
CTAAGGTCCCCCTCTTTGGTCTGCGACGTATGCGGTATTAGCTACCGTTTCCAGTAGTTATCCCCCTCCATCA
GGCAGTTTCCCAGACATTACTCACCCGTCCGCCACTCGTCAGCAAACAGCAAGCTGCTTCCTGTTACCGTTCG
ACTGCATGTGTTAGGCCTGCCGCCAGCGTCAATCTGAGCCAGGATCAAACTCT
>371
CTGCTGCCGCCCGTAGGAGTCTGGACCGTGTCTCAGTTCCAGTGTGGCTGGTCATCCTCTCAGACCAGCTAGG
GATCGTCGCCTAGGTGAGCCGTTACCCCACCTACTAGCTAATCCCATCTGGGCACATCCGATGGCAAGAGGCC
CTAAGGTCCCCCTCTTTGTCTTGCGACGTTATGCGGTATTAGCTACCGTTTCCAGTAGTTATCCCCCTCCATC
AGGCAGTTTTCCCAGACATTACTCACCCGTCCGCCACTCGTCAGCAAAGCAGCAAGCTGCTTCCTGTTACCGT
TCGACTGCATGTGTTAGGCCTGCCGCCAGCGTTCAATCTGAGCCAGGATCAAACTCT
>372
CTGCTGCCGCCCGTAGGAGACTGGACCGTGTCTCAGTTCCAGTGTGGCTGGTCATCCTCTCAGACCAGCTAGG
GATCGTCGCCTAGGTGAGCCGTTACCCCACCTACTAGCTAATTCCCATCTGGGCACATCCGATGGCAAGAGGC
CCTAAGGTCCCCCTCTTTGTCTTGCGACGTTATGCGGTATTAGCTACCGTTTCCAGTAGTTATCCCCCTCCAT
CAGGCAGTTTCCCAGACATTACTCACCCGTCCGCCACTCGTCAGCAAAGCAGCAAGCTGCTTCTGTTACCGTT
CGACTGCATGTGTTAGGCCTGCCGCCAGCGTTCAATCGAGCCAGGATCAAACTCT
>373
CTGCTGCCGCCCGTAGGAGTCTGGACCGTGTCTCAGTTCCAGTGTGGCTGGTCATCCTCTCAGACCAGCTAGG
GATCGTCGCCTAGGTGAGCCGTTACCCCACCTACTAGCTAATCCCATCTGGGCACATCCGATGGCAAGAGGCC
CTAAGGTCCCCCTCTTGTCTGCGACGTTATGCGGTATTAGCTACCGTTTCCAGTAGTTATCCCCTCCATCGGG
CAGTTTCCCAGACATTACTCACCCGTCCGCCACTCGTCAGCAAGCAGCAAGCTGCTTCCTGTTACCGTTCGAC
TGCATGTGTTAGGCCTGCCGCCAGCGTCAATCTGAGCCAGGATCAACTCT
>374
CTGCTGCCGCCCGTAGGAGTCTGGACCGTGTCTCAGTTCCAGTGTGGCTGGTCATCCTCTCAGACCAGCTAGG
GATCGTCGCCTAGGTGAGCCGTTACCCCACCTACTAGCTAATCCCATCTGGGCACATCCGATGGCAAGAGGCC
CTAAGGTCCCCCTCTTGTCTGCGACGTATGCGGTATTAGCTACCGTTTCCAGTAGTTATCCCCCTCCATCGGG
CAGTTTCCCAGACATTACTCACCCGTCCGCCACTCGTCAGCAAAGCAGCAAGCTGCTTCCTGTTACCGTTCGA
CTGCATGTGTTAGGCCTGCCGCCAGCGTCAATCTGAGCCAGGATCAACTCT
>375
CTGCTGCCGCCCGTAGGAGTCTGGACCGTGTCTCAGTTCCAGTGTGGCTGGTCATCCTCTCAGACCAGCTAGG
GATCGTCGCCTAGGTGAGCCGTTACCCCACCTACTAGCTAATCCCATCTGGGCACATCCGATGGCAAGAGGCC
CTAAGGTCCCCCTCTTTGTCTGCGACGTATGCGGTATTAGCTACCGTTTCCAGTAGTTATCCCCCTCCATCAG
GCAGTTTCCCAGACATTACTCACCCGTCCGCCACTCGTCAGCAAACAGCAAGCTGCTTCCTGTTACCGTTCGA
CTGCATGTGTTAGGCCTGCCGCCAGCGTCAATCTG
>376
CTGCTGCCGCCCGTAGGAGTCTGGACCGTGTCTCAGTTCCAGTGTGGCTGGTCATCCTCTCAGACCAGCTAGG
GATCGTCGCCTAGGTGAGCCGTTACCCCACCTACTAGCTAATCCCATCTGGGCACATCCGATGGCAAGAGGCC
CTAAGGTCCCCCTCTTTGTCTTGCGACGTATGCGGTATTAGCTACCGTTTCCAGTAGTTATCCCCCTCCATCA
GGCAGTTTTCCCAGACATTACTCACCCGTCCGCCACTCGTCAGCAAAGCAGCAAGCTGCTTCCTGTTACCGTT
CGACTGCATGTGTTAGGCCTGCCGCCAGCGTCAATCTGAGCCAGGATCAAACTCT
>377
CTGCTGCCGCCCGTAGGAGTCTGGACCGTGTCTCAGTTCCAGTGTGGCTGGTCATCCTCTCAGACCAGCTAGG
GATCGTCGCCTAGGTGAGCCGTTACCCCACCTACTAGCTAATCCCATCTGGGCACATCCGATGGCAAGAGGCC
CTAAGGTCCCCCTCTTGTCTTGCGACGTATGCGGTATTAGCTACCGTTTCCAGTAGTTATCCCCTCCATCGGG
CAGTTTCCCAGACATTACTCACCCGTCCGCCACTCGTCAGCAAAGCAGCAAGCTGCTTCCTGTTACCGTTCGA
CTGCATGTGTTAGGCCTGCCGCCAGCGTCAATCTGAGCCAGGATCAAACTCT
>378
CTGCTGCCGCCCGTAGGAGTCTGGACCGTGTCTCAGTTCCAGTGTGGCTGGTCATCCTCTCAGACCAGCTAGG
GATCGTCGCCTAGGTGAGCCGTTACCCCACCTACTAGCTAATCCCATCTGGGCACATCCGATGGCAAGAGGCC
CTAAGGTCCCCCTCTTTGTCTGCGACGTATGCGGTATTAGCTACCGTTTCCAGTAGTTATCCCCCTCCATCAG

GCAGTTTCCCAGACATTACTCACCCGTCCGCCACTCGTCAGCAAACAGCAAGCTGCTTCCTGTTACCGTTCGA
CTGCATGTGTTAGGCCTGCCGCCAGCGTCAATCTGAGCCAGGATCAAACTCT
>379
CTGCTGCCGCCCGTAGGAGTCTGGACCGTGTCTCAGTTCCAGTGTGGCTGGTCATCCTCTCAGACCAGCTAGG
GATCGTCGCCTAGGTGAGCCGTTACCCCACCTACTAGCTAATCCCATCTGGGCACATCCGATGGCAAGAGGCC
CTAAGGTCCCCCTCTTGGTCTTGCGACGTATGCGGTATTAGCTACCGTTTCCAGTAGTTATCCCCCTCCATCA
GGCAGTTTCCCAGACATTACTCACCCGTCCGCCACTCGTCAGCAAAGCAGCAAGCTGCTTCCTGTTACCGTTC
GACTGCATGTGTTAGGCCTGCCGCCAGCGTCAATCTGAGCCAGGATCAAACTCT
>380
CTGCTGCCGCCCGTAGGAGTCTGGACCGTGTCTCAGTTCCAGTGTGGCTGGTCATCCTCTCAGACCAGCTAGG
GATCGTCGCCTAGGTGAGCCGTTACCCCACCTACTAGCTAATCCCATCTGGGCACATCCGATGGCAAGAGGCC
CTAAGGTCCCCCTCTTGTCTTGCGACGTATGCGGTATTAGCTACCGTTTCCAGTAGTTATCCCCCTCCATCGG
GCAGTTTCCCAGACATTACTCACCCGTCCGCCACTCGTCAGCAAAGCAGCAAGCTGCTTCCTGTTACCGTTCG
ACTCGCATGTGTTAGGCCTGCCGCCAGCGTCAATCTGAGCCAGGATCAAACTCT
>381
CTGCTGCCGCCCGTAGGAGTCTGGACCGTGTCTCAGTTCCAGTGTGGCTGGTCATCCTCTCAGACCAGCTAGG
GATCGTCGCCTAGGTGAGCCGTTACCCCACCTACTAGCTAATCCCATCTGGGCACATCCGATGGCAAGAGGCC
CTAAGGTCCCCCTCTTGTCTTGCGACGTATGCGGTATTAGCTACCGTTTCCAGTAGTTATCCCCCTCCATCAG
GCAGTTTCCCAGACATTACTCACCCGTCCGCCACTCGTCAGCAAAGCAGCAAGCTGCTTCCTGTTACCGTTCG
ACTGCATGTGTTAGGCCTGCCGCCAGCGTCAATCTGAGCCAGGATCAAACTCT
>382
CTGCTGCCGCCCGTAGGAGTCTGGACCGTGTCTCAGTTCCAGTGTGGCTGGTCATCCTCTCAGACCAGCTAGG
GATCGTCGCCTAGGTGAGCCGTTACCCCACCTACTAGCTAATCCCATCTGGGCACATCCCGATGGCAAGAGGC
CCTAAGGTCCCCCTCTTGTCTGCGACGTATGCGGTATTAGCTACCGTTTCCAGTAGTTATCCCCCTCCATCAG
GCAGTTTCCCAGACATTACTCACCGTCCGCCACTCGTCAGCAAACAGCAAGCTGCTTCCTGTTACCGTTCGAC
TGCATGTGTTAGGCCTGACCGCCA
>383
CTGCTGCCGCCCGTAGGAGTCTGGACCGTGTCTCAGTTCCAGTGTGGCTGGTCATCCTCTCAGACCAGCTAGG
GATCGTCGCCTAGGTGAGCCGTTACCCCACCTACTAGCTAATCCCATCTGGGCACATCCGATGGCAAGAGGCC
CTAAGGTCCCCCTCTTGTCTTGCGACGTATGCGGTATTAGCTACCGTTTCCAGTAGTTATCCCCCTCCATCAG
GCAGTTTCCCAGACATACTCACCGTCCGCCACTCGTCAGCAAAGCAGCAAGCTGCTTCTGTTACCGTTCGACT
GCATGTGTTAGGCCTGCCGCCAGCGTCAACTCT
>384
CTGCTGCCGCCCGTAGGAGTCTGGACCGTGTCTCAGTTCCAGTGTGGCTGGTCATCCTCTCAGACCAGCTAGG
GATCGTCGCCTAGGTGAGCCGTTACCCCACCTACTAGCTAATCCCATCTGGGCACATCCGATGGCAAGAGGCC
CTAAGGTCCCCCTCTTTGTCTTGCGACGTTATGCGGTATTAGCTACCGTTTCCAGTAGTTATCCCCCTCCATC
AGGCAGTTTCCCAGACATTACTCACCCGTCCGCCACTCGTCAGCAAAACAGCAAGCTGCTTCCTGTTACCGTT
CGACTGCATGTGTTAGGCCTGCCGCCAGCGTCAATCTGAGCCAGGATCAAACTCT
>385
CTGCTGCCGCCCGTAGGAGTCTGGACCGTGTCTCAGTTCCAGTGTGGCTGGTCATCCTCTCAGACCAGCTAGG
GATCGTCGCCTAGGTGAGCCGTTACCCCACCTACTAGCTAATCCCATCTGGGCACATCCGATGGCAAGAGGCC
CTAAGGTCCCCCTCTTTGTCTTGCGACGTTATGCGGTATTAGCTACCGTTTCCAGTAGTTATCCCCCTCCATC
AGGCAGTTTCCCAGACATTACTCACCCGTCCGCCACTCGTCAGCAAAACAGCAAGCTGCTTCCTGTTACCGTT
CGACTGCATGTGTTAGGCCTGCCGCCAGCGTCAATCTGAGCCAGGATCAAACTCT
>386
CTGCTGCCGCCCGTAGGAGTCTGGACCGTGTCTCAGTTCCAGTGTGGCTGGTCATCCTCTCAGACCAGCTAGG
GATCGTCGCCTAGGTGAGCCGTTACCCCACCTACTAGCTAATCCCATCTGGGCACATCCGATGGCAAGAGGCC
CTAAGGTCCCCCTCTTGGTCTGCGACGTATGCGGTATTAGCTACCGTTCCAGTAGTTATCCCCGTCCATCGGG
CAGTTTCCCAGACATTACTCACCCTCCGCCACTCGTCAGCAAAGCAGCAAGCTGCTTCCTGTTACCGTTCGAC
TGCATGTGTTAGGCCTGCCGCCAGCGTCAATCTGAGCCAGGATCAAACTCT
>387
CTGCTGCCGCCCGTAGGAGTCTGGACCGTGTCTCAGTTCCAGTGTGGCTGGTCATCCTCTCAGACCAGCTAGG
GATCGTCGCCTAGGTGAGCCGTTACCCCACCTACTAGCTAATCCCATCTGGGCACATCCGATGGCAAGAGGCC
CTAAGGTCCCCCTCTTTGTCTTGCGACGTTATGCGGTATTAGCTACCGTTTCCAGTAGTTATCCCCCTCCATC

AGGCAGTTTCCCAGACATTACTCACCCGTCCGCCACTCGTCAGCAAAGCAGCAAGCTGCTTCCTGTTACCGTT
CGACTGCATGTGTTAGGCCTGCCGCCAGCGTTCAATCTGAGCCAGGATCAAACTCT
>388
CTGCTGCCGCCCGTAGGAGTCTGGACCGTGTCTCAGTTCCAGTGTGGCTGGTCATCCTCTCAGACCAGCTAGG
GATCGTCGCCTAGGTGAGCCGTTACCCCACCTACTAGCTAATCCCATCTGGGCACATCCGATGGCAAGAGGCC
CTAAGGTCCCCCTCTTTGTCTTGCGACGTTATGCGGTATTAGCTACCGTTTCCAGTAGTTATTCCCCTCCATC
GGGCAGTTTCCCAGACATTACTCACCCGTCCGCCACTCGTCAGCAAAGCAGCAAGCTGCTTCCTGTTACCGTT
CGACTTGCATGTGTTAGGCCTGCCGCCAGCGTCAACCTGAGCCAGGATCGAAACTCT
>389
CTGCTGCCGCCGTAGGAGTCTGGACCGTGTCTCAGTTCCAGTGTGGCTGGTCATCCTCTCAGACCAGCTAGGG
ATCGTCGCCTAGGTGAGCCGTTACCCCACCTACTAGCTAATCCCATCTGGGCACATCCGATGGCAAGAGGCCC
TAAGGTCCCCCTCTTTGGTCTTGCGACGTTATGCGGTATTAGCTACCGTTTCCAGTAGTTATCCCCCTCCATC
AGGCAGTTTCCCAGACATTACTCACCCGTCCGCCACTCGTCAGCAAAGCAGCAAGCTGCTTTCTGTTACCGTT
CGACTGCATGTGTTAGGCCTGCCGCCAGCGTCAATCTGAGCCAGGATCAAACTCT
>390
CTGCTGCCGCCCGTTGGAGTCTGGACCGTGTCTCAGTTCCAGTGTGGCTGGTCATCCTCTCAGACCAGCTAGG
GATCGTCGCCTAGGTGAGCCGTTACCCCACCTACTAGCTAATCCCATCTGGGCACATCCGATGGCAAGAGGCC
CTAAGGTCCCCCTCTTTGTCTGCGACGTATGCGGTATTAGCTACCGTTTCCAGTAGTTATCCCCCTCCATCGG
GCAGTTTCCCAGACATTACTCACCCGTCCGCCACTCGTCAGCAAAGCAGCAAGCTGCTTCCTGTTACCGTTCG
ACTGCATGTGTTAGGCCTGCCGCCAGCGTCAATCTGAGCCAGGATCAAACTCT
>391
CTGCTGCCGCCCGTAGGAGTCTGGACCGTGTCTCAGTTCCAGTGTGGCTGGTCATCCTCTCAGACCAGCTAGG
GATCGTCGCCTAGGTGAGCCGTTACCCCACCTACTAGCTAATCCCATCTGGGCACATCCGATGGCAAGAGGCC
CTAAGGTCCCCCTCTTTGTCTTGCGACGTTATGCGGTATTAGCTACCGTTTCCAGTAGTTATCCCCCTCCATC
AGGCAGTTTTCCCAGACATTACTCACCCGTCCGCCACTCGTCAGCAAAGCAGCAAGCTGCTTCCTGTTACCGT
TCGACTGCATGTGTTAGGCCTGCCGCCAGCGTTCAATCTGAGCCAGGATCAAACTCT
>392
CTGCTGCCGCCCGTAGGAGTCTGGACCGTGTCTCAGTTCCAGTGTGGCTGGTCATCCTCTCAGACCAGCTAGG
GATCGTCGCCTAGGTGAGCCGTTACCCCACCTACTAGCTAATCCCATCTGGGCACATCCGATGGCAAGAGGCC
CTAAGGTCCCCCTCTTTGTCTTGCGACGTATGCGGTATTAGCTACCGTTTCCAGTAGTTATCCCCCTCCATCA
GGCAGTTTCCCAGACATTACTCACCCGTCCGCCACTCGTCAGCAAAGCAGCAAGCTGCTTCCTGTTACCGTTC
GACTGCATGTGTTAGGCCTGCCGCCAGCGTTCAATCTGAGCCAGGATCAAACTCT
>393
CTGCTGCCGCCCGTAGGAGTCTGGACCGTGTCTCAGTTCCAGTGTGGCTGGTCATCCTCTCAGACCAGCTAGG
GATCGTCGCCTAGGTGAGCCGTTACCCCACCTACTAGCTAATCCCATCTGGGCACATCCGATGGCAAGAGGCC
CTAAGGTCCCCCTCTTGTCTGCGACGTTATGCGGTATTAGCTACCGTTCCAGTAGTTATCCCCTCCATCGGGC
AGTTTCCCAGACATTACTCACCCGTCCGCCACTCGTCAGCAAAGCAGCAAGCTGCTTCCTGTTACCGTTCGAC
TGCATGTGTTAGGCCTGCCGCCAGCGTCAATCTGAGCCAGGATCAAACTCT
>394
CTGCTGCCGCCCGTAGGAGTCTGGACCGTGTCTCAGTTCCAGTGTGGCTGGTCATCCTCTCAGACCAGCTAGG
GATCGTCGCCTAGGTGAGCCGTTACCCCCACCTACTAGCTAATTCCCATCTGGGCACATCCGATTGGCAAGAG
GCCCCTAAGGTCCCCCTCTTTGTCTTGCGACGTTATGCGGTATTAGCTTACCGTTTCCAGTAGTTATCCCCTT
CCCATCAGGCAGTTTCCCCAGACATTACTCACCCGTCCGCCACTCGTCAGCAAAGCAGCAAGCTGCTTTCCTG
TTTACCCGTTCGACTTGCATGTGTTAGGCCTGCCGCCAGCGTTCAATCTGAGCCAGGATCAAACTCT
>395
CTGCTGCCGCCCGTAGGAGTCTGGACCGTGTCTCAGTTCCAGTGTGGCTGGTCATCCTCTCAGACCAGCTAGG
GATCGTCGCCTAGGTGAGCCGTTTACCCCACCTACTAGCTAATCCCATCTGGGCACATCCGATGGCAAGAGGC
CCTAAGGTCCCCCTCTTTGTCTTGCGACGTATGCGGTATTAGCTACCGTTTCCAGTAGTTATCCCCCTCCATC
AGGCAGTTTCCCAGACATTACTCACCCGTCCGCCACTCGTCAGCAAAGCAGCAAGCTGCTTCCTGTTACCGTT
CGACTGCATGTGTTAGGCCTGCCGCCAGCGTTCAATCTGAGCCAGGATCAAACTCT
>396
CTGCTGCCGCCCGTAGGAGTCTGGACCGTGTCTCAGTTCCAGTGTGGCTGGTCATCCTCTCAGACCAGCTAGG
GATCGTCGCCTAGGTGAGCCGTTACCCCACCTACTAGCTAACCCCATCTGGGCACATCCGATGGCAAGAGGCC
CTAAGGTCCCCCTCTTTGGTCTTGCGACGTTATGCGGTATTAGCTACCGTTTCCAGTAGTTATCCCCCTCCAT

CAGGCAGTTTCCCAGACATTACTCACCCGTCCGCCACTCGTCAGCAAAGCAGCAAGCTGCTTCCTGTTACCGT
CCGACTGCATGTGTTAGGCCTGCCGCCAGCGTTCAATCTGAGCCAGGATCAAACTCT
>397
CTGCCGCCCGTAGGAGTCTGGACCGTGTCTCAGTTCCAGTGTGGCTGGTCGTCCTCTCAGACCAGCTAGGGAT
CGTCGCCTAGGTGAGCCGTTACCCCACCTACTAGCTAATCCCATCTGGGCACATCCGATGGCAAGAGGCCCTA
AGGTCCCCCTCTTTGTCTTGCGACGTTATGCGGTATTAGCTACCGTTTCCAGTAGTTATCCCCCTCCATCGGG
CAGTTTCCCAGACATTACTCACCCGTCCGCCACTCGTCAGCAAAGCAGCAAGCTGCTTCCTGTTACCGTTCGA
CTGCATGTGTTAGGCCTGCCGCCAGCGTCAATCTGAGCCAGGATCAAACTCT
>398
CTGCTGCCGCCCGTAGGAGTCTGGACCGTGTCTCAGTTCCAGTGTGGCTGGTCATCCTCTCAGACCAGCTAGG
GATCGTCGCCTAGGTGAGCCGTTACCCCACCTACTAGCTAATCCCATCTGGGCACATCCGATGGCAAGAGGCC
CTAAGGTCCCCCCTCTTTGTCTTGCGACGTTATGCGGTATTAGCTACCGTTTCCAGTAGTTATCCCCCTCCAT
CAGGCAGTTTCCCAGACATTACTCACCCGTCCGCCACTCGTCAGCAAAACAGCAAGCTGCTTCCTGTTACCGT
TCGACTTGCATGTGCTAGGCCTGCCGCCAGCGTCAATCTGAGCCAGGATCAAACTCT
>399
CTGCTGCCGCCCGTAGGAGTCTGGACCGTGTCTCAGTTCCAGTGTGGCTGGTCATCCTCTCAGACCAGCTAGG
GATCGTCGCCTAGGTGAGCCGTTACCCCACCTACTAGCTAATCCCATCTGGGCACATCCGATGGCAAGAGGCC
CTAAGGTCCCCCTCTTTGGTCTTGCGACGTTATGCGGTATTAGCTACCGTTTCCAGTAGTTATCCCCCTCCAT
CGGGCAGTTTCCCAGACATTACTCACCCGTCCGCCACTCGTCAGCAAAGCAGCAAGCTGCTTCCTGTTACCGT
TCGACTTGCATGTGTCAGGCCTGCCGCCAGCGTCAATCTGAGCCAGGATCAAACTCT
>400
CTGCTGCCGCCCGTAGGAGTCTGGACCGTGTCTCAGTTCCAGTGTGGCTGGTCATCCTCTCAGACCAGCTAGG
GATCGTCGCCTAGGTGAGCCGTTTACCCCACCTACTAGCTAATCCCATCTGGGCACATCCGATGGCAAGAGGC
CCTAAGGTCCCCCTCTTTGTCTTGCGACGTTATGCGGTATTAGCTACCGTTTCCAGTAGTTATCCCCCTCCAT
CAGGCAGTTTCCCAGACATTACTCACCCGTCCGCCACTCGTCAGCAAAGCAGCAAGCTGCTTCCTGTTACCGT
TCGACTTGCATGTGTTAGGCCTGCCGCCAGCGTTCAATCTGAGCCAGGATCAAACTCT
>401
TGCTGCCGCCCGTAGGAGTCTGGACCGTGTCTCAGTTTCCAGTGTGGCTGGTCATCCTCTCAGACCAGCTAGG
GATCGTCGCCTAGGTGAGCCGTTACCCCACTACTAGCTAATCCCATCTGGGCACATCCGATGGCAAGAGGCCC
TAAGGTCCCCCTCTTTGGTCTTGCGACGTTATGCGGTATTAGCTACCGTTTCCAGTAGTTATCCCCCTCCATC
AGGCAGTTTCCCAGACATTACTCACCCGTCCGCCACTCGTCAGCAAAGCAGCAAGCTGCTTTCTGTTTACCGT
TCGACTGCATGTGTTAGGCCTGCCGCCAGCGTTCAATCTGAGCCAGGATCAAACTCT
>402
CTGCTGCCGCCCGTAGGAGTCTGGACCGTGTCTCAGTTCCAGTGTGGCTGGTCATCCTCTCAGACCAGCTAGG
GATCGTCGCCTAGGTGAGCCGTTACCCCACCTACTAGCTAATCCCATCTGGGCACATCCGATGGCAAGAGGCC
CTAAGGTCCCCCTCTTGGTCTTGCGACGTATGCGGTATTAGCTACCGTTTCCAGTAGTTATCCCCCTCCATCG
GGCAGTTTCCCAGACATTACTCACCCGTCCGCCACTCGTCAGCAAAGCAGCAAGCTGCTTCCTGTTACCGTTC
GACTGCATGTGTTAGGCCTGCCGCCAGCGTCAATCTGAGCCAGGATCAAACTCT
>403
CTGCTGCCGCCCGTAGGAGTCTGGACCGTGTCTCAGTTCCAGTGTGGCTGGTCATCCTCTCAGACCAGCTAGG
GATCGTCGCCTAGGTGAGCCGTTACCCCACCTACTAGCTAATCCCATCTGGGCACATCCGATGGCAAGAGGCC
CTAAGGTCCCCCTCTTTGGTCTTGCGACGTATGCGGTATTAGCTACCGTTTCCAGTAGTTATCCCCCTCCATC
GGGCAGTTTCCCAGACATTACTCACCCGTCCGCCACTCGTCAGCAAAGCAGCAAGCTGCTTCCTGTTACCGTT
CGACTGCATGTGTTAGGCCTGCCGCCAGCGTTCAATCTGAGCCAGGATCAAACTCT
>404
CTGCTGCCGCCCGTAGGAGTCTGGACCGTGTCTCAGTTCCAGTGTGGCTGGTCATCCTCTCAGACCAGCTAGG
GATCGTCGCCTAGGTGAGCCGTTTACCCCACCTACTAGCTAATCCCATCTGGGCACATCCGATGGCAAGAGGC
CCTAAGGTCCCCCTCTTTGGTCTTGCGACGTATGCGGTATTAGCTACCGTTTCCAGTAGTTATCCCCCTCCAT
CAGGCAGTTTCCCAGACATTACTCACCCGTCCGCCACTCGTCAGCAAAGCAGCAAGCTGCTTCCTGTTACCGT
TCGACTGCATGTGTTAGGCCTGCCGCCAGCGTCAATCTGAGCCAGGATCAAACTCT
>405
CTGCTGCCGCCCGTAGGAGTCTGGACCGTGTCTCAGTTCCAGTGTGGCTGGTCATCCTCTCAGACCAGCTAGG
GATCGTCGCCTAGGTGAGCCGTTACCCACCTACTAGCTAATCCCATCTGGGCACATCCGATGGCAAGAGGCCC
TAAGGTCCCCCTCTTGTCTTGCGACGTTATGCGGTATTAGCTACCGTTTCCAGTAGTTATCCCCCTCCATCAG

GCAGTTTCCCAGACATTACTCACCCGTCCGCCACTCGTCAGCAAAGCAGCAAGCTGCTTCCTGTTACCGTTCG
ACTGCATGTGTTAGGCCTGCCGCCAGCCGTTCAATCTGAGCCAGGACA
>406
CTGCTGCCGCCCGTAGGAGTCTGGACCGTGTCTCAGTTCCAGTGTGGCTGGTCATCCTCTCAGACCAGCTAGG
GATCGTCGCCTAGGTGAGCCGTTACCCCACCTACTAGCTAATCCCATCTGGGCACATCCGATGGCAAGAGGCC
CTAAGGTCCCCCTCTTTGTCTTGCGACGTATGCGGTATTAGCTACCGTTTCCAGTAGTTATCCCCCTCCATCA
GGCAGTTTCCCAGACATTACTCACCCGTCCGCCACTCGTCAGCAAAGCAGCAAGCTGCTTCTGTTACCGTTCG
ACTGCATGTGTTAGGCCTGCCGCCAGCGTTCAATCTGAGCCAGGATCAAACTCT
>407
CTGCTGCCGCCCGTAGGAATCTGGACCGTGTCTCAGTTCCAGTGTGGCTGGTCATCCTCTCAGACCAGCTAGG
GATCGTCGCCTAGGTGAGCCGTTACCCCACCTACTAGCTAATCCCATCTGGGCACATCCGATGGCAAGAGGCC
CTAAGGTCCCCCTCTTTGTCTTGCGACGTATGCGGTATTAGCTACCGTTTCCAGTAGTTATCCCCCTCCATCA
GGCAGTTTCCCAGACATTACTCACCCGTCCGCCACTCGTCAGCAAGCAGCAAGCTGCTTCTGTTACCGTTCGA
CTGCATGTGTTAGGCCTGCCGCCAGCGTCAATCTGAGCCAGGATCAAACTCT
>408
CTGCTGCCGCCCGTAGGAGTCTGGACCGTGTCTCAGTTCCAGTGTGGCTGGTCATCCTCTCAGACCAGCTAGG
GATCGTCGCCTAGGTGAGCCGTTACCCCACCTACTAGCTAATCCCCATCTGGGCACATCCGATGGCAAGAGGC
CCTAAGGTCCCCCTCTTGTCTGCGACGTATGCGGTATTAGCTACCGTTTCCAGTAGTTATCCCCCTCCATCGG
GCAGTTTCCCAGACATTACTCACCCGTCCGCCACTCGTCAGCAAGCAGCAAGCTGCTTCCTGTTACCGTTCGA
CTGCATGTGTTAGGCCTGCCGCCAGCGTCAATCTGAGCCAGGATCAAACTCT
>409
CTGCTGCCGCCCGTAGGAGTCTGGACCGTGTCTCAGTTCCAGTGTGGCTGGTCATCCCCTCAGACCAGCTAGG
GATCGTCGCCTAGGTGAGCCGTTACCCCACCTACTAGCTAATCCCATCTGGGCACATCCGATGGCAAGAGGCC
CTAAGGTCCCCCTCTTTGTCTTGCGACGTTATGCGGTATTAGCTACCGTTTCCAGTAGTTATCCCCCTCCATC
GGGCAGTTTTCCCAGACATTACTCACCCGTCCGCCACTCGTCAGCAAAGCAGCAAGCTGCTTCCTGTTACCGT
TCGACTGCATGTGTTAGGCCTGCCGCCAGCGTCAATCTGAGCCAGGATCAAACTCT
>410
TGCTGCCGCCCGTAGGAGTCTGGACCGTGTCTCAGTTCCAGTGTGGCTGGTCATCCTCTCAGACCAGCTAGGG
ATCGTCGCCTAGGTGAGCCGTTACCCCACCTACTAGCTAATCCCATCTGGGCACATCCGATGGCAAGAGGCCC
TAAGGTCCCCCCTTGTCTTGCGACGTATGCGGTATTAGCTACCGTTTCCAGTAGTTATCCCCCTCCATCAGGC
AGTTTCCCAGACATTACTCACCCGTCCGCCACTCGTCAGCAAAGCAGCAAGCTGCTTCCTGTTACCGTTCGAC
TGCATGTGTTAGGGCCTGACCGCCAGCGTTCAATCTGAGCCAGGATCAAACTCT
>411
CTGCTGCCGCCCGTAGGAGTCTGGACCGTGTCTCAGTTCCAGTGTGGCTGGTCATCCTCTCAGACCAGCTAGG
GATCGTCGCCTAGGTGAGCCGTTACCCCACCTACTAGCTAATCCCATCTGGGCACATCCGATGGCAAGAGGCC
CTAAGGTCCCCCTCTTGTCTGCGACGTATGCGGTATTAGCTACCGTTTCCAGTAGTTATCCCCCTCCATCGGG
CAGTTTCCCAGACATTACTCACCCGTCCGCCACTCGTCAGCAAAGCAGCAAGCTGCTTCCTGTTACCGTTCGA
CTGCATGTGTTAGGCCTGCCGCCAGCGTCAATCTGAGCCAGGATCAAACTCT
>412
CTGCTGCCGCCCGTAGGAGTCTGGACCGTGTCTCAGTTCCAGTGTGGCTGGTCATCCTCTCAGCCCCCCAGCT
AGGGATCGTCGCCTAGGTGAGCCGTTACCCCACCTACTAGCTAATCCCATCTGGGCACATCCGATGGCAAGAG
GCCCTAAGGTCCCCCTCTTGTCTTGCGACGTATGCGGTATTAGCTACCGTTTCCAGTAGTTATCCCCCTCCAT
CAGGCAGTTTCCCAGACATTACTCACCCGTCCGCCACTCGTCAGCAAAGCAGCAAGCTGCTTTCTGTTACCGT
TCGACTTGCATGTGTTAGGCCTGCCGCCAGCGTCAATCTGAGCCAGGATCAAACTCT
>413
CTGCTGCCGCCCGTAGGAGTCTGGACCGTGTCTCAGTTCCAGTGTGGCTGGTCATCCTCTCAGACCAGCTAGG
GATCGTCGCCTAGGTGAGCCGTTACCCCACCTACTAGCTAATCCCATCTGGGCACATCCGATGGCAAGAGGCC
CTAAGGTCCCCCTCTTTGGTCTTGCGACGTTATGCGGTATTAGCTACCGTTTCCAGTAGTTATCCCCCTCCAT
CGGGCAGTTTTCCCAGACATTACTCACCCGTCCGCCACTCGTCAGCAAAGCAGCAAGCTGCTTCCTGTTACCG
TTCGACTGCATGTGTTAGGCCTGCCGCCAGCGTCAATCTGAGCCAGGATCAAACTCT
>414
CTGCTGCCGCCCGTAGGAGTCTGGACCCGTGTCTCAGTTCCAGTGTGGCTGGTCATCCTCTCAGACCAGCTAG
GGATCGTCGCCTAGGTGAGCCGTTTACCCCACCTACTGGCTAATCCCATCTGGGCACATCCGATGGCAAGAGG
CCCCTAAGGTCCCCTCTTGTCTGCGACGTATGCGGTATTAGCTACCGTTTCCAGTAGTTATCCCCCTTCCATC

AGGCAGTTTTCCCAGACATTACTCACCCGTCCGCCACTCGTCAGCAAAGCAGCAAGCTGCTTCCTGTTACCGT
TCGACTGCATGTGTTAGGCCTGCCGCCAGCGTCAATCTGAGCCAGGATCAAACTCT
>415
CTGCTGCCGCCCGTAGGAGTCTGGACCGTGTCTCAGTTCCAGTGTGGCTGGTCATCCTCTCAGACCAGCTAGG
GGTCGTCGCCTAGGTGAGCCGTTACCCCACCTACTAGCTAATCCCATCTGGGCACATCCGATGGCAAGAGGCC
CTAAGGTCCCCCTCTTTGTCTGCGACGTTATGCGGTATTAGCTACCGTTTCCAGTAGTTATCCCCCTCCATCG
GGCAGTTTTCCCAGACATTACTCACCCGTCCGCCACTCGTCAGCAAAGCAGCAAGCTGCTTCCTGTTACCGTT
CGACTGCATGTGTTAGGCCTGCCGCCAGCGTCAATCTGAGCCAGGATCAAACTCT
>416
CTGCTGCCGCCCGTAGGAGTCTGGACCGTGTCTCAGTTCCAGTGTGGCTGGTCATCCTCTCAGACCAGCTAGG
GATCGTCGCCTAGGTGAGCCGTTACCCCACCTACTAGCTAATCCCATCTGGGCACATCCGATGGCAAGAGGCC
CTAAGGTCCCCCTCTTTGTCTGCGACGTATGCGGTATTAGCTACCGTTTCCAGTAGTTATCCCCCTCCATCGG
GCAGTTTCCCAGACATTACTCACCCGTCCGCCACTCGTCAGCAAAGCAGCAAGCTGCTTCCTGTTACCGTTCG
ACTGCATGTGTTAGGCCTGCCGCCAGCGTCAATCTGAGCCAGGATCAAACTCT
>417
CTGCTGCCGCCCGTAGGAGTCTGGACCGTGTCTCAGTTCCAGTGTGGCTGGTCATCCTCTCAGACCAGCTAGG
GATCGTCGCCTAGGTGAGCCGTTACCCCACTTACTAGCTAATCCCATCTGGGCACATCCGATGGCAAGAGGCC
CTAAGGTCCCCCTCTTTGTCTTGCGACGTTATGCGGTATTAGCTACCGTTTCCAGTAGTTATCCCCCTCCATC
AGGCAGTTTTCCCAGACATTACTCACCCGTCCGCCACTCGTCAGCAAAGCAGCAAGCTGCTTCCTGTTACCGT
TCGACTGCATGTGTTAGGCCTGCCGCCAGCGTCAATCTGAGCCAGGATCAAACTCT
>418
CTGCTGCCGCCCGTAGGAGTCTGGACCGTGTCTCAGTTCCAGTGTGGCTGGTCATCCTCTCAGACCAGCTAGG
GATCGTCGCCTAGGTGAGCCGTTACCCCACCTACTAGCTAATCCCATCTGGGCACATCCGATGGCAAGAGGCC
CTAAGGTCCCCCTCTTTGGTCTGCGACGTTATGCGGTATTAGCTACCGCTTCAGTAGTTATCCCCCTCCATCA
GGCAGTTTCCCAGACATTACTCACCCGTCCGCCACTCGTCAGCAAAACAGCAAGCTGCTTCCTGTTACCGTTC
GACTGCATGTGTTAGGCCTGCCGCCAGCGTTCGAATCTGAGCCAGGATCAAACTCT
>419
CTGCTGCCGCCCGTAGGAGTCTGGACCGTGTCTCAGTTCCAGTGTGGCTGGTCATCCTCTCAGACCAGCTAGG
GATCGTCGCCTAGGTGAGCCGTTACCCCACCTACTAGCTAATCCCATCTGGGCACATCCGATGGCAAGAGGCC
CTAAGGTCCCCCTCTTGTCTGCGACGTATGCGGTATTAGCTACCGTTTCCAGTAGTTATCCCCCTCCATCGGG
CAGTTTCCCAGACATTACTCACCCGTCCGCCACTCGTCAGCAAAGCAGCAAGCTGCTTCCTGTTACCGTTCGA
CTGCATGTGTTAGGCCTGCCGCCAGCGTCAATCTGAGCCAGGATCAAACTCT
>420
CTGCTGCCGCCCGTAGGAGTCTGGACCGTGTCTCAGTTCCAGTGTGGCTGGTCATCCTCTCAGACCAGCTAGG
GATCGTCGCCTAGGTGAGCCGTTACCCCACCTACTAGCTAATCCCATCTGGGCACATCCGATGGCAAGAGGCC
CTAAGGTCCCCCTCTTGTCTTGCGACGTATGCGGTATTAGCTACCGTTTCCAGTAGTTATCCCCCTCCATCAG
GCAGTTTCCCAGACATTACTAACCCGTCCGCCACTCGTCAGCAAACAGCAAGCTGCTTCCTGTTACCGTTCGA
CTGCATGTGTTAGGCCTGCCGCCAGCGTCAATCTGAGCCAGGATCAAACTCT
>421
CTGCTGCCGCCCGTAGGAGTCTGGACCGTGTCTCAGTTCCAGTGTGGCTGGTCATCCTCTCAGACAGCTAGGG
ATCGTCGCCTAGGTGAGCCGTTACCCCACCTACTAGCTAATCCCATCTGGGCACATCCGATGGCAAGAGGCCC
TAAGGTCCCCCTCTTGTCTTGCGACGTATGCGGTATTAGCTACCGTTTCCAGTAGTTATCCCCCTCCATCAGG
CAGTTTTCCCAGACATTACTCACCCGTCCGCCACTCGTCAGCAAAGCAGCAAGCTGCTTCCTGTTACCGTTCG
ACTGCATGTGTTAGGCCTGCCGCCAGCGTTCAATCTGAGCCAGGATCAAACTCT
>422
CTGCTGCCGCCCGTAGGAGTCTGGACCGTGTCTCAGTTCCAGTGTGGCTGGTCATCCTCTCAGACCAGCTAGG
GATCGTCGCCTAGGTGAGCCGTTACCCCACCTACTAGCTAATCCCATCTGGGCACATCCGATGGCAAGAGGCC
CTAAGGTCCCCCTCTTTGTCTTGCGACGTATGCGGTATTAGCTACCGTTTCCAGTAGTTATCCCCCTCCATCG
GGCAGTTTTCCCAGACATTACTCACCCGTCCGCCGCTCGTCAGCAAAGCAGCAAGCTGCTTCCTGTTACCGTT
CGACTGCATGTGTTAGGCCTGCCGCCAGCGTCAATCTGAGCCAGGATCAAACTCT
>423
CTGCTGCCGCCCGTAGGAGTCTGGACCGTGTCTCAGTTCCAGTGTGGCTGGTCATCCTCTCAGACCAGCTAGG
GATCGTCGCCTAGGTGAGCCGTTACCCCACCTACTAGCTAATCCCATCTGGGCACATCCGATGGCAAGAGGCC
CTAAGGTCCCCCTCTTGTCTTGCGACGTATGCGGTATTAGCTACCGTTTCCAGTAGTTATCCCACCTCATCAG

GCAGTTTCCCAGACATTACTCACCCGTCCGCCACTCGTCAGCAAAGCAGCAAGCTGCTTCCTGTTACCGTTCG
ACTGCATGTGTTAGGCCTGACCGCCAGCGTCAATCTGAGCCAGGATCAAACTCT
>424
CTGTTGCCGCCCGTAGGAGTCTGGACCGTGTCTCAGTTCCAGTGTGGCTGGTCATCCTCTCAGACAGCTAGGG
ATCGTCGCCTAGGTGAGCCGTTACCCCACCTACTAGCTAATCCCATCTGGGCACATCCGATGGCAAGAGGCCC
TAAGGTCCCCCTCTTGTCTGCGACGTTATGCGGTATTAGCTACCGTTTCCAGTAGTTATCCCCCTCCATCGGG
CAGTTTCCCAGACATTACTCACCCGTCCGCCACTCGTCAGCAAAGCAGCAAGCTGCTTCCTGTTACCGTTCGA
CTGCATGTGTTAGGCCTGCCGCCAGCGTCAATCTGAGCCAGGATCAAACTCT
>425
CTGCTGCCGCCCGTAGGAGTCTGGACCGTGTCTCAGTTCCAGTGTGGCTGGTCATCCTCTCAGACCAGCTAGG
GATCGTCGCCTAGGTGAGCCGTTACCCCACCTACTAGCTAATCCCATCTGGGCACATCCGATGGCAAGAGGCC
CTAAGGTCCCCCTCTTTGTCTTGCGACGTATGCGGTATTAGCTACCGTTTCCAGTAGTTATCCCCCTCCATCA
GGCAGTTTCCCAGACATTACTCACCCGTCCGCCACTCGTCAGCAAAGCAGCAAGCTGCTTCCTGTTACCGTTC
GACTGCATGTGTTAGGCCTGCCGCCAGCGTCAATCTGAGCCAGGATCAAACTCT
>426
CTGCTGCCGCCCGTAGGAGTCTGGACCGTGTCTCAGTTCCAGTGTGGCTGGTCATCCTCTCAGACCAGCTAGG
GATCGTCGCCTAGGTGAGCCGTTACCCCACCTACTAGCTAATCCCATCTGGGCACGTCCGATGGCAAGAGGCC
CTAAGGTCCCCCTCTTGTCTTGCGACGTATGCGGTATTAGCTACCGTTTCCAGTAGTTATCCCCCTCCATCGG
GCAGTTTCCCAGACATTACTCACCCGTCCGCCACTCGTCAGCAAAGCAGCAAGCTGCTTTCTGTTACCGTTTG
ACTGCATGTGTTAGGCCTGCCGCCAGCGTCAATCTGAGCCAGGATCAACTCT
>427
CTGCTGCCGCCCGTAGGAGTCTGGACCGTGTCTCAGTTCCAGTGTGGCTGGTCATCCTCTCAGACCAGCTAGG
GATCGTCGCCTAGGTGAGCCGTAACCCCACCTACTAGCTAATCCCATCTGGGCACATCCGATGGCAAGAGGCC
CTAAGGTCCCCCTCTTGTCTTGCGACGTATGCGGTATTAGCTACCGTTTCCAGTAGTTATCCCCTCCATCAGG
CAGTTTCCCAGACATTACTCACCCGTCCGCCACTCGTCAGCAAAGCAGCAAGCTGCTTCCTGTTACCGTTCGA
ACTGCATGTGTTAGGCCTGCCGCCAGCGTCAATCTGAGCCAGGATCAAACTCT
>428
CTGCTGCCGCCCGTAGGAGTCTGGACCGTGTCTCAGTTCCAGTGTGGCTGGTCATCCTCTCAGACCAGCTAGG
GATCGTCGCCTAGGTGAGCCGTTACCCCACCTACTAGCTAATCCCATCTGGGCACATCCGATGGCAAGAGGCC
CTAAGGTCCCCCTCTTTGTCTGCGACGTTATGCGGTATTAGCTACCGTTTCCAGTAGTTATCCCCCTCCATCA
GGCAGTTTCCCAGACATTACTCACCCGTCCGCCACTCGTCAGCAAACAGCAAGCTGCTTCCTGTTACCGTTCG
ACTGCATGTGTTAGGCCTGCCGCCAGCGTTCAATCTGAGCCAGGATCAAACTCT
>429
TGCTGCCGCCCGTAGGAGTCTGGACCGTGTCTCAGTTCCAGTGTGGCTGGTCATCCTCTCAGACCAGCTAGGG
ATCGTCGCCTAGGTGAGCCGTTACCCCACCTACTAGCTAATCCCATCTGGGCACATCCGATGGCAAGAGGCCC
TAAGGTCCCCCTCTTTGTCTTGCGACGTTATGCGGTATTAGCTACCGTTTCCAGTAGTTATCCCCCTCCATCA
GGCAGTTTCCCAGACATTACTCACCCGTCCGCCACTCGTCAGCAAAGCAGCAAGCTGCTTCCTGTTACCGTTC
GACTGCATGTGTTAGGCCTGCCGCCAGCGTCAATCTGAGCCAGGATCAAACTCT
>430
CTGCTGCCGCCCGTAGGAGTCTGGACCGTGTCTCAGTTCCAGTGTGGCTGGTCATCCTCTCAGACCAGCTAGG
GATCGTCGCCTAGGTGAGCCGTTACCCCACCTACTAGCTAATCCCATCTGGGCACATCCGATGGCAAGAGGCC
CTAAGGTCCCCCTCTTTGGTCTTGCGACGTATGCGGTATTAGCTACCGTTTCCAGTAGTTATCCCCCTCCATC
AGGCAGCTTCCCAGACATTACTCACCCGTCCGCCACTCGTCAGCAAAGCAGCAAGCTGCTTCCTGTTACCGTT
CGACTGCATGTGTTAGGCCTGCCGCCAGCGTTCAATCTGAGCCAGGATCAAACTCT
>431
CTGCTGCCGCCCGTAGGAGTCTGGACCGTGTCTCAGTTCCAGTGTGGCTGGTCATCCTCTCAGACCAGCTAGG
GATCGTCGCCTAGGTGAGCCGTTACCCCACCTACTAGCTAATCCCATCTGGGCACATCCGATGGCAAGAGGCC
CTAAGGTCCCCCTCTTTGTCTTGCGACGTATGCGGTATTAGCTACCGTTTCCAGTAGTTATCCCCCTCCATCG
GGCAGTTTTCCCAGACATTACTCACCCGTCCGCCACTCGTCAGCAAAAGCAGCAAGCTGCTTCCTGTTACCGT
TCGACTGCATGTGTTAGGCCTGCCGCCAGCGTAATCTGAGCCAGGATCAAACTCT
>432
CTGCTGCCGCCCGTAGGAGTCTGGACCGTGTCTCAGTTCCAGTGTAGCTGGTCATCCTCTCAGACCAGCTAGG
GATCGTCGCCTAGGTGAGCCGTTACCCCACCTACTAGCTAATCCCATCTGGGCACATCCGATGGCAAGAGGCC
CTAAGGTCCCCCTCTTTGTCTTGCGACGTTATGCGGTATTAGCTACCGTTTCCAGTAGTTATCCCCCTCCATC

AGGCAGTTTCCCAGACATTACTCACCCGTCCGCCACTCGTCAGCAAAGCAGCAAGCTGCTTTCTGTTACCGTT
CGACTGCATGTGTTAGGCCTGCCGCCAGCGTTCAATCTGAGCCAGGATCAAACTCT
>433
TGCTGCCGCCCGTAGGAGTCTGGACCGTGTCTCAGTTCCAGTGTGGCTGGTCATCCTCTCAGACAGCTAGGGA
TCGTCGCCTAGGTGAGCCGTTACCCCACCTACTAGCTAATCCCATCTGGGCACATCCGATGGCAAGAGGCCCT
AAGGTCCCCCTCTTTGTCTTGCGACGTTATGCGGTATTAGCTACCGTTTCCAGTAGTTATCCCCCTCCATCGG
GCAGTTTCCCAGACATTACTCACCCGTCCGCCACTCGTCAGCAAAGCAGCAAGCTGCTTCCTGTTACCGTTCG
ACTGCATGTGTTAGGCCTGCCGCCAGCGTCAATCTGAGCCAGGATCAAACTCT
>434
CTGCTGCCGCCCGTAGGAGTCTGGACCGTGTCTCAGTTCCAGTGTGGCTGGTCATCCTCTCAGACCAGCTAGG
GATCGTCGCCTAGGTGAGCCGTTACCCCACCTACTAGCTAATCCCATCTGGGCACATCCGATGGCAAGAGGCC
CTAAGGTCCCCCTCTTTGTCTTGCGACGTTATGCGGTATTAGCTACCGTTTCCAGTAGTTATCCCCCTCCATC
GGGCAGTTTCCCAGACATTACTCACCCGTCCGCCACTCGTCAGCAAAGCAGCAAGCTGCTTCCTGTTACCGTT
CGACTGCATGTGTTAGGCCTGCCGCCAGCGTCAATCTGAGCCAGGATCAAACTCT
>435
CTGCTGCCGCCCGTAGGAGTCTGGACCGTGTCTCAGTTCCAGTGTGGCTGGTCATCCTCTCAGACCAGCTAGG
GATCGTCGCCTAGGTGAGCCGTTACCCCACCTACTAGCTAATCCCATCTGGGCACATCCGATGGCAAGAGGCC
CTAAGGTCCCCCTCTTTGGTCTTGCGACGTATGCGGTATTAGCTACCGTTTCCAGTAGTTATCCCCCTCCATC
AGGCAGTTTCCCAGACATTACTCACCCGTCCGCCACTCGTCAGCAAAGCAGCAAGCTGCTTCTGTTACCGTTC
GACTGCATGTGTTAGGCCTGCCGCCAGCGTCAATCTGAGCCAGGATCAAACTCT
>436
CTGCTGCCGCCCGTAGGAGTCTGGACCGTGTCTCAGTTCCAGTGTGGCTGGTCATCCTCTCAGACCAGCTAGG
GATCGTCGCCTAGGTGAGCCGTTACCCCACCTACTAGCTAATCCCATCTGGGCACATCCGATGGCAAGAGGCC
CTAAGGTCCCCCTCTTTGTCTTGCGACGTTATGCGGTATTAGCTACCGTTTCCAGTAGTTATCCCCCTCCATC
AGGCAGTTTCCCAGACATTACTCACCCGTCCGCCACTCGTCAGCAAAGCAGCAAGCTGCTTCCTGTTACCGTT
CGACTGCATGTGTTAGGCCTGCCGCCAGCGTTCAATCTGAGCCAGGATCAAACTCT
>437
CTGCTGCCGCCCGTAGGAGTCTGGACCGTGTCTCAGTTCCAGTGTGGCTGGCCATCCTCTCAGACCAGCTAGG
GATCGTCGCCTAGGTGAGCCGTTACCCCACCTACTAGCTAATCCCATCTGGGCACATCCGATGGCAAGAGGCC
CTAAGGTCCCCCTCTTTGTCTGCGACGTATGCGGTATTAGCTACCGTTTCCAGTAGTTATCCCCCTCCATCAG
GCAGTTTCCCAGACATTACTCACCCGTCCGCCACTCGTCAGCAAACAGCAAGCTGCTTCCTGTTACCGTTCGA
CTGCATGTGTTAGGCCTGCCGCCAGCGTTCAATCTGAGCCAGGATCAAACTCT
>438
CTGCTGCCGCCCGTAGGAGTCTGGACCGTGTCTCAGTTCCAGTGTGGCTGGTCATCCTCTCAGACCAGCTAGG
GATCGTCGCCTAGGTGAGCCGTTACCCCACCTACTAGCTAATCCCATCTGGGCACATCCGATGGCAAGAGGCC
CTAAGGTCCCCCTCTTTGTCTTGCGACGTTATGCGGTATTAGCTACCGTTTCCAGTAGTTATCCCCCTCCATC
AGGCAGTTTCCCAGACATTACTCACCCGTCCGCCACTCGTCAGCAAAGCAGCAAGCTGCTTCCTGTTACCGTT
CGACTGCATGTGTTAGGCCTGCCGCCAGCCGTCAATCTGAGCAG
>439
CTGTTGCCGCCCGTAGGAGTCTGGACCGTGCCTCAGTTCCAGTGTGGCTGGTCATCCTCTCAGACCAGCTAGG
GATCGTCGCCTAGGTGAGCCGTTACCCCACCTACTAGCTAATCCCATCTGGGCACATCCGATGGCAAGAGGCC
CTAAGGTCCCCCCCTTGTCTTGCGACGTATGCGGTATTAGCTACCGTTTCCAGTAGTTATCCCCCTCCATCGG
GCAGTTTCCCAGACATACTCACCCGTCCGCCACTCGTCAGCAAAGCAGCAAGCTGCTTCCTGTTACCGTTCGA
CTGCATGTGTTAGGCCTGCCGCCAGCGTCAATCTGAGCCAGGATCAAACTCT
>440
CTGCTGCCGCCCGTAGGAGTCTGGACCGTGTCTCAGTTCCAGTGTGGCTGGTCATCCTCTCAGACCAGCTAGG
GATCGTCGCCTAGGTGAGCCGTTACCCCACCTACTAGCTAATCCCATCTGGGCACATCCGATGGCAAGAGGCC
CTAAGGTCCCCCTCTTTGTCTTGCGACGTTATGCGGTATTAGCTACCGTTTCCAGTAGTTATCCCCCTCCATC
GGGCAGTTTCCCAGACATTACTCACCCGTCCGCCACTCGTCAGCAAAGCAGCAAGCTGCTTCTGTTACCGTTC
GACTGCATGTGTTAGGCCTGCCGCCAGCGTTCAATCTGAGCCAGGATCAAACTCT
>441
CTGCTGCCGCCCGTAGGAGTCTGGACCGTGTCTCAGTTCCAGTGTGGCTGGTCATCCTCTCAGACCAGCTAGG
GATCGTCGCCTAGGTGAGCCGTTACCCCACCTACTAGCTAATCCCATCTGGGCACATCCGATGGCAAGAGGCC
CTAAGGTCCCCCTCTTTGTCTTGCGACGTATGCGGTATTAGCTACCGTTTCCAGTAGTTATCCCCCTCCATCG

GGCAGTTTCCCAGACATTACTCACCCGTCCGCCACTCGTCAGCAAAGCAGCAAGCTGCTTCCTGTTACCGTTC
GACTGCATGTGTTAGGCCTGCCGCCAGCGTCAATCTGAGCCAGGATCAAACTCT
>442
CTGCTGCCGCCCGTAGGAGTCTGGACCGTGTCTCAGTTCCAGTGTGGCTGGTCATCCTCTCAGACCAGCTAGG
GATCGTCGCCTAGGTGAGCCGTTACCCCACCTACTAGCTAATCCCATCTGGGCACATCCGATGGCAAGAGGCC
CTAAGGTCCCCCTCTTTGTCTTGCGACGTTATGCGGTATTAGCTACCGTTTCCAGTAGTTATCCCCCTCCATC
GGGCAGTTTTCCCAGACATTACTCACCCGTCCGCCACTCGTCAGCAAAGCAGCAAGCTGCTTCCTGTTACCGT
TCGACTGCATGTGTTAGGCCTGCCGCCAGCGTTCAATCTGAGCCAGGATCAAACTCT
>443
CTGCTGCCGCCCGTAGGAGTCTGGACCGTGTCTCAGTTCCAGTGTGGCTGGTCATCCTCTCAGACCAGCTAGG
GATCGTCGCCTAGGTGAGCCGTTACCCCACCTACTAGCTAATCCCATCTGGGCACATCCGATGGCAAGAGGCC
CTAAGGTCCCCCTCTTTGGTCTTGCGACGTATGCGGTATTAGCCACCGTTTCCAGTAGTTATCCCCCTCCATC
AGGCAGTTTCCCAGACATTACTCACCCGTCCGCCACTCGTCAGCAAAGCAGCAAGCTGCTTCCTGTTACCGTT
CGACTGCATGTGTTAGGCCTGCCGCCAGCGTCAATCTGAGCCAGGATCAAACTCT
>444
CTGCTGCCGCCCGTAGGAGTCTGGACCGTGTCTCAGTTCCAGTGTGGCTGGTCATCCTCTCAGACCAGCTAGG
GATCGTCGCCTAGGTGAGCCGTTACCCCACCTACTAGCTAATCCCATCTGGGCACATCCGGTGGCAAGAGGCC
CTGAGGTCCCCCTCTTTGGTCTTGCGACGTTATGCGGTATTAGCTACCGTTTCCAGTAGTTATCCCCTCCATC
GGGCAGTTTCCCAGACATTACTCACCCGTCCGCCACTCGTCAGCAAAGCAGCAAGCTGCTTCCTGTTACCGTT
CGACTGCATGTGTTAGGCCTGCCGCCAGCGTCAATCTGAGCCAGGATCAAACTCT
>445
CTGCTGCCGCCCGTAGGAGTCTGGACCGTGTCTCAGTTCCAGTGTGGCTGGTCATCCTCTCAGACCAGCTAGG
GATCGTCGCCTAGGTGAGCCGTTACCCCACCTACTAGCTAATCCCATCTGGGCACATCCGATGGCAAGAGGCC
CTAAGGTCCCCCTCTTGTCTTGCGACGTTATGCGGTATTAGCTACCGTTTCCAGTAGTTATCCCCCTCCATCA
GGCAGTTTCCCAGACATTACTCACCCGTCCGCCACTCGTCAGCAAAGCAGCAAGCTGCTTCCTGTTACCGTTC
GACTGCATGTGTTAGGCCTGCCGCCAGCGTCAATCTGAGCCAGGATCAAACTCT
>446
TGCTGCCGCCCGTAGGAGTCTGGACCGTGTCTCAGTTCCAGTGTGGCTGGTCATCCTCTCAGACAGCTAGGGA
TCGTCGCCTAGGTGAGCCGTTACCCCACCTACTAGCTAATCCCATCTGGGCACATCCGATGGCAAGAGGCCCT
AAGGTCCCCCTCTTGTCTTGCGACGTTATGCGGTATTAGCTACCGTTTCCAGTAGTTATCCCCCTCCATCGGG
CAGTTTCCCAGACATTACTCACCCGTCCGCCACTCGTCAGCAAAGCAGCAAGCTGCTTTCTGTTACCGTTCGA
CTGCATGTGTTAGGCCTGCCGCCAGCGTCAATCTGAGCCAGGATCAAACTCT
>447
CTGCTGCCGCCCGTAGGAGTCTGGACCGTGTCTCAGTTCCAGTGTGGCTGGTCATCCTCTCAGACCAGCTAGG
GATCGTCGCCTAGGTGAGCCGTTACCCCACCTACTAGCTAATCCCATCTGGGCACATCCGATGGCAAGAGGCC
CTAAGGTCCCCCTCTTGTCTGCGACGTTATGCGGTATTAGCTACCGTTTCCAGTAGTTATCCCCCTCCATCGG
GCAGTTTCCCAGACATTACTCACCCTCCGCCACTCGTCAGCAAAGCAGCAAGCTGCTTCCTGTTACCGTTCGA
CTGCATGTGTTAGGCCTGCCGCCAGCGTCAATCTGAGCCAGGATCAAACTCT
>448
CTGCTGCCGCCCGTAGGAGTCTGGACCGTGTCTCAGTTCCAGTGTGGCTGGTCATCCTCTCAGACCAGCTAGG
GATCGTCGCCTAGGTGAGCCGTTACCCCACCTACTAGCTAATCCCATCTGGGCACATCCGATGGCAAGAGGCC
CTAAGGTCCCCCTCTTTGGTCTTGCGACGTTATGCGGTATTAGCTACCGTTTCCAGTAGTTATCCCCCTCCAT
CAGGCAGTTTCCCAGACATTACTCACCCGTCCGCCACTCGTCAGCAAAGCAGCAAGCTGCTTCCTGTTACCGT
TCGACTGCATGTGTTAGGCCTGCCGCCAGCGTCAATCTGAGCCAGGATCAAACTCT
>449
CTGCTGCCGCCCGTAGGAGTCTGGACCGTGTCTCAGTTCCAGTGTGGCTGGTCATCCTCTCAGACCAGCTAGG
GATCGTCGCCTAGGTGAGCCGTTACCCCACCTACTAGCTAATCCCATCTGGGCACATCCGATGGCAAGAGGCC
CTAAGGTCCCCCTCTTTGTCTTGCGACGTATGCGGTATTAGCTACCGTTTCCAGTAGTTATCCCCCTCCATCA
GGCAGTTTCCCAGACATTACTCACCCGTCCGCCACTCGTCAGCAAAGCAGCAAGCTGCTTCCTGTTACCGTTC
GACTGCATGTGTTAGGCCTGCCGCCAGCGTCAATCTGAGCCAGGATCAACTCT
>450
CTGCTGCCGCCCGTAGGAGTCTGGACCGTGTCTCAGTTCCAGTGTGGCTGGTCATCCTCTCAGACCAGCTAGG
GATCGTCGCCTAGGTGAGCCGTTACCCCCACCTACTAGCTAATCCCATCTGGGCACATCCGATGGCAAGAGGC
CCTAAGGTCCCCCTCTTTGGTCTTGCGACGTATGCGGTATTAGCTACCGTTTCCAGTAGTTATCCCCCTCCAT

CGGGCAGTTTTCCCCAGACATTACTCACCCGTCCGCCACTCGTCAGCAAAGCAGCAAGCTGCTTCCTGTTACC
GTTCGACTGCATGTGTTAGGCCTGCCGCCAGCGTCAATCTGAGCCAGGATCAAACTCT
>451
CTGCTGCCGCCCGTAGGAGTCTGGACCGTGTCTCAGTTCCAGTGTGGCTGGTCATCCTCTCAGACCTGCTAGG
GATTGTCGCCTAGGTGAGCCGTTACCCCACCTACTAGCTAATCCCATCTGGGCACATCCGATGGCAAGAGGCC
CTAAGGTCCCCCTCTTTGGTCTTGCGACGTATGCGGTATTAGCTACCGTTTCCGGTAGTTATCCCCCTCCATC
GGGCAGTTTCCCAGACATACTCACCCGTCCGCCACTCGTCAGCAAAGCAGCAAGCTGCGTCCTGTTACCGTTC
GACTGCATGTGTTAGGCCTGCCGCCAGCGTCAATCTGAGCCAGGATCAAACTCT
>452
CTGCTGCCGCCCGTAGGAGTCTGGACCGTGTCTCAGTTCCAGTGTGGCTGGTCATCCTCTCAGACAGCTAGGG
ATCGTCGCCTAGGTGAGCCGTTACCCCACCTACTAGCTAATCCCATCTGGGCACATCCGATGGCAAGAGGCCC
TAAGGTCCCCCTCTTGTCTTGCGACGTATGCGGTATTAGCTACCGTTTCCAGTAGTTATCCCCCTCCATCAGG
CAGTTTCCCAGACATTACTCACCCGTCCGCCACTCGTCAGCAAAGCAGCAAGCTGCTTCTGTTACCGTTCGAC
TGCATGTGTTAGGCCTGCCGCCAGCGTTCAATCTGAGCCAGGACAAACTCT
>453
CTGCTGCCGCCCGTAGGAGTCTGGACCGTGTCTCAGTTCCAGTGTGGCTGGTCATCCTCTCAGACCAGCTAGG
GATCGTCGCCTAGGTGAGCCGTTACCCCACCTACTAGCTAATCCCATCTGGGCACATCCGATGGCAAGAGGCC
CTAAGGTCCCCCTCTTTGTCTTGCGACGTATGCGGTATTAGCTACCGTTTCCAGTAGTTATCCCCCTCCATCA
GGCAGTTTCCCAGACATTACTCACCCGTCCGCCACTCGTCAGCAAAGCAGCAAGCTGCTTCCTGTTACCGTTC
GACTGCATGTGTTAGGCCTGCCGCCAGCGTCAATCTGAGCCAGGATCAAACTCT
>454
CTGCTGCCGCCCGTAGGAGTCTGGACCGTGTCTCAGTTCCAGTGTGGCTGGTCATCCTCTCAGACCAGCTAGG
GATCGTCGCCTAGGTGAGCCGTTACCCCACCTACTAGCTAATCCCATCTGGGCACATCCGATGGCAAGAGGCC
CTAAGGTCCCCCTCTTGTCTGCGACGTATGCGGTATTAGCTACCGTTTCCAGTAGTTATCCCCCTCCATCAGG
CAGTTTTCCCAGACATTACTCACCCGTCCGCCACTCGTCAGCAAAGCAGCAAGCTGCTTCCTGTTACCGTTCG
ACTGCATGTGTTAGGCCTGCCGCCAGCGTCAATCTGAGCCAGGATCAAACTCT
>455
CTGCTGCCGCCCGTAGGAGCCTGGACTGTGTCTCAGTTCCAGTGTGGCTGGTCATCCTCTCAGACCAGCTAGG
GATCGTCGCCTAGGTGAGCCGTTACCCCACCTACTAGCTAATCCCATCTGGGCACATCCGATGGCAAGAGGCC
CTAAGGTCCCCCTCTTGTCTTGCGACGTATGCGGTATTAGCTACCGTTTCCAGTAGTTATCCCCCTCCATCAG
GCAGTTTCCCAGACATTACTCACCCGTCCGCCACTCGTCGCAAACAGCAAGCTGCTTCCTGTTACCGTTCGAC
TGCATGTGTTAGGCCTGCCGCCAGCGTCAATCTGAGCCAGGATCAAACTCT
>456
CTGCTGCCGCCCGTAGGAGTCTGGACCGTGTCTCAGTTCCAGTGTGGCTGGTCATCCTCTCAGACCAGCTAGG
GATCGTCGCCTAGGTGAGCCGTTACCCCACCTACTAGCTAATCCCATCTGGGCACATCCGATGGCAAGAGGCC
CTAAGGTCCCCCTCTTTGTCTGCGACGTATGCGGTATTAGCTACCGTTTCCAGTAGTTATCCCCCTCCATCGG
GCAGTTTCCCAGACATTACTCACCCGTCCGCCACTCGTCAGCAAAGCAGCAAGCTGCTTCCTGTTACCGTTCG
ACTGCATGTGTTAGGCCTGCCGCCAGCGTCAATCTGAGCCAGGATCAACTCT
>457
CTGCTGCCGCCCGTAGGAGTCTGGACCGTGTCTCAGTTCCAGTGTGGCTGGTCATCCTCTCAGACCAGCTAGG
GATCGTCGCCTAGGTGAGCCGTTACCCCCACCTACTAGCTAATCCCATCTGGGCACATCCGATGGCAAGAGGC
CCTAAGGTCCCCCTCTTGTCTTGCGACGTATGCGGTATTAGCTACCGTTTCCAGTAGTTATCCCCCTCCATCA
GGCAGTTTCCCCAGACATTACTCACCCGTCCGCCACTCGTCAGCAAAGCAGCAAGCTGCTTCCTGTTACCGTT
CGACTGCATGTGTTAGGCCTGCCGCCAGCGTCAATCTGAGCCAGGATCAAACTCT
>458
CTGCTGCCGCCCGTAGGAGTCTGGACCGTGTCTCAGTTCCAGTGTGGCTGGTCATCCTCTCAGACCAGCTAGG
GATCGTCGCCTAGGTGAGCCGTTACCCCACCTACTAGCTAATCCCATCTGGGCACATCCGATGGCAAGAGGCC
CTAAGGTCCCCCTCTTTGGTCTTGCGACGTTATGCGGTATTAGCTACCGTTTCCAGTAGTTATCCCCTCCATC
GGGCAGTTTCCCAGACATTACTCACCCGTCCGCCACTCGTCAGCAAAGCAGCAAGCTGCTTCCTGTTACCGTT
CGACTGCATGTGTTAGGCCTGCCGCCAGCGTCAATCTGAGCCAGGATCAAACTCT
>459
CTGCTGCCGCCCGTAGGAGTCTGGACCGTGTCGCTAGTACTCAGTGTGGCTGGTCATCCTCTCAGACCAGCTA
GGGATCGTCGCCTAGGTGAGCCGTTACCCCACCTACTAGCTAATCCCATCTGGGCACATCCGATGGCAAGAGG
CCCTAAGGTCCCCCTCTTTGGTCTTGCGACGTTATGCGGTATTAGCTACCGTTTCCAGTAGTTATCCCCCTCC

ATCAGGCAGTTTCCCAGACATTACTCACCCGTCCGCCACTCGTCAGCAAAAGCAGCAAAGCTGCTTCCTGTTA
CCGTTCGACTGCATGTGTTAGGCCTGCCGCCAGCGTCAATGAG
>460
CTGCTGCCGCCCGTAGGAGTCTGGACCGTGTCTCAGTTCCAGTGTGGCTGGTCATCCTCTCAGACCAGCTAGG
GATCGTCGCCTAGGTGAGCCGTTACCCCACCTACTAGCTAATCCCATCTGGGCACATCCGATGGCAAGAGGCC
CTAAGGTCCCCCTCTTTGGTCTTGCGACGTTATGCGGTATTAGCTACCGTTTCCAGTAGTTATCCCCCTCCAT
CAGGCAGTTTCCCAGACATTACTCACCCGTCCGCCACTCGTCAGCAAAGCAGCAAGCTGCTTCCTGTTACCGT
TCGACTGCATGTGTTAGGCCTGCCGCCAGTGTTCAATCTGAGCCAGGATCAAACTCT
>461
CTGCTGCCGCCCGTAGGAGTCTGGACCGTGTCTCAGTTCCAGTGTGGCTGGTCATCCTCTCAGACCAGCTAGG
GATCGTCGCCTAGGTGAGCCGTTACCCCACCTACTAGCTAATCCCATCTGGGCACATCCGATGGCAAGAGGCC
CTAAGGTCCCCCTCTTGTCTGCGACGTATGCGGTATTAGCTACCGTTTCCAGTAGTTATCCCCCTCCATCAGG
CAGTTTTCCCAGACATTACTCACCCGTCCGCCACTCGTCAGCAAAGCAGCAAGCTGCTTCCTGTTACCGTTCG
ACTGCATGTGTTAGGCCTGCCGCCAGCGTCAATCTAGCCAGG
>462
TGCTGCCGCCCGTAGGAGTCTGGACCGTGTCTCAGTTCCAGTGTGGCTGGTCATCCTCTCAGACCAGCTAGGG
ATCGTCGCCTAGGTGAGCCGTTACCCCACCTACTACGCTAATCCCATCTGGGCACACCCGATGGCAAGAGGCC
CTAAGGTCCCCCTCTTTGGTCTTGCGGCGTTATGCGGTATTAGCTACCGTTTCCAGTAGTTATCCCCCTCCAT
CAGGCAGTTTCCCAGACATTACTCACCCGTCCGCCACTCGTCAGCAAAGCAGCAAGCTGCTTCCTGTTACCGT
TCGACTTGCATGTGTTAGGCCTGCCGCCAGCGTTCAATCTGAGCCAGGATCAAACTCT
>463
CTGCTGCCGCCCGTAGGAGTCTGGACCGTGTCTCAGTTCCAGTGTGGCTGGTCATCCTCTCAGACCAGCTAGG
GATCGTCGCCTAGGTGAGCCGTTACCCCACCTACTAGCTAATCCCATCTGGGCACATCCGATGGCAAGAGGCC
CTAAGGTCCCCCTCTTTGGTCTTGCGACGTTATGCGGTATTAGCTACCGTTTCCAGTAGTTATCCCCCTCCAT
CAGGCAGTTTCCCAGACATTACTCACCCGTCCGCCACTCGTCAGCAAACAGCAAGCTGCTTCCTGTTACCGTT
CGACTGCATGTGTTAGGCCTGCCGCCAGCGTTCAATCTGAGCCAGGATCAAACTCT
>464
CTGCTGCCGCCCGTAGGAGTCTGGACCGTGTCTCAGTTCCAGTGTGGCTGGTCATCCTCTCAGACCAGCTAGG
GATCGTCGCCTAGGTGAGCCGTTACCCCACCTACTAGCTAATCCCATCTGGGCACATCCGATGGCAAGAGGCC
CTAAGGTCCCCCTCTTTGGTCTTGCGACGTTATGCGGTATTAGCTACCGTTTCCAGTAGTTATCCCCCTCCAT
CGGGCAGTTTCCCAGACATTACTCACCCGTCCGCCACTCGTCAGCAAAGCAACAAGCTGCTTCCTGTTACCGT
TCGACTGCATGTGTTAGGCCTGCCGCCAGCGCTCAATCTGAGCCAGGATCAAACTCT
>465
CTGCTGCCGCCCGTAGGAGTCTGGACCGTGTCTCAGTTCCAGTGTGGCTGGTCATCCTCTCAGACCAGCTAGG
GATCGTCGCCTAGGTGAGCCGTTACCCCACCTACTAGCTAATCCCATCTGGGCACATCCGATGGCAAGAGGCC
CTAAGGTCCCCCTCTTTGTCTGCGACGTATGCGGTATTAGCTACCGTTTCCAGTAGTTATCCCCCTCCATCAG
GCAGTTTCCCAGACATTACTCACCCGTCCGCCACTCGTCAGCAAACAGCAAGCTGCTTCCTGTTACCGTTCGA
CTGCATGTGTTAGGCCTGCCGCCAGCGTCAATCTGAG
>466
CTGCTGCCGCCCGTAGGAGTCTGGACCGTGTCTCAGTTCCAGTGTGGCTGGTCATCCTCTCAGACCAGCTAGG
GATCGTCGCCTAGGTGAGCCGTTACCCCACCTACTAGCTAATCCCATCTGGGCACATCCCGATGGCAAGAGGC
CCTAAGGTCCCCCCCTTTGTCTTGCGACGTATGCGGTATTAGCTACCGTTTCCAGTAGCTATCCCCCTCCATC
AGGCAGTTTTCCCAGACATTACTCACCCGTCCGCCACTCGTCAGCAAAGCAGCAAGCTGCTTTCTGTTACCGT
TCGACTGCATGTGTTAGGCCTGCCGCCAGCGTCAATCTGAGCCAGGATCAAACTCT
>467
CTGCTGCCGCCCGTAGGAGTCTGGACCGTGTCTCAGTTCCAGTGTGGCTGGTCATCCTCTCAGACCAGCTAGG
GATCGTCGCCTAGGTGAGCCGTTTACCCCACCTACTAGCTAATCCCATCTGGGCACATCCGATGGCAAGAGGC
CCTAAGGTCCCCCTCTTTGTCTTGCGACGTTATGCGGTATTAGCTACCGTTCCAGTAGTTATCCCCCTCCATC
GGGCAGTTTCCCAGACATTACTCACCCGTCCGCCACTCGTCAACAAGCAGCAAGCTGCTTCCTGTTACCGTTC
GACTGCATGTGTTAGGCCTGCCGCCAGCGTCAATCTGAGCCAGGATCAAACTCT
>468
CTGCTGCCGCCCGTAGGAGTCTGGACCGTGTCTCAGTTCCAGTGTGGCTGGTCATCCTCTCAGACCAGCTAGG
GATCGTCGCCTAGGTGAGCCGTTACCCCACCTACTAGCTAACCCCATCTGGGCACATCCGATGGCAAGAGGCC
CTAAGGTCCCCCTCTTGGTCTTGCGACGTATGCGGTATTAGCTACCGTTTCCAGTAGTTATCCCCCTCCATCA

GGCAGTTTCCCAGACATTACTCACCCGTCCGCCACTCGTCAGCAAAGCAGCAAGCTGCTTCCTGTTACCGTTC
GACTGCATGTGTTAGGCCTGCCGCCAGCGTCAATCTGAGCCAGGATCAAACTCT
>469
CTGCTGCCGCCCGTAGGAGTCTGGACCGTGTCTCAGTTCCAGTGTGGCTGGTCATCCTCTCAGACCAGCTAGG
GATCGTCGCCTAGGTGAGCCGTTACCCCACCTACTAGCTAATCCCATCTGGGCACATCCGATGGCAAGAGGCC
CTAAGGTCCCCCTCTTGTCTTGCGACGTTATGCGGTATTAGCTACCGTTTCCAGTAGTTATCCCCCTCCATCA
GGCAGTTTCCCAGACATTACTCACCCGTCCGCCACTCGTCAGCAAAGCAGCAAGCTGCTTCCTGTTACCGTTC
GACTGCATGTGTTAGGCCTGCCGCCAGCGTCAATCTGAGCCAGGATCAAACTCT
>470
CTGCTGCCGCCCGTAGGAGTCTGGACCGTGTCTCAGTTCCAGTGTGGCTGGTCATCCTCTCAGACCAGCTAGG
GATCGTCGCCTAGGTGAGCCGTTACCCCACCTACTAGCTAATCCCATCTGGGCACATCCGATGGCAAGAGGCC
CTAAGGTCCCCCTCTTTGTCTTGCGACGTTATGCGGTATTAGCTACCGTTTCCAGTAGTTATCCCCCTCCATC
AGGCAGTTTCCCAGACATTACTCACCCGTCCGCCACTCGTCAGCAAAGCAGCAAGCTGCTTCCTGTTACCGTT
CGACTGCATGTGTTAGGCCTGCCGCCAGCGTTCAATCTGAGCCAGGATCAAACTCT
>471
CTGCTGCCGCCCGTAGGAGTCTGGACCGTGTCTCAGTTCCAGTGTGGCTGGTCATCCTCTCAGACCAGCTAGG
GATCGTCGCCTAGGTGAGCCGTTACCCCACCTACTAGCTAATCCCATCTGGGCACATCCGATGGCAAGAGGCC
CTAAGGTCCCCCCTCTTTGGTCTGCGACGTTATGCGGTATTAGCTACCGTTTCCAGTAGTTATCCCCCTCCAT
CGGGCAGTTTCCCAGACATTACTCACCCGTCCGCCACTCGTCAGCAAAGCAGCAAGCTGCTTCCTGTTACCGT
TCGACTGCATGTGTTAGGCCTGCCGCCAGCGTCAATCTGAGCCAGGATCAAACTCT
>472
CTGCTGCCGCCCGTAGGAGTCTGGACCGTGTCTCAGTTCCAGTGTGGCTGGTCATCCTCTCAGACCAGCTAGG
GATCGTCGCCTAGGTGAGCCGTTACCCCACCTACTAGCTAATCCCATCTGGGCACATCCGATGGCAAGAGGCC
CTAAGGTCCCCCTCTTGGTCTTGCGACGTTATGCGGTATTAGCTACCGTTTCCAGTAGTTATCCCCTCCATCA
GGCAGTTTCCCAGACATTACTCACCCGTCCGCCACTCGTCAGCAAAGCAGCAAGCTGCTTCTGTTACCGTTCG
ACTGCATGTGTTAGGCCTGCCGCCAGCGTTCAATCTGAGCCAGGATCAAACTCT
>473
CTGCTGCCGCCCGTAGGAGTCTGGACCGTGTCTCAGTTCCAGTGTGGCTGGTCATCCTCTCAGACCAGCTAGG
GATCGTCGCCTAGGTGAGCCGTTACCCCACCTACTAGCTAATCCCATCTGGGCACATCCGATGGCAAGAGGCC
CTAAGGTCCCCCTCTTTGTCTTGCGACGTATGCGGTATTAGCTACCGTTCCAGTAGTTATCCCCCTCCATCAG
GCAGTTTCCCAGACATTACTCACCCGTCCGCCACTCGTCAGCAAACAGCAAGCTGCTTCCTGTTACCGTTCGA
CTGCATGTGTTAGGCCTGCCGCCAGCGTCAATCTGAGCCAGGATCAAACTGT
>474
CTGCTGCCGCCCGTAGGAGTCTGGACCGTGTCTCAGTTCCAGTGTGGCTGGTCATCCTCTCAGACCAGCTAGG
GATCGTCGCCTAGGTGAGCCGTTACCCCACCTACTAGCTAATCCCATCTGGGCACATCCGATGGCAAGAGGCC
CTAAGGTCCCCCTCTTTGTCTGCGACGTTATGCGGTATTAGCTACCGTTTCCAGTAGTTATCCCCCTCCATCG
GGCAGTTTCCCAGACATTACTCACCCGTCCGCCACTCGTCAGCAAAGCAGCAAGCTGCTTCCTGTTACCGTTC
GACTGCATGTGTTAGGCCTGCCGCCAGCGTCAATCTGAGCCAGGATCAAACTCT
>475
CTGCTGCCGCCCGTAGGAGTCTGGACCGTGTCTCAGTTCCAGTGTGGCTGGTCATCCTCTCAGACCAGCTAGG
GATCGTCGCCTAGGTGAGCCGTTACCCCACCTACTAGCTAATCCCATCTGGGCACATCCGATGGCAAGAGGCC
CTAAGGTCCCCCTCTTTGGTCTTGCGACGTTATGCGGTATTAGCTACCGTTTCCAGTAGTTATCCCCCTCCAT
CAGGCAGTTTCCCAGACATTACTCACCCGTCCGCCACTCGTCAGCAAAGCAGCAAGCTGCTTCCTGTTACCGT
TCGACTTGCATGTGTTAGGCCTGCCGCCAGCGTTCAATCTGAGCCAGGATCAAACTCT
>476
CTGCTGCCGCCCGTAGGAGTCTGGACCGTGTCTCAGTTCCAGTGTGGCTGGTCATCCTCTCAGACCAGCTAGG
GATCGTCGCCTAGGTGAGCCGTTTACCCCACCTACTAGCTAATCCCATCTGGGCACATCCGATGGCAAGAGGC
CCTAAGGTCCCCCTCTTTGTCTTGCGACGTTATGCGGTATTAGCTACCGTTTCCAGTAGTTATCCCCCTCCAT
CAGGCAGTTTCCCAGACATTACTCACCCGTCCGCCACTCGTCAGCAAAGCAGCAAGCTGCTTCCGGTTACCGT
TCGACTGCATGTGTTAGGCCTGCCGCCAGCGTTCAATCTGAGCCAGGATCAAACTCT
>477
CTGCTGCCGCCCGTAGGAGTCTGGACCGTGTCTCAGTTCCAGTGTGGCTGGTCATCCTCTCAGACCAGCTAGG
GATCGTCGCCTAGGTGAGCCGTTTACCCCACCTACTAGCTAATCCCATCTGGGCACATCCGATGGCAAGAGGC
CCTAAGGTCCCCCTCTTGTCTTGCGACGTTATGCGGTATTAGCTACCGTTTCCAGTAGTTATCCCCCTCCATC

AGGCAGTTTCCCAGACAGTACTCACCCGTCCGCCACTCGTCAGCAAAACAGCAAGCTGCTTCCTGTTACCGTT
CGACTGCATGTGTTAGGCCTGCCGCCAGCGTTCAATCTGAAGCCAGGATCAAACTCT
>478
CTGCTGCCGCCCGTAGGAGTCTGGACCGTGTCTCAGTTCCAGTGTGGCTGGTCATCCTCTCAGACCAGCTAGG
GATCGTCGCCTAGGTGAGCCGTTACCCCACCTACTAGCTAATCCCATCTGGGCACATCCGATGGCAAGAGGCC
CTAAGGTCCCCCTCTTTGTCTTGCGACGTTATGCGGTATTAGCTACCGTTTCCAGTAGTTATCCCCCTCCATC
AGGCAGTTTCCCAGACATTACTCACCCGTCCGCCACTCGTCAGCAAACAGCAAGCTGCTTCCTGTTACCGTTC
GACTGCATGTGTTAGGCCTGCCGCCAGCGTTCAATCTGAGCCAGGATCAAACTCT
>479
TGCTGCCGCCCGTAGGAGTCTGGACCGTGTCTCAGTTCCAGTGTGGCTGGTCATCCTCTCAGACAGCTAGGGA
TCGTCGCCTAGGTGAGCCGTTACCCCACTACTAGCTAATCCCATCTGGGCACATCCGATGGCAAGAGGCCCTA
AGGTCCCCCTCTTTGGTCTTGCGACGTTATGCGGTATTAGCTACCGTTTCCAGTAGTTATCCCCCTCCATCAG
GCAGTTTCCCAGACATTACTCACCCGTCCGCCACTCGTCAGCAAAGCAGCAAGCTGCTTCCTGTTACCGTTCG
ACTGCATGTGTTAGGCCTGCCGCCAGCGTCAATCTGAGCCAGGATCAAACTCT
>480
CTGCTGCCGCCCGTAGGAGTCTGGACCGTGTCTCAGTTCCAGTGTGGCTGGCCATCCTCTCAGACCAGCTAGG
GATCGTCGCCTAGGTGAGCCGTTACCCCACCTACTAGCTAATCCCATCTGGGCACATCCGATGGCAAGAGGCC
CTAAGGTCCCCCTCTTGTCTGCGACGTATGCGGTATTAGCTACCGTTCCAGTAGTTATCCCCTCCATCGGGCA
GTTTCCCAGACATTACTCACCCGTCCGCCACTCGTCAGCAAAGCAGCAAGCTGCTTCCTGTTACCGTTCGACT
GCATGTGTTAGGCCTGCCGCCAGCGTCAATCTGAGCCAGGATCAAACTCT
>481
TGCTGCCGCCCGTAGGAGTCTGGACCGTGTCTCAGTTCCAGTGTGGCTGGTCATCCTCTCAGACAGCTAGGGA
TCGTCGCCTAGGTGAGCCGTTACCCCACTACTAGCTAATCCCCATGTGGCACATCCGATGGCAAGAGGCCCTA
AGGTCCCCCTCTTTGTCTTGCGACGTATGCGGTATTAGCTACCGTTTCCAGTAGTTATCCCCCTCCATCGGGC
AGTTTCCCAGACATTACTCACCCGTCCGCCACTCGTCAGCAAAGCAGCAAGCTGCTTCCTGTTACCGTTCGAC
TGCATGTGTTAGGCCTGCCGCCAGCGTCAATCTGAGCCAGGATCAAACTCT
>482
CTGCTGCCGCCCGTAGGAGTCTGGACCGTGTCTCAGTTCCAGTGTGGCTGGTCATCCTCTCAGACCAGCTAGG
GATCGTCGCCTAGGTGAGCCGTTACCCCACCTACTAGCTAATCCCATCTGGGCACATCCGATGGCAAGAGGCC
CTAAGGTCCCCCTCTTTGTCTGCGACGTATGCGGTATTAGCTACCGTTTCCAGTAGTTATCCCCTCCATCGGG
CAGTTTCCCAGACATTACTCACCCGTCCGCCACTCGTCAGCAGAGCAGCAAGCTGCTTCTGTTACCGTTCGAC
TGCATGTGTTAGGCCTGCCGCCAGCGTCAATCTGAGCCAGGATCAAACTCT
>483
CTGCTGCCGCCCGTAGGAGTCTGGACCGTGTCTCAGTTCCAGTGTGGCTGGTCATCCTCTCAGACCAGCTAGG
GATCGTCGCCTAGGTGAGCCGTTACCCCACCTACTAGCTAATCCCATCTGGGCACATCCGATGGCAAGAGGCC
CTAAGGTCCCCCTCTTTGGTCTTGCGACGTATGCGGTATTAGCTACCGTTTCCAGTAGTTATCCCCCTCCATC
AGGCAGTTTCCCAGACATTACTCACCCGTCCGCCACTCGTCAGCAAACAGCAAGCTGCTTCCTGTTACCGTTC
GACTGCATGTGTTAGGCCTGCCGCCAGCGTCAATCTGAGCCAGGATCAAACTCT
>484
CTGCTGCCGCCCGTAGGAGTCTGGACCGTGTCTCAGTTCCAGTGTGGCTGGTCATCCTCTCAGACCAGCTAGG
GATCGTCGCCTAGGTGAGCCGTTACCCCACCTACTAGCTAATCCCATCTGGGCACATCCGATGGCAAGAGGCC
CTAAGGTCCCCCTCTTTGTCTGCGACGTATGCGGTATTAGCTACCGTTTCCAGTAGTTATCCCCCTCCATCGG
GCAGTTTCCCAGACATTACTCACCCGTCCGCCACTCGTCAGCAAAGCAGCAAGCTGCTTCCTGTTACCGTTCG
ACTGCATGTGTTAGGCCTGCCGCCAGCGTCAATCTGAGCCAGGATCAAACTCT
>485
CTGCTGCCGCCCGTAGGAGTCTGGACCGTGTCTCAGTTCCAGTGTGGCTGGTCATCCTCTCAGACCAGCTAGG
GATCGTCGCCTAGGTGAGCCGTTACCCCACCTACTAGCTAATCCCATCTGGGCACATCCGATGGCAAGAGGCC
CTAAGGTCCCCCTCTTGTCTTGCGACGTATGCGGTATTAGCTACCGTTTCCAGTAGTTATCCCCTCCATCGGG
CAGTTTCCCAGACATTACTCACCCGTCCGCCACTCGTCAGCAAAGCAGCAAGCTGCTTCCTGTTACCGTTCGA
CTGCATGTGTTAGGCCTGCCGCCAGCGTCAATCTGAGCCAGGATCAAACTCT
>486
CTGCTGCCGCCCGTAGGAGTCTGGACCGTGTCTCAGTTCCAGTGTGGCTGGTCATCCTCTCAGACCAGCTAGG
GATCGTCGCCTAGGTGAGCCGTTACCCCACCTACTAGCTAATCCCATCTGGGCACATCCGATGGCAAGAGGCC
CTAAGGTCCCCCTCTTGTCTTGCGACGTATGCGGTATTAGCTACCGTTTCCAGTAGTTATCCCCCTCCATCGG

GCAGTTTCCCAGACATTACTCACCCGTCCGCCACTCGTCAGCAAAGCAGCAAGCTGCTTCCTGTTACCGTTCG
ACTGCATGTGTTAGGCCTGCCGCCAGCGTCAATCTGAGCCAAGGATCAAACTCT
>487
CTGCTGCCGCCCGTAGGAGTCTGGACCGTGTCTCAGTTCCAGTGTGGCTGGTCATCCTCTCAGACCAGCTAGG
GATCGTCGCCTAGGTGAGCCGTTACCCCACCTACTAGCTAATCCCATCTGGGCACATCCGATGGCAAGAGGCC
CTAAGGTCCCCCTCTTTGTCTTGCGACGTTATGCGGTATTAGCTACCGTTTCCAGTAGTTATCCCCCTCCATC
GGGCAGTTTTCCCAGACATTACTCACCCGTCCGCCACTCGTCAGCAAAGCAGCAAGCTGCTTCCTGTTACCGT
TCGACTGCATGTGTTAGGCCTGCCGCCAGCGTCAATCTGAGCCAGGATCAAACTCT
>488
CTGCTGCCGCCCGTAGGAGTCTGGACCGTGTCTCAGTTCCAGTGTGGCTGGTCATCCTCTCAGACCAGCTAGG
GATCGTCGCCTAGGTGAGCCGTTACCCCACCTACTAGCTAATCCCATCTGGGCACATCCGATGGCAAGAGGCC
CTAAGGTCCCCCTCTTGTCTTGCGACGTATGCGGTATTAGCTACCGTTTCCAGTAGTTATCCCCCTCCATCAG
GCAGTTTCCCAGACATTACTCACCCGTCCGCCACTCGTCAGCAAAGCAGCAAGCTGCTTTCTGTTACCGTTCG
ACTGCATGTGTTAGGCCTGCCGCCAGCGTCAATCTGAGCCAGGATCAAACTCT
>489
CTGCTGCCGCCCGTAGGAGTCTGGACCGTGTCTCAGTTCCAGTGTGGCTGGTCATCCTCTCAGACCAGCTAGG
GATCGTCGCCTAGGTGAGCCGTTCACCCCCACTACTAGCTAATCCCATCTGGGCACATCCGATGGCAAGAGGC
CCTAAGGTCCCCTCTTGTCTTGCGACGTATGCGGTATTAGCTACCGTTTCCAGTAGTTATCCCCCTCCATCAG
GCAGTTTCCCAGACATTACTCACCCGTCCGCCACTCGTCAGCAAGCAGCAAGCTGCTTCTGTTACCGTTCGAC
TGCATGTGTTAGGCCTGCCGCCAGCGTTCAATCTGAGCCAGGATCAAACTCT
>490
TTGCTGCCGCCCGTAGGAGTCTGGACCGTGTCTCAGTTCCAGTGTGGCTGGTCATCCTCTCAGACCAGCTAGG
GATCGTCGCCTAGGTGAGCCGTTACCCCACCTACTAGCTAATCCCATCTGGGCACATCCGATGGCAAGAGGCC
CTAAGGTCCCCCTCTTTGTCTTGCGACGTATGCGGTATTAGCTACCGTTTCCAGTAGTTATCCCCCTCCATCG
GGCAGTTTTCCCAGACATTACTCACCCGTCCGCCACTCGTCAGCAAAGCAGCAAGCTGCTTCCTGTTACCGTT
CGACTGCATGTGTTAGGCCTGCCGCCAGCGTCAATCTGAGCCAGGATCAAACTCT
>491
CTGCTGCCGCCCGTAGGAGTCTGGACCGTGTCTCAGTTCCAGTGTGGCTGGTCATCCTCTCAGACCAGCTAGG
GATCGTCGCCTAGGTGAGCCGTTACCCCACCTACTAGCTAATCCCATCTGGGCACATCCGATGGCAAGAGGCC
CTAAGGTCCCCCTCTCTGTCTTGCGACGTTATGCGGTATTAGCTACCGTTTCCAGTAGTTATCCCCCTCCATC
AGGCAGTTTCCCAGACATTACTCACCCGTCCGCCACTCGTCAGCAAAGCAGCAAGCTGCTTCCTGTTACCGTT
CGACTGCATGTGTTAGGCCTGCCGCCAGCGTCAATCTGAGCCAGGATCAAACTCT
>492
TGCTGCCGCCCGTAGGAGTCTGGACCGTGTCTCAGTTCCAGTGTGGCTGGTCATCCTCTCAGACCAGCTAGGG
ATCGTCGCCTAGGTGAGCCGTTACCCCACCTACTAGCTAATCCCATCTGGGCACATCCGATGGCAAGAGGCCC
TAAGGTCCCCCTCTTTGGTCTTGCGACGTTATGCGGTATTAGCTACCGTTTCCAGTAGTTATCCCCCTCCATC
GGGCAGTCTCCCAGACATTACTCACCCGTCCGCCACTCGTCAGCAAAGCAGCAAGCTGCTTCCTGTTACCGTT
CGACTGCATGTGTTAGGCCTGCCGCCAGCGTTCAATCTGAGCCAGGATCAAACTCT
>493
CTGCTGCCGCCCGTAGGAGTCTGGACCGTGTCTCAGTTCCAGTGTGGCTGGTCATCCTCTCAGACAGCTAGGG
ATCGTCGCCTAGGTGAGCCGTTACCCCACCTACTAGCTAATCCCATCTGGGCACATCCGATGGCAAGAGGCCC
TAAGGTCCCCCTCTTTGGTCTGCGGCGTTATGCGGTATTAGCTACCGTTTCCAGTAGTTATCCCCCTCCATCA
GGCAGTTTCCCAGACATTACTCACCCGTCCGCCACTCGTCAGCAAAGCAGCAAGCTGCTTCCTGTTACCGTTC
GACTGCATGTGTTAGGCCGCC
>494
CTGCTGCCGCCCGTAGGAGTCTGGACCGTGTCTCAGTTCCAGTGTGGCTGGTCATCCTCTCAGACCAGCTAGG
GATCGTCGCCTAGGTGAGCCGTTACCCCACCTACTAGCTAATCCCATCTGGGCACATCCGATGGCAAGAGGCC
CTAAGGTCCCCCTCTTGTCTTGCGACGTTATGCGGTATTAGCTACCGTTTCCAGTAGTTATCCCCCTCCATCA
GGCAGTTTTCCCAGACATTACTCACCCGTCCGCCACTCGTCAGCAAACAGCAAGCTGCTTCCTGTTACCGTTC
GACTGCATGTGTTAGGCCTGCCGCCAGCGTTCAATCTGAGCCAGGATCAAACTCT
>495
CTGCTGCCGCCCGTAGGAGTCTGGACCGTGTCTCAGTTCCAGTGTGGCTGGTCATCCCCTCAGACCAGCTAGG
GATCGTCGCCTAGGTGAGCCGTTACCCCACCTACTAGCTAATCCCATCTGGGCACATCCGATGGCAAGAGGCC
CTAAGGTCCCCCTCTTTGGTCTTGCGACGTTATGCGGTATTAGCTACCGTTTCCAGTAGTTATCCCCCTCCAT

CGGGCAGTTTCCCAGACATTACTCACCCGTCCGCCACTCGTCAGCAAAGCAGCAAGCTGCTTCCTGTTACCGT
TCGACTTGCATGCGTTAGGCCTGCCGCCAGCGTTCAATCTGAGCCAGGATCAAACTCT
>496
TGCTGCCGCCCGTAGGAGTCTGGACCGTGTCTCAGTTCCAGTGTGGCTGGTCATCCTCTCAGACCAGCTAGGG
ATCGTCGCCTAGGTGAGCCGTTACCCCACCTACTAGCTAATCCCATCTGGGCACATCCGATGGCAAGAGGCCC
TAAGGTCCCCCTCTTTGTCTTGCGACGTTATGCGGTATTAGCTACCGTTTCCAGTAGTTATCCCCCTCCATCG
GGCAGTTTCCCAGACATTACTCACCCGTCCGCCACTCGTCAGCAAAGCAGCAAGCTGCTTCCTGTTACCGTTC
GACTGCATGTGTTAGGCCTGCCGCCAGCGTCAATCTGAGCCAGGATCAAACTCT
>497
CTGCTGCCGCCCGTAGGAGTCTGGACCGTGTCTCAGTTCCAGTGTGGCTGGTCATCCTCTCAGACCAGCTAGG
GATCGTCGCCTAGGTGAGCCGTTACCCCACCTACTAGCTAATCCCATCTGGGCACATCCGATGGCAAGAGGCC
CTAAGGTCCCCCTCTTTGGTCTTGCGACGTTATGCGGTATTAGCTACCGTTTCCAGTAGTTATCCCCCTCCAT
CGGGCAGTTTTCCCAGACATTACTCACCCGTCCGCCACTCGTCAGCAAAGCAGCAAGCTGCTTCCTGTTACCG
TTCGACTGCATGTGTTAGGCCTGCCGCCAGCGTTCAATCTGAGCCAGGATCAAACTCT
>498
CTGCTGCCGCCCGTAGGAGTCTGGACCGTGTCTCAGTTCCAGTGTGGCTGGTCATCCTCTCAGACCAGCTAGG
GATCGTCGCCTAGGTGAGCCGTTACCCCACCTACTAGCTAATCCCATCTGGGCACATCCGATGGCAAGAGGCC
CTAAGGTCCCCCTCTTTGGTCTTGCGACGTATGCGGTATTAGCTACCGTTTCCAGTAGTTATCCCCCTCCATC
AGGCAGTTTCCCAGACATTACTCACCCGTCCGCCACTCGTCAGCAAAGCAGCAAGCTGCTTCTGTTACCGTTC
GACTGCATGTGTTAGGCCTGCCGCCAGCGTTCAATCTGAGCCAGGACAACTCT
>499
CTGCTGCCGCCCGTAGGAGTCTGGACCGTGTCTCAGTTCCAGTGTGGCTGGTCATCCTCTCAGACCAGCTAGG
GATCGTCGCCTAGGTGAGCCGTTACCCCACCTACTAGCTAATCCCATCTGGGCACATCCGATGGCAAGAGGCC
CTAAGGTCCCCCTCTTTGTCTTGCGACGTATGCGGTATTAGCTACCGTTTCCAGTAGTTATCCCCCTCCATCG
GGCAGTTTCCCAGACATTACTCACCCGTCCGCCACTCGTCAGCAAAGCAGCAAGCTGCTTCCTGTTACCGTTC
GACTTGCATGTGTTAGGCCTGCCGCCAGCGTCAATCTGAGCCAGGATCAAACTCT
>500
CTGCTGCCGCCCGTAGGAGTCTGGACCGTGTCTCAGTTCCAGTGTGGCTGGTCATCCTCTCAGACCAGCTAGG
GATCGTCGCCTAGGTGAGCCGTTACCCCACCTACTAGCTAATCCCATCTGGGCACATCCGATGGCAAGAGGCC
CTAAGGTCCCCCTCTTTGGTCTTGCGACGTTATGCGGTATTAGCTACCGTTTCCAGTAGTTATCCCCCTCCAT
CAGGCAGTTTCCCAGACATTACTCACCCGTCCGCCACTCGTCAGCAAAGCAGCAAGCTGCTTCCTGTTACCGT
TCGACTGCATGTGTTAGGCCTGCCGCCAGCGTTCAATCTGAGCCAGGATCAAACTCT
>501
CTGCTGCCGCCCGTAGGAGTCTGGACCGTGTCTCAGTTCCAGTGTGGCTGGTCATCCTCTCAGACCAGCTAGG
GATCGTCGCCTAGGTGAGCCGTTACCCCACCTACTAGCTAATCCCATCTGGGCACATCCGATGGCAAGAGGCC
CTAAGGTCCCCCTCTTGTCTGCGACGTTATGCGGTATTAGCTACCGTTTCCAGTAGTTATCCCCCTCCATCGG
GCAGTTTCCCAGACATTACTCACCCGTCCGCCACTCGTCAGCAAAGCAGCAAGCTGCTTCCTGTTACCGTTCG
ACTGCATGTGTTAGGCCTGCCGCCAGCGTCAATCTGAGACAGGATCAACTCT
>502
CTGCTGCCGCCCGTAGGAGTCTGGACCGTGTCTCAGTTCCAGTGTGGCTGGTCATCCTCTCAGACCAGCTAGG
GATCGTCGCCTAGGTGAGCCGTTACCCCACCTACTAGCTAATCCCATCTGGGCACATCCGATGGCAAGAGGCC
CTAAGGTCCCCCTCTTTGTCTTGCGACGTATGCGGTATTAGCTACCGTTTCCAGTAGTTATCCCCCTCCATCA
GGCAGTTTCCCAGACATTACTCACCCGTCCGCCACTCGTCAGCAAAGCAGCAAGCTGCTTCCTGTTACCGTTC
GACTGCATGTGTTAGGCCTGCCGCCAGCGTCAATCTGAGCCAGGATCAAACTCT
>503
CTGCTGCCGCCCGTAGGAGTCTGGACCGTGTCTCAGTTCCAGTGTGGCTGGTCATCCTCTCAGACCAGCTAGG
GATCGTCGCCTAGGTGAGCCGTTACCCCACCTACTAGCTAATCCCATCTGGGCGCATCCGATGGCAAGAGGCC
CTAAGGTCCCCCTCTTTGGTCTTGCGACGTTATGCGGTATTAGCTACCGTTTCCAGTAGTTATCCCCCTCCAT
CAGGCAGTTTCCCAGACATTACTCACCCGTCCGCCACTCGTCAGCAAAGCAGCAAGCTGCTTCCTGTTACCGT
TCGACTGCATGTGTTAGGCCTGCCGCCAGCGTCAATCTGAGCCAGGATCAAACTCT
>504
CTGCTGCCGCCCGTAGGAGTCTGGACCGTGTCTCAGTTCCAGTGTGGCTGGTCATCCTCTCAGACCAGCTAGG
GATCGTCGCCTAGGTGAGCCGTTACCCCACCTACTAGCTAATCCCATCTGGGCACATCCGATGGCAAGAGGCC
CTAAGGTCCCCCTCTTTGGTCTTGCGACGTTGTGCGGTATTAGCTACCGTTTCCAGTAGTTATCCCCCTCCAT

CAGGCAGTTTCCCAGACATTACTCACCCGTCCGCCACTCGTCAGCAAACAGCAAGCTGCTTCCTGTTACCGTT
CGACTGCATGTGTTAGGCCTGCCGCCAGCGTCAATCTGAGCCAGGATCAAACTCT
>505
CTGCTGCCGCCCGTAGGAGTCTGGACCGTGTCTCAGTTCCAGTGTGGCTGGTCATCCTCTCAGACCAGCTAGG
GATCGTCGCCTAGGTGAGCCGTTACCCCACCTATAGCTAATCCCATCTGGGCACATCCGATGGCAAGAGGCCC
TAAGGTCCCCTCTTTGTCTGCGACGTATGCGGTATTAGCTACCGTTTCCAGTAGTTATCCCCCTCCATCAGGC
AGTTTCCCAGACATACTCACCCGTCCGCCACTCGTCAGCAAAGCAGCAAGCTGCTTCCTGTTACCGTTCGACC
TGCATGTG
>506
CTGCTGCCGCCCGTAGGAGTCTGGACCGTGTCTCAGTTCCAGTGTGGCTGGTCATCCTCTCAGACCAGCTAGG
GATCGTCGCCTAGGTGAGCCGTTACCCCACCTACTAGCTAATCCCATCTGGGCACATCCGATGGCAAGAGGCC
CTAAGGTCCCCCTCTTTGTCTTGCGACGTTATGCGGTATTAGCTACCGTTTCCAGTAGTTATCCCCCTCCATC
GGGCAGTTTCCCAGACATTACTCACCCGTCCGCCACTCGTCAGCAAAGCAGCAAGCTGCTTCCTGTTACCGTT
CGACTGCATGTGTTAGGCCTGCCGCCAGCGTCAATCTGAGCCAGGATCAAACTCT
>507
CTGCTGCCGCCCGTAGGAGTCTGGACCGTGTCTCAGTTCCAGTGTGGCTGGTCATCCTCTCAGACCAGCTAGG
GATCGTCGCCTAGGTGAGCCGTTTACCCCACCTACTAGCTAATCCCATCTGGGCACATCCGATGGCAAGAGGC
CCTAAGGTCCCCCTCTTTGGTCTTGCGACGTTATGCGGTATTAGCTACCGTTTCCAGTAGTTATCCCCCTCCA
TCAGGCAGTTTCCCAGACATTACTCACCCGTCCGCCACTCGTCAGCAAAGCAGCAAGCTGCTTTCTGTTACCG
TTCGACTGCATGTGTTAGGCCTGCCGCCAGCGTCAATCTGAGCCAGGATCAAACTCT
>508
CTGCTGCCGCCCGTAGGAGTCTGGACCGTGTCTCAGTTCCAGTGTGGCTGGTCATCCTCTCAGACCAGCTAGG
GATCGTCGCCTAGGTGAGCCGTTACCCCACCTACTAGCTAATCCCATCTGGGCACATCCGATGGCAAGAGGCC
CTAAGGTCCCCCTCTTTGTCTTGCGACGTTATGCGGTATTAGCTACCGTTTCCAGTAGTTATCCCCCTCCATC
AGGCAGTTTCCCAGACATTACTCACCCGTCCGCCACTCGTCAGCAAAGCAGCAAGCTGCTTCCTGTTACCGTT
CGACTGCATGTGTTAGGCCTGCCGCCAGCGTCAATCTGAGCCAGGATCAAACTCT
>509
CTGCTGCCGCCCGTAGGAGTCTGGACCATGTCTCAGTTCCAGTGTGGCTGGTCATCCTCTCAGACCAGCTAGG
GATCGTCGCCTAGGTGAGCCGTTACCCCACCTACTAGCTAATCCCATCTGGGCACATCCGATGGCAAGAGGCC
CTAAGGTCCCCCTCTTTGGTCTTGCGACGTTATGCGGTATTAGCTACCGTTTCCAGTAGTTATCCCCCTCCAT
CAGGCAGTTTTCCCAGACACTACTCACCCGTCCGCCACTCGTCAGCAAAGCAGCAAGCTGCTTCCTGTTACCG
TTCGACTGCATGTGTTAGGCCTGCCGCCAGCGTCAATCTGAGCCCAGGATCAAACTCT
>510
CTGCTGCCGCCCGTAGGAGTCTGGACCGTGTCTCAGTTCCAGTGTGGCTGGTCATCCTCTCAGACAGCTAGGG
ATCGTCGCCTAGGTGAGCCGTTACCCCACCTACTAGCTAATCCCATCTGGGCACATCCGATGGCAAGAGGCCC
TAAGGTCCCCCTCTTTGTCTTGCGACGTATGCGGTATTAGCTACCGTTCCAGTAGTTATCCCCCTCATCGGGC
AGTTTCCCAGACATTACTCACCCGTCCGCCACTCGTCAGCAAAGCAGCAAGCTGCTTCCTGTTACCGTTCGAC
TGCATGTGTTAGGCCTGCCGCCAGCGTCAATCTGAGCCAGGATCAAACTCT
>511
CTGCTGCCGCCCGTAGGAGTCTGGACCGTGTCTCAGTTCCAGTGTGGCTGGTCATCCTCTCAGACCAGCTAGG
GATCGTCGCCTAGGTGAGCCGTTACCCCACCTACTAGCTAATCCCATCTGGGCACATCCGATGGCAAGAGGCC
CTAAGGTCCCCCTCTTTGTCTTGCGACGTATGCGGTATTAGCTACCGTTTCCAGTAGTTATCCCCCTCCATCA
GGCAGTTTCCCAGACATTACTCACCCGTCCGCCACTCGTCAGCAAAGCAGCAAGCTGCTTCCTGTTACCGTTC
GACTGCATGTGTTAGGCCTGCCGCCAGCGTCAATCTGAGCCAGGATCAAACTCT
>512
CTGCTGCCGCCCGTAGGAGTCTGGACCGTGTCTCAGTTCCAGTGTGGCTGGTCATCCTCTCAGACCAGCTAGG
GATCGTCGCCTAGGTGAGCCGTTACCCCACCTACTAGCTAATCCCATCTGGGCACATCCCGATGGCAAGAGGC
CCTAAGGTCCCCCTCTTGGTCTGCGACGTATGCGGTATTAGCTACCGTTTCCAGTAGTTATCCCCCTCCATCA
GGCAGCTTCCCAGACATTACTCACCCGTCCGCCACTCGTCAGCAAAGCAGCAAGCTGCTTCCTGTTACCGTTC
GACTGCATGTGTTAGGCCT
>513
CTGCTGCCGCCCGTAGGAGTCTGGACCGTGTCTCAGTTCCAGTGTGGCTGGTCATCCTCTCAGACCAGCTAGG
GATCGTCGCCTAGGTGAGCCGTTACCCCACCTACTAACTAATCCCATCTGGGCACATCCCGATGGCAAGAGGC
CCTAAGGTCCCCCTCTTGTCTTGCGACGTTATGCGGTATTAGCTACCGTTTCCAGTAGTTATCCCCCTCCATC

GGGCAGTTTCCCAGACATTACTCACCCGTCCGCCACTCGTCAGCAAAGCAGCAAGCTGCTTCCTGTTACCGTT
CGACTGCATGTGTTAGGCCTGCCGCCAGCGTCAATCTGAGCCAGGATCAAACTCT
>514
CTGCTGCCGCCCGTAGGAGTCTGGACCGTGTCTCAGTTCCAGTGTGGCTGGTCATCCTCTCAGACCAGCTAGG
GATCGTCGCCTAGGTGAGCCGTTACCCCACCTACTAGCTAATCCCATCTGGGCACATCCCGATGGCAAGAGGC
CCTAAGGTCCCCCTCTTTGTCTTGCGACGTATGCGGTATTAGCTACCGTTTCCAGTAGTTATCCCCCTCCATC
GGGCAGTTTCCCAGACATTACTCACCCGTCCGCCACTCGTCAGCAAAGCAGCAAGCTGCTTCCTGTTACCGTT
CGACTGCATGTGTTAGGCCTGCCGCCAGCGTCAATCTGAGCCAGGATCAAACTCT
>515
TGCTGCCGCCCGTAGGAGTCTGGACCGTGTCTCAGTTCCAGTGTGGCTGGTCATCCTCTCAGACCAGCTAGGG
ATCGTCGCCTAGGTGAGCCGTTACCCCACCTACTAGCTAATCCCATCTGGGCACATCCGATGGCAAGAGGCCC
TAAGGTCCCCCTCTTTGGTCTGCGACGTTATGCGGTATTAGCTACCGCTTCCAGTAGTTATCCCCCTCCATCA
GGCAGTTTCCCAGACATTACTCACCCGTCCGCCACTCGTCAGCAAAGCAGCAAGCTGCTTCCTGTTACCGTTC
GACTGCATGTGTTAGGCCTGCCGCCAGCGTCAATCTGAGCCAGGATCAAACTCT
>516
CTGCTGCCGCCCGTAGGAGTCTGGACCGTGTCTCAGTTCCAGTGTGGCTGGTCATCCTCTCAGACCAGCTAGG
GATCGTCGCCTAGGTGAGCCGTTACCCCACCTACTAGCTAATCCCATCTGGGCACATCCGATGGCAAGAGGCC
CTAAGGTCCCCCTCTTTGGTCTTGCGACGTTATGCGGTATTAGCTACCGTTTCCAGTAGTTATCCCCCTCCAT
CAGGCAGTTTCCCAGACATTACTCACCCGTCCGCCACTCGTCAGCAAAGCAGCAAGCTGCTTCTGTTACCGTT
CGACTGCATGTGTTAGGCCTGCCGCCAGCGTTCAATCTGAGCCAGGATCAAACTCT
>517
CTGCTGCCGCCCGTAGGAGTCTGGACCGTGTCTCAGTTCCAGTGTGGCTGGTCATCCTCTCAGACCAGCTAGG
GATCGTCGCCTAGGTGAGCCGTTACCCCACCTACTAGCTAATCCCATCTGGGCACATCCGATGGCAAGAGGCC
CTAAGGTCCCCCTCTTTGTCTTGCGACGTTATGCGGTATTAGCTACCGTTTCCAGTAGTTATCCCCCTCCATC
AGGCAGTTTCCCAGACATTACTCACCCGTCCGCCACTCGTCAGCAAAGCAGCAAGCTGCTTCCTGTTACCGTT
CGACTGCATGTGTTAGGCCTGCCGCCAGCGTCAATCTGAGCCAGGATCAAACTCT
>518
CTGCTGCCGCCCGAGAGTCTGGACCGTGTCTCAGTTCCAGTGTGGCTGGTCATCCTCTCAGACCAGCTAGGGA
TCGTCGCCTAGGTGAGCCGTTACCCCACCTACTAGCTAATCCCATCTGGGCACATCCGATGGCAAGAGGCCCT
AAGGTCCCCCTCTTTGTCTGCGACGTATGCGGTATTAGCTACCGTTTCCAGTAGTTATCCCCCTCCATCGGGC
AGTTTCCCAGACATTACTCACCCGTCCGCCACTCGTCAGCAAAGCAGCAAGCTGCTTCCTGTTACCGTTCGAC
TGCATGTGTTAGGCCTGCCGCCAGCGTCAATCTGAGCCAGGATCAAACTCT
>519
CTGCTGCCGCCCGTAGGAGTCTGGACCGTGTCTCAGTTCCAGTGTGGCTGGTCATCCTCTCAGACCAGCTAGG
GATCGTCGCCTAGGTGAGCCGTTACCCCACCTACTAGCTAATCCCATCTGGGCACATCCGATGGCAAGAGGCC
CTAAGGTCCCCCTCTTGGTCTGCGACGTATGCGGTATTAGCTACCGTTTCCAGTAGTTATCCCCCTCCATCAG
GCAGTTTCCCAGACATTACTCACCCGTCCGCCACTCGTCAGCAAAGCAGCAAGCTGCTCCTGTTACCGTTCGA
CTGCATGTGTTAGGCCTGCCGCCAGCGTCAATCTGAGCCAGGATCAAACTCT
>520
CTGCTGCCGCCCGTAGGAGTCTGGACCGTGTCTCAGTTCCAGTGTGGCTGGTCATCCTCTCAGACCAGCTAGG
GATCGCCGCCTAGGTGAGCCGTTACCCCACCTACTAGCTAATCCCATCTGGGCACATCCCGATGGCAAGAGGC
CCTAAGGTCCCCCTCTTGTCTGCGACGTATGCGGTATTAGCTACCGTTTCCAGTAGTTATCCCCCTCCATCGG
GCAGTTTCCCAGACACTACTCACCCGTCCGCCACTCGTCAGCAAGCAGCAAGCTGCTTCCTGTTACCGTTCGA
CTGCATGTGTTAGGCCTGCCGCCAGCGTCAATCTGAGCCAGGATCAAACTCT
>521
CTGCTGCCGCCCGTAGGAGTCTGGACCGTGTCTCAGTTCCAGTGTGGCTGGTCATCCTCTCAGACCAGCTAGG
GATCGTCGCCTAGGTGAGCCGTTACCCCACCTACTAGCTAATCCCATCTGGGCACATCCGATGGCAAGAGGCC
CTAAGGTCCCCCTCTTTGGTCTTGCGATGTTATGCGGTATTAGCTACCGTTTCCAGTAGTTATCCCCCTCCAT
CGGGCAGTTTCCCAGACATTACTCACCCGTCCGCCACTCGTCAGCAAAGCAGCAAGCTGCTTCCTGTTACCGT
TCGACTGCATGTGTTAGGCCTGCCGCCAGCGTCAATCTGAGCCAGGATCAAACTCT
>522
CTGCTGCCGCCCGTAGGAGTCTGGACCGTGTCTCAGTTCCAGTGTGGCTGGTCATCCTCTCAGACCAGCTAGG
GATCGTCGCCTAGGTGAGCCGTTACCCCACCTACTAGCTAATCCCATCTGGGCACATCCGATGGCAAGAGGCC
CTAAGGTCCCCCTCTTGTCTGCGACGTTATGCGGTATTAGCTACCGTTTCCAGTAGTTATCCCCCTCCATCAG

GCAGTTTTCCCAGACATTACTCACCCGTCCGCCACTCGTCAGCAAACAGCAAGCTGCTTCCTGTTACCGTTCG
ACTGCATGTGTTAGGCCTGCCGCCAGCGTTCAATCTGAGCCAGGATCAAACTCT
>523
CTGCTGCCGCCCGTAGGAGTCTGGACCGTGTCTCAGTTCCAGTGTGGCTGGTCATCCTCTCAGACCAGCTAGG
GATCGTCGCCTAGGTGAGCCGTTACCCCACCTACTAGCTAATCCCATCCGGGCACATCCGATGGCAAGAGGCC
CGAAGGTCCCCCTCTTTGTCTGCGACATTATGCGGTATTAGCTACCGTTTCCAGTAGTTATCCCCCTCCATCA
GGCAGTTTTCCCAGACATTACTCACCCGTCCGCCACTCGTCAGCGACAGCAAGCTGTTTCCTGTTACCGTTCG
ACTGCATGTGTTAGGCCTGCCGCCAGCGTCAATCTGAGCCAGGATCAAACTCT
>524
CTGCTGCCGCCCGTAGGAGTCTGGACCGTGTCTCAGTTTCCAGTGTGGCTGGTCATCCTCTCAGACCAGCTAG
GGATCGTCGCCTAGGTGAGCCGTTGCCCCACCTACTAGCTAATCCCATCTGGGCACATCCGATGGCAAGAGGC
CCCTAAGGTCCCCTCTTTGTCTGCGACGTATGCGGTATTAGCTACCGTTTCCAGTAGTTATCCCCCTCCATCG
GGCAGTTTCCCCAGACATTACTCACCCGTCCGCCACTCGTCAGCAAAGCAGCAAGCTGCTTCCTGTTACCGTT
CGACTGCATGTGTTAGGCCTGCCGCCAGCGTCAATCTGAGCCAGGATCAAACTCT
>525
CTGCTGCCGCCCGTAGGAGTCTGGACCGTGTCTCAGTTCCAGTGTGGCTGGTCATCCTCTCAGACCAGCTAGG
GATCGTCGCCTAGGTGAGCCGTTACCCCACCTACTAGCTAATCCCATCTGGGCACGTCCGATGGCAAGAGGCC
CTAAGGTCCCCCTCTTTGGTCTTGCGACGTATGCGGTATTAGCTACCGTTTCCAGTAGTTATCCCCCTCCATC
GGGCAGTTTCCCAGACATTACTCACCCGTCCGCCACTCGTCAGCAAAGCAGCAAGCTGCTTCCTGTTACCGTT
CGACTGCATGTGTTAGGCCTGCCGCCAGCGTCAATCTGAGCCAGGATCAAACTCT
>526
CTGCTGCCGCCCGTAGGAGTCTGGACCGTGTCTCAGTTCCAGTGTGGCTGGTCATCCTCTCAGACCAGCTAGG
GATCGTCGCCTAGGTGAGCCGTTACCCCACCTACTAGCTAATCCCATCTGGGCACATCCGATGGCAAGAGGCC
CTAAGGCCCCCCTCTTGTCTTGCGACGTATGCGGTATTAGCTACCGTTTCCAAGTAGTTATCCCCCTCCATCA
GGCAGTTTCCCAGAACATACTCACCCGTCCGCCACTCGTCAGCAAAGCAGCAAGCTGCTTCCTGTTACCGTTC
GAACTGCATGTGTTAGGCCTGCCGCCAGCATTCAATCTGAGCC
>527
CTGCTGCCGCCCGTAGGAGTCTGGACCGTGTCTCAGTTCCAGTGTGGCTGGTCATCCTCTCAGACAGCTAGGG
ATCGTCGCCTAGGTGAGCCGTTACCCCACCTACTAGCTAATCCCATCTGGGCACATCCGATGGCAAGAGGCCC
TAAGGTCCCCCTCTTGTCTTGCGACGTATGCGGTATTAGCTACCGTTTCCAGTAGTTATCCCCCTCCATCAGG
CAGTTTCCCAGACATTACTCACCCGTCCGCCACTCGTCAGCAAAGCAGCAAGCTGCTTCTGTTACCGTTCGAC
TGCATGTGTTAGGCCTGACCGCCAGCAGTCAATCTGAGCCAGGATCAAACTCGT
>528
CTGCTGCCGCCCGTAGGAGTCTGGACCGTGTCTCAGTTCCAGTGTGGCTGGTCATCCTCTCAGACCAGCTAGG
GATCGTCGCCTAGGTGAGCCGTTACCCCACCTACTAGCTAATCCCATCTGGGCACATCCGATGGCAAGAGGCC
CTAAGGTCCCCCTCTTTGTCTTGCGACGTTATGCGGTATTAGCTACCGTTTCCAGTAGTTATCCCCCTCCATC
GGGCAGTTTTCCCAGACATTACTCACCCGTCCGCCACTCGTCAGCAAAGCAGCAAACTGCTTCCTGTTACCGT
TCGACTGCATGTGTTAGGCCTGCCGCCAGCGTCAATCTGAGCCAGGATCAAACTCT
>529
CTGCTGCCGCCCGTAGGAGTCTGGACCGTGTCTCAGTTCCAGTGTGGCTGGTCATCCTCTCAGACCAGCTAGG
GATCGTCGCCTAGGTGAGCCGTTACCCCACCTACTAGCTAATCCCATCTGGGCACATCCGATGGCAAGAGGCC
CTAAGGTCCCCCTCTTTGTCTGCGACGTTATGCGGTATTAGCTACCGTTTCCAGTAGTTATCCCCCTCCATCA
GGCAGTTTCCCAGACATTACTCACCCGTCCGCCACTCGTCAGCAAAGCAGCAAGCTGCTTCTGTTACCGTTCG
ACTGCATGTGTTAGGCCTGCCGCCAGCGTTCAATCTGAGCCAGGATCAAACTCT
>530
CTGCTGCCGCCCGTAGGAGTCTGGACCGTGTCTCAGTTCCAGTGTGGCTGGTCATCCTCTCAGACCAGCTAGG
GATCGTCGCCTAGGTGAGCCGTTACCCCACCTACTAGCTAATCCCATCTGGGCACATCCGATGGCAAGAGGCC
CTAAGGTCCCCCCTCTTTGGTCTTGCGACGTTATGCGGTATTAGCTACCGTTTCCAGTAGTTATCCCCCTCCA
TCAGGCAGTTTCCCAGACATTACTCACCCGTCCGCCACTCGTCAGCAAAGCAGCAAGCTGCTTCCTGTTACCG
TTCGACTGCATGTGTTAGGCCTGCCGCCAGCGTTCAATCTGAGCCAGGATCAAACTCT
>531
CTGCTGCCGCCCGTAGGAGTCTGGACCGTGTCTCAGTTCCAGTGTGGCTGGTCATCCTCTCAGACCAGCTAGG
GATCGTCGCCTAGGTGAGCCGTTACCCCACCTACTAGCTAATCCCATCTGGGCACATCCGATGGCAAGAGGCC
CTAAGGTCCCCCTCTTTGTCTTGCGACGTTATGCGGTATTAGCTACCGTTTCCAGTAGTTATCCCCCTCCATC

AGGCAGTTTCCCAGACATTACTCACCCGTCCGCCACTCGTCAGCAAAGCAGCAAGCTGCTTCCTGTTACCGTT
CGACTGCATGTGTTAGGCCTGCCGCCAGCGTTCAATCTGAGCCAGGATCAAACTCT
>532
CTGCTGCCGCCCGTAGGAGTCTGGACCGTGTCTCAGTTCCAGTGTGGCTGGTCATCCTCTCAGACCAGCTAGG
GATCGTCGCCTAGGTGAGCCGTTACCCCACCTACTAGCTAATCCCATCTGGGCACATCCGATGGCAAGAGGCC
CTAAGGTCCCCCTCTTTGTCTGCGACGTATGCGGTATTAGCTACCGTTTCCAGTAGTTATCCCCCTCCATCAG
GCAGTTTTCCCAGACATTACTCACCCGTCCGCCACTCGTCAGCAAAGCAGCAAGCTGCTTCTGTTACCGTTCG
ACTGCATGTGTTAGGCCTGCCGCCAGCGTTCAATCTGAGCCAGGACAAACTCT
>533
CTGCTGCCGCCCGTAGGAGTCTGGACCGTGTCTCAGTTCCAGTGTGGCTGGTCATCCTCTCAGACCAGCTAGG
GATCGTCGCCTAGGTGAGCCGTTACCCCACCTACTAGCTAATCCCATCTGGGCACATCCGATGGCAAGAGGCC
CTAAGGTCCCCCTCTTTGGTCTTGCGACGTTATGCGGTATTAGCTACCGTTTCCAGTAGTTATCCCCCTCCAT
CAGGCAGTTTTCCCAGACATTACTCACCCGTCCGCCACTCGTCAGCAAAGCAGCAAGCTGCTTTCTGTTACCG
TTCGACTGCATGTGTTAGGCCTGCCGCCAGCGTTCAATCTGAGCCAGGATCAAACTCT
>534
CTGCTGCCGCCCGTAGGAGTCTGGACCGTGTCTCAGTTCCAGTGTGGCTGGTCATCCTCTCAGACCAGCTAGG
GATCGTCGCCTAGGTGAGCCGTTACCCCACCTACTAGCTAATCCCATCTGGGCACATCCGATGGCAAGAGGCC
CTAAGGTCCCCCCTCTTTGGTCTTGCGACGTTATGCGGTATTAGCTACCGTTTCCAGTAGTTATCCCCCTCCA
TCGGGCAGTTTCCCAGACATTACTCACCCGTCCGCCACTCGTCAGCAAAGCAGCAAGCTGCTTCCTGTTACCG
TTCGACTGCATGTGTTAGGCCTGCCGCCAGCGTTCAATCTGAGCCAGGATCAAACTCT
>535
CTGCTGCCGCCCGTAGGAGTCTGGACCGTGTCTCAGTTCCAGTGTGGCTGGTCATCCTCTCAGACCAGCTAGG
GATCGTCGCCTAGGTGAGCCGTTACCCCACCTACTAGCTAATCCCATCTGGGCACATCCGATGGCAAGAGGCC
CTAAGGTCCCCCTCTTTGGTCTGCGACGTTATGCGGTATTAGCTACCGTTTCCAGTAGTTATCCCCCTCCATC
GGGCAGTTTTCCCAGACATTACTCACCCGTCCGCCACTCGTCAGCAAAGCAGCAAGCTGCTTCCTGTTACCGT
TCGACTGCATGTGTTAGGCCTGCCGCCAGCGTCAATCTGAGCCAGGATCAAACTCT
>536
CTGCTGCCGCCCGTAGGAGTCTGGACCGTGTCTCAGTTCCAGTGTGGCTGGTCATCCTCTCAGACCAGCTAGG
GATCGTCGCCTAGGTGAGCCGTTACCCCACCTACTAGCTAATCCCATCTGGGCACATCCGATGGCAAGAGGCC
CTAAGGTCCCCCTCTTTGTCTTGCGACGTATGCGGTATTAGCTACCGTTTCCAGTAGTTATCCCCCTCCATCA
GGCAGTTTCCCAGACATTACTCACCCGTCCGCCACTCGTCAGCAAAGCAGCAAGCTGCTTCTGTTACCGTTCG
ACTTGCATGTGTTAGGCCTGCCGCCAGCGTCAATCTGAGCCAGGATCAAACTCT
>537
CTGCTGCCGCCCGTAGGAGTCTGGACCGTGTCTCAGTTCCAGTGTGGCTGGTCATCCTCTCAGACCAGCTAGG
GATCGTCGCCTAGGTGAGCCGTTACCCCACCTACTAGCTAATCCCATCTGGGCACATCCGATGGCAAGAGGCC
CTAAGGTCCCCCTCTTGTCTTGCGACGTATGCGGTATTAGCTACCGTTTCCAGTAGTTATCCCCCTCCATCAG
GCAGTTTCCCAGACATTACTCACCCGTCCGCCACTCGTCAGCAAAGCAGCAAGCTGCTTCTGTTACCGTTCGA
CTGCATGTGTTAGGCCTGCCGCCAGCGTCAATCTGAGCCAGGATCAAACTCT
>538
CTGCTGCCGCCCGTAGGAGTCTGGACCGTGTCTCAGTTCCAGTGTGGCTGGTCATCCTCTCAGACCAGCTAGG
GATCGTCGCCTAGGTGAGCCGTTACCCCACCTACTAGCTAATCCCATCTGGGCACATCCGATGGCAAGAGGCC
CTAAGGTCCCCCTCTTTGTCTTGCGACGTTATGCGGTATTAGCTACCGTTTCCAGTAGTTATCCCCCTCCATC
GGGCAGTTTCCCAGACATTACTCACCCGTCCGCCACTCGTCAGCAAAGCAGCAAGCTGCTTCCTGTCACCGTT
CGACTGCATGTGTTAGGCCTGCCGCCAGCGTCAATCTGAGCCAGGATCAAACTCT
>539
CTGCTGCCGCCCGTAGGAGTCTGGACCGTGTCTCAGTTCCAGTGTGGCTGGTCATCCTCTCAGACCAGCTAGG
GATCGTCGCCTAGGTGAGCCGTTACCCCACCTACTAACTAATCCCATCTGGGCACATCCGATGGCAAGAGGCC
CTAAGGTCCCCCTCTTTGGTCTTGCGGACGTTATGCGGTATTAGCTACCGTTTCCAGTAGTTATCCCCCTCCA
TCAGGCAGTTTCCCAGACATTACCCACCCGTCCGCCACTCGTCAGCAAAGCAGCAAGCTGCTTTCTGTTACCG
TTCGACTGCATGTGTTAGGCCTGCCGCCAGCGTCAATCTGAGCCAGGATCAAACTCT
>540
CTGCTGCCGCCCGTAGGAGTCTGGACCGTGTCTCAGTTCCAGTGTGGCTGGTCATCCTCTCAGACCAGCTAGG
GATCGTCGCCTAGGTGAGCCGTTACCCCACCTACTAGCTAATCCCATCTGGGCACATCCGATGGCAAGAGGCC
CTAAGGTCCCCCTCTTTGGTCTTGCGACGTTATGCGGTATTAGCTACCGTTTCCAGTAGTTATCCCCCTCCAT

CAGGCAGCTTCCCAGACATTACTCACCCGTCCGCCACTCGTCAGCAAAGCAGCAAGCTGCTTCTGTTACCGTT
CGACTTGCATGTGTTAGGCCTGCCGCCAGCGTTCAATCTGAGCCAGGATCAAACTCT
>541
CTGCTGCCGCCCGTAGGAGTCTGGACCGTGTCTCAGTTCCAGTGTGGCTGGTCATCCTCTCAGACCAGCTAGG
GATCGTCGCCTAGGTGAGCCGTTACCCCACCTACTAGCTAATCCCATCTGGGCACATCCGATGGCAAGAGGCC
CTAAGGTCCCCCTCTTTGGTCTGCGACGTTATGCGGTATTAGCTACCGTTTCCAGTAGTTATCCCCCTCCATC
GGGCAGTTTCCCAGACATTACTCACCCGTCCGCCACTCGTCAGCAAAGCAGCAAGCTGCTTCCTGTTACCGTT
CGACTGCATGTGTTAGGCCTGCCGCCAGCGTCAATCTGAGCCAGGATCAAACTCT
>542
CTGCTGCCGCCCGTAGGAGTCTGGACCGTGTCTCAGTTCCAGTGTGGCTGGTCATCCTCTCAGACCAGCTAGG
GATCGTCGCCTAGGTGAGCCGTTACCCCACCTACTAGCTAATCCCATCTGGGCACATCCGATGGCAAGAGGCC
CTAAGGTCCCCCCTCTTTGTCTTGCGACGTTATGCGGTATTAGCTACCGTTTCCAGTAGTTATCCCCCTCCAT
CGGGCAGTTTTCCCAGACGTACTCACCCGTCCGCCACTCGTCAGCAAAGCAGCAAGCTGCTTCCTGTTACCGT
TCGACTGCATGTGTTAGGCCTGCCGCCAGCGTCAATCTGAGCCAGGATCAAACTCT
>543
CTGCTGCCGCCCGTAGGAGTCTGGACCGTGTCTCAGTTCCAGTGTGGCTGGTCATCCTCTCAGACCAGCTAGG
GATCGTCGCCTAGGTGAGCCGTTACCCCACCTACTAGCTAATCCCATCTGGGCACATCCGATGGCAAGAGGCC
CTAAGGTCCCCCTCTTTGTCTTGCGACGTTATGCGGTATTAGCTACCGTTTCCAGTAGTTATCCCCCTCCATC
GGGCAGTTCCCAGACATTACTCACCCGTCCGCCACTCGTCAGCAAAGCAGCAAGCTGCTTCCTGTTACCGTTC
GACTGCATGTGTTAGGCCTGCCGCCAGCGTCAATCTGAGCCAGGGTCAAACTCT
>544
CTGCTGCCGCCCGTAGGAGTCTGGACCGTGTCTCAGTTCCAGTGTGGCTGGTCATCCTCTCAGACCAGCTAGG
GATCGTCGCCTAGGTGAGCCGTTACCCCACCTACTAGCTAATCCCATCTGGGCACATCCGATGGCAAGAGGCC
CTAAGGTCCCCCTCTTTGTCTTGCGACGTTATGCGGTATTAGCTACCGTTTCCAGTAGTTATCCCCCTCCATC
AGGCAGTTTCCCAGACATTACTCACCCGTCCGCCACTCGTCAGCAAAGCAGCAAGCTGCTTCCTGTTACCGTT
CGACTGCATGTGTTAGGCCTGCCGCCAGCGTCAATCTGAGCCAGGATCAAACTCT
>545
CTGCTGTCGCCCGTAGGAGTCTGGACCGTGTCTCAGTTCCAGTGTGGCTGGTCATCCTCTCAGACCAGCTAGG
GATCGTCGCCTAGGTGAGCCGTTACCCCACCTACTAGCTAATCCCATCTGGGCACATCCGATGGCAAGAGGCC
CTAAGGTCCCCCTCTTGTCTGCGACGTATGCGGTATTAGCTACCGTTTCCAGTAGTTATCCCCCTCCATCGGG
CAGTTTCCCAGACATTACTCACCCGTCCGCCACTCGTCAGCAAAGCAGCAAGCTGCTTCCTGTTACCGTTCGA
CTGCAATGTGTTAGGCCTGCCGCCAGCGTCAATCTGAGCCAGGATCAACTCT
>546
CTGCTGCCGCCCGTAGGAGTCTGGACCGTGTCTCAGTTCCAGTGTGGCTGGTCATCCTCTCAGACCAGCTAGG
GATCGTCGCCTAGGTGAGCCGTTACCCCACCTACTAGCTAATCCCATCTGGGCACATCCGATGGCAAGAGGCC
CTAAGGTCCCCCCTCTTTGGTCTTGCGACGTTATGCGGTATTAGCTACCGTTTCCAGTAGTTATCCCCCTCCA
TCGGGCAGTTTTCCCAGACATTACTCACCCGTCCGCCACTCGTCAGCAAAGCAGCAAGCTGCTTCCTGTTACC
GTTCGACTGCATGTGTTAGGCCTGCCGCCAGCGTCAATCTGAGCCAGGATCAAACTCT
>547
CTGCTGCCGCCCGTAGGAGTCTGGACCGTGTCTCAGTTCCAGTGTGGCTGGTCATCCTCTCAGACAGCTAGGG
ATCGTCGCCTAGGTGAGCGTTACCCCACCTACTAGCTAATCCCATCTGGGCACATCCGATGGCAAGGGCCCTA
AGGTCCCCCTCTTTGGTCTTGCGACGTTATGCGGTATTAGCTACCGTTTCCAGTAGTTATCCCCCTCCATCAG
GCAGTTTCCCAGACATTACTCACCCGTCCGCCACTCGTCAGCAAAGCAGCAAGCTGCTTCCTGTTGCCGTTCG
ACTGCATGTGTTAGGCTGCCGCCAGCGTCAATCTGAGCAGGATCAAACTCT
>548
CTGCTGCCGCCCGTAGGAGTCTGGACCGTGTCTCAGTTCCAGTGTGGCTGGTCATCCTCTCAGACCAGCTAGG
GATCGTCGCCTAGGTGAGCCGTTACCCCACCTACTAGCTAATCCCATCTGGGCACATCCGATGGCAAGAGGCC
CTAAGGTCCCCCTCTTTGGTCTGCGACGTTATGCGGTATTAGCTACCGTTTCCAGTAGTTATCCCCCTCCATC
GGGCAGTTTCCCAGACATTACTCACCCGTCCGCCACTCGTCAGCAAAGCAGCAAGCTGCTTCCTGTTACCGTT
CGACTGCATGTGTTAGGCCTGCCGCCAGCGTCAATCTGAGCCAGGATCAAACTCT
>549
CTGCTGCCGCCCGTAGGAGTCTGGACCGTGTCTCAGTTCCAGTGTGGCTGGTCATCCTCTCAGACCAGCTAGG
GATCGTCGCCTAGGTGAGCCGTTACCCCACCTACTAGCTAATCCCATCTGGGCACATCCGATGGCAAGAGGCC
CTAAGGTCCCCCTCTTTGTCTTGCGACGTTATGCGGTATTAGCTACCGTTTCCAGTAGTTATCCCCCTCCATC

AGGCAGTTTCCCAGACATTACTCACCCGTCCGCCACTCGTCAGCAAAGCAGCAAGCTGCTTCCTGTTACCGTT
CGACTGCATGTGTTAGGCCTGCCGCCAGCGTCAATCTGAGCCAGAATCAAACTCT
>550
CTGCTGCCGCCCGTAGGAGTCTGGACCGTGTCTCAGTTCCAGTGTGGCTGGTCATCCTCTCAGACCAGCTAGG
GATCGTCGCCTAGGTGAGCCGTTACCCCACCTACTAGCTAATCCCATCTGGGCACATCCGATGGCAAGAGGCC
CTAAGGTCCCCCTCTTTGGTCTTGCGACGTTATGCGGTATTAGCTACCGTTTCCAGTAGTTATCCCCCTCCAT
CAGGCAGTTTCCCAGACATTACTCACCCGTCCGCCACTCGTCAGCAAAGCAGCAAGCTGCCTCCTGTTACCGT
TCGACTGCATGTGTTAGGCCTGCCGCCAGCGTTCAATCTGAGCCAGGATCAAACTCT
>551
CTGCTGCCGCCCGTAGGAGTCTGGACCGTGTCTCAGTTCCAGTGTGGCCGGTCATCCTCTCAGACCAGCTAGG
GATCGTCGCCTAGGTGAGCCGTTACCCCACCTACTAGCTAATCCCATCTGGGCACATCCGATGGCAAGAGGCC
CTAAGGTCCCCCTCTTTGGTCTGCGACGTATGCGGTATTAGCTACCGTTTCCAGTAGTTATCCCCCTCCATCA
GGCAGTTTCCCAGACATTACTCACCCGTCCGCCACTCGTCAGCAAACAGCAAGCTGCTTCCTGTTACCGTTCG
ACTGCATGTGTTAGGCCTGCCGCCAGCGTTCAATCTGAGCCAGGATCAAACTCT
>552
CTGCTGCCGCCCGTAGGAGTCTGGACCGTGTCTCAATTCCAGTGTGGCTGGTCATCCTCTCAGACCAGCTAGG
GATCGTCGCCTAGGTGAGCCGTTACCCCACCTACTAGCTAATCCCATCTGGGCACATCCGATGGCAAGAGGCC
CTAAGGTCCCCCTCTTTGTCTTGCGACGTTATGCGGTATTAGCTACCGTTTCCAGTAGTTATCCCCCTCCATC
AGGCAGTTTCCCAGACATTACTCACCCGTCCGCCACTCGTCAGCAAAGCAGCAAGCTGCTTCCTGTTACCGTT
CGACTGCATGTGGTTAGGCCT
>553
CTGCTGCCGCCCGTAGGAGTCTGGACCGTGTCTCAGTTCCAGTGTGGCTGGTCATCCTCTCAGACCAGCTAGG
GATCGTCGCCTAGGTGAGCCGTTACCCCACCTACTAGCTAATCCCATCTGGGCACATCCGATGGCAAGAGGCC
CTAAGGTCCCCCTCTTTGGTCTTGCGACGTTATGCGGTATTAGCTACCGTTTCCAGTAGTTATCCCCCTCCAT
CGGGCAGTTTCCCAGACATTACTCACCCGTCCGCCACTCGTCAGCAAAGCAGCAAGCTGCTTCCTGTTACCGT
TCGACTGCATGTGTTAGGCCTGCCGCCAGCGTTCAATCTGAGCCAGGATCAAACTCT
>554
CTGCTGCCGCCCGTAGGAGTCTGGACCGTGTCTCAGTTCCAGTGTGGCTGGTCATCCTCTCAGACCAGCTAGG
GATCGTCGCCTAGGTGAGCCGTTACCCCACCTACTAGCTAATCCCATCTGGGCACATCCGATGGCAAGAGGCC
CTAAGGTCCCCCTCTTGTCTTGCGACGTATGCGGTATTAGCTACCGTTTCCAGTAGTTATCCCCCTCCATCAG
GCAGTTTCCCAGACATTACTCACCCGTCCGCCACTCGTCAGCAAAGCAGCAAGCTGCTTCTGTTACCGTCGAC
TGCATGTCGTTAGGCCTGCCGCCAGCCGTTCAATCTGAAGCCAGGATCAAACTCGT
>555
CTGCTGCCGCCCGTAGGAGTCTGGACCGTGTCTCAGTTCCAGTGTGGCTGGTCATCCTCTCAGACCAGCTAGG
GATCGTCGCCTAGGTGAGCCGTTACCCCACCTACTAGCTAACCCCATCTGGGCACATCCGATGGCAAGAGGCC
CTAAGGTCCCCTCTTTGTCTTGCGACGTATGCGGTATTAGCTACCGTTTCCAGTAGTTATCCCCTCCATCGGG
CAGTTTCCCAGACATTACTCACCCGTCCGCCACTCGTCAGCAAAGCAGCAAGCTGCTTCCTGTTACCGTTCGA
CTGCATGTGTTAGGCCTGCCGCCAGCGTCAATCTGAGCCAGGATCAAACTCT
>556
CTGCTGCCGCCCGTAGGAGTCTGGACCGTGTCTCAGTTCCAGTGTGGCTGGTCATCCTCTCAGACCAGCTAGG
GATCGTCGCCTAGGTGAGCCGTTACCCCACCTACTAGCTAATCCCATCTGGGCACATCCGATGGCAAGAGGCC
CTAAGGTCCCCCTCTTTGGTCTTGCGACGTTATGCGGTATTAGCTACCGTTTCCAGTAGTTATCCCCCTCCAT
CAGGCAGTTTCCCAGACATTACTCACCCGTCCGCCACTCGTCAGCAAAGCAGCAAGCTGCTTCCTGTTACCGT
TCGACTGCATGTGTTAGGCCTGCCGCCAGCGTTCAATCTGAGCCAGGATCAAACTCT
>557
CTGCTGCCGCCCGTAGGAGTCTGGACCGTGTCTCAGTTCCAGTGTGGCTGGTCATCCTCTCAGACCAGCTAGG
GATCGTCGCCTAGGTGAGCCGTTACCCCACCTACTAGCTAATCCCATCTGGGCACATCCGATGGCAAGAGGCC
CTAAGGTCCCCCTCTTGTCTTGCGACGTATGCGGTATTAGCTACCGTTTCCAGTAGTTATCCCCCTCCATCAG
GCAGTTTTCCCAGACATTACTCACCCGTCCGCCACTCGTCAGCAAAGCAGCAAGCTGCTTCCTGTTACCGTTC
GACTGCATGTGTTAGGCCTGCCGCCAGCGTTCAATCTGAGCCAGGATCAAACTCT
>558
CTGCTGCCGCCCGTAGGAGTCTGGACCGTGTCTCAGTTCCAGTGTGGCTGGACATCCTCTCAGACCAGCTAGG
GATCGTCGCCTAGGTGAACCGTTACCCCACCTACTAGCTAATCCCATCTGGGCACATCCGATGGCAAGAGGCC
CTAAGGTCCCCCTCTTTGGTCTTGCGACGTATGCGGTATTAGCTACCGTTTCCAGTAGTTATCCCCCTCCATC

AGGCAGTTTCCCAGACATTACCCACCCGTCCGCCACTCGTCAGCAAAGCAGCAAGCTGCTTCCTGTTACCGTT
CGACTGCATGTGTTAGGCCTGCCGCCAGCGTCAATCTGAGCCAGGATCAAACTCT
>559
CTGCTGCCGCCCGTAGGAGTCTGGACCGTGTCTCAGTTCCAGTGTGGCTGGTCATCCTCTCAGACCAGCTAGG
GATCGTCGCCTAGGTGAGCCGTTACCCCACCTACTAGCTAATCCCATCTGGGCACATCCGATGGCAAGAGGCC
CTAAGGTCCCCCTCTTGTCTGCGACGTATGCGGTATTAGCTACCGTTTCCAGTAGTTCTCCCCTCCATCGGGC
AGTTTCCCAGACATTACTCACCCGTCCGCCACTCGTCAGCAAGCAGCAAGCTGCTTCCTGTTACCGTTCGACT
GCATGTGTTAGGCCTGCCGCCAGCGTCAATCTGAGCCAGGATCAAACTCT
>560
CTGCTGCCGCCCGTAGGAGTCTGGACCGTGTCTCAGTTCCAGTGTGGCTGGTCATCCTCTCAGACCAGCTAGG
GATCGTCGCCTAGGTGAGCCGTTACCCCACCTACTAGCTAATCCCATCTGGGCACATCCGATGGCAAGAGGCC
CTAAGGTCCCCCCTCTTTGTCTTGCGACGTTATGCGGTATTAGCTACCGTTTCCAGTAGTTATCCCCCTCCAT
CGGGCAGTTTTCCCAGACATTACTCACCCGTCCGCCACTCGTCAGCAAAGCAGCAAGCTGCTTCCTGTTACCG
TTCGACTGCATGTGTTAGGCCTGCCGCCAGCGTTCAATCTGAGCCAGGATCAAACTCT
>561
CTGCTGCCGCCCGTAGGAGTCTGGACCGTGTCTCAGTTCCAGTGTGGCTGGTCATCCTCTCAGACCAGCTAGG
GATCGTCGCCTAGGTGAGCCGTTACCCCACCTACTAGCTAATCCCATCTGGGCACATCCGATGGCAAGAGGCC
CTAAGGTCCCCCTCTTGTCTGCGACGTATGCGGTATTAGCTACCGTTTCCAGTAGTTTATCCCCCTCCATCAG
GCAGTTTCCCAGACATTACTCACCCGTCCGCCACTCGTCAGCAAAGCAGCAAGCTGCTTCCTGTTACCGTTCG
ACTGCATGTGTTAGGCCTGCCGCCAGCGTCAATCTGAGCCAGGATCAAACTCT
>562
CTGCTGCCGCCCGTAGGAGTCTGGACCGTGTCTCAGTTCCAGTGTGGCTGGTCATCCTCTCAGACCAGCTAGG
GATCGTCGCCTAGGTGAGCCGTTACCCCACCTACTAGCTAATCCCATCTGGGCACATCCGATGGCAAGAGGCC
CTAAGGTCCCCCTCTTTGTCTTGCGACGTATGCGGTATTAGCTACCGTTTCCAGTAGTTATCCCCCTCCATTA
GGCAGTTTCCCAGACATTACTCACCCGTCCGCCACTCGTCAGCAAAGCAGCAAGCTGCTTCTGTTACCGTTCG
ACTGCATGTGTTAGGCCTGCCGCCAGCGTTCAATCTGAGCCAGGATCAAACTCT
>563
CTGCTGCCGCCCGTAGGAGTCTGGACCGTGTCTCAGTTCCAGTGTGGCTGGTCATCCTCTCAGACCAGCTAGG
GATCGTCGCCTAGGTGAGCCGTTACCCCACCTACTAGCTAATCCCATCTGGGCACATCCGATGGCAAGAGGCC
CTAAGGTCCCCCTCTTTGGCCTTGCGACGTATGCGGTATTAGCTACCGTTTCCAGTAGTTATCCCCCTCCATC
AGGCAGTTTCCCAGACATTACTCACCCGTCCGCCACTCGTCAGCAAGCAGCAAGCTGCTTCCTGTTACCGTTC
GACTGCATGTGTTAGGCCTGCCGCCAGCGTCAATCTGAGCCAGGATCAAACTCT
>564
CTGCTGCCGCCCGTAGGAGTCTGGACCGTGTCTCAGTTCCAGTGTGGCTGGTCATCCTCTCAGACCAGCTAGG
GATCGTCGCCTAGGTGAGCCGTTACCCCACCTACTAGCTAATCCCATCTGGGCACATCCGATGGCAAGAGGCC
CTAAGGTCCCCCTCTTGTCTTGCGACGTATGCGGTATTAGCTACCGTTTCCAGTAGTTATCCCCCTCCAACAG
GCAGTTTCCCAGACATTACTCACCCGTCCGCCACTCGTCAGCAAGCAGCAAGCTGCTTCTGTTACCGTTCGAC
TGCATGTGTTAGGCCTGCCGCCAGCGTCGATCTGAGCCAGGATCAAACTCT
>565
CTGCTGCCGCCCGTAGGAGTCTGGACCGTGTCTCAGTTCCAGTGTGGCTGGTCATCCTCTCAGACCAGCTAGG
GATCGTCGCCTAGGTGAGCCGTTACCCCACCTACTAGCTAATCCCATCTGGGCACATCCGATGGCAAGAGGCC
CTAAGGTCCCCCTCTTGTCTTGCGACGTATGCGGTATTAGCTACCGTTTCCAGTAGTTATCCCCCTCCATCGG
GCAGTTTCCCAGACATTACTCACCCGTCCGCCACTCGTCAGCAAAGCAGCAAGCTGCTTCCTGTTACCGTTCG
ACTGCATGTGTTAGGCCTGCCGCCAGCGTCAATCTGAGCCAGGATCAAACTCT
>566
CTGCTGCCGCCCGTAGGAGTCTGGACCGTGTCTCAGTTCCAATGTGGCTGGTCGTCCTCTCAGACCAGCTAGG
GATCGTCGCCTAGGTGAGCCGTTACCCCACCTACTAGCTAATCCCATCTGGGCACACCCGATGGCAAGAGGCC
CTAAGGTCCCCCTCTTTGTCTTGCGACGTATGCGGTATTAGCTACCGTTTCCAGTAGTTATCCCCCTCCATCA
GGCGGTTTCCCAGACATTACTCACCCGTCCGCCACTCGTCAGCAAAGCAGCAAGCTGCTTCTGTTACCGTTCG
ACTGCATGTGTTAGGCCTGCCGCCAGCGTCAATCTGAGCCAGGATCAAACTCT
>567
CTGCTGCCGCCCGTAGGAGTCTGGACCGTGTCTCAGTTCCAGTGTGGCTGGTCATCCTCTCAGACCAGCTAGG
GATCGTCGCCTAGGTGAGCCGTTACCCCACCTACTAGCTAATCCCATCTGGGCACATCCGATGGCAAGAGGCC
CTAAGGTCCCCCTCTTTGGTCTTGTGACGTTATGCGGTATTAGCTACCGTTTCCAGTAGTTATCCCCCTCCAT

CGGGCAGTTTCCCAGACATTACTCACCCGTCCGCCACTCGTCAGCAAAGCAGCAAGCTGCTTCCTGTTACCGT
TCGACTTGCATGTGTTAGGCCTGCCGCCAGCGTCAATCTGAGCCAGGATCAAACTCT
>568
CTGCTGCCGCCCGTAGGAGTCTGGACCGTGTCTCAGTTCCAGTGTGGCTGGTCATCCTCTCAGACAGCTAGGG
ATCGTCGCCTAGGTGAGCCGTTACCCCACCTACTAGCTAATCCCATCTGGGCACATCCGATGGCAAGAGGCCC
TAAGGTCCCCCTCTTGTCTTGCGACGTATGCGGTATTAGCTACCGTTTCCAGTAGTTATCCCCCTCCATCAGG
CAGTTTCCCAGACATTACTCACCCGTCCGCCACTCGTCAGCAAGCAGCAAGCTGCTTCTGTTACCGTTCGACT
GCATGTGTTAGGCCTGCCGCCAGCGTCAATCTGAGCCAGGATCAACTCT
>569
CTGCTGCCGCCCGTAGGAGTCTGGACCGTGCCTCAGTTCCAGTGTGGCTGGTCATCCTCTCAGACCAGCTAGG
GATCGTCGCCTAGGTGAGCCGTTACCCCACCTACTAGCTAATCCCATCTGGGCACATCCGATGGCAAGAGGCC
CTAAGGTCCCCCTCTTGTCTTGCGACGTATGCGGTATTAGCTACCGTTTCCAGTAGTTATCCCCCTCCATCGG
GCAGTTTCCCAGACATTACTCACCCGTCCGCCACTCGTCAGCAAAGCAGCAAGCTGCTTCCTGTTACCGTTCG
ACTGCCATGTGTTAGGCCTGCCGCCAGCGTCAATCTGAGCCAGGATCAACTCT
>570
CTGCTGCCGCCCGTAGGAGTCTGGACCGTGTCTCAGTTCCAGTGTGGCTGGTCATCCTCTCAGACCAGCTAGG
GATCGTCGCCTAGGTGAGCCGTTACCCCACCTACTAGCTAATCCCATCTGGGCACATCCGATGGCAAGAGGCC
CTAAGGTCCCCCTCTTGTCTGCGACGTATGCGGTATTAGCTACCGTTTCCAGTAGTTATCCCCCTCCATCGGG
CAGTTTCCCAGACATTACTCACCCGTCCCGCCACTCGTCAGCAAGCAGCAAGCTGCTTCCTGTTACCGTTCGA
CTGCATGTGTTAGGCCTGCCGCCAGCGTCAATCTGAGCCAGGATCAAACTCT
>571
CTGCTGCCGCCCGTAGGAGTCTGGACCGTGTCTCAGTTCCAGTGTGGCTGGTCATCCTCTCAGACCAGCTAGG
GATCGTCGCCTAGGTGAGCCGTTACCCCACCTACTAGCTAATCCCATCTGGGCACATCCGATGGCAAGAGGCC
CTAAGGTCCCCCCTCTTTGGTCTTGCGACGTTATGCGGTATTAGCTACCGTTTCCAGTAGTTATCCCCCTCCA
TCAGGCAGTTTCCCAGACATTACTCACCCGTCCGCCACTCGTCAGCAAACAGCAAGCTGCTTCCTGTTACCGT
TCGACTGCATGTGTTAGGCCTGCCGCCAGCGTCAATCTGAGCCAGGATCAAACTCT
>572
TGCTGCCGCCCGTAGGAGTCTGGACCGTGTCTCAGTTCCAGTGTGGCTGGTCATCCTCTCAGACCAGCTAGGG
ATCGTCGCCTAGGTGAGCCGTTACCCCACCTACTAGCTAATCCCATCTGGGCACATCCGATGGCAAGAGGCCC
TAAGGTCCCCCTCTTTGGTCTTGCGACGTATGCGGTATTAGCTACCGTTTCCAGTAGTTATCCCCCTCCATCA
GGCAGTTTTCCCAGACATTACTCACCCGTCCGCCACTCGTCAGCAAAGCAGCAAGCTGCTTCCTGTTACCGTT
CGACTGCATGTGTTAGGCCTGCCGCCAGCGTCAATCTGAGCCAGGATCAAACTCT
>573
CTGCTGCCGCCCGTAGGAGTCTGGACCGTGTCTCAGTTCCAGTGTGGCTGGTCATCCTCTCAGACCAGCTAGG
GATCGTCGCCTAGGTGAGCCGTTACCCCACCTACTAGCTAATCCCATCTGGGCACATCCGATGGCAAGAGGCC
CTAAGGTCCCCCTCTTGTCTTGCGACGTTATGCGGTATTAGCTACCGTTTCCAGTAGTTATCCCCCTCCATCG
GGCAGTTTCCCAGACATTACTCACCCGTCCGCCACTCGTCAGCAAGCAGCAAGCTGCTTCCTGTTACCGTTCG
ACTGCATGTGTTAGGCCTGCCGCCAGCGTCAATCTGAGCCAGGATCAACTCT
>574
CTGCTGCCGCCCGTAGGAGTCTGGACCGTGTCTCAGTTCCAGTGTGGCTGGTCATCCTCTCAGACCAGCTAGG
GATCGTCGCCTAGGTGAGCCGTTACCCCACCTACCTAGCTAATCCCATCTGGGCACATCCGATGGCAAGAGGC
CCTAAGGTCCCCCTCTTGTCTTGCGACGTATGCGGTATTAGCTACCGTTTCCAGTAGTTATCCCCCTCCATCA
GGCAGTTTCCCAGACATTACTCACCCGTCCGCCACTCGTCAGCAAAGCAGCAAGCTGCTTTCTGTTACCGTTC
GACTGCATGTGTTAGGCCTGCCGCCAGCGTTCAATCTGAGCCAGGATCAAACCTCT
>575
CTGCTGCCGCCCGTAGGAGTCTGGACCGTGTCTCAGTTCCAGTGTGGCTGGTCATCCTCTCAGACCAGCTAGG
GATCGTCGCCTAGGTGAGCCGTTACCCCACCTACTAGCTAATCCCATCTGGGCACATCCGATGGCAAGAGGCC
CTAAGGTCCCCCTCTTTGGTCTTGCGACGTTATGCGGTATTAGCTACCGTTTCCAGTAGTTATCCCCTCCATC
GGGCAGTTTCCCAGACATTACTCACCCGTCCGCCACTCGTCAGCAAAGCAGCAAGCTGCTTCCTGTTACCGTT
CGACTGCATGTGTTAGGCCTGCCGCCAGCGTCAATCTGAGCCAGGATCAAACTCT
>576
CTGCTGCCGCCCGTAGGAGTCTGGACCGTGTCTCAGTTCCAGTGTGGCTGGTCATCCTCTCAGACCAGCTAGG
GATCGTCGCCTAGGTGAGCCGTTACCCCACCTACTAGCTAATCCCATCTGGGCACATCCCGATGGCAAGAGGC
CCTAAGGTCCCCCTCTTTGGTCTTGCGACGTTATGCGGTATTAGCTACCGTTTCCAGTAGTTATCCCCCTCCA

TCGGGCAGTTTTCCCAGACATTACTCACCCGTCCGCCACTCGTCAGCAAAGCAGCAAGCTGCTTCCTGTTACC
GTTCGACTGCATGTGTTAGGCCTGCCGCCAGCGTTCAATCTGAGCCAGGATCAAACTCT
>577
CTGCTGCCGCCCGTAGGAGTCTGGACCGTGTCTCAGTTCCAGTGTGGCTGGTCATCCTCTCAGACCAGCTAGG
GATCGTCGCCTAGGTGAGCCGTTACCCCACCTACTAGCTAATCCCATCTGGGCACATCCGATGGCAAGAGGCC
CTAAGGTCCCCCTCTTTGGTCTTGCGACGTTATGCGGTATTAGCTACCGTTTCCAGTAGTTATCCCCCTCCAT
CGGGCAGTTTTCCCAGACATTACTCACCCGTCCGCCACTCGTCAGCAAAGCAGCAAGCTGCTTCCTGTTACCG
TTCGACTGCATGTGTTAGGCCTGCCGCCAGCGTCAATCTGAGCCAGGATCAAACTCT
>578
CTGCTGCCGCCCGTAGGAGTCTGGACCGTGTCTCAGTTCCAGTGTGGCTGGTCATCCTCTCAGACCAGCTAGG
GATCGTCGCCTAGGTGAGCCGTTACCCCACCTACTAGCTAATCCCATCTGGGCACATCCGATGGCAAGAGGCC
CTAAGGTCCCCCCTCTTTGGTCTTGCGACGTTATGCGGTATTAGCTACCGTTTCCAGTAGTTATCCCCCTCCA
TCGGGCAGTTTTCCCAGACATTACTCACCCGTCCGCCACTCGTCAGCAAAGCAGCAAGCTGCTTCCTGTTACC
GTTCGACTGCATGTGTTAGGCCTGCCGCCAGCGTTCAATCTGAGCCAGGATCAAACTCT
>579
CTGCTGCCGCCCGTAGGAGTCTGGACCGTGTCTCAGTTCCAGTGTGGCTGGTCATCCTCTCAGACCAGCTAGG
GATCGTCGCCTAGGTGAGCCGTTACCCCACCTACTAGCTAATCCCATCTGGGCACATCCGATGGCAAGAGGCC
CTAAGGTCCCCCTCTTGTCTTGCGACGTCTGCGGTATTAGCTACCGTTTCCAGTAGTTATCCCCCTCCATCAG
GCAGTTTCCCAGACATACTCACCCGTCCGCCACTCGTCAGCAAACAGCAAGCTGCTTCCTGTTACCGTTCGAA
CTGCATGTGTTAGGCCTGCCGCC
>580
CTGCTGCCGCCCGTAGGAGTCTGGACCGTGTCTCAGTTCCAGTGTGGCTGGTCATCCTCTCAGACCAGCTAGG
GATCGTCGCCTAGGTGAGCCGTTACCCCACCTACTAGCTAATCCCATCTGGGCACATCCGATGGCAAGAGGCC
CTAAGGTCCCCCTCTTTGTCTTGCGACGTTATGCGGTATTAGCTACCGTTTCCAGTAGTTATCCCCCTCCATC
GGGCAGTTTTCCCAGACATTACTCACCCGTCCGCCACTCGTCAGCAAAGCAGCAAGCTGCTTCCTGTTACCGT
TCGACTGCATGTGTTAGGCCTGCCGCCAGCGTCAATCTGAGCCAGGATCAAACTCT
>581
CTGCTGCCACCCGTAGGAGTCTGGACCGTGTCTCAGTTCCAGTGTGGCTGGTCATCCTCTCAGACCAGCTAGG
GATCGTCGCCTAGGTGAGCCGTTACCCCACCTACTAGCTAATCCCATCTGGGCACATCCGATGGCAAGAGGCC
CTAAGGTCCCCCTCTTTGGTCTTGCGACGTTATGCGGTATTAGCTACCGTTTCCAGTAGTTATCCCCCTCCAT
CAGGCAGTTTTCCCAGACATTACTCACCCGTCCGCCACTCGTCAGCAAAGCAGCAAGCTGCTTCCTGTTACCG
TTCGACTGCATGTGTTAGGCCTGCCGCCAGCGTCAATCTGAGCCAGGATCAAACTCT
>582
CTGCTGCCGCCCGTAGGAGTCTGGACCGTGTCTCAGTTCCAGTGTGGCTGGTCATCCTCTCAGACCAGCTAGG
GATCGTCGCCTAGGTGAGCCGTTACCCCACCTACTAGCTAATCCCATCTGGGCACATCCGATGGCAAGAGGCC
CTAAGGTCCCCCTCTTGTCTTGCGACGTATGCGGTATTAGCTACCGTTTCCAGTAGTTATCCCCTCCATCGGG
CAGTTTCCCAGACATTACTCACCCGTCCGCCACTCGTCAGCAAAGCAGCAAGCTGCTTCCTGTTACCGTTCGA
CTGCAATGTGTTAGGCCTGCCGCCAGCGTCAATCTGAGCCAAGATCAAACTCT
>583
CTGCTGCCGCCCGTAGGAGTCTGGACCGTGTCTCAGTTCCAGTGTGGCTGGTCATCCTCTCAGACCAGCTAGG
GATCGTCGCCTAGGTGAGCCGTTACCCCACCTACTAGCTAATCCCATCTGGGCACATCCGATGGCAAGAGGCC
CTAAGGTCCCCCTCTTTGGTCTTGCGACGTTATGCGGTATTAGCTACCGTTTCCAGTAGTTATCCCCCTCCAT
CGGGCAGTTTTCCCAGACATTACTCACCCGTCCGCCACTCGTCAGCAAAGCAGCAAGCTGCTTCCTGTTACCG
TTCGACTGCATGTGTTAGGCCTGCCGCCAGCGTCAATCTGAGCCAGGATCAAACTCT
>584
CTGCTGCCGCCCGTAGGAGTCTGGACCGTGTCTCAGTTCCAGTGTGGCTGGTCATCCTCTCAGACCAGCTAGG
GATCGTCGCCTAGGTGAGCCGTTACCCCACCTACTAGCTAATCCCATCTGGGCACATCCGATGGCAAGAGGCC
CTAAGGTCCCCCTCTTTGGTCTTGCGACGTATGCGGTATTAGCTACCGTTTCCAGTAGTTATCCCCCTCCATC
GGGCAGTTTCCCAGACATTACTCACCCGTCCGCCACTCGTCAGCAAAGCAGCAAGCTGCTTCCCGTTACCGTT
CGACTGCATGTGTTAGGCCTGCCGCCAGCGTCAATCTGAGCCAGGATCAAACTCT
>585
CTGCTGCCGCCCGTAGGAGTCTGGACCGTGTCTCAGTTCCAGTGTGGCTGGTCATCCTCTCAGACCAGCTAGG
GATCGTCGCCTAGGTGAGCCGTTTACCCCACCTACTAGCTAATCCCATCTGGGCACATCCGATGGCAAGAGGC
CCTAAGGTCCCCCCTCTTTGGTCTTGCGACGTTATGCGGTATTAGCTACCGTTTCCAGTAGTTATCCCCCTCC

ATCAGGCAGTTTCCCAGACATTACTCACCCGTCCGCCACTCGTCAGCAAAACAGCAAGCTGCTTCCTGTTACC
GTTCGACTGCATGTGTTAGGCCTGCCGCCAGCGTTCAATCTGAGCCAGGATCAAACTCT
>586
CTGCTGCCGCCCGTAGGAGTCTGGACCGTGTCTCAGTTCCAGTGTGGCTGGTCATCCTCTCAGACCAGCTAGG
GATCGTCGCCTAGGTGAGCCGTTACCCCACCTACTAGCTAATCCCATCTGGGCACATCCGATGGCAAGAGGCC
CTAAGGTCCCCCTCTTTGTCTTGCGACGTTATGCGGTATTAGCTACCGTTTCCAGTAGTTATCCCCCTCCATC
AGGCAGTTTCCCAGACATTACTCACCCGTCCGCCACTCGTCAGCAAAGCAGCAAGCTGCTTCCTGTTACCGTT
CGACTGCATGTGTTAGGCCTGCCGCCAGCGTTCAATCTGAGCCAGGACAAACTCT
>587
CTGCTGCCGCCCGTAGGAGTCTGGACCGTGTCTCAGTTCCAGTGTGGCTGGTCATCCTCTCAGACCAGCTAGG
GATCGTCGCCTAGGTGAGCCGTTACCCCACCTACTAGCTAATCCCATCTGGGCACATCCGATGGCAAGAGGCC
CTAAGGTCCCCCTCTTTGTCTTGCGACGTATGCGGTATTAGCTACCGTTTCCAGTAGTTATCCCCCTCCATCG
GGCAGTTTTCCCAGACATTACTCACCCGTCCGCCACTCGTCAGCAAAACGCAGCAAGCTGCTTCCTGTTACCG
TTCGACTGCATGTGTTAGGCCTGCCGCCAGCGTCAATCTGAGCCAGGATCAAACTCT
>588
CCTGCTGCCGCCCGTAGGAGTCTGGACCGTGTCTCAGTTCCAGTGTGGCTGGTCATCCTCTCAGACCAGCTAG
GGATCGTCGCCTAGGTGAGCCGTTACCCCACCTACTAGCTAATCCCATCTGGGCACATCCGATGGCAAGAGGC
CCTAAAGTCCCCCTCTTTGTCTTGCGACGTATGCGGTATTAGCTACCGTTTCCAGTAGTTATCCCCCTCCATC
AGGCAGTTTCCCAGACATTACTCACCCGTCCGCCACTCGTCAGCAAAGCAGCAAGCTGCTTCCTGTTACCGTT
CGACTGCATGTGTTAGGCCTGCCGCCAGCGTCAATCTGAGCCAGGATCAAACTCT
>589
CTGCTGCCGCCCGTAGGAGTCTGGACCGTGTCTCAGTTCCAGTGTGGCTGGTCATCCTCTCAGACCAGCTAGG
GATCGTCGCCTAGGTGAGCCGTTTACCCCACCTACTAGCTAATCCCATCTGGGCACATCCGATGGCAAGAGGC
CCTAAGGTCCCCCTCTTTGTCTTGCGACGTATGCGGTATTAGCTACCGTTTCCAGTAGTTATCCCCCTCCATC
AGGCAGTTTCCCAGACATTACTCACCCGTCCGCCACTCGTCAGCAAAGCAGCAAGCTGCTTTCTGTTACCGTT
CGACTGCATGTGTTAGGCCTGCCCGCCAGCGTTCAATCTGAGCCAGGATCAAACTCT
>590
CTGCTGCCGCCCGTAGGAGTCTGGACCGTGTCTCAGTTCCAGTGTGGCTGGTCATCCTCTCGGACAGCTAGGG
ATCGTCGCCTAGGTGAGCCGTTACCCCACCTACTAGCTAATCCCATCTGGGCACATCCCGATGGCAAGAGGCC
CTAAGGTCCCCCTCTTTGGTCTTGCGGACGTTATGCGGTATTAGCTACCGTTTCCAGTAGTTATCCCCCTCCA
TCAGGCAGTTTCCCCAGACATTACTCACCTGTCCGCCACTCGTCAGCAAAGCAGCAAGCTGCTTCTGTTACCG
TTCGACTGCATGTGTTAGGCCTGCCGCCAGCGTCAATCTGAGCCAGGATCAAACGTC
>591
CTGCTGCCGCCCGTAGGAGTCTGGACCGTGTCTCAGTTCCAGTGTGGCTGGTCATCCTCTCAGACCAGCTAGG
GATCGTCGCCTAGGTGAGCCGTTACTCCACCTACTAGCTAATCCCATCTGGGCACATCCGATGGCAAGAGGCC
CTAAGGTCCCCCTCTTTGTCTTGCGACGTATGCGGTATTAGCTACCGTTTCCAGTAGTTATCCCCCTCCATCG
GGCAGTTTCCCAGACATTACTCACCCGTCCGCCACTCGTCAGCAAAGCAGCAAGCTGCTTCCTGTTACCGTTC
GACTGCATGTGTTAGGCCTGCCGCCAGCGTCAATCTGAGCCAGGATCAAACTCT
>592
CTGCTGCCGCCCGTAGGAGTCTGGACCGTGTCTCAGTTCCAGTGTGGCTGGTCATCCTCTCAGACCAGCTAGG
GATCGTCGCCTAGGTGAGCCGTTACCCCACCTACTAGCTAATCCCATCTGGGCACATCCGATGGCAAGAGGCC
CTAAGGTCCCCCTCTTGTCTTGCGACGTTATGCGGTATTAGCTACCGTTTCCAGTAGTTATCCCCCTCCATCA
GGCAGTTTCCCAGACATTACTCACCCGTCCGCCACTCGTCAGCAAAGCAGCAAGCTGCTTCCTGTTACCGTTC
GACTGCATGTGTTAGGCCTGCCGCCAGCGTTCAATCTGAGCCAGGATCAAACTCT
>593
CTGCTGCCGCCCGTAGGAGTCTGGACCGTGTCTCAGTTCCAGTGTGGCTGGTCATCCTCTCAGACCAGCTAGG
GATCGTCGCCTAGGTGAGCCGTTACCCCACCTACTAGCTAATCCCATCTGGGCACATCCGATGGCAAGAGGCC
CTAAGGTCCCCCTCTTTGGTCTTGCGACGTTATGCGGTATTAGCTACCGTTTCCAGTAGTTATCCCCCTCCAT
CGGGCAGTTTCCCAGACATTACTCACCCGTCCGCCACTCGTCAGCAAAGCAGCAAGCTGCTTCCTGTTACCGT
TCGACTGCATGTGTTAGGCCTGCCGCCAGCGTTCAATCTGAGCCAGGATCAAACTCT
>594
CTGCTGCCGCCCGTAGGAGTCTGGACCGTGTCTCAGTTCCAGTGTGGCTGGTCATCCTCTCAGACCAGCTAGG
GATCGTCGCCTAGGTGAGCCGTTACCCCACCTACTAGCTAATCCCATCTGGGCACATCCGATGGCAAGAGGCC
CTAAGGTCCCCCTCTTTGGTCTTGCGACGTTATGCGGTATTAGCTACCGTTTCCAGTAGTTATCCCCCTCCAT

CAGGCAGTTTCCCAGACATTACTCACCCGTCCGCCACTCGTCAGCAAAGCAGCAAGCTGCTTCCTGTTACCGT
TCGACTTGCATGTGTTAGGCCTGCCGCCAGCGTCAATCTGAGCCAGGATCAAACTCT
>595
CTGCTGCCGCCCGTAGGAGTCTGGACCGTGTCTCAGTTCCAGTGTGGCTGGTCATCCTCTCAGACCAGCTAGG
GATCGTCGCCTAGGTGAGCCGTTACCCCACCTACTAGCTAATCCCATCTGGGCACATCCGATGGCAAGAGGCC
CTAAGGTCCCCCTCTTTGGTCTTGCGACGTTATGCGGTATTAGCTACCGTTTCCAGTAGTTATCCCCCTCCAT
CAGGCAGTTTCCCAGACATTACTCACCCGTCCGCCACTCGTCAGCAAAACAGCAAGCTGCTTCCTGTTACCGT
TCGACTGCATGTGTTAGGCCTGCCGCCAGCGTTCAATCTGAGCCAGGATCAAACTCT
>596
CTGCTGCCGCCCGTAGGAGTCTGGACCGTGTCTCAGTTCCAGTGTGGCTGGTCATCCTCTCAGACCAGCTAGG
GATCGTCGCCTAGGTGAGCCGTTACCCCACCTACTAGCTAATCCCATCTGGGCACATCCGATGGCAAGAGGCC
CTAAGGTCCCCCTCTTTGTCTTGCGACGTTATGCGGTATTAGCTACCGTTCCAGTAGTTATCCCCCTCCATCG
AGGCCAGTTTCCCAGACATTACCTCACCCGTCCGCCACTCGTCAGCAAACAGCAAGCTGCTTCCTGTTACCGT
TCGACTGCATGTGTTAGGCCTGCCGCCAGCGTTCAATCTGAGCCAGGATCAACTCT
>597
CTGCTGCCGCCCGTAGGAGTCTGGACCGTGTCTCAGTTCCAGTGTGGCTGGTCATCCTCTCAGACCAGCTAGG
GATCGTCGCCTAGGTGAGCCGTTACCCCACCTACTAGCTAATCCCATCTGGGCACATCCGATGGCAAGAGGCC
CTAAGGTCCCCCTCTTTGTCTTGCGACGCTATGCGGTATTAGCTACCGTTTCCAGTAGTTATCCCCCTCCATC
AGGCGGTTTCCCAGACATTACTCACCCGTCCGCCACTCGTCAGCAAACAGCAAGCTGCTTCCTGTTACCGTTC
GACTGCATGTGTTAGGCCTGCCGCCAGCGTCAATCTGAGCCAGGATCAAACTCT
>598
CTGCTGCCGCCCGTAGGAGTCTGGACCGTGTCTCAGTTCCAGTGTGGCTGGTCATCCCCTCAGACCAGCTAGG
GATCGTCGCCTAGGTGAGCCGTTACCCCACCTACTAGCTAATCCCATCTGGGCACATCCGATGGCAAGAGGCC
CTAAGGTCCCCCTCTTTGGTCTTGCGACGTTATGCGGTATTAGCTACCGTTTCCAGTAGTTATCCCCCTCCAT
CAGGCAGTTTCCCAGACATTACTCACCCGTCCGCCACTCGTCAGCAAAGCAGCAAGCTGCTTCCTGTTACTGT
TCGACTGCATGTGTTAGGCCTGCCGCCAGCGTCAATCCGAGCCAGGATCAAACTCT
>599
CTGCTGCCGCCCGTAGGAGTCTGGACCGTGTCTCAGTTCCAGTGTGGCTGGTCATCCTCTCAGACCAGCTAGG
GATCGTCGCCTAGGTGAGCCGTTACCCCACCTATAGCTAATCCCATCTGGGCACATCCGATGGCAAGAGGCCC
TAAGGTCCCCCTCTTTGTCTTGCGACGTATGCGGTATTAGCTACCGTTTCCAGTAGTTATCCCCCTCCATCAG
GCAGTTTCCCAGACATTACTCACCCGTCCGCCACTCGTCAGCAAACAGCAAGCTGCTTCCTGTTACCGTTCGA
CTGCATGTGTTAGGCCTGCCGCCAGCGTCAATCTGAGCCAGGATCAAACTCT
>600
CTGCTGCCGCCCGTAGGAGTCTGGACCGTGTCTCAGTTCCAGTGTGGCTGGTCATCCTCTCAGACCAGCTAGG
GATCGTCGCCTAGGTGAGCCGTTACCCCACCTACTAGCTAATCCCATCTGGGCACATCCGATGGCAAGAGGCC
CTAAGGTCCCCCTCTTTGGTCTTGCGACGTATGCGGTATTAGCTACCGTTTCCAGTAGTTATCCCCCTCCATC
AGGCAGTTTCCCAGACATTACTCACCCGTCCGCCACTCGTCAGCAAAACAGCAAGCTGCTTCCTGTTACCGTT
CGACTGCATGTGTTAGGCCTGCCGCCAGCGTCAATCTGAGCCAGGATCAAACTCT
>601
CTGCTGCCGCCCGTAGGAGTCTGGACCGTGTCTCAGTCCCAGTGTGGCTGGTCATCCTCTCAGACCAGCTAGG
GATCGTCGCCTAGGTGAGCCGTTACCCCACCTACTAGCTAATCCCATCTGGGCACATCCGATGGCAAGAGGCC
CTAAGGTCCCCCTCTTTGTCTGCGACGTATGCGGTATTAGCTACCGTTTCCAGTAGTTATCCCCCTCCATCAG
GCAGTTTCCCAGACATCACTCACCCGTCCGCCACTCGTCAGCAAACAGCAAGCTGCTTCCTGTTACCGTTCGA
CTGCATGTGTTAGGCCTGCCGCCAGCGTCAATCTGAGCCAGGATCAAACTCT
>602
CTGCTGCCGCCCGTAGGAGTCTGGACCGTGTCTCAGTTCCAGTGTGGCTGGTCATCCTCTCAGACCAGCTAGG
GATCGTCGCCTAGGTGAGCCGTTACCCCACCTACTAGCTAATTCCCATCTGGGCACATCCGATGGCAAGAGGC
CCTAAGGTCCCCCTCTTGTCTTGCGACGTTATGCGGTATTAGCTACCGTTTCCAGTAGTTATCCCCCTTCCAT
CGGGCAGTTTCCCAGACATTACTTCACCCGTCCGCCACTCGTCAGCAAACAGCAAGCTGCTTTCCTGTTACCG
TTCGACTTGCATGTGTTAGGCCTGCCGCCAGCGTCAATCTGAGCCAGGATCAAACTCT
>603
CTGCTGCCGCCCGTAGGAGTCTGGACCGTGTCTCAGTTCCAGTGTGGCTGGTCGTCCTCTCAGACCAGCTAGG
GATCGTCGCCTAGGTGAGCCGTTACCCCACCTACTAGCTAATCCCATCTGGGCACATCCGATGGCAAGAGGCC
CTAAGGTCCCCTCTTGTCTTGCGACGTTATGCGGTATTAGCTACCGTTTCCAGTAGTTATCCCCCTCCATCAG

GCAGTTTCCCAGACATTACTCACCCGTCCGCCACTCGTCAGCAAACAGCAAGCTGCTTCCTGTTTACCGTTCG
ACTTGCATGTGTTAGGCCTGCCGCCAGCGTTCAATCTGAGCCAGGATCAAACTCT
>604
CTGCTGCCGCCCGTAGGAGTCTGGACCGTGTCTCAGTTCCAGTGTGGCTGGTCATCCTCTCAGACCAGCTAGG
GATCGTCGCCTAGGTGAGCCGTTACCCCCACCTACTAGCTAATTCCCATCTGGGCACATCCGATTGGCAAGAG
GCCCTAAGGTCCCCCCTCTTTGTCTTGCGACGTTATGCGGTATTAGCTACCGTTTCCAGTAGTTATCCCCTCC
CATCAGCAGTTTCCCCAGACATTACTCACCCGTCCGCCACTCGTCAGCAAAGCAGCAAGCTTGCTTCCTGTTA
CCGTTCGACTGCATGTGTTAGGCCTGCCCGCCAGCGTTCAATCTGAGCCCAGGATCAACTCT
>605
CTGCTGCCGCCCGTAGGAGTCTGGACCGTGTCTCAGTTCCAGTGTGGCTGGTCATCCTCCCAGACCAGCTAGG
GATCGTCGCCTAGGTGAGCCGTTACCCCACCTACTAGCTAATCCCATCTGGGCACATCCGATGGCAAGAGGCC
CTAAGGTCCCCCTCTTTGGTCTTGCGACGTATGCGGTATTAGCTACCGTTTCCAGTAGTTATCCCCCTCCATC
GGGCAGTTTTCCCCAGACATTACTCACCCGTCCGCCACTCGTCAGCAAAGCAGCAAGCTGCTTCCTGTTACCG
TTCGACTGCATGTGTTAGGCCTGCCGCCAGCGTCAATCTGAGCCAGGATCAAACTCT
>606
CTGCTGCCGCCCGTAGGAGTCTGGACCGTGTCTCAGTTCCAGTGTGGCTGGTCATCCTCTCAGACCAGCTAGG
GATCGTCGCCTAGGTGAGCCGTTACCCCACCTACTAGCTAATCCCATCTGGGCACATCCGATGGCAAGAGGCC
CTAAGGTCCCCCTCTTGTCTGCGACGTATGCGGTATTAGCTACCGTTTCCAGTAGTTATCCCCCTCCATCAGG
CAGTTTCCCAGACATTACTCACCCGTCCGCCACTCGTCAGCAAACAGCAAGCTGCTTCCTGTTACCGTTCGAC
TGCATGTGTTAGGCCTGACCGCCAGCGTTCAATCTGAGCCAGGATCAAACTCT
>607
CTGCTGCCGCCCGTAGGAGTCTGGACCGTGTCTCAGTTCCAGTGTGGCTGGTCATCCTCTCAGACCAGCTAGG
GATCGTCGCCTAGGTGAGCCGTTACCCCACCTACTAGCTAATCCCATCTGGGCACATCCGATGGCAAGAGGCC
CTAAGGTCCCCCTCTTTGGTCTTGCGACGTTATGCGGTATTAGCTACCGTTTCCAGTAGTTATCCCCCTCCAT
CGGGCAGTTTTCCCAGACATTACTCACCCGTCCGCCACTCGTCAGCAAAGCAGCAAGCTGCTTCCTGTTACCG
TTCGACTGCATGTGTTAGGCCTGCCGCCAGCGTCAATCTGAGCCAGGATCAAACTCT
>608
CTGCTGCCGCCCGTAGGAGTCTGGACCGTGTCTCAGTTCCAGTGTGGCTGGTCATCCTCTCAGACCAGCTAGG
GATCGTCGCCTAGGTGAGCCGTTACCCCACCTACTAGCTAATCCCATCTGGGCACATCCGATGGCAAGAGGCC
CTAAGGTCCCCCTCTTTGTCTTGCGACGTTATGCGGTATTAGCTACCGTTTCCAGTAGTTATCCCCCTCCATC
AGGCAGTTTCCCAGACATTACTCACCCGTCCGCCACTCGTCAGCAAACAGCAAGCTGCTTCCTGTTACCGTTC
GACTGCATGTGTTAGGCCTGCCGCCAGCGTCAATCTGAGCCAGGATCAAACTCT
>609
CTGCTGCCGCCCGTAGGAGTCTGGACCGTGTCTCAGTTCCAGTGTGGCTGGTCATCCTCTCAGACCAGCTAGG
GATCGTCGCCTAGGTGAGCCGTTACCCCACCTACTAGCTAATCCCATCTGGGCACATCCGATGGCAAGAGGCC
CTAAGGTCCCCCTCTTGTCTTGCGACGTTATGCGGTATTAGCTACCGTTTCCAGTAGTTATCCCCCTCCATCG
GGCAGTTTCCCAGACATTACTCACCCTCCGCCACTCGTCAGCAAAGCAGCAAGCTGCTTCCTGTTACCGTTCG
ACTGCATGTGTTAGGCCTGCCGCCAGCGTCAATCTGAGCCAGGATCAAACTCT
>610
CTGCTGCCGCCCGTAGGAGTCTGGACCGTGTCTCAGTTCCAGTGTGGCTGGTCATCCTCTCAGACCAGCTAGG
GATCGTCGCCTAGGTGAGCCGTTACCCCACCTACTAGCTAACCCTCCTGGTGCACATCCGATGGCAAGAGGCC
CTAAGGTCCCCCTCTTGTCTTGCGACGTATGCGGTATTAGCTACCGTTTCCAGTAGTTATCCCCCTCCATCAG
GCAGTTTCCCAGACATTACTCACCCGTCCGCCACTCGTCAGCAAAGCAGCAAGCTGCTTCCTGTTACCGTTCG
ACTGCATGTGTTAGGCCTGCCGCCAGCGTCAATCTGAGCCAGGATCAAACTCT
>611
CTGCTGCCGCCCGTAGGAGTCTGGACCGTGTCTCAGTTCCAGTGTGGCTGGTCATCCTCTCAGACCAGCTAGG
GATCGTCGCCTAGGTGAGCCGTTACCCCACCTACTAGCTAATCCCATCTGGGCACATCCGATGGCAAGAGGCC
CTAAGGTCCCCCTCTTTGTCTGCGACGTATGCGGTATTAGCTACCGTTTCCAGTAGTTATCCCCCTCCATCGG
GCAGTTTCCCAGACATTACTCACCCGTCCGCCACTCGTCAGCAAAGCAGCAAGCTGCTTCCTGTTACCGTTCG
GACTGCATGTGTTAGGCCTGCCGCCAGCGTCAATCTGAGCCAGAATCAAACTCT
>612
CTGCTGCCGCCCGTAGGAGTCTGGACCGTGTCTCAGTTCCAGTGTAGCTGGTCATCCTCTCAGACCAGCTAGG
GATCGTCGCCTAGGTGAGCCGTTACCCCACCTACTAGCTAATCCCATCTGGGCACATCCGATGGCAAGAGGCC
CTAAGGTCCCCCTCTTTGTCTTGCGACGTTATGCGGTATTAGCTACCGTTCCAGTAGTTATCCCCCTCCATCA

GGCAGTTTCCCAGACATTACTCACCCGTCCGCCACTCGTCAGCAAAGCAGCAAGCTGCTTCCTGTTACCGTTC
GACTGCATGTGTTAGGCCTGCCGCCAGCGTCAATCTGAGCCAGGATCAAACTCT
>613
CTGCTGCCGCCCGTAGGAGTCTGGACCGTGTCTCAGTTCCAGTGTGGCTGGTCATCCTCTCAGACCAGCTAGG
GATCGTCGCCTAGGTGAGCCGTTACCCCACCTACTAGCTAATCCCATCTGGGCACATCCGATGGCAAGAGGCC
CTAAGGTCCCCCTCTTGTCTTGCGACGTATGCGGTATTAGCTACCGTTTCCAGTAGTTATCCCCCTCCATCAG
GCAGTTTCCCAGACATTACTCACCCGTCCGCCACTCGTCAGCAAAGCAGCAAGCTGCTTCCTGTTACCGTTCG
ACTGCATGTGTTAGGCCTGCCGCCAGCGTCAATCTGAGCCAGGATCAAACTCT
>614
CTGCTGCCGCCCGTAGGAGTCTGGACCGTGTCTCAGTTCCAGTGTGGCTGGTCATCCTCTCAGACCAGCTAGG
GATCGTCGCCTAGGTGAGCCGTTACCCCACCTACTAGCTAATCCCATCTGGGCACATCCGATGGCAAGAGGCC
CTAAGGTCCCCCTCTTGTCTTGCGACGTTCTGCGGTATTAGCTACCGTTTCCAGTAGTTATCCCCCTCCATCA
GGCAGTTTCCCAGACATTACTCACCCGTCCGCCACTCGTCAGCAAAGCAGCAAGCTGCTTCCTGTTACCGTTC
GACTGCATGTGTTAGGCCTGACCGC
>615
CTGCTGCCGCCCGTAGGAGTCTGGACCGTGTCTCAGTTCCAGTGTGGCTGGTCATCCTCTCAGACAGCTAGGG
ATCGTCGCCTAGGTGAGCCGTTACCCCACCTACTAGCTAATCCCATCTGGGCACATCCGATGGCAAGAGGCCC
TAAGGTCCCCCTCTTGTCTGCGACGTATGCGGTATTAGCTACCGTTTCCAGTAGTATCCCCCTCCATCAGGCA
GTTTTCCCAGACATACTCACCCGTCCGCCACTCGTCAGCAAAAGCAGCAAGCTGCTTCCTGTTACCGTTCGAA
CTGCATGTGTTAGGCCTGCCGCCAGCGTCAATCTGAAGCCAG
>616
CTGCTGCCGCCCGTAGGAGTCTGGACCGTGTCTCAGTTCCAGTGTGGCTGGTCATCCTCTCAGACCAGCTAGG
GATCGTCGCCTAGGTGAGCCGTTACCCCACCTACTAGCTAATCCCATCTGGGCACATCCGATGGCAAGAGGCC
CTAAGGTCCCCCTCTTTGTCTTGCGACGTTATGCGGTATTAGCTACCGTTTCCAGTAGTTATCCCCCTCCATC
AGGCAGTTTTCCCAGACATTACTCACCCGTCCGCCACTCGTCAGCAAAGCAGCAAGCTGCTTCCTGTTACCGT
TCGACTGCATGTGTTAGGCCTGCCGCCAGCGTCAATCTGAGCCAGGATCAAACTCT
>617
CTGCTGCCGCCCGTAGGAGTCTGGACCGTGTCTCAGTTCCAGTGTGGCTGGTCATCCTCTCAGACCAGCTAGG
GATCGTCGCCTAGGTGAGCCGTTACCCCACCTACTAGCTAATCCCATCTGGGCACATCCGATGGCAAGAGGCC
CTAAGGTCCCCCTCTTGTCTTGCGACGTATGCGGTATTAGCTACCGTTTCCAGTAGTTATCCCCCTCCATCGG
GCAGTTTCCCAGACATTACTCACCCGTCCGCCACTCGTCAGCAAAGCAGCAAGCTGCTTCCTGTTACCGTTCG
ACTGCATGTGTTAGGCCTGCCGCCAGCGTCAATCTGAGCCAGGATCAAACTCT
>618
CTGCTGCCGCCCGTAGGAGTCTGGACCGTGTCTCAGTTCCAGTGCGGCTGGTCATCCTCTCAGACCAGCTAGG
GATCGTCGCCTAGGTGAGCCGTTACCCCACCTACTAGCTAATCCCATCTGGGCACATCCCGATGGCAAGAGGC
CCTAAGGTCCCCCTCTTTGTCTTGCGACGTTATGCGGTATTAGCTACCGTTTCCAGTAGTTATCCCCCTCCAT
CGGGCAGTTTTCCCAGACATTACTCACCCGTCCGCCACTCGTCAGCAAAGCAGCAAGCTGCTTCCTGTTACCG
TTCGACTGCATGTGTTAGGCCTGCCGCCAGCGTTCAATCTGAGCCAGGATCAAACTCT
>619
CTGCTGCCGCCCGTAGGAGTCTGGACCGTGTCTCAGTTCCAGTGTGGCTGGTCATCCTCTCAGACCAGCTAGG
GATCGTCGCCTAGGTGAGCCGTTACCCCACCTACTAGCTAATCCCATCTGGGCACATCCGATGGCAAGAGGCC
CTAAGGTCCCCCTCTTTGGTCTTGCGACGTTATGCGGTATTAGCTACCGTTTCCAGTAGTTATCCCCCTCCAT
CGGGCAGTTTCCCAGACATTACTCACCCGTCCGCCACTCGTCAGCAAAGCAGCAAGCTGCTTCCTGTTACCGT
TCGACTGCATGTGTTAGGCCTGCCGCCAGCGTCAATCTGAGCCAGGATCAAACTCT
>620
CTGCTGCCGCCCGTAGGAGTCTGGACCGTGTCTCAGTTCCAGTGTGGCTGGTCATCCTCTCAGACCAGCTAGG
GATCGTCGCCTAGGTGAGCCGTTACCCCACCTACTAGCTAATCCCATCTGGGCACATCCGATGGCAAGAGGCC
CTAAGGTCCCCCTCTTTGTCTTGCGACGTTATGCGGTATTAGCTACCGTTTCCAGTAGTTATCCCCCTCCATC
GGGCAGTTTTCCCAGACATTACTCACCCGTCCGCCACTCGTCAGCAAAAGCAGCAAGCTGCTTCCTGTTACCG
TTCGACCTGCATGTGTTAGGCCTGCCGCCAGCGTCAATCTGAGCCAGGATCAAACTCT
>621
CTGCTGCCGCCCGTAGGAGTCTGGACCGTGTCTCAGTTCCAGTGTGGCTGGTCATCCTCTCAGACCAGCTAGG
GATCGTCGCCTAGGTGAGCCGTTACCCCACCTACTAGCTAATCCCATCTGGGCACATCCGATGGCAAGAGGCC
CTAAGGTCCCCCTCTTTGGTCTGCGACGTATGCGGTATTAGCTACCGTTTCCAGTAGTTATCCCCCTCCATCG

GGCAGTTTCCCAGACATTACTCACCCGTCCGCCACTCGTCAGCAAAGCAGCAAGCTGCTTCCTGTTACCGTTC
GACTGCATGTGTTAGGCCTGCCGCCAGCGTCAATCTGAGCCAGGATCAAACTCT
>622
CTGCTGCCGCCCGTAGGAGTCTGGACCGTGTCTCAGTTCCAGTGTGGCTGGTCATCCTCTCAGACCAGCTAGG
GATCGTCGCCTAGGTGAGCCGTTACCCCACCTACTAGCTAATCCCATCTGGGCACATCCCGATGGCAAGAGGC
CCTAAGGTCCCCCTCTTTGTCTGCGACGTATGCGGTATTAGCTACCGTTTCCAGTACGTTATCCCCCTCATCG
GGCAGTTTCCCAGACATTACTCACCCGTCCGCCACTCGTCAGCAAAGCAGCAAGCTGCTTCCTGTTACCGTTC
GACTGCAATGTGTTAGGCCTGCCGCCAGCGTCAATCTGAGCCAGGATCAACTCT
>623
CTGCTGCCGCCCGTAGGAGTCTGGACCGTGTCTCAGTTCCAGTGTGGCTGGTCATCCTCTCAGACCAGCTAGG
GATCGTCGCCTAGGTGAGCCGTTACCCCACCTACTAGCTAATCCCATCTGGGCACATCCGATGGCAAGAGGCC
CTAAGGTCCCCCTCTTGTCTTGCGACGTTATGCGGTATTAGCTACCGTTTCCAGTAGTTATCCCCCTCCATCG
GGCAGTTTCCCAGACATTACTCACCCGTCCGCCACTCGTCAGCAAGCAGCAAGCTGCTTCCTGTTACCGTTCG
ACTGCATGTGTTAGGCCTGCCGCCAGCGTCAATCTGAGCCAGGATCAAACTCT
>624
CTGCTGCCGCCCGTAGGAGTCTGGACCGTGTCTCAGTTCCAGTGTGGCTGGTCATCCTCTCAGACAGCTAGGG
ATCGTCGCCTAGGTGAGCCGTTACCCCACCTACTAGCTAATCCCATCTGGGCACATCCGATGGCAAGAGGCCC
TAAGGTCCCCCTCTTGTCTTGCGACGTATGCGGTATTAGCTACCGTTTCCAGTAGTTATCCCCCTCCATCAGG
CAGTTTCCCAGACATTACTCACCCGTCCGCCACTCGTCAGCAAAGCAGCAAGCTGCTTCTGTTACCGTTCGAC
TGCATGTGTTAGGCCTGCCGCCAGCGTTCAATCTGAGCCAG
>625
CTGCTGCCGCCCGTAGGAGTCTGGACCGTGTCTCAGTTCCAGTGTGGCTGGTCATCCTCTCAGACCAGCTAGG
GATCGTCGCCTAGGTGAGCCGTTACCCCACCTACTAGCTAATCCCATCTGGGCACATCCGATGGCAAGAGGCC
CTAAGGTCCCCCTCTTTGTCTTGCGACGTATGCGGTATTAGCTACCGTTTCCAGTAGTTATCCCCCTCCATCG
GGCAGTTTCCCAGACATTACTCACCCGTCCGCCACTCGTCAGCAAAGCAGCAAGCTGCTTCCTGTTACCGTTC
GACTGCATGTGTTAGGCCTGCCGCCAGCGTCAATCTGAGCCAGGATCAAACTCT
>626
CTGCTGCCGCCCGTAGGAGTCTGGACCGTGTCTCAGTTCCAGTGTGGCTGGTCATCCTCTCAGACCAGCTAGG
GATCGTCGCCTAGGTGAGCCGTTACCCCACCTACTAGCTAATCCCATCTGGGCACATCCGATGGCAAGAGGCC
CTAAGGTCCCCCTCTTTGTCTTGCGACGTATGCGGTATTAGCTACCGTTTCCAGTAGTTTATCCCCCTCCATC
AGGCAGTTTCCCAGACATTACTCACCCGTCCGCCACTCGTCAGCAAAACAGCAAGCTGCTTCCTGTTACCGTT
CGACTGCATGTGTTAGGCCTGCCGCCAGCGTCAATCTGAGCCAGGATCAAACTCT
>627
CTGCTGCCGCCCGTAGGAGTCTGGACCGTGTCTCAGTTCCAGTGTGGCTGGTCATCCTCTCAGACCAGCTAGG
GATCGTCGCCTAGGTGAGCCGTTACCCCACCTACTAGCTAATCCCATCTGGGCACATCCGATGGCAAGAGGCC
CTAAGGTCCCCCTCTTTGTCTTGCGACGTTATGCGGTATTAGCTACCGTTTCCAGTAGTTATCCCCCTCCATC
AGGCAGTTTCCCAGACATTACTCACCCGTCCGCCACTCGTCAGCAAAGCAGCAAGCTGCTTCTGTTACCGTTC
GACTGCATGTGTTAGGCTTGCCGCCAGCGTTCAATCTGAGCCAGGATCAAACTCT
>628
TGCTGCCGCCCGTAGGAGTCTGGACCGTGTCTCAGTTCCAGTGTGGCTGGTCATCCTCTCAGACAGCTAGGGA
TCGTCGCCTAGGTGAGCCGTTACCCCACTACTAGCTAATCCCATCTGGGCACATCCGATGGCAAGAGGCCCTA
AGGTCCCCCTCTTGTCTTGCGACGTATGCGGTATTAGCTACCGTTTCCAGTAGTTATCCCCCTCCATCAGGCA
GTTTCCCAGACATTACTCACCCGTCCGCCACTCGTCAGCAAAGCAGCAAGCTGCTTCCTGTTACCGTTCGACT
GCATGTGTTAGGCCTGCCGCCAGCGTTCAATCTGAGCCAGGATCAAACTCT
>629
CTGCTGCCGCCCGTAGGAGTCTGGACCGTGTCTCAGTTCCAGTGTGGCTGGTCATCCTCTCAGACCAGCTAGG
GATCGTCGCCTAGGTGAGCCGTTTACCCCACCTACTAGCTAATCCCATCTGGGCACATCCCGATGGCAAGAGG
CCCTAAGGTCCCCCTCTTTGTCTTGCGACGTTCTGCGGTATTAGCTACCGTTTCCAGTAGTTATCCCCCTCCA
TCAGGCAGTTCCCCAGACATTACTCACCCGTCCGCCACTCGTCAGCAAAGCAGCAAGCTGCTTCTGTTACCGT
TCGACTGCATGTGTTAGGCCTGCCGCCAGCGTTCAATCTGAGCCAGGACAAACTCT
>630
CTGCTGCCGCCCGTAGGAGTCTGGACCGTGTCTCAGTTCCAGTGTGGCTGGTCATCCTCTCAGACCAGCTAGG
GATCGTCGCCTAGGTGAGCCGTTTACCCCACCTACTAGCTAATCCCATCTGGGCACATCCGATGGCAAGAGGC
CCTAAGGTCCCCCTCTTTGTCTTGCGACGTTATGCGGTATTAGCTACCGTTTCCAGTAGTTATCCCCCTCCAT

CGGGCAGTTTCCCAGACATTACTCACCCGTCCGCCACTCGTCAGCAAAGCAGCAAGCTGCTTCCTGTTACCGT
TCGACTGCATGTGTTAGGCCTGCCGCCAGCGTCAATCTGAGCCAGGATCAAACTCT
>631
CTGCTGCCGCCCGTAGGAGTCTGGACCGTGTCTCAGTTCCAGTGTGGCTGGTCATCCTCTCAGACCAGCTAGG
GATCGTCGCCTAGGTGAGCCGTTACCCCACCTACTAGCTAATCCCATCTGGGCACATCCGATGGCAAGAGGCC
CTAAGGTCCCCCTCTTTGTCTTGCGACGTTATGCGGTATTAGCTACCGTTTCCAGTAGTTATCCCCCTCCATC
AGGCAGTTTCCCAGACATTACTCACCCGTCCGCCACTCGTCAGCAAAGCAGCAAGCTGCTTCTGTTACCGTTC
GACTGCATGTGTTAGGCCTGCCGCCAGCGTTCAATCTGAGCCAGGACAAACTCT
>632
CTGCTGCCGCCCGTAGGAGTCTGGACCGTGTCTCAGTTCCAGTGTGGCTGGTCATCCTCTCAGACCAGCTAGG
GATCGTCGCCTAGGTGAGCCGTTACCCCACCTACTAGCTAATCCCATCTGGGCACATCCGATGGCAAGAGGCC
CTAAGGTCCCCCTCTTTGTCTTGCGACGTTATGCGGTATTAGCTACCGTTTCCAGTAGTTATCCCCCTCCATC
AGGCAGTTTTCCCAGACATTACTCACCCGTCCGCCACTCGTCAGCAAAGCAGCAAGCTGCTTCCTGTTACCGT
TCGACTGCATGTGTTAGGCCTGCCGCCAGCGTTCAATCTGAGCCAGGATCAAACTCT
>633
CTGCTGCCGCCCGTAGGAGTCTGGACCGTGTCTCAGTTCCAGTGTGGCTGGTCATCCTCTCAGACCAGCTAGG
GATCGTCGCCTAGGTGAGCCGTTACCCCACCTACTAGCTAATCCCATCTGGGCACATCCGATGGCAAGAGGCC
CTAAGGTCCCCCTCTTTGTCTTGCGACGTTATGCGGTATTAGCTACCGTTTCCAGTAGTTATCCCTCCTCATC
GGGCAGTTTCCCAGACATTACTCACCCGTCCGCCACTCGTCAGCAAAGCAGCAAGCTGCTTCCTGTTACCGTT
CGACTGCATGTGTTAGGCCTGCCGCCAGCGTCAATCTGAGCCAGGATCAAACTCT
>634
CTGCTGCCGCCCGTAGGAGTCTGGACCGTGTCTCAGTTCCAGTGTGGCTGGTCATCCTCTCAGACCAGCTAGG
GATCGTCGCCTAGGTGAGCCGTTACCCCACCTACTAGCTAATCCCATCTGGGCACATCCGATGGCAAGAGGCC
CTAAGGTCCCCCCTCTTTGGTCTTGCGACGTTATGCGGTATTAGCTACCGTTTCCAGTAGTTATCCCCCTCCA
TCAGGCAGTTTCCCAGACATTACTCACCCGTCCGCCACTCGTCAGCAAAGCAGCAAGCTGCTTCTGTTACCGT
TCGACTGCATGTGTTAGGCCTGCCGCCAGCGTCAATCTGAGCCAGGATCAAACTCT
>635
CTGCTGCCGCCCGTAGGAGTCTGGACCGTGTCTCAGTTCCAGTGTGGCTGGTCATCCTCTCAGACCAGCTAGG
GATCGTCGCCTAGGTGAGCCGTTACCCCACCTACTAGCTAATCCCATCTGGGCACATCCGATGGCAAGAGGCC
CTAAGGTCCCCCTCTTTGGTCTTGCGACGTTATGCGGTATTAGCTACCGTTTCCAGTAGTTATCCCCCTCCAT
CAGGCAGTTTCCCAGACATTACTCACCCGTCCGCCACTCGTCAGCAAAAGCAGCAAGCTGCTTCCTGTTACCG
TTCGACTGCATGTGTTAGGCCTGCCGCCAGCGTCAATCTGAGCCAGGATCAAACTCT
>636
CTGCTGCCGCCCGTAGGAGTCTGGACCGTGTCTCAGTTCCAGTGTGGCTGGTCATCCTCTCAGACCAGCTAGG
GATCGTCGCCTAGGTGAGCCGTTACCCCACCTACTAGCTAATCCCATCTGGGCACATCCGATGGCAAGAGGCC
CTAAGGTCCCCCTCTTTGGTCTTGCGACGTTATGCGGTATTAGCTACCGTTTCCAGTAGTTATCCCCCTCCAT
CGGGCAGTTTCCCAGACATTACTCACCCGTCCGCCACTCGTCAGCAAAGCAGCAAGCTGCTTCCTGTTACCGT
TCGACTGCATGTGTTAGGCCTGCCGCCAGCGTCAATCTGAGCCAGGATCAAACTCT
>637
CTGCTGCCGCCCGTAGGAGTCTGGACCGTGTCTCAGTTCCAGTGTGGCTGGTCATCCTCTCAGACCAGCTAGG
GATCGTCGCCTAGGTGAGCCGTTACCCCACCTACTAGCTAATCCCATCTGGGCACATCCGATGGCAAGAGGCC
CTAAGGTCCCCCTCTTTGTCTGCGACGTTATGCGGTATTAGCTACCGTTTCCAGTAGTTATCCCCCTCCATCG
GGCAGTTTTCCCAGACATTACTCACCCGTCCGCCACTCGTCAGCAAAGCAGCAAGCTGCTTCCTGTTACCGTT
CGACTGCATGTGTTAGGCCTGCCGCCAGCGTCAATCTGAGCCAGGATCAAACTCT
>638
TGCTGCCGCCCGTAGGAGTCTGGACCGTGTCTCAGTTCCAGTGTGGCTGGTCATCCTCTCAGACCAGCTAGGG
ATCGTCGCCTAGGTGAGCCGTTACCCTACCTACTAGCTAATCCCATCTGGGCACATCCGATGGCAAGAGGCCC
TAAGGTCCCCCTCTTTGGTCTTGCGACGTTATGCGGTATTAGCTACCGTTTCCAGTAGTTATCCCCCTCCATC
AGGCAGTTTCCCAGACATTACTCACCCGTCCGCCACTCGTCAGCAAAGCAGCAAGCTGCTTCTGTTACCGTTC
GACTGCATGTGTTAGGCCTGCCACCAGCGTTCAATCTGAGCCAGGATCAAACTCT
>639
CTGCTGCCGCCCGTAGGAGTCTGGACCGTGTCTCAGTTCCAGTGTGGCTGGTCATCCTCTCAGACCAGCTAGG
GATCGTCGCCTAGGTGAGCCGTTACCCCACCTACTAGCTAATCCCATCTGGGCACATCCGATGGCAAGAGGCC
CTAAGGTCCCCCTCTTTGGTCTTGCGACGTTATGCGGTATTAGCTACCGTTTCCAGTAGTTATCCCCCTCCAT

CGGGCAGTTTCCCAGACATTACTCACCCGTCCGCCACTCGTCAGCAAAGCAGCAAGCTGCTTCCTGTTACCGT
TCGACTGCATGTGTTAGGCCTGCCGCCAGCGTCAATCTGAGCCAGATCAAACTCT
>640
CTGCTGCCGCCCGTAGGAGTCTGGACCGTGTCTCAGTTCCAGTGTGGCTGGTCATCCTCTCAGACCAGCTAGG
GATCGTCGCCTAGGTGAGCCGTTACCCCACCTACTAGCTAATCCCATCTGGGCACATCCGATGGCAAGAGGCC
CTAAGGACCCCCTCTTTGTCTTGCGACGTTATGCGGTATTAGCTACCGTTTCCAGTAGTTATCCCCCTCCATC
AGGCAGTTTTCCCAGACATTACTCACCCGTCCGCCACTCGTCAGCAAAGCAGCAAGCTGCTTCCTGTTACCGT
TCGACTGCATGTGTTAGGCCTGCCGCCAGCGTTCAATCTGAGCCAGGATCAAACTCT
>641
CTGCTGCCGCCCGTAGGAGTCTGGACCGTGTCTCAGTTCCAGTGTGGCTGGTCATCCTCTCAGACCAGCTAGG
GATCGTCGCCTAGGTGAGCCGTTACCCCACCTACTAGCTAATCCCATCTGGGCACATCCGATGGCAAGAGGCC
CTAAGGTCCCCCTCTCTGTCTTGCGACGTTATGCGGTATTAGCTACCGTTTCCAGTAGTTATCCCCCTCCATC
AGGCAGTTTCCCAGACATTACTCACCCGTCCGCCACTCGTCAGCAAACAGCAAGCTGCTTCCTGTTACCGTTC
GACTGCATGTGTTAGGCCTGCCGCCAGCGTCAATCTGAGCCAGGATCAAACTCT
>642
CTGCTGCCGCCCGTAGGAGTCTGGACCGTGTCTCAGTTCCAGTGTGGCTGGTCATCCTCTCAGACCAGCTAGG
GATCGTCGCCTAGGTGAGCCGTTACCCCACCTACTAGCTAATCCCATCTGGGCACATCCGATGGCAAGAGGCC
CTAAGGTCCCCCTCTTTGGTCTTGCGACGTATGCGGTATTAGCTACCGTTTCCAGTAGTTATCCCCCTCCATC
AGGCAGTTTTCCCCAGACATTACTCACCCGTCCGCCACTCGTCAGCAAAAGCAGCAAGCTGCTTCCTGTTACC
GTTCGACTGCATGTGTTAGGCCTGCCGCCAGCGTCAATCTGAGCCAGGATCAAACTCT
>643
CTGCTGCCCCCCGTAGGAGTCTGGACCGTGTCTCAGTTCCAGTGTGGCTGGTCATCCTCTCAGACCAGCTAGGG
ATCGTCGCCTAGGTGAGCCGTTACCCCACCTACTAGCTAATCCCATCTGGGCACATCCGATGGCAAGAGGCCC
TAAGGTCCCCCTCTTTGGTCTGCGACGTATGCGGTATTAGCTACCGTTTCCAGTAGTTATCCCCCTCCATCGG
GGCAGTTTCCCAGACATTACTCACCCGTCCGCCACTCGTCAGCAAAGCAGCAAGCTGCTTCCTGTTACCGTTC
GACTGCATGTGTTAGGCCTGCCGCCAGCGTCAATCTGAGCCAGGATCAAACTCT
>644
CTGCTGCCGCCCGTAGGAGTCTGGACCGTGTCTCAGTTCCAGTGTGGCTGGTCATCCTCTCAGACCAGCTAGG
GATCGTCGCCTAGGTGAGCCGTTACCCCACCTACTAGCTAATCCCATCTGGGCACATCCGATGGCAAGAGGCC
CTAAGGTCCCCCTCTTTGTCTTGCGACGTATGCGGTATTAGCTACCGTTTCCAGTAGTTATCCCCCTCCATCA
GGCAGTTTCCCAGACATTACTCACCCGTCCGCCACTCGTCAGCAAAGCAGCAAGCTGCTTCTGTTACCGTTCG
ACTGCATGTGTTAGGCCTGCCGCCAGCGTCAATCTGAGCCAGGATCAAACTTCT
>645
CTGCTGCCGCCCGTAGGAGTCTGGACCGTGTCTCAGTTCCAGTGTGGCTGGTCATCCTCTCAGACCAGCTAGG
GATCGTCGCCTAGGTGAGCCGTTACCCCACCTACTAGCTAATCCCATCTGGGCACATCCGATGGCAAGAGGCC
CTAAGGTCCCCCTCTTTGTCTTGCGACGTATGCGGTATTAGCTACCGTTTCCAGTAGTTATCCCCCTCCATCA
GGCAGTTTCCCAGACATTACTCACCCGTCCGCCACTCGTCAGCAAAGCAGCAAGCTGCTTTCTGTTACCGTTC
GACTGCATGTGTTAGGCCTGCCGCCAGCGTTAATCTGAGCCAGGATCAAACTCT
>646
CTGCTGCCGCCCGTAGGAGTCTGGACCGTGTCTCAGTTCCAGTGTGGCTGGTCATCCTCTCAGACCAGCTAGG
GATCGTCGCCTAGGTGAGCCGTTACCCCACCTACTAGCTAATCCCATCTGGGCACATCCGATGGCAAGAGGCC
CTAAGGTCCCCCTCTTGTCTTGCGACGTATGCGGTATTAGCTACCGTTTCCAGTAGTTATCCCCCTCCATCGG
GCGGTTTCCCAGACATTACTCACTCGTCCGCCACTCGTCAGCAAAGCAGCAAGCTGCTTCCTGTTACCGTTCG
ACTGCATGTGTTAGGCCTGCCGCCAGCGTCAATCTGAGCCAGGATCAAACTCT
>647
CTGCTGCCGCCCGTAGGAGTCTGGACCGTGTCTCAGTTCCAGTGTGGCTGGTCATCCTCTCAGACCAGCTAGG
GATCGTCGCCTAGGTGAGCCGTTACCCCACCTACTAGCTAATCCCATCTGGGCACATCCGATGGCAAGAGGCC
CTAAGGTCCCCCTCTTGTCTGCGACGTATGCGGTATTAGCTACCGTTTCCAGTAGTTATCCCCCTCCATCAGG
CAGTTTCCCAGACATTACTCACCCGTCCGCCACTCGTCAGCAAAGCAGCAAGCTGCTTCCTGTTACCGTTCGA
CTGCATGTGTTAGGCCTGCCGCCAGCGTCAATCTGAGCCAGGATCAAACTCT
>648
CTGCTGCCGCCCGTAGGAGTCTGGACCGTGTCTCAGTTCCAGTGTGGCTGGTCATCCTCTCAGACCAGCTAGG
GATCGTCGCCTAGGTGAGCCGTTACCCCACCTACTAGCTAATCCCATCTGGGCACATCCGATGGCAAGAGGCC
CTAAGGTCCCCCTCTTTGGTCTTGCGACGTTATGCGGTATTAGCTACCGTTTCCAGTAGTTATCCCCCTCCAT

CGGGCAGTTTTCCCAGACATTACTCACCCGTCCGCCACTCGTCAGCAAAGCAGCAAGCTGCTTCCTGTTACCG
TTCGACTGCATGTGTTAGGCCTGCCGCCAGCGTCAATCTGAGCCAGGATCAAACTCT
>649
CTGCTGCCGCCCGTAGGAGTCTGGACCGTGTCTCAGTTCCAGTGTGGCTGGTCATCCTCTCAGACCAGCTAGG
GATCGTCGCCTAGGTGAGCCGTTACCCCACCTACTAGCTAATCCCATCTGGGCACATCCGATGGCAAGAGGCC
CTAAGGTCCCCCTCTTTGTCTGCGACGTATGCGGTATTAGCTACCGTTTCCAGTAGTTATCCCCCTCCATCGG
GCAGTTTTCCCAGACATTACTCACCCGTCCGCCACTCGTCAGCAAAGCAGCAAGCTGCTTCCTGTTACCGTTC
GACTGCATGTGTTAGGCCTGCCGCCAGCGTCAATCTGAGCCAGGATCAAACTCT
>650
CTGCTGCCGCCCGTAGGAGTCTGGACCGTGTCTCAGTTCCAGTGTGGCTGGTCATCCTCTCAGACCAGCTAGG
GATCGTCGCCTAGGTGAGCCGTTACCCCACCTACTAGCTAATCCCATCTGGCACATCCGATGGCAAGAGGCCC
TAAGGTCCCCCTCTTGTCTTGCGACGTATGCGGTATTAGCTACCGTTTCCAGTAGTTATCCCCCTCCATCAGG
CAGTTTCCCAGACATTACTCACCCGTCCGCCACTCGTCAGCAAAGCAGCAAGCTGCTTTCTGTTACCGTTCGA
CTGCATGTGTTAGGCCTCGACCGC
>651
CTGCTGCCGCCCGTAGGAGTCTGGACCGTGTCTCAGTTCCAGTGTGGCTGGTCATCCTCTCAGACCAGCTAGG
GATCGTCGCCTAGGTGAGCCGTTACCCCACCTACTAGCTAATCCCATCTGGGCACATCCGATGGCAAGAGGCC
CTAAGGTCCCCCTCTTGTCTTGCGACGTATGCGGTATTAGCTACCGTTTCCAGTAGTTATCCCCCTCCATCAG
GCAGTTCCCAGACATTACTCACCCGTCCGCCACTCGTCAGCAAAGCAGCAAGCTGCTTCCTGTTACCGTTCGA
CTGCATGTGTTAGGCCTGCCGCCAGCGTTCAATCTGAGCCAGGATCAAACTCT
>652
CTGCTGCCGCCCGTAGGAGTCTGGACCGTGTCTCAGTTCCAGTGTGGCTGGTCATCCTCTCAGACCAGCTAGG
GATCGTCGCCTAGGTGAGCCGTTACCCCACCTACTAGCTAATCCCATCTGGGCACATCCGATGGCAAGAGGCC
CTAAGGTCCCCCTCTTTGGTCTTGCGACGTTATGCGGTATTAGCTACCGTTTCCAGTAGTTATCCCCCTCCAT
CAGGCAGTTTCCCAGACTTACTCACCCGTCCGCCACTCGTCAGCAAAGCAGCAAGCTGCTTCCTGTTACCGTT
CGACTGCATGTGTTAGGCCTGCCGCCAGCGTCAATCTGAGCCAGGATCAAACTCT
>653
CTGCTGCCGCCCGTAGGGGTCTGGACCGTGTCTCAGTTCCAGTGTGGCTGGTCATCCTCTCAGACCAGCTAGG
GATCGTCGCCTAGGTGAGCCGTTACCCCACCTACTAGCTAATCCCATCTGGGCACATCCGATGGCAAGAGGCC
CTAAGGTCCCCCTCTTTGTCTTGCGACGTTATGCGGTATTAGCTACCGTTTCCAGTAGTTATCCCCCTCCATC
AGGCAGTTTCCCAGACATTACTCACCCGTCCGCCACTCGTCAGCAAAGCAGCAAGCTGCTTCTGTTACCGTTC
GACTGCATGTGTTAGGCCTGCCGCCAGCGTTCAATCTGAGCCAGGATCAAACTCT
>654
CTGCTGCCGCCCGTAGGAGTCTGGACCGTGTCTCAGTTCCAGTGTGGCTGGTCATCCTCTCAGACCAGCTAGG
GATCGTCGCCTAGGTGAGCCGTTACCCCACCTACTAGCTAATCCCATCTGGGCACATCCGATGGCAAGAGGCC
CTAAGGTCCCCCTCTTTGTCTTGCGACGTTATGCGGTATTAGCTACCGTTTCCAGTAGTTATCCCCCTCCATC
AGGCAGTTTTCCCCAGACATTACTCACCCGTCCGCCACTCGTCAGCAGAGCAGCAAGCTGCTTTCCTGTTACC
GTTCGACTGCATGTGTTGGGCCTGCCGCCAGCGTCAATCTGAGCCAGGATCAAACTCT
>655
CTGCTGCCGCCCGTAGGAGTCTGGACCGTGTCTCAGTTCCAGTGTGGCTGGTCATCCTCTCAGACCAGCTAGG
GATCGTCGCCTAGGTGAGCCGTTACCCCACCTACTAGCTAATCCCATCTGGGCACATCCGATGGCAAGAGGCC
CTAAGGTCCCCCTCTTTGTCTGCGACGTATGCGGTATTAGCTACCGTTTCCAGTAGTTATCCCCCTCCATCGG
GCAGTTTTCCCAGACATTACTCACCCGTCCGCCACTCGTCAGCAAAGCAGCAAGCTGCTTCCTGTTACCGTTC
GACTGCATGTGTTAGGCCTGCCGCCAGCGTCAATCTGAGCCAGGATCAAACTCT
>656
CTGCTGCCGCCCGTAGGAGTCTGGACCGTGTCTCAGTTCCAGTGTGGCTGGTCATCCTCTCAGACCAGCTAGG
GATCGTCGCCTAGGTGAGCCGTTACCCCACCTACTAGCTAATCCCATCTGGGCACATCCCGATGGCAAGAGGC
CCTAAGGTCCCCCTCTTTGGTCTTGCGACGTTATGCGGTATTAGCTACCGTTTCCAGTAGTTATCCCCCTCCA
TCGGGCAGTTTTCCCAGACATTACTCACCCGTCCGCCACTCGTCAGCAAAGCAGCAAGCTGCTTCCTGTTACC
GTTCGACTGCATGTGTTAGGCCTGCCGCCAGCGTTCAATCTGAGCCAGGATCAAACTCT
>657
CTGCTGCCGCCCGTAGGAGTCTGGACCGTGTCTCAGTTCCAGTGTGGCTGGTCATCCTCTCAGACCAGCTAGG
GATCGTCGCCTAGGTGAGCCGTTACCCCACCTACTAGCTAATCCCATCTGGGCACATCCGATGGCAAGAGGCC
CTAAGGTCCCCCTCTTTGGTCTTGCGACGTTATGCGGTATTAGCTACCGTTTCCAGTAGTTATCCCCCTCCAT

CAGGCAGTTTTCCCAGACATTACTCACCCGTCCGCCACTCGTCAGCAAAGCAGCAAGCTGCTTTCTGTTACCG
TTCGACTGCATGTGTTAGGCCTGCCGCCAGCGTCAATCTGAGCCAGGATCAAACTCT
>658
CTGCTGCCGCCCGTAGGAGTCTGGACCGTGTCTCAGTTCCAGTGTGGCTGGTCATCCTCTCAGACCAGCTAGG
GATCGTCGCCTAGGTGAGCCGTTACCCCACCTACTAGCTAATCCCATCTGGGCACATCCGATGGCAAGAGGCC
CTAAGGTCCCCCTCTTTGTCTTGCGACGTTATGCGGTATTAGCTACCGTTTCCAGTAGTTATCCCCCTCCATC
AGGCAGTTTCCCAGACATTACTCACCCGTCCGCCACTCGTCAGCAAAACAGCAAGCTGCTTCCTGTTACCGTT
CGACTTGCATGTGTTAGGCCTGCCGCCAGCGTCAATCTGAGCCAGGATCAAACTCT
>659
CTGCTGCCGCCCGTAGGAGTCTGGACCGTGTCTCAGTTCCAGTGTGGCTGGTCATCCTCTCAGACCAGCTAGG
GATCGTCGCCTAGGTGAGCCGTTACCCCACCTACTAGCTAATCCCATCTGGGCACATCCGATGGCAAGAGGCC
CTAAGGTCCCCCTCTTTGGTCTTGCGACGTTATGCGGTATTAGCTACCGTTTCCAGTAGTTATCCCCCTCCAT
CGGGCAGTTTTCCCAGACATTACTCACCCGTCCGCCACTCGTCAGCAAAGCAGCAAGCTGCTTCCTGTTACCG
TTCGACTGCATGTGTTAGGCCTGCCGCCAGCGTTCAATCTGAGCCAGGATCAAACTCT
>660
CTGCTGCCGCCCGTAGGAGTCTGGACCGTGTCTCAGTTCCAGTGTGGCTGGTCATCCTCTCAGACCAGCTAGG
GATCGTCGCCTAGGTGAGCCGTTACCCCACCTACTAGCTAATCCCATCTGGGCACATCCGATGGCAAGAGGCC
CTAAGGTCCCCCTCTTTGTCTGCGACGTTATGCGGTATTAGCTACCGTTTCCAGTAGTTATCCCCCTCCATCA
GGCAGTTTCCCAGACATTACTCACCCGTCCGCCACTCGTCAGCAAAGCAGCAAGCTGCTTCCTGTTACCGTTC
GACTGCATGTGTTAGGCCTGCCGCCAGCGTTCAATCTGAAGCCAGGATCAAACTCT
>661
CTGCTGCCGCCCGTAGGAGTCTGGACCGTGTCTCAGTTCCAGTGTGGCTGGTCATCCTCTCAGACCAGCTAGG
GATCGTCGCCTAGGTGAGCCGTTACCCCACCTACTAGCTAATCCCATCTGGGCACATCCGATGGCAAGAGGCC
CTAAGGTCCCCCTCTTTGGTCTTGCGACGTTATGCGGTATTAGCTACCGTTTCCAGCAGTTATCCCCCTCCAT
CGGGCAGTTTCCCAGACATTACTCACCCGTCCGCCACTCGTCAGCAAAGCAGCAAGCTGCTTCCTGTTACCGT
TCGACTGCATGTGTTAGGCCTGCCGCCAGCGTCAATCTGAGCCAGGATCAAACTCT
>662
CTGCTGCCGCCCGTAGGAGTCTGGACCGTGTCTCAGTTCCAGTGTGGCTGGTCATCCTCTCAGACCAGCTAGG
GATCGTCGCCTAGGTGAGCCGTTACCCCACCTACTAGCTAATCCCATCTGGGCACATCCGATGGCAAGAGGCC
CTAAGGTCCCCCTCTTGTCTGCGACGTTATGCGGTATTAGCTACCGTTTCCAGTAGTTATCCCCCTCCATCAG
GCAGTTTCCCAGACATTACTCACCCGTCCGCCACTCGTCAGCAAACAGCAAGCTGCTTCCTGTTACCGTTCGA
ACTGCGTGTGTTAGGCCTGCCGCCAGCCGTCAATCGAGCCAGGATCAAACGTCT
>663
CTGCTGCCGCCCGTAGGAGTCTGGACCGTGTCTCAGTTCCAGTGTGGCTGGTCATCCTCTCAGACCAGCTAGG
GATCGTCGCCTAGGTGAGCCGTTACCCCACCTACTAGCTAATCCCATCTGGGCACATCCGATGGCAAGAGGCC
CTAAGGTCCCCTCTTGTCTTGCGACGTATGCGGTATTAGCTACCGTTTCCAGTAGTTATCCCCCTCCATCGGG
CAGTTTCCCAGACATACTCACCCGTCCGCCACTCGTCAGCAAGCAGCAAGCTGCTTCCGTTACCGTTCGATGC
ATGTGTTAGGCTGCCGCCAGCGTAATCTGAGCCAGGATCAACTC
>664
CTGCTGCCGCCCGTAGGAGTCTGGACCGTGTCTCAGTTCCAGTGTGGCTGGTCATCCTCTCAGACCAGCTAGG
GATCGTCGCCTAGGTGAGCCGTTACCCCACCTACTAGCTAATCCCATCTGGGCACATCCGATGGCAAGAGGCC
CTAAGGTCCCCCTCTTGTCTTGCGACGTTATGCGGTATTAGCTACCGTTTCCAGTAGTTATCCCCCTCCATCA
GGCAGTTTCCCAGACATTACTCACCCGTCCGCCACTCGTCAGCAAAGCAGCAAGCTGCTTCCTGTTACCGTTC
GACTGCATGTGTTAGGCCTGCCGCCAGCGTCAATCTGAGCCAGGATCAAACTCT
>665
CTGCTGCCGCCCGTAGGAGTCTGGACCGTGTCTCAGTTCCAGTGTGGCTGGTCATCCTCTCAGACCAGCTAGG
GATCGTCGCCTAGGTGAGCCGTTACCCCACCTACTAGCTAATCCCATCTGGGCACATCCGATGGCAAGAGGCC
CTAAGGTCCCCCTCTTTGTCTTGCGACGTTATGCGGTATTAGCTACCGTTTCCAGTAGTTATCCCCCTCCATC
AGGCAGTTTCCCAGACATTACTCACCCGACCGCCACTCGTCAGCGAACAGCAAGCTGTTTCCTGTTACCGTTC
GACTCGCATGTGTTGGGCCTGCCGCCAGCGTCAATCTGAGCCAGGATCAAACTCT
>666
CTGCTGCCGCCCGTAGGAGTCTGGACCGTGTCTCAGTTCCAGTGTGGCTGGTCATCCTCTCAGACCAGCTAGG
GATCGTCGCCTAGGTGAGCCGTTACCCCACCTACTAGCTAATCCCATCTGGGCACATCCGATGGCAAGAGGCC
CTAAGGTCCCCCTCTTTGTCTTGCGACGTTATGCGGTATTAGCTACCGTTTCCAGTAGTTATCCCCCTCCATC

AGGCAGTTTCCCAGACATTACTCACCCGTCCGCCACTCGTCAGCAAAGCAGCAAGCTGCTTCCTGTTACCGTT
CGACTGCATGTGTTAGGCCTGCCGCCAGCGTCAATCTGAGCCAGGATCAAACTCT
>667
CTGCTGCCGCCCGTAGGAGTCTGGACCGTGTCTCAGTTCCAGTGTGGCTGGTCATCCTCTCAGACCAGCTAGG
GATCGTCGCCTAGGTGAGCCGTTACCCCACCTACTAGCTAATCCCATCTGGGCACATCCGATGGCAAGAGGCC
CTAAGGTCCCCCTCTTTGTCTTGCGACGTTATGCGGTATTAGCTACCGTTCCAGTAGTCTACCTCCCCATAGG
CAGTTTCCCAGACATTACTCACCCGTCCGCCACTCGTCAGCAAACAGCAAGCTGCTTCCTGTTACCGTTCGAC
TGCATGTGTTAGGCCTGCCGCCAGCGTTCAATCGAGCCAGGATCAAACTGT
>668
CTGCTGCCGCCCGTAGGAGTCTGGACCGTGTCTCAGTTCCAGTGTGGCTGGTCATCCTCTCAGACCAGCTAGG
GATCGTCGCCTAGGTGAGCCGTTACCCCACCTACTAGCTAATCCCATCTGGGCACATCCGATGGCAAGAGGCC
CTAAGGTCCCCCTCTTTGGTCTTGCGACGTTATGCGGTATTAGCTACCGTTTCCAGTAGTTATCCCCCTCCAT
CAGGCAGTTTCCCAGACATTACTCACCCGTCCGCCACTCGTCAGCAAAGCAGCAAGCTGCTTCCTGTTACCGT
TCGACTGCATGTGTTAGGCCTGCCGCCAGCGTTCAATCTGAGCCAGGATCAAACTCT
>669
CTGCTGCCGCCCGTAGGAGTCTGGACCGTGTCTCAGTTCCAGTGTGGCTGGTCATCCTCTCAGACAGCTAGGG
ATCGTCGCCTAGGTGAGCCGTTACCCCACCTACTAGCTAACCCCATCTGGGCACATCCGATGGCAAGAGGCCC
TAAGGTCCCCCTCTTTGGTCTTGCGACGTATGCGGTATTAGCTACCGTTTCCAGTAGTTATCCCCCTCCATCA
GGCAGTTTCCCAGACATTACTCACCCGTCCGCCACTCGTCAGCAAACAGCAAGCTGCTTCCTGTTACCGTTCG
ACTGCATGTGTTAGGCCTGCCGCCAGCGTCGAATCTG
>670
CTGCTGCCGCCCGTAGGAGTCTGGACCGTGTCTCAGTTCCAGTGTGGCTGGTCATCCTCTCAGACCAGCTAGG
GATCGTCGCCTAGGTGAGCCGTTACCCCACCTACTAGCTAATCCCATCTGGCACATCCGATGGCAAGAGGCCC
TAAGGTCCCCCTCTTGTCTGCGACGTATGCGGTATTAGCTACCGTTTCCAGTAGTTCTCCCCTCCATCGGGCA
GTTCCCAGACATTACTCACCCGTCCGCCACTCTCAGCAAGCAGCAAGCTGCTTCCTGTTACCGTTCGACTGCA
TGTGTTAGGCCTGCCGCCAGCGTCAATCTGAGCCAGGATCAACTCT
>671
CTGCTGCCGCCCGTAGGAGTCTGGACCGTGTCTCAGTTCCAGTGTGGCTGGTCATCCTCTCAGACCAGCTAGG
GATCGTCGCCTAGGTGAGCCGTTACCCCACCTACTAGCTAATCCCATCTGGGCACATCCGATGGCAAGAGGCC
CTAAGGTCCCCCTCTTGTCTTGCGACGTTATGCGGTATTAGCTACCGTTTCCAGTAGTTATCCCCTCCATCGG
GCAGTTTCCCAGACATTACTCACCGTCCGCCACTCGTCAGCAAAGCAGCAAGCTGCTTCCTGTTACCGTTCGA
CTGCATGTGTTAGGCCTGCCGCCAGCGTCAATCTGAGCCAGGATCAAACTCT
>672
CTGCTGCCGCCCGTAGGAGTCTGGACCGTGTCTCAGTTCCAGTGTGGCTGGTCATCCTCTCAGACCAGCTAGG
GATCGTCGCCTAGGTGAGCCGTTACCCCACCTACTAGCTAATCCCATCTGGGCACATCCGATGGCAAGAGGCC
CTAAGGTCCCTCTCTTGTCTGCGACGTATGCGGTATTAGCTACCGTTTCCAGTAGTTATCCCCCTCCATCGGG
CAGTTTCCCAGACATTACTCACCCGTCCGCCACTCGTCAGCAAGCAGCAAGCTGCTTCCTGTTACCGTTCGAC
TGCATGTGTTAGGCCTGCCGCCAGCGTCAATCTGAGCCAGGATCAAACTCT
>673
CTGCTGCCGCCCGTAGGAGTCTGGACCGTGTCTCAGTTCCAGTGTGGCTGGTCATCCTCTCAGACCAGCTAGG
GATCGTCGCCTAGGTGAGCCGTTTACCCCACCTACTAGCTAATCCCATCTGGGCACATCCGATGGCAAGAGGC
CCTAAGGTCCCCCTCTTGTCTGCGACGTATGCGGTATTAGCTACCGTTTCCAGTAGTTATCCCCTCCATCGGG
CAGTTTCCCAGACATACTCACCCGTCCGCCACTCGTCAGCAAAGCAGCAAGCTGCTTCCTGTTACCGTTCGAC
TGCATGTGTTAGGCCTGCCGCCAGCGTCAATCTGAGCCAGGATCAACTCT
>674
CTGCTGCCGCCCGTAGGAGTCTGGACCGTGTCTCAGTTCCAGTGTGGCTGGTCATCCTCTCAGACCAGCTAGG
GATCGTCGCCTAGGTGAGCCGTTACCCCACCTACTAGCTAATCCCATCTGGGCACATCCGATGGCAAGAGGCC
CTAAGGTCCCCCTCTTGTCTTGCGACGTTATGCGGTATTAGCTACCGTTTCCAGTAGTTATCCCCCTCCATCG
GGCAGTTTCCCAGACATTACTCACCCGTCCGCCACTCGTCAGCAAAGCAGCAAGCTGCTTCCTGTTACCGTTC
GACTGCATGTGTTAGGCCTGCCGCCAGCGTTCAATCTGAGCCAGGATCAAACTCT
>675
CTGCTGCCGCCCGTAGGAGTCTGGACCGTGTCTCAGTTCCAGTGTGGCTGGTCATCCTCTCAGACCAGCTAGG
GATCGTCGCCTAGGTGAGCCGTTACCCCACCTACTAGCTAATCCCATCTGGGCACATCCGATGGCAAGAGGCC
CTAAGGTCCCCCCTCTTTGGTCTTGCGACGTATGCGGTATTAGCTACCGTTTCCAGTAGTTATCCCCCTCCAT

CAGGCAGTTTTCCCAGACATTACTCACCCGTCCGCCACTCGTCAGCAAAGCAGCAAGCTGCTTCCTGTTACCG
TTCGACTGCATGTGTTAGGCCTGCCGCCAGCGTCAATCTGAGCCAGGATCAAACTCT
>676
TGCTGCCGCCCGTAGGAGTCTGGACCGTGTCTCAGTTCCAGTGTGGCTGGTCATCCTCTCAGACCAGCTAGGG
ATCGTCGCCTAGGTGAGCCGTTACCCCACCTACTAGCTAATCCCATCTGGGCACATCCGATGGCAAGAGGCCC
TAAGGTCCCCCTCTTTGTCTTGCGACGTTATGCGGTATTAGCTACCGTTTCCAGTAGTTATCCCCCTCCATCA
GGCAGTTTTCCCAGACATTACTCACCCGTCCGCCACTCGTCAGCAAAGCAGCAAGCTGCTTCCTGTTACCGTT
CGACTGCATGTGTTAGGCCTGCCGCCAGCGTCAATCTGAGCCAGGATCAAACTCT
>677
CTGCTGCCGCCCGTAGGAGTCTGGACCGTGTCTCAGTTCCAGTGTGGCTGGTCATCCTCTCAGACCAGCTAGG
GATCGTCGCCTAGGTGAGCCGTTACCCCACCTACTAGCTAATCCCATCTGGGCACGTCCGATGGCAAGAGGCC
CTAAGGTCCCCTCCTTGTCTGCGACGTATGCGGTATTAGCTACCGTTTCCAGTAGTTATCCCCCTCCATCAGG
CAGTTTCCCAGACATTACTCACCCGTCCGCCACTCGTCAGCAAGCAGCAAGCTGCTTCCTGTTACCGTTCGAC
TGCATGTGTTAGGCCTGCCGCCAAGCGTTCAATCTGAGCCAGGACAAACTCT
>678
CTGCTGCCGCCCGTAGGAGTCTGGACCGTGTCTCAGTTCCAGTGTGGCTGGTCATCCTCTCAGACCAGCTAGG
GATCGTCGCCTAGGTGAGCCGTTACCCCACCTACTAGCTAATCCCATCTGGGCACATCCGATGGCAAGAGGCC
CTAAGGTCCCCCTCTTGTCTGCGACGTATGCGGTATTAGCTACCGTTTCCAGTAGTTATCCCCCTCCATCAGG
CAGTTTTCCCAGACATTACTCACCCGTCCGCCACTCGTCAGCAAACAGCAAGCTGCTTCCTGTTACCGTTCGA
CTGCATGTGTTAGGCCTGCCGCCAGCGTTCAATCGAGCC
>679
CTGCTGCCGCCCGTAGGAGTCTGGACCGTGTCTCAGTTCCAGTGTGGCTGGTCATCCTCTCAGACCAGCTAGG
GATCGTCGCCTAGGTGAGCCGTTACCCCACCTACTAGCTAATCCCATCTGGGCACATCCGATGGCAAGAGGCC
CTAAGGTCCCCCTCTTTGTCTGCGACGTTATGCGGTATTAGCTACCGTTTCCAGTAGTTATCCCCCTCCATCA
GGCAGTTTTCCCAGACATTACTCACCCGTCCGCCACTCGTCAGCAAAGCAGCAAGCTGCTTTCTGTTACCGTT
CGACTGCATGTGTTAGGCCTGCCGCCAGCGTCAATCTGAGCCAGGGTCAAACTCT
>680
CTGCTGCCGCCCGTAGGAGTCTGGACCGTGTCTCAGTTCCAGTGTGGCTGGTCATCCTCTCAGACCAGCTAGG
GATCGTCGCCTAGGTGAGCCGTTACCCCACCTACTAGCTAATCCCATCTGGGCACATCCGATGGCAAGAGGCC
CTAAGGTCCCCCCCTTGTCTTGCGACGTATGCGGTATTAGCTACCGTTTCCAGTAGTTATCCCCCTCCATCGG
GCAGTTTCCCAGACATTACCCACCCGTCCGCCCACTCGTCAGCAAAGCAGCAAGCTGCTTCCTGTTACCGTTC
GACTGCATGTGTTAGGCCTGCCGCCAGCGTCAATCTGAGCCAGGATCAAACTCT
>681
CTGCTGCCGCCCGTAGGAGTCTGGACCGTGTCTCGGTTCCAGTGTGGCTGGTCATCCTCTCAGACAGCTAGGG
ATCGTCGCCTAGGTGAGCCGTTACCCCACCTACTAGCTAATCCCATCTGGGCACATCCGATGGCAAGAGGCCC
TAAGGTCCCCCTCTTGTCTGCGACGTTATGCGGTATTAGCTACCGTTTCCAGTAGTTATCCCCCTCCATCAGG
CAGTTTCCCACGACATTACTCACCCGTCCGCCACTCGTCAGCAAAGCAGCAAGCTGCTTCTGTTACCGTTCGA
CTGCATGTCGTTAGGCCTGCCCGCCAGCGTTCAATCTGAAGCCAGGACAAACCTCTT
>682
CTGCTGCCGCCCGTAGGAGTCTGGACCGTGTCTCAGTTCCAGTGTGGCTGGTCATCCTCTCAGACCAGCTAGG
GATCGTCGCCTAGGTGAGCCGTTACCCCACCTACTAGCTAATCCCATCTGGGCACATCCGATGGCAAGAGGCC
CTAAGGTCCCCCTCTTTGTCTGCGACGTATGCGGTATTAGCTACCGTTTCCAGTAGTTATCCCCCTCCATCGG
GCAGTTTCCCAGACATTACTCACCCGTCCGCCACTCGTCAGCAAAGCAGCAAGCTGCTTCCTGTTACCGTTCG
ACTGCATGTGTTAGGCCTGCCGCCAGCGTCAATCTGAGCCAGGATCAAACTCT
>683
CTGCTGCCGCCCGTAGGAGTCTGGACCGTGTCTCAGTTCCAGTGTGGCTGGTCATCCTCTCAGACCAGCTAGG
GATCGTCGCCTAGGTGAGCCGTTACCCCACCTACTAGCTAATCCCATCTGGGCACATCCGATGGCAAGAGGCC
CTAAGGTCCCCCTCTTGTCTGCGACGTTATGCGGTATTAGCTACCGTTTCCAGTAGTTATCCCCCTCCATCGG
GCAGTTTTCCCAGACATACTCACCCGTCCGCCACTCGTCAGCAAAAGCAGCAAGCTGCTTCCTGTTACCGTTC
GACTGCATGTGTTAGGCCTGCCGCCAGCGTCAATCTGAGCCAGGATCAAACTCT
>684
CTGCTGCCGCCCGTAGGAGTCTGGACCGTGTCTCAGTTCCAGTGTGGCTGGTCATCCTCTCAGACCAGCTAGG
GATCGTCGCCTAGGTGAGCCGTTACCCCACCTACTAGCTAATCCCATCTGGGCACATCCGATGGCAAGAGGCC
CTAAGGTCCCCCTCTTTGGTCTTGCGACGTTATGCGGTATTAGCTACCGTTTCCAGTAGTTATCCCCCTCCAT

CAGGCAGTTTCCCAGACATTACTCACCCGTCCGCCACTCGTCAGCAAAGCAGCAAGCTGCTTCTGTTACCGTT
CGACTGCATGTGTTAGGCCTGCCGCCAGCGTCAATCTGAGCCAGGATCAAACTCT
>685
CTGCTGCCGCCCGTAGGAGTCTGGACCGTGTCTCAGTTCCAGTGTGGCTGGTCATCCTCTCAGACCAGCTAGG
GATCGTCGCCTAGGTGAGCCGTTACCCCACCTACTAGCTAATCCCATCTGGGCACATCCGATGGCAAGAGGCC
CTAAGGTCCCCCTCTTTGGTCTTGCGACGTATGCGGTATTAGCTACCGTTTCCAGTAGTCATCCCCCTCCATC
AGGCAGTTTCCCAGACATTACTCACCCGTCCGCCACTCGTCAGCAAAGCAGCAAGCTGCTTCCTGTTACCGTT
CGACTGCATGTGTTAGGCCTGCCGCCAGCGTCAATCTGAGCCAGGATCAAACTCT
>686
CTGCTGCCGCCCGTAGGAGTCTGGACCGTGTCTCAGTTCCAGTGTGGCTGGTCATCCTCTCAGACCAGCTAGG
GATCGTCGCCTAGGTGAGCCGTTCACCCCACCTACTAGCTAATCCCATCTGGGCACATCCCGATGGCAAGAGG
CCCTAAGGTCCCCCTCTTTGGTCTTGCGACGTTATGCGGTATTAGCTACCGTTTCCAGTAGTTATCCCCCTCC
ATCGGGGCAGTTTTCCCAGACATTACTCACCCGTCCGCCACTCGTCAGCAAAGCAGCAAGCTGCTTCCTGTTA
CCGTTCGACTGCATGTGTTAGGCCTGCCGCCAGCGTCAATCTGAGCCAGGATCAAACTCT
>687
CTGCTGCCGCCCGTAGGAGTCTGGACCGTGTCTCAGTTCCAGTGTGGCTGGTCATCCTCTCAGACCAGCTAGG
GATCGTCGCCTAGGTGAGCCGTTACCCCACCTACTAGCTAATCCCATCTGGGCACATCCCGATGGCAAGAGGC
CCTAAGGTCCCCCTCTTGGTCTTGCGACGTATGCGGTATTAGCTACCGTTTCCAGTAGTTATCCCCCTCCATC
GGGCAGTTTCCCCAGACATTACTCACCCGTCCGCCACTCGTCAGCAAAGCAGCAAGCTGCTTCCTGTTACCGT
TCGACTGCATGTGTTAGGCCTGCCGCCAGCGTCAATCTGAGCCAGGATCAAACTCT
>688
TGCTGCCGCCCGAGGAGTCTGGACCGTGTCTCAGTTCCAGTGTGGCTGGTCATCCTCTCAGACCAGCTAGGGA
TCGTCGCCTAGGTGAGCCGTTACCCCACCTACTAGCTAATCCCATCTGGGCACATCCCGATGGCAAGAGGCCC
TAAGGTCCCCCTCTTGTCTTGCGACGTATGCGGTATTAGCTACCGTTTCCAGTAGTTATCCCCCTCCATCGGG
CAGTTTCCCAGACATTACTCACCCGTCCGCCACTCTCAGCAAGCAGCAAGCTGCTTCCTGTTACCGTTCGACT
GCATGTGTTAGGCCTGCCGCCAGCGTCAATCTGAGCCAGGATCAAACTCT
>689
TGCTGCCGCCCGTAGGAGTCTGGACCGTGTCTCAGTTCCAGTGTGGCTGGTCATCCTCTCAGACCAGCTAGGG
ATCGTCGCCTAGGTGAGCCGTTACCCCACCTACTACGCTAATCCATCTGGGCACATCCGATGGCAAGAGGCCC
TAAGGTCCCCCTCTTTGTCTTGCGACGTTATGCGGTATTAGCTACCGTTTCCAGTAGTTATCCCCCTCCATCG
GGCAGTTTCCCAGACATTACTCACCCGTCCGCCACTCGTCAGCAAAGCAGCAAGCTGCTTCCTGTTACCGTTC
GACTGCATGTGTTAGGCCTGCCGCCAGCGTCAATCTGAGCCAGGATCAAACTCT
>690
TGCTGCCGCCCGTAGGAGTCTGGACCGTGTCTCAGTTCCAGTGTGGCTGGTCATCCTCTCAGACCAGCTAGGG
ATCGTCGCCTAGGTGAGCCGTTACCCCACCTACTAGCTAATCCCATCTGGGCACATCCGATGGCAAGAGGCCC
TAAGGTCCCCCTCTTTGTCTTGCGACGTTATGCGGTATTAGCTACCGTTTCCAGTAGTTATCCCCCTCCATCA
GGCAGTTTCCCAGACATTACTCACCCGTCCGCCACTCGTCAGCAAAGCAGCAAGCTGCTTCCTGTTACCGTTC
GACTGCATGTGTTAGGCCTGCCGCCAGCGTTCAATCTGAGCCAGGATCAAACTCT
>691
CTGCTGCCGCCCGTAGGAGTCTGGACCGTGTCTCAGTTCCAGTGTGGCTGGTCATCCTCTCAGACCAGCTAGG
GATCGTCGCCTAGGTGAGCCGTTACCCCACCTACTAGCTAATCCCATCTGGGCACATCCGATGGCAAGAGGCC
CTAAGGTCCCCCTCTTCGGTCTTGCGACGTATGCGGTATTAGCTACCGTTTCCAGTAGTTATCCCCCTCCATC
GGGCAGTTTCCCAGACATTACTCACCCGTCCGCCACTCGTCAGCAAAGCAGCAAGCTGCTTCCTGTTACCGTT
CGACTGCATGTGTTAGGCCTGCCGCCAGCGTCAATCTGAGCCAGGATCAAACTCT
>692
CTGCTGCCGCCCGTAGGAGTCTGGACCGTGTCTCAGTTCCAGTGTGGCTGGTCATCCCCTCAGACCAGCTAGG
GATCGTCGCCTAGGTGAGCCGTTACCCCACCTACTAGCTAATCCCATCTGGGCACATCCGATGGCAAGAGGCC
CTAAGGTCCCCCTCTTTGGTCTTGCGACGTTATGCGGTATTAGCTACCGTTTCCAGTAGTTATCCCCCTCCAT
CGGGCAGTTTTCCCAGACATTACTCACCCGTCCGCCACTCGTCAGCAAAGCAGCAAGCTGCTTCCTGTTACCG
TTCGACTGCATGTGTTAGGCCTGCCGCCAGCGTCAATCTGAGCCAGGATCAAACTCT
>693
CTGCTGCCGCCCGTAGGAGTCTGGACCGTGTCTCAGTTCCAGTGTGGCTGGTCATCCTCTCAGACCAGCTAGG
GATTGTCGCCTAGGTGAGCCGTTACCCCACCTACTAGCTAATCCCATCTGGGCACATCCGATGGCAAGAGGCC
CTAAGGTCCCCCTCTTGTCTTGCGACGTTATGCGGTATTAGCTACCGTTTCCAGTAGTTATCCCCCTCCATCG

GGCAGTTTCCCAGACATTACTCACCCGTCCGCCACTCGTCAGCAAAGCAGCAAGCTGCTTCCTGTTACCGTTC
GACTGCATGTGTTAGGCCTGCCGCCAGCGTCAATCTGAGCCAGGATCAAACTCT
>694
CTGCTGCCGCCCGTAGGAGTCTGGACCGTGTCTCAGTTCCAGTGTGGCTGGTCATCCTCTCAGACCAGCTAGG
GATCGTCGCCTAGGTGAGCCGTTACCCCACCTACTAGCTAATCCCATCTGGGCACATCCGATGGCAAGAGGCC
CTAAGGTCCCCCTCTCTGTCTTGCGACGTATGCGGTATTAGCTACCGTTTCCAGTAGTTATCCCCCTCCATCA
GGCAGTTTCCCAGACATTACTCACCGTCCGCCACTCGTCAGCAAAACAGCAAGCTGCTTCCTGTTACCGTTCG
ACTGCATGTGTTAGGCCTGCCGCCAGCGTCAATCTGAAGCCAGGATCAAACTCT
>695
CTGCTGCCGCCCGTAGGAGTCTGGACCGTGTCTCAGTTCCAGTGTGGCTGGTCATCCTCTCAGACCAGCTAGG
GATCGTCGCCTAGGTGAGCCGTTACCCCACCTACTAGCTAATCCCATCTGGGCACATCCGATGGCAAGAGGCC
CTAAGGTCCCCCTCTTTGGTCTTGCGACGTTATGCGGTATTAGCTATCGTTTCCAGTAGTTATCCCCCTCCAT
CGGGCAGTTTCCCAGACATTACTCACCCGTCCGCCACTCGTCAGCAAAGCAGCAAGCTGCTTTCTGTTACCGT
TCGACTGCATGTGTTAGGCCTGCCGCCAGCGTCAATCTGAGCCAGGATCAAACTCT
>696
CTGCTGCCGCCCGTAGGAGTCTGGACCGTGTCTCAGTTCCAGTGTGGCTGGTCATCCTCTCAGACCAGCTAGG
GATCGTCGCCTAGGTGAGCCGTTACCCCACCTACTAGCTAATCCCATCTGGGCACATCCGATGGCAAGAGGCC
CTAAGGTCCCCCTCTTGGTCTTGCGACGTTATGCGGTATTAGCTACCGTTTCCAGTAGTTATCCCCCTCCATC
AGGCAGTTTCCCAGACATTACTCACCCGTCCGCCACTCGTCAGCAAACAGCAAGCTGCTTCCTGTTACCGTTC
GACTGCATGTGTTAGGCCTGCCGCCAGCGTCAATCTGAGCCAGGATCAAACTCT
>697
CTGCTGCCGCCCGTAGGAGTCTGGACCGTGTCTCAGTTCCAGTGTGGCTGGTCATCCTCTCAGACCAGCTAGG
GATCGTCGCCTAGGTGAGCCGTTACCCCACCTACTAGCTAATCCCATCTGGGCACATCCGATGGCAAGAGGCC
CTAAGGTCCCCCTCTTTGTCTTGCGACGTATGCGGTATTAGCTACCGTTTCCAGTAGTTATCCCCCTCCATCG
GGCAGTTTCCCAGACATTACTCACCCGTCCCGCCACTCGTCAGCAAAGCAGCAAGCTGCTTCCTGTTACCGTT
CGACTGCATGTGTTAGGCCTGCCGCCAGCGTCAATCTGAGCCAGGATCAAACTCT
>698
CTGCTGCCGCCCGTAGGAGTCTGGACCGTGTCTCAGTTCCAGTGTGGCTGGTCATCCTCTCAGACCAGCTAGG
GATCGTCGCCTAGGTGAGCTGTTACCCCACCTACTAGCTAATCCCATCTGGGCACATCCGATGGCAAGAGGCC
CTAAGGTCCCCCTCTTTGGTCTGCGACGTATGCGGTATTAGCTACCGTTTCCAGTAGTTATCCCCCTCCATCG
GGCAGTTCCCCAGACATTACTCACCCGTCCGCCACTCGTCAGCAAAAGCAGCAAGCTGCTTCCTGTTACCGTT
CGACTGCATGTGTTAGGCCTGCCGCCAGCGTCAATCTGAGCCAGGATCAAACTCT
>699
CTGCTGCCGCCCGTAGGAGTCTGGACCGTGTCTCAGTTCCAGTGTGGCTGGTCATCCTCTCAGACCAGCTAGG
GATCGTCGCCTAGGTGAGCCGTTACCCCACCTACTAGCTAATCCCATCTGGGCACATCCGATGGCAAGAGGCC
CTAAGGTCCCCCTCTTTGTCTTGCGACGTTATGCGGTATTAGCTACCGTTTCCAGTAGTTATCCCCCTCCATC
AGGCAGTTTCCCAGACATTACTCACCCGTCCGCCACTCGTCAGCAAAGCAGCAAGCTGCTTCCTGTTACCGTT
CGACTTGCATGTGTTAGGCCTGCCGCCAGCGTTCAATCTGAGCCAGGATCAAACTCT
>700
CTGCTGCCGCCCGTAGGAGTCTGGACCGTGTCTCAGTTCCAGTGTGGCTGGTCATCCTCTCAGACCAGCTAGG
GATCGTCGCCTAGGTGAGCCGTTACCCCACCTACTAGCTAATCCCATCTGGGCACATCCGATGGCAAGAGGCC
CTAAGGTCCCCCTCTTTGTCTTGCGACGTTATGCGGTATTAGCTACCGTTTCCAGTAGTTATCCCCCTCCATC
AGGCAGTTTCCCAGACATTACTCACCCGTCCGCCACTCGTCAGCAAAGCAGTAAGCTGCTTCCTGTTACCGTT
CGACTGCATGTGTTAGGCCTGCCGCCAGCGTCAATCTGAGCCAGGATCAAACTCT
>701
CTGCTGCCGCCCGTAGGAGTCTGGACCGTGTCTCAGTTCCAGTGTGGCTGGTCATCCTCTCAGACCAGCTAGG
GATCGTCGCCTAGGTGAGCCGTTACCCCACCTACTAGCTAATCCCATCTGGGCACATCCGATGGCAAGAGGCC
CTAAGGTCCCCCTCTTTGGTTTGCGACGTTACTGCGGTATTAGCTACCGTTTCCAGTAGTTATCCCCCTCCAT
CAGGCAGTTTCCCAGACATTACTCACCCGTCCGCCACTCGTCAGCAAAGCAGCAAGCTGCTTCTGTTACCGTT
CGACTGCATGTCGTTAGGCCTGCCGCCAGCGTCAATCTGAGCCAGGATCAAACCTTCT
>702
CTGCTGCTCGCCCGTAGGAGTCTGGACCGTGTCTCAGTTCCAGTGTGGCTGGTCATCCTCTCAGACAGCTAGG
GATCGTCGCCTAGGTGAGCCGTTACCCCACCTACTAGCTAATCCCATCTGGGCACATCCGATGGCAAGAGGCC
CTAAGGTCCCCCTCTTTGTCTTGCGACGTTATGCGGTATTAGCTACCGTTTCCAGTAGTTATCCCCCTCCATC

GGGCAGTTTTCCCAGACATTACTCACCCGTCCGCCACTCGTCAGCAAAGCAGCAAGCTGCTTCCTGTTACCGT
TCGAACTGCATGTGTTAGGCCTGCCGCCAGCGTCAATCTGAGCCAGGATCAAACTCT
>703
CTGCTGCCGCCCGTAGGAGTCTGGACCGTGTCTCAGTTCCAGTGTGGCTGGTCATCCTCTCAGACCAGCTAGG
GATCGTCGCCTAGGTGAGCCGTTACCCCACCTACTAGCTAATCCCATCTGGGCACATCCGATGGCAAGAGGCC
CTAAGGTCCCCCTCTTTGGTCTTGCGACGTTATGCGGTATTAGCTACCGTTTCCAGTAGTTATCCCCCTCCAT
CAGGCAGTTTCCCAGACATTACTCACCCGTCCGCCACTCGTCAGCAAAGCAGCAAGCTGCTTCTGTTACCGTT
CGACTGCATGTGTTAGGCCTGCCGCCAGCGTCAATCTGAGCCAGGATCAAACTCT
>704
CTGCTGCCGCCCGTAGGAGTCTGGACCGTGTCTCAGTTCCAGTGTGGCTGGTCATCCTCTCAGACCAGCTAGG
GATCGTCGCCTAGGTGAGCCGTTACCCCACCTACTAGCTAATCCCATCTGGGCACATCCGATGGCAAGAGGCC
CTAAGGTCCCCCTCTTTGGTCTTGCGACGTTATGCGGTATTAGCTACCGTTTCCAGTAGTTATCCCCCTCCAT
CGGGCAGTTTCCCAGACATTACTCACCCGTCCGCCACTCGTCAGCAAAGCAGCAAGCTGCTTCCTGTTACCGT
TCGACTGCATGTGTTAGGCCTGCCGCCAGCGTCAATCTGAGCCAGGATCAAACTCT
>705
CTGCTGCCGCCCGTAGGAGTCTGGACCGTGTCTCAGTTCCAGTGTGGCTGGTCATCCTCTCAGACCAGCTAGG
GATCGTCGCCTAGGTGAGCCGTTACCCCACCTACTAGCTAATCCCATCTGGGCACATCCGATGGCAAGAGGCC
CTAAGGTCCCCCTCTTTGGTCTTGCGACGTTATGCGGTATTAGCTACCGTTTCCAGTAGTTATCCCCCTCCAT
CAGGCAGTTTCCCAGACATTACTCACCCGTCCGCCACTCGTCAGCAAAGCAGCAAGCTGTTTTCTGTTACCGT
TCGACTGCATGTGTTAGGCCTGCCGCCAGCGTCAATCTGAGCCAGGATCAAACTCT
>706
CTGCTGCCGCCCGTAGGAGTCTGGACCGTGTCTCAGTTCCAGTGTGGCTGGTCATCCTCTCAGACCAGCTAGG
GATCGTCGCCTAGGTGAGCCGTTACCCCACCTACTAGCTAATCCCATCTGGGCACATCCGATGGCAAGAGGCC
CTAAGGTCCCCCTCTTTGGTCTGCGACGTTATGCGGTATTAGCTACCGTTTCCAGTAGTTATCCCCCTCCATC
GGGCAGTTTTCCCAGACATTACTCACCCGTCCGCCACTCGTCAGCAAAGCAGCAAGCTGCTTCCTGTTACCGT
TCGACTGCATGTGTTAGGCCTGCCGCCAGCGTTCAATCTGAGCCAGGATCAAACTCT
>707
CTGCTGCCGCCCGTAGGAGTCTGGACCGTGTCTCAGTTCCAGTGTGGCTGGTCATCCTCTCAGACCAGCTAGG
ATCGTCGCCTAGGTGAGCCGTTACCCCACCTACTAGCTAATCCCATCTGGGCACATCCGATGGCAAGAGGCCC
TAAGGTCCCCTCTTGTCTTGCGACGTATGCGGTATTAGCTACCGTTTCCAGTAGTTACTCCCCTCCATGGGCA
GTTTCCCAGACATACTCACCCGTCCGCCACTCGTCAGCAAAGCAGCAAGCTGCTTCCTGTTACCGTTCGACTG
CATGTGTTAG
>708
CTGCTGCCGCCCGTAGGAGTCTGGACCGTGTCTCAGTTCCAGTGTGGCTGGTCATCCTCTCAGACCAGCTAGG
GATCGTCGCCTAGGTGAGCCGTTACCCCACCTACTAGCTAATCCCATCTGGGCACATCCGATGGCAAGAGGCC
CTAAGGTCCCCCTCTTTGGTCTTGCGACGCTATGCGGTATTAGCTACCGTTTCCAGTAGTTATCCCCCTCCAT
CAGGCAGTTTCCCAGACATTACTCACCCGTCCGCCACTCGTCAGCAAAGCAGCAAGCTGCTTCTGTTACCGTT
CGACTGCATGTGTTAGGCCTGACCGCCAGCGTTCACTCAGCCAGGATCAAAACTGATCAACCGCTGCCCATAG
TGA
>709
CTGCTGCCGCCCGTAGGAGTCTGGACCGTGTCTCAGTTCCAGTGTGGCTGGTCATCCTCTCAGACCAGCTAGG
GATCGTCGCCTAGGTGAGCCGTTACCCCACCTACTAGCTAATCCCATCTGGGCACATCCGATGGCAAGAGGCC
CTAAGGTCCCCCTCTTTGGTCTTGCGACGTTATGCGGTATTAGCTACCGTTTCCAGTAGTTATCCCCCTCCAT
CAGGCAGTTTCCCAGACATTACTCACCCGTCCGCCACTCGTCAGCAAAGCAGCAAGCTGCTTCCTGTTACCGT
TCGACTGCATGTGTTAGGCCTGCCGCCAGCGTCAATCTGAGCCAGGATCAAACTCT
>710
CTGCTGCCGCCCGTAGGAGTCTGGACCGTGTCTCAGTTCCAGTGTGGCTGGTCATCCTCTCAGACCAGCTAGG
GATCGTCGCCTAGGTGAGCCGTTACCCCACCTACTAGCTAATCCCATCTGGGCACATCCGATGGCAAGAGGCC
CTAAGGTCCCCCTCTTTGGTCTTGCGACGTATGCGGTATTAGCTACCGTTTCCAGTAGTTATCCCCCTCCATC
GGGCAGTTTCCCAGACATTACTCACCCGTCCGCCACTCGTCAGCAAAGCAGCAAGCTGCTTCCTGTTACCGTT
CGACTGCATGTGTTAGGCCTGCCGCCAGCGTCAATCTGAGCCAGGATCAAACTCT
>711
CTGCTGCCGCCCGTAGGAGTCTGGACCGTGTCTCAGTTCCAGTGTGGCTGGTCATCCTCTCAGACCAGCTAGG
GATCGTCGCCTAGGTGAGCCGTTACCCCACCTACTAGCTAATCCCATCTGGGCACATCCGATGGCAAGAGGCC

CTAAGGTCCCCCTCTTTGTCTTGCGACGTTATGCGGTATTAGCTACCGTTTCCAGTAGTTATCCCCCTCCATC
AGGCAGTTTCCCAGACATTACTCACCCGTCCGCCACTCGTCAGCAAAGCAGCAAGCTGCTTTCCTGTTACCGT
TCGACTTGCATGTGTTAGGCCTGCCGCCAGCGTTCAATCTGAGCCAGGATCAAACTCT
>712
CTGCTGCCGCCCGTAGGAGTCTGGACCGTGTCTCAGTTCCAGTGTGGCTGGTCATCCTCTCAGACCAGCTAGG
GATCGTCGCCTAGGTGAGCCGTTACCCCACCTACTAGCTAATCCCATCTGGGCACATCCGATGGCAAGAGGCC
CTAAGGTCCCCCTCTTGTCTTGCGACGTATGCGGTATTAGCTACCGTTTCCAGTAGTTATCCCCCTCCATCGG
GCAGTTTCCCAGACATTACTCACCCGTCCGCCACTCGTCAGCAAGCAGCAAGCTGCTTCCTGTTACCGTTCGA
CTGCATGTGTTAGGCCTGCCGCCAGCGTCAATCTGAGCCAGGATCAACTCT
>713
CTGCTGCCGCCCGTAGGAGTCTGGACCGTGTCTCAGTTCCAGTGTGGCTGGTCATCCTCTCAGACCAGCTAGG
GATCGTCGCCTAGGTGAGCCGTTACCCCACCTACTAGCTAATCCCATCTGGGCACATCCGATGGCAAGAGGCC
CTAAGGTCCCCCTCTTGTCTTGCGACGTTATGCGGTATTAGCTACCGTTTCCAGTAGTTATCCCCCTCCATCA
GGCAGTTTTCCCAGACATTACTCACCCGTCCGCCACTCGTCAGCAAAGCAGCAAGCTGCTTCCTGTTACCGTT
CGACTGCATGTGTTAGGCCTGCCGCCAGCGTCAATCTGAGCCAGGATCAAACTCT
>714
GCTGCCGCCCGTAGGAGTCTGGACCGTGTCTCAGTTCCAGTGTGGCTGGTCATCCTCTCAGACCAGCTAGGGA
TCGTCGCCTAGGTGAGCCGTTACCCCACCTACTAGCTAATCCCATCTGGGCACATCCGATGGCAAGAGGCCCT
AAGGTCCCCCTCTTTGGTCTTGCGACGTTATGCGGTATTAGCTACCGTTTCCAGTAGTTATCCCCCTCCATCG
GGCAGTTTCCCGGACATTACTCACCCGTCCGCCACTCGTCAGCAAAGCAGCAAGCTGCTTCCTGTTACCGTTC
GACTGCATGTGTTAGGCCTGCCGCCAGCGTTCAATCTGAGCCAGGATCAAACTCT
>715
CTGCTGCCGCCCGTAGGAGTCTGGACCGTGTCTCAGTTCCGATGTGGCTGGTCATCCTCTCAGACCAGCTAGG
GATCGTCGCCTAGGTGAGCCGTTACCCCACCTACTAGCTAATCCCATCTGGGCACATCCGATGGCAAGAGGCC
CTAAGGTCCCCCTCTTTGTCTGCGACGTTATGCGGTATTAGCTACCGTTTCCAGTAGTTATCCCCCCTCCATC
AGGCAGTTTTCCCAGACATTACTCACCCGTCCGCCACTCGTCAGCAAAGCAGCAAGCTGCTTCTGTTACCGTT
CGACTGCATGTGTTAGGCCTGCCGCCAGCGTCAATCTGAGCCAGGATCAAACTCT
>716
CTGCTGCCGCCCGTAGGAGTCTGGACCGTGTCTCAGTTCCAGTGTGGCTGGTCATCCTCTCAGACCAGCTAGG
GATCGTCGCCTAGGTGAGCCGTTACCCCACCTACTAGCTAATCCCATCTGGGCATATCCGATGGCAAGAGGCC
CTAAGGTCCCCCTCTTGTCTTGCGACGTATGCGGTATTAGCTACCGTTTCCAGTAGTTATCCCCCTCCATCAG
GCAGTTTCCCAGACATTACTCACCGTCCGCCACTCGTCAGCAAAGCAGCAAGCTGCTTCCTGTTACCGTTCGA
CTGCATGTGTTTAGGCCTG
>717
CTGCTGCCCCCGTAGGAGTCTGGACCGTGTCTCAGTTCCAGTGTGGCTGGTCATCCTCTCAGACCAGCTAGGG
ATCGTCGCCTAGGTGAGCCGTTACCCCACCTACTAGCTAATCCCATCTGGGCACATCCGATGGCAAGAGGCCC
TAAGGTCCCCCTCTTTGGTCTTGCGACGTTATGCGGTATTAGCTACCGTTTCCAGTAGTTATCCCCCTCCATC
GGGCAGTTTCCCAGACATTACTCACCCGTCCGCCACTCGTCAGCAAAGCAGCAAGCTGCTTCCTGTTACCGTT
CGACTGCATGTGTTAGGCCTGCCGCCAGCGTCAATCGGAGCCAGGATCAAACTCT
>718
CTGCTGCCGCCCGTAGGAGTCTGGACCGTGTCTCAGTTCCAGTGTGGCTGGTCATCCTCTCAGACCAGCTAGG
GATCGTCGCCTAGGTGAGCCGTTACCCCACCTACTAGCTAATCCCATCTGGGCACATCCGATGGCAAGAGGCC
CTAAGGTCCCCCTCTTTGGTCTTGCGACGTATGCGGTATTAGCTACCGTTTCCAGTAGTTATCCCCCTCCATC
AGGCAGTTTCCCAGACATTACTCACCCGTCCGCCACTCGTCAGCAAACAGCAAGCTGCTTCCTGTTACCGTTC
GACTGCATGTGTTAGGCCTGCCGCCAGCGTCAATCTGAGCCAGGATCAAACTCT
>719
CTGCTGCCGCCCGTAGGAGTCTGGACCGTGTCTCAGTTCCAGTGTGGCTGGTCATCCTCTCAGACCAGCTAGG
GATCGTCGCCTAGGTGAGCCGTTACCCCACCTACTAGCTAATCCCATCTGGGCACATCCGATGGCAAGAGGCC
CTAAGGTCCCCCTCTTTGGTCTTGCGACGTTATGCGGTATTAGCTACCGTTTCCAGTAGTTATCCCCCTCCAT
CAGGCAGTTTCCCAGACATTACTCACCCGTCCGCCACTCGTCAGCAAAGCAGCAAGCTGCTTCTGTTACCGTT
CGACTGCATGTGTTAGGCCTGCCGCCAGCGTTCAATCTGAGCCAGGATCAAACTCT
>720
CTGCTGCCGCCCGTAGGGTAGTGTGACCGTGTCTCAGTTCCAGTGTGGCTGGTCATCCTCTCAGACCAGCTAG
GGATCGTCGCCTAGGTGAGCCGTTACCCCACCTACTAGCTAATCCCATCTGGGCACATCCGATGGCAAGAGGC

CCTAAGGTCCCCCTCTTTGTCTTGCGACGTTATGCGGTATTAGCTACCGTTTCCAGTAGTTATCCCCCTCCAT
CAGGCAGTTTCCCAGACATTACTCACCCGTCCGCCACTCGTCAGCAAAGCAGCAAGCTGCTTCCTGTTACCGT
TCGACTGCATGTGTTAGGCCTGCCGCCAGCGTCAATCTGAGCCAGGATCAAACTCT
>721
CTGCTGCCGCCCGTAGGAGTCTGGACCGTGTCTCAGTTCCAGTGTGGCTGGTCATCCTCTCAGACCAGCTAGG
GATCGTCGCCTAGGTGAGCCGTTACCCCACCTACTAGCTAATCCCATCTGGGCACATCCGATGGCAAGAGGCC
CTAAGGTCCCCCTCTTTGTCTTGCGACGTATGCGGTATTAGCTACCGTTTCCAGTAGTTATCCCCCTCCATCG
GGCAGTTTCCCAGACATTACTCACCCGTCCGCCACTCGTCAGCAAAGCAGCAAGCTGCTTCCTGTTACCGTTC
GACTACATGTGTTAGGCCTGCCGCCAGCGTTCAATCTGAGCCAGGATCAAACTCT
>722
CTGCTGCCGCCCGTAGGAGTCTGGACCGTGTCTCAGTTCCAGTGTGGCTGGTCATCCTCTCAGACAGCTAGGG
ATCGTCGCCTAGGTGAGCCGTTACCCCACCTACTAGCTAATCCCATCTGGGCACATCCGATGGCAAGAGGCCC
TAAGGTCCCCCTCTTTGGTCTTGCGACGTATGCGGTATTAGCTACCGTTTCCAGTAGTTATCCCCCTCCCATC
AGGCAGTTTCCCAGACATTACTCACCCGTCCGCCACTCGTCAGCAAAGCAGCAAGCTGCTTTCTGTTACCGTT
CGACTGCATGTGTTAGGCCTGCCGCCAGCGTTCAATCTGAGCCAGGATCAAACTCT
>723
CTGCTGCCGCCCGTAGGAGTCTGGACCGTGTCTCAGTTCCAGTGTGGCTGGTCATCCTCTCAGACCAGCTAGG
GATCGTCGCCTAGGTGAGCCGTTACCCCACCTACTAGCTAATCCCATCTGGGCACATCCGATGGCAAGAGGCC
CTAAGGTCCCCCTCTTGTCTTGCGACGTTATGCGGTATTAGCTACCGTTTCCAGTAGTTATCCCCCTCCATCA
GGCAGTTTCCCAGACATTACTCACCCGTCCGCCACTCGTCAGCAAAAGCAGCAAGCTGCTTCTGTTACCGTTC
GACTGCATGTGTTAGGCCTGCCGCCAGCGTCAATCTGAGCCAGGATCAAACTCT
>724
CTGCTGCCGCCCGTAGGAGTCTGGACCGTGTCTCAGTTCCAGTGTGGCTGGTCATCCTCTCAGACCAGCTAGG
GATCGTCGCCTAGGTGAGCCGTTACCCCACCTACTAGCTAATCCCATCTGGGCACATCCGATGGCAAGAGGCC
CTAAGGTCCCCCCTCTTTGTCTTGCGACGTTATGCGGTATTAGCTACCGTTTCCAGTAGTTATCCCCCTCCAT
CGGGCAGTTTCCCAGACATTACTCACCCGTCCGCCACTCGTCAGCAAAGCAGCAAGCTGCTTCCTGTTACCGT
TCGACTGCATGTGTTAGGCCTGCCGCCAGCGTCAATCTGAGCCAGGATCAAACTCT
>725
CTGCTGCCGCCCGTAGGAGTCTGGACCGTGTCTCAGTTCCAGTGTGGCTGGTCATCCTCTCAGACCAGCTAGG
GATCGTCGCCTAGGTGAGCCGTTACCCCACCTACTAGCTAATCCCATCTGGGCACATCCGATGGCAAGAGGCC
CTAAGGTCCCCCTCTTGTCTTGCGACGTTATGCGGTATTAGCTACCGTTTCCAGTAGTTATCCCCCTCCATCA
GGCAGTTTCCCAGACATTACTCACCCGTCCGCCACTCGTCAGCAAAGCAGCAAGCTGCTTCCTGTTACCGTTC
GACTGCATGTGTTAGGCCTGCCGCCAGCGTCAATCTGAGCCAGGATCAAACCTCT
>726
CTGCTGCCGCCCGTAGGAGTCTGGACCGTGTCTCAGTTCCAGTGTGGCTGGTCATCCTCTCAGACCAGCTAGG
GATCGTCGCCTAGGTGAGCCGTTACCCCACCTACTAGCTAATCCCATCTGGGCACATCCGATGGCAAGAGGCC
CTAAGGTCCCCCTCTTGTCTGCGACGTATGCGGTATTAGCTACCGTTTCCAGTAGTATCCCCCTCCATCGGGC
AGTTTCCCAGACATACTCACCCCGTCCGCCACTCGTCAGCAAAGCAGCAAGCTGCTTCCTGTTACCGTTCGAC
TGCATGTGTTAGGCCTGCCGCCAGCGTCAATCTGAGCCAGGACAAACTCT
>727
CTGCTGCCGCCCGTAGGAGTCTGGACCGTGTCTCAGTTCCAGTGTGGCTGGTCATCCTCTCAGACCAGCTAGG
GATCGTCGCCTAGGTGAGCCGTTACCCCACCTACTAGCTAATCCCATCTGGGCACATCCGATGGCAAGAGGCC
CTAAGGTCCCCCTCTTTGGTCTTGCGACGTTATGCGGTATTAGCTACCGTTTCCAGTAGTTATCCCCCCTCCA
TCAGGCAGTTTCCCAGACATTACTCACCCGTCCGCCACTCGTCAGCAAAACAGCAAGCTGCTTCCTGTTACCG
TTCGACTGCATGTGTTAGGCCTGCCGCCAGCGTTCAATCTGAGCCAGGATCAAACTCT
>728
CTGCTGCCGCCCGTAGGAGTCTGGACCGTGTCTCAGTTCCAGTGTGGCTGGTCATCCTCTCAGACCAGCTAGG
GATCGTCGCCTAGGTGAGCCGTTACCCCACCTACTAGCTAATCCCATCTGGGCACATCCGATGGCAAGAGGCC
CTAAGGTCCCCCTCTTTGGTCTGCGACGTTATGCGGTATTAGCTACCGTTTCCAGTAGTTATCCCCCTCCATC
GGGCAGTTTCCCAGACATTACTCATCCGTCCGCCACTCGTCAGCAAGGCAGCAAGCTGCTTCCTGTTACCGTT
CGACTGCATGTGTTAGGCCTGCCGCCAGCGTTCAATCTGAGCCAGGATCAAACTCT
>729
CTGCTGCCGCCCGTAGGAGTCTGGACCGTGTCTCAGTTCCAGTGTGGCTGGTCATCCTCTCAGACCAGCTAGG
GATCGTCGCCTAGGTGAGCCGTTACCCCACCTACTAGCTAATCCCATCTGGGCACATCCGATGGCAAGAGGCC

CTAAGGTCCCCCCTCTTTGGTCTTGCGACGTTATGCGGTATTAGCTACCGTTTCCAGTAGTTATCCCCCTCCA
TCGGGCAGTTTCCCAGACATTACTCACCCGTCCGCCACTCGTCAGCAAAGCAGCGAGCTGCTTCCTGTTACCG
TTCGACTGCATGTGTTAGGCCTGCCGCCAGCGTCAATCTGAGCCAGGATCAAACTCT
>730
CTGCTGCCGCCCGTAGGAGTCTGGACCGTGTCTCAGTTCCAGTGTGGCTGGTCATCCTCTCAGACCAGCTAGG
GATCGTCGCCTAGGTGAGCCGTTTACCCCACCTACTAGCTAATCCCATCTGGGCACATCCGATGGCAAGAGGC
CCTAAGGTCCCCCTCTTGTCTTGCGACGTATGCGGTATTAGCTACCGTTTCCAGTAGTTATCCCCCTCCATCA
GGCAGTTTCCCAGACATTACTCACCCGTCCGCCACTCGTCAGCAAAGCAGCAAGCTGCTTCCTGTTACCGTTC
GACTGCATGTGTTAGGCCTGCCGCCAGCGTTCAATCTGAGCCAGGATCAAACTCT
>731
CTGCTGCCGCCCGTAGGAGTCTGGACCGTGTCTCAGTTCCAGTGTGGCTGGTCATCCTCTCAGACCAGCTAGG
GATCGTCGCCTAGGTGAGCCGTTACCCCACCTACTAGCTAATCCCATCTGGGCACATCCGATGGCAAGAGGCC
CTAAGGTCCCCCTCTTTGTCTTGCGACGTTATGCGGTATTAGCTACCGTTTCCAGTAGTTATCCCCCTCCATC
AGGCAGTTTCCCAGACATTACTCACCCGTCCGCCACTCGCCAGCAAAGCAGCAAGCTGCTTTCTGTTACCGTT
CGACTGCATGTGTTAGGCCTGCCGCCAGCGTCAATCTGAGCCAGGATCAAACTCT
>732
CTGCTGCCGCCGTAGGAGTCTGGACCGTGTCTCAGTTCCAGTGTGGCTGGTCATCCTCTCAGACCAGCTAGGA
ATCGTCGCCTAGGTGAGCCGTTACCCCACCTACTAGCTAATCCCATCTGGGCACATCCGATGGCAAGAGGCCC
TAAGGTCCCCCTCTTTGGTCTTGCGACGTTATGCGGTATTAGCTACCGTTTCCAGTAGTTATCCCCCTCCATC
GGGCAGTTTCCCAGACATTACTCACCCGTCCGCCACTCGTCAGCAAAGCAGCAAGCTGCTTCCTGTTACCGTT
CGACTGCATGTGTTAGGCCTGCCGCCAGCGTCAATCTGAGCCAGGATCAAACTCT
>733
CTGCTGCCGCCCGTAGGAGTCTGGACCGTGTCTCAGCTCCAGTGTGGCTGGTCATCCTCTCAGACCAGCTAGG
GATCGTCGCCTAGGTGAGCCGTTACCCCACCTACTAGCTAATCCCATCTGGGCACATCCGATGGCAAGAGGCC
CTAAGGTCCCCCTCTTTGTCTTGCGACGTTATGCGGTATTAGCTACCGTTTCCAGTAGTTATCCCCCTCCATC
AGGCAGTTTCCCAGACATTACTCACCCGTCCGCCACTCGTCAGCAAAGCAGCAAGCTGCTTCCTGTTACCGTT
CGACTGCATGTGTTAGGCCTGCCGCCAGCGTTCAATCTGAGCCAGGATCAAACTCT
>734
CTGCTGCCGCCCGTAGGAGTCTGGACCGTGTCTCAGTTCCAGTGTGGCTGGTCATCCTCTCAGACCAGCTAGG
GATCGTCGCCTAGGTGAGCCGTTACCCCACCTACTAGCTAATCCCATCTGGGCACATCCGATGGCAAGAGGCC
CTAAGGTCCCCCTCTTTGTCTTGCGACGTTATGCGGTATTAGCTACCGTTCCAGTAGTTATCCCCCTCCATCA
GGCAGTTTCCCAGACATTACTCACCCGTCCGCCACTCGTCAGCAAAGCAGCAAGCTGCTTCTGTTACCGTTCG
ACTGCATGTGTTAGGCCTGCCG
>735
CTGCTGCCGCCCGTAGGAGTCTGGACCGTGTCTCAGTTCCAGTGTGGCTGGTCATCCTCTCAGACCAGCTAGG
GATCGTCGCCTAGGTGAGCCGTTACCCCACCTACTAGCTAATCCCATCTGGGCACATCCGATGGCAAGAGGCC
CTAAGGTCCCCTCTTGTCTGCGACGTATGCGGTATTAGCTACCGTTTCCAGTAGTTATCCCCCTCCATCAGGC
AGTTTCCCCAGACATTACTCACCCGTCCGCCACTCGTCAGCAAAGCAGCAAGCTGCTTCCTGTTACCGTTCGA
CTGCATGTGTTAGGCCTGCCGCCAGCGTCAATCTGAGCCAGGATCAAACTCT
>736
CTGCTGCCGCCCGTAGGAGTCTGGACCGTGTCTCAGTTTCCAGTGTGGCTGGTCATCCTCTCAGACCAGCTAG
GGATCGTCGCCTAGGTGAGCCCGTTACCCCACCTACTAGCTAATTCCCATCTGGGCACATCCGATGGCAAGAG
GCCCCTAAGGTCCCCCTCTTTGGTCTTGCGACGTATGCGGTATTAGCTACCGTTTCCAGTAGTTATCCCCCTC
CATCGGGCAGTTTTCCCAGACATTACTCACCCGTCCCGCCACTCGTCAGCAAAGCAGCAAGCTGCTTCCTGTT
ACCCGTTCGACTGCATTGTGTTAGGCCTGCCCGCCAGCGTCAATCTGAGCCCAGGATCAAACTCT
>737
CTGCTGCCGCCCGTAGGAGTCTGGACCGTGTCTCAGTTCCAGTGTGGCTGGTCATCCTCTCAGACCAGCTAGG
GATCGTCGCCTAGGTGAGCCGTTACCCCACCTACTAGCTAATCCCATCTGGGCACATCCGATGGCAAGAGGCC
CTAAGGTCCCCCTCTTGTCTTGCGACGTTATGCGGTATTAGCTACCGTTTCCAGTAGTTATCCCCCTCCATCA
GGCAGTTTCCCAGACATTACTCACCCGTCCGCCACTCGTCAGCAAAGCAGCAAGCTGCTTCCTGTTATCGTTC
GACTTGCATGTGTTAGGCCTGCCGCCAGCGTCAATCTGAGCCAGGATCAAACTCT
>738
CTGCTGCCGCCCGTAGGAGTCTGGACCGTGTCTCAGTTCCAGTGTGGCTGGTCATCCTCTCAGACCAGCTAGG
GATCGTCGCCTAGGTGAGCCGTTACCCCACCTACTAGCTAATCCCATCTGGGCACATCCGATAGCAAGAGGCC

CTAAGGTCCCCCTCTTGGTCCTGCGACGTTATTGCGGTATTAGCTACCGTTTCCAGTAGTTATCCCCCTCCAT
CAGGCAGTTTCCCAGACATTACTCACCCGTCCGCCACTCGTCAGCAAAGCAGCAAGCTGCTTCTGTTACCGTT
CGACTGCATGTGTAGGCCTGCCGCCAGCGTTCAATCTGAGCCAGGATCAACTCT
>739
CTGCTGCCGCCCGTAGGAGTCTGGACCGTGTCTCAGTTCCAGTGTGGCTGGTCATCCTCTCAGACCAGCTAGG
GATCGTCGCCTAGGTGAGCCGTTACCCCACCTACTAGCTAATCCCATCTGGGCACATCCGATGGCAAGAGGCC
CTAAGGTCCCCCTCTTTGGTCTTGCGACGTATGCGGTATTAGCTACCGTTTCCAGTAGTTATCCCCCTCCATC
AGGCAGTTTCCCAGACATTACTCACCCGTCCGCCACTCGTCAGCAAAGCAGCAAGCTGCTTCCTGTTACCGTT
CGACTGCATGTGTTAGGCCTGCCGCCAGCGTTCAATCTGAGCCAGGATCAAACTCT
>740
CTGCTGCCGCCCGTAGGAGTCTGGACCGTGTCTCAGTTCCAGTGTGGCTGGTCATCCTCTCAGACCAGCTAGG
GATCGTCGCCTAGGTGAGCCGTTTACCCCACCTACTAGCTAATCCCATCTGGGCACATCCGATGGCAAGAGGC
CCTAAGGTCCCCCTCTTGTCTTGCGACGTATGCGGTATTAGCTACCGTTTCCAGTAGTTATCCCCCTCCATCA
GGCAGTTTCCCAGGCATTACTCACCCGTCCGCCACTCGTCAGCAAAGCAGCAAGCTGCTTCCTGTTACCGCTC
GACTGCATGTGTTAGGCCTGCCGCCAGCGTCAATCTGAGCCAGGATCAAACTCT
>741
CTGCTGCCGCCCGTAGGAGTCTGGACCGTGTCTCAGTTCCAGTGTGGCTGGTCATCCTCTCAGACCAGCTAGG
GATCGTCGCCTAGGTGAGCCGTTACCCCACCTACTAGCTAATCCCATCTGGGCACATCCGATGGCAAGAGGCC
CTAAGGTCCCCCTCTTTGTCTTGCGACGTTATGCGGTATTAGCTACCGTTTCCAGTAGTTATCCCCCTCCATC
AGGCAGTTTTCCCAGACATTACTCACCCGTCCGCCACTCTCAGCAAAGCAGCAAGCTGCTTCCTGTTACCGTT
CGACTGCATGTGTTAGGCCTGCCGCCAGCGTCAATCTGAGCCAGGATCAAACTCT
>742
CTGCTGCCGCCCGTAGGAGTCTGGACCGTGTCTCAGTTCCAGTGTGGCTGGTCATCCTCTCAGACCAGCTAGG
GATCGTCGCCTAGGTGAGCCGTTACCCCACCTACTAGCTAATCCCATCTGGGCACATCCGATGGCAGGAGGCC
CTAAGGTCCCCCTCTTTGTCTTGCGACGTATGCGGTATTAGCTACCGTTTCCAGTAGTTATCCCCCTCCATCG
GGCAGTTTCCCAGACATTACTCACCCGTCCGCCACTCGTCAGCAAAGCAGCAAGCTGCTTCCTGTTACCGTTC
GACTGCATGTGTCAGGCCTGCCGCCAGCGTCAATCTGAGCCAGGATCAAACTCT
>743
CTGCTGCCGCCCGTAGGAGTCTGGACCGTGTCTCAGTTCCAGTGTGGCTGGTCATCCTCTCAGACCAGCTAGG
GATCGTCGCCTAGGTGAGCCGTTACCCCACCTACTAGCTAATCCCATCTGGGCACATCCGATGGCAAGAGGCC
CTAAGGTCCCCCTCTTGTCTTGCGACGTATGCGGTATTAGCTACCTTCGTTCAGTAGTTATCCCCCTCCATCA
GGCAGTTTCCCAGACATTACTCACCCGTCCGCCACTCGTCAGCAAACAGCAAGCTGCTTCCTGTTACCGTTCG
ACTGCATGTGTTAGGCCTGCCGCCAGCGTCAACTC
>744
CTGCTGCCGCCCGTAGGAGTCTGGACCGTGTCTCAGTTCCAGTGTGGCTGGTCATCCTCTCAGACCAGCTAGG
GATCGTCGCCTAGGTGAGCCGTTACCCCACCTACTAGCTAATCCCATCTGGGCACATCCGATGGCAAGAGGCC
CTAAGGTCCCCCTCTTTGGTCTTGCGACGTTATGCGGTATTAGCTACCGTTTCCAGTAGTTATCCCCCTCCAT
CGGGCAGTTTTCCCAGACATTACTCACCCGTCCGCCACTCGTCAGCAAAGCAGCAAGCTGCTTCCTATTACCG
TTCGACTGCATGTGTTAGGCCTGCCGCCAGCGTCAATCTGAGCCAGGATCAAACTCT
>745
CTGCTGCCGCCCGTAGGAGTCTGGACCGTGTCTCAGTTCCAGTGTGGCTGGTCATCCTCTCAGACCAGCTAGG
GATCGTCGCCTAGGTGAGCCGTTACCCCACCTACTAGCTAATCCCATCTGGGCACATCCGATGGCAAGAGGCC
CTAAGGTCCCCCTCTTGTCTGCGACGTAATGCGGTATTAGCTACCGTTTCCAGTAGTTATCCCCCTCCATCAG
GCAGTTTTCCCAGACATTACTCACCCGTCCGCCACTCGTCAGCAAACAGCAAGCTGCTTCCTGTTACCGTTCG
ACTGCATGTGTTAGGCCTGCCGCCAGCGTCAATCTGAGCCAGGATCAAACTCT
>746
CTGCTGCCGCCCGTAGGAGTCTGGACCGTGTCTCAGTTCCAGTGTGGCTGGTCATCCTCTCAGACCAGCTAGG
GATCGTCGCCTAGGTGAGCCGTTACCCCACCTACTAGCTAATCCCATCTGGGCACATCCGATGGCAAGAGGCC
CTAAGGTCCCCCTCTTTGGTCTTGCGGACGTTATGCGGTATTAGCTACCGTTTCCAGTAGTTATCCCCCTCCA
TCAGGCAGTTTCCCAGACATTACTCACCCGTCCGCCACTCGTCAGCAAAGCAGCAAGCTGCTTCCTGTTACCG
TTCGACTGCATGTGTTGGCCTGCCGCCAGCGTCAATCTGAGCCAGGATCAAACTCT
>747
CTGCTGCCGCCCGTAGGAGTCTGGACCGTGTCTCAGTTCCAGTGTGGCTGGTCATCCTCTCAGACCAGCTAGG
GATCGTCGCCTAGGTGAGCCGTTACCCCACCTACTAGCTAATCCCATCTGGGCACATCCGATGGCAAGAGGCC

CTAAGGTCCCCCTCTTGGTCTTGCGATGTTATGCGGTATTAGCTACCGTTTCCAGTAGTTGTCCCCCTCCATC
AGGCAGTTTCCCAGACATTACTCACCCGTCCGCCACTCGTCAGCAAAGCAGCAAGCTGCTTCCTGTTACCGTT
CGACTGCATGTGTTAGGCCTGCCGCCAGCCGTCAATCTGAGCCAGGATCAAACTCT
>748
CTGCTGCCGCCCGTAGGAGTCTGGACCGTGTCTCAGTTCCAGTGTGGCTGGTCATCCTCTCAGACCAGCTAGG
GATCGTCGCCTAGGTGAGCCGTTACCCCACCTACTAGCTAATCCCATCTGGGCACATCCGATGGCAAGAGGCC
CTAAGGTCCCCCTCTTGTCTTGCGACGTATGCGGTATTAGCTACCGTTTCCAGTAGTTATCCCCCTCCATCAG
GCAGTTTCCCAGACATTACTCACCCGTCCGCCACTCGTCAGCAAAGCAGCAAGCTGCTTCCTGTTACCGTCGA
CTGCATGTGTTAGGCCTGCCGCCAGCGTCAATCTGAGCCAGGATCAACTCT
>749
CTGCTGCCGCCCGTAGGAGTCTGGACCGTGTCTCAGTTCCAGTGTGGCTGGTCATCCTCTCAGACCAGCTAGG
GATCGTCGCCTAGGTGAGCCGTTACCCCACCTACTAGCTAATCCCATCTGGGCACATCCGATGGCAAGAGGCC
CTAAGGTCCCCCTCTTTGTCTTGCGACGTTATGCGGTATTAGCTACCGTTTCCAGTAGTTATCCCCCTCCATC
GGGCAGTTTCCCAGACATTACTCACCCGTCCGCCACTCGTCAGCAAAGCAGCAAGCTGCTTCCTGTTACCGTT
CGACTGCATGTGTTAGGCCTGCCGCCAGCGTTCAATCTGAGCCAGGACAAACTCT
>750
CTGCTGCCGCCCGTAGGAGTCTGGACCGTGTCTCAGTTCCAGTGTGGCTGGTCATCCTCTCAGTCCAGCTAGG
GATCGTCGCCTAGGTGGCACTATCCACCCTACTAGCTAATCCCATCTGGGCACATCCGATGGCAAGAGGCCCT
AAGGTCCCCCTCTTTGGTCTTGCGACGTTATGCGGTATTAGCTACCGTTTCCAGTAGTTATCCCCCTCCATCA
GGCAGTTTCCCAGACATTACTCACCCGTCCGCCACTCGTCAGCAAAGCAGCAAGCTGCTTCCTGTTACCGTTC
GACTGCATGTGTTAGGCCTGCCGCCAGCG
>751
CTGCTGCCGCCCGTAGGAGTCTGGACCGTGTCTCAGTTCCAGTGTGGCTGGTCATCCTCTCAGACCAGCTAGG
GATCGTCGCCTAGGTGAGCCGTTACCCCACCTACTAGCTAATCCCATCTGGGCACATCCGATGGCAAGAGGCC
CTAAGGTCCCCCTCTTTGGTCTTGCGACGTATGCGGTATTAGCTACCGTTTCCAGTAGTTATCCCCCTCCATC
AGGCAGTTTTCCCAGACATTACTCACCCGTCCGCCACTCGTCAGCAAAAGCAGCAAGCTGCTTCCTGTTACCG
TTCGACTGCATGTGTTAGGCCTGCCGCCGACGTCAATCTGAGCCAGGATCAAACTCT
>752
CTGCTGCCGCCCGTAGGAGTCTGGACCGTGTCTCAGTTCCAGTGTGGCTGGTCATCCTCTCAGACCAGCTAGG
GATCGTCGCCTAGGTGAGCCGTTACCCCACCTACTAGCTAATCCCATCTGGGCACATCCGATGGCAAGAGGCC
CTAAAGGTCCCCCTCTTTGTCTTGCGACGTTATGCGGTATTAGCTACCGTTTCCAGTAGTTATCCCCCTCCAT
CGGGCAGTTTCCCAGACATTACTCACCCGTCCGCCACTCGTCAGCAAAGCAGCAAGCTGCTTCCTGTTACCGT
TCGACTGCATGTGTTAGGCCTGCCGCCAGCGTCAATCTGAGCCAGATCAAACTCT
>753
CTGCTGCCGCCCGTAGGAGTCTGGACCGTGTCTCAGTTCCAGTGTGGCTGGTCATCCTCTCAGACCAGCTAGG
GATCGTCGCCTAGGTGAGCTGTTACCCCACCTACTAGCTAATCCCATCTGGGCACATCCGATGGCAAGAGGCC
CTAAGGTCCCCCTCTTTGGTCTTGCGACGTTATGCGGTATTAGCTACCGTTTCCAGTAGTTATCCCCCTCCAT
CAGGCAGTTTCCCAGACATTACTCACCCGTCCGCCACTCGTCAGCAAAGCAGCAAGCTGCTTCCTGTTACCGT
TCGACTGCATGTGTTAGGCCTGCCGCCAGCGTCAATCTGAGCCAGGATCAAACTCT
>754
CTGCTGCCGCCCGTAGGAGTCTGGACCGTGTCTCAGTCCAGTGTGGCTGGTCATCCTCTCAGACAGCTAGGGA
TCGTCGCCTAGGTGAGCCGTTACCCCACCTACTAGCTAATCCCATCTGGGCACATCCGATGGCAAGAGGCCCT
AAGGTCCCCCTCTTGTCTTGCGACGTATGCGGTATTAGCTACCGTTTCCAGTAGTTATCCCCCTCCATCGGGC
AGTTTCCCAGACATTACTCACCCGTCCGCCACTCGTCAGCAAAGCAGCAAGCTGCTTCCTGTTACCGTTCGAC
TGCATGTGTTAGGCCTGCCGCCAGCGTCAATCTGAGCCAGGATCAAACTCT
>755
CTGCTGCCGCCCGTAGGAGTCTGGACCGTGTCTCAGTTCCAGTGTGGCTGGTCATCCTCTCAGACCAGCTAGG
GATCGTCGCCTAGGTGGCGCTGTCCACCCTACTAGCTAATCCCATCTGGGCACATCCGATGGCAAGAGGCCCT
AAGGTCCCCCTCTTTGGTCTTGCGACGTTATGCGGTATTAGCTACCGTTTCCAGTAGTTATCCCCCTCCATCA
GGCAGTTTCCCAGACATTACTCACCCGTCCGCCACTCGTCAGCAAAGCAGCAAGCTGCTTTCTGTTACCGTTC
GACTGCATGTGTTAGGCCTGCCGCCAGCGTTCAATCTGAGCCAGGATCAAACTCT
>756
CTGCTGCCGCCCGTAGGAGTCTGGACCGTGTCTCAGTTCCAGTGTGGCTGGTCATCCTCTCAGACCAGCTAGG
GATCGTCGCCTAGGTGAGCCGTTACCCCACCTACTAGCTAATCCCATCTGGGCACATCCGATGGCAAGAGGCC

CTAAGGTCCCCCTCTTTGTCTTGCGACGTATGCGGTATTAGCCACCGTTTCCAGTAGTTATCCCCCTCCATCG
GGCAGTTTCCCAGACATTACTCACCCGTCCGCCACTCGTCAGCAAAGCAGCAAGCTGCTTCCAGTTACCGTTC
GACTGCATGTGTTAGGCCTGCCGCCAGCGTTCAATCTGAGCCAGGATCAAACTCT
>757
CTGCTGCCGCCCGTAGGAGTCTGGACCGTGTCTCAGTTCCAGTGTGGCTGGTCATCCTCTCAGACCAGCTAGG
GATCGTCGCCTAGGTGAGCCGTTACCCCACCTACTAGCTAATCCCATCTGGGCACATCCGATGGCAAGAGGCC
CTAAGGTCCCCCTCTTTGGTCTTGCGACGTATGCGGTATTAGCTACCGTTTCCAGTAGTTATCCCCCTCCATC
AGGCAGTTTCCCAGACATTACTCACCCGTCCGCCACTCGTCAGCAAAACAGCAAGCTGCTTCCTGTTACCGTT
CGACTGCATGTGTTAGGCCTGCCGCCAGCGTCAATCTGAGCCAGGATCAAACTCT
>758
CTGCTGCCGCCCGTAGGAGTCTGGACCGTGTCTCAGTTCCAGTGTGGCTGGTCATCCTCTCAGACCAGCTAGG
GATCGTCGCCTAGGTGAGCCGTTACCCCACCTACTAGCTAATCCCATCTGGGCACATCCGATGGCAAGAGGCC
CTAAGGTCCCCCTCTTTGTCTTGCGACGTTATGCGGTATTAGCTACCGTTTCCAGTAGTTATCCCCCTCCATC
AGGCAGTTTCCCAGACATTACTCACCCGTCCGCCACTCGTCAGCAAAACAGCAAGCTGCTTCCTGTTACCGTT
CGACTGCATGTGTTAGGCCTGCCGCCAGCGTTCAATCTGAGCCAGGATCAAACTCT
>759
CTGCTGCCGCCCGTAGGAGTCTGGACCGTGTCTCAGTTCCAGTGTGGCTGGTCATCCTCTCAGACCAGCTAGG
GATCGTCGCCTAGGTGAGCCGTTACCCCACCTACTAGCTAATCCCATCTGGGCACATCCGATGGCAAGAGGCC
CTAAGGTCCCCCTCTTGTCTTGCGACGTTATGCGGTATTAGCTACCGTTTCCAGTAGTTATCCCCCTCCATCG
GGCAGTTTTCCCAGACATTACTCACCCGTCCGCCACTCGTCAGCAAAGCAGCAAGCTGCTTCCTGTTACCGTT
CGACTGCATGTGTTAGGCCTGCCGCCAGCGTTCAATCTGAGCCAGGATCAAACTCT
>760
CTGCTGCCGCCCGTAGGAGTCTGGACCGTGTCTCAGTTCCAGTGTGGCTGGTCATCCTCTCAGACCAGCTAGG
GATCGTCGCCTAGGTGAGCCGTTACCCCACCTACTAGCTAATCCCATCTGGGCACATCCGATGGCAAGAGGCC
CTAAGGTCCCCCTCTTTGGTCTTGCGACGTTATGCGGTATTAGCTACCGTTTCCAGTAGTTATCCCCCTCCAT
CAGGCAGTTTCCCAGACATTACTCACCCGTCCGCCACTCGTCAGCAAACAGCAAGCTGCTTCCTGTTACCGTT
CGACTGCATGTGTTAGGCCTGCCGCCAGCGTCAATCTGAGCCAGGATCAAACTCT
>761
CTGCTGCCGCCCGTAGGAGTCTGGACCGTGTCTCAGTTCCAGTGTGGCTGGTCATCCTCTCAGACCAGCTAGG
GATCGTCGCCTAGGTGAGCCGTTACCCCACCTACTAGCTAATCCCATCTGGGCACATCCGATGGCAAGAGGCC
CTAAGGTCCCCCTCTTTGGTCTTGCAACGTTATGCGGTATTAGCTACCGTTTCCAGTAGTTATCCCCCTCCAT
CGGGCAGTTTCCCAGACATTACTCACCCGTCCGCCACTCGTCAGCAAAGCAGCAGGCTGCTTCCTGTTACCGT
TCGACTGCATGTGTTAGGCCTGCCGCCAGCGTTCAATCTGAGCCAGGATCAAACTCT
>762
CTGCTGCCGCCCGTAGGAGTCTGGACCGTGTCTCAGTTCCAGTGTGGCTGGTCATCCTCTCAGACCAGCTAGG
GATCGTCGCCTAGGTGAGCCGTTACCCCACCTACTAGCTAATCCCATCTGGGCACATCCGATGGCAAGAGGCC
CTAAGGTCCCCCTCTTTGGTCTTGCGACGTTATGCGGTATTAGCTACCGTTTCCAGTAGTTATCCCCCTCCAT
CAGGCAGTTTCCCAGACATTACTCACCCGTCCGCCACTCGTCAGCAAAACAGCAAGCTGCTTCCTGTTACCGT
TCGACTGCATGTGTTAGGCCTGCCGCCAGCGTCAATCTGAGCCAGGATCAAACTCT
>763
CTGCTGCCGCCCGTAGGAGTCTGGACCGTGTCTCAGTTCCAGTGTGGCTGGTCATCCTCTCAGACCAGCTAGG
GATCGTCGCCTAGGTGAGCCGTTACCCCACCTACTAGCTAATCCCATCTGGGCACATCCGATGGCAAGAGGCC
CTAAGGTCCCCCTCTTTGGTCTTGCGACGTTATGCGGTATTAGCTACCGTTTCCAGTAGTTATCCCCCTCCAT
CGGGCAGTTTCCCAGACATTACTCACCCGTCCGCCACTCGTCAGCAAAGCAGCAAGCTGCTTCCTGTTACCGT
TCGACTGCATGTGTTAGGCCTGCCGCCAGCGTCAATCTGAGCCAGGATCAAACTCT
>764
CTGCTGCCGCCCGTAGGAGTCTGGACCGTGTCTCAGTTCCAGTGTGGCTGGTCATCCTCTCAGACCAGCTAGG
GATCGTCGCCTAGGTGAGCCGTTACCCCACCTACTAGCTAATCCCATCTGGGCACATCCGATGGCAAGAGGCC
CTAAGGTCCCCCTCTTTGGTCTTGCGACGTTATGCGGTATTAGCTACCGTTTCCAGTAGTTATCCCCCTCCAT
CAGGCAGTTTCCCAGACATTACTCACCCGTCCGCCACTCGTCAGCAAACAGCAAGCTGCTTCCTGTTACCGTT
CGACTGCATGTGTTAGGCCTGCCGCCAGCGTCAATCTGAGCCAGGATCAAACTCT
>765
CTGCTGCCGCCCGTAGGAGTCTGGACCGTGTCTCAGTTCCAGTGTGGCTGGTCATCCTCTCAGACCAGCTAGG
GATCGTCGCCTAGGTGAGCCGTTACCCCACCTACTAGCTAATCCCATCTGGGCACATCCGATGGCAAGAGGCC

CTAAGGTCCCCCTCTTTGGTCTTGCGACGTTATGCGGTATTAGCTACCGTTTCCAGTAGTTATCCCCCTCCAT
CAGGCAGTTTCCCAGACATTACTCACCCGTCCGCCACTCGTCAGCAAAGCAGCAAGCTGCTTTCTGTTACCGT
TCGACTGCATGTGTTAGGCCTGCCGCCAGCGTTCAATCTGAGCCAGGATCAAACTCT
>766
CTGCTGCCGCCCGTAGGAGTCTGGACCGTGTCTCAGTTCCAGTGTGGCTGGTCATCCTCTCAGACCAGCTAGG
GATCGTCGCCTAGGTGAGCCGTTACCCCACCTACTAGCTAATCCCATCTGGGCACATCCGATGGCAAGAGGCC
CTAAGGTCCCCCTCTTTGTCTTGCGACGTTATGCGGTATTAGCTACCGTTTCCAGTAGTTATCCCCCTCCATC
GGGCAGTTTCCCAGACATTACTCACCCGTCCGCCACTCGTCAGCAAAGCAGCAAGCTGCTTCCTGTTACCGTT
CGACTGCATGTGTTAGGCCTGCCGCCAGCGTCAATCTGAGCCAGGATCAAACTCT
>767
CTGCTGCCGCCCGTAGGAGTCTGGACCGTGTCTCAGTTCCAGTGTGGCTGGTCATCCTCTCAGACCAGCTAGG
GATCGTCGCCTAGGTGAGCCGTTACCCCACCTACTAGCTAATCCCATCTGGGCACATCCGATGGCAAGAGGCC
CTAAGGTCCCCCTCTTTGTCTTGCGACGTTATGCGGTATTAGCTACCGTTTCCAGTAGTTATCCCCCTCCATC
AGGCAGTTTTCCCAGACATTACTCACCCGTCCGCCACTCGTCAGCAAAGCAGCAAGCTGCTTCTGTTACCGTC
GACTGCATGTGTTAGGCCTGCCGCCAGCGTCAATCTGAGCCAGGATAA
>768
CTGCTGCCGCCCGTAGGAGTCTGGACCGTGTCTCAGTTCCAGTGTGGCTGGTCATCCTCTCAGACCAGCTAGG
GATCGTCGCCTAGGTGAGCCGTTACCCCACCTACTAGCTAATCCCATCTGGGCACATCCGATGGCAAGAGGCC
CTAAGGTCCCCCTCTTTGGTCTTGCGACGTTATGCGGTATTAGCTACCGTTTCCAGTAGTTATCCCCCCTCAT
CAGGCAGTTTCCCAGACATTACTCACCCGTCCGCCACTCGTCAGCAAAGCAGCAAGCTGCTTCCTGTTACCGT
TCGACTGCATGTGTTAGGCCTGCCGCCAGCGTCAATCTGAGCCAGGATCAAACTCT
>769
CTGCTGCCGCCCGTAGGAGTCTGGACCGTGTCTCAGTTCCAGTGTGGCTGGTCATCCTCTCAGACCAGCTAGG
GATCGTCGCCTAGGTGAGCCGTTACCCCACCTACTAGCTAATCCCATCTGGGCACATCCGATGGCAAGAGGCC
CTAAGGTCCCCCTCTTTGGTCTTGCGACGTTATGCGGTATTAGCTACCGTTTCCAGTAGTTATCCCCCTCCAT
CGTGCAGTTCCCCAGACATTACTCACCCGTCCGCCACTCGTCAGCAAAGCAGCAAGCTGCTTCCTGTTACCGT
TCGACTGCATGTGTTAGGCCTGCCGCCAGCGTTCAATCTGAGCCAGGATCAAACTCT
>770
CTGCTGCCGCCCGTAGGAGTCTGGACCGTGTCTCAGTTCCAGTGTGGCTGGTCATCCTCTCAGACCAGCTAGG
GATCGTCGCCTAGGTGAGCCGTTTACCCCACCTACTAGCTAATCCCATCTGGGCACATCCGATGGCAAGAGGC
CCTAAGGTCCCCCTCTTTGTCTTGCGACGTTATGCGGTATTAGCTACCGTTTCCAGTAGTTATCCCCCTCCAT
CAGGCAGTTTCCCAGACATTACTCACCCGTCCGCCACTCGTCAGCAAAGCAGCAAGCTGCTTCCTGTTACCGT
TCGACTGCATGTGTTAGGCCTGCCGCCAGCGTTCAATCTGAGCCAGGACAAACTCT
>771
CTGCTGCCGCCCGTAGGAGTCTGGACCGTGTCTCAGTTCCAGTGTGGCTGGTCATCCTCTCAGACCAGCTAGG
GATCGTCGCCTAGGTGAGCCGTTACCCCACCTACTAGCTAATCCCATCTGGGCACATCCGATGGCAAGAGGCC
CTAAGGTCCCCCTCTTTGGTCTGCGACGTTATGCGGTATTAGCTACCGTTTCCAGTAGTTATCCCCCTCCATC
GGGCAGTTTCCCAGACATTACTCACCCGTCCGCCACTCGTCAGCAAAGCAGCAAGCTGCTTCCTGTTACCGTT
CGACTGCATGTGTTAGGCCTGCCGCCAGCGTTCAATCTGAGCCAGGATCAAACTCT
>772
CTGCTGCCGCCCGTAGGAGTCTGGTCCGTGTCTCAGTTCCAGTGTGGCTGGTCATCCTCTCAGACCAGCTAGG
GATCGTCGCCTAGGTGAGCCGTTACCCCACCTACTAGCTAATCCCATCTGGGCACATCCGATGGCAAGAGGCC
CTAAGGTCCCCCTCTTGGTCTTGCGACGTTATGCGGTATTAGCTACCGTTTCCAGTAGTTATCCCCCTCCATC
GGGCAGTTTCCCAGACATTACTCACCCGTCCGCCACTCGTCAGCAAAGCAGCAAGCTGCTTCCTGTTTACCGT
TCGACTGCATGTGTTAGGCCTGCCGCCAGCGTTCAATCTGAGCCAGGATCAAACTCT
>773
CTGCTGCCGCCCGTAGGAGTCTGGACCGTGTCTCAGTTCCAGTGTGGCTGGTCATCCTCTCAGACCAGCTAGG
GATCGTCGCCTAGGTGAGCCGTTACCCCACCTACTAGCTAATCCCATCTGGGCACATCCGATGGCAAGAGGCC
CTAAGGTCCCCCTCTTTGGTCTTGCGACGTTATGCGGTGTTAGCTACCGTTTCCAGTAGTTATCCCCCTCCAT
CGGGCAGTTTTCCCAGACATTACTCACCCGTCCGCCACTCGTCAGCAAAGCAGCAAGCTGCTTCCTGTTACCG
TTCGACTGCATGTGTTAGGCCTGCCGCCAGCGTCAATCTGAGCCAGGATCAAACTCT
>774
CTGCTGCCGCCCGTAGGAGTCTGGACCGTGTCTCAGTTCCAGTGTGGCTGGTCATCCTCTCAGACCAGCTAGG
GATCGTCGCCTAGGTGAGCCGTTACCCCACCTACTAGCTAATCCCATCTGGGCACATCCGATGGCAAGAGGCC

CTAAGGTCCCCCTCTTTGGTCTTGCGACGTTATGCGGTATTAGCTACCGTTTCCAGTAGTTATCCCCCTCCAT
CAGGCAGTTTCCCAGACATTACTCACCCGTCCGCCACTCGTCAGCAAAGCAGCAAGCTGCTTCCTGTTACCGT
TCGACTGCATGTGTTAGGCCTGCCGCCAGCGTTCAATCTGAGCCAGGATCAAACTCT
>775
CTGCTGCCGCCCGTAGGAGTCTGGACCGTGTCTCAGTTCAGTGTGGCTGGTCATCCTCTCAGACCAGCTAGGG
ATCGTCGCCTAGGTGAGCCGTTACCCCACCTACTAGCTAATCCCATCTGGGCACATCCGATGGCAAGAGGCCC
TAAGGTCCCCCTCTTTGGTCTTGCGACGTATGCGGTATTAGCTACCGTTTCCAGTAGTTATCCCCTCCATCGG
GCAGTTTCCCAGACATTACTCACCCGTCCGCCACTCGTCAGCAAAGCAGCAAGCTGCTTCCTGTTACCGTTCG
ACTGCATGTGTTAGGCCTGCCGCCAGCGTCAATCTGAGCCAGGATCAAACTCT
>776
CTGCTGCCGCCCGTAGGAGTCTGGACCGTGTCTCAGTTCCAGTGTGGCTGGTCATCCTCTCAGACAGCTAGGG
ATCGTCGCCTAGGTGAGCCGTTACCCCACCTACTAGCTAATCCCATCTGGGCACATCCGATGGCAAGAGGCCC
TAAGGTCCCCCTCTTGTCTTGCGACGTATGCGGTATTAGCTACCGTTTCCAGTAGTTATCCCCCTCCATCAGG
CAGTTTCCCAGACATTACTCACCCGTCCGCCACTCGTCAGCAAACAGCAAGCTGCTTCCTGTTACCGTTCGAC
TTGCATGTGTTAGGCCTGCCGCCAGCGTCAATCTGAGCCAGGATCAAACTTCT
>777
CTGCTGCCGCCCGTAGGAGTCTGGACCGTGTCTCAGTTCCAGTGTGGCTGGTCATCCTCTCAGACCAGCTAGG
GATCGTCGCCTAGGTGAGCCGTTACCCCACCTACTAGCTAATCCCATCTGGGCACATCCGATGGCAAGAGGCC
CTAAGGTCCCCCTCTTTGGTCTTGCGACGTATGCGGTATTAGCTACCGTTTCCAGTAGTTATCCCCCCTCCAT
CAGGCAGTTTTCCCCAGACATTACTCACCCGTCCGCCACTCGTCAGCAAACAGCAAGCTGCTTCCTGTTACCG
TTCGACTGCATGTGTTAGGCCTGCCGCCAGCGTCAATCTGAGCCAGGATCAAACTCT
>778
CTGCTGCCGCCCGTAGGAGTCTGGACCGTGTCTCAGTTCCAGTGTGGCTGGTCATCCTCTCAGACCAGCTAGG
GATCGTCGCCTAGGTGAGCCGTTACCCCACCTACTAGCTAATCCCATCTGGGCACATCCGATGGCAAGAGGCC
CTAAGGTCCCCCTCTTTGGTCTGCGACGTATGCGGTATTAGCTACCGTTTCCAGTAGTTATCCCCTCCATCGG
GCAGTTTCCCAGACATTACTCACCCGTCCGCCACTCGTCAGCAAAGCAGCAAGCTGCTTCCTGTTACCGTTCG
ACTGCATGTGTTAGGCCTGCCGCCAGCGTCAATCTGAGCCAGGATCAAACTCT
>779
CTGCTGCCGCCCGTAGGAGTCTGGACCGTGTCTCAGTTCCAGTGTGGCTGGTCATCCTCTCAGACCAGCTAGG
GATCGTCACCTAGGTGAGCCGTTACCCCACCTACTAGCTAATCCCATCTGGGCACATCCGATGGCAAGAGGCC
CTAAGGTCCCCCTCTTTGGTCTTGCGACGTATGCGGTATTAGCTACCGTTTCCAGTAGTTATCCCCCTCCATC
AGGCAGTTTCCCAGACATTACTCACCCGTCCGCCACTCGTCAGCAAAACAGCAAGCTGCTTCCTGTTACCGTT
CGACTGCATGTGTTAGGCCTGCCGCCAGCGTTCAATCTGAGCCAGGATCAAACTCT
>780
CTGCTGCCGCCCGTAGGAGTCTGGACCGTGTCTCAGTTCCAGTGTGGCTGGTCATCCTCTCAGACCAGCTAGG
GATCGTCGCCTAGGTGAGCCGTTACCCCACCTACTAGCTAATCCCATCTGGGCACATCCGATGGCAAGAGGCC
CTAAGGTCCCCCTCTTGTCTGCGACGTATGCGGTATTAGCTACCGTTTCCAGTAGTTATCCCCCTCCATCGGG
CAGTTTTCCCAGACATTACTCACCCGTCCGCCACTCGTCAGCAAAGCAGCAAGCTGCTTCCTGTTACCGTTCG
ACTGCATGTGTTAGGCCTGCCGCCAGCGTCAATCTGAGCCAGGATCAAACTCT
>781
CTGCTGCCGCCCGTAGGAGTCTGGACCGTGTCTCAGTTCCAGTGTGGCTGGTCATCCTCTCAGACCAGCTAGG
GATCGTCGCCTAGGTGAGCCGTTACCCCACCTACTAGCTAATCCCATCTGGGCACATCCGATGGCAAGAGGCC
CTAAGGTCCCCCTCTTGGTCTGCGACGTATGCGGTATTAGCTACCGTTTCCAGTAGTTATCCCCTCCATCGGG
CAGTTTCCCAGACATTACTCACCCGTCCGCCACTCGTCAGCAAAGCAGCAAGCTGCTTCCTGTTACCGTCGAC
TGCATGTGTTAGGCCTGCCGCCAGCGTCAATCTGACGCAAGGATCAAA
>782
CTGCTGCCGCCCGTAGGAGTCTGGACCGTGTCTCAGTTCCAGTGTGGCTGGTCATCCTCTCAGGCCAGCTAGG
GATCGTCGCCTAGGTGAGCCGTTACCCCACCTACTAGCTAATCCCATCTGGGCACATCCGATGGCAAGAGGCC
CTAAGGTCCCCCTCTTTGGTCTGCGACGTAAGCGGTATTAGCTACCGTTTCCAGTAGTTATCCCCCTCCATCA
GGCAGTTTCCCAGACATTACACACCCGTCCGCCACTCGTCAGCAAAGCAGCAAGCTGCTCCCTGTTACCGTTC
GACTGCATGTGTTAGGCCTGCCGCCAGCGTCAATCTGAGCCAGGATCAAACTCT
>783
CTGCTGCCGCCCGTAGGAGTCTGGACCGTGTCTCAGTTCCAGTGTGGCTGGTCATCCTCTCAGACCAGCTAGG
GATCGTCGCCTAGGTGAGCCGTTACCCCACCTACTAGCTAATCCCATCTGGGCACATCCGATGGCAAGAGGCC

CTAAGGTCCCCCTCTTTGGTCTTGCGACGTTATGCGGTATTAGCTACCGTTTCCAGTAGTTATCCCCCTCCAT
CAGGCAGTTTCCCAGACATTACTCACCCGTCCGCCACTCGTCAGCAAAGCAGCAAGCTGCTTCCTGTTACCGT
TCGACTGCATGTGTTAGGCCTGCCGCCAGCGTCCAATCTGAGCCAGGATCAAACTCT
>784
CTGCTGCCGCCCGTAGGAGTCTGGACCGTGTCTCAGTTCCAGTGTGGCTGGTCATCCTCTCAGACCAGCTAGG
GATCGTCGCCTAGGTGAGCCGTTACCCCACCTACTAGCTAATCCCATCTGGGCACATCCGATGGCAAGAGGCC
CTAAGGTCCCCCTCTTGTCTTGCGACGTTATGCGGTATTAGCTACCGTTTCCAGTAGTTATCCCCCTCCATCA
GGCAGTTTCCCAGACATACTCACCCGTCCCGCCACTCGTCAGCAAACAGCAAGCTGCTTCCTGTTACCGTTCG
ACTGCATGTGTTAGGCCTGCCGCCAGCGTTCAATCTGAGCCAGGATCAAACTCT
>785
CTGCTGCCGCCCGTAGGAGTCTGGACCGTGTCTCAGTTCCAGTGTGGCTGGTCATCCTCTCAGACCAGCTAGG
GATCGTCGCCTAGGTGAGCCGTTACCCCACCTACTAGCTAATCCCATCTGGGCACATCCGATGGCAAGAGGCC
CTAAGGTCCCCCTCTTGTCTTGCGACGTATGCGGTATTAGCTACCGTTTCCAGTAGTTATCCCCCTCCATCAG
GCAGTTTCCCAGACATTACTCACCCGTCCGCCACTCGTCAGCAAAGCAGCAAGCTGCTTCCTGTTACCGTTCG
ACTGCATGTGTTAGGCCTGCCGCCAGCGTCAATCTGAGCCAGGATCAAACTCT
>786
CTGCTGCCGCCCGTAGGAGTCTGGACCGTGTCTCAGTTCCAGTGTGGCTGGTCATCCTCTCAGACCAGCTAGG
GATCGTCGCCTAGGTGAGCCGTTACCCCACCTACTAGCTAATCCCATCTGGGCACATCCGATGGCAAGAGGCC
CTAAGGTCCCCCTCTTTGTCTTGCGACGTATGCGGTATTAGCTACCGTTTCCAGTAGTTATCCCCCTCCATCA
GGCAGTTTCCCAGACATTACTCACCCGTCCGCCACTCGTCAGCAAAGCAGCAAGCTGCTTCCTGTTACCGTTC
GACTGCATGTGTTAGGCCTGCCGCCAGCGTTCAATCTGAGCCAGGACAAACTCT
>787
CTGCTGCCGCCCGTAGGAGTCTGGACCGTGTCTCAGTTCCAGTGTGGCTGGTCATCCTCTCAGACCAGCTAGG
GATCGTCGCCTAGGTGAGCCGTTACCCCACCTACTAGCTAATCCCATCTGGGCACATCCCGATGGCAAGAGGC
CCTAAGGTCCCCCCTCTTTGGTCTTGCGACGTATGCGGTATTAGCTACCGTTTCCAGTAGTTATCCCCCTCCA
TCAGGCAGTTTTCCCAGACATTACTCACCCGTCCGCCACTCGTCAGCAAAGCAGCAAGCTGCTTCCTGTTACC
GTTCGACTGCATGTGTTAGGCCTGCCGCCAGCGTTCAATCTGAGCCAGGATCAAACTCT
>788
CTGCTGCCGCCCGTAGGAGTCTGGACCGTGTCTCAGTTCCAGTGTGGCTGGTCATCCTCTCAGACCAGCTAGG
GATCGTCGCCTAGGTGAGCCGTTACCCCACCTACTAGCTAATCCCATCTGGGCACATCCGATGGCAAGAGGCC
CTAAGGTCCCCCTCTTGTCTTGCGACGTTATGCGGTATTAGCTACCGTTTCCAGTAGTTATCCCCCTCCATCA
GGCAGTTTCCCAGACATTACTCACCCGTCCGCCACTCGTCAGCAAAGCAGCAAGCTGCTTCCTGTTACCGTTC
GACTGCATGTGTTAGGCCTGCCGCCAGCGTCAATCTGAGCCAGGATCAAACTCT
>789
CTGCTGCCGCCCGTAGGAGTCTGGACCGTGTCTCAGTTCCAGTGTGGCTGGTCATCCTCTCAGACCAGCTAGG
GATCGTCGCCTAGGTGAGCCGTTACCCCACCTACTAGCTAATCCCATCTGGGCACATCCGATGGCAAGAGGCC
CTAAGGTCCCCCTCTTGGTCTTGCGACGTTATGCGGTATTAGCTACCGTTTCCAGTAGTTATCCCCCTCCATC
AGGCAGTTTCCCAGACATTACTCACCCGTCCGCCACTCGTCAGCAAAGCAGCAAGCTGCTTCCTGTTACCGTT
CGACTGCATGTGTTAGGCCTGCCGCCAGCGTTCAATCTGAGCCAGGACAACTCT
>790
CTGCTGCCGCCCGTAGGAGTCTGGACCGTGTCTCAGTTCCAGTGTGGCTGGTCATCCTCTCAGACCAGCTAGG
GATCGTCGCCTAGGTGAGCCGTTACCCCACCTACTAGCTAATCCCACCTGGGCACATCCGATGGCAAGAGGCC
CTAAGGTCCCCCTCTTTGTCTTGCGACGTTATGCGGTATTAGCTACCGTTTCCAGTAGTTATCCCCCTCCATC
GGGCAGTTTCCCAGACATTACTCACCCGTCCGCCACTCGTCAGCAAAGCAGCAAGCTGCTTCCTGTTACCGTT
CGACTGCACGTGTTAGGCCTGCCGCCAGCGTCAATCTGAGCCAGGATCAAACTCT
>791
CTGCTGCCGCCCGTAGGAGTCTGGACCGTGTCTCAGTTCCAGTGTGGCTGGTCATCCTCTCAGACAGCTAGGG
ATCGTCGCCTAGGTGAGCCGTTACCCCACCTACTAGCTAATCCCATCTGGGCACATCCGATGGCAAGAGGCCC
TAAGGTCCCCTCTTTGTCTGCGACGTTATGCGGTATTAGCTACCGTTTCCAGTTAGTTATCCCCCTCCATCAG
GCAGTTTCCCAGACATTACTCACCCGTCCGCCACTCGTCAGCAAAGCAGCAAGCTGCTTTCCTGTTACCGTTC
GAACTGCA
>792
CTGCTGCCGCCCGTAGGAGTCTGGACCGTGTCTCAGTTCCAGTGTGGCTGGTCATCCTCTCAGACCAGCTAGG
GATCGTCGCCTAGGTGAGCCGTTACCCCACCTACTAGCTAATCCCATCTGGGCACATCCGATGGCAAGAGGCC

CTAAGGTCCCCCTCTTGTCTTGCGACGTATGCGGTATTAGCTACCGTTTCCAGTAGTTATCCCCCTCCATCGG
GCGGTTTCCCAGACATTACTCACCCGTCCGCCACTCGTCAGCAAAGCAGCAAGCTGCTCCTGTTACCGTTCGA
CTGCATGTGTTAGGCCTGCCGCCAGCGTCAATCTGAGCCAGGATCAAACTCT
>793
CTGCTGCCGCCCGTAGGAGTCTGGACCGTGTCTCAGTTCCAGTGTGGCTGGTCATCCTCTCAGACCAGCTAGG
GATCGTCGCCTAGGTGAGCCGTTACCCCACCTACTAGCTAATCCCATCTGGGCACATCCGATGGCAAGAGGCC
CTAAGGTCCCCCTCTTTGGTCTTGCGACGTTATGCGGTATTAGCTACCGTTTCCAGTAGTTATCCCCCTCCAT
CAGGCAGTTTCCCAGACATTACTCACCCGTCCGCCACTCGTCAGCAAAGCAGCAAGCTGCTTCTGTTACCGTT
CGACTGCATGTGTTAGGCCTGCCGCCAGCGTCAATCTGAGCCAGGATCAAACTCT
>794
CTGCTGCCGCCCGTAGGAGTCTGGACCGTGTCTCAGTTCCAGTGTGGCTGGTCATCCTCTCAGACCAGCTAGG
GATCGTCGCCTAGGTGAGCCGTACCCCACCTACTAGCTAATCCCATCTGGGCACATCCGATGGCAAGAGGCCC
TAAGGTCCCCCTCTTTGGTCTTGCGACGTTATGCGGTATTAGCTACCGTTTCCAGTAGTTATCCCCCTCCAT
CAGGCAGTTTCCCAGACATTACTCACCCGTCCGCCACTCGTCAGCAAAGCAGCAAGCTGCTTCCTGTTACCGT
TCGACTGCATGTGTTAGGCCTGCCGCCAGCGTCAATCTGAGCCAGGATCAAACTCT
>795
CTGCTGCCGCCCGTAGGAGTCTGGACCGTGTCTCAGTCCCAGTGTGGCTGGTCATCCTCTCAGACCAGCTAGG
GATCGTCGCCTAGGTGAGCCGTTACCCCACCTACTAGCTAATCCCATCTGGGCACATCCGATGGCAAGAGGCC
CTAAGGTCCCCCTCTTTGGTCTGCGACGTATGCGGTATTAGCTACCGTTTCCAGTAGTTATCCCCCTCCATCA
GGCAGTTTCCCAGACATTACTCACCCGTCCGCCACTCGTCAGCAAAGCAGCAAGCTGCTTCCTGTTACCGTTC
GACTGCATGTGTTAGGCCTGCCGCCAGCGTTCAATCTGAGCCAGGATCAAACTCT
>796
CTGCTGCCGCCCGTAGGAGTCTGGACCGTGTCTCAGTTCCAGTGTGGCTGGTCATCCTCTCAGACCAGCTAGG
GATCGTCGCCTAGGTGAGCCGTTACCCCACCTACTAGCTAATCCCATCTGGGCACATTCGATGGCAAGAGGCC
CTAAGGTCCCCCTCTTTGTCTTGCGACGTATGCGGTATTAGCTACCGTTTCCAGTAGTTATCCCCCTCCATCA
GGCAGTTTCTCAGACATACTCACCCGTCCGCCACTCGTCAGCAAAGCAGCAAGCTGCTTCCTGTTACCGTTCG
ACTTGCATGTGTTAGGCCTGCCGCCAGCGTCAATCTGAGCCAGGATCAAACTCT
>797
CTGCTGCCGCCCGTAGGAGTCTGGACCGTGTCTCAGTTCCAGTGTGGCTGGTCATCCTCTCAGACCAGCTAGG
GATCGTCGCCTAGGTGAGCCGTTTACCCCACCTACTAGCTAATCCCATCTGGGCACATCCGATGGCAAGAGGC
CCTAAGGTCCCCCTCTTTGTCTTGCGACGTATGCGGTATTAGCTACCGTTTCCAGTAGTTATCCCCTCCATCG
GGCAGTTTCCCAGACATTACTCACCCGTCCGCCACTCGTCAGCAAAGCAGCAAGCTGCTTCCTGTTACCGTTC
GACTGCATGTGTTAGGCCTGCCGCCAGCGTCAATCTGAGCCAGGATCAAACTCT
>798
CTGCTGCCGCCCGTAGGAGTCTGGACCGTGTCTCAGTTCCAGTGTGGCTGGTCATCCTCTCAGACCAGCTAGG
GATCGTCGCCTAGGTGAGCCGTTACCCCACCTACTAGCTAATCCCATCTGGGCACATCCGATGGCAAGAGGCC
CTAAGGTCCCCCTCTTTGGTCTTGCGACGTTATGCGGTATTAGCTACCGTTTCCAGTAGTTATCCCCCTCCAT
CAGGCAGTTTCCCAGACATTACTCACCCGTCCGCCACTCGTCAGCAAAGCAGCAAGCTGCTTCCTGTTACCGT
TCGACTGCATGTGTTAGGCCTGCCGCCAGCGTTCAATCTGAGCCAGGATCAAACTCT
>799
CTGCTGCCGCCCGTAGGAGTCTGGACCGTGTCTCAGTTCCAGTGTGGCTGGTCATCCTCTCAGACCAGCTAGG
GATCGTCGCCTAGGTGAGCCGTTACCCCACCTACTAGCTAATCCCATCTGGGCACATCCGATGGCAAGAGGCC
CTAAGGTCCCCCTCTTTGTCTTGCGACGTTATGCGGTATTAGCTACCGTTTCCAGTAGTTATCCCCCTCCATC
AGGCAGTTTTCCCAGACATTACTCACCCGTCCGCCACTCGTCAGCAAACAGCAAGCTGCTTCCTGTTACCGTT
CGACTGCATGTGTTAGGCCTGCCGCCAGCGTTCAATCTGAGCCAGGATCAAACTCT
>800
CTGCTGCCGCCCGTAGGAGTCTGGACCGTGTCTCAGTTCCAGTGTGGCTGGTCATCCTCTCAGACCAGCTAGG
GATCGTCGCCTAGGTGAGCCGTTACCCCACCTACTAGCTAATCCCATCTGGGCACATCCGATGGCAAGAGGCC
CTAAGGTCCCCCTCTTGTCTTGCGACGTATGCGGTATTAGCTACCGTTTCCAGTAGTTATCCCCCTCCATCGG
GCAGTTTCCCAGACATTACTCACCCGTCCGCCACTCGTCAGCAAAGCAGCAAGCTGCTTCCTGTTACCGTTCG
ACTCGCATGTGTTAGGCCTGCCGCCAGCGTCAATC
>801
CTGCTGCCGCCCGTAGGAGTCTGGACCGTGTCTCAGTTCCAGTGTGGCTGGTCATCCTCTCAGACCAGCTAGG
GATCGTCGCCTAGGTGAGCCGTTACCCCACCTACTAGCTAATCCCATCTGGGCACATCCGATGGCAAGAGGCC

CTAAGGTCCCCCTCTTGTCTTGCGACGTATGCGGTATTAGCTACCGTTTCCAGTAGTTATCCCCCTCCATCAG
GCAGTTTCCCAGACATTACTCACCCGTCCGCCACTCGTCAGCAAAGCAGCAAGCTGCTTCCTGTTACCGTTCG
ACTGCATGTGTTAGGCCTGCCGCCAGCGTCAATCTGAGCCAGGATCAAACTCT
>802
CTGCTGCCGCCCGTAGGAGTCTGGACCGTGTCTCAGTTCCAGTGTGGCTGGTCATCCTCTCAGACCAGCTAGG
GATCGTCGCCTAGGTGAGCCGTTACCCCACCTACTAGCTAATCCCATCTGGGCACATCCGATGGCAAGAGGCC
CTAAGGTCCCCCCTCTTTGGTCTTGCGACGTTATGCGGTATTAGCTACCGTTTCCAGTAGTTATCCCCCCTCC
ATCAGGCAGTTTCCCAGACATTACTCACCCGTCCGCCACTCGTCAGCAAAGCAGCAAGCTGCTTCTGTTACCG
TTCGACTGCATGTGTTAGGCCTGCCGCCAGCGTCAATCTGAGCCAGGATCAAACTCT
>803
CTGCTGCCGCCCGTAGGAGTCTGGACCGTGTCTCAGTTCCAGTGTGGCTGGTCATCCTCTCAGACCAGCTAGG
GATCGTCGCCTAGGTGAGCCGTTACCCCACCTACTAGCTAATCCCATCTGGGCACATCCGATGGCAAGAGGCC
CTAAGGTCCCCCTCTTTGGTCTTGCGACGTTATGCGGTATTAGCTACCGTTTCCAGTAGTTATCCCCCTCCAT
CAGGCAGTTTCCCAGACATTACTCACCCGTCCGCCACTCGTCAGCAAACAGCAAGCTGCTTCCTGTTACCGTT
CGACTGCATGTGTTAGGCCTGCCGCCAGCGTCAATCTGAGCCAGGATCAAACTCT
>804
CTGCTGCCGCCCGTAGGAGTCTGGACCGTGTCTCAGTTCCAGTGTGGCTGGTCATCCTCTCAGACCAGCTAGG
GATCGTCGCCTAGGTGAGCCGTTACCCCACCTACTAGCTAATCCCATCTGGGCACATCCGATGGCAAGAGGCC
CTAAGGTCCCCCTCTTTGGTCTTGCGACGTATGCGGTATTAGCTACCGTTTCCAGTAGTTATCCCCCTCCATC
GGGCAGTTTCCCAGACATTACTCACCCGTCCGCCACTCGTCAGCAAAGCAGCAAGCTGCTTCCTGTTACCGTT
CGACTGCATGTGTTAGGCCTGCCGCCAGCGTCAATCTGAGCCAGGATCAAACTCT
>805
CTGCTGCCGCCCGTAGGAGTCTGGACCGTGTCTCAGTTCCAGTGTGGCTGGTCATCCTCTCAGACCAGCTAGG
GATCGTCGCCTAGGTGAGCCGTTACCCCACCTACTAGCTAATCCCATCTGGGCACATCCGATGGCAAGAGGCC
CTAAGGTCCCCCTCTTGTCTGCGACGTATGCGGTATTAGCTACCGTTTCCAGTAGTTATCCCCCTCCATCAGG
CAGTTTCCCAGACATACTCACCCGTCCGCCACTCGTCAGCAAAGCAGCAAGCTGCTTCCTGTTACCGTTCGAC
TGCATGTGTTAGGCCTGCCGCCAGCGTCAATCTGAGCCAGGATCAAACTCT
>806
CTGCTGCCGCCCGTAGGAGTCTGGACCGTGTCTCAGTTCCAGTGTGGCTGGTCATCCTCTCAGACCAGCTAGG
GATCGTCGCCTAGGTGAGCCGTTACCCCACCTACTAGCTAATCCCATCTGGGCACATCCGATGGCAAGAGGCC
CTAAGGTCCCCCTCTTGTCTGCGACGTTATGCGGTATTAGCTACCGTTTCCAGTAGTTATCCCCCTCCATCAG
GCAGTTTCCCAGACATTACTCACCCGTCCGCCACTCGTCAGCAAAGCAGCAAGCTGCTTCCTGTTACCGTTCG
ACTGCATGTGTTAGGCCTGCCGCCAGCGTCAATCTGAGCCAGGATCAAACTCT
>807
CTGCTGCCGCCCGTAGGAGTCTGGACCGTGTCTCAGTTCCAGTGTGGCTGGTCATCCTCTCAGACCAGCTAGG
GATCGTCGCCTAGGTGAGCCGTTACCCCACCTACTAGCTAATCCCATCTGGGCACATCCGATGGCAAGAGGCC
CTAAGGTCCCCCTCTTTGTCTTGCGACGTTATGCGGTATTAGCTACCGTTTCCAGTAGTTATCCCCCTCCATC
AGGCAGTTTCCCAGACATTACTCACCCGTCCGCCACTCGTCAGCAAAGCAGCAAGCTGCTTCCTGTTACCGTT
CGACTGCATGTGTTAGGCCTGCCGCCAGCCGTCAATCTGAGCCAGGATCAAACTCT
>808
CTGCTGCCGCCCGTAGGGGTCTGGACCGTGTCTCAGTTCCGAGTGTGGCTGGTCATCCTCTCAGACCAGCTAG
GGATCGTCGCCTAGGTGAGCCGTTACCCCACCTACTAGCTAATCCCATCTGGGCACATCCGATGGCAAGAGGC
CCTAAGGTCCCCCTCTTTGTCTTGCGACGTATGCGGTATTAGCTACCGTTTCCAGTAGTTATCCCCCTCCATC
AGGCAGTTTCCCAGACATTACTCACCCGTCCGCCACTCGTCAGCAAAGCAGCAAGCTGCTTCCTGTTACCGTT
CGACTGCATGTGTTAGGCCTGCCGCCAGCGTCAATCTGAGCCAGGATCAAACTCT
>809
CTGCTGCCGCCCGTAGGAGTCTGGACCGTGTCTCAGTTCCAGTGTGGCTGGTCATCCTCTCAGACCAGCTAGG
GATCGTCGCCTAGGTGAGCCGTTACCCCACCTACTAGCTAATCCCATCTGGGCACATCCGATGGCAAGAGGCC
CTAAGGTCCCCCTCTTTGTCTGCGACGTATGCGGTATTAGCTACCGTTTCCAGTAGTTATCCCCCTCCATCAG
GCAGTTTCCCAGACATTACTCACCCGTCCGCCACTCGTCAGCAAACAGCAAGCTGCTTCCTGTTACCGTTCGA
CTGCATGTGTTAGGCCTGCCGCCAGCGTCAATCTGAAGCCAGGATCAAACTTCT
>810
CTGCTGCCGCCCGTAGGAGTCTGGACCGTGTCTCAGTTCCAGTGTGGCTGGTCATCCTCTCAGACGGCTAGGG
ATCGTCGCCTAGGTGAGCCGTTACCCCACCTACTAGCTAATCCCATCTGGGCACATCCGATGGCAAGAGGCCC

TAAGGTCCCCCTCTTGTCTTGCGACGTCATGCGGTATTAGCTACCGTTTCCAGTAGTTATCCCCCTCCATCAG
GCAGTTTTCCCAGACATTACTCACCCGTCCGCCACTCGTCAGCAAACAGCAAGCTGCTTCCTGTTACCGTTCG
ACTGCATGTGTTAGGCCTGCCGCCAGCGTCAATCTGAGCCAGGATCAAACTCT
>811
CTGCTGCCGCCCGTAGGAGTCTGGACCGTGTCTCAGTTCCAGTGTGGCTGGTCATCCTCTCAGACCAGCTAGG
GATCGTCGCCTAGGTGAGCCGTTACCCCACCTACTAGCTAATCCCATCTGGGCACATCCGATGGCAAGAGGCC
CTAAGGTCCCCCTCTTTGGTCTTGCGACGTATGCGGTATTAGCTACCGTTTCCAGTAGTTATCCCCCTCCATC
AGGCAGTTTCCCAGACATTACTCACCCGTCCGCCACTCGTCAGCAAAGCAGCAAGCTGCTTCCTGTTACCGTC
GACTGCATGTGTTAGGCCTGCCGCCAGCGTCAATCTGAGCCAGGATCAAACTCT
>812
CTGCTGCCGCCCGTAGGAGTCTGGACCGTGTCTCAGTTCCAGTGTGGCTGGTCATCCTCTCAGACCAGCTAGG
GATCGTCGCCTAGGTGAGCCGTTACCCCACCTACTAGCTAATCCCATCTGGGCACATCCGATGGCAAGAGGCC
CTAAGGTCCCCCTCTTTGTCTTGCGACGTATGCGGTATTAGCTACCGTTTCCAGTAGTTATCCCCCTCCATCG
GGCAGTTTCCCAGACATTACTCACCCGTCCGCCACTCGTCAGCAAAGCAGCAAGCTGCTTCCTGTTACCGTTC
GACTGCATGTGTTAGGCCTGCCGCCAGCGTCAATCTGAGCCAGGATCAAACTCT
>813
CTGCTGCCGCCCGTAGGAGTCTGGACCGTGTCTCAGTTCCAGTGTGGCTGGTCATCCTCTCAGACCAGCTAGG
GATCGTCGCCTAGGTGAGCCGTTACCCCACCTACTAGCTAATCCCATCTGGGCACATCCGATGGCAAGAGGCC
CTAAGGTCCCCCTCTTTGGTCTGCGACGTTATGCGGTATTAGCTACCGCTTCCAGTAGTTATCCCCCTCCATC
AGGCAGTTTCCCAGACATTACTCACCCGTCCGCCACTCGTCAGCAAAGCAGCAAGCTGCTTCTGTTACCGTTC
GACTGCATGTGTTAGGCCTGCCGCCAGCGTTCAATCTGAGCCAGGATCAAACTCT
>814
CTGCTGCCGCCCGTAGGAGTCTGGACCGTGTCTCAGTTCCAGTGTGGCTGGTCATCCTCTCAGACCAGCTAGG
GATCGTCGCCTAGGTGAGCCGTTACCCCACCTACTAGCTAATCCCATCTGGGCACATCCGATGGCAAGAGGCC
CTAAGGTCCCCCTCTTTGTCTTGCGACGTTATGCGGTATTAGCTACCGTTTCCAGTAGTTATCCCCCTCCATC
AGGCAGTTTTCCCAGACATTACTCACCCGTCCGCCACTCGTCAGCAAAGCAGCAAGCTGCTTTCTGTTACCGT
TCGACTGCATGTGTTAGGCCTGCCGCCAGCGTTCAATCTGAGCCAGGATCAAACTCT
>815
CTGCTGCCGCCCGTAGGAGTCTGGACCGTGTCTCAGTTCCAGTGTGGCTGGTCATCCTCTCAGACCAGCTAGG
GATCGTCGCCTAGGTGAGCCGTTACCCCACCTACTAGCTAATCCCATCTGGGCACATCCGATGGCAAGAGGCC
CTAAGGTCCCCCTCTTTGGTCTTGCGACGTTATGCGGTATTAGCTACCGTTTCCAGTAGTTATCCCCCTCCAT
CAGGCAGTTTCCCCAGACATTACTCACCCGTCCGCCACTCGTCAGCAAAACAGCAAGCTGCTTCCTGTTACCG
TTCGACTGCATATGTTAGGCCTGCCGCCAGCGTCAATCTGAGCCAGGATCAAACTCT
>816
CTGCTGCCGCCCGTAGGAGTCTGGACCGTGTCTCAGTTCCAGTGTGGCTGGTCATCCTCTCAGACCAGCTAGG
GATCGTCGCCTAGGTGAGCCGTTACCCCTCCTACTAGCTAATCCCATCTGGGCACATCCGATGGCAAGAGGCC
CTAAGGTCCCCCTCTTTGGTCTTGCGACGTTATGCGGTATTAGCTACCGTTTCCAGTAGTTATCCCCCTCCAT
CGGGCAGTTTCCCAGACATTACTCACCCGTCCGCCACTCGTCAGCAAAGCAGCAAGCTGCTTCCTGTTACCGT
TCGACTGCATGTGTTAGGCCTGCCGCCAGCGTCAATCTGAGCCAGGATCAAACTCT
>817
CTGCTGCCGCCCGTAGGAGTCTGGACCGTGTCTCAGTTCCAGTGTGGCTGGTCATCCTCTCAGACCAGCTAGG
GATCGTCGCCTAGGTGAGCCGTTACCCCACCTACTAGCTAATCCCATCTGGGCACATCCGATGGCAAGAGGCC
CTAAGGTCCCCCTCTTTGTCTTGCGACGTATGCGGTATTAGCTACCGTTTCCAGTAGTTATCCCCCTCCATCA
GGCAGTTTCCCAGACATTACTCACCCGTCCGCCACTCGTCAGCAAAGCAGCAAGCTGCTTCCTGTTACCGTTC
GACTGCATGTGTTAGGCCTGCCGCCAGCGTCAATCTGAGCCAGGATCAAACTAT
>818
CTGCTGCCGCCCGTAGGAGTCTGGACCGTGTCTCAGTTCCAGTGTGGCTGGTCATCCTCTCAGACCAGCTAGG
GATCGTCGCCTAGGTGAGCCGTTACCCCACCTACTAGCTAATCCCATCTGGGCACATCCGATGGCAAGAGGCC
CTAAGGTCCCCCTCTTTGGTCTTGCGACGTTATGCGGTATTAGCTACCGTTTCCAGTAGTTATCCCCCTCCAT
CGGGCAGTTTCCCAGACATTACTCACCCGTCCACCACTCGTCAGCAAAGCAGCAAGCTGCTTCCTGTTACCGT
TCGACTGCATGTGTTAGGCCTGCCGCCAGCGTTCAATCTGAGCCAGGATCAAACTCT
>819
CTGCTGCCGCCCGTAGGAGTCTGGACCGTGTCTCAGTTCCAGTGTGGCTGGTCATCCTCTCAGACCAGCTAGG
GATCGTCGCCTAGGTGAGCCGTTACCCCACCTACTAGCTAATCCCATCTGGGCACATCCGATGGCAAGAGGCC

CTAAGGTCCCCCTCTTTGTCTTGCGACGTTATGCGGTATTAGCTACCGTTTCCAGTAGTTATCCCCCTCCATC
AGGCAGTTTCCCAGACATTACTCACCCGTCCGCCACTCGTCAGCAAAGCAGCAAGCTGCTTCTGTTACCGTTC
GACTGCATGTGTTAGGCCTGCCGCCAGCCGTTCAATCTGAGCCAGGACAAACTCT
>820
CTGCTGCCGCCCGTAGGAGTCTGGACCGTGTCTCAGTTCCAGTGTGGCTGGTCATCCTCTCAGACCAGCTAGG
GATCGTCGCCTAGGTGAGCCGTTACCCCACCTACTAGCTAATCCCATCTGGGCACATCCGATGGCAAGAGGCC
CTAAGGTCCCCCTCTTTGGTCTTGCGAACGTTATGCGGTATTAGCTACCGTTTCCAGTAGTTATCCCCCTCCA
TCGGGCAGTTTCCCAGACATTACTCACCCGTCCGCCACTCGTCAGCAAAGCAGCAAGCTGCTTCCTGTTACCG
TTCGACTGCATGTGTTAGGCCTGCCGCCAGCGTTCAATCTGAGCCAGGATCAAACTCT
>821
CTGCTGCCGCCCGTAGGAGTCTGGACCGTGTCTCAGTTCCAGTGTGGCTGGTCATCCTCTCAGACCAGCTAGG
GATCGTCGCCTAGGTGAGCCGTTACCCCACCTACTAGCTAATCCCATCTGGGCACATCCGATGGCAAGAGGCC
CTAAGGTCCCCCTCTTTGTCTTGCGACGTTATGCGGTATTAGCTACCGTTTCCAGTAGTTATCCCCCTCCATC
AGGCAGTTTCCCAGACATTACTCACCCGTCCGCCACTCGTCAGCAAAGCAGCAAGCTGCTTCCTGTTACCGTT
CGACTGCATGTGTTAGGCCTGACCGCCAGCGTTCAATCTGAGCCAGGACAACTAT
>822
CTGCTGCCGCCCGTAGGAGTCTGGACCGTGTCTCAGTTCCAGTGTGGCTGGTCATCCTCTCAGACCAGCTAGG
GATCGTCGCCTAGGTGAGCCGTTACCCCACCTACTAGCTAATCCCATCTGGGCACATCCGATGGCAAGAGGCC
CTAAGGTCCCCCTCTTTGTCTGCGACGTATGCGGTATTAGCTACCGTTTCCAGTAGTTATCCCCCTCCATCAG
GCAGTTTCCCAGACATTACTCACCCGTCCGCCACTCGTCAGCAAAACAGCAAGCTGCTTCCTGTTACCGTTCG
ACTGCATGTGTTAGGCCTGCCGCCAGCGTCAATCTGAGCCAGGATCAAACTCT
>823
CTGCTGCCGCCCGTAGGAGTCTGGACCGTGTCTCAGTTCCAGTGTGGCTGGTCATCCTCTCAGACCAGCTAGG
GATCGTCGCCTAGGTGAGCCGTTACCCCACCTACTAGCTAATCCCATCTGGGCACATCCGATGGCAAGAGGCC
CTAAGGTCCCCCTCTTTGTCTTGCGACGTTATGCGGTATTAGCTACCGTTTCCAGTAGTTATCCCCCTCCATC
GGGCAGTTTCCCAGACATTACTCACCCGTCCGCCACTCGTCAGCAAAGCAGCAAGCTGCTTCCTGTTACCGTT
CGACTGCATGTGTTAGGCCTGCCGCCAGCGTCAATCTGAGCCAGGATCAAACTCT
>824
CTGCTGCCGCCCGTAGGAGTCTGGACCGTGTCTCAGTTCCAGTGTGGCTGGTCATCCTCTCAGACCAGCTAGG
GATCGTCGCCTAGGTGAGCCGTTACCCCACCTACTAGCTAATCCCATCTGGGCACATCCGATGGCAAGAGGCC
CTAAGGTCCCCCTCTTGTCTTGCGACGTATGCGGTATTAGCTACCGTTTCCAGTAGTTATCCCCCTCCATCGG
GCAGTTTCCCAGACATTACTCACCCGTCCGCCACTCGTCAGCAAAGCAGCAAGCTGCTTCCTGTTACCGTTCG
ACTGCATGTGTTAGGCCTGCCGCCAGCGTCAATCTGAGCCAGGATCAAACTCT
>825
CTGCTGCCGCCCGTAGGAGTCTGGACCGTGTCTCAGTTCCAGTGTGGCTGGTCATCCTCTCAGACCAGCTAGG
GATCGTCGCCTAGGTGAGCCGTTACCCCACCTACTAGCTAATCCCATCTGGGCACATCCGATGGCAAGAGGCC
CTAAGGTCCCCCTCTTTGGTCTGCGACGTATGCGGTATTAGCTACCGTCTCCAGTAGTTATCCCCCTCCATCG
GGCAGTTTCCCAGACATTACTCACCCGTCCGCCACTCGTCAGCAAAGCAGCAGGCTGCTTCCTGTTACCGTTC
GACTGCATGTGTTAGGCCTGCCGCCAGCGTCAATCTGAGCCAGGATCAAACTCT
>826
CTGCTGCCGCCCGTAGGAGTCTGGACCGTGTCTCAGTTCCAGTGTGGCTGGTCATCCTCTCAGACCAGCTAGG
GATCGTCGCCTAGGTGAGCCGTTACCCCACCTACTAGCTAATCCCATCTGGGCACATCCGATGGCAAGAGGCC
CTAAGGTCCCCCCTCTTTGTCTTGCGACGTTATGCGGTATTAGCTACCGTTTCCAGTAGTTATCCCCCCTCCA
TCAGGCAGTTTCCCAGACATTACTCACCCGTCCGCCACTCGTCAGCAAAGCAGCAAGCTGCTTCTGTTACCGT
TCGACTGCATGTGTTAGGCCTGCCGCCAGCGTTCAATCTGAGCCAGGATCAAACCTCT
>827
CTGCTGCCGCCCGTAGGAGTCTGGACCGTGTCTCAGTTCCAGTGTGGCTGGTCATCCTCTCAGACCAGCTAGG
GATCGTCGCCTAGGTGAGCCGTTACCCCACCTACTAGCTAATCCCATCTGGGCACATCCGATGGCGAGAGGCC
CTAAGGTCCCCCTCTTTGTCTGCGACGTTATGCGGTATTAGCTACCGTTTCCAGTAGTTATCCCCCTCCATCA
GGCAGTTTCCCAGACATTACTCACCCGTCCGCCACTCGTCAGCAAACAGCAAGCTGCTTCCTGTTACCGTTCG
ACTGCATGTGTTAGGCCTGCCGCCAGCGTTCAATCTGAGCCAGGATCAAACTCT
>828
CTGCTGCCGCCCGTAGGAGTCTGGACCGTGTCTCAGTTCCAGTGTGGCTGGTCATCCTCTCAGACCAGCTAGG
GATCGTCGCCTAGGTGAGCCGTTACCCCACCTACTAGCTAATCCCATCTGGGCACATCCGATGGCAAGAGGCC

CTAAGGTCCCCCTCTTTGGTCTTGCGACGTTATGCGGTATTAGCTACCGTTTCCAGTAGTTATCCCCCTCCAT
CAGGCAGTTTCCCAGACATTACTCACCCGTCCGCCACTCGTCAGCAAAACAGCAAGCTGCTTCCTGTTACCGT
TCGACTTGCATTGTGTTAGGCCTGCCGCCAGCGTCAATCTGAGCCAGGATCAAACTCT
>829
CTGCTGCCGCCCGTAGGAGTCTGGACCGTGTCTCAGTTCCAGTGTGGCTGGTCATCCTCTCAGACCAGCTAGG
GATCGTCGCCTAGGTGAGCCGTTACCCCACCTACCAGCTAATCCCATCTGGGCACATCCGAGGGCAAGAGGCC
CGAAGGTCCCCCTCTTGGTCTTGCGACGTATGCGGTATTAGCTACCGTTTCCAGTAGTTATCCCCCTCCATCA
GGCAGTTTCCCAGACATTACTCACCCGTCCGCCACTCGTCAGCGAACAGCAAGCTGCTTCCTGTTACCGTTCG
ACTGCATGTGTTAGGCCTGCCGCCAGCGTCATCTGAAGCCAGGATCAAACTCT
>830
CTGCTGCCGCCCGTAGGAGTCTGGACCGTGTCTCAGTTTCCAGTGTGGCTGGTCATCCTCTCAGACCAGCTAG
GGATCGTCGCCTAGGTGAGCCGTTACCCCACCTACTAGCTAATCCCATCTGGGCACATCCCGATGGCAAGAGG
CCCTAAGGTCCCCCTCTTTGGTCTGCGACGTATGCGGTATTAGCTACCGTTTCCAGTAGTTATCCCCCTCCAT
CAGGCAGTTTTCCCAGACATTACTCACCCGTCCGCCACTCGTCAGCAAAGCAGCAAGCTGCTTCCTGTTACCG
TTCGACTGCATGTGTTAGGCCTGCCGCCAGCGTCAATCTGAGCCAGGATCAAACTCT
>831
CTGCTGCCGCCCGTAGGAGTCTGGACCGTGTCTCAGTTCCAGTGTGGCTGGTCATCCTCTCAGACCAGCTAGG
GATCGTCGCCTAGGTGAGCCGTTTACCCCACCTACTAGCTAATCCCATCTGGGCACATCCGATGGCAAGAGGC
CCTAAGGTCCCCCTCTTTGTCTGCGACGTATGCGGTATTAGCTACCGTTTCCAGTAGTTATCCCCCTCCATCA
GGCAGTTTTCCCAGACATTACTCACCCGTCCGCCACTCGTCAGCAAAGCAGCAAGCTGCTTCTGTTACCGTTC
GACTGCATGCGTTAGGCCTGCCGCCAGCGTCAATCTGAGCCAGGATCAAACTCT
>832
CTGCTGCCGCCCGTAGGAGTCTGGACCGTGTCTCAGTTCCAGTGTGGCTGGTCATCCTCTCAGACCAGCTAGG
GATCGTCGCCTAGGTGAGCCGTTTACCCCACCTACTAGCTAATCCCATCTGGGCACATCCGATGGCAAGAGGC
CCTAAGGTCCCCCTCTTGTCTTGCGACGTTATGCGGTATTAGCTACCGTTTCCAGTAGTTATCCCCCTCCATC
AGGCAGTTTCCCAGACATTACTCACCCGTCCGCCACTCGTCAGCAAACAGCAAGCTGCTTCCTGTTACCGTTC
GACTGCATGTGTTAGGCCTGCCGCCAGCGTTCAATCTGAGCCAGGATCAAACTCT
>833
CTGCTGCCGCCCGTAGGAGTCTGGACCGTGTCTCAGTTCCAGTGTGGCTGGTCATCCTCTCAGACCAGCTAGG
GATCGTCGCCTAGGTGAGCCGTTACCCCACCTACTAGCTAATCCCATCTGGGCACATCCGATGGCAAGAGGCC
CTAAGGTCCCCCTCTTTGGTCTTGCGACGTTATGCGGTATTAGCTACCGTTTCCAGTAGTTATCCCCCTCCAT
CAGGCAGTTTCCCAGACATTACTCACCCGTCCGCCACTCGTCAGCAAAACAGCAAGCTGCTTCCTGTTACCGT
TCGACTGCATGTGTTAGGCCTGCCGCCAGCGTTCAATCTGAGCCAGGATCAAACTCT
>834
CTGCTGCCGCCCGTAGGAGTCTGGACCGTGTCTCAGTTCCAGTGTGGCTGGTCATCCTCTCAGACCAGCTAGG
GATCGTCGCCTAGGTGAGCCGTTACCCCACCTACTAGCTAATCCCATCTGGGCACATCCGATGGCAAGAGGCC
CTAAGGTCCCCCTCTTTGTCTTGCGACGTTATGCGGTATTAGCTACCGTTTCCAGTAGTTTATCCCCCTCCAT
CGGGCAGTTTCCCAGACATTACTCACCCGTCCGCCACTCGTCAGCAAAGCAGCAAGCTGCTTCCTGTTACCGT
TCGACTGCATGTGTTAGGCCTGCCGCCAGCGTTCAATCTGAGCCAGGATCAAACTCT
>835
CTGCTGCCGCCCGTAGGAGTCTGGACCGTGTCTCAGTTCCAGTGTGGCTGGTCATCCTCTCAGACCAGCTAGG
GATCGTCGCCTAGGTGAGCCGTTACCCCACCTACTAGCTAATCCCATCTGGGCACATCCGATGGCAAGAGGCC
CTAAGGTCCCCCTCTTGTCTTGCGACGTTATGCGGTATTAGCTACCGTTTCCAGTAGTTATCCCCCTCCATCA
GGCAGTTTCCCAGACATTACTCACCCGTCCGCCACTCGTCAGCAAAGCAGCAAGCTGCTTCCTGTTACCGTTC
GACTGCATGTGTTAGGCCTGCCGCCAGCGTCAATCTGAGCCAGGATCAACTCT
>836
CTGCTGCCGCCCGTAGGAGTCTGGACCGTGTCTCAGTTCCAGTGTGGCTGGTCATCCTCTCAGACCAGCTAGG
GATCGTCGCCTAGGTGAGCCGTTACCCCACCTACTAGCTAATCCCATCTGGGCACATCCGATGGCAAGAGGCC
CTAAGGTCCCCCTCTTTGGTCTTGCGACGTATGCGGTATTAGCTACCGTTTCCAGTAGTTATCCCCCTCCATC
GGGCAGTTTTCCCAGACATTACTCACCCGTCCGCCACTCGTCAGCAAAGCAGCAAGCTGCTTCCTGTTACCGT
TCGACTGCATGTGTTAGGCCTGCCGCCAGCGTCAATCTGAGCCAGGATCAAACTCT
>837
CTGCTGCCGCCCGTAGGAGTCTGGACCGTGTCTCAGTTCCAGTGTGGCTGGTCATCCTCTCAGACCAGCTAGG
GATCGTCGCCTAGGTGAGCCGTTACCCCACCTACTAGCTAATCCCATCTGGGCACATCCGATGGCAAGAGGCC

CTAAGGTCCCCCTCTTGTCTGCGACGTATGCGGTATTAGCTACCGTTTCCAGTAGTTATCCCCCTCCATCGGG
CAGTTTCCCAGACATTACTCACCCGTCCGCCACTCGTCAGCAAGCAGCAAGCTGCCTCCTGTTACCGTTCGAC
TGCATGTGTTAGGCCTGCCGCCAGCGTCAATCTGAGCCAGGATCAAACTCT
>838
CTGCTGCCGCCCGTAGGAGTCTGGACCGTGTCTCAGTTCCAGTGTGGCTGGTCATCCTCTCAGACCAGCTAGG
GATCGTCGCCTAGGTGAGCCGTTACCCCACCTACTAGCTAATCCCATCTGGGCACATCCGATGGCAAGAGGCC
CTAAGGTCCCCCTCTTGTCTGCGACGTATGCGGTATTAGCTACCGTTTCCAGTAGTTATCCCCCTCCATCAGG
CAGTTTTCCCAGACATTACTCACCCGTCCGCCACTCGTCAGCAAAGCAGCAAGCTGCTTCCTGTTACCGTTCG
ACTGCATGTGTTAGGCCTGCCGCCAGCGTTCAATCTGAGCCAGGATCAAACTCT
>839
CTGCTGCGCCCGTAGGAGTCTGGACCGTGTCTCAGTTCCAGTGTGGCTGGTCATCCTCTCAGACCAGCTAGGG
ATCGTCGCCTAGGTGAGCCGTTACCCCACCTACTAGCTAATCCCATCTGGGCACATCCGATGGCAAGAGGCCC
TAAGGTCCCCCTCTTTGTCTTGCGACGTATGCGGTATTAGCTACCGTTTCCAGTAGTTATCCCCCTCCATCAG
GCAGTTTCCCAGACATTACTCACCCGTCCGCCACTCGTCAGCAAAGCAGCAAGCTGCTTCTGTTACCGTTCGA
CTGCATGTGTTAGGCCTGCCGCCAGCGTCAATCTGAAGCCAGGATCAACTCT
>840
CTGCTGCCGCCCGTAGGAGTCTGGACCGTGTCTCAGTTCCAGTGTGGCTGGTCATCCTCTCAGACCAGCTAGG
GATCGTCGCCTAGGTGAGCCGTTACCCCACCTACTAGCTAATCCCATCTGGGCACATCCGATGGCAAGAGGCC
CTAAGGTCCCCCTCTTTGTCTGCGACGTTATGCGGTATTAGCTACCGTTTCCAGTAGTTATCCCCCTCCATCG
GGCAGTTTCCCAGACATTACTCACCCGTCCGCCACTCGCCAGCAAAGCAGCAAGCTGCTTCCTGTTACCGTTC
GACTGCATGTGTTAGGCCTGCCGCCAGCGTCAATCTGAGCCAGGATCAAACTCT
>841
TGCTGCCGCCCGTAGGAGTCTGGACCGTGTCTCAGTTCCAGTGTGGCTGGTCATCCTCTCAGACAGCTAGGGA
TCGTCGCCTAGGTGAGCCGTTACCCCACCTACTAGCTAATCCCATCTGGGCACATCCGATGGCAAGAGGCCCT
AAGGTCCCCCTCTTTGGTCTTGCGACGTTATGCGGTATTAGCTACCGTTTCCAGTAGTTATCCCCCTCCATCA
GGCAGTTTCCCAGACATTACTCACCCGTCCGCCACTCGTCAGCAAAGCAGCAAGCTGCTTCCTGTTACCGTTC
GACTGCATGTGTTAGGCCTGCCGCCAGCGTCAATCTGAGCCAGGATCAAACTCT
>842
CTGCTGCCGCCCGTAGGAGTCTGGACCGTGTCTCAGTTCCAGTGTGGCTGGTCATCCTCTCAGACCAGCTAGG
GATCGTCGCCTAGGTGAGCCGTTACCCCACCTACTAGCTAATCCCATCTGGGCACATCCGATGGCAAGAGGCC
CTAAGGTCCCCCTCTTGTCTTGCGACGTTATGCGGTATTAGCTACCGTTCCAGTAGTTATCCCCCTCCATCAG
GCAGTTTCCCAGACATTACTCACCCGTCCGCCACTCGTCAGCAAACAGCAAGCTGCTTCCTGTTACCGTTCGA
CTGCATGTGTTAGGCCTGCCGCCAGCGTCAATCTGAGCCAGGATCAAACTCT
>843
CTGCTGCCGCCCGTAGGAGTCTGGACCGTGTCTCAGTTCCAGTGTGGCTGGTCATCCTCTCAGACCAGCTAGG
GATCGTCGCCTAGGTGAGCCGTTACCCCACCTACTAGCTAATCCCATCTGGGCACATCCGATGGCAAGAGGCC
CTAAGGTCCCCCTCTTTGGTCTTGCGACGTTATGCGGTATTAGCTACCGTTTCCAGTAGTTATCCCCCTCCAT
CGGGCAGTTTTCCCAGACATTACTCACCCGTCCGCCACTCGTCAGCAAAGCAGCAAGCTGCTTCCTGTTACCG
TTCGACTGCATGTGTTAGGCCTGCCGCCAGCGTTCAATCTGAGCCAGGATCAAACTCT
>844
CTGCTGCCGCCCGTAGGAGTCTGGACCGTGTCTCAGTTCCAGTGTGGCTGGTCATCCTCTCAGACCAGCTAGG
GATCGTCGCCTAGGTGAGCCGTTACCCCACCTACTAGCTAATCCCATCTGGGCACATCCGATGGCAAGAGGCC
CTAAGGTCCCCCTCTTTGTCTGCGACGTTATGCGGTATTAGCTACCGTTTCCAGTAGTTATCCCCCTCCATCG
GGCAGTTTCCCAGACATTACTCACCCGTCCGCCACTCGTCAGCAAAGCAGCAAGCTGCTTCCTGTTACCGTTC
GACTGCATGTGTTAGGCCTGCCGCCAGCGTCAATCTGAGCCAGGATCAAACTCT
>845
CTGCTGCCGCCCGTAGGAGTCTGGACCGTGTCTCAGTTCCAGTGTGGCTGGTCATCCTCTCAGACCAGCTAGG
GATCGTCGCCTAGGTGAGCCGTTACCCCACCTACTAGCTAATCCCATCTGGGCACATCCGATGGCAAGAGGCC
CTAAGGTCCCCCTCTTTGTCTTGCGACGTTATGCGGTATTAGCTACCGTTTCCAGTAGTTATCCCCCTCCATC
GGGCAGTTTCCCAGACATTACTCACCCGTCCGCCACTCGTCAGCAAGCAGCAAGCTGCTTCCTGTTACCGTTC
GACTGCATGTGTTAGGCCTGCCGCCAGCGTCAATCTGAGCCAGAATCAAACTCT
>846
CTGCTGCCGCCCGTAGGAGTCTGGACCGTGTCTCAGTTCCAGTGTGGCTGGTCATCCTCTCAGACCAGCTAGG
GATCGTCGCCTAGGTGAGCCGTTACCCCACCTACTAGCTAATCCCATCTGGGCACATCCGATGGCAAGAGGCC

CTAAGGTCCCCCCCTTGTCTTGCGACGTATGCGGTATTAGCTACCGTTTCCAGTAGTTATCCCCCTCCATCAG
GCAGTTTCCCCAGACATTACTCACCCGTCCGCCACTCGTCAGCAAAGCAGCAAGCTGCTTCCTGTTACCGTTC
GACTGCATGTGTTAGGCCTGCCGCCAGCGTTCAATCTGAGCCAGGACAAACTCT
>847
CTGCTGCCGCCCGTAGGAGTCTGGACCGTGTCTCAGTTCCAGTGTGGCTGGTCATCCTCTCAGACAGCTAGGG
ATCGTCGCCTAGGTGAGCCGTTACCCCACCTACTAGCTAATCCCATCTGGGCACATCCGATGGCAAGAGGCCC
TAAGGTCCCCCTCTTTGTCTTGCGACGTTATGCGGTATTAGCTACCGTTTCCAGTAGTTATCCCCCTCCATCA
GGCAGTTTCCCAGACATACTCACCCGTCCGCCACTCGTCAGCAAACAGCAAGCTGCTTCCTGTTACCGTTCGA
CTGCATGTGTTAGGCCTGCCGCCAGCGTCAATCGAGCC
>848
CTGCTGCCGCCCGTAGGAGTCTGGACCGTGTCTCAGTTCCAGTGTGGCTGGTCATCCTCTCAGACCAGCTAGG
GATCGTCGCCTAGGTGAGCCGTTACCCCACCTACTAGCTAATCCCATCTGGGCACATCCGATGGCAAGAGGCC
CTAAGGTCCCCCTCTTTGTCTTGCGACGTTATGCGGTATTAGCTACCGTTTCCAGTAGTTATCCCCCTCCATC
GGGCAGTTTCCCAGACATTACTCACCCGTCCGCCACTCGTCAGCAAAGCAGCAAGCTGCTTCCTGTTACCGTT
CGACTGCATGTGTTAGGCCTGCCGCCAGCGTCAATCTGAGCCAGGATCAAACTCT
>849
CTGCTGCCGCCCGTAGGAGTCTGGACCGTGTCTCAGTTCCAGTGTGGCTGGTCATCCTCTCAGACCAGCTAGG
GATCGTCGCCTAGGTGAGCCGTTACCCCACCTACTAGCTAATCCCATCTGGGCACATCCGATGGCAAGAGGCC
CTAAGGTCCCCCTCTTTGTCTTGCGACGTTATGCGGTATTAGCTACCGTTTCCAGTAGTTATCCCCCTCCATC
AGGCAGTTTCCCAGACATTACTCACCCGTCCGCCACTCGTCAGCAAAGCAGCAAGCTGCTTCCTGTTACCGTT
CGACTGCATGTGTTAGGCCTGCCGCCAGCGTTCAATCTGAGCCAGGACAAACTCT
>850
CTGCTGCCGCCCGTAGGAGTCTGGACCGTGTCTCAGTTCCAGTGTGGCTGGTCATCCTCTCAGACCAGCTAGG
GATCGTCGCCTAGGTGAGCCGTTACCCCACCTACTAGCTAATCCCATCTGGGCACATCCGATGGCAAGAGGCC
CTAAGGTCCCCCTCTTTGTCTTGCGACGTTATGCGGTATTAGCTACCGTTCCAGTAGTTATCCCCCTCCATCA
GGCAGTTTCCCAGACATTACTCACCCGTCCGCCACTCGTCAGCAAAGCAGCAAGCTGCTTCCTGTTACCGTTC
GACTGCATGTGTTAGGCCTGCCGCCAGCGTTCAATCTGAGCCAGGACAAAGCTGT
>851
CTGCTGCCGCCCGTAGGAGTCTGGACCGTGTCTCAGTTCCAGTGTGGCTGGTCATCCTCTCAGACCAGCTAGG
GATCGTCGCCTAGGTGAGCCGTTACCCCACCTACTAGCTAATCCCATCTGGGCACATCCGATGGCAAGAGGCC
CTAAGGTCCCCCTCTTTGTCTTGCGACGTTATGCGGTATTAGCTACCGTTTCCAGTAGTTATCCCCCTCCATC
AGGCAGTTTCCCAGACATTACTCACCCGTCCGCCACTCGTCAGCAAAGCAGCAAGCTGCTTTCTGTTACCGTT
CGACTGCATGTGTTAGGCCTGCCGCCAGCGTTCAATCTGAGCCAGGATCAAACTCT
>852
CTGCTGCCGCCCGTAGGAGTCTGGACCGTGTCTCAGTTCCAGTGTGGCTGGTCATCCTCTCAGACCAGCTAGG
GATCGTCGCCTAGGTGAGCCGTTACCCCACCTACTAGCTAATCCCATCTGGGCACATCCGATGGCAAGAGGCC
CTAAGGTCCCCCTCTTTGTCTTGCGACGTTATGCGGTATTAGCTACCGTTTCCAGTAGTTATCCCCCTCCATC
GGGCAGTTTCCCAGACATTACTCACCCGTCCGCCACTCGTCAGCAAAGCAGCAAGCTGCTTCCTGTTACCGTT
CGACTGCATGTGTTAGGCCTGCCGCCAGCGTCAATCTGAGCCAGGATCAAACTCT
>853
CTGCTGCCGCCCGTAGGAGTCTGGACCGTGTCTCAGTTCCAGTGTGGCTGGTCATCCTCTCAGACCAGCTAGG
GATCGTCGCCTAGGTGAGCCGTTCACCCACCTACTAGCTAATCCCATCTGGGCACATCCCGATGGCAAGAGGC
CCTAAGGTCCCCCTCTTGTCTGCGACGTTATGCGGTATTAGCTACCGTTCCAGTAGTTATCCCCCTCCATCGG
GCAGTTTCCCAGACATTACTCACCCGTCCGCCACTCGTCAGCAAAGCAGCAAGCTGCTTCCTGTTACCGTCGA
CTGCATGTGTTAGGCCTGCCGCCAGCGCATCATC
>854
CTGCTGCCGCCCGTAGGAGTCTGGACCGTGTCTCAGTTCCAGTGTGGCTGGTCATCCTCTCAGACCAGCTAGG
GATCGTCGCCTAGGTGAGCCGTTACCCCACCTACTAGCTAATCCCATCTGGGCACATCCGATGGCAAGAGGCC
CTAAGGTCCCCCTCTTTGTCTTGCGACGTTATGCGGTATTAGCTACCGTTTCCAGTAGTTATCCCCCTCCATC
GGGCAGTTTCCCAGACATTACTCACCCGTCCGCCACTCGTCAGCAAAGCAGCAAGCTGCTTCCTGCTACCGTT
CGACTGCATGTGTTAGGCCTGCCGCCAGCGTCAATCTGAGCCAGGATCAAACTCT
>855
CTGCTGCCGCCCGTAGGAGTCTGGACCGTGTCTCAGTTCCAGTGTGGCTGGTCATCCTCTCAGACCAGCTAGG
GATCGTCGCCTAGGTGAGCCGTTACCCCACCTACTAGCTAATCCCATCTGGGCACATCCGATGGCAAGAGGCC

CTAAGGTCCCCCCTCTTTGGTCTTGCGACGTTATGCGGTATTAGCTACCGTTTCCAGTAGTTATCCCCCCTCC
ATCAGGCAGTTTCCCAGACATTACTCACCCGTCCGCCACTCGTCAGCAAAGCAGCAAGCTGCTTCCTGTTACC
GTTCGACTGCATGTGTTAGGCCTGCCGCCAGCGTCAATCTGAGCCAGGATCAAACTCT
>856
CTGCTGCCGCCCGTAGGAGTCTGGACCGTGTCTCAGTTCCAGTGTGGCTGGTCATCCTCTCAGACCAGCTAGG
GATCGTCGCCTAGGTGAGCCGTTACCCCACCTACTAGCTAATCCCATCTGGGCACATCCGATGGCAAGAGGCC
CTAAGGTCCCCCTCTTTGTCTTGCGACGTTATGCGGTATTAGCTACCGTTTCCAGTAGTTATCCCCCTCCATC
GGGCAGTTTCCCAGACATTACTCACCCGTCCGCCACTCGTCAGCAAAGCAGCAAGCTGCTTCCTGTTACCGTT
CGACTGCATGTGTTAGGCCTGCCGCCAGCGTCAATCTGAGCCAGGATCAAACTCT
>857
CTGCTGCCGCCCGTAGGATTCTGGACCGTGTCTCAGTTCCAGTGTGGCTGGTCATCCTCTCAGACCAGCTAGG
GATCGTCGCCTAGGTGAGCCGTTACCCCACCTACTAGCTAATCCCATCTGGGCACATCCGATGGCAAGGGGCC
CTAAGGTCCCCCTCTTGTCTGCGACGTTATGCGGTATTAGCTACCGTTTCCAGTAGTTATCCCCCTCCATCAG
GCAGTTTCCCAGACATTACTCACCCGTCCGCCACTCGTCAGCAAAGCAGCAAGCTGCTTCCTGTACCGTTCGA
CTGCGTGTGTTAGGCCTGCCGCCAGCGTCAATCG
>858
CTGCTGCCGCCCGTAGGAGTCTGGACCGTGTCTCAGTTCCAGTGTGGCTGGTCATCCTCTCAGACCAGCTAGG
GATCGTCGCCTAGGTGAGCCGTTACCCCACCTACTAGCTAATCCCATCTGGGCACATCCGATGGCAAGAGGCC
CTAAGGTCCCCCCTCTTTGGTCTTGCGACGTTATGCGGTATTAGCTACCGTTTCCAGTAGTTATCCCCCTCCA
TCAGGCAGTTTCCCAGACATTACTCACCCGTCCGCCACTCGTCAGCAAAGCAGCAAGCTGCTTCCTGTTACCG
TTCGACTGCATGTGTTAGGCCTGCCGCCAGCGTCAATCTGAGCCAGGATCAAACTCT
>859
CTGCTGCCGCCCGTAGGAGTCTGGACCGTGTCTCAGTTCCAGTGTGGCTGGTCATCCTCTCAGACCAGCTAGG
GATCGTCGCCTAGGTGAGCCGTTACCCCACCTACTAGCTAATCCCATCTGGGCACATCCGATGGCAAGAGGCC
CTAAGGTCCCCCTCTTTGTCTTGCGACGTTATGCGGTATTAGCTACCGTTTCCAGTAGTTATCCCCCTCCATC
AGGCAGTTTCCCAGACATTACTCACCCGTCCGCCACTCGTCAGCAAAGCAGCAAGCTGCTTCTGTTACCGTTC
GACTGCATGTGTTAGGCCTGCCGCCAGCGTCAATCTGAGCCAGGATCAAACTGT
>860
CTGCTGCCGCCCGTAGGAGTCTGGACCGTGTCTCAGTTCCAGTGTGGCTGGTCATCCTCTCAGACAGCTGGGG
ATCGTCGCCTAGGTGAGCCGTTACCCCACCTACTAGCTAATCCCATCTGGGCACATCCGATGGCAAGAGGCCC
TAAGGTCCCCCTCTTTGGTCTTGCGACGTTATGCGGTATTAGCTACCGTTTCCAGTAGTTATCCCCCTCCATC
GGGCAGTTTTCCCAGACATTACTCACCCGTCCGCCACTCGTCAGCAAAGCAGCAAGCTGCTTCCTGTTACCGT
TCGACTGCATGTGTTAGGCCTGCCGCCAGCGTCAATCTGAGCCAGGATCAAACTCT
>861
CTGCTGCCGCCCGTAGGAGTCTGGACCGTGTCTCAGTTCCAGTGTGGCTGGTCATCCTCTCAGACCAGCTAGG
GATCGTCGCCTAGGTGAGCCGTTTACCCCACCTACTAGCTAATCCCATCTGGGCACATCCGATGGCAAGAGGC
CCTAAGGTCCCCCTCTTTGTCTTGCGACGTTATGCGGTATTAGCTACCGTTTCCAGTAGTTATCCCCCTCCAT
CAGGCAGTTTCCCCAGACATTACTCACCCGTCCGCCACTCGTCAGCAAAGCAGCAAGCTGCTTCCTGTTACCG
TTCGACTGCATGTGTTAGGCCTGCCGCCAGCGTCAATCTGAGCCAGAATCAAACTCT
>862
CTGCTGCCGCCCGTAGGAGTCTGGACCGTGTCTCAGTTCCAGTGTGGCTGGTCATCCTCTCAGACCAGCTAGG
GATCGTCGCCTAGGTGAGCCGTTACCCCACCTACTAGCTAATCCCATCTGGGCACATCCGATGGCAAGAGGCC
CGAAGGTCCCCCTCTTGGTCTTGCGACGTTATGCGGTATTAGCTACCGTTTCCAGTAGTTATCCCCCTCCATC
AGGCAGTTTCCCAGACATTACTCACCCGTCCGCCACTCGTCAGCGAAACAGCAAGCTGCTTCCTGTTACCGTT
CGACTGCATGTGTTAGGCCTGCCGCCAGCGTCAATCTGAGCCAGGATCAAACTCT
>863
CTGCTGCCGCCCGTAGGAGTCTGGACCGTGTCTCAGTTCCAGTGTGGCTGGTCATCCTCTCAGACCAGCTAGG
GATCGTCGCCTAGGTGAGCCGTTACCCCACCTACTAGCTAATCCCATCTGGGCACATCCGATGGCAAGAGGCC
CTAAGGTCCCCCTCTTGGTCTTGCGACGTTATGCGGTATTAGCTACCGTTTCCAGTAGTTATCCCCCTCCATC
AGGCAGTTTCCCAGACATTACTCACCCGTCCGCCACTCGTCAGCAAAGCAGCAGGCTGCTTCTGTTACCGTTC
GACTGCATGTGTTAGGCCTGCCGCCAGCGTTCAATCTGAGCCAGGATCAAACTCT
>864
CTGCTGCCGCCCGTAGGAGTCTGGACCGTGTCTCAGTTCCAGTGTGGCTGGTCATCCTCTCAGACCAGCTAGG
GATCGTCGCCTAGGTGAGCCGTTACCCCACCTACTAGCTAATCCCATCTGGGCACATCCGATGGCAAGAGGCC

CTAAGGTCCCCCTCTTTGTCTTGCGACGTATGCGGTATTAGCTACCGTTTCCAGTAGTTATCCCCCTCCATCA
GGCAGTTTCCCAGACATTACTCACCCGTCCGCCACTCGTCAGCAAAGCAGCAAGCTGCTTCCTGTTACCGTTC
GACTGCATGTGTTAGGCCTGCCGCCAGCGTCAATCTGAGCCAGGATCAAACTCT
>865
CTGCTGCCGCCCGTAGGAGTCTGGACCGTGTCTCAGTTCCAGTGTGGCTGGTCATCCTCTCAGACCAGCTAGG
GATCGTCGCCTAGGTGAGCCGTTACCCCACCTACTAGCTAATCCCATCTGGGCACATCCCGATGGCAAGAGGC
CCTAAGGTCCCCCTCTTTGTCTTGCGACGTTATGCGGTATTAGCTACCGTTTCCAGTAGTTATCCCCCTCCAT
CAGGCAGTTTTCCCAGACATTACTCACCCGTCCGCCACTCGTCAGCAAACAGCAAGCTGCTTCCTGTTACCGT
TCGACTGCATGTGTAGGCCTGCCGCCAGCGTCAATCTGAGCCAGGATCAAACTCT
>866
CTGCTGCCGCCCGTAGGAGTCTGGACCGTGTCTCAGTTCCAGTGTGGCTGGTCATCCTCTCAGACCAGCTAGG
GATCGTCGCCTAGGTGAGCCGTTACCCCACCTACTAGCTAATCCCATCTGGGCACATCCGATGGCAAGAGGCC
CTAAGGTCCCCCTCTTGTCTGCGACGTATGCGGTATTAGCTACCGTTTCCAGTAGTTATCCCCCTCCATCAGG
CAGTTTCTCAGACATTACTCACCCGTCCGCCACTCGTCAGCAAAGCAGCAAGCTGCTTCTGTTACCGTTCGAC
TGCAATGTGTTAGGCCTGCCGCCAGCGTCAATCTGAGCCAGGATCAAACTCT
>867
CTGCTGCCGCCCGTAGGAGTCTGGACCGTGTCTCAGTTCCAGTGTGGCTGGTCATCCTCTCAGACCAGCTAGG
GATCGTCGCCTAGGTGAGCCGTTACCCCACCTACTAGCTAATCCCATCTGGGCACATCCGATGGCAAGAGGCC
CTAAGGTCCCCCTCTTGTCTTGCGACGTTATGCGGTATTAGCTACCGTTTCCAGTAGTTATCCCCCTCCATCA
GGCAGTTTCCCAGACATTACTCACCCGTCCGCCACTCGTCAGCAAAGCAGCAAGCTGCTTCCTGTTACCGTTC
GACTGCATGTGTTAGGCCTGCCGCCAGCGTCAATCTGAGCCAGGATCAAACTCT
>868
CTGCTGCCGCCCGTAGGAGTCTGGACCGTGTCTCAGTTCCAGTGTGGCTGGTCATCCTCTCAGACCAGCTAGG
GATCGTCGCCTAGGTGAGCCGTTACCCCACCTACTAGCTAATCCCATCTGGGCACATCCGATGGCAAGAGGCC
CTAAGGTCCCCCTCTTTGGTCTTGCGACGTTATGCGGTATTAGCTACCGTTTCCAGTAGTTATCCCCCTCCAT
CAGGCAGTTTCCCAGACATTACTCACCCGTCCGCCACTCGTCAGCAAAGCAGCAAGCTGCTTTCTGTTACCGT
TCGACTGCTGTGTTAGGCCTGCCGCCAGCGGTCAATCTGAGCCAGGATCAAACTCT
>869
CTGCTGCCGCCCGTAGGAGTCTGGACCGTGTCTCAGTTCCAGTGTGGCTGGTCATCCTCTCAGACCAGCTAGG
GATCGTCGCCTAGGTGAGCCGTTACCCCACCTACTAGCTAATCCCATCTGGGCACATCCGATGGCAAGAGGCC
CTAAGGTCCCCCTCTTTGTCTTGCGACGTTATGCGGTATTAGCTACTTCTCAGTAGTTATCCCCCTCCATCAG
GCAGTTTCCCAGACATTACTCACCCGTCCGCCACTCGTCAGCAAACAGCAAGCTGCTTCCTGTTACCGTTCGA
CTGCATGTGTAGGCCTGCCGCCAGCGTTCGAATCTGAG
>870
CTGCTGCCGCCCGTAGGAGTCTGGACCGTGTCTCAGTTCCAGTGTGGCTGGTCATCCTCTCAGACCAGCTAGG
GATCGTCGCCTAGGTGAGCCGTTACCCCACCTACTAGCTAATCCCACTGGGCACATCCGATGGCAAGAGGCCC
TAAGGTCCCCCTCTTTGTCTTGCGACGTTATGCGGTATTAGCTACCGTTTCCAGTAGTTATCCCCCTCCATCG
GGCAGTTTCCCAGACATTACTCACCCGTCCGCCACTCGTCAGCAAAGCAGCAAGCTGCTTCCTGTTACCGTTC
GACTGCATGTGTTAGGCCTGCCGCCAGCGTCAATCTGAGCCAGGATCAAACTCT
>871
CTGCTGCCGCCCGTAGGAGTCTGGACCGTGTCTCAGTTCCAGTGTGGCTGGTCATCCTCTCAGACCAGCTAGG
GATCGTCGCCTAGGTGAGCCGTTACCCCACCTACTAGCTAATCCCATCTGGGCACATCCGATGGCAAGAGGCC
CTAAGGTCCCCCTCTTTGTCTTGCGACGTTATGCGGTATTAGCTACCGTTTCCAGTAGTTATCCCCCTCCATC
AGGCAGTTTCCCAGACATTACTCACCCGTCCGCCACTCGTCAGCAAAGCAGCAAGCTGCTTCCTGTTACCGTT
CGACTGCATGTGTTAGGCCTGCCGCCAGCGTCAATCTGAGCCAGGATCAAACTCT
>872
CTGCTGCCGCCCGTAGGAGTCTGGACCGTGTCTCAGTTCCAGTGTGGCTGGTCATCCTCTCAGACAGCTAGGG
ATCGTCGCCTAGGTGAGCCGTTACCCCACCTACTAGCTAATCCCATCTGGGCACATCCGATGGCAAGAGGCCC
TAAGGTCCCCCTCTTGTCTTGCGACGTTATGCGGTATTAGCTACCGTTTCCAGTAGTTATCCCCCTCCATCAG
GCAGTTTCCCAGACATTACTCACCCGTCCGCCACTCGTCAGCAAAGCAGCAAGCTGCTTCCTGTTACCGTTCG
ACTGCATGTGTTAGGCCTGCCGCCAGCGTCAATCTGAGCCAGGATCAAACTCT
>873
CTGCTGCCGCCCGTAGGAGTCTGGACCGTGTCTCAGTTCCAGTGTGGCTGGTCATCCTCTCAGACCAGCTAGG
GATCGTCGCCTAGGTGAGCCGTTACCCCACCTACTAGCTAATCCCATCTGGGCACATCCGACGGCAAGAGGCC

CTAAGGTCCCCCTCTTTGTCTTGCGACGTTATGCGGTATTAGCTACCGTTTCCAGTAGTTATCCCCCTCCATC
AGGCAGTTTCCCAGACATTACTCACCCGTCCGCCACTCGTCAGCAAAGCAGCAAGCTGCTTCCTGTTACCGTT
CGACTGCATGTGTTAGGCCTGCCGCCAGCGTCAATCTGAGCCAGGATCAAACTCT
>874
CTGCTGCCGCCCGTAGGAGTCTGGACCGTGTCTCAGTTCCAGTGTGGCTGGTCATCCTCTCAGACCAGCTAGG
GATCGTCGCCTAGGTGAGCCGTTACCCCACCTACTAGCTAATCCCATCTGGGCACATCCGATGGCAAGAGGCC
CTAAGGTCCCCCTCTTGTCTGCGACGTATGCGGTATTAGCTACCGTTTCCAGTAGTTATCCCCTCCATCGGGC
AGTTTCCCAGACATACTCACCCGTCCGCCACTCGTCCGCAAAGCAGCAAGCTGCTTCCTGTTACCGTCGACTG
CATGTGTTAGGCCTGCCGCCAGCGTCAATCTGAGCCAGGATCAAACTCGT
>875
CTGCTGCCGCCCGTAGGAGTCTGGACCGTGTCTCAGTTCCAGTGTGGCTGGTCATCCTCTCAGACCAGCTAGG
GATCGTCGCCTAGGTGAGCCGTTACCCCACCTACTAGCTAATCCCATCTGGGCACATCCGATGGCAAGAGGCC
CTAAGGTCCCCCTCTTTGGTCTTGCGACGTTATGCGGTATTAGCTACCGTTTCCAGTAGTTATCCCCCTCCAT
CGGGCAGTTTTCCCAGACATTACTCACCCGTCCGCCACTCGTCAGCAAAGCAGCAAGCTGCTTCCTGTTACCG
TTCGACTGCATGTGTTAGGCCTGCCGCCAGCGTTCAATCTGAGCCAGGATCAAACTCT
>876
CTGCTGCCGCCCGTAGGAGTCTGGACCGTGTCTCAGTTCCAGTGTGGCTGGTCATCCTCTCAGACCAGCTAGG
GATCGTCGCCTAGGTGAGCCGTTACCCCACCTACTAGCTAATCCCATCTGGGCACATCCGATGGCAAGAGGCC
CTAAGGTCCCCCTCTTTGTCTTGCGACGTTATGCGGTATTAGCTACCGTTTCCAGTAGTTATCCCCCTCCATC
GGGCAGTTTCCCAGACATTACTCACCCGTCCGCCACTCGTCAGCAAAGCAGCAAGCTGCTTCCTGTTACCGTT
CGACTGCATGTGTTAGGCCTGCCGCCAGCGTCAATCTGAGCCAGGATCAAACTCT
>877
CTGCTGCCGCCCGTAGGAGTCTGGACCGTGTCTCAGTTCCAGTGTGGCTGGTCCTCCTCTCAGACCAGCTAGG
GATCGTCGCCTAGGTGAGCCGTTACCCCACCTACTAGCTAATCCCATCTGGGCACATCCGATGGCAAGAGGCC
CTAAGGTCCCCCTCTTTGGTCTTGCGACGTATGCGGTATTAGCTACGTTCCTCAGTAGTTATCCCCCTCCCAT
CAGGCAGTTTCCCAGACATTACTCACCCGTCCGCCACTCGTCAGCAAAGCAGCAAGCTGCTTCTGTTACCGTT
CGACTGCATGTGTTAGGCCTGCCGCCAGCGCTCAATCTGAGCCAGGATCAAACTCT
>878
CTGCTGCCGCCCGTAGGAGTCTGGACCGTGTCTCAGTTCCAGTGTGGCTGGTCATCCTCTCAGACCAGCTAGG
GATCGTCGCCTAGGTGAGCCGTTACCCCACCCACTAGCTAATCCCATCTGGGCACATCCGATGGCAAGAGGCC
CTAAGGTCCCCCTCTTGTCTTGCGACGTATGCGGTATTAGCTACCGTTTCCAGTAGTTATCCCCCTCCATCGG
GCAGTTTCCCAGACATACTCACCCGTCCGCCACTCGTCAGCAAAGCAGCAAGCTGCTTCCTGTTACCCGTTCG
ACTGCAATGTGTTAGGCCTGCCGCCAGCGTCAATCTGAGCCAGGATCAAACTCT
>879
CTGCTGCCGCCCGTAGGAGTCTGGACCGTGTCTCAGTTCCAGTGTGGCTGGTCATCCTCTCAGACCAGCTAGG
GATCGTCGCCTAGGTGAGCCGTTACCCCACCTACTAGCTAATCCCATCTGGGCACATCCGATGGCAAGAGGCC
CTAAGGTCCCCCTCTTTGGTCTGCGACGTATGCGGTATTAGCTACCGTTTCCAGTAGTTATCCCCCTCCATCG
GGCAGTTTCCCAGACATTACTCACCCGTCCGCCACTCGTCAGCAAAGCAGCAAGCTGCTTCCTGTTACCGTTC
GACTGCATGTGTTAGGCCTGCCGCCAGCGTCAATCTGAGCCAGGATCAAACTCT
>880
CTGCTGCCGCCCGTAGGAGTCTGGACCGTGTCTCAGTTCCAGTGTGGCTGGTCATCCTCTCAGACCAGCTAGG
GATCGTCGCCTAGGTGAGCCGTTACCCCACCTACTAGCTAATCCCATCTGGGCACATCCGATGGCAAGAGGCC
CTAAGGTCCCCCTCTTGTCTTGCGACGTATGCGGTATTAGCTACCGTTTCCAGTAGTTATCCCCCACCATCGG
GCAGTTTCCCAGACATTACTCACCCGTCCGCCACTCGTCAGCAAAGCAGCAAGCTGCTTCCTGTTACCGTTCG
ACTGCATGTGTTAGGCCTGCCGCCAGCGTCAATCTGAGCCAGGATCAACTCT
>881
CTGCTGCCGCCCGTAGGAGTCTGGACCGTGTCTCAGTTCCAGTGTGGCTGGTCATCCTCTCAGACCAGCTAGG
GATCGTCGCCTAGGTGAGCCGTTACCCCACCTACTAGCTAATCCCATCTGGGCACATCCGATGGCAAGAGGCC
CTAAGGTCCCCCTCTTTGTCTTGCGACGTTATGCGGTATTAGCTACCGTTTCCAGTAGTTATCCCCCTCCATC
AGGCAGTTTCCCAGACATTACTCACCCGTCCGCCACTCGTCAGCAAACAGCAAGCTGCTTCTGTTACCGTTCG
ACTGCATGTGTTAGGCCTGCCGCCAGCGTTCAATCTGAGCCAGGATCAAACTCT
>882
CTGCTGCCGCCCGTAGGAGTCTGGACCGTGTCTCAGTTCCAGTGTGGCTGGTCATCCACTCAGACCAGCTAGG
GATCGTCGCCTAGGTGAGCCGTTACCCCCACCTACTAGCTAATCCCATCTGGGCACATCCGATGGCAAGAGGC

CCTAAGGTCCCCCCTCTTTGTCTTGCGACGTTATGCGGTATTAGCTACCGTTTCCAGTAGTTATCCCCCTCCA
TCAGGCAGTTTCCCAGACATTACTCACCCGTCCGCCACTCGTTAGCAAAACAGCAAGCTGCTTTCCTGTTTAC
CGTTCGACTGCATGTGTTAGGCCTGCCGCCAGCGTTCAATCTGAGCCAGGATCAAACTCT
>883
CTGCTGCCGCCCGTAGGAGTCTGGACCGTGTCTCAGTTCCAGTGTGGCTGGTCATCCTCTCAGACCAGCTAGG
GATCGTCGCCTAGGTGAGCCGTTACCCCACCTACTAGCTAATCCCATCTGGGCACATCCGATGGCAAGAGGCC
CTAAGGTCCCCCTCTTTGGTCTTGCGACGTTATGCGGTATTAGCTACCGTTTCCAGTAGTTATCCCCCCTCCA
TCAGGCAGTTTTCCCAGACATTACTCACCCGTCCGCCACTCGTCAGCAAAGCAGCAAGCTGCTTCCTGTTACC
GTTCGACTGCATGTGTTAGGCCTGCCGCCAGCGTCAATCTGAGCCAGGATCAAACTCT
>884
TGCTGCCGCCCGTAGGAGTCTGGACCGTGTCTCAGTTCCAGTGTGGCTGGTCATCCTCTCAGACAGCTAGGGA
TCGTCGCCTAGGTGAGCCGTTACCCCACCTACTAGCTAATCCCATCTGGGCACATCCGATGGCAAGAGGCCCT
AAGGTCCCCCTCTTTGGTCTTGCGACGTTATGCGGTATTAGCTACCGTTTCCAGTAGTTATCCCCCTCCATCG
GGCAGTTTCCCAGACATTACTCACCCGTCCGCCACTCGTCAGCAAAGCAGCAAGCTGCTTCCTGTTACCGTTC
GACTGCATGTGTTAGGCCTGCCGCCAGCGTCAATCTGAGCCAGGATCAAACTCT
>885
CTGCTGCCGCCCGTAGGAGTCTGGACCGTGTCTCAGTTCCAGTGTGGCTGGTCATCCTCTCAGACCAGCTAGG
GATCGTCGCCTAGGTGAGCCGTTACCCCACCTACTAGCTAATCCCATCTGGGCACATCCGATGGCAAGAGGCC
CTAAGGTCCCCCTCTTGTCTTGCGACGTTATGCGGTATTAGCTACCGTTTCCAGTAGTTATCCCCCTCCATCA
GGCAGTTTCCCACGACATTACTCACCCGTCCGCCACTCGTCAGCAAAGCAGCAAGCTGCTTCTGTTACCGTTC
GACTGCATGTGTTAGGCCTGACCGCCAGCGTTCAATCGAGCCA
>886
CTGCTGCCGCCCGTAGGAGTCTGGACCGTGTCTCAGTTCCAGTGTGGCTGGTCATCCTCTCAGACCAGCTAGG
GATCGTCGCCTAGGTGAGCCGTTACCCCACCTACTAGCTAATCCCATCTGGGCACATCCGATGGCAAGAGGCC
CTAAGGTCCCCCTCTTGTCTTGCGACGTTATGCGGTATTAGCTACCGTTTCCAGTAGTTATCCCCCTCCATCG
GGCAGTTTCCCAGACATTACTCACCCGTCCGCCACTCGTCAGCAAAGCAGCAAGCTGCTTCCTGTTACCGTTC
GACTGCATGTGTTAGGCCTGCCGCCAGCGTCAATCTGAGCCAGGATCAAACTCT
>887
CTGCTGCCGCCCGTAGGAGTCTGGACCGTGTCTCAGTTCCAGTGTGGCTGGTCATCCTCTCAGACCAGCTAGG
GATCGTCGCCTAGGTGAGCCGTTACCCCACCTACTAGCTAATCCCATCTGGGCACATCCGATGGCAAGAGGCC
CTAAGGTCCCCCTCTTGTCTTGCGACGTTATGCGGTATTAGCTACCGTTTCCAGTAGTTATCCCCCTCCATCA
GGCAGTTTCCCAGACATTACTCACCCGTCCGCCACTCGTCAGCAAGCAGCAAGCTGCTTCTGTTACCGTTCGA
CTGCATGTGTTAGGCCTGCCGCCAGCGTTCAATCTGAGCCAGGATCAATCT
>888
CTGCTGCCGCCCGTAGGAGTCTGGACCGTGTCTCAGTTCCAGTGTGGCTGGTCATCCTCTCAGACCAGCTAGG
GATCGTCGCCTAGGTGAGCCGTTACCCCACTACTAGCTAATCCCATCTGGGCACATCCGATGGCAAGAGGCCC
TAAGGTCCCCCTCCTTGTCTGCGACGTATGCGGTATTAGCTACCGTTTCCAGTAGTTATCCCCTCCCATCGGG
CAGTTTCCCAGACATTACTCACCCGTCCGCCACTCGTCAGCAAAGCAGCAAGCTGCTTCCTGTTACCGTTCGA
CTGCATGTGTTAGGCCTGCCGCCAGCGTCAATCTGAGCCAGGATCAAACTCT
>889
CTGCTGCCGCCCGTAGGAGTCTGGACCGTGTCTCAGTTCCAGTGTGGCTGGTCATCCTCTCAGACCAGCTAGG
GATCGTCGCCTAGGTGAGCCGTTACCCCACCTACTAGCTAATCCCATCTGGGCACATCCGATGGCAAGAGGCC
CTAAGGTCCCCCTCTTTGGTCTTGCGACGTATGCGGTATTAGCTACCGTTTCCAGTAGTTATCCCCCTCCATC
AGGCAGTTTCCCAGACATTACTCACCCGTCCGCCACTCGTCAGCAAAGCAGCAAGCTGCTTCCTGTTACCGTC
GACTGCATGTGTTAGGCCTGCCGCCAGCGTCAATCTGAGCCAGGATCAAACTCT
>890
CTGCTGCCGCCCGTAGGAGTCTGGACCGTGTCTCAGTTTCCAGTGTGGCTGGTCATCCTCTCAGACCAGCTAG
GGATCGTCGCCTAGGTGAGCCGTTACCCCACCTACTAGCTAATCCCATCTGGGCACATCCGATGGCAAGAGGC
CCTAAGGTCCCCCTCTTGTCTGCGACGTTATGCGGTATTAGCTACCGTTTCCAGTAGTTATCCCCCTCCATCG
GGCAGTTTTCCCAGACATTACTCACCCGTCCGCCACTCGTCAGCAAAGCAGCAAGCTGCTTCCTGTTACCGTT
CGACTGCATGTGTTAGGCCTGCCGCCAGCGTCAATCTGAGCCAGGATCAAACTCT
>891
CTGCTGCCGCCCGTAGGAGTCTGGACCGTGTCTCAGTTCCAGTGTGGCTGGTCATCCTCTCAGACCAGCTAGG
GATCGTCGCCTAGGTGAGCCGTTACCCACCTACTAGCTAATCCCATCTGGGCACATCCGATGGCAAGAGGCCC

TAAGGTCCCCTCTTGGTCTTGCGACGTTATGCGGTATTAGCTACCGTTTCCAGTAGTTATCCCCTCCATCAGG
CAGTTTCCCAGACATTACTCACCCGTCCGCCACTCGTCAGCAAAGCAGCAAGCTGCTTCCTGTTACCGTTCGA
CTGCATGTGTTAGGCCTGCCGCCAGCGTTCAATCTGAGCCAGGATCAAACTCT
>892
CTGCTGCCGCCCGTAGGAGTCTGGACCGTGTCTCAGTTCCAGTGTGGCTGGTCATCCTCTCAGACCAGCTAGG
GATCGTCGCCTAGGTGAGCCGTTACCCCACCTACTAGCTAATCCCATCTGGGCACATCCGATGGCAAGAGGCC
CTAAGGTCCCCCTCTTGTCTTGCGACGTTATGCGGTATTAGCTACCGTTTCCAGTAGTTATCCCCTCCATCGG
GCAGTTTCCCAGACATTACTCACCCGTCCGCCACTCGTCAGCAAAGCAGCAAGCTGCTTCCTGTTACCGTTCG
ACTGCATGTGTTAGGCCTGCCGCCAGCGTTCAATCTGAGCCAGGATCAAACTCT
>893
CTGCTGCCGCCCGTAGGAGTCTGGACCGTGTCTCAGTTCCAGTGTGGCTGGTCATCCTCTCAGACCAGCTAGG
GATCGTCGCCTAGGTGAGCCGTTACCCCACCTACTAGCTAATCCCATCTGGGCACATCCGATGGCAAGAGGCC
CTAAGGTCCCCCTCTTGTCTTGCGACGTTATGCGGTATTAGCTACCGTTTCCAGTAGTTATCCCCCTCCATCA
GGCAGTTTCCCAGACATTACTCACCCGTCCGCCACTCGTCGGCAGAGCAGCAAGCTGCTTCCTGTTTACCGTT
CGACTTGCATGTGTTAGGCCTGCCGCCAGCGTTCAATCTGAGCCAGGATCAAACTCT
>894
CTGCTGCCGCCCGTAGGAGTCTGGACCGTGTCTCAGTTCCAGTGTGGCTGGTCATCCTCTCAGATCAGCTAGG
GATCGTCGCCTAGGTGAGCCGTTACCCCACCTACTAGCTAATCCCATCTGGGCACATCCCGATGGCAAGAGGC
CCTAAGGTCCCCCTCTTTGTCTTGCGACGTTATGCGGTATTAGCTACCATTCCAGTAGTTATCCCCCTCCATC
AGGCAGTTTCCCAGACATTACTCACCCGTCCGCCACTCGTCAGCAAAGCAGCAAGCTGCTTCTGTTACCGTTC
GACTGCATGTGTTAGGCCTGACCGCCAGCGTTCAATCTGAGCCAGGATCAAACTCT
>895
CTGCTGCCGCCCGTAGGAGTCTGGACCGTGTCTCAGTTCCAGTGTGGCTGGTCATCCTCTCAGACCAGCTAGG
GGATCGTCGCCTAGGTGAGCCGTTACCCCACCTATAGCTAATCCCATCTGGGCACATCCGATGGCAAGAGGCC
CTAAGGTCCCCCTCTTTGTCTTGCGACGTATGCGGTATTAGCTACCGTTTCCAGTAGTTTATCCCCCTCCATA
GGCAGTTTCCCAGACATTACTCACCCGTCCGCCACTCGTCAGCAAAACAGCAAGCTGCTTCCTGTTACCGTTC
GACTGCATGTGTTAGGCCTGCCGCCAGCGTCAATCTGAGCCAGGATCAAACTCT
>896
CTGCTGCCGCCCGTAGGAGTCTGGACCGTGTCTCAGTTCCAGTGTGGCTGGTCATCCTCTCAGACCAGCTAGG
GATCGTCGCCTAGGTGAGCCGTTACCCCACCTACTAGCTAATCCCATCTGGGCACATCCGATGGCAAGAGGCC
CTAAGGTCCCCCTCTTTGGTCTTGCGACGTTATGCGGTATTAGCTACCGTTTCCAGTAGTTATCCCCCTCCAT
CAGGCCGTTTTCCCAGACATTACTCACCCGTCCGCCACTCGTCAGCAAAGCAGCAAGCTGCTTCCTGTTACCG
TTCGACTGCATGTGTTAGGCCTGCCGCCAGCGTCAATCTGAGCCAGGATCAAACTCT
>897
CTGCTGCCGCCCGTAGGAGTCTGGACCGTGTCTCAGTTCCAGTGTGGCTGGTCATCCTCTCAGACCAGCTAGG
GATCGTCGCCTAGGTGAGCCGTTACCCCACCTACTAGCTAATCCCATCTGGGCACATCCGATGGCAAGAGGCC
CTAAGGTCCCCCTCTTTGTCTTGCGACGTTATGCGGTATTAGCTACCGTTTCCAGTAGTTATCCCCCTCCATC
GGGCAGTTTCCCAGACATTACTCACCCGTCCGCCACTCGTCAGCAAAGCAGCAAGCTGCTTCCTGTTACCGTT
CGACTGCATGTGTTAGGCCTGCCGCCAGCGTCAATCTGAGCCAGGATCAAACTCT
>898
CTGCTGCCGCCCGTAGGAGTCTGGACCGTGTCTCAGTTCCAGTGTGGCTGGTCATCCTCTCAGACCAGCTAGG
GATCGTCGCCTAGGTGAGCCGTTACCCCACCTACTAGCTAATCCCATCTGGGCACATCCGATGGCAAGAGGCC
CTAAGGTCCCCCTCTTGTCTTGCGACGTATGCGGTATTAGCTACCGTTTCCAGTAGTTATCCCCCTCCATCAG
GCAGTTTTCCCAGACATTACTCACCCGTCCGCCACTCGTCAGCAAAGCAGCAAGCTGCTTCCTGTTACCGTTC
GACTGCATGTGTTAGGCCTGCCGCCAGCGTCAATCTGAGCCAGGATCAAACTCT
>899
CTGCTGCCGCCCGTAGGAGTCTGGACCGTGTCTCAGTTCCAGTGTGGCTGGTCATCCTCTCAGACCAGCTAGG
GATCGTCGCCTAGGTGAGCCGTTACCCCACCTACTAGCTAATCCCATCTGGGCACATCCGATGGCAAGAGGCC
CTAAGGTCCCCCTCTTTGTCTTGCGACGTATGCGGTATTAGCTACCGTTTCCAGTAGTTATCCCCCCTCCATC
AGGCAGTTTTCCCAGACATTACTCACCCGTCCGCCACTCGTCAGCAAAGCAGCAAGCTGCTTCCTGTTACCGT
TCGACTGCATGTGTTAGGCCTGCCGCCAGCGTTCAATCTGAGCCAGGATCAAACTCT
>900
CTGCTGCCGCCCGTAGGAGTCTGGACCGTGTCTCAGTTCCAGTGTGGCTGGTCATCCTCTCAGACCAGCTAGG
GATCGTCGCCTAGGTGAGCCGTTACCCCACCTACTAGCTAATCCCATCTGGGCACATCCGATGGCAAGAGGCC

CTAAGGTCCCCCTCTTGTCTTGCGACGTATGCGGTATTAGCTACCGTTTCCAGTAGTTATCCCCCTCCATCGG
GCAGTTTCCCAGACATTACTCACCCGTCCGCCATTCGTCAGCAAAGCAGCAAGCTGCTTCCTGTTACCGTTCG
ACTGCATGTGTTAGGCCTGCCGCCAGCGTCAATCTGAGCCAGGATCAAACTCT
>901
CTGCTGCCGCCCGTAGGAGTCTGGACCGTGTCTCAGTTCCAGTGTGGCTGGTCATCCTCTCAGACCAGCTAGG
GATCGTCGCCTAGGTGAGCCGTTACCCCACCTACTAGCTAATCCCATCTGGGCACATCCGATGGCAAGAGGCC
CTAAGGTCCCCCTCTTTGGTCTTGCGACGTTATGCGGTATTAGCTACCGTTTCCAGTAGTTATCCCCCTCCAT
CAGGCAGTTTCCCAGACATTACTCACCCGTCCGCCACTCGTCAGCAAACAGCAAGCTGCTTCCTGTTACCGTT
CGACTGCATGTGTTAGGCCTGCCGCCAGCGTTCAATCTGGACCAGGATCAAACTCT
>902
CTGCTGCCGCCCGTAGGAGTCTGGACCGTGTCTCAGTTCCAGTGTGGCTGGTCATCCTCTCAGACCAGCTAGG
GATCGTCACCTAGGTGAGCCGTTACCCCACCTACTAGCTAATCCCATCTGGGCACATCCGATGGCAAGAGGCC
CTAAGGTCCCCCTCTTGTCTTGCGACGTATGCGGTATTAGCTACCGTTTCCAGTAGTTATCCCCCTCCATCGG
GCAGTTTCCCAGACATTACTCACCCGTCCGCCACTCGTCAGCAAAGCAGCAAGCTGCTTCCTGTTACCGTTCG
ACTGCATGCGTTAGGCCTGCCGCCAGCGTCAATCTGAGCCAGGATCAAACTCT
>903
CTGCTGCCGCCCGTAGGAGTCTGGACCGTGTCTCAGTTCCAGTGTGGCTGGTCATCCTCTCAGACCAGCTAGG
GATCGTCGCCTAGGTGAGCCGTTACCCCACCTACTAGCTAATCCCATCTGGGCACATCCGATGGCAAGAGGCC
CTAAGGTCCCCCTCTTGTCTTGCGACGTATGCGGTATTAGCTACCGTTTCCAGTAGTTATCCCCCTCCATCAG
GCAGTTTCCCAGACATTACTCACCCGTCCGCCACTCGTCAGCAAAGCAGCAAGCTGCTTTCTGTTACCGTTCG
ACTGCATGTGTTAGGCCTGCCGCCAGCGTTCAATCTGAGCCAGGACAACTCT
>904
CTGCTGCCGCCCGTAGGAGTCTGGACCGTGTCTCAGTTCCAGTGTGGCTGGTCATCCTCTCAGACCAGCTAGG
GATCGTCGCCTAGGTGAGCCGTTACCCCACCTACTAGCTAATCCCATCTGGGCACATCCGATGGCAAGAGGCC
CTAAGGTCCCCCTCTTTGGTCTTGCGACGTATGCGGTATTAGCTACCGTTTCCAGTAGTTATCCCCCTCCATC
GGGCAGTTTCCCAGACATTACTCACCCGTCCGCCACTCGTCAGCAAAGCAGCAAGCTGCTTCCTGTTACCGTT
CGACTGCATGTGTTAGGCCTGCCGCCAGCGTCAATCTGAGCCAAGATCAAACTCT
>905
TGCTGCCGCCCGTAGGAGTCTGGACCGTGTCTCAGTTCCAGTGTGGCTGGTCATCCTCTCAGACCAGCTAGGG
ATCGTCGCCTAGGTGAGCCGTTACCCCACCTACTAGCTAATCCCATCTGGGCACATCCGATGGCAAGAGGCCC
TAAGGTCCCCCTCTTTGTCTTGCGACGTATGCGGTATTAGCTACCGTTTCCAGTAGTTATCCCCCTCCATCAG
GCAGTTTCCCAGACATTACTCACCCGTCCGCCTCTCGTCAGCAAACAGCAAGCTGCTTCCTGTTACCGTTCGA
CTGCATGTGTTAGGCCTGCCGCCAGCGTCAATCTGAGCCAGGATCAAACTCT
>906
CTGCTGCCGCCCGTAGGAGTCTGGACCGTGTCTCAGTTCCAGTGTGGCTGGTCATCCTCTCAGACCAGCTAGG
GATCGTCGCCTAGGTGAGCCGTTACCCCACCTACTAGCTAATCCCATCTGGGCACATCCGATGGCAAGAGGCC
CTAAGGTCCCCCTCTTTGGTCTTGCGACGTTATGCGGTATTAGCTACCGTTTCCAGTAGTTATCCCCCTCCAT
CGGGCAGTTTCCCAGACATTACTCACCCGTCCGCCACTCGTCAGCAAAGCAGCAAGCTGCTTCCTGTTACCGT
TCGACTCGCATGTGTTAGGCCTGCCGCCAGCGTCAATCTGAGCCAGGATCAAACTCT
>907
CTGCTGCCGCCCGTAGGAGTCTGGACCGTGTCTCAGTTCCAGTGTGGCTGGTCATCCTCTCAGACCAGCTAGG
GATCGTCGCCTAGGTGAGCCGTTACCCCACCTACTAGCTAATCCCATCTGGGCACATCCGATGGCAAGAGGCC
CTAAGGTCCCCCTCTTTGTCTTGCGACGTTATGCGGTATTAGCTACCGTTTCCAGTAGTTATCCCCCTCCATC
AGGCAGTTTCCCAGACATTACTCACCCGTCCGCCACTCGTCAGCAAAGCAGCAAGCTGCTTCCTGTTACCGTT
CGACTGCATGTGTTAGGCCTGCCGCCAGCGTTCAATCTGAGCCAGGACAAACTCT
>908
CTGCTGCCGCCCGTAGGAGTCTGGACCGTGTCTCAGTTCCAGTGTGGCTGGTCATCCTCTCAGACCAGCTAGG
GATCGTCGCCTAGGTGAGCCGTTACCCCACCTACTAGCTAATCCCATCTGGGCACATCCGATGGCAAGAGGCC
CTAAGGTCCCCCTCTTGTCTTGCGACGTTATGCGGTATTAGCTACCGTTTCCAGTAGTTATCCCCCTCCATCA
GGCAGTTTCCCAGACATTACTCACCCGTCCGCCACTCGTCAGCAAAGCAGCAAGCTGCTTCTGTTACCGTTCG
ACTGCATGTGTTAGGCCTGCCGCCAGCGTTCAATCTGAGCCAGGATCAAACTCT
>909
CTGCTGCCGCCCGTAGGAGTCTGGACCGTGTCTCAGTTCCAGTGTGGCTGGTCATCCTCTCAGACCAGCTAGG
GATCGTCGCCTAGGTGAGCCGTTACCCCACCTACTAGCTAATCCCATCTGGGCACATCCGATGGCAAGAGGCC

CTAAGGTCCCCCTCTTTGTCTTGCGACGTTATGCGGTATTAGCTACCGTTTCCAGTAGTTATCCCCCTCCATC
AGGCAGTTTTCCCAGACATTACTCACCCGTCCGCCACTCGTCAGCAAAGCAGCAAGCTGCTTCTGTTACCGTT
CGACTGCATGTGTTAGGCCTGCCGCCAGCGTCAATCTGAGCCAGGATCAAACTCT
>910
CTGCTGCCGCCCGTAGGAGTCTGGACCGTGTCTCAGTTCCAGTGTGGCTGGTCATCCTCTCAGACCAGCTAGG
GATCGTCGCCTAGGTGAGCCGTTACCCCACCTACTAGCTAATCCCATCTGGGCACATCCGATGGCAAGAGGCC
CTAAGGTCCCCCTCTTTGGTCTTGCGACGTATGCGGTATTAGCTACCGTTTCCAGTAGTTATCCCCCTCCATC
AGGCAGTTTCCCAGACATTACTCACCCGTCCGCCACTCGTCAGCAAAGCAGCAAGCTGCTTCCTGTTACCGTT
CGACTGCATGTGTTAGGCCTGCCGCCAGCGTTCAATCTGAGCCAGGATCAAACTCT
>911
CTGCTGCCGCCCGTAGGAGTCTGGACCGTGTCTCAGTTCCAGTGTGGCTGGTCATCCTCTCAGACCAGCTAGG
GATCGTCGCCTAGGTGAGCCGTTACCCCACCTACTAGCTAATCCCATCTGGGCACATCCGATGGCAAGAGGCC
CTAAGGTCCCCCTCTTGTCTTGCGACGTTATGCGGTATTAGCTACCGTTTCCAGTAGTTATCCCCCTCCATCG
GGCAGTTTCCCAGACATTACTCACCCGTCCGCCACTCGTCAGCAAAGCAGCAAGCTGCTTCCTGTTACCGTTC
GACTGCATGTGTTAGGCCTGCCGCCAGCGTCAATCTGAGCCAGGATCAACTCT
>912
CTGCTGCCGCCCGTAGGAGTCTGGACCGTGTCTCAGTTCCAGTGTGGCTGGTCATCCTCTCAGACCAGCTAGG
GATCGTCGCCTAGGTGAGCCGTTACCCCACCTACTAGCTAATCCCATCTGGGCACATCCGATGGCAAGAGGCC
CTAAGGTCCCCCTCTTTGGTCTTGCGACGTTATGCGGTATTAGCTACCGTTTCCAGTAGTTATCCCCCTCCAT
CAGGCAGTTTCCCAGACATTACTCACCCGTCCGCCACTCGTCAGCAAAGCAGCAAGCTGCTTCCTGTTACCGT
TCGACTGCATGTGTTAGGCCTGCCGCCAGCGTCAATCTGAGCCAGGATCAAACTCT
>913
CTGCTGCCGCCCGTAGGAGTCTGGACCGTGTCTCAGTTCCAGTGTGGCTGGTCATCCTCTCAGACCAGCTAGG
GATCGTCGCCTAGGTGAGCCGTTACCCCACCTACTAGCTAATCCCATCTGGGCACATCCGATGGCAAGAGGCC
CTAAGGTCCCCCTCTTTGGTCTTGCGACGTTATGCGGTATTAGCTACCGTTTCCAGTAGTTATCCCCCTCCAT
CAGGCAGTTTCCCAGACATTACTCACCCGTCCGCCACTCGTCAGCAAAGCAGCAAGCTGCTTCTGTTACCGTT
CGACTGCATGTGTTAGGCCTGCCGCCGAGCA
>914
CTGCTGCCGCCCGTAGGAGTCTGGACCGTGTCTCAGTTCCAGTGTGGCTGGTCATCCTCTCAGACCAGCTAGG
GATCGTCGCCTAGGTGAGCCGTTACCCCACCTACTAGCTAATCCCATCTGGGCACATCCGATGGCAAGAGGCC
CTAAGGTCCCCCTCTTTGGTCTGCGACGTTATGCGGTATTAGCTACCGTTTCCAGTAGTTATCCCCCTCCATC
AGGCAGTTTTCCCCAGACATTACTCACCCGTCCGCCACTCGTCAGCAAAACAGCAAGCTGCTTCCTGTTACCG
TTCGACTGCATGTGTTAGGCCTGCCGCCAGCGTTCAATCTGAGCCAGGATCAAACTCT
>915
CTGCTGCCGCCCGTAGGAGTCTGGACCGTGTCTCAGTTCCAGTGTGGCTGGTCATCCTCTCAGACCAGCTAGG
GATCGTCGCCTAGGTGAGCCGTTACCCCACCTACTAGCTAATCCCATCTGGGCACATCCGATGGCAAGAGGCC
CTAAGGTCCCCCTCTTTGTCTTGCGACGTATGCGGTATTAGCTACCGTTTCCAGTAGTTATCCCCCTCCATCA
GGCAGTTTTCCCAGACATTACTCACCCGTCCGCCACTCGTCAGCAAAACAGCAAGCTGCTTCCTGTTACCGTT
CGACTGCATGTGTTAGGCCTGCCGCCAGCGTTCAATCTGAGCCAGGATCAAACTCT
>916
CTGCTGCCGCCCGTAGGAGTCTGGACCGTGTCTCAGTTCCAGTGTGGCTGGTCATCCTCTCAGACCAGCTAGG
GATCGTCGCCTAGGTGAGCCGTTACCCCACCTACTAGCTAATCCCATCTGGGCACATCCGATGGCAAGAGGCC
CTAAGGTCCCCCCTCTTTGGTCTTGCGACGTTATGCGGTATTAGCTACCGTTTCCAGTAGTTATCCCCCTCCA
TCAGGCAGTTTCCCAGACATTACTCACCCGTCCGCCACTCGTCAGCAAAGCAGCAAGCTGCTTCCTGTTACCG
TTCGACTGCATGTGTTAGGCCTGCCGCCAGCGTTCAATCTGAGCCAGGATCAAACTCT
>917
CTGCTGCCGCCCGTAGGAGTCTGGACCGTGTCTCAGTTCCAGTGTGGCTGGTCATCCTCTCAGACCAGCTAGG
GATCGTCGCCTAGGTGAGCCGTTACCCCACCTACTAGCTAATCCCATCTGGGCACATCCGATGGCAAGAGGCC
CTAAGGTCCCCCTCTTTGGTCTTGCGACGTTATGCGGTATTAGCTACCGTTTCCAGTAGTTATCCCCCTCCAT
CAGGCAGTTTCCCCAGACATTACTCACCCGTCCGCCACTCGTCAGCAAAGCAGCAAGCTGCTTCCTGTTACCG
TTCGACTGCATGTGTTAGGCCTGCCGCCAGCGTTCAATCTGAGCCAGGATCAAACTCT
>918
CTGCTGCCGCCCGTAGGAGTCTGGACCGTGTCTCAGTTCCAGTGTGACTGGTCATCCTCTCAGACCAGCTAGG
GATCGTCGCCTAGGTGAGCCGGTACCCCACCTACTAGCTAATCCCATCTGGGCACATCCGATGGCAAGAGGCC

CTAAGGTCCCCCTCTTTGTCTTGCGACGTATGCGGTATTAGCTACCGTTTCCAGTAGTTATCCCCCTCCATCA
GGCAGTTTCCCAGACATTACTCACCCGTCCGCCACTCGTCAGCAAAGCAGCAAGCTGCTTCCTGTTACCGTTC
GACTGCATGTGTTAGGCTGCCGCCAGCGTCAACTGAGCCAGGATCAAACTCT
>919
CTGCTGCCGCCCGTAGGAGTCTGGACCGTGTCTCAGTTCCAGTGTGGCTGGTCATCCTCTCAGACCAGCTAGG
GATCGTCGCCTAGGTGAGCCGTTACCCCACCTACTAGCTAATCCCATCTGGGCACATCCGATGGCAAGAGGCC
CTAAGGTCCCCCTCTTTGGTCTTGCGACGTTATGCGGTATTAGCTACCGTTTCCAGTAGTTATCCCCCTCCAT
CGGGCAGTTTCCCAGACATTACTCACCCGTCCGCCACTCGTCAGCAAAGCAGCAAGCTGCTTCCTGTTACCGT
TCGACTGCATGTGTTAGGCCTGCCGCCAGCGTCAATCTGAGCCAGGATCAAACTCT
>920
CTGCTGCCGCCCGTAGGAGTCTGGACCGTGTCTCAGTTCCAGTGTGGCTGGTCATCCTCTCAGACAGCTAGGG
ATCGTCGCCTAGGTGAGCCGTTACCCCACCTACTAGCTAATCCCATCTGGGCACATCCGATGGCAAGAGGCCC
TAAGGTCCCCCTCTTTGTCTTGCGACGTTATGCGGTATTAGCTACCGTTTCCAGTAGTTATCCCCCTCCATCA
GGCAGTTTCCCAGACATTACTCACCCGTCTGCCACTCGTCAGCAAAACAGCAAGCTGCTTCCTGTTACCGTTC
GACTGCATGTGTTAGGCCTGCCGCCAGCGTCAATCGAGCCAGGATCAA
>921
CTGCTGCCGCCCGTAGGAGTCTGGACCGTGTCTCAGTTCCAGTGTGGCTGGTCATCCTCTCAGACCAGCTAGG
GATCGTCGCCTAGGTGAGCCGTTACCCCACCTACTAGCTAATCCCATCTGGGCACATCCGATGGCAAGAGGCC
CTAAGGTCCCCCTCTTTGTCTTGCGACGTTATGCGGTATTAGCTACCGTTTCCAGTAGTTATCCCCCTCCATC
AGGCAGTTTCCCAGACATTACTCACCCGTCCGCCACTCGTCAGCAAAGCAGCAAGCTGCTTCTGTTACCGTTC
GACTGCATGTGTTAGGCCTGCCGCCAGCGTCAATCTGAAGCCAGGATTCAAACCTCT
>922
CTGCTGCCGCCCGTAGGAGTCTGGACCGTGTCTCAGTTCCAGTGTGGCTGGTCATCCTCTCAGACCAGCTAGG
GATCGTCGCCTAGGTGAGCCGTTACCCCACCTACTAGCTAATCCCATCTGGGCACATCCGATGGCAGGAGGCC
CTAAGGTCCCCCTCTTTGGTCTTGCGACGTTATGCGGTATTAGCTACCGTTTCCAGTAGTTATCCCCCTCCAT
CAGGCAGTTTCCCAGACATTACTCACCCGTCCGCCACTCGTCAGCAAACAGCAAGCTGCTTCCTGTTACCGTT
CGACTGCATGTGTTAGGCCTGCCGCCAGCGTCAATCTGAGCCAGGATCAAACTCT
>923
CTGCTGCCGCCCGTAGGAGTCTGGACCGTGTCTCAGTTCCAGTGTGGCTGGTCATCCTCTCAGACCAGCTAGG
GATCGTCGCCTAGGTGAGCCGTTACCCCACCTACTAGCTAATCCCATCTGGGCACATCCGATGGCAAGAGGCC
CTAAGGTCCCCCTCTTGTCTTGCGACGTTATGCGGTATTAGCTACCGTTTCCAGTAGTTATCCCCCTCCATCA
GGCAGTTTCCCAGACATTACTCACCCGTCCGCCACTCGTCAGCAAAGCAGCAAGCTGCTTTCTGTTACCGTTC
GACTGCATGTGTTAGGCC
>924
CTGCTGCCGCCCGTAGGAGTCTGGACCGTGTCTCAGTTCCAGTGTGGCTGGTCATCCTCTCAGACCAGCTAGG
GATCGTCGCCTAGGTGAGCCGTTACCCCACCTACTAGCTAATCCCATCTGGGCACATCCGATGGCAAGGGGCC
CTAAGGTCCCCCTCTTTGTCTTGCGACGTTATGCGGTATTAGCTACCGTTTCCAGTAGTTATCCCCTCCATCG
GGCAGTTCCCAGACATTACTCACCCGTCCGCCACTCGTCAGCAAAGCAGCAAGCTGCTTCCTGTTACCGTTCG
ACTGCAATGTGTTAGGCCTGCCGCCAGCGTCAATCTGAGCCAGGATAACTCT
>925
CTGCTGCCGCCCGTAGGAGTCTGGACCGTGTCTCAGTTCCAGTGTGGCTGGTCATCCTCTCAGACCAGCTAGG
GATCGTCGCCTAGGTGAGCCGTTACCCCACCTACTAGCTAATCCCATCTGGGCACATCCGATGGCAAGAGGCC
CTAAGGTCCCCCTCTTGTCTTGCGACGTTATGCGGTATTAGCTACCGTTCCAGTAGTTATCCCCCTCCATCAG
GCAGTTTCCCAGACATTGCTCACCCGTCCGCCACTCGTCAGCAAAACAGCAAGCTGCTTCCTGTTACCGTTCG
ACTGCATGTGTAGGCCTGACCGCCAGCGTTCAATCGAGCCAGGATCAAACTCT
>926
CTGCTGCCGCCCGTAGGAGTCTGGACCGTGTCTCAGTTTCCAGTGTGGCTGGTCATCCTCTCAGACCAGCTAG
GGATCGTCGCCTAGGTGAGCCGTTACCCCACCTACTAGCTAATCCCATCTGGGCACATCCGATGGCAAGAGGC
CCTAAGGTCCCCCTCTTTGTCTTGCGACGTTATGCGGTATTAGCTACCGTTTCCAGTAGTTATCCCCCTCCAT
CAGGCAGTTTTCCCAGACATTACTCACCCGTCCGCCACTCGTCAGCAAAGCAGCAAGCTGCTTCTGTTACCGT
TCGACTGCATGTGTTAGGCCTGCCGCCAGCGTTCAATCTGAGCCAGGATCAAACTCT
>927
CTGCTGCCGCCCGTAGGAGTCTGGACCGTGTCTCAGTTCCAGTGTGGCTGGTCATCCTCTCAGACCAGCTAGG
GATCGTCGCCTAGGTGAGCCGTTACCCCACCTACTAGCTAATCCCATCTGGGCACATCCGATGGCAAGAGGCC

CTAAGGTCCCCCCTCTTTGGTCTTGCGACGTTATGCGGTATTAGCTACCGTTTCCAGTAGTTATCCCCCTCCA
TCGGGCAGTTTTCCCAGACATTACTCACCCGTCCGCCACTCGTCAGCAAAGCAGCAAGCTGCTTCCTGTTACC
GTTCGACTGCATGTGTTAGGCCTGCCGCCAGCGTCAATCTGAGCCAGGATCAAACTCT
>928
CTGCTGCCGCCCGTAGGAGTCTGGACCGTGTCTCAGTTCCAGTGTGGCTGGTCATCCTCTCAGACCAGCTAGG
GATCGTCGCCTAGGTGAGCCGTTACCCCACCTACTAGCTAATCCCATCTGGGCACATCCGATGGCAAGAGGCC
CTAAGGTCCCCCTCTTTGTCTTGCGACGTTATGCGGTATTAGCTACCGTTTCCAGTAGTTATCCCCCTCCATC
AGGCAGTTTCCCAGACATTACTCACCCGTCCGCCACTCGTCAGCAAACAGCAAGCTGCTTCCTGTTACCGTTC
GACTGCATGTGTTAGGCCTGCCGCCAGCGTTCAATCTGAGCCAGGATCAAACTCT
>929
CTGCTGCCGCCCGTAGGAGTCTGGACCGTGTCTCAGTTCCAGTGTGGCTGGTCATCCTCTCAGACCAGCTAGG
GATCGTCGCCTAGGTGAGCCGTTACCCCACCTACTAGCTAATCCCATCTGGGCACATCCGATGGCAAGAGGCC
CTAAGGTCCCCCTCTTGTCTTGCGACGTATGCGGTATTAGCTACCGTTCCAGTAGTTATCCCCTCCATCGGGC
AGTTTCCCAGACATTACTCACCCGTCCGCCACTCGTCAGCAAAGCAGCAAGCTGCTTCCTGTTACCGTTCGAC
TGCATGTGTTAGGCCTGCCGCCAGCGTCAATCTGAGCCAGGATCAAACTCT
>930
CTGCTGCCGCCCGTAGGAGTCTGGACCGTGTCTCAGTTCCAGTGTGGCTGGTCATCCTCTCAGACCAGCTAGG
GATCGTCGCCTAGGTGAGCCGTTACCCCACCTACTAGCTAATCCCATCTGGGCACATCCGATGGCAAGAGGCC
CTAAGGTCCCCCTCTTTGTCTTGCGACGTATGCGGTATTAGCTACCGTTTCCAGTAGTTATCCCCTCCATCAG
GCAGTTTTCCCAGACATTACTCACCCGTCCGCCACTCGTCAGCAAACAGCAAGCTGCTTCCTGTTACCGTTCG
ACTGCATGTGTTAGGCCTGCCGCCAGCGTCAATCTGAGCCAGGATCAAACTCT
>931
CTGCTGCCGCCCGTAGGAGTCTGGACCGTGTCTCAGTTCCAGTGTGGCTGGTCATCCTCTCAGACCAGCTAGG
GATCGTCGCCTAGGTGAGCCGTTACCCCACCTACTAGCTAATCCCATCTGGGCACATCCGATGGCAAGAGGCC
CTAAGGTCCCCCTCTTTGTCTTGCGACGTTATGCGGTATTAGCTACCGTTTCCAGTAGTTATCCCCCTCCATC
AGGCAGTTTCCCAGACATTACTCACCCGTCCGCCACTCGTCAGCAAAGCAGCAAGCTGCTTCCTGTTACCGTT
CGACTGCATGTGTTAGGCCTGCCGCCAGCGTCAATCTGAGCCAGGATCAAACTCT
>932
CTGCTGCCGCCCGTAGGAGTCTGGACCGTGTCTCAGTTCCAGTGTGGCTGGTCATCCTCTCAGACCAGCTAGG
GATCGTCGCCTAGGTGAGCCGTTACCCCACCTACTAGCTAATCCCATCTGGGCACATCCGATGGCAAGAGGCC
CTAAGGTCCCCCTCTTGTCTTGCGACGTATGCGGTATTAGCTACCGTTTCCAGTAGTTATCCCCCTCCATCGG
GCAGTTTCCCAGACATTACTCACCCGTCCGCCACTCGTCAGCAAAGCAGCAAGCTGCTTCCTGTTACCGTTCG
ACTGCATGTGTTAGGCCTGCCGCCAGCGTCAATCTGAGCCAGGATCAAACTCT
>933
CTGCTGCCGCCCGTAGGAGTCTGGACCGTGTCTCAGTTCCAGTGTGGCTGGTCATCCTCTCAGACCAGCTAGG
GATCGTCGCCTAGGTGAGCCGTTACCCCACCTACTAGCTAATCCCATCTGGGCACATCCGATGGCAAGAGGCC
CTAAGGTCCCCCTCTTTGTCTTGCGACGTATGCGGTATTAGCTACCGTTTCCAGTAGTTATCCCCCTCCATCA
GGCAGTTTCCCAGACATTACTCACCCGTCCGCCACTCGTCAGCAAAGCAGCAAGCTGCTTCCTGTTACCGTTC
GACTGCATGTGTTAGGCCTGCCGCCAGCGTTCAATCTGAGCCAGGACAAACTCT
>934
CTGCTGCCGCCCGTAGGAGTCTGGACCGTGTCTCAGTTCCAGTGTGGCTGGTCATCCTCTCAGACCAGCTAGG
GATCGTCGCCTAGGTGAGCCGTTACCCCACCTACTAGCTAATCCCATCTGGGCACATCCGATGGCAAGAGGCC
CTAAGGTCCCCCTCTTTGTCTTGCGACGTTATGCGGTATTAGCTACCGTTTCCAGTAGTTATCCCCCTCCATC
GGGCAGTTTTCCCAGACATTACTCACCCGTCCGCCACTCGTCAGCAAAGCAGCAAGCTGCTTCCTGTTACCGT
TCGACTGCATGTGTTAGGCCTGCCGCCAGCGTCAATCTGAGCCAGGATCAAACTCT
>935
CTGCTGCCGCCCGTAGGAGTCTGGACCGTGTCTCAGTTCCAGTGTGGCTGGTCATCCTCTCAGACCAGCTAGG
GACCGTCGCCTAGGTGGGCCGTTACCCCACCTACTAGCTAATCCCATCTGGGCACATCCGATGGCAAGAGGCC
CTAAGGTCCCCCTCTTTGGTCTTGCGACGTTATGCGGTATTAGCTACCGTTTCCAGTAGTTATCCCCCCTCCAT
CGGGCAGTTTCCCAGACATTACTCACCCGTCCGCCACTCGTCAGCAAAGCAGCAAGCTGCTTCCTGTTACCGT
CCGACTGCATGTGTTAGGCCTGCCGCCAGCGTCAATCTGAGCCAGGATCAAACTCT
>936
CTGCCGCCGCCCGTAGGAGTCTGGACCGTGTCTCAGTTCCAGTGTGGCTGGTCATCCTCTCAGACCAGCTAGG
GATCGTCGCCTAGGTGAGCCGTTACCCCACCTACTAGCTAATCCCATCTGGGCACATCCGATGGCAAGAGGCC

CTAAGGTCCCCCTCTTTGTCTTGCGACGTATGCGGTATTAGCTACCGTTTCCAGTAGTTATCCCCCTCCATCA
GGCAGTTTCCCAGACATTACTCACCCGTCCGCCACTCGTCAGCAAAGCAGCAAGCTGCTTCTGTTACCGTTCG
ACTGCATGTGTTAGGCCTGCCGCCAGCGTTCAATCTGAGCCAGGACAAACTCT
>937
CTGCTGCCGCCCGTAGGAGTCTGGACCGTGTCTCAGTTCCAGTGTGGCTGGTCATCCTCTCAGACCAGCTAGG
GATCGTCGCCTAGGTGAGCCGTTACCCCACCTACTAGCTAATCCCATCTGGGCACATCCGATGGCAAGAGGCC
CTAAGGTCCCCCTCTTTGTCTGCGACGTTATGCGGTATTAGCTACCGTTTCCAGTAGTTATCCCCTCCATCAG
GCAGTTTTCCCACGACATTACTCACCGTCCGCCACTCGTCAGCAAACAGCAAGCTGCTTCCTGTTACCGTTCG
AACTGCATGTCGTTAGGCCTGCCGCCAGCGTTCGAACTCGAGCCAGG
>938
CTGCTGCCGCCCGTAGGAGTCTGGACCGTGTCTCAGTTCCAGTGTGGCTGGTCATCCTCTCAGACCAGCTAGG
GATCGTCGCCTAGGTGAGCCGTTACCCCACCTACTAGCTAATCCCATCTGGGCACATCCGATGGCAAGAGGCC
CTAAGGTCCCCCTCTTTGTCTGCGACGTTATGCGGTATTAGCTACCGTTTCCAGTAGTTATTCCCCTCCAACA
GGCAGTTTCCCAGACATTACTCACCCGTCCGCCACTCGTCAGCAAACAGCAAGCTGCTTCCTGTTACCGTTCG
ACTGCATGTGTTGGGCCTGCCGCCAGCGTCAATCTGAGCCAGGATCAAACTCT
>939
CTGCTGCCGCCCGTAGGAGTCTGGACCGTGTCTCAGTTCCAGTGTGGCTGGTCATCCTCTCAGACCAGCTAGG
GATCGTCGCCTAGGTGAGCCGTTACCCCACCTACTAGCTAATCCCATCTGGGCACATCCGATGGCAAGAGGCC
CTAAGGTCCCCCTCTTGTCTTGCGACGTTATGCGGTATTAGCTACCGTTTCCAGTAGTTATCCCCCTCCATCG
GGCAGTTTCCCAGACATTACTCACCCGTCCGCCACTCGTCAGCAAAGCAGCAAGCTGCTTCCTGTTACCGTTC
GACTGCATGTGTTAGCTGCCGCCAGCGTCAATCTGAGCCAGGATCAAACTCT
>940
CTGCTGCCGCCCGTAGGAGTCTGGACCGTGTCTCAGTTCCAGTGTGGCTGGTCATCCTCTCAGACCAGCTAGG
GATCGTCGCCTAGGTGAGCCGTTACCCCACCTACTAGCTAATCCCATCTGGGCACATCCGATGGCAAGAGGCC
CTAAGGTCCCCCTCTTGTCTTGCGACGTTATGCGGTATTAGCTACCGTTTCCAGTAGTTATCCCCCTCCATCA
GGCAGTTTCCCAGACATTACTCACCCGTCCGCCACTCGTCAGCAAAGCAGCAAGCTGCTTCCTGTTACCGTCG
ACTGCATGTGTTAGGCCTGCCGCCAGCGTTCAATCTGAGCCAGGATCAAACTCT
>941
CTGCTGCCGCCCGTAGGAGTCTGGACCGTGTCTCAGTTCCAGTGTGGCTGGTCATCCTCTCAGACCAGCTAGG
GATCGTCGCCTAGGTGAGCCGTTACCCCACCTACTAGCTAATCCCATCTGGGCACATCCGATGGCAAGAGGCC
CTAAGGTCCCCCTCTTTGTCTGCGACGTTATGCGGTATTAGCTACCGTTCCAGTAGTTATCCCCCTCCATCAG
GCAGTTTCCCAGACATTACTCACCCGTCCGCCACTCGTCAGCAAACAGCAAGCTGCTTCCTGTTACCGTTCGA
CTGCATGTGTTAGGCCTGCCGCCAGCGTCAACCTGAGCCAGGATCAAACTCT
>942
CTGCTGCCGCCCGTAGGAGTCTGGACCGTGTCTCAGTTCCAGTGTGGCTGGTCATCCTCTCAGACCAGCTAGG
GATCGTCGCCTAGGTGAGCCGTTACCCCACCTACTAGCTAATCCCATCTGGGCACATCCGATGGCAAGAGGCC
CTAAGGTCCCCCTCTTGGTCTTGCGACGTTATGCGGTATTAGCTACCGTTTCCAGTAGTTATCCCCCTCCATC
AGGCAGTTTCCCAGACATTACTCACCCGTCCGCCACTCGTCAGCAAAGCAGCAAGCTGCTTCCTGTTACCGTT
CGACTGCATGTGTTAGGCCTGCCGCCAGCGTCAATCTGAGCCAGGATCAAACTCT
>943
CTGCTGCCGCCCGTAGGAGTCTGGACCGTGTCTCAGTTCCAGTGTGGCTGGTCATCCTCTCAGACCAGCTAGG
GATCGTCGCCTAGGTGAGCCGTTACCCCACCTACTAGCTAATCCCATCTGGGCACATCCGATGGCAAGAGGCC
CTAAGGTCCCCCTCTTTGGTCTTGCGACGTTATGCGGTATTAGCTACCGTTTCCAGTAGTTATCCCCCTCATC
GGGCAGTTTCCCAGACATTACTCACCCGTCCGCCACTCGTCAGCAAAGCAGCAAGCTGCTTCCTGTTACCGTT
CGACTTGCATGTGTTAGGCCTGCCGCCAGCGTCAATCTGAGCCAGGATCAAACTCT
>944
CTGCTGCCGCCCGTAGGAGTCTGGACCGTGTCTCAGTTCCAGTGTGGCTGGTCATCCTCTCAGACCAGCTAGG
GATCGTCGCCTAGGTGAGCCGTTACCCCACCTACTAGCTAATCCCATCTGGGCACATCCGATGGCAAGAGGCC
CTAAGGTCCCCCTCTTTGGTCTTGCGACGTTATGCGGTATTAGCTACCGTTTCCAGTAGTTATCCCCCTCCAT
CGGGCAGTTTCCCAGACATTACTCACCCGTCCGCCACTCGTCAGCAAAGCAGCAAGCTGCTTCCTGTTACCGT
TCGACTGCATGTGTTAGGCCTGCCGCCAGCGTCAATCTGAGCCAGGATCAAACTCT
>945
CTGCTGCCGCCCGTAGGAGTCTGGACCGTGTCTCAGTTCCAGTGTGGCTGGTCATCCTCTCAGACCAGCTAGG
GATCGTCGCCTAGGTGAGCCGTTACCCCACCTACTAGCTAATCCCATCTGGGCACATCCGATGGCAAGAGGCC

CTAAGGTCCCCTCTTTAGTCTTGCGACGTATGCGGTATTAGCTACCGTTTCCAGTAGTTATCCCCCTCCATCA
GGCAGTTTCCCAGACATTACTCACCCGTCCGCCACTCGTCAGCAAAGCAGCAAGCTGCTTCCTGTTACCGTTC
GACTGCATGTGTTAGGCCTGCCGCCAGCGTTCAATCGAGCCAGGATCA
>946
CTGCTGCCGCCCGTAGGAGTCTGGACCGTGTCTCAGTTCCAGTGTGGCTGGTCATCCTCTCAGACCAGCTAGG
GATCGTCGCCTAGGTGAGCCGTTACCCCACTTACTAGCTAATCCCATCTGGGCACATCCGATGGCAAGAGGCC
CTAAGGTCCCCCTCTTTGTCTTGCGACGTTATGCGGTATTAGCTACCGTTTCCAGTAGTTATCCCCCTCCATC
AGGCAGTTTCCCAGACATTACTCACCCGTCCGCCACTCGTCAGCAAAACGCAAGCTGCTTCCTGTTACCGTTC
GACTGCATGTGTTAGGCCTGCCGCCAGCGTTCAATCTGAGCCAGGATCAAACTCT
>947
CTGCTGCCGCCCGTAGGAGTCTGGACCGTGTCTCAGTTCCAGTGTGGCTGGTCATCCTCTCAGACCAGCTAGG
GATCGTCGCCTAGGTGAGCCGTTACCCCACCTACTAGCTAATCCCATCTGGGCACATCCGATGGCAAGAGGCC
CTAAGGTCCCCCTCTTGGTCTCGCGACGTATGCGGTATTAGCTACCGTTTCCAGTAGTTATCCCCCTCCATCA
GGCAGTTTCCCAGACATTACTCACCCGTCCGCCACTCGTCAGCAAAGCAGCAAGCTGCTTCCTGTTACCGTTC
GACTGCATGTGTTAGGCCTGCCGCCAGCGTTCAATCTGAGCCAGGACAAACTCT
>948
CTGCTGCCGCCCGTAGGAGTCTGGACCGTGTCTCAGTTCCAGTGTGGCTGGTCATCCTCTCAGACCAGCTAGG
GATCGTCGCCTAGGTGAGCCGTTACCCCACCTACTAGCTAATCCCATCTGGGCACATCCGATGGCAAGAGGCC
CTAAGGTCCCCCTCTTGTCTGCGACGTATGCGGTATTAGCTACCGTTTCCAGTAGTTATCCCCCTCCATCGGG
CAGTTTCCCAGACATTACTCACCCGTCCGCCACTCGTCAGCAAGGCAGCAAGCTGCTTCCTGTTACCGTTCGA
CTGCATGTGTTAGGCCTGCCGCCAGCGTCAATCTGAGCCAGGATCAAACTCT
>949
CTGCTGCCGCCCGTAGGAGTCTGGACCGTGTCTCAGTTCCGAGTGTGGCTGGTCATCCTCTCAGACCAGCTAG
GGATCGTCGCCTAGAGTAGCCGTTACCCCACCTACTAGCTAATCCCATCTGGGCACATCCGATGGCAAGAGGC
CCTAAGGTCCCCCTCTTGTCTTGCGACGTATGCGGTATTAGCTACTTCGTTCAGTAGTTATCCCCCTCCATCA
GGCAGTTTCCCAGACATTACTCACCCGTCCGCCACTCGTCGCAAAGCAGCAAGCTGCTTCTGTTACCGTTCGA
CTGCATGTGTTAGGCCTGCCGCCAGCGTCAATCTGAAGGCCAGGATCAAACGTCT
>950
CTGCTGTCGCCCGTAGGAGTCTGGACCGTGTCTCAGTTCCAGTGTGGCTGGTCATCCTCTCAGACCAGCTAGG
GATCGTCGCCTAGGTGAGCCGTTACCCCACCTACTAGCTAATCCCATCTGGGCACATCCGATGGCAAGAGGCC
CTAAGGTCCCCCTCTTGTCTGCGACGTATGCGGTATTAGCTACCGTTTCCAGTAGTTATCCCCCTCCATCAGG
CAGTTTCCCAGACATTACTCACCCGTCCGCCACTCGTCAGCAAACAGCAAGCTGCTTCCTGTTACCGTTCGAC
TGCATGTGTTAGGCCTGCCGCCAGCGTCAATCTGAGCCAGGATCAAACCTCT
>951
TGCTGCCCCCGTAGGAGTCTGGACCGTGTCTCAGTTCCAGTGTGGCTGGTCATCCTCTCAGACAGCTAGGGAT
CGTCGCCTAGGTGAGCCGTTACCCCACCTACTAGCTAATCCCATCTGGGCACATCCGATGGCAAGAGGCCCTA
AGGTCCCCCTCTTGTCTGCGACGTTATGCGGTATTAGCTACCGTTCCAGTAGTTATCCCCCTCCATCAGGCAG
TTTCCCAGACATTACTCACCCGTCCGCCACTCGTCAGCAAAGCAGCAAGCTGCTTCCTGTTACCGTTCGAACT
GCATGTGTTAGGCCTGCCGCCAGCGTTCAATCGAGCCAGGATCAAAA
>952
CTGCTGCCGCCCGTAGGAGTCTGGACCGTGTCTCAGTTCCAGTGTGGCTGGTCATCCTCTCAGACCAGCTAGG
GATCGTCGCCTAGGTGAGCCGTTACCCCACCTACTAGCTAATCCCATCTGGGCACATCCGATGGCAAGAGGCC
CTAAAGGTCCCCCTCTTTGTCTTGCGACGTTATGCGGTATTAGCTACCGTTCCAGTAGTTATCCCCCTCCATC
AGGCAGTTTCCCAGACATTACTCACCCGTCCGCCACTCGTCAGCAAAGCAGCAAGCTGCTTCCTGTTACCGTT
CGACTGCATGTGTTAGGCCTGCCGCCAGCGTCAATCGAGCCA
>953
CTGCTGCCGCCCGTAGGAGTCTGGACCGTGTCTCAGTTCCAGTGTGGCTGGTCATCCTCTCAGACCAGCTAGG
GATCGTCGCCTAGGTGAGCCGTTACCCCACCTACTAGCTAATCCCATCTGGGCACATCCGATGGCAAGAGGCC
CGAAGGTCCCCCTCTTGGTCTTGCGACATTATGCGGTATTAGCTACCGTTTCCAGTAGTTATTCCCCCTCCAT
TCAGGCAGTTTCCCAGACATTACTCACCCGTCCGCCACTCGTCAGCGACAGCAAGCTGTTTCCTGTTACCGTT
CGACTGCATGTGTTAGGCCTGCCGCCAGCGTTCAATCTGAGCCAGGATCAAACTCT
>954
CTGCTGCCGCCCGTAGGAGTCTGGACCGTGTCTCAGTTCCAGTGTGGCTGGTCATCCTCTCAGACCAGCTAGG
GATCGTCGCCTAGGTGAGCCGTTACCCCACCTACTAGCTAATCCCATCTGGGCACATCCGATGGCAAGAGGCC

CTAAGGTCCCCCTCTTGGTCTTGCGACGTTATGCGGTATTAGCTACCGTTTCCAGTAGTTATCCCCCTCCACC
AGGCAGTTTCCCAGACATTACTCACCCGTCCGCCACTCGTCAGCAAAGCAGCAAGCTGCTTTCCTGTTACCGT
CGACTGCATGTGTTAGGCCTGCCGCCAGCGTTCAATCTGAGCCAGGATCAAACTCT
>955
CTGCTGCCGCCCGTAGGAGTCTGGACCGTGTCTCAGTTCCAGTGTGGCTGGTCATCCTCTCAGACCAGCTAGG
GATCGTCGCCTAGGTGAGCCGTTACCCCACCTACTAGCTAATCCCATCTGGGCACATCCGATGGCAAGAGGCC
CTAAGGTCCCCCTCTTGGTCTTGCGACGTACGCGGTATTAGCTACCGTTTCCAGTAGTTATCCCCCTCCATCA
GGCAGTTTCCCAGACATTACTCACCCGTCCGCCACTCGTCAGCAAAGCAGCAAGCTGCTTCCTGTTACCGTTC
GACTGCATGTGTTAGGCCTGCCGCCAGCGTCAATCTGAGCCAGGATCAAACTCT
>956
CTGCTGCCGCCCGTAGGAGTCTGGACCGTGTCTCAGTTCCAGTGTGGCTGGTCATCCTCTCAGACCAGCTAGG
GATCGTCGCCTAGGTGAGCCGTTACCCCACCTACTAGCTAATCCCATCTGGGCACATCCGATGGCAAGAGGCC
CTAAGGTCCCCCTCTTGGCCTTGCGACGTTATGCGGTATAGCTACCGTTTCCAGTAGTTATCCCCCTCCATCA
GGCAGTTTCCCAGACATTACTCACCCGTCCGCCACTCGTCAGCAAACAGCAAGCTGCTTCCTGTTACCGTTCG
ACTGCATGTGTTAGGCCTGCCGCCAGCGTCAATCTGAGCCAGGATCAAACTCT
>957
CTGCTGCCGCCCGTAGGAGTCTGGACCGTGTCTCAGTTCCAGTGTGGCTGGTCATCCTCTCAGACCAGCTAGG
GATCGTCGCCTAGGTGAGCCGTTACCCCACCTACTAGCTAATCCCATCTGGGCACATCCGATGGCAAGAGGCC
CTAAGGTCCCCCTCTTGTCTTGCGACGTATGCGGTATTAGCTACCGTTTCCAGTAGTTATCCCCCCCCATCAG
GCAGTTCCCAGACATTACTCACCCGTCCGCCACTCGTCAGCAAAGCAGCAAGCTGCCTCTGTTACCGTTCGAC
GTCGCATGTGTTAGGCCTGCCGCCAAGCGTCAAT
>958
CTGCTGCCGCCCGTAGGAGTCTGGACCGTGTCTCAGTTCCAGTGTGGCCGGTCATCCTCTCAGACCAGCTAGG
GATCGTCGCCTAGGTGAGCCGTTACCCCACCCTACTAGCTAATCCCATCTGGGCACATCCGATGGCAAGAGGC
CCTAAGGTCCCCCTCTTTGTCTTGCGACGTTATGCGGTATTAGCTACCGTTTCCAGTAGTTATCCCCCTCCAT
CGGGCAGTTTCCCAGACATTACTCACCCGTCCGCCACCTCAGCAAAGCAGCAAGCTGCTTCCTGTTACCGTTC
GACTGCATGTGTTAGGCCTGCCGCCAGCGTCAATCTGAGCCAGGATCAAACTCT
>959
CTGCTGCCGCCCGTAGGAGTCTGGACCGTGTCTCAGTTCCAGTGTGGCTGGTCATCCTCTCAGACCAGCTAGG
GATCGTCGCCTAGGTGAGCCGTTACCCCACCTACTAGCTAATCCCATCTGGGCACATCCGATGGCAAGAGGCC
CTAAGGTCCCCCTCTTTGTCTGCGACGTATGCGGTATTAGCTACCGTTTCCAGTAGTTATCCCCCCTCCATCG
GGCAGTTTCCCAGACATTACTCACCCGTCCGCCACTCGTCAGCAAAGCAGCAAGCTGCTTCCTGTTACCGTTC
GACTGCATGTGTTAGGCCTGCCGCCAGCGTCAATCTGAGCCAGGATCAAACTCT
>960
CTGCTGCCGCCCGTAGGAGTCTGGACCGTGTCTCAGTTCCAGTGTGGCTGGTCATCCTCTCAGACCAGCTAGG
GATCGTCGCCTAGGTGAGCCGTTACCCCACCTACTAGCTAATCCCATCTGGGCACATCCGATGGCAAGAGGCC
CTAAGGTCCCCCTCTTTGGTCTTGCGACGTTATGCGGTATTAGCTACCGTTTCCAGTAGTTATCCCCCTCCAT
CGGGCAGTTTTCCCAGACATTACTCACCCGTCTGCCACTCGTCAGCAAAGCAGCAAGCTGCTTCCTGTTACCG
TTCGACTGCATGTGTTAGGCCTGCCGCCAGCGTCAATCTGAGCCAGGATCAAACTCT
>961
CTGCTGCCGCCCGTAGGAGTCTGGACCGTGTCTCAGTTCCAGTGTGGCTGGTCATCCTCTCAGACCAGCTAGG
GATCGTCGCCTAGGTGAGCCGTTACCCCACCTACTAGCTAATCCCATCTGGGCACATCCGATGGCAAGAGGCC
CTAAGGTCCCCCTCTTTGTCTTGCGACGTTATGCGGTATTAGCTACCGTTTCCAGTAGTTATCCCCCTCCATC
AGGCAGTTTCCCAGACATTACTCACCCGTCCGCCACTCGTCAGCAAAGCAGCAAGCTGCTTCCTGTTACCGTT
CGACTGCATGTGTTAGGCCTGCCGCCAGCCGTTCAATCTGAGCCAGGATCAAACTCT
>962
CTGCTGCCGCCCGTAGGAGTCTGGACCGTGTCTCAGTTCCAGTGTGGCTGGTCATCCTCTCAGGCCAGCTAGG
GATCGTCGCCTAGGTGAGCCGTTACCCCACCTACTAGCTAATCCCATCTGGGCACATCCGATGGCAAGAGGCC
CTAAGGTCCCCCTCTTGTCTTGCGACGTTATGCGGTATTAGCTACCGTTTCCAGTAGTTATCCCCCTCCATCA
GGCAGTTTCCCAGACATTACTCACCCGTCCGCCACTCGTCAGCAAAGCAGCAAGCTGCTTCTGTTACCGTTCG
ACTGCATGTGTTAGGCCTGCCGCCAGCGTCAATCTGAGCCAGGATCAAACTCT
>963
CTGCTGCCGCCCGTAGGAGTCTGGACCGTGTCTCAGTTCCAGTGTGGCTGGTCATCCTCTCAGACCAGCTAGG
GATCGTCGCCTAGGTGAGCCGTTACCCCACCTACTAGCTAATCCCATCTGGGCACATCCGATGGCAAGAGGCC

CTAAGGTCCCCCTCTTGTCTTGCGACGTTATGCGGTATTAGCTACCGTTTCCAGTAGTTATCCCCCTCCATCA
GGCAGTTTCCCAGACATTACTCACCCGTCCGCCACTCGTCAGCAAAGCAGCAAGCTGCTTCCTGTTACCGTTC
GACTGCATGTGTTAGGCCTGCCGCCAGCGTCAATCTGAG
>964
CTGCTGCCGCCCGTAGGAGTCTGGACCGTGTCTCAGTTCCAGTGTGGCTGGTCATCCTCTCAGACCAGCTAGG
GATCGTCGCCTAGGTGAGCCGTTACCCCACCTACTAGCTAATCCCATCTGGGCACATCCGATGGCAAGAGGCC
CTAGGGTCCCCCTCTTGTCTTGCGACGTTATGCGGTATTAGCTACCGTTTCCAGTAGTTATCCCCCTCCATCA
GGCAGTTTCCCAGACATTACTCACCCGTCCGCCACTCGTCAACAAAGCAGCAAGCTGCTTCCTGTTACCGTTC
GACTGCATGTGTTAGGCCTGCCGCCAGCGTCAATCTGAGCCAGGATCAAACTCT
>965
CTGCTGCCGCCCGTAGGAGTCTGGACCGTGTCTCAGTTCCAGTGTGGCTGGTCATCCTCTCAGACCAGCTAGG
GATCGTCGCCTAGGTGAGCCGTTACCCCACCTACTAGCTAATCCCATCTGGGCACATCCGATGGCAAGAGGCC
CTAAGGTCCCCCTCTTTGTCTTGCGACGTTATGCGGTATTAGCTACCGTTTCCAGTAGTTATCCCCCTCCATC
AGGCAGTTTCCCAGACATTACTCACCCGTCCGCCACTCGTCAGCAGAGCAGCAAGCTGCTCCCTGTTACCGTT
CGACTGCATGTGTTAGGCCTGCCGCCAGCGTCAATCTGAGCCAGGATCAAACTCT
>966
CTGCTGCCGCCCGTAGGAGTCTGGACCGTGTCTCAGTTCCAGTGTGGCTGGTCATCCTCTCAGACCAGCTAGG
GATCGTCGCCTAGGTGAGCCGTTACCCCACCTACTAGCTAATCCCATCTGGGCACATCCGATGGCAAGAGGCC
CTAAGGTCCCCCTCTTTGGTCTTGCGACGTTATGCGGTATTAGCTACCGTTTCCAGTAGTTATCCCCCTCCAT
CGGGCAGTTTCCCAGACATTACTCACCCGTCCGCCACTCGTCAGCAAAGCAGCAAGCTGCTTCCTGTTACCGT
TCGACTGCATGTGTTAGGCCTGCCGCCAGCGTCAATCTGAGCCAGGATCAAACTCT
>967
CTGCTGCCGCCCGTAGGAGTCTGGACCGTGTCTCAGTTCCAGTGTGGCTGGTCATCCTCTCAGACCAGCTAGG
GATCGTCGCCTAGGTGAGCCGTTACCCCACCTACTAGCTAATCCCATCTGGGCACATCCGATGGCAAGAGGCC
CTAAGGTCCCCCCTCTTTGGTCTTGCGACGTTATGCGGTATTAGCTACCGTTTCCAGTAGTTATCCCCCTCCA
TCGGGCAGTTTTCCCAGACATTACTCACCCGTCCGCCACTCGTCAGCAAAAGCAGCAAGCTGCTTCCTGTTAC
CGTTCGACTGCATGTGTTAGGCCTGCCGCCAGCGTCAATCTGAGCCAGGATCAAACTCT
>968
CTGCTGCCGCCCGTAGGAGTCTGGACCGTGTCTCAGTTCCAGTGTGGCTGGTCATCCTCTCAGACCAGCTAGG
GATCGTCGCCTAGGTGAGCCGTTACCCCACCTACTAGCTAATCCCATCTGGGCACATCCGATGGCAAGAGGCC
CTAAGGTCCCCCTCTTGTCTTGCGACGTTATGCGGTATTAGCTACCGTTTCCAGTAGTTATCCCCCTCCATCG
GGCAGTTTCCCAGACATTACTCACCCGTCCGCCACTCGTCAGCAAAGCAGCAAGCTGCTTCCTGTTACCGTTC
GACTGCATGTGTTAGGCCTGCCGCCAGCGTCAATCTGAGCCAGGATCAAACTCT
>969
CTGCTGCCGCCCGTAGGAGTCTGGACCGTGTCTCAGTTCCAGTGTGGCTGGTCACCCTCTCAGACCAGCTAGG
GATCGTCGCCTAGGTGAGCCGTTACCCCACCTACTAGCTAATCCCATCTGGGCACATCCGATGGCAAGAGGCC
TAGGGTCCCCCTCTTGTCTTGCGACGTTATGCGGTATTAGCTACCGTTTCCAGTAGTTATCCCCCTCCATCAG
GCAGTTTCCCAGACATTACTCACCCGTCCGCCACTCGTCAGCAAAGCAGCAAGCTGCTTCCTGTTACCGTTCG
ACTGCATGTGTTAGGCCTGCCGCCAGCGTCAATCTGAAGCCAGGATCAAACTCT
>970
CTGCTGCGCCCGTAGGAGTCTGGACCGTGTCTCAGTTCCAGTGTGGCTGGTCATCCTCTCAGACCGATAGGGA
TCGTCGCCTAGGTGAGCCGTTACCCCACCTATAGCTAATCCCATCTGGGCACATCCGATGGCAAGAGGCCCTA
AGGTCCCCCTCTTTGTCTGCGACGTTATGCGGTATTAGCTACCGTTCCAGTAGTTATCCCCTCCATCAGGCAG
TTTCCCAGACATTACTCACCCGTCCGCCACTCGTCAGCAAACAGCAAGCTGCTTCCTGTTACCGTTCGAACTG
CATGTGTTAGGCCTGCCGCCAGCGTTCAAT
>971
CTGCTGCCGCCCGTAGGAGTCTGGACCGTGTCTCAGTTCCAGTGTGGCTGGTCATCCTCTCAGACCAGCTAGG
GATCGTCGCCTAGGTGAGCCGTTACCCCACCTACTAGCTAATCCCATCTGGGCACATCCGATGGCAAGGGGCC
CTAAGGTCCCCCTCTTTGTCTTGCGACGTTATGCGGTATTAGCTACCGTTTCCAGTAGTTATCCCCTCCATCG
GGCAGTTTCCCAGACATTACTCACCCCGTCCGCCACTCGTCAGCAAAGCAGCAAGCTGCTTCCTGTTACCGTT
CGACTGCATGTGTTAGGCCTGCCGCCAGCGTCAATCTGAGCCAGGATCAAACTCT
>972
CTGCTGCCGCCCGTAGGAGTCTGGACCGTGTCTCAGTTCCAGTGTGGCTGGTCATCCTCTCAGACCAGCTAGG
GATCGCCGCCTAGGTGAGCCGTTACCCCACCTACTAGCTAATCCCATCTGGGCACATCCGATGGCAAGAGGCC

CTAAGGTCCCCCTCTTTGTCTTGCGACGTTATGCGGTATTAGCTACCGTTTCCAGTAGTTATCCCCCTCCATC
GGGCAGTTTCCCAGACATTACTCACCCGTCCGCCACTCGTCAGCAAAGCAGCAAGCTGCTTCCTGTTACCGTT
CGACTGCATGTGTTAGGCCTGCCGCCAGCGTCAATCTGAGCCAGGATCAAACTCT
>973
CTGCTGCCGCCCGTAGGAGTCTGGACCGTGTCTCAGTTCCAGTGTGGCTGGTCATCCTCTCAGACCAGCTAGG
GATCGTCGCCTAGGTGAGCCGTTACCCCACCTACTAGCTAATCCCATCTGGGCACATCCGATGGCAAGAGGCC
CTAAGGTCCCCCTCTTGTCTGCGACGTTATGCGGTATTAGCTACCGTTCCAGTAGTTATCCCCTCCATCAGGC
AGTTTCCCAGACATTACTCACCGTCCGCCACTCGTCAGCAAACAGCAAGCTGCTTCCTGTTACCGTTCGACTG
CATGTGTTAGGCCTGACCGCCAGCGTCAATCTG
>974
TGCTGCCGCCCGTAGGAGTCTGGACCGTGTCTCAGTTCCAGTGTGGCTGGTCATCCTCTCAGACCAGCTAGGG
ATCGTCGCCTAGGTGAGCCGTTACCCCACCTACTAGCTAATCCCATCTGGGCACATCCGATGGCAAGAGGCCC
TAAGGTCCCCCTCTTTGGTCTTGCGACGTTATGCGGTATTAGCTACCGTTTCCAGTAGTTATCCCCCTCCATC
AGGCAGTTTCCCAGACATTACTCACCCGTCCGCCACTCGTCAGCAAACAGCAAGCTGCTTCCTGTTACCGTTC
GACTGCATGTGTTAGGCCTGCCGCCAGCGTTCAATCTGAGCCAGGATCAAACGTCT
>975
CTGCTGCCGCCCGTAGGAGTCTGGACCGTGTCTCAGTTCCAGTGTGGCTGGTCATCCTCTCAGACCAGCTAGG
GATTGTCGCCTAGGTGAGCCGTTACCCCACCTACTAGCTAATCCCATCTGGGCACATCCGATGGCAAGAGGCC
CTAAGGTCCCCCTCTTGTCTTGCGAACGTTATGCGGTATTAGCTACCGTTTCCAGTAGTTATCCCCCTCCATC
AGGCAGTTTCCCAGACATTACTCACCCGTCCGCCACTCGTCAGCAAAGCAGCAAGCTGCTTCCTGTTACCGTT
CGACTGCATGTGTTAGGCCTGCCGCCAGCGTCAATCTGAGCCAGGATCAAACTCT
>976
CTGCTGCCGCCCGTAGGAGTCTGGACCGTGTCTCAGTTCCAGTGTGGCTGGTCATCCTCTCAGACCAGCTAGG
GATCGTCGCCTAGGTGAGCCGTTACCCCACCTACTAGCTAATCCCATCTGGGCACATCCGATGGCAAGAGGCC
CTAAGGTCCCCCTCTTTGTCTTGCGACGTTATGCGGTATTAGCTACCGTTTCCAGTAGTTATCCCCCTCCATC
AGGCAGTTTCCCAGACATTACTCACCCGTCCGCCACTCGTCAGCAAAGCAGCAAGCTGCTTCCTGTTACCGTT
CGACTGCATGTGTTAGGCCTGCCGCCAGCGTCAATCTGAGCCAGGATCAAACTCT
>977
CTGCTGCCGCCCGTAGGAGTCTGGACCGTGTCTCAGTTCCAGTGTGGCTGGTCATCCTCTCAGACCAGCTAGG
GATCGTCGCCTAGGTGAGCCGTTACCCCACCTACTAGCTAATCCCATCTGGGCACATCCGATGGCAAGAGGCC
CTAAGGTCCCCCTCTTGTCTGCGACGTTATGCGGTATTAGCTACCGTTTCCAGTAGTTATCCCCCTCCATCGG
GCAGTTTCCCAGTCATTACTCACCCGTCCCGCCACTCGTCAGCAAAGCAGCAAGCTGCTTCCTGTTACCCGTT
CGACTGCATGTGTTAGGCCTGCCGCCAGCGTCAATCTGAGCCAGGATCAAACTCT
>978
CTGCTGCCGCCCGTAGGAGTCTGGACCGTGTCTCAGTTCCAGTGTGGCTGGTCATCCTCTCAGACCAGCTAGG
GATCGTCGCCTAGGTGAGCCGTTACCCCACCTACTAGCTAATCCCATCTGGGCACATCCGATGGCAAGAGGCC
CTAAGGTCCCCCTCTTTGGTTCTTGCGACGTTATGCGGTATTAGCTACCGTTTCCAGTAGTTATCCCCTCCAT
CGGGCAGTTTCCCAGACATTACTCACCCGTCCGCCACTCGTCAGCAAAGCAGCAAGCTGCTTTCCTGTTACCG
TTCGACTGCATGTGTTAGGCCTGCCGCCAGCGTTCAATCTGAGCCAGGATCAAACTCT
>979
CTGCTGCCGCCCGTAGGAGTCTGGACCGTGTCTCTGTTCCAGTGTGGCTGGTCATCCTCTCAGACCAGCTAGG
GATCGTCGCCTAGGTGAGCCGTTACCCCACCTACTAGCTAATCCCATCTGGGCCACATCCGATGGCAAGAGGC
CCTAAGGTCCCCCTCTTGTCTTGCGACGTTATGCGGTATTAGCTACCGTTTCCAGTAGTTATCCCCCTCCATC
AGGCAGTTTTCCCAGACATTACTCACCCGTCCGCCACTCGTCAGCCAAGCAGCAAGCTGCTTCTGTTACCGTT
CGACTGCATGTGTTAGGCCTGCCGCCAGCCGTCAATC
>980
CTGCTGCCGCCCGTAGGAGTCTGGACCGTGTCTCAGTTCCAGTGTGGCTGGTCATCCTCTCAGACCAGCTAGG
GATCGTCGCCTAGGCGAGCCGTTACCCCACCTACTAGCTAATCCCATCTGGGCACATCCGATGGCAAGAGGCC
CTAAGGTCCCCCTCTTTGGTCTTGCGACGTTCTGCGGTATTAGCTACCGTTTCCAGTAGTTATCCCCCTCCAT
CAGGCAGTTTCCCCAGACATTACTCGCCCGTCCGCCACTCGTCAGCAAAGCAGCAAGCTGCTTCCTGTTACCG
TTCGACTGCATGTGTTAGGCCTGCCGCCAGCGTTCAATCTGAGCCAGGATCAAACTCT
>981
CTGCTGCCGCCCGTAGGAGTCTGGACCGTGTCTCAGTTCCAGTGTGGCTGGTCATCCTCTCAGACCAGCTAGG
GATCGTCGCCTAGGTGAGCCGTTACCCCACCTACTAGCTAATCCCATCTGGGCACATCCGATGGCAAGAGGCC

CTAAGGTCCCCCTCTTTGGTCTTGCGACGTTATGCGGTATTAGCTACCGTTTCCAGTAGTTATCCCCCTCCAT
CAGGCAGTTTCCCAGACATTACTCACCCGTCCGCCACTCGTCAGCAAAGCAGCAAGCTGCTTCCTGTTACCGT
TCGACTTGCATGTGTTAGGCCTGCCGCCAGCGTCAATCTGAGCCAGGATCAAACTCT
>982
CTGCTGCCGCCCGTAGGAGTCTGGACCGTGTCTCAGTTCCAGTGTGGCTGGTCATCCTCTCAGACCAGCTAGG
GATCGTCGCCTAGGTGAGCCGTTACCCCACCTACTAGCTAATCCCATCTGGGCACATCCGATGGCAAGAGGCC
CTAAGGTCCCCCTCTTTGTCTGCGACGTATGCGGTATTAGCTACCGTTCCAGTAGTTATCCCCCTCCATCAGG
CAGTTTCCCAGACATTACTCACCCGTCCGCCACTCGTCAGCAAAGCAGCAAGCTGCTTCCTGTTACCGTTCGA
CTGCATGTGTTAGGCCTGCCGCCAGCGTCAATCTGAGCCAGGATCAAACTCT
>983
CTGCTGCCGCCCGTAGGAGTCTGGACCGTGTCTCAGTTCCAGTGTGGCTGGTCATCCTCTCAGACCAGCTAGG
GATCGTCGCCTAGGTGAGCCGTTACCCCACCTACTAGCTAATCCCATCTGGGCACATCCCGATGGCAAGAGGC
CCTAAGGTCCCCCTCTTGTCTGCGACGTATGCGGTATTAGCTACCGTTTCCAGTAGTTATCCCCCTCCATCGG
GCAGTTCCCAGACATTACTCACCCGTCCGCCACTCGTCAGCAAAGCAGCAAGCTGCTTCCTGTTACCGTTCGA
CTGCATGTGTTAGGCCTGCCGCCAGCGTCAATCTGAGCCAGGATCAAACTCT
>984
CTGCTGCCGCCCGTAGGAGTCTGGACCGTGTCTCAGTTCCAGTGTGGCTGGTCATCCTCTCAGACCAGCTAGG
GATCGTCGCCTAGGTGAGCCGTTACCCCACCTACTAGCTAATCCCATCTGGGCACATCCGATGGCAAGAGGCC
CTAAGGTCCCCCTCTTGTCTGCGACGTATGCGGTATTAGCTACCGTTTCCAGTAGTTATCCCCTCCATCAGGC
AGTTTCCCAGACATTACTCACCCGTCCGCCACTCGTCAGCAAAGCAGCAAGCTGCTTCCTGTTACCGTTCGAC
TGCATGTGTTAGGCCTGCCGCCAGCGTCAATCTGAGCCAGGATCAAACTCT
>985
CTGCTGCCGCCCGTAGGAGTCTGGACCGTGTCTCAGTTCCAGTGTGGCTGGTCATCCTCTCAGACCAGCTAGG
GATCGTCGCCTAGGTGAGCCGTTACCCCACCTACTAGCTAATCCCATCTGGGCACATCCGATGGCAAGAGGCC
CTAAGGTCCCCCTCTTGGTCTTGCGACGTTATGCGGTATTAGCTACCGTTTCCAGTAGTTATCCCCCTCCATC
GGGCAGTTTCCCAGACATTACTCACCCGTCCGCCACTCGTCAGCAAAGCAGCAAGCTGCTTCCTGTACCGTTC
GACTGCATGTGTTAGGCCTGCCGCCAGCGTCAATCTGAGCCAGGATCAAACTCT
>986
CTGCTGCCGCCCGTAGGAGTCTGGACCGTGTCTCAGTTCCAGTGTGGCTGGTCATCCTCTCAGACCAGCTAGG
GATCGTCGCCTAGGTGAGCCGTTACCCCACCTACTAGCTAATCCCATCGTGGGCACATCCGATGACAAGAGGC
CCTAAGGTCCCCCCTCTTTGGTCTTGCGACGTATGCGGTATTAGCTACCGTTTCCAGTAGTTATCCCCCTCCA
TCAGGCAGTTTCCCAGACATTACTCACCCGTCCGCCACTCGTCAGCAAAGCAGCAAGCTGCTTCCTGTTACCG
TTCGACTGCATGTGTTAGGCCTGCCGCCAGCGTTCAATCTGAGCCAGGATCAAACTCT
>987
CTGCTGCCGCCCGTAGGAGTCTGGACCGTGTCTCAGTTCCAGTGTGGCTGGTCATCCTCCCAGACCAGCTAGG
GATCGTCGCCTAGGTGAGCCGTTACCCCACCTACTAGCTAATCCCATCTGGGCACATCCGATGGCAAGAGGCC
CTAAGGTCCCCCTCTTGGTCTTGCGACGTATGCGGTATTAGCTACCGTTTCCAGTAGTTATCCCCCTCCATCA
GGCAGTTTCCCAGACATTACTCACCCGTCCGCCACTCGTCAGCAAGCAGCAAGCTGCTTCTGTTACCGTTCGA
CTGCATGTGTTAGGCCTGCCGCCAGCGTCAATCTGAGCCAGGATCAAACTCT
>988
CTGCTGCCGCCCGTAGGAGTCTGGACCGTGTCTCAGTTCCAGTGTGGCTGGTCATCCTCTCAGACCAGCTAGG
GATCGTCGCCTAGGTGAGCCGTTACCCCACCTACTAGCTAATCCCATCTGGGCACATCCGATGGCAAGAGGCC
CTAAGGTCCCCCTCTTGTCTTGCGACGTTATGCGGTATTAGCTACCGTTTCCAGTAGTTATCCCCCTCCATCA
GGCAGTTTCCCAGACATTACTCACCCGTCCGCCACTCGTCAGCAAAACAGCAAGCTGCTTCCTGTTACCGTTC
GACTGCATGTGTTAGGCCTGCCGCCAGCGTCAATCTGAGCCAGGATCAAACTCT
>989
CTGCTGCCGCCCGTAGGAGTCTGGACCGTGTCTCAGTTCCAGTGTGGCTGGTCATCCTCTCAGACCAGCTAGG
GATCGTCGCCTAGGTGAGCCGTTACCCCACCTACTAGCTAATCCCATCTGGGCACATCCGATGGCAAGAGGCC
CTAAGGTCCCCCTCTTTGGTCTGCGACGTTATGCGGTATTAGCTACCGTTTCCAGTAGTTATCCCCCTCCATC
AGGCAGTTTCCCAGACATTACTCACCCGTCCGCCACTCGTCAGCAAAAGCAGCAAGCTGCTTCCTGTTACCGT
TCGACTGCATGTGTTAGGCCTGCCGCCAGCGTCAATCTGAGCCAGGATCAAACTCT
>990
CTGCTGCCGCCCGTAGGAGTCTGGACCGTGTCTCAGTTCCAGTGTGGCTGGTCATCCTCTCAGACCAGCTAGG
GATCGTCGCCTAGGTGAGCCGTTACCCCACCTACTAGCTAATCCCATCTGGGCACATCCGATGGCAAGAGGCC

CTAAGGTCCCCCTCTTTGGTCTTGCGACGTTATGCGGTATTAGCTACCGTTTCCAGTAGTTATCCCCCTCCAT
CAGGCAGTTTCCCAGACATTACTCACCCGTCCGCCACTCGTCAGCAAAGCAGCAAGCTGCTTTCTGTTACCGT
TCGACTGCATGTGTTAGGCCTGCCGCCAGCGTCAATCTGAGCCAGGATCAAACTCT
>991
CTGCTGCCGCCCGTAGGAGTCTGGACCGTGTCTCAGTTCCAGTGTGGCTGGTCATCCTCTCAGACCAGCTAGG
GATCGTCGCCTAGGTGAGCCGTTACCCCACCTACTAGCTAATCCCATCTGGGCACATCCGATGGCAAGAGGCC
CTAAGGTCCCCCTCTTTGTCTTGCGACGTATGCGGTATTAGCTACCGTTTCCAGTAGTTATCCCCCTCCATCG
GGCAGTTTCCCAGACATTGCTCACCCGTCCGCCACTCGTCAGCAAAGCAGCAAGCTGCTTCCTGTTACCGTTC
GACTGCATGTGTTAGGCCTGCCGCCAGCGTCAATCTGAGCCAGGATCAAACTCT
>992
CTGCTGCCGCCCGTAGGAGTCTGGACCGTGTCTCAGTTCCAGTGTGGCTGGTCATCCTCTCAGACCAGCTAGG
GATCGTCGCCTAGGTGAGCCGTTACCCCACCTACTAGCTAATCCCATCTGGGCACATCCGATGGCAAGAGGCC
CTAAGGTCCCCCTCTTGTCTGCGACGTTATGCGGTATTAGCTACCGTTTCCAGTAGTTATCCCCCTCCATCAG
GCAGTTTCCCAGACATTACTCACCGTCCGCCACTCGTCAGCAAACAGCAAGCTGCTTCCTGTTACCGTTCGAC
TGCATGTGTTAGGCCTGC
>993
CTGCTGCCGCCCGTAGGAGTCTGGACCGTGTCTCAGTTCCAGTGTGGCTGGTCATCCTCTCAGACCAGCTAGG
GATCGTCGCCTAGGTGAGCCGTTACCCCACCTACTAGCTAATCCCATCTGGGCACATCCGATGGCAAGAGGCC
CTAAGGTCCCCCTCTTGTCTTGCGACGTTATGCGGTATTAGCTACCGTTTCCAGTAGTTATCCCCCTCCATCA
GGCAGTTTCCCAGACATTACTCACCCGTCCGCCACTCGTCAGCAAAGCAGCAAGCTGCTTCCTGTTACCGTTC
GACTGCATGTGTTAGGCCTGCCGCCAGCGTCAATCTGAGCCAGGATCAAACTCT
>994
CTGCTGCCGCCCGTAGGAGTCTGGACCGTGTCTCAGTTCCAGTGTGGCTGGTCATCCTCTCAGACCAGCTAGG
GATCGTCGCCTAGGTGAGCCGTTACCCCACCTACTAGCTAATCCCATCTGGGCACATCCGATGGCAAGAGGCC
CTAAGGTCCCCCTCTTTGTCTTGCGACGTTATGCGGTATTAGCTACCGTTTCCAGTAGTTATCCCCCTCCATC
AGGCAGTTTCCCAGACATTACTCACCCGTCCGCCACTCGTCAGCAAAGCAGCAAGCTGCTTCCTGTTACCGTT
CGACTGCATGTCGTTAGGCCTGCCGCCAGCGTTCAATCTGAGCCAGGATCAAACTCT
>995
CTGCTGCCGCCCGTAGGAGTCTGGACCGTGTCTCAGTTCCAGTGTGGCTGGTCATCCTCTCAGACCAGCTAGG
GATCGTCGCCTAGGTGAGCCGTTACCCCACCTACTAGCTAATCCCATCTGGGCACATCCGATGGCAAGAGGCC
CTAAGGTCCCCCTCTTTGGTCTCGCGACGTTATGCGGTATTAGCTACCGTTTCCAGTAGTTATCCCCCTCCAT
CGGGCAGTTTCCCAGACATTACTCACCCGTCCGCCACTCGTCAGCAAAGCAGCAAGCTGCTTCCTGTTACCGT
TCGACTGCATGTGTTAGGCCTGCCGCCAGCGTTCAATCTGAGCCAGGATCAAACTCT
>996
CTGCTGCCGCCCGTAGGAGTCTGGACCGTGTCTCAGTTCCAGTGTGGCTGGTCATCCTCTCAGACCAGCTAGG
GATCGTCGCCTAGGTGAGCCGTTACCCCACCTACTAGCTAATCCCATCTGGGCACATCCGATGGCAAGAGGCC
CTAAGGTCCCCCTCTTGTCTTGCGACGTTATGCGGTATTAGCTACCGTTTCCAGTAGTTATCCCCCTCCATCA
GGCAGTTTCCCAGACATTACTCACCCGTCCGCCACTCGTCAGCAAAACAGCAAGCTGCTTCCTGTTACCGTTC
GACTGCATGTGTTAGGCCTGCCGCCAGCGTTCAATCTGAGCCAGGAT
>997
CTGCTGCCGCCCGTAGGAGTCTGGACCGTGTCTCAGTTCCAGTGTGGCTGGTCATCCTCTCAGACCAGCTAGG
GATCGTCGCCTAGGTGAGCCGTTACCCCACCTACTAGCTAATCCCATCTGGGCACATCCGATGGCAAGAGGCC
CTAAGGTCCCCCTCTTTGGTCTGCGTCGTTATGCGGTATTAGCTACCGTTTCCAGTAGTTATCCCCCTCCATC
AGGCAGTTTCCCAGACATTACTCACCCGTCCGCCACTCGTCAGCAAAACAGCAAGCTGCTTCCTGTTACCGTT
CGACTGCATGTGTTAGGCCTGCCGCCAGCGTCAATCTGAGCCAGGATCAAACTCT
>998
CTGCTGCCGCCCGTAGGAGTCTGGACCGTGTCACAGTTCCAGTGTGGCTGGTCATCCTCTCAGACCAGCTAGG
GATCGTCGCCTAGGTGAGCCGTTACCCCACCTACTAGCTAATCCCATCTGGGCACATCCGATGGCAAGAGGCC
CTAAGGTCCCCCTCTTTGTCTTGCGACGTTATGCGGTATTAGCTACCGTTTCCAGTAGTTATCCCCCTCCATC
AGGCAGTTTCCCAGACATTACTCACCCGTCCGCCACTCGTCAGCAAAGCAGCAAGCTGCTTCCTGTTACCGTT
CGACTGCATGTGTTAGGCCTGCCGCCAGCGTCAATCTGAGCCAGGATCAAACTCT
>999
CTGCTGCCGCCCGTAGGAGTCTGGACCGTGTCTCAGTTCCAGTGTGGCTGGTCATCCTCTCAGACCAGCTAGG
GATCGTCGCCTAGGTGAGCCGTTACCCCACCTACTAGCTAATCCCATCTGGGCACATCCGATGGCAAGAGGCC

CTAAGGTCCCCCTCTTTGGTCTTGCGACGTTATGCGGTATTAGCTACCGTTTCCAGTAGTTATCCCCCTCCAT
CAGGCAGTTTCCCAGACATTACTCACCCGTCCGCCACTCGTCAGCAAAGCAGCAAGCTGCTTTCTGTTACCGT
TCGACTGCATGTGTTAGGCCTGCCGCCAGCGTTCAATCTGAGCCAGGATCAAACTCT
>1000
CTGCTGCCGCCCGTAGGAGTCTGGACCGTGTCTCAGTTCCAGTGTGGCTGGTCATCCTCTCAGACCAGCTAGG
GATCGTCGCCTAGGTGAGCCGTTACCCCACCTACTAGCTAATCCCATCTGGGCACATCCGATGGCAAGAGGCC
CTAAGGTCCCCCTCTTGTCTGCGACGTATGCGGTATCAGTACCGTTTCCAGTAGTTACTCCCCTCCATCGGGC
AGTTTCCCAGACATACTCACCCGTCCGCCACTCGTCAGCAAAGCAGCGAGCTGCTTCTGTTACCGTTCGACTG
CATGTGTTAGGCCTGCCGCCAGCGTCAATCTGAGCAGGATAAACTC
>1001
CTGCTGCCGCCCGTAGGAGTCTGGACCGTGTCTCAGTTCCAGTGTGGCTGGTCATCCTCTCAGACCAGCTAGG
GATCGTCGCCTAGGTGAGCCGTTACCCCACCTACTAGCTAATCCCATCTGGGCACATCCGATGGCAAGAGGCC
CTAAGGTCCCCCTCTTTGTCTTGCGACGTTATGCGGTATTAGCTACCGTTTCCAGTAGTTATCCCCCTCCATC
GGGCAGTTTTCCCAGACATTACTCACCCGTCCGCCACTCGTCAGCAAAGCAGCAAGCTGCTTCCCGTTACCGT
TCGACTGCATGTGTTAGGCCTGCCGCCAGCGTTCAATCTGAGCCAGGATCAAACTCT
>1002
CTGCTGCCGCCCGTAGGAGTCTGGACCGTGTCTCAGTTCCAGTGTGGCTGGTCATCCTCTCAGACCAGCTAGG
GATCGTCGCCTAGGTGAGCCGTTACCCCACCTACTAGCTAATCCCATCTGGGCACATCCGATGGCAAGAGGCC
CTAAGGTCCCCCTCTTTGGTCTTGCGACGTTATGCGGTATTAGCTACCGTTTCCAGTAGTTATCCCCCTCCAT
CGGGCAGTTTCCCAGACATTACTCACCCGTCCGCCACTCGTCAGCAAAGCAGCAAGCTGCTTCCTGTTACCGT
TCGACTGCATGTGTTAGGCCTGCCGCCAGCGTCAATCTGAGCCAGGATCAAACTCT
>1003
CTGCTGCCGCCCGTAGGAGTCTGGACCGTGCCTCAGTTCCAGTGTGGCTGGTCATCCTCTCAGACCAGCTAGG
GATCGTCGCCTAGGTGAGCCGTTACCCCACCTACTAGCTAATCCCATCTGGGCACATCCGATGGCAAGAGGCC
CTAAGGTCCCCCTCTTTGTCTTGCGACGTTATGCGGTATTAGCTACCGTTTCCAGTAGTTATCCCCCTCCATC
GGGCAGTTTTCCCAGACATTACTCACCCGTCCGCCACTCGTCAGCAAAGCAGCAAGCTGCTTCCTGTTACCGT
TCGACTGCATGTGTTAGGCCTGCCGCCAGCGTTCAATCTGAGCCAGGATCAAACTCT
>1004
CTGCTGCCGCCCGTAGGAGTCTGGACCGTGTCTCAGTTCCAGTGTGGCTGGTCATCCTCTCAGACAGCTAGGG
ATCGTCGCCTAGGTGAGCCGTTACCCCACCTACTAGCTAATCCCATCTGGGCACATCCGATGGCAAGAGGCCC
TAAGGTCCCCCTCTTTGGTCTTGCGACGTTATGCGGTATTAGCTACCGTTTCCAGTAGTTATCCCCCTCCATC
AGGCAGTTTCCCAGACATTACTCACCCGTCCGCCACTCGTCAGCAAAGCAGCAAGCTGCTTTCTGTTACCGTT
CGACTGCATGTGTTAGGCCTGCCGCCAGCGTCAATCTGAGCCAGGATCAAACTCT
>1005
CTGCTGCCGCCCGTAGGAGTCTGGACCGTGTCTCAGTTCCAGTGTGGCTGGTCATCCTCTCAGACCAGCTAGG
GATCGTCGCCTAGGTGAGCCGTTACCCCACCTACTAGCTAATCCCATCTGGGCACATCCGATGGCAAGAGGCC
CTAAGGTCCCCCTCTTTGTCTTGCGACGTTATGCGGTATTAGCTACCGTTTCCAGTAGTTATCCCCCTCCATC
AGGCAGTTTTCCCAGACATTACTCACCCGTCCGCCACTCGTCAGCAAAGCAGCAAGCTGCTTTCTGTTACCGT
TCGACTGCATGTGTTAGGCCTGCCGCCAGCGTTCAATCTGAGCCAGGATCAAACTCT
>1006
CTGCTGCCGCCCGTAGGAGTCTGGACCGTGTCTCAGTTCCAGTGTGGCTGGTCATCCTCTCAGACCAGCTAGG
GATCGTCGCCTAGGTGAGCCGTTACCCCACCTACTAGCTAATCCCATCTGGGCACATCCGATGGCAAGAGGCC
CTAAGGTCCCCCCTCTTTGTCTTGCGACGTTATGCGGTATTAGCTACCGTTTCCAGTAGTTATCCCCCTCCAT
CGGGCAGTTTCCCAGACATTACTCACCCGTCCGCCACTCGTCAGCAAAGCAGCAAGCTGCTTCCTGTTACCGT
TCGACTGCATGTGTTAGGCCTGCCGCCAGCGTTCAATCTGAGCCAGGATCAAACTCT
>1007
CTGCTGCCGCCCGTAGGAGTCTGGACCGTGTCTCAGTTCCAGTGTGGCTGGTCATCCTCTCAGACCAGCTAGG
GATCGTCGCCTAGGTGAGCCGTTACCCCACCTACTAGCTAATCCCATCTGGGCACATCCGATGGCAAGAGGCC
CTAAGGTCCCCCTCTTTGTCTTGCGACGTTATGCGGTATTAGCTACCGTTTCCAGTAGTTATCCCCCTCCATC
AGGCAGTTTCCCAGACATTACTCACCCGTCCGCCACTCGTCAGCAAAGCAGCAAGCTGCTTCCTGTTACCGTT
CGACTGCATGTGTTAGGCCTGCCGCCAGCGTTCAATCTGAGCCAGGATCAAACTCT
>1008
CTGCTGCCGCCCGTAGGAGTCTGGACCGTGTCTCAGTTCCAGTGTGGCTGGTCATCCTCTCAGACCAGCTAGG
GATCGTCGCCTAGGTGAGCCGTTACCCCACCTACTAGCTAATCCCATCTGGGCACATCCGATGGCAAGAGGCC

CTAAGGTCCCCCTCTTTGGTCTTGCGACGTTATGCGGTATTAGCTACCGTTTCCAGTAGTTATCCCCCTCCAT
CAGGCAGTTTCCCAGACATTACTCACCCGTCCGCCACTCGTCAGCAAAGCAGCAAGCTGCTTCCTGTTACCGT
TCGACTTGCATGTGTTAGGCCTGCCGCCAGCGTTCAATCTGAGCCAGGATCAAACTCT
>1009
CTGCTGCCGCCCGTAGGAGTCTGGACCGTGTCTCAGTTCCAGTGTGGCTGGTCATCCTCTCAGACCAGCTAGG
GATCGTCGCCTAGGTGAGCCGTTACCCCACCTACTAGCTAATCCCATCTGGGCACATCCGATGGCAAGAGGCC
CTAAGGTCCCCCTCTTGTCTTGCGACGTTATGCGGTATTAGCTACCGTTTCCAGTAGTTATCCCCCTCCATCA
GGCAGTTTCCCAGACATTACTCACCCGTCCGCCACTCGTCAGCAAAGCAGCAAGCTGCTTCCTGTTACCGTTC
GACTGCATGTGTTAGGCCTGCCGCCAGCGTTCAATCTGAG
>1010
TGCTGCCGCCCGTAGGAGTCTGGACCGTGTCTCAGTTCCAGTGTGGCTGGTCATCCTCTCAGACCAGCTAGGG
ATCGTCGCCTAGGTGAGCCGTTACCCCACCTACTAGCTAATCCCATCTGGGCACATCCGATGGCAAGAGGCCC
TAAGGTCCCCCTCTTTGGTCTTGCGACGTTATGCGGTATTAGCTACCGTTTCCAGTAGTTATCCCCCTCCATC
AGGCAGTTTCCCAGACATTACTCACCCGTCCGCCACTCGTCAGCAAAGCAGCAAGCTGCTTTCTGTTACCGTT
CGACTGCATGTGTTAGGCCTGCCGCCAGCGTTCAATCTGAGCCAGGATCAAACTCT
>1011
CTGCTGCCGCCCGTAGGAGTCTGGACCGTGTCTCAGTTCCAGTGTGGCTGGTCATCCTCTCAGACCAGCTAGG
GATCGTCGCCTAGGTGAGCCGTTACCCCACCTACTAGCTAATCCCATCTGGGCACATCCGATGGCAAGAGGCC
CTAAGGTCCCCCTCTTTGTCTGCGACGTTATGCGGTATTAGCTACCGTTTCCAGTAGTTATCCCCCTCCATCA
GGCAGTTTCCCAGACATTACTCACCCGTCCGCCACTCGTCAGCAAAGCAGCAAGCTGCTTCTGTTACCGTTCG
ACTGCATGTGTTAGGCCTGCCGCCAGCGTTCAATGAGCCAGGACAAACCTT
>1012
CTGCTGCCGCCCGTAGGAGTCTGGACCGTGTCTCAGTTCCAGTGTGGCTGGTCATCCTCTCAGACCAGCTAGG
GATCGTCGCCTAGGTGAGCCGTTACCCCACCTACTAGCTAATCCCATCTGGGCACATCCGATGGCAAGAGGCC
CTAAGGTCCCCCTCTTTGTCTGCGACGTATGCGGTATTAGCTACCGTTTCCAGTAGTTATCCCCCTCCATCAG
GCAGTTTCCCAGACATTACTCACCCGTCCGCCACTCGTCAGCAAAGCAGCAAGCTGCTTCTGTTACCGTTCGA
CTGCATGTGTTAGGCCTGCCGCCAGCGTTCAATCTGAGCCAG
>1013
CTGCTGCCGCCCGTAGGAGTCTGGACCGTGTCTCAGTTCCAGTGTGGCTGGTCATCCTCTCAGACCAGCTAGG
GATCGTCGCCTAGGTGAGCCGTTACCCCACCTACTAGCTAATCCCATCTGGGCACATCCGATGGCAAGAGGCC
CTAAGGTCCCCCTCTTTGTCTTGCGACGTTATGCGGTATTAGCTACCGTTTCCAGTAGTTATCCCCCTCCATC
AGGCAGTTTCCCAGACATTACTCACCCGTCCGCCACTCGTCAGCAAAGCAGCAAGCTGCTTCCTGTTACCGTT
CGACTGCATGTGTTAGGCCTGCCGCCAGCGTCAATCTGAGCCAGGATCAAACTTGT
>1014
CTGCTGCCGCCCGTAGGAGTCTGGACCGTGTCTCAGTTCCAGTGTGGCTGGTCATCCTCTCAGACCAGCTAGG
GATCGTCGCCTAGGTGAGCCGTTACCCCACCTACTAGCTAATCCCATCTGGGCACATCCGATGGCAAGAGGCC
CTAAGGTCCCCCTCTTTGTCTGCGACGTATGCGGTATTAGCTACCGTTTCCAGTAGTTATCCCCCTCCATCGG
GCAGTTTCCCAGACATTACTCACCCGTCCGCCACTCGTCAGCAAAACAGCAAGCTGCTTCCTGTTACCGTTCG
ACTGCATGTGTTAGGCCTGCCGCCAGCGTCAATCTGAGCCAGGATCAAACTCT
>1015
CTGCTGCCGCCCGTAGGAGTCTGGACCGTGTCTCAGTTCCAGTGTGGCTGGTCATCCTCTCGGACCAGCTAGG
GATCGTCGCCTAGGTGAGCCGTTACCCCACCTACTAGCTAATCTCATCTGGGCACATCCGATGGCAAGAGGCC
CTAAGGTCCCCCCTCTTTGGTCTTGCGACGTTATGCGGTATTAGCTACCGTTTCCAGTAGTTATCCCCCTCCA
TCGGGCAGTTTCCCAGACATTACTCACCCGTCCGCCACTCGTCAGCAAAGCAGCAGGCTGCTTCCTGTTACCG
TTCGACTGCATGTGTTAGGCCTGCCGCCAGCGTCAATCTGAGCCAGGATCAAACTCT
>1016
CTGCTGCCGCCCGTAGGAGTCTGGACCGTGTCTCAGTTCCAGTGTGGCTGGTCATCCTCTCAGACCAGCTAGG
ACGTCGCTAGGTGAGCCGTTACCCCACCTACTAGCTAATCCCATCCGGTGCACATCCGATGGCAAGAGGCCCT
AAGGTCCCCCTCTTTGGTCTTGCGACGTTATGCGGTATTAGCTACCGTTTCCAGTAGTTATCCCCCTCATCGG
GCAGTTTCCCAGACATTACTCACCCGTCCGCCACTCGTCAGCAAAGCAGCAAGCTGCTTCCTGTTACCGTTCG
ACTGCATGTGTTAGGCCTGCCGTCAGCGTTCAATCTGAGCCAGGATCAAACTCT
>1017
CTGCTGCCGCCCGTAGGAGTCTGGACCGTGTCTCAGTTCCAGTGTGGCTGGTCATCCTCTCAGACCAGCTAGG
GATCGTCGCCTAGGTGAGCCGTTACCCCACCTACTAGCTAATCCCATCTGGGCACATCCGATGGCAAGAGGCC

CTAAGGTCCCCCTCTTGTCTTGCGACGTATGCGGTATTAGCTACCGTTTCCAGTAGTTATCCCCCTCCATCGG
GCAGTTTCCCAGACATTACTCACCCGTCCGCCACTCGTCAGCAAAGCAGCAAGCTGCTTCCTGTTACCGTTCG
ACTGCATGTGTTAGGCCTGCCGCCAGCGTCAATCTGAGCCAGGATCAAACTCT
>1018
CTGCTGCCGCCCGTAGGAGTCTGGACCGTGTCTCAGTTCCAGTGTGGCTGGTCATCCTCTCAGACCAGCTAGG
GATCGTCGCCTAGGTGAGCCGTTACCCCACCTACTAGCTAATCCCATCTGGGCACGTCCGATGGCAAGAGGCC
CTAAGGTCCCCCTCTTTGGTCTGCGACGTTATGCGGTATTAGCTACCGTTTCCAGTAGTTATCCCCCTCCATC
AGGCAGTTTTCCCAGACATTACTCACCCGTCCGCCACTCGTCAGCAAAGCAGCAAGCTGCTTCCTGTTACCGT
TCGACTGCATGTGTTAGGCCTGCCGCCAGCGTCAATCTGAGCCAGGATCAAACTCT
>1019
CTGCTGCCGCCCGTAGGAGTCTGGACCGTGTCTCAGTTCCAGTGTGGCTGGTCATCCTCTCAGACCAGCTAGG
GATCGTCGCCTAGGTGAGCCGTTACCCCACCTACTAGCTAATCCCATCTGGGCACATCCGATGGCAAGAGGCC
CTAAGGTCCCCTCTTGTCTGCGACGTATGCGGTATTAGCTACCGTTTCCAGTAGTTATCCCCCTCCATCGGGC
AGTTTCCCAGACATTACTCACCCGTCCGCCACTCGTCAGCAAAGCAGCAAGTGCTTCCTGTTACCGTTCGACT
GCATGTGTTAGGCCTGCCGCCAGCGTCAAACTGAGCCAGGATCAACTCT
>1020
CTGCTGCCGCCCGTAGGAGTCTGGACCGTGTCTCAGTTCCAGTGTGGCTGGTCATCCTCTCAGACCAGCTAGG
GATCGTCGCCTAGGTGAGCCGTTACCCCTCCTACTAGCTAATCCCATCTGGGCACATCCGGTGGCAAGAGGCC
CTAAGGTCCCCCTCTTTGGTCTTGCGACGTATGCGGTATTAGCTACCGTTTCCAGTAGTTATCCCCCTCCATC
AGGCAGTTTCCCAGACATTACTCACCCGTCCGCCACTCTCAGCAAAGCAGCAAGCTGCTTCCTGTTACCGTTC
GACTGCATGTGTTAGGCCTGCCGCCAGCGTCAATCTGAG
>1021
CTGCTGCCGCCCGTAGGAGTCTGGACCGTGTCTCAGTTCCAGTGTGGCTGGTCATCCTCTCAGACCAGCTAGG
GATCGTCGCCTAGGTGAGCCGTTACCCCACCTACTAGCTAATCCCATCTGGGCACATCCGATGGCAAGAGGCC
CTAAGGTCCCCCTCTTGTCTGCGACGTATGCGGTATTAGCTACCGTTTCCAGTAGTTATCCCCCTCCATCAGG
CAGTTTCCCAGACATTACTCACCCGTCCGCCACTCGTCAGCAAAGCAGCAAGCTGCTTCCTGTTACCGTTCGA
CTGCATGTGTTAGGCCTGCCGCCAGCCGTCAATCTGAGCCAGGATCAAACTCT
>1022
CTGCTGCCGCCCGTAGGAGTCTGGACCGTGTCTCAGTTCCAGTGTGGCTGGTCATCCTCTCAGACCAGCTAGG
GATCGTCGCCTAGGTGAGCCGTTACCCCACCTACTAGCTAATCCCATCTGGGCACATCCGATGGCAAGAGGCC
CTAAGGTCCCCCTCTTTGTCTTGCGACATTATGCGGTATTAGCTACCGTTTCCAGTAGTTATCCCCCTCCATC
AGGCAGTTTCCCAGACATTACTCACCCGTCCGCCACTCGTCAGCAAAGCAGCAAGCTGCTTCTGTTACCGTTC
GACTGCATGTGTTGGGCCTGCCGCCAGCGTTCAATCTGAGCCAGGATCAAACTCT
>1023
CTGCTGCCGCCCGTAGGAGTCTGGACCGTGTCTCAGTTCCAGTGTGGCTGGTCATCCTCTCAGACCAGCTAGG
GATCGTCGCCTAGGTGAGCCGTTACCCCACCTACTAGCTAATCCCATCTGGGCACATCCGATGGCAAGAGGCC
CTAAGGTCCCCCTCTTTGGTCTTGCGACGTATGCGGTATTAGCTACCGTTTCCAGTAGTTATCCCCCTCCATC
GGGCAGTTTCCCAGACATTACTCACCCGTCCGCCACTCGTCAGCAAAGCAGCAAGCTGCTTCCTGTTACCGTT
CGACTGCATGTGTTAGGCCTGCCGCCAGCGTCAATCTGAGCCAGGATCAAACTCT
>1024
CTGCTGCCGCCCGTAGGAGTCTGGACCGTGTCTCAGTTCCAGTGTGGCTGGTCATCCTCTCAGACCAGCTAGG
GATCGTCGCCTAGGTGAGCCGTTACCCCACCTACTAGCTAATCCCATCTGGGCACATCCGATGGCAAGAGGCC
CTAAGGTCCCCCTCTTGTCTTGCGACGTATGCGGTATTAGCTACCGTTTCCAGTAGTTATCCCCCTCCATCGG
GCAGTTTCCCAGACATTACTCACCCGTCCGCCACTCGTCAGCAAAGCAGCAAGCTGCTTCCTGTTACCGTTCG
ACTGCATGTGTTAGGCCTGCCGCCAGCGTCAATCTGAGCCAGGATCAAACTCT
>1025
CTGCTGCCGCCCGTAGGAGTCTGGACCGTGTCTCAGTTCCAGTGTGGCTGGTCATCCTCTCAGACCAGCTAGG
GATCGTCGCCTAGGTGAGCCGCTACCCCACCTACTAGCTAATCCCATCTGGGCACATCCGATGGCAAGAGGCC
CTAAGGTCCCCCTCTTTGGTCTTGCGACGTATGCGGTATTAGCTACCGTTTCCAGTAGTTATCCCCCTCCATC
AGGCAGTTTCCCAGACATTACTCACCCGTCCGCCACTCGTCAGCAAAGCAGCAAGCTGCTTCCTGTTACCGTT
CGACTGCATGTGTTAGGCCTGCCGCCAGCGTCAATCTGAGCCAGGATCAAACTCT
>1026
CTGCTGCCGCCCGTAGGAGTCTGGACCGTGTCTCAGTTCCAGTGTGGCTGGTCATCCTCTCAGACCAGCTAGG
GATCGTCGCCTAGGTGAGCCGTTACCCCACCTACTAGCTAATCCCATCTGGGCACATCCGATGGCAAGAGGCC

CTAAGGTCCCCCTCTTTGTCTGCGACGTTATGCGGTATTAGCTACCGTTTCCAGTAGTTATCCCCCTCCATCG
GGCAGTTTCCCAGACATTACTCACCCGTCCGCCACTCGTCAGCAAAGCAGCAAGCTGCTTCCTGTTACCGTTC
GACTGCATGTGTTAGGCCTGCCGCCAGCGTCAATCTGAGCCAGGATCAAACTCT
>1027
CTGCTGCCGCCCGTAGGAGTCTGGACCGTGTCTCAGTTCCAGTGTGGCTGGTCATCCTCTCAGACCAGCTAGG
GATCGTCGCCTAGGTGAGCCGTTACCCCACCTACTAGCTAATCCCATCTGGGCACATCCGATGGCAAGAGGCC
CTAAGGTCCCCCTCTTGTCTGCGACGTTATGCGGTATTAGCTACCGTTTCCAGTAGTTATCCCCCTCCATCAG
GCAGTTTCCCAGACATTACTCACCCGTCCGCCACTCGTCAGCAAAGCAGCAAGCTGCTTCCTGTTACCGTTCG
ACTGCATGTGTTAGGCCTGCCGCCAGCGTTCAATCTGAGCCAGGATCAAACTCT
>1028
CTGCTGCCGCCCGTAGGAGTCTGGACCGTGTCTCAGTTCCAGTGTGGCTGGTCATCCTCTCAGACCAGCTAGG
GATCGTCGCCTAGGTGAGCCGTTACCCCACCTACTAGCTAATCCCATCTGGGCACATCCGATGGCAAGAGGCC
CTAGGTCCCCCTCTTGTCTTGCGACGTTATGCGGTATTAGCTACCGTTTCCAGTAGTTATCCCCCTCCATCGG
GCAGTTTCCCAGACATTACTCACCCGTCCGCCACTCGTCAGCAAGCAGCAAGCTGCTTCCTGTTACCGTTCGA
CTGCATGTGTTAGGCCTGCCGCCAGCGTCAATCTGAGCCAGGATCAAACTCT
>1029
CTGCTGCCGCCCGTAGGAGTCTGGACCGTGTCTCAGTTCCAGTGTGGCTGGTCATCCTCTCAGACCAGCTAGG
GATCGTCGCCTAGGTGAGCCCGTTACCCCACCTACTAGCTAATCCCATCTGGGCACATCCGATGGCAAGACGG
CCCTAAGGTCCCCCTCTTTGTCTTGCGACGTTATGCGGTATTAGCTACCGTTCCAGTAGTTATCCCCCTCCAT
CGGGCAGTTCCCAGACACTACTCACCCGTCCGCCACTCGTCAGCAAGCAGCAAGCTGCTCCTGTTACCGTTCG
ACTGCATGTGTTAGGCCTGCCGCCAGCGTCAATCTGAGCCAGGATCAACTCT
>1030
CTGCTGCCGCCCGTAGGAGTCTGGACCGTGTCTCAGTTCCAGTGTGGCTGGTCATCCTCTCAGACCAGCTAGG
GATCGTCGCCTAGGTGAGCCGTTACCCCACCTACTAGCTAATCCCATCTGGGCACATCCGATGGCAAGAGGCC
CTAAGGTCCCCCTCTTGTCTTGCGACGTTATGCGGTATTAGCTACCGTTTCCAGTAGTTATCCCCCTCCATCG
GGCAGTTTCCCAGACATTACTCACCCGTCCGCCACTCGTCAGCAAAGCAGCAAGCTGCTTCCTGTTACCGTTC
GACTGCATGTGTTAGGCCTGCCGCCAGCGTCAATCTGAGCCAGGATCAAACTCT
>1031
CTGCTGCCGCCCGTAGGAGTCTGGACCGTGTCTCAGTTCCAGTGTGGCTGGTCATCCTCTCAGACCAGCTAGG
GATCGTCGCCTAGGTGAGCCGTTACCCCACCTACTAGCTAATCCCATCTGGGCACATCCGATGGCAAGAGGCC
CTAAGGTCCCCCTCTTGTCTTGCGACGTATGCGGTATTACGCTACCGTTTCCAGTAGTTATCCCCCTCCATCG
GGCAGTTTCCCAGACATTACTCACCCGTCCGCCACTCGTCAGCAAAGCAGCAAGCTGCTTCCTGTTACCGTTC
GACTGCATGTGTTAGGCCTGCCGCCAGCGTCAATCTGAGCCAGGATCAAACTCT
>1032
CTGCTGCCGCCCGTAGGAGTCTGGACCGTGTCTCAGTTCCAGTGTGGCTGGTCATCCTCTCAGACCAGCTAGG
GATCGTCGCCTAGGTGAGCCGTTACCCCACCTACTAGCTAATCCCATCTGGGCACATCCGATGGCAAGAGGCC
CTAAGGTCCCCCTCTTGTCTTGCGACGTATGCGGTATTAGCTACCGTTTCCAGTAGTTATCCCCCTCCATCAG
GCAGTTTCCCAGACATTACTCACCCGTCCGCCACTCGTCAGCAAAGCAGCAAGCTGCTTCCTGTTACCGTTCG
ACTGCATGTGTTAGGCCTGCCGCCAGCGTCAATCTGAGCCAGGATCAAACTCT
>1033
CTGCTGCCGCCCGTAGGAGTCTGGACCGTGTCTCAGTTCCAGTGTGGCTGGTCATCCTCTCAGACCAGCTAGG
GATCGTCGCCTAGGTGAGCCGTTACCCCACCTACTAGCTAATCCCATCTGGGCACATCCGATGGCAAGAGGCC
CTAAGGTCCCCCTCTTGTCTTGCGACGTTATGCGGTATTAGCTACCGTTTCCAGTAGTTATCCCCTCCATCGG
GCAGTTTCCCAGACATTACTCACCCGTCCGCCACTCGTCAGCAAAGCAGCAAGCTGCTTCCTGTTACCGTTCG
ACTGCATGTGTTAGGCCTGCCGCCAGCGTCAATCTGAGCCAGGATCAAACTCT
>1034
CTGCCGCCGCCCGTAGGAGTCTGGACCGTGTCTCAGTTCCAGTGTGGCTGGTCATCCTCTCAGACCAGCTAGG
GATCGTCGCCTAGGTGAGCCGTTACCCCACCTACTAGCTAATCCCATCTGGGCACATCCGATGGCAAGAGGCC
CTAAGGTCCCCCTCTTTGTCTTGCGACGTTATGCGGTATTAGCTACCGTTTCCAGTAGTTATCCCCCTCCATC
AGGCAGTTTCCCAGACATTACTCACCCGTCCGCCACTCGTCAGCAAAGCAGCAAGCTGCTTTCTGTTACCGTT
CGACTGCATGTGTTAGGCCTGCCGCCAGCGTCAATCTGAGCCAGGATCAAACTCT
>1035
CTGCTGCCGCCCGTAGGAGTCTGGACCGTGTCTCAGTTCCAGTGTGGCTGGTCATCCTCTCAGACCAGCTAGG
GATCGTCGCCTAGGTGAGCCGTTACCCCACCTACTAGCTAATCCCATCTGGGCACATCCGATGGCAAGAGGCC

CTAAGGTCCCCCTCTTTGGTCTTGCGACGTTATGCGGTATTAGCTACCGTTTCCAGTAGTTATCCCCCTCCAT
CGGGCAGTTTCCCAGACATTACTCACCCGTCCGCCACTCGTCAGCAAAGCAGCAAGCTGCTTCCTGTTACCGT
TCGACTGCATGTGTTAGGCCTGCCGCCAGCGTCAATCTGAGCCAGGATCAAACTCT
>1036
CTGCTGCCGCCCGTAGGAGTCTGGACCGTGTCTCAGTTCCAGTGTGGCTGGGTCATCCTCTCAGACCAGCTAG
GGATCGTCGCCTAGGTGAGCCGTTACCCCACCTACTAGCTAATCCCATCTGGGCACATCCGATGGCAAGAGGC
CCTAAGGTCCCCCTCTTTGGTCTTGCGACGTTATGCGGTATTAGCTACCGTTTCCAGTAGTTATCCCCCTCCA
TCGGGCAGTTTTCCCAGACATTACTCACCCGTCCGCCACTCGTCAGCAAAGCAGCAAGCTGCTTCCTGTTACC
GTTCGACTGCATGTGTTAGGCCTGCCGCCAGCGTTCAATCTGAGCCAGGATCAAACTCT
>1037
CTGCTGCCGCCCGTAGGAGTCTGGACCGTGTCTCAGTTCCAGTGTGGCTGGTCATCCTCTCAGACCAGCTAGG
GATCGTCGCCTAGGTGAGCCGTTACCCCACCTACTAGCTAATCCCATCTGGGCACATCCGATGGCAAGAGGCC
CTAAGGTCCCCCTCTTTGGTCTGCGACGTTATGCGGTATTAGCTACCGTTTCCAGTAGTTATCCCCCTCCATC
GGGCAGTTTCCCAGACATTACTCACCCGTCCGCCACTCGTCAGCAAAGCAGCAAGCTGCTTCCTGTTACCGTT
CGACTGCATGTGTTAGGCCTGCCGCCAGCGTCAATCTGAGCCAGGATCAAACTCT
>1038
CTGCTGCCGCCCGTAGGAGTCTGGACCGTGTCTCAGTTCCAGTGTGGCTGGTCATCCTCTCAGACCAGCTAGG
GATCGTCGCCTAGGTGAGCCGTTACCCCACCTGCTAGCTAATCCCATCTGGGCACATCCGATGGCAAGAGGCC
CTAAGGTCCCCCTCTTTGGTCTTGCGACGTTATGCGGTATTAGCTACCGTTTCCAGTAGTTATCCCCCTCCAT
CGGGCAGTTTCCCAGACATTACTCACCCGTCCGCCACTCGTCAGCAAAGCAGCAAGCTGCTTCCTGTTACCGT
TCGACTGCATGTGTTAGGCCTGCCGCCAGCGTCAATCTGAGCCAGGATCAACTCT
>1039
CTGCTGCCGCCCGTAGGAGTCTGGACCGTGTCTCAGTTCCAGTGTGGCTGGTCATCCTCTCAGACCAGCTATG
GATCGTCGCCTAGGTGAGCCGTTACCCCACCTACTAGCTAATCCCATCTGGGCACATCCGATGGCAAGAGGCC
CTAAGGTCCCCCTCTTTGGTCTTGCGACGTATGCGGTATTAGCTACCGTTTCCAGTAGTTATCCCCCTCCATC
AGGCAGTTTCCCAGACATTACTCACCCGTCCGCCACTCGTCAGCAAAGCAGCAAGCTGCTTCTGTTACCGTTC
GACTGCATGTGTTAGGCCTGCCCGCCAGCGTTCAATCTGAGCCAGGATCAAACCTCT
>1040
CTGCTGCCGCCCGTAGGAGTCTGGACCGTGTCTCAGTTCCAGTGTGGCTGGTCATCCTCTCAGACCAGCTAGG
GATCGTCGCCTAGGTGAGCCGTTACCCCACCTACTAGCTAATCCCATCTGGGCACATCCGATGGCAAGAGGCC
CTAAGGTCCCCCTCTTTGTCTGCGACGTATGCGGTATTAGCTACCGTTTCCAGTAGTTATCCCCCTCCATCGG
GCAGTTTCCCAGACATTACTCACCCGTCCGCCACTCGTCAGCAAAGCAGCAAGCTGCTTCCTGTTACCGTTCG
ACTGCATGTGTTAGGCCTGCCGCCAGCGTCAATCTGAGCCAGGATCAAACTCT
>1041
CTGCTGCCGCCCGTAGGAGTCTGGACCGTGTCTCAGTTCCAGTGTGGCTGGTCATCCTCTCAGACCAGCTAGG
GATCGTCGCCTAGGTGAGCCGTTACCCCACCTACTAGCTAATCCCATCTGGGCACATCCGATGGCAAGAGGCC
ATAAGGTCCCCCCTCTTTGGTCTTGCGACGTTATGCGGTATTAGCTACCGTTTCCAGTAGTTATCCCCCTCCA
TCGGGCAGTTTCCCAGACATTACTCACCCGTCCGCCACTCGTCAGCAAAGCAGCAAGCTGCTTCCTGTTACCG
TTCGACTGCATGTGTTAGGCCTGCCGCCAGCGTCAATCTGAGCCAGGATCAAACTCT
>1042
CTGCTGCCGCCCGTAGGAGTCTGGACCGTGTCTCAGTTCCAGTGTGGCTGGTCATCCTCTCAGACCAGCTAGG
GATCGTCGCCTAGGTGAGCCGTTACCCCACCTACTAGCTAATCCCATCTGGGCACATCCGATGGCAAGAGGCC
CTAAGGTCCCCCCTCTTTGTCTTGCGACGTTATGCGGTATTAGCTACCGTTCCAGTAGTTATCCCCCTCCATC
AGGCAGTTTCCCAGACATTACTCACCCGTCCGCCACTCGTCAGCAAAGCAGCAAGCTGCTTCCTGTTACCGTT
CGACTGCATGTGTTAGGCCTGCCGCCAGCCGTTCAATCTGAAGCCAGGATCAAACCATCT
>1043
CTGCTGCCGCCCGTAGGAGTCTGGACCGTGTCTCAGTTCCAGTGTGGCTGGTCATCCTCTCAGACCAGCTAGG
GATCGTCGCCTAGGTGAGCCGTTACCCCACCTACTAGCTAATCCCATCTGGGCACATCCGATGGCAAGAGGCC
CTAAGGTCCCCCTCTTTGGTCTTGCGAACGTTATGCGGTATTAGCTACCGTTTCCAGTAGTTATCCCCCTCCA
TCAGGCAGTTTCCCCAGACATTACTCACCCGTCCGCCACTCGTCAGCAAAGCAGCCAAGCTGCTTCCTGTTAC
CGTTCGACTGCATGTGTTAGGCCTGACCGCCAGCGTTCACTCGAACCAGGTCAA
>1044
CTGCTGCCGCCCGTAGGAGTCTGGGCCGTGTCTCAGTTCCAGTGTGGCTGGTCATCCTCTCAGACCAGCTAGG
GATCGTCGCCTAGGTGAGCCGTTACCCCACCTACTAGCTAATCCCATCTGGGCACATCCGATGGCAAGAGGCC

CTAAGGTCCCCCTCTTGTCTTGCGACGTTATGCGGTATTAGCTACCGTTTCCAGTAGTTATCCCCCTCCATCG
GGCAGTTTTCCCAGACATTACTCACCCGTCCGCCACTCGTCAGCAAGCAGCAAGCTGCTTCCTGTTACCGTTC
GACTGCATGTGTTAGGCCTGCCGCCAGCGTCAATCTGAGCCAAGGATCAAACTCT
>1045
CTGCTGTCGCCCGTAGGAGTCTGGACCGTGTCTCAGTTTCCAGTGTGGCTGGTCATTCCTCTCAGACCAGCTA
GGGATCGTCGCCTAGGTGAGCCGTTTACCCCACCTACTAGCTAATCCCATCTGGGCACATCCGATGGCAAGAG
GCCCTAAGGTCCCCCTCTTGTCTGCGACGTATGCGGTATTAGCTACCGTTTCCAGTAGTTATCCCCTCCATCG
GGCAGTTTCCCAGACATTACTCACCCGTCCGCCACTCGTCAGCAAAGCAGCAAGCTGCTTCCTGTTACCGTTC
GGCTTGCATGTGTTAGGCCTGCCGCCAGCGTTCAATCTGAGCCAGGATCAAACTCT
>1046
CTGCTGCCGCCCGTAGGAGTCTGGACCGTGTCTCAGTTCCAGTGTGGCTGGTCATCCTCTCAGACCAGCTAGG
GATCGTCGCCTAGGTGAGCCGTTACCCCACCTACTAGCTAATCCCATCTGGGCACATCCGATGGCAAGAGGCC
CTAAGGTCCCCCTCTTTGGTCTGCGACGTATGCGGTATTAGCTACCGTTTCCAGTAGTTATCCCCCTCCATCG
GGCAGTTTCCCAGACATTACTCACCCGTCCGCCACTCGTCAGCAAAGCAGCAAGCTGCTTCCTGTTACCGTTC
GACTGCATGTGTTAGGCCTGCCGCCAGCGTCAATCTGAGCCAGGATCAAACTCT
>1047
CTGCTGCCGCCCGTAGGAGTCTGGACCGTGTCTCAGTTCCAGTGTGGCTGGTCATCCTCTCAGACCAGCTAGG
GATCGTCGCCTAGGTGAGCCGTTACCCCACCTACTAGCTAATCCCATCTGGGCACATCCGATGGCAAGAGGCC
CTAAGGTCCCCCTCTTGTCTTGCGACGTTATGCGGTATTAGCTACCGTTTCCAGTAGTTATCCCCCTCCATCA
GGCAGTTTCCCAGACATTACTCACCCGTCCGCCACTCGTCAGCAAACAGCAAGCTGCTTCCTGTTACCGTTCG
ACTGCATGTGTTAGGCCTGCCGCCAGCGTCAATCTGAGCCAGGATCAAACTCT
>1048
CTGCTGCCGCCCGTAGGAGTCTGGACCGTGTCTCAGTTCCAGTGTGGCTGGTCATCCTCTCAGACCAGCTAGG
GATCGTCGCCTAGGTGAGCCGTTACCCCACCTACTAGCTAATCCCATCTGGGCACATCCGATGGCAAGAGGCC
CGAAGGTCCCCCTCTTTGGTCTTCGACATTATGCGGTATTAGCTACCGTTTCCAGTAGTTATCCCCCTCCATC
AGGCAGTTTCCCAGACATTACTCACCCGTCCGCCACTCGTCAGCAAAGCAGCAAGCTGCTTCTGTTACCGTTC
GACTGCATGTGTTAGGCCTGCCGCCAGCGTCAATCTGAGCCAGGATCAAACTCT
>1049
CTGCTGCCGCCCGTAGGAGTCTGGACCGTGTCTCAGTTCCAGTGTGGCTGGTCATCCTCTCAGACCAGCTAGG
GATCGTCGCCTAGGTGAGCCGTTACCCCACCTACTAGCTAATCCCATCTGGGCACATCCGATGGCAAGAGGCC
CTAAGGTCCCCCTCTTTGGTCTTGCGACGTATGCGGTATTAGCTACCGTTTCCAGTAGTTATCCCCCTCCATC
AGGCAGTTTCCCAGACATTACTCACCCGTCCGCCACTCGTCAGCAAAGCAGCAAGCTGCTTTCTGTTACCGTT
CGACTGCATGTGTTAGGCCTGCCGCCAGCGTCAATCTGAGCCAGGATCAAACTCT
>1050
CTGCTGCCGCCCGTAGGAGTCTGGACCGTGTCTCAGTTCCAGTGTGGCTGGTCATCCTCCCAGACCAGCTAGG
GATCGTCGCCTAGGTGAGCCGTTACCCCACCTACTAGCTAATCCCATCTGGGCACATCCGATGGCAAGAGGCC
CTAAGGTCCCCCTCTTGGTCTTGCGACGTATGCGGTATTAGCTACCGTTTCCAGTAGTTATCCCCCTCCATCG
GGCAGTTTCCCAGACATTACTCACCCGTCCGCCACTCGTCAGCAAAAGCAGCAAGCTGCTTCCTGTTACCGTT
CGACTGCATGTGTTAGGCCTGCCGCCAGCGTCAATCTGAGCCAGGATCAAACTCT
>1051
TGCTGCCGCCCGTAGGAGTCTGGACCGTGTCTCAGTTCCAGTGTGGCTGGTCATCCTCTCAGACCAGCTAGGG
ATCGTCGCCTAGGTGAGCCGTTACCCCCACCTACTAGCTAATCCCATCTGGGCACATCCGATGGCAAGAGGCC
CTAAGGTCCCCCTCTTTGTCTTGCGACGTTATGCGGTATTAGCTACCGTTTCCAGTAGTTATCCCCCTCCATC
GGGCAGTTTCCCAGACATTACTCACCCGTCCGCCACTCGTCAGCAAAGCAGCAAGCTGCTTCCTGTTACCGTT
CGACTGCAATGTGTTAGGCCTGCCGCCAGCGTCAATCTGAGCCAGGATCAAACTCT
>1052
CTGCTGCCGCCCGTAGGAGTCTGGAACCGTGTCTCAGTTCCAGTGTGGCTGGTCATCCTCTCAGACCAGCTAG
GGATCGTCGCCTAGGTGAGCCGTTACCCCACCTACTAGCTAATCCCATCTGGGCACATCCGATGGCAAGAGGC
CCTAAGGTCCCCCTCTTTGTCTGCGACGTTATGCGGTATTAGCTACCGTTTCCAGTAGTTATCCCCCTCCATC
AGGCAGTTTTCCCAGACATTACTCACCCGTCCGCCACTCGTCAGCAAAGCAGCAAGCTGCTTCCTGTTACCGT
TCGACTGCATGTGTTAGGCCTGCCGCCAGCCGTC
>1053
CTGCTGCCGCCCGTAGGAGTCTGGACCGTGTCTCAGTTCCAGTGTGGCTGGTCATCCTCTCAGACCAGCTAGG
GATCGTCGCCTAGGTGAGCCGTTACCCCACCTACTAGCTAATCCCATCTGGGCACATCCGATGGCAAGAGGCC

CTAAGGTCCCCCTCTTTGTCTTGCGACGTTATGCGGTATTAGCTACCGTTTCCAGTAGTTATCCCCCTCCATC
AGGCAGTTTCCCAGACATTACTCACCCGTCCGCCACTCGTCAGCAAAGCAGCAAGCTGCTTCCTGTTACCGTT
CGACTGCATGTGTTAGGCCTGACCGCCAGCGTCAT
>1054
CTGCTGCCGCCCGTAGGAGTCTGGACCGTGTCTCAGTTCCAGTGTGGCTGGTCATCCTCTCAGACCAGCTAGG
GATCGTCGCCTAGGTGAGCCGTTACCCCACCTACTAGCTAATCCCATCTGGGCACATCCGATGGCAAGAGGCC
CTAAGGTCCCCCTCTTGTCTTGCGACGTTATGCGGTATTAGCTACCGTTTCCAGTAGTTATCCCCCTCCATCA
GGCAGTTTCCCAGACATTACTCACCCGTCCGCCACTCATCAGCAAAGCAGCAAGCTGCTTCCTGTTACCGTTC
GACTGCATGTCGTTAGGCCTGCCGCCAGCGTCAATCTG
>1055
CTGCTGCCGCCCGTAGGAGTCTGGACCGTGTCTCAGTTCCAGTGTGGCTGGTCATCCTCTCAGACAGCTAGGG
ATCGTCGCCTAGGTGAGCCGTTACCCCACCTACTAGCTAATCCCATCTGGGCACATCCGATGGCAAGAGGCCC
TAAGGTCCCCCTCTTGTCTTGCGACGTTATGCGGTATTAGCTACCGTTTCCAGTAGTTATCCCCCTCCATCAG
GCAGTTTTCCCAGACATTACTCACCCGTCCGCCACTCGTCAGCAAAGCAGCAAGCTGCTTTCTGTTACCGTTC
GACTGCATGTGTTAGGCCTGCCCGCCAGCGTTCAATCTGAGCCAGGATCAAACTCT
>1056
CTGCTGCCGCCCGTAGGAGTCTGGACCGTGTCTCAGTTCCAGTGTGGCTGGTCATCCTCTCAGACCAGCTAGG
GATCGTCGCCTAGGTGAGCCGTTACCCCACCTACTAGCTAATCCCATCTGGGCACATCCGATGGCAAGAGGCC
CTAAGGTCCCCCCTCTTTGGTCTTGCGACGTTATGCGGTATTAGCTACCGTTTCCAGTAGTTATCCCCCTCCA
TCGGGCAGTTTTCCCAGACATTACTCACCCGTCCGCCACTCGTCAGCAAAGCAGCAAGCTGCTTCCTGTTACC
GTTCGACTGCATGTGTTAGGCCTGCCGCCAGCGTTCAATCTGAGCCAGGATCAAACACT
>1057
CTGCTGCCGCCCGTAGGAGTCTGGACCGTGTCTCAGTTCCAGTGTGGCTGGTCATCCTCTCAGACCAGCTAGG
GATCGTCGCCTAGGTGAGCCGTTACCCCACCTACTAGCTAATCCCATCTGGGCACATCCGATGGCAAGAGGCC
CTAAGGTCCCCCTCTTTGGTCTTGCGACGTTATGCGGTATTAGCTACCGTTTCCAGTAGTTATCCCCCTCCAT
CAGGCAGTTTTCCCAGACATTACTCACCCGTCCGCCACTCGTCAGCAAAGCAGCAAGCTGCTTCCTGTTACCG
TTCGACTGCATGTGTTAGGCCTGCCGCCAGCGTCAATCTGAGCCAGGATCAAACTCT
>1058
CTGCTGCCGCCCGTAGGAGTCTGGACCGTGTCTCAGTTCCAGTGTGGCTGGTCATCCTCTCAGACCAGCTAGG
GATCGTCGCCTAGGTGAGCCGTTACCCCACCTACTAGCTAATCCCATCTGGGCACATCCGATGGCGAGAGGCC
CTAAGGTCCCCCTCTTTGTCTTGCGACGTTATGCGGTATTAGCTACCGTTTCCAGTAGTTATCCCCCTCCATC
AGGCAGTTTCCCAGACATTACTCACCCGTCCGCCACTCGTCAGCAAACAGCAAGCTGCTTCCTGTTACCGTTC
GACTTGCATGTGTTAGGCCTGCCGCCAGCGTCAATCTGAGCCAGGATCAAACTCT
>1059
CTGCTGCCGCCCGTAGGAGTCTGGACCGTGTCTCAGTTCCAGTGTGGCTGGTCATCCTCTCAGACCAGCCAGG
GATCGTCGCCTAGGTGAGCCGTTACCCCACCTATAGCTAATCCCATCTGGGCACATCCGATGGCAAGAGGCCC
TAAGGTCCCCCTCTTTGGTCTTGCGATCGTTATGCGGTATTAGCTACCGTTTCCAGTAGTTATCCCCCTCCAT
CAGGCAGTTTCCCAGACATTACTCACCCGTCCGCCACTCGTCAGCAAAGCAGCAAGCTGCTTCTGTTACCGTT
CGGCTGCATGTGTTAGGCCTGCCGCCAGCGTCAATCTGAGCCAG
>1060
CTGCTGCCGCCCGTAGGAGTCTGGACCGTGTCTCAGTTCCAGTGTGGCTGGTCATCCTCTCAGACCAGCTAGG
GATCGTCGCCTAGGTGAGCCGTTACCCCACCTACTAGCTAATCCCATCTGGGCACATCCGATGGCAAGAGGCC
CTAAGGTCCCCCTCTTTGTCTTGCGACGTTATGCGGTATTAGCTACCGTTTCCAGTAGTTATCCCCCTCCATC
AGGCAGTTTCCCAGACATTACTCACCCGTCCGCCACTCGTCAGCAAAGCAGCAAGCTGCTTCTGTTACCGTTC
GACTGCATGTGTTAGGCCTGCCGCCAGCGTTCAATCGAGCCAGGATCAAACTCT
>1061
CTGCTGCCGCCCGTAGGAGTCTGGACCGTGTCTCAGTTCCAGTGTGGCTGGTCATCCTCTCAGACCAGCTAGG
GATCGTCGCCTAGGTGAGCCGTTACCCCACCTACTAGCTAATCCCATCTGGGCACATCCGATGGCAAGAGGCC
CTAAGGTCCCCCTCTTTGGTCTTGCGACGTTATGCGGTATTAGCTACCGTTTCCAGTAGTTATCCCCCTCCAT
CAGGCAGTTTCCCAGACATTACTCACCCGTCCGCCACTCGTCAGCAAAGCAGCAAGCTGCTTCTGTTACCGTT
CGACTGCATGTGTTAGGCCTGCCGCCAGCGTCAATCTGAGCCAGGATCAAACTCT
>1062
CTGCTGCCGCCCGTAGGAGTCTGGACCGTGTCTCAGTTCCAGTGTGGCTGGTCATCCTCTCAGACCAGCTAGG
GATCGTCGCCTAGGTGAGCCGTTACCCCACCTACTAGCTAATCCCATCTGGGCACATCCGATGGCAAGAGGCC

CTAAGGTCCCCCTCTTGTCTTGCGACGTTATGCGGTATTAGCTACCGTTTCCAGTAGTTATCCCCCTCCATCA
GGCAGTTTCCCAGACATTACTCACCCGTCCGCCACTCGTCAGCAAAGCAGCAAGCTGCTTCTGTTACCGTCGA
CTGCATGTGTTAGGCCTGCCGCCAGCGTCAATCTGAGCCAGGATCAAACTCT
>1063
CTGCTGCCGCCCGTAGGAGTCTGGACCGTGTCTCAGTTCCAGTGTGGCTGGTCATCCTCTCAGACCAGCTAGG
GATCGTCGCCTAGGTGAGCCGTTACCCCACCTACTAGCTAATCCCATCTGGGCACATCCGATGGCAAGAGGCC
CTAAGGTCCCCCTCTTTGTCTGCGACGTATGCGGTATTAGCTACCGTTTCCAGTAGTTATCCCCCTCCATCAG
GCAGTTTCCCAGACATTACTCACCCGTCCGCCACTCGTCAGCAAAGCAGCAAGCTGCTTCCTGTTACCGTTCG
ACTGCATGTGTTAGGCCTGCCGCCAGCGTCAATCTGAGCCAGGATCAAACTCT
>1064
CTGCTGCCGCCCGTAGGAGTCTGGACCGTGTCTCAGTTCCAGTGTGGCTGGTCATCCTCTCAGACCAGCTAGG
GATCGTCGCCTAGGTGAGCCGTTACCCCACCTACTAGCTAATCCCGTCTGGGCACATCCGATGGCAAGAGGCC
CTAAGGTCCCCCTCTTGTCTTGCGACGTATGCGGTATTAGCTACCGTTTCCAGTAGTTATCCCCCTCCATCAG
GCAGTTTCCCAGACATTACTCACCCGTCCGCCACTCGTCAGCAAACAGCAAGCTGCTTCCTGTTACCGTTCGA
CTGCATGTGTTAGGCCTGCCGCCAGCGTCAATCTGAGCCAGGATCAAACTCT
>1065
CTGCTGCCGCCCGTAGGAGTCTGGACCGTGTCTCAGTTCCAGTGTGGCTGGTCATCCTCTCAGACCAGCTAGG
GATCGTCGCCTAGGTGAGCCGTTACCCCACCTACTAGCTAATCCCATCTGGGCACATCCGATGGCAAGAGGCC
CTAAGGTCCCCCTCTTTGTCTGCGACGTTATGCGGTATTAGCTACCGTTCCAGTAGTTATCCCCCTCCATCAG
GCAGTTTCCCAGACATTACTCACCCGTCCGCCACTCGTCAGCAAAGCAGCAAGCTGCTTCCTGTTACCGTTCG
ACTGCATGTGTTAGGCCTGCCGCCAGCGTCAATCTGAGCCAGGATCAAACTCT
>1066
CTGCTGCCGCCCGTAGGAGTCTGGACCGTGTCTCAGTTCCAGTGTGGCTGGTCATCCTCTCAGACCAGCTAGG
GATCGTCGCCTAGGTGAGCCGTTTACCCCACCTACTAGCTAATCCCATCTGGGCACATCCGATGGCAAGAGGC
CCTAAGGTCCCCCTCTTTGTCTTGCGACGTATGCGGTATTAGCTACCGTTTCCAGTAGTTATCCCCCTCCATC
AGGCAGTTTCCCAGACATTACTCACCCGTCCGCCACTCGTCAGCAAAGCAGCAAGCTGCTTCCTGTTACCGTT
CGACTGCATGTGTTAGGCCTGCCGCCAGCGTTCAATCTGAGCCAGGATCAAACTCT
>1067
CTGCTGCCGCCCGTAGGAGTCTGGACCGTGTCTCAGTTCCAGTGTGGCTGGTCATCCTCTCAGACCAGCTAGG
GATCGTCGCCTAGGTGAGCCGTTACCCCACCTACTAGCTAATCCCATCTGGGCACATCCGATGGCAAGAGGCC
CTAAGGTCCCCCTCTTTGTCTTGCGACGTTATGCGGTATTAGCTACCGTTTCCAGTAGTTATCCCCCCTCCAT
CGAGACATTTCCCAGACATTACTCACCCGTCCGCCACTCGTCAGCAAAGCAGCAAGCTGCTTCCTGTTACCGT
TCGACTGCATGTGTTAGGCCTGCCGCCAGCGTTCAATCTGAGCCAGGACAAACTCT
>1068
CTGCTGCCGCCCGTAGGAGTCTGGACCGTGTCTCAGTTCCAGTGTGGCTGGTCATCCTCTCAGACCAGCTAGG
GATCGTCGCCTAGGTGAGCCGTTACCCCACCTACTAGCTAATCCCATCTGGGCACATCCCGATGGCAAGAGGC
CCTAAGGTCCCCCTCTTGTCTTGCGACGTTATGCGGTATTAGCTACCGTTTCCAGTAGTTATCCCCCTCCATC
AGGCAGTTTCCCAGACACTACTCACCCGTCCGCCACTCGTCAGCAAAGCAGCAAGCTGCTTCCTGTTACCGTT
CGACTGCATGTGTTAGGCCTGCCGCCAGCGTCAATCTGAGCCAGGATCAAACTCT
>1069
CTGCTGCCGCCCGTAGGAGTCTGGACCGTGTCTCAGTTCCAGTGTGGCTGGTCATCCTCTCAGACCAGCTAGG
GATCGTCGCCTAGGTGAGCCGTTACCCCACCTACTAGCTAATCCCATCTGGGCACATCCGATGGCAAGAGGCC
CTAAGGTCCCCCTCTTTGTCTTGCGACGTATGCGGTATTAGCTACCGTTTCCAGTAGTTATCCCCCTCCATCG
GGCAGTTTCCCAGACATTACTCACCCGTCCGCCACTCGTCAGCAAAGCAGCAAGCTGCCTCCTGTTACCGTTC
GACTGCATGTGTTAGGCCTGCCGCCAGCGTCAATCTGAGCCAGGATCAAACTCT
>1070
CTGCTGCCGCCCGTAGGAGTCTGGACCGTGTCTCAGTTCCAGTGTGGCTGGTCATCCTCTCAGACCAGCTAGG
GATCGTCGCCTAGGTGAGCCGTTACCCCACCTACTAGCTAATCCCATCTGGGCACATCCGATGGCAAGAGGCC
CTAAGGTCCCCCTCTTTGTCTTGCGAACGTTATGCGGTATTAGCTACCGTTTCCAGTAGTTATCCCCCTCCAT
CAGGCAGTTTCCCAGACATTACTCACCCGTCCGCCACTCGTCAGCAAAGCAGCAAGCTGCTTCCTGTTACCGT
TCGACTGCATGTGTTAGGCCTGCCGCCAGCGTTCAATCTGAGCCAGGATCAAACTCT
>1071
CTGCTGCCGCCCGTAGGAGTCTGGACCGTGTCTCAGTTCCAGTGTGGCTGGTCATCCTCTCAGACAGCTAGGG
ATCGTCGCCTAGGTGAGCCGTTACCCCACCTACTAGCTAATCCCATCTGGGCACATCCGATGGCAAGAGGCCC

TAAGGTCCCCCTCTTGGTCTTGCGACGTATGCGGTATTAGCTACCGTTTCCAGTAGTTATCCCCCTCCATCAG
GCAGTTTCCCAGACATTACTCACCCGTCCGCCACTCGTCAGCAAAGCAGCAAGCTGCTTCCTGTTACCGTTCG
ACTGCATGTGTTAGGCCTGCCGCCAGCGTCAATCTGAGCCAGGATCAAACTCT
>1072
CTGCTGCCGCCCGTAGGAGTCTGGACCGTGTCTCAGCTCCAGTGTGGCTGGTCATCCTCTCAGACCAGCTAGG
GATCGTCGCCTAGGTGAGCCGTTACCCCACCTACTAGCTAATCCCATCTTGGCACATCCGATGGCAAGAGGCC
CTAAGGTCCCCTCTTTGGTCTTGCGACGTTATGCGGTATTAGCTACCGTTTCCAGTAGTTATCCCCCTCCATC
GAGGCAGTTTCCCAGACATTACTCACCCGTCCGCCACTCGTCAGCAAAGCAGCAAGCTGCTTCCTGTTACCGT
TCGACTGCATGTGGTTAGGCCTGCCGCCAGCGTCAATCTGAGCCAGGATCAAACTGT
>1073
CTGCTGCCGCCCGTAGGAGTCTGGACCGTGTCTCAGTTCCAGTGTGGCTGGTCATCCTCTCAGACCAGCTAGG
GATCGTCGCCTAGGTGAGCCGTTACCCCACCTACTAGCTAATCCCATCTGGGCACATCCGATGGCAAGAGGCC
CTAAGGTCCCCCTCTTGTCTTGCGACGTTATGCGGTATTAGCTACCGTTTCCAGTAGTTATCCCCCTCCATCA
GGCAGTTTCCCAGACATTACTCACCCGTCCGCCACTCGTCAGCAAAGCAGCAAGCTGCTTCCTGTTACCGTTC
GACTGCATGTGTTAGGCCTGCCGCCAGCGTCAATCTGAAGCCAGGATCAAACTCT
>1074
CTGCTGCCGCCCGTAGGAGTCTGGACCGTGTCTCAGTTCCAGTGTGGCTGGTCATCCTCTCAGACCAGCTAGG
GATCGTCGCCTAGGTGAGCCGTTACCCCACCTACTAGCTAATCCCATCTGGGCACATCCGATGGCAAGAGGCC
CTAAGGTCCCCCTCTTTGGTCTTGCGACGTTATGCGGTATTAGCTACCGTTTCCAGTAGTTATCCCCCTCCAT
CAGGCAGTTTCCCAGACATTACTCACCCGTCCGCCACTCGTCAGCAAAGCAGCAAGCTGCTTTCTGTTACCGT
TCGACTGCATGTGTTAGGCCTGCCGCCAGCGTTCAATCTGAGCCAGGATCAAACTCT
>1075
CTGCTGCCGCCCGTAGGAGTCTGGACCGTGTCTCAGTTCCAGTGTGGCTGGTCATCCTCTCAGACCAGCTAGG
GATCGTCGCCTAGGTGAGCCGTTACCCCACCTACTAGCTAATCCCATCTGGGCACATCCGATGGCAAGAGGCC
CTAAGGTCCCCCTCTTGTCTTGCGACGTTATGCGGTATTAGCTACCGTTTCCAGTAGTTATCCCCCTCCATCG
GGCAGTTTCCCAGACATTACTCACCCGTCCGCCACTCGTCAGCAAAGCAGCAAGCTGCTTCCTGTTACCGTTC
GACTGCATGTGTTAGGCCTGCCGCCAGCGTCAATCTGAGCCAGGATCAAACTCT
>1076
CTGCTGCCGCCCGTAGGAGTCTGGACCGTGTCTCAGTTCCAGTGTGGCTGGTCATCCTCTCAGACCAGCTAGG
GATCGTCGCCTAGGTGAGCCGTTACCCCACCTACTAGCTAATCCCATCTGGGCACATCCGATGGCAAGAGGCC
CTAAGGTCCCCCTCTTTGTCTTGCGACGTTATGCGGTATTAGCTACCGTTTCCAGTAGTTATCCCCCTCCATC
AGGCAGTTTCCCAGACATTACTCACCCGTCCGCCACTCGTCAGCAAAGCAGCAAGCTGCTTCTGTTACCGTTC
GACTGCATGTGTTAGGCCTGCCGCCAGCGTCAATCTGAGCCAGGATCAAACTCT
>1077
CTGCTGCCGCCCGTAGGAGTCTGGACCGTGTCTCAGTTCCAGTGTGGCTGGTCATCCTCTCAGACCAGCTAGG
GATCGTCGCCTAGGTGAGCCGTTACCCCACCTACTAGCTAATCCCATCTGGGCACATCCGATGGCAAGAGGCC
CTAAGGTCCCCCTCTTTGGTCTTGCGACGTATGCGGTATTAGCTACCGTTTCCAGTAGTTATCCCCCTCCATC
AGGCAGTTTTCCCAGACATTACTCACCCGTCCGCCACTCGTCAGCAAAGCAGCAAGCTGCTTCCTGTTACCGT
TCGACTGCATGTGTTAGGCCTGCCGCCAGCGTCAATCTGAGCCAGGATCAAACTCT
>1078
CTGCTGCCGCCCGTAGGAGTCTGGACCGTGTCTCAGTTCCAGTGTGGCTGGTCATCCTCCCAGACCAGCTAGG
GATCGTCGCCTAGGTGAGCCGTTACCCCACCTACTAGCTAATCCCATCTGGGCACATCCGATGGCAAGAGGCC
CTAAGGTCCCCCTCTTGGTCTTGCGACGTTATGCGGTATTAGCTACCGTTTCCAGTAGTTATCCCCCTCCATC
AGGCAGTTTCCCAGACATTACTCACCCGTCCGCCACTCGTCAGCAAAGCAGCAAGCTGCTTCCTGTTACCGTT
CGACTGCATGTGTTAGGCCTGCCGCCAGCGTTCAATCTGAGCCAGGATTCAAACTCT
>1079
CTGCTGCCGCCCGTAGGAGTCTGGACCGTGTCTCAGTTCCAGTGTGGCTGGTCATCCTCTCAGACCAGCTAGG
GATCGTCGCCTAGGTGAGCCGTTTACCCCACCTACTAGCTAATCCCATCTGGGCACATCCGATGGCAAGAGGC
CCTAAGGTCCCCCTCTTTGGTCTGCGACGTTATGCGGTATTAGCTACCGTTTCCAGTAGTTATCCCCCTCCAT
CAGGCAGTTTCCCAGACATTACTCACCCGTCCGCCACTCGTCAGCAAAGCAGCAAGCTGCTTCCTGTTACCGT
TCGACTGCATGTGTTAGGCCTGCCGCCAGCGTCAATCTGAGCCAGGATCAAACTCT
>1080
CTGCTGCCGCCCGTAGGAGTCTGGACCGTGTCTCAGTTCCAGTGTGGCTGGTCATCCTCTCAGACCAGCTAGG
GATCGTCGCCTAGGTGAGCCGTTACCCCACCTACTAGCTAATCCCATCTGGGCACATCCCGGTGGCAAGAGGC

CCTAAGGTCCCCCTCTTGTCTGCGACGTATGCGGTATTAGCTACCGTTTCCAGTAGTTATCCCCCTCCATCAG
GCAGTTTCCCAGACATTACTCACCCGTCCGCCACTCGTCAGCAAAGCAGCAAGCTGCTTCCTGTTACCGTTCG
ACTGCATGTGTTAGGCCTGCCGCCAGCGTTCAATCTGAGCCAGGACAAACTGT
>1081
CTGCTGCCGCCCGTAGGAGTCTGGACCGTGTCTCAGTTCCAGTGTGGCTGGTCGTCTCTCAGACCAGCTAGGG
ATCGTCGCCTAGGTGAGCCGTTACCCACCCTACTAGCTAATCCCATCTGGGCACATCCGATGGCAAGAGGCCC
TAAGGTCCCCCTCTTTGGTCTTGCGACGTTATGCGGTATTAGCTACCGTTTCCAGTAGTTATCCCCCTCCATC
AGGCAGTTTCCCAGACATTACTCACCCGTCCACCACTCGTCAAGCAAACAGCAAGCTGCTTCCTGTTACCGTT
CGACTGCATGTGTTAGGCCTGCCGCCAGCGTTCAATCTGAGCCAGGATCAAACTCT
>1082
CTGCTGCCGCCCGTAGGAGTCTGGACCGTGTCTCAGTTCCAGTGTGGCTGGTCATCCTCTCAGACCAGCTAGG
GATCGTCGCCTAGGTGAGCCGTTACCCCACCTACTAGCTAATCCCATCTGGGCACATCCGATGGCAAGAGGCC
CTAAGGTCCCCCTCTTGGTCTGCGACGTATGCGGTATTAGCTACCGTCTCCAGTAGTTATCCCCCTCCATCGG
GCAGTTTTCCCAGACATTACTCACCCGTCCGCCACTCGTCAGCAAAGCAGCAAGCTGCTTCCTGTTACCGTTC
GACTGCATGTGTTAGGCCTGCCGCCAGCGTCAATCTGAGCCAGGATCAAACTCT
>1083
CTGCTGCCGCCCGTAGGAGTCTGGACCGTGTCTCAGTTCCAGTGTGGCTGGTCATCCTCTCAGACCAGCTAGG
GATCGTCGCCTAGGTGAGCCGTTACCCCACCTACTAGCTAATCCCATCTGGGCACATCCGATGGCAAGAGGCC
CTAAGGTCCCCCTCTTTGGTCTTGCGACGTATGCGGTATTAGCTACCGTTTCCAGTAGTTATCCCCCTCCATC
GGGCAGTTTCCCAGACATTACTCACCCGTCCGCCACTCGTCAGCAAAGCAGCAAGCTGCTTCCTGTTACCGTT
CGACTGCATGTGTTAGGCCTGCCGCCAGCGTCAATCTGAGCCAGGATCAAACTCT
>1084
CTGCTGCCGCCCGTAGGAGTCTGGACCGTGTCTCAGTTCCAGTGTGGCTGGTCATCCTCTCAGACCAGCTAGG
GATCGTCGCCTAGGTGAGCCGTTACCCCACCTACTAGCTAATCCCATCTGGGCACATCCGATGGCAAGAGGCC
CTAAGGTCCCCCTCTTTGGTCTTGCGACGTTATGCGGTATTAGCTACCGTTTCCAGTAGTTATCCCCCTCCAT
CAGGCAGTTTCCCAGACATTACTCACCCGTCCGCCACTCGTCAGCAAAGCAGCAAGCTGCTTCCTGTTACCGT
TCGACTGCATGTGTTAGGCCTGCCGCCAGCGTCAATCTGAGCCAGGATCAAACTCT
>1085
CTGCTGCCGCCCGTAGGAGTCTGGACCGTGTCTCAGTTCCAGTGTGGCTGGTCATCCTCTCAGACCAGCTAGG
GATCGTCGCCTAGGTGAGCCGTTACCCCACCTACTAGCTAATCCCATCTGGGCACATCCGATGGCAAGAGGCC
CTAAGGTCCCCCTCTTGTCTTGCGACGTTATGCGGTATTAGCTACCGTTTCCAGTAGTTATCCCCCTCCATCA
GGCAGTTTCCCAGACATTACTCGCCCGTCCGCCACTCGTCAGCAAGCAGCAAGCTGCTTCCTGTTACCGTCGA
CTGCATGTGTTAGGCCTGCCGCCAGCGTTCAATCTGAGCCAGGATCAAACTCT
>1086
CTGCTGCCGCCCGTAGGAGTCTGGACCGTGTCTCAGTTCCAGTGTGGCTGGTCATCCTCTCAGACCAGCTAGG
GATCGTCGCCTAGGTGAGCCGTTACCCCACCTACTAGCTAATCCCATCTGGGCACATCCGATGGCAAGAGGCC
CTAAGGTCCCCCTCTTTGTCTTGCGACGTTATGCGGTATTAGCTACCGTTTCCAGTAGTTATCCCCCTCCATC
AGGCAGTTTCCCAGACATTACTCACCCGTCCGCCACTCGTCAGCAAAGCAGCAAGCTGCCTCCTGTTACCGTT
CGACTGCATGTGTTAGGCCTGCCGCCAGCGTCAATCTGAGCCAGGATCAAACTCT
>1087
CTGCTGCCGCCCGTAGGAGTCTGGACCGTGTCTCAGTTCCAGTGTGGCTGGTCATCCTCTCAGACCAGCTAGG
GATCGTCGCCTAGGTGAGCCGTTACCCCACCTACTAGCTAATCCCATCTGGGCACATCCGATGGCAAGAGGCC
CTAAGGTCCCCCTCCTTGTCTTGCGACGTTATGCGGTATTAGCTACCGTTTCCAGTAGTTATCCCCCTCCATC
AGGCAGTTTCCCAGACATTACTCACCCGTCCGCCACTCGTCAGCAAACAGCAAGCTGCTTCCTGTTACCGTTC
GACTGCATGTGTTAGGCCTGCCGCCAGCGTCAATCTGAGCCAGGATCAAACTCT
>1088
CTGCTGCCGCCCGTAGGAGTCTGGACCGTGTCTCAGTTCCAGTGTGGCTGGTCATCCTCTCAGACCAGCTAGG
GATCGTCGCCTAGGTGAGCCGTTACCCCACCTACTAGCTAATCCCATCTGGGCACATCCGATGGCAAGAGGCC
CTAAGGTCCCCCTCTTGGTCTTGCGACGTTATGCGGTATTAGCTACCGTTTCCAGTAGTTATCCCCCTCCATC
GGGCAGTTTCCCAGACATTACTCACCCGTCCGCCACTCGTCAGCAAAGCAGCAAGCTGCTTCCTGTTACCGTT
CGACTGCATGTGTTAGGCCTGCCGCCAGCGTCAATCTGAGCCAGGATCAAACTCT
>1089
CTGCTGCCGCCCGTAGGAGTCTGGACCGTGTCTCAGTTCCAGTGTGGCTGGTCATCCTCTCAGACCAGCTAGG
GATCGTCGCCTAGGTGAGCCGTTACCCCACCTACTAGCTAATCCCATCTGGGCACATCCGATGGCAAGAGGCC

CTAAGGTCCCCCTCTTTGTCTTGCGACGTTATGCGGTATTAGCTACCGTTTCCAGTAGTTATCCCCCTCCATC
AGGCAGTTTCCCAGACATTACTCACCCGTCCGCCACTCGTCAGCAAACAGCAAGCTGCTTCCTGTTACCGTTC
GACTGCATGTGTTAGGCCTGCCGCCAGCGTCAATCTGAGCCAGGATCAAACTCT
>1090
CTGCTGCCGCCCGTAGGAGTCTGGACCGTGTCTCAGTTCCAGTGTGGCTGGTCATCCTCTCAGACCAGCTAGG
GATCGTCGCCTAGGTGAGCCGTTACCCCACCTACTAGCTAATCCCATCTGGGCACATCCGATGGCAAGAGACC
CTAAGGTCCCCCTCTTTGGTCTGCGACGTATGCGGTATTAGCTACCGTTTCCAGTAGTTATCCCCCTCCATCG
GGCAGTTTCCCAGACATTACTCACCCGTCCGCCACTCGTCAGCAAAGCAGCAAGCTGCTTCCTGTTACCGTTC
GACTGCATGTGTTAGGCCTGCCGCCAGCGTCAATTGAGCCAGGACTCAA
>1091
CTGCTGCCGCCCGTAGGAGTCTGGACCGTGTCTCAGTTCCAGTGTGGCTGGTCATCCTCTCAGACCAGCTAGG
GATCGTCGCCTAGGTGAGCCGTTACCCCACCTACTAGCTAATCCCATCTGGGCACATCCGATGGCAAGAGGCC
CTAAGGTCCCCCTCTTTGTCTGCGACGTTATGCGGTATTAGCTACCGTTTCCAGTAGTTATCCCCCTCCATCG
GGCAGTTTCCCAGACATTACTCACCCGTCCGCCACTCGTCAGCAAAGCAGCAAGCTGCTTCCTGTTACCGTTC
GACTGCATGTGTTAGGCCTGCCGCCAGCGTCAATCTGAGCCAGGATCAAACTCT
>1092
CTGCTGCCGCCCGTAGGAGTCTGGACCGTGTCTCAGTTCCAGTGTGGCTGGTCATCCTCTCAGACCAGCTAGG
GATCGTCGCCTAGGTGAGCCGTTACCCCACCTACTAGCTAATCCCATCTGGGCACATCCGATGGCAAGAGGCC
CTAAGGTCCCCCTCTTTGTCTTGCGACGTTATGCGGTATTAGCTACCGTTTCCAGTAGTTATCCCCCTCCATC
AGGCAGTTTCCCAGACATTACTCACCCGTCCGCCACTCGTCAGCAAAGCAGCAAGCTGCTTTCTGTTACCGTT
CGACTGCATGTGTTAGGCCTGCCGCCAGCGTCAATCTGAGCCAGGATCAAACTCT
>1093
CTGCTGCCGCCCGTAGGAGTCTGGACCGTGTCTCAGTTCCAGTGTGGCTGGTCATCCTCCTCAGACCAGCTAG
GGATCGTCGCCTAGGTGAGCCGTTACCCCACCTACTAGCTAATCCCATCTGGGCACATCCGATGGCAAGAGGC
CCTAAGGTCCCCCTCTTTGTCTTGCGACGTTATGCGGTATTAGCTACCGTTTCCAGTAGTTATCCCCCTCCAT
CAGGCAGTTTCCCAGACATTACTCACCCGTCCGCCACTCGTCAGCAAGCAGCGAGCTGCTTTCTGTTACCGTT
CGACTTGCATGTGTTAGGCCTGCCGCCAGCGTTCAATCTGAGCCAGGATCAAACTCT
>1094
CTGCTGCCGCCCGTAGGAGTCTGGACCGTGTCTCAGTCCTACGTGGTGCTGGTCATCCTCTCAGACCAGCTAG
GGATCGTCGCCTAGGTGAGCCGTTACCCCACCTACTAGCTAATCCCATCTGGGCACATCCGATGGCAAGAGGC
CCTAAGGTCCCCCTCTTTGGTCTTGCGACGTTATGCGGTATTAGCTACCGTTTCCAGTAGTTATCCCCCTCCA
TCAGGCAGTTTCCCAGACATTACTCACCCGTCCGCCACTCGTCAGCAAAGCAGCAAGCTGCTTCCTGTTACCG
TCGACTGCATGTGTTAGGCCTGCCGCCAGCGTCAATCTGAGCCAGGATCAAACTCT
>1095
CTGCTGCCGCCCGTAGGAGTCTGGACCGTGTCTCAGTTCCAGTGTGGCTGGTCATCCTCTCAGACCAGCTAGG
GATCGTCGCCTAGGTGAGCCGTTACCCCACCTACTAGCTAATCCCATCTGGGCACATCCGATGGCAAGAGGCC
CTAAGGTCCCCCTCTTTGGTCTTGCGACGTTATGCGGTATTAGCTACCGTTTCCAGTAGTTATCCCCCTCCAT
CAGGCAGTTTCCCAGACATTACTCACCCGTCCGCCACTCGTCAGCAAAGCAGCAAGCTGCTTTCTGTTACCGT
TCGACTGCATGTGTTAGGCCTGCCGCCAGCGTTCAACCTGAAG
>1096
CTGCTGCCGCCCGTAGGAGTCTGGACCGTGTCTCAGTTCCAGTGTGGCTGGTCATCCTCTCAGACCAGCTAGG
GATCGTCGCCTAGGTGAGCCGTTACCCCACCTACTAGCTAATCCCATCTGGGCACATCCGATGGCAAGAGGCC
CTAAGGTCCCCCTCTTGGTCTTGCGACGTTATGCGGTATTAGCTACCGTCTCCAGTACGTTATCCCCCTCCAT
CGGGCAGTTTCCCAGACATTACTCACCCGTCCGCCACTCCGTCAGCAAAGCAGCAAGCTGCTTCCGTTACCGT
TCGATGCATGTGTTAGGCCTGCCGCCAGCGTCAATCTGAGCCAGGATCAACTCT
>1097
CTGCTGCCGCCCGTAGGAGTCTGGACCGTGTCTCAGTTCCAGTGTGGCTGGTCATCCTCTCAGACCAGCTAGG
GATCGTCGCCTAGGTGAGCCGTTACCCCACCTACTAGCTAATCCCATCTGGGCACATCCGATGGCAAGAGGCC
CGAAGGTCCCCCTCCTTGTCTTGCGACGTATGCGGTATTAGCTACCGTTTCCAGTAGTTATCCCCCTCCATCA
GGCAGTTTCCCAGACATTACTCACCCGTCCGCCACTCGTCAGCGACAGCAAGCTGTTCCTGTTACCGTTCGAC
TGCATGTGTTAGGCCTGCCGCCAGCGTCAATCTGAGCCAGGATCAAACTCT
>1098
CTGCTGCCGCCCGTAGGAGTCTGGACCGTGTCTCAGTTCCAGTGTGGCTGGTCATCCTCTCAGACCAGCTAGG
GATCGTCGCCTAGGTGAGCCGTAACCCCACCTACTAGCTAATCCCATCTGGGCACATCCGATGGCAAGAGGCC

CTAAGGTCCCCCTCTTTGGTCTTGCGACGTTATGCGGTATTAGCTACCGTTTCCAGTAGTTATCCCCCTCCAT
CGGGCAGTTTCCCAAACATTACTCACCCGTCCGCCACTCGTCAGCAAAGCAGCAAGCTGCTTCCTGTTACCGT
TCGACTTGCATGTGTTAGGCCTGCCGCCAGCGTCAATCTGAGCCAGGATCAAACTCT
>1099
CTGCTGCCGCCCGTAGGAGTCTGGACCGTGTCTCAGTTCCAGTGTGGCTGGTCATCCTCTCAGACAGCTAGGG
ATCGTCGCCTAGGTGAGCCGTTACCCCACCTACTAGCTAATCCCATCTGGGCACATCCGATGGCAAGAGGCCC
TAAGGTCCCCCTCTTTGTCTTGCGACGTTATGCGGTATTAGCTACCGTTTCCAGTAGTTATCCCCCTCATCAG
GCAGTTTCCCAGACATTACTCACCCGTCCGCCACTCGTCAGCAAAGCAGCAAGCTGCTTCCTGTTACCGTTCG
AACTGCATGTGTTAGGCTGCCGCCAGCGTCAATCTGAGCAGGATAAACATCT
>1100
CTGCTGCCGCCCGTAGGAGTCTGGACCGTGTCTCAGTTCCAGTGTGGCTGGTCATCCTCTCAGACCAGCTAGG
GATCGTCGCCTAGGTGAGCCGTTACCCCACCTACTAGCTAATCCCATCTGGGCACATCCGATGGCAAGAGGCC
CTAAGGTCCCCCTCTTTGGTCTTGCGACGTTATGCGGTATTAGCTACCGTTTCCAGTAGTTATCCCCCTCCAT
CAGGCAGTTTCCCAGACATTACTCACCCGTCCGCCACTCGTCAGCAAAACAGCAAGCTGCTTCCTGTTACCGT
TCGACTTGCATGTGTTAGGCCTGCCGCCAGCGTCAATCTGAGCCAGGATCAAACTCT
>1101
CTGCTGCCGCCCGTAGGAGTCTGGACCGTGTCTCAGTTCCAGTGTGGCTGGTCATCCTCTCAGACCAGCTAGG
GATCGTCGCCTAGGTGAGCCGTTACCCCACCTACTAGCTAATCCCATCTGGGCACATCCGATGGCAAGAGGCC
CTAAGGTCCCCCTCTTTGTCTGCGACGTTATGCGGTATTAGCTACCGTTTCCAGTAGTTATCCCCCTCCATCG
GGCAGTTTCCCAGACATTACTCACCCGTCCGCCACTCGTCAGCAAAGCAGCAAGCTGCTTCCTGTTACCGTTC
GACTGCATGTGTTAGGCCTGCCGCCAGCGTCAATCTGAGCCAGGATCAAACTCT
>1102
CTGCTGCCGCCCGTAGGAGTCTGGACCGTGTCTCAGTTCCAGTGTGGCTGGTCATCCTCTCAGACCAGCTAGG
GATCGTCGCCTAGGTGAGCCGTTACCCCACCTACTAGCTAATCCCATCTGGGCACATCCGATGGCAAGAGGCC
CTAAGGTCCCCCTCTTTGGTCTTGCGACGTTATGCGGTATTAGCTACCGTTTCCAGTAGTTATCCCCCTCCAT
CGGGCAGTTTCCCAGACATTACTCACCCGTCCGCCACTCGTCAGCAAAGCAGCAAGCTGCTTCCTGTTACCGT
TCGACTGCATGTGTTAGGCCTGCCGCCAGCGTCAATCTGAGCCAGGATCAAACTCT
>1103
CTGCTGCCGCCCGTAGGAGTCTGGACCGTGTCTCAGTTCCAGTGTGGCTGGTCATCCTCTCAGACCAGCTAGG
GATCGTCGCCTAGGTGAGCCGTTACCCCACCTACTAGCTAATCCCATCTGGGCACATCCGATGGCAAGAGGCC
CTAAGGTCCCCCTCTTTGGTCTGCGACGTTATGCGGTATTAGCTACCGTTTCCAGTAGTTATCCCCCTCCATC
GGGCAGTTTCCCAGACATTACTCACCCGTCCGCCACTCGTCAGCAAAGCAGCAAGCTGCTTCCTGTTACCGTT
CGACTGCATGTGTTAGGCCTGCCGCCAGCGTCAATCTGAGCCAGGATCAAACTCT
>1104
CTGCTGCCGCCCGTAGGAGTCTGGACCGTGTCTCAGTTCCAGTGTGGCTGGTCATCCTCTCAGACCAGCTAGG
GATCGTCGCCTAGGTGAGCCGTTACCCCACCTACTAGCTAATCCCATCTGGGCACATCCGATGGCAAGAGGCC
CTAAGGTCCCCCTCTTTGTCTTGCGACGTTATGCGGTATTAGCTACCGTTTCCAGTAGTTATCCCCCTCCATC
AGGCAGTTTCCCAGACATTACTCACCCGTCCGCCACTCGTCAGCAAAGCAGCAAGCTGCTTCCTGTTACCGTT
CGACTGCATGTGTTAGGCCTGCCGCCAGCGTTCAATCTGAGCCAGGACAAACTCT
>1105
CTGCTGCCGCCCGTAGGAGTCTGGACCGTGTCTCAGTTCCAGTGTGGCTGGTCATCCTCTCAGACCAGCTAGG
GATCGTCGCCTAGGTGAGCCGTTACCCCACCTACTAGCTAATCCCATCTGGGCACATCCGATGGCAAGAGGCC
CTAAGGTCCCCCTCTTTGGTCTTGCGACGTTATGCGGTATTAGCTACCGTTCCAGTAGTTATCCCCCTCCATC
GGGCAGTTTCCCAGACATTACTCACCCGTCCGCCACTCGTCAGCAAAGCAGCAAGCTGCTTCCTGTTACCGTT
CGACTGCATGTGTTAGGCCTGCCGCCAGCGTCAATCTGAGCCAGGATCAAACTCT
>1106
CTGCTGCCGCCCGTAGGAGTCTGGACCGTGTCTCAGTTCCAGTGTGGCTGGTCATCCTCTCAGACCAGCTAGG
GATCGTCGCCTAGGTGAGCCGTTACCCCACCTACTAGCTAATCCCATCTGGGCACATCCGATGGCAAGAGGCC
CTAAGGTCCCCCTCTTGTCTGCGACGTTATGCGGTATTAGCTACCGTTTCCAGTAGTTATCCCCCTCCATCGG
GCAGTTTCCCAGACATTACTCACCCGTCCGCCACTCGTCAGCAAAGCAGCAGCTGCTTCCTGTTACCGTTCGA
CTGCATGTGTTAGGCCTGCCGCCAGCGTCAATCTGAGCCAAGATCAAACTCT
>1107
CTGCTGCCGCCCGTAGGAGTCTGGACCGTGTCTCAGTTCCAGTGCGGCTGGTCATCCTCTCAGACCAGCTAGG
GATCGTCGCCTAGGTGAGCCGTTACCCCACCTACTAGCTAATCCCATCTGGGCACATCCGATGGCAAGAGGCC

CTAAGGTCCCCCTCTTGTCTGCGACGTATGCGGTATTAGCTACCGTTTCCAGTAGTTATCCCCCTCCATCAGG
CAGTTTCCCAGACATTACTCACCCGTCCGCCACTCGTCAGCAAAGCAGCAAGCTGCTTCCTGTTACCGTTCGA
CTGCATGTGTTAGGCCTGCCGCCAGCGTCAATCTGAGCCAGGATCAAACTCT
>1108
CTGCTGCCGCTCGTAGGAGTCTGGACCGTGTCTCAGTTCCAGTGTGGCTGGTCATCCTCTCAGACCAGCTAGG
GATCGTCGCCTAGGTGAGCCGTTACCCCACCTACTAGCTAATCCCATCTGGGCACATCCGATGGCAAGAGGCC
CTAAGGTCCCCCTCTTTGGTCTTGCGACGTTATGCGGTATTAGCTACCGTTTCCAGTAGTTATCCCCCTCCAT
CGGGCAGTTTCCCAGACATTACTCACCCGTCCGCCACTCGTCAGCAAAGCAGCAAGCTGCTTCCTGTTACCGT
TCGACTGCATGTGTTAGGCCTGCCGCCAGCGTCAATCTGAGCCAGGATCAAACTCT
>1109
CTGCTGCCGCCCGTAGGAGTCTGGACCGTGTCTCAGTTCCAGTGTGGCTGGTCATCCTCTCAGACCAGCTAGG
GATCGTCGCCTAGGTGAGCCGTTACCCCACCTACTAGCTAATCCCATCTGGGCACATCCGATGGCAAGAGGCC
CTAAGGTCCCCCTCTTTGTCTGCGACGTATGCGGTATTAGCTACCGTTTCCAGTAGTTATCCCCCTCCATCAG
GCAGTTTCCCCAGACATTACTCACCCGTCCGCCACTCGTCAGCAAAACAGCAAGCTGCTTCCTGTTACCGTTC
GACTGCATGTGTTAGGCCTGCCGCCAGCGTCAACTCGAAGCC
>1110
CTGCTGCCGCCCGTAGGAGTCTGGACCGTGTCTCAGTTTCCAGTGTGGCTGGTCATCCTCTCAGACCAGCTAG
GGATCGTCGCCTAGGTGAGCGTCACCCACTACTAGCTAATCCCATCTGGGCACATCCGATGGCAAGAGGCCCT
AAGGTCCCCCTCTTTGGTCTTGCGACGTTATGCGGTATTAGCTACCGTTTCCAGTAGTTATCCCCTCCATCGG
GCAGTTTTCCCAGACATTACTCACCCGTCCGCCACTCGTCAGCAAGCAGCAAGCTGCTTCCTGTTACCGTTCG
ACTGCATGTGTTAGGCCTGCCGCCAGCGTCAATCTGAGCCAGGATCAAACTCT
>1111
CTGCTGCCGCCCGTAGGAGTCTGGACCGTGTCTCAGTTCCAGTGTGGCTGGTCATCCTCTCAGACCAGCTAGG
GATCGTCGCCTAGGTGAGCCGTTACCCCACCTACTAGCTAATCCCATCTGGGCACATCCGATGGCAAGAGGCC
CTAAGGTCCCCCTCTTTGTCTTGCGACGTATGCGGTATTAGCTACCGTTTCCAGTAGTTATCCCCTCCATCGG
GCAGTTTTCCCAGACATTACTCACCCGTCCGCCACTCGTCAGCAAAGCAGCAAGCTGCTTCCTGTTACCGTTC
GACTGCATGTGTTAGGCCTGCCGCCAGCGTTCAATCTGAGCCAGGATCAAACTCT
>1112
CTGCTGCCGCCCGTAGGAGTCTGGACCGTGTCTCAGTTCCAGTGTGGCTGGTCATCCTCTCAGACCAGCTAGG
GATCGTCGCCTAGGTGAGCCGTTACCCCACCTACTAGCTAATCCCCATCTGGGCACATCCGATGGCAAGAGGC
CCTAAGGTCCCCCTCTTGTCTTGCGACGTATGCGGTATTAGCTACCGTTTCCAGTAGTTATCCCCCTCCATCA
GGCAGTTTCCCAGACATTACTCACCCGTCCGCCACTCGTCAGCAAAGCAGCAAGCTGCTTTCTGTTACCGTTC
GACTGCATGTGTTAGGCCTGCCGCCAGCGTCAATCTGAGCCAGGATCAAACTCT
>1113
TGCTGCCGCCCGTAGGAGTCTGGACCGTGTCTCAGTTCCAGTGTGGCTGGTCATCCTCTCAGACCAGCTAGGG
ATCGTCGCCTAGGTGAGCCGCTACCCCACCTACTAGCTAATCCCATCTGGGCACATCCGATGGCAAGAGGCCC
TAAGGTCCCCCTCTTTGGTCTTGCGACGTTATGCGGTATTAGCTACCGTTTCCAGTAGTTATCCCCCTCCATC
AGGCAGTTTCCCAGACATTACTCACCCGTCCGCCACTCGTCAGCAAAACAGCAAGCTGCTTCCTGTTACCGTT
CGACTGCATGTGTTAGGCCTGCCGCCAGCGTTCAATCTGAGCCAGGATCAAACTCT
>1114
CTGCTGCCGCCCGTAGGAGTCTGGACCGTGTCTCAGTTCCAGTGTGGCTGGTCATCCTCTCAGACCAGCCAGG
GATCGTCGCCTAGGTGAGCCGTTACCCCACCTACTAGCTAATCCCATCTGGGCACATCCGATGGCAAGAGGCC
CTAAGGTCCCCCTCTTTGGTCTTGCGACGTTATGCGGTATTAGCTACCGTTTCCAGTAGTTATCCCCCTCCAT
CGGGCAGTTTCCCAGACATTACTCACCCGTCCGCCACTCGTCAGCAAAGCAGCAAGCTGCTTCCTGTTACCGT
TCGACTGCATGCGTAGGCCTGCCGCCAGCGTCAATCTGAGCCAGGATCAAACTCT
>1115
CTGCTGCCGCCCGTAGGAGTCTGGACCGTGTCTCAGTTCCAGTGTGGACTGGTCATCCTCTCAGACCAGCTAG
GGATCGTCGCCTAGGTGAGCCGTTACCCCACCTACTAGCTAATCCCATCTGGGCACATCCGATGGCAAGAGGC
CCTAAGGTCCCCCTCTTTGTCTTGCGACGTATGCGGTATTAGCTACCGTTTCCAGTAGTTATCCCCCTCCATC
AGGCAGTTTCCCCAGACATTACTCACCCGTCCGCCACTCGTCAGCAAAGCAGCAAGCTGCTTCCTGTTACCGT
TCGACTGCATGTGTTAGGCCTGCCGCCAGCGTCAATCTGAGCCAGGATCAAACTCT
>1116
CTGCTGCCGCCCGTAGGAGTCTGGACCGTGTCTCAGTTCCAGTGTGGCTGGTCATCCTCTCAGACCAGCTAGG
GATCGTCGCCTAGGTGAGCCGTTACCCCACCTACTAGCTAATCCCATCTGGGCACATCCGATGGCAAGAGGCC

CTAAGGTCCCCCTCTTTGGTCTTGCGACGTTATGCGGTATTAGCTACCGTTTCCAGTAGTTATCCCCCTCCAT
CGGGCAGTTTCCCAGACATTACTCACCCGTCCGCCACTCGTCAGCAAAGCAGCAAGCTGCTTCCTGTTACCGT
TCGACTGCACGTGTTAGGCCTGCCGCCAGCGTCAATCTGAGCCAGGATCAAACTCT
>1117
CTGCTGCCGCCCGTAGGAGTCTGGACCGTGTCTCAGTTCCAGTGTGGCTGGTCATCCTCTCAGACCAGCTAGG
GATCGTCGCCTAGGTGAGCCGTTACCCCACCTACTAGCTAATCCCATCTGGGCACATCCGATGGCAGGAGGCC
CTAAGGTCCCCCTCTTTGGTCTTGCGACGTTATGCGGTATTAGCTACCGTTCCCAGTAGTTATCCCCCTCCAT
CAGGCAGTTTCCCAGACATTACTCACCCGTCCGCCACTCTCAGCAAAGCAGCAAGCTGCTTCCTGTTACCGTT
CGACTGCATGTGTTAGGCCTGCCGCCAGCGTCAATCTGAGCCAGGATCAAACTCT
>1118
CTGCTGCCGCCCGTAGGAGTCTGGACCGTGTCTCAGTTCCAGTGTGGCTGGTCATCCTCTCAGACCAGCTAGG
GATCGTCGCCTAGGTGAGCCGTTACCCCACCTACTAGCTAATCCCATCTGGGCACATCCGATGGCAAGAGGCC
CTAAGGTCCCCCTCTTGTCTTGCGAACGTATGCGGTATTAGCTACCGTTTCCAGTAGTTATCCCCCTCCATCG
GGCAGTTCCCAGACATACTCACCCGTCCGCCACTCGTCAGCAAAGCAGCAAGCTGCTTTCTGTTACCGTTCGA
CTGCATGTGTTAGGCCTGCCGCCAAGCGTCAATCTGAGCCAGGATCAAACTCT
>1119
CTGCTGCCGCCCGTAGGAGTCTGGACCGTGTCTCAGTTCCAGTGTGGCTGGTCATCCTCTCAGACAGCTAGGG
ATCGTCGCCTAGGTGAGCCGTTACCCCACCTACTAGCTAATCCCATCTGGGCACATCCGATGGCAAGAGGCCC
TAAGGTCCCCCTCTTGGTCTGCGACGTATGCGGTATTAGCTACCGTTTCCAGTAGTTATCCCCTCCATCGGGC
AGTTTCCCAGACATTACTCACCCGTCCGCCACCTCAGCAAAGCAGCAAGCTGCTTCCTGTTACCGTTCGACTG
CATGTGTTAGGCCTGCCGCCAGCGTCAATCTGAGCCAGGATCAACTCT
>1120
CTGCTGCCGCCCGTAGGAGTCTGGACCGTGTCTCAGTTCCAGTGTGGCTGGTCATCCTCTCAGACCAGCTAGG
GATCGTCGCCTAGGTGAGCCGTTACCCCACCTACTAGCTAATCCCATCTGGGCACATCCGATGGCAAGAGGCC
CTAAGGTCCCCCTCTTGGTCTCGCGACGTATGCGGTATTAGCTACCGTTTCCAGTAGTTATCCCCCTCCATCA
GGCAGTTTCCCAGACATTACTCACCCGTCCGCCACTCGCAGCAAAGCAGCAAGCTGCTTCCTGTTACCGTTCG
ACTGCATGTGTTAGGCCTGCCGCCAGCGTTCAATCTGAAGCCAGGATCAAACTCT
>1121
CTGCTGCCGCCCGTAGGAGTCTGGACCGTGTCTCAGTTCCAGTGTGGCTGGTCATCCTCTCAGACCAGCTAGG
GATCGTCGCCTAGGTGAGCCGTTACCCCACCTACTAGCTAATCCCATCTGGGCACATCCGATGGCAAGAGGCC
CTAAGGTCCCCCTCTTGTCTGCGACGTATGCGGTATTAGCTACCGTTTCCAGTAGTTATCCCCCTCCATCGGG
CAGTTTCCCAGACATTACTCACCCGTCCGCCACTCGTCAGCAAAGCAGCAAGCCTGCTTCCTGTTACCGTTCG
ACTGCATGTGTTAGGCCTGCCGCCAGCGTCAATCTGAGCCAGGATCAACTCT
>1122
CTGCTGCCGCCCGTAGGAGTCTGGACCGTGTCTCAGTTCCAGTGTGGCTGGTCATCCTCTCAGACCAGCTAGG
GATCGTCGCCTAGGTGAGCCGTTACCCCACCTACTAGCTAATCCCATCTGGGCACATCCGATGGCAAGAGGCC
CTAAGGTCCCCCTCTTTGTCTTGCGACGTATGCGGTATTAGCTACCGTTTCCAGTAGTTATCCCCCTCCATCA
GGCAGTTTCCCAGACATTACTCACCCGTCCGCCACTCGTCAGCAAAGCAGCAAGCTGCTTCTGTTACCGTTCG
ACTGCATGTGTTAGGCCTGCC
>1123
CTGCTGCCGCCCGTAGGAGTCTGGACCGTGTCTCAGTTCCAGTGTGGCTGGTCATCCTCTCAGACCAGCTAGG
GATCGTCGCCTAGGTGAGCCGTTACCCCACCTACTAGCTAATCCCATCTGGGCACATCCGATGGCAAGAGGCC
CTAAGGTCCCCCTCTTTGTCTTGCGACGTTATGCGGTATTAGCTACCGTTTCCAGTAGTTATCCCCCTCCATC
AGGCAGTTTCCCAGACATTACTCACCCGTCCGCCACTCGTCAGCAAAGCAGCAAGCTGCTTCCTGTTACCGTT
CGACTGCATGTGTTAGGCCTGCCGCCAGCGTCAATCTGAGCCAGGATCAAACTCT
>1124
CTGCTGCCGCCCGTAGGAGTCTGGACCGTGTCTCAGTTCCAGTGTGGCTGGTCATCCTCTCAGACCAGCTAGG
GATCGTCGCCTAGGTGAGCCGTTACCCCACCTACTAGCTAATCCCATCTGGGCACATCCGATGGCAAGAGGCC
CTAAGGTCCCCCTCTTTGGTCTTGCAACGTTATGCGGTATTAGCTACCGTTCCAGTAGTTATCCCCTCCATCG
GGCAGTTTCCCAGACATTACTCACCCGTCCGCCACTCGTCAGCAAAGCAGCAAGCTGCTTCCTGTTACCGTTC
GACTGCATGTGTTAGGCCTGCCGCCAGCGTTCAATCTGAGCCAGGATCAAACTCT
>1125
CTGCTGCCGCCCGTAGGAGTCTGGACCGTGTCTCAGTTCCAGTGTGGCTGGTCATCCTCTCAGACCAGCTAGG
GATCGTCGCCTAGGTGAGCCGTTACCCCACCTACTAGCTAATCCCATCTGGGCACATCCGATGGCAAGAGGCC

CTAAGGTCCCCCTCTTTGTCTTGCGACGTTATGCGGTATTAGCTACCGTTTCCAGTAGTTATCCCCCTCCATC
AGGCAGTTTCCCAGACATTACTCACCCGTCCGCCACTCGTCAGCAAACAGCAAGCTGCTTCCTGTTACCGTTC
GACTGCATGTGTTAGGCCTGCCGCCAGCGTTCAATCTGAGCCAGGATCAAACTCT
>1126
CTGCTGCCGCCCGTAGGAGTCTGGACCGTGTCTCAGTTCCAGTGTGGCTGGTCATCCTCTCAGACCAGCTAGG
GATCGTCGCCTAGGTGAGCCGTTACCCCACCTACTAGCTAATCCCATCTGGGCACATCCGATGGCAAGAGGCC
CTAAGGTCCCCCTCTTTGTCTTGCGACGTTATGCGGTATTAGCTACCGTTTCCAGTAGTTATCCCCCTCCATC
AGGCAGTTTCCCAGACATTACTCACCCGTCCGCCACTCGTCAGCAAAACAGCAAGCTGCTTCCTGTTACCGTT
CGACTGCATGTGTTAGGCCTGCCGCCAGCGTTCAATCTGAGCCAGGATCAAACTCT
>1127
CTGCTGCCGCCCGTAGGAGTCTGGACCGTGTCTCAGTTCCAGTGTGGCTGGTCATCCTCTCAGACCAGCTAGG
GATCGTCGCCTAGGTGAGCCGTTACCCCACCTACTAGCTAATCCCATCTGGGCACATCCGATGGCAAGAGGCC
CTAAGGTCCCCCTCTTTGTCTGCGACGTTATGCGGTATTAGCTACCGTTTCCAGTAGTTATCCCCCTCCATCG
GGCAGTTTCCCAGACATTACTCACCCGTCCGCCACTCGTCAGCAAAGCAGCAAGCTGCTTCCTGTTACCGTTC
GACTGCATGTGTTAGGCCTGCCGCCAGCGTCAATCTGAGCCAGGATCAAACTCT
>1128
CTGCTGCCGCCCGTAGGAGTCTGGACCGTGTCTCAGTTCCAGTGTGGCTGGTCATCCTCTCAGACCAGCTAGG
GATCGTCGCCTAGGTGAGCCGTTACCCCACCTACTAGCTAATCCCATCTGGGCACATCCGATGGCAAGAGGCC
CTAAGGTCCCCCTCTTTGTCTTGCGACGTTATGCGGTATTAGCTACCGTTTCCAGTAGTTATCCCCCTCCATC
GGGCAGTTTTCCCAGACATTACTCACCCGTCCGCCACTCGTCAGCAAAGCAGCAAGCTGCTTCCTGTTACCGT
TCGACTGCATGTGTTAGGCCTGCCGCCAGCGTCAATCTGAGCCAGGATCAAACTCT
>1129
CTGCTGCCGCCCGTAGGAGTCTGGACCGTGTCTCAGTTCCAGTGTGGCTGGTCATCCTCTCAGACCAGCTAGG
GATCGTCGCCTAGGTGAGCCGTTACCCCACCTACTAGCTAATCCCATCTGGGCACATCCGATGGCAAGAGGCC
CTAAGGTCCCCCTCTTTGTCTTGCGACGTTATGCGGTATTAGCTACCGTTTCCAGTAGTTATCCCCCTCCATC
AGGCAGTTTCCCAGACATTACTCACCCGTCCGCCACTCGTCAGCAAAGCAGCAAGCTGCTTCCTGTTACCGTT
CGACTTGCATGTGTTAGGCCTGCCGCCAGCGTCAATCTGAGCCAGGATCAAACTCT
>1130
CTGCTGCCGCCCGTAGGAGTCTGGACCGTGTCTCAGTTCCAGTGTGGCTGGTCATCCTCTCAGACCAGCTAGG
GATCGTCGCCTAGGTGAGCCGTTACCCCACCTACTAGCTAATCCCATCTGGGCACATCCGATGGCAAGAGGCC
CTAAGGTCCCCCTCTTTGGTCTTGCGACGTTATGCGGTATTAGCTACCGTTCCAGTAGTTATCCCCCCTCCAT
CAGGCAGTTTCCCAGACATTACTCACCCGTCCGCCACTCGTCAGCAAAACAGCAAGCTGCTTCCTGTTACCGT
TCGACTGCATGTGTTAGGCCTGCCGCCAGCGTCAATCTGAGCCAGGATCAAACTCT
>1131
CTGCTGCCGCCCGTAGGAGTCTGGACCGTGTCTCAGTTCCAGTGTGGCTGGTCATCCTCTCAGACCAGCTAGG
GATCGTCGCCTAGGTGAGCCGTTACCCCACCTACTAGCTAATCCCATCTGGGCACATCCGATGGCAAGAGGCC
CTAAGGTCCCCCTCTTGTCTTGCGACGTTATGCGGTATTAGCTACCGTTTCCAGTAGTTATCCCCCTCCATCA
GGCAGTTTCCCAGACATTACTCACCCGTCCGCCACTCGTCAGCAAAGCAGCAAGCCGCTTCCTGTTACCGTTC
GACTGCATGTGTTAGGCCTGCCGCCAGCGTCAATCTGAGCCAGGATCAAACTCT
>1132
CTGCTGCCGCCCGTAGGAGTCTGGACCGTGTCTCAGTTCCAGTGTGGCTGGTCATCCTCTCAGACCAGCTAGG
GATCGTCGCCTAGGTGAGCCGTTACCCCACCTACTAGCTAATCCCATCTGGGCACATCCGATGGCAAGAGGCC
CGAAGGTCCCCCTCTTTGGTCTTGCGACATTATGCGGTATTAGCTACCGTTTCCAGTAGTTATCCCCCTCCAT
CAGGCAGTTTCCCAGACATTACTCACCCGTCCGCCACTCGTCAGCGACAGCAAGCTGTTCCTGTTACCGTTCG
ACTGCATGTGTTAGGCTGCCGCCAGCGTCAATCTGAGCCAGGATAAACTCT
>1133
CTGCTGCCGCCCGTAGGAGTCTGGACCGTGTCTCAGTTCCAGTGCGGCTGGTCATCCTCTCAGACCAGCTAGG
GATCGTCGCCTAGGTGAGCCGTTCACCCCACTACTAGCTAATCCCATCTGGGCACATCCGATGGCAAGAGGCC
CTAAGGTCCCCCTCTTTGGTCTTGCGACGTTATGCGGTATTAGCTACCGTTTCCAGTAGTTATCCCCCTCCAT
CAGGCAGTTTCCCAGACATTACTCACCCGTCCGCCACTCGTCAGCAAAGCAGCAAGCTGCTTCCTGTTACCGT
TCGACTGCATGTGTTAGGCCTGCCGCCAGCGTTCAATCTGAAGCCAGGATCAAACTCT
>1134
CTGCTGCCCCCGTAGGAGTCTGGACCGTGTCTCAGTTCCAGTGTGGCTGGTCATCCTCTCAGACCAGCTAGGG
ATCGTCGCCTAGGTGAGCCGTTACCCCACCTACTAGCTAATCCCATCTGGGCACATCCGATGGCAAGAGGCCC

TAAGGTCCCCCTCTTTGGTCTTGCGACGTTATGCGGTATTAGCTACCGTTTCCAGTAGTTATCCCCCTCCATC
AGGCAGTTTCCCAGACATTACTCACCCGTCCGCCACTCGTCAGCAAAGCAGCAAGCTGCTTCCTGTTACCGTT
CGACTGCATGTGTTAGGCCTGCCGCCAGCGTCAATCTGAGCCAGGATCAAACTCT
>1135
CTGCTGCCGCCCGTAGGAGTCTGGACCGTGTCTCAGTTCCAGTGTGGCTGGTCATCCTCTCAGACCAGCTAGG
GATCGTCGCCTAGGTGAGCCGTTACCCCACCTACTAGCTAATCCCATCTGGGCACATCCGATGGCAAGAGGCC
CTAAGGTCCCCCTCTTTGTCTTGCGACGTTATGCGGTATTAGCTACCGTTTCCAGTAGTTATCCCCCTCCATC
AGGCAGTTTCCCAGACATTACTCACCCGTCCGCCACTCGTCAGCAAAGCAGCAAGCTGCTTCTGTTACCGTTC
GACTGCATGTGTTAGGCCTGCCGCCAGCGTCAATCTGAGCCAGGATCAAACTCT
>1136
CTGCTGCCGCCCGTAGGAGTCTGGACCGTGTCTCAGTTCCAGTGTGGCTGGTCATCCTCTCAGACCAGCTAGG
GATCGTCGCCTAGGTGAGCCGTTACCCCACCTACTAGCTAATCCCATCTGGGCACATCCGATGGCAAGAGGCC
CTAAGGTCCCCCTCTTTGGTCTTGCGACGTTATGCGGTATTAGCTACCGTTTCCAGTAGTTATCCCCCTCCAT
CAGGCAGTTTCCCAGACATTACTCACCCGTCCGCCACTCGTCAGCAAAAGCAGCAAGCTGCTTCCTGTTACCG
TTCGACTGCATGTGTTAGGCCTGCCGCCAAGCGTCAATCTGAGCCAGGATAAACTCT
>1137
CTGCTGCCGCCCGTAGGAGTCTGGACCGTGTCTCAGTTCCAGTGTGGCTGGTCATCCTCTCAGACCAGCTAGG
GATCGTCGCCAAGGTGAGCCGTTACCCCACCTACTAGCTAATCCCATCTGGGCACATCCGATGGCAAGAGGCC
CTAAGGTCCCCCTCTTTGTCTTGCGACGTATGCGGTATTAGCTACCGTTTCCAGTAGTTATCCCCCTCCATCG
GGCAGTTCCCAGACATTACTCACCCGTCCGCCACTCGTCAGCAAAGCAGCAAGCTGCTTCCTGTTACCGTTCG
ACTGCATGTGTTAGGCCTGCCGCCAAGCGTCAATCTGAGCCAGGATCAAACTCT
>1138
CTGCTGCCGCCCGTAGGAGTCTGGACCGTGTCTCAGTTCCAGTGTGGCTGGTCATCCTCTCAGACCAGCTAGG
GATCGTCGCCTAGGTGAGCCGTTACCCCACCTACTAGCTAATCCCATCTGGGCACATCCGATGGCAAGAGGCC
CTAAGGTCCCCCTCTTTGTCTGCGACGTTATGCGGTATTAGCTACCGTTTCCAGTAGTTATCCCCCTCCATCG
GGCAGTTTCCCAGACATTACTCACCCGTCCGCCACTCGTCAGCAAAGCAGCAAGCTGCTTCCTGTTACCGTTC
GACTGCATGTGTTAGGCCTGCCGCCAGCGTCAATCTGAGCCAGGATCAAACTCT
>1139
CTGCTGCCGCCCGTAGGAGTCTGGACCGTGTCTCAGTTCCAGTGTGGCTGGTCATCCTCTCAGACCAGCTAGG
GATCGTCGCCTAGGTGAGCCGTTACCCCACCTACTAGCTAATCCCATCTGGGCACATCCGATGGCAAGAGGCC
CTAAGGTCCCCCTCTTGTCTTGCGACGTATGCGGTATTAGCTACCGTTTCCAGTAGTTATCCCCCTCCATCGG
GCAGTTTCCCAGACATTACTCACCCGTCCGCCACTCGTCAGCAAAGCAGCAAGCTGCTTCCTGTTACCGTTCG
ACTGCATGTGTTAGGCCTGCCGCCAGCGTCAATCTGAGCCAGGATCAAACTCT
>1140
CTGCTGCCGCCCGTAGGAGTCTGGACCGTGTCTCAGTTCCAGTGTGGCTGGTCATCCTCTCAGACCAGCTAGG
GATCGTCGCCTAGGTGAGCCGTTACCCCACCTACTAGCTAATCCCATCTGGGCACATCCGATGGCAAGAGGCC
CTAAGGTCCCCCTCTTTGTCTTGCGACGTTATGCGGTATTAGCTACCGTTTCCAGTAGTTATCCCCCACCATC
AGGCAGTTTCCCAGACATTACTCACCCGTCCGCCACTCGTCAGCAAAGCAGCAAGCTGCTTCCTGTTACCGTT
CGACTGCATGTGTTAGGCTGCCGCCAGCGGTCAACTCTGAA
>1141
CTGCTGCCGCCCGTAGGAGTCTGGACCGTGTCTCAGTTCCAGTGTGGCTGGTCATCCTCTCAGACCAGCTAGG
GATCGTCGCCTAGGTGAGCCGTTACCCCCACCTACTAGCTAATTCCCATCTGGGCACATCCGATTGGCAAGAG
GCCCTAAGGTCCCCCTCTTGGTCTTGCGACATTATTGCGGTATTAGCTACCGTTTCCAGTAGTTATCCCCCTC
CATCAGGCAGTTTTCCCCAGACATTACTCACCCGTCCGCCACTCGTCAGCAAAGCAGCAAGCTGCTTCTGTTA
CCGTTCGACTGCATGTGTTAGGCCTGCCGCCAGCCGTTCAATCTGAGCCAGGATCAAACTCT
>1142
CTGCTGCCGCCCGTAGGAGTCTGGACCGTGTCTCAGTTTCCAGTGTGGCTGGTCATCCTCTCAGACCAGCTAG
GGATCGTCGCCTAGGTGAGCCGTTACCCCACCTACTAGCTAATCCCATCTGGGCACATCCGATGGCAAGAGGC
CCTAAGGTCCCCCTCTTTGTCTTGCGACGTTATGCGGTATTAGCTACCGTTTCCAGTAGTTTATCCCCCTCCA
TCGGGCAGTTTTTCCCAGACATTACTCACCCGTCCGCCACTCGTCAGCAAGCAGCAAGCTGCTTCCTGTTACC
GTTCGACTGCATGTGTTAGGCCTGCCGCCAGCGTTCAATCTGAGCCAGGATCAAACTCT
>1143
CTGCTGCCGCCCGTAGGAGTCTGGACCGTGTCTCAGTTCCAGTGTGGCTGGTCATCCTCTCAGACCAGCTAGG
GATCGTCGCCTAGGTGAGCCGTTACCCCACCTACTAGCTAATCCCATCTGGGCACATCCGATGGCAAGAGGCC

CTAAGGTCCCCCTCTTTGTCTTGCGACGTTATGCGGTATTAGCTACCGTTTCCAGTAGTTATCCCCTCCATCG
GGCAGTTTCCCAGACATTACTCACCCGTCCGCCACTCGTCAGCAAGCAGCAAGCTGCTTCCTGTTACCGTTCG
ACTGCATGTGTTAGGCCTGCCGCCAGCGTCAATCTGAGCCAGGATCAAACTCT
>1144
CTGCTGCCGCCCGTAGGAGTCTGGACCGTGTCTCAGTTCCAGTGTGGCTGGTCATCCTCTCAGACCAGCTAGG
GATCGTCGCCTAGGTGAGCCGTTACCCCACCTACTAGCTAATCCCATCTGGGCACATCCGATGGCAAGAGGCC
CGAAGGTCCCCCTCTTTGGTCTGCGACATTATGCGGTATTAGCTACCGTTTCCAGTAGTTATCCCCCTCCATC
AGGCAGTTTTCCCAGACATTACTCACCCGTCCGCCACTCGTCAGCAAAGCAGCAAGCTGCTTCTGTTACCGTT
CGACTGCATGTGTTAGCCTGCCGCCAGCGTTCAATCTGAGCCAGGATCAAACTCT
>1145
CTGCTGCCGCCCGTAGGAGTCTGGACCGTGTCGCTACTCGTCAGTGTGGCTGGTCATCCTCTCAGACCAGCTA
GGGATCGTCGCCTAGGTGAGCCGTTACCCCACCTACTAGCTAATCCCATCTGGGCACATCCGATGGCAAGAGG
CCCTAAGGTCCCCCTCTTTGGTCTTGCGACGTTATGCGGTATTAGCTACCGTTTCCAGTAGTTATCCCCCTCC
ATCGGGCAGTTTCCCAGACATTACTCACCCGTCCGCCACTCGTCAGCAAAGCAGCAAGCTGCTTCCTGTTACC
GTTCGACTTGCATGTGTTAGGCCTGCCGCCAGCGTTCAATCTGAGCCAGGATCAAACTCT
>1146
TGCTGCCGCCCGTAGGAGTCTGGACCGTGTCTCAGTTCCAGTGTGGCTGGTCATCCTCTCAGACCAGCTAGGG
ATCGTCGCCTAGGTGAGCCGTTACCCCACCTACTAGCTAATCCCATCTGGGCACATCCGATGGCAAGAGGCCC
TAAGGTCCCCCTCTTTGGTCTTGCGACGTTATGCGGTATTAGCTACCGTTTCCAGTAGTTATCCCCCTCCATC
GGGCAGTTTTCCCAGACATTACTCACCCGTCCGCCACTCGTCAGCAAAGCAGCAAGCTGCTTCCTGTTACCGT
TCGACTGCATGTGTTAGGCCTGCCGCCAGCGTCAATCTGAGCCAGGATCAAACTCT
>1147
CTGCTGCCGCCCGTAGGAGTCTGGACCGTGTCTCAGTTCCAGTGTGGCTGGTCATCCTCTCAGACCAGCTAGG
GATCGTCGCCTAGGTGAGCCGTTACCCCACCTACTAGCTAATCCCATCTGGGCACATCCGATGGCAAGAGGCC
CTAAGGTCCCCCTCTTTGGTCTTGCGACGTTATGCGGTATTAGCTACCGTTTCCAGTAGTTATCCCCTCCATC
AGGCAGTTTCCCAGACATTACTCACCCGTCCGCCACTCGTCAGCAAAGCAGCAAGCTGCTTCTGTTACCGTTC
GACTGCATGTGTTAGGCCTGCCGCCAGCGTTCAATCTGAGCCAGGATCAAACTCT
>1148
CTGCTGCCGCCCGTAGGAGTCTGGACCGTGTCTCAGTTCCAGTGTGGCTGGTCATCCTCTCAGACCAGCTAGG
GATCGTCGCCTAGGTGAGCCGTTACCCCACCTACTAGCTAATCCCATCTGGGCACATCCGATGGCAAGAGGCC
CTAAGGTCCCCCTCTTGTCTGCGACGTATGCGGTATTAGCTACCGTTTCCCAGTAGTTATCCCCCTCCATCAG
GCAGTTTCCCACGACATTACTCACCCGTCCCGCCACTCGTCAGCCAAAGCAGCAAGCTGCTTCCTGTTACCCG
TTCGACTTGCCATGTCGTTAGGCCTGCCGCCAGCCGGTTCAACTCCGAAGGCC
>1149
CTGCTGCCGCCCGTAGGAGTCTGGACCGTGTCTCAGTTCCAGTGTGGCTGGTCATCCTCTCAGACCAGCTAGG
GATCGTCGCCTAGGTGAGCCGTTACCCCACCTACTAGCTAATCCCATCTGGGCACATCCGATGGCAAGAGGCC
CTAAGGTCCCCCTCTTTGTCTGCGACGTATGCGGTATTAGCTACCGTTTCCAGTAGTTATCCCCCTCCATCAG
GCAGTTTCCCAGACATACTCACCCGTCCGCCACTCGTCAGCAAAGCAGCAAGCTGCTTCCTGTTACCGTTCGA
ACTGCATGTGTTAGGCTGCCGCCAGCGTACAATGAGCCAGGATCAAACTCT
>1150
CTGCTGCCGCCCGTAGGAGTCTGGACCGTGTCTCAGTTCCAGTGTGGCTGGTCATCCTCTCAGACCAGCTAGG
GATCGTCGCCTAGGTGAGCCGTTACCCCACCTACTAGCTAATCCCATCTGGGCACATCCGATGGCAAGAGGCC
CTAAGGTCCCCCTCTTTGTCTGCGACGTTATGCGGTATTAGCTACCGTTTCCAGTAGTTATCCCCCTCCATCG
GGCAGTTTTCCCAGACATTACTCACCCGTCCGCCACTCGTCAGCAAAGCAGCAAGCTGCTTCCTGTTACCGTT
CGACTGCATGTGTTAGGCCTGCCGCCAGCGTCAATCTGAGCCAGGATCAAACTCT
>1151
CTGCTGCCGCCCGTAGGAGTCTGGACCGTGTCTCAGTTCCAGTGTGGCTGGTCATCCTCTCAGACCAGCTAGG
GATCGTCGCCTAGGTGAGCCGTTACCCCACCTACTAGCTAATCCCATCTGGGCACATCCGATGGCAAGAGGCC
CTAAGGTCCCCCTCTTTGGTCTTGCGACGTTATGCGGTATTAGCTACCGTTTCCAGTAGTTATCCCCCTCCAT
CGGGCAGTTTTCCCAGACATTACTCACCCGTCCGCCACTCGTCAGCAAAGCAGCAAGCTGCTTCCTGTTACCG
TTCGACTGCGTGTGTTAGGCCTGCCGCCAGCGTCAATCTGAGCCAGGATCAAACTCT
>1152
CTGCTGCCGCCCGTAGGAGTCTGGACCGTGTCTCAGTTCCAGTGTGGCTGGTCATCCTCTCAGACCAGCTAGG
GATCGTCGCCTAGGTGAGCCGTTACCCCACCTACTAGCTAATCCCATCTGGGCACATCCGATGGCGAGAGGCC

CTAAGGTCCCCCTCTTGTCTGCGACGTATGCGGTATTAGCTACCGTTTCCAGTAGTTATCCCCCTCCATCAGG
CAGTTTCCCAGACATTACTCACCCGTCCGCCACTCGTCAGCAAAGCAGCAAGCTGCTTCTGTTACCGTTCGAC
TGCAATGTGTTAGGCCTGCCGCCAGCGTTCAT
>1153
TGCTGCCGCCCGTAGGAGTCTGGACCGTGTCTCAGTTCCAGTGTGGCTGGTCATCCTCTCAGACCAGCTAGGG
ATCGTCGCCTAGGTGAGCCGTTACCCCACCTACTAGCTAATCCCATCTGGGCACATCCGATGGCAAGAGGCCC
TAAGGTCCCCCTCTTTGTCTTGCGACGTATGCGGTATTAGCTACCGTTTCCAGTAGTTATCCCCCTCCATCGG
GCAGTTTCCCAGACATTACTCACCCGTCCGCCACTCGTCAGCAAAGCAGCAAGCTGCTTCCTGTTACCGTTCG
ACTGCATGTGTTAGGCCTGCCGCCAGCGTCAATCTGAGCCAGGATCAAACTCT
>1154
CTGCTGCCGCCCGTAGGAGTCTGGACCGTGTCTCAGTTCCAGTGTGGCTGGTCATCCTCTCAGACCAGCTAGG
GATCGTCGCCTAGGTGAGCCGTTACCCCACCTACTAGCTAATCCCATCTGGGCACATCCGATGGCAAGAGGCC
CTAAGGTCCCCCTCTTTGGTCTTGCGACGTTATGCGGTATTAGCTACCGTTCCAGTAGTTATCCCCCTCCATC
AGGCAGTTTCCCAGACATTACTCACCCGTCCGCCACTCGTCAGCAAAGCAGCAAGCTGCTTCCTGTTACCGTT
CGACTGCATGTGTTAGGCCTGCCGCCAGCGTCAATCTGAGCCAGGATCAAACTTCT
>1155
CTGCTGCCGCCCGTAGGAGTCTGGACCGTGTCTCAGTTCCAGTGTGGCTGGTCATCCTCTCAGACCAGCTAGG
GATCGTCGCCTAGGTGAGCCGTTACCCCACCTACTAGCTAATCCCATCTGGGCACATCCGATGGCAAGAGGCC
CTAAGGTCCCCCTCTTTGTCTTGCGACGTTATGCGGTATTAGCTACCGTTTCCAGTAGTTATCCCCCTCCATC
AGGCAGTTTCCCAGACATTACTCACCCGTCCGCCACTCGTCAGCAAAGCAGCAAGCTGCTTCCTGTTACCGTT
CAACTGCATGTGTTAGGCCTGCCGCCAGCGTCAATCTGAGCCAGGATCAAACTCT
>1156
CTGCTGCCGCCCGTAGGAGTCTGGACCGTGTCTCAGTTCCAGTGTGGCTGGTCATCCTCTCAGACCAGCTAGG
GATCGTCGCCTAGGTGAGCCGTTACCCCACCTACTAGCTAATCCCATCCGGGCACATCCGATGGCAAGAGGCC
CTAAGGTCCCCCTCTTTGGTCTTGCGACGTTATGCGGTATTAGCTACCGTTTCCAGTAGTTATCCCCCTCCAT
CGGGCAGTTTCCCAGACATTACTCACCCGTCCGCCACTCGTCAGCAAAGCAGCAAGCTGCTTCCTGTTACCGT
TCGACTGCATGTGTTAGGCCTGCCGCCAGCGTCAATCTGAGCCAGGATCAAACTCT
>1157
CTGCTGCCGCCCGTAGGAGTCTGGACCGTGTCTCAGTTCCAGTGTGGCTGGTCATCCTCTCAGACCAGCTAGG
GGTCGTCGCCTAGGTGAGCCGTTACCCCACCTACTAGCTAATCCCATCTGGGCACATCCGATGGCAAGAGGCC
CTAAGGTCCCCCTCTTTGTCTTGCGACGTTATGCGGTATTAGCTACCGTTCCAGTAGTTATCCCCCTCCATCA
GGCAGTTTCCCAGACATTACTCACCCGTCCGCCACTCGTCAGCAAAGCAGCAAGCTGCTTCCTGTTACCGTTC
GACTGCATGTGTTAGGCCTGCCGCCAGCGTCAATCTGAGCCAGGATCAAACTCT
>1158
CTGCTGCCGCCCTAGGAGTCTGGACCGTGTCTCAGTTCCAGTGTGGCTGGTCATCCTCTCAGACCAGCTAGGG
ATCGTCGCCTAGGTGAGCCGTTACCCCACCTACTAGCTAATCCCATCTGGGCACATCCGATGGCAAGAGGCCC
TAAGGTCCCCCTCTTTGGTCTGCGACGTTATGCGGTATTAGCTACCGTTTCCAGTAGTTATCCCCCTCCATCA
GGCAGTTTCCCAGACATTACTCACCCGTCCGCCACTCGTCAGCAAAGCAGCAAGCTGCTTCCTGTTACCGTTC
GACTGCATGTGTTAGGCCTGCCGCCAGCGTCAATCTGAGCCAGGATCAAACTCT
>1159
CTGCTGCCGCCCGTAGGAGTCTGGACCGTGTCTCAGTTCCAGTGTGGCTGGTCATCCTCTCAGACCAGCTAGG
GATCGTCGCCTAGGTGAGCCGTTACCCCACCTACTAGCTAATCCCATCTGGGCACATCCGATGGCAAGAGGCC
CTAAGGTCCCCCTCTTTGTCTTGCGACGTATGCGGTATTAGCTACCGTTTCCAGTAGTTATCCCCCTCCATCG
GGCAGTTTTCCCAGACATTACTCACCCGTCCGCCACTCGTCAGCAAAGCAGCAAGCTGCTTCCTGTTACCGTT
CGACTGCATGTGTTAGGCCTGCCGCCAGCGTCAATCTGAGCCAGGATCAAACTCT
>1160
CTGCTGCCGCCCGTAGGAGTCTGGACCGCGTCTCAGTTCCAGTGTGGCTGGTCATCCTCTCAGACCAGCTAGG
GATCGTCGCCTAGGTGAGCCGTTACCCACCTACTAGCTAATCCCATCTGGGCACATCCGATGGCAAGAGGCCC
TAAGGTCCCCCTCTTGTCTTGCGACGTATGCGGTATTAGCTACCGTTTCCAGTAGTTATCCCCTCCATCGGGC
AGTTTCCCAGACATTACTCACCCGTCCGCCACTCGTCAGCAAAGCAGCAAGCTGCTTCCTGTTACCGTTCCGA
CTGCAATGTGTTAGGCCTGCCGCCAGCGTCAATCTGAGCCAGGATCAAACTCT
>1161
CTGCTGCCGCCCGTAGGAGTCTGGACCGTGTCTCAGTTCCAGTGTGGCTGGTCATCCTCTCAGACCAGCTAGG
GATCGTCGCCTAGGTGAGCCGTTACCCCACCTACTAGCTAATCCCATCTGGGCACATCCCGATGGCAAGAGGC

CCTAAGGTCCCCCTCTTGTCTTGCGACGTATGCGGTATTACGCTACCGTTTCCAGTAGTTATCCCCCTCCATC
GGGCAGTTTCCCAGACATTACTCACCCGTCCGCCACTCGTCAGCAAAGCAGCAAGCTGCTTCCTGTTACCGTT
CGACTGCATGTGTTAGGCCTGCCGCCAGCGTCAATCTGAGCCAGGATCAAACTCT
>1162
CTGCTGCCGCCCGTAGGAGTCTGGACCGTGTCTCAGTTCCAGTGTGGCTGGTCATCCTCTCAGACCAGCTAGG
GATCGTCGCCTAGGTGAGCCGTTACCCCACCTACTAGCTAATCCCATCTGGGCACATCCGATGGCAAGAGGCC
CTAAGGTCCCCCTCTTTGGTCGTGCGACGTTATGCGGTATTAGCTACCGTTTCCAGTAGTTATCCCCCTCCAT
CAGGCAGTTTCCCAGACATTACTCACCCGTCCGCCCACGTCAGCAAAGCAGCAAGCTGCTTCCTGTTACCGTT
CGACTTGCATGTGTTAGGCCTGCCGCCAGCGTCAATCTGAGCCAGGATCAAACTCT
>1163
CTGCTGCCGCCCGTAGGAGTCTGGACCGTGTCTCAGTTCCAGTGTGGCTGGTCATCCTCTCAGACCAGCTAGG
GATCGTCGCCTAGGTGAGCCGTTACCCCACCTACTAGCTAATCCCATCTGGGCACATCCGATGGCAAGAGGCC
CTAAGGTCCCCCTCTTTGGTCTTGCGACGTTATGCGGTATTAGCTACCGTTCCAGTAGTTATCCCCCTCCATC
AGGCAGTTTCCCAGACATTACTCACCCGTCCGCCACTCGTCAGCAAAGCAGCAAGCTGCTTCCTGTTACCGTT
CGACTGCATGTGTTAGGCCTGCCGCCAGCGTTCAATCTGAAGCCAGGATCAAACGTCT
>1164
CTGCTGCCGCCCGTAGGAGTCTGGACCGTGTCTCAGTTCCAGTGTGGCTGGTCATCCTCTCAGACCAGCTAGG
GATCGTCGCCTAGGTGAGCCGTTACCCCACCTACTAGCTAATCCCATCTGGGCACATCCGATGGCAAGAGGCC
CTAAGGTCCCCCTCTTTGTCTGCGACGTATGCGGTATTAGCTACCGTTTCCAGTAGTTATCCCCCTCCATCAG
GCAGTTTCCCAGACATTACTCACCCGTCCGCCACTCGTCAGCAAAGCAGCAAGCTGCTCCCTGTTACCGTTCG
ACTGCATGTGTTAGGCCTGCCGCCAGCGATCAATCTGAGCCAGGATCAAACTCT
>1165
CTGCTGCCGCCCGTAGGAGTCTGGACCGTGTCTCAGTTCCAGTGTGGCTGGTCATCCTCTCAGACCAGCTAGG
GATCGTCGCCTAGGTGAGCCGTTACCCCACCTTCTAGCTAATCCCATCTGGGCACATCCGATGGCAAGAGGCC
CTAAGGTCCCTCTCTTTGTCTTGCGACGTATGCGGTATTAGCTACCGTTTCCAGTAGTTATCCCCTCCATCAG
GCAGTTTCCCAGACATTACTCACCCGTCCGCCACTCGTCAGCAAACAGCAAGCTGCTTCCTGTTACCGTTCGA
CTGCATGTGTTAGGCCTGCCGCCAGCGTCAATCGAGCCAGGATCAAACTTAT
>1166
CTGCTGCCGCCCGTAGGAGTCTGGACCGTGTCTCGTACTCAGTGTGGCTGGTCATCCTCTCAGACCAGCGAGG
GATCGTCGCCTAGGTGAGCCGTTACCCCACCTACTAGCTAATCCCATCTGGGCACATCCGATGGCAAGAGGCC
CTAAGGTCCCCCTCTTTGGTCTTGCGACGTTATGCGGTATTAGCTACCGTTTCCAGTAGTTATCCCCCTCATC
GGGCAGTTTCCCAGACATTACTCACCCGTCCGCCACTCGTCAGCAAAGCAGCAAGCTGCTTCCTGTTACCGTT
CGACTGCATGTGTTAGGCCTGCCGCCAGCGTCAATCTGAGCCAGGATCAAACTCT
>1167
CTGCTGCCGCCCGTAGGAGTCTGGACCGTGTCCCAGTTCCAGTGTGGCTGGTCATCCTCTCAGACCAGCTAGG
GATCGTCGCCTAGGTGAGCCGTTACCCCACCTACTAGCTAATCCCATCTGGGCACATCCGATGGCAAGAGGCC
CTAAGGTTCCCCTCTTTGTCTTGCGACGTTATGCGGTATTAGCTACCGTTTCCAGTAGTTATCCCCCTCCATC
AGGCAGTTTCCCAGACATTACTCACCCGTCCGCCACTCGTCAGCAAAGCAGCAAGCTGCTTTCTGTTACCGTT
CGACTGCATGTGTTAGGCCTGCCGCCAGCGTCAATCTGAGCCAGGATCAAACTCT
>1168
CTGCTGCCGCCCGTAGGAGTCTGGACCGTGTCTCAGTTCCAGTGTGGCTGGTCATCCTCTCAGACCAGCTAGG
GATCGTCGCCTAGGTGAGCCGTTACCCCACCTACTAGCTAATCCCATCTGGGCACATCCGATGGCAAGAGGCC
CTAAGGTCCCCCTCTTTGTCTTGCGACGTTATGCGGTATTAGCTACCGTTCCAGTAGTTATCCCCCTCCATCG
GGCAGTTTCCCAGACATTACTCACCCGTCCGCCACTCGTCAGCAAAGCAGCAAGCTGCTTCCTGATACCGTTC
GACTGCATGTGTTAGGCCTGCCGCCAGCGTCAATCTGAGCCAGGATCAAACTCT
>1169
CTGCTGCCGCCCGTAGGAGTCTGGACCGTGTCTCAGTTCCAGTGTGGCTGGTCATCCTCTCAGACCAGCTAGG
GATCGTCGCCTAGGTGAGCCGTTACCCCACCTACTAGCTAATCCCATCTGGGCACATCCGATGGCAAGAGGCC
CTAAGGTCCCCCTCTTTGTCTTGCGACGTTATGCGGTATTAGCTACCGTTCCAGTAGTTATCCCCCTCCATCA
GGCAGTTTCCCAGACATTACTCACCCGTCCGCCACTCGTCAGCAAAGCAGCAAGCTGCTTCTGTTACCGTTCG
ACTGCATGTGTTAGGCCTGCCGCCAGCGTTCAATCTGAGCCAGGATCAAACTCT
>1170
CTGCTGCCGCCCGTAGGAGTCTGGACCGTGTCTCAGTTCCAGTGTGGCTGGTCATCCTCTCAGACCAGCTAGG
GATCGTCGCCTAGGTGAGCCGTTACCCCACCTACTAGCTAATCCCGTCTGGGCACATCCGATGGCAAGAGGCC

CTAAGGTCCCCCTCTTTGTCTTGCGACGTTATGCGGTATTAGCTACCGTTTCCAGTAGTTATCCCCCTCCATC
AGGCAGTTTCCCAGACATTACTCACCCGTCCGCCACTCGTCAGCAAAGCAGCAAGCTGCTTCCTGTTACCGTT
CGACTTGCATGTGTTAGGCCTGCCGCCAGCGTCAATCTGAGCCAGGATCAAACTGT
>1171
CTGCTGCCGCCCGTAGGAGTCTGGACCGTGTCTCAGTTCCAGTGTGGCTGGTCATCCTCTCAGACCAGCTAGG
GATCGTCGCCTAGGTGAGCCGTTACCCCACCTACTAGCTAATCCCATCTGGGCACATCCGATGGCAAGAGGCC
CTAAGGTCCCCCCTCTTTGGTCTTGCGACGTTATGCGGTATTAGCTACCGTTTCCAGTAGTTATCCCCCTCCA
TCAGGCAGTTTCCCCAGACATTACTCACCCGTCCGCCACTCGTCAGCAAAGCAGCAAGCTGCTTCCTGTTACC
GTTCGACTGCATGTGTTAGGCCTGCCGCCAGCGTTCAATCTGAGCCAGGATCAAACTCT
>1172
CTGCTGCCGCCCGTAGGAGTCTGGACCGTGTCTCAGTTCCAGTGTGGCTGGTCATCCTCTCAGACCAGCTAGG
GATCGTCGCCTAGGTGAGCCGTTACCCCACCTACTAGCTAATCCCATCTGGGCACATCCGATGGCAAGAGGCC
CTAAGGTCCCCCTCTTGTGTTGCGACGTATGCGGTATTAGCTACCGTTTCCAGTAGTTATCCCCTCCATCGGG
CAGTTTCCCAGACATTACTCACCCGTCCGCCACTCGTCAGCAAGCAGCAAGCTGCTTCCTGTACCGTTCGATG
CATGTGTTAGGCTGCCGCCAGCGTCAATCTGAGCCAGGATCAAACTCT
>1173
CTGCTGCCGCCCGTAGGAGTCTGGACCGTGTCTCAGTTCCAGTGTGGCTGGTCATCCTCTCAGACCAGCTAGG
GATCGTCGCCTAGGTGAGCCGTTACCCCACCTACTAGCTAATCCCATCTGGGCACATCCGATGGCAAGAGGCC
CTAAGGTCCCCCTCTTTGGTCTGCGACGTTATGCGGTATTAGCTACCGTTTCCAGTAGTTATCCCCCTCCATC
GGGCAGTTTCCCAGACATTACTCACCCGTCCGCCACTCGTCAGCAAAGCAGCAAGCTGCTTCCTGTTACCGTT
CGACTGCATGTGTTAGGCCTGCCGCCAGCGTCAATCTGAGCCAGGATCAAACTCT
>1174
CTGCTGCCGCCCTAGGAGTCTGGACCGTGTCTCAGTTCCAGTGTGGCTGGTCATCCTCTCAGACCAGCTAGGG
ATCGTCGCCTAGGTGAGCCGTTACCCCACCTACTAGCTAATCCCATCTGGGCACATCCGATGGCAAGAGGCCC
TAAGGTCCCCCTCTTGTCTTGCGACGTTATGCGGTATTAGCTACCGTTTCCAGTAGTTATCCCCCTCCATCAG
GCAGTTTCCCAGACATTACTCACCCGTCCGCCACTCGTCAGCAAAGCAGCAAGCTGCTTCCTGTTACCGTTCG
ACTGCATGCGTTAGGCCTGCCGCAGCGT
>1175
CTGCTGCCGCCCGTAGGAGTCTGGACCGTGTCTCAGTTCCAGTGTGGCTGGTCATCCTCTCAGACCAGCTAGG
GATCGTCGCCTAGGTGAGCCGTTACCCCACCTACTAGCTAATCCCATCTGGGCACATCCGATGGCAAGAGGCC
CTAAGGTCCCCCTCTTGTCTTGCGACGTTATGCGGTATTAGCTACCGTTTCCAGTAGTTATCCCCCTCCATCG
GGCGATGTTCCCAGACATTACTCACCCGTCCGCCACTCGCAGCAAGCAGCAAGCTGCTTCCTGTTACCGTTCG
ACTGCATGTGTTAGGCCTGCCGCCAGCGTCAATCTGAGCCAGGATCAACTCT
>1176
CTGCTGCCGCCCGTAGGAGTCTGGACCGTGTCTCAGTTTCCAGTGTGGCTGGTCATCCTCTCAGACCAGCTAG
GGATCGTCGCCTAGGTGAGCCGTTTACCCCACCTACTAGCTAATCCCATCTGGGCACATCCGATGGCAAGAGG
CCCTAAGGTCCCCCTCTTGGTCTTGCGACGTTATGCGGTATTAGCTACCGTTTCCAGTAGTTTATCCCCCTCC
ATCAGGCAGTTTTCCCAGACATTACTCACCCGTCCGCCACTCTCAGCAAGCAGCAAGCTGCTTCCTGTTACCG
TTCGACTGCATGTGTTAG
>1177
CTGCTGCCGCCCGTAGGAGTCTGGACCGTGTCTCAGTTTCCAGTGTGGCTGGTCATCCTCTCAGACCAGCTAG
GGATCGTCGCCTAGGTGAGCCGTTACCCCACCTACTAGCTAATCCCATCTGGGCACATCCGATGGCAAGAGGC
CCTAAGGTCCCCCTCTTTGTCTTGCGACGTATGCGGTATTAGCTACCGTTTCCAGTAGTTATCCCCCTCCATC
AGGCAGTTTTCCCAGACATTACTCACCCGTCCGCCACTCGTCAGCAAAGCAGCAAGCTGCTTCCTGTTACCGT
TCGACTGCATGTGTTTAGGCCTGCCGCCAGCGTCAATCTGAGCCAGGATCAAACTCT
>1178
CTGCTGCCGCCCGTAGGAGTCTGGACCGTGTCTCAGTTCCAGTGTGGCTGGTCATCCTCTCAGACCAGCTAGG
GATCGTCGCCTAGGTGAGCCGTTACCCCACCTACTAGCTAATCCCATCTGGGCACATCCGATGGCAAGAGGCC
CTAAGGTCCCCCTCTTGTCTTGCGACGTATGCGGTATTAGCTACCGTTTCCAGTAGTTATCCCCCTCCATCAG
GCAGTTTCCCAGACATTACTCACCCGTCCGCCACTCGTCAGCAAAGCAGCAAGCTGCTTCTGTTACCGTTCGA
CTGCATGTGTTAGGCCTGCCGCCAGCGTTCAATCTGAGCCAGGACAAACTGT
>1179
CTGCTGCCGCCCGTAGGAGTCTGGACCGTGTCTCAGTTCCAGTGTGGCTGGTCATCCTCTCAGACCAGCTAGG
GATCGTCGCCTAGGTGAGCCGTTACCCCACCTACTAGCTAATCCCATCTGGGCACATCCGATGGCAAGAGGCC

CTAAGGTCCCCCTCTTGTCTGCGACGTTATGCGGTATTAGCTACCGTTTCCAGTAGTTATCCCCCTCCATCGG
GCAGTTTCCCAGACATTACTCACCCGTCCGCCACTCGTCAGCAAAGCAGCAAGCTGCTTCCTGTTACCGCTCG
ACTGCATGTGTTAGGCCTGCCGCCAGCGTCAATCTGAGCCAGGATCAAACTCT
>1180
CTGCTGCCGCCCGTAGGAGTCTGGACCGTGTCTCAGTTCCAGTGTGGCTGGTCATCCTCTCAGACCAGCTAGG
GATCGTCGCCTAGGTGAGCCGTTACCCCACCTACTAGCTAATCCCATCTGGGCACATCCGATGGCAAGAGGCC
CTAAGGTCCCCCTCTTTGGTCTTGCGACGTTATGCGGTATTAGCTACCGTTTCCAGTAGTTATCCCCCTCCAT
CAGGCAGTTTCCCAGACATTACTCACCCGTCCGCCACTCGTCAGCAAAGCAGCAAGCTGCTTCCTGTTACCGT
TCGACTGCATGTGTTAGGCCTGCCGCCAGCGTCAATCTGAGCCAGGATCAAACTCT
>1181
CTGCTGCCGCCCGTAGGAGTCTGGACCGTGTCTCAGTTCCAGTGTGGCTGGTCATCCTCTCAGACCAGCTAGG
GATCGTCGCCTAGGTGAGCCGTTACCCCACCTACTAGCTAATCCCATCTGGGCACATCCGATGGCAAGAGGCC
CTAAGGTCCCCCTCTTGTCTTGCGACGTATGCGGTATTAGCTACCGTTTCCAGTAGTTATCCCCCTCCATCAG
GCAGTTTCCCAGACATTACTCACCCGTCCGCCACTCGTCAGCAAACAGCAAGCTGCTTCCTGTTACCGTTCGA
ACTGCATGTGTTA
>1182
CTGCTGCCGCCCGTAGGAGTCTGGACCGTGTCTCAGTTCCAGTGTGGCTGGTCATCCTCTCAGACCAGCTAGG
GATCGTCGCCTAGGTGAGCCGTTACCCCACCTACTAGCTAATCCCATCTGGGCACATCCGATGGCAAGAGGCC
CTAAGGTCCCCCTCTTTGGTCTTGCAACGTTATGCGGTATTAGCTACCGTTTCCAGTAGTTATCCCCCTCCAT
CAGGCAGTTTCCCAGACATTACTCACCCGTCCGCCACTCGTCAGCAAAAGCAGCAAGCTGCTTCTGTTACCGT
TCGACTGCATGTGTTAGGCCTGCCGCCAAGCGTCAAT
>1183
CTGCTGCCGCCCGTAGGAGTCTGGACCGTGTCTCAGTTCCAGTGTGGCTGGTCATCCTCTCAGACCAGCTAGG
GATCGTCGCCTAGGTGAGCCGTTACCCCACCTACTAGCTAATCCCATCTGGGCACATCCGATGGCAAGAGGCC
CTAAGGTCCCCCTCTTGTCTGCGACGTTATGCGGTATTAGCTACCGTTTCCAGTAGTTATCCCCCTCCATCGG
GCAGTTTCCCGGACATTACTCACCCGTCCGCCACTCGTCAGCAAAGCAGCAAGCTGCTTCCTGTTACCGTTCG
ACTGCATGTGTTAGGCCTGCCGCCAGCGTCAATCTGAGCCAGGATCAAACTCT
>1184
CTGCTGCCGCCCGTAGGAGTCTGGACCGTGTCTCAGTTCCAGTGTGGCTGGTCATCCTCTCAGACCAGCTAGG
GATCGTCGCCTAGGTGAGCCGTTACCCCACCTACTAGCTAATCCCATCTGGGCACATCCGATGGCAAGAGGCC
CTAAGGTCCCCCTCTTGGTCTTGCGACGTTATGCGGTATTAGCTACCGTTTCCAGTAGTTATCCCCTCCATCG
GGCAGTTTCCCAGACATTACTCACCCGTCCGCCACTCGTCAGCAAAGCAGCAAGCTGCTTCCTGTTACCGTTC
GACTGCATGTGTTAGGCCTGCCGCCAGCGTCAATCTGAGCCAGGATCAAACTCT
>1185
CTGCTGCCGCCCGTAGGAGTCTGGACCGTGTCTCAGTTCCAGTGTGGCTGGTCATCCTCTCAGACCAGCTAGG
GATCGTCGCCTAGGTGAGCCGTTACCCCACCTACTAGCTATTCCCATCTGGGCACATCCGATGGCAAGAGGCC
CTAAGGTCCCCCTCTTGTCTGCGACGTATGCGGTATTAGCTACCGTTTCCAGTAGTTATCCCCCTCCATCGGG
CAGTTACCCAGACATTACTCACCCGTCCGCCACTCGTCAGCAAAGCAGCAAGCTGCTTCCTGTTACCGTTCGA
CTGCATGTGTTAGGCCTGCCACCAGCGTTCAATCTGAGCCAGGATCAAACTCT
>1186
CTGCTGCCGCCCGTAGGAGTCTGGACCGTGTCTCAGTTCCAGTGTGGCTGGTCATCCTCTCAGACCAGCTAGG
GATCGTCGCCTAGGTGAGCCGTTACCCCACCTACTAGCTAATCCCATCTGGGCACATCCGATGGCAAGAGGCC
CTAAGGTCCCCCTCTTGTCTGCGACGTATGCGGTATTAGCTACCGTTTCCAGTAGTTATCCCCCTCCATCGGG
CAGTTTCCCAGACATTACTCACCCGTCCGCCACTCGTCAGCAAGCAGCAAGCTGCTTCCTGTTACCGTTCGAC
TGCATGTGTTAGGCCTGCCGCCAGCGTCAATCTGAGCCAGGATCAACTCT
>1187
CTGCTGCCGCCCGTAGGAGTCTGGACCGTGTCTCAGTTCCAGTGTGGCTGGTCATCCTCTCAGACCAGCTAGG
GATCGTCGCCTAGGTGAGCCGTTACCCCACCTACTAGCTAATCCCATCTGGGCACATCCGATGGCAAGAGGCC
CTAAGGTCCCCCTCTTTGGTCTTGCGACGTTATGCGGTATTAGCTACCGTTTCCAGTAGTTATCCCCCTCCAT
CAGGCAGTTTCCCCAGACATTACTCACCCGTCCGCCACTCGTCAGCAAAGCAGCAAGCTGCTTTCTGTTACCG
TTCGACTGCATGTGTTAGGCCTGCCGCCAGCGTCAATCTGAGCCAGGATCAAACTCT
>1188
CTGCTGCCGCCCGTAGGAGTCTGGACCGTGTCTCAGTTCCAGTGTGGCTGGTCATCCTCTCAGACCAGCTAGG
GATCGTCGCCTAGGTGAGCCGTTACCCCACCTACTAGCTAATCCCATCTGGGCACATCCGATGGCAAGAGGCC

CTAAGGTCCCCCTCTTGTCTGCGACGTATGCGGTATTAGCTACCGTTCCAGTAGTATCCCCTCCATCAGGCAG
TTTCCCAGACATTACTCACCCGTCCGCCACTCGTCAGCAAAGCAGCAAGCTGCTTCTGTTACCGTTCGACTGC
ATGTGTTAGGCCTGCCGCCAGCGTCAATCTGAGCCAGGATCAACTCT
>1189
CTGCTGTCGCCCGTAGGAGTCTGGACCGTGTCTCAGTTCCAGTGTGGCTGGTCATCCTCTCAGACCAGCTAGG
GATCGTCGCCTAGGTGAGCCGTTACCCCACCTACTAGCTAATCCCATCTGGGCACATCCGATGGCAAGAGGCC
CTAAGGTCCCCCTCTTTGTCTGCGACGTATGCGGTATTAGCTACCGTTTCCAGTAGTTATCCCCCTCCATCAG
GCAGTTTTCCCCAGACATTACTCACCCGTCCGCCACTCGTCAGCAAACAGCAAGCTGCTTCCTGTTACCGTTC
GACTGCATGTGTTAGGCCTGCCGCCAGCGTCAATCTGAGCCAGGATCAAACTCT
>1190
CTGCTGCCGCCCGTAGGAGTCTGGACCGTGTCTCAGTTCCAGTGTGGCTGGTCATCCTCTCAGACCAGCTAGG
GATCGTCGCCTAGGTGAGCCGTTACCCCACCTACTAGCTAATCCCATCTGGGCACATCCGATGGCAAGAGGCC
CTAAGGTCCCCCTCTTTGTCTTGCGACGTTATGCGGTATTAGCTACCGTTTCCAGTAGTTATCCCCCTCCATC
AGGCAGTTTCCCAGACATTACTCACCCGTCCGCCACTCGTCAGCAAAGCAGCAAGCTGCTTCCTGTTACCGTT
CGACTGCATGTGTTAGGCCTGCCGCCAGCGTTCGACTCGAAGCC
>1191
CTGCTGCCGCCCGTAGGAGTCTGGACCGTGTCTCAGTTCCAGTGTGGCTGGTCATCCTCTCAGACCAGCTAGG
GATCGTCGCCTAGGTGAGCCGTTACCCCACCTACTAGCTAATCCCATCTGGGCACATCCGATGGCAAGAGGCC
CTAAGGTCCCCCTCTTTGGTCTTGCGACGTTATGCGGTATTAGCTACCGTTCCCAGTAGTAATCCCCCCTCCA
TCAGGCAGTTTCCCAGACATTACTCACCCGTCCGCCACTCGTCAGCAAAGCAGCAAGCTGCTTCCTGTTACCC
GTTCGACTGCATGTGTTAGGCCTGCCGCCAGCGTCAATCTGAGCCAGGATCAAACTCT
>1192
CTGCTGCCGCCCGTAGGAGTCTGGACCGTGTCTCAGTTCCAGTGTGGCTGGTCATCCTCTCAGACCAGCTAGG
GATCGTCGCCTAGGTGAGCCGTTACCCCACCTACTAGCTAATCCCATCTGGGCACATCCGATGGCAAGAGGCC
CTAAGGTCCCCCTCTTTGTCTTGCGACGTATGCGGTATTAGCTACCGTTTCCAGTAGTTATCCCCCCTCCATC
AGGCAGTTTCCCAGACATTACTCACCCGTCCGCCACTCGTCAGCAAAACAGCAAGCTGCTTCCTGTTACCGTT
CGACTGCATGTGTTAGGCCTGCCGCCAGCGTCAATCTGAGCCAGGATCAAACTCT
>1193
CTGCTGCCCCCGTAGGAGTCTGGACCGTGTCTCAGTTCCAGTGTGGCTGGTCATCCTCTCAGACCAGCTAGGG
ATCGTCGCCTAGGTGAGCCGTTACCCCACCTACTAGCTAATCCCATCTGGGCACATCCGATGGCAAGAGGCCC
TAAGGTCCCCCTCTTTGGTCTTGCGACGTTATGCGGTATTAGCTACCGTTTCCAGTAGTTATCCCCCTCCATC
GGGCAGTTTCCCAGACATTACTCACCCGTCCGCCACTCGTCAGCAAAGCAGCAAGCTGCTTCCCGTTACCGTT
CGACTGCATGTGTTAGGCCTGCCGCCAGCGTCAATCTGAGCCAGGATCAAACTCT
>1194
CTGCTGCCGCCCGTAGGAGTCTGGACCGTGTCTCAGTTCCAGTGTGGCTGGTCATCCTCTCAGACCAGCTAGG
GATCGTCGCCTAGGTGAGCCGTTACCCCACCTACTAGCTAATCCCATCTGGGCACATCCGATGGCAAGAGGCC
CTAAGGTCCCCCTCTTGTCTTGCGACGTTATGCGGTATTAGCTACCGTTTCCAGTAGTTATCCCCCTCCATCA
GGCAGTTTCCCAGACATTACTCACCCGTCCGCCACTCGTCAGCAAAGCAGCAAGCTGCTTCCTGTTACCGTCG
ACTTGCATGTGTTAGGCCTGCCGCCAGCCGTTCAATCTGAAGCCAGGATCAAACTCT
>1195
CTGCTGCCGCCCGTAGGAGTCTGGACCGTGTCTCAGTTCCAGTGTGGCTGGTCATCCTCTCAGACCAGCTAGG
GATCGTCGCCTAGGTGAGCCGTTACCCCACCTACTAGCTAATCCCATCTGGGCACATCCGATGGCAAGAGGCC
CTAAGGTCCCCCTCTTTGTCTTGCGACGTTATGCGGTATTAGCTACCGTTTCCAGTAGTTATCCCCCTCCATC
AGGCAGTTTCCCAGACATTACTCACCCGTCCGCCACTCGTCAGCAAAGCAGCAAGCTGCTTTCTGTTACCGTT
CGAACTGCATGTGTTAGGCCTGCCGCCAGCGTCAATCTGAGCCAGGATCAAACGTCT
>1196
CTGCTGCCGCCCGTAGGAGTCTGGACCGTGTCTCAGTTCCAGTGTGGCTGGTCATCCTCTCAGACCAGCTAGG
GATCGTCGCCTAGGTGAGCCGTTACCCCACCTACTAGCTAATCCCATCTGGGCACATCCGATGGCAAGAGGCC
CTAAGGTCCCCCTCTTTGTCTTGCGACGTTATGCGGTATTAGCTACCGTTTCCAGTAGTTATCCCCCTCCATC
GGGCAGCTTCCCAGACATTACTCACCCGTCCGCCACTCGTCAGCAAAGCAGCAAGCTGCTTCCTGTTACCGTT
CGACTGCATGTGTTAGGCCTGCCGCCAGCGTTCAATCTGAGCCAGGATCAAACTCT
>1197
CTGCTGCCGCCCGTAGGAGTCTGGACCGTGTCTCAGTTCCAGTGTGGCTGGTCATCCTCTCAGACCAGCTAGG
GATCGTCGCCTAGGTGAGCCGTTACCCCACCTACTAGCTAATCCCATCTGGGCACATCCGATGGCAAGAGGCC

CTAAGGTCCCCCTCTTGTCTGCGACGTATGCGGTATTAGCTACCGTTTCCAGTAGTTATCCCCCTCCATCGGG
CAGTTTCCCAGACATTACTCACCCGTCCGCCACTCGTCAGCAAAGCAGCAAGCTGCTTCCTGTTACCGTCGAC
TGCATGTGTTAGGCCTGCCGCCAGCGTCAATCTGAGCCAGGATCAAACTCT
>1198
TGCTGCCGCCCGTAGGAGTCTGGACCGTGTCTCAGTTCCAGTGTGGCTGGTCATCCTCTCAGACCAGCTAGGG
GATCGTCGCCTAGGTGAGCCGTTACCCCCACCTACTAGCTAATCCCATCTGGGCACATCCGATGGCAAGAGGC
CCTAAGGTCCCCCTCTTTGGTCTTGCGACGTTATGCGGTATTAGCTACCGTTTCCAGTAGTTATCCCCCTCCA
TCGGGCAGTTTCCCAGACATTACTCACCCGTCCGCCACTCGTCAGCAAAGCAGCAAGCTGCTTCCTGTTACCG
TTCGACTGCATGTGTTAGGCCTGCCGCCAGCGTCAATCTGAGCCAGGATCAAACTCT
>1199
CTGCTGCCGCCCGTAGGAGTCTGGACCGTGTCTCAGTTCCAGTGTGGCTGGTCATCCTCTCAGACCAGCTAGG
GATCGTCGCCTAGGTGAGCCGTTACCCCACCTACTAGCTAATCCCATCTGGGCACATCCGATGGCAAGAGGCC
CTAAGGTCCCCCTCTTTGGTCTTGCGACGTTATGCGGTATTAGCTACCGTTTCCAGTAGTTATCCCCCTCCAT
CAGGCAGTTTCCCAGACATTACTCACCCGTCCGCCACTCGTCAGCAAAGCAGCAAGCTGCTTCTGTTACCGTT
CGACTGCATGTGTTAGGCTGCCGCCAGCGTTCAATCTGAGCCAGATCA
>1200
CTGCTGCCGCCCGTAGGAGTCTGGACCGTGTCTCAGTTCCAGTGTGGCTGGTCATCCTCTCAGACCAGCTAGG
GATCGTCGCCTAGGTGAGCCGTTACCCCACCTACTAGCTAATCCCATCTGGGCACATCCGATGGCAAGAGGCC
CTAAGGTCCCCCTCTTTGGTCTGCGACGTTATGCGGTATTAGCTACCGTTTCCAGTAGTTATCCCCCTCCATC
AGGCAGTTTCCCAGACATTACTCACCCGTCCGCCACTCGTCAGCAAAACAGCAAGCTGCTTCCTGTTACCGTT
CGACTGCATGTGTTAGGCCTGCCGCCAGCGTTCAATCTGAGCCAGGATCAAACCTCT
>1201
CTGCTGCCGCCCGTAGGAGTCTGGACCGTGTCTCAGTTCCAGTGTGGCTGGTCATCCTCTCAGACCAGCTAGG
GATCGTCGCCTAGGTGAGCCGTTACCCCACCTACTAGCTAATCCCATCTGGGCACATCCGATGGCAAGAGGCC
CTAAGGTCCCCCTCTTGTCTGCGACGTATGCGGTATTAGCTACCGTTTCCAGTAGTTATCCCCCTCCATCGGG
CAGTTTCCCAGACATACTCACCCGTCCGCCACTCTCAGCAAAGCAGCAAGCTGCTCCTGTTACCGTTCGACTG
CATGTGTTAGGCCTGCCGCCAGCGTCAATCTGAGCCAGGATCAAACTCT
>1202
CTGCTGCCGCCCGTAGGAGTCTGGACCGTGTCTCAGTTCCAGTGTGGCTGGTCATCCTCTCAGACCAGCTAGG
GATCGTCGCCTAGGTGAGCCGTTACCCCACCTACTAGCTAATCCCATCTGGGCACATCCGATGGCAAGAGGCC
CTAAGGTCCCCCTCTTTGGTCTTGCGAACGTTATGCGGTATTAGCTACCGTTTCCAGTAGTATCCCCTCCCAT
CAGGCAGTTTCCCAGACATTACTCACCCGTCCGCCACTCGTCAGCAAAGCAGCAAGCTGCTTTCTGTTACCGT
TCGACTGCATGTGTTAGGCCTGACCGCCAGCGTTCATCGAGCCAGGTCAAAAG
>1203
CTGCTGCCGCCCGTAGGAGTCTGGACCGTGTCTCAGTTCCAGTGTGGCTGGTCATCCCCTCAGACCAGCTAGG
GATCGTCGCCTAGGTGAGCCATTACCCCACCTACTAGCTAATCCCATCTGGGCACATCCGATGGCAAGAGGCC
CTAAGGTCCCCCTCTTTGTCTTGCGACGTTATGCGGTATTAGCTACCGTTTCCAGTAGTTATCCCCCTCCATC
GGGCAGTTTCCCAGACATTACTCACCCGTCCGCCACTCGTCAGCAAAGCAGCAAGCTGCTTCCTGTTACCGTT
CGACTGCATGTGTTAGGCCTGCCGCCAGCGTCAATCTGAGCCAGGATCAAACTCT
>1204
CTGCTGCCGCCCGTAGGAGTCTGGACCGTGTCTCAGTTCCAGTGTGGCTGGTCATCCTCTCAGACCAGCTAGG
GATCGTCGCCTAGGTGAGCCGTTACCCCACCTACTAGCTAATCCCATCTGGGCACATCCGATGGCAAGAGGCC
CTAAGGTCCCCCTCTTTGGTCTTGCGACGTTATGCGGTATTAGCTACCGTTTCCAGTAGTTATCCCCCTCCAT
CAGGCAGTTTCCCAGACATTACTCACCCGTCCGCCACTCGTCAGCAAAACAGCAAGCTGCTTCCTGTTACCGT
TCGACTGCATGTGTTAGGCCTGCCGCCAGCGTCAATCTGAGCCAGGATCAAACTCT
>1205
CTGCTGCCGCCCGTAGGAGTCTGGACCGTGTCTCAGTTCCAGTGTGGCTGGTCATCCTCTCAGACCAGCTAGG
GATCGTCGCCTAGGTGAGCCGTTACCCCACCTACTAGCTAATCCCATCTGGGCACATCCGATGGCAAGAGGCC
CTAAGGTCCCCCTCTTTGTCTTGCGACGTATGCGGTATTAGCTACCGTTTCCAGTAGTTATCCCCCTCCATCA
GGCAGTTTCCCAGACATTACTCACCCGTCCGCCACTCGTCAGCAAAGCGGCAAGCTGCTTCCTGTTACCGTTC
GACTGCATGTGTTAGGCCTGCCGCCAGCGTCAATCTGAGCCAGGATCAAACGTCT
>1206
CTGCTGCCGCCCGTAGGAGTCTGGACCGTGTCTCAGTTCCAGTGTGGCTGGTCATCCTCTCAGACCAGCTAGG
GATCGTCGCCTAGGTGAGCCGTTACCCCACCTACTAGCTAATCCCATCTGGGCACATCCGATGGCAAGAGGCC

CTAAGGTCCCCCTCTTTGTCTGCGACGTTATGCGGTATTAGCTACCGTTTCCAGTAGTTATCCCCCTCCATCG
GGCAGTTTCCCAGACATTACTCACCCGTCCGCCACTCGTCAGCAAAGCAGCAAGCTGCTTCCTGTTACCGTTC
GACTGCATGTGTTAGGCCTGCCGCCAGCGTCAATCTGAGCCAGGATCAAACTCT
>1207
TGTGCCGCCCGTAGGAGTCTGGACCGTGTCTCAGTTCCAGTGTGGCTGGTCATCCTCTCAGACCAGCTAGGGA
TCGTCGCCTAGGTGAGCCGTTACCCCACCTACTAGCTAATCCCATCTGGGCACATCCCGATGGCAAGAGGCCC
TAAAGGTCCCCCTCTTTGGTCTTGCGACGTTATGCGGGTATTAGCTACCGTTTCCAGTAGTTATCCCCCTCCA
TCGGGCAGTTTCCCAGACATTACTCACCCGTCCGCCACTCGTCAGCAAAGCAGCAAGCTGCTTCCGTTACCGT
TCGATGCATGTGTTAGGCCGCCGCCAGCGTCAATCTGAGCCAGGATCAAACTCT
>1208
CTGCTGCCGCCCGTAGGAGTCTGGACCGTGTCTCAGTTCCAGTGTGGCTGGTCATCCTCTCAGACCAGCTAGG
GATCGTCGCATAGGTGAGCCGTTACCCCACCTACTAGCTAATCCCATCTGGGCACATCCGATGGCAAGAGGCC
CTAAGGTCCCCCTCTTGTCTTGCGACGTATGCGGTATTAGCTACCGTTTCCAGTAGTTATCCCCCTCCATCAG
GCAGTTTCCCAGACATTACTCACCCGTCCCGCCACTCGTCAGCAAAGCAGCAAGCTGCTTCTGTTACCGTTCG
ACTGCATGTGTTAGGCCTGCCGCCAGCCGTTCAATCTGAGCCAGGACAAACGTCT
>1209
CTGCTGCCGCCCGTAGGAGTCTGGACCGTGTCTCAGTTCCAGTGCGGCTGGTCATCCTCTCAGACCAGCTAGG
GATCGTCGCCTAGGTGAGCCGTTACCCCACCTACTAGCTAATCCCATCTGGGCACATCCGATGGCAAGAGGCC
CTAAGGTCCCCCTCTTTGTCTTGCGACGTTATGCGGTATTAGCTACCGTTTCCAGTAGTTATCCCCCTCCATC
AGGCAGTTTCCCAGACATTACTCACCCGTCCGCCACTCGTCAGCAAAGCAGCAAGCTGCTTCTGTTACCGTTC
GACTGCATGTGTTAGGCCTGCCGCCAGCGTCAATCTGAGCCAGGATCAAACTCT
>1210
CTGCTGCCGCCCGTAGGAGTCTGGACCGTGTCTCAGTTCCAGTGTGGCTGGTCATCCTCTCAGACCAGCTAGG
GATCGTCGCCTAGGTGAGCCGTTACCCCACCTACTAGCTAATCCCATCTGGGCACATCCGATGGCAAGAGGCC
CTAAGGTCCCCCTCTTGTCTTGCGACGTTATGCGGTATTAGCTACCGTTCCAGTAGTTATCCCCCTCCATCAG
GCAGTTTCCCAGACATTACTCACCCGTCCGCCACTCGTCAGCAAAGCAGCAAGCTGCTTTCTGTTACCGTTCG
ACTGCATGTGTTAGGCCTGCCGCCAGCGTCAATCGAGCCAGGTCAAAGC
>1211
CTGCTGCCGCCCGTAGGAGTCTGGACCGTGTCTCAGTTCCAGTGTGGCTGGTCATCCTCTCAGACCAGCTAGG
GATCGTCGCCTAGGTGAGCCGTTACCCCACCTACTAGCTAATCCCATCTGGGCACATCCGATGGCAAGAGGCC
CTAAGGTCCCCCTCTTTGGTCTGCGACGTTATGCGGTATTAGCTACCGTTTCCAGTAGTTATCCCCCTCCATC
AGGCAGTTTCCCAGACATTACTCACCCGTCCGCCACTCGTCAGCAAAGCAGCAAGCTGCTTCCTGTTACCGTT
CGACTGCATGTGTTAGGCCTGCCGCCAGCGTTCAATCTGAAGCCAGGATCAAACCGTCT
>1212
TGCTGCCGCCCGTAGGAGTCTGGACCGTGTCTCAGTTCCAGTGTGGCTGGTCATCCTCTCAGACCAGCTAGGG
ATCGTCGCCTAGGTGAGCCGTTACCCCACCTACTAGCTAATCCCATCTGGGCACATCCGATGGCAAGAGGCCC
TAAGGTCCCCCTCTTTGTCTTGCGACGTTATGCGGTATTAGCTACCGTTTCCAGTAGTTATCCCCCTCCATCA
GGCAGTTTCCCAGACATTACTCACCCGTCCGCCACTCGTCAGCAAAGCAGCAAGCTGCTTCCTGTTACCGTTC
GACTGCATGTGTTTAGGCCTGCCGCCAGCGGTTCAA
>1213
CTGCTGCCGCCCGTAGGAGTCTGGACCGTGTCTCAGTTCCAGTGTGGCTGGTCATCCTCTCAGACCAGCTAGG
GATCGTCGCCTAGGTGAGCCGTTTACCCCACCTACTAGCTAATCCCATCTGGGCACATCCGATTGGCAAGAGG
CCCTAAGGTCCCCCTCTTTGTCTTGCGACGTTATGCGGTATTAGCTACCGTTTCCAGTAGTTTATCCCCTCCA
TCGGGCAGTTTTCCCAGACATTACTCACCCGTCCGCCACTCGTCAGCAAGCAGCAAGCTGCTTCCTGTTACCG
TTCGACTGCATGTGTTAGGCCTGCCGCCAGCGTCAATCTGAGCCAGGATCAAACTCT
>1214
CTGCTGCCGCCCGTAGGAGTCTGGACCGTGTCTCAGTTCCAGTGTGGCTGGTCATCCTCTCAGACCAGCTAGG
GATCGTCGCCTAGGTGAGCCGTTTACCCCACCTACTAGCTAATCCCATCTGGGCACATCCGATGGCAAGAGGC
CCTAAGGTCCCCCTCTTTGGTCTTGCGACGTTATGCGGTATTAGCTACCGTTTCCAGTAGTTATCCCCTCCAT
CGGGCAGTTTCCCAGACATTACTCACCCGTCCGCCACTCGTCAGCAAAGCAGCAAGCTGCTTCCTGTTACCGT
TCGACTGCATGTGTTAGGCCTGCCGCCAGCGTCAATCTGAGCCAGGATCAAACTCT
>1215
CTGCTGCCGCCCGTAGGAGTCTGGACCGTGTCTCAGTTCCAGTGTGGCTGGTCATCCTCTCAGACCAGCTAGG
GATCGTCGCCTAGGTGAGCCGTTACCCCACCTACTAGCTAATCCCATCTGGGCACATCCGATGGCAAGAGGCC

CTAAGGTCCCCCTCTTGTCTTGCGACGTTATGCGGTATTAGCTACCGTTTCCAGTAGTTATCCCCCTCCATCA
GGCAGTTTTCCCAGACATTACTCACCCGTCCGCCACTCGTCAGCAAAGCAGCAAGCTGCTTCTGTTACCGTTC
GACTGCATGTGTTAGGCCTGCCGCCAGCGTTCAATCTGAGCCAGGATCAAACTCT
>1216
CTGCTGCCGCCCGTAGGAGTCTGGACCGTGTCTCAGTTCCAGTGTGGCTGGTCATCCTCTCAGACCAGCTAGG
GATCGTCGCCTAGGTGAGCCGTTACCCCACCTACTAGCTAATCCCATCTGGGCACATCCGATGGCAAGAGGCC
CTAAGGTCCCCCTCTTTGTCTTGCGACGTTATGCGGTATTAGCTACCGTTTCCAGTAGTTATCCCCCTCCATC
AGGCAGTTTCCCAGACATTACTCACCCGTCCGCCACTCGTCAGCAAAGCAGCAAGCTGCTTCCTGTTACCGTT
CGACTGCATGTGTTAGGCCTGCCGCCAGCGTCAATCTGAGCCAGGATCAACTCT
>1217
CTGCTGCCGCCCGTAGGAGTCTGGACCGTGTCTCAGTTCCAGTGTGGCTGGTCATCCTCTCAGACCAGCTAGG
GATCGTCGCCTAGGTGAGCCGTTACCCCACCTACTAGCTAATCCCATCTGGGCACATCCGATGGCAAGAGGCC
CTAAGGTCCCCCTCTTTGTCTTGCGACGTATGCGGTATTAGCTACCGTTTCCAGTAGTTATCCCCCTCCATCG
GGCAGTTTCCCAGACATTACTCACCCGTCCGCCACTCGTCAGCAAAGCAGCAAGCTGCTTCCTGTTACCGTTC
GACTGCATGTGTTAGGCCTGCCGCCAGCGTCAATCTGAGCCAGGATCAAACTCT
>1218
CTGCTGCCGCCCGTAGGAGTCTGGACCGTATCTCAGTTCCAGTGTGGCTGGTCATCCTCTCAGACCAGCTAGG
GATCGTCGCCTAGGTGAGCCGTTACCCCACCTACTAGCCAATCCCATCTGGGCACATCCGATGGCAAGAGGCC
[truncated: 450,311 more chars]
